# Comparative structural insights and functional analysis for the distinct unbound states of Human AGO proteins

**DOI:** 10.1038/s41598-025-91849-5

**Published:** 2025-03-19

**Authors:** Panos Kakoulidis, Eleni I. Theotoki, Vasiliki I. Pantazopoulou, Ioannis S. Vlachos, Ioannis Z. Emiris, Dimitrios J. Stravopodis, Ema Anastasiadou

**Affiliations:** 1 Department of Informatics and Telecommunications, National and Kapodistrian University of Athens, 16122 Athens, Greece; 2 Center of Basic Research, Biomedical Research Foundation of the Academy of Athens, 4 Soranou Ephessiou St, 11527 Athens, Greece; 3 Section of Cell Biology and Biophysics, Department of Biology, School of Science, National and Kapodistrian University of Athens, 15701 Athens, Greece; 4 Department of Pathology, Medical School, National and Kapodistrian University of Athens, 11527 Athens, Greece; 5 Broad Institute of MIT and Harvard, Merkin Building, 415 Main St, Cambridge, MA 02142 USA; 6 Cancer Research Institute, Beth Israel Deaconess Medical Center, 330 Brookline Avenue, Boston, MA 02215 USA; 7 Department of Pathology, Beth Israel Deaconess Medical Center, 330 Brookline Avenue, Boston, MA 02215 USA; 8 Harvard Medical School, 25 Shattuck Street, Boston, MA 02115 USA; 9 Spatial Technologies Unit, Harvard Medical School Initiative for RNA Medicine, Beth Israel Deaconess Medical Center, 330 Brookline Avenue, Dana BuildingBoston, MA 02215 USA; 10 ATHENA Research Center, Aigialias & Chalepa, 15125 Marousi, Greece; 11 Department of Health Science, Higher Colleges of Technology (HCT), Academic City Campus, 17155 Dubai, United Arab Emirates

**Keywords:** Molecular biology, RNAi, Structural biology, Molecular modelling, Data integration, Computational chemistry, Mitosis, Protein function predictions, Software

## Abstract

The four human Argonaute (AGO) proteins, critical in RNA interference and gene regulation, exhibit high sequence and structural similarity but differ functionally. We investigated the underexplored structural relationships of these paralogs through microsecond-scale molecular dynamics simulations. Our findings reveal that AGO proteins adopt similar, yet unsynchronized, open-close states. We observed similar and unique local conformations, interdomain distances and intramolecular interactions. Conformational differences at GW182/ZSWIM8 interaction sites and in catalytic/pseudo-catalytic tetrads were minimal. Tetrads display conserved movements, interacting with distant miRNA binding residues. We pinpointed long common protein subsequences with consistent molecular movement but varying solvent accessibility per AGO. We observed diverse conformational patterns at the post-transcriptional sites of the AGOs, except for AGO4. By combining simulation data with large datasets of experimental structures and AlphaFold’s predictions, we identified proteins with genomic and proteomic similarities. Some of the identified proteins operate in the mitosis pathway, sharing mitosis-related interactors and miRNA targets. Additionally, we suggest that AGOs interact with a mitosis initiator, zinc ion, by predicting potential binding sites and detecting structurally similar proteins with the same function. These findings further advance our understanding for the human AGO protein family and their role in central cellular processes.

## Introduction

AGO proteins constitute a subfamily of the Argonaute protein family, which are highly conserved and widely expressed proteins across species, from prokaryotes to eukaryotes. Each eukaryotic AGO protein encompasses an N-terminal (N) domain, a Piwi/Argonaute/Zwille (PAZ) domain, a Middle (MID) domain and a P-element Induced Wimpy testis (PIWI) domain. They also include domain linkers such as L1 that links N-terminal with PAZ and L2 that links PAZ with MID. Most of the domains are present in all prokaryotic AGOs, despite having low sequence similarity with the eukaryotic ones^1^. This structural conservation is not random since the entire AGO protein family regulates the expression of genes, a critical and fundamental biological function across organisms. AGO proteins compose RNA-induced silencing complexes (RISCs) in conjunction with other proteins and small RNAs to mediate post-transcriptional gene silencing. In humans, microRNAs (miRNAs) bind to AGO proteins and consist of approximately 22 nucleotides. MiRNA guides RISC to mRNA targets, leading to translation inhibition or mRNA degradation. The miRNA-mRNA binding relies on the base complementarity between a subsequence of the guide miRNA called seed region that resides in its 5’-end and a subsequence of the target mRNA called microRNA response elements (MREs) that is usually located in the Untranslated Region of its 3’-end (3’-UTR). The 5’-end of miRNA binds to the MID domain of AGO protein, supported by a supplementary binding between the 3’-end and the PAZ domain. The bound miRNAs interact with all AGO linkers and domains via protein secondary structure folds^2–6^.

The human AGO protein family consists of AGO1, AGO2, AGO3 and AGO4 and each AGO member includes approximately 860 amino acids (aa). AGOs share a high conservation in gene/protein sequence and protein structure, each of them with overlapping or distinct functions per biological pathway^7,8^, justifying the existence of the four distinguished members. For instance, AGO2 and AGO3 possess a metal-coordinating catalytic tetrad (DEDH) in their PIWI domain that enables them to perform RNA slicing^9^. In contrast, AGO1 and AGO4 have not been observed yet to have such a function, although they retain the second catalytic residue (“glutamate finger”) and thus playing other subsidiary roles^1,2^. Another distinction is evident on miRNA sorting mechanisms, such as the dependence of AGO3’s slicing activity on miRNA length, presumably reflecting the impact of flanking regions for a sufficient target cleavage and the preferential miRNA loading on the four AGOs in a competitive setting^9,10^. Also, each human AGO protein displays a different pattern in post-transcriptional modifications and interacts with a unique subset of proteins. At the genomic level, *AGO1, AGO3, AGO4* genes reside in a consecutive order within the p-arm of Chromosome 1, whereas *AGO2* gene resides in the q-arm of Chromosome 8, near a telomeric region^11^. Despite these differences, research into their unique functions might result to contradictory findings such as AGO2’s antiviral activity against (SARS-CoV-2)^12^ but not against other viruses^13^.

Here, we employ molecular dynamics (MD) simulations to comparatively explore the unbound states of the four AGO proteins. MD simulations provide atom-level insights on a protein structure and generate different conformational states in a time-scaled computation of molecular motions. As we initially attempted to study the proteins using AGO antibodies, we realized that extended research at such level of information is prohibitive in terms of resources, time or experimental feasibility. Previous MD studies have focused primarily on mutated or bound states of AGO2 to investigate active sites^14,15^ and nucleic acid binding^3,4,16–25^. Due to the prioritization of the catalytically active AGO2, research on the other AGOs lags behind. In this study, we focus on comparing the four human AGOs by their whole structures, their domains and their interaction sites on the microsecond scale. Our results reveal distinct molecular motions and accessibility for each AGO protein despite their high resemblance of structure and sequence. Also, we extend our analysis on human AGO proteins by predicting binding patterns on refined structures and utilizing the simulation data to identify structurally similar human protein structures. Based on the structural relationships with other proteins, we present potential links with mitosis and zinc ions, which are linked with structural stability and mitosis regulation. Extending our knowledge on the human AGO paralogs will allow us to disambiguate their RNA binding function and more importantly, the mechanism of miRNA targeting. The presented atomistic and functional details of human AGOs provide critical information for drug design, mutagenesis studies and the development of improved experimental methods that are applicable beyond our species due to the AGO family’s omnipresence in all living organisms.

## Results

### The varying conformational stability of the unbound human AGOs

**Fig. 1:**
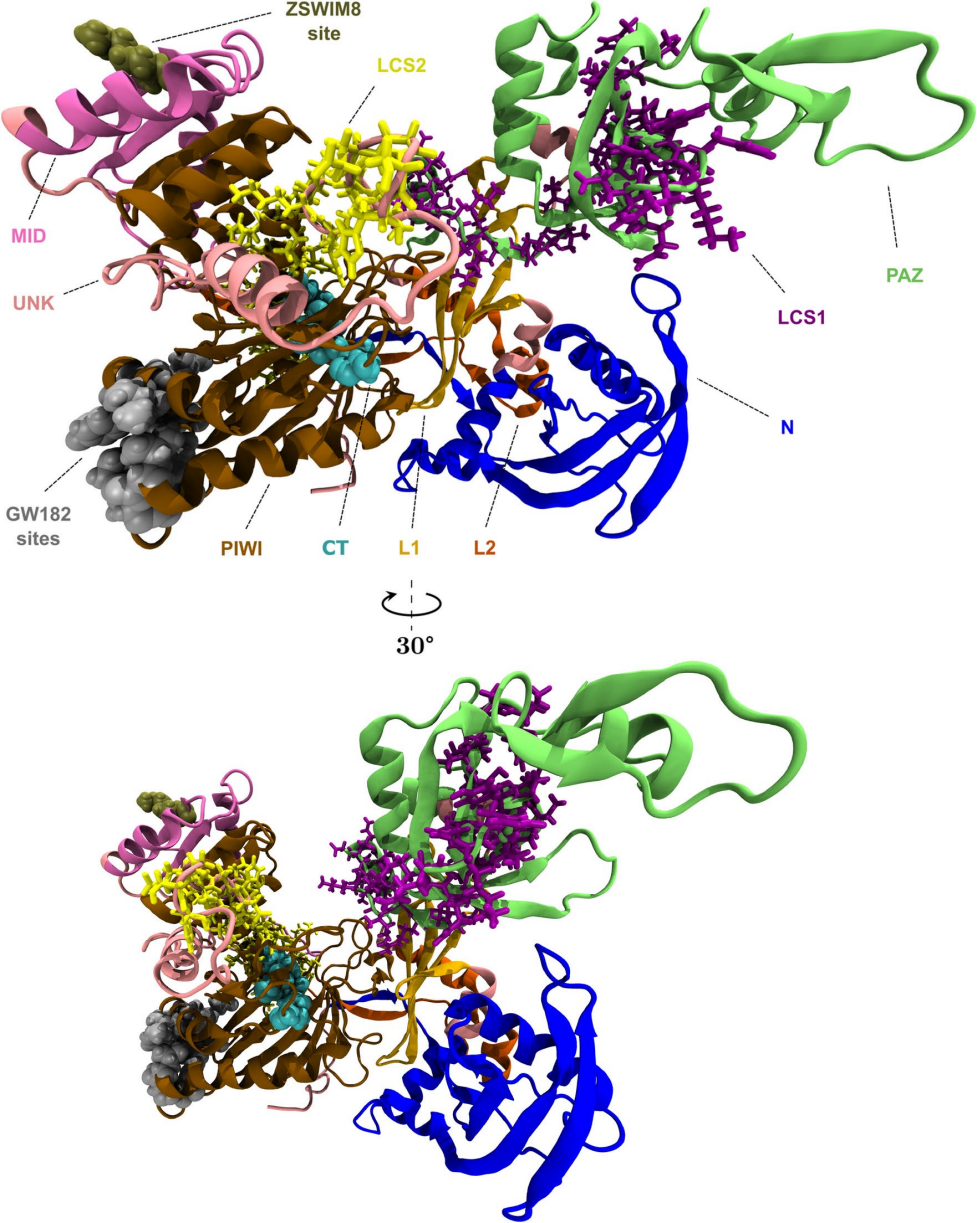
Domains, binding sites and conserved segments of AGO proteins annotated on human AGO2 protein structure. The AGO2 structure (PDB ID: 4Z4D) is displayed in New Cartoon graphical representation, the binding sites are displayed in Van der Waals graphical representation and the conserved segments are displayed in Licorice graphical representation. Domains: N domain in blue (amino acid [aa] positions: 36–166), L1 linker in light orange (aa positions: 175–227), PAZ domain in light green (aa positions: 235–370), L2 linker in orange (aa positions: 374–420), MID domain in light purple (aa positions: 429–510), PIWI domain in brown (aa positions: 517–818), residues not belonging to a specific domain are represented by ‘UNK’ (unknown domain origin) in pink. Binding sites: catalytic tetrad (CT) in light blue (aa positions: 597, 737, 669, 807), ZSWIM8-related in dark gold (aa position: 493), GW182-related in gray (aa positions: 590, 620, 651, 653, 654, 657, 659, 660, 694, 695, 698). Conserved segments: Longest common subsequence 1 (LCS1) in purple (aa positions: 322–365), LCS2 in yellow (aa positions: 782–829). The area between MID-PIWI and PAZ-N constitutes the nucleic acid binding channel, where the CT sites reside. The structure was preprocessed with Schrödinger Maestro suite.

The free state of AGO proteins is the universal starting point that determines their ability to form complexes and perform their functions. In three independent (R1, R2, R3) simulations per AGO protein (12 μs in total), we compared the four human AGO proteins in their unbound states by leveraging their available experimentally solved structures. We selected crystal structures with high resolution/minimum missing residues to ensure a realistic simulation. We utilized the solved structures of human AGO1 bound to endogenous Sf9 RNA (PDB entry: 4KRE^26^) and human AGO2 bound to t1-G target RNA (PDB entry: 4Z4D^27^) that fulfill our quality criteria (Fig. 1). For the remaining two AGOs, we used the only available experimental structures of AGO3 (PDB entry: 5VM9^9^) and AGO4 (PDB entry: 6OON^28^). We assessed AGO3 to be the most mobile protein and AGO4 as the most rigid, based on RMSD (Root Mean Square Deviation) measurements (Figures S1, S2, Supplementary Data 2). The global conformational transitions of the proteins reflect the open-closed conformation states of the nucleic acid binding channel, as previously documented for AGO2-AGO3^19,25^ (Figure S2). Averaged Root Mean Square Fluctuations (RMSF) measurements designate AGO1 and AGO4 as the least mobile proteins among the four, with AGO3 being the most flexible (Supplementary Data 3). Concerning the level of compactness, AGO4 seems to be the most compact, whereas AGO3 and AGO2 appear to be the least compact structures (Figure S3).

**Fig. 2:**
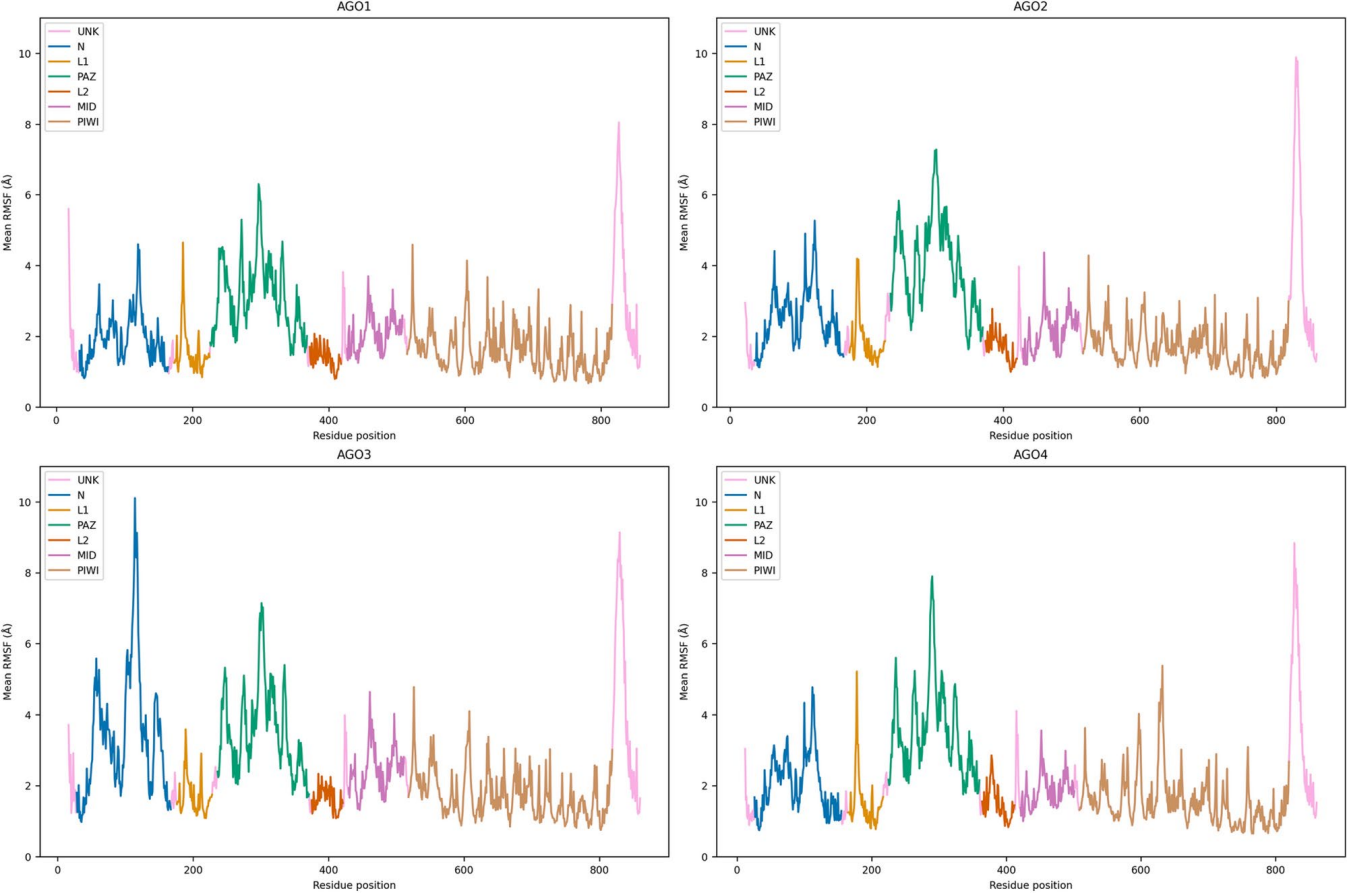
Mean Root Mean Square Fluctuation analysis per AGO for R1, R2, R3 replicas. Domains are represented with distinct colors in the RMSF curve. Amino acid positions that do not belong to a specific domain are labeled as ‘UNK’ (unknown domain origin). The coloring matches the one used in Fig. 1 for convenience.

Considering the domains, we observed distinct and similar motions, based on statistical tests on domain-based RMSD with p-value < 0.001 (Figure S4, Supplementary Data 2). The N domain of AGO3 demonstrates the largest and most variable conformational alterations. We observed that the trajectories of all AGO N domains exhibit statistically significant differences (see “Evaluation of the measurements on simulated data” in “Methods”). According to the statistical tests (Supplementary Data 2), the PIWI domains of AGO2 and AGO4 exhibit similar molecular motions to those of AGO1 PIWI, while all three differ from the motions of AGO3’s PIWI domain. Based on the Root Mean Square Fluctuations (RMSF) computations, PIWI demonstrates decreased movement compared to N, PAZ and MID domains (Fig. 2, Table S1).The MID domain of AGO3 also shows statistically significant differences in RMSD compared to the equivalent domains of AGO1 and AGO4. Regarding the PAZ domain, the RMSD trajectories of the AGO1 and AGO3 domains are assessed to be different with statistical significance. In general, PAZ domain’s RMSD trajectories have large mean values compared to the trajectories of the other domains per AGO, except for AGO3. This is also shown by the corresponding RMSF curves (Fig. 2). There are differences between the RMSD distributions of linkers as AGO3 L1 and L2 distributions differ from those of AGO2. Also, AGO3 L1 distribution is distant from the AGO1 L1 distribution. The AGO4 L2 RMSD values are statistically significantly different from the corresponding measurements of AGO1 and AGO3 L2 domains. The findings on the domains complement the global ones, providing further insight into the disordered AGO3^9^ and the conformationally stable AGO4, while they reveal less obvious differences in the domains of the catalytic AGO2.

We scrutinized the flexibility of specific amino acids in AGO proteins using RMSF measurements, focusing on critical RNA interference (RNAi) pathway interaction sites, post-transcriptional sites, and common segments (Fig. 1). We found that each catalytic tetrad^26^ of AGO2-AGO3 and pseudo-catalytic tetrad^26,28^ of AGO1-AGO4 (CT sites) follows a similar limited mobility pattern, with an average Frechet distance of ~ 0.6 Å between their RMSF curves (Figure S5). The first and third CT residues were observed to be less flexible than the second residue in the tetrads of all AGOs. We analyzed the interaction sites of AGOs with Glycine-tryptophan repeat–containing protein of 182 kDa (GW182, also known as trinucleotide repeat containing 6, TNRC6) and Zinc Finger SWIM-Type Containing 8 (ZSWIM8). GW182 binds to several AGO proteins, controlling mRNA degradation^29^ and ZSWIM8 is involved with target-directed miRNA degradation (TDMD)^30,31^. Our analysis yielded minimal differences in the molecular movements of the AGO-GW182/ZSWIM8 interaction sites^29,31^ among the AGO structures (Figure S6, Table S2, Supplementary Data 3). We found that the fluctuations of post-transcriptional modification (PTM) sites^32^ range approximately from 1 to 9 Å in all AGOs except for AGO4, where RMSF varies within a ~ 1–3 Å interval (Tables S3-6). We identified a long common protein subsequence in all human AGO isoforms (LCS1) and another in AGO2-AGO3 (LCS2), both located in the nucleic acid binding channel (Fig. 1) with ~ 80% gene coding sequence (CDS) identity (Table S7, Supplementary Data 4). There are minimal differences in secondary protein structure (Figure S7) and there are no statistically significant conformational changes in this area per AGO, having ~ 1.3 Å of mean Frechet distance between RMSF curves and ~ 1.2 Å of averaged mean RMSF (Figure S8, Supplementary Data 3). LCS sequences are conserved in AGO proteins of other organisms belonging to different taxa (Supplementary Data 5), such as *Drosophila melanogaster* AGO1, which contains both subsequences with ~ 1–2 mismatches (Supplementary Data 6).

The interaction networks between AGOs and water molecules are crucial for RISC assembly and miRNA binding^28^. We computed the Solvent Accessible Surface Area (SASA) for each AGO and we came across well-defined variations, especially in the domain level. In the context of whole structures, we observed that AGO3 is the most solvent accessible among the four AGOs, while the others displayed limited difference with no statistical significance (Figure S9, Supplementary Data 7). We found that PIWI and PAZ domains are the most accessible domains in each AGO with high variance. The solvent accessibility of AGO1/AGO3 L2 and AGO1/AGO4 L2 pairs displayed statistically significant difference (Figure S10, Supplementary Data 7). We also assessed the exposure to solvent of specific sites and segments. The solvent accessibility of AGO-GW182/ZSWIM8 interaction sites (Figures S11, S12) and the fourth CT site exhibited limited variance (Figure S13). All the CT sites demonstrated a noisy exposure pattern in the range of ~ 1–4 signal-to-noise ratio (SNR), particularly at the first and third positions, which had the highest noise levels (~ 0.6–2 SNR) (Figures S13-16, Supplementary Data 7). The second and fourth CT amino acids were consistently the most accessible to solvent per AGO (Figures S13-16). Concerning segments, the exposure of LCS1 and LCS2 varied between AGOs, although the segments have identical sequences (Figures S17-18, Supplementary Data 7). For instance, the pairs AGO1 LCS1/AGO2 LCS1 and AGO2 LCS2/AGO3 LCS2 have statistically significantly different solvent exposures.

**Fig. 3:**
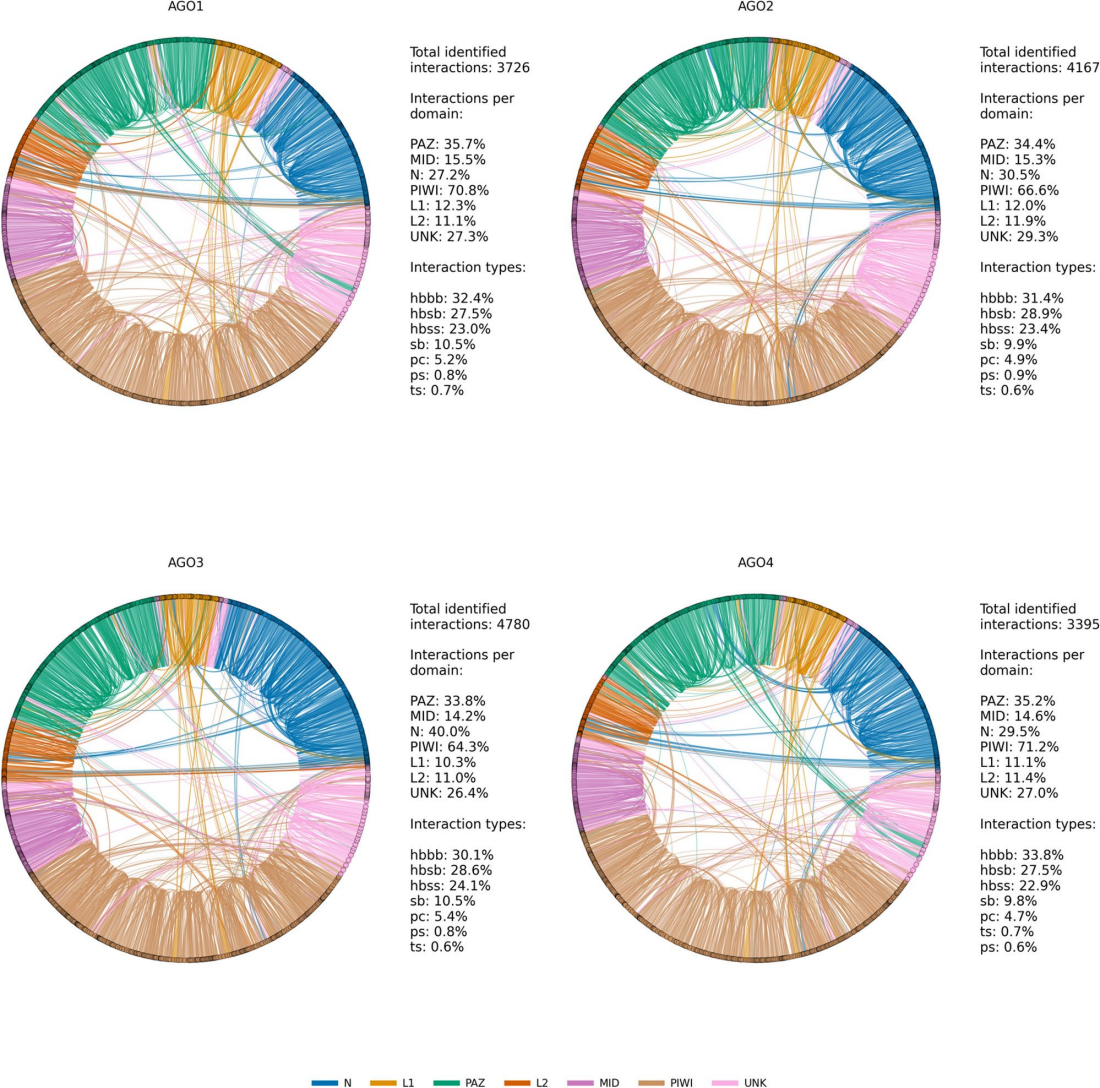
Weak interaction pattern per AGO protein. Each pattern includes all unique occurrences of five types of weak interaction in the clustered trajectories of R1, R2, R3 replicas. The types are: hydrogen bonds (hbbb: backbone to backbone, hbsb: side-chain to backbone, hbss: side-chain to side-chain), salt bridges (sb), pi-cation (pc), pi-stacking (ps), t-stacking (ts). Amino acid positions that do not belong to a specific domain are labeled as ‘UNK’ (unknown domain origin). The coloring matches the one used in Fig. 1 for convenience.

To further probe the stability of the four AGO proteins, we analyzed the formation of chemical bonds in each trajectory. Hydrogen bonds^33^ and intermolecular interactions involving aromatic rings are factors of protein stabilization^34^, whereas salt bridges can either disrupt or maintain conformational stability depending on their location within the structure^35,36^. We quantified the presence of hydrogen bonds throughout each simulation unveiling the lower hydrogen bond count of AGO3 and confirming once again its disordered nature (Figure S19). We also clustered the trajectories (see Methods) and identified unique occurrences of hydrogen bonds, salt bridges, pi-stacking, pi-cation and t-stacking interactions^37^ (Supplementary Data 8). AGO3 possesses the largest repertoire of weak interactions, probably due to its increased flexibility. Each domain of the AGOs exhibits a different pattern of interdomain and intradomain weak interactions of all four types (Fig. 3). Upon examining the CT sites of each AGO, we noticed a conserved interaction pattern (Supplementary Data 8). Each residue in the CT site interacts with the other tetrad residues or their flanking/proximal residues, except for the second residue, which does not interact with the fourth one. We found a lysine (K7xx) approximately 40 aa away from the third CT amino acid (AGO1 K707, AGO2 K709, AGO3 K710, AGO4 K711), which interacts with all the residues of the tetrad in each AGO apart from the second one. K7xx interacts only with the second residue of the AGO4 CT site. Also, the second CT residue of AGO2 and AGO4 interacts with an arginine (R7xx) flanking to K7xx (AGO2 R710, AGO4 R712). AGO2 K709 and R710 have been reported as miRNA binding sites^38,39^.

### The conformational differences of human AGO proteins

**Fig. 4:**
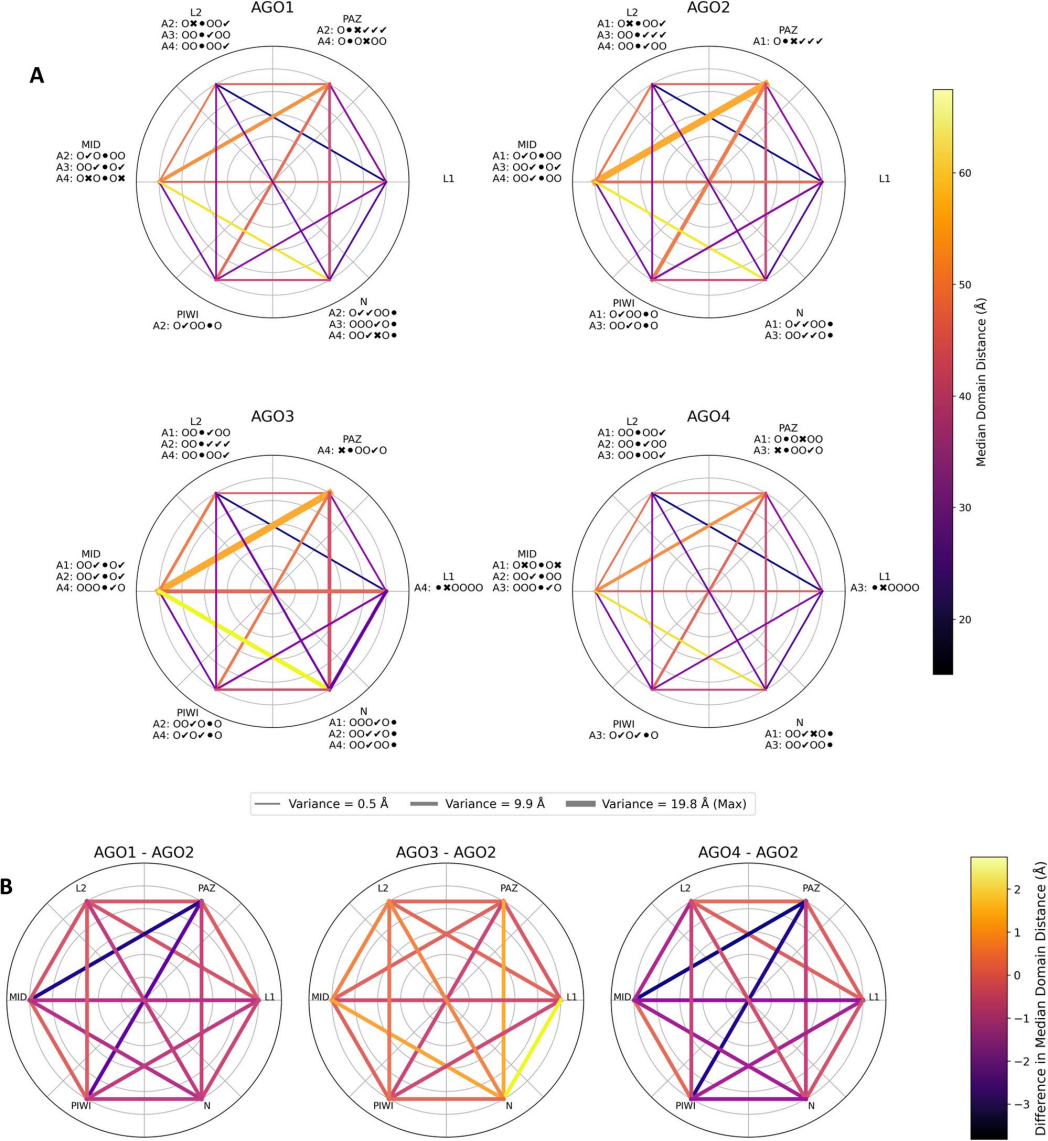
Median domain distances per AGO protein. (**A**) Median domain distances measured between the centers of masses of the domains from the trajectories of the R1, R2, R3 replicas. The thickness of the lines illustrates the variance of the measures and the bright coloring refers to high values. Each domain label includes annotations about the consistent statistical significance or insignificance of the inter-domain distances across R1, R2, R3: a) A1-A4: AGO1-AGO4, b) the sequence of 6 symbols refers to the domains in this order: [L1, PAZ, L2, MID, PIWI, N] c) O = placeholder, ⚫ = current domain, ✔ = significant, ✖ = insignificant. (**B**) The differences in the median domain distances between the catalytic AGO2 and the other AGOs.

**Fig. 5.**
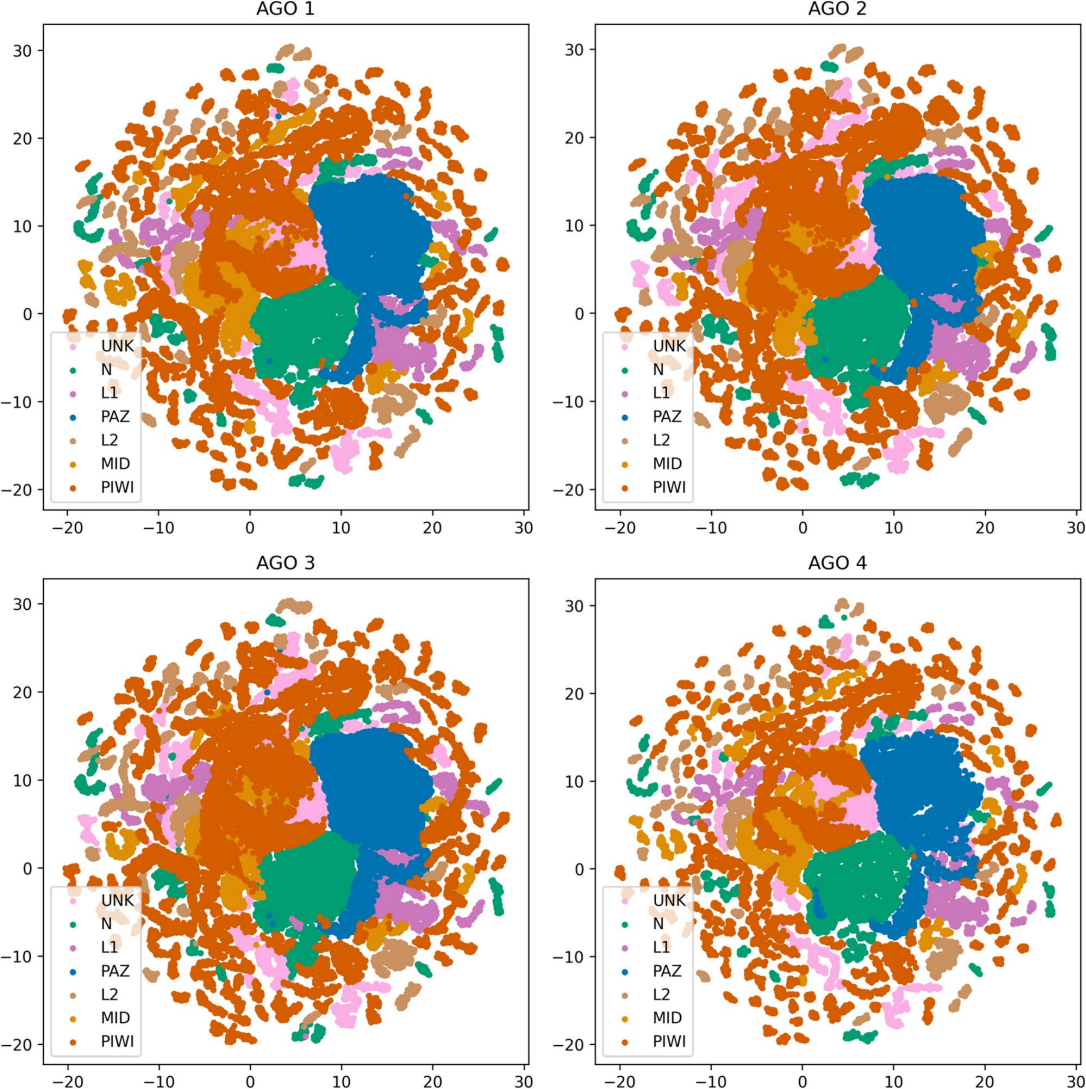
2D Projection of the conformational space of the four human AGO proteins with UMAP algorithm for R1, R2, R3 replicas. The projected data derive from the atomic Cartesian coordinates of the medoids’ alpha carbons in the clustered trajectories. Amino acid positions that do not belong to a specific domain are labeled as ‘UNK’ (unknown domain origin). The coloring matches the one used in Fig. 1 for convenience.

Interdomain distances convey information on the folding of multi-domain proteins and the interactions between the domains^40^. Linkers regulate the domain interactions to avoid disruptions or enable the activation of an individual domain function^41,42^. We computed the distances between the domains/linkers of each AGO to spot the differences in their positioning that could potentially affect their miRNA binding capabilities (Supplementary Data 9). The distances between MID, PAZ and N domains are the largest measured inter-domain distances because of open-closed states. The most statistically significantly different inter-domain distances are the ones that involve the L2 (Fig. 4A). AGO2 and AGO3 have more variable domain distances compared to AGO1 and AGO4 (Fig. 4A). Examining the differences between the AGOs with AGO2 as a reference, we notice that AGO1 and AGO4 are more restricted on their domain motions, especially in the distance between PAZ and MID domains (Fig. 4B). By collectively projecting the medoids of all the clustered trajectories to two dimensions (Figure S20), we observed that each AGO has unique local domain motions that are interchangeable, maintaining a similar overall conformational profile (Fig. 5). AGO3, once again, appears as the most mobile among the four with a wider conformational space (Figure S20). Differences in interdomain distances may highlight distinct and non-overlapping functional modes of the AGO proteins.

**Fig. 6:**
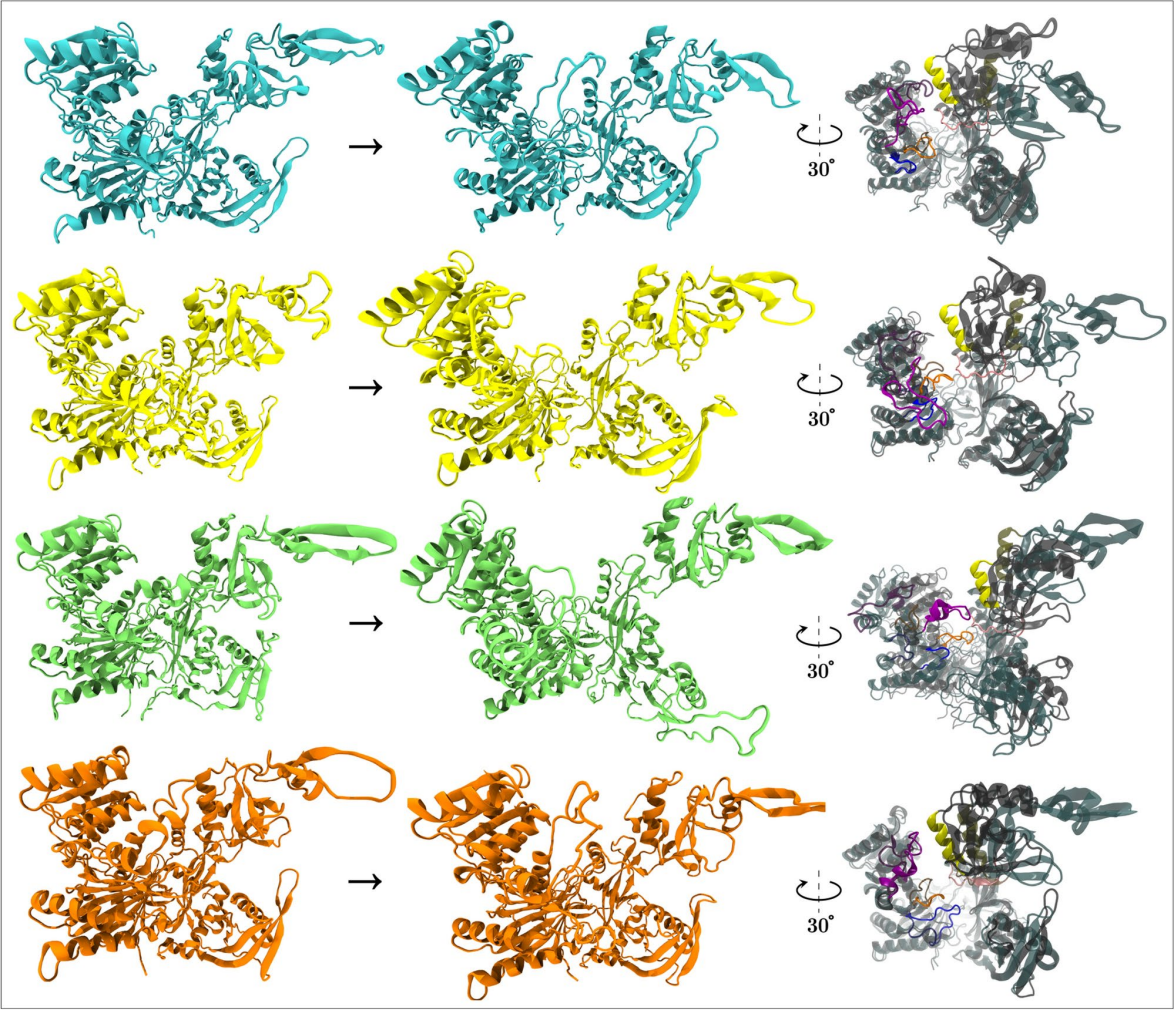
Observed closed-open states and interacting elements in the nucleic acid binding channels per human AGO. In the first row, there are the states of AGO1 (PDB ID: 4KRE) in cyan. Second row is dedicated to AGO2 (PDB ID: 4Z4D) states in yellow. Third and last row refer to AGO3 (lime, PDB ID: 5VM9) and AGO4 (orange, PDB ID: 6OON) states respectively. The first column includes the closed states and the middle column depicts the open states. The last column on the right depicts the superposition of open (gray glass representation) and closed (cyan glass representation) states per AGO with the interacting elements highlighted in different colors. AGO1: (1) yellow is H1 at 250–261 (2) pink is L1 at 346–355 positions 3) orange is SL1 at 599–608 positions (4) blue is SL2 at 630–638 positions (5) purple is LL1 at 816–836 positions AGO2: (1) yellow is H1 at 251–263 (2) pink is L1 at 347–357 positions (3) orange is SL1 at 601–610 positions 4) blue is SL2 at 633–640 positions 5) purple is LL1 at 818–839 positions. AGO3: (1) yellow is H1 at 252–264 (2) pink is L1 at 348–358 positions 3) orange is SL1 at 601–610 positions (4) blue is SL2 at 634–641 positions (5) purple is LL1 at 819–838 positions. AGO4: (1) yellow is H1 at 242–254 (2) pink is L1 at 337–347 positions (3) orange is SL1 at 592–601 positions (4) blue is SL2 at 625–635 positions 5) purple is LL1 at 820–840 positions. The displayed conformations are selected among the medoids of all clustered trajectories based on the minimum or maximum center-of-mass distance between PAZ and MID per AGO.

Our manual inspection of the trajectories (Supplementary Movies 1–12) provided extended information on global conformation states and interactions of secondary structure folds within the nucleic acid binding channel. We identified the open and closed states of the AGO proteins (Fig. 6), confirming previous research on AGO2^18,25^. These states resemble a pincer movement with the MID and PAZ domains moving over 50 Å apart. The open state exposes the miRNA binding interface and may accommodate water molecule accumulation forming LAKE (loop-associated key estuary) clusters^28^. The time delay and the extent of the open-to-closed state transition vary among AGO proteins (Figure S21, Supplementary Data 12). The only pair that consistently demonstrated synchronization in their PAZ-MID distance trajectories are AGO1 and AGO3 (Figure S22). Another finding was the presence of different secondary structure folds within the pincer’s jaws, affecting the flexibility of the nucleic acid binding channel. A common interaction seen in AGO1, AGO3 and AGO4 is a long loop (Long Loop 1—LL1) approaching a PAZ helix (Helix 1—H1) (Fig. 6). However, such a case was not detected in any of the three replicates of AGO2. LL1 does not belong to any AGO domain and is located just after the PIWI domain, yet it resides in an area that belongs to the Ribonuclease H folding superfamily according to InterPro^43^. In all AGOs, there is a common interaction between a pair of loops in PIWI (Short Loop 1—SL1) and PAZ (Loop 1—L1) (Fig. 6). In AGO4, SL1 also interacts with an additional PIWI loop (Short Loop 2—SL2), forming a network of interactions (Fig. 6). These interactions contribute to the folding motions of each AGO protein, which resemble the opening/closing of a book. These mechanistic details highlight the local structural differences among the AGOs in terms of morphology and nucleic acid binding channel accessibility.

### The functional aspects of human AGO proteins

The synthesis of similar molecules with distinct properties in nature is an energy-consuming process for cells; the predominant reason for their existence might be the fine-tuning of their common functions. Comparisons between available protein–protein interaction data of each AGO protein yielded that human AGO proteins have both common and unique interactors that shape their functional profile (Supplementary Data 10). Our simulations are in accordance with this binding distinction, as they show that the unbound human AGO proteins do not adopt identical conformational paths. Seeking additional evidence, we scanned the preprocessed AGO structures for binding sites using PocketMiner^44^, a deep learning model that is trained on simulated cryptic pocket opening events. The predicted binding sites stretch outwards the nucleic acid binding channel to every domain and vary per AGO (Figure S23), further supporting our findings on the different open/closed conformational states of the AGO protein structures (Fig. 6). The overall interfacing patterns differ enough to allow a unique interaction and include not only flexible loops, such as parts of LL1, but also more conformationally stable areas.

**Fig. 7:**
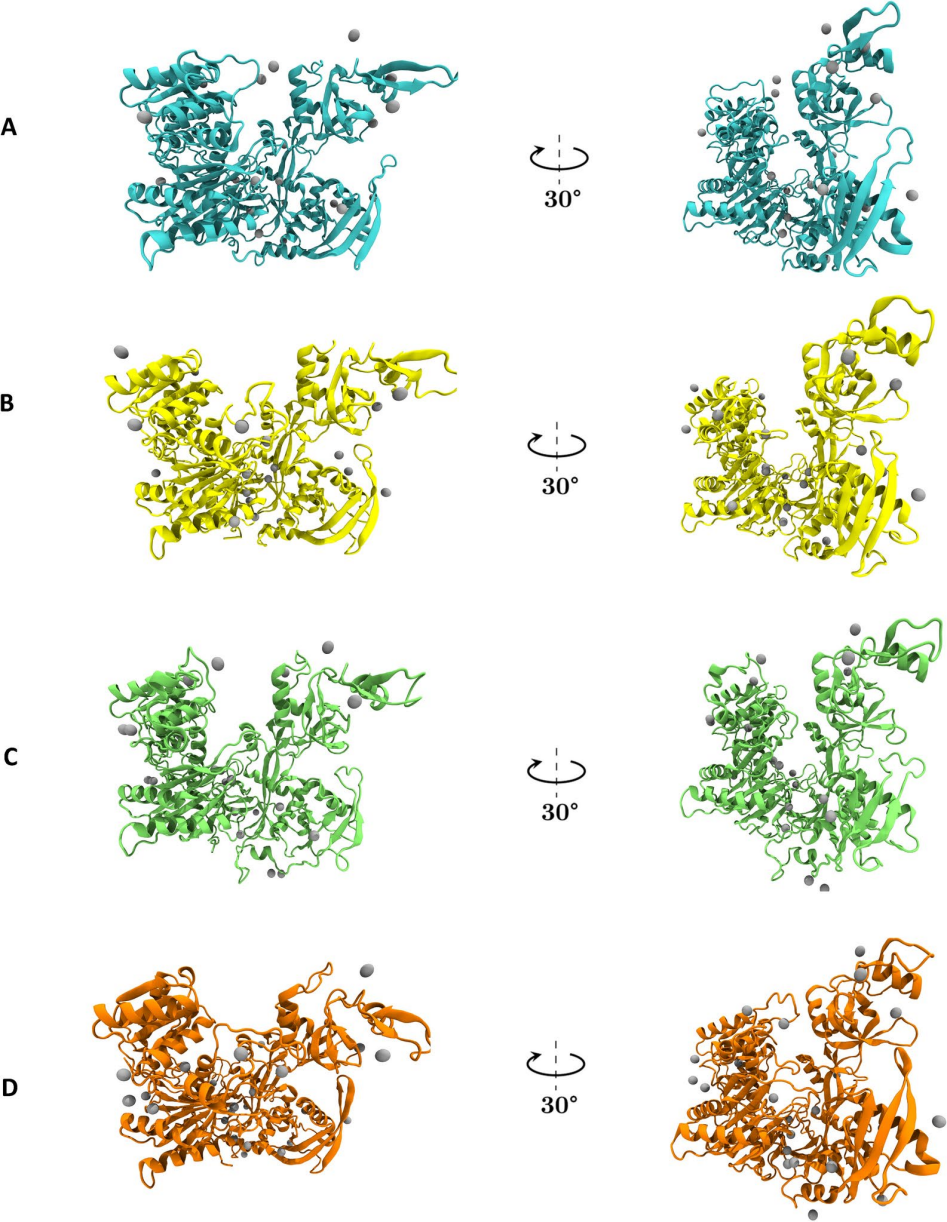
Predicted zinc ion binding sites of the four human AGO proteins. The binding areas of zinc ions as predicted by Metal3D. Zinc ions are depicted as gray spheres. (**A**) AGO1 (PDB ID: 4KRE), cyan color (**B**) AGO2 (PDB ID: 4Z4D), yellow color (**C**) AGO3 (PDB ID: 5VM9), lime color (**D**) AGO4 (PDB ID: 6OON), orange color. The structures were preprocessed with Schrödinger Maestro suite. The coloring matches the one used in Fig. 6 for convenience.

We investigated potential links between the human AGO family and mitosis, given that human AGO2 is a chromatin modulator^45^ and it is essential for cellular division^46^. Our analyses utilized the data generated by the structure refinement processes and simulations. We searched for putative zinc ion binding sites in the preprocessed AGO structures using Metal3D^47^. A mechanism of mitosis initiation relies on the flow of zinc ions (Zn^2+^) into the cell as mediated by the heteromer of Zinc-regulated transporter, Iron-regulated transporter-like Protein 6 (ZIP6) and ZIP10 transmembrane proteins^48^. Zinc ion interactions stabilize protein structures^49^ and could be essential for the functions of AGOs, as these proteins consist of highly flexible subunits according to our simulations. We observed that there is a divergent predicted binding pattern for each AGO, especially in the case of catalytically active AGO2-AGO3, which harbor more putative zinc ions in their nucleic acid binding channels (Fig. 7). As function is connected to structure, we performed whole structure comparisons via Machaon^50^ for each preprocessed AGO structure, scanning two large, refined datasets of human protein structures: ~ 183,000 PDB chains from RCSB^51^ (HL2 –Human protein list 2, Supplementary Data 11) and ~ 24,000 human protein structures from AlphaFoldDB^52^ (AF4 – AlphaFold version 4 *Homo sapiens* dataset). Machaon identifies structurally similar proteins to a reference protein using alignment-free metrics and builds a multi-level profile for the protein by integrating third-party data and established metrics. We included the medoids of the reference protein’s clustered trajectories in the search space per each session, enhancing the selection of the most structurally similar proteins from each dataset. All results yielded by Machaon for each AGO are available in Supplementary Data 12. We focused on the meta-analysis results that associate the reference protein with the biological properties of the identified similar structures. The results for all AGOs in all search sessions involving both HL2 and AF4 datasets separately indicate a zinc ion binding function, suggesting a potential link between zinc ion abundance and the stability of AGOs.

**Fig. 8:**
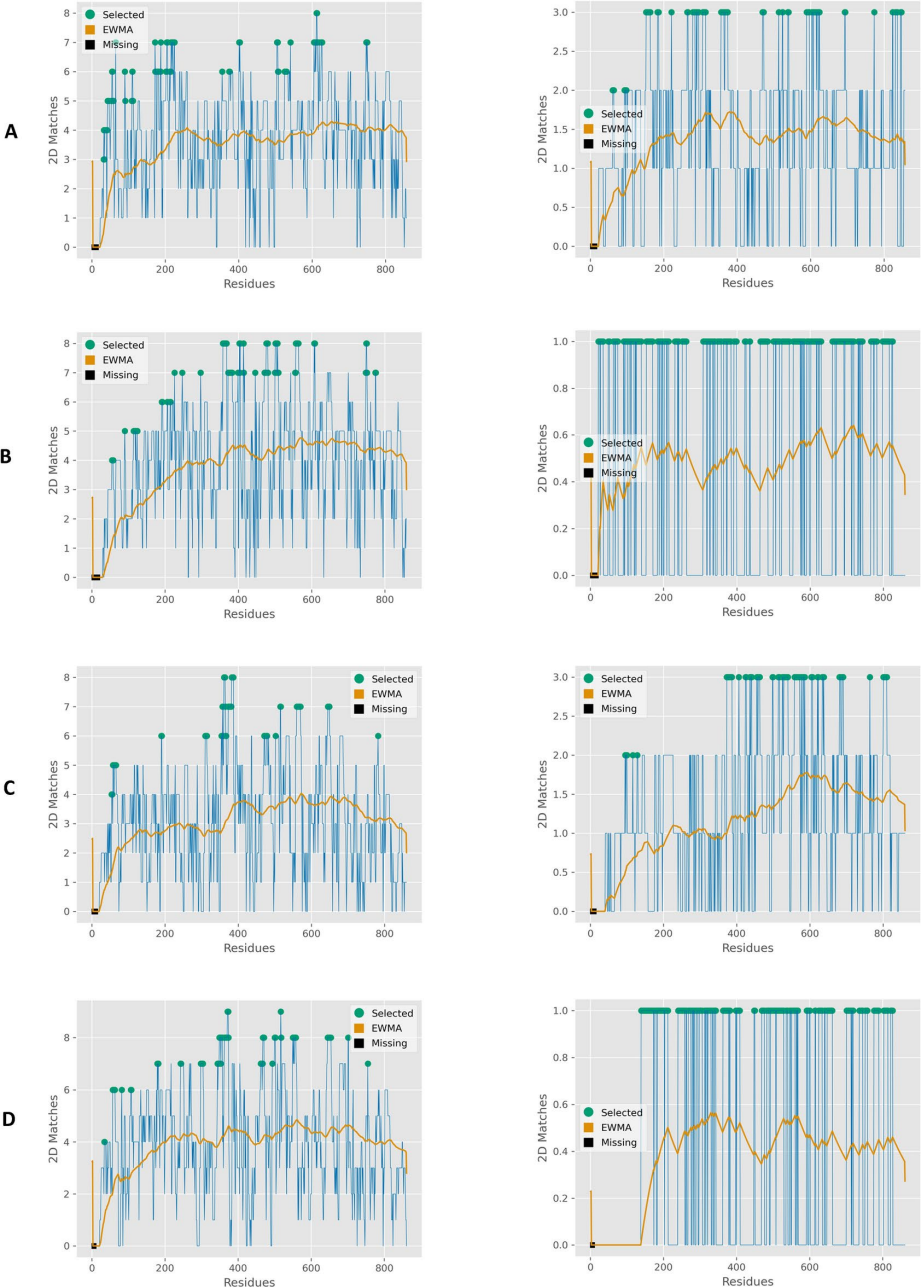
Secondary protein structure alignments between the four human AGO proteins and structurally similar proteins with functions related to mitosis. The detection and alignment of the structurally similar proteins were conducted with Machaon. On the left are the meta-analysis results for comparing an AGO protein with more than 180,000 experimentally derived protein structures and on the right are the comparisons with AlphaFold predicted structures for *Homo Sapiens* species (v4). The feature extraction utilized the refined versions of the metrics (see Methods). The clustered conformations of each AGO were included in each search session for improving the clustering formation (see Methods). The reference structures were preprocessed with Schrödinger Maestro suite. (**A**) AGO1 (PDB ID: 4KRE) (**B**) AGO2 (PDB ID: 4Z4D) (**C**) AGO3 (PDB ID: 5VM9) (**D**) AGO4 (PDB ID: 6OON).

Machaon’s meta-analysis yielded mitosis-related proteins, which are structurally similar to AGOs (see “Extended structural and functional analysis” section in “Methods”). Transitional endoplasmic reticulum ATPase (VCP)^53^ and DNA polymerase alpha catalytic subunit (POLA1) appear in top places in the HL2 results of every AGO protein. The identified proteins have ~ 40% 2D / ~ 20% 3D protein structural similarity and ~ 40% similarity in gene coding sequence. These proteins share common protein interactors not only among themselves but also with the AGO proteins. For instance, Tripartite motif-containing protein 67 (TRIM67), a protein that operates in pathways related to mitosis^54^, interacts with AGO1, AGO2, AGO3 and their commonly associated proteins. There is also varying similarity in the untranslated regions between the coding genes of the AGOs and these common proteins, all of them being common targets of miRNAs related to mitosis^55–57^ according to TarBase^58^ (Supplementary Data 13). Other mitosis-related proteins in top places of the results are epidermal growth factor receptor (EGFR)^59,60^, UBX domain-containing protein 6 (UBXN6) and Transcription initiation factor TFIID subunit 2 (TAF2). We noticed that 2D protein structure alignments of mitosis-related proteins peaked around the area of PIWI (Fig. 8), thus we performed constrained comparisons between AGO2 PIWI and domains of the proteins in HL2 dataset. Kinesin-like protein (KIF11)^61^ scored the highest in this search session with 38.7% 5-UTR and 43% CDS sequence identities. Once again, we observed TRIM67 as the top common interactor of AGO2 and the mitosis-related proteins in the domain search results. Concerning the meta-analysis of comparisons between the AGO structures and the proteins in the results on AF4 dataset, we observed that Probable RNA-binding protein 46 (RBM46) and Serine/threonine-protein kinase PLK3 were ranked in top places in the results of more than one AGO. RBM46, PLK3 and AGO genes are common targets of miRNA families which are linked with mitosis^55,56,62,63^ (Supplementary Data 14). These findings strongly suggest a potential involvement of human AGO proteins in pathways linked to mitosis.

## Discussion

In this work, we performed structural analysis of the human AGO family to gain deeper insights into their similarities and differences. Past relevant analyses have emphasized miRNA loading and the distinction between the bound and unbound states of AGO2, as the structures of AGO3 and AGO4 became recently available. Genomic studies have solidified the distinction of the AGOs in expression under different conditions or tissues, affecting the biological operations of the organism or causing disease^7^. To complement previous findings, we performed molecular dynamics simulations of each AGO unbound state to investigate their mechanistic details. We analyzed the generated data and we found that the human AGO proteins differ in conformational stability and accessibility at whole structure and segment levels, offering a previously unexplored perspective.

Our analysis unraveled structural differences between the AGOs in the nucleic acid binding channel, supporting the notion of preferential miRNA loading. Thorough understanding of miRNA sorting could provide valuable insights into the RNAi mechanism and could have a diagnostic and therapeutic value^64^. Differential miRNA sorting has been consistently detected in fruit fly and *C. elegans*. In *D. melanogaster*, most mature miRNAs associate with AGO1, while most antisense miRNA* sequences either degraded or associated with AGO2^65,66^. In *C. elegans*, discrete miRNA is associated with different AGO-like proteins^67^. In humans, no characterized sorting system exists, and it has been thought that small RNAs randomly bind to the four AGOs based on miRNA populations in the cell^10,68–70^. Exosomal miRNA sorting is not only controlled by cell activation-dependent changes of miRNA target abundance^71^ but also by human AGO2^72^. Knockout of AGO2 could decrease the abundance of some exosomal miRNAs, suggesting that the existence of this specific AGO member leads to the selection of specific miRNAs. Alu element-derived repeat-inducing RNAs, mirtrons and Agotrons demonstrate a preferential loading to the human AGOs^2^. AGO1- and AGO2-biased miRNAs derive mostly from 3p (antisense) and 5p (sense) strands of pre-miRNA respectively^73^. Also, the slicing capabilities of AGO2-AGO3 rely on different miRNA lengths^2,74,75^ and there are reports of specific activating miRNAs per AGO^76^. Thus, evidence suggests a non-characterized system that would allow specific AGOs to load a specific sub-cohort of small RNAs. This could be achieved either autonomously or after an interaction with additional components, contributing to the discrimination of these sub-cohorts.

We detected conformational differences at the global and domain levels that impact miRNA binding. We found that AGO3 is the most conformationally unstable AGO protein, especially its N domain^77^, which is essential for mRNA cleavage^1^. We observed that L1-L2 linkers move differently per AGO and this inequality could propagate to the whole structure. L2 joins the two lobes of the AGO protein (Fig. 1) and greatly affects the pool of global available conformations. In our simulations, we witnessed different inter-domain distances and weak interaction patterns as well as global open-closing states with different width^9^ and time lag. All these points of differentiation could contribute to a mechanism for competitive and/or adaptive RNA loading. Depending on the environmental conditions and the cell type, there is a specialized gene regulation, which could potentially be achieved in biophysical terms. The repertoire of gene regulation adjustments before the miRNA/mRNA binding to AGOs greatly increases if we also consider the RNA editing^78^ of miRNA^79^/mRNA transcripts, the post-transcriptional modifications of RISC-associated proteins^2,7^ and the sponging effect of lncRNAs^80^. Despite this complexity, our investigations on the projected aligned medoids showcased an equilibrium within a common conformational space among the four AGOs, where there is an exchange of local molecular motions. Also, some parts of the AGOs conserve their molecular motion pattern, such as the ZSWIM8, GW182 interaction sites that initiate degradation processes, suggesting that these mechanisms are not AGO-specific. LCS1/LCS2 have high structure similarity resulting from synonymous codons, which can alter protein folding by affecting translational elongation rates^81^. Interestingly, only the AGOs with known slicing function possess LCS2, yet this segment has different solvent exposure per AGO.

Regarding mRNA processing, we examined the molecular motions of the catalytic tetrads in AGO2-AGO3 slicers and the pseudo-catalytic ones of AGO1-AGO4. The second CT residue is between SL1 and SL2, which do not interact in the same manner in each AGO and potentially regulate the accessibility to the first and second residues of the tetrad. The local conformation state of the ‘plugged’ glutamate finger in the second position of the tetrad is adopted during RISC assembly. This arrangement is essential not only for the RNA cleavage function of AGO2 but also for the silencing activity of all human AGO proteins^1,2^. The last residue of the tetrad resides in LL1, where we distinguished a unique behavior in AGO2 of avoiding interactions with H1, thus exposing more the fourth residue of the tetrad and the nucleic acid binding channel overall. The equivalent residues in AGO1 and AGO4 are arginines, which are more hydrophilic than histidines^82^ and have stronger affinity for guanine, a binding that constitutes the highest affinity among amino-acid/base interactions^83^. Our data revealed inter-residue interactions in CT and interactions between CT and two distant residues (K7xx, R7xx) that are observed in more than one AGO. K7xx and R7xx are adjacent to some of the GW182 binding sites (residues 694, 695 and 698 in AGO2), which reside in a flexible part of the protein (Fig. 1). We speculate that GW182-AGO binding could affect CT-K7xx-R7xx interactions and induce conformational changes in the area of CT during the course of the mRNA degradation pathway^29^. We should also consider the post-transcriptional modifications such the cases of AGO1 and AGO3, where we found some PTMs with low RMSF (~ 1–2 Å), which reside within the area of their tetrads (621, 626 positions for AGO1 and 682 for AGO3).

The presence of four members in the human AGO protein family has not been thoroughly explored from a biochemical perspective to integrate existing knowledge from genomics. AGO proteins and their guides exhibit a varying profile in expression within the human tissues^7,84,85^, which differ in pH. Our results suggest that zinc ions bind to AGO proteins, leading not only to the enhanced stabilization of the latter, but also to the alteration of their respective unique isoelectric points (pI)^86^. Variations in acidity occurring in tumor microenvironment (TME) and cancer progression, showcase the importance of this chemical property. To the best of our knowledge, there is no documented association between human AGO proteins and zinc ions, which requires further investigation. AGOs recognize the 5′-phosphate of their guide small RNAs depending on metal ions and there is a recently documented presence of a zinc ion in piRNA recognition by Drosophilla Piwi protein^87^. Also, considering the reports on the dysregulation of miRNAs due to zinc deficiency^88^, our data propose an essential role of zinc ions on gene regulation as well as on the regulation of each AGO member’s function. Potential Zn^2+^-AGO interactions might occur during mitosis initiation since AGOs can be found within GW bodies^46^, which operate in the G2 phase.

The regulation of cell division is a demanding biological process due to the required balance of the mRNAs, mitotic dynamics and organelle separation. Mitosis has increased spatio-temporal local needs of specific molecules and most likely this becomes more demanding during its last phase the cytokinesis. There is a link between AGO proteins and mitosis, as being established by reports on the mitotic regulation function of *Drosophila melanogaster* AGO1^89^, the regulation of mitotic spindle assembly by *Trypanosoma brucei* AGO1^90^, the requirement of an Argonaute protein for mitosis *in C. elegans*^91^ and the connection of Plant AGO1^92^/bacterial AGOs^93^ with cell division. Human AGO2 has been detected in the midbody arms, co-localizing with other proteins that operate in the RNAi pathway^46^ and distinct microRNA activity has been documented for every phase^56^. All the above suggest that AGOs have a direct link with local translation/cell plasticity and may play an essential role to the local phenomena of cell mitosis^94^. However, the exact activity of AGO2 in this context remains to be revealed and as for the rest of the AGOs, they have not been associated yet with this biological process. We found indications that all four human AGO proteins could be involved in mitosis, as we identified common interactors with structurally similar proteins that operate in relevant pathways such as mitotic phase transition, hinting potential agonist or antagonist roles for the AGOs. Also, the coding genes of the AGOs and mitosis-related proteins share parts of sequence and miRNA targets that could support a co-regulated or antagonizing gene expression pattern. These data could be utilized for further investigation to uncover the specific role of AGO proteins in each phase of cell division.

Our study would greatly benefit from the existence of experimental structures modeling wild-type apo forms with higher resolution and residue coverage. However, AGO proteins are overly flexible molecules with disordered regions^2,6^ and their structural determination is quite a challenging experimental task. For instance, there is only one experimental structure available for each of AGO3 and AGO4 and these have been determined in recent years. To address limitations in existing raw data, we employed established methods of structure preparation relevant to our study. Also, we investigated the AGO proteins in a multi-faceted approach leveraging multiple microsecond-scaled trajectories for searching structurally similar proteins to form strong hypotheses for the AGOs’ properties. These proposals primarily rely on the integration of established third-party experimental data, complemented by state-of-the-art predictions. Our combined results provide reliable targets for future experimental research such as the transplantation of structural parts between AGOs, mutagenesis studies, or drug design for gene expression control.

Gene regulation could be controlled in multiple levels by the distinct structural and chemical properties of the AGO proteins. We demonstrated the conformational differences of the AGO structures at global and domain level and the unique pattern of weak interactions in each AGO protein. These data enabled us to examine specific conserved segments, binding sites and PTMs. We proposed that AGO proteins interact with zinc ions, which are connected with a mitosis initiation pathway. We associated all AGO proteins with known actors in the mitotic cell division pathway via meta-analysis of structural comparisons with large human protein datasets, including also our MD simulation data and established third-party data. In conclusion, the results of our in-silico approach suggest that although human AGOs exhibit high structural similarity, they do not necessarily adopt strictly similar conformations and binding patterns, allowing them to possess individual and fine-tuned common functions. Our functional analysis provides indications that further disambiguate the role of the AGOs in mitosis. The generated data and meta-analysis results will promote the research of the human AGOs. Moreover, the developed methodology and software could be employed to analyze homologs or other groups of related proteins.

## Methods

### Structure preparation

The starting AGO structures for the replica 1 (R1), replica 2 (R2) groups of simulations were selected from PDB-REDO Databank^95^ which contains optimized versions of PDB entries. We chose the AGO1 structure in 4KRE.A^26^ (resolution 1.75 Å) and the AGO2 structure in 4Z4D.A^27^ (resolution 1.60 Å) due to the high resolution and completeness among the various structural models available for AGO1/AGO2 proteins (Tables S8, S9). For AGO3/AGO4 proteins, we selected the recent and only available structures, which were 5VM9.A^9^ (resolution 2.60 Å) and 6OON.A^28^ (resolution 1.90 Å) (Table S9). We reverted the mutation of the 4Z4D.A structure in residue position 387, converting aspartic acid to serine using Schrödinger Maestro Suite^96^. The selected structures were refined according to the Protein Preparation Wizard’s protocol in Maestro: ligands and water molecules were removed, missing residues were positioned by Prime^97^, protonation states in physiological pH (7.4) were determined by PropKa^98^ and there was a short and constrained energy minimization step of the protein’s heavy atoms by OPLS force field^99^. For the replica 3 (R3) group of simulations, we used an alternative structure refinement strategy using PDBFixer^100^ for generating the missing residues and APBS-PDB2PQR^101^ for further refinement and the determination of the protonation state. We used the raw PDB files^51^ for AGO1, AGO3, AGO4 and the PDB-REDO optimized PDB file for AGO2 aiming for the best result with PDBFixer. Also, we used Modeller^102^ on the AGO2 structure to convert the aspartic acid in position 387 to serine for R3. We evaluated all the refined structures via Ramachadran^103^ and Janin^104^ plots (Figures S24-S26). PDB visualizations and trajectory movies are created with VMD^105^. Table S10 encompasses detailed information for the prepared structures.

### Molecular dynamics

We performed the three independent simulation instances (R1, R2, R3) per AGO protein with GROMACS^106^(2020.2 version for R1 replicas, 2021 version for R2 replicas, 2024 version for R3 replicas) and DES-Amber force field^107^ (default for R1, DES-AMBER_SF1.0 for R2-R3). DES-AMBER is a modern force field with state-of-the-art accuracy on single-chain proteins. The simulations reach the microsecond time scale, allowing for the effective simulation of helix, loop and domain motions^108^. We generated R1, R2, R3 replicate groups independently on different hardware, simulation package versions and either force field configuration or structure preparation to emphasize the independence of the results from the originating setup. The structures were solvated with TIP4P-D model^109^ in simulation boxes of dodecahedral shape. The distance (-d parameter) between the solute and the box was set to 2 nm, aiming for simulations of high quality. We neutralized the charges of each system prior to its simulation by introducing Cl^−^ counterions explicitly in the solvent, replacing water molecules. The energy of the systems was minimized for 0.05 ns or until the threshold of 1000 kJ/mol/nm (Figure S27). Afterwards, each system was equilibrated at 300 K with restraints in a canonical ensemble (NVT) for 2 ns (Figure S28) and unrestrained in an isothermal–isobaric ensemble (NPT) for 10 ns (Figure S29). Bonds constraints were applied with the linear constraint solver (LINCS), long-range electrostatic forces were computed with particle mesh Ewald method (PME) and non-bonded interactions were evaluated by a Verlet cut-off scheme. After equilibration, we moved on the production phase where each system was simulated for 1020 ns, except for AGO1 R1 replicate that was simulated for 1010 ns. V-rescale thermostat and Parrinello-Rahman barostat were set with nsttcouple = 20 and nstpcouple = 20. The time step was 0.002 ps and the pressure was applied isotropically. We kept 1 μs from each trajectory, discarding the rest of the initial nanoseconds as additional equilibration. This truncation was performed by monitoring the increase of the Exponentially Weighted Moving Average (EWMA) of RMSD from zero to a state of periodic motion with limited upward trend (Figure S2). The EWMA of radius of gyration serves as another assessment for the convergence of the simulations and quantifies the extent to which a structure maintains its compactness throughout its trajectory (Figure S3). For each trajectory, each molecule’s center of mass is placed inside the simulation box, where the whole system is centered into (-pbc mol & -center options for trjconv command). The details of the simulated systems are available in Tables S11 and S12.

### Analysis of the simulations

We analyzed the trajectories with GROMACS^106^ and calculated: a) RMSD, SASA for whole structures/domains b) radius of gyration, hydrogen bonds, RMSF for whole structures and c) RMSF for specific sites. The analysis required to maintain mapping between original residue positions and renumbered positions, which were introduced by the GROMACS package. We measured the center-of-mass distances between all pairs of domains with MDAnalysis^110^ for each trajectory. EWMAs for the distance measurements and their parameters (mean, median, variance) were computed with Pandas Python and Numpy packages. We computed the time delay between pairs of inter-domain distances with time-lagged cross correlation function of SciPy package. The inter-domain distance trajectories were smoothed using an EWMA with a span of 10% of the data sequence length (100,000 data points, 1 per 10 picoseconds [ps]) and normalized to capture the inflection points of the largest motions. We used k-Means NANI^111^ algorithm for the clustering of trajectories. K-means NANI is a modern and efficient clustering method that provides metrics on the clustering result. We selected the number of clusters by the Davies-Bouldin score, with a preference for an average inter-cluster mean square deviation of 4 Å. We computed hydrogen bonds, salt-bridges, pi-cation/pi-stacking/t-stacking interactions with the GetContacts software package using the medoids of the clusters.

### Two-dimensional projections of trajectories’ medoids

For each set of replicas (R1, R2, R3), the alpha carbons of the medoids in the clustered trajectories of the four AGOs were aligned with BioPython^112^ and MDAnalysis, based on the pairwise alignments between AGO1 and AGO2/AGO3/AGO4 protein sequences that were generated by EMBOSS Needle^113^ in ClustalW format. The Cartesian values of the alpha carbon atoms in the aligned medoids were projected to two dimensions with Uniform Manifold Approximation and Projection (UMAP)^114^ Python package (min_dist = 1, n_neighbours = 1000).

### Evaluation of the measurements on simulated data

We assessed the statistical significance of the domain RMSD and SASA measurements using the Kolmogorov–Smirnov test (K-test) by comparing pairs of EWMAs on 100,000 data points (1 point per 10 ps) per simulation. The span of each EWMA is the 10% of the source data sequence length, reducing the noise and improving SNR as computed by SciPy Package. We compared the EWMAs of the inter-domain distances (span = 1000, 100,000 data points) conducting the K-test. We employed the Wilcoxon signed rank t to compare domain and LCS1/LCS2 RMSF measurements. Both types of statistical tests used were non-parametric. The comparisons were evaluated as significant only by fulfilling the following criteria in all simulation replicates: K-test metric > 0.55 (< 0.45 for insignificance) or non-zero large Wilcoxon rank sum, accompanied by a p-value < 0.001. The N domain of AGO3, documented for its high flexibility, was used as a sanity check for our measurements.

### Longest common sequence segments of AGOs

LCS1, LCS2 sites were traced in AGO protein sequences retrieved from UniProt^115^. We compared each pair of them individually and all four together to identify longest common subsequences. We extracted the secondary structure of the segments by the preprocessed PDB chains and we performed pairwise 2D alignments via Machaon’s API. We plotted the alignments with Biotite^116^ and a custom score matrix for coloring the matches (Table S13). We retrieved the underlying CDS segments of the LCS segments by querying Ensembl BioMart^117^ using UniProt identifiers. We aligned these CDS segments with Clustal Omega^118^ for LCS1 and EMBOSS Needle^113^ for LCS2. We found similar protein segments in other AGO proteins of other species via PSI-BLAST^119^. We conducted pairwise alignments between the sequences of *Drosophila melanogaster* AGO1-AGO2 proteins from FlyBase^120^ and the human ones using EMBOSS Needle.

### Extended structural and functional analysis

We employed Metal3D^47^, a deep learning model with state-of-the-art zinc ion binding site prediction, to predict zinc ion binding sites in the preprocessed AGO protein structures used in R1, R2. PocketMiner^44^, a highly performant and accurate deep learning model that predicts opening cryptic pockets, was applied to the same starting structures. We identified structurally similar structures to the preprocessed AGO protein structures by whole structure searches and constrained structural searches for AGO2 PIWI domain via Machaon^50^. We set Machaon to extract features from a PDB dataset that was refined with PDBFixer^100^ and APBS-PDB2PQR^101^. Similarly, we refined the AlphaFold dataset with APBS-PDB2PQR. The energy of the structures in both datasets was minimized with OpenMM^100^ (AMBER ff14SB forcefield^121^ and LangevinIntegrator set with temperature = 300 K temperature, frictionCoeff = 1/ps, stepSize = 0.002*ps). For every search, we included the medoids of the clustered trajectories of the reference protein in the search space until the formation of the clusters. During Machaon’s evaluation/meta-analysis steps, we discarded the simulated conformations from the pool of candidates. We extended the meta-analysis module of Machaon to pinpoint common interactors between the interactors of the reference protein and those of the structurally similar proteins by integrating data from BioGRID^122^. Also, we implemented the option of explicitly selecting the organisms from which the candidate proteins are derived. We chose *Homo Sapiens* for all structural comparisons with Machaon. A table with the methods used for each task is available in the supporting information (Table S14).

## Supplementary Information

Supplementary Information 1.

Supplementary Information 2.

Supplementary Information 3.

Supplementary Information 4.

Supplementary Information 5.

Supplementary Information 6.

Supplementary Video 1.

Supplementary Video 2.

Supplementary Video 3.

Supplementary Video 4.

Supplementary Information 7.

Supplementary Video 5.

Supplementary Video 6.

Supplementary Video 7.

Supplementary Video 8.

Supplementary Video 9.

Supplementary Video 10.

Supplementary Video 11.

Supplementary Video 12.

Supplementary Information 8.

Supplementary Information 9.

Supplementary Information 10.

Supplementary Information 11.

Supplementary Information 12.

Supplementary Information 13.

Supplementary Information 14.

Supplementary Information 15.

Supplementary Information 16.

## Data Availability

Source data from all figures and tables are available in Supplementary Data 1. Machaon’s third-party source datasets are retrieved in September 2023: PDB files (https://www.rcsb.org/), UniProt ID mapping resources (https://ftp.uniprot.org/pub/databases/uniprot/current_release/knowledgebase/idmapping), RefSeq sequences (ftp://ftp.ncbi.nlm.nih.gov/refseq/), BioGID protein–protein interactions ( https://downloads.thebiogrid.org/BioGRID/). AlphaFold v4 human proteins dataset was retrieved from https://ftp.ebi.ac.uk/pub/databases/alphafold/latest/UP000005640_9606_HUMAN_v4.tar. The trajectory files and the scripts of their analysis are available at Zenodo: https://doi.org/10.5281/zenodo.10553483 (version 1: R1, R2 trajectories, version 2: R3 trajectory and analysis scripts). DES-Amber force field can be accessed here: https://github.com/paulrobustelli/Force-Fields.

## Code availability

The code of GROMACS is available at https://github.com/gromacs/gromacs . The code of the GetContacts library can be found here: https://getcontacts.github.io/. The trajectories were analyzed and visualized with the following Python packages: HoloViews ( https://holoviews.org/), Matplotlib (https://matplotlib.org/), MDAnalysis ( https://www.mdanalysis.org/), Pandas (https://github.com/pandas-dev/pandas), SciPy ( https://www.scipy.org/ ), Seaborn (https://seaborn.pydata.org/), UMAP (https://github.com/lmcinnes/umap). The clustering of the trajectories relied on K-means NANI of the M-DANCE package: https://github.com/mqcomplab/MDANCE. The source code of the methods employed in the extended analysis: PocketMiner—https://github.com/Mickdub/gvp/tree/pocket_pred , Metal3D—https://github.com/lcbc-epfl/metal-site-prediction . The meta-analysis extension of Machaon for PPI is available to the community via MachaonWeb at https://machaonweb.com . Machaon’s source code is available at https://github.com/anastasiadoulab/machaon. PDBFixer (https://github.com/openmm/pdbfixer), PDB2PQR (https://github.com/Electrostatics/pdb2pqr) and OpenMM (https://github.com/openmm/openmm) were employed for structure refinement.
