# Supplementary material for: Comparative structural insights and functional analysis for the distinct unbound states of Human AGO proteins
Source: Sci Rep. 2025 Mar 19;15:9432. doi: 10.1038/s41598-025-91849-5 (PMC11923369; doi:10.1038/s41598-025-91849-5)
Supplement: Supplementary file 11 — Supplementary Information 7. [file 41598_2025_91849_MOESM11_ESM.docx]

**Supplementary Movie 1:**

The R1 trajectory of AGO1 structure, which is colored in cyan. The coloring matches the one used in Figures 6-7 for convenience.

**Supplementary Movie 2:**

The R2 trajectory of AGO1 structure, which is colored in cyan. The coloring matches the one used in Figures 6-7 for convenience.

**Supplementary Movie 3:**

The R3 trajectory of AGO1 structure, which is colored in cyan. The coloring matches the one used in Figures 6-7 for convenience.

**Supplementary Movie 4:**

The R1 trajectory of AGO2 structure, which is colored in yellow. The coloring matches the one used in Figures 6-7 for convenience.

**Supplementary Movie 5:**

The R2 trajectory of AGO2 structure, which is colored in yellow. The coloring matches the one used in Figures 6-7 for convenience.

**Supplementary Movie 6:**

The R3 trajectory of AGO2 structure, which is colored in yellow. The coloring matches the one used in Figures 6-7 for convenience.

**Supplementary Movie 7:**

The R1 trajectory of AGO3 structure, which is colored in green. The coloring matches the one used in Figures 6-7 for convenience.

**Supplementary Movie 8:**

The R2 trajectory of AGO3 structure, which is colored in green. The coloring matches the one used in Figures 6-7 for convenience.

**Supplementary Movie 9:**

The R3 trajectory of AGO3 structure, which is colored in green. The coloring matches the one used in Figures 6-7 for convenience.

**Supplementary Movie 10:**

The R1 trajectory of AGO4 structure, which is colored in orange. The coloring matches the one used in Figures 6-7 for convenience.

**Supplementary Movie 11:**

The R2 trajectory of AGO4 structure, which is colored in orange. The coloring matches the one used in Figures 6-7 for convenience.

**Supplementary Movie 12:**

The R3 trajectory of AGO4 structure, which is colored in orange. The coloring matches the one used in Figures 6-7 for convenience.
