# Supplementary material for: Comparative structural insights and functional analysis for the distinct unbound states of Human AGO proteins
Source: Sci Rep. 2025 Mar 19;15:9432. doi: 10.1038/s41598-025-91849-5 (PMC11923369; doi:10.1038/s41598-025-91849-5)
Supplement: Supplementary file 20 — Supplementary Information 8. [file 41598_2025_91849_MOESM20_ESM.pdf]

Supplementary Tables

Protein	Domain	Mean Root Mean Square Fluctuation (Å)	Rescaled to [0,1]
AGO1	N	1.94083	0.316669
AGO1	L1	1.574962	0.109311
AGO1	PAZ	3.146515	1
AGO1	L2	1.382092	0
AGO1	MID	2.08498	0.398367
AGO1	PIWI	1.586864	0.116056
AGO2	N	2.51358	0.356807
AGO2	L1	1.859824	0.075467
AGO2	PAZ	4.008186	1
AGO2	L2	1.694525	0.004332
AGO2	MID	2.120341	0.187579
AGO2	PIWI	1.684459	0
AGO3	N	3.407233	0.919555
AGO3	L1	1.686069	0.041854
AGO3	PAZ	3.564985	1
AGO3	L2	1.603993	0
AGO3	MID	2.440626	0.426638
AGO3	PIWI	1.710064	0.054091
AGO4	N	2.061326	0.274964
AGO4	L1	1.532075	0.014452
AGO4	PAZ	3.534297	1
AGO4	L2	1.502714	0
AGO4	MID	1.839572	0.165811
AGO4	PIWI	1.554612	0.025546

Table S1. Mean root mean square fluctuations (RMSF) for the domains of AGOs. Average RMSF (Å) of the residues of AGO domains for R1, R2, R3 replicas.

Protein	Residue position	Mean Root Mean Square Fluctuation (Å)
AGO1	491	0.671
AGO2	493	0.675
AGO3	494	0.666
AGO4	485	0.749

Table S2. Mean root mean square fluctuations (RMSF) for the ZSWIM8 interaction sites of AGOs. Average RMSF (Å) of the residues of AGOs that interact with ZSWIM8 for R1, R2, R3 replicas.

Modification	Mean RMSF (Å)
K246-ub	4.415
Y336-p	2.985
K382-ub	1.933
K398-ub	1.892
K438-ac	1.557
K458-ub	3.694
K477-ub	2.08
T524-p	3.16
K552-ub	2.782
S621-p	1.208
T626-p	1.335
Y696	1.573
K718-ub	2.26
S822-p	5.909
S826-p	8.05
S829-p	6.38

Table S3. Mean root mean square fluctuations (RMSF) for the post-transcriptional modification sites (PTM) of AGO1. Average RMSF (Å) of the PTMs retrieved from PhosphoSitePlus, for R1, R2, R3 replicas.

Modification	Mean RMSF (Å)
K62-ub	3.334
K91-ub	2.527
S153-p	2.31
T158	1.575

Modification	Mean RMSF (Å)
S171	1.607
K248-ub	5.585
S253-p	4.548
R255	4.26
T303-p	6.591
T307-p	5.033
K317-ac	5.66
Y338-p	3.948
T357-p	3.027
K381-ub	2.318
S385-p	1.975
S387-p	1.999
T390-p	2.029
Y393-p	1.809
K402-sm	1.875
K425-ub	2.77
K440-ub	1.573
K468-ub	2.704
T526-p	3.051
Y529-p	2.049
K550-ub	2.977
K566-ub	1.557
S672-p	1.71
K720-ub	2.351
K726-ub	2.652
Y749-p	0.905
S752-p	1.03
T759-p	1.609
S760-p	1.333
S798-p	1.115
S824-p	5.264
S828-p	9.166
T830-p	9.059
S831-p	9.786
S834-p	7.106
K844-ub	2.814

Table S4. Mean root mean square fluctuations (RMSF) for the post-transcriptional modification sites (PTM) of AGO2. Average RMSF (Å) of the PTMs retrieved from PhosphoSitePlus, for R1, R2, R3 replicas.

Modification	Mean RMSF (Å)
K28-ub	1.798
K89-ub	3.106
K174-ub	1.899
Y175	1.542
Y189-p	3.587
T275-p	5.106
Y317-p	4.49
Y323-p	3.5
Y339-p	3.201
T445-p	1.422
K480-ub	2.39
T527-p	3.299
Y682-p	2.327
Y683-p	2.593
Y705-p	1.099
S825-p	6.977
S829-p	8.607
S832-p	8.201
S835-p	6.709

Table S5. Mean root mean square fluctuations (RMSF) for the post-transcriptional modification sites (PTM) of AGO3. Average RMSF (Å) of the PTMs retrieved from PhosphoSitePlus, for R1, R2, R3 replicas.

Modification	Mean RMSF (Å)
Y47-p	1.573
T108-p	3.046
Y328-p	3.496
K374-ub	1.792
K460-ub	2.339
K471-ub	2.086
T503	1.947
T518-p	2.44
K525-ub	1.458
K542	2.728
K542	2.616
K546-ub	1.265
K558-ub	1.164

Modification	Mean RMSF (Å)
K562	0.855
K562-sm	1.573
S798-p	3.046

Table S6. Mean root mean square fluctuations (RMSF) for the post-transcriptional modification sites (PTM) of AGO4. Average RMSF (Å) of the PTMs retrieved from PhosphoSitePlus, for R1, R2, R3 replicas.

	Protein sequence	Residue range		Location
**LCS1**	YPHLPCLQVGQEQKHTYLPLEVCNIVAGQRCIKKLTDNQTSTMI	AGO1	320-363	PAZ domain (end)
		AGO2	322-365	
		AGO3	323-366	
		AGO4	312-355	
**LCS2**	LTYQLCHTYVRCTRSVSIPAPAYYAHLVAFRARYHLVDKEHDSAEGSH	AGO2	782-829	PIWI domain (end)
		AGO3	783-830	

Table S7. Longest common subsequences of AGOs.

PDB ID	Resolution (Å)	R-Value Free	Mutations	UNIPROT Sequence Mismatches	Missing residues (Unmodeled)	Residues with zero occupancy atoms	Year of release	Bound
4F3T	2.25	0.254	No	No [Notes: unmodeled expression tag in -1, 0 positions]	1-24, 122-128, 188-190, 247-249, 275-277, 605-608, 823-838 [Total: 60]	52, 54, 62, 64, 65, 69, 70, 73, 80, 83, 85, 86, 90, 97, 112, 114-117, 119, 127, 129, 133, 151, 157, 160, 207, 212, 241, 248, 257, 260, 266, 276, 277, 299, 302, 332, 333, 355, 404, 423-425, 438, 447, 460, 465, 468, 472, 493, 525, 554, 637, 673, 675, 678, 699, 819, 820, 837, 839, 840, 844 [Total: 64]	2012	miR-20a
4OLA	2.30	0.253	Yes: 387	Yes: 387	1-22, 120-125, 152-153, 186-188, 245-246, 271-276, 334-335, 602-607, 818-838 [Total: 70]	-	2014	guide RNA fragment
4OLB	2.90	0.249	Yes: 387	Yes: 387	1-21, 120-125, 152-153, 186-188, 245-246, 272-275, 334-335, 603-606, 819-839 [Total: 65]	-	2014	guide RNA fragment

PDB ID	Resolution (Å)	R-Value Free	Mutations	UNIPROT Sequence Mismatches	Missing residues (Unmodeled)	Residues with zero occupancy atoms	Year of release	Bound
4W5N	2.90	0.253	Yes: 387	Yes: 387	1-21, 150-153, 246, 273-275, 603-605, 818-838 [Total: 53]	-	2014	guide RNA
4W5O	1.80	0.197	Yes: 387	Yes: 387	1-21, 89-90, 121-126, 270-275, 297-305, 822-835 [Total: 58]	-	2014	guide RNA & target mRNA
4W5Q	3.10	0.233	Yes: 387	Yes: 387	1-21, 121-126, 270-275, 297-303, 822-837 [Total: 56]	-	2014	guide RNA & target mRNA
4W5R	2.50	0.234	Yes: 387	Yes: 387	1-21, 88-89, 121-126, 270-275, 297-303, 822-835 [Total: 56]	-	2014	guide RNA & target mRNA
4W5T	2.50	0.215	Yes: 387	Yes: 387	1-21, 121-126, 272-275, 297-303, 822-835 [Total: 52]	-	2014	guide RNA & target mRNA
4Z4C	2.30	0.219	Yes: 387	Yes: 387	1-21, 89-90, 121-126, 270-275, 297-305, 822-835 [Total: 58]	-	2015	guide miRNA & target mRNA

PDB ID	Resolution (Å)	R-Value Free	Mutations	UNIPROT Sequence Mismatches	Missing residues (Unmodeled)	Residues with zero occupancy atoms	Year of release	Bound
4Z4D	1.60	0.189	Yes: 387	Yes: 387	1-21, 121-126, 270-275, 296-304, 822-835 [Total: 56]	-	2015	guide miRNA & target mRNA
4Z4E	1.80	0.185	Yes: 387	Yes: 387	1-21, 121-125, 270-277, 297-305, 822-835 [Total: 57]	-	2015	guide miRNA & target mRNA
4Z4F	2.80	0.233	Yes: 387	Yes: 387	1-21, 64-65, 121-126, 270-275, 296-304, 822-835 [Total: 58]	-	2015	guide miRNA & target mRNA
4Z4G	2.70	0.224	Yes: 387	Yes: 387	1-21, 89-90, 121-126, 270-275, 295-305, 822-835 [Total: 60]	-	2015	guide miRNA & target mRNA
4Z4H	2.50	0.211	Yes: 387,481	Yes: 387, 481	1-21, 89, 121-126, 270-275, 297-305, 822-835 [Total: 57]	-	2015	guide miRNA & target mRNA
4Z4I	2.80	0.233	Yes: 387,481	Yes: 387, 481	1-21, 89-90, 121-126, 270-276, 297-305, 822-835 [Total: 57]	-	2015	guide miRNA & target mRNA

PDB ID	Resolution (Å)	R-Value Free	Mutations	UNIPROT Sequence Mismatches	Missing residues (Unmodeled)	Residues with zero occupancy atoms	Year of release	Bound
5JS1	2.50	0.247	Yes: 387	Yes: 387	1-22, 121-125, 152-153, 186-188, 272-276, 603-605, 820-837 [Total: 58]	-	2016	siRNA
5JS2	2.95	0.261	Yes: 387	Yes: 387	1-22, 121-124, 152-153, 186-188, 272-277, 603-606, 820-837 [Total: 58]	-	2016	siRNA
5KI6	2.15	0.270	Yes: 387	Yes: 387	1-21, 120-125, 152-153, 186-189, 271-276, 354, 818-838 [Total: 61]	-	2016	Guide RNA
5T7B	2.53	0.238	Yes: 599, 671	Yes: 599, 671 [Notes: unmodeled expression tag in -1, 0 positions]	1-24, 123-128, 188-190, 247-249, 275-277, 605-608, 823-838 [Total: 59]	-	2016	Guide RNA
5WEA	3.12	0.282	Yes: 365, 387	Yes: 365, 387	1-22, 120-126, 151-153, 186-194, 238-248, 271-276, 332-334, 355-361 [Total: 68]	-	2017	miRNA

PDB ID	Resolution (Å)	R-Value Free	Mutations	UNIPROT Sequence Mismatches	Missing residues (Unmodeled)	Residues with zero occupancy atoms	Year of release	Bound
6CBD	2.20	0.215	Yes: 387	Yes: 387	1-21, 86, 89-90, 109-110, 121-126, 246-247, 270-277, 297-305, 331-336, 822-835 [Total: 71]	-	2018	guide RNA & target RNA
6MDZ	3.40	0.264	Yes: 387, 669, 824, 828, 831, 834	Yes: 387, 669, 824, 828, 831, 834	1-21, 89-90, 121-126, 273-275, 603-606, 819-837 [Total: 55]	-	2019	miR-122 & target RNA
6MFN	2.50	0.247	Yes: 387, 669, 824, 828, 831, 834	Yes: 387, 669, 824, 828, 831, 834	1-21, 121-126, 187-189, 273-275, 603-607, 820-837 [Total: 56]	-	2019	miR-27a & target RNA
6MFR	3.60	0.283	Yes: 387, 669, 824, 828, 831, 834	Yes: 387, 669, 824, 828, 831, 834	1-21, 89-90, 121-126, 273-275, 603-606, 817-837 [Total: 57]	-		
6N4O	2.90	0.25273-2757	Yes: 387, 669, 824, 828, 831, 834	Yes: 387, 669, 824, 828, 831, 834	1-21, 121-125, 186-189, 247-250, 273-275, 296-302, 333, 820-837 [Total: 63]	-	2019	miR-122 & target RNA

PDB ID	Resolution (Å)	R-Value Free	Mutations	UNIPROT Sequence Mismatches	Missing residues (Unmodeled)	Residues with zero occupancy atoms	Year of release	Bound
6NIT	3.80	0.94	Yes: 387, 669, 824, 828, 831, 834	Yes: 387, 669, 824, 828, 831, 834	1-21, 64-65, 87-90, 121-126, 273-275, 603-606, 817-837 [Total: 61]	-	2019	miR-122 & target RNA
7KI3	3.00	0.276	Yes: 387, 824, 828, 831, 834	Yes: 387, 824, 828, 831, 834	1-21, 296-306, 334-338, 820-837 [Total:55]	-	2021	miR-122 & target RNA
8D6J	2.50	0.284	No	Yes: 387, 602, 603, 605-608, 824, 828, 831, 834 [Sequence conflicts]	1-22, 121-124, 152, 271-275, 673, 821-836 [Total: 49]	-	2023	miR122
8D71	2.50	0.280	No	Yes: 387, 824, 828, 831, 834 [Sequence conflicts]	1-22, 123, 274-275, 603-605, 818-838 [Total: 49]	-	2023	miR122

Table S8. Currently available experimental structural models of AGO2. The selected model is highlighted in blue. The information is retrieved from Uniprot and RCSB PDB.

Protein	PDB ID	Resolution (Å)	R-Value Free	Mutations	UNIPROT Sequence Mismatches	Missing residues (Unmodeled)	Residues with zero occupancy atoms	Year of release	Bound
**AGO1**	**4KRE**	**1.75**	**0.202**	**No**	**No [Notes: unmodeled expression tag in 1 position]**	**1-18, 83-84, 109, 118-124, 241-244, 273-274, 331-334, 602-605, 819-835 [Total: 59]**	**-**	**2013**	**Sf9 RNA**

Protein	PDB ID	Resolution (Å)	R-Value Free	Mutations	UNIPROT Sequence Mismatches	Missing residues (Unmodeled)	Residues with zero occupancy atoms	Year of release	Bound
AGO1	4KRF	2.10	0.217	No	No [Notes: unmodeled expression tag in 1 position]	1-17, 120-122, 273-274, 603-605, 820-835 [Total: 41]	-	2013	let7
AGO1	4KXT	2.29	0.228	No	No [Notes: unmodeled expression tag in 1-5 positions]	1-26, 118-131, 824-839 [Total: 56]	-	2013	guide RNA
AGO1	5W6V	2.83	0.245	No	No [Notes: unmodeled expression tag in 1-2 positions]	1-22, 121-122, 243-245, 273-275, 296-304, 331-333, 604-606, 820-837 [Total: 63]	-	2017	GW182 motif & guide RNA
**AGO3**	**5VM9**	**3.28**	**0.239**	**No**	**No [Notes: unmodeled expression tag in 1-2 positions]**	**1-17, 113-121, 130-136, 143-159, 248-249, 276-278, 301-307, 607-609, 676, 823-839 [Total: 83]**	**-**	**2017**	**RNA segments**
**AGO4**	**6OON**	**1.90**	**0.202**	**No**	**No [Notes: unmodeled expression tag in 1-2 positions]**	**1-13, 112-117, 236-239, 262-268, 289-295, 323-328, 378-383, 597-600, 628-637, 826-840 [Total: 78]**	**647**	**2019**	**guide RNA**

Table S9. Currently available experimental structural models of AGO1, AGO3 and AGO4. The selected model are highlighted in blue. The information is retrieved from Uniprot and RCSB PDB.

Protein	PDB ID	Included residues after preparation	Edited residues
AGO1	4KRE.A	18-857	-
AGO2	4Z4D.A	22-859	Converted ASP387 to SER387
AGO3	5VM9.A	16-860	-
AGO4	6OON.A	12-861	-

Table S10. The resulting structures after structural preparation for both R1/R2 and R3 workflows

	AGO1	AGO2	AGO3	AGO4
All protein atoms	13522	13424	13532	13533
Protein atoms excluding hydrogens	6733	6692	6751	6753
C_α atoms	840	838	845	850
Protein backbone atoms	2520	2514	2535	2550
Protein main chain atoms	3361	3353	3381	3401
Protein main chain atoms excluding C_β	4149	4141	4174	4195
Protein main chain atoms including backbone amide hydrogens and hydrogens on the N-terminus	4149	4142	4173	4199
Protein side chain atoms	9373	9282	9359	9334
Protein side chain atoms excluding all hydrogens	3372	3339	3370	3352
Water molecules	272620	269872	271296	251048
Ions (Cl^-)	30	32	29	26
Total atoms in the system	286172	283328	284857	264607

Table S11. Setup for each simulated system for R1, R2 replicas.

	AGO1	AGO2	AGO3	AGO4
All protein atoms	13522	13422	13531	13533
Protein atoms excluding hydrogens	6733	6692	6751	6753
C_α atoms	840	838	845	850
Protein backbone atoms	2520	2514	2535	2550
Protein main chain atoms	3361	3353	3381	3401
Protein main chain atoms excluding C_β	4149	4141	4174	4195
Protein main chain atoms including backbone amide hydrogens and hydrogens on the N-terminus	4149	4142	4173	4199
Protein side chain atoms	9373	9280	9358	9334
Protein side chain atoms excluding all hydrogens	3372	3339	3370	3352
Water molecules	275124	273892	268532	268304

	AGO1	AGO2	AGO3	AGO4
Ions (Cl^-)	30	30	28	26
Total atoms in the system	288676	287344	282091	281863

Table S12. Setup for each simulated system for R3 replica.

	H	B	E	G	I	T	S	P	.	*	-
**H**	2	-1	-1	1	1	0	-1	-1	-1	-1	-1
**B**	-1	2	0	-1	-1	-1	-1	-1	-1	-1	-1
**E**	-1	0	2	-1	-1	-1	-1	-1	-1	-1	-1
**G**	1	-1	-1	2	1	0	-1	-1	-1	-1	-1
**I**	1	-1	-1	1	2	0	-1	-1	-1	-1	-1
**T**	0	-1	-1	0	0	2	-1	-1	-1	-1	-1
**S**	-1	-1	-1	-1	-1	-1	2	-1	-1	-1	-1
**P**	-1	-1	-1	-1	-1	-1	-1	2	-1	-1	-1
**.**	-1	-1	-1	-1	-1	-1	-1	-1	2	-1	-1
*****	-1	-1	-1	-1	-1	-1	-1	-1	-1	2	-1
**-**	-1	-1	-1	-1	-1	-1	-1	-1	-1	-1	-1

Table S13. Custom scoring matrix for plotting 2D pairwise alignments of LCS1, LCS2. The scoring only affects the coloring tone of the plotted matches between DSSP symbols. Helices (G,H,I) have a matching score of 1. E, B match also with score of 1. G,H,I have a matching score of 0 with T. The rest of the relationships are scored by a negative score.

Task	Method
Sequence similarity	PSI-BLAST
Sequence alignment	EMBOSS Needle
Structure preparation	Schrodinger Maestro for R1, R2 and PDBFixer, Modeller, PDB2PQR for R3
Molecular dynamics	GROMACS with DES-Amber forcefield
Trajectory clustering	K-Means NANI
Trajectory analysis	MDAnalysis, Pandas, SciPy, UMAP Python packages
Weak interactions analysis	GetContacts
Structural similarity & functional meta-analysis	Machaon
Structural search space refinement for Machaon	PDBFixer, PDB2PQR, OpenMM
Zinc-ion binding sites predictions	Metal3D
Binding pockets prediction	PocketMiner

Table S14. The methods and software packages that were employed for each task of this study.

Supplementary Figures

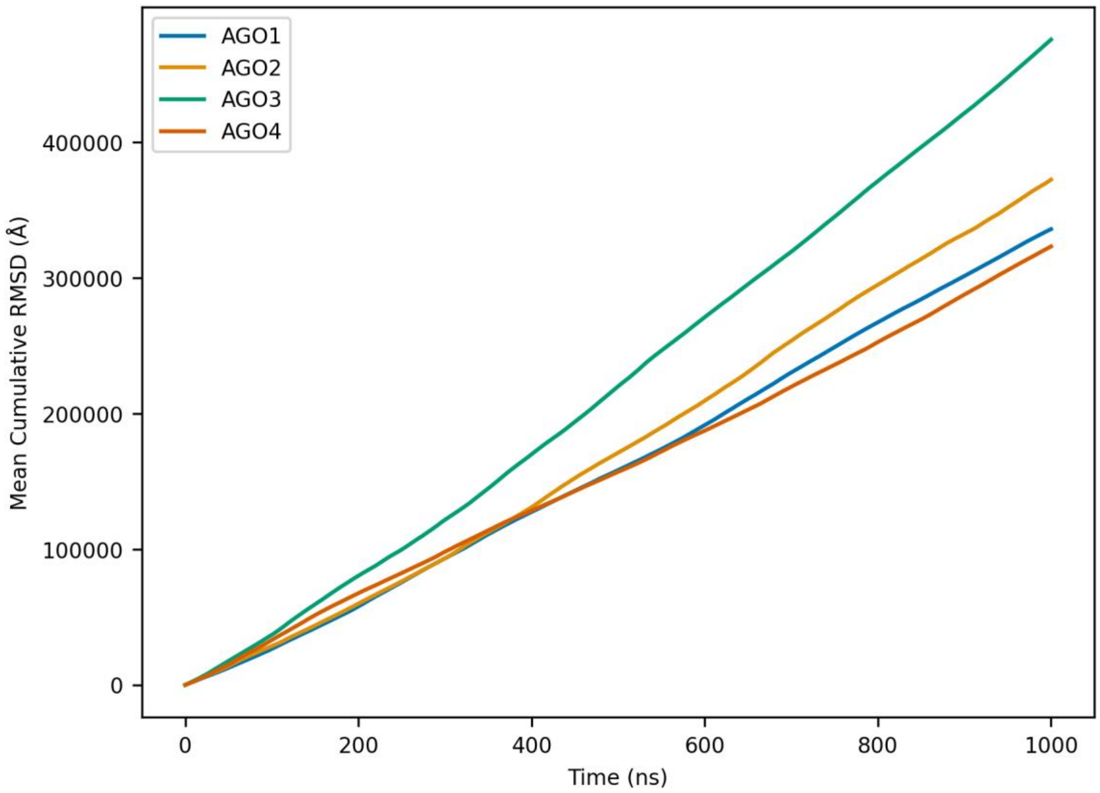

Figure S1. Mean cumulative root mean square deviation of the AGOs. Mean cumulative root mean square deviation (RMSD) (Å) from the initial conformation per AGO protein, measured by alpha-carbon RMSD from R1, R2, R3 replicates. The RMSD was computed by the alpha carbons of the protein.

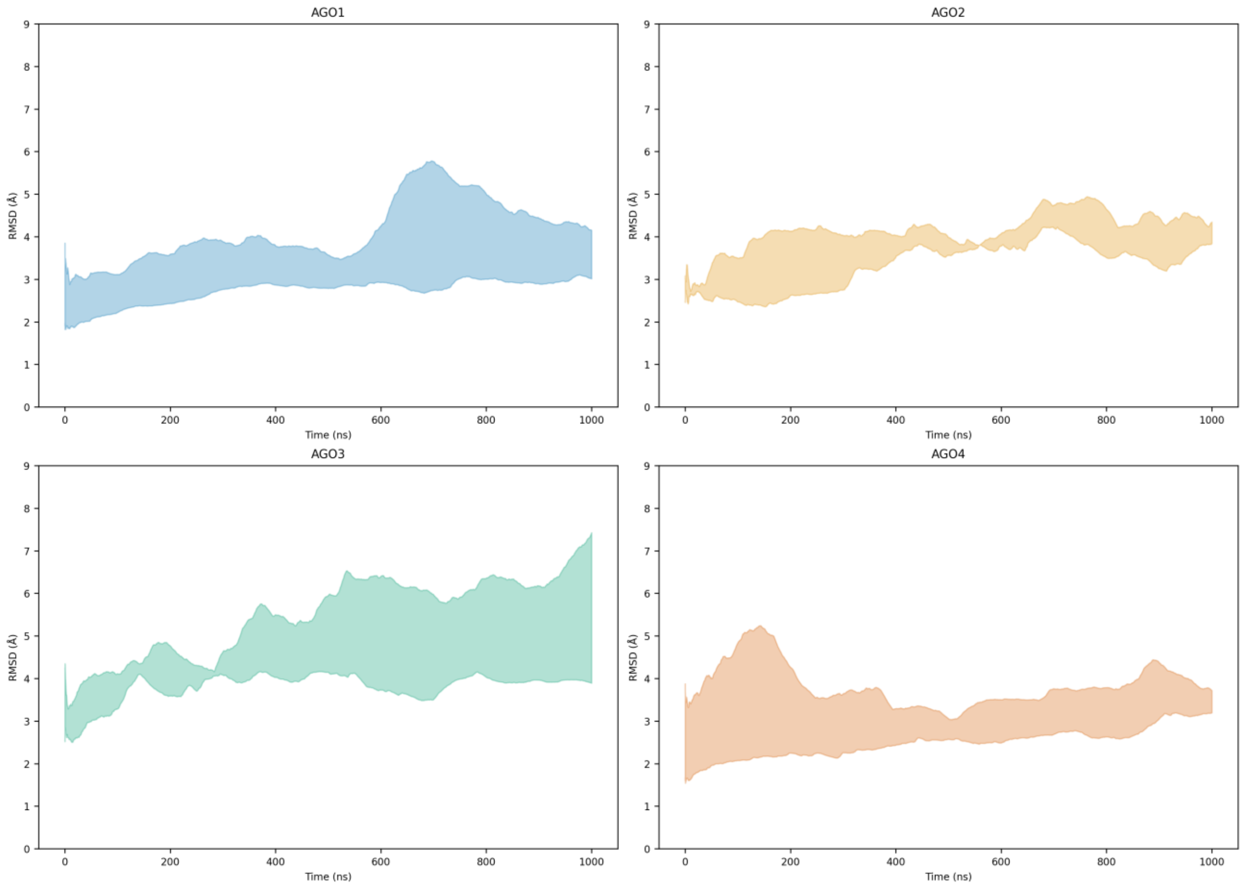

Figure S2. Intervals of RMSD values per AGO. (A-B) Intervals based on the min/max values of Exponentially Weighted Moving Averages (EWMA, span=10000) of RMSD (Å) per AGO protein for R1, R2, R3 replicas. The RMSD was computed by the alpha carbons of the protein.

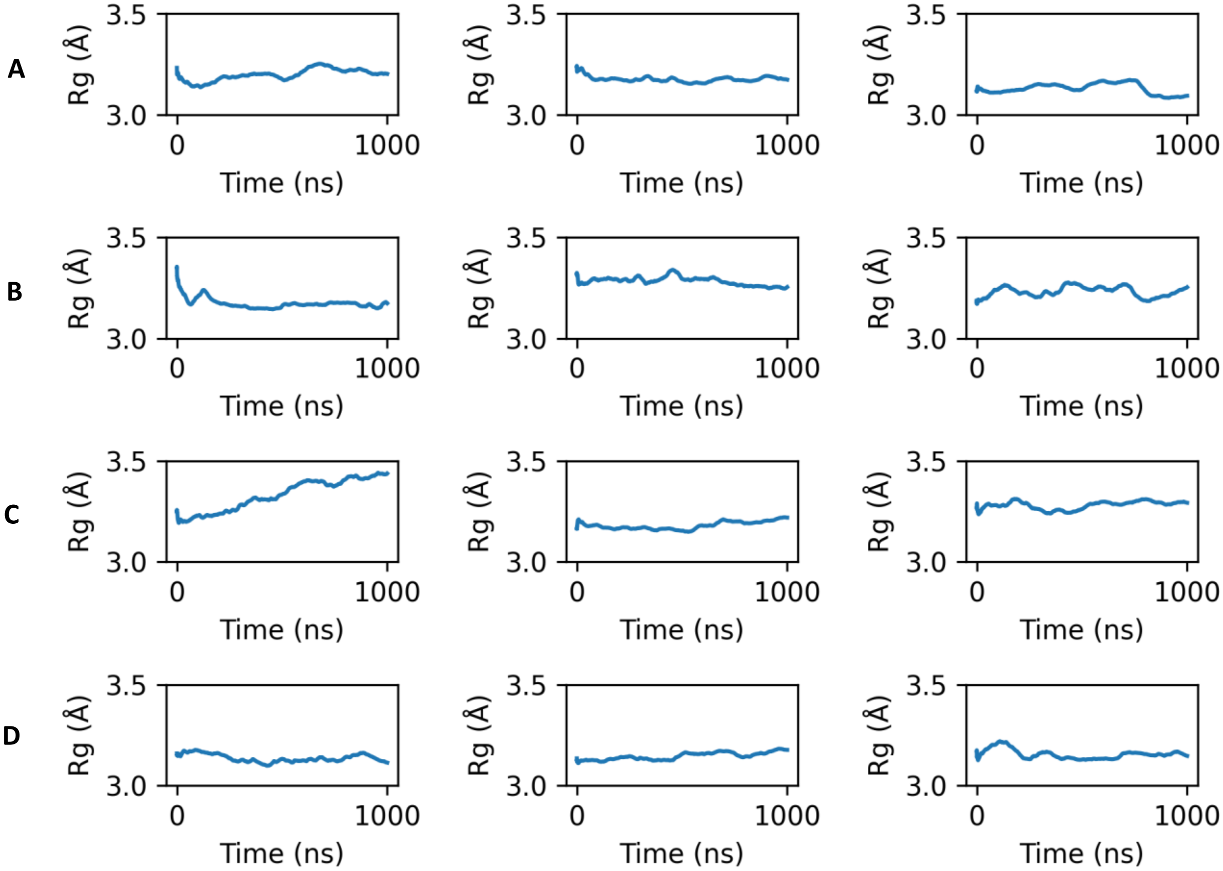

Figure S3. Radius of gyration (Rg) per simulation and AGO. (A-D) Exponentially Weighted Moving Averages (EWMA, span=10000) of Rg (Å) per AGO protein for R1, R2, R3 replicas (left to right). **(A)** AGO1 **(B)** AGO2 **(C)** AGO3 **(D)** AGO4

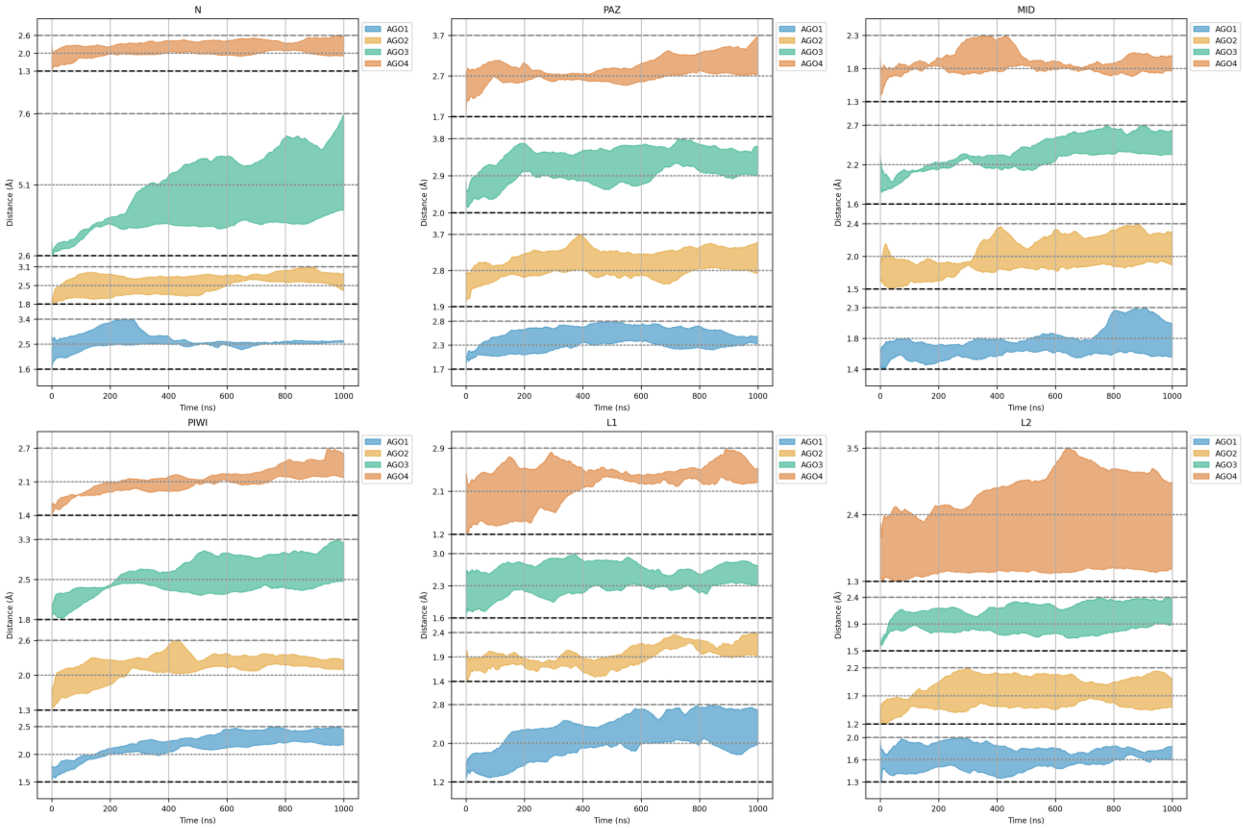

Figure S4. Intervals of domain-level RMSD values of the AGOs. Intervals based on the min/max values of Exponentially Weighted Moving Averages (EWMA) (span=10000) of RMSD (Å) per AGO protein and domain for R1, R2, R3 replicas.

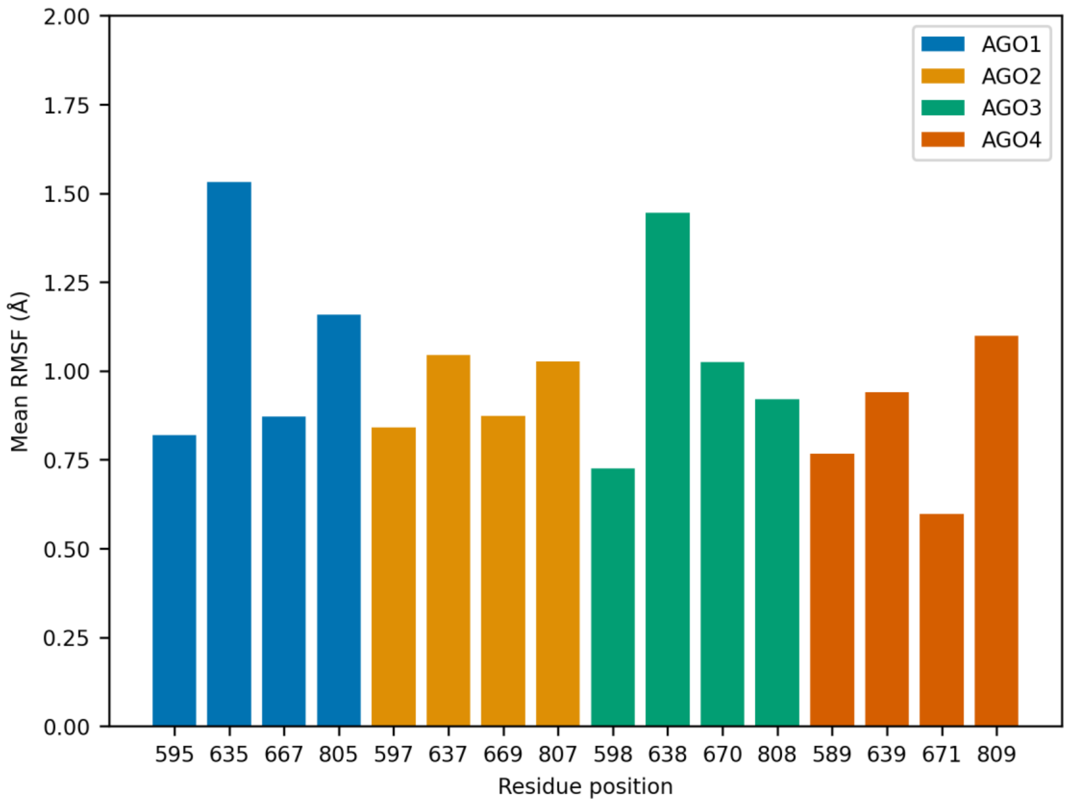

Figure S5. Mean root mean square fluctuations (RMSF) of the tetrads in AGOs. Average RMSF (Å) of the residues in the positions of DEDH catalytic tetrads of AGO2-AGO3 and pseudo-catalytic ones of AGO1-AGO4 for R1, R2**,** R3 replicas.

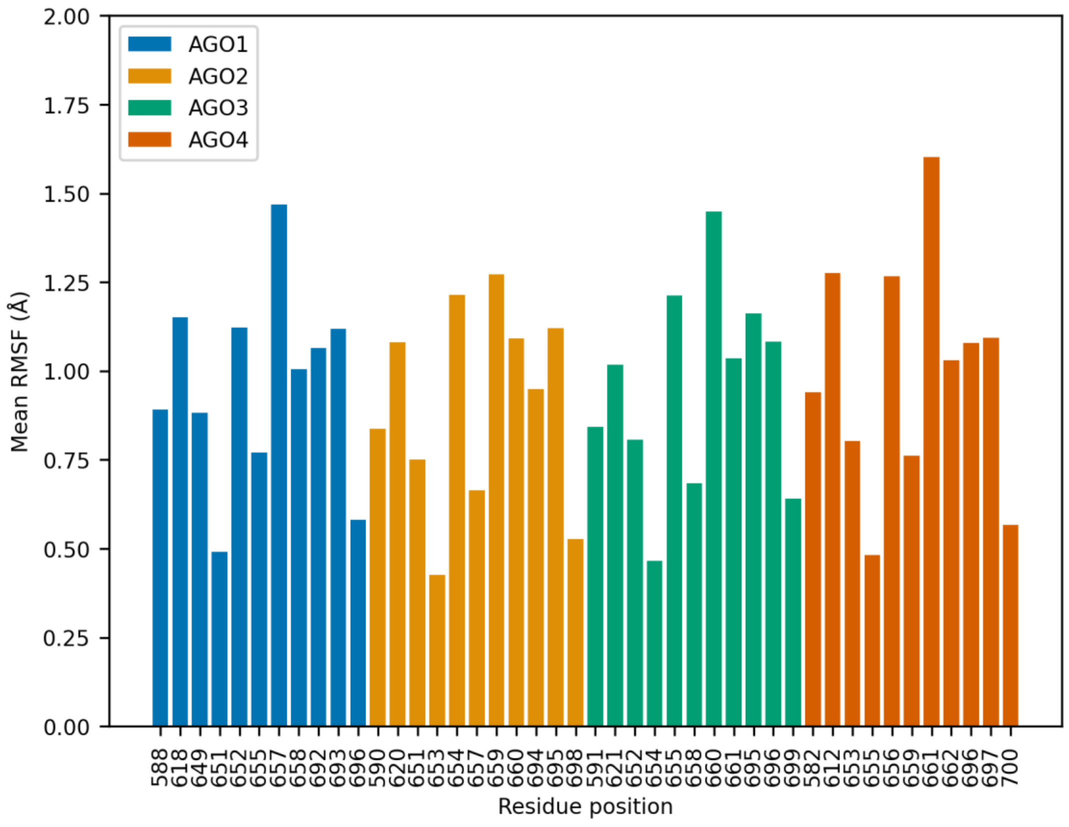

Figure S6. Mean root mean square fluctuations (RMSF) of the GW182 interaction sites of AGOs. Average RMSF (Å) of the residues of AGOs that interact with GW182 for R1, R2, R3 replicas**.**

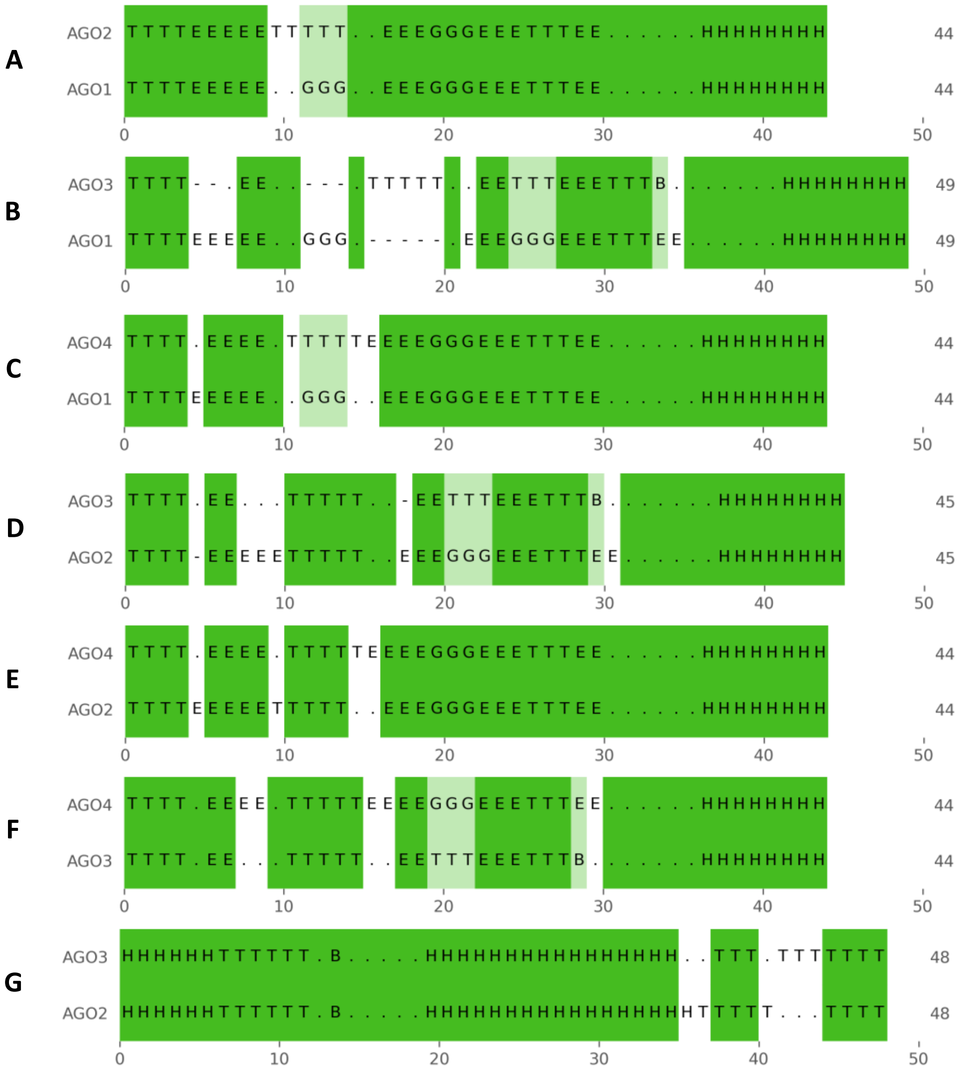

Figure S7. Pairwise secondary structure alignments for long common subsequences LCS1, LCS2. (A-F) LCS1 2D alignments and **(G)** LCS2 2D alignments. Coloring designates a matching 2D fold and a lighter color tone refers to a close classification relationship between the two compared folds (see Table S8).

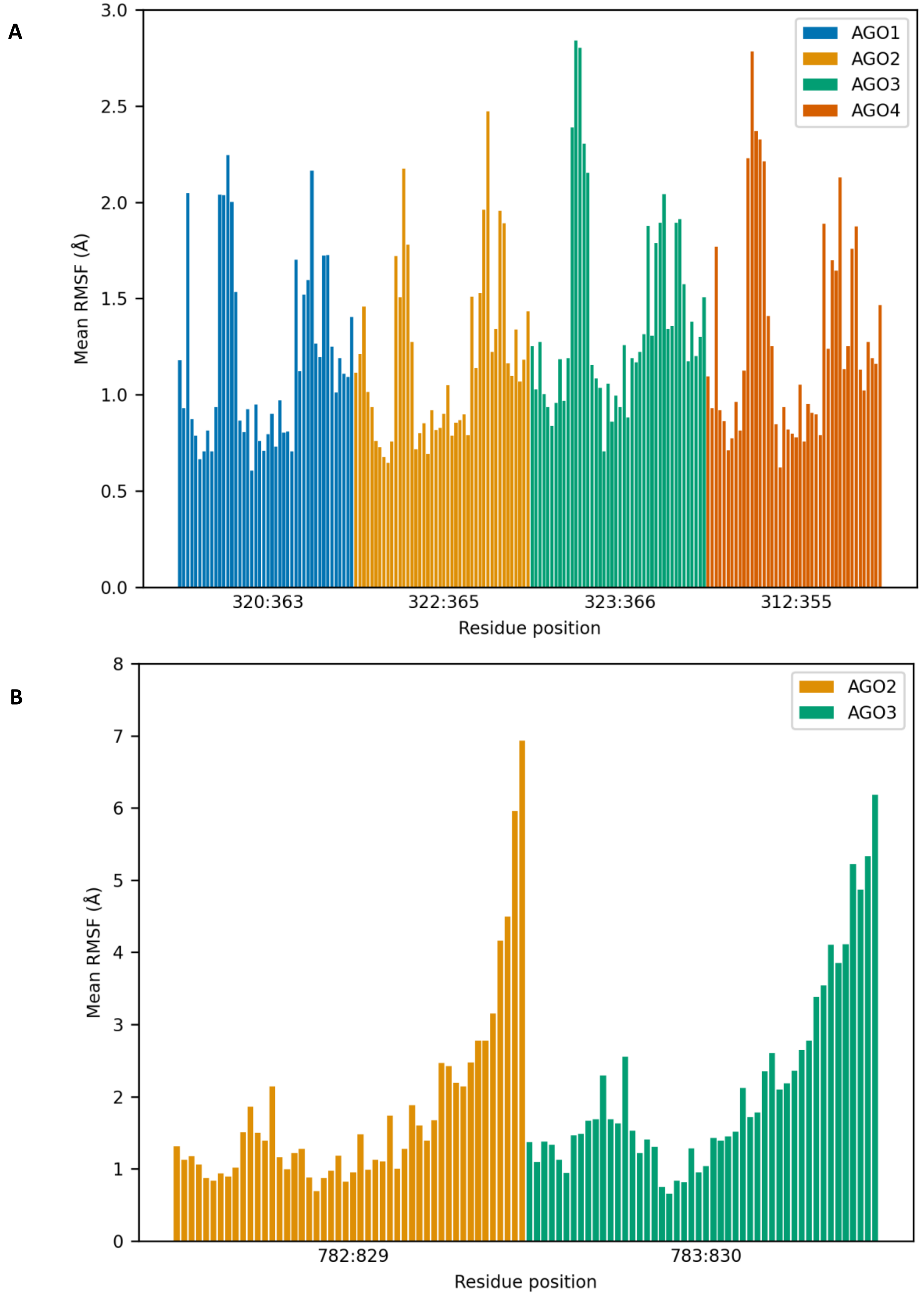

Figure S8. Mean root mean square fluctuations (RMSF) for long common subsequences LCS1, LCS2 of AGOs. Average RMSF (Å) of the residues that reside in LCS1 **(A)** and LCS2 **(B)** for R1, R2, R3 replicas.

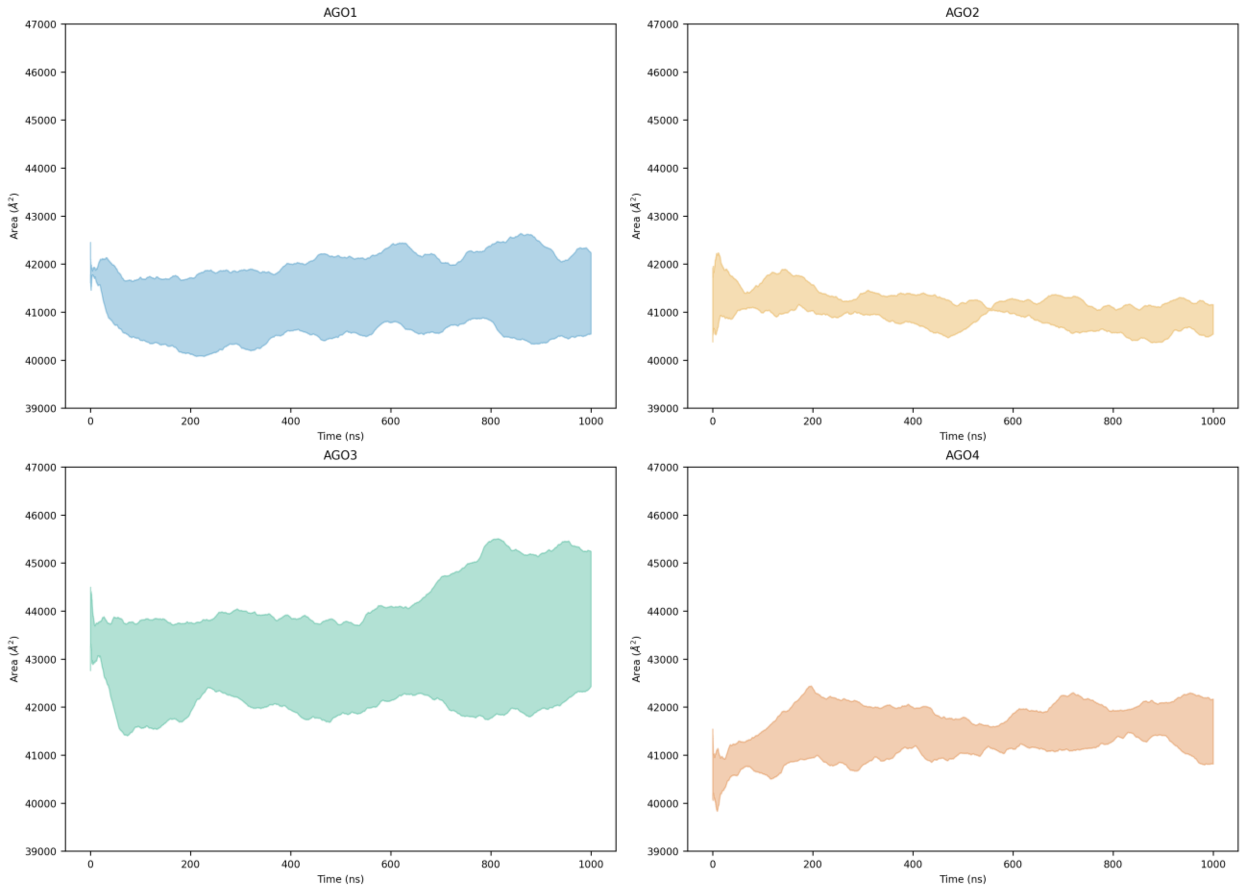

Figure S9. Intervals as yielded by Surface Accessible Area Surface (SASA) analysis per AGO. Intervals based on the min/max values of Exponentially Weighted Moving Averages (EWMA, span=10000) of SASA (Å^2) per AGO protein for R1, R2, R3 replicas.

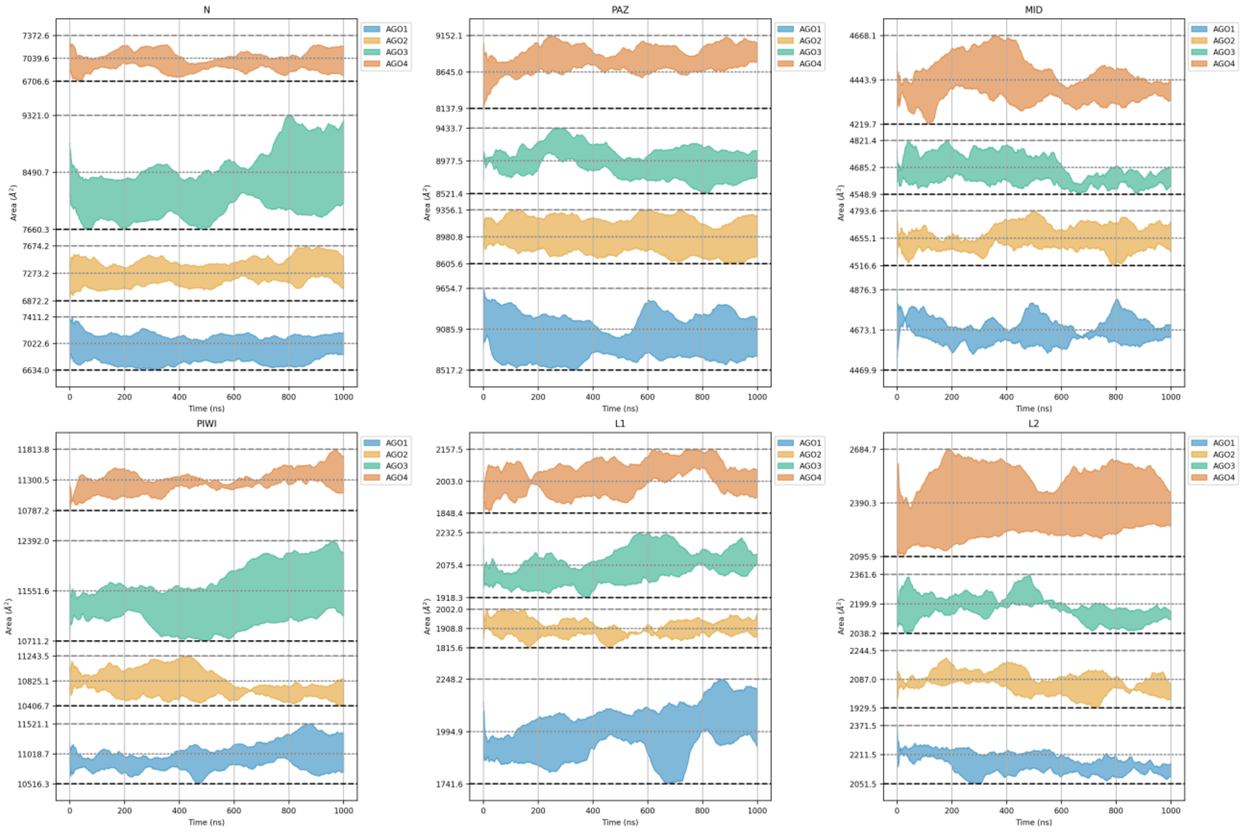

Figure S10. Intervals of domain-level SASA values per AGO. Intervals based on the min/max values of Exponentially Weighted Moving Averages (EWMA) (span=10000) of SASA (Å^2) per AGO protein and domain for R1, R2, R3 replicas.

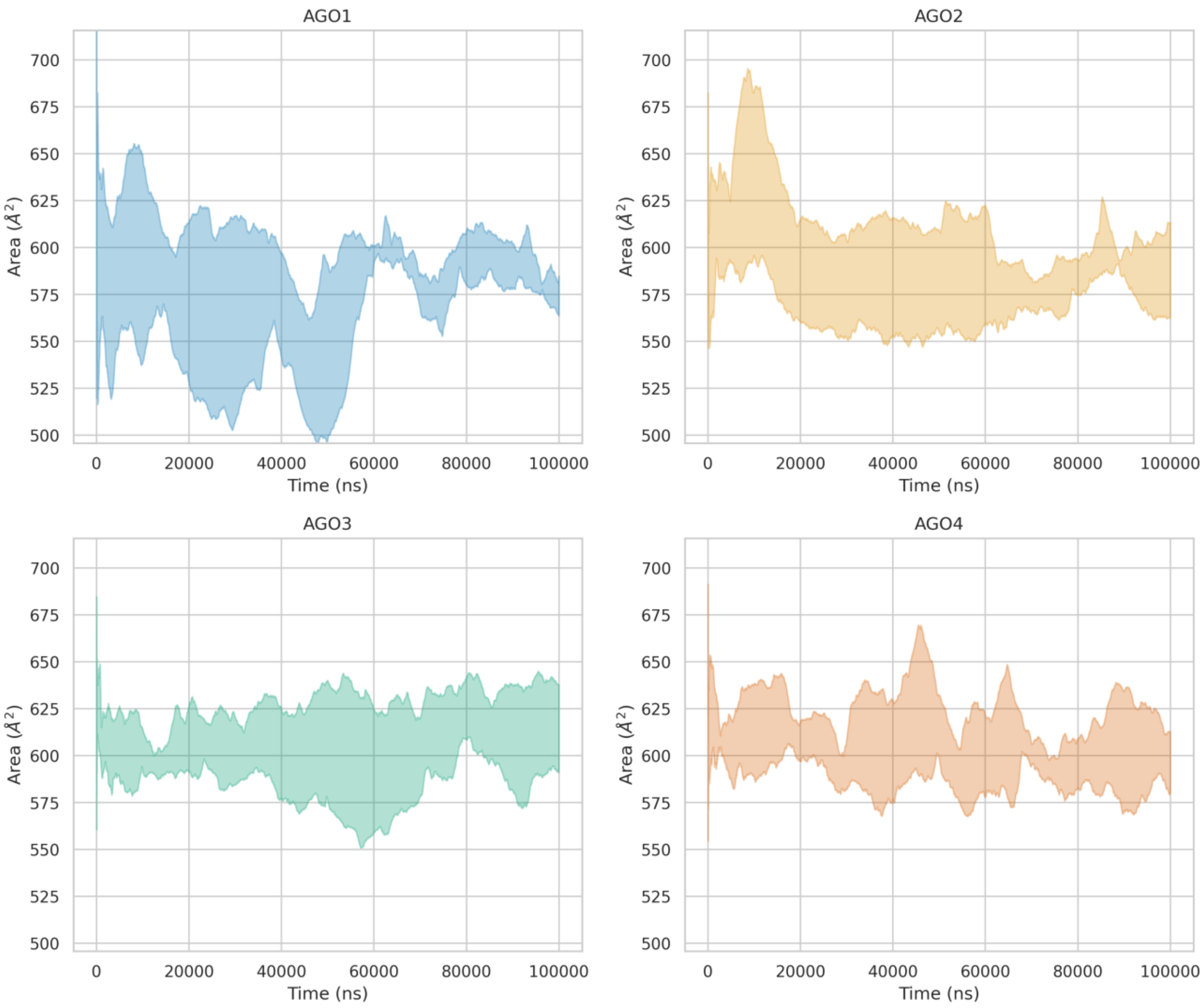

Figure S11. Intervals of SASA values for the GW182 binding sites per AGO. Intervals based on the min/max values of Exponentially Weighted Moving Averages (EWMA) (span=10000) of SASA (Å^2) per AGO protein and GW182 sites for R1, R2, R3 replicas.

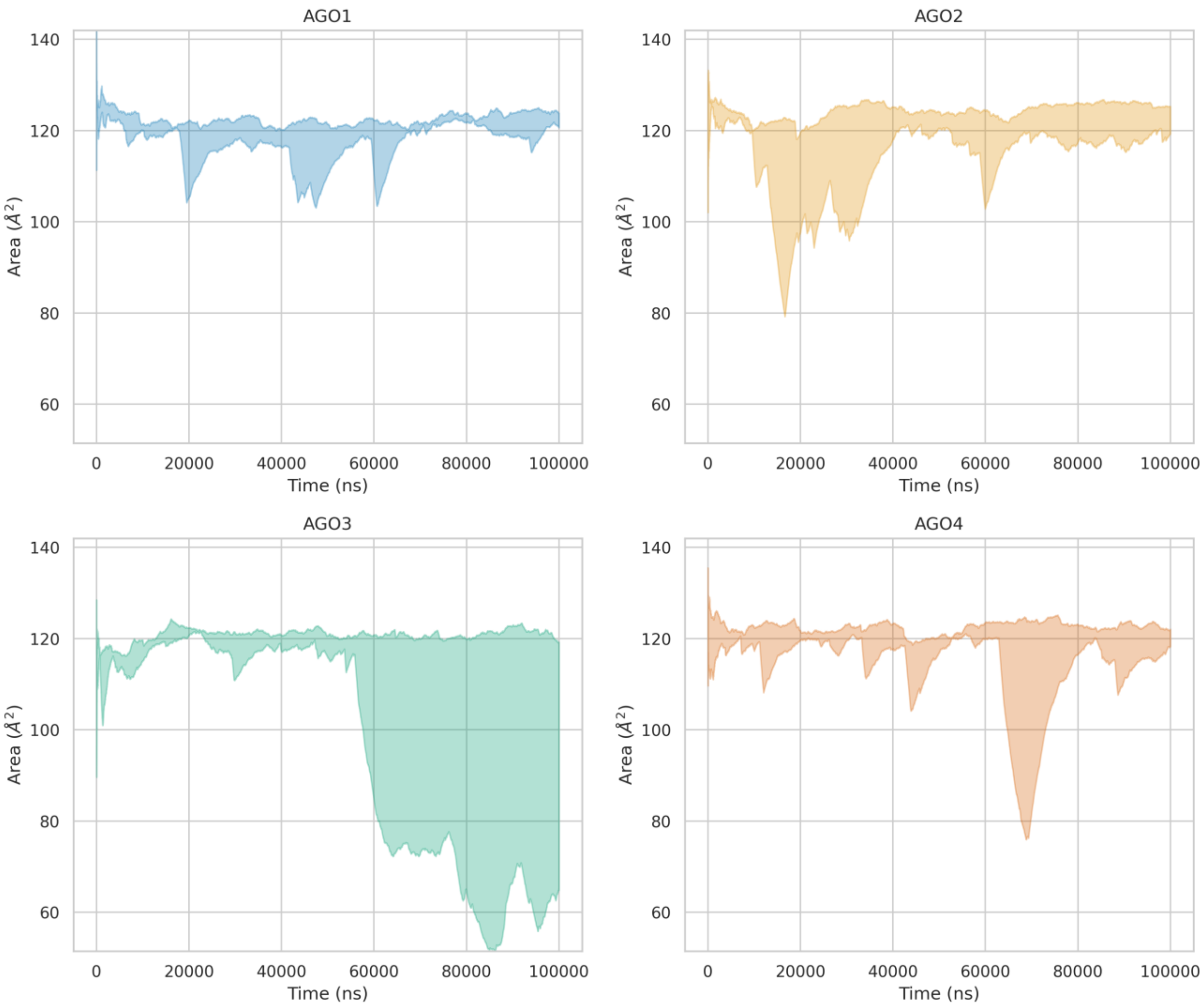

Figure S12. Intervals of SASA values for the ZSWIM8 binding site per AGO. Intervals based on the min/max values of Exponentially Weighted Moving Averages (EWMA) (span=10000) of SASA (Å^2) per AGO protein and GW182 sites for R1, R2, R3 replicas.

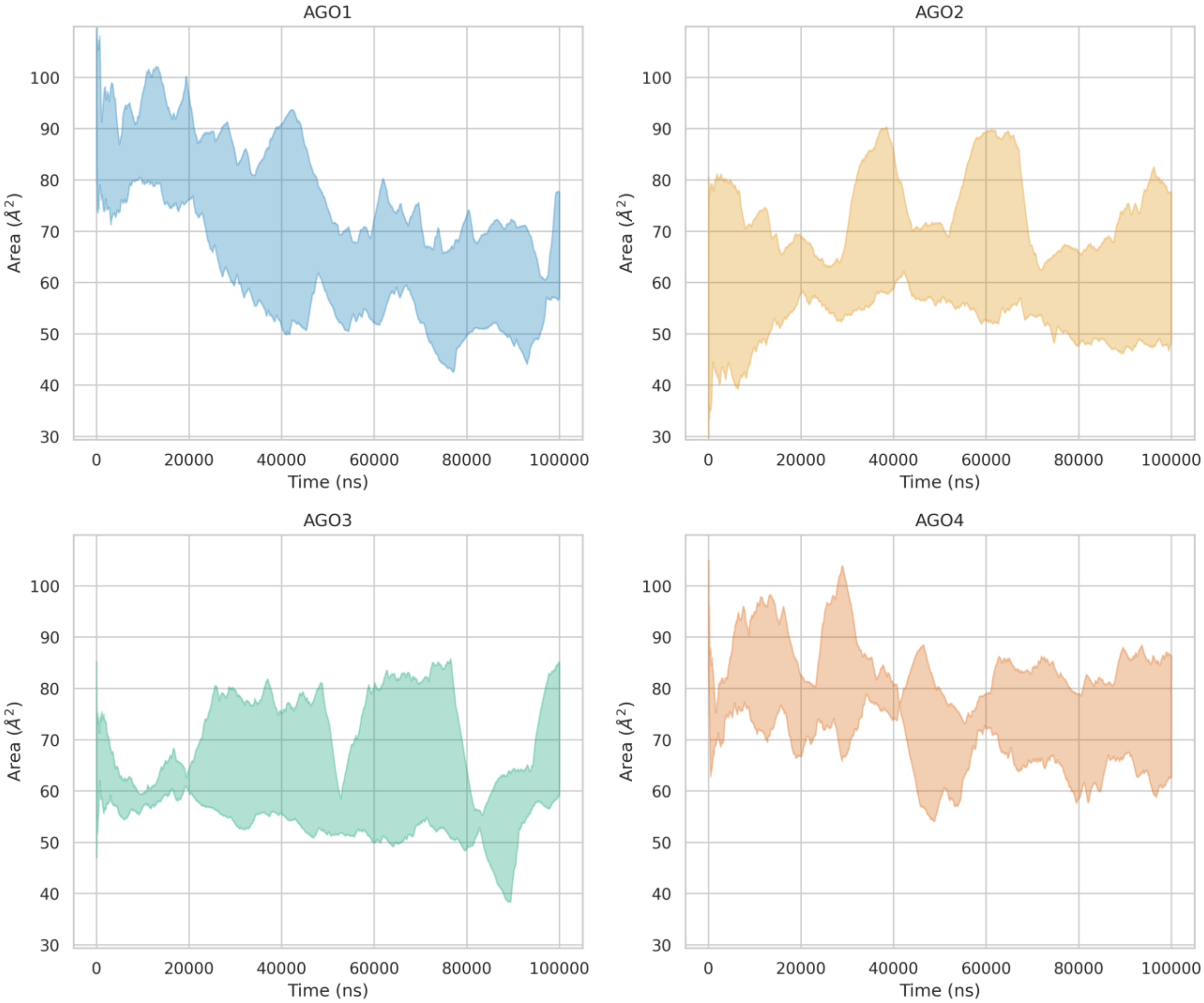

Figure S13. Intervals of SASA values for the fourth amino acid in the catalytic tetrad of AGO2, AGO3 or pseudo-catalytic tetrad of AGO1, AGO4. Intervals based on the min/max values of Exponentially Weighted Moving Averages (EWMA) (span=10000) of SASA (Å^2) per AGO protein for R1, R2, R3 replicas.

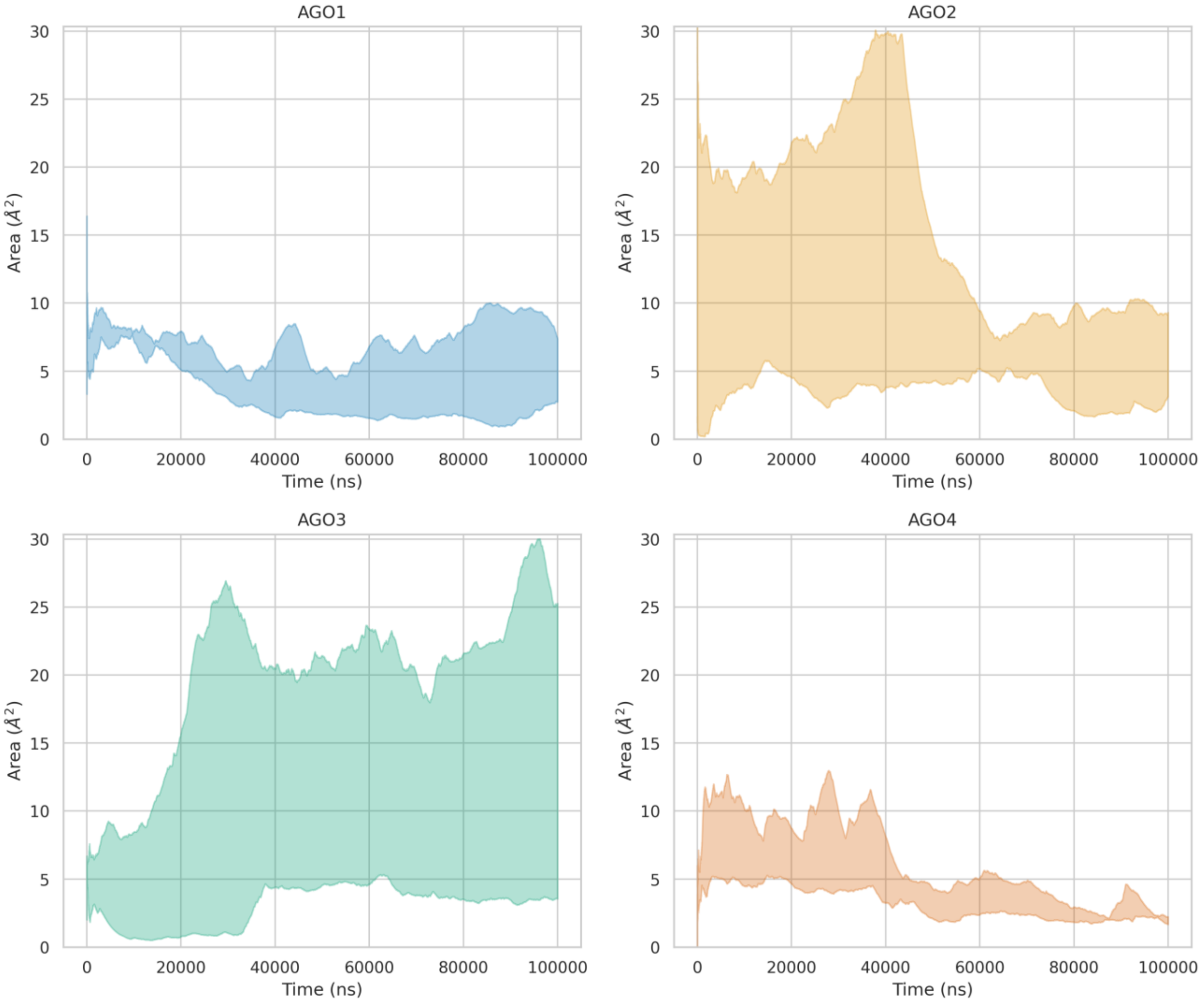

Figure S14. Intervals of SASA values for the first amino acid in the catalytic tetrad of AGO2, AGO3 or pseudo-catalytic tetrad of AGO1, AGO4. Intervals based on the min/max values of Exponentially Weighted Moving Averages (EWMA) (span=10000) of SASA (Å^2) per AGO protein for R1, R2, R3 replicas.

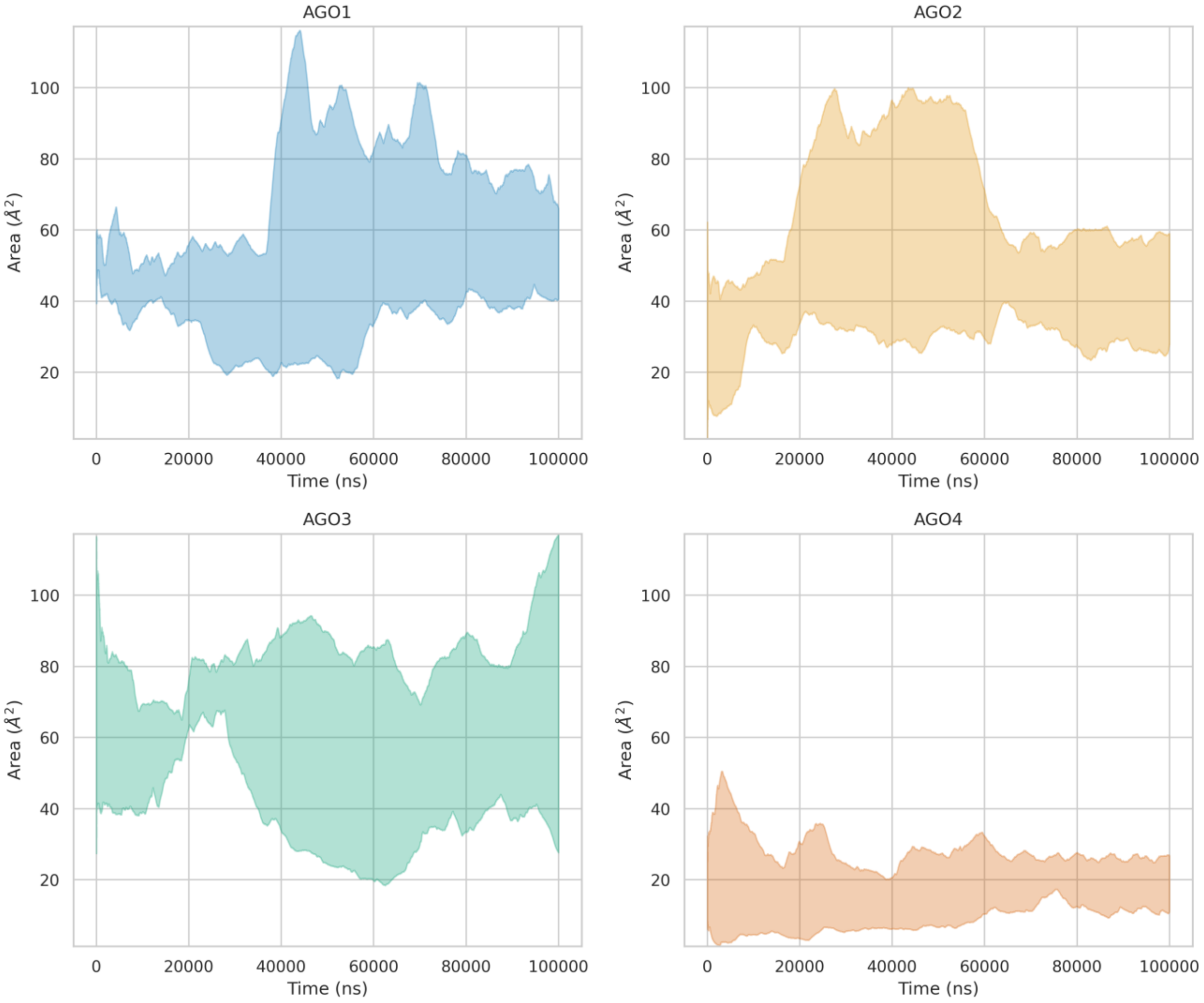

Figure S15. Intervals of SASA values for the second amino acid in the catalytic tetrad of AGO2, AGO3 or pseudo-catalytic tetrad of AGO1, AGO4. Intervals based on the min/max values of Exponentially Weighted Moving Averages (EWMA) (span=10000) of SASA (Å^2) per AGO protein for R1, R2, R3 replicas.

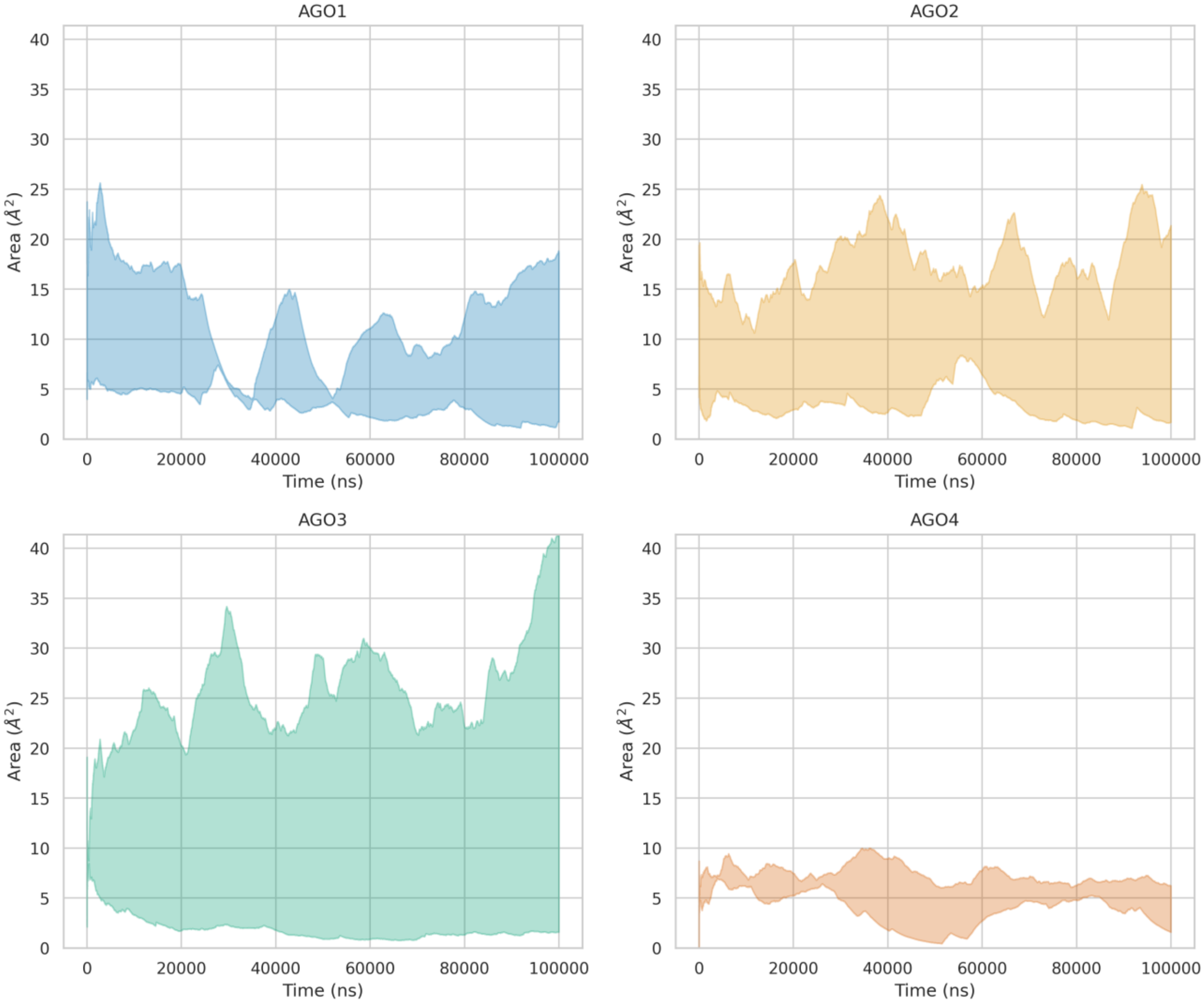

Figure S16. Intervals of SASA values for the third amino acid in the catalytic tetrad of AGO2, AGO3 or pseudo-catalytic tetrad of AGO1, AGO4. Intervals based on the min/max values of Exponentially Weighted Moving Averages (EWMA) (span=10000) of SASA (Å^2) per AGO protein for R1, R2, R3 replicas.

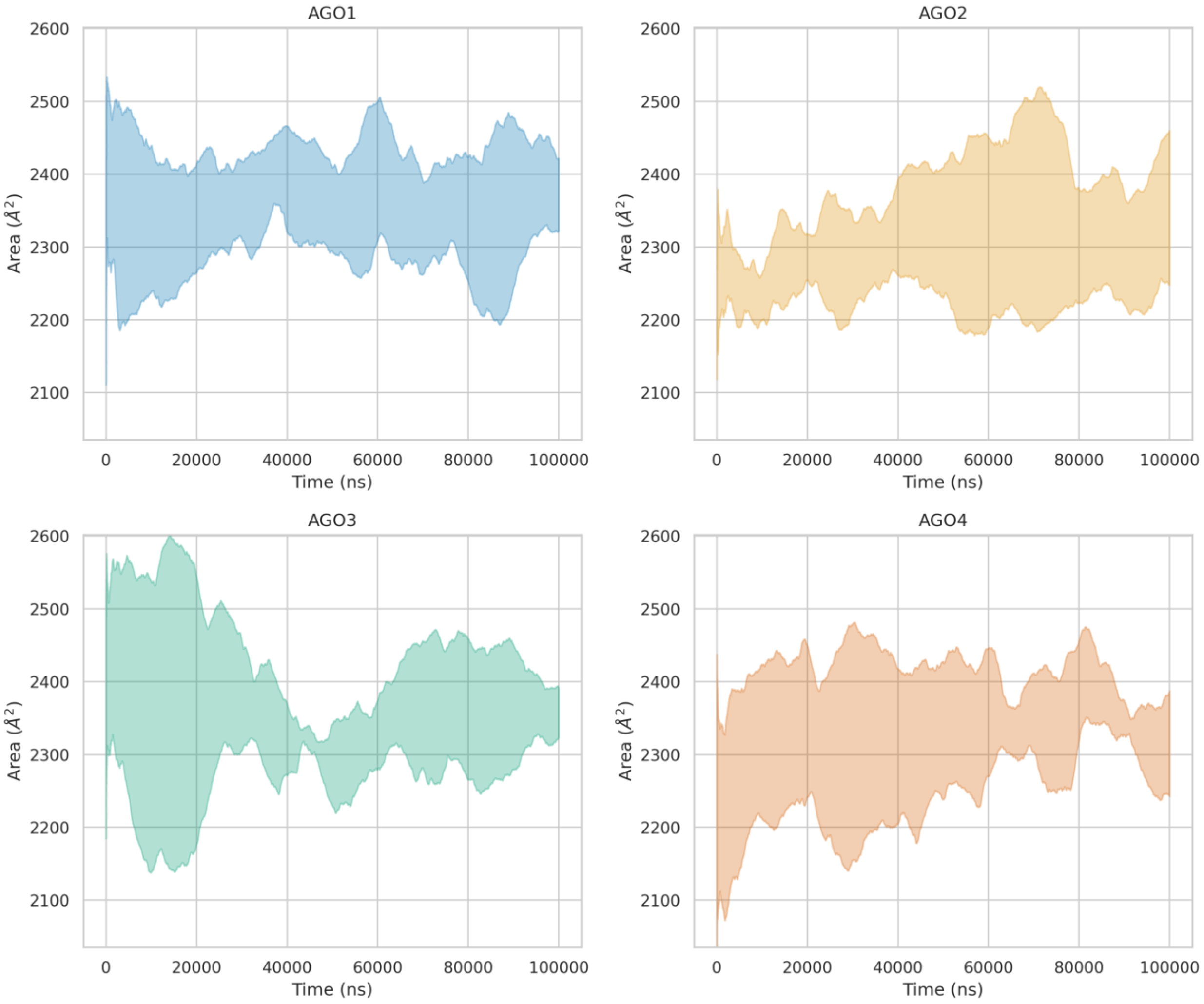

Figure S17. Intervals of SASA values for LCS1 per AGO. Intervals based on the min/max values of Exponentially Weighted Moving Averages (EWMA) (span=10000) of SASA (Å^2) per AGO protein and LCS1 segments for R1, R2, R3 replicas.

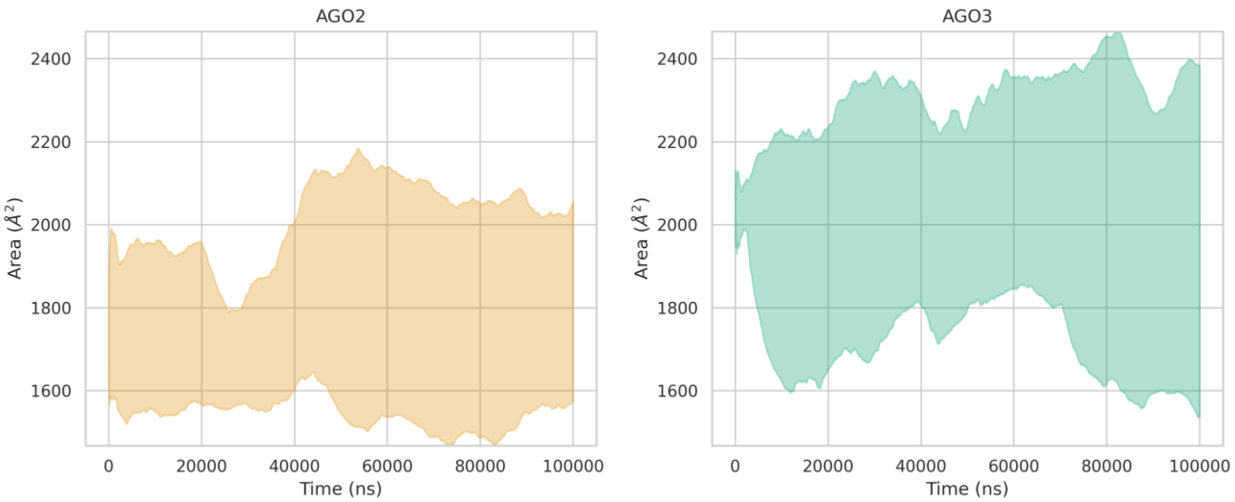

Figure S18. Intervals of SASA values for LCS2 per AGO. Intervals based on the min/max values of Exponentially Weighted Moving Averages (EWMA) (span=10000) of SASA ($Å^2$) per AGO protein and LCS2 segments for R1, R2, R3 replicas.

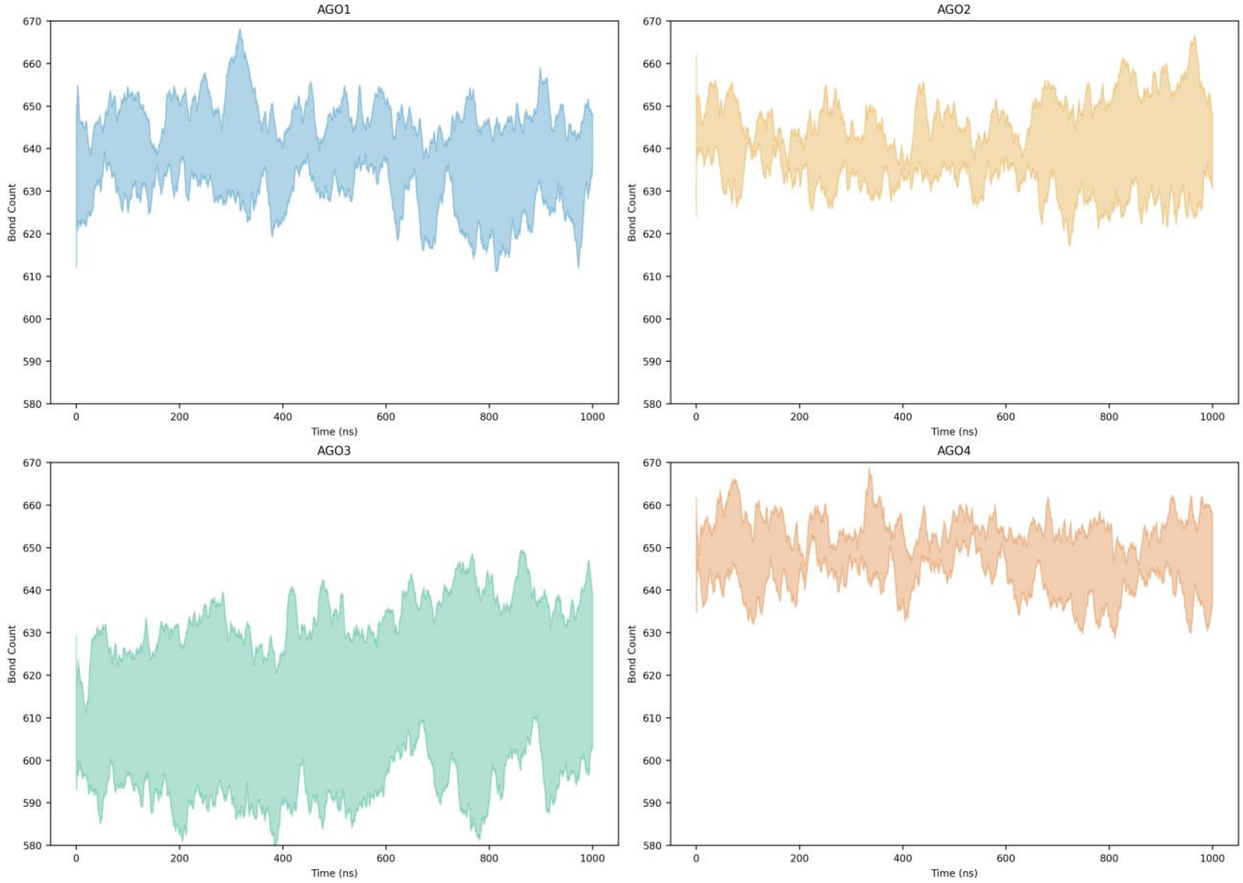

Figure S19. Intervals of hydrogen bond count per AGO. Intervals based on the min/max values of Exponentially Weighted Moving Averages (EWMA) (span=1000) of hydrogen bonds per AGO protein for R1, R2, R3 replicas.

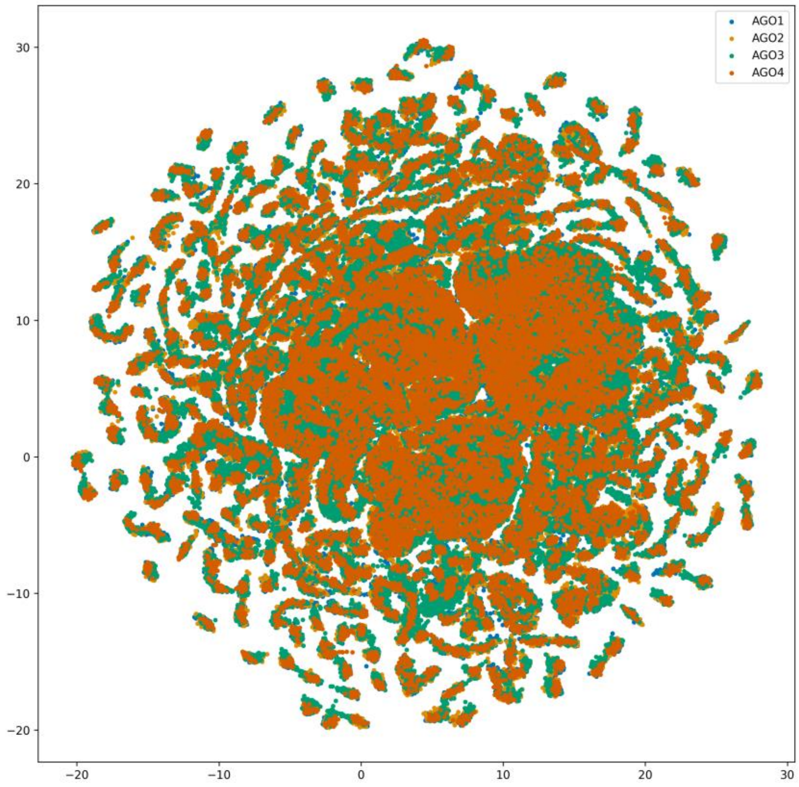

Figure S20. Overlapping 2D projection of the conformational spaces of the human AGO proteins. The projections were generated via UMAP algorithm by fitting all the atomic Cartesian coordinates of the medoids in the clustered trajectories in R1, R2, R3 replicas.

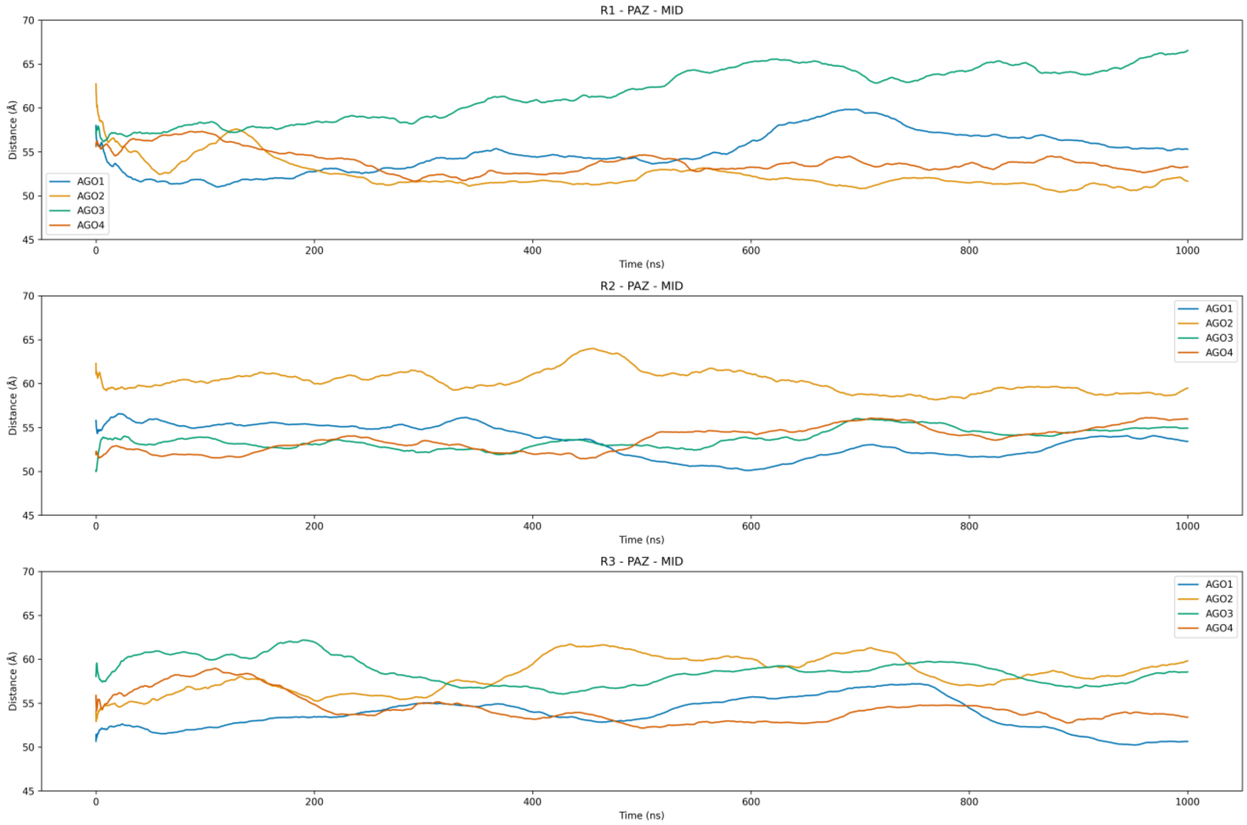

Figure S21. PAZ-MID domain distances per AGO. Exponentially Weighted Moving Averages (EWMA) (span=10000) of PAZ-MID domain distances (Å) per AGO protein for R1, R2, R3 replicas. The distances are measured between the centers of masses of the two domains. These curves represent the distance between the two lobes of each AGO throughout the simulations. We observe that the PAZ-MIZ distance transitions to higher or lower values are not identical for all AGOs. These transitions may impact the accessibility of the nucleic acid binding channel and therefore the function of the AGO.

A

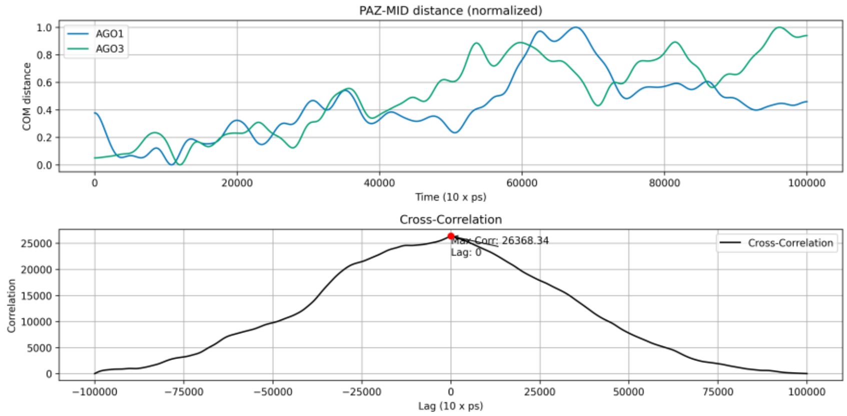

B

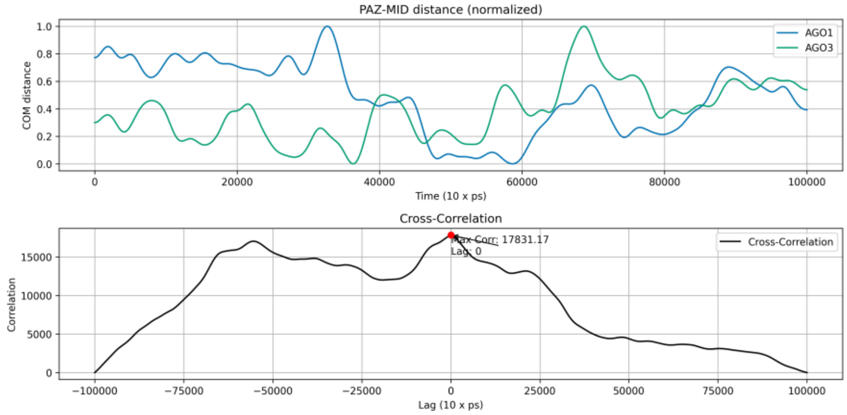

C

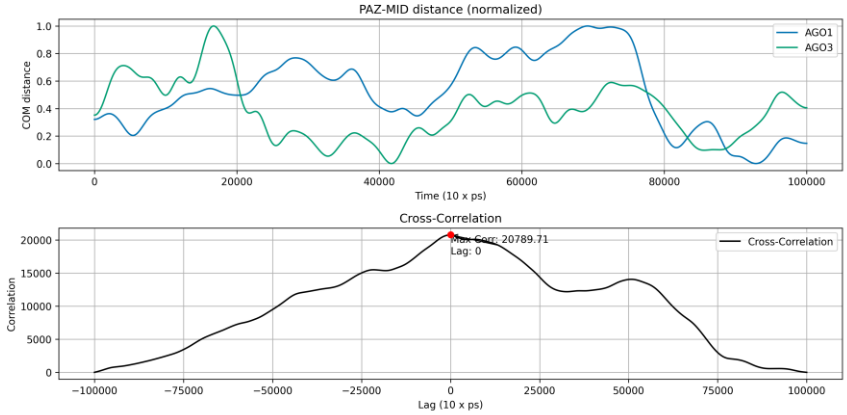

Figure S22. Time-lags of PAZ-MID domain distance of AGO1 and AGO3. Exponentially Weighted Moving Averages (EWMA) (span=10000) of PAZ-MID domain distances (Å) per AGO protein for **(A)** R1, **(B)** R2, **(C)** R3 replicas. The distances are normalized and measured between the centers of masses of the two domains. This analysis focuses on probing the synchronization of the PAZ-MID distances' oscillations. The delay of the transitions from an open to a closed state could possibly affect all stages of an AGO's lifecycle such as translation, modification, trafficking, binding and degradation. The plot shows that the oscillations of PAZ-MID distances of AGO1 and AGO3 exhibit high synchronization.

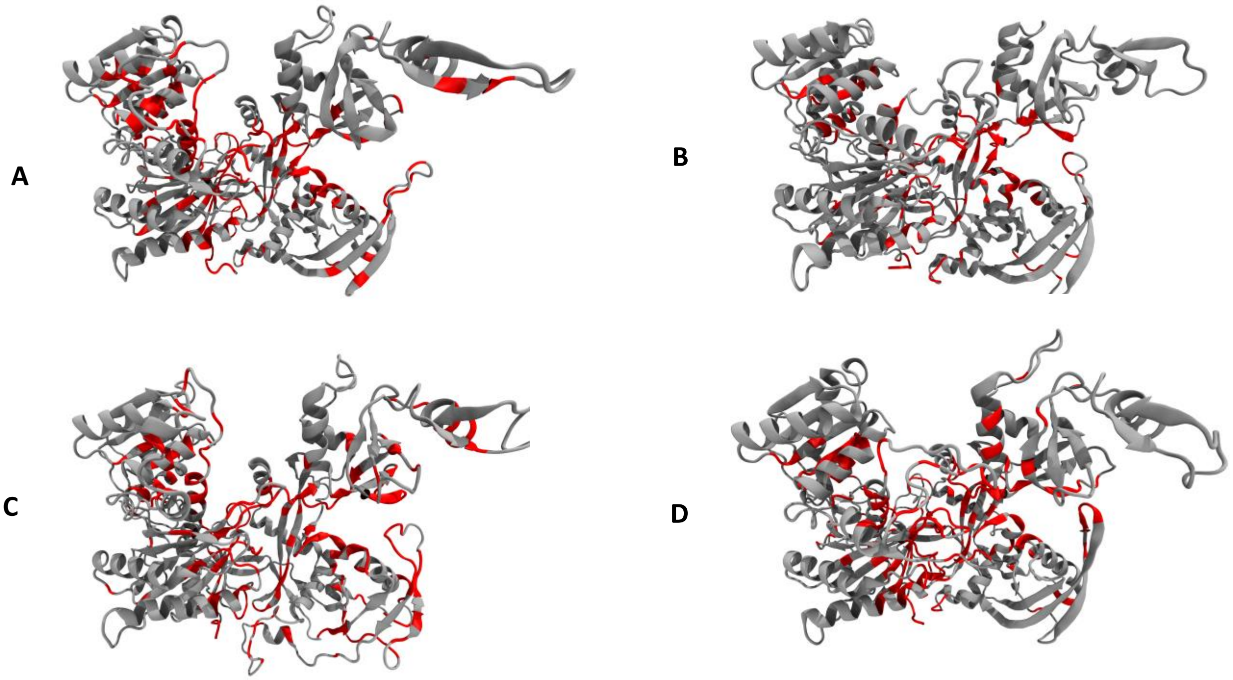

Figure S23. Cryptic pocket predictions of the four human AGO proteins. The interface areas of the AGOs as predicted by PocketMiner. Areas with a binding probability higher than 70% are depicted in color. The reference structures were preprocessed with Schrodinger Maestro suite. **(A)** AGO1 (PDB ID: 4KRE) **(B)** AGO2 (PDB ID: 4Z4D) **(C)** AGO3 (PDB ID: 5VM9) **(D)** AGO4 (PDB ID: 6OON). In each structure, we notice that the predictions do not highlight identical areas of cryptic pockets. This is in accordance with our observations of different open-close states in the molecular dynamics simulations. Thus, the combination of these findings suggests that each AGO does not have the same accessibility in the nucleic binding channel and consequently not the same binding capabilities.

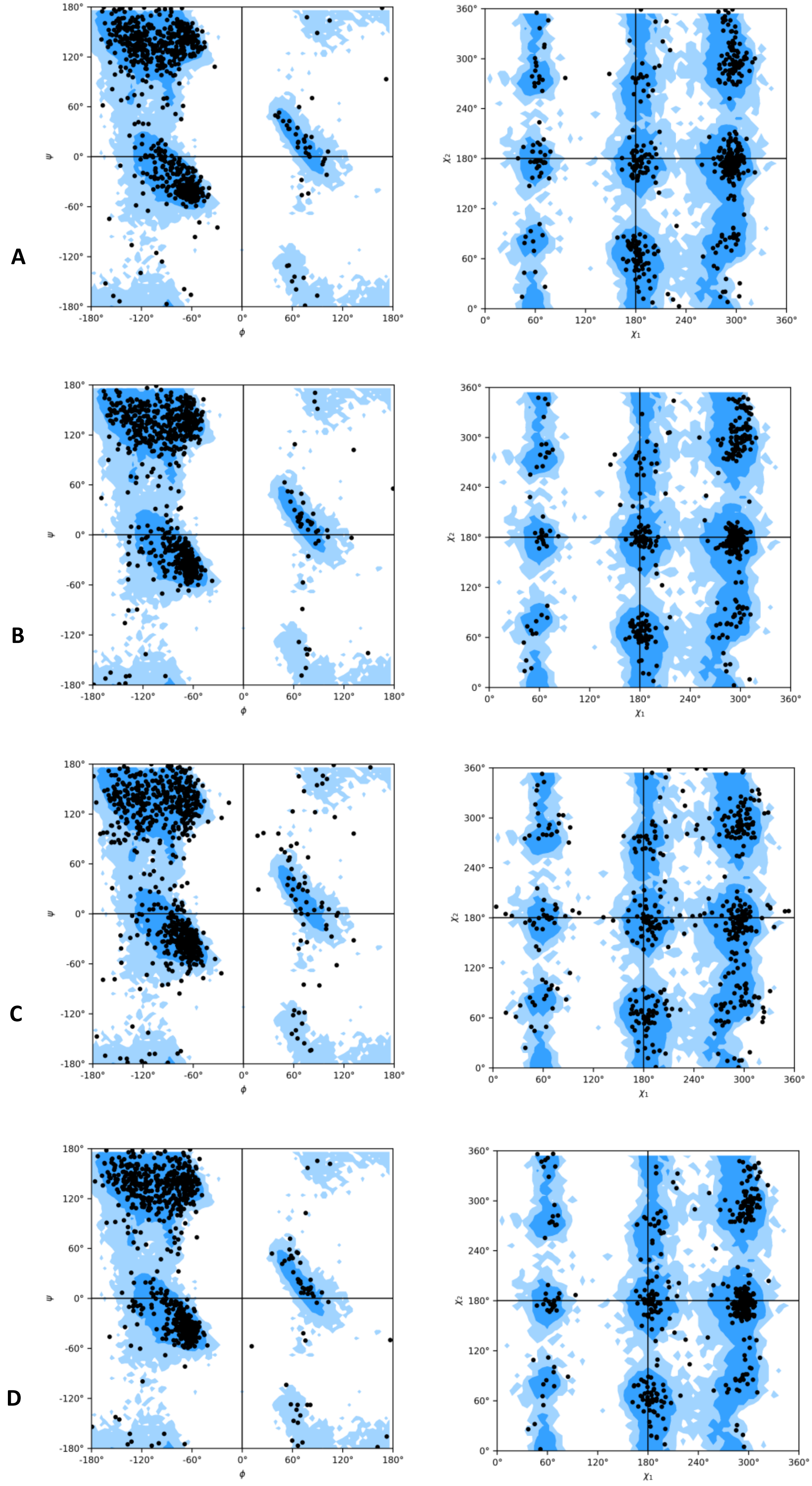
A
B
C
D
ψ
ϕ
χ2
χ1
180°
120°
60°
0°
-60°
-120°
-180°
360°
300°
240°

Figure S24. The distributions of the dihedral angles in the preprocessed AGO structures for R1, R2. The left column contains Ramachandran plots and the right one includes Janin plots. **(A)** Plots for preprocessed 4KRE.A PDB chain. **(B)** Plots for preprocessed 4Z4D.A PDB chain. **(C)** Plots for preprocessed 5VM9.A PDB chain. **(D)** Plots for preprocessed 6OON.A PDB chain.

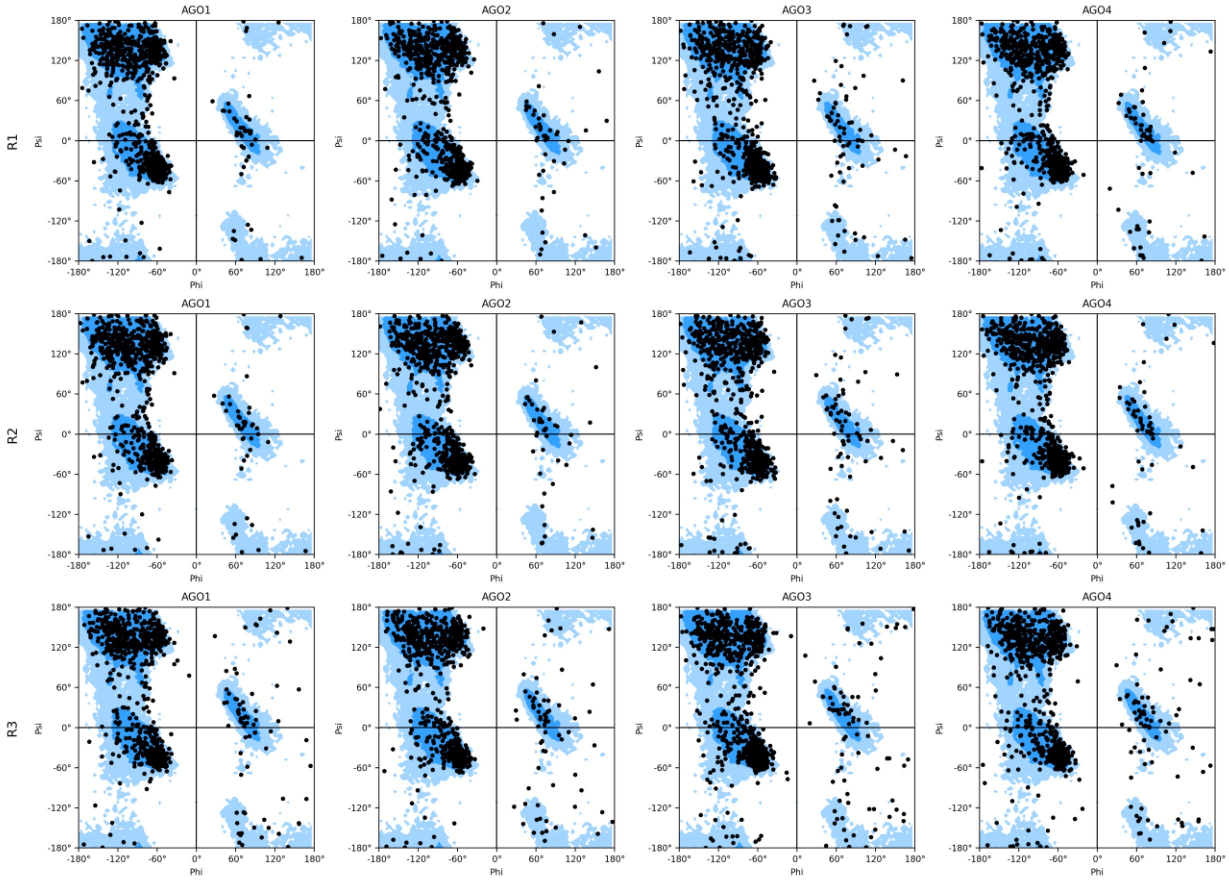

Figure S25. The distributions of the phi-psi angles in each simulated protein after energy minimization step for each simulation of R1,R2,R3. Ramachandran plots for each simulation instance and AGO structure.

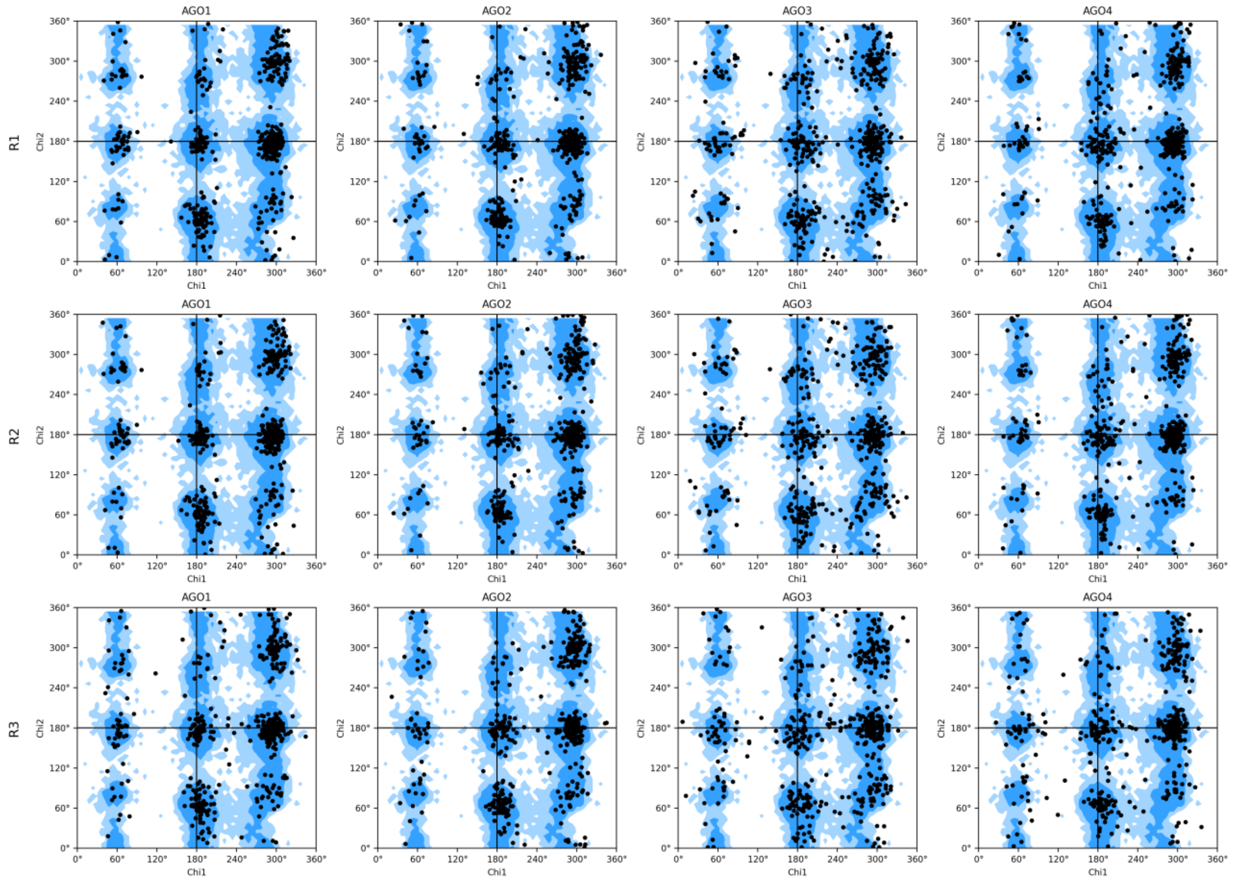

Figure S26. The distributions of the chi1-chi2 angles in each simulated protein after energy minimization step for each simulation of R1, R2, R3 replicates. Janin plots for each simulation instance and AGO structure.

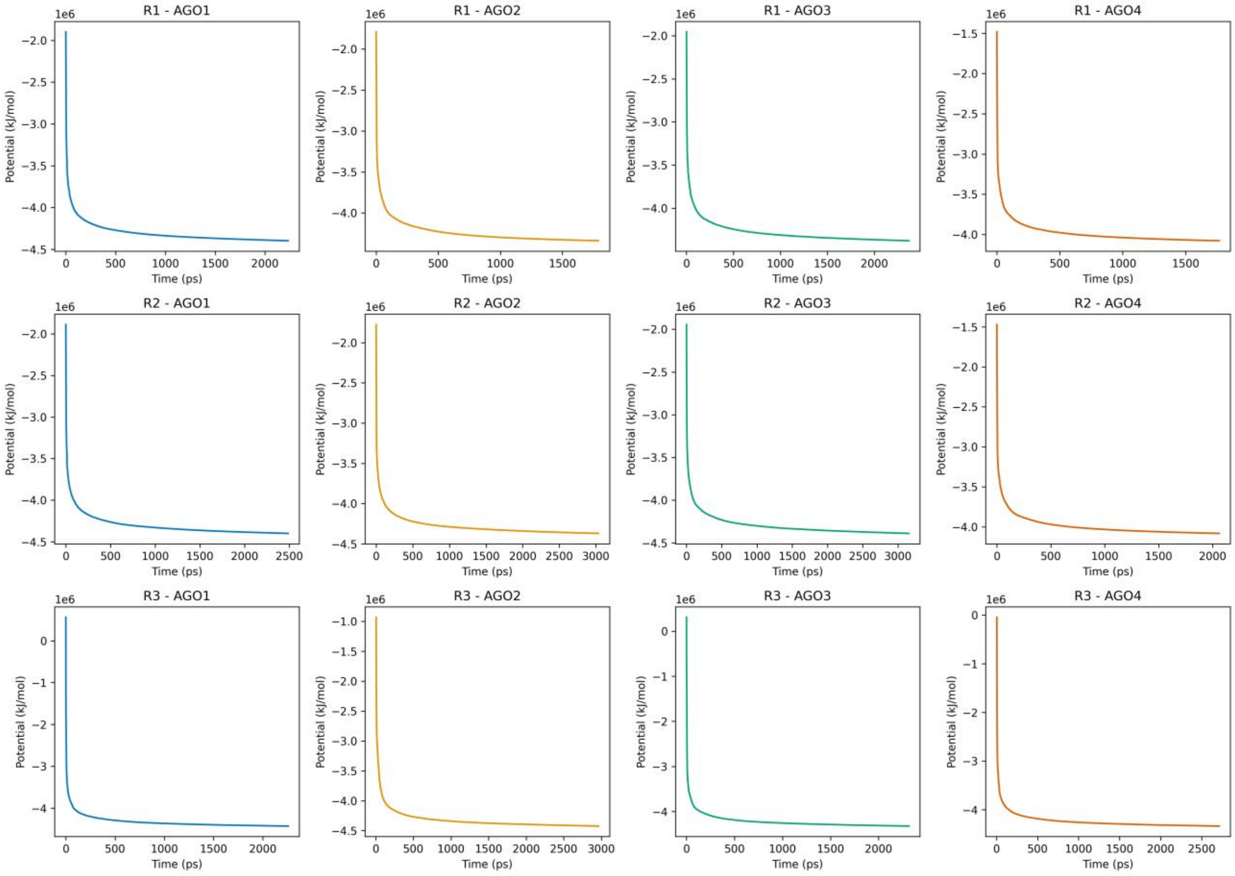

Figure S27. Potential after energy minimization step for each simulation of R1, R2, R3 replicates.

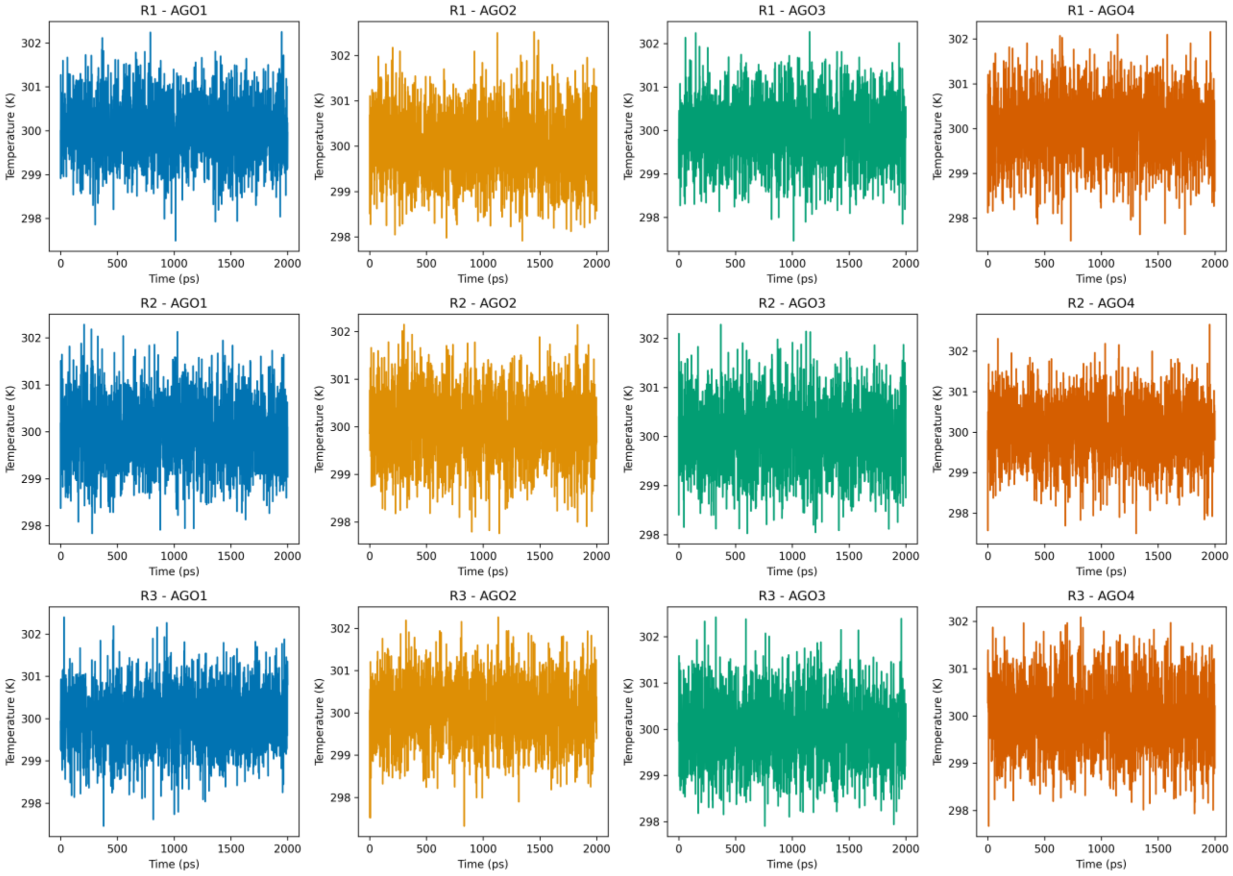

Figure S28. Temperature after NVT equilibration step for each simulation of R1, R2, R3 replicates.

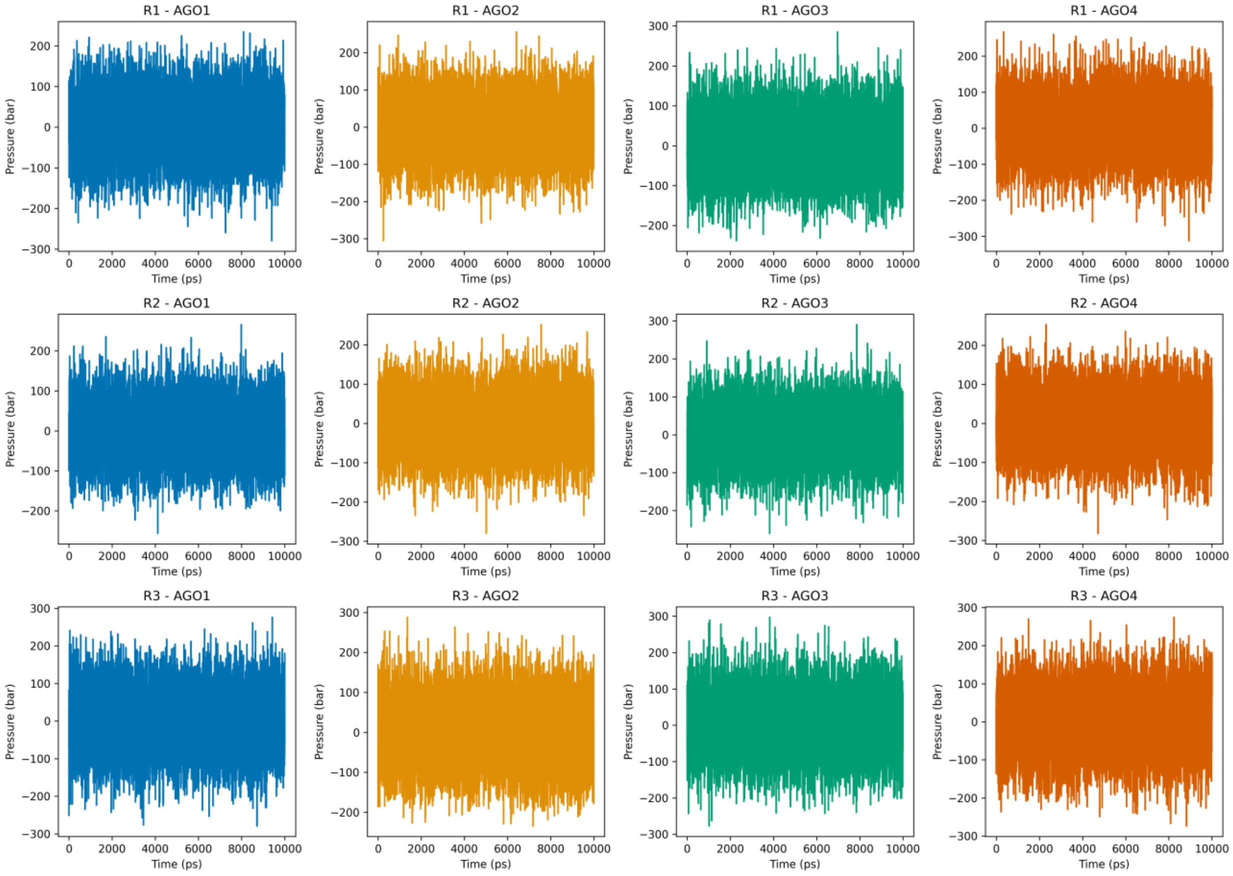

Figure S29. Pressure after NPT equilibration step for each simulation of R1, R2, R3 replicates.
