# Supplementary material for: Comparative structural insights and functional analysis for the distinct unbound states of Human AGO proteins
Source: Sci Rep. 2025 Mar 19;15:9432. doi: 10.1038/s41598-025-91849-5 (PMC11923369; doi:10.1038/s41598-025-91849-5)
Supplement: Supplementary file 24 — Supplementary Information 12. [file 41598_2025_91849_MOESM24_ESM.zip › 4KREp_A_mdwhole_AF4REF/candidates/4KREp_A-merged-enriched_eval_report.html]

 

# Structural Comparison Report for 4KREp\_A - whole structures (total: 100)

---

1

- **Protein name:** Protein argonaute-4
- **Organism:** Homo sapiens
- **Uniprot Accession Number:** Q9HCK5
- **Protein sequence length:** 861 aa
- **1D identity (%):** 82.64
- **1D identity (%) [Gaps excluded]:** 84.79
- **1D identity - Alignment Gaps:** 22
- **1D aligned content (<aminoacid>:%):** {'M': 2.36, 'E': 4.59, 'A': 5.98, 'G': 7.09, 'P': 7.09, 'F': 4.17, 'Q': 6.68, 'R': 6.4, 'T': 5.84, 'V': 8.48, 'K': 6.12, 'I': 5.29, 'L': 7.23, 'N': 2.5, 'D': 4.59, 'Y': 4.45, 'H': 3.2, 'S': 4.45, 'W': 0.83, 'C': 2.64}
- **Common reported functions (%):** 33.33
- **Common reported locations (%):** 20.0
- **Common reported processes (%):** 12.5

- **AF ID:** Q9HCK5
- **Chain:** A
- **Protein length:** 861 aa
- **Resolution:** N/A
- **b-phipsi:** 0.000416
- **w-rdist:** 0.173039
- **t-alpha:** 0.042335
- **Chemical similarity (Tanimoto Index) (%):** 99.62
- **1D identity (%) [PDB]:** 82.41
- **1D identity (%) [Gaps excluded][PDB]:** 85.07
- **1D identity - Alignment Gaps [PDB]:** 27
- **1D aligned content [PDB] (<aminoacid>:%):** {'F': 4.21, 'Q': 6.74, 'P': 6.74, 'R': 6.46, 'G': 7.02, 'T': 5.9, 'V': 8.57, 'K': 6.18, 'I': 5.34, 'L': 7.3, 'A': 5.9, 'N': 2.53, 'D': 4.63, 'Y': 4.49, 'H': 3.23, 'E': 4.49, 'M': 2.25, 'S': 4.49, 'W': 0.84, 'C': 2.67}
- **2D identity (%) [PDB]:** 92.06
- **2D identity (%) [Gaps excluded][PDB]:** 96.15
- **2D identity - Alignment Gaps [PDB]:** 37
- **2D aligned content [PDB] (<2D-fold>:%):** {'.': 20.5, 'E': 27.25, 'T': 18.62, 'H': 29.75, 'I': 0.62, 'G': 1.88, 'b': 0.12, 'B': 1.25}
- **3D similarity (TM-Score) (%) [PDB]:** 97.55

- **Gene name:** AGO4
- **Entrez ID:** 419629
- **RefSeq ID:** NM\_017629
- **Transcript sequence length:** 7272
- **5-UTR|CDS|3-UTR identity (%):** 34.74 | 70.22 | 25.12
- **5-UTR|CDS|3-UTR identity (%) [Gaps excluded]:** 78.72 | 77.88 | 76.1
- **5-UTR|CDS|3-UTR identity [Alignment Gaps]:** 238 | 272 | 7610
- **5-UTR aligned content (<base>:%):** {'C': 40.54, 'T': 7.43, 'G': 46.62, 'A': 5.41}
- **CDS aligned content (<base>:%):** {'A': 26.35, 'T': 22.23, 'G': 26.35, 'C': 25.06}
- **3-UTR aligned content (<base>:%):** {'A': 27.83, 'G': 21.77, 'C': 15.84, 'T': 34.56}

**Uniprot Description:**  
  
 Required for RNA-mediated gene silencing (RNAi). Binds to short RNAs such as microRNAs (miRNAs) and represses the translation of mRNAs which are complementary to them. Lacks endonuclease activity and does not appear to cleave target mRNAs. Also required for RNA-directed transcription and replication of the human hapatitis delta virus (HDV).   
  
Interacts with EIF4B, IMP8, PRMT5, TNRC6A and TNRC6B (PubMed:19167051). Interacts with ZFP36 (PubMed:15766526).   
  
 **Gene Ontology Information:**

Molecular Function

- miRNA binding
- endoribonuclease activity
- single-stranded RNA binding

Location

- P-body
- RISC complex

Biological process

- miRNA mediated inhibition of translation
- mRNA catabolic process

---

2

- **Protein name:** Protein argonaute-2
- **Organism:** Homo sapiens
- **Uniprot Accession Number:** Q9UKV8
- **Protein sequence length:** 859 aa
- **1D identity (%):** 82.67
- **1D identity (%) [Gaps excluded]:** 83.06
- **1D identity - Alignment Gaps:** 4
- **1D aligned content (<aminoacid>:%):** {'M': 2.39, 'G': 7.03, 'A': 6.19, 'P': 6.47, 'Q': 6.61, 'F': 4.36, 'R': 6.33, 'T': 6.05, 'I': 5.91, 'K': 6.33, 'L': 7.17, 'N': 2.67, 'E': 4.92, 'D': 4.78, 'Y': 3.66, 'H': 3.23, 'C': 2.81, 'V': 8.02, 'S': 4.22, 'W': 0.84}
- **Common reported functions (%):** 33.33
- **Common reported locations (%):** 60.0
- **Common reported processes (%):** 25.0

- **AF ID:** Q9UKV8
- **Chain:** A
- **Protein length:** 859 aa
- **Resolution:** N/A
- **b-phipsi:** 0.000936
- **w-rdist:** 0.087145
- **t-alpha:** 0.013314
- **Chemical similarity (Tanimoto Index) (%):** 100.0
- **1D identity (%) [PDB]:** 81.6
- **1D identity (%) [Gaps excluded][PDB]:** 84.43
- **1D identity - Alignment Gaps [PDB]:** 29
- **1D aligned content [PDB] (<aminoacid>:%):** {'F': 4.4, 'P': 6.24, 'R': 6.38, 'G': 6.95, 'T': 6.1, 'I': 6.1, 'K': 6.38, 'L': 7.38, 'A': 5.82, 'N': 2.7, 'E': 4.96, 'D': 4.82, 'Y': 3.69, 'H': 3.26, 'C': 2.84, 'V': 8.09, 'M': 2.27, 'Q': 6.52, 'S': 4.26, 'W': 0.85}
- **2D identity (%) [PDB]:** 91.44
- **2D identity (%) [Gaps excluded][PDB]:** 94.61
- **2D identity - Alignment Gaps [PDB]:** 29
- **2D aligned content [PDB] (<2D-fold>:%):** {'.': 20.0, 'E': 27.09, 'T': 18.86, 'H': 30.13, 'I': 0.63, 'G': 1.9, 'b': 0.13, 'B': 1.27}
- **3D similarity (TM-Score) (%) [PDB]:** 97.07

- **Gene name:** AGO2
- **Entrez ID:** 404130
- **RefSeq ID:** NM\_012154
- **Transcript sequence length:** 14595
- **5-UTR|CDS|3-UTR identity (%):** 38.94 | 72.39 | 44.15
- **5-UTR|CDS|3-UTR identity (%) [Gaps excluded]:** 77.19 | 77.64 | 74.09
- **5-UTR|CDS|3-UTR identity [Alignment Gaps]:** 112 | 184 | 5751
- **5-UTR aligned content (<base>:%):** {'C': 44.32, 'T': 3.41, 'G': 50.0, 'A': 2.27}
- **CDS aligned content (<base>:%):** {'A': 24.94, 'T': 19.3, 'G': 27.78, 'C': 27.98}
- **3-UTR aligned content (<base>:%):** {'C': 19.16, 'T': 33.93, 'G': 22.0, 'A': 24.91}

**Uniprot Description:**  
  
 Required for RNA-mediated gene silencing (RNAi) by the RNA-induced silencing complex (RISC). The 'minimal RISC' appears to include AGO2 bound to a short guide RNA such as a microRNA (miRNA) or short interfering RNA (siRNA). These guide RNAs direct RISC to complementary mRNAs that are targets for RISC-mediated gene silencing. The precise mechanism of gene silencing depends on the degree of complementarity between the miRNA or siRNA and its target. Binding of RISC to a perfectly complementary mRNA generally results in silencing due to endonucleolytic cleavage of the mRNA specifically by AGO2. Binding of RISC to a partially complementary mRNA results in silencing through inhibition of translation, and this is independent of endonuclease activity. May inhibit translation initiation by binding to the 7-methylguanosine cap, thereby preventing the recruitment of the translation initiation factor eIF4-E. May also inhibit translation initiation via interaction with EIF6, which itself binds to the 60S ribosomal subunit and prevents its association with the 40S ribosomal subunit. The inhibition of translational initiation leads to the accumulation of the affected mRNA in cytoplasmic processing bodies (P-bodies), where mRNA degradation may subsequently occur. In some cases RISC-mediated translational repression is also observed for miRNAs that perfectly match the 3' untranslated region (3'-UTR). Can also up-regulate the translation of specific mRNAs under certain growth conditions. Binds to the AU element of the 3'-UTR of the TNF (TNF-alpha) mRNA and up-regulates translation under conditions of serum starvation. Also required for transcriptional gene silencing (TGS), in which short RNAs known as antigene RNAs or agRNAs direct the transcriptional repression of complementary promoter regions.   
  
Interacts with DICER1 through its Piwi domain and with TARBP2 during assembly of the RNA-induced silencing complex (RISC). Together, DICER1, AGO2 and TARBP2 constitute the trimeric RISC loading complex (RLC), or micro-RNA (miRNA) loading complex (miRLC). Within the RLC/miRLC, DICER1 and TARBP2 are required to process precursor miRNAs (pre-miRNAs) to mature miRNAs and then load them onto AGO2. AGO2 bound to the mature miRNA constitutes the minimal RISC and may subsequently dissociate from DICER1 and TARBP2. Note however that the term RISC has also been used to describe the trimeric RLC/miRLC. The formation of RISC complexes containing siRNAs rather than miRNAs appears to occur independently of DICER1. Interacts with AGO1. Also interacts with DDB1, DDX5, DDX6, DDX20, DHX30, DHX36, DDX47, DHX9, ELAVL, FXR1, GEMIN4, HNRNPF, IGF2BP1, ILF3, IMP8, MATR3, PABPC1, PRMT5, P4HA1, P4HB, RBM4, SART3, TNRC6A, TNRC6B, UPF1 and YBX1. Interacts with the P-body components DCP1A and XRN1. Associates with polysomes and messenger ribonucleoproteins (mNRPs). Interacts with RBM4; the interaction is modulated under stress-induced conditions, occurs under both cell proliferation and differentiation conditions and in an RNA- and phosphorylation-independent manner. Interacts with LIMD1, WTIP and AJUBA. Interacts with TRIM71; the interaction increases in presence of RNA (PubMed:23125361). Interacts with APOBEC3G in an RNA-dependent manner. Interacts with APOBEC3A, APOBEC3C, APOBEC3F and APOBEC3H. Interacts with DICER1, TARBP2, EIF6, MOV10 and RPL7A (60S ribosome subunit); they form a large RNA-induced silencing complex (RISC) (PubMed:17507929, PubMed:24726324). Interacts with FMR1 (PubMed:14703574). Interacts with ZFP36 (PubMed:15766526). Found in a complex, composed of AGO2, CHD7 and FAM172A (By similarity). Interacts with RC3H1; the interaction is RNA independent (PubMed:25697406). Interacts with SND1 (PubMed:14508492, PubMed:28546213). Interacts with SYT11 (By similarity). Interacts with CLNK (PubMed:26009488). Interacts with GARRE1 (PubMed:29395067).   
  
 **Gene Ontology Information:**

Molecular Function

- endoribonuclease activity, cleaving siRNA-paired mRNA
- metal ion binding
- miRNA binding
- RNA 7-methylguanosine cap binding
- endoribonuclease activity
- single-stranded RNA binding
- siRNA binding

Location

- mRNA cap binding complex
- nucleus
- P-body
- polysome
- ribonucleoprotein complex
- RISC complex
- RISC-loading complex

Biological process

- miRNA mediated inhibition of translation
- mRNA cleavage involved in gene silencing by miRNA
- negative regulation of translational initiation
- pre-miRNA processing
- regulation of transcription, DNA-templated

---

3

- **Protein name:** Methionine synthase reductase
- **Organism:** Homo sapiens
- **Uniprot Accession Number:** Q9UBK8
- **Protein sequence length:** 698 aa
- **1D identity (%):** 12.49
- **1D identity (%) [Gaps excluded]:** 24.71
- **1D identity - Alignment Gaps:** 511
- **1D aligned content (<aminoacid>:%):** {'L': 10.85, 'Q': 8.53, 'G': 10.08, 'E': 4.65, 'C': 1.55, 'V': 7.75, 'I': 0.78, 'D': 6.98, 'T': 5.43, 'P': 10.85, 'N': 1.55, 'R': 6.2, 'W': 1.55, 'K': 5.43, 'F': 3.88, 'A': 5.43, 'Y': 2.33, 'S': 2.33, 'H': 3.88}
- **Common reported functions (%):** 0.0
- **Common reported locations (%):** 10.0
- **Common reported processes (%):** 0.0

- **AF ID:** Q9UBK8
- **Chain:** A
- **Protein length:** 698 aa
- **Resolution:** N/A
- **b-phipsi:** 0.004623
- **w-rdist:** 0.350139
- **t-alpha:** 0.00073
- **Chemical similarity (Tanimoto Index) (%):** 99.55
- **1D identity (%) [PDB]:** 3.21
- **1D identity (%) [Gaps excluded][PDB]:** 62.67
- **1D identity - Alignment Gaps [PDB]:** 1388
- **1D aligned content [PDB] (<aminoacid>:%):** {'L': 17.02, 'A': 6.38, 'T': 6.38, 'Q': 8.51, 'K': 12.77, 'V': 12.77, 'I': 6.38, 'G': 8.51, 'H': 4.26, 'R': 2.13, 'S': 6.38, 'F': 4.26, 'P': 4.26}
- **2D identity (%) [PDB]:** 36.79
- **2D identity (%) [Gaps excluded][PDB]:** 91.82
- **2D identity - Alignment Gaps [PDB]:** 658
- **2D aligned content [PDB] (<2D-fold>:%):** {'E': 25.99, 'T': 19.06, 'H': 41.09, '.': 13.37, 'B': 0.5}
- **3D similarity (TM-Score) (%) [PDB]:** 20.21

- **Gene name:** MTRR
- **Entrez ID:** 507991
- **RefSeq ID:** NM\_024010
- **Transcript sequence length:** 3219
- **5-UTR|CDS|3-UTR identity (%):** 22.07 | 44.3 | 6.15
- **5-UTR|CDS|3-UTR identity (%) [Gaps excluded]:** 70.15 | 74.96 | 74.59
- **5-UTR|CDS|3-UTR identity [Alignment Gaps]:** 146 | 1227 | 10068
- **5-UTR aligned content (<base>:%):** {'A': 14.89, 'G': 44.68, 'T': 21.28, 'C': 19.15}
- **CDS aligned content (<base>:%):** {'G': 25.21, 'A': 26.79, 'C': 24.98, 'T': 23.02}
- **3-UTR aligned content (<base>:%):** {'A': 30.96, 'C': 15.11, 'T': 39.56, 'G': 14.37}

**Uniprot Description:**  
  
 Key enzyme in methionine and folate homeostasis responsible for the reactivation of methionine synthase (MTR/MS) activity by catalyzing the reductive methylation of MTR-bound cob(II)alamin (PubMed:17892308). Cobalamin (vitamin B12) forms a complex with MTR to serve as an intermediary in methyl transfer reactions that cycles between MTR-bound methylcob(III)alamin and MTR bound-cob(I)alamin forms, and occasional oxidative escape of the cob(I)alamin intermediate during the catalytic cycle leads to the inactive cob(II)alamin species (Probable). The processing of cobalamin in the cytosol occurs in a multiprotein complex composed of at least MMACHC, MMADHC, MTRR and MTR which may contribute to shuttle safely and efficiently cobalamin towards MTR in order to produce methionine (PubMed:27771510). Also necessary for the utilization of methyl groups from the folate cycle, thereby affecting transgenerational epigenetic inheritance (By similarity). Also acts as a molecular chaperone for methionine synthase by stabilizing apoMTR and incorporating methylcob(III)alamin into apoMTR to form the holoenzyme (PubMed:16769880). Also serves as an aquacob(III)alamin reductase by reducing aquacob(III)alamin to cob(II)alamin; this reduction leads to stimulation of the conversion of apoMTR and aquacob(III)alamin to MTR holoenzyme (PubMed:16769880).   
  
Forms a multiprotein complex with MMACHC, MMADHC AND MTR.   
  
 **Gene Ontology Information:**

Molecular Function

- [methionine synthase] reductase activity
- flavin adenine dinucleotide binding
- FMN binding
- oxidoreductase activity, oxidizing metal ions, NAD or NADP as acceptor

Location

- cytosol

Biological process

- DNA methylation
- folic acid metabolic process
- homocysteine metabolic process
- methionine biosynthetic process

---

4

- **Protein name:** Protein argonaute-3
- **Organism:** Homo sapiens
- **Uniprot Accession Number:** Q9H9G7
- **Protein sequence length:** 860 aa
- **1D identity (%):** 84.06
- **1D identity (%) [Gaps excluded]:** 85.55
- **1D identity - Alignment Gaps:** 15
- **1D aligned content (<aminoacid>:%):** {'M': 2.34, 'E': 4.67, 'G': 7.01, 'A': 6.18, 'Q': 6.32, 'P': 6.73, 'R': 6.59, 'T': 5.91, 'K': 6.04, 'I': 5.49, 'L': 7.55, 'N': 2.34, 'F': 4.12, 'V': 8.52, 'D': 5.08, 'Y': 4.26, 'C': 2.61, 'H': 3.16, 'S': 4.26, 'W': 0.82}
- **Common reported functions (%):** 66.67
- **Common reported locations (%):** 70.0
- **Common reported processes (%):** 62.5

- **AF ID:** Q9H9G7
- **Chain:** A
- **Protein length:** 860 aa
- **Resolution:** N/A
- **b-phipsi:** 0.001097
- **w-rdist:** 0.047396
- **t-alpha:** 0.041034
- **Chemical similarity (Tanimoto Index) (%):** 100.0
- **1D identity (%) [PDB]:** 83.14
- **1D identity (%) [Gaps excluded][PDB]:** 86.33
- **1D identity - Alignment Gaps [PDB]:** 32
- **1D aligned content [PDB] (<aminoacid>:%):** {'P': 6.81, 'R': 6.67, 'G': 6.67, 'T': 5.97, 'K': 6.11, 'I': 5.56, 'L': 7.64, 'A': 5.97, 'N': 2.36, 'F': 4.17, 'V': 8.61, 'D': 5.14, 'Y': 4.31, 'E': 4.58, 'C': 2.64, 'M': 2.22, 'Q': 6.25, 'H': 3.19, 'S': 4.31, 'W': 0.83}
- **2D identity (%) [PDB]:** 90.54
- **2D identity (%) [Gaps excluded][PDB]:** 94.24
- **2D identity - Alignment Gaps [PDB]:** 34
- **2D aligned content [PDB] (<2D-fold>:%):** {'.': 20.38, 'E': 27.77, 'T': 18.22, 'H': 30.06, 'I': 0.64, 'G': 1.53, 'b': 0.13, 'B': 1.27}
- **3D similarity (TM-Score) (%) [PDB]:** 97.47

- **Gene name:** AGO3
- **Entrez ID:** 192669
- **RefSeq ID:** N/A
- **Sequence length:** N/A
- **5-UTR|CDS|3-UTR identity (%):** N/A | N/A | N/A
- **5-UTR|CDS|3-UTR identity (%) [Gaps excluded]:** N/A | N/A | N/A
- **5-UTR|CDS|3-UTR identity [Alignment Gaps]:** N/A | N/A | N/A
- **5-UTR aligned content (<base>:%):** N/A
- **CDS aligned content (<base>:%):** N/A
- **3-UTR aligned content (<base>:%):** N/A

**Uniprot Description:**  
  
 Required for RNA-mediated gene silencing (RNAi). Binds to short RNAs such as microRNAs (miRNAs) and represses the translation of mRNAs which are complementary to them. Proposed to be involved in stabilization of small RNA derivates (siRNA) derived from processed RNA polymerase III-transcribed Alu repeats containing a DR2 retinoic acid response element (RARE) in stem cells and in the subsequent siRNA-dependent degradation of a subset of RNA polymerase II-transcribed coding mRNAs by recruiting a mRNA decapping complex involving EDC4. Possesses RNA slicer activity but only on select RNAs bearing 5'- and 3'-flanking sequences to the region of guide-target complementarity (PubMed:29040713).   
  
Interacts with EIF4B, IMP8, PRMT5 and TNRC6B. Interacts with APOBEC3F, APOBEC3G and APOBEC3H. Interacts with EDC4.   
  
 **Gene Ontology Information:**

Molecular Function

- double-stranded RNA binding
- endoribonuclease activity, cleaving miRNA-paired mRNA
- metal ion binding
- miRNA binding
- RNA binding
- endoribonuclease activity
- single-stranded RNA binding

Location

- condensed nuclear chromosome
- cytoplasm
- cytoplasmic ribonucleoprotein granule
- cytosol
- membrane
- nucleoplasm
- P-body
- RISC complex
- RISC-loading complex

Biological process

- production of miRNAs involved in gene silencing by miRNA
- miRNA mediated inhibition of translation
- mRNA catabolic process
- positive regulation of gene expression
- positive regulation of NIK/NF-kappaB signaling
- pre-miRNA processing
- regulation of stem cell proliferation
- small RNA loading onto RISC
- RNA secondary structure unwinding

---

5

- **Protein name:** Endoplasmic reticulum aminopeptidase 1
- **Organism:** Homo sapiens
- **Uniprot Accession Number:** Q9NZ08
- **Protein sequence length:** 941 aa
- **1D identity (%):** 16.22
- **1D identity (%) [Gaps excluded]:** 22.05
- **1D identity - Alignment Gaps:** 274
- **1D aligned content (<aminoacid>:%):** {'V': 8.93, 'F': 4.17, 'P': 8.93, 'T': 11.31, 'L': 11.31, 'D': 4.17, 'N': 4.76, 'E': 4.76, 'Y': 5.95, 'H': 2.38, 'I': 5.36, 'G': 8.93, 'S': 4.76, 'A': 4.17, 'Q': 3.57, 'K': 2.98, 'C': 1.19, 'R': 1.19, 'W': 0.6, 'M': 0.6}
- **Common reported functions (%):** 0.0
- **Common reported locations (%):** 20.0
- **Common reported processes (%):** 0.0

- **AF ID:** Q9NZ08
- **Chain:** A
- **Protein length:** 941 aa
- **Resolution:** N/A
- **b-phipsi:** 0.006038
- **w-rdist:** 0.349721
- **t-alpha:** 0.00073
- **Chemical similarity (Tanimoto Index) (%):** 96.63
- **1D identity (%) [PDB]:** 3.11
- **1D identity (%) [Gaps excluded][PDB]:** 69.74
- **1D identity - Alignment Gaps [PDB]:** 1629
- **1D aligned content [PDB] (<aminoacid>:%):** {'M': 1.89, 'V': 5.66, 'L': 13.21, 'I': 11.32, 'F': 5.66, 'Y': 7.55, 'K': 5.66, 'S': 1.89, 'T': 7.55, 'P': 5.66, 'D': 5.66, 'G': 3.77, 'E': 5.66, 'H': 1.89, 'R': 7.55, 'A': 1.89, 'C': 1.89, 'Q': 3.77, 'N': 1.89}
- **2D identity (%) [PDB]:** 39.53
- **2D identity (%) [Gaps excluded][PDB]:** 87.55
- **2D identity - Alignment Gaps [PDB]:** 673
- **2D aligned content [PDB] (<2D-fold>:%):** {'.': 17.94, 'E': 31.34, 'T': 16.49, 'G': 2.27, 'H': 31.96}
- **3D similarity (TM-Score) (%) [PDB]:** 26.19

- **Gene name:** ERAP1
- **Entrez ID:** 51752
- **RefSeq ID:** N/A
- **Sequence length:** N/A
- **5-UTR|CDS|3-UTR identity (%):** N/A | N/A | N/A
- **5-UTR|CDS|3-UTR identity (%) [Gaps excluded]:** N/A | N/A | N/A
- **5-UTR|CDS|3-UTR identity [Alignment Gaps]:** N/A | N/A | N/A
- **5-UTR aligned content (<base>:%):** N/A
- **CDS aligned content (<base>:%):** N/A
- **3-UTR aligned content (<base>:%):** N/A

**Uniprot Description:**  
  
 Aminopeptidase that plays a central role in peptide trimming, a step required for the generation of most HLA class I-binding peptides. Peptide trimming is essential to customize longer precursor peptides to fit them to the correct length required for presentation on MHC class I molecules. Strongly prefers substrates 9-16 residues long. Rapidly degrades 13-mer to a 9-mer and then stops. Preferentially hydrolyzes the residue Leu and peptides with a hydrophobic C-terminus, while it has weak activity toward peptides with charged C-terminus. May play a role in the inactivation of peptide hormones. May be involved in the regulation of blood pressure through the inactivation of angiotensin II and/or the generation of bradykinin in the kidney.   
  
Monomer. May also exist as a heterodimer; with ERAP2. Interacts with RBMX.   
  
 **Gene Ontology Information:**

Molecular Function

- aminopeptidase activity
- endopeptidase activity
- interleukin-1, type II receptor binding
- interleukin-6 receptor binding
- metalloaminopeptidase activity
- metalloexopeptidase activity
- peptide binding
- zinc ion binding

Location

- cytoplasm
- cytosol
- endoplasmic reticulum
- endoplasmic reticulum lumen
- endoplasmic reticulum membrane
- extracellular exosome
- extracellular region
- extracellular space
- membrane

Biological process

- adaptive immune response
- angiogenesis
- antigen processing and presentation of endogenous peptide antigen via MHC class I
- antigen processing and presentation of peptide antigen via MHC class I
- fat cell differentiation
- membrane protein ectodomain proteolysis
- peptide catabolic process
- positive regulation of angiogenesis
- proteolysis
- regulation of blood pressure
- regulation of innate immune response
- response to bacterium

---

6

- **Protein name:** Cytosolic phospholipase A2 zeta
- **Organism:** Homo sapiens
- **Uniprot Accession Number:** Q68DD2
- **Protein sequence length:** 849 aa
- **1D identity (%):** 15.54
- **1D identity (%) [Gaps excluded]:** 24.03
- **1D identity - Alignment Gaps:** 366
- **1D aligned content (<aminoacid>:%):** {'A': 4.97, 'P': 12.42, 'L': 14.29, 'R': 8.07, 'G': 12.42, 'K': 2.48, 'N': 1.24, 'D': 4.35, 'Y': 4.35, 'V': 6.21, 'E': 4.35, 'F': 3.73, 'Q': 8.07, 'I': 2.48, 'T': 3.11, 'S': 3.73, 'H': 1.24, 'C': 2.48}
- **Common reported functions (%):** 0.0
- **Common reported locations (%):** 10.0
- **Common reported processes (%):** 0.0

- **AF ID:** Q68DD2
- **Chain:** A
- **Protein length:** 849 aa
- **Resolution:** N/A
- **b-phipsi:** 0.000873
- **w-rdist:** 0.219049
- **t-alpha:** 0.062044
- **Chemical similarity (Tanimoto Index) (%):** 99.62
- **1D identity (%) [PDB]:** 1.88
- **1D identity (%) [Gaps excluded][PDB]:** 86.11
- **1D identity - Alignment Gaps [PDB]:** 1617
- **1D aligned content [PDB] (<aminoacid>:%):** {'W': 3.23, 'L': 16.13, 'A': 16.13, 'I': 3.23, 'V': 12.9, 'S': 9.68, 'R': 6.45, 'H': 6.45, 'E': 6.45, 'G': 3.23, 'Q': 6.45, 'P': 6.45, 'D': 3.23}
- **2D identity (%) [PDB]:** 43.92
- **2D identity (%) [Gaps excluded][PDB]:** 89.73
- **2D identity - Alignment Gaps [PDB]:** 579
- **2D aligned content [PDB] (<2D-fold>:%):** {'.': 17.07, 'E': 32.73, 'T': 22.49, 'H': 27.11, 'G': 0.6}
- **3D similarity (TM-Score) (%) [PDB]:** 22.37

- **Gene name:** PLA2G4F
- **Entrez ID:** 255189
- **RefSeq ID:** NM\_213600
- **Transcript sequence length:** 5587
- **5-UTR|CDS|3-UTR identity (%):** 23.93 | 47.68 | 15.56
- **5-UTR|CDS|3-UTR identity (%) [Gaps excluded]:** 84.85 | 76.11 | 76.81
- **5-UTR|CDS|3-UTR identity [Alignment Gaps]:** 168 | 1200 | 9133
- **5-UTR aligned content (<base>:%):** {'A': 10.71, 'G': 44.64, 'T': 7.14, 'C': 37.5}
- **CDS aligned content (<base>:%):** {'A': 21.02, 'G': 29.18, 'C': 31.79, 'T': 18.02}
- **3-UTR aligned content (<base>:%):** {'C': 24.8, 'A': 21.21, 'G': 27.95, 'T': 26.04}

**Uniprot Description:**  
  
 Has calcium-dependent phospholipase and lysophospholipase activities with a potential role in membrane lipid remodeling and biosynthesis of lipid mediators (PubMed:29158256). Preferentially hydrolyzes the ester bond of the fatty acyl group attached at sn-2 position of phospholipids (phospholipase A2 activity) (PubMed:29158256). Selectively hydrolyzes sn-2 arachidonoyl group from membrane phospholipids, providing the precursor for eicosanoid biosynthesis (PubMed:29158256). In myocardial mitochondria, plays a major role in arachidonate release that is metabolically channeled to the formation of cardioprotective eicosanoids, epoxyeicosatrienoates (EETs) (PubMed:29158256). N/A   
  
 **Gene Ontology Information:**

Molecular Function

- calcium ion binding
- calcium-dependent phospholipase A2 activity
- calcium-dependent phospholipid binding
- lysophospholipase activity
- phospholipase A1 activity

Location

- cytosol
- mitochondrion
- ruffle membrane
- vesicle

Biological process

- arachidonic acid secretion
- cellular response to antibiotic
- cellular response to organic cyclic compound
- glycerophospholipid catabolic process
- phosphatidylglycerol acyl-chain remodeling
- prostaglandin biosynthetic process

---

7

- **Protein name:** Histone H4 transcription factor
- **Organism:** Homo sapiens
- **Uniprot Accession Number:** Q9BQA5
- **Protein sequence length:** 517 aa
- **1D identity (%):** 8.86
- **1D identity (%) [Gaps excluded]:** 24.05
- **1D identity - Alignment Gaps:** 634
- **1D aligned content (<aminoacid>:%):** {'P': 13.48, 'K': 5.62, 'R': 6.74, 'N': 3.37, 'E': 6.74, 'Q': 7.87, 'H': 6.74, 'G': 10.11, 'D': 3.37, 'W': 1.12, 'A': 3.37, 'S': 7.87, 'L': 5.62, 'I': 3.37, 'Y': 3.37, 'V': 2.25, 'F': 2.25, 'C': 4.49, 'T': 2.25}
- **Common reported functions (%):** 0.0
- **Common reported locations (%):** 10.0
- **Common reported processes (%):** 0.0

- **AF ID:** Q9BQA5
- **Chain:** A
- **Protein length:** 517 aa
- **Resolution:** N/A
- **b-phipsi:** 0.005745
- **w-rdist:** 0.198816
- **t-alpha:** 0.005109
- **Chemical similarity (Tanimoto Index) (%):** 95.71
- **1D identity (%) [PDB]:** 1.58
- **1D identity (%) [Gaps excluded][PDB]:** 77.78
- **1D identity - Alignment Gaps [PDB]:** 1303
- **1D aligned content [PDB] (<aminoacid>:%):** {'P': 9.52, 'F': 14.29, 'E': 4.76, 'D': 4.76, 'Y': 14.29, 'L': 9.52, 'C': 4.76, 'S': 14.29, 'H': 4.76, 'G': 9.52, 'T': 4.76, 'V': 4.76}
- **2D identity (%) [PDB]:** 38.3
- **2D identity (%) [Gaps excluded][PDB]:** 86.33
- **2D identity - Alignment Gaps [PDB]:** 523
- **2D aligned content [PDB] (<2D-fold>:%):** {'.': 31.67, 'E': 13.89, 'H': 38.89, 'T': 14.72, 'G': 0.83}
- **3D similarity (TM-Score) (%) [PDB]:** 18.75

- **Gene name:** HINFP
- **Entrez ID:** 511965
- **RefSeq ID:** N/A
- **Sequence length:** N/A
- **5-UTR|CDS|3-UTR identity (%):** N/A | N/A | N/A
- **5-UTR|CDS|3-UTR identity (%) [Gaps excluded]:** N/A | N/A | N/A
- **5-UTR|CDS|3-UTR identity [Alignment Gaps]:** N/A | N/A | N/A
- **5-UTR aligned content (<base>:%):** N/A
- **CDS aligned content (<base>:%):** N/A
- **3-UTR aligned content (<base>:%):** N/A

**Uniprot Description:**  
  
 Transcriptional repressor that binds to the consensus sequence 5'-CGGACGTT-3' and to the RB1 promoter. Transcriptional activator that promotes histone H4 gene transcription at the G1/S phase transition in conjunction with NPAT. Also activates transcription of the ATM and PRKDC genes. Autoregulates its expression by associating with its own promoter.   
  
Binds MBD2 and a histone deacetylase complex. Interacts with NPAT.   
  
 **Gene Ontology Information:**

Molecular Function

- DNA-binding transcription factor activity, RNA polymerase II-specific
- metal ion binding
- RNA polymerase II cis-regulatory region sequence-specific DNA binding

Location

- nucleus

Biological process

- anatomical structure development
- regulation of transcription by RNA polymerase II

---

8

- **Protein name:** Mediator of RNA polymerase II transcription subunit 16
- **Organism:** Homo sapiens
- **Uniprot Accession Number:** Q9Y2X0
- **Protein sequence length:** 877 aa
- **1D identity (%):** 13.55
- **1D identity (%) [Gaps excluded]:** 19.97
- **1D identity - Alignment Gaps:** 332
- **1D aligned content (<aminoacid>:%):** {'R': 5.71, 'P': 13.57, 'G': 9.29, 'V': 5.71, 'F': 3.57, 'D': 7.14, 'I': 2.86, 'W': 1.43, 'L': 13.57, 'S': 5.0, 'H': 2.86, 'A': 3.57, 'T': 7.86, 'Y': 2.14, 'C': 5.0, 'N': 0.71, 'K': 4.29, 'Q': 3.57, 'E': 2.14}
- **Common reported functions (%):** 0.0
- **Common reported locations (%):** 20.0
- **Common reported processes (%):** 0.0

- **AF ID:** Q9Y2X0
- **Chain:** A
- **Protein length:** 877 aa
- **Resolution:** N/A
- **b-phipsi:** 0.000383
- **w-rdist:** 0.366508
- **t-alpha:** 0.048202
- **Chemical similarity (Tanimoto Index) (%):** 96.49
- **1D identity (%) [PDB]:** 1.85
- **1D identity (%) [Gaps excluded][PDB]:** 81.58
- **1D identity - Alignment Gaps [PDB]:** 1641
- **1D aligned content [PDB] (<aminoacid>:%):** {'R': 6.45, 'P': 12.9, 'A': 9.68, 'M': 6.45, 'N': 3.23, 'I': 12.9, 'V': 6.45, 'T': 3.23, 'F': 6.45, 'Y': 3.23, 'K': 6.45, 'Q': 6.45, 'E': 6.45, 'L': 3.23, 'D': 6.45}
- **2D identity (%) [PDB]:** 40.9
- **2D identity (%) [Gaps excluded][PDB]:** 88.91
- **2D identity - Alignment Gaps [PDB]:** 635
- **2D aligned content [PDB] (<2D-fold>:%):** {'.': 17.46, 'E': 25.57, 'T': 21.83, 'H': 34.51, 'G': 0.62}
- **3D similarity (TM-Score) (%) [PDB]:** 23.64

- **Gene name:** MED16
- **Entrez ID:** 10025
- **RefSeq ID:** NM\_005481
- **Transcript sequence length:** 2892
- **5-UTR|CDS|3-UTR identity (%):** 36.61 | 47.28 | 0.67
- **5-UTR|CDS|3-UTR identity (%) [Gaps excluded]:** 75.23 | 75.13 | 77.66
- **5-UTR|CDS|3-UTR identity [Alignment Gaps]:** 115 | 1208 | 10773
- **5-UTR aligned content (<base>:%):** {'G': 50.0, 'C': 31.71, 'T': 10.98, 'A': 7.32}
- **CDS aligned content (<base>:%):** {'A': 21.16, 'T': 16.87, 'G': 29.33, 'C': 32.64}
- **3-UTR aligned content (<base>:%):** {'C': 35.62, 'G': 26.03, 'T': 23.29, 'A': 15.07}

**Uniprot Description:**  
  
 Component of the Mediator complex, a coactivator involved in the regulated transcription of nearly all RNA polymerase II-dependent genes. Mediator functions as a bridge to convey information from gene-specific regulatory proteins to the basal RNA polymerase II transcription machinery. Mediator is recruited to promoters by direct interactions with regulatory proteins and serves as a scaffold for the assembly of a functional preinitiation complex with RNA polymerase II and the general transcription factors.   
  
Component of the Mediator complex, which is composed of MED1, MED4, MED6, MED7, MED8, MED9, MED10, MED11, MED12, MED13, MED13L, MED14, MED15, MED16, MED17, MED18, MED19, MED20, MED21, MED22, MED23, MED24, MED25, MED26, MED27, MED29, MED30, MED31, CCNC, CDK8 and CDC2L6/CDK11. The MED12, MED13, CCNC and CDK8 subunits form a distinct module termed the CDK8 module. Mediator containing the CDK8 module is less active than Mediator lacking this module in supporting transcriptional activation. Individual preparations of the Mediator complex lacking one or more distinct subunits have been variously termed ARC, CRSP, DRIP, PC2, SMCC and TRAP.   
  
 **Gene Ontology Information:**

Molecular Function

- thyroid hormone receptor binding
- vitamin D receptor binding
- transcription coactivator activity

Location

- core mediator complex
- mediator complex
- membrane
- nucleoplasm
- nucleus

Biological process

- positive regulation of transcription, DNA-templated
- positive regulation of transcription elongation from RNA polymerase II promoter
- positive regulation of transcription initiation from RNA polymerase II promoter
- regulation of transcription by RNA polymerase II
- RNA polymerase II preinitiation complex assembly
- transcription by RNA polymerase II

---

9

- **Protein name:** Evolutionarily conserved signaling intermediate in Toll pathway, mitochondrial
- **Organism:** Homo sapiens
- **Uniprot Accession Number:** Q9BQ95
- **Protein sequence length:** 431 aa
- **1D identity (%):** 8.3
- **1D identity (%) [Gaps excluded]:** 30.43
- **1D identity - Alignment Gaps:** 736
- **1D aligned content (<aminoacid>:%):** {'M': 2.38, 'G': 5.95, 'A': 9.52, 'L': 7.14, 'P': 16.67, 'F': 5.95, 'Q': 7.14, 'R': 8.33, 'I': 5.95, 'Y': 5.95, 'E': 5.95, 'D': 3.57, 'V': 4.76, 'H': 4.76, 'K': 2.38, 'S': 1.19, 'W': 1.19, 'T': 1.19}
- **Common reported functions (%):** 0.0
- **Common reported locations (%):** 40.0
- **Common reported processes (%):** 0.0

- **AF ID:** Q9BQ95
- **Chain:** A
- **Protein length:** 431 aa
- **Resolution:** N/A
- **b-phipsi:** 0.006028
- **w-rdist:** 0.276995
- **t-alpha:** 0.003663
- **Chemical similarity (Tanimoto Index) (%):** 99.17
- **1D identity (%) [PDB]:** 1.36
- **1D identity (%) [Gaps excluded][PDB]:** 80.95
- **1D identity - Alignment Gaps [PDB]:** 1229
- **1D aligned content [PDB] (<aminoacid>:%):** {'S': 17.65, 'W': 5.88, 'R': 5.88, 'L': 17.65, 'H': 5.88, 'A': 5.88, 'V': 11.76, 'G': 5.88, 'Q': 5.88, 'I': 5.88, 'P': 11.76}
- **2D identity (%) [PDB]:** 27.0
- **2D identity (%) [Gaps excluded][PDB]:** 94.68
- **2D identity - Alignment Gaps [PDB]:** 707
- **2D aligned content [PDB] (<2D-fold>:%):** {'.': 20.97, 'G': 3.37, 'T': 24.34, 'H': 40.82, 'E': 10.49}
- **3D similarity (TM-Score) (%) [PDB]:** 17.04

- **Gene name:** ECSIT
- **Entrez ID:** 507245
- **RefSeq ID:** NM\_016581
- **Transcript sequence length:** 1647
- **5-UTR|CDS|3-UTR identity (%):** 27.11 | 27.36 | 1.51
- **5-UTR|CDS|3-UTR identity (%) [Gaps excluded]:** 72.62 | 78.16 | 77.36
- **5-UTR|CDS|3-UTR identity [Alignment Gaps]:** 141 | 1912 | 10654
- **5-UTR aligned content (<base>:%):** {'C': 27.87, 'T': 11.48, 'G': 49.18, 'A': 11.48}
- **CDS aligned content (<base>:%):** {'A': 22.24, 'T': 17.52, 'G': 30.43, 'C': 29.81}
- **3-UTR aligned content (<base>:%):** {'T': 25.61, 'C': 26.83, 'G': 31.71, 'A': 15.85}

**Uniprot Description:**  
  
 Adapter protein of the Toll-like and IL-1 receptor signaling pathway that is involved in the activation of NF-kappa-B via MAP3K1. Promotes proteolytic activation of MAP3K1. Involved in the BMP signaling pathway. Required for normal embryonic development (By similarity).   
  
Interacts with MAP3K1, SMAD4 and TRAF6. Interacts with SMAD1 only after BMP4-treatment (By similarity). Part of the mitochondrial complex I assembly/MCIA complex that comprises at least the core subunits TMEM126B, NDUFAF1, ECSIT and ACAD9 and complement subunits such as COA1 and TMEM186 (PubMed:32320651). Interacts with NDUFAF1 (PubMed:17344420). Interacts with ACAD9 (PubMed:20816094). Interacts with TRIM59 (By similarity). Interacts with TMEM70 and TMEM242 (PubMed:33753518).   
  
 **Gene Ontology Information:**

Molecular Function

- molecular adaptor activity

Location

- cytoplasm
- cytosol
- mitochondrion
- nucleoplasm
- nucleus

Biological process

- innate immune response
- regulation of oxidoreductase activity
- regulation of protein complex stability
- toll-like receptor 4 signaling pathway

---

10

- **Protein name:** Ribosome-releasing factor 2, mitochondrial
- **Organism:** Homo sapiens
- **Uniprot Accession Number:** Q969S9
- **Protein sequence length:** 779 aa
- **1D identity (%):** 14.76
- **1D identity (%) [Gaps excluded]:** 24.63
- **1D identity - Alignment Gaps:** 410
- **1D aligned content (<aminoacid>:%):** {'M': 1.99, 'L': 14.57, 'S': 5.3, 'V': 5.96, 'A': 5.3, 'P': 8.61, 'G': 11.26, 'R': 4.64, 'H': 1.32, 'K': 6.62, 'I': 4.64, 'D': 5.3, 'T': 5.96, 'Y': 1.99, 'F': 3.31, 'Q': 3.31, 'E': 5.3, 'C': 1.32, 'N': 3.31}
- **Common reported functions (%):** 0.0
- **Common reported locations (%):** 0.0
- **Common reported processes (%):** 0.0

- **AF ID:** Q969S9
- **Chain:** A
- **Protein length:** 779 aa
- **Resolution:** N/A
- **b-phipsi:** 0.00628
- **w-rdist:** 0.365705
- **t-alpha:** 0.000731
- **Chemical similarity (Tanimoto Index) (%):** 98.57
- **1D identity (%) [PDB]:** 3.42
- **1D identity (%) [Gaps excluded][PDB]:** 77.94
- **1D identity - Alignment Gaps [PDB]:** 1483
- **1D aligned content [PDB] (<aminoacid>:%):** {'L': 13.21, 'P': 5.66, 'I': 9.43, 'G': 7.55, 'N': 1.89, 'E': 7.55, 'R': 7.55, 'V': 9.43, 'D': 5.66, 'F': 1.89, 'K': 3.77, 'A': 9.43, 'S': 5.66, 'H': 1.89, 'Q': 3.77, 'M': 1.89, 'Y': 1.89, 'T': 1.89}
- **2D identity (%) [PDB]:** 43.68
- **2D identity (%) [Gaps excluded][PDB]:** 90.51
- **2D identity - Alignment Gaps [PDB]:** 565
- **2D aligned content [PDB] (<2D-fold>:%):** {'.': 14.47, 'E': 35.22, 'T': 17.4, 'H': 31.66, 'G': 1.26}
- **3D similarity (TM-Score) (%) [PDB]:** 20.32

- **Gene name:** GFM2
- **Entrez ID:** 527467
- **RefSeq ID:** N/A
- **Sequence length:** N/A
- **5-UTR|CDS|3-UTR identity (%):** N/A | N/A | N/A
- **5-UTR|CDS|3-UTR identity (%) [Gaps excluded]:** N/A | N/A | N/A
- **5-UTR|CDS|3-UTR identity [Alignment Gaps]:** N/A | N/A | N/A
- **5-UTR aligned content (<base>:%):** N/A
- **CDS aligned content (<base>:%):** N/A
- **3-UTR aligned content (<base>:%):** N/A

**Uniprot Description:**  
  
 Mitochondrial GTPase that mediates the disassembly of ribosomes from messenger RNA at the termination of mitochondrial protein biosynthesis. Acts in collaboration with MRRF. GTP hydrolysis follows the ribosome disassembly and probably occurs on the ribosome large subunit. Not involved in the GTP-dependent ribosomal translocation step during translation elongation. N/A   
  
 **Gene Ontology Information:**

Molecular Function

- GTP binding
- GTPase activity

Location

- mitochondrion

Biological process

- mitochondrial translation
- ribosome disassembly

---

11

- **Protein name:** Zinc finger protein 793
- **Organism:** Homo sapiens
- **Uniprot Accession Number:** Q6ZN11
- **Protein sequence length:** 406 aa
- **1D identity (%):** 7.89
- **1D identity (%) [Gaps excluded]:** 20.51
- **1D identity - Alignment Gaps:** 561
- **1D aligned content (<aminoacid>:%):** {'P': 13.89, 'Q': 4.17, 'R': 13.89, 'T': 5.56, 'Y': 1.39, 'S': 4.17, 'L': 5.56, 'V': 5.56, 'G': 6.94, 'A': 5.56, 'C': 6.94, 'I': 2.78, 'D': 2.78, 'E': 6.94, 'K': 4.17, 'H': 5.56, 'F': 2.78, 'N': 1.39}
- **Common reported functions (%):** 0.0
- **Common reported locations (%):** 10.0
- **Common reported processes (%):** 0.0

- **AF ID:** Q6ZN11
- **Chain:** A
- **Protein length:** 406 aa
- **Resolution:** N/A
- **b-phipsi:** 0.015115
- **w-rdist:** 0.239161
- **t-alpha:** 0.00146
- **Chemical similarity (Tanimoto Index) (%):** 96.12
- **1D identity (%) [PDB]:** 1.31
- **1D identity (%) [Gaps excluded][PDB]:** 64.0
- **1D identity - Alignment Gaps [PDB]:** 1196
- **1D aligned content [PDB] (<aminoacid>:%):** {'I': 12.5, 'E': 6.25, 'L': 18.75, 'S': 12.5, 'R': 6.25, 'F': 6.25, 'Y': 6.25, 'K': 12.5, 'T': 12.5, 'P': 6.25}
- **2D identity (%) [PDB]:** 30.3
- **2D identity (%) [Gaps excluded][PDB]:** 90.71
- **2D identity - Alignment Gaps [PDB]:** 622
- **2D aligned content [PDB] (<2D-fold>:%):** {'.': 26.15, 'H': 59.01, 'T': 8.48, 'E': 6.36}
- **3D similarity (TM-Score) (%) [PDB]:** 14.78

- **Gene name:** ZNF793
- **Entrez ID:** 390927
- **RefSeq ID:** N/A
- **Sequence length:** N/A
- **5-UTR|CDS|3-UTR identity (%):** N/A | N/A | N/A
- **5-UTR|CDS|3-UTR identity (%) [Gaps excluded]:** N/A | N/A | N/A
- **5-UTR|CDS|3-UTR identity [Alignment Gaps]:** N/A | N/A | N/A
- **5-UTR aligned content (<base>:%):** N/A
- **CDS aligned content (<base>:%):** N/A
- **3-UTR aligned content (<base>:%):** N/A

**Uniprot Description:**  
  
 May be involved in transcriptional regulation. N/A   
  
 **Gene Ontology Information:**

Molecular Function

- DNA-binding transcription factor activity, RNA polymerase II-specific
- metal ion binding
- RNA polymerase II cis-regulatory region sequence-specific DNA binding

Location

- nucleus

Biological process

- regulation of transcription by RNA polymerase II

---

12

- **Protein name:** Ankyrin repeat domain-containing protein 13D
- **Organism:** Homo sapiens
- **Uniprot Accession Number:** Q6ZTN6
- **Protein sequence length:** 605 aa
- **1D identity (%):** 11.18
- **1D identity (%) [Gaps excluded]:** 20.62
- **1D identity - Alignment Gaps:** 434
- **1D aligned content (<aminoacid>:%):** {'P': 14.15, 'G': 9.43, 'L': 16.98, 'R': 10.38, 'V': 6.6, 'W': 1.89, 'H': 2.83, 'A': 2.83, 'I': 2.83, 'E': 6.6, 'T': 7.55, 'Q': 4.72, 'K': 3.77, 'C': 0.94, 'S': 2.83, 'N': 1.89, 'F': 1.89, 'Y': 0.94, 'D': 0.94}
- **Common reported functions (%):** 0.0
- **Common reported locations (%):** 10.0
- **Common reported processes (%):** 0.0

- **AF ID:** Q6ZTN6
- **Chain:** A
- **Protein length:** 518 aa
- **Resolution:** N/A
- **b-phipsi:** 0.001672
- **w-rdist:** 0.642884
- **t-alpha:** 0.0
- **Chemical similarity (Tanimoto Index) (%):** 97.85
- **1D identity (%) [PDB]:** 2.44
- **1D identity (%) [Gaps excluded][PDB]:** 71.11
- **1D identity - Alignment Gaps [PDB]:** 1268
- **1D aligned content [PDB] (<aminoacid>:%):** {'M': 3.12, 'V': 15.62, 'Q': 6.25, 'F': 6.25, 'K': 9.38, 'G': 12.5, 'D': 6.25, 'R': 6.25, 'Y': 3.12, 'N': 6.25, 'I': 3.12, 'T': 6.25, 'L': 3.12, 'P': 6.25, 'E': 6.25}
- **2D identity (%) [PDB]:** 40.64
- **2D identity (%) [Gaps excluded][PDB]:** 89.83
- **2D identity - Alignment Gaps [PDB]:** 512
- **2D aligned content [PDB] (<2D-fold>:%):** {'H': 42.37, '.': 16.58, 'E': 25.26, 'T': 15.53, 'B': 0.26}
- **3D similarity (TM-Score) (%) [PDB]:** 15.51

- **Gene name:** ANKRD13D
- **Entrez ID:** 338692
- **RefSeq ID:** NM\_207354
- **Transcript sequence length:** 2128
- **5-UTR|CDS|3-UTR identity (%):** 43.88 | 41.78 | 0.86
- **5-UTR|CDS|3-UTR identity (%) [Gaps excluded]:** 77.61 | 76.98 | 73.81
- **5-UTR|CDS|3-UTR identity [Alignment Gaps]:** 103 | 1332 | 10723
- **5-UTR aligned content (<base>:%):** {'G': 50.0, 'C': 36.54, 'T': 6.73, 'A': 6.73}
- **CDS aligned content (<base>:%):** {'T': 17.01, 'G': 29.33, 'C': 31.88, 'A': 21.77}
- **3-UTR aligned content (<base>:%):** {'G': 33.33, 'C': 19.35, 'T': 22.58, 'A': 24.73}

**Uniprot Description:**  
  
 Ubiquitin-binding protein that specifically recognizes and binds 'Lys-63'-linked ubiquitin. Does not bind 'Lys-48'-linked ubiquitin. Positively regulates the internalization of ligand-activated EGFR by binding to the Ub moiety of ubiquitinated EGFR at the cell membrane.   
  
Interacts with EGFR (ubiquitinated); the interaction is direct and may regulate EGFR internalization.   
  
 **Gene Ontology Information:**

Molecular Function

- ubiquitin-dependent protein binding

Location

- cytoplasm
- late endosome
- perinuclear region of cytoplasm
- plasma membrane

Biological process

- negative regulation of receptor internalization

---

13

- **Protein name:** Pseudouridylate synthase 7 homolog
- **Organism:** Homo sapiens
- **Uniprot Accession Number:** Q96PZ0
- **Protein sequence length:** 661 aa
- **1D identity (%):** 12.85
- **1D identity (%) [Gaps excluded]:** 22.94
- **1D identity - Alignment Gaps:** 428
- **1D aligned content (<aminoacid>:%):** {'A': 4.0, 'L': 11.2, 'V': 11.2, 'P': 11.2, 'E': 4.0, 'G': 8.0, 'S': 1.6, 'M': 1.6, 'D': 5.6, 'T': 4.8, 'F': 1.6, 'R': 4.8, 'Q': 4.0, 'K': 9.6, 'Y': 4.8, 'I': 4.8, 'N': 6.4, 'C': 0.8}
- **Common reported functions (%):** 16.67
- **Common reported locations (%):** 10.0
- **Common reported processes (%):** 0.0

- **AF ID:** Q96PZ0
- **Chain:** A
- **Protein length:** 661 aa
- **Resolution:** N/A
- **b-phipsi:** 0.001003
- **w-rdist:** 0.302039
- **t-alpha:** 0.052555
- **Chemical similarity (Tanimoto Index) (%):** 99.24
- **1D identity (%) [PDB]:** 2.92
- **1D identity (%) [Gaps excluded][PDB]:** 67.74
- **1D identity - Alignment Gaps [PDB]:** 1377
- **1D aligned content [PDB] (<aminoacid>:%):** {'E': 9.52, 'V': 16.67, 'Y': 4.76, 'H': 2.38, 'F': 4.76, 'K': 14.29, 'P': 7.14, 'I': 11.9, 'G': 7.14, 'N': 4.76, 'A': 2.38, 'L': 4.76, 'R': 4.76, 'S': 2.38, 'W': 2.38}
- **2D identity (%) [PDB]:** 39.81
- **2D identity (%) [Gaps excluded][PDB]:** 91.23
- **2D identity - Alignment Gaps [PDB]:** 589
- **2D aligned content [PDB] (<2D-fold>:%):** {'.': 17.55, 'T': 20.91, 'H': 42.07, 'E': 19.47}
- **3D similarity (TM-Score) (%) [PDB]:** 20.07

- **Gene name:** PUS7
- **Entrez ID:** 615644
- **RefSeq ID:** N/A
- **Sequence length:** N/A
- **5-UTR|CDS|3-UTR identity (%):** N/A | N/A | N/A
- **5-UTR|CDS|3-UTR identity (%) [Gaps excluded]:** N/A | N/A | N/A
- **5-UTR|CDS|3-UTR identity [Alignment Gaps]:** N/A | N/A | N/A
- **5-UTR aligned content (<base>:%):** N/A
- **CDS aligned content (<base>:%):** N/A
- **3-UTR aligned content (<base>:%):** N/A

**Uniprot Description:**  
  
 Pseudouridylate synthase that catalyzes pseudouridylation of RNAs (PubMed:28073919, PubMed:29628141, PubMed:30778726). Acts as a regulator of protein synthesis in embryonic stem cells by mediating pseudouridylation of RNA fragments derived from tRNAs (tRFs): pseudouridylated tRFs inhibit translation by targeting the translation initiation complex (PubMed:29628141). Also catalyzes pseudouridylation of mRNAs: mediates pseudouridylation of mRNAs with the consensus sequence 5'-UGUAG-3' (PubMed:28073919). In addition to mRNAs and tRNAs, binds other types of RNAs, such as snRNAs, Y RNAs and vault RNAs, suggesting that it can catalyze pseudouridylation of many RNA types (PubMed:29628141). N/A   
  
 **Gene Ontology Information:**

Molecular Function

- pseudouridine synthase activity
- RNA binding

Location

- nucleus

Biological process

- mRNA processing
- mRNA pseudouridine synthesis
- negative regulation of translation
- pseudouridine synthesis
- regulation of hematopoietic stem cell differentiation
- regulation of mesoderm development
- RNA splicing
- tRNA pseudouridine synthesis

---

14

- **Protein name:** Piwi-like protein 4
- **Organism:** Homo sapiens
- **Uniprot Accession Number:** Q7Z3Z4
- **Protein sequence length:** 852 aa
- **1D identity (%):** 22.41
- **1D identity (%) [Gaps excluded]:** 28.38
- **1D identity - Alignment Gaps:** 201
- **1D aligned content (<aminoacid>:%):** {'M': 0.93, 'A': 4.67, 'G': 8.88, 'P': 8.41, 'V': 8.88, 'Q': 4.67, 'R': 7.94, 'I': 3.74, 'K': 6.54, 'L': 9.81, 'N': 2.8, 'F': 4.67, 'D': 5.61, 'Y': 6.07, 'H': 1.87, 'T': 5.14, 'E': 3.27, 'S': 3.27, 'W': 1.4, 'C': 1.4}
- **Common reported functions (%):** 0.0
- **Common reported locations (%):** 30.0
- **Common reported processes (%):** 0.0

- **AF ID:** Q7Z3Z4
- **Chain:** A
- **Protein length:** 852 aa
- **Resolution:** N/A
- **b-phipsi:** 0.002384
- **w-rdist:** 0.203397
- **t-alpha:** 0.021626
- **Chemical similarity (Tanimoto Index) (%):** 99.4
- **1D identity (%) [PDB]:** 4.78
- **1D identity (%) [Gaps excluded][PDB]:** 51.39
- **1D identity - Alignment Gaps [PDB]:** 1404
- **1D aligned content [PDB] (<aminoacid>:%):** {'I': 6.76, 'D': 8.11, 'L': 12.16, 'S': 5.41, 'Y': 9.46, 'R': 6.76, 'Q': 5.41, 'K': 2.7, 'T': 6.76, 'P': 8.11, 'G': 5.41, 'V': 9.46, 'F': 2.7, 'E': 1.35, 'N': 2.7, 'C': 1.35, 'H': 1.35, 'A': 4.05}
- **2D identity (%) [PDB]:** 67.53
- **2D identity (%) [Gaps excluded][PDB]:** 90.72
- **2D identity - Alignment Gaps [PDB]:** 248
- **2D aligned content [PDB] (<2D-fold>:%):** {'.': 15.88, 'E': 31.3, 'T': 17.71, 'H': 32.82, 'G': 1.37, 'B': 0.92}
- **3D similarity (TM-Score) (%) [PDB]:** 75.71

- **Gene name:** PIWIL4
- **Entrez ID:** 143689
- **RefSeq ID:** NM\_152431
- **Transcript sequence length:** 3139
- **5-UTR|CDS|3-UTR identity (%):** 35.92 | 46.12 | 2.41
- **5-UTR|CDS|3-UTR identity (%) [Gaps excluded]:** 73.95 | 72.93 | 76.68
- **5-UTR|CDS|3-UTR identity [Alignment Gaps]:** 126 | 1179 | 10566
- **5-UTR aligned content (<base>:%):** {'G': 32.95, 'C': 37.5, 'A': 13.64, 'T': 15.91}
- **CDS aligned content (<base>:%):** {'A': 25.22, 'T': 23.33, 'G': 25.56, 'C': 25.9}
- **3-UTR aligned content (<base>:%):** {'T': 35.74, 'G': 19.39, 'C': 10.65, 'A': 34.22}

**Uniprot Description:**  
  
 Plays a central role during spermatogenesis by repressing transposable elements and preventing their mobilization, which is essential for the germline integrity (By similarity). Acts via the piRNA metabolic process, which mediates the repression of transposable elements during meiosis by forming complexes composed of piRNAs and Piwi proteins and governs the methylation and subsequent repression of transposons (By similarity). Directly binds piRNAs, a class of 24 to 30 nucleotide RNAs that are generated by a Dicer-independent mechanism and are primarily derived from transposons and other repeated sequence elements (By similarity). Associates with secondary piRNAs antisense and PIWIL2/MILI is required for such association (By similarity). The piRNA process acts upstream of known mediators of DNA methylation (By similarity). Does not show endonuclease activity (By similarity). Plays a key role in the piRNA amplification loop, also named ping-pong amplification cycle, by acting as a 'slicer-incompetent' component that loads cleaved piRNAs from the 'slicer-competent' component PIWIL2 and target them on genomic transposon loci in the nucleus (By similarity). May be involved in the chromatin-modifying pathway by inducing 'Lys-9' methylation of histone H3 at some loci (PubMed:17544373). In addition to its role in germline, PIWIL4 also plays a role in the regulation of somatic cells activities. Plays a role in pancreatic beta cell function and insulin secretion (By similarity). Involved in maintaining cell morphology and functional integrity of retinal epithelial through Akt/GSK3alpha/beta signaling pathway (PubMed:28025795). When overexpressed, acts as an oncogene by inhibition of apoptosis and promotion of cells proliferation in tumors (PubMed:22483988).   
  
Interacts with PRMT5 and WDR77. Interacts (when methylated on arginine residues) with TDRD1, TDRKH/TDRD2 and TDRD9. Interacts with MOV10L1 (By similarity). Interacts with TEX15 and SPOCD1 (By similarity).   
  
 **Gene Ontology Information:**

Molecular Function

- piRNA binding
- endoribonuclease activity

Location

- cytoplasm
- mitochondrion
- nucleoplasm
- nucleus
- P granule
- piP-body

Biological process

- epithelial structure maintenance
- genetic imprinting
- meiotic cell cycle
- regulation of translation
- retrotransposon silencing by heterochromatin formation
- gene silencing by RNA
- secondary piRNA processing
- spermatogenesis

---

15

- **Protein name:** Methylcrotonoyl-CoA carboxylase subunit alpha, mitochondrial
- **Organism:** Homo sapiens
- **Uniprot Accession Number:** Q96RQ3
- **Protein sequence length:** 725 aa
- **1D identity (%):** 12.63
- **1D identity (%) [Gaps excluded]:** 20.67
- **1D identity - Alignment Gaps:** 382
- **1D aligned content (<aminoacid>:%):** {'V': 8.87, 'H': 4.84, 'P': 6.45, 'R': 7.26, 'Y': 4.03, 'T': 4.03, 'G': 12.1, 'E': 8.87, 'K': 7.26, 'A': 4.84, 'S': 3.23, 'M': 3.23, 'Q': 5.65, 'L': 4.03, 'F': 3.23, 'D': 5.65, 'C': 0.81, 'I': 3.23, 'W': 0.81, 'N': 1.61}
- **Common reported functions (%):** 0.0
- **Common reported locations (%):** 10.0
- **Common reported processes (%):** 0.0

- **AF ID:** Q96RQ3
- **Chain:** A
- **Protein length:** 725 aa
- **Resolution:** N/A
- **b-phipsi:** 0.007636
- **w-rdist:** 0.368436
- **t-alpha:** 0.00219
- **Chemical similarity (Tanimoto Index) (%):** 99.02
- **1D identity (%) [PDB]:** 2.44
- **1D identity (%) [Gaps excluded][PDB]:** 72.55
- **1D identity - Alignment Gaps [PDB]:** 1463
- **1D aligned content [PDB] (<aminoacid>:%):** {'R': 8.11, 'K': 13.51, 'L': 8.11, 'Q': 5.41, 'T': 8.11, 'S': 8.11, 'M': 2.7, 'I': 8.11, 'A': 5.41, 'P': 2.7, 'E': 10.81, 'N': 5.41, 'Y': 5.41, 'G': 2.7, 'V': 2.7, 'D': 2.7}
- **2D identity (%) [PDB]:** 42.67
- **2D identity (%) [Gaps excluded][PDB]:** 86.99
- **2D identity - Alignment Gaps [PDB]:** 535
- **2D aligned content [PDB] (<2D-fold>:%):** {'.': 22.1, 'E': 25.45, 'H': 33.26, 'T': 18.75, 'B': 0.45}
- **3D similarity (TM-Score) (%) [PDB]:** 22.0

- **Gene name:** MCCC1
- **Entrez ID:** 56922
- **RefSeq ID:** NM\_020166
- **Transcript sequence length:** 2454
- **5-UTR|CDS|3-UTR identity (%):** 16.59 | 40.49 | 1.28
- **5-UTR|CDS|3-UTR identity (%) [Gaps excluded]:** 80.43 | 75.8 | 83.73
- **5-UTR|CDS|3-UTR identity [Alignment Gaps]:** 177 | 1474 | 10711
- **5-UTR aligned content (<base>:%):** {'G': 51.35, 'T': 10.81, 'A': 8.11, 'C': 29.73}
- **CDS aligned content (<base>:%):** {'A': 28.57, 'T': 21.39, 'G': 26.7, 'C': 23.34}
- **3-UTR aligned content (<base>:%):** {'A': 30.22, 'C': 18.71, 'T': 34.53, 'G': 16.55}

**Uniprot Description:**  
  
 Biotin-attachment subunit of the 3-methylcrotonyl-CoA carboxylase, an enzyme that catalyzes the conversion of 3-methylcrotonyl-CoA to 3-methylglutaconyl-CoA, a critical step for leucine and isovaleric acid catabolism.   
  
Probably a dodecamer composed of six biotin-containing alpha subunits (MCCC1) and six beta (MCCC2) subunits (PubMed:17360195). Interacts (via the biotin carboxylation domain) with SIRT4 (PubMed:23438705).   
  
 **Gene Ontology Information:**

Molecular Function

- ATP binding
- biotin binding
- biotin carboxylase activity
- metal ion binding
- methylcrotonoyl-CoA carboxylase activity

Location

- 3-methylcrotonyl-CoA carboxylase complex, mitochondrial
- cytosol
- methylcrotonoyl-CoA carboxylase complex
- mitochondrial matrix
- mitochondrion

Biological process

- biotin metabolic process
- branched-chain amino acid catabolic process
- leucine catabolic process

---

16

- **Protein name:** Cytosolic phospholipase A2 delta
- **Organism:** Homo sapiens
- **Uniprot Accession Number:** Q86XP0
- **Protein sequence length:** 818 aa
- **1D identity (%):** 18.2
- **1D identity (%) [Gaps excluded]:** 25.54
- **1D identity - Alignment Gaps:** 281
- **1D aligned content (<aminoacid>:%):** {'P': 8.99, 'G': 9.55, 'Y': 3.37, 'T': 4.49, 'V': 6.18, 'L': 14.61, 'N': 0.56, 'R': 5.06, 'D': 7.3, 'K': 5.62, 'S': 5.62, 'W': 1.12, 'Q': 6.18, 'E': 6.18, 'F': 4.49, 'A': 5.62, 'C': 1.12, 'M': 1.12, 'I': 2.25, 'H': 0.56}
- **Common reported functions (%):** 0.0
- **Common reported locations (%):** 10.0
- **Common reported processes (%):** 0.0

- **AF ID:** Q86XP0
- **Chain:** A
- **Protein length:** 818 aa
- **Resolution:** N/A
- **b-phipsi:** 0.001452
- **w-rdist:** 0.330555
- **t-alpha:** 0.017518
- **Chemical similarity (Tanimoto Index) (%):** 96.56
- **1D identity (%) [PDB]:** 2.17
- **1D identity (%) [Gaps excluded][PDB]:** 74.47
- **1D identity - Alignment Gaps [PDB]:** 1564
- **1D aligned content [PDB] (<aminoacid>:%):** {'M': 2.86, 'A': 2.86, 'C': 5.71, 'Q': 11.43, 'V': 8.57, 'K': 8.57, 'N': 11.43, 'T': 2.86, 'S': 8.57, 'P': 5.71, 'L': 14.29, 'G': 5.71, 'I': 2.86, 'H': 2.86, 'R': 2.86, 'F': 2.86}
- **2D identity (%) [PDB]:** 47.19
- **2D identity (%) [Gaps excluded][PDB]:** 89.84
- **2D identity - Alignment Gaps [PDB]:** 516
- **2D aligned content [PDB] (<2D-fold>:%):** {'.': 16.96, 'E': 31.77, 'T': 19.49, 'H': 30.41, 'G': 1.36}
- **3D similarity (TM-Score) (%) [PDB]:** 23.88

- **Gene name:** PLA2G4D
- **Entrez ID:** 283748
- **RefSeq ID:** NM\_178034
- **Transcript sequence length:** 4266
- **5-UTR|CDS|3-UTR identity (%):** 26.78 | 46.82 | 10.98
- **5-UTR|CDS|3-UTR identity (%) [Gaps excluded]:** 83.12 | 76.24 | 78.79
- **5-UTR|CDS|3-UTR identity [Alignment Gaps]:** 162 | 1227 | 9465
- **5-UTR aligned content (<base>:%):** {'A': 14.06, 'G': 45.31, 'C': 29.69, 'T': 10.94}
- **CDS aligned content (<base>:%):** {'A': 22.23, 'T': 18.27, 'G': 28.95, 'C': 30.56}
- **3-UTR aligned content (<base>:%):** {'G': 19.72, 'T': 37.12, 'C': 21.62, 'A': 21.54}

**Uniprot Description:**  
  
 Calcium-dependent phospholipase A2 that selectively hydrolyzes glycerophospholipids in the sn-2 position (PubMed:14709560). Has a preference for linoleic acid at the sn-2 position (PubMed:14709560). N/A   
  
 **Gene Ontology Information:**

Molecular Function

- calcium ion binding
- calcium-dependent phospholipase A2 activity
- calcium-dependent phospholipid binding
- phospholipase A1 activity

Location

- cytosol
- membrane

Biological process

- fatty acid metabolic process
- glycerophospholipid catabolic process
- phosphatidylglycerol acyl-chain remodeling
- phosphatidylinositol acyl-chain remodeling

---

17

- **Protein name:** F-box/LRR-repeat protein 7
- **Organism:** Homo sapiens
- **Uniprot Accession Number:** Q9UJT9
- **Protein sequence length:** 491 aa
- **1D identity (%):** 9.84
- **1D identity (%) [Gaps excluded]:** 25.81
- **1D identity - Alignment Gaps:** 604
- **1D aligned content (<aminoacid>:%):** {'G': 9.38, 'D': 4.17, 'V': 10.42, 'S': 3.12, 'K': 4.17, 'A': 5.21, 'Q': 4.17, 'L': 15.62, 'R': 7.29, 'P': 8.33, 'I': 3.12, 'H': 2.08, 'C': 6.25, 'Y': 5.21, 'N': 3.12, 'E': 3.12, 'T': 4.17, 'F': 1.04}
- **Common reported functions (%):** 0.0
- **Common reported locations (%):** 10.0
- **Common reported processes (%):** 0.0

- **AF ID:** Q9UJT9
- **Chain:** A
- **Protein length:** 491 aa
- **Resolution:** N/A
- **b-phipsi:** 0.006002
- **w-rdist:** 0.549913
- **t-alpha:** 0.0
- **Chemical similarity (Tanimoto Index) (%):** 98.5
- **1D identity (%) [PDB]:** 2.09
- **1D identity (%) [Gaps excluded][PDB]:** 72.97
- **1D identity - Alignment Gaps [PDB]:** 1257
- **1D aligned content [PDB] (<aminoacid>:%):** {'G': 11.11, 'E': 7.41, 'K': 11.11, 'D': 3.7, 'R': 7.41, 'V': 14.81, 'S': 14.81, 'I': 3.7, 'L': 18.52, 'A': 7.41}
- **2D identity (%) [PDB]:** 25.7
- **2D identity (%) [Gaps excluded][PDB]:** 88.33
- **2D identity - Alignment Gaps [PDB]:** 731
- **2D aligned content [PDB] (<2D-fold>:%):** {'.': 17.74, 'T': 27.17, 'G': 1.13, 'H': 46.79, 'E': 7.17}
- **3D similarity (TM-Score) (%) [PDB]:** 17.5

- **Gene name:** FBXL7
- **Entrez ID:** 569430
- **RefSeq ID:** N/A
- **Sequence length:** N/A
- **5-UTR|CDS|3-UTR identity (%):** N/A | N/A | N/A
- **5-UTR|CDS|3-UTR identity (%) [Gaps excluded]:** N/A | N/A | N/A
- **5-UTR|CDS|3-UTR identity [Alignment Gaps]:** N/A | N/A | N/A
- **5-UTR aligned content (<base>:%):** N/A
- **CDS aligned content (<base>:%):** N/A
- **3-UTR aligned content (<base>:%):** N/A

**Uniprot Description:**  
  
 Substrate recognition component of a SCF (SKP1-CUL1-F-box protein) E3 ubiquitin-protein ligase complex (PubMed:25778398). During mitosis, it mediates the ubiquitination and subsequent proteasomal degradation of AURKA, causing mitotic arrest (By similarity). It also regulates mitochondrial function by mediating the ubiquitination and proteasomal degradation of the apoptosis inhibitor BIRC5 (PubMed:25778398, PubMed:28218735).   
  
Part of the SCF (SKP1-CUL1-F-box) E3 ubiquitin-protein ligase complex SCF(FBXL7) composed of CUL1, SKP1, RBX1 and FBXL7 (By similarity). Interacts with AURKA; interaction takes place during mitosis but not in interphase (By similarity). Interacts with BIRC5; this interaction allows BIRC5 to be polyubiquitinated by the SCF(FBXL7) E3 ubiquitin-protein ligase complex (PubMed:28218735).   
  
 **Gene Ontology Information:**

Molecular Function   
  
N/A

Location

- cytoplasm
- microtubule organizing center
- SCF ubiquitin ligase complex

Biological process

- protein ubiquitination
- SCF-dependent proteasomal ubiquitin-dependent protein catabolic process

---

18

- **Protein name:** Interferon-induced, double-stranded RNA-activated protein kinase
- **Organism:** Homo sapiens
- **Uniprot Accession Number:** P19525
- **Protein sequence length:** 551 aa
- **1D identity (%):** 9.73
- **1D identity (%) [Gaps excluded]:** 19.91
- **1D identity - Alignment Gaps:** 484
- **1D aligned content (<aminoacid>:%):** {'M': 1.09, 'F': 7.61, 'Y': 6.52, 'K': 13.04, 'P': 6.52, 'R': 5.43, 'V': 5.43, 'I': 4.35, 'G': 6.52, 'E': 6.52, 'A': 5.43, 'L': 8.7, 'S': 7.61, 'C': 2.17, 'T': 5.43, 'Q': 3.26, 'N': 1.09, 'D': 3.26}
- **Common reported functions (%):** 33.33
- **Common reported locations (%):** 30.0
- **Common reported processes (%):** 0.0

- **AF ID:** P19525
- **Chain:** A
- **Protein length:** 551 aa
- **Resolution:** N/A
- **b-phipsi:** 0.005716
- **w-rdist:** 0.512484
- **t-alpha:** 0.000731
- **Chemical similarity (Tanimoto Index) (%):** 98.94
- **1D identity (%) [PDB]:** 1.77
- **1D identity (%) [Gaps excluded][PDB]:** 70.59
- **1D identity - Alignment Gaps [PDB]:** 1323
- **1D aligned content [PDB] (<aminoacid>:%):** {'M': 4.17, 'F': 4.17, 'N': 4.17, 'T': 12.5, 'Y': 4.17, 'S': 4.17, 'G': 12.5, 'Q': 4.17, 'I': 12.5, 'L': 12.5, 'K': 8.33, 'V': 8.33, 'R': 4.17, 'D': 4.17}
- **2D identity (%) [PDB]:** 38.14
- **2D identity (%) [Gaps excluded][PDB]:** 90.31
- **2D identity - Alignment Gaps [PDB]:** 565
- **2D aligned content [PDB] (<2D-fold>:%):** {'.': 16.09, 'T': 18.5, 'H': 39.41, 'E': 25.2, 'G': 0.8}
- **3D similarity (TM-Score) (%) [PDB]:** 18.17

- **Gene name:** EIF2AK2
- **Entrez ID:** 5610
- **RefSeq ID:** N/A
- **Sequence length:** N/A
- **5-UTR|CDS|3-UTR identity (%):** N/A | N/A | N/A
- **5-UTR|CDS|3-UTR identity (%) [Gaps excluded]:** N/A | N/A | N/A
- **5-UTR|CDS|3-UTR identity [Alignment Gaps]:** N/A | N/A | N/A
- **5-UTR aligned content (<base>:%):** N/A
- **CDS aligned content (<base>:%):** N/A
- **3-UTR aligned content (<base>:%):** N/A

**Uniprot Description:**  
  
 IFN-induced dsRNA-dependent serine/threonine-protein kinase that phosphorylates the alpha subunit of eukaryotic translation initiation factor 2 (EIF2S1/eIF-2-alpha) and plays a key role in the innate immune response to viral infection (PubMed:18835251, PubMed:19189853, PubMed:19507191, PubMed:21072047, PubMed:21123651, PubMed:22381929, PubMed:22948139, PubMed:23229543). Inhibits viral replication via the integrated stress response (ISR): EIF2S1/eIF-2-alpha phosphorylation in response to viral infection converts EIF2S1/eIF-2-alpha in a global protein synthesis inhibitor, resulting to a shutdown of cellular and viral protein synthesis, while concomitantly initiating the preferential translation of ISR-specific mRNAs, such as the transcriptional activator ATF4 (PubMed:19189853, PubMed:21123651, PubMed:22948139, PubMed:23229543). Exerts its antiviral activity on a wide range of DNA and RNA viruses including hepatitis C virus (HCV), hepatitis B virus (HBV), measles virus (MV) and herpes simplex virus 1 (HHV-1) (PubMed:11836380, PubMed:19189853, PubMed:19840259, PubMed:20171114, PubMed:21710204, PubMed:23115276, PubMed:23399035). Also involved in the regulation of signal transduction, apoptosis, cell proliferation and differentiation: phosphorylates other substrates including p53/TP53, PPP2R5A, DHX9, ILF3, IRS1 and the HHV-1 viral protein US11 (PubMed:11836380, PubMed:19229320, PubMed:22214662). In addition to serine/threonine-protein kinase activity, also has tyrosine-protein kinase activity and phosphorylates CDK1 at 'Tyr-4' upon DNA damage, facilitating its ubiquitination and proteasomal degradation (PubMed:20395957). Either as an adapter protein and/or via its kinase activity, can regulate various signaling pathways (p38 MAP kinase, NF-kappa-B and insulin signaling pathways) and transcription factors (JUN, STAT1, STAT3, IRF1, ATF3) involved in the expression of genes encoding pro-inflammatory cytokines and IFNs (PubMed:22948139, PubMed:23084476, PubMed:23372823). Activates the NF-kappa-B pathway via interaction with IKBKB and TRAF family of proteins and activates the p38 MAP kinase pathway via interaction with MAP2K6 (PubMed:10848580, PubMed:15121867, PubMed:15229216). Can act as both a positive and negative regulator of the insulin signaling pathway (ISP) (PubMed:20685959). Negatively regulates ISP by inducing the inhibitory phosphorylation of insulin receptor substrate 1 (IRS1) at 'Ser-312' and positively regulates ISP via phosphorylation of PPP2R5A which activates FOXO1, which in turn up-regulates the expression of insulin receptor substrate 2 (IRS2) (PubMed:20685959). Can regulate NLRP3 inflammasome assembly and the activation of NLRP3, NLRP1, AIM2 and NLRC4 inflammasomes (PubMed:22801494). Plays a role in the regulation of the cytoskeleton by binding to gelsolin (GSN), sequestering the protein in an inactive conformation away from actin (By similarity).   
  
Homodimer (PubMed:16179258, PubMed:31246429). Interacts with STRBP (By similarity). Interacts with DNAJC3. Forms a complex with FANCA, FANCC, FANCG and HSP70. Interacts with ADAR/ADAR1. Interacts with IRS1 (By similarity). The inactive form interacts with NCK1 and GSN. Interacts (via the kinase catalytic domain) with STAT3 (via SH2 domain), TRAF2 (C-terminus), TRAF5 (C-terminus) and TRAF6 (C-terminus). Interacts with MAP2K6, IKBKB/IKKB, NPM1, TARBP2, NLRP1, NLRP3, NLRC4 and AIM2. Interacts (via DRBM 1 domain) with DUS2L (via DRBM domain). Interacts with DHX9 (via N-terminus) and this interaction is dependent upon activation of the kinase. Interacts with EIF2S1/EIF-2ALPHA; this interaction induces a conformational change in EIF2S1 and its phosphorylation by EIF2AK2 (PubMed:16179258).   
  
 **Gene Ontology Information:**

Molecular Function

- ATP binding
- double-stranded RNA binding
- eukaryotic translation initiation factor 2alpha kinase activity
- identical protein binding
- kinase activity
- non-membrane spanning protein tyrosine kinase activity
- protein kinase activity
- protein phosphatase regulator activity
- protein serine kinase activity
- protein serine/threonine kinase activity
- RNA binding

Location

- cytoplasm
- cytosol
- membrane
- nucleus
- perinuclear region of cytoplasm
- ribosome

Biological process

- antiviral innate immune response
- cellular response to amino acid starvation
- defense response to virus
- endoplasmic reticulum unfolded protein response
- negative regulation of apoptotic process
- negative regulation of cell population proliferation
- negative regulation of osteoblast proliferation
- negative regulation of translation
- negative regulation of viral genome replication
- positive regulation of chemokine production
- positive regulation of cytokine production
- positive regulation of MAPK cascade
- positive regulation of NF-kappaB transcription factor activity
- positive regulation of NIK/NF-kappaB signaling
- positive regulation of stress-activated MAPK cascade
- protein autophosphorylation
- protein phosphorylation
- regulation of hematopoietic progenitor cell differentiation
- regulation of hematopoietic stem cell differentiation
- regulation of hematopoietic stem cell proliferation
- regulation of NLRP3 inflammasome complex assembly
- response to interferon-alpha
- response to virus
- translation

---

19

- **Protein name:** Muskelin
- **Organism:** Homo sapiens
- **Uniprot Accession Number:** Q9UL63
- **Protein sequence length:** 735 aa
- **1D identity (%):** 16.26
- **1D identity (%) [Gaps excluded]:** 23.14
- **1D identity - Alignment Gaps:** 278
- **1D aligned content (<aminoacid>:%):** {'M': 0.66, 'A': 8.55, 'G': 10.53, 'P': 7.89, 'L': 7.89, 'V': 7.24, 'D': 5.92, 'E': 2.63, 'R': 3.95, 'K': 6.58, 'N': 3.95, 'T': 5.92, 'F': 2.63, 'I': 5.92, 'S': 5.92, 'W': 0.66, 'H': 1.97, 'Y': 3.29, 'Q': 3.95, 'C': 3.95}
- **Common reported functions (%):** 0.0
- **Common reported locations (%):** 30.0
- **Common reported processes (%):** 0.0

- **AF ID:** Q9UL63
- **Chain:** A
- **Protein length:** 735 aa
- **Resolution:** N/A
- **b-phipsi:** 0.001455
- **w-rdist:** 0.24384
- **t-alpha:** 0.057099
- **Chemical similarity (Tanimoto Index) (%):** 99.4
- **1D identity (%) [PDB]:** 2.09
- **1D identity (%) [Gaps excluded][PDB]:** 71.11
- **1D identity - Alignment Gaps [PDB]:** 1485
- **1D aligned content [PDB] (<aminoacid>:%):** {'M': 6.25, 'L': 15.62, 'H': 3.12, 'E': 3.12, 'S': 12.5, 'G': 9.38, 'I': 3.12, 'V': 9.38, 'P': 9.38, 'Q': 3.12, 'A': 3.12, 'D': 3.12, 'R': 6.25, 'Y': 3.12, 'T': 3.12, 'F': 6.25}
- **2D identity (%) [PDB]:** 42.06
- **2D identity (%) [Gaps excluded][PDB]:** 89.11
- **2D identity - Alignment Gaps [PDB]:** 565
- **2D aligned content [PDB] (<2D-fold>:%):** {'.': 23.56, 'E': 32.44, 'T': 19.11, 'H': 21.56, 'B': 0.44, 'G': 2.89}
- **3D similarity (TM-Score) (%) [PDB]:** 22.8

- **Gene name:** MKLN1
- **Entrez ID:** 4289
- **RefSeq ID:** NM\_013255
- **Transcript sequence length:** 11136
- **5-UTR|CDS|3-UTR identity (%):** 8.41 | 41.42 | 43.91
- **5-UTR|CDS|3-UTR identity (%) [Gaps excluded]:** 78.26 | 75.45 | 74.14
- **5-UTR|CDS|3-UTR identity [Alignment Gaps]:** 191 | 1422 | 5053
- **5-UTR aligned content (<base>:%):** {'G': 55.56, 'C': 33.33, 'A': 5.56, 'T': 5.56}
- **CDS aligned content (<base>:%):** {'A': 30.47, 'T': 22.89, 'G': 24.89, 'C': 21.75}
- **3-UTR aligned content (<base>:%):** {'C': 16.16, 'G': 18.86, 'A': 29.56, 'T': 35.42}

**Uniprot Description:**  
  
 Component of the CTLH E3 ubiquitin-protein ligase complex that selectively accepts ubiquitin from UBE2H and mediates ubiquitination and subsequent proteasomal degradation of the transcription factor HBP1 (PubMed:29911972). Required for internalization of the GABA receptor GABRA1 from the cell membrane via endosomes and subsequent GABRA1 degradation (By similarity). Acts as a mediator of cell spreading and cytoskeletal responses to the extracellular matrix component THBS1 (PubMed:18710924).   
  
Homodimer; may form higher oligomers (By similarity). Identified in the CTLH complex that contains GID4, RANBP9 and/or RANBP10, MKLN1, MAEA, RMND5A (or alternatively its paralog RMND5B), GID8, ARMC8, WDR26 and YPEL5 (PubMed:17467196, PubMed:29911972). Within this complex, MAEA, RMND5A (or alternatively its paralog RMND5B), GID8, WDR26, and RANBP9 and/or RANBP10 form the catalytic core, while GID4, MKLN1, ARMC8 and YPEL5 have ancillary roles (PubMed:29911972). Interacts with RANBP9 (PubMed:18710924). Part of a complex consisting of RANBP9, MKLN1 and GID8 (PubMed:12559565). Interacts with GABRA1. Interacts with the C-terminal tail of PTGER3 (By similarity).   
  
 **Gene Ontology Information:**

Molecular Function

- identical protein binding
- protein homodimerization activity

Location

- cell cortex
- cytoplasm
- cytosol
- nucleoplasm
- postsynapse
- ruffle
- ubiquitin ligase complex

Biological process

- actin cytoskeleton reorganization
- cell-matrix adhesion
- regulation of cell shape
- regulation of receptor internalization
- signal transduction

---

20

- **Protein name:** Centrosomal protein of 76 kDa
- **Organism:** Homo sapiens
- **Uniprot Accession Number:** Q8TAP6
- **Protein sequence length:** 659 aa
- **1D identity (%):** 11.41
- **1D identity (%) [Gaps excluded]:** 25.86
- **1D identity - Alignment Gaps:** 588
- **1D aligned content (<aminoacid>:%):** {'M': 0.83, 'P': 14.17, 'A': 5.0, 'L': 8.33, 'Q': 6.67, 'R': 10.0, 'G': 5.0, 'I': 5.0, 'V': 8.33, 'K': 4.17, 'E': 6.67, 'Y': 5.0, 'F': 5.0, 'D': 1.67, 'T': 3.33, 'C': 5.0, 'N': 2.5, 'S': 2.5, 'H': 0.83}
- **Common reported functions (%):** 0.0
- **Common reported locations (%):** 10.0
- **Common reported processes (%):** 0.0

- **AF ID:** Q8TAP6
- **Chain:** A
- **Protein length:** 659 aa
- **Resolution:** N/A
- **b-phipsi:** 0.00038
- **w-rdist:** 0.5307
- **t-alpha:** 0.042335
- **Chemical similarity (Tanimoto Index) (%):** 99.02
- **1D identity (%) [PDB]:** 2.19
- **1D identity (%) [Gaps excluded][PDB]:** 80.0
- **1D identity - Alignment Gaps [PDB]:** 1419
- **1D aligned content [PDB] (<aminoacid>:%):** {'M': 3.12, 'L': 6.25, 'N': 6.25, 'I': 6.25, 'D': 3.12, 'V': 9.38, 'S': 6.25, 'T': 9.38, 'A': 6.25, 'F': 9.38, 'Q': 6.25, 'P': 9.38, 'C': 3.12, 'R': 9.38, 'E': 3.12, 'K': 3.12}
- **2D identity (%) [PDB]:** 41.94
- **2D identity (%) [Gaps excluded][PDB]:** 86.89
- **2D identity - Alignment Gaps [PDB]:** 523
- **2D aligned content [PDB] (<2D-fold>:%):** {'.': 21.23, 'H': 35.38, 'T': 19.34, 'E': 23.35, 'G': 0.71}
- **3D similarity (TM-Score) (%) [PDB]:** 20.32

- **Gene name:** CEP76
- **Entrez ID:** 79959
- **RefSeq ID:** NM\_024899
- **Transcript sequence length:** 2895
- **5-UTR|CDS|3-UTR identity (%):** 44.26 | 41.86 | 4.29
- **5-UTR|CDS|3-UTR identity (%) [Gaps excluded]:** 75.0 | 76.04 | 74.56
- **5-UTR|CDS|3-UTR identity [Alignment Gaps]:** 100 | 1350 | 10305
- **5-UTR aligned content (<base>:%):** {'A': 11.11, 'T': 6.48, 'C': 32.41, 'G': 50.0}
- **CDS aligned content (<base>:%):** {'A': 28.0, 'T': 24.5, 'G': 24.26, 'C': 23.23}
- **3-UTR aligned content (<base>:%):** {'G': 16.42, 'C': 10.02, 'A': 34.33, 'T': 39.23}

**Uniprot Description:**  
  
 Centrosomal protein involved in regulation of centriole duplication. Required to limit centriole duplication to once per cell cycle by preventing centriole reduplication.   
  
Interacts with CCP110 and CEP97.   
  
 **Gene Ontology Information:**

Molecular Function   
  
N/A

Location

- centriole
- centrosome
- cytosol
- protein-containing complex

Biological process

- regulation of centriole replication

---

21

- **Protein name:** E3 ubiquitin-protein ligase HECTD3
- **Organism:** Homo sapiens
- **Uniprot Accession Number:** Q5T447
- **Protein sequence length:** 861 aa
- **1D identity (%):** 14.74
- **1D identity (%) [Gaps excluded]:** 24.88
- **1D identity - Alignment Gaps:** 440
- **1D aligned content (<aminoacid>:%):** {'A': 3.14, 'G': 10.69, 'P': 15.72, 'L': 7.55, 'E': 5.66, 'C': 2.52, 'R': 4.4, 'V': 9.43, 'K': 5.03, 'Q': 5.66, 'D': 9.43, 'F': 3.77, 'W': 1.26, 'H': 1.89, 'S': 3.14, 'Y': 3.14, 'T': 3.77, 'I': 1.89, 'N': 1.89}
- **Common reported functions (%):** 0.0
- **Common reported locations (%):** 0.0
- **Common reported processes (%):** 0.0

- **AF ID:** Q5T447
- **Chain:** A
- **Protein length:** 861 aa
- **Resolution:** N/A
- **b-phipsi:** 0.003585
- **w-rdist:** 0.291201
- **t-alpha:** 0.015328
- **Chemical similarity (Tanimoto Index) (%):** 99.4
- **1D identity (%) [PDB]:** 3.53
- **1D identity (%) [Gaps excluded][PDB]:** 64.77
- **1D identity - Alignment Gaps [PDB]:** 1525
- **1D aligned content [PDB] (<aminoacid>:%):** {'M': 1.75, 'A': 3.51, 'P': 7.02, 'S': 5.26, 'R': 15.79, 'V': 10.53, 'Q': 3.51, 'E': 8.77, 'D': 7.02, 'L': 8.77, 'Y': 5.26, 'I': 5.26, 'K': 7.02, 'F': 3.51, 'T': 3.51, 'H': 1.75, 'G': 1.75}
- **2D identity (%) [PDB]:** 42.0
- **2D identity (%) [Gaps excluded][PDB]:** 92.29
- **2D identity - Alignment Gaps [PDB]:** 637
- **2D aligned content [PDB] (<2D-fold>:%):** {'.': 18.94, 'E': 24.44, 'H': 37.27, 'T': 17.31, 'G': 1.83, 'B': 0.2}
- **3D similarity (TM-Score) (%) [PDB]:** 25.91

- **Gene name:** HECTD3
- **Entrez ID:** 79654
- **RefSeq ID:** NM\_024602
- **Transcript sequence length:** 3597
- **5-UTR|CDS|3-UTR identity (%):** 18.22 | 46.18 | 5.63
- **5-UTR|CDS|3-UTR identity (%) [Gaps excluded]:** 74.55 | 75.8 | 74.85
- **5-UTR|CDS|3-UTR identity [Alignment Gaps]:** 170 | 1278 | 10121
- **5-UTR aligned content (<base>:%):** {'G': 34.15, 'C': 39.02, 'T': 17.07, 'A': 9.76}
- **CDS aligned content (<base>:%):** {'A': 21.79, 'T': 18.28, 'G': 31.19, 'C': 28.74}
- **3-UTR aligned content (<base>:%):** {'G': 18.99, 'C': 31.17, 'T': 28.9, 'A': 20.94}

**Uniprot Description:**  
  
 E3 ubiquitin ligases accepts ubiquitin from an E2 ubiquitin-conjugating enzyme in the form of a thioester and then directly transfers the ubiquitin to targeted substrates. Mediates ubiquitination of TRIOBP and its subsequent proteasomal degradation, thus facilitating cell cycle progression by regulating the turn-over of TRIOBP. Mediates also ubiquitination of STX8 (By similarity).   
  
Interacts with TRIOBP. Interacts with STX8 (By similarity).   
  
 **Gene Ontology Information:**

Molecular Function

- syntaxin binding
- ubiquitin-protein transferase activity

Location

- perinuclear region of cytoplasm

Biological process

- proteasome-mediated ubiquitin-dependent protein catabolic process
- protein ubiquitination

---

22

- **Protein name:** Alpha-mannosidase 2
- **Organism:** Homo sapiens
- **Uniprot Accession Number:** Q16706
- **Protein sequence length:** 1144 aa
- **1D identity (%):** 15.53
- **1D identity (%) [Gaps excluded]:** 24.11
- **1D identity - Alignment Gaps:** 433
- **1D aligned content (<aminoacid>:%):** {'M': 3.17, 'G': 6.88, 'Y': 3.7, 'L': 9.52, 'P': 7.41, 'R': 4.76, 'I': 6.88, 'A': 3.17, 'E': 4.23, 'H': 3.7, 'F': 5.82, 'V': 7.94, 'T': 4.23, 'S': 5.29, 'K': 8.99, 'W': 1.59, 'D': 4.76, 'C': 1.06, 'N': 4.23, 'Q': 2.65}
- **Common reported functions (%):** 0.0
- **Common reported locations (%):** 0.0
- **Common reported processes (%):** 0.0

- **AF ID:** Q16706
- **Chain:** A
- **Protein length:** 1144 aa
- **Resolution:** N/A
- **b-phipsi:** 0.000631
- **w-rdist:** 0.407687
- **t-alpha:** 0.048905
- **Chemical similarity (Tanimoto Index) (%):** 97.2
- **1D identity (%) [PDB]:** 3.76
- **1D identity (%) [Gaps excluded][PDB]:** 74.74
- **1D identity - Alignment Gaps [PDB]:** 1794
- **1D aligned content [PDB] (<aminoacid>:%):** {'E': 7.04, 'G': 7.04, 'P': 2.82, 'Q': 8.45, 'I': 11.27, 'H': 4.23, 'L': 11.27, 'A': 5.63, 'R': 4.23, 'D': 7.04, 'T': 9.86, 'Y': 2.82, 'V': 4.23, 'F': 5.63, 'K': 2.82, 'N': 2.82, 'S': 2.82}
- **2D identity (%) [PDB]:** 36.98
- **2D identity (%) [Gaps excluded][PDB]:** 90.61
- **2D identity - Alignment Gaps [PDB]:** 834
- **2D aligned content [PDB] (<2D-fold>:%):** {'.': 21.31, 'T': 25.14, 'H': 32.82, 'E': 17.66, 'G': 2.88, 'B': 0.19}
- **3D similarity (TM-Score) (%) [PDB]:** 26.79

- **Gene name:** MAN2A1
- **Entrez ID:** 4124
- **RefSeq ID:** NM\_002372
- **Transcript sequence length:** 6553
- **5-UTR|CDS|3-UTR identity (%):** 33.4 | 43.58 | 16.0
- **5-UTR|CDS|3-UTR identity (%) [Gaps excluded]:** 81.86 | 74.47 | 76.87
- **5-UTR|CDS|3-UTR identity [Alignment Gaps]:** 296 | 1599 | 8816
- **5-UTR aligned content (<base>:%):** {'C': 32.34, 'T': 12.57, 'G': 46.11, 'A': 8.98}
- **CDS aligned content (<base>:%):** {'A': 27.86, 'T': 23.69, 'G': 24.52, 'C': 23.93}
- **3-UTR aligned content (<base>:%):** {'A': 29.76, 'T': 37.79, 'G': 17.63, 'C': 14.82}

**Uniprot Description:**  
  
 Catalyzes the first committed step in the biosynthesis of complex N-glycans. It controls conversion of high mannose to complex N-glycans; the final hydrolytic step in the N-glycan maturation pathway.   
  
Homodimer; disulfide-linked.   
  
 **Gene Ontology Information:**

Molecular Function

- alpha-mannosidase activity
- carbohydrate binding
- hydrolase activity, hydrolyzing N-glycosyl compounds
- mannosyl-oligosaccharide 1,3-1,6-alpha-mannosidase activity
- metal ion binding
- protein homodimerization activity

Location

- cis-Golgi network
- extracellular exosome
- Golgi medial cisterna
- Golgi membrane
- membrane

Biological process

- in utero embryonic development
- liver development
- lung alveolus development
- mannose metabolic process
- mitochondrion organization
- N-glycan processing
- positive regulation of neurogenesis
- protein deglycosylation
- protein glycosylation
- respiratory gaseous exchange by respiratory system
- retina morphogenesis in camera-type eye
- vacuole organization
- viral protein processing

---

23

- **Protein name:** Tyrosine-protein phosphatase non-receptor type 11
- **Organism:** Homo sapiens
- **Uniprot Accession Number:** Q06124
- **Protein sequence length:** 593 aa
- **1D identity (%):** 13.11
- **1D identity (%) [Gaps excluded]:** 24.6
- **1D identity - Alignment Gaps:** 442
- **1D aligned content (<aminoacid>:%):** {'F': 3.23, 'I': 3.23, 'V': 7.26, 'D': 5.65, 'P': 7.26, 'K': 10.48, 'H': 3.23, 'Q': 4.84, 'G': 11.29, 'Y': 5.65, 'A': 2.42, 'E': 6.45, 'S': 3.23, 'R': 8.06, 'L': 8.06, 'M': 0.81, 'T': 3.23, 'C': 3.23, 'N': 2.42}
- **Common reported functions (%):** 0.0
- **Common reported locations (%):** 20.0
- **Common reported processes (%):** 0.0

- **AF ID:** Q06124
- **Chain:** A
- **Protein length:** 593 aa
- **Resolution:** N/A
- **b-phipsi:** 0.003022
- **w-rdist:** 0.645113
- **t-alpha:** 0.001462
- **Chemical similarity (Tanimoto Index) (%):** 99.24
- **1D identity (%) [PDB]:** 2.31
- **1D identity (%) [Gaps excluded][PDB]:** 66.67
- **1D identity - Alignment Gaps [PDB]:** 1337
- **1D aligned content [PDB] (<aminoacid>:%):** {'P': 6.25, 'I': 9.38, 'T': 6.25, 'V': 12.5, 'G': 3.12, 'S': 9.38, 'A': 6.25, 'R': 15.62, 'Q': 9.38, 'E': 6.25, 'D': 3.12, 'M': 3.12, 'L': 6.25, 'K': 3.12}
- **2D identity (%) [PDB]:** 38.88
- **2D identity (%) [Gaps excluded][PDB]:** 85.49
- **2D identity - Alignment Gaps [PDB]:** 537
- **2D aligned content [PDB] (<2D-fold>:%):** {'.': 16.97, 'H': 35.77, 'T': 21.15, 'E': 24.54, 'G': 1.57}
- **3D similarity (TM-Score) (%) [PDB]:** 21.29

- **Gene name:** PTPN11
- **Entrez ID:** 395815
- **RefSeq ID:** N/A
- **Sequence length:** N/A
- **5-UTR|CDS|3-UTR identity (%):** N/A | N/A | N/A
- **5-UTR|CDS|3-UTR identity (%) [Gaps excluded]:** N/A | N/A | N/A
- **5-UTR|CDS|3-UTR identity [Alignment Gaps]:** N/A | N/A | N/A
- **5-UTR aligned content (<base>:%):** N/A
- **CDS aligned content (<base>:%):** N/A
- **3-UTR aligned content (<base>:%):** N/A

**Uniprot Description:**  
  
 Acts downstream of various receptor and cytoplasmic protein tyrosine kinases to participate in the signal transduction from the cell surface to the nucleus (PubMed:10655584, PubMed:18559669, PubMed:18829466, PubMed:26742426, PubMed:28074573). Positively regulates MAPK signal transduction pathway (PubMed:28074573). Dephosphorylates GAB1, ARHGAP35 and EGFR (PubMed:28074573). Dephosphorylates ROCK2 at 'Tyr-722' resulting in stimulation of its RhoA binding activity (PubMed:18559669). Dephosphorylates CDC73 (PubMed:26742426). Dephosphorylates SOX9 on tyrosine residues, leading to inactivate SOX9 and promote ossification (By similarity).   
  
Interacts with phosphorylated LIME1 and BCAR3. Interacts with SHB and INPP5D/SHIP1 (By similarity). Interacts with MILR1 (tyrosine-phosphorylated). Interacts with FLT1 (tyrosine-phosphorylated), FLT3 (tyrosine-phosphorylated), FLT4 (tyrosine-phosphorylated), KIT and GRB2. Interacts with PDGFRA (tyrosine phosphorylated). Interacts (via SH2 domain) with TEK/TIE2 (tyrosine phosphorylated) (By similarity). Interacts with PTPNS1 and CD84. Interacts with phosphorylated SIT1 and MPZL1. Interacts with FCRL4, FCRL6 and ANKHD1. Interacts with KIR2DL1; the interaction is enhanced by ARRB2. Interacts with GAB2. Interacts with TERT; the interaction retains TERT in the nucleus. Interacts with PECAM1 and FER. Interacts with EPHA2 (activated); participates in PTK2/FAK1 dephosphorylation in EPHA2 downstream signaling. Interacts with ROS1; mediates PTPN11 phosphorylation. Interacts with PDGFRB (tyrosine phosphorylated); this interaction increases the PTPN11 phosphatase activity. Interacts with GAREM1 isoform 1 (tyrosine phosphorylated); the interaction increases MAPK/ERK activity and does not affect the GRB2/SOS complex formation. Interacts with CDC73 (PubMed:26742426). Interacts with CEACAM1 (via cytoplasmic domain); this interaction depends on the monomer/dimer equilibrium and is phosphorylation-dependent (By similarity). Interacts with MPIG6B (via ITIM motif) (PubMed:23112346). Interacts with SIGLEC10 (By similarity). Interacts with FCRL3 (via phosphorylated ITIM motifs) (PubMed:11162587, PubMed:19843936).   
  
 **Gene Ontology Information:**

Molecular Function

- cell adhesion molecule binding
- non-membrane spanning protein tyrosine phosphatase activity
- protein tyrosine phosphatase activity
- receptor tyrosine kinase binding

Location

- cytoplasm
- nucleus

Biological process

- cellular response to epidermal growth factor stimulus
- cellular response to organic substance
- negative regulation of chondrocyte differentiation
- positive regulation of ERK1 and ERK2 cascade
- positive regulation of ossification
- protein dephosphorylation

---

24

- **Protein name:** Endogenous retrovirus group K member 21 Env polyprotein
- **Organism:** Homo sapiens
- **Uniprot Accession Number:** P61565
- **Protein sequence length:** 698 aa
- **1D identity (%):** 15.71
- **1D identity (%) [Gaps excluded]:** 26.33
- **1D identity - Alignment Gaps:** 393
- **1D aligned content (<aminoacid>:%):** {'E': 3.92, 'K': 6.54, 'P': 9.15, 'R': 4.58, 'N': 5.23, 'M': 1.96, 'V': 12.42, 'T': 5.88, 'L': 9.8, 'I': 7.84, 'S': 5.88, 'A': 3.27, 'Y': 1.96, 'W': 0.65, 'H': 0.65, 'C': 4.58, 'G': 5.23, 'Q': 5.88, 'F': 2.61, 'D': 1.96}
- **Common reported functions (%):** 0.0
- **Common reported locations (%):** 0.0
- **Common reported processes (%):** 0.0

- **AF ID:** P61565
- **Chain:** A
- **Protein length:** 698 aa
- **Resolution:** N/A
- **b-phipsi:** 0.000216
- **w-rdist:** 1.066071
- **t-alpha:** 0.002195
- **Chemical similarity (Tanimoto Index) (%):** 96.84
- **1D identity (%) [PDB]:** 1.53
- **1D identity (%) [Gaps excluded][PDB]:** 69.7
- **1D identity - Alignment Gaps [PDB]:** 1472
- **1D aligned content [PDB] (<aminoacid>:%):** {'H': 4.35, 'P': 17.39, 'A': 13.04, 'G': 13.04, 'K': 8.7, 'V': 17.39, 'S': 8.7, 'M': 4.35, 'D': 4.35, 'R': 4.35, 'T': 4.35}
- **2D identity (%) [PDB]:** 40.54
- **2D identity (%) [Gaps excluded][PDB]:** 92.13
- **2D identity - Alignment Gaps [PDB]:** 598
- **2D aligned content [PDB] (<2D-fold>:%):** {'.': 20.55, 'H': 40.65, 'T': 19.4, 'E': 17.78, 'B': 0.23, 'G': 1.39}
- **3D similarity (TM-Score) (%) [PDB]:** 17.51

- **Gene name:** ERVK-21
- **Entrez ID:** N/A
- **RefSeq ID:** N/A
- **Sequence length:** N/A
- **5-UTR|CDS|3-UTR identity (%):** N/A | N/A | N/A
- **5-UTR|CDS|3-UTR identity (%) [Gaps excluded]:** N/A | N/A | N/A
- **5-UTR|CDS|3-UTR identity [Alignment Gaps]:** N/A | N/A | N/A
- **5-UTR aligned content (<base>:%):** N/A
- **CDS aligned content (<base>:%):** N/A
- **3-UTR aligned content (<base>:%):** N/A

**Uniprot Description:**  
  
 Retroviral envelope proteins mediate receptor recognition and membrane fusion during early infection. Endogenous envelope proteins may have kept, lost or modified their original function during evolution. This endogenous envelope protein has lost its original fusogenic properties.   
  
The surface (SU) and transmembrane (TM) proteins form a heterodimer. SU and TM are attached by noncovalent interactions or by a labile interchain disulfide bond (By similarity).   
  
 **Gene Ontology Information:**

Molecular Function

- structural molecule activity

Location

- plasma membrane

Biological process   
  
N/A

---

25

- **Protein name:** TBC1 domain family member 17
- **Organism:** Homo sapiens
- **Uniprot Accession Number:** Q9HA65
- **Protein sequence length:** 648 aa
- **1D identity (%):** 14.23
- **1D identity (%) [Gaps excluded]:** 24.77
- **1D identity - Alignment Gaps:** 407
- **1D aligned content (<aminoacid>:%):** {'V': 10.29, 'K': 5.15, 'Y': 2.21, 'D': 7.35, 'R': 5.88, 'E': 3.68, 'H': 2.94, 'P': 11.76, 'G': 8.82, 'F': 3.68, 'W': 0.74, 'I': 1.47, 'A': 5.15, 'L': 10.29, 'S': 7.35, 'Q': 2.94, 'T': 6.62, 'C': 2.21, 'N': 1.47}
- **Common reported functions (%):** 0.0
- **Common reported locations (%):** 10.0
- **Common reported processes (%):** 0.0

- **AF ID:** Q9HA65
- **Chain:** A
- **Protein length:** 648 aa
- **Resolution:** N/A
- **b-phipsi:** 0.009372
- **w-rdist:** 0.494813
- **t-alpha:** 0.0
- **Chemical similarity (Tanimoto Index) (%):** 99.55
- **1D identity (%) [PDB]:** 2.29
- **1D identity (%) [Gaps excluded][PDB]:** 73.33
- **1D identity - Alignment Gaps [PDB]:** 1398
- **1D aligned content [PDB] (<aminoacid>:%):** {'M': 3.03, 'V': 6.06, 'E': 9.09, 'L': 18.18, 'I': 6.06, 'F': 6.06, 'K': 6.06, 'S': 3.03, 'T': 6.06, 'R': 6.06, 'P': 6.06, 'G': 3.03, 'Q': 3.03, 'H': 3.03, 'Y': 3.03, 'A': 6.06, 'D': 3.03, 'C': 3.03}
- **2D identity (%) [PDB]:** 36.38
- **2D identity (%) [Gaps excluded][PDB]:** 90.4
- **2D identity - Alignment Gaps [PDB]:** 634
- **2D aligned content [PDB] (<2D-fold>:%):** {'.': 17.1, 'T': 22.28, 'E': 14.51, 'H': 43.78, 'G': 2.33}
- **3D similarity (TM-Score) (%) [PDB]:** 19.82

- **Gene name:** TBC1D17
- **Entrez ID:** 79735
- **RefSeq ID:** NM\_024682
- **Transcript sequence length:** 2095
- **5-UTR|CDS|3-UTR identity (%):** 7.98 | 43.5 | 0.74
- **5-UTR|CDS|3-UTR identity (%) [Gaps excluded]:** 77.27 | 76.84 | 77.67
- **5-UTR|CDS|3-UTR identity [Alignment Gaps]:** 191 | 1281 | 10743
- **5-UTR aligned content (<base>:%):** {'A': 5.88, 'G': 47.06, 'T': 17.65, 'C': 29.41}
- **CDS aligned content (<base>:%):** {'A': 19.78, 'T': 18.38, 'G': 28.12, 'C': 33.72}
- **3-UTR aligned content (<base>:%):** {'C': 36.25, 'G': 26.25, 'A': 20.0, 'T': 17.5}

**Uniprot Description:**  
  
 Probable RAB GTPase-activating protein that inhibits RAB8A/B function. Reduces Rab8 recruitment to tubules emanating from the endocytic recycling compartment (ERC) and inhibits Rab8-mediated endocytic trafficking, such as that of transferrin receptor (TfR) (PubMed:22854040). Involved in regulation of autophagy.   
  
Interacts with OPTN; this interaction mediates TBC1D17 transient association with Rab8.   
  
 **Gene Ontology Information:**

Molecular Function

- GTPase activator activity

Location

- autophagosome
- cytosol
- recycling endosome

Biological process

- activation of GTPase activity
- autophagy
- protein transport
- retrograde transport, endosome to Golgi

---

26

- **Protein name:** Beta-1,4 N-acetylgalactosaminyltransferase 2
- **Organism:** Homo sapiens
- **Uniprot Accession Number:** Q8NHY0
- **Protein sequence length:** 566 aa
- **1D identity (%):** 12.62
- **1D identity (%) [Gaps excluded]:** 23.59
- **1D identity - Alignment Gaps:** 431
- **1D aligned content (<aminoacid>:%):** {'G': 9.4, 'V': 6.84, 'A': 5.98, 'R': 8.55, 'T': 5.98, 'P': 9.4, 'S': 4.27, 'L': 11.97, 'W': 1.71, 'F': 4.27, 'H': 2.56, 'M': 0.85, 'K': 5.98, 'Q': 6.84, 'E': 2.56, 'C': 2.56, 'D': 2.56, 'Y': 3.42, 'I': 3.42, 'N': 0.85}
- **Common reported functions (%):** 0.0
- **Common reported locations (%):** 0.0
- **Common reported processes (%):** 0.0

- **AF ID:** Q8NHY0
- **Chain:** A
- **Protein length:** 566 aa
- **Resolution:** N/A
- **b-phipsi:** 0.000511
- **w-rdist:** 0.650812
- **t-alpha:** 0.027737
- **Chemical similarity (Tanimoto Index) (%):** 97.49
- **1D identity (%) [PDB]:** 2.04
- **1D identity (%) [Gaps excluded][PDB]:** 82.35
- **1D identity - Alignment Gaps [PDB]:** 1338
- **1D aligned content [PDB] (<aminoacid>:%):** {'L': 17.86, 'C': 3.57, 'K': 7.14, 'I': 3.57, 'V': 14.29, 'G': 10.71, 'N': 7.14, 'P': 7.14, 'H': 3.57, 'Q': 7.14, 'R': 3.57, 'S': 3.57, 'F': 7.14, 'A': 3.57}
- **2D identity (%) [PDB]:** 39.21
- **2D identity (%) [Gaps excluded][PDB]:** 90.8
- **2D identity - Alignment Gaps [PDB]:** 558
- **2D aligned content [PDB] (<2D-fold>:%):** {'.': 20.26, 'T': 14.81, 'H': 37.66, 'E': 27.27}
- **3D similarity (TM-Score) (%) [PDB]:** 19.21

- **Gene name:** B4GALNT2
- **Entrez ID:** 124872
- **RefSeq ID:** N/A
- **Sequence length:** N/A
- **5-UTR|CDS|3-UTR identity (%):** N/A | N/A | N/A
- **5-UTR|CDS|3-UTR identity (%) [Gaps excluded]:** N/A | N/A | N/A
- **5-UTR|CDS|3-UTR identity [Alignment Gaps]:** N/A | N/A | N/A
- **5-UTR aligned content (<base>:%):** N/A
- **CDS aligned content (<base>:%):** N/A
- **3-UTR aligned content (<base>:%):** N/A

**Uniprot Description:**  
  
 Involved in the synthesis of the Sd(a) antigen (Sia-alpha2,3-[GalNAc-beta1,4]Gal-beta1,4-GlcNAc), a carbohydrate determinant expressed on erythrocytes, the colonic mucosa and other tissues. Transfers a beta-1,4-linked GalNAc to the galactose residue of an alpha-2,3-sialylated chain. N/A   
  
 **Gene Ontology Information:**

Molecular Function

- acetylgalactosaminyltransferase activity

Location

- Golgi membrane
- membrane

Biological process

- lipid glycosylation
- negative regulation of cell-cell adhesion
- oligosaccharide biosynthetic process
- protein N-linked glycosylation via asparagine
- UDP-N-acetylgalactosamine metabolic process
- UDP-N-acetylglucosamine metabolic process

---

27

- **Protein name:** 5-oxoprolinase
- **Organism:** Homo sapiens
- **Uniprot Accession Number:** O14841
- **Protein sequence length:** 1288 aa
- **1D identity (%):** 15.83
- **1D identity (%) [Gaps excluded]:** 24.73
- **1D identity - Alignment Gaps:** 471
- **1D aligned content (<aminoacid>:%):** {'G': 14.01, 'V': 7.25, 'F': 4.35, 'P': 10.63, 'K': 2.42, 'L': 11.59, 'A': 8.21, 'N': 1.45, 'Y': 3.38, 'D': 5.8, 'H': 2.9, 'R': 6.28, 'T': 4.83, 'E': 3.86, 'I': 4.35, 'W': 0.97, 'S': 2.9, 'M': 0.48, 'Q': 3.86, 'C': 0.48}
- **Common reported functions (%):** 0.0
- **Common reported locations (%):** 10.0
- **Common reported processes (%):** 0.0

- **AF ID:** O14841
- **Chain:** A
- **Protein length:** 1288 aa
- **Resolution:** N/A
- **b-phipsi:** 0.003688
- **w-rdist:** 0.318233
- **t-alpha:** 0.009489
- **Chemical similarity (Tanimoto Index) (%):** 99.25
- **1D identity (%) [PDB]:** 2.78
- **1D identity (%) [Gaps excluded][PDB]:** 73.08
- **1D identity - Alignment Gaps [PDB]:** 1972
- **1D aligned content [PDB] (<aminoacid>:%):** {'G': 8.77, 'I': 8.77, 'L': 7.02, 'V': 14.04, 'P': 8.77, 'Q': 3.51, 'R': 8.77, 'S': 8.77, 'A': 7.02, 'F': 1.75, 'D': 3.51, 'H': 3.51, 'K': 1.75, 'T': 1.75, 'M': 1.75, 'Y': 3.51, 'C': 1.75, 'E': 5.26}
- **2D identity (%) [PDB]:** 42.16
- **2D identity (%) [Gaps excluded][PDB]:** 90.95
- **2D identity - Alignment Gaps [PDB]:** 780
- **2D aligned content [PDB] (<2D-fold>:%):** {'.': 15.82, 'E': 28.71, 'T': 20.55, 'H': 33.61, 'G': 0.98, 'B': 0.33}
- **3D similarity (TM-Score) (%) [PDB]:** 28.28

- **Gene name:** OPLAH
- **Entrez ID:** 26873
- **RefSeq ID:** NM\_017570
- **Transcript sequence length:** 4020
- **5-UTR|CDS|3-UTR identity (%):** 28.57 | 42.75 | 0.4
- **5-UTR|CDS|3-UTR identity (%) [Gaps excluded]:** 78.05 | 76.33 | 86.0
- **5-UTR|CDS|3-UTR identity [Alignment Gaps]:** 142 | 1845 | 10783
- **5-UTR aligned content (<base>:%):** {'G': 42.19, 'C': 39.06, 'A': 14.06, 'T': 4.69}
- **CDS aligned content (<base>:%):** {'A': 19.63, 'T': 17.46, 'G': 30.45, 'C': 32.46}
- **3-UTR aligned content (<base>:%):** {'G': 37.21, 'A': 27.91, 'C': 23.26, 'T': 11.63}

**Uniprot Description:**  
  
 Catalyzes the cleavage of 5-oxo-L-proline to form L-glutamate coupled to the hydrolysis of ATP to ADP and inorganic phosphate.   
  
Homodimer.   
  
 **Gene Ontology Information:**

Molecular Function

- 5-oxoprolinase (ATP-hydrolyzing) activity
- ATP binding
- identical protein binding

Location

- cytosol

Biological process

- glutathione metabolic process

---

28

- **Protein name:** Adenylyl cyclase-associated protein 1
- **Organism:** Homo sapiens
- **Uniprot Accession Number:** Q01518
- **Protein sequence length:** 475 aa
- **1D identity (%):** 9.04
- **1D identity (%) [Gaps excluded]:** 22.57
- **1D identity - Alignment Gaps:** 570
- **1D aligned content (<aminoacid>:%):** {'A': 5.81, 'R': 2.33, 'E': 3.49, 'S': 3.49, 'D': 8.14, 'P': 10.47, 'Y': 3.49, 'Q': 5.81, 'F': 4.65, 'L': 10.47, 'I': 12.79, 'G': 9.3, 'C': 2.33, 'H': 2.33, 'V': 3.49, 'K': 8.14, 'T': 2.33, 'W': 1.16}
- **Common reported functions (%):** 0.0
- **Common reported locations (%):** 10.0
- **Common reported processes (%):** 0.0

- **AF ID:** Q01518
- **Chain:** A
- **Protein length:** 475 aa
- **Resolution:** N/A
- **b-phipsi:** 0.003199
- **w-rdist:** 0.329961
- **t-alpha:** 0.012565
- **Chemical similarity (Tanimoto Index) (%):** 99.62
- **1D identity (%) [PDB]:** 2.11
- **1D identity (%) [Gaps excluded][PDB]:** 71.05
- **1D identity - Alignment Gaps [PDB]:** 1239
- **1D aligned content [PDB] (<aminoacid>:%):** {'G': 11.11, 'D': 3.7, 'L': 14.81, 'A': 3.7, 'Q': 11.11, 'C': 3.7, 'V': 7.41, 'K': 14.81, 'N': 11.11, 'T': 7.41, 'P': 3.7, 'S': 3.7, 'I': 3.7}
- **2D identity (%) [PDB]:** 31.01
- **2D identity (%) [Gaps excluded][PDB]:** 88.56
- **2D identity - Alignment Gaps [PDB]:** 633
- **2D aligned content [PDB] (<2D-fold>:%):** {'H': 52.65, 'T': 16.56, '.': 13.25, 'G': 0.99, 'E': 16.56}
- **3D similarity (TM-Score) (%) [PDB]:** 18.16

- **Gene name:** CAP1
- **Entrez ID:** 10487
- **RefSeq ID:** N/A
- **Sequence length:** N/A
- **5-UTR|CDS|3-UTR identity (%):** N/A | N/A | N/A
- **5-UTR|CDS|3-UTR identity (%) [Gaps excluded]:** N/A | N/A | N/A
- **5-UTR|CDS|3-UTR identity [Alignment Gaps]:** N/A | N/A | N/A
- **5-UTR aligned content (<base>:%):** N/A
- **CDS aligned content (<base>:%):** N/A
- **3-UTR aligned content (<base>:%):** N/A

**Uniprot Description:**  
  
 Directly regulates filament dynamics and has been implicated in a number of complex developmental and morphological processes, including mRNA localization and the establishment of cell polarity.   
  
Homodimer. Binds actin monomers.   
  
 **Gene Ontology Information:**

Molecular Function

- actin binding
- adenylate cyclase binding

Location

- azurophil granule lumen
- cortical actin cytoskeleton
- cytoplasm
- extracellular exosome
- extracellular region
- focal adhesion
- plasma membrane

Biological process

- actin filament organization
- activation of adenylate cyclase activity
- ameboidal-type cell migration
- cAMP-mediated signaling
- cell morphogenesis
- establishment or maintenance of cell polarity
- receptor-mediated endocytosis
- signal transduction

---

29

- **Protein name:** Poly(A) polymerase beta
- **Organism:** Homo sapiens
- **Uniprot Accession Number:** Q9NRJ5
- **Protein sequence length:** 637 aa
- **1D identity (%):** 13.56
- **1D identity (%) [Gaps excluded]:** 23.27
- **1D identity - Alignment Gaps:** 394
- **1D aligned content (<aminoacid>:%):** {'P': 10.94, 'Q': 3.91, 'G': 6.25, 'V': 10.16, 'I': 3.91, 'L': 11.72, 'E': 7.03, 'K': 7.81, 'F': 4.69, 'R': 5.47, 'D': 4.69, 'T': 3.12, 'W': 1.56, 'H': 3.12, 'M': 0.78, 'A': 3.12, 'N': 4.69, 'Y': 3.12, 'S': 3.91}
- **Common reported functions (%):** 16.67
- **Common reported locations (%):** 20.0
- **Common reported processes (%):** 0.0

- **AF ID:** Q9NRJ5
- **Chain:** A
- **Protein length:** 636 aa
- **Resolution:** N/A
- **b-phipsi:** 0.000437
- **w-rdist:** 0.344785
- **t-alpha:** 0.183211
- **Chemical similarity (Tanimoto Index) (%):** 99.02
- **1D identity (%) [PDB]:** 1.95
- **1D identity (%) [Gaps excluded][PDB]:** 71.79
- **1D identity - Alignment Gaps [PDB]:** 1398
- **1D aligned content [PDB] (<aminoacid>:%):** {'L': 10.71, 'E': 7.14, 'N': 3.57, 'I': 10.71, 'A': 10.71, 'R': 7.14, 'C': 3.57, 'K': 7.14, 'T': 14.29, 'D': 7.14, 'S': 7.14, 'M': 3.57, 'P': 3.57, 'Q': 3.57}
- **2D identity (%) [PDB]:** 39.15
- **2D identity (%) [Gaps excluded][PDB]:** 90.99
- **2D identity - Alignment Gaps [PDB]:** 588
- **2D aligned content [PDB] (<2D-fold>:%):** {'.': 18.32, 'T': 22.03, 'H': 42.82, 'B': 0.5, 'E': 13.86, 'G': 2.48}
- **3D similarity (TM-Score) (%) [PDB]:** 18.13

- **Gene name:** PAPOLB
- **Entrez ID:** 56903
- **RefSeq ID:** NM\_020144
- **Transcript sequence length:** 4293
- **5-UTR|CDS|3-UTR identity (%):** 42.03 | 40.2 | 12.77
- **5-UTR|CDS|3-UTR identity (%) [Gaps excluded]:** 73.89 | 73.55 | 76.56
- **5-UTR|CDS|3-UTR identity [Alignment Gaps]:** 119 | 1346 | 9270
- **5-UTR aligned content (<base>:%):** {'A': 9.48, 'G': 52.59, 'C': 29.31, 'T': 8.62}
- **CDS aligned content (<base>:%):** {'A': 28.33, 'G': 24.81, 'C': 24.31, 'T': 22.55}
- **3-UTR aligned content (<base>:%):** {'T': 34.69, 'G': 17.17, 'A': 30.33, 'C': 17.8}

**Uniprot Description:**  
  
 N/A   
  
Interacts with GSG1.   
  
 **Gene Ontology Information:**

Molecular Function

- ATP binding
- metal ion binding
- RNA adenylyltransferase activity
- RNA binding

Location

- cytoplasm
- nucleus

Biological process

- mRNA polyadenylation
- RNA polyadenylation

---

30

- **Protein name:** E3 ubiquitin-protein ligase SMURF2
- **Organism:** Homo sapiens
- **Uniprot Accession Number:** Q9HAU4
- **Protein sequence length:** 748 aa
- **1D identity (%):** 13.98
- **1D identity (%) [Gaps excluded]:** 22.75
- **1D identity - Alignment Gaps:** 383
- **1D aligned content (<aminoacid>:%):** {'P': 10.79, 'G': 11.51, 'K': 4.32, 'L': 14.39, 'D': 3.6, 'V': 6.47, 'Q': 6.47, 'H': 0.72, 'Y': 4.32, 'T': 2.88, 'R': 7.91, 'I': 6.47, 'S': 2.16, 'F': 5.76, 'W': 0.72, 'A': 2.16, 'E': 4.32, 'N': 4.32, 'C': 0.72}
- **Common reported functions (%):** 0.0
- **Common reported locations (%):** 40.0
- **Common reported processes (%):** 0.0

- **AF ID:** Q9HAU4
- **Chain:** A
- **Protein length:** 748 aa
- **Resolution:** N/A
- **b-phipsi:** 0.004169
- **w-rdist:** 0.207324
- **t-alpha:** 0.045256
- **Chemical similarity (Tanimoto Index) (%):** 99.02
- **1D identity (%) [PDB]:** 2.22
- **1D identity (%) [Gaps excluded][PDB]:** 62.96
- **1D identity - Alignment Gaps [PDB]:** 1480
- **1D aligned content [PDB] (<aminoacid>:%):** {'S': 8.82, 'N': 8.82, 'L': 11.76, 'C': 2.94, 'K': 8.82, 'V': 11.76, 'G': 8.82, 'I': 5.88, 'Q': 8.82, 'R': 2.94, 'A': 8.82, 'D': 2.94, 'T': 5.88, 'P': 2.94}
- **2D identity (%) [PDB]:** 43.21
- **2D identity (%) [Gaps excluded][PDB]:** 86.74
- **2D identity - Alignment Gaps [PDB]:** 532
- **2D aligned content [PDB] (<2D-fold>:%):** {'.': 20.52, 'E': 21.83, 'T': 17.47, 'H': 37.12, 'G': 2.84, 'B': 0.22}
- **3D similarity (TM-Score) (%) [PDB]:** 23.2

- **Gene name:** SMURF2
- **Entrez ID:** 64750
- **RefSeq ID:** NM\_022739
- **Transcript sequence length:** 6240
- **5-UTR|CDS|3-UTR identity (%):** 37.41 | 44.79 | 20.06
- **5-UTR|CDS|3-UTR identity (%) [Gaps excluded]:** 82.91 | 73.76 | 75.42
- **5-UTR|CDS|3-UTR identity [Alignment Gaps]:** 242 | 1203 | 8343
- **5-UTR aligned content (<base>:%):** {'A': 9.09, 'G': 46.06, 'C': 37.58, 'T': 7.27}
- **CDS aligned content (<base>:%):** {'T': 23.76, 'G': 24.85, 'C': 22.45, 'A': 28.94}
- **3-UTR aligned content (<base>:%):** {'C': 15.48, 'A': 29.3, 'G': 18.86, 'T': 36.36}

**Uniprot Description:**  
  
 E3 ubiquitin-protein ligase which accepts ubiquitin from an E2 ubiquitin-conjugating enzyme in the form of a thioester and then directly transfers the ubiquitin to targeted substrates (PubMed:11016919). Interacts with SMAD7 to trigger SMAD7-mediated transforming growth factor beta/TGF-beta receptor ubiquitin-dependent degradation, thereby downregulating TGF-beta signaling (PubMed:11163210, PubMed:12717440). In addition, interaction with SMAD7 activates autocatalytic degradation, which is prevented by interaction with AIMP1 (PubMed:18448069). Also forms a stable complex with TGF-beta receptor-mediated phosphorylated SMAD1, SMAD2 and SMAD3, and targets SMAD1 and SMAD2 for ubiquitination and proteasome-mediated degradation (PubMed:11016919, PubMed:11158580, PubMed:11389444). SMAD2 may recruit substrates, such as SNON, for ubiquitin-dependent degradation (PubMed:11389444). Negatively regulates TGFB1-induced epithelial-mesenchymal transition and myofibroblast differentiation (PubMed:30696809).   
  
Interacts (via WW domains) with SMAD1 (PubMed:11158580). Interacts (via WW domains) with SMAD2 (via PY-motif) (PubMed:11158580, PubMed:11389444). Interacts (via WW domains) with SMAD3 (via PY-motif) (PubMed:11158580, PubMed:11389444). Interacts with SMAD6 (PubMed:11158580). Interacts with SMAD7 (via PY-motif) and TGFBR1; SMAD7 recruits SMURF2 to the TGF-beta receptor and regulates its degradation (PubMed:11163210, PubMed:11158580, PubMed:33673144, PubMed:16061177, PubMed:16641086). Does not interact with SMAD4; SMAD4 lacks a PY-motif (PubMed:11158580). Interacts with AIMP1 (PubMed:18448069). Interacts with SNON (PubMed:11389444). Interacts with STAMBP and RNF11 (PubMed:14562029, PubMed:14755250). May interact with NDFIP1 and NDFIP2; this interaction induces the E3 ubiquitin-protein ligase activity. Interacts with TTC3 (Probable).   
  
 **Gene Ontology Information:**

Molecular Function

- identical protein binding
- SMAD binding
- ubiquitin protein ligase activity
- ubiquitin-protein transferase activity

Location

- cytoplasm
- cytosol
- membrane raft
- nuclear speck
- nucleoplasm
- nucleus
- plasma membrane
- ubiquitin ligase complex

Biological process

- negative regulation of BMP signaling pathway
- negative regulation of transcription, DNA-templated
- negative regulation of transcription by RNA polymerase II
- negative regulation of transforming growth factor beta receptor signaling pathway
- positive regulation of canonical Wnt signaling pathway
- positive regulation of trophoblast cell migration
- proteasome-mediated ubiquitin-dependent protein catabolic process
- protein ubiquitination
- regulation of transforming growth factor beta receptor signaling pathway
- ubiquitin-dependent protein catabolic process
- ubiquitin-dependent SMAD protein catabolic process
- Wnt signaling pathway, planar cell polarity pathway

---

31

- **Protein name:** Ribosomal protein S6 kinase alpha-2
- **Organism:** Homo sapiens
- **Uniprot Accession Number:** Q15349
- **Protein sequence length:** 733 aa
- **1D identity (%):** 14.37
- **1D identity (%) [Gaps excluded]:** 23.15
- **1D identity - Alignment Gaps:** 372
- **1D aligned content (<aminoacid>:%):** {'M': 2.13, 'A': 4.26, 'F': 5.67, 'R': 9.93, 'L': 8.51, 'E': 7.8, 'I': 3.55, 'D': 4.26, 'H': 1.42, 'P': 10.64, 'Q': 5.67, 'K': 7.8, 'Y': 2.84, 'V': 7.09, 'T': 4.96, 'G': 5.67, 'S': 4.26, 'W': 0.71, 'C': 1.42, 'N': 1.42}
- **Common reported functions (%):** 0.0
- **Common reported locations (%):** 40.0
- **Common reported processes (%):** 0.0

- **AF ID:** Q15349
- **Chain:** A
- **Protein length:** 733 aa
- **Resolution:** N/A
- **b-phipsi:** 0.005207
- **w-rdist:** 0.474504
- **t-alpha:** 0.00292
- **Chemical similarity (Tanimoto Index) (%):** 99.02
- **1D identity (%) [PDB]:** 3.69
- **1D identity (%) [Gaps excluded][PDB]:** 65.48
- **1D identity - Alignment Gaps [PDB]:** 1405
- **1D aligned content [PDB] (<aminoacid>:%):** {'M': 1.82, 'D': 9.09, 'A': 7.27, 'P': 7.27, 'S': 5.45, 'T': 3.64, 'V': 9.09, 'R': 9.09, 'Q': 5.45, 'E': 5.45, 'I': 9.09, 'Y': 7.27, 'L': 10.91, 'K': 5.45, 'G': 1.82, 'H': 1.82}
- **2D identity (%) [PDB]:** 39.42
- **2D identity (%) [Gaps excluded][PDB]:** 90.57
- **2D identity - Alignment Gaps [PDB]:** 619
- **2D aligned content [PDB] (<2D-fold>:%):** {'.': 18.06, 'H': 43.06, 'T': 16.2, 'E': 19.21, 'G': 2.78, 'B': 0.69}
- **3D similarity (TM-Score) (%) [PDB]:** 23.73

- **Gene name:** RPS6KA2
- **Entrez ID:** 6196
- **RefSeq ID:** NM\_021135
- **Transcript sequence length:** 5832
- **5-UTR|CDS|3-UTR identity (%):** 48.5 | 40.52 | 18.14
- **5-UTR|CDS|3-UTR identity (%) [Gaps excluded]:** 72.07 | 76.64 | 74.53
- **5-UTR|CDS|3-UTR identity [Alignment Gaps]:** 87 | 1504 | 8653
- **5-UTR aligned content (<base>:%):** {'C': 37.21, 'T': 7.75, 'G': 48.84, 'A': 6.2}
- **CDS aligned content (<base>:%):** {'A': 24.9, 'G': 28.38, 'C': 27.69, 'T': 19.03}
- **3-UTR aligned content (<base>:%):** {'C': 24.24, 'G': 24.24, 'T': 30.27, 'A': 21.25}

**Uniprot Description:**  
  
 Serine/threonine-protein kinase that acts downstream of ERK (MAPK1/ERK2 and MAPK3/ERK1) signaling and mediates mitogenic and stress-induced activation of transcription factors, regulates translation, and mediates cellular proliferation, survival, and differentiation. May function as tumor suppressor in epithelial ovarian cancer cells.   
  
Forms a complex with either MAPK1/ERK2 or MAPK3/ERK1 in quiescent cells. Transiently dissociates following mitogenic stimulation (By similarity). Interacts with FBXO5; cooperate to induce the metaphase arrest of early blastomeres; increases and stabilizes interaction of FBXO5 with CDC20 (By similarity).   
  
 **Gene Ontology Information:**

Molecular Function

- ATP binding
- magnesium ion binding
- protein serine kinase activity
- protein serine/threonine kinase activity
- protein serine/threonine/tyrosine kinase activity
- ribosomal protein S6 kinase activity

Location

- cytoplasm
- cytosol
- meiotic spindle
- nucleoplasm
- nucleus
- synapse

Biological process

- brain renin-angiotensin system
- cardiac muscle cell apoptotic process
- cellular response to carbohydrate stimulus
- chemical synaptic transmission
- heart contraction
- heart development
- intracellular signal transduction
- negative regulation of cell cycle
- negative regulation of cell population proliferation
- negative regulation of meiotic nuclear division
- oocyte maturation
- peptidyl-serine phosphorylation
- positive regulation of apoptotic process
- positive regulation of gene expression
- regulation of protein processing
- signal transduction

---

32

- **Protein name:** RNA exonuclease 5
- **Organism:** Homo sapiens
- **Uniprot Accession Number:** Q96IC2
- **Protein sequence length:** 774 aa
- **1D identity (%):** 17.04
- **1D identity (%) [Gaps excluded]:** 26.05
- **1D identity - Alignment Gaps:** 341
- **1D aligned content (<aminoacid>:%):** {'P': 8.33, 'G': 10.71, 'R': 4.76, 'V': 6.55, 'K': 7.74, 'A': 4.76, 'D': 4.76, 'E': 5.95, 'L': 13.69, 'H': 4.17, 'Y': 2.98, 'S': 3.57, 'W': 0.6, 'F': 4.17, 'Q': 4.76, 'C': 4.17, 'N': 1.19, 'T': 4.76, 'I': 2.38}
- **Common reported functions (%):** 16.67
- **Common reported locations (%):** 10.0
- **Common reported processes (%):** 0.0

- **AF ID:** Q96IC2
- **Chain:** A
- **Protein length:** 774 aa
- **Resolution:** N/A
- **b-phipsi:** 0.004048
- **w-rdist:** 0.277346
- **t-alpha:** 0.037879
- **Chemical similarity (Tanimoto Index) (%):** 99.55
- **1D identity (%) [PDB]:** 2.77
- **1D identity (%) [Gaps excluded][PDB]:** 67.19
- **1D identity - Alignment Gaps [PDB]:** 1486
- **1D aligned content [PDB] (<aminoacid>:%):** {'G': 9.3, 'A': 6.98, 'V': 11.63, 'T': 9.3, 'H': 4.65, 'P': 11.63, 'D': 6.98, 'S': 9.3, 'I': 2.33, 'R': 6.98, 'C': 2.33, 'Q': 4.65, 'E': 4.65, 'L': 4.65, 'F': 2.33, 'K': 2.33}
- **2D identity (%) [PDB]:** 44.77
- **2D identity (%) [Gaps excluded][PDB]:** 90.81
- **2D identity - Alignment Gaps [PDB]:** 548
- **2D aligned content [PDB] (<2D-fold>:%):** {'.': 22.11, 'E': 20.45, 'T': 16.32, 'H': 41.12}
- **3D similarity (TM-Score) (%) [PDB]:** 25.01

- **Gene name:** REXO5
- **Entrez ID:** 532864
- **RefSeq ID:** N/A
- **Sequence length:** N/A
- **5-UTR|CDS|3-UTR identity (%):** N/A | N/A | N/A
- **5-UTR|CDS|3-UTR identity (%) [Gaps excluded]:** N/A | N/A | N/A
- **5-UTR|CDS|3-UTR identity [Alignment Gaps]:** N/A | N/A | N/A
- **5-UTR aligned content (<base>:%):** N/A
- **CDS aligned content (<base>:%):** N/A
- **3-UTR aligned content (<base>:%):** N/A

**Uniprot Description:**  
  
 N/A N/A   
  
 **Gene Ontology Information:**

Molecular Function

- exonuclease activity
- RNA binding

Location

- nucleolus
- nucleus

Biological process   
  
N/A

---

33

- **Protein name:** Dermatan-sulfate epimerase
- **Organism:** Homo sapiens
- **Uniprot Accession Number:** Q9UL01
- **Protein sequence length:** 958 aa
- **1D identity (%):** 15.08
- **1D identity (%) [Gaps excluded]:** 21.22
- **1D identity - Alignment Gaps:** 307
- **1D aligned content (<aminoacid>:%):** {'T': 4.38, 'G': 10.0, 'P': 11.88, 'I': 4.38, 'L': 11.88, 'Y': 1.88, 'E': 3.75, 'V': 5.0, 'H': 2.5, 'K': 5.0, 'D': 5.62, 'S': 3.75, 'W': 0.62, 'A': 6.88, 'M': 1.25, 'Q': 5.62, 'N': 5.0, 'R': 5.62, 'F': 4.38, 'C': 0.62}
- **Common reported functions (%):** 0.0
- **Common reported locations (%):** 0.0
- **Common reported processes (%):** 0.0

- **AF ID:** Q9UL01
- **Chain:** A
- **Protein length:** 958 aa
- **Resolution:** N/A
- **b-phipsi:** 0.008453
- **w-rdist:** 0.391405
- **t-alpha:** 0.002195
- **Chemical similarity (Tanimoto Index) (%):** 96.33
- **1D identity (%) [PDB]:** 3.57
- **1D identity (%) [Gaps excluded][PDB]:** 67.03
- **1D identity - Alignment Gaps [PDB]:** 1616
- **1D aligned content [PDB] (<aminoacid>:%):** {'M': 3.28, 'D': 4.92, 'H': 3.28, 'P': 6.56, 'S': 3.28, 'R': 8.2, 'A': 4.92, 'V': 4.92, 'E': 6.56, 'I': 11.48, 'Y': 8.2, 'L': 9.84, 'Q': 6.56, 'K': 4.92, 'T': 6.56, 'F': 4.92, 'C': 1.64}
- **2D identity (%) [PDB]:** 33.54
- **2D identity (%) [Gaps excluded][PDB]:** 92.08
- **2D identity - Alignment Gaps [PDB]:** 838
- **2D aligned content [PDB] (<2D-fold>:%):** {'.': 16.29, 'H': 45.7, 'T': 23.08, 'G': 1.36, 'E': 13.57}
- **3D similarity (TM-Score) (%) [PDB]:** 23.3

- **Gene name:** DSE
- **Entrez ID:** 29940
- **RefSeq ID:** N/A
- **Sequence length:** N/A
- **5-UTR|CDS|3-UTR identity (%):** N/A | N/A | N/A
- **5-UTR|CDS|3-UTR identity (%) [Gaps excluded]:** N/A | N/A | N/A
- **5-UTR|CDS|3-UTR identity [Alignment Gaps]:** N/A | N/A | N/A
- **5-UTR aligned content (<base>:%):** N/A
- **CDS aligned content (<base>:%):** N/A
- **3-UTR aligned content (<base>:%):** N/A

**Uniprot Description:**  
  
 Converts D-glucuronic acid to L-iduronic acid (IdoUA) residues. Plays an important role in the biosynthesis of the glycosaminoglycan/mucopolysaccharide dermatan sulfate. N/A   
  
 **Gene Ontology Information:**

Molecular Function

- chondroitin-glucuronate 5-epimerase activity
- metal ion binding

Location

- cytoplasmic vesicle membrane
- endoplasmic reticulum
- endoplasmic reticulum membrane
- Golgi apparatus
- Golgi membrane

Biological process

- chondroitin sulfate biosynthetic process
- chondroitin sulfate metabolic process
- dermatan sulfate biosynthetic process
- dermatan sulfate metabolic process
- heparan sulfate proteoglycan biosynthetic process

---

34

- **Protein name:** Nitric oxide-associated protein 1
- **Organism:** Homo sapiens
- **Uniprot Accession Number:** Q8NC60
- **Protein sequence length:** 698 aa
- **1D identity (%):** 14.03
- **1D identity (%) [Gaps excluded]:** 25.64
- **1D identity - Alignment Gaps:** 455
- **1D aligned content (<aminoacid>:%):** {'P': 10.64, 'R': 5.67, 'A': 4.26, 'L': 10.64, 'E': 7.8, 'H': 2.13, 'S': 2.13, 'Y': 4.96, 'V': 8.51, 'F': 4.96, 'G': 9.22, 'K': 6.38, 'Q': 7.8, 'C': 2.84, 'D': 3.55, 'N': 2.84, 'I': 3.55, 'W': 0.71, 'T': 0.71, 'M': 0.71}
- **Common reported functions (%):** 0.0
- **Common reported locations (%):** 0.0
- **Common reported processes (%):** 0.0

- **AF ID:** Q8NC60
- **Chain:** A
- **Protein length:** 698 aa
- **Resolution:** N/A
- **b-phipsi:** 0.003209
- **w-rdist:** 0.299198
- **t-alpha:** 0.038686
- **Chemical similarity (Tanimoto Index) (%):** 98.87
- **1D identity (%) [PDB]:** 2.43
- **1D identity (%) [Gaps excluded][PDB]:** 64.29
- **1D identity - Alignment Gaps [PDB]:** 1426
- **1D aligned content [PDB] (<aminoacid>:%):** {'L': 16.67, 'P': 5.56, 'I': 13.89, 'E': 5.56, 'A': 8.33, 'R': 5.56, 'D': 8.33, 'K': 11.11, 'Y': 2.78, 'G': 5.56, 'T': 5.56, 'V': 5.56, 'H': 2.78, 'S': 2.78}
- **2D identity (%) [PDB]:** 37.43
- **2D identity (%) [Gaps excluded][PDB]:** 91.07
- **2D identity - Alignment Gaps [PDB]:** 642
- **2D aligned content [PDB] (<2D-fold>:%):** {'.': 25.74, 'T': 18.14, 'H': 34.8, 'B': 0.25, 'E': 19.36, 'G': 1.72}
- **3D similarity (TM-Score) (%) [PDB]:** 21.43

- **Gene name:** NOA1
- **Entrez ID:** 539773
- **RefSeq ID:** NM\_032313
- **Transcript sequence length:** 2218
- **5-UTR|CDS|3-UTR identity (%):** 7.04 | 42.49 | 0.64
- **5-UTR|CDS|3-UTR identity (%) [Gaps excluded]:** 71.43 | 76.18 | 75.82
- **5-UTR|CDS|3-UTR identity [Alignment Gaps]:** 192 | 1355 | 10741
- **5-UTR aligned content (<base>:%):** {'C': 46.67, 'T': 26.67, 'G': 20.0, 'A': 6.67}
- **CDS aligned content (<base>:%):** {'A': 23.04, 'G': 29.42, 'C': 29.95, 'T': 17.59}
- **3-UTR aligned content (<base>:%):** {'A': 44.93, 'C': 15.94, 'G': 4.35, 'T': 34.78}

**Uniprot Description:**  
  
 Involved in regulation of mitochondrial protein translation and respiration. Plays a role in mitochondria-mediated cell death. May act as a scaffolding protein or stabilizer of respiratory chain supercomplexes. Binds GTP.   
  
Homodimer or multimer (By similarity). Interacts with mitochondrial complex I, DAP3, MRPL12 and MRPS27.   
  
 **Gene Ontology Information:**

Molecular Function

- GTP binding

Location

- mitochondrial inner membrane

Biological process

- apoptotic process
- mitochondrial ribosome assembly
- mitochondrion organization
- regulation of cellular respiration

---

35

- **Protein name:** Bardet-Biedl syndrome 2 protein
- **Organism:** Homo sapiens
- **Uniprot Accession Number:** Q9BXC9
- **Protein sequence length:** 721 aa
- **1D identity (%):** 14.52
- **1D identity (%) [Gaps excluded]:** 24.11
- **1D identity - Alignment Gaps:** 392
- **1D aligned content (<aminoacid>:%):** {'V': 8.39, 'H': 2.8, 'P': 5.59, 'Y': 2.1, 'D': 7.69, 'G': 11.89, 'N': 3.5, 'T': 4.9, 'A': 9.09, 'S': 2.8, 'L': 10.49, 'R': 8.39, 'F': 4.2, 'W': 0.7, 'E': 4.2, 'I': 3.5, 'K': 4.9, 'C': 1.4, 'Q': 2.8, 'M': 0.7}
- **Common reported functions (%):** 0.0
- **Common reported locations (%):** 10.0
- **Common reported processes (%):** 0.0

- **AF ID:** Q9BXC9
- **Chain:** A
- **Protein length:** 721 aa
- **Resolution:** N/A
- **b-phipsi:** 0.003836
- **w-rdist:** 0.301145
- **t-alpha:** 0.028528
- **Chemical similarity (Tanimoto Index) (%):** 99.55
- **1D identity (%) [PDB]:** 3.3
- **1D identity (%) [Gaps excluded][PDB]:** 62.82
- **1D identity - Alignment Gaps [PDB]:** 1405
- **1D aligned content [PDB] (<aminoacid>:%):** {'I': 8.16, 'P': 8.16, 'H': 2.04, 'R': 6.12, 'S': 10.2, 'V': 8.16, 'F': 2.04, 'Q': 8.16, 'L': 8.16, 'A': 8.16, 'G': 6.12, 'D': 4.08, 'K': 6.12, 'M': 4.08, 'E': 6.12, 'Y': 4.08}
- **2D identity (%) [PDB]:** 40.8
- **2D identity (%) [Gaps excluded][PDB]:** 90.52
- **2D identity - Alignment Gaps [PDB]:** 591
- **2D aligned content [PDB] (<2D-fold>:%):** {'.': 14.35, 'E': 30.98, 'T': 18.0, 'H': 36.45, 'B': 0.23}
- **3D similarity (TM-Score) (%) [PDB]:** 22.15

- **Gene name:** BBS2
- **Entrez ID:** 259187
- **RefSeq ID:** NM\_031885
- **Transcript sequence length:** 2704
- **5-UTR|CDS|3-UTR identity (%):** 47.81 | 44.76 | 2.13
- **5-UTR|CDS|3-UTR identity (%) [Gaps excluded]:** 74.15 | 74.07 | 76.82
- **5-UTR|CDS|3-UTR identity [Alignment Gaps]:** 81 | 1194 | 10595
- **5-UTR aligned content (<base>:%):** {'G': 39.45, 'C': 42.2, 'A': 5.5, 'T': 12.84}
- **CDS aligned content (<base>:%):** {'T': 24.2, 'G': 25.91, 'C': 24.28, 'A': 25.61}
- **3-UTR aligned content (<base>:%):** {'T': 41.81, 'G': 18.1, 'A': 31.47, 'C': 8.62}

**Uniprot Description:**  
  
 The BBSome complex is thought to function as a coat complex required for sorting of specific membrane proteins to the primary cilia. The BBSome complex is required for ciliogenesis but is dispensable for centriolar satellite function. This ciliogenic function is mediated in part by the Rab8 GDP/GTP exchange factor, which localizes to the basal body and contacts the BBSome. Rab8(GTP) enters the primary cilium and promotes extension of the ciliary membrane. Firstly the BBSome associates with the ciliary membrane and binds to RAB3IP/Rabin8, the guanosyl exchange factor (GEF) for Rab8 and then the Rab8-GTP localizes to the cilium and promotes docking and fusion of carrier vesicles to the base of the ciliary membrane. The BBSome complex, together with the LTZL1, controls SMO ciliary trafficking and contributes to the sonic hedgehog (SHH) pathway regulation. Required for proper BBSome complex assembly and its ciliary localization.   
  
Part of BBSome complex, that contains BBS1, BBS2, BBS4, BBS5, BBS7, BBS8/TTC8, BBS9 and BBIP10. Interacts (via C-terminus) with BBS7. Interacts (via coiled coil domain) with MKKS. Interacts with CCDC28B and ALDOB. Interacts with DLEC1 (PubMed:33144677).   
  
 **Gene Ontology Information:**

Molecular Function   
  
N/A

Location

- BBSome
- ciliary basal body
- ciliary membrane
- cytoplasm
- membrane
- motile cilium
- neuron projection

Biological process

- cilium assembly
- determination of left/right symmetry
- gastrulation
- intracellular transport
- Kupffer's vesicle development
- melanosome transport
- non-motile cilium assembly
- photoreceptor cell maintenance
- pigment granule aggregation in cell center
- regulation of eye photoreceptor cell development

---

36

- **Protein name:** Angiopoietin-related protein 6
- **Organism:** Homo sapiens
- **Uniprot Accession Number:** Q8NI99
- **Protein sequence length:** 470 aa
- **1D identity (%):** 8.42
- **1D identity (%) [Gaps excluded]:** 23.23
- **1D identity - Alignment Gaps:** 621
- **1D aligned content (<aminoacid>:%):** {'G': 13.41, 'K': 1.22, 'W': 1.22, 'A': 8.54, 'C': 3.66, 'R': 7.32, 'L': 18.29, 'V': 6.1, 'Q': 4.88, 'P': 9.76, 'S': 2.44, 'D': 8.54, 'M': 1.22, 'H': 3.66, 'F': 2.44, 'T': 2.44, 'E': 2.44, 'Y': 2.44}
- **Common reported functions (%):** 0.0
- **Common reported locations (%):** 0.0
- **Common reported processes (%):** 0.0

- **AF ID:** Q8NI99
- **Chain:** A
- **Protein length:** 470 aa
- **Resolution:** N/A
- **b-phipsi:** 0.009029
- **w-rdist:** 0.61147
- **t-alpha:** 0.0
- **Chemical similarity (Tanimoto Index) (%):** 96.99
- **1D identity (%) [PDB]:** 1.56
- **1D identity (%) [Gaps excluded][PDB]:** 68.97
- **1D identity - Alignment Gaps [PDB]:** 1252
- **1D aligned content [PDB] (<aminoacid>:%):** {'M': 5.0, 'R': 15.0, 'L': 10.0, 'A': 5.0, 'S': 15.0, 'T': 5.0, 'P': 15.0, 'V': 5.0, 'G': 10.0, 'E': 5.0, 'Y': 5.0, 'H': 5.0}
- **2D identity (%) [PDB]:** 28.29
- **2D identity (%) [Gaps excluded][PDB]:** 92.81
- **2D identity - Alignment Gaps [PDB]:** 698
- **2D aligned content [PDB] (<2D-fold>:%):** {'.': 11.97, 'H': 50.7, 'T': 20.07, 'E': 15.85, 'G': 1.06, 'B': 0.35}
- **3D similarity (TM-Score) (%) [PDB]:** 18.68

- **Gene name:** ANGPTL6
- **Entrez ID:** 83854
- **RefSeq ID:** N/A
- **Sequence length:** N/A
- **5-UTR|CDS|3-UTR identity (%):** N/A | N/A | N/A
- **5-UTR|CDS|3-UTR identity (%) [Gaps excluded]:** N/A | N/A | N/A
- **5-UTR|CDS|3-UTR identity [Alignment Gaps]:** N/A | N/A | N/A
- **5-UTR aligned content (<base>:%):** N/A
- **CDS aligned content (<base>:%):** N/A
- **3-UTR aligned content (<base>:%):** N/A

**Uniprot Description:**  
  
 May play a role in the wound healing process. May promote epidermal proliferation, remodeling and regeneration. May promote the chemotactic activity of endothelial cells and induce neovascularization. May counteract high-fat diet-induced obesity and related insulin resistance through increased energy expenditure. N/A   
  
 **Gene Ontology Information:**

Molecular Function

- signaling receptor binding

Location

- collagen-containing extracellular matrix
- extracellular exosome
- extracellular space
- secretory granule

Biological process

- angiogenesis
- cell differentiation

---

37

- **Protein name:** Calcium-activated chloride channel regulator 4
- **Organism:** Homo sapiens
- **Uniprot Accession Number:** Q14CN2
- **Protein sequence length:** 919 aa
- **1D identity (%):** 18.51
- **1D identity (%) [Gaps excluded]:** 25.03
- **1D identity - Alignment Gaps:** 266
- **1D aligned content (<aminoacid>:%):** {'G': 9.52, 'L': 7.94, 'Q': 2.65, 'I': 5.82, 'K': 6.35, 'N': 4.23, 'F': 5.29, 'E': 6.35, 'D': 3.7, 'Y': 2.12, 'R': 4.23, 'V': 6.88, 'P': 10.58, 'A': 7.41, 'T': 5.82, 'W': 1.59, 'S': 4.76, 'M': 1.06, 'C': 1.59, 'H': 2.12}
- **Common reported functions (%):** 0.0
- **Common reported locations (%):** 0.0
- **Common reported processes (%):** 0.0

- **AF ID:** Q14CN2
- **Chain:** A
- **Protein length:** 919 aa
- **Resolution:** N/A
- **b-phipsi:** 0.006249
- **w-rdist:** 0.220689
- **t-alpha:** 0.027007
- **Chemical similarity (Tanimoto Index) (%):** 96.33
- **1D identity (%) [PDB]:** 3.61
- **1D identity (%) [Gaps excluded][PDB]:** 60.61
- **1D identity - Alignment Gaps [PDB]:** 1561
- **1D aligned content [PDB] (<aminoacid>:%):** {'L': 8.33, 'C': 1.67, 'K': 3.33, 'I': 13.33, 'N': 1.67, 'V': 11.67, 'P': 11.67, 'H': 1.67, 'R': 6.67, 'F': 3.33, 'Q': 3.33, 'D': 5.0, 'T': 5.0, 'A': 5.0, 'G': 5.0, 'S': 6.67, 'Y': 3.33, 'E': 3.33}
- **2D identity (%) [PDB]:** 39.82
- **2D identity (%) [Gaps excluded][PDB]:** 89.65
- **2D identity - Alignment Gaps [PDB]:** 677
- **2D aligned content [PDB] (<2D-fold>:%):** {'.': 20.0, 'E': 28.04, 'T': 22.89, 'H': 27.42, 'G': 1.24, 'B': 0.41}
- **3D similarity (TM-Score) (%) [PDB]:** 22.95

- **Gene name:** CLCA4
- **Entrez ID:** N/A
- **RefSeq ID:** NM\_012128
- **Transcript sequence length:** 3211
- **5-UTR|CDS|3-UTR identity (%):** 9.82 | 45.31 | 2.59
- **5-UTR|CDS|3-UTR identity (%) [Gaps excluded]:** 70.97 | 73.67 | 76.15
- **5-UTR|CDS|3-UTR identity [Alignment Gaps]:** 193 | 1296 | 10494
- **5-UTR aligned content (<base>:%):** {'G': 36.36, 'A': 27.27, 'C': 31.82, 'T': 4.55}
- **CDS aligned content (<base>:%):** {'T': 23.67, 'G': 22.89, 'A': 30.03, 'C': 23.41}
- **3-UTR aligned content (<base>:%):** {'A': 43.77, 'T': 34.88, 'G': 13.88, 'C': 7.47}

**Uniprot Description:**  
  
 May be involved in mediating calcium-activated chloride conductance. N/A   
  
 **Gene Ontology Information:**

Molecular Function

- chloride channel activity
- intracellular calcium activated chloride channel activity
- ligand-gated ion channel activity
- metal ion binding
- metallopeptidase activity

Location

- apical plasma membrane
- extracellular region
- plasma membrane

Biological process

- chloride transport
- proteolysis

---

38

- **Protein name:** Adenylyl cyclase-associated protein 2
- **Organism:** Homo sapiens
- **Uniprot Accession Number:** P40123
- **Protein sequence length:** 477 aa
- **1D identity (%):** 9.31
- **1D identity (%) [Gaps excluded]:** 22.62
- **1D identity - Alignment Gaps:** 556
- **1D aligned content (<aminoacid>:%):** {'A': 6.82, 'S': 9.09, 'P': 13.64, 'E': 5.68, 'F': 3.41, 'K': 9.09, 'D': 7.95, 'M': 2.27, 'R': 2.27, 'G': 6.82, 'Q': 6.82, 'I': 10.23, 'H': 1.14, 'L': 4.55, 'V': 5.68, 'Y': 1.14, 'C': 1.14, 'N': 1.14, 'W': 1.14}
- **Common reported functions (%):** 0.0
- **Common reported locations (%):** 10.0
- **Common reported processes (%):** 0.0

- **AF ID:** P40123
- **Chain:** A
- **Protein length:** 477 aa
- **Resolution:** N/A
- **b-phipsi:** 0.007281
- **w-rdist:** 0.439679
- **t-alpha:** 0.001462
- **Chemical similarity (Tanimoto Index) (%):** 99.47
- **1D identity (%) [PDB]:** 2.35
- **1D identity (%) [Gaps excluded][PDB]:** 73.17
- **1D identity - Alignment Gaps [PDB]:** 1235
- **1D aligned content [PDB] (<aminoacid>:%):** {'A': 16.67, 'R': 13.33, 'L': 10.0, 'V': 6.67, 'F': 3.33, 'H': 10.0, 'D': 3.33, 'K': 6.67, 'E': 3.33, 'S': 6.67, 'G': 10.0, 'I': 3.33, 'Q': 3.33, 'P': 3.33}
- **2D identity (%) [PDB]:** 30.04
- **2D identity (%) [Gaps excluded][PDB]:** 91.69
- **2D identity - Alignment Gaps [PDB]:** 667
- **2D aligned content [PDB] (<2D-fold>:%):** {'H': 55.37, '.': 13.09, 'G': 2.35, 'T': 13.42, 'E': 15.77}
- **3D similarity (TM-Score) (%) [PDB]:** 16.7

- **Gene name:** CAP2
- **Entrez ID:** 10486
- **RefSeq ID:** NM\_006366
- **Transcript sequence length:** 2925
- **5-UTR|CDS|3-UTR identity (%):** 33.86 | 31.46 | 7.95
- **5-UTR|CDS|3-UTR identity (%) [Gaps excluded]:** 73.91 | 76.12 | 75.15
- **5-UTR|CDS|3-UTR identity [Alignment Gaps]:** 136 | 1706 | 9835
- **5-UTR aligned content (<base>:%):** {'G': 54.12, 'C': 27.06, 'T': 5.88, 'A': 12.94}
- **CDS aligned content (<base>:%):** {'A': 27.32, 'T': 22.08, 'G': 25.57, 'C': 25.03}
- **3-UTR aligned content (<base>:%):** {'C': 16.59, 'T': 36.61, 'G': 15.1, 'A': 31.69}

**Uniprot Description:**  
  
 May have a regulatory bifunctional role. N/A   
  
 **Gene Ontology Information:**

Molecular Function

- actin binding
- adenylate cyclase binding
- identical protein binding

Location

- cytoplasm
- plasma membrane
- postsynaptic density

Biological process

- actin filament organization
- activation of adenylate cyclase activity
- cAMP-mediated signaling
- cell morphogenesis
- establishment or maintenance of cell polarity
- presynaptic actin cytoskeleton organization
- signal transduction

---

39

- **Protein name:** Alpha-mannosidase 2x
- **Organism:** Homo sapiens
- **Uniprot Accession Number:** P49641
- **Protein sequence length:** 1150 aa
- **1D identity (%):** 15.22
- **1D identity (%) [Gaps excluded]:** 22.44
- **1D identity - Alignment Gaps:** 385
- **1D aligned content (<aminoacid>:%):** {'M': 1.65, 'G': 11.54, 'A': 4.4, 'Y': 4.95, 'L': 11.54, 'V': 6.04, 'P': 9.34, 'R': 8.24, 'I': 1.1, 'N': 3.85, 'E': 4.4, 'K': 3.85, 'F': 3.3, 'D': 7.14, 'T': 3.3, 'S': 4.4, 'W': 1.1, 'Q': 4.4, 'H': 3.85, 'C': 1.65}
- **Common reported functions (%):** 0.0
- **Common reported locations (%):** 0.0
- **Common reported processes (%):** 0.0

- **AF ID:** P49641
- **Chain:** A
- **Protein length:** 1150 aa
- **Resolution:** N/A
- **b-phipsi:** 0.000614
- **w-rdist:** 0.487342
- **t-alpha:** 0.064234
- **Chemical similarity (Tanimoto Index) (%):** 97.34
- **1D identity (%) [PDB]:** 2.33
- **1D identity (%) [Gaps excluded][PDB]:** 78.95
- **1D identity - Alignment Gaps [PDB]:** 1876
- **1D aligned content [PDB] (<aminoacid>:%):** {'T': 4.44, 'V': 8.89, 'R': 8.89, 'Q': 6.67, 'P': 6.67, 'E': 6.67, 'I': 11.11, 'D': 4.44, 'L': 15.56, 'S': 4.44, 'M': 2.22, 'F': 6.67, 'Y': 4.44, 'K': 2.22, 'G': 4.44, 'H': 2.22}
- **2D identity (%) [PDB]:** 35.63
- **2D identity (%) [Gaps excluded][PDB]:** 92.59
- **2D identity - Alignment Gaps [PDB]:** 884
- **2D aligned content [PDB] (<2D-fold>:%):** {'.': 19.73, 'H': 33.98, 'E': 19.73, 'T': 23.63, 'G': 2.93}
- **3D similarity (TM-Score) (%) [PDB]:** 26.91

- **Gene name:** MAN2A2
- **Entrez ID:** 4122
- **RefSeq ID:** N/A
- **Sequence length:** N/A
- **5-UTR|CDS|3-UTR identity (%):** N/A | N/A | N/A
- **5-UTR|CDS|3-UTR identity (%) [Gaps excluded]:** N/A | N/A | N/A
- **5-UTR|CDS|3-UTR identity [Alignment Gaps]:** N/A | N/A | N/A
- **5-UTR aligned content (<base>:%):** N/A
- **CDS aligned content (<base>:%):** N/A
- **3-UTR aligned content (<base>:%):** N/A

**Uniprot Description:**  
  
 Catalyzes the first committed step in the biosynthesis of complex N-glycans. It controls conversion of high mannose to complex N-glycans; the final hydrolytic step in the N-glycan maturation pathway.   
  
Homodimer; disulfide-linked (By similarity). Interacts with MGAT4D (By similarity).   
  
 **Gene Ontology Information:**

Molecular Function

- alpha-mannosidase activity
- carbohydrate binding
- hydrolase activity, hydrolyzing N-glycosyl compounds
- mannosyl-oligosaccharide 1,3-1,6-alpha-mannosidase activity
- metal ion binding

Location

- Golgi membrane

Biological process

- mannose metabolic process
- N-glycan processing
- protein deglycosylation
- protein glycosylation

---

40

- **Protein name:** Protein mono-ADP-ribosyltransferase PARP15
- **Organism:** Homo sapiens
- **Uniprot Accession Number:** Q460N3
- **Protein sequence length:** 678 aa
- **1D identity (%):** 13.26
- **1D identity (%) [Gaps excluded]:** 25.0
- **1D identity - Alignment Gaps:** 471
- **1D aligned content (<aminoacid>:%):** {'P': 8.27, 'L': 9.02, 'S': 4.51, 'E': 4.51, 'G': 8.27, 'V': 12.78, 'N': 3.01, 'R': 5.26, 'A': 6.02, 'C': 3.01, 'K': 4.51, 'M': 0.75, 'D': 3.76, 'Y': 4.51, 'Q': 4.51, 'T': 6.02, 'I': 3.01, 'F': 6.02, 'H': 2.26}
- **Common reported functions (%):** 0.0
- **Common reported locations (%):** 20.0
- **Common reported processes (%):** 0.0

- **AF ID:** Q460N3
- **Chain:** A
- **Protein length:** 678 aa
- **Resolution:** N/A
- **b-phipsi:** 0.005892
- **w-rdist:** 0.253722
- **t-alpha:** 0.028467
- **Chemical similarity (Tanimoto Index) (%):** 96.34
- **1D identity (%) [PDB]:** 4.32
- **1D identity (%) [Gaps excluded][PDB]:** 58.1
- **1D identity - Alignment Gaps [PDB]:** 1308
- **1D aligned content [PDB] (<aminoacid>:%):** {'M': 1.64, 'A': 4.92, 'C': 1.64, 'N': 6.56, 'V': 14.75, 'S': 8.2, 'Q': 4.92, 'T': 4.92, 'L': 9.84, 'K': 3.28, 'G': 8.2, 'I': 6.56, 'F': 4.92, 'D': 3.28, 'H': 1.64, 'P': 6.56, 'E': 4.92, 'Y': 1.64, 'R': 1.64}
- **2D identity (%) [PDB]:** 41.72
- **2D identity (%) [Gaps excluded][PDB]:** 88.87
- **2D identity - Alignment Gaps [PDB]:** 548
- **2D aligned content [PDB] (<2D-fold>:%):** {'.': 15.08, 'E': 27.15, 'T': 20.19, 'H': 36.89, 'G': 0.7}
- **3D similarity (TM-Score) (%) [PDB]:** 23.55

- **Gene name:** PARP15
- **Entrez ID:** 165631
- **RefSeq ID:** NM\_152615
- **Transcript sequence length:** 4377
- **5-UTR|CDS|3-UTR identity (%):** 19.92 | 28.38 | 16.21
- **5-UTR|CDS|3-UTR identity (%) [Gaps excluded]:** 65.28 | 75.02 | 75.58
- **5-UTR|CDS|3-UTR identity [Alignment Gaps]:** 164 | 1809 | 8906
- **5-UTR aligned content (<base>:%):** {'T': 21.28, 'A': 25.53, 'G': 27.66, 'C': 25.53}
- **CDS aligned content (<base>:%):** {'A': 29.66, 'T': 22.64, 'G': 23.61, 'C': 24.09}
- **3-UTR aligned content (<base>:%):** {'A': 25.46, 'T': 37.0, 'C': 18.5, 'G': 19.04}

**Uniprot Description:**  
  
 Mono-ADP-ribosyltransferase that mediates mono-ADP-ribosylation of target proteins (PubMed:16061477, PubMed:25043379, PubMed:25635049). Acts as a negative regulator of transcription (PubMed:16061477). N/A   
  
 **Gene Ontology Information:**

Molecular Function

- NAD+ ADP-ribosyltransferase activity
- NAD+ binding
- protein ADP-ribosylase activity
- nucleotidyltransferase activity
- transcription corepressor activity

Location

- cytoplasm
- nucleus

Biological process

- negative regulation of gene expression
- negative regulation of transcription by RNA polymerase II
- protein poly-ADP-ribosylation

---

41

- **Protein name:** Insulin-like growth factor 2 mRNA-binding protein 1
- **Organism:** Homo sapiens
- **Uniprot Accession Number:** Q9NZI8
- **Protein sequence length:** 577 aa
- **1D identity (%):** 14.3
- **1D identity (%) [Gaps excluded]:** 25.29
- **1D identity - Alignment Gaps:** 398
- **1D aligned content (<aminoacid>:%):** {'G': 9.16, 'S': 4.58, 'V': 6.87, 'P': 7.63, 'A': 6.11, 'K': 12.21, 'Y': 2.29, 'Q': 6.11, 'D': 4.58, 'T': 3.05, 'E': 7.63, 'H': 3.82, 'R': 6.11, 'L': 5.34, 'C': 2.29, 'N': 3.82, 'I': 6.87, 'F': 1.53}
- **Common reported functions (%):** 0.0
- **Common reported locations (%):** 40.0
- **Common reported processes (%):** 0.0

- **AF ID:** Q9NZI8
- **Chain:** A
- **Protein length:** 577 aa
- **Resolution:** N/A
- **b-phipsi:** 0.006044
- **w-rdist:** 0.264984
- **t-alpha:** 0.021898
- **Chemical similarity (Tanimoto Index) (%):** 99.4
- **1D identity (%) [PDB]:** 2.95
- **1D identity (%) [Gaps excluded][PDB]:** 64.52
- **1D identity - Alignment Gaps [PDB]:** 1293
- **1D aligned content [PDB] (<aminoacid>:%):** {'M': 2.5, 'L': 7.5, 'N': 5.0, 'V': 7.5, 'S': 5.0, 'A': 5.0, 'F': 5.0, 'K': 12.5, 'I': 7.5, 'E': 12.5, 'D': 5.0, 'R': 7.5, 'P': 2.5, 'T': 5.0, 'Q': 5.0, 'G': 2.5, 'H': 2.5}
- **2D identity (%) [PDB]:** 40.04
- **2D identity (%) [Gaps excluded][PDB]:** 90.99
- **2D identity - Alignment Gaps [PDB]:** 551
- **2D aligned content [PDB] (<2D-fold>:%):** {'.': 13.2, 'E': 25.63, 'T': 13.71, 'H': 45.43, 'G': 2.03}
- **3D similarity (TM-Score) (%) [PDB]:** 19.25

- **Gene name:** IGF2BP1
- **Entrez ID:** 395953
- **RefSeq ID:** NM\_006546
- **Transcript sequence length:** 8796
- **5-UTR|CDS|3-UTR identity (%):** 37.77 | 35.97 | 34.2
- **5-UTR|CDS|3-UTR identity (%) [Gaps excluded]:** 71.72 | 74.34 | 74.93
- **5-UTR|CDS|3-UTR identity [Alignment Gaps]:** 178 | 1534 | 6540
- **5-UTR aligned content (<base>:%):** {'C': 39.44, 'G': 43.66, 'A': 6.34, 'T': 10.56}
- **CDS aligned content (<base>:%):** {'A': 26.38, 'T': 17.31, 'G': 27.69, 'C': 28.62}
- **3-UTR aligned content (<base>:%):** {'C': 20.02, 'A': 25.27, 'T': 32.03, 'G': 22.67}

**Uniprot Description:**  
  
 RNA-binding factor that recruits target transcripts to cytoplasmic protein-RNA complexes (mRNPs). This transcript 'caging' into mRNPs allows mRNA transport and transient storage. It also modulates the rate and location at which target transcripts encounter the translational apparatus and shields them from endonuclease attacks or microRNA-mediated degradation. Plays a direct role in the transport and translation of transcripts required for axonal regeneration in adult sensory neurons (By similarity). Regulates localized beta-actin/ACTB mRNA translation, a crucial process for cell polarity, cell migration and neurite outgrowth. Co-transcriptionally associates with the ACTB mRNA in the nucleus. This binding involves a conserved 54-nucleotide element in the ACTB mRNA 3'-UTR, known as the 'zipcode'. The RNP thus formed is exported to the cytoplasm, binds to a motor protein and is transported along the cytoskeleton to the cell periphery. During transport, prevents ACTB mRNA from being translated into protein. When the RNP complex reaches its destination near the plasma membrane, IGF2BP1 is phosphorylated. This releases the mRNA, allowing ribosomal 40S and 60S subunits to assemble and initiate ACTB protein synthesis. Monomeric ACTB then assembles into the subcortical actin cytoskeleton (By similarity). During neuronal development, key regulator of neurite outgrowth, growth cone guidance and neuronal cell migration, presumably through the spatiotemporal fine tuning of protein synthesis, such as that of ACTB (By similarity). May regulate mRNA transport to activated synapses (By similarity). Binds to and stabilizes ABCB1/MDR-1 mRNA (By similarity). During interstinal wound repair, interacts with and stabilizes PTGS2 transcript. PTGS2 mRNA stabilization may be crucial for colonic mucosal wound healing (By similarity). Binds to the 3'-UTR of IGF2 mRNA by a mechanism of cooperative and sequential dimerization and regulates IGF2 mRNA subcellular localization and translation. Binds to MYC mRNA, in the coding region instability determinant (CRD) of the open reading frame (ORF), hence prevents MYC cleavage by endonucleases and possibly microRNA targeting to MYC-CRD. Binds to the 3'-UTR of CD44 mRNA and stabilizes it, hence promotes cell adhesion and invadopodia formation in cancer cells. Binds to the oncofetal H19 transcript and to the neuron-specific TAU mRNA and regulates their localizations. Binds to and stabilizes BTRC/FBW1A mRNA. Binds to the adenine-rich autoregulatory sequence (ARS) located in PABPC1 mRNA and represses its translation. PABPC1 mRNA-binding is stimulated by PABPC1 protein. Prevents BTRC/FBW1A mRNA degradation by disrupting microRNA-dependent interaction with AGO2. Promotes the directed movement of tumor-derived cells by fine-tuning intracellular signaling networks. Binds to MAPK4 3'-UTR and inhibits its translation. Interacts with PTEN transcript open reading frame (ORF) and prevents mRNA decay. This combined action on MAPK4 (down-regulation) and PTEN (up-regulation) antagonizes HSPB1 phosphorylation, consequently it prevents G-actin sequestration by phosphorylated HSPB1, allowing F-actin polymerization. Hence enhances the velocity of cell migration and stimulates directed cell migration by PTEN-modulated polarization. Interacts with Hepatitis C virus (HCV) 5'-UTR and 3'-UTR and specifically enhances translation at the HCV IRES, but not 5'-cap-dependent translation, possibly by recruiting eIF3. Interacts with HIV-1 GAG protein and blocks the formation of infectious HIV-1 particles. Reduces HIV-1 assembly by inhibiting viral RNA packaging, as well as assembly and processing of GAG protein on cellular membranes. During cellular stress, such as oxidative stress or heat shock, stabilizes target mRNAs that are recruited to stress granules, including CD44, IGF2, MAPK4, MYC, PTEN, RAPGEF2 and RPS6KA5 transcripts.   
  
Can form homodimers and heterodimers with IGF2BP1 and IGF2BP3. Component of the coding region determinant (CRD)-mediated complex, composed of DHX9, HNRNPU, IGF2BP1, SYNCRIP and YBX1. During HCV infection, identified in a HCV IRES-mediated translation complex, at least composed of EIF3C, IGF2BP1, RPS3 and HCV RNA-replicon. Interacts (via the KH domains) with HIV-1 GAG (via the second zinc finger motif of NC). Associates (via the RRM domains and KH domains) with HIV-1 particles. Identified in a mRNP complex, composed of at least DHX9, DDX3X, ELAVL1, HNRNPU, IGF2BP1, ILF3, PABPC1, PCBP2, PTBP2, STAU1, STAU2, SYNCRIP and YBX1. Identified in a IGF2BP1-dependent mRNP granule complex containing untranslated mRNAs. Interacts with DHX9, ELAVL2, HNRNPA2B1, HNRNPC, HNRNPH1, HNRNPU, IGF2BP2, ILF2, and YBX1. Interacts with FMR1. Component of a multisubunit autoregulatory RNP complex (ARC), at least composed of IGF2BP1, PABPC1 and CSDE1/UNR. Directly interacts with PABPC1. Component of a TAU mRNP complex, at least composed of IGF2BP1, ELAVL4 and G3BP. Interacts with ELAVL4 in an RNA-dependent manner. Associates with microtubules and polysomes. Interacts with AGO1 and AGO2.   
  
 **Gene Ontology Information:**

Molecular Function

- mRNA 3'-UTR binding
- mRNA binding
- N6-methyladenosine-containing RNA binding

Location

- CRD-mediated mRNA stability complex
- cytoplasm
- cytoplasmic stress granule
- cytosol
- filopodium
- growth cone
- lamellipodium
- nucleus
- P-body
- perinuclear region of cytoplasm

Biological process

- CRD-mediated mRNA stabilization
- mRNA transport
- negative regulation of translation
- nervous system development
- positive regulation of neuron projection development
- regulation of gene expression

---

42

- **Protein name:** Pre-mRNA-splicing factor ATP-dependent RNA helicase DHX15
- **Organism:** Homo sapiens
- **Uniprot Accession Number:** O43143
- **Protein sequence length:** 795 aa
- **1D identity (%):** N/A
- **1D identity (%) [Gaps excluded]:** N/A
- **1D identity - Alignment Gaps:** N/A
- **1D aligned content (<aminoacid>:%):** N/A
- **Common reported functions (%):** N/A
- **Common reported locations (%):** N/A
- **Common reported processes (%):** N/A

- **AF ID:** O43143
- **Chain:** A
- **Protein length:** 795 aa
- **Resolution:** N/A
- **b-phipsi:** 0.010402
- **w-rdist:** 0.363988
- **t-alpha:** 0.003663
- **Chemical similarity (Tanimoto Index) (%):** N/A
- **1D identity (%) [PDB]:** N/A
- **1D identity (%) [Gaps excluded][PDB]:** N/A
- **1D identity - Alignment Gaps [PDB]:** N/A
- **1D aligned content [PDB] (<aminoacid>:%):** N/A
- **2D identity (%) [PDB]:** N/A
- **2D identity (%) [Gaps excluded][PDB]:** N/A
- **2D identity - Alignment Gaps [PDB]:** N/A
- **2D aligned content [PDB] (<2D-fold>:%):** N/A
- **3D similarity (TM-Score) (%) [PDB]:** N/A

- **Gene name:** DHX15
- **Entrez ID:** 1665
- **RefSeq ID:** N/A
- **Sequence length:** N/A
- **5-UTR|CDS|3-UTR identity (%):** N/A | N/A | N/A
- **5-UTR|CDS|3-UTR identity (%) [Gaps excluded]:** N/A | N/A | N/A
- **5-UTR|CDS|3-UTR identity [Alignment Gaps]:** N/A | N/A | N/A
- **5-UTR aligned content (<base>:%):** N/A
- **CDS aligned content (<base>:%):** N/A
- **3-UTR aligned content (<base>:%):** N/A

**Uniprot Description:**  
  
 Pre-mRNA processing factor involved in disassembly of spliceosomes after the release of mature mRNA. In cooperation with TFIP11 seem to be involved in the transition of the U2, U5 and U6 snRNP-containing IL complex to the snRNP-free IS complex leading to efficient debranching and turnover of excised introns.   
  
Interacts with SSB/La (PubMed:12458796). Component of the U11/U12 snRNPs that are part of the U12-type spliceosome (PubMed:15146077). Identified in the Intron Large (IL) complex, a post-mRNA release spliceosomal complex containing the excised intron, U2, U5 and U6 snRNPs, and splicing factors; the association may be transient. Interacts with TFIP11; indicative for a recruitment to the IL complex (PubMed:19103666). Interacts with GPATCH2 (PubMed:19432882).   
  
 **Gene Ontology Information:**

Molecular Function

- ATP binding
- ATPase activity
- RNA-dependent ATPase activity
- double-stranded RNA binding
- helicase activity
- RNA binding
- RNA helicase activity

Location

- nuclear speck
- nucleolus
- nucleoplasm
- nucleus
- spliceosomal complex
- U12-type spliceosomal complex

Biological process

- antiviral innate immune response
- defense response to bacterium
- defense response to virus
- mRNA processing
- mRNA splicing, via spliceosome
- positive regulation of I-kappaB kinase/NF-kappaB signaling
- response to alkaloid
- response to toxic substance
- RNA splicing

---

43

- **Protein name:** PX domain-containing protein kinase-like protein
- **Organism:** Homo sapiens
- **Uniprot Accession Number:** Q7Z7A4
- **Protein sequence length:** 578 aa
- **1D identity (%):** 9.9
- **1D identity (%) [Gaps excluded]:** 21.32
- **1D identity - Alignment Gaps:** 525
- **1D aligned content (<aminoacid>:%):** {'F': 5.15, 'P': 10.31, 'K': 10.31, 'L': 12.37, 'V': 5.15, 'T': 5.15, 'A': 6.19, 'Q': 6.19, 'E': 2.06, 'N': 4.12, 'D': 3.09, 'I': 5.15, 'G': 6.19, 'M': 2.06, 'R': 6.19, 'C': 4.12, 'Y': 3.09, 'S': 3.09}
- **Common reported functions (%):** 0.0
- **Common reported locations (%):** 30.0
- **Common reported processes (%):** 0.0

- **AF ID:** Q7Z7A4
- **Chain:** A
- **Protein length:** 578 aa
- **Resolution:** N/A
- **b-phipsi:** 0.004152
- **w-rdist:** 0.581409
- **t-alpha:** 0.00365
- **Chemical similarity (Tanimoto Index) (%):** 97.4
- **1D identity (%) [PDB]:** 2.1
- **1D identity (%) [Gaps excluded][PDB]:** 76.32
- **1D identity - Alignment Gaps [PDB]:** 1342
- **1D aligned content [PDB] (<aminoacid>:%):** {'A': 3.45, 'T': 10.34, 'V': 6.9, 'R': 13.79, 'Q': 6.9, 'I': 17.24, 'L': 10.34, 'S': 6.9, 'M': 3.45, 'E': 3.45, 'F': 6.9, 'K': 6.9, 'P': 3.45}
- **2D identity (%) [PDB]:** 39.9
- **2D identity (%) [Gaps excluded][PDB]:** 92.29
- **2D identity - Alignment Gaps [PDB]:** 562
- **2D aligned content [PDB] (<2D-fold>:%):** {'.': 16.2, 'E': 17.47, 'T': 12.91, 'H': 53.42}
- **3D similarity (TM-Score) (%) [PDB]:** 21.13

- **Gene name:** PXK
- **Entrez ID:** 54899
- **RefSeq ID:** N/A
- **Sequence length:** N/A
- **5-UTR|CDS|3-UTR identity (%):** N/A | N/A | N/A
- **5-UTR|CDS|3-UTR identity (%) [Gaps excluded]:** N/A | N/A | N/A
- **5-UTR|CDS|3-UTR identity [Alignment Gaps]:** N/A | N/A | N/A
- **5-UTR aligned content (<base>:%):** N/A
- **CDS aligned content (<base>:%):** N/A
- **3-UTR aligned content (<base>:%):** N/A

**Uniprot Description:**  
  
 Binds to and modulates brain Na,K-ATPase subunits ATP1B1 and ATP1B3 and may thereby participate in the regulation of electrical excitability and synaptic transmission. May not display kinase activity. N/A   
  
 **Gene Ontology Information:**

Molecular Function

- actin binding
- phosphatidylinositol binding

Location

- centriolar satellite
- cytoplasm
- cytosol
- early endosome
- extrinsic component of endosome membrane
- late endosome
- nucleus
- plasma membrane
- protein-containing complex

Biological process

- early endosome to late endosome transport
- endosome to lysosome transport
- inflammatory response
- modulation of chemical synaptic transmission
- negative regulation of ATPase activity
- negative regulation of ion transport
- protein targeting to lysosome

---

44

- **Protein name:** Calcium-activated chloride channel regulator 1
- **Organism:** Homo sapiens
- **Uniprot Accession Number:** A8K7I4
- **Protein sequence length:** 914 aa
- **1D identity (%):** 14.87
- **1D identity (%) [Gaps excluded]:** 25.34
- **1D identity - Alignment Gaps:** 461
- **1D aligned content (<aminoacid>:%):** {'F': 4.22, 'K': 9.04, 'G': 8.43, 'N': 3.61, 'Y': 4.22, 'I': 8.43, 'V': 6.63, 'T': 7.83, 'D': 4.22, 'S': 2.41, 'L': 12.05, 'R': 4.82, 'E': 4.22, 'H': 1.81, 'P': 4.82, 'Q': 6.02, 'C': 2.41, 'W': 1.2, 'A': 3.61}
- **Common reported functions (%):** 0.0
- **Common reported locations (%):** 0.0
- **Common reported processes (%):** 0.0

- **AF ID:** A8K7I4
- **Chain:** A
- **Protein length:** 914 aa
- **Resolution:** N/A
- **b-phipsi:** 0.008677
- **w-rdist:** 0.28976
- **t-alpha:** 0.007299
- **Chemical similarity (Tanimoto Index) (%):** 94.35
- **1D identity (%) [PDB]:** 2.79
- **1D identity (%) [Gaps excluded][PDB]:** 70.15
- **1D identity - Alignment Gaps [PDB]:** 1620
- **1D aligned content [PDB] (<aminoacid>:%):** {'G': 8.51, 'A': 6.38, 'D': 4.26, 'V': 12.77, 'P': 6.38, 'M': 2.13, 'F': 2.13, 'H': 2.13, 'K': 6.38, 'N': 2.13, 'T': 10.64, 'S': 4.26, 'L': 12.77, 'Q': 6.38, 'I': 8.51, 'Y': 2.13, 'R': 2.13}
- **2D identity (%) [PDB]:** 41.08
- **2D identity (%) [Gaps excluded][PDB]:** 90.16
- **2D identity - Alignment Gaps [PDB]:** 656
- **2D aligned content [PDB] (<2D-fold>:%):** {'.': 22.02, 'E': 25.25, 'T': 25.86, 'H': 26.26, 'G': 0.61}
- **3D similarity (TM-Score) (%) [PDB]:** 23.42

- **Gene name:** CLCA1
- **Entrez ID:** 1179
- **RefSeq ID:** N/A
- **Sequence length:** N/A
- **5-UTR|CDS|3-UTR identity (%):** N/A | N/A | N/A
- **5-UTR|CDS|3-UTR identity (%) [Gaps excluded]:** N/A | N/A | N/A
- **5-UTR|CDS|3-UTR identity [Alignment Gaps]:** N/A | N/A | N/A
- **5-UTR aligned content (<base>:%):** N/A
- **CDS aligned content (<base>:%):** N/A
- **3-UTR aligned content (<base>:%):** N/A

**Uniprot Description:**  
  
 May be involved in mediating calcium-activated chloride conductance. May play critical roles in goblet cell metaplasia, mucus hypersecretion, cystic fibrosis and AHR. May be involved in the regulation of mucus production and/or secretion by goblet cells. Involved in the regulation of tissue inflammation in the innate immune response. May play a role as a tumor suppressor. Induces MUC5AC. N/A   
  
 **Gene Ontology Information:**

Molecular Function

- chloride channel activity
- intracellular calcium activated chloride channel activity
- metal ion binding
- metalloendopeptidase activity

Location

- extracellular space
- microvillus
- plasma membrane
- zymogen granule membrane

Biological process

- calcium ion transport
- cellular response to hypoxia
- ion transmembrane transport
- proteolysis

---

45

- **Protein name:** Piwi-like protein 1
- **Organism:** Homo sapiens
- **Uniprot Accession Number:** Q96J94
- **Protein sequence length:** 861 aa
- **1D identity (%):** 21.97
- **1D identity (%) [Gaps excluded]:** 28.15
- **1D identity - Alignment Gaps:** 212
- **1D aligned content (<aminoacid>:%):** {'M': 2.83, 'A': 2.83, 'G': 12.74, 'Y': 6.6, 'P': 8.96, 'F': 3.77, 'R': 8.96, 'L': 9.91, 'N': 3.77, 'D': 6.13, 'Q': 5.66, 'H': 1.89, 'T': 5.19, 'V': 6.6, 'I': 2.36, 'W': 1.42, 'S': 2.83, 'K': 2.83, 'E': 3.3, 'C': 1.42}
- **Common reported functions (%):** 16.67
- **Common reported locations (%):** 20.0
- **Common reported processes (%):** 0.0

- **AF ID:** Q96J94
- **Chain:** A
- **Protein length:** 861 aa
- **Resolution:** N/A
- **b-phipsi:** 0.006719
- **w-rdist:** 0.14168
- **t-alpha:** 0.041034
- **Chemical similarity (Tanimoto Index) (%):** 99.4
- **1D identity (%) [PDB]:** 4.99
- **1D identity (%) [Gaps excluded][PDB]:** 56.12
- **1D identity - Alignment Gaps [PDB]:** 1423
- **1D aligned content [PDB] (<aminoacid>:%):** {'M': 1.28, 'V': 7.69, 'R': 7.69, 'E': 1.28, 'L': 6.41, 'I': 5.13, 'Q': 5.13, 'F': 6.41, 'Y': 10.26, 'K': 3.85, 'S': 3.85, 'T': 7.69, 'P': 8.97, 'D': 7.69, 'G': 6.41, 'N': 1.28, 'A': 5.13, 'H': 2.56, 'C': 1.28}
- **2D identity (%) [PDB]:** 65.62
- **2D identity (%) [Gaps excluded][PDB]:** 90.49
- **2D identity - Alignment Gaps [PDB]:** 271
- **2D aligned content [PDB] (<2D-fold>:%):** {'.': 18.14, 'E': 31.78, 'T': 15.5, 'H': 32.56, 'G': 1.4, 'B': 0.62}
- **3D similarity (TM-Score) (%) [PDB]:** 75.53

- **Gene name:** PIWIL1
- **Entrez ID:** 9271
- **RefSeq ID:** N/A
- **Sequence length:** N/A
- **5-UTR|CDS|3-UTR identity (%):** N/A | N/A | N/A
- **5-UTR|CDS|3-UTR identity (%) [Gaps excluded]:** N/A | N/A | N/A
- **5-UTR|CDS|3-UTR identity [Alignment Gaps]:** N/A | N/A | N/A
- **5-UTR aligned content (<base>:%):** N/A
- **CDS aligned content (<base>:%):** N/A
- **3-UTR aligned content (<base>:%):** N/A

**Uniprot Description:**  
  
 Endoribonuclease that plays a central role in postnatal germ cells by repressing transposable elements and preventing their mobilization, which is essential for the germline integrity. Acts via the piRNA metabolic process, which mediates the repression of transposable elements during meiosis by forming complexes composed of piRNAs and Piwi proteins and governs the methylation and subsequent repression of transposons. Directly binds methylated piRNAs, a class of 24 to 30 nucleotide RNAs that are generated by a Dicer-independent mechanism and are primarily derived from transposons and other repeated sequence elements. Strongly prefers a uridine in the first position of their guide (g1U preference, also named 1U-bias). Not involved in the piRNA amplification loop, also named ping-pong amplification cycle. Acts as an endoribonuclease that cleaves transposon messenger RNAs. Besides their function in transposable elements repression, piRNAs are probably involved in other processes during meiosis such as translation regulation. Probable component of some RISC complex, which mediates RNA cleavage and translational silencing. Also plays a role in the formation of chromatoid bodies and is required for some miRNAs stability. Required to sequester RNF8 in the cytoplasm until late spermatogenesis; RNF8 being released upon ubiquitination and degradation of PIWIL1.   
  
Interacts (via Piwi domain) with DICER1, suggesting that it forms ribonucleoprotein RISC complexes; this interaction is regulated by HSP90AB1 activity. Interacts with MAEL, KIF17, PABPC1, PRMT5 and WDR77. Interacts (when methylated on arginine residues) with TDRD1, TDRKH/TDRD2, RNF17/TDRD4, TDRD6, TDRD7 and TDRD9. Interacts with CLOCK. Interacts with MOV10L1. Interacts with ANAPC10; interaction oly takes place following piRNA-binding. Interacts with RNF8; leading to sequester RNF8 in the cytoplasm. Interacts with TEX19 (By similarity).   
  
 **Gene Ontology Information:**

Molecular Function

- metal ion binding
- mRNA binding
- mRNA cap binding complex binding
- piRNA binding
- polysome binding
- protein kinase binding
- endoribonuclease activity
- single-stranded RNA binding

Location

- chromatoid body
- cytoplasm
- dense body
- nucleus
- P granule

Biological process

- meiotic cell cycle
- piRNA-mediated retrotransposon silencing by heterochromatin formation
- primary piRNA processing
- regulation of translation
- gene silencing by RNA
- sperm chromatin condensation
- spermatid development
- spermatogenesis

---

46

- **Protein name:** Probable RNA-binding protein 46
- **Organism:** Homo sapiens
- **Uniprot Accession Number:** Q8TBY0
- **Protein sequence length:** 533 aa
- **1D identity (%):** 10.21
- **1D identity (%) [Gaps excluded]:** 20.04
- **1D identity - Alignment Gaps:** 452
- **1D aligned content (<aminoacid>:%):** {'E': 8.51, 'G': 12.77, 'A': 6.38, 'L': 10.64, 'M': 2.13, 'R': 5.32, 'F': 3.19, 'P': 12.77, 'V': 5.32, 'Y': 3.19, 'K': 6.38, 'I': 5.32, 'C': 4.26, 'T': 3.19, 'Q': 4.26, 'W': 1.06, 'N': 3.19, 'S': 1.06, 'H': 1.06}
- **Common reported functions (%):** 0.0
- **Common reported locations (%):** 20.0
- **Common reported processes (%):** 0.0

- **AF ID:** Q8TBY0
- **Chain:** A
- **Protein length:** 533 aa
- **Resolution:** N/A
- **b-phipsi:** 0.005759
- **w-rdist:** 0.302584
- **t-alpha:** 0.017075
- **Chemical similarity (Tanimoto Index) (%):** 98.28
- **1D identity (%) [PDB]:** 1.79
- **1D identity (%) [Gaps excluded][PDB]:** 70.59
- **1D identity - Alignment Gaps [PDB]:** 1305
- **1D aligned content [PDB] (<aminoacid>:%):** {'G': 8.33, 'T': 4.17, 'V': 12.5, 'K': 16.67, 'I': 12.5, 'L': 4.17, 'N': 4.17, 'Y': 8.33, 'F': 4.17, 'P': 8.33, 'D': 12.5, 'E': 4.17}
- **2D identity (%) [PDB]:** 34.92
- **2D identity (%) [Gaps excluded][PDB]:** 88.66
- **2D identity - Alignment Gaps [PDB]:** 597
- **2D aligned content [PDB] (<2D-fold>:%):** {'.': 25.29, 'H': 35.47, 'E': 25.29, 'T': 13.95}
- **3D similarity (TM-Score) (%) [PDB]:** 19.45

- **Gene name:** RBM46
- **Entrez ID:** 166863
- **RefSeq ID:** N/A
- **Sequence length:** N/A
- **5-UTR|CDS|3-UTR identity (%):** N/A | N/A | N/A
- **5-UTR|CDS|3-UTR identity (%) [Gaps excluded]:** N/A | N/A | N/A
- **5-UTR|CDS|3-UTR identity [Alignment Gaps]:** N/A | N/A | N/A
- **5-UTR aligned content (<base>:%):** N/A
- **CDS aligned content (<base>:%):** N/A
- **3-UTR aligned content (<base>:%):** N/A

**Uniprot Description:**  
  
 N/A N/A   
  
 **Gene Ontology Information:**

Molecular Function

- mRNA binding

Location

- cytoplasm
- nucleus

Biological process

- cell cycle switching, mitotic to meiotic cell cycle
- female meiotic nuclear division
- male meiotic nuclear division
- mRNA stabilization
- oogenesis
- spermatid differentiation
- spermatogenesis
- trophectodermal cell differentiation

---

47

- **Protein name:** Testicular spindle-associated protein SHCBP1L
- **Organism:** Homo sapiens
- **Uniprot Accession Number:** Q9BZQ2
- **Protein sequence length:** 653 aa
- **1D identity (%):** 11.23
- **1D identity (%) [Gaps excluded]:** 21.26
- **1D identity - Alignment Gaps:** 466
- **1D aligned content (<aminoacid>:%):** {'S': 4.5, 'G': 8.11, 'T': 5.41, 'L': 8.11, 'V': 11.71, 'A': 9.91, 'R': 3.6, 'K': 5.41, 'P': 8.11, 'E': 3.6, 'D': 7.21, 'Q': 3.6, 'N': 2.7, 'I': 6.31, 'C': 3.6, 'F': 3.6, 'Y': 1.8, 'H': 2.7}
- **Common reported functions (%):** 0.0
- **Common reported locations (%):** 10.0
- **Common reported processes (%):** 0.0

- **AF ID:** Q9BZQ2
- **Chain:** A
- **Protein length:** 653 aa
- **Resolution:** N/A
- **b-phipsi:** 0.000967
- **w-rdist:** 0.665709
- **t-alpha:** 0.013869
- **Chemical similarity (Tanimoto Index) (%):** 95.99
- **1D identity (%) [PDB]:** 2.21
- **1D identity (%) [Gaps excluded][PDB]:** 72.73
- **1D identity - Alignment Gaps [PDB]:** 1405
- **1D aligned content [PDB] (<aminoacid>:%):** {'A': 9.38, 'P': 9.38, 'D': 3.12, 'R': 6.25, 'K': 9.38, 'S': 3.12, 'N': 3.12, 'L': 9.38, 'I': 9.38, 'Q': 6.25, 'E': 6.25, 'F': 3.12, 'G': 6.25, 'V': 6.25, 'M': 3.12, 'T': 6.25}
- **2D identity (%) [PDB]:** 38.61
- **2D identity (%) [Gaps excluded][PDB]:** 91.22
- **2D identity - Alignment Gaps [PDB]:** 605
- **2D aligned content [PDB] (<2D-fold>:%):** {'.': 16.79, 'T': 24.2, 'H': 36.3, 'B': 0.25, 'E': 20.0, 'G': 2.47}
- **3D similarity (TM-Score) (%) [PDB]:** 20.69

- **Gene name:** SHCBP1L
- **Entrez ID:** 81626
- **RefSeq ID:** NM\_030933
- **Transcript sequence length:** 2113
- **5-UTR|CDS|3-UTR identity (%):** 11.74 | 38.47 | 0.84
- **5-UTR|CDS|3-UTR identity (%) [Gaps excluded]:** 75.76 | 73.82 | 79.82
- **5-UTR|CDS|3-UTR identity [Alignment Gaps]:** 180 | 1460 | 10713
- **5-UTR aligned content (<base>:%):** {'A': 4.0, 'C': 40.0, 'G': 44.0, 'T': 12.0}
- **CDS aligned content (<base>:%):** {'A': 28.9, 'T': 22.76, 'G': 28.05, 'C': 20.29}
- **3-UTR aligned content (<base>:%):** {'A': 32.97, 'G': 10.99, 'C': 13.19, 'T': 42.86}

**Uniprot Description:**  
  
 Testis-specific spindle-associated factor that plays a role in spermatogenesis. In association with HSPA2, participates in the maintenance of spindle integrity during meiosis in male germ cells.   
  
Interacts with HSPA2; this interaction may promote the recruitment of HSPA2 to the spindle.   
  
 **Gene Ontology Information:**

Molecular Function   
  
N/A

Location

- cytoplasm
- meiotic spindle

Biological process

- cell differentiation
- male meiosis cytokinesis
- positive regulation of chromosome organization
- spermatogenesis

---

48

- **Protein name:** Gasdermin-C
- **Organism:** Homo sapiens
- **Uniprot Accession Number:** Q9BYG8
- **Protein sequence length:** 508 aa
- **1D identity (%):** 10.59
- **1D identity (%) [Gaps excluded]:** 24.57
- **1D identity - Alignment Gaps:** 543
- **1D aligned content (<aminoacid>:%):** {'S': 5.94, 'N': 2.97, 'L': 13.86, 'E': 5.94, 'G': 6.93, 'K': 3.96, 'D': 9.9, 'T': 3.96, 'V': 9.9, 'A': 2.97, 'R': 4.95, 'W': 1.98, 'Y': 4.95, 'I': 5.94, 'P': 7.92, 'F': 1.98, 'H': 0.99, 'Q': 2.97, 'C': 1.98}
- **Common reported functions (%):** 0.0
- **Common reported locations (%):** 20.0
- **Common reported processes (%):** 0.0

- **AF ID:** Q9BYG8
- **Chain:** A
- **Protein length:** 508 aa
- **Resolution:** N/A
- **b-phipsi:** 0.005063
- **w-rdist:** 0.737822
- **t-alpha:** 0.000731
- **Chemical similarity (Tanimoto Index) (%):** 99.55
- **1D identity (%) [PDB]:** 1.99
- **1D identity (%) [Gaps excluded][PDB]:** 66.67
- **1D identity - Alignment Gaps [PDB]:** 1270
- **1D aligned content [PDB] (<aminoacid>:%):** {'P': 7.69, 'E': 11.54, 'R': 11.54, 'V': 7.69, 'D': 3.85, 'T': 3.85, 'I': 11.54, 'G': 7.69, 'F': 3.85, 'K': 7.69, 'L': 7.69, 'A': 7.69, 'S': 3.85, 'M': 3.85}
- **2D identity (%) [PDB]:** 34.08
- **2D identity (%) [Gaps excluded][PDB]:** 91.78
- **2D identity - Alignment Gaps [PDB]:** 618
- **2D aligned content [PDB] (<2D-fold>:%):** {'.': 14.33, 'H': 51.04, 'T': 13.43, 'E': 21.19}
- **3D similarity (TM-Score) (%) [PDB]:** 20.03

- **Gene name:** GSDMC
- **Entrez ID:** 56169
- **RefSeq ID:** NM\_031415
- **Transcript sequence length:** 2450
- **5-UTR|CDS|3-UTR identity (%):** 25.72 | 33.61 | 1.77
- **5-UTR|CDS|3-UTR identity (%) [Gaps excluded]:** 78.54 | 75.39 | 79.42
- **5-UTR|CDS|3-UTR identity [Alignment Gaps]:** 421 | 1611 | 10642
- **5-UTR aligned content (<base>:%):** {'A': 11.8, 'C': 31.06, 'T': 14.29, 'G': 42.86}
- **CDS aligned content (<base>:%):** {'A': 27.84, 'G': 24.77, 'C': 24.46, 'T': 22.93}
- **3-UTR aligned content (<base>:%):** {'G': 27.98, 'C': 11.92, 'T': 29.02, 'A': 31.09}

**Uniprot Description:**  
  
 Gasdermin-C
This form constitutes the precursor of the pore-forming protein: upon cleavage, the released N-terminal moiety (Gasdermin-C, N-terminal) binds to membranes and forms pores, triggering pyroptosis.   
  
Gasdermin-C, N-terminal
Homooligomer; homooligomeric ring-shaped pore complex containing 27-28 subunits when inserted in the membrane.   
  
 **Gene Ontology Information:**

Molecular Function

- phosphatidylinositol-4,5-bisphosphate binding
- phosphatidylinositol-4-phosphate binding
- phosphatidylserine binding

Location

- cytoplasm
- cytosol
- plasma membrane

Biological process

- defense response to bacterium
- pyroptosis

---

49

- **Protein name:** Epididymis-specific alpha-mannosidase
- **Organism:** Homo sapiens
- **Uniprot Accession Number:** Q9Y2E5
- **Protein sequence length:** 1009 aa
- **1D identity (%):** 16.15
- **1D identity (%) [Gaps excluded]:** 21.47
- **1D identity - Alignment Gaps:** 264
- **1D aligned content (<aminoacid>:%):** {'L': 10.47, 'P': 11.05, 'R': 6.4, 'G': 15.7, 'I': 3.49, 'F': 2.91, 'V': 10.47, 'D': 6.4, 'Y': 4.07, 'E': 2.91, 'W': 0.58, 'A': 5.23, 'S': 2.33, 'T': 5.81, 'N': 2.33, 'H': 5.23, 'Q': 4.07, 'K': 0.58}
- **Common reported functions (%):** 0.0
- **Common reported locations (%):** 0.0
- **Common reported processes (%):** 0.0

- **AF ID:** Q9Y2E5
- **Chain:** A
- **Protein length:** 1009 aa
- **Resolution:** N/A
- **b-phipsi:** 0.001505
- **w-rdist:** 0.325157
- **t-alpha:** 0.059551
- **Chemical similarity (Tanimoto Index) (%):** 96.83
- **1D identity (%) [PDB]:** 3.11
- **1D identity (%) [Gaps excluded][PDB]:** 67.9
- **1D identity - Alignment Gaps [PDB]:** 1687
- **1D aligned content [PDB] (<aminoacid>:%):** {'R': 9.09, 'P': 5.45, 'S': 9.09, 'H': 7.27, 'V': 7.27, 'W': 1.82, 'F': 3.64, 'A': 12.73, 'E': 3.64, 'Q': 7.27, 'I': 5.45, 'L': 9.09, 'T': 3.64, 'Y': 3.64, 'C': 1.82, 'D': 1.82, 'G': 5.45, 'N': 1.82}
- **2D identity (%) [PDB]:** 38.27
- **2D identity (%) [Gaps excluded][PDB]:** 91.56
- **2D identity - Alignment Gaps [PDB]:** 759
- **2D aligned content [PDB] (<2D-fold>:%):** {'.': 22.29, 'E': 19.08, 'T': 23.29, 'H': 34.74, 'G': 0.6}
- **3D similarity (TM-Score) (%) [PDB]:** 25.81

- **Gene name:** MAN2B2
- **Entrez ID:** 23324
- **RefSeq ID:** NM\_015274
- **Transcript sequence length:** 5129
- **5-UTR|CDS|3-UTR identity (%):** 8.92 | 46.82 | 11.93
- **5-UTR|CDS|3-UTR identity (%) [Gaps excluded]:** 86.36 | 76.47 | 76.0
- **5-UTR|CDS|3-UTR identity [Alignment Gaps]:** 191 | 1372 | 9400
- **5-UTR aligned content (<base>:%):** {'A': 10.53, 'C': 47.37, 'T': 10.53, 'G': 31.58}
- **CDS aligned content (<base>:%):** {'A': 21.97, 'T': 17.62, 'G': 28.0, 'C': 32.41}
- **3-UTR aligned content (<base>:%):** {'G': 23.83, 'C': 22.93, 'T': 24.89, 'A': 28.35}

**Uniprot Description:**  
  
 N/A N/A   
  
 **Gene Ontology Information:**

Molecular Function

- alpha-mannosidase activity
- carbohydrate binding
- metal ion binding

Location

- extracellular exosome
- lysosomal lumen
- lysosome

Biological process

- mannose metabolic process
- oligosaccharide catabolic process

---

50

- **Protein name:** Spermatogenesis-associated protein 16
- **Organism:** Homo sapiens
- **Uniprot Accession Number:** Q9BXB7
- **Protein sequence length:** 569 aa
- **1D identity (%):** 10.75
- **1D identity (%) [Gaps excluded]:** 25.65
- **1D identity - Alignment Gaps:** 584
- **1D aligned content (<aminoacid>:%):** {'M': 2.78, 'A': 7.41, 'G': 9.26, 'S': 6.48, 'P': 6.48, 'I': 2.78, 'T': 5.56, 'K': 10.19, 'L': 6.48, 'N': 3.7, 'E': 6.48, 'F': 6.48, 'D': 4.63, 'R': 8.33, 'W': 2.78, 'Q': 3.7, 'H': 1.85, 'Y': 3.7, 'V': 0.93}
- **Common reported functions (%):** 0.0
- **Common reported locations (%):** 0.0
- **Common reported processes (%):** 0.0

- **AF ID:** Q9BXB7
- **Chain:** A
- **Protein length:** 569 aa
- **Resolution:** N/A
- **b-phipsi:** 0.012493
- **w-rdist:** 0.517759
- **t-alpha:** 0.00073
- **Chemical similarity (Tanimoto Index) (%):** 99.24
- **1D identity (%) [PDB]:** 3.19
- **1D identity (%) [Gaps excluded][PDB]:** 68.25
- **1D identity - Alignment Gaps [PDB]:** 1283
- **1D aligned content [PDB] (<aminoacid>:%):** {'I': 9.3, 'K': 11.63, 'L': 9.3, 'D': 4.65, 'N': 4.65, 'Q': 6.98, 'T': 9.3, 'M': 2.33, 'A': 6.98, 'R': 11.63, 'S': 4.65, 'E': 6.98, 'Y': 4.65, 'V': 2.33, 'P': 2.33, 'G': 2.33}
- **2D identity (%) [PDB]:** 34.11
- **2D identity (%) [Gaps excluded][PDB]:** 92.37
- **2D identity - Alignment Gaps [PDB]:** 649
- **2D aligned content [PDB] (<2D-fold>:%):** {'.': 19.66, 'T': 15.1, 'H': 58.97, 'E': 5.41, 'G': 0.85}
- **3D similarity (TM-Score) (%) [PDB]:** 18.28

- **Gene name:** SPATA16
- **Entrez ID:** 83893
- **RefSeq ID:** NM\_031955
- **Transcript sequence length:** 2074
- **5-UTR|CDS|3-UTR identity (%):** 36.18 | 35.22 | 1.4
- **5-UTR|CDS|3-UTR identity (%) [Gaps excluded]:** 75.42 | 74.33 | 76.38
- **5-UTR|CDS|3-UTR identity [Alignment Gaps]:** 128 | 1566 | 10638
- **5-UTR aligned content (<base>:%):** {'G': 53.93, 'C': 25.84, 'A': 11.24, 'T': 8.99}
- **CDS aligned content (<base>:%):** {'A': 33.11, 'T': 19.75, 'G': 24.24, 'C': 22.9}
- **3-UTR aligned content (<base>:%):** {'T': 27.63, 'C': 21.05, 'A': 38.82, 'G': 12.5}

**Uniprot Description:**  
  
 Essential for spermiogenesis and male fertility (By similarity). Involved in the formation of sperm acrosome during spermatogenesis. N/A   
  
 **Gene Ontology Information:**

Molecular Function   
  
N/A

Location

- acrosomal vesicle
- Golgi apparatus

Biological process

- spermatid development
- spermatogenesis

---

51

- **Protein name:** Phosphoribosylformylglycinamidine synthase
- **Organism:** Homo sapiens
- **Uniprot Accession Number:** O15067
- **Protein sequence length:** 1338 aa
- **1D identity (%):** 16.32
- **1D identity (%) [Gaps excluded]:** 28.57
- **1D identity - Alignment Gaps:** 599
- **1D aligned content (<aminoacid>:%):** {'V': 10.09, 'H': 2.19, 'P': 12.28, 'R': 5.7, 'E': 6.14, 'Q': 4.82, 'K': 3.51, 'G': 10.09, 'D': 6.14, 'S': 3.51, 'A': 8.77, 'F': 3.95, 'L': 9.65, 'W': 0.88, 'N': 2.63, 'I': 2.19, 'T': 4.39, 'C': 1.32, 'Y': 0.88, 'M': 0.88}
- **Common reported functions (%):** 0.0
- **Common reported locations (%):** 20.0
- **Common reported processes (%):** 0.0

- **AF ID:** O15067
- **Chain:** A
- **Protein length:** 1338 aa
- **Resolution:** N/A
- **b-phipsi:** 0.004066
- **w-rdist:** 0.274735
- **t-alpha:** 0.052555
- **Chemical similarity (Tanimoto Index) (%):** 99.47
- **1D identity (%) [PDB]:** 2.32
- **1D identity (%) [Gaps excluded][PDB]:** 75.38
- **1D identity - Alignment Gaps [PDB]:** 2048
- **1D aligned content [PDB] (<aminoacid>:%):** {'M': 2.04, 'L': 12.24, 'H': 4.08, 'E': 8.16, 'A': 8.16, 'S': 10.2, 'G': 14.29, 'Q': 4.08, 'I': 2.04, 'P': 10.2, 'V': 8.16, 'R': 8.16, 'Y': 2.04, 'T': 2.04, 'W': 2.04, 'F': 2.04}
- **2D identity (%) [PDB]:** 42.03
- **2D identity (%) [Gaps excluded][PDB]:** 90.45
- **2D identity - Alignment Gaps [PDB]:** 796
- **2D aligned content [PDB] (<2D-fold>:%):** {'.': 16.16, 'E': 28.16, 'H': 34.88, 'T': 19.36, 'G': 1.44}
- **3D similarity (TM-Score) (%) [PDB]:** 31.62

- **Gene name:** PFAS
- **Entrez ID:** 5198
- **RefSeq ID:** NM\_012393
- **Transcript sequence length:** 5369
- **5-UTR|CDS|3-UTR identity (%):** 24.22 | 42.38 | 7.71
- **5-UTR|CDS|3-UTR identity (%) [Gaps excluded]:** 71.26 | 76.35 | 76.94
- **5-UTR|CDS|3-UTR identity [Alignment Gaps]:** 169 | 1915 | 9851
- **5-UTR aligned content (<base>:%):** {'A': 16.13, 'C': 37.1, 'G': 30.65, 'T': 16.13}
- **CDS aligned content (<base>:%):** {'A': 20.83, 'T': 18.64, 'G': 30.76, 'C': 29.77}
- **3-UTR aligned content (<base>:%):** {'C': 26.07, 'T': 31.4, 'G': 21.21, 'A': 21.33}

**Uniprot Description:**  
  
 Phosphoribosylformylglycinamidine synthase involved in the purines biosynthetic pathway. Catalyzes the ATP-dependent conversion of formylglycinamide ribonucleotide (FGAR) and glutamine to yield formylglycinamidine ribonucleotide (FGAM) and glutamate. N/A   
  
 **Gene Ontology Information:**

Molecular Function

- ATP binding
- metal ion binding
- phosphoribosylformylglycinamidine synthase activity

Location

- cytoplasm
- cytosol
- extracellular exosome

Biological process

- 'de novo' AMP biosynthetic process
- 'de novo' IMP biosynthetic process
- 'de novo' XMP biosynthetic process
- anterior head development
- glutamine metabolic process
- GMP biosynthetic process
- purine nucleotide biosynthetic process
- purine ribonucleoside monophosphate biosynthetic process
- response to xenobiotic stimulus

---

52

- **Protein name:** Advillin
- **Organism:** Homo sapiens
- **Uniprot Accession Number:** O75366
- **Protein sequence length:** 819 aa
- **1D identity (%):** 14.67
- **1D identity (%) [Gaps excluded]:** 26.86
- **1D identity - Alignment Gaps:** 492
- **1D aligned content (<aminoacid>:%):** {'P': 8.81, 'L': 8.81, 'F': 5.03, 'A': 8.18, 'G': 9.43, 'I': 4.4, 'K': 9.43, 'N': 2.52, 'E': 6.92, 'D': 5.66, 'H': 2.52, 'C': 1.26, 'Y': 3.14, 'V': 5.66, 'Q': 4.4, 'T': 6.29, 'R': 3.77, 'W': 0.63, 'S': 1.89, 'M': 1.26}
- **Common reported functions (%):** 0.0
- **Common reported locations (%):** 10.0
- **Common reported processes (%):** 0.0

- **AF ID:** O75366
- **Chain:** A
- **Protein length:** 819 aa
- **Resolution:** N/A
- **b-phipsi:** 0.003412
- **w-rdist:** 0.671811
- **t-alpha:** 0.00365
- **Chemical similarity (Tanimoto Index) (%):** 98.28
- **1D identity (%) [PDB]:** 2.57
- **1D identity (%) [Gaps excluded][PDB]:** 67.21
- **1D identity - Alignment Gaps [PDB]:** 1537
- **1D aligned content [PDB] (<aminoacid>:%):** {'P': 4.88, 'L': 7.32, 'E': 7.32, 'V': 2.44, 'N': 7.32, 'I': 9.76, 'A': 12.2, 'G': 4.88, 'R': 4.88, 'K': 7.32, 'T': 4.88, 'Q': 7.32, 'S': 9.76, 'M': 4.88, 'D': 2.44, 'F': 2.44}
- **2D identity (%) [PDB]:** 45.18
- **2D identity (%) [Gaps excluded][PDB]:** 88.91
- **2D identity - Alignment Gaps [PDB]:** 541
- **2D aligned content [PDB] (<2D-fold>:%):** {'.': 15.9, 'E': 28.57, 'T': 22.94, 'H': 31.19, 'B': 0.2, 'G': 1.21}
- **3D similarity (TM-Score) (%) [PDB]:** 24.97

- **Gene name:** AVIL
- **Entrez ID:** 10677
- **RefSeq ID:** N/A
- **Sequence length:** N/A
- **5-UTR|CDS|3-UTR identity (%):** N/A | N/A | N/A
- **5-UTR|CDS|3-UTR identity (%) [Gaps excluded]:** N/A | N/A | N/A
- **5-UTR|CDS|3-UTR identity [Alignment Gaps]:** N/A | N/A | N/A
- **5-UTR aligned content (<base>:%):** N/A
- **CDS aligned content (<base>:%):** N/A
- **3-UTR aligned content (<base>:%):** N/A

**Uniprot Description:**  
  
 Ca(2+)-regulated actin-binding protein which plays an important role in actin bundling (PubMed:29058690). May have a unique function in the morphogenesis of neuronal cells which form ganglia. Required for SREC1-mediated regulation of neurite-like outgrowth. Plays a role in regenerative sensory axon outgrowth and remodeling processes after peripheral injury in neonates. Involved in the formation of long fine actin-containing filopodia-like structures in fibroblast. Plays a role in ciliogenesis. In podocytes, controls lamellipodia formation through the regulation of EGF-induced diacylglycerol generation by PLCE1 and ARP2/3 complex assembly (PubMed:29058690).   
  
Associates (via C-terminus) with F-actin (PubMed:15096633, PubMed:29058690). Interacts with SCARF1 (By similarity). Interacts with PLCE1 (PubMed:29058690). Interacts with ACTR2 and ACTR3; associates with the ARP2/3 complex (PubMed:29058690).   
  
 **Gene Ontology Information:**

Molecular Function

- actin binding
- actin filament binding
- Arp2/3 complex binding
- phosphatidylinositol-4,5-bisphosphate binding

Location

- actin cytoskeleton
- actin filament
- axon
- cell projection
- cytoplasm
- focal adhesion
- lamellipodium
- neuron projection

Biological process

- actin filament organization
- actin filament severing
- actin polymerization or depolymerization
- barbed-end actin filament capping
- cilium assembly
- nervous system development
- positive regulation of lamellipodium assembly
- positive regulation of neuron projection development
- regulation of diacylglycerol biosynthetic process

---

53

- **Protein name:** Cytosolic phospholipase A2 beta
- **Organism:** Homo sapiens
- **Uniprot Accession Number:** P0C869
- **Protein sequence length:** 781 aa
- **1D identity (%):** 17.2
- **1D identity (%) [Gaps excluded]:** 23.28
- **1D identity - Alignment Gaps:** 246
- **1D aligned content (<aminoacid>:%):** {'V': 8.64, 'L': 11.11, 'P': 10.49, 'D': 4.32, 'Y': 4.32, 'C': 3.7, 'R': 5.56, 'Q': 6.17, 'F': 5.56, 'I': 1.85, 'K': 4.94, 'G': 8.64, 'E': 3.7, 'S': 6.79, 'H': 3.7, 'A': 4.94, 'W': 1.23, 'T': 3.09, 'N': 1.23}
- **Common reported functions (%):** 0.0
- **Common reported locations (%):** 10.0
- **Common reported processes (%):** 0.0

- **AF ID:** P0C869
- **Chain:** A
- **Protein length:** 781 aa
- **Resolution:** N/A
- **b-phipsi:** 0.00163
- **w-rdist:** 0.390724
- **t-alpha:** 0.007353
- **Chemical similarity (Tanimoto Index) (%):** 97.92
- **1D identity (%) [PDB]:** 2.69
- **1D identity (%) [Gaps excluded][PDB]:** 72.41
- **1D identity - Alignment Gaps [PDB]:** 1505
- **1D aligned content [PDB] (<aminoacid>:%):** {'A': 2.38, 'S': 4.76, 'H': 4.76, 'Q': 16.67, 'T': 4.76, 'F': 4.76, 'L': 19.05, 'G': 4.76, 'V': 9.52, 'E': 4.76, 'K': 9.52, 'Y': 4.76, 'N': 4.76, 'P': 2.38, 'C': 2.38}
- **2D identity (%) [PDB]:** 46.86
- **2D identity (%) [Gaps excluded][PDB]:** 90.25
- **2D identity - Alignment Gaps [PDB]:** 513
- **2D aligned content [PDB] (<2D-fold>:%):** {'.': 17.8, 'E': 30.6, 'T': 22.6, 'H': 28.2, 'G': 0.8}
- **3D similarity (TM-Score) (%) [PDB]:** 25.17

- **Gene name:** PLA2G4B
- **Entrez ID:** 100137049; 8681
- **RefSeq ID:** NM\_005090
- **Transcript sequence length:** 3355
- **5-UTR|CDS|3-UTR identity (%):** 11.87 | 46.4 | 1.67
- **5-UTR|CDS|3-UTR identity (%) [Gaps excluded]:** 96.3 | 74.97 | 75.83
- **5-UTR|CDS|3-UTR identity [Alignment Gaps]:** 192 | 1345 | 10626
- **5-UTR aligned content (<base>:%):** {'A': 15.38, 'G': 53.85, 'C': 30.77}
- **CDS aligned content (<base>:%):** {'A': 21.37, 'T': 18.32, 'G': 28.88, 'C': 31.44}
- **3-UTR aligned content (<base>:%):** {'T': 24.73, 'G': 25.82, 'C': 30.77, 'A': 18.68}

**Uniprot Description:**  
  
 Calcium-dependent phospholipase A1 and A2 and lysophospholipase that may play a role in membrane phospholipid remodeling. N/A   
  
 **Gene Ontology Information:**

Molecular Function

- calcium ion binding
- calcium-dependent phospholipase A2 activity
- calcium-dependent phospholipid binding
- lysophospholipase activity
- phosphatidyl phospholipase B activity
- phospholipase A1 activity
- phospholipase A2 activity

Location

- cytosol
- early endosome membrane
- extracellular region
- mitochondrial inner membrane

Biological process

- arachidonic acid metabolic process
- calcium-mediated signaling
- glycerophospholipid catabolic process
- inflammatory response
- parturition
- phosphatidylcholine acyl-chain remodeling
- phosphatidylethanolamine acyl-chain remodeling
- phosphatidylglycerol acyl-chain remodeling

---

54

- **Protein name:** Ras GTPase-activating protein 4
- **Organism:** Homo sapiens
- **Uniprot Accession Number:** O43374
- **Protein sequence length:** 803 aa
- **1D identity (%):** 17.34
- **1D identity (%) [Gaps excluded]:** 24.31
- **1D identity - Alignment Gaps:** 278
- **1D aligned content (<aminoacid>:%):** {'R': 10.12, 'K': 4.76, 'I': 4.17, 'G': 8.93, 'P': 9.52, 'D': 3.57, 'T': 4.17, 'E': 8.93, 'V': 5.95, 'S': 6.55, 'W': 1.79, 'L': 11.9, 'A': 4.17, 'C': 2.98, 'Q': 5.95, 'H': 1.79, 'N': 2.38, 'Y': 1.79, 'F': 0.6}
- **Common reported functions (%):** 0.0
- **Common reported locations (%):** 10.0
- **Common reported processes (%):** 0.0

- **AF ID:** O43374
- **Chain:** A
- **Protein length:** 803 aa
- **Resolution:** N/A
- **b-phipsi:** 0.002792
- **w-rdist:** 0.214661
- **t-alpha:** 0.096878
- **Chemical similarity (Tanimoto Index) (%):** 98.95
- **1D identity (%) [PDB]:** 2.27
- **1D identity (%) [Gaps excluded][PDB]:** 66.67
- **1D identity - Alignment Gaps [PDB]:** 1535
- **1D aligned content [PDB] (<aminoacid>:%):** {'E': 5.56, 'P': 8.33, 'R': 2.78, 'H': 2.78, 'L': 16.67, 'T': 13.89, 'S': 5.56, 'G': 5.56, 'Q': 8.33, 'I': 5.56, 'V': 13.89, 'K': 8.33, 'A': 2.78}
- **2D identity (%) [PDB]:** 44.69
- **2D identity (%) [Gaps excluded][PDB]:** 88.57
- **2D identity - Alignment Gaps [PDB]:** 541
- **2D aligned content [PDB] (<2D-fold>:%):** {'.': 18.44, 'E': 29.71, 'T': 19.26, 'H': 30.74, 'G': 1.84}
- **3D similarity (TM-Score) (%) [PDB]:** 22.6

- **Gene name:** RASA4
- **Entrez ID:** 10156
- **RefSeq ID:** NM\_006989
- **Transcript sequence length:** 5604
- **5-UTR|CDS|3-UTR identity (%):** 23.04 | 45.78 | 17.53
- **5-UTR|CDS|3-UTR identity (%) [Gaps excluded]:** 79.37 | 74.54 | 73.21
- **5-UTR|CDS|3-UTR identity [Alignment Gaps]:** 154 | 1216 | 8558
- **5-UTR aligned content (<base>:%):** {'A': 6.0, 'G': 34.0, 'C': 50.0, 'T': 10.0}
- **CDS aligned content (<base>:%):** {'T': 16.29, 'G': 32.02, 'C': 30.84, 'A': 20.86}
- **3-UTR aligned content (<base>:%):** {'G': 25.85, 'C': 23.57, 'A': 23.21, 'T': 27.37}

**Uniprot Description:**  
  
 Ca(2+)-dependent Ras GTPase-activating protein, that switches off the Ras-MAPK pathway following a stimulus that elevates intracellular calcium. Functions as an adaptor for Cdc42 and Rac1 during FcR-mediated phagocytosis. N/A   
  
 **Gene Ontology Information:**

Molecular Function

- GTPase activator activity
- metal ion binding
- phospholipid binding

Location

- cytosol
- plasma membrane

Biological process

- cellular response to calcium ion
- intracellular signal transduction
- negative regulation of GTPase activity
- negative regulation of Ras protein signal transduction

---

55

- **Protein name:** BTB/POZ domain-containing protein 9
- **Organism:** Homo sapiens
- **Uniprot Accession Number:** Q96Q07
- **Protein sequence length:** 612 aa
- **1D identity (%):** 13.17
- **1D identity (%) [Gaps excluded]:** 24.04
- **1D identity - Alignment Gaps:** 429
- **1D aligned content (<aminoacid>:%):** {'S': 5.6, 'R': 6.4, 'F': 3.2, 'T': 7.2, 'E': 4.0, 'I': 8.0, 'H': 3.2, 'G': 8.0, 'V': 7.2, 'P': 8.8, 'Q': 5.6, 'Y': 4.8, 'L': 7.2, 'A': 4.8, 'K': 4.0, 'D': 4.0, 'N': 4.8, 'C': 2.4, 'W': 0.8}
- **Common reported functions (%):** 0.0
- **Common reported locations (%):** 10.0
- **Common reported processes (%):** 0.0

- **AF ID:** Q96Q07
- **Chain:** A
- **Protein length:** 612 aa
- **Resolution:** N/A
- **b-phipsi:** 0.005631
- **w-rdist:** 0.653134
- **t-alpha:** 0.00219
- **Chemical similarity (Tanimoto Index) (%):** 99.47
- **1D identity (%) [PDB]:** 2.64
- **1D identity (%) [Gaps excluded][PDB]:** 71.15
- **1D identity - Alignment Gaps [PDB]:** 1348
- **1D aligned content [PDB] (<aminoacid>:%):** {'M': 5.41, 'L': 8.11, 'N': 2.7, 'I': 8.11, 'D': 8.11, 'V': 13.51, 'A': 5.41, 'T': 5.41, 'Y': 2.7, 'Q': 8.11, 'E': 10.81, 'R': 5.41, 'S': 2.7, 'F': 2.7, 'K': 2.7, 'G': 2.7, 'H': 2.7, 'C': 2.7}
- **2D identity (%) [PDB]:** 33.49
- **2D identity (%) [Gaps excluded][PDB]:** 93.47
- **2D identity - Alignment Gaps [PDB]:** 686
- **2D aligned content [PDB] (<2D-fold>:%):** {'.': 19.55, 'T': 17.88, 'H': 41.62, 'E': 17.88, 'G': 2.51, 'B': 0.56}
- **3D similarity (TM-Score) (%) [PDB]:** 20.31

- **Gene name:** BTBD9
- **Entrez ID:** 505504
- **RefSeq ID:** N/A
- **Sequence length:** N/A
- **5-UTR|CDS|3-UTR identity (%):** N/A | N/A | N/A
- **5-UTR|CDS|3-UTR identity (%) [Gaps excluded]:** N/A | N/A | N/A
- **5-UTR|CDS|3-UTR identity [Alignment Gaps]:** N/A | N/A | N/A
- **5-UTR aligned content (<base>:%):** N/A
- **CDS aligned content (<base>:%):** N/A
- **3-UTR aligned content (<base>:%):** N/A

**Uniprot Description:**  
  
 N/A N/A   
  
 **Gene Ontology Information:**

Molecular Function   
  
N/A

Location

- cytoplasm

Biological process

- adult locomotory behavior
- circadian behavior
- modulation of chemical synaptic transmission

---

56

- **Protein name:** Polynucleotide 5'-hydroxyl-kinase NOL9
- **Organism:** Homo sapiens
- **Uniprot Accession Number:** Q5SY16
- **Protein sequence length:** 702 aa
- **1D identity (%):** 13.39
- **1D identity (%) [Gaps excluded]:** 21.23
- **1D identity - Alignment Gaps:** 353
- **1D aligned content (<aminoacid>:%):** {'D': 3.12, 'K': 7.81, 'T': 5.47, 'L': 12.5, 'R': 8.59, 'S': 7.03, 'W': 1.56, 'A': 3.91, 'G': 7.03, 'Q': 6.25, 'P': 10.16, 'F': 2.34, 'V': 4.69, 'I': 4.69, 'E': 3.91, 'H': 1.56, 'N': 3.91, 'Y': 3.91, 'C': 1.56}
- **Common reported functions (%):** 16.67
- **Common reported locations (%):** 10.0
- **Common reported processes (%):** 0.0

- **AF ID:** Q5SY16
- **Chain:** A
- **Protein length:** 702 aa
- **Resolution:** N/A
- **b-phipsi:** 0.003217
- **w-rdist:** 0.316173
- **t-alpha:** 0.047445
- **Chemical similarity (Tanimoto Index) (%):** 99.4
- **1D identity (%) [PDB]:** 2.56
- **1D identity (%) [Gaps excluded][PDB]:** 66.67
- **1D identity - Alignment Gaps [PDB]:** 1428
- **1D aligned content [PDB] (<aminoacid>:%):** {'G': 10.53, 'V': 7.89, 'P': 5.26, 'E': 10.53, 'Q': 7.89, 'L': 7.89, 'H': 5.26, 'Y': 7.89, 'A': 2.63, 'R': 5.26, 'I': 10.53, 'K': 5.26, 'D': 5.26, 'T': 2.63, 'C': 2.63, 'N': 2.63}
- **2D identity (%) [PDB]:** 39.76
- **2D identity (%) [Gaps excluded][PDB]:** 91.24
- **2D identity - Alignment Gaps [PDB]:** 606
- **2D aligned content [PDB] (<2D-fold>:%):** {'.': 24.59, 'H': 30.68, 'T': 18.03, 'E': 24.59, 'G': 2.11}
- **3D similarity (TM-Score) (%) [PDB]:** 21.9

- **Gene name:** NOL9
- **Entrez ID:** 523474
- **RefSeq ID:** N/A
- **Sequence length:** N/A
- **5-UTR|CDS|3-UTR identity (%):** N/A | N/A | N/A
- **5-UTR|CDS|3-UTR identity (%) [Gaps excluded]:** N/A | N/A | N/A
- **5-UTR|CDS|3-UTR identity [Alignment Gaps]:** N/A | N/A | N/A
- **5-UTR aligned content (<base>:%):** N/A
- **CDS aligned content (<base>:%):** N/A
- **3-UTR aligned content (<base>:%):** N/A

**Uniprot Description:**  
  
 Polynucleotide 5'-kinase involved in rRNA processing. The kinase activity is required for the processing of the 32S precursor into 5.8S and 28S rRNAs, more specifically for the generation of the major 5.8S(S) form. In vitro, has both DNA and RNA 5'-kinase activities. Probably binds RNA.   
  
Interacts with PELP1, WDR18 and SENP3.   
  
 **Gene Ontology Information:**

Molecular Function

- ATP binding
- ATP-dependent polydeoxyribonucleotide 5'-hydroxyl-kinase activity
- polynucleotide 5'-hydroxyl-kinase activity
- RNA binding

Location

- nucleolus
- nucleus

Biological process

- cleavage in ITS2 between 5.8S rRNA and LSU-rRNA of tricistronic rRNA transcript (SSU-rRNA, 5.8S rRNA, LSU-rRNA)
- maturation of 5.8S rRNA
- phosphorylation
- RNA processing

---

57

- **Protein name:** Probable ATP-dependent RNA helicase DDX53
- **Organism:** Homo sapiens
- **Uniprot Accession Number:** Q86TM3
- **Protein sequence length:** 631 aa
- **1D identity (%):** 13.09
- **1D identity (%) [Gaps excluded]:** 23.45
- **1D identity - Alignment Gaps:** 422
- **1D aligned content (<aminoacid>:%):** {'E': 1.6, 'K': 8.0, 'G': 9.6, 'R': 4.8, 'P': 8.0, 'H': 3.2, 'Q': 6.4, 'S': 5.6, 'C': 3.2, 'F': 4.8, 'N': 4.0, 'T': 3.2, 'A': 4.0, 'Y': 2.4, 'D': 8.8, 'I': 8.0, 'W': 1.6, 'L': 8.8, 'V': 4.0}
- **Common reported functions (%):** 16.67
- **Common reported locations (%):** 20.0
- **Common reported processes (%):** 0.0

- **AF ID:** Q86TM3
- **Chain:** A
- **Protein length:** 631 aa
- **Resolution:** N/A
- **b-phipsi:** 0.004385
- **w-rdist:** 0.288214
- **t-alpha:** 0.0474
- **Chemical similarity (Tanimoto Index) (%):** 99.17
- **1D identity (%) [PDB]:** 2.32
- **1D identity (%) [Gaps excluded][PDB]:** 64.71
- **1D identity - Alignment Gaps [PDB]:** 1369
- **1D aligned content [PDB] (<aminoacid>:%):** {'M': 3.03, 'K': 6.06, 'S': 3.03, 'Y': 6.06, 'N': 9.09, 'L': 6.06, 'P': 12.12, 'F': 3.03, 'I': 9.09, 'D': 6.06, 'V': 9.09, 'G': 9.09, 'Q': 6.06, 'R': 6.06, 'A': 3.03, 'T': 3.03}
- **2D identity (%) [PDB]:** 43.58
- **2D identity (%) [Gaps excluded][PDB]:** 89.42
- **2D identity - Alignment Gaps [PDB]:** 507
- **2D aligned content [PDB] (<2D-fold>:%):** {'.': 22.74, 'E': 17.63, 'H': 46.87, 'T': 12.76}
- **3D similarity (TM-Score) (%) [PDB]:** 22.84

- **Gene name:** DDX53
- **Entrez ID:** 168400
- **RefSeq ID:** NM\_182699
- **Transcript sequence length:** 3630
- **5-UTR|CDS|3-UTR identity (%):** 24.89 | 36.88 | 9.47
- **5-UTR|CDS|3-UTR identity (%) [Gaps excluded]:** 74.36 | 73.54 | 76.12
- **5-UTR|CDS|3-UTR identity [Alignment Gaps]:** 155 | 1518 | 9703
- **5-UTR aligned content (<base>:%):** {'A': 18.97, 'T': 18.97, 'C': 34.48, 'G': 27.59}
- **CDS aligned content (<base>:%):** {'A': 31.79, 'G': 26.27, 'T': 21.1, 'C': 20.84}
- **3-UTR aligned content (<base>:%):** {'A': 29.74, 'T': 38.8, 'C': 13.92, 'G': 17.54}

**Uniprot Description:**  
  
 N/A N/A   
  
 **Gene Ontology Information:**

Molecular Function

- ATP binding
- ATPase activity
- RNA binding
- RNA helicase activity

Location

- cytosol
- intracellular membrane-bounded organelle
- nucleolus
- nucleoplasm

Biological process   
  
N/A

---

58

- **Protein name:** Heat shock 70 kDa protein 1-like
- **Organism:** Homo sapiens
- **Uniprot Accession Number:** P34931
- **Protein sequence length:** 641 aa
- **1D identity (%):** 12.92
- **1D identity (%) [Gaps excluded]:** 20.17
- **1D identity - Alignment Gaps:** 328
- **1D aligned content (<aminoacid>:%):** {'A': 5.08, 'Y': 5.08, 'V': 7.63, 'G': 13.56, 'E': 8.47, 'F': 4.24, 'K': 9.32, 'T': 4.24, 'P': 5.93, 'I': 5.93, 'L': 9.32, 'N': 1.69, 'Q': 4.24, 'H': 0.85, 'R': 4.24, 'D': 6.78, 'C': 1.69, 'M': 0.85, 'S': 0.85}
- **Common reported functions (%):** 0.0
- **Common reported locations (%):** 30.0
- **Common reported processes (%):** 0.0

- **AF ID:** P34931
- **Chain:** A
- **Protein length:** 641 aa
- **Resolution:** N/A
- **b-phipsi:** 0.006093
- **w-rdist:** 0.612503
- **t-alpha:** 0.00219
- **Chemical similarity (Tanimoto Index) (%):** 98.8
- **1D identity (%) [PDB]:** 2.81
- **1D identity (%) [Gaps excluded][PDB]:** 71.43
- **1D identity - Alignment Gaps [PDB]:** 1369
- **1D aligned content [PDB] (<aminoacid>:%):** {'A': 12.5, 'T': 7.5, 'D': 7.5, 'Q': 5.0, 'E': 10.0, 'I': 7.5, 'S': 2.5, 'L': 10.0, 'K': 5.0, 'N': 2.5, 'Y': 2.5, 'F': 2.5, 'G': 10.0, 'V': 7.5, 'M': 2.5, 'R': 2.5, 'P': 2.5}
- **2D identity (%) [PDB]:** 45.33
- **2D identity (%) [Gaps excluded][PDB]:** 89.74
- **2D identity - Alignment Gaps [PDB]:** 487
- **2D aligned content [PDB] (<2D-fold>:%):** {'.': 15.02, 'E': 27.8, 'T': 13.45, 'H': 41.93, 'G': 1.79}
- **3D similarity (TM-Score) (%) [PDB]:** 18.37

- **Gene name:** HSPA1L
- **Entrez ID:** 540190
- **RefSeq ID:** N/A
- **Sequence length:** N/A
- **5-UTR|CDS|3-UTR identity (%):** N/A | N/A | N/A
- **5-UTR|CDS|3-UTR identity (%) [Gaps excluded]:** N/A | N/A | N/A
- **5-UTR|CDS|3-UTR identity [Alignment Gaps]:** N/A | N/A | N/A
- **5-UTR aligned content (<base>:%):** N/A
- **CDS aligned content (<base>:%):** N/A
- **3-UTR aligned content (<base>:%):** N/A

**Uniprot Description:**  
  
 Molecular chaperone implicated in a wide variety of cellular processes, including protection of the proteome from stress, folding and transport of newly synthesized polypeptides, activation of proteolysis of misfolded proteins and the formation and dissociation of protein complexes. Plays a pivotal role in the protein quality control system, ensuring the correct folding of proteins, the re-folding of misfolded proteins and controlling the targeting of proteins for subsequent degradation. This is achieved through cycles of ATP binding, ATP hydrolysis and ADP release, mediated by co-chaperones. The affinity for polypeptides is regulated by its nucleotide bound state. In the ATP-bound form, it has a low affinity for substrate proteins. However, upon hydrolysis of the ATP to ADP, it undergoes a conformational change that increases its affinity for substrate proteins. It goes through repeated cycles of ATP hydrolysis and nucleotide exchange, which permits cycles of substrate binding and release (PubMed:26865365). Positive regulator of PRKN translocation to damaged mitochondria (PubMed:24270810).   
  
Interacts with PRKN.   
  
 **Gene Ontology Information:**

Molecular Function

- ATP binding
- ATPase activity
- ATP-dependent protein folding chaperone
- heat shock protein binding
- protein folding chaperone
- ubiquitin protein ligase binding
- unfolded protein binding

Location

- cell body
- COP9 signalosome
- cytoplasm
- cytosol
- nucleus
- zona pellucida receptor complex

Biological process

- binding of sperm to zona pellucida
- chaperone cofactor-dependent protein refolding
- positive regulation of protein targeting to mitochondrion
- protein refolding

---

59

- **Protein name:** Threonine--tRNA ligase 1, cytoplasmic
- **Organism:** Homo sapiens
- **Uniprot Accession Number:** P26639
- **Protein sequence length:** 723 aa
- **1D identity (%):** 14.33
- **1D identity (%) [Gaps excluded]:** 22.37
- **1D identity - Alignment Gaps:** 346
- **1D aligned content (<aminoacid>:%):** {'S': 2.9, 'P': 7.25, 'G': 12.32, 'E': 7.25, 'K': 7.97, 'L': 6.52, 'N': 3.62, 'I': 5.07, 'V': 5.07, 'Q': 4.35, 'Y': 5.8, 'C': 3.62, 'A': 5.8, 'T': 4.35, 'H': 5.07, 'F': 4.35, 'D': 4.35, 'R': 4.35}
- **Common reported functions (%):** 0.0
- **Common reported locations (%):** 10.0
- **Common reported processes (%):** 0.0

- **AF ID:** P26639
- **Chain:** A
- **Protein length:** 723 aa
- **Resolution:** N/A
- **b-phipsi:** 0.005185
- **w-rdist:** 0.732811
- **t-alpha:** 0.00146
- **Chemical similarity (Tanimoto Index) (%):** 98.65
- **1D identity (%) [PDB]:** 3.21
- **1D identity (%) [Gaps excluded][PDB]:** 71.64
- **1D identity - Alignment Gaps [PDB]:** 1429
- **1D aligned content [PDB] (<aminoacid>:%):** {'F': 4.17, 'G': 10.42, 'K': 10.42, 'P': 6.25, 'N': 2.08, 'I': 10.42, 'Y': 2.08, 'T': 4.17, 'V': 10.42, 'L': 10.42, 'E': 8.33, 'R': 4.17, 'D': 2.08, 'S': 6.25, 'W': 2.08, 'Q': 4.17, 'A': 2.08}
- **2D identity (%) [PDB]:** 44.2
- **2D identity (%) [Gaps excluded][PDB]:** 88.65
- **2D identity - Alignment Gaps [PDB]:** 523
- **2D aligned content [PDB] (<2D-fold>:%):** {'.': 12.61, 'E': 30.65, 'T': 15.0, 'H': 41.09, 'G': 0.65}
- **3D similarity (TM-Score) (%) [PDB]:** 25.82

- **Gene name:** TARS1
- **Entrez ID:** 8626539
- **RefSeq ID:** NM\_152295
- **Transcript sequence length:** 2672
- **5-UTR|CDS|3-UTR identity (%):** 28.39 | 43.12 | 2.3
- **5-UTR|CDS|3-UTR identity (%) [Gaps excluded]:** 67.68 | 74.79 | 75.53
- **5-UTR|CDS|3-UTR identity [Alignment Gaps]:** 137 | 1302 | 10539
- **5-UTR aligned content (<base>:%):** {'G': 28.36, 'T': 22.39, 'A': 10.45, 'C': 38.81}
- **CDS aligned content (<base>:%):** {'A': 29.49, 'T': 22.4, 'G': 26.55, 'C': 21.57}
- **3-UTR aligned content (<base>:%):** {'G': 20.4, 'A': 35.2, 'T': 33.6, 'C': 10.8}

**Uniprot Description:**  
  
 Catalyzes the attachment of threonine to tRNA(Thr) in a two-step reaction: threonine is first activated by ATP to form Thr-AMP and then transferred to the acceptor end of tRNA(Thr) (PubMed:25824639, PubMed:31374204). Also edits incorrectly charged tRNA(Thr) via its editing domain, at the post-transfer stage (By similarity).   
  
Homodimer.   
  
 **Gene Ontology Information:**

Molecular Function

- ATP binding
- threonine-tRNA ligase activity

Location

- cytoplasm
- phagocytic vesicle

Biological process

- threonyl-tRNA aminoacylation

---

60

- **Protein name:** Ras GTPase-activating protein 4B
- **Organism:** Homo sapiens
- **Uniprot Accession Number:** C9J798
- **Protein sequence length:** 803 aa
- **1D identity (%):** 16.96
- **1D identity (%) [Gaps excluded]:** 23.67
- **1D identity - Alignment Gaps:** 274
- **1D aligned content (<aminoacid>:%):** {'R': 10.37, 'K': 4.88, 'I': 3.66, 'G': 8.54, 'P': 9.15, 'D': 3.66, 'T': 4.27, 'E': 9.15, 'V': 5.49, 'S': 6.71, 'W': 1.83, 'L': 12.2, 'A': 4.27, 'C': 3.05, 'Q': 6.1, 'H': 1.83, 'N': 2.44, 'Y': 1.83, 'F': 0.61}
- **Common reported functions (%):** 0.0
- **Common reported locations (%):** 10.0
- **Common reported processes (%):** 0.0

- **AF ID:** C9J798
- **Chain:** A
- **Protein length:** 803 aa
- **Resolution:** N/A
- **b-phipsi:** 0.002806
- **w-rdist:** 0.256322
- **t-alpha:** 0.087302
- **Chemical similarity (Tanimoto Index) (%):** 98.95
- **1D identity (%) [PDB]:** 2.27
- **1D identity (%) [Gaps excluded][PDB]:** 66.67
- **1D identity - Alignment Gaps [PDB]:** 1535
- **1D aligned content [PDB] (<aminoacid>:%):** {'E': 5.56, 'P': 8.33, 'R': 2.78, 'H': 2.78, 'L': 16.67, 'T': 13.89, 'S': 5.56, 'G': 5.56, 'Q': 8.33, 'I': 5.56, 'V': 13.89, 'K': 8.33, 'A': 2.78}
- **2D identity (%) [PDB]:** 45.4
- **2D identity (%) [Gaps excluded][PDB]:** 89.01
- **2D identity - Alignment Gaps [PDB]:** 533
- **2D aligned content [PDB] (<2D-fold>:%):** {'.': 18.02, 'E': 29.35, 'T': 18.83, 'H': 31.98, 'G': 1.82}
- **3D similarity (TM-Score) (%) [PDB]:** 22.8

- **Gene name:** RASA4B
- **Entrez ID:** N/A
- **RefSeq ID:** N/A
- **Sequence length:** N/A
- **5-UTR|CDS|3-UTR identity (%):** N/A | N/A | N/A
- **5-UTR|CDS|3-UTR identity (%) [Gaps excluded]:** N/A | N/A | N/A
- **5-UTR|CDS|3-UTR identity [Alignment Gaps]:** N/A | N/A | N/A
- **5-UTR aligned content (<base>:%):** N/A
- **CDS aligned content (<base>:%):** N/A
- **3-UTR aligned content (<base>:%):** N/A

**Uniprot Description:**  
  
 Ca(2+)-dependent Ras GTPase-activating protein, that may play a role in the Ras-MAPK pathway. N/A   
  
 **Gene Ontology Information:**

Molecular Function

- GTPase activator activity
- metal ion binding
- phospholipid binding

Location

- cytosol
- plasma membrane

Biological process

- cellular response to calcium ion
- intracellular signal transduction
- negative regulation of Ras protein signal transduction
- regulation of GTPase activity

---

61

- **Protein name:** Cytosolic phospholipase A2 epsilon
- **Organism:** Homo sapiens
- **Uniprot Accession Number:** Q3MJ16
- **Protein sequence length:** 868 aa
- **1D identity (%):** 16.67
- **1D identity (%) [Gaps excluded]:** 23.77
- **1D identity - Alignment Gaps:** 303
- **1D aligned content (<aminoacid>:%):** {'Q': 5.33, 'A': 2.37, 'P': 8.88, 'G': 6.51, 'T': 4.14, 'N': 2.37, 'F': 5.92, 'D': 2.96, 'R': 8.88, 'E': 4.14, 'V': 8.28, 'S': 6.51, 'W': 1.18, 'L': 10.65, 'I': 4.14, 'H': 2.37, 'Y': 4.73, 'C': 4.14, 'K': 6.51}
- **Common reported functions (%):** 0.0
- **Common reported locations (%):** 10.0
- **Common reported processes (%):** 0.0

- **AF ID:** Q3MJ16
- **Chain:** A
- **Protein length:** 868 aa
- **Resolution:** N/A
- **b-phipsi:** 0.00099
- **w-rdist:** 0.554243
- **t-alpha:** 0.041825
- **Chemical similarity (Tanimoto Index) (%):** 99.55
- **1D identity (%) [PDB]:** 1.98
- **1D identity (%) [Gaps excluded][PDB]:** 78.57
- **1D identity - Alignment Gaps [PDB]:** 1624
- **1D aligned content [PDB] (<aminoacid>:%):** {'E': 12.12, 'S': 6.06, 'R': 6.06, 'M': 3.03, 'K': 6.06, 'N': 6.06, 'A': 6.06, 'Y': 3.03, 'L': 9.09, 'P': 6.06, 'I': 9.09, 'Q': 6.06, 'G': 6.06, 'V': 9.09, 'D': 6.06}
- **2D identity (%) [PDB]:** 45.76
- **2D identity (%) [Gaps excluded][PDB]:** 89.93
- **2D identity - Alignment Gaps [PDB]:** 556
- **2D aligned content [PDB] (<2D-fold>:%):** {'.': 17.76, 'E': 31.27, 'T': 24.13, 'H': 25.48, 'G': 1.35}
- **3D similarity (TM-Score) (%) [PDB]:** 23.01

- **Gene name:** PLA2G4E
- **Entrez ID:** 123745
- **RefSeq ID:** N/A
- **Sequence length:** N/A
- **5-UTR|CDS|3-UTR identity (%):** N/A | N/A | N/A
- **5-UTR|CDS|3-UTR identity (%) [Gaps excluded]:** N/A | N/A | N/A
- **5-UTR|CDS|3-UTR identity [Alignment Gaps]:** N/A | N/A | N/A
- **5-UTR aligned content (<base>:%):** N/A
- **CDS aligned content (<base>:%):** N/A
- **3-UTR aligned content (<base>:%):** N/A

**Uniprot Description:**  
  
 Calcium-dependent N-acyltransferase involved in the biosynthesis of N-acyl ethanolamines (NAEs) in the brain (PubMed:29447909). Transfers the sn-1 fatty acyl chain of phosphatidylcholine (fatty acyl donor) to the amine group of phosphatidylethanolamine (fatty acyl acceptor) to generate N-acyl phosphatidylethanolamine (NAPE). Similarly can use plasmenylethanolamine as a fatty acyl acceptor to form N-acyl plasmenylethanolamine (N-Acyl-PlsEt). Both NAPE and N-Acyl-PlsEt can serve as precursors of bioactive NAEs like N-arachidonoyl phosphatidylethanolamine also called anandamide (PubMed:29447909, PubMed:30517655). Has weak phospholipase A2 and lysophospholipase activities (By similarity). Regulates intracellular membrane trafficking that requires modulation of membrane curvature as it occurs by enrichment in lysophospholipids. Promotes tubule formation involved in clathrin-independent endocytotic trafficking and cargo recycling (By similarity). N/A   
  
 **Gene Ontology Information:**

Molecular Function

- calcium ion binding
- calcium-dependent phospholipase A2 activity
- calcium-dependent phospholipid binding
- N-acyltransferase activity
- phosphatidylinositol-3,4,5-trisphosphate binding
- phosphatidylinositol-3,4-bisphosphate binding
- phosphatidylinositol-3,5-bisphosphate binding
- phosphatidylinositol-3-phosphate binding
- phosphatidylinositol-4,5-bisphosphate binding
- phosphatidylinositol-4-phosphate binding
- phosphatidylinositol-5-phosphate binding
- phospholipase A1 activity

Location

- cytosol
- early endosome membrane
- lysosomal membrane
- plasma membrane

Biological process

- glycerophospholipid catabolic process
- N-acylphosphatidylethanolamine metabolic process
- phosphatidylinositol acyl-chain remodeling
- positive regulation of endocytic recycling

---

62

- **Protein name:** Retinoic acid receptor RXR-gamma
- **Organism:** Homo sapiens
- **Uniprot Accession Number:** P48443
- **Protein sequence length:** 463 aa
- **1D identity (%):** 7.34
- **1D identity (%) [Gaps excluded]:** 17.3
- **1D identity - Alignment Gaps:** 534
- **1D aligned content (<aminoacid>:%):** {'G': 16.18, 'H': 1.47, 'S': 4.41, 'P': 10.29, 'R': 4.41, 'A': 11.76, 'I': 4.41, 'N': 1.47, 'D': 5.88, 'T': 4.41, 'C': 5.88, 'F': 2.94, 'K': 2.94, 'L': 10.29, 'Y': 4.41, 'Q': 2.94, 'E': 1.47, 'V': 2.94, 'M': 1.47}
- **Common reported functions (%):** 0.0
- **Common reported locations (%):** 0.0
- **Common reported processes (%):** 12.5

- **AF ID:** P48443
- **Chain:** A
- **Protein length:** 463 aa
- **Resolution:** N/A
- **b-phipsi:** 0.009543
- **w-rdist:** 0.530927
- **t-alpha:** 0.001462
- **Chemical similarity (Tanimoto Index) (%):** 98.57
- **1D identity (%) [PDB]:** 2.8
- **1D identity (%) [Gaps excluded][PDB]:** 67.31
- **1D identity - Alignment Gaps [PDB]:** 1199
- **1D aligned content [PDB] (<aminoacid>:%):** {'P': 8.57, 'A': 5.71, 'G': 8.57, 'T': 8.57, 'S': 8.57, 'M': 2.86, 'D': 5.71, 'H': 2.86, 'Y': 2.86, 'C': 2.86, 'R': 5.71, 'V': 2.86, 'Q': 8.57, 'E': 8.57, 'I': 5.71, 'L': 8.57, 'K': 2.86}
- **2D identity (%) [PDB]:** 27.43
- **2D identity (%) [Gaps excluded][PDB]:** 94.54
- **2D identity - Alignment Gaps [PDB]:** 717
- **2D aligned content [PDB] (<2D-fold>:%):** {'.': 14.44, 'E': 4.33, 'H': 67.51, 'T': 13.72}
- **3D similarity (TM-Score) (%) [PDB]:** 16.12

- **Gene name:** RXRG
- **Entrez ID:** N/A
- **RefSeq ID:** NM\_006917
- **Transcript sequence length:** 1966
- **5-UTR|CDS|3-UTR identity (%):** 47.6 | 31.46 | 2.01
- **5-UTR|CDS|3-UTR identity (%) [Gaps excluded]:** 73.71 | 76.54 | 76.04
- **5-UTR|CDS|3-UTR identity [Alignment Gaps]:** 96 | 1698 | 10588
- **5-UTR aligned content (<base>:%):** {'A': 16.28, 'C': 28.68, 'G': 43.41, 'T': 11.63}
- **CDS aligned content (<base>:%):** {'A': 25.47, 'G': 25.8, 'T': 19.63, 'C': 29.11}
- **3-UTR aligned content (<base>:%):** {'C': 26.03, 'A': 26.94, 'G': 17.35, 'T': 29.68}

**Uniprot Description:**  
  
 Receptor for retinoic acid. Retinoic acid receptors bind as heterodimers to their target response elements in response to their ligands, all-trans or 9-cis retinoic acid, and regulate gene expression in various biological processes. The RAR/RXR heterodimers bind to the retinoic acid response elements (RARE) composed of tandem 5'-AGGTCA-3' sites known as DR1-DR5. The high affinity ligand for RXRs is 9-cis retinoic acid (By similarity).   
  
Homodimer (By similarity). Heterodimer with a RAR molecule (PubMed:28167758). Binds DNA preferentially as a RAR/RXR heterodimer (PubMed:28167758). Interacts with RARA (PubMed:28167758).   
  
 **Gene Ontology Information:**

Molecular Function

- DNA-binding transcription factor activity
- nuclear receptor activity
- steroid hormone receptor activity
- retinoic acid-responsive element binding
- zinc ion binding

Location

- RNA polymerase II transcription regulator complex

Biological process

- anatomical structure development
- cell differentiation
- positive regulation of transcription, DNA-templated
- positive regulation of transcription by RNA polymerase II
- response to retinoic acid
- retinoic acid receptor signaling pathway

---

63

- **Protein name:** Zinc finger protein 514
- **Organism:** Homo sapiens
- **Uniprot Accession Number:** Q96K75
- **Protein sequence length:** 400 aa
- **1D identity (%):** 9.45
- **1D identity (%) [Gaps excluded]:** 22.83
- **1D identity - Alignment Gaps:** 521
- **1D aligned content (<aminoacid>:%):** {'D': 2.38, 'E': 4.76, 'S': 4.76, 'L': 7.14, 'P': 8.33, 'M': 2.38, 'G': 16.67, 'R': 5.95, 'I': 4.76, 'T': 5.95, 'W': 2.38, 'K': 10.71, 'Q': 3.57, 'A': 2.38, 'H': 7.14, 'V': 2.38, 'C': 2.38, 'N': 1.19, 'F': 2.38, 'Y': 2.38}
- **Common reported functions (%):** 0.0
- **Common reported locations (%):** 10.0
- **Common reported processes (%):** 0.0

- **AF ID:** Q96K75
- **Chain:** A
- **Protein length:** 400 aa
- **Resolution:** N/A
- **b-phipsi:** 0.018452
- **w-rdist:** 0.24056
- **t-alpha:** 0.005839
- **Chemical similarity (Tanimoto Index) (%):** 98.49
- **1D identity (%) [PDB]:** 2.98
- **1D identity (%) [Gaps excluded][PDB]:** 53.85
- **1D identity - Alignment Gaps [PDB]:** 1110
- **1D aligned content [PDB] (<aminoacid>:%):** {'R': 8.57, 'E': 2.86, 'L': 17.14, 'N': 2.86, 'F': 2.86, 'Q': 8.57, 'I': 8.57, 'S': 5.71, 'A': 5.71, 'M': 5.71, 'P': 2.86, 'K': 8.57, 'G': 5.71, 'D': 2.86, 'H': 2.86, 'T': 5.71, 'V': 2.86}
- **2D identity (%) [PDB]:** 28.78
- **2D identity (%) [Gaps excluded][PDB]:** 89.4
- **2D identity - Alignment Gaps [PDB]:** 636
- **2D aligned content [PDB] (<2D-fold>:%):** {'.': 27.78, 'G': 1.11, 'H': 53.7, 'T': 12.96, 'E': 4.44}
- **3D similarity (TM-Score) (%) [PDB]:** 15.37

- **Gene name:** ZNF514
- **Entrez ID:** 84874
- **RefSeq ID:** N/A
- **Sequence length:** N/A
- **5-UTR|CDS|3-UTR identity (%):** N/A | N/A | N/A
- **5-UTR|CDS|3-UTR identity (%) [Gaps excluded]:** N/A | N/A | N/A
- **5-UTR|CDS|3-UTR identity [Alignment Gaps]:** N/A | N/A | N/A
- **5-UTR aligned content (<base>:%):** N/A
- **CDS aligned content (<base>:%):** N/A
- **3-UTR aligned content (<base>:%):** N/A

**Uniprot Description:**  
  
 May be involved in transcriptional regulation. N/A   
  
 **Gene Ontology Information:**

Molecular Function

- DNA-binding transcription factor activity, RNA polymerase II-specific
- metal ion binding
- RNA polymerase II cis-regulatory region sequence-specific DNA binding

Location

- nucleus

Biological process

- regulation of transcription by RNA polymerase II

---

64

- **Protein name:** Protein maelstrom homolog
- **Organism:** Homo sapiens
- **Uniprot Accession Number:** Q96JY0
- **Protein sequence length:** 434 aa
- **1D identity (%):** 9.26
- **1D identity (%) [Gaps excluded]:** 21.01
- **1D identity - Alignment Gaps:** 501
- **1D aligned content (<aminoacid>:%):** {'P': 14.46, 'R': 7.23, 'V': 8.43, 'Q': 4.82, 'A': 6.02, 'D': 1.2, 'E': 4.82, 'K': 7.23, 'W': 1.2, 'G': 8.43, 'S': 4.82, 'L': 9.64, 'H': 1.2, 'F': 3.61, 'C': 3.61, 'I': 4.82, 'N': 3.61, 'T': 3.61, 'Y': 1.2}
- **Common reported functions (%):** 0.0
- **Common reported locations (%):** 20.0
- **Common reported processes (%):** 0.0

- **AF ID:** Q96JY0
- **Chain:** A
- **Protein length:** 434 aa
- **Resolution:** N/A
- **b-phipsi:** 0.012846
- **w-rdist:** 0.465018
- **t-alpha:** 0.00146
- **Chemical similarity (Tanimoto Index) (%):** 99.17
- **1D identity (%) [PDB]:** 2.03
- **1D identity (%) [Gaps excluded][PDB]:** 59.52
- **1D identity - Alignment Gaps [PDB]:** 1190
- **1D aligned content [PDB] (<aminoacid>:%):** {'M': 8.0, 'L': 12.0, 'A': 16.0, 'V': 16.0, 'I': 4.0, 'P': 12.0, 'Q': 4.0, 'D': 4.0, 'S': 12.0, 'T': 4.0, 'G': 4.0, 'F': 4.0}
- **2D identity (%) [PDB]:** 30.47
- **2D identity (%) [Gaps excluded][PDB]:** 91.22
- **2D identity - Alignment Gaps [PDB]:** 636
- **2D aligned content [PDB] (<2D-fold>:%):** {'T': 16.49, '.': 28.18, 'H': 46.39, 'G': 1.03, 'E': 7.56, 'B': 0.34}
- **3D similarity (TM-Score) (%) [PDB]:** 15.58

- **Gene name:** MAEL
- **Entrez ID:** 84944
- **RefSeq ID:** NM\_032858
- **Transcript sequence length:** 1737
- **5-UTR|CDS|3-UTR identity (%):** 22.73 | 29.48 | 2.18
- **5-UTR|CDS|3-UTR identity (%) [Gaps excluded]:** 75.76 | 76.15 | 79.0
- **5-UTR|CDS|3-UTR identity [Alignment Gaps]:** 154 | 1759 | 10582
- **5-UTR aligned content (<base>:%):** {'A': 8.0, 'C': 32.0, 'G': 54.0, 'T': 6.0}
- **CDS aligned content (<base>:%):** {'A': 28.84, 'T': 23.17, 'G': 23.52, 'C': 24.47}
- **3-UTR aligned content (<base>:%):** {'G': 17.3, 'T': 38.82, 'C': 10.13, 'A': 33.76}

**Uniprot Description:**  
  
 Plays a central role during spermatogenesis by repressing transposable elements and preventing their mobilization, which is essential for the germline integrity. Acts via the piRNA metabolic process, which mediates the repression of transposable elements during meiosis by forming complexes composed of piRNAs and Piwi proteins and governs the methylation and subsequent repression of transposons. Its association with piP-bodies suggests a participation in the secondary piRNAs metabolic process. Required for the localization of germ-cell factors to the meiotic nuage (By similarity).   
  
Interacts with SMARCB1, SIN3B and DDX4. Interacts with piRNA-associated proteins TDRD1, PIWIL1 and PIWIL2 (By similarity). Interacts with TEX19 (By similarity).   
  
 **Gene Ontology Information:**

Molecular Function

- sequence-specific DNA binding

Location

- autosome
- chromatin
- chromatoid body
- cytoplasm
- male germ cell nucleus
- nucleus
- P granule
- perinuclear region of cytoplasm
- piP-body
- XY body

Biological process

- cell morphogenesis
- ectopic germ cell programmed cell death
- genetic imprinting
- fertilization
- homologous chromosome pairing at meiosis
- intrinsic apoptotic signaling pathway in response to DNA damage
- male meiotic nuclear division
- negative regulation of apoptotic process
- negative regulation of developmental process
- negative regulation of transcription, DNA-templated
- negative regulation of reproductive process
- negative regulation of transcription by RNA polymerase II
- piRNA metabolic process
- regulation of gene silencing by miRNA
- regulation of organ growth
- gene silencing by RNA
- spermatogenesis

---

65

- **Protein name:** Calpain-7
- **Organism:** Homo sapiens
- **Uniprot Accession Number:** Q9Y6W3
- **Protein sequence length:** 813 aa
- **1D identity (%):** 16.0
- **1D identity (%) [Gaps excluded]:** 23.52
- **1D identity - Alignment Gaps:** 318
- **1D aligned content (<aminoacid>:%):** {'M': 1.89, 'A': 6.29, 'G': 8.18, 'Y': 4.4, 'Q': 5.03, 'H': 1.89, 'V': 8.18, 'K': 10.06, 'P': 10.69, 'E': 5.03, 'F': 5.66, 'D': 6.29, 'N': 2.52, 'L': 5.66, 'S': 1.89, 'I': 3.77, 'T': 6.29, 'R': 4.4, 'C': 1.26, 'W': 0.63}
- **Common reported functions (%):** 0.0
- **Common reported locations (%):** 20.0
- **Common reported processes (%):** 0.0

- **AF ID:** Q9Y6W3
- **Chain:** A
- **Protein length:** 813 aa
- **Resolution:** N/A
- **b-phipsi:** 0.000505
- **w-rdist:** 0.591192
- **t-alpha:** 0.083004
- **Chemical similarity (Tanimoto Index) (%):** 99.62
- **1D identity (%) [PDB]:** 3.37
- **1D identity (%) [Gaps excluded][PDB]:** 67.09
- **1D identity - Alignment Gaps [PDB]:** 1495
- **1D aligned content [PDB] (<aminoacid>:%):** {'M': 1.89, 'A': 5.66, 'T': 11.32, 'V': 20.75, 'Q': 5.66, 'K': 11.32, 'N': 5.66, 'S': 5.66, 'L': 7.55, 'C': 1.89, 'G': 5.66, 'I': 5.66, 'P': 3.77, 'F': 1.89, 'D': 1.89, 'Y': 1.89, 'R': 1.89}
- **2D identity (%) [PDB]:** 33.92
- **2D identity (%) [Gaps excluded][PDB]:** 90.67
- **2D identity - Alignment Gaps [PDB]:** 753
- **2D aligned content [PDB] (<2D-fold>:%):** {'.': 21.08, 'H': 32.84, 'T': 20.34, 'E': 24.26, 'G': 1.47}
- **3D similarity (TM-Score) (%) [PDB]:** 22.45

- **Gene name:** CAPN7
- **Entrez ID:** 23473
- **RefSeq ID:** NM\_014296
- **Transcript sequence length:** 4348
- **5-UTR|CDS|3-UTR identity (%):** 45.61 | 45.63 | 10.0
- **5-UTR|CDS|3-UTR identity (%) [Gaps excluded]:** 73.03 | 75.0 | 75.05
- **5-UTR|CDS|3-UTR identity [Alignment Gaps]:** 107 | 1246 | 9545
- **5-UTR aligned content (<base>:%):** {'G': 47.69, 'A': 9.23, 'C': 35.38, 'T': 7.69}
- **CDS aligned content (<base>:%):** {'A': 30.1, 'T': 23.42, 'G': 23.69, 'C': 22.8}
- **3-UTR aligned content (<base>:%):** {'T': 36.42, 'G': 17.08, 'A': 33.15, 'C': 13.35}

**Uniprot Description:**  
  
 Calcium-regulated non-lysosomal thiol-protease. N/A   
  
 **Gene Ontology Information:**

Molecular Function

- calcium-dependent cysteine-type endopeptidase activity
- cysteine-type endopeptidase activity
- endopeptidase activity
- MIT domain binding

Location

- centrosome
- cytosol
- extracellular exosome
- nucleus

Biological process

- positive regulation of epithelial cell migration
- proteolysis
- self proteolysis

---

66

- **Protein name:** TGF-beta receptor type-2
- **Organism:** Homo sapiens
- **Uniprot Accession Number:** P37173
- **Protein sequence length:** 567 aa
- **1D identity (%):** 11.86
- **1D identity (%) [Gaps excluded]:** 23.33
- **1D identity - Alignment Gaps:** 464
- **1D aligned content (<aminoacid>:%):** {'G': 8.04, 'R': 9.82, 'W': 0.89, 'H': 2.68, 'S': 3.57, 'P': 8.04, 'N': 2.68, 'D': 7.14, 'T': 7.14, 'F': 5.36, 'C': 6.25, 'Q': 4.46, 'V': 6.25, 'K': 3.57, 'Y': 1.79, 'E': 4.46, 'L': 12.5, 'I': 2.68, 'A': 2.68}
- **Common reported functions (%):** 0.0
- **Common reported locations (%):** 10.0
- **Common reported processes (%):** 0.0

- **AF ID:** P37173
- **Chain:** A
- **Protein length:** 567 aa
- **Resolution:** N/A
- **b-phipsi:** 0.000811
- **w-rdist:** 0.557405
- **t-alpha:** 0.069477
- **Chemical similarity (Tanimoto Index) (%):** 96.69
- **1D identity (%) [PDB]:** 2.13
- **1D identity (%) [Gaps excluded][PDB]:** 64.44
- **1D identity - Alignment Gaps [PDB]:** 1317
- **1D aligned content [PDB] (<aminoacid>:%):** {'D': 10.34, 'N': 6.9, 'Q': 6.9, 'S': 13.79, 'M': 3.45, 'I': 6.9, 'A': 3.45, 'P': 6.9, 'R': 3.45, 'E': 6.9, 'L': 6.9, 'K': 10.34, 'Y': 3.45, 'F': 3.45, 'G': 3.45, 'V': 3.45}
- **2D identity (%) [PDB]:** 41.68
- **2D identity (%) [Gaps excluded][PDB]:** 90.11
- **2D identity - Alignment Gaps [PDB]:** 517
- **2D aligned content [PDB] (<2D-fold>:%):** {'.': 23.94, 'E': 20.45, 'T': 22.44, 'H': 32.17, 'G': 0.75, 'B': 0.25}
- **3D similarity (TM-Score) (%) [PDB]:** 22.75

- **Gene name:** TGFBR2
- **Entrez ID:** 7048
- **RefSeq ID:** NM\_003242
- **Transcript sequence length:** 4530
- **5-UTR|CDS|3-UTR identity (%):** 44.83 | 35.81 | 13.65
- **5-UTR|CDS|3-UTR identity (%) [Gaps excluded]:** 80.79 | 75.51 | 75.78
- **5-UTR|CDS|3-UTR identity [Alignment Gaps]:** 142 | 1562 | 9286
- **5-UTR aligned content (<base>:%):** {'A': 6.29, 'C': 37.76, 'T': 9.79, 'G': 46.15}
- **CDS aligned content (<base>:%):** {'A': 26.22, 'T': 19.27, 'G': 26.32, 'C': 28.2}
- **3-UTR aligned content (<base>:%):** {'C': 18.82, 'T': 33.51, 'G': 18.5, 'A': 29.17}

**Uniprot Description:**  
  
 Transmembrane serine/threonine kinase forming with the TGF-beta type I serine/threonine kinase receptor, TGFBR1, the non-promiscuous receptor for the TGF-beta cytokines TGFB1, TGFB2 and TGFB3. Transduces the TGFB1, TGFB2 and TGFB3 signal from the cell surface to the cytoplasm and thus regulates a plethora of physiological and pathological processes including cell cycle arrest in epithelial and hematopoietic cells, control of mesenchymal cell proliferation and differentiation, wound healing, extracellular matrix production, immunosuppression and carcinogenesis. The formation of the receptor complex composed of 2 TGFBR1 and 2 TGFBR2 molecules symmetrically bound to the cytokine dimer results in the phosphorylation and activation of TGFBR1 by the constitutively active TGFBR2. Activated TGFBR1 phosphorylates SMAD2 which dissociates from the receptor and interacts with SMAD4. The SMAD2-SMAD4 complex is subsequently translocated to the nucleus where it modulates the transcription of the TGF-beta-regulated genes. This constitutes the canonical SMAD-dependent TGF-beta signaling cascade. Also involved in non-canonical, SMAD-independent TGF-beta signaling pathways.   
  
Homodimer. Heterohexamer; TGFB1, TGFB2 and TGFB3 homodimeric ligands assemble a functional receptor composed of two TGFBR1 and TGFBR2 heterodimers to form a ligand-receptor heterohexamer. The respective affinity of TGFRB1 and TGFRB2 for the ligands may modulate the kinetics of assembly of the receptor and may explain the different biological activities of TGFB1, TGFB2 and TGFB3. Interacts with DAXX. Interacts with DYNLT4. Interacts with ZFYVE9; ZFYVE9 recruits SMAD2 and SMAD3 to the TGF-beta receptor. Interacts with and is activated by SCUBE3; this interaction does not affect TGFB1-binding to TGFBR2. Interacts with VPS39; this interaction is independent of the receptor kinase activity and of the presence of TGF-beta. Interacts with CLU (PubMed:8555189).   
  
 **Gene Ontology Information:**

Molecular Function

- activin binding
- activin-activated receptor activity
- ATP binding
- glycosaminoglycan binding
- kinase activator activity
- metal ion binding
- molecular adaptor activity
- SMAD binding
- transforming growth factor beta binding
- transforming growth factor beta-activated receptor activity
- transforming growth factor beta receptor activity, type II
- transmembrane receptor protein serine/threonine kinase activity
- type I transforming growth factor beta receptor binding
- type III transforming growth factor beta receptor binding

Location

- caveola
- cytosol
- external side of plasma membrane
- extracellular region
- extracellular space
- membrane
- membrane raft
- plasma membrane
- receptor complex
- transforming growth factor beta ligand-receptor complex

Biological process

- activation of protein kinase activity
- aorta morphogenesis
- aortic valve morphogenesis
- apoptotic process
- artery morphogenesis
- atrioventricular valve morphogenesis
- blood vessel development
- brain development
- branching involved in blood vessel morphogenesis
- bronchus morphogenesis
- cardiac left ventricle morphogenesis
- cellular response to growth factor stimulus
- embryonic cranial skeleton morphogenesis
- embryonic hemopoiesis
- endocardial cushion fusion
- gastrulation
- growth plate cartilage chondrocyte growth
- heart development
- heart looping
- in utero embryonic development
- inferior endocardial cushion morphogenesis
- Langerhans cell differentiation
- lens development in camera-type eye
- lens fiber cell apoptotic process
- lung lobe morphogenesis
- mammary gland morphogenesis
- membranous septum morphogenesis
- miRNA transport
- myeloid dendritic cell differentiation
- Notch signaling pathway
- outflow tract morphogenesis
- outflow tract septum morphogenesis
- pathway-restricted SMAD protein phosphorylation
- peptidyl-serine phosphorylation
- peptidyl-threonine phosphorylation
- positive regulation of angiogenesis
- positive regulation of B cell tolerance induction
- positive regulation of CD4-positive, alpha-beta T cell proliferation
- positive regulation of cell population proliferation
- positive regulation of epithelial cell migration
- positive regulation of epithelial to mesenchymal transition
- positive regulation of epithelial to mesenchymal transition involved in endocardial cushion formation
- positive regulation of mesenchymal cell proliferation
- positive regulation of NK T cell differentiation
- positive regulation of pathway-restricted SMAD protein phosphorylation
- positive regulation of reactive oxygen species metabolic process
- positive regulation of SMAD protein signal transduction
- positive regulation of T cell tolerance induction
- positive regulation of tolerance induction to self antigen
- protein phosphorylation
- regulation of cell population proliferation
- regulation of gene expression
- regulation of stem cell proliferation
- response to cholesterol
- response to xenobiotic stimulus
- roof of mouth development
- secondary palate development
- smoothened signaling pathway
- trachea formation
- transforming growth factor beta receptor signaling pathway
- tricuspid valve morphogenesis
- vasculogenesis
- ventricular septum morphogenesis

---

67

- **Protein name:** Protein O-linked-mannose beta-1,2-N-acetylglucosaminyltransferase 1
- **Organism:** Homo sapiens
- **Uniprot Accession Number:** Q8WZA1
- **Protein sequence length:** 660 aa
- **1D identity (%):** 13.33
- **1D identity (%) [Gaps excluded]:** 25.63
- **1D identity - Alignment Gaps:** 479
- **1D aligned content (<aminoacid>:%):** {'D': 6.02, 'I': 3.01, 'K': 5.26, 'P': 12.03, 'N': 3.01, 'Q': 6.02, 'G': 8.27, 'R': 6.77, 'V': 6.02, 'A': 6.77, 'L': 9.77, 'E': 5.26, 'H': 3.01, 'Y': 3.76, 'W': 3.01, 'F': 3.01, 'S': 3.76, 'C': 1.5, 'T': 3.76}
- **Common reported functions (%):** 0.0
- **Common reported locations (%):** 0.0
- **Common reported processes (%):** 0.0

- **AF ID:** Q8WZA1
- **Chain:** A
- **Protein length:** 660 aa
- **Resolution:** N/A
- **b-phipsi:** 0.002214
- **w-rdist:** 0.27541
- **t-alpha:** 0.09781
- **Chemical similarity (Tanimoto Index) (%):** 96.19
- **1D identity (%) [PDB]:** 2.34
- **1D identity (%) [Gaps excluded][PDB]:** 75.56
- **1D identity - Alignment Gaps [PDB]:** 1410
- **1D aligned content [PDB] (<aminoacid>:%):** {'D': 8.82, 'P': 11.76, 'I': 5.88, 'F': 2.94, 'G': 17.65, 'K': 2.94, 'V': 11.76, 'T': 2.94, 'R': 8.82, 'L': 5.88, 'A': 2.94, 'Y': 2.94, 'N': 5.88, 'Q': 2.94, 'W': 2.94, 'M': 2.94}
- **2D identity (%) [PDB]:** 43.84
- **2D identity (%) [Gaps excluded][PDB]:** 89.27
- **2D identity - Alignment Gaps [PDB]:** 512
- **2D aligned content [PDB] (<2D-fold>:%):** {'.': 19.95, 'H': 35.83, 'T': 21.77, 'E': 21.09, 'G': 1.36}
- **3D similarity (TM-Score) (%) [PDB]:** 19.72

- **Gene name:** POMGNT1
- **Entrez ID:** N/A
- **RefSeq ID:** N/A
- **Sequence length:** N/A
- **5-UTR|CDS|3-UTR identity (%):** N/A | N/A | N/A
- **5-UTR|CDS|3-UTR identity (%) [Gaps excluded]:** N/A | N/A | N/A
- **5-UTR|CDS|3-UTR identity [Alignment Gaps]:** N/A | N/A | N/A
- **5-UTR aligned content (<base>:%):** N/A
- **CDS aligned content (<base>:%):** N/A
- **3-UTR aligned content (<base>:%):** N/A

**Uniprot Description:**  
  
 Participates in O-mannosyl glycosylation by catalyzing the addition of N-acetylglucosamine to O-linked mannose on glycoproteins (PubMed:11709191, PubMed:27493216, PubMed:28512129). Catalyzes the synthesis of the GlcNAc(beta1-2)Man(alpha1-)O-Ser/Thr moiety on alpha-dystroglycan and other O-mannosylated proteins, providing the necessary basis for the addition of further carbohydrate moieties (PubMed:11709191, PubMed:27493216). Is specific for alpha linked terminal mannose and does not have MGAT3, MGAT4, MGAT5, MGAT7 or MGAT8 activity.   
  
Interacts with DAG1 (via O-linked mannose moiety) (PubMed:27493216). Interacts (via transmembrane domain) with FKTN; the interaction is direct and is required for normal location in Golgi membranes (PubMed:17034757).   
  
 **Gene Ontology Information:**

Molecular Function

- acetylglucosaminyltransferase activity
- beta-1,3-galactosyl-O-glycosyl-glycoprotein beta-1,3-N-acetylglucosaminyltransferase activity
- manganese ion binding

Location

- Golgi membrane
- membrane

Biological process

- O-glycan processing
- protein O-linked glycosylation

---

68

- **Protein name:** Insulin-like growth factor 2 mRNA-binding protein 3
- **Organism:** Homo sapiens
- **Uniprot Accession Number:** O00425
- **Protein sequence length:** 579 aa
- **1D identity (%):** 13.38
- **1D identity (%) [Gaps excluded]:** 24.36
- **1D identity - Alignment Gaps:** 418
- **1D aligned content (<aminoacid>:%):** {'N': 4.03, 'P': 12.9, 'I': 10.48, 'F': 4.03, 'D': 4.03, 'K': 8.06, 'G': 8.87, 'Y': 1.61, 'E': 4.84, 'R': 5.65, 'W': 0.81, 'L': 8.06, 'V': 7.26, 'Q': 5.65, 'A': 8.06, 'T': 2.42, 'S': 2.42, 'H': 0.81}
- **Common reported functions (%):** 16.67
- **Common reported locations (%):** 40.0
- **Common reported processes (%):** 0.0

- **AF ID:** O00425
- **Chain:** A
- **Protein length:** 579 aa
- **Resolution:** N/A
- **b-phipsi:** 0.00847
- **w-rdist:** 0.303998
- **t-alpha:** 0.015328
- **Chemical similarity (Tanimoto Index) (%):** 99.4
- **1D identity (%) [PDB]:** 2.18
- **1D identity (%) [Gaps excluded][PDB]:** 66.67
- **1D identity - Alignment Gaps [PDB]:** 1329
- **1D aligned content [PDB] (<aminoacid>:%):** {'M': 3.33, 'E': 10.0, 'V': 10.0, 'L': 3.33, 'I': 10.0, 'R': 6.67, 'N': 3.33, 'D': 3.33, 'Q': 10.0, 'P': 6.67, 'K': 13.33, 'T': 6.67, 'S': 3.33, 'F': 3.33, 'G': 6.67}
- **2D identity (%) [PDB]:** 40.97
- **2D identity (%) [Gaps excluded][PDB]:** 93.3
- **2D identity - Alignment Gaps [PDB]:** 553
- **2D aligned content [PDB] (<2D-fold>:%):** {'E': 25.81, 'T': 13.4, '.': 15.14, 'H': 44.67, 'G': 0.74, 'B': 0.25}
- **3D similarity (TM-Score) (%) [PDB]:** 20.53

- **Gene name:** IGF2BP3
- **Entrez ID:** 10643
- **RefSeq ID:** NM\_006547
- **Transcript sequence length:** 4274
- **5-UTR|CDS|3-UTR identity (%):** 27.29 | 36.24 | 12.22
- **5-UTR|CDS|3-UTR identity (%) [Gaps excluded]:** 75.32 | 74.98 | 74.34
- **5-UTR|CDS|3-UTR identity [Alignment Gaps]:** 278 | 1538 | 9312
- **5-UTR aligned content (<base>:%):** {'A': 13.45, 'C': 32.77, 'G': 40.34, 'T': 13.45}
- **CDS aligned content (<base>:%):** {'T': 20.39, 'G': 24.93, 'C': 25.49, 'A': 29.19}
- **3-UTR aligned content (<base>:%):** {'A': 29.66, 'G': 17.84, 'C': 16.3, 'T': 36.2}

**Uniprot Description:**  
  
 RNA-binding factor that may recruit target transcripts to cytoplasmic protein-RNA complexes (mRNPs). This transcript 'caging' into mRNPs allows mRNA transport and transient storage. It also modulates the rate and location at which target transcripts encounter the translational apparatus and shields them from endonuclease attacks or microRNA-mediated degradation. Preferentially binds to N6-methyladenosine (m6A)-containing mRNAs and increases their stability (PubMed:29476152). Binds to the 3'-UTR of CD44 mRNA and stabilizes it, hence promotes cell adhesion and invadopodia formation in cancer cells. Binds to beta-actin/ACTB and MYC transcripts. Increases MYC mRNA stability by binding to the coding region instability determinant (CRD) and binding is enhanced by m6A-modification of the CRD (PubMed:29476152). Binds to the 5'-UTR of the insulin-like growth factor 2 (IGF2) mRNAs.   
  
Can form homooligomers and heterooligomers with IGF2BP1 and IGF2BP3 in an RNA-dependent manner (PubMed:23640942). Interacts with IGF2BP1 (PubMed:17289661). Interacts with ELAVL1, DHX9, HNRNPU, MATR3 and PABPC1 (PubMed:23640942, PubMed:29476152).   
  
 **Gene Ontology Information:**

Molecular Function

- mRNA 3'-UTR binding
- mRNA 5'-UTR binding
- N6-methyladenosine-containing RNA binding
- RNA binding
- translation regulator activity

Location

- cytoplasm
- cytoplasmic stress granule
- cytosol
- nucleus
- P-body

Biological process

- anatomical structure morphogenesis
- CRD-mediated mRNA stabilization
- mRNA transport
- negative regulation of translation
- nervous system development
- regulation of cytokine production
- regulation of gene expression
- translation

---

69

- **Protein name:** Zinc finger and SCAN domain-containing protein 4
- **Organism:** Homo sapiens
- **Uniprot Accession Number:** Q8NAM6
- **Protein sequence length:** 433 aa
- **1D identity (%):** 7.81
- **1D identity (%) [Gaps excluded]:** 19.57
- **1D identity - Alignment Gaps:** 554
- **1D aligned content (<aminoacid>:%):** {'A': 1.39, 'G': 8.33, 'E': 6.94, 'P': 12.5, 'Q': 8.33, 'R': 6.94, 'F': 4.17, 'D': 2.78, 'Y': 6.94, 'H': 4.17, 'K': 5.56, 'L': 5.56, 'V': 5.56, 'N': 2.78, 'S': 5.56, 'T': 6.94, 'I': 1.39, 'C': 4.17}
- **Common reported functions (%):** 0.0
- **Common reported locations (%):** 10.0
- **Common reported processes (%):** 0.0

- **AF ID:** Q8NAM6
- **Chain:** A
- **Protein length:** 433 aa
- **Resolution:** N/A
- **b-phipsi:** 0.018865
- **w-rdist:** 0.296876
- **t-alpha:** 0.004399
- **Chemical similarity (Tanimoto Index) (%):** 99.09
- **1D identity (%) [PDB]:** 2.7
- **1D identity (%) [Gaps excluded][PDB]:** 62.26
- **1D identity - Alignment Gaps [PDB]:** 1167
- **1D aligned content [PDB] (<aminoacid>:%):** {'Q': 12.12, 'C': 3.03, 'R': 3.03, 'E': 9.09, 'V': 3.03, 'L': 3.03, 'F': 6.06, 'D': 6.06, 'K': 9.09, 'S': 9.09, 'G': 6.06, 'P': 9.09, 'A': 6.06, 'M': 3.03, 'H': 3.03, 'N': 3.03, 'T': 3.03, 'Y': 3.03}
- **2D identity (%) [PDB]:** 34.93
- **2D identity (%) [Gaps excluded][PDB]:** 89.64
- **2D identity - Alignment Gaps [PDB]:** 559
- **2D aligned content [PDB] (<2D-fold>:%):** {'.': 33.12, 'T': 17.81, 'H': 45.31, 'E': 3.75}
- **3D similarity (TM-Score) (%) [PDB]:** 16.38

- **Gene name:** ZSCAN4
- **Entrez ID:** N/A
- **RefSeq ID:** N/A
- **Sequence length:** N/A
- **5-UTR|CDS|3-UTR identity (%):** N/A | N/A | N/A
- **5-UTR|CDS|3-UTR identity (%) [Gaps excluded]:** N/A | N/A | N/A
- **5-UTR|CDS|3-UTR identity [Alignment Gaps]:** N/A | N/A | N/A
- **5-UTR aligned content (<base>:%):** N/A
- **CDS aligned content (<base>:%):** N/A
- **3-UTR aligned content (<base>:%):** N/A

**Uniprot Description:**  
  
 Embryonic stem (ES) cell-specific transcription factor required to regulate ES cell pluripotency. Binds telomeres and plays a key role in genomic stability in ES cells by regulating telomere elongation. Acts as an activator of spontaneous telomere sister chromatid exchange (T-SCE) and telomere elongation in undifferentiated ES cells (By similarity). N/A   
  
 **Gene Ontology Information:**

Molecular Function

- DNA binding
- metal ion binding

Location

- chromosome, telomeric region
- nucleus

Biological process

- telomere maintenance via telomere lengthening

---

70

- **Protein name:** Serine protease FAM111A
- **Organism:** Homo sapiens
- **Uniprot Accession Number:** Q96PZ2
- **Protein sequence length:** 611 aa
- **1D identity (%):** 12.21
- **1D identity (%) [Gaps excluded]:** 22.94
- **1D identity - Alignment Gaps:** 448
- **1D aligned content (<aminoacid>:%):** {'K': 11.11, 'V': 9.4, 'M': 0.85, 'T': 5.13, 'E': 8.55, 'S': 5.98, 'G': 10.26, 'Q': 6.84, 'P': 5.13, 'Y': 5.13, 'L': 4.27, 'N': 2.56, 'A': 2.56, 'R': 5.13, 'F': 4.27, 'I': 5.13, 'C': 2.56, 'D': 5.13}
- **Common reported functions (%):** 0.0
- **Common reported locations (%):** 30.0
- **Common reported processes (%):** 0.0

- **AF ID:** Q96PZ2
- **Chain:** A
- **Protein length:** 611 aa
- **Resolution:** N/A
- **b-phipsi:** 0.002769
- **w-rdist:** 0.293473
- **t-alpha:** 0.085579
- **Chemical similarity (Tanimoto Index) (%):** 98.94
- **1D identity (%) [PDB]:** 1.55
- **1D identity (%) [Gaps excluded][PDB]:** 73.33
- **1D identity - Alignment Gaps [PDB]:** 1391
- **1D aligned content [PDB] (<aminoacid>:%):** {'S': 9.09, 'Q': 18.18, 'T': 4.55, 'P': 4.55, 'L': 13.64, 'E': 9.09, 'V': 9.09, 'A': 4.55, 'Y': 9.09, 'F': 4.55, 'K': 9.09, 'N': 4.55}
- **2D identity (%) [PDB]:** 37.62
- **2D identity (%) [Gaps excluded][PDB]:** 90.82
- **2D identity - Alignment Gaps [PDB]:** 601
- **2D aligned content [PDB] (<2D-fold>:%):** {'.': 16.58, 'E': 26.94, 'T': 22.54, 'H': 32.9, 'G': 0.78, 'B': 0.26}
- **3D similarity (TM-Score) (%) [PDB]:** 21.36

- **Gene name:** FAM111A
- **Entrez ID:** 63901
- **RefSeq ID:** NM\_198847
- **Transcript sequence length:** 3473
- **5-UTR|CDS|3-UTR identity (%):** 25.0 | 36.5 | 8.96
- **5-UTR|CDS|3-UTR identity (%) [Gaps excluded]:** 69.88 | 73.81 | 76.17
- **5-UTR|CDS|3-UTR identity [Alignment Gaps]:** 149 | 1526 | 9756
- **5-UTR aligned content (<base>:%):** {'G': 36.21, 'C': 34.48, 'A': 10.34, 'T': 18.97}
- **CDS aligned content (<base>:%):** {'T': 22.05, 'G': 24.68, 'C': 20.78, 'A': 32.49}
- **3-UTR aligned content (<base>:%):** {'G': 18.67, 'A': 27.95, 'T': 34.21, 'C': 19.17}

**Uniprot Description:**  
  
 Single-stranded DNA-binding serine protease that mediates the proteolytic cleavage of covalent DNA-protein cross-links (DPCs) during DNA synthesis, thereby playing a key role in maintaining genomic integrity (PubMed:32165630). DPCs are highly toxic DNA lesions that interfere with essential chromatin transactions, such as replication and transcription, and which are induced by reactive agents, such as UV light or formaldehyde (PubMed:32165630). Protects replication fork from stalling by removing DPCs, such as covalently trapped topoisomerase 1 (TOP1) adducts on DNA lesion, or poly(ADP-ribose) polymerase 1 (PARP1)-DNA complexes trapped by PARP inhibitors (PubMed:32165630). Required for PCNA loading on replication sites (PubMed:24561620). Promotes S-phase entry and DNA synthesis (PubMed:24561620).   
  
Interacts (via PIP-box) with PCNA; then interaction is direct.   
  
 **Gene Ontology Information:**

Molecular Function

- peptidase activity
- single-stranded DNA binding

Location

- chromatin
- cytoplasm
- fibrillar center
- nucleoplasm
- nucleus

Biological process

- cellular response to DNA damage stimulus
- DNA replication
- negative regulation of viral genome replication
- protein autoprocessing
- protein-DNA covalent cross-linking repair
- proteolysis
- replication fork processing

---

71

- **Protein name:** Aminopeptidase N
- **Organism:** Homo sapiens
- **Uniprot Accession Number:** P15144
- **Protein sequence length:** 967 aa
- **1D identity (%):** 16.04
- **1D identity (%) [Gaps excluded]:** 22.87
- **1D identity - Alignment Gaps:** 320
- **1D aligned content (<aminoacid>:%):** {'F': 4.07, 'G': 6.98, 'I': 4.07, 'V': 9.3, 'Y': 5.81, 'E': 2.91, 'P': 9.88, 'A': 6.98, 'K': 2.91, 'R': 6.98, 'L': 10.47, 'D': 5.81, 'H': 1.16, 'T': 8.14, 'S': 3.49, 'N': 5.23, 'Q': 3.49, 'C': 1.16, 'W': 1.16}
- **Common reported functions (%):** 0.0
- **Common reported locations (%):** 10.0
- **Common reported processes (%):** 0.0

- **AF ID:** P15144
- **Chain:** A
- **Protein length:** 967 aa
- **Resolution:** N/A
- **b-phipsi:** 0.012597
- **w-rdist:** 0.2671
- **t-alpha:** 0.009489
- **Chemical similarity (Tanimoto Index) (%):** 96.69
- **1D identity (%) [PDB]:** 2.16
- **1D identity (%) [Gaps excluded][PDB]:** 79.17
- **1D identity - Alignment Gaps [PDB]:** 1711
- **1D aligned content [PDB] (<aminoacid>:%):** {'M': 5.26, 'K': 5.26, 'N': 7.89, 'A': 7.89, 'S': 2.63, 'Y': 5.26, 'L': 7.89, 'P': 7.89, 'Q': 7.89, 'F': 2.63, 'G': 10.53, 'V': 7.89, 'D': 5.26, 'I': 5.26, 'R': 5.26, 'T': 2.63, 'W': 2.63}
- **2D identity (%) [PDB]:** 39.6
- **2D identity (%) [Gaps excluded][PDB]:** 87.72
- **2D identity - Alignment Gaps [PDB]:** 683
- **2D aligned content [PDB] (<2D-fold>:%):** {'.': 16.63, 'E': 29.61, 'T': 19.68, 'G': 1.83, 'H': 32.25}
- **3D similarity (TM-Score) (%) [PDB]:** 26.26

- **Gene name:** ANPEP
- **Entrez ID:** 404191
- **RefSeq ID:** N/A
- **Sequence length:** N/A
- **5-UTR|CDS|3-UTR identity (%):** N/A | N/A | N/A
- **5-UTR|CDS|3-UTR identity (%) [Gaps excluded]:** N/A | N/A | N/A
- **5-UTR|CDS|3-UTR identity [Alignment Gaps]:** N/A | N/A | N/A
- **5-UTR aligned content (<base>:%):** N/A
- **CDS aligned content (<base>:%):** N/A
- **3-UTR aligned content (<base>:%):** N/A

**Uniprot Description:**  
  
 Broad specificity aminopeptidase which plays a role in the final digestion of peptides generated from hydrolysis of proteins by gastric and pancreatic proteases. Also involved in the processing of various peptides including peptide hormones, such as angiotensin III and IV, neuropeptides, and chemokines. May also be involved the cleavage of peptides bound to major histocompatibility complex class II molecules of antigen presenting cells. May have a role in angiogenesis and promote cholesterol crystallization. May have a role in amino acid transport by acting as binding partner of amino acid transporter SLC6A19 and regulating its activity (By similarity).   
  
Homodimer. Interacts with SLC6A19 (By similarity).   
  
 **Gene Ontology Information:**

Molecular Function

- metalloaminopeptidase activity
- peptide binding
- virus receptor activity
- zinc ion binding

Location

- cytoplasm
- plasma membrane

Biological process

- angiogenesis
- cell differentiation
- peptide catabolic process
- proteolysis

---

72

- **Protein name:** TBC1 domain family member 3K
- **Organism:** Homo sapiens
- **Uniprot Accession Number:** A0A087X1G2
- **Protein sequence length:** 549 aa
- **1D identity (%):** 11.18
- **1D identity (%) [Gaps excluded]:** 23.14
- **1D identity - Alignment Gaps:** 490
- **1D aligned content (<aminoacid>:%):** {'M': 1.89, 'G': 12.26, 'R': 11.32, 'K': 6.6, 'P': 15.09, 'N': 3.77, 'E': 4.72, 'V': 5.66, 'D': 7.55, 'I': 3.77, 'F': 4.72, 'L': 8.49, 'A': 2.83, 'S': 2.83, 'C': 4.72, 'T': 2.83, 'W': 0.94}
- **Common reported functions (%):** 0.0
- **Common reported locations (%):** 0.0
- **Common reported processes (%):** 0.0

- **AF ID:** A0A087X1G2
- **Chain:** A
- **Protein length:** 549 aa
- **Resolution:** N/A
- **b-phipsi:** 0.011781
- **w-rdist:** 0.535088
- **t-alpha:** 0.001462
- **Chemical similarity (Tanimoto Index) (%):** 98.58
- **1D identity (%) [PDB]:** 2.14
- **1D identity (%) [Gaps excluded][PDB]:** 78.38
- **1D identity - Alignment Gaps [PDB]:** 1315
- **1D aligned content [PDB] (<aminoacid>:%):** {'D': 3.45, 'V': 10.34, 'E': 6.9, 'P': 10.34, 'F': 3.45, 'R': 6.9, 'L': 13.79, 'K': 6.9, 'N': 3.45, 'T': 6.9, 'G': 10.34, 'Q': 3.45, 'I': 10.34, 'A': 3.45}
- **2D identity (%) [PDB]:** 32.68
- **2D identity (%) [Gaps excluded][PDB]:** 90.0
- **2D identity - Alignment Gaps [PDB]:** 649
- **2D aligned content [PDB] (<2D-fold>:%):** {'H': 57.36, 'T': 18.92, '.': 20.42, 'G': 0.9, 'E': 2.4}
- **3D similarity (TM-Score) (%) [PDB]:** 16.96

- **Gene name:** TBC1D3K
- **Entrez ID:** 101060351
- **RefSeq ID:** N/A
- **Sequence length:** N/A
- **5-UTR|CDS|3-UTR identity (%):** N/A | N/A | N/A
- **5-UTR|CDS|3-UTR identity (%) [Gaps excluded]:** N/A | N/A | N/A
- **5-UTR|CDS|3-UTR identity [Alignment Gaps]:** N/A | N/A | N/A
- **5-UTR aligned content (<base>:%):** N/A
- **CDS aligned content (<base>:%):** N/A
- **3-UTR aligned content (<base>:%):** N/A

**Uniprot Description:**  
  
 Acts as a GTPase activating protein for RAB5. Does not act on RAB4 or RAB11 (By similarity). N/A   
  
 **Gene Ontology Information:**

Molecular Function

- GTPase activator activity

Location

- plasma membrane

Biological process

- activation of GTPase activity

---

73

- **Protein name:** E3 ubiquitin-protein ligase XIAP
- **Organism:** Homo sapiens
- **Uniprot Accession Number:** P98170
- **Protein sequence length:** 497 aa
- **1D identity (%):** 9.2
- **1D identity (%) [Gaps excluded]:** 21.32
- **1D identity - Alignment Gaps:** 538
- **1D aligned content (<aminoacid>:%):** {'M': 1.15, 'P': 11.49, 'L': 8.05, 'G': 11.49, 'F': 5.75, 'H': 3.45, 'V': 6.9, 'W': 2.3, 'D': 3.45, 'K': 5.75, 'C': 5.75, 'T': 4.6, 'S': 4.6, 'R': 4.6, 'A': 4.6, 'E': 4.6, 'I': 4.6, 'N': 2.3, 'Y': 2.3, 'Q': 2.3}
- **Common reported functions (%):** 0.0
- **Common reported locations (%):** 40.0
- **Common reported processes (%):** 0.0

- **AF ID:** P98170
- **Chain:** A
- **Protein length:** 497 aa
- **Resolution:** N/A
- **b-phipsi:** 0.013517
- **w-rdist:** 0.272351
- **t-alpha:** 0.007353
- **Chemical similarity (Tanimoto Index) (%):** 99.47
- **1D identity (%) [PDB]:** 2.9
- **1D identity (%) [Gaps excluded][PDB]:** 62.71
- **1D identity - Alignment Gaps [PDB]:** 1219
- **1D aligned content [PDB] (<aminoacid>:%):** {'M': 2.7, 'T': 2.7, 'V': 10.81, 'P': 5.41, 'A': 10.81, 'I': 8.11, 'Q': 5.41, 'Y': 5.41, 'G': 10.81, 'R': 5.41, 'N': 5.41, 'D': 2.7, 'K': 10.81, 'F': 5.41, 'E': 5.41, 'C': 2.7}
- **2D identity (%) [PDB]:** 38.8
- **2D identity (%) [Gaps excluded][PDB]:** 89.6
- **2D identity - Alignment Gaps [PDB]:** 529
- **2D aligned content [PDB] (<2D-fold>:%):** {'.': 23.76, 'T': 25.97, 'H': 42.82, 'E': 6.35, 'G': 0.83, 'B': 0.28}
- **3D similarity (TM-Score) (%) [PDB]:** 18.85

- **Gene name:** XIAP
- **Entrez ID:** 331
- **RefSeq ID:** N/A
- **Sequence length:** N/A
- **5-UTR|CDS|3-UTR identity (%):** N/A | N/A | N/A
- **5-UTR|CDS|3-UTR identity (%) [Gaps excluded]:** N/A | N/A | N/A
- **5-UTR|CDS|3-UTR identity [Alignment Gaps]:** N/A | N/A | N/A
- **5-UTR aligned content (<base>:%):** N/A
- **CDS aligned content (<base>:%):** N/A
- **3-UTR aligned content (<base>:%):** N/A

**Uniprot Description:**  
  
 Multi-functional protein which regulates not only caspases and apoptosis, but also modulates inflammatory signaling and immunity, copper homeostasis, mitogenic kinase signaling, cell proliferation, as well as cell invasion and metastasis. Acts as a direct caspase inhibitor. Directly bind to the active site pocket of CASP3 and CASP7 and obstructs substrate entry. Inactivates CASP9 by keeping it in a monomeric, inactive state. Acts as an E3 ubiquitin-protein ligase regulating NF-kappa-B signaling and the target proteins for its E3 ubiquitin-protein ligase activity include: RIPK1, CASP3, CASP7, CASP8, CASP9, MAP3K2/MEKK2, DIABLO/SMAC, AIFM1, CCS and BIRC5/survivin. Ubiquitinion of CCS leads to enhancement of its chaperone activity toward its physiologic target, SOD1, rather than proteasomal degradation. Ubiquitinion of MAP3K2/MEKK2 and AIFM1 does not lead to proteasomal degradation. Plays a role in copper homeostasis by ubiquitinationg COMMD1 and promoting its proteasomal degradation. Can also function as E3 ubiquitin-protein ligase of the NEDD8 conjugation pathway, targeting effector caspases for neddylation and inactivation. Regulates the BMP signaling pathway and the SMAD and MAP3K7/TAK1 dependent pathways leading to NF-kappa-B and JNK activation. Acts as an important regulator of innate immune signaling via regulation of Nodlike receptors (NLRs). Protects cells from spontaneous formation of the ripoptosome, a large multi-protein complex that has the capability to kill cancer cells in a caspase-dependent and caspase-independent manner. Suppresses ripoptosome formation by ubiquitinating RIPK1 and CASP8. Acts as a positive regulator of Wnt signaling and ubiquitinates TLE1, TLE2, TLE3, TLE4 and AES. Ubiquitination of TLE3 results in inhibition of its interaction with TCF7L2/TCF4 thereby allowing efficient recruitment and binding of the transcriptional coactivator beta-catenin to TCF7L2/TCF4 that is required to initiate a Wnt-specific transcriptional program.   
  
Monomer, and homodimer. Interacts with DIABLO/SMAC and with PRSS25; these interactions inhibit apoptotic suppressor activity. Interacts with TAB1/MAP3K7IP1 and AIFM1. Interaction with SMAC hinders binding of TAB1/MAP3K7IP1 and AIFM1. Interacts with TCF25 and COMMD1. Interacts with SEPTIN4 isoform 6, but not with other SEPTIN4 isoforms. Interacts with RIP1, RIP2, RIP3, RIP4, CCS and USP19. Interacts (via BIR 2 domain and BIR 3 domain) with HAX1 (via C-terminus) and this interaction blocks ubiquitination of XIAP/BIRC4. Interacts with the monomeric form of BIRC5/survivin. Interacts with TLE3 and TCF7L2/TCF4. Interacts (via BIR 3 and RING domains) with PDCL3 (PubMed:19012568).   
  
 **Gene Ontology Information:**

Molecular Function

- cysteine-type endopeptidase inhibitor activity
- cysteine-type endopeptidase inhibitor activity involved in apoptotic process
- endopeptidase regulator activity
- identical protein binding
- metal ion binding
- protein serine/threonine kinase binding
- ubiquitin protein ligase activity
- ubiquitin-protein transferase activity

Location

- cytoplasm
- cytosol
- nucleoplasm
- nucleus

Biological process

- copper ion homeostasis
- cellular response to DNA damage stimulus
- inhibition of cysteine-type endopeptidase activity
- inhibition of cysteine-type endopeptidase activity involved in apoptotic process
- negative regulation of apoptotic process
- negative regulation of cysteine-type endopeptidase activity involved in apoptotic process
- negative regulation of tumor necrosis factor-mediated signaling pathway
- neuron apoptotic process
- nucleotide-binding oligomerization domain containing 2 signaling pathway
- positive regulation of canonical Wnt signaling pathway
- positive regulation of I-kappaB kinase/NF-kappaB signaling
- positive regulation of JNK cascade
- positive regulation of protein linear polyubiquitination
- positive regulation of protein ubiquitination
- positive regulation of type I interferon production
- protein K63-linked ubiquitination
- regulation of apoptosis involved in tissue homeostasis
- regulation of apoptotic process
- regulation of BMP signaling pathway
- regulation of cell cycle
- regulation of cell population proliferation
- regulation of inflammatory response
- regulation of innate immune response
- regulation of nucleotide-binding oligomerization domain containing signaling pathway
- Wnt signaling pathway

---

74

- **Protein name:** Zinc finger protein 79
- **Organism:** Homo sapiens
- **Uniprot Accession Number:** Q15937
- **Protein sequence length:** 498 aa
- **1D identity (%):** 9.69
- **1D identity (%) [Gaps excluded]:** 21.88
- **1D identity - Alignment Gaps:** 523
- **1D aligned content (<aminoacid>:%):** {'M': 1.1, 'E': 5.49, 'P': 12.09, 'L': 3.3, 'G': 15.38, 'A': 4.4, 'R': 7.69, 'F': 4.4, 'W': 1.1, 'I': 3.3, 'V': 3.3, 'T': 6.59, 'K': 6.59, 'N': 2.2, 'H': 8.79, 'Q': 2.2, 'S': 4.4, 'D': 1.1, 'Y': 4.4, 'C': 2.2}
- **Common reported functions (%):** 0.0
- **Common reported locations (%):** 10.0
- **Common reported processes (%):** 0.0

- **AF ID:** Q15937
- **Chain:** A
- **Protein length:** 498 aa
- **Resolution:** N/A
- **b-phipsi:** 0.008637
- **w-rdist:** 0.703879
- **t-alpha:** 0.00146
- **Chemical similarity (Tanimoto Index) (%):** 96.25
- **1D identity (%) [PDB]:** 1.21
- **1D identity (%) [Gaps excluded][PDB]:** 80.0
- **1D identity - Alignment Gaps [PDB]:** 1298
- **1D aligned content [PDB] (<aminoacid>:%):** {'M': 6.25, 'L': 18.75, 'E': 12.5, 'V': 18.75, 'S': 6.25, 'G': 6.25, 'I': 6.25, 'P': 12.5, 'Q': 6.25, 'A': 6.25}
- **2D identity (%) [PDB]:** 33.37
- **2D identity (%) [Gaps excluded][PDB]:** 91.34
- **2D identity - Alignment Gaps [PDB]:** 622
- **2D aligned content [PDB] (<2D-fold>:%):** {'.': 29.36, 'T': 18.65, 'H': 43.43, 'E': 8.56}
- **3D similarity (TM-Score) (%) [PDB]:** 14.54

- **Gene name:** ZNF79
- **Entrez ID:** 7633
- **RefSeq ID:** NM\_007135
- **Transcript sequence length:** 2078
- **5-UTR|CDS|3-UTR identity (%):** 37.29 | 33.01 | 1.18
- **5-UTR|CDS|3-UTR identity (%) [Gaps excluded]:** 79.29 | 75.89 | 80.5
- **5-UTR|CDS|3-UTR identity [Alignment Gaps]:** 223 | 1643 | 10680
- **5-UTR aligned content (<base>:%):** {'A': 12.1, 'C': 29.94, 'T': 10.83, 'G': 47.13}
- **CDS aligned content (<base>:%):** {'A': 27.6, 'G': 27.4, 'C': 27.4, 'T': 17.6}
- **3-UTR aligned content (<base>:%):** {'C': 18.75, 'A': 35.16, 'G': 23.44, 'T': 22.66}

**Uniprot Description:**  
  
 May be involved in transcriptional regulation. N/A   
  
 **Gene Ontology Information:**

Molecular Function

- DNA-binding transcription repressor activity, RNA polymerase II-specific
- metal ion binding
- RNA polymerase II transcription regulatory region sequence-specific DNA binding

Location

- nucleus

Biological process

- negative regulation of transcription by RNA polymerase II

---

75

- **Protein name:** Anaphase-promoting complex subunit 4
- **Organism:** Homo sapiens
- **Uniprot Accession Number:** Q9UJX5
- **Protein sequence length:** 808 aa
- **1D identity (%):** 14.93
- **1D identity (%) [Gaps excluded]:** 23.49
- **1D identity - Alignment Gaps:** 371
- **1D aligned content (<aminoacid>:%):** {'S': 7.24, 'R': 4.61, 'E': 3.29, 'I': 4.61, 'P': 7.24, 'A': 5.26, 'L': 11.84, 'V': 7.89, 'G': 6.58, 'W': 2.63, 'F': 6.58, 'M': 1.32, 'T': 4.61, 'Y': 6.58, 'N': 3.95, 'K': 4.61, 'H': 1.97, 'C': 2.63, 'D': 1.97, 'Q': 4.61}
- **Common reported functions (%):** 0.0
- **Common reported locations (%):** 30.0
- **Common reported processes (%):** 0.0

- **AF ID:** Q9UJX5
- **Chain:** A
- **Protein length:** 808 aa
- **Resolution:** N/A
- **b-phipsi:** 0.002462
- **w-rdist:** 0.728817
- **t-alpha:** 0.004399
- **Chemical similarity (Tanimoto Index) (%):** 99.17
- **1D identity (%) [PDB]:** 3.03
- **1D identity (%) [Gaps excluded][PDB]:** 76.19
- **1D identity - Alignment Gaps [PDB]:** 1522
- **1D aligned content [PDB] (<aminoacid>:%):** {'R': 4.17, 'V': 4.17, 'Q': 8.33, 'P': 4.17, 'E': 12.5, 'I': 12.5, 'D': 6.25, 'L': 14.58, 'S': 4.17, 'F': 6.25, 'K': 8.33, 'T': 4.17, 'Y': 4.17, 'G': 4.17, 'A': 2.08}
- **2D identity (%) [PDB]:** 39.36
- **2D identity (%) [Gaps excluded][PDB]:** 91.15
- **2D identity - Alignment Gaps [PDB]:** 654
- **2D aligned content [PDB] (<2D-fold>:%):** {'.': 18.1, 'E': 31.35, 'T': 22.96, 'H': 26.27, 'G': 1.32}
- **3D similarity (TM-Score) (%) [PDB]:** 17.87

- **Gene name:** ANAPC4
- **Entrez ID:** 29945
- **RefSeq ID:** NM\_013367
- **Transcript sequence length:** 2635
- **5-UTR|CDS|3-UTR identity (%):** 29.15 | 45.63 | 0.8
- **5-UTR|CDS|3-UTR identity (%) [Gaps excluded]:** 79.27 | 74.9 | 75.0
- **5-UTR|CDS|3-UTR identity [Alignment Gaps]:** 141 | 1239 | 10707
- **5-UTR aligned content (<base>:%):** {'C': 29.23, 'T': 7.69, 'G': 53.85, 'A': 9.23}
- **CDS aligned content (<base>:%):** {'A': 28.68, 'T': 25.43, 'G': 25.98, 'C': 19.9}
- **3-UTR aligned content (<base>:%):** {'T': 36.78, 'A': 36.78, 'G': 19.54, 'C': 6.9}

**Uniprot Description:**  
  
 Component of the anaphase promoting complex/cyclosome (APC/C), a cell cycle-regulated E3 ubiquitin ligase that controls progression through mitosis and the G1 phase of the cell cycle (PubMed:18485873). The APC/C complex acts by mediating ubiquitination and subsequent degradation of target proteins: it mainly mediates the formation of 'Lys-11'-linked polyubiquitin chains and, to a lower extent, the formation of 'Lys-48'- and 'Lys-63'-linked polyubiquitin chains (PubMed:18485873). The APC/C complex catalyzes assembly of branched 'Lys-11'-/'Lys-48'-linked branched ubiquitin chains on target proteins (PubMed:29033132).   
  
The mammalian APC/C is composed at least of 14 distinct subunits ANAPC1, ANAPC2, CDC27/APC3, ANAPC4, ANAPC5, CDC16/APC6, ANAPC7, CDC23/APC8, ANAPC10, ANAPC11, CDC26/APC12, ANAPC13, ANAPC15 and ANAPC16 that assemble into a complex of at least 19 chains with a combined molecular mass of around 1.2 MDa; APC/C interacts with FZR1 and FBXO5 (PubMed:25043029, PubMed:26083744, PubMed:27259151, PubMed:9469815). In the context of the APC/C complex, directly interacts with UBE2S (PubMed:27259151). Interacts with FBXO43.   
  
 **Gene Ontology Information:**

Molecular Function

- protein phosphatase binding
- ubiquitin-protein transferase activity

Location

- anaphase-promoting complex
- cytosol
- nuclear periphery
- nucleoplasm
- nucleus

Biological process

- anaphase-promoting complex-dependent catabolic process
- cell cycle
- cell division
- protein K11-linked ubiquitination
- regulation of meiotic cell cycle
- regulation of mitotic cell cycle
- regulation of mitotic metaphase/anaphase transition

---

76

- **Protein name:** RasGAP-activating-like protein 1
- **Organism:** Homo sapiens
- **Uniprot Accession Number:** O95294
- **Protein sequence length:** 804 aa
- **1D identity (%):** 15.35
- **1D identity (%) [Gaps excluded]:** 24.61
- **1D identity - Alignment Gaps:** 385
- **1D aligned content (<aminoacid>:%):** {'A': 8.28, 'V': 8.28, 'D': 5.73, 'C': 1.91, 'E': 3.82, 'P': 12.74, 'G': 10.83, 'Y': 3.18, 'T': 5.1, 'L': 9.55, 'F': 1.27, 'I': 2.55, 'K': 6.37, 'S': 4.46, 'W': 0.64, 'R': 8.92, 'Q': 4.46, 'H': 1.91}
- **Common reported functions (%):** 0.0
- **Common reported locations (%):** 10.0
- **Common reported processes (%):** 0.0

- **AF ID:** O95294
- **Chain:** A
- **Protein length:** 804 aa
- **Resolution:** N/A
- **b-phipsi:** 0.004832
- **w-rdist:** 0.222225
- **t-alpha:** 0.096
- **Chemical similarity (Tanimoto Index) (%):** 98.88
- **1D identity (%) [PDB]:** 2.26
- **1D identity (%) [Gaps excluded][PDB]:** 73.47
- **1D identity - Alignment Gaps [PDB]:** 1546
- **1D aligned content [PDB] (<aminoacid>:%):** {'A': 11.11, 'R': 8.33, 'V': 11.11, 'Y': 2.78, 'L': 8.33, 'D': 11.11, 'E': 5.56, 'H': 2.78, 'S': 11.11, 'I': 2.78, 'G': 5.56, 'N': 2.78, 'P': 2.78, 'Q': 8.33, 'K': 2.78, 'T': 2.78}
- **2D identity (%) [PDB]:** 42.37
- **2D identity (%) [Gaps excluded][PDB]:** 90.82
- **2D identity - Alignment Gaps [PDB]:** 598
- **2D aligned content [PDB] (<2D-fold>:%):** {'.': 16.63, 'E': 28.84, 'T': 18.32, 'H': 34.32, 'G': 1.89}
- **3D similarity (TM-Score) (%) [PDB]:** 22.88

- **Gene name:** RASAL1
- **Entrez ID:** 8437
- **RefSeq ID:** N/A
- **Sequence length:** N/A
- **5-UTR|CDS|3-UTR identity (%):** N/A | N/A | N/A
- **5-UTR|CDS|3-UTR identity (%) [Gaps excluded]:** N/A | N/A | N/A
- **5-UTR|CDS|3-UTR identity [Alignment Gaps]:** N/A | N/A | N/A
- **5-UTR aligned content (<base>:%):** N/A
- **CDS aligned content (<base>:%):** N/A
- **3-UTR aligned content (<base>:%):** N/A

**Uniprot Description:**  
  
 Probable inhibitory regulator of the Ras-cyclic AMP pathway (PubMed:9751798). Plays a role in dendrite formation by melanocytes (PubMed:23999003). N/A   
  
 **Gene Ontology Information:**

Molecular Function

- GTPase activator activity
- metal ion binding
- phospholipid binding

Location

- cytosol

Biological process

- cell differentiation
- cellular response to calcium ion
- intracellular signal transduction
- negative regulation of Ras protein signal transduction
- positive regulation of dendrite extension
- regulation of GTPase activity
- signal transduction

---

77

- **Protein name:** Nucleolar protein 11
- **Organism:** Homo sapiens
- **Uniprot Accession Number:** Q9H8H0
- **Protein sequence length:** 719 aa
- **1D identity (%):** 13.07
- **1D identity (%) [Gaps excluded]:** 22.82
- **1D identity - Alignment Gaps:** 428
- **1D aligned content (<aminoacid>:%):** {'A': 3.82, 'V': 9.92, 'P': 6.11, 'L': 13.74, 'D': 4.58, 'T': 5.34, 'G': 6.11, 'R': 6.87, 'W': 2.29, 'F': 0.76, 'I': 6.87, 'N': 2.29, 'K': 9.16, 'E': 6.11, 'Y': 1.53, 'Q': 5.34, 'S': 4.58, 'C': 3.82, 'H': 0.76}
- **Common reported functions (%):** 0.0
- **Common reported locations (%):** 0.0
- **Common reported processes (%):** 0.0

- **AF ID:** Q9H8H0
- **Chain:** A
- **Protein length:** 719 aa
- **Resolution:** N/A
- **b-phipsi:** 0.000913
- **w-rdist:** 1.197169
- **t-alpha:** 0.00219
- **Chemical similarity (Tanimoto Index) (%):** 98.65
- **1D identity (%) [PDB]:** 2.25
- **1D identity (%) [Gaps excluded][PDB]:** 75.56
- **1D identity - Alignment Gaps [PDB]:** 1469
- **1D aligned content [PDB] (<aminoacid>:%):** {'P': 8.82, 'E': 8.82, 'G': 2.94, 'L': 14.71, 'Q': 8.82, 'I': 14.71, 'Y': 2.94, 'R': 5.88, 'A': 2.94, 'K': 8.82, 'D': 2.94, 'T': 5.88, 'V': 5.88, 'H': 2.94, 'F': 2.94}
- **2D identity (%) [PDB]:** 40.54
- **2D identity (%) [Gaps excluded][PDB]:** 89.51
- **2D identity - Alignment Gaps [PDB]:** 587
- **2D aligned content [PDB] (<2D-fold>:%):** {'.': 15.86, 'E': 23.22, 'T': 20.69, 'H': 38.16, 'G': 2.07}
- **3D similarity (TM-Score) (%) [PDB]:** 19.47

- **Gene name:** NOL11
- **Entrez ID:** 517743
- **RefSeq ID:** NM\_015462
- **Transcript sequence length:** 2844
- **5-UTR|CDS|3-UTR identity (%):** 3.24 | 43.78 | 4.09
- **5-UTR|CDS|3-UTR identity (%) [Gaps excluded]:** 77.78 | 74.71 | 77.34
- **5-UTR|CDS|3-UTR identity [Alignment Gaps]:** 207 | 1262 | 10339
- **5-UTR aligned content (<base>:%):** {'G': 42.86, 'T': 42.86, 'A': 14.29}
- **CDS aligned content (<base>:%):** {'T': 23.75, 'G': 23.9, 'C': 21.87, 'A': 30.49}
- **3-UTR aligned content (<base>:%):** {'A': 25.06, 'T': 34.68, 'C': 19.46, 'G': 20.81}

**Uniprot Description:**  
  
 Ribosome biogenesis factor. May be required for both optimal rDNA transcription and small subunit (SSU) pre-rRNA processing at sites A', A0, 1 and 2b.   
  
Interacts with UTP4. Interacts with FBL/fibrillarin in a transcription-dependent manner (PubMed:22916032). May associate with the proposed t-UTP subcomplex of the SSU processome containing at least UTP4, WDR43, HEATR1, UTP15, WDR75.   
  
 **Gene Ontology Information:**

Molecular Function   
  
N/A

Location

- nucleolus
- t-UTP complex

Biological process

- maturation of SSU-rRNA
- positive regulation of transcription of nucleolar large rRNA by RNA polymerase I

---

78

- **Protein name:** Ubiquitin-associated and SH3 domain-containing protein B
- **Organism:** Homo sapiens
- **Uniprot Accession Number:** Q8TF42
- **Protein sequence length:** 649 aa
- **1D identity (%):** 13.47
- **1D identity (%) [Gaps excluded]:** 28.02
- **1D identity - Alignment Gaps:** 528
- **1D aligned content (<aminoacid>:%):** {'M': 1.46, 'Q': 6.57, 'P': 10.95, 'G': 11.68, 'Y': 2.92, 'N': 3.65, 'D': 1.46, 'A': 5.11, 'L': 8.03, 'S': 6.57, 'V': 7.3, 'F': 5.84, 'K': 7.3, 'I': 2.19, 'C': 3.65, 'E': 4.38, 'T': 4.38, 'H': 2.92, 'R': 2.92, 'W': 0.73}
- **Common reported functions (%):** 0.0
- **Common reported locations (%):** 20.0
- **Common reported processes (%):** 0.0

- **AF ID:** Q8TF42
- **Chain:** A
- **Protein length:** 649 aa
- **Resolution:** N/A
- **b-phipsi:** 0.001636
- **w-rdist:** 0.372635
- **t-alpha:** 0.051095
- **Chemical similarity (Tanimoto Index) (%):** 99.02
- **1D identity (%) [PDB]:** 2.21
- **1D identity (%) [Gaps excluded][PDB]:** 78.05
- **1D identity - Alignment Gaps [PDB]:** 1407
- **1D aligned content [PDB] (<aminoacid>:%):** {'A': 3.12, 'Q': 9.38, 'P': 9.38, 'I': 9.38, 'F': 3.12, 'M': 3.12, 'C': 3.12, 'E': 12.5, 'V': 6.25, 'L': 9.38, 'D': 6.25, 'R': 6.25, 'N': 3.12, 'S': 3.12, 'T': 3.12, 'K': 6.25, 'G': 3.12}
- **2D identity (%) [PDB]:** 39.65
- **2D identity (%) [Gaps excluded][PDB]:** 91.56
- **2D identity - Alignment Gaps [PDB]:** 589
- **2D aligned content [PDB] (<2D-fold>:%):** {'.': 26.46, 'H': 31.55, 'T': 19.66, 'E': 19.9, 'G': 2.18, 'B': 0.24}
- **3D similarity (TM-Score) (%) [PDB]:** 23.99

- **Gene name:** UBASH3B
- **Entrez ID:** 84959
- **RefSeq ID:** NM\_032873
- **Transcript sequence length:** 6865
- **5-UTR|CDS|3-UTR identity (%):** 43.35 | 39.89 | 26.36
- **5-UTR|CDS|3-UTR identity (%) [Gaps excluded]:** 76.92 | 74.52 | 75.93
- **5-UTR|CDS|3-UTR identity [Alignment Gaps]:** 151 | 1400 | 7468
- **5-UTR aligned content (<base>:%):** {'T': 12.0, 'G': 44.67, 'C': 35.33, 'A': 8.0}
- **CDS aligned content (<base>:%):** {'A': 24.71, 'T': 20.72, 'G': 26.46, 'C': 28.12}
- **3-UTR aligned content (<base>:%):** {'A': 31.51, 'C': 15.02, 'G': 19.5, 'T': 33.96}

**Uniprot Description:**  
  
 Interferes with CBL-mediated down-regulation and degradation of receptor-type tyrosine kinases. Promotes accumulation of activated target receptors, such as T-cell receptors and EGFR, on the cell surface. Exhibits tyrosine phosphatase activity toward several substrates including EGFR, FAK, SYK, and ZAP70. Down-regulates proteins that are dually modified by both protein tyrosine phosphorylation and ubiquitination.   
  
Homodimer. Interacts with JAK2 (in vitro) (By similarity). Interacts with CBL (PubMed:15159412). Part of a complex containing CBL and activated EGFR (PubMed:15159412). Interacts with ubiquitin and with mono-ubiquitinated proteins (PubMed:15159412). Interacts with ZAP70 (ubiquitinated form) (PubMed:26903241).   
  
 **Gene Ontology Information:**

Molecular Function

- identical protein binding
- phosphoprotein binding
- protein tyrosine phosphatase activity
- ubiquitin protein ligase binding

Location

- cytoplasm
- nucleus

Biological process

- collagen-activated tyrosine kinase receptor signaling pathway
- negative regulation of bone resorption
- negative regulation of osteoclast differentiation
- negative regulation of platelet aggregation
- negative regulation of protein kinase activity
- negative regulation of signal transduction
- peptidyl-tyrosine dephosphorylation
- platelet aggregation
- regulation of osteoclast differentiation
- regulation of protein binding
- regulation of release of sequestered calcium ion into cytosol

---

79

- **Protein name:** Tyrosine-protein phosphatase non-receptor type 6
- **Organism:** Homo sapiens
- **Uniprot Accession Number:** P29350
- **Protein sequence length:** 595 aa
- **1D identity (%):** 15.02
- **1D identity (%) [Gaps excluded]:** 25.89
- **1D identity - Alignment Gaps:** 386
- **1D aligned content (<aminoacid>:%):** {'F': 3.62, 'D': 6.52, 'E': 5.8, 'K': 7.97, 'P': 6.52, 'R': 7.25, 'N': 2.17, 'V': 5.07, 'H': 2.17, 'Q': 7.97, 'G': 12.32, 'Y': 4.35, 'T': 6.52, 'S': 3.62, 'L': 7.97, 'A': 2.9, 'I': 4.35, 'C': 0.72, 'W': 0.72, 'M': 1.45}
- **Common reported functions (%):** 0.0
- **Common reported locations (%):** 40.0
- **Common reported processes (%):** 0.0

- **AF ID:** P29350
- **Chain:** A
- **Protein length:** 595 aa
- **Resolution:** N/A
- **b-phipsi:** 0.00661
- **w-rdist:** 0.524736
- **t-alpha:** 0.004399
- **Chemical similarity (Tanimoto Index) (%):** 99.1
- **1D identity (%) [PDB]:** 3.05
- **1D identity (%) [Gaps excluded][PDB]:** 73.68
- **1D identity - Alignment Gaps [PDB]:** 1321
- **1D aligned content [PDB] (<aminoacid>:%):** {'M': 4.76, 'R': 4.76, 'H': 2.38, 'L': 19.05, 'K': 9.52, 'Y': 4.76, 'S': 7.14, 'G': 7.14, 'Q': 9.52, 'P': 7.14, 'T': 2.38, 'A': 4.76, 'E': 2.38, 'V': 9.52, 'C': 2.38, 'N': 2.38}
- **2D identity (%) [PDB]:** 42.34
- **2D identity (%) [Gaps excluded][PDB]:** 87.21
- **2D identity - Alignment Gaps [PDB]:** 497
- **2D aligned content [PDB] (<2D-fold>:%):** {'T': 22.74, '.': 13.45, 'H': 33.99, 'E': 27.63, 'G': 2.2}
- **3D similarity (TM-Score) (%) [PDB]:** 22.14

- **Gene name:** PTPN6
- **Entrez ID:** 5777
- **RefSeq ID:** N/A
- **Sequence length:** N/A
- **5-UTR|CDS|3-UTR identity (%):** N/A | N/A | N/A
- **5-UTR|CDS|3-UTR identity (%) [Gaps excluded]:** N/A | N/A | N/A
- **5-UTR|CDS|3-UTR identity [Alignment Gaps]:** N/A | N/A | N/A
- **5-UTR aligned content (<base>:%):** N/A
- **CDS aligned content (<base>:%):** N/A
- **3-UTR aligned content (<base>:%):** N/A

**Uniprot Description:**  
  
 Modulates signaling by tyrosine phosphorylated cell surface receptors such as KIT and the EGF receptor/EGFR. The SH2 regions may interact with other cellular components to modulate its own phosphatase activity against interacting substrates. Together with MTUS1, induces UBE2V2 expression upon angiotensin II stimulation. Plays a key role in hematopoiesis.   
  
Monomer. Interacts with MTUS1 (By similarity). Interacts with MILR1 (tyrosine-phosphorylated) (By similarity). Interacts with KIT (By similarity). Interacts with SIRPA/PTPNS1 (PubMed:9712903). Interacts with LILRB1 and LILRB2 (PubMed:9285411, PubMed:9842885). Interacts with FCRL2 and FCRL4 (PubMed:11162587, PubMed:14597715). Interacts with FCRL3 and FCRL6 (tyrosine phosphorylated form) (PubMed:20933011, PubMed:11162587, PubMed:19843936). Interacts with CD84 (PubMed:11414741). Interacts with CD300LF (PubMed:15184070). Interacts with CDK2 (PubMed:21262353). Interacts with KIR2DL1; the interaction is enhanced by ARRB2 (PubMed:18604210). Interacts (via SH2 1 domain) with ROS1; the interaction is direct and promotes ROS1 dephosphorylation (PubMed:11266449). Interacts with EGFR; inhibits EGFR-dependent activation of MAPK/ERK (PubMed:21258366). Interacts with LYN (PubMed:10574931). Interacts with the tyrosine phosphorylated form of PDPK1 (PubMed:19591923). Interacts with CEACAM1 (via cytoplasmic domain); this interaction depends on the monomer/dimer equilibrium and is phosphorylation-dependent (By similarity). Interacts with MPIG6B (via ITIM motif) (PubMed:23112346). Interacts with moesin/MSN.   
  
 **Gene Ontology Information:**

Molecular Function

- cell adhesion molecule binding
- non-membrane spanning protein tyrosine phosphatase activity
- phosphorylation-dependent protein binding
- phosphotyrosine residue binding
- protein kinase binding
- protein tyrosine phosphatase activity
- SH2 domain binding
- SH3 domain binding
- transmembrane receptor protein tyrosine phosphatase activity

Location

- alpha-beta T cell receptor complex
- cell-cell junction
- cytoplasm
- cytosol
- extracellular exosome
- extracellular region
- membrane
- nucleolus
- nucleoplasm
- nucleus
- protein-containing complex
- specific granule lumen
- tertiary granule lumen

Biological process

- B cell receptor signaling pathway
- cell differentiation
- cytokine-mediated signaling pathway
- epididymis development
- G protein-coupled receptor signaling pathway
- hematopoietic progenitor cell differentiation
- intracellular signal transduction
- MAPK cascade
- megakaryocyte development
- mitotic cell cycle
- natural killer cell mediated cytotoxicity
- negative regulation of cell population proliferation
- negative regulation of humoral immune response mediated by circulating immunoglobulin
- negative regulation of inflammatory response to wounding
- negative regulation of interleukin-6 production
- negative regulation of MAP kinase activity
- negative regulation of mast cell activation involved in immune response
- negative regulation of peptidyl-tyrosine phosphorylation
- negative regulation of T cell proliferation
- negative regulation of T cell receptor signaling pathway
- negative regulation of tumor necrosis factor production
- peptidyl-tyrosine dephosphorylation
- peptidyl-tyrosine phosphorylation
- platelet aggregation
- platelet formation
- positive regulation of cell adhesion mediated by integrin
- positive regulation of cell population proliferation
- positive regulation of phosphatidylinositol 3-kinase signaling
- protein dephosphorylation
- regulation of apoptotic process
- regulation of B cell differentiation
- regulation of ERK1 and ERK2 cascade
- regulation of G1/S transition of mitotic cell cycle
- regulation of release of sequestered calcium ion into cytosol
- regulation of type I interferon-mediated signaling pathway
- T cell costimulation
- T cell proliferation
- T cell receptor signaling pathway

---

80

- **Protein name:** RNA-binding protein 45
- **Organism:** Homo sapiens
- **Uniprot Accession Number:** Q8IUH3
- **Protein sequence length:** 476 aa
- **1D identity (%):** 11.46
- **1D identity (%) [Gaps excluded]:** 25.18
- **1D identity - Alignment Gaps:** 499
- **1D aligned content (<aminoacid>:%):** {'M': 1.9, 'S': 2.86, 'A': 4.76, 'G': 9.52, 'P': 12.38, 'K': 9.52, 'L': 5.71, 'F': 3.81, 'D': 4.76, 'I': 4.76, 'V': 8.57, 'R': 9.52, 'E': 4.76, 'Q': 3.81, 'T': 2.86, 'Y': 3.81, 'H': 0.95, 'C': 0.95, 'N': 4.76}
- **Common reported functions (%):** 16.67
- **Common reported locations (%):** 40.0
- **Common reported processes (%):** 0.0

- **AF ID:** Q8IUH3
- **Chain:** A
- **Protein length:** 476 aa
- **Resolution:** N/A
- **b-phipsi:** 0.005776
- **w-rdist:** 0.253606
- **t-alpha:** 0.078102
- **Chemical similarity (Tanimoto Index) (%):** 96.41
- **1D identity (%) [PDB]:** 1.63
- **1D identity (%) [Gaps excluded][PDB]:** 84.0
- **1D identity - Alignment Gaps [PDB]:** 1266
- **1D aligned content [PDB] (<aminoacid>:%):** {'R': 9.52, 'F': 9.52, 'P': 14.29, 'T': 4.76, 'I': 14.29, 'Y': 9.52, 'G': 9.52, 'V': 4.76, 'Q': 9.52, 'L': 9.52, 'H': 4.76}
- **2D identity (%) [PDB]:** 39.09
- **2D identity (%) [Gaps excluded][PDB]:** 91.14
- **2D identity - Alignment Gaps [PDB]:** 526
- **2D aligned content [PDB] (<2D-fold>:%):** {'.': 29.53, 'E': 23.12, 'T': 18.38, 'H': 28.13, 'G': 0.84}
- **3D similarity (TM-Score) (%) [PDB]:** 18.04

- **Gene name:** RBM45
- **Entrez ID:** 129831
- **RefSeq ID:** N/A
- **Sequence length:** N/A
- **5-UTR|CDS|3-UTR identity (%):** N/A | N/A | N/A
- **5-UTR|CDS|3-UTR identity (%) [Gaps excluded]:** N/A | N/A | N/A
- **5-UTR|CDS|3-UTR identity [Alignment Gaps]:** N/A | N/A | N/A
- **5-UTR aligned content (<base>:%):** N/A
- **CDS aligned content (<base>:%):** N/A
- **3-UTR aligned content (<base>:%):** N/A

**Uniprot Description:**  
  
 RNA-binding protein with binding specificity for poly(C). May play an important role in neural development. N/A   
  
 **Gene Ontology Information:**

Molecular Function

- identical protein binding
- RNA binding

Location

- cytoplasm
- nucleoplasm
- nucleus
- ribonucleoprotein complex

Biological process

- cell differentiation
- nervous system development

---

81

- **Protein name:** Xanthine dehydrogenase/oxidase
- **Organism:** Homo sapiens
- **Uniprot Accession Number:** P47989
- **Protein sequence length:** 1333 aa
- **1D identity (%):** 15.18
- **1D identity (%) [Gaps excluded]:** 24.73
- **1D identity - Alignment Gaps:** 524
- **1D aligned content (<aminoacid>:%):** {'M': 0.49, 'A': 7.77, 'G': 13.11, 'S': 4.85, 'L': 5.83, 'P': 10.19, 'I': 4.37, 'N': 3.4, 'E': 3.4, 'Y': 3.4, 'C': 2.43, 'V': 6.8, 'F': 6.8, 'K': 8.25, 'W': 0.49, 'T': 8.25, 'R': 3.88, 'Q': 1.94, 'H': 2.43, 'D': 1.94}
- **Common reported functions (%):** 0.0
- **Common reported locations (%):** 0.0
- **Common reported processes (%):** 0.0

- **AF ID:** P47989
- **Chain:** A
- **Protein length:** 1333 aa
- **Resolution:** N/A
- **b-phipsi:** 0.004339
- **w-rdist:** 0.297631
- **t-alpha:** 0.072993
- **Chemical similarity (Tanimoto Index) (%):** 99.25
- **1D identity (%) [PDB]:** 2.32
- **1D identity (%) [Gaps excluded][PDB]:** 80.33
- **1D identity - Alignment Gaps [PDB]:** 2051
- **1D aligned content [PDB] (<aminoacid>:%):** {'T': 12.24, 'Y': 2.04, 'L': 14.29, 'Q': 6.12, 'I': 6.12, 'V': 18.37, 'G': 6.12, 'K': 8.16, 'A': 4.08, 'E': 2.04, 'R': 2.04, 'M': 2.04, 'C': 4.08, 'N': 6.12, 'S': 4.08, 'P': 2.04}
- **2D identity (%) [PDB]:** 42.05
- **2D identity (%) [Gaps excluded][PDB]:** 90.57
- **2D identity - Alignment Gaps [PDB]:** 795
- **2D aligned content [PDB] (<2D-fold>:%):** {'.': 15.06, 'E': 29.65, 'T': 19.87, 'H': 34.46, 'G': 0.48, 'B': 0.48}
- **3D similarity (TM-Score) (%) [PDB]:** 29.07

- **Gene name:** XDH
- **Entrez ID:** 280960
- **RefSeq ID:** NM\_000379
- **Transcript sequence length:** 5715
- **5-UTR|CDS|3-UTR identity (%):** 18.26 | 42.75 | 9.71
- **5-UTR|CDS|3-UTR identity (%) [Gaps excluded]:** 71.19 | 77.75 | 76.71
- **5-UTR|CDS|3-UTR identity [Alignment Gaps]:** 171 | 1940 | 9660
- **5-UTR aligned content (<base>:%):** {'C': 30.95, 'G': 33.33, 'A': 19.05, 'T': 16.67}
- **CDS aligned content (<base>:%):** {'A': 24.0, 'T': 19.65, 'G': 28.28, 'C': 28.07}
- **3-UTR aligned content (<base>:%):** {'A': 29.98, 'G': 21.14, 'T': 31.28, 'C': 17.6}

**Uniprot Description:**  
  
 Key enzyme in purine degradation. Catalyzes the oxidation of hypoxanthine to xanthine. Catalyzes the oxidation of xanthine to uric acid. Contributes to the generation of reactive oxygen species. Has also low oxidase activity towards aldehydes (in vitro).   
  
Homodimer. Interacts with BTN1A1 (By similarity).   
  
 **Gene Ontology Information:**

Molecular Function

- 2 iron, 2 sulfur cluster binding
- FAD binding
- flavin adenine dinucleotide binding
- iron ion binding
- molybdenum ion binding
- molybdopterin cofactor binding
- protein homodimerization activity
- xanthine dehydrogenase activity
- xanthine oxidase activity

Location

- extracellular space
- peroxisome
- xanthine dehydrogenase complex

Biological process

- xanthine catabolic process

---

82

- **Protein name:** Alpha-1,6-mannosylglycoprotein 6-beta-N-acetylglucosaminyltransferase B
- **Organism:** Homo sapiens
- **Uniprot Accession Number:** Q3V5L5
- **Protein sequence length:** 792 aa
- **1D identity (%):** 14.02
- **1D identity (%) [Gaps excluded]:** 24.96
- **1D identity - Alignment Gaps:** 463
- **1D aligned content (<aminoacid>:%):** {'P': 12.84, 'D': 2.7, 'C': 2.03, 'R': 8.11, 'F': 4.73, 'G': 14.19, 'A': 6.76, 'L': 11.49, 'M': 1.35, 'H': 3.38, 'E': 5.41, 'Q': 4.73, 'W': 1.35, 'N': 3.38, 'K': 6.76, 'V': 2.7, 'T': 2.7, 'Y': 2.03, 'I': 2.03, 'S': 1.35}
- **Common reported functions (%):** 0.0
- **Common reported locations (%):** 0.0
- **Common reported processes (%):** 0.0

- **AF ID:** Q3V5L5
- **Chain:** A
- **Protein length:** 792 aa
- **Resolution:** N/A
- **b-phipsi:** 0.009935
- **w-rdist:** 0.299978
- **t-alpha:** 0.024087
- **Chemical similarity (Tanimoto Index) (%):** 96.99
- **1D identity (%) [PDB]:** 1.95
- **1D identity (%) [Gaps excluded][PDB]:** 68.89
- **1D identity - Alignment Gaps [PDB]:** 1542
- **1D aligned content [PDB] (<aminoacid>:%):** {'M': 3.23, 'L': 12.9, 'H': 3.23, 'A': 12.9, 'V': 6.45, 'S': 12.9, 'Q': 6.45, 'I': 3.23, 'P': 16.13, 'D': 3.23, 'R': 6.45, 'G': 6.45, 'F': 3.23, 'E': 3.23}
- **2D identity (%) [PDB]:** 39.51
- **2D identity (%) [Gaps excluded][PDB]:** 91.28
- **2D identity - Alignment Gaps [PDB]:** 646
- **2D aligned content [PDB] (<2D-fold>:%):** {'.': 22.22, 'H': 49.78, 'T': 16.22, 'G': 2.67, 'B': 0.22, 'E': 8.89}
- **3D similarity (TM-Score) (%) [PDB]:** 23.72

- **Gene name:** MGAT5B
- **Entrez ID:** 146664
- **RefSeq ID:** NM\_198955
- **Transcript sequence length:** 4068
- **5-UTR|CDS|3-UTR identity (%):** 25.76 | 46.35 | 8.18
- **5-UTR|CDS|3-UTR identity (%) [Gaps excluded]:** 67.82 | 75.83 | 76.76
- **5-UTR|CDS|3-UTR identity [Alignment Gaps]:** 142 | 1226 | 9998
- **5-UTR aligned content (<base>:%):** {'G': 25.42, 'C': 38.98, 'T': 22.03, 'A': 13.56}
- **CDS aligned content (<base>:%):** {'A': 19.9, 'T': 17.24, 'G': 29.34, 'C': 33.52}
- **3-UTR aligned content (<base>:%):** {'A': 16.83, 'T': 25.25, 'C': 31.48, 'G': 26.45}

**Uniprot Description:**  
  
 Glycosyltransferase that acts on alpha-linked mannose of N-glycans and O-mannosyl glycans. Catalyzes the transfer of N-acetylglucosamine (GlcNAc) to the beta 1-6 linkage of the mannose residue of GlcNAc-beta1,2-Man-alpha on both the alpha1,3- and alpha1,6-linked mannose arms in the core structure of N-glycan. Also acts on the GlcNAc-beta1,2-Man-alpha1-Ser/Thr moiety, forming a 2,6-branched structure in brain O-mannosyl glycan. Plays an active role in modulating integrin and laminin-dependent adhesion and migration of neuronal cells via its activity in the O-mannosyl glycan pathway. N/A   
  
 **Gene Ontology Information:**

Molecular Function

- alpha-1,6-mannosylglycoprotein 6-beta-N-acetylglucosaminyltransferase activity
- manganese ion binding

Location

- Golgi apparatus
- Golgi membrane

Biological process

- protein N-linked glycosylation
- protein O-linked glycosylation via serine

---

83

- **Protein name:** Mitochondrial ribonuclease P catalytic subunit
- **Organism:** Homo sapiens
- **Uniprot Accession Number:** O15091
- **Protein sequence length:** 583 aa
- **1D identity (%):** 10.81
- **1D identity (%) [Gaps excluded]:** 23.78
- **1D identity - Alignment Gaps:** 540
- **1D aligned content (<aminoacid>:%):** {'T': 6.54, 'K': 8.41, 'P': 9.35, 'L': 12.15, 'G': 7.48, 'H': 1.87, 'Y': 4.67, 'C': 4.67, 'I': 6.54, 'Q': 4.67, 'R': 5.61, 'N': 1.87, 'A': 4.67, 'S': 3.74, 'E': 4.67, 'D': 5.61, 'V': 2.8, 'M': 0.93, 'F': 3.74}
- **Common reported functions (%):** 0.0
- **Common reported locations (%):** 10.0
- **Common reported processes (%):** 0.0

- **AF ID:** O15091
- **Chain:** A
- **Protein length:** 583 aa
- **Resolution:** N/A
- **b-phipsi:** 0.012751
- **w-rdist:** 0.218583
- **t-alpha:** 0.035037
- **Chemical similarity (Tanimoto Index) (%):** 98.57
- **1D identity (%) [PDB]:** 2.54
- **1D identity (%) [Gaps excluded][PDB]:** 74.47
- **1D identity - Alignment Gaps [PDB]:** 1329
- **1D aligned content [PDB] (<aminoacid>:%):** {'L': 22.86, 'K': 14.29, 'N': 8.57, 'T': 5.71, 'Y': 2.86, 'S': 2.86, 'I': 8.57, 'V': 8.57, 'P': 8.57, 'G': 2.86, 'E': 2.86, 'R': 2.86, 'D': 2.86, 'Q': 2.86, 'C': 2.86}
- **2D identity (%) [PDB]:** 36.59
- **2D identity (%) [Gaps excluded][PDB]:** 93.27
- **2D identity - Alignment Gaps [PDB]:** 621
- **2D aligned content [PDB] (<2D-fold>:%):** {'.': 19.25, 'T': 16.58, 'H': 58.02, 'E': 4.81, 'G': 0.8, 'B': 0.53}
- **3D similarity (TM-Score) (%) [PDB]:** 20.8

- **Gene name:** PRORP
- **Entrez ID:** 9692
- **RefSeq ID:** N/A
- **Sequence length:** N/A
- **5-UTR|CDS|3-UTR identity (%):** N/A | N/A | N/A
- **5-UTR|CDS|3-UTR identity (%) [Gaps excluded]:** N/A | N/A | N/A
- **5-UTR|CDS|3-UTR identity [Alignment Gaps]:** N/A | N/A | N/A
- **5-UTR aligned content (<base>:%):** N/A
- **CDS aligned content (<base>:%):** N/A
- **3-UTR aligned content (<base>:%):** N/A

**Uniprot Description:**  
  
 Catalytic ribonuclease component of mitochondrial ribonuclease P, a complex composed of TRMT10C/MRPP1, HSD17B10/MRPP2 and PRORP/MRPP3, which cleaves tRNA molecules in their 5'-ends (PubMed:18984158, PubMed:25953853). The presence of TRMT10C/MRPP1, HSD17B10/MRPP2 is required to catalyze tRNA molecules in their 5'-ends (PubMed:25953853).   
  
Catalytic component of mitochondrial ribonuclease P, a complex composed of TRMT10C/MRPP1, HSD17B10/MRPP2 and PRORP/MRPP3 (PubMed:18984158).   
  
 **Gene Ontology Information:**

Molecular Function

- metal ion binding
- ribonuclease P activity

Location

- mitochondrial matrix
- mitochondrial nucleoid
- mitochondrial ribonuclease P complex
- mitochondrion
- nucleoplasm

Biological process

- mitochondrial tRNA 5'-end processing
- tRNA 5'-leader removal

---

84

- **Protein name:** TAF5-like RNA polymerase II p300/CBP-associated factor-associated factor 65 kDa subunit 5L
- **Organism:** Homo sapiens
- **Uniprot Accession Number:** O75529
- **Protein sequence length:** 589 aa
- **1D identity (%):** 11.09
- **1D identity (%) [Gaps excluded]:** 22.25
- **1D identity - Alignment Gaps:** 484
- **1D aligned content (<aminoacid>:%):** {'V': 5.61, 'Y': 6.54, 'K': 5.61, 'P': 6.54, 'L': 12.15, 'Q': 6.54, 'N': 5.61, 'I': 5.61, 'C': 4.67, 'D': 8.41, 'S': 4.67, 'T': 4.67, 'G': 2.8, 'A': 5.61, 'F': 3.74, 'R': 6.54, 'H': 2.8, 'W': 0.93, 'E': 0.93}
- **Common reported functions (%):** 0.0
- **Common reported locations (%):** 10.0
- **Common reported processes (%):** 0.0

- **AF ID:** O75529
- **Chain:** A
- **Protein length:** 589 aa
- **Resolution:** N/A
- **b-phipsi:** 0.003495
- **w-rdist:** 0.382065
- **t-alpha:** 0.032847
- **Chemical similarity (Tanimoto Index) (%):** 97.33
- **1D identity (%) [PDB]:** 2.92
- **1D identity (%) [Gaps excluded][PDB]:** 65.57
- **1D identity - Alignment Gaps [PDB]:** 1307
- **1D aligned content [PDB] (<aminoacid>:%):** {'K': 5.0, 'Q': 17.5, 'Y': 7.5, 'L': 12.5, 'P': 10.0, 'H': 5.0, 'V': 2.5, 'E': 5.0, 'T': 10.0, 'I': 5.0, 'A': 5.0, 'G': 2.5, 'R': 2.5, 'D': 2.5, 'N': 2.5, 'S': 5.0}
- **2D identity (%) [PDB]:** 28.66
- **2D identity (%) [Gaps excluded][PDB]:** 95.45
- **2D identity - Alignment Gaps [PDB]:** 769
- **2D aligned content [PDB] (<2D-fold>:%):** {'H': 38.73, 'T': 18.41, '.': 20.32, 'E': 22.54}
- **3D similarity (TM-Score) (%) [PDB]:** 20.94

- **Gene name:** TAF5L
- **Entrez ID:** 60926380
- **RefSeq ID:** N/A
- **Sequence length:** N/A
- **5-UTR|CDS|3-UTR identity (%):** N/A | N/A | N/A
- **5-UTR|CDS|3-UTR identity (%) [Gaps excluded]:** N/A | N/A | N/A
- **5-UTR|CDS|3-UTR identity [Alignment Gaps]:** N/A | N/A | N/A
- **5-UTR aligned content (<base>:%):** N/A
- **CDS aligned content (<base>:%):** N/A
- **3-UTR aligned content (<base>:%):** N/A

**Uniprot Description:**  
  
 Functions as a component of the PCAF complex. The PCAF complex is capable of efficiently acetylating histones in a nucleosomal context. The PCAF complex could be considered as the human version of the yeast SAGA complex (Probable). With TAF6L, acts as an epigenetic regulator essential for somatic reprogramming. Regulates target genes through H3K9ac deposition and MYC recruitment which trigger MYC regulatory network to orchestrate gene expression programs to control embryonic stem cell state (By similarity).   
  
The PCAF complex is composed of a number of TBP-associated factors (TAFS), such as TAF5, TAF5L, TAF6, TAF6L, TAF9, TAF10 and TAF12, PCAF, and also PCAF-associated factors (PAFs), such as TADA2L/ADA2, TADA3L/ADA3 and SPT3. Component of the STAGA transcription coactivator-HAT complex, at least composed of SUPT3H, GCN5L2, TAF5L, TAF6L, SUPT7L, TADA3L, TAD1L, TAF10, TAF12, TRRAP and TAF9.   
  
 **Gene Ontology Information:**

Molecular Function

- ATP binding
- metal ion binding
- threonine-tRNA ligase activity
- tRNA binding

Location

- cytoplasm

Biological process

- threonyl-tRNA aminoacylation

---

85

- **Protein name:** Ribosomal protein S6 kinase alpha-6
- **Organism:** Homo sapiens
- **Uniprot Accession Number:** Q9UK32
- **Protein sequence length:** 745 aa
- **1D identity (%):** 14.64
- **1D identity (%) [Gaps excluded]:** 22.47
- **1D identity - Alignment Gaps:** 338
- **1D aligned content (<aminoacid>:%):** {'L': 11.27, 'P': 7.75, 'Q': 7.04, 'R': 3.52, 'G': 8.45, 'N': 4.23, 'V': 6.34, 'D': 7.04, 'H': 2.82, 'E': 4.93, 'K': 9.86, 'F': 4.93, 'Y': 2.82, 'A': 4.23, 'T': 5.63, 'I': 5.63, 'S': 2.11, 'C': 0.7, 'M': 0.7}
- **Common reported functions (%):** 0.0
- **Common reported locations (%):** 30.0
- **Common reported processes (%):** 0.0

- **AF ID:** Q9UK32
- **Chain:** A
- **Protein length:** 745 aa
- **Resolution:** N/A
- **b-phipsi:** 0.005413
- **w-rdist:** 0.43923
- **t-alpha:** 0.005874
- **Chemical similarity (Tanimoto Index) (%):** 99.02
- **1D identity (%) [PDB]:** 4.02
- **1D identity (%) [Gaps excluded][PDB]:** 65.22
- **1D identity - Alignment Gaps [PDB]:** 1401
- **1D aligned content [PDB] (<aminoacid>:%):** {'E': 11.67, 'V': 15.0, 'P': 8.33, 'I': 10.0, 'H': 3.33, 'Y': 5.0, 'D': 3.33, 'K': 8.33, 'N': 1.67, 'R': 5.0, 'Q': 5.0, 'F': 3.33, 'G': 5.0, 'T': 1.67, 'L': 8.33, 'S': 1.67, 'M': 1.67, 'A': 1.67}
- **2D identity (%) [PDB]:** 42.28
- **2D identity (%) [Gaps excluded][PDB]:** 90.67
- **2D identity - Alignment Gaps [PDB]:** 577
- **2D aligned content [PDB] (<2D-fold>:%):** {'.': 22.76, 'T': 17.94, 'E': 17.07, 'H': 40.26, 'G': 1.97}
- **3D similarity (TM-Score) (%) [PDB]:** 24.73

- **Gene name:** RPS6KA6
- **Entrez ID:** 27330
- **RefSeq ID:** N/A
- **Sequence length:** N/A
- **5-UTR|CDS|3-UTR identity (%):** N/A | N/A | N/A
- **5-UTR|CDS|3-UTR identity (%) [Gaps excluded]:** N/A | N/A | N/A
- **5-UTR|CDS|3-UTR identity [Alignment Gaps]:** N/A | N/A | N/A
- **5-UTR aligned content (<base>:%):** N/A
- **CDS aligned content (<base>:%):** N/A
- **3-UTR aligned content (<base>:%):** N/A

**Uniprot Description:**  
  
 Constitutively active serine/threonine-protein kinase that exhibits growth-factor-independent kinase activity and that may participate in p53/TP53-dependent cell growth arrest signaling and play an inhibitory role during embryogenesis.   
  
Forms a complex with MAPK3/ERK1 but not with MAPK9 or MAPK14 in serum-starved cells.   
  
 **Gene Ontology Information:**

Molecular Function

- ATP binding
- magnesium ion binding
- protein kinase activity
- protein serine kinase activity
- ribosomal protein S6 kinase activity

Location

- cytoplasm
- cytosol
- fibrillar center
- mitochondrion
- nucleolus
- nucleoplasm

Biological process

- central nervous system development
- DNA damage response, signal transduction by p53 class mediator resulting in transcription of p21 class mediator
- negative regulation of embryonic development
- negative regulation of ERK1 and ERK2 cascade
- negative regulation of mesoderm development
- peptidyl-serine phosphorylation
- signal transduction

---

86

- **Protein name:** 5'-AMP-activated protein kinase catalytic subunit alpha-1
- **Organism:** Homo sapiens
- **Uniprot Accession Number:** Q13131
- **Protein sequence length:** 559 aa
- **1D identity (%):** 11.61
- **1D identity (%) [Gaps excluded]:** 21.66
- **1D identity - Alignment Gaps:** 428
- **1D aligned content (<aminoacid>:%):** {'R': 9.35, 'K': 5.61, 'T': 1.87, 'V': 9.35, 'I': 4.67, 'G': 8.41, 'Q': 3.74, 'N': 3.74, 'L': 11.21, 'P': 12.15, 'H': 2.8, 'E': 2.8, 'C': 2.8, 'D': 7.48, 'S': 3.74, 'F': 1.87, 'M': 1.87, 'Y': 2.8, 'W': 0.93, 'A': 2.8}
- **Common reported functions (%):** 0.0
- **Common reported locations (%):** 40.0
- **Common reported processes (%):** 0.0

- **AF ID:** Q13131
- **Chain:** A
- **Protein length:** 559 aa
- **Resolution:** N/A
- **b-phipsi:** 0.003768
- **w-rdist:** 0.376346
- **t-alpha:** 0.037093
- **Chemical similarity (Tanimoto Index) (%):** 99.24
- **1D identity (%) [PDB]:** 2.84
- **1D identity (%) [Gaps excluded][PDB]:** 60.32
- **1D identity - Alignment Gaps [PDB]:** 1273
- **1D aligned content [PDB] (<aminoacid>:%):** {'M': 5.26, 'R': 10.53, 'L': 7.89, 'S': 10.53, 'T': 2.63, 'P': 7.89, 'G': 5.26, 'E': 5.26, 'V': 7.89, 'W': 5.26, 'F': 2.63, 'H': 2.63, 'Q': 5.26, 'A': 10.53, 'K': 5.26, 'I': 2.63, 'D': 2.63}
- **2D identity (%) [PDB]:** 39.92
- **2D identity (%) [Gaps excluded][PDB]:** 95.63
- **2D identity - Alignment Gaps [PDB]:** 575
- **2D aligned content [PDB] (<2D-fold>:%):** {'.': 22.08, 'E': 19.04, 'T': 12.44, 'H': 44.16, 'G': 2.28}
- **3D similarity (TM-Score) (%) [PDB]:** 20.59

- **Gene name:** PRKAA1
- **Entrez ID:** 5562
- **RefSeq ID:** NM\_206907
- **Transcript sequence length:** 5299
- **5-UTR|CDS|3-UTR identity (%):** 45.71 | 36.2 | 18.96
- **5-UTR|CDS|3-UTR identity (%) [Gaps excluded]:** 73.2 | 75.53 | 76.2
- **5-UTR|CDS|3-UTR identity [Alignment Gaps]:** 92 | 1549 | 8548
- **5-UTR aligned content (<base>:%):** {'A': 6.25, 'C': 42.86, 'T': 7.14, 'G': 43.75}
- **CDS aligned content (<base>:%):** {'A': 31.1, 'T': 23.68, 'G': 23.96, 'C': 21.26}
- **3-UTR aligned content (<base>:%):** {'A': 29.33, 'C': 14.64, 'T': 39.94, 'G': 16.08}

**Uniprot Description:**  
  
 Catalytic subunit of AMP-activated protein kinase (AMPK), an energy sensor protein kinase that plays a key role in regulating cellular energy metabolism. In response to reduction of intracellular ATP levels, AMPK activates energy-producing pathways and inhibits energy-consuming processes: inhibits protein, carbohydrate and lipid biosynthesis, as well as cell growth and proliferation. AMPK acts via direct phosphorylation of metabolic enzymes, and by longer-term effects via phosphorylation of transcription regulators. Also acts as a regulator of cellular polarity by remodeling the actin cytoskeleton; probably by indirectly activating myosin. Regulates lipid synthesis by phosphorylating and inactivating lipid metabolic enzymes such as ACACA, ACACB, GYS1, HMGCR and LIPE; regulates fatty acid and cholesterol synthesis by phosphorylating acetyl-CoA carboxylase (ACACA and ACACB) and hormone-sensitive lipase (LIPE) enzymes, respectively. Regulates insulin-signaling and glycolysis by phosphorylating IRS1, PFKFB2 and PFKFB3. AMPK stimulates glucose uptake in muscle by increasing the translocation of the glucose transporter SLC2A4/GLUT4 to the plasma membrane, possibly by mediating phosphorylation of TBC1D4/AS160. Regulates transcription and chromatin structure by phosphorylating transcription regulators involved in energy metabolism such as CRTC2/TORC2, FOXO3, histone H2B, HDAC5, MEF2C, MLXIPL/ChREBP, EP300, HNF4A, p53/TP53, SREBF1, SREBF2 and PPARGC1A. Acts as a key regulator of glucose homeostasis in liver by phosphorylating CRTC2/TORC2, leading to CRTC2/TORC2 sequestration in the cytoplasm. In response to stress, phosphorylates 'Ser-36' of histone H2B (H2BS36ph), leading to promote transcription. Acts as a key regulator of cell growth and proliferation by phosphorylating TSC2, RPTOR and ATG1/ULK1: in response to nutrient limitation, negatively regulates the mTORC1 complex by phosphorylating RPTOR component of the mTORC1 complex and by phosphorylating and activating TSC2. In response to nutrient limitation, promotes autophagy by phosphorylating and activating ATG1/ULK1. In that process also activates WDR45 (PubMed:28561066). In response to nutrient limitation, phosphorylates transcription factor FOXO3 promoting FOXO3 mitochondrial import (By similarity). AMPK also acts as a regulator of circadian rhythm by mediating phosphorylation of CRY1, leading to destabilize it. May regulate the Wnt signaling pathway by phosphorylating CTNNB1, leading to stabilize it. Also has tau-protein kinase activity: in response to amyloid beta A4 protein (APP) exposure, activated by CAMKK2, leading to phosphorylation of MAPT/TAU; however the relevance of such data remains unclear in vivo. Also phosphorylates CFTR, EEF2K, KLC1, NOS3 and SLC12A1.   
  
AMPK is a heterotrimer of an alpha catalytic subunit (PRKAA1 or PRKAA2), a beta (PRKAB1 or PRKAB2) and a gamma non-catalytic subunits (PRKAG1, PRKAG2 or PRKAG3). Interacts with FNIP1 and FNIP2.   
  
 **Gene Ontology Information:**

Molecular Function

- [acetyl-CoA carboxylase] kinase activity
- [hydroxymethylglutaryl-CoA reductase (NADPH)] kinase activity
- AMP-activated protein kinase activity
- ATP binding
- cAMP-dependent protein kinase activity
- chromatin binding
- histone H2BS36 kinase activity
- metal ion binding
- protein kinase activity
- protein serine kinase activity
- protein serine/threonine kinase activity
- tau protein binding
- tau-protein kinase activity

Location

- apical plasma membrane
- axon
- chromatin
- cytoplasm
- cytosol
- dendrite
- neuronal cell body
- nuclear speck
- nucleoplasm
- nucleotide-activated protein kinase complex
- nucleus

Biological process

- autophagy
- bile acid and bile salt transport
- bile acid signaling pathway
- CAMKK-AMPK signaling cascade
- cellular response to calcium ion
- cellular response to ethanol
- cellular response to glucose starvation
- cellular response to glucose stimulus
- cellular response to hydrogen peroxide
- cellular response to hypoxia
- cellular response to nutrient levels
- cellular response to organonitrogen compound
- cellular response to oxidative stress
- cellular response to prostaglandin E stimulus
- cellular response to xenobiotic stimulus
- cholesterol biosynthetic process
- cold acclimation
- energy homeostasis
- fatty acid biosynthetic process
- fatty acid homeostasis
- fatty acid oxidation
- glucose homeostasis
- glucose metabolic process
- intracellular signal transduction
- lipid biosynthetic process
- lipid droplet disassembly
- motor behavior
- negative regulation of apoptotic process
- negative regulation of gene expression
- negative regulation of glucosylceramide biosynthetic process
- negative regulation of hepatocyte apoptotic process
- negative regulation of insulin receptor signaling pathway
- negative regulation of lipid catabolic process
- negative regulation of TOR signaling
- negative regulation of tubulin deacetylation
- neuron cellular homeostasis
- positive regulation of adipose tissue development
- positive regulation of autophagy
- positive regulation of cell population proliferation
- positive regulation of cholesterol biosynthetic process
- positive regulation of transcription, DNA-templated
- positive regulation of gene expression
- positive regulation of glycolytic process
- positive regulation of mitochondrial transcription
- positive regulation of peptidyl-lysine acetylation
- positive regulation of cellular protein localization
- positive regulation of protein targeting to mitochondrion
- positive regulation of skeletal muscle tissue development
- protein localization to lipid droplet
- protein phosphorylation
- regulation of bile acid secretion
- regulation of circadian rhythm
- regulation of microtubule cytoskeleton organization
- regulation of peptidyl-serine phosphorylation
- regulation of stress granule assembly
- regulation of vesicle-mediated transport
- response to activity
- response to caffeine
- response to estrogen
- response to gamma radiation
- response to hypoxia
- response to UV
- rhythmic process
- signal transduction
- Wnt signaling pathway

---

87

- **Protein name:** Kelch repeat and BTB domain-containing protein 2
- **Organism:** Homo sapiens
- **Uniprot Accession Number:** Q8IY47
- **Protein sequence length:** 623 aa
- **1D identity (%):** 13.3
- **1D identity (%) [Gaps excluded]:** 25.29
- **1D identity - Alignment Gaps:** 460
- **1D aligned content (<aminoacid>:%):** {'Q': 5.43, 'E': 3.88, 'L': 9.3, 'T': 7.75, 'D': 8.53, 'K': 6.2, 'C': 3.1, 'R': 6.2, 'H': 3.1, 'N': 6.2, 'V': 7.75, 'I': 5.43, 'A': 4.65, 'S': 3.88, 'Y': 5.43, 'G': 4.65, 'P': 3.88, 'F': 3.1, 'M': 0.78, 'W': 0.78}
- **Common reported functions (%):** 0.0
- **Common reported locations (%):** 0.0
- **Common reported processes (%):** 0.0

- **AF ID:** Q8IY47
- **Chain:** A
- **Protein length:** 623 aa
- **Resolution:** N/A
- **b-phipsi:** 0.003415
- **w-rdist:** 0.361823
- **t-alpha:** 0.052228
- **Chemical similarity (Tanimoto Index) (%):** 98.87
- **1D identity (%) [PDB]:** 2.93
- **1D identity (%) [Gaps excluded][PDB]:** 62.12
- **1D identity - Alignment Gaps [PDB]:** 1331
- **1D aligned content [PDB] (<aminoacid>:%):** {'T': 4.88, 'D': 7.32, 'E': 7.32, 'Q': 4.88, 'I': 7.32, 'L': 7.32, 'C': 2.44, 'H': 7.32, 'V': 4.88, 'S': 12.2, 'A': 7.32, 'Y': 4.88, 'F': 2.44, 'K': 2.44, 'G': 9.76, 'N': 2.44, 'R': 2.44, 'P': 2.44}
- **2D identity (%) [PDB]:** 33.4
- **2D identity (%) [Gaps excluded][PDB]:** 93.51
- **2D identity - Alignment Gaps [PDB]:** 693
- **2D aligned content [PDB] (<2D-fold>:%):** {'.': 20.83, 'H': 37.78, 'T': 20.0, 'E': 18.61, 'G': 2.5, 'B': 0.28}
- **3D similarity (TM-Score) (%) [PDB]:** 21.76

- **Gene name:** KBTBD2
- **Entrez ID:** 25948
- **RefSeq ID:** N/A
- **Sequence length:** N/A
- **5-UTR|CDS|3-UTR identity (%):** N/A | N/A | N/A
- **5-UTR|CDS|3-UTR identity (%) [Gaps excluded]:** N/A | N/A | N/A
- **5-UTR|CDS|3-UTR identity [Alignment Gaps]:** N/A | N/A | N/A
- **5-UTR aligned content (<base>:%):** N/A
- **CDS aligned content (<base>:%):** N/A
- **3-UTR aligned content (<base>:%):** N/A

**Uniprot Description:**  
  
 N/A N/A   
  
 **Gene Ontology Information:**

Molecular Function   
  
N/A

Location   
  
N/A

Biological process

- gene expression
- glucose metabolic process
- lipid metabolic process
- phosphatidylinositol 3-kinase signaling
- protein ubiquitination
- response to insulin

---

88

- **Protein name:** Cilia- and flagella-associated protein 77
- **Organism:** Homo sapiens
- **Uniprot Accession Number:** Q6ZQR2
- **Protein sequence length:** 320 aa
- **1D identity (%):** 8.6
- **1D identity (%) [Gaps excluded]:** 27.3
- **1D identity - Alignment Gaps:** 613
- **1D aligned content (<aminoacid>:%):** {'E': 3.9, 'G': 6.49, 'L': 9.09, 'Q': 7.79, 'V': 6.49, 'T': 6.49, 'S': 6.49, 'C': 1.3, 'N': 1.3, 'R': 9.09, 'F': 2.6, 'P': 9.09, 'I': 2.6, 'A': 5.19, 'H': 7.79, 'Y': 6.49, 'K': 3.9, 'D': 2.6, 'W': 1.3}
- **Common reported functions (%):** 0.0
- **Common reported locations (%):** 0.0
- **Common reported processes (%):** 0.0

- **AF ID:** Q6ZQR2
- **Chain:** A
- **Protein length:** 320 aa
- **Resolution:** N/A
- **b-phipsi:** 0.009087
- **w-rdist:** 0.788264
- **t-alpha:** 0.00073
- **Chemical similarity (Tanimoto Index) (%):** 98.8
- **1D identity (%) [PDB]:** 2.79
- **1D identity (%) [Gaps excluded][PDB]:** 65.96
- **1D identity - Alignment Gaps [PDB]:** 1066
- **1D aligned content [PDB] (<aminoacid>:%):** {'E': 6.45, 'A': 9.68, 'S': 6.45, 'G': 6.45, 'Q': 6.45, 'P': 16.13, 'V': 9.68, 'L': 9.68, 'D': 3.23, 'M': 3.23, 'R': 9.68, 'T': 3.23, 'F': 3.23, 'Y': 3.23, 'H': 3.23}
- **2D identity (%) [PDB]:** 24.64
- **2D identity (%) [Gaps excluded][PDB]:** 82.4
- **2D identity - Alignment Gaps [PDB]:** 626
- **2D aligned content [PDB] (<2D-fold>:%):** {'H': 40.45, '.': 37.73, 'T': 18.18, 'E': 1.36, 'G': 2.27}
- **3D similarity (TM-Score) (%) [PDB]:** 10.5

- **Gene name:** CFAP77
- **Entrez ID:** 389799
- **RefSeq ID:** NM\_207417
- **Transcript sequence length:** 1833
- **5-UTR|CDS|3-UTR identity (%):** 19.0 | 22.36 | 4.56
- **5-UTR|CDS|3-UTR identity (%) [Gaps excluded]:** 79.25 | 78.34 | 73.49
- **5-UTR|CDS|3-UTR identity [Alignment Gaps]:** 168 | 2023 | 10274
- **5-UTR aligned content (<base>:%):** {'C': 42.86, 'G': 35.71, 'A': 14.29, 'T': 7.14}
- **CDS aligned content (<base>:%):** {'A': 23.7, 'T': 16.9, 'G': 28.91, 'C': 30.49}
- **3-UTR aligned content (<base>:%):** {'C': 28.86, 'T': 32.26, 'G': 17.23, 'A': 21.64}

**Uniprot Description:**  
  
 Microtubule inner protein (MIP) part of the dynein-decorated doublet microtubules (DMTs) in cilia axoneme, which is required for motile cilia beating. N/A   
  
 **Gene Ontology Information:**

Molecular Function   
  
N/A

Location

- axonemal microtubule

Biological process   
  
N/A

---

89

- **Protein name:** TBC1 domain family member 3H
- **Organism:** Homo sapiens
- **Uniprot Accession Number:** P0C7X1
- **Protein sequence length:** 549 aa
- **1D identity (%):** 10.73
- **1D identity (%) [Gaps excluded]:** 22.42
- **1D identity - Alignment Gaps:** 496
- **1D aligned content (<aminoacid>:%):** {'A': 8.82, 'R': 10.78, 'I': 4.9, 'K': 5.88, 'S': 5.88, 'Y': 2.94, 'N': 0.98, 'G': 10.78, 'T': 5.88, 'V': 5.88, 'D': 4.9, 'M': 0.98, 'W': 1.96, 'Q': 3.92, 'E': 0.98, 'F': 0.98, 'P': 10.78, 'L': 8.82, 'C': 2.94, 'H': 0.98}
- **Common reported functions (%):** 0.0
- **Common reported locations (%):** 0.0
- **Common reported processes (%):** 0.0

- **AF ID:** P0C7X1
- **Chain:** A
- **Protein length:** 549 aa
- **Resolution:** N/A
- **b-phipsi:** 0.014275
- **w-rdist:** 0.51718
- **t-alpha:** 0.001462
- **Chemical similarity (Tanimoto Index) (%):** 98.58
- **1D identity (%) [PDB]:** 2.14
- **1D identity (%) [Gaps excluded][PDB]:** 78.38
- **1D identity - Alignment Gaps [PDB]:** 1315
- **1D aligned content [PDB] (<aminoacid>:%):** {'D': 3.45, 'V': 10.34, 'E': 6.9, 'P': 10.34, 'F': 3.45, 'R': 6.9, 'L': 13.79, 'K': 6.9, 'N': 3.45, 'T': 6.9, 'G': 10.34, 'Q': 3.45, 'I': 10.34, 'A': 3.45}
- **2D identity (%) [PDB]:** 33.97
- **2D identity (%) [Gaps excluded][PDB]:** 87.63
- **2D identity - Alignment Gaps [PDB]:** 613
- **2D aligned content [PDB] (<2D-fold>:%):** {'.': 18.53, 'H': 54.12, 'T': 25.0, 'E': 2.35}
- **3D similarity (TM-Score) (%) [PDB]:** 16.78

- **Gene name:** TBC1D3H
- **Entrez ID:** 729877
- **RefSeq ID:** N/A
- **Sequence length:** N/A
- **5-UTR|CDS|3-UTR identity (%):** N/A | N/A | N/A
- **5-UTR|CDS|3-UTR identity (%) [Gaps excluded]:** N/A | N/A | N/A
- **5-UTR|CDS|3-UTR identity [Alignment Gaps]:** N/A | N/A | N/A
- **5-UTR aligned content (<base>:%):** N/A
- **CDS aligned content (<base>:%):** N/A
- **3-UTR aligned content (<base>:%):** N/A

**Uniprot Description:**  
  
 Acts as a GTPase activating protein for RAB5. Does not act on RAB4 or RAB11 (By similarity). N/A   
  
 **Gene Ontology Information:**

Molecular Function

- GTPase activator activity

Location

- plasma membrane

Biological process

- activation of GTPase activity

---

90

- **Protein name:** Endoplasmic reticulum aminopeptidase 2
- **Organism:** Homo sapiens
- **Uniprot Accession Number:** Q6P179
- **Protein sequence length:** 960 aa
- **1D identity (%):** 14.64
- **1D identity (%) [Gaps excluded]:** 24.3
- **1D identity - Alignment Gaps:** 451
- **1D aligned content (<aminoacid>:%):** {'M': 1.2, 'H': 3.61, 'P': 9.04, 'L': 12.05, 'S': 4.22, 'F': 6.02, 'G': 7.23, 'R': 7.23, 'W': 1.81, 'V': 4.22, 'K': 10.24, 'T': 6.02, 'Y': 3.01, 'Q': 4.82, 'C': 2.41, 'A': 4.22, 'D': 3.01, 'I': 3.61, 'N': 3.01, 'E': 3.01}
- **Common reported functions (%):** 0.0
- **Common reported locations (%):** 10.0
- **Common reported processes (%):** 0.0

- **AF ID:** Q6P179
- **Chain:** A
- **Protein length:** 960 aa
- **Resolution:** N/A
- **b-phipsi:** 0.007121
- **w-rdist:** 0.25133
- **t-alpha:** 0.066423
- **Chemical similarity (Tanimoto Index) (%):** 96.12
- **1D identity (%) [PDB]:** 2.59
- **1D identity (%) [Gaps excluded][PDB]:** 75.0
- **1D identity - Alignment Gaps [PDB]:** 1680
- **1D aligned content [PDB] (<aminoacid>:%):** {'L': 13.33, 'P': 6.67, 'Q': 4.44, 'I': 11.11, 'H': 6.67, 'Y': 4.44, 'A': 6.67, 'R': 6.67, 'K': 6.67, 'E': 2.22, 'D': 4.44, 'G': 6.67, 'T': 11.11, 'V': 4.44, 'S': 2.22, 'N': 2.22}
- **2D identity (%) [PDB]:** 38.13
- **2D identity (%) [Gaps excluded][PDB]:** 89.93
- **2D identity - Alignment Gaps [PDB]:** 728
- **2D aligned content [PDB] (<2D-fold>:%):** {'.': 16.39, 'E': 29.46, 'T': 15.98, 'B': 0.21, 'H': 36.1, 'G': 1.87}
- **3D similarity (TM-Score) (%) [PDB]:** 27.12

- **Gene name:** ERAP2
- **Entrez ID:** 64167
- **RefSeq ID:** NM\_022350
- **Transcript sequence length:** 5131
- **5-UTR|CDS|3-UTR identity (%):** 23.22 | 45.57 | 11.93
- **5-UTR|CDS|3-UTR identity (%) [Gaps excluded]:** 64.58 | 74.89 | 76.21
- **5-UTR|CDS|3-UTR identity [Alignment Gaps]:** 171 | 1353 | 9423
- **5-UTR aligned content (<base>:%):** {'A': 17.74, 'G': 35.48, 'C': 29.03, 'T': 17.74}
- **CDS aligned content (<base>:%):** {'T': 22.54, 'G': 23.87, 'C': 25.71, 'A': 27.87}
- **3-UTR aligned content (<base>:%):** {'A': 32.11, 'T': 32.33, 'G': 19.28, 'C': 16.28}

**Uniprot Description:**  
  
 Aminopeptidase that plays a central role in peptide trimming, a step required for the generation of most HLA class I-binding peptides. Peptide trimming is essential to customize longer precursor peptides to fit them to the correct length required for presentation on MHC class I molecules. Preferentially hydrolyzes the basic residues Arg and Lys.   
  
Heterodimer with ERAP1.   
  
 **Gene Ontology Information:**

Molecular Function

- aminopeptidase activity
- endopeptidase activity
- metalloaminopeptidase activity
- metallopeptidase activity
- peptide binding
- zinc ion binding

Location

- cytoplasm
- endoplasmic reticulum lumen
- endoplasmic reticulum membrane

Biological process

- adaptive immune response
- antigen processing and presentation of endogenous peptide antigen via MHC class I
- antigen processing and presentation of peptide antigen via MHC class I
- peptide catabolic process
- proteolysis
- regulation of blood pressure

---

91

- **Protein name:** Tripartite motif-containing protein 46
- **Organism:** Homo sapiens
- **Uniprot Accession Number:** Q7Z4K8
- **Protein sequence length:** 759 aa
- **1D identity (%):** 14.69
- **1D identity (%) [Gaps excluded]:** 22.64
- **1D identity - Alignment Gaps:** 344
- **1D aligned content (<aminoacid>:%):** {'G': 11.81, 'E': 4.86, 'C': 4.17, 'V': 7.64, 'Q': 4.86, 'P': 11.11, 'T': 7.64, 'D': 5.56, 'R': 8.33, 'K': 4.17, 'L': 11.11, 'H': 3.47, 'A': 3.47, 'Y': 4.17, 'F': 0.69, 'S': 4.17, 'I': 1.39, 'W': 0.69, 'N': 0.69}
- **Common reported functions (%):** 0.0
- **Common reported locations (%):** 0.0
- **Common reported processes (%):** 0.0

- **AF ID:** Q7Z4K8
- **Chain:** A
- **Protein length:** 759 aa
- **Resolution:** N/A
- **b-phipsi:** 0.00069
- **w-rdist:** 1.46316
- **t-alpha:** 0.001462
- **Chemical similarity (Tanimoto Index) (%):** 99.17
- **1D identity (%) [PDB]:** 2.86
- **1D identity (%) [Gaps excluded][PDB]:** 70.97
- **1D identity - Alignment Gaps [PDB]:** 1475
- **1D aligned content [PDB] (<aminoacid>:%):** {'M': 2.27, 'E': 9.09, 'V': 13.64, 'L': 11.36, 'R': 9.09, 'N': 2.27, 'I': 2.27, 'Q': 4.55, 'P': 9.09, 'T': 11.36, 'S': 6.82, 'K': 4.55, 'G': 2.27, 'H': 2.27, 'C': 6.82, 'A': 2.27}
- **2D identity (%) [PDB]:** 33.73
- **2D identity (%) [Gaps excluded][PDB]:** 90.55
- **2D identity - Alignment Gaps [PDB]:** 731
- **2D aligned content [PDB] (<2D-fold>:%):** {'T': 25.7, 'H': 40.97, '.': 13.23, 'E': 19.34, 'G': 0.76}
- **3D similarity (TM-Score) (%) [PDB]:** 16.25

- **Gene name:** TRIM46
- **Entrez ID:** 80128
- **RefSeq ID:** NM\_025058
- **Transcript sequence length:** 3178
- **5-UTR|CDS|3-UTR identity (%):** 27.35 | 44.47 | 5.05
- **5-UTR|CDS|3-UTR identity (%) [Gaps excluded]:** 77.91 | 76.09 | 77.68
- **5-UTR|CDS|3-UTR identity [Alignment Gaps]:** 159 | 1300 | 10187
- **5-UTR aligned content (<base>:%):** {'G': 47.76, 'C': 35.82, 'A': 5.97, 'T': 10.45}
- **CDS aligned content (<base>:%):** {'T': 17.97, 'G': 30.41, 'C': 31.63, 'A': 19.99}
- **3-UTR aligned content (<base>:%):** {'G': 22.55, 'T': 29.82, 'C': 35.45, 'A': 12.18}

**Uniprot Description:**  
  
 Microtubule-associated protein that is involved in the formation of parallel microtubule bundles linked by cross-bridges in the proximal axon. Required for the uniform orientation and maintenance of the parallel microtubule fascicles, which are important for efficient cargo delivery and trafficking in axons. Thereby also required for proper axon specification, the establishment of neuronal polarity and proper neuronal migration.   
  
Interacts with TUBB3 and TUBA4A.   
  
 **Gene Ontology Information:**

Molecular Function

- zinc ion binding

Location

- axon cytoplasm
- axon initial segment
- cytoskeleton
- main axon
- proximal neuron projection

Biological process

- anterograde synaptic vesicle transport
- axonogenesis
- microtubule bundle formation
- negative regulation of axon extension
- neuron migration
- positive regulation of anterograde dense core granule transport
- protein localization to axon
- regulation of protein localization

---

92

- **Protein name:** Tyrosine-protein kinase Fyn
- **Organism:** Homo sapiens
- **Uniprot Accession Number:** P06241
- **Protein sequence length:** 537 aa
- **1D identity (%):** 10.73
- **1D identity (%) [Gaps excluded]:** 23.02
- **1D identity - Alignment Gaps:** 508
- **1D aligned content (<aminoacid>:%):** {'G': 10.78, 'V': 5.88, 'K': 10.78, 'L': 8.82, 'Y': 2.94, 'D': 3.92, 'P': 10.78, 'H': 0.98, 'T': 4.9, 'E': 1.96, 'F': 2.94, 'S': 3.92, 'W': 0.98, 'I': 9.8, 'Q': 4.9, 'A': 5.88, 'R': 5.88, 'N': 1.96, 'M': 0.98, 'C': 0.98}
- **Common reported functions (%):** 0.0
- **Common reported locations (%):** 20.0
- **Common reported processes (%):** 0.0

- **AF ID:** P06241
- **Chain:** A
- **Protein length:** 537 aa
- **Resolution:** N/A
- **b-phipsi:** 0.014078
- **w-rdist:** 0.7139
- **t-alpha:** 0.0
- **Chemical similarity (Tanimoto Index) (%):** 99.25
- **1D identity (%) [PDB]:** 1.78
- **1D identity (%) [Gaps excluded][PDB]:** 80.0
- **1D identity - Alignment Gaps [PDB]:** 1317
- **1D aligned content [PDB] (<aminoacid>:%):** {'A': 8.33, 'G': 8.33, 'Q': 12.5, 'T': 8.33, 'R': 8.33, 'P': 4.17, 'S': 4.17, 'Y': 12.5, 'L': 8.33, 'W': 4.17, 'D': 8.33, 'N': 4.17, 'F': 4.17, 'E': 4.17}
- **2D identity (%) [PDB]:** 32.08
- **2D identity (%) [Gaps excluded][PDB]:** 89.29
- **2D identity - Alignment Gaps [PDB]:** 649
- **2D aligned content [PDB] (<2D-fold>:%):** {'.': 13.54, 'E': 27.69, 'T': 21.85, 'H': 34.15, 'G': 2.77}
- **3D similarity (TM-Score) (%) [PDB]:** 18.45

- **Gene name:** FYN
- **Entrez ID:** 2534
- **RefSeq ID:** N/A
- **Sequence length:** N/A
- **5-UTR|CDS|3-UTR identity (%):** N/A | N/A | N/A
- **5-UTR|CDS|3-UTR identity (%) [Gaps excluded]:** N/A | N/A | N/A
- **5-UTR|CDS|3-UTR identity [Alignment Gaps]:** N/A | N/A | N/A
- **5-UTR aligned content (<base>:%):** N/A
- **CDS aligned content (<base>:%):** N/A
- **3-UTR aligned content (<base>:%):** N/A

**Uniprot Description:**  
  
 Non-receptor tyrosine-protein kinase that plays a role in many biological processes including regulation of cell growth and survival, cell adhesion, integrin-mediated signaling, cytoskeletal remodeling, cell motility, immune response and axon guidance. Inactive FYN is phosphorylated on its C-terminal tail within the catalytic domain. Following activation by PKA, the protein subsequently associates with PTK2/FAK1, allowing PTK2/FAK1 phosphorylation, activation and targeting to focal adhesions. Involved in the regulation of cell adhesion and motility through phosphorylation of CTNNB1 (beta-catenin) and CTNND1 (delta-catenin). Regulates cytoskeletal remodeling by phosphorylating several proteins including the actin regulator WAS and the microtubule-associated proteins MAP2 and MAPT. Promotes cell survival by phosphorylating AGAP2/PIKE-A and preventing its apoptotic cleavage. Participates in signal transduction pathways that regulate the integrity of the glomerular slit diaphragm (an essential part of the glomerular filter of the kidney) by phosphorylating several slit diaphragm components including NPHS1, KIRREL1 and TRPC6. Plays a role in neural processes by phosphorylating DPYSL2, a multifunctional adapter protein within the central nervous system, ARHGAP32, a regulator for Rho family GTPases implicated in various neural functions, and SNCA, a small pre-synaptic protein. Participates in the downstream signaling pathways that lead to T-cell differentiation and proliferation following T-cell receptor (TCR) stimulation. Phosphorylates PTK2B/PYK2 in response to T-cell receptor activation. Also participates in negative feedback regulation of TCR signaling through phosphorylation of PAG1, thereby promoting interaction between PAG1 and CSK and recruitment of CSK to lipid rafts. CSK maintains LCK and FYN in an inactive form. Promotes CD28-induced phosphorylation of VAV1. In mast cells, phosphorylates CLNK after activation of immunoglobulin epsilon receptor signaling (By similarity).   
  
Interacts (via its SH3 domain) with PIK3R1 and PRMT8. Interacts with FYB1, PAG1, and SH2D1A. Interacts with CD79A (tyrosine-phosphorylated form); the interaction increases FYN activity. Interacts (via SH2 domain) with CSF1R (tyrosine phosphorylated) (By similarity). Interacts with TOM1L1 (phosphorylated form). Interacts with KDR (tyrosine phosphorylated). Interacts (via SH3 domain) with KLHL2 (via N-terminus) (By similarity). Interacts with SH2D1A and SLAMF1. Interacts with ITCH; the interaction phosphorylates ITCH and negatively regulates its activity. Interacts with FASLG. Interacts with RUNX3. Interacts with KIT. Interacts with EPHA8; possible downstream effector of EPHA8 in regulation of cell adhesion. Interacts with PTK2/FAK1; this interaction leads to PTK2/FAK1 phosphorylation and activation. Interacts with CAV1; this interaction couples integrins to the Ras-ERK pathway. Interacts with UNC119. Interacts (via SH2 domain) with PTPRH (phosphorylated form) (By similarity). Interacts with PTPRO (phosphorylated form) (By similarity). Interacts with PTPRB (phosphorylated form) (By similarity). Interacts with FYB2 (PubMed:27335501). Interacts with DSCAM (By similarity). Interacts with SKAP1 and FYB1; this interaction promotes the phosphorylation of CLNK (By similarity).   
  
 **Gene Ontology Information:**

Molecular Function

- alpha-tubulin binding
- ATP binding
- disordered domain specific binding
- enzyme binding
- ephrin receptor binding
- growth factor receptor binding
- identical protein binding
- metal ion binding
- non-membrane spanning protein tyrosine kinase activity
- phospholipase activator activity
- phospholipase binding
- protein tyrosine kinase activity
- scaffold protein binding
- signaling receptor binding
- tau protein binding
- tau-protein kinase activity
- ion channel binding
- type 5 metabotropic glutamate receptor binding

Location

- actin filament
- cell body
- cytosol
- dendrite
- endosome
- extrinsic component of cytoplasmic side of plasma membrane
- glial cell projection
- membrane raft
- mitochondrion
- nucleus
- perikaryon
- perinuclear endoplasmic reticulum
- perinuclear region of cytoplasm
- plasma membrane
- postsynaptic density
- Schaffer collateral - CA1 synapse

Biological process

- activated T cell proliferation
- adaptive immune response
- axon guidance
- calcium ion transport
- cell differentiation
- cellular response to amyloid-beta
- cellular response to glycine
- cellular response to hydrogen peroxide
- cellular response to L-glutamate
- cellular response to platelet-derived growth factor stimulus
- cellular response to transforming growth factor beta stimulus
- dendrite morphogenesis
- dendritic spine maintenance
- detection of mechanical stimulus involved in sensory perception of pain
- ephrin receptor signaling pathway
- Fc-gamma receptor signaling pathway involved in phagocytosis
- feeding behavior
- forebrain development
- heart process
- I-kappaB kinase/NF-kappaB signaling
- innate immune response
- intracellular signal transduction
- learning
- leukocyte migration
- modulation of chemical synaptic transmission
- negative regulation of dendritic spine maintenance
- negative regulation of gene expression
- negative regulation of hydrogen peroxide biosynthetic process
- negative regulation of inflammatory response to antigenic stimulus
- negative regulation of oxidative stress-induced intrinsic apoptotic signaling pathway
- negative regulation of protein catabolic process
- negative regulation of protein ubiquitination
- neuron migration
- peptidyl-tyrosine phosphorylation
- positive regulation of cysteine-type endopeptidase activity
- positive regulation of I-kappaB kinase/NF-kappaB signaling
- positive regulation of neuron death
- positive regulation of neuron projection development
- positive regulation of protein localization to membrane
- positive regulation of protein localization to nucleus
- positive regulation of protein targeting to membrane
- positive regulation of tyrosine phosphorylation of STAT protein
- protein autophosphorylation
- protein catabolic process
- protein phosphorylation
- protein ubiquitination
- regulation of calcium ion import across plasma membrane
- regulation of cell shape
- regulation of glutamate receptor signaling pathway
- regulation of peptidyl-tyrosine phosphorylation
- response to amyloid-beta
- response to ethanol
- response to singlet oxygen
- stimulatory C-type lectin receptor signaling pathway
- T cell costimulation
- T cell receptor signaling pathway
- transmembrane receptor protein tyrosine kinase signaling pathway
- vascular endothelial growth factor receptor signaling pathway

---

93

- **Protein name:** Ribosomal protein S6 kinase alpha-4
- **Organism:** Homo sapiens
- **Uniprot Accession Number:** O75676
- **Protein sequence length:** 772 aa
- **1D identity (%):** 13.97
- **1D identity (%) [Gaps excluded]:** 24.53
- **1D identity - Alignment Gaps:** 447
- **1D aligned content (<aminoacid>:%):** {'M': 1.38, 'L': 11.72, 'F': 4.83, 'G': 10.34, 'K': 6.21, 'D': 2.76, 'Y': 4.14, 'V': 7.59, 'R': 8.28, 'E': 5.52, 'Q': 6.9, 'H': 2.07, 'N': 2.07, 'T': 1.38, 'I': 1.38, 'A': 5.52, 'W': 2.07, 'P': 10.34, 'S': 4.14, 'C': 1.38}
- **Common reported functions (%):** 0.0
- **Common reported locations (%):** 40.0
- **Common reported processes (%):** 12.5

- **AF ID:** O75676
- **Chain:** A
- **Protein length:** 772 aa
- **Resolution:** N/A
- **b-phipsi:** 0.010741
- **w-rdist:** 0.336353
- **t-alpha:** 0.008759
- **Chemical similarity (Tanimoto Index) (%):** 99.17
- **1D identity (%) [PDB]:** 1.33
- **1D identity (%) [Gaps excluded][PDB]:** 63.64
- **1D identity - Alignment Gaps [PDB]:** 1546
- **1D aligned content [PDB] (<aminoacid>:%):** {'Q': 23.81, 'Y': 14.29, 'F': 4.76, 'K': 4.76, 'L': 23.81, 'P': 4.76, 'G': 4.76, 'E': 9.52, 'H': 4.76, 'T': 4.76}
- **2D identity (%) [PDB]:** 42.31
- **2D identity (%) [Gaps excluded][PDB]:** 88.85
- **2D identity - Alignment Gaps [PDB]:** 572
- **2D aligned content [PDB] (<2D-fold>:%):** {'.': 22.08, 'E': 15.15, 'T': 18.18, 'H': 41.77, 'G': 2.6, 'B': 0.22}
- **3D similarity (TM-Score) (%) [PDB]:** 24.09

- **Gene name:** RPS6KA4
- **Entrez ID:** 8986
- **RefSeq ID:** NM\_003942
- **Transcript sequence length:** 3128
- **5-UTR|CDS|3-UTR identity (%):** 25.35 | 43.92 | 4.27
- **5-UTR|CDS|3-UTR identity (%) [Gaps excluded]:** 72.97 | 74.92 | 76.85
- **5-UTR|CDS|3-UTR identity [Alignment Gaps]:** 139 | 1303 | 10340
- **5-UTR aligned content (<base>:%):** {'G': 42.59, 'C': 46.3, 'A': 9.26, 'T': 1.85}
- **CDS aligned content (<base>:%):** {'A': 19.31, 'T': 16.2, 'G': 31.38, 'C': 33.12}
- **3-UTR aligned content (<base>:%):** {'C': 29.49, 'A': 16.03, 'T': 29.06, 'G': 25.43}

**Uniprot Description:**  
  
 Serine/threonine-protein kinase that is required for the mitogen or stress-induced phosphorylation of the transcription factors CREB1 and ATF1 and for the regulation of the transcription factor RELA, and that contributes to gene activation by histone phosphorylation and functions in the regulation of inflammatory genes. Phosphorylates CREB1 and ATF1 in response to mitogenic or stress stimuli such as UV-C irradiation, epidermal growth factor (EGF) and anisomycin. Plays an essential role in the control of RELA transcriptional activity in response to TNF. Phosphorylates 'Ser-10' of histone H3 in response to mitogenics, stress stimuli and EGF, which results in the transcriptional activation of several immediate early genes, including proto-oncogenes c-fos/FOS and c-jun/JUN. May also phosphorylate 'Ser-28' of histone H3. Mediates the mitogen- and stress-induced phosphorylation of high mobility group protein 1 (HMGN1/HMG14). In lipopolysaccharide-stimulated primary macrophages, acts downstream of the Toll-like receptor TLR4 to limit the production of pro-inflammatory cytokines. Functions probably by inducing transcription of the MAP kinase phosphatase DUSP1 and the anti-inflammatory cytokine interleukin 10 (IL10), via CREB1 and ATF1 transcription factors.   
  
Forms a complex with either MAPK1/ERK2 or MAPK3/ERK1 in quiescent cells which transiently dissociates following mitogenic stimulation. Also associates with MAPK14/p38-alpha. Activated RPS6KA4 associates with and phosphorylates the NF-kappa-B p65 subunit RELA.   
  
 **Gene Ontology Information:**

Molecular Function

- ATP binding
- histone kinase activity (H3-S10 specific)
- histone H3S28 kinase activity
- magnesium ion binding
- protein serine kinase activity
- protein serine/threonine kinase activity
- ribosomal protein S6 kinase activity

Location

- cytoplasm
- cytosol
- nucleoplasm
- nucleus
- synapse

Biological process

- inflammatory response
- interleukin-1-mediated signaling pathway
- intracellular signal transduction
- negative regulation of cytokine production
- peptidyl-serine phosphorylation
- positive regulation of CREB transcription factor activity
- positive regulation of NF-kappaB transcription factor activity
- positive regulation of transcription by RNA polymerase II
- post-translational protein modification
- protein phosphorylation
- regulation of transcription, DNA-templated

---

94

- **Protein name:** Leucine-rich repeat transmembrane neuronal protein 2
- **Organism:** Homo sapiens
- **Uniprot Accession Number:** O43300
- **Protein sequence length:** 516 aa
- **1D identity (%):** 12.95
- **1D identity (%) [Gaps excluded]:** 24.32
- **1D identity - Alignment Gaps:** 419
- **1D aligned content (<aminoacid>:%):** {'G': 6.9, 'L': 13.79, 'Y': 2.59, 'V': 4.31, 'P': 6.9, 'A': 6.03, 'Q': 5.17, 'F': 4.31, 'K': 5.17, 'H': 3.45, 'N': 4.31, 'S': 6.03, 'T': 5.17, 'E': 5.17, 'D': 6.03, 'R': 8.62, 'W': 0.86, 'I': 4.31, 'C': 0.86}
- **Common reported functions (%):** 0.0
- **Common reported locations (%):** 0.0
- **Common reported processes (%):** 0.0

- **AF ID:** O43300
- **Chain:** A
- **Protein length:** 516 aa
- **Resolution:** N/A
- **b-phipsi:** 0.007229
- **w-rdist:** 0.780469
- **t-alpha:** 0.00146
- **Chemical similarity (Tanimoto Index) (%):** 96.91
- **1D identity (%) [PDB]:** 1.66
- **1D identity (%) [Gaps excluded][PDB]:** 70.97
- **1D identity - Alignment Gaps [PDB]:** 1294
- **1D aligned content [PDB] (<aminoacid>:%):** {'G': 4.55, 'R': 9.09, 'C': 4.55, 'K': 9.09, 'L': 4.55, 'T': 13.64, 'D': 9.09, 'N': 4.55, 'Q': 9.09, 'S': 4.55, 'I': 4.55, 'A': 13.64, 'P': 4.55, 'E': 4.55}
- **2D identity (%) [PDB]:** 33.88
- **2D identity (%) [Gaps excluded][PDB]:** 86.39
- **2D identity - Alignment Gaps [PDB]:** 592
- **2D aligned content [PDB] (<2D-fold>:%):** {'.': 29.39, 'T': 28.18, 'H': 27.27, 'E': 13.03, 'G': 1.82, 'B': 0.3}
- **3D similarity (TM-Score) (%) [PDB]:** 15.95

- **Gene name:** LRRTM2
- **Entrez ID:** 26045
- **RefSeq ID:** NM\_015564
- **Transcript sequence length:** 6064
- **5-UTR|CDS|3-UTR identity (%):** 35.08 | 32.14 | 22.95
- **5-UTR|CDS|3-UTR identity (%) [Gaps excluded]:** 77.78 | 73.68 | 73.95
- **5-UTR|CDS|3-UTR identity [Alignment Gaps]:** 241 | 1659 | 7848
- **5-UTR aligned content (<base>:%):** {'A': 15.58, 'C': 34.42, 'T': 14.29, 'G': 35.71}
- **CDS aligned content (<base>:%):** {'T': 23.36, 'G': 22.2, 'C': 25.16, 'A': 29.28}
- **3-UTR aligned content (<base>:%):** {'T': 33.77, 'A': 33.12, 'C': 16.19, 'G': 16.92}

**Uniprot Description:**  
  
 Involved in the development and maintenance of excitatory synapses in the vertebrate nervous system. Regulates surface expression of AMPA receptors and instructs the development of functional glutamate release sites. Acts as a ligand for the presynaptic receptors NRXN1-A and NRXN1-B (By similarity).   
  
Interacts with DLG4. Interacts with neurexin NRXN1; interaction is mediated by heparan sulfate glycan modification on neurexin.   
  
 **Gene Ontology Information:**

Molecular Function

- neurexin family protein binding

Location

- excitatory synapse
- extracellular space
- GABA-ergic synapse
- glutamatergic synapse
- hippocampal mossy fiber to CA3 synapse
- postsynaptic density membrane
- postsynaptic specialization membrane
- Schaffer collateral - CA1 synapse

Biological process

- long-term synaptic potentiation
- negative regulation of receptor internalization
- positive regulation of synapse assembly
- regulation of postsynaptic density assembly
- synapse organization

---

95

- **Protein name:** Protein transport protein Sec23B
- **Organism:** Homo sapiens
- **Uniprot Accession Number:** Q15437
- **Protein sequence length:** 767 aa
- **1D identity (%):** 13.43
- **1D identity (%) [Gaps excluded]:** 21.41
- **1D identity - Alignment Gaps:** 372
- **1D aligned content (<aminoacid>:%):** {'F': 2.99, 'Q': 5.22, 'V': 7.46, 'R': 6.72, 'W': 0.75, 'A': 5.97, 'T': 7.46, 'C': 2.99, 'P': 11.19, 'S': 1.49, 'K': 6.72, 'N': 3.73, 'L': 10.45, 'E': 2.99, 'I': 3.73, 'G': 11.19, 'M': 1.49, 'Y': 2.24, 'D': 4.48, 'H': 0.75}
- **Common reported functions (%):** 0.0
- **Common reported locations (%):** 10.0
- **Common reported processes (%):** 0.0

- **AF ID:** Q15437
- **Chain:** A
- **Protein length:** 767 aa
- **Resolution:** N/A
- **b-phipsi:** 0.001089
- **w-rdist:** 0.62846
- **t-alpha:** 0.050614
- **Chemical similarity (Tanimoto Index) (%):** 99.1
- **1D identity (%) [PDB]:** 2.31
- **1D identity (%) [Gaps excluded][PDB]:** 72.0
- **1D identity - Alignment Gaps [PDB]:** 1507
- **1D aligned content [PDB] (<aminoacid>:%):** {'M': 2.78, 'A': 8.33, 'T': 2.78, 'C': 2.78, 'V': 13.89, 'N': 11.11, 'K': 8.33, 'S': 5.56, 'Q': 5.56, 'L': 16.67, 'I': 11.11, 'G': 8.33, 'F': 2.78}
- **2D identity (%) [PDB]:** 39.78
- **2D identity (%) [Gaps excluded][PDB]:** 89.11
- **2D identity - Alignment Gaps [PDB]:** 615
- **2D aligned content [PDB] (<2D-fold>:%):** {'.': 16.74, 'H': 40.5, 'E': 23.3, 'T': 18.1, 'B': 0.68, 'G': 0.68}
- **3D similarity (TM-Score) (%) [PDB]:** 22.1

- **Gene name:** SEC23B
- **Entrez ID:** 10483
- **RefSeq ID:** N/A
- **Sequence length:** N/A
- **5-UTR|CDS|3-UTR identity (%):** N/A | N/A | N/A
- **5-UTR|CDS|3-UTR identity (%) [Gaps excluded]:** N/A | N/A | N/A
- **5-UTR|CDS|3-UTR identity [Alignment Gaps]:** N/A | N/A | N/A
- **5-UTR aligned content (<base>:%):** N/A
- **CDS aligned content (<base>:%):** N/A
- **3-UTR aligned content (<base>:%):** N/A

**Uniprot Description:**  
  
 Component of the coat protein complex II (COPII) which promotes the formation of transport vesicles from the endoplasmic reticulum (ER). The coat has two main functions, the physical deformation of the endoplasmic reticulum membrane into vesicles and the selection of cargo molecules for their transport to the Golgi complex.   
  
COPII is composed of at least five proteins: the Sec23/24 complex, the Sec13/31 complex and Sar1 (By similarity). Interacts with SAR1A (PubMed:26522472).   
  
 **Gene Ontology Information:**

Molecular Function

- GTPase activator activity
- zinc ion binding

Location

- COPII vesicle coat
- cytosol
- endomembrane system
- endoplasmic reticulum
- endoplasmic reticulum exit site
- endoplasmic reticulum membrane
- perinuclear region of cytoplasm

Biological process

- COPII-coated vesicle cargo loading
- intracellular protein transport

---

96

- **Protein name:** SUMO-activating enzyme subunit 2
- **Organism:** Homo sapiens
- **Uniprot Accession Number:** Q9UBT2
- **Protein sequence length:** 640 aa
- **1D identity (%):** 12.83
- **1D identity (%) [Gaps excluded]:** 21.35
- **1D identity - Alignment Gaps:** 373
- **1D aligned content (<aminoacid>:%):** {'A': 10.83, 'L': 6.67, 'G': 10.0, 'R': 5.0, 'E': 2.5, 'V': 8.33, 'D': 12.5, 'S': 2.5, 'P': 9.17, 'H': 0.83, 'M': 0.83, 'C': 4.17, 'K': 9.17, 'T': 2.5, 'F': 4.17, 'Y': 1.67, 'Q': 3.33, 'I': 5.0, 'N': 0.83}
- **Common reported functions (%):** 0.0
- **Common reported locations (%):** 20.0
- **Common reported processes (%):** 0.0

- **AF ID:** Q9UBT2
- **Chain:** A
- **Protein length:** 640 aa
- **Resolution:** N/A
- **b-phipsi:** 0.006171
- **w-rdist:** 0.26604
- **t-alpha:** 0.072262
- **Chemical similarity (Tanimoto Index) (%):** 98.8
- **1D identity (%) [PDB]:** 2.45
- **1D identity (%) [Gaps excluded][PDB]:** 66.04
- **1D identity - Alignment Gaps [PDB]:** 1374
- **1D aligned content [PDB] (<aminoacid>:%):** {'L': 8.57, 'P': 5.71, 'E': 8.57, 'N': 5.71, 'I': 8.57, 'V': 2.86, 'A': 11.43, 'G': 2.86, 'Q': 5.71, 'K': 11.43, 'T': 8.57, 'D': 8.57, 'S': 8.57, 'R': 2.86}
- **2D identity (%) [PDB]:** 39.21
- **2D identity (%) [Gaps excluded][PDB]:** 93.59
- **2D identity - Alignment Gaps [PDB]:** 606
- **2D aligned content [PDB] (<2D-fold>:%):** {'.': 18.34, 'T': 22.0, 'H': 42.05, 'E': 13.69, 'G': 3.67, 'B': 0.24}
- **3D similarity (TM-Score) (%) [PDB]:** 21.25

- **Gene name:** UBA2
- **Entrez ID:** 10054
- **RefSeq ID:** NM\_005499
- **Transcript sequence length:** 4005
- **5-UTR|CDS|3-UTR identity (%):** 17.29 | 43.4 | 11.93
- **5-UTR|CDS|3-UTR identity (%) [Gaps excluded]:** 72.55 | 74.29 | 75.71
- **5-UTR|CDS|3-UTR identity [Alignment Gaps]:** 163 | 1207 | 9353
- **5-UTR aligned content (<base>:%):** {'G': 37.84, 'C': 48.65, 'T': 13.51}
- **CDS aligned content (<base>:%):** {'A': 29.84, 'T': 22.38, 'G': 26.67, 'C': 21.11}
- **3-UTR aligned content (<base>:%):** {'A': 25.06, 'G': 20.45, 'T': 34.19, 'C': 20.3}

**Uniprot Description:**  
  
 The heterodimer acts as an E1 ligase for SUMO1, SUMO2, SUMO3, and probably SUMO4. It mediates ATP-dependent activation of SUMO proteins followed by formation of a thioester bond between a SUMO protein and a conserved active site cysteine residue on UBA2/SAE2.   
  
Heterodimer of SAE1 and UBA2/SAE2. The heterodimer corresponds to the two domains that are encoded on a single polypeptide chain in ubiquitin-activating enzyme E1. Interacts with UBE2I.   
  
 **Gene Ontology Information:**

Molecular Function

- ATP binding
- magnesium ion binding
- protein heterodimerization activity
- small protein activating enzyme binding
- SUMO activating enzyme activity
- SUMO binding
- transferase activity
- ubiquitin-like protein conjugating enzyme binding

Location

- cytoplasm
- nucleoplasm
- SUMO activating enzyme complex

Biological process

- positive regulation of protein sumoylation
- protein sumoylation

---

97

- **Protein name:** Protein arginine N-methyltransferase 7
- **Organism:** Homo sapiens
- **Uniprot Accession Number:** Q9NVM4
- **Protein sequence length:** 692 aa
- **1D identity (%):** 14.36
- **1D identity (%) [Gaps excluded]:** 23.03
- **1D identity - Alignment Gaps:** 359
- **1D aligned content (<aminoacid>:%):** {'F': 2.19, 'P': 13.14, 'G': 8.03, 'V': 12.41, 'E': 6.57, 'D': 8.03, 'Y': 2.92, 'H': 2.92, 'K': 4.38, 'T': 5.11, 'A': 3.65, 'I': 5.11, 'R': 6.57, 'L': 6.57, 'W': 1.46, 'C': 2.92, 'Q': 2.92, 'N': 2.19, 'S': 2.19, 'M': 0.73}
- **Common reported functions (%):** 0.0
- **Common reported locations (%):** 20.0
- **Common reported processes (%):** 0.0

- **AF ID:** Q9NVM4
- **Chain:** A
- **Protein length:** 692 aa
- **Resolution:** N/A
- **b-phipsi:** 0.001184
- **w-rdist:** 0.691023
- **t-alpha:** 0.036309
- **Chemical similarity (Tanimoto Index) (%):** 99.17
- **1D identity (%) [PDB]:** 1.88
- **1D identity (%) [Gaps excluded][PDB]:** 68.29
- **1D identity - Alignment Gaps [PDB]:** 1450
- **1D aligned content [PDB] (<aminoacid>:%):** {'K': 7.14, 'I': 10.71, 'N': 7.14, 'V': 10.71, 'G': 10.71, 'L': 7.14, 'P': 10.71, 'H': 7.14, 'Q': 10.71, 'S': 3.57, 'A': 7.14, 'F': 3.57, 'D': 3.57}
- **2D identity (%) [PDB]:** 40.11
- **2D identity (%) [Gaps excluded][PDB]:** 86.6
- **2D identity - Alignment Gaps [PDB]:** 562
- **2D aligned content [PDB] (<2D-fold>:%):** {'.': 15.71, 'E': 33.33, 'H': 26.67, 'T': 22.38, 'G': 1.67, 'B': 0.24}
- **3D similarity (TM-Score) (%) [PDB]:** 25.19

- **Gene name:** PRMT7
- **Entrez ID:** 514202
- **RefSeq ID:** N/A
- **Sequence length:** N/A
- **5-UTR|CDS|3-UTR identity (%):** N/A | N/A | N/A
- **5-UTR|CDS|3-UTR identity (%) [Gaps excluded]:** N/A | N/A | N/A
- **5-UTR|CDS|3-UTR identity [Alignment Gaps]:** N/A | N/A | N/A
- **5-UTR aligned content (<base>:%):** N/A
- **CDS aligned content (<base>:%):** N/A
- **3-UTR aligned content (<base>:%):** N/A

**Uniprot Description:**  
  
 Arginine methyltransferase that can both catalyze the formation of omega-N monomethylarginine (MMA) and symmetrical dimethylarginine (sDMA), with a preference for the formation of MMA. Specifically mediates the symmetrical dimethylation of arginine residues in the small nuclear ribonucleoproteins Sm D1 (SNRPD1) and Sm D3 (SNRPD3); such methylation being required for the assembly and biogenesis of snRNP core particles. Specifically mediates the symmetric dimethylation of histone H4 'Arg-3' to form H4R3me2s. Plays a role in gene imprinting by being recruited by CTCFL at the H19 imprinted control region (ICR) and methylating histone H4 to form H4R3me2s, possibly leading to recruit DNA methyltransferases at these sites. May also play a role in embryonic stem cell (ESC) pluripotency. Also able to mediate the arginine methylation of histone H2A and myelin basic protein (MBP) in vitro; the relevance of such results is however unclear in vivo.   
  
Homodimer and heterodimer (By similarity). Interacts with CTCFL (By similarity). Interacts with PRMT5 and SNRPD3.   
  
 **Gene Ontology Information:**

Molecular Function

- histone methyltransferase activity (H4-R3 specific)
- protein-arginine omega-N monomethyltransferase activity
- protein-arginine omega-N symmetric methyltransferase activity

Location

- cytosol
- nucleus

Biological process

- cell differentiation
- peptidyl-arginine methylation
- regulation of gene expression by genetic imprinting
- spliceosomal snRNP assembly

---

98

- **Protein name:** Putative E3 ubiquitin-protein ligase UBR7
- **Organism:** Homo sapiens
- **Uniprot Accession Number:** Q8N806
- **Protein sequence length:** 425 aa
- **1D identity (%):** 7.6
- **1D identity (%) [Gaps excluded]:** 21.49
- **1D identity - Alignment Gaps:** 612
- **1D aligned content (<aminoacid>:%):** {'M': 1.39, 'A': 4.17, 'P': 6.94, 'V': 2.78, 'D': 12.5, 'E': 5.56, 'S': 4.17, 'G': 1.39, 'K': 15.28, 'R': 4.17, 'Y': 2.78, 'T': 4.17, 'H': 2.78, 'F': 1.39, 'L': 8.33, 'C': 6.94, 'N': 5.56, 'Q': 6.94, 'W': 1.39, 'I': 1.39}
- **Common reported functions (%):** 0.0
- **Common reported locations (%):** 0.0
- **Common reported processes (%):** 0.0

- **AF ID:** Q8N806
- **Chain:** A
- **Protein length:** 425 aa
- **Resolution:** N/A
- **b-phipsi:** 0.006093
- **w-rdist:** 0.421416
- **t-alpha:** 0.005874
- **Chemical similarity (Tanimoto Index) (%):** 99.17
- **1D identity (%) [PDB]:** 1.2
- **1D identity (%) [Gaps excluded][PDB]:** 75.0
- **1D identity - Alignment Gaps [PDB]:** 1225
- **1D aligned content [PDB] (<aminoacid>:%):** {'M': 6.67, 'C': 6.67, 'V': 6.67, 'L': 13.33, 'R': 6.67, 'N': 6.67, 'I': 6.67, 'D': 13.33, 'E': 6.67, 'Q': 6.67, 'K': 6.67, 'T': 6.67, 'S': 6.67}
- **2D identity (%) [PDB]:** 27.67
- **2D identity (%) [Gaps excluded][PDB]:** 84.89
- **2D identity - Alignment Gaps [PDB]:** 643
- **2D aligned content [PDB] (<2D-fold>:%):** {'.': 26.14, 'H': 46.97, 'T': 14.02, 'E': 10.61, 'G': 2.27}
- **3D similarity (TM-Score) (%) [PDB]:** 14.91

- **Gene name:** UBR7
- **Entrez ID:** N/A
- **RefSeq ID:** NM\_175748
- **Transcript sequence length:** 3494
- **5-UTR|CDS|3-UTR identity (%):** 10.36 | 29.02 | 12.95
- **5-UTR|CDS|3-UTR identity (%) [Gaps excluded]:** 85.19 | 73.61 | 74.95
- **5-UTR|CDS|3-UTR identity [Alignment Gaps]:** 195 | 1718 | 9171
- **5-UTR aligned content (<base>:%):** {'G': 52.17, 'C': 26.09, 'A': 13.04, 'T': 8.7}
- **CDS aligned content (<base>:%):** {'T': 22.72, 'G': 27.95, 'C': 20.05, 'A': 29.28}
- **3-UTR aligned content (<base>:%):** {'A': 22.42, 'G': 22.35, 'T': 32.73, 'C': 22.49}

**Uniprot Description:**  
  
 E3 ubiquitin-protein ligase which is a component of the N-end rule pathway. Recognizes and binds to proteins bearing specific N-terminal residues that are destabilizing according to the N-end rule, leading to their ubiquitination and subsequent degradation. N/A   
  
 **Gene Ontology Information:**

Molecular Function

- ubiquitin protein ligase activity
- zinc ion binding

Location   
  
N/A

Biological process

- protein ubiquitination

---

99

- **Protein name:** Mannosyl-oligosaccharide glucosidase
- **Organism:** Homo sapiens
- **Uniprot Accession Number:** Q13724
- **Protein sequence length:** 837 aa
- **1D identity (%):** 15.98
- **1D identity (%) [Gaps excluded]:** 23.09
- **1D identity - Alignment Gaps:** 308
- **1D aligned content (<aminoacid>:%):** {'M': 1.25, 'G': 15.0, 'A': 8.75, 'P': 15.0, 'V': 7.5, 'R': 5.62, 'L': 13.12, 'F': 3.75, 'Y': 3.75, 'K': 3.12, 'T': 5.0, 'W': 3.12, 'H': 3.12, 'Q': 2.5, 'D': 3.12, 'S': 1.88, 'E': 1.88, 'N': 1.88, 'I': 0.62}
- **Common reported functions (%):** 0.0
- **Common reported locations (%):** 0.0
- **Common reported processes (%):** 0.0

- **AF ID:** Q13724
- **Chain:** A
- **Protein length:** 837 aa
- **Resolution:** N/A
- **b-phipsi:** 0.008015
- **w-rdist:** 0.511184
- **t-alpha:** 0.005109
- **Chemical similarity (Tanimoto Index) (%):** 98.28
- **1D identity (%) [PDB]:** 3.1
- **1D identity (%) [Gaps excluded][PDB]:** 76.92
- **1D identity - Alignment Gaps [PDB]:** 1547
- **1D aligned content [PDB] (<aminoacid>:%):** {'A': 8.0, 'T': 2.0, 'R': 14.0, 'S': 4.0, 'P': 6.0, 'D': 6.0, 'Q': 6.0, 'I': 6.0, 'L': 8.0, 'K': 4.0, 'Y': 4.0, 'N': 6.0, 'E': 4.0, 'F': 2.0, 'G': 8.0, 'V': 8.0, 'M': 2.0, 'W': 2.0}
- **2D identity (%) [PDB]:** 44.74
- **2D identity (%) [Gaps excluded][PDB]:** 90.45
- **2D identity - Alignment Gaps [PDB]:** 567
- **2D aligned content [PDB] (<2D-fold>:%):** {'.': 18.33, 'H': 35.46, 'T': 20.52, 'E': 22.51, 'G': 2.39, 'B': 0.8}
- **3D similarity (TM-Score) (%) [PDB]:** 22.19

- **Gene name:** MOGS
- **Entrez ID:** 5142394
- **RefSeq ID:** NM\_006302
- **Transcript sequence length:** 2867
- **5-UTR|CDS|3-UTR identity (%):** 42.79 | 43.78 | 1.21
- **5-UTR|CDS|3-UTR identity (%) [Gaps excluded]:** 75.4 | 74.2 | 78.11
- **5-UTR|CDS|3-UTR identity [Alignment Gaps]:** 96 | 1338 | 10703
- **5-UTR aligned content (<base>:%):** {'G': 50.53, 'C': 31.58, 'A': 11.58, 'T': 6.32}
- **CDS aligned content (<base>:%):** {'A': 19.87, 'G': 30.44, 'C': 31.28, 'T': 18.4}
- **3-UTR aligned content (<base>:%):** {'A': 19.7, 'G': 23.48, 'C': 23.48, 'T': 33.33}

**Uniprot Description:**  
  
 Cleaves the distal alpha 1,2-linked glucose residue from the Glc(3)Man(9)GlcNAc(2) oligosaccharide precursor in a highly specific manner. N/A   
  
 **Gene Ontology Information:**

Molecular Function

- DNA binding
- DNA-directed DNA polymerase activity
- nucleotide binding

Location   
  
N/A

Biological process

- DNA replication
- viral DNA genome replication

---

100

- **Protein name:** Zinc finger protein 114
- **Organism:** Homo sapiens
- **Uniprot Accession Number:** Q8NC26
- **Protein sequence length:** 417 aa
- **1D identity (%):** 8.16
- **1D identity (%) [Gaps excluded]:** 24.53
- **1D identity - Alignment Gaps:** 638
- **1D aligned content (<aminoacid>:%):** {'D': 6.41, 'S': 5.13, 'V': 6.41, 'F': 5.13, 'P': 8.97, 'Y': 2.56, 'A': 6.41, 'T': 8.97, 'C': 7.69, 'K': 7.69, 'L': 6.41, 'R': 8.97, 'H': 8.97, 'G': 5.13, 'E': 1.28, 'N': 1.28, 'Q': 1.28, 'I': 1.28}
- **Common reported functions (%):** 0.0
- **Common reported locations (%):** 10.0
- **Common reported processes (%):** 0.0

- **AF ID:** Q8NC26
- **Chain:** A
- **Protein length:** 417 aa
- **Resolution:** N/A
- **b-phipsi:** 0.002478
- **w-rdist:** 0.178076
- **t-alpha:** 0.239416
- **Chemical similarity (Tanimoto Index) (%):** 96.99
- **1D identity (%) [PDB]:** 1.55
- **1D identity (%) [Gaps excluded][PDB]:** 65.52
- **1D identity - Alignment Gaps [PDB]:** 1199
- **1D aligned content [PDB] (<aminoacid>:%):** {'S': 5.26, 'V': 15.79, 'D': 5.26, 'A': 10.53, 'R': 15.79, 'L': 5.26, 'M': 5.26, 'Y': 5.26, 'P': 10.53, 'G': 5.26, 'F': 10.53, 'E': 5.26}
- **2D identity (%) [PDB]:** 23.01
- **2D identity (%) [Gaps excluded][PDB]:** 91.3
- **2D identity - Alignment Gaps [PDB]:** 751
- **2D aligned content [PDB] (<2D-fold>:%):** {'.': 31.6, 'G': 1.3, 'H': 52.81, 'T': 9.96, 'E': 4.33}
- **3D similarity (TM-Score) (%) [PDB]:** 13.47

- **Gene name:** ZNF114
- **Entrez ID:** 163071
- **RefSeq ID:** NM\_153608
- **Transcript sequence length:** 2499
- **5-UTR|CDS|3-UTR identity (%):** 31.7 | 29.01 | 3.85
- **5-UTR|CDS|3-UTR identity (%) [Gaps excluded]:** 84.85 | 76.95 | 73.06
- **5-UTR|CDS|3-UTR identity [Alignment Gaps]:** 332 | 1778 | 10395
- **5-UTR aligned content (<base>:%):** {'A': 13.1, 'C': 32.14, 'T': 11.31, 'G': 43.45}
- **CDS aligned content (<base>:%):** {'T': 19.69, 'C': 23.31, 'A': 32.25, 'G': 24.76}
- **3-UTR aligned content (<base>:%):** {'A': 35.93, 'G': 24.11, 'T': 24.35, 'C': 15.6}

**Uniprot Description:**  
  
 May be involved in transcriptional regulation. N/A   
  
 **Gene Ontology Information:**

Molecular Function

- DNA-binding transcription factor activity, RNA polymerase II-specific
- identical protein binding
- metal ion binding
- RNA polymerase II cis-regulatory region sequence-specific DNA binding

Location

- extracellular exosome
- nucleus

Biological process

- regulation of transcription, DNA-templated

---
