# Supplementary material for: Comparative structural insights and functional analysis for the distinct unbound states of Human AGO proteins
Source: Sci Rep. 2025 Mar 19;15:9432. doi: 10.1038/s41598-025-91849-5 (PMC11923369; doi:10.1038/s41598-025-91849-5)
Supplement: Supplementary file 24 — Supplementary Information 12. [file 41598_2025_91849_MOESM24_ESM.zip › 4KREp_A_mdwhole_AF4REF/candidates/4KREp_A-merged-h-enriched_report.html]

 

# Structural Comparison Report for 4KREp\_A - whole structures (total: 100)

---

0

- **AF ID:** AF-Q9H9G7-F1-model-v4 | **Chain:** A
- **b-phipsi:** 0.0010968103034356
- **w-rdist:** 0.0473955288326407
- **t-alpha:** 0.0410335958026029

---

---

1

- **AF ID:** AF-Q9NZ08-F1-model-v4 | **Chain:** A
- **b-phipsi:** 0.0060381124708424
- **w-rdist:** 0.3497212898670014
- **t-alpha:** 0.0007298270463003

---

---

2

- **AF ID:** AF-Q68DD2-F1-model-v4 | **Chain:** A
- **b-phipsi:** 0.0008731647376385
- **w-rdist:** 0.2190491441134769
- **t-alpha:** 0.0620435921263067

---

---

3

- **AF ID:** AF-Q9Y2X0-F1-model-v4 | **Chain:** A
- **b-phipsi:** 0.0003830591833502
- **w-rdist:** 0.3665082565098729
- **t-alpha:** 0.0482019055190958

---

---

4

- **AF ID:** AF-Q6ZN11-F1-model-v4 | **Chain:** A
- **b-phipsi:** 0.0151145785568739
- **w-rdist:** 0.2391606493014683
- **t-alpha:** 0.0014597092068022

---

---

5

- **AF ID:** AF-Q6ZTN6-F1-model-v4 | **Chain:** A
- **b-phipsi:** 0.0016721359662489
- **w-rdist:** 0.6428840344993882
- **t-alpha:** 0.0

---

---

6

- **AF ID:** AF-Q7Z3Z4-F1-model-v4 | **Chain:** A
- **b-phipsi:** 0.0023840053768281
- **w-rdist:** 0.2033971750884409
- **t-alpha:** 0.0216257249425573

---

---

7

- **AF ID:** AF-Q96RQ3-F1-model-v4 | **Chain:** A
- **b-phipsi:** 0.0076364565306422
- **w-rdist:** 0.368435967667157
- **t-alpha:** 0.0021896458253802

---

---

8

- **AF ID:** AF-Q86XP0-F1-model-v4 | **Chain:** A
- **b-phipsi:** 0.0014517199230324
- **w-rdist:** 0.330555074781289
- **t-alpha:** 0.0175180821197378

---

---

9

- **AF ID:** AF-P19525-F1-model-v4 | **Chain:** A
- **b-phipsi:** 0.0057156461662916
- **w-rdist:** 0.5124837582891691
- **t-alpha:** 0.0007307814170889

---

---

10

- **AF ID:** AF-Q9UL63-F1-model-v4 | **Chain:** A
- **b-phipsi:** 0.0014548175009959
- **w-rdist:** 0.2438400992419745
- **t-alpha:** 0.0570987886882312

---

---

11

- **AF ID:** AF-Q8TAP6-F1-model-v4 | **Chain:** A
- **b-phipsi:** 0.0003802029505623
- **w-rdist:** 0.5306996793983226
- **t-alpha:** 0.0423354827531265

---

---

12

- **AF ID:** AF-Q5T447-F1-model-v4 | **Chain:** A
- **b-phipsi:** 0.0035850422787007
- **w-rdist:** 0.2912012832162159
- **t-alpha:** 0.0153283513243391

---

---

13

- **AF ID:** AF-Q16706-F1-model-v4 | **Chain:** A
- **b-phipsi:** 0.0006309441763766
- **w-rdist:** 0.4076872403167024
- **t-alpha:** 0.0489048903395892

---

---

14

- **AF ID:** AF-P61565-F1-model-v4 | **Chain:** A
- **b-phipsi:** 0.0002162086799721
- **w-rdist:** 1.0660705997724096
- **t-alpha:** 0.002194902533056

---

---

15

- **AF ID:** AF-Q9HA65-F1-model-v4 | **Chain:** A
- **b-phipsi:** 0.0093718540687799
- **w-rdist:** 0.4948129624364749
- **t-alpha:** 0.0

---

---

16

- **AF ID:** AF-Q8NHY0-F1-model-v4 | **Chain:** A
- **b-phipsi:** 0.0005105635558549
- **w-rdist:** 0.6508121807869264
- **t-alpha:** 0.0277371931127072

---

---

17

- **AF ID:** AF-O14841-F1-model-v4 | **Chain:** A
- **b-phipsi:** 0.0036879324202503
- **w-rdist:** 0.3182327983044591
- **t-alpha:** 0.0094889742856814

---

---

18

- **AF ID:** AF-Q01518-F1-model-v4 | **Chain:** A
- **b-phipsi:** 0.0031989877376582
- **w-rdist:** 0.3299608570530466
- **t-alpha:** 0.0125647391185939

---

---

19

- **AF ID:** AF-Q9NRJ5-F1-model-v4 | **Chain:** A
- **b-phipsi:** 0.0004368254237582
- **w-rdist:** 0.3447848318380939
- **t-alpha:** 0.1832110413152596

---

---

20

- **AF ID:** AF-Q9HAU4-F1-model-v4 | **Chain:** A
- **b-phipsi:** 0.0041691031668369
- **w-rdist:** 0.207324367540024
- **t-alpha:** 0.0452556070301213

---

---

21

- **AF ID:** AF-Q15349-F1-model-v4 | **Chain:** A
- **b-phipsi:** 0.0052068760765145
- **w-rdist:** 0.4745042871949803
- **t-alpha:** 0.0029196362453518

---

---

22

- **AF ID:** AF-Q9UL01-F1-model-v4 | **Chain:** A
- **b-phipsi:** 0.008452537584962
- **w-rdist:** 0.3914053683398837
- **t-alpha:** 0.002194902533056

---

---

23

- **AF ID:** AF-Q8NI99-F1-model-v4 | **Chain:** A
- **b-phipsi:** 0.009029364859538
- **w-rdist:** 0.6114701476785901
- **t-alpha:** 0.0

---

---

24

- **AF ID:** AF-P40123-F1-model-v4 | **Chain:** A
- **b-phipsi:** 0.0072813836489104
- **w-rdist:** 0.4396786495045962
- **t-alpha:** 0.0014620968756575

---

---

25

- **AF ID:** AF-P49641-F1-model-v4 | **Chain:** A
- **b-phipsi:** 0.0006140024653658
- **w-rdist:** 0.4873417687739382
- **t-alpha:** 0.0642335893644507

---

---

26

- **AF ID:** AF-Q460N3-F1-model-v4 | **Chain:** A
- **b-phipsi:** 0.0058915365842895
- **w-rdist:** 0.2537221629549929
- **t-alpha:** 0.0284671564505767

---

---

27

- **AF ID:** AF-O43143-F1-model-v4 | **Chain:** A
- **b-phipsi:** 0.01040186415315
- **w-rdist:** 0.3639878383702604
- **t-alpha:** 0.0036630800678474

---

---

28

- **AF ID:** AF-Q7Z7A4-F1-model-v4 | **Chain:** A
- **b-phipsi:** 0.0041515316676992
- **w-rdist:** 0.5814085546726753
- **t-alpha:** 0.0036496798094789

---

---

29

- **AF ID:** AF-A8K7I4-F1-model-v4 | **Chain:** A
- **b-phipsi:** 0.0086765831626286
- **w-rdist:** 0.2897602142222059
- **t-alpha:** 0.0072992307962134

---

---

30

- **AF ID:** AF-Q96J94-F1-model-v4 | **Chain:** A
- **b-phipsi:** 0.0067187863218571
- **w-rdist:** 0.1416800876176836
- **t-alpha:** 0.0410335958026029

---

---

31

- **AF ID:** AF-Q8TBY0-F1-model-v4 | **Chain:** A
- **b-phipsi:** 0.0057588111913705
- **w-rdist:** 0.3025836055810361
- **t-alpha:** 0.0170751996684577

---

---

32

- **AF ID:** AF-Q9BZQ2-F1-model-v4 | **Chain:** A
- **b-phipsi:** 0.0009668923948145
- **w-rdist:** 0.6657086003023913
- **t-alpha:** 0.0138687321491499

---

---

33

- **AF ID:** AF-Q9BYG8-F1-model-v4 | **Chain:** A
- **b-phipsi:** 0.005063272525478
- **w-rdist:** 0.7378221925696524
- **t-alpha:** 0.0007307814170889

---

---

34

- **AF ID:** AF-Q9Y2E5-F1-model-v4 | **Chain:** A
- **b-phipsi:** 0.0015051408209955
- **w-rdist:** 0.325157028788994
- **t-alpha:** 0.0595513803978347

---

---

35

- **AF ID:** AF-Q9BXB7-F1-model-v4 | **Chain:** A
- **b-phipsi:** 0.0124934575060942
- **w-rdist:** 0.517758599439876
- **t-alpha:** 0.0007298270463003

---

---

36

- **AF ID:** AF-O15067-F1-model-v4 | **Chain:** A
- **b-phipsi:** 0.0040655576948775
- **w-rdist:** 0.27473532668292
- **t-alpha:** 0.052554868726995

---

---

37

- **AF ID:** AF-O75366-F1-model-v4 | **Chain:** A
- **b-phipsi:** 0.0034115477489951
- **w-rdist:** 0.6718105361387106
- **t-alpha:** 0.0036496798094789

---

---

38

- **AF ID:** AF-P0C869-F1-model-v4 | **Chain:** A
- **b-phipsi:** 0.0016297898363481
- **w-rdist:** 0.3907235331496774
- **t-alpha:** 0.0073530278154354

---

---

39

- **AF ID:** AF-O43374-F1-model-v4 | **Chain:** A
- **b-phipsi:** 0.0027915641782217
- **w-rdist:** 0.2146612660510597
- **t-alpha:** 0.0968778049427547

---

---

40

- **AF ID:** AF-Q86TM3-F1-model-v4 | **Chain:** A
- **b-phipsi:** 0.0043852919187933
- **w-rdist:** 0.2882143210655885
- **t-alpha:** 0.0474004981600588

---

---

41

- **AF ID:** AF-C9J798-F1-model-v4 | **Chain:** A
- **b-phipsi:** 0.0028063897379396
- **w-rdist:** 0.2563218896810988
- **t-alpha:** 0.0873018129196776

---

---

42

- **AF ID:** AF-Q3MJ16-F1-model-v4 | **Chain:** A
- **b-phipsi:** 0.0009904599626414
- **w-rdist:** 0.5542429226529162
- **t-alpha:** 0.0418251637726221

---

---

43

- **AF ID:** AF-Q96K75-F1-model-v4 | **Chain:** A
- **b-phipsi:** 0.01845161902866
- **w-rdist:** 0.2405599774302477
- **t-alpha:** 0.0058391635426686

---

---

44

- **AF ID:** AF-Q96JY0-F1-model-v4 | **Chain:** A
- **b-phipsi:** 0.0128457360574514
- **w-rdist:** 0.465017920017768
- **t-alpha:** 0.0014597092068022

---

---

45

- **AF ID:** AF-Q9Y6W3-F1-model-v4 | **Chain:** A
- **b-phipsi:** 0.0005048859904156
- **w-rdist:** 0.5911917257779645
- **t-alpha:** 0.0830039517924496

---

---

46

- **AF ID:** AF-P37173-F1-model-v4 | **Chain:** A
- **b-phipsi:** 0.0008112932526835
- **w-rdist:** 0.5574047038821384
- **t-alpha:** 0.0694769169631865

---

---

47

- **AF ID:** AF-O00425-F1-model-v4 | **Chain:** A
- **b-phipsi:** 0.0084696030865584
- **w-rdist:** 0.3039984052850126
- **t-alpha:** 0.0153283513243391

---

---

48

- **AF ID:** AF-Q96PZ2-F1-model-v4 | **Chain:** A
- **b-phipsi:** 0.0027689110563137
- **w-rdist:** 0.2934730534489749
- **t-alpha:** 0.0855787620207846

---

---

49

- **AF ID:** AF-A0A087X1G2-F1-model-v4 | **Chain:** A
- **b-phipsi:** 0.01178112804553
- **w-rdist:** 0.535088239998369
- **t-alpha:** 0.0014620968756575

---

---

50

- **AF ID:** AF-P98170-F1-model-v4 | **Chain:** A
- **b-phipsi:** 0.0135172099033531
- **w-rdist:** 0.2723512647395505
- **t-alpha:** 0.0073530278154354

---

---

51

- **AF ID:** AF-Q15937-F1-model-v4 | **Chain:** A
- **b-phipsi:** 0.0086371763038098
- **w-rdist:** 0.7038794893596165
- **t-alpha:** 0.0014597092068022

---

---

52

- **AF ID:** AF-Q9UJX5-F1-model-v4 | **Chain:** A
- **b-phipsi:** 0.0024622330964529
- **w-rdist:** 0.7288173038690174
- **t-alpha:** 0.0043989330665894

---

---

53

- **AF ID:** AF-O95294-F1-model-v4 | **Chain:** A
- **b-phipsi:** 0.0048315872897498
- **w-rdist:** 0.2222251016637016
- **t-alpha:** 0.095999985496791

---

---

54

- **AF ID:** AF-Q8TF42-F1-model-v4 | **Chain:** A
- **b-phipsi:** 0.0016364681796517
- **w-rdist:** 0.3726353342627674
- **t-alpha:** 0.0510948593011042

---

---

55

- **AF ID:** AF-P29350-F1-model-v4 | **Chain:** A
- **b-phipsi:** 0.0066097262661145
- **w-rdist:** 0.5247360846405141
- **t-alpha:** 0.0043989330665894

---

---

56

- **AF ID:** AF-Q8IUH3-F1-model-v4 | **Chain:** A
- **b-phipsi:** 0.0057755552726935
- **w-rdist:** 0.2536061625319976
- **t-alpha:** 0.0781018927757748

---

---

57

- **AF ID:** AF-Q3V5L5-F1-model-v4 | **Chain:** A
- **b-phipsi:** 0.0099353025505298
- **w-rdist:** 0.2999776908736934
- **t-alpha:** 0.0240873273423209

---

---

58

- **AF ID:** AF-O15091-F1-model-v4 | **Chain:** A
- **b-phipsi:** 0.0127513225713664
- **w-rdist:** 0.2185832214993818
- **t-alpha:** 0.035036511315305

---

---

59

- **AF ID:** AF-Q9UK32-F1-model-v4 | **Chain:** A
- **b-phipsi:** 0.0054129914979712
- **w-rdist:** 0.4392301987473027
- **t-alpha:** 0.0058736968826307

---

---

60

- **AF ID:** AF-Q13131-F1-model-v4 | **Chain:** A
- **b-phipsi:** 0.0037675527381664
- **w-rdist:** 0.3763462645458072
- **t-alpha:** 0.0370931750792566

---

---

61

- **AF ID:** AF-Q8IY47-F1-model-v4 | **Chain:** A
- **b-phipsi:** 0.003414926939008
- **w-rdist:** 0.3618226071198236
- **t-alpha:** 0.0522276825687215

---

---

62

- **AF ID:** AF-Q6ZQR2-F1-model-v4 | **Chain:** A
- **b-phipsi:** 0.0090873961598402
- **w-rdist:** 0.788263719916835
- **t-alpha:** 0.0007298270463003

---

---

63

- **AF ID:** AF-P0C7X1-F1-model-v4 | **Chain:** A
- **b-phipsi:** 0.0142750230994999
- **w-rdist:** 0.5171796613716435
- **t-alpha:** 0.0014620968756575

---

---

64

- **AF ID:** AF-Q6P179-F1-model-v4 | **Chain:** A
- **b-phipsi:** 0.0071212017070825
- **w-rdist:** 0.2513296699926155
- **t-alpha:** 0.0664230173941995

---

---

65

- **AF ID:** AF-Q7Z4K8-F1-model-v4 | **Chain:** A
- **b-phipsi:** 0.000689617697657
- **w-rdist:** 1.4631598619348734
- **t-alpha:** 0.0014620968756575

---

---

66

- **AF ID:** AF-P06241-F1-model-v4 | **Chain:** A
- **b-phipsi:** 0.0140782875123862
- **w-rdist:** 0.7138997317981913
- **t-alpha:** 0.0

---

---

67

- **AF ID:** AF-O75676-F1-model-v4 | **Chain:** A
- **b-phipsi:** 0.0107411671139756
- **w-rdist:** 0.3363534534139124
- **t-alpha:** 0.0087590123987277

---

---

68

- **AF ID:** AF-O43300-F1-model-v4 | **Chain:** A
- **b-phipsi:** 0.0072294833039345
- **w-rdist:** 0.7804690452784253
- **t-alpha:** 0.0014597092068022

---

---

69

- **AF ID:** AF-Q15437-F1-model-v4 | **Chain:** A
- **b-phipsi:** 0.0010888888443386
- **w-rdist:** 0.6284596715388369
- **t-alpha:** 0.0506138163692111

---

---

70

- **AF ID:** AF-Q9UBT2-F1-model-v4 | **Chain:** A
- **b-phipsi:** 0.0061713822711717
- **w-rdist:** 0.266040269763397
- **t-alpha:** 0.0722624044746096

---

---

71

- **AF ID:** AF-Q8N806-F1-model-v4 | **Chain:** A
- **b-phipsi:** 0.0060929720281122
- **w-rdist:** 0.4214160442754247
- **t-alpha:** 0.0058736968826307

---

---

72

- **AF ID:** AF-Q8NC26-F1-model-v4 | **Chain:** A
- **b-phipsi:** 0.0024777770149246
- **w-rdist:** 0.1780760079161555
- **t-alpha:** 0.2394163921955769

---

---

73

- **AF ID:** AF-Q9UK97-F1-model-v4 | **Chain:** A
- **b-phipsi:** 0.0007969085334394
- **w-rdist:** 0.8167937041605775
- **t-alpha:** 0.0442074641360952

---

---

74

- **AF ID:** AF-Q9BZH6-F1-model-v4 | **Chain:** A
- **b-phipsi:** 0.000862654019149
- **w-rdist:** 0.9405145489442928
- **t-alpha:** 0.0073530278154354

---

---

75

- **AF ID:** AF-Q09013-F1-model-v4 | **Chain:** A
- **b-phipsi:** 0.009149877013126
- **w-rdist:** 0.2887467173676233
- **t-alpha:** 0.0481749154992192

---

---

76

- **AF ID:** AF-Q3KNW1-F1-model-v4 | **Chain:** A
- **b-phipsi:** 0.011548366899415
- **w-rdist:** 0.4735216804175859
- **t-alpha:** 0.0043792969344855

---

---

77

- **AF ID:** AF-Q9BW92-F1-model-v4 | **Chain:** A
- **b-phipsi:** 0.0053417868345026
- **w-rdist:** 0.5520569156472555
- **t-alpha:** 0.0065694124035411

---

---

78

- **AF ID:** AF-Q96NY9-F1-model-v4 | **Chain:** A
- **b-phipsi:** 0.0119185186682905
- **w-rdist:** 0.1792841611342331
- **t-alpha:** 0.0628394789167019

---

---

79

- **AF ID:** AF-Q96JB8-F1-model-v4 | **Chain:** A
- **b-phipsi:** 0.006821390367795
- **w-rdist:** 0.9332820600755708
- **t-alpha:** 0.0007298270463003

---

---

80

- **AF ID:** AF-P10075-F1-model-v4 | **Chain:** A
- **b-phipsi:** 0.012165169184827
- **w-rdist:** 0.5321337794586072
- **t-alpha:** 0.0036630800678474

---

---

81

- **AF ID:** AF-Q6ZV50-F1-model-v4 | **Chain:** A
- **b-phipsi:** 0.0186488875034474
- **w-rdist:** 0.2567905733979096
- **t-alpha:** 0.0148148020346907

---

---

82

- **AF ID:** AF-Q902F9-F1-model-v4 | **Chain:** A
- **b-phipsi:** 0.00016127501054
- **w-rdist:** 1.046297717401112
- **t-alpha:** 0.0248176303394369

---

---

83

- **AF ID:** AF-Q9UIF7-F1-model-v4 | **Chain:** A
- **b-phipsi:** 0.0130886559836935
- **w-rdist:** 0.3430510412898052
- **t-alpha:** 0.01094855486171

---

---

84

- **AF ID:** AF-P35626-F1-model-v4 | **Chain:** A
- **b-phipsi:** 0.0141792046230993
- **w-rdist:** 0.387473015345541
- **t-alpha:** 0.0051358048547096

---

---

85

- **AF ID:** AF-Q5T2T1-F1-model-v4 | **Chain:** A
- **b-phipsi:** 0.0028929152572814
- **w-rdist:** 0.7992068569604205
- **t-alpha:** 0.0051358048547096

---

---

86

- **AF ID:** AF-Q9NVM9-F1-model-v4 | **Chain:** A
- **b-phipsi:** 0.0048362090320187
- **w-rdist:** 1.0158966120617745
- **t-alpha:** 0.0007307814170889

---

---

87

- **AF ID:** AF-Q96ME1-F1-model-v4 | **Chain:** A
- **b-phipsi:** 0.0048299271900046
- **w-rdist:** 0.4041877420292917
- **t-alpha:** 0.0285284596970611

---

---

88

- **AF ID:** AF-P10253-F1-model-v4 | **Chain:** A
- **b-phipsi:** 0.0139550072023652
- **w-rdist:** 0.3174673025868696
- **t-alpha:** 0.0254491879579255

---

---

89

- **AF ID:** AF-Q96EX3-F1-model-v4 | **Chain:** A
- **b-phipsi:** 0.0168031112857227
- **w-rdist:** 0.7033824175891171
- **t-alpha:** 0.0007307814170889

---

---

90

- **AF ID:** AF-Q8N653-F1-model-v4 | **Chain:** A
- **b-phipsi:** 0.0040587349487597
- **w-rdist:** 0.912844767020438
- **t-alpha:** 0.0036496798094789

---

---

91

- **AF ID:** AF-Q16394-F1-model-v4 | **Chain:** A
- **b-phipsi:** 0.004372895163623
- **w-rdist:** 0.3973749147973184
- **t-alpha:** 0.0418251637726221

---

---

92

- **AF ID:** AF-O15259-F1-model-v4 | **Chain:** A
- **b-phipsi:** 0.0045076810769
- **w-rdist:** 0.2538642494960827
- **t-alpha:** 0.1919703346256052

---

---

93

- **AF ID:** AF-Q658Y4-F1-model-v4 | **Chain:** A
- **b-phipsi:** 0.010919481818504
- **w-rdist:** 0.2477484733189863
- **t-alpha:** 0.0810216958060698

---

---

94

- **AF ID:** AF-D6RBQ6-F1-model-v4 | **Chain:** A
- **b-phipsi:** 0.0173679213448151
- **w-rdist:** 0.7126436931625577
- **t-alpha:** 0.0007307814170889

---

---

95

- **AF ID:** AF-Q86UR1-F1-model-v4 | **Chain:** A
- **b-phipsi:** 0.0113467292545109
- **w-rdist:** 0.7175907050453332
- **t-alpha:** 0.0029282444105804

---

---

96

- **AF ID:** AF-Q16819-F1-model-v4 | **Chain:** A
- **b-phipsi:** 0.0120387296883108
- **w-rdist:** 0.2696260416736785
- **t-alpha:** 0.0636648718565564

---

---

97

- **AF ID:** AF-Q14181-F1-model-v4 | **Chain:** A
- **b-phipsi:** 0.0011475185954166
- **w-rdist:** 0.8306084666095694
- **t-alpha:** 0.0347433877716041

---

---

98

- **AF ID:** AF-Q5JTZ5-F1-model-v4 | **Chain:** A
- **b-phipsi:** 0.012396678202016
- **w-rdist:** 0.5931363250132718
- **t-alpha:** 0.0043792969344855

---

---

99

- **AF ID:** AF-Q86VW2-F1-model-v4 | **Chain:** A
- **b-phipsi:** 0.0141545784004771
- **w-rdist:** 0.4577158406212103
- **t-alpha:** 0.0051094444648316

---

---
