# Supplementary material for: Comparative structural insights and functional analysis for the distinct unbound states of Human AGO proteins
Source: Sci Rep. 2025 Mar 19;15:9432. doi: 10.1038/s41598-025-91849-5 (PMC11923369; doi:10.1038/s41598-025-91849-5)
Supplement: Supplementary file 24 — Supplementary Information 12. [file 41598_2025_91849_MOESM24_ESM.zip › 4KREp_A_mdwhole_AF4REF/candidates/4KREp_A-merged-notenriched_report.html]

 

# Structural Comparison Report for 4KREp\_A - whole structures (total: 580)

---

1

- **AF ID:** AF-Q9UL18-F1-model-v4 | **Chain:** A
- **b-phipsi:** 0.0003939709503112
- **w-rdist:** 0.0502730094573728
- **t-alpha:** 0.0316268163453545

---

---

2

- **AF ID:** AF-Q9HCK5-F1-model-v4 | **Chain:** A
- **b-phipsi:** 0.0004163230240476
- **w-rdist:** 0.1730393605103452
- **t-alpha:** 0.0423354827531265

---

---

3

- **AF ID:** AF-Q9UKV8-F1-model-v4 | **Chain:** A
- **b-phipsi:** 0.0009358773473456
- **w-rdist:** 0.0871451573874778
- **t-alpha:** 0.0133138831004895

---

---

4

- **AF ID:** AF-Q9UBK8-F1-model-v4 | **Chain:** A
- **b-phipsi:** 0.0046231956219998
- **w-rdist:** 0.3501388103377091
- **t-alpha:** 0.0007298270463003

---

---

5

- **AF ID:** AF-Q9H9G7-F1-model-v4 | **Chain:** A
- **b-phipsi:** 0.0010968103034356
- **w-rdist:** 0.0473955288326407
- **t-alpha:** 0.0410335958026029

---

---

6

- **AF ID:** AF-Q9NZ08-F1-model-v4 | **Chain:** A
- **b-phipsi:** 0.0060381124708424
- **w-rdist:** 0.3497212898670014
- **t-alpha:** 0.0007298270463003

---

---

7

- **AF ID:** AF-Q68DD2-F1-model-v4 | **Chain:** A
- **b-phipsi:** 0.0008731647376385
- **w-rdist:** 0.2190491441134769
- **t-alpha:** 0.0620435921263067

---

---

8

- **AF ID:** AF-Q9BQA5-F1-model-v4 | **Chain:** A
- **b-phipsi:** 0.0057449617486512
- **w-rdist:** 0.1988157270694643
- **t-alpha:** 0.0051094444648316

---

---

9

- **AF ID:** AF-Q9BQ95-F1-model-v4 | **Chain:** A
- **b-phipsi:** 0.0060276394201072
- **w-rdist:** 0.276995318519683
- **t-alpha:** 0.0036630800678474

---

---

10

- **AF ID:** AF-Q9Y2X0-F1-model-v4 | **Chain:** A
- **b-phipsi:** 0.0003830591833502
- **w-rdist:** 0.3665082565098729
- **t-alpha:** 0.0482019055190958

---

---

11

- **AF ID:** AF-Q969S9-F1-model-v4 | **Chain:** A
- **b-phipsi:** 0.0062800128192865
- **w-rdist:** 0.3657045227832043
- **t-alpha:** 0.0007307814170889

---

---

12

- **AF ID:** AF-Q6ZN11-F1-model-v4 | **Chain:** A
- **b-phipsi:** 0.0151145785568739
- **w-rdist:** 0.2391606493014683
- **t-alpha:** 0.0014597092068022

---

---

13

- **AF ID:** AF-Q6ZTN6-F1-model-v4 | **Chain:** A
- **b-phipsi:** 0.0016721359662489
- **w-rdist:** 0.6428840344993882
- **t-alpha:** 0.0

---

---

14

- **AF ID:** AF-Q96PZ0-F1-model-v4 | **Chain:** A
- **b-phipsi:** 0.0010033442773642
- **w-rdist:** 0.3020394134398053
- **t-alpha:** 0.052554868726995

---

---

15

- **AF ID:** AF-Q7Z3Z4-F1-model-v4 | **Chain:** A
- **b-phipsi:** 0.0023840053768281
- **w-rdist:** 0.2033971750884409
- **t-alpha:** 0.0216257249425573

---

---

16

- **AF ID:** AF-Q96RQ3-F1-model-v4 | **Chain:** A
- **b-phipsi:** 0.0076364565306422
- **w-rdist:** 0.368435967667157
- **t-alpha:** 0.0021896458253802

---

---

17

- **AF ID:** AF-Q86XP0-F1-model-v4 | **Chain:** A
- **b-phipsi:** 0.0014517199230324
- **w-rdist:** 0.330555074781289
- **t-alpha:** 0.0175180821197378

---

---

18

- **AF ID:** AF-Q9UJT9-F1-model-v4 | **Chain:** A
- **b-phipsi:** 0.0060021531061447
- **w-rdist:** 0.5499131088762762
- **t-alpha:** 0.0

---

---

19

- **AF ID:** AF-P19525-F1-model-v4 | **Chain:** A
- **b-phipsi:** 0.0057156461662916
- **w-rdist:** 0.5124837582891691
- **t-alpha:** 0.0007307814170889

---

---

20

- **AF ID:** AF-Q9UL63-F1-model-v4 | **Chain:** A
- **b-phipsi:** 0.0014548175009959
- **w-rdist:** 0.2438400992419745
- **t-alpha:** 0.0570987886882312

---

---

21

- **AF ID:** AF-Q8TAP6-F1-model-v4 | **Chain:** A
- **b-phipsi:** 0.0003802029505623
- **w-rdist:** 0.5306996793983226
- **t-alpha:** 0.0423354827531265

---

---

22

- **AF ID:** AF-Q5T447-F1-model-v4 | **Chain:** A
- **b-phipsi:** 0.0035850422787007
- **w-rdist:** 0.2912012832162159
- **t-alpha:** 0.0153283513243391

---

---

23

- **AF ID:** AF-Q16706-F1-model-v4 | **Chain:** A
- **b-phipsi:** 0.0006309441763766
- **w-rdist:** 0.4076872403167024
- **t-alpha:** 0.0489048903395892

---

---

24

- **AF ID:** AF-P61565-F1-model-v4 | **Chain:** A
- **b-phipsi:** 0.0002162086799721
- **w-rdist:** 1.0660705997724096
- **t-alpha:** 0.002194902533056

---

---

25

- **AF ID:** AF-Q06124-F1-model-v4 | **Chain:** A
- **b-phipsi:** 0.0030221805021547
- **w-rdist:** 0.645112602530479
- **t-alpha:** 0.0014620968756575

---

---

26

- **AF ID:** AF-Q9HA65-F1-model-v4 | **Chain:** A
- **b-phipsi:** 0.0093718540687799
- **w-rdist:** 0.4948129624364749
- **t-alpha:** 0.0

---

---

27

- **AF ID:** AF-Q8NHY0-F1-model-v4 | **Chain:** A
- **b-phipsi:** 0.0005105635558549
- **w-rdist:** 0.6508121807869264
- **t-alpha:** 0.0277371931127072

---

---

28

- **AF ID:** AF-O14841-F1-model-v4 | **Chain:** A
- **b-phipsi:** 0.0036879324202503
- **w-rdist:** 0.3182327983044591
- **t-alpha:** 0.0094889742856814

---

---

29

- **AF ID:** AF-Q01518-F1-model-v4 | **Chain:** A
- **b-phipsi:** 0.0031989877376582
- **w-rdist:** 0.3299608570530466
- **t-alpha:** 0.0125647391185939

---

---

30

- **AF ID:** AF-Q9NRJ5-F1-model-v4 | **Chain:** A
- **b-phipsi:** 0.0004368254237582
- **w-rdist:** 0.3447848318380939
- **t-alpha:** 0.1832110413152596

---

---

31

- **AF ID:** AF-Q9HAU4-F1-model-v4 | **Chain:** A
- **b-phipsi:** 0.0041691031668369
- **w-rdist:** 0.207324367540024
- **t-alpha:** 0.0452556070301213

---

---

32

- **AF ID:** AF-Q15349-F1-model-v4 | **Chain:** A
- **b-phipsi:** 0.0052068760765145
- **w-rdist:** 0.4745042871949803
- **t-alpha:** 0.0029196362453518

---

---

33

- **AF ID:** AF-Q9UL01-F1-model-v4 | **Chain:** A
- **b-phipsi:** 0.008452537584962
- **w-rdist:** 0.3914053683398837
- **t-alpha:** 0.002194902533056

---

---

34

- **AF ID:** AF-Q96IC2-F1-model-v4 | **Chain:** A
- **b-phipsi:** 0.0040484238161746
- **w-rdist:** 0.2773457452506451
- **t-alpha:** 0.0378787774827127

---

---

35

- **AF ID:** AF-Q8NI99-F1-model-v4 | **Chain:** A
- **b-phipsi:** 0.009029364859538
- **w-rdist:** 0.6114701476785901
- **t-alpha:** 0.0

---

---

36

- **AF ID:** AF-Q9BXC9-F1-model-v4 | **Chain:** A
- **b-phipsi:** 0.0038355435986638
- **w-rdist:** 0.3011449305724684
- **t-alpha:** 0.0285284596970611

---

---

37

- **AF ID:** AF-P40123-F1-model-v4 | **Chain:** A
- **b-phipsi:** 0.0072813836489104
- **w-rdist:** 0.4396786495045962
- **t-alpha:** 0.0014620968756575

---

---

38

- **AF ID:** AF-Q8NC60-F1-model-v4 | **Chain:** A
- **b-phipsi:** 0.0032089611184012
- **w-rdist:** 0.2991980228367118
- **t-alpha:** 0.0386857767018733

---

---

39

- **AF ID:** AF-Q14CN2-F1-model-v4 | **Chain:** A
- **b-phipsi:** 0.0062485253884154
- **w-rdist:** 0.2206886694166967
- **t-alpha:** 0.0270072581572047

---

---

40

- **AF ID:** AF-Q460N3-F1-model-v4 | **Chain:** A
- **b-phipsi:** 0.0058915365842895
- **w-rdist:** 0.2537221629549929
- **t-alpha:** 0.0284671564505767

---

---

41

- **AF ID:** AF-P49641-F1-model-v4 | **Chain:** A
- **b-phipsi:** 0.0006140024653658
- **w-rdist:** 0.4873417687739382
- **t-alpha:** 0.0642335893644507

---

---

42

- **AF ID:** AF-Q9NZI8-F1-model-v4 | **Chain:** A
- **b-phipsi:** 0.0060436461940543
- **w-rdist:** 0.2649840249585621
- **t-alpha:** 0.0218975903112057

---

---

43

- **AF ID:** AF-O43143-F1-model-v4 | **Chain:** A
- **b-phipsi:** 0.01040186415315
- **w-rdist:** 0.3639878383702604
- **t-alpha:** 0.0036630800678474

---

---

44

- **AF ID:** AF-Q7Z7A4-F1-model-v4 | **Chain:** A
- **b-phipsi:** 0.0041515316676992
- **w-rdist:** 0.5814085546726753
- **t-alpha:** 0.0036496798094789

---

---

45

- **AF ID:** AF-A8K7I4-F1-model-v4 | **Chain:** A
- **b-phipsi:** 0.0086765831626286
- **w-rdist:** 0.2897602142222059
- **t-alpha:** 0.0072992307962134

---

---

46

- **AF ID:** AF-Q8TBY0-F1-model-v4 | **Chain:** A
- **b-phipsi:** 0.0057588111913705
- **w-rdist:** 0.3025836055810361
- **t-alpha:** 0.0170751996684577

---

---

47

- **AF ID:** AF-Q96J94-F1-model-v4 | **Chain:** A
- **b-phipsi:** 0.0067187863218571
- **w-rdist:** 0.1416800876176836
- **t-alpha:** 0.0410335958026029

---

---

48

- **AF ID:** AF-Q9BZQ2-F1-model-v4 | **Chain:** A
- **b-phipsi:** 0.0009668923948145
- **w-rdist:** 0.6657086003023913
- **t-alpha:** 0.0138687321491499

---

---

49

- **AF ID:** AF-Q9BYG8-F1-model-v4 | **Chain:** A
- **b-phipsi:** 0.005063272525478
- **w-rdist:** 0.7378221925696524
- **t-alpha:** 0.0007307814170889

---

---

50

- **AF ID:** AF-Q9Y2E5-F1-model-v4 | **Chain:** A
- **b-phipsi:** 0.0015051408209955
- **w-rdist:** 0.325157028788994
- **t-alpha:** 0.0595513803978347

---

---

51

- **AF ID:** AF-Q9BXB7-F1-model-v4 | **Chain:** A
- **b-phipsi:** 0.0124934575060942
- **w-rdist:** 0.517758599439876
- **t-alpha:** 0.0007298270463003

---

---

52

- **AF ID:** AF-O75366-F1-model-v4 | **Chain:** A
- **b-phipsi:** 0.0034115477489951
- **w-rdist:** 0.6718105361387106
- **t-alpha:** 0.0036496798094789

---

---

53

- **AF ID:** AF-O15067-F1-model-v4 | **Chain:** A
- **b-phipsi:** 0.0040655576948775
- **w-rdist:** 0.27473532668292
- **t-alpha:** 0.052554868726995

---

---

54

- **AF ID:** AF-P0C869-F1-model-v4 | **Chain:** A
- **b-phipsi:** 0.0016297898363481
- **w-rdist:** 0.3907235331496774
- **t-alpha:** 0.0073530278154354

---

---

55

- **AF ID:** AF-Q96Q07-F1-model-v4 | **Chain:** A
- **b-phipsi:** 0.0056305911953787
- **w-rdist:** 0.653134471783065
- **t-alpha:** 0.0021896458253802

---

---

56

- **AF ID:** AF-O43374-F1-model-v4 | **Chain:** A
- **b-phipsi:** 0.0027915641782217
- **w-rdist:** 0.2146612660510597
- **t-alpha:** 0.0968778049427547

---

---

57

- **AF ID:** AF-Q5SY16-F1-model-v4 | **Chain:** A
- **b-phipsi:** 0.0032166172400522
- **w-rdist:** 0.3161725360025341
- **t-alpha:** 0.0474449492167272

---

---

58

- **AF ID:** AF-Q86TM3-F1-model-v4 | **Chain:** A
- **b-phipsi:** 0.0043852919187933
- **w-rdist:** 0.2882143210655885
- **t-alpha:** 0.0474004981600588

---

---

59

- **AF ID:** AF-P34931-F1-model-v4 | **Chain:** A
- **b-phipsi:** 0.0060930730128734
- **w-rdist:** 0.6125030624528522
- **t-alpha:** 0.0021896458253802

---

---

60

- **AF ID:** AF-P26639-F1-model-v4 | **Chain:** A
- **b-phipsi:** 0.005185499719163
- **w-rdist:** 0.7328111946259366
- **t-alpha:** 0.0014597092068022

---

---

61

- **AF ID:** AF-C9J798-F1-model-v4 | **Chain:** A
- **b-phipsi:** 0.0028063897379396
- **w-rdist:** 0.2563218896810988
- **t-alpha:** 0.0873018129196776

---

---

62

- **AF ID:** AF-Q3MJ16-F1-model-v4 | **Chain:** A
- **b-phipsi:** 0.0009904599626414
- **w-rdist:** 0.5542429226529162
- **t-alpha:** 0.0418251637726221

---

---

63

- **AF ID:** AF-P48443-F1-model-v4 | **Chain:** A
- **b-phipsi:** 0.0095425969354183
- **w-rdist:** 0.5309265060726726
- **t-alpha:** 0.0014620968756575

---

---

64

- **AF ID:** AF-Q96K75-F1-model-v4 | **Chain:** A
- **b-phipsi:** 0.01845161902866
- **w-rdist:** 0.2405599774302477
- **t-alpha:** 0.0058391635426686

---

---

65

- **AF ID:** AF-Q96JY0-F1-model-v4 | **Chain:** A
- **b-phipsi:** 0.0128457360574514
- **w-rdist:** 0.465017920017768
- **t-alpha:** 0.0014597092068022

---

---

66

- **AF ID:** AF-Q9Y6W3-F1-model-v4 | **Chain:** A
- **b-phipsi:** 0.0005048859904156
- **w-rdist:** 0.5911917257779645
- **t-alpha:** 0.0830039517924496

---

---

67

- **AF ID:** AF-P37173-F1-model-v4 | **Chain:** A
- **b-phipsi:** 0.0008112932526835
- **w-rdist:** 0.5574047038821384
- **t-alpha:** 0.0694769169631865

---

---

68

- **AF ID:** AF-O00425-F1-model-v4 | **Chain:** A
- **b-phipsi:** 0.0084696030865584
- **w-rdist:** 0.3039984052850126
- **t-alpha:** 0.0153283513243391

---

---

69

- **AF ID:** AF-Q8WZA1-F1-model-v4 | **Chain:** A
- **b-phipsi:** 0.0022144587124245
- **w-rdist:** 0.2754095151532624
- **t-alpha:** 0.0978097207620083

---

---

70

- **AF ID:** AF-Q8NAM6-F1-model-v4 | **Chain:** A
- **b-phipsi:** 0.0188653465124877
- **w-rdist:** 0.2968762241630902
- **t-alpha:** 0.0043989330665894

---

---

71

- **AF ID:** AF-Q96PZ2-F1-model-v4 | **Chain:** A
- **b-phipsi:** 0.0027689110563137
- **w-rdist:** 0.2934730534489749
- **t-alpha:** 0.0855787620207846

---

---

72

- **AF ID:** AF-P15144-F1-model-v4 | **Chain:** A
- **b-phipsi:** 0.0125970429555477
- **w-rdist:** 0.2670998360922035
- **t-alpha:** 0.0094889742856814

---

---

73

- **AF ID:** AF-A0A087X1G2-F1-model-v4 | **Chain:** A
- **b-phipsi:** 0.01178112804553
- **w-rdist:** 0.535088239998369
- **t-alpha:** 0.0014620968756575

---

---

74

- **AF ID:** AF-Q15937-F1-model-v4 | **Chain:** A
- **b-phipsi:** 0.0086371763038098
- **w-rdist:** 0.7038794893596165
- **t-alpha:** 0.0014597092068022

---

---

75

- **AF ID:** AF-P98170-F1-model-v4 | **Chain:** A
- **b-phipsi:** 0.0135172099033531
- **w-rdist:** 0.2723512647395505
- **t-alpha:** 0.0073530278154354

---

---

76

- **AF ID:** AF-Q9UJX5-F1-model-v4 | **Chain:** A
- **b-phipsi:** 0.0024622330964529
- **w-rdist:** 0.7288173038690174
- **t-alpha:** 0.0043989330665894

---

---

77

- **AF ID:** AF-O95294-F1-model-v4 | **Chain:** A
- **b-phipsi:** 0.0048315872897498
- **w-rdist:** 0.2222251016637016
- **t-alpha:** 0.095999985496791

---

---

78

- **AF ID:** AF-Q9H8H0-F1-model-v4 | **Chain:** A
- **b-phipsi:** 0.0009128196228252
- **w-rdist:** 1.1971688571116028
- **t-alpha:** 0.0021896458253802

---

---

79

- **AF ID:** AF-P29350-F1-model-v4 | **Chain:** A
- **b-phipsi:** 0.0066097262661145
- **w-rdist:** 0.5247360846405141
- **t-alpha:** 0.0043989330665894

---

---

80

- **AF ID:** AF-Q8TF42-F1-model-v4 | **Chain:** A
- **b-phipsi:** 0.0016364681796517
- **w-rdist:** 0.3726353342627674
- **t-alpha:** 0.0510948593011042

---

---

81

- **AF ID:** AF-Q3V5L5-F1-model-v4 | **Chain:** A
- **b-phipsi:** 0.0099353025505298
- **w-rdist:** 0.2999776908736934
- **t-alpha:** 0.0240873273423209

---

---

82

- **AF ID:** AF-Q9UK32-F1-model-v4 | **Chain:** A
- **b-phipsi:** 0.0054129914979712
- **w-rdist:** 0.4392301987473027
- **t-alpha:** 0.0058736968826307

---

---

83

- **AF ID:** AF-Q8IUH3-F1-model-v4 | **Chain:** A
- **b-phipsi:** 0.0057755552726935
- **w-rdist:** 0.2536061625319976
- **t-alpha:** 0.0781018927757748

---

---

84

- **AF ID:** AF-P47989-F1-model-v4 | **Chain:** A
- **b-phipsi:** 0.0043386129893487
- **w-rdist:** 0.2976306536322454
- **t-alpha:** 0.0729927817404325

---

---

85

- **AF ID:** AF-O15091-F1-model-v4 | **Chain:** A
- **b-phipsi:** 0.0127513225713664
- **w-rdist:** 0.2185832214993818
- **t-alpha:** 0.035036511315305

---

---

86

- **AF ID:** AF-O75529-F1-model-v4 | **Chain:** A
- **b-phipsi:** 0.003495308232727
- **w-rdist:** 0.3820647507330804
- **t-alpha:** 0.0328465096023253

---

---

87

- **AF ID:** AF-Q6ZQR2-F1-model-v4 | **Chain:** A
- **b-phipsi:** 0.0090873961598402
- **w-rdist:** 0.788263719916835
- **t-alpha:** 0.0007298270463003

---

---

88

- **AF ID:** AF-Q13131-F1-model-v4 | **Chain:** A
- **b-phipsi:** 0.0037675527381664
- **w-rdist:** 0.3763462645458072
- **t-alpha:** 0.0370931750792566

---

---

89

- **AF ID:** AF-P0C7X1-F1-model-v4 | **Chain:** A
- **b-phipsi:** 0.0142750230994999
- **w-rdist:** 0.5171796613716435
- **t-alpha:** 0.0014620968756575

---

---

90

- **AF ID:** AF-Q8IY47-F1-model-v4 | **Chain:** A
- **b-phipsi:** 0.003414926939008
- **w-rdist:** 0.3618226071198236
- **t-alpha:** 0.0522276825687215

---

---

91

- **AF ID:** AF-Q6P179-F1-model-v4 | **Chain:** A
- **b-phipsi:** 0.0071212017070825
- **w-rdist:** 0.2513296699926155
- **t-alpha:** 0.0664230173941995

---

---

92

- **AF ID:** AF-Q7Z4K8-F1-model-v4 | **Chain:** A
- **b-phipsi:** 0.000689617697657
- **w-rdist:** 1.4631598619348734
- **t-alpha:** 0.0014620968756575

---

---

93

- **AF ID:** AF-P06241-F1-model-v4 | **Chain:** A
- **b-phipsi:** 0.0140782875123862
- **w-rdist:** 0.7138997317981913
- **t-alpha:** 0.0

---

---

94

- **AF ID:** AF-O43300-F1-model-v4 | **Chain:** A
- **b-phipsi:** 0.0072294833039345
- **w-rdist:** 0.7804690452784253
- **t-alpha:** 0.0014597092068022

---

---

95

- **AF ID:** AF-O75676-F1-model-v4 | **Chain:** A
- **b-phipsi:** 0.0107411671139756
- **w-rdist:** 0.3363534534139124
- **t-alpha:** 0.0087590123987277

---

---

96

- **AF ID:** AF-Q15437-F1-model-v4 | **Chain:** A
- **b-phipsi:** 0.0010888888443386
- **w-rdist:** 0.6284596715388369
- **t-alpha:** 0.0506138163692111

---

---

97

- **AF ID:** AF-Q9UBT2-F1-model-v4 | **Chain:** A
- **b-phipsi:** 0.0061713822711717
- **w-rdist:** 0.266040269763397
- **t-alpha:** 0.0722624044746096

---

---

98

- **AF ID:** AF-Q9NVM4-F1-model-v4 | **Chain:** A
- **b-phipsi:** 0.0011842635574245
- **w-rdist:** 0.6910231182341523
- **t-alpha:** 0.036308661472808

---

---

99

- **AF ID:** AF-Q8N806-F1-model-v4 | **Chain:** A
- **b-phipsi:** 0.0060929720281122
- **w-rdist:** 0.4214160442754247
- **t-alpha:** 0.0058736968826307

---

---

100

- **AF ID:** AF-Q13724-F1-model-v4 | **Chain:** A
- **b-phipsi:** 0.0080151072731036
- **w-rdist:** 0.5111840822334743
- **t-alpha:** 0.0051094444648316

---

---

101

- **AF ID:** AF-Q8NC26-F1-model-v4 | **Chain:** A
- **b-phipsi:** 0.0024777770149246
- **w-rdist:** 0.1780760079161555
- **t-alpha:** 0.2394163921955769

---

---

102

- **AF ID:** AF-Q9BZH6-F1-model-v4 | **Chain:** A
- **b-phipsi:** 0.000862654019149
- **w-rdist:** 0.9405145489442928
- **t-alpha:** 0.0073530278154354

---

---

103

- **AF ID:** AF-Q9UK97-F1-model-v4 | **Chain:** A
- **b-phipsi:** 0.0007969085334394
- **w-rdist:** 0.8167937041605775
- **t-alpha:** 0.0442074641360952

---

---

104

- **AF ID:** AF-Q09013-F1-model-v4 | **Chain:** A
- **b-phipsi:** 0.009149877013126
- **w-rdist:** 0.2887467173676233
- **t-alpha:** 0.0481749154992192

---

---

105

- **AF ID:** AF-Q96HM7-F1-model-v4 | **Chain:** A
- **b-phipsi:** 0.003052203821162
- **w-rdist:** 0.7054121670141322
- **t-alpha:** 0.0058736968826307

---

---

106

- **AF ID:** AF-Q3KNW1-F1-model-v4 | **Chain:** A
- **b-phipsi:** 0.011548366899415
- **w-rdist:** 0.4735216804175859
- **t-alpha:** 0.0043792969344855

---

---

107

- **AF ID:** AF-Q07075-F1-model-v4 | **Chain:** A
- **b-phipsi:** 0.0115477641339231
- **w-rdist:** 0.2240128746168212
- **t-alpha:** 0.0620435921263067

---

---

108

- **AF ID:** AF-Q9BW92-F1-model-v4 | **Chain:** A
- **b-phipsi:** 0.0053417868345026
- **w-rdist:** 0.5520569156472555
- **t-alpha:** 0.0065694124035411

---

---

109

- **AF ID:** AF-Q96NY9-F1-model-v4 | **Chain:** A
- **b-phipsi:** 0.0119185186682905
- **w-rdist:** 0.1792841611342331
- **t-alpha:** 0.0628394789167019

---

---

110

- **AF ID:** AF-Q96JB8-F1-model-v4 | **Chain:** A
- **b-phipsi:** 0.006821390367795
- **w-rdist:** 0.9332820600755708
- **t-alpha:** 0.0007298270463003

---

---

111

- **AF ID:** AF-P10075-F1-model-v4 | **Chain:** A
- **b-phipsi:** 0.012165169184827
- **w-rdist:** 0.5321337794586072
- **t-alpha:** 0.0036630800678474

---

---

112

- **AF ID:** AF-Q6ZV50-F1-model-v4 | **Chain:** A
- **b-phipsi:** 0.0186488875034474
- **w-rdist:** 0.2567905733979096
- **t-alpha:** 0.0148148020346907

---

---

113

- **AF ID:** AF-Q902F9-F1-model-v4 | **Chain:** A
- **b-phipsi:** 0.00016127501054
- **w-rdist:** 1.046297717401112
- **t-alpha:** 0.0248176303394369

---

---

114

- **AF ID:** AF-Q9UIF7-F1-model-v4 | **Chain:** A
- **b-phipsi:** 0.0130886559836935
- **w-rdist:** 0.3430510412898052
- **t-alpha:** 0.01094855486171

---

---

115

- **AF ID:** AF-P35626-F1-model-v4 | **Chain:** A
- **b-phipsi:** 0.0141792046230993
- **w-rdist:** 0.387473015345541
- **t-alpha:** 0.0051358048547096

---

---

116

- **AF ID:** AF-Q5T2T1-F1-model-v4 | **Chain:** A
- **b-phipsi:** 0.0028929152572814
- **w-rdist:** 0.7992068569604205
- **t-alpha:** 0.0051358048547096

---

---

117

- **AF ID:** AF-P13639-F1-model-v4 | **Chain:** A
- **b-phipsi:** 0.0042799070510158
- **w-rdist:** 0.385607383454371
- **t-alpha:** 0.0458015158382385

---

---

118

- **AF ID:** AF-Q9NSD9-F1-model-v4 | **Chain:** A
- **b-phipsi:** 0.0006922482798253
- **w-rdist:** 0.7254171332591921
- **t-alpha:** 0.1057306140816347

---

---

119

- **AF ID:** AF-Q9NVM9-F1-model-v4 | **Chain:** A
- **b-phipsi:** 0.0048362090320187
- **w-rdist:** 1.0158966120617745
- **t-alpha:** 0.0007307814170889

---

---

120

- **AF ID:** AF-Q53T94-F1-model-v4 | **Chain:** A
- **b-phipsi:** 0.0137996523392708
- **w-rdist:** 0.605002227000302
- **t-alpha:** 0.0029282444105804

---

---

121

- **AF ID:** AF-Q9Y6U3-F1-model-v4 | **Chain:** A
- **b-phipsi:** 0.0118362498821613
- **w-rdist:** 0.3640854727287324
- **t-alpha:** 0.0118171139231386

---

---

122

- **AF ID:** AF-O00754-F1-model-v4 | **Chain:** A
- **b-phipsi:** 0.0045109754780275
- **w-rdist:** 0.3920275849282998
- **t-alpha:** 0.0394537506304994

---

---

123

- **AF ID:** AF-Q96EX3-F1-model-v4 | **Chain:** A
- **b-phipsi:** 0.0168031112857227
- **w-rdist:** 0.7033824175891171
- **t-alpha:** 0.0007307814170889

---

---

124

- **AF ID:** AF-Q96ME1-F1-model-v4 | **Chain:** A
- **b-phipsi:** 0.0048299271900046
- **w-rdist:** 0.4041877420292917
- **t-alpha:** 0.0285284596970611

---

---

125

- **AF ID:** AF-P10253-F1-model-v4 | **Chain:** A
- **b-phipsi:** 0.0139550072023652
- **w-rdist:** 0.3174673025868696
- **t-alpha:** 0.0254491879579255

---

---

126

- **AF ID:** AF-Q9Y2F9-F1-model-v4 | **Chain:** A
- **b-phipsi:** 0.0023522500501069
- **w-rdist:** 0.3813033299701223
- **t-alpha:** 0.0787400536666942

---

---

127

- **AF ID:** AF-Q9BQ52-F1-model-v4 | **Chain:** A
- **b-phipsi:** 0.0025254595854174
- **w-rdist:** 0.3406073050744325
- **t-alpha:** 0.1372265998230157

---

---

128

- **AF ID:** AF-P19447-F1-model-v4 | **Chain:** A
- **b-phipsi:** 0.0037284041797411
- **w-rdist:** 0.4040017023533689
- **t-alpha:** 0.043412095966399

---

---

129

- **AF ID:** AF-Q8N653-F1-model-v4 | **Chain:** A
- **b-phipsi:** 0.0040587349487597
- **w-rdist:** 0.912844767020438
- **t-alpha:** 0.0036496798094789

---

---

130

- **AF ID:** AF-Q16394-F1-model-v4 | **Chain:** A
- **b-phipsi:** 0.004372895163623
- **w-rdist:** 0.3973749147973184
- **t-alpha:** 0.0418251637726221

---

---

131

- **AF ID:** AF-O15259-F1-model-v4 | **Chain:** A
- **b-phipsi:** 0.0045076810769
- **w-rdist:** 0.2538642494960827
- **t-alpha:** 0.1919703346256052

---

---

132

- **AF ID:** AF-D6RBQ6-F1-model-v4 | **Chain:** A
- **b-phipsi:** 0.0173679213448151
- **w-rdist:** 0.7126436931625577
- **t-alpha:** 0.0007307814170889

---

---

133

- **AF ID:** AF-Q658Y4-F1-model-v4 | **Chain:** A
- **b-phipsi:** 0.010919481818504
- **w-rdist:** 0.2477484733189863
- **t-alpha:** 0.0810216958060698

---

---

134

- **AF ID:** AF-P09327-F1-model-v4 | **Chain:** A
- **b-phipsi:** 0.003508355211593
- **w-rdist:** 0.3891386420688577
- **t-alpha:** 0.0611931441077286

---

---

135

- **AF ID:** AF-Q86UR1-F1-model-v4 | **Chain:** A
- **b-phipsi:** 0.0113467292545109
- **w-rdist:** 0.7175907050453332
- **t-alpha:** 0.0029282444105804

---

---

136

- **AF ID:** AF-Q16819-F1-model-v4 | **Chain:** A
- **b-phipsi:** 0.0120387296883108
- **w-rdist:** 0.2696260416736785
- **t-alpha:** 0.0636648718565564

---

---

137

- **AF ID:** AF-Q14181-F1-model-v4 | **Chain:** A
- **b-phipsi:** 0.0011475185954166
- **w-rdist:** 0.8306084666095694
- **t-alpha:** 0.0347433877716041

---

---

138

- **AF ID:** AF-Q05655-F1-model-v4 | **Chain:** A
- **b-phipsi:** 0.0047012484709664
- **w-rdist:** 0.3685146464429891
- **t-alpha:** 0.0708026304911424

---

---

139

- **AF ID:** AF-Q5JTZ5-F1-model-v4 | **Chain:** A
- **b-phipsi:** 0.012396678202016
- **w-rdist:** 0.5931363250132718
- **t-alpha:** 0.0043792969344855

---

---

140

- **AF ID:** AF-Q86VW2-F1-model-v4 | **Chain:** A
- **b-phipsi:** 0.0141545784004771
- **w-rdist:** 0.4577158406212103
- **t-alpha:** 0.0051094444648316

---

---

141

- **AF ID:** AF-A6NFN9-F1-model-v4 | **Chain:** A
- **b-phipsi:** 0.0070566584954032
- **w-rdist:** 0.6663246027859808
- **t-alpha:** 0.0058391635426686

---

---

142

- **AF ID:** AF-P50747-F1-model-v4 | **Chain:** A
- **b-phipsi:** 0.0090818022154561
- **w-rdist:** 0.3379841603704875
- **t-alpha:** 0.058394017848627

---

---

143

- **AF ID:** AF-Q6DHY5-F1-model-v4 | **Chain:** A
- **b-phipsi:** 0.0180818346413241
- **w-rdist:** 0.5343769409980232
- **t-alpha:** 0.0029282444105804

---

---

144

- **AF ID:** AF-O00303-F1-model-v4 | **Chain:** A
- **b-phipsi:** 0.0010705758418368
- **w-rdist:** 0.6385343621759336
- **t-alpha:** 0.1012863867690643

---

---

145

- **AF ID:** AF-P55055-F1-model-v4 | **Chain:** A
- **b-phipsi:** 0.0254920885182795
- **w-rdist:** 0.5210555736104907
- **t-alpha:** 0.0007307814170889

---

---

146

- **AF ID:** AF-P84022-F1-model-v4 | **Chain:** A
- **b-phipsi:** 0.000392464716379
- **w-rdist:** 0.7162792752538398
- **t-alpha:** 0.2038662972579192

---

---

147

- **AF ID:** AF-A5YM72-F1-model-v4 | **Chain:** A
- **b-phipsi:** 0.0099641586824145
- **w-rdist:** 0.3854461148541861
- **t-alpha:** 0.0178305927380209

---

---

148

- **AF ID:** AF-Q9H6W3-F1-model-v4 | **Chain:** A
- **b-phipsi:** 0.0032185606202064
- **w-rdist:** 0.38611628623751
- **t-alpha:** 0.080292034788115

---

---

149

- **AF ID:** AF-Q8NA19-F1-model-v4 | **Chain:** A
- **b-phipsi:** 0.0084981299621598
- **w-rdist:** 0.3981273371697795
- **t-alpha:** 0.0182480702564928

---

---

150

- **AF ID:** AF-Q96BZ4-F1-model-v4 | **Chain:** A
- **b-phipsi:** 0.0002724587826976
- **w-rdist:** 0.7634177142133385
- **t-alpha:** 0.165957360961179

---

---

151

- **AF ID:** AF-Q8NFF5-F1-model-v4 | **Chain:** A
- **b-phipsi:** 0.0041614536550193
- **w-rdist:** 0.2967410329151446
- **t-alpha:** 0.2080295884426151

---

---

152

- **AF ID:** AF-Q02156-F1-model-v4 | **Chain:** A
- **b-phipsi:** 0.0131871472986864
- **w-rdist:** 0.3153211679003083
- **t-alpha:** 0.0481749154992192

---

---

153

- **AF ID:** AF-Q9P2G3-F1-model-v4 | **Chain:** A
- **b-phipsi:** 0.0046800510855655
- **w-rdist:** 0.3840582393573234
- **t-alpha:** 0.0694769169631865

---

---

154

- **AF ID:** AF-Q8TC57-F1-model-v4 | **Chain:** A
- **b-phipsi:** 0.0010636754570585
- **w-rdist:** 0.6062352378255398
- **t-alpha:** 0.1312966452446498

---

---

155

- **AF ID:** AF-Q9H4B4-F1-model-v4 | **Chain:** A
- **b-phipsi:** 0.0092527234296466
- **w-rdist:** 0.3720826827106717
- **t-alpha:** 0.0416056097301684

---

---

156

- **AF ID:** AF-Q9BRP7-F1-model-v4 | **Chain:** A
- **b-phipsi:** 0.0011570992680728
- **w-rdist:** 0.4766578073978536
- **t-alpha:** 0.1512608079233615

---

---

157

- **AF ID:** AF-P07237-F1-model-v4 | **Chain:** A
- **b-phipsi:** 0.0020980452693278
- **w-rdist:** 0.3063984073576239
- **t-alpha:** 0.2851782279610129

---

---

158

- **AF ID:** AF-P14735-F1-model-v4 | **Chain:** A
- **b-phipsi:** 0.0063884105743595
- **w-rdist:** 0.3660400518596678
- **t-alpha:** 0.0664230173941995

---

---

159

- **AF ID:** AF-O43776-F1-model-v4 | **Chain:** A
- **b-phipsi:** 0.0012429927087299
- **w-rdist:** 0.7304843107739436
- **t-alpha:** 0.0628394789167019

---

---

160

- **AF ID:** AF-Q9NXP7-F1-model-v4 | **Chain:** A
- **b-phipsi:** 0.002783551026683
- **w-rdist:** 0.8021191034302485
- **t-alpha:** 0.0072992307962134

---

---

161

- **AF ID:** AF-P52849-F1-model-v4 | **Chain:** A
- **b-phipsi:** 0.0062379857955726
- **w-rdist:** 0.7556600137992061
- **t-alpha:** 0.0051358048547096

---

---

162

- **AF ID:** AF-Q9UBI4-F1-model-v4 | **Chain:** A
- **b-phipsi:** 0.0008276452030966
- **w-rdist:** 0.6926737505462183
- **t-alpha:** 0.1629883663299385

---

---

163

- **AF ID:** AF-Q9N2K0-F1-model-v4 | **Chain:** A
- **b-phipsi:** 0.001524871255154
- **w-rdist:** 0.7683728689805234
- **t-alpha:** 0.020104517651075

---

---

164

- **AF ID:** AF-O15033-F1-model-v4 | **Chain:** A
- **b-phipsi:** 0.0013731382547711
- **w-rdist:** 0.7160174487978579
- **t-alpha:** 0.0546574059878011

---

---

165

- **AF ID:** AF-Q15916-F1-model-v4 | **Chain:** A
- **b-phipsi:** 0.0166090124071387
- **w-rdist:** 0.3188859352666925
- **t-alpha:** 0.036308661472808

---

---

166

- **AF ID:** AF-A0A1W2PPF3-F1-model-v4 | **Chain:** A
- **b-phipsi:** 0.0201839634180857
- **w-rdist:** 0.7423864922338561
- **t-alpha:** 0.0007298270463003

---

---

167

- **AF ID:** AF-O95567-F1-model-v4 | **Chain:** A
- **b-phipsi:** 0.0159754614017066
- **w-rdist:** 0.8267954951192358
- **t-alpha:** 0.0007307814170889

---

---

168

- **AF ID:** AF-Q6PJQ5-F1-model-v4 | **Chain:** A
- **b-phipsi:** 0.0049058349832261
- **w-rdist:** 0.3630694102874132
- **t-alpha:** 0.1066236145665033

---

---

169

- **AF ID:** AF-Q8N323-F1-model-v4 | **Chain:** A
- **b-phipsi:** 0.0008380652431836
- **w-rdist:** 0.8326122518221927
- **t-alpha:** 0.0942490624608394

---

---

170

- **AF ID:** AF-Q9HB09-F1-model-v4 | **Chain:** A
- **b-phipsi:** 0.0133894231878926
- **w-rdist:** 0.2330884963568429
- **t-alpha:** 0.0970802370411048

---

---

171

- **AF ID:** AF-P55786-F1-model-v4 | **Chain:** A
- **b-phipsi:** 0.0066653607901174
- **w-rdist:** 0.4103320257598898
- **t-alpha:** 0.0324038473531247

---

---

172

- **AF ID:** AF-O94923-F1-model-v4 | **Chain:** A
- **b-phipsi:** 0.0159423011983397
- **w-rdist:** 0.3043662968102666
- **t-alpha:** 0.0445251834570807

---

---

173

- **AF ID:** AF-Q8IZ07-F1-model-v4 | **Chain:** A
- **b-phipsi:** 0.0041589503227142
- **w-rdist:** 0.4066417752765599
- **t-alpha:** 0.0693428042269768

---

---

174

- **AF ID:** AF-Q08380-F1-model-v4 | **Chain:** A
- **b-phipsi:** 0.0030281985893962
- **w-rdist:** 0.3823260511899835
- **t-alpha:** 0.1183672531842279

---

---

175

- **AF ID:** AF-P00747-F1-model-v4 | **Chain:** A
- **b-phipsi:** 0.0187190222576501
- **w-rdist:** 0.2749592200646505
- **t-alpha:** 0.0426178307838962

---

---

176

- **AF ID:** AF-Q15796-F1-model-v4 | **Chain:** A
- **b-phipsi:** 0.0009787326748882
- **w-rdist:** 0.6204675520445143
- **t-alpha:** 0.1729450994583095

---

---

177

- **AF ID:** AF-P31321-F1-model-v4 | **Chain:** A
- **b-phipsi:** 0.0020655598066982
- **w-rdist:** 0.3924833833431698
- **t-alpha:** 0.1147276925961973

---

---

178

- **AF ID:** AF-Q8NCR0-F1-model-v4 | **Chain:** A
- **b-phipsi:** 0.0010052987907019
- **w-rdist:** 0.5253806321796726
- **t-alpha:** 0.2386979447608388

---

---

179

- **AF ID:** AF-Q9BQS7-F1-model-v4 | **Chain:** A
- **b-phipsi:** 0.0078730054819364
- **w-rdist:** 0.3729380455060541
- **t-alpha:** 0.0605840753082309

---

---

180

- **AF ID:** AF-Q06187-F1-model-v4 | **Chain:** A
- **b-phipsi:** 0.0041560753638295
- **w-rdist:** 0.4069805141038957
- **t-alpha:** 0.0703125681220877

---

---

181

- **AF ID:** AF-Q13490-F1-model-v4 | **Chain:** A
- **b-phipsi:** 0.0160528631112668
- **w-rdist:** 0.3655036686518185
- **t-alpha:** 0.0189780962148271

---

---

182

- **AF ID:** AF-Q99717-F1-model-v4 | **Chain:** A
- **b-phipsi:** 0.0012075184030442
- **w-rdist:** 0.5853428573248199
- **t-alpha:** 0.1445283142236533

---

---

183

- **AF ID:** AF-Q3SY69-F1-model-v4 | **Chain:** A
- **b-phipsi:** 0.007396883446821
- **w-rdist:** 0.8960936138083863
- **t-alpha:** 0.0036496798094789

---

---

184

- **AF ID:** AF-Q6R6M4-F1-model-v4 | **Chain:** A
- **b-phipsi:** 0.0182659152019967
- **w-rdist:** 0.6738662943961058
- **t-alpha:** 0.0029282444105804

---

---

185

- **AF ID:** AF-P26599-F1-model-v4 | **Chain:** A
- **b-phipsi:** 0.035405354601956
- **w-rdist:** 0.5617520813598751
- **t-alpha:** 0.0007298270463003

---

---

186

- **AF ID:** AF-Q8N6K7-F1-model-v4 | **Chain:** A
- **b-phipsi:** 0.0279964746758983
- **w-rdist:** 0.2670729164674553
- **t-alpha:** 0.0133138831004895

---

---

187

- **AF ID:** AF-Q86V97-F1-model-v4 | **Chain:** A
- **b-phipsi:** 0.0012854873772286
- **w-rdist:** 0.8810787986690161
- **t-alpha:** 0.0386659655318466

---

---

188

- **AF ID:** AF-Q92935-F1-model-v4 | **Chain:** A
- **b-phipsi:** 0.0044966148698388
- **w-rdist:** 0.4013889422564591
- **t-alpha:** 0.0736678509011601

---

---

189

- **AF ID:** AF-P07992-F1-model-v4 | **Chain:** A
- **b-phipsi:** 0.0005522051460263
- **w-rdist:** 0.7022417416068981
- **t-alpha:** 0.2948961267842704

---

---

190

- **AF ID:** AF-Q06278-F1-model-v4 | **Chain:** A
- **b-phipsi:** 0.0053110211746227
- **w-rdist:** 0.3392516307311249
- **t-alpha:** 0.1503652472933854

---

---

191

- **AF ID:** AF-Q9H9S5-F1-model-v4 | **Chain:** A
- **b-phipsi:** 0.0006494987056368
- **w-rdist:** 0.6610691570349373
- **t-alpha:** 0.3097514409214357

---

---

192

- **AF ID:** AF-Q969P6-F1-model-v4 | **Chain:** A
- **b-phipsi:** 0.013756600397571
- **w-rdist:** 0.2859612526944377
- **t-alpha:** 0.0847192952571229

---

---

193

- **AF ID:** AF-P35858-F1-model-v4 | **Chain:** A
- **b-phipsi:** 0.0179343722085405
- **w-rdist:** 0.6816751544493855
- **t-alpha:** 0.0036496798094789

---

---

194

- **AF ID:** AF-Q14147-F1-model-v4 | **Chain:** A
- **b-phipsi:** 0.0129281174609234
- **w-rdist:** 1.012413736311707
- **t-alpha:** 0.0

---

---

195

- **AF ID:** AF-P0DPD6-F1-model-v4 | **Chain:** A
- **b-phipsi:** 0.0282907570871512
- **w-rdist:** 0.5892229258755317
- **t-alpha:** 0.0014597092068022

---

---

196

- **AF ID:** AF-P0CF97-F1-model-v4 | **Chain:** A
- **b-phipsi:** 0.0363558497108146
- **w-rdist:** 0.2356953160936439
- **t-alpha:** 0.0087590123987277

---

---

197

- **AF ID:** AF-Q8IVL6-F1-model-v4 | **Chain:** A
- **b-phipsi:** 0.0186952119674824
- **w-rdist:** 0.3504742488163603
- **t-alpha:** 0.0204377723355742

---

---

198

- **AF ID:** AF-Q8WTU0-F1-model-v4 | **Chain:** A
- **b-phipsi:** 0.0047689515026682
- **w-rdist:** 0.8541888038082031
- **t-alpha:** 0.0058736968826307

---

---

199

- **AF ID:** AF-Q8N3Y3-F1-model-v4 | **Chain:** A
- **b-phipsi:** 0.0098372421074526
- **w-rdist:** 0.3649341297131953
- **t-alpha:** 0.0694769169631865

---

---

200

- **AF ID:** AF-Q9GZT8-F1-model-v4 | **Chain:** A
- **b-phipsi:** 0.0007659669805724
- **w-rdist:** 0.7155107192118026
- **t-alpha:** 0.2557286090305555

---

---

201

- **AF ID:** AF-Q2TBF2-F1-model-v4 | **Chain:** A
- **b-phipsi:** 0.0118806807389373
- **w-rdist:** 0.3799188147242717
- **t-alpha:** 0.0430653698396612

---

---

202

- **AF ID:** AF-Q68CQ4-F1-model-v4 | **Chain:** A
- **b-phipsi:** 0.0069632518079613
- **w-rdist:** 0.3458870047399824
- **t-alpha:** 0.1167877305104161

---

---

203

- **AF ID:** AF-P49759-F1-model-v4 | **Chain:** A
- **b-phipsi:** 0.0243577502365445
- **w-rdist:** 0.6972400852221597
- **t-alpha:** 0.0014597092068022

---

---

204

- **AF ID:** AF-P58335-F1-model-v4 | **Chain:** A
- **b-phipsi:** 0.00154062355089
- **w-rdist:** 1.38041115793177
- **t-alpha:** 0.0014597092068022

---

---

205

- **AF ID:** AF-Q9Y6F1-F1-model-v4 | **Chain:** A
- **b-phipsi:** 0.00062010393978
- **w-rdist:** 0.7613602054866302
- **t-alpha:** 0.2465881593411327

---

---

206

- **AF ID:** AF-A8K5M9-F1-model-v4 | **Chain:** A
- **b-phipsi:** 0.0043386042434567
- **w-rdist:** 0.302644932396351
- **t-alpha:** 0.3022814889156082

---

---

207

- **AF ID:** AF-Q9H0J9-F1-model-v4 | **Chain:** A
- **b-phipsi:** 0.001453696546952
- **w-rdist:** 0.4016809211134656
- **t-alpha:** 0.1986000504091436

---

---

208

- **AF ID:** AF-Q9UL40-F1-model-v4 | **Chain:** A
- **b-phipsi:** 0.0116691156424553
- **w-rdist:** 0.271887777930523
- **t-alpha:** 0.1454848740897429

---

---

209

- **AF ID:** AF-P06396-F1-model-v4 | **Chain:** A
- **b-phipsi:** 0.0058384681367537
- **w-rdist:** 0.3756774388792642
- **t-alpha:** 0.1138212479791738

---

---

210

- **AF ID:** AF-Q00973-F1-model-v4 | **Chain:** A
- **b-phipsi:** 0.0014608656967928
- **w-rdist:** 0.549250497181849
- **t-alpha:** 0.130363252467047

---

---

211

- **AF ID:** AF-P11150-F1-model-v4 | **Chain:** A
- **b-phipsi:** 0.0005666494315591
- **w-rdist:** 0.7433742464910483
- **t-alpha:** 0.2900187997113126

---

---

212

- **AF ID:** AF-Q8IUF8-F1-model-v4 | **Chain:** A
- **b-phipsi:** 0.0006149378904551
- **w-rdist:** 0.7746891299923061
- **t-alpha:** 0.2661737836891764

---

---

213

- **AF ID:** AF-Q9BSQ5-F1-model-v4 | **Chain:** A
- **b-phipsi:** 0.0189337593920729
- **w-rdist:** 0.7560990643603023
- **t-alpha:** 0.0021896458253802

---

---

214

- **AF ID:** AF-Q9UKJ1-F1-model-v4 | **Chain:** A
- **b-phipsi:** 0.0192106137655452
- **w-rdist:** 0.8468856597847987
- **t-alpha:** 0.0007307814170889

---

---

215

- **AF ID:** AF-Q16671-F1-model-v4 | **Chain:** A
- **b-phipsi:** 0.0069810154274683
- **w-rdist:** 0.3874396181007234
- **t-alpha:** 0.0745098508994681

---

---

216

- **AF ID:** AF-Q9NXZ2-F1-model-v4 | **Chain:** A
- **b-phipsi:** 0.0068749269389316
- **w-rdist:** 0.3454653298914256
- **t-alpha:** 0.1437950563327696

---

---

217

- **AF ID:** AF-O43781-F1-model-v4 | **Chain:** A
- **b-phipsi:** 0.0247050012428382
- **w-rdist:** 0.693574852836698
- **t-alpha:** 0.0014620968756575

---

---

218

- **AF ID:** AF-Q9Y6M1-F1-model-v4 | **Chain:** A
- **b-phipsi:** 0.0184854668916564
- **w-rdist:** 0.3013676619548999
- **t-alpha:** 0.0627735967823235

---

---

219

- **AF ID:** AF-Q07869-F1-model-v4 | **Chain:** A
- **b-phipsi:** 0.0227796564157454
- **w-rdist:** 0.6314413059041731
- **t-alpha:** 0.0029282444105804

---

---

220

- **AF ID:** AF-P0CB48-F1-model-v4 | **Chain:** A
- **b-phipsi:** 0.0295728187943925
- **w-rdist:** 0.2888224626536086
- **t-alpha:** 0.0246822773765587

---

---

221

- **AF ID:** AF-P0C860-F1-model-v4 | **Chain:** A
- **b-phipsi:** 0.0098060396594932
- **w-rdist:** 0.906028354725078
- **t-alpha:** 0.0036630800678474

---

---

222

- **AF ID:** AF-Q96HU1-F1-model-v4 | **Chain:** A
- **b-phipsi:** 0.0433068993872395
- **w-rdist:** 0.3529076559256336
- **t-alpha:** 0.0051358048547096

---

---

223

- **AF ID:** AF-P54646-F1-model-v4 | **Chain:** A
- **b-phipsi:** 0.0055536383637247
- **w-rdist:** 0.4101791672244688
- **t-alpha:** 0.0770439234728341

---

---

224

- **AF ID:** AF-Q9Y6Y0-F1-model-v4 | **Chain:** A
- **b-phipsi:** 0.0154409167621084
- **w-rdist:** 0.2806193272508653
- **t-alpha:** 0.1072992501854066

---

---

225

- **AF ID:** AF-P23743-F1-model-v4 | **Chain:** A
- **b-phipsi:** 0.012270251524278
- **w-rdist:** 0.3757050994484794
- **t-alpha:** 0.0603716916996117

---

---

226

- **AF ID:** AF-Q8NFW8-F1-model-v4 | **Chain:** A
- **b-phipsi:** 0.000984663450954
- **w-rdist:** 0.7154886237745318
- **t-alpha:** 0.228699704072582

---

---

227

- **AF ID:** AF-Q9Y4C4-F1-model-v4 | **Chain:** A
- **b-phipsi:** 0.0093181182274901
- **w-rdist:** 0.8528614449912669
- **t-alpha:** 0.0051094444648316

---

---

228

- **AF ID:** AF-Q9UQF0-F1-model-v4 | **Chain:** A
- **b-phipsi:** 0.0032671037263292
- **w-rdist:** 1.1107098582292103
- **t-alpha:** 0.0043792969344855

---

---

229

- **AF ID:** AF-Q13505-F1-model-v4 | **Chain:** A
- **b-phipsi:** 0.0149229077133933
- **w-rdist:** 0.848705314460637
- **t-alpha:** 0.0029282444105804

---

---

230

- **AF ID:** AF-P58317-F1-model-v4 | **Chain:** A
- **b-phipsi:** 0.0309328761137844
- **w-rdist:** 0.357008255337099
- **t-alpha:** 0.0066130905937114

---

---

231

- **AF ID:** AF-Q9Y6W6-F1-model-v4 | **Chain:** A
- **b-phipsi:** 0.0102189676893938
- **w-rdist:** 0.3813399413182545
- **t-alpha:** 0.0711493829927376

---

---

232

- **AF ID:** AF-P16885-F1-model-v4 | **Chain:** A
- **b-phipsi:** 0.0005764916827369
- **w-rdist:** 0.9201357759496492
- **t-alpha:** 0.1562043815098039

---

---

233

- **AF ID:** AF-Q96JF0-F1-model-v4 | **Chain:** A
- **b-phipsi:** 0.0192670751405345
- **w-rdist:** 0.6687421381819227
- **t-alpha:** 0.0043792969344855

---

---

234

- **AF ID:** AF-O15499-F1-model-v4 | **Chain:** A
- **b-phipsi:** 0.0078975138284695
- **w-rdist:** 0.285805507681502
- **t-alpha:** 0.2779849214617771

---

---

235

- **AF ID:** AF-O43462-F1-model-v4 | **Chain:** A
- **b-phipsi:** 0.0203118396370677
- **w-rdist:** 0.5783341826575932
- **t-alpha:** 0.0051094444648316

---

---

236

- **AF ID:** AF-P17028-F1-model-v4 | **Chain:** A
- **b-phipsi:** 0.0200307785797984
- **w-rdist:** 0.3698785044098523
- **t-alpha:** 0.0208645945567758

---

---

237

- **AF ID:** AF-P31751-F1-model-v4 | **Chain:** A
- **b-phipsi:** 0.0008326141792367
- **w-rdist:** 0.8478724922054327
- **t-alpha:** 0.1810346730489735

---

---

238

- **AF ID:** AF-O43847-F1-model-v4 | **Chain:** A
- **b-phipsi:** 0.0107016569893816
- **w-rdist:** 0.3225435203604602
- **t-alpha:** 0.1489048727530237

---

---

239

- **AF ID:** AF-P49619-F1-model-v4 | **Chain:** A
- **b-phipsi:** 0.0159610738511085
- **w-rdist:** 0.2723905271309142
- **t-alpha:** 0.1255468052397776

---

---

240

- **AF ID:** AF-Q8NFJ9-F1-model-v4 | **Chain:** A
- **b-phipsi:** 0.0090715659640463
- **w-rdist:** 0.9358340127407864
- **t-alpha:** 0.0043989330665894

---

---

241

- **AF ID:** AF-P0CH99-F1-model-v4 | **Chain:** A
- **b-phipsi:** 0.0176487420901163
- **w-rdist:** 0.2617255625212317
- **t-alpha:** 0.1201962945629606

---

---

242

- **AF ID:** AF-O43791-F1-model-v4 | **Chain:** A
- **b-phipsi:** 0.0014466300245695
- **w-rdist:** 0.4633799280230284
- **t-alpha:** 0.2431944905478436

---

---

243

- **AF ID:** AF-P38435-F1-model-v4 | **Chain:** A
- **b-phipsi:** 0.0234415219137858
- **w-rdist:** 0.3445129052940983
- **t-alpha:** 0.0277572859037011

---

---

244

- **AF ID:** AF-Q8NBF2-F1-model-v4 | **Chain:** A
- **b-phipsi:** 0.0186607793718634
- **w-rdist:** 0.3698822581182221
- **t-alpha:** 0.0378787774827127

---

---

245

- **AF ID:** AF-Q7L190-F1-model-v4 | **Chain:** A
- **b-phipsi:** 0.0215362411844736
- **w-rdist:** 0.7981960810536881
- **t-alpha:** 0.0014620968756575

---

---

246

- **AF ID:** AF-Q9H7Z6-F1-model-v4 | **Chain:** A
- **b-phipsi:** 0.000491930881352
- **w-rdist:** 0.8531769475422638
- **t-alpha:** 0.2803741353843718

---

---

247

- **AF ID:** AF-Q8WTU2-F1-model-v4 | **Chain:** A
- **b-phipsi:** 0.0476054763979362
- **w-rdist:** 0.4979827922682326
- **t-alpha:** 0.002194902533056

---

---

248

- **AF ID:** AF-Q3SY52-F1-model-v4 | **Chain:** A
- **b-phipsi:** 0.0153538895981525
- **w-rdist:** 0.7914530535462142
- **t-alpha:** 0.0043989330665894

---

---

249

- **AF ID:** AF-Q9Y6K8-F1-model-v4 | **Chain:** A
- **b-phipsi:** 0.0306702186698679
- **w-rdist:** 0.2625864225196804
- **t-alpha:** 0.0481749154992192

---

---

250

- **AF ID:** AF-P12956-F1-model-v4 | **Chain:** A
- **b-phipsi:** 0.0076624631291829
- **w-rdist:** 0.7837192769640933
- **t-alpha:** 0.0073530278154354

---

---

251

- **AF ID:** AF-Q9UK28-F1-model-v4 | **Chain:** A
- **b-phipsi:** 0.0154469212247329
- **w-rdist:** 1.03748475348097
- **t-alpha:** 0.0007307814170889

---

---

252

- **AF ID:** AF-P23378-F1-model-v4 | **Chain:** A
- **b-phipsi:** 0.0149135383081754
- **w-rdist:** 0.3847685063788151
- **t-alpha:** 0.052554868726995

---

---

253

- **AF ID:** AF-Q96RU8-F1-model-v4 | **Chain:** A
- **b-phipsi:** 0.0006887650166153
- **w-rdist:** 0.9543452996219198
- **t-alpha:** 0.156118378599569

---

---

254

- **AF ID:** AF-Q7LFX5-F1-model-v4 | **Chain:** A
- **b-phipsi:** 0.019991283709114
- **w-rdist:** 0.4998661195182102
- **t-alpha:** 0.0066130905937114

---

---

255

- **AF ID:** AF-Q9H1B5-F1-model-v4 | **Chain:** A
- **b-phipsi:** 0.007347620372048
- **w-rdist:** 0.3541986630002807
- **t-alpha:** 0.1948908108919698

---

---

256

- **AF ID:** AF-P29597-F1-model-v4 | **Chain:** A
- **b-phipsi:** 0.0014625206698525
- **w-rdist:** 0.6851511778983178
- **t-alpha:** 0.1423353966321134

---

---

257

- **AF ID:** AF-Q9NRM6-F1-model-v4 | **Chain:** A
- **b-phipsi:** 0.0012521864698328
- **w-rdist:** 0.8590968062631053
- **t-alpha:** 0.1012863867690643

---

---

258

- **AF ID:** AF-P49005-F1-model-v4 | **Chain:** A
- **b-phipsi:** 0.000487405236194
- **w-rdist:** 0.954115791874736
- **t-alpha:** 0.223214581067924

---

---

259

- **AF ID:** AF-Q99988-F1-model-v4 | **Chain:** A
- **b-phipsi:** 0.000872637276915
- **w-rdist:** 0.7421312456563683
- **t-alpha:** 0.3185757809955751

---

---

260

- **AF ID:** AF-Q9Y243-F1-model-v4 | **Chain:** A
- **b-phipsi:** 0.0010284113147602
- **w-rdist:** 0.8710581063639715
- **t-alpha:** 0.1416666910534196

---

---

261

- **AF ID:** AF-P58215-F1-model-v4 | **Chain:** A
- **b-phipsi:** 0.0288339118539701
- **w-rdist:** 0.3418555879857743
- **t-alpha:** 0.0269866903238209

---

---

262

- **AF ID:** AF-Q9HCG7-F1-model-v4 | **Chain:** A
- **b-phipsi:** 0.0126099460992776
- **w-rdist:** 0.3811626988409722
- **t-alpha:** 0.0781018927757748

---

---

263

- **AF ID:** AF-O95786-F1-model-v4 | **Chain:** A
- **b-phipsi:** 0.0227482627558627
- **w-rdist:** 0.3402059618599238
- **t-alpha:** 0.0467149921820611

---

---

264

- **AF ID:** AF-Q5JRX3-F1-model-v4 | **Chain:** A
- **b-phipsi:** 0.00968095888824
- **w-rdist:** 0.3885364569412384
- **t-alpha:** 0.0970802370411048

---

---

265

- **AF ID:** AF-Q9NXT0-F1-model-v4 | **Chain:** A
- **b-phipsi:** 0.0248632114357934
- **w-rdist:** 0.7238090863278706
- **t-alpha:** 0.0036496798094789

---

---

266

- **AF ID:** AF-Q14697-F1-model-v4 | **Chain:** A
- **b-phipsi:** 0.0118369675978816
- **w-rdist:** 0.3896810805276541
- **t-alpha:** 0.0812944145256238

---

---

267

- **AF ID:** AF-Q6ZN28-F1-model-v4 | **Chain:** A
- **b-phipsi:** 0.0007933934922803
- **w-rdist:** 0.9602023866553976
- **t-alpha:** 0.17445227210315

---

---

268

- **AF ID:** AF-P08697-F1-model-v4 | **Chain:** A
- **b-phipsi:** 0.0013460798226337
- **w-rdist:** 0.7752991904263518
- **t-alpha:** 0.1445283142236533

---

---

269

- **AF ID:** AF-Q13087-F1-model-v4 | **Chain:** A
- **b-phipsi:** 0.0009416308569623
- **w-rdist:** 0.8803674473368317
- **t-alpha:** 0.2199467857211037

---

---

270

- **AF ID:** AF-Q15036-F1-model-v4 | **Chain:** A
- **b-phipsi:** 0.0009525378318475
- **w-rdist:** 1.0144448249539424
- **t-alpha:** 0.112013220624304

---

---

271

- **AF ID:** AF-Q9H489-F1-model-v4 | **Chain:** A
- **b-phipsi:** 0.0256069596593166
- **w-rdist:** 0.8093703355081205
- **t-alpha:** 0.0021896458253802

---

---

272

- **AF ID:** AF-P51795-F1-model-v4 | **Chain:** A
- **b-phipsi:** 0.0315856887934118
- **w-rdist:** 0.615742586401641
- **t-alpha:** 0.0043792969344855

---

---

273

- **AF ID:** AF-Q01432-F1-model-v4 | **Chain:** A
- **b-phipsi:** 0.0212709500832342
- **w-rdist:** 0.6139691501263835
- **t-alpha:** 0.0065694124035411

---

---

274

- **AF ID:** AF-Q96LQ0-F1-model-v4 | **Chain:** A
- **b-phipsi:** 0.0466167760170973
- **w-rdist:** 0.6955350350181362
- **t-alpha:** 0.0021896458253802

---

---

275

- **AF ID:** AF-Q8TA94-F1-model-v4 | **Chain:** A
- **b-phipsi:** 0.0184985116171569
- **w-rdist:** 0.7677687493495906
- **t-alpha:** 0.0051358048547096

---

---

276

- **AF ID:** AF-Q06203-F1-model-v4 | **Chain:** A
- **b-phipsi:** 0.0007847011730247
- **w-rdist:** 1.0171423118081655
- **t-alpha:** 0.1580726629625284

---

---

277

- **AF ID:** AF-Q6IPX1-F1-model-v4 | **Chain:** A
- **b-phipsi:** 0.0210229685286514
- **w-rdist:** 0.5706295212547947
- **t-alpha:** 0.0072992307962134

---

---

278

- **AF ID:** AF-Q8IYR2-F1-model-v4 | **Chain:** A
- **b-phipsi:** 0.0317468871874511
- **w-rdist:** 0.0779845456728259
- **t-alpha:** 0.1012863867690643

---

---

279

- **AF ID:** AF-Q3MJ62-F1-model-v4 | **Chain:** A
- **b-phipsi:** 0.0180949386551525
- **w-rdist:** 0.3309669328452208
- **t-alpha:** 0.1156354068678819

---

---

280

- **AF ID:** AF-Q9NPH2-F1-model-v4 | **Chain:** A
- **b-phipsi:** 0.0014003501233505
- **w-rdist:** 0.8120559963170818
- **t-alpha:** 0.1312966452446498

---

---

281

- **AF ID:** AF-O60476-F1-model-v4 | **Chain:** A
- **b-phipsi:** 0.0216356192327804
- **w-rdist:** 0.5899610968518371
- **t-alpha:** 0.0066130905937114

---

---

282

- **AF ID:** AF-Q96K49-F1-model-v4 | **Chain:** A
- **b-phipsi:** 0.0217796251293592
- **w-rdist:** 0.896329874820208
- **t-alpha:** 0.0021896458253802

---

---

283

- **AF ID:** AF-P63252-F1-model-v4 | **Chain:** A
- **b-phipsi:** 0.0011139312272574
- **w-rdist:** 0.9612357902425984
- **t-alpha:** 0.1156354068678819

---

---

284

- **AF ID:** AF-Q9UBS4-F1-model-v4 | **Chain:** A
- **b-phipsi:** 0.0012841769643514
- **w-rdist:** 0.7653016039112672
- **t-alpha:** 0.2342341413527844

---

---

285

- **AF ID:** AF-Q9BWW7-F1-model-v4 | **Chain:** A
- **b-phipsi:** 0.0322301004625035
- **w-rdist:** 0.3536761022243244
- **t-alpha:** 0.0324038473531247

---

---

286

- **AF ID:** AF-A8MX76-F1-model-v4 | **Chain:** A
- **b-phipsi:** 0.0014757189454351
- **w-rdist:** 0.7196796300821401
- **t-alpha:** 0.1590527016415781

---

---

287

- **AF ID:** AF-P34903-F1-model-v4 | **Chain:** A
- **b-phipsi:** 0.0059872197658675
- **w-rdist:** 1.2168860556842804
- **t-alpha:** 0.0036630800678474

---

---

288

- **AF ID:** AF-O43155-F1-model-v4 | **Chain:** A
- **b-phipsi:** 0.0102894800970482
- **w-rdist:** 0.9270821471776116
- **t-alpha:** 0.0065694124035411

---

---

289

- **AF ID:** AF-O95460-F1-model-v4 | **Chain:** A
- **b-phipsi:** 0.0060242992414484
- **w-rdist:** 1.3462844424931355
- **t-alpha:** 0.002194902533056

---

---

290

- **AF ID:** AF-Q96GY3-F1-model-v4 | **Chain:** A
- **b-phipsi:** 0.0174942591869776
- **w-rdist:** 0.367483089749684
- **t-alpha:** 0.0942490624608394

---

---

291

- **AF ID:** AF-Q9BSL1-F1-model-v4 | **Chain:** A
- **b-phipsi:** 0.0360087015046594
- **w-rdist:** 0.2903506388698395
- **t-alpha:** 0.0603716916996117

---

---

292

- **AF ID:** AF-O60304-F1-model-v4 | **Chain:** A
- **b-phipsi:** 0.022337997121235
- **w-rdist:** 0.8928970422255762
- **t-alpha:** 0.002194902533056

---

---

293

- **AF ID:** AF-Q96HY7-F1-model-v4 | **Chain:** A
- **b-phipsi:** 0.0126003266010438
- **w-rdist:** 0.3946896918971635
- **t-alpha:** 0.0951239129512884

---

---

294

- **AF ID:** AF-P57764-F1-model-v4 | **Chain:** A
- **b-phipsi:** 0.0008424222004779
- **w-rdist:** 0.8932303534577426
- **t-alpha:** 0.2998104879513312

---

---

295

- **AF ID:** AF-P59910-F1-model-v4 | **Chain:** A
- **b-phipsi:** 0.0004274977207783
- **w-rdist:** 0.8893051103666805
- **t-alpha:** 0.4652406860676175

---

---

296

- **AF ID:** AF-P27694-F1-model-v4 | **Chain:** A
- **b-phipsi:** 0.016067864864492
- **w-rdist:** 0.733363844194262
- **t-alpha:** 0.0072992307962134

---

---

297

- **AF ID:** AF-A8MPY1-F1-model-v4 | **Chain:** A
- **b-phipsi:** 0.0051580593314727
- **w-rdist:** 1.3361823966358162
- **t-alpha:** 0.0036496798094789

---

---

298

- **AF ID:** AF-Q53FZ2-F1-model-v4 | **Chain:** A
- **b-phipsi:** 0.0010035877006849
- **w-rdist:** 0.8887341442286787
- **t-alpha:** 0.2420674935610343

---

---

299

- **AF ID:** AF-P28288-F1-model-v4 | **Chain:** A
- **b-phipsi:** 0.0512606676837093
- **w-rdist:** 0.8229740311248577
- **t-alpha:** 0.0007307814170889

---

---

300

- **AF ID:** AF-P06681-F1-model-v4 | **Chain:** A
- **b-phipsi:** 0.006120274151455
- **w-rdist:** 0.3941766075744552
- **t-alpha:** 0.2243075415760307

---

---

301

- **AF ID:** AF-Q99675-F1-model-v4 | **Chain:** A
- **b-phipsi:** 0.0012229434132146
- **w-rdist:** 0.7257734952874587
- **t-alpha:** 0.3457760617909846

---

---

302

- **AF ID:** AF-Q8N1L9-F1-model-v4 | **Chain:** A
- **b-phipsi:** 0.0183724253604754
- **w-rdist:** 1.1804769383149671
- **t-alpha:** 0.0

---

---

303

- **AF ID:** AF-Q6J4K2-F1-model-v4 | **Chain:** A
- **b-phipsi:** 0.0829648135482008
- **w-rdist:** 0.7328183411293563
- **t-alpha:** 0.0014597092068022

---

---

304

- **AF ID:** AF-Q969Y0-F1-model-v4 | **Chain:** A
- **b-phipsi:** 0.0009308357572448
- **w-rdist:** 0.8603323480767775
- **t-alpha:** 0.3160424853180514

---

---

305

- **AF ID:** AF-O00330-F1-model-v4 | **Chain:** A
- **b-phipsi:** 0.0226472627043293
- **w-rdist:** 0.930599696920914
- **t-alpha:** 0.002194902533056

---

---

306

- **AF ID:** AF-Q9NYK6-F1-model-v4 | **Chain:** A
- **b-phipsi:** 0.0341348560910103
- **w-rdist:** 0.3558371531239613
- **t-alpha:** 0.0410335958026029

---

---

307

- **AF ID:** AF-Q7Z7J5-F1-model-v4 | **Chain:** A
- **b-phipsi:** 0.0185722947993272
- **w-rdist:** 0.3580998070162137
- **t-alpha:** 0.1093117061739596

---

---

308

- **AF ID:** AF-Q5RL73-F1-model-v4 | **Chain:** A
- **b-phipsi:** 0.0219385068357779
- **w-rdist:** 0.9546690237559236
- **t-alpha:** 0.0021896458253802

---

---

309

- **AF ID:** AF-P54578-F1-model-v4 | **Chain:** A
- **b-phipsi:** 0.0012978029408596
- **w-rdist:** 0.9415187688291294
- **t-alpha:** 0.1285009290470284

---

---

310

- **AF ID:** AF-Q16581-F1-model-v4 | **Chain:** A
- **b-phipsi:** 0.0251508530766474
- **w-rdist:** 0.8826214079064394
- **t-alpha:** 0.0029196362453518

---

---

311

- **AF ID:** AF-Q8NHX9-F1-model-v4 | **Chain:** A
- **b-phipsi:** 0.0852881637003503
- **w-rdist:** 0.7464610816926456
- **t-alpha:** 0.0014597092068022

---

---

312

- **AF ID:** AF-Q8TBX8-F1-model-v4 | **Chain:** A
- **b-phipsi:** 0.0007952925657028
- **w-rdist:** 0.9326757704708584
- **t-alpha:** 0.357779958722014

---

---

313

- **AF ID:** AF-Q8N987-F1-model-v4 | **Chain:** A
- **b-phipsi:** 0.0241358677114349
- **w-rdist:** 0.3274286563719515
- **t-alpha:** 0.0951239129512884

---

---

314

- **AF ID:** AF-Q9HB96-F1-model-v4 | **Chain:** A
- **b-phipsi:** 0.0304750293859107
- **w-rdist:** 0.3250925283867982
- **t-alpha:** 0.0729927817404325

---

---

315

- **AF ID:** AF-O95561-F1-model-v4 | **Chain:** A
- **b-phipsi:** 0.0155769003990643
- **w-rdist:** 0.3695751151100139
- **t-alpha:** 0.1445283142236533

---

---

316

- **AF ID:** AF-Q86X67-F1-model-v4 | **Chain:** A
- **b-phipsi:** 0.0006789538826463
- **w-rdist:** 0.9373171207132832
- **t-alpha:** 0.4421053954695855

---

---

317

- **AF ID:** AF-P19113-F1-model-v4 | **Chain:** A
- **b-phipsi:** 0.0132905499084616
- **w-rdist:** 0.3705295960191161
- **t-alpha:** 0.1861315485044292

---

---

318

- **AF ID:** AF-P31267-F1-model-v4 | **Chain:** A
- **b-phipsi:** 0.0187291558380915
- **w-rdist:** 0.8244299586234227
- **t-alpha:** 0.0058736968826307

---

---

319

- **AF ID:** AF-Q96MR7-F1-model-v4 | **Chain:** A
- **b-phipsi:** 0.0182590358566962
- **w-rdist:** 0.2581076814133577
- **t-alpha:** 0.3326849711230111

---

---

320

- **AF ID:** AF-P49910-F1-model-v4 | **Chain:** A
- **b-phipsi:** 0.0227232109903158
- **w-rdist:** 0.7378615695871076
- **t-alpha:** 0.0058736968826307

---

---

321

- **AF ID:** AF-P24046-F1-model-v4 | **Chain:** A
- **b-phipsi:** 0.0006404169946904
- **w-rdist:** 1.2628539536668333
- **t-alpha:** 0.1138212479791738

---

---

322

- **AF ID:** AF-P59826-F1-model-v4 | **Chain:** A
- **b-phipsi:** 0.0011642100342371
- **w-rdist:** 0.8511072594849817
- **t-alpha:** 0.2936735267766412

---

---

323

- **AF ID:** AF-Q9HAH1-F1-model-v4 | **Chain:** A
- **b-phipsi:** 0.011420427913326
- **w-rdist:** 1.2388921349778528
- **t-alpha:** 0.0029282444105804

---

---

324

- **AF ID:** AF-O94886-F1-model-v4 | **Chain:** A
- **b-phipsi:** 0.0795079836900414
- **w-rdist:** 0.5829662903846462
- **t-alpha:** 0.0043989330665894

---

---

325

- **AF ID:** AF-A8MXV6-F1-model-v4 | **Chain:** A
- **b-phipsi:** 0.0197159453566785
- **w-rdist:** 1.071056056603817
- **t-alpha:** 0.0014620968756575

---

---

326

- **AF ID:** AF-Q6GPH6-F1-model-v4 | **Chain:** A
- **b-phipsi:** 0.0272288430551305
- **w-rdist:** 0.377245227020476
- **t-alpha:** 0.0546574059878011

---

---

327

- **AF ID:** AF-Q3LFD5-F1-model-v4 | **Chain:** A
- **b-phipsi:** 0.0007967853120587
- **w-rdist:** 1.0829384952862824
- **t-alpha:** 0.2113174449262456

---

---

328

- **AF ID:** AF-Q9Y625-F1-model-v4 | **Chain:** A
- **b-phipsi:** 0.0405461409581694
- **w-rdist:** 0.9826411085264514
- **t-alpha:** 0.0007298270463003

---

---

329

- **AF ID:** AF-Q03154-F1-model-v4 | **Chain:** A
- **b-phipsi:** 0.0006906474495006
- **w-rdist:** 0.9967737282406588
- **t-alpha:** 0.3951122956901294

---

---

330

- **AF ID:** AF-P53794-F1-model-v4 | **Chain:** A
- **b-phipsi:** 0.0819512943177124
- **w-rdist:** 0.6872462518639324
- **t-alpha:** 0.0036630800678474

---

---

331

- **AF ID:** AF-Q96LR2-F1-model-v4 | **Chain:** A
- **b-phipsi:** 0.0161931033335135
- **w-rdist:** 0.3711390783399676
- **t-alpha:** 0.1512608079233615

---

---

332

- **AF ID:** AF-Q96KJ4-F1-model-v4 | **Chain:** A
- **b-phipsi:** 0.0253751774080733
- **w-rdist:** 0.6285063787993775
- **t-alpha:** 0.0073530278154354

---

---

333

- **AF ID:** AF-Q14449-F1-model-v4 | **Chain:** A
- **b-phipsi:** 0.0167689932934661
- **w-rdist:** 0.4086833097282039
- **t-alpha:** 0.0912409767262552

---

---

334

- **AF ID:** AF-Q96JB6-F1-model-v4 | **Chain:** A
- **b-phipsi:** 0.0244913798308874
- **w-rdist:** 0.3377710655321315
- **t-alpha:** 0.1247951360850041

---

---

335

- **AF ID:** AF-P0DMS9-F1-model-v4 | **Chain:** A
- **b-phipsi:** 0.0003281199168346
- **w-rdist:** 1.0326674572999048
- **t-alpha:** 0.4605544556575509

---

---

336

- **AF ID:** AF-Q9H5J8-F1-model-v4 | **Chain:** A
- **b-phipsi:** 0.0108177747377821
- **w-rdist:** 1.2260621365426374
- **t-alpha:** 0.0036630800678474

---

---

337

- **AF ID:** AF-P49748-F1-model-v4 | **Chain:** A
- **b-phipsi:** 0.0367476032336942
- **w-rdist:** 0.785278305985352
- **t-alpha:** 0.0036630800678474

---

---

338

- **AF ID:** AF-Q13426-F1-model-v4 | **Chain:** A
- **b-phipsi:** 0.0103174649374013
- **w-rdist:** 1.2409011740345717
- **t-alpha:** 0.0036630800678474

---

---

339

- **AF ID:** AF-P10266-F1-model-v4 | **Chain:** A
- **b-phipsi:** 0.0008528240564299
- **w-rdist:** 1.2499514752457828
- **t-alpha:** 0.1102187159977201

---

---

340

- **AF ID:** AF-Q96MM7-F1-model-v4 | **Chain:** A
- **b-phipsi:** 0.0138719433935444
- **w-rdist:** 0.9561956242111788
- **t-alpha:** 0.0066130905937114

---

---

341

- **AF ID:** AF-Q9UIW0-F1-model-v4 | **Chain:** A
- **b-phipsi:** 0.022362468684445
- **w-rdist:** 1.0613030328455764
- **t-alpha:** 0.0014620968756575

---

---

342

- **AF ID:** AF-A2RU67-F1-model-v4 | **Chain:** A
- **b-phipsi:** 0.0269411893202222
- **w-rdist:** 0.7061188878324997
- **t-alpha:** 0.0066130905937114

---

---

343

- **AF ID:** AF-Q6XYB7-F1-model-v4 | **Chain:** A
- **b-phipsi:** 0.0086851754229028
- **w-rdist:** 0.3924223467944904
- **t-alpha:** 0.2998104879513312

---

---

344

- **AF ID:** AF-O95872-F1-model-v4 | **Chain:** A
- **b-phipsi:** 0.031101459104022
- **w-rdist:** 0.906266651892493
- **t-alpha:** 0.0029282444105804

---

---

345

- **AF ID:** AF-P08833-F1-model-v4 | **Chain:** A
- **b-phipsi:** 0.0013357035341135
- **w-rdist:** 0.7753794840265875
- **t-alpha:** 0.4080167576763944

---

---

346

- **AF ID:** AF-P10600-F1-model-v4 | **Chain:** A
- **b-phipsi:** 0.0011546058739765
- **w-rdist:** 0.7916726628276238
- **t-alpha:** 0.4891305195462965

---

---

347

- **AF ID:** AF-Q9NY47-F1-model-v4 | **Chain:** A
- **b-phipsi:** 0.0010510921484529
- **w-rdist:** 1.0566122640248623
- **t-alpha:** 0.1897806913930422

---

---

348

- **AF ID:** AF-P78395-F1-model-v4 | **Chain:** A
- **b-phipsi:** 0.0148698164346806
- **w-rdist:** 0.4097643755595774
- **t-alpha:** 0.1331679050161578

---

---

349

- **AF ID:** AF-P20592-F1-model-v4 | **Chain:** A
- **b-phipsi:** 0.0189560718074949
- **w-rdist:** 1.2696293391165423
- **t-alpha:** 0.0007298270463003

---

---

350

- **AF ID:** AF-P78508-F1-model-v4 | **Chain:** A
- **b-phipsi:** 0.0013382254414382
- **w-rdist:** 0.7951628889122362
- **t-alpha:** 0.3838383478012463

---

---

351

- **AF ID:** AF-Q9Y334-F1-model-v4 | **Chain:** A
- **b-phipsi:** 0.0154338579632833
- **w-rdist:** 0.3876362933939741
- **t-alpha:** 0.1583940949914124

---

---

352

- **AF ID:** AF-O95170-F1-model-v4 | **Chain:** A
- **b-phipsi:** 0.0058684281981004
- **w-rdist:** 1.157090421555619
- **t-alpha:** 0.0073530278154354

---

---

353

- **AF ID:** AF-Q7Z4H8-F1-model-v4 | **Chain:** A
- **b-phipsi:** 0.0011290811263402
- **w-rdist:** 0.8924952691995536
- **t-alpha:** 0.3880447721603595

---

---

354

- **AF ID:** AF-Q86U38-F1-model-v4 | **Chain:** A
- **b-phipsi:** 0.0755427462751532
- **w-rdist:** 0.3863256916102761
- **t-alpha:** 0.0211679073923163

---

---

355

- **AF ID:** AF-Q92670-F1-model-v4 | **Chain:** A
- **b-phipsi:** 0.0342914164155845
- **w-rdist:** 0.3419566863707111
- **t-alpha:** 0.0890514862251048

---

---

356

- **AF ID:** AF-Q8IV36-F1-model-v4 | **Chain:** A
- **b-phipsi:** 0.0410654725200037
- **w-rdist:** 0.3901425926040065
- **t-alpha:** 0.0386659655318466

---

---

357

- **AF ID:** AF-Q6P161-F1-model-v4 | **Chain:** A
- **b-phipsi:** 0.0035971672152395
- **w-rdist:** 0.4062518543362287
- **t-alpha:** 0.6789217302123349

---

---

358

- **AF ID:** AF-Q7Z3H0-F1-model-v4 | **Chain:** A
- **b-phipsi:** 0.0214515365556999
- **w-rdist:** 0.3544132378797587
- **t-alpha:** 0.1510950327523956

---

---

359

- **AF ID:** AF-Q96B86-F1-model-v4 | **Chain:** A
- **b-phipsi:** 0.001333059792576
- **w-rdist:** 0.7929666223880931
- **t-alpha:** 0.4451480112011774

---

---

360

- **AF ID:** AF-O60832-F1-model-v4 | **Chain:** A
- **b-phipsi:** 0.02368360856488
- **w-rdist:** 0.7260075895216669
- **t-alpha:** 0.0073530278154354

---

---

361

- **AF ID:** AF-Q15822-F1-model-v4 | **Chain:** A
- **b-phipsi:** 0.0010057600688447
- **w-rdist:** 1.3165331151098851
- **t-alpha:** 0.0847192952571229

---

---

362

- **AF ID:** AF-Q6UXV4-F1-model-v4 | **Chain:** A
- **b-phipsi:** 0.01900282061168
- **w-rdist:** 0.953034723077823
- **t-alpha:** 0.0058391635426686

---

---

363

- **AF ID:** AF-Q9H4T2-F1-model-v4 | **Chain:** A
- **b-phipsi:** 0.0168466664899261
- **w-rdist:** 0.4006260291034419
- **t-alpha:** 0.1388198251143049

---

---

364

- **AF ID:** AF-Q6EMK4-F1-model-v4 | **Chain:** A
- **b-phipsi:** 0.0155510124901954
- **w-rdist:** 1.043899021546149
- **t-alpha:** 0.0058391635426686

---

---

365

- **AF ID:** AF-P13056-F1-model-v4 | **Chain:** A
- **b-phipsi:** 0.0177979106897993
- **w-rdist:** 1.1117062086741352
- **t-alpha:** 0.0036630800678474

---

---

366

- **AF ID:** AF-Q99689-F1-model-v4 | **Chain:** A
- **b-phipsi:** 0.0211809037836501
- **w-rdist:** 1.1990773683644131
- **t-alpha:** 0.0014597092068022

---

---

367

- **AF ID:** AF-Q96Q05-F1-model-v4 | **Chain:** A
- **b-phipsi:** 0.0008520581929965
- **w-rdist:** 1.579879979798692
- **t-alpha:** 0.09197074326617

---

---

368

- **AF ID:** AF-Q13946-F1-model-v4 | **Chain:** A
- **b-phipsi:** 0.053261811806965
- **w-rdist:** 0.7037843458604723
- **t-alpha:** 0.0058391635426686

---

---

369

- **AF ID:** AF-O95704-F1-model-v4 | **Chain:** A
- **b-phipsi:** 0.023180538718734
- **w-rdist:** 0.3671144220379232
- **t-alpha:** 0.1369294736439956

---

---

370

- **AF ID:** AF-Q9Y5X1-F1-model-v4 | **Chain:** A
- **b-phipsi:** 0.0169441176326886
- **w-rdist:** 0.4072777788663917
- **t-alpha:** 0.1357666572455418

---

---

371

- **AF ID:** AF-P63132-F1-model-v4 | **Chain:** A
- **b-phipsi:** 0.0005606752834258
- **w-rdist:** 1.254472740564606
- **t-alpha:** 0.2379563014178145

---

---

372

- **AF ID:** AF-Q7Z304-F1-model-v4 | **Chain:** A
- **b-phipsi:** 0.0332976784631121
- **w-rdist:** 0.3515506701074896
- **t-alpha:** 0.1066236145665033

---

---

373

- **AF ID:** AF-P51798-F1-model-v4 | **Chain:** A
- **b-phipsi:** 0.0245541806751007
- **w-rdist:** 0.4091403859235052
- **t-alpha:** 0.0693428042269768

---

---

374

- **AF ID:** AF-Q9H2W6-F1-model-v4 | **Chain:** A
- **b-phipsi:** 0.0014776029225136
- **w-rdist:** 0.7715031700127067
- **t-alpha:** 0.3979591031927509

---

---

375

- **AF ID:** AF-Q9NQZ5-F1-model-v4 | **Chain:** A
- **b-phipsi:** 0.0013321336554975
- **w-rdist:** 0.8358476249619022
- **t-alpha:** 0.4528103751143975

---

---

376

- **AF ID:** AF-P01127-F1-model-v4 | **Chain:** A
- **b-phipsi:** 0.0148205640073589
- **w-rdist:** 0.3846910657157286
- **t-alpha:** 0.2888051065657671

---

---

377

- **AF ID:** AF-Q9Y6H8-F1-model-v4 | **Chain:** A
- **b-phipsi:** 0.0220062372736921
- **w-rdist:** 1.2053394858432325
- **t-alpha:** 0.0014597092068022

---

---

378

- **AF ID:** AF-P11926-F1-model-v4 | **Chain:** A
- **b-phipsi:** 0.001492111656586
- **w-rdist:** 0.7876606617360756
- **t-alpha:** 0.3686314544145967

---

---

379

- **AF ID:** AF-Q9NQ86-F1-model-v4 | **Chain:** A
- **b-phipsi:** 0.0012799909727992
- **w-rdist:** 1.4515491136048964
- **t-alpha:** 0.0569339726158755

---

---

380

- **AF ID:** AF-Q96C57-F1-model-v4 | **Chain:** A
- **b-phipsi:** 0.0536915970833052
- **w-rdist:** 1.0565220072186012
- **t-alpha:** 0.0007298270463003

---

---

381

- **AF ID:** AF-Q86XR7-F1-model-v4 | **Chain:** A
- **b-phipsi:** 0.0012658919456728
- **w-rdist:** 0.8260961776832952
- **t-alpha:** 0.5256128268560598

---

---

382

- **AF ID:** AF-Q96KB5-F1-model-v4 | **Chain:** A
- **b-phipsi:** 0.0004378276571426
- **w-rdist:** 1.0769874401825807
- **t-alpha:** 0.4907510766758922

---

---

383

- **AF ID:** AF-Q9NR12-F1-model-v4 | **Chain:** A
- **b-phipsi:** 0.023466608344105
- **w-rdist:** 0.8442695111043015
- **t-alpha:** 0.0066130905937114

---

---

384

- **AF ID:** AF-Q5SWX8-F1-model-v4 | **Chain:** A
- **b-phipsi:** 0.0013288910952676
- **w-rdist:** 0.999591195393848
- **t-alpha:** 0.2454545226020776

---

---

385

- **AF ID:** AF-A0A494C086-F1-model-v4 | **Chain:** A
- **b-phipsi:** 0.0154248432215461
- **w-rdist:** 1.139579230456161
- **t-alpha:** 0.0051094444648316

---

---

386

- **AF ID:** AF-O94832-F1-model-v4 | **Chain:** A
- **b-phipsi:** 0.0157725084144793
- **w-rdist:** 1.592469833931204
- **t-alpha:** 0.0014597092068022

---

---

387

- **AF ID:** AF-Q9NRN7-F1-model-v4 | **Chain:** A
- **b-phipsi:** 0.0001730300067484
- **w-rdist:** 1.1436401538887124
- **t-alpha:** 0.4747040097207244

---

---

388

- **AF ID:** AF-O14772-F1-model-v4 | **Chain:** A
- **b-phipsi:** 0.0015300064961811
- **w-rdist:** 0.8838460656159548
- **t-alpha:** 0.2500000328268428

---

---

389

- **AF ID:** AF-Q04741-F1-model-v4 | **Chain:** A
- **b-phipsi:** 0.0491095775988594
- **w-rdist:** 1.0186054100471025
- **t-alpha:** 0.0014597092068022

---

---

390

- **AF ID:** AF-Q96RQ1-F1-model-v4 | **Chain:** A
- **b-phipsi:** 0.0007334585843306
- **w-rdist:** 1.0764950604078949
- **t-alpha:** 0.4390760476964592

---

---

391

- **AF ID:** AF-Q96QD9-F1-model-v4 | **Chain:** A
- **b-phipsi:** 0.0085198392452784
- **w-rdist:** 1.208846254368564
- **t-alpha:** 0.0066130905937114

---

---

392

- **AF ID:** AF-Q63ZY6-F1-model-v4 | **Chain:** A
- **b-phipsi:** 0.0012037045483174
- **w-rdist:** 0.983119979302768
- **t-alpha:** 0.3262346865359695

---

---

393

- **AF ID:** AF-Q08AH1-F1-model-v4 | **Chain:** A
- **b-phipsi:** 0.0012658981426802
- **w-rdist:** 0.9540494543675522
- **t-alpha:** 0.3300972957653796

---

---

394

- **AF ID:** AF-Q9HD26-F1-model-v4 | **Chain:** A
- **b-phipsi:** 0.0277878785631042
- **w-rdist:** 0.844659000286451
- **t-alpha:** 0.0058736968826307

---

---

395

- **AF ID:** AF-Q86YL5-F1-model-v4 | **Chain:** A
- **b-phipsi:** 0.0149685191098624
- **w-rdist:** 0.4067497680933945
- **t-alpha:** 0.223214581067924

---

---

396

- **AF ID:** AF-Q7RTS3-F1-model-v4 | **Chain:** A
- **b-phipsi:** 0.0424447059822659
- **w-rdist:** 1.046067403675642
- **t-alpha:** 0.0014597092068022

---

---

397

- **AF ID:** AF-Q99797-F1-model-v4 | **Chain:** A
- **b-phipsi:** 0.0443753918672703
- **w-rdist:** 0.8364350868667004
- **t-alpha:** 0.0043989330665894

---

---

398

- **AF ID:** AF-Q9NUJ1-F1-model-v4 | **Chain:** A
- **b-phipsi:** 0.0008617865038944
- **w-rdist:** 1.0289134679671688
- **t-alpha:** 0.4715360139374722

---

---

399

- **AF ID:** AF-Q9UMW8-F1-model-v4 | **Chain:** A
- **b-phipsi:** 0.0010482315084857
- **w-rdist:** 1.0654689603876706
- **t-alpha:** 0.2900187997113126

---

---

400

- **AF ID:** AF-Q9UQG0-F1-model-v4 | **Chain:** A
- **b-phipsi:** 0.0013568373771373
- **w-rdist:** 1.2497023896182415
- **t-alpha:** 0.0737226325244466

---

---

401

- **AF ID:** AF-O60909-F1-model-v4 | **Chain:** A
- **b-phipsi:** 0.0015312042287712
- **w-rdist:** 0.8282304008131776
- **t-alpha:** 0.3391985559617581

---

---

402

- **AF ID:** AF-O60928-F1-model-v4 | **Chain:** A
- **b-phipsi:** 0.0014752024273591
- **w-rdist:** 0.8460716891942545
- **t-alpha:** 0.3564358916585775

---

---

403

- **AF ID:** AF-Q6UW63-F1-model-v4 | **Chain:** A
- **b-phipsi:** 0.001312891591106
- **w-rdist:** 0.8837453108263762
- **t-alpha:** 0.4482032613745739

---

---

404

- **AF ID:** AF-P46060-F1-model-v4 | **Chain:** A
- **b-phipsi:** 0.0309430858083615
- **w-rdist:** 0.3913426648318981
- **t-alpha:** 0.0736678509011601

---

---

405

- **AF ID:** AF-Q14654-F1-model-v4 | **Chain:** A
- **b-phipsi:** 0.0014623451875807
- **w-rdist:** 1.011516037726314
- **t-alpha:** 0.1800174790223048

---

---

406

- **AF ID:** AF-Q96GL9-F1-model-v4 | **Chain:** A
- **b-phipsi:** 0.0199055306368286
- **w-rdist:** 0.3216700685349713
- **t-alpha:** 0.3922765965372021

---

---

407

- **AF ID:** AF-Q7Z3S7-F1-model-v4 | **Chain:** A
- **b-phipsi:** 0.0012822611877868
- **w-rdist:** 1.0592971469624717
- **t-alpha:** 0.2328465826983856

---

---

408

- **AF ID:** AF-O95800-F1-model-v4 | **Chain:** A
- **b-phipsi:** 0.0186751179265799
- **w-rdist:** 1.0597806800961986
- **t-alpha:** 0.0051358048547096

---

---

409

- **AF ID:** AF-Q86XR2-F1-model-v4 | **Chain:** A
- **b-phipsi:** 0.0285857072196733
- **w-rdist:** 0.3917537939084772
- **t-alpha:** 0.0839416535012711

---

---

410

- **AF ID:** AF-Q7Z392-F1-model-v4 | **Chain:** A
- **b-phipsi:** 0.0015374608818407
- **w-rdist:** 1.5430287031111047
- **t-alpha:** 0.0321168830372178

---

---

411

- **AF ID:** AF-P50454-F1-model-v4 | **Chain:** A
- **b-phipsi:** 0.0009504595605916
- **w-rdist:** 0.9875061608089336
- **t-alpha:** 0.5273131409781351

---

---

412

- **AF ID:** AF-E9PJI5-F1-model-v4 | **Chain:** A
- **b-phipsi:** 0.0162270033820203
- **w-rdist:** 1.1584363989918172
- **t-alpha:** 0.0051094444648316

---

---

413

- **AF ID:** AF-Q9UHR4-F1-model-v4 | **Chain:** A
- **b-phipsi:** 0.0119238820020425
- **w-rdist:** 1.17699144572629
- **t-alpha:** 0.0065694124035411

---

---

414

- **AF ID:** AF-O60486-F1-model-v4 | **Chain:** A
- **b-phipsi:** 0.0008577405218243
- **w-rdist:** 1.2493775291509304
- **t-alpha:** 0.2350365667031186

---

---

415

- **AF ID:** AF-Q96AY2-F1-model-v4 | **Chain:** A
- **b-phipsi:** 0.0010680310270853
- **w-rdist:** 1.1733477080657124
- **t-alpha:** 0.2094889858268971

---

---

416

- **AF ID:** AF-Q5H8A4-F1-model-v4 | **Chain:** A
- **b-phipsi:** 0.0611315587644494
- **w-rdist:** 0.6706693864457518
- **t-alpha:** 0.0072992307962134

---

---

417

- **AF ID:** AF-P31314-F1-model-v4 | **Chain:** A
- **b-phipsi:** 0.0222569712156524
- **w-rdist:** 1.0375274055812185
- **t-alpha:** 0.0051094444648316

---

---

418

- **AF ID:** AF-P34949-F1-model-v4 | **Chain:** A
- **b-phipsi:** 0.0009784568737996
- **w-rdist:** 1.051214350010904
- **t-alpha:** 0.4270834012940876

---

---

419

- **AF ID:** AF-Q9H2S6-F1-model-v4 | **Chain:** A
- **b-phipsi:** 0.0010708046757995
- **w-rdist:** 0.995473085221342
- **t-alpha:** 0.4558984859647033

---

---

420

- **AF ID:** AF-P22735-F1-model-v4 | **Chain:** A
- **b-phipsi:** 0.0205319423738937
- **w-rdist:** 0.4104403421747116
- **t-alpha:** 0.1226277150743757

---

---

421

- **AF ID:** AF-O94776-F1-model-v4 | **Chain:** A
- **b-phipsi:** 0.0008473838219313
- **w-rdist:** 1.0997776581604517
- **t-alpha:** 0.4328464777471188

---

---

422

- **AF ID:** AF-P38117-F1-model-v4 | **Chain:** A
- **b-phipsi:** 0.000188336014809
- **w-rdist:** 1.1585730546703303
- **t-alpha:** 0.5657145845131406

---

---

423

- **AF ID:** AF-O14832-F1-model-v4 | **Chain:** A
- **b-phipsi:** 0.0009113159300678
- **w-rdist:** 1.0553998081339502
- **t-alpha:** 0.4715360139374722

---

---

424

- **AF ID:** AF-Q8TAM6-F1-model-v4 | **Chain:** A
- **b-phipsi:** 0.0630391552910133
- **w-rdist:** 1.1273435098851918
- **t-alpha:** 0.0

---

---

425

- **AF ID:** AF-Q969E3-F1-model-v4 | **Chain:** A
- **b-phipsi:** 0.0249960270062341
- **w-rdist:** 0.3263240991651878
- **t-alpha:** 0.2912348672325333

---

---

426

- **AF ID:** AF-Q8N8U2-F1-model-v4 | **Chain:** A
- **b-phipsi:** 0.0350636565876635
- **w-rdist:** 0.8812021427968033
- **t-alpha:** 0.0051358048547096

---

---

427

- **AF ID:** AF-Q96SW2-F1-model-v4 | **Chain:** A
- **b-phipsi:** 0.001380460904456
- **w-rdist:** 1.014898427979992
- **t-alpha:** 0.2720522999082921

---

---

428

- **AF ID:** AF-P0CG43-F1-model-v4 | **Chain:** A
- **b-phipsi:** 0.0246016025388639
- **w-rdist:** 0.9451560138283044
- **t-alpha:** 0.0058736968826307

---

---

429

- **AF ID:** AF-Q8WUH2-F1-model-v4 | **Chain:** A
- **b-phipsi:** 0.0094908653494493
- **w-rdist:** 1.4806103583464436
- **t-alpha:** 0.0051358048547096

---

---

430

- **AF ID:** AF-Q9NP66-F1-model-v4 | **Chain:** A
- **b-phipsi:** 0.0253532895304735
- **w-rdist:** 1.124643345911846
- **t-alpha:** 0.0029282444105804

---

---

431

- **AF ID:** AF-Q9Y6R7-F6-model-v4 | **Chain:** A
- **b-phipsi:** 0.0257388376524974
- **w-rdist:** 1.2411339862800046
- **t-alpha:** 0.0014597092068022

---

---

432

- **AF ID:** AF-Q8IV08-F1-model-v4 | **Chain:** A
- **b-phipsi:** 0.001336706919413
- **w-rdist:** 0.967511974326505
- **t-alpha:** 0.3672655620337721

---

---

433

- **AF ID:** AF-Q9Y485-F4-model-v4 | **Chain:** A
- **b-phipsi:** 0.0008372473135519
- **w-rdist:** 1.220164376869705
- **t-alpha:** 0.3116789334965493

---

---

434

- **AF ID:** AF-Q8WZ19-F1-model-v4 | **Chain:** A
- **b-phipsi:** 0.0011749901846619
- **w-rdist:** 1.0188927547964584
- **t-alpha:** 0.3936927280547244

---

---

435

- **AF ID:** AF-Q9Y4L5-F1-model-v4 | **Chain:** A
- **b-phipsi:** 0.0257244023188765
- **w-rdist:** 0.3802127096152264
- **t-alpha:** 0.1502939397071412

---

---

436

- **AF ID:** AF-Q96QS6-F1-model-v4 | **Chain:** A
- **b-phipsi:** 0.0012608222848102
- **w-rdist:** 1.0637350849997556
- **t-alpha:** 0.2900187997113126

---

---

437

- **AF ID:** AF-Q9NSG2-F1-model-v4 | **Chain:** A
- **b-phipsi:** 0.2067187829838229
- **w-rdist:** 1.078518402788331
- **t-alpha:** 0.0

---

---

438

- **AF ID:** AF-Q9BXS4-F1-model-v4 | **Chain:** A
- **b-phipsi:** 0.0226597690346129
- **w-rdist:** 1.1035543815531326
- **t-alpha:** 0.0043792969344855

---

---

439

- **AF ID:** AF-Q07866-F1-model-v4 | **Chain:** A
- **b-phipsi:** 0.0996590132474186
- **w-rdist:** 1.0428476747274944
- **t-alpha:** 0.0014597092068022

---

---

440

- **AF ID:** AF-O95780-F1-model-v4 | **Chain:** A
- **b-phipsi:** 0.0117301973182341
- **w-rdist:** 1.2854574004698331
- **t-alpha:** 0.0058736968826307

---

---

441

- **AF ID:** AF-P0C6A0-F1-model-v4 | **Chain:** A
- **b-phipsi:** 0.0760201961973618
- **w-rdist:** 0.3176761007244826
- **t-alpha:** 0.1474036756193304

---

---

442

- **AF ID:** AF-P62995-F1-model-v4 | **Chain:** A
- **b-phipsi:** 0.1077912414594943
- **w-rdist:** 1.1547509767178106
- **t-alpha:** 0.0

---

---

443

- **AF ID:** AF-Q68CJ6-F1-model-v4 | **Chain:** A
- **b-phipsi:** 0.0560517512593622
- **w-rdist:** 0.3454524085700901
- **t-alpha:** 0.1378737142433073

---

---

444

- **AF ID:** AF-Q96MI6-F1-model-v4 | **Chain:** A
- **b-phipsi:** 0.0009586375548377
- **w-rdist:** 1.0728214784367087
- **t-alpha:** 0.4826842704060508

---

---

445

- **AF ID:** AF-Q9NYA1-F1-model-v4 | **Chain:** A
- **b-phipsi:** 0.0008745790030053
- **w-rdist:** 1.1621072688102745
- **t-alpha:** 0.4330548794062239

---

---

446

- **AF ID:** AF-Q8TDJ6-F4-model-v4 | **Chain:** A
- **b-phipsi:** 0.0010446944524036
- **w-rdist:** 1.2264418038359854
- **t-alpha:** 0.2664231605904708

---

---

447

- **AF ID:** AF-Q9BQ24-F1-model-v4 | **Chain:** A
- **b-phipsi:** 0.0007000143840476
- **w-rdist:** 1.1367846140232272
- **t-alpha:** 0.6446578484271672

---

---

448

- **AF ID:** AF-Q9HBX9-F1-model-v4 | **Chain:** A
- **b-phipsi:** 0.0214488542133218
- **w-rdist:** 1.0350735681171943
- **t-alpha:** 0.0066130905937114

---

---

449

- **AF ID:** AF-Q8IYN6-F1-model-v4 | **Chain:** A
- **b-phipsi:** 0.0215683168803896
- **w-rdist:** 0.311965079006429
- **t-alpha:** 0.539325770392604

---

---

450

- **AF ID:** AF-Q8TF45-F1-model-v4 | **Chain:** A
- **b-phipsi:** 0.0186957198840706
- **w-rdist:** 1.3149701618227354
- **t-alpha:** 0.0036630800678474

---

---

451

- **AF ID:** AF-O15524-F1-model-v4 | **Chain:** A
- **b-phipsi:** 0.0013498169390949
- **w-rdist:** 0.9079988827528545
- **t-alpha:** 0.6348451025682076

---

---

452

- **AF ID:** AF-Q9NZM6-F1-model-v4 | **Chain:** A
- **b-phipsi:** 0.0603305973554477
- **w-rdist:** 0.8752585617276968
- **t-alpha:** 0.0058391635426686

---

---

453

- **AF ID:** AF-Q08462-F1-model-v4 | **Chain:** A
- **b-phipsi:** 0.0533060338801221
- **w-rdist:** 1.2457808686633003
- **t-alpha:** 0.0007298270463003

---

---

454

- **AF ID:** AF-A6NCN8-F1-model-v4 | **Chain:** A
- **b-phipsi:** 0.0355515093734045
- **w-rdist:** 0.3433162017625927
- **t-alpha:** 0.2465881593411327

---

---

455

- **AF ID:** AF-Q9NXX6-F1-model-v4 | **Chain:** A
- **b-phipsi:** 0.0348581602635304
- **w-rdist:** 1.2863634198073883
- **t-alpha:** 0.0007307814170889

---

---

456

- **AF ID:** AF-Q53FT3-F1-model-v4 | **Chain:** A
- **b-phipsi:** 0.0007812566718388
- **w-rdist:** 1.094004940269636
- **t-alpha:** 0.8315509056837473

---

---

457

- **AF ID:** AF-P38936-F1-model-v4 | **Chain:** A
- **b-phipsi:** 0.0155937028872343
- **w-rdist:** 0.3790289353490427
- **t-alpha:** 0.5324386148895914

---

---

458

- **AF ID:** AF-Q92979-F1-model-v4 | **Chain:** A
- **b-phipsi:** 0.0002930319334151
- **w-rdist:** 1.2088418802657477
- **t-alpha:** 0.7631919903212132

---

---

459

- **AF ID:** AF-P0C7M4-F1-model-v4 | **Chain:** A
- **b-phipsi:** 0.0326487256935472
- **w-rdist:** 0.9459011057247302
- **t-alpha:** 0.0065694124035411

---

---

460

- **AF ID:** AF-P0C866-F1-model-v4 | **Chain:** A
- **b-phipsi:** 0.0014142259699094
- **w-rdist:** 0.9816213401363764
- **t-alpha:** 0.4482032613745739

---

---

461

- **AF ID:** AF-Q9NVU7-F1-model-v4 | **Chain:** A
- **b-phipsi:** 0.0650199332950605
- **w-rdist:** 1.0126002749314376
- **t-alpha:** 0.0036630800678474

---

---

462

- **AF ID:** AF-P0C2L3-F1-model-v4 | **Chain:** A
- **b-phipsi:** 0.0161584691173974
- **w-rdist:** 0.4025517067727521
- **t-alpha:** 0.416753081395566

---

---

463

- **AF ID:** AF-Q8IZF2-F1-model-v4 | **Chain:** A
- **b-phipsi:** 0.0008643590380774
- **w-rdist:** 1.544024266699262
- **t-alpha:** 0.2671528548954336

---

---

464

- **AF ID:** AF-A1A4G5-F1-model-v4 | **Chain:** A
- **b-phipsi:** 0.0338193914450068
- **w-rdist:** 0.3524172069141676
- **t-alpha:** 0.2875943972208628

---

---

465

- **AF ID:** AF-Q99445-F1-model-v4 | **Chain:** A
- **b-phipsi:** 0.0010975097935928
- **w-rdist:** 0.9763826499310644
- **t-alpha:** 1.333901503498478

---

---

466

- **AF ID:** AF-Q9BQQ7-F1-model-v4 | **Chain:** A
- **b-phipsi:** 0.001023387303433
- **w-rdist:** 1.0706756359993743
- **t-alpha:** 0.640718660769992

---

---

467

- **AF ID:** AF-Q9Y2L5-F1-model-v4 | **Chain:** A
- **b-phipsi:** 0.0008514781601402
- **w-rdist:** 1.5638776670849777
- **t-alpha:** 0.289050950076283

---

---

468

- **AF ID:** AF-O14508-F1-model-v4 | **Chain:** A
- **b-phipsi:** 0.000765224735057
- **w-rdist:** 1.192257004459484
- **t-alpha:** 0.6768671188423523

---

---

469

- **AF ID:** AF-Q92934-F1-model-v4 | **Chain:** A
- **b-phipsi:** 0.0341788589854688
- **w-rdist:** 0.3338775673064176
- **t-alpha:** 0.3470992886363773

---

---

470

- **AF ID:** AF-P18509-F1-model-v4 | **Chain:** A
- **b-phipsi:** 0.0543906258556187
- **w-rdist:** 0.3021097764668872
- **t-alpha:** 0.3010444712249329

---

---

471

- **AF ID:** AF-Q9UHY8-F1-model-v4 | **Chain:** A
- **b-phipsi:** 0.0231246817592113
- **w-rdist:** 1.1195394590058474
- **t-alpha:** 0.0058391635426686

---

---

472

- **AF ID:** AF-P48553-F1-model-v4 | **Chain:** A
- **b-phipsi:** 0.0004680904010248
- **w-rdist:** 1.4311696785889938
- **t-alpha:** 0.4781015300832141

---

---

473

- **AF ID:** AF-Q8WXI8-F1-model-v4 | **Chain:** A
- **b-phipsi:** 0.0009589964859336
- **w-rdist:** 1.07621105594558
- **t-alpha:** 0.7211055442874423

---

---

474

- **AF ID:** AF-Q9BZM4-F1-model-v4 | **Chain:** A
- **b-phipsi:** 0.0014440143493159
- **w-rdist:** 0.9423632291828716
- **t-alpha:** 0.5801616637669966

---

---

475

- **AF ID:** AF-Q8IUI8-F1-model-v4 | **Chain:** A
- **b-phipsi:** 0.0010264496818411
- **w-rdist:** 1.2800192869292206
- **t-alpha:** 0.3035203043148384

---

---

476

- **AF ID:** AF-Q75V66-F1-model-v4 | **Chain:** A
- **b-phipsi:** 0.0653212814834325
- **w-rdist:** 0.8361593192660695
- **t-alpha:** 0.0072992307962134

---

---

477

- **AF ID:** AF-Q7RTR0-F1-model-v4 | **Chain:** A
- **b-phipsi:** 0.0360410437167707
- **w-rdist:** 0.9873161148086358
- **t-alpha:** 0.0058736968826307

---

---

478

- **AF ID:** AF-Q5VYX0-F1-model-v4 | **Chain:** A
- **b-phipsi:** 0.0012329586846491
- **w-rdist:** 1.1611781270787136
- **t-alpha:** 0.3782697589468184

---

---

479

- **AF ID:** AF-Q15389-F1-model-v4 | **Chain:** A
- **b-phipsi:** 0.0122303839352893
- **w-rdist:** 1.625079052520712
- **t-alpha:** 0.0058736968826307

---

---

480

- **AF ID:** AF-Q8TC36-F1-model-v4 | **Chain:** A
- **b-phipsi:** 0.0013961032848704
- **w-rdist:** 1.3333204281880375
- **t-alpha:** 0.1454848740897429

---

---

481

- **AF ID:** AF-Q8N4Q0-F1-model-v4 | **Chain:** A
- **b-phipsi:** 0.0013915895656654
- **w-rdist:** 1.0875970435028464
- **t-alpha:** 0.3497541520557985

---

---

482

- **AF ID:** AF-Q9H7B2-F1-model-v4 | **Chain:** A
- **b-phipsi:** 0.0014435632476328
- **w-rdist:** 1.0151721004790473
- **t-alpha:** 0.4731184577804364

---

---

483

- **AF ID:** AF-P51164-F1-model-v4 | **Chain:** A
- **b-phipsi:** 0.0015056374662024
- **w-rdist:** 0.9392812327684136
- **t-alpha:** 0.5324386148895914

---

---

484

- **AF ID:** AF-Q04760-F1-model-v4 | **Chain:** A
- **b-phipsi:** 0.0008086845617578
- **w-rdist:** 1.1843089496031682
- **t-alpha:** 0.7363752907291139

---

---

485

- **AF ID:** AF-Q9NVS9-F1-model-v4 | **Chain:** A
- **b-phipsi:** 0.0013772833392121
- **w-rdist:** 1.032082732354041
- **t-alpha:** 0.4842907346914542

---

---

486

- **AF ID:** AF-Q96DA2-F1-model-v4 | **Chain:** A
- **b-phipsi:** 0.0007963224338163
- **w-rdist:** 1.1716015213382756
- **t-alpha:** 0.9001384756579907

---

---

487

- **AF ID:** AF-O95279-F1-model-v4 | **Chain:** A
- **b-phipsi:** 0.0342487358340067
- **w-rdist:** 1.0020373834824936
- **t-alpha:** 0.0065694124035411

---

---

488

- **AF ID:** AF-P49802-F1-model-v4 | **Chain:** A
- **b-phipsi:** 0.0510164516625926
- **w-rdist:** 0.8532635452239243
- **t-alpha:** 0.0073530278154354

---

---

489

- **AF ID:** AF-Q86U90-F1-model-v4 | **Chain:** A
- **b-phipsi:** 0.0009094710156832
- **w-rdist:** 1.180544991657536
- **t-alpha:** 0.6155659141754322

---

---

490

- **AF ID:** AF-Q15413-F3-model-v4 | **Chain:** A
- **b-phipsi:** 0.0014560023320545
- **w-rdist:** 1.0856039760903686
- **t-alpha:** 0.314598058647044

---

---

491

- **AF ID:** AF-Q96PP4-F1-model-v4 | **Chain:** A
- **b-phipsi:** 0.0261806291764397
- **w-rdist:** 1.1275033755470962
- **t-alpha:** 0.0051358048547096

---

---

492

- **AF ID:** AF-Q5VY09-F1-model-v4 | **Chain:** A
- **b-phipsi:** 0.0468674093613885
- **w-rdist:** 1.0451530454195737
- **t-alpha:** 0.0051094444648316

---

---

493

- **AF ID:** AF-P48547-F1-model-v4 | **Chain:** A
- **b-phipsi:** 0.0443074188458633
- **w-rdist:** 0.925993262628612
- **t-alpha:** 0.0072992307962134

---

---

494

- **AF ID:** AF-Q8WZ82-F1-model-v4 | **Chain:** A
- **b-phipsi:** 0.0005114835842966
- **w-rdist:** 1.2718039397820102
- **t-alpha:** 0.7385790836341046

---

---

495

- **AF ID:** AF-Q8IX05-F1-model-v4 | **Chain:** A
- **b-phipsi:** 0.0006983882085702
- **w-rdist:** 1.2231191447918834
- **t-alpha:** 0.8896554308523608

---

---

496

- **AF ID:** AF-Q96HA8-F1-model-v4 | **Chain:** A
- **b-phipsi:** 0.0004493401999592
- **w-rdist:** 1.2647604141227002
- **t-alpha:** 0.9855073275777456

---

---

497

- **AF ID:** AF-P18621-F1-model-v4 | **Chain:** A
- **b-phipsi:** 0.0012345677272174
- **w-rdist:** 1.0221129729221077
- **t-alpha:** 0.8291054087747902

---

---

498

- **AF ID:** AF-A0A5S8K742-F1-model-v4 | **Chain:** A
- **b-phipsi:** 0.0012254354574154
- **w-rdist:** 1.3408007804596858
- **t-alpha:** 0.2615105072902588

---

---

499

- **AF ID:** AF-P43308-F1-model-v4 | **Chain:** A
- **b-phipsi:** 0.001341905084948
- **w-rdist:** 0.9653617224736184
- **t-alpha:** 0.8922657948626411

---

---

500

- **AF ID:** AF-P78509-F6-model-v4 | **Chain:** A
- **b-phipsi:** 0.0425725849317096
- **w-rdist:** 1.6095516913125234
- **t-alpha:** 0.0007298270463003

---

---

501

- **AF ID:** AF-P07911-F1-model-v4 | **Chain:** A
- **b-phipsi:** 0.0416638811530469
- **w-rdist:** 1.40485119042117
- **t-alpha:** 0.0014597092068022

---

---

502

- **AF ID:** AF-O94812-F1-model-v4 | **Chain:** A
- **b-phipsi:** 0.0191884427003941
- **w-rdist:** 1.5580022900764685
- **t-alpha:** 0.0043989330665894

---

---

503

- **AF ID:** AF-P55160-F1-model-v4 | **Chain:** A
- **b-phipsi:** 0.1661546813933934
- **w-rdist:** 1.2842343616809735
- **t-alpha:** 0.0007298270463003

---

---

504

- **AF ID:** AF-Q8NCU7-F1-model-v4 | **Chain:** A
- **b-phipsi:** 0.0309362394421733
- **w-rdist:** 1.094927247753427
- **t-alpha:** 0.0058391635426686

---

---

505

- **AF ID:** AF-Q14764-F1-model-v4 | **Chain:** A
- **b-phipsi:** 0.0013983450099325
- **w-rdist:** 1.662406546871745
- **t-alpha:** 0.1167877305104161

---

---

506

- **AF ID:** AF-Q2WGJ8-F1-model-v4 | **Chain:** A
- **b-phipsi:** 0.0013732753799578
- **w-rdist:** 1.010833682473086
- **t-alpha:** 0.6955449091007295

---

---

507

- **AF ID:** AF-Q99435-F1-model-v4 | **Chain:** A
- **b-phipsi:** 0.0335491508099735
- **w-rdist:** 1.4038403302891562
- **t-alpha:** 0.0021896458253802

---

---

508

- **AF ID:** AF-P17024-F1-model-v4 | **Chain:** A
- **b-phipsi:** 0.027338156711645
- **w-rdist:** 1.1970393476712722
- **t-alpha:** 0.0051094444648316

---

---

509

- **AF ID:** AF-Q00059-F1-model-v4 | **Chain:** A
- **b-phipsi:** 0.0475856419058346
- **w-rdist:** 0.3852717534267236
- **t-alpha:** 0.1975523096759199

---

---

510

- **AF ID:** AF-Q8N8N7-F1-model-v4 | **Chain:** A
- **b-phipsi:** 0.0012974949095973
- **w-rdist:** 1.1639465189601066
- **t-alpha:** 0.4300629549057742

---

---

511

- **AF ID:** AF-Q32M78-F1-model-v4 | **Chain:** A
- **b-phipsi:** 0.019369517519409
- **w-rdist:** 1.1887546786355232
- **t-alpha:** 0.0072992307962134

---

---

512

- **AF ID:** AF-P45381-F1-model-v4 | **Chain:** A
- **b-phipsi:** 0.0011341120975565
- **w-rdist:** 1.1782994690485182
- **t-alpha:** 0.5205326591048633

---

---

513

- **AF ID:** AF-Q16613-F1-model-v4 | **Chain:** A
- **b-phipsi:** 0.0011467014986553
- **w-rdist:** 1.1155067585702056
- **t-alpha:** 0.6768671188423523

---

---

514

- **AF ID:** AF-Q5W186-F1-model-v4 | **Chain:** A
- **b-phipsi:** 0.0015326924758166
- **w-rdist:** 0.8998822371195174
- **t-alpha:** 0.9655670390081146

---

---

515

- **AF ID:** AF-P06340-F1-model-v4 | **Chain:** A
- **b-phipsi:** 0.001462123761564
- **w-rdist:** 0.9563933630933475
- **t-alpha:** 0.7496809556115014

---

---

516

- **AF ID:** AF-P00403-F1-model-v4 | **Chain:** A
- **b-phipsi:** 0.0013921806385948
- **w-rdist:** 1.059665555445502
- **t-alpha:** 0.5307267554534134

---

---

517

- **AF ID:** AF-Q86U42-F1-model-v4 | **Chain:** A
- **b-phipsi:** 0.0751663924554316
- **w-rdist:** 1.1123203338990555
- **t-alpha:** 0.0036630800678474

---

---

518

- **AF ID:** AF-Q6NSJ5-F1-model-v4 | **Chain:** A
- **b-phipsi:** 0.0200004815066175
- **w-rdist:** 1.4012725513226965
- **t-alpha:** 0.0051358048547096

---

---

519

- **AF ID:** AF-Q712K3-F1-model-v4 | **Chain:** A
- **b-phipsi:** 0.0015567493410368
- **w-rdist:** 0.9849467404707192
- **t-alpha:** 0.5324386148895914

---

---

520

- **AF ID:** AF-Q8TAQ5-F1-model-v4 | **Chain:** A
- **b-phipsi:** 0.0294969590509477
- **w-rdist:** 1.5592621470673975
- **t-alpha:** 0.0029196362453518

---

---

521

- **AF ID:** AF-Q5VZT2-F1-model-v4 | **Chain:** A
- **b-phipsi:** 0.0210743087038146
- **w-rdist:** 0.409765979798027
- **t-alpha:** 0.4065713159111985

---

---

522

- **AF ID:** AF-Q9UI47-F1-model-v4 | **Chain:** A
- **b-phipsi:** 0.195679176933112
- **w-rdist:** 0.9276463950909998
- **t-alpha:** 0.0065694124035411

---

---

523

- **AF ID:** AF-Q7Z2X4-F1-model-v4 | **Chain:** A
- **b-phipsi:** 0.0013550566390504
- **w-rdist:** 1.058598529364127
- **t-alpha:** 0.6768671188423523

---

---

524

- **AF ID:** AF-A3KMH1-F1-model-v4 | **Chain:** A
- **b-phipsi:** 0.001117505954201
- **w-rdist:** 1.3749301654634116
- **t-alpha:** 0.3686131813352493

---

---

525

- **AF ID:** AF-Q8TDW0-F1-model-v4 | **Chain:** A
- **b-phipsi:** 0.014906026473373
- **w-rdist:** 1.4209115947006443
- **t-alpha:** 0.0073530278154354

---

---

526

- **AF ID:** AF-O15031-F1-model-v4 | **Chain:** A
- **b-phipsi:** 0.0008849796901954
- **w-rdist:** 1.533474391023203
- **t-alpha:** 0.4562046720418089

---

---

527

- **AF ID:** AF-Q9NQT4-F1-model-v4 | **Chain:** A
- **b-phipsi:** 0.0010274046944511
- **w-rdist:** 1.2372467867933
- **t-alpha:** 0.6023393159731623

---

---

528

- **AF ID:** AF-Q12765-F1-model-v4 | **Chain:** A
- **b-phipsi:** 0.001336263746421
- **w-rdist:** 1.1461606473264956
- **t-alpha:** 0.492374820627441

---

---

529

- **AF ID:** AF-P59095-F1-model-v4 | **Chain:** A
- **b-phipsi:** 0.000951561403877
- **w-rdist:** 1.2476864292056613
- **t-alpha:** 0.7061026812932953

---

---

530

- **AF ID:** AF-P0C024-F1-model-v4 | **Chain:** A
- **b-phipsi:** 0.0009135699049495
- **w-rdist:** 1.270908922005559
- **t-alpha:** 0.6686968613398787

---

---

531

- **AF ID:** AF-Q6ZTR6-F1-model-v4 | **Chain:** A
- **b-phipsi:** 0.0275227433815812
- **w-rdist:** 0.4067554949205871
- **t-alpha:** 0.3035203043148384

---

---

532

- **AF ID:** AF-O95873-F1-model-v4 | **Chain:** A
- **b-phipsi:** 0.0367334563860883
- **w-rdist:** 1.370185729939226
- **t-alpha:** 0.0029282444105804

---

---

533

- **AF ID:** AF-P20340-F1-model-v4 | **Chain:** A
- **b-phipsi:** 0.0012580999562195
- **w-rdist:** 1.1103526593070776
- **t-alpha:** 0.7723158525733587

---

---

534

- **AF ID:** AF-Q9HAV7-F1-model-v4 | **Chain:** A
- **b-phipsi:** 0.0015357043301032
- **w-rdist:** 1.0313304782765498
- **t-alpha:** 0.6525937755490394

---

---

535

- **AF ID:** AF-Q8IV77-F1-model-v4 | **Chain:** A
- **b-phipsi:** 0.0379460473112844
- **w-rdist:** 1.2513780156623526
- **t-alpha:** 0.0043792969344855

---

---

536

- **AF ID:** AF-Q96A22-F1-model-v4 | **Chain:** A
- **b-phipsi:** 0.0530579218010631
- **w-rdist:** 0.3268185812242783
- **t-alpha:** 0.522222230344618

---

---

537

- **AF ID:** AF-Q08257-F1-model-v4 | **Chain:** A
- **b-phipsi:** 0.0013479457279006
- **w-rdist:** 1.1749651548855884
- **t-alpha:** 0.5138125961351625

---

---

538

- **AF ID:** AF-Q9NQR4-F1-model-v4 | **Chain:** A
- **b-phipsi:** 0.0011596660468095
- **w-rdist:** 1.256378746541025
- **t-alpha:** 0.5188471359099591

---

---

539

- **AF ID:** AF-Q8N8Q3-F1-model-v4 | **Chain:** A
- **b-phipsi:** 0.0014112421894802
- **w-rdist:** 1.09580529751686
- **t-alpha:** 0.5893270990924859

---

---

540

- **AF ID:** AF-Q6IPT4-F1-model-v4 | **Chain:** A
- **b-phipsi:** 0.0015398010132168
- **w-rdist:** 1.0722807015009612
- **t-alpha:** 0.4907510766758922

---

---

541

- **AF ID:** AF-O75223-F1-model-v4 | **Chain:** A
- **b-phipsi:** 0.0009093336011913
- **w-rdist:** 1.3097675214563511
- **t-alpha:** 0.8315509056837473

---

---

542

- **AF ID:** AF-Q13137-F1-model-v4 | **Chain:** A
- **b-phipsi:** 0.0214146862314397
- **w-rdist:** 1.932339608075729
- **t-alpha:** 0.0051094444648316

---

---

543

- **AF ID:** AF-P00492-F1-model-v4 | **Chain:** A
- **b-phipsi:** 0.0010950552529717
- **w-rdist:** 1.2576170910507856
- **t-alpha:** 0.674816619069684

---

---

544

- **AF ID:** AF-Q9Y5E1-F1-model-v4 | **Chain:** A
- **b-phipsi:** 0.0231766802496738
- **w-rdist:** 1.8094397828315
- **t-alpha:** 0.0043989330665894

---

---

545

- **AF ID:** AF-P41219-F1-model-v4 | **Chain:** A
- **b-phipsi:** 0.1069779579815183
- **w-rdist:** 1.6750455939357631
- **t-alpha:** 0.0007307814170889

---

---

546

- **AF ID:** AF-P0CAP1-F1-model-v4 | **Chain:** A
- **b-phipsi:** 0.0825486613429408
- **w-rdist:** 1.996009207656729
- **t-alpha:** 0.0007307814170889

---

---

547

- **AF ID:** AF-P19235-F1-model-v4 | **Chain:** A
- **b-phipsi:** 0.0297093499908687
- **w-rdist:** 1.398316179318836
- **t-alpha:** 0.0051094444648316

---

---

548

- **AF ID:** AF-Q9UMY4-F1-model-v4 | **Chain:** A
- **b-phipsi:** 0.0013273210163995
- **w-rdist:** 1.1257469707155736
- **t-alpha:** 1.0948010003099893

---

---

549

- **AF ID:** AF-Q5JT25-F1-model-v4 | **Chain:** A
- **b-phipsi:** 0.0014245005288508
- **w-rdist:** 1.1773165997067463
- **t-alpha:** 0.5324386148895914

---

---

550

- **AF ID:** AF-Q96DT5-F16-model-v4 | **Chain:** A
- **b-phipsi:** 0.0907701105684875
- **w-rdist:** 1.5945067986206896
- **t-alpha:** 0.0014620968756575

---

---

551

- **AF ID:** AF-P51160-F1-model-v4 | **Chain:** A
- **b-phipsi:** 0.0410873518572108
- **w-rdist:** 1.3595375914014527
- **t-alpha:** 0.0043989330665894

---

---

552

- **AF ID:** AF-Q63HN8-F21-model-v4 | **Chain:** A
- **b-phipsi:** 0.0570626273863737
- **w-rdist:** 1.0710256008996246
- **t-alpha:** 0.0072992307962134

---

---

553

- **AF ID:** AF-O43264-F1-model-v4 | **Chain:** A
- **b-phipsi:** 0.1227071311900831
- **w-rdist:** 1.4306315665101037
- **t-alpha:** 0.0029196362453518

---

---

554

- **AF ID:** AF-Q9NW61-F1-model-v4 | **Chain:** A
- **b-phipsi:** 0.0012066173635511
- **w-rdist:** 1.2648034947219398
- **t-alpha:** 0.8073879938481423

---

---

555

- **AF ID:** AF-Q9GZZ8-F1-model-v4 | **Chain:** A
- **b-phipsi:** 0.0341622111622615
- **w-rdist:** 0.37673743786349
- **t-alpha:** 0.727616851097366

---

---

556

- **AF ID:** AF-Q14532-F1-model-v4 | **Chain:** A
- **b-phipsi:** 0.076158857507557
- **w-rdist:** 1.6618043147628048
- **t-alpha:** 0.0029196362453518

---

---

557

- **AF ID:** AF-Q9P225-F11-model-v4 | **Chain:** A
- **b-phipsi:** 0.0542379780409458
- **w-rdist:** 1.593372994105649
- **t-alpha:** 0.0036630800678474

---

---

558

- **AF ID:** AF-Q8N4B5-F1-model-v4 | **Chain:** A
- **b-phipsi:** 0.0330089804933218
- **w-rdist:** 1.2308239551393985
- **t-alpha:** 0.0073530278154354

---

---

559

- **AF ID:** AF-Q8IVF4-F14-model-v4 | **Chain:** A
- **b-phipsi:** 0.0827539080433874
- **w-rdist:** 1.584153083167651
- **t-alpha:** 0.0036496798094789

---

---

560

- **AF ID:** AF-Q6ZRQ5-F1-model-v4 | **Chain:** A
- **b-phipsi:** 0.162369564594442
- **w-rdist:** 1.5016446202762457
- **t-alpha:** 0.0036496798094789

---

---

561

- **AF ID:** AF-Q8IWA4-F1-model-v4 | **Chain:** A
- **b-phipsi:** 0.076166894043789
- **w-rdist:** 1.626872943066728
- **t-alpha:** 0.0036496798094789

---

---

562

- **AF ID:** AF-Q9Y4D7-F1-model-v4 | **Chain:** A
- **b-phipsi:** 0.0015062708440062
- **w-rdist:** 1.512066924031232
- **t-alpha:** 0.3854011313169918

---

---

563

- **AF ID:** AF-Q7RTV5-F1-model-v4 | **Chain:** A
- **b-phipsi:** 0.0014039630289328
- **w-rdist:** 1.2669138692422837
- **t-alpha:** 0.6934489596953388

---

---

564

- **AF ID:** AF-P55290-F1-model-v4 | **Chain:** A
- **b-phipsi:** 0.0275416570129047
- **w-rdist:** 1.6082785613743134
- **t-alpha:** 0.0065694124035411

---

---

565

- **AF ID:** AF-Q9HBG7-F1-model-v4 | **Chain:** A
- **b-phipsi:** 0.0260332298738343
- **w-rdist:** 1.5133828268748348
- **t-alpha:** 0.0072992307962134

---

---

566

- **AF ID:** AF-Q9NRW3-F1-model-v4 | **Chain:** A
- **b-phipsi:** 0.0013564371999976
- **w-rdist:** 1.295100759895428
- **t-alpha:** 0.7586652983627977

---

---

567

- **AF ID:** AF-Q9Y6W8-F1-model-v4 | **Chain:** A
- **b-phipsi:** 0.0015193254196593
- **w-rdist:** 1.1848177413952392
- **t-alpha:** 1.1306382143892066

---

---

568

- **AF ID:** AF-Q0VFZ6-F1-model-v4 | **Chain:** A
- **b-phipsi:** 0.2437940068361034
- **w-rdist:** 1.6736750943008327
- **t-alpha:** 0.0036630800678474

---

---

569

- **AF ID:** AF-Q9H9E3-F1-model-v4 | **Chain:** A
- **b-phipsi:** 0.1928851024273684
- **w-rdist:** 1.4531204613772892
- **t-alpha:** 0.0051094444648316

---

---

570

- **AF ID:** AF-Q6P4E1-F1-model-v4 | **Chain:** A
- **b-phipsi:** 0.0415121087641483
- **w-rdist:** 1.6721167684699323
- **t-alpha:** 0.0058736968826307

---

---

571

- **AF ID:** AF-Q03001-F21-model-v4 | **Chain:** A
- **b-phipsi:** 0.3974765604287153
- **w-rdist:** 1.974532868166104
- **t-alpha:** 0.0036630800678474

---

---

572

- **AF ID:** AF-Q9NYC9-F15-model-v4 | **Chain:** A
- **b-phipsi:** 0.0776877856449073
- **w-rdist:** 1.6027925085492043
- **t-alpha:** 0.0051358048547096

---

---

573

- **AF ID:** AF-Q14CZ8-F1-model-v4 | **Chain:** A
- **b-phipsi:** 0.0514740248314526
- **w-rdist:** 1.3773973363454586
- **t-alpha:** 0.0072992307962134

---

---

574

- **AF ID:** AF-P60983-F1-model-v4 | **Chain:** A
- **b-phipsi:** 0.0014863315511452
- **w-rdist:** 1.3332035919319238
- **t-alpha:** 0.9912795991649817

---

---

575

- **AF ID:** AF-Q8NCM8-F12-model-v4 | **Chain:** A
- **b-phipsi:** 0.0657671793028384
- **w-rdist:** 1.590502783591381
- **t-alpha:** 0.0072992307962134

---

---

576

- **AF ID:** AF-Q5VST9-F16-model-v4 | **Chain:** A
- **b-phipsi:** 0.0516644166749528
- **w-rdist:** 1.9083577013101296
- **t-alpha:** 0.0072992307962134

---

---

577

- **AF ID:** AF-Q16352-F1-model-v4 | **Chain:** A
- **b-phipsi:** 0.1395705244533484
- **w-rdist:** 1.6770174955333583
- **t-alpha:** 0.0072992307962134

---

---

578

- **AF ID:** AF-Q8NCM8-F14-model-v4 | **Chain:** A
- **b-phipsi:** 0.0911797763323
- **w-rdist:** 1.6101569688812838
- **t-alpha:** 0.0073530278154354

---

---

579

- **AF ID:** AF-Q96CV9-F1-model-v4 | **Chain:** A
- **b-phipsi:** 0.1397057727022799
- **w-rdist:** 1.783691439276858
- **t-alpha:** 0.0072992307962134

---

---

580

- **AF ID:** AF-Q9Y2D4-F1-model-v4 | **Chain:** A
- **b-phipsi:** 0.1636434933441171
- **w-rdist:** 1.7240345548992717
- **t-alpha:** 0.0072992307962134

---

---
