# Supplementary material for: Comparative structural insights and functional analysis for the distinct unbound states of Human AGO proteins
Source: Sci Rep. 2025 Mar 19;15:9432. doi: 10.1038/s41598-025-91849-5 (PMC11923369; doi:10.1038/s41598-025-91849-5)
Supplement: Supplementary file 24 — Supplementary Information 12. [file 41598_2025_91849_MOESM24_ESM.zip › 4KREp_A_mdwhole_AF4REF/go/4KREp_A_mitot_mitosis_7bedd80c00a949cdb7afa7a9f8f82984-pres_report.html]

 

# Structural Comparison Report for 4KREp\_A - whole structures (total: 3)

---

1

- **Protein name:** Probable RNA-binding protein 46
- **Organism:** Homo sapiens
- **Uniprot Accession Number:** Q8TBY0
- **Protein sequence length:** 533 aa
- **1D identity (%):** 10.21
- **1D identity (%) [Gaps excluded]:** 20.04
- **1D identity - Alignment Gaps:** 452
- **1D aligned content (<aminoacid>:%):** {'E': 8.51, 'G': 12.77, 'A': 6.38, 'L': 10.64, 'M': 2.13, 'R': 5.32, 'F': 3.19, 'P': 12.77, 'V': 5.32, 'Y': 3.19, 'K': 6.38, 'I': 5.32, 'C': 4.26, 'T': 3.19, 'Q': 4.26, 'W': 1.06, 'N': 3.19, 'S': 1.06, 'H': 1.06}
- **Common reported functions (%):** 0.0
- **Common reported locations (%):** 20.0
- **Common reported processes (%):** 0.0

- **AF ID:** Q8TBY0
- **Chain:** A
- **Protein length:** 533 aa
- **Resolution:** N/A
- **b-phipsi ():** 0.005759
- **w-rdist ():** 0.302584
- **t-alpha ():** 0.017075
- **Chemical similarity (Tanimoto Index) (%):** 98.28
- **1D identity (%) [PDB]:** 1.79
- **1D identity (%) [Gaps excluded][PDB]:** 70.59
- **1D identity - Alignment Gaps [PDB]:** 1305
- **1D aligned content [PDB] (<aminoacid>:%):** {'G': 8.33, 'T': 4.17, 'V': 12.5, 'K': 16.67, 'I': 12.5, 'L': 4.17, 'N': 4.17, 'Y': 8.33, 'F': 4.17, 'P': 8.33, 'D': 12.5, 'E': 4.17}
- **2D identity (%) [PDB]:** 34.92
- **2D identity (%) [Gaps excluded][PDB]:** 88.66
- **2D identity - Alignment Gaps [PDB]:** 597
- **2D aligned content [PDB] (<2D-fold>:%):** {'.': 25.29, 'H': 35.47, 'E': 25.29, 'T': 13.95}
- **3D similarity (TM-Score) (%) [PDB]:** 19.45

- **Gene name:** RBM46
- **Entrez ID:** 16686300
- **RefSeq ID:** N/A
- **Sequence length:** N/A
- **5-UTR|CDS|3-UTR identity (%):** N/A | N/A | N/A
- **5-UTR|CDS|3-UTR identity (%) [Gaps excluded]:** N/A | N/A | N/A
- **5-UTR|CDS|3-UTR identity [Alignment Gaps]:** N/A | N/A | N/A
- **5-UTR aligned content (<base>:%):** N/A
- **CDS aligned content (<base>:%):** N/A
- **3-UTR aligned content (<base>:%):** N/A

**Uniprot Description:**  
  
 N/A N/A   
  
 **Gene Ontology Information:**

Molecular Function

- mRNA binding

Location

- cytoplasm
- nucleus

Biological process

- cell cycle switching, mitotic to meiotic cell cycle
- female meiotic nuclear division
- male meiotic nuclear division
- mRNA stabilization
- oogenesis
- spermatid differentiation
- spermatogenesis
- trophectodermal cell differentiation

---

2

- **Protein name:** Anaphase-promoting complex subunit 4
- **Organism:** Homo sapiens
- **Uniprot Accession Number:** Q9UJX5
- **Protein sequence length:** 808 aa
- **1D identity (%):** 14.93
- **1D identity (%) [Gaps excluded]:** 23.49
- **1D identity - Alignment Gaps:** 371
- **1D aligned content (<aminoacid>:%):** {'S': 7.24, 'R': 4.61, 'E': 3.29, 'I': 4.61, 'P': 7.24, 'A': 5.26, 'L': 11.84, 'V': 7.89, 'G': 6.58, 'W': 2.63, 'F': 6.58, 'M': 1.32, 'T': 4.61, 'Y': 6.58, 'N': 3.95, 'K': 4.61, 'H': 1.97, 'C': 2.63, 'D': 1.97, 'Q': 4.61}
- **Common reported functions (%):** 0.0
- **Common reported locations (%):** 30.0
- **Common reported processes (%):** 0.0

- **AF ID:** Q9UJX5
- **Chain:** A
- **Protein length:** 808 aa
- **Resolution:** N/A
- **b-phipsi ():** 0.002462
- **w-rdist ():** 0.728817
- **t-alpha ():** 0.004399
- **Chemical similarity (Tanimoto Index) (%):** 99.17
- **1D identity (%) [PDB]:** 3.03
- **1D identity (%) [Gaps excluded][PDB]:** 76.19
- **1D identity - Alignment Gaps [PDB]:** 1522
- **1D aligned content [PDB] (<aminoacid>:%):** {'R': 4.17, 'V': 4.17, 'Q': 8.33, 'P': 4.17, 'E': 12.5, 'I': 12.5, 'D': 6.25, 'L': 14.58, 'S': 4.17, 'F': 6.25, 'K': 8.33, 'T': 4.17, 'Y': 4.17, 'G': 4.17, 'A': 2.08}
- **2D identity (%) [PDB]:** 39.36
- **2D identity (%) [Gaps excluded][PDB]:** 91.15
- **2D identity - Alignment Gaps [PDB]:** 654
- **2D aligned content [PDB] (<2D-fold>:%):** {'.': 18.1, 'E': 31.35, 'T': 22.96, 'H': 26.27, 'G': 1.32}
- **3D similarity (TM-Score) (%) [PDB]:** 17.87

- **Gene name:** ANAPC4
- **Entrez ID:** 2994500
- **RefSeq ID:** NM\_013367
- **Transcript sequence length:** 2635
- **5-UTR|CDS|3-UTR identity (%):** 29.15 | 45.63 | 0.8
- **5-UTR|CDS|3-UTR identity (%) [Gaps excluded]:** 79.27 | 74.9 | 75.0
- **5-UTR|CDS|3-UTR identity [Alignment Gaps]:** 141 | 1239 | 10707
- **5-UTR aligned content (<base>:%):** {'C': 29.23, 'T': 7.69, 'G': 53.85, 'A': 9.23}
- **CDS aligned content (<base>:%):** {'A': 28.68, 'T': 25.43, 'G': 25.98, 'C': 19.9}
- **3-UTR aligned content (<base>:%):** {'T': 36.78, 'A': 36.78, 'G': 19.54, 'C': 6.9}

**Uniprot Description:**  
  
 Component of the anaphase promoting complex/cyclosome (APC/C), a cell cycle-regulated E3 ubiquitin ligase that controls progression through mitosis and the G1 phase of the cell cycle (PubMed:18485873). The APC/C complex acts by mediating ubiquitination and subsequent degradation of target proteins: it mainly mediates the formation of 'Lys-11'-linked polyubiquitin chains and, to a lower extent, the formation of 'Lys-48'- and 'Lys-63'-linked polyubiquitin chains (PubMed:18485873). The APC/C complex catalyzes assembly of branched 'Lys-11'-/'Lys-48'-linked branched ubiquitin chains on target proteins (PubMed:29033132).   
  
The mammalian APC/C is composed at least of 14 distinct subunits ANAPC1, ANAPC2, CDC27/APC3, ANAPC4, ANAPC5, CDC16/APC6, ANAPC7, CDC23/APC8, ANAPC10, ANAPC11, CDC26/APC12, ANAPC13, ANAPC15 and ANAPC16 that assemble into a complex of at least 19 chains with a combined molecular mass of around 1.2 MDa; APC/C interacts with FZR1 and FBXO5 (PubMed:25043029, PubMed:26083744, PubMed:27259151, PubMed:9469815). In the context of the APC/C complex, directly interacts with UBE2S (PubMed:27259151). Interacts with FBXO43.   
  
 **Gene Ontology Information:**

Molecular Function

- protein phosphatase binding
- ubiquitin-protein transferase activity

Location

- anaphase-promoting complex
- cytosol
- nuclear periphery
- nucleoplasm
- nucleus

Biological process

- anaphase-promoting complex-dependent catabolic process
- cell cycle
- cell division
- protein K11-linked ubiquitination
- regulation of meiotic cell cycle
- regulation of mitotic cell cycle
- regulation of mitotic metaphase/anaphase transition

---

3

- **Protein name:** Tyrosine-protein phosphatase non-receptor type 6
- **Organism:** Homo sapiens
- **Uniprot Accession Number:** P29350
- **Protein sequence length:** 595 aa
- **1D identity (%):** 15.02
- **1D identity (%) [Gaps excluded]:** 25.89
- **1D identity - Alignment Gaps:** 386
- **1D aligned content (<aminoacid>:%):** {'F': 3.62, 'D': 6.52, 'E': 5.8, 'K': 7.97, 'P': 6.52, 'R': 7.25, 'N': 2.17, 'V': 5.07, 'H': 2.17, 'Q': 7.97, 'G': 12.32, 'Y': 4.35, 'T': 6.52, 'S': 3.62, 'L': 7.97, 'A': 2.9, 'I': 4.35, 'C': 0.72, 'W': 0.72, 'M': 1.45}
- **Common reported functions (%):** 0.0
- **Common reported locations (%):** 40.0
- **Common reported processes (%):** 0.0

- **AF ID:** P29350
- **Chain:** A
- **Protein length:** 595 aa
- **Resolution:** N/A
- **b-phipsi ():** 0.00661
- **w-rdist ():** 0.524736
- **t-alpha ():** 0.004399
- **Chemical similarity (Tanimoto Index) (%):** 99.1
- **1D identity (%) [PDB]:** 3.05
- **1D identity (%) [Gaps excluded][PDB]:** 73.68
- **1D identity - Alignment Gaps [PDB]:** 1321
- **1D aligned content [PDB] (<aminoacid>:%):** {'M': 4.76, 'R': 4.76, 'H': 2.38, 'L': 19.05, 'K': 9.52, 'Y': 4.76, 'S': 7.14, 'G': 7.14, 'Q': 9.52, 'P': 7.14, 'T': 2.38, 'A': 4.76, 'E': 2.38, 'V': 9.52, 'C': 2.38, 'N': 2.38}
- **2D identity (%) [PDB]:** 42.34
- **2D identity (%) [Gaps excluded][PDB]:** 87.21
- **2D identity - Alignment Gaps [PDB]:** 497
- **2D aligned content [PDB] (<2D-fold>:%):** {'T': 22.74, '.': 13.45, 'H': 33.99, 'E': 27.63, 'G': 2.2}
- **3D similarity (TM-Score) (%) [PDB]:** 22.14

- **Gene name:** PTPN6
- **Entrez ID:** 577700
- **RefSeq ID:** N/A
- **Sequence length:** N/A
- **5-UTR|CDS|3-UTR identity (%):** N/A | N/A | N/A
- **5-UTR|CDS|3-UTR identity (%) [Gaps excluded]:** N/A | N/A | N/A
- **5-UTR|CDS|3-UTR identity [Alignment Gaps]:** N/A | N/A | N/A
- **5-UTR aligned content (<base>:%):** N/A
- **CDS aligned content (<base>:%):** N/A
- **3-UTR aligned content (<base>:%):** N/A

**Uniprot Description:**  
  
 Modulates signaling by tyrosine phosphorylated cell surface receptors such as KIT and the EGF receptor/EGFR. The SH2 regions may interact with other cellular components to modulate its own phosphatase activity against interacting substrates. Together with MTUS1, induces UBE2V2 expression upon angiotensin II stimulation. Plays a key role in hematopoiesis.   
  
Monomer. Interacts with MTUS1 (By similarity). Interacts with MILR1 (tyrosine-phosphorylated) (By similarity). Interacts with KIT (By similarity). Interacts with SIRPA/PTPNS1 (PubMed:9712903). Interacts with LILRB1 and LILRB2 (PubMed:9285411, PubMed:9842885). Interacts with FCRL2 and FCRL4 (PubMed:11162587, PubMed:14597715). Interacts with FCRL3 and FCRL6 (tyrosine phosphorylated form) (PubMed:20933011, PubMed:11162587, PubMed:19843936). Interacts with CD84 (PubMed:11414741). Interacts with CD300LF (PubMed:15184070). Interacts with CDK2 (PubMed:21262353). Interacts with KIR2DL1; the interaction is enhanced by ARRB2 (PubMed:18604210). Interacts (via SH2 1 domain) with ROS1; the interaction is direct and promotes ROS1 dephosphorylation (PubMed:11266449). Interacts with EGFR; inhibits EGFR-dependent activation of MAPK/ERK (PubMed:21258366). Interacts with LYN (PubMed:10574931). Interacts with the tyrosine phosphorylated form of PDPK1 (PubMed:19591923). Interacts with CEACAM1 (via cytoplasmic domain); this interaction depends on the monomer/dimer equilibrium and is phosphorylation-dependent (By similarity). Interacts with MPIG6B (via ITIM motif) (PubMed:23112346). Interacts with moesin/MSN.   
  
 **Gene Ontology Information:**

Molecular Function

- cell adhesion molecule binding
- non-membrane spanning protein tyrosine phosphatase activity
- phosphorylation-dependent protein binding
- phosphotyrosine residue binding
- protein kinase binding
- protein tyrosine phosphatase activity
- SH2 domain binding
- SH3 domain binding
- transmembrane receptor protein tyrosine phosphatase activity

Location

- alpha-beta T cell receptor complex
- cell-cell junction
- cytoplasm
- cytosol
- extracellular exosome
- extracellular region
- membrane
- nucleolus
- nucleoplasm
- nucleus
- protein-containing complex
- specific granule lumen
- tertiary granule lumen

Biological process

- B cell receptor signaling pathway
- cell differentiation
- cytokine-mediated signaling pathway
- epididymis development
- G protein-coupled receptor signaling pathway
- hematopoietic progenitor cell differentiation
- intracellular signal transduction
- MAPK cascade
- megakaryocyte development
- mitotic cell cycle
- natural killer cell mediated cytotoxicity
- negative regulation of cell population proliferation
- negative regulation of humoral immune response mediated by circulating immunoglobulin
- negative regulation of inflammatory response to wounding
- negative regulation of interleukin-6 production
- negative regulation of MAP kinase activity
- negative regulation of mast cell activation involved in immune response
- negative regulation of peptidyl-tyrosine phosphorylation
- negative regulation of T cell proliferation
- negative regulation of T cell receptor signaling pathway
- negative regulation of tumor necrosis factor production
- peptidyl-tyrosine dephosphorylation
- peptidyl-tyrosine phosphorylation
- platelet aggregation
- platelet formation
- positive regulation of cell adhesion mediated by integrin
- positive regulation of cell population proliferation
- positive regulation of phosphatidylinositol 3-kinase signaling
- protein dephosphorylation
- regulation of apoptotic process
- regulation of B cell differentiation
- regulation of ERK1 and ERK2 cascade
- regulation of G1/S transition of mitotic cell cycle
- regulation of release of sequestered calcium ion into cytosol
- regulation of type I interferon-mediated signaling pathway
- T cell costimulation
- T cell proliferation
- T cell receptor signaling pathway

---
