# Supplementary material for: Comparative structural insights and functional analysis for the distinct unbound states of Human AGO proteins
Source: Sci Rep. 2025 Mar 19;15:9432. doi: 10.1038/s41598-025-91849-5 (PMC11923369; doi:10.1038/s41598-025-91849-5)
Supplement: Supplementary file 24 — Supplementary Information 12. [file 41598_2025_91849_MOESM24_ESM.zip › 4KREp_A_mdwhole_HL2REF/candidates/4KREp_A-merged-h-enriched_report.html]

 

# Structural Comparison Report for 4KREp\_A - whole structures (total: 80)

---

0

- **PDB ID:** 4Z4E | **Chain:** A
- **b-phipsi:** 0.0015266965524345
- **w-rdist:** 0.1461022058763982
- **t-alpha:** 0.0073530278154354

---

---

1

- **PDB ID:** 5IZ5 | **Chain:** B
- **b-phipsi:** 0.0039675777398058
- **w-rdist:** 0.401810249471191
- **t-alpha:** 0.0007307814170889

---

---

2

- **PDB ID:** 4QCL | **Chain:** A
- **b-phipsi:** 0.0090671595990991
- **w-rdist:** 0.1621979184043103
- **t-alpha:** 0.0043792969344855

---

---

3

- **PDB ID:** 8ASC | **Chain:** O
- **b-phipsi:** 0.0012806387629723
- **w-rdist:** 0.3725195550250375
- **t-alpha:** 0.0116786539344329

---

---

4

- **PDB ID:** 6MGQ | **Chain:** A
- **b-phipsi:** 0.0081076385400508
- **w-rdist:** 0.1654902314180817
- **t-alpha:** 0.0073530278154354

---

---

5

- **PDB ID:** 7RZI | **Chain:** B
- **b-phipsi:** 0.007905420847295
- **w-rdist:** 0.4290884723608169
- **t-alpha:** 0.0

---

---

6

- **PDB ID:** 4DUR | **Chain:** A
- **b-phipsi:** 0.0193546436841978
- **w-rdist:** 0.1417496500175507
- **t-alpha:** 0.0014620968756575

---

---

7

- **PDB ID:** 7ZWA | **Chain:** A
- **b-phipsi:** 0.0045835575502272
- **w-rdist:** 0.3881744934260059
- **t-alpha:** 0.0088364585901983

---

---

8

- **PDB ID:** 3ZD6 | **Chain:** A
- **b-phipsi:** 0.0221774098670269
- **w-rdist:** 0.2901545603375102
- **t-alpha:** 0.0

---

---

9

- **PDB ID:** 5GVC | **Chain:** A
- **b-phipsi:** 0.0068068764247712
- **w-rdist:** 0.458143749499335
- **t-alpha:** 0.002194902533056

---

---

10

- **PDB ID:** 6EJA | **Chain:** A
- **b-phipsi:** 0.0024061196614294
- **w-rdist:** 0.5785655626249655
- **t-alpha:** 0.0036630800678474

---

---

11

- **PDB ID:** 5C1B | **Chain:** C
- **b-phipsi:** 0.0087543259550295
- **w-rdist:** 0.1868816868427378
- **t-alpha:** 0.01094855486171

---

---

12

- **PDB ID:** 6MZC | **Chain:** B
- **b-phipsi:** 0.0168105137903268
- **w-rdist:** 0.1908299470251563
- **t-alpha:** 0.0051358048547096

---

---

13

- **PDB ID:** 4R08 | **Chain:** D
- **b-phipsi:** 0.0114253181767265
- **w-rdist:** 0.3854336224601717
- **t-alpha:** 0.0036496798094789

---

---

14

- **PDB ID:** 5JTV | **Chain:** E
- **b-phipsi:** 0.0028802024559202
- **w-rdist:** 0.6458115798370089
- **t-alpha:** 0.0058391635426686

---

---

15

- **PDB ID:** 1N52 | **Chain:** A
- **b-phipsi:** 0.0878529153831904
- **w-rdist:** 0.2951015337319208
- **t-alpha:** 0.002194902533056

---

---

16

- **PDB ID:** 7SOL | **Chain:** C
- **b-phipsi:** 0.004892163791082
- **w-rdist:** 0.3301417728603333
- **t-alpha:** 0.067152979131726

---

---

17

- **PDB ID:** 3TON | **Chain:** A
- **b-phipsi:** 0.0051977022389986
- **w-rdist:** 0.6196935070994957
- **t-alpha:** 0.0051358048547096

---

---

18

- **PDB ID:** 6OON | **Chain:** A
- **b-phipsi:** 0.0096204195739037
- **w-rdist:** 0.0448533414195819
- **t-alpha:** 0.0410335958026029

---

---

19

- **PDB ID:** 7JN7 | **Chain:** A
- **b-phipsi:** 0.0077482562608604
- **w-rdist:** 0.6837457848277255
- **t-alpha:** 0.0007298270463003

---

---

20

- **PDB ID:** 7ABI | **Chain:** r
- **b-phipsi:** 0.0162559003241073
- **w-rdist:** 0.2652964022524349
- **t-alpha:** 0.0080290976816657

---

---

21

- **PDB ID:** 3F8S | **Chain:** B
- **b-phipsi:** 0.0067588660442362
- **w-rdist:** 0.7908210230322221
- **t-alpha:** 0.0007307814170889

---

---

22

- **PDB ID:** 2E1Q | **Chain:** A
- **b-phipsi:** 0.0077072006818306
- **w-rdist:** 0.3344900308194809
- **t-alpha:** 0.0284671564505767

---

---

23

- **PDB ID:** 6AHU | **Chain:** B
- **b-phipsi:** 0.0214178117517286
- **w-rdist:** 0.3030603176985635
- **t-alpha:** 0.0066130905937114

---

---

24

- **PDB ID:** 8A7D | **Chain:** C
- **b-phipsi:** 0.0228618353839349
- **w-rdist:** 0.2963777055782466
- **t-alpha:** 0.0065694124035411

---

---

25

- **PDB ID:** 6DDQ | **Chain:** A
- **b-phipsi:** 0.0082256914449526
- **w-rdist:** 0.6824730150494973
- **t-alpha:** 0.0007307814170889

---

---

26

- **PDB ID:** 7SVO | **Chain:** A
- **b-phipsi:** 0.00531982619442
- **w-rdist:** 0.609038311398768
- **t-alpha:** 0.0072992307962134

---

---

27

- **PDB ID:** 6ZYM | **Chain:** B
- **b-phipsi:** 0.0057689511822424
- **w-rdist:** 0.3803731297393054
- **t-alpha:** 0.0402431252841868

---

---

28

- **PDB ID:** 6PYR | **Chain:** A
- **b-phipsi:** 0.0143720911171205
- **w-rdist:** 0.4556948743959526
- **t-alpha:** 0.002194902533056

---

---

29

- **PDB ID:** 3W1B | **Chain:** A
- **b-phipsi:** 0.0030516082296551
- **w-rdist:** 0.4287515124438537
- **t-alpha:** 0.0474004981600588

---

---

30

- **PDB ID:** 2FJU | **Chain:** B
- **b-phipsi:** 0.0053034614219495
- **w-rdist:** 0.7595513450215365
- **t-alpha:** 0.0043792969344855

---

---

31

- **PDB ID:** 8P4E | **Chain:** O
- **b-phipsi:** 0.0021926475888307
- **w-rdist:** 0.5194837788786166
- **t-alpha:** 0.0262171618622435

---

---

32

- **PDB ID:** 6U23 | **Chain:** O
- **b-phipsi:** 0.0155950569706046
- **w-rdist:** 0.3310542127561603
- **t-alpha:** 0.0088364585901983

---

---

33

- **PDB ID:** 6R25 | **Chain:** K
- **b-phipsi:** 0.0068495365059536
- **w-rdist:** 0.5101343452385987
- **t-alpha:** 0.009579955775703

---

---

34

- **PDB ID:** 5VNE | **Chain:** B
- **b-phipsi:** 0.0051945048209056
- **w-rdist:** 0.6832217486552135
- **t-alpha:** 0.0073530278154354

---

---

35

- **PDB ID:** 3LPO | **Chain:** B
- **b-phipsi:** 0.0088765846921009
- **w-rdist:** 0.6294868556663125
- **t-alpha:** 0.0029282444105804

---

---

36

- **PDB ID:** 6C02 | **Chain:** A
- **b-phipsi:** 0.0033083329508168
- **w-rdist:** 0.5111478182859857
- **t-alpha:** 0.0269866903238209

---

---

37

- **PDB ID:** 3IHP | **Chain:** A
- **b-phipsi:** 0.010549376579409
- **w-rdist:** 0.368892326919952
- **t-alpha:** 0.0178305927380209

---

---

38

- **PDB ID:** 3L4G | **Chain:** H
- **b-phipsi:** 0.0017645124951361
- **w-rdist:** 0.7214858084914312
- **t-alpha:** 0.0118171139231386

---

---

39

- **PDB ID:** 7NSK | **Chain:** B
- **b-phipsi:** 0.0120757362355008
- **w-rdist:** 0.6033072491749103
- **t-alpha:** 0.0014620968756575

---

---

40

- **PDB ID:** 2C11 | **Chain:** D
- **b-phipsi:** 0.0100174836067555
- **w-rdist:** 0.7439620905486566
- **t-alpha:** 0.0007298270463003

---

---

41

- **PDB ID:** 8SBJ | **Chain:** A
- **b-phipsi:** 0.0114167149743223
- **w-rdist:** 0.5366626111772286
- **t-alpha:** 0.0036496798094789

---

---

42

- **PDB ID:** 3FBY | **Chain:** C
- **b-phipsi:** 0.0477568312891555
- **w-rdist:** 0.3262366235329539
- **t-alpha:** 0.0072992307962134

---

---

43

- **PDB ID:** 8FCN | **Chain:** A
- **b-phipsi:** 0.0104988853193558
- **w-rdist:** 0.1575149942415842
- **t-alpha:** 0.0810216958060698

---

---

44

- **PDB ID:** 4AH6 | **Chain:** B
- **b-phipsi:** 0.0058938009135648
- **w-rdist:** 0.3584700286473714
- **t-alpha:** 0.126644897239454

---

---

45

- **PDB ID:** 5VM9 | **Chain:** A
- **b-phipsi:** 0.0128183479856162
- **w-rdist:** 0.0730912512857967
- **t-alpha:** 0.0628394789167019

---

---

46

- **PDB ID:** 5LS6 | **Chain:** J
- **b-phipsi:** 0.0108608627772584
- **w-rdist:** 0.7397735685267779
- **t-alpha:** 0.0

---

---

47

- **PDB ID:** 7ZSC | **Chain:** D
- **b-phipsi:** 0.0051119312298462
- **w-rdist:** 0.4614100224868614
- **t-alpha:** 0.0442074641360952

---

---

48

- **PDB ID:** 5O9Z | **Chain:** B
- **b-phipsi:** 0.0084733264455363
- **w-rdist:** 0.3380018323930703
- **t-alpha:** 0.0506138163692111

---

---

49

- **PDB ID:** 2VXO | **Chain:** A
- **b-phipsi:** 0.0084614774525197
- **w-rdist:** 0.3894469582387004
- **t-alpha:** 0.0300754907253892

---

---

50

- **PDB ID:** 1SC7 | **Chain:** A
- **b-phipsi:** 0.0216007438359304
- **w-rdist:** 0.3937984614590088
- **t-alpha:** 0.0066130905937114

---

---

51

- **PDB ID:** 7T3B | **Chain:** A
- **b-phipsi:** 0.0044610296569389
- **w-rdist:** 0.5314788600755569
- **t-alpha:** 0.0372262107185461

---

---

52

- **PDB ID:** 7DVQ | **Chain:** C
- **b-phipsi:** 0.0056155339136092
- **w-rdist:** 0.5502599277641719
- **t-alpha:** 0.0208645945567758

---

---

53

- **PDB ID:** 7WVJ | **Chain:** B
- **b-phipsi:** 0.0122325395114933
- **w-rdist:** 0.6775309214948072
- **t-alpha:** 0.0021896458253802

---

---

54

- **PDB ID:** 4NGE | **Chain:** A
- **b-phipsi:** 0.0137106948775211
- **w-rdist:** 0.5028017133442028
- **t-alpha:** 0.0058391635426686

---

---

55

- **PDB ID:** 3CDZ | **Chain:** A
- **b-phipsi:** 0.0438801103211926
- **w-rdist:** 0.3702459179905237
- **t-alpha:** 0.0072992307962134

---

---

56

- **PDB ID:** 3KRW | **Chain:** A
- **b-phipsi:** 0.0150581935059202
- **w-rdist:** 0.3307316322006382
- **t-alpha:** 0.0262171618622435

---

---

57

- **PDB ID:** 6Y0F | **Chain:** C
- **b-phipsi:** 0.0068851174102683
- **w-rdist:** 0.7939054393956338
- **t-alpha:** 0.0058736968826307

---

---

58

- **PDB ID:** 3BDL | **Chain:** A
- **b-phipsi:** 0.0019024777395061
- **w-rdist:** 0.4976707155600286
- **t-alpha:** 0.135987099964115

---

---

59

- **PDB ID:** 6GYR | **Chain:** B
- **b-phipsi:** 0.007781468156227
- **w-rdist:** 0.4287507170432029
- **t-alpha:** 0.0394537506304994

---

---

60

- **PDB ID:** 8IF3 | **Chain:** A
- **b-phipsi:** 0.0071537522639396
- **w-rdist:** 0.5128395273873659
- **t-alpha:** 0.0223883978326699

---

---

61

- **PDB ID:** 7BG0 | **Chain:** A
- **b-phipsi:** 0.0131444483679187
- **w-rdist:** 0.3908589524302357
- **t-alpha:** 0.020104517651075

---

---

62

- **PDB ID:** 7SFC | **Chain:** A
- **b-phipsi:** 0.0071815934154308
- **w-rdist:** 0.424569797051101
- **t-alpha:** 0.0635035960950682

---

---

63

- **PDB ID:** 5UZ0 | **Chain:** A
- **b-phipsi:** 0.00747792315044
- **w-rdist:** 0.7535923826222958
- **t-alpha:** 0.0072992307962134

---

---

64

- **PDB ID:** 7AUA | **Chain:** A
- **b-phipsi:** 0.0074483941569596
- **w-rdist:** 0.4109266789134524
- **t-alpha:** 0.0678101173054155

---

---

65

- **PDB ID:** 5MHL | **Chain:** A
- **b-phipsi:** 0.0167316945333825
- **w-rdist:** 0.3139183163177882
- **t-alpha:** 0.0331824489470444

---

---

66

- **PDB ID:** 5DYH | **Chain:** B
- **b-phipsi:** 0.0077361220387648
- **w-rdist:** 0.4320981844956871
- **t-alpha:** 0.0498085626203308

---

---

67

- **PDB ID:** 4ENZ | **Chain:** A
- **b-phipsi:** 0.0168803633421573
- **w-rdist:** 0.5501809732240217
- **t-alpha:** 0.0036496798094789

---

---

68

- **PDB ID:** 6JT0 | **Chain:** B
- **b-phipsi:** 0.0232216014217424
- **w-rdist:** 0.4129231775995897
- **t-alpha:** 0.0073530278154354

---

---

69

- **PDB ID:** 6H0G | **Chain:** D
- **b-phipsi:** 0.0235272458720541
- **w-rdist:** 0.5078632667946059
- **t-alpha:** 0.0043989330665894

---

---

70

- **PDB ID:** 7U6E | **Chain:** E
- **b-phipsi:** 0.0158833092045217
- **w-rdist:** 0.4364307350350556
- **t-alpha:** 0.0110700407352846

---

---

71

- **PDB ID:** 4ZG6 | **Chain:** A
- **b-phipsi:** 0.0102623217708496
- **w-rdist:** 0.4705656701233642
- **t-alpha:** 0.0239164285917981

---

---

72

- **PDB ID:** 3EH1 | **Chain:** A
- **b-phipsi:** 0.0018124169365006
- **w-rdist:** 0.7554429741570876
- **t-alpha:** 0.0522276825687215

---

---

73

- **PDB ID:** 4DB1 | **Chain:** B
- **b-phipsi:** 0.0205435228568811
- **w-rdist:** 0.4683280485672662
- **t-alpha:** 0.0080944703406706

---

---

74

- **PDB ID:** 6KAM | **Chain:** D
- **b-phipsi:** 0.0008624138718353
- **w-rdist:** 0.7173399337433565
- **t-alpha:** 0.174957521031396

---

---

75

- **PDB ID:** 6U7G | **Chain:** A
- **b-phipsi:** 0.012110994517728
- **w-rdist:** 0.6148156192375813
- **t-alpha:** 0.009579955775703

---

---

76

- **PDB ID:** 3B2D | **Chain:** A
- **b-phipsi:** 0.0138166367474365
- **w-rdist:** 0.4356588132488542
- **t-alpha:** 0.0293011937554052

---

---

77

- **PDB ID:** 8DP0 | **Chain:** A
- **b-phipsi:** 0.0154133384276223
- **w-rdist:** 0.4698256594755919
- **t-alpha:** 0.0218975903112057

---

---

78

- **PDB ID:** 4ASI | **Chain:** C
- **b-phipsi:** 0.0105070983837728
- **w-rdist:** 0.7214020098381139
- **t-alpha:** 0.0102189826802625

---

---

79

- **PDB ID:** 7QTT | **Chain:** n
- **b-phipsi:** 0.0244595047917531
- **w-rdist:** 0.2156166620446509
- **t-alpha:** 0.1452554889642807

---

---
