# Supplementary material for: Comparative structural insights and functional analysis for the distinct unbound states of Human AGO proteins
Source: Sci Rep. 2025 Mar 19;15:9432. doi: 10.1038/s41598-025-91849-5 (PMC11923369; doi:10.1038/s41598-025-91849-5)
Supplement: Supplementary file 24 — Supplementary Information 12. [file 41598_2025_91849_MOESM24_ESM.zip › 4KREp_A_mdwhole_HL2REF/candidates/4KREp_A-merged-notenriched_report.html]

 

# Structural Comparison Report for 4KREp\_A - whole structures (total: 708)

---

1

- **PDB ID:** 4Z4E | **Chain:** A
- **b-phipsi:** 0.0015266965524345
- **w-rdist:** 0.1461022058763982
- **t-alpha:** 0.0073530278154354

---

---

2

- **PDB ID:** 4W5N | **Chain:** A
- **b-phipsi:** 0.0042970334702519
- **w-rdist:** 0.1719970809195241
- **t-alpha:** 0.0058736968826307

---

---

3

- **PDB ID:** 4W5Q | **Chain:** A
- **b-phipsi:** 0.0051083730463051
- **w-rdist:** 0.1728170026082534
- **t-alpha:** 0.0051094444648316

---

---

4

- **PDB ID:** 4W5T | **Chain:** A
- **b-phipsi:** 0.0048449357955406
- **w-rdist:** 0.1211912723178414
- **t-alpha:** 0.01094855486171

---

---

5

- **PDB ID:** 8D71 | **Chain:** A
- **b-phipsi:** 0.0048668412246206
- **w-rdist:** 0.2424580661201727
- **t-alpha:** 0.002194902533056

---

---

6

- **PDB ID:** 5IZ5 | **Chain:** B
- **b-phipsi:** 0.0039675777398058
- **w-rdist:** 0.401810249471191
- **t-alpha:** 0.0007307814170889

---

---

7

- **PDB ID:** 4W5R | **Chain:** A
- **b-phipsi:** 0.0054240322669591
- **w-rdist:** 0.1153120059418452
- **t-alpha:** 0.0178305927380209

---

---

8

- **PDB ID:** 4KXT | **Chain:** A
- **b-phipsi:** 0.0028268243767481
- **w-rdist:** 0.0834411400421236
- **t-alpha:** 0.029197147494578

---

---

9

- **PDB ID:** 7NFC | **Chain:** G
- **b-phipsi:** 0.0021701848938172
- **w-rdist:** 0.3539905317103813
- **t-alpha:** 0.0088364585901983

---

---

10

- **PDB ID:** 4Z4H | **Chain:** A
- **b-phipsi:** 0.0024246365823159
- **w-rdist:** 0.1454566914560177
- **t-alpha:** 0.0343063240734486

---

---

11

- **PDB ID:** 4Z4G | **Chain:** A
- **b-phipsi:** 0.0042560491091995
- **w-rdist:** 0.155005670766033
- **t-alpha:** 0.0240873273423209

---

---

12

- **PDB ID:** 8ASC | **Chain:** O
- **b-phipsi:** 0.0012806387629723
- **w-rdist:** 0.3725195550250375
- **t-alpha:** 0.0116786539344329

---

---

13

- **PDB ID:** 4QCL | **Chain:** A
- **b-phipsi:** 0.0090671595990991
- **w-rdist:** 0.1621979184043103
- **t-alpha:** 0.0043792969344855

---

---

14

- **PDB ID:** 3ZIM | **Chain:** A
- **b-phipsi:** 0.0129758435519069
- **w-rdist:** 0.1308608432811218
- **t-alpha:** 0.0021896458253802

---

---

15

- **PDB ID:** 4OLA | **Chain:** A
- **b-phipsi:** 0.0031228796109463
- **w-rdist:** 0.2357815661918286
- **t-alpha:** 0.0167881324733683

---

---

16

- **PDB ID:** 6MGQ | **Chain:** A
- **b-phipsi:** 0.0081076385400508
- **w-rdist:** 0.1654902314180817
- **t-alpha:** 0.0073530278154354

---

---

17

- **PDB ID:** 7ZWA | **Chain:** A
- **b-phipsi:** 0.0045835575502272
- **w-rdist:** 0.3881744934260059
- **t-alpha:** 0.0088364585901983

---

---

18

- **PDB ID:** 4Z4D | **Chain:** A
- **b-phipsi:** 0.0061052228852203
- **w-rdist:** 0.1512218713430206
- **t-alpha:** 0.0262171618622435

---

---

19

- **PDB ID:** 4Z4I | **Chain:** A
- **b-phipsi:** 0.0040335673967296
- **w-rdist:** 0.15293402908317
- **t-alpha:** 0.0355252350918542

---

---

20

- **PDB ID:** 4F3T | **Chain:** A
- **b-phipsi:** 0.0050394015705826
- **w-rdist:** 0.11013759297616
- **t-alpha:** 0.0408757514567565

---

---

21

- **PDB ID:** 6CBD | **Chain:** A
- **b-phipsi:** 0.0030890495736064
- **w-rdist:** 0.187403327747466
- **t-alpha:** 0.0246822773765587

---

---

22

- **PDB ID:** 4W5O | **Chain:** A
- **b-phipsi:** 0.0046139364391804
- **w-rdist:** 0.1546308434306182
- **t-alpha:** 0.035036511315305

---

---

23

- **PDB ID:** 8ASC | **Chain:** E
- **b-phipsi:** 0.0029314085146358
- **w-rdist:** 0.3749600655229853
- **t-alpha:** 0.0140635813350504

---

---

24

- **PDB ID:** 7ZT6 | **Chain:** B
- **b-phipsi:** 0.0031311191432428
- **w-rdist:** 0.432864528414056
- **t-alpha:** 0.0094889742856814

---

---

25

- **PDB ID:** 5GVC | **Chain:** A
- **b-phipsi:** 0.0068068764247712
- **w-rdist:** 0.458143749499335
- **t-alpha:** 0.002194902533056

---

---

26

- **PDB ID:** 6PYS | **Chain:** A
- **b-phipsi:** 0.0119657523195993
- **w-rdist:** 0.1197912598975207
- **t-alpha:** 0.0072992307962134

---

---

27

- **PDB ID:** 7NFC | **Chain:** C
- **b-phipsi:** 0.0089211304956095
- **w-rdist:** 0.2606942446421239
- **t-alpha:** 0.002194902533056

---

---

28

- **PDB ID:** 7NFC | **Chain:** B
- **b-phipsi:** 0.0041587019304064
- **w-rdist:** 0.3260087346276992
- **t-alpha:** 0.0178305927380209

---

---

29

- **PDB ID:** 7RZI | **Chain:** B
- **b-phipsi:** 0.007905420847295
- **w-rdist:** 0.4290884723608169
- **t-alpha:** 0.0

---

---

30

- **PDB ID:** 6EJA | **Chain:** A
- **b-phipsi:** 0.0024061196614294
- **w-rdist:** 0.5785655626249655
- **t-alpha:** 0.0036630800678474

---

---

31

- **PDB ID:** 5JS1 | **Chain:** A
- **b-phipsi:** 0.0040355148829862
- **w-rdist:** 0.1150766613962996
- **t-alpha:** 0.0613135828266362

---

---

32

- **PDB ID:** 4TUU | **Chain:** A
- **b-phipsi:** 0.0108506251716868
- **w-rdist:** 0.1303461170566309
- **t-alpha:** 0.01094855486171

---

---

33

- **PDB ID:** 4DUR | **Chain:** A
- **b-phipsi:** 0.0193546436841978
- **w-rdist:** 0.1417496500175507
- **t-alpha:** 0.0014620968756575

---

---

34

- **PDB ID:** 8EZA | **Chain:** A
- **b-phipsi:** 0.0056682966785476
- **w-rdist:** 0.3919205688222712
- **t-alpha:** 0.0138687321491499

---

---

35

- **PDB ID:** 6MGQ | **Chain:** B
- **b-phipsi:** 0.0113758544956583
- **w-rdist:** 0.0837773069016121
- **t-alpha:** 0.0124087975246982

---

---

36

- **PDB ID:** 8ASC | **Chain:** F
- **b-phipsi:** 0.0031174385933673
- **w-rdist:** 0.4966481303776029
- **t-alpha:** 0.0088364585901983

---

---

37

- **PDB ID:** 6B3Q | **Chain:** B
- **b-phipsi:** 0.0083634104377933
- **w-rdist:** 0.4389794006983369
- **t-alpha:** 0.0007307814170889

---

---

38

- **PDB ID:** 6ZHA | **Chain:** C
- **b-phipsi:** 0.0087048262760725
- **w-rdist:** 0.4156813670788027
- **t-alpha:** 0.0014620968756575

---

---

39

- **PDB ID:** 5C1B | **Chain:** C
- **b-phipsi:** 0.0087543259550295
- **w-rdist:** 0.1868816868427378
- **t-alpha:** 0.01094855486171

---

---

40

- **PDB ID:** 8D6J | **Chain:** A
- **b-phipsi:** 0.0024747945160059
- **w-rdist:** 0.1842276677135335
- **t-alpha:** 0.0518248614636667

---

---

41

- **PDB ID:** 5JS2 | **Chain:** A
- **b-phipsi:** 0.0056603876304776
- **w-rdist:** 0.12523622853912
- **t-alpha:** 0.0627735967823235

---

---

42

- **PDB ID:** 6ERF | **Chain:** E
- **b-phipsi:** 0.0036881204897269
- **w-rdist:** 0.4422357953964919
- **t-alpha:** 0.0145985211566161

---

---

43

- **PDB ID:** 7R9Y | **Chain:** A
- **b-phipsi:** 0.0163672785706327
- **w-rdist:** 0.1683351091040176
- **t-alpha:** 0.0036496798094789

---

---

44

- **PDB ID:** 8EZA | **Chain:** J
- **b-phipsi:** 0.0053878929363235
- **w-rdist:** 0.4006351129360193
- **t-alpha:** 0.0182480702564928

---

---

45

- **PDB ID:** 4KRF | **Chain:** A
- **b-phipsi:** 0.0026484238294918
- **w-rdist:** 0.1020285479845138
- **t-alpha:** 0.1131384481440944

---

---

46

- **PDB ID:** 7ZYG | **Chain:** A
- **b-phipsi:** 0.0076718084876409
- **w-rdist:** 0.4408420203914016
- **t-alpha:** 0.0051094444648316

---

---

47

- **PDB ID:** 5JTV | **Chain:** E
- **b-phipsi:** 0.0028802024559202
- **w-rdist:** 0.6458115798370089
- **t-alpha:** 0.0058391635426686

---

---

48

- **PDB ID:** 6MGQ | **Chain:** C
- **b-phipsi:** 0.0086981675739514
- **w-rdist:** 0.2548201006413522
- **t-alpha:** 0.0094889742856814

---

---

49

- **PDB ID:** 7ZWA | **Chain:** B
- **b-phipsi:** 0.0035243769406465
- **w-rdist:** 0.4501884916148149
- **t-alpha:** 0.0160582219856566

---

---

50

- **PDB ID:** 4TV3 | **Chain:** A
- **b-phipsi:** 0.0124771636872386
- **w-rdist:** 0.128747219394129
- **t-alpha:** 0.0138687321491499

---

---

51

- **PDB ID:** 5JTV | **Chain:** G
- **b-phipsi:** 0.0033370469054796
- **w-rdist:** 0.6437888352731985
- **t-alpha:** 0.0066130905937114

---

---

52

- **PDB ID:** 3TON | **Chain:** A
- **b-phipsi:** 0.0051977022389986
- **w-rdist:** 0.6196935070994957
- **t-alpha:** 0.0051358048547096

---

---

53

- **PDB ID:** 4Z4C | **Chain:** A
- **b-phipsi:** 0.0078008871764396
- **w-rdist:** 0.16395349061261
- **t-alpha:** 0.029197147494578

---

---

54

- **PDB ID:** 6MFN | **Chain:** A
- **b-phipsi:** 0.0032756358409374
- **w-rdist:** 0.3117306943171667
- **t-alpha:** 0.0474449492167272

---

---

55

- **PDB ID:** 4R08 | **Chain:** D
- **b-phipsi:** 0.0114253181767265
- **w-rdist:** 0.3854336224601717
- **t-alpha:** 0.0036496798094789

---

---

56

- **PDB ID:** 7SVO | **Chain:** A
- **b-phipsi:** 0.00531982619442
- **w-rdist:** 0.609038311398768
- **t-alpha:** 0.0072992307962134

---

---

57

- **PDB ID:** 6B70 | **Chain:** B
- **b-phipsi:** 0.0109044979033463
- **w-rdist:** 0.4279247193276403
- **t-alpha:** 0.0014597092068022

---

---

58

- **PDB ID:** 1JEY | **Chain:** A
- **b-phipsi:** 0.0019293855231692
- **w-rdist:** 0.5575197467874368
- **t-alpha:** 0.0125647391185939

---

---

59

- **PDB ID:** 8ONE | **Chain:** A
- **b-phipsi:** 0.0058834962244448
- **w-rdist:** 0.7914007106480122
- **t-alpha:** 0.0014597092068022

---

---

60

- **PDB ID:** 2FJU | **Chain:** B
- **b-phipsi:** 0.0053034614219495
- **w-rdist:** 0.7595513450215365
- **t-alpha:** 0.0043792969344855

---

---

61

- **PDB ID:** 7Z6O | **Chain:** B
- **b-phipsi:** 0.0025550204279347
- **w-rdist:** 0.4231866169214531
- **t-alpha:** 0.0321168830372178

---

---

62

- **PDB ID:** 6ERG | **Chain:** B
- **b-phipsi:** 0.0026750632113274
- **w-rdist:** 0.2674682812877991
- **t-alpha:** 0.058394017848627

---

---

63

- **PDB ID:** 5W6V | **Chain:** A
- **b-phipsi:** 0.007299849635509
- **w-rdist:** 0.0480016471813546
- **t-alpha:** 0.0700727235417066

---

---

64

- **PDB ID:** 7SVM | **Chain:** B
- **b-phipsi:** 0.0047924367720043
- **w-rdist:** 0.6115808645620524
- **t-alpha:** 0.0087590123987277

---

---

65

- **PDB ID:** 7N2M | **Chain:** A
- **b-phipsi:** 0.0112069254379016
- **w-rdist:** 0.1272469051524368
- **t-alpha:** 0.0226277946303061

---

---

66

- **PDB ID:** 3N56 | **Chain:** B
- **b-phipsi:** 0.0075913607419006
- **w-rdist:** 0.4406745655727152
- **t-alpha:** 0.0094889742856814

---

---

67

- **PDB ID:** 8AG4 | **Chain:** B
- **b-phipsi:** 0.0034313046282815
- **w-rdist:** 0.4064815368232283
- **t-alpha:** 0.035036511315305

---

---

68

- **PDB ID:** 6ERG | **Chain:** E
- **b-phipsi:** 0.0025848895614163
- **w-rdist:** 0.2698532929711568
- **t-alpha:** 0.0605840753082309

---

---

69

- **PDB ID:** 5KI6 | **Chain:** A
- **b-phipsi:** 0.0063100222942972
- **w-rdist:** 0.2616204065776599
- **t-alpha:** 0.0430653698396612

---

---

70

- **PDB ID:** 3F8S | **Chain:** B
- **b-phipsi:** 0.0067588660442362
- **w-rdist:** 0.7908210230322221
- **t-alpha:** 0.0007307814170889

---

---

71

- **PDB ID:** 8SZP | **Chain:** B
- **b-phipsi:** 0.0137080196321889
- **w-rdist:** 0.3839095728819436
- **t-alpha:** 0.0021896458253802

---

---

72

- **PDB ID:** 6MZC | **Chain:** B
- **b-phipsi:** 0.0168105137903268
- **w-rdist:** 0.1908299470251563
- **t-alpha:** 0.0051358048547096

---

---

73

- **PDB ID:** 8AG5 | **Chain:** B
- **b-phipsi:** 0.0081301990430004
- **w-rdist:** 0.4610323661243504
- **t-alpha:** 0.0058736968826307

---

---

74

- **PDB ID:** 6TRW | **Chain:** C
- **b-phipsi:** 0.0068586522117542
- **w-rdist:** 0.6173433024691491
- **t-alpha:** 0.0051358048547096

---

---

75

- **PDB ID:** 5VNE | **Chain:** B
- **b-phipsi:** 0.0051945048209056
- **w-rdist:** 0.6832217486552135
- **t-alpha:** 0.0073530278154354

---

---

76

- **PDB ID:** 6R25 | **Chain:** K
- **b-phipsi:** 0.0068495365059536
- **w-rdist:** 0.5101343452385987
- **t-alpha:** 0.009579955775703

---

---

77

- **PDB ID:** 1NU8 | **Chain:** A
- **b-phipsi:** 0.0060808356371026
- **w-rdist:** 0.7944291133076992
- **t-alpha:** 0.0021896458253802

---

---

78

- **PDB ID:** 2P8S | **Chain:** A
- **b-phipsi:** 0.0065702534391805
- **w-rdist:** 0.7922665725503555
- **t-alpha:** 0.0021896458253802

---

---

79

- **PDB ID:** 3ZD6 | **Chain:** A
- **b-phipsi:** 0.0221774098670269
- **w-rdist:** 0.2901545603375102
- **t-alpha:** 0.0

---

---

80

- **PDB ID:** 7AD7 | **Chain:** A
- **b-phipsi:** 0.0027066301175605
- **w-rdist:** 0.2396467688058965
- **t-alpha:** 0.0711493829927376

---

---

81

- **PDB ID:** 8AG4 | **Chain:** A
- **b-phipsi:** 0.004809725836874
- **w-rdist:** 0.5650203430778917
- **t-alpha:** 0.0118171139231386

---

---

82

- **PDB ID:** 6OON | **Chain:** A
- **b-phipsi:** 0.0096204195739037
- **w-rdist:** 0.0448533414195819
- **t-alpha:** 0.0410335958026029

---

---

83

- **PDB ID:** 6B3Q | **Chain:** b
- **b-phipsi:** 0.0092583558771981
- **w-rdist:** 0.4442552096972814
- **t-alpha:** 0.0051094444648316

---

---

84

- **PDB ID:** 3L4G | **Chain:** H
- **b-phipsi:** 0.0017645124951361
- **w-rdist:** 0.7214858084914312
- **t-alpha:** 0.0118171139231386

---

---

85

- **PDB ID:** 7A3L | **Chain:** C
- **b-phipsi:** 0.0050415065068048
- **w-rdist:** 0.5458716821762288
- **t-alpha:** 0.0138687321491499

---

---

86

- **PDB ID:** 6B1E | **Chain:** B
- **b-phipsi:** 0.0067615686980559
- **w-rdist:** 0.795885341921654
- **t-alpha:** 0.0

---

---

87

- **PDB ID:** 2QJR | **Chain:** A
- **b-phipsi:** 0.0073101462120645
- **w-rdist:** 0.7867262968674937
- **t-alpha:** 0.0007307814170889

---

---

88

- **PDB ID:** 6EOT | **Chain:** G
- **b-phipsi:** 0.0081480566786535
- **w-rdist:** 0.594780270572101
- **t-alpha:** 0.0007307814170889

---

---

89

- **PDB ID:** 2IIT | **Chain:** A
- **b-phipsi:** 0.0057346177010899
- **w-rdist:** 0.7953971345278352
- **t-alpha:** 0.0029282444105804

---

---

90

- **PDB ID:** 7KI3 | **Chain:** A
- **b-phipsi:** 0.0054212573115643
- **w-rdist:** 0.4271664847429192
- **t-alpha:** 0.0299266744605297

---

---

91

- **PDB ID:** 7JN7 | **Chain:** A
- **b-phipsi:** 0.0077482562608604
- **w-rdist:** 0.6837457848277255
- **t-alpha:** 0.0007298270463003

---

---

92

- **PDB ID:** 6TRW | **Chain:** B
- **b-phipsi:** 0.0073977823167957
- **w-rdist:** 0.6305730621891614
- **t-alpha:** 0.0036630800678474

---

---

93

- **PDB ID:** 7SGL | **Chain:** B
- **b-phipsi:** 0.0071159629089559
- **w-rdist:** 0.400057928931409
- **t-alpha:** 0.0239164285917981

---

---

94

- **PDB ID:** 6ZYM | **Chain:** B
- **b-phipsi:** 0.0057689511822424
- **w-rdist:** 0.3803731297393054
- **t-alpha:** 0.0402431252841868

---

---

95

- **PDB ID:** 6EJD | **Chain:** A
- **b-phipsi:** 0.001574662540743
- **w-rdist:** 0.5389047452140457
- **t-alpha:** 0.0231516400799582

---

---

96

- **PDB ID:** 6TEU | **Chain:** A
- **b-phipsi:** 0.0061580507444843
- **w-rdist:** 0.8017215718023853
- **t-alpha:** 0.0007307814170889

---

---

97

- **PDB ID:** 7KI3 | **Chain:** D
- **b-phipsi:** 0.0119573643868445
- **w-rdist:** 0.16032125521324
- **t-alpha:** 0.0216257249425573

---

---

98

- **PDB ID:** 8ASC | **Chain:** B
- **b-phipsi:** 0.0036928014953877
- **w-rdist:** 0.5096266402487796
- **t-alpha:** 0.0208645945567758

---

---

99

- **PDB ID:** 6EOP | **Chain:** B
- **b-phipsi:** 0.0074596852485888
- **w-rdist:** 0.616077181160095
- **t-alpha:** 0.0043989330665894

---

---

100

- **PDB ID:** 7SVO | **Chain:** C
- **b-phipsi:** 0.0073422673078183
- **w-rdist:** 0.6214282371927149
- **t-alpha:** 0.0051094444648316

---

---

101

- **PDB ID:** 3MDJ | **Chain:** C
- **b-phipsi:** 0.0140576181835127
- **w-rdist:** 0.4211192957882859
- **t-alpha:** 0.0014620968756575

---

---

102

- **PDB ID:** 6ERF | **Chain:** G
- **b-phipsi:** 0.0032055690326512
- **w-rdist:** 0.4802440826974335
- **t-alpha:** 0.0248176303394369

---

---

103

- **PDB ID:** 6N4O | **Chain:** A
- **b-phipsi:** 0.0078595704481958
- **w-rdist:** 0.3024498941414086
- **t-alpha:** 0.0239164285917981

---

---

104

- **PDB ID:** 6TEX | **Chain:** A
- **b-phipsi:** 0.0066227171131914
- **w-rdist:** 0.7987037446791934
- **t-alpha:** 0.0007307814170889

---

---

105

- **PDB ID:** 8D96 | **Chain:** C
- **b-phipsi:** 0.0051621702584621
- **w-rdist:** 0.4026066921746822
- **t-alpha:** 0.0426178307838962

---

---

106

- **PDB ID:** 7LT3 | **Chain:** A
- **b-phipsi:** 0.0089053747802316
- **w-rdist:** 0.3294226092496613
- **t-alpha:** 0.0155670635003994

---

---

107

- **PDB ID:** 6I2X | **Chain:** A
- **b-phipsi:** 0.0024401491850084
- **w-rdist:** 0.2489062503958447
- **t-alpha:** 0.087591155387261

---

---

108

- **PDB ID:** 3W1B | **Chain:** A
- **b-phipsi:** 0.0030516082296551
- **w-rdist:** 0.4287515124438537
- **t-alpha:** 0.0474004981600588

---

---

109

- **PDB ID:** 7ZYG | **Chain:** B
- **b-phipsi:** 0.0035802074290379
- **w-rdist:** 0.4177155055212449
- **t-alpha:** 0.0474449492167272

---

---

110

- **PDB ID:** 6ERF | **Chain:** C
- **b-phipsi:** 0.0047741467267334
- **w-rdist:** 0.4783611787483461
- **t-alpha:** 0.0239164285917981

---

---

111

- **PDB ID:** 5T4B | **Chain:** B
- **b-phipsi:** 0.0066542943783874
- **w-rdist:** 0.7923892788845359
- **t-alpha:** 0.0043792969344855

---

---

112

- **PDB ID:** 8P4E | **Chain:** O
- **b-phipsi:** 0.0021926475888307
- **w-rdist:** 0.5194837788786166
- **t-alpha:** 0.0262171618622435

---

---

113

- **PDB ID:** 7A3J | **Chain:** C
- **b-phipsi:** 0.0088245025436739
- **w-rdist:** 0.5094559274649146
- **t-alpha:** 0.0043792969344855

---

---

114

- **PDB ID:** 6BFC | **Chain:** B
- **b-phipsi:** 0.0100191664697572
- **w-rdist:** 0.4434130794754997
- **t-alpha:** 0.0065694124035411

---

---

115

- **PDB ID:** 4OLB | **Chain:** A
- **b-phipsi:** 0.007576789769875
- **w-rdist:** 0.1411310654457884
- **t-alpha:** 0.0781018927757748

---

---

116

- **PDB ID:** 8ASC | **Chain:** P
- **b-phipsi:** 0.0050895300227189
- **w-rdist:** 0.4920669767211683
- **t-alpha:** 0.0223883978326699

---

---

117

- **PDB ID:** 7AXZ | **Chain:** B
- **b-phipsi:** 0.0063339724826381
- **w-rdist:** 0.4865229256701008
- **t-alpha:** 0.0193454927164979

---

---

118

- **PDB ID:** 3HAC | **Chain:** B
- **b-phipsi:** 0.0075441413549399
- **w-rdist:** 0.7929190030037234
- **t-alpha:** 0.0

---

---

119

- **PDB ID:** 6DDQ | **Chain:** A
- **b-phipsi:** 0.0082256914449526
- **w-rdist:** 0.6824730150494973
- **t-alpha:** 0.0007307814170889

---

---

120

- **PDB ID:** 7A3L | **Chain:** A
- **b-phipsi:** 0.0061967671812129
- **w-rdist:** 0.5492949103515157
- **t-alpha:** 0.0148148020346907

---

---

121

- **PDB ID:** 6GVH | **Chain:** A
- **b-phipsi:** 0.0118596267335544
- **w-rdist:** 0.1152109348264806
- **t-alpha:** 0.0386857767018733

---

---

122

- **PDB ID:** 7A3K | **Chain:** C
- **b-phipsi:** 0.0106697082982042
- **w-rdist:** 0.5305182510161506
- **t-alpha:** 0.0007307814170889

---

---

123

- **PDB ID:** 7ZVT | **Chain:** B
- **b-phipsi:** 0.0015253605721672
- **w-rdist:** 0.4137099320485575
- **t-alpha:** 0.0627735967823235

---

---

124

- **PDB ID:** 4FLH | **Chain:** A
- **b-phipsi:** 0.0314388970258082
- **w-rdist:** 0.2292285395423934
- **t-alpha:** 0.0007298270463003

---

---

125

- **PDB ID:** 6EOT | **Chain:** A
- **b-phipsi:** 0.0060198277862963
- **w-rdist:** 0.6139366824398529
- **t-alpha:** 0.0124087975246982

---

---

126

- **PDB ID:** 6EJ7 | **Chain:** A
- **b-phipsi:** 0.0010078268069324
- **w-rdist:** 0.5622077094619765
- **t-alpha:** 0.0269866903238209

---

---

127

- **PDB ID:** 2QOE | **Chain:** A
- **b-phipsi:** 0.0067703932845
- **w-rdist:** 0.7948428753773439
- **t-alpha:** 0.0036630800678474

---

---

128

- **PDB ID:** 6C02 | **Chain:** A
- **b-phipsi:** 0.0033083329508168
- **w-rdist:** 0.5111478182859857
- **t-alpha:** 0.0269866903238209

---

---

129

- **PDB ID:** 2E1Q | **Chain:** A
- **b-phipsi:** 0.0077072006818306
- **w-rdist:** 0.3344900308194809
- **t-alpha:** 0.0284671564505767

---

---

130

- **PDB ID:** 2QT9 | **Chain:** A
- **b-phipsi:** 0.0071973117616704
- **w-rdist:** 0.7891056120938362
- **t-alpha:** 0.0043989330665894

---

---

131

- **PDB ID:** 3NOX | **Chain:** A
- **b-phipsi:** 0.0074688066315068
- **w-rdist:** 0.7850633658385959
- **t-alpha:** 0.0036630800678474

---

---

132

- **PDB ID:** 6ERF | **Chain:** B
- **b-phipsi:** 0.0047026151446853
- **w-rdist:** 0.4427483069955125
- **t-alpha:** 0.0408757514567565

---

---

133

- **PDB ID:** 2QKY | **Chain:** D
- **b-phipsi:** 0.0061656958354652
- **w-rdist:** 0.8015473839673778
- **t-alpha:** 0.0036630800678474

---

---

134

- **PDB ID:** 7ABI | **Chain:** r
- **b-phipsi:** 0.0162559003241073
- **w-rdist:** 0.2652964022524349
- **t-alpha:** 0.0080290976816657

---

---

135

- **PDB ID:** 2BGR | **Chain:** B
- **b-phipsi:** 0.0071000075947619
- **w-rdist:** 0.782768394471362
- **t-alpha:** 0.0065694124035411

---

---

136

- **PDB ID:** 6TRX | **Chain:** B
- **b-phipsi:** 0.0050434425385688
- **w-rdist:** 0.6148253387730882
- **t-alpha:** 0.0155670635003994

---

---

137

- **PDB ID:** 3HIZ | **Chain:** A
- **b-phipsi:** 0.0155307640771455
- **w-rdist:** 0.3771961170955666
- **t-alpha:** 0.0058391635426686

---

---

138

- **PDB ID:** 5WOB | **Chain:** H
- **b-phipsi:** 0.0071245147307788
- **w-rdist:** 0.3915748489416472
- **t-alpha:** 0.0357662262619218

---

---

139

- **PDB ID:** 3L4G | **Chain:** F
- **b-phipsi:** 0.0016778906721311
- **w-rdist:** 0.706774951540803
- **t-alpha:** 0.0193454927164979

---

---

140

- **PDB ID:** 8AYH | **Chain:** A
- **b-phipsi:** 0.0037493493262308
- **w-rdist:** 0.2486678541551362
- **t-alpha:** 0.0985396895399577

---

---

141

- **PDB ID:** 8EZB | **Chain:** A
- **b-phipsi:** 0.0059412479286354
- **w-rdist:** 0.3843856962516117
- **t-alpha:** 0.0518248614636667

---

---

142

- **PDB ID:** 7SOL | **Chain:** C
- **b-phipsi:** 0.004892163791082
- **w-rdist:** 0.3301417728603333
- **t-alpha:** 0.067152979131726

---

---

143

- **PDB ID:** 6GVG | **Chain:** A
- **b-phipsi:** 0.0130791378365829
- **w-rdist:** 0.1249098211413021
- **t-alpha:** 0.035036511315305

---

---

144

- **PDB ID:** 6GVF | **Chain:** A
- **b-phipsi:** 0.0154672981136316
- **w-rdist:** 0.1118688734613787
- **t-alpha:** 0.0277371931127072

---

---

145

- **PDB ID:** 6BFC | **Chain:** b
- **b-phipsi:** 0.0092978091989853
- **w-rdist:** 0.4463153144764664
- **t-alpha:** 0.0087590123987277

---

---

146

- **PDB ID:** 6EOT | **Chain:** I
- **b-phipsi:** 0.0055342877321286
- **w-rdist:** 0.613421600657787
- **t-alpha:** 0.0155670635003994

---

---

147

- **PDB ID:** 6C01 | **Chain:** A
- **b-phipsi:** 0.0027198783379398
- **w-rdist:** 0.5114076759150271
- **t-alpha:** 0.0316268163453545

---

---

148

- **PDB ID:** 7K6N | **Chain:** A
- **b-phipsi:** 0.0157603315979016
- **w-rdist:** 0.1062948496033748
- **t-alpha:** 0.0270072581572047

---

---

149

- **PDB ID:** 3VJM | **Chain:** B
- **b-phipsi:** 0.0076007623428666
- **w-rdist:** 0.7900919240629835
- **t-alpha:** 0.0029196362453518

---

---

150

- **PDB ID:** 5JPN | **Chain:** B
- **b-phipsi:** 0.0048356461409091
- **w-rdist:** 0.3467757809579773
- **t-alpha:** 0.0664230173941995

---

---

151

- **PDB ID:** 7K71 | **Chain:** A
- **b-phipsi:** 0.0182166710222249
- **w-rdist:** 0.3125356276187309
- **t-alpha:** 0.0065694124035411

---

---

152

- **PDB ID:** 6MFR | **Chain:** A
- **b-phipsi:** 0.0072366154628438
- **w-rdist:** 0.4312229420509489
- **t-alpha:** 0.0262773522597838

---

---

153

- **PDB ID:** 2BGR | **Chain:** A
- **b-phipsi:** 0.0065547388344139
- **w-rdist:** 0.7858534251660735
- **t-alpha:** 0.0087590123987277

---

---

154

- **PDB ID:** 3E4Z | **Chain:** B
- **b-phipsi:** 0.0077608823782745
- **w-rdist:** 0.4558020074414185
- **t-alpha:** 0.0153283513243391

---

---

155

- **PDB ID:** 3VJL | **Chain:** A
- **b-phipsi:** 0.0073051163393838
- **w-rdist:** 0.7878807905586193
- **t-alpha:** 0.0051358048547096

---

---

156

- **PDB ID:** 5C1B | **Chain:** A
- **b-phipsi:** 0.0098626094475988
- **w-rdist:** 0.2162128920394614
- **t-alpha:** 0.0255474760956377

---

---

157

- **PDB ID:** 4AY2 | **Chain:** A
- **b-phipsi:** 0.0152566827499727
- **w-rdist:** 0.3374934113243322
- **t-alpha:** 0.0080290976816657

---

---

158

- **PDB ID:** 8AG5 | **Chain:** A
- **b-phipsi:** 0.001700953672302
- **w-rdist:** 0.535342252832611
- **t-alpha:** 0.0355252350918542

---

---

159

- **PDB ID:** 5C1B | **Chain:** D
- **b-phipsi:** 0.0095380828609982
- **w-rdist:** 0.2122801212036357
- **t-alpha:** 0.0270072581572047

---

---

160

- **PDB ID:** 5I7U | **Chain:** B
- **b-phipsi:** 0.0066088220785414
- **w-rdist:** 0.7880717922311199
- **t-alpha:** 0.0080944703406706

---

---

161

- **PDB ID:** 7ZT6 | **Chain:** A
- **b-phipsi:** 0.003812518694613
- **w-rdist:** 0.5825434632596334
- **t-alpha:** 0.0223883978326699

---

---

162

- **PDB ID:** 6MZM | **Chain:** B
- **b-phipsi:** 0.026389523579386
- **w-rdist:** 0.2341878567585477
- **t-alpha:** 0.0036630800678474

---

---

163

- **PDB ID:** 6PYR | **Chain:** A
- **b-phipsi:** 0.0143720911171205
- **w-rdist:** 0.4556948743959526
- **t-alpha:** 0.002194902533056

---

---

164

- **PDB ID:** 1R9N | **Chain:** A
- **b-phipsi:** 0.0074815794377973
- **w-rdist:** 0.791677994587198
- **t-alpha:** 0.0036496798094789

---

---

165

- **PDB ID:** 6TEC | **Chain:** A
- **b-phipsi:** 0.0048887500149894
- **w-rdist:** 0.7913210717540029
- **t-alpha:** 0.01094855486171

---

---

166

- **PDB ID:** 4FAD | **Chain:** A
- **b-phipsi:** 0.0272864360874197
- **w-rdist:** 0.1535867576690965
- **t-alpha:** 0.01094855486171

---

---

167

- **PDB ID:** 7BAG | **Chain:** B
- **b-phipsi:** 0.0008034800064782
- **w-rdist:** 1.3623122070215203
- **t-alpha:** 0.0118171139231386

---

---

168

- **PDB ID:** 4FWE | **Chain:** A
- **b-phipsi:** 0.0076154445537219
- **w-rdist:** 0.4618531900930668
- **t-alpha:** 0.0175180821197378

---

---

169

- **PDB ID:** 4RE9 | **Chain:** B
- **b-phipsi:** 0.0063422934487333
- **w-rdist:** 0.4669821704128303
- **t-alpha:** 0.0284671564505767

---

---

170

- **PDB ID:** 7DVQ | **Chain:** C
- **b-phipsi:** 0.0056155339136092
- **w-rdist:** 0.5502599277641719
- **t-alpha:** 0.0208645945567758

---

---

171

- **PDB ID:** 6Y0F | **Chain:** C
- **b-phipsi:** 0.0068851174102683
- **w-rdist:** 0.7939054393956338
- **t-alpha:** 0.0058736968826307

---

---

172

- **PDB ID:** 6ZHA | **Chain:** B
- **b-phipsi:** 0.0132692458706143
- **w-rdist:** 0.3514609706451297
- **t-alpha:** 0.0103244826675092

---

---

173

- **PDB ID:** 7A3I | **Chain:** C
- **b-phipsi:** 0.012278638814264
- **w-rdist:** 0.4786528171715806
- **t-alpha:** 0.0036496798094789

---

---

174

- **PDB ID:** 6GVI | **Chain:** A
- **b-phipsi:** 0.0129845202729073
- **w-rdist:** 0.1355897672759409
- **t-alpha:** 0.040145908618359

---

---

175

- **PDB ID:** 6ERF | **Chain:** F
- **b-phipsi:** 0.0044415098270382
- **w-rdist:** 0.4599267188948843
- **t-alpha:** 0.0452556070301213

---

---

176

- **PDB ID:** 3VJK | **Chain:** B
- **b-phipsi:** 0.0079184948267682
- **w-rdist:** 0.7860236745402746
- **t-alpha:** 0.0036496798094789

---

---

177

- **PDB ID:** 3LPO | **Chain:** B
- **b-phipsi:** 0.0088765846921009
- **w-rdist:** 0.6294868556663125
- **t-alpha:** 0.0029282444105804

---

---

178

- **PDB ID:** 8ASC | **Chain:** L
- **b-phipsi:** 0.0031185940811384
- **w-rdist:** 0.4787615221136183
- **t-alpha:** 0.043412095966399

---

---

179

- **PDB ID:** 6U23 | **Chain:** O
- **b-phipsi:** 0.0155950569706046
- **w-rdist:** 0.3310542127561603
- **t-alpha:** 0.0088364585901983

---

---

180

- **PDB ID:** 6EJ9 | **Chain:** A
- **b-phipsi:** 0.0010853214623571
- **w-rdist:** 0.558185234622221
- **t-alpha:** 0.0378787774827127

---

---

181

- **PDB ID:** 5UL1 | **Chain:** A
- **b-phipsi:** 0.0089351254278136
- **w-rdist:** 0.5262125510284531
- **t-alpha:** 0.0066130905937114

---

---

182

- **PDB ID:** 8EZB | **Chain:** J
- **b-phipsi:** 0.0068130511646355
- **w-rdist:** 0.3760710291057556
- **t-alpha:** 0.053284378152949

---

---

183

- **PDB ID:** 1JEY | **Chain:** B
- **b-phipsi:** 0.0055923338074109
- **w-rdist:** 0.4828796414416673
- **t-alpha:** 0.0306567185378972

---

---

184

- **PDB ID:** 6G6W | **Chain:** A
- **b-phipsi:** 0.0162352336654108
- **w-rdist:** 0.4264898068050154
- **t-alpha:** 0.0043792969344855

---

---

185

- **PDB ID:** 6ERH | **Chain:** B
- **b-phipsi:** 0.0083809900001442
- **w-rdist:** 0.2970323247258843
- **t-alpha:** 0.0335766519568103

---

---

186

- **PDB ID:** 6AHU | **Chain:** B
- **b-phipsi:** 0.0214178117517286
- **w-rdist:** 0.3030603176985635
- **t-alpha:** 0.0066130905937114

---

---

187

- **PDB ID:** 5T4F | **Chain:** B
- **b-phipsi:** 0.0058718727480841
- **w-rdist:** 0.7943066282720269
- **t-alpha:** 0.0088364585901983

---

---

188

- **PDB ID:** 8A7D | **Chain:** C
- **b-phipsi:** 0.0228618353839349
- **w-rdist:** 0.2963777055782466
- **t-alpha:** 0.0065694124035411

---

---

189

- **PDB ID:** 5UZ0 | **Chain:** A
- **b-phipsi:** 0.00747792315044
- **w-rdist:** 0.7535923826222958
- **t-alpha:** 0.0072992307962134

---

---

190

- **PDB ID:** 6TES | **Chain:** A
- **b-phipsi:** 0.0061917871091023
- **w-rdist:** 0.7985021603166966
- **t-alpha:** 0.0072992307962134

---

---

191

- **PDB ID:** 3W2T | **Chain:** A
- **b-phipsi:** 0.0074386156356221
- **w-rdist:** 0.7936438828056442
- **t-alpha:** 0.0043989330665894

---

---

192

- **PDB ID:** 5C1B | **Chain:** F
- **b-phipsi:** 0.008637685367797
- **w-rdist:** 0.1849536259974265
- **t-alpha:** 0.0489048903395892

---

---

193

- **PDB ID:** 4Z4F | **Chain:** A
- **b-phipsi:** 0.0081991771746305
- **w-rdist:** 0.1731819503548787
- **t-alpha:** 0.0603716916996117

---

---

194

- **PDB ID:** 7ZSC | **Chain:** D
- **b-phipsi:** 0.0051119312298462
- **w-rdist:** 0.4614100224868614
- **t-alpha:** 0.0442074641360952

---

---

195

- **PDB ID:** 8SBJ | **Chain:** A
- **b-phipsi:** 0.0114167149743223
- **w-rdist:** 0.5366626111772286
- **t-alpha:** 0.0036496798094789

---

---

196

- **PDB ID:** 6OAC | **Chain:** A
- **b-phipsi:** 0.0270113328848098
- **w-rdist:** 0.2150944718525149
- **t-alpha:** 0.0065694124035411

---

---

197

- **PDB ID:** 6ERF | **Chain:** D
- **b-phipsi:** 0.0080207166357241
- **w-rdist:** 0.4290339860795426
- **t-alpha:** 0.0211679073923163

---

---

198

- **PDB ID:** 5I7U | **Chain:** A
- **b-phipsi:** 0.0076548656991418
- **w-rdist:** 0.7901022086931692
- **t-alpha:** 0.0043989330665894

---

---

199

- **PDB ID:** 3IHP | **Chain:** A
- **b-phipsi:** 0.010549376579409
- **w-rdist:** 0.368892326919952
- **t-alpha:** 0.0178305927380209

---

---

200

- **PDB ID:** 7ABI | **Chain:** R
- **b-phipsi:** 0.0150512819280006
- **w-rdist:** 0.2545436785236252
- **t-alpha:** 0.0138687321491499

---

---

201

- **PDB ID:** 4L2Y | **Chain:** A
- **b-phipsi:** 0.0078446885030247
- **w-rdist:** 0.4996247291721827
- **t-alpha:** 0.013138501865866

---

---

202

- **PDB ID:** 5UBT | **Chain:** A
- **b-phipsi:** 0.016348403842224
- **w-rdist:** 0.4383323436989504
- **t-alpha:** 0.0036630800678474

---

---

203

- **PDB ID:** 5UBR | **Chain:** A
- **b-phipsi:** 0.0193210050599201
- **w-rdist:** 0.1247089030816309
- **t-alpha:** 0.0255474760956377

---

---

204

- **PDB ID:** 1N52 | **Chain:** A
- **b-phipsi:** 0.0878529153831904
- **w-rdist:** 0.2951015337319208
- **t-alpha:** 0.002194902533056

---

---

205

- **PDB ID:** 6PYU | **Chain:** A
- **b-phipsi:** 0.0131610730106777
- **w-rdist:** 0.4335868426749642
- **t-alpha:** 0.0073530278154354

---

---

206

- **PDB ID:** 3IHP | **Chain:** B
- **b-phipsi:** 0.0115832242741381
- **w-rdist:** 0.3867522057489833
- **t-alpha:** 0.0140635813350504

---

---

207

- **PDB ID:** 6TY5 | **Chain:** A
- **b-phipsi:** 0.023711587229119
- **w-rdist:** 0.3330423288629252
- **t-alpha:** 0.0051094444648316

---

---

208

- **PDB ID:** 3VJM | **Chain:** A
- **b-phipsi:** 0.0063522810613863
- **w-rdist:** 0.7871078645316049
- **t-alpha:** 0.0118171139231386

---

---

209

- **PDB ID:** 3O95 | **Chain:** D
- **b-phipsi:** 0.0074773718517801
- **w-rdist:** 0.7953700701021109
- **t-alpha:** 0.0036630800678474

---

---

210

- **PDB ID:** 5C18 | **Chain:** C
- **b-phipsi:** 0.009263941176638
- **w-rdist:** 0.1795826522708984
- **t-alpha:** 0.0459850450846748

---

---

211

- **PDB ID:** 5F98 | **Chain:** A
- **b-phipsi:** 0.0139668203258139
- **w-rdist:** 0.5421591982247289
- **t-alpha:** 0.0

---

---

212

- **PDB ID:** 1J2E | **Chain:** A
- **b-phipsi:** 0.0062492837890377
- **w-rdist:** 0.7893432012194035
- **t-alpha:** 0.0118171139231386

---

---

213

- **PDB ID:** 2C11 | **Chain:** D
- **b-phipsi:** 0.0100174836067555
- **w-rdist:** 0.7439620905486566
- **t-alpha:** 0.0007298270463003

---

---

214

- **PDB ID:** 3C43 | **Chain:** A
- **b-phipsi:** 0.0076113590201972
- **w-rdist:** 0.7949291809708624
- **t-alpha:** 0.0036630800678474

---

---

215

- **PDB ID:** 6FXR | **Chain:** A
- **b-phipsi:** 0.0064079937768823
- **w-rdist:** 0.7969920157947692
- **t-alpha:** 0.0080944703406706

---

---

216

- **PDB ID:** 3W5O | **Chain:** B
- **b-phipsi:** 0.004367749472386
- **w-rdist:** 0.4777980831153304
- **t-alpha:** 0.0474004981600588

---

---

217

- **PDB ID:** 7AAV | **Chain:** r
- **b-phipsi:** 0.0171183802326114
- **w-rdist:** 0.2475225692702646
- **t-alpha:** 0.0124087975246982

---

---

218

- **PDB ID:** 6EOT | **Chain:** B
- **b-phipsi:** 0.0079468993818965
- **w-rdist:** 0.6155417166359046
- **t-alpha:** 0.0080944703406706

---

---

219

- **PDB ID:** 3G0D | **Chain:** A
- **b-phipsi:** 0.0083857180575962
- **w-rdist:** 0.792577146511689
- **t-alpha:** 0.0014620968756575

---

---

220

- **PDB ID:** 8EXL | **Chain:** A
- **b-phipsi:** 0.0110715960894368
- **w-rdist:** 0.1240901431508654
- **t-alpha:** 0.0627735967823235

---

---

221

- **PDB ID:** 6EOS | **Chain:** E
- **b-phipsi:** 0.0152419214804522
- **w-rdist:** 0.4311773396043867
- **t-alpha:** 0.0066130905937114

---

---

222

- **PDB ID:** 5LS6 | **Chain:** J
- **b-phipsi:** 0.0108608627772584
- **w-rdist:** 0.7397735685267779
- **t-alpha:** 0.0

---

---

223

- **PDB ID:** 7T3B | **Chain:** A
- **b-phipsi:** 0.0044610296569389
- **w-rdist:** 0.5314788600755569
- **t-alpha:** 0.0372262107185461

---

---

224

- **PDB ID:** 8BFU | **Chain:** A
- **b-phipsi:** 0.0145611674520559
- **w-rdist:** 0.112725658296881
- **t-alpha:** 0.0459850450846748

---

---

225

- **PDB ID:** 4R07 | **Chain:** D
- **b-phipsi:** 0.0130399118194298
- **w-rdist:** 0.3851171447895203
- **t-alpha:** 0.013138501865866

---

---

226

- **PDB ID:** 3EIO | **Chain:** A
- **b-phipsi:** 0.0077801477628442
- **w-rdist:** 0.7996933509546474
- **t-alpha:** 0.0014597092068022

---

---

227

- **PDB ID:** 6ERF | **Chain:** H
- **b-phipsi:** 0.0035209954184032
- **w-rdist:** 0.4587538499819001
- **t-alpha:** 0.0598540584701086

---

---

228

- **PDB ID:** 3LPP | **Chain:** C
- **b-phipsi:** 0.0076274950223736
- **w-rdist:** 0.6359622710969228
- **t-alpha:** 0.009579955775703

---

---

229

- **PDB ID:** 5UY8 | **Chain:** A
- **b-phipsi:** 0.008132861952
- **w-rdist:** 0.7531626389532664
- **t-alpha:** 0.0058391635426686

---

---

230

- **PDB ID:** 7SVM | **Chain:** C
- **b-phipsi:** 0.00683723670565
- **w-rdist:** 0.6192841921968978
- **t-alpha:** 0.0170751996684577

---

---

231

- **PDB ID:** 3SWW | **Chain:** A
- **b-phipsi:** 0.0073436838095634
- **w-rdist:** 0.7879678869658316
- **t-alpha:** 0.0080944703406706

---

---

232

- **PDB ID:** 7JIU | **Chain:** A
- **b-phipsi:** 0.0122393256711392
- **w-rdist:** 0.121160809481375
- **t-alpha:** 0.0569339726158755

---

---

233

- **PDB ID:** 7NSK | **Chain:** B
- **b-phipsi:** 0.0120757362355008
- **w-rdist:** 0.6033072491749103
- **t-alpha:** 0.0014620968756575

---

---

234

- **PDB ID:** 8IF3 | **Chain:** A
- **b-phipsi:** 0.0071537522639396
- **w-rdist:** 0.5128395273873659
- **t-alpha:** 0.0223883978326699

---

---

235

- **PDB ID:** 3W1G | **Chain:** A
- **b-phipsi:** 0.0031789048088207
- **w-rdist:** 0.4434951546303687
- **t-alpha:** 0.0669784550437508

---

---

236

- **PDB ID:** 8BCY | **Chain:** A
- **b-phipsi:** 0.0161861144407863
- **w-rdist:** 0.4109447712437809
- **t-alpha:** 0.0073530278154354

---

---

237

- **PDB ID:** 3D4L | **Chain:** A
- **b-phipsi:** 0.0076315107388809
- **w-rdist:** 0.7939436689444568
- **t-alpha:** 0.0051094444648316

---

---

238

- **PDB ID:** 4DUR | **Chain:** B
- **b-phipsi:** 0.0209964035883713
- **w-rdist:** 0.1188858369421114
- **t-alpha:** 0.0277572859037011

---

---

239

- **PDB ID:** 7P7P | **Chain:** B
- **b-phipsi:** 0.0114538624889177
- **w-rdist:** 0.6015795214816023
- **t-alpha:** 0.0029282444105804

---

---

240

- **PDB ID:** 4DTT | **Chain:** A
- **b-phipsi:** 0.0071514154366125
- **w-rdist:** 0.467875573250494
- **t-alpha:** 0.0299266744605297

---

---

241

- **PDB ID:** 7P7P | **Chain:** A
- **b-phipsi:** 0.0147141420016946
- **w-rdist:** 0.4999485750207305
- **t-alpha:** 0.0029196362453518

---

---

242

- **PDB ID:** 7SVM | **Chain:** A
- **b-phipsi:** 0.0064740545988927
- **w-rdist:** 0.6088356698054721
- **t-alpha:** 0.0216257249425573

---

---

243

- **PDB ID:** 1JEQ | **Chain:** B
- **b-phipsi:** 0.010783907794134
- **w-rdist:** 0.4397159006921292
- **t-alpha:** 0.013138501865866

---

---

244

- **PDB ID:** 7K6O | **Chain:** A
- **b-phipsi:** 0.0142199842725376
- **w-rdist:** 0.170605102294233
- **t-alpha:** 0.0299266744605297

---

---

245

- **PDB ID:** 5Y7J | **Chain:** D
- **b-phipsi:** 0.0072828780423756
- **w-rdist:** 0.7997435808320101
- **t-alpha:** 0.0051358048547096

---

---

246

- **PDB ID:** 5C18 | **Chain:** A
- **b-phipsi:** 0.0103280882615365
- **w-rdist:** 0.191436062243368
- **t-alpha:** 0.0437952703033026

---

---

247

- **PDB ID:** 3KWF | **Chain:** B
- **b-phipsi:** 0.0070610586616748
- **w-rdist:** 0.7863623610027592
- **t-alpha:** 0.0110700407352846

---

---

248

- **PDB ID:** 8EXV | **Chain:** A
- **b-phipsi:** 0.0123147163746189
- **w-rdist:** 0.1301825938226964
- **t-alpha:** 0.0576639954602209

---

---

249

- **PDB ID:** 2E1Q | **Chain:** B
- **b-phipsi:** 0.0077531035063943
- **w-rdist:** 0.3293172363247956
- **t-alpha:** 0.0503648629317097

---

---

250

- **PDB ID:** 5ZID | **Chain:** B
- **b-phipsi:** 0.0092558095863338
- **w-rdist:** 0.778833181779836
- **t-alpha:** 0.0029196362453518

---

---

251

- **PDB ID:** 2QTB | **Chain:** A
- **b-phipsi:** 0.008003398580678
- **w-rdist:** 0.7956810337031093
- **t-alpha:** 0.0029196362453518

---

---

252

- **PDB ID:** 2VXO | **Chain:** A
- **b-phipsi:** 0.0084614774525197
- **w-rdist:** 0.3894469582387004
- **t-alpha:** 0.0300754907253892

---

---

253

- **PDB ID:** 4N8D | **Chain:** B
- **b-phipsi:** 0.0060549696331186
- **w-rdist:** 0.7850278985176652
- **t-alpha:** 0.0170751996684577

---

---

254

- **PDB ID:** 6Y0F | **Chain:** B
- **b-phipsi:** 0.0055654579791759
- **w-rdist:** 0.8012998511762273
- **t-alpha:** 0.0103244826675092

---

---

255

- **PDB ID:** 7WUB | **Chain:** G
- **b-phipsi:** 0.0104151434187948
- **w-rdist:** 0.2759880850360971
- **t-alpha:** 0.0306567185378972

---

---

256

- **PDB ID:** 4RAL | **Chain:** B
- **b-phipsi:** 0.0077050447503385
- **w-rdist:** 0.4477502961926386
- **t-alpha:** 0.0270072581572047

---

---

257

- **PDB ID:** 1RWQ | **Chain:** A
- **b-phipsi:** 0.0075086440026805
- **w-rdist:** 0.7868705603337562
- **t-alpha:** 0.0087590123987277

---

---

258

- **PDB ID:** 4DSZ | **Chain:** B
- **b-phipsi:** 0.0076612293285274
- **w-rdist:** 0.8013783804941071
- **t-alpha:** 0.0036496798094789

---

---

259

- **PDB ID:** 8FCQ | **Chain:** A
- **b-phipsi:** 0.0086659056173061
- **w-rdist:** 0.1805602190524249
- **t-alpha:** 0.0627735967823235

---

---

260

- **PDB ID:** 4PNZ | **Chain:** A
- **b-phipsi:** 0.0087664668430467
- **w-rdist:** 0.790877275113125
- **t-alpha:** 0.0029282444105804

---

---

261

- **PDB ID:** 6ERG | **Chain:** A
- **b-phipsi:** 0.0015532033921463
- **w-rdist:** 0.5793812522712836
- **t-alpha:** 0.0482019055190958

---

---

262

- **PDB ID:** 6MQ3 | **Chain:** B
- **b-phipsi:** 0.0064255583229491
- **w-rdist:** 0.4624394560750338
- **t-alpha:** 0.0481749154992192

---

---

263

- **PDB ID:** 4LKO | **Chain:** A
- **b-phipsi:** 0.0072721943486144
- **w-rdist:** 0.7944699876534348
- **t-alpha:** 0.0080290976816657

---

---

264

- **PDB ID:** 3TON | **Chain:** B
- **b-phipsi:** 0.0038597488546571
- **w-rdist:** 0.6220344761006338
- **t-alpha:** 0.0308503701641407

---

---

265

- **PDB ID:** 5CJO | **Chain:** A
- **b-phipsi:** 0.0063769509887073
- **w-rdist:** 0.4667882965579772
- **t-alpha:** 0.0459850450846748

---

---

266

- **PDB ID:** 6EJB | **Chain:** A
- **b-phipsi:** 0.0015187821193541
- **w-rdist:** 0.5482536126288083
- **t-alpha:** 0.0554699020381697

---

---

267

- **PDB ID:** 1SC7 | **Chain:** A
- **b-phipsi:** 0.0216007438359304
- **w-rdist:** 0.3937984614590088
- **t-alpha:** 0.0066130905937114

---

---

268

- **PDB ID:** 6C01 | **Chain:** B
- **b-phipsi:** 0.0040173006788629
- **w-rdist:** 0.5011225715519116
- **t-alpha:** 0.0506138163692111

---

---

269

- **PDB ID:** 3F8S | **Chain:** A
- **b-phipsi:** 0.0069345621351719
- **w-rdist:** 0.793420555796559
- **t-alpha:** 0.0103244826675092

---

---

270

- **PDB ID:** 5C1B | **Chain:** B
- **b-phipsi:** 0.0074059394680287
- **w-rdist:** 0.2094368562539616
- **t-alpha:** 0.0970802370411048

---

---

271

- **PDB ID:** 5VM9 | **Chain:** A
- **b-phipsi:** 0.0128183479856162
- **w-rdist:** 0.0730912512857967
- **t-alpha:** 0.0628394789167019

---

---

272

- **PDB ID:** 7DVQ | **Chain:** c
- **b-phipsi:** 0.005682311806327
- **w-rdist:** 0.5546823800454544
- **t-alpha:** 0.0308503701641407

---

---

273

- **PDB ID:** 8IF4 | **Chain:** A
- **b-phipsi:** 0.0072277490679861
- **w-rdist:** 0.4773961811157571
- **t-alpha:** 0.0300754907253892

---

---

274

- **PDB ID:** 1X70 | **Chain:** B
- **b-phipsi:** 0.0079938137620749
- **w-rdist:** 0.7920552528405803
- **t-alpha:** 0.0051358048547096

---

---

275

- **PDB ID:** 3OC0 | **Chain:** A
- **b-phipsi:** 0.0067672797325973
- **w-rdist:** 0.790568446000255
- **t-alpha:** 0.013138501865866

---

---

276

- **PDB ID:** 6TY5 | **Chain:** B
- **b-phipsi:** 0.0243200875547788
- **w-rdist:** 0.3433622682228473
- **t-alpha:** 0.0066130905937114

---

---

277

- **PDB ID:** 3G0C | **Chain:** A
- **b-phipsi:** 0.0068582810690721
- **w-rdist:** 0.792759967581537
- **t-alpha:** 0.0110700407352846

---

---

278

- **PDB ID:** 8FCL | **Chain:** A
- **b-phipsi:** 0.0139339938512997
- **w-rdist:** 0.1595647650662127
- **t-alpha:** 0.0437952703033026

---

---

279

- **PDB ID:** 3IBE | **Chain:** A
- **b-phipsi:** 0.0234733374341212
- **w-rdist:** 0.1540063957757953
- **t-alpha:** 0.0233575447719152

---

---

280

- **PDB ID:** 5M6U | **Chain:** A
- **b-phipsi:** 0.0178897128051354
- **w-rdist:** 0.4363474851955097
- **t-alpha:** 0.0065694124035411

---

---

281

- **PDB ID:** 7ZXS | **Chain:** A
- **b-phipsi:** 0.0069796555135612
- **w-rdist:** 0.6638189326768269
- **t-alpha:** 0.0178305927380209

---

---

282

- **PDB ID:** 5F9H | **Chain:** I
- **b-phipsi:** 0.0177835600776819
- **w-rdist:** 0.5340669850480335
- **t-alpha:** 0.0

---

---

283

- **PDB ID:** 7RC9 | **Chain:** A
- **b-phipsi:** 0.0206350615035391
- **w-rdist:** 0.4011046857884365
- **t-alpha:** 0.0072992307962134

---

---

284

- **PDB ID:** 7SVO | **Chain:** B
- **b-phipsi:** 0.0055685305372956
- **w-rdist:** 0.6042594101360702
- **t-alpha:** 0.0300754907253892

---

---

285

- **PDB ID:** 8FCN | **Chain:** D
- **b-phipsi:** 0.0083866018875254
- **w-rdist:** 0.1672536781903251
- **t-alpha:** 0.087591155387261

---

---

286

- **PDB ID:** 3QBJ | **Chain:** B
- **b-phipsi:** 0.0085742149259341
- **w-rdist:** 0.7892928255707204
- **t-alpha:** 0.0051094444648316

---

---

287

- **PDB ID:** 2RGU | **Chain:** A
- **b-phipsi:** 0.008771565949997
- **w-rdist:** 0.7974416822936383
- **t-alpha:** 0.0007307814170889

---

---

288

- **PDB ID:** 3L4G | **Chain:** J
- **b-phipsi:** 0.0019146603063907
- **w-rdist:** 0.7025347680718163
- **t-alpha:** 0.0372262107185461

---

---

289

- **PDB ID:** 5C1B | **Chain:** E
- **b-phipsi:** 0.0073412300680124
- **w-rdist:** 0.2568649375503349
- **t-alpha:** 0.0861312290118383

---

---

290

- **PDB ID:** 4FXG | **Chain:** A
- **b-phipsi:** 0.0424755799758968
- **w-rdist:** 0.3613383161819605
- **t-alpha:** 0.0036630800678474

---

---

291

- **PDB ID:** 3L4G | **Chain:** D
- **b-phipsi:** 0.0018293549846619
- **w-rdist:** 0.7100418141977272
- **t-alpha:** 0.0378787774827127

---

---

292

- **PDB ID:** 6EOP | **Chain:** A
- **b-phipsi:** 0.006384311010734
- **w-rdist:** 0.6203883619646169
- **t-alpha:** 0.0246822773765587

---

---

293

- **PDB ID:** 2G48 | **Chain:** A
- **b-phipsi:** 0.0068896800493472
- **w-rdist:** 0.4635868758104824
- **t-alpha:** 0.0445251834570807

---

---

294

- **PDB ID:** 5Y7H | **Chain:** A
- **b-phipsi:** 0.00648939093706
- **w-rdist:** 0.7949724440124212
- **t-alpha:** 0.0124087975246982

---

---

295

- **PDB ID:** 5WOB | **Chain:** c
- **b-phipsi:** 0.00850281629458
- **w-rdist:** 0.4434532323504542
- **t-alpha:** 0.0233575447719152

---

---

296

- **PDB ID:** 6V9U | **Chain:** A
- **b-phipsi:** 0.0189122370448566
- **w-rdist:** 0.3870295215856443
- **t-alpha:** 0.0094889742856814

---

---

297

- **PDB ID:** 7NSK | **Chain:** A
- **b-phipsi:** 0.0118380631204684
- **w-rdist:** 0.5160135616907655
- **t-alpha:** 0.0073530278154354

---

---

298

- **PDB ID:** 7WVJ | **Chain:** B
- **b-phipsi:** 0.0122325395114933
- **w-rdist:** 0.6775309214948072
- **t-alpha:** 0.0021896458253802

---

---

299

- **PDB ID:** 4NGE | **Chain:** A
- **b-phipsi:** 0.0137106948775211
- **w-rdist:** 0.5028017133442028
- **t-alpha:** 0.0058391635426686

---

---

300

- **PDB ID:** 2QJR | **Chain:** B
- **b-phipsi:** 0.0099820363929777
- **w-rdist:** 0.793975783808622
- **t-alpha:** 0.0007298270463003

---

---

301

- **PDB ID:** 8EXU | **Chain:** A
- **b-phipsi:** 0.0126969832036846
- **w-rdist:** 0.1247135875744794
- **t-alpha:** 0.0656935549704063

---

---

302

- **PDB ID:** 7OZ7 | **Chain:** B
- **b-phipsi:** 0.0195999157658188
- **w-rdist:** 0.5244642609494661
- **t-alpha:** 0.0

---

---

303

- **PDB ID:** 7A3L | **Chain:** B
- **b-phipsi:** 0.0071963211626789
- **w-rdist:** 0.5914305500641952
- **t-alpha:** 0.0218975903112057

---

---

304

- **PDB ID:** 4BPB | **Chain:** A
- **b-phipsi:** 0.0119732569889666
- **w-rdist:** 0.3590359712898371
- **t-alpha:** 0.0223883978326699

---

---

305

- **PDB ID:** 4KZ0 | **Chain:** A
- **b-phipsi:** 0.0224143963574116
- **w-rdist:** 0.1504339719340216
- **t-alpha:** 0.0299266744605297

---

---

306

- **PDB ID:** 6XOV | **Chain:** A
- **b-phipsi:** 0.0132092034871313
- **w-rdist:** 0.3824135572914783
- **t-alpha:** 0.0182480702564928

---

---

307

- **PDB ID:** 1JEQ | **Chain:** A
- **b-phipsi:** 0.0066873273048689
- **w-rdist:** 0.7502911656843608
- **t-alpha:** 0.0197076730907748

---

---

308

- **PDB ID:** 3OPM | **Chain:** C
- **b-phipsi:** 0.00854279596855
- **w-rdist:** 0.8035240055311939
- **t-alpha:** 0.0014597092068022

---

---

309

- **PDB ID:** 7WVJ | **Chain:** A
- **b-phipsi:** 0.0127770839178265
- **w-rdist:** 0.6790784667392421
- **t-alpha:** 0.0014620968756575

---

---

310

- **PDB ID:** 6GYR | **Chain:** B
- **b-phipsi:** 0.007781468156227
- **w-rdist:** 0.4287507170432029
- **t-alpha:** 0.0394537506304994

---

---

311

- **PDB ID:** 3L4G | **Chain:** N
- **b-phipsi:** 0.0019600079346677
- **w-rdist:** 0.7170929511230041
- **t-alpha:** 0.040145908618359

---

---

312

- **PDB ID:** 7LN5 | **Chain:** D
- **b-phipsi:** 0.0106272280067569
- **w-rdist:** 0.7876986609125601
- **t-alpha:** 0.0029196362453518

---

---

313

- **PDB ID:** 3TOP | **Chain:** A
- **b-phipsi:** 0.0075060313028594
- **w-rdist:** 0.6243818226850854
- **t-alpha:** 0.0155670635003994

---

---

314

- **PDB ID:** 1NU6 | **Chain:** A
- **b-phipsi:** 0.0057497078836463
- **w-rdist:** 0.7833112663803085
- **t-alpha:** 0.0223883978326699

---

---

315

- **PDB ID:** 1R9N | **Chain:** C
- **b-phipsi:** 0.009403019036083
- **w-rdist:** 0.7882753087773777
- **t-alpha:** 0.0036630800678474

---

---

316

- **PDB ID:** 3EIO | **Chain:** B
- **b-phipsi:** 0.0093690612699548
- **w-rdist:** 0.7936586664421181
- **t-alpha:** 0.0021896458253802

---

---

317

- **PDB ID:** 3CCB | **Chain:** D
- **b-phipsi:** 0.0072865775563261
- **w-rdist:** 0.802529265121978
- **t-alpha:** 0.0073530278154354

---

---

318

- **PDB ID:** 4KRE | **Chain:** A
- **b-phipsi:** 0.0099658460050939
- **w-rdist:** 0.0268686836495369
- **t-alpha:** 0.1284670285521387

---

---

319

- **PDB ID:** 7YBU | **Chain:** D
- **b-phipsi:** 0.0089155813758374
- **w-rdist:** 0.4641640806813737
- **t-alpha:** 0.0185875182467154

---

---

320

- **PDB ID:** 7WVF | **Chain:** B
- **b-phipsi:** 0.0141528673211955
- **w-rdist:** 0.6784463929390914
- **t-alpha:** 0.0

---

---

321

- **PDB ID:** 6TRX | **Chain:** C
- **b-phipsi:** 0.0063572019460826
- **w-rdist:** 0.6035940273836743
- **t-alpha:** 0.0300754907253892

---

---

322

- **PDB ID:** 4FWF | **Chain:** A
- **b-phipsi:** 0.0091093145047613
- **w-rdist:** 0.4802686299765647
- **t-alpha:** 0.0153283513243391

---

---

323

- **PDB ID:** 2FJP | **Chain:** A
- **b-phipsi:** 0.007550607172102
- **w-rdist:** 0.7990695725411233
- **t-alpha:** 0.0066130905937114

---

---

324

- **PDB ID:** 5CJO | **Chain:** a
- **b-phipsi:** 0.0068883189659753
- **w-rdist:** 0.4655225384782934
- **t-alpha:** 0.0481749154992192

---

---

325

- **PDB ID:** 5WOB | **Chain:** e
- **b-phipsi:** 0.0084304553277145
- **w-rdist:** 0.4193551043704682
- **t-alpha:** 0.035036511315305

---

---

326

- **PDB ID:** 7WVE | **Chain:** B
- **b-phipsi:** 0.0121217038817274
- **w-rdist:** 0.6840296144824605
- **t-alpha:** 0.0029282444105804

---

---

327

- **PDB ID:** 7AXZ | **Chain:** A
- **b-phipsi:** 0.0015903275129636
- **w-rdist:** 0.5862716534786362
- **t-alpha:** 0.0570987886882312

---

---

328

- **PDB ID:** 2ONC | **Chain:** D
- **b-phipsi:** 0.0078133600653128
- **w-rdist:** 0.7945509661753483
- **t-alpha:** 0.0066130905937114

---

---

329

- **PDB ID:** 4AH6 | **Chain:** B
- **b-phipsi:** 0.0058938009135648
- **w-rdist:** 0.3584700286473714
- **t-alpha:** 0.126644897239454

---

---

330

- **PDB ID:** 3KWF | **Chain:** A
- **b-phipsi:** 0.0072937885008812
- **w-rdist:** 0.8010718040839612
- **t-alpha:** 0.0080290976816657

---

---

331

- **PDB ID:** 5IUD | **Chain:** J
- **b-phipsi:** 0.0069940905922707
- **w-rdist:** 0.3420649413385231
- **t-alpha:** 0.0912409767262552

---

---

332

- **PDB ID:** 2WBY | **Chain:** A
- **b-phipsi:** 0.0072895421854179
- **w-rdist:** 0.4712627019713499
- **t-alpha:** 0.0372262107185461

---

---

333

- **PDB ID:** 4Q5V | **Chain:** E
- **b-phipsi:** 0.0081294401075587
- **w-rdist:** 0.1727250190547304
- **t-alpha:** 0.1116786836949257

---

---

334

- **PDB ID:** 3LPP | **Chain:** A
- **b-phipsi:** 0.0076738423480033
- **w-rdist:** 0.6390147280585121
- **t-alpha:** 0.0140635813350504

---

---

335

- **PDB ID:** 6HP8 | **Chain:** B
- **b-phipsi:** 0.0090872161758354
- **w-rdist:** 0.6155633758924063
- **t-alpha:** 0.0087590123987277

---

---

336

- **PDB ID:** 7R7T | **Chain:** D
- **b-phipsi:** 0.0185354292381004
- **w-rdist:** 0.5336022248960752
- **t-alpha:** 0.0014597092068022

---

---

337

- **PDB ID:** 5WOB | **Chain:** b
- **b-phipsi:** 0.007491821265626
- **w-rdist:** 0.4419736927510063
- **t-alpha:** 0.0452556070301213

---

---

338

- **PDB ID:** 3NOX | **Chain:** B
- **b-phipsi:** 0.0081545354904098
- **w-rdist:** 0.7893816695040327
- **t-alpha:** 0.0073530278154354

---

---

339

- **PDB ID:** 5C18 | **Chain:** F
- **b-phipsi:** 0.0136141855839827
- **w-rdist:** 0.179316545498281
- **t-alpha:** 0.0416056097301684

---

---

340

- **PDB ID:** 5CU5 | **Chain:** A
- **b-phipsi:** 0.0095423959278005
- **w-rdist:** 0.4151533753276544
- **t-alpha:** 0.0262171618622435

---

---

341

- **PDB ID:** 2JBU | **Chain:** B
- **b-phipsi:** 0.0069199410507903
- **w-rdist:** 0.4591470438055078
- **t-alpha:** 0.0518248614636667

---

---

342

- **PDB ID:** 2WK3 | **Chain:** A
- **b-phipsi:** 0.0066980837958856
- **w-rdist:** 0.4748805288889211
- **t-alpha:** 0.049634873047397

---

---

343

- **PDB ID:** 5O9Z | **Chain:** B
- **b-phipsi:** 0.0084733264455363
- **w-rdist:** 0.3380018323930703
- **t-alpha:** 0.0506138163692111

---

---

344

- **PDB ID:** 8FCN | **Chain:** A
- **b-phipsi:** 0.0104988853193558
- **w-rdist:** 0.1575149942415842
- **t-alpha:** 0.0810216958060698

---

---

345

- **PDB ID:** 7PVN | **Chain:** A
- **b-phipsi:** 0.0073403603955715
- **w-rdist:** 0.7703303015057772
- **t-alpha:** 0.0153283513243391

---

---

346

- **PDB ID:** 8EXO | **Chain:** A
- **b-phipsi:** 0.0127305233380912
- **w-rdist:** 0.1182719011855382
- **t-alpha:** 0.0781018927757748

---

---

347

- **PDB ID:** 6TRW | **Chain:** A
- **b-phipsi:** 0.0070853464630469
- **w-rdist:** 0.6095960340144719
- **t-alpha:** 0.0246822773765587

---

---

348

- **PDB ID:** 6EOP | **Chain:** C
- **b-phipsi:** 0.0093087945419308
- **w-rdist:** 0.629090552914715
- **t-alpha:** 0.0080290976816657

---

---

349

- **PDB ID:** 7LT3 | **Chain:** J
- **b-phipsi:** 0.0157948437354256
- **w-rdist:** 0.3214407515460427
- **t-alpha:** 0.0197076730907748

---

---

350

- **PDB ID:** 7BG0 | **Chain:** A
- **b-phipsi:** 0.0131444483679187
- **w-rdist:** 0.3908589524302357
- **t-alpha:** 0.020104517651075

---

---

351

- **PDB ID:** 8FCN | **Chain:** E
- **b-phipsi:** 0.0082844831799954
- **w-rdist:** 0.1636517258721118
- **t-alpha:** 0.1313861965388669

---

---

352

- **PDB ID:** 3FBY | **Chain:** C
- **b-phipsi:** 0.0477568312891555
- **w-rdist:** 0.3262366235329539
- **t-alpha:** 0.0072992307962134

---

---

353

- **PDB ID:** 5T4F | **Chain:** A
- **b-phipsi:** 0.0086176415918957
- **w-rdist:** 0.7985710400930963
- **t-alpha:** 0.0036496798094789

---

---

354

- **PDB ID:** 3LJ3 | **Chain:** A
- **b-phipsi:** 0.0239679449175249
- **w-rdist:** 0.174763384982042
- **t-alpha:** 0.0204377723355742

---

---

355

- **PDB ID:** 5UZ0 | **Chain:** D
- **b-phipsi:** 0.0072438661546217
- **w-rdist:** 0.7578926533727789
- **t-alpha:** 0.0175180821197378

---

---

356

- **PDB ID:** 2E1Q | **Chain:** D
- **b-phipsi:** 0.0092738826203063
- **w-rdist:** 0.3284340824618911
- **t-alpha:** 0.0452556070301213

---

---

357

- **PDB ID:** 6MDZ | **Chain:** A
- **b-phipsi:** 0.0039832147275091
- **w-rdist:** 0.4494492418012445
- **t-alpha:** 0.0956206337488771

---

---

358

- **PDB ID:** 5UY8 | **Chain:** D
- **b-phipsi:** 0.0067286836260019
- **w-rdist:** 0.7576399133269722
- **t-alpha:** 0.0218975903112057

---

---

359

- **PDB ID:** 3D4L | **Chain:** B
- **b-phipsi:** 0.0072622923106713
- **w-rdist:** 0.7937890718018003
- **t-alpha:** 0.0118171139231386

---

---

360

- **PDB ID:** 5IFW | **Chain:** B
- **b-phipsi:** 0.007564081035575
- **w-rdist:** 0.7571107510040196
- **t-alpha:** 0.0138687321491499

---

---

361

- **PDB ID:** 5WOB | **Chain:** B
- **b-phipsi:** 0.0074462159752137
- **w-rdist:** 0.4400986538575948
- **t-alpha:** 0.0503648629317097

---

---

362

- **PDB ID:** 8OW2 | **Chain:** A
- **b-phipsi:** 0.0138594839596746
- **w-rdist:** 0.1998387387208283
- **t-alpha:** 0.0372262107185461

---

---

363

- **PDB ID:** 4PNZ | **Chain:** B
- **b-phipsi:** 0.0082292043864035
- **w-rdist:** 0.7940215993898072
- **t-alpha:** 0.0065694124035411

---

---

364

- **PDB ID:** 7R9V | **Chain:** A
- **b-phipsi:** 0.0182605026988217
- **w-rdist:** 0.1755356533317906
- **t-alpha:** 0.0285284596970611

---

---

365

- **PDB ID:** 7K6M | **Chain:** A
- **b-phipsi:** 0.0167322272984826
- **w-rdist:** 0.1660860878519553
- **t-alpha:** 0.0386857767018733

---

---

366

- **PDB ID:** 6JT0 | **Chain:** B
- **b-phipsi:** 0.0232216014217424
- **w-rdist:** 0.4129231775995897
- **t-alpha:** 0.0073530278154354

---

---

367

- **PDB ID:** 2HHA | **Chain:** A
- **b-phipsi:** 0.0081071459428307
- **w-rdist:** 0.7966712091279551
- **t-alpha:** 0.0058736968826307

---

---

368

- **PDB ID:** 6I2X | **Chain:** C
- **b-phipsi:** 0.0320498537062727
- **w-rdist:** 0.348914789925301
- **t-alpha:** 0.0066130905937114

---

---

369

- **PDB ID:** 5C19 | **Chain:** E
- **b-phipsi:** 0.0139007631112543
- **w-rdist:** 0.1398160145658332
- **t-alpha:** 0.064963575889908

---

---

370

- **PDB ID:** 7SFC | **Chain:** A
- **b-phipsi:** 0.0071815934154308
- **w-rdist:** 0.424569797051101
- **t-alpha:** 0.0635035960950682

---

---

371

- **PDB ID:** 3CWW | **Chain:** A
- **b-phipsi:** 0.0074652171074598
- **w-rdist:** 0.4701988858967219
- **t-alpha:** 0.0372262107185461

---

---

372

- **PDB ID:** 6ZHE | **Chain:** G
- **b-phipsi:** 0.0133586584937449
- **w-rdist:** 0.3568227164009824
- **t-alpha:** 0.0223883978326699

---

---

373

- **PDB ID:** 7NFE | **Chain:** B
- **b-phipsi:** 0.0106403174036277
- **w-rdist:** 0.3389365222287895
- **t-alpha:** 0.0370931750792566

---

---

374

- **PDB ID:** 5T4H | **Chain:** B
- **b-phipsi:** 0.0072380998283224
- **w-rdist:** 0.7914070627217723
- **t-alpha:** 0.0140635813350504

---

---

375

- **PDB ID:** 8P4F | **Chain:** O
- **b-phipsi:** 0.0018833180188085
- **w-rdist:** 0.5181914246440251
- **t-alpha:** 0.0753535367540028

---

---

376

- **PDB ID:** 8FCO | **Chain:** B
- **b-phipsi:** 0.0117065884365274
- **w-rdist:** 0.4411881938910359
- **t-alpha:** 0.0182480702564928

---

---

377

- **PDB ID:** 6U23 | **Chain:** K
- **b-phipsi:** 0.0124028992399114
- **w-rdist:** 0.3319589164689585
- **t-alpha:** 0.0285284596970611

---

---

378

- **PDB ID:** 5UKJ | **Chain:** A
- **b-phipsi:** 0.0108622433965741
- **w-rdist:** 0.4978629037859555
- **t-alpha:** 0.013138501865866

---

---

379

- **PDB ID:** 4OVU | **Chain:** A
- **b-phipsi:** 0.0108584460899748
- **w-rdist:** 0.4085350514339786
- **t-alpha:** 0.0269866903238209

---

---

380

- **PDB ID:** 7PVN | **Chain:** B
- **b-phipsi:** 0.0079535550119126
- **w-rdist:** 0.7601460916308994
- **t-alpha:** 0.0110700407352846

---

---

381

- **PDB ID:** 4GS8 | **Chain:** B
- **b-phipsi:** 0.0070249462746606
- **w-rdist:** 0.4624768080045515
- **t-alpha:** 0.0547444112487871

---

---

382

- **PDB ID:** 7YBU | **Chain:** F
- **b-phipsi:** 0.0100026478743636
- **w-rdist:** 0.4625258894507817
- **t-alpha:** 0.0193454927164979

---

---

383

- **PDB ID:** 7OP0 | **Chain:** A
- **b-phipsi:** 0.0018463171502529
- **w-rdist:** 0.6225295527773336
- **t-alpha:** 0.0562044536492789

---

---

384

- **PDB ID:** 6ERF | **Chain:** A
- **b-phipsi:** 0.0023990947739755
- **w-rdist:** 0.5076933618147772
- **t-alpha:** 0.0795901497283129

---

---

385

- **PDB ID:** 5C18 | **Chain:** D
- **b-phipsi:** 0.0110523223868223
- **w-rdist:** 0.1963863048091024
- **t-alpha:** 0.058394017848627

---

---

386

- **PDB ID:** 5T4B | **Chain:** A
- **b-phipsi:** 0.0074459930920846
- **w-rdist:** 0.7995447597732908
- **t-alpha:** 0.0088364585901983

---

---

387

- **PDB ID:** 6TEZ | **Chain:** A
- **b-phipsi:** 0.0044057673884473
- **w-rdist:** 0.7955805939278477
- **t-alpha:** 0.0231516400799582

---

---

388

- **PDB ID:** 7WV3 | **Chain:** A
- **b-phipsi:** 0.0157532761671275
- **w-rdist:** 0.7036108593240763
- **t-alpha:** 0.0

---

---

389

- **PDB ID:** 1J2E | **Chain:** B
- **b-phipsi:** 0.0088872914002166
- **w-rdist:** 0.7887535781741364
- **t-alpha:** 0.0072992307962134

---

---

390

- **PDB ID:** 7LM2 | **Chain:** A
- **b-phipsi:** 0.015284820290481
- **w-rdist:** 0.4012807707933917
- **t-alpha:** 0.0160582219856566

---

---

391

- **PDB ID:** 7U6E | **Chain:** E
- **b-phipsi:** 0.0158833092045217
- **w-rdist:** 0.4364307350350556
- **t-alpha:** 0.0110700407352846

---

---

392

- **PDB ID:** 7OR4 | **Chain:** A
- **b-phipsi:** 0.0142051072540124
- **w-rdist:** 0.595441713736012
- **t-alpha:** 0.0043792969344855

---

---

393

- **PDB ID:** 6XRM | **Chain:** A
- **b-phipsi:** 0.0197733933708163
- **w-rdist:** 0.1537744772901385
- **t-alpha:** 0.0437952703033026

---

---

394

- **PDB ID:** 2JID | **Chain:** B
- **b-phipsi:** 0.0080014965166781
- **w-rdist:** 0.7975606317936424
- **t-alpha:** 0.0066130905937114

---

---

395

- **PDB ID:** 3G0D | **Chain:** C
- **b-phipsi:** 0.0097107133032787
- **w-rdist:** 0.7899330132806448
- **t-alpha:** 0.0051358048547096

---

---

396

- **PDB ID:** 4LTE | **Chain:** A
- **b-phipsi:** 0.0078537887186814
- **w-rdist:** 0.4697423096433048
- **t-alpha:** 0.0321168830372178

---

---

397

- **PDB ID:** 6ICZ | **Chain:** C
- **b-phipsi:** 0.008441534004381
- **w-rdist:** 0.3970016512475053
- **t-alpha:** 0.0482019055190958

---

---

398

- **PDB ID:** 6ICZ | **Chain:** c
- **b-phipsi:** 0.008441534004381
- **w-rdist:** 0.3970016512475053
- **t-alpha:** 0.0482019055190958

---

---

399

- **PDB ID:** 3SWW | **Chain:** B
- **b-phipsi:** 0.0080093604154623
- **w-rdist:** 0.788834516355482
- **t-alpha:** 0.0103244826675092

---

---

400

- **PDB ID:** 5DYH | **Chain:** B
- **b-phipsi:** 0.0077361220387648
- **w-rdist:** 0.4320981844956871
- **t-alpha:** 0.0498085626203308

---

---

401

- **PDB ID:** 3W3G | **Chain:** A
- **b-phipsi:** 0.0140030591302641
- **w-rdist:** 0.3969619498256374
- **t-alpha:** 0.0197076730907748

---

---

402

- **PDB ID:** 2E1Q | **Chain:** C
- **b-phipsi:** 0.0088419301195401
- **w-rdist:** 0.3239983905544468
- **t-alpha:** 0.058394017848627

---

---

403

- **PDB ID:** 4ENZ | **Chain:** A
- **b-phipsi:** 0.0168803633421573
- **w-rdist:** 0.5501809732240217
- **t-alpha:** 0.0036496798094789

---

---

404

- **PDB ID:** 1R9M | **Chain:** C
- **b-phipsi:** 0.0073928033210947
- **w-rdist:** 0.7984457453128053
- **t-alpha:** 0.0103244826675092

---

---

405

- **PDB ID:** 7A3G | **Chain:** A
- **b-phipsi:** 0.0098988394659625
- **w-rdist:** 0.5156474101608043
- **t-alpha:** 0.0145985211566161

---

---

406

- **PDB ID:** 5IXC | **Chain:** A
- **b-phipsi:** 0.0169723115239475
- **w-rdist:** 0.4203418416053109
- **t-alpha:** 0.0118171139231386

---

---

407

- **PDB ID:** 2HHA | **Chain:** B
- **b-phipsi:** 0.0066037092905933
- **w-rdist:** 0.7936507490163187
- **t-alpha:** 0.0193454927164979

---

---

408

- **PDB ID:** 6BYZ | **Chain:** B
- **b-phipsi:** 0.0077109635080688
- **w-rdist:** 0.4585841627697036
- **t-alpha:** 0.0430653698396612

---

---

409

- **PDB ID:** 6EOO | **Chain:** A
- **b-phipsi:** 0.0089869050458404
- **w-rdist:** 0.5397492865846761
- **t-alpha:** 0.0155670635003994

---

---

410

- **PDB ID:** 7K1N | **Chain:** B
- **b-phipsi:** 0.0150722759994815
- **w-rdist:** 0.6460476686436923
- **t-alpha:** 0.002194902533056

---

---

411

- **PDB ID:** 7LMZ | **Chain:** E
- **b-phipsi:** 0.0115939145848702
- **w-rdist:** 0.7567596349573559
- **t-alpha:** 0.0051094444648316

---

---

412

- **PDB ID:** 7AAV | **Chain:** R
- **b-phipsi:** 0.0165371083782546
- **w-rdist:** 0.2499812182982103
- **t-alpha:** 0.0248176303394369

---

---

413

- **PDB ID:** 3CDZ | **Chain:** A
- **b-phipsi:** 0.0438801103211926
- **w-rdist:** 0.3702459179905237
- **t-alpha:** 0.0072992307962134

---

---

414

- **PDB ID:** 6U23 | **Chain:** G
- **b-phipsi:** 0.0162238549491865
- **w-rdist:** 0.3218430217090138
- **t-alpha:** 0.0223883978326699

---

---

415

- **PDB ID:** 5O9Z | **Chain:** b
- **b-phipsi:** 0.0126888739624517
- **w-rdist:** 0.3905710317557844
- **t-alpha:** 0.0254491879579255

---

---

416

- **PDB ID:** 2OPH | **Chain:** B
- **b-phipsi:** 0.0089068530528551
- **w-rdist:** 0.7888617335152227
- **t-alpha:** 0.0073530278154354

---

---

417

- **PDB ID:** 6MQ3 | **Chain:** A
- **b-phipsi:** 0.0067805235689872
- **w-rdist:** 0.4624867414399678
- **t-alpha:** 0.0627735967823235

---

---

418

- **PDB ID:** 7AUA | **Chain:** A
- **b-phipsi:** 0.0074483941569596
- **w-rdist:** 0.4109266789134524
- **t-alpha:** 0.0678101173054155

---

---

419

- **PDB ID:** 4PF7 | **Chain:** B
- **b-phipsi:** 0.0073882589002932
- **w-rdist:** 0.4304315894869007
- **t-alpha:** 0.0627735967823235

---

---

420

- **PDB ID:** 5ISM | **Chain:** B
- **b-phipsi:** 0.0068459748472552
- **w-rdist:** 0.7960632071194143
- **t-alpha:** 0.0155670635003994

---

---

421

- **PDB ID:** 6EOT | **Chain:** D
- **b-phipsi:** 0.0111388315768706
- **w-rdist:** 0.6192759308691869
- **t-alpha:** 0.0080290976816657

---

---

422

- **PDB ID:** 5J6S | **Chain:** A
- **b-phipsi:** 0.0110360092715259
- **w-rdist:** 0.5265271263841084
- **t-alpha:** 0.0124087975246982

---

---

423

- **PDB ID:** 4GSC | **Chain:** A
- **b-phipsi:** 0.0071439588962564
- **w-rdist:** 0.4641218168470016
- **t-alpha:** 0.0562044536492789

---

---

424

- **PDB ID:** 6AS7 | **Chain:** A
- **b-phipsi:** 0.0064291882048948
- **w-rdist:** 0.424078689520943
- **t-alpha:** 0.0992701436974832

---

---

425

- **PDB ID:** 6V9U | **Chain:** B
- **b-phipsi:** 0.0263157174681916
- **w-rdist:** 0.3441755856946575
- **t-alpha:** 0.0102189826802625

---

---

426

- **PDB ID:** 6MDZ | **Chain:** B
- **b-phipsi:** 0.006953523186949
- **w-rdist:** 0.4097618165704417
- **t-alpha:** 0.0905106578984824

---

---

427

- **PDB ID:** 5WOB | **Chain:** f
- **b-phipsi:** 0.0102009224996877
- **w-rdist:** 0.3983813940350487
- **t-alpha:** 0.0364964556675917

---

---

428

- **PDB ID:** 5WEA | **Chain:** A
- **b-phipsi:** 0.0157440622682156
- **w-rdist:** 0.1816294270784352
- **t-alpha:** 0.0430653698396612

---

---

429

- **PDB ID:** 6BF7 | **Chain:** B
- **b-phipsi:** 0.0143080704814496
- **w-rdist:** 0.3963623913831006
- **t-alpha:** 0.0204377723355742

---

---

430

- **PDB ID:** 5WOB | **Chain:** a
- **b-phipsi:** 0.0073170150597249
- **w-rdist:** 0.4631362774614954
- **t-alpha:** 0.0518248614636667

---

---

431

- **PDB ID:** 5DXU | **Chain:** A
- **b-phipsi:** 0.0136058842722302
- **w-rdist:** 0.4392472441466591
- **t-alpha:** 0.0160582219856566

---

---

432

- **PDB ID:** 2QOE | **Chain:** B
- **b-phipsi:** 0.00663381591692
- **w-rdist:** 0.7949632858315213
- **t-alpha:** 0.0185875182467154

---

---

433

- **PDB ID:** 7AYQ | **Chain:** A
- **b-phipsi:** 0.0165219760266904
- **w-rdist:** 0.5583014228919423
- **t-alpha:** 0.0036630800678474

---

---

434

- **PDB ID:** 4E36 | **Chain:** A
- **b-phipsi:** 0.0092539618964356
- **w-rdist:** 0.4299069143876856
- **t-alpha:** 0.0347433877716041

---

---

435

- **PDB ID:** 3HGZ | **Chain:** A
- **b-phipsi:** 0.0072548159404083
- **w-rdist:** 0.4680822977883175
- **t-alpha:** 0.0518248614636667

---

---

436

- **PDB ID:** 3EH1 | **Chain:** A
- **b-phipsi:** 0.0018124169365006
- **w-rdist:** 0.7554429741570876
- **t-alpha:** 0.0522276825687215

---

---

437

- **PDB ID:** 6EDS | **Chain:** A
- **b-phipsi:** 0.007743299116726
- **w-rdist:** 0.4653519275634008
- **t-alpha:** 0.0416056097301684

---

---

438

- **PDB ID:** 3OFI | **Chain:** A
- **b-phipsi:** 0.0067979017157313
- **w-rdist:** 0.4756271842353148
- **t-alpha:** 0.0598540584701086

---

---

439

- **PDB ID:** 5WOB | **Chain:** A
- **b-phipsi:** 0.0072610247626244
- **w-rdist:** 0.4631456780915927
- **t-alpha:** 0.0562044536492789

---

---

440

- **PDB ID:** 2BUB | **Chain:** A
- **b-phipsi:** 0.008738570756881
- **w-rdist:** 0.7970035543614093
- **t-alpha:** 0.0058736968826307

---

---

441

- **PDB ID:** 6B1O | **Chain:** B
- **b-phipsi:** 0.0072497218682398
- **w-rdist:** 0.7915838842922303
- **t-alpha:** 0.0178305927380209

---

---

442

- **PDB ID:** 2BUB | **Chain:** B
- **b-phipsi:** 0.0087417336485372
- **w-rdist:** 0.7919964169934226
- **t-alpha:** 0.0080290976816657

---

---

443

- **PDB ID:** 8FCN | **Chain:** B
- **b-phipsi:** 0.0098140698854918
- **w-rdist:** 0.1598117919635663
- **t-alpha:** 0.1240879206199325

---

---

444

- **PDB ID:** 7MDO | **Chain:** B
- **b-phipsi:** 0.0268333973398691
- **w-rdist:** 0.5116053339204749
- **t-alpha:** 0.0

---

---

445

- **PDB ID:** 3KRW | **Chain:** A
- **b-phipsi:** 0.0150581935059202
- **w-rdist:** 0.3307316322006382
- **t-alpha:** 0.0262171618622435

---

---

446

- **PDB ID:** 2G5P | **Chain:** B
- **b-phipsi:** 0.0075555962932493
- **w-rdist:** 0.7974486351719055
- **t-alpha:** 0.01094855486171

---

---

447

- **PDB ID:** 4ANV | **Chain:** A
- **b-phipsi:** 0.02032086220516
- **w-rdist:** 0.1678087370221602
- **t-alpha:** 0.0394160818999413

---

---

448

- **PDB ID:** 4JH0 | **Chain:** B
- **b-phipsi:** 0.0074334179501701
- **w-rdist:** 0.7862756560352295
- **t-alpha:** 0.0185875182467154

---

---

449

- **PDB ID:** 3G0B | **Chain:** C
- **b-phipsi:** 0.00693842236966
- **w-rdist:** 0.8053663357783257
- **t-alpha:** 0.0133138831004895

---

---

450

- **PDB ID:** 6KYV | **Chain:** L
- **b-phipsi:** 0.0132061260490816
- **w-rdist:** 0.6575467963292977
- **t-alpha:** 0.0051358048547096

---

---

451

- **PDB ID:** 3ML9 | **Chain:** A
- **b-phipsi:** 0.025325860813599
- **w-rdist:** 0.1723237320755838
- **t-alpha:** 0.0255474760956377

---

---

452

- **PDB ID:** 5T8F | **Chain:** A
- **b-phipsi:** 0.0156772403441769
- **w-rdist:** 0.4505286099676088
- **t-alpha:** 0.0124087975246982

---

---

453

- **PDB ID:** 4FXK | **Chain:** B
- **b-phipsi:** 0.0079351889349951
- **w-rdist:** 0.3413407249139158
- **t-alpha:** 0.0751822857042021

---

---

454

- **PDB ID:** 7MLK | **Chain:** A
- **b-phipsi:** 0.0189659135579712
- **w-rdist:** 0.1188448236224258
- **t-alpha:** 0.0620435921263067

---

---

455

- **PDB ID:** 5ISM | **Chain:** A
- **b-phipsi:** 0.0070345494469474
- **w-rdist:** 0.7905139122674913
- **t-alpha:** 0.0208645945567758

---

---

456

- **PDB ID:** 4JBS | **Chain:** A
- **b-phipsi:** 0.0104564124686836
- **w-rdist:** 0.4083400634947715
- **t-alpha:** 0.0370931750792566

---

---

457

- **PDB ID:** 2YPU | **Chain:** A
- **b-phipsi:** 0.007910842566144
- **w-rdist:** 0.4670941082796775
- **t-alpha:** 0.040145908618359

---

---

458

- **PDB ID:** 7BW8 | **Chain:** A
- **b-phipsi:** 0.0258592031136492
- **w-rdist:** 0.4698068891917514
- **t-alpha:** 0.0036496798094789

---

---

459

- **PDB ID:** 6OCO | **Chain:** A
- **b-phipsi:** 0.0180939579912431
- **w-rdist:** 0.4433394777590759
- **t-alpha:** 0.01094855486171

---

---

460

- **PDB ID:** 3C45 | **Chain:** A
- **b-phipsi:** 0.0069203891144849
- **w-rdist:** 0.7949087663162363
- **t-alpha:** 0.0182480702564928

---

---

461

- **PDB ID:** 6TE3 | **Chain:** A
- **b-phipsi:** 0.0068553820283641
- **w-rdist:** 0.7967189944966225
- **t-alpha:** 0.0178305927380209

---

---

462

- **PDB ID:** 7K1D | **Chain:** A
- **b-phipsi:** 0.006732519119839
- **w-rdist:** 0.4672168102905145
- **t-alpha:** 0.067152979131726

---

---

463

- **PDB ID:** 3QZ2 | **Chain:** B
- **b-phipsi:** 0.0082010997451082
- **w-rdist:** 0.4502180214182999
- **t-alpha:** 0.0430653698396612

---

---

464

- **PDB ID:** 8FCO | **Chain:** F
- **b-phipsi:** 0.0134881274488107
- **w-rdist:** 0.1490070733722817
- **t-alpha:** 0.0854012245476465

---

---

465

- **PDB ID:** 3HAB | **Chain:** B
- **b-phipsi:** 0.0069712956317766
- **w-rdist:** 0.7927276333785851
- **t-alpha:** 0.020104517651075

---

---

466

- **PDB ID:** 7JIS | **Chain:** A
- **b-phipsi:** 0.0140218674660114
- **w-rdist:** 0.4156049496387997
- **t-alpha:** 0.0204377723355742

---

---

467

- **PDB ID:** 2P8S | **Chain:** B
- **b-phipsi:** 0.0065013457066295
- **w-rdist:** 0.7969577661951666
- **t-alpha:** 0.020104517651075

---

---

468

- **PDB ID:** 6MFR | **Chain:** B
- **b-phipsi:** 0.0077585029861974
- **w-rdist:** 0.4218233424778504
- **t-alpha:** 0.0620435921263067

---

---

469

- **PDB ID:** 7WUB | **Chain:** E
- **b-phipsi:** 0.0248436985237474
- **w-rdist:** 0.4949552641512723
- **t-alpha:** 0.0029282444105804

---

---

470

- **PDB ID:** 3KRX | **Chain:** A
- **b-phipsi:** 0.0136887458052647
- **w-rdist:** 0.3224672960653355
- **t-alpha:** 0.0355252350918542

---

---

471

- **PDB ID:** 5HE1 | **Chain:** A
- **b-phipsi:** 0.0143765238321313
- **w-rdist:** 0.3117438488332669
- **t-alpha:** 0.0331824489470444

---

---

472

- **PDB ID:** 7AD6 | **Chain:** B
- **b-phipsi:** 0.0353583310389307
- **w-rdist:** 0.2479804785821529
- **t-alpha:** 0.0138687321491499

---

---

473

- **PDB ID:** 5VM9 | **Chain:** C
- **b-phipsi:** 0.0134322548393383
- **w-rdist:** 0.1529850433810552
- **t-alpha:** 0.0838610257695162

---

---

474

- **PDB ID:** 3O9V | **Chain:** C
- **b-phipsi:** 0.008308133062997
- **w-rdist:** 0.7903657951094336
- **t-alpha:** 0.0110700407352846

---

---

475

- **PDB ID:** 7MYO | **Chain:** A
- **b-phipsi:** 0.0108796985438074
- **w-rdist:** 0.2716557671983944
- **t-alpha:** 0.058394017848627

---

---

476

- **PDB ID:** 3HAB | **Chain:** A
- **b-phipsi:** 0.0070070169338978
- **w-rdist:** 0.7978343732258789
- **t-alpha:** 0.0163203672213534

---

---

477

- **PDB ID:** 5F98 | **Chain:** G
- **b-phipsi:** 0.0139212518842051
- **w-rdist:** 0.56511903574855
- **t-alpha:** 0.0073530278154354

---

---

478

- **PDB ID:** 8G1U | **Chain:** M
- **b-phipsi:** 0.0008055481969474
- **w-rdist:** 0.5598649290873433
- **t-alpha:** 0.1066236145665033

---

---

479

- **PDB ID:** 3CST | **Chain:** A
- **b-phipsi:** 0.017911540449017
- **w-rdist:** 0.1740643038863646
- **t-alpha:** 0.0452556070301213

---

---

480

- **PDB ID:** 5C19 | **Chain:** C
- **b-phipsi:** 0.010975660188746
- **w-rdist:** 0.2663349520538799
- **t-alpha:** 0.0591240390843355

---

---

481

- **PDB ID:** 8FCL | **Chain:** F
- **b-phipsi:** 0.0117670396554144
- **w-rdist:** 0.1495750824169021
- **t-alpha:** 0.129196921332773

---

---

482

- **PDB ID:** 3C45 | **Chain:** B
- **b-phipsi:** 0.007895072757781
- **w-rdist:** 0.7922606632024429
- **t-alpha:** 0.0125647391185939

---

---

483

- **PDB ID:** 7A3I | **Chain:** B
- **b-phipsi:** 0.0153685570219426
- **w-rdist:** 0.5061817467652142
- **t-alpha:** 0.0094889742856814

---

---

484

- **PDB ID:** 3N57 | **Chain:** B
- **b-phipsi:** 0.0085980963817372
- **w-rdist:** 0.4527274711899467
- **t-alpha:** 0.040145908618359

---

---

485

- **PDB ID:** 3BJM | **Chain:** A
- **b-phipsi:** 0.0083372738577878
- **w-rdist:** 0.7910117940254957
- **t-alpha:** 0.0110700407352846

---

---

486

- **PDB ID:** 5DXT | **Chain:** A
- **b-phipsi:** 0.0174527287942772
- **w-rdist:** 0.1168780407329723
- **t-alpha:** 0.0715325243705191

---

---

487

- **PDB ID:** 5FTJ | **Chain:** A
- **b-phipsi:** 0.0199360053238868
- **w-rdist:** 0.1574479700162955
- **t-alpha:** 0.0518248614636667

---

---

488

- **PDB ID:** 3BDL | **Chain:** A
- **b-phipsi:** 0.0019024777395061
- **w-rdist:** 0.4976707155600286
- **t-alpha:** 0.135987099964115

---

---

489

- **PDB ID:** 2G47 | **Chain:** A
- **b-phipsi:** 0.0076879611780446
- **w-rdist:** 0.4696397755172574
- **t-alpha:** 0.0481749154992192

---

---

490

- **PDB ID:** 6BYZ | **Chain:** A
- **b-phipsi:** 0.0073973928085647
- **w-rdist:** 0.4643527961725683
- **t-alpha:** 0.0591240390843355

---

---

491

- **PDB ID:** 6QZW | **Chain:** B
- **b-phipsi:** 0.0089751574496526
- **w-rdist:** 0.6050844604537455
- **t-alpha:** 0.0160582219856566

---

---

492

- **PDB ID:** 5WOB | **Chain:** g
- **b-phipsi:** 0.0094189102904639
- **w-rdist:** 0.4110885228599991
- **t-alpha:** 0.0452556070301213

---

---

493

- **PDB ID:** 5AWC | **Chain:** C
- **b-phipsi:** 0.0211015568415005
- **w-rdist:** 0.3088631009333121
- **t-alpha:** 0.0218975903112057

---

---

494

- **PDB ID:** 6BKF | **Chain:** A
- **b-phipsi:** 0.0132269489936517
- **w-rdist:** 0.4839538319668121
- **t-alpha:** 0.0133138831004895

---

---

495

- **PDB ID:** 4R08 | **Chain:** C
- **b-phipsi:** 0.0145656998036028
- **w-rdist:** 0.3744025416987315
- **t-alpha:** 0.0270072581572047

---

---

496

- **PDB ID:** 8AYH | **Chain:** C
- **b-phipsi:** 0.0351909407404735
- **w-rdist:** 0.3440645075870182
- **t-alpha:** 0.01094855486171

---

---

497

- **PDB ID:** 8SW0 | **Chain:** A
- **b-phipsi:** 0.01013202894043
- **w-rdist:** 0.2945708398200898
- **t-alpha:** 0.0627735967823235

---

---

498

- **PDB ID:** 6ERH | **Chain:** C
- **b-phipsi:** 0.0082674404822994
- **w-rdist:** 0.5434734595329715
- **t-alpha:** 0.0262171618622435

---

---

499

- **PDB ID:** 2YKG | **Chain:** A
- **b-phipsi:** 0.0155135623918599
- **w-rdist:** 0.3167637030998184
- **t-alpha:** 0.0328465096023253

---

---

500

- **PDB ID:** 5Y3R | **Chain:** A
- **b-phipsi:** 0.0158153470130757
- **w-rdist:** 0.3963403573920715
- **t-alpha:** 0.0223883978326699

---

---

501

- **PDB ID:** 2QTB | **Chain:** B
- **b-phipsi:** 0.0077453469586827
- **w-rdist:** 0.7926552090267088
- **t-alpha:** 0.0140635813350504

---

---

502

- **PDB ID:** 6H0G | **Chain:** D
- **b-phipsi:** 0.0235272458720541
- **w-rdist:** 0.5078632667946059
- **t-alpha:** 0.0043989330665894

---

---

503

- **PDB ID:** 7WUB | **Chain:** J
- **b-phipsi:** 0.0121276591971009
- **w-rdist:** 0.274403176044784
- **t-alpha:** 0.0518248614636667

---

---

504

- **PDB ID:** 8EZB | **Chain:** K
- **b-phipsi:** 0.0088842209839206
- **w-rdist:** 0.3610602015894438
- **t-alpha:** 0.0627735967823235

---

---

505

- **PDB ID:** 8FCO | **Chain:** C
- **b-phipsi:** 0.0132060401865873
- **w-rdist:** 0.2153139758971039
- **t-alpha:** 0.0562044536492789

---

---

506

- **PDB ID:** 8B5R | **Chain:** B
- **b-phipsi:** 0.0123944065685888
- **w-rdist:** 0.8567076316726178
- **t-alpha:** 0.0

---

---

507

- **PDB ID:** 7LN1 | **Chain:** E
- **b-phipsi:** 0.0082116843216078
- **w-rdist:** 0.7600061018720131
- **t-alpha:** 0.0153283513243391

---

---

508

- **PDB ID:** 7MXA | **Chain:** A
- **b-phipsi:** 0.0006728647894181
- **w-rdist:** 0.5469554821887839
- **t-alpha:** 0.1388198251143049

---

---

509

- **PDB ID:** 3N57 | **Chain:** A
- **b-phipsi:** 0.0076408133945495
- **w-rdist:** 0.4729970328121665
- **t-alpha:** 0.0489048903395892

---

---

510

- **PDB ID:** 8CYI | **Chain:** A
- **b-phipsi:** 0.000999038681939
- **w-rdist:** 0.54228272212823
- **t-alpha:** 0.1369294736439956

---

---

511

- **PDB ID:** 3G0D | **Chain:** D
- **b-phipsi:** 0.007980633805128
- **w-rdist:** 0.7981720145865516
- **t-alpha:** 0.0102189826802625

---

---

512

- **PDB ID:** 4ZG6 | **Chain:** A
- **b-phipsi:** 0.0102623217708496
- **w-rdist:** 0.4705656701233642
- **t-alpha:** 0.0239164285917981

---

---

513

- **PDB ID:** 6U7G | **Chain:** A
- **b-phipsi:** 0.012110994517728
- **w-rdist:** 0.6148156192375813
- **t-alpha:** 0.009579955775703

---

---

514

- **PDB ID:** 4ASI | **Chain:** C
- **b-phipsi:** 0.0105070983837728
- **w-rdist:** 0.7214020098381139
- **t-alpha:** 0.0102189826802625

---

---

515

- **PDB ID:** 4EZJ | **Chain:** A
- **b-phipsi:** 0.0247927662628121
- **w-rdist:** 0.1678717601790153
- **t-alpha:** 0.035036511315305

---

---

516

- **PDB ID:** 8G1U | **Chain:** I
- **b-phipsi:** 0.0013797563232897
- **w-rdist:** 0.5497351063338713
- **t-alpha:** 0.1174551848221758

---

---

517

- **PDB ID:** 7WVE | **Chain:** A
- **b-phipsi:** 0.011958868290023
- **w-rdist:** 0.6862518680086336
- **t-alpha:** 0.0080944703406706

---

---

518

- **PDB ID:** 4EZL | **Chain:** A
- **b-phipsi:** 0.0194912578758581
- **w-rdist:** 0.1601204990016556
- **t-alpha:** 0.0547444112487871

---

---

519

- **PDB ID:** 8FCQ | **Chain:** D
- **b-phipsi:** 0.0111299231431714
- **w-rdist:** 0.2165739554369694
- **t-alpha:** 0.0708026304911424

---

---

520

- **PDB ID:** 4DB1 | **Chain:** B
- **b-phipsi:** 0.0205435228568811
- **w-rdist:** 0.4683280485672662
- **t-alpha:** 0.0080944703406706

---

---

521

- **PDB ID:** 6JT1 | **Chain:** B
- **b-phipsi:** 0.0159788967883086
- **w-rdist:** 0.4128536809477245
- **t-alpha:** 0.0204377723355742

---

---

522

- **PDB ID:** 2A5U | **Chain:** A
- **b-phipsi:** 0.0233405214591627
- **w-rdist:** 0.1552714107427335
- **t-alpha:** 0.0489048903395892

---

---

523

- **PDB ID:** 7VPQ | **Chain:** A
- **b-phipsi:** 0.0131296239412034
- **w-rdist:** 0.6144942309318152
- **t-alpha:** 0.0087590123987277

---

---

524

- **PDB ID:** 5WOB | **Chain:** F
- **b-phipsi:** 0.0092044744696043
- **w-rdist:** 0.3989316460155298
- **t-alpha:** 0.0540143931905516

---

---

525

- **PDB ID:** 4KR0 | **Chain:** A
- **b-phipsi:** 0.0074492747594182
- **w-rdist:** 0.7943533192702725
- **t-alpha:** 0.0175180821197378

---

---

526

- **PDB ID:** 5WOB | **Chain:** G
- **b-phipsi:** 0.0095015452294259
- **w-rdist:** 0.4111608157561887
- **t-alpha:** 0.0481749154992192

---

---

527

- **PDB ID:** 7MDM | **Chain:** C
- **b-phipsi:** 0.0278643655312869
- **w-rdist:** 0.4141488816840221
- **t-alpha:** 0.0088364585901983

---

---

528

- **PDB ID:** 4N8E | **Chain:** B
- **b-phipsi:** 0.0077708871402165
- **w-rdist:** 0.7855503747006181
- **t-alpha:** 0.0185875182467154

---

---

529

- **PDB ID:** 4OVV | **Chain:** A
- **b-phipsi:** 0.0089850164176165
- **w-rdist:** 0.3909361443250626
- **t-alpha:** 0.0587327133805999

---

---

530

- **PDB ID:** 7MEZ | **Chain:** A
- **b-phipsi:** 0.0121648455872632
- **w-rdist:** 0.4425403896579383
- **t-alpha:** 0.0248176303394369

---

---

531

- **PDB ID:** 8EFD | **Chain:** A
- **b-phipsi:** 0.0163187166857581
- **w-rdist:** 0.4190621630650369
- **t-alpha:** 0.0193454927164979

---

---

532

- **PDB ID:** 6ID0 | **Chain:** c
- **b-phipsi:** 0.0081776399531928
- **w-rdist:** 0.4514665013752574
- **t-alpha:** 0.0490049264750351

---

---

533

- **PDB ID:** 7AU2 | **Chain:** B
- **b-phipsi:** 0.0076533328044851
- **w-rdist:** 0.4050137538495875
- **t-alpha:** 0.0838610257695162

---

---

534

- **PDB ID:** 3SE6 | **Chain:** A
- **b-phipsi:** 0.0113660057764745
- **w-rdist:** 0.4535606424199272
- **t-alpha:** 0.0262171618622435

---

---

535

- **PDB ID:** 6DDO | **Chain:** A
- **b-phipsi:** 0.0144309968125758
- **w-rdist:** 0.6906287268898025
- **t-alpha:** 0.0051358048547096

---

---

536

- **PDB ID:** 4DWK | **Chain:** A
- **b-phipsi:** 0.0081486186398061
- **w-rdist:** 0.4673337933355136
- **t-alpha:** 0.0437952703033026

---

---

537

- **PDB ID:** 4PES | **Chain:** B
- **b-phipsi:** 0.008886197188369
- **w-rdist:** 0.4335385974596006
- **t-alpha:** 0.0481749154992192

---

---

538

- **PDB ID:** 3HHM | **Chain:** A
- **b-phipsi:** 0.0125008107750795
- **w-rdist:** 0.4247600267444945
- **t-alpha:** 0.0277572859037011

---

---

539

- **PDB ID:** 2IIV | **Chain:** B
- **b-phipsi:** 0.0081041306373941
- **w-rdist:** 0.7955846549528702
- **t-alpha:** 0.0110700407352846

---

---

540

- **PDB ID:** 8SW1 | **Chain:** A
- **b-phipsi:** 0.0104711954582026
- **w-rdist:** 0.2923425914169921
- **t-alpha:** 0.064963575889908

---

---

541

- **PDB ID:** 3H44 | **Chain:** B
- **b-phipsi:** 0.0089120312494361
- **w-rdist:** 0.4564974687163822
- **t-alpha:** 0.0394160818999413

---

---

542

- **PDB ID:** 5VLR | **Chain:** A
- **b-phipsi:** 0.0170353864757001
- **w-rdist:** 0.4393256014230728
- **t-alpha:** 0.0153283513243391

---

---

543

- **PDB ID:** 5FUR | **Chain:** I
- **b-phipsi:** 0.0166096977452465
- **w-rdist:** 0.1900886571970773
- **t-alpha:** 0.0482019055190958

---

---

544

- **PDB ID:** 3PRZ | **Chain:** A
- **b-phipsi:** 0.0202607440537331
- **w-rdist:** 0.154412945427343
- **t-alpha:** 0.0591240390843355

---

---

545

- **PDB ID:** 1RWQ | **Chain:** B
- **b-phipsi:** 0.0088650583861281
- **w-rdist:** 0.7971354608497445
- **t-alpha:** 0.0080290976816657

---

---

546

- **PDB ID:** 6EDS | **Chain:** B
- **b-phipsi:** 0.0075750626659288
- **w-rdist:** 0.4575742011702427
- **t-alpha:** 0.0627735967823235

---

---

547

- **PDB ID:** 4L72 | **Chain:** A
- **b-phipsi:** 0.0087700470555809
- **w-rdist:** 0.792785980579676
- **t-alpha:** 0.0103244826675092

---

---

548

- **PDB ID:** 7MXC | **Chain:** A
- **b-phipsi:** 0.0012427980889123
- **w-rdist:** 0.5521550397972377
- **t-alpha:** 0.1294311683533731

---

---

549

- **PDB ID:** 4FA6 | **Chain:** A
- **b-phipsi:** 0.0217253674508713
- **w-rdist:** 0.1563148783761914
- **t-alpha:** 0.0554744316333071

---

---

550

- **PDB ID:** 8EOK | **Chain:** G
- **b-phipsi:** 0.0306689697757455
- **w-rdist:** 0.329637817387002
- **t-alpha:** 0.0138687321491499

---

---

551

- **PDB ID:** 3R7R | **Chain:** A
- **b-phipsi:** 0.029135912772396
- **w-rdist:** 0.1575857563790819
- **t-alpha:** 0.0364964556675917

---

---

552

- **PDB ID:** 7AYR | **Chain:** B
- **b-phipsi:** 0.0121692677604878
- **w-rdist:** 0.4506319821604696
- **t-alpha:** 0.0240873273423209

---

---

553

- **PDB ID:** 7PG6 | **Chain:** A
- **b-phipsi:** 0.0091670284479508
- **w-rdist:** 0.5376715716692826
- **t-alpha:** 0.0226277946303061

---

---

554

- **PDB ID:** 2OQI | **Chain:** B
- **b-phipsi:** 0.0075212514805681
- **w-rdist:** 0.8001118257255087
- **t-alpha:** 0.0138687321491499

---

---

555

- **PDB ID:** 7KKE | **Chain:** A
- **b-phipsi:** 0.0158574979785047
- **w-rdist:** 0.1694793071189629
- **t-alpha:** 0.0627735967823235

---

---

556

- **PDB ID:** 6U23 | **Chain:** C
- **b-phipsi:** 0.01648787522543
- **w-rdist:** 0.3267059144285534
- **t-alpha:** 0.0308503701641407

---

---

557

- **PDB ID:** 6UXY | **Chain:** A
- **b-phipsi:** 0.0008230785732921
- **w-rdist:** 0.547555386319287
- **t-alpha:** 0.1759658673306416

---

---

558

- **PDB ID:** 6WKR | **Chain:** C
- **b-phipsi:** 0.0100121498196966
- **w-rdist:** 0.8470799362390834
- **t-alpha:** 0.0051094444648316

---

---

559

- **PDB ID:** 3G0B | **Chain:** D
- **b-phipsi:** 0.0081046863054963
- **w-rdist:** 0.8039539216285472
- **t-alpha:** 0.009579955775703

---

---

560

- **PDB ID:** 7MXN | **Chain:** A
- **b-phipsi:** 0.0006366231506349
- **w-rdist:** 0.5532664124396014
- **t-alpha:** 0.156118378599569

---

---

561

- **PDB ID:** 3O95 | **Chain:** C
- **b-phipsi:** 0.0079316631275912
- **w-rdist:** 0.7983699619303939
- **t-alpha:** 0.0116786539344329

---

---

562

- **PDB ID:** 5MHL | **Chain:** A
- **b-phipsi:** 0.0167316945333825
- **w-rdist:** 0.3139183163177882
- **t-alpha:** 0.0331824489470444

---

---

563

- **PDB ID:** 6UXX | **Chain:** A
- **b-phipsi:** 0.0009013525879763
- **w-rdist:** 0.5463876915498543
- **t-alpha:** 0.1975523096759199

---

---

564

- **PDB ID:** 7B2M | **Chain:** A
- **b-phipsi:** 0.0292809005531357
- **w-rdist:** 0.4036038081152346
- **t-alpha:** 0.0103244826675092

---

---

565

- **PDB ID:** 4L3T | **Chain:** B
- **b-phipsi:** 0.0122086609960235
- **w-rdist:** 0.5030982119863645
- **t-alpha:** 0.0178305927380209

---

---

566

- **PDB ID:** 7RLH | **Chain:** E
- **b-phipsi:** 0.0133125601993143
- **w-rdist:** 0.1692094521537786
- **t-alpha:** 0.0810216958060698

---

---

567

- **PDB ID:** 5F9H | **Chain:** A
- **b-phipsi:** 0.0120440113996968
- **w-rdist:** 0.5830778010967596
- **t-alpha:** 0.0125647391185939

---

---

568

- **PDB ID:** 6RLL | **Chain:** A
- **b-phipsi:** 0.0009798051212449
- **w-rdist:** 0.5524847763823072
- **t-alpha:** 0.1502939397071412

---

---

569

- **PDB ID:** 2OGZ | **Chain:** B
- **b-phipsi:** 0.0076717288413794
- **w-rdist:** 0.7945339384847504
- **t-alpha:** 0.0160582219856566

---

---

570

- **PDB ID:** 3TL5 | **Chain:** A
- **b-phipsi:** 0.0254394219423434
- **w-rdist:** 0.1588302318128849
- **t-alpha:** 0.0445251834570807

---

---

571

- **PDB ID:** 4GSC | **Chain:** B
- **b-phipsi:** 0.0077727194648767
- **w-rdist:** 0.4548331642102415
- **t-alpha:** 0.0613135828266362

---

---

572

- **PDB ID:** 3L4G | **Chain:** B
- **b-phipsi:** 0.0016121598636239
- **w-rdist:** 0.7252848378913258
- **t-alpha:** 0.0745098508994681

---

---

573

- **PDB ID:** 7S1P | **Chain:** A
- **b-phipsi:** 0.0011553926831107
- **w-rdist:** 0.5525084733211953
- **t-alpha:** 0.1464438734176729

---

---

574

- **PDB ID:** 1N1M | **Chain:** A
- **b-phipsi:** 0.0090202729137094
- **w-rdist:** 0.7963066348528336
- **t-alpha:** 0.0080944703406706

---

---

575

- **PDB ID:** 3SWR | **Chain:** A
- **b-phipsi:** 0.0103639636377668
- **w-rdist:** 0.5346286068024325
- **t-alpha:** 0.0211679073923163

---

---

576

- **PDB ID:** 7M05 | **Chain:** C
- **b-phipsi:** 0.0012861927241178
- **w-rdist:** 0.5426948582073138
- **t-alpha:** 0.1800174790223048

---

---

577

- **PDB ID:** 1U8E | **Chain:** A
- **b-phipsi:** 0.009133786481262
- **w-rdist:** 0.8048101377530251
- **t-alpha:** 0.0066130905937114

---

---

578

- **PDB ID:** 5FA5 | **Chain:** A
- **b-phipsi:** 0.0015859045501983
- **w-rdist:** 0.5515288250085293
- **t-alpha:** 0.130363252467047

---

---

579

- **PDB ID:** 6CKC | **Chain:** A
- **b-phipsi:** 0.0008264014893608
- **w-rdist:** 0.5590947302307704
- **t-alpha:** 0.1483653765329244

---

---

580

- **PDB ID:** 6U7F | **Chain:** A
- **b-phipsi:** 0.0113113567096717
- **w-rdist:** 0.6009279171251916
- **t-alpha:** 0.0140635813350504

---

---

581

- **PDB ID:** 5EMJ | **Chain:** A
- **b-phipsi:** 0.0013072662697717
- **w-rdist:** 0.547607540768073
- **t-alpha:** 0.1522290382566056

---

---

582

- **PDB ID:** 7MXG | **Chain:** C
- **b-phipsi:** 0.0008112705287321
- **w-rdist:** 0.5524307131938175
- **t-alpha:** 0.1769762014421934

---

---

583

- **PDB ID:** 5AZ5 | **Chain:** C
- **b-phipsi:** 0.0219191853088964
- **w-rdist:** 0.4241683558664089
- **t-alpha:** 0.0145985211566161

---

---

584

- **PDB ID:** 7TZ7 | **Chain:** A
- **b-phipsi:** 0.0113123261796865
- **w-rdist:** 0.523689181396632
- **t-alpha:** 0.0197076730907748

---

---

585

- **PDB ID:** 5MHL | **Chain:** B
- **b-phipsi:** 0.0113342734714776
- **w-rdist:** 0.2937831785848879
- **t-alpha:** 0.0644914133791398

---

---

586

- **PDB ID:** 8FCR | **Chain:** A
- **b-phipsi:** 0.0132107342206777
- **w-rdist:** 0.2254296768229527
- **t-alpha:** 0.0613135828266362

---

---

587

- **PDB ID:** 7R53 | **Chain:** A
- **b-phipsi:** 0.0241080728763376
- **w-rdist:** 0.3389684262427082
- **t-alpha:** 0.0197076730907748

---

---

588

- **PDB ID:** 2C11 | **Chain:** B
- **b-phipsi:** 0.0108707339985248
- **w-rdist:** 0.7418154928874554
- **t-alpha:** 0.0110700407352846

---

---

589

- **PDB ID:** 7M05 | **Chain:** A
- **b-phipsi:** 0.0013164550698963
- **w-rdist:** 0.543633547539615
- **t-alpha:** 0.2038662972579192

---

---

590

- **PDB ID:** 8FCM | **Chain:** A
- **b-phipsi:** 0.0117868219094933
- **w-rdist:** 0.2663544257485036
- **t-alpha:** 0.0656935549704063

---

---

591

- **PDB ID:** 1W1I | **Chain:** A
- **b-phipsi:** 0.0087096815048556
- **w-rdist:** 0.7916491121476754
- **t-alpha:** 0.013138501865866

---

---

592

- **PDB ID:** 7NUP | **Chain:** A
- **b-phipsi:** 0.0126698988718309
- **w-rdist:** 0.4983172818269739
- **t-alpha:** 0.0185875182467154

---

---

593

- **PDB ID:** 7PFS | **Chain:** A
- **b-phipsi:** 0.0120952119059709
- **w-rdist:** 0.4814625174123652
- **t-alpha:** 0.0216257249425573

---

---

594

- **PDB ID:** 3LPP | **Chain:** B
- **b-phipsi:** 0.009295317651846
- **w-rdist:** 0.646490633705876
- **t-alpha:** 0.0170751996684577

---

---

595

- **PDB ID:** 7RC9 | **Chain:** B
- **b-phipsi:** 0.0196028252729479
- **w-rdist:** 0.4026275957462859
- **t-alpha:** 0.0204377723355742

---

---

596

- **PDB ID:** 6K1S | **Chain:** A
- **b-phipsi:** 0.0013962301311121
- **w-rdist:** 0.5522007653494596
- **t-alpha:** 0.1590527016415781

---

---

597

- **PDB ID:** 7MXG | **Chain:** A
- **b-phipsi:** 0.0012202596305794
- **w-rdist:** 0.5627174162276136
- **t-alpha:** 0.1512608079233615

---

---

598

- **PDB ID:** 3L17 | **Chain:** A
- **b-phipsi:** 0.0252003809260166
- **w-rdist:** 0.1501848067975043
- **t-alpha:** 0.0554744316333071

---

---

599

- **PDB ID:** 4DB1 | **Chain:** A
- **b-phipsi:** 0.0165630111795896
- **w-rdist:** 0.4621982029927995
- **t-alpha:** 0.0155670635003994

---

---

600

- **PDB ID:** 4GQB | **Chain:** A
- **b-phipsi:** 0.0013011280206714
- **w-rdist:** 0.5576473344346073
- **t-alpha:** 0.1689422960399142

---

---

601

- **PDB ID:** 1W1I | **Chain:** C
- **b-phipsi:** 0.0088468140997404
- **w-rdist:** 0.7986546304303603
- **t-alpha:** 0.0094889742856814

---

---

602

- **PDB ID:** 8SZP | **Chain:** A
- **b-phipsi:** 0.0132093394886723
- **w-rdist:** 0.492840515028992
- **t-alpha:** 0.0185875182467154

---

---

603

- **PDB ID:** 1R9M | **Chain:** D
- **b-phipsi:** 0.0084352080768605
- **w-rdist:** 0.7971084103118974
- **t-alpha:** 0.0116786539344329

---

---

604

- **PDB ID:** 3DPD | **Chain:** A
- **b-phipsi:** 0.0246978422128303
- **w-rdist:** 0.1893479919725733
- **t-alpha:** 0.0357662262619218

---

---

605

- **PDB ID:** 7Z61 | **Chain:** A
- **b-phipsi:** 0.0245087570484088
- **w-rdist:** 0.1922968050913528
- **t-alpha:** 0.0357662262619218

---

---

606

- **PDB ID:** 4GB9 | **Chain:** A
- **b-phipsi:** 0.0301252111706338
- **w-rdist:** 0.1730916091660685
- **t-alpha:** 0.035036511315305

---

---

607

- **PDB ID:** 4HVB | **Chain:** A
- **b-phipsi:** 0.0278376435855245
- **w-rdist:** 0.1537541225385528
- **t-alpha:** 0.0503648629317097

---

---

608

- **PDB ID:** 7MDM | **Chain:** A
- **b-phipsi:** 0.0116606140407621
- **w-rdist:** 0.292135589244916
- **t-alpha:** 0.0693428042269768

---

---

609

- **PDB ID:** 3W3L | **Chain:** C
- **b-phipsi:** 0.0202047319990117
- **w-rdist:** 0.3377636808219068
- **t-alpha:** 0.0284671564505767

---

---

610

- **PDB ID:** 2OGZ | **Chain:** A
- **b-phipsi:** 0.0083776494329042
- **w-rdist:** 0.8004842696658852
- **t-alpha:** 0.0110700407352846

---

---

611

- **PDB ID:** 8BH3 | **Chain:** T
- **b-phipsi:** 0.027922840821294
- **w-rdist:** 0.4463263076214246
- **t-alpha:** 0.0088364585901983

---

---

612

- **PDB ID:** 6RU5 | **Chain:** B
- **b-phipsi:** 0.0008405427276633
- **w-rdist:** 0.6944209559860808
- **t-alpha:** 0.1160584082911944

---

---

613

- **PDB ID:** 1E8Y | **Chain:** A
- **b-phipsi:** 0.0212279021480761
- **w-rdist:** 0.1741972025223719
- **t-alpha:** 0.0547444112487871

---

---

614

- **PDB ID:** 2CHZ | **Chain:** A
- **b-phipsi:** 0.018187617038456
- **w-rdist:** 0.1606350066731394
- **t-alpha:** 0.0715325243705191

---

---

615

- **PDB ID:** 3B2D | **Chain:** A
- **b-phipsi:** 0.0138166367474365
- **w-rdist:** 0.4356588132488542
- **t-alpha:** 0.0293011937554052

---

---

616

- **PDB ID:** 3O3U | **Chain:** N
- **b-phipsi:** 0.0006225253388873
- **w-rdist:** 0.7279206832618059
- **t-alpha:** 0.1192811334068133

---

---

617

- **PDB ID:** 3ZVV | **Chain:** A
- **b-phipsi:** 0.0242053958720576
- **w-rdist:** 0.156467385878094
- **t-alpha:** 0.0598540584701086

---

---

618

- **PDB ID:** 5C18 | **Chain:** E
- **b-phipsi:** 0.0117088249893096
- **w-rdist:** 0.2827119991587405
- **t-alpha:** 0.0744524674147246

---

---

619

- **PDB ID:** 8GHR | **Chain:** A
- **b-phipsi:** 0.0013117245602982
- **w-rdist:** 0.6535042301974391
- **t-alpha:** 0.126644897239454

---

---

620

- **PDB ID:** 8FCP | **Chain:** A
- **b-phipsi:** 0.0114637290079273
- **w-rdist:** 0.2748305376327881
- **t-alpha:** 0.0795618367211374

---

---

621

- **PDB ID:** 3APF | **Chain:** A
- **b-phipsi:** 0.0237589291766634
- **w-rdist:** 0.1631376079340346
- **t-alpha:** 0.0613135828266362

---

---

622

- **PDB ID:** 4PS8 | **Chain:** A
- **b-phipsi:** 0.01965940910649
- **w-rdist:** 0.1636991330564259
- **t-alpha:** 0.0722624044746096

---

---

623

- **PDB ID:** 3ZW3 | **Chain:** A
- **b-phipsi:** 0.0249880706221425
- **w-rdist:** 0.1490825405832395
- **t-alpha:** 0.067152979131726

---

---

624

- **PDB ID:** 5XGJ | **Chain:** A
- **b-phipsi:** 0.0133652585531105
- **w-rdist:** 0.4797778152035603
- **t-alpha:** 0.0233575447719152

---

---

625

- **PDB ID:** 3OAW | **Chain:** A
- **b-phipsi:** 0.0222220267761885
- **w-rdist:** 0.1511724682647013
- **t-alpha:** 0.0832115711346694

---

---

626

- **PDB ID:** 6XRL | **Chain:** A
- **b-phipsi:** 0.0232883833430554
- **w-rdist:** 0.1569580873698674
- **t-alpha:** 0.0729927817404325

---

---

627

- **PDB ID:** 8DP0 | **Chain:** A
- **b-phipsi:** 0.0154133384276223
- **w-rdist:** 0.4698256594755919
- **t-alpha:** 0.0218975903112057

---

---

628

- **PDB ID:** 6XRN | **Chain:** A
- **b-phipsi:** 0.0176543924624377
- **w-rdist:** 0.1601999751122514
- **t-alpha:** 0.0970802370411048

---

---

629

- **PDB ID:** 3MJW | **Chain:** A
- **b-phipsi:** 0.0226811399958769
- **w-rdist:** 0.1654541401566851
- **t-alpha:** 0.0678829313183804

---

---

630

- **PDB ID:** 6KAM | **Chain:** D
- **b-phipsi:** 0.0008624138718353
- **w-rdist:** 0.7173399337433565
- **t-alpha:** 0.174957521031396

---

---

631

- **PDB ID:** 5OQ4 | **Chain:** A
- **b-phipsi:** 0.0173297403751488
- **w-rdist:** 0.1684488827015282
- **t-alpha:** 0.0854012245476465

---

---

632

- **PDB ID:** 3NZU | **Chain:** A
- **b-phipsi:** 0.0188041697887117
- **w-rdist:** 0.1762793099429601
- **t-alpha:** 0.0700727235417066

---

---

633

- **PDB ID:** 3APC | **Chain:** A
- **b-phipsi:** 0.0235964251973774
- **w-rdist:** 0.154145481967386
- **t-alpha:** 0.0781018927757748

---

---

634

- **PDB ID:** 4XX5 | **Chain:** A
- **b-phipsi:** 0.0162533384795982
- **w-rdist:** 0.1704619199470389
- **t-alpha:** 0.0992701436974832

---

---

635

- **PDB ID:** 7RLH | **Chain:** A
- **b-phipsi:** 0.0171759568259072
- **w-rdist:** 0.1864786216985101
- **t-alpha:** 0.0729927817404325

---

---

636

- **PDB ID:** 2CHW | **Chain:** A
- **b-phipsi:** 0.0273884094245982
- **w-rdist:** 0.1753126591641133
- **t-alpha:** 0.049634873047397

---

---

637

- **PDB ID:** 5EDS | **Chain:** A
- **b-phipsi:** 0.0234525819015525
- **w-rdist:** 0.1478750330229853
- **t-alpha:** 0.0941608854281319

---

---

638

- **PDB ID:** 3PRE | **Chain:** A
- **b-phipsi:** 0.0232193129789258
- **w-rdist:** 0.1595515809848547
- **t-alpha:** 0.0788321322144196

---

---

639

- **PDB ID:** 4EZK | **Chain:** A
- **b-phipsi:** 0.0261947626603234
- **w-rdist:** 0.1583351158722381
- **t-alpha:** 0.0664230173941995

---

---

640

- **PDB ID:** 4FHJ | **Chain:** A
- **b-phipsi:** 0.024122209430142
- **w-rdist:** 0.1473762981246011
- **t-alpha:** 0.0941608854281319

---

---

641

- **PDB ID:** 8FCL | **Chain:** C
- **b-phipsi:** 0.0124175641709017
- **w-rdist:** 0.2017818956074849
- **t-alpha:** 0.1474452578505956

---

---

642

- **PDB ID:** 7RLH | **Chain:** D
- **b-phipsi:** 0.0184051093523624
- **w-rdist:** 0.1785151914467229
- **t-alpha:** 0.0795618367211374

---

---

643

- **PDB ID:** 4FJY | **Chain:** A
- **b-phipsi:** 0.0199983777470179
- **w-rdist:** 0.1678500846059397
- **t-alpha:** 0.087591155387261

---

---

644

- **PDB ID:** 6C1S | **Chain:** A
- **b-phipsi:** 0.0244543684312207
- **w-rdist:** 0.1958487340792846
- **t-alpha:** 0.0562044536492789

---

---

645

- **PDB ID:** 3SD5 | **Chain:** A
- **b-phipsi:** 0.0294812229405839
- **w-rdist:** 0.1530579542480126
- **t-alpha:** 0.0708026304911424

---

---

646

- **PDB ID:** 3R7Q | **Chain:** A
- **b-phipsi:** 0.0245976152494378
- **w-rdist:** 0.157446523775546
- **t-alpha:** 0.0817518496585396

---

---

647

- **PDB ID:** 7RLH | **Chain:** B
- **b-phipsi:** 0.0156215556342566
- **w-rdist:** 0.1825421853542916
- **t-alpha:** 0.1175185950756378

---

---

648

- **PDB ID:** 1E8Z | **Chain:** A
- **b-phipsi:** 0.0242250521027732
- **w-rdist:** 0.1575660216076282
- **t-alpha:** 0.0861312290118383

---

---

649

- **PDB ID:** 4ANX | **Chain:** A
- **b-phipsi:** 0.0232472245840455
- **w-rdist:** 0.2188059781504125
- **t-alpha:** 0.0591240390843355

---

---

650

- **PDB ID:** 8FCT | **Chain:** F
- **b-phipsi:** 0.0142512196090698
- **w-rdist:** 0.2246435068984109
- **t-alpha:** 0.1014602199307292

---

---

651

- **PDB ID:** 4WWO | **Chain:** A
- **b-phipsi:** 0.0246668277114889
- **w-rdist:** 0.1538830061971164
- **t-alpha:** 0.0956206337488771

---

---

652

- **PDB ID:** 8FCM | **Chain:** B
- **b-phipsi:** 0.0149481412259398
- **w-rdist:** 0.1959618931226324
- **t-alpha:** 0.1233570449311542

---

---

653

- **PDB ID:** 6AUD | **Chain:** A
- **b-phipsi:** 0.0289386270574228
- **w-rdist:** 0.1948458889605273
- **t-alpha:** 0.0510948593011042

---

---

654

- **PDB ID:** 6T3C | **Chain:** A
- **b-phipsi:** 0.0252498837859851
- **w-rdist:** 0.1504253246362595
- **t-alpha:** 0.0985396895399577

---

---

655

- **PDB ID:** 3DBS | **Chain:** A
- **b-phipsi:** 0.022695287250391
- **w-rdist:** 0.1780391985094235
- **t-alpha:** 0.0781018927757748

---

---

656

- **PDB ID:** 3L13 | **Chain:** A
- **b-phipsi:** 0.0237802095252838
- **w-rdist:** 0.2064949887599458
- **t-alpha:** 0.0627735967823235

---

---

657

- **PDB ID:** 5T23 | **Chain:** A
- **b-phipsi:** 0.0236519160955762
- **w-rdist:** 0.1741133587772738
- **t-alpha:** 0.0751822857042021

---

---

658

- **PDB ID:** 4FHK | **Chain:** A
- **b-phipsi:** 0.0243151697791289
- **w-rdist:** 0.1436827617789473
- **t-alpha:** 0.1197076528051555

---

---

659

- **PDB ID:** 4J6I | **Chain:** A
- **b-phipsi:** 0.0272023782172523
- **w-rdist:** 0.1677254060631489
- **t-alpha:** 0.0708026304911424

---

---

660

- **PDB ID:** 4DK5 | **Chain:** A
- **b-phipsi:** 0.0251089650620967
- **w-rdist:** 0.1467853368027204
- **t-alpha:** 0.1175185950756378

---

---

661

- **PDB ID:** 4PS7 | **Chain:** A
- **b-phipsi:** 0.0229113472616434
- **w-rdist:** 0.1646258303411152
- **t-alpha:** 0.1109484600168524

---

---

662

- **PDB ID:** 3PS6 | **Chain:** A
- **b-phipsi:** 0.0261791224751676
- **w-rdist:** 0.1508666634417135
- **t-alpha:** 0.1109484600168524

---

---

663

- **PDB ID:** 5G55 | **Chain:** A
- **b-phipsi:** 0.0231414516755186
- **w-rdist:** 0.1637389530534408
- **t-alpha:** 0.1131384481440944

---

---

664

- **PDB ID:** 7RLJ | **Chain:** L
- **b-phipsi:** 0.0179975166734936
- **w-rdist:** 0.2089357528025506
- **t-alpha:** 0.0970802370411048

---

---

665

- **PDB ID:** 3L08 | **Chain:** A
- **b-phipsi:** 0.0239155125037426
- **w-rdist:** 0.1927185649815372
- **t-alpha:** 0.0708026304911424

---

---

666

- **PDB ID:** 4PS3 | **Chain:** A
- **b-phipsi:** 0.0234502780334213
- **w-rdist:** 0.1687016349136982
- **t-alpha:** 0.1007293602259888

---

---

667

- **PDB ID:** 3L16 | **Chain:** A
- **b-phipsi:** 0.0257882530807749
- **w-rdist:** 0.1676075253582388
- **t-alpha:** 0.0839416535012711

---

---

668

- **PDB ID:** 7JWZ | **Chain:** A
- **b-phipsi:** 0.0315866855540247
- **w-rdist:** 0.1613016555329635
- **t-alpha:** 0.0781018927757748

---

---

669

- **PDB ID:** 3TJP | **Chain:** A
- **b-phipsi:** 0.0265613986558629
- **w-rdist:** 0.1544740785181016
- **t-alpha:** 0.1080291886428159

---

---

670

- **PDB ID:** 4URK | **Chain:** A
- **b-phipsi:** 0.0221525172849024
- **w-rdist:** 0.1759597161510755
- **t-alpha:** 0.1036493968305367

---

---

671

- **PDB ID:** 5KAE | **Chain:** A
- **b-phipsi:** 0.0234026691875654
- **w-rdist:** 0.1759452736812433
- **t-alpha:** 0.0956206337488771

---

---

672

- **PDB ID:** 5C19 | **Chain:** A
- **b-phipsi:** 0.0150798456619269
- **w-rdist:** 0.2242943967898939
- **t-alpha:** 0.1372265998230157

---

---

673

- **PDB ID:** 3QAR | **Chain:** A
- **b-phipsi:** 0.0264911118482752
- **w-rdist:** 0.1801136694327119
- **t-alpha:** 0.0722624044746096

---

---

674

- **PDB ID:** 4XZ4 | **Chain:** A
- **b-phipsi:** 0.0279415543035692
- **w-rdist:** 0.1639521355403305
- **t-alpha:** 0.0868612061966194

---

---

675

- **PDB ID:** 4WWP | **Chain:** A
- **b-phipsi:** 0.0209029951043785
- **w-rdist:** 0.205014801408431
- **t-alpha:** 0.0963501924934129

---

---

676

- **PDB ID:** 3T8M | **Chain:** A
- **b-phipsi:** 0.0292422195153708
- **w-rdist:** 0.1727749382934151
- **t-alpha:** 0.0737226325244466

---

---

677

- **PDB ID:** 7RLJ | **Chain:** K
- **b-phipsi:** 0.0191237996293434
- **w-rdist:** 0.2078044566110194
- **t-alpha:** 0.1087585508850761

---

---

678

- **PDB ID:** 2V4L | **Chain:** A
- **b-phipsi:** 0.0203318823351316
- **w-rdist:** 0.2103616075584614
- **t-alpha:** 0.0985396895399577

---

---

679

- **PDB ID:** 3NZS | **Chain:** A
- **b-phipsi:** 0.0259270372266636
- **w-rdist:** 0.1600912239805987
- **t-alpha:** 0.1160584082911944

---

---

680

- **PDB ID:** 7RLJ | **Chain:** B
- **b-phipsi:** 0.0162272657134616
- **w-rdist:** 0.2191150271081947
- **t-alpha:** 0.1445250397065645

---

---

681

- **PDB ID:** 3APD | **Chain:** A
- **b-phipsi:** 0.0283399910315177
- **w-rdist:** 0.1725167131230494
- **t-alpha:** 0.0824814643431366

---

---

682

- **PDB ID:** 6GQ7 | **Chain:** A
- **b-phipsi:** 0.023935428942403
- **w-rdist:** 0.1757414019155012
- **t-alpha:** 0.1014602199307292

---

---

683

- **PDB ID:** 4G11 | **Chain:** A
- **b-phipsi:** 0.0243570605919048
- **w-rdist:** 0.2260602479802668
- **t-alpha:** 0.0678829313183804

---

---

684

- **PDB ID:** 4HLE | **Chain:** A
- **b-phipsi:** 0.0183451858448212
- **w-rdist:** 0.1885664624429021
- **t-alpha:** 0.1496348281350508

---

---

685

- **PDB ID:** 3QAQ | **Chain:** A
- **b-phipsi:** 0.0223385490268716
- **w-rdist:** 0.2481572429678181
- **t-alpha:** 0.0795618367211374

---

---

686

- **PDB ID:** 3QJZ | **Chain:** A
- **b-phipsi:** 0.0343877899767645
- **w-rdist:** 0.2065948524461103
- **t-alpha:** 0.0642335893644507

---

---

687

- **PDB ID:** 4FUL | **Chain:** A
- **b-phipsi:** 0.0267235168526154
- **w-rdist:** 0.1769166992068789
- **t-alpha:** 0.0861312290118383

---

---

688

- **PDB ID:** 8FCR | **Chain:** B
- **b-phipsi:** 0.0168038987067144
- **w-rdist:** 0.2132054246435182
- **t-alpha:** 0.1598543638747036

---

---

689

- **PDB ID:** 8FCQ | **Chain:** F
- **b-phipsi:** 0.0167852867483199
- **w-rdist:** 0.2240440390225465
- **t-alpha:** 0.1452554889642807

---

---

690

- **PDB ID:** 7RLJ | **Chain:** E
- **b-phipsi:** 0.0181396914861316
- **w-rdist:** 0.2259766256580341
- **t-alpha:** 0.1248171264087285

---

---

691

- **PDB ID:** 5G2N | **Chain:** A
- **b-phipsi:** 0.0290164351118527
- **w-rdist:** 0.1663094363709176
- **t-alpha:** 0.1065687372842305

---

---

692

- **PDB ID:** 7RLJ | **Chain:** H
- **b-phipsi:** 0.0202419139260194
- **w-rdist:** 0.2214548861096227
- **t-alpha:** 0.1131384481440944

---

---

693

- **PDB ID:** 2CHX | **Chain:** A
- **b-phipsi:** 0.023911705340524
- **w-rdist:** 0.1912281247606125
- **t-alpha:** 0.1065687372842305

---

---

694

- **PDB ID:** 3L54 | **Chain:** A
- **b-phipsi:** 0.0214570103806428
- **w-rdist:** 0.1885237218509499
- **t-alpha:** 0.1379556468436567

---

---

695

- **PDB ID:** 7RLJ | **Chain:** J
- **b-phipsi:** 0.0183410555133196
- **w-rdist:** 0.2098539531772982
- **t-alpha:** 0.1510950327523956

---

---

696

- **PDB ID:** 2A4Z | **Chain:** A
- **b-phipsi:** 0.0318124386488418
- **w-rdist:** 0.1610584260944607
- **t-alpha:** 0.1116786836949257

---

---

697

- **PDB ID:** 4ANU | **Chain:** A
- **b-phipsi:** 0.0289126449802262
- **w-rdist:** 0.2227128918616162
- **t-alpha:** 0.067152979131726

---

---

698

- **PDB ID:** 3S2A | **Chain:** A
- **b-phipsi:** 0.0205515248493663
- **w-rdist:** 0.2184450225735756
- **t-alpha:** 0.1182478050534847

---

---

699

- **PDB ID:** 6T3B | **Chain:** A
- **b-phipsi:** 0.0284627623612345
- **w-rdist:** 0.171277175370998
- **t-alpha:** 0.1072992501854066

---

---

700

- **PDB ID:** 3ENE | **Chain:** A
- **b-phipsi:** 0.0241575622935603
- **w-rdist:** 0.21810229320529
- **t-alpha:** 0.0941608854281319

---

---

701

- **PDB ID:** 4F1S | **Chain:** A
- **b-phipsi:** 0.0265544538660833
- **w-rdist:** 0.228400798955613
- **t-alpha:** 0.0744524674147246

---

---

702

- **PDB ID:** 7RLJ | **Chain:** I
- **b-phipsi:** 0.0217096499912725
- **w-rdist:** 0.2150221113941854
- **t-alpha:** 0.1262769574199462

---

---

703

- **PDB ID:** 7RLJ | **Chain:** F
- **b-phipsi:** 0.0227774863307095
- **w-rdist:** 0.2105898411899143
- **t-alpha:** 0.1233570449311542

---

---

704

- **PDB ID:** 4ANW | **Chain:** A
- **b-phipsi:** 0.0288861726946091
- **w-rdist:** 0.2317739932553337
- **t-alpha:** 0.0708026304911424

---

---

705

- **PDB ID:** 7JX0 | **Chain:** A
- **b-phipsi:** 0.0360396433156356
- **w-rdist:** 0.1734936675783321
- **t-alpha:** 0.1094894513224573

---

---

706

- **PDB ID:** 7K1J | **Chain:** C
- **b-phipsi:** 0.0278531379844812
- **w-rdist:** 0.2316378261963607
- **t-alpha:** 0.080292034788115

---

---

707

- **PDB ID:** 4FJZ | **Chain:** A
- **b-phipsi:** 0.0331525649488527
- **w-rdist:** 0.1840575369484055
- **t-alpha:** 0.1218977888030892

---

---

708

- **PDB ID:** 7QTT | **Chain:** n
- **b-phipsi:** 0.0244595047917531
- **w-rdist:** 0.2156166620446509
- **t-alpha:** 0.1452554889642807

---

---
