# Supplementary material for: Comparative structural insights and functional analysis for the distinct unbound states of Human AGO proteins
Source: Sci Rep. 2025 Mar 19;15:9432. doi: 10.1038/s41598-025-91849-5 (PMC11923369; doi:10.1038/s41598-025-91849-5)
Supplement: Supplementary file 24 — Supplementary Information 12. [file 41598_2025_91849_MOESM24_ESM.zip › 4KREp_A_mdwhole_HL2REF/go/4KREp_A_mitot_mitosis_de7538da1f7844b2a2310efe4413d0e5-pres_report.html]

 

# Structural Comparison Report for 4KREp\_A - whole structures (total: 8)

---

1

- **Protein name:** DNA polymerase alpha catalytic subunit
- **Organism:** Homo sapiens
- **Uniprot Accession Number:** P09884
- **Protein sequence length:** 1462 aa
- **1D identity (%):** 14.26
- **1D identity (%) [Gaps excluded]:** 25.48
- **1D identity - Alignment Gaps:** 655
- **1D aligned content (<aminoacid>:%):** {'P': 8.02, 'S': 4.72, 'G': 9.91, 'R': 4.72, 'K': 9.43, 'F': 3.3, 'D': 7.55, 'Y': 4.72, 'V': 8.49, 'T': 6.13, 'A': 2.83, 'W': 1.42, 'E': 6.13, 'Q': 4.25, 'H': 1.89, 'L': 8.02, 'N': 2.36, 'I': 3.3, 'C': 2.36, 'M': 0.47}
- **Common reported functions (%):** 0.0
- **Common reported locations (%):** 30.0
- **Common reported processes (%):** 0.0

- **PDB ID:** 4QCL
- **Chain:** A
- **Crystallized protein length:** 865 aa
- **Resolution:** 2.2 Å
- **b-phipsi ():** 0.009067
- **w-rdist ():** 0.162198
- **t-alpha ():** 0.004379
- **Chemical similarity (Tanimoto Index) (%):** 67.7
- **1D identity (%) [PDB]:** 2.93
- **1D identity (%) [Gaps excluded][PDB]:** 71.64
- **1D identity - Alignment Gaps [PDB]:** 1574
- **1D aligned content [PDB] (<aminoacid>:%):** {'Q': 8.33, 'T': 8.33, 'I': 12.5, 'K': 4.17, 'A': 12.5, 'P': 10.42, 'D': 4.17, 'E': 4.17, 'S': 2.08, 'R': 8.33, 'L': 8.33, 'M': 2.08, 'Y': 4.17, 'F': 2.08, 'V': 4.17, 'G': 4.17}
- **2D identity (%) [PDB]:** 39.57
- **2D identity (%) [Gaps excluded][PDB]:** 84.9
- **2D identity - Alignment Gaps [PDB]:** 622
- **2D aligned content [PDB] (<2D-fold>:%):** {'.': 19.52, 'E': 29.07, 'T': 6.94, 'H': 42.52, 'I': 1.08, 'B': 0.22, 'G': 0.65}
- **3D similarity (TM-Score) (%) [PDB]:** 25.05

- **Gene name:** POLA1
- **Entrez ID:** 5422
- **RefSeq ID:** NM\_016937
- **Transcript sequence length:** 5469
- **5-UTR|CDS|3-UTR identity (%):** 14.35 | 39.89 | 6.16
- **5-UTR|CDS|3-UTR identity (%) [Gaps excluded]:** 75.61 | 77.05 | 74.5
- **5-UTR|CDS|3-UTR identity [Alignment Gaps]:** 175 | 2245 | 10047
- **5-UTR aligned content (<base>:%):** {'G': 58.06, 'C': 25.81, 'A': 6.45, 'T': 9.68}
- **CDS aligned content (<base>:%):** {'A': 27.57, 'T': 22.51, 'G': 26.01, 'C': 23.91}
- **3-UTR aligned content (<base>:%):** {'G': 21.48, 'A': 28.15, 'T': 32.15, 'C': 18.22}

**Uniprot Description:**  
  
 Catalytic subunit of the DNA polymerase alpha complex (also known as the alpha DNA polymerase-primase complex) which plays an essential role in the initiation of DNA synthesis. During the S phase of the cell cycle, the DNA polymerase alpha complex (composed of a catalytic subunit POLA1, a regulatory subunit POLA2 and two primase subunits PRIM1 and PRIM2) is recruited to DNA at the replicative forks via direct interactions with MCM10 and WDHD1. The primase subunit of the polymerase alpha complex initiates DNA synthesis by oligomerising short RNA primers on both leading and lagging strands. These primers are initially extended by the polymerase alpha catalytic subunit and subsequently transferred to polymerase delta and polymerase epsilon for processive synthesis on the lagging and leading strand, respectively. The reason this transfer occurs is because the polymerase alpha has limited processivity and lacks intrinsic 3' exonuclease activity for proofreading error, and therefore is not well suited for replicating long complexes. In the cytosol, responsible for a substantial proportion of the physiological concentration of cytosolic RNA:DNA hybrids, which are necessary to prevent spontaneous activation of type I interferon responses (PubMed:27019227).   
  
Component of the alpha DNA polymerase complex (also known as the alpha DNA polymerase-primase complex) consisting of four subunits: the catalytic subunit POLA1, the regulatory subunit POLA2, and the primase complex subunits PRIM1 and PRIM2 respectively (PubMed:9705292). Interacts with PARP1; this interaction functions as part of the control of replication fork progression (PubMed:9518481). Interacts with MCM10 and WDHD1; these interactions recruit the polymerase alpha complex to the pre-replicative complex bound to DNA (PubMed:19608746). Interacts with RPA1; this interaction stabilizes the replicative complex and reduces the misincorporation rate of DNA polymerase alpha by acting as a fidelity clamp (PubMed:9214288).   
  
 **Gene Ontology Information:**

Molecular Function

- chromatin binding
- DNA binding
- DNA replication origin binding
- DNA-directed DNA polymerase activity
- nucleotide binding
- protein kinase binding
- single-stranded DNA binding
- zinc ion binding

Location

- alpha DNA polymerase:primase complex
- cytosol
- nuclear envelope
- nuclear matrix
- nucleolus
- nucleoplasm
- nucleus

Biological process

- DNA repair
- DNA replication
- DNA replication initiation
- DNA replication, synthesis of RNA primer
- DNA strand elongation involved in DNA replication
- DNA synthesis involved in DNA repair
- double-strand break repair via nonhomologous end joining
- lagging strand elongation
- leading strand elongation
- mitotic DNA replication initiation
- regulation of type I interferon production

---

2

- **Protein name:** Transitional endoplasmic reticulum ATPase
- **Organism:** Homo sapiens
- **Uniprot Accession Number:** P55072
- **Protein sequence length:** 806 aa
- **1D identity (%):** 14.1
- **1D identity (%) [Gaps excluded]:** 20.53
- **1D identity - Alignment Gaps:** 309
- **1D aligned content (<aminoacid>:%):** {'D': 9.35, 'K': 5.04, 'R': 10.79, 'N': 2.16, 'V': 5.04, 'P': 8.63, 'T': 4.32, 'L': 8.63, 'G': 9.35, 'S': 2.88, 'Q': 4.32, 'F': 5.04, 'Y': 2.16, 'C': 1.44, 'E': 2.88, 'I': 7.91, 'M': 1.44, 'A': 7.91, 'H': 0.72}
- **Common reported functions (%):** 16.67
- **Common reported locations (%):** 40.0
- **Common reported processes (%):** 0.0

- **PDB ID:** 5C1B
- **Chain:** C
- **Crystallized protein length:** 724 aa
- **Resolution:** 3.08 Å
- **b-phipsi ():** 0.008754
- **w-rdist ():** 0.186882
- **t-alpha ():** 0.010949
- **Chemical similarity (Tanimoto Index) (%):** 74.99
- **1D identity (%) [PDB]:** 2.1
- **1D identity (%) [Gaps excluded][PDB]:** 78.05
- **1D identity - Alignment Gaps [PDB]:** 1484
- **1D aligned content [PDB] (<aminoacid>:%):** {'L': 18.75, 'Q': 6.25, 'I': 3.12, 'V': 12.5, 'P': 6.25, 'G': 9.38, 'K': 12.5, 'T': 12.5, 'A': 3.12, 'R': 3.12, 'M': 3.12, 'N': 3.12, 'S': 6.25}
- **2D identity (%) [PDB]:** 39.72
- **2D identity (%) [Gaps excluded][PDB]:** 85.69
- **2D identity - Alignment Gaps [PDB]:** 574
- **2D aligned content [PDB] (<2D-fold>:%):** {'.': 18.35, 'E': 26.35, 'H': 44.71, 'T': 6.82, 'B': 0.94, 'G': 2.82}
- **3D similarity (TM-Score) (%) [PDB]:** 22.69

- **Gene name:** VCP
- **Entrez ID:** N/A
- **RefSeq ID:** NM\_007126
- **Transcript sequence length:** 3746
- **5-UTR|CDS|3-UTR identity (%):** 42.02 | 45.89 | 6.32
- **5-UTR|CDS|3-UTR identity (%) [Gaps excluded]:** 72.47 | 74.86 | 75.14
- **5-UTR|CDS|3-UTR identity [Alignment Gaps]:** 129 | 1223 | 10034
- **5-UTR aligned content (<base>:%):** {'A': 9.3, 'T': 10.08, 'C': 35.66, 'G': 44.96}
- **CDS aligned content (<base>:%):** {'A': 23.52, 'T': 21.38, 'G': 30.28, 'C': 24.83}
- **3-UTR aligned content (<base>:%):** {'G': 25.0, 'T': 30.2, 'C': 20.66, 'A': 24.13}

**Uniprot Description:**  
  
 Necessary for the fragmentation of Golgi stacks during mitosis and for their reassembly after mitosis. Involved in the formation of the transitional endoplasmic reticulum (tER). The transfer of membranes from the endoplasmic reticulum to the Golgi apparatus occurs via 50-70 nm transition vesicles which derive from part-rough, part-smooth transitional elements of the endoplasmic reticulum (tER). Vesicle budding from the tER is an ATP-dependent process. The ternary complex containing UFD1, VCP and NPLOC4 binds ubiquitinated proteins and is necessary for the export of misfolded proteins from the ER to the cytoplasm, where they are degraded by the proteasome. The NPLOC4-UFD1-VCP complex regulates spindle disassembly at the end of mitosis and is necessary for the formation of a closed nuclear envelope. Regulates E3 ubiquitin-protein ligase activity of RNF19A. Component of the VCP/p97-AMFR/gp78 complex that participates in the final step of the sterol-mediated ubiquitination and endoplasmic reticulum-associated degradation (ERAD) of HMGCR. Involved in endoplasmic reticulum stress-induced pre-emptive quality control, a mechanism that selectively attenuates the translocation of newly synthesized proteins into the endoplasmic reticulum and reroutes them to the cytosol for proteasomal degradation (PubMed:26565908). Plays a role in the regulation of stress granules (SGs) clearance process upon arsenite-induced response (PubMed:29804830). Also involved in DNA damage response: recruited to double-strand breaks (DSBs) sites in a RNF8- and RNF168-dependent manner and promotes the recruitment of TP53BP1 at DNA damage sites (PubMed:22020440, PubMed:22120668). Recruited to stalled replication forks by SPRTN: may act by mediating extraction of DNA polymerase eta (POLH) to prevent excessive translesion DNA synthesis and limit the incidence of mutations induced by DNA damage (PubMed:23042607, PubMed:23042605). Together with SPRTN metalloprotease, involved in the repair of covalent DNA-protein cross-links (DPCs) during DNA synthesis (PubMed:32152270). Involved in interstrand cross-link repair in response to replication stress by mediating unloading of the ubiquitinated CMG helicase complex (By similarity). Required for cytoplasmic retrotranslocation of stressed/damaged mitochondrial outer-membrane proteins and their subsequent proteasomal degradation (PubMed:16186510, PubMed:21118995). Essential for the maturation of ubiquitin-containing autophagosomes and the clearance of ubiquitinated protein by autophagy (PubMed:20104022, PubMed:27753622). Acts as a negative regulator of type I interferon production by interacting with DDX58/RIG-I: interaction takes place when DDX58/RIG-I is ubiquitinated via 'Lys-63'-linked ubiquitin on its CARD domains, leading to recruit RNF125 and promote ubiquitination and degradation of DDX58/RIG-I (PubMed:26471729). May play a role in the ubiquitin-dependent sorting of membrane proteins to lysosomes where they undergo degradation (PubMed:21822278). May more particularly play a role in caveolins sorting in cells (PubMed:21822278, PubMed:23335559). By controlling the steady-state expression of the IGF1R receptor, indirectly regulates the insulin-like growth factor receptor signaling pathway (PubMed:26692333).   
  
Homohexamer. Forms a ring-shaped particle of 12.5 nm diameter, that displays 6-fold radial symmetry. Part of a ternary complex containing STX5A, NSFL1C and VCP. NSFL1C forms a homotrimer that binds to one end of a VCP homohexamer. The complex binds to membranes enriched in phosphatidylethanolamine-containing lipids and promotes Golgi membrane fusion. Binds to a heterodimer of NPLOC4 and UFD1, binding to this heterodimer inhibits Golgi-membrane fusion (PubMed:26471729). Interaction with VCIP135 leads to dissociation of the complex via ATP hydrolysis by VCP. Part of a ternary complex containing NPLOC4, UFD1 and VCP. Interacts with NSFL1C-like protein p37; the complex has membrane fusion activity and is required for Golgi and endoplasmic reticulum biogenesis. Interacts with SELENOS and SYVN1, as well as with DERL1, DERL2 and DERL3; which probably transfer misfolded proteins from the ER to VCP. Interacts with SVIP. Component of a complex required to couple retrotranslocation, ubiquitination and deglycosylation composed of NGLY1, SAKS1, AMFR, VCP and RAD23B. Directly interacts with UBXN4 and RNF19A. Interacts with CASR. Interacts with UBE4B and YOD1. Interacts with clathrin. Interacts with RNF103. Interacts with TRIM13 and TRIM21. Component of a VCP/p97-AMFR/gp78 complex that participates in the final step of the endoplasmic reticulum-associated degradation (ERAD) of HMGCR. Interacts directly with AMFR/gp78 (via its VIM). Interacts with RHBDD1 (via C-terminal domain). Interacts with SPRTN; leading to recruitment to stalled replication forks (PubMed:23042607, PubMed:23042605). Interacts with WASHC5. Interacts with UBOX5. Interacts (via N-terminus) with UBXN7, UBXN8, and probably several other UBX domain-containing proteins (via UBX domains); the interactions are mutually exclusive with VIM-dependent interactions such as those with AMFR and SELENOS. Forms a complex with UBQLN1 and UBXN4. Interacts (via the PIM motif) with RNF31 (via the PUB domain) (PubMed:24726327). Interacts with DDX58/RIG-I and RNF125; interaction takes place when DDX58/RIG-I is ubiquitinated via 'Lys-63'-linked ubiquitin on its CARD domains, leading to recruit RNF125 and promote ubiquitination and degradation of DDX58/RIG-I (PubMed:26471729). Interacts with BAG6 (PubMed:21636303). Interacts with UBXN10 (PubMed:26389662). Interacts with UBXN6; the interaction with UBXN6 is direct and competitive with UFD1 (PubMed:19174149, PubMed:19275885). Forms a ternary complex with CAV1 and UBXN6 (PubMed:21822278, PubMed:18656546, PubMed:19174149). Interacts with PLAA, UBXN6 and YOD1; may form a complex involved in macroautophagy (PubMed:27753622). Interacts with ANKZF1 (PubMed:28302725). Interacts with ubiquitin-binding protein FAF1 (PubMed:26842564). Interacts with ZFAND2B (via VIM motif); the interaction is direct (PubMed:24160817, PubMed:26337389). Interacts with ZFAND1 (via its ubiquitin-like region); this interaction occurs in an arsenite-dependent manner (PubMed:29804830). Interacts with CCDC47 (By similarity). Interacts with UBAC2 (By similarity). Interacts with LMBR1L (PubMed:31073040). Interacts with ATXN3 (PubMed:30455355). Interacts with TEX264; bridging VCP to covalent DNA-protein cross-links (DPCs) (PubMed:32152270).   
  
 **Gene Ontology Information:**

Molecular Function

- ADP binding
- ATP binding
- ATPase activity
- BAT3 complex binding
- deubiquitinase activator activity
- identical protein binding
- K48-linked polyubiquitin modification-dependent protein binding
- lipid binding
- MHC class I protein binding
- polyubiquitin modification-dependent protein binding
- protein domain specific binding
- protein phosphatase binding
- RNA binding
- ubiquitin protein ligase binding
- ubiquitin-dependent protein binding
- ubiquitin-like protein ligase binding
- ubiquitin-specific protease binding

Location

- ATPase complex
- azurophil granule lumen
- cytoplasm
- cytoplasmic stress granule
- cytosol
- Derlin-1 retrotranslocation complex
- endoplasmic reticulum
- endoplasmic reticulum membrane
- extracellular exosome
- extracellular region
- ficolin-1-rich granule lumen
- glutamatergic synapse
- intracellular membrane-bounded organelle
- lipid droplet
- nucleoplasm
- nucleus
- perinuclear region of cytoplasm
- proteasome complex
- protein-containing complex
- secretory granule lumen
- site of double-strand break
- VCP-NPL4-UFD1 AAA ATPase complex
- VCP-NSFL1C complex

Biological process

- activation of cysteine-type endopeptidase activity involved in apoptotic process
- aggresome assembly
- ATP metabolic process
- autophagosome maturation
- autophagy
- cellular response to arsenite ion
- cellular response to heat
- cellular response to DNA damage stimulus
- DNA repair
- double-strand break repair
- endoplasmic reticulum stress-induced pre-emptive quality control
- endoplasmic reticulum to Golgi vesicle-mediated transport
- endoplasmic reticulum unfolded protein response
- endosome to lysosome transport via multivesicular body sorting pathway
- ER-associated misfolded protein catabolic process
- ERAD pathway
- establishment of protein localization
- flavin adenine dinucleotide catabolic process
- interstrand cross-link repair
- macroautophagy
- mitotic spindle disassembly
- NADH metabolic process
- negative regulation of protein localization to chromatin
- negative regulation of smoothened signaling pathway
- positive regulation of ATP biosynthetic process
- positive regulation of canonical Wnt signaling pathway
- positive regulation of Lys63-specific deubiquitinase activity
- positive regulation of mitochondrial membrane potential
- positive regulation of oxidative phosphorylation
- positive regulation of proteasomal ubiquitin-dependent protein catabolic process
- positive regulation of protein catabolic process
- positive regulation of protein K63-linked deubiquitination
- positive regulation of protein-containing complex assembly
- proteasomal protein catabolic process
- proteasome-mediated ubiquitin-dependent protein catabolic process
- protein ubiquitination
- protein-DNA covalent cross-linking repair
- regulation of aerobic respiration
- regulation of apoptotic process
- regulation of protein localization to chromatin
- regulation of synapse organization
- retrograde protein transport, ER to cytosol
- stress granule disassembly
- translesion synthesis
- ubiquitin-dependent ERAD pathway
- viral genome replication

---

3

- **Protein name:** Transcription initiation factor TFIID subunit 2
- **Organism:** Homo sapiens
- **Uniprot Accession Number:** Q6P1X5
- **Protein sequence length:** 1199 aa
- **1D identity (%):** 16.14
- **1D identity (%) [Gaps excluded]:** 24.78
- **1D identity - Alignment Gaps:** 434
- **1D aligned content (<aminoacid>:%):** {'P': 7.46, 'L': 12.94, 'V': 6.47, 'F': 6.47, 'R': 7.46, 'I': 4.48, 'K': 5.97, 'E': 6.47, 'C': 2.49, 'D': 4.48, 'N': 3.48, 'Y': 2.49, 'A': 5.97, 'G': 5.47, 'S': 4.98, 'W': 1.0, 'H': 3.48, 'M': 1.0, 'T': 2.49, 'Q': 4.98}
- **Common reported functions (%):** 0.0
- **Common reported locations (%):** 20.0
- **Common reported processes (%):** 0.0

- **PDB ID:** 6MZC
- **Chain:** B
- **Crystallized protein length:** 968 aa
- **Resolution:** 4.5 Å
- **b-phipsi ():** 0.016811
- **w-rdist ():** 0.19083
- **t-alpha ():** 0.005136
- **Chemical similarity (Tanimoto Index) (%):** 99.55
- **1D identity (%) [PDB]:** 2.94
- **1D identity (%) [Gaps excluded][PDB]:** 68.92
- **1D identity - Alignment Gaps [PDB]:** 1660
- **1D aligned content [PDB] (<aminoacid>:%):** {'E': 7.84, 'P': 7.84, 'L': 11.76, 'T': 9.8, 'Q': 9.8, 'R': 7.84, 'V': 9.8, 'I': 1.96, 'K': 7.84, 'H': 5.88, 'C': 3.92, 'A': 3.92, 'S': 1.96, 'F': 3.92, 'Y': 3.92, 'N': 1.96}
- **2D identity (%) [PDB]:** 36.95
- **2D identity (%) [Gaps excluded][PDB]:** 85.85
- **2D identity - Alignment Gaps [PDB]:** 720
- **2D aligned content [PDB] (<2D-fold>:%):** {'.': 20.77, 'E': 20.56, 'T': 12.42, 'H': 45.18, 'G': 0.64, 'B': 0.43}
- **3D similarity (TM-Score) (%) [PDB]:** 27.49

- **Gene name:** TAF2
- **Entrez ID:** 6873
- **RefSeq ID:** NM\_003184
- **Transcript sequence length:** 5027
- **5-UTR|CDS|3-UTR identity (%):** 49.66 | 42.69 | 6.66
- **5-UTR|CDS|3-UTR identity (%) [Gaps excluded]:** 75.77 | 75.7 | 74.62
- **5-UTR|CDS|3-UTR identity [Alignment Gaps]:** 102 | 1750 | 10011
- **5-UTR aligned content (<base>:%):** {'A': 12.24, 'T': 10.88, 'G': 46.26, 'C': 30.61}
- **CDS aligned content (<base>:%):** {'T': 22.94, 'G': 24.58, 'C': 23.35, 'A': 29.13}
- **3-UTR aligned content (<base>:%):** {'G': 15.16, 'A': 30.33, 'C': 14.34, 'T': 40.16}

**Uniprot Description:**  
  
 Transcription factor TFIID is one of the general factors required for accurate and regulated initiation by RNA polymerase II. TFIID is a multimeric protein complex that plays a central role in mediating promoter responses to various activators and repressors. It requires core promoter-specific cofactors for productive transcription stimulation. TAF2 stabilizes TFIID binding to core promoter.   
  
Component of transcription factor TFIID which is composed of TBP and a number of TBP-associated factors. Interacts with TAF2C1. Component of the TFTC-HAT complex.   
  
 **Gene Ontology Information:**

Molecular Function

- chromatin binding
- RNA polymerase II general transcription initiation factor activity
- transcription regulatory region sequence-specific DNA binding

Location

- nucleoplasm
- nucleus
- transcription factor TFIID complex
- transcription factor TFTC complex

Biological process

- G2/M transition of mitotic cell cycle
- histone H3 acetylation
- monoubiquitinated histone deubiquitination
- monoubiquitinated histone H2A deubiquitination
- mRNA transcription by RNA polymerase II
- positive regulation of transcription, DNA-templated
- positive regulation of transcription initiation from RNA polymerase II promoter
- protein phosphorylation
- regulation of DNA repair
- regulation of transcription by RNA polymerase II
- response to organic cyclic compound
- RNA polymerase II preinitiation complex assembly
- transcription initiation from RNA polymerase II promoter

---

4

- **Protein name:** UBX domain-containing protein 6
- **Organism:** Homo sapiens
- **Uniprot Accession Number:** Q9BZV1
- **Protein sequence length:** 441 aa
- **1D identity (%):** 10.33
- **1D identity (%) [Gaps excluded]:** 29.12
- **1D identity - Alignment Gaps:** 618
- **1D aligned content (<aminoacid>:%):** {'Q': 7.07, 'E': 5.05, 'F': 4.04, 'G': 10.1, 'K': 6.06, 'P': 9.09, 'A': 8.08, 'R': 9.09, 'N': 3.03, 'V': 7.07, 'Y': 4.04, 'T': 3.03, 'L': 10.1, 'C': 2.02, 'S': 4.04, 'I': 2.02, 'H': 2.02, 'D': 4.04}
- **Common reported functions (%):** 16.67
- **Common reported locations (%):** 40.0
- **Common reported processes (%):** 0.0

- **PDB ID:** 8FCN
- **Chain:** A
- **Crystallized protein length:** 721 aa
- **Resolution:** 2.95 Å
- **b-phipsi ():** 0.010499
- **w-rdist ():** 0.157515
- **t-alpha ():** 0.081022
- **Chemical similarity (Tanimoto Index) (%):** 79.7
- **1D identity (%) [PDB]:** 2.07
- **1D identity (%) [Gaps excluded][PDB]:** 73.81
- **1D identity - Alignment Gaps [PDB]:** 1459
- **1D aligned content [PDB] (<aminoacid>:%):** {'L': 19.35, 'Q': 6.45, 'I': 3.23, 'V': 12.9, 'P': 6.45, 'G': 9.68, 'K': 12.9, 'T': 12.9, 'A': 3.23, 'R': 3.23, 'M': 3.23, 'N': 3.23, 'S': 3.23}
- **2D identity (%) [PDB]:** 42.97
- **2D identity (%) [Gaps excluded][PDB]:** 90.16
- **2D identity - Alignment Gaps [PDB]:** 547
- **2D aligned content [PDB] (<2D-fold>:%):** {'.': 13.81, 'E': 24.5, 'H': 44.1, 'T': 16.04, 'G': 1.34, 'B': 0.22}
- **3D similarity (TM-Score) (%) [PDB]:** 2.26

- **Gene name:** UBXN6
- **Entrez ID:** 80700
- **RefSeq ID:** N/A
- **Sequence length:** N/A
- **5-UTR|CDS|3-UTR identity (%):** N/A | N/A | N/A
- **5-UTR|CDS|3-UTR identity (%) [Gaps excluded]:** N/A | N/A | N/A
- **5-UTR|CDS|3-UTR identity [Alignment Gaps]:** N/A | N/A | N/A
- **5-UTR aligned content (<base>:%):** N/A
- **CDS aligned content (<base>:%):** N/A
- **3-UTR aligned content (<base>:%):** N/A

**Uniprot Description:**  
  
 May negatively regulate the ATPase activity of VCP, an ATP-driven segregase that associates with different cofactors to control a wide variety of cellular processes (PubMed:26475856). As a cofactor of VCP, it may play a role in the transport of CAV1 to lysosomes for degradation (PubMed:21822278, PubMed:23335559). It may also play a role in endoplasmic reticulum-associated degradation (ERAD) of misfolded proteins (PubMed:19275885). Together with VCP and other cofactors, it may play a role in macroautophagy, regulating for instance the clearance of damaged lysosomes (PubMed:27753622).   
  
Interacts with VCP through the PUB domain (via C-terminus) and VIM motif (via N-terminus); the interaction is direct (PubMed:18656546, PubMed:19174149, PubMed:21896481, PubMed:21822278, PubMed:26475856). Forms a ternary complex with CAV1 and VCP (PubMed:21822278). Interacts with SYVN1 (PubMed:18656546). Interacts with HERPUD1 (PubMed:18656546). Interacts with VCPKMT (PubMed:23349634). May interact with DERL1 (PubMed:19275885). Interacts with PLAA, VCP and YOD1; may form a complex involved in macroautophagy (PubMed:27753622). Interacts with LMAN1 (PubMed:22337587).   
  
 **Gene Ontology Information:**

Molecular Function

- ADP binding
- ATP binding
- ATPase activity
- BAT3 complex binding
- deubiquitinase activator activity
- identical protein binding
- K48-linked polyubiquitin modification-dependent protein binding
- lipid binding
- MHC class I protein binding
- polyubiquitin modification-dependent protein binding
- protein domain specific binding
- protein phosphatase binding
- RNA binding
- ubiquitin protein ligase binding
- ubiquitin-like protein ligase binding
- ubiquitin-specific protease binding

Location

- ATPase complex
- azurophil granule lumen
- cytoplasm
- cytoplasmic stress granule
- cytosol
- Derlin-1 retrotranslocation complex
- endoplasmic reticulum
- endoplasmic reticulum membrane
- extracellular exosome
- extracellular region
- ficolin-1-rich granule lumen
- glutamatergic synapse
- intracellular membrane-bounded organelle
- lipid droplet
- nucleoplasm
- nucleus
- perinuclear region of cytoplasm
- proteasome complex
- protein-containing complex
- secretory granule lumen
- site of double-strand break
- VCP-NPL4-UFD1 AAA ATPase complex
- VCP-NSFL1C complex

Biological process

- activation of cysteine-type endopeptidase activity involved in apoptotic process
- aggresome assembly
- ATP metabolic process
- autophagosome maturation
- autophagy
- cellular response to arsenite ion
- cellular response to DNA damage stimulus
- cellular response to heat
- DNA repair
- double-strand break repair
- endoplasmic reticulum stress-induced pre-emptive quality control
- endoplasmic reticulum to Golgi vesicle-mediated transport
- endoplasmic reticulum unfolded protein response
- endosome to lysosome transport via multivesicular body sorting pathway
- ER-associated misfolded protein catabolic process
- ERAD pathway
- establishment of protein localization
- flavin adenine dinucleotide catabolic process
- interstrand cross-link repair
- macroautophagy
- mitotic spindle disassembly
- NADH metabolic process
- negative regulation of smoothened signaling pathway
- positive regulation of ATP biosynthetic process
- positive regulation of canonical Wnt signaling pathway
- positive regulation of Lys63-specific deubiquitinase activity
- positive regulation of mitochondrial membrane potential
- positive regulation of oxidative phosphorylation
- positive regulation of proteasomal ubiquitin-dependent protein catabolic process
- positive regulation of protein catabolic process
- positive regulation of protein K63-linked deubiquitination
- positive regulation of protein-containing complex assembly
- proteasomal protein catabolic process
- proteasome-mediated ubiquitin-dependent protein catabolic process
- protein ubiquitination
- protein-DNA covalent cross-linking repair
- regulation of aerobic respiration
- regulation of apoptotic process
- regulation of protein localization to chromatin
- regulation of synapse organization
- retrograde protein transport, ER to cytosol
- stress granule disassembly
- translesion synthesis
- ubiquitin-dependent ERAD pathway
- viral genome replication

---

5

- **Protein name:** Histone-lysine N-methyltransferase EZH2
- **Organism:** Homo sapiens
- **Uniprot Accession Number:** Q15910
- **Protein sequence length:** 746 aa
- **1D identity (%):** 16.44
- **1D identity (%) [Gaps excluded]:** 23.85
- **1D identity - Alignment Gaps:** 295
- **1D aligned content (<aminoacid>:%):** {'K': 12.18, 'P': 9.62, 'R': 8.33, 'V': 5.77, 'E': 8.97, 'F': 4.49, 'D': 6.41, 'I': 5.77, 'S': 2.56, 'L': 7.69, 'H': 2.56, 'G': 5.13, 'N': 5.13, 'A': 3.21, 'Y': 3.21, 'C': 3.21, 'Q': 2.56, 'T': 2.56, 'M': 0.64}
- **Common reported functions (%):** 0.0
- **Common reported locations (%):** 20.0
- **Common reported processes (%):** 0.0

- **PDB ID:** 5LS6
- **Chain:** J
- **Crystallized protein length:** 570 aa
- **Resolution:** 3.47 Å
- **b-phipsi ():** 0.010861
- **w-rdist ():** 0.739774
- **t-alpha ():** 0.0
- **Chemical similarity (Tanimoto Index) (%):** N/A
- **1D identity (%) [PDB]:** 2.79
- **1D identity (%) [Gaps excluded][PDB]:** 70.37
- **1D identity - Alignment Gaps [PDB]:** 1307
- **1D aligned content [PDB] (<aminoacid>:%):** {'G': 5.26, 'I': 5.26, 'Q': 5.26, 'T': 7.89, 'S': 5.26, 'P': 5.26, 'Y': 10.53, 'V': 7.89, 'L': 7.89, 'D': 2.63, 'F': 2.63, 'A': 15.79, 'E': 2.63, 'H': 2.63, 'R': 10.53, 'C': 2.63}
- **2D identity (%) [PDB]:** 28.05
- **2D identity (%) [Gaps excluded][PDB]:** 83.43
- **2D identity - Alignment Gaps [PDB]:** 703
- **2D aligned content [PDB] (<2D-fold>:%):** {'H': 51.52, 'T': 10.1, 'E': 19.87, '.': 17.85, 'G': 0.67}
- **3D similarity (TM-Score) (%) [PDB]:** 21.46

- **Gene name:** EZH2
- **Entrez ID:** 2146
- **RefSeq ID:** N/A
- **Sequence length:** N/A
- **5-UTR|CDS|3-UTR identity (%):** N/A | N/A | N/A
- **5-UTR|CDS|3-UTR identity (%) [Gaps excluded]:** N/A | N/A | N/A
- **5-UTR|CDS|3-UTR identity [Alignment Gaps]:** N/A | N/A | N/A
- **5-UTR aligned content (<base>:%):** N/A
- **CDS aligned content (<base>:%):** N/A
- **3-UTR aligned content (<base>:%):** N/A

**Uniprot Description:**  
  
 Polycomb group (PcG) protein. Catalytic subunit of the PRC2/EED-EZH2 complex, which methylates 'Lys-9' (H3K9me) and 'Lys-27' (H3K27me) of histone H3, leading to transcriptional repression of the affected target gene. Able to mono-, di- and trimethylate 'Lys-27' of histone H3 to form H3K27me1, H3K27me2 and H3K27me3, respectively. Displays a preference for substrates with less methylation, loses activity when progressively more methyl groups are incorporated into H3K27, H3K27me0 > H3K27me1 > H3K27me2 (PubMed:22323599, PubMed:30923826). Compared to EZH1-containing complexes, it is more abundant in embryonic stem cells and plays a major role in forming H3K27me3, which is required for embryonic stem cell identity and proper differentiation. The PRC2/EED-EZH2 complex may also serve as a recruiting platform for DNA methyltransferases, thereby linking two epigenetic repression systems. Genes repressed by the PRC2/EED-EZH2 complex include HOXC8, HOXA9, MYT1, CDKN2A and retinoic acid target genes. EZH2 can also methylate non-histone proteins such as the transcription factor GATA4 and the nuclear receptor RORA. Regulates the circadian clock via histone methylation at the promoter of the circadian genes. Essential for the CRY1/2-mediated repression of the transcriptional activation of PER1/2 by the CLOCK-ARNTL/BMAL1 heterodimer; involved in the di and trimethylation of 'Lys-27' of histone H3 on PER1/2 promoters which is necessary for the CRY1/2 proteins to inhibit transcription.   
  
Component of the PRC2/EED-EZH2 complex, which includes EED, EZH2, SUZ12, RBBP4 and RBBP7 and possibly AEBP2. The minimum components required for methyltransferase activity of the PRC2/EED-EZH2 complex are EED, EZH2 and SUZ12. The PRC2 complex may also interact with DNMT1, DNMT3A, DNMT3B and PHF1 via the EZH2 subunit and with SIRT1 via the SUZ12 subunit. Interacts with HDAC1 and HDAC2. Binds ATRX via the SET domain (Probable). Interacts with PRAME. Interacts with CDYL. Interacts with CLOCK, ARNTL/BMAL1 and CRY1 (By similarity). Interacts with DNMT3L; the interaction is direct (By similarity). Interacts with EZHIP; the interaction blocks EZH2 methyltransferase activity (PubMed:30923826, PubMed:31086175, PubMed:31451685). Interacts with ZNF263; recruited to the SIX3 promoter along with other proteins involved in chromatin modification and transcriptional corepression where it contributes to transcriptional repression (PubMed:32051553).   
  
 **Gene Ontology Information:**

Molecular Function

- chromatin binding
- chromatin DNA binding
- histone methyltransferase activity (H3-K27 specific)
- histone H3K27 trimethyltransferase activity
- histone methyltransferase activity
- lncRNA binding
- primary miRNA binding
- promoter-specific chromatin binding
- protein-lysine N-methyltransferase activity
- ribonucleoprotein complex binding
- RNA polymerase II cis-regulatory region sequence-specific DNA binding
- RNA polymerase II core promoter sequence-specific DNA binding
- transcription corepressor activity
- transcription corepressor binding

Location

- chromatin
- chromatin silencing complex
- chromosome, telomeric region
- ESC/E(Z) complex
- nucleoplasm
- nucleus
- pericentric heterochromatin
- pronucleus
- synapse

Biological process

- B cell differentiation
- cardiac muscle hypertrophy in response to stress
- cellular response to hydrogen peroxide
- cellular response to trichostatin A
- cerebellar cortex development
- chromatin organization
- DNA methylation
- facultative heterochromatin formation
- G1 to G0 transition
- G1/S transition of mitotic cell cycle
- hepatocyte homeostasis
- heterochromatin assembly
- hippocampus development
- histone H3-K27 methylation
- keratinocyte differentiation
- liver regeneration
- negative regulation of cytokine production involved in inflammatory response
- negative regulation of DNA-binding transcription factor activity
- negative regulation of transcription, DNA-templated
- negative regulation of G1/S transition of mitotic cell cycle
- negative regulation of gene expression, epigenetic
- negative regulation of keratinocyte differentiation
- negative regulation of retinoic acid receptor signaling pathway
- negative regulation of stem cell differentiation
- negative regulation of striated muscle cell differentiation
- negative regulation of transcription by RNA polymerase II
- negative regulation of transcription elongation from RNA polymerase II promoter
- positive regulation of cell cycle G1/S phase transition
- positive regulation of cell population proliferation
- positive regulation of dendrite development
- positive regulation of epithelial to mesenchymal transition
- positive regulation of GTPase activity
- positive regulation of MAP kinase activity
- positive regulation of protein serine/threonine kinase activity
- protein localization to chromatin
- regulation of circadian rhythm
- regulation of transcription, DNA-templated
- regulation of gliogenesis
- regulation of kidney development
- response to estradiol
- response to tetrachloromethane
- rhythmic process
- skeletal muscle satellite cell maintenance involved in skeletal muscle regeneration
- stem cell differentiation
- subtelomeric heterochromatin assembly
- synaptic transmission, GABAergic

---

6

- **Protein name:** DNA damage-binding protein 1
- **Organism:** Homo sapiens
- **Uniprot Accession Number:** Q16531
- **Protein sequence length:** 1140 aa
- **1D identity (%):** 15.58
- **1D identity (%) [Gaps excluded]:** 23.16
- **1D identity - Alignment Gaps:** 391
- **1D aligned content (<aminoacid>:%):** {'V': 8.6, 'A': 4.3, 'T': 5.91, 'L': 12.37, 'Y': 4.3, 'P': 10.22, 'K': 5.38, 'E': 4.84, 'F': 3.23, 'G': 14.52, 'N': 2.15, 'D': 4.84, 'R': 5.38, 'I': 3.23, 'S': 4.3, 'M': 1.08, 'H': 2.15, 'Q': 2.69, 'C': 0.54}
- **Common reported functions (%):** 0.0
- **Common reported locations (%):** 30.0
- **Common reported processes (%):** 0.0

- **PDB ID:** 6H0G
- **Chain:** D
- **Crystallized protein length:** 825 aa
- **Resolution:** 4.25 Å
- **b-phipsi ():** 0.023527
- **w-rdist ():** 0.507863
- **t-alpha ():** 0.004399
- **Chemical similarity (Tanimoto Index) (%):** 99.17
- **1D identity (%) [PDB]:** 3.33
- **1D identity (%) [Gaps excluded][PDB]:** 71.62
- **1D identity - Alignment Gaps [PDB]:** 1518
- **1D aligned content [PDB] (<aminoacid>:%):** {'K': 7.55, 'N': 1.89, 'I': 7.55, 'Y': 3.77, 'V': 15.09, 'T': 1.89, 'A': 9.43, 'E': 5.66, 'P': 5.66, 'G': 5.66, 'D': 1.89, 'R': 7.55, 'F': 3.77, 'S': 9.43, 'L': 9.43, 'Q': 1.89, 'M': 1.89}
- **2D identity (%) [PDB]:** 34.58
- **2D identity (%) [Gaps excluded][PDB]:** 82.52
- **2D identity - Alignment Gaps [PDB]:** 682
- **2D aligned content [PDB] (<2D-fold>:%):** {'.': 22.66, 'E': 51.72, 'T': 15.52, 'G': 0.74, 'B': 0.49, 'H': 8.87}
- **3D similarity (TM-Score) (%) [PDB]:** 25.41

- **Gene name:** DDB1
- **Entrez ID:** 1642
- **RefSeq ID:** NM\_001923
- **Transcript sequence length:** 4245
- **5-UTR|CDS|3-UTR identity (%):** 35.68 | 44.76 | 3.69
- **5-UTR|CDS|3-UTR identity (%) [Gaps excluded]:** 78.9 | 76.35 | 73.19
- **5-UTR|CDS|3-UTR identity [Alignment Gaps]:** 132 | 1591 | 10404
- **5-UTR aligned content (<base>:%):** {'G': 46.51, 'C': 33.72, 'A': 10.47, 'T': 9.3}
- **CDS aligned content (<base>:%):** {'A': 23.88, 'T': 20.74, 'G': 28.12, 'C': 27.25}
- **3-UTR aligned content (<base>:%):** {'C': 25.0, 'A': 17.57, 'G': 26.24, 'T': 31.19}

**Uniprot Description:**  
  
 Protein, which is both involved in DNA repair and protein ubiquitination, as part of the UV-DDB complex and DCX (DDB1-CUL4-X-box) complexes, respectively (PubMed:15448697, PubMed:14739464, PubMed:16260596, PubMed:16482215, PubMed:17079684, PubMed:16407242, PubMed:16407252, PubMed:16940174). Core component of the UV-DDB complex (UV-damaged DNA-binding protein complex), a complex that recognizes UV-induced DNA damage and recruit proteins of the nucleotide excision repair pathway (the NER pathway) to initiate DNA repair (PubMed:15448697, PubMed:16260596, PubMed:16407242, PubMed:16940174). The UV-DDB complex preferentially binds to cyclobutane pyrimidine dimers (CPD), 6-4 photoproducts (6-4 PP), apurinic sites and short mismatches (PubMed:15448697, PubMed:16260596, PubMed:16407242, PubMed:16940174). Also functions as a component of numerous distinct DCX (DDB1-CUL4-X-box) E3 ubiquitin-protein ligase complexes which mediate the ubiquitination and subsequent proteasomal degradation of target proteins (PubMed:14739464, PubMed:16407252, PubMed:16482215, PubMed:17079684, PubMed:25043012, PubMed:25108355, PubMed:18332868, PubMed:18381890, PubMed:19966799, PubMed:22118460, PubMed:28886238). The functional specificity of the DCX E3 ubiquitin-protein ligase complex is determined by the variable substrate recognition component recruited by DDB1 (PubMed:14739464, PubMed:16407252, PubMed:16482215, PubMed:17079684, PubMed:25043012, PubMed:25108355, PubMed:18332868, PubMed:18381890, PubMed:19966799, PubMed:22118460). DCX(DDB2) (also known as DDB1-CUL4-ROC1, CUL4-DDB-ROC1 and CUL4-DDB-RBX1) may ubiquitinate histone H2A, histone H3 and histone H4 at sites of UV-induced DNA damage (PubMed:16678110, PubMed:17041588, PubMed:16473935, PubMed:18593899). The ubiquitination of histones may facilitate their removal from the nucleosome and promote subsequent DNA repair (PubMed:16678110, PubMed:17041588, PubMed:16473935, PubMed:18593899). DCX(DDB2) also ubiquitinates XPC, which may enhance DNA-binding by XPC and promote NER (PubMed:15882621). DCX(DTL) plays a role in PCNA-dependent polyubiquitination of CDT1 and MDM2-dependent ubiquitination of TP53 in response to radiation-induced DNA damage and during DNA replication (PubMed:17041588). DCX(ERCC8) (the CSA complex) plays a role in transcription-coupled repair (TCR) (PubMed:12732143). The DDB1-CUL4A-DTL E3 ligase complex regulates the circadian clock function by mediating the ubiquitination and degradation of CRY1 (PubMed:26431207). DDB1-mediated CRY1 degradation promotes FOXO1 protein stability and FOXO1-mediated gluconeogenesis in the liver (By similarity).   
  
Component of the UV-DDB complex which includes DDB1 and DDB2; the heterodimer dimerizes to give rise to a heterotetramer when bound to damaged DNA (PubMed:9632823, PubMed:16223728, PubMed:16527807, PubMed:19109893, PubMed:22822215). The UV-DDB complex interacts with monoubiquitinated histone H2A and binds to XPC via the DDB2 subunit (PubMed:16473935). Component of numerous DCX (DDB1-CUL4-X-box) E3 ubiquitin-protein ligase complexes which consist of a core of DDB1, CUL4A or CUL4B and RBX1 (PubMed:11673459, PubMed:12732143, PubMed:15882621, PubMed:16678110, PubMed:18593899, PubMed:28886238, PubMed:28437394, PubMed:28302793, PubMed:31693891, PubMed:31686031, PubMed:31819272, PubMed:31693911). DDB1 may recruit specific substrate targeting subunits to the DCX complex (PubMed:11673459, PubMed:12732143, PubMed:15882621, PubMed:18593899, PubMed:28886238). These substrate targeting subunits are generally known as DCAF (DDB1- and CUL4-associated factor) or CDW (CUL4-DDB1-associated WD40-repeat) proteins (PubMed:17079684, PubMed:16949367, PubMed:18606781, PubMed:19608861, PubMed:16964240, PubMed:19966799). Interacts with AMBRA1, ATG16L1, BTRC, CRBN, DCAF1, DCAF4, DCAF5, DCAF6, DCAF7, DCAF8, DCAF9, DCAF10, DCAF11, DCAF12, DCAF15, DCAF16, DCAF17, DDA1, DET1, DTL, ERCC8, FBXW5, FBXW8, GRWD1, KATNB1, NLE1, NUP43, PAFAH1B1, PHIP, PWP1, RBBP4, RBBP5, RBBP7, COP1, SNRNP40, DCAF1, WDR5, WDR5B, WDR12, WDR26, WDR39, WDR42, WDR53, WDR59, WDR61, WSB1, WSB2, LRWD1 and WDTC1 (PubMed:14739464, PubMed:17079684, PubMed:16949367, PubMed:17041588, PubMed:18606781, PubMed:22935713, PubMed:23478445, PubMed:22118460, PubMed:25043012, PubMed:25108355). DCX complexes may associate with the COP9 signalosome, and this inhibits the E3 ubiquitin-protein ligase activity of the complex (PubMed:15448697, PubMed:16260596). Interacts with NF2, TSC1 and TSC2 (PubMed:18332868, PubMed:18381890). Interacts with AGO1 and AGO2 (PubMed:17932509). Associates with the E3 ligase complex containing DYRK2, EDD/UBR5, DDB1 and DCAF1 proteins (EDVP complex) (PubMed:19287380). Interacts directly with DYRK2 (PubMed:19287380). DCX(DTL) complex interacts with FBXO11; does not ubiquitinate and degradate FBXO11 (PubMed:19287380). Interacts with TRPC4AP (PubMed:19966799). Interacts with CRY1 and CRY2 (By similarity). The DDB1-CUL4A complex interacts with CRY1 (PubMed:26431207). May also interact with DCUN1D1, DCUN1D2, DCUN1D3 and DCUN1D5 (PubMed:26906416).   
  
 **Gene Ontology Information:**

Molecular Function

- cullin family protein binding
- damaged DNA binding
- DNA binding
- protein-containing complex binding
- protein-macromolecule adaptor activity
- ubiquitin ligase complex scaffold activity
- WD40-repeat domain binding

Location

- chromosome, telomeric region
- Cul4-RING E3 ubiquitin ligase complex
- Cul4A-RING E3 ubiquitin ligase complex
- Cul4B-RING E3 ubiquitin ligase complex
- cytoplasm
- extracellular exosome
- extracellular space
- nucleolus
- nucleoplasm
- nucleus
- protein-containing complex
- site of double-strand break

Biological process

- apoptotic process
- biological process involved in interaction with symbiont
- cellular response to UV
- cellular response to DNA damage stimulus
- DNA repair
- ectopic germ cell programmed cell death
- epigenetic programming in the zygotic pronuclei
- negative regulation of apoptotic process
- negative regulation of developmental process
- negative regulation of reproductive process
- nucleotide-excision repair
- positive regulation by virus of viral protein levels in host cell
- positive regulation of gluconeogenesis
- positive regulation of protein catabolic process
- positive regulation of viral genome replication
- proteasomal protein catabolic process
- proteasome-mediated ubiquitin-dependent protein catabolic process
- protein ubiquitination
- regulation of circadian rhythm
- regulation of mitotic cell cycle phase transition
- rhythmic process
- spindle assembly involved in female meiosis
- ubiquitin-dependent protein catabolic process
- UV-damage excision repair
- viral release from host cell
- Wnt signaling pathway

---

7

- **Protein name:** Insulin receptor
- **Organism:** Homo sapiens
- **Uniprot Accession Number:** P06213
- **Protein sequence length:** 1382 aa
- **1D identity (%):** 14.76
- **1D identity (%) [Gaps excluded]:** 26.08
- **1D identity - Alignment Gaps:** 621
- **1D aligned content (<aminoacid>:%):** {'M': 0.95, 'G': 10.9, 'A': 3.79, 'L': 9.95, 'P': 12.32, 'I': 5.69, 'E': 5.69, 'H': 3.32, 'D': 6.16, 'Y': 3.79, 'N': 3.79, 'V': 6.64, 'F': 3.32, 'R': 5.21, 'K': 4.74, 'W': 1.9, 'Q': 2.37, 'T': 4.74, 'C': 2.37, 'S': 2.37}
- **Common reported functions (%):** 0.0
- **Common reported locations (%):** 0.0
- **Common reported processes (%):** 0.0

- **PDB ID:** 7U6E
- **Chain:** E
- **Crystallized protein length:** 584 aa
- **Resolution:** 3.0 Å
- **b-phipsi ():** 0.015883
- **w-rdist ():** 0.436431
- **t-alpha ():** 0.01107
- **Chemical similarity (Tanimoto Index) (%):** 83.93
- **1D identity (%) [PDB]:** 1.88
- **1D identity (%) [Gaps excluded][PDB]:** 70.27
- **1D identity - Alignment Gaps [PDB]:** 1343
- **1D aligned content [PDB] (<aminoacid>:%):** {'V': 7.69, 'E': 3.85, 'G': 7.69, 'L': 19.23, 'Q': 11.54, 'I': 15.38, 'Y': 11.54, 'R': 3.85, 'D': 7.69, 'K': 3.85, 'P': 3.85, 'T': 3.85}
- **2D identity (%) [PDB]:** 35.74
- **2D identity (%) [Gaps excluded][PDB]:** 84.56
- **2D identity - Alignment Gaps [PDB]:** 575
- **2D aligned content [PDB] (<2D-fold>:%):** {'.': 18.26, 'E': 34.83, 'T': 33.43, 'B': 0.28, 'H': 12.36, 'G': 0.84}
- **3D similarity (TM-Score) (%) [PDB]:** 14.84

- **Gene name:** INSR
- **Entrez ID:** 3643
- **RefSeq ID:** NM\_001079817
- **Transcript sequence length:** 9427
- **5-UTR|CDS|3-UTR identity (%):** 31.89 | 41.76 | 27.08
- **5-UTR|CDS|3-UTR identity (%) [Gaps excluded]:** 82.04 | 76.51 | 74.87
- **5-UTR|CDS|3-UTR identity [Alignment Gaps]:** 324 | 1995 | 7320
- **5-UTR aligned content (<base>:%):** {'A': 5.92, 'C': 35.5, 'T': 10.06, 'G': 48.52}
- **CDS aligned content (<base>:%):** {'A': 23.94, 'T': 19.74, 'G': 27.97, 'C': 28.35}
- **3-UTR aligned content (<base>:%):** {'C': 18.36, 'A': 26.86, 'G': 23.0, 'T': 31.79}

**Uniprot Description:**  
  
 Receptor tyrosine kinase which mediates the pleiotropic actions of insulin. Binding of insulin leads to phosphorylation of several intracellular substrates, including, insulin receptor substrates (IRS1, 2, 3, 4), SHC, GAB1, CBL and other signaling intermediates. Each of these phosphorylated proteins serve as docking proteins for other signaling proteins that contain Src-homology-2 domains (SH2 domain) that specifically recognize different phosphotyrosine residues, including the p85 regulatory subunit of PI3K and SHP2. Phosphorylation of IRSs proteins lead to the activation of two main signaling pathways: the PI3K-AKT/PKB pathway, which is responsible for most of the metabolic actions of insulin, and the Ras-MAPK pathway, which regulates expression of some genes and cooperates with the PI3K pathway to control cell growth and differentiation. Binding of the SH2 domains of PI3K to phosphotyrosines on IRS1 leads to the activation of PI3K and the generation of phosphatidylinositol-(3, 4, 5)-triphosphate (PIP3), a lipid second messenger, which activates several PIP3-dependent serine/threonine kinases, such as PDPK1 and subsequently AKT/PKB. The net effect of this pathway is to produce a translocation of the glucose transporter SLC2A4/GLUT4 from cytoplasmic vesicles to the cell membrane to facilitate glucose transport. Moreover, upon insulin stimulation, activated AKT/PKB is responsible for: anti-apoptotic effect of insulin by inducing phosphorylation of BAD; regulates the expression of gluconeogenic and lipogenic enzymes by controlling the activity of the winged helix or forkhead (FOX) class of transcription factors. Another pathway regulated by PI3K-AKT/PKB activation is mTORC1 signaling pathway which regulates cell growth and metabolism and integrates signals from insulin. AKT mediates insulin-stimulated protein synthesis by phosphorylating TSC2 thereby activating mTORC1 pathway. The Ras/RAF/MAP2K/MAPK pathway is mainly involved in mediating cell growth, survival and cellular differentiation of insulin. Phosphorylated IRS1 recruits GRB2/SOS complex, which triggers the activation of the Ras/RAF/MAP2K/MAPK pathway. In addition to binding insulin, the insulin receptor can bind insulin-like growth factors (IGFI and IGFII). Isoform Short has a higher affinity for IGFII binding. When present in a hybrid receptor with IGF1R, binds IGF1. PubMed:12138094 shows that hybrid receptors composed of IGF1R and INSR isoform Long are activated with a high affinity by IGF1, with low affinity by IGF2 and not significantly activated by insulin, and that hybrid receptors composed of IGF1R and INSR isoform Short are activated by IGF1, IGF2 and insulin. In contrast, PubMed:16831875 shows that hybrid receptors composed of IGF1R and INSR isoform Long and hybrid receptors composed of IGF1R and INSR isoform Short have similar binding characteristics, both bind IGF1 and have a low affinity for insulin. In adipocytes, inhibits lipolysis (By similarity).   
  
Tetramer of 2 alpha and 2 beta chains linked by disulfide bonds. The alpha chains carry the insulin-binding regions, while the beta chains carry the kinase domain. Forms a hybrid receptor with IGF1R, the hybrid is a tetramer consisting of 1 alpha chain and 1 beta chain of INSR and 1 alpha chain and 1 beta chain of IGF1R. Interacts with SORBS1 but dissociates from it following insulin stimulation. Binds SH2B2. Activated form of INSR interacts (via Tyr-999) with the PTB/PID domains of IRS1 and SHC1. The sequences surrounding the phosphorylated NPXY motif contribute differentially to either IRS1 or SHC1 recognition. Interacts (via tyrosines in the C-terminus) with IRS2 (via PTB domain and 591-786 AA); the 591-786 would be the primary anchor of IRS2 to INSR while the PTB domain would have a stabilizing action on the interaction with INSR. Interacts with the SH2 domains of the 85 kDa regulatory subunit of PI3K (PIK3R1) in vitro, when autophosphorylated on tyrosine residues. Interacts with SOCS7. Interacts (via the phosphorylated Tyr-999), with SOCS3. Interacts (via the phosphorylated Tyr-1185, Tyr-1189, Tyr-1190) with SOCS1. Interacts with CAV2 (tyrosine-phosphorylated form); the interaction is increased with 'Tyr-27'phosphorylation of CAV2 (By similarity). Interacts with ARRB2 (By similarity). Interacts with GRB10; this interaction blocks the association between IRS1/IRS2 and INSR, significantly reduces insulin-stimulated tyrosine phosphorylation of IRS1 and IRS2 and thus decreases insulin signaling. Interacts with GRB7. Interacts with PDPK1. Interacts (via Tyr-1190) with GRB14 (via BPS domain); this interaction protects the tyrosines in the activation loop from dephosphorylation, but promotes dephosphorylation of Tyr-999, this results in decreased interaction with, and phosphorylation of, IRS1. Interacts (via subunit alpha) with ENPP1 (via 485-599 AA); this interaction blocks autophosphorylation. Interacts with PTPRE; this interaction is dependent of Tyr-1185, Tyr-1189 and Tyr-1190 of the INSR. Interacts with STAT5B (via SH2 domain). Interacts with PTPRF. Interacts with ATIC; ATIC together with PRKAA2/AMPK2 and HACD3/PTPLAD1 is proposed to be part of a signaling netwok regulating INSR autophosphorylation and endocytosis (By similarity). Interacts with the cone snail venom insulin Con-Ins G1 (PubMed:27617429). Interacts with the insulin receptor SORL1; this interaction strongly increases its surface exposure, hence strengthens insulin signal reception (PubMed:27322061). Interacts (tyrosine phosphorylated) with CCDC88A/GIV (via SH2-like region); binding requires autophosphorylation of the INSR C-terminal region (PubMed:25187647). Interacts with GNAI3; the interaction is probably mediated by CCDC88A/GIV (PubMed:25187647). Interacts with LMBRD1 (By similarity).   
  
 **Gene Ontology Information:**

Molecular Function

- amyloid-beta binding
- ATP binding
- cargo receptor activity
- GTP binding
- identical protein binding
- insulin binding
- insulin-activated receptor activity
- insulin receptor substrate binding
- insulin-like growth factor I binding
- insulin-like growth factor II binding
- insulin-like growth factor receptor binding
- phosphatidylinositol 3-kinase binding
- protein domain specific binding
- protein tyrosine kinase activity
- protein-containing complex binding
- PTB domain binding
- structural molecule activity

Location

- axon
- caveola
- dendrite membrane
- endosome membrane
- external side of plasma membrane
- extracellular exosome
- insulin receptor complex
- late endosome
- lysosome
- membrane
- neuronal cell body membrane
- plasma membrane
- receptor complex

Biological process

- activation of protein kinase activity
- activation of protein kinase B activity
- adrenal gland development
- amyloid-beta clearance
- carbohydrate metabolic process
- cellular response to growth factor stimulus
- cellular response to insulin stimulus
- dendritic spine maintenance
- epidermis development
- exocrine pancreas development
- G protein-coupled receptor signaling pathway
- glucose homeostasis
- heart morphogenesis
- insulin receptor signaling pathway
- learning
- male gonad development
- male sex determination
- memory
- neuron projection maintenance
- peptidyl-tyrosine autophosphorylation
- peptidyl-tyrosine phosphorylation
- positive regulation of cell migration
- positive regulation of cell population proliferation
- positive regulation of developmental growth
- positive regulation of transcription, DNA-templated
- positive regulation of glucose import
- positive regulation of glycogen biosynthetic process
- positive regulation of glycolytic process
- positive regulation of kinase activity
- positive regulation of MAP kinase activity
- positive regulation of MAPK cascade
- positive regulation of meiotic cell cycle
- positive regulation of mitotic nuclear division
- positive regulation of nitric oxide biosynthetic process
- positive regulation of phosphatidylinositol 3-kinase signaling
- positive regulation of protein kinase B signaling
- positive regulation of protein phosphorylation
- positive regulation of protein-containing complex disassembly
- positive regulation of receptor internalization
- positive regulation of respiratory burst
- protein autophosphorylation
- protein phosphorylation
- receptor internalization
- receptor-mediated endocytosis
- regulation of transcription, DNA-templated
- regulation of embryonic development
- regulation of female gonad development
- transmembrane receptor protein tyrosine kinase signaling pathway
- transport across blood-brain barrier
- viral entry into host cell

---

8

- **Protein name:** CD180 antigen
- **Organism:** Homo sapiens
- **Uniprot Accession Number:** Q99467
- **Protein sequence length:** 661 aa
- **1D identity (%):** 12.36
- **1D identity (%) [Gaps excluded]:** 21.94
- **1D identity - Alignment Gaps:** 424
- **1D aligned content (<aminoacid>:%):** {'F': 5.83, 'K': 5.0, 'I': 8.33, 'Y': 1.67, 'E': 5.83, 'P': 7.5, 'D': 4.17, 'N': 4.17, 'T': 5.0, 'G': 8.33, 'S': 5.83, 'L': 15.83, 'V': 3.33, 'H': 4.17, 'R': 1.67, 'W': 0.83, 'A': 5.0, 'C': 3.33, 'Q': 4.17}
- **Common reported functions (%):** 0.0
- **Common reported locations (%):** 10.0
- **Common reported processes (%):** 0.0

- **PDB ID:** 3B2D
- **Chain:** A
- **Crystallized protein length:** 601 aa
- **Resolution:** 2.8 Å
- **b-phipsi ():** 0.013817
- **w-rdist ():** 0.435659
- **t-alpha ():** 0.029301
- **Chemical similarity (Tanimoto Index) (%):** 79.22
- **1D identity (%) [PDB]:** 1.27
- **1D identity (%) [Gaps excluded][PDB]:** 72.0
- **1D identity - Alignment Gaps [PDB]:** 1391
- **1D aligned content [PDB] (<aminoacid>:%):** {'Q': 11.11, 'V': 16.67, 'K': 22.22, 'N': 11.11, 'T': 5.56, 'S': 5.56, 'L': 22.22, 'G': 5.56}
- **2D identity (%) [PDB]:** 29.2
- **2D identity (%) [Gaps excluded][PDB]:** 84.82
- **2D identity - Alignment Gaps [PDB]:** 703
- **2D aligned content [PDB] (<2D-fold>:%):** {'.': 31.95, 'E': 29.39, 'T': 28.75, 'G': 3.19, 'H': 6.39, 'B': 0.32}
- **3D similarity (TM-Score) (%) [PDB]:** 21.65

- **Gene name:** CD180
- **Entrez ID:** 4064
- **RefSeq ID:** NM\_005582
- **Transcript sequence length:** 5388
- **5-UTR|CDS|3-UTR identity (%):** 25.36 | 40.22 | 18.67
- **5-UTR|CDS|3-UTR identity (%) [Gaps excluded]:** 73.68 | 74.19 | 76.98
- **5-UTR|CDS|3-UTR identity [Alignment Gaps]:** 181 | 1384 | 8575
- **5-UTR aligned content (<base>:%):** {'C': 37.14, 'G': 27.14, 'A': 15.71, 'T': 20.0}
- **CDS aligned content (<base>:%):** {'A': 26.73, 'T': 25.0, 'G': 22.29, 'C': 25.99}
- **3-UTR aligned content (<base>:%):** {'T': 31.5, 'C': 21.1, 'G': 21.24, 'A': 26.16}

**Uniprot Description:**  
  
 May cooperate with MD-1 and TLR4 to mediate the innate immune response to bacterial lipopolysaccharide (LPS) in B-cells. Leads to NF-kappa-B activation. Also involved in the life/death decision of B-cells (By similarity).   
  
M-shaped tetramer of two CD180-LY86 heterodimers.   
  
 **Gene Ontology Information:**

Molecular Function   
  
N/A

Location

- extracellular matrix
- extracellular space
- mitotic spindle
- nucleolus
- nucleoplasm
- plasma membrane

Biological process

- B cell proliferation involved in immune response
- cellular response to lipopolysaccharide
- inflammatory response
- innate immune response
- lipopolysaccharide-mediated signaling pathway
- positive regulation of lipopolysaccharide-mediated signaling pathway

---
