# Supplementary material for: Comparative structural insights and functional analysis for the distinct unbound states of Human AGO proteins
Source: Sci Rep. 2025 Mar 19;15:9432. doi: 10.1038/s41598-025-91849-5 (PMC11923369; doi:10.1038/s41598-025-91849-5)
Supplement: Supplementary file 24 — Supplementary Information 12. [file 41598_2025_91849_MOESM24_ESM.zip › 4Z4Dp_A_mddomain_HL2REF/candidates/4Z4Dp_A_Piwi-merged-enriched_eval_report.html]

 

# Structural Comparison Report for 4Z4Dp\_A\_Piwi - domains (total: 37)

---

1

- **Protein name:** 5'-AMP-activated protein kinase catalytic subunit alpha-2
- **Organism:** Homo sapiens
- **Uniprot Accession Number:** P54646
- **Protein sequence length:** 552 aa
- **1D identity (%):** 11.14
- **1D identity (%) [Gaps excluded]:** 20.04
- **1D identity - Alignment Gaps:** 403
- **1D aligned content (<aminoacid>:%):** {'E': 3.96, 'Q': 3.96, 'K': 5.94, 'R': 10.89, 'V': 9.9, 'G': 11.88, 'T': 2.97, 'L': 9.9, 'P': 11.88, 'H': 2.97, 'C': 2.97, 'S': 3.96, 'A': 2.97, 'D': 3.96, 'F': 4.95, 'M': 1.98, 'N': 0.99, 'W': 0.99, 'Y': 1.98, 'I': 0.99}
- **Common reported functions (%):** 0.0
- **Common reported locations (%):** 35.71
- **Common reported processes (%):** 0.0

- **PDB ID:** 4CFF
- **Chain:** E
- **Crystallized protein length:** 299 aa
- **Resolution:** 3.92 Å
- **Associated domain:** Protein-kinase
- **b-phipsi:** 0.008113
- **w-rdist:** 0.097836
- **t-alpha:** 0.0
- **Chemical similarity (Tanimoto Index) (%):** 80.28
- **1D identity (%) [PDB]:** 1.52
- **1D identity (%) [Gaps excluded][PDB]:** 77.27
- **1D identity - Alignment Gaps [PDB]:** 1093
- **1D aligned content [PDB] (<aminoacid>:%):** {'L': 5.88, 'V': 35.29, 'I': 5.88, 'P': 11.76, 'T': 11.76, 'Y': 5.88, 'A': 5.88, 'E': 5.88, 'R': 5.88, 'D': 5.88}
- **2D identity (%) [PDB]:** 18.98
- **2D identity (%) [Gaps excluded][PDB]:** 81.4
- **2D identity - Alignment Gaps [PDB]:** 707
- **2D aligned content [PDB] (<2D-fold>:%):** {'H': 54.86, '.': 13.71, 'E': 18.29, 'T': 11.43, 'G': 1.71}
- **3D similarity (TM-Score) (%) [PDB]:** 15.34

- **Gene name:** PRKAA2
- **Entrez ID:** 5571
- **RefSeq ID:** NM\_006252
- **Transcript sequence length:** 9355
- **5-UTR|CDS|3-UTR identity (%):** 42.11 | 33.12 | 36.17
- **5-UTR|CDS|3-UTR identity (%) [Gaps excluded]:** 82.35 | 76.05 | 75.53
- **5-UTR|CDS|3-UTR identity [Alignment Gaps]:** 65 | 1667 | 6874
- **5-UTR aligned content (<base>:%):** {'G': 62.5, 'T': 5.36, 'A': 1.79, 'C': 30.36}
- **CDS aligned content (<base>:%):** {'A': 29.04, 'T': 22.29, 'G': 25.87, 'C': 22.8}
- **3-UTR aligned content (<base>:%):** {'T': 38.87, 'G': 17.29, 'C': 15.38, 'A': 28.46}

**Uniprot Description:**  
  
 Catalytic subunit of AMP-activated protein kinase (AMPK), an energy sensor protein kinase that plays a key role in regulating cellular energy metabolism. In response to reduction of intracellular ATP levels, AMPK activates energy-producing pathways and inhibits energy-consuming processes: inhibits protein, carbohydrate and lipid biosynthesis, as well as cell growth and proliferation. AMPK acts via direct phosphorylation of metabolic enzymes, and by longer-term effects via phosphorylation of transcription regulators. Also acts as a regulator of cellular polarity by remodeling the actin cytoskeleton; probably by indirectly activating myosin. Regulates lipid synthesis by phosphorylating and inactivating lipid metabolic enzymes such as ACACA, ACACB, GYS1, HMGCR and LIPE; regulates fatty acid and cholesterol synthesis by phosphorylating acetyl-CoA carboxylase (ACACA and ACACB) and hormone-sensitive lipase (LIPE) enzymes, respectively. Regulates insulin-signaling and glycolysis by phosphorylating IRS1, PFKFB2 and PFKFB3. Involved in insulin receptor/INSR internalization (PubMed:25687571). AMPK stimulates glucose uptake in muscle by increasing the translocation of the glucose transporter SLC2A4/GLUT4 to the plasma membrane, possibly by mediating phosphorylation of TBC1D4/AS160. Regulates transcription and chromatin structure by phosphorylating transcription regulators involved in energy metabolism such as CRTC2/TORC2, FOXO3, histone H2B, HDAC5, MEF2C, MLXIPL/ChREBP, EP300, HNF4A, p53/TP53, SREBF1, SREBF2 and PPARGC1A. Acts as a key regulator of glucose homeostasis in liver by phosphorylating CRTC2/TORC2, leading to CRTC2/TORC2 sequestration in the cytoplasm. In response to stress, phosphorylates 'Ser-36' of histone H2B (H2BS36ph), leading to promote transcription. Acts as a key regulator of cell growth and proliferation by phosphorylating TSC2, RPTOR and ATG1/ULK1: in response to nutrient limitation, negatively regulates the mTORC1 complex by phosphorylating RPTOR component of the mTORC1 complex and by phosphorylating and activating TSC2. In response to nutrient limitation, promotes autophagy by phosphorylating and activating ATG1/ULK1. In that process also activates WDR45 (PubMed:28561066). AMPK also acts as a regulator of circadian rhythm by mediating phosphorylation of CRY1, leading to destabilize it. May regulate the Wnt signaling pathway by phosphorylating CTNNB1, leading to stabilize it. Also phosphorylates CFTR, EEF2K, KLC1, NOS3 and SLC12A1. Plays an important role in the differential regulation of pro-autophagy (composed of PIK3C3, BECN1, PIK3R4 and UVRAG or ATG14) and non-autophagy (composed of PIK3C3, BECN1 and PIK3R4) complexes, in response to glucose starvation. Can inhibit the non-autophagy complex by phosphorylating PIK3C3 and can activate the pro-autophagy complex by phosphorylating BECN1 (By similarity).   
  
AMPK is a heterotrimer of an alpha catalytic subunit (PRKAA1 or PRKAA2), a beta (PRKAB1 or PRKAB2) and a gamma non-catalytic subunits (PRKAG1, PRKAG2 or PRKAG3). Interacts with FNIP1 and FNIP2. Associates with internalized insulin receptor/INSR complexes on Golgi/endosomal membranes; PRKAA2/AMPK2 together with ATIC and HACD3/PTPLAD1 is proposed to be part of a signaling network regulating INSR autophosphorylation and endocytosis (PubMed:25687571).   
  
 **Gene Ontology Information:**

Molecular Function

- ADP binding
- AMP binding
- ATP binding
- cAMP-dependent protein kinase activity
- cAMP-dependent protein kinase regulator activity
- protein kinase activity
- protein kinase binding
- protein kinase regulator activity

Location

- cytoplasm
- cytosol
- membrane
- nucleoplasm
- nucleotide-activated protein kinase complex
- nucleus

Biological process

- cellular response to nutrient levels
- fatty acid biosynthetic process
- import into nucleus
- positive regulation of gene expression
- positive regulation of protein kinase activity
- protein phosphorylation
- regulation of catalytic activity
- regulation of glycolytic process
- signal transduction
- spermatogenesis

---

2

- **Protein name:** Kinesin-like protein KIF11
- **Organism:** Homo sapiens
- **Uniprot Accession Number:** P52732
- **Protein sequence length:** 1056 aa
- **1D identity (%):** 15.27
- **1D identity (%) [Gaps excluded]:** 21.2
- **1D identity - Alignment Gaps:** 311
- **1D aligned content (<aminoacid>:%):** {'M': 1.18, 'S': 4.12, 'G': 5.29, 'P': 5.29, 'R': 5.29, 'T': 9.41, 'K': 7.65, 'D': 8.82, 'I': 4.71, 'Y': 2.94, 'E': 7.65, 'F': 2.94, 'L': 14.71, 'V': 4.71, 'A': 3.53, 'N': 2.94, 'Q': 4.12, 'H': 2.35, 'C': 2.35}
- **Common reported functions (%):** 0.0
- **Common reported locations (%):** 21.43
- **Common reported processes (%):** 0.0

- **PDB ID:** 3HQD
- **Chain:** B
- **Crystallized protein length:** 350 aa
- **Resolution:** 2.19 Å
- **Associated domain:** Kinesin-motor
- **b-phipsi:** 0.001376
- **w-rdist:** 0.126258
- **t-alpha:** 0.003027
- **Chemical similarity (Tanimoto Index) (%):** 78.39
- **1D identity (%) [PDB]:** 1.91
- **1D identity (%) [Gaps excluded][PDB]:** 62.86
- **1D identity - Alignment Gaps [PDB]:** 1118
- **1D aligned content [PDB] (<aminoacid>:%):** {'K': 4.55, 'N': 13.64, 'Q': 4.55, 'R': 9.09, 'T': 13.64, 'L': 27.27, 'V': 9.09, 'G': 9.09, 'I': 4.55, 'P': 4.55}
- **2D identity (%) [PDB]:** 24.51
- **2D identity (%) [Gaps excluded][PDB]:** 83.33
- **2D identity - Alignment Gaps [PDB]:** 648
- **2D aligned content [PDB] (<2D-fold>:%):** {'.': 14.22, 'E': 36.89, 'T': 6.22, 'H': 41.33, 'G': 1.33}
- **3D similarity (TM-Score) (%) [PDB]:** 15.34

- **Gene name:** KIF11
- **Entrez ID:** 3832
- **RefSeq ID:** NM\_004523
- **Transcript sequence length:** 5016
- **5-UTR|CDS|3-UTR identity (%):** 38.74 | 43.03 | 8.51
- **5-UTR|CDS|3-UTR identity (%) [Gaps excluded]:** 81.67 | 72.39 | 76.16
- **5-UTR|CDS|3-UTR identity [Alignment Gaps]:** 133 | 1463 | 10777
- **5-UTR aligned content (<base>:%):** {'G': 42.86, 'T': 7.14, 'C': 48.98, 'A': 1.02}
- **CDS aligned content (<base>:%):** {'A': 31.89, 'T': 21.13, 'G': 24.81, 'C': 22.16}
- **3-UTR aligned content (<base>:%):** {'T': 38.66, 'C': 15.6, 'A': 28.88, 'G': 16.86}

**Uniprot Description:**  
  
 Motor protein required for establishing a bipolar spindle during mitosis (PubMed:19001501). Required in non-mitotic cells for transport of secretory proteins from the Golgi complex to the cell surface (PubMed:23857769).   
  
Interacts with the thyroid hormone receptor in the presence of thyroid hormone. Component of a large chromatin remodeling complex, at least composed of MYSM1, PCAF, RBM10 and KIF11/TRIP5. Interacts (via C-terminus) with the kinase NEK6 in both interphase and mitosis.   
  
 **Gene Ontology Information:**

Molecular Function

- ATP binding
- microtubule binding
- microtubule motor activity
- ATP-dependent microtubule motor activity, plus-end-directed
- protein kinase binding

Location

- cytosol
- kinesin complex
- membrane
- microtubule
- mitotic spindle
- nucleus
- protein-containing complex
- spindle
- spindle pole

Biological process

- cell division
- microtubule-based movement
- mitotic cell cycle
- mitotic centrosome separation
- mitotic spindle assembly
- mitotic spindle organization
- regulation of mitotic centrosome separation
- spindle elongation
- spindle organization

---

3

- **Protein name:** Chitinase-3-like protein 2
- **Organism:** Homo sapiens
- **Uniprot Accession Number:** Q15782
- **Protein sequence length:** 390 aa
- **1D identity (%):** 9.25
- **1D identity (%) [Gaps excluded]:** 23.58
- **1D identity - Alignment Gaps:** 545
- **1D aligned content (<aminoacid>:%):** {'G': 12.05, 'S': 6.02, 'L': 15.66, 'W': 3.61, 'A': 8.43, 'F': 4.82, 'C': 1.2, 'I': 2.41, 'E': 3.61, 'D': 6.02, 'V': 7.23, 'K': 6.02, 'P': 7.23, 'R': 4.82, 'T': 4.82, 'Q': 3.61, 'N': 2.41}
- **Common reported functions (%):** 0.0
- **Common reported locations (%):** 0.0
- **Common reported processes (%):** 0.0

- **PDB ID:** 4AY1
- **Chain:** L
- **Crystallized protein length:** 364 aa
- **Resolution:** 1.95 Å
- **Associated domain:** GH18
- **b-phipsi:** 0.010348
- **w-rdist:** 0.077985
- **t-alpha:** 0.002016
- **Chemical similarity (Tanimoto Index) (%):** 84.26
- **1D identity (%) [PDB]:** 2.07
- **1D identity (%) [Gaps excluded][PDB]:** 55.81
- **1D identity - Alignment Gaps [PDB]:** 1116
- **1D aligned content [PDB] (<aminoacid>:%):** {'K': 8.33, 'V': 16.67, 'I': 4.17, 'S': 16.67, 'L': 25.0, 'G': 4.17, 'R': 8.33, 'F': 4.17, 'E': 4.17, 'Q': 4.17, 'D': 4.17}
- **2D identity (%) [PDB]:** 25.4
- **2D identity (%) [Gaps excluded][PDB]:** 86.45
- **2D identity - Alignment Gaps [PDB]:** 656
- **2D aligned content [PDB] (<2D-fold>:%):** {'E': 30.08, '.': 11.02, 'G': 5.08, 'T': 12.29, 'H': 41.53}
- **3D similarity (TM-Score) (%) [PDB]:** 17.78

- **Gene name:** CHI3L2
- **Entrez ID:** 1117
- **RefSeq ID:** NM\_004000
- **Transcript sequence length:** 1416
- **5-UTR|CDS|3-UTR identity (%):** 9.38 | 27.49 | 1.28
- **5-UTR|CDS|3-UTR identity (%) [Gaps excluded]:** 75.0 | 74.98 | 76.77
- **5-UTR|CDS|3-UTR identity [Alignment Gaps]:** 112 | 1739 | 11718
- **5-UTR aligned content (<base>:%):** {'G': 50.0, 'A': 16.67, 'C': 25.0, 'T': 8.33}
- **CDS aligned content (<base>:%):** {'T': 19.07, 'G': 27.55, 'A': 25.56, 'C': 27.81}
- **3-UTR aligned content (<base>:%):** {'A': 21.05, 'G': 25.0, 'T': 30.92, 'C': 23.03}

**Uniprot Description:**  
  
 Lectin that binds chitooligosaccharides and other glycans with high affinity, but not heparin. Has no chitinase activity. N/A   
  
 **Gene Ontology Information:**

Molecular Function

- carbohydrate binding
- chitin binding
- hydrolase activity

Location

- extracellular region
- extracellular space

Biological process

- carbohydrate metabolic process
- chitin catabolic process

---

4

- **Protein name:** Casein kinase II subunit alpha
- **Organism:** Homo sapiens
- **Uniprot Accession Number:** P68400
- **Protein sequence length:** 391 aa
- **1D identity (%):** 9.32
- **1D identity (%) [Gaps excluded]:** 24.07
- **1D identity - Alignment Gaps:** 552
- **1D aligned content (<aminoacid>:%):** {'P': 9.52, 'R': 5.95, 'V': 16.67, 'E': 7.14, 'D': 7.14, 'W': 1.19, 'Q': 1.19, 'G': 7.14, 'F': 3.57, 'I': 3.57, 'L': 9.52, 'N': 3.57, 'S': 1.19, 'T': 4.76, 'K': 4.76, 'C': 1.19, 'M': 2.38, 'H': 1.19, 'A': 5.95, 'Y': 2.38}
- **Common reported functions (%):** 0.0
- **Common reported locations (%):** 21.43
- **Common reported processes (%):** 0.0

- **PDB ID:** 7ZY8
- **Chain:** A
- **Crystallized protein length:** 325 aa
- **Resolution:** 1.85 Å
- **Associated domain:** Protein-kinase
- **b-phipsi:** 0.006161
- **w-rdist:** 0.282454
- **t-alpha:** 0.001006
- **Chemical similarity (Tanimoto Index) (%):** 98.71
- **1D identity (%) [PDB]:** 1.49
- **1D identity (%) [Gaps excluded][PDB]:** 70.83
- **1D identity - Alignment Gaps [PDB]:** 1115
- **1D aligned content [PDB] (<aminoacid>:%):** {'G': 5.88, 'R': 17.65, 'I': 11.76, 'K': 5.88, 'L': 5.88, 'T': 23.53, 'D': 5.88, 'Q': 5.88, 'M': 5.88, 'A': 11.76}
- **2D identity (%) [PDB]:** 25.7
- **2D identity (%) [Gaps excluded][PDB]:** 84.19
- **2D identity - Alignment Gaps [PDB]:** 619
- **2D aligned content [PDB] (<2D-fold>:%):** {'.': 17.03, 'T': 23.58, 'E': 17.47, 'H': 40.61, 'G': 1.31}
- **3D similarity (TM-Score) (%) [PDB]:** 16.05

- **Gene name:** CSNK2A1
- **Entrez ID:** 1457
- **RefSeq ID:** NM\_177560
- **Transcript sequence length:** 12657
- **5-UTR|CDS|3-UTR identity (%):** 22.74 | 16.92 | 43.57
- **5-UTR|CDS|3-UTR identity (%) [Gaps excluded]:** 80.33 | 73.88 | 75.35
- **5-UTR|CDS|3-UTR identity [Alignment Gaps]:** 309 | 2100 | 6241
- **5-UTR aligned content (<base>:%):** {'G': 47.96, 'T': 8.16, 'C': 40.82, 'A': 3.06}
- **CDS aligned content (<base>:%):** {'A': 25.16, 'T': 23.64, 'G': 26.9, 'C': 24.3}
- **3-UTR aligned content (<base>:%):** {'C': 20.18, 'G': 22.11, 'A': 26.06, 'T': 31.65}

**Uniprot Description:**  
  
 Catalytic subunit of a constitutively active serine/threonine-protein kinase complex that phosphorylates a large number of substrates containing acidic residues C-terminal to the phosphorylated serine or threonine (PubMed:11239457, PubMed:11704824, PubMed:16193064, PubMed:19188443, PubMed:20625391, PubMed:22406621, PubMed:24962073). Regulates numerous cellular processes, such as cell cycle progression, apoptosis and transcription, as well as viral infection (PubMed:12631575, PubMed:19387552, PubMed:19387551). May act as a regulatory node which integrates and coordinates numerous signals leading to an appropriate cellular response (PubMed:12631575, PubMed:19387552, PubMed:19387551). During mitosis, functions as a component of the p53/TP53-dependent spindle assembly checkpoint (SAC) that maintains cyclin-B-CDK1 activity and G2 arrest in response to spindle damage (PubMed:11704824, PubMed:19188443). Also required for p53/TP53-mediated apoptosis, phosphorylating 'Ser-392' of p53/TP53 following UV irradiation. Can also negatively regulate apoptosis (PubMed:11239457). Phosphorylates the caspases CASP9 and CASP2 and the apoptotic regulator NOL3 (PubMed:16193064). Phosphorylation protects CASP9 from cleavage and activation by CASP8, and inhibits the dimerization of CASP2 and activation of CASP8 (PubMed:16193064). Regulates transcription by direct phosphorylation of RNA polymerases I, II, III and IV. Also phosphorylates and regulates numerous transcription factors including NF-kappa-B, STAT1, CREB1, IRF1, IRF2, ATF1, ATF4, SRF, MAX, JUN, FOS, MYC and MYB (PubMed:19387550, PubMed:12631575, PubMed:19387552, PubMed:19387551, PubMed:23123191). Phosphorylates Hsp90 and its co-chaperones FKBP4 and CDC37, which is essential for chaperone function (PubMed:19387550). Mediates sequential phosphorylation of FNIP1, promoting its gradual interaction with Hsp90, leading to activate both kinase and non-kinase client proteins of Hsp90 (PubMed:30699359). Regulates Wnt signaling by phosphorylating CTNNB1 and the transcription factor LEF1 (PubMed:19387549). Acts as an ectokinase that phosphorylates several extracellular proteins (PubMed:19387550, PubMed:12631575, PubMed:19387552, PubMed:19387551). During viral infection, phosphorylates various proteins involved in the viral life cycles of EBV, HSV, HBV, HCV, HIV, CMV and HPV (PubMed:19387550, PubMed:12631575, PubMed:19387552, PubMed:19387551). Phosphorylates PML at 'Ser-565' and primes it for ubiquitin-mediated degradation (PubMed:20625391, PubMed:22406621). Plays an important role in the circadian clock function by phosphorylating ARNTL/BMAL1 at 'Ser-90' which is pivotal for its interaction with CLOCK and which controls CLOCK nuclear entry (By similarity). Phosphorylates CCAR2 at 'Thr-454' in gastric carcinoma tissue (PubMed:24962073).   
  
Heterotetramer composed of two catalytic subunits (alpha chain and/or alpha' chain) and two regulatory subunits (beta chains). The tetramer can exist as a combination of 2 alpha/2 beta, 2 alpha'/2 beta or 1 alpha/1 alpha'/2 beta subunits. Also part of a CK2-SPT16-SSRP1 complex composed of SSRP1, SUPT16H, CSNK2A1, CSNK2A2 and CSNK2B, which forms following UV irradiation. Interacts with RNPS1. Interacts with SNAI1. Interacts with PML (isoform PML-12). Interacts with CCAR2.   
  
 **Gene Ontology Information:**

Molecular Function

- ATP binding
- Hsp90 protein binding
- identical protein binding
- kinase activity
- protein serine kinase activity
- protein serine/threonine kinase activity

Location

- cytosol
- nucleoplasm
- nucleus
- NuRD complex
- plasma membrane
- PML body
- protein kinase CK2 complex
- Sin3 complex

Biological process

- apoptotic process
- cell cycle
- chaperone-mediated protein folding
- symbiont-mediated disruption of host cell PML body
- negative regulation of apoptotic signaling pathway
- negative regulation of cysteine-type endopeptidase activity involved in apoptotic process
- negative regulation of translation
- negative regulation of ubiquitin-dependent protein catabolic process
- peptidyl-serine phosphorylation
- peptidyl-threonine phosphorylation
- positive regulation of cell growth
- positive regulation of cell population proliferation
- positive regulation of protein catabolic process
- positive regulation of Wnt signaling pathway
- protein phosphorylation
- protein stabilization
- regulation of cell cycle
- regulation of chromosome separation
- rhythmic process
- signal transduction
- Wnt signaling pathway

---

5

- **Protein name:** Protein arginine N-methyltransferase 5
- **Organism:** Homo sapiens
- **Uniprot Accession Number:** O14744
- **Protein sequence length:** 637 aa
- **1D identity (%):** 11.56
- **1D identity (%) [Gaps excluded]:** 21.25
- **1D identity - Alignment Gaps:** 442
- **1D aligned content (<aminoacid>:%):** {'A': 5.36, 'S': 4.46, 'G': 8.04, 'C': 3.57, 'V': 6.25, 'D': 4.46, 'P': 11.61, 'K': 5.36, 'R': 9.82, 'L': 9.82, 'I': 3.57, 'F': 4.46, 'E': 5.36, 'H': 2.68, 'T': 4.46, 'W': 1.79, 'Q': 4.46, 'Y': 1.79, 'M': 0.89, 'N': 1.79}
- **Common reported functions (%):** 0.0
- **Common reported locations (%):** 28.57
- **Common reported processes (%):** 0.0

- **PDB ID:** 5EMM
- **Chain:** A
- **Crystallized protein length:** 625 aa
- **Resolution:** 2.37 Å
- **Associated domain:** SAM-dependent-MTase-PRMT-type
- **b-phipsi:** 0.00533
- **w-rdist:** 0.413078
- **t-alpha:** 0.0
- **Chemical similarity (Tanimoto Index) (%):** 78.98
- **1D identity (%) [PDB]:** 4.19
- **1D identity (%) [Gaps excluded][PDB]:** 72.5
- **1D identity - Alignment Gaps [PDB]:** 1303
- **1D aligned content [PDB] (<aminoacid>:%):** {'R': 8.62, 'V': 12.07, 'D': 1.72, 'T': 5.17, 'L': 10.34, 'G': 8.62, 'A': 6.9, 'Q': 8.62, 'C': 3.45, 'M': 1.72, 'K': 5.17, 'N': 6.9, 'P': 8.62, 'S': 3.45, 'I': 3.45, 'F': 1.72, 'H': 1.72, 'Y': 1.72}
- **2D identity (%) [PDB]:** 26.21
- **2D identity (%) [Gaps excluded][PDB]:** 91.41
- **2D identity - Alignment Gaps [PDB]:** 811
- **2D aligned content [PDB] (<2D-fold>:%):** {'.': 16.78, 'E': 27.85, 'H': 38.59, 'T': 13.42, 'G': 3.02, 'B': 0.34}
- **3D similarity (TM-Score) (%) [PDB]:** 24.7

- **Gene name:** PRMT5
- **Entrez ID:** 10419
- **RefSeq ID:** NM\_006109
- **Transcript sequence length:** 2304
- **5-UTR|CDS|3-UTR identity (%):** 6.87 | 41.77 | 2.04
- **5-UTR|CDS|3-UTR identity (%) [Gaps excluded]:** 81.82 | 75.41 | 73.64
- **5-UTR|CDS|3-UTR identity [Alignment Gaps]:** 120 | 1290 | 11603
- **5-UTR aligned content (<base>:%):** {'G': 77.78, 'C': 11.11, 'A': 11.11}
- **CDS aligned content (<base>:%):** {'A': 23.18, 'T': 20.12, 'G': 28.56, 'C': 28.15}
- **3-UTR aligned content (<base>:%):** {'C': 24.28, 'G': 20.99, 'A': 23.46, 'T': 31.28}

**Uniprot Description:**  
  
 Arginine methyltransferase that can both catalyze the formation of omega-N monomethylarginine (MMA) and symmetrical dimethylarginine (sDMA), with a preference for the formation of MMA (PubMed:10531356, PubMed:11152681, PubMed:11747828, PubMed:12411503, PubMed:15737618, PubMed:17709427, PubMed:20159986, PubMed:20810653, PubMed:21258366, PubMed:21917714, PubMed:22269951, PubMed:21081503). Specifically mediates the symmetrical dimethylation of arginine residues in the small nuclear ribonucleoproteins Sm D1 (SNRPD1) and Sm D3 (SNRPD3); such methylation being required for the assembly and biogenesis of snRNP core particles (PubMed:12411503, PubMed:11747828, PubMed:17709427). Methylates SUPT5H and may regulate its transcriptional elongation properties (PubMed:12718890). Mono- and dimethylates arginine residues of myelin basic protein (MBP) in vitro. May play a role in cytokine-activated transduction pathways. Negatively regulates cyclin E1 promoter activity and cellular proliferation. Methylates histone H2A and H4 'Arg-3' during germ cell development. Methylates histone H3 'Arg-8', which may repress transcription. Methylates the Piwi proteins (PIWIL1, PIWIL2 and PIWIL4), methylation of Piwi proteins being required for the interaction with Tudor domain-containing proteins and subsequent localization to the meiotic nuage (By similarity). Methylates RPS10. Attenuates EGF signaling through the MAPK1/MAPK3 pathway acting at 2 levels. First, monomethylates EGFR; this enhances EGFR 'Tyr-1197' phosphorylation and PTPN6 recruitment, eventually leading to reduced SOS1 phosphorylation (PubMed:21917714, PubMed:21258366). Second, methylates RAF1 and probably BRAF, hence destabilizing these 2 signaling proteins and reducing their catalytic activity (PubMed:21917714). Required for induction of E-selectin and VCAM-1, on the endothelial cells surface at sites of inflammation. Methylates HOXA9 (PubMed:22269951). Methylates and regulates SRGAP2 which is involved in cell migration and differentiation (PubMed:20810653). Acts as a transcriptional corepressor in CRY1-mediated repression of the core circadian component PER1 by regulating the H4R3 dimethylation at the PER1 promoter (By similarity). Methylates GM130/GOLGA2, regulating Golgi ribbon formation (PubMed:20421892). Methylates H4R3 in genes involved in glioblastomagenesis in a CHTOP- and/or TET1-dependent manner (PubMed:25284789). Symmetrically methylates POLR2A, a modification that allows the recruitment to POLR2A of proteins including SMN1/SMN2 and SETX. This is required for resolving RNA-DNA hybrids created by RNA polymerase II, that form R-loop in transcription terminal regions, an important step in proper transcription termination (PubMed:26700805). Along with LYAR, binds the promoter of gamma-globin HBG1/HBG2 and represses its expression (PubMed:25092918). Symmetrically methylates NCL (PubMed:21081503). Methylates TP53; methylation might possibly affect TP53 target gene specificity (PubMed:19011621). Involved in spliceosome maturation and mRNA splicing in prophase I spermatocytes through the catalysis of the symmetrical arginine dimethylation of SNRPB (small nuclear ribonucleoprotein-associated protein) and the interaction with tudor domain-containing protein TDRD6 (By similarity).   
  
Forms, at least, homodimers and homotetramers (PubMed:11152681). Component of the methylosome complex, composed of PRMT5, WDR77 and CLNS1A (PubMed:21081503). Found in a complex composed of PRMT5, WDR77 and RIOK1 (PubMed:21081503). RIOK1 and CLNS1A associate with PRMT5 in a mutually exclusive fashion, which allows the recruitment of distinct methylation substrates, such as nucleolin/NCL and Sm proteins, respectively (PubMed:21081503). Interacts with PRDM1 (By similarity). Identified in a complex composed of methylosome and PRMT1 and ERH (PubMed:25284789). Interacts with EGFR; methylates EGFR and stimulates EGFR-mediated ERK activation. Interacts with HOXA9. Interacts with SRGAP2. Found in a complex with COPRS, RUNX1 and CBFB. Interacts with CHTOP; the interaction symmetrically methylates CHTOP, but seems to require the presence of PRMT1 (PubMed:25284789). Interacts with EPB41L3; this modulates methylation of target proteins. Component of a high molecular weight E2F-pocket protein complex, CERC (cyclin E1 repressor complex). Associates with SWI/SNF remodeling complexes containing SMARCA2 and SMARCA4. Interacts with JAK2, SSTR1, SUPT5H, BRAF and with active RAF1. Interacts with LSM11, PRMT7 and SNRPD3 (PubMed:17709427, PubMed:16087681). Interacts with COPRS; promoting its recruitment on histone H4. Interacts with CLNS1A/pICln (PubMed:21081503, PubMed:9556550). Identified in a complex with CLNS1A/pICln and Sm proteins. Interacts with RPS10 (PubMed:20159986). Interacts with WDR77. Interacts with IWS1. Interacts with CRY1. Interacts with POLR2A (PubMed:26700805). Interacts with SMN1/SMN2 (PubMed:26700805). Interacts with LYAR; this interaction is direct (PubMed:25092918). Interacts with STRAP (PubMed:19011621). Interacts with TP53 in response to DNA damage; the interaction is STRAP dependent (PubMed:19011621). Interacts with TDRD6 (By similarity).   
  
 **Gene Ontology Information:**

Molecular Function

- E-box binding
- histone-arginine N-methyltransferase activity
- histone methyltransferase activity (H4-R3 specific)
- identical protein binding
- methyl-CpG binding
- methyltransferase activity
- p53 binding
- protein heterodimerization activity
- protein-arginine N-methyltransferase activity
- protein-arginine omega-N symmetric methyltransferase activity
- ribonucleoprotein complex binding
- transcription corepressor activity

Location

- chromatin
- cytoplasm
- cytosol
- Golgi apparatus
- histone methyltransferase complex
- methylosome
- nucleoplasm
- nucleus

Biological process

- circadian regulation of gene expression
- DNA-templated transcription, termination
- endothelial cell activation
- Golgi ribbon formation
- histone arginine methylation
- histone H4-R3 methylation
- liver regeneration
- negative regulation of cell differentiation
- peptidyl-arginine methylation
- peptidyl-arginine N-methylation
- positive regulation of adenylate cyclase-inhibiting dopamine receptor signaling pathway
- positive regulation of mRNA splicing, via spliceosome
- positive regulation of oligodendrocyte differentiation
- regulation of DNA methylation
- regulation of transcription, DNA-templated
- regulation of ERK1 and ERK2 cascade
- regulation of mitotic nuclear division
- regulation of signal transduction by p53 class mediator
- spliceosomal snRNP assembly

---

6

- **Protein name:** Mitogen-activated protein kinase 10
- **Organism:** Homo sapiens
- **Uniprot Accession Number:** P53779
- **Protein sequence length:** 464 aa
- **1D identity (%):** 12.74
- **1D identity (%) [Gaps excluded]:** 25.23
- **1D identity - Alignment Gaps:** 435
- **1D aligned content (<aminoacid>:%):** {'S': 4.46, 'L': 8.93, 'V': 9.82, 'Q': 6.25, 'D': 4.46, 'H': 2.68, 'M': 1.79, 'F': 3.57, 'G': 7.14, 'R': 3.57, 'P': 12.5, 'A': 7.14, 'Y': 3.57, 'I': 2.68, 'K': 8.04, 'E': 4.46, 'C': 4.46, 'N': 2.68, 'T': 0.89, 'W': 0.89}
- **Common reported functions (%):** 0.0
- **Common reported locations (%):** 28.57
- **Common reported processes (%):** 0.0

- **PDB ID:** 7S1N
- **Chain:** A
- **Crystallized protein length:** 346 aa
- **Resolution:** 2.11 Å
- **Associated domain:** Protein-kinase
- **b-phipsi:** 0.009242
- **w-rdist:** 0.208685
- **t-alpha:** 0.001007
- **Chemical similarity (Tanimoto Index) (%):** 78.73
- **1D identity (%) [PDB]:** 2.62
- **1D identity (%) [Gaps excluded][PDB]:** 75.0
- **1D identity - Alignment Gaps [PDB]:** 1106
- **1D aligned content [PDB] (<aminoacid>:%):** {'V': 20.0, 'G': 6.67, 'D': 3.33, 'T': 10.0, 'L': 16.67, 'Q': 6.67, 'M': 3.33, 'K': 10.0, 'N': 13.33, 'P': 3.33, 'C': 3.33, 'I': 3.33}
- **2D identity (%) [PDB]:** 29.57
- **2D identity (%) [Gaps excluded][PDB]:** 83.55
- **2D identity - Alignment Gaps [PDB]:** 566
- **2D aligned content [PDB] (<2D-fold>:%):** {'.': 20.08, 'E': 19.69, 'T': 11.2, 'H': 45.56, 'G': 3.47}
- **3D similarity (TM-Score) (%) [PDB]:** 15.66

- **Gene name:** MAPK10
- **Entrez ID:** 5602
- **RefSeq ID:** N/A
- **Sequence length:** N/A
- **5-UTR|CDS|3-UTR identity (%):** N/A | N/A | N/A
- **5-UTR|CDS|3-UTR identity (%) [Gaps excluded]:** N/A | N/A | N/A
- **5-UTR|CDS|3-UTR identity [Alignment Gaps]:** N/A | N/A | N/A
- **5-UTR aligned content (<base>:%):** N/A
- **CDS aligned content (<base>:%):** N/A
- **3-UTR aligned content (<base>:%):** N/A

**Uniprot Description:**  
  
 Serine/threonine-protein kinase involved in various processes such as neuronal proliferation, differentiation, migration and programmed cell death. Extracellular stimuli such as proinflammatory cytokines or physical stress stimulate the stress-activated protein kinase/c-Jun N-terminal kinase (SAP/JNK) signaling pathway. In this cascade, two dual specificity kinases MAP2K4/MKK4 and MAP2K7/MKK7 phosphorylate and activate MAPK10/JNK3. In turn, MAPK10/JNK3 phosphorylates a number of transcription factors, primarily components of AP-1 such as JUN and ATF2 and thus regulates AP-1 transcriptional activity. Plays regulatory roles in the signaling pathways during neuronal apoptosis. Phosphorylates the neuronal microtubule regulator STMN2. Acts in the regulation of the amyloid-beta precursor protein/APP signaling during neuronal differentiation by phosphorylating APP. Participates also in neurite growth in spiral ganglion neurons. Phosphorylates the CLOCK-ARNTL/BMAL1 heterodimer and plays a role in the photic regulation of the circadian clock (PubMed:22441692). Phosphorylates JUND and this phosphorylation is inhibited in the presence of MEN1 (PubMed:22327296).   
  
Interacts with MAPKBP1 (By similarity). Interacts with MAPK8IP1/JIP-1 and MAPK8IP3/JIP-3/JSAP1 (By similarity). Interacts with SPAG9/MAPK8IP4/JIP4 (PubMed:15693750). Interacts with HDAC9 (PubMed:16611996). Interacts with ARRB2; the interaction enhances MAPK10 activation by MAP3K5 (PubMed:18435604). Interacts with SARM1 (By similarity). Interacts with JUND; interaction is inhibited in the presence of MEN1 (PubMed:22327296).   
  
 **Gene Ontology Information:**

Molecular Function

- ATP binding
- JUN kinase activity
- MAP kinase kinase activity
- protein serine kinase activity

Location

- cytoplasm
- cytosol
- mitochondrion
- nucleoplasm
- nucleus
- plasma membrane

Biological process

- cellular senescence
- Fc-epsilon receptor signaling pathway
- JNK cascade
- protein phosphorylation
- regulation of circadian rhythm
- regulation of DNA-binding transcription factor activity
- response to light stimulus
- rhythmic process
- signal transduction

---

7

- **Protein name:** Protein argonaute-3
- **Organism:** Homo sapiens
- **Uniprot Accession Number:** Q9H9G7
- **Protein sequence length:** 860 aa
- **1D identity (%):** 79.52
- **1D identity (%) [Gaps excluded]:** 81.29
- **1D identity - Alignment Gaps:** 19
- **1D aligned content (<aminoacid>:%):** {'M': 2.17, 'G': 7.09, 'A': 6.8, 'P': 6.8, 'R': 6.66, 'T': 6.37, 'I': 5.21, 'K': 5.35, 'L': 7.67, 'N': 2.32, 'F': 4.49, 'D': 4.78, 'Y': 3.62, 'E': 4.78, 'C': 2.75, 'V': 8.54, 'Q': 6.08, 'H': 3.47, 'S': 4.34, 'W': 0.72}
- **Common reported functions (%):** 46.67
- **Common reported locations (%):** 57.14
- **Common reported processes (%):** 23.81

- **PDB ID:** 5VM9
- **Chain:** C
- **Crystallized protein length:** 785 aa
- **Resolution:** 3.28 Å
- **Associated domain:** Piwi
- **b-phipsi:** 0.007425
- **w-rdist:** 0.02604
- **t-alpha:** 0.036217
- **Chemical similarity (Tanimoto Index) (%):** 100.0
- **1D identity (%) [PDB]:** 76.93
- **1D identity (%) [Gaps excluded][PDB]:** 81.8
- **1D identity - Alignment Gaps [PDB]:** 50
- **1D aligned content [PDB] (<aminoacid>:%):** {'P': 6.65, 'R': 6.96, 'G': 6.03, 'T': 6.8, 'I': 5.56, 'K': 5.41, 'L': 8.19, 'A': 6.65, 'N': 2.32, 'F': 4.79, 'D': 4.48, 'Y': 3.71, 'E': 4.17, 'C': 2.94, 'V': 9.12, 'M': 2.01, 'Q': 6.03, 'H': 3.4, 'S': 4.02, 'W': 0.77}
- **2D identity (%) [PDB]:** 65.52
- **2D identity (%) [Gaps excluded][PDB]:** 75.36
- **2D identity - Alignment Gaps [PDB]:** 114
- **2D aligned content [PDB] (<2D-fold>:%):** {'.': 18.74, 'E': 32.22, 'T': 10.16, 'H': 36.43, 'B': 1.93, 'G': 0.53}
- **3D similarity (TM-Score) (%) [PDB]:** 89.83

- **Gene name:** AGO3
- **Entrez ID:** 192669
- **RefSeq ID:** N/A
- **Sequence length:** N/A
- **5-UTR|CDS|3-UTR identity (%):** N/A | N/A | N/A
- **5-UTR|CDS|3-UTR identity (%) [Gaps excluded]:** N/A | N/A | N/A
- **5-UTR|CDS|3-UTR identity [Alignment Gaps]:** N/A | N/A | N/A
- **5-UTR aligned content (<base>:%):** N/A
- **CDS aligned content (<base>:%):** N/A
- **3-UTR aligned content (<base>:%):** N/A

**Uniprot Description:**  
  
 Required for RNA-mediated gene silencing (RNAi). Binds to short RNAs such as microRNAs (miRNAs) and represses the translation of mRNAs which are complementary to them. Proposed to be involved in stabilization of small RNA derivates (siRNA) derived from processed RNA polymerase III-transcribed Alu repeats containing a DR2 retinoic acid response element (RARE) in stem cells and in the subsequent siRNA-dependent degradation of a subset of RNA polymerase II-transcribed coding mRNAs by recruiting a mRNA decapping complex involving EDC4. Possesses RNA slicer activity but only on select RNAs bearing 5'- and 3'-flanking sequences to the region of guide-target complementarity (PubMed:29040713).   
  
Interacts with EIF4B, IMP8, PRMT5 and TNRC6B. Interacts with APOBEC3F, APOBEC3G and APOBEC3H. Interacts with EDC4.   
  
 **Gene Ontology Information:**

Molecular Function

- double-stranded RNA binding
- endoribonuclease activity, cleaving miRNA-paired mRNA
- metal ion binding
- miRNA binding
- RNA binding
- endoribonuclease activity
- single-stranded RNA binding

Location

- condensed nuclear chromosome
- cytoplasm
- cytoplasmic ribonucleoprotein granule
- cytosol
- membrane
- nucleoplasm
- P-body
- RISC complex
- RISC-loading complex

Biological process

- production of miRNAs involved in gene silencing by miRNA
- miRNA mediated inhibition of translation
- mRNA catabolic process
- positive regulation of gene expression
- positive regulation of NIK/NF-kappaB signaling
- pre-miRNA processing
- regulation of stem cell proliferation
- small RNA loading onto RISC
- RNA secondary structure unwinding

---

8

- **Protein name:** Serotransferrin
- **Organism:** Homo sapiens
- **Uniprot Accession Number:** P02787
- **Protein sequence length:** 698 aa
- **1D identity (%):** 11.82
- **1D identity (%) [Gaps excluded]:** 24.41
- **1D identity - Alignment Gaps:** 541
- **1D aligned content (<aminoacid>:%):** {'M': 1.61, 'R': 4.84, 'V': 8.06, 'G': 9.68, 'A': 8.06, 'W': 1.61, 'F': 4.84, 'H': 3.23, 'S': 4.03, 'P': 7.26, 'K': 7.26, 'D': 9.68, 'Q': 3.23, 'C': 5.65, 'E': 4.84, 'L': 6.45, 'N': 4.03, 'I': 2.42, 'Y': 0.81, 'T': 2.42}
- **Common reported functions (%):** 0.0
- **Common reported locations (%):** 7.14
- **Common reported processes (%):** 0.0

- **PDB ID:** 6D05
- **Chain:** C
- **Crystallized protein length:** 679 aa
- **Resolution:** 3.8 Å
- **Associated domain:** Transferrin-like-1
- **b-phipsi:** 0.015299
- **w-rdist:** 0.151558
- **t-alpha:** 0.0
- **Chemical similarity (Tanimoto Index) (%):** 68.63
- **1D identity (%) [PDB]:** 1.69
- **1D identity (%) [Gaps excluded][PDB]:** 62.5
- **1D identity - Alignment Gaps [PDB]:** 1437
- **1D aligned content [PDB] (<aminoacid>:%):** {'V': 16.0, 'E': 8.0, 'T': 4.0, 'L': 12.0, 'P': 8.0, 'G': 8.0, 'K': 8.0, 'D': 8.0, 'F': 4.0, 'S': 8.0, 'I': 4.0, 'H': 4.0, 'A': 4.0, 'R': 4.0}
- **2D identity (%) [PDB]:** 32.52
- **2D identity (%) [Gaps excluded][PDB]:** 86.06
- **2D identity - Alignment Gaps [PDB]:** 685
- **2D aligned content [PDB] (<2D-fold>:%):** {'.': 19.27, 'H': 49.44, 'T': 10.61, 'E': 19.83, 'G': 0.84}
- **3D similarity (TM-Score) (%) [PDB]:** 21.9

- **Gene name:** TF
- **Entrez ID:** 7018
- **RefSeq ID:** N/A
- **Sequence length:** N/A
- **5-UTR|CDS|3-UTR identity (%):** N/A | N/A | N/A
- **5-UTR|CDS|3-UTR identity (%) [Gaps excluded]:** N/A | N/A | N/A
- **5-UTR|CDS|3-UTR identity [Alignment Gaps]:** N/A | N/A | N/A
- **5-UTR aligned content (<base>:%):** N/A
- **CDS aligned content (<base>:%):** N/A
- **3-UTR aligned content (<base>:%):** N/A

**Uniprot Description:**  
  
 Transferrins are iron binding transport proteins which can bind two Fe(3+) ions in association with the binding of an anion, usually bicarbonate. It is responsible for the transport of iron from sites of absorption and heme degradation to those of storage and utilization. Serum transferrin may also have a further role in stimulating cell proliferation.   
  
Monomer.   
  
 **Gene Ontology Information:**

Molecular Function

- ferric iron binding
- ferrous iron binding
- iron chaperone activity
- transferrin receptor binding

Location

- apical plasma membrane
- basal part of cell
- basal plasma membrane
- blood microparticle
- cell surface
- clathrin-coated endocytic vesicle membrane
- clathrin-coated pit
- cytoplasmic vesicle
- early endosome
- endocytic vesicle
- endoplasmic reticulum lumen
- endosome membrane
- extracellular exosome
- extracellular region
- extracellular space
- HFE-transferrin receptor complex
- late endosome
- perinuclear region of cytoplasm
- plasma membrane
- recycling endosome
- secretory granule lumen
- vesicle

Biological process

- actin filament organization
- antibacterial humoral response
- cellular response to iron ion
- ERK1 and ERK2 cascade
- cellular iron ion homeostasis
- iron ion transmembrane transport
- iron ion transport
- osteoclast differentiation
- positive regulation of bone resorption
- positive regulation of cell motility
- positive regulation of transcription, DNA-templated
- positive regulation of phosphorylation
- positive regulation of receptor-mediated endocytosis
- regulation of iron ion transport
- regulation of protein stability
- retina homeostasis
- SMAD protein signal transduction

---

9

- **Protein name:** Glycogen synthase kinase-3 beta
- **Organism:** Homo sapiens
- **Uniprot Accession Number:** P49841
- **Protein sequence length:** 420 aa
- **1D identity (%):** 9.89
- **1D identity (%) [Gaps excluded]:** 22.62
- **1D identity - Alignment Gaps:** 501
- **1D aligned content (<aminoacid>:%):** {'G': 9.09, 'E': 6.82, 'C': 3.41, 'V': 9.09, 'D': 7.95, 'T': 7.95, 'A': 5.68, 'P': 7.95, 'R': 5.68, 'S': 4.55, 'F': 4.55, 'L': 6.82, 'K': 3.41, 'M': 1.14, 'H': 3.41, 'Y': 2.27, 'N': 3.41, 'Q': 2.27, 'I': 4.55}
- **Common reported functions (%):** 0.0
- **Common reported locations (%):** 35.71
- **Common reported processes (%):** 0.0

- **PDB ID:** 6H0U
- **Chain:** A
- **Crystallized protein length:** 348 aa
- **Resolution:** 2.3 Å
- **Associated domain:** Protein-kinase
- **b-phipsi:** 0.0068
- **w-rdist:** 0.314828
- **t-alpha:** 0.001007
- **Chemical similarity (Tanimoto Index) (%):** 84.07
- **1D identity (%) [PDB]:** 3.66
- **1D identity (%) [Gaps excluded][PDB]:** 62.12
- **1D identity - Alignment Gaps [PDB]:** 1054
- **1D aligned content [PDB] (<aminoacid>:%):** {'K': 4.88, 'V': 19.51, 'P': 9.76, 'G': 4.88, 'D': 4.88, 'R': 9.76, 'S': 9.76, 'I': 2.44, 'L': 9.76, 'E': 4.88, 'T': 7.32, 'H': 2.44, 'Y': 2.44, 'F': 4.88, 'N': 2.44}
- **2D identity (%) [PDB]:** 22.58
- **2D identity (%) [Gaps excluded][PDB]:** 78.49
- **2D identity - Alignment Gaps [PDB]:** 656
- **2D aligned content [PDB] (<2D-fold>:%):** {'E': 26.92, '.': 18.75, 'T': 9.62, 'H': 42.79, 'B': 0.48, 'G': 1.44}
- **3D similarity (TM-Score) (%) [PDB]:** 15.96

- **Gene name:** GSK3B
- **Entrez ID:** 2932
- **RefSeq ID:** NM\_002093
- **Transcript sequence length:** 7782
- **5-UTR|CDS|3-UTR identity (%):** 11.44 | 28.03 | 26.23
- **5-UTR|CDS|3-UTR identity (%) [Gaps excluded]:** 92.06 | 74.62 | 74.19
- **5-UTR|CDS|3-UTR identity [Alignment Gaps]:** 888 | 1762 | 8289
- **5-UTR aligned content (<base>:%):** {'C': 45.69, 'T': 10.34, 'G': 40.52, 'A': 3.45}
- **CDS aligned content (<base>:%):** {'A': 26.8, 'T': 20.23, 'G': 26.3, 'C': 26.68}
- **3-UTR aligned content (<base>:%):** {'C': 18.23, 'A': 26.52, 'G': 18.91, 'T': 36.34}

**Uniprot Description:**  
  
 Constitutively active protein kinase that acts as a negative regulator in the hormonal control of glucose homeostasis, Wnt signaling and regulation of transcription factors and microtubules, by phosphorylating and inactivating glycogen synthase (GYS1 or GYS2), EIF2B, CTNNB1/beta-catenin, APC, AXIN1, DPYSL2/CRMP2, JUN, NFATC1/NFATC, MAPT/TAU and MACF1. Requires primed phosphorylation of the majority of its substrates. In skeletal muscle, contributes to insulin regulation of glycogen synthesis by phosphorylating and inhibiting GYS1 activity and hence glycogen synthesis. May also mediate the development of insulin resistance by regulating activation of transcription factors. Regulates protein synthesis by controlling the activity of initiation factor 2B (EIF2BE/EIF2B5) in the same manner as glycogen synthase. In Wnt signaling, GSK3B forms a multimeric complex with APC, AXIN1 and CTNNB1/beta-catenin and phosphorylates the N-terminus of CTNNB1 leading to its degradation mediated by ubiquitin/proteasomes. Phosphorylates JUN at sites proximal to its DNA-binding domain, thereby reducing its affinity for DNA. Phosphorylates NFATC1/NFATC on conserved serine residues promoting NFATC1/NFATC nuclear export, shutting off NFATC1/NFATC gene regulation, and thereby opposing the action of calcineurin. Phosphorylates MAPT/TAU on 'Thr-548', decreasing significantly MAPT/TAU ability to bind and stabilize microtubules. MAPT/TAU is the principal component of neurofibrillary tangles in Alzheimer disease. Plays an important role in ERBB2-dependent stabilization of microtubules at the cell cortex. Phosphorylates MACF1, inhibiting its binding to microtubules which is critical for its role in bulge stem cell migration and skin wound repair. Probably regulates NF-kappa-B (NFKB1) at the transcriptional level and is required for the NF-kappa-B-mediated anti-apoptotic response to TNF-alpha (TNF/TNFA). Negatively regulates replication in pancreatic beta-cells, resulting in apoptosis, loss of beta-cells and diabetes. Through phosphorylation of the anti-apoptotic protein MCL1, may control cell apoptosis in response to growth factors deprivation. Phosphorylates MUC1 in breast cancer cells, decreasing the interaction of MUC1 with CTNNB1/beta-catenin. Is necessary for the establishment of neuronal polarity and axon outgrowth. Phosphorylates MARK2, leading to inhibit its activity. Phosphorylates SIK1 at 'Thr-182', leading to sustain its activity. Phosphorylates ZC3HAV1 which enhances its antiviral activity. Phosphorylates SNAI1, leading to its BTRC-triggered ubiquitination and proteasomal degradation. Phosphorylates SFPQ at 'Thr-687' upon T-cell activation. Phosphorylates NR1D1 st 'Ser-55' and 'Ser-59' and stabilizes it by protecting it from proteasomal degradation. Regulates the circadian clock via phosphorylation of the major clock components including ARNTL/BMAL1, CLOCK and PER2 (PubMed:19946213, PubMed:28903391). Phosphorylates CLOCK AT 'Ser-427' and targets it for proteasomal degradation (PubMed:19946213). Phosphorylates ARNTL/BMAL1 at 'Ser-17' and 'Ser-21' and primes it for ubiquitination and proteasomal degradation (PubMed:28903391). Phosphorylates OGT at 'Ser-3' or 'Ser-4' which positively regulates its activity. Phosphorylates MYCN in neuroblastoma cells which may promote its degradation (PubMed:24391509). Regulates the circadian rhythmicity of hippocampal long-term potentiation and ARNTL/BMLA1 and PER2 expression (By similarity). Acts as a regulator of autophagy by mediating phosphorylation of KAT5/TIP60 under starvation conditions, leading to activate KAT5/TIP60 acetyltransferase activity and promote acetylation of key autophagy regulators, such as ULK1 and RUBCNL/Pacer (PubMed:30704899). Negatively regulates extrinsic apoptotic signaling pathway via death domain receptors. Promotes the formation of an anti-apoptotic complex, made of DDX3X, BRIC2 and GSK3B, at death receptors, including TNFRSF10B. The anti-apoptotic function is most effective with weak apoptotic signals and can be overcome by stronger stimulation (PubMed:18846110).   
  
Monomer. Interacts with ARRB2, DISC1 and ZBED3 (By similarity). Interacts with CABYR, MMP2, MUC1, NIN and PRUNE1. Interacts with AXIN1; the interaction mediates hyperphosphorylation of CTNNB1 leading to its ubiquitination and destruction. Interacts with and phosphorylates SNAI1. Interacts with DNM1L (via a C-terminal domain). Found in a complex composed of MACF1, APC, AXIN1, CTNNB1 and GSK3B (By similarity). Interacts with SGK3. Interacts with DAB2IP (via C2 domain); the interaction stimulates GSK3B kinase activation. Interacts (via C2 domain) with PPP2CA. Interacts with the CLOCK-ARNTL/BMAL1 heterodimer (PubMed:19946213). Interacts with the ARNTL/BMAL1 (PubMed:28903391). Interacts with CTNND2 (PubMed:19706605). Interacts with NCYM (PubMed:24391509). The complex composed, at least, of APC, CTNNB1 and GSK3B interacts with JPT1; the interaction requires the inactive form of GSK3B (phosphorylated at 'Ser-9') (PubMed:25169422). Forms a complex composed of PRKAR2A or PRKAR2B, GSK3B and GSKIP through GSKIP interaction; facilitates PKA-induced phosphorylation and regulates GSK3B activity (PubMed:27484798, PubMed:20007971, PubMed:25920809). Interacts with GSKIP (PubMed:16981698). Interacts with GID8 (PubMed:28829046). Interacts with PIWIL2 (By similarity). Interacts with LMBR1L (PubMed:31073040). Interacts with DDX3X (PubMed:18846110). Interacts with BIRC2 (PubMed:18846110). Interacts with TNFRSF10B; TNFRSF10B stimulation inhibits GSK3B kinase activity (PubMed:18846110).   
  
 **Gene Ontology Information:**

Molecular Function

- ATP binding
- beta-catenin binding
- dynactin binding
- kinase activity
- NF-kappaB binding
- p53 binding
- protease binding
- protein kinase A catalytic subunit binding
- protein kinase activity
- protein kinase binding
- protein serine kinase activity
- protein serine/threonine kinase activity
- RNA polymerase II-specific DNA-binding transcription factor binding
- tau protein binding
- tau-protein kinase activity
- ubiquitin protein ligase binding

Location

- axon
- beta-catenin destruction complex
- centrosome
- cytoplasm
- cytosol
- dendrite
- glutamatergic synapse
- mitochondrion
- nucleoplasm
- nucleus
- plasma membrane
- postsynapse
- presynapse
- Wnt signalosome

Biological process

- beta-catenin destruction complex disassembly
- cellular response to amyloid-beta
- cellular response to interleukin-3
- cellular response to retinoic acid
- circadian rhythm
- dopamine receptor signaling pathway
- epithelial to mesenchymal transition
- ER overload response
- establishment of cell polarity
- excitatory postsynaptic potential
- extrinsic apoptotic signaling pathway
- extrinsic apoptotic signaling pathway in absence of ligand
- glycogen metabolic process
- heart valve development
- hippocampus development
- insulin receptor signaling pathway
- intracellular signal transduction
- maintenance of cell polarity
- negative regulation of apoptotic process
- negative regulation of calcineurin-NFAT signaling cascade
- negative regulation of canonical Wnt signaling pathway
- negative regulation of canonical Wnt signaling pathway involved in osteoblast differentiation
- negative regulation of dopaminergic neuron differentiation
- negative regulation of extrinsic apoptotic signaling pathway via death domain receptors
- negative regulation of gene expression
- negative regulation of glycogen (starch) synthase activity
- negative regulation of glycogen biosynthetic process
- negative regulation of mesenchymal stem cell differentiation
- negative regulation of phosphoprotein phosphatase activity
- negative regulation of protein acetylation
- negative regulation of protein binding
- negative regulation of protein localization to nucleus
- negative regulation of protein-containing complex assembly
- negative regulation of type B pancreatic cell development
- neuron projection development
- neuron projection organization
- peptidyl-serine phosphorylation
- peptidyl-threonine phosphorylation
- positive regulation of autophagy
- positive regulation of cell differentiation
- positive regulation of cell-matrix adhesion
- positive regulation of cilium assembly
- positive regulation of gene expression
- positive regulation of GTPase activity
- positive regulation of mitochondrial outer membrane permeabilization involved in apoptotic signaling pathway
- positive regulation of mitochondrion organization
- positive regulation of proteasomal ubiquitin-dependent protein catabolic process
- positive regulation of protein binding
- positive regulation of protein catabolic process
- positive regulation of protein export from nucleus
- positive regulation of protein localization to centrosome
- positive regulation of protein localization to cilium
- positive regulation of protein ubiquitination
- positive regulation of protein-containing complex assembly
- presynaptic modulation of chemical synaptic transmission
- proteasome-mediated ubiquitin-dependent protein catabolic process
- protein autophosphorylation
- protein phosphorylation
- regulation of axon extension
- regulation of axonogenesis
- regulation of cellular response to heat
- regulation of circadian rhythm
- regulation of dendrite morphogenesis
- regulation of long-term synaptic potentiation
- regulation of microtubule anchoring at centrosome
- regulation of microtubule cytoskeleton organization
- regulation of microtubule-based process
- regulation of neuron projection development
- regulation of protein export from nucleus
- superior temporal gyrus development
- viral protein processing

---

10

- **Protein name:** Kinesin-like protein KIF9
- **Organism:** Homo sapiens
- **Uniprot Accession Number:** Q9HAQ2
- **Protein sequence length:** 790 aa
- **1D identity (%):** 14.76
- **1D identity (%) [Gaps excluded]:** 23.7
- **1D identity - Alignment Gaps:** 383
- **1D aligned content (<aminoacid>:%):** {'M': 0.67, 'K': 10.0, 'F': 4.67, 'P': 6.0, 'D': 5.33, 'G': 7.33, 'L': 10.0, 'W': 0.67, 'S': 5.33, 'H': 3.33, 'A': 4.67, 'E': 8.0, 'T': 5.33, 'V': 6.0, 'Y': 2.0, 'I': 4.0, 'Q': 6.0, 'R': 7.33, 'C': 0.67, 'N': 2.67}
- **Common reported functions (%):** 0.0
- **Common reported locations (%):** 7.14
- **Common reported processes (%):** 0.0

- **PDB ID:** 3NWN
- **Chain:** A
- **Crystallized protein length:** 308 aa
- **Resolution:** 2.0 Å
- **Associated domain:** Kinesin-motor
- **b-phipsi:** 0.013041
- **w-rdist:** 0.223031
- **t-alpha:** 0.0
- **Chemical similarity (Tanimoto Index) (%):** N/A
- **1D identity (%) [PDB]:** 1.27
- **1D identity (%) [Gaps excluded][PDB]:** 73.68
- **1D identity - Alignment Gaps [PDB]:** 1084
- **1D aligned content [PDB] (<aminoacid>:%):** {'Q': 14.29, 'T': 14.29, 'V': 21.43, 'A': 7.14, 'F': 7.14, 'K': 14.29, 'D': 7.14, 'R': 7.14, 'L': 7.14}
- **2D identity (%) [PDB]:** 25.8
- **2D identity (%) [Gaps excluded][PDB]:** 91.87
- **2D identity - Alignment Gaps [PDB]:** 630
- **2D aligned content [PDB] (<2D-fold>:%):** {'.': 8.85, 'E': 31.42, 'T': 21.68, 'H': 35.4, 'G': 2.65}
- **3D similarity (TM-Score) (%) [PDB]:** 15.67

- **Gene name:** KIF9
- **Entrez ID:** 64147
- **RefSeq ID:** N/A
- **Sequence length:** N/A
- **5-UTR|CDS|3-UTR identity (%):** N/A | N/A | N/A
- **5-UTR|CDS|3-UTR identity (%) [Gaps excluded]:** N/A | N/A | N/A
- **5-UTR|CDS|3-UTR identity [Alignment Gaps]:** N/A | N/A | N/A
- **5-UTR aligned content (<base>:%):** N/A
- **CDS aligned content (<base>:%):** N/A
- **3-UTR aligned content (<base>:%):** N/A

**Uniprot Description:**  
  
 Essential for normal male fertility and for progressive motility of spermatozoa.   
  
Interacts with HYDIN.   
  
 **Gene Ontology Information:**

Molecular Function

- ATP binding
- ATPase activity
- identical protein binding
- microtubule binding
- microtubule motor activity

Location

- cytoplasm
- kinesin complex
- microtubule
- podosome
- sperm flagellum
- vesicle

Biological process

- extracellular matrix disassembly
- microtubule-based movement
- organelle disassembly
- regulation of flagellated sperm motility
- regulation of podosome assembly

---

11

- **Protein name:** Atlastin-1
- **Organism:** Homo sapiens
- **Uniprot Accession Number:** Q8WXF7
- **Protein sequence length:** 558 aa
- **1D identity (%):** 10.9
- **1D identity (%) [Gaps excluded]:** 20.61
- **1D identity - Alignment Gaps:** 437
- **1D aligned content (<aminoacid>:%):** {'M': 1.98, 'R': 5.94, 'N': 3.96, 'S': 3.96, 'T': 5.94, 'E': 5.94, 'V': 4.95, 'K': 3.96, 'D': 7.92, 'H': 4.95, 'L': 6.93, 'A': 6.93, 'G': 10.89, 'I': 5.94, 'P': 8.91, 'Q': 3.96, 'F': 3.96, 'C': 0.99, 'W': 0.99, 'Y': 0.99}
- **Common reported functions (%):** 0.0
- **Common reported locations (%):** 7.14
- **Common reported processes (%):** 0.0

- **PDB ID:** 4IDP
- **Chain:** B
- **Crystallized protein length:** 407 aa
- **Resolution:** 2.59 Å
- **Associated domain:** GB1-RHD3-type-G
- **b-phipsi:** 0.001876
- **w-rdist:** 0.407972
- **t-alpha:** 0.003027
- **Chemical similarity (Tanimoto Index) (%):** 72.86
- **1D identity (%) [PDB]:** 2.56
- **1D identity (%) [Gaps excluded][PDB]:** 68.89
- **1D identity - Alignment Gaps [PDB]:** 1164
- **1D aligned content [PDB] (<aminoacid>:%):** {'K': 16.13, 'A': 3.23, 'P': 6.45, 'V': 9.68, 'D': 6.45, 'S': 6.45, 'E': 9.68, 'L': 3.23, 'T': 9.68, 'Q': 3.23, 'R': 6.45, 'F': 3.23, 'I': 6.45, 'G': 6.45, 'C': 3.23}
- **2D identity (%) [PDB]:** 31.95
- **2D identity (%) [Gaps excluded][PDB]:** 86.94
- **2D identity - Alignment Gaps [PDB]:** 580
- **2D aligned content [PDB] (<2D-fold>:%):** {'.': 13.99, 'E': 21.16, 'T': 9.22, 'H': 54.27, 'G': 1.02, 'B': 0.34}
- **3D similarity (TM-Score) (%) [PDB]:** 15.68

- **Gene name:** ATL1
- **Entrez ID:** 51062
- **RefSeq ID:** NM\_181598
- **Transcript sequence length:** 2512
- **5-UTR|CDS|3-UTR identity (%):** 55.8 | 36.35 | 4.07
- **5-UTR|CDS|3-UTR identity (%) [Gaps excluded]:** 70.0 | 74.66 | 75.27
- **5-UTR|CDS|3-UTR identity [Alignment Gaps]:** 28 | 1464 | 11323
- **5-UTR aligned content (<base>:%):** {'G': 40.26, 'T': 7.79, 'C': 46.75, 'A': 5.19}
- **CDS aligned content (<base>:%):** {'A': 30.47, 'C': 22.47, 'G': 25.94, 'T': 21.12}
- **3-UTR aligned content (<base>:%):** {'T': 40.04, 'G': 13.14, 'C': 13.55, 'A': 33.26}

**Uniprot Description:**  
  
 GTPase tethering membranes through formation of trans-homooligomers and mediating homotypic fusion of endoplasmic reticulum membranes. Functions in endoplasmic reticulum tubular network biogenesis (PubMed:27619977). May also regulate Golgi biogenesis. May regulate axonal development.   
  
Monomer as apoprotein and in the GDP-bound form. Homodimer in the GTP-bound form. Interacts (via N-terminal region) with MAP4K4 (via CNH regulatory domain). Interacts with REEP5, RTN3 and RTN4 (via the transmembrane region). Interacts with SPAST; interaction is direct. May interact with TMED2. Interacts with REEP1. Interacts with CPT1C. Interacts with ARL6IP1 (By similarity). Interacts with ZFYVE27 (PubMed:23969831).   
  
 **Gene Ontology Information:**

Molecular Function

- GTP binding
- GTPase activity
- identical protein binding

Location

- axon
- endoplasmic reticulum
- endoplasmic reticulum membrane
- endoplasmic reticulum tubular network
- endoplasmic reticulum tubular network membrane
- Golgi apparatus
- Golgi cis cisterna
- Golgi membrane
- membrane

Biological process

- axonogenesis
- endoplasmic reticulum organization
- endoplasmic reticulum tubular network membrane organization
- protein homooligomerization

---

12

- **Protein name:** Kinesin-1 heavy chain
- **Organism:** Homo sapiens
- **Uniprot Accession Number:** P33176
- **Protein sequence length:** 963 aa
- **1D identity (%):** 17.23
- **1D identity (%) [Gaps excluded]:** 22.56
- **1D identity - Alignment Gaps:** 244
- **1D aligned content (<aminoacid>:%):** {'M': 0.56, 'A': 6.74, 'P': 3.37, 'Q': 6.74, 'G': 7.87, 'Y': 2.25, 'F': 2.25, 'K': 11.8, 'T': 6.74, 'S': 3.93, 'I': 5.06, 'D': 4.49, 'E': 6.74, 'R': 6.74, 'V': 7.87, 'L': 11.8, 'C': 1.12, 'N': 1.12, 'H': 2.81}
- **Common reported functions (%):** 0.0
- **Common reported locations (%):** 14.29
- **Common reported processes (%):** 0.0

- **PDB ID:** 3J8X
- **Chain:** K
- **Crystallized protein length:** 316 aa
- **Resolution:** 5.0 Å
- **Associated domain:** Kinesin-motor
- **b-phipsi:** 0.004635
- **w-rdist:** 0.296499
- **t-alpha:** 0.003027
- **Chemical similarity (Tanimoto Index) (%):** 86.81
- **1D identity (%) [PDB]:** 2.82
- **1D identity (%) [Gaps excluded][PDB]:** 57.41
- **1D identity - Alignment Gaps [PDB]:** 1046
- **1D aligned content [PDB] (<aminoacid>:%):** {'A': 6.45, 'S': 9.68, 'D': 6.45, 'R': 6.45, 'Q': 6.45, 'E': 9.68, 'I': 6.45, 'K': 6.45, 'L': 9.68, 'N': 3.23, 'P': 6.45, 'Y': 3.23, 'V': 6.45, 'F': 3.23, 'T': 3.23, 'G': 6.45}
- **2D identity (%) [PDB]:** 22.91
- **2D identity (%) [Gaps excluded][PDB]:** 84.55
- **2D identity - Alignment Gaps [PDB]:** 662
- **2D aligned content [PDB] (<2D-fold>:%):** {'.': 18.75, 'E': 32.21, 'H': 43.75, 'B': 0.48, 'T': 4.81}
- **3D similarity (TM-Score) (%) [PDB]:** 20.73

- **Gene name:** KIF5B
- **Entrez ID:** 3799
- **RefSeq ID:** NM\_004521
- **Transcript sequence length:** 5866
- **5-UTR|CDS|3-UTR identity (%):** 24.4 | 43.6 | 13.89
- **5-UTR|CDS|3-UTR identity (%) [Gaps excluded]:** 89.52 | 73.7 | 77.49
- **5-UTR|CDS|3-UTR identity [Alignment Gaps]:** 331 | 1404 | 10030
- **5-UTR aligned content (<base>:%):** {'G': 45.95, 'T': 9.01, 'C': 42.34, 'A': 2.7}
- **CDS aligned content (<base>:%):** {'A': 31.02, 'T': 20.48, 'G': 26.08, 'C': 22.41}
- **3-UTR aligned content (<base>:%):** {'T': 39.66, 'G': 15.79, 'A': 31.94, 'C': 12.61}

**Uniprot Description:**  
  
 Microtubule-dependent motor required for normal distribution of mitochondria and lysosomes. Can induce formation of neurite-like membrane protrusions in non-neuronal cells in a ZFYVE27-dependent manner (By similarity). Regulates centrosome and nuclear positioning during mitotic entry. During the G2 phase of the cell cycle in a BICD2-dependent manner, antagonizes dynein function and drives the separation of nuclei and centrosomes (PubMed:20386726). Required for anterograde axonal transportation of MAPK8IP3/JIP3 which is essential for MAPK8IP3/JIP3 function in axon elongation (By similarity). Through binding with PLEKHM2 and ARL8B, directs lysosome movement toward microtubule plus ends (Probable). Involved in NK cell-mediated cytotoxicity. Drives the polarization of cytolytic granules and microtubule-organizing centers (MTOCs) toward the immune synapse between effector NK lymphocytes and target cells (PubMed:24088571).   
  
Oligomer composed of two heavy chains and two light chains. Interacts with GRIP1 and PPP1R42 (By similarity). Interacts with SYBU (PubMed:15459722). Interacts with JAKMIP1 (PubMed:17532644). Interacts with PLEKHM2 (PubMed:15905402). Interacts with ECPAS (PubMed:20682791). Interacts with ZFYVE27 (By similarity). Found in a complex with OGT, RHOT1, RHOT2 and TRAK1 (PubMed:24995978). Interacts with APP (via cytoplasmic domain) (PubMed:23011729).   
  
 **Gene Ontology Information:**

Molecular Function

- ATP binding
- ATPase activity
- cadherin binding
- identical protein binding
- microtubule binding
- microtubule motor activity
- ATP-dependent microtubule motor activity, plus-end-directed
- protein-containing complex binding

Location

- axon cytoplasm
- centriolar satellite
- ciliary rootlet
- cytosol
- dendrite cytoplasm
- kinesin complex
- lysosomal membrane
- membrane
- microtubule
- mitochondrion
- perinuclear region of cytoplasm
- phagocytic vesicle
- vesicle

Biological process

- anterograde axonal protein transport
- anterograde dendritic transport of neurotransmitter receptor complex
- anterograde neuronal dense core vesicle transport
- axon guidance
- cellular response to interferon-gamma
- centrosome localization
- cytoplasm organization
- lysosome localization
- microtubule-based movement
- mitochondrion transport along microtubule
- mitocytosis
- natural killer cell mediated cytotoxicity
- plus-end-directed vesicle transport along microtubule
- positive regulation of potassium ion transport
- positive regulation of protein localization to plasma membrane
- positive regulation of synaptic transmission, GABAergic
- regulation of membrane potential
- retrograde neuronal dense core vesicle transport
- stress granule disassembly
- synaptic vesicle transport
- vesicle transport along microtubule

---

13

- **Protein name:** Pyruvate kinase
- **Organism:** Homo sapiens
- **Uniprot Accession Number:** Q16716
- **Protein sequence length:** 566 aa
- **1D identity (%):** 12.91
- **1D identity (%) [Gaps excluded]:** 24.8
- **1D identity - Alignment Gaps:** 449
- **1D aligned content (<aminoacid>:%):** {'C': 3.31, 'G': 8.26, 'Q': 4.96, 'R': 7.44, 'V': 14.05, 'P': 8.26, 'A': 8.26, 'L': 11.57, 'H': 4.13, 'S': 2.48, 'T': 4.96, 'I': 6.61, 'E': 5.79, 'Y': 1.65, 'D': 2.48, 'K': 1.65, 'F': 4.13}
- **Common reported functions (%):** 0.0
- **Common reported locations (%):** 21.43
- **Common reported processes (%):** 0.0

- **PDB ID:** 5SC9
- **Chain:** F
- **Crystallized protein length:** 431 aa
- **Resolution:** 1.69 Å
- **Associated domain:** PK
- **b-phipsi:** 0.013453
- **w-rdist:** 0.085795
- **t-alpha:** 0.00503
- **Chemical similarity (Tanimoto Index) (%):** 72.95
- **1D identity (%) [PDB]:** 2.71
- **1D identity (%) [Gaps excluded][PDB]:** 64.71
- **1D identity - Alignment Gaps [PDB]:** 1168
- **1D aligned content [PDB] (<aminoacid>:%):** {'S': 6.06, 'T': 9.09, 'I': 9.09, 'A': 12.12, 'R': 15.15, 'Q': 3.03, 'E': 9.09, 'L': 6.06, 'D': 6.06, 'P': 3.03, 'Y': 3.03, 'F': 3.03, 'G': 6.06, 'V': 9.09}
- **2D identity (%) [PDB]:** 29.72
- **2D identity (%) [Gaps excluded][PDB]:** 85.37
- **2D identity - Alignment Gaps [PDB]:** 614
- **2D aligned content [PDB] (<2D-fold>:%):** {'.': 13.57, 'H': 56.07, 'T': 9.29, 'E': 20.0, 'G': 1.07}
- **3D similarity (TM-Score) (%) [PDB]:** 17.63

- **Gene name:** N/A
- **Entrez ID:** 5313
- **RefSeq ID:** N/A
- **Sequence length:** N/A
- **5-UTR|CDS|3-UTR identity (%):** N/A | N/A | N/A
- **5-UTR|CDS|3-UTR identity (%) [Gaps excluded]:** N/A | N/A | N/A
- **5-UTR|CDS|3-UTR identity [Alignment Gaps]:** N/A | N/A | N/A
- **5-UTR aligned content (<base>:%):** N/A
- **CDS aligned content (<base>:%):** N/A
- **3-UTR aligned content (<base>:%):** N/A

**Uniprot Description:**  
  
 N/A N/A   
  
 **Gene Ontology Information:**

Molecular Function

- ATP binding
- kinase activity
- magnesium ion binding
- monosaccharide binding
- potassium ion binding
- pyruvate kinase activity

Location

- cytoplasm
- cytosol
- extracellular exosome

Biological process

- cellular response to epinephrine stimulus
- cellular response to insulin stimulus
- glycolytic process
- phosphorylation
- pyruvate biosynthetic process
- response to ATP
- response to cAMP
- response to glucose
- response to hypoxia
- response to metal ion
- response to nutrient

---

14

- **Protein name:** Protein cereblon
- **Organism:** Homo sapiens
- **Uniprot Accession Number:** Q96SW2
- **Protein sequence length:** 442 aa
- **1D identity (%):** 8.49
- **1D identity (%) [Gaps excluded]:** 18.72
- **1D identity - Alignment Gaps:** 489
- **1D aligned content (<aminoacid>:%):** {'G': 6.58, 'E': 10.53, 'A': 5.26, 'H': 3.95, 'L': 10.53, 'P': 11.84, 'S': 3.95, 'F': 2.63, 'T': 7.89, 'V': 6.58, 'M': 1.32, 'Q': 9.21, 'I': 3.95, 'C': 2.63, 'K': 5.26, 'N': 1.32, 'R': 3.95, 'D': 2.63}
- **Common reported functions (%):** 6.67
- **Common reported locations (%):** 28.57
- **Common reported processes (%):** 0.0

- **PDB ID:** 6H0F
- **Chain:** B
- **Crystallized protein length:** 374 aa
- **Resolution:** 3.25 Å
- **Associated domain:** Lon-N-terminal
- **b-phipsi:** 0.002079
- **w-rdist:** 0.786815
- **t-alpha:** 0.003018
- **Chemical similarity (Tanimoto Index) (%):** 86.0
- **1D identity (%) [PDB]:** 2.65
- **1D identity (%) [Gaps excluded][PDB]:** 75.61
- **1D identity - Alignment Gaps [PDB]:** 1130
- **1D aligned content [PDB] (<aminoacid>:%):** {'G': 6.45, 'R': 9.68, 'T': 6.45, 'P': 3.23, 'V': 12.9, 'Q': 12.9, 'N': 3.23, 'F': 3.23, 'I': 9.68, 'E': 6.45, 'K': 3.23, 'A': 9.68, 'C': 6.45, 'H': 3.23, 'L': 3.23}
- **2D identity (%) [PDB]:** 31.31
- **2D identity (%) [Gaps excluded][PDB]:** 86.92
- **2D identity - Alignment Gaps [PDB]:** 570
- **2D aligned content [PDB] (<2D-fold>:%):** {'T': 11.47, 'E': 39.78, '.': 15.41, 'H': 30.11, 'G': 3.23}
- **3D similarity (TM-Score) (%) [PDB]:** 16.93

- **Gene name:** CRBN
- **Entrez ID:** 51185
- **RefSeq ID:** NM\_016302
- **Transcript sequence length:** 2187
- **5-UTR|CDS|3-UTR identity (%):** 2.36 | 27.78 | 4.16
- **5-UTR|CDS|3-UTR identity (%) [Gaps excluded]:** 75.0 | 73.51 | 75.98
- **5-UTR|CDS|3-UTR identity [Alignment Gaps]:** 123 | 1765 | 11418
- **5-UTR aligned content (<base>:%):** {'A': 33.33, 'G': 33.33, 'C': 33.33}
- **CDS aligned content (<base>:%):** {'A': 29.57, 'T': 21.07, 'G': 26.4, 'C': 22.97}
- **3-UTR aligned content (<base>:%):** {'A': 35.59, 'G': 12.72, 'T': 38.17, 'C': 13.52}

**Uniprot Description:**  
  
 Substrate recognition component of a DCX (DDB1-CUL4-X-box) E3 protein ligase complex that mediates the ubiquitination and subsequent proteasomal degradation of target proteins, such as MEIS2 (Probable). Normal degradation of key regulatory proteins is required for normal limb outgrowth and expression of the fibroblast growth factor FGF8 (PubMed:20223979, PubMed:24328678, PubMed:25043012, PubMed:25108355). Maintains presynaptic glutamate release and consequently cognitive functions, such as memory and learning, by negatively regulating large-conductance calcium-activated potassium (BK) channels in excitatory neurons (PubMed:18414909, PubMed:29530986). Likely to function by regulating the assembly and neuronal surface expression of BK channels via its interaction with KCNT1 (PubMed:18414909). May also be involved in regulating anxiety-like behaviors via a BK channel-independent mechanism (By similarity).   
  
Interacts with KCNT1 (By similarity). Component of a DCX (DDB1-CUL4-X-box) protein ligase complex, at least composed of CRBN, CUL4A, DDB1 and RBX1. Interacts directly with DDB1 (PubMed:25043012, PubMed:25108355). Interacts (in pomalidomide-bound form) with IKZF1 and IKZF3 (PubMed:24328678).   
  
 **Gene Ontology Information:**

Molecular Function

- metal ion binding
- ion channel binding

Location

- Cul4A-RING E3 ubiquitin ligase complex
- cytoplasm
- cytosol
- membrane
- nucleus
- perinuclear region of cytoplasm

Biological process

- locomotory exploration behavior
- negative regulation of large conductance calcium-activated potassium channel activity
- negative regulation of protein-containing complex assembly
- positive regulation of protein-containing complex assembly
- positive regulation of Wnt signaling pathway
- proteasome-mediated ubiquitin-dependent protein catabolic process
- protein ubiquitination

---

15

- **Protein name:** Beta-secretase 1
- **Organism:** Homo sapiens
- **Uniprot Accession Number:** P56817
- **Protein sequence length:** 501 aa
- **1D identity (%):** 9.17
- **1D identity (%) [Gaps excluded]:** 20.38
- **1D identity - Alignment Gaps:** 516
- **1D aligned content (<aminoacid>:%):** {'A': 6.98, 'P': 15.12, 'G': 10.47, 'R': 5.81, 'L': 4.65, 'F': 5.81, 'E': 5.81, 'M': 3.49, 'Y': 3.49, 'N': 2.33, 'H': 3.49, 'Q': 5.81, 'D': 2.33, 'K': 5.81, 'V': 5.81, 'W': 1.16, 'S': 4.65, 'C': 2.33, 'I': 2.33, 'T': 2.33}
- **Common reported functions (%):** 0.0
- **Common reported locations (%):** 14.29
- **Common reported processes (%):** 0.0

- **PDB ID:** 5MXD
- **Chain:** A
- **Crystallized protein length:** 390 aa
- **Resolution:** 2.52 Å
- **Associated domain:** Peptidase-A1
- **b-phipsi:** 0.015547
- **w-rdist:** 0.043499
- **t-alpha:** 0.005056
- **Chemical similarity (Tanimoto Index) (%):** 89.66
- **1D identity (%) [PDB]:** 0.99
- **1D identity (%) [Gaps excluded][PDB]:** 85.71
- **1D identity - Alignment Gaps [PDB]:** 1195
- **1D aligned content [PDB] (<aminoacid>:%):** {'E': 8.33, 'V': 8.33, 'G': 25.0, 'K': 8.33, 'S': 8.33, 'N': 8.33, 'I': 8.33, 'A': 8.33, 'T': 16.67}
- **2D identity (%) [PDB]:** 28.1
- **2D identity (%) [Gaps excluded][PDB]:** 82.05
- **2D identity - Alignment Gaps [PDB]:** 599
- **2D aligned content [PDB] (<2D-fold>:%):** {'T': 16.8, '.': 16.02, 'E': 47.66, 'B': 1.17, 'G': 4.69, 'H': 13.67}
- **3D similarity (TM-Score) (%) [PDB]:** 17.08

- **Gene name:** BACE1
- **Entrez ID:** 23621
- **RefSeq ID:** NM\_138973
- **Transcript sequence length:** 5628
- **5-UTR|CDS|3-UTR identity (%):** 23.64 | 31.22 | 18.56
- **5-UTR|CDS|3-UTR identity (%) [Gaps excluded]:** 85.83 | 76.51 | 73.57
- **5-UTR|CDS|3-UTR identity [Alignment Gaps]:** 334 | 1631 | 9408
- **5-UTR aligned content (<base>:%):** {'C': 44.95, 'T': 10.09, 'G': 42.2, 'A': 2.75}
- **CDS aligned content (<base>:%):** {'A': 19.3, 'G': 31.05, 'C': 31.16, 'T': 18.49}
- **3-UTR aligned content (<base>:%):** {'G': 19.83, 'A': 29.08, 'C': 19.66, 'T': 31.43}

**Uniprot Description:**  
  
 Responsible for the proteolytic processing of the amyloid precursor protein (APP). Cleaves at the N-terminus of the A-beta peptide sequence, between residues 671 and 672 of APP, leads to the generation and extracellular release of beta-cleaved soluble APP, and a corresponding cell-associated C-terminal fragment which is later released by gamma-secretase (PubMed:10656250, PubMed:10677483, PubMed:20354142). Cleaves CHL1 (By similarity).   
  
Monomer. Interacts (via DXXLL motif) with GGA1, GGA2 and GGA3 (via their VHS domain); the interaction highly increases when BACE1 is phosphorylated at Ser-498 (PubMed:14567678, PubMed:15886016). Interacts with RTN1; RTN2; RTN3 and RTN4; the interaction leads to inhibition of amyloid precursor protein processing (PubMed:15286784, PubMed:16965550, PubMed:16979658). Interacts with SNX6 (PubMed:20354142). Interacts with PCSK9 (PubMed:18660751). Interacts with NAT8 and NAT8B (PubMed:19011241). Interacts with BIN1 (PubMed:27179792). Interacts (via extracellular domain) with ADAM10 (via extracellular domain) (By similarity). Interacts with SORL1; this interaction may affect binding with APP and hence reduce APP cleavage (PubMed:16407538).   
  
 **Gene Ontology Information:**

Molecular Function

- amyloid-beta binding
- aspartic-type endopeptidase activity
- beta-aspartyl-peptidase activity
- endopeptidase activity
- enzyme binding
- peptidase activity

Location

- axon
- cell surface
- dendrite
- early endosome
- endoplasmic reticulum lumen
- endosome
- endosome membrane
- Golgi apparatus
- Golgi-associated vesicle lumen
- hippocampal mossy fiber to CA3 synapse
- late endosome
- lysosome
- membrane
- membrane raft
- multivesicular body
- neuronal cell body
- plasma membrane
- recycling endosome
- synaptic vesicle
- trans-Golgi network

Biological process

- amyloid fibril formation
- amyloid precursor protein catabolic process
- amyloid-beta formation
- amyloid-beta metabolic process
- cellular response to amyloid-beta
- cellular response to copper ion
- cellular response to manganese ion
- detection of mechanical stimulus involved in sensory perception of pain
- membrane protein ectodomain proteolysis
- positive regulation of neuron apoptotic process
- prepulse inhibition
- presynaptic modulation of chemical synaptic transmission
- protein processing
- proteolysis
- response to lead ion
- signaling receptor ligand precursor processing

---

16

- **Protein name:** Lymphokine-activated killer T-cell-originated protein kinase
- **Organism:** Homo sapiens
- **Uniprot Accession Number:** Q96KB5
- **Protein sequence length:** 322 aa
- **1D identity (%):** 5.8
- **1D identity (%) [Gaps excluded]:** 18.25
- **1D identity - Alignment Gaps:** 611
- **1D aligned content (<aminoacid>:%):** {'M': 1.92, 'T': 5.77, 'P': 13.46, 'L': 11.54, 'S': 3.85, 'K': 7.69, 'I': 3.85, 'Q': 3.85, 'G': 7.69, 'V': 5.77, 'D': 11.54, 'Y': 5.77, 'A': 9.62, 'E': 3.85, 'N': 1.92, 'R': 1.92}
- **Common reported functions (%):** 0.0
- **Common reported locations (%):** 7.14
- **Common reported processes (%):** 0.0

- **PDB ID:** 5J0A
- **Chain:** A
- **Crystallized protein length:** 299 aa
- **Resolution:** 2.74 Å
- **Associated domain:** Protein-kinase
- **b-phipsi:** 0.012773
- **w-rdist:** 0.196343
- **t-alpha:** 0.003018
- **Chemical similarity (Tanimoto Index) (%):** 97.77
- **1D identity (%) [PDB]:** 1.16
- **1D identity (%) [Gaps excluded][PDB]:** 72.22
- **1D identity - Alignment Gaps [PDB]:** 1101
- **1D aligned content [PDB] (<aminoacid>:%):** {'T': 7.69, 'R': 7.69, 'S': 15.38, 'V': 7.69, 'I': 7.69, 'P': 7.69, 'A': 23.08, 'Y': 15.38, 'L': 7.69}
- **2D identity (%) [PDB]:** 18.12
- **2D identity (%) [Gaps excluded][PDB]:** 80.0
- **2D identity - Alignment Gaps [PDB]:** 717
- **2D aligned content [PDB] (<2D-fold>:%):** {'E': 13.1, '.': 22.02, 'H': 52.98, 'T': 10.71, 'B': 1.19}
- **3D similarity (TM-Score) (%) [PDB]:** 12.56

- **Gene name:** PBK
- **Entrez ID:** 55872
- **RefSeq ID:** N/A
- **Sequence length:** N/A
- **5-UTR|CDS|3-UTR identity (%):** N/A | N/A | N/A
- **5-UTR|CDS|3-UTR identity (%) [Gaps excluded]:** N/A | N/A | N/A
- **5-UTR|CDS|3-UTR identity [Alignment Gaps]:** N/A | N/A | N/A
- **5-UTR aligned content (<base>:%):** N/A
- **CDS aligned content (<base>:%):** N/A
- **3-UTR aligned content (<base>:%):** N/A

**Uniprot Description:**  
  
 Phosphorylates MAP kinase p38. Seems to be active only in mitosis. May also play a role in the activation of lymphoid cells. When phosphorylated, forms a complex with TP53, leading to TP53 destabilization and attenuation of G2/M checkpoint during doxorubicin-induced DNA damage.   
  
Interacts with DLG1 and TP53.   
  
 **Gene Ontology Information:**

Molecular Function

- ATP binding
- MAP kinase kinase activity
- protein serine kinase activity
- protein serine/threonine kinase activity
- protein tyrosine kinase activity

Location

- nucleus

Biological process

- cellular response to UV
- mitotic cell cycle
- negative regulation of inflammatory response
- negative regulation of proteasomal ubiquitin-dependent protein catabolic process
- negative regulation of protein phosphorylation
- negative regulation of stress-activated MAPK cascade
- peptidyl-serine phosphorylation
- proteasome-mediated ubiquitin-dependent protein catabolic process
- stress-activated MAPK cascade

---

17

- **Protein name:** Cyclin-dependent kinase 9
- **Organism:** Homo sapiens
- **Uniprot Accession Number:** P50750
- **Protein sequence length:** 372 aa
- **1D identity (%):** N/A
- **1D identity (%) [Gaps excluded]:** N/A
- **1D identity - Alignment Gaps:** N/A
- **1D aligned content (<aminoacid>:%):** N/A
- **Common reported functions (%):** N/A
- **Common reported locations (%):** N/A
- **Common reported processes (%):** N/A

- **PDB ID:** 6CYT
- **Chain:** A
- **Crystallized protein length:** 318 aa
- **Resolution:** 3.5 Å
- **Associated domain:** Protein-kinase
- **b-phipsi:** 0.012857
- **w-rdist:** 0.227912
- **t-alpha:** 0.002012
- **Chemical similarity (Tanimoto Index) (%):** N/A
- **1D identity (%) [PDB]:** N/A
- **1D identity (%) [Gaps excluded][PDB]:** N/A
- **1D identity - Alignment Gaps [PDB]:** N/A
- **1D aligned content [PDB] (<aminoacid>:%):** N/A
- **2D identity (%) [PDB]:** N/A
- **2D identity (%) [Gaps excluded][PDB]:** N/A
- **2D identity - Alignment Gaps [PDB]:** N/A
- **2D aligned content [PDB] (<2D-fold>:%):** N/A
- **3D similarity (TM-Score) (%) [PDB]:** N/A

- **Gene name:** CDK9
- **Entrez ID:** 1025
- **RefSeq ID:** N/A
- **Sequence length:** N/A
- **5-UTR|CDS|3-UTR identity (%):** N/A | N/A | N/A
- **5-UTR|CDS|3-UTR identity (%) [Gaps excluded]:** N/A | N/A | N/A
- **5-UTR|CDS|3-UTR identity [Alignment Gaps]:** N/A | N/A | N/A
- **5-UTR aligned content (<base>:%):** N/A
- **CDS aligned content (<base>:%):** N/A
- **3-UTR aligned content (<base>:%):** N/A

**Uniprot Description:**  
  
 Protein kinase involved in the regulation of transcription (PubMed:10574912, PubMed:10757782, PubMed:11145967, PubMed:11575923, PubMed:11809800, PubMed:11884399, PubMed:14701750, PubMed:16109376, PubMed:16109377, PubMed:20930849, PubMed:28426094). Member of the cyclin-dependent kinase pair (CDK9/cyclin-T) complex, also called positive transcription elongation factor b (P-TEFb), which facilitates the transition from abortive to productive elongation by phosphorylating the CTD (C-terminal domain) of the large subunit of RNA polymerase II (RNAP II) POLR2A, SUPT5H and RDBP (PubMed:10574912, PubMed:10757782, PubMed:11145967, PubMed:11575923, PubMed:11809800, PubMed:11884399, PubMed:14701750, PubMed:16109376, PubMed:16109377, PubMed:20930849, PubMed:28426094). This complex is inactive when in the 7SK snRNP complex form (PubMed:10574912, PubMed:10757782, PubMed:11145967, PubMed:11575923, PubMed:11809800, PubMed:11884399, PubMed:14701750, PubMed:16109376, PubMed:16109377, PubMed:20930849, PubMed:28426094). Phosphorylates EP300, MYOD1, RPB1/POLR2A and AR and the negative elongation factors DSIF and NELF (PubMed:9857195, PubMed:10912001, PubMed:11112772, PubMed:12037670, PubMed:20081228, PubMed:20980437, PubMed:21127351). Regulates cytokine inducible transcription networks by facilitating promoter recognition of target transcription factors (e.g. TNF-inducible RELA/p65 activation and IL-6-inducible STAT3 signaling) (PubMed:17956865, PubMed:18362169). Promotes RNA synthesis in genetic programs for cell growth, differentiation and viral pathogenesis (PubMed:10393184, PubMed:11112772). P-TEFb is also involved in cotranscriptional histone modification, mRNA processing and mRNA export (PubMed:15564463, PubMed:19575011, PubMed:19844166). Modulates a complex network of chromatin modifications including histone H2B monoubiquitination (H2Bub1), H3 lysine 4 trimethylation (H3K4me3) and H3K36me3; integrates phosphorylation during transcription with chromatin modifications to control co-transcriptional histone mRNA processing (PubMed:15564463, PubMed:19575011, PubMed:19844166). The CDK9/cyclin-K complex has also a kinase activity towards CTD of RNAP II and can substitute for CDK9/cyclin-T P-TEFb in vitro (PubMed:21127351). Replication stress response protein; the CDK9/cyclin-K complex is required for genome integrity maintenance, by promoting cell cycle recovery from replication arrest and limiting single-stranded DNA amount in response to replication stress, thus reducing the breakdown of stalled replication forks and avoiding DNA damage (PubMed:20493174). In addition, probable function in DNA repair of isoform 2 via interaction with KU70/XRCC6 (PubMed:20493174). Promotes cardiac myocyte enlargement (PubMed:20081228). RPB1/POLR2A phosphorylation on 'Ser-2' in CTD activates transcription (PubMed:21127351). AR phosphorylation modulates AR transcription factor promoter selectivity and cell growth. DSIF and NELF phosphorylation promotes transcription by inhibiting their negative effect (PubMed:9857195, PubMed:10912001, PubMed:11112772). The phosphorylation of MYOD1 enhances its transcriptional activity and thus promotes muscle differentiation (PubMed:12037670).   
  
Component of the super elongation complex (SEC), at least composed of EAF1, EAF2, CDK9, MLLT3/AF9, AFF (AFF1 or AFF4), the P-TEFb complex and ELL (ELL, ELL2 or ELL3). Associates with CCNT1/cyclin-T1, CCNT2/cyclin-T2 (isoform A and isoform B) or CCNK/cyclin-K to form active P-TEFb. P-TEFb forms a complex with AFF4/AF5Q31 and is part of the super elongation complex (SEC). Component of a complex which is composed of at least 5 members: HTATSF1/Tat-SF1, P-TEFb complex, RNA pol II, SUPT5H and NCL/nucleolin. Associates with UBR5 and forms a transcription regulatory complex composed of CDK9, RNAP II, UBR5 and TFIIS/TCEA1 that can stimulate target gene transcription (e.g. gamma fibrinogen/FGG) by recruiting their promoters. Component of the 7SK snRNP inactive complex which is composed of at least 8 members: P-TEFb (composed of CDK9 and CCNT1/cyclin-T1), HEXIM1, HEXIM2, LARP7, BCDIN3, SART3 proteins and 7SK and U6 snRNAs. This inactive 7SK snRNP complex can also interact with NCOR1 and HDAC3, probably to regulate CDK9 acetylation. Release of P-TEFb from P-TEFb/7SK snRNP complex requires both PP2B to transduce calcium Ca(2+) signaling in response to stimuli (e.g. UV or hexamethylene bisacetamide (HMBA)) and PPP1CA to dephosphorylate Thr-186. This released P-TEFb remains inactive in the pre-initiation complex with BRD4 until new Thr-186 phosphorylation occurs after the synthesis of a short RNA (PubMed:10393184, PubMed:10574912, PubMed:12037670, PubMed:11884399, PubMed:12065898, PubMed:12718890, PubMed:15965233, PubMed:16109376, PubMed:17452463, PubMed:17643375, PubMed:18249148, PubMed:18483222, PubMed:18566585, PubMed:20159561, PubMed:20471948, PubMed:21127351, PubMed:21779453, PubMed:22195968, PubMed:9491887). Interacts with BRD4; to target chromatin binding (PubMed:16109376, PubMed:16109377, PubMed:18483222). Interacts with JMJD6 (PubMed:24360279). Interacts with activated nuclear STAT3 and RELA/p65 (PubMed:17956865, PubMed:18362169). Binds to AR and MYOD1 (PubMed:12037670, PubMed:20980437). Forms a complex composed of CDK9, CCNT1/cyclin-T1, EP300 and GATA4 that stimulates hypertrophy in cardiomyocytes (PubMed:20081228). The large PER complex involved in the repression of transcriptional termination is composed of at least PER2, CDK9, DDX5, DHX9, NCBP1 and POLR2A (By similarity). Interacts with HSF1 (PubMed:27189267). Interacts with TBX21 (By similarity). Isoform 3: binds to KU70/XRCC6 (PubMed:20535204). Interacts with WDR43 (By similarity).   
  
 **Gene Ontology Information:**

Molecular Function

- 7SK snRNA binding
- ATP binding
- chromatin binding
- cyclin-dependent protein serine/threonine kinase activity
- DNA binding
- kinase activity
- protein kinase activity
- protein kinase binding
- protein serine kinase activity
- protein serine/threonine kinase activity
- RNA polymerase II cis-regulatory region sequence-specific DNA binding
- RNA polymerase II CTD heptapeptide repeat kinase activity
- transcription coactivator binding

Location

- cyclin/CDK positive transcription elongation factor complex
- cytoplasmic ribonucleoprotein granule
- membrane
- nucleoplasm
- nucleus
- P-TEFb complex
- PML body
- transcription elongation factor complex

Biological process

- cell population proliferation
- cellular response to cytokine stimulus
- DNA repair
- negative regulation of protein localization to chromatin
- nucleus localization
- positive regulation by host of viral transcription
- positive regulation of protein localization to chromatin
- positive regulation of transcription by RNA polymerase II
- positive regulation of transcription elongation from RNA polymerase II promoter
- protein phosphorylation
- regulation of cell cycle
- regulation of DNA repair
- regulation of mRNA 3'-end processing
- regulation of muscle cell differentiation
- replication fork processing
- transcription by RNA polymerase II
- transcription elongation from RNA polymerase II promoter
- transcription elongation-coupled chromatin remodeling
- transcription initiation from RNA polymerase II promoter

---

18

- **Protein name:** Lactotransferrin
- **Organism:** Homo sapiens
- **Uniprot Accession Number:** P02788
- **Protein sequence length:** 710 aa
- **1D identity (%):** 13.52
- **1D identity (%) [Gaps excluded]:** 26.81
- **1D identity - Alignment Gaps:** 517
- **1D aligned content (<aminoacid>:%):** {'L': 10.64, 'A': 7.8, 'G': 9.93, 'R': 8.51, 'S': 3.55, 'V': 9.93, 'P': 9.22, 'C': 4.96, 'F': 4.26, 'I': 2.84, 'Y': 1.42, 'K': 7.8, 'E': 4.26, 'T': 2.84, 'N': 2.84, 'D': 5.67, 'W': 0.71, 'M': 0.71, 'Q': 2.13}
- **Common reported functions (%):** 0.0
- **Common reported locations (%):** 21.43
- **Common reported processes (%):** 0.0

- **PDB ID:** 1FCK
- **Chain:** A
- **Crystallized protein length:** 691 aa
- **Resolution:** 2.2 Å
- **Associated domain:** Transferrin-like-1
- **b-phipsi:** 0.00874
- **w-rdist:** 0.097045
- **t-alpha:** 0.043022
- **Chemical similarity (Tanimoto Index) (%):** 97.06
- **1D identity (%) [PDB]:** 2.8
- **1D identity (%) [Gaps excluded][PDB]:** 64.06
- **1D identity - Alignment Gaps [PDB]:** 1401
- **1D aligned content [PDB] (<aminoacid>:%):** {'R': 9.76, 'S': 14.63, 'V': 12.2, 'P': 4.88, 'A': 14.63, 'Y': 7.32, 'L': 7.32, 'D': 7.32, 'K': 4.88, 'E': 2.44, 'G': 2.44, 'T': 4.88, 'Q': 4.88, 'H': 2.44}
- **2D identity (%) [PDB]:** 39.39
- **2D identity (%) [Gaps excluded][PDB]:** 84.02
- **2D identity - Alignment Gaps [PDB]:** 553
- **2D aligned content [PDB] (<2D-fold>:%):** {'.': 15.85, 'E': 23.17, 'H': 45.12, 'T': 13.17, 'G': 2.68}
- **3D similarity (TM-Score) (%) [PDB]:** 22.27

- **Gene name:** LTF
- **Entrez ID:** 4057
- **RefSeq ID:** NM\_002343
- **Transcript sequence length:** 2721
- **5-UTR|CDS|3-UTR identity (%):** 16.18 | 40.83 | 2.97
- **5-UTR|CDS|3-UTR identity (%) [Gaps excluded]:** 75.86 | 75.02 | 77.73
- **5-UTR|CDS|3-UTR identity [Alignment Gaps]:** 107 | 1391 | 11522
- **5-UTR aligned content (<base>:%):** {'G': 31.82, 'A': 4.55, 'C': 54.55, 'T': 9.09}
- **CDS aligned content (<base>:%):** {'A': 24.16, 'G': 29.53, 'T': 19.02, 'C': 27.29}
- **3-UTR aligned content (<base>:%):** {'A': 21.91, 'C': 27.53, 'G': 24.16, 'T': 26.4}

**Uniprot Description:**  
  
 Transferrins are iron binding transport proteins which can bind two Fe(3+) ions in association with the binding of an anion, usually bicarbonate.   
  
Monomer. Found in a complex with LTF, CLU, EPPIN and SEMG1.   
  
 **Gene Ontology Information:**

Molecular Function

- cysteine-type endopeptidase inhibitor activity
- DNA binding
- heparin binding
- iron ion binding
- lipopolysaccharide binding
- membrane destabilizing activity
- protein serine/threonine kinase activator activity
- serine-type endopeptidase activity

Location

- cell surface
- cytoplasm
- early endosome
- extracellular exosome
- extracellular region
- extracellular space
- nucleus
- phagocytic vesicle lumen
- plasma membrane
- protein-containing complex
- recycling endosome
- secretory granule
- specific granule
- specific granule lumen
- tertiary granule lumen

Biological process

- antibacterial humoral response
- antifungal humoral response
- antimicrobial humoral immune response mediated by antimicrobial peptide
- bone morphogenesis
- cytolysis by host of symbiont cells
- defense response to Gram-negative bacterium
- defense response to Gram-positive bacterium
- humoral immune response
- innate immune response
- innate immune response in mucosa
- iron ion transmembrane transport
- iron ion transport
- killing of cells of other organism
- negative regulation by host of viral process
- negative regulation of apoptotic process
- negative regulation of ATPase activity
- negative regulation of cysteine-type endopeptidase activity
- negative regulation of lipopolysaccharide-mediated signaling pathway
- negative regulation of membrane potential
- negative regulation of osteoclast development
- negative regulation of single-species biofilm formation in or on host organism
- negative regulation of tumor necrosis factor (ligand) superfamily member 11 production
- negative regulation of viral genome replication
- negative regulation of viral process
- ossification
- positive regulation of bone mineralization involved in bone maturation
- positive regulation of chondrocyte proliferation
- positive regulation of I-kappaB kinase/NF-kappaB signaling
- positive regulation of NF-kappaB transcription factor activity
- positive regulation of osteoblast differentiation
- positive regulation of osteoblast proliferation
- positive regulation of protein serine/threonine kinase activity
- positive regulation of toll-like receptor 4 signaling pathway
- proteolysis
- regulation of cytokine production
- regulation of tumor necrosis factor production
- retina homeostasis

---

19

- **Protein name:** Chitotriosidase-1
- **Organism:** Homo sapiens
- **Uniprot Accession Number:** Q13231
- **Protein sequence length:** 466 aa
- **1D identity (%):** 10.62
- **1D identity (%) [Gaps excluded]:** 25.19
- **1D identity - Alignment Gaps:** 539
- **1D aligned content (<aminoacid>:%):** {'S': 5.05, 'W': 3.03, 'L': 11.11, 'Q': 10.1, 'R': 6.06, 'P': 8.08, 'A': 10.1, 'G': 8.08, 'N': 1.01, 'E': 4.04, 'F': 6.06, 'K': 3.03, 'I': 2.02, 'D': 5.05, 'T': 8.08, 'V': 4.04, 'H': 1.01, 'Y': 2.02, 'C': 2.02}
- **Common reported functions (%):** 0.0
- **Common reported locations (%):** 0.0
- **Common reported processes (%):** 0.0

- **PDB ID:** 6JJR
- **Chain:** A
- **Crystallized protein length:** 370 aa
- **Resolution:** 1.83 Å
- **Associated domain:** GH18
- **b-phipsi:** 0.011134
- **w-rdist:** 0.060761
- **t-alpha:** 0.041928
- **Chemical similarity (Tanimoto Index) (%):** 85.02
- **1D identity (%) [PDB]:** 1.61
- **1D identity (%) [Gaps excluded][PDB]:** 70.37
- **1D identity - Alignment Gaps [PDB]:** 1154
- **1D aligned content [PDB] (<aminoacid>:%):** {'A': 21.05, 'L': 10.53, 'V': 10.53, 'R': 5.26, 'H': 10.53, 'D': 5.26, 'K': 5.26, 'E': 5.26, 'S': 10.53, 'G': 10.53, 'T': 5.26}
- **2D identity (%) [PDB]:** 27.49
- **2D identity (%) [Gaps excluded][PDB]:** 89.44
- **2D identity - Alignment Gaps [PDB]:** 640
- **2D aligned content [PDB] (<2D-fold>:%):** {'E': 28.74, 'T': 14.17, 'G': 3.54, '.': 12.99, 'H': 40.55}
- **3D similarity (TM-Score) (%) [PDB]:** 17.47

- **Gene name:** CHIT1
- **Entrez ID:** 1118
- **RefSeq ID:** N/A
- **Sequence length:** N/A
- **5-UTR|CDS|3-UTR identity (%):** N/A | N/A | N/A
- **5-UTR|CDS|3-UTR identity (%) [Gaps excluded]:** N/A | N/A | N/A
- **5-UTR|CDS|3-UTR identity [Alignment Gaps]:** N/A | N/A | N/A
- **5-UTR aligned content (<base>:%):** N/A
- **CDS aligned content (<base>:%):** N/A
- **3-UTR aligned content (<base>:%):** N/A

**Uniprot Description:**  
  
 Degrades chitin, chitotriose and chitobiose. May participate in the defense against nematodes and other pathogens. Isoform 3 has no enzymatic activity.   
  
Monomer.   
  
 **Gene Ontology Information:**

Molecular Function

- chitin binding
- chitinase activity
- endochitinase activity
- hydrolase activity, hydrolyzing O-glycosyl compounds

Location

- extracellular region
- extracellular space
- lysosome
- specific granule lumen
- tertiary granule lumen

Biological process

- chitin catabolic process
- immune response
- polysaccharide catabolic process
- polysaccharide digestion
- response to bacterium

---

20

- **Protein name:** von Willebrand factor
- **Organism:** Homo sapiens
- **Uniprot Accession Number:** P04275
- **Protein sequence length:** 2813 aa
- **1D identity (%):** 9.36
- **1D identity (%) [Gaps excluded]:** 31.02
- **1D identity - Alignment Gaps:** 1970
- **1D aligned content (<aminoacid>:%):** {'M': 1.89, 'G': 9.47, 'A': 4.55, 'L': 10.23, 'P': 9.09, 'Y': 2.65, 'F': 4.17, 'R': 7.2, 'I': 2.65, 'D': 6.82, 'C': 6.06, 'E': 3.41, 'T': 5.3, 'V': 7.58, 'S': 4.55, 'Q': 5.68, 'H': 1.89, 'K': 4.17, 'N': 2.27, 'W': 0.38}
- **Common reported functions (%):** 0.0
- **Common reported locations (%):** 0.0
- **Common reported processes (%):** 0.0

- **PDB ID:** 7WN3
- **Chain:** E
- **Crystallized protein length:** 711 aa
- **Resolution:** 3.29 Å
- **Associated domain:** VWFD-2
- **b-phipsi:** 0.008081
- **w-rdist:** 0.449531
- **t-alpha:** 0.002012
- **Chemical similarity (Tanimoto Index) (%):** 76.38
- **1D identity (%) [PDB]:** 2.74
- **1D identity (%) [Gaps excluded][PDB]:** 74.55
- **1D identity - Alignment Gaps [PDB]:** 1439
- **1D aligned content [PDB] (<aminoacid>:%):** {'S': 12.2, 'V': 12.2, 'P': 2.44, 'A': 14.63, 'Y': 2.44, 'L': 7.32, 'F': 2.44, 'R': 2.44, 'H': 7.32, 'D': 4.88, 'E': 4.88, 'G': 7.32, 'T': 4.88, 'Q': 9.76, 'N': 2.44, 'M': 2.44}
- **2D identity (%) [PDB]:** 26.19
- **2D identity (%) [Gaps excluded][PDB]:** 82.57
- **2D identity - Alignment Gaps [PDB]:** 803
- **2D aligned content [PDB] (<2D-fold>:%):** {'.': 18.18, 'E': 43.51, 'T': 17.86, 'H': 20.13, 'B': 0.32}
- **3D similarity (TM-Score) (%) [PDB]:** 19.39

- **Gene name:** VWF
- **Entrez ID:** N/A
- **RefSeq ID:** NM\_000552
- **Transcript sequence length:** 8830
- **5-UTR|CDS|3-UTR identity (%):** 31.84 | 24.88 | 0.78
- **5-UTR|CDS|3-UTR identity (%) [Gaps excluded]:** 77.27 | 83.58 | 79.49
- **5-UTR|CDS|3-UTR identity [Alignment Gaps]:** 157 | 5966 | 11792
- **5-UTR aligned content (<base>:%):** {'C': 34.12, 'T': 14.12, 'G': 49.41, 'A': 2.35}
- **CDS aligned content (<base>:%):** {'A': 23.52, 'T': 18.22, 'G': 29.25, 'C': 29.01}
- **3-UTR aligned content (<base>:%):** {'G': 29.03, 'C': 34.41, 'T': 22.58, 'A': 13.98}

**Uniprot Description:**  
  
 Important in the maintenance of hemostasis, it promotes adhesion of platelets to the sites of vascular injury by forming a molecular bridge between sub-endothelial collagen matrix and platelet-surface receptor complex GPIb-IX-V. Also acts as a chaperone for coagulation factor VIII, delivering it to the site of injury, stabilizing its heterodimeric structure and protecting it from premature clearance from plasma.   
  
Multimeric. Interacts with F8.   
  
 **Gene Ontology Information:**

Molecular Function   
  
N/A

Location   
  
N/A

Biological process   
  
N/A

---

21

- **Protein name:** Mitogen-activated protein kinase 14
- **Organism:** Homo sapiens
- **Uniprot Accession Number:** Q16539
- **Protein sequence length:** 360 aa
- **1D identity (%):** 9.0
- **1D identity (%) [Gaps excluded]:** 24.24
- **1D identity - Alignment Gaps:** 559
- **1D aligned content (<aminoacid>:%):** {'R': 10.0, 'P': 7.5, 'L': 8.75, 'N': 3.75, 'V': 10.0, 'C': 3.75, 'D': 3.75, 'K': 8.75, 'I': 7.5, 'H': 5.0, 'Y': 2.5, 'G': 6.25, 'T': 5.0, 'A': 2.5, 'S': 1.25, 'F': 1.25, 'E': 1.25, 'M': 2.5, 'W': 2.5, 'Q': 6.25}
- **Common reported functions (%):** 0.0
- **Common reported locations (%):** 28.57
- **Common reported processes (%):** 4.76

- **PDB ID:** 5WJJ
- **Chain:** A
- **Crystallized protein length:** 338 aa
- **Resolution:** 1.6 Å
- **Associated domain:** Protein-kinase
- **b-phipsi:** 0.011611
- **w-rdist:** 0.277601
- **t-alpha:** 0.002012
- **Chemical similarity (Tanimoto Index) (%):** 83.02
- **1D identity (%) [PDB]:** 1.56
- **1D identity (%) [Gaps excluded][PDB]:** 66.67
- **1D identity - Alignment Gaps [PDB]:** 1125
- **1D aligned content [PDB] (<aminoacid>:%):** {'R': 5.56, 'P': 22.22, 'V': 16.67, 'F': 5.56, 'Q': 5.56, 'I': 5.56, 'L': 5.56, 'G': 5.56, 'A': 11.11, 'D': 5.56, 'K': 11.11}
- **2D identity (%) [PDB]:** 24.48
- **2D identity (%) [Gaps excluded][PDB]:** 86.54
- **2D identity - Alignment Gaps [PDB]:** 659
- **2D aligned content [PDB] (<2D-fold>:%):** {'E': 22.67, 'T': 10.67, 'H': 44.89, '.': 19.11, 'G': 2.67}
- **3D similarity (TM-Score) (%) [PDB]:** 15.21

- **Gene name:** MAPK14
- **Entrez ID:** 1432
- **RefSeq ID:** NM\_139014
- **Transcript sequence length:** 4143
- **5-UTR|CDS|3-UTR identity (%):** 30.7 | 21.95 | 15.05
- **5-UTR|CDS|3-UTR identity (%) [Gaps excluded]:** 89.34 | 74.44 | 75.14
- **5-UTR|CDS|3-UTR identity [Alignment Gaps]:** 233 | 1908 | 9833
- **5-UTR aligned content (<base>:%):** {'C': 43.12, 'T': 8.26, 'G': 46.79, 'A': 1.83}
- **CDS aligned content (<base>:%):** {'A': 27.27, 'T': 21.89, 'G': 27.95, 'C': 22.9}
- **3-UTR aligned content (<base>:%):** {'G': 20.97, 'T': 34.32, 'A': 26.05, 'C': 18.65}

**Uniprot Description:**  
  
 Serine/threonine kinase which acts as an essential component of the MAP kinase signal transduction pathway. MAPK14 is one of the four p38 MAPKs which play an important role in the cascades of cellular responses evoked by extracellular stimuli such as proinflammatory cytokines or physical stress leading to direct activation of transcription factors. Accordingly, p38 MAPKs phosphorylate a broad range of proteins and it has been estimated that they may have approximately 200 to 300 substrates each. Some of the targets are downstream kinases which are activated through phosphorylation and further phosphorylate additional targets. RPS6KA5/MSK1 and RPS6KA4/MSK2 can directly phosphorylate and activate transcription factors such as CREB1, ATF1, the NF-kappa-B isoform RELA/NFKB3, STAT1 and STAT3, but can also phosphorylate histone H3 and the nucleosomal protein HMGN1. RPS6KA5/MSK1 and RPS6KA4/MSK2 play important roles in the rapid induction of immediate-early genes in response to stress or mitogenic stimuli, either by inducing chromatin remodeling or by recruiting the transcription machinery. On the other hand, two other kinase targets, MAPKAPK2/MK2 and MAPKAPK3/MK3, participate in the control of gene expression mostly at the post-transcriptional level, by phosphorylating ZFP36 (tristetraprolin) and ELAVL1, and by regulating EEF2K, which is important for the elongation of mRNA during translation. MKNK1/MNK1 and MKNK2/MNK2, two other kinases activated by p38 MAPKs, regulate protein synthesis by phosphorylating the initiation factor EIF4E2. MAPK14 interacts also with casein kinase II, leading to its activation through autophosphorylation and further phosphorylation of TP53/p53. In the cytoplasm, the p38 MAPK pathway is an important regulator of protein turnover. For example, CFLAR is an inhibitor of TNF-induced apoptosis whose proteasome-mediated degradation is regulated by p38 MAPK phosphorylation. In a similar way, MAPK14 phosphorylates the ubiquitin ligase SIAH2, regulating its activity towards EGLN3. MAPK14 may also inhibit the lysosomal degradation pathway of autophagy by interfering with the intracellular trafficking of the transmembrane protein ATG9. Another function of MAPK14 is to regulate the endocytosis of membrane receptors by different mechanisms that impinge on the small GTPase RAB5A. In addition, clathrin-mediated EGFR internalization induced by inflammatory cytokines and UV irradiation depends on MAPK14-mediated phosphorylation of EGFR itself as well as of RAB5A effectors. Ectodomain shedding of transmembrane proteins is regulated by p38 MAPKs as well. In response to inflammatory stimuli, p38 MAPKs phosphorylate the membrane-associated metalloprotease ADAM17. Such phosphorylation is required for ADAM17-mediated ectodomain shedding of TGF-alpha family ligands, which results in the activation of EGFR signaling and cell proliferation. Another p38 MAPK substrate is FGFR1. FGFR1 can be translocated from the extracellular space into the cytosol and nucleus of target cells, and regulates processes such as rRNA synthesis and cell growth. FGFR1 translocation requires p38 MAPK activation. In the nucleus, many transcription factors are phosphorylated and activated by p38 MAPKs in response to different stimuli. Classical examples include ATF1, ATF2, ATF6, ELK1, PTPRH, DDIT3, TP53/p53 and MEF2C and MEF2A. The p38 MAPKs are emerging as important modulators of gene expression by regulating chromatin modifiers and remodelers. The promoters of several genes involved in the inflammatory response, such as IL6, IL8 and IL12B, display a p38 MAPK-dependent enrichment of histone H3 phosphorylation on 'Ser-10' (H3S10ph) in LPS-stimulated myeloid cells. This phosphorylation enhances the accessibility of the cryptic NF-kappa-B-binding sites marking promoters for increased NF-kappa-B recruitment. Phosphorylates CDC25B and CDC25C which is required for binding to 14-3-3 proteins and leads to initiation of a G2 delay after ultraviolet radiation. Phosphorylates TIAR following DNA damage, releasing TIAR from GADD45A mRNA and preventing mRNA degradation. The p38 MAPKs may also have kinase-independent roles, which are thought to be due to the binding to targets in the absence of phosphorylation. Protein O-Glc-N-acylation catalyzed by the OGT is regulated by MAPK14, and, although OGT does not seem to be phosphorylated by MAPK14, their interaction increases upon MAPK14 activation induced by glucose deprivation. This interaction may regulate OGT activity by recruiting it to specific targets such as neurofilament H, stimulating its O-Glc-N-acylation. Required in mid-fetal development for the growth of embryo-derived blood vessels in the labyrinth layer of the placenta. Also plays an essential role in developmental and stress-induced erythropoiesis, through regulation of EPO gene expression. Isoform MXI2 activation is stimulated by mitogens and oxidative stress and only poorly phosphorylates ELK1 and ATF2. Isoform EXIP may play a role in the early onset of apoptosis. Phosphorylates S100A9 at 'Thr-113'.   
  
Component of a signaling complex containing at least AKAP13, PKN1, MAPK14, ZAK and MAP2K3. Within this complex, AKAP13 interacts directly with PKN1, which in turn recruits MAPK14, MAP2K3 and ZAK (PubMed:21224381). Binds to a kinase interaction motif within the protein tyrosine phosphatase, PTPRR (By similarity). This interaction retains MAPK14 in the cytoplasm and prevents nuclear accumulation (By similarity). Interacts with SPAG9 and GADD45A (By similarity). Interacts with CDC25B, CDC25C, DUSP1, DUSP10, DUSP16, NP60, SUPT20H and TAB1. Interacts with casein kinase II subunits CSNK2A1 and CSNK2B. Interacts with PPM1D. Interacts with CDK5RAP3; recruits PPM1D to MAPK14 and may regulate its dephosphorylation (PubMed:21283629).   
  
 **Gene Ontology Information:**

Molecular Function

- ATP binding
- enzyme binding
- MAP kinase activity
- MAP kinase kinase activity
- mitogen-activated protein kinase p38 binding
- NFAT protein binding
- protein phosphatase binding
- protein serine kinase activity
- protein serine/threonine kinase activity

Location

- cytoplasm
- cytosol
- extracellular region
- ficolin-1-rich granule lumen
- glutamatergic synapse
- mitochondrion
- nuclear speck
- nucleoplasm
- nucleus
- secretory granule lumen
- spindle pole

Biological process

- 3'-UTR-mediated mRNA stabilization
- angiogenesis
- apoptotic process
- bone development
- cartilage condensation
- cell morphogenesis
- cell surface receptor signaling pathway
- cellular response to ionizing radiation
- cellular response to lipopolysaccharide
- cellular response to lipoteichoic acid
- cellular response to tumor necrosis factor
- cellular response to UV-B
- cellular response to vascular endothelial growth factor stimulus
- cellular response to virus
- cellular senescence
- chemotaxis
- chondrocyte differentiation
- DNA damage checkpoint
- fatty acid oxidation
- glucose import
- glucose metabolic process
- intracellular signal transduction
- lipopolysaccharide-mediated signaling pathway
- negative regulation of canonical Wnt signaling pathway
- negative regulation of hippo signaling
- negative regulation of inflammatory response to antigenic stimulus
- osteoblast differentiation
- osteoclast differentiation
- p38MAPK cascade
- peptidyl-serine phosphorylation
- placenta development
- platelet activation
- positive regulation of brown fat cell differentiation
- positive regulation of cardiac muscle cell proliferation
- positive regulation of cyclase activity
- positive regulation of erythrocyte differentiation
- positive regulation of gene expression
- positive regulation of glucose import
- positive regulation of interleukin-12 production
- positive regulation of muscle cell differentiation
- positive regulation of myoblast differentiation
- positive regulation of myoblast fusion
- positive regulation of myotube differentiation
- positive regulation of protein import into nucleus
- positive regulation of reactive oxygen species metabolic process
- positive regulation of transcription by RNA polymerase II
- regulation of cytokine production involved in inflammatory response
- regulation of ossification
- regulation of synaptic membrane adhesion
- regulation of transcription by RNA polymerase II
- response to dietary excess
- response to insulin
- response to muramyl dipeptide
- response to muscle stretch
- signal transduction
- signal transduction in response to DNA damage
- skeletal muscle tissue development
- stem cell differentiation
- stress-activated MAPK cascade
- stress-activated protein kinase signaling cascade
- stress-induced premature senescence
- striated muscle cell differentiation
- transcription by RNA polymerase II
- transmembrane receptor protein serine/threonine kinase signaling pathway
- vascular endothelial growth factor receptor signaling pathway

---

22

- **Protein name:** Glucosylceramidase
- **Organism:** Homo sapiens
- **Uniprot Accession Number:** B2R6A7
- **Protein sequence length:** 536 aa
- **1D identity (%):** 10.27
- **1D identity (%) [Gaps excluded]:** 24.51
- **1D identity - Alignment Gaps:** 571
- **1D aligned content (<aminoacid>:%):** {'F': 5.94, 'S': 4.95, 'E': 4.95, 'K': 1.98, 'P': 13.86, 'L': 9.9, 'T': 7.92, 'G': 10.89, 'I': 3.96, 'V': 5.94, 'C': 2.97, 'N': 1.98, 'Y': 2.97, 'R': 4.95, 'D': 3.96, 'A': 6.93, 'M': 0.99, 'Q': 1.98, 'W': 0.99, 'H': 1.98}
- **Common reported functions (%):** 0.0
- **Common reported locations (%):** 7.14
- **Common reported processes (%):** 0.0

- **PDB ID:** 6Q1N
- **Chain:** B
- **Crystallized protein length:** 494 aa
- **Resolution:** 2.53 Å
- **Associated domain:** Glyco-hydro-30
- **b-phipsi:** 0.011393
- **w-rdist:** 0.101463
- **t-alpha:** 0.010061
- **Chemical similarity (Tanimoto Index) (%):** 82.93
- **1D identity (%) [PDB]:** 2.17
- **1D identity (%) [Gaps excluded][PDB]:** 70.0
- **1D identity - Alignment Gaps [PDB]:** 1253
- **1D aligned content [PDB] (<aminoacid>:%):** {'A': 7.14, 'F': 7.14, 'P': 10.71, 'G': 7.14, 'T': 3.57, 'S': 3.57, 'R': 3.57, 'L': 7.14, 'Q': 3.57, 'N': 3.57, 'E': 3.57, 'I': 14.29, 'K': 7.14, 'D': 7.14, 'Y': 7.14, 'H': 3.57}
- **2D identity (%) [PDB]:** 27.06
- **2D identity (%) [Gaps excluded][PDB]:** 84.26
- **2D identity - Alignment Gaps [PDB]:** 685
- **2D aligned content [PDB] (<2D-fold>:%):** {'.': 19.78, 'E': 36.63, 'T': 10.26, 'H': 33.33}
- **3D similarity (TM-Score) (%) [PDB]:** 19.66

- **Gene name:** N/A
- **Entrez ID:** 2629
- **RefSeq ID:** N/A
- **Sequence length:** N/A
- **5-UTR|CDS|3-UTR identity (%):** N/A | N/A | N/A
- **5-UTR|CDS|3-UTR identity (%) [Gaps excluded]:** N/A | N/A | N/A
- **5-UTR|CDS|3-UTR identity [Alignment Gaps]:** N/A | N/A | N/A
- **5-UTR aligned content (<base>:%):** N/A
- **CDS aligned content (<base>:%):** N/A
- **3-UTR aligned content (<base>:%):** N/A

**Uniprot Description:**  
  
 N/A N/A   
  
 **Gene Ontology Information:**

Molecular Function

- galactosylceramidase activity
- glucosylceramidase activity
- glucosyltransferase activity
- scavenger receptor binding
- signaling receptor binding
- steryl-beta-glucosidase activity

Location

- endoplasmic reticulum
- extracellular exosome
- Golgi apparatus
- lysosomal lumen
- lysosomal membrane
- lysosome
- trans-Golgi network

Biological process

- antigen processing and presentation
- autophagosome organization
- autophagy
- beta-glucoside catabolic process
- brain morphogenesis
- cell maturation
- cellular response to starvation
- cellular response to tumor necrosis factor
- ceramide biosynthetic process
- cerebellar Purkinje cell layer formation
- cholesterol metabolic process
- determination of adult lifespan
- establishment of skin barrier
- glucosylceramide catabolic process
- hematopoietic stem cell proliferation
- homeostasis of number of cells
- lipid glycosylation
- lipid storage
- lymphocyte migration
- lysosome organization
- microglia differentiation
- microglial cell proliferation
- motor behavior
- negative regulation of inflammatory response
- negative regulation of interleukin-6 production
- negative regulation of MAP kinase activity
- negative regulation of neuron apoptotic process
- negative regulation of neuron death
- negative regulation of protein-containing complex assembly
- neuromuscular process
- neuron apoptotic process
- positive regulation of autophagy of mitochondrion in response to mitochondrial depolarization
- positive regulation of neuronal action potential
- positive regulation of proteasomal ubiquitin-dependent protein catabolic process
- positive regulation of protein dephosphorylation
- positive regulation of protein lipidation
- positive regulation of protein metabolic process
- positive regulation of protein-containing complex disassembly
- positive regulation of proteolysis involved in cellular protein catabolic process
- proteasome-mediated ubiquitin-dependent protein catabolic process
- pyramidal neuron differentiation
- regulation of lysosomal protein catabolic process
- regulation of macroautophagy
- regulation of protein metabolic process
- regulation of TOR signaling
- respiratory electron transport chain
- response to dexamethasone
- response to estrogen
- response to pH
- response to testosterone
- response to thyroid hormone
- sphingosine biosynthetic process
- T cell differentiation in thymus
- termination of signal transduction

---

23

- **Protein name:** NADPH--cytochrome P450 reductase
- **Organism:** Homo sapiens
- **Uniprot Accession Number:** P16435
- **Protein sequence length:** 677 aa
- **1D identity (%):** 14.6
- **1D identity (%) [Gaps excluded]:** 23.35
- **1D identity - Alignment Gaps:** 354
- **1D aligned content (<aminoacid>:%):** {'G': 12.32, 'V': 7.97, 'T': 5.07, 'S': 2.9, 'I': 2.9, 'L': 10.87, 'P': 5.8, 'F': 7.25, 'Q': 7.25, 'R': 9.42, 'M': 2.17, 'N': 2.9, 'D': 4.35, 'A': 2.9, 'K': 5.8, 'H': 3.62, 'Y': 0.72, 'E': 5.8}
- **Common reported functions (%):** 0.0
- **Common reported locations (%):** 14.29
- **Common reported processes (%):** 0.0

- **PDB ID:** 3QFR
- **Chain:** A
- **Crystallized protein length:** 604 aa
- **Resolution:** 2.4 Å
- **Associated domain:** FAD-binding-FR-type
- **b-phipsi:** 0.002207
- **w-rdist:** 0.729996
- **t-alpha:** 0.00404
- **Chemical similarity (Tanimoto Index) (%):** 75.1
- **1D identity (%) [PDB]:** 1.77
- **1D identity (%) [Gaps excluded][PDB]:** 75.76
- **1D identity - Alignment Gaps [PDB]:** 1377
- **1D aligned content [PDB] (<aminoacid>:%):** {'S': 12.0, 'G': 8.0, 'R': 12.0, 'L': 12.0, 'P': 12.0, 'F': 4.0, 'E': 4.0, 'I': 4.0, 'A': 4.0, 'D': 4.0, 'V': 12.0, 'M': 4.0, 'H': 4.0, 'T': 4.0}
- **2D identity (%) [PDB]:** 33.52
- **2D identity (%) [Gaps excluded][PDB]:** 87.72
- **2D identity - Alignment Gaps [PDB]:** 645
- **2D aligned content [PDB] (<2D-fold>:%):** {'.': 18.29, 'H': 45.14, 'T': 14.0, 'E': 21.14, 'B': 0.57, 'G': 0.86}
- **3D similarity (TM-Score) (%) [PDB]:** 22.33

- **Gene name:** POR
- **Entrez ID:** 5447
- **RefSeq ID:** N/A
- **Sequence length:** N/A
- **5-UTR|CDS|3-UTR identity (%):** N/A | N/A | N/A
- **5-UTR|CDS|3-UTR identity (%) [Gaps excluded]:** N/A | N/A | N/A
- **5-UTR|CDS|3-UTR identity [Alignment Gaps]:** N/A | N/A | N/A
- **5-UTR aligned content (<base>:%):** N/A
- **CDS aligned content (<base>:%):** N/A
- **3-UTR aligned content (<base>:%):** N/A

**Uniprot Description:**  
  
 This enzyme is required for electron transfer from NADP to cytochrome P450 in microsomes. It can also provide electron transfer to heme oxygenase and cytochrome B5. N/A   
  
 **Gene Ontology Information:**

Molecular Function

- flavin adenine dinucleotide binding
- FMN binding
- NADP binding
- NADPH-hemoprotein reductase activity

Location

- cytosol
- endoplasmic reticulum membrane
- intracellular membrane-bounded organelle
- membrane

Biological process

- cellular organofluorine metabolic process
- electron transport chain
- positive regulation of monooxygenase activity
- response to hormone
- xenobiotic metabolic process

---

24

- **Protein name:** Cyclin-dependent kinase 4
- **Organism:** Homo sapiens
- **Uniprot Accession Number:** P11802
- **Protein sequence length:** 303 aa
- **1D identity (%):** 6.99
- **1D identity (%) [Gaps excluded]:** 25.91
- **1D identity - Alignment Gaps:** 668
- **1D aligned content (<aminoacid>:%):** {'A': 10.94, 'P': 12.5, 'E': 1.56, 'G': 12.5, 'T': 1.56, 'V': 6.25, 'K': 7.81, 'R': 7.81, 'H': 1.56, 'L': 10.94, 'S': 4.69, 'D': 3.12, 'Y': 1.56, 'M': 1.56, 'I': 3.12, 'W': 3.12, 'Q': 6.25, 'C': 1.56, 'F': 1.56}
- **Common reported functions (%):** 0.0
- **Common reported locations (%):** 28.57
- **Common reported processes (%):** 0.0

- **PDB ID:** 3G33
- **Chain:** A
- **Crystallized protein length:** 291 aa
- **Resolution:** 3.0 Å
- **Associated domain:** Protein-kinase
- **b-phipsi:** 0.002192
- **w-rdist:** 0.228603
- **t-alpha:** 0.026859
- **Chemical similarity (Tanimoto Index) (%):** 99.09
- **1D identity (%) [PDB]:** 1.92
- **1D identity (%) [Gaps excluded][PDB]:** 63.64
- **1D identity - Alignment Gaps [PDB]:** 1063
- **1D aligned content [PDB] (<aminoacid>:%):** {'R': 9.52, 'Y': 4.76, 'P': 14.29, 'V': 14.29, 'G': 19.05, 'F': 4.76, 'A': 4.76, 'S': 14.29, 'C': 4.76, 'Q': 4.76, 'L': 4.76}
- **2D identity (%) [PDB]:** 18.35
- **2D identity (%) [Gaps excluded][PDB]:** 86.8
- **2D identity - Alignment Gaps [PDB]:** 735
- **2D aligned content [PDB] (<2D-fold>:%):** {'E': 18.71, '.': 25.73, 'T': 15.2, 'H': 40.35}
- **3D similarity (TM-Score) (%) [PDB]:** 13.9

- **Gene name:** CDK4
- **Entrez ID:** 1019
- **RefSeq ID:** NM\_000075
- **Transcript sequence length:** 1865
- **5-UTR|CDS|3-UTR identity (%):** 48.52 | 21.24 | 4.1
- **5-UTR|CDS|3-UTR identity (%) [Gaps excluded]:** 72.57 | 76.75 | 73.21
- **5-UTR|CDS|3-UTR identity [Alignment Gaps]:** 56 | 1978 | 11342
- **5-UTR aligned content (<base>:%):** {'A': 3.66, 'G': 43.9, 'C': 45.12, 'T': 7.32}
- **CDS aligned content (<base>:%):** {'A': 22.72, 'T': 19.1, 'G': 29.26, 'C': 28.92}
- **3-UTR aligned content (<base>:%):** {'C': 17.07, 'A': 30.69, 'G': 19.92, 'T': 32.32}

**Uniprot Description:**  
  
 Ser/Thr-kinase component of cyclin D-CDK4 (DC) complexes that phosphorylate and inhibit members of the retinoblastoma (RB) protein family including RB1 and regulate the cell-cycle during G(1)/S transition. Phosphorylation of RB1 allows dissociation of the transcription factor E2F from the RB/E2F complexes and the subsequent transcription of E2F target genes which are responsible for the progression through the G(1) phase. Hypophosphorylates RB1 in early G(1) phase. Cyclin D-CDK4 complexes are major integrators of various mitogenenic and antimitogenic signals. Also phosphorylates SMAD3 in a cell-cycle-dependent manner and represses its transcriptional activity. Component of the ternary complex, cyclin D/CDK4/CDKN1B, required for nuclear translocation and activity of the cyclin D-CDK4 complex.   
  
Component of the D-CDK4 complex, composed of CDK4 and some D-type G1 cyclin (CCND1, CCND2 or CCND3). Interacts directly in the complex with CCND1, CCND2 or CCND3. Interacts with SEI1 and ZNF655. Forms a ternary complex, cyclin D-CDK4-CDKN1B, involved in modulating CDK4 enzymatic activity. Interacts directly with CDKN1B (phosphorylated on 'Tyr-88' and 'Tyr-89'); the interaction allows assembly of the cyclin D-CDK4 complex, Thr-172 phosphorylation, nuclear translocation and enhances the cyclin D-CDK4 complex activity. CDK4 activity is either inhibited or enhanced depending on stoichiometry of complex. The non-tyrosine-phosphorylated form of CDKN1B prevents T-loop phosphorylation of CDK4 producing inactive CDK4. Interacts (unphosphorylated form) with CDK2. Also forms ternary complexes with CDKN1A or CDKN2A. Interacts directly with CDKN1A (via its N-terminal); the interaction promotes the assembly of the cyclin D-CDK4 complex, its nuclear translocation and promotes the cyclin D-dependent enzyme activity of CDK4. Interacts with CCND1; the interaction is prevented with the binding of CCND1 to INSM1 during cell cycle progression. Probably forms a complex composed of chaperones HSP90 and HSP70, co-chaperones CDC37, PPP5C, TSC1 and client protein TSC2, CDK4, AKT, RAF1 and NR3C1; this complex does not contain co-chaperones STIP1/HOP and PTGES3/p23 (PubMed:29127155). Interacts with CEBPA (when phosphorylated) (PubMed:15107404). Interacts with FNIP1 and FNIP2 (PubMed:27353360).   
  
 **Gene Ontology Information:**

Molecular Function

- ATP binding
- cyclin binding
- cyclin-dependent protein serine/threonine kinase activity
- cyclin-dependent protein serine/threonine kinase regulator activity
- protein serine kinase activity

Location

- bicellular tight junction
- chromatin
- cyclin D1-CDK4 complex
- cyclin D2-CDK4 complex
- cyclin D3-CDK4 complex
- cyclin-dependent protein kinase holoenzyme complex
- cytoplasm
- cytosol
- nuclear membrane
- nucleolus
- nucleoplasm
- nucleus
- transcription regulator complex

Biological process

- cell division
- cellular response to interleukin-4
- cellular response to ionomycin
- cellular response to lipopolysaccharide
- cellular response to phorbol 13-acetate 12-myristate
- G1/S transition of mitotic cell cycle
- positive regulation of cell population proliferation
- positive regulation of fibroblast proliferation
- positive regulation of G2/M transition of mitotic cell cycle
- protein phosphorylation
- regulation of cell cycle
- regulation of G2/M transition of mitotic cell cycle
- regulation of gene expression
- regulation of transcription initiation from RNA polymerase II promoter
- regulation of type B pancreatic cell proliferation
- response to organic substance
- response to xenobiotic stimulus
- signal transduction

---

25

- **Protein name:** Phosphatidylinositol 5-phosphate 4-kinase type-2 alpha
- **Organism:** Homo sapiens
- **Uniprot Accession Number:** P48426
- **Protein sequence length:** 406 aa
- **1D identity (%):** 8.1
- **1D identity (%) [Gaps excluded]:** 23.24
- **1D identity - Alignment Gaps:** 611
- **1D aligned content (<aminoacid>:%):** {'A': 7.89, 'G': 7.89, 'L': 13.16, 'S': 7.89, 'H': 3.95, 'F': 7.89, 'W': 1.32, 'P': 5.26, 'K': 5.26, 'I': 3.95, 'V': 6.58, 'C': 3.95, 'E': 5.26, 'Q': 1.32, 'T': 5.26, 'Y': 2.63, 'N': 3.95, 'D': 5.26, 'R': 1.32}
- **Common reported functions (%):** 0.0
- **Common reported locations (%):** 14.29
- **Common reported processes (%):** 0.0

- **PDB ID:** 6UX9
- **Chain:** A
- **Crystallized protein length:** 316 aa
- **Resolution:** 1.71 Å
- **Associated domain:** PIPK
- **b-phipsi:** 0.007036
- **w-rdist:** 0.213928
- **t-alpha:** 0.010061
- **Chemical similarity (Tanimoto Index) (%):** N/A
- **1D identity (%) [PDB]:** 1.5
- **1D identity (%) [Gaps excluded][PDB]:** 77.27
- **1D identity - Alignment Gaps [PDB]:** 1113
- **1D aligned content [PDB] (<aminoacid>:%):** {'N': 5.88, 'L': 23.53, 'Y': 5.88, 'A': 5.88, 'P': 17.65, 'I': 5.88, 'D': 5.88, 'K': 5.88, 'V': 5.88, 'E': 11.76, 'G': 5.88}
- **2D identity (%) [PDB]:** 26.07
- **2D identity (%) [Gaps excluded][PDB]:** 85.24
- **2D identity - Alignment Gaps [PDB]:** 615
- **2D aligned content [PDB] (<2D-fold>:%):** {'.': 17.32, 'H': 43.29, 'G': 1.3, 'E': 28.14, 'T': 9.96}
- **3D similarity (TM-Score) (%) [PDB]:** 14.77

- **Gene name:** PIP4K2A
- **Entrez ID:** 5305
- **RefSeq ID:** N/A
- **Sequence length:** N/A
- **5-UTR|CDS|3-UTR identity (%):** N/A | N/A | N/A
- **5-UTR|CDS|3-UTR identity (%) [Gaps excluded]:** N/A | N/A | N/A
- **5-UTR|CDS|3-UTR identity [Alignment Gaps]:** N/A | N/A | N/A
- **5-UTR aligned content (<base>:%):** N/A
- **CDS aligned content (<base>:%):** N/A
- **3-UTR aligned content (<base>:%):** N/A

**Uniprot Description:**  
  
 Catalyzes the phosphorylation of phosphatidylinositol 5-phosphate (PtdIns5P) on the fourth hydroxyl of the myo-inositol ring, to form phosphatidylinositol 4,5-bisphosphate (PtdIns(4,5)P2) (PubMed:9367159, PubMed:23326584). Has both ATP- and GTP-dependent kinase activities (PubMed:26774281). May exert its function by regulating the levels of PtdIns5P, which functions in the cytosol by increasing AKT activity and in the nucleus signals through ING2 (PubMed:18364242). May regulate the pool of cytosolic PtdIns5P in response to the activation of tyrosine phosphorylation (By similarity). Required for lysosome-peroxisome membrane contacts and intracellular cholesterol transport through modulating peroxisomal PtdIns(4,5)P2 level (PubMed:29353240). In collaboration with PIP4K2B, has a role in mediating autophagy in times of nutrient stress (By similarity). Required for autophagosome-lysosome fusion and the regulation of cellular lipid metabolism (PubMed:31091439). May be involved in thrombopoiesis, and the terminal maturation of megakaryocytes and regulation of their size (By similarity). Negatively regulates insulin signaling through a catalytic-independent mechanism (PubMed:31091439). PIP4Ks interact with PIP5Ks and suppress PIP5K-mediated PtdIns(4,5)P2 synthesis and insulin-dependent conversion to PtdIns(3,4,5)P3 (PubMed:31091439).   
  
Homodimer (PubMed:32130941). Interacts with PIP4K2B; the interaction may regulate localization to the nucleus (PubMed:20583997). Probably interacts with PIP5K1A; the interaction inhibits PIP5K1A kinase activity (By similarity).   
  
 **Gene Ontology Information:**

Molecular Function

- 1-phosphatidylinositol-4-phosphate 5-kinase activity
- 1-phosphatidylinositol-5-phosphate 4-kinase activity
- ATP binding
- protein homodimerization activity

Location

- autophagosome
- cytosol
- lysosome
- nucleoplasm
- photoreceptor inner segment
- photoreceptor outer segment
- plasma membrane

Biological process

- 1-phosphatidyl-1D-myo-inositol 4,5-bisphosphate biosynthetic process
- autophagosome-lysosome fusion
- megakaryocyte development
- negative regulation of insulin receptor signaling pathway
- phosphatidylinositol phosphorylation
- phosphorylation
- positive regulation of autophagosome assembly
- regulation of autophagy
- vesicle-mediated cholesterol transport

---

26

- **Protein name:** Mitogen-activated protein kinase 1
- **Organism:** Homo sapiens
- **Uniprot Accession Number:** P28482
- **Protein sequence length:** 360 aa
- **1D identity (%):** 7.73
- **1D identity (%) [Gaps excluded]:** 23.59
- **1D identity - Alignment Gaps:** 617
- **1D aligned content (<aminoacid>:%):** {'A': 5.63, 'P': 9.86, 'G': 7.04, 'F': 4.23, 'R': 5.63, 'N': 4.23, 'I': 7.04, 'K': 8.45, 'C': 1.41, 'E': 7.04, 'Y': 5.63, 'L': 8.45, 'D': 5.63, 'T': 2.82, 'S': 7.04, 'H': 1.41, 'V': 4.23, 'W': 1.41, 'M': 1.41, 'Q': 1.41}
- **Common reported functions (%):** 0.0
- **Common reported locations (%):** 28.57
- **Common reported processes (%):** 0.0

- **PDB ID:** 4QTE
- **Chain:** A
- **Crystallized protein length:** 348 aa
- **Resolution:** 1.5 Å
- **Associated domain:** Protein-kinase
- **b-phipsi:** 0.010652
- **w-rdist:** 0.251454
- **t-alpha:** 0.003027
- **Chemical similarity (Tanimoto Index) (%):** 87.08
- **1D identity (%) [PDB]:** 2.27
- **1D identity (%) [Gaps excluded][PDB]:** 65.0
- **1D identity - Alignment Gaps [PDB]:** 1106
- **1D aligned content [PDB] (<aminoacid>:%):** {'P': 7.69, 'G': 7.69, 'E': 7.69, 'K': 7.69, 'R': 7.69, 'V': 3.85, 'I': 3.85, 'S': 11.54, 'C': 3.85, 'L': 19.23, 'Q': 7.69, 'H': 3.85, 'D': 3.85, 'A': 3.85}
- **2D identity (%) [PDB]:** 26.43
- **2D identity (%) [Gaps excluded][PDB]:** 80.55
- **2D identity - Alignment Gaps [PDB]:** 600
- **2D aligned content [PDB] (<2D-fold>:%):** {'.': 16.53, 'E': 21.61, 'T': 10.59, 'H': 49.58, 'B': 0.42, 'G': 1.27}
- **3D similarity (TM-Score) (%) [PDB]:** 15.06

- **Gene name:** MAPK1
- **Entrez ID:** 5594
- **RefSeq ID:** NM\_138957
- **Transcript sequence length:** 1514
- **5-UTR|CDS|3-UTR identity (%):** 38.96 | 26.14 | 1.07
- **5-UTR|CDS|3-UTR identity (%) [Gaps excluded]:** 82.2 | 74.55 | 74.85
- **5-UTR|CDS|3-UTR identity [Alignment Gaps]:** 131 | 1761 | 11737
- **5-UTR aligned content (<base>:%):** {'G': 45.36, 'C': 44.33, 'A': 3.09, 'T': 7.22}
- **CDS aligned content (<base>:%):** {'A': 27.22, 'T': 21.3, 'C': 27.79, 'G': 23.7}
- **3-UTR aligned content (<base>:%):** {'T': 42.97, 'G': 11.72, 'C': 7.81, 'A': 37.5}

**Uniprot Description:**  
  
 Serine/threonine kinase which acts as an essential component of the MAP kinase signal transduction pathway. MAPK1/ERK2 and MAPK3/ERK1 are the 2 MAPKs which play an important role in the MAPK/ERK cascade. They participate also in a signaling cascade initiated by activated KIT and KITLG/SCF. Depending on the cellular context, the MAPK/ERK cascade mediates diverse biological functions such as cell growth, adhesion, survival and differentiation through the regulation of transcription, translation, cytoskeletal rearrangements. The MAPK/ERK cascade plays also a role in initiation and regulation of meiosis, mitosis, and postmitotic functions in differentiated cells by phosphorylating a number of transcription factors. About 160 substrates have already been discovered for ERKs. Many of these substrates are localized in the nucleus, and seem to participate in the regulation of transcription upon stimulation. However, other substrates are found in the cytosol as well as in other cellular organelles, and those are responsible for processes such as translation, mitosis and apoptosis. Moreover, the MAPK/ERK cascade is also involved in the regulation of the endosomal dynamics, including lysosome processing and endosome cycling through the perinuclear recycling compartment (PNRC); as well as in the fragmentation of the Golgi apparatus during mitosis. The substrates include transcription factors (such as ATF2, BCL6, ELK1, ERF, FOS, HSF4 or SPZ1), cytoskeletal elements (such as CANX, CTTN, GJA1, MAP2, MAPT, PXN, SORBS3 or STMN1), regulators of apoptosis (such as BAD, BTG2, CASP9, DAPK1, IER3, MCL1 or PPARG), regulators of translation (such as EIF4EBP1) and a variety of other signaling-related molecules (like ARHGEF2, DCC, FRS2 or GRB10). Protein kinases (such as RAF1, RPS6KA1/RSK1, RPS6KA3/RSK2, RPS6KA2/RSK3, RPS6KA6/RSK4, SYK, MKNK1/MNK1, MKNK2/MNK2, RPS6KA5/MSK1, RPS6KA4/MSK2, MAPKAPK3 or MAPKAPK5) and phosphatases (such as DUSP1, DUSP4, DUSP6 or DUSP16) are other substrates which enable the propagation the MAPK/ERK signal to additional cytosolic and nuclear targets, thereby extending the specificity of the cascade. Mediates phosphorylation of TPR in response to EGF stimulation. May play a role in the spindle assembly checkpoint. Phosphorylates PML and promotes its interaction with PIN1, leading to PML degradation. Phosphorylates CDK2AP2 (By similarity).   
  
Binds both upstream activators and downstream substrates in multimolecular complexes. This interaction inhibits its tyrosine-kinase activity. Interacts with ADAM15, ARHGEF2, ARRB2, DAPK1 (via death domain), HSF4, IER3, IPO7, DUSP6, NISCH, SGK1, and isoform 1 of NEK2. Interacts (via phosphorylated form) with TPR (via C-terminal region and phosphorylated form); the interaction requires dimerization of MAPK1/ERK2 and increases following EGF stimulation (PubMed:18794356). Interacts (phosphorylated form) with CAV2 ('Tyr-19'-phosphorylated form); the interaction, promoted by insulin, leads to nuclear location and MAPK1 activation. Interacts with MORG1, PEA15 and MKNK2 (By similarity). MKNK2 isoform 1 binding prevents from dephosphorylation and inactivation (By similarity). Interacts with DCC (By similarity). The phosphorylated form interacts with PML (isoform PML-4). Interacts with STYX. Interacts with CDK2AP2. Interacts with CAVIN4 (By similarity). Interacts with DUSP7; the interaction enhances DUSP7 phosphatase activity (PubMed:9788880). Interacts with GIT1; this interaction is necessary for MAPK1 localization to focal adhesions (By similarity). Interacts with ZNF263 (PubMed:32051553).   
  
 **Gene Ontology Information:**

Molecular Function

- ATP binding
- DNA binding
- identical protein binding
- MAP kinase activity
- MAP kinase kinase activity
- phosphatase binding
- phosphotyrosine residue binding
- protein serine kinase activity
- protein serine/threonine kinase activity
- RNA polymerase II CTD heptapeptide repeat kinase activity

Location

- azurophil granule lumen
- caveola
- cytoplasm
- cytoskeleton
- cytosol
- early endosome
- endoplasmic reticulum lumen
- extracellular region
- ficolin-1-rich granule lumen
- focal adhesion
- Golgi apparatus
- late endosome
- microtubule organizing center
- mitochondrion
- mitotic spindle
- nucleoplasm
- nucleus
- plasma membrane
- pseudopodium
- synapse

Biological process

- androgen receptor signaling pathway
- apoptotic process
- B cell receptor signaling pathway
- Bergmann glial cell differentiation
- cardiac neural crest cell development involved in heart development
- caveolin-mediated endocytosis
- cell cycle
- cell surface receptor signaling pathway
- cellular response to amino acid starvation
- cellular response to cadmium ion
- cellular response to reactive oxygen species
- cellular response to tumor necrosis factor
- chemical synaptic transmission
- chemotaxis
- cytosine metabolic process
- cellular response to DNA damage stimulus
- ERBB signaling pathway
- ERK1 and ERK2 cascade
- face development
- insulin receptor signaling pathway
- insulin-like growth factor receptor signaling pathway
- intracellular signal transduction
- labyrinthine layer blood vessel development
- learning or memory
- lipopolysaccharide-mediated signaling pathway
- long-term synaptic potentiation
- lung morphogenesis
- mammary gland epithelial cell proliferation
- negative regulation of cell differentiation
- outer ear morphogenesis
- peptidyl-serine phosphorylation
- peptidyl-threonine phosphorylation
- positive regulation of macrophage chemotaxis
- positive regulation of macrophage proliferation
- positive regulation of peptidyl-threonine phosphorylation
- positive regulation of telomerase activity
- positive regulation of telomere capping
- positive regulation of telomere maintenance via telomerase
- progesterone receptor signaling pathway
- protein phosphorylation
- regulation of cellular pH
- regulation of cytoskeleton organization
- regulation of early endosome to late endosome transport
- regulation of Golgi inheritance
- regulation of ossification
- regulation of protein stability
- regulation of stress-activated MAPK cascade
- response to epidermal growth factor
- response to exogenous dsRNA
- response to nicotine
- signal transduction
- steroid hormone mediated signaling pathway
- stress-activated MAPK cascade
- T cell receptor signaling pathway
- thymus development
- thyroid gland development
- trachea formation

---

27

- **Protein name:** 3-oxoacyl-[acyl-carrier-protein] synthase, mitochondrial
- **Organism:** Homo sapiens
- **Uniprot Accession Number:** Q9NWU1
- **Protein sequence length:** 459 aa
- **1D identity (%):** 10.41
- **1D identity (%) [Gaps excluded]:** 22.65
- **1D identity - Alignment Gaps:** 488
- **1D aligned content (<aminoacid>:%):** {'C': 2.13, 'T': 7.45, 'S': 4.26, 'R': 3.19, 'I': 7.45, 'L': 7.45, 'V': 8.51, 'G': 14.89, 'D': 3.19, 'K': 4.26, 'N': 3.19, 'F': 2.13, 'A': 9.57, 'H': 5.32, 'Q': 2.13, 'M': 1.06, 'P': 8.51, 'E': 4.26, 'Y': 1.06}
- **Common reported functions (%):** 0.0
- **Common reported locations (%):** 7.14
- **Common reported processes (%):** 0.0

- **PDB ID:** 2C9H
- **Chain:** A
- **Crystallized protein length:** 426 aa
- **Resolution:** 1.8 Å
- **Associated domain:** Ketosynthase-family-3-KS3
- **b-phipsi:** 0.009856
- **w-rdist:** 0.157169
- **t-alpha:** 0.009137
- **Chemical similarity (Tanimoto Index) (%):** 88.31
- **1D identity (%) [PDB]:** 2.21
- **1D identity (%) [Gaps excluded][PDB]:** 61.36
- **1D identity - Alignment Gaps [PDB]:** 1177
- **1D aligned content [PDB] (<aminoacid>:%):** {'L': 14.81, 'P': 7.41, 'E': 11.11, 'V': 3.7, 'N': 3.7, 'A': 14.81, 'G': 3.7, 'Q': 3.7, 'I': 7.41, 'K': 3.7, 'T': 3.7, 'D': 7.41, 'S': 11.11, 'R': 3.7}
- **2D identity (%) [PDB]:** 27.48
- **2D identity (%) [Gaps excluded][PDB]:** 85.39
- **2D identity - Alignment Gaps [PDB]:** 649
- **2D aligned content [PDB] (<2D-fold>:%):** {'.': 17.49, 'E': 26.24, 'T': 9.51, 'H': 44.11, 'G': 2.28, 'B': 0.38}
- **3D similarity (TM-Score) (%) [PDB]:** 19.98

- **Gene name:** OXSM
- **Entrez ID:** 54995
- **RefSeq ID:** N/A
- **Sequence length:** N/A
- **5-UTR|CDS|3-UTR identity (%):** N/A | N/A | N/A
- **5-UTR|CDS|3-UTR identity (%) [Gaps excluded]:** N/A | N/A | N/A
- **5-UTR|CDS|3-UTR identity [Alignment Gaps]:** N/A | N/A | N/A
- **5-UTR aligned content (<base>:%):** N/A
- **CDS aligned content (<base>:%):** N/A
- **3-UTR aligned content (<base>:%):** N/A

**Uniprot Description:**  
  
 May play a role in the biosynthesis of lipoic acid as well as longer chain fatty acids required for optimal mitochondrial function. N/A   
  
 **Gene Ontology Information:**

Molecular Function

- 3-oxoacyl-[acyl-carrier-protein] synthase activity

Location

- cytosol
- mitochondrion

Biological process

- acyl-CoA metabolic process
- fatty acid biosynthetic process
- medium-chain fatty acid biosynthetic process
- short-chain fatty acid biosynthetic process

---

28

- **Protein name:** Septin-2
- **Organism:** Homo sapiens
- **Uniprot Accession Number:** Q15019
- **Protein sequence length:** 361 aa
- **1D identity (%):** 7.59
- **1D identity (%) [Gaps excluded]:** 23.49
- **1D identity - Alignment Gaps:** 624
- **1D aligned content (<aminoacid>:%):** {'S': 2.86, 'K': 8.57, 'N': 2.86, 'T': 4.29, 'Y': 1.43, 'V': 10.0, 'E': 8.57, 'F': 2.86, 'M': 1.43, 'D': 7.14, 'L': 7.14, 'P': 4.29, 'G': 8.57, 'I': 7.14, 'Q': 7.14, 'A': 1.43, 'C': 4.29, 'R': 5.71, 'H': 4.29}
- **Common reported functions (%):** 0.0
- **Common reported locations (%):** 0.0
- **Common reported processes (%):** 0.0

- **PDB ID:** 6UPR
- **Chain:** B
- **Crystallized protein length:** 270 aa
- **Resolution:** 2.3 Å
- **Associated domain:** Septin-type-G
- **b-phipsi:** 0.005333
- **w-rdist:** 0.274449
- **t-alpha:** 0.010163
- **Chemical similarity (Tanimoto Index) (%):** 75.94
- **1D identity (%) [PDB]:** 1.0
- **1D identity (%) [Gaps excluded][PDB]:** 91.67
- **1D identity - Alignment Gaps [PDB]:** 1084
- **1D aligned content [PDB] (<aminoacid>:%):** {'G': 18.18, 'K': 18.18, 'P': 9.09, 'I': 9.09, 'A': 18.18, 'V': 18.18, 'S': 9.09}
- **2D identity (%) [PDB]:** 20.86
- **2D identity (%) [Gaps excluded][PDB]:** 81.42
- **2D identity - Alignment Gaps [PDB]:** 656
- **2D aligned content [PDB] (<2D-fold>:%):** {'.': 15.76, 'E': 26.63, 'H': 46.74, 'T': 9.24, 'G': 1.63}
- **3D similarity (TM-Score) (%) [PDB]:** 14.75

- **Gene name:** SEPTIN2
- **Entrez ID:** 23176
- **RefSeq ID:** N/A
- **Sequence length:** N/A
- **5-UTR|CDS|3-UTR identity (%):** N/A | N/A | N/A
- **5-UTR|CDS|3-UTR identity (%) [Gaps excluded]:** N/A | N/A | N/A
- **5-UTR|CDS|3-UTR identity [Alignment Gaps]:** N/A | N/A | N/A
- **5-UTR aligned content (<base>:%):** N/A
- **CDS aligned content (<base>:%):** N/A
- **3-UTR aligned content (<base>:%):** N/A

**Uniprot Description:**  
  
 Filament-forming cytoskeletal GTPase. Forms a filamentous structure with SEPTIN12, SEPTIN6, SEPTIN2 and probably SEPTIN4 at the sperm annulus which is required for the structural integrity and motility of the sperm tail during postmeiotic differentiation (PubMed:25588830). Required for normal organization of the actin cytoskeleton. Plays a role in the biogenesis of polarized columnar-shaped epithelium by maintaining polyglutamylated microtubules, thus facilitating efficient vesicle transport, and by impeding MAP4 binding to tubulin. Required for the progression through mitosis. Forms a scaffold at the midplane of the mitotic splindle required to maintain CENPE localization at kinetochores and consequently chromosome congression. During anaphase, may be required for chromosome segregation and spindle elongation. Plays a role in ciliogenesis and collective cell movements. In cilia, required for the integrity of the diffusion barrier at the base of the primary cilium that prevents diffusion of transmembrane proteins between the cilia and plasma membranes: probably acts by regulating the assembly of the tectonic-like complex (also named B9 complex) by localizing TMEM231 protein. May play a role in the internalization of 2 intracellular microbial pathogens, Listeria monocytogenes and Shigella flexneri.   
  
Septins polymerize into heterooligomeric protein complexes that form filaments, and associate with cellular membranes, actin filaments and microtubules (PubMed:17637674, PubMed:25588830, Ref.36). GTPase activity is required for filament formation. Filaments are assembled from asymmetrical heterotrimers, composed of SEPTIN2, SEPTIN6 and SEPTIN7 that associate head-to-head to form a hexameric unit (PubMed:16093351, PubMed:16914550). Interaction between SEPTIN2 and SEPTIN7 seems indirect. Interacts with SEPTIN5 (By similarity). Interaction with SEPTIN4 not detected (By similarity). Interacts with SEPTIN9 (PubMed:19145258). Component of a septin core octameric complex consisting of SEPTIN12, SEPTIN7, SEPTIN6 and SEPTIN2 or SEPTIN4 in the order 12-7-6-2-2-6-7-12 or 12-7-6-4-4-6-7-12 and located in the sperm annulus (PubMed:16093351, PubMed:16914550). Interacts with MAP4. Interacts with DZIP1L (PubMed:28530676).   
  
 **Gene Ontology Information:**

Molecular Function

- GTP binding
- GTPase activity
- molecular adaptor activity

Location

- axon
- cell division site
- microtubule cytoskeleton
- presynapse
- septin complex
- septin ring
- synaptic vesicle membrane

Biological process

- cytoskeleton-dependent cytokinesis
- regulation of intracellular protein transport
- regulation of protein stability
- regulation of SNARE complex assembly

---

29

- **Protein name:** Myotubularin-related protein 2
- **Organism:** Homo sapiens
- **Uniprot Accession Number:** Q13614
- **Protein sequence length:** 643 aa
- **1D identity (%):** 12.72
- **1D identity (%) [Gaps excluded]:** 24.08
- **1D identity - Alignment Gaps:** 464
- **1D aligned content (<aminoacid>:%):** {'M': 1.6, 'G': 6.4, 'P': 9.6, 'A': 6.4, 'D': 4.8, 'R': 9.6, 'V': 9.6, 'E': 6.4, 'K': 5.6, 'L': 6.4, 'T': 5.6, 'S': 4.8, 'F': 4.0, 'W': 1.6, 'I': 3.2, 'C': 3.2, 'Q': 4.0, 'Y': 3.2, 'H': 3.2, 'N': 0.8}
- **Common reported functions (%):** 0.0
- **Common reported locations (%):** 42.86
- **Common reported processes (%):** 0.0

- **PDB ID:** 1M7R
- **Chain:** B
- **Crystallized protein length:** 513 aa
- **Resolution:** 2.6 Å
- **Associated domain:** Myotubularin-phosphatase
- **b-phipsi:** 0.027762
- **w-rdist:** 0.082446
- **t-alpha:** 0.005056
- **Chemical similarity (Tanimoto Index) (%):** 99.39
- **1D identity (%) [PDB]:** 2.69
- **1D identity (%) [Gaps excluded][PDB]:** 68.63
- **1D identity - Alignment Gaps [PDB]:** 1249
- **1D aligned content [PDB] (<aminoacid>:%):** {'E': 8.57, 'L': 11.43, 'P': 8.57, 'G': 2.86, 'K': 8.57, 'D': 8.57, 'R': 8.57, 'I': 5.71, 'V': 5.71, 'S': 11.43, 'Q': 2.86, 'A': 5.71, 'F': 2.86, 'T': 2.86, 'M': 2.86, 'H': 2.86}
- **2D identity (%) [PDB]:** 31.51
- **2D identity (%) [Gaps excluded][PDB]:** 85.44
- **2D identity - Alignment Gaps [PDB]:** 623
- **2D aligned content [PDB] (<2D-fold>:%):** {'.': 17.36, 'T': 10.93, 'E': 22.19, 'G': 2.89, 'H': 46.3, 'B': 0.32}
- **3D similarity (TM-Score) (%) [PDB]:** 24.43

- **Gene name:** MTMR2
- **Entrez ID:** 8898
- **RefSeq ID:** N/A
- **Sequence length:** N/A
- **5-UTR|CDS|3-UTR identity (%):** N/A | N/A | N/A
- **5-UTR|CDS|3-UTR identity (%) [Gaps excluded]:** N/A | N/A | N/A
- **5-UTR|CDS|3-UTR identity [Alignment Gaps]:** N/A | N/A | N/A
- **5-UTR aligned content (<base>:%):** N/A
- **CDS aligned content (<base>:%):** N/A
- **3-UTR aligned content (<base>:%):** N/A

**Uniprot Description:**  
  
 Phosphatase that acts on lipids with a phosphoinositol headgroup. Has phosphatase activity towards phosphatidylinositol 3-phosphate and phosphatidylinositol 3,5-bisphosphate (PubMed:11733541, PubMed:12668758, PubMed:21372139, PubMed:14690594). Binds phosphatidylinositol 4-phosphate, phosphatidylinositol 5-phosphate, phosphatidylinositol 3,5-bisphosphate and phosphatidylinositol 3,4,5-trisphosphate (By similarity). Stabilizes SBF2/MTMR13 at the membranes (By similarity). Specifically in peripheral nerves, stabilizes SBF2/MTMR13 protein (By similarity).   
  
Homodimer (via coiled-coil domain) (PubMed:12668758, PubMed:15998640). Heterotetramer consisting of one MTMR2 dimer and one SBF2/MTMR13 dimer (PubMed:15998640). Heterodimer with SBF1/MTMR5 (PubMed:12668758, PubMed:21372139). Heterodimer with MTMR12 (PubMed:12847286).   
  
 **Gene Ontology Information:**

Molecular Function

- identical protein binding
- phosphatidylinositol-3,5-bisphosphate 3-phosphatase activity
- phosphatidylinositol-3-phosphatase activity
- protein tyrosine/serine/threonine phosphatase activity

Location

- axon
- cytoplasm
- cytosol
- dendrite
- dendritic spine
- early endosome membrane
- extracellular exosome
- intracellular membrane-bounded organelle
- membrane
- nucleus
- perinuclear region of cytoplasm
- postsynaptic density
- synaptic membrane
- synaptic vesicle
- vacuolar membrane

Biological process

- dendritic spine maintenance
- myelin assembly
- negative regulation of endocytosis
- negative regulation of excitatory postsynaptic potential
- negative regulation of myelination
- negative regulation of receptor catabolic process
- negative regulation of receptor internalization
- neuron development
- phosphatidylinositol biosynthetic process
- phosphatidylinositol dephosphorylation
- positive regulation of early endosome to late endosome transport
- protein dephosphorylation
- regulation of phosphatidylinositol dephosphorylation

---

30

- **Protein name:** Casein kinase I isoform epsilon
- **Organism:** Homo sapiens
- **Uniprot Accession Number:** P49674
- **Protein sequence length:** 416 aa
- **1D identity (%):** 10.15
- **1D identity (%) [Gaps excluded]:** 25.91
- **1D identity - Alignment Gaps:** 557
- **1D aligned content (<aminoacid>:%):** {'M': 1.08, 'L': 11.83, 'G': 11.83, 'K': 7.53, 'I': 6.45, 'V': 4.3, 'C': 1.08, 'H': 3.23, 'S': 9.68, 'Q': 2.15, 'P': 8.6, 'E': 5.38, 'N': 1.08, 'F': 6.45, 'D': 3.23, 'Y': 3.23, 'R': 6.45, 'T': 3.23, 'A': 3.23}
- **Common reported functions (%):** 6.67
- **Common reported locations (%):** 35.71
- **Common reported processes (%):** 0.0

- **PDB ID:** 4HOK
- **Chain:** S
- **Crystallized protein length:** 286 aa
- **Resolution:** 2.77 Å
- **Associated domain:** Protein-kinase
- **b-phipsi:** 0.015256
- **w-rdist:** 0.301673
- **t-alpha:** 0.001006
- **Chemical similarity (Tanimoto Index) (%):** 98.87
- **1D identity (%) [PDB]:** 2.11
- **1D identity (%) [Gaps excluded][PDB]:** 63.89
- **1D identity - Alignment Gaps [PDB]:** 1053
- **1D aligned content [PDB] (<aminoacid>:%):** {'R': 4.35, 'V': 8.7, 'G': 21.74, 'K': 8.7, 'N': 4.35, 'I': 8.7, 'T': 4.35, 'H': 8.7, 'P': 4.35, 'E': 4.35, 'F': 4.35, 'C': 4.35, 'S': 8.7, 'Q': 4.35}
- **2D identity (%) [PDB]:** 20.4
- **2D identity (%) [Gaps excluded][PDB]:** 82.51
- **2D identity - Alignment Gaps [PDB]:** 679
- **2D aligned content [PDB] (<2D-fold>:%):** {'T': 10.33, 'E': 20.11, 'H': 50.0, '.': 18.48, 'B': 1.09}
- **3D similarity (TM-Score) (%) [PDB]:** 14.55

- **Gene name:** CSNK1E
- **Entrez ID:** 102800317; 1454
- **RefSeq ID:** NM\_152221
- **Transcript sequence length:** 2817
- **5-UTR|CDS|3-UTR identity (%):** 33.89 | 25.39 | 6.87
- **5-UTR|CDS|3-UTR identity (%) [Gaps excluded]:** 91.07 | 77.22 | 76.43
- **5-UTR|CDS|3-UTR identity [Alignment Gaps]:** 189 | 1935 | 10996
- **5-UTR aligned content (<base>:%):** {'G': 50.98, 'C': 45.1, 'A': 1.96, 'T': 1.96}
- **CDS aligned content (<base>:%):** {'A': 20.08, 'T': 15.44, 'G': 28.83, 'C': 35.66}
- **3-UTR aligned content (<base>:%):** {'G': 25.9, 'A': 16.27, 'C': 27.95, 'T': 29.88}

**Uniprot Description:**  
  
 Casein kinases are operationally defined by their preferential utilization of acidic proteins such as caseins as substrates (Probable). Participates in Wnt signaling (PubMed:12556519, PubMed:23413191). Phosphorylates DVL1 (PubMed:12556519). Phosphorylates DVL2 (PubMed:23413191). Phosphorylates NEDD9/HEF1 (By similarity). Central component of the circadian clock (PubMed:16790549). In balance with PP1, determines the circadian period length, through the regulation of the speed and rhythmicity of PER1 and PER2 phosphorylation (PubMed:15917222, PubMed:16790549). Controls PER1 and PER2 nuclear transport and degradation (By similarity). Inhibits cytokine-induced granuloytic differentiation (PubMed:15070676).   
  
Monomer (PubMed:23106386). Component of the circadian core oscillator, which includes the CRY proteins, CLOCK, or NPAS2, ARTNL/BMAL1 or ARTNL2/BMAL2, CSNK1D and/or CSNK1E, TIMELESS and the PER proteins (By similarity). Interacts with PER1 (PubMed:10790862). Interacts with ANKRD6 (By similarity). Interacts with DBNDD2 (PubMed:16618118). Interacts with LRP5 and LRP6 (PubMed:16513652). Interacts with SOCS3 (PubMed:15070676). Interacts with SNAI1 (via zinc fingers) (PubMed:20305697). Interacts with DDX3X; this interaction greatly enhances CSNK1E affinity for ATP and DVL2 phosphorylation, but inhibits DDX3X ATPase/helicase activity. In the presence of RNA, the interaction is decreased (PubMed:23413191, PubMed:29222110).   
  
 **Gene Ontology Information:**

Molecular Function

- ATP binding
- protein kinase activity
- protein serine kinase activity
- protein serine/threonine kinase activity
- RNA binding

Location

- cytoplasm
- cytosol
- growth cone
- neuronal cell body
- nucleoplasm
- nucleus
- ribonucleoprotein complex

Biological process

- canonical Wnt signaling pathway
- cellular response to nerve growth factor stimulus
- circadian behavior
- circadian regulation of gene expression
- DNA repair
- endocytosis
- negative regulation of protein binding
- negative regulation of Wnt signaling pathway
- peptidyl-serine phosphorylation
- positive regulation of amyloid-beta formation
- positive regulation of canonical Wnt signaling pathway
- positive regulation of non-canonical Wnt signaling pathway
- positive regulation of proteasomal ubiquitin-dependent protein catabolic process
- positive regulation of Wnt-mediated midbrain dopaminergic neuron differentiation
- protein localization
- protein phosphorylation
- regulation of circadian rhythm
- regulation of protein localization
- signal transduction

---

31

- **Protein name:** Chitinase-3-like protein 1
- **Organism:** Homo sapiens
- **Uniprot Accession Number:** P36222
- **Protein sequence length:** 383 aa
- **1D identity (%):** N/A
- **1D identity (%) [Gaps excluded]:** N/A
- **1D identity - Alignment Gaps:** N/A
- **1D aligned content (<aminoacid>:%):** N/A
- **Common reported functions (%):** N/A
- **Common reported locations (%):** N/A
- **Common reported processes (%):** N/A

- **PDB ID:** 1HJV
- **Chain:** B
- **Crystallized protein length:** 362 aa
- **Resolution:** 2.75 Å
- **Associated domain:** GH18
- **b-phipsi:** 0.014532
- **w-rdist:** 0.055741
- **t-alpha:** 0.045216
- **Chemical similarity (Tanimoto Index) (%):** N/A
- **1D identity (%) [PDB]:** N/A
- **1D identity (%) [Gaps excluded][PDB]:** N/A
- **1D identity - Alignment Gaps [PDB]:** N/A
- **1D aligned content [PDB] (<aminoacid>:%):** N/A
- **2D identity (%) [PDB]:** N/A
- **2D identity (%) [Gaps excluded][PDB]:** N/A
- **2D identity - Alignment Gaps [PDB]:** N/A
- **2D aligned content [PDB] (<2D-fold>:%):** N/A
- **3D similarity (TM-Score) (%) [PDB]:** N/A

- **Gene name:** CHI3L1
- **Entrez ID:** 1116
- **RefSeq ID:** N/A
- **Sequence length:** N/A
- **5-UTR|CDS|3-UTR identity (%):** N/A | N/A | N/A
- **5-UTR|CDS|3-UTR identity (%) [Gaps excluded]:** N/A | N/A | N/A
- **5-UTR|CDS|3-UTR identity [Alignment Gaps]:** N/A | N/A | N/A
- **5-UTR aligned content (<base>:%):** N/A
- **CDS aligned content (<base>:%):** N/A
- **3-UTR aligned content (<base>:%):** N/A

**Uniprot Description:**  
  
 Carbohydrate-binding lectin with a preference for chitin. Has no chitinase activity. May play a role in tissue remodeling and in the capacity of cells to respond to and cope with changes in their environment. Plays a role in T-helper cell type 2 (Th2) inflammatory response and IL-13-induced inflammation, regulating allergen sensitization, inflammatory cell apoptosis, dendritic cell accumulation and M2 macrophage differentiation. Facilitates invasion of pathogenic enteric bacteria into colonic mucosa and lymphoid organs. Mediates activation of AKT1 signaling pathway and subsequent IL8 production in colonic epithelial cells. Regulates antibacterial responses in lung by contributing to macrophage bacterial killing, controlling bacterial dissemination and augmenting host tolerance. Also regulates hyperoxia-induced injury, inflammation and epithelial apoptosis in lung.   
  
Monomer.   
  
 **Gene Ontology Information:**

Molecular Function

- carbohydrate binding
- chitin binding
- extracellular matrix structural constituent

Location

- cytoplasm
- endoplasmic reticulum
- extracellular exosome
- extracellular matrix
- extracellular region
- extracellular space
- perinuclear region of cytoplasm
- specific granule lumen

Biological process

- activation of NF-kappaB-inducing kinase activity
- apoptotic process
- carbohydrate metabolic process
- cartilage development
- cellular response to tumor necrosis factor
- chitin catabolic process
- inflammatory response
- lung development
- positive regulation of angiogenesis
- positive regulation of ERK1 and ERK2 cascade
- positive regulation of interleukin-8 production
- positive regulation of peptidyl-threonine phosphorylation
- positive regulation of protein kinase B signaling
- response to interleukin-1
- response to interleukin-6
- response to mechanical stimulus
- response to tumor necrosis factor

---

32

- **Protein name:** Serine/threonine-protein kinase Nek2
- **Organism:** Homo sapiens
- **Uniprot Accession Number:** P51955
- **Protein sequence length:** 445 aa
- **1D identity (%):** 10.21
- **1D identity (%) [Gaps excluded]:** 23.66
- **1D identity - Alignment Gaps:** 518
- **1D aligned content (<aminoacid>:%):** {'R': 10.75, 'I': 3.23, 'Q': 3.23, 'V': 7.53, 'L': 16.13, 'D': 2.15, 'S': 4.3, 'E': 5.38, 'K': 10.75, 'H': 3.23, 'T': 5.38, 'G': 5.38, 'P': 10.75, 'N': 3.23, 'M': 1.08, 'A': 4.3, 'F': 1.08, 'C': 2.15}
- **Common reported functions (%):** 6.67
- **Common reported locations (%):** 28.57
- **Common reported processes (%):** 0.0

- **PDB ID:** 2XK4
- **Chain:** A
- **Crystallized protein length:** 252 aa
- **Resolution:** 2.1 Å
- **Associated domain:** Protein-kinase
- **b-phipsi:** 0.017608
- **w-rdist:** 0.353253
- **t-alpha:** 0.0
- **Chemical similarity (Tanimoto Index) (%):** 89.18
- **1D identity (%) [PDB]:** 1.21
- **1D identity (%) [Gaps excluded][PDB]:** 65.0
- **1D identity - Alignment Gaps [PDB]:** 1054
- **1D aligned content [PDB] (<aminoacid>:%):** {'S': 23.08, 'R': 15.38, 'Y': 7.69, 'V': 7.69, 'L': 15.38, 'N': 7.69, 'F': 7.69, 'D': 7.69, 'E': 7.69}
- **2D identity (%) [PDB]:** 19.53
- **2D identity (%) [Gaps excluded][PDB]:** 85.71
- **2D identity - Alignment Gaps [PDB]:** 688
- **2D aligned content [PDB] (<2D-fold>:%):** {'.': 17.82, 'E': 19.54, 'T': 13.22, 'H': 47.7, 'G': 1.72}
- **3D similarity (TM-Score) (%) [PDB]:** 13.63

- **Gene name:** NEK2
- **Entrez ID:** 4751
- **RefSeq ID:** N/A
- **Sequence length:** N/A
- **5-UTR|CDS|3-UTR identity (%):** N/A | N/A | N/A
- **5-UTR|CDS|3-UTR identity (%) [Gaps excluded]:** N/A | N/A | N/A
- **5-UTR|CDS|3-UTR identity [Alignment Gaps]:** N/A | N/A | N/A
- **5-UTR aligned content (<base>:%):** N/A
- **CDS aligned content (<base>:%):** N/A
- **3-UTR aligned content (<base>:%):** N/A

**Uniprot Description:**  
  
 Protein kinase which is involved in the control of centrosome separation and bipolar spindle formation in mitotic cells and chromatin condensation in meiotic cells. Regulates centrosome separation (essential for the formation of bipolar spindles and high-fidelity chromosome separation) by phosphorylating centrosomal proteins such as CROCC, CEP250 and NINL, resulting in their displacement from the centrosomes. Regulates kinetochore microtubule attachment stability in mitosis via phosphorylation of NDC80. Involved in regulation of mitotic checkpoint protein complex via phosphorylation of CDC20 and MAD2L1. Plays an active role in chromatin condensation during the first meiotic division through phosphorylation of HMGA2. Phosphorylates: PPP1CC; SGO1; NECAB3 and NPM1. Essential for localization of MAD2L1 to kinetochore and MAPK1 and NPM1 to the centrosome. Phosphorylates CEP68 and CNTLN directly or indirectly (PubMed:24554434). NEK2-mediated phosphorylation of CEP68 promotes CEP68 dissociation from the centrosome and its degradation at the onset of mitosis (PubMed:25704143). Involved in the regulation of centrosome disjunction (PubMed:26220856).   
  
Isoform 1, isoform 2 and isoform 4 form homo- and heterodimers. Interacts with NECAB3 and HMGA2 (By similarity). Isoform 1 interacts with CDC20, CTNB1, MAD1L1, MAPK, NEK11, NPM1, NDC80, PCNT and SGO1 (PubMed:14978040, PubMed:15358203, PubMed:15388344, PubMed:15161910, PubMed:17621308, PubMed:18086858, PubMed:18297113, PubMed:20599736, PubMed:20034488). Isoform 1 interacts with STK3/MST2 (via SARAH domain) and SAV1 (via SARAH domain) (PubMed:21076410). Isoform 1 and isoform 2 interact with MAD2L1 (PubMed:20034488). Isoform 1 and isoform 4 interact with PPP1CA and PPP1CC (PubMed:15659832, PubMed:17283141). Interacts with CEP68; the interaction leads to phosphorylation of CEP68. Interacts with CNTLN; the interaction leads to phosphorylation of CNTLN (PubMed:24554434). Isoform 1 interacts with CEP85 (PubMed:26220856).   
  
 **Gene Ontology Information:**

Molecular Function

- ATP binding
- metal ion binding
- protein kinase activity
- protein phosphatase binding
- protein serine kinase activity
- protein serine/threonine kinase activity

Location

- centrosome
- condensed nuclear chromosome
- cytoplasm
- cytosol
- kinetochore
- microtubule
- midbody
- nucleolus
- nucleoplasm
- nucleus
- protein-containing complex
- spindle pole

Biological process

- blastocyst development
- cell division
- centrosome separation
- chromosome segregation
- meiotic cell cycle
- mitotic cell cycle
- mitotic spindle assembly
- negative regulation of centriole-centriole cohesion
- negative regulation of DNA binding
- positive regulation of telomerase activity
- positive regulation of telomere capping
- positive regulation of telomere maintenance via telomerase
- protein autophosphorylation
- protein phosphorylation
- regulation of attachment of spindle microtubules to kinetochore
- regulation of mitotic centrosome separation
- regulation of mitotic nuclear division
- spindle assembly

---

33

- **Protein name:** Acidic mammalian chitinase
- **Organism:** Homo sapiens
- **Uniprot Accession Number:** Q9BZP6
- **Protein sequence length:** 476 aa
- **1D identity (%):** 9.85
- **1D identity (%) [Gaps excluded]:** 27.71
- **1D identity - Alignment Gaps:** 635
- **1D aligned content (<aminoacid>:%):** {'L': 10.31, 'T': 5.15, 'G': 14.43, 'Q': 10.31, 'F': 2.06, 'A': 8.25, 'D': 7.22, 'P': 8.25, 'H': 2.06, 'E': 3.09, 'K': 2.06, 'N': 5.15, 'R': 2.06, 'S': 8.25, 'V': 5.15, 'I': 3.09, 'Y': 1.03, 'W': 1.03, 'C': 1.03}
- **Common reported functions (%):** 0.0
- **Common reported locations (%):** 7.14
- **Common reported processes (%):** 0.0

- **PDB ID:** 2YBU
- **Chain:** E
- **Crystallized protein length:** 376 aa
- **Resolution:** 2.25 Å
- **Associated domain:** GH18
- **b-phipsi:** 0.014636
- **w-rdist:** 0.166266
- **t-alpha:** 0.005056
- **Chemical similarity (Tanimoto Index) (%):** 84.09
- **1D identity (%) [PDB]:** 2.94
- **1D identity (%) [Gaps excluded][PDB]:** 58.62
- **1D identity - Alignment Gaps [PDB]:** 1098
- **1D aligned content [PDB] (<aminoacid>:%):** {'K': 8.82, 'N': 11.76, 'Q': 11.76, 'T': 5.88, 'P': 11.76, 'S': 2.94, 'L': 8.82, 'V': 8.82, 'G': 8.82, 'I': 5.88, 'F': 2.94, 'A': 8.82, 'D': 2.94}
- **2D identity (%) [PDB]:** 27.33
- **2D identity (%) [Gaps excluded][PDB]:** 86.3
- **2D identity - Alignment Gaps [PDB]:** 630
- **2D aligned content [PDB] (<2D-fold>:%):** {'E': 28.17, 'T': 14.68, '.': 11.51, 'H': 43.25, 'G': 2.38}
- **3D similarity (TM-Score) (%) [PDB]:** 17.25

- **Gene name:** CHIA
- **Entrez ID:** 27159
- **RefSeq ID:** N/A
- **Sequence length:** N/A
- **5-UTR|CDS|3-UTR identity (%):** N/A | N/A | N/A
- **5-UTR|CDS|3-UTR identity (%) [Gaps excluded]:** N/A | N/A | N/A
- **5-UTR|CDS|3-UTR identity [Alignment Gaps]:** N/A | N/A | N/A
- **5-UTR aligned content (<base>:%):** N/A
- **CDS aligned content (<base>:%):** N/A
- **3-UTR aligned content (<base>:%):** N/A

**Uniprot Description:**  
  
 Degrades chitin and chitotriose. May participate in the defense against nematodes, fungi and other pathogens. Plays a role in T-helper cell type 2 (Th2) immune response. Contributes to the response to IL-13 and inflammation in response to IL-13. Stimulates chemokine production by pulmonary epithelial cells. Protects lung epithelial cells against apoptosis and promotes phosphorylation of AKT1. Its function in the inflammatory response and in protecting cells against apoptosis is inhibited by allosamidin, suggesting that the function of this protein depends on carbohydrate binding.   
  
Interacts with EGFR.   
  
 **Gene Ontology Information:**

Molecular Function

- chitin binding
- chitinase activity
- kinase binding

Location

- cytoplasm
- extracellular region
- extracellular space

Biological process

- apoptotic process
- chitin catabolic process
- chitin metabolic process
- immune system process
- polysaccharide catabolic process
- polysaccharide digestion
- positive regulation of chemokine production
- production of molecular mediator involved in inflammatory response

---

34

- **Protein name:** Beta-secretase 2
- **Organism:** Homo sapiens
- **Uniprot Accession Number:** Q9Y5Z0
- **Protein sequence length:** 518 aa
- **1D identity (%):** 10.72
- **1D identity (%) [Gaps excluded]:** 27.32
- **1D identity - Alignment Gaps:** 601
- **1D aligned content (<aminoacid>:%):** {'G': 11.32, 'A': 5.66, 'P': 12.26, 'L': 7.55, 'T': 9.43, 'N': 2.83, 'F': 1.89, 'M': 2.83, 'D': 5.66, 'Y': 0.94, 'E': 6.6, 'I': 2.83, 'K': 2.83, 'V': 9.43, 'R': 3.77, 'W': 1.89, 'S': 7.55, 'Q': 2.83, 'C': 1.89}
- **Common reported functions (%):** 0.0
- **Common reported locations (%):** 7.14
- **Common reported processes (%):** 4.76

- **PDB ID:** 3ZKM
- **Chain:** B
- **Crystallized protein length:** 375 aa
- **Resolution:** 1.85 Å
- **Associated domain:** Peptidase-A1
- **b-phipsi:** 0.025591
- **w-rdist:** 0.136751
- **t-alpha:** 0.006073
- **Chemical similarity (Tanimoto Index) (%):** 96.46
- **1D identity (%) [PDB]:** 1.78
- **1D identity (%) [Gaps excluded][PDB]:** 61.76
- **1D identity - Alignment Gaps [PDB]:** 1146
- **1D aligned content [PDB] (<aminoacid>:%):** {'F': 14.29, 'G': 9.52, 'S': 4.76, 'R': 4.76, 'T': 4.76, 'I': 23.81, 'N': 4.76, 'P': 9.52, 'K': 4.76, 'Y': 9.52, 'E': 4.76, 'L': 4.76}
- **2D identity (%) [PDB]:** 25.91
- **2D identity (%) [Gaps excluded][PDB]:** 84.86
- **2D identity - Alignment Gaps [PDB]:** 646
- **2D aligned content [PDB] (<2D-fold>:%):** {'.': 17.01, 'T': 16.6, 'E': 50.62, 'G': 2.49, 'H': 12.86, 'B': 0.41}
- **3D similarity (TM-Score) (%) [PDB]:** 18.33

- **Gene name:** BACE2
- **Entrez ID:** 25825
- **RefSeq ID:** NM\_138992
- **Transcript sequence length:** 8398
- **5-UTR|CDS|3-UTR identity (%):** 50.7 | 24.69 | 34.65
- **5-UTR|CDS|3-UTR identity (%) [Gaps excluded]:** 80.9 | 75.21 | 75.63
- **5-UTR|CDS|3-UTR identity [Alignment Gaps]:** 53 | 1907 | 7057
- **5-UTR aligned content (<base>:%):** {'A': 1.39, 'C': 51.39, 'G': 41.67, 'T': 5.56}
- **CDS aligned content (<base>:%):** {'T': 19.12, 'G': 30.39, 'C': 27.1, 'A': 23.4}
- **3-UTR aligned content (<base>:%):** {'C': 20.58, 'G': 22.16, 'T': 32.53, 'A': 24.73}

**Uniprot Description:**  
  
 Responsible for the proteolytic processing of the amyloid precursor protein (APP). Cleaves APP, between residues 690 and 691, leading to the generation and extracellular release of beta-cleaved soluble APP, and a corresponding cell-associated C-terminal fragment which is later released by gamma-secretase. It has also been shown that it can cleave APP between residues 671 and 672. Responsible also for the proteolytic processing of CLTRN in pancreatic beta cells (PubMed:21907142).   
  
Monomer. Interacts ith RTN3 and RTN4.   
  
 **Gene Ontology Information:**

Molecular Function

- aspartic-type endopeptidase activity

Location

- endoplasmic reticulum
- endosome
- Golgi apparatus
- melanosome membrane
- membrane
- plasma membrane
- trans-Golgi network

Biological process

- amyloid-beta metabolic process
- glucose homeostasis
- melanosome organization
- membrane protein ectodomain proteolysis
- negative regulation of amyloid precursor protein biosynthetic process
- peptide hormone processing
- protein processing
- proteolysis

---

35

- **Protein name:** Mucin-2
- **Organism:** Homo sapiens
- **Uniprot Accession Number:** Q02817
- **Protein sequence length:** 5289 aa
- **1D identity (%):** 5.0
- **1D identity (%) [Gaps excluded]:** 31.44
- **1D identity - Alignment Gaps:** 4462
- **1D aligned content (<aminoacid>:%):** {'G': 12.45, 'L': 5.28, 'A': 3.77, 'F': 4.15, 'P': 13.58, 'D': 4.91, 'T': 12.83, 'I': 2.26, 'H': 5.66, 'Y': 2.26, 'E': 3.02, 'K': 4.53, 'C': 5.66, 'V': 6.42, 'W': 0.75, 'R': 2.26, 'S': 2.26, 'Q': 4.91, 'N': 1.89, 'M': 1.13}
- **Common reported functions (%):** 0.0
- **Common reported locations (%):** 0.0
- **Common reported processes (%):** 0.0

- **PDB ID:** 7PP6
- **Chain:** D
- **Crystallized protein length:** 1148 aa
- **Resolution:** 3.4 Å
- **Associated domain:** VWFD-3
- **b-phipsi:** 0.021823
- **w-rdist:** 0.155146
- **t-alpha:** 0.007092
- **Chemical similarity (Tanimoto Index) (%):** 76.42
- **1D identity (%) [PDB]:** 3.87
- **1D identity (%) [Gaps excluded][PDB]:** 70.19
- **1D identity - Alignment Gaps [PDB]:** 1784
- **1D aligned content [PDB] (<aminoacid>:%):** {'N': 4.11, 'P': 4.11, 'A': 10.96, 'G': 5.48, 'T': 6.85, 'V': 5.48, 'D': 9.59, 'K': 2.74, 'E': 4.11, 'F': 2.74, 'L': 9.59, 'S': 9.59, 'H': 9.59, 'R': 5.48, 'Y': 4.11, 'Q': 5.48}
- **2D identity (%) [PDB]:** 32.31
- **2D identity (%) [Gaps excluded][PDB]:** 82.83
- **2D identity - Alignment Gaps [PDB]:** 874
- **2D aligned content [PDB] (<2D-fold>:%):** {'.': 19.87, 'E': 36.72, 'T': 18.57, 'H': 23.33, 'G': 1.3, 'B': 0.22}
- **3D similarity (TM-Score) (%) [PDB]:** 19.22

- **Gene name:** MUC2
- **Entrez ID:** 4583
- **RefSeq ID:** N/A
- **Sequence length:** N/A
- **5-UTR|CDS|3-UTR identity (%):** N/A | N/A | N/A
- **5-UTR|CDS|3-UTR identity (%) [Gaps excluded]:** N/A | N/A | N/A
- **5-UTR|CDS|3-UTR identity [Alignment Gaps]:** N/A | N/A | N/A
- **5-UTR aligned content (<base>:%):** N/A
- **CDS aligned content (<base>:%):** N/A
- **3-UTR aligned content (<base>:%):** N/A

**Uniprot Description:**  
  
 Coats the epithelia of the intestines and other mucus membrane-containing organs to provide a protective, lubricating barrier against particles and infectious agents at mucosal surfaces (PubMed:17058067, PubMed:19432394, PubMed:33031746). Major constituent of the colon mucus, which is mainly formed by large polymeric networks of MUC2 secreted by goblet cells that cover the exposed surfaces of intestine (PubMed:19432394, PubMed:33031746). MUC2 networks form hydrogels that guard the underlying epithelium from pathogens and other hazardous matter entering from the outside world, while permitting nutrient absorption and gas exchange (PubMed:33031746, PubMed:36206754). Acts as a divalent copper chaperone that protects intestinal cells from copper toxicity and facilitates nutritional copper unptake into cells (PubMed:36206754). Binds both Cu(2+) and its reduced form, Cu(1+), at two juxtaposed binding sites: Cu(2+), once reduced to Cu(1+) by vitamin C (ascorbate) or other dietary antioxidants, transits to the other binding site (PubMed:36206754). MUC2-bound Cu(1+) is protected from oxidation in aerobic environments, and can be released for nutritional delivery to cells (PubMed:36206754). Mucin gels store antimicrobial molecules that participate in innate immunity (PubMed:33031746). Mucin glycoproteins also house and feed the microbiome, lubricate tissue surfaces, and may facilitate the removal of contaminants and waste products from the body (PubMed:33031746). Goblet cells synthesize two forms of MUC2 mucin that differ in branched chain O-glycosylation and the site of production in the colon: a (1) 'thick' mucus that wraps the microbiota to form fecal pellets is produced in the proximal, ascending colon (By similarity). 'Thick' mucus transits along the descending colon and is lubricated by a (2) 'thin' MUC2 mucus produced in the distal colon which adheres to the 'thick' mucus (By similarity).   
  
Homomultimer; disulfide-linked (PubMed:12374796, PubMed:31310764, PubMed:33031746, PubMed:35377815). The N- and C-terminus mediate their assembly into higher order structures to form filaments (PubMed:33031746, PubMed:35377815). The CTCK domains of two polypeptides associate in the endoplasmic reticulum to generate intermolecularly disulfide-bonded dimers (By similarity). These dimers progress to the Golgi apparatus, which is a more acidic environment than the endoplasmic reticulum (PubMed:33031746). Under acidic conditions, the N-termini form non-covalent intermolecular interactions that juxtapose assemblies of the third VWD domain (VWD3) from different CTCK-linked dimers (PubMed:33031746). The VWD3 assemblies then become disulfide bonded to one another to produce long, disulfide-linked polymers that remain highly compact until secretion (PubMed:33031746). Interacts with FCGBP (PubMed:19432394). Interacts with AGR2; disulfide-linked (PubMed:19359471).   
  
 **Gene Ontology Information:**

Molecular Function

- cupric ion binding
- cuprous ion binding

Location

- collagen-containing extracellular matrix
- extracellular matrix
- extracellular space
- Golgi lumen
- inner mucus layer
- outer mucus layer
- plasma membrane

Biological process

- detoxification of copper ion
- host-mediated regulation of intestinal microbiota composition
- maintenance of gastrointestinal epithelium
- mucus secretion

---

36

- **Protein name:** E3 ubiquitin-protein ligase CBL
- **Organism:** Homo sapiens
- **Uniprot Accession Number:** P22681
- **Protein sequence length:** 906 aa
- **1D identity (%):** 16.11
- **1D identity (%) [Gaps excluded]:** 23.6
- **1D identity - Alignment Gaps:** 333
- **1D aligned content (<aminoacid>:%):** {'K': 2.96, 'G': 9.47, 'S': 7.69, 'I': 3.55, 'L': 8.88, 'F': 3.55, 'H': 3.55, 'P': 14.2, 'E': 5.33, 'C': 4.73, 'V': 5.33, 'Q': 4.73, 'D': 5.92, 'Y': 2.37, 'R': 4.14, 'T': 5.33, 'A': 5.92, 'W': 0.59, 'M': 1.18, 'N': 0.59}
- **Common reported functions (%):** 0.0
- **Common reported locations (%):** 7.14
- **Common reported processes (%):** 0.0

- **PDB ID:** 1FBV
- **Chain:** A
- **Crystallized protein length:** 388 aa
- **Resolution:** 2.9 Å
- **Associated domain:** Cbl-PTB
- **b-phipsi:** 0.029616
- **w-rdist:** 0.115765
- **t-alpha:** 0.008048
- **Chemical similarity (Tanimoto Index) (%):** N/A
- **1D identity (%) [PDB]:** 2.45
- **1D identity (%) [Gaps excluded][PDB]:** 65.91
- **1D identity - Alignment Gaps [PDB]:** 1138
- **1D aligned content [PDB] (<aminoacid>:%):** {'P': 13.79, 'G': 10.34, 'D': 6.9, 'K': 6.9, 'V': 10.34, 'L': 17.24, 'T': 3.45, 'E': 3.45, 'R': 3.45, 'I': 3.45, 'F': 6.9, 'S': 3.45, 'C': 3.45, 'Q': 3.45, 'A': 3.45}
- **2D identity (%) [PDB]:** 28.14
- **2D identity (%) [Gaps excluded][PDB]:** 86.09
- **2D identity - Alignment Gaps [PDB]:** 622
- **2D aligned content [PDB] (<2D-fold>:%):** {'.': 15.38, 'H': 60.77, 'T': 11.92, 'G': 3.46, 'E': 8.46}
- **3D similarity (TM-Score) (%) [PDB]:** 15.86

- **Gene name:** CBL
- **Entrez ID:** N/A
- **RefSeq ID:** NM\_005188
- **Transcript sequence length:** 11168
- **5-UTR|CDS|3-UTR identity (%):** 43.94 | 45.39 | 37.87
- **5-UTR|CDS|3-UTR identity (%) [Gaps excluded]:** 78.38 | 73.55 | 76.06
- **5-UTR|CDS|3-UTR identity [Alignment Gaps]:** 58 | 1255 | 6790
- **5-UTR aligned content (<base>:%):** {'C': 56.9, 'T': 12.07, 'G': 29.31, 'A': 1.72}
- **CDS aligned content (<base>:%):** {'A': 24.26, 'T': 20.43, 'G': 24.8, 'C': 30.51}
- **3-UTR aligned content (<base>:%):** {'C': 20.52, 'A': 23.16, 'T': 35.09, 'G': 21.23}

**Uniprot Description:**  
  
 Adapter protein that functions as a negative regulator of many signaling pathways that are triggered by activation of cell surface receptors. Acts as an E3 ubiquitin-protein ligase, which accepts ubiquitin from specific E2 ubiquitin-conjugating enzymes, and then transfers it to substrates promoting their degradation by the proteasome (PubMed:17094949). Ubiquitinates SPRY2 (PubMed:17094949, PubMed:17974561). Ubiquitinates EGFR (PubMed:17974561). Recognizes activated receptor tyrosine kinases, including KIT, FLT1, FGFR1, FGFR2, PDGFRA, PDGFRB, CSF1R, EPHA8 and KDR and terminates signaling. Recognizes membrane-bound HCK, SRC and other kinases of the SRC family and mediates their ubiquitination and degradation. Participates in signal transduction in hematopoietic cells. Plays an important role in the regulation of osteoblast differentiation and apoptosis. Essential for osteoclastic bone resorption. The 'Tyr-731' phosphorylated form induces the activation and recruitment of phosphatidylinositol 3-kinase to the cell membrane in a signaling pathway that is critical for osteoclast function. May be functionally coupled with the E2 ubiquitin-protein ligase UB2D3. In association with CBLB, required for proper feedback inhibition of ciliary platelet-derived growth factor receptor-alpha (PDGFRA) signaling pathway via ubiquitination and internalization of PDGFRA (By similarity).   
  
Forms homodimers; IFT20 promotes the formation of stable homodimers (PubMed:29237719). Interacts (phosphorylated at Tyr-731) with PIK3R1. Associates with NCK via its SH3 domain. The phosphorylated C-terminus interacts with CD2AP via its second SH3 domain. Binds to UBE2L3. Interacts with adapters SLA, SLA2 and with the phosphorylated C-terminus of SH2B2. Interacts with EGFR, SYK and ZAP70 via the highly conserved Cbl-N region. Also interacts with SORBS1 and INPPL1/SHIP2. Interacts with phosphorylated LAT2 (By similarity). Interacts with CBLB (PubMed:29237719). Interacts with ALK, AXL, BLK, FGR and FGFR2. Interacts with CSF1R, EPHB1, FLT1, KDR, PDGFRA and PDGFRB; regulates receptor degradation through ubiquitination. Interacts with HCK and LYN. Interacts with ATX2 (By similarity). Interacts with TEK/TIE2 (tyrosine phosphorylated). Interacts with SH3KBP1 and this interaction is inhibited in the presence of SHKBP1 (By similarity). Interacts with SIGLEC10 (By similarity). Interacts with IFT20 (PubMed:29237719). Interacts with SPRY2; the interaction inhibits CBL-mediated ubiquitination of EGFR (PubMed:17974561).   
  
 **Gene Ontology Information:**

Molecular Function

- cadherin binding
- calcium ion binding
- ephrin receptor binding
- phosphatidylinositol 3-kinase regulatory subunit binding
- phosphotyrosine residue binding
- receptor tyrosine kinase binding
- SH3 domain binding
- ubiquitin protein ligase activity
- ubiquitin-protein transferase activity

Location

- cilium
- cytosol
- flotillin complex
- focal adhesion
- Golgi apparatus
- growth cone
- membrane raft
- perinuclear region of cytoplasm
- plasma membrane

Biological process

- cellular response to nerve growth factor stimulus
- cellular response to oxygen-glucose deprivation
- cellular response to platelet-derived growth factor stimulus
- cytokine-mediated signaling pathway
- cellular response to DNA damage stimulus
- entry of bacterium into host cell
- male gonad development
- mast cell degranulation
- negative regulation of apoptotic process
- negative regulation of epidermal growth factor receptor signaling pathway
- negative regulation of epidermal growth factor-activated receptor activity
- positive regulation of epidermal growth factor receptor signaling pathway
- positive regulation of phosphatidylinositol 3-kinase signaling
- positive regulation of receptor-mediated endocytosis
- protein autoubiquitination
- protein monoubiquitination
- protein polyubiquitination
- protein ubiquitination
- regulation of platelet-derived growth factor receptor-alpha signaling pathway
- regulation of Rap protein signal transduction
- response to activity
- response to ethanol
- response to gamma radiation
- response to starvation
- response to testosterone
- signal transduction
- ubiquitin-dependent protein catabolic process

---

37

- **Protein name:** Ceruloplasmin
- **Organism:** Homo sapiens
- **Uniprot Accession Number:** P00450
- **Protein sequence length:** 1065 aa
- **1D identity (%):** 17.23
- **1D identity (%) [Gaps excluded]:** 26.21
- **1D identity - Alignment Gaps:** 398
- **1D aligned content (<aminoacid>:%):** {'I': 5.5, 'G': 12.5, 'A': 5.0, 'T': 6.5, 'E': 4.5, 'D': 7.5, 'H': 4.0, 'P': 6.5, 'R': 6.0, 'F': 5.0, 'K': 6.0, 'V': 8.0, 'N': 3.0, 'L': 6.5, 'Y': 3.0, 'C': 3.0, 'S': 4.0, 'M': 0.5, 'Q': 3.0}
- **Common reported functions (%):** 0.0
- **Common reported locations (%):** 7.14
- **Common reported processes (%):** 0.0

- **PDB ID:** 4EJX
- **Chain:** A
- **Crystallized protein length:** 1034 aa
- **Resolution:** 4.69 Å
- **Associated domain:** F5-8-type-A-3
- **b-phipsi:** 0.027729
- **w-rdist:** 0.106666
- **t-alpha:** 0.014286
- **Chemical similarity (Tanimoto Index) (%):** 74.82
- **1D identity (%) [PDB]:** 2.96
- **1D identity (%) [Gaps excluded][PDB]:** 66.25
- **1D identity - Alignment Gaps [PDB]:** 1713
- **1D aligned content [PDB] (<aminoacid>:%):** {'K': 7.55, 'E': 9.43, 'I': 3.77, 'G': 7.55, 'V': 11.32, 'T': 11.32, 'H': 7.55, 'Q': 9.43, 'R': 7.55, 'Y': 1.89, 'P': 5.66, 'A': 1.89, 'S': 3.77, 'C': 1.89, 'D': 1.89, 'L': 7.55}
- **2D identity (%) [PDB]:** 34.94
- **2D identity (%) [Gaps excluded][PDB]:** 84.49
- **2D identity - Alignment Gaps [PDB]:** 777
- **2D aligned content [PDB] (<2D-fold>:%):** {'.': 19.22, 'E': 43.41, 'T': 21.38, 'H': 14.04, 'G': 1.94}
- **3D similarity (TM-Score) (%) [PDB]:** 28.55

- **Gene name:** CP
- **Entrez ID:** 1356
- **RefSeq ID:** NM\_000096
- **Transcript sequence length:** 4452
- **5-UTR|CDS|3-UTR identity (%):** 12.23 | 43.29 | 6.57
- **5-UTR|CDS|3-UTR identity (%) [Gaps excluded]:** 68.0 | 74.37 | 73.08
- **5-UTR|CDS|3-UTR identity [Alignment Gaps]:** 114 | 1526 | 10943
- **5-UTR aligned content (<base>:%):** {'C': 41.18, 'T': 23.53, 'G': 35.29}
- **CDS aligned content (<base>:%):** {'A': 29.16, 'T': 20.68, 'G': 25.87, 'C': 24.29}
- **3-UTR aligned content (<base>:%):** {'T': 32.03, 'A': 40.0, 'G': 13.92, 'C': 14.05}

**Uniprot Description:**  
  
 Ceruloplasmin is a blue, copper-binding (6-7 atoms per molecule) glycoprotein. It has ferroxidase activity oxidizing Fe(2+) to Fe(3+) without releasing radical oxygen species. It is involved in iron transport across the cell membrane. Provides Cu(2+) ions for the ascorbate-mediated deaminase degradation of the heparan sulfate chains of GPC1. May also play a role in fetal lung development or pulmonary antioxidant defense (By similarity). N/A   
  
 **Gene Ontology Information:**

Molecular Function

- copper ion binding
- ferroxidase activity
- oxidoreductase activity
- chaperone binding

Location

- blood microparticle
- endoplasmic reticulum lumen
- extracellular exosome
- extracellular region
- extracellular space
- lysosomal membrane
- plasma membrane

Biological process

- copper ion transport
- cellular iron ion homeostasis
- iron ion transport

---
