# Supplementary material for: Comparative structural insights and functional analysis for the distinct unbound states of Human AGO proteins
Source: Sci Rep. 2025 Mar 19;15:9432. doi: 10.1038/s41598-025-91849-5 (PMC11923369; doi:10.1038/s41598-025-91849-5)
Supplement: Supplementary file 24 — Supplementary Information 12. [file 41598_2025_91849_MOESM24_ESM.zip › 4Z4Dp_A_mddomain_HL2REF/candidates/4Z4Dp_A_Piwi-merged-notenriched_report.html]

 

# Structural Comparison Report for 4Z4Dp\_A\_Piwi - domains (total: 800)

---

1

- **PDB ID:** 5MXD | **Chain:** A
- **b-phipsi:** 0.0155468448395744
- **w-rdist:** 0.0434985891141304
- **t-alpha:** 0.005055767262688

---

---

2

- **PDB ID:** 4ZSQ | **Chain:** B
- **b-phipsi:** 0.015677784840094
- **w-rdist:** 0.0537287103483591
- **t-alpha:** 0.0050303674430436

---

---

3

- **PDB ID:** 7MYU | **Chain:** A
- **b-phipsi:** 0.0169597529026005
- **w-rdist:** 0.0578605903372952
- **t-alpha:** 0.0

---

---

4

- **PDB ID:** 2Q11 | **Chain:** A
- **b-phipsi:** 0.0218867580622917
- **w-rdist:** 0.0462500989955005
- **t-alpha:** 0.0

---

---

5

- **PDB ID:** 3RTH | **Chain:** A
- **b-phipsi:** 0.02229242258275
- **w-rdist:** 0.04272101428178
- **t-alpha:** 0.001007110036042

---

---

6

- **PDB ID:** 3KMY | **Chain:** A
- **b-phipsi:** 0.0203190725650428
- **w-rdist:** 0.0411912014510472
- **t-alpha:** 0.0050303674430436

---

---

7

- **PDB ID:** 4DJX | **Chain:** A
- **b-phipsi:** 0.0171162770169002
- **w-rdist:** 0.0586900052239221
- **t-alpha:** 0.0030181563299565

---

---

8

- **PDB ID:** 6JT3 | **Chain:** A
- **b-phipsi:** 0.0269721794786759
- **w-rdist:** 0.041997782351828
- **t-alpha:** 0.0020161899393582

---

---

9

- **PDB ID:** 5HTZ | **Chain:** B
- **b-phipsi:** 0.0144077144375042
- **w-rdist:** 0.0416400698062847
- **t-alpha:** 0.02213297304878

---

---

10

- **PDB ID:** 4J1E | **Chain:** A
- **b-phipsi:** 0.0270556800340895
- **w-rdist:** 0.0455586203356168
- **t-alpha:** 0.001007110036042

---

---

11

- **PDB ID:** 4AY1 | **Chain:** L
- **b-phipsi:** 0.0103477298326194
- **w-rdist:** 0.0779849754731027
- **t-alpha:** 0.0020161899393582

---

---

12

- **PDB ID:** 1XN3 | **Chain:** C
- **b-phipsi:** 0.0140910120256962
- **w-rdist:** 0.0758343845712583
- **t-alpha:** 0.001007110036042

---

---

13

- **PDB ID:** 5VM9 | **Chain:** C
- **b-phipsi:** 0.0074245320931729
- **w-rdist:** 0.0260403504272094
- **t-alpha:** 0.0362172476666218

---

---

14

- **PDB ID:** 6JSG | **Chain:** A
- **b-phipsi:** 0.0240767911670499
- **w-rdist:** 0.0407518035041913
- **t-alpha:** 0.005055767262688

---

---

15

- **PDB ID:** 3MSJ | **Chain:** A
- **b-phipsi:** 0.0267961318183504
- **w-rdist:** 0.048406758139932
- **t-alpha:** 0.001007110036042

---

---

16

- **PDB ID:** 6UVV | **Chain:** A
- **b-phipsi:** 0.0178543838256912
- **w-rdist:** 0.0463020106963286
- **t-alpha:** 0.0110661838165213

---

---

17

- **PDB ID:** 5HTZ | **Chain:** A
- **b-phipsi:** 0.0144525208502314
- **w-rdist:** 0.0574269700874286
- **t-alpha:** 0.012220058253459

---

---

18

- **PDB ID:** 7D2X | **Chain:** A
- **b-phipsi:** 0.0269151588356969
- **w-rdist:** 0.0456196693469085
- **t-alpha:** 0.0030272436213401

---

---

19

- **PDB ID:** 6UWV | **Chain:** A
- **b-phipsi:** 0.0171457119015997
- **w-rdist:** 0.0673881712257393
- **t-alpha:** 0.002011889761613

---

---

20

- **PDB ID:** 4AY1 | **Chain:** K
- **b-phipsi:** 0.0069509015329004
- **w-rdist:** 0.07697244751813
- **t-alpha:** 0.0060363072918738

---

---

21

- **PDB ID:** 5MCO | **Chain:** A
- **b-phipsi:** 0.0230913885211096
- **w-rdist:** 0.0566175703726424
- **t-alpha:** 0.0010061554017259

---

---

22

- **PDB ID:** 4P8X | **Chain:** A
- **b-phipsi:** 0.0116147217215586
- **w-rdist:** 0.0875549534978793
- **t-alpha:** 0.0010061554017259

---

---

23

- **PDB ID:** 4FRS | **Chain:** A
- **b-phipsi:** 0.0173428877716691
- **w-rdist:** 0.0696671689149403
- **t-alpha:** 0.002011889761613

---

---

24

- **PDB ID:** 5HU1 | **Chain:** A
- **b-phipsi:** 0.0209892646258718
- **w-rdist:** 0.0606508872276296
- **t-alpha:** 0.002011889761613

---

---

25

- **PDB ID:** 7N66 | **Chain:** A
- **b-phipsi:** 0.0240791442799444
- **w-rdist:** 0.0472094934396308
- **t-alpha:** 0.0060363072918738

---

---

26

- **PDB ID:** 5HDX | **Chain:** A
- **b-phipsi:** 0.0164161394968173
- **w-rdist:** 0.0544176311322951
- **t-alpha:** 0.0140843739056586

---

---

27

- **PDB ID:** 4CFF | **Chain:** E
- **b-phipsi:** 0.0081134377733349
- **w-rdist:** 0.0978355457187933
- **t-alpha:** 0.0

---

---

28

- **PDB ID:** 2VA5 | **Chain:** A
- **b-phipsi:** 0.0251203497006016
- **w-rdist:** 0.0464478588106582
- **t-alpha:** 0.0060363072918738

---

---

29

- **PDB ID:** 4YBI | **Chain:** A
- **b-phipsi:** 0.017805807931139
- **w-rdist:** 0.0600334639313453
- **t-alpha:** 0.0060363072918738

---

---

30

- **PDB ID:** 3MSK | **Chain:** A
- **b-phipsi:** 0.024945184080114
- **w-rdist:** 0.0569909264028423
- **t-alpha:** 0.002011889761613

---

---

31

- **PDB ID:** 4IVT | **Chain:** A
- **b-phipsi:** 0.0313757755610019
- **w-rdist:** 0.0421334610208691
- **t-alpha:** 0.0030272436213401

---

---

32

- **PDB ID:** 7MYI | **Chain:** B
- **b-phipsi:** 0.0164689330258456
- **w-rdist:** 0.0737281530831261
- **t-alpha:** 0.0030181563299565

---

---

33

- **PDB ID:** 4X7I | **Chain:** A
- **b-phipsi:** 0.0173187434460344
- **w-rdist:** 0.0704686681179212
- **t-alpha:** 0.0030181563299565

---

---

34

- **PDB ID:** 5QD0 | **Chain:** C
- **b-phipsi:** 0.0220286676917025
- **w-rdist:** 0.0576477193809219
- **t-alpha:** 0.0030272436213401

---

---

35

- **PDB ID:** 4ZSR | **Chain:** A
- **b-phipsi:** 0.0185379129220078
- **w-rdist:** 0.0427607248307072
- **t-alpha:** 0.0160964977574751

---

---

36

- **PDB ID:** 2Q15 | **Chain:** A
- **b-phipsi:** 0.0258153143024309
- **w-rdist:** 0.0466684411217042
- **t-alpha:** 0.0070422936000285

---

---

37

- **PDB ID:** 5F01 | **Chain:** A
- **b-phipsi:** 0.026087573353247
- **w-rdist:** 0.0392134264050365
- **t-alpha:** 0.0091371600897414

---

---

38

- **PDB ID:** 3CID | **Chain:** A
- **b-phipsi:** 0.0165505579169068
- **w-rdist:** 0.0765089207321803
- **t-alpha:** 0.0030181563299565

---

---

39

- **PDB ID:** 4FSL | **Chain:** B
- **b-phipsi:** 0.0175526235263219
- **w-rdist:** 0.0706617308360814
- **t-alpha:** 0.0030272436213401

---

---

40

- **PDB ID:** 4TRY | **Chain:** B
- **b-phipsi:** 0.0192840965537925
- **w-rdist:** 0.0697748578040317
- **t-alpha:** 0.0010061554017259

---

---

41

- **PDB ID:** 6WNY | **Chain:** A
- **b-phipsi:** 0.0299517369351572
- **w-rdist:** 0.045033462819846
- **t-alpha:** 0.0050303674430436

---

---

42

- **PDB ID:** 5HU1 | **Chain:** B
- **b-phipsi:** 0.0149494595337349
- **w-rdist:** 0.0779602911791275
- **t-alpha:** 0.0050303674430436

---

---

43

- **PDB ID:** 4IVS | **Chain:** A
- **b-phipsi:** 0.0322172279125963
- **w-rdist:** 0.0458066537084738
- **t-alpha:** 0.0030272436213401

---

---

44

- **PDB ID:** 4J0V | **Chain:** A
- **b-phipsi:** 0.0295230435276637
- **w-rdist:** 0.0452149268884118
- **t-alpha:** 0.005055767262688

---

---

45

- **PDB ID:** 5HU0 | **Chain:** A
- **b-phipsi:** 0.014888047823977
- **w-rdist:** 0.049089430067692
- **t-alpha:** 0.0291750635565768

---

---

46

- **PDB ID:** 5QD8 | **Chain:** B
- **b-phipsi:** 0.0331086523717516
- **w-rdist:** 0.0456547289484998
- **t-alpha:** 0.0030181563299565

---

---

47

- **PDB ID:** 4I0H | **Chain:** A
- **b-phipsi:** 0.0321310940781922
- **w-rdist:** 0.0529683872162891
- **t-alpha:** 0.001007110036042

---

---

48

- **PDB ID:** 3HQD | **Chain:** B
- **b-phipsi:** 0.0013761789127475
- **w-rdist:** 0.1262582743781032
- **t-alpha:** 0.0030272436213401

---

---

49

- **PDB ID:** 7MYJ | **Chain:** F
- **b-phipsi:** 0.004614601921315
- **w-rdist:** 0.1090756462060585
- **t-alpha:** 0.0030272436213401

---

---

50

- **PDB ID:** 6PZ4 | **Chain:** A
- **b-phipsi:** 0.0290875268258755
- **w-rdist:** 0.0383155821674877
- **t-alpha:** 0.0091371600897414

---

---

51

- **PDB ID:** 4Z4H | **Chain:** A
- **b-phipsi:** 0.0018097685022291
- **w-rdist:** 0.1630199995389748
- **t-alpha:** 0.0030181563299565

---

---

52

- **PDB ID:** 5HDU | **Chain:** A
- **b-phipsi:** 0.01456721363468
- **w-rdist:** 0.0539108662912337
- **t-alpha:** 0.0281690295641283

---

---

53

- **PDB ID:** 5SC9 | **Chain:** F
- **b-phipsi:** 0.0134528190972949
- **w-rdist:** 0.0857950153316614
- **t-alpha:** 0.0050303674430436

---

---

54

- **PDB ID:** 2P83 | **Chain:** A
- **b-phipsi:** 0.0431046247942312
- **w-rdist:** 0.0422764118705731
- **t-alpha:** 0.0

---

---

55

- **PDB ID:** 3LPJ | **Chain:** A
- **b-phipsi:** 0.0155932090452148
- **w-rdist:** 0.0816007936661723
- **t-alpha:** 0.0040402750029742

---

---

56

- **PDB ID:** 5SCL | **Chain:** H
- **b-phipsi:** 0.0125156309594704
- **w-rdist:** 0.0702205116987723
- **t-alpha:** 0.0140843739056586

---

---

57

- **PDB ID:** 4Z4G | **Chain:** A
- **b-phipsi:** 0.0065051814081296
- **w-rdist:** 0.1897992521424759
- **t-alpha:** 0.002011889761613

---

---

58

- **PDB ID:** 4DV9 | **Chain:** A
- **b-phipsi:** 0.0349180818796282
- **w-rdist:** 0.0348668199060068
- **t-alpha:** 0.0050303674430436

---

---

59

- **PDB ID:** 3L5C | **Chain:** B
- **b-phipsi:** 0.0179136029297204
- **w-rdist:** 0.0779678835023745
- **t-alpha:** 0.001007110036042

---

---

60

- **PDB ID:** 4TRW | **Chain:** A
- **b-phipsi:** 0.0175043980502178
- **w-rdist:** 0.0629441860140347
- **t-alpha:** 0.0100605125377637

---

---

61

- **PDB ID:** 3CIC | **Chain:** A
- **b-phipsi:** 0.0168654874496012
- **w-rdist:** 0.087583316951857
- **t-alpha:** 0.0

---

---

62

- **PDB ID:** 4HA5 | **Chain:** A
- **b-phipsi:** 0.0155584481488505
- **w-rdist:** 0.0431163648085809
- **t-alpha:** 0.0392351918850506

---

---

63

- **PDB ID:** 4TRZ | **Chain:** A
- **b-phipsi:** 0.0162973570979809
- **w-rdist:** 0.064017979869278
- **t-alpha:** 0.0130785989784187

---

---

64

- **PDB ID:** 3OHF | **Chain:** B
- **b-phipsi:** 0.017632076916529
- **w-rdist:** 0.0825670614196748
- **t-alpha:** 0.0

---

---

65

- **PDB ID:** 5SCK | **Chain:** H
- **b-phipsi:** 0.0172264559621572
- **w-rdist:** 0.0664960741333062
- **t-alpha:** 0.0090543980992385

---

---

66

- **PDB ID:** 4J1K | **Chain:** A
- **b-phipsi:** 0.0223865411595174
- **w-rdist:** 0.0363316833023127
- **t-alpha:** 0.0174001876755087

---

---

67

- **PDB ID:** 7S1N | **Chain:** A
- **b-phipsi:** 0.0092415787994062
- **w-rdist:** 0.2086853701591551
- **t-alpha:** 0.001007110036042

---

---

68

- **PDB ID:** 5EMM | **Chain:** A
- **b-phipsi:** 0.0053299524292043
- **w-rdist:** 0.4130782207279386
- **t-alpha:** 0.0

---

---

69

- **PDB ID:** 3L5E | **Chain:** A
- **b-phipsi:** 0.0168110062708159
- **w-rdist:** 0.0531052900923038
- **t-alpha:** 0.0231389553638574

---

---

70

- **PDB ID:** 7ZY8 | **Chain:** A
- **b-phipsi:** 0.0061609573909186
- **w-rdist:** 0.2824537564022286
- **t-alpha:** 0.0010061554017259

---

---

71

- **PDB ID:** 5QCW | **Chain:** B
- **b-phipsi:** 0.0278012951942751
- **w-rdist:** 0.0554359830689838
- **t-alpha:** 0.0050303674430436

---

---

72

- **PDB ID:** 5CU0 | **Chain:** A
- **b-phipsi:** 0.0095779431182656
- **w-rdist:** 0.2656434278965263
- **t-alpha:** 0.0010061554017259

---

---

73

- **PDB ID:** 3K5C | **Chain:** B
- **b-phipsi:** 0.0256524008365934
- **w-rdist:** 0.0555387793133857
- **t-alpha:** 0.0070422936000285

---

---

74

- **PDB ID:** 4P8V | **Chain:** A
- **b-phipsi:** 0.0145925953693702
- **w-rdist:** 0.0843160302358471
- **t-alpha:** 0.0060363072918738

---

---

75

- **PDB ID:** 4Z4E | **Chain:** A
- **b-phipsi:** 0.0034809000309159
- **w-rdist:** 0.1477279781842629
- **t-alpha:** 0.0040402750029742

---

---

76

- **PDB ID:** 6H0U | **Chain:** A
- **b-phipsi:** 0.0068000468082401
- **w-rdist:** 0.3148284332211333
- **t-alpha:** 0.001007110036042

---

---

77

- **PDB ID:** 5SC9 | **Chain:** H
- **b-phipsi:** 0.0140984153493563
- **w-rdist:** 0.0691619544276443
- **t-alpha:** 0.0150906633237464

---

---

78

- **PDB ID:** 3NWN | **Chain:** A
- **b-phipsi:** 0.0130405015708005
- **w-rdist:** 0.2230313678232616
- **t-alpha:** 0.0

---

---

79

- **PDB ID:** 1XN2 | **Chain:** D
- **b-phipsi:** 0.0183929826473237
- **w-rdist:** 0.0747287383337629
- **t-alpha:** 0.0030272436213401

---

---

80

- **PDB ID:** 4FSE | **Chain:** E
- **b-phipsi:** 0.0191397972590851
- **w-rdist:** 0.0713769225842211
- **t-alpha:** 0.004023997168737

---

---

81

- **PDB ID:** 7MYI | **Chain:** A
- **b-phipsi:** 0.0163295360986819
- **w-rdist:** 0.0624702921410865
- **t-alpha:** 0.0171028438612663

---

---

82

- **PDB ID:** 3DV1 | **Chain:** B
- **b-phipsi:** 0.0319290791149709
- **w-rdist:** 0.0511009622542747
- **t-alpha:** 0.0050303674430436

---

---

83

- **PDB ID:** 3I4B | **Chain:** B
- **b-phipsi:** 0.0103745013392133
- **w-rdist:** 0.3116077489487351
- **t-alpha:** 0.0

---

---

84

- **PDB ID:** 2ZHR | **Chain:** B
- **b-phipsi:** 0.0151852537517472
- **w-rdist:** 0.1092766946995712
- **t-alpha:** 0.0

---

---

85

- **PDB ID:** 5EMJ | **Chain:** A
- **b-phipsi:** 0.0059734957852666
- **w-rdist:** 0.4110210769388464
- **t-alpha:** 0.002011889761613

---

---

86

- **PDB ID:** 4ZSM | **Chain:** A
- **b-phipsi:** 0.0175949953070761
- **w-rdist:** 0.0624243827811834
- **t-alpha:** 0.0130785989784187

---

---

87

- **PDB ID:** 5SCA | **Chain:** H
- **b-phipsi:** 0.0144048060151144
- **w-rdist:** 0.0713485539880769
- **t-alpha:** 0.0140843739056586

---

---

88

- **PDB ID:** 4AY1 | **Chain:** C
- **b-phipsi:** 0.0134566836483281
- **w-rdist:** 0.07901916592493
- **t-alpha:** 0.0101626241883787

---

---

89

- **PDB ID:** 6JWA | **Chain:** A
- **b-phipsi:** 0.0106569811497858
- **w-rdist:** 0.2588715299279084
- **t-alpha:** 0.002011889761613

---

---

90

- **PDB ID:** 5SC8 | **Chain:** H
- **b-phipsi:** 0.0161981670636474
- **w-rdist:** 0.0667872749410233
- **t-alpha:** 0.0140843739056586

---

---

91

- **PDB ID:** 5EZZ | **Chain:** A
- **b-phipsi:** 0.0330624534050828
- **w-rdist:** 0.0433976932912139
- **t-alpha:** 0.0070922351376341

---

---

92

- **PDB ID:** 6H0F | **Chain:** B
- **b-phipsi:** 0.0020788782755681
- **w-rdist:** 0.7868146780752001
- **t-alpha:** 0.0030181563299565

---

---

93

- **PDB ID:** 4J0P | **Chain:** A
- **b-phipsi:** 0.0331109201245784
- **w-rdist:** 0.0521128125572652
- **t-alpha:** 0.004023997168737

---

---

94

- **PDB ID:** 3J8X | **Chain:** K
- **b-phipsi:** 0.0046354595002959
- **w-rdist:** 0.2964993219584484
- **t-alpha:** 0.0030272436213401

---

---

95

- **PDB ID:** 5QCZ | **Chain:** A
- **b-phipsi:** 0.034528097826505
- **w-rdist:** 0.0545559183867552
- **t-alpha:** 0.0020161899393582

---

---

96

- **PDB ID:** 3UQX | **Chain:** A
- **b-phipsi:** 0.0302157795119382
- **w-rdist:** 0.0488922709259128
- **t-alpha:** 0.0080483244942393

---

---

97

- **PDB ID:** 4IDP | **Chain:** B
- **b-phipsi:** 0.0018761167946485
- **w-rdist:** 0.4079723205366136
- **t-alpha:** 0.0030272436213401

---

---

98

- **PDB ID:** 7WN3 | **Chain:** E
- **b-phipsi:** 0.0080805968552729
- **w-rdist:** 0.4495308913014346
- **t-alpha:** 0.002011889761613

---

---

99

- **PDB ID:** 6CYT | **Chain:** A
- **b-phipsi:** 0.0128565139481091
- **w-rdist:** 0.2279115000495777
- **t-alpha:** 0.002011889761613

---

---

100

- **PDB ID:** 4AY1 | **Chain:** H
- **b-phipsi:** 0.0115963813758423
- **w-rdist:** 0.0784567866910227
- **t-alpha:** 0.0132515540606032

---

---

101

- **PDB ID:** 3L38 | **Chain:** A
- **b-phipsi:** 0.0352935140320318
- **w-rdist:** 0.0440015588804685
- **t-alpha:** 0.0060363072918738

---

---

102

- **PDB ID:** 3TPP | **Chain:** A
- **b-phipsi:** 0.0320571970671753
- **w-rdist:** 0.0578149107597527
- **t-alpha:** 0.0030181563299565

---

---

103

- **PDB ID:** 4R95 | **Chain:** A
- **b-phipsi:** 0.0187585173647166
- **w-rdist:** 0.0699800332217479
- **t-alpha:** 0.0060363072918738

---

---

104

- **PDB ID:** 6D05 | **Chain:** C
- **b-phipsi:** 0.0152993041640769
- **w-rdist:** 0.1515581306288897
- **t-alpha:** 0.0

---

---

105

- **PDB ID:** 4FRS | **Chain:** B
- **b-phipsi:** 0.0170807970901708
- **w-rdist:** 0.0895920328249322
- **t-alpha:** 0.0020161899393582

---

---

106

- **PDB ID:** 5WJJ | **Chain:** A
- **b-phipsi:** 0.0116114279640274
- **w-rdist:** 0.2776012055943559
- **t-alpha:** 0.002011889761613

---

---

107

- **PDB ID:** 3PI5 | **Chain:** B
- **b-phipsi:** 0.0337595911270716
- **w-rdist:** 0.0570172348912327
- **t-alpha:** 0.002011889761613

---

---

108

- **PDB ID:** 2WF1 | **Chain:** A
- **b-phipsi:** 0.0266871961410103
- **w-rdist:** 0.0552104170553268
- **t-alpha:** 0.0090543980992385

---

---

109

- **PDB ID:** 3NSH | **Chain:** A
- **b-phipsi:** 0.0370973626010136
- **w-rdist:** 0.0520550347881422
- **t-alpha:** 0.0020161899393582

---

---

110

- **PDB ID:** 2OHN | **Chain:** A
- **b-phipsi:** 0.0277257977935577
- **w-rdist:** 0.0542529054516845
- **t-alpha:** 0.0081136984001406

---

---

111

- **PDB ID:** 5SCF | **Chain:** A
- **b-phipsi:** 0.0122791873349239
- **w-rdist:** 0.0394646406225206
- **t-alpha:** 0.0965797171599183

---

---

112

- **PDB ID:** 5SCH | **Chain:** A
- **b-phipsi:** 0.0125785496985938
- **w-rdist:** 0.0372511482641472
- **t-alpha:** 0.0995978005518705

---

---

113

- **PDB ID:** 5HU0 | **Chain:** B
- **b-phipsi:** 0.0167870380179418
- **w-rdist:** 0.0707176236347616
- **t-alpha:** 0.0110661838165213

---

---

114

- **PDB ID:** 3SKG | **Chain:** D
- **b-phipsi:** 0.019954160289479
- **w-rdist:** 0.073369994437075
- **t-alpha:** 0.004023997168737

---

---

115

- **PDB ID:** 5J0A | **Chain:** A
- **b-phipsi:** 0.0127726354053896
- **w-rdist:** 0.1963428348131249
- **t-alpha:** 0.0030181563299565

---

---

116

- **PDB ID:** 3CIB | **Chain:** A
- **b-phipsi:** 0.0193482808602317
- **w-rdist:** 0.0820127063188828
- **t-alpha:** 0.0010061554017259

---

---

117

- **PDB ID:** 5QD0 | **Chain:** B
- **b-phipsi:** 0.0271902336608521
- **w-rdist:** 0.0576027239452187
- **t-alpha:** 0.0070422936000285

---

---

118

- **PDB ID:** 3LNK | **Chain:** A
- **b-phipsi:** 0.0146249331739321
- **w-rdist:** 0.0781278135975372
- **t-alpha:** 0.0111900946877709

---

---

119

- **PDB ID:** 2QMG | **Chain:** B
- **b-phipsi:** 0.0159825835441242
- **w-rdist:** 0.0892046624092562
- **t-alpha:** 0.0050303674430436

---

---

120

- **PDB ID:** 1XN2 | **Chain:** C
- **b-phipsi:** 0.0189882264252128
- **w-rdist:** 0.081459925211752
- **t-alpha:** 0.002011889761613

---

---

121

- **PDB ID:** 4QTE | **Chain:** A
- **b-phipsi:** 0.0106524831059793
- **w-rdist:** 0.2514542123188192
- **t-alpha:** 0.0030272436213401

---

---

122

- **PDB ID:** 4FRI | **Chain:** A
- **b-phipsi:** 0.0318918349544161
- **w-rdist:** 0.0473265875822144
- **t-alpha:** 0.0090543980992385

---

---

123

- **PDB ID:** 3LPJ | **Chain:** B
- **b-phipsi:** 0.0151382621498871
- **w-rdist:** 0.1011961266648216
- **t-alpha:** 0.0030272436213401

---

---

124

- **PDB ID:** 3L58 | **Chain:** B
- **b-phipsi:** 0.0141876107675212
- **w-rdist:** 0.1113726459842069
- **t-alpha:** 0.0030272436213401

---

---

125

- **PDB ID:** 7MYU | **Chain:** B
- **b-phipsi:** 0.0171273433085091
- **w-rdist:** 0.0771017205840557
- **t-alpha:** 0.0070922351376341

---

---

126

- **PDB ID:** 5SC9 | **Chain:** C
- **b-phipsi:** 0.0118153607003869
- **w-rdist:** 0.0631766357859855
- **t-alpha:** 0.0311871353768524

---

---

127

- **PDB ID:** 5SCE | **Chain:** H
- **b-phipsi:** 0.0148429434330434
- **w-rdist:** 0.0686488549475879
- **t-alpha:** 0.0191146395025525

---

---

128

- **PDB ID:** 4LXK | **Chain:** B
- **b-phipsi:** 0.0337614406583498
- **w-rdist:** 0.0574064939759445
- **t-alpha:** 0.0020161899393582

---

---

129

- **PDB ID:** 3GCU | **Chain:** A
- **b-phipsi:** 0.0100514705369295
- **w-rdist:** 0.2577845691132897
- **t-alpha:** 0.004023997168737

---

---

130

- **PDB ID:** 6UVP | **Chain:** A
- **b-phipsi:** 0.0206870329988724
- **w-rdist:** 0.0767293916650588
- **t-alpha:** 0.0030181563299565

---

---

131

- **PDB ID:** 4ZHX | **Chain:** E
- **b-phipsi:** 0.0040465601656927
- **w-rdist:** 0.1674813658200115
- **t-alpha:** 0.006072766330031

---

---

132

- **PDB ID:** 5SCD | **Chain:** F
- **b-phipsi:** 0.015646396871896
- **w-rdist:** 0.0862861641161133
- **t-alpha:** 0.0070422936000285

---

---

133

- **PDB ID:** 5QDB | **Chain:** B
- **b-phipsi:** 0.0291917934499587
- **w-rdist:** 0.0506726674132575
- **t-alpha:** 0.0100605125377637

---

---

134

- **PDB ID:** 3ODZ | **Chain:** X
- **b-phipsi:** 0.0059855747412663
- **w-rdist:** 0.2264480661676813
- **t-alpha:** 0.005055767262688

---

---

135

- **PDB ID:** 5OTD | **Chain:** B
- **b-phipsi:** 0.0074253066709395
- **w-rdist:** 0.2746126642463547
- **t-alpha:** 0.0040402750029742

---

---

136

- **PDB ID:** 6UVY | **Chain:** A
- **b-phipsi:** 0.0178040310795294
- **w-rdist:** 0.0632584339667769
- **t-alpha:** 0.0142855519524551

---

---

137

- **PDB ID:** 3WOW | **Chain:** A
- **b-phipsi:** 0.008362935545976
- **w-rdist:** 0.2633908893883379
- **t-alpha:** 0.0040402750029742

---

---

138

- **PDB ID:** 4IDQ | **Chain:** C
- **b-phipsi:** 0.0038397352611843
- **w-rdist:** 0.4231746603100076
- **t-alpha:** 0.0040402750029742

---

---

139

- **PDB ID:** 3QFR | **Chain:** A
- **b-phipsi:** 0.0022065302237552
- **w-rdist:** 0.7299959202209995
- **t-alpha:** 0.0040402750029742

---

---

140

- **PDB ID:** 4EH6 | **Chain:** A
- **b-phipsi:** 0.0125402858415482
- **w-rdist:** 0.2285937042702079
- **t-alpha:** 0.0030272436213401

---

---

141

- **PDB ID:** 5MPJ | **Chain:** B
- **b-phipsi:** 0.0059049320910754
- **w-rdist:** 0.3077213072124833
- **t-alpha:** 0.0040402750029742

---

---

142

- **PDB ID:** 5SCH | **Chain:** H
- **b-phipsi:** 0.0129155381544425
- **w-rdist:** 0.0652035086968318
- **t-alpha:** 0.0281690295641283

---

---

143

- **PDB ID:** 4BXN | **Chain:** B
- **b-phipsi:** 0.0128442653434936
- **w-rdist:** 0.1502665835013625
- **t-alpha:** 0.0040402750029742

---

---

144

- **PDB ID:** 3LPI | **Chain:** A
- **b-phipsi:** 0.0205377110662247
- **w-rdist:** 0.0813307466638187
- **t-alpha:** 0.001007110036042

---

---

145

- **PDB ID:** 4H3F | **Chain:** A
- **b-phipsi:** 0.0215865449322316
- **w-rdist:** 0.0477677395238171
- **t-alpha:** 0.02213297304878

---

---

146

- **PDB ID:** 2QK5 | **Chain:** A
- **b-phipsi:** 0.0187163601007396
- **w-rdist:** 0.0631271535931578
- **t-alpha:** 0.0130785989784187

---

---

147

- **PDB ID:** 2XAE | **Chain:** B
- **b-phipsi:** 0.0101562868410801
- **w-rdist:** 0.0780250654289325
- **t-alpha:** 0.0184427898172117

---

---

148

- **PDB ID:** 3IW8 | **Chain:** A
- **b-phipsi:** 0.0077283053523203
- **w-rdist:** 0.2708480399297432
- **t-alpha:** 0.0050303674430436

---

---

149

- **PDB ID:** 2HIZ | **Chain:** A
- **b-phipsi:** 0.0494725138707799
- **w-rdist:** 0.0389959821769333
- **t-alpha:** 0.0050303674430436

---

---

150

- **PDB ID:** 4H3G | **Chain:** A
- **b-phipsi:** 0.0178674369367584
- **w-rdist:** 0.044277250964236
- **t-alpha:** 0.038229225245195

---

---

151

- **PDB ID:** 5CLM | **Chain:** A
- **b-phipsi:** 0.0302575440172812
- **w-rdist:** 0.0641225212716637
- **t-alpha:** 0.0020161899393582

---

---

152

- **PDB ID:** 6HKY | **Chain:** B
- **b-phipsi:** 0.0136406227055692
- **w-rdist:** 0.1342713689195198
- **t-alpha:** 0.0040402750029742

---

---

153

- **PDB ID:** 5SCC | **Chain:** H
- **b-phipsi:** 0.0141405871824173
- **w-rdist:** 0.0660337292347648
- **t-alpha:** 0.0261569749948251

---

---

154

- **PDB ID:** 3F88 | **Chain:** B
- **b-phipsi:** 0.0143722994335463
- **w-rdist:** 0.3047011644311427
- **t-alpha:** 0.001007110036042

---

---

155

- **PDB ID:** 4FRK | **Chain:** A
- **b-phipsi:** 0.0318017103800855
- **w-rdist:** 0.0535999226808658
- **t-alpha:** 0.0070922351376341

---

---

156

- **PDB ID:** 5JS1 | **Chain:** A
- **b-phipsi:** 0.0107050194209507
- **w-rdist:** 0.3771038122809562
- **t-alpha:** 0.0030272436213401

---

---

157

- **PDB ID:** 5HE7 | **Chain:** A
- **b-phipsi:** 0.0183696567658397
- **w-rdist:** 0.0651966025615909
- **t-alpha:** 0.0130785989784187

---

---

158

- **PDB ID:** 3LNK | **Chain:** B
- **b-phipsi:** 0.0189255526024319
- **w-rdist:** 0.0892599426316364
- **t-alpha:** 0.0010061554017259

---

---

159

- **PDB ID:** 5SCG | **Chain:** H
- **b-phipsi:** 0.0141148953581186
- **w-rdist:** 0.0721485800060273
- **t-alpha:** 0.0201205697607655

---

---

160

- **PDB ID:** 6DMI | **Chain:** A
- **b-phipsi:** 0.0326511028642839
- **w-rdist:** 0.0497278266441975
- **t-alpha:** 0.0081136984001406

---

---

161

- **PDB ID:** 5ORK | **Chain:** A
- **b-phipsi:** 0.0059296611155974
- **w-rdist:** 0.2917784077365444
- **t-alpha:** 0.005055767262688

---

---

162

- **PDB ID:** 2X2R | **Chain:** C
- **b-phipsi:** 0.0151673811188735
- **w-rdist:** 0.3190345613568887
- **t-alpha:** 0.0

---

---

163

- **PDB ID:** 1YM4 | **Chain:** B
- **b-phipsi:** 0.0266967915575492
- **w-rdist:** 0.0553677765377867
- **t-alpha:** 0.0111900946877709

---

---

164

- **PDB ID:** 5CU2 | **Chain:** B
- **b-phipsi:** 0.0106437097219768
- **w-rdist:** 0.279083331819026
- **t-alpha:** 0.0040402750029742

---

---

165

- **PDB ID:** 7MYR | **Chain:** A
- **b-phipsi:** 0.0158572981928399
- **w-rdist:** 0.0584470524880583
- **t-alpha:** 0.0331991995154452

---

---

166

- **PDB ID:** 4IDQ | **Chain:** A
- **b-phipsi:** 0.005137646403967
- **w-rdist:** 0.4126909337725038
- **t-alpha:** 0.005055767262688

---

---

167

- **PDB ID:** 6JJR | **Chain:** A
- **b-phipsi:** 0.011133992391698
- **w-rdist:** 0.0607605722794358
- **t-alpha:** 0.0419284993347488

---

---

168

- **PDB ID:** 2OAH | **Chain:** A
- **b-phipsi:** 0.0435940102054281
- **w-rdist:** 0.042503507985576
- **t-alpha:** 0.0060363072918738

---

---

169

- **PDB ID:** 2G94 | **Chain:** A
- **b-phipsi:** 0.0176059066569226
- **w-rdist:** 0.079472911161342
- **t-alpha:** 0.0070422936000285

---

---

170

- **PDB ID:** 4R8Y | **Chain:** A
- **b-phipsi:** 0.0196609954754533
- **w-rdist:** 0.0742162703114919
- **t-alpha:** 0.0060363072918738

---

---

171

- **PDB ID:** 4IDQ | **Chain:** B
- **b-phipsi:** 0.0071273237841467
- **w-rdist:** 0.4140873341125923
- **t-alpha:** 0.0050303674430436

---

---

172

- **PDB ID:** 4HOK | **Chain:** S
- **b-phipsi:** 0.0152560665480882
- **w-rdist:** 0.3016729030502667
- **t-alpha:** 0.0010061554017259

---

---

173

- **PDB ID:** 6DHC | **Chain:** A
- **b-phipsi:** 0.036043502306866
- **w-rdist:** 0.0594602931190429
- **t-alpha:** 0.001007110036042

---

---

174

- **PDB ID:** 3RU1 | **Chain:** A
- **b-phipsi:** 0.0392904274890724
- **w-rdist:** 0.0508739090418112
- **t-alpha:** 0.0040402750029742

---

---

175

- **PDB ID:** 5TOL | **Chain:** A
- **b-phipsi:** 0.0389384285230036
- **w-rdist:** 0.0495847792543774
- **t-alpha:** 0.0050303674430436

---

---

176

- **PDB ID:** 3G9N | **Chain:** A
- **b-phipsi:** 0.0133250718846709
- **w-rdist:** 0.2082375021412651
- **t-alpha:** 0.0040402750029742

---

---

177

- **PDB ID:** 4Z4F | **Chain:** A
- **b-phipsi:** 0.0054972999034296
- **w-rdist:** 0.1619638517833448
- **t-alpha:** 0.0081136984001406

---

---

178

- **PDB ID:** 1HJV | **Chain:** B
- **b-phipsi:** 0.0145321921050792
- **w-rdist:** 0.055741153073313
- **t-alpha:** 0.045215734453575

---

---

179

- **PDB ID:** 5MO5 | **Chain:** B
- **b-phipsi:** 0.0050464966859153
- **w-rdist:** 0.2820958903294975
- **t-alpha:** 0.006072766330031

---

---

180

- **PDB ID:** 2HIZ | **Chain:** B
- **b-phipsi:** 0.0517149028426075
- **w-rdist:** 0.0467868611169468
- **t-alpha:** 0.004023997168737

---

---

181

- **PDB ID:** 5YGX | **Chain:** A
- **b-phipsi:** 0.0264184855005221
- **w-rdist:** 0.0415692324622804
- **t-alpha:** 0.023686980845901

---

---

182

- **PDB ID:** 3MH0 | **Chain:** A
- **b-phipsi:** 0.0146647704255824
- **w-rdist:** 0.267769183831367
- **t-alpha:** 0.0030181563299565

---

---

183

- **PDB ID:** 3K5F | **Chain:** B
- **b-phipsi:** 0.0283707548799456
- **w-rdist:** 0.037609998013071
- **t-alpha:** 0.0215828567205209

---

---

184

- **PDB ID:** 6D03 | **Chain:** D
- **b-phipsi:** 0.0153926810403015
- **w-rdist:** 0.1541975002815137
- **t-alpha:** 0.0030272436213401

---

---

185

- **PDB ID:** 7D2V | **Chain:** A
- **b-phipsi:** 0.0326067039526216
- **w-rdist:** 0.0456529345835742
- **t-alpha:** 0.0120723738025678

---

---

186

- **PDB ID:** 3HQD | **Chain:** A
- **b-phipsi:** 0.0003591649494162
- **w-rdist:** 0.1441427059619902
- **t-alpha:** 0.0101626241883787

---

---

187

- **PDB ID:** 4R93 | **Chain:** A
- **b-phipsi:** 0.0219772546664024
- **w-rdist:** 0.0760057365335862
- **t-alpha:** 0.004023997168737

---

---

188

- **PDB ID:** 6BFX | **Chain:** A
- **b-phipsi:** 0.0182309100460566
- **w-rdist:** 0.0784818861469212
- **t-alpha:** 0.006072766330031

---

---

189

- **PDB ID:** 2P4J | **Chain:** A
- **b-phipsi:** 0.0166801157882959
- **w-rdist:** 0.0801755152894301
- **t-alpha:** 0.0100605125377637

---

---

190

- **PDB ID:** 4IB5 | **Chain:** B
- **b-phipsi:** 0.0073655306823923
- **w-rdist:** 0.2748265958566639
- **t-alpha:** 0.006072766330031

---

---

191

- **PDB ID:** 2ONL | **Chain:** C
- **b-phipsi:** 0.0046354601244822
- **w-rdist:** 0.3662176651219412
- **t-alpha:** 0.006072766330031

---

---

192

- **PDB ID:** 5SCC | **Chain:** F
- **b-phipsi:** 0.0161088956509615
- **w-rdist:** 0.0867074189538054
- **t-alpha:** 0.0090543980992385

---

---

193

- **PDB ID:** 4LXA | **Chain:** B
- **b-phipsi:** 0.0308630039709329
- **w-rdist:** 0.0512827974733529
- **t-alpha:** 0.0110661838165213

---

---

194

- **PDB ID:** 3UQR | **Chain:** B
- **b-phipsi:** 0.0265795140471816
- **w-rdist:** 0.0635010139936815
- **t-alpha:** 0.0070922351376341

---

---

195

- **PDB ID:** 4L7J | **Chain:** A
- **b-phipsi:** 0.0295994901401869
- **w-rdist:** 0.0504864474946485
- **t-alpha:** 0.0130785989784187

---

---

196

- **PDB ID:** 4X60 | **Chain:** A
- **b-phipsi:** 0.0049236912899349
- **w-rdist:** 0.4145270884763654
- **t-alpha:** 0.006072766330031

---

---

197

- **PDB ID:** 3KMX | **Chain:** B
- **b-phipsi:** 0.0159164209639394
- **w-rdist:** 0.0759040799371767
- **t-alpha:** 0.0150906633237464

---

---

198

- **PDB ID:** 5QCS | **Chain:** C
- **b-phipsi:** 0.0319258465163149
- **w-rdist:** 0.0618743837615397
- **t-alpha:** 0.0040402750029742

---

---

199

- **PDB ID:** 3IXK | **Chain:** A
- **b-phipsi:** 0.0258518249576801
- **w-rdist:** 0.0586306291816511
- **t-alpha:** 0.0111900946877709

---

---

200

- **PDB ID:** 5SCI | **Chain:** H
- **b-phipsi:** 0.0137089679139681
- **w-rdist:** 0.0700263722345371
- **t-alpha:** 0.0261569749948251

---

---

201

- **PDB ID:** 4AY1 | **Chain:** F
- **b-phipsi:** 0.0126451678599834
- **w-rdist:** 0.0826425833148445
- **t-alpha:** 0.0163601067821594

---

---

202

- **PDB ID:** 6JT4 | **Chain:** A
- **b-phipsi:** 0.0326759612481958
- **w-rdist:** 0.0460372786513773
- **t-alpha:** 0.012220058253459

---

---

203

- **PDB ID:** 4LXK | **Chain:** C
- **b-phipsi:** 0.0377302064293619
- **w-rdist:** 0.0417281608135412
- **t-alpha:** 0.0100605125377637

---

---

204

- **PDB ID:** 6SFJ | **Chain:** A
- **b-phipsi:** 0.0100352610190219
- **w-rdist:** 0.2599598792407041
- **t-alpha:** 0.006072766330031

---

---

205

- **PDB ID:** 3ZSG | **Chain:** A
- **b-phipsi:** 0.0139548149505767
- **w-rdist:** 0.2784146834684906
- **t-alpha:** 0.004023997168737

---

---

206

- **PDB ID:** 1HJW | **Chain:** B
- **b-phipsi:** 0.0121665210104761
- **w-rdist:** 0.0603125988697392
- **t-alpha:** 0.045215734453575

---

---

207

- **PDB ID:** 1HJX | **Chain:** D
- **b-phipsi:** 0.0115774828674834
- **w-rdist:** 0.0691714863347307
- **t-alpha:** 0.0311202652104596

---

---

208

- **PDB ID:** 3L59 | **Chain:** A
- **b-phipsi:** 0.0183007620481263
- **w-rdist:** 0.0484573063565904
- **t-alpha:** 0.0362172476666218

---

---

209

- **PDB ID:** 2P83 | **Chain:** C
- **b-phipsi:** 0.0507233920437344
- **w-rdist:** 0.0462778901179807
- **t-alpha:** 0.0050303674430436

---

---

210

- **PDB ID:** 7WN6 | **Chain:** E
- **b-phipsi:** 0.0082521080383254
- **w-rdist:** 0.4549632209082045
- **t-alpha:** 0.005055767262688

---

---

211

- **PDB ID:** 6BFD | **Chain:** A
- **b-phipsi:** 0.0150065274676839
- **w-rdist:** 0.0933445550154657
- **t-alpha:** 0.0090543980992385

---

---

212

- **PDB ID:** 2HIZ | **Chain:** C
- **b-phipsi:** 0.0492826911358584
- **w-rdist:** 0.0468534363338258
- **t-alpha:** 0.0050303674430436

---

---

213

- **PDB ID:** 5SCI | **Chain:** G
- **b-phipsi:** 0.0170150421275189
- **w-rdist:** 0.0596249672317129
- **t-alpha:** 0.0281690295641283

---

---

214

- **PDB ID:** 2P4J | **Chain:** C
- **b-phipsi:** 0.0157258229970945
- **w-rdist:** 0.0717321516690718
- **t-alpha:** 0.0201205697607655

---

---

215

- **PDB ID:** 5HE5 | **Chain:** A
- **b-phipsi:** 0.0217542581082467
- **w-rdist:** 0.0534585263645345
- **t-alpha:** 0.02213297304878

---

---

216

- **PDB ID:** 6Q1N | **Chain:** B
- **b-phipsi:** 0.0113930441238428
- **w-rdist:** 0.1014626799003265
- **t-alpha:** 0.0100605125377637

---

---

217

- **PDB ID:** 2QMD | **Chain:** B
- **b-phipsi:** 0.0170682760438904
- **w-rdist:** 0.0956215739026827
- **t-alpha:** 0.0040402750029742

---

---

218

- **PDB ID:** 4QP3 | **Chain:** A
- **b-phipsi:** 0.0053592114458717
- **w-rdist:** 0.2734325180478839
- **t-alpha:** 0.0081136984001406

---

---

219

- **PDB ID:** 4TRY | **Chain:** C
- **b-phipsi:** 0.0212596931479766
- **w-rdist:** 0.0648175364705662
- **t-alpha:** 0.0120723738025678

---

---

220

- **PDB ID:** 2VKM | **Chain:** B
- **b-phipsi:** 0.0159539770996851
- **w-rdist:** 0.0973861529430526
- **t-alpha:** 0.0060363072918738

---

---

221

- **PDB ID:** 6OPI | **Chain:** A
- **b-phipsi:** 0.0108185729376781
- **w-rdist:** 0.2588209905527167
- **t-alpha:** 0.006072766330031

---

---

222

- **PDB ID:** 5HE4 | **Chain:** A
- **b-phipsi:** 0.0206958933054709
- **w-rdist:** 0.0550314719734157
- **t-alpha:** 0.0231389553638574

---

---

223

- **PDB ID:** 3KMY | **Chain:** B
- **b-phipsi:** 0.0181122445003541
- **w-rdist:** 0.074761782646163
- **t-alpha:** 0.0100605125377637

---

---

224

- **PDB ID:** 4J0Y | **Chain:** A
- **b-phipsi:** 0.025767734140088
- **w-rdist:** 0.0466141464964142
- **t-alpha:** 0.023686980845901

---

---

225

- **PDB ID:** 5SC9 | **Chain:** G
- **b-phipsi:** 0.0116161367088895
- **w-rdist:** 0.0647302512883138
- **t-alpha:** 0.040241637205524

---

---

226

- **PDB ID:** 5CU0 | **Chain:** B
- **b-phipsi:** 0.0070734041388578
- **w-rdist:** 0.2816048256017845
- **t-alpha:** 0.0070922351376341

---

---

227

- **PDB ID:** 5QD7 | **Chain:** B
- **b-phipsi:** 0.0246455150872836
- **w-rdist:** 0.059039263320303
- **t-alpha:** 0.0130785989784187

---

---

228

- **PDB ID:** 1M4H | **Chain:** A
- **b-phipsi:** 0.0155461536560291
- **w-rdist:** 0.0937171260670425
- **t-alpha:** 0.0081136984001406

---

---

229

- **PDB ID:** 5QCX | **Chain:** A
- **b-phipsi:** 0.0321596826796984
- **w-rdist:** 0.0571013155632908
- **t-alpha:** 0.0070922351376341

---

---

230

- **PDB ID:** 4IDQ | **Chain:** D
- **b-phipsi:** 0.0040447560702455
- **w-rdist:** 0.4249423608060468
- **t-alpha:** 0.0070922351376341

---

---

231

- **PDB ID:** 4R92 | **Chain:** A
- **b-phipsi:** 0.0184204429807674
- **w-rdist:** 0.0650877061979079
- **t-alpha:** 0.0160964977574751

---

---

232

- **PDB ID:** 3LPI | **Chain:** B
- **b-phipsi:** 0.0140579382347703
- **w-rdist:** 0.097289755999858
- **t-alpha:** 0.0091371600897414

---

---

233

- **PDB ID:** 3K3B | **Chain:** A
- **b-phipsi:** 0.010681358055466
- **w-rdist:** 0.1377583799572017
- **t-alpha:** 0.0090543980992385

---

---

234

- **PDB ID:** 1SGZ | **Chain:** C
- **b-phipsi:** 0.0176568361448974
- **w-rdist:** 0.0597054573830139
- **t-alpha:** 0.0251509582443987

---

---

235

- **PDB ID:** 4FS4 | **Chain:** B
- **b-phipsi:** 0.0169180698093023
- **w-rdist:** 0.0572139002230974
- **t-alpha:** 0.0352112405915889

---

---

236

- **PDB ID:** 4IMY | **Chain:** A
- **b-phipsi:** 0.0151304136245402
- **w-rdist:** 0.2495851130702077
- **t-alpha:** 0.004023997168737

---

---

237

- **PDB ID:** 6EJ3 | **Chain:** A
- **b-phipsi:** 0.0221011180142245
- **w-rdist:** 0.072256036480916
- **t-alpha:** 0.0060363072918738

---

---

238

- **PDB ID:** 3SKG | **Chain:** E
- **b-phipsi:** 0.0237565442478051
- **w-rdist:** 0.0751636178830356
- **t-alpha:** 0.0040402750029742

---

---

239

- **PDB ID:** 4IDP | **Chain:** A
- **b-phipsi:** 0.0043557873524473
- **w-rdist:** 0.400938104458191
- **t-alpha:** 0.0080483244942393

---

---

240

- **PDB ID:** 5OT6 | **Chain:** B
- **b-phipsi:** 0.0080153365810997
- **w-rdist:** 0.2736001091619768
- **t-alpha:** 0.0080483244942393

---

---

241

- **PDB ID:** 4H1E | **Chain:** B
- **b-phipsi:** 0.0185006260195775
- **w-rdist:** 0.0620212532692659
- **t-alpha:** 0.0201205697607655

---

---

242

- **PDB ID:** 4HA5 | **Chain:** B
- **b-phipsi:** 0.0146964646805131
- **w-rdist:** 0.0705815969994467
- **t-alpha:** 0.0251509582443987

---

---

243

- **PDB ID:** 4H3I | **Chain:** B
- **b-phipsi:** 0.0200275652101334
- **w-rdist:** 0.0595971651391493
- **t-alpha:** 0.0191146395025525

---

---

244

- **PDB ID:** 5SCB | **Chain:** F
- **b-phipsi:** 0.0117841096354655
- **w-rdist:** 0.0890647724349601
- **t-alpha:** 0.0150906633237464

---

---

245

- **PDB ID:** 3LFB | **Chain:** A
- **b-phipsi:** 0.0105831045657751
- **w-rdist:** 0.247052782074873
- **t-alpha:** 0.0070922351376341

---

---

246

- **PDB ID:** 4FSL | **Chain:** E
- **b-phipsi:** 0.0189405054028898
- **w-rdist:** 0.0722435121114622
- **t-alpha:** 0.0100605125377637

---

---

247

- **PDB ID:** 5HDV | **Chain:** A
- **b-phipsi:** 0.0207796097577498
- **w-rdist:** 0.0468850389660608
- **t-alpha:** 0.0321931693669585

---

---

248

- **PDB ID:** 4J0T | **Chain:** A
- **b-phipsi:** 0.0284459379867499
- **w-rdist:** 0.0407198919085576
- **t-alpha:** 0.023686980845901

---

---

249

- **PDB ID:** 4DJU | **Chain:** A
- **b-phipsi:** 0.0181822847823183
- **w-rdist:** 0.060518775573063
- **t-alpha:** 0.0231389553638574

---

---

250

- **PDB ID:** 2QK5 | **Chain:** B
- **b-phipsi:** 0.0173986251147236
- **w-rdist:** 0.0934134151325102
- **t-alpha:** 0.0050303674430436

---

---

251

- **PDB ID:** 4DI2 | **Chain:** A
- **b-phipsi:** 0.0397591597064779
- **w-rdist:** 0.0472788850486703
- **t-alpha:** 0.0070922351376341

---

---

252

- **PDB ID:** 6DHC | **Chain:** B
- **b-phipsi:** 0.0318114854956387
- **w-rdist:** 0.0531400235505279
- **t-alpha:** 0.0110661838165213

---

---

253

- **PDB ID:** 4H3J | **Chain:** A
- **b-phipsi:** 0.018569038916555
- **w-rdist:** 0.0575020873793198
- **t-alpha:** 0.0251509582443987

---

---

254

- **PDB ID:** 2C9H | **Chain:** A
- **b-phipsi:** 0.0098557943590038
- **w-rdist:** 0.1571690699779033
- **t-alpha:** 0.0091371600897414

---

---

255

- **PDB ID:** 6TLE | **Chain:** B
- **b-phipsi:** 0.0097566790634172
- **w-rdist:** 0.2331849147925145
- **t-alpha:** 0.0081136984001406

---

---

256

- **PDB ID:** 4DJW | **Chain:** A
- **b-phipsi:** 0.0180539314833962
- **w-rdist:** 0.050742177430225
- **t-alpha:** 0.038229225245195

---

---

257

- **PDB ID:** 5QD7 | **Chain:** A
- **b-phipsi:** 0.0318435003943046
- **w-rdist:** 0.0618816451067599
- **t-alpha:** 0.005055767262688

---

---

258

- **PDB ID:** 2YBU | **Chain:** E
- **b-phipsi:** 0.0146355168353275
- **w-rdist:** 0.1662658920552068
- **t-alpha:** 0.005055767262688

---

---

259

- **PDB ID:** 3K5F | **Chain:** C
- **b-phipsi:** 0.0371160517712052
- **w-rdist:** 0.0590158931045196
- **t-alpha:** 0.0030272436213401

---

---

260

- **PDB ID:** 4J0Z | **Chain:** A
- **b-phipsi:** 0.028435518513773
- **w-rdist:** 0.045464998001256
- **t-alpha:** 0.0215828567205209

---

---

261

- **PDB ID:** 5QDB | **Chain:** C
- **b-phipsi:** 0.0214106658683393
- **w-rdist:** 0.0581758037513904
- **t-alpha:** 0.0191146395025525

---

---

262

- **PDB ID:** 7DCZ | **Chain:** A
- **b-phipsi:** 0.030426869824225
- **w-rdist:** 0.0429558133355707
- **t-alpha:** 0.0194869465164286

---

---

263

- **PDB ID:** 4ZSP | **Chain:** A
- **b-phipsi:** 0.0163141787638009
- **w-rdist:** 0.0948779357495975
- **t-alpha:** 0.0070422936000285

---

---

264

- **PDB ID:** 6UX9 | **Chain:** A
- **b-phipsi:** 0.0070359540844017
- **w-rdist:** 0.2139280287618614
- **t-alpha:** 0.0100605125377637

---

---

265

- **PDB ID:** 2QMG | **Chain:** A
- **b-phipsi:** 0.0167494439818126
- **w-rdist:** 0.0771444734281283
- **t-alpha:** 0.0140843739056586

---

---

266

- **PDB ID:** 3N4L | **Chain:** A
- **b-phipsi:** 0.0293372492374598
- **w-rdist:** 0.0589916054376419
- **t-alpha:** 0.0091371600897414

---

---

267

- **PDB ID:** 4DJV | **Chain:** B
- **b-phipsi:** 0.0163164234848991
- **w-rdist:** 0.0516249054209525
- **t-alpha:** 0.0513078913478914

---

---

268

- **PDB ID:** 3OHF | **Chain:** A
- **b-phipsi:** 0.0202026630554588
- **w-rdist:** 0.0919960590990919
- **t-alpha:** 0.002011889761613

---

---

269

- **PDB ID:** 5OTH | **Chain:** A
- **b-phipsi:** 0.0051706277294045
- **w-rdist:** 0.2795397296588881
- **t-alpha:** 0.0091371600897414

---

---

270

- **PDB ID:** 4J1F | **Chain:** A
- **b-phipsi:** 0.026773133411348
- **w-rdist:** 0.0480470542752043
- **t-alpha:** 0.0226336461702254

---

---

271

- **PDB ID:** 5QCQ | **Chain:** C
- **b-phipsi:** 0.0346917177741491
- **w-rdist:** 0.0455310634854694
- **t-alpha:** 0.0130785989784187

---

---

272

- **PDB ID:** 5QCV | **Chain:** B
- **b-phipsi:** 0.0264236967301775
- **w-rdist:** 0.0555642149361746
- **t-alpha:** 0.0160964977574751

---

---

273

- **PDB ID:** 5HD0 | **Chain:** A
- **b-phipsi:** 0.0195178951276531
- **w-rdist:** 0.0420487907074914
- **t-alpha:** 0.0432593630846658

---

---

274

- **PDB ID:** 4K8S | **Chain:** A
- **b-phipsi:** 0.0422633683464019
- **w-rdist:** 0.0605592187006922
- **t-alpha:** 0.0010061554017259

---

---

275

- **PDB ID:** 5SCA | **Chain:** G
- **b-phipsi:** 0.011729907708825
- **w-rdist:** 0.0692077015115096
- **t-alpha:** 0.0362172476666218

---

---

276

- **PDB ID:** 5O8U | **Chain:** A
- **b-phipsi:** 0.0082025132276905
- **w-rdist:** 0.2478077964185157
- **t-alpha:** 0.0091371600897414

---

---

277

- **PDB ID:** 3PG3 | **Chain:** A
- **b-phipsi:** 0.0147165214313305
- **w-rdist:** 0.2812498313535765
- **t-alpha:** 0.0040402750029742

---

---

278

- **PDB ID:** 4YBI | **Chain:** B
- **b-phipsi:** 0.0177173457105002
- **w-rdist:** 0.0465229999336732
- **t-alpha:** 0.0482898778152505

---

---

279

- **PDB ID:** 4DJW | **Chain:** B
- **b-phipsi:** 0.0170611761449546
- **w-rdist:** 0.045320977000003
- **t-alpha:** 0.06136824019931

---

---

280

- **PDB ID:** 3VV6 | **Chain:** A
- **b-phipsi:** 0.0357784235785153
- **w-rdist:** 0.0484428875589874
- **t-alpha:** 0.0100605125377637

---

---

281

- **PDB ID:** 4ZSP | **Chain:** B
- **b-phipsi:** 0.0187240311994111
- **w-rdist:** 0.0732752932177867
- **t-alpha:** 0.0110661838165213

---

---

282

- **PDB ID:** 5SCF | **Chain:** F
- **b-phipsi:** 0.0159434822131339
- **w-rdist:** 0.0844698255412232
- **t-alpha:** 0.0130785989784187

---

---

283

- **PDB ID:** 4FS4 | **Chain:** A
- **b-phipsi:** 0.0173930059731118
- **w-rdist:** 0.0614577845579915
- **t-alpha:** 0.0281690295641283

---

---

284

- **PDB ID:** 5CVH | **Chain:** B
- **b-phipsi:** 0.0100680051204821
- **w-rdist:** 0.3014448336542417
- **t-alpha:** 0.0070922351376341

---

---

285

- **PDB ID:** 4H3G | **Chain:** B
- **b-phipsi:** 0.0180965829434948
- **w-rdist:** 0.0766011101699855
- **t-alpha:** 0.0110661838165213

---

---

286

- **PDB ID:** 3UQW | **Chain:** A
- **b-phipsi:** 0.0469530612087383
- **w-rdist:** 0.0482316049406425
- **t-alpha:** 0.0060363072918738

---

---

287

- **PDB ID:** 6OHD | **Chain:** A
- **b-phipsi:** 0.0174513043255378
- **w-rdist:** 0.2435416642641336
- **t-alpha:** 0.0010061554017259

---

---

288

- **PDB ID:** 5ORK | **Chain:** B
- **b-phipsi:** 0.007199318692704
- **w-rdist:** 0.2998242786933238
- **t-alpha:** 0.0090543980992385

---

---

289

- **PDB ID:** 2OHM | **Chain:** A
- **b-phipsi:** 0.0264583839062165
- **w-rdist:** 0.0476201014128852
- **t-alpha:** 0.0247423777489943

---

---

290

- **PDB ID:** 3CIB | **Chain:** B
- **b-phipsi:** 0.0168845492700288
- **w-rdist:** 0.0989460168902427
- **t-alpha:** 0.005055767262688

---

---

291

- **PDB ID:** 4TRW | **Chain:** C
- **b-phipsi:** 0.0213291763338335
- **w-rdist:** 0.0929861214480259
- **t-alpha:** 0.0010061554017259

---

---

292

- **PDB ID:** 3DV1 | **Chain:** A
- **b-phipsi:** 0.0358903134108362
- **w-rdist:** 0.0540900026222378
- **t-alpha:** 0.0070922351376341

---

---

293

- **PDB ID:** 3QBH | **Chain:** B
- **b-phipsi:** 0.038381135553106
- **w-rdist:** 0.052461264582032
- **t-alpha:** 0.0070422936000285

---

---

294

- **PDB ID:** 5MO5 | **Chain:** A
- **b-phipsi:** 0.005235496252058
- **w-rdist:** 0.2800858863196152
- **t-alpha:** 0.0100605125377637

---

---

295

- **PDB ID:** 3O8U | **Chain:** A
- **b-phipsi:** 0.0151384015913841
- **w-rdist:** 0.2202803090685215
- **t-alpha:** 0.005055767262688

---

---

296

- **PDB ID:** 5SCK | **Chain:** D
- **b-phipsi:** 0.0161113276720398
- **w-rdist:** 0.0689520303498147
- **t-alpha:** 0.0251509582443987

---

---

297

- **PDB ID:** 6JSN | **Chain:** A
- **b-phipsi:** 0.0296436675434809
- **w-rdist:** 0.0323638672691901
- **t-alpha:** 0.0251509582443987

---

---

298

- **PDB ID:** 5HDZ | **Chain:** A
- **b-phipsi:** 0.0175473319508341
- **w-rdist:** 0.0576474318111187
- **t-alpha:** 0.0342052238987842

---

---

299

- **PDB ID:** 1SGZ | **Chain:** D
- **b-phipsi:** 0.0184462929266944
- **w-rdist:** 0.053406669849769
- **t-alpha:** 0.0362172476666218

---

---

300

- **PDB ID:** 3MI9 | **Chain:** A
- **b-phipsi:** 0.0114596828657094
- **w-rdist:** 0.2281522016573146
- **t-alpha:** 0.0090543980992385

---

---

301

- **PDB ID:** 5MMR | **Chain:** B
- **b-phipsi:** 0.010137331266455
- **w-rdist:** 0.2689958304767937
- **t-alpha:** 0.0090543980992385

---

---

302

- **PDB ID:** 3L5B | **Chain:** A
- **b-phipsi:** 0.0179376974952922
- **w-rdist:** 0.0391660807474738
- **t-alpha:** 0.06136824019931

---

---

303

- **PDB ID:** 6UPR | **Chain:** B
- **b-phipsi:** 0.0053331772835252
- **w-rdist:** 0.2744486892340068
- **t-alpha:** 0.0101626241883787

---

---

304

- **PDB ID:** 6NV7 | **Chain:** C
- **b-phipsi:** 0.0312489867590223
- **w-rdist:** 0.0649203594336983
- **t-alpha:** 0.005055767262688

---

---

305

- **PDB ID:** 2IWY | **Chain:** B
- **b-phipsi:** 0.0092175012570295
- **w-rdist:** 0.162926616099553
- **t-alpha:** 0.0110661838165213

---

---

306

- **PDB ID:** 5B0X | **Chain:** A
- **b-phipsi:** 0.0085294645998768
- **w-rdist:** 0.2952238565856382
- **t-alpha:** 0.0090543980992385

---

---

307

- **PDB ID:** 2WCG | **Chain:** B
- **b-phipsi:** 0.0055593627377081
- **w-rdist:** 0.0855711746556671
- **t-alpha:** 0.0251509582443987

---

---

308

- **PDB ID:** 4AY1 | **Chain:** A
- **b-phipsi:** 0.0115717289769781
- **w-rdist:** 0.0854704440829997
- **t-alpha:** 0.0215828567205209

---

---

309

- **PDB ID:** 6TRL | **Chain:** A
- **b-phipsi:** 0.016452724211452
- **w-rdist:** 0.3088164752244968
- **t-alpha:** 0.0020161899393582

---

---

310

- **PDB ID:** 1HJX | **Chain:** A
- **b-phipsi:** 0.0100966289024012
- **w-rdist:** 0.0627945676380515
- **t-alpha:** 0.0552016840097893

---

---

311

- **PDB ID:** 3IVI | **Chain:** B
- **b-phipsi:** 0.0288545608259992
- **w-rdist:** 0.0801531876209941
- **t-alpha:** 0.0

---

---

312

- **PDB ID:** 5QD6 | **Chain:** B
- **b-phipsi:** 0.0304914271928497
- **w-rdist:** 0.0527298436824012
- **t-alpha:** 0.0140843739056586

---

---

313

- **PDB ID:** 3KEN | **Chain:** A
- **b-phipsi:** 0.0131580267500322
- **w-rdist:** 0.206242544265278
- **t-alpha:** 0.0081136984001406

---

---

314

- **PDB ID:** 4AY1 | **Chain:** I
- **b-phipsi:** 0.0097276289118538
- **w-rdist:** 0.0796457716831708
- **t-alpha:** 0.0279214734263297

---

---

315

- **PDB ID:** 4H3I | **Chain:** A
- **b-phipsi:** 0.0180595421780196
- **w-rdist:** 0.0456326955611054
- **t-alpha:** 0.0513078913478914

---

---

316

- **PDB ID:** 5QCU | **Chain:** A
- **b-phipsi:** 0.0343905813229228
- **w-rdist:** 0.0518788690996899
- **t-alpha:** 0.0101626241883787

---

---

317

- **PDB ID:** 3L5D | **Chain:** A
- **b-phipsi:** 0.0206176540525395
- **w-rdist:** 0.0376514338235383
- **t-alpha:** 0.0462778608457075

---

---

318

- **PDB ID:** 3OHH | **Chain:** B
- **b-phipsi:** 0.0175558407270915
- **w-rdist:** 0.0801451671656205
- **t-alpha:** 0.0111900946877709

---

---

319

- **PDB ID:** 3INH | **Chain:** A
- **b-phipsi:** 0.0295752676008738
- **w-rdist:** 0.0401159880120891
- **t-alpha:** 0.0257998410196613

---

---

320

- **PDB ID:** 5SCL | **Chain:** F
- **b-phipsi:** 0.0175887661616518
- **w-rdist:** 0.1040405957848122
- **t-alpha:** 0.004023997168737

---

---

321

- **PDB ID:** 4K8S | **Chain:** B
- **b-phipsi:** 0.0314833896923384
- **w-rdist:** 0.0446760709858819
- **t-alpha:** 0.0205341202479885

---

---

322

- **PDB ID:** 5SCF | **Chain:** H
- **b-phipsi:** 0.0139526350162247
- **w-rdist:** 0.0657699239137024
- **t-alpha:** 0.0412475676983714

---

---

323

- **PDB ID:** 4X7I | **Chain:** B
- **b-phipsi:** 0.0198509919782547
- **w-rdist:** 0.0830457952269577
- **t-alpha:** 0.0060363072918738

---

---

324

- **PDB ID:** 2QMD | **Chain:** A
- **b-phipsi:** 0.0171484337759359
- **w-rdist:** 0.0752724108725778
- **t-alpha:** 0.0160964977574751

---

---

325

- **PDB ID:** 4DJV | **Chain:** A
- **b-phipsi:** 0.020043762596581
- **w-rdist:** 0.0523196239124724
- **t-alpha:** 0.0331991995154452

---

---

326

- **PDB ID:** 3LPK | **Chain:** B
- **b-phipsi:** 0.0153133499816111
- **w-rdist:** 0.0851088736827193
- **t-alpha:** 0.0153215732989637

---

---

327

- **PDB ID:** 4ZSQ | **Chain:** A
- **b-phipsi:** 0.0176128930531271
- **w-rdist:** 0.066539242813794
- **t-alpha:** 0.0231389553638574

---

---

328

- **PDB ID:** 5UYU | **Chain:** A
- **b-phipsi:** 0.0256004726368523
- **w-rdist:** 0.0590370170207522
- **t-alpha:** 0.0153215732989637

---

---

329

- **PDB ID:** 4L7H | **Chain:** A
- **b-phipsi:** 0.028286369430502
- **w-rdist:** 0.0645179247913843
- **t-alpha:** 0.0090543980992385

---

---

330

- **PDB ID:** 4DJY | **Chain:** A
- **b-phipsi:** 0.0185812465054631
- **w-rdist:** 0.0876310529153849
- **t-alpha:** 0.0060363072918738

---

---

331

- **PDB ID:** 4J17 | **Chain:** A
- **b-phipsi:** 0.032598139760743
- **w-rdist:** 0.051375996251976
- **t-alpha:** 0.0132515540606032

---

---

332

- **PDB ID:** 2G94 | **Chain:** D
- **b-phipsi:** 0.0150191984027261
- **w-rdist:** 0.0714929514883598
- **t-alpha:** 0.0291750635565768

---

---

333

- **PDB ID:** 3LPK | **Chain:** A
- **b-phipsi:** 0.0179172525030372
- **w-rdist:** 0.0872896454418204
- **t-alpha:** 0.0080483244942393

---

---

334

- **PDB ID:** 2OF0 | **Chain:** A
- **b-phipsi:** 0.0281733261668754
- **w-rdist:** 0.0507241237663163
- **t-alpha:** 0.0215828567205209

---

---

335

- **PDB ID:** 5SCD | **Chain:** G
- **b-phipsi:** 0.0132693371246402
- **w-rdist:** 0.0660350227771828
- **t-alpha:** 0.0432593630846658

---

---

336

- **PDB ID:** 2XK4 | **Chain:** A
- **b-phipsi:** 0.017607838176385
- **w-rdist:** 0.35325336243357
- **t-alpha:** 0.0

---

---

337

- **PDB ID:** 3S2O | **Chain:** A
- **b-phipsi:** 0.0314808738589592
- **w-rdist:** 0.0530808079860796
- **t-alpha:** 0.0142855519524551

---

---

338

- **PDB ID:** 4R91 | **Chain:** A
- **b-phipsi:** 0.016231349418058
- **w-rdist:** 0.0801923495168433
- **t-alpha:** 0.0171028438612663

---

---

339

- **PDB ID:** 3CGO | **Chain:** A
- **b-phipsi:** 0.0171801214835364
- **w-rdist:** 0.1923282880725374
- **t-alpha:** 0.0030272436213401

---

---

340

- **PDB ID:** 4FSL | **Chain:** A
- **b-phipsi:** 0.0185368772829898
- **w-rdist:** 0.0593787667412865
- **t-alpha:** 0.0281690295641283

---

---

341

- **PDB ID:** 4AY1 | **Chain:** G
- **b-phipsi:** 0.0113282915924106
- **w-rdist:** 0.0793178796060791
- **t-alpha:** 0.0289856519083209

---

---

342

- **PDB ID:** 5SC8 | **Chain:** D
- **b-phipsi:** 0.0166577752114296
- **w-rdist:** 0.072107754472023
- **t-alpha:** 0.0231389553638574

---

---

343

- **PDB ID:** 3L5C | **Chain:** A
- **b-phipsi:** 0.0179072977988464
- **w-rdist:** 0.0500931344860584
- **t-alpha:** 0.0482898778152505

---

---

344

- **PDB ID:** 6TA3 | **Chain:** K
- **b-phipsi:** 0.0006627086846613
- **w-rdist:** 0.1617653817219928
- **t-alpha:** 0.0153215732989637

---

---

345

- **PDB ID:** 3K5G | **Chain:** B
- **b-phipsi:** 0.0438562689021035
- **w-rdist:** 0.058206051239902
- **t-alpha:** 0.004023997168737

---

---

346

- **PDB ID:** 2OHQ | **Chain:** A
- **b-phipsi:** 0.0186395647032106
- **w-rdist:** 0.0584677745886935
- **t-alpha:** 0.0300519144382522

---

---

347

- **PDB ID:** 2OHK | **Chain:** A
- **b-phipsi:** 0.0286278258747565
- **w-rdist:** 0.0512554723205992
- **t-alpha:** 0.0205341202479885

---

---

348

- **PDB ID:** 4DH6 | **Chain:** A
- **b-phipsi:** 0.041078589734797
- **w-rdist:** 0.0553304978905582
- **t-alpha:** 0.0060363072918738

---

---

349

- **PDB ID:** 4GID | **Chain:** B
- **b-phipsi:** 0.0155998071520741
- **w-rdist:** 0.0830319465225055
- **t-alpha:** 0.018108730237069

---

---

350

- **PDB ID:** 1YM4 | **Chain:** C
- **b-phipsi:** 0.0320618979401394
- **w-rdist:** 0.0746721410208398
- **t-alpha:** 0.002011889761613

---

---

351

- **PDB ID:** 5HD0 | **Chain:** B
- **b-phipsi:** 0.0206879240126367
- **w-rdist:** 0.0634598376200854
- **t-alpha:** 0.0201205697607655

---

---

352

- **PDB ID:** 1HJW | **Chain:** A
- **b-phipsi:** 0.0100913246898705
- **w-rdist:** 0.0602740597154259
- **t-alpha:** 0.081610524354118

---

---

353

- **PDB ID:** 1XN2 | **Chain:** A
- **b-phipsi:** 0.0171970467483322
- **w-rdist:** 0.0894582349721977
- **t-alpha:** 0.0100605125377637

---

---

354

- **PDB ID:** 1HJX | **Chain:** C
- **b-phipsi:** 0.0152800880362947
- **w-rdist:** 0.0672974471430563
- **t-alpha:** 0.0364964556675917

---

---

355

- **PDB ID:** 6UVV | **Chain:** B
- **b-phipsi:** 0.0158299000106847
- **w-rdist:** 0.0880060494301869
- **t-alpha:** 0.0142855519524551

---

---

356

- **PDB ID:** 5SCJ | **Chain:** A
- **b-phipsi:** 0.0132136929281986
- **w-rdist:** 0.0572610585898311
- **t-alpha:** 0.0925551162529434

---

---

357

- **PDB ID:** 2QZK | **Chain:** A
- **b-phipsi:** 0.0325625119420757
- **w-rdist:** 0.0489771187131727
- **t-alpha:** 0.0171028438612663

---

---

358

- **PDB ID:** 6UVP | **Chain:** B
- **b-phipsi:** 0.0185244032774793
- **w-rdist:** 0.1038286240073935
- **t-alpha:** 0.0030272436213401

---

---

359

- **PDB ID:** 3BUG | **Chain:** A
- **b-phipsi:** 0.0459653251858089
- **w-rdist:** 0.0405964195462692
- **t-alpha:** 0.012220058253459

---

---

360

- **PDB ID:** 3SKF | **Chain:** B
- **b-phipsi:** 0.0174481859705378
- **w-rdist:** 0.0744253543582911
- **t-alpha:** 0.0191146395025525

---

---

361

- **PDB ID:** 1ZYJ | **Chain:** A
- **b-phipsi:** 0.0188495724912357
- **w-rdist:** 0.2391609786150653
- **t-alpha:** 0.0

---

---

362

- **PDB ID:** 6QDZ | **Chain:** A
- **b-phipsi:** 0.017769996005335
- **w-rdist:** 0.2251867483296596
- **t-alpha:** 0.0030181563299565

---

---

363

- **PDB ID:** 5SCJ | **Chain:** F
- **b-phipsi:** 0.0182915404030056
- **w-rdist:** 0.0889861639888124
- **t-alpha:** 0.0080483244942393

---

---

364

- **PDB ID:** 2ZHV | **Chain:** A
- **b-phipsi:** 0.0271429752525837
- **w-rdist:** 0.0514164933333112
- **t-alpha:** 0.0247423777489943

---

---

365

- **PDB ID:** 3RTM | **Chain:** A
- **b-phipsi:** 0.0302402289708843
- **w-rdist:** 0.0421447279388267
- **t-alpha:** 0.026859374808243

---

---

366

- **PDB ID:** 6UWP | **Chain:** A
- **b-phipsi:** 0.0170345457595697
- **w-rdist:** 0.0496995019483335
- **t-alpha:** 0.0684105993302228

---

---

367

- **PDB ID:** 4AY1 | **Chain:** B
- **b-phipsi:** 0.0087134536648681
- **w-rdist:** 0.0754155680950181
- **t-alpha:** 0.0397492001139665

---

---

368

- **PDB ID:** 4ZSM | **Chain:** B
- **b-phipsi:** 0.0162873171720321
- **w-rdist:** 0.0706089515999615
- **t-alpha:** 0.0281690295641283

---

---

369

- **PDB ID:** 1YM2 | **Chain:** B
- **b-phipsi:** 0.0297202425871001
- **w-rdist:** 0.0663703408249647
- **t-alpha:** 0.0080483244942393

---

---

370

- **PDB ID:** 4FGX | **Chain:** A
- **b-phipsi:** 0.0406704099606839
- **w-rdist:** 0.0501056430246611
- **t-alpha:** 0.0100605125377637

---

---

371

- **PDB ID:** 5KQF | **Chain:** A
- **b-phipsi:** 0.0368439521979262
- **w-rdist:** 0.0503136171212953
- **t-alpha:** 0.012220058253459

---

---

372

- **PDB ID:** 4LXM | **Chain:** B
- **b-phipsi:** 0.0426639911103978
- **w-rdist:** 0.0614679724929234
- **t-alpha:** 0.0030272436213401

---

---

373

- **PDB ID:** 5SCI | **Chain:** F
- **b-phipsi:** 0.0219857573425815
- **w-rdist:** 0.0959486223231593
- **t-alpha:** 0.002011889761613

---

---

374

- **PDB ID:** 3KMX | **Chain:** A
- **b-phipsi:** 0.0221338004638479
- **w-rdist:** 0.0493685540034612
- **t-alpha:** 0.0364964556675917

---

---

375

- **PDB ID:** 6NW3 | **Chain:** C
- **b-phipsi:** 0.0321919151254856
- **w-rdist:** 0.0740763596632561
- **t-alpha:** 0.0030181563299565

---

---

376

- **PDB ID:** 5SCD | **Chain:** H
- **b-phipsi:** 0.0135329763386713
- **w-rdist:** 0.0773109073583637
- **t-alpha:** 0.0301810994667925

---

---

377

- **PDB ID:** 1M4H | **Chain:** B
- **b-phipsi:** 0.0164768194786176
- **w-rdist:** 0.0890659223797486
- **t-alpha:** 0.0132515540606032

---

---

378

- **PDB ID:** 5KI6 | **Chain:** A
- **b-phipsi:** 0.0096148271573864
- **w-rdist:** 0.0728274037952416
- **t-alpha:** 0.0432593630846658

---

---

379

- **PDB ID:** 5SC9 | **Chain:** D
- **b-phipsi:** 0.0153230091735358
- **w-rdist:** 0.0777288143139886
- **t-alpha:** 0.0251509582443987

---

---

380

- **PDB ID:** 5QD2 | **Chain:** B
- **b-phipsi:** 0.028534910739755
- **w-rdist:** 0.0656182350828273
- **t-alpha:** 0.0100605125377637

---

---

381

- **PDB ID:** 2QMF | **Chain:** A
- **b-phipsi:** 0.0208584059638031
- **w-rdist:** 0.0937250273803061
- **t-alpha:** 0.004023997168737

---

---

382

- **PDB ID:** 2WJO | **Chain:** A
- **b-phipsi:** 0.0238726960529775
- **w-rdist:** 0.0612482430940744
- **t-alpha:** 0.0194869465164286

---

---

383

- **PDB ID:** 6JSE | **Chain:** A
- **b-phipsi:** 0.0212699913338195
- **w-rdist:** 0.0479415836682096
- **t-alpha:** 0.0419284993347488

---

---

384

- **PDB ID:** 3IVH | **Chain:** A
- **b-phipsi:** 0.0371318851056897
- **w-rdist:** 0.0686356366868218
- **t-alpha:** 0.002011889761613

---

---

385

- **PDB ID:** 6D03 | **Chain:** C
- **b-phipsi:** 0.0124928222123588
- **w-rdist:** 0.1538886673801475
- **t-alpha:** 0.0130785989784187

---

---

386

- **PDB ID:** 5SC8 | **Chain:** F
- **b-phipsi:** 0.0162473321951652
- **w-rdist:** 0.0873390106258598
- **t-alpha:** 0.0150906633237464

---

---

387

- **PDB ID:** 3SKF | **Chain:** A
- **b-phipsi:** 0.0184716006948549
- **w-rdist:** 0.0769162161440476
- **t-alpha:** 0.0140843739056586

---

---

388

- **PDB ID:** 3HV5 | **Chain:** A
- **b-phipsi:** 0.0178088238762443
- **w-rdist:** 0.2615311666678995
- **t-alpha:** 0.0030181563299565

---

---

389

- **PDB ID:** 5SCF | **Chain:** G
- **b-phipsi:** 0.0143752906975433
- **w-rdist:** 0.066389883585376
- **t-alpha:** 0.0452715564866297

---

---

390

- **PDB ID:** 3SKG | **Chain:** A
- **b-phipsi:** 0.0209811461963645
- **w-rdist:** 0.0818076091384494
- **t-alpha:** 0.0080483244942393

---

---

391

- **PDB ID:** 4DVF | **Chain:** A
- **b-phipsi:** 0.0282781529120303
- **w-rdist:** 0.0652879101224658
- **t-alpha:** 0.0110661838165213

---

---

392

- **PDB ID:** 5YGY | **Chain:** A
- **b-phipsi:** 0.0259567182450426
- **w-rdist:** 0.0471116557001047
- **t-alpha:** 0.0343393687124173

---

---

393

- **PDB ID:** 6Q1P | **Chain:** B
- **b-phipsi:** 0.0086052264850905
- **w-rdist:** 0.0861972374568253
- **t-alpha:** 0.0291750635565768

---

---

394

- **PDB ID:** 5QD4 | **Chain:** B
- **b-phipsi:** 0.035407902213718
- **w-rdist:** 0.0548878027927875
- **t-alpha:** 0.0111900946877709

---

---

395

- **PDB ID:** 2F3F | **Chain:** C
- **b-phipsi:** 0.0284428267454887
- **w-rdist:** 0.0490657901798588
- **t-alpha:** 0.0261569749948251

---

---

396

- **PDB ID:** 2OHS | **Chain:** A
- **b-phipsi:** 0.0325852610385579
- **w-rdist:** 0.0548034570139025
- **t-alpha:** 0.0142855519524551

---

---

397

- **PDB ID:** 4I0I | **Chain:** A
- **b-phipsi:** 0.0307676569103166
- **w-rdist:** 0.0605380004586271
- **t-alpha:** 0.0120723738025678

---

---

398

- **PDB ID:** 7MYR | **Chain:** B
- **b-phipsi:** 0.0210794808728557
- **w-rdist:** 0.071787923430841
- **t-alpha:** 0.0132515540606032

---

---

399

- **PDB ID:** 3IXJ | **Chain:** A
- **b-phipsi:** 0.0324168001942032
- **w-rdist:** 0.0578096309061009
- **t-alpha:** 0.012220058253459

---

---

400

- **PDB ID:** 4P8W | **Chain:** A
- **b-phipsi:** 0.0181214858860814
- **w-rdist:** 0.085691129202207
- **t-alpha:** 0.0101626241883787

---

---

401

- **PDB ID:** 4DJY | **Chain:** B
- **b-phipsi:** 0.0166201869464512
- **w-rdist:** 0.0800995742603248
- **t-alpha:** 0.0201205697607655

---

---

402

- **PDB ID:** 5SCA | **Chain:** F
- **b-phipsi:** 0.0166357410899785
- **w-rdist:** 0.0865961623526693
- **t-alpha:** 0.0150906633237464

---

---

403

- **PDB ID:** 5QCS | **Chain:** A
- **b-phipsi:** 0.0258442652735849
- **w-rdist:** 0.0549624564250467
- **t-alpha:** 0.0261569749948251

---

---

404

- **PDB ID:** 5SC8 | **Chain:** B
- **b-phipsi:** 0.0156261746413068
- **w-rdist:** 0.0885310462289401
- **t-alpha:** 0.018108730237069

---

---

405

- **PDB ID:** 4GID | **Chain:** A
- **b-phipsi:** 0.0228678241069748
- **w-rdist:** 0.077778023455568
- **t-alpha:** 0.0081136984001406

---

---

406

- **PDB ID:** 1XN2 | **Chain:** B
- **b-phipsi:** 0.0195167274453885
- **w-rdist:** 0.0824406373397871
- **t-alpha:** 0.0100605125377637

---

---

407

- **PDB ID:** 4J1I | **Chain:** A
- **b-phipsi:** 0.0299561205451136
- **w-rdist:** 0.0403838540317589
- **t-alpha:** 0.0321912006166056

---

---

408

- **PDB ID:** 4R91 | **Chain:** B
- **b-phipsi:** 0.0217199904589194
- **w-rdist:** 0.08554828062635
- **t-alpha:** 0.006072766330031

---

---

409

- **PDB ID:** 3CKP | **Chain:** C
- **b-phipsi:** 0.0196103916764474
- **w-rdist:** 0.1324155325744183
- **t-alpha:** 0.0030181563299565

---

---

410

- **PDB ID:** 5QCP | **Chain:** A
- **b-phipsi:** 0.0264689504156458
- **w-rdist:** 0.0613838978895997
- **t-alpha:** 0.0184427898172117

---

---

411

- **PDB ID:** 7D5A | **Chain:** A
- **b-phipsi:** 0.0383643518114653
- **w-rdist:** 0.0451775978818334
- **t-alpha:** 0.0163601067821594

---

---

412

- **PDB ID:** 6BFE | **Chain:** A
- **b-phipsi:** 0.0165634821135472
- **w-rdist:** 0.0944706199918455
- **t-alpha:** 0.012220058253459

---

---

413

- **PDB ID:** 5SCB | **Chain:** D
- **b-phipsi:** 0.0184182667039107
- **w-rdist:** 0.070360405710363
- **t-alpha:** 0.02213297304878

---

---

414

- **PDB ID:** 5F00 | **Chain:** A
- **b-phipsi:** 0.0276840012970604
- **w-rdist:** 0.040065578931713
- **t-alpha:** 0.0397492001139665

---

---

415

- **PDB ID:** 2ZDZ | **Chain:** A
- **b-phipsi:** 0.0560891740968817
- **w-rdist:** 0.050542418365846
- **t-alpha:** 0.0091371600897414

---

---

416

- **PDB ID:** 3OOZ | **Chain:** A
- **b-phipsi:** 0.0354692599262606
- **w-rdist:** 0.0471180453274572
- **t-alpha:** 0.0184427898172117

---

---

417

- **PDB ID:** 4FSE | **Chain:** B
- **b-phipsi:** 0.0212830040198788
- **w-rdist:** 0.1080956440625318
- **t-alpha:** 0.0020161899393582

---

---

418

- **PDB ID:** 4TRY | **Chain:** A
- **b-phipsi:** 0.0196651227460434
- **w-rdist:** 0.0697121683594688
- **t-alpha:** 0.0194869465164286

---

---

419

- **PDB ID:** 1XN3 | **Chain:** B
- **b-phipsi:** 0.0155762760636692
- **w-rdist:** 0.0757698953149169
- **t-alpha:** 0.0301810994667925

---

---

420

- **PDB ID:** 5SCG | **Chain:** F
- **b-phipsi:** 0.0147232308777027
- **w-rdist:** 0.0893689398481737
- **t-alpha:** 0.0211265191128235

---

---

421

- **PDB ID:** 4AY1 | **Chain:** E
- **b-phipsi:** 0.0114110492871212
- **w-rdist:** 0.0786855952876016
- **t-alpha:** 0.0375784171587227

---

---

422

- **PDB ID:** 5QCY | **Chain:** B
- **b-phipsi:** 0.0422760956243884
- **w-rdist:** 0.0635358910625838
- **t-alpha:** 0.004023997168737

---

---

423

- **PDB ID:** 4FSL | **Chain:** D
- **b-phipsi:** 0.0211045554529775
- **w-rdist:** 0.0654767295147708
- **t-alpha:** 0.02213297304878

---

---

424

- **PDB ID:** 6JSF | **Chain:** A
- **b-phipsi:** 0.0475471538520845
- **w-rdist:** 0.0466777294565104
- **t-alpha:** 0.012220058253459

---

---

425

- **PDB ID:** 2QMF | **Chain:** B
- **b-phipsi:** 0.015955420590737
- **w-rdist:** 0.1049979450306727
- **t-alpha:** 0.0111900946877709

---

---

426

- **PDB ID:** 5SCK | **Chain:** F
- **b-phipsi:** 0.0159692339706143
- **w-rdist:** 0.0861705335569004
- **t-alpha:** 0.0201205697607655

---

---

427

- **PDB ID:** 4XXS | **Chain:** A
- **b-phipsi:** 0.0383429197074248
- **w-rdist:** 0.0446732903959594
- **t-alpha:** 0.0184427898172117

---

---

428

- **PDB ID:** 6CBD | **Chain:** A
- **b-phipsi:** 0.001373224678086
- **w-rdist:** 0.1637469364249406
- **t-alpha:** 0.0215828567205209

---

---

429

- **PDB ID:** 5QCO | **Chain:** B
- **b-phipsi:** 0.0310817785608355
- **w-rdist:** 0.0635808983002983
- **t-alpha:** 0.0110661838165213

---

---

430

- **PDB ID:** 4P8U | **Chain:** A
- **b-phipsi:** 0.0121350291481953
- **w-rdist:** 0.0846461434626771
- **t-alpha:** 0.0301810994667925

---

---

431

- **PDB ID:** 5I3W | **Chain:** A
- **b-phipsi:** 0.0276634455592181
- **w-rdist:** 0.0560496181335188
- **t-alpha:** 0.023686980845901

---

---

432

- **PDB ID:** 5SCJ | **Chain:** D
- **b-phipsi:** 0.0171773656478586
- **w-rdist:** 0.0700375608386857
- **t-alpha:** 0.0291750635565768

---

---

433

- **PDB ID:** 4X2L | **Chain:** A
- **b-phipsi:** 0.0274502813752868
- **w-rdist:** 0.0475280025486899
- **t-alpha:** 0.0343393687124173

---

---

434

- **PDB ID:** 5QD1 | **Chain:** C
- **b-phipsi:** 0.0273476276818464
- **w-rdist:** 0.0565148575624505
- **t-alpha:** 0.0241449510645854

---

---

435

- **PDB ID:** 5QD6 | **Chain:** A
- **b-phipsi:** 0.0232777519996627
- **w-rdist:** 0.0633007469016742
- **t-alpha:** 0.0215828567205209

---

---

436

- **PDB ID:** 3NSH | **Chain:** B
- **b-phipsi:** 0.0334928928468596
- **w-rdist:** 0.0356659121279131
- **t-alpha:** 0.0281690295641283

---

---

437

- **PDB ID:** 4L7G | **Chain:** A
- **b-phipsi:** 0.0388424541066994
- **w-rdist:** 0.0408445444765776
- **t-alpha:** 0.0211265191128235

---

---

438

- **PDB ID:** 5SCB | **Chain:** H
- **b-phipsi:** 0.0151658572375143
- **w-rdist:** 0.067989856424559
- **t-alpha:** 0.0442657220407964

---

---

439

- **PDB ID:** 3HW1 | **Chain:** B
- **b-phipsi:** 0.0297742242752143
- **w-rdist:** 0.0587221410802554
- **t-alpha:** 0.0171028438612663

---

---

440

- **PDB ID:** 5SCJ | **Chain:** H
- **b-phipsi:** 0.0140731681830316
- **w-rdist:** 0.0723351272071826
- **t-alpha:** 0.0422534769714337

---

---

441

- **PDB ID:** 5SCL | **Chain:** G
- **b-phipsi:** 0.016914938136242
- **w-rdist:** 0.0619263408805038
- **t-alpha:** 0.0462778608457075

---

---

442

- **PDB ID:** 3KN0 | **Chain:** A
- **b-phipsi:** 0.0223997080542153
- **w-rdist:** 0.0785426994847375
- **t-alpha:** 0.0100605125377637

---

---

443

- **PDB ID:** 5SCD | **Chain:** D
- **b-phipsi:** 0.020241698196958
- **w-rdist:** 0.0678228877551989
- **t-alpha:** 0.02213297304878

---

---

444

- **PDB ID:** 5SCE | **Chain:** G
- **b-phipsi:** 0.0166934903498309
- **w-rdist:** 0.0682733574873194
- **t-alpha:** 0.0372232431947987

---

---

445

- **PDB ID:** 2OHP | **Chain:** A
- **b-phipsi:** 0.0439292388934261
- **w-rdist:** 0.0493519133737924
- **t-alpha:** 0.0130785989784187

---

---

446

- **PDB ID:** 3R2F | **Chain:** E
- **b-phipsi:** 0.0176840227498002
- **w-rdist:** 0.0916628045480673
- **t-alpha:** 0.0120723738025678

---

---

447

- **PDB ID:** 5SCH | **Chain:** G
- **b-phipsi:** 0.0132521811284509
- **w-rdist:** 0.0706616445539433
- **t-alpha:** 0.0492955855664358

---

---

448

- **PDB ID:** 4LXA | **Chain:** C
- **b-phipsi:** 0.0445036910724166
- **w-rdist:** 0.0594712838320463
- **t-alpha:** 0.0060363072918738

---

---

449

- **PDB ID:** 3L5D | **Chain:** B
- **b-phipsi:** 0.0174556267284346
- **w-rdist:** 0.0565432705118911
- **t-alpha:** 0.0563379260275018

---

---

450

- **PDB ID:** 5DQC | **Chain:** B
- **b-phipsi:** 0.0388955748792743
- **w-rdist:** 0.0733947708536441
- **t-alpha:** 0.001007110036042

---

---

451

- **PDB ID:** 5QCO | **Chain:** A
- **b-phipsi:** 0.0307867109801458
- **w-rdist:** 0.057291012937147
- **t-alpha:** 0.0184427898172117

---

---

452

- **PDB ID:** 4FSE | **Chain:** D
- **b-phipsi:** 0.0213492041451358
- **w-rdist:** 0.0717653054943706
- **t-alpha:** 0.0160964977574751

---

---

453

- **PDB ID:** 2WF3 | **Chain:** A
- **b-phipsi:** 0.0263614916432423
- **w-rdist:** 0.0597736717021724
- **t-alpha:** 0.0231389553638574

---

---

454

- **PDB ID:** 5QCY | **Chain:** A
- **b-phipsi:** 0.0251891989633097
- **w-rdist:** 0.0645668796364297
- **t-alpha:** 0.0194869465164286

---

---

455

- **PDB ID:** 3L58 | **Chain:** A
- **b-phipsi:** 0.0213236685744962
- **w-rdist:** 0.0766118556068975
- **t-alpha:** 0.0132515540606032

---

---

456

- **PDB ID:** 1XN3 | **Chain:** D
- **b-phipsi:** 0.0171632298364131
- **w-rdist:** 0.0791933172553182
- **t-alpha:** 0.02213297304878

---

---

457

- **PDB ID:** 4W5R | **Chain:** A
- **b-phipsi:** 0.0081501481560042
- **w-rdist:** 0.1043395509177576
- **t-alpha:** 0.023686980845901

---

---

458

- **PDB ID:** 4R8Y | **Chain:** B
- **b-phipsi:** 0.0181032749245249
- **w-rdist:** 0.0915194061481334
- **t-alpha:** 0.0110661838165213

---

---

459

- **PDB ID:** 5SCI | **Chain:** D
- **b-phipsi:** 0.0171200866002012
- **w-rdist:** 0.0684023869516459
- **t-alpha:** 0.0352112405915889

---

---

460

- **PDB ID:** 5SCG | **Chain:** G
- **b-phipsi:** 0.0124272856003389
- **w-rdist:** 0.0672385786397535
- **t-alpha:** 0.0674043846886078

---

---

461

- **PDB ID:** 5QD4 | **Chain:** C
- **b-phipsi:** 0.0369288505396644
- **w-rdist:** 0.0590435601242616
- **t-alpha:** 0.0100605125377637

---

---

462

- **PDB ID:** 5SCL | **Chain:** D
- **b-phipsi:** 0.0175657498366143
- **w-rdist:** 0.0646414607213286
- **t-alpha:** 0.0392351918850506

---

---

463

- **PDB ID:** 4H3F | **Chain:** B
- **b-phipsi:** 0.0167408178167243
- **w-rdist:** 0.0890581530439966
- **t-alpha:** 0.018108730237069

---

---

464

- **PDB ID:** 5SCE | **Chain:** B
- **b-phipsi:** 0.0167112724690537
- **w-rdist:** 0.0982402567115803
- **t-alpha:** 0.0130785989784187

---

---

465

- **PDB ID:** 5SCJ | **Chain:** B
- **b-phipsi:** 0.018706877683182
- **w-rdist:** 0.0872479293502353
- **t-alpha:** 0.0120723738025678

---

---

466

- **PDB ID:** 2Q11 | **Chain:** B
- **b-phipsi:** 0.0170030820253884
- **w-rdist:** 0.0626564434252802
- **t-alpha:** 0.0482898778152505

---

---

467

- **PDB ID:** 1M7R | **Chain:** B
- **b-phipsi:** 0.027762297355981
- **w-rdist:** 0.0824460146646682
- **t-alpha:** 0.005055767262688

---

---

468

- **PDB ID:** 5SCH | **Chain:** F
- **b-phipsi:** 0.0160844615069577
- **w-rdist:** 0.0913939051830812
- **t-alpha:** 0.0194869465164286

---

---

469

- **PDB ID:** 3L5B | **Chain:** B
- **b-phipsi:** 0.0180616214344492
- **w-rdist:** 0.0624348100733773
- **t-alpha:** 0.040241637205524

---

---

470

- **PDB ID:** 5HDZ | **Chain:** B
- **b-phipsi:** 0.0193157882844098
- **w-rdist:** 0.0660872923154594
- **t-alpha:** 0.0281690295641283

---

---

471

- **PDB ID:** 4FSE | **Chain:** A
- **b-phipsi:** 0.0216252855443847
- **w-rdist:** 0.0856540012409041
- **t-alpha:** 0.0091371600897414

---

---

472

- **PDB ID:** 7D36 | **Chain:** A
- **b-phipsi:** 0.0355989860870654
- **w-rdist:** 0.0449825736212886
- **t-alpha:** 0.0247423777489943

---

---

473

- **PDB ID:** 1XN3 | **Chain:** A
- **b-phipsi:** 0.0182778247897526
- **w-rdist:** 0.0616364909349693
- **t-alpha:** 0.0412475676983714

---

---

474

- **PDB ID:** 5HE5 | **Chain:** B
- **b-phipsi:** 0.0169942063868123
- **w-rdist:** 0.0962201491375558
- **t-alpha:** 0.0132515540606032

---

---

475

- **PDB ID:** 4H3J | **Chain:** B
- **b-phipsi:** 0.0178369306896083
- **w-rdist:** 0.0813545775161328
- **t-alpha:** 0.0191146395025525

---

---

476

- **PDB ID:** 3K5C | **Chain:** C
- **b-phipsi:** 0.0302031145178255
- **w-rdist:** 0.0476032400088043
- **t-alpha:** 0.0321931693669585

---

---

477

- **PDB ID:** 8D6J | **Chain:** A
- **b-phipsi:** 0.0018678912604541
- **w-rdist:** 0.2754206409806353
- **t-alpha:** 0.02213297304878

---

---

478

- **PDB ID:** 6E3Z | **Chain:** A
- **b-phipsi:** 0.0353665656827895
- **w-rdist:** 0.0805812766954106
- **t-alpha:** 0.0010061554017259

---

---

479

- **PDB ID:** 1HJV | **Chain:** D
- **b-phipsi:** 0.0146002855172198
- **w-rdist:** 0.0621849820554923
- **t-alpha:** 0.099557427953085

---

---

480

- **PDB ID:** 5QCY | **Chain:** C
- **b-phipsi:** 0.0200144427801202
- **w-rdist:** 0.0828599255034007
- **t-alpha:** 0.0130785989784187

---

---

481

- **PDB ID:** 6NV9 | **Chain:** B
- **b-phipsi:** 0.0495569620500988
- **w-rdist:** 0.0551457964858526
- **t-alpha:** 0.0100605125377637

---

---

482

- **PDB ID:** 3L59 | **Chain:** B
- **b-phipsi:** 0.0174520607525463
- **w-rdist:** 0.0793258056467817
- **t-alpha:** 0.0231389553638574

---

---

483

- **PDB ID:** 3ZMG | **Chain:** A
- **b-phipsi:** 0.0322640248108776
- **w-rdist:** 0.0416666337224562
- **t-alpha:** 0.0354166098692967

---

---

484

- **PDB ID:** 5HE7 | **Chain:** B
- **b-phipsi:** 0.0250065594036492
- **w-rdist:** 0.1182962924775879
- **t-alpha:** 0.002011889761613

---

---

485

- **PDB ID:** 5QD8 | **Chain:** C
- **b-phipsi:** 0.0258407454027802
- **w-rdist:** 0.0568494796586651
- **t-alpha:** 0.0311871353768524

---

---

486

- **PDB ID:** 5SCE | **Chain:** D
- **b-phipsi:** 0.0157559695506647
- **w-rdist:** 0.0739064729506306
- **t-alpha:** 0.0392351918850506

---

---

487

- **PDB ID:** 1W50 | **Chain:** A
- **b-phipsi:** 0.0355123442694567
- **w-rdist:** 0.0376799981280855
- **t-alpha:** 0.0291750635565768

---

---

488

- **PDB ID:** 5QDC | **Chain:** B
- **b-phipsi:** 0.0445953825482474
- **w-rdist:** 0.0635690561826945
- **t-alpha:** 0.005055767262688

---

---

489

- **PDB ID:** 5QDD | **Chain:** C
- **b-phipsi:** 0.0440334353282963
- **w-rdist:** 0.0557496917560222
- **t-alpha:** 0.0110661838165213

---

---

490

- **PDB ID:** 4DI2 | **Chain:** C
- **b-phipsi:** 0.0251731740942695
- **w-rdist:** 0.0869790223143345
- **t-alpha:** 0.0070422936000285

---

---

491

- **PDB ID:** 8DF1 | **Chain:** C
- **b-phipsi:** 0.0132533094435409
- **w-rdist:** 0.065954799693702
- **t-alpha:** 0.0947135439792254

---

---

492

- **PDB ID:** 5QD9 | **Chain:** B
- **b-phipsi:** 0.0343059699406458
- **w-rdist:** 0.0666294673264918
- **t-alpha:** 0.0090543980992385

---

---

493

- **PDB ID:** 2G94 | **Chain:** C
- **b-phipsi:** 0.0164465749744212
- **w-rdist:** 0.0780884105663511
- **t-alpha:** 0.0301810994667925

---

---

494

- **PDB ID:** 4GID | **Chain:** C
- **b-phipsi:** 0.019130201388997
- **w-rdist:** 0.0954864189303024
- **t-alpha:** 0.0091371600897414

---

---

495

- **PDB ID:** 3CID | **Chain:** B
- **b-phipsi:** 0.0173361796926517
- **w-rdist:** 0.1009781613481836
- **t-alpha:** 0.012220058253459

---

---

496

- **PDB ID:** 5QDA | **Chain:** C
- **b-phipsi:** 0.0278960394326342
- **w-rdist:** 0.043943135841154
- **t-alpha:** 0.0452715564866297

---

---

497

- **PDB ID:** 1SGZ | **Chain:** B
- **b-phipsi:** 0.0177109630054507
- **w-rdist:** 0.0605762022245152
- **t-alpha:** 0.0503017573499313

---

---

498

- **PDB ID:** 8DF1 | **Chain:** D
- **b-phipsi:** 0.0134256855653871
- **w-rdist:** 0.0656516652742356
- **t-alpha:** 0.0983427491668371

---

---

499

- **PDB ID:** 3K5C | **Chain:** A
- **b-phipsi:** 0.0337627051577166
- **w-rdist:** 0.0542290108891725
- **t-alpha:** 0.0205341202479885

---

---

500

- **PDB ID:** 1HKJ | **Chain:** A
- **b-phipsi:** 0.0132619388309569
- **w-rdist:** 0.076670085614959
- **t-alpha:** 0.0463157782490606

---

---

501

- **PDB ID:** 4J1H | **Chain:** A
- **b-phipsi:** 0.0478252471354256
- **w-rdist:** 0.0513548664716985
- **t-alpha:** 0.0132515540606032

---

---

502

- **PDB ID:** 6BFD | **Chain:** B
- **b-phipsi:** 0.0161708812937074
- **w-rdist:** 0.1083368026833335
- **t-alpha:** 0.0142855519524551

---

---

503

- **PDB ID:** 2IS0 | **Chain:** A
- **b-phipsi:** 0.0392908560103241
- **w-rdist:** 0.0516883986738183
- **t-alpha:** 0.0163601067821594

---

---

504

- **PDB ID:** 5SCK | **Chain:** G
- **b-phipsi:** 0.0133458791348973
- **w-rdist:** 0.0732346068977353
- **t-alpha:** 0.0533200381607938

---

---

505

- **PDB ID:** 4J1C | **Chain:** A
- **b-phipsi:** 0.0327112729067554
- **w-rdist:** 0.0414907419124389
- **t-alpha:** 0.0364964556675917

---

---

506

- **PDB ID:** 5HDV | **Chain:** B
- **b-phipsi:** 0.0217377098195886
- **w-rdist:** 0.0946991764608757
- **t-alpha:** 0.0070422936000285

---

---

507

- **PDB ID:** 5SCH | **Chain:** B
- **b-phipsi:** 0.0149109342709355
- **w-rdist:** 0.102304356288412
- **t-alpha:** 0.0201205697607655

---

---

508

- **PDB ID:** 5SCK | **Chain:** B
- **b-phipsi:** 0.016674703907158
- **w-rdist:** 0.0928325045138835
- **t-alpha:** 0.0191146395025525

---

---

509

- **PDB ID:** 6UVY | **Chain:** B
- **b-phipsi:** 0.018116355204164
- **w-rdist:** 0.0917232906262004
- **t-alpha:** 0.0132515540606032

---

---

510

- **PDB ID:** 3CIC | **Chain:** B
- **b-phipsi:** 0.0186511256688987
- **w-rdist:** 0.0891710750758147
- **t-alpha:** 0.0132515540606032

---

---

511

- **PDB ID:** 4D83 | **Chain:** C
- **b-phipsi:** 0.024938852319023
- **w-rdist:** 0.130208199656102
- **t-alpha:** 0.0030181563299565

---

---

512

- **PDB ID:** 1WAW | **Chain:** A
- **b-phipsi:** 0.016348684064508
- **w-rdist:** 0.059796010649491
- **t-alpha:** 0.0887186656530896

---

---

513

- **PDB ID:** 5HDX | **Chain:** B
- **b-phipsi:** 0.0197438628856642
- **w-rdist:** 0.0642730400838452
- **t-alpha:** 0.0352112405915889

---

---

514

- **PDB ID:** 3L3A | **Chain:** A
- **b-phipsi:** 0.0397097433444558
- **w-rdist:** 0.0550092749638134
- **t-alpha:** 0.0142855519524551

---

---

515

- **PDB ID:** 6NV9 | **Chain:** C
- **b-phipsi:** 0.0375313937413085
- **w-rdist:** 0.0553958149388439
- **t-alpha:** 0.0160964977574751

---

---

516

- **PDB ID:** 3G33 | **Chain:** A
- **b-phipsi:** 0.0021917086132592
- **w-rdist:** 0.228603155856278
- **t-alpha:** 0.026859374808243

---

---

517

- **PDB ID:** 5SDT | **Chain:** H
- **b-phipsi:** 0.0146862890138077
- **w-rdist:** 0.0879722535498107
- **t-alpha:** 0.0301810994667925

---

---

518

- **PDB ID:** 3L5F | **Chain:** A
- **b-phipsi:** 0.0179085423342026
- **w-rdist:** 0.065996272319836
- **t-alpha:** 0.0412475676983714

---

---

519

- **PDB ID:** 5SCC | **Chain:** G
- **b-phipsi:** 0.0158351169607292
- **w-rdist:** 0.0666366160147344
- **t-alpha:** 0.0583501114605293

---

---

520

- **PDB ID:** 5SCC | **Chain:** D
- **b-phipsi:** 0.0176977710618705
- **w-rdist:** 0.0686138762118642
- **t-alpha:** 0.038229225245195

---

---

521

- **PDB ID:** 5QCW | **Chain:** A
- **b-phipsi:** 0.039747109048815
- **w-rdist:** 0.05988615400693
- **t-alpha:** 0.0101626241883787

---

---

522

- **PDB ID:** 5QCV | **Chain:** C
- **b-phipsi:** 0.0291739901210311
- **w-rdist:** 0.0544748854765474
- **t-alpha:** 0.0311871353768524

---

---

523

- **PDB ID:** 3IN4 | **Chain:** A
- **b-phipsi:** 0.0339788887852629
- **w-rdist:** 0.064961123957535
- **t-alpha:** 0.0111900946877709

---

---

524

- **PDB ID:** 5SCI | **Chain:** B
- **b-phipsi:** 0.0174023681715201
- **w-rdist:** 0.0937662130068553
- **t-alpha:** 0.0160964977574751

---

---

525

- **PDB ID:** 2NTR | **Chain:** A
- **b-phipsi:** 0.0443322103619831
- **w-rdist:** 0.0463147888275091
- **t-alpha:** 0.0205341202479885

---

---

526

- **PDB ID:** 5QDC | **Chain:** A
- **b-phipsi:** 0.0302348083969217
- **w-rdist:** 0.0584044336461834
- **t-alpha:** 0.023686980845901

---

---

527

- **PDB ID:** 5SCA | **Chain:** D
- **b-phipsi:** 0.0166200587670564
- **w-rdist:** 0.0710061873450885
- **t-alpha:** 0.0432593630846658

---

---

528

- **PDB ID:** 5SCG | **Chain:** D
- **b-phipsi:** 0.0154088278554647
- **w-rdist:** 0.0774709016986396
- **t-alpha:** 0.0412475676983714

---

---

529

- **PDB ID:** 3L5E | **Chain:** B
- **b-phipsi:** 0.0171335219357452
- **w-rdist:** 0.0716498433632166
- **t-alpha:** 0.0392351918850506

---

---

530

- **PDB ID:** 6OD6 | **Chain:** A
- **b-phipsi:** 0.0305391591819225
- **w-rdist:** 0.0439565571530191
- **t-alpha:** 0.0430220993447727

---

---

531

- **PDB ID:** 1YM4 | **Chain:** A
- **b-phipsi:** 0.0203558888981979
- **w-rdist:** 0.0694455336705213
- **t-alpha:** 0.0279214734263297

---

---

532

- **PDB ID:** 2VIE | **Chain:** A
- **b-phipsi:** 0.0266125500647234
- **w-rdist:** 0.0626673536932537
- **t-alpha:** 0.0261569749948251

---

---

533

- **PDB ID:** 2P8H | **Chain:** A
- **b-phipsi:** 0.0330802070812178
- **w-rdist:** 0.08074192975847
- **t-alpha:** 0.0040402750029742

---

---

534

- **PDB ID:** 2QU3 | **Chain:** A
- **b-phipsi:** 0.0373343044209512
- **w-rdist:** 0.0483295130285211
- **t-alpha:** 0.0247423777489943

---

---

535

- **PDB ID:** 3TPR | **Chain:** A
- **b-phipsi:** 0.043025622296571
- **w-rdist:** 0.0477506271163968
- **t-alpha:** 0.0205341202479885

---

---

536

- **PDB ID:** 4TRZ | **Chain:** B
- **b-phipsi:** 0.0165007894667349
- **w-rdist:** 0.0704486610909524
- **t-alpha:** 0.0462778608457075

---

---

537

- **PDB ID:** 5HE4 | **Chain:** B
- **b-phipsi:** 0.0208619312599717
- **w-rdist:** 0.1071091333606654
- **t-alpha:** 0.0070422936000285

---

---

538

- **PDB ID:** 4OLA | **Chain:** A
- **b-phipsi:** 0.0035430293671152
- **w-rdist:** 0.0911133849974047
- **t-alpha:** 0.0462778608457075

---

---

539

- **PDB ID:** 5SCE | **Chain:** F
- **b-phipsi:** 0.01608489282277
- **w-rdist:** 0.0996464597343652
- **t-alpha:** 0.0201205697607655

---

---

540

- **PDB ID:** 3L5F | **Chain:** B
- **b-phipsi:** 0.0174153217373006
- **w-rdist:** 0.0750106050344791
- **t-alpha:** 0.0342052238987842

---

---

541

- **PDB ID:** 5QDD | **Chain:** B
- **b-phipsi:** 0.0380745603530421
- **w-rdist:** 0.048497638503366
- **t-alpha:** 0.0241449510645854

---

---

542

- **PDB ID:** 4TRZ | **Chain:** C
- **b-phipsi:** 0.027455195149543
- **w-rdist:** 0.0557856701429769
- **t-alpha:** 0.0362172476666218

---

---

543

- **PDB ID:** 5SCH | **Chain:** D
- **b-phipsi:** 0.0184543053051707
- **w-rdist:** 0.0756373259695338
- **t-alpha:** 0.0271629994054913

---

---

544

- **PDB ID:** 5QCT | **Chain:** A
- **b-phipsi:** 0.0275595739508133
- **w-rdist:** 0.0784554673765148
- **t-alpha:** 0.0110661838165213

---

---

545

- **PDB ID:** 8DF1 | **Chain:** B
- **b-phipsi:** 0.014553664491686
- **w-rdist:** 0.0672644401997511
- **t-alpha:** 0.0887186656530896

---

---

546

- **PDB ID:** 2VNN | **Chain:** A
- **b-phipsi:** 0.0223708917224373
- **w-rdist:** 0.0594467139442003
- **t-alpha:** 0.0392351918850506

---

---

547

- **PDB ID:** 5SCB | **Chain:** G
- **b-phipsi:** 0.0156393966986339
- **w-rdist:** 0.0736720492167749
- **t-alpha:** 0.0482898778152505

---

---

548

- **PDB ID:** 4KE0 | **Chain:** C
- **b-phipsi:** 0.0422128073015901
- **w-rdist:** 0.0763647700055563
- **t-alpha:** 0.0030181563299565

---

---

549

- **PDB ID:** 6UWP | **Chain:** B
- **b-phipsi:** 0.0197349847816737
- **w-rdist:** 0.0617447599407252
- **t-alpha:** 0.0442657220407964

---

---

550

- **PDB ID:** 5SCF | **Chain:** D
- **b-phipsi:** 0.0161293557576883
- **w-rdist:** 0.0716341806339088
- **t-alpha:** 0.0492955855664358

---

---

551

- **PDB ID:** 3R2F | **Chain:** D
- **b-phipsi:** 0.017116168622732
- **w-rdist:** 0.1040424479438108
- **t-alpha:** 0.0150906633237464

---

---

552

- **PDB ID:** 3IXJ | **Chain:** B
- **b-phipsi:** 0.0292731216035291
- **w-rdist:** 0.0693554978190983
- **t-alpha:** 0.0150906633237464

---

---

553

- **PDB ID:** 3CKR | **Chain:** C
- **b-phipsi:** 0.0247789039484382
- **w-rdist:** 0.1217819799871617
- **t-alpha:** 0.0040402750029742

---

---

554

- **PDB ID:** 4LXK | **Chain:** A
- **b-phipsi:** 0.0416676883068196
- **w-rdist:** 0.0426679013930924
- **t-alpha:** 0.026859374808243

---

---

555

- **PDB ID:** 5QCX | **Chain:** B
- **b-phipsi:** 0.0284287123593009
- **w-rdist:** 0.0540693091199231
- **t-alpha:** 0.038229225245195

---

---

556

- **PDB ID:** 5V0N | **Chain:** A
- **b-phipsi:** 0.034155205032608
- **w-rdist:** 0.0779070467774865
- **t-alpha:** 0.005055767262688

---

---

557

- **PDB ID:** 1FKN | **Chain:** A
- **b-phipsi:** 0.017422879815136
- **w-rdist:** 0.1076390206392367
- **t-alpha:** 0.0140843739056586

---

---

558

- **PDB ID:** 5SDT | **Chain:** F
- **b-phipsi:** 0.015819670385763
- **w-rdist:** 0.0854111252152019
- **t-alpha:** 0.0342052238987842

---

---

559

- **PDB ID:** 2OHT | **Chain:** A
- **b-phipsi:** 0.0279752757891701
- **w-rdist:** 0.0444998746779902
- **t-alpha:** 0.0563233187944307

---

---

560

- **PDB ID:** 4KE0 | **Chain:** A
- **b-phipsi:** 0.0331832785566015
- **w-rdist:** 0.046380986072189
- **t-alpha:** 0.0364964556675917

---

---

561

- **PDB ID:** 4H1E | **Chain:** A
- **b-phipsi:** 0.0205205145441057
- **w-rdist:** 0.0678017563523936
- **t-alpha:** 0.0321931693669585

---

---

562

- **PDB ID:** 6NV7 | **Chain:** A
- **b-phipsi:** 0.0390220634722187
- **w-rdist:** 0.0525133555371451
- **t-alpha:** 0.0215828567205209

---

---

563

- **PDB ID:** 5SCJ | **Chain:** G
- **b-phipsi:** 0.0188892154566021
- **w-rdist:** 0.059126550448637
- **t-alpha:** 0.0563379260275018

---

---

564

- **PDB ID:** 5QCX | **Chain:** C
- **b-phipsi:** 0.032341164383161
- **w-rdist:** 0.0582774187779957
- **t-alpha:** 0.0241449510645854

---

---

565

- **PDB ID:** 6UWV | **Chain:** B
- **b-phipsi:** 0.018048437727303
- **w-rdist:** 0.085514721876252
- **t-alpha:** 0.0231389553638574

---

---

566

- **PDB ID:** 4BEK | **Chain:** A
- **b-phipsi:** 0.0289691565377194
- **w-rdist:** 0.055440992250304
- **t-alpha:** 0.0364964556675917

---

---

567

- **PDB ID:** 4RCE | **Chain:** A
- **b-phipsi:** 0.0390726024650077
- **w-rdist:** 0.0473363779255251
- **t-alpha:** 0.026859374808243

---

---

568

- **PDB ID:** 2VKM | **Chain:** A
- **b-phipsi:** 0.0165206381056823
- **w-rdist:** 0.0832359979754163
- **t-alpha:** 0.0331991995154452

---

---

569

- **PDB ID:** 4DJU | **Chain:** B
- **b-phipsi:** 0.0167358280033464
- **w-rdist:** 0.0674678326034648
- **t-alpha:** 0.0563379260275018

---

---

570

- **PDB ID:** 5SCG | **Chain:** C
- **b-phipsi:** 0.0140646754363052
- **w-rdist:** 0.0762767574285406
- **t-alpha:** 0.0603620848752721

---

---

571

- **PDB ID:** 5MCQ | **Chain:** A
- **b-phipsi:** 0.022691616769741
- **w-rdist:** 0.0853474805986373
- **t-alpha:** 0.0132515540606032

---

---

572

- **PDB ID:** 3VV8 | **Chain:** A
- **b-phipsi:** 0.0455323560627979
- **w-rdist:** 0.0563540429543419
- **t-alpha:** 0.0142855519524551

---

---

573

- **PDB ID:** 2G94 | **Chain:** B
- **b-phipsi:** 0.0174917934825374
- **w-rdist:** 0.0835070395261816
- **t-alpha:** 0.0271629994054913

---

---

574

- **PDB ID:** 5SDT | **Chain:** D
- **b-phipsi:** 0.017777822386547
- **w-rdist:** 0.0719803730491931
- **t-alpha:** 0.0392351918850506

---

---

575

- **PDB ID:** 2VKM | **Chain:** D
- **b-phipsi:** 0.0159775726904036
- **w-rdist:** 0.1012235054814787
- **t-alpha:** 0.0231389553638574

---

---

576

- **PDB ID:** 1SGZ | **Chain:** A
- **b-phipsi:** 0.0174828382496788
- **w-rdist:** 0.0666166525747625
- **t-alpha:** 0.0533200381607938

---

---

577

- **PDB ID:** 7F1D | **Chain:** A
- **b-phipsi:** 0.0279638433410349
- **w-rdist:** 0.0497035807651786
- **t-alpha:** 0.0482898778152505

---

---

578

- **PDB ID:** 4D88 | **Chain:** A
- **b-phipsi:** 0.029122683027888
- **w-rdist:** 0.0911764118211453
- **t-alpha:** 0.0060363072918738

---

---

579

- **PDB ID:** 4LXM | **Chain:** A
- **b-phipsi:** 0.044303431820122
- **w-rdist:** 0.0613277385488755
- **t-alpha:** 0.0111900946877709

---

---

580

- **PDB ID:** 5HDU | **Chain:** B
- **b-phipsi:** 0.0207475836108011
- **w-rdist:** 0.0843162259651021
- **t-alpha:** 0.018108730237069

---

---

581

- **PDB ID:** 4AY1 | **Chain:** J
- **b-phipsi:** 0.0131119613275809
- **w-rdist:** 0.0772623435842574
- **t-alpha:** 0.0665237071513429

---

---

582

- **PDB ID:** 2WF2 | **Chain:** A
- **b-phipsi:** 0.0298056821663501
- **w-rdist:** 0.0538730901743863
- **t-alpha:** 0.038229225245195

---

---

583

- **PDB ID:** 1W51 | **Chain:** A
- **b-phipsi:** 0.032119716617408
- **w-rdist:** 0.062785171322597
- **t-alpha:** 0.0215828567205209

---

---

584

- **PDB ID:** 3QBH | **Chain:** A
- **b-phipsi:** 0.0445662651561438
- **w-rdist:** 0.0540831812417871
- **t-alpha:** 0.0184427898172117

---

---

585

- **PDB ID:** 8DF1 | **Chain:** E
- **b-phipsi:** 0.0158864333583911
- **w-rdist:** 0.0666312740250139
- **t-alpha:** 0.0971304585383399

---

---

586

- **PDB ID:** 2VNM | **Chain:** A
- **b-phipsi:** 0.028282824701869
- **w-rdist:** 0.0618626530795475
- **t-alpha:** 0.0291750635565768

---

---

587

- **PDB ID:** 6E3Z | **Chain:** B
- **b-phipsi:** 0.0225039648192825
- **w-rdist:** 0.1178769936478111
- **t-alpha:** 0.0070422936000285

---

---

588

- **PDB ID:** 6BFW | **Chain:** A
- **b-phipsi:** 0.015997464837702
- **w-rdist:** 0.0981911344198525
- **t-alpha:** 0.0251509582443987

---

---

589

- **PDB ID:** 3MSJ | **Chain:** B
- **b-phipsi:** 0.0378011775899157
- **w-rdist:** 0.0535663500579739
- **t-alpha:** 0.0247423777489943

---

---

590

- **PDB ID:** 3INE | **Chain:** A
- **b-phipsi:** 0.032310271153155
- **w-rdist:** 0.0646504149962514
- **t-alpha:** 0.0194869465164286

---

---

591

- **PDB ID:** 4A28 | **Chain:** B
- **b-phipsi:** 0.0284720054438074
- **w-rdist:** 0.152097152564303
- **t-alpha:** 0.0030181563299565

---

---

592

- **PDB ID:** 6YZH | **Chain:** A
- **b-phipsi:** 0.0203017194488786
- **w-rdist:** 0.1687603853515014
- **t-alpha:** 0.0081136984001406

---

---

593

- **PDB ID:** 4RCD | **Chain:** A
- **b-phipsi:** 0.0394441652944229
- **w-rdist:** 0.0515402159443127
- **t-alpha:** 0.0247423777489943

---

---

594

- **PDB ID:** 5SDT | **Chain:** C
- **b-phipsi:** 0.0146117625361035
- **w-rdist:** 0.0723485866744743
- **t-alpha:** 0.0744468317020319

---

---

595

- **PDB ID:** 3OHH | **Chain:** A
- **b-phipsi:** 0.0152063452332701
- **w-rdist:** 0.0825849130418202
- **t-alpha:** 0.0442657220407964

---

---

596

- **PDB ID:** 2F3E | **Chain:** A
- **b-phipsi:** 0.029363726795167
- **w-rdist:** 0.0581127992872454
- **t-alpha:** 0.0332642337077917

---

---

597

- **PDB ID:** 1FCK | **Chain:** A
- **b-phipsi:** 0.0087399735652263
- **w-rdist:** 0.0970452859109997
- **t-alpha:** 0.0430220993447727

---

---

598

- **PDB ID:** 2Q11 | **Chain:** C
- **b-phipsi:** 0.0237926950678728
- **w-rdist:** 0.0514160360649796
- **t-alpha:** 0.0694167426287164

---

---

599

- **PDB ID:** 5SCD | **Chain:** B
- **b-phipsi:** 0.0192094918204641
- **w-rdist:** 0.0927802062404684
- **t-alpha:** 0.0160964977574751

---

---

600

- **PDB ID:** 3RSV | **Chain:** A
- **b-phipsi:** 0.0356330578182109
- **w-rdist:** 0.0575891013335631
- **t-alpha:** 0.0226336461702254

---

---

601

- **PDB ID:** 3FKT | **Chain:** A
- **b-phipsi:** 0.04076799607755
- **w-rdist:** 0.0444713263311291
- **t-alpha:** 0.0311871353768524

---

---

602

- **PDB ID:** 4B7B | **Chain:** A
- **b-phipsi:** 0.0183755818378174
- **w-rdist:** 0.1676620977826682
- **t-alpha:** 0.0111900946877709

---

---

603

- **PDB ID:** 4EXG | **Chain:** A
- **b-phipsi:** 0.0284327012458511
- **w-rdist:** 0.0487925967005369
- **t-alpha:** 0.0543260470581381

---

---

604

- **PDB ID:** 4EWO | **Chain:** A
- **b-phipsi:** 0.0330176765686665
- **w-rdist:** 0.0537771443285112
- **t-alpha:** 0.0331991995154452

---

---

605

- **PDB ID:** 5SDT | **Chain:** B
- **b-phipsi:** 0.0168568925313631
- **w-rdist:** 0.090466919461403
- **t-alpha:** 0.0281690295641283

---

---

606

- **PDB ID:** 7PP6 | **Chain:** D
- **b-phipsi:** 0.0218229074013605
- **w-rdist:** 0.1551458148582763
- **t-alpha:** 0.0070922351376341

---

---

607

- **PDB ID:** 4DPF | **Chain:** A
- **b-phipsi:** 0.0389921473268825
- **w-rdist:** 0.0493895262420494
- **t-alpha:** 0.0281690295641283

---

---

608

- **PDB ID:** 3TPL | **Chain:** A
- **b-phipsi:** 0.027935530846003
- **w-rdist:** 0.0429438166656727
- **t-alpha:** 0.074594394652218

---

---

609

- **PDB ID:** 7B1E | **Chain:** A
- **b-phipsi:** 0.0266276488469841
- **w-rdist:** 0.0463976711409233
- **t-alpha:** 0.0764586075718078

---

---

610

- **PDB ID:** 5SC8 | **Chain:** G
- **b-phipsi:** 0.013127685542405
- **w-rdist:** 0.0903394724045791
- **t-alpha:** 0.0442657220407964

---

---

611

- **PDB ID:** 4GID | **Chain:** D
- **b-phipsi:** 0.0211518233031211
- **w-rdist:** 0.0951296927695901
- **t-alpha:** 0.0130785989784187

---

---

612

- **PDB ID:** 1SQY | **Chain:** A
- **b-phipsi:** 0.0082190185728548
- **w-rdist:** 0.0870459545423545
- **t-alpha:** 0.0619656060250684

---

---

613

- **PDB ID:** 5SCC | **Chain:** C
- **b-phipsi:** 0.0131806801627764
- **w-rdist:** 0.0768533135732536
- **t-alpha:** 0.0824948847783451

---

---

614

- **PDB ID:** 3RSX | **Chain:** A
- **b-phipsi:** 0.0252696302746911
- **w-rdist:** 0.0665559097585583
- **t-alpha:** 0.0311202652104596

---

---

615

- **PDB ID:** 5SCJ | **Chain:** C
- **b-phipsi:** 0.013293087988273
- **w-rdist:** 0.0773225998444854
- **t-alpha:** 0.0744468317020319

---

---

616

- **PDB ID:** 5QD9 | **Chain:** C
- **b-phipsi:** 0.0216632198439735
- **w-rdist:** 0.0895164450577761
- **t-alpha:** 0.0150906633237464

---

---

617

- **PDB ID:** 5QD2 | **Chain:** A
- **b-phipsi:** 0.0259338720049674
- **w-rdist:** 0.0667615186277525
- **t-alpha:** 0.0300519144382522

---

---

618

- **PDB ID:** 5I3V | **Chain:** A
- **b-phipsi:** 0.0399593314562351
- **w-rdist:** 0.0494847101095366
- **t-alpha:** 0.0279214734263297

---

---

619

- **PDB ID:** 4BFD | **Chain:** A
- **b-phipsi:** 0.0396212733886693
- **w-rdist:** 0.0482097667314712
- **t-alpha:** 0.0300519144382522

---

---

620

- **PDB ID:** 2QP8 | **Chain:** B
- **b-phipsi:** 0.0182867241724611
- **w-rdist:** 0.1022389393427941
- **t-alpha:** 0.0153215732989637

---

---

621

- **PDB ID:** 4R92 | **Chain:** B
- **b-phipsi:** 0.024143605282442
- **w-rdist:** 0.0830496646820631
- **t-alpha:** 0.0160964977574751

---

---

622

- **PDB ID:** 2F3F | **Chain:** B
- **b-phipsi:** 0.0323187204673888
- **w-rdist:** 0.0519131617592415
- **t-alpha:** 0.0392351918850506

---

---

623

- **PDB ID:** 3UQP | **Chain:** A
- **b-phipsi:** 0.0407822892791689
- **w-rdist:** 0.0439508334905241
- **t-alpha:** 0.0343393687124173

---

---

624

- **PDB ID:** 3VG1 | **Chain:** A
- **b-phipsi:** 0.0292258518279679
- **w-rdist:** 0.1012987996478001
- **t-alpha:** 0.0050303674430436

---

---

625

- **PDB ID:** 2F3E | **Chain:** B
- **b-phipsi:** 0.0329240492468246
- **w-rdist:** 0.0844676257957266
- **t-alpha:** 0.0070422936000285

---

---

626

- **PDB ID:** 2P4J | **Chain:** B
- **b-phipsi:** 0.021488970195448
- **w-rdist:** 0.094176364890342
- **t-alpha:** 0.0132515540606032

---

---

627

- **PDB ID:** 3ZKM | **Chain:** B
- **b-phipsi:** 0.0255914289745905
- **w-rdist:** 0.1367511913219856
- **t-alpha:** 0.006072766330031

---

---

628

- **PDB ID:** 1WB0 | **Chain:** A
- **b-phipsi:** 0.0150468779717673
- **w-rdist:** 0.0820380613937861
- **t-alpha:** 0.0507400688245485

---

---

629

- **PDB ID:** 5EZX | **Chain:** A
- **b-phipsi:** 0.0444279417980703
- **w-rdist:** 0.0429283492613363
- **t-alpha:** 0.0331991995154452

---

---

630

- **PDB ID:** 6NV9 | **Chain:** A
- **b-phipsi:** 0.0381370386181011
- **w-rdist:** 0.0609008234379658
- **t-alpha:** 0.0184427898172117

---

---

631

- **PDB ID:** 4R93 | **Chain:** B
- **b-phipsi:** 0.0217335376076718
- **w-rdist:** 0.077538863789781
- **t-alpha:** 0.0251509582443987

---

---

632

- **PDB ID:** 5QCT | **Chain:** C
- **b-phipsi:** 0.029744650071615
- **w-rdist:** 0.0572617394089759
- **t-alpha:** 0.038229225245195

---

---

633

- **PDB ID:** 4GMI | **Chain:** A
- **b-phipsi:** 0.0237929916353242
- **w-rdist:** 0.0539564343365744
- **t-alpha:** 0.0704223021796839

---

---

634

- **PDB ID:** 5SDT | **Chain:** G
- **b-phipsi:** 0.0135638557391825
- **w-rdist:** 0.09044037892283
- **t-alpha:** 0.0452715564866297

---

---

635

- **PDB ID:** 3DM6 | **Chain:** A
- **b-phipsi:** 0.0364184413186553
- **w-rdist:** 0.0996254319980793
- **t-alpha:** 0.0

---

---

636

- **PDB ID:** 4DUS | **Chain:** A
- **b-phipsi:** 0.0322155678667866
- **w-rdist:** 0.0672594429114077
- **t-alpha:** 0.0194869465164286

---

---

637

- **PDB ID:** 2VKM | **Chain:** C
- **b-phipsi:** 0.0170271140411352
- **w-rdist:** 0.0917979063944548
- **t-alpha:** 0.0291750635565768

---

---

638

- **PDB ID:** 7CJ2 | **Chain:** B
- **b-phipsi:** 0.0266103847374271
- **w-rdist:** 0.0872805382402535
- **t-alpha:** 0.0130785989784187

---

---

639

- **PDB ID:** 5QDA | **Chain:** A
- **b-phipsi:** 0.0294234093892827
- **w-rdist:** 0.0560121476501121
- **t-alpha:** 0.0419284993347488

---

---

640

- **PDB ID:** 3IN3 | **Chain:** A
- **b-phipsi:** 0.0460171051389391
- **w-rdist:** 0.0775699167939423
- **t-alpha:** 0.0040402750029742

---

---

641

- **PDB ID:** 5QD7 | **Chain:** C
- **b-phipsi:** 0.0230552502882734
- **w-rdist:** 0.0642374138316131
- **t-alpha:** 0.0412475676983714

---

---

642

- **PDB ID:** 5QDB | **Chain:** A
- **b-phipsi:** 0.0301422534762663
- **w-rdist:** 0.0494372970040107
- **t-alpha:** 0.0518519454679657

---

---

643

- **PDB ID:** 4K8S | **Chain:** C
- **b-phipsi:** 0.0364837984730179
- **w-rdist:** 0.0409406574688922
- **t-alpha:** 0.045215734453575

---

---

644

- **PDB ID:** 2XFI | **Chain:** A
- **b-phipsi:** 0.0274259145191399
- **w-rdist:** 0.0509356799104902
- **t-alpha:** 0.0633803735522946

---

---

645

- **PDB ID:** 4WTU | **Chain:** A
- **b-phipsi:** 0.0348182367748083
- **w-rdist:** 0.0603681048361092
- **t-alpha:** 0.023686980845901

---

---

646

- **PDB ID:** 5QCP | **Chain:** B
- **b-phipsi:** 0.0247864769932162
- **w-rdist:** 0.0590328458666154
- **t-alpha:** 0.0492955855664358

---

---

647

- **PDB ID:** 6DHC | **Chain:** C
- **b-phipsi:** 0.0356170237089258
- **w-rdist:** 0.0626820917581483
- **t-alpha:** 0.0201205697607655

---

---

648

- **PDB ID:** 3K3B | **Chain:** B
- **b-phipsi:** 0.0129529040842336
- **w-rdist:** 0.1038840849836189
- **t-alpha:** 0.0386624986159414

---

---

649

- **PDB ID:** 5SCE | **Chain:** C
- **b-phipsi:** 0.0138475445980007
- **w-rdist:** 0.0804950271490858
- **t-alpha:** 0.0684105993302228

---

---

650

- **PDB ID:** 6D04 | **Chain:** D
- **b-phipsi:** 0.0181178821771439
- **w-rdist:** 0.1606614705723419
- **t-alpha:** 0.0142855519524551

---

---

651

- **PDB ID:** 4DPI | **Chain:** A
- **b-phipsi:** 0.0209148595645689
- **w-rdist:** 0.0571604257045867
- **t-alpha:** 0.0865192111806096

---

---

652

- **PDB ID:** 6T13 | **Chain:** A
- **b-phipsi:** 0.010076407515395
- **w-rdist:** 0.0884515672955839
- **t-alpha:** 0.0674043846886078

---

---

653

- **PDB ID:** 4FRJ | **Chain:** A
- **b-phipsi:** 0.0334776050777614
- **w-rdist:** 0.055251574308981
- **t-alpha:** 0.0343393687124173

---

---

654

- **PDB ID:** 5SCC | **Chain:** B
- **b-phipsi:** 0.0156589347180766
- **w-rdist:** 0.1022604840384569
- **t-alpha:** 0.0301810994667925

---

---

655

- **PDB ID:** 3S7M | **Chain:** A
- **b-phipsi:** 0.033111108446025
- **w-rdist:** 0.0754969737435104
- **t-alpha:** 0.012220058253459

---

---

656

- **PDB ID:** 4XKX | **Chain:** A
- **b-phipsi:** 0.0354792851742866
- **w-rdist:** 0.0731250575680254
- **t-alpha:** 0.0111900946877709

---

---

657

- **PDB ID:** 4LXA | **Chain:** A
- **b-phipsi:** 0.0497022000786666
- **w-rdist:** 0.0592972541000211
- **t-alpha:** 0.0153215732989637

---

---

658

- **PDB ID:** 1NWR | **Chain:** A
- **b-phipsi:** 0.0166886626375765
- **w-rdist:** 0.0815257589602039
- **t-alpha:** 0.0441178429367157

---

---

659

- **PDB ID:** 4K9H | **Chain:** C
- **b-phipsi:** 0.0363927025087602
- **w-rdist:** 0.0679682534290052
- **t-alpha:** 0.0140843739056586

---

---

660

- **PDB ID:** 2WCG | **Chain:** A
- **b-phipsi:** 0.0072131304794289
- **w-rdist:** 0.0907970675185584
- **t-alpha:** 0.0694167426287164

---

---

661

- **PDB ID:** 2GM1 | **Chain:** A
- **b-phipsi:** 0.0217248930059948
- **w-rdist:** 0.1432020626650147
- **t-alpha:** 0.0101626241883787

---

---

662

- **PDB ID:** 5SCA | **Chain:** C
- **b-phipsi:** 0.0158738286735068
- **w-rdist:** 0.0748813954510961
- **t-alpha:** 0.0724347245856311

---

---

663

- **PDB ID:** 4B78 | **Chain:** A
- **b-phipsi:** 0.0230776840560169
- **w-rdist:** 0.0551647317444543
- **t-alpha:** 0.0824948847783451

---

---

664

- **PDB ID:** 5QDA | **Chain:** B
- **b-phipsi:** 0.02920999581493
- **w-rdist:** 0.050878997575638
- **t-alpha:** 0.0593563782252406

---

---

665

- **PDB ID:** 1NWS | **Chain:** B
- **b-phipsi:** 0.0177389385258701
- **w-rdist:** 0.069328782954722
- **t-alpha:** 0.0608324157938926

---

---

666

- **PDB ID:** 5QD6 | **Chain:** C
- **b-phipsi:** 0.0344639252294095
- **w-rdist:** 0.0513754941243774
- **t-alpha:** 0.040241637205524

---

---

667

- **PDB ID:** 5SCL | **Chain:** C
- **b-phipsi:** 0.0160744289293411
- **w-rdist:** 0.0745519819357096
- **t-alpha:** 0.0744468317020319

---

---

668

- **PDB ID:** 2VIY | **Chain:** A
- **b-phipsi:** 0.0264973374456082
- **w-rdist:** 0.055810647056997
- **t-alpha:** 0.0603620848752721

---

---

669

- **PDB ID:** 5QD9 | **Chain:** A
- **b-phipsi:** 0.0219142226350138
- **w-rdist:** 0.0607005290063594
- **t-alpha:** 0.059701445364084

---

---

670

- **PDB ID:** 3UQR | **Chain:** A
- **b-phipsi:** 0.0316763323295484
- **w-rdist:** 0.0717825511745447
- **t-alpha:** 0.0194869465164286

---

---

671

- **PDB ID:** 1NWS | **Chain:** D
- **b-phipsi:** 0.0180220261855029
- **w-rdist:** 0.0669466532801977
- **t-alpha:** 0.0711207806002658

---

---

672

- **PDB ID:** 3R2F | **Chain:** A
- **b-phipsi:** 0.0198918815269292
- **w-rdist:** 0.1005474485546751
- **t-alpha:** 0.0174001876755087

---

---

673

- **PDB ID:** 2QP8 | **Chain:** A
- **b-phipsi:** 0.0205165033088205
- **w-rdist:** 0.0991615450862548
- **t-alpha:** 0.0171028438612663

---

---

674

- **PDB ID:** 5QD1 | **Chain:** B
- **b-phipsi:** 0.034425399280846
- **w-rdist:** 0.0548036334063736
- **t-alpha:** 0.038229225245195

---

---

675

- **PDB ID:** 3IXK | **Chain:** C
- **b-phipsi:** 0.035135244893883
- **w-rdist:** 0.1034237122095963
- **t-alpha:** 0.0030181563299565

---

---

676

- **PDB ID:** 1LCF | **Chain:** A
- **b-phipsi:** 0.0141669917712024
- **w-rdist:** 0.100929368171159
- **t-alpha:** 0.0419284993347488

---

---

677

- **PDB ID:** 4WKF | **Chain:** A
- **b-phipsi:** 0.018852693548986
- **w-rdist:** 0.0637201276911063
- **t-alpha:** 0.0804347333893407

---

---

678

- **PDB ID:** 4K9H | **Chain:** A
- **b-phipsi:** 0.0408604879321915
- **w-rdist:** 0.0634111554976455
- **t-alpha:** 0.018108730237069

---

---

679

- **PDB ID:** 5SC8 | **Chain:** C
- **b-phipsi:** 0.01685769580954
- **w-rdist:** 0.0722448911085199
- **t-alpha:** 0.0754525056331989

---

---

680

- **PDB ID:** 3CKR | **Chain:** B
- **b-phipsi:** 0.0193499827340487
- **w-rdist:** 0.096829848078263
- **t-alpha:** 0.0205341202479885

---

---

681

- **PDB ID:** 4DVF | **Chain:** B
- **b-phipsi:** 0.0268941434375942
- **w-rdist:** 0.0560796958490485
- **t-alpha:** 0.062374337083412

---

---

682

- **PDB ID:** 4RCF | **Chain:** A
- **b-phipsi:** 0.0427709663888749
- **w-rdist:** 0.0570611568808887
- **t-alpha:** 0.0247423777489943

---

---

683

- **PDB ID:** 3PI5 | **Chain:** C
- **b-phipsi:** 0.0348647604520377
- **w-rdist:** 0.0679662375824791
- **t-alpha:** 0.0191146395025525

---

---

684

- **PDB ID:** 2OHL | **Chain:** A
- **b-phipsi:** 0.024438924288397
- **w-rdist:** 0.0593341150689688
- **t-alpha:** 0.059701445364084

---

---

685

- **PDB ID:** 1XS7 | **Chain:** D
- **b-phipsi:** 0.022134649688222
- **w-rdist:** 0.0935882583074785
- **t-alpha:** 0.0174001876755087

---

---

686

- **PDB ID:** 5QD3 | **Chain:** B
- **b-phipsi:** 0.0322834415766078
- **w-rdist:** 0.0566442013886101
- **t-alpha:** 0.0412475676983714

---

---

687

- **PDB ID:** 1NWU | **Chain:** A
- **b-phipsi:** 0.0187718566694895
- **w-rdist:** 0.0668380082873843
- **t-alpha:** 0.0653805729706817

---

---

688

- **PDB ID:** 6EQM | **Chain:** A
- **b-phipsi:** 0.032083743895285
- **w-rdist:** 0.0479595248530585
- **t-alpha:** 0.062374337083412

---

---

689

- **PDB ID:** 4A51 | **Chain:** A
- **b-phipsi:** 0.0270635852607559
- **w-rdist:** 0.1637716227756153
- **t-alpha:** 0.0070922351376341

---

---

690

- **PDB ID:** 1NWR | **Chain:** D
- **b-phipsi:** 0.0170547923830755
- **w-rdist:** 0.0805796850244906
- **t-alpha:** 0.0496303685128725

---

---

691

- **PDB ID:** 1HJX | **Chain:** B
- **b-phipsi:** 0.0163724749558148
- **w-rdist:** 0.0744432842587821
- **t-alpha:** 0.093509430468313

---

---

692

- **PDB ID:** 3DV1 | **Chain:** C
- **b-phipsi:** 0.0326377710154729
- **w-rdist:** 0.0619021928418632
- **t-alpha:** 0.0311871353768524

---

---

693

- **PDB ID:** 4DJX | **Chain:** B
- **b-phipsi:** 0.0193101878913197
- **w-rdist:** 0.0800929749055181
- **t-alpha:** 0.0372232431947987

---

---

694

- **PDB ID:** 3IVI | **Chain:** C
- **b-phipsi:** 0.0328206584937439
- **w-rdist:** 0.1536020734184754
- **t-alpha:** 0.0030272436213401

---

---

695

- **PDB ID:** 5SCF | **Chain:** B
- **b-phipsi:** 0.0141444269153843
- **w-rdist:** 0.095719400549831
- **t-alpha:** 0.0482898778152505

---

---

696

- **PDB ID:** 5QD5 | **Chain:** C
- **b-phipsi:** 0.0208900975825936
- **w-rdist:** 0.075904782554577
- **t-alpha:** 0.038229225245195

---

---

697

- **PDB ID:** 5QCW | **Chain:** C
- **b-phipsi:** 0.0384116598537501
- **w-rdist:** 0.0668676350763894
- **t-alpha:** 0.0171028438612663

---

---

698

- **PDB ID:** 3UQU | **Chain:** A
- **b-phipsi:** 0.0292205626279173
- **w-rdist:** 0.1083444239065141
- **t-alpha:** 0.0070922351376341

---

---

699

- **PDB ID:** 2ZHT | **Chain:** A
- **b-phipsi:** 0.0331858245346035
- **w-rdist:** 0.0651924799287826
- **t-alpha:** 0.0257998410196613

---

---

700

- **PDB ID:** 3K5F | **Chain:** A
- **b-phipsi:** 0.0401003365301569
- **w-rdist:** 0.0551938636247546
- **t-alpha:** 0.0301810994667925

---

---

701

- **PDB ID:** 1NWT | **Chain:** D
- **b-phipsi:** 0.0219301652428678
- **w-rdist:** 0.0626330650941819
- **t-alpha:** 0.0653805729706817

---

---

702

- **PDB ID:** 5SCG | **Chain:** B
- **b-phipsi:** 0.0176394077658968
- **w-rdist:** 0.0946287436110612
- **t-alpha:** 0.0301810994667925

---

---

703

- **PDB ID:** 1NWU | **Chain:** D
- **b-phipsi:** 0.0204910120924819
- **w-rdist:** 0.0633626425447476
- **t-alpha:** 0.07692324021217

---

---

704

- **PDB ID:** 3BUH | **Chain:** A
- **b-phipsi:** 0.0535734175612119
- **w-rdist:** 0.0561394990869577
- **t-alpha:** 0.0247423777489943

---

---

705

- **PDB ID:** 3N4L | **Chain:** B
- **b-phipsi:** 0.0312626040888018
- **w-rdist:** 0.0682575673184317
- **t-alpha:** 0.026859374808243

---

---

706

- **PDB ID:** 5QD5 | **Chain:** B
- **b-phipsi:** 0.0327863424454295
- **w-rdist:** 0.061820781894055
- **t-alpha:** 0.0331991995154452

---

---

707

- **PDB ID:** 5QCZ | **Chain:** C
- **b-phipsi:** 0.034776145139162
- **w-rdist:** 0.055998967492548
- **t-alpha:** 0.0392351918850506

---

---

708

- **PDB ID:** 3KN0 | **Chain:** B
- **b-phipsi:** 0.0165484416326683
- **w-rdist:** 0.0973141929841866
- **t-alpha:** 0.0375784171587227

---

---

709

- **PDB ID:** 4R95 | **Chain:** B
- **b-phipsi:** 0.0200694114449139
- **w-rdist:** 0.0960979072492027
- **t-alpha:** 0.02213297304878

---

---

710

- **PDB ID:** 5QCZ | **Chain:** B
- **b-phipsi:** 0.028145623204903
- **w-rdist:** 0.0566324482305022
- **t-alpha:** 0.06136824019931

---

---

711

- **PDB ID:** 6BFW | **Chain:** B
- **b-phipsi:** 0.0185233759272913
- **w-rdist:** 0.0739985586230597
- **t-alpha:** 0.052313985604153

---

---

712

- **PDB ID:** 5QD5 | **Chain:** A
- **b-phipsi:** 0.0380315414988462
- **w-rdist:** 0.0577725357591043
- **t-alpha:** 0.0311202652104596

---

---

713

- **PDB ID:** 3H0B | **Chain:** C
- **b-phipsi:** 0.0224526409652003
- **w-rdist:** 0.1357614079488039
- **t-alpha:** 0.0132515540606032

---

---

714

- **PDB ID:** 2IRZ | **Chain:** A
- **b-phipsi:** 0.0439456049402171
- **w-rdist:** 0.0684464949700496
- **t-alpha:** 0.0140843739056586

---

---

715

- **PDB ID:** 5T1W | **Chain:** A
- **b-phipsi:** 0.0494508841679079
- **w-rdist:** 0.0673934329911861
- **t-alpha:** 0.0132515540606032

---

---

716

- **PDB ID:** 5SCA | **Chain:** B
- **b-phipsi:** 0.0163608706633237
- **w-rdist:** 0.0917040253079088
- **t-alpha:** 0.0452715564866297

---

---

717

- **PDB ID:** 1B0L | **Chain:** A
- **b-phipsi:** 0.0143628132390964
- **w-rdist:** 0.0944889089248345
- **t-alpha:** 0.0552016840097893

---

---

718

- **PDB ID:** 1FBV | **Chain:** A
- **b-phipsi:** 0.0296156884359769
- **w-rdist:** 0.1157647956157855
- **t-alpha:** 0.0080483244942393

---

---

719

- **PDB ID:** 1YM2 | **Chain:** A
- **b-phipsi:** 0.0356691624226104
- **w-rdist:** 0.0660227725016899
- **t-alpha:** 0.023686980845901

---

---

720

- **PDB ID:** 2WF4 | **Chain:** A
- **b-phipsi:** 0.0267997676761518
- **w-rdist:** 0.0595659523664192
- **t-alpha:** 0.0593563782252406

---

---

721

- **PDB ID:** 6OD6 | **Chain:** B
- **b-phipsi:** 0.0309631641491841
- **w-rdist:** 0.0983529443842433
- **t-alpha:** 0.0090543980992385

---

---

722

- **PDB ID:** 1II6 | **Chain:** B
- **b-phipsi:** 0.0243862986901011
- **w-rdist:** 0.1512259690698048
- **t-alpha:** 0.012220058253459

---

---

723

- **PDB ID:** 6BFX | **Chain:** B
- **b-phipsi:** 0.0174993815051111
- **w-rdist:** 0.092555461805783
- **t-alpha:** 0.0375784171587227

---

---

724

- **PDB ID:** 5I3Y | **Chain:** A
- **b-phipsi:** 0.0272359968663587
- **w-rdist:** 0.0620985488151987
- **t-alpha:** 0.0518519454679657

---

---

725

- **PDB ID:** 4K9H | **Chain:** B
- **b-phipsi:** 0.0392936693314873
- **w-rdist:** 0.0516095406478311
- **t-alpha:** 0.0408375348842262

---

---

726

- **PDB ID:** 3TPL | **Chain:** C
- **b-phipsi:** 0.0315098056195927
- **w-rdist:** 0.070256206174964
- **t-alpha:** 0.026859374808243

---

---

727

- **PDB ID:** 5QD1 | **Chain:** A
- **b-phipsi:** 0.0321892766072028
- **w-rdist:** 0.0570399029485072
- **t-alpha:** 0.0474184781355553

---

---

728

- **PDB ID:** 5QD8 | **Chain:** A
- **b-phipsi:** 0.0352872919860072
- **w-rdist:** 0.0574684591065888
- **t-alpha:** 0.0386624986159414

---

---

729

- **PDB ID:** 5QCU | **Chain:** B
- **b-phipsi:** 0.027581415454938
- **w-rdist:** 0.063806685447669
- **t-alpha:** 0.0462778608457075

---

---

730

- **PDB ID:** 4D8C | **Chain:** B
- **b-phipsi:** 0.0229998201692697
- **w-rdist:** 0.1156346580299283
- **t-alpha:** 0.0142855519524551

---

---

731

- **PDB ID:** 1HJV | **Chain:** C
- **b-phipsi:** 0.0191412843890963
- **w-rdist:** 0.0682567916645519
- **t-alpha:** 0.0734339085116317

---

---

732

- **PDB ID:** 5QCR | **Chain:** C
- **b-phipsi:** 0.0417936940598942
- **w-rdist:** 0.0498507778071438
- **t-alpha:** 0.0412475676983714

---

---

733

- **PDB ID:** 3QI1 | **Chain:** A
- **b-phipsi:** 0.0220030174037444
- **w-rdist:** 0.0674308740991772
- **t-alpha:** 0.0543260470581381

---

---

734

- **PDB ID:** 3DUY | **Chain:** A
- **b-phipsi:** 0.0397515144714614
- **w-rdist:** 0.0517625619808059
- **t-alpha:** 0.0408375348842262

---

---

735

- **PDB ID:** 7CJ2 | **Chain:** A
- **b-phipsi:** 0.031719907634993
- **w-rdist:** 0.0808824830177504
- **t-alpha:** 0.0174001876755087

---

---

736

- **PDB ID:** 3TPJ | **Chain:** A
- **b-phipsi:** 0.0443343989627273
- **w-rdist:** 0.0583506173822835
- **t-alpha:** 0.0271629994054913

---

---

737

- **PDB ID:** 3ZKN | **Chain:** B
- **b-phipsi:** 0.0260736032872492
- **w-rdist:** 0.137375952334718
- **t-alpha:** 0.012220058253459

---

---

738

- **PDB ID:** 5QD2 | **Chain:** C
- **b-phipsi:** 0.0227176559906785
- **w-rdist:** 0.0802892427256486
- **t-alpha:** 0.0321931693669585

---

---

739

- **PDB ID:** 2OHU | **Chain:** A
- **b-phipsi:** 0.0401239091240363
- **w-rdist:** 0.0528739445362517
- **t-alpha:** 0.0397492001139665

---

---

740

- **PDB ID:** 5QCR | **Chain:** B
- **b-phipsi:** 0.0302084132272975
- **w-rdist:** 0.0578478203109309
- **t-alpha:** 0.0543260470581381

---

---

741

- **PDB ID:** 6OD6 | **Chain:** C
- **b-phipsi:** 0.0340644570049075
- **w-rdist:** 0.0481460829884908
- **t-alpha:** 0.0674043846886078

---

---

742

- **PDB ID:** 3MSL | **Chain:** A
- **b-phipsi:** 0.0333436216976329
- **w-rdist:** 0.0660394300658141
- **t-alpha:** 0.0300519144382522

---

---

743

- **PDB ID:** 5QD0 | **Chain:** A
- **b-phipsi:** 0.0396765390086652
- **w-rdist:** 0.0635864291822985
- **t-alpha:** 0.0247423777489943

---

---

744

- **PDB ID:** 5SCD | **Chain:** C
- **b-phipsi:** 0.0167930449191962
- **w-rdist:** 0.0804146964926791
- **t-alpha:** 0.0714288072460622

---

---

745

- **PDB ID:** 3DV5 | **Chain:** C
- **b-phipsi:** 0.0273971945385464
- **w-rdist:** 0.0672205391681856
- **t-alpha:** 0.0432593630846658

---

---

746

- **PDB ID:** 5QCU | **Chain:** C
- **b-phipsi:** 0.0364346351294735
- **w-rdist:** 0.0688076522108822
- **t-alpha:** 0.02213297304878

---

---

747

- **PDB ID:** 5QD3 | **Chain:** A
- **b-phipsi:** 0.0298548054717614
- **w-rdist:** 0.0638394736086704
- **t-alpha:** 0.0430220993447727

---

---

748

- **PDB ID:** 5QD3 | **Chain:** C
- **b-phipsi:** 0.024126286901576
- **w-rdist:** 0.070500349852806
- **t-alpha:** 0.0452715564866297

---

---

749

- **PDB ID:** 6ZE8 | **Chain:** A
- **b-phipsi:** 0.0150392401015277
- **w-rdist:** 0.0921976845915934
- **t-alpha:** 0.0653805729706817

---

---

750

- **PDB ID:** 5MXD | **Chain:** B
- **b-phipsi:** 0.0286058149244687
- **w-rdist:** 0.1430219362867644
- **t-alpha:** 0.0100605125377637

---

---

751

- **PDB ID:** 4A1Z | **Chain:** B
- **b-phipsi:** 0.0298939895278744
- **w-rdist:** 0.1059538424528973
- **t-alpha:** 0.0100605125377637

---

---

752

- **PDB ID:** 2F3F | **Chain:** A
- **b-phipsi:** 0.0382127582953818
- **w-rdist:** 0.0545196983371777
- **t-alpha:** 0.0430220993447727

---

---

753

- **PDB ID:** 3IXK | **Chain:** B
- **b-phipsi:** 0.0348409078489716
- **w-rdist:** 0.0740013184588088
- **t-alpha:** 0.0201205697607655

---

---

754

- **PDB ID:** 3UQR | **Chain:** C
- **b-phipsi:** 0.0363609510828745
- **w-rdist:** 0.1230321281736777
- **t-alpha:** 0.0040402750029742

---

---

755

- **PDB ID:** 1NWR | **Chain:** C
- **b-phipsi:** 0.015341567298302
- **w-rdist:** 0.0919585840625003
- **t-alpha:** 0.0653805729706817

---

---

756

- **PDB ID:** 5SCH | **Chain:** C
- **b-phipsi:** 0.0163613004689305
- **w-rdist:** 0.0851008879899178
- **t-alpha:** 0.0714288072460622

---

---

757

- **PDB ID:** 3RTN | **Chain:** A
- **b-phipsi:** 0.0354457172024753
- **w-rdist:** 0.0668167256561178
- **t-alpha:** 0.026859374808243

---

---

758

- **PDB ID:** 4I0I | **Chain:** B
- **b-phipsi:** 0.0461470887449065
- **w-rdist:** 0.0448436954975051
- **t-alpha:** 0.0503017573499313

---

---

759

- **PDB ID:** 5ENK | **Chain:** A
- **b-phipsi:** 0.0415930778892143
- **w-rdist:** 0.0721236134672194
- **t-alpha:** 0.0150906633237464

---

---

760

- **PDB ID:** 3PI5 | **Chain:** A
- **b-phipsi:** 0.0383059842125129
- **w-rdist:** 0.0649599611398682
- **t-alpha:** 0.026859374808243

---

---

761

- **PDB ID:** 4KE1 | **Chain:** A
- **b-phipsi:** 0.0299485381997769
- **w-rdist:** 0.0795287500806608
- **t-alpha:** 0.023686980845901

---

---

762

- **PDB ID:** 5QD4 | **Chain:** A
- **b-phipsi:** 0.0363748227559718
- **w-rdist:** 0.0569674799104395
- **t-alpha:** 0.0430220993447727

---

---

763

- **PDB ID:** 5SCI | **Chain:** C
- **b-phipsi:** 0.0189657375000242
- **w-rdist:** 0.0747840289869818
- **t-alpha:** 0.062374337083412

---

---

764

- **PDB ID:** 5I3X | **Chain:** A
- **b-phipsi:** 0.0325416439717138
- **w-rdist:** 0.0669787659665616
- **t-alpha:** 0.0332642337077917

---

---

765

- **PDB ID:** 1HKI | **Chain:** A
- **b-phipsi:** 0.0129111732935121
- **w-rdist:** 0.1033687469077492
- **t-alpha:** 0.0688172240723723

---

---

766

- **PDB ID:** 1NWS | **Chain:** C
- **b-phipsi:** 0.0197783891389514
- **w-rdist:** 0.0683923245124612
- **t-alpha:** 0.101995546264151

---

---

767

- **PDB ID:** 5J3X | **Chain:** D
- **b-phipsi:** 0.0174240350391591
- **w-rdist:** 0.0937118687745637
- **t-alpha:** 0.0441178429367157

---

---

768

- **PDB ID:** 3K5D | **Chain:** C
- **b-phipsi:** 0.0356738810374677
- **w-rdist:** 0.1131041487554365
- **t-alpha:** 0.005055767262688

---

---

769

- **PDB ID:** 5DQC | **Chain:** C
- **b-phipsi:** 0.0275059784030863
- **w-rdist:** 0.0920199203697165
- **t-alpha:** 0.0201205697607655

---

---

770

- **PDB ID:** 6Q1N | **Chain:** A
- **b-phipsi:** 0.0178464106245935
- **w-rdist:** 0.1035703464382893
- **t-alpha:** 0.0342052238987842

---

---

771

- **PDB ID:** 2VA6 | **Chain:** A
- **b-phipsi:** 0.0322700378749109
- **w-rdist:** 0.0896092356334862
- **t-alpha:** 0.0142855519524551

---

---

772

- **PDB ID:** 6JK6 | **Chain:** A
- **b-phipsi:** 0.0172148409159651
- **w-rdist:** 0.0868785016683494
- **t-alpha:** 0.0574466500591699

---

---

773

- **PDB ID:** 6FGY | **Chain:** A
- **b-phipsi:** 0.029276774481826
- **w-rdist:** 0.066080292190975
- **t-alpha:** 0.0452715564866297

---

---

774

- **PDB ID:** 1NWT | **Chain:** A
- **b-phipsi:** 0.019414535679767
- **w-rdist:** 0.0783776735240375
- **t-alpha:** 0.054082748085932

---

---

775

- **PDB ID:** 5SCK | **Chain:** C
- **b-phipsi:** 0.0182946332707118
- **w-rdist:** 0.0772545576331454
- **t-alpha:** 0.0704223021796839

---

---

776

- **PDB ID:** 1BKA | **Chain:** A
- **b-phipsi:** 0.0155924205543122
- **w-rdist:** 0.097576798095694
- **t-alpha:** 0.0574466500591699

---

---

777

- **PDB ID:** 3QBH | **Chain:** C
- **b-phipsi:** 0.0376334984873595
- **w-rdist:** 0.0514760860609864
- **t-alpha:** 0.0553320103347625

---

---

778

- **PDB ID:** 5QCQ | **Chain:** A
- **b-phipsi:** 0.0330633074511233
- **w-rdist:** 0.0564974674286478
- **t-alpha:** 0.0574466500591699

---

---

779

- **PDB ID:** 4FCO | **Chain:** A
- **b-phipsi:** 0.0336197510652408
- **w-rdist:** 0.1013918205212012
- **t-alpha:** 0.0090543980992385

---

---

780

- **PDB ID:** 4EJX | **Chain:** A
- **b-phipsi:** 0.0277294887999573
- **w-rdist:** 0.1066659610087908
- **t-alpha:** 0.0142855519524551

---

---

781

- **PDB ID:** 2F3E | **Chain:** C
- **b-phipsi:** 0.0352106345784573
- **w-rdist:** 0.0830188997733207
- **t-alpha:** 0.0150906633237464

---

---

782

- **PDB ID:** 3S7L | **Chain:** A
- **b-phipsi:** 0.0345831600575343
- **w-rdist:** 0.0747936339479692
- **t-alpha:** 0.0231389553638574

---

---

783

- **PDB ID:** 5T1U | **Chain:** A
- **b-phipsi:** 0.0336340213857778
- **w-rdist:** 0.0649857984554839
- **t-alpha:** 0.0375784171587227

---

---

784

- **PDB ID:** 5QCV | **Chain:** A
- **b-phipsi:** 0.0332556961765579
- **w-rdist:** 0.0563447736460561
- **t-alpha:** 0.0574466500591699

---

---

785

- **PDB ID:** 3BRA | **Chain:** A
- **b-phipsi:** 0.0449697616599385
- **w-rdist:** 0.0736201655214939
- **t-alpha:** 0.0150906633237464

---

---

786

- **PDB ID:** 2VIJ | **Chain:** A
- **b-phipsi:** 0.031100988267693
- **w-rdist:** 0.0598481602421643
- **t-alpha:** 0.0563379260275018

---

---

787

- **PDB ID:** 4ZSR | **Chain:** B
- **b-phipsi:** 0.0185911598174962
- **w-rdist:** 0.0831708573302287
- **t-alpha:** 0.0513078913478914

---

---

788

- **PDB ID:** 6C2I | **Chain:** A
- **b-phipsi:** 0.0339571817619796
- **w-rdist:** 0.071028055389605
- **t-alpha:** 0.0279214734263297

---

---

789

- **PDB ID:** 3HVG | **Chain:** A
- **b-phipsi:** 0.0266046348152902
- **w-rdist:** 0.070939005770878
- **t-alpha:** 0.0472836358422945

---

---

790

- **PDB ID:** 5KR8 | **Chain:** A
- **b-phipsi:** 0.0457669754562308
- **w-rdist:** 0.0637647649010648
- **t-alpha:** 0.0257998410196613

---

---

791

- **PDB ID:** 4LXM | **Chain:** C
- **b-phipsi:** 0.0390737731562322
- **w-rdist:** 0.063912425211166
- **t-alpha:** 0.0301810994667925

---

---

792

- **PDB ID:** 2XFK | **Chain:** A
- **b-phipsi:** 0.0381478040293921
- **w-rdist:** 0.0587155581108347
- **t-alpha:** 0.0412475676983714

---

---

793

- **PDB ID:** 3VF3 | **Chain:** A
- **b-phipsi:** 0.0397646424647191
- **w-rdist:** 0.1084211362689344
- **t-alpha:** 0.0050303674430436

---

---

794

- **PDB ID:** 1LFG | **Chain:** A
- **b-phipsi:** 0.0149977072852331
- **w-rdist:** 0.1007419520594681
- **t-alpha:** 0.0676690861025068

---

---

795

- **PDB ID:** 3MSJ | **Chain:** C
- **b-phipsi:** 0.032346566564832
- **w-rdist:** 0.0625092949658085
- **t-alpha:** 0.0492955855664358

---

---

796

- **PDB ID:** 2P4J | **Chain:** D
- **b-phipsi:** 0.0213629495080318
- **w-rdist:** 0.0908538372511401
- **t-alpha:** 0.0362172476666218

---

---

797

- **PDB ID:** 4B77 | **Chain:** A
- **b-phipsi:** 0.0191033278216232
- **w-rdist:** 0.103148889407885
- **t-alpha:** 0.0331991995154452

---

---

798

- **PDB ID:** 6NW3 | **Chain:** A
- **b-phipsi:** 0.0368379639839201
- **w-rdist:** 0.0505832539344219
- **t-alpha:** 0.0734339085116317

---

---

799

- **PDB ID:** 1LG2 | **Chain:** A
- **b-phipsi:** 0.0227352224835339
- **w-rdist:** 0.0673554777970612
- **t-alpha:** 0.0899123067133345

---

---

800

- **PDB ID:** 1NWR | **Chain:** B
- **b-phipsi:** 0.0198380696256824
- **w-rdist:** 0.0832259424449664
- **t-alpha:** 0.0507400688245485

---

---
