# Supplementary material for: Comparative structural insights and functional analysis for the distinct unbound states of Human AGO proteins
Source: Sci Rep. 2025 Mar 19;15:9432. doi: 10.1038/s41598-025-91849-5 (PMC11923369; doi:10.1038/s41598-025-91849-5)
Supplement: Supplementary file 24 — Supplementary Information 12. [file 41598_2025_91849_MOESM24_ESM.zip › 4Z4Dp_A_mddomain_HL2REF/go/4Z4Dp_A_Piwi_mitot_mitosis_e8649561789a4b21afe18d44787a2120-pres_report.html]

 

# Structural Comparison Report for 4Z4Dp\_A\_Piwi - domains (total: 6)

---

1

- **Protein name:** Kinesin-like protein KIF11
- **Organism:** Homo sapiens
- **Uniprot Accession Number:** P52732
- **Protein sequence length:** 1056 aa
- **1D identity (%):** 15.27
- **1D identity (%) [Gaps excluded]:** 21.2
- **1D identity - Alignment Gaps:** 311
- **1D aligned content (<aminoacid>:%):** {'M': 1.18, 'S': 4.12, 'G': 5.29, 'P': 5.29, 'R': 5.29, 'T': 9.41, 'K': 7.65, 'D': 8.82, 'I': 4.71, 'Y': 2.94, 'E': 7.65, 'F': 2.94, 'L': 14.71, 'V': 4.71, 'A': 3.53, 'N': 2.94, 'Q': 4.12, 'H': 2.35, 'C': 2.35}
- **Common reported functions (%):** 0.0
- **Common reported locations (%):** 21.43
- **Common reported processes (%):** 0.0

- **PDB ID:** 3HQD
- **Chain:** B
- **Crystallized protein length:** 350 aa
- **Resolution:** 2.19 Å
- **Associated domain:** Kinesin-motor
- **b-phipsi ():** 0.001376
- **w-rdist ():** 0.126258
- **t-alpha ():** 0.003027
- **Chemical similarity (Tanimoto Index) (%):** 78.39
- **1D identity (%) [PDB]:** 1.91
- **1D identity (%) [Gaps excluded][PDB]:** 62.86
- **1D identity - Alignment Gaps [PDB]:** 1118
- **1D aligned content [PDB] (<aminoacid>:%):** {'K': 4.55, 'N': 13.64, 'Q': 4.55, 'R': 9.09, 'T': 13.64, 'L': 27.27, 'V': 9.09, 'G': 9.09, 'I': 4.55, 'P': 4.55}
- **2D identity (%) [PDB]:** 24.51
- **2D identity (%) [Gaps excluded][PDB]:** 83.33
- **2D identity - Alignment Gaps [PDB]:** 648
- **2D aligned content [PDB] (<2D-fold>:%):** {'.': 14.22, 'E': 36.89, 'T': 6.22, 'H': 41.33, 'G': 1.33}
- **3D similarity (TM-Score) (%) [PDB]:** 15.34

- **Gene name:** KIF11
- **Entrez ID:** 383200
- **RefSeq ID:** NM\_004523
- **Transcript sequence length:** 5016
- **5-UTR|CDS|3-UTR identity (%):** 38.74 | 43.03 | 8.51
- **5-UTR|CDS|3-UTR identity (%) [Gaps excluded]:** 81.67 | 72.39 | 76.16
- **5-UTR|CDS|3-UTR identity [Alignment Gaps]:** 133 | 1463 | 10777
- **5-UTR aligned content (<base>:%):** {'G': 42.86, 'T': 7.14, 'C': 48.98, 'A': 1.02}
- **CDS aligned content (<base>:%):** {'A': 31.89, 'T': 21.13, 'G': 24.81, 'C': 22.16}
- **3-UTR aligned content (<base>:%):** {'T': 38.66, 'C': 15.6, 'A': 28.88, 'G': 16.86}

**Uniprot Description:**  
  
 Motor protein required for establishing a bipolar spindle during mitosis (PubMed:19001501). Required in non-mitotic cells for transport of secretory proteins from the Golgi complex to the cell surface (PubMed:23857769).   
  
Interacts with the thyroid hormone receptor in the presence of thyroid hormone. Component of a large chromatin remodeling complex, at least composed of MYSM1, PCAF, RBM10 and KIF11/TRIP5. Interacts (via C-terminus) with the kinase NEK6 in both interphase and mitosis.   
  
 **Gene Ontology Information:**

Molecular Function

- ATP binding
- microtubule binding
- microtubule motor activity
- ATP-dependent microtubule motor activity, plus-end-directed
- protein kinase binding

Location

- cytosol
- kinesin complex
- membrane
- microtubule
- mitotic spindle
- nucleus
- protein-containing complex
- spindle
- spindle pole

Biological process

- cell division
- microtubule-based movement
- mitotic cell cycle
- mitotic centrosome separation
- mitotic spindle assembly
- mitotic spindle organization
- regulation of mitotic centrosome separation
- spindle elongation
- spindle organization

---

2

- **Protein name:** Protein arginine N-methyltransferase 5
- **Organism:** Homo sapiens
- **Uniprot Accession Number:** O14744
- **Protein sequence length:** 637 aa
- **1D identity (%):** 11.56
- **1D identity (%) [Gaps excluded]:** 21.25
- **1D identity - Alignment Gaps:** 442
- **1D aligned content (<aminoacid>:%):** {'A': 5.36, 'S': 4.46, 'G': 8.04, 'C': 3.57, 'V': 6.25, 'D': 4.46, 'P': 11.61, 'K': 5.36, 'R': 9.82, 'L': 9.82, 'I': 3.57, 'F': 4.46, 'E': 5.36, 'H': 2.68, 'T': 4.46, 'W': 1.79, 'Q': 4.46, 'Y': 1.79, 'M': 0.89, 'N': 1.79}
- **Common reported functions (%):** 0.0
- **Common reported locations (%):** 28.57
- **Common reported processes (%):** 0.0

- **PDB ID:** 5EMM
- **Chain:** A
- **Crystallized protein length:** 625 aa
- **Resolution:** 2.37 Å
- **Associated domain:** SAM-dependent-MTase-PRMT-type
- **b-phipsi ():** 0.00533
- **w-rdist ():** 0.413078
- **t-alpha ():** 0.0
- **Chemical similarity (Tanimoto Index) (%):** 78.98
- **1D identity (%) [PDB]:** 4.19
- **1D identity (%) [Gaps excluded][PDB]:** 72.5
- **1D identity - Alignment Gaps [PDB]:** 1303
- **1D aligned content [PDB] (<aminoacid>:%):** {'R': 8.62, 'V': 12.07, 'D': 1.72, 'T': 5.17, 'L': 10.34, 'G': 8.62, 'A': 6.9, 'Q': 8.62, 'C': 3.45, 'M': 1.72, 'K': 5.17, 'N': 6.9, 'P': 8.62, 'S': 3.45, 'I': 3.45, 'F': 1.72, 'H': 1.72, 'Y': 1.72}
- **2D identity (%) [PDB]:** 26.21
- **2D identity (%) [Gaps excluded][PDB]:** 91.41
- **2D identity - Alignment Gaps [PDB]:** 811
- **2D aligned content [PDB] (<2D-fold>:%):** {'.': 16.78, 'E': 27.85, 'H': 38.59, 'T': 13.42, 'G': 3.02, 'B': 0.34}
- **3D similarity (TM-Score) (%) [PDB]:** 24.7

- **Gene name:** PRMT5
- **Entrez ID:** 1041900
- **RefSeq ID:** NM\_006109
- **Transcript sequence length:** 2304
- **5-UTR|CDS|3-UTR identity (%):** 6.87 | 41.77 | 2.04
- **5-UTR|CDS|3-UTR identity (%) [Gaps excluded]:** 81.82 | 75.41 | 73.64
- **5-UTR|CDS|3-UTR identity [Alignment Gaps]:** 120 | 1290 | 11603
- **5-UTR aligned content (<base>:%):** {'G': 77.78, 'C': 11.11, 'A': 11.11}
- **CDS aligned content (<base>:%):** {'A': 23.18, 'T': 20.12, 'G': 28.56, 'C': 28.15}
- **3-UTR aligned content (<base>:%):** {'C': 24.28, 'G': 20.99, 'A': 23.46, 'T': 31.28}

**Uniprot Description:**  
  
 Arginine methyltransferase that can both catalyze the formation of omega-N monomethylarginine (MMA) and symmetrical dimethylarginine (sDMA), with a preference for the formation of MMA (PubMed:10531356, PubMed:11152681, PubMed:11747828, PubMed:12411503, PubMed:15737618, PubMed:17709427, PubMed:20159986, PubMed:20810653, PubMed:21258366, PubMed:21917714, PubMed:22269951, PubMed:21081503). Specifically mediates the symmetrical dimethylation of arginine residues in the small nuclear ribonucleoproteins Sm D1 (SNRPD1) and Sm D3 (SNRPD3); such methylation being required for the assembly and biogenesis of snRNP core particles (PubMed:12411503, PubMed:11747828, PubMed:17709427). Methylates SUPT5H and may regulate its transcriptional elongation properties (PubMed:12718890). Mono- and dimethylates arginine residues of myelin basic protein (MBP) in vitro. May play a role in cytokine-activated transduction pathways. Negatively regulates cyclin E1 promoter activity and cellular proliferation. Methylates histone H2A and H4 'Arg-3' during germ cell development. Methylates histone H3 'Arg-8', which may repress transcription. Methylates the Piwi proteins (PIWIL1, PIWIL2 and PIWIL4), methylation of Piwi proteins being required for the interaction with Tudor domain-containing proteins and subsequent localization to the meiotic nuage (By similarity). Methylates RPS10. Attenuates EGF signaling through the MAPK1/MAPK3 pathway acting at 2 levels. First, monomethylates EGFR; this enhances EGFR 'Tyr-1197' phosphorylation and PTPN6 recruitment, eventually leading to reduced SOS1 phosphorylation (PubMed:21917714, PubMed:21258366). Second, methylates RAF1 and probably BRAF, hence destabilizing these 2 signaling proteins and reducing their catalytic activity (PubMed:21917714). Required for induction of E-selectin and VCAM-1, on the endothelial cells surface at sites of inflammation. Methylates HOXA9 (PubMed:22269951). Methylates and regulates SRGAP2 which is involved in cell migration and differentiation (PubMed:20810653). Acts as a transcriptional corepressor in CRY1-mediated repression of the core circadian component PER1 by regulating the H4R3 dimethylation at the PER1 promoter (By similarity). Methylates GM130/GOLGA2, regulating Golgi ribbon formation (PubMed:20421892). Methylates H4R3 in genes involved in glioblastomagenesis in a CHTOP- and/or TET1-dependent manner (PubMed:25284789). Symmetrically methylates POLR2A, a modification that allows the recruitment to POLR2A of proteins including SMN1/SMN2 and SETX. This is required for resolving RNA-DNA hybrids created by RNA polymerase II, that form R-loop in transcription terminal regions, an important step in proper transcription termination (PubMed:26700805). Along with LYAR, binds the promoter of gamma-globin HBG1/HBG2 and represses its expression (PubMed:25092918). Symmetrically methylates NCL (PubMed:21081503). Methylates TP53; methylation might possibly affect TP53 target gene specificity (PubMed:19011621). Involved in spliceosome maturation and mRNA splicing in prophase I spermatocytes through the catalysis of the symmetrical arginine dimethylation of SNRPB (small nuclear ribonucleoprotein-associated protein) and the interaction with tudor domain-containing protein TDRD6 (By similarity).   
  
Forms, at least, homodimers and homotetramers (PubMed:11152681). Component of the methylosome complex, composed of PRMT5, WDR77 and CLNS1A (PubMed:21081503). Found in a complex composed of PRMT5, WDR77 and RIOK1 (PubMed:21081503). RIOK1 and CLNS1A associate with PRMT5 in a mutually exclusive fashion, which allows the recruitment of distinct methylation substrates, such as nucleolin/NCL and Sm proteins, respectively (PubMed:21081503). Interacts with PRDM1 (By similarity). Identified in a complex composed of methylosome and PRMT1 and ERH (PubMed:25284789). Interacts with EGFR; methylates EGFR and stimulates EGFR-mediated ERK activation. Interacts with HOXA9. Interacts with SRGAP2. Found in a complex with COPRS, RUNX1 and CBFB. Interacts with CHTOP; the interaction symmetrically methylates CHTOP, but seems to require the presence of PRMT1 (PubMed:25284789). Interacts with EPB41L3; this modulates methylation of target proteins. Component of a high molecular weight E2F-pocket protein complex, CERC (cyclin E1 repressor complex). Associates with SWI/SNF remodeling complexes containing SMARCA2 and SMARCA4. Interacts with JAK2, SSTR1, SUPT5H, BRAF and with active RAF1. Interacts with LSM11, PRMT7 and SNRPD3 (PubMed:17709427, PubMed:16087681). Interacts with COPRS; promoting its recruitment on histone H4. Interacts with CLNS1A/pICln (PubMed:21081503, PubMed:9556550). Identified in a complex with CLNS1A/pICln and Sm proteins. Interacts with RPS10 (PubMed:20159986). Interacts with WDR77. Interacts with IWS1. Interacts with CRY1. Interacts with POLR2A (PubMed:26700805). Interacts with SMN1/SMN2 (PubMed:26700805). Interacts with LYAR; this interaction is direct (PubMed:25092918). Interacts with STRAP (PubMed:19011621). Interacts with TP53 in response to DNA damage; the interaction is STRAP dependent (PubMed:19011621). Interacts with TDRD6 (By similarity).   
  
 **Gene Ontology Information:**

Molecular Function

- E-box binding
- histone-arginine N-methyltransferase activity
- histone methyltransferase activity (H4-R3 specific)
- identical protein binding
- methyl-CpG binding
- methyltransferase activity
- p53 binding
- protein heterodimerization activity
- protein-arginine N-methyltransferase activity
- protein-arginine omega-N symmetric methyltransferase activity
- ribonucleoprotein complex binding
- transcription corepressor activity

Location

- chromatin
- cytoplasm
- cytosol
- Golgi apparatus
- histone methyltransferase complex
- methylosome
- nucleoplasm
- nucleus

Biological process

- circadian regulation of gene expression
- DNA-templated transcription, termination
- endothelial cell activation
- Golgi ribbon formation
- histone arginine methylation
- histone H4-R3 methylation
- liver regeneration
- negative regulation of cell differentiation
- peptidyl-arginine methylation
- peptidyl-arginine N-methylation
- positive regulation of adenylate cyclase-inhibiting dopamine receptor signaling pathway
- positive regulation of mRNA splicing, via spliceosome
- positive regulation of oligodendrocyte differentiation
- regulation of DNA methylation
- regulation of transcription, DNA-templated
- regulation of ERK1 and ERK2 cascade
- regulation of mitotic nuclear division
- regulation of signal transduction by p53 class mediator
- spliceosomal snRNP assembly

---

3

- **Protein name:** Lymphokine-activated killer T-cell-originated protein kinase
- **Organism:** Homo sapiens
- **Uniprot Accession Number:** Q96KB5
- **Protein sequence length:** 322 aa
- **1D identity (%):** 5.8
- **1D identity (%) [Gaps excluded]:** 18.25
- **1D identity - Alignment Gaps:** 611
- **1D aligned content (<aminoacid>:%):** {'M': 1.92, 'T': 5.77, 'P': 13.46, 'L': 11.54, 'S': 3.85, 'K': 7.69, 'I': 3.85, 'Q': 3.85, 'G': 7.69, 'V': 5.77, 'D': 11.54, 'Y': 5.77, 'A': 9.62, 'E': 3.85, 'N': 1.92, 'R': 1.92}
- **Common reported functions (%):** 0.0
- **Common reported locations (%):** 7.14
- **Common reported processes (%):** 0.0

- **PDB ID:** 5J0A
- **Chain:** A
- **Crystallized protein length:** 299 aa
- **Resolution:** 2.74 Å
- **Associated domain:** Protein-kinase
- **b-phipsi ():** 0.012773
- **w-rdist ():** 0.196343
- **t-alpha ():** 0.003018
- **Chemical similarity (Tanimoto Index) (%):** 97.77
- **1D identity (%) [PDB]:** 1.16
- **1D identity (%) [Gaps excluded][PDB]:** 72.22
- **1D identity - Alignment Gaps [PDB]:** 1101
- **1D aligned content [PDB] (<aminoacid>:%):** {'T': 7.69, 'R': 7.69, 'S': 15.38, 'V': 7.69, 'I': 7.69, 'P': 7.69, 'A': 23.08, 'Y': 15.38, 'L': 7.69}
- **2D identity (%) [PDB]:** 18.12
- **2D identity (%) [Gaps excluded][PDB]:** 80.0
- **2D identity - Alignment Gaps [PDB]:** 717
- **2D aligned content [PDB] (<2D-fold>:%):** {'E': 13.1, '.': 22.02, 'H': 52.98, 'T': 10.71, 'B': 1.19}
- **3D similarity (TM-Score) (%) [PDB]:** 12.56

- **Gene name:** PBK
- **Entrez ID:** 5587200
- **RefSeq ID:** N/A
- **Sequence length:** N/A
- **5-UTR|CDS|3-UTR identity (%):** N/A | N/A | N/A
- **5-UTR|CDS|3-UTR identity (%) [Gaps excluded]:** N/A | N/A | N/A
- **5-UTR|CDS|3-UTR identity [Alignment Gaps]:** N/A | N/A | N/A
- **5-UTR aligned content (<base>:%):** N/A
- **CDS aligned content (<base>:%):** N/A
- **3-UTR aligned content (<base>:%):** N/A

**Uniprot Description:**  
  
 Phosphorylates MAP kinase p38. Seems to be active only in mitosis. May also play a role in the activation of lymphoid cells. When phosphorylated, forms a complex with TP53, leading to TP53 destabilization and attenuation of G2/M checkpoint during doxorubicin-induced DNA damage.   
  
Interacts with DLG1 and TP53.   
  
 **Gene Ontology Information:**

Molecular Function

- ATP binding
- MAP kinase kinase activity
- protein serine kinase activity
- protein serine/threonine kinase activity
- protein tyrosine kinase activity

Location

- nucleus

Biological process

- cellular response to UV
- mitotic cell cycle
- negative regulation of inflammatory response
- negative regulation of proteasomal ubiquitin-dependent protein catabolic process
- negative regulation of protein phosphorylation
- negative regulation of stress-activated MAPK cascade
- peptidyl-serine phosphorylation
- proteasome-mediated ubiquitin-dependent protein catabolic process
- stress-activated MAPK cascade

---

4

- **Protein name:** Cyclin-dependent kinase 4
- **Organism:** Homo sapiens
- **Uniprot Accession Number:** P11802
- **Protein sequence length:** 303 aa
- **1D identity (%):** 6.99
- **1D identity (%) [Gaps excluded]:** 25.91
- **1D identity - Alignment Gaps:** 668
- **1D aligned content (<aminoacid>:%):** {'A': 10.94, 'P': 12.5, 'E': 1.56, 'G': 12.5, 'T': 1.56, 'V': 6.25, 'K': 7.81, 'R': 7.81, 'H': 1.56, 'L': 10.94, 'S': 4.69, 'D': 3.12, 'Y': 1.56, 'M': 1.56, 'I': 3.12, 'W': 3.12, 'Q': 6.25, 'C': 1.56, 'F': 1.56}
- **Common reported functions (%):** 0.0
- **Common reported locations (%):** 28.57
- **Common reported processes (%):** 0.0

- **PDB ID:** 3G33
- **Chain:** A
- **Crystallized protein length:** 291 aa
- **Resolution:** 3.0 Å
- **Associated domain:** Protein-kinase
- **b-phipsi ():** 0.002192
- **w-rdist ():** 0.228603
- **t-alpha ():** 0.026859
- **Chemical similarity (Tanimoto Index) (%):** 99.09
- **1D identity (%) [PDB]:** 1.92
- **1D identity (%) [Gaps excluded][PDB]:** 63.64
- **1D identity - Alignment Gaps [PDB]:** 1063
- **1D aligned content [PDB] (<aminoacid>:%):** {'R': 9.52, 'Y': 4.76, 'P': 14.29, 'V': 14.29, 'G': 19.05, 'F': 4.76, 'A': 4.76, 'S': 14.29, 'C': 4.76, 'Q': 4.76, 'L': 4.76}
- **2D identity (%) [PDB]:** 18.35
- **2D identity (%) [Gaps excluded][PDB]:** 86.8
- **2D identity - Alignment Gaps [PDB]:** 735
- **2D aligned content [PDB] (<2D-fold>:%):** {'E': 18.71, '.': 25.73, 'T': 15.2, 'H': 40.35}
- **3D similarity (TM-Score) (%) [PDB]:** 13.9

- **Gene name:** CDK4
- **Entrez ID:** 101900
- **RefSeq ID:** NM\_000075
- **Transcript sequence length:** 1865
- **5-UTR|CDS|3-UTR identity (%):** 48.52 | 21.24 | 4.1
- **5-UTR|CDS|3-UTR identity (%) [Gaps excluded]:** 72.57 | 76.75 | 73.21
- **5-UTR|CDS|3-UTR identity [Alignment Gaps]:** 56 | 1978 | 11342
- **5-UTR aligned content (<base>:%):** {'A': 3.66, 'G': 43.9, 'C': 45.12, 'T': 7.32}
- **CDS aligned content (<base>:%):** {'A': 22.72, 'T': 19.1, 'G': 29.26, 'C': 28.92}
- **3-UTR aligned content (<base>:%):** {'C': 17.07, 'A': 30.69, 'G': 19.92, 'T': 32.32}

**Uniprot Description:**  
  
 Ser/Thr-kinase component of cyclin D-CDK4 (DC) complexes that phosphorylate and inhibit members of the retinoblastoma (RB) protein family including RB1 and regulate the cell-cycle during G(1)/S transition. Phosphorylation of RB1 allows dissociation of the transcription factor E2F from the RB/E2F complexes and the subsequent transcription of E2F target genes which are responsible for the progression through the G(1) phase. Hypophosphorylates RB1 in early G(1) phase. Cyclin D-CDK4 complexes are major integrators of various mitogenenic and antimitogenic signals. Also phosphorylates SMAD3 in a cell-cycle-dependent manner and represses its transcriptional activity. Component of the ternary complex, cyclin D/CDK4/CDKN1B, required for nuclear translocation and activity of the cyclin D-CDK4 complex.   
  
Component of the D-CDK4 complex, composed of CDK4 and some D-type G1 cyclin (CCND1, CCND2 or CCND3). Interacts directly in the complex with CCND1, CCND2 or CCND3. Interacts with SEI1 and ZNF655. Forms a ternary complex, cyclin D-CDK4-CDKN1B, involved in modulating CDK4 enzymatic activity. Interacts directly with CDKN1B (phosphorylated on 'Tyr-88' and 'Tyr-89'); the interaction allows assembly of the cyclin D-CDK4 complex, Thr-172 phosphorylation, nuclear translocation and enhances the cyclin D-CDK4 complex activity. CDK4 activity is either inhibited or enhanced depending on stoichiometry of complex. The non-tyrosine-phosphorylated form of CDKN1B prevents T-loop phosphorylation of CDK4 producing inactive CDK4. Interacts (unphosphorylated form) with CDK2. Also forms ternary complexes with CDKN1A or CDKN2A. Interacts directly with CDKN1A (via its N-terminal); the interaction promotes the assembly of the cyclin D-CDK4 complex, its nuclear translocation and promotes the cyclin D-dependent enzyme activity of CDK4. Interacts with CCND1; the interaction is prevented with the binding of CCND1 to INSM1 during cell cycle progression. Probably forms a complex composed of chaperones HSP90 and HSP70, co-chaperones CDC37, PPP5C, TSC1 and client protein TSC2, CDK4, AKT, RAF1 and NR3C1; this complex does not contain co-chaperones STIP1/HOP and PTGES3/p23 (PubMed:29127155). Interacts with CEBPA (when phosphorylated) (PubMed:15107404). Interacts with FNIP1 and FNIP2 (PubMed:27353360).   
  
 **Gene Ontology Information:**

Molecular Function

- ATP binding
- cyclin binding
- cyclin-dependent protein serine/threonine kinase activity
- cyclin-dependent protein serine/threonine kinase regulator activity
- protein serine kinase activity

Location

- bicellular tight junction
- chromatin
- cyclin D1-CDK4 complex
- cyclin D2-CDK4 complex
- cyclin D3-CDK4 complex
- cyclin-dependent protein kinase holoenzyme complex
- cytoplasm
- cytosol
- nuclear membrane
- nucleolus
- nucleoplasm
- nucleus
- transcription regulator complex

Biological process

- cell division
- cellular response to interleukin-4
- cellular response to ionomycin
- cellular response to lipopolysaccharide
- cellular response to phorbol 13-acetate 12-myristate
- G1/S transition of mitotic cell cycle
- positive regulation of cell population proliferation
- positive regulation of fibroblast proliferation
- positive regulation of G2/M transition of mitotic cell cycle
- protein phosphorylation
- regulation of cell cycle
- regulation of G2/M transition of mitotic cell cycle
- regulation of gene expression
- regulation of transcription initiation from RNA polymerase II promoter
- regulation of type B pancreatic cell proliferation
- response to organic substance
- response to xenobiotic stimulus
- signal transduction

---

5

- **Protein name:** Mitogen-activated protein kinase 1
- **Organism:** Homo sapiens
- **Uniprot Accession Number:** P28482
- **Protein sequence length:** 360 aa
- **1D identity (%):** 7.73
- **1D identity (%) [Gaps excluded]:** 23.59
- **1D identity - Alignment Gaps:** 617
- **1D aligned content (<aminoacid>:%):** {'A': 5.63, 'P': 9.86, 'G': 7.04, 'F': 4.23, 'R': 5.63, 'N': 4.23, 'I': 7.04, 'K': 8.45, 'C': 1.41, 'E': 7.04, 'Y': 5.63, 'L': 8.45, 'D': 5.63, 'T': 2.82, 'S': 7.04, 'H': 1.41, 'V': 4.23, 'W': 1.41, 'M': 1.41, 'Q': 1.41}
- **Common reported functions (%):** 0.0
- **Common reported locations (%):** 28.57
- **Common reported processes (%):** 0.0

- **PDB ID:** 4QTE
- **Chain:** A
- **Crystallized protein length:** 348 aa
- **Resolution:** 1.5 Å
- **Associated domain:** Protein-kinase
- **b-phipsi ():** 0.010652
- **w-rdist ():** 0.251454
- **t-alpha ():** 0.003027
- **Chemical similarity (Tanimoto Index) (%):** 87.08
- **1D identity (%) [PDB]:** 2.27
- **1D identity (%) [Gaps excluded][PDB]:** 65.0
- **1D identity - Alignment Gaps [PDB]:** 1106
- **1D aligned content [PDB] (<aminoacid>:%):** {'P': 7.69, 'G': 7.69, 'E': 7.69, 'K': 7.69, 'R': 7.69, 'V': 3.85, 'I': 3.85, 'S': 11.54, 'C': 3.85, 'L': 19.23, 'Q': 7.69, 'H': 3.85, 'D': 3.85, 'A': 3.85}
- **2D identity (%) [PDB]:** 26.43
- **2D identity (%) [Gaps excluded][PDB]:** 80.55
- **2D identity - Alignment Gaps [PDB]:** 600
- **2D aligned content [PDB] (<2D-fold>:%):** {'.': 16.53, 'E': 21.61, 'T': 10.59, 'H': 49.58, 'B': 0.42, 'G': 1.27}
- **3D similarity (TM-Score) (%) [PDB]:** 15.06

- **Gene name:** MAPK1
- **Entrez ID:** 559400
- **RefSeq ID:** NM\_138957
- **Transcript sequence length:** 1514
- **5-UTR|CDS|3-UTR identity (%):** 38.96 | 26.14 | 1.07
- **5-UTR|CDS|3-UTR identity (%) [Gaps excluded]:** 82.2 | 74.55 | 74.85
- **5-UTR|CDS|3-UTR identity [Alignment Gaps]:** 131 | 1761 | 11737
- **5-UTR aligned content (<base>:%):** {'G': 45.36, 'C': 44.33, 'A': 3.09, 'T': 7.22}
- **CDS aligned content (<base>:%):** {'A': 27.22, 'T': 21.3, 'C': 27.79, 'G': 23.7}
- **3-UTR aligned content (<base>:%):** {'T': 42.97, 'G': 11.72, 'C': 7.81, 'A': 37.5}

**Uniprot Description:**  
  
 Serine/threonine kinase which acts as an essential component of the MAP kinase signal transduction pathway. MAPK1/ERK2 and MAPK3/ERK1 are the 2 MAPKs which play an important role in the MAPK/ERK cascade. They participate also in a signaling cascade initiated by activated KIT and KITLG/SCF. Depending on the cellular context, the MAPK/ERK cascade mediates diverse biological functions such as cell growth, adhesion, survival and differentiation through the regulation of transcription, translation, cytoskeletal rearrangements. The MAPK/ERK cascade plays also a role in initiation and regulation of meiosis, mitosis, and postmitotic functions in differentiated cells by phosphorylating a number of transcription factors. About 160 substrates have already been discovered for ERKs. Many of these substrates are localized in the nucleus, and seem to participate in the regulation of transcription upon stimulation. However, other substrates are found in the cytosol as well as in other cellular organelles, and those are responsible for processes such as translation, mitosis and apoptosis. Moreover, the MAPK/ERK cascade is also involved in the regulation of the endosomal dynamics, including lysosome processing and endosome cycling through the perinuclear recycling compartment (PNRC); as well as in the fragmentation of the Golgi apparatus during mitosis. The substrates include transcription factors (such as ATF2, BCL6, ELK1, ERF, FOS, HSF4 or SPZ1), cytoskeletal elements (such as CANX, CTTN, GJA1, MAP2, MAPT, PXN, SORBS3 or STMN1), regulators of apoptosis (such as BAD, BTG2, CASP9, DAPK1, IER3, MCL1 or PPARG), regulators of translation (such as EIF4EBP1) and a variety of other signaling-related molecules (like ARHGEF2, DCC, FRS2 or GRB10). Protein kinases (such as RAF1, RPS6KA1/RSK1, RPS6KA3/RSK2, RPS6KA2/RSK3, RPS6KA6/RSK4, SYK, MKNK1/MNK1, MKNK2/MNK2, RPS6KA5/MSK1, RPS6KA4/MSK2, MAPKAPK3 or MAPKAPK5) and phosphatases (such as DUSP1, DUSP4, DUSP6 or DUSP16) are other substrates which enable the propagation the MAPK/ERK signal to additional cytosolic and nuclear targets, thereby extending the specificity of the cascade. Mediates phosphorylation of TPR in response to EGF stimulation. May play a role in the spindle assembly checkpoint. Phosphorylates PML and promotes its interaction with PIN1, leading to PML degradation. Phosphorylates CDK2AP2 (By similarity).   
  
Binds both upstream activators and downstream substrates in multimolecular complexes. This interaction inhibits its tyrosine-kinase activity. Interacts with ADAM15, ARHGEF2, ARRB2, DAPK1 (via death domain), HSF4, IER3, IPO7, DUSP6, NISCH, SGK1, and isoform 1 of NEK2. Interacts (via phosphorylated form) with TPR (via C-terminal region and phosphorylated form); the interaction requires dimerization of MAPK1/ERK2 and increases following EGF stimulation (PubMed:18794356). Interacts (phosphorylated form) with CAV2 ('Tyr-19'-phosphorylated form); the interaction, promoted by insulin, leads to nuclear location and MAPK1 activation. Interacts with MORG1, PEA15 and MKNK2 (By similarity). MKNK2 isoform 1 binding prevents from dephosphorylation and inactivation (By similarity). Interacts with DCC (By similarity). The phosphorylated form interacts with PML (isoform PML-4). Interacts with STYX. Interacts with CDK2AP2. Interacts with CAVIN4 (By similarity). Interacts with DUSP7; the interaction enhances DUSP7 phosphatase activity (PubMed:9788880). Interacts with GIT1; this interaction is necessary for MAPK1 localization to focal adhesions (By similarity). Interacts with ZNF263 (PubMed:32051553).   
  
 **Gene Ontology Information:**

Molecular Function

- ATP binding
- DNA binding
- identical protein binding
- MAP kinase activity
- MAP kinase kinase activity
- phosphatase binding
- phosphotyrosine residue binding
- protein serine kinase activity
- protein serine/threonine kinase activity
- RNA polymerase II CTD heptapeptide repeat kinase activity

Location

- azurophil granule lumen
- caveola
- cytoplasm
- cytoskeleton
- cytosol
- early endosome
- endoplasmic reticulum lumen
- extracellular region
- ficolin-1-rich granule lumen
- focal adhesion
- Golgi apparatus
- late endosome
- microtubule organizing center
- mitochondrion
- mitotic spindle
- nucleoplasm
- nucleus
- plasma membrane
- pseudopodium
- synapse

Biological process

- androgen receptor signaling pathway
- apoptotic process
- B cell receptor signaling pathway
- Bergmann glial cell differentiation
- cardiac neural crest cell development involved in heart development
- caveolin-mediated endocytosis
- cell cycle
- cell surface receptor signaling pathway
- cellular response to amino acid starvation
- cellular response to cadmium ion
- cellular response to reactive oxygen species
- cellular response to tumor necrosis factor
- chemical synaptic transmission
- chemotaxis
- cytosine metabolic process
- cellular response to DNA damage stimulus
- ERBB signaling pathway
- ERK1 and ERK2 cascade
- face development
- insulin receptor signaling pathway
- insulin-like growth factor receptor signaling pathway
- intracellular signal transduction
- labyrinthine layer blood vessel development
- learning or memory
- lipopolysaccharide-mediated signaling pathway
- long-term synaptic potentiation
- lung morphogenesis
- mammary gland epithelial cell proliferation
- negative regulation of cell differentiation
- outer ear morphogenesis
- peptidyl-serine phosphorylation
- peptidyl-threonine phosphorylation
- positive regulation of macrophage chemotaxis
- positive regulation of macrophage proliferation
- positive regulation of peptidyl-threonine phosphorylation
- positive regulation of telomerase activity
- positive regulation of telomere capping
- positive regulation of telomere maintenance via telomerase
- progesterone receptor signaling pathway
- protein phosphorylation
- regulation of cellular pH
- regulation of cytoskeleton organization
- regulation of early endosome to late endosome transport
- regulation of Golgi inheritance
- regulation of ossification
- regulation of protein stability
- regulation of stress-activated MAPK cascade
- response to epidermal growth factor
- response to exogenous dsRNA
- response to nicotine
- signal transduction
- steroid hormone mediated signaling pathway
- stress-activated MAPK cascade
- T cell receptor signaling pathway
- thymus development
- thyroid gland development
- trachea formation

---

6

- **Protein name:** Serine/threonine-protein kinase Nek2
- **Organism:** Homo sapiens
- **Uniprot Accession Number:** P51955
- **Protein sequence length:** 445 aa
- **1D identity (%):** 10.21
- **1D identity (%) [Gaps excluded]:** 23.66
- **1D identity - Alignment Gaps:** 518
- **1D aligned content (<aminoacid>:%):** {'R': 10.75, 'I': 3.23, 'Q': 3.23, 'V': 7.53, 'L': 16.13, 'D': 2.15, 'S': 4.3, 'E': 5.38, 'K': 10.75, 'H': 3.23, 'T': 5.38, 'G': 5.38, 'P': 10.75, 'N': 3.23, 'M': 1.08, 'A': 4.3, 'F': 1.08, 'C': 2.15}
- **Common reported functions (%):** 6.67
- **Common reported locations (%):** 28.57
- **Common reported processes (%):** 0.0

- **PDB ID:** 2XK4
- **Chain:** A
- **Crystallized protein length:** 252 aa
- **Resolution:** 2.1 Å
- **Associated domain:** Protein-kinase
- **b-phipsi ():** 0.017608
- **w-rdist ():** 0.353253
- **t-alpha ():** 0.0
- **Chemical similarity (Tanimoto Index) (%):** 89.18
- **1D identity (%) [PDB]:** 1.21
- **1D identity (%) [Gaps excluded][PDB]:** 65.0
- **1D identity - Alignment Gaps [PDB]:** 1054
- **1D aligned content [PDB] (<aminoacid>:%):** {'S': 23.08, 'R': 15.38, 'Y': 7.69, 'V': 7.69, 'L': 15.38, 'N': 7.69, 'F': 7.69, 'D': 7.69, 'E': 7.69}
- **2D identity (%) [PDB]:** 19.53
- **2D identity (%) [Gaps excluded][PDB]:** 85.71
- **2D identity - Alignment Gaps [PDB]:** 688
- **2D aligned content [PDB] (<2D-fold>:%):** {'.': 17.82, 'E': 19.54, 'T': 13.22, 'H': 47.7, 'G': 1.72}
- **3D similarity (TM-Score) (%) [PDB]:** 13.63

- **Gene name:** NEK2
- **Entrez ID:** 475100
- **RefSeq ID:** N/A
- **Sequence length:** N/A
- **5-UTR|CDS|3-UTR identity (%):** N/A | N/A | N/A
- **5-UTR|CDS|3-UTR identity (%) [Gaps excluded]:** N/A | N/A | N/A
- **5-UTR|CDS|3-UTR identity [Alignment Gaps]:** N/A | N/A | N/A
- **5-UTR aligned content (<base>:%):** N/A
- **CDS aligned content (<base>:%):** N/A
- **3-UTR aligned content (<base>:%):** N/A

**Uniprot Description:**  
  
 Protein kinase which is involved in the control of centrosome separation and bipolar spindle formation in mitotic cells and chromatin condensation in meiotic cells. Regulates centrosome separation (essential for the formation of bipolar spindles and high-fidelity chromosome separation) by phosphorylating centrosomal proteins such as CROCC, CEP250 and NINL, resulting in their displacement from the centrosomes. Regulates kinetochore microtubule attachment stability in mitosis via phosphorylation of NDC80. Involved in regulation of mitotic checkpoint protein complex via phosphorylation of CDC20 and MAD2L1. Plays an active role in chromatin condensation during the first meiotic division through phosphorylation of HMGA2. Phosphorylates: PPP1CC; SGO1; NECAB3 and NPM1. Essential for localization of MAD2L1 to kinetochore and MAPK1 and NPM1 to the centrosome. Phosphorylates CEP68 and CNTLN directly or indirectly (PubMed:24554434). NEK2-mediated phosphorylation of CEP68 promotes CEP68 dissociation from the centrosome and its degradation at the onset of mitosis (PubMed:25704143). Involved in the regulation of centrosome disjunction (PubMed:26220856).   
  
Isoform 1, isoform 2 and isoform 4 form homo- and heterodimers. Interacts with NECAB3 and HMGA2 (By similarity). Isoform 1 interacts with CDC20, CTNB1, MAD1L1, MAPK, NEK11, NPM1, NDC80, PCNT and SGO1 (PubMed:14978040, PubMed:15358203, PubMed:15388344, PubMed:15161910, PubMed:17621308, PubMed:18086858, PubMed:18297113, PubMed:20599736, PubMed:20034488). Isoform 1 interacts with STK3/MST2 (via SARAH domain) and SAV1 (via SARAH domain) (PubMed:21076410). Isoform 1 and isoform 2 interact with MAD2L1 (PubMed:20034488). Isoform 1 and isoform 4 interact with PPP1CA and PPP1CC (PubMed:15659832, PubMed:17283141). Interacts with CEP68; the interaction leads to phosphorylation of CEP68. Interacts with CNTLN; the interaction leads to phosphorylation of CNTLN (PubMed:24554434). Isoform 1 interacts with CEP85 (PubMed:26220856).   
  
 **Gene Ontology Information:**

Molecular Function

- ATP binding
- metal ion binding
- protein kinase activity
- protein phosphatase binding
- protein serine kinase activity
- protein serine/threonine kinase activity

Location

- centrosome
- condensed nuclear chromosome
- cytoplasm
- cytosol
- kinetochore
- microtubule
- midbody
- nucleolus
- nucleoplasm
- nucleus
- protein-containing complex
- spindle pole

Biological process

- blastocyst development
- cell division
- centrosome separation
- chromosome segregation
- meiotic cell cycle
- mitotic cell cycle
- mitotic spindle assembly
- negative regulation of centriole-centriole cohesion
- negative regulation of DNA binding
- positive regulation of telomerase activity
- positive regulation of telomere capping
- positive regulation of telomere maintenance via telomerase
- protein autophosphorylation
- protein phosphorylation
- regulation of attachment of spindle microtubules to kinetochore
- regulation of mitotic centrosome separation
- regulation of mitotic nuclear division
- spindle assembly

---
