# Supplementary material for: Comparative structural insights and functional analysis for the distinct unbound states of Human AGO proteins
Source: Sci Rep. 2025 Mar 19;15:9432. doi: 10.1038/s41598-025-91849-5 (PMC11923369; doi:10.1038/s41598-025-91849-5)
Supplement: Supplementary file 24 — Supplementary Information 12. [file 41598_2025_91849_MOESM24_ESM.zip › 4Z4Dp_A_mdwhole_AF4REF/candidates/4Z4Dp_A-merged-enriched_eval_report.html]

 

# Structural Comparison Report for 4Z4Dp\_A - whole structures (total: 100)

---

1

- **Protein name:** Protein argonaute-1
- **Organism:** Homo sapiens
- **Uniprot Accession Number:** Q9UL18
- **Protein sequence length:** 857 aa
- **1D identity (%):** 82.58
- **1D identity (%) [Gaps excluded]:** 83.16
- **1D identity - Alignment Gaps:** 6
- **1D aligned content (<aminoacid>:%):** {'A': 6.19, 'G': 7.03, 'P': 6.61, 'Q': 6.61, 'F': 4.36, 'R': 6.33, 'T': 6.05, 'I': 5.91, 'K': 6.33, 'L': 7.17, 'N': 2.67, 'E': 4.92, 'D': 4.78, 'Y': 3.66, 'H': 3.23, 'C': 2.81, 'V': 8.02, 'M': 2.25, 'S': 4.22, 'W': 0.84}
- **Common reported functions (%):** 40.0
- **Common reported locations (%):** 71.43
- **Common reported processes (%):** 28.57

- **AF ID:** Q9UL18
- **Chain:** A
- **Protein length:** 857 aa
- **Resolution:** N/A
- **b-phipsi:** 0.000307
- **w-rdist:** 0.241401
- **t-alpha:** 0.033886
- **Chemical similarity (Tanimoto Index) (%):** 100.0
- **1D identity (%) [PDB]:** 81.98
- **1D identity (%) [Gaps excluded][PDB]:** 84.43
- **1D identity - Alignment Gaps [PDB]:** 25
- **1D aligned content [PDB] (<aminoacid>:%):** {'F': 4.4, 'P': 6.24, 'R': 6.38, 'G': 6.95, 'T': 6.1, 'I': 6.1, 'K': 6.38, 'L': 7.38, 'A': 5.82, 'N': 2.7, 'E': 4.96, 'D': 4.82, 'Y': 3.69, 'H': 3.26, 'C': 2.84, 'V': 8.09, 'M': 2.27, 'Q': 6.52, 'S': 4.26, 'W': 0.85}
- **2D identity (%) [PDB]:** 91.96
- **2D identity (%) [Gaps excluded][PDB]:** 94.27
- **2D identity - Alignment Gaps [PDB]:** 21
- **2D aligned content [PDB] (<2D-fold>:%):** {'.': 19.77, 'B': 1.65, 'E': 27.38, 'T': 18.5, 'H': 30.29, 'G': 2.28, 'b': 0.13}
- **3D similarity (TM-Score) (%) [PDB]:** 96.6

- **Gene name:** AGO1
- **Entrez ID:** 26523
- **RefSeq ID:** NM\_012199
- **Transcript sequence length:** 13712
- **5-UTR|CDS|3-UTR identity (%):** 42.22 | 75.21 | 44.87
- **5-UTR|CDS|3-UTR identity (%) [Gaps excluded]:** 82.61 | 77.64 | 73.98
- **5-UTR|CDS|3-UTR identity [Alignment Gaps]:** 110 | 82 | 5587
- **5-UTR aligned content (<base>:%):** {'T': 3.16, 'G': 49.47, 'C': 44.21, 'A': 3.16}
- **CDS aligned content (<base>:%):** {'A': 24.94, 'T': 19.3, 'G': 27.78, 'C': 27.98}
- **3-UTR aligned content (<base>:%):** {'A': 25.3, 'G': 22.05, 'C': 18.97, 'T': 33.68}

**Uniprot Description:**  
  
 Required for RNA-mediated gene silencing (RNAi). Binds to short RNAs such as microRNAs (miRNAs) or short interfering RNAs (siRNAs), and represses the translation of mRNAs which are complementary to them. Lacks endonuclease activity and does not appear to cleave target mRNAs. Also required for transcriptional gene silencing (TGS) of promoter regions which are complementary to bound short antigene RNAs (agRNAs).   
  
Interacts with DDB1, DDX5, DDX6, DHX30, DHX36, DDX47, DICER1, AGO2, ELAVL1, HNRNPF, IGF2BP1, ILF3, IMP8, MATR3, MOV10, PABPC1, PRMT5, RBM4, SART3, TNRC6B, UPF1 and YBX1. Associates with polysomes and messenger ribonucleoproteins (mNRPs). Interacts with LIMD1, WTIP and AJUBA. Interacts with APOBEC3F, APOBEC3G and APOBEC3H.   
  
 **Gene Ontology Information:**

Molecular Function

- core promoter sequence-specific DNA binding
- double-stranded RNA binding
- miRNA binding
- RNA binding
- RNA polymerase II complex binding
- single-stranded RNA binding

Location

- cytoplasm
- cytoplasmic ribonucleoprotein granule
- cytosol
- nucleoplasm
- nucleus
- P-body
- polysome
- ribonucleoprotein complex
- RISC complex
- RISC-loading complex

Biological process

- production of miRNAs involved in gene silencing by miRNA
- miRNA mediated inhibition of translation
- negative regulation of angiogenesis
- nuclear-transcribed mRNA catabolic process
- positive regulation of transcription by RNA polymerase II
- pre-miRNA processing
- small RNA loading onto RISC
- RNA secondary structure unwinding

---

2

- **Protein name:** Protein argonaute-3
- **Organism:** Homo sapiens
- **Uniprot Accession Number:** Q9H9G7
- **Protein sequence length:** 860 aa
- **1D identity (%):** 79.52
- **1D identity (%) [Gaps excluded]:** 81.29
- **1D identity - Alignment Gaps:** 19
- **1D aligned content (<aminoacid>:%):** {'M': 2.17, 'G': 7.09, 'A': 6.8, 'P': 6.8, 'R': 6.66, 'T': 6.37, 'I': 5.21, 'K': 5.35, 'L': 7.67, 'N': 2.32, 'F': 4.49, 'D': 4.78, 'Y': 3.62, 'E': 4.78, 'C': 2.75, 'V': 8.54, 'Q': 6.08, 'H': 3.47, 'S': 4.34, 'W': 0.72}
- **Common reported functions (%):** 46.67
- **Common reported locations (%):** 57.14
- **Common reported processes (%):** 23.81

- **AF ID:** Q9H9G7
- **Chain:** A
- **Protein length:** 860 aa
- **Resolution:** N/A
- **b-phipsi:** 0.000233
- **w-rdist:** 0.227214
- **t-alpha:** 0.043313
- **Chemical similarity (Tanimoto Index) (%):** 100.0
- **1D identity (%) [PDB]:** 79.24
- **1D identity (%) [Gaps excluded][PDB]:** 82.67
- **1D identity - Alignment Gaps [PDB]:** 36
- **1D aligned content [PDB] (<aminoacid>:%):** {'P': 6.7, 'R': 6.84, 'G': 6.84, 'T': 6.4, 'I': 5.24, 'K': 5.53, 'L': 7.86, 'A': 6.4, 'N': 2.33, 'F': 4.51, 'D': 4.8, 'Y': 3.64, 'E': 4.8, 'C': 2.77, 'V': 8.59, 'M': 2.04, 'Q': 6.11, 'H': 3.49, 'S': 4.37, 'W': 0.73}
- **2D identity (%) [PDB]:** 91.59
- **2D identity (%) [Gaps excluded][PDB]:** 95.78
- **2D identity - Alignment Gaps [PDB]:** 38
- **2D aligned content [PDB] (<2D-fold>:%):** {'.': 19.12, 'B': 1.64, 'E': 27.3, 'T': 20.13, 'H': 29.81, 'G': 1.89, 'b': 0.13}
- **3D similarity (TM-Score) (%) [PDB]:** 95.67

- **Gene name:** AGO3
- **Entrez ID:** 192669
- **RefSeq ID:** N/A
- **Sequence length:** N/A
- **5-UTR|CDS|3-UTR identity (%):** N/A | N/A | N/A
- **5-UTR|CDS|3-UTR identity (%) [Gaps excluded]:** N/A | N/A | N/A
- **5-UTR|CDS|3-UTR identity [Alignment Gaps]:** N/A | N/A | N/A
- **5-UTR aligned content (<base>:%):** N/A
- **CDS aligned content (<base>:%):** N/A
- **3-UTR aligned content (<base>:%):** N/A

**Uniprot Description:**  
  
 Required for RNA-mediated gene silencing (RNAi). Binds to short RNAs such as microRNAs (miRNAs) and represses the translation of mRNAs which are complementary to them. Proposed to be involved in stabilization of small RNA derivates (siRNA) derived from processed RNA polymerase III-transcribed Alu repeats containing a DR2 retinoic acid response element (RARE) in stem cells and in the subsequent siRNA-dependent degradation of a subset of RNA polymerase II-transcribed coding mRNAs by recruiting a mRNA decapping complex involving EDC4. Possesses RNA slicer activity but only on select RNAs bearing 5'- and 3'-flanking sequences to the region of guide-target complementarity (PubMed:29040713).   
  
Interacts with EIF4B, IMP8, PRMT5 and TNRC6B. Interacts with APOBEC3F, APOBEC3G and APOBEC3H. Interacts with EDC4.   
  
 **Gene Ontology Information:**

Molecular Function

- double-stranded RNA binding
- endoribonuclease activity, cleaving miRNA-paired mRNA
- metal ion binding
- miRNA binding
- RNA binding
- endoribonuclease activity
- single-stranded RNA binding

Location

- condensed nuclear chromosome
- cytoplasm
- cytoplasmic ribonucleoprotein granule
- cytosol
- membrane
- nucleoplasm
- P-body
- RISC complex
- RISC-loading complex

Biological process

- production of miRNAs involved in gene silencing by miRNA
- miRNA mediated inhibition of translation
- mRNA catabolic process
- positive regulation of gene expression
- positive regulation of NIK/NF-kappaB signaling
- pre-miRNA processing
- regulation of stem cell proliferation
- small RNA loading onto RISC
- RNA secondary structure unwinding

---

3

- **Protein name:** Histone H4 transcription factor
- **Organism:** Homo sapiens
- **Uniprot Accession Number:** Q9BQA5
- **Protein sequence length:** 517 aa
- **1D identity (%):** 10.76
- **1D identity (%) [Gaps excluded]:** 24.58
- **1D identity - Alignment Gaps:** 538
- **1D aligned content (<aminoacid>:%):** {'P': 11.65, 'K': 5.83, 'R': 3.88, 'N': 2.91, 'E': 10.68, 'H': 4.85, 'Q': 3.88, 'G': 9.71, 'S': 3.88, 'W': 1.94, 'C': 6.8, 'L': 4.85, 'D': 4.85, 'F': 4.85, 'A': 2.91, 'V': 3.88, 'T': 5.83, 'I': 2.91, 'M': 2.91, 'Y': 0.97}
- **Common reported functions (%):** 6.67
- **Common reported locations (%):** 7.14
- **Common reported processes (%):** 0.0

- **AF ID:** Q9BQA5
- **Chain:** A
- **Protein length:** 517 aa
- **Resolution:** N/A
- **b-phipsi:** 0.003609
- **w-rdist:** 0.243673
- **t-alpha:** 0.002913
- **Chemical similarity (Tanimoto Index) (%):** 95.71
- **1D identity (%) [PDB]:** 1.9
- **1D identity (%) [Gaps excluded][PDB]:** 67.57
- **1D identity - Alignment Gaps [PDB]:** 1281
- **1D aligned content [PDB] (<aminoacid>:%):** {'M': 4.0, 'P': 12.0, 'K': 16.0, 'V': 4.0, 'E': 12.0, 'G': 4.0, 'D': 4.0, 'R': 4.0, 'F': 4.0, 'S': 12.0, 'I': 4.0, 'W': 4.0, 'L': 8.0, 'Q': 4.0, 'A': 4.0}
- **2D identity (%) [PDB]:** 38.73
- **2D identity (%) [Gaps excluded][PDB]:** 89.27
- **2D identity - Alignment Gaps [PDB]:** 535
- **2D aligned content [PDB] (<2D-fold>:%):** {'.': 28.69, 'E': 14.75, 'H': 38.52, 'T': 16.94, 'B': 0.27, 'G': 0.82}
- **3D similarity (TM-Score) (%) [PDB]:** 18.95

- **Gene name:** HINFP
- **Entrez ID:** 511965
- **RefSeq ID:** N/A
- **Sequence length:** N/A
- **5-UTR|CDS|3-UTR identity (%):** N/A | N/A | N/A
- **5-UTR|CDS|3-UTR identity (%) [Gaps excluded]:** N/A | N/A | N/A
- **5-UTR|CDS|3-UTR identity [Alignment Gaps]:** N/A | N/A | N/A
- **5-UTR aligned content (<base>:%):** N/A
- **CDS aligned content (<base>:%):** N/A
- **3-UTR aligned content (<base>:%):** N/A

**Uniprot Description:**  
  
 Transcriptional repressor that binds to the consensus sequence 5'-CGGACGTT-3' and to the RB1 promoter. Transcriptional activator that promotes histone H4 gene transcription at the G1/S phase transition in conjunction with NPAT. Also activates transcription of the ATM and PRKDC genes. Autoregulates its expression by associating with its own promoter.   
  
Binds MBD2 and a histone deacetylase complex. Interacts with NPAT.   
  
 **Gene Ontology Information:**

Molecular Function

- DNA-binding transcription factor activity, RNA polymerase II-specific
- metal ion binding
- RNA polymerase II cis-regulatory region sequence-specific DNA binding

Location

- nucleus

Biological process

- anatomical structure development
- regulation of transcription by RNA polymerase II

---

4

- **Protein name:** Protein argonaute-4
- **Organism:** Homo sapiens
- **Uniprot Accession Number:** Q9HCK5
- **Protein sequence length:** 861 aa
- **1D identity (%):** 77.38
- **1D identity (%) [Gaps excluded]:** 79.39
- **1D identity - Alignment Gaps:** 22
- **1D aligned content (<aminoacid>:%):** {'P': 7.42, 'F': 4.45, 'R': 6.23, 'G': 7.27, 'T': 6.08, 'I': 5.19, 'L': 7.57, 'A': 6.08, 'N': 2.37, 'K': 5.49, 'D': 4.75, 'Y': 3.86, 'H': 3.12, 'E': 4.75, 'V': 8.16, 'M': 2.08, 'Q': 6.68, 'S': 4.75, 'W': 0.89, 'C': 2.82}
- **Common reported functions (%):** 20.0
- **Common reported locations (%):** 14.29
- **Common reported processes (%):** 4.76

- **AF ID:** Q9HCK5
- **Chain:** A
- **Protein length:** 861 aa
- **Resolution:** N/A
- **b-phipsi:** 0.000321
- **w-rdist:** 0.324692
- **t-alpha:** 0.040058
- **Chemical similarity (Tanimoto Index) (%):** 99.62
- **1D identity (%) [PDB]:** 77.84
- **1D identity (%) [Gaps excluded][PDB]:** 80.17
- **1D identity - Alignment Gaps [PDB]:** 25
- **1D aligned content [PDB] (<aminoacid>:%):** {'F': 4.47, 'P': 7.0, 'R': 6.26, 'G': 7.3, 'T': 6.11, 'I': 5.22, 'L': 7.6, 'A': 6.11, 'N': 2.38, 'K': 5.51, 'D': 4.77, 'Y': 3.87, 'H': 3.13, 'E': 4.77, 'V': 8.2, 'M': 2.09, 'Q': 6.71, 'S': 4.77, 'W': 0.89, 'C': 2.83}
- **2D identity (%) [PDB]:** 91.13
- **2D identity (%) [Gaps excluded][PDB]:** 95.19
- **2D identity - Alignment Gaps [PDB]:** 37
- **2D aligned content [PDB] (<2D-fold>:%):** {'.': 19.09, 'B': 1.64, 'E': 27.43, 'T': 19.6, 'H': 30.21, 'G': 1.9, 'b': 0.13}
- **3D similarity (TM-Score) (%) [PDB]:** 95.34

- **Gene name:** AGO4
- **Entrez ID:** 419629
- **RefSeq ID:** NM\_017629
- **Transcript sequence length:** 7272
- **5-UTR|CDS|3-UTR identity (%):** 25.74 | 69.33 | 21.24
- **5-UTR|CDS|3-UTR identity (%) [Gaps excluded]:** 87.5 | 74.74 | 75.77
- **5-UTR|CDS|3-UTR identity [Alignment Gaps]:** 288 | 194 | 9091
- **5-UTR aligned content (<base>:%):** {'G': 46.67, 'T': 5.71, 'C': 45.71, 'A': 1.9}
- **CDS aligned content (<base>:%):** {'A': 26.26, 'T': 20.99, 'G': 27.02, 'C': 25.73}
- **3-UTR aligned content (<base>:%):** {'G': 22.74, 'T': 33.92, 'C': 17.7, 'A': 25.64}

**Uniprot Description:**  
  
 Required for RNA-mediated gene silencing (RNAi). Binds to short RNAs such as microRNAs (miRNAs) and represses the translation of mRNAs which are complementary to them. Lacks endonuclease activity and does not appear to cleave target mRNAs. Also required for RNA-directed transcription and replication of the human hapatitis delta virus (HDV).   
  
Interacts with EIF4B, IMP8, PRMT5, TNRC6A and TNRC6B (PubMed:19167051). Interacts with ZFP36 (PubMed:15766526).   
  
 **Gene Ontology Information:**

Molecular Function

- miRNA binding
- endoribonuclease activity
- single-stranded RNA binding

Location

- P-body
- RISC complex

Biological process

- miRNA mediated inhibition of translation
- mRNA catabolic process

---

5

- **Protein name:** Alpha-mannosidase 2
- **Organism:** Homo sapiens
- **Uniprot Accession Number:** Q16706
- **Protein sequence length:** 1144 aa
- **1D identity (%):** 13.98
- **1D identity (%) [Gaps excluded]:** 23.3
- **1D identity - Alignment Gaps:** 501
- **1D aligned content (<aminoacid>:%):** {'K': 9.71, 'L': 10.29, 'F': 6.29, 'I': 6.29, 'D': 6.29, 'P': 6.86, 'R': 8.0, 'E': 4.57, 'H': 4.57, 'V': 7.43, 'G': 5.14, 'N': 4.0, 'W': 1.71, 'Q': 3.43, 'A': 2.29, 'M': 1.71, 'Y': 3.43, 'C': 1.14, 'T': 4.57, 'S': 2.29}
- **Common reported functions (%):** 6.67
- **Common reported locations (%):** 14.29
- **Common reported processes (%):** 0.0

- **AF ID:** Q16706
- **Chain:** A
- **Protein length:** 1144 aa
- **Resolution:** N/A
- **b-phipsi:** 0.000576
- **w-rdist:** 0.286697
- **t-alpha:** 0.046613
- **Chemical similarity (Tanimoto Index) (%):** 97.2
- **1D identity (%) [PDB]:** 2.95
- **1D identity (%) [Gaps excluded][PDB]:** 66.67
- **1D identity - Alignment Gaps [PDB]:** 1814
- **1D aligned content [PDB] (<aminoacid>:%):** {'Q': 8.93, 'G': 7.14, 'A': 3.57, 'S': 3.57, 'V': 17.86, 'P': 7.14, 'H': 1.79, 'L': 17.86, 'K': 7.14, 'N': 7.14, 'Y': 1.79, 'I': 3.57, 'D': 1.79, 'T': 7.14, 'M': 1.79, 'R': 1.79}
- **2D identity (%) [PDB]:** 36.96
- **2D identity (%) [Gaps excluded][PDB]:** 92.24
- **2D identity - Alignment Gaps [PDB]:** 848
- **2D aligned content [PDB] (<2D-fold>:%):** {'.': 22.37, 'T': 25.05, 'H': 33.08, 'E': 17.21, 'G': 1.72, 'B': 0.57}
- **3D similarity (TM-Score) (%) [PDB]:** 25.28

- **Gene name:** MAN2A1
- **Entrez ID:** 4124
- **RefSeq ID:** NM\_002372
- **Transcript sequence length:** 6553
- **5-UTR|CDS|3-UTR identity (%):** 21.37 | 42.41 | 13.38
- **5-UTR|CDS|3-UTR identity (%) [Gaps excluded]:** 86.89 | 72.92 | 75.01
- **5-UTR|CDS|3-UTR identity [Alignment Gaps]:** 374 | 1591 | 10121
- **5-UTR aligned content (<base>:%):** {'C': 42.45, 'T': 7.55, 'G': 47.17, 'A': 2.83}
- **CDS aligned content (<base>:%):** {'A': 28.33, 'T': 22.26, 'G': 24.92, 'C': 24.49}
- **3-UTR aligned content (<base>:%):** {'A': 29.85, 'C': 14.14, 'T': 39.5, 'G': 16.5}

**Uniprot Description:**  
  
 Catalyzes the first committed step in the biosynthesis of complex N-glycans. It controls conversion of high mannose to complex N-glycans; the final hydrolytic step in the N-glycan maturation pathway.   
  
Homodimer; disulfide-linked.   
  
 **Gene Ontology Information:**

Molecular Function

- alpha-mannosidase activity
- carbohydrate binding
- hydrolase activity, hydrolyzing N-glycosyl compounds
- mannosyl-oligosaccharide 1,3-1,6-alpha-mannosidase activity
- metal ion binding
- protein homodimerization activity

Location

- cis-Golgi network
- extracellular exosome
- Golgi medial cisterna
- Golgi membrane
- membrane

Biological process

- in utero embryonic development
- liver development
- lung alveolus development
- mannose metabolic process
- mitochondrion organization
- N-glycan processing
- positive regulation of neurogenesis
- protein deglycosylation
- protein glycosylation
- respiratory gaseous exchange by respiratory system
- retina morphogenesis in camera-type eye
- vacuole organization
- viral protein processing

---

6

- **Protein name:** Ubiquitin-associated and SH3 domain-containing protein B
- **Organism:** Homo sapiens
- **Uniprot Accession Number:** Q8TF42
- **Protein sequence length:** 649 aa
- **1D identity (%):** 12.5
- **1D identity (%) [Gaps excluded]:** 21.9
- **1D identity - Alignment Gaps:** 412
- **1D aligned content (<aminoacid>:%):** {'G': 10.83, 'P': 10.0, 'R': 5.0, 'L': 13.33, 'Y': 2.5, 'W': 2.5, 'S': 7.5, 'V': 5.0, 'D': 4.17, 'Q': 4.17, 'F': 5.83, 'K': 5.0, 'E': 5.83, 'H': 1.67, 'A': 4.17, 'C': 3.33, 'N': 2.5, 'T': 3.33, 'I': 2.5, 'M': 0.83}
- **Common reported functions (%):** 0.0
- **Common reported locations (%):** 14.29
- **Common reported processes (%):** 0.0

- **AF ID:** Q8TF42
- **Chain:** A
- **Protein length:** 649 aa
- **Resolution:** N/A
- **b-phipsi:** 0.000459
- **w-rdist:** 0.353034
- **t-alpha:** 0.048798
- **Chemical similarity (Tanimoto Index) (%):** 99.02
- **1D identity (%) [PDB]:** 2.52
- **1D identity (%) [Gaps excluded][PDB]:** 60.0
- **1D identity - Alignment Gaps [PDB]:** 1367
- **1D aligned content [PDB] (<aminoacid>:%):** {'A': 8.33, 'G': 11.11, 'P': 8.33, 'L': 8.33, 'R': 2.78, 'V': 13.89, 'Q': 8.33, 'S': 5.56, 'K': 5.56, 'N': 2.78, 'I': 2.78, 'D': 2.78, 'T': 2.78, 'F': 8.33, 'E': 8.33}
- **2D identity (%) [PDB]:** 39.42
- **2D identity (%) [Gaps excluded][PDB]:** 88.84
- **2D identity - Alignment Gaps [PDB]:** 573
- **2D aligned content [PDB] (<2D-fold>:%):** {'.': 23.21, 'H': 33.09, 'T': 21.48, 'E': 21.23, 'G': 0.74, 'B': 0.25}
- **3D similarity (TM-Score) (%) [PDB]:** 23.52

- **Gene name:** UBASH3B
- **Entrez ID:** 84959
- **RefSeq ID:** NM\_032873
- **Transcript sequence length:** 6865
- **5-UTR|CDS|3-UTR identity (%):** 30.42 | 40.76 | 21.68
- **5-UTR|CDS|3-UTR identity (%) [Gaps excluded]:** 82.11 | 74.42 | 74.25
- **5-UTR|CDS|3-UTR identity [Alignment Gaps]:** 209 | 1324 | 9027
- **5-UTR aligned content (<base>:%):** {'C': 43.56, 'T': 6.93, 'G': 47.52, 'A': 1.98}
- **CDS aligned content (<base>:%):** {'A': 24.06, 'G': 27.66, 'C': 29.92, 'T': 18.36}
- **3-UTR aligned content (<base>:%):** {'A': 29.58, 'C': 15.99, 'G': 18.37, 'T': 36.06}

**Uniprot Description:**  
  
 Interferes with CBL-mediated down-regulation and degradation of receptor-type tyrosine kinases. Promotes accumulation of activated target receptors, such as T-cell receptors and EGFR, on the cell surface. Exhibits tyrosine phosphatase activity toward several substrates including EGFR, FAK, SYK, and ZAP70. Down-regulates proteins that are dually modified by both protein tyrosine phosphorylation and ubiquitination.   
  
Homodimer. Interacts with JAK2 (in vitro) (By similarity). Interacts with CBL (PubMed:15159412). Part of a complex containing CBL and activated EGFR (PubMed:15159412). Interacts with ubiquitin and with mono-ubiquitinated proteins (PubMed:15159412). Interacts with ZAP70 (ubiquitinated form) (PubMed:26903241).   
  
 **Gene Ontology Information:**

Molecular Function

- identical protein binding
- phosphoprotein binding
- protein tyrosine phosphatase activity
- ubiquitin protein ligase binding

Location

- cytoplasm
- nucleus

Biological process

- collagen-activated tyrosine kinase receptor signaling pathway
- negative regulation of bone resorption
- negative regulation of osteoclast differentiation
- negative regulation of platelet aggregation
- negative regulation of protein kinase activity
- negative regulation of signal transduction
- peptidyl-tyrosine dephosphorylation
- platelet aggregation
- regulation of osteoclast differentiation
- regulation of protein binding
- regulation of release of sequestered calcium ion into cytosol

---

7

- **Protein name:** E3 ubiquitin-protein ligase HECTD3
- **Organism:** Homo sapiens
- **Uniprot Accession Number:** Q5T447
- **Protein sequence length:** 861 aa
- **1D identity (%):** 16.3
- **1D identity (%) [Gaps excluded]:** 23.31
- **1D identity - Alignment Gaps:** 304
- **1D aligned content (<aminoacid>:%):** {'G': 10.91, 'P': 9.7, 'F': 5.45, 'R': 6.67, 'Y': 2.42, 'K': 4.85, 'V': 9.7, 'E': 6.06, 'L': 12.12, 'D': 7.27, 'T': 6.06, 'S': 5.45, 'W': 1.21, 'M': 1.82, 'H': 0.61, 'N': 1.21, 'Q': 4.24, 'A': 3.03, 'I': 0.61, 'C': 0.61}
- **Common reported functions (%):** 0.0
- **Common reported locations (%):** 0.0
- **Common reported processes (%):** 0.0

- **AF ID:** Q5T447
- **Chain:** A
- **Protein length:** 861 aa
- **Resolution:** N/A
- **b-phipsi:** 0.002012
- **w-rdist:** 0.125048
- **t-alpha:** 0.01311
- **Chemical similarity (Tanimoto Index) (%):** 99.4
- **1D identity (%) [PDB]:** 2.38
- **1D identity (%) [Gaps excluded][PDB]:** 66.1
- **1D identity - Alignment Gaps [PDB]:** 1581
- **1D aligned content [PDB] (<aminoacid>:%):** {'S': 10.26, 'L': 17.95, 'A': 7.69, 'R': 7.69, 'P': 10.26, 'E': 5.13, 'T': 7.69, 'I': 2.56, 'D': 2.56, 'V': 5.13, 'H': 2.56, 'M': 2.56, 'Y': 2.56, 'F': 5.13, 'G': 7.69, 'N': 2.56}
- **2D identity (%) [PDB]:** 40.46
- **2D identity (%) [Gaps excluded][PDB]:** 93.01
- **2D identity - Alignment Gaps [PDB]:** 669
- **2D aligned content [PDB] (<2D-fold>:%):** {'.': 16.49, 'H': 39.87, 'T': 19.0, 'E': 23.17, 'G': 1.25, 'B': 0.21}
- **3D similarity (TM-Score) (%) [PDB]:** 27.22

- **Gene name:** HECTD3
- **Entrez ID:** 79654
- **RefSeq ID:** NM\_024602
- **Transcript sequence length:** 3597
- **5-UTR|CDS|3-UTR identity (%):** 38.28 | 47.02 | 4.85
- **5-UTR|CDS|3-UTR identity (%) [Gaps excluded]:** 74.24 | 74.74 | 76.07
- **5-UTR|CDS|3-UTR identity [Alignment Gaps]:** 62 | 1176 | 11294
- **5-UTR aligned content (<base>:%):** {'G': 38.78, 'C': 51.02, 'T': 10.2}
- **CDS aligned content (<base>:%):** {'A': 22.74, 'T': 17.44, 'G': 31.32, 'C': 28.5}
- **3-UTR aligned content (<base>:%):** {'G': 21.88, 'T': 26.32, 'C': 30.77, 'A': 21.03}

**Uniprot Description:**  
  
 E3 ubiquitin ligases accepts ubiquitin from an E2 ubiquitin-conjugating enzyme in the form of a thioester and then directly transfers the ubiquitin to targeted substrates. Mediates ubiquitination of TRIOBP and its subsequent proteasomal degradation, thus facilitating cell cycle progression by regulating the turn-over of TRIOBP. Mediates also ubiquitination of STX8 (By similarity).   
  
Interacts with TRIOBP. Interacts with STX8 (By similarity).   
  
 **Gene Ontology Information:**

Molecular Function

- syntaxin binding
- ubiquitin-protein transferase activity

Location

- perinuclear region of cytoplasm

Biological process

- proteasome-mediated ubiquitin-dependent protein catabolic process
- protein ubiquitination

---

8

- **Protein name:** Evolutionarily conserved signaling intermediate in Toll pathway, mitochondrial
- **Organism:** Homo sapiens
- **Uniprot Accession Number:** Q9BQ95
- **Protein sequence length:** 431 aa
- **1D identity (%):** 10.4
- **1D identity (%) [Gaps excluded]:** 21.93
- **1D identity - Alignment Gaps:** 460
- **1D aligned content (<aminoacid>:%):** {'T': 4.4, 'L': 7.69, 'C': 2.2, 'G': 8.79, 'S': 5.49, 'R': 10.99, 'A': 7.69, 'E': 6.59, 'P': 10.99, 'V': 6.59, 'D': 6.59, 'Q': 6.59, 'K': 2.2, 'I': 2.2, 'F': 2.2, 'Y': 3.3, 'M': 1.1, 'H': 3.3, 'N': 1.1}
- **Common reported functions (%):** 0.0
- **Common reported locations (%):** 28.57
- **Common reported processes (%):** 0.0

- **AF ID:** Q9BQ95
- **Chain:** A
- **Protein length:** 431 aa
- **Resolution:** N/A
- **b-phipsi:** 0.002339
- **w-rdist:** 0.312411
- **t-alpha:** 0.005861
- **Chemical similarity (Tanimoto Index) (%):** 99.17
- **1D identity (%) [PDB]:** 1.77
- **1D identity (%) [Gaps excluded][PDB]:** 84.62
- **1D identity - Alignment Gaps [PDB]:** 1217
- **1D aligned content [PDB] (<aminoacid>:%):** {'A': 9.09, 'L': 18.18, 'G': 4.55, 'R': 9.09, 'P': 13.64, 'V': 13.64, 'F': 4.55, 'E': 4.55, 'T': 4.55, 'I': 4.55, 'Q': 4.55, 'D': 4.55, 'S': 4.55}
- **2D identity (%) [PDB]:** 28.09
- **2D identity (%) [Gaps excluded][PDB]:** 94.83
- **2D identity - Alignment Gaps [PDB]:** 689
- **2D aligned content [PDB] (<2D-fold>:%):** {'.': 20.36, 'T': 25.82, 'H': 41.45, 'G': 2.18, 'E': 10.18}
- **3D similarity (TM-Score) (%) [PDB]:** 17.0

- **Gene name:** ECSIT
- **Entrez ID:** 507245
- **RefSeq ID:** NM\_016581
- **Transcript sequence length:** 1647
- **5-UTR|CDS|3-UTR identity (%):** 40.29 | 27.79 | 1.35
- **5-UTR|CDS|3-UTR identity (%) [Gaps excluded]:** 66.67 | 71.94 | 73.18
- **5-UTR|CDS|3-UTR identity [Alignment Gaps]:** 55 | 1716 | 11703
- **5-UTR aligned content (<base>:%):** {'C': 33.93, 'T': 8.93, 'G': 53.57, 'A': 3.57}
- **CDS aligned content (<base>:%):** {'T': 15.57, 'G': 30.63, 'C': 33.85, 'A': 19.95}
- **3-UTR aligned content (<base>:%):** {'T': 23.6, 'C': 27.33, 'G': 31.06, 'A': 18.01}

**Uniprot Description:**  
  
 Adapter protein of the Toll-like and IL-1 receptor signaling pathway that is involved in the activation of NF-kappa-B via MAP3K1. Promotes proteolytic activation of MAP3K1. Involved in the BMP signaling pathway. Required for normal embryonic development (By similarity).   
  
Interacts with MAP3K1, SMAD4 and TRAF6. Interacts with SMAD1 only after BMP4-treatment (By similarity). Part of the mitochondrial complex I assembly/MCIA complex that comprises at least the core subunits TMEM126B, NDUFAF1, ECSIT and ACAD9 and complement subunits such as COA1 and TMEM186 (PubMed:32320651). Interacts with NDUFAF1 (PubMed:17344420). Interacts with ACAD9 (PubMed:20816094). Interacts with TRIM59 (By similarity). Interacts with TMEM70 and TMEM242 (PubMed:33753518).   
  
 **Gene Ontology Information:**

Molecular Function

- molecular adaptor activity

Location

- cytoplasm
- cytosol
- mitochondrion
- nucleoplasm
- nucleus

Biological process

- innate immune response
- regulation of oxidoreductase activity
- regulation of protein complex stability
- toll-like receptor 4 signaling pathway

---

9

- **Protein name:** Piwi-like protein 4
- **Organism:** Homo sapiens
- **Uniprot Accession Number:** Q7Z3Z4
- **Protein sequence length:** 852 aa
- **1D identity (%):** 22.44
- **1D identity (%) [Gaps excluded]:** 27.95
- **1D identity - Alignment Gaps:** 187
- **1D aligned content (<aminoacid>:%):** {'S': 5.16, 'G': 9.39, 'A': 3.29, 'P': 8.45, 'F': 5.63, 'D': 5.63, 'K': 6.1, 'L': 9.86, 'N': 2.82, 'Y': 5.16, 'R': 6.57, 'H': 1.88, 'E': 3.76, 'V': 7.98, 'T': 6.57, 'I': 4.23, 'Q': 4.69, 'W': 1.41, 'C': 1.41}
- **Common reported functions (%):** 6.67
- **Common reported locations (%):** 21.43
- **Common reported processes (%):** 4.76

- **AF ID:** Q7Z3Z4
- **Chain:** A
- **Protein length:** 852 aa
- **Resolution:** N/A
- **b-phipsi:** 0.001031
- **w-rdist:** 0.260227
- **t-alpha:** 0.023863
- **Chemical similarity (Tanimoto Index) (%):** 99.4
- **1D identity (%) [PDB]:** 6.12
- **1D identity (%) [Gaps excluded][PDB]:** 54.71
- **1D identity - Alignment Gaps [PDB]:** 1350
- **1D aligned content [PDB] (<aminoacid>:%):** {'S': 5.38, 'A': 7.53, 'V': 11.83, 'G': 5.38, 'M': 1.08, 'D': 7.53, 'H': 4.3, 'R': 7.53, 'C': 2.15, 'T': 7.53, 'Q': 7.53, 'E': 1.08, 'I': 5.38, 'Y': 6.45, 'K': 2.15, 'P': 5.38, 'L': 6.45, 'F': 2.15, 'N': 3.23}
- **2D identity (%) [PDB]:** 67.54
- **2D identity (%) [Gaps excluded][PDB]:** 87.76
- **2D identity - Alignment Gaps [PDB]:** 220
- **2D aligned content [PDB] (<2D-fold>:%):** {'.': 15.19, 'E': 31.63, 'T': 18.6, 'H': 33.18, 'G': 0.47, 'B': 0.93}
- **3D similarity (TM-Score) (%) [PDB]:** 75.29

- **Gene name:** PIWIL4
- **Entrez ID:** 143689
- **RefSeq ID:** NM\_152431
- **Transcript sequence length:** 3139
- **5-UTR|CDS|3-UTR identity (%):** 38.2 | 46.83 | 2.4
- **5-UTR|CDS|3-UTR identity (%) [Gaps excluded]:** 68.0 | 71.72 | 78.85
- **5-UTR|CDS|3-UTR identity [Alignment Gaps]:** 78 | 1079 | 11589
- **5-UTR aligned content (<base>:%):** {'G': 38.24, 'T': 16.18, 'C': 42.65, 'A': 2.94}
- **CDS aligned content (<base>:%):** {'A': 28.64, 'T': 21.29, 'G': 25.34, 'C': 24.73}
- **3-UTR aligned content (<base>:%):** {'T': 36.24, 'G': 19.16, 'A': 33.45, 'C': 11.15}

**Uniprot Description:**  
  
 Plays a central role during spermatogenesis by repressing transposable elements and preventing their mobilization, which is essential for the germline integrity (By similarity). Acts via the piRNA metabolic process, which mediates the repression of transposable elements during meiosis by forming complexes composed of piRNAs and Piwi proteins and governs the methylation and subsequent repression of transposons (By similarity). Directly binds piRNAs, a class of 24 to 30 nucleotide RNAs that are generated by a Dicer-independent mechanism and are primarily derived from transposons and other repeated sequence elements (By similarity). Associates with secondary piRNAs antisense and PIWIL2/MILI is required for such association (By similarity). The piRNA process acts upstream of known mediators of DNA methylation (By similarity). Does not show endonuclease activity (By similarity). Plays a key role in the piRNA amplification loop, also named ping-pong amplification cycle, by acting as a 'slicer-incompetent' component that loads cleaved piRNAs from the 'slicer-competent' component PIWIL2 and target them on genomic transposon loci in the nucleus (By similarity). May be involved in the chromatin-modifying pathway by inducing 'Lys-9' methylation of histone H3 at some loci (PubMed:17544373). In addition to its role in germline, PIWIL4 also plays a role in the regulation of somatic cells activities. Plays a role in pancreatic beta cell function and insulin secretion (By similarity). Involved in maintaining cell morphology and functional integrity of retinal epithelial through Akt/GSK3alpha/beta signaling pathway (PubMed:28025795). When overexpressed, acts as an oncogene by inhibition of apoptosis and promotion of cells proliferation in tumors (PubMed:22483988).   
  
Interacts with PRMT5 and WDR77. Interacts (when methylated on arginine residues) with TDRD1, TDRKH/TDRD2 and TDRD9. Interacts with MOV10L1 (By similarity). Interacts with TEX15 and SPOCD1 (By similarity).   
  
 **Gene Ontology Information:**

Molecular Function

- piRNA binding
- endoribonuclease activity

Location

- cytoplasm
- mitochondrion
- nucleoplasm
- nucleus
- P granule
- piP-body

Biological process

- epithelial structure maintenance
- genetic imprinting
- meiotic cell cycle
- regulation of translation
- retrotransposon silencing by heterochromatin formation
- gene silencing by RNA
- secondary piRNA processing
- spermatogenesis

---

10

- **Protein name:** Ribosomal protein S6 kinase alpha-2
- **Organism:** Homo sapiens
- **Uniprot Accession Number:** Q15349
- **Protein sequence length:** 733 aa
- **1D identity (%):** 16.14
- **1D identity (%) [Gaps excluded]:** 25.36
- **1D identity - Alignment Gaps:** 354
- **1D aligned content (<aminoacid>:%):** {'K': 6.37, 'R': 7.64, 'F': 5.1, 'L': 12.1, 'E': 6.37, 'I': 5.73, 'D': 5.73, 'H': 3.18, 'P': 5.73, 'Q': 7.01, 'Y': 3.82, 'V': 7.64, 'A': 3.82, 'T': 5.73, 'G': 5.1, 'S': 3.82, 'C': 1.91, 'N': 3.18}
- **Common reported functions (%):** 0.0
- **Common reported locations (%):** 28.57
- **Common reported processes (%):** 0.0

- **AF ID:** Q15349
- **Chain:** A
- **Protein length:** 733 aa
- **Resolution:** N/A
- **b-phipsi:** 0.003181
- **w-rdist:** 0.482672
- **t-alpha:** 0.000728
- **Chemical similarity (Tanimoto Index) (%):** 99.02
- **1D identity (%) [PDB]:** 2.57
- **1D identity (%) [Gaps excluded][PDB]:** 69.64
- **1D identity - Alignment Gaps [PDB]:** 1459
- **1D aligned content [PDB] (<aminoacid>:%):** {'D': 5.13, 'R': 10.26, 'K': 10.26, 'V': 12.82, 'F': 5.13, 'L': 15.38, 'Y': 2.56, 'A': 5.13, 'M': 2.56, 'E': 5.13, 'P': 2.56, 'G': 5.13, 'I': 2.56, 'W': 2.56, 'S': 7.69, 'Q': 2.56, 'H': 2.56}
- **2D identity (%) [PDB]:** 40.55
- **2D identity (%) [Gaps excluded][PDB]:** 91.89
- **2D identity - Alignment Gaps [PDB]:** 609
- **2D aligned content [PDB] (<2D-fold>:%):** {'.': 17.19, 'H': 46.15, 'T': 18.55, 'E': 16.52, 'G': 1.36, 'B': 0.23}
- **3D similarity (TM-Score) (%) [PDB]:** 23.01

- **Gene name:** RPS6KA2
- **Entrez ID:** 6196
- **RefSeq ID:** NM\_021135
- **Transcript sequence length:** 5832
- **5-UTR|CDS|3-UTR identity (%):** 41.32 | 45.33 | 17.29
- **5-UTR|CDS|3-UTR identity (%) [Gaps excluded]:** 85.47 | 76.32 | 74.62
- **5-UTR|CDS|3-UTR identity [Alignment Gaps]:** 125 | 1218 | 9534
- **5-UTR aligned content (<base>:%):** {'C': 42.0, 'T': 8.0, 'G': 48.0, 'A': 2.0}
- **CDS aligned content (<base>:%):** {'G': 29.85, 'A': 24.19, 'C': 28.38, 'T': 17.57}
- **3-UTR aligned content (<base>:%):** {'C': 24.93, 'G': 24.32, 'T': 29.73, 'A': 21.02}

**Uniprot Description:**  
  
 Serine/threonine-protein kinase that acts downstream of ERK (MAPK1/ERK2 and MAPK3/ERK1) signaling and mediates mitogenic and stress-induced activation of transcription factors, regulates translation, and mediates cellular proliferation, survival, and differentiation. May function as tumor suppressor in epithelial ovarian cancer cells.   
  
Forms a complex with either MAPK1/ERK2 or MAPK3/ERK1 in quiescent cells. Transiently dissociates following mitogenic stimulation (By similarity). Interacts with FBXO5; cooperate to induce the metaphase arrest of early blastomeres; increases and stabilizes interaction of FBXO5 with CDC20 (By similarity).   
  
 **Gene Ontology Information:**

Molecular Function

- ATP binding
- magnesium ion binding
- protein serine kinase activity
- protein serine/threonine kinase activity
- protein serine/threonine/tyrosine kinase activity
- ribosomal protein S6 kinase activity

Location

- cytoplasm
- cytosol
- meiotic spindle
- nucleoplasm
- nucleus
- synapse

Biological process

- brain renin-angiotensin system
- cardiac muscle cell apoptotic process
- cellular response to carbohydrate stimulus
- chemical synaptic transmission
- heart contraction
- heart development
- intracellular signal transduction
- negative regulation of cell cycle
- negative regulation of cell population proliferation
- negative regulation of meiotic nuclear division
- oocyte maturation
- peptidyl-serine phosphorylation
- positive regulation of apoptotic process
- positive regulation of gene expression
- regulation of protein processing
- signal transduction

---

11

- **Protein name:** Adenylyl cyclase-associated protein 2
- **Organism:** Homo sapiens
- **Uniprot Accession Number:** P40123
- **Protein sequence length:** 477 aa
- **1D identity (%):** 8.69
- **1D identity (%) [Gaps excluded]:** 20.05
- **1D identity - Alignment Gaps:** 528
- **1D aligned content (<aminoacid>:%):** {'A': 7.41, 'S': 4.94, 'P': 11.11, 'E': 4.94, 'V': 6.17, 'F': 3.7, 'D': 7.41, 'L': 7.41, 'I': 8.64, 'G': 7.41, 'T': 1.23, 'H': 4.94, 'Q': 8.64, 'M': 1.23, 'N': 3.7, 'K': 4.94, 'Y': 1.23, 'R': 2.47, 'C': 1.23, 'W': 1.23}
- **Common reported functions (%):** 0.0
- **Common reported locations (%):** 7.14
- **Common reported processes (%):** 0.0

- **AF ID:** P40123
- **Chain:** A
- **Protein length:** 477 aa
- **Resolution:** N/A
- **b-phipsi:** 0.00421
- **w-rdist:** 0.336579
- **t-alpha:** 0.003655
- **Chemical similarity (Tanimoto Index) (%):** 99.47
- **1D identity (%) [PDB]:** 2.11
- **1D identity (%) [Gaps excluded][PDB]:** 77.14
- **1D identity - Alignment Gaps [PDB]:** 1245
- **1D aligned content [PDB] (<aminoacid>:%):** {'M': 3.7, 'N': 3.7, 'I': 7.41, 'V': 11.11, 'S': 7.41, 'A': 11.11, 'T': 3.7, 'F': 7.41, 'Y': 3.7, 'K': 11.11, 'Q': 7.41, 'P': 3.7, 'E': 11.11, 'C': 3.7, 'D': 3.7}
- **2D identity (%) [PDB]:** 29.57
- **2D identity (%) [Gaps excluded][PDB]:** 90.43
- **2D identity - Alignment Gaps [PDB]:** 667
- **2D aligned content [PDB] (<2D-fold>:%):** {'H': 56.31, '.': 9.9, 'G': 2.05, 'T': 13.65, 'E': 18.09}
- **3D similarity (TM-Score) (%) [PDB]:** 17.8

- **Gene name:** CAP2
- **Entrez ID:** 10486
- **RefSeq ID:** NM\_006366
- **Transcript sequence length:** 2925
- **5-UTR|CDS|3-UTR identity (%):** 41.95 | 28.31 | 7.45
- **5-UTR|CDS|3-UTR identity (%) [Gaps excluded]:** 68.87 | 75.0 | 76.15
- **5-UTR|CDS|3-UTR identity [Alignment Gaps]:** 68 | 1814 | 10870
- **5-UTR aligned content (<base>:%):** {'G': 47.95, 'C': 43.84, 'A': 4.11, 'T': 4.11}
- **CDS aligned content (<base>:%):** {'A': 29.94, 'T': 20.36, 'G': 23.76, 'C': 25.94}
- **3-UTR aligned content (<base>:%):** {'C': 16.5, 'T': 38.68, 'A': 31.88, 'G': 12.93}

**Uniprot Description:**  
  
 May have a regulatory bifunctional role. N/A   
  
 **Gene Ontology Information:**

Molecular Function

- actin binding
- adenylate cyclase binding
- identical protein binding

Location

- cytoplasm
- plasma membrane
- postsynaptic density

Biological process

- actin filament organization
- activation of adenylate cyclase activity
- cAMP-mediated signaling
- cell morphogenesis
- establishment or maintenance of cell polarity
- presynaptic actin cytoskeleton organization
- signal transduction

---

12

- **Protein name:** Adenylyl cyclase-associated protein 1
- **Organism:** Homo sapiens
- **Uniprot Accession Number:** Q01518
- **Protein sequence length:** 475 aa
- **1D identity (%):** 9.53
- **1D identity (%) [Gaps excluded]:** 24.93
- **1D identity - Alignment Gaps:** 596
- **1D aligned content (<aminoacid>:%):** {'D': 4.35, 'Q': 5.43, 'V': 13.04, 'G': 6.52, 'H': 4.35, 'M': 1.09, 'R': 3.26, 'Y': 4.35, 'P': 7.61, 'A': 3.26, 'K': 7.61, 'E': 4.35, 'T': 3.26, 'L': 8.7, 'C': 4.35, 'N': 5.43, 'S': 5.43, 'W': 1.09, 'F': 3.26, 'I': 3.26}
- **Common reported functions (%):** 0.0
- **Common reported locations (%):** 14.29
- **Common reported processes (%):** 0.0

- **AF ID:** Q01518
- **Chain:** A
- **Protein length:** 475 aa
- **Resolution:** N/A
- **b-phipsi:** 0.001918
- **w-rdist:** 0.232863
- **t-alpha:** 0.014782
- **Chemical similarity (Tanimoto Index) (%):** 99.62
- **1D identity (%) [PDB]:** 2.11
- **1D identity (%) [Gaps excluded][PDB]:** 77.14
- **1D identity - Alignment Gaps [PDB]:** 1243
- **1D aligned content [PDB] (<aminoacid>:%):** {'V': 18.52, 'S': 18.52, 'I': 7.41, 'K': 3.7, 'Q': 3.7, 'A': 11.11, 'L': 14.81, 'D': 7.41, 'G': 3.7, 'P': 7.41, 'T': 3.7}
- **2D identity (%) [PDB]:** 29.48
- **2D identity (%) [Gaps excluded][PDB]:** 91.85
- **2D identity - Alignment Gaps [PDB]:** 675
- **2D aligned content [PDB] (<2D-fold>:%):** {'H': 53.24, 'T': 15.02, '.': 10.92, 'G': 2.05, 'E': 18.77}
- **3D similarity (TM-Score) (%) [PDB]:** 18.58

- **Gene name:** CAP1
- **Entrez ID:** 10487
- **RefSeq ID:** N/A
- **Sequence length:** N/A
- **5-UTR|CDS|3-UTR identity (%):** N/A | N/A | N/A
- **5-UTR|CDS|3-UTR identity (%) [Gaps excluded]:** N/A | N/A | N/A
- **5-UTR|CDS|3-UTR identity [Alignment Gaps]:** N/A | N/A | N/A
- **5-UTR aligned content (<base>:%):** N/A
- **CDS aligned content (<base>:%):** N/A
- **3-UTR aligned content (<base>:%):** N/A

**Uniprot Description:**  
  
 Directly regulates filament dynamics and has been implicated in a number of complex developmental and morphological processes, including mRNA localization and the establishment of cell polarity.   
  
Homodimer. Binds actin monomers.   
  
 **Gene Ontology Information:**

Molecular Function

- actin binding
- adenylate cyclase binding

Location

- azurophil granule lumen
- cortical actin cytoskeleton
- cytoplasm
- extracellular exosome
- extracellular region
- focal adhesion
- plasma membrane

Biological process

- actin filament organization
- activation of adenylate cyclase activity
- ameboidal-type cell migration
- cAMP-mediated signaling
- cell morphogenesis
- establishment or maintenance of cell polarity
- receptor-mediated endocytosis
- signal transduction

---

13

- **Protein name:** Zinc finger protein 793
- **Organism:** Homo sapiens
- **Uniprot Accession Number:** Q6ZN11
- **Protein sequence length:** 406 aa
- **1D identity (%):** 9.41
- **1D identity (%) [Gaps excluded]:** 23.48
- **1D identity - Alignment Gaps:** 541
- **1D aligned content (<aminoacid>:%):** {'E': 5.88, 'P': 7.06, 'K': 11.76, 'D': 3.53, 'W': 2.35, 'S': 5.88, 'L': 7.06, 'V': 5.88, 'R': 5.88, 'G': 9.41, 'A': 4.71, 'C': 9.41, 'Q': 2.35, 'M': 1.18, 'F': 3.53, 'I': 2.35, 'H': 4.71, 'T': 4.71, 'N': 1.18, 'Y': 1.18}
- **Common reported functions (%):** 6.67
- **Common reported locations (%):** 7.14
- **Common reported processes (%):** 0.0

- **AF ID:** Q6ZN11
- **Chain:** A
- **Protein length:** 406 aa
- **Resolution:** N/A
- **b-phipsi:** 0.010119
- **w-rdist:** 0.336462
- **t-alpha:** 0.000729
- **Chemical similarity (Tanimoto Index) (%):** 96.12
- **1D identity (%) [PDB]:** 1.73
- **1D identity (%) [Gaps excluded][PDB]:** 77.78
- **1D identity - Alignment Gaps [PDB]:** 1190
- **1D aligned content [PDB] (<aminoacid>:%):** {'M': 9.52, 'I': 9.52, 'R': 14.29, 'A': 4.76, 'P': 4.76, 'D': 4.76, 'Q': 4.76, 'E': 9.52, 'S': 14.29, 'K': 4.76, 'L': 4.76, 'F': 4.76, 'N': 4.76, 'T': 4.76}
- **2D identity (%) [PDB]:** 28.48
- **2D identity (%) [Gaps excluded][PDB]:** 94.12
- **2D identity - Alignment Gaps [PDB]:** 666
- **2D aligned content [PDB] (<2D-fold>:%):** {'.': 24.63, 'G': 2.21, 'H': 53.68, 'T': 14.34, 'E': 5.15}
- **3D similarity (TM-Score) (%) [PDB]:** 13.6

- **Gene name:** ZNF793
- **Entrez ID:** 390927
- **RefSeq ID:** N/A
- **Sequence length:** N/A
- **5-UTR|CDS|3-UTR identity (%):** N/A | N/A | N/A
- **5-UTR|CDS|3-UTR identity (%) [Gaps excluded]:** N/A | N/A | N/A
- **5-UTR|CDS|3-UTR identity [Alignment Gaps]:** N/A | N/A | N/A
- **5-UTR aligned content (<base>:%):** N/A
- **CDS aligned content (<base>:%):** N/A
- **3-UTR aligned content (<base>:%):** N/A

**Uniprot Description:**  
  
 May be involved in transcriptional regulation. N/A   
  
 **Gene Ontology Information:**

Molecular Function

- DNA-binding transcription factor activity, RNA polymerase II-specific
- metal ion binding
- RNA polymerase II cis-regulatory region sequence-specific DNA binding

Location

- nucleus

Biological process

- regulation of transcription by RNA polymerase II

---

14

- **Protein name:** Mediator of RNA polymerase II transcription subunit 16
- **Organism:** Homo sapiens
- **Uniprot Accession Number:** Q9Y2X0
- **Protein sequence length:** 877 aa
- **1D identity (%):** 14.71
- **1D identity (%) [Gaps excluded]:** 20.95
- **1D identity - Alignment Gaps:** 304
- **1D aligned content (<aminoacid>:%):** {'R': 8.67, 'P': 11.33, 'G': 6.67, 'L': 15.33, 'Y': 2.67, 'D': 7.33, 'I': 2.67, 'W': 1.33, 'C': 4.67, 'S': 7.33, 'V': 4.0, 'A': 3.33, 'H': 2.67, 'F': 3.33, 'E': 2.67, 'K': 3.33, 'T': 5.33, 'Q': 6.67, 'M': 0.67}
- **Common reported functions (%):** 0.0
- **Common reported locations (%):** 21.43
- **Common reported processes (%):** 0.0

- **AF ID:** Q9Y2X0
- **Chain:** A
- **Protein length:** 877 aa
- **Resolution:** N/A
- **b-phipsi:** 0.000654
- **w-rdist:** 0.340917
- **t-alpha:** 0.050497
- **Chemical similarity (Tanimoto Index) (%):** 96.49
- **1D identity (%) [PDB]:** 3.54
- **1D identity (%) [Gaps excluded][PDB]:** 75.32
- **1D identity - Alignment Gaps [PDB]:** 1561
- **1D aligned content [PDB] (<aminoacid>:%):** {'M': 3.45, 'P': 10.34, 'L': 15.52, 'I': 3.45, 'G': 6.9, 'K': 6.9, 'V': 8.62, 'T': 5.17, 'D': 1.72, 'F': 1.72, 'S': 13.79, 'W': 1.72, 'Q': 1.72, 'A': 5.17, 'H': 3.45, 'R': 5.17, 'E': 3.45, 'Y': 1.72}
- **2D identity (%) [PDB]:** 39.4
- **2D identity (%) [Gaps excluded][PDB]:** 90.04
- **2D identity - Alignment Gaps [PDB]:** 671
- **2D aligned content [PDB] (<2D-fold>:%):** {'.': 15.53, 'E': 27.23, 'T': 21.7, 'H': 33.62, 'G': 1.91}
- **3D similarity (TM-Score) (%) [PDB]:** 23.23

- **Gene name:** MED16
- **Entrez ID:** 10025
- **RefSeq ID:** NM\_005481
- **Transcript sequence length:** 2892
- **5-UTR|CDS|3-UTR identity (%):** 43.75 | 43.81 | 0.76
- **5-UTR|CDS|3-UTR identity (%) [Gaps excluded]:** 80.46 | 73.75 | 71.65
- **5-UTR|CDS|3-UTR identity [Alignment Gaps]:** 73 | 1328 | 11772
- **5-UTR aligned content (<base>:%):** {'A': 4.29, 'G': 47.14, 'C': 44.29, 'T': 4.29}
- **CDS aligned content (<base>:%):** {'A': 18.7, 'T': 15.7, 'G': 31.96, 'C': 33.64}
- **3-UTR aligned content (<base>:%):** {'C': 32.97, 'G': 34.07, 'T': 16.48, 'A': 16.48}

**Uniprot Description:**  
  
 Component of the Mediator complex, a coactivator involved in the regulated transcription of nearly all RNA polymerase II-dependent genes. Mediator functions as a bridge to convey information from gene-specific regulatory proteins to the basal RNA polymerase II transcription machinery. Mediator is recruited to promoters by direct interactions with regulatory proteins and serves as a scaffold for the assembly of a functional preinitiation complex with RNA polymerase II and the general transcription factors.   
  
Component of the Mediator complex, which is composed of MED1, MED4, MED6, MED7, MED8, MED9, MED10, MED11, MED12, MED13, MED13L, MED14, MED15, MED16, MED17, MED18, MED19, MED20, MED21, MED22, MED23, MED24, MED25, MED26, MED27, MED29, MED30, MED31, CCNC, CDK8 and CDC2L6/CDK11. The MED12, MED13, CCNC and CDK8 subunits form a distinct module termed the CDK8 module. Mediator containing the CDK8 module is less active than Mediator lacking this module in supporting transcriptional activation. Individual preparations of the Mediator complex lacking one or more distinct subunits have been variously termed ARC, CRSP, DRIP, PC2, SMCC and TRAP.   
  
 **Gene Ontology Information:**

Molecular Function

- thyroid hormone receptor binding
- vitamin D receptor binding
- transcription coactivator activity

Location

- core mediator complex
- mediator complex
- membrane
- nucleoplasm
- nucleus

Biological process

- positive regulation of transcription, DNA-templated
- positive regulation of transcription elongation from RNA polymerase II promoter
- positive regulation of transcription initiation from RNA polymerase II promoter
- regulation of transcription by RNA polymerase II
- RNA polymerase II preinitiation complex assembly
- transcription by RNA polymerase II

---

15

- **Protein name:** Ribosome-releasing factor 2, mitochondrial
- **Organism:** Homo sapiens
- **Uniprot Accession Number:** Q969S9
- **Protein sequence length:** 779 aa
- **1D identity (%):** 14.12
- **1D identity (%) [Gaps excluded]:** 24.62
- **1D identity - Alignment Gaps:** 444
- **1D aligned content (<aminoacid>:%):** {'L': 12.24, 'A': 6.8, 'S': 4.08, 'P': 8.16, 'V': 9.52, 'I': 4.76, 'G': 8.16, 'R': 6.8, 'C': 3.4, 'H': 2.04, 'K': 3.4, 'M': 0.68, 'D': 4.08, 'T': 8.84, 'F': 2.04, 'E': 5.44, 'Q': 5.44, 'N': 2.72, 'Y': 1.36}
- **Common reported functions (%):** 0.0
- **Common reported locations (%):** 0.0
- **Common reported processes (%):** 0.0

- **AF ID:** Q969S9
- **Chain:** A
- **Protein length:** 779 aa
- **Resolution:** N/A
- **b-phipsi:** 0.00424
- **w-rdist:** 0.355822
- **t-alpha:** 0.002922
- **Chemical similarity (Tanimoto Index) (%):** 98.57
- **1D identity (%) [PDB]:** 3.72
- **1D identity (%) [Gaps excluded][PDB]:** 66.28
- **1D identity - Alignment Gaps [PDB]:** 1445
- **1D aligned content [PDB] (<aminoacid>:%):** {'I': 8.77, 'F': 1.75, 'G': 8.77, 'D': 7.02, 'V': 10.53, 'R': 8.77, 'N': 1.75, 'L': 14.04, 'A': 5.26, 'P': 7.02, 'K': 5.26, 'E': 5.26, 'T': 5.26, 'S': 8.77, 'Q': 1.75}
- **2D identity (%) [PDB]:** 43.66
- **2D identity (%) [Gaps excluded][PDB]:** 89.79
- **2D identity - Alignment Gaps [PDB]:** 559
- **2D aligned content [PDB] (<2D-fold>:%):** {'.': 13.68, 'E': 35.37, 'T': 17.89, 'H': 31.79, 'G': 1.26}
- **3D similarity (TM-Score) (%) [PDB]:** 23.47

- **Gene name:** GFM2
- **Entrez ID:** 527467
- **RefSeq ID:** N/A
- **Sequence length:** N/A
- **5-UTR|CDS|3-UTR identity (%):** N/A | N/A | N/A
- **5-UTR|CDS|3-UTR identity (%) [Gaps excluded]:** N/A | N/A | N/A
- **5-UTR|CDS|3-UTR identity [Alignment Gaps]:** N/A | N/A | N/A
- **5-UTR aligned content (<base>:%):** N/A
- **CDS aligned content (<base>:%):** N/A
- **3-UTR aligned content (<base>:%):** N/A

**Uniprot Description:**  
  
 Mitochondrial GTPase that mediates the disassembly of ribosomes from messenger RNA at the termination of mitochondrial protein biosynthesis. Acts in collaboration with MRRF. GTP hydrolysis follows the ribosome disassembly and probably occurs on the ribosome large subunit. Not involved in the GTP-dependent ribosomal translocation step during translation elongation. N/A   
  
 **Gene Ontology Information:**

Molecular Function

- GTP binding
- GTPase activity

Location

- mitochondrion

Biological process

- mitochondrial translation
- ribosome disassembly

---

16

- **Protein name:** Pseudouridylate synthase 7 homolog
- **Organism:** Homo sapiens
- **Uniprot Accession Number:** Q96PZ0
- **Protein sequence length:** 661 aa
- **1D identity (%):** 13.8
- **1D identity (%) [Gaps excluded]:** 24.91
- **1D identity - Alignment Gaps:** 436
- **1D aligned content (<aminoacid>:%):** {'E': 6.67, 'K': 11.85, 'D': 6.67, 'P': 5.93, 'V': 5.93, 'L': 12.59, 'G': 10.37, 'R': 2.96, 'S': 5.19, 'M': 2.96, 'H': 2.22, 'F': 3.7, 'T': 5.93, 'A': 2.96, 'I': 3.7, 'Q': 4.44, 'Y': 2.96, 'N': 1.48, 'W': 0.74, 'C': 0.74}
- **Common reported functions (%):** 6.67
- **Common reported locations (%):** 7.14
- **Common reported processes (%):** 0.0

- **AF ID:** Q96PZ0
- **Chain:** A
- **Protein length:** 661 aa
- **Resolution:** N/A
- **b-phipsi:** 0.000186
- **w-rdist:** 0.419134
- **t-alpha:** 0.050255
- **Chemical similarity (Tanimoto Index) (%):** 99.24
- **1D identity (%) [PDB]:** 1.92
- **1D identity (%) [Gaps excluded][PDB]:** 73.68
- **1D identity - Alignment Gaps [PDB]:** 1423
- **1D aligned content [PDB] (<aminoacid>:%):** {'G': 7.14, 'A': 10.71, 'V': 17.86, 'E': 7.14, 'P': 7.14, 'R': 7.14, 'H': 3.57, 'L': 14.29, 'K': 7.14, 'N': 3.57, 'T': 7.14, 'Y': 3.57, 'Q': 3.57}
- **2D identity (%) [PDB]:** 40.1
- **2D identity (%) [Gaps excluded][PDB]:** 92.29
- **2D identity - Alignment Gaps [PDB]:** 591
- **2D aligned content [PDB] (<2D-fold>:%):** {'.': 16.71, 'T': 21.96, 'H': 40.1, 'E': 20.76, 'G': 0.48}
- **3D similarity (TM-Score) (%) [PDB]:** 19.27

- **Gene name:** PUS7
- **Entrez ID:** 615644
- **RefSeq ID:** N/A
- **Sequence length:** N/A
- **5-UTR|CDS|3-UTR identity (%):** N/A | N/A | N/A
- **5-UTR|CDS|3-UTR identity (%) [Gaps excluded]:** N/A | N/A | N/A
- **5-UTR|CDS|3-UTR identity [Alignment Gaps]:** N/A | N/A | N/A
- **5-UTR aligned content (<base>:%):** N/A
- **CDS aligned content (<base>:%):** N/A
- **3-UTR aligned content (<base>:%):** N/A

**Uniprot Description:**  
  
 Pseudouridylate synthase that catalyzes pseudouridylation of RNAs (PubMed:28073919, PubMed:29628141, PubMed:30778726). Acts as a regulator of protein synthesis in embryonic stem cells by mediating pseudouridylation of RNA fragments derived from tRNAs (tRFs): pseudouridylated tRFs inhibit translation by targeting the translation initiation complex (PubMed:29628141). Also catalyzes pseudouridylation of mRNAs: mediates pseudouridylation of mRNAs with the consensus sequence 5'-UGUAG-3' (PubMed:28073919). In addition to mRNAs and tRNAs, binds other types of RNAs, such as snRNAs, Y RNAs and vault RNAs, suggesting that it can catalyze pseudouridylation of many RNA types (PubMed:29628141). N/A   
  
 **Gene Ontology Information:**

Molecular Function

- pseudouridine synthase activity
- RNA binding

Location

- nucleus

Biological process

- mRNA processing
- mRNA pseudouridine synthesis
- negative regulation of translation
- pseudouridine synthesis
- regulation of hematopoietic stem cell differentiation
- regulation of mesoderm development
- RNA splicing
- tRNA pseudouridine synthesis

---

17

- **Protein name:** Advillin
- **Organism:** Homo sapiens
- **Uniprot Accession Number:** O75366
- **Protein sequence length:** 819 aa
- **1D identity (%):** 15.7
- **1D identity (%) [Gaps excluded]:** 27.2
- **1D identity - Alignment Gaps:** 450
- **1D aligned content (<aminoacid>:%):** {'P': 7.78, 'A': 7.19, 'F': 6.59, 'R': 5.39, 'K': 8.38, 'N': 2.4, 'E': 5.99, 'D': 5.39, 'I': 4.19, 'C': 1.8, 'V': 5.39, 'Q': 5.39, 'H': 1.2, 'G': 10.78, 'Y': 2.4, 'L': 9.58, 'W': 0.6, 'S': 3.59, 'M': 1.8, 'T': 4.19}
- **Common reported functions (%):** 0.0
- **Common reported locations (%):** 7.14
- **Common reported processes (%):** 0.0

- **AF ID:** O75366
- **Chain:** A
- **Protein length:** 819 aa
- **Resolution:** N/A
- **b-phipsi:** 0.00189
- **w-rdist:** 0.610344
- **t-alpha:** 0.001457
- **Chemical similarity (Tanimoto Index) (%):** 98.28
- **1D identity (%) [PDB]:** 2.3
- **1D identity (%) [Gaps excluded][PDB]:** 75.51
- **1D identity - Alignment Gaps [PDB]:** 1559
- **1D aligned content [PDB] (<aminoacid>:%):** {'M': 2.7, 'P': 8.11, 'L': 10.81, 'I': 5.41, 'G': 10.81, 'D': 5.41, 'K': 10.81, 'V': 10.81, 'E': 5.41, 'T': 2.7, 'R': 5.41, 'F': 5.41, 'S': 10.81, 'Q': 2.7, 'H': 2.7}
- **2D identity (%) [PDB]:** 45.79
- **2D identity (%) [Gaps excluded][PDB]:** 88.5
- **2D identity - Alignment Gaps [PDB]:** 527
- **2D aligned content [PDB] (<2D-fold>:%):** {'.': 13.8, 'E': 28.8, 'T': 26.8, 'H': 28.8, 'G': 1.8}
- **3D similarity (TM-Score) (%) [PDB]:** 25.02

- **Gene name:** AVIL
- **Entrez ID:** 10677
- **RefSeq ID:** N/A
- **Sequence length:** N/A
- **5-UTR|CDS|3-UTR identity (%):** N/A | N/A | N/A
- **5-UTR|CDS|3-UTR identity (%) [Gaps excluded]:** N/A | N/A | N/A
- **5-UTR|CDS|3-UTR identity [Alignment Gaps]:** N/A | N/A | N/A
- **5-UTR aligned content (<base>:%):** N/A
- **CDS aligned content (<base>:%):** N/A
- **3-UTR aligned content (<base>:%):** N/A

**Uniprot Description:**  
  
 Ca(2+)-regulated actin-binding protein which plays an important role in actin bundling (PubMed:29058690). May have a unique function in the morphogenesis of neuronal cells which form ganglia. Required for SREC1-mediated regulation of neurite-like outgrowth. Plays a role in regenerative sensory axon outgrowth and remodeling processes after peripheral injury in neonates. Involved in the formation of long fine actin-containing filopodia-like structures in fibroblast. Plays a role in ciliogenesis. In podocytes, controls lamellipodia formation through the regulation of EGF-induced diacylglycerol generation by PLCE1 and ARP2/3 complex assembly (PubMed:29058690).   
  
Associates (via C-terminus) with F-actin (PubMed:15096633, PubMed:29058690). Interacts with SCARF1 (By similarity). Interacts with PLCE1 (PubMed:29058690). Interacts with ACTR2 and ACTR3; associates with the ARP2/3 complex (PubMed:29058690).   
  
 **Gene Ontology Information:**

Molecular Function

- actin binding
- actin filament binding
- Arp2/3 complex binding
- phosphatidylinositol-4,5-bisphosphate binding

Location

- actin cytoskeleton
- actin filament
- axon
- cell projection
- cytoplasm
- focal adhesion
- lamellipodium
- neuron projection

Biological process

- actin filament organization
- actin filament severing
- actin polymerization or depolymerization
- barbed-end actin filament capping
- cilium assembly
- nervous system development
- positive regulation of lamellipodium assembly
- positive regulation of neuron projection development
- regulation of diacylglycerol biosynthetic process

---

18

- **Protein name:** Dermatan-sulfate epimerase
- **Organism:** Homo sapiens
- **Uniprot Accession Number:** Q9UL01
- **Protein sequence length:** 958 aa
- **1D identity (%):** 14.21
- **1D identity (%) [Gaps excluded]:** 20.35
- **1D identity - Alignment Gaps:** 323
- **1D aligned content (<aminoacid>:%):** {'P': 9.21, 'Y': 3.29, 'D': 6.58, 'E': 4.61, 'N': 3.95, 'H': 1.97, 'F': 5.92, 'T': 4.61, 'L': 11.18, 'K': 6.58, 'S': 4.61, 'W': 0.66, 'A': 7.24, 'I': 3.95, 'M': 1.32, 'G': 9.87, 'C': 1.32, 'Q': 4.61, 'R': 5.26, 'V': 3.29}
- **Common reported functions (%):** 6.67
- **Common reported locations (%):** 0.0
- **Common reported processes (%):** 0.0

- **AF ID:** Q9UL01
- **Chain:** A
- **Protein length:** 958 aa
- **Resolution:** N/A
- **b-phipsi:** 0.006341
- **w-rdist:** 0.314309
- **t-alpha:** 0.004389
- **Chemical similarity (Tanimoto Index) (%):** 96.33
- **1D identity (%) [PDB]:** 2.53
- **1D identity (%) [Gaps excluded][PDB]:** 73.33
- **1D identity - Alignment Gaps [PDB]:** 1676
- **1D aligned content [PDB] (<aminoacid>:%):** {'M': 2.27, 'K': 4.55, 'V': 9.09, 'Q': 4.55, 'R': 2.27, 'T': 9.09, 'P': 13.64, 'L': 11.36, 'S': 4.55, 'I': 9.09, 'N': 4.55, 'G': 6.82, 'F': 4.55, 'A': 9.09, 'D': 2.27, 'H': 2.27}
- **2D identity (%) [PDB]:** 34.38
- **2D identity (%) [Gaps excluded][PDB]:** 91.63
- **2D identity - Alignment Gaps [PDB]:** 816
- **2D aligned content [PDB] (<2D-fold>:%):** {'.': 14.92, 'H': 47.22, 'T': 26.06, 'G': 1.34, 'E': 10.24, 'B': 0.22}
- **3D similarity (TM-Score) (%) [PDB]:** 20.8

- **Gene name:** DSE
- **Entrez ID:** 29940
- **RefSeq ID:** N/A
- **Sequence length:** N/A
- **5-UTR|CDS|3-UTR identity (%):** N/A | N/A | N/A
- **5-UTR|CDS|3-UTR identity (%) [Gaps excluded]:** N/A | N/A | N/A
- **5-UTR|CDS|3-UTR identity [Alignment Gaps]:** N/A | N/A | N/A
- **5-UTR aligned content (<base>:%):** N/A
- **CDS aligned content (<base>:%):** N/A
- **3-UTR aligned content (<base>:%):** N/A

**Uniprot Description:**  
  
 Converts D-glucuronic acid to L-iduronic acid (IdoUA) residues. Plays an important role in the biosynthesis of the glycosaminoglycan/mucopolysaccharide dermatan sulfate. N/A   
  
 **Gene Ontology Information:**

Molecular Function

- chondroitin-glucuronate 5-epimerase activity
- metal ion binding

Location

- cytoplasmic vesicle membrane
- endoplasmic reticulum
- endoplasmic reticulum membrane
- Golgi apparatus
- Golgi membrane

Biological process

- chondroitin sulfate biosynthetic process
- chondroitin sulfate metabolic process
- dermatan sulfate biosynthetic process
- dermatan sulfate metabolic process
- heparan sulfate proteoglycan biosynthetic process

---

19

- **Protein name:** 5-oxoprolinase
- **Organism:** Homo sapiens
- **Uniprot Accession Number:** O14841
- **Protein sequence length:** 1288 aa
- **1D identity (%):** 16.26
- **1D identity (%) [Gaps excluded]:** 25.45
- **1D identity - Alignment Gaps:** 473
- **1D aligned content (<aminoacid>:%):** {'M': 0.94, 'S': 3.76, 'G': 12.68, 'A': 7.51, 'P': 9.39, 'D': 5.16, 'T': 6.57, 'R': 7.98, 'H': 3.76, 'L': 9.39, 'K': 1.88, 'E': 3.29, 'V': 9.39, 'Q': 5.63, 'F': 4.69, 'I': 2.82, 'C': 2.35, 'Y': 2.35, 'N': 0.47}
- **Common reported functions (%):** 0.0
- **Common reported locations (%):** 7.14
- **Common reported processes (%):** 0.0

- **AF ID:** O14841
- **Chain:** A
- **Protein length:** 1288 aa
- **Resolution:** N/A
- **b-phipsi:** 0.002271
- **w-rdist:** 0.316907
- **t-alpha:** 0.007283
- **Chemical similarity (Tanimoto Index) (%):** 99.25
- **1D identity (%) [PDB]:** 3.47
- **1D identity (%) [Gaps excluded][PDB]:** 65.42
- **1D identity - Alignment Gaps [PDB]:** 1912
- **1D aligned content [PDB] (<aminoacid>:%):** {'R': 7.14, 'G': 11.43, 'T': 7.14, 'V': 15.71, 'A': 11.43, 'Q': 8.57, 'K': 2.86, 'L': 11.43, 'I': 4.29, 'N': 1.43, 'P': 8.57, 'F': 1.43, 'D': 1.43, 'H': 1.43, 'S': 1.43, 'Y': 1.43, 'C': 1.43, 'E': 1.43}
- **2D identity (%) [PDB]:** 42.26
- **2D identity (%) [Gaps excluded][PDB]:** 92.64
- **2D identity - Alignment Gaps [PDB]:** 794
- **2D aligned content [PDB] (<2D-fold>:%):** {'.': 16.21, 'E': 27.88, 'T': 20.42, 'H': 33.39, 'B': 0.65, 'G': 1.46}
- **3D similarity (TM-Score) (%) [PDB]:** 27.26

- **Gene name:** OPLAH
- **Entrez ID:** 26873
- **RefSeq ID:** NM\_017570
- **Transcript sequence length:** 4020
- **5-UTR|CDS|3-UTR identity (%):** 39.72 | 42.2 | 0.4
- **5-UTR|CDS|3-UTR identity (%) [Gaps excluded]:** 70.89 | 76.7 | 81.36
- **5-UTR|CDS|3-UTR identity [Alignment Gaps]:** 62 | 1871 | 11830
- **5-UTR aligned content (<base>:%):** {'G': 41.07, 'A': 3.57, 'C': 50.0, 'T': 5.36}
- **CDS aligned content (<base>:%):** {'A': 18.8, 'T': 16.58, 'G': 31.79, 'C': 32.82}
- **3-UTR aligned content (<base>:%):** {'G': 29.17, 'A': 18.75, 'T': 16.67, 'C': 35.42}

**Uniprot Description:**  
  
 Catalyzes the cleavage of 5-oxo-L-proline to form L-glutamate coupled to the hydrolysis of ATP to ADP and inorganic phosphate.   
  
Homodimer.   
  
 **Gene Ontology Information:**

Molecular Function

- 5-oxoprolinase (ATP-hydrolyzing) activity
- ATP binding
- identical protein binding

Location

- cytosol

Biological process

- glutathione metabolic process

---

20

- **Protein name:** PX domain-containing protein kinase-like protein
- **Organism:** Homo sapiens
- **Uniprot Accession Number:** Q7Z7A4
- **Protein sequence length:** 578 aa
- **1D identity (%):** 12.82
- **1D identity (%) [Gaps excluded]:** 23.38
- **1D identity - Alignment Gaps:** 419
- **1D aligned content (<aminoacid>:%):** {'F': 3.36, 'P': 10.92, 'K': 5.88, 'L': 10.08, 'V': 6.72, 'T': 4.2, 'A': 6.72, 'Q': 7.56, 'E': 8.4, 'S': 4.2, 'D': 4.2, 'I': 2.52, 'G': 8.4, 'M': 0.84, 'R': 5.88, 'C': 1.68, 'Y': 3.36, 'N': 3.36, 'H': 1.68}
- **Common reported functions (%):** 0.0
- **Common reported locations (%):** 21.43
- **Common reported processes (%):** 0.0

- **AF ID:** Q7Z7A4
- **Chain:** A
- **Protein length:** 578 aa
- **Resolution:** N/A
- **b-phipsi:** 0.002487
- **w-rdist:** 0.589204
- **t-alpha:** 0.001457
- **Chemical similarity (Tanimoto Index) (%):** 97.4
- **1D identity (%) [PDB]:** 3.73
- **1D identity (%) [Gaps excluded][PDB]:** 65.79
- **1D identity - Alignment Gaps [PDB]:** 1264
- **1D aligned content [PDB] (<aminoacid>:%):** {'M': 6.0, 'P': 12.0, 'L': 14.0, 'I': 6.0, 'G': 8.0, 'R': 6.0, 'K': 8.0, 'E': 6.0, 'V': 6.0, 'D': 2.0, 'S': 12.0, 'C': 2.0, 'F': 4.0, 'T': 4.0, 'Q': 2.0, 'Y': 2.0}
- **2D identity (%) [PDB]:** 39.31
- **2D identity (%) [Gaps excluded][PDB]:** 90.44
- **2D identity - Alignment Gaps [PDB]:** 558
- **2D aligned content [PDB] (<2D-fold>:%):** {'.': 21.39, 'E': 16.24, 'T': 14.18, 'H': 48.2}
- **3D similarity (TM-Score) (%) [PDB]:** 19.87

- **Gene name:** PXK
- **Entrez ID:** 54899
- **RefSeq ID:** N/A
- **Sequence length:** N/A
- **5-UTR|CDS|3-UTR identity (%):** N/A | N/A | N/A
- **5-UTR|CDS|3-UTR identity (%) [Gaps excluded]:** N/A | N/A | N/A
- **5-UTR|CDS|3-UTR identity [Alignment Gaps]:** N/A | N/A | N/A
- **5-UTR aligned content (<base>:%):** N/A
- **CDS aligned content (<base>:%):** N/A
- **3-UTR aligned content (<base>:%):** N/A

**Uniprot Description:**  
  
 Binds to and modulates brain Na,K-ATPase subunits ATP1B1 and ATP1B3 and may thereby participate in the regulation of electrical excitability and synaptic transmission. May not display kinase activity. N/A   
  
 **Gene Ontology Information:**

Molecular Function

- actin binding
- phosphatidylinositol binding

Location

- centriolar satellite
- cytoplasm
- cytosol
- early endosome
- extrinsic component of endosome membrane
- late endosome
- nucleus
- plasma membrane
- protein-containing complex

Biological process

- early endosome to late endosome transport
- endosome to lysosome transport
- inflammatory response
- modulation of chemical synaptic transmission
- negative regulation of ATPase activity
- negative regulation of ion transport
- protein targeting to lysosome

---

21

- **Protein name:** Methionine synthase reductase
- **Organism:** Homo sapiens
- **Uniprot Accession Number:** Q9UBK8
- **Protein sequence length:** 698 aa
- **1D identity (%):** 12.21
- **1D identity (%) [Gaps excluded]:** 24.0
- **1D identity - Alignment Gaps:** 507
- **1D aligned content (<aminoacid>:%):** {'R': 5.56, 'L': 10.32, 'Q': 7.14, 'G': 9.52, 'E': 3.97, 'C': 2.38, 'V': 8.73, 'I': 0.79, 'D': 7.14, 'T': 7.94, 'P': 8.73, 'K': 5.56, 'W': 1.59, 'F': 5.56, 'A': 7.14, 'H': 3.97, 'S': 2.38, 'Y': 1.59}
- **Common reported functions (%):** 0.0
- **Common reported locations (%):** 7.14
- **Common reported processes (%):** 0.0

- **AF ID:** Q9UBK8
- **Chain:** A
- **Protein length:** 698 aa
- **Resolution:** N/A
- **b-phipsi:** 0.003921
- **w-rdist:** 0.452584
- **t-alpha:** 0.001459
- **Chemical similarity (Tanimoto Index) (%):** 99.55
- **1D identity (%) [PDB]:** 3.2
- **1D identity (%) [Gaps excluded][PDB]:** 68.12
- **1D identity - Alignment Gaps [PDB]:** 1398
- **1D aligned content [PDB] (<aminoacid>:%):** {'M': 2.13, 'A': 6.38, 'T': 8.51, 'Q': 8.51, 'K': 10.64, 'V': 12.77, 'L': 10.64, 'I': 8.51, 'G': 8.51, 'N': 4.26, 'P': 8.51, 'F': 4.26, 'H': 2.13, 'S': 4.26}
- **2D identity (%) [PDB]:** 37.71
- **2D identity (%) [Gaps excluded][PDB]:** 89.87
- **2D identity - Alignment Gaps [PDB]:** 628
- **2D aligned content [PDB] (<2D-fold>:%):** {'.': 13.24, 'E': 25.0, 'T': 20.34, 'H': 41.42}
- **3D similarity (TM-Score) (%) [PDB]:** 22.09

- **Gene name:** MTRR
- **Entrez ID:** 507991
- **RefSeq ID:** NM\_024010
- **Transcript sequence length:** 3219
- **5-UTR|CDS|3-UTR identity (%):** 23.78 | 43.09 | 5.92
- **5-UTR|CDS|3-UTR identity (%) [Gaps excluded]:** 66.67 | 74.21 | 79.93
- **5-UTR|CDS|3-UTR identity [Alignment Gaps]:** 92 | 1241 | 11159
- **5-UTR aligned content (<base>:%):** {'G': 52.94, 'C': 32.35, 'T': 14.71}
- **CDS aligned content (<base>:%):** {'A': 27.61, 'T': 22.82, 'G': 23.84, 'C': 25.73}
- **3-UTR aligned content (<base>:%):** {'A': 31.42, 'G': 13.32, 'T': 39.69, 'C': 15.57}

**Uniprot Description:**  
  
 Key enzyme in methionine and folate homeostasis responsible for the reactivation of methionine synthase (MTR/MS) activity by catalyzing the reductive methylation of MTR-bound cob(II)alamin (PubMed:17892308). Cobalamin (vitamin B12) forms a complex with MTR to serve as an intermediary in methyl transfer reactions that cycles between MTR-bound methylcob(III)alamin and MTR bound-cob(I)alamin forms, and occasional oxidative escape of the cob(I)alamin intermediate during the catalytic cycle leads to the inactive cob(II)alamin species (Probable). The processing of cobalamin in the cytosol occurs in a multiprotein complex composed of at least MMACHC, MMADHC, MTRR and MTR which may contribute to shuttle safely and efficiently cobalamin towards MTR in order to produce methionine (PubMed:27771510). Also necessary for the utilization of methyl groups from the folate cycle, thereby affecting transgenerational epigenetic inheritance (By similarity). Also acts as a molecular chaperone for methionine synthase by stabilizing apoMTR and incorporating methylcob(III)alamin into apoMTR to form the holoenzyme (PubMed:16769880). Also serves as an aquacob(III)alamin reductase by reducing aquacob(III)alamin to cob(II)alamin; this reduction leads to stimulation of the conversion of apoMTR and aquacob(III)alamin to MTR holoenzyme (PubMed:16769880).   
  
Forms a multiprotein complex with MMACHC, MMADHC AND MTR.   
  
 **Gene Ontology Information:**

Molecular Function

- [methionine synthase] reductase activity
- flavin adenine dinucleotide binding
- FMN binding
- oxidoreductase activity, oxidizing metal ions, NAD or NADP as acceptor

Location

- cytosol

Biological process

- DNA methylation
- folic acid metabolic process
- homocysteine metabolic process
- methionine biosynthetic process

---

22

- **Protein name:** F-box/LRR-repeat protein 7
- **Organism:** Homo sapiens
- **Uniprot Accession Number:** Q9UJT9
- **Protein sequence length:** 491 aa
- **1D identity (%):** 11.43
- **1D identity (%) [Gaps excluded]:** 22.94
- **1D identity - Alignment Gaps:** 452
- **1D aligned content (<aminoacid>:%):** {'M': 0.97, 'Y': 1.94, 'E': 4.85, 'K': 3.88, 'V': 6.8, 'H': 3.88, 'T': 8.74, 'L': 14.56, 'P': 5.83, 'G': 5.83, 'R': 7.77, 'S': 7.77, 'Q': 4.85, 'F': 2.91, 'C': 6.8, 'W': 1.94, 'N': 0.97, 'D': 4.85, 'A': 2.91, 'I': 1.94}
- **Common reported functions (%):** 0.0
- **Common reported locations (%):** 7.14
- **Common reported processes (%):** 0.0

- **AF ID:** Q9UJT9
- **Chain:** A
- **Protein length:** 491 aa
- **Resolution:** N/A
- **b-phipsi:** 0.002471
- **w-rdist:** 0.510315
- **t-alpha:** 0.00219
- **Chemical similarity (Tanimoto Index) (%):** 98.5
- **1D identity (%) [PDB]:** 2.33
- **1D identity (%) [Gaps excluded][PDB]:** 75.0
- **1D identity - Alignment Gaps [PDB]:** 1249
- **1D aligned content [PDB] (<aminoacid>:%):** {'G': 10.0, 'S': 3.33, 'E': 10.0, 'F': 3.33, 'Q': 6.67, 'L': 13.33, 'H': 10.0, 'A': 6.67, 'R': 6.67, 'I': 10.0, 'K': 6.67, 'T': 6.67, 'V': 6.67}
- **2D identity (%) [PDB]:** 26.61
- **2D identity (%) [Gaps excluded][PDB]:** 84.59
- **2D identity - Alignment Gaps [PDB]:** 693
- **2D aligned content [PDB] (<2D-fold>:%):** {'.': 12.27, 'T': 32.71, 'H': 45.72, 'G': 1.12, 'E': 8.18}
- **3D similarity (TM-Score) (%) [PDB]:** 16.97

- **Gene name:** FBXL7
- **Entrez ID:** 569430
- **RefSeq ID:** N/A
- **Sequence length:** N/A
- **5-UTR|CDS|3-UTR identity (%):** N/A | N/A | N/A
- **5-UTR|CDS|3-UTR identity (%) [Gaps excluded]:** N/A | N/A | N/A
- **5-UTR|CDS|3-UTR identity [Alignment Gaps]:** N/A | N/A | N/A
- **5-UTR aligned content (<base>:%):** N/A
- **CDS aligned content (<base>:%):** N/A
- **3-UTR aligned content (<base>:%):** N/A

**Uniprot Description:**  
  
 Substrate recognition component of a SCF (SKP1-CUL1-F-box protein) E3 ubiquitin-protein ligase complex (PubMed:25778398). During mitosis, it mediates the ubiquitination and subsequent proteasomal degradation of AURKA, causing mitotic arrest (By similarity). It also regulates mitochondrial function by mediating the ubiquitination and proteasomal degradation of the apoptosis inhibitor BIRC5 (PubMed:25778398, PubMed:28218735).   
  
Part of the SCF (SKP1-CUL1-F-box) E3 ubiquitin-protein ligase complex SCF(FBXL7) composed of CUL1, SKP1, RBX1 and FBXL7 (By similarity). Interacts with AURKA; interaction takes place during mitosis but not in interphase (By similarity). Interacts with BIRC5; this interaction allows BIRC5 to be polyubiquitinated by the SCF(FBXL7) E3 ubiquitin-protein ligase complex (PubMed:28218735).   
  
 **Gene Ontology Information:**

Molecular Function   
  
N/A

Location

- cytoplasm
- microtubule organizing center
- SCF ubiquitin ligase complex

Biological process

- protein ubiquitination
- SCF-dependent proteasomal ubiquitin-dependent protein catabolic process

---

23

- **Protein name:** Insulin-like growth factor 2 mRNA-binding protein 1
- **Organism:** Homo sapiens
- **Uniprot Accession Number:** Q9NZI8
- **Protein sequence length:** 577 aa
- **1D identity (%):** 13.73
- **1D identity (%) [Gaps excluded]:** 24.85
- **1D identity - Alignment Gaps:** 414
- **1D aligned content (<aminoacid>:%):** {'G': 10.24, 'S': 3.15, 'V': 8.66, 'P': 7.87, 'L': 6.3, 'K': 11.81, 'F': 3.15, 'C': 3.15, 'E': 7.87, 'I': 4.72, 'T': 3.15, 'H': 2.36, 'R': 6.3, 'Q': 7.09, 'A': 5.51, 'Y': 1.57, 'N': 2.36, 'D': 3.94, 'M': 0.79}
- **Common reported functions (%):** 0.0
- **Common reported locations (%):** 28.57
- **Common reported processes (%):** 0.0

- **AF ID:** Q9NZI8
- **Chain:** A
- **Protein length:** 577 aa
- **Resolution:** N/A
- **b-phipsi:** 0.00337
- **w-rdist:** 0.232682
- **t-alpha:** 0.019665
- **Chemical similarity (Tanimoto Index) (%):** 99.4
- **1D identity (%) [PDB]:** 3.27
- **1D identity (%) [Gaps excluded][PDB]:** 62.86
- **1D identity - Alignment Gaps [PDB]:** 1275
- **1D aligned content [PDB] (<aminoacid>:%):** {'R': 9.09, 'K': 9.09, 'I': 4.55, 'S': 4.55, 'G': 11.36, 'P': 9.09, 'Q': 4.55, 'A': 9.09, 'V': 13.64, 'E': 4.55, 'M': 4.55, 'L': 9.09, 'N': 2.27, 'T': 4.55}
- **2D identity (%) [PDB]:** 41.47
- **2D identity (%) [Gaps excluded][PDB]:** 93.12
- **2D identity - Alignment Gaps [PDB]:** 543
- **2D aligned content [PDB] (<2D-fold>:%):** {'.': 13.58, 'E': 28.15, 'H': 45.93, 'T': 11.6, 'G': 0.74}
- **3D similarity (TM-Score) (%) [PDB]:** 17.41

- **Gene name:** IGF2BP1
- **Entrez ID:** 395953
- **RefSeq ID:** NM\_006546
- **Transcript sequence length:** 8796
- **5-UTR|CDS|3-UTR identity (%):** 30.39 | 33.99 | 32.01
- **5-UTR|CDS|3-UTR identity (%) [Gaps excluded]:** 87.3 | 75.45 | 74.32
- **5-UTR|CDS|3-UTR identity [Alignment Gaps]:** 236 | 1634 | 7397
- **5-UTR aligned content (<base>:%):** {'C': 43.64, 'T': 10.91, 'G': 42.73, 'A': 2.73}
- **CDS aligned content (<base>:%):** {'A': 27.79, 'G': 29.87, 'C': 26.51, 'T': 15.83}
- **3-UTR aligned content (<base>:%):** {'C': 23.03, 'A': 23.11, 'G': 23.13, 'T': 30.73}

**Uniprot Description:**  
  
 RNA-binding factor that recruits target transcripts to cytoplasmic protein-RNA complexes (mRNPs). This transcript 'caging' into mRNPs allows mRNA transport and transient storage. It also modulates the rate and location at which target transcripts encounter the translational apparatus and shields them from endonuclease attacks or microRNA-mediated degradation. Plays a direct role in the transport and translation of transcripts required for axonal regeneration in adult sensory neurons (By similarity). Regulates localized beta-actin/ACTB mRNA translation, a crucial process for cell polarity, cell migration and neurite outgrowth. Co-transcriptionally associates with the ACTB mRNA in the nucleus. This binding involves a conserved 54-nucleotide element in the ACTB mRNA 3'-UTR, known as the 'zipcode'. The RNP thus formed is exported to the cytoplasm, binds to a motor protein and is transported along the cytoskeleton to the cell periphery. During transport, prevents ACTB mRNA from being translated into protein. When the RNP complex reaches its destination near the plasma membrane, IGF2BP1 is phosphorylated. This releases the mRNA, allowing ribosomal 40S and 60S subunits to assemble and initiate ACTB protein synthesis. Monomeric ACTB then assembles into the subcortical actin cytoskeleton (By similarity). During neuronal development, key regulator of neurite outgrowth, growth cone guidance and neuronal cell migration, presumably through the spatiotemporal fine tuning of protein synthesis, such as that of ACTB (By similarity). May regulate mRNA transport to activated synapses (By similarity). Binds to and stabilizes ABCB1/MDR-1 mRNA (By similarity). During interstinal wound repair, interacts with and stabilizes PTGS2 transcript. PTGS2 mRNA stabilization may be crucial for colonic mucosal wound healing (By similarity). Binds to the 3'-UTR of IGF2 mRNA by a mechanism of cooperative and sequential dimerization and regulates IGF2 mRNA subcellular localization and translation. Binds to MYC mRNA, in the coding region instability determinant (CRD) of the open reading frame (ORF), hence prevents MYC cleavage by endonucleases and possibly microRNA targeting to MYC-CRD. Binds to the 3'-UTR of CD44 mRNA and stabilizes it, hence promotes cell adhesion and invadopodia formation in cancer cells. Binds to the oncofetal H19 transcript and to the neuron-specific TAU mRNA and regulates their localizations. Binds to and stabilizes BTRC/FBW1A mRNA. Binds to the adenine-rich autoregulatory sequence (ARS) located in PABPC1 mRNA and represses its translation. PABPC1 mRNA-binding is stimulated by PABPC1 protein. Prevents BTRC/FBW1A mRNA degradation by disrupting microRNA-dependent interaction with AGO2. Promotes the directed movement of tumor-derived cells by fine-tuning intracellular signaling networks. Binds to MAPK4 3'-UTR and inhibits its translation. Interacts with PTEN transcript open reading frame (ORF) and prevents mRNA decay. This combined action on MAPK4 (down-regulation) and PTEN (up-regulation) antagonizes HSPB1 phosphorylation, consequently it prevents G-actin sequestration by phosphorylated HSPB1, allowing F-actin polymerization. Hence enhances the velocity of cell migration and stimulates directed cell migration by PTEN-modulated polarization. Interacts with Hepatitis C virus (HCV) 5'-UTR and 3'-UTR and specifically enhances translation at the HCV IRES, but not 5'-cap-dependent translation, possibly by recruiting eIF3. Interacts with HIV-1 GAG protein and blocks the formation of infectious HIV-1 particles. Reduces HIV-1 assembly by inhibiting viral RNA packaging, as well as assembly and processing of GAG protein on cellular membranes. During cellular stress, such as oxidative stress or heat shock, stabilizes target mRNAs that are recruited to stress granules, including CD44, IGF2, MAPK4, MYC, PTEN, RAPGEF2 and RPS6KA5 transcripts.   
  
Can form homodimers and heterodimers with IGF2BP1 and IGF2BP3. Component of the coding region determinant (CRD)-mediated complex, composed of DHX9, HNRNPU, IGF2BP1, SYNCRIP and YBX1. During HCV infection, identified in a HCV IRES-mediated translation complex, at least composed of EIF3C, IGF2BP1, RPS3 and HCV RNA-replicon. Interacts (via the KH domains) with HIV-1 GAG (via the second zinc finger motif of NC). Associates (via the RRM domains and KH domains) with HIV-1 particles. Identified in a mRNP complex, composed of at least DHX9, DDX3X, ELAVL1, HNRNPU, IGF2BP1, ILF3, PABPC1, PCBP2, PTBP2, STAU1, STAU2, SYNCRIP and YBX1. Identified in a IGF2BP1-dependent mRNP granule complex containing untranslated mRNAs. Interacts with DHX9, ELAVL2, HNRNPA2B1, HNRNPC, HNRNPH1, HNRNPU, IGF2BP2, ILF2, and YBX1. Interacts with FMR1. Component of a multisubunit autoregulatory RNP complex (ARC), at least composed of IGF2BP1, PABPC1 and CSDE1/UNR. Directly interacts with PABPC1. Component of a TAU mRNP complex, at least composed of IGF2BP1, ELAVL4 and G3BP. Interacts with ELAVL4 in an RNA-dependent manner. Associates with microtubules and polysomes. Interacts with AGO1 and AGO2.   
  
 **Gene Ontology Information:**

Molecular Function

- mRNA 3'-UTR binding
- mRNA binding
- N6-methyladenosine-containing RNA binding

Location

- CRD-mediated mRNA stability complex
- cytoplasm
- cytoplasmic stress granule
- cytosol
- filopodium
- growth cone
- lamellipodium
- nucleus
- P-body
- perinuclear region of cytoplasm

Biological process

- CRD-mediated mRNA stabilization
- mRNA transport
- negative regulation of translation
- nervous system development
- positive regulation of neuron projection development
- regulation of gene expression

---

24

- **Protein name:** Heat shock 70 kDa protein 1-like
- **Organism:** Homo sapiens
- **Uniprot Accession Number:** P34931
- **Protein sequence length:** 641 aa
- **1D identity (%):** 12.62
- **1D identity (%) [Gaps excluded]:** 22.37
- **1D identity - Alignment Gaps:** 418
- **1D aligned content (<aminoacid>:%):** {'A': 4.96, 'Y': 3.31, 'V': 9.09, 'G': 11.57, 'E': 9.09, 'N': 2.48, 'F': 6.61, 'K': 7.44, 'T': 8.26, 'P': 6.61, 'I': 4.13, 'D': 4.13, 'L': 9.09, 'R': 4.96, 'Q': 2.48, 'H': 0.83, 'S': 2.48, 'C': 1.65, 'M': 0.83}
- **Common reported functions (%):** 0.0
- **Common reported locations (%):** 21.43
- **Common reported processes (%):** 0.0

- **AF ID:** P34931
- **Chain:** A
- **Protein length:** 641 aa
- **Resolution:** N/A
- **b-phipsi:** 0.003848
- **w-rdist:** 0.646564
- **t-alpha:** 0.0
- **Chemical similarity (Tanimoto Index) (%):** 98.8
- **1D identity (%) [PDB]:** 4.12
- **1D identity (%) [Gaps excluded][PDB]:** 60.64
- **1D identity - Alignment Gaps [PDB]:** 1291
- **1D aligned content [PDB] (<aminoacid>:%):** {'T': 3.51, 'A': 8.77, 'I': 5.26, 'G': 8.77, 'D': 5.26, 'K': 3.51, 'V': 15.79, 'E': 8.77, 'L': 8.77, 'R': 7.02, 'F': 1.75, 'S': 8.77, 'P': 8.77, 'Q': 1.75, 'M': 1.75, 'C': 1.75}
- **2D identity (%) [PDB]:** 44.16
- **2D identity (%) [Gaps excluded][PDB]:** 90.52
- **2D identity - Alignment Gaps [PDB]:** 509
- **2D aligned content [PDB] (<2D-fold>:%):** {'.': 10.48, 'E': 26.88, 'T': 13.9, 'H': 48.06, 'G': 0.68}
- **3D similarity (TM-Score) (%) [PDB]:** 21.93

- **Gene name:** HSPA1L
- **Entrez ID:** 540190
- **RefSeq ID:** N/A
- **Sequence length:** N/A
- **5-UTR|CDS|3-UTR identity (%):** N/A | N/A | N/A
- **5-UTR|CDS|3-UTR identity (%) [Gaps excluded]:** N/A | N/A | N/A
- **5-UTR|CDS|3-UTR identity [Alignment Gaps]:** N/A | N/A | N/A
- **5-UTR aligned content (<base>:%):** N/A
- **CDS aligned content (<base>:%):** N/A
- **3-UTR aligned content (<base>:%):** N/A

**Uniprot Description:**  
  
 Molecular chaperone implicated in a wide variety of cellular processes, including protection of the proteome from stress, folding and transport of newly synthesized polypeptides, activation of proteolysis of misfolded proteins and the formation and dissociation of protein complexes. Plays a pivotal role in the protein quality control system, ensuring the correct folding of proteins, the re-folding of misfolded proteins and controlling the targeting of proteins for subsequent degradation. This is achieved through cycles of ATP binding, ATP hydrolysis and ADP release, mediated by co-chaperones. The affinity for polypeptides is regulated by its nucleotide bound state. In the ATP-bound form, it has a low affinity for substrate proteins. However, upon hydrolysis of the ATP to ADP, it undergoes a conformational change that increases its affinity for substrate proteins. It goes through repeated cycles of ATP hydrolysis and nucleotide exchange, which permits cycles of substrate binding and release (PubMed:26865365). Positive regulator of PRKN translocation to damaged mitochondria (PubMed:24270810).   
  
Interacts with PRKN.   
  
 **Gene Ontology Information:**

Molecular Function

- ATP binding
- ATPase activity
- ATP-dependent protein folding chaperone
- heat shock protein binding
- protein folding chaperone
- ubiquitin protein ligase binding
- unfolded protein binding

Location

- cell body
- COP9 signalosome
- cytoplasm
- cytosol
- nucleus
- zona pellucida receptor complex

Biological process

- binding of sperm to zona pellucida
- chaperone cofactor-dependent protein refolding
- positive regulation of protein targeting to mitochondrion
- protein refolding

---

25

- **Protein name:** Zinc finger protein SNAI3
- **Organism:** Homo sapiens
- **Uniprot Accession Number:** Q3KNW1
- **Protein sequence length:** 292 aa
- **1D identity (%):** 7.29
- **1D identity (%) [Gaps excluded]:** 23.44
- **1D identity - Alignment Gaps:** 605
- **1D aligned content (<aminoacid>:%):** {'L': 6.25, 'V': 6.25, 'P': 14.06, 'Y': 6.25, 'G': 4.69, 'C': 6.25, 'I': 1.56, 'D': 6.25, 'S': 4.69, 'A': 3.12, 'H': 10.94, 'R': 7.81, 'T': 7.81, 'E': 3.12, 'M': 1.56, 'F': 1.56, 'K': 6.25, 'Q': 1.56}
- **Common reported functions (%):** 0.0
- **Common reported locations (%):** 7.14
- **Common reported processes (%):** 0.0

- **AF ID:** Q3KNW1
- **Chain:** A
- **Protein length:** 292 aa
- **Resolution:** N/A
- **b-phipsi:** 0.006851
- **w-rdist:** 0.371078
- **t-alpha:** 0.002185
- **Chemical similarity (Tanimoto Index) (%):** 96.19
- **1D identity (%) [PDB]:** 1.53
- **1D identity (%) [Gaps excluded][PDB]:** 94.44
- **1D identity - Alignment Gaps [PDB]:** 1094
- **1D aligned content [PDB] (<aminoacid>:%):** {'M': 5.88, 'P': 17.65, 'L': 17.65, 'R': 5.88, 'D': 5.88, 'K': 5.88, 'E': 17.65, 'V': 5.88, 'T': 5.88, 'G': 11.76}
- **2D identity (%) [PDB]:** 18.49
- **2D identity (%) [Gaps excluded][PDB]:** 86.0
- **2D identity - Alignment Gaps [PDB]:** 730
- **2D aligned content [PDB] (<2D-fold>:%):** {'.': 33.72, 'T': 25.58, 'E': 8.14, 'H': 32.56}
- **3D similarity (TM-Score) (%) [PDB]:** 10.74

- **Gene name:** SNAI3
- **Entrez ID:** 333929
- **RefSeq ID:** NM\_178310
- **Transcript sequence length:** 1740
- **5-UTR|CDS|3-UTR identity (%):** 49.24 | 20.82 | 4.08
- **5-UTR|CDS|3-UTR identity (%) [Gaps excluded]:** 67.71 | 76.38 | 74.89
- **5-UTR|CDS|3-UTR identity [Alignment Gaps]:** 36 | 1977 | 11342
- **5-UTR aligned content (<base>:%):** {'G': 40.0, 'C': 55.38, 'T': 3.08, 'A': 1.54}
- **CDS aligned content (<base>:%):** {'A': 17.67, 'T': 15.55, 'C': 38.52, 'G': 28.27}
- **3-UTR aligned content (<base>:%):** {'G': 28.43, 'A': 19.02, 'T': 21.06, 'C': 31.49}

**Uniprot Description:**  
  
 Seems to inhibit myoblast differentiation. Transcriptional repressor of E-box-dependent transactivation of downstream myogenic bHLHs genes. Binds preferentially to the canonical E-box sequences 5'-CAGGTG-3' and 5'-CACCTG-3' (By similarity). N/A   
  
 **Gene Ontology Information:**

Molecular Function

- copper ion binding
- DNA-binding transcription factor activity, RNA polymerase II-specific
- DNA-binding transcription repressor activity, RNA polymerase II-specific
- RNA polymerase II cis-regulatory region sequence-specific DNA binding
- sequence-specific double-stranded DNA binding

Location

- nucleus
- transcription regulator complex

Biological process

- regulation of transcription, DNA-templated

---

26

- **Protein name:** Methylcrotonoyl-CoA carboxylase subunit alpha, mitochondrial
- **Organism:** Homo sapiens
- **Uniprot Accession Number:** Q96RQ3
- **Protein sequence length:** 725 aa
- **1D identity (%):** 14.85
- **1D identity (%) [Gaps excluded]:** 22.61
- **1D identity - Alignment Gaps:** 328
- **1D aligned content (<aminoacid>:%):** {'A': 6.34, 'P': 7.75, 'R': 9.86, 'T': 3.52, 'K': 7.75, 'I': 2.11, 'L': 4.93, 'V': 10.56, 'Q': 5.63, 'D': 5.63, 'Y': 2.82, 'E': 9.15, 'S': 3.52, 'H': 4.93, 'F': 4.23, 'G': 7.04, 'C': 2.11, 'M': 1.41, 'N': 0.7}
- **Common reported functions (%):** 6.67
- **Common reported locations (%):** 7.14
- **Common reported processes (%):** 0.0

- **AF ID:** Q96RQ3
- **Chain:** A
- **Protein length:** 725 aa
- **Resolution:** N/A
- **b-phipsi:** 0.007435
- **w-rdist:** 0.442477
- **t-alpha:** 0.0
- **Chemical similarity (Tanimoto Index) (%):** 99.02
- **1D identity (%) [PDB]:** 2.86
- **1D identity (%) [Gaps excluded][PDB]:** 70.49
- **1D identity - Alignment Gaps [PDB]:** 1441
- **1D aligned content [PDB] (<aminoacid>:%):** {'M': 9.3, 'A': 13.95, 'S': 6.98, 'D': 9.3, 'R': 6.98, 'Q': 2.33, 'E': 6.98, 'I': 9.3, 'K': 2.33, 'F': 4.65, 'N': 4.65, 'Y': 4.65, 'V': 2.33, 'T': 6.98, 'G': 4.65, 'L': 2.33, 'P': 2.33}
- **2D identity (%) [PDB]:** 42.37
- **2D identity (%) [Gaps excluded][PDB]:** 86.21
- **2D identity - Alignment Gaps [PDB]:** 533
- **2D aligned content [PDB] (<2D-fold>:%):** {'.': 18.69, 'E': 27.7, 'H': 32.88, 'T': 20.5, 'B': 0.23}
- **3D similarity (TM-Score) (%) [PDB]:** 24.04

- **Gene name:** MCCC1
- **Entrez ID:** 56922
- **RefSeq ID:** NM\_020166
- **Transcript sequence length:** 2454
- **5-UTR|CDS|3-UTR identity (%):** 30.71 | 42.02 | 1.24
- **5-UTR|CDS|3-UTR identity (%) [Gaps excluded]:** 69.64 | 73.34 | 78.31
- **5-UTR|CDS|3-UTR identity [Alignment Gaps]:** 71 | 1292 | 11730
- **5-UTR aligned content (<base>:%):** {'G': 48.72, 'T': 15.38, 'C': 35.9}
- **CDS aligned content (<base>:%):** {'A': 26.99, 'T': 19.98, 'G': 29.03, 'C': 24.0}
- **3-UTR aligned content (<base>:%):** {'A': 32.43, 'C': 17.57, 'T': 32.43, 'G': 17.57}

**Uniprot Description:**  
  
 Biotin-attachment subunit of the 3-methylcrotonyl-CoA carboxylase, an enzyme that catalyzes the conversion of 3-methylcrotonyl-CoA to 3-methylglutaconyl-CoA, a critical step for leucine and isovaleric acid catabolism.   
  
Probably a dodecamer composed of six biotin-containing alpha subunits (MCCC1) and six beta (MCCC2) subunits (PubMed:17360195). Interacts (via the biotin carboxylation domain) with SIRT4 (PubMed:23438705).   
  
 **Gene Ontology Information:**

Molecular Function

- ATP binding
- biotin binding
- biotin carboxylase activity
- metal ion binding
- methylcrotonoyl-CoA carboxylase activity

Location

- 3-methylcrotonyl-CoA carboxylase complex, mitochondrial
- cytosol
- methylcrotonoyl-CoA carboxylase complex
- mitochondrial matrix
- mitochondrion

Biological process

- biotin metabolic process
- branched-chain amino acid catabolic process
- leucine catabolic process

---

27

- **Protein name:** E3 ubiquitin-protein ligase SMURF2
- **Organism:** Homo sapiens
- **Uniprot Accession Number:** Q9HAU4
- **Protein sequence length:** 748 aa
- **1D identity (%):** 15.66
- **1D identity (%) [Gaps excluded]:** 25.12
- **1D identity - Alignment Gaps:** 373
- **1D aligned content (<aminoacid>:%):** {'P': 10.97, 'G': 8.39, 'K': 3.23, 'V': 6.45, 'C': 3.87, 'D': 3.87, 'R': 9.03, 'L': 12.9, 'F': 5.16, 'Y': 2.58, 'S': 2.58, 'T': 8.39, 'N': 1.94, 'I': 7.74, 'Q': 5.81, 'H': 3.23, 'E': 1.94, 'A': 1.29, 'M': 0.65}
- **Common reported functions (%):** 0.0
- **Common reported locations (%):** 28.57
- **Common reported processes (%):** 4.76

- **AF ID:** Q9HAU4
- **Chain:** A
- **Protein length:** 748 aa
- **Resolution:** N/A
- **b-phipsi:** 0.002558
- **w-rdist:** 0.19056
- **t-alpha:** 0.042972
- **Chemical similarity (Tanimoto Index) (%):** 99.02
- **1D identity (%) [PDB]:** 2.68
- **1D identity (%) [Gaps excluded][PDB]:** 74.55
- **1D identity - Alignment Gaps [PDB]:** 1476
- **1D aligned content [PDB] (<aminoacid>:%):** {'K': 9.76, 'V': 9.76, 'D': 4.88, 'G': 9.76, 'N': 2.44, 'L': 17.07, 'Y': 2.44, 'T': 4.88, 'A': 7.32, 'P': 7.32, 'R': 4.88, 'E': 7.32, 'S': 4.88, 'I': 2.44, 'W': 2.44, 'Q': 2.44}
- **2D identity (%) [PDB]:** 44.77
- **2D identity (%) [Gaps excluded][PDB]:** 88.2
- **2D identity - Alignment Gaps [PDB]:** 518
- **2D aligned content [PDB] (<2D-fold>:%):** {'.': 17.83, 'E': 24.84, 'T': 19.32, 'H': 36.73, 'G': 1.27}
- **3D similarity (TM-Score) (%) [PDB]:** 24.46

- **Gene name:** SMURF2
- **Entrez ID:** 64750
- **RefSeq ID:** NM\_022739
- **Transcript sequence length:** 6240
- **5-UTR|CDS|3-UTR identity (%):** 25.64 | 41.3 | 18.29
- **5-UTR|CDS|3-UTR identity (%) [Gaps excluded]:** 88.0 | 73.28 | 74.26
- **5-UTR|CDS|3-UTR identity [Alignment Gaps]:** 304 | 1347 | 9346
- **5-UTR aligned content (<base>:%):** {'C': 43.64, 'T': 10.0, 'G': 43.64, 'A': 2.73}
- **CDS aligned content (<base>:%):** {'A': 29.18, 'T': 21.18, 'G': 26.27, 'C': 23.37}
- **3-UTR aligned content (<base>:%):** {'C': 14.68, 'A': 30.11, 'G': 17.2, 'T': 38.01}

**Uniprot Description:**  
  
 E3 ubiquitin-protein ligase which accepts ubiquitin from an E2 ubiquitin-conjugating enzyme in the form of a thioester and then directly transfers the ubiquitin to targeted substrates (PubMed:11016919). Interacts with SMAD7 to trigger SMAD7-mediated transforming growth factor beta/TGF-beta receptor ubiquitin-dependent degradation, thereby downregulating TGF-beta signaling (PubMed:11163210, PubMed:12717440). In addition, interaction with SMAD7 activates autocatalytic degradation, which is prevented by interaction with AIMP1 (PubMed:18448069). Also forms a stable complex with TGF-beta receptor-mediated phosphorylated SMAD1, SMAD2 and SMAD3, and targets SMAD1 and SMAD2 for ubiquitination and proteasome-mediated degradation (PubMed:11016919, PubMed:11158580, PubMed:11389444). SMAD2 may recruit substrates, such as SNON, for ubiquitin-dependent degradation (PubMed:11389444). Negatively regulates TGFB1-induced epithelial-mesenchymal transition and myofibroblast differentiation (PubMed:30696809).   
  
Interacts (via WW domains) with SMAD1 (PubMed:11158580). Interacts (via WW domains) with SMAD2 (via PY-motif) (PubMed:11158580, PubMed:11389444). Interacts (via WW domains) with SMAD3 (via PY-motif) (PubMed:11158580, PubMed:11389444). Interacts with SMAD6 (PubMed:11158580). Interacts with SMAD7 (via PY-motif) and TGFBR1; SMAD7 recruits SMURF2 to the TGF-beta receptor and regulates its degradation (PubMed:11163210, PubMed:11158580, PubMed:33673144, PubMed:16061177, PubMed:16641086). Does not interact with SMAD4; SMAD4 lacks a PY-motif (PubMed:11158580). Interacts with AIMP1 (PubMed:18448069). Interacts with SNON (PubMed:11389444). Interacts with STAMBP and RNF11 (PubMed:14562029, PubMed:14755250). May interact with NDFIP1 and NDFIP2; this interaction induces the E3 ubiquitin-protein ligase activity. Interacts with TTC3 (Probable).   
  
 **Gene Ontology Information:**

Molecular Function

- identical protein binding
- SMAD binding
- ubiquitin protein ligase activity
- ubiquitin-protein transferase activity

Location

- cytoplasm
- cytosol
- membrane raft
- nuclear speck
- nucleoplasm
- nucleus
- plasma membrane
- ubiquitin ligase complex

Biological process

- negative regulation of BMP signaling pathway
- negative regulation of transcription, DNA-templated
- negative regulation of transcription by RNA polymerase II
- negative regulation of transforming growth factor beta receptor signaling pathway
- positive regulation of canonical Wnt signaling pathway
- positive regulation of trophoblast cell migration
- proteasome-mediated ubiquitin-dependent protein catabolic process
- protein ubiquitination
- regulation of transforming growth factor beta receptor signaling pathway
- ubiquitin-dependent protein catabolic process
- ubiquitin-dependent SMAD protein catabolic process
- Wnt signaling pathway, planar cell polarity pathway

---

28

- **Protein name:** Zinc finger protein 114
- **Organism:** Homo sapiens
- **Uniprot Accession Number:** Q8NC26
- **Protein sequence length:** 417 aa
- **1D identity (%):** 8.74
- **1D identity (%) [Gaps excluded]:** 26.67
- **1D identity - Alignment Gaps:** 646
- **1D aligned content (<aminoacid>:%):** {'D': 5.95, 'S': 4.76, 'V': 7.14, 'F': 4.76, 'P': 8.33, 'Y': 2.38, 'L': 8.33, 'A': 4.76, 'T': 9.52, 'C': 8.33, 'K': 7.14, 'R': 8.33, 'H': 10.71, 'E': 2.38, 'G': 4.76, 'N': 1.19, 'Q': 1.19}
- **Common reported functions (%):** 6.67
- **Common reported locations (%):** 14.29
- **Common reported processes (%):** 0.0

- **AF ID:** Q8NC26
- **Chain:** A
- **Protein length:** 417 aa
- **Resolution:** N/A
- **b-phipsi:** 0.000357
- **w-rdist:** 0.279503
- **t-alpha:** 0.236708
- **Chemical similarity (Tanimoto Index) (%):** 96.99
- **1D identity (%) [PDB]:** 1.71
- **1D identity (%) [Gaps excluded][PDB]:** 70.0
- **1D identity - Alignment Gaps [PDB]:** 1195
- **1D aligned content [PDB] (<aminoacid>:%):** {'L': 9.52, 'A': 9.52, 'R': 9.52, 'E': 9.52, 'C': 4.76, 'K': 14.29, 'D': 4.76, 'Q': 9.52, 'G': 4.76, 'T': 9.52, 'V': 9.52, 'H': 4.76}
- **2D identity (%) [PDB]:** 18.77
- **2D identity (%) [Gaps excluded][PDB]:** 85.02
- **2D identity - Alignment Gaps [PDB]:** 801
- **2D aligned content [PDB] (<2D-fold>:%):** {'.': 27.46, 'G': 1.55, 'H': 49.22, 'T': 15.54, 'E': 6.22}
- **3D similarity (TM-Score) (%) [PDB]:** 13.3

- **Gene name:** ZNF114
- **Entrez ID:** 163071
- **RefSeq ID:** NM\_153608
- **Transcript sequence length:** 2499
- **5-UTR|CDS|3-UTR identity (%):** 20.89 | 27.8 | 3.96
- **5-UTR|CDS|3-UTR identity (%) [Gaps excluded]:** 86.4 | 73.04 | 74.41
- **5-UTR|CDS|3-UTR identity [Alignment Gaps]:** 392 | 1720 | 11344
- **5-UTR aligned content (<base>:%):** {'C': 42.59, 'T': 9.26, 'G': 45.37, 'A': 2.78}
- **CDS aligned content (<base>:%):** {'T': 17.88, 'G': 27.33, 'C': 23.45, 'A': 31.35}
- **3-UTR aligned content (<base>:%):** {'A': 35.23, 'T': 25.11, 'G': 20.68, 'C': 18.99}

**Uniprot Description:**  
  
 May be involved in transcriptional regulation. N/A   
  
 **Gene Ontology Information:**

Molecular Function

- DNA-binding transcription factor activity, RNA polymerase II-specific
- identical protein binding
- metal ion binding
- RNA polymerase II cis-regulatory region sequence-specific DNA binding

Location

- extracellular exosome
- nucleus

Biological process

- regulation of transcription, DNA-templated

---

29

- **Protein name:** Insulin-like growth factor 2 mRNA-binding protein 3
- **Organism:** Homo sapiens
- **Uniprot Accession Number:** O00425
- **Protein sequence length:** 579 aa
- **1D identity (%):** 12.49
- **1D identity (%) [Gaps excluded]:** 22.24
- **1D identity - Alignment Gaps:** 404
- **1D aligned content (<aminoacid>:%):** {'G': 11.3, 'P': 11.3, 'S': 4.35, 'L': 8.7, 'F': 2.61, 'V': 5.22, 'C': 2.61, 'E': 8.7, 'K': 9.57, 'I': 5.22, 'H': 4.35, 'R': 6.09, 'Q': 5.22, 'Y': 1.74, 'A': 3.48, 'D': 3.48, 'T': 2.61, 'M': 0.87, 'N': 2.61}
- **Common reported functions (%):** 6.67
- **Common reported locations (%):** 28.57
- **Common reported processes (%):** 4.76

- **AF ID:** O00425
- **Chain:** A
- **Protein length:** 579 aa
- **Resolution:** N/A
- **b-phipsi:** 0.004127
- **w-rdist:** 0.247519
- **t-alpha:** 0.01311
- **Chemical similarity (Tanimoto Index) (%):** 99.4
- **1D identity (%) [PDB]:** 2.18
- **1D identity (%) [Gaps excluded][PDB]:** 68.18
- **1D identity - Alignment Gaps [PDB]:** 1329
- **1D aligned content [PDB] (<aminoacid>:%):** {'F': 6.67, 'V': 10.0, 'E': 10.0, 'L': 6.67, 'D': 3.33, 'S': 6.67, 'Q': 13.33, 'P': 3.33, 'T': 10.0, 'K': 13.33, 'I': 6.67, 'G': 6.67, 'R': 3.33}
- **2D identity (%) [PDB]:** 41.73
- **2D identity (%) [Gaps excluded][PDB]:** 93.59
- **2D identity - Alignment Gaps [PDB]:** 543
- **2D aligned content [PDB] (<2D-fold>:%):** {'.': 14.46, 'E': 27.94, 'H': 44.36, 'T': 12.5, 'G': 0.74}
- **3D similarity (TM-Score) (%) [PDB]:** 18.04

- **Gene name:** IGF2BP3
- **Entrez ID:** 10643
- **RefSeq ID:** NM\_006547
- **Transcript sequence length:** 4274
- **5-UTR|CDS|3-UTR identity (%):** 22.56 | 39.91 | 10.84
- **5-UTR|CDS|3-UTR identity (%) [Gaps excluded]:** 74.58 | 74.73 | 75.06
- **5-UTR|CDS|3-UTR identity [Alignment Gaps]:** 272 | 1312 | 10497
- **5-UTR aligned content (<base>:%):** {'G': 43.18, 'T': 13.64, 'C': 38.64, 'A': 4.55}
- **CDS aligned content (<base>:%):** {'T': 19.57, 'A': 27.94, 'C': 25.98, 'G': 26.51}
- **3-UTR aligned content (<base>:%):** {'A': 33.38, 'G': 16.17, 'C': 14.29, 'T': 36.17}

**Uniprot Description:**  
  
 RNA-binding factor that may recruit target transcripts to cytoplasmic protein-RNA complexes (mRNPs). This transcript 'caging' into mRNPs allows mRNA transport and transient storage. It also modulates the rate and location at which target transcripts encounter the translational apparatus and shields them from endonuclease attacks or microRNA-mediated degradation. Preferentially binds to N6-methyladenosine (m6A)-containing mRNAs and increases their stability (PubMed:29476152). Binds to the 3'-UTR of CD44 mRNA and stabilizes it, hence promotes cell adhesion and invadopodia formation in cancer cells. Binds to beta-actin/ACTB and MYC transcripts. Increases MYC mRNA stability by binding to the coding region instability determinant (CRD) and binding is enhanced by m6A-modification of the CRD (PubMed:29476152). Binds to the 5'-UTR of the insulin-like growth factor 2 (IGF2) mRNAs.   
  
Can form homooligomers and heterooligomers with IGF2BP1 and IGF2BP3 in an RNA-dependent manner (PubMed:23640942). Interacts with IGF2BP1 (PubMed:17289661). Interacts with ELAVL1, DHX9, HNRNPU, MATR3 and PABPC1 (PubMed:23640942, PubMed:29476152).   
  
 **Gene Ontology Information:**

Molecular Function

- mRNA 3'-UTR binding
- mRNA 5'-UTR binding
- N6-methyladenosine-containing RNA binding
- RNA binding
- translation regulator activity

Location

- cytoplasm
- cytoplasmic stress granule
- cytosol
- nucleus
- P-body

Biological process

- anatomical structure morphogenesis
- CRD-mediated mRNA stabilization
- mRNA transport
- negative regulation of translation
- nervous system development
- regulation of cytokine production
- regulation of gene expression
- translation

---

30

- **Protein name:** Ribosomal protein S6 kinase alpha-4
- **Organism:** Homo sapiens
- **Uniprot Accession Number:** O75676
- **Protein sequence length:** 772 aa
- **1D identity (%):** 16.6
- **1D identity (%) [Gaps excluded]:** 25.51
- **1D identity - Alignment Gaps:** 345
- **1D aligned content (<aminoacid>:%):** {'E': 7.32, 'D': 3.05, 'S': 7.32, 'C': 3.05, 'V': 4.88, 'L': 15.85, 'G': 14.63, 'Y': 1.83, 'F': 4.27, 'H': 4.27, 'T': 3.66, 'Q': 7.32, 'K': 1.83, 'R': 4.88, 'N': 1.83, 'P': 6.71, 'A': 4.88, 'I': 2.44}
- **Common reported functions (%):** 0.0
- **Common reported locations (%):** 28.57
- **Common reported processes (%):** 4.76

- **AF ID:** O75676
- **Chain:** A
- **Protein length:** 772 aa
- **Resolution:** N/A
- **b-phipsi:** 0.005546
- **w-rdist:** 0.287987
- **t-alpha:** 0.006555
- **Chemical similarity (Tanimoto Index) (%):** 99.17
- **1D identity (%) [PDB]:** 2.58
- **1D identity (%) [Gaps excluded][PDB]:** 68.97
- **1D identity - Alignment Gaps [PDB]:** 1494
- **1D aligned content [PDB] (<aminoacid>:%):** {'G': 10.0, 'D': 2.5, 'K': 7.5, 'V': 12.5, 'F': 5.0, 'R': 7.5, 'L': 15.0, 'Y': 2.5, 'A': 2.5, 'M': 2.5, 'E': 7.5, 'T': 2.5, 'I': 5.0, 'S': 12.5, 'Q': 2.5, 'P': 2.5}
- **2D identity (%) [PDB]:** 42.83
- **2D identity (%) [Gaps excluded][PDB]:** 89.27
- **2D identity - Alignment Gaps [PDB]:** 566
- **2D aligned content [PDB] (<2D-fold>:%):** {'.': 19.1, 'E': 13.52, 'H': 46.14, 'T': 19.1, 'B': 0.21, 'G': 1.93}
- **3D similarity (TM-Score) (%) [PDB]:** 24.16

- **Gene name:** RPS6KA4
- **Entrez ID:** 8986
- **RefSeq ID:** NM\_003942
- **Transcript sequence length:** 3128
- **5-UTR|CDS|3-UTR identity (%):** 42.19 | 46.5 | 3.8
- **5-UTR|CDS|3-UTR identity (%) [Gaps excluded]:** 73.97 | 73.59 | 76.55
- **5-UTR|CDS|3-UTR identity [Alignment Gaps]:** 55 | 1105 | 11429
- **5-UTR aligned content (<base>:%):** {'G': 38.89, 'C': 59.26, 'T': 1.85}
- **CDS aligned content (<base>:%):** {'T': 15.33, 'G': 33.74, 'A': 19.13, 'C': 31.81}
- **3-UTR aligned content (<base>:%):** {'T': 24.29, 'A': 16.85, 'G': 31.29, 'C': 27.57}

**Uniprot Description:**  
  
 Serine/threonine-protein kinase that is required for the mitogen or stress-induced phosphorylation of the transcription factors CREB1 and ATF1 and for the regulation of the transcription factor RELA, and that contributes to gene activation by histone phosphorylation and functions in the regulation of inflammatory genes. Phosphorylates CREB1 and ATF1 in response to mitogenic or stress stimuli such as UV-C irradiation, epidermal growth factor (EGF) and anisomycin. Plays an essential role in the control of RELA transcriptional activity in response to TNF. Phosphorylates 'Ser-10' of histone H3 in response to mitogenics, stress stimuli and EGF, which results in the transcriptional activation of several immediate early genes, including proto-oncogenes c-fos/FOS and c-jun/JUN. May also phosphorylate 'Ser-28' of histone H3. Mediates the mitogen- and stress-induced phosphorylation of high mobility group protein 1 (HMGN1/HMG14). In lipopolysaccharide-stimulated primary macrophages, acts downstream of the Toll-like receptor TLR4 to limit the production of pro-inflammatory cytokines. Functions probably by inducing transcription of the MAP kinase phosphatase DUSP1 and the anti-inflammatory cytokine interleukin 10 (IL10), via CREB1 and ATF1 transcription factors.   
  
Forms a complex with either MAPK1/ERK2 or MAPK3/ERK1 in quiescent cells which transiently dissociates following mitogenic stimulation. Also associates with MAPK14/p38-alpha. Activated RPS6KA4 associates with and phosphorylates the NF-kappa-B p65 subunit RELA.   
  
 **Gene Ontology Information:**

Molecular Function

- ATP binding
- histone kinase activity (H3-S10 specific)
- histone H3S28 kinase activity
- magnesium ion binding
- protein serine kinase activity
- protein serine/threonine kinase activity
- ribosomal protein S6 kinase activity

Location

- cytoplasm
- cytosol
- nucleoplasm
- nucleus
- synapse

Biological process

- inflammatory response
- interleukin-1-mediated signaling pathway
- intracellular signal transduction
- negative regulation of cytokine production
- peptidyl-serine phosphorylation
- positive regulation of CREB transcription factor activity
- positive regulation of NF-kappaB transcription factor activity
- positive regulation of transcription by RNA polymerase II
- post-translational protein modification
- protein phosphorylation
- regulation of transcription, DNA-templated

---

31

- **Protein name:** Zinc phosphodiesterase ELAC protein 2
- **Organism:** Homo sapiens
- **Uniprot Accession Number:** Q9BQ52
- **Protein sequence length:** 826 aa
- **1D identity (%):** 14.85
- **1D identity (%) [Gaps excluded]:** 23.77
- **1D identity - Alignment Gaps:** 389
- **1D aligned content (<aminoacid>:%):** {'M': 0.65, 'S': 5.84, 'A': 8.44, 'G': 11.69, 'Q': 5.84, 'P': 9.74, 'R': 5.84, 'D': 2.6, 'T': 8.44, 'L': 9.09, 'Y': 1.3, 'E': 3.9, 'C': 3.9, 'V': 6.49, 'H': 5.19, 'F': 1.95, 'I': 1.95, 'K': 3.9, 'N': 3.25}
- **Common reported functions (%):** 6.67
- **Common reported locations (%):** 7.14
- **Common reported processes (%):** 0.0

- **AF ID:** Q9BQ52
- **Chain:** A
- **Protein length:** 826 aa
- **Resolution:** N/A
- **b-phipsi:** 0.000514
- **w-rdist:** 0.329026
- **t-alpha:** 0.134742
- **Chemical similarity (Tanimoto Index) (%):** 99.62
- **1D identity (%) [PDB]:** 1.72
- **1D identity (%) [Gaps excluded][PDB]:** 82.35
- **1D identity - Alignment Gaps [PDB]:** 1596
- **1D aligned content [PDB] (<aminoacid>:%):** {'G': 17.86, 'N': 7.14, 'I': 7.14, 'L': 7.14, 'P': 14.29, 'Q': 10.71, 'R': 3.57, 'F': 7.14, 'V': 7.14, 'A': 7.14, 'D': 7.14, 'H': 3.57}
- **2D identity (%) [PDB]:** 47.18
- **2D identity (%) [Gaps excluded][PDB]:** 87.95
- **2D identity - Alignment Gaps [PDB]:** 502
- **2D aligned content [PDB] (<2D-fold>:%):** {'.': 12.92, 'H': 40.12, 'T': 19.96, 'E': 26.81, 'B': 0.2}
- **3D similarity (TM-Score) (%) [PDB]:** 23.75

- **Gene name:** ELAC2
- **Entrez ID:** 101145069
- **RefSeq ID:** NM\_173717
- **Transcript sequence length:** 3764
- **5-UTR|CDS|3-UTR identity (%):** 35.04 | 43.76 | 5.89
- **5-UTR|CDS|3-UTR identity (%) [Gaps excluded]:** 68.57 | 74.73 | 75.18
- **5-UTR|CDS|3-UTR identity [Alignment Gaps]:** 67 | 1322 | 11192
- **5-UTR aligned content (<base>:%):** {'T': 10.42, 'G': 62.5, 'C': 27.08}
- **CDS aligned content (<base>:%):** {'A': 23.14, 'T': 17.69, 'G': 29.8, 'C': 29.37}
- **3-UTR aligned content (<base>:%):** {'G': 24.34, 'T': 30.35, 'C': 21.4, 'A': 23.92}

**Uniprot Description:**  
  
 Zinc phosphodiesterase, which displays mitochondrial tRNA 3'-processing endonuclease activity. Involved in tRNA maturation, by removing a 3'-trailer from precursor tRNA (PubMed:21593607). Associates with mitochondrial DNA complexes at the nucleoids to initiate RNA processing and ribosome assembly (PubMed:24703694).   
  
Homodimer (By similarity). Interacts with PTCD1.   
  
 **Gene Ontology Information:**

Molecular Function

- 3'-tRNA processing endoribonuclease activity
- metal ion binding

Location

- mitochondrial nucleoid
- mitochondrion
- nucleus

Biological process

- mitochondrial tRNA 3'-end processing

---

32

- **Protein name:** BTB/POZ domain-containing protein 9
- **Organism:** Homo sapiens
- **Uniprot Accession Number:** Q96Q07
- **Protein sequence length:** 612 aa
- **1D identity (%):** 14.17
- **1D identity (%) [Gaps excluded]:** 27.18
- **1D identity - Alignment Gaps:** 463
- **1D aligned content (<aminoacid>:%):** {'S': 9.49, 'F': 2.19, 'T': 6.57, 'E': 5.11, 'I': 3.65, 'H': 5.11, 'G': 5.11, 'V': 8.76, 'P': 8.03, 'Q': 5.11, 'Y': 3.65, 'L': 8.03, 'A': 5.84, 'K': 5.11, 'D': 5.84, 'M': 1.46, 'W': 1.46, 'N': 1.46, 'R': 5.11, 'C': 2.92}
- **Common reported functions (%):** 0.0
- **Common reported locations (%):** 7.14
- **Common reported processes (%):** 0.0

- **AF ID:** Q96Q07
- **Chain:** A
- **Protein length:** 612 aa
- **Resolution:** N/A
- **b-phipsi:** 0.005168
- **w-rdist:** 0.59699
- **t-alpha:** 0.0
- **Chemical similarity (Tanimoto Index) (%):** 99.47
- **1D identity (%) [PDB]:** 2.71
- **1D identity (%) [Gaps excluded][PDB]:** 79.17
- **1D identity - Alignment Gaps [PDB]:** 1354
- **1D aligned content [PDB] (<aminoacid>:%):** {'M': 2.63, 'P': 10.53, 'L': 15.79, 'R': 5.26, 'D': 5.26, 'V': 10.53, 'E': 7.89, 'G': 5.26, 'K': 7.89, 'I': 2.63, 'F': 2.63, 'S': 13.16, 'W': 2.63, 'C': 2.63, 'A': 5.26}
- **2D identity (%) [PDB]:** 27.54
- **2D identity (%) [Gaps excluded][PDB]:** 94.21
- **2D identity - Alignment Gaps [PDB]:** 794
- **2D aligned content [PDB] (<2D-fold>:%):** {'T': 19.42, '.': 16.18, 'H': 41.1, 'E': 21.68, 'B': 0.65, 'G': 0.97}
- **3D similarity (TM-Score) (%) [PDB]:** 19.21

- **Gene name:** BTBD9
- **Entrez ID:** 505504
- **RefSeq ID:** N/A
- **Sequence length:** N/A
- **5-UTR|CDS|3-UTR identity (%):** N/A | N/A | N/A
- **5-UTR|CDS|3-UTR identity (%) [Gaps excluded]:** N/A | N/A | N/A
- **5-UTR|CDS|3-UTR identity [Alignment Gaps]:** N/A | N/A | N/A
- **5-UTR aligned content (<base>:%):** N/A
- **CDS aligned content (<base>:%):** N/A
- **3-UTR aligned content (<base>:%):** N/A

**Uniprot Description:**  
  
 N/A N/A   
  
 **Gene Ontology Information:**

Molecular Function   
  
N/A

Location

- cytoplasm

Biological process

- adult locomotory behavior
- circadian behavior
- modulation of chemical synaptic transmission

---

33

- **Protein name:** 1-phosphatidylinositol 4,5-bisphosphate phosphodiesterase delta-1
- **Organism:** Homo sapiens
- **Uniprot Accession Number:** P51178
- **Protein sequence length:** 756 aa
- **1D identity (%):** 15.51
- **1D identity (%) [Gaps excluded]:** 26.95
- **1D identity - Alignment Gaps:** 435
- **1D aligned content (<aminoacid>:%):** {'G': 7.55, 'R': 8.18, 'F': 4.4, 'T': 4.4, 'E': 5.03, 'L': 13.21, 'V': 8.18, 'S': 3.77, 'W': 1.26, 'Y': 2.52, 'K': 3.77, 'Q': 6.29, 'D': 8.18, 'I': 3.14, 'H': 4.4, 'C': 1.89, 'P': 9.43, 'N': 2.52, 'A': 1.89}
- **Common reported functions (%):** 6.67
- **Common reported locations (%):** 0.0
- **Common reported processes (%):** 0.0

- **AF ID:** P51178
- **Chain:** A
- **Protein length:** 756 aa
- **Resolution:** N/A
- **b-phipsi:** 0.000465
- **w-rdist:** 0.666464
- **t-alpha:** 0.014033
- **Chemical similarity (Tanimoto Index) (%):** 98.8
- **1D identity (%) [PDB]:** 2.53
- **1D identity (%) [Gaps excluded][PDB]:** 75.0
- **1D identity - Alignment Gaps [PDB]:** 1490
- **1D aligned content [PDB] (<aminoacid>:%):** {'M': 2.56, 'L': 7.69, 'N': 2.56, 'I': 7.69, 'D': 5.13, 'S': 7.69, 'T': 7.69, 'A': 5.13, 'F': 7.69, 'Y': 2.56, 'K': 12.82, 'Q': 10.26, 'C': 2.56, 'V': 5.13, 'E': 7.69, 'P': 2.56, 'G': 2.56}
- **2D identity (%) [PDB]:** 34.66
- **2D identity (%) [Gaps excluded][PDB]:** 90.91
- **2D identity - Alignment Gaps [PDB]:** 714
- **2D aligned content [PDB] (<2D-fold>:%):** {'.': 20.25, 'H': 40.0, 'E': 17.0, 'T': 20.0, 'G': 2.25, 'B': 0.5}
- **3D similarity (TM-Score) (%) [PDB]:** 24.28

- **Gene name:** PLCD1
- **Entrez ID:** 403811
- **RefSeq ID:** NM\_006225
- **Transcript sequence length:** 2651
- **5-UTR|CDS|3-UTR identity (%):** 46.43 | 44.28 | 1.68
- **5-UTR|CDS|3-UTR identity (%) [Gaps excluded]:** 72.22 | 73.87 | 77.22
- **5-UTR|CDS|3-UTR identity [Alignment Gaps]:** 50 | 1215 | 11647
- **5-UTR aligned content (<base>:%):** {'T': 10.77, 'A': 3.08, 'G': 38.46, 'C': 47.69}
- **CDS aligned content (<base>:%):** {'T': 16.75, 'G': 29.56, 'C': 31.05, 'A': 22.64}
- **3-UTR aligned content (<base>:%):** {'C': 32.0, 'T': 23.5, 'G': 26.0, 'A': 18.5}

**Uniprot Description:**  
  
 The production of the second messenger molecules diacylglycerol (DAG) and inositol 1,4,5-trisphosphate (IP3) is mediated by activated phosphatidylinositol-specific phospholipase C enzymes (PubMed:9188725). Essential for trophoblast and placental development (By similarity). Binds phosphatidylinositol 4,5-bisphosphate (PubMed:7890667, PubMed:9188725).   
  
Interacts with TGM2.   
  
 **Gene Ontology Information:**

Molecular Function

- ATP binding
- cytokine binding
- growth factor binding
- metal ion binding
- protease binding
- protein homodimerization activity
- SH2 domain binding
- stem cell factor receptor activity
- transmembrane receptor protein tyrosine kinase activity

Location

- cell-cell junction
- external side of plasma membrane
- extracellular space
- fibrillar center
- receptor complex

Biological process

- actin cytoskeleton reorganization
- B cell differentiation
- cell chemotaxis
- cellular response to thyroid hormone stimulus
- cytokine-mediated signaling pathway
- detection of mechanical stimulus involved in sensory perception of sound
- digestive tract development
- ectopic germ cell programmed cell death
- embryonic hemopoiesis
- erythrocyte differentiation
- erythropoietin-mediated signaling pathway
- Fc receptor signaling pathway
- glycosphingolipid metabolic process
- hematopoietic progenitor cell differentiation
- hematopoietic stem cell migration
- immature B cell differentiation
- inflammatory response
- intracellular signal transduction
- Kit signaling pathway
- lamellipodium assembly
- lymphoid progenitor cell differentiation
- male gonad development
- mast cell chemotaxis
- mast cell degranulation
- mast cell differentiation
- mast cell proliferation
- megakaryocyte development
- melanocyte adhesion
- melanocyte differentiation
- melanocyte migration
- myeloid progenitor cell differentiation
- negative regulation of developmental process
- negative regulation of programmed cell death
- negative regulation of reproductive process
- ovarian follicle development
- peptidyl-tyrosine phosphorylation
- pigmentation
- positive regulation of cell migration
- positive regulation of dendritic cell cytokine production
- positive regulation of DNA-binding transcription factor activity
- positive regulation of MAP kinase activity
- positive regulation of mast cell cytokine production
- positive regulation of mast cell proliferation
- positive regulation of receptor signaling pathway via JAK-STAT
- positive regulation of tyrosine phosphorylation of STAT protein
- positive regulation of vascular associated smooth muscle cell differentiation
- protein autophosphorylation
- regulation of cell shape
- response to radiation
- spermatid development
- spermatogenesis
- stem cell differentiation
- T cell differentiation

---

34

- **Protein name:** Endoplasmic reticulum aminopeptidase 1
- **Organism:** Homo sapiens
- **Uniprot Accession Number:** Q9NZ08
- **Protein sequence length:** 941 aa
- **1D identity (%):** 15.27
- **1D identity (%) [Gaps excluded]:** 21.28
- **1D identity - Alignment Gaps:** 296
- **1D aligned content (<aminoacid>:%):** {'F': 5.0, 'P': 9.38, 'T': 11.88, 'N': 4.38, 'E': 5.0, 'H': 2.5, 'I': 5.62, 'L': 11.25, 'G': 9.38, 'K': 4.38, 'V': 6.25, 'S': 5.0, 'R': 3.12, 'A': 3.75, 'Y': 3.75, 'Q': 3.12, 'D': 3.12, 'C': 2.5, 'M': 0.62}
- **Common reported functions (%):** 0.0
- **Common reported locations (%):** 28.57
- **Common reported processes (%):** 4.76

- **AF ID:** Q9NZ08
- **Chain:** A
- **Protein length:** 941 aa
- **Resolution:** N/A
- **b-phipsi:** 0.005717
- **w-rdist:** 0.447102
- **t-alpha:** 0.001459
- **Chemical similarity (Tanimoto Index) (%):** 96.63
- **1D identity (%) [PDB]:** 2.99
- **1D identity (%) [Gaps excluded][PDB]:** 70.83
- **1D identity - Alignment Gaps [PDB]:** 1635
- **1D aligned content [PDB] (<aminoacid>:%):** {'M': 1.96, 'V': 7.84, 'L': 9.8, 'I': 9.8, 'F': 7.84, 'Y': 5.88, 'K': 9.8, 'S': 3.92, 'T': 5.88, 'P': 3.92, 'D': 3.92, 'H': 1.96, 'E': 7.84, 'R': 7.84, 'A': 1.96, 'C': 1.96, 'Q': 3.92, 'G': 1.96, 'N': 1.96}
- **2D identity (%) [PDB]:** 38.8
- **2D identity (%) [Gaps excluded][PDB]:** 88.56
- **2D identity - Alignment Gaps [PDB]:** 695
- **2D aligned content [PDB] (<2D-fold>:%):** {'.': 16.46, 'E': 32.08, 'T': 18.33, 'G': 1.88, 'H': 31.25}
- **3D similarity (TM-Score) (%) [PDB]:** 25.31

- **Gene name:** ERAP1
- **Entrez ID:** 51752
- **RefSeq ID:** N/A
- **Sequence length:** N/A
- **5-UTR|CDS|3-UTR identity (%):** N/A | N/A | N/A
- **5-UTR|CDS|3-UTR identity (%) [Gaps excluded]:** N/A | N/A | N/A
- **5-UTR|CDS|3-UTR identity [Alignment Gaps]:** N/A | N/A | N/A
- **5-UTR aligned content (<base>:%):** N/A
- **CDS aligned content (<base>:%):** N/A
- **3-UTR aligned content (<base>:%):** N/A

**Uniprot Description:**  
  
 Aminopeptidase that plays a central role in peptide trimming, a step required for the generation of most HLA class I-binding peptides. Peptide trimming is essential to customize longer precursor peptides to fit them to the correct length required for presentation on MHC class I molecules. Strongly prefers substrates 9-16 residues long. Rapidly degrades 13-mer to a 9-mer and then stops. Preferentially hydrolyzes the residue Leu and peptides with a hydrophobic C-terminus, while it has weak activity toward peptides with charged C-terminus. May play a role in the inactivation of peptide hormones. May be involved in the regulation of blood pressure through the inactivation of angiotensin II and/or the generation of bradykinin in the kidney.   
  
Monomer. May also exist as a heterodimer; with ERAP2. Interacts with RBMX.   
  
 **Gene Ontology Information:**

Molecular Function

- aminopeptidase activity
- endopeptidase activity
- interleukin-1, type II receptor binding
- interleukin-6 receptor binding
- metalloaminopeptidase activity
- metalloexopeptidase activity
- peptide binding
- zinc ion binding

Location

- cytoplasm
- cytosol
- endoplasmic reticulum
- endoplasmic reticulum lumen
- endoplasmic reticulum membrane
- extracellular exosome
- extracellular region
- extracellular space
- membrane

Biological process

- adaptive immune response
- angiogenesis
- antigen processing and presentation of endogenous peptide antigen via MHC class I
- antigen processing and presentation of peptide antigen via MHC class I
- fat cell differentiation
- membrane protein ectodomain proteolysis
- peptide catabolic process
- positive regulation of angiogenesis
- proteolysis
- regulation of blood pressure
- regulation of innate immune response
- response to bacterium

---

35

- **Protein name:** Zinc finger MYND domain-containing protein 15
- **Organism:** Homo sapiens
- **Uniprot Accession Number:** Q9H091
- **Protein sequence length:** 742 aa
- **1D identity (%):** 16.54
- **1D identity (%) [Gaps excluded]:** 24.46
- **1D identity - Alignment Gaps:** 309
- **1D aligned content (<aminoacid>:%):** {'M': 1.27, 'G': 11.39, 'R': 7.59, 'D': 6.96, 'L': 12.03, 'T': 6.96, 'F': 4.43, 'K': 4.43, 'V': 6.96, 'A': 3.8, 'P': 11.39, 'S': 5.7, 'E': 5.7, 'I': 1.27, 'C': 2.53, 'Q': 2.53, 'H': 2.53, 'W': 0.63, 'Y': 1.27, 'N': 0.63}
- **Common reported functions (%):** 6.67
- **Common reported locations (%):** 7.14
- **Common reported processes (%):** 0.0

- **AF ID:** Q9H091
- **Chain:** A
- **Protein length:** 742 aa
- **Resolution:** N/A
- **b-phipsi:** 0.000602
- **w-rdist:** 0.711277
- **t-alpha:** 0.006555
- **Chemical similarity (Tanimoto Index) (%):** 99.47
- **1D identity (%) [PDB]:** 3.25
- **1D identity (%) [Gaps excluded][PDB]:** 66.22
- **1D identity - Alignment Gaps [PDB]:** 1432
- **1D aligned content [PDB] (<aminoacid>:%):** {'E': 2.04, 'K': 8.16, 'R': 4.08, 'V': 12.24, 'D': 4.08, 'T': 8.16, 'L': 12.24, 'G': 12.24, 'M': 2.04, 'A': 4.08, 'C': 2.04, 'Q': 6.12, 'P': 14.29, 'S': 2.04, 'I': 4.08, 'F': 2.04}
- **2D identity (%) [PDB]:** 41.67
- **2D identity (%) [Gaps excluded][PDB]:** 85.69
- **2D identity - Alignment Gaps [PDB]:** 546
- **2D aligned content [PDB] (<2D-fold>:%):** {'.': 23.93, 'H': 35.21, 'T': 23.02, 'E': 17.16, 'G': 0.68}
- **3D similarity (TM-Score) (%) [PDB]:** 26.77

- **Gene name:** ZMYND15
- **Entrez ID:** 84225
- **RefSeq ID:** NM\_032265
- **Transcript sequence length:** 2307
- **5-UTR|CDS|3-UTR identity (%):** 27.97 | 41.89 | 0.65
- **5-UTR|CDS|3-UTR identity (%) [Gaps excluded]:** 80.0 | 74.14 | 73.33
- **5-UTR|CDS|3-UTR identity [Alignment Gaps]:** 93 | 1304 | 11807
- **5-UTR aligned content (<base>:%):** {'G': 50.0, 'A': 2.5, 'T': 12.5, 'C': 35.0}
- **CDS aligned content (<base>:%):** {'A': 21.34, 'G': 30.73, 'T': 16.08, 'C': 31.85}
- **3-UTR aligned content (<base>:%):** {'A': 49.35, 'T': 23.38, 'G': 15.58, 'C': 11.69}

**Uniprot Description:**  
  
 Acts as a transcriptional repressor through interaction with histone deacetylases (HDACs). May be important for spermiogenesis.   
  
Interacts with HDAC1, HDAC3, HDAC6 and, to a lesser extent, with HDAC7.   
  
 **Gene Ontology Information:**

Molecular Function

- histone deacetylase binding
- metal ion binding

Location

- cytoplasm
- male germ cell nucleus

Biological process

- negative regulation of transcription, DNA-templated
- spermatid development

---

36

- **Protein name:** Protein mono-ADP-ribosyltransferase PARP15
- **Organism:** Homo sapiens
- **Uniprot Accession Number:** Q460N3
- **Protein sequence length:** 678 aa
- **1D identity (%):** 14.59
- **1D identity (%) [Gaps excluded]:** 22.91
- **1D identity - Alignment Gaps:** 341
- **1D aligned content (<aminoacid>:%):** {'A': 8.76, 'G': 6.57, 'L': 8.03, 'P': 5.84, 'E': 4.38, 'V': 9.49, 'R': 5.84, 'S': 5.11, 'K': 10.95, 'N': 4.38, 'Y': 2.92, 'Q': 3.65, 'D': 2.92, 'T': 9.49, 'C': 2.92, 'H': 2.19, 'F': 3.65, 'I': 2.19, 'M': 0.73}
- **Common reported functions (%):** 0.0
- **Common reported locations (%):** 14.29
- **Common reported processes (%):** 0.0

- **AF ID:** Q460N3
- **Chain:** A
- **Protein length:** 678 aa
- **Resolution:** N/A
- **b-phipsi:** 0.004397
- **w-rdist:** 0.214814
- **t-alpha:** 0.02622
- **Chemical similarity (Tanimoto Index) (%):** 96.34
- **1D identity (%) [PDB]:** 2.48
- **1D identity (%) [Gaps excluded][PDB]:** 56.25
- **1D identity - Alignment Gaps [PDB]:** 1388
- **1D aligned content [PDB] (<aminoacid>:%):** {'A': 5.56, 'P': 5.56, 'V': 16.67, 'I': 8.33, 'E': 13.89, 'L': 5.56, 'F': 2.78, 'S': 2.78, 'Q': 5.56, 'D': 2.78, 'T': 8.33, 'G': 5.56, 'K': 5.56, 'R': 5.56, 'Y': 2.78, 'N': 2.78}
- **2D identity (%) [PDB]:** 38.15
- **2D identity (%) [Gaps excluded][PDB]:** 92.12
- **2D identity - Alignment Gaps [PDB]:** 628
- **2D aligned content [PDB] (<2D-fold>:%):** {'.': 12.96, 'E': 32.27, 'T': 20.54, 'H': 34.23}
- **3D similarity (TM-Score) (%) [PDB]:** 23.8

- **Gene name:** PARP15
- **Entrez ID:** 165631
- **RefSeq ID:** NM\_152615
- **Transcript sequence length:** 4377
- **5-UTR|CDS|3-UTR identity (%):** 20.36 | 28.29 | 14.03
- **5-UTR|CDS|3-UTR identity (%) [Gaps excluded]:** 61.82 | 73.6 | 74.55
- **5-UTR|CDS|3-UTR identity [Alignment Gaps]:** 112 | 1741 | 10135
- **5-UTR aligned content (<base>:%):** {'T': 17.65, 'C': 32.35, 'A': 5.88, 'G': 44.12}
- **CDS aligned content (<base>:%):** {'A': 32.38, 'T': 21.0, 'G': 22.5, 'C': 24.12}
- **3-UTR aligned content (<base>:%):** {'A': 24.77, 'T': 38.58, 'G': 17.92, 'C': 18.72}

**Uniprot Description:**  
  
 Mono-ADP-ribosyltransferase that mediates mono-ADP-ribosylation of target proteins (PubMed:16061477, PubMed:25043379, PubMed:25635049). Acts as a negative regulator of transcription (PubMed:16061477). N/A   
  
 **Gene Ontology Information:**

Molecular Function

- NAD+ ADP-ribosyltransferase activity
- NAD+ binding
- protein ADP-ribosylase activity
- nucleotidyltransferase activity
- transcription corepressor activity

Location

- cytoplasm
- nucleus

Biological process

- negative regulation of gene expression
- negative regulation of transcription by RNA polymerase II
- protein poly-ADP-ribosylation

---

37

- **Protein name:** Glutamyl-tRNA(Gln) amidotransferase subunit B, mitochondrial
- **Organism:** Homo sapiens
- **Uniprot Accession Number:** O75879
- **Protein sequence length:** 557 aa
- **1D identity (%):** 11.12
- **1D identity (%) [Gaps excluded]:** 25.0
- **1D identity - Alignment Gaps:** 544
- **1D aligned content (<aminoacid>:%):** {'A': 5.5, 'P': 10.09, 'G': 6.42, 'F': 3.67, 'R': 4.59, 'D': 5.5, 'T': 5.5, 'I': 5.5, 'N': 2.75, 'V': 8.26, 'E': 5.5, 'K': 9.17, 'Y': 2.75, 'L': 11.01, 'S': 4.59, 'H': 1.83, 'M': 1.83, 'Q': 5.5}
- **Common reported functions (%):** 0.0
- **Common reported locations (%):** 0.0
- **Common reported processes (%):** 0.0

- **AF ID:** O75879
- **Chain:** A
- **Protein length:** 557 aa
- **Resolution:** N/A
- **b-phipsi:** 0.000668
- **w-rdist:** 0.430207
- **t-alpha:** 0.036416
- **Chemical similarity (Tanimoto Index) (%):** 99.02
- **1D identity (%) [PDB]:** 3.76
- **1D identity (%) [Gaps excluded][PDB]:** 75.76
- **1D identity - Alignment Gaps [PDB]:** 1263
- **1D aligned content [PDB] (<aminoacid>:%):** {'A': 12.0, 'V': 10.0, 'G': 2.0, 'S': 2.0, 'P': 4.0, 'N': 2.0, 'R': 12.0, 'C': 2.0, 'T': 4.0, 'Q': 10.0, 'I': 8.0, 'D': 2.0, 'L': 14.0, 'E': 8.0, 'Y': 2.0, 'F': 2.0, 'K': 4.0}
- **2D identity (%) [PDB]:** 42.2
- **2D identity (%) [Gaps excluded][PDB]:** 93.76
- **2D identity - Alignment Gaps [PDB]:** 529
- **2D aligned content [PDB] (<2D-fold>:%):** {'.': 17.73, 'E': 20.44, 'T': 17.98, 'H': 42.36, 'G': 1.48}
- **3D similarity (TM-Score) (%) [PDB]:** 20.17

- **Gene name:** GATB
- **Entrez ID:** 5188
- **RefSeq ID:** NM\_004564
- **Transcript sequence length:** 2369
- **5-UTR|CDS|3-UTR identity (%):** 13.39 | 36.7 | 3.55
- **5-UTR|CDS|3-UTR identity (%) [Gaps excluded]:** 68.0 | 73.87 | 74.87
- **5-UTR|CDS|3-UTR identity [Alignment Gaps]:** 102 | 1430 | 11420
- **5-UTR aligned content (<base>:%):** {'C': 47.06, 'T': 11.76, 'G': 29.41, 'A': 11.76}
- **CDS aligned content (<base>:%):** {'A': 26.27, 'T': 19.27, 'G': 28.0, 'C': 26.46}
- **3-UTR aligned content (<base>:%):** {'A': 27.23, 'T': 27.93, 'G': 22.54, 'C': 22.3}

**Uniprot Description:**  
  
 Allows the formation of correctly charged Gln-tRNA(Gln) through the transamidation of misacylated Glu-tRNA(Gln) in the mitochondria. The reaction takes place in the presence of glutamine and ATP through an activated gamma-phospho-Glu-tRNA(Gln).   
  
Subunit of the heterotrimeric GatCAB amidotransferase (AdT) complex, composed of A (QRSL1), B (GATB) and C (GATC) subunits.   
  
 **Gene Ontology Information:**

Molecular Function

- ATP binding
- glutaminyl-tRNA synthase (glutamine-hydrolyzing) activity

Location

- glutamyl-tRNA(Gln) amidotransferase complex
- mitochondrion

Biological process

- glutaminyl-tRNAGln biosynthesis via transamidation
- mitochondrial translation

---

38

- **Protein name:** TBC1 domain family member 17
- **Organism:** Homo sapiens
- **Uniprot Accession Number:** Q9HA65
- **Protein sequence length:** 648 aa
- **1D identity (%):** 12.94
- **1D identity (%) [Gaps excluded]:** 22.59
- **1D identity - Alignment Gaps:** 409
- **1D aligned content (<aminoacid>:%):** {'K': 4.03, 'Y': 0.81, 'L': 7.26, 'R': 6.45, 'V': 7.26, 'E': 5.65, 'H': 2.42, 'G': 9.68, 'D': 8.06, 'T': 6.45, 'P': 12.9, 'W': 0.81, 'S': 9.68, 'Q': 3.23, 'A': 5.65, 'F': 4.03, 'C': 3.23, 'N': 1.61, 'I': 0.81}
- **Common reported functions (%):** 0.0
- **Common reported locations (%):** 7.14
- **Common reported processes (%):** 0.0

- **AF ID:** Q9HA65
- **Chain:** A
- **Protein length:** 648 aa
- **Resolution:** N/A
- **b-phipsi:** 0.005462
- **w-rdist:** 0.430232
- **t-alpha:** 0.00219
- **Chemical similarity (Tanimoto Index) (%):** 99.55
- **1D identity (%) [PDB]:** 2.01
- **1D identity (%) [Gaps excluded][PDB]:** 67.44
- **1D identity - Alignment Gaps [PDB]:** 1400
- **1D aligned content [PDB] (<aminoacid>:%):** {'E': 6.9, 'G': 6.9, 'D': 6.9, 'R': 6.9, 'I': 6.9, 'V': 10.34, 'S': 10.34, 'C': 3.45, 'L': 17.24, 'A': 10.34, 'H': 3.45, 'P': 3.45, 'F': 3.45, 'Q': 3.45}
- **2D identity (%) [PDB]:** 32.82
- **2D identity (%) [Gaps excluded][PDB]:** 91.58
- **2D identity - Alignment Gaps [PDB]:** 702
- **2D aligned content [PDB] (<2D-fold>:%):** {'.': 13.93, 'T': 21.45, 'E': 14.48, 'H': 47.63, 'G': 2.51}
- **3D similarity (TM-Score) (%) [PDB]:** 20.31

- **Gene name:** TBC1D17
- **Entrez ID:** 79735
- **RefSeq ID:** NM\_024682
- **Transcript sequence length:** 2095
- **5-UTR|CDS|3-UTR identity (%):** 12.6 | 40.68 | 0.72
- **5-UTR|CDS|3-UTR identity (%) [Gaps excluded]:** 72.73 | 72.72 | 82.69
- **5-UTR|CDS|3-UTR identity [Alignment Gaps]:** 105 | 1279 | 11806
- **5-UTR aligned content (<base>:%):** {'G': 50.0, 'T': 12.5, 'C': 37.5}
- **CDS aligned content (<base>:%):** {'A': 19.31, 'T': 16.85, 'G': 30.91, 'C': 32.94}
- **3-UTR aligned content (<base>:%):** {'C': 37.21, 'G': 26.74, 'A': 15.12, 'T': 20.93}

**Uniprot Description:**  
  
 Probable RAB GTPase-activating protein that inhibits RAB8A/B function. Reduces Rab8 recruitment to tubules emanating from the endocytic recycling compartment (ERC) and inhibits Rab8-mediated endocytic trafficking, such as that of transferrin receptor (TfR) (PubMed:22854040). Involved in regulation of autophagy.   
  
Interacts with OPTN; this interaction mediates TBC1D17 transient association with Rab8.   
  
 **Gene Ontology Information:**

Molecular Function

- GTPase activator activity

Location

- autophagosome
- cytosol
- recycling endosome

Biological process

- activation of GTPase activity
- autophagy
- protein transport
- retrograde transport, endosome to Golgi

---

39

- **Protein name:** Protein maelstrom homolog
- **Organism:** Homo sapiens
- **Uniprot Accession Number:** Q96JY0
- **Protein sequence length:** 434 aa
- **1D identity (%):** 9.28
- **1D identity (%) [Gaps excluded]:** 26.65
- **1D identity - Alignment Gaps:** 625
- **1D aligned content (<aminoacid>:%):** {'P': 8.99, 'R': 6.74, 'F': 3.37, 'G': 7.87, 'Y': 6.74, 'K': 4.49, 'A': 4.49, 'E': 3.37, 'T': 3.37, 'L': 12.36, 'V': 8.99, 'N': 3.37, 'S': 7.87, 'I': 4.49, 'Q': 2.25, 'D': 4.49, 'H': 4.49, 'C': 2.25}
- **Common reported functions (%):** 0.0
- **Common reported locations (%):** 14.29
- **Common reported processes (%):** 4.76

- **AF ID:** Q96JY0
- **Chain:** A
- **Protein length:** 434 aa
- **Resolution:** N/A
- **b-phipsi:** 0.00782
- **w-rdist:** 0.446927
- **t-alpha:** 0.000729
- **Chemical similarity (Tanimoto Index) (%):** 99.17
- **1D identity (%) [PDB]:** 1.77
- **1D identity (%) [Gaps excluded][PDB]:** 84.62
- **1D identity - Alignment Gaps [PDB]:** 1220
- **1D aligned content [PDB] (<aminoacid>:%):** {'P': 4.55, 'N': 4.55, 'R': 9.09, 'Y': 9.09, 'C': 4.55, 'A': 4.55, 'T': 4.55, 'V': 9.09, 'E': 4.55, 'I': 9.09, 'Q': 9.09, 'D': 4.55, 'L': 13.64, 'K': 4.55, 'S': 4.55}
- **2D identity (%) [PDB]:** 28.78
- **2D identity (%) [Gaps excluded][PDB]:** 93.65
- **2D identity - Alignment Gaps [PDB]:** 674
- **2D aligned content [PDB] (<2D-fold>:%):** {'T': 19.29, '.': 23.21, 'H': 48.57, 'G': 1.07, 'E': 7.86}
- **3D similarity (TM-Score) (%) [PDB]:** 17.59

- **Gene name:** MAEL
- **Entrez ID:** 84944
- **RefSeq ID:** NM\_032858
- **Transcript sequence length:** 1737
- **5-UTR|CDS|3-UTR identity (%):** 31.62 | 29.71 | 2.02
- **5-UTR|CDS|3-UTR identity (%) [Gaps excluded]:** 67.19 | 74.46 | 75.08
- **5-UTR|CDS|3-UTR identity [Alignment Gaps]:** 72 | 1669 | 11605
- **5-UTR aligned content (<base>:%):** {'C': 41.86, 'T': 6.98, 'G': 48.84, 'A': 2.33}
- **CDS aligned content (<base>:%):** {'A': 27.76, 'T': 22.79, 'G': 25.09, 'C': 24.36}
- **3-UTR aligned content (<base>:%):** {'G': 16.6, 'T': 40.25, 'A': 30.29, 'C': 12.86}

**Uniprot Description:**  
  
 Plays a central role during spermatogenesis by repressing transposable elements and preventing their mobilization, which is essential for the germline integrity. Acts via the piRNA metabolic process, which mediates the repression of transposable elements during meiosis by forming complexes composed of piRNAs and Piwi proteins and governs the methylation and subsequent repression of transposons. Its association with piP-bodies suggests a participation in the secondary piRNAs metabolic process. Required for the localization of germ-cell factors to the meiotic nuage (By similarity).   
  
Interacts with SMARCB1, SIN3B and DDX4. Interacts with piRNA-associated proteins TDRD1, PIWIL1 and PIWIL2 (By similarity). Interacts with TEX19 (By similarity).   
  
 **Gene Ontology Information:**

Molecular Function

- sequence-specific DNA binding

Location

- autosome
- chromatin
- chromatoid body
- cytoplasm
- male germ cell nucleus
- nucleus
- P granule
- perinuclear region of cytoplasm
- piP-body
- XY body

Biological process

- cell morphogenesis
- ectopic germ cell programmed cell death
- genetic imprinting
- fertilization
- homologous chromosome pairing at meiosis
- intrinsic apoptotic signaling pathway in response to DNA damage
- male meiotic nuclear division
- negative regulation of apoptotic process
- negative regulation of developmental process
- negative regulation of transcription, DNA-templated
- negative regulation of reproductive process
- negative regulation of transcription by RNA polymerase II
- piRNA metabolic process
- regulation of gene silencing by miRNA
- regulation of organ growth
- gene silencing by RNA
- spermatogenesis

---

40

- **Protein name:** Ankyrin repeat domain-containing protein 13D
- **Organism:** Homo sapiens
- **Uniprot Accession Number:** Q6ZTN6
- **Protein sequence length:** 605 aa
- **1D identity (%):** 12.12
- **1D identity (%) [Gaps excluded]:** 23.45
- **1D identity - Alignment Gaps:** 466
- **1D aligned content (<aminoacid>:%):** {'A': 5.98, 'G': 7.69, 'P': 11.97, 'L': 11.97, 'R': 9.4, 'V': 5.98, 'W': 1.71, 'H': 3.42, 'I': 2.56, 'E': 7.69, 'T': 9.4, 'Q': 9.4, 'D': 1.71, 'K': 3.42, 'C': 0.85, 'S': 3.42, 'N': 1.71, 'F': 0.85, 'M': 0.85}
- **Common reported functions (%):** 0.0
- **Common reported locations (%):** 7.14
- **Common reported processes (%):** 0.0

- **AF ID:** Q6ZTN6
- **Chain:** A
- **Protein length:** 518 aa
- **Resolution:** N/A
- **b-phipsi:** 0.00118
- **w-rdist:** 0.700339
- **t-alpha:** 0.00219
- **Chemical similarity (Tanimoto Index) (%):** 97.85
- **1D identity (%) [PDB]:** 3.17
- **1D identity (%) [Gaps excluded][PDB]:** 66.13
- **1D identity - Alignment Gaps [PDB]:** 1232
- **1D aligned content [PDB] (<aminoacid>:%):** {'M': 2.44, 'V': 9.76, 'K': 4.88, 'E': 9.76, 'T': 7.32, 'D': 2.44, 'R': 7.32, 'L': 7.32, 'Q': 9.76, 'P': 7.32, 'S': 4.88, 'A': 7.32, 'F': 4.88, 'H': 2.44, 'G': 2.44, 'I': 7.32, 'W': 2.44}
- **2D identity (%) [PDB]:** 39.49
- **2D identity (%) [Gaps excluded][PDB]:** 89.86
- **2D identity - Alignment Gaps [PDB]:** 528
- **2D aligned content [PDB] (<2D-fold>:%):** {'H': 44.35, '.': 16.4, 'E': 23.66, 'T': 15.59}
- **3D similarity (TM-Score) (%) [PDB]:** 15.97

- **Gene name:** ANKRD13D
- **Entrez ID:** 338692
- **RefSeq ID:** NM\_207354
- **Transcript sequence length:** 2128
- **5-UTR|CDS|3-UTR identity (%):** 44.75 | 40.03 | 0.79
- **5-UTR|CDS|3-UTR identity (%) [Gaps excluded]:** 77.88 | 75.03 | 75.81
- **5-UTR|CDS|3-UTR identity [Alignment Gaps]:** 77 | 1338 | 11792
- **5-UTR aligned content (<base>:%):** {'G': 43.21, 'C': 50.62, 'T': 3.7, 'A': 2.47}
- **CDS aligned content (<base>:%):** {'A': 22.04, 'T': 15.85, 'G': 30.31, 'C': 31.79}
- **3-UTR aligned content (<base>:%):** {'G': 40.43, 'C': 22.34, 'A': 19.15, 'T': 18.09}

**Uniprot Description:**  
  
 Ubiquitin-binding protein that specifically recognizes and binds 'Lys-63'-linked ubiquitin. Does not bind 'Lys-48'-linked ubiquitin. Positively regulates the internalization of ligand-activated EGFR by binding to the Ub moiety of ubiquitinated EGFR at the cell membrane.   
  
Interacts with EGFR (ubiquitinated); the interaction is direct and may regulate EGFR internalization.   
  
 **Gene Ontology Information:**

Molecular Function

- ubiquitin-dependent protein binding

Location

- cytoplasm
- late endosome
- perinuclear region of cytoplasm
- plasma membrane

Biological process

- negative regulation of receptor internalization

---

41

- **Protein name:** Threonine--tRNA ligase 1, cytoplasmic
- **Organism:** Homo sapiens
- **Uniprot Accession Number:** P26639
- **Protein sequence length:** 723 aa
- **1D identity (%):** 14.36
- **1D identity (%) [Gaps excluded]:** 22.64
- **1D identity - Alignment Gaps:** 354
- **1D aligned content (<aminoacid>:%):** {'E': 6.47, 'S': 2.88, 'P': 9.35, 'G': 10.79, 'K': 7.19, 'L': 8.63, 'I': 3.6, 'V': 6.47, 'Q': 5.04, 'Y': 4.32, 'C': 2.88, 'A': 4.32, 'T': 5.04, 'F': 5.04, 'D': 4.32, 'H': 3.6, 'N': 2.16, 'R': 6.47, 'M': 1.44}
- **Common reported functions (%):** 0.0
- **Common reported locations (%):** 7.14
- **Common reported processes (%):** 0.0

- **AF ID:** P26639
- **Chain:** A
- **Protein length:** 723 aa
- **Resolution:** N/A
- **b-phipsi:** 0.003904
- **w-rdist:** 0.681213
- **t-alpha:** 0.000729
- **Chemical similarity (Tanimoto Index) (%):** 98.65
- **1D identity (%) [PDB]:** 2.65
- **1D identity (%) [Gaps excluded][PDB]:** 74.07
- **1D identity - Alignment Gaps [PDB]:** 1453
- **1D aligned content [PDB] (<aminoacid>:%):** {'M': 2.5, 'P': 7.5, 'I': 10.0, 'G': 5.0, 'R': 7.5, 'L': 15.0, 'E': 7.5, 'V': 7.5, 'T': 5.0, 'K': 7.5, 'S': 7.5, 'C': 2.5, 'A': 7.5, 'D': 2.5, 'F': 2.5, 'Q': 2.5}
- **2D identity (%) [PDB]:** 41.57
- **2D identity (%) [Gaps excluded][PDB]:** 91.39
- **2D identity - Alignment Gaps [PDB]:** 585
- **2D aligned content [PDB] (<2D-fold>:%):** {'.': 12.78, 'E': 31.17, 'T': 15.7, 'H': 39.69, 'G': 0.67}
- **3D similarity (TM-Score) (%) [PDB]:** 25.42

- **Gene name:** TARS1
- **Entrez ID:** 8626539
- **RefSeq ID:** NM\_152295
- **Transcript sequence length:** 2672
- **5-UTR|CDS|3-UTR identity (%):** 49.65 | 41.53 | 2.08
- **5-UTR|CDS|3-UTR identity (%) [Gaps excluded]:** 66.98 | 74.63 | 77.09
- **5-UTR|CDS|3-UTR identity [Alignment Gaps]:** 37 | 1354 | 11620
- **5-UTR aligned content (<base>:%):** {'G': 38.03, 'T': 9.86, 'C': 49.3, 'A': 2.82}
- **CDS aligned content (<base>:%):** {'A': 29.57, 'T': 20.82, 'G': 27.13, 'C': 22.48}
- **3-UTR aligned content (<base>:%):** {'T': 38.15, 'A': 39.36, 'C': 6.83, 'G': 15.66}

**Uniprot Description:**  
  
 Catalyzes the attachment of threonine to tRNA(Thr) in a two-step reaction: threonine is first activated by ATP to form Thr-AMP and then transferred to the acceptor end of tRNA(Thr) (PubMed:25824639, PubMed:31374204). Also edits incorrectly charged tRNA(Thr) via its editing domain, at the post-transfer stage (By similarity).   
  
Homodimer.   
  
 **Gene Ontology Information:**

Molecular Function

- ATP binding
- threonine-tRNA ligase activity

Location

- cytoplasm
- phagocytic vesicle

Biological process

- threonyl-tRNA aminoacylation

---

42

- **Protein name:** Cytosolic phospholipase A2 zeta
- **Organism:** Homo sapiens
- **Uniprot Accession Number:** Q68DD2
- **Protein sequence length:** 849 aa
- **1D identity (%):** 15.69
- **1D identity (%) [Gaps excluded]:** 23.26
- **1D identity - Alignment Gaps:** 332
- **1D aligned content (<aminoacid>:%):** {'M': 0.62, 'P': 11.25, 'G': 10.0, 'R': 5.62, 'K': 3.12, 'N': 1.88, 'D': 3.12, 'Y': 5.62, 'L': 14.37, 'I': 2.5, 'V': 8.12, 'F': 5.0, 'Q': 7.5, 'H': 1.25, 'T': 3.12, 'A': 5.62, 'E': 5.0, 'S': 2.5, 'C': 3.75}
- **Common reported functions (%):** 0.0
- **Common reported locations (%):** 7.14
- **Common reported processes (%):** 0.0

- **AF ID:** Q68DD2
- **Chain:** A
- **Protein length:** 849 aa
- **Resolution:** N/A
- **b-phipsi:** 0.001067
- **w-rdist:** 0.283534
- **t-alpha:** 0.059723
- **Chemical similarity (Tanimoto Index) (%):** 99.62
- **1D identity (%) [PDB]:** 2.92
- **1D identity (%) [Gaps excluded][PDB]:** 62.67
- **1D identity - Alignment Gaps [PDB]:** 1537
- **1D aligned content [PDB] (<aminoacid>:%):** {'M': 2.13, 'K': 6.38, 'R': 8.51, 'C': 4.26, 'V': 12.77, 'T': 6.38, 'A': 6.38, 'S': 2.13, 'Q': 14.89, 'F': 4.26, 'P': 4.26, 'L': 10.64, 'E': 4.26, 'G': 4.26, 'D': 2.13, 'Y': 4.26, 'I': 2.13}
- **2D identity (%) [PDB]:** 46.49
- **2D identity (%) [Gaps excluded][PDB]:** 89.43
- **2D identity - Alignment Gaps [PDB]:** 533
- **2D aligned content [PDB] (<2D-fold>:%):** {'.': 17.83, 'E': 33.14, 'T': 21.32, 'H': 26.55, 'G': 1.16}
- **3D similarity (TM-Score) (%) [PDB]:** 21.98

- **Gene name:** PLA2G4F
- **Entrez ID:** 255189
- **RefSeq ID:** NM\_213600
- **Transcript sequence length:** 5587
- **5-UTR|CDS|3-UTR identity (%):** 37.14 | 44.15 | 14.86
- **5-UTR|CDS|3-UTR identity (%) [Gaps excluded]:** 70.27 | 74.49 | 75.66
- **5-UTR|CDS|3-UTR identity [Alignment Gaps]:** 66 | 1312 | 9966
- **5-UTR aligned content (<base>:%):** {'A': 1.92, 'G': 48.08, 'C': 44.23, 'T': 5.77}
- **CDS aligned content (<base>:%):** {'A': 20.89, 'T': 17.93, 'G': 29.89, 'C': 31.29}
- **3-UTR aligned content (<base>:%):** {'A': 20.62, 'G': 27.51, 'C': 26.48, 'T': 25.39}

**Uniprot Description:**  
  
 Has calcium-dependent phospholipase and lysophospholipase activities with a potential role in membrane lipid remodeling and biosynthesis of lipid mediators (PubMed:29158256). Preferentially hydrolyzes the ester bond of the fatty acyl group attached at sn-2 position of phospholipids (phospholipase A2 activity) (PubMed:29158256). Selectively hydrolyzes sn-2 arachidonoyl group from membrane phospholipids, providing the precursor for eicosanoid biosynthesis (PubMed:29158256). In myocardial mitochondria, plays a major role in arachidonate release that is metabolically channeled to the formation of cardioprotective eicosanoids, epoxyeicosatrienoates (EETs) (PubMed:29158256). N/A   
  
 **Gene Ontology Information:**

Molecular Function

- calcium ion binding
- calcium-dependent phospholipase A2 activity
- calcium-dependent phospholipid binding
- lysophospholipase activity
- phospholipase A1 activity

Location

- cytosol
- mitochondrion
- ruffle membrane
- vesicle

Biological process

- arachidonic acid secretion
- cellular response to antibiotic
- cellular response to organic cyclic compound
- glycerophospholipid catabolic process
- phosphatidylglycerol acyl-chain remodeling
- prostaglandin biosynthetic process

---

43

- **Protein name:** Piwi-like protein 1
- **Organism:** Homo sapiens
- **Uniprot Accession Number:** Q96J94
- **Protein sequence length:** 861 aa
- **1D identity (%):** 21.87
- **1D identity (%) [Gaps excluded]:** 28.55
- **1D identity - Alignment Gaps:** 228
- **1D aligned content (<aminoacid>:%):** {'G': 11.27, 'P': 9.39, 'F': 5.16, 'R': 8.45, 'D': 6.1, 'S': 3.76, 'L': 9.86, 'N': 2.35, 'Y': 5.63, 'Q': 5.63, 'H': 2.35, 'T': 4.69, 'K': 3.76, 'V': 7.04, 'I': 3.29, 'M': 1.88, 'W': 0.94, 'E': 4.23, 'C': 1.41, 'A': 2.82}
- **Common reported functions (%):** 20.0
- **Common reported locations (%):** 14.29
- **Common reported processes (%):** 4.76

- **AF ID:** Q96J94
- **Chain:** A
- **Protein length:** 861 aa
- **Resolution:** N/A
- **b-phipsi:** 0.004231
- **w-rdist:** 0.161129
- **t-alpha:** 0.043313
- **Chemical similarity (Tanimoto Index) (%):** 99.4
- **1D identity (%) [PDB]:** 4.94
- **1D identity (%) [Gaps excluded][PDB]:** 55.4
- **1D identity - Alignment Gaps [PDB]:** 1421
- **1D aligned content [PDB] (<aminoacid>:%):** {'M': 1.3, 'V': 7.79, 'R': 7.79, 'E': 1.3, 'L': 5.19, 'I': 5.19, 'Q': 5.19, 'F': 6.49, 'Y': 10.39, 'K': 3.9, 'S': 3.9, 'T': 7.79, 'P': 7.79, 'D': 7.79, 'G': 6.49, 'N': 1.3, 'A': 5.19, 'H': 3.9, 'C': 1.3}
- **2D identity (%) [PDB]:** 65.85
- **2D identity (%) [Gaps excluded][PDB]:** 89.32
- **2D identity - Alignment Gaps [PDB]:** 257
- **2D aligned content [PDB] (<2D-fold>:%):** {'.': 17.29, 'E': 31.31, 'T': 16.98, 'H': 32.71, 'G': 0.93, 'B': 0.78}
- **3D similarity (TM-Score) (%) [PDB]:** 76.88

- **Gene name:** PIWIL1
- **Entrez ID:** 9271
- **RefSeq ID:** N/A
- **Sequence length:** N/A
- **5-UTR|CDS|3-UTR identity (%):** N/A | N/A | N/A
- **5-UTR|CDS|3-UTR identity (%) [Gaps excluded]:** N/A | N/A | N/A
- **5-UTR|CDS|3-UTR identity [Alignment Gaps]:** N/A | N/A | N/A
- **5-UTR aligned content (<base>:%):** N/A
- **CDS aligned content (<base>:%):** N/A
- **3-UTR aligned content (<base>:%):** N/A

**Uniprot Description:**  
  
 Endoribonuclease that plays a central role in postnatal germ cells by repressing transposable elements and preventing their mobilization, which is essential for the germline integrity. Acts via the piRNA metabolic process, which mediates the repression of transposable elements during meiosis by forming complexes composed of piRNAs and Piwi proteins and governs the methylation and subsequent repression of transposons. Directly binds methylated piRNAs, a class of 24 to 30 nucleotide RNAs that are generated by a Dicer-independent mechanism and are primarily derived from transposons and other repeated sequence elements. Strongly prefers a uridine in the first position of their guide (g1U preference, also named 1U-bias). Not involved in the piRNA amplification loop, also named ping-pong amplification cycle. Acts as an endoribonuclease that cleaves transposon messenger RNAs. Besides their function in transposable elements repression, piRNAs are probably involved in other processes during meiosis such as translation regulation. Probable component of some RISC complex, which mediates RNA cleavage and translational silencing. Also plays a role in the formation of chromatoid bodies and is required for some miRNAs stability. Required to sequester RNF8 in the cytoplasm until late spermatogenesis; RNF8 being released upon ubiquitination and degradation of PIWIL1.   
  
Interacts (via Piwi domain) with DICER1, suggesting that it forms ribonucleoprotein RISC complexes; this interaction is regulated by HSP90AB1 activity. Interacts with MAEL, KIF17, PABPC1, PRMT5 and WDR77. Interacts (when methylated on arginine residues) with TDRD1, TDRKH/TDRD2, RNF17/TDRD4, TDRD6, TDRD7 and TDRD9. Interacts with CLOCK. Interacts with MOV10L1. Interacts with ANAPC10; interaction oly takes place following piRNA-binding. Interacts with RNF8; leading to sequester RNF8 in the cytoplasm. Interacts with TEX19 (By similarity).   
  
 **Gene Ontology Information:**

Molecular Function

- metal ion binding
- mRNA binding
- mRNA cap binding complex binding
- piRNA binding
- polysome binding
- protein kinase binding
- endoribonuclease activity
- single-stranded RNA binding

Location

- chromatoid body
- cytoplasm
- dense body
- nucleus
- P granule

Biological process

- meiotic cell cycle
- piRNA-mediated retrotransposon silencing by heterochromatin formation
- primary piRNA processing
- regulation of translation
- gene silencing by RNA
- sperm chromatin condensation
- spermatid development
- spermatogenesis

---

44

- **Protein name:** Muskelin
- **Organism:** Homo sapiens
- **Uniprot Accession Number:** Q9UL63
- **Protein sequence length:** 735 aa
- **1D identity (%):** 16.05
- **1D identity (%) [Gaps excluded]:** 23.12
- **1D identity - Alignment Gaps:** 288
- **1D aligned content (<aminoacid>:%):** {'A': 8.61, 'G': 8.61, 'P': 7.95, 'Y': 2.65, 'S': 5.3, 'L': 8.61, 'N': 3.31, 'D': 7.28, 'E': 1.99, 'R': 3.31, 'I': 4.64, 'V': 7.28, 'K': 6.62, 'T': 6.62, 'F': 5.3, 'W': 1.32, 'H': 2.65, 'Q': 3.97, 'C': 3.31, 'M': 0.66}
- **Common reported functions (%):** 0.0
- **Common reported locations (%):** 21.43
- **Common reported processes (%):** 0.0

- **AF ID:** Q9UL63
- **Chain:** A
- **Protein length:** 735 aa
- **Resolution:** N/A
- **b-phipsi:** 0.002589
- **w-rdist:** 0.220003
- **t-alpha:** 0.059413
- **Chemical similarity (Tanimoto Index) (%):** 99.4
- **1D identity (%) [PDB]:** 2.35
- **1D identity (%) [Gaps excluded][PDB]:** 85.71
- **1D identity - Alignment Gaps [PDB]:** 1489
- **1D aligned content [PDB] (<aminoacid>:%):** {'T': 11.11, 'Y': 2.78, 'L': 11.11, 'P': 2.78, 'E': 8.33, 'V': 5.56, 'C': 5.56, 'N': 5.56, 'I': 5.56, 'Q': 8.33, 'K': 8.33, 'D': 5.56, 'S': 5.56, 'M': 2.78, 'R': 2.78, 'A': 8.33}
- **2D identity (%) [PDB]:** 42.54
- **2D identity (%) [Gaps excluded][PDB]:** 89.17
- **2D identity - Alignment Gaps [PDB]:** 557
- **2D aligned content [PDB] (<2D-fold>:%):** {'.': 20.53, 'E': 33.55, 'T': 22.74, 'H': 21.63, 'B': 0.22, 'G': 1.32}
- **3D similarity (TM-Score) (%) [PDB]:** 22.72

- **Gene name:** MKLN1
- **Entrez ID:** 4289
- **RefSeq ID:** NM\_013255
- **Transcript sequence length:** 11136
- **5-UTR|CDS|3-UTR identity (%):** 14.17 | 44.07 | 39.32
- **5-UTR|CDS|3-UTR identity (%) [Gaps excluded]:** 75.0 | 73.03 | 75.32
- **5-UTR|CDS|3-UTR identity [Alignment Gaps]:** 103 | 1184 | 6530
- **5-UTR aligned content (<base>:%):** {'G': 55.56, 'C': 38.89, 'A': 5.56}
- **CDS aligned content (<base>:%):** {'A': 29.79, 'T': 23.33, 'G': 24.54, 'C': 22.34}
- **3-UTR aligned content (<base>:%):** {'C': 16.24, 'T': 37.29, 'G': 17.72, 'A': 28.75}

**Uniprot Description:**  
  
 Component of the CTLH E3 ubiquitin-protein ligase complex that selectively accepts ubiquitin from UBE2H and mediates ubiquitination and subsequent proteasomal degradation of the transcription factor HBP1 (PubMed:29911972). Required for internalization of the GABA receptor GABRA1 from the cell membrane via endosomes and subsequent GABRA1 degradation (By similarity). Acts as a mediator of cell spreading and cytoskeletal responses to the extracellular matrix component THBS1 (PubMed:18710924).   
  
Homodimer; may form higher oligomers (By similarity). Identified in the CTLH complex that contains GID4, RANBP9 and/or RANBP10, MKLN1, MAEA, RMND5A (or alternatively its paralog RMND5B), GID8, ARMC8, WDR26 and YPEL5 (PubMed:17467196, PubMed:29911972). Within this complex, MAEA, RMND5A (or alternatively its paralog RMND5B), GID8, WDR26, and RANBP9 and/or RANBP10 form the catalytic core, while GID4, MKLN1, ARMC8 and YPEL5 have ancillary roles (PubMed:29911972). Interacts with RANBP9 (PubMed:18710924). Part of a complex consisting of RANBP9, MKLN1 and GID8 (PubMed:12559565). Interacts with GABRA1. Interacts with the C-terminal tail of PTGER3 (By similarity).   
  
 **Gene Ontology Information:**

Molecular Function

- identical protein binding
- protein homodimerization activity

Location

- cell cortex
- cytoplasm
- cytosol
- nucleoplasm
- postsynapse
- ruffle
- ubiquitin ligase complex

Biological process

- actin cytoskeleton reorganization
- cell-matrix adhesion
- regulation of cell shape
- regulation of receptor internalization
- signal transduction

---

45

- **Protein name:** Leucine-rich repeat transmembrane neuronal protein 2
- **Organism:** Homo sapiens
- **Uniprot Accession Number:** O43300
- **Protein sequence length:** 516 aa
- **1D identity (%):** 11.29
- **1D identity (%) [Gaps excluded]:** 22.25
- **1D identity - Alignment Gaps:** 449
- **1D aligned content (<aminoacid>:%):** {'G': 3.88, 'L': 15.53, 'M': 0.97, 'Y': 4.85, 'V': 3.88, 'P': 4.85, 'A': 4.85, 'Q': 5.83, 'F': 6.8, 'D': 5.83, 'H': 3.88, 'N': 6.8, 'S': 4.85, 'T': 5.83, 'K': 3.88, 'R': 6.8, 'W': 0.97, 'E': 4.85, 'I': 2.91, 'C': 1.94}
- **Common reported functions (%):** 0.0
- **Common reported locations (%):** 0.0
- **Common reported processes (%):** 0.0

- **AF ID:** O43300
- **Chain:** A
- **Protein length:** 516 aa
- **Resolution:** N/A
- **b-phipsi:** 0.003366
- **w-rdist:** 0.735312
- **t-alpha:** 0.000729
- **Chemical similarity (Tanimoto Index) (%):** 96.91
- **1D identity (%) [PDB]:** 2.21
- **1D identity (%) [Gaps excluded][PDB]:** 67.44
- **1D identity - Alignment Gaps [PDB]:** 1268
- **1D aligned content [PDB] (<aminoacid>:%):** {'L': 20.69, 'G': 6.9, 'M': 3.45, 'A': 3.45, 'T': 6.9, 'Q': 10.34, 'N': 13.79, 'R': 6.9, 'P': 10.34, 'S': 3.45, 'K': 6.9, 'I': 3.45, 'V': 3.45}
- **2D identity (%) [PDB]:** 30.91
- **2D identity (%) [Gaps excluded][PDB]:** 88.32
- **2D identity - Alignment Gaps [PDB]:** 652
- **2D aligned content [PDB] (<2D-fold>:%):** {'.': 21.94, 'H': 30.97, 'T': 33.23, 'E': 12.58, 'G': 0.97, 'B': 0.32}
- **3D similarity (TM-Score) (%) [PDB]:** 16.66

- **Gene name:** LRRTM2
- **Entrez ID:** 26045
- **RefSeq ID:** NM\_015564
- **Transcript sequence length:** 6064
- **5-UTR|CDS|3-UTR identity (%):** 16.97 | 34.86 | 18.3
- **5-UTR|CDS|3-UTR identity (%) [Gaps excluded]:** 68.81 | 73.33 | 74.76
- **5-UTR|CDS|3-UTR identity [Alignment Gaps]:** 333 | 1469 | 9693
- **5-UTR aligned content (<base>:%):** {'C': 40.0, 'G': 44.0, 'T': 12.0, 'A': 4.0}
- **CDS aligned content (<base>:%):** {'A': 27.77, 'G': 23.57, 'T': 20.59, 'C': 28.07}
- **3-UTR aligned content (<base>:%):** {'T': 35.93, 'C': 17.24, 'A': 32.57, 'G': 14.26}

**Uniprot Description:**  
  
 Involved in the development and maintenance of excitatory synapses in the vertebrate nervous system. Regulates surface expression of AMPA receptors and instructs the development of functional glutamate release sites. Acts as a ligand for the presynaptic receptors NRXN1-A and NRXN1-B (By similarity).   
  
Interacts with DLG4. Interacts with neurexin NRXN1; interaction is mediated by heparan sulfate glycan modification on neurexin.   
  
 **Gene Ontology Information:**

Molecular Function

- neurexin family protein binding

Location

- excitatory synapse
- extracellular space
- GABA-ergic synapse
- glutamatergic synapse
- hippocampal mossy fiber to CA3 synapse
- postsynaptic density membrane
- postsynaptic specialization membrane
- Schaffer collateral - CA1 synapse

Biological process

- long-term synaptic potentiation
- negative regulation of receptor internalization
- positive regulation of synapse assembly
- regulation of postsynaptic density assembly
- synapse organization

---

46

- **Protein name:** Alpha-1,6-mannosylglycoprotein 6-beta-N-acetylglucosaminyltransferase B
- **Organism:** Homo sapiens
- **Uniprot Accession Number:** Q3V5L5
- **Protein sequence length:** 792 aa
- **1D identity (%):** 13.41
- **1D identity (%) [Gaps excluded]:** 21.78
- **1D identity - Alignment Gaps:** 393
- **1D aligned content (<aminoacid>:%):** {'P': 10.95, 'V': 5.84, 'R': 6.57, 'F': 5.84, 'G': 11.68, 'A': 5.11, 'L': 11.68, 'E': 5.84, 'K': 5.84, 'S': 2.19, 'H': 2.92, 'I': 1.46, 'Q': 7.3, 'D': 3.65, 'W': 1.46, 'C': 3.65, 'M': 2.19, 'T': 2.92, 'N': 1.46, 'Y': 1.46}
- **Common reported functions (%):** 0.0
- **Common reported locations (%):** 0.0
- **Common reported processes (%):** 0.0

- **AF ID:** Q3V5L5
- **Chain:** A
- **Protein length:** 792 aa
- **Resolution:** N/A
- **b-phipsi:** 0.006563
- **w-rdist:** 0.178434
- **t-alpha:** 0.02185
- **Chemical similarity (Tanimoto Index) (%):** 96.99
- **1D identity (%) [PDB]:** 3.29
- **1D identity (%) [Gaps excluded][PDB]:** 62.2
- **1D identity - Alignment Gaps [PDB]:** 1466
- **1D aligned content [PDB] (<aminoacid>:%):** {'M': 3.92, 'L': 9.8, 'I': 5.88, 'G': 9.8, 'R': 9.8, 'D': 3.92, 'K': 5.88, 'V': 7.84, 'E': 5.88, 'T': 5.88, 'P': 7.84, 'F': 7.84, 'S': 7.84, 'Q': 1.96, 'A': 1.96, 'H': 1.96, 'C': 1.96}
- **2D identity (%) [PDB]:** 41.44
- **2D identity (%) [Gaps excluded][PDB]:** 88.46
- **2D identity - Alignment Gaps [PDB]:** 590
- **2D aligned content [PDB] (<2D-fold>:%):** {'.': 21.96, 'H': 41.3, 'T': 22.61, 'G': 1.96, 'E': 12.17}
- **3D similarity (TM-Score) (%) [PDB]:** 24.18

- **Gene name:** MGAT5B
- **Entrez ID:** 146664
- **RefSeq ID:** NM\_198955
- **Transcript sequence length:** 4068
- **5-UTR|CDS|3-UTR identity (%):** 34.21 | 46.7 | 8.01
- **5-UTR|CDS|3-UTR identity (%) [Gaps excluded]:** 66.67 | 73.26 | 73.75
- **5-UTR|CDS|3-UTR identity [Alignment Gaps]:** 74 | 1104 | 10811
- **5-UTR aligned content (<base>:%):** {'G': 36.54, 'C': 48.08, 'A': 1.92, 'T': 13.46}
- **CDS aligned content (<base>:%):** {'A': 18.85, 'T': 16.39, 'G': 30.94, 'C': 33.83}
- **3-UTR aligned content (<base>:%):** {'A': 18.31, 'T': 24.07, 'C': 30.25, 'G': 27.37}

**Uniprot Description:**  
  
 Glycosyltransferase that acts on alpha-linked mannose of N-glycans and O-mannosyl glycans. Catalyzes the transfer of N-acetylglucosamine (GlcNAc) to the beta 1-6 linkage of the mannose residue of GlcNAc-beta1,2-Man-alpha on both the alpha1,3- and alpha1,6-linked mannose arms in the core structure of N-glycan. Also acts on the GlcNAc-beta1,2-Man-alpha1-Ser/Thr moiety, forming a 2,6-branched structure in brain O-mannosyl glycan. Plays an active role in modulating integrin and laminin-dependent adhesion and migration of neuronal cells via its activity in the O-mannosyl glycan pathway. N/A   
  
 **Gene Ontology Information:**

Molecular Function

- alpha-1,6-mannosylglycoprotein 6-beta-N-acetylglucosaminyltransferase activity
- manganese ion binding

Location

- Golgi apparatus
- Golgi membrane

Biological process

- protein N-linked glycosylation
- protein O-linked glycosylation via serine

---

47

- **Protein name:** Exosome complex exonuclease RRP44
- **Organism:** Homo sapiens
- **Uniprot Accession Number:** Q9Y2L1
- **Protein sequence length:** 958 aa
- **1D identity (%):** 16.73
- **1D identity (%) [Gaps excluded]:** 23.01
- **1D identity - Alignment Gaps:** 287
- **1D aligned content (<aminoacid>:%):** {'M': 0.57, 'A': 5.11, 'G': 7.95, 'P': 8.52, 'S': 4.55, 'Q': 2.84, 'D': 5.11, 'R': 9.66, 'V': 6.82, 'K': 7.39, 'Y': 2.27, 'T': 6.82, 'E': 5.68, 'W': 1.14, 'L': 8.52, 'N': 1.7, 'I': 7.39, 'F': 2.84, 'H': 3.41, 'C': 1.7}
- **Common reported functions (%):** 6.67
- **Common reported locations (%):** 28.57
- **Common reported processes (%):** 0.0

- **AF ID:** Q9Y2L1
- **Chain:** A
- **Protein length:** 958 aa
- **Resolution:** N/A
- **b-phipsi:** 0.002812
- **w-rdist:** 0.342991
- **t-alpha:** 0.011654
- **Chemical similarity (Tanimoto Index) (%):** 99.47
- **1D identity (%) [PDB]:** 2.23
- **1D identity (%) [Gaps excluded][PDB]:** 76.47
- **1D identity - Alignment Gaps [PDB]:** 1694
- **1D aligned content [PDB] (<aminoacid>:%):** {'M': 2.56, 'V': 7.69, 'R': 7.69, 'E': 7.69, 'L': 10.26, 'Q': 7.69, 'F': 5.13, 'Y': 2.56, 'K': 10.26, 'S': 5.13, 'T': 5.13, 'P': 2.56, 'I': 10.26, 'D': 2.56, 'G': 2.56, 'H': 5.13, 'A': 5.13}
- **2D identity (%) [PDB]:** 41.96
- **2D identity (%) [Gaps excluded][PDB]:** 87.31
- **2D identity - Alignment Gaps [PDB]:** 630
- **2D aligned content [PDB] (<2D-fold>:%):** {'.': 17.09, 'E': 19.65, 'T': 23.18, 'H': 36.74, 'G': 2.95, 'B': 0.39}
- **3D similarity (TM-Score) (%) [PDB]:** 24.41

- **Gene name:** DIS3
- **Entrez ID:** 22894
- **RefSeq ID:** NM\_014953
- **Transcript sequence length:** 10571
- **5-UTR|CDS|3-UTR identity (%):** 21.29 | 42.8 | 35.3
- **5-UTR|CDS|3-UTR identity (%) [Gaps excluded]:** 82.5 | 71.24 | 74.42
- **5-UTR|CDS|3-UTR identity [Alignment Gaps]:** 115 | 1361 | 6958
- **5-UTR aligned content (<base>:%):** {'G': 57.58, 'T': 15.15, 'C': 21.21, 'A': 6.06}
- **CDS aligned content (<base>:%):** {'A': 30.09, 'T': 20.97, 'G': 25.09, 'C': 23.85}
- **3-UTR aligned content (<base>:%):** {'C': 18.21, 'T': 33.9, 'A': 29.49, 'G': 18.39}

**Uniprot Description:**  
  
 Putative catalytic component of the RNA exosome complex which has 3'->5' exoribonuclease activity and participates in a multitude of cellular RNA processing and degradation events. In the nucleus, the RNA exosome complex is involved in proper maturation of stable RNA species such as rRNA, snRNA and snoRNA, in the elimination of RNA processing by-products and non-coding 'pervasive' transcripts, such as antisense RNA species and promoter-upstream transcripts (PROMPTs), and of mRNAs with processing defects, thereby limiting or excluding their export to the cytoplasm. The RNA exosome may be involved in Ig class switch recombination (CSR) and/or Ig variable region somatic hypermutation (SHM) by targeting AICDA deamination activity to transcribed dsDNA substrates. In the cytoplasm, the RNA exosome complex is involved in general mRNA turnover and specifically degrades inherently unstable mRNAs containing AU-rich elements (AREs) within their 3' untranslated regions, and in RNA surveillance pathways, preventing translation of aberrant mRNAs. It seems to be involved in degradation of histone mRNA. DIS3 has both 3'-5' exonuclease and endonuclease activities.   
  
Component of the RNA exosome complex (PubMed:29906447). The catalytically inactive RNA exosome core (Exo-9) complex is believed to associate with catalytic subunits EXOSC10, and DIS3 or DIS3L in cytoplasmic- and nuclear-specific RNA exosome complex forms.   
  
 **Gene Ontology Information:**

Molecular Function

- 3'-5'-exoribonuclease activity
- endonuclease activity
- guanyl-nucleotide exchange factor activity
- RNA binding

Location

- cytoplasmic exosome (RNase complex)
- cytosol
- exosome (RNase complex)
- membrane
- nuclear exosome (RNase complex)
- nucleoplasm
- nucleus

Biological process

- CUT catabolic process
- exonucleolytic catabolism of deadenylated mRNA
- RNA catabolic process
- RNA processing
- rRNA catabolic process
- rRNA processing

---

48

- **Protein name:** Putative E3 ubiquitin-protein ligase UBR7
- **Organism:** Homo sapiens
- **Uniprot Accession Number:** Q8N806
- **Protein sequence length:** 425 aa
- **1D identity (%):** 7.96
- **1D identity (%) [Gaps excluded]:** 23.1
- **1D identity - Alignment Gaps:** 626
- **1D aligned content (<aminoacid>:%):** {'M': 3.95, 'A': 5.26, 'P': 9.21, 'V': 3.95, 'E': 2.63, 'D': 9.21, 'S': 3.95, 'G': 6.58, 'K': 9.21, 'R': 6.58, 'Y': 2.63, 'T': 2.63, 'H': 1.32, 'F': 3.95, 'L': 9.21, 'C': 7.89, 'I': 2.63, 'Q': 6.58, 'N': 2.63}
- **Common reported functions (%):** 0.0
- **Common reported locations (%):** 0.0
- **Common reported processes (%):** 0.0

- **AF ID:** Q8N806
- **Chain:** A
- **Protein length:** 425 aa
- **Resolution:** N/A
- **b-phipsi:** 0.002895
- **w-rdist:** 0.348672
- **t-alpha:** 0.008076
- **Chemical similarity (Tanimoto Index) (%):** 99.17
- **1D identity (%) [PDB]:** 1.87
- **1D identity (%) [Gaps excluded][PDB]:** 67.65
- **1D identity - Alignment Gaps [PDB]:** 1195
- **1D aligned content [PDB] (<aminoacid>:%):** {'A': 8.7, 'G': 8.7, 'Q': 4.35, 'L': 13.04, 'V': 26.09, 'P': 4.35, 'K': 8.7, 'Y': 4.35, 'E': 4.35, 'D': 4.35, 'T': 4.35, 'M': 4.35, 'C': 4.35}
- **2D identity (%) [PDB]:** 27.13
- **2D identity (%) [Gaps excluded][PDB]:** 86.71
- **2D identity - Alignment Gaps [PDB]:** 661
- **2D aligned content [PDB] (<2D-fold>:%):** {'.': 24.14, 'H': 47.13, 'T': 16.09, 'E': 11.49, 'G': 1.15}
- **3D similarity (TM-Score) (%) [PDB]:** 15.57

- **Gene name:** UBR7
- **Entrez ID:** N/A
- **RefSeq ID:** NM\_175748
- **Transcript sequence length:** 3494
- **5-UTR|CDS|3-UTR identity (%):** 18.32 | 28.54 | 10.63
- **5-UTR|CDS|3-UTR identity (%) [Gaps excluded]:** 75.0 | 74.84 | 76.56
- **5-UTR|CDS|3-UTR identity [Alignment Gaps]:** 99 | 1728 | 10638
- **5-UTR aligned content (<base>:%):** {'G': 58.33, 'C': 29.17, 'A': 4.17, 'T': 8.33}
- **CDS aligned content (<base>:%):** {'A': 29.36, 'G': 30.61, 'C': 20.08, 'T': 19.95}
- **3-UTR aligned content (<base>:%):** {'A': 24.45, 'G': 20.49, 'T': 36.79, 'C': 18.28}

**Uniprot Description:**  
  
 E3 ubiquitin-protein ligase which is a component of the N-end rule pathway. Recognizes and binds to proteins bearing specific N-terminal residues that are destabilizing according to the N-end rule, leading to their ubiquitination and subsequent degradation. N/A   
  
 **Gene Ontology Information:**

Molecular Function

- ubiquitin protein ligase activity
- zinc ion binding

Location   
  
N/A

Biological process

- protein ubiquitination

---

49

- **Protein name:** Pre-mRNA-splicing factor ATP-dependent RNA helicase DHX15
- **Organism:** Homo sapiens
- **Uniprot Accession Number:** O43143
- **Protein sequence length:** 795 aa
- **1D identity (%):** N/A
- **1D identity (%) [Gaps excluded]:** N/A
- **1D identity - Alignment Gaps:** N/A
- **1D aligned content (<aminoacid>:%):** N/A
- **Common reported functions (%):** N/A
- **Common reported locations (%):** N/A
- **Common reported processes (%):** N/A

- **AF ID:** O43143
- **Chain:** A
- **Protein length:** 795 aa
- **Resolution:** N/A
- **b-phipsi:** 0.007069
- **w-rdist:** 0.328879
- **t-alpha:** 0.005861
- **Chemical similarity (Tanimoto Index) (%):** N/A
- **1D identity (%) [PDB]:** N/A
- **1D identity (%) [Gaps excluded][PDB]:** N/A
- **1D identity - Alignment Gaps [PDB]:** N/A
- **1D aligned content [PDB] (<aminoacid>:%):** N/A
- **2D identity (%) [PDB]:** N/A
- **2D identity (%) [Gaps excluded][PDB]:** N/A
- **2D identity - Alignment Gaps [PDB]:** N/A
- **2D aligned content [PDB] (<2D-fold>:%):** N/A
- **3D similarity (TM-Score) (%) [PDB]:** N/A

- **Gene name:** DHX15
- **Entrez ID:** 1665
- **RefSeq ID:** N/A
- **Sequence length:** N/A
- **5-UTR|CDS|3-UTR identity (%):** N/A | N/A | N/A
- **5-UTR|CDS|3-UTR identity (%) [Gaps excluded]:** N/A | N/A | N/A
- **5-UTR|CDS|3-UTR identity [Alignment Gaps]:** N/A | N/A | N/A
- **5-UTR aligned content (<base>:%):** N/A
- **CDS aligned content (<base>:%):** N/A
- **3-UTR aligned content (<base>:%):** N/A

**Uniprot Description:**  
  
 Pre-mRNA processing factor involved in disassembly of spliceosomes after the release of mature mRNA. In cooperation with TFIP11 seem to be involved in the transition of the U2, U5 and U6 snRNP-containing IL complex to the snRNP-free IS complex leading to efficient debranching and turnover of excised introns.   
  
Interacts with SSB/La (PubMed:12458796). Component of the U11/U12 snRNPs that are part of the U12-type spliceosome (PubMed:15146077). Identified in the Intron Large (IL) complex, a post-mRNA release spliceosomal complex containing the excised intron, U2, U5 and U6 snRNPs, and splicing factors; the association may be transient. Interacts with TFIP11; indicative for a recruitment to the IL complex (PubMed:19103666). Interacts with GPATCH2 (PubMed:19432882).   
  
 **Gene Ontology Information:**

Molecular Function

- ATP binding
- ATPase activity
- RNA-dependent ATPase activity
- double-stranded RNA binding
- helicase activity
- RNA binding
- RNA helicase activity

Location

- nuclear speck
- nucleolus
- nucleoplasm
- nucleus
- spliceosomal complex
- U12-type spliceosomal complex

Biological process

- antiviral innate immune response
- defense response to bacterium
- defense response to virus
- mRNA processing
- mRNA splicing, via spliceosome
- positive regulation of I-kappaB kinase/NF-kappaB signaling
- response to alkaloid
- response to toxic substance
- RNA splicing

---

50

- **Protein name:** Nitric oxide-associated protein 1
- **Organism:** Homo sapiens
- **Uniprot Accession Number:** Q8NC60
- **Protein sequence length:** 698 aa
- **1D identity (%):** 14.73
- **1D identity (%) [Gaps excluded]:** 26.3
- **1D identity - Alignment Gaps:** 439
- **1D aligned content (<aminoacid>:%):** {'L': 12.24, 'P': 9.52, 'R': 6.12, 'Q': 6.12, 'Y': 2.04, 'V': 8.16, 'E': 6.12, 'G': 10.88, 'F': 4.08, 'K': 4.08, 'S': 3.4, 'D': 2.04, 'H': 4.76, 'C': 2.72, 'A': 4.08, 'N': 2.72, 'T': 4.76, 'I': 5.44, 'W': 0.68}
- **Common reported functions (%):** 0.0
- **Common reported locations (%):** 0.0
- **Common reported processes (%):** 0.0

- **AF ID:** Q8NC60
- **Chain:** A
- **Protein length:** 698 aa
- **Resolution:** N/A
- **b-phipsi:** 0.003816
- **w-rdist:** 0.293537
- **t-alpha:** 0.036416
- **Chemical similarity (Tanimoto Index) (%):** 98.87
- **1D identity (%) [PDB]:** 3.12
- **1D identity (%) [Gaps excluded][PDB]:** 76.67
- **1D identity - Alignment Gaps [PDB]:** 1416
- **1D aligned content [PDB] (<aminoacid>:%):** {'P': 8.7, 'A': 2.17, 'S': 4.35, 'Q': 15.22, 'F': 2.17, 'L': 15.22, 'G': 6.52, 'V': 13.04, 'C': 2.17, 'T': 2.17, 'R': 4.35, 'Y': 4.35, 'E': 4.35, 'K': 6.52, 'H': 2.17, 'N': 2.17, 'I': 4.35}
- **2D identity (%) [PDB]:** 37.83
- **2D identity (%) [Gaps excluded][PDB]:** 86.32
- **2D identity - Alignment Gaps [PDB]:** 600
- **2D aligned content [PDB] (<2D-fold>:%):** {'.': 22.03, 'T': 23.02, 'H': 36.14, 'E': 18.07, 'G': 0.74}
- **3D similarity (TM-Score) (%) [PDB]:** 21.96

- **Gene name:** NOA1
- **Entrez ID:** 539773
- **RefSeq ID:** NM\_032313
- **Transcript sequence length:** 2218
- **5-UTR|CDS|3-UTR identity (%):** 9.7 | 32.63 | 0.61
- **5-UTR|CDS|3-UTR identity (%) [Gaps excluded]:** 92.86 | 75.84 | 76.6
- **5-UTR|CDS|3-UTR identity [Alignment Gaps]:** 120 | 1863 | 11800
- **5-UTR aligned content (<base>:%):** {'C': 61.54, 'G': 23.08, 'A': 7.69, 'T': 7.69}
- **CDS aligned content (<base>:%):** {'A': 21.56, 'T': 15.84, 'G': 28.96, 'C': 33.65}
- **3-UTR aligned content (<base>:%):** {'G': 11.11, 'C': 22.22, 'A': 36.11, 'T': 30.56}

**Uniprot Description:**  
  
 Involved in regulation of mitochondrial protein translation and respiration. Plays a role in mitochondria-mediated cell death. May act as a scaffolding protein or stabilizer of respiratory chain supercomplexes. Binds GTP.   
  
Homodimer or multimer (By similarity). Interacts with mitochondrial complex I, DAP3, MRPL12 and MRPS27.   
  
 **Gene Ontology Information:**

Molecular Function

- GTP binding

Location

- mitochondrial inner membrane

Biological process

- apoptotic process
- mitochondrial ribosome assembly
- mitochondrion organization
- regulation of cellular respiration

---

51

- **Protein name:** Zinc finger protein 79
- **Organism:** Homo sapiens
- **Uniprot Accession Number:** Q15937
- **Protein sequence length:** 498 aa
- **1D identity (%):** 10.43
- **1D identity (%) [Gaps excluded]:** 24.26
- **1D identity - Alignment Gaps:** 541
- **1D aligned content (<aminoacid>:%):** {'M': 2.02, 'G': 11.11, 'L': 6.06, 'P': 9.09, 'A': 4.04, 'R': 5.05, 'F': 5.05, 'C': 7.07, 'E': 7.07, 'S': 6.06, 'I': 2.02, 'V': 5.05, 'N': 2.02, 'Q': 8.08, 'K': 4.04, 'Y': 2.02, 'H': 9.09, 'T': 4.04, 'D': 1.01}
- **Common reported functions (%):** 6.67
- **Common reported locations (%):** 7.14
- **Common reported processes (%):** 0.0

- **AF ID:** Q15937
- **Chain:** A
- **Protein length:** 498 aa
- **Resolution:** N/A
- **b-phipsi:** 0.005701
- **w-rdist:** 0.646769
- **t-alpha:** 0.000729
- **Chemical similarity (Tanimoto Index) (%):** 96.25
- **1D identity (%) [PDB]:** 1.37
- **1D identity (%) [Gaps excluded][PDB]:** 78.26
- **1D identity - Alignment Gaps [PDB]:** 1290
- **1D aligned content [PDB] (<aminoacid>:%):** {'F': 16.67, 'K': 11.11, 'P': 11.11, 'D': 5.56, 'G': 11.11, 'T': 11.11, 'S': 5.56, 'R': 5.56, 'Q': 5.56, 'A': 5.56, 'N': 5.56, 'E': 5.56}
- **2D identity (%) [PDB]:** 28.88
- **2D identity (%) [Gaps excluded][PDB]:** 87.35
- **2D identity - Alignment Gaps [PDB]:** 672
- **2D aligned content [PDB] (<2D-fold>:%):** {'.': 28.62, 'T': 21.38, 'H': 41.72, 'E': 8.28}
- **3D similarity (TM-Score) (%) [PDB]:** 14.38

- **Gene name:** ZNF79
- **Entrez ID:** 7633
- **RefSeq ID:** NM\_007135
- **Transcript sequence length:** 2078
- **5-UTR|CDS|3-UTR identity (%):** 24.69 | 33.86 | 1.17
- **5-UTR|CDS|3-UTR identity (%) [Gaps excluded]:** 81.45 | 75.0 | 84.76
- **5-UTR|CDS|3-UTR identity [Alignment Gaps]:** 285 | 1541 | 11735
- **5-UTR aligned content (<base>:%):** {'T': 8.91, 'G': 47.52, 'C': 41.58, 'A': 1.98}
- **CDS aligned content (<base>:%):** {'A': 27.13, 'T': 15.98, 'G': 28.71, 'C': 28.18}
- **3-UTR aligned content (<base>:%):** {'C': 19.42, 'T': 20.14, 'G': 26.62, 'A': 33.81}

**Uniprot Description:**  
  
 May be involved in transcriptional regulation. N/A   
  
 **Gene Ontology Information:**

Molecular Function

- DNA-binding transcription repressor activity, RNA polymerase II-specific
- metal ion binding
- RNA polymerase II transcription regulatory region sequence-specific DNA binding

Location

- nucleus

Biological process

- negative regulation of transcription by RNA polymerase II

---

52

- **Protein name:** Apoptosis-resistant E3 ubiquitin protein ligase 1
- **Organism:** Homo sapiens
- **Uniprot Accession Number:** O15033
- **Protein sequence length:** 823 aa
- **1D identity (%):** 15.58
- **1D identity (%) [Gaps excluded]:** 26.48
- **1D identity - Alignment Gaps:** 436
- **1D aligned content (<aminoacid>:%):** {'G': 4.85, 'T': 7.27, 'A': 6.06, 'F': 6.67, 'E': 7.27, 'L': 12.73, 'R': 6.06, 'I': 3.64, 'D': 3.03, 'K': 6.06, 'P': 9.7, 'V': 8.48, 'S': 4.24, 'H': 2.42, 'Y': 1.82, 'Q': 3.03, 'C': 2.42, 'N': 4.24}
- **Common reported functions (%):** 0.0
- **Common reported locations (%):** 14.29
- **Common reported processes (%):** 0.0

- **AF ID:** O15033
- **Chain:** A
- **Protein length:** 823 aa
- **Resolution:** N/A
- **b-phipsi:** 0.000179
- **w-rdist:** 0.660731
- **t-alpha:** 0.056967
- **Chemical similarity (Tanimoto Index) (%):** 99.4
- **1D identity (%) [PDB]:** 3.27
- **1D identity (%) [Gaps excluded][PDB]:** 71.23
- **1D identity - Alignment Gaps [PDB]:** 1515
- **1D aligned content [PDB] (<aminoacid>:%):** {'M': 3.85, 'R': 7.69, 'S': 3.85, 'F': 3.85, 'N': 1.92, 'D': 3.85, 'Y': 3.85, 'V': 9.62, 'E': 5.77, 'G': 9.62, 'I': 7.69, 'K': 5.77, 'T': 7.69, 'Q': 3.85, 'P': 5.77, 'L': 3.85, 'A': 7.69, 'W': 1.92, 'H': 1.92}
- **2D identity (%) [PDB]:** 43.45
- **2D identity (%) [Gaps excluded][PDB]:** 86.82
- **2D identity - Alignment Gaps [PDB]:** 553
- **2D aligned content [PDB] (<2D-fold>:%):** {'H': 32.22, 'T': 23.49, '.': 13.93, 'E': 28.27, 'G': 1.87, 'B': 0.21}
- **3D similarity (TM-Score) (%) [PDB]:** 21.33

- **Gene name:** AREL1
- **Entrez ID:** 9870
- **RefSeq ID:** N/A
- **Sequence length:** N/A
- **5-UTR|CDS|3-UTR identity (%):** N/A | N/A | N/A
- **5-UTR|CDS|3-UTR identity (%) [Gaps excluded]:** N/A | N/A | N/A
- **5-UTR|CDS|3-UTR identity [Alignment Gaps]:** N/A | N/A | N/A
- **5-UTR aligned content (<base>:%):** N/A
- **CDS aligned content (<base>:%):** N/A
- **3-UTR aligned content (<base>:%):** N/A

**Uniprot Description:**  
  
 E3 ubiquitin-protein ligase which accepts ubiquitin from an E2 ubiquitin-conjugating enzyme in the form of a thioester and then directly transfers the ubiquitin to targeted substrates. Inhibits apoptosis by ubiquitinating and targeting for degradation a number of proapoptotic proteins including DIABLO/SMAC, HTRA2 and SEPT4/ARTS which are released from the mitochondrion into the cytosol following apoptotic stimulation (PubMed:23479728). Modulates pulmonary inflammation by targeting SOCS2 for ubiquitination and subsequent degradation by the proteasome (PubMed:31578312).   
  
Interacts with SOCS2.   
  
 **Gene Ontology Information:**

Molecular Function

- ubiquitin protein ligase activity
- ubiquitin-protein transferase activity

Location

- cytoplasm
- cytosol

Biological process

- apoptotic process
- negative regulation of apoptotic process
- protein K11-linked ubiquitination
- protein K33-linked ubiquitination
- protein polyubiquitination
- protein ubiquitination
- regulation of inflammatory response
- ubiquitin-dependent protein catabolic process

---

53

- **Protein name:** Aspartate--tRNA ligase, mitochondrial
- **Organism:** Homo sapiens
- **Uniprot Accession Number:** Q6PI48
- **Protein sequence length:** 645 aa
- **1D identity (%):** 14.6
- **1D identity (%) [Gaps excluded]:** 25.69
- **1D identity - Alignment Gaps:** 414
- **1D aligned content (<aminoacid>:%):** {'P': 6.43, 'S': 7.14, 'W': 0.71, 'L': 12.14, 'Q': 7.86, 'R': 9.29, 'E': 7.86, 'V': 7.14, 'C': 3.57, 'G': 6.43, 'H': 3.57, 'Y': 3.57, 'T': 3.57, 'I': 3.57, 'A': 5.0, 'F': 3.57, 'K': 2.14, 'M': 1.43, 'D': 3.57, 'N': 1.43}
- **Common reported functions (%):** 0.0
- **Common reported locations (%):** 0.0
- **Common reported processes (%):** 0.0

- **AF ID:** Q6PI48
- **Chain:** A
- **Protein length:** 645 aa
- **Resolution:** N/A
- **b-phipsi:** 0.000821
- **w-rdist:** 0.498116
- **t-alpha:** 0.016752
- **Chemical similarity (Tanimoto Index) (%):** 97.55
- **1D identity (%) [PDB]:** 1.95
- **1D identity (%) [Gaps excluded][PDB]:** 63.64
- **1D identity - Alignment Gaps [PDB]:** 1395
- **1D aligned content [PDB] (<aminoacid>:%):** {'E': 10.71, 'L': 14.29, 'V': 14.29, 'T': 3.57, 'G': 7.14, 'D': 7.14, 'R': 3.57, 'F': 3.57, 'S': 14.29, 'I': 3.57, 'W': 3.57, 'Q': 3.57, 'A': 3.57, 'H': 3.57, 'P': 3.57}
- **2D identity (%) [PDB]:** 47.15
- **2D identity (%) [Gaps excluded][PDB]:** 87.84
- **2D identity - Alignment Gaps [PDB]:** 447
- **2D aligned content [PDB] (<2D-fold>:%):** {'.': 20.22, 'H': 35.6, 'T': 20.22, 'E': 23.52, 'B': 0.44}
- **3D similarity (TM-Score) (%) [PDB]:** 21.32

- **Gene name:** DARS2
- **Entrez ID:** 538772
- **RefSeq ID:** NM\_018122
- **Transcript sequence length:** 3336
- **5-UTR|CDS|3-UTR identity (%):** 18.89 | 39.93 | 4.6
- **5-UTR|CDS|3-UTR identity (%) [Gaps excluded]:** 86.18 | 76.08 | 76.03
- **5-UTR|CDS|3-UTR identity [Alignment Gaps]:** 438 | 1408 | 11277
- **5-UTR aligned content (<base>:%):** {'G': 41.51, 'T': 10.38, 'C': 44.34, 'A': 3.77}
- **CDS aligned content (<base>:%):** {'A': 27.3, 'T': 21.05, 'G': 26.12, 'C': 25.53}
- **3-UTR aligned content (<base>:%):** {'A': 34.42, 'T': 37.32, 'G': 14.31, 'C': 13.95}

**Uniprot Description:**  
  
 N/A   
  
Homodimer.   
  
 **Gene Ontology Information:**

Molecular Function

- aspartate-tRNA ligase activity
- ATP binding
- nucleic acid binding

Location

- mitochondrial matrix
- mitochondrion
- organelle membrane

Biological process

- aspartyl-tRNA aminoacylation
- mitochondrial asparaginyl-tRNA aminoacylation

---

54

- **Protein name:** Threonine--tRNA ligase, mitochondrial
- **Organism:** Homo sapiens
- **Uniprot Accession Number:** Q9BW92
- **Protein sequence length:** 718 aa
- **1D identity (%):** 15.03
- **1D identity (%) [Gaps excluded]:** 23.26
- **1D identity - Alignment Gaps:** 339
- **1D aligned content (<aminoacid>:%):** {'Y': 1.39, 'C': 3.47, 'R': 8.33, 'V': 3.47, 'F': 4.86, 'L': 13.89, 'A': 3.47, 'P': 12.5, 'G': 9.03, 'K': 2.78, 'D': 4.86, 'S': 6.25, 'E': 4.17, 'T': 8.33, 'Q': 5.56, 'H': 4.17, 'N': 1.39, 'I': 2.08}
- **Common reported functions (%):** 0.0
- **Common reported locations (%):** 0.0
- **Common reported processes (%):** 0.0

- **AF ID:** Q9BW92
- **Chain:** A
- **Protein length:** 718 aa
- **Resolution:** N/A
- **b-phipsi:** 0.003817
- **w-rdist:** 0.477895
- **t-alpha:** 0.00437
- **Chemical similarity (Tanimoto Index) (%):** 98.65
- **1D identity (%) [PDB]:** 3.78
- **1D identity (%) [Gaps excluded][PDB]:** 73.68
- **1D identity - Alignment Gaps [PDB]:** 1404
- **1D aligned content [PDB] (<aminoacid>:%):** {'L': 10.71, 'W': 1.79, 'D': 3.57, 'R': 8.93, 'F': 3.57, 'S': 12.5, 'E': 5.36, 'Q': 8.93, 'T': 5.36, 'C': 1.79, 'V': 7.14, 'I': 1.79, 'P': 1.79, 'Y': 3.57, 'A': 12.5, 'H': 5.36, 'G': 3.57, 'K': 1.79}
- **2D identity (%) [PDB]:** 42.95
- **2D identity (%) [Gaps excluded][PDB]:** 89.13
- **2D identity - Alignment Gaps [PDB]:** 544
- **2D aligned content [PDB] (<2D-fold>:%):** {'.': 13.97, 'H': 36.81, 'T': 18.18, 'E': 30.38, 'G': 0.67}
- **3D similarity (TM-Score) (%) [PDB]:** 24.53

- **Gene name:** TARS2
- **Entrez ID:** 80222
- **RefSeq ID:** NM\_025150
- **Transcript sequence length:** 2727
- **5-UTR|CDS|3-UTR identity (%):** 14.62 | 44.56 | 2.88
- **5-UTR|CDS|3-UTR identity (%) [Gaps excluded]:** 65.52 | 75.44 | 74.51
- **5-UTR|CDS|3-UTR identity [Alignment Gaps]:** 101 | 1219 | 11500
- **5-UTR aligned content (<base>:%):** {'G': 57.89, 'T': 15.79, 'A': 10.53, 'C': 15.79}
- **CDS aligned content (<base>:%):** {'A': 21.7, 'T': 19.97, 'G': 29.16, 'C': 29.16}
- **3-UTR aligned content (<base>:%):** {'G': 23.48, 'C': 22.32, 'T': 19.13, 'A': 35.07}

**Uniprot Description:**  
  
 Catalyzes the attachment of threonine to tRNA(Thr) in a two-step reaction: threonine is first activated by ATP to form Thr-AMP and then transferred to the acceptor end of tRNA(Thr). Also edits incorrectly charged tRNA(Thr) via its editing domain.   
  
Homodimer.   
  
 **Gene Ontology Information:**

Molecular Function

- aminoacyl-tRNA editing activity
- ATP binding
- protein homodimerization activity
- threonine-tRNA ligase activity

Location

- mitochondrial matrix

Biological process

- mitochondrial threonyl-tRNA aminoacylation
- threonyl-tRNA aminoacylation

---

55

- **Protein name:** Angiopoietin-related protein 6
- **Organism:** Homo sapiens
- **Uniprot Accession Number:** Q8NI99
- **Protein sequence length:** 470 aa
- **1D identity (%):** 10.87
- **1D identity (%) [Gaps excluded]:** 25.25
- **1D identity - Alignment Gaps:** 529
- **1D aligned content (<aminoacid>:%):** {'M': 1.98, 'R': 11.88, 'L': 13.86, 'Q': 5.94, 'A': 9.9, 'F': 2.97, 'T': 1.98, 'G': 11.88, 'E': 3.96, 'P': 6.93, 'V': 3.96, 'C': 3.96, 'D': 4.95, 'H': 6.93, 'I': 0.99, 'S': 3.96, 'Y': 3.96}
- **Common reported functions (%):** 0.0
- **Common reported locations (%):** 7.14
- **Common reported processes (%):** 0.0

- **AF ID:** Q8NI99
- **Chain:** A
- **Protein length:** 470 aa
- **Resolution:** N/A
- **b-phipsi:** 0.004904
- **w-rdist:** 0.580117
- **t-alpha:** 0.00219
- **Chemical similarity (Tanimoto Index) (%):** 96.99
- **1D identity (%) [PDB]:** 1.4
- **1D identity (%) [Gaps excluded][PDB]:** 72.0
- **1D identity - Alignment Gaps [PDB]:** 1258
- **1D aligned content [PDB] (<aminoacid>:%):** {'G': 11.11, 'S': 5.56, 'E': 16.67, 'Q': 16.67, 'V': 5.56, 'L': 22.22, 'H': 5.56, 'A': 5.56, 'C': 5.56, 'K': 5.56}
- **2D identity (%) [PDB]:** 28.22
- **2D identity (%) [Gaps excluded][PDB]:** 92.79
- **2D identity - Alignment Gaps [PDB]:** 698
- **2D aligned content [PDB] (<2D-fold>:%):** {'.': 9.89, 'H': 50.88, 'T': 20.49, 'E': 16.96, 'G': 1.06, 'B': 0.71}
- **3D similarity (TM-Score) (%) [PDB]:** 19.52

- **Gene name:** ANGPTL6
- **Entrez ID:** 83854
- **RefSeq ID:** N/A
- **Sequence length:** N/A
- **5-UTR|CDS|3-UTR identity (%):** N/A | N/A | N/A
- **5-UTR|CDS|3-UTR identity (%) [Gaps excluded]:** N/A | N/A | N/A
- **5-UTR|CDS|3-UTR identity [Alignment Gaps]:** N/A | N/A | N/A
- **5-UTR aligned content (<base>:%):** N/A
- **CDS aligned content (<base>:%):** N/A
- **3-UTR aligned content (<base>:%):** N/A

**Uniprot Description:**  
  
 May play a role in the wound healing process. May promote epidermal proliferation, remodeling and regeneration. May promote the chemotactic activity of endothelial cells and induce neovascularization. May counteract high-fat diet-induced obesity and related insulin resistance through increased energy expenditure. N/A   
  
 **Gene Ontology Information:**

Molecular Function

- signaling receptor binding

Location

- collagen-containing extracellular matrix
- extracellular exosome
- extracellular space
- secretory granule

Biological process

- angiogenesis
- cell differentiation

---

56

- **Protein name:** Interferon-induced, double-stranded RNA-activated protein kinase
- **Organism:** Homo sapiens
- **Uniprot Accession Number:** P19525
- **Protein sequence length:** 551 aa
- **1D identity (%):** 11.86
- **1D identity (%) [Gaps excluded]:** 26.14
- **1D identity - Alignment Gaps:** 530
- **1D aligned content (<aminoacid>:%):** {'D': 8.7, 'S': 5.22, 'A': 1.74, 'F': 5.22, 'Y': 2.61, 'V': 4.35, 'L': 12.17, 'P': 4.35, 'R': 7.83, 'I': 6.09, 'G': 8.7, 'K': 11.3, 'N': 4.35, 'E': 6.09, 'H': 1.74, 'C': 1.74, 'Q': 4.35, 'T': 2.61, 'M': 0.87}
- **Common reported functions (%):** 13.33
- **Common reported locations (%):** 28.57
- **Common reported processes (%):** 4.76

- **AF ID:** P19525
- **Chain:** A
- **Protein length:** 551 aa
- **Resolution:** N/A
- **b-phipsi:** 0.005031
- **w-rdist:** 0.484336
- **t-alpha:** 0.002922
- **Chemical similarity (Tanimoto Index) (%):** 98.94
- **1D identity (%) [PDB]:** 1.62
- **1D identity (%) [Gaps excluded][PDB]:** 66.67
- **1D identity - Alignment Gaps [PDB]:** 1323
- **1D aligned content [PDB] (<aminoacid>:%):** {'M': 4.55, 'F': 4.55, 'N': 4.55, 'T': 13.64, 'Y': 4.55, 'Q': 4.55, 'V': 22.73, 'I': 4.55, 'L': 4.55, 'P': 4.55, 'G': 9.09, 'K': 9.09, 'R': 4.55, 'D': 4.55}
- **2D identity (%) [PDB]:** 31.46
- **2D identity (%) [Gaps excluded][PDB]:** 90.25
- **2D identity - Alignment Gaps [PDB]:** 671
- **2D aligned content [PDB] (<2D-fold>:%):** {'.': 11.73, 'H': 46.6, 'E': 26.23, 'T': 15.43}
- **3D similarity (TM-Score) (%) [PDB]:** 19.6

- **Gene name:** EIF2AK2
- **Entrez ID:** 5610
- **RefSeq ID:** N/A
- **Sequence length:** N/A
- **5-UTR|CDS|3-UTR identity (%):** N/A | N/A | N/A
- **5-UTR|CDS|3-UTR identity (%) [Gaps excluded]:** N/A | N/A | N/A
- **5-UTR|CDS|3-UTR identity [Alignment Gaps]:** N/A | N/A | N/A
- **5-UTR aligned content (<base>:%):** N/A
- **CDS aligned content (<base>:%):** N/A
- **3-UTR aligned content (<base>:%):** N/A

**Uniprot Description:**  
  
 IFN-induced dsRNA-dependent serine/threonine-protein kinase that phosphorylates the alpha subunit of eukaryotic translation initiation factor 2 (EIF2S1/eIF-2-alpha) and plays a key role in the innate immune response to viral infection (PubMed:18835251, PubMed:19189853, PubMed:19507191, PubMed:21072047, PubMed:21123651, PubMed:22381929, PubMed:22948139, PubMed:23229543). Inhibits viral replication via the integrated stress response (ISR): EIF2S1/eIF-2-alpha phosphorylation in response to viral infection converts EIF2S1/eIF-2-alpha in a global protein synthesis inhibitor, resulting to a shutdown of cellular and viral protein synthesis, while concomitantly initiating the preferential translation of ISR-specific mRNAs, such as the transcriptional activator ATF4 (PubMed:19189853, PubMed:21123651, PubMed:22948139, PubMed:23229543). Exerts its antiviral activity on a wide range of DNA and RNA viruses including hepatitis C virus (HCV), hepatitis B virus (HBV), measles virus (MV) and herpes simplex virus 1 (HHV-1) (PubMed:11836380, PubMed:19189853, PubMed:19840259, PubMed:20171114, PubMed:21710204, PubMed:23115276, PubMed:23399035). Also involved in the regulation of signal transduction, apoptosis, cell proliferation and differentiation: phosphorylates other substrates including p53/TP53, PPP2R5A, DHX9, ILF3, IRS1 and the HHV-1 viral protein US11 (PubMed:11836380, PubMed:19229320, PubMed:22214662). In addition to serine/threonine-protein kinase activity, also has tyrosine-protein kinase activity and phosphorylates CDK1 at 'Tyr-4' upon DNA damage, facilitating its ubiquitination and proteasomal degradation (PubMed:20395957). Either as an adapter protein and/or via its kinase activity, can regulate various signaling pathways (p38 MAP kinase, NF-kappa-B and insulin signaling pathways) and transcription factors (JUN, STAT1, STAT3, IRF1, ATF3) involved in the expression of genes encoding pro-inflammatory cytokines and IFNs (PubMed:22948139, PubMed:23084476, PubMed:23372823). Activates the NF-kappa-B pathway via interaction with IKBKB and TRAF family of proteins and activates the p38 MAP kinase pathway via interaction with MAP2K6 (PubMed:10848580, PubMed:15121867, PubMed:15229216). Can act as both a positive and negative regulator of the insulin signaling pathway (ISP) (PubMed:20685959). Negatively regulates ISP by inducing the inhibitory phosphorylation of insulin receptor substrate 1 (IRS1) at 'Ser-312' and positively regulates ISP via phosphorylation of PPP2R5A which activates FOXO1, which in turn up-regulates the expression of insulin receptor substrate 2 (IRS2) (PubMed:20685959). Can regulate NLRP3 inflammasome assembly and the activation of NLRP3, NLRP1, AIM2 and NLRC4 inflammasomes (PubMed:22801494). Plays a role in the regulation of the cytoskeleton by binding to gelsolin (GSN), sequestering the protein in an inactive conformation away from actin (By similarity).   
  
Homodimer (PubMed:16179258, PubMed:31246429). Interacts with STRBP (By similarity). Interacts with DNAJC3. Forms a complex with FANCA, FANCC, FANCG and HSP70. Interacts with ADAR/ADAR1. Interacts with IRS1 (By similarity). The inactive form interacts with NCK1 and GSN. Interacts (via the kinase catalytic domain) with STAT3 (via SH2 domain), TRAF2 (C-terminus), TRAF5 (C-terminus) and TRAF6 (C-terminus). Interacts with MAP2K6, IKBKB/IKKB, NPM1, TARBP2, NLRP1, NLRP3, NLRC4 and AIM2. Interacts (via DRBM 1 domain) with DUS2L (via DRBM domain). Interacts with DHX9 (via N-terminus) and this interaction is dependent upon activation of the kinase. Interacts with EIF2S1/EIF-2ALPHA; this interaction induces a conformational change in EIF2S1 and its phosphorylation by EIF2AK2 (PubMed:16179258).   
  
 **Gene Ontology Information:**

Molecular Function

- ATP binding
- double-stranded RNA binding
- eukaryotic translation initiation factor 2alpha kinase activity
- identical protein binding
- kinase activity
- non-membrane spanning protein tyrosine kinase activity
- protein kinase activity
- protein phosphatase regulator activity
- protein serine kinase activity
- protein serine/threonine kinase activity
- RNA binding

Location

- cytoplasm
- cytosol
- membrane
- nucleus
- perinuclear region of cytoplasm
- ribosome

Biological process

- antiviral innate immune response
- cellular response to amino acid starvation
- defense response to virus
- endoplasmic reticulum unfolded protein response
- negative regulation of apoptotic process
- negative regulation of cell population proliferation
- negative regulation of osteoblast proliferation
- negative regulation of translation
- negative regulation of viral genome replication
- positive regulation of chemokine production
- positive regulation of cytokine production
- positive regulation of MAPK cascade
- positive regulation of NF-kappaB transcription factor activity
- positive regulation of NIK/NF-kappaB signaling
- positive regulation of stress-activated MAPK cascade
- protein autophosphorylation
- protein phosphorylation
- regulation of hematopoietic progenitor cell differentiation
- regulation of hematopoietic stem cell differentiation
- regulation of hematopoietic stem cell proliferation
- regulation of NLRP3 inflammasome complex assembly
- response to interferon-alpha
- response to virus
- translation

---

57

- **Protein name:** Calcium-activated chloride channel regulator 1
- **Organism:** Homo sapiens
- **Uniprot Accession Number:** A8K7I4
- **Protein sequence length:** 914 aa
- **1D identity (%):** 15.41
- **1D identity (%) [Gaps excluded]:** 26.62
- **1D identity - Alignment Gaps:** 473
- **1D aligned content (<aminoacid>:%):** {'F': 6.36, 'K': 5.78, 'G': 9.25, 'N': 2.89, 'Y': 1.16, 'I': 6.94, 'E': 6.36, 'T': 10.98, 'L': 5.78, 'D': 5.2, 'S': 4.62, 'P': 7.51, 'V': 6.36, 'H': 1.73, 'R': 4.05, 'Q': 6.36, 'C': 2.89, 'A': 4.62, 'W': 1.16}
- **Common reported functions (%):** 6.67
- **Common reported locations (%):** 0.0
- **Common reported processes (%):** 0.0

- **AF ID:** A8K7I4
- **Chain:** A
- **Protein length:** 914 aa
- **Resolution:** N/A
- **b-phipsi:** 0.009215
- **w-rdist:** 0.344302
- **t-alpha:** 0.005098
- **Chemical similarity (Tanimoto Index) (%):** 94.35
- **1D identity (%) [PDB]:** 1.81
- **1D identity (%) [Gaps excluded][PDB]:** 81.58
- **1D identity - Alignment Gaps [PDB]:** 1676
- **1D aligned content [PDB] (<aminoacid>:%):** {'T': 6.45, 'E': 6.45, 'F': 6.45, 'D': 12.9, 'Y': 3.23, 'L': 9.68, 'C': 3.23, 'S': 16.13, 'A': 3.23, 'G': 6.45, 'I': 6.45, 'R': 6.45, 'H': 3.23, 'V': 3.23, 'W': 3.23, 'N': 3.23}
- **2D identity (%) [PDB]:** 41.47
- **2D identity (%) [Gaps excluded][PDB]:** 89.21
- **2D identity - Alignment Gaps [PDB]:** 640
- **2D aligned content [PDB] (<2D-fold>:%):** {'.': 20.97, 'E': 25.0, 'T': 27.22, 'H': 26.41, 'B': 0.4}
- **3D similarity (TM-Score) (%) [PDB]:** 22.37

- **Gene name:** CLCA1
- **Entrez ID:** 1179
- **RefSeq ID:** N/A
- **Sequence length:** N/A
- **5-UTR|CDS|3-UTR identity (%):** N/A | N/A | N/A
- **5-UTR|CDS|3-UTR identity (%) [Gaps excluded]:** N/A | N/A | N/A
- **5-UTR|CDS|3-UTR identity [Alignment Gaps]:** N/A | N/A | N/A
- **5-UTR aligned content (<base>:%):** N/A
- **CDS aligned content (<base>:%):** N/A
- **3-UTR aligned content (<base>:%):** N/A

**Uniprot Description:**  
  
 May be involved in mediating calcium-activated chloride conductance. May play critical roles in goblet cell metaplasia, mucus hypersecretion, cystic fibrosis and AHR. May be involved in the regulation of mucus production and/or secretion by goblet cells. Involved in the regulation of tissue inflammation in the innate immune response. May play a role as a tumor suppressor. Induces MUC5AC. N/A   
  
 **Gene Ontology Information:**

Molecular Function

- chloride channel activity
- intracellular calcium activated chloride channel activity
- metal ion binding
- metalloendopeptidase activity

Location

- extracellular space
- microvillus
- plasma membrane
- zymogen granule membrane

Biological process

- calcium ion transport
- cellular response to hypoxia
- ion transmembrane transport
- proteolysis

---

58

- **Protein name:** Testicular spindle-associated protein SHCBP1L
- **Organism:** Homo sapiens
- **Uniprot Accession Number:** Q9BZQ2
- **Protein sequence length:** 653 aa
- **1D identity (%):** 12.76
- **1D identity (%) [Gaps excluded]:** 27.01
- **1D identity - Alignment Gaps:** 542
- **1D aligned content (<aminoacid>:%):** {'M': 3.05, 'S': 3.82, 'G': 8.4, 'P': 9.92, 'A': 6.87, 'R': 4.58, 'T': 5.34, 'L': 8.4, 'E': 6.11, 'C': 3.05, 'V': 8.4, 'D': 6.87, 'K': 9.92, 'Y': 2.29, 'F': 3.82, 'W': 0.76, 'I': 3.05, 'H': 3.05, 'Q': 0.76, 'N': 1.53}
- **Common reported functions (%):** 0.0
- **Common reported locations (%):** 7.14
- **Common reported processes (%):** 0.0

- **AF ID:** Q9BZQ2
- **Chain:** A
- **Protein length:** 653 aa
- **Resolution:** N/A
- **b-phipsi:** 0.000756
- **w-rdist:** 0.61123
- **t-alpha:** 0.011654
- **Chemical similarity (Tanimoto Index) (%):** 95.99
- **1D identity (%) [PDB]:** 1.57
- **1D identity (%) [Gaps excluded][PDB]:** 82.14
- **1D identity - Alignment Gaps [PDB]:** 1435
- **1D aligned content [PDB] (<aminoacid>:%):** {'M': 4.35, 'P': 13.04, 'L': 13.04, 'I': 8.7, 'R': 8.7, 'D': 8.7, 'V': 13.04, 'E': 13.04, 'G': 4.35, 'F': 4.35, 'K': 4.35, 'S': 4.35}
- **2D identity (%) [PDB]:** 38.43
- **2D identity (%) [Gaps excluded][PDB]:** 90.34
- **2D identity - Alignment Gaps [PDB]:** 601
- **2D aligned content [PDB] (<2D-fold>:%):** {'.': 13.93, 'T': 25.37, 'H': 36.57, 'E': 21.89, 'G': 2.24}
- **3D similarity (TM-Score) (%) [PDB]:** 21.27

- **Gene name:** SHCBP1L
- **Entrez ID:** 81626
- **RefSeq ID:** NM\_030933
- **Transcript sequence length:** 2113
- **5-UTR|CDS|3-UTR identity (%):** 19.53 | 39.16 | 0.75
- **5-UTR|CDS|3-UTR identity (%) [Gaps excluded]:** 78.12 | 72.35 | 82.41
- **5-UTR|CDS|3-UTR identity [Alignment Gaps]:** 96 | 1352 | 11790
- **5-UTR aligned content (<base>:%):** {'C': 44.0, 'G': 48.0, 'T': 8.0}
- **CDS aligned content (<base>:%):** {'A': 27.47, 'T': 21.4, 'G': 30.5, 'C': 20.62}
- **3-UTR aligned content (<base>:%):** {'A': 43.82, 'G': 8.99, 'T': 39.33, 'C': 7.87}

**Uniprot Description:**  
  
 Testis-specific spindle-associated factor that plays a role in spermatogenesis. In association with HSPA2, participates in the maintenance of spindle integrity during meiosis in male germ cells.   
  
Interacts with HSPA2; this interaction may promote the recruitment of HSPA2 to the spindle.   
  
 **Gene Ontology Information:**

Molecular Function   
  
N/A

Location

- cytoplasm
- meiotic spindle

Biological process

- cell differentiation
- male meiosis cytokinesis
- positive regulation of chromosome organization
- spermatogenesis

---

59

- **Protein name:** Fermitin family homolog 3
- **Organism:** Homo sapiens
- **Uniprot Accession Number:** Q86UX7
- **Protein sequence length:** 667 aa
- **1D identity (%):** 12.78
- **1D identity (%) [Gaps excluded]:** 23.33
- **1D identity - Alignment Gaps:** 446
- **1D aligned content (<aminoacid>:%):** {'A': 3.17, 'K': 7.94, 'P': 7.14, 'S': 2.38, 'T': 4.76, 'E': 8.73, 'I': 3.97, 'G': 8.73, 'Q': 8.73, 'R': 9.52, 'F': 5.56, 'H': 3.17, 'L': 11.9, 'C': 0.79, 'W': 1.59, 'D': 2.38, 'V': 7.14, 'Y': 1.59, 'N': 0.79}
- **Common reported functions (%):** 0.0
- **Common reported locations (%):** 14.29
- **Common reported processes (%):** 0.0

- **AF ID:** Q86UX7
- **Chain:** A
- **Protein length:** 667 aa
- **Resolution:** N/A
- **b-phipsi:** 0.000594
- **w-rdist:** 0.600042
- **t-alpha:** 0.038578
- **Chemical similarity (Tanimoto Index) (%):** 98.28
- **1D identity (%) [PDB]:** 2.05
- **1D identity (%) [Gaps excluded][PDB]:** 76.92
- **1D identity - Alignment Gaps [PDB]:** 1427
- **1D aligned content [PDB] (<aminoacid>:%):** {'M': 6.67, 'I': 6.67, 'A': 10.0, 'R': 10.0, 'S': 13.33, 'P': 6.67, 'Q': 3.33, 'E': 10.0, 'L': 3.33, 'F': 6.67, 'T': 3.33, 'D': 6.67, 'V': 6.67, 'G': 3.33, 'K': 3.33}
- **2D identity (%) [PDB]:** 42.8
- **2D identity (%) [Gaps excluded][PDB]:** 90.29
- **2D identity - Alignment Gaps [PDB]:** 537
- **2D aligned content [PDB] (<2D-fold>:%):** {'T': 24.03, 'E': 23.57, '.': 13.27, 'H': 35.7, 'G': 3.43}
- **3D similarity (TM-Score) (%) [PDB]:** 23.26

- **Gene name:** FERMT3
- **Entrez ID:** 83706
- **RefSeq ID:** N/A
- **Sequence length:** N/A
- **5-UTR|CDS|3-UTR identity (%):** N/A | N/A | N/A
- **5-UTR|CDS|3-UTR identity (%) [Gaps excluded]:** N/A | N/A | N/A
- **5-UTR|CDS|3-UTR identity [Alignment Gaps]:** N/A | N/A | N/A
- **5-UTR aligned content (<base>:%):** N/A
- **CDS aligned content (<base>:%):** N/A
- **3-UTR aligned content (<base>:%):** N/A

**Uniprot Description:**  
  
 Plays a central role in cell adhesion in hematopoietic cells (PubMed:19234463, PubMed:26359933). Acts by activating the integrin beta-1-3 (ITGB1, ITGB2 and ITGB3) (By similarity). Required for integrin-mediated platelet adhesion and leukocyte adhesion to endothelial cells (PubMed:19234460). Required for activation of integrin beta-2 (ITGB2) in polymorphonuclear granulocytes (PMNs) (By similarity).   
  
Interacts with ITGB1, ITGB2 and ITGB3 (via cytoplasmic tails).   
  
 **Gene Ontology Information:**

Molecular Function

- integrin binding
- lipid binding

Location

- cell projection
- cell-substrate junction
- extracellular exosome
- extracellular region
- membrane
- platelet alpha granule lumen
- podosome

Biological process

- cell-matrix adhesion
- integrin activation
- integrin-mediated signaling pathway
- leukocyte cell-cell adhesion
- platelet aggregation
- positive regulation of cell migration
- regulation of cell-cell adhesion mediated by integrin
- substrate adhesion-dependent cell spreading

---

60

- **Protein name:** Tyrosine-protein phosphatase non-receptor type 11
- **Organism:** Homo sapiens
- **Uniprot Accession Number:** Q06124
- **Protein sequence length:** 593 aa
- **1D identity (%):** 12.1
- **1D identity (%) [Gaps excluded]:** 22.35
- **1D identity - Alignment Gaps:** 432
- **1D aligned content (<aminoacid>:%):** {'F': 3.51, 'I': 1.75, 'L': 9.65, 'P': 6.14, 'K': 9.65, 'V': 7.02, 'H': 4.39, 'Q': 6.14, 'G': 10.53, 'D': 5.26, 'A': 2.63, 'C': 5.26, 'S': 2.63, 'E': 7.89, 'R': 7.02, 'Y': 3.51, 'T': 4.39, 'N': 1.75, 'M': 0.88}
- **Common reported functions (%):** 0.0
- **Common reported locations (%):** 14.29
- **Common reported processes (%):** 0.0

- **AF ID:** Q06124
- **Chain:** A
- **Protein length:** 593 aa
- **Resolution:** N/A
- **b-phipsi:** 0.001473
- **w-rdist:** 0.679201
- **t-alpha:** 0.003655
- **Chemical similarity (Tanimoto Index) (%):** 99.24
- **1D identity (%) [PDB]:** 2.09
- **1D identity (%) [Gaps excluded][PDB]:** 64.44
- **1D identity - Alignment Gaps [PDB]:** 1341
- **1D aligned content [PDB] (<aminoacid>:%):** {'V': 10.34, 'G': 3.45, 'S': 6.9, 'A': 13.79, 'P': 3.45, 'N': 3.45, 'R': 13.79, 'Y': 3.45, 'T': 3.45, 'Q': 10.34, 'H': 3.45, 'E': 6.9, 'I': 3.45, 'D': 3.45, 'L': 6.9, 'K': 3.45}
- **2D identity (%) [PDB]:** 40.56
- **2D identity (%) [Gaps excluded][PDB]:** 85.06
- **2D identity - Alignment Gaps [PDB]:** 507
- **2D aligned content [PDB] (<2D-fold>:%):** {'.': 11.2, 'H': 36.9, 'T': 24.43, 'E': 25.7, 'G': 1.53, 'B': 0.25}
- **3D similarity (TM-Score) (%) [PDB]:** 20.9

- **Gene name:** PTPN11
- **Entrez ID:** 395815
- **RefSeq ID:** N/A
- **Sequence length:** N/A
- **5-UTR|CDS|3-UTR identity (%):** N/A | N/A | N/A
- **5-UTR|CDS|3-UTR identity (%) [Gaps excluded]:** N/A | N/A | N/A
- **5-UTR|CDS|3-UTR identity [Alignment Gaps]:** N/A | N/A | N/A
- **5-UTR aligned content (<base>:%):** N/A
- **CDS aligned content (<base>:%):** N/A
- **3-UTR aligned content (<base>:%):** N/A

**Uniprot Description:**  
  
 Acts downstream of various receptor and cytoplasmic protein tyrosine kinases to participate in the signal transduction from the cell surface to the nucleus (PubMed:10655584, PubMed:18559669, PubMed:18829466, PubMed:26742426, PubMed:28074573). Positively regulates MAPK signal transduction pathway (PubMed:28074573). Dephosphorylates GAB1, ARHGAP35 and EGFR (PubMed:28074573). Dephosphorylates ROCK2 at 'Tyr-722' resulting in stimulation of its RhoA binding activity (PubMed:18559669). Dephosphorylates CDC73 (PubMed:26742426). Dephosphorylates SOX9 on tyrosine residues, leading to inactivate SOX9 and promote ossification (By similarity).   
  
Interacts with phosphorylated LIME1 and BCAR3. Interacts with SHB and INPP5D/SHIP1 (By similarity). Interacts with MILR1 (tyrosine-phosphorylated). Interacts with FLT1 (tyrosine-phosphorylated), FLT3 (tyrosine-phosphorylated), FLT4 (tyrosine-phosphorylated), KIT and GRB2. Interacts with PDGFRA (tyrosine phosphorylated). Interacts (via SH2 domain) with TEK/TIE2 (tyrosine phosphorylated) (By similarity). Interacts with PTPNS1 and CD84. Interacts with phosphorylated SIT1 and MPZL1. Interacts with FCRL4, FCRL6 and ANKHD1. Interacts with KIR2DL1; the interaction is enhanced by ARRB2. Interacts with GAB2. Interacts with TERT; the interaction retains TERT in the nucleus. Interacts with PECAM1 and FER. Interacts with EPHA2 (activated); participates in PTK2/FAK1 dephosphorylation in EPHA2 downstream signaling. Interacts with ROS1; mediates PTPN11 phosphorylation. Interacts with PDGFRB (tyrosine phosphorylated); this interaction increases the PTPN11 phosphatase activity. Interacts with GAREM1 isoform 1 (tyrosine phosphorylated); the interaction increases MAPK/ERK activity and does not affect the GRB2/SOS complex formation. Interacts with CDC73 (PubMed:26742426). Interacts with CEACAM1 (via cytoplasmic domain); this interaction depends on the monomer/dimer equilibrium and is phosphorylation-dependent (By similarity). Interacts with MPIG6B (via ITIM motif) (PubMed:23112346). Interacts with SIGLEC10 (By similarity). Interacts with FCRL3 (via phosphorylated ITIM motifs) (PubMed:11162587, PubMed:19843936).   
  
 **Gene Ontology Information:**

Molecular Function

- cell adhesion molecule binding
- non-membrane spanning protein tyrosine phosphatase activity
- protein tyrosine phosphatase activity
- receptor tyrosine kinase binding

Location

- cytoplasm
- nucleus

Biological process

- cellular response to epidermal growth factor stimulus
- cellular response to organic substance
- negative regulation of chondrocyte differentiation
- positive regulation of ERK1 and ERK2 cascade
- positive regulation of ossification
- protein dephosphorylation

---

61

- **Protein name:** HERV-H\_2q24.3 provirus ancestral Env polyprotein
- **Organism:** Homo sapiens
- **Uniprot Accession Number:** Q9N2K0
- **Protein sequence length:** 584 aa
- **1D identity (%):** 10.53
- **1D identity (%) [Gaps excluded]:** 22.15
- **1D identity - Alignment Gaps:** 513
- **1D aligned content (<aminoacid>:%):** {'I': 2.91, 'F': 4.85, 'P': 17.48, 'N': 4.85, 'T': 6.8, 'M': 0.97, 'L': 16.5, 'V': 2.91, 'W': 1.94, 'C': 3.88, 'S': 7.77, 'H': 2.91, 'E': 2.91, 'D': 2.91, 'G': 3.88, 'Q': 9.71, 'K': 2.91, 'R': 1.94, 'A': 1.94}
- **Common reported functions (%):** 0.0
- **Common reported locations (%):** 0.0
- **Common reported processes (%):** 0.0

- **AF ID:** Q9N2K0
- **Chain:** A
- **Protein length:** 584 aa
- **Resolution:** N/A
- **b-phipsi:** 0.000536
- **w-rdist:** 0.727553
- **t-alpha:** 0.022338
- **Chemical similarity (Tanimoto Index) (%):** 97.12
- **1D identity (%) [PDB]:** 2.25
- **1D identity (%) [Gaps excluded][PDB]:** 72.09
- **1D identity - Alignment Gaps [PDB]:** 1336
- **1D aligned content [PDB] (<aminoacid>:%):** {'Y': 3.23, 'F': 3.23, 'D': 6.45, 'R': 9.68, 'L': 22.58, 'P': 6.45, 'H': 3.23, 'C': 6.45, 'V': 3.23, 'G': 3.23, 'E': 3.23, 'N': 6.45, 'I': 6.45, 'K': 6.45, 'Q': 3.23, 'T': 3.23, 'A': 3.23}
- **2D identity (%) [PDB]:** 33.14
- **2D identity (%) [Gaps excluded][PDB]:** 89.58
- **2D identity - Alignment Gaps [PDB]:** 654
- **2D aligned content [PDB] (<2D-fold>:%):** {'.': 16.28, 'H': 39.83, 'T': 19.19, 'E': 24.71}
- **3D similarity (TM-Score) (%) [PDB]:** 16.77

- **Gene name:** N/A
- **Entrez ID:** N/A
- **RefSeq ID:** N/A
- **Sequence length:** N/A
- **5-UTR|CDS|3-UTR identity (%):** N/A | N/A | N/A
- **5-UTR|CDS|3-UTR identity (%) [Gaps excluded]:** N/A | N/A | N/A
- **5-UTR|CDS|3-UTR identity [Alignment Gaps]:** N/A | N/A | N/A
- **5-UTR aligned content (<base>:%):** N/A
- **CDS aligned content (<base>:%):** N/A
- **3-UTR aligned content (<base>:%):** N/A

**Uniprot Description:**  
  
 Retroviral envelope proteins mediate receptor recognition and membrane fusion during early infection. Endogenous envelope proteins may have kept, lost or modified their original function during evolution. This endogenous envelope protein has lost its original fusogenic properties but has immunosuppressive properties in vivo.   
  
The surface (SU) and transmembrane (TM) proteins form a heterodimer. SU and TM are attached by noncovalent interactions or by a labile interchain disulfide bond (By similarity).   
  
 **Gene Ontology Information:**

Molecular Function   
  
N/A

Location

- plasma membrane

Biological process

- syncytium formation by plasma membrane fusion

---

62

- **Protein name:** RNA exonuclease 5
- **Organism:** Homo sapiens
- **Uniprot Accession Number:** Q96IC2
- **Protein sequence length:** 774 aa
- **1D identity (%):** 16.49
- **1D identity (%) [Gaps excluded]:** 23.41
- **1D identity - Alignment Gaps:** 283
- **1D aligned content (<aminoacid>:%):** {'P': 6.96, 'G': 10.76, 'V': 8.86, 'L': 12.66, 'A': 5.7, 'R': 3.8, 'F': 4.43, 'Y': 1.9, 'S': 3.8, 'C': 4.43, 'H': 3.8, 'Q': 6.33, 'E': 4.43, 'D': 1.9, 'T': 6.33, 'K': 8.23, 'N': 1.27, 'I': 3.8, 'M': 0.63}
- **Common reported functions (%):** 6.67
- **Common reported locations (%):** 7.14
- **Common reported processes (%):** 0.0

- **AF ID:** Q96IC2
- **Chain:** A
- **Protein length:** 774 aa
- **Resolution:** N/A
- **b-phipsi:** 0.001149
- **w-rdist:** 0.347295
- **t-alpha:** 0.040151
- **Chemical similarity (Tanimoto Index) (%):** 99.55
- **1D identity (%) [PDB]:** 2.85
- **1D identity (%) [Gaps excluded][PDB]:** 66.67
- **1D identity - Alignment Gaps [PDB]:** 1480
- **1D aligned content [PDB] (<aminoacid>:%):** {'M': 2.27, 'T': 4.55, 'D': 4.55, 'V': 9.09, 'L': 6.82, 'Q': 11.36, 'P': 6.82, 'S': 6.82, 'I': 11.36, 'G': 6.82, 'K': 11.36, 'R': 6.82, 'N': 2.27, 'F': 4.55, 'E': 4.55}
- **2D identity (%) [PDB]:** 43.9
- **2D identity (%) [Gaps excluded][PDB]:** 89.62
- **2D identity - Alignment Gaps [PDB]:** 552
- **2D aligned content [PDB] (<2D-fold>:%):** {'.': 19.16, 'E': 19.58, 'T': 16.84, 'H': 44.42}
- **3D similarity (TM-Score) (%) [PDB]:** 25.24

- **Gene name:** REXO5
- **Entrez ID:** 532864
- **RefSeq ID:** N/A
- **Sequence length:** N/A
- **5-UTR|CDS|3-UTR identity (%):** N/A | N/A | N/A
- **5-UTR|CDS|3-UTR identity (%) [Gaps excluded]:** N/A | N/A | N/A
- **5-UTR|CDS|3-UTR identity [Alignment Gaps]:** N/A | N/A | N/A
- **5-UTR aligned content (<base>:%):** N/A
- **CDS aligned content (<base>:%):** N/A
- **3-UTR aligned content (<base>:%):** N/A

**Uniprot Description:**  
  
 N/A N/A   
  
 **Gene Ontology Information:**

Molecular Function

- exonuclease activity
- RNA binding

Location

- nucleolus
- nucleus

Biological process   
  
N/A

---

63

- **Protein name:** Gypsy retrotransposon integrase-like protein 1
- **Organism:** Homo sapiens
- **Uniprot Accession Number:** Q9NXP7
- **Protein sequence length:** 522 aa
- **1D identity (%):** 11.29
- **1D identity (%) [Gaps excluded]:** 23.98
- **1D identity - Alignment Gaps:** 497
- **1D aligned content (<aminoacid>:%):** {'R': 6.6, 'S': 5.66, 'N': 0.94, 'D': 5.66, 'T': 5.66, 'G': 8.49, 'I': 7.55, 'K': 5.66, 'F': 2.83, 'C': 3.77, 'E': 4.72, 'H': 6.6, 'L': 9.43, 'A': 9.43, 'V': 5.66, 'P': 3.77, 'Y': 3.77, 'Q': 3.77}
- **Common reported functions (%):** 0.0
- **Common reported locations (%):** 0.0
- **Common reported processes (%):** 0.0

- **AF ID:** Q9NXP7
- **Chain:** A
- **Protein length:** 522 aa
- **Resolution:** N/A
- **b-phipsi:** 0.000928
- **w-rdist:** 0.758064
- **t-alpha:** 0.005098
- **Chemical similarity (Tanimoto Index) (%):** 99.02
- **1D identity (%) [PDB]:** 1.88
- **1D identity (%) [Gaps excluded][PDB]:** 75.76
- **1D identity - Alignment Gaps [PDB]:** 1294
- **1D aligned content [PDB] (<aminoacid>:%):** {'V': 16.0, 'T': 4.0, 'L': 8.0, 'P': 4.0, 'E': 4.0, 'G': 4.0, 'K': 8.0, 'D': 8.0, 'R': 4.0, 'I': 8.0, 'F': 4.0, 'S': 12.0, 'W': 4.0, 'C': 4.0, 'A': 4.0, 'H': 4.0}
- **2D identity (%) [PDB]:** 34.62
- **2D identity (%) [Gaps excluded][PDB]:** 90.93
- **2D identity - Alignment Gaps [PDB]:** 610
- **2D aligned content [PDB] (<2D-fold>:%):** {'.': 19.94, 'H': 43.7, 'T': 21.7, 'E': 14.37, 'B': 0.29}
- **3D similarity (TM-Score) (%) [PDB]:** 17.81

- **Gene name:** GIN1
- **Entrez ID:** 504972
- **RefSeq ID:** N/A
- **Sequence length:** N/A
- **5-UTR|CDS|3-UTR identity (%):** N/A | N/A | N/A
- **5-UTR|CDS|3-UTR identity (%) [Gaps excluded]:** N/A | N/A | N/A
- **5-UTR|CDS|3-UTR identity [Alignment Gaps]:** N/A | N/A | N/A
- **5-UTR aligned content (<base>:%):** N/A
- **CDS aligned content (<base>:%):** N/A
- **3-UTR aligned content (<base>:%):** N/A

**Uniprot Description:**  
  
 N/A N/A   
  
 **Gene Ontology Information:**

Molecular Function

- nucleic acid binding

Location   
  
N/A

Biological process

- DNA integration

---

64

- **Protein name:** Probable ATP-dependent RNA helicase DDX53
- **Organism:** Homo sapiens
- **Uniprot Accession Number:** Q86TM3
- **Protein sequence length:** 631 aa
- **1D identity (%):** 13.06
- **1D identity (%) [Gaps excluded]:** 23.45
- **1D identity - Alignment Gaps:** 424
- **1D aligned content (<aminoacid>:%):** {'E': 4.0, 'K': 4.0, 'G': 12.8, 'R': 8.8, 'P': 8.0, 'H': 2.4, 'Q': 6.4, 'S': 5.6, 'C': 3.2, 'F': 1.6, 'V': 8.0, 'T': 4.8, 'N': 1.6, 'I': 8.8, 'A': 2.4, 'D': 7.2, 'W': 0.8, 'L': 8.8, 'Y': 0.8}
- **Common reported functions (%):** 6.67
- **Common reported locations (%):** 14.29
- **Common reported processes (%):** 0.0

- **AF ID:** Q86TM3
- **Chain:** A
- **Protein length:** 631 aa
- **Resolution:** N/A
- **b-phipsi:** 0.002223
- **w-rdist:** 0.316819
- **t-alpha:** 0.049694
- **Chemical similarity (Tanimoto Index) (%):** 99.17
- **1D identity (%) [PDB]:** 3.13
- **1D identity (%) [Gaps excluded][PDB]:** 68.75
- **1D identity - Alignment Gaps [PDB]:** 1341
- **1D aligned content [PDB] (<aminoacid>:%):** {'M': 4.55, 'P': 6.82, 'L': 13.64, 'I': 9.09, 'G': 4.55, 'R': 9.09, 'D': 6.82, 'V': 13.64, 'E': 6.82, 'F': 4.55, 'K': 2.27, 'S': 4.55, 'Q': 4.55, 'A': 4.55, 'H': 4.55}
- **2D identity (%) [PDB]:** 42.5
- **2D identity (%) [Gaps excluded][PDB]:** 88.66
- **2D identity - Alignment Gaps [PDB]:** 517
- **2D aligned content [PDB] (<2D-fold>:%):** {'.': 20.14, 'E': 18.01, 'H': 49.05, 'T': 12.8}
- **3D similarity (TM-Score) (%) [PDB]:** 22.85

- **Gene name:** DDX53
- **Entrez ID:** 168400
- **RefSeq ID:** NM\_182699
- **Transcript sequence length:** 3630
- **5-UTR|CDS|3-UTR identity (%):** 30.13 | 37.54 | 8.89
- **5-UTR|CDS|3-UTR identity (%) [Gaps excluded]:** 68.12 | 74.25 | 75.46
- **5-UTR|CDS|3-UTR identity [Alignment Gaps]:** 87 | 1470 | 10672
- **5-UTR aligned content (<base>:%):** {'G': 42.55, 'C': 46.81, 'A': 2.13, 'T': 8.51}
- **CDS aligned content (<base>:%):** {'A': 32.17, 'G': 26.25, 'C': 21.68, 'T': 19.89}
- **3-UTR aligned content (<base>:%):** {'A': 31.69, 'G': 16.17, 'T': 39.59, 'C': 12.55}

**Uniprot Description:**  
  
 N/A N/A   
  
 **Gene Ontology Information:**

Molecular Function

- ATP binding
- ATPase activity
- RNA binding
- RNA helicase activity

Location

- cytosol
- intracellular membrane-bounded organelle
- nucleolus
- nucleoplasm

Biological process   
  
N/A

---

65

- **Protein name:** 1-phosphatidylinositol 4,5-bisphosphate phosphodiesterase delta-3
- **Organism:** Homo sapiens
- **Uniprot Accession Number:** Q8N3E9
- **Protein sequence length:** 789 aa
- **1D identity (%):** 14.48
- **1D identity (%) [Gaps excluded]:** 23.22
- **1D identity - Alignment Gaps:** 382
- **1D aligned content (<aminoacid>:%):** {'M': 2.04, 'G': 9.52, 'P': 10.88, 'A': 6.8, 'D': 6.8, 'T': 6.12, 'R': 10.88, 'K': 2.72, 'Y': 5.44, 'H': 1.36, 'V': 4.76, 'Q': 4.76, 'F': 5.44, 'L': 10.2, 'W': 0.68, 'S': 2.72, 'E': 4.76, 'N': 2.04, 'C': 1.36, 'I': 0.68}
- **Common reported functions (%):** 6.67
- **Common reported locations (%):** 14.29
- **Common reported processes (%):** 0.0

- **AF ID:** Q8N3E9
- **Chain:** A
- **Protein length:** 789 aa
- **Resolution:** N/A
- **b-phipsi:** 0.000699
- **w-rdist:** 0.461018
- **t-alpha:** 0.048091
- **Chemical similarity (Tanimoto Index) (%):** 99.17
- **1D identity (%) [PDB]:** 2.96
- **1D identity (%) [Gaps excluded][PDB]:** 64.79
- **1D identity - Alignment Gaps [PDB]:** 1485
- **1D aligned content [PDB] (<aminoacid>:%):** {'L': 8.7, 'C': 4.35, 'R': 13.04, 'S': 6.52, 'V': 8.7, 'I': 2.17, 'P': 4.35, 'A': 19.57, 'D': 6.52, 'K': 2.17, 'E': 4.35, 'G': 4.35, 'H': 4.35, 'T': 4.35, 'Q': 6.52}
- **2D identity (%) [PDB]:** 38.39
- **2D identity (%) [Gaps excluded][PDB]:** 90.12
- **2D identity - Alignment Gaps [PDB]:** 655
- **2D aligned content [PDB] (<2D-fold>:%):** {'.': 19.18, 'T': 19.86, 'H': 38.36, 'E': 20.09, 'G': 2.05, 'B': 0.46}
- **3D similarity (TM-Score) (%) [PDB]:** 22.79

- **Gene name:** PLCD3
- **Entrez ID:** 113026
- **RefSeq ID:** NM\_133373
- **Transcript sequence length:** 6132
- **5-UTR|CDS|3-UTR identity (%):** 50.71 | 45.92 | 18.33
- **5-UTR|CDS|3-UTR identity (%) [Gaps excluded]:** 77.17 | 73.65 | 74.62
- **5-UTR|CDS|3-UTR identity [Alignment Gaps]:** 48 | 1148 | 9413
- **5-UTR aligned content (<base>:%):** {'T': 5.63, 'G': 39.44, 'C': 53.52, 'A': 1.41}
- **CDS aligned content (<base>:%):** {'T': 15.21, 'C': 32.64, 'G': 31.86, 'A': 20.29}
- **3-UTR aligned content (<base>:%):** {'G': 27.88, 'C': 27.01, 'T': 24.3, 'A': 20.8}

**Uniprot Description:**  
  
 Hydrolyzes the phosphatidylinositol 4,5-bisphosphate (PIP2) to generate 2 second messenger molecules diacylglycerol (DAG) and inositol 1,4,5-trisphosphate (IP3). DAG mediates the activation of protein kinase C (PKC), while IP3 releases Ca(2+) from intracellular stores. Essential for trophoblast and placental development. May participate in cytokinesis by hydrolyzing PIP2 at the cleavage furrow (PubMed:10336610). Regulates neurite outgrowth through the inhibition of RhoA/Rho kinase signaling (By similarity). N/A   
  
 **Gene Ontology Information:**

Molecular Function

- metal ion binding
- phosphatidylinositol phospholipase C activity

Location

- cleavage furrow
- cytoplasm
- nucleoplasm
- plasma membrane

Biological process

- angiogenesis
- intracellular signal transduction
- labyrinthine layer blood vessel development
- lipid catabolic process
- regulation of cell population proliferation

---

66

- **Protein name:** Calpain-7
- **Organism:** Homo sapiens
- **Uniprot Accession Number:** Q9Y6W3
- **Protein sequence length:** 813 aa
- **1D identity (%):** 15.59
- **1D identity (%) [Gaps excluded]:** 23.61
- **1D identity - Alignment Gaps:** 342
- **1D aligned content (<aminoacid>:%):** {'A': 5.1, 'R': 5.73, 'G': 9.55, 'Q': 6.37, 'E': 3.82, 'H': 1.27, 'K': 9.55, 'P': 8.28, 'V': 8.92, 'T': 5.73, 'F': 5.73, 'D': 5.73, 'N': 1.91, 'L': 7.01, 'S': 4.46, 'I': 5.73, 'W': 1.27, 'C': 1.27, 'Y': 2.55}
- **Common reported functions (%):** 0.0
- **Common reported locations (%):** 21.43
- **Common reported processes (%):** 0.0

- **AF ID:** Q9Y6W3
- **Chain:** A
- **Protein length:** 813 aa
- **Resolution:** N/A
- **b-phipsi:** 0.000372
- **w-rdist:** 0.536624
- **t-alpha:** 0.085375
- **Chemical similarity (Tanimoto Index) (%):** 99.62
- **1D identity (%) [PDB]:** 2.06
- **1D identity (%) [Gaps excluded][PDB]:** 70.21
- **1D identity - Alignment Gaps [PDB]:** 1557
- **1D aligned content [PDB] (<aminoacid>:%):** {'A': 12.12, 'T': 9.09, 'R': 12.12, 'D': 9.09, 'E': 9.09, 'I': 3.03, 'S': 9.09, 'K': 6.06, 'L': 6.06, 'F': 6.06, 'N': 3.03, 'P': 3.03, 'M': 3.03, 'V': 6.06, 'Q': 3.03}
- **2D identity (%) [PDB]:** 32.92
- **2D identity (%) [Gaps excluded][PDB]:** 91.74
- **2D identity - Alignment Gaps [PDB]:** 779
- **2D aligned content [PDB] (<2D-fold>:%):** {'.': 21.75, 'H': 42.5, 'T': 13.5, 'E': 20.25, 'B': 0.5, 'G': 1.5}
- **3D similarity (TM-Score) (%) [PDB]:** 21.73

- **Gene name:** CAPN7
- **Entrez ID:** 23473
- **RefSeq ID:** NM\_014296
- **Transcript sequence length:** 4348
- **5-UTR|CDS|3-UTR identity (%):** 35.58 | 44.77 | 9.35
- **5-UTR|CDS|3-UTR identity (%) [Gaps excluded]:** 86.36 | 72.09 | 77.61
- **5-UTR|CDS|3-UTR identity [Alignment Gaps]:** 157 | 1174 | 10632
- **5-UTR aligned content (<base>:%):** {'G': 47.37, 'A': 3.16, 'C': 45.26, 'T': 4.21}
- **CDS aligned content (<base>:%):** {'A': 30.14, 'T': 20.84, 'G': 25.16, 'C': 23.86}
- **3-UTR aligned content (<base>:%):** {'T': 37.79, 'G': 14.51, 'A': 35.13, 'C': 12.57}

**Uniprot Description:**  
  
 Calcium-regulated non-lysosomal thiol-protease. N/A   
  
 **Gene Ontology Information:**

Molecular Function

- calcium-dependent cysteine-type endopeptidase activity
- cysteine-type endopeptidase activity
- endopeptidase activity
- MIT domain binding

Location

- centrosome
- cytosol
- extracellular exosome
- nucleus

Biological process

- positive regulation of epithelial cell migration
- proteolysis
- self proteolysis

---

67

- **Protein name:** Serine/threonine-protein kinase PLK3
- **Organism:** Homo sapiens
- **Uniprot Accession Number:** Q9H4B4
- **Protein sequence length:** 646 aa
- **1D identity (%):** 12.26
- **1D identity (%) [Gaps excluded]:** 21.23
- **1D identity - Alignment Gaps:** 403
- **1D aligned content (<aminoacid>:%):** {'A': 5.98, 'F': 5.13, 'P': 12.82, 'L': 10.26, 'E': 4.27, 'D': 4.27, 'R': 9.4, 'T': 3.42, 'G': 8.55, 'K': 5.13, 'Y': 3.42, 'V': 7.69, 'S': 4.27, 'Q': 4.27, 'H': 4.27, 'C': 3.42, 'N': 1.71, 'I': 0.85, 'W': 0.85}
- **Common reported functions (%):** 0.0
- **Common reported locations (%):** 28.57
- **Common reported processes (%):** 0.0

- **AF ID:** Q9H4B4
- **Chain:** A
- **Protein length:** 646 aa
- **Resolution:** N/A
- **b-phipsi:** 0.005512
- **w-rdist:** 0.249785
- **t-alpha:** 0.03933
- **Chemical similarity (Tanimoto Index) (%):** 97.33
- **1D identity (%) [PDB]:** 2.44
- **1D identity (%) [Gaps excluded][PDB]:** 74.47
- **1D identity - Alignment Gaps [PDB]:** 1390
- **1D aligned content [PDB] (<aminoacid>:%):** {'E': 8.57, 'L': 14.29, 'T': 5.71, 'P': 5.71, 'G': 5.71, 'K': 8.57, 'R': 2.86, 'I': 5.71, 'F': 2.86, 'V': 11.43, 'S': 11.43, 'C': 2.86, 'A': 8.57, 'H': 2.86, 'Q': 2.86}
- **2D identity (%) [PDB]:** 41.97
- **2D identity (%) [Gaps excluded][PDB]:** 87.53
- **2D identity - Alignment Gaps [PDB]:** 522
- **2D aligned content [PDB] (<2D-fold>:%):** {'.': 18.29, 'T': 18.53, 'H': 40.14, 'E': 22.33, 'G': 0.71}
- **3D similarity (TM-Score) (%) [PDB]:** 21.37

- **Gene name:** PLK3
- **Entrez ID:** 1263
- **RefSeq ID:** NM\_004073
- **Transcript sequence length:** 2340
- **5-UTR|CDS|3-UTR identity (%):** 40.43 | 43.66 | 1.73
- **5-UTR|CDS|3-UTR identity (%) [Gaps excluded]:** 78.08 | 75.24 | 76.3
- **5-UTR|CDS|3-UTR identity [Alignment Gaps]:** 68 | 1201 | 11660
- **5-UTR aligned content (<base>:%):** {'G': 54.39, 'C': 45.61}
- **CDS aligned content (<base>:%):** {'A': 19.78, 'T': 17.45, 'G': 29.38, 'C': 33.39}
- **3-UTR aligned content (<base>:%):** {'G': 21.36, 'C': 30.58, 'A': 16.02, 'T': 32.04}

**Uniprot Description:**  
  
 Serine/threonine-protein kinase involved in cell cycle regulation, response to stress and Golgi disassembly. Polo-like kinases act by binding and phosphorylating proteins are that already phosphorylated on a specific motif recognized by the POLO box domains. Phosphorylates ATF2, BCL2L1, CDC25A, CDC25C, CHEK2, HIF1A, JUN, p53/TP53, p73/TP73, PTEN, TOP2A and VRK1. Involved in cell cycle regulation: required for entry into S phase and cytokinesis. Phosphorylates BCL2L1, leading to regulate the G2 checkpoint and progression to cytokinesis during mitosis. Plays a key role in response to stress: rapidly activated upon stress stimulation, such as ionizing radiation, reactive oxygen species (ROS), hyperosmotic stress, UV irradiation and hypoxia. Involved in DNA damage response and G1/S transition checkpoint by phosphorylating CDC25A, p53/TP53 and p73/TP73. Phosphorylates p53/TP53 in response to reactive oxygen species (ROS), thereby promoting p53/TP53-mediated apoptosis. Phosphorylates CHEK2 in response to DNA damage, promoting the G2/M transition checkpoint. Phosphorylates the transcription factor p73/TP73 in response to DNA damage, leading to inhibit p73/TP73-mediated transcriptional activation and pro-apoptotic functions. Phosphorylates HIF1A and JUN is response to hypoxia. Phosphorylates ATF2 following hyperosmotic stress in corneal epithelium. Also involved in Golgi disassembly during the cell cycle: part of a MEK1/MAP2K1-dependent pathway that induces Golgi fragmentation during mitosis by mediating phosphorylation of VRK1. May participate in endomitotic cell cycle, a form of mitosis in which both karyokinesis and cytokinesis are interrupted and is a hallmark of megakaryocyte differentiation, via its interaction with CIB1.   
  
Interacts (via the POLO-box domain) with CIB1; leading to inhibit PLK3 kinase activity. Interacts with GOLGB1.   
  
 **Gene Ontology Information:**

Molecular Function

- ATP binding
- p53 binding
- protein serine kinase activity
- protein serine/threonine kinase activity

Location

- centrosome
- cytoplasm
- dendrite
- Golgi stack
- kinetochore
- neuronal cell body
- nucleolus
- nucleoplasm
- nucleus
- spindle pole

Biological process

- apoptotic process
- cytoplasmic microtubule organization
- cellular response to DNA damage stimulus
- DNA damage response, signal transduction by p53 class mediator resulting in cell cycle arrest
- endomitotic cell cycle
- G1/S transition of mitotic cell cycle
- G2/M transition of mitotic cell cycle
- Golgi disassembly
- mitotic G1/S transition checkpoint
- mitotic spindle organization
- negative regulation of apoptotic process
- negative regulation of transcription by RNA polymerase II
- positive regulation of chaperone-mediated autophagy
- positive regulation of intracellular protein transport
- positive regulation of proteasomal ubiquitin-dependent protein catabolic process involved in cellular response to hypoxia
- protein kinase B signaling
- protein phosphorylation
- regulation of cell division
- regulation of cytokinesis
- regulation of signal transduction by p53 class mediator
- response to osmotic stress
- response to radiation
- response to reactive oxygen species

---

68

- **Protein name:** Bardet-Biedl syndrome 2 protein
- **Organism:** Homo sapiens
- **Uniprot Accession Number:** Q9BXC9
- **Protein sequence length:** 721 aa
- **1D identity (%):** 13.45
- **1D identity (%) [Gaps excluded]:** 23.44
- **1D identity - Alignment Gaps:** 428
- **1D aligned content (<aminoacid>:%):** {'M': 1.48, 'L': 13.33, 'P': 5.19, 'R': 7.41, 'K': 7.41, 'G': 11.11, 'F': 4.44, 'S': 6.67, 'V': 8.89, 'A': 8.15, 'D': 5.93, 'W': 0.74, 'N': 1.48, 'E': 3.7, 'T': 4.44, 'H': 2.22, 'C': 0.74, 'Y': 1.48, 'Q': 2.96, 'I': 2.22}
- **Common reported functions (%):** 0.0
- **Common reported locations (%):** 14.29
- **Common reported processes (%):** 0.0

- **AF ID:** Q9BXC9
- **Chain:** A
- **Protein length:** 721 aa
- **Resolution:** N/A
- **b-phipsi:** 0.005202
- **w-rdist:** 0.287773
- **t-alpha:** 0.030781
- **Chemical similarity (Tanimoto Index) (%):** 99.55
- **1D identity (%) [PDB]:** 4.46
- **1D identity (%) [Gaps excluded][PDB]:** 65.0
- **1D identity - Alignment Gaps [PDB]:** 1359
- **1D aligned content [PDB] (<aminoacid>:%):** {'L': 10.77, 'P': 3.08, 'G': 7.69, 'R': 12.31, 'V': 9.23, 'F': 4.62, 'Q': 10.77, 'A': 6.15, 'D': 6.15, 'K': 3.08, 'S': 4.62, 'N': 1.54, 'Y': 3.08, 'H': 3.08, 'I': 6.15, 'E': 4.62, 'T': 3.08}
- **2D identity (%) [PDB]:** 41.99
- **2D identity (%) [Gaps excluded][PDB]:** 91.06
- **2D identity - Alignment Gaps [PDB]:** 575
- **2D aligned content [PDB] (<2D-fold>:%):** {'.': 15.4, 'E': 31.25, 'T': 16.96, 'H': 36.16, 'B': 0.22}
- **3D similarity (TM-Score) (%) [PDB]:** 21.22

- **Gene name:** BBS2
- **Entrez ID:** 259187
- **RefSeq ID:** NM\_031885
- **Transcript sequence length:** 2704
- **5-UTR|CDS|3-UTR identity (%):** 49.13 | 43.62 | 2.19
- **5-UTR|CDS|3-UTR identity (%) [Gaps excluded]:** 73.28 | 75.16 | 80.37
- **5-UTR|CDS|3-UTR identity [Alignment Gaps]:** 57 | 1260 | 11612
- **5-UTR aligned content (<base>:%):** {'A': 3.53, 'G': 42.35, 'C': 45.88, 'T': 8.24}
- **CDS aligned content (<base>:%):** {'A': 25.88, 'T': 21.91, 'G': 27.02, 'C': 25.19}
- **3-UTR aligned content (<base>:%):** {'G': 14.89, 'T': 39.31, 'A': 34.73, 'C': 11.07}

**Uniprot Description:**  
  
 The BBSome complex is thought to function as a coat complex required for sorting of specific membrane proteins to the primary cilia. The BBSome complex is required for ciliogenesis but is dispensable for centriolar satellite function. This ciliogenic function is mediated in part by the Rab8 GDP/GTP exchange factor, which localizes to the basal body and contacts the BBSome. Rab8(GTP) enters the primary cilium and promotes extension of the ciliary membrane. Firstly the BBSome associates with the ciliary membrane and binds to RAB3IP/Rabin8, the guanosyl exchange factor (GEF) for Rab8 and then the Rab8-GTP localizes to the cilium and promotes docking and fusion of carrier vesicles to the base of the ciliary membrane. The BBSome complex, together with the LTZL1, controls SMO ciliary trafficking and contributes to the sonic hedgehog (SHH) pathway regulation. Required for proper BBSome complex assembly and its ciliary localization.   
  
Part of BBSome complex, that contains BBS1, BBS2, BBS4, BBS5, BBS7, BBS8/TTC8, BBS9 and BBIP10. Interacts (via C-terminus) with BBS7. Interacts (via coiled coil domain) with MKKS. Interacts with CCDC28B and ALDOB. Interacts with DLEC1 (PubMed:33144677).   
  
 **Gene Ontology Information:**

Molecular Function   
  
N/A

Location

- BBSome
- ciliary basal body
- ciliary membrane
- cytoplasm
- membrane
- motile cilium
- neuron projection

Biological process

- cilium assembly
- determination of left/right symmetry
- gastrulation
- intracellular transport
- Kupffer's vesicle development
- melanosome transport
- non-motile cilium assembly
- photoreceptor cell maintenance
- pigment granule aggregation in cell center
- regulation of eye photoreceptor cell development

---

69

- **Protein name:** Xanthine dehydrogenase/oxidase
- **Organism:** Homo sapiens
- **Uniprot Accession Number:** P47989
- **Protein sequence length:** 1333 aa
- **1D identity (%):** 14.43
- **1D identity (%) [Gaps excluded]:** 23.82
- **1D identity - Alignment Gaps:** 538
- **1D aligned content (<aminoacid>:%):** {'M': 0.51, 'A': 7.61, 'L': 8.12, 'P': 9.14, 'Q': 2.03, 'K': 6.09, 'F': 7.11, 'T': 10.15, 'G': 10.15, 'N': 3.55, 'Y': 4.06, 'C': 2.54, 'V': 5.58, 'E': 4.57, 'W': 0.51, 'D': 4.57, 'R': 4.06, 'S': 2.54, 'I': 3.55, 'H': 3.55}
- **Common reported functions (%):** 0.0
- **Common reported locations (%):** 0.0
- **Common reported processes (%):** 0.0

- **AF ID:** P47989
- **Chain:** A
- **Protein length:** 1333 aa
- **Resolution:** N/A
- **b-phipsi:** 0.00228
- **w-rdist:** 0.282034
- **t-alpha:** 0.070648
- **Chemical similarity (Tanimoto Index) (%):** 99.25
- **1D identity (%) [PDB]:** 3.58
- **1D identity (%) [Gaps excluded][PDB]:** 70.48
- **1D identity - Alignment Gaps [PDB]:** 1961
- **1D aligned content [PDB] (<aminoacid>:%):** {'L': 9.46, 'V': 17.57, 'I': 2.7, 'G': 8.11, 'K': 5.41, 'T': 10.81, 'A': 9.46, 'E': 1.35, 'R': 6.76, 'M': 4.05, 'Q': 4.05, 'N': 4.05, 'S': 2.7, 'P': 5.41, 'F': 2.7, 'D': 2.7, 'H': 1.35, 'C': 1.35}
- **2D identity (%) [PDB]:** 41.73
- **2D identity (%) [Gaps excluded][PDB]:** 90.92
- **2D identity - Alignment Gaps [PDB]:** 805
- **2D aligned content [PDB] (<2D-fold>:%):** {'.': 13.04, 'E': 28.99, 'T': 21.9, 'H': 35.1, 'B': 0.48, 'G': 0.48}
- **3D similarity (TM-Score) (%) [PDB]:** 29.36

- **Gene name:** XDH
- **Entrez ID:** 280960
- **RefSeq ID:** NM\_000379
- **Transcript sequence length:** 5715
- **5-UTR|CDS|3-UTR identity (%):** 24.0 | 41.79 | 8.15
- **5-UTR|CDS|3-UTR identity (%) [Gaps excluded]:** 67.92 | 76.77 | 74.27
- **5-UTR|CDS|3-UTR identity [Alignment Gaps]:** 97 | 1942 | 10851
- **5-UTR aligned content (<base>:%):** {'C': 33.33, 'G': 50.0, 'A': 2.78, 'T': 13.89}
- **CDS aligned content (<base>:%):** {'A': 23.98, 'T': 18.75, 'G': 28.47, 'C': 28.8}
- **3-UTR aligned content (<base>:%):** {'A': 32.33, 'G': 18.03, 'C': 16.62, 'T': 33.03}

**Uniprot Description:**  
  
 Key enzyme in purine degradation. Catalyzes the oxidation of hypoxanthine to xanthine. Catalyzes the oxidation of xanthine to uric acid. Contributes to the generation of reactive oxygen species. Has also low oxidase activity towards aldehydes (in vitro).   
  
Homodimer. Interacts with BTN1A1 (By similarity).   
  
 **Gene Ontology Information:**

Molecular Function

- 2 iron, 2 sulfur cluster binding
- FAD binding
- flavin adenine dinucleotide binding
- iron ion binding
- molybdenum ion binding
- molybdopterin cofactor binding
- protein homodimerization activity
- xanthine dehydrogenase activity
- xanthine oxidase activity

Location

- extracellular space
- peroxisome
- xanthine dehydrogenase complex

Biological process

- xanthine catabolic process

---

70

- **Protein name:** Mannosyl-oligosaccharide glucosidase
- **Organism:** Homo sapiens
- **Uniprot Accession Number:** Q13724
- **Protein sequence length:** 837 aa
- **1D identity (%):** 14.63
- **1D identity (%) [Gaps excluded]:** 25.65
- **1D identity - Alignment Gaps:** 464
- **1D aligned content (<aminoacid>:%):** {'G': 13.29, 'R': 7.59, 'P': 13.29, 'A': 11.39, 'D': 3.8, 'V': 9.49, 'L': 11.39, 'W': 1.9, 'H': 2.53, 'C': 0.63, 'F': 5.06, 'S': 1.9, 'T': 4.43, 'K': 3.16, 'E': 2.53, 'Y': 2.53, 'N': 1.27, 'Q': 3.16, 'I': 0.63}
- **Common reported functions (%):** 0.0
- **Common reported locations (%):** 0.0
- **Common reported processes (%):** 0.0

- **AF ID:** Q13724
- **Chain:** A
- **Protein length:** 837 aa
- **Resolution:** N/A
- **b-phipsi:** 0.005858
- **w-rdist:** 0.550983
- **t-alpha:** 0.002913
- **Chemical similarity (Tanimoto Index) (%):** 98.28
- **1D identity (%) [PDB]:** 3.99
- **1D identity (%) [Gaps excluded][PDB]:** 66.32
- **1D identity - Alignment Gaps [PDB]:** 1485
- **1D aligned content [PDB] (<aminoacid>:%):** {'D': 3.17, 'G': 9.52, 'R': 3.17, 'K': 1.59, 'L': 15.87, 'T': 4.76, 'A': 6.35, 'P': 11.11, 'V': 11.11, 'E': 1.59, 'I': 3.17, 'S': 11.11, 'Q': 3.17, 'F': 7.94, 'H': 1.59, 'W': 1.59, 'N': 1.59, 'Y': 1.59}
- **2D identity (%) [PDB]:** 45.81
- **2D identity (%) [Gaps excluded][PDB]:** 89.75
- **2D identity - Alignment Gaps [PDB]:** 543
- **2D aligned content [PDB] (<2D-fold>:%):** {'.': 17.13, 'H': 35.63, 'T': 22.64, 'E': 22.64, 'G': 1.77, 'B': 0.2}
- **3D similarity (TM-Score) (%) [PDB]:** 22.87

- **Gene name:** MOGS
- **Entrez ID:** 5142394
- **RefSeq ID:** NM\_006302
- **Transcript sequence length:** 2867
- **5-UTR|CDS|3-UTR identity (%):** 48.12 | 42.94 | 1.11
- **5-UTR|CDS|3-UTR identity (%) [Gaps excluded]:** 75.49 | 74.85 | 76.44
- **5-UTR|CDS|3-UTR identity [Alignment Gaps]:** 58 | 1380 | 11758
- **5-UTR aligned content (<base>:%):** {'G': 51.95, 'C': 40.26, 'T': 5.19, 'A': 2.6}
- **CDS aligned content (<base>:%):** {'A': 18.13, 'T': 17.19, 'C': 32.52, 'G': 32.16}
- **3-UTR aligned content (<base>:%):** {'A': 24.06, 'G': 24.06, 'C': 19.55, 'T': 32.33}

**Uniprot Description:**  
  
 Cleaves the distal alpha 1,2-linked glucose residue from the Glc(3)Man(9)GlcNAc(2) oligosaccharide precursor in a highly specific manner. N/A   
  
 **Gene Ontology Information:**

Molecular Function

- DNA binding
- DNA-directed DNA polymerase activity
- nucleotide binding

Location   
  
N/A

Biological process

- DNA replication
- viral DNA genome replication

---

71

- **Protein name:** Cytosolic phospholipase A2 delta
- **Organism:** Homo sapiens
- **Uniprot Accession Number:** Q86XP0
- **Protein sequence length:** 818 aa
- **1D identity (%):** 14.55
- **1D identity (%) [Gaps excluded]:** 24.05
- **1D identity - Alignment Gaps:** 413
- **1D aligned content (<aminoacid>:%):** {'L': 15.79, 'P': 13.16, 'Q': 5.26, 'G': 7.89, 'A': 5.26, 'F': 3.29, 'T': 3.29, 'K': 5.26, 'D': 5.26, 'I': 1.97, 'Y': 3.29, 'R': 5.92, 'E': 5.92, 'V': 5.26, 'S': 6.58, 'C': 1.32, 'N': 1.32, 'H': 2.63, 'W': 1.32}
- **Common reported functions (%):** 0.0
- **Common reported locations (%):** 14.29
- **Common reported processes (%):** 0.0

- **AF ID:** Q86XP0
- **Chain:** A
- **Protein length:** 818 aa
- **Resolution:** N/A
- **b-phipsi:** 0.00244
- **w-rdist:** 0.367088
- **t-alpha:** 0.015295
- **Chemical similarity (Tanimoto Index) (%):** 96.56
- **1D identity (%) [PDB]:** 3.14
- **1D identity (%) [Gaps excluded][PDB]:** 75.76
- **1D identity - Alignment Gaps [PDB]:** 1524
- **1D aligned content [PDB] (<aminoacid>:%):** {'Q': 10.0, 'L': 16.0, 'V': 16.0, 'I': 4.0, 'P': 8.0, 'G': 8.0, 'K': 6.0, 'T': 10.0, 'E': 2.0, 'D': 2.0, 'M': 2.0, 'A': 2.0, 'C': 2.0, 'R': 4.0, 'S': 2.0, 'N': 6.0}
- **2D identity (%) [PDB]:** 47.08
- **2D identity (%) [Gaps excluded][PDB]:** 88.04
- **2D identity - Alignment Gaps [PDB]:** 502
- **2D aligned content [PDB] (<2D-fold>:%):** {'.': 18.31, 'E': 31.3, 'T': 18.7, 'H': 30.51, 'G': 1.18}
- **3D similarity (TM-Score) (%) [PDB]:** 23.56

- **Gene name:** PLA2G4D
- **Entrez ID:** 283748
- **RefSeq ID:** NM\_178034
- **Transcript sequence length:** 4266
- **5-UTR|CDS|3-UTR identity (%):** 39.44 | 46.31 | 9.08
- **5-UTR|CDS|3-UTR identity (%) [Gaps excluded]:** 63.64 | 75.12 | 76.6
- **5-UTR|CDS|3-UTR identity [Alignment Gaps]:** 54 | 1195 | 10714
- **5-UTR aligned content (<base>:%):** {'G': 41.07, 'A': 5.36, 'T': 12.5, 'C': 41.07}
- **CDS aligned content (<base>:%):** {'A': 21.34, 'T': 16.22, 'G': 29.87, 'C': 32.57}
- **3-UTR aligned content (<base>:%):** {'G': 21.58, 'T': 35.9, 'C': 23.12, 'A': 19.4}

**Uniprot Description:**  
  
 Calcium-dependent phospholipase A2 that selectively hydrolyzes glycerophospholipids in the sn-2 position (PubMed:14709560). Has a preference for linoleic acid at the sn-2 position (PubMed:14709560). N/A   
  
 **Gene Ontology Information:**

Molecular Function

- calcium ion binding
- calcium-dependent phospholipase A2 activity
- calcium-dependent phospholipid binding
- phospholipase A1 activity

Location

- cytosol
- membrane

Biological process

- fatty acid metabolic process
- glycerophospholipid catabolic process
- phosphatidylglycerol acyl-chain remodeling
- phosphatidylinositol acyl-chain remodeling

---

72

- **Protein name:** Ectonucleotide pyrophosphatase/phosphodiesterase family member 3
- **Organism:** Homo sapiens
- **Uniprot Accession Number:** O14638
- **Protein sequence length:** 875 aa
- **1D identity (%):** 15.95
- **1D identity (%) [Gaps excluded]:** 24.67
- **1D identity - Alignment Gaps:** 372
- **1D aligned content (<aminoacid>:%):** {'E': 5.95, 'T': 7.14, 'L': 10.71, 'K': 4.17, 'C': 1.79, 'A': 5.36, 'S': 6.55, 'R': 7.74, 'F': 5.95, 'G': 7.14, 'W': 0.6, 'M': 1.19, 'N': 2.98, 'D': 3.57, 'Q': 5.95, 'P': 8.33, 'H': 4.17, 'Y': 3.57, 'I': 1.19, 'V': 5.95}
- **Common reported functions (%):** 0.0
- **Common reported locations (%):** 0.0
- **Common reported processes (%):** 0.0

- **AF ID:** O14638
- **Chain:** A
- **Protein length:** 875 aa
- **Resolution:** N/A
- **b-phipsi:** 0.000673
- **w-rdist:** 0.471332
- **t-alpha:** 0.058267
- **Chemical similarity (Tanimoto Index) (%):** 96.63
- **1D identity (%) [PDB]:** 3.84
- **1D identity (%) [Gaps excluded][PDB]:** 62.63
- **1D identity - Alignment Gaps [PDB]:** 1515
- **1D aligned content [PDB] (<aminoacid>:%):** {'M': 4.84, 'F': 1.61, 'R': 3.23, 'L': 9.68, 'N': 8.06, 'T': 6.45, 'A': 6.45, 'G': 9.68, 'V': 12.9, 'I': 4.84, 'P': 11.29, 'K': 6.45, 'D': 4.84, 'C': 1.61, 'Q': 4.84, 'S': 1.61, 'H': 1.61}
- **2D identity (%) [PDB]:** 43.12
- **2D identity (%) [Gaps excluded][PDB]:** 87.61
- **2D identity - Alignment Gaps [PDB]:** 583
- **2D aligned content [PDB] (<2D-fold>:%):** {'.': 18.38, 'H': 34.95, 'T': 27.07, 'E': 17.58, 'G': 1.21, 'B': 0.81}
- **3D similarity (TM-Score) (%) [PDB]:** 22.35

- **Gene name:** ENPP3
- **Entrez ID:** 529405
- **RefSeq ID:** NM\_005021
- **Transcript sequence length:** 3165
- **5-UTR|CDS|3-UTR identity (%):** 20.0 | 43.29 | 2.26
- **5-UTR|CDS|3-UTR identity (%) [Gaps excluded]:** 64.0 | 74.63 | 73.44
- **5-UTR|CDS|3-UTR identity [Alignment Gaps]:** 110 | 1384 | 11604
- **5-UTR aligned content (<base>:%):** {'C': 34.38, 'T': 21.88, 'G': 37.5, 'A': 6.25}
- **CDS aligned content (<base>:%):** {'A': 28.17, 'T': 23.13, 'G': 24.18, 'C': 24.53}
- **3-UTR aligned content (<base>:%):** {'T': 45.39, 'A': 36.16, 'C': 11.81, 'G': 6.64}

**Uniprot Description:**  
  
 Hydrolase that metabolizes extracellular nucleotides, including ATP, GTP, UTP and CTP (PubMed:29717535). Limits mast cell and basophil responses during inflammation and during the chronic phases of allergic responses by eliminating the extracellular ATP that functions as signaling molecule and activates basophils and mast cells and induces the release of inflammatory cytokines. Metabolizes extracellular ATP in the lumen of the small intestine, and thereby prevents ATP-induced apoptosis of intestinal plasmacytoid dendritic cells (By similarity). Has also alkaline phosphodiesterase activity (PubMed:11342463).   
  
Monomer and homodimer.   
  
 **Gene Ontology Information:**

Molecular Function

- calcium ion binding
- nucleic acid binding
- nucleoside-triphosphate diphosphatase activity
- phosphodiesterase I activity
- zinc ion binding

Location

- apical plasma membrane
- external side of plasma membrane
- extracellular region
- perinuclear region of cytoplasm

Biological process

- ATP metabolic process
- basophil activation involved in immune response
- negative regulation of inflammatory response
- negative regulation of mast cell activation involved in immune response
- negative regulation of mast cell proliferation
- nucleoside triphosphate catabolic process
- phosphate ion homeostasis
- pyrimidine nucleotide metabolic process

---

73

- **Protein name:** Exostosin-1
- **Organism:** Homo sapiens
- **Uniprot Accession Number:** Q16394
- **Protein sequence length:** 746 aa
- **1D identity (%):** 13.63
- **1D identity (%) [Gaps excluded]:** 20.34
- **1D identity - Alignment Gaps:** 317
- **1D aligned content (<aminoacid>:%):** {'K': 3.82, 'R': 8.4, 'F': 6.11, 'S': 6.11, 'C': 2.29, 'L': 6.87, 'G': 7.63, 'P': 9.92, 'V': 8.4, 'D': 7.63, 'H': 2.29, 'E': 3.82, 'Q': 3.82, 'N': 3.82, 'I': 3.05, 'Y': 5.34, 'T': 6.11, 'M': 0.76, 'A': 2.29, 'W': 1.53}
- **Common reported functions (%):** 6.67
- **Common reported locations (%):** 7.14
- **Common reported processes (%):** 0.0

- **AF ID:** Q16394
- **Chain:** A
- **Protein length:** 746 aa
- **Resolution:** N/A
- **b-phipsi:** 0.003088
- **w-rdist:** 0.322427
- **t-alpha:** 0.044106
- **Chemical similarity (Tanimoto Index) (%):** 96.62
- **1D identity (%) [PDB]:** 2.14
- **1D identity (%) [Gaps excluded][PDB]:** 73.33
- **1D identity - Alignment Gaps [PDB]:** 1494
- **1D aligned content [PDB] (<aminoacid>:%):** {'F': 3.03, 'R': 12.12, 'A': 12.12, 'H': 12.12, 'E': 6.06, 'D': 6.06, 'S': 12.12, 'G': 6.06, 'T': 6.06, 'N': 3.03, 'Q': 6.06, 'L': 6.06, 'K': 3.03, 'V': 6.06}
- **2D identity (%) [PDB]:** 39.17
- **2D identity (%) [Gaps excluded][PDB]:** 89.81
- **2D identity - Alignment Gaps [PDB]:** 622
- **2D aligned content [PDB] (<2D-fold>:%):** {'H': 40.28, 'T': 23.84, '.': 16.2, 'E': 18.29, 'G': 1.39}
- **3D similarity (TM-Score) (%) [PDB]:** 23.94

- **Gene name:** EXT1
- **Entrez ID:** 2131
- **RefSeq ID:** NM\_000127
- **Transcript sequence length:** 8243
- **5-UTR|CDS|3-UTR identity (%):** 14.14 | 43.4 | 26.5
- **5-UTR|CDS|3-UTR identity (%) [Gaps excluded]:** 90.98 | 74.48 | 74.9
- **5-UTR|CDS|3-UTR identity [Alignment Gaps]:** 663 | 1271 | 8168
- **5-UTR aligned content (<base>:%):** {'T': 6.31, 'G': 45.05, 'C': 45.05, 'A': 3.6}
- **CDS aligned content (<base>:%):** {'T': 19.97, 'C': 28.74, 'A': 25.57, 'G': 25.72}
- **3-UTR aligned content (<base>:%):** {'G': 18.99, 'A': 28.52, 'T': 34.46, 'C': 18.04}

**Uniprot Description:**  
  
 Glycosyltransferase forming with EXT2 the heterodimeric heparan sulfate polymerase which catalyzes the elongation of the heparan sulfate glycan backbone (PubMed:10639137, PubMed:22660413, PubMed:36402845, PubMed:36593275, PubMed:9620772). Glycan backbone extension consists in the alternating transfer of (1->4)-beta-D-GlcA and (1->4)-alpha-D-GlcNAc residues from their respective UDP-sugar donors. Both EXT1 and EXT2 are required for the full activity of the polymerase since EXT1 bears the N-acetylglucosaminyl-proteoglycan 4-beta-glucuronosyltransferase activity within the complex while EXT2 carries the glucuronosyl-N-acetylglucosaminyl-proteoglycan 4-alpha-N-acetylglucosaminyltransferase activity (PubMed:36402845, PubMed:36593275). Heparan sulfate proteoglycans are ubiquitous components of the extracellular matrix and play an important role in tissue homeostasis and signaling (PubMed:10639137, PubMed:11391482, PubMed:22660413, PubMed:9620772).   
  
Part of the heparan sulfate polymerase, a dimeric complex composed of EXT1 and EXT2 (PubMed:10639137, PubMed:10679296, PubMed:36402845, PubMed:36593275). Could also form homooligomeric complexes (PubMed:10679296). Interacts with NDST1 (PubMed:35137078).   
  
 **Gene Ontology Information:**

Molecular Function

- acetylglucosaminyltransferase activity
- glucuronosyl-N-acetylglucosaminyl-proteoglycan 4-alpha-N-acetylglucosaminyltransferase activity
- glucuronosyltransferase activity
- transferase activity, transferring glycosyl groups
- heparan sulfate N-acetylglucosaminyltransferase activity
- metal ion binding
- N-acetylglucosaminyl-proteoglycan 4-beta-glucuronosyltransferase activity
- protein heterodimerization activity
- protein homodimerization activity

Location

- endoplasmic reticulum
- endoplasmic reticulum membrane
- Golgi apparatus
- Golgi membrane
- membrane
- synapse

Biological process

- antigen processing and presentation
- axon guidance
- basement membrane organization
- blood vessel remodeling
- BMP signaling pathway
- bone resorption
- canonical Wnt signaling pathway
- cartilage development involved in endochondral bone morphogenesis
- cell adhesion mediated by integrin
- cell fate commitment
- cellular response to virus
- chondrocyte hypertrophy
- chondrocyte proliferation
- chondroitin sulfate metabolic process
- collagen fibril organization
- cranial skeletal system development
- dendrite self-avoidance
- dendritic cell migration
- developmental growth involved in morphogenesis
- embryonic skeletal joint development
- endochondral bone growth
- endochondral ossification
- endoderm development
- epithelial tube branching involved in lung morphogenesis
- fear response
- fibroblast growth factor receptor signaling pathway
- fluid transport
- gastrulation
- gene expression
- glandular epithelial cell differentiation
- glomerular basement membrane development
- glycosaminoglycan biosynthetic process
- hair follicle morphogenesis
- heart contraction
- heart field specification
- hematopoietic stem cell differentiation
- hematopoietic stem cell homeostasis
- hematopoietic stem cell migration to bone marrow
- heparan sulfate proteoglycan biosynthetic process
- heparan sulfate proteoglycan biosynthetic process, polysaccharide chain biosynthetic process
- heparin biosynthetic process
- hypersensitivity
- leukocyte tethering or rolling
- limb joint morphogenesis
- lymphocyte adhesion to endothelial cell of high endothelial venule
- lymphocyte migration into lymphoid organs
- mesenchymal cell differentiation involved in bone development
- mesoderm development
- motor behavior
- multicellular organism growth
- multicellular organismal water homeostasis
- neural crest cell differentiation
- olfactory bulb development
- optic nerve development
- ossification
- ossification involved in bone maturation
- perichondral bone morphogenesis
- glomerular visceral epithelial cell differentiation
- polysaccharide biosynthetic process
- protein catabolic process
- protein glycosylation
- protein-containing complex assembly
- regulation of blood pressure
- response to heparin
- response to leukemia inhibitory factor
- response to light intensity
- sebaceous gland development
- signal transduction
- skeletal system development
- smoothened signaling pathway involved in lung development
- social behavior
- sodium ion homeostasis
- stem cell division
- stomach development
- sulfation
- sweat gland development
- synaptic transmission, glutamatergic
- tight junction organization
- TNFSF11-mediated signaling pathway
- vacuole organization
- vasodilation
- vocalization behavior
- wound healing

---

74

- **Protein name:** MAGUK p55 subfamily member 7
- **Organism:** Homo sapiens
- **Uniprot Accession Number:** Q5T2T1
- **Protein sequence length:** 576 aa
- **1D identity (%):** 12.6
- **1D identity (%) [Gaps excluded]:** 22.57
- **1D identity - Alignment Gaps:** 407
- **1D aligned content (<aminoacid>:%):** {'P': 8.62, 'T': 8.62, 'G': 6.9, 'A': 4.31, 'E': 6.03, 'W': 0.86, 'F': 5.17, 'S': 2.59, 'L': 6.03, 'K': 10.34, 'Y': 1.72, 'Q': 5.17, 'D': 8.62, 'R': 6.03, 'H': 2.59, 'V': 6.9, 'I': 5.17, 'C': 1.72, 'N': 2.59}
- **Common reported functions (%):** 0.0
- **Common reported locations (%):** 7.14
- **Common reported processes (%):** 0.0

- **AF ID:** Q5T2T1
- **Chain:** A
- **Protein length:** 576 aa
- **Resolution:** N/A
- **b-phipsi:** 0.000667
- **w-rdist:** 0.75418
- **t-alpha:** 0.007337
- **Chemical similarity (Tanimoto Index) (%):** 98.72
- **1D identity (%) [PDB]:** 2.4
- **1D identity (%) [Gaps excluded][PDB]:** 84.62
- **1D identity - Alignment Gaps [PDB]:** 1336
- **1D aligned content [PDB] (<aminoacid>:%):** {'E': 9.09, 'L': 9.09, 'A': 6.06, 'I': 12.12, 'R': 12.12, 'K': 12.12, 'D': 6.06, 'Q': 6.06, 'G': 3.03, 'T': 9.09, 'F': 6.06, 'V': 3.03, 'H': 3.03, 'N': 3.03}
- **2D identity (%) [PDB]:** 34.93
- **2D identity (%) [Gaps excluded][PDB]:** 91.07
- **2D identity - Alignment Gaps [PDB]:** 630
- **2D aligned content [PDB] (<2D-fold>:%):** {'.': 20.73, 'H': 42.02, 'T': 16.81, 'E': 20.45}
- **3D similarity (TM-Score) (%) [PDB]:** 17.68

- **Gene name:** MPP7
- **Entrez ID:** 143098
- **RefSeq ID:** N/A
- **Sequence length:** N/A
- **5-UTR|CDS|3-UTR identity (%):** N/A | N/A | N/A
- **5-UTR|CDS|3-UTR identity (%) [Gaps excluded]:** N/A | N/A | N/A
- **5-UTR|CDS|3-UTR identity [Alignment Gaps]:** N/A | N/A | N/A
- **5-UTR aligned content (<base>:%):** N/A
- **CDS aligned content (<base>:%):** N/A
- **3-UTR aligned content (<base>:%):** N/A

**Uniprot Description:**  
  
 Acts as an important adapter that promotes epithelial cell polarity and tight junction formation via its interaction with DLG1. Involved in the assembly of protein complexes at sites of cell-cell contact.   
  
Heterodimer; able to heterodimerize via its C-terminal L27 domain with LIN7A, LIN7B and LIN7C. Forms a tripartite complex composed of DLG1, MPP7 and LIN7 (LIN7A or LIN7C). Interacts with DLG1 via its N-terminal L27 domain. Interacts with PALS1 and PATJ.   
  
 **Gene Ontology Information:**

Molecular Function

- cadherin binding
- molecular adaptor activity
- protein domain specific binding
- signaling adaptor activity

Location

- adherens junction
- bicellular tight junction
- cell cortex
- cell junction
- cell-cell junction
- lateral plasma membrane
- MPP7-DLG1-LIN7 complex
- nucleoplasm
- plasma membrane

Biological process

- bicellular tight junction assembly
- establishment of cell polarity
- positive regulation of protein-containing complex assembly
- protein localization to adherens junction

---

75

- **Protein name:** Protein kinase C theta type
- **Organism:** Homo sapiens
- **Uniprot Accession Number:** Q04759
- **Protein sequence length:** 706 aa
- **1D identity (%):** 14.62
- **1D identity (%) [Gaps excluded]:** 23.91
- **1D identity - Alignment Gaps:** 377
- **1D aligned content (<aminoacid>:%):** {'C': 5.63, 'S': 3.52, 'Q': 4.93, 'A': 4.93, 'E': 7.75, 'P': 7.75, 'M': 2.82, 'Y': 2.82, 'F': 6.34, 'G': 10.56, 'W': 1.41, 'N': 3.52, 'T': 3.52, 'V': 4.23, 'K': 7.75, 'R': 4.93, 'H': 3.52, 'L': 8.45, 'I': 3.52, 'D': 2.11}
- **Common reported functions (%):** 6.67
- **Common reported locations (%):** 7.14
- **Common reported processes (%):** 0.0

- **AF ID:** Q04759
- **Chain:** A
- **Protein length:** 706 aa
- **Resolution:** N/A
- **b-phipsi:** 0.00385
- **w-rdist:** 0.343291
- **t-alpha:** 0.024035
- **Chemical similarity (Tanimoto Index) (%):** 99.02
- **1D identity (%) [PDB]:** 1.79
- **1D identity (%) [Gaps excluded][PDB]:** 72.97
- **1D identity - Alignment Gaps [PDB]:** 1470
- **1D aligned content [PDB] (<aminoacid>:%):** {'M': 3.7, 'K': 7.41, 'N': 14.81, 'V': 7.41, 'T': 11.11, 'P': 11.11, 'Q': 3.7, 'L': 18.52, 'C': 3.7, 'I': 7.41, 'G': 7.41, 'R': 3.7}
- **2D identity (%) [PDB]:** 42.27
- **2D identity (%) [Gaps excluded][PDB]:** 89.31
- **2D identity - Alignment Gaps [PDB]:** 552
- **2D aligned content [PDB] (<2D-fold>:%):** {'.': 21.67, 'E': 26.64, 'T': 23.02, 'H': 27.09, 'G': 1.35, 'B': 0.23}
- **3D similarity (TM-Score) (%) [PDB]:** 26.15

- **Gene name:** PRKCQ
- **Entrez ID:** 5588
- **RefSeq ID:** N/A
- **Sequence length:** N/A
- **5-UTR|CDS|3-UTR identity (%):** N/A | N/A | N/A
- **5-UTR|CDS|3-UTR identity (%) [Gaps excluded]:** N/A | N/A | N/A
- **5-UTR|CDS|3-UTR identity [Alignment Gaps]:** N/A | N/A | N/A
- **5-UTR aligned content (<base>:%):** N/A
- **CDS aligned content (<base>:%):** N/A
- **3-UTR aligned content (<base>:%):** N/A

**Uniprot Description:**  
  
 Calcium-independent, phospholipid- and diacylglycerol (DAG)-dependent serine/threonine-protein kinase that mediates non-redundant functions in T-cell receptor (TCR) signaling, including T-cells activation, proliferation, differentiation and survival, by mediating activation of multiple transcription factors such as NF-kappa-B, JUN, NFATC1 and NFATC2. In TCR-CD3/CD28-co-stimulated T-cells, is required for the activation of NF-kappa-B and JUN, which in turn are essential for IL2 production, and participates in the calcium-dependent NFATC1 and NFATC2 transactivation (PubMed:21964608). Mediates the activation of the canonical NF-kappa-B pathway (NFKB1) by direct phosphorylation of CARD11 on several serine residues, inducing CARD11 association with lipid rafts and recruitment of the BCL10-MALT1 complex, which then activates IKK complex, resulting in nuclear translocation and activation of NFKB1. May also play an indirect role in activation of the non-canonical NF-kappa-B (NFKB2) pathway. In the signaling pathway leading to JUN activation, acts by phosphorylating the mediator STK39/SPAK and may not act through MAP kinases signaling. Plays a critical role in TCR/CD28-induced NFATC1 and NFATC2 transactivation by participating in the regulation of reduced inositol 1,4,5-trisphosphate generation and intracellular calcium mobilization. After costimulation of T-cells through CD28 can phosphorylate CBLB and is required for the ubiquitination and subsequent degradation of CBLB, which is a prerequisite for the activation of TCR. During T-cells differentiation, plays an important role in the development of T-helper 2 (Th2) cells following immune and inflammatory responses, and, in the development of inflammatory autoimmune diseases, is necessary for the activation of IL17-producing Th17 cells. May play a minor role in Th1 response. Upon TCR stimulation, mediates T-cell protective survival signal by phosphorylating BAD, thus protecting T-cells from BAD-induced apoptosis, and by up-regulating BCL-X(L)/BCL2L1 levels through NF-kappa-B and JUN pathways. In platelets, regulates signal transduction downstream of the ITGA2B, CD36/GP4, F2R/PAR1 and F2RL3/PAR4 receptors, playing a positive role in 'outside-in' signaling and granule secretion signal transduction. May relay signals from the activated ITGA2B receptor by regulating the uncoupling of WASP and WIPF1, thereby permitting the regulation of actin filament nucleation and branching activity of the Arp2/3 complex. May mediate inhibitory effects of free fatty acids on insulin signaling by phosphorylating IRS1, which in turn blocks IRS1 tyrosine phosphorylation and downstream activation of the PI3K/AKT pathway. Phosphorylates MSN (moesin) in the presence of phosphatidylglycerol or phosphatidylinositol. Phosphorylates PDPK1 at 'Ser-504' and 'Ser-532' and negatively regulates its ability to phosphorylate PKB/AKT1. Phosphorylates CCDC88A/GIV and inhibits its guanine nucleotide exchange factor activity (PubMed:23509302).   
  
Part of a lipid raft complex composed at least of BCL10, CARD11, MALT1 and IKBKB (PubMed:16356855). Interacts with GLRX3 (via N-terminus) (PubMed:10636891). Interacts with ECT2 (PubMed:15254234). Interacts with CCDC88A/GIV; the interaction leads to phosphorylation of CCDC88A and inhibition of its guanine nucleotide exchange factor activity (PubMed:23509302). Interacts with PRKCH upstream open reading frame 2; the interaction leads to inhibition of kinase activity (PubMed:34593629). Interacts with CD28 (PubMed:21964608).   
  
 **Gene Ontology Information:**

Molecular Function

- ATP binding
- calcium-dependent protein kinase C activity
- metal ion binding
- protein kinase activity
- protein kinase C activity
- protein serine kinase activity
- protein serine/threonine kinase activity

Location

- aggresome
- centriolar satellite
- cytosol
- immunological synapse
- plasma membrane

Biological process

- axon guidance
- CD4-positive, alpha-beta T cell proliferation
- cell chemotaxis
- Fc-epsilon receptor signaling pathway
- inflammatory response
- intracellular signal transduction
- membrane protein ectodomain proteolysis
- negative regulation of insulin receptor signaling pathway
- negative regulation of T cell apoptotic process
- peptidyl-serine phosphorylation
- positive regulation of CD4-positive, alpha-beta T cell proliferation
- positive regulation of interleukin-17 production
- positive regulation of interleukin-2 production
- positive regulation of interleukin-4 production
- positive regulation of NF-kappaB transcription factor activity
- positive regulation of T cell activation
- positive regulation of T-helper 17 type immune response
- positive regulation of T-helper 2 cell activation
- positive regulation of telomerase activity
- positive regulation of telomere capping
- positive regulation of telomere maintenance via telomerase
- protein phosphorylation
- regulation of cell growth
- regulation of transcription, DNA-templated
- regulation of platelet aggregation

---

76

- **Protein name:** Rho guanine nucleotide exchange factor 25
- **Organism:** Homo sapiens
- **Uniprot Accession Number:** Q86VW2
- **Protein sequence length:** 580 aa
- **1D identity (%):** 10.56
- **1D identity (%) [Gaps excluded]:** 25.12
- **1D identity - Alignment Gaps:** 587
- **1D aligned content (<aminoacid>:%):** {'G': 13.08, 'C': 4.67, 'Y': 2.8, 'A': 5.61, 'S': 5.61, 'P': 6.54, 'D': 1.87, 'K': 6.54, 'H': 1.87, 'L': 14.02, 'V': 3.74, 'Q': 7.48, 'E': 4.67, 'T': 4.67, 'R': 9.35, 'M': 1.87, 'F': 2.8, 'I': 2.8}
- **Common reported functions (%):** 0.0
- **Common reported locations (%):** 14.29
- **Common reported processes (%):** 0.0

- **AF ID:** Q86VW2
- **Chain:** A
- **Protein length:** 580 aa
- **Resolution:** N/A
- **b-phipsi:** 0.010962
- **w-rdist:** 0.380451
- **t-alpha:** 0.002913
- **Chemical similarity (Tanimoto Index) (%):** 96.62
- **1D identity (%) [PDB]:** 2.25
- **1D identity (%) [Gaps excluded][PDB]:** 72.09
- **1D identity - Alignment Gaps [PDB]:** 1332
- **1D aligned content [PDB] (<aminoacid>:%):** {'R': 6.45, 'K': 9.68, 'V': 9.68, 'E': 6.45, 'L': 12.9, 'T': 3.23, 'P': 6.45, 'G': 9.68, 'D': 3.23, 'I': 6.45, 'S': 12.9, 'C': 3.23, 'Q': 3.23, 'A': 6.45}
- **2D identity (%) [PDB]:** 33.24
- **2D identity (%) [Gaps excluded][PDB]:** 90.79
- **2D identity - Alignment Gaps [PDB]:** 658
- **2D aligned content [PDB] (<2D-fold>:%):** {'.': 11.59, 'T': 16.23, 'H': 57.1, 'E': 13.33, 'G': 1.74}
- **3D similarity (TM-Score) (%) [PDB]:** 19.47

- **Gene name:** ARHGEF25
- **Entrez ID:** 115557
- **RefSeq ID:** NM\_182947
- **Transcript sequence length:** 2554
- **5-UTR|CDS|3-UTR identity (%):** 21.37 | 39.43 | 1.74
- **5-UTR|CDS|3-UTR identity (%) [Gaps excluded]:** 84.03 | 73.99 | 77.04
- **5-UTR|CDS|3-UTR identity [Alignment Gaps]:** 349 | 1317 | 11699
- **5-UTR aligned content (<base>:%):** {'T': 6.0, 'G': 44.0, 'C': 49.0, 'A': 1.0}
- **CDS aligned content (<base>:%):** {'A': 22.3, 'T': 18.35, 'G': 31.56, 'C': 27.79}
- **3-UTR aligned content (<base>:%):** {'C': 21.63, 'T': 30.77, 'G': 32.21, 'A': 15.38}

**Uniprot Description:**  
  
 May play a role in actin cytoskeleton reorganization in different tissues since its activation induces formation of actin stress fibers. It works as a guanine nucleotide exchange factor for Rho family of small GTPases. Links specifically G alpha q/11-coupled receptors to RHOA activation. May be an important regulator of processes involved in axon and dendrite formation. In neurons seems to be an exchange factor primarily for RAC1. Involved in skeletal myogenesis (By similarity).   
  
Interacts (via the DH domain) with BVES (via the C-terminus cytoplasmic tail) (By similarity). Interacts with activated GNAQ and GNA11. Interacts with RHOA, CDC42 and RAC1.   
  
 **Gene Ontology Information:**

Molecular Function

- guanyl-nucleotide exchange factor activity

Location

- cytoplasm
- cytosol
- extrinsic component of membrane
- myofibril
- plasma membrane
- sarcomere

Biological process

- axon guidance
- regulation of small GTPase mediated signal transduction

---

77

- **Protein name:** Beta-1,4 N-acetylgalactosaminyltransferase 2
- **Organism:** Homo sapiens
- **Uniprot Accession Number:** Q8NHY0
- **Protein sequence length:** 566 aa
- **1D identity (%):** 12.63
- **1D identity (%) [Gaps excluded]:** 24.64
- **1D identity - Alignment Gaps:** 459
- **1D aligned content (<aminoacid>:%):** {'S': 5.04, 'F': 5.04, 'V': 8.4, 'R': 5.88, 'T': 4.2, 'P': 5.88, 'G': 12.61, 'A': 5.88, 'C': 3.36, 'L': 12.61, 'W': 1.68, 'H': 3.36, 'K': 5.04, 'Q': 6.72, 'E': 3.36, 'D': 4.2, 'Y': 3.36, 'I': 2.52, 'N': 0.84}
- **Common reported functions (%):** 0.0
- **Common reported locations (%):** 7.14
- **Common reported processes (%):** 0.0

- **AF ID:** Q8NHY0
- **Chain:** A
- **Protein length:** 566 aa
- **Resolution:** N/A
- **b-phipsi:** 0.000816
- **w-rdist:** 0.594488
- **t-alpha:** 0.025492
- **Chemical similarity (Tanimoto Index) (%):** 97.49
- **1D identity (%) [PDB]:** 2.13
- **1D identity (%) [Gaps excluded][PDB]:** 67.44
- **1D identity - Alignment Gaps [PDB]:** 1318
- **1D aligned content [PDB] (<aminoacid>:%):** {'M': 3.45, 'P': 10.34, 'L': 13.79, 'G': 3.45, 'R': 3.45, 'D': 6.9, 'V': 17.24, 'E': 6.9, 'T': 3.45, 'K': 6.9, 'I': 3.45, 'S': 3.45, 'C': 3.45, 'Q': 3.45, 'A': 6.9, 'H': 3.45}
- **2D identity (%) [PDB]:** 38.9
- **2D identity (%) [Gaps excluded][PDB]:** 90.52
- **2D identity - Alignment Gaps [PDB]:** 560
- **2D aligned content [PDB] (<2D-fold>:%):** {'.': 19.11, 'T': 16.75, 'H': 38.22, 'E': 25.92}
- **3D similarity (TM-Score) (%) [PDB]:** 19.16

- **Gene name:** B4GALNT2
- **Entrez ID:** 124872
- **RefSeq ID:** N/A
- **Sequence length:** N/A
- **5-UTR|CDS|3-UTR identity (%):** N/A | N/A | N/A
- **5-UTR|CDS|3-UTR identity (%) [Gaps excluded]:** N/A | N/A | N/A
- **5-UTR|CDS|3-UTR identity [Alignment Gaps]:** N/A | N/A | N/A
- **5-UTR aligned content (<base>:%):** N/A
- **CDS aligned content (<base>:%):** N/A
- **3-UTR aligned content (<base>:%):** N/A

**Uniprot Description:**  
  
 Involved in the synthesis of the Sd(a) antigen (Sia-alpha2,3-[GalNAc-beta1,4]Gal-beta1,4-GlcNAc), a carbohydrate determinant expressed on erythrocytes, the colonic mucosa and other tissues. Transfers a beta-1,4-linked GalNAc to the galactose residue of an alpha-2,3-sialylated chain. N/A   
  
 **Gene Ontology Information:**

Molecular Function

- acetylgalactosaminyltransferase activity

Location

- Golgi membrane
- membrane

Biological process

- lipid glycosylation
- negative regulation of cell-cell adhesion
- oligosaccharide biosynthetic process
- protein N-linked glycosylation via asparagine
- UDP-N-acetylgalactosamine metabolic process
- UDP-N-acetylglucosamine metabolic process

---

78

- **Protein name:** Alpha-mannosidase 2C1
- **Organism:** Homo sapiens
- **Uniprot Accession Number:** Q9NTJ4
- **Protein sequence length:** 1040 aa
- **1D identity (%):** 16.68
- **1D identity (%) [Gaps excluded]:** 26.01
- **1D identity - Alignment Gaps:** 415
- **1D aligned content (<aminoacid>:%):** {'P': 8.81, 'R': 7.25, 'E': 5.7, 'V': 8.81, 'F': 6.22, 'G': 9.84, 'T': 5.18, 'L': 10.36, 'D': 3.63, 'W': 1.55, 'Q': 6.22, 'H': 4.15, 'S': 3.63, 'C': 1.55, 'K': 4.66, 'M': 2.07, 'A': 5.18, 'I': 2.59, 'N': 1.55, 'Y': 1.04}
- **Common reported functions (%):** 6.67
- **Common reported locations (%):** 14.29
- **Common reported processes (%):** 0.0

- **AF ID:** Q9NTJ4
- **Chain:** A
- **Protein length:** 1040 aa
- **Resolution:** N/A
- **b-phipsi:** 0.004009
- **w-rdist:** 0.349873
- **t-alpha:** 0.017791
- **Chemical similarity (Tanimoto Index) (%):** 97.92
- **1D identity (%) [PDB]:** 3.18
- **1D identity (%) [Gaps excluded][PDB]:** 67.86
- **1D identity - Alignment Gaps [PDB]:** 1710
- **1D aligned content [PDB] (<aminoacid>:%):** {'V': 8.77, 'G': 5.26, 'Q': 8.77, 'E': 7.02, 'T': 10.53, 'L': 7.02, 'C': 1.75, 'N': 5.26, 'I': 5.26, 'A': 8.77, 'K': 3.51, 'D': 3.51, 'S': 5.26, 'R': 8.77, 'F': 3.51, 'P': 5.26, 'Y': 1.75}
- **2D identity (%) [PDB]:** 38.53
- **2D identity (%) [Gaps excluded][PDB]:** 88.42
- **2D identity - Alignment Gaps [PDB]:** 738
- **2D aligned content [PDB] (<2D-fold>:%):** {'.': 15.67, 'E': 25.99, 'T': 27.18, 'H': 30.36, 'B': 0.2, 'G': 0.6}
- **3D similarity (TM-Score) (%) [PDB]:** 25.75

- **Gene name:** MAN2C1
- **Entrez ID:** 4123
- **RefSeq ID:** NM\_006715
- **Transcript sequence length:** 3261
- **5-UTR|CDS|3-UTR identity (%):** 12.31 | 44.68 | 0.6
- **5-UTR|CDS|3-UTR identity (%) [Gaps excluded]:** 76.19 | 75.52 | 72.73
- **5-UTR|CDS|3-UTR identity [Alignment Gaps]:** 109 | 1463 | 11804
- **5-UTR aligned content (<base>:%):** {'G': 68.75, 'A': 6.25, 'C': 25.0}
- **CDS aligned content (<base>:%):** {'A': 20.36, 'T': 17.74, 'G': 30.61, 'C': 31.29}
- **3-UTR aligned content (<base>:%):** {'G': 25.0, 'C': 13.89, 'T': 37.5, 'A': 23.61}

**Uniprot Description:**  
  
 Cleaves alpha 1,2-, alpha 1,3-, and alpha 1,6-linked mannose residues from glycoproteins. Involved in the degradation of free oligosaccharides in the cytoplasm. N/A   
  
 **Gene Ontology Information:**

Molecular Function

- alpha-mannosidase activity
- carbohydrate binding
- metal ion binding

Location

- cytosol
- nucleoplasm

Biological process

- mannose metabolic process
- oligosaccharide catabolic process

---

79

- **Protein name:** Polynucleotide 5'-hydroxyl-kinase NOL9
- **Organism:** Homo sapiens
- **Uniprot Accession Number:** Q5SY16
- **Protein sequence length:** 702 aa
- **1D identity (%):** 13.79
- **1D identity (%) [Gaps excluded]:** 24.64
- **1D identity - Alignment Gaps:** 441
- **1D aligned content (<aminoacid>:%):** {'G': 10.14, 'L': 14.49, 'A': 4.35, 'P': 12.32, 'I': 5.07, 'R': 7.25, 'D': 2.17, 'N': 2.9, 'E': 2.17, 'V': 5.07, 'F': 4.35, 'S': 9.42, 'C': 3.62, 'H': 2.17, 'K': 5.07, 'T': 5.07, 'Y': 0.72, 'Q': 2.9, 'M': 0.72}
- **Common reported functions (%):** 6.67
- **Common reported locations (%):** 7.14
- **Common reported processes (%):** 0.0

- **AF ID:** Q5SY16
- **Chain:** A
- **Protein length:** 702 aa
- **Resolution:** N/A
- **b-phipsi:** 0.002739
- **w-rdist:** 0.341961
- **t-alpha:** 0.045156
- **Chemical similarity (Tanimoto Index) (%):** 99.4
- **1D identity (%) [PDB]:** 2.64
- **1D identity (%) [Gaps excluded][PDB]:** 65.0
- **1D identity - Alignment Gaps [PDB]:** 1420
- **1D aligned content [PDB] (<aminoacid>:%):** {'M': 5.13, 'P': 10.26, 'I': 5.13, 'Q': 7.69, 'G': 7.69, 'A': 5.13, 'S': 2.56, 'V': 15.38, 'F': 2.56, 'H': 2.56, 'L': 12.82, 'K': 5.13, 'N': 2.56, 'Y': 2.56, 'T': 5.13, 'E': 2.56, 'R': 2.56, 'C': 2.56}
- **2D identity (%) [PDB]:** 41.12
- **2D identity (%) [Gaps excluded][PDB]:** 90.25
- **2D identity - Alignment Gaps [PDB]:** 576
- **2D aligned content [PDB] (<2D-fold>:%):** {'.': 24.6, 'H': 29.66, 'T': 20.69, 'E': 23.68, 'G': 1.38}
- **3D similarity (TM-Score) (%) [PDB]:** 21.45

- **Gene name:** NOL9
- **Entrez ID:** 523474
- **RefSeq ID:** N/A
- **Sequence length:** N/A
- **5-UTR|CDS|3-UTR identity (%):** N/A | N/A | N/A
- **5-UTR|CDS|3-UTR identity (%) [Gaps excluded]:** N/A | N/A | N/A
- **5-UTR|CDS|3-UTR identity [Alignment Gaps]:** N/A | N/A | N/A
- **5-UTR aligned content (<base>:%):** N/A
- **CDS aligned content (<base>:%):** N/A
- **3-UTR aligned content (<base>:%):** N/A

**Uniprot Description:**  
  
 Polynucleotide 5'-kinase involved in rRNA processing. The kinase activity is required for the processing of the 32S precursor into 5.8S and 28S rRNAs, more specifically for the generation of the major 5.8S(S) form. In vitro, has both DNA and RNA 5'-kinase activities. Probably binds RNA.   
  
Interacts with PELP1, WDR18 and SENP3.   
  
 **Gene Ontology Information:**

Molecular Function

- ATP binding
- ATP-dependent polydeoxyribonucleotide 5'-hydroxyl-kinase activity
- polynucleotide 5'-hydroxyl-kinase activity
- RNA binding

Location

- nucleolus
- nucleus

Biological process

- cleavage in ITS2 between 5.8S rRNA and LSU-rRNA of tricistronic rRNA transcript (SSU-rRNA, 5.8S rRNA, LSU-rRNA)
- maturation of 5.8S rRNA
- phosphorylation
- RNA processing

---

80

- **Protein name:** Ras GTPase-activating protein 4
- **Organism:** Homo sapiens
- **Uniprot Accession Number:** O43374
- **Protein sequence length:** 803 aa
- **1D identity (%):** 17.69
- **1D identity (%) [Gaps excluded]:** 23.26
- **1D identity - Alignment Gaps:** 226
- **1D aligned content (<aminoacid>:%):** {'L': 10.78, 'E': 7.19, 'P': 8.98, 'D': 4.79, 'I': 2.4, 'C': 4.19, 'V': 6.59, 'K': 4.19, 'T': 2.99, 'G': 10.18, 'F': 1.2, 'Y': 2.4, 'R': 9.58, 'A': 6.59, 'H': 2.99, 'S': 7.19, 'Q': 5.39, 'W': 1.2, 'N': 0.6, 'M': 0.6}
- **Common reported functions (%):** 6.67
- **Common reported locations (%):** 7.14
- **Common reported processes (%):** 0.0

- **AF ID:** O43374
- **Chain:** A
- **Protein length:** 803 aa
- **Resolution:** N/A
- **b-phipsi:** 0.001594
- **w-rdist:** 0.281857
- **t-alpha:** 0.09928
- **Chemical similarity (Tanimoto Index) (%):** 98.95
- **1D identity (%) [PDB]:** 2.52
- **1D identity (%) [Gaps excluded][PDB]:** 78.43
- **1D identity - Alignment Gaps [PDB]:** 1539
- **1D aligned content [PDB] (<aminoacid>:%):** {'M': 2.5, 'L': 15.0, 'P': 10.0, 'I': 5.0, 'G': 7.5, 'R': 7.5, 'D': 5.0, 'K': 10.0, 'V': 10.0, 'E': 5.0, 'F': 2.5, 'S': 10.0, 'W': 2.5, 'C': 2.5, 'Q': 2.5, 'H': 2.5}
- **2D identity (%) [PDB]:** 45.83
- **2D identity (%) [Gaps excluded][PDB]:** 88.24
- **2D identity - Alignment Gaps [PDB]:** 519
- **2D aligned content [PDB] (<2D-fold>:%):** {'.': 18.18, 'E': 27.47, 'T': 19.19, 'H': 34.55, 'G': 0.61}
- **3D similarity (TM-Score) (%) [PDB]:** 22.08

- **Gene name:** RASA4
- **Entrez ID:** 10156
- **RefSeq ID:** NM\_006989
- **Transcript sequence length:** 5604
- **5-UTR|CDS|3-UTR identity (%):** 35.94 | 47.12 | 15.61
- **5-UTR|CDS|3-UTR identity (%) [Gaps excluded]:** 69.7 | 75.46 | 72.86
- **5-UTR|CDS|3-UTR identity [Alignment Gaps]:** 62 | 1154 | 9715
- **5-UTR aligned content (<base>:%):** {'G': 34.78, 'C': 60.87, 'T': 2.17, 'A': 2.17}
- **CDS aligned content (<base>:%):** {'A': 20.79, 'T': 15.12, 'G': 30.8, 'C': 33.29}
- **3-UTR aligned content (<base>:%):** {'G': 25.75, 'C': 26.32, 'T': 26.17, 'A': 21.76}

**Uniprot Description:**  
  
 Ca(2+)-dependent Ras GTPase-activating protein, that switches off the Ras-MAPK pathway following a stimulus that elevates intracellular calcium. Functions as an adaptor for Cdc42 and Rac1 during FcR-mediated phagocytosis. N/A   
  
 **Gene Ontology Information:**

Molecular Function

- GTPase activator activity
- metal ion binding
- phospholipid binding

Location

- cytosol
- plasma membrane

Biological process

- cellular response to calcium ion
- intracellular signal transduction
- negative regulation of GTPase activity
- negative regulation of Ras protein signal transduction

---

81

- **Protein name:** Calcium-activated chloride channel regulator 4
- **Organism:** Homo sapiens
- **Uniprot Accession Number:** Q14CN2
- **Protein sequence length:** 919 aa
- **1D identity (%):** 17.37
- **1D identity (%) [Gaps excluded]:** 25.59
- **1D identity - Alignment Gaps:** 340
- **1D aligned content (<aminoacid>:%):** {'G': 7.07, 'F': 5.98, 'I': 6.52, 'K': 7.07, 'L': 7.61, 'N': 3.8, 'E': 4.89, 'D': 5.98, 'P': 11.41, 'M': 1.09, 'V': 6.52, 'T': 6.52, 'C': 2.17, 'H': 2.72, 'A': 5.98, 'R': 3.26, 'S': 6.52, 'Y': 1.09, 'Q': 2.72, 'W': 1.09}
- **Common reported functions (%):** 6.67
- **Common reported locations (%):** 0.0
- **Common reported processes (%):** 0.0

- **AF ID:** Q14CN2
- **Chain:** A
- **Protein length:** 919 aa
- **Resolution:** N/A
- **b-phipsi:** 0.006752
- **w-rdist:** 0.302532
- **t-alpha:** 0.024763
- **Chemical similarity (Tanimoto Index) (%):** 96.33
- **1D identity (%) [PDB]:** 3.67
- **1D identity (%) [Gaps excluded][PDB]:** 64.89
- **1D identity - Alignment Gaps [PDB]:** 1569
- **1D aligned content [PDB] (<aminoacid>:%):** {'L': 9.84, 'C': 1.64, 'K': 4.92, 'I': 11.48, 'N': 3.28, 'V': 13.11, 'P': 11.48, 'G': 3.28, 'R': 6.56, 'F': 3.28, 'Q': 3.28, 'D': 4.92, 'T': 3.28, 'A': 6.56, 'S': 4.92, 'Y': 3.28, 'H': 1.64, 'E': 3.28}
- **2D identity (%) [PDB]:** 39.98
- **2D identity (%) [Gaps excluded][PDB]:** 89.15
- **2D identity - Alignment Gaps [PDB]:** 669
- **2D aligned content [PDB] (<2D-fold>:%):** {'.': 20.62, 'E': 27.22, 'T': 21.86, 'H': 28.87, 'B': 0.82, 'G': 0.62}
- **3D similarity (TM-Score) (%) [PDB]:** 23.9

- **Gene name:** CLCA4
- **Entrez ID:** N/A
- **RefSeq ID:** NM\_012128
- **Transcript sequence length:** 3211
- **5-UTR|CDS|3-UTR identity (%):** 10.42 | 42.44 | 2.31
- **5-UTR|CDS|3-UTR identity (%) [Gaps excluded]:** 60.0 | 74.25 | 75.82
- **5-UTR|CDS|3-UTR identity [Alignment Gaps]:** 119 | 1456 | 11569
- **5-UTR aligned content (<base>:%):** {'G': 53.33, 'A': 13.33, 'C': 33.33}
- **CDS aligned content (<base>:%):** {'A': 30.17, 'T': 22.4, 'G': 24.34, 'C': 23.09}
- **3-UTR aligned content (<base>:%):** {'A': 45.29, 'C': 7.61, 'T': 34.42, 'G': 12.68}

**Uniprot Description:**  
  
 May be involved in mediating calcium-activated chloride conductance. N/A   
  
 **Gene Ontology Information:**

Molecular Function

- chloride channel activity
- intracellular calcium activated chloride channel activity
- ligand-gated ion channel activity
- metal ion binding
- metallopeptidase activity

Location

- apical plasma membrane
- extracellular region
- plasma membrane

Biological process

- chloride transport
- proteolysis

---

82

- **Protein name:** Phosphoribosylformylglycinamidine synthase
- **Organism:** Homo sapiens
- **Uniprot Accession Number:** O15067
- **Protein sequence length:** 1338 aa
- **1D identity (%):** 16.59
- **1D identity (%) [Gaps excluded]:** 27.7
- **1D identity - Alignment Gaps:** 551
- **1D aligned content (<aminoacid>:%):** {'P': 8.77, 'K': 4.82, 'L': 9.21, 'Q': 5.7, 'E': 5.26, 'Y': 1.32, 'F': 6.58, 'D': 5.7, 'G': 10.09, 'V': 8.77, 'R': 5.26, 'S': 3.95, 'H': 3.95, 'A': 7.46, 'W': 1.32, 'N': 2.63, 'I': 0.88, 'T': 4.82, 'C': 3.07, 'M': 0.44}
- **Common reported functions (%):** 6.67
- **Common reported locations (%):** 21.43
- **Common reported processes (%):** 0.0

- **AF ID:** O15067
- **Chain:** A
- **Protein length:** 1338 aa
- **Resolution:** N/A
- **b-phipsi:** 0.002024
- **w-rdist:** 0.348192
- **t-alpha:** 0.050255
- **Chemical similarity (Tanimoto Index) (%):** 99.47
- **1D identity (%) [PDB]:** 3.67
- **1D identity (%) [Gaps excluded][PDB]:** 71.7
- **1D identity - Alignment Gaps [PDB]:** 1964
- **1D aligned content [PDB] (<aminoacid>:%):** {'R': 11.84, 'V': 13.16, 'E': 6.58, 'I': 3.95, 'Q': 3.95, 'H': 2.63, 'F': 2.63, 'K': 3.95, 'G': 6.58, 'D': 5.26, 'P': 9.21, 'N': 1.32, 'L': 11.84, 'T': 3.95, 'M': 1.32, 'S': 9.21, 'A': 1.32, 'Y': 1.32}
- **2D identity (%) [PDB]:** 42.49
- **2D identity (%) [Gaps excluded][PDB]:** 91.32
- **2D identity - Alignment Gaps [PDB]:** 794
- **2D aligned content [PDB] (<2D-fold>:%):** {'.': 16.64, 'E': 29.48, 'H': 33.76, 'T': 19.18, 'G': 0.95}
- **3D similarity (TM-Score) (%) [PDB]:** 31.83

- **Gene name:** PFAS
- **Entrez ID:** 5198
- **RefSeq ID:** NM\_012393
- **Transcript sequence length:** 5369
- **5-UTR|CDS|3-UTR identity (%):** 43.79 | 41.6 | 6.15
- **5-UTR|CDS|3-UTR identity (%) [Gaps excluded]:** 64.42 | 75.82 | 77.73
- **5-UTR|CDS|3-UTR identity [Alignment Gaps]:** 49 | 1923 | 11188
- **5-UTR aligned content (<base>:%):** {'A': 2.99, 'C': 50.75, 'G': 35.82, 'T': 10.45}
- **CDS aligned content (<base>:%):** {'A': 19.7, 'T': 19.3, 'G': 30.76, 'C': 30.25}
- **3-UTR aligned content (<base>:%):** {'C': 25.44, 'G': 21.95, 'A': 21.29, 'T': 31.33}

**Uniprot Description:**  
  
 Phosphoribosylformylglycinamidine synthase involved in the purines biosynthetic pathway. Catalyzes the ATP-dependent conversion of formylglycinamide ribonucleotide (FGAR) and glutamine to yield formylglycinamidine ribonucleotide (FGAM) and glutamate. N/A   
  
 **Gene Ontology Information:**

Molecular Function

- ATP binding
- metal ion binding
- phosphoribosylformylglycinamidine synthase activity

Location

- cytoplasm
- cytosol
- extracellular exosome

Biological process

- 'de novo' AMP biosynthetic process
- 'de novo' IMP biosynthetic process
- 'de novo' XMP biosynthetic process
- anterior head development
- glutamine metabolic process
- GMP biosynthetic process
- purine nucleotide biosynthetic process
- purine ribonucleoside monophosphate biosynthetic process
- response to xenobiotic stimulus

---

83

- **Protein name:** Zinc finger protein 514
- **Organism:** Homo sapiens
- **Uniprot Accession Number:** Q96K75
- **Protein sequence length:** 400 aa
- **1D identity (%):** 8.87
- **1D identity (%) [Gaps excluded]:** 22.41
- **1D identity - Alignment Gaps:** 545
- **1D aligned content (<aminoacid>:%):** {'E': 5.0, 'P': 5.0, 'R': 8.75, 'M': 2.5, 'F': 7.5, 'I': 3.75, 'G': 12.5, 'K': 10.0, 'V': 3.75, 'S': 8.75, 'A': 2.5, 'H': 5.0, 'L': 5.0, 'T': 6.25, 'W': 1.25, 'N': 1.25, 'Y': 5.0, 'C': 5.0, 'Q': 1.25}
- **Common reported functions (%):** 6.67
- **Common reported locations (%):** 7.14
- **Common reported processes (%):** 0.0

- **AF ID:** Q96K75
- **Chain:** A
- **Protein length:** 400 aa
- **Resolution:** N/A
- **b-phipsi:** 0.01343
- **w-rdist:** 0.357478
- **t-alpha:** 0.003642
- **Chemical similarity (Tanimoto Index) (%):** 98.49
- **1D identity (%) [PDB]:** 1.65
- **1D identity (%) [Gaps excluded][PDB]:** 76.92
- **1D identity - Alignment Gaps [PDB]:** 1186
- **1D aligned content [PDB] (<aminoacid>:%):** {'M': 5.0, 'T': 15.0, 'D': 5.0, 'V': 10.0, 'G': 15.0, 'L': 10.0, 'Q': 5.0, 'S': 5.0, 'I': 5.0, 'R': 5.0, 'N': 5.0, 'K': 5.0, 'A': 10.0}
- **2D identity (%) [PDB]:** 31.53
- **2D identity (%) [Gaps excluded][PDB]:** 93.59
- **2D identity - Alignment Gaps [PDB]:** 614
- **2D aligned content [PDB] (<2D-fold>:%):** {'H': 57.19, '.': 25.34, 'T': 11.3, 'E': 6.16}
- **3D similarity (TM-Score) (%) [PDB]:** 15.07

- **Gene name:** ZNF514
- **Entrez ID:** 84874
- **RefSeq ID:** N/A
- **Sequence length:** N/A
- **5-UTR|CDS|3-UTR identity (%):** N/A | N/A | N/A
- **5-UTR|CDS|3-UTR identity (%) [Gaps excluded]:** N/A | N/A | N/A
- **5-UTR|CDS|3-UTR identity [Alignment Gaps]:** N/A | N/A | N/A
- **5-UTR aligned content (<base>:%):** N/A
- **CDS aligned content (<base>:%):** N/A
- **3-UTR aligned content (<base>:%):** N/A

**Uniprot Description:**  
  
 May be involved in transcriptional regulation. N/A   
  
 **Gene Ontology Information:**

Molecular Function

- DNA-binding transcription factor activity, RNA polymerase II-specific
- metal ion binding
- RNA polymerase II cis-regulatory region sequence-specific DNA binding

Location

- nucleus

Biological process

- regulation of transcription by RNA polymerase II

---

84

- **Protein name:** Adenine DNA glycosylase
- **Organism:** Homo sapiens
- **Uniprot Accession Number:** Q9UIF7
- **Protein sequence length:** 546 aa
- **1D identity (%):** 11.9
- **1D identity (%) [Gaps excluded]:** 23.52
- **1D identity - Alignment Gaps:** 461
- **1D aligned content (<aminoacid>:%):** {'P': 12.61, 'L': 8.11, 'M': 0.9, 'G': 9.01, 'K': 4.5, 'C': 1.8, 'A': 7.21, 'E': 6.31, 'V': 11.71, 'H': 3.6, 'F': 2.7, 'W': 2.7, 'Q': 8.11, 'R': 4.5, 'D': 3.6, 'Y': 1.8, 'T': 7.21, 'N': 0.9, 'S': 2.7}
- **Common reported functions (%):** 6.67
- **Common reported locations (%):** 14.29
- **Common reported processes (%):** 0.0

- **AF ID:** Q9UIF7
- **Chain:** A
- **Protein length:** 546 aa
- **Resolution:** N/A
- **b-phipsi:** 0.007752
- **w-rdist:** 0.314219
- **t-alpha:** 0.00874
- **Chemical similarity (Tanimoto Index) (%):** 99.62
- **1D identity (%) [PDB]:** 3.9
- **1D identity (%) [Gaps excluded][PDB]:** 66.23
- **1D identity - Alignment Gaps [PDB]:** 1230
- **1D aligned content [PDB] (<aminoacid>:%):** {'A': 7.84, 'S': 3.92, 'E': 3.92, 'H': 1.96, 'K': 1.96, 'N': 3.92, 'G': 9.8, 'L': 19.61, 'Q': 9.8, 'V': 11.76, 'P': 9.8, 'R': 3.92, 'D': 1.96, 'T': 3.92, 'C': 3.92, 'I': 1.96}
- **2D identity (%) [PDB]:** 36.61
- **2D identity (%) [Gaps excluded][PDB]:** 90.7
- **2D identity - Alignment Gaps [PDB]:** 588
- **2D aligned content [PDB] (<2D-fold>:%):** {'.': 19.67, 'T': 18.56, 'H': 49.03, 'G': 0.83, 'E': 11.63, 'B': 0.28}
- **3D similarity (TM-Score) (%) [PDB]:** 19.29

- **Gene name:** MUTYH
- **Entrez ID:** 4595
- **RefSeq ID:** N/A
- **Sequence length:** N/A
- **5-UTR|CDS|3-UTR identity (%):** N/A | N/A | N/A
- **5-UTR|CDS|3-UTR identity (%) [Gaps excluded]:** N/A | N/A | N/A
- **5-UTR|CDS|3-UTR identity [Alignment Gaps]:** N/A | N/A | N/A
- **5-UTR aligned content (<base>:%):** N/A
- **CDS aligned content (<base>:%):** N/A
- **3-UTR aligned content (<base>:%):** N/A

**Uniprot Description:**  
  
 Involved in oxidative DNA damage repair. Initiates repair of A\*oxoG to C\*G by removing the inappropriately paired adenine base from the DNA backbone. Possesses both adenine and 2-OH-A DNA glycosylase activities. N/A   
  
 **Gene Ontology Information:**

Molecular Function

- 4 iron, 4 sulfur cluster binding
- 8-oxo-7,8-dihydroguanine DNA N-glycosylase activity
- adenine/guanine mispair binding
- DNA N-glycosylase activity
- metal ion binding
- MutSalpha complex binding
- oxidized purine DNA binding
- purine-specific mismatch base pair DNA N-glycosylase activity

Location

- mitochondrion
- nucleoplasm
- nucleus

Biological process

- base-excision repair
- depurination
- DNA repair
- mismatch repair
- negative regulation of necroptotic process

---

85

- **Protein name:** Leucine-zipper-like transcriptional regulator 1
- **Organism:** Homo sapiens
- **Uniprot Accession Number:** Q8N653
- **Protein sequence length:** 840 aa
- **1D identity (%):** 15.28
- **1D identity (%) [Gaps excluded]:** 23.76
- **1D identity - Alignment Gaps:** 369
- **1D aligned content (<aminoacid>:%):** {'G': 9.49, 'K': 6.96, 'P': 8.23, 'D': 5.7, 'F': 5.7, 'S': 2.53, 'H': 6.96, 'C': 5.06, 'V': 8.86, 'I': 3.16, 'L': 8.23, 'W': 1.9, 'A': 4.43, 'Y': 3.8, 'T': 4.43, 'Q': 4.43, 'E': 3.16, 'N': 1.9, 'R': 3.8, 'M': 1.27}
- **Common reported functions (%):** 0.0
- **Common reported locations (%):** 0.0
- **Common reported processes (%):** 0.0

- **AF ID:** Q8N653
- **Chain:** A
- **Protein length:** 840 aa
- **Resolution:** N/A
- **b-phipsi:** 0.002984
- **w-rdist:** 0.878527
- **t-alpha:** 0.001457
- **Chemical similarity (Tanimoto Index) (%):** 98.57
- **1D identity (%) [PDB]:** 2.65
- **1D identity (%) [Gaps excluded][PDB]:** 76.79
- **1D identity - Alignment Gaps [PDB]:** 1566
- **1D aligned content [PDB] (<aminoacid>:%):** {'M': 2.33, 'R': 11.63, 'E': 9.3, 'L': 13.95, 'Q': 11.63, 'F': 9.3, 'K': 6.98, 'T': 2.33, 'I': 13.95, 'Y': 2.33, 'A': 4.65, 'D': 2.33, 'P': 2.33, 'V': 4.65, 'H': 2.33}
- **2D identity (%) [PDB]:** 39.3
- **2D identity (%) [Gaps excluded][PDB]:** 90.0
- **2D identity - Alignment Gaps [PDB]:** 658
- **2D aligned content [PDB] (<2D-fold>:%):** {'.': 21.57, 'E': 27.23, 'T': 15.03, 'G': 0.65, 'H': 35.29, 'B': 0.22}
- **3D similarity (TM-Score) (%) [PDB]:** 24.56

- **Gene name:** LZTR1
- **Entrez ID:** 8216
- **RefSeq ID:** NM\_006767
- **Transcript sequence length:** 4282
- **5-UTR|CDS|3-UTR identity (%):** 38.41 | 47.59 | 9.08
- **5-UTR|CDS|3-UTR identity (%) [Gaps excluded]:** 82.81 | 74.64 | 75.45
- **5-UTR|CDS|3-UTR identity [Alignment Gaps]:** 74 | 1129 | 10656
- **5-UTR aligned content (<base>:%):** {'T': 13.21, 'G': 49.06, 'C': 35.85, 'A': 1.89}
- **CDS aligned content (<base>:%):** {'A': 20.63, 'T': 16.45, 'G': 30.88, 'C': 32.03}
- **3-UTR aligned content (<base>:%):** {'G': 29.27, 'C': 29.73, 'T': 22.73, 'A': 18.27}

**Uniprot Description:**  
  
 Substrate-specific adapter of a BCR (BTB-CUL3-RBX1) E3 ubiquitin-protein ligase complex that mediates ubiquitination of Ras (K-Ras/KRAS, N-Ras/NRAS and H-Ras/HRAS) (PubMed:30442762, PubMed:30442766, PubMed:30481304). Is a negative regulator of RAS-MAPK signaling that acts by controlling Ras levels and decreasing Ras association with membranes (PubMed:30442762, PubMed:30442766, PubMed:30481304).   
  
Homodimer (PubMed:30442762). Component of the BCR(LZTR1) E3 ubiquitin ligase complex, at least composed of CUL3, LZTR1 and RBX1 (PubMed:30442762, PubMed:30442766). Interacts with Ras (K-Ras/KRAS, N-Ras/NRAS and H-Ras/HRAS) (PubMed:30442762). Interacts with RAF1 (PubMed:30368668). Interacts with SHOC2 (PubMed:30368668). Interacts with PPP1CB (PubMed:30368668).   
  
 **Gene Ontology Information:**

Molecular Function

- small GTPase binding

Location

- Cul3-RING ubiquitin ligase complex
- endomembrane system
- Golgi apparatus
- recycling endosome membrane

Biological process

- negative regulation of Ras protein signal transduction
- protein ubiquitination

---

86

- **Protein name:** Zinc finger and SCAN domain-containing protein 4
- **Organism:** Homo sapiens
- **Uniprot Accession Number:** Q8NAM6
- **Protein sequence length:** 433 aa
- **1D identity (%):** 9.72
- **1D identity (%) [Gaps excluded]:** 23.67
- **1D identity - Alignment Gaps:** 540
- **1D aligned content (<aminoacid>:%):** {'L': 5.62, 'I': 7.87, 'A': 6.74, 'R': 7.87, 'E': 4.49, 'S': 5.62, 'F': 4.49, 'Y': 1.12, 'K': 3.37, 'D': 1.12, 'V': 7.87, 'G': 12.36, 'W': 1.12, 'T': 6.74, 'Q': 10.11, 'P': 5.62, 'N': 3.37, 'H': 3.37, 'C': 1.12}
- **Common reported functions (%):** 6.67
- **Common reported locations (%):** 7.14
- **Common reported processes (%):** 0.0

- **AF ID:** Q8NAM6
- **Chain:** A
- **Protein length:** 433 aa
- **Resolution:** N/A
- **b-phipsi:** 0.012478
- **w-rdist:** 0.280617
- **t-alpha:** 0.006598
- **Chemical similarity (Tanimoto Index) (%):** 99.09
- **1D identity (%) [PDB]:** 2.54
- **1D identity (%) [Gaps excluded][PDB]:** 63.27
- **1D identity - Alignment Gaps [PDB]:** 1173
- **1D aligned content [PDB] (<aminoacid>:%):** {'L': 12.9, 'E': 9.68, 'C': 3.23, 'N': 3.23, 'A': 16.13, 'R': 12.9, 'I': 6.45, 'T': 9.68, 'D': 3.23, 'Q': 6.45, 'S': 12.9, 'M': 3.23}
- **2D identity (%) [PDB]:** 24.95
- **2D identity (%) [Gaps excluded][PDB]:** 86.32
- **2D identity - Alignment Gaps [PDB]:** 701
- **2D aligned content [PDB] (<2D-fold>:%):** {'.': 22.76, 'T': 22.36, 'H': 54.07, 'E': 0.81}
- **3D similarity (TM-Score) (%) [PDB]:** 16.12

- **Gene name:** ZSCAN4
- **Entrez ID:** N/A
- **RefSeq ID:** N/A
- **Sequence length:** N/A
- **5-UTR|CDS|3-UTR identity (%):** N/A | N/A | N/A
- **5-UTR|CDS|3-UTR identity (%) [Gaps excluded]:** N/A | N/A | N/A
- **5-UTR|CDS|3-UTR identity [Alignment Gaps]:** N/A | N/A | N/A
- **5-UTR aligned content (<base>:%):** N/A
- **CDS aligned content (<base>:%):** N/A
- **3-UTR aligned content (<base>:%):** N/A

**Uniprot Description:**  
  
 Embryonic stem (ES) cell-specific transcription factor required to regulate ES cell pluripotency. Binds telomeres and plays a key role in genomic stability in ES cells by regulating telomere elongation. Acts as an activator of spontaneous telomere sister chromatid exchange (T-SCE) and telomere elongation in undifferentiated ES cells (By similarity). N/A   
  
 **Gene Ontology Information:**

Molecular Function

- DNA binding
- metal ion binding

Location

- chromosome, telomeric region
- nucleus

Biological process

- telomere maintenance via telomere lengthening

---

87

- **Protein name:** Spermatogenesis-associated protein 16
- **Organism:** Homo sapiens
- **Uniprot Accession Number:** Q9BXB7
- **Protein sequence length:** 569 aa
- **1D identity (%):** 13.26
- **1D identity (%) [Gaps excluded]:** 21.93
- **1D identity - Alignment Gaps:** 352
- **1D aligned content (<aminoacid>:%):** {'D': 5.93, 'G': 4.24, 'S': 5.93, 'R': 8.47, 'N': 2.54, 'P': 9.32, 'K': 12.71, 'I': 6.78, 'M': 5.93, 'Q': 5.93, 'E': 3.39, 'L': 9.32, 'Y': 1.69, 'V': 5.08, 'F': 3.39, 'C': 1.69, 'A': 3.39, 'T': 2.54, 'H': 0.85, 'W': 0.85}
- **Common reported functions (%):** 0.0
- **Common reported locations (%):** 0.0
- **Common reported processes (%):** 0.0

- **AF ID:** Q9BXB7
- **Chain:** A
- **Protein length:** 569 aa
- **Resolution:** N/A
- **b-phipsi:** 0.010505
- **w-rdist:** 0.519583
- **t-alpha:** 0.001459
- **Chemical similarity (Tanimoto Index) (%):** 99.24
- **1D identity (%) [PDB]:** 2.57
- **1D identity (%) [Gaps excluded][PDB]:** 74.47
- **1D identity - Alignment Gaps [PDB]:** 1313
- **1D aligned content [PDB] (<aminoacid>:%):** {'D': 2.86, 'A': 8.57, 'S': 11.43, 'R': 8.57, 'L': 11.43, 'P': 11.43, 'T': 5.71, 'Q': 2.86, 'V': 5.71, 'H': 2.86, 'Y': 2.86, 'F': 2.86, 'E': 5.71, 'C': 2.86, 'N': 2.86, 'G': 8.57, 'W': 2.86}
- **2D identity (%) [PDB]:** 34.7
- **2D identity (%) [Gaps excluded][PDB]:** 92.45
- **2D identity - Alignment Gaps [PDB]:** 639
- **2D aligned content [PDB] (<2D-fold>:%):** {'.': 18.87, 'T': 13.24, 'H': 62.82, 'E': 5.07}
- **3D similarity (TM-Score) (%) [PDB]:** 18.97

- **Gene name:** SPATA16
- **Entrez ID:** 83893
- **RefSeq ID:** NM\_031955
- **Transcript sequence length:** 2074
- **5-UTR|CDS|3-UTR identity (%):** 43.33 | 38.17 | 1.19
- **5-UTR|CDS|3-UTR identity (%) [Gaps excluded]:** 79.59 | 75.38 | 73.96
- **5-UTR|CDS|3-UTR identity [Alignment Gaps]:** 82 | 1406 | 11717
- **5-UTR aligned content (<base>:%):** {'G': 47.44, 'C': 43.59, 'A': 2.56, 'T': 6.41}
- **CDS aligned content (<base>:%):** {'A': 31.28, 'G': 26.49, 'T': 19.96, 'C': 22.26}
- **3-UTR aligned content (<base>:%):** {'A': 35.92, 'T': 27.46, 'G': 17.61, 'C': 19.01}

**Uniprot Description:**  
  
 Essential for spermiogenesis and male fertility (By similarity). Involved in the formation of sperm acrosome during spermatogenesis. N/A   
  
 **Gene Ontology Information:**

Molecular Function   
  
N/A

Location

- acrosomal vesicle
- Golgi apparatus

Biological process

- spermatid development
- spermatogenesis

---

88

- **Protein name:** Alpha-mannosidase 2x
- **Organism:** Homo sapiens
- **Uniprot Accession Number:** P49641
- **Protein sequence length:** 1150 aa
- **1D identity (%):** 15.83
- **1D identity (%) [Gaps excluded]:** 23.49
- **1D identity - Alignment Gaps:** 391
- **1D aligned content (<aminoacid>:%):** {'M': 1.05, 'G': 10.0, 'A': 3.68, 'P': 12.11, 'R': 8.42, 'F': 5.26, 'L': 11.05, 'Q': 4.74, 'D': 6.32, 'I': 4.21, 'E': 2.11, 'V': 7.37, 'K': 4.21, 'Y': 3.16, 'T': 4.74, 'W': 1.58, 'H': 3.68, 'S': 3.68, 'C': 1.58, 'N': 1.05}
- **Common reported functions (%):** 6.67
- **Common reported locations (%):** 0.0
- **Common reported processes (%):** 0.0

- **AF ID:** P49641
- **Chain:** A
- **Protein length:** 1150 aa
- **Resolution:** N/A
- **b-phipsi:** 0.000838
- **w-rdist:** 0.389124
- **t-alpha:** 0.061908
- **Chemical similarity (Tanimoto Index) (%):** 97.34
- **1D identity (%) [PDB]:** 2.6
- **1D identity (%) [Gaps excluded][PDB]:** 79.37
- **1D identity - Alignment Gaps [PDB]:** 1862
- **1D aligned content [PDB] (<aminoacid>:%):** {'I': 6.0, 'F': 8.0, 'V': 12.0, 'K': 2.0, 'W': 2.0, 'S': 14.0, 'L': 14.0, 'Q': 4.0, 'A': 4.0, 'D': 4.0, 'G': 4.0, 'R': 8.0, 'P': 10.0, 'E': 2.0, 'T': 4.0, 'Y': 2.0}
- **2D identity (%) [PDB]:** 37.17
- **2D identity (%) [Gaps excluded][PDB]:** 90.02
- **2D identity - Alignment Gaps [PDB]:** 826
- **2D aligned content [PDB] (<2D-fold>:%):** {'.': 19.31, 'H': 32.5, 'T': 28.68, 'E': 17.59, 'G': 1.72, 'B': 0.19}
- **3D similarity (TM-Score) (%) [PDB]:** 25.65

- **Gene name:** MAN2A2
- **Entrez ID:** 4122
- **RefSeq ID:** N/A
- **Sequence length:** N/A
- **5-UTR|CDS|3-UTR identity (%):** N/A | N/A | N/A
- **5-UTR|CDS|3-UTR identity (%) [Gaps excluded]:** N/A | N/A | N/A
- **5-UTR|CDS|3-UTR identity [Alignment Gaps]:** N/A | N/A | N/A
- **5-UTR aligned content (<base>:%):** N/A
- **CDS aligned content (<base>:%):** N/A
- **3-UTR aligned content (<base>:%):** N/A

**Uniprot Description:**  
  
 Catalyzes the first committed step in the biosynthesis of complex N-glycans. It controls conversion of high mannose to complex N-glycans; the final hydrolytic step in the N-glycan maturation pathway.   
  
Homodimer; disulfide-linked (By similarity). Interacts with MGAT4D (By similarity).   
  
 **Gene Ontology Information:**

Molecular Function

- alpha-mannosidase activity
- carbohydrate binding
- hydrolase activity, hydrolyzing N-glycosyl compounds
- mannosyl-oligosaccharide 1,3-1,6-alpha-mannosidase activity
- metal ion binding

Location

- Golgi membrane

Biological process

- mannose metabolic process
- N-glycan processing
- protein deglycosylation
- protein glycosylation

---

89

- **Protein name:** Mini-chromosome maintenance complex-binding protein
- **Organism:** Homo sapiens
- **Uniprot Accession Number:** Q9BTE3
- **Protein sequence length:** 642 aa
- **1D identity (%):** 13.33
- **1D identity (%) [Gaps excluded]:** 24.71
- **1D identity - Alignment Gaps:** 449
- **1D aligned content (<aminoacid>:%):** {'P': 13.08, 'S': 6.15, 'H': 5.38, 'Q': 8.46, 'F': 5.38, 'E': 4.62, 'V': 4.62, 'Y': 2.31, 'K': 7.69, 'L': 6.92, 'N': 4.62, 'R': 6.92, 'C': 3.08, 'I': 5.38, 'D': 4.62, 'A': 3.85, 'G': 4.62, 'T': 2.31}
- **Common reported functions (%):** 0.0
- **Common reported locations (%):** 21.43
- **Common reported processes (%):** 0.0

- **AF ID:** Q9BTE3
- **Chain:** A
- **Protein length:** 642 aa
- **Resolution:** N/A
- **b-phipsi:** 0.000769
- **w-rdist:** 0.574686
- **t-alpha:** 0.046613
- **Chemical similarity (Tanimoto Index) (%):** 99.4
- **1D identity (%) [PDB]:** 3.17
- **1D identity (%) [Gaps excluded][PDB]:** 73.77
- **1D identity - Alignment Gaps [PDB]:** 1358
- **1D aligned content [PDB] (<aminoacid>:%):** {'P': 8.89, 'G': 6.67, 'D': 4.44, 'K': 8.89, 'V': 13.33, 'E': 6.67, 'L': 17.78, 'T': 2.22, 'F': 2.22, 'S': 11.11, 'I': 4.44, 'A': 6.67, 'H': 2.22, 'R': 4.44}
- **2D identity (%) [PDB]:** 40.93
- **2D identity (%) [Gaps excluded][PDB]:** 87.69
- **2D identity - Alignment Gaps [PDB]:** 538
- **2D aligned content [PDB] (<2D-fold>:%):** {'.': 13.8, 'H': 40.68, 'T': 21.31, 'E': 23.97, 'B': 0.24}
- **3D similarity (TM-Score) (%) [PDB]:** 20.33

- **Gene name:** MCMBP
- **Entrez ID:** 514863
- **RefSeq ID:** NM\_024834
- **Transcript sequence length:** 4222
- **5-UTR|CDS|3-UTR identity (%):** 40.38 | 40.03 | 10.19
- **5-UTR|CDS|3-UTR identity (%) [Gaps excluded]:** 88.43 | 73.77 | 74.84
- **5-UTR|CDS|3-UTR identity [Alignment Gaps]:** 144 | 1337 | 10584
- **5-UTR aligned content (<base>:%):** {'T': 8.41, 'G': 42.06, 'C': 46.73, 'A': 2.8}
- **CDS aligned content (<base>:%):** {'A': 27.69, 'T': 21.54, 'G': 25.13, 'C': 25.64}
- **3-UTR aligned content (<base>:%):** {'A': 30.18, 'G': 17.53, 'T': 37.23, 'C': 15.05}

**Uniprot Description:**  
  
 Associated component of the MCM complex that acts as a regulator of DNA replication. Binds to the MCM complex during late S phase and promotes the disassembly of the MCM complex from chromatin, thereby acting as a key regulator of pre-replication complex (pre-RC) unloading from replicated DNA. Can dissociate the MCM complex without addition of ATP; probably acts by destabilizing interactions of each individual subunits of the MCM complex. Required for sister chromatid cohesion.   
  
Interacts with the MCM complex: associates with the MCM3-7 complex which lacks MCM2, while it does not interact with the MCM complex when MCM2 is present (MCM2-7 complex). Interacts with the RPA complex, when composed of all RPA1, RPA2 and RPA3 components, but not with RPA1 or RPA2 alone.   
  
 **Gene Ontology Information:**

Molecular Function

- chromatin binding

Location

- cell junction
- cytosol
- MCM complex
- nucleoplasm
- nucleus

Biological process

- cell division
- DNA-dependent DNA replication
- sister chromatid cohesion

---

90

- **Protein name:** Protein ANKUB1
- **Organism:** Homo sapiens
- **Uniprot Accession Number:** A6NFN9
- **Protein sequence length:** 502 aa
- **1D identity (%):** 11.33
- **1D identity (%) [Gaps excluded]:** 24.19
- **1D identity - Alignment Gaps:** 493
- **1D aligned content (<aminoacid>:%):** {'R': 2.86, 'S': 5.71, 'F': 4.76, 'E': 4.76, 'V': 6.67, 'Y': 4.76, 'K': 11.43, 'L': 6.67, 'G': 5.71, 'C': 3.81, 'D': 4.76, 'T': 7.62, 'A': 6.67, 'I': 2.86, 'P': 9.52, 'W': 0.95, 'Q': 7.62, 'H': 1.9, 'N': 0.95}
- **Common reported functions (%):** 0.0
- **Common reported locations (%):** 0.0
- **Common reported processes (%):** 0.0

- **AF ID:** A6NFN9
- **Chain:** A
- **Protein length:** 502 aa
- **Resolution:** N/A
- **b-phipsi:** 0.005222
- **w-rdist:** 0.615838
- **t-alpha:** 0.003642
- **Chemical similarity (Tanimoto Index) (%):** 99.55
- **1D identity (%) [PDB]:** 2.17
- **1D identity (%) [Gaps excluded][PDB]:** 59.57
- **1D identity - Alignment Gaps [PDB]:** 1246
- **1D aligned content [PDB] (<aminoacid>:%):** {'T': 7.14, 'V': 14.29, 'E': 7.14, 'A': 7.14, 'Y': 7.14, 'F': 3.57, 'K': 10.71, 'H': 3.57, 'L': 17.86, 'P': 7.14, 'Q': 10.71, 'C': 3.57}
- **2D identity (%) [PDB]:** 38.71
- **2D identity (%) [Gaps excluded][PDB]:** 87.8
- **2D identity - Alignment Gaps [PDB]:** 520
- **2D aligned content [PDB] (<2D-fold>:%):** {'E': 15.83, 'T': 16.94, 'H': 46.11, '.': 19.17, 'G': 1.67, 'B': 0.28}
- **3D similarity (TM-Score) (%) [PDB]:** 17.7

- **Gene name:** ANKUB1
- **Entrez ID:** 389161
- **RefSeq ID:** NM\_001315506
- **Transcript sequence length:** 2222
- **5-UTR|CDS|3-UTR identity (%):** 31.55 | 31.74 | 2.97
- **5-UTR|CDS|3-UTR identity (%) [Gaps excluded]:** 59.63 | 75.6 | 74.37
- **5-UTR|CDS|3-UTR identity [Alignment Gaps]:** 97 | 1671 | 11461
- **5-UTR aligned content (<base>:%):** {'G': 41.54, 'T': 15.38, 'C': 40.0, 'A': 3.08}
- **CDS aligned content (<base>:%):** {'A': 29.21, 'T': 21.55, 'G': 24.62, 'C': 24.62}
- **3-UTR aligned content (<base>:%):** {'T': 30.51, 'G': 22.32, 'C': 22.32, 'A': 24.86}

**Uniprot Description:**  
  
 N/A N/A   
  
 **Gene Ontology Information:**

Molecular Function   
  
N/A

Location   
  
N/A

Biological process   
  
N/A

---

91

- **Protein name:** Procollagen galactosyltransferase 2
- **Organism:** Homo sapiens
- **Uniprot Accession Number:** Q8IYK4
- **Protein sequence length:** 626 aa
- **1D identity (%):** 12.33
- **1D identity (%) [Gaps excluded]:** 24.01
- **1D identity - Alignment Gaps:** 477
- **1D aligned content (<aminoacid>:%):** {'R': 6.61, 'P': 14.05, 'A': 4.96, 'L': 11.57, 'S': 3.31, 'E': 4.96, 'H': 3.31, 'T': 2.48, 'I': 4.96, 'D': 9.09, 'K': 8.26, 'V': 4.96, 'N': 1.65, 'G': 5.79, 'F': 4.13, 'W': 1.65, 'C': 1.65, 'Y': 3.31, 'Q': 2.48, 'M': 0.83}
- **Common reported functions (%):** 0.0
- **Common reported locations (%):** 0.0
- **Common reported processes (%):** 0.0

- **AF ID:** Q8IYK4
- **Chain:** A
- **Protein length:** 626 aa
- **Resolution:** N/A
- **b-phipsi:** 0.000127
- **w-rdist:** 0.683719
- **t-alpha:** 0.0984
- **Chemical similarity (Tanimoto Index) (%):** 96.54
- **1D identity (%) [PDB]:** 2.62
- **1D identity (%) [Gaps excluded][PDB]:** 68.52
- **1D identity - Alignment Gaps [PDB]:** 1356
- **1D aligned content [PDB] (<aminoacid>:%):** {'M': 2.7, 'K': 8.11, 'N': 10.81, 'V': 5.41, 'Q': 5.41, 'R': 5.41, 'T': 8.11, 'P': 16.22, 'L': 16.22, 'S': 2.7, 'C': 2.7, 'I': 5.41, 'G': 2.7, 'A': 2.7, 'D': 2.7, 'H': 2.7}
- **2D identity (%) [PDB]:** 43.05
- **2D identity (%) [Gaps excluded][PDB]:** 88.52
- **2D identity - Alignment Gaps [PDB]:** 506
- **2D aligned content [PDB] (<2D-fold>:%):** {'.': 19.15, 'E': 19.86, 'H': 39.95, 'T': 20.8, 'B': 0.24}
- **3D similarity (TM-Score) (%) [PDB]:** 20.2

- **Gene name:** COLGALT2
- **Entrez ID:** 23127
- **RefSeq ID:** NM\_015101
- **Transcript sequence length:** 5179
- **5-UTR|CDS|3-UTR identity (%):** 29.41 | 39.19 | 14.57
- **5-UTR|CDS|3-UTR identity (%) [Gaps excluded]:** 88.71 | 74.77 | 75.7
- **5-UTR|CDS|3-UTR identity [Alignment Gaps]:** 250 | 1393 | 10033
- **5-UTR aligned content (<base>:%):** {'C': 45.45, 'T': 5.45, 'G': 45.45, 'A': 3.64}
- **CDS aligned content (<base>:%):** {'G': 27.46, 'C': 27.72, 'T': 18.13, 'A': 26.68}
- **3-UTR aligned content (<base>:%):** {'A': 22.82, 'G': 21.71, 'C': 20.66, 'T': 34.81}

**Uniprot Description:**  
  
 Beta-galactosyltransferase that transfers beta-galactose to hydroxylysine residues of collagen. N/A   
  
 **Gene Ontology Information:**

Molecular Function

- procollagen galactosyltransferase activity

Location

- endoplasmic reticulum lumen

Biological process

- collagen fibril organization

---

92

- **Protein name:** Nuclear receptor-binding protein
- **Organism:** Homo sapiens
- **Uniprot Accession Number:** Q9UHY1
- **Protein sequence length:** 535 aa
- **1D identity (%):** 11.8
- **1D identity (%) [Gaps excluded]:** 22.55
- **1D identity - Alignment Gaps:** 436
- **1D aligned content (<aminoacid>:%):** {'G': 9.26, 'Q': 10.19, 'V': 9.26, 'S': 5.56, 'A': 5.56, 'P': 8.33, 'E': 11.11, 'I': 5.56, 'C': 2.78, 'N': 4.63, 'F': 0.93, 'R': 3.7, 'L': 7.41, 'K': 4.63, 'T': 4.63, 'H': 3.7, 'D': 1.85, 'Y': 0.93}
- **Common reported functions (%):** 0.0
- **Common reported locations (%):** 21.43
- **Common reported processes (%):** 0.0

- **AF ID:** Q9UHY1
- **Chain:** A
- **Protein length:** 535 aa
- **Resolution:** N/A
- **b-phipsi:** 0.001047
- **w-rdist:** 0.413123
- **t-alpha:** 0.032331
- **Chemical similarity (Tanimoto Index) (%):** 99.17
- **1D identity (%) [PDB]:** 1.63
- **1D identity (%) [Gaps excluded][PDB]:** 84.62
- **1D identity - Alignment Gaps [PDB]:** 1321
- **1D aligned content [PDB] (<aminoacid>:%):** {'M': 4.55, 'K': 9.09, 'R': 13.64, 'Y': 4.55, 'V': 9.09, 'N': 4.55, 'T': 9.09, 'P': 9.09, 'A': 4.55, 'H': 4.55, 'Q': 13.64, 'L': 4.55, 'E': 4.55, 'S': 4.55}
- **2D identity (%) [PDB]:** 43.1
- **2D identity (%) [Gaps excluded][PDB]:** 92.01
- **2D identity - Alignment Gaps [PDB]:** 497
- **2D aligned content [PDB] (<2D-fold>:%):** {'.': 18.66, 'H': 41.29, 'T': 18.66, 'E': 20.4, 'B': 0.25, 'G': 0.75}
- **3D similarity (TM-Score) (%) [PDB]:** 17.83

- **Gene name:** NRBP1
- **Entrez ID:** 29959
- **RefSeq ID:** NM\_013392
- **Transcript sequence length:** 2176
- **5-UTR|CDS|3-UTR identity (%):** 47.33 | 36.05 | 2.3
- **5-UTR|CDS|3-UTR identity (%) [Gaps excluded]:** 72.45 | 75.54 | 76.82
- **5-UTR|CDS|3-UTR identity [Alignment Gaps]:** 52 | 1482 | 11619
- **5-UTR aligned content (<base>:%):** {'G': 54.93, 'C': 35.21, 'A': 4.23, 'T': 5.63}
- **CDS aligned content (<base>:%):** {'A': 26.32, 'T': 18.69, 'G': 28.18, 'C': 26.81}
- **3-UTR aligned content (<base>:%):** {'G': 30.91, 'C': 30.55, 'T': 25.45, 'A': 13.09}

**Uniprot Description:**  
  
 May play a role in subcellular trafficking between the endoplasmic reticulum and Golgi apparatus through interactions with the Rho-type GTPases. Binding to the NS3 protein of dengue virus type 2 appears to subvert this activity into the alteration of the intracellular membrane structure associated with flaviviral replication.   
  
Homodimer. Binds to MLF1, recruiting a serine kinase which phosphorylates both itself and MLF1. Phosphorylated MLF1 binds to YWHAZ and is retained in the cytoplasm (By similarity).   
  
 **Gene Ontology Information:**

Molecular Function

- ATP binding
- protein homodimerization activity
- protein serine/threonine kinase activity

Location

- cell cortex
- cytoplasm
- endomembrane system
- lamellipodium
- membrane
- nucleoplasm

Biological process

- endoplasmic reticulum to Golgi vesicle-mediated transport
- protein phosphorylation

---

93

- **Protein name:** TGF-beta receptor type-2
- **Organism:** Homo sapiens
- **Uniprot Accession Number:** P37173
- **Protein sequence length:** 567 aa
- **1D identity (%):** 12.47
- **1D identity (%) [Gaps excluded]:** 23.39
- **1D identity - Alignment Gaps:** 434
- **1D aligned content (<aminoacid>:%):** {'L': 12.93, 'W': 1.72, 'I': 5.17, 'P': 7.76, 'K': 8.62, 'S': 5.17, 'V': 7.76, 'F': 2.59, 'C': 5.17, 'T': 8.62, 'Q': 4.31, 'E': 5.17, 'A': 3.45, 'H': 4.31, 'N': 1.72, 'G': 6.9, 'R': 5.17, 'D': 3.45}
- **Common reported functions (%):** 6.67
- **Common reported locations (%):** 14.29
- **Common reported processes (%):** 4.76

- **AF ID:** P37173
- **Chain:** A
- **Protein length:** 567 aa
- **Resolution:** N/A
- **b-phipsi:** 0.000668
- **w-rdist:** 0.498165
- **t-alpha:** 0.071819
- **Chemical similarity (Tanimoto Index) (%):** 96.69
- **1D identity (%) [PDB]:** 2.74
- **1D identity (%) [Gaps excluded][PDB]:** 68.52
- **1D identity - Alignment Gaps [PDB]:** 1297
- **1D aligned content [PDB] (<aminoacid>:%):** {'M': 2.7, 'R': 5.41, 'L': 13.51, 'I': 10.81, 'S': 5.41, 'T': 5.41, 'F': 8.11, 'K': 5.41, 'P': 5.41, 'Y': 5.41, 'V': 5.41, 'E': 10.81, 'Q': 2.7, 'H': 2.7, 'A': 5.41, 'D': 2.7, 'G': 2.7}
- **2D identity (%) [PDB]:** 33.2
- **2D identity (%) [Gaps excluded][PDB]:** 90.21
- **2D identity - Alignment Gaps [PDB]:** 649
- **2D aligned content [PDB] (<2D-fold>:%):** {'.': 15.84, 'E': 22.58, 'T': 20.23, 'H': 41.35}
- **3D similarity (TM-Score) (%) [PDB]:** 22.06

- **Gene name:** TGFBR2
- **Entrez ID:** 7048
- **RefSeq ID:** NM\_003242
- **Transcript sequence length:** 4530
- **5-UTR|CDS|3-UTR identity (%):** 35.76 | 36.94 | 13.42
- **5-UTR|CDS|3-UTR identity (%) [Gaps excluded]:** 84.43 | 73.65 | 76.36
- **5-UTR|CDS|3-UTR identity [Alignment Gaps]:** 166 | 1422 | 10117
- **5-UTR aligned content (<base>:%):** {'T': 7.77, 'G': 41.75, 'C': 49.51, 'A': 0.97}
- **CDS aligned content (<base>:%):** {'G': 28.18, 'T': 17.55, 'C': 28.94, 'A': 25.33}
- **3-UTR aligned content (<base>:%):** {'T': 36.37, 'C': 18.15, 'G': 15.73, 'A': 29.75}

**Uniprot Description:**  
  
 Transmembrane serine/threonine kinase forming with the TGF-beta type I serine/threonine kinase receptor, TGFBR1, the non-promiscuous receptor for the TGF-beta cytokines TGFB1, TGFB2 and TGFB3. Transduces the TGFB1, TGFB2 and TGFB3 signal from the cell surface to the cytoplasm and thus regulates a plethora of physiological and pathological processes including cell cycle arrest in epithelial and hematopoietic cells, control of mesenchymal cell proliferation and differentiation, wound healing, extracellular matrix production, immunosuppression and carcinogenesis. The formation of the receptor complex composed of 2 TGFBR1 and 2 TGFBR2 molecules symmetrically bound to the cytokine dimer results in the phosphorylation and activation of TGFBR1 by the constitutively active TGFBR2. Activated TGFBR1 phosphorylates SMAD2 which dissociates from the receptor and interacts with SMAD4. The SMAD2-SMAD4 complex is subsequently translocated to the nucleus where it modulates the transcription of the TGF-beta-regulated genes. This constitutes the canonical SMAD-dependent TGF-beta signaling cascade. Also involved in non-canonical, SMAD-independent TGF-beta signaling pathways.   
  
Homodimer. Heterohexamer; TGFB1, TGFB2 and TGFB3 homodimeric ligands assemble a functional receptor composed of two TGFBR1 and TGFBR2 heterodimers to form a ligand-receptor heterohexamer. The respective affinity of TGFRB1 and TGFRB2 for the ligands may modulate the kinetics of assembly of the receptor and may explain the different biological activities of TGFB1, TGFB2 and TGFB3. Interacts with DAXX. Interacts with DYNLT4. Interacts with ZFYVE9; ZFYVE9 recruits SMAD2 and SMAD3 to the TGF-beta receptor. Interacts with and is activated by SCUBE3; this interaction does not affect TGFB1-binding to TGFBR2. Interacts with VPS39; this interaction is independent of the receptor kinase activity and of the presence of TGF-beta. Interacts with CLU (PubMed:8555189).   
  
 **Gene Ontology Information:**

Molecular Function

- activin binding
- activin-activated receptor activity
- ATP binding
- glycosaminoglycan binding
- kinase activator activity
- metal ion binding
- molecular adaptor activity
- SMAD binding
- transforming growth factor beta binding
- transforming growth factor beta-activated receptor activity
- transforming growth factor beta receptor activity, type II
- transmembrane receptor protein serine/threonine kinase activity
- type I transforming growth factor beta receptor binding
- type III transforming growth factor beta receptor binding

Location

- caveola
- cytosol
- external side of plasma membrane
- extracellular region
- extracellular space
- membrane
- membrane raft
- plasma membrane
- receptor complex
- transforming growth factor beta ligand-receptor complex

Biological process

- activation of protein kinase activity
- aorta morphogenesis
- aortic valve morphogenesis
- apoptotic process
- artery morphogenesis
- atrioventricular valve morphogenesis
- blood vessel development
- brain development
- branching involved in blood vessel morphogenesis
- bronchus morphogenesis
- cardiac left ventricle morphogenesis
- cellular response to growth factor stimulus
- embryonic cranial skeleton morphogenesis
- embryonic hemopoiesis
- endocardial cushion fusion
- gastrulation
- growth plate cartilage chondrocyte growth
- heart development
- heart looping
- in utero embryonic development
- inferior endocardial cushion morphogenesis
- Langerhans cell differentiation
- lens development in camera-type eye
- lens fiber cell apoptotic process
- lung lobe morphogenesis
- mammary gland morphogenesis
- membranous septum morphogenesis
- miRNA transport
- myeloid dendritic cell differentiation
- Notch signaling pathway
- outflow tract morphogenesis
- outflow tract septum morphogenesis
- pathway-restricted SMAD protein phosphorylation
- peptidyl-serine phosphorylation
- peptidyl-threonine phosphorylation
- positive regulation of angiogenesis
- positive regulation of B cell tolerance induction
- positive regulation of CD4-positive, alpha-beta T cell proliferation
- positive regulation of cell population proliferation
- positive regulation of epithelial cell migration
- positive regulation of epithelial to mesenchymal transition
- positive regulation of epithelial to mesenchymal transition involved in endocardial cushion formation
- positive regulation of mesenchymal cell proliferation
- positive regulation of NK T cell differentiation
- positive regulation of pathway-restricted SMAD protein phosphorylation
- positive regulation of reactive oxygen species metabolic process
- positive regulation of SMAD protein signal transduction
- positive regulation of T cell tolerance induction
- positive regulation of tolerance induction to self antigen
- protein phosphorylation
- regulation of cell population proliferation
- regulation of gene expression
- regulation of stem cell proliferation
- response to cholesterol
- response to xenobiotic stimulus
- roof of mouth development
- secondary palate development
- smoothened signaling pathway
- trachea formation
- transforming growth factor beta receptor signaling pathway
- tricuspid valve morphogenesis
- vasculogenesis
- ventricular septum morphogenesis

---

94

- **Protein name:** Protein PALS2
- **Organism:** Homo sapiens
- **Uniprot Accession Number:** Q9NZW5
- **Protein sequence length:** 540 aa
- **1D identity (%):** 12.26
- **1D identity (%) [Gaps excluded]:** 23.69
- **1D identity - Alignment Gaps:** 445
- **1D aligned content (<aminoacid>:%):** {'N': 4.42, 'L': 9.73, 'T': 4.42, 'P': 8.85, 'G': 11.5, 'I': 5.31, 'K': 10.62, 'S': 1.77, 'H': 2.65, 'V': 8.85, 'E': 4.42, 'D': 5.31, 'A': 4.42, 'Y': 2.65, 'Q': 4.42, 'R': 6.19, 'F': 3.54, 'W': 0.88}
- **Common reported functions (%):** 0.0
- **Common reported locations (%):** 14.29
- **Common reported processes (%):** 0.0

- **AF ID:** Q9NZW5
- **Chain:** A
- **Protein length:** 540 aa
- **Resolution:** N/A
- **b-phipsi:** 0.000994
- **w-rdist:** 0.449124
- **t-alpha:** 0.039364
- **Chemical similarity (Tanimoto Index) (%):** 97.91
- **1D identity (%) [PDB]:** 1.48
- **1D identity (%) [Gaps excluded][PDB]:** 71.43
- **1D identity - Alignment Gaps [PDB]:** 1322
- **1D aligned content [PDB] (<aminoacid>:%):** {'Q': 15.0, 'V': 5.0, 'L': 10.0, 'H': 5.0, 'E': 15.0, 'A': 10.0, 'I': 10.0, 'R': 5.0, 'K': 10.0, 'D': 5.0, 'G': 5.0, 'T': 5.0}
- **2D identity (%) [PDB]:** 33.83
- **2D identity (%) [Gaps excluded][PDB]:** 89.18
- **2D identity - Alignment Gaps [PDB]:** 620
- **2D aligned content [PDB] (<2D-fold>:%):** {'T': 19.53, '.': 17.75, 'H': 37.57, 'E': 23.37, 'G': 1.78}
- **3D similarity (TM-Score) (%) [PDB]:** 18.71

- **Gene name:** PALS2
- **Entrez ID:** 51678
- **RefSeq ID:** N/A
- **Sequence length:** N/A
- **5-UTR|CDS|3-UTR identity (%):** N/A | N/A | N/A
- **5-UTR|CDS|3-UTR identity (%) [Gaps excluded]:** N/A | N/A | N/A
- **5-UTR|CDS|3-UTR identity [Alignment Gaps]:** N/A | N/A | N/A
- **5-UTR aligned content (<base>:%):** N/A
- **CDS aligned content (<base>:%):** N/A
- **3-UTR aligned content (<base>:%):** N/A

**Uniprot Description:**  
  
 N/A   
  
Interacts with CADM1 (By similarity). Interacts with the LIN7 proteins.   
  
 **Gene Ontology Information:**

Molecular Function   
  
N/A

Location

- cell-cell junction
- extracellular exosome
- membrane
- plasma membrane

Biological process

- protein-containing complex assembly

---

95

- **Protein name:** Mitochondrial 10-formyltetrahydrofolate dehydrogenase
- **Organism:** Homo sapiens
- **Uniprot Accession Number:** Q3SY69
- **Protein sequence length:** 923 aa
- **1D identity (%):** 18.0
- **1D identity (%) [Gaps excluded]:** 27.18
- **1D identity - Alignment Gaps:** 362
- **1D aligned content (<aminoacid>:%):** {'M': 2.07, 'A': 6.74, 'L': 5.18, 'P': 10.88, 'G': 9.33, 'T': 8.81, 'I': 6.74, 'K': 6.74, 'F': 5.18, 'D': 2.59, 'H': 1.04, 'R': 3.63, 'V': 8.81, 'E': 4.66, 'Q': 4.66, 'S': 3.63, 'N': 4.15, 'W': 1.55, 'Y': 2.07, 'C': 1.55}
- **Common reported functions (%):** 0.0
- **Common reported locations (%):** 14.29
- **Common reported processes (%):** 0.0

- **AF ID:** Q3SY69
- **Chain:** A
- **Protein length:** 923 aa
- **Resolution:** N/A
- **b-phipsi:** 0.003943
- **w-rdist:** 0.860367
- **t-alpha:** 0.001457
- **Chemical similarity (Tanimoto Index) (%):** 98.28
- **1D identity (%) [PDB]:** 3.35
- **1D identity (%) [Gaps excluded][PDB]:** 62.22
- **1D identity - Alignment Gaps [PDB]:** 1581
- **1D aligned content [PDB] (<aminoacid>:%):** {'P': 5.36, 'D': 7.14, 'G': 7.14, 'T': 7.14, 'I': 14.29, 'K': 10.71, 'A': 1.79, 'F': 3.57, 'M': 1.79, 'Y': 3.57, 'H': 1.79, 'E': 8.93, 'L': 8.93, 'R': 5.36, 'V': 10.71, 'Q': 1.79}
- **2D identity (%) [PDB]:** 46.93
- **2D identity (%) [Gaps excluded][PDB]:** 89.44
- **2D identity - Alignment Gaps [PDB]:** 549
- **2D aligned content [PDB] (<2D-fold>:%):** {'.': 16.61, 'E': 22.69, 'T': 19.37, 'H': 40.04, 'G': 1.11, 'B': 0.18}
- **3D similarity (TM-Score) (%) [PDB]:** 22.4

- **Gene name:** ALDH1L2
- **Entrez ID:** 160428
- **RefSeq ID:** N/A
- **Sequence length:** N/A
- **5-UTR|CDS|3-UTR identity (%):** N/A | N/A | N/A
- **5-UTR|CDS|3-UTR identity (%) [Gaps excluded]:** N/A | N/A | N/A
- **5-UTR|CDS|3-UTR identity [Alignment Gaps]:** N/A | N/A | N/A
- **5-UTR aligned content (<base>:%):** N/A
- **CDS aligned content (<base>:%):** N/A
- **3-UTR aligned content (<base>:%):** N/A

**Uniprot Description:**  
  
 Mitochondrial 10-formyltetrahydrofolate dehydrogenase that catalyzes the NADP(+)-dependent conversion of 10-formyltetrahydrofolate to tetrahydrofolate and carbon dioxide. N/A   
  
 **Gene Ontology Information:**

Molecular Function

- aldehyde dehydrogenase (NAD+) activity
- formyltetrahydrofolate dehydrogenase activity
- hydroxymethyl-, formyl- and related transferase activity

Location

- extracellular exosome
- mitochondrial matrix
- mitochondrion
- nucleoplasm

Biological process

- 10-formyltetrahydrofolate catabolic process
- biosynthetic process
- fatty acid beta-oxidation
- folic acid metabolic process
- NADPH regeneration
- one-carbon metabolic process

---

96

- **Protein name:** mRNA (2'-O-methyladenosine-N(6)-)-methyltransferase
- **Organism:** Homo sapiens
- **Uniprot Accession Number:** Q9H4Z3
- **Protein sequence length:** 704 aa
- **1D identity (%):** 15.91
- **1D identity (%) [Gaps excluded]:** 24.19
- **1D identity - Alignment Gaps:** 323
- **1D aligned content (<aminoacid>:%):** {'P': 15.33, 'R': 12.0, 'G': 8.0, 'T': 4.0, 'S': 7.33, 'D': 4.0, 'H': 4.67, 'E': 6.67, 'K': 6.0, 'C': 2.67, 'N': 1.33, 'F': 2.0, 'Q': 2.0, 'V': 7.33, 'A': 6.0, 'L': 7.33, 'I': 2.0, 'M': 0.67, 'Y': 0.67}
- **Common reported functions (%):** 0.0
- **Common reported locations (%):** 7.14
- **Common reported processes (%):** 4.76

- **AF ID:** Q9H4Z3
- **Chain:** A
- **Protein length:** 704 aa
- **Resolution:** N/A
- **b-phipsi:** 0.00439
- **w-rdist:** 0.482453
- **t-alpha:** 0.005827
- **Chemical similarity (Tanimoto Index) (%):** 98.57
- **1D identity (%) [PDB]:** 2.97
- **1D identity (%) [Gaps excluded][PDB]:** 73.33
- **1D identity - Alignment Gaps [PDB]:** 1422
- **1D aligned content [PDB] (<aminoacid>:%):** {'A': 13.64, 'D': 4.55, 'V': 13.64, 'T': 6.82, 'P': 11.36, 'G': 6.82, 'K': 6.82, 'I': 4.55, 'S': 2.27, 'M': 4.55, 'R': 11.36, 'Y': 2.27, 'C': 2.27, 'H': 2.27, 'E': 2.27, 'L': 2.27, 'F': 2.27}
- **2D identity (%) [PDB]:** 36.88
- **2D identity (%) [Gaps excluded][PDB]:** 92.06
- **2D identity - Alignment Gaps [PDB]:** 660
- **2D aligned content [PDB] (<2D-fold>:%):** {'.': 19.21, 'H': 45.57, 'E': 16.75, 'T': 18.23, 'B': 0.25}
- **3D similarity (TM-Score) (%) [PDB]:** 22.5

- **Gene name:** PCIF1
- **Entrez ID:** 553360
- **RefSeq ID:** N/A
- **Sequence length:** N/A
- **5-UTR|CDS|3-UTR identity (%):** N/A | N/A | N/A
- **5-UTR|CDS|3-UTR identity (%) [Gaps excluded]:** N/A | N/A | N/A
- **5-UTR|CDS|3-UTR identity [Alignment Gaps]:** N/A | N/A | N/A
- **5-UTR aligned content (<base>:%):** N/A
- **CDS aligned content (<base>:%):** N/A
- **3-UTR aligned content (<base>:%):** N/A

**Uniprot Description:**  
  
 Cap-specific adenosine methyltransferase that catalyzes formation of N(6),2'-O-dimethyladenosine cap (m6A(m)) by methylating the adenosine at the second transcribed position of capped mRNAs (PubMed:30467178, PubMed:30487554, PubMed:31279658, PubMed:31279659, PubMed:33428944). Recruited to the early elongation complex of RNA polymerase II (RNAPII) via interaction with POLR2A and mediates formation of m6A(m) co-transcriptionally (PubMed:30467178).   
  
Interacts with POLR2A; interacts with the phosphorylated C-terminal domain (CTD) of POLR2A.   
  
 **Gene Ontology Information:**

Molecular Function

- mRNA (2'-O-methyladenosine-N6-)-methyltransferase activity
- RNA polymerase II C-terminal domain binding
- S-adenosyl-L-methionine binding

Location

- nucleus

Biological process

- mRNA methylation
- positive regulation of translation

---

97

- **Protein name:** Cilia- and flagella-associated protein 77
- **Organism:** Homo sapiens
- **Uniprot Accession Number:** Q6ZQR2
- **Protein sequence length:** 320 aa
- **1D identity (%):** 9.14
- **1D identity (%) [Gaps excluded]:** 27.65
- **1D identity - Alignment Gaps:** 593
- **1D aligned content (<aminoacid>:%):** {'G': 9.88, 'W': 1.23, 'C': 1.23, 'P': 8.64, 'R': 11.11, 'T': 7.41, 'S': 2.47, 'E': 6.17, 'D': 3.7, 'K': 7.41, 'A': 6.17, 'V': 8.64, 'Q': 7.41, 'L': 7.41, 'N': 1.23, 'I': 2.47, 'F': 2.47, 'M': 1.23, 'H': 2.47, 'Y': 1.23}
- **Common reported functions (%):** 0.0
- **Common reported locations (%):** 0.0
- **Common reported processes (%):** 0.0

- **AF ID:** Q6ZQR2
- **Chain:** A
- **Protein length:** 320 aa
- **Resolution:** N/A
- **b-phipsi:** 0.006256
- **w-rdist:** 0.741707
- **t-alpha:** 0.001459
- **Chemical similarity (Tanimoto Index) (%):** 98.8
- **1D identity (%) [PDB]:** 1.77
- **1D identity (%) [Gaps excluded][PDB]:** 80.0
- **1D identity - Alignment Gaps [PDB]:** 1108
- **1D aligned content [PDB] (<aminoacid>:%):** {'F': 5.0, 'Q': 10.0, 'L': 20.0, 'H': 10.0, 'E': 15.0, 'I': 10.0, 'R': 5.0, 'A': 5.0, 'K': 10.0, 'D': 5.0, 'P': 5.0}
- **2D identity (%) [PDB]:** 25.11
- **2D identity (%) [Gaps excluded][PDB]:** 85.88
- **2D identity - Alignment Gaps [PDB]:** 634
- **2D aligned content [PDB] (<2D-fold>:%):** {'H': 38.22, '.': 37.33, 'T': 20.44, 'E': 2.67, 'G': 1.33}
- **3D similarity (TM-Score) (%) [PDB]:** 12.43

- **Gene name:** CFAP77
- **Entrez ID:** 389799
- **RefSeq ID:** NM\_207417
- **Transcript sequence length:** 1833
- **5-UTR|CDS|3-UTR identity (%):** 28.47 | 22.99 | 4.48
- **5-UTR|CDS|3-UTR identity (%) [Gaps excluded]:** 76.47 | 76.34 | 77.3
- **5-UTR|CDS|3-UTR identity [Alignment Gaps]:** 86 | 1903 | 11305
- **5-UTR aligned content (<base>:%):** {'C': 46.15, 'G': 46.15, 'T': 5.13, 'A': 2.56}
- **CDS aligned content (<base>:%):** {'A': 20.13, 'T': 14.54, 'C': 35.62, 'G': 29.71}
- **3-UTR aligned content (<base>:%):** {'C': 29.93, 'T': 29.74, 'G': 19.52, 'A': 20.82}

**Uniprot Description:**  
  
 Microtubule inner protein (MIP) part of the dynein-decorated doublet microtubules (DMTs) in cilia axoneme, which is required for motile cilia beating. N/A   
  
 **Gene Ontology Information:**

Molecular Function   
  
N/A

Location

- axonemal microtubule

Biological process   
  
N/A

---

98

- **Protein name:** Retinoic acid receptor RXR-gamma
- **Organism:** Homo sapiens
- **Uniprot Accession Number:** P48443
- **Protein sequence length:** 463 aa
- **1D identity (%):** 9.99
- **1D identity (%) [Gaps excluded]:** 21.38
- **1D identity - Alignment Gaps:** 480
- **1D aligned content (<aminoacid>:%):** {'K': 6.67, 'H': 2.22, 'P': 12.22, 'S': 6.67, 'T': 7.78, 'D': 1.11, 'V': 8.89, 'G': 7.78, 'N': 2.22, 'L': 8.89, 'A': 3.33, 'Q': 4.44, 'E': 6.67, 'I': 2.22, 'C': 5.56, 'F': 5.56, 'Y': 2.22, 'R': 4.44, 'W': 1.11}
- **Common reported functions (%):** 0.0
- **Common reported locations (%):** 0.0
- **Common reported processes (%):** 4.76

- **AF ID:** P48443
- **Chain:** A
- **Protein length:** 463 aa
- **Resolution:** N/A
- **b-phipsi:** 0.007768
- **w-rdist:** 0.462894
- **t-alpha:** 0.003655
- **Chemical similarity (Tanimoto Index) (%):** 98.57
- **1D identity (%) [PDB]:** 2.64
- **1D identity (%) [Gaps excluded][PDB]:** 62.26
- **1D identity - Alignment Gaps [PDB]:** 1195
- **1D aligned content [PDB] (<aminoacid>:%):** {'P': 9.09, 'A': 12.12, 'G': 9.09, 'S': 6.06, 'M': 3.03, 'D': 3.03, 'H': 3.03, 'N': 3.03, 'R': 6.06, 'Y': 3.03, 'C': 3.03, 'V': 3.03, 'Q': 9.09, 'E': 6.06, 'I': 6.06, 'L': 9.09, 'K': 3.03, 'T': 3.03}
- **2D identity (%) [PDB]:** 31.4
- **2D identity (%) [Gaps excluded][PDB]:** 91.29
- **2D identity - Alignment Gaps [PDB]:** 635
- **2D aligned content [PDB] (<2D-fold>:%):** {'.': 16.78, 'T': 20.39, 'E': 3.95, 'H': 57.89, 'G': 0.99}
- **3D similarity (TM-Score) (%) [PDB]:** 17.59

- **Gene name:** RXRG
- **Entrez ID:** N/A
- **RefSeq ID:** NM\_006917
- **Transcript sequence length:** 1966
- **5-UTR|CDS|3-UTR identity (%):** 33.47 | 32.66 | 1.84
- **5-UTR|CDS|3-UTR identity (%) [Gaps excluded]:** 74.11 | 76.73 | 72.28
- **5-UTR|CDS|3-UTR identity [Alignment Gaps]:** 136 | 1600 | 11623
- **5-UTR aligned content (<base>:%):** {'C': 40.96, 'A': 4.82, 'G': 45.78, 'T': 8.43}
- **CDS aligned content (<base>:%):** {'T': 18.79, 'G': 27.25, 'A': 23.96, 'C': 30.0}
- **3-UTR aligned content (<base>:%):** {'C': 20.55, 'A': 28.31, 'G': 20.09, 'T': 31.05}

**Uniprot Description:**  
  
 Receptor for retinoic acid. Retinoic acid receptors bind as heterodimers to their target response elements in response to their ligands, all-trans or 9-cis retinoic acid, and regulate gene expression in various biological processes. The RAR/RXR heterodimers bind to the retinoic acid response elements (RARE) composed of tandem 5'-AGGTCA-3' sites known as DR1-DR5. The high affinity ligand for RXRs is 9-cis retinoic acid (By similarity).   
  
Homodimer (By similarity). Heterodimer with a RAR molecule (PubMed:28167758). Binds DNA preferentially as a RAR/RXR heterodimer (PubMed:28167758). Interacts with RARA (PubMed:28167758).   
  
 **Gene Ontology Information:**

Molecular Function

- DNA-binding transcription factor activity
- nuclear receptor activity
- steroid hormone receptor activity
- retinoic acid-responsive element binding
- zinc ion binding

Location

- RNA polymerase II transcription regulator complex

Biological process

- anatomical structure development
- cell differentiation
- positive regulation of transcription, DNA-templated
- positive regulation of transcription by RNA polymerase II
- response to retinoic acid
- retinoic acid receptor signaling pathway

---

99

- **Protein name:** Gasdermin-C
- **Organism:** Homo sapiens
- **Uniprot Accession Number:** Q9BYG8
- **Protein sequence length:** 508 aa
- **1D identity (%):** 10.29
- **1D identity (%) [Gaps excluded]:** 21.4
- **1D identity - Alignment Gaps:** 479
- **1D aligned content (<aminoacid>:%):** {'E': 9.47, 'I': 3.16, 'V': 6.32, 'K': 6.32, 'F': 3.16, 'D': 8.42, 'R': 5.26, 'P': 7.37, 'T': 3.16, 'S': 6.32, 'A': 4.21, 'L': 13.68, 'G': 7.37, 'W': 1.05, 'Q': 7.37, 'Y': 4.21, 'C': 2.11, 'H': 1.05}
- **Common reported functions (%):** 0.0
- **Common reported locations (%):** 14.29
- **Common reported processes (%):** 0.0

- **AF ID:** Q9BYG8
- **Chain:** A
- **Protein length:** 508 aa
- **Resolution:** N/A
- **b-phipsi:** 0.002995
- **w-rdist:** 0.783235
- **t-alpha:** 0.002922
- **Chemical similarity (Tanimoto Index) (%):** 99.55
- **1D identity (%) [PDB]:** 1.75
- **1D identity (%) [Gaps excluded][PDB]:** 74.19
- **1D identity - Alignment Gaps [PDB]:** 1284
- **1D aligned content [PDB] (<aminoacid>:%):** {'M': 4.35, 'P': 13.04, 'I': 4.35, 'Q': 13.04, 'G': 8.7, 'F': 8.7, 'K': 4.35, 'Y': 8.7, 'A': 8.7, 'D': 4.35, 'S': 4.35, 'E': 4.35, 'R': 4.35, 'L': 4.35, 'T': 4.35}
- **2D identity (%) [PDB]:** 35.18
- **2D identity (%) [Gaps excluded][PDB]:** 92.45
- **2D identity - Alignment Gaps [PDB]:** 604
- **2D aligned content [PDB] (<2D-fold>:%):** {'.': 13.7, 'H': 48.4, 'T': 15.16, 'E': 21.87, 'G': 0.87}
- **3D similarity (TM-Score) (%) [PDB]:** 18.56

- **Gene name:** GSDMC
- **Entrez ID:** 56169
- **RefSeq ID:** NM\_031415
- **Transcript sequence length:** 2450
- **5-UTR|CDS|3-UTR identity (%):** 15.99 | 31.88 | 1.84
- **5-UTR|CDS|3-UTR identity (%) [Gaps excluded]:** 78.57 | 74.23 | 84.23
- **5-UTR|CDS|3-UTR identity [Alignment Gaps]:** 493 | 1639 | 11673
- **5-UTR aligned content (<base>:%):** {'C': 40.4, 'T': 12.12, 'G': 43.43, 'A': 4.04}
- **CDS aligned content (<base>:%):** {'A': 28.49, 'T': 21.18, 'G': 26.09, 'C': 24.24}
- **3-UTR aligned content (<base>:%):** {'G': 20.09, 'C': 15.53, 'T': 31.51, 'A': 32.88}

**Uniprot Description:**  
  
 Gasdermin-C
This form constitutes the precursor of the pore-forming protein: upon cleavage, the released N-terminal moiety (Gasdermin-C, N-terminal) binds to membranes and forms pores, triggering pyroptosis.   
  
Gasdermin-C, N-terminal
Homooligomer; homooligomeric ring-shaped pore complex containing 27-28 subunits when inserted in the membrane.   
  
 **Gene Ontology Information:**

Molecular Function

- phosphatidylinositol-4,5-bisphosphate binding
- phosphatidylinositol-4-phosphate binding
- phosphatidylserine binding

Location

- cytoplasm
- cytosol
- plasma membrane

Biological process

- defense response to bacterium
- pyroptosis

---

100

- **Protein name:** RasGAP-activating-like protein 1
- **Organism:** Homo sapiens
- **Uniprot Accession Number:** O95294
- **Protein sequence length:** 804 aa
- **1D identity (%):** 15.32
- **1D identity (%) [Gaps excluded]:** 25.0
- **1D identity - Alignment Gaps:** 399
- **1D aligned content (<aminoacid>:%):** {'L': 12.66, 'E': 5.7, 'P': 10.13, 'D': 5.06, 'C': 2.53, 'V': 7.59, 'G': 12.03, 'Y': 2.53, 'K': 5.06, 'T': 3.8, 'S': 5.06, 'Q': 8.23, 'R': 8.23, 'A': 5.06, 'F': 1.9, 'W': 1.27, 'N': 1.9, 'I': 0.63, 'H': 0.63}
- **Common reported functions (%):** 6.67
- **Common reported locations (%):** 7.14
- **Common reported processes (%):** 0.0

- **AF ID:** O95294
- **Chain:** A
- **Protein length:** 804 aa
- **Resolution:** N/A
- **b-phipsi:** 0.00206
- **w-rdist:** 0.307195
- **t-alpha:** 0.0984
- **Chemical similarity (Tanimoto Index) (%):** 98.88
- **1D identity (%) [PDB]:** 1.87
- **1D identity (%) [Gaps excluded][PDB]:** 75.0
- **1D identity - Alignment Gaps [PDB]:** 1562
- **1D aligned content [PDB] (<aminoacid>:%):** {'M': 3.33, 'L': 6.67, 'N': 3.33, 'D': 10.0, 'V': 13.33, 'S': 10.0, 'A': 6.67, 'T': 3.33, 'F': 6.67, 'Y': 3.33, 'K': 10.0, 'P': 3.33, 'I': 3.33, 'E': 6.67, 'Q': 6.67, 'R': 3.33}
- **2D identity (%) [PDB]:** 43.3
- **2D identity (%) [Gaps excluded][PDB]:** 88.85
- **2D identity - Alignment Gaps [PDB]:** 566
- **2D aligned content [PDB] (<2D-fold>:%):** {'.': 15.69, 'E': 29.71, 'T': 20.08, 'H': 33.89, 'G': 0.63}
- **3D similarity (TM-Score) (%) [PDB]:** 24.0

- **Gene name:** RASAL1
- **Entrez ID:** 8437
- **RefSeq ID:** N/A
- **Sequence length:** N/A
- **5-UTR|CDS|3-UTR identity (%):** N/A | N/A | N/A
- **5-UTR|CDS|3-UTR identity (%) [Gaps excluded]:** N/A | N/A | N/A
- **5-UTR|CDS|3-UTR identity [Alignment Gaps]:** N/A | N/A | N/A
- **5-UTR aligned content (<base>:%):** N/A
- **CDS aligned content (<base>:%):** N/A
- **3-UTR aligned content (<base>:%):** N/A

**Uniprot Description:**  
  
 Probable inhibitory regulator of the Ras-cyclic AMP pathway (PubMed:9751798). Plays a role in dendrite formation by melanocytes (PubMed:23999003). N/A   
  
 **Gene Ontology Information:**

Molecular Function

- GTPase activator activity
- metal ion binding
- phospholipid binding

Location

- cytosol

Biological process

- cell differentiation
- cellular response to calcium ion
- intracellular signal transduction
- negative regulation of Ras protein signal transduction
- positive regulation of dendrite extension
- regulation of GTPase activity
- signal transduction

---
