# Supplementary material for: Comparative structural insights and functional analysis for the distinct unbound states of Human AGO proteins
Source: Sci Rep. 2025 Mar 19;15:9432. doi: 10.1038/s41598-025-91849-5 (PMC11923369; doi:10.1038/s41598-025-91849-5)
Supplement: Supplementary file 24 — Supplementary Information 12. [file 41598_2025_91849_MOESM24_ESM.zip › 4Z4Dp_A_mdwhole_AF4REF/candidates/4Z4Dp_A-merged-h-enriched_report.html]

 

# Structural Comparison Report for 4Z4Dp\_A - whole structures (total: 100)

---

0

- **AF ID:** AF-Q9UL18-F1-model-v4 | **Chain:** A
- **b-phipsi:** 0.0003066883135033
- **w-rdist:** 0.2414011170953403
- **t-alpha:** 0.0338857136971153

---

---

1

- **AF ID:** AF-Q9H9G7-F1-model-v4 | **Chain:** A
- **b-phipsi:** 0.0002325967977732
- **w-rdist:** 0.2272140051911793
- **t-alpha:** 0.0433130906697325

---

---

2

- **AF ID:** AF-Q16706-F1-model-v4 | **Chain:** A
- **b-phipsi:** 0.0005764987810473
- **w-rdist:** 0.2866969025230809
- **t-alpha:** 0.0466131781632361

---

---

3

- **AF ID:** AF-Q8TF42-F1-model-v4 | **Chain:** A
- **b-phipsi:** 0.0004591038273828
- **w-rdist:** 0.3530341349448036
- **t-alpha:** 0.0487983623453289

---

---

4

- **AF ID:** AF-Q5T447-F1-model-v4 | **Chain:** A
- **b-phipsi:** 0.0020124814544824
- **w-rdist:** 0.1250475819111011
- **t-alpha:** 0.0131099992438439

---

---

5

- **AF ID:** AF-Q7Z3Z4-F1-model-v4 | **Chain:** A
- **b-phipsi:** 0.0010308215497335
- **w-rdist:** 0.2602270777763357
- **t-alpha:** 0.0238627234462787

---

---

6

- **AF ID:** AF-Q15349-F1-model-v4 | **Chain:** A
- **b-phipsi:** 0.0031808232338456
- **w-rdist:** 0.4826721997624241
- **t-alpha:** 0.0007283954918235

---

---

7

- **AF ID:** AF-P40123-F1-model-v4 | **Chain:** A
- **b-phipsi:** 0.0042100057616756
- **w-rdist:** 0.3365787471877647
- **t-alpha:** 0.0036549441753579

---

---

8

- **AF ID:** AF-Q01518-F1-model-v4 | **Chain:** A
- **b-phipsi:** 0.0019179877394586
- **w-rdist:** 0.232862702747337
- **t-alpha:** 0.0147818972725322

---

---

9

- **AF ID:** AF-Q6ZN11-F1-model-v4 | **Chain:** A
- **b-phipsi:** 0.010119332571359
- **w-rdist:** 0.336461673011481
- **t-alpha:** 0.0007288726764216

---

---

10

- **AF ID:** AF-Q9Y2X0-F1-model-v4 | **Chain:** A
- **b-phipsi:** 0.0006540061423279
- **w-rdist:** 0.3409170187502385
- **t-alpha:** 0.0504970964456714

---

---

11

- **AF ID:** AF-O75366-F1-model-v4 | **Chain:** A
- **b-phipsi:** 0.0018895590721252
- **w-rdist:** 0.6103435241245627
- **t-alpha:** 0.0014568440116902

---

---

12

- **AF ID:** AF-Q9UL01-F1-model-v4 | **Chain:** A
- **b-phipsi:** 0.0063405358476029
- **w-rdist:** 0.3143093020434866
- **t-alpha:** 0.004389354417605

---

---

13

- **AF ID:** AF-O14841-F1-model-v4 | **Chain:** A
- **b-phipsi:** 0.0022705853567763
- **w-rdist:** 0.3169074966484093
- **t-alpha:** 0.0072833804367331

---

---

14

- **AF ID:** AF-Q7Z7A4-F1-model-v4 | **Chain:** A
- **b-phipsi:** 0.0024869909773614
- **w-rdist:** 0.5892044925053923
- **t-alpha:** 0.0014568440116902

---

---

15

- **AF ID:** AF-Q3KNW1-F1-model-v4 | **Chain:** A
- **b-phipsi:** 0.0068505572928429
- **w-rdist:** 0.3710780999405203
- **t-alpha:** 0.0021848670241468

---

---

16

- **AF ID:** AF-Q96RQ3-F1-model-v4 | **Chain:** A
- **b-phipsi:** 0.0074347470562844
- **w-rdist:** 0.4424766538404435
- **t-alpha:** 0.0

---

---

17

- **AF ID:** AF-Q9HAU4-F1-model-v4 | **Chain:** A
- **b-phipsi:** 0.0025582991345086
- **w-rdist:** 0.1905597379585445
- **t-alpha:** 0.0429718680332931

---

---

18

- **AF ID:** AF-Q8NC26-F1-model-v4 | **Chain:** A
- **b-phipsi:** 0.000356967053683
- **w-rdist:** 0.2795029796716288
- **t-alpha:** 0.2367084387255091

---

---

19

- **AF ID:** AF-O00425-F1-model-v4 | **Chain:** A
- **b-phipsi:** 0.004126510224361
- **w-rdist:** 0.2475192168276953
- **t-alpha:** 0.0131099992438439

---

---

20

- **AF ID:** AF-O75676-F1-model-v4 | **Chain:** A
- **b-phipsi:** 0.0055462909778492
- **w-rdist:** 0.2879867832640781
- **t-alpha:** 0.0065550134155867

---

---

21

- **AF ID:** AF-Q9NZ08-F1-model-v4 | **Chain:** A
- **b-phipsi:** 0.0057167306902904
- **w-rdist:** 0.4471017878761965
- **t-alpha:** 0.0014587541408541

---

---

22

- **AF ID:** AF-Q9H091-F1-model-v4 | **Chain:** A
- **b-phipsi:** 0.0006017139735541
- **w-rdist:** 0.7112768607270422
- **t-alpha:** 0.0065550134155867

---

---

23

- **AF ID:** AF-Q460N3-F1-model-v4 | **Chain:** A
- **b-phipsi:** 0.0043972934414173
- **w-rdist:** 0.2148140264615601
- **t-alpha:** 0.0262200978972946

---

---

24

- **AF ID:** AF-O75879-F1-model-v4 | **Chain:** A
- **b-phipsi:** 0.0006677332420321
- **w-rdist:** 0.4302073861233585
- **t-alpha:** 0.036416391876046

---

---

25

- **AF ID:** AF-Q9HA65-F1-model-v4 | **Chain:** A
- **b-phipsi:** 0.0054616787489352
- **w-rdist:** 0.4302324415165067
- **t-alpha:** 0.0021896458253802

---

---

26

- **AF ID:** AF-Q96JY0-F1-model-v4 | **Chain:** A
- **b-phipsi:** 0.0078197713547741
- **w-rdist:** 0.4469270525686398
- **t-alpha:** 0.0007288726764216

---

---

27

- **AF ID:** AF-Q6ZTN6-F1-model-v4 | **Chain:** A
- **b-phipsi:** 0.0011798042362088
- **w-rdist:** 0.7003385349343969
- **t-alpha:** 0.0021896458253802

---

---

28

- **AF ID:** AF-Q68DD2-F1-model-v4 | **Chain:** A
- **b-phipsi:** 0.0010669735979758
- **w-rdist:** 0.283534365650539
- **t-alpha:** 0.0597231737029493

---

---

29

- **AF ID:** AF-Q96J94-F1-model-v4 | **Chain:** A
- **b-phipsi:** 0.0042312013965369
- **w-rdist:** 0.161129481858615
- **t-alpha:** 0.0433130906697325

---

---

30

- **AF ID:** AF-Q9UL63-F1-model-v4 | **Chain:** A
- **b-phipsi:** 0.002588793135836
- **w-rdist:** 0.220002540272522
- **t-alpha:** 0.0594134606378968

---

---

31

- **AF ID:** AF-O43300-F1-model-v4 | **Chain:** A
- **b-phipsi:** 0.0033656760644481
- **w-rdist:** 0.7353118763821469
- **t-alpha:** 0.0007288726764216

---

---

32

- **AF ID:** AF-Q3V5L5-F1-model-v4 | **Chain:** A
- **b-phipsi:** 0.0065632410055385
- **w-rdist:** 0.1784341883601119
- **t-alpha:** 0.0218498381101377

---

---

33

- **AF ID:** AF-Q9Y2L1-F1-model-v4 | **Chain:** A
- **b-phipsi:** 0.0028120019946062
- **w-rdist:** 0.3429913086807502
- **t-alpha:** 0.011653569134763

---

---

34

- **AF ID:** AF-Q8N806-F1-model-v4 | **Chain:** A
- **b-phipsi:** 0.0028946051857684
- **w-rdist:** 0.3486717173004471
- **t-alpha:** 0.0080762040238695

---

---

35

- **AF ID:** AF-O43143-F1-model-v4 | **Chain:** A
- **b-phipsi:** 0.0070687491050394
- **w-rdist:** 0.3288788143183344
- **t-alpha:** 0.0058607467412064

---

---

36

- **AF ID:** AF-Q15937-F1-model-v4 | **Chain:** A
- **b-phipsi:** 0.005700844600732
- **w-rdist:** 0.6467686143441221
- **t-alpha:** 0.0007288726764216

---

---

37

- **AF ID:** AF-O15033-F1-model-v4 | **Chain:** A
- **b-phipsi:** 0.0001790186060663
- **w-rdist:** 0.6607314685895532
- **t-alpha:** 0.0569667321740288

---

---

38

- **AF ID:** AF-Q9BW92-F1-model-v4 | **Chain:** A
- **b-phipsi:** 0.0038171275749441
- **w-rdist:** 0.4778945967650733
- **t-alpha:** 0.0043701973936818

---

---

39

- **AF ID:** AF-Q8NI99-F1-model-v4 | **Chain:** A
- **b-phipsi:** 0.0049043390449329
- **w-rdist:** 0.5801165434274829
- **t-alpha:** 0.0021896458253802

---

---

40

- **AF ID:** AF-P19525-F1-model-v4 | **Chain:** A
- **b-phipsi:** 0.0050308277013414
- **w-rdist:** 0.4843364547647412
- **t-alpha:** 0.0029220273949486

---

---

41

- **AF ID:** AF-A8K7I4-F1-model-v4 | **Chain:** A
- **b-phipsi:** 0.0092153687135678
- **w-rdist:** 0.3443020268967584
- **t-alpha:** 0.0050984212340619

---

---

42

- **AF ID:** AF-Q9BZQ2-F1-model-v4 | **Chain:** A
- **b-phipsi:** 0.0007562863597093
- **w-rdist:** 0.6112300151029483
- **t-alpha:** 0.011653569134763

---

---

43

- **AF ID:** AF-Q86UX7-F1-model-v4 | **Chain:** A
- **b-phipsi:** 0.0005937517805737
- **w-rdist:** 0.6000422467498301
- **t-alpha:** 0.0385778104072074

---

---

44

- **AF ID:** AF-Q9N2K0-F1-model-v4 | **Chain:** A
- **b-phipsi:** 0.0005356855461661
- **w-rdist:** 0.7275527246016726
- **t-alpha:** 0.0223381852496014

---

---

45

- **AF ID:** AF-Q86TM3-F1-model-v4 | **Chain:** A
- **b-phipsi:** 0.0022229814935704
- **w-rdist:** 0.3168191759715351
- **t-alpha:** 0.0496939342883562

---

---

46

- **AF ID:** AF-Q8N3E9-F1-model-v4 | **Chain:** A
- **b-phipsi:** 0.0006990186097381
- **w-rdist:** 0.4610178658390951
- **t-alpha:** 0.0480914507615701

---

---

47

- **AF ID:** AF-Q9Y6W3-F1-model-v4 | **Chain:** A
- **b-phipsi:** 0.0003718395327692
- **w-rdist:** 0.5366244752050346
- **t-alpha:** 0.0853753468743623

---

---

48

- **AF ID:** AF-Q9H4B4-F1-model-v4 | **Chain:** A
- **b-phipsi:** 0.0055116885652779
- **w-rdist:** 0.2497847911477329
- **t-alpha:** 0.0393298454728356

---

---

49

- **AF ID:** AF-Q86XP0-F1-model-v4 | **Chain:** A
- **b-phipsi:** 0.0024402545807624
- **w-rdist:** 0.3670877089562501
- **t-alpha:** 0.0152949457801805

---

---

50

- **AF ID:** AF-Q16394-F1-model-v4 | **Chain:** A
- **b-phipsi:** 0.0030875247654264
- **w-rdist:** 0.3224269055254203
- **t-alpha:** 0.0441063918932529

---

---

51

- **AF ID:** AF-Q5T2T1-F1-model-v4 | **Chain:** A
- **b-phipsi:** 0.0006668754822545
- **w-rdist:** 0.7541804982952017
- **t-alpha:** 0.0073366962737499

---

---

52

- **AF ID:** AF-Q04759-F1-model-v4 | **Chain:** A
- **b-phipsi:** 0.0038502741964588
- **w-rdist:** 0.3432908775635467
- **t-alpha:** 0.0240350781259224

---

---

53

- **AF ID:** AF-Q86VW2-F1-model-v4 | **Chain:** A
- **b-phipsi:** 0.0109622102440941
- **w-rdist:** 0.3804506943663119
- **t-alpha:** 0.0029134192830806

---

---

54

- **AF ID:** AF-Q8NHY0-F1-model-v4 | **Chain:** A
- **b-phipsi:** 0.0008155775641725
- **w-rdist:** 0.594488439764107
- **t-alpha:** 0.0254917294284022

---

---

55

- **AF ID:** AF-Q9NTJ4-F1-model-v4 | **Chain:** A
- **b-phipsi:** 0.0040094948776726
- **w-rdist:** 0.3498729971703247
- **t-alpha:** 0.0177907956813867

---

---

56

- **AF ID:** AF-O43374-F1-model-v4 | **Chain:** A
- **b-phipsi:** 0.0015941302092716
- **w-rdist:** 0.281857134440261
- **t-alpha:** 0.0992795788492999

---

---

57

- **AF ID:** AF-O15067-F1-model-v4 | **Chain:** A
- **b-phipsi:** 0.0020240751194128
- **w-rdist:** 0.3481920953462453
- **t-alpha:** 0.0502551818524676

---

---

58

- **AF ID:** AF-Q96K75-F1-model-v4 | **Chain:** A
- **b-phipsi:** 0.0134297143720729
- **w-rdist:** 0.357478014322832
- **t-alpha:** 0.0036415440256147

---

---

59

- **AF ID:** AF-Q9UIF7-F1-model-v4 | **Chain:** A
- **b-phipsi:** 0.0077515402109007
- **w-rdist:** 0.3142194774777452
- **t-alpha:** 0.0087397720309874

---

---

60

- **AF ID:** AF-Q8N653-F1-model-v4 | **Chain:** A
- **b-phipsi:** 0.0029837745014629
- **w-rdist:** 0.8785268036146492
- **t-alpha:** 0.0014568440116902

---

---

61

- **AF ID:** AF-Q9BXB7-F1-model-v4 | **Chain:** A
- **b-phipsi:** 0.0105049420779871
- **w-rdist:** 0.5195828102537532
- **t-alpha:** 0.0014587541408541

---

---

62

- **AF ID:** AF-P49641-F1-model-v4 | **Chain:** A
- **b-phipsi:** 0.0008378236730446
- **w-rdist:** 0.3891243083991874
- **t-alpha:** 0.0619083860998908

---

---

63

- **AF ID:** AF-A6NFN9-F1-model-v4 | **Chain:** A
- **b-phipsi:** 0.0052218789764719
- **w-rdist:** 0.615837825135609
- **t-alpha:** 0.0036415440256147

---

---

64

- **AF ID:** AF-Q8IYK4-F1-model-v4 | **Chain:** A
- **b-phipsi:** 0.000126657664718
- **w-rdist:** 0.6837187327758599
- **t-alpha:** 0.0983998372896508

---

---

65

- **AF ID:** AF-Q9UHY1-F1-model-v4 | **Chain:** A
- **b-phipsi:** 0.0010469760437791
- **w-rdist:** 0.4131229866030809
- **t-alpha:** 0.0323309912234826

---

---

66

- **AF ID:** AF-P37173-F1-model-v4 | **Chain:** A
- **b-phipsi:** 0.0006678049967396
- **w-rdist:** 0.4981645968350738
- **t-alpha:** 0.0718186926297554

---

---

67

- **AF ID:** AF-Q9NZW5-F1-model-v4 | **Chain:** A
- **b-phipsi:** 0.0009935617215847
- **w-rdist:** 0.4491236973794088
- **t-alpha:** 0.0393640418205993

---

---

68

- **AF ID:** AF-Q3SY69-F1-model-v4 | **Chain:** A
- **b-phipsi:** 0.0039434315632253
- **w-rdist:** 0.8603669333884739
- **t-alpha:** 0.0014568440116902

---

---

69

- **AF ID:** AF-Q6ZQR2-F1-model-v4 | **Chain:** A
- **b-phipsi:** 0.0062560505946834
- **w-rdist:** 0.7417069042512422
- **t-alpha:** 0.0014587541408541

---

---

70

- **AF ID:** AF-Q9BYG8-F1-model-v4 | **Chain:** A
- **b-phipsi:** 0.0029950272427551
- **w-rdist:** 0.7832353614791551
- **t-alpha:** 0.0029220273949486

---

---

71

- **AF ID:** AF-O95294-F1-model-v4 | **Chain:** A
- **b-phipsi:** 0.0020597790941192
- **w-rdist:** 0.3071952770108285
- **t-alpha:** 0.0983998372896508

---

---

72

- **AF ID:** AF-Q9NXH9-F1-model-v4 | **Chain:** A
- **b-phipsi:** 0.0010043165317791
- **w-rdist:** 0.5232902773990337
- **t-alpha:** 0.0246270663199015

---

---

73

- **AF ID:** AF-Q9Y2E5-F1-model-v4 | **Chain:** A
- **b-phipsi:** 0.0008884216162606
- **w-rdist:** 0.4273546844619379
- **t-alpha:** 0.0618714226546988

---

---

74

- **AF ID:** AF-Q5JTZ5-F1-model-v4 | **Chain:** A
- **b-phipsi:** 0.0113891210624455
- **w-rdist:** 0.5359682123755116
- **t-alpha:** 0.0021848670241468

---

---

75

- **AF ID:** AF-Q96JB8-F1-model-v4 | **Chain:** A
- **b-phipsi:** 0.0032429518311675
- **w-rdist:** 0.9004176631043326
- **t-alpha:** 0.0014587541408541

---

---

76

- **AF ID:** AF-Q9H6W3-F1-model-v4 | **Chain:** A
- **b-phipsi:** 0.0028343760052904
- **w-rdist:** 0.3250819344203794
- **t-alpha:** 0.077931746040353

---

---

77

- **AF ID:** AF-Q6NUM6-F1-model-v4 | **Chain:** A
- **b-phipsi:** 0.0071696606896772
- **w-rdist:** 0.417714599733502
- **t-alpha:** 0.0058266934612722

---

---

78

- **AF ID:** AF-Q09013-F1-model-v4 | **Chain:** A
- **b-phipsi:** 0.00673114318516
- **w-rdist:** 0.3122734161616992
- **t-alpha:** 0.0458847982169747

---

---

79

- **AF ID:** AF-Q8NA19-F1-model-v4 | **Chain:** A
- **b-phipsi:** 0.0063791606537925
- **w-rdist:** 0.3592782377117982
- **t-alpha:** 0.0160233389937762

---

---

80

- **AF ID:** AF-O15259-F1-model-v4 | **Chain:** A
- **b-phipsi:** 0.0023579591307223
- **w-rdist:** 0.1720848408264949
- **t-alpha:** 0.1893660442320035

---

---

81

- **AF ID:** AF-A0A087X1G2-F1-model-v4 | **Chain:** A
- **b-phipsi:** 0.0080424083104011
- **w-rdist:** 0.5609837492119722
- **t-alpha:** 0.0036549441753579

---

---

82

- **AF ID:** AF-Q8IY47-F1-model-v4 | **Chain:** A
- **b-phipsi:** 0.0044407059730991
- **w-rdist:** 0.3372460929795613
- **t-alpha:** 0.0545316885212077

---

---

83

- **AF ID:** AF-Q9BQS7-F1-model-v4 | **Chain:** A
- **b-phipsi:** 0.0076970520360819
- **w-rdist:** 0.2692708188389689
- **t-alpha:** 0.0582668457273456

---

---

84

- **AF ID:** AF-P0C7X1-F1-model-v4 | **Chain:** A
- **b-phipsi:** 0.0094691812807584
- **w-rdist:** 0.50920055388851
- **t-alpha:** 0.0036549441753579

---

---

85

- **AF ID:** AF-C9J798-F1-model-v4 | **Chain:** A
- **b-phipsi:** 0.0029370363098615
- **w-rdist:** 0.3287344208525272
- **t-alpha:** 0.0896826187952655

---

---

86

- **AF ID:** AF-Q9UJX5-F1-model-v4 | **Chain:** A
- **b-phipsi:** 0.0018342075040473
- **w-rdist:** 0.6848330801471858
- **t-alpha:** 0.0065982109973949

---

---

87

- **AF ID:** AF-Q92562-F1-model-v4 | **Chain:** A
- **b-phipsi:** 0.0011704336034589
- **w-rdist:** 0.2993597449417604
- **t-alpha:** 0.1507652002007882

---

---

88

- **AF ID:** AF-Q658Y4-F1-model-v4 | **Chain:** A
- **b-phipsi:** 0.0076142851566125
- **w-rdist:** 0.1809325273307173
- **t-alpha:** 0.0786598128498576

---

---

89

- **AF ID:** AF-Q96NY9-F1-model-v4 | **Chain:** A
- **b-phipsi:** 0.0062497877206543
- **w-rdist:** 0.296695775513759
- **t-alpha:** 0.0651667209447612

---

---

90

- **AF ID:** AF-P35858-F1-model-v4 | **Chain:** A
- **b-phipsi:** 0.0131446652442243
- **w-rdist:** 0.6377010139207502
- **t-alpha:** 0.0014568440116902

---

---

91

- **AF ID:** AF-Q9UBT2-F1-model-v4 | **Chain:** A
- **b-phipsi:** 0.0030811237314507
- **w-rdist:** 0.3506266636387898
- **t-alpha:** 0.0699196593590019

---

---

92

- **AF ID:** AF-Q9BSQ5-F1-model-v4 | **Chain:** A
- **b-phipsi:** 0.0135926152283923
- **w-rdist:** 0.7109856222425024
- **t-alpha:** 0.0

---

---

93

- **AF ID:** AF-P50747-F1-model-v4 | **Chain:** A
- **b-phipsi:** 0.0064986879068654
- **w-rdist:** 0.3172814914589946
- **t-alpha:** 0.0560815732405199

---

---

94

- **AF ID:** AF-Q2NL67-F1-model-v4 | **Chain:** A
- **b-phipsi:** 0.000484484409048
- **w-rdist:** 0.7139439124022491
- **t-alpha:** 0.0975966865124746

---

---

95

- **AF ID:** AF-Q9H0J9-F1-model-v4 | **Chain:** A
- **b-phipsi:** 0.0004652080392326
- **w-rdist:** 0.4807038764037619
- **t-alpha:** 0.2012245600058226

---

---

96

- **AF ID:** AF-Q7Z4K8-F1-model-v4 | **Chain:** A
- **b-phipsi:** 0.000462315557246
- **w-rdist:** 1.4537677052906823
- **t-alpha:** 0.0036549441753579

---

---

97

- **AF ID:** AF-Q8NFF5-F1-model-v4 | **Chain:** A
- **b-phipsi:** 0.0024719891117185
- **w-rdist:** 0.2222047513206378
- **t-alpha:** 0.2053902107995835

---

---

98

- **AF ID:** AF-P29350-F1-model-v4 | **Chain:** A
- **b-phipsi:** 0.0042591278331811
- **w-rdist:** 0.5504215305057711
- **t-alpha:** 0.0065982109973949

---

---

99

- **AF ID:** AF-Q8IVL6-F1-model-v4 | **Chain:** A
- **b-phipsi:** 0.0139602974334313
- **w-rdist:** 0.2852311170796359
- **t-alpha:** 0.0182082568765367

---

---
