# Supplementary material for: Comparative structural insights and functional analysis for the distinct unbound states of Human AGO proteins
Source: Sci Rep. 2025 Mar 19;15:9432. doi: 10.1038/s41598-025-91849-5 (PMC11923369; doi:10.1038/s41598-025-91849-5)
Supplement: Supplementary file 24 — Supplementary Information 12. [file 41598_2025_91849_MOESM24_ESM.zip › 4Z4Dp_A_mdwhole_AF4REF/candidates/4Z4Dp_A-merged-notenriched_report.html]

 

# Structural Comparison Report for 4Z4Dp\_A - whole structures (total: 607)

---

1

- **AF ID:** AF-Q9UKV8-F1-model-v4 | **Chain:** A
- **b-phipsi:** 0.0001086267780307
- **w-rdist:** 0.2592605461549681
- **t-alpha:** 0.0155326816144203

---

---

2

- **AF ID:** AF-Q9UL18-F1-model-v4 | **Chain:** A
- **b-phipsi:** 0.0003066883135033
- **w-rdist:** 0.2414011170953403
- **t-alpha:** 0.0338857136971153

---

---

3

- **AF ID:** AF-Q9H9G7-F1-model-v4 | **Chain:** A
- **b-phipsi:** 0.0002325967977732
- **w-rdist:** 0.2272140051911793
- **t-alpha:** 0.0433130906697325

---

---

4

- **AF ID:** AF-Q9BQA5-F1-model-v4 | **Chain:** A
- **b-phipsi:** 0.0036085707396194
- **w-rdist:** 0.2436731661417707
- **t-alpha:** 0.0029134192830806

---

---

5

- **AF ID:** AF-Q9HCK5-F1-model-v4 | **Chain:** A
- **b-phipsi:** 0.0003211657497325
- **w-rdist:** 0.3246924841095752
- **t-alpha:** 0.0400581238241422

---

---

6

- **AF ID:** AF-Q16706-F1-model-v4 | **Chain:** A
- **b-phipsi:** 0.0005764987810473
- **w-rdist:** 0.2866969025230809
- **t-alpha:** 0.0466131781632361

---

---

7

- **AF ID:** AF-Q5T447-F1-model-v4 | **Chain:** A
- **b-phipsi:** 0.0020124814544824
- **w-rdist:** 0.1250475819111011
- **t-alpha:** 0.0131099992438439

---

---

8

- **AF ID:** AF-Q8TF42-F1-model-v4 | **Chain:** A
- **b-phipsi:** 0.0004591038273828
- **w-rdist:** 0.3530341349448036
- **t-alpha:** 0.0487983623453289

---

---

9

- **AF ID:** AF-Q9BQ95-F1-model-v4 | **Chain:** A
- **b-phipsi:** 0.0023386543279668
- **w-rdist:** 0.3124109051506253
- **t-alpha:** 0.0058607467412064

---

---

10

- **AF ID:** AF-Q7Z3Z4-F1-model-v4 | **Chain:** A
- **b-phipsi:** 0.0010308215497335
- **w-rdist:** 0.2602270777763357
- **t-alpha:** 0.0238627234462787

---

---

11

- **AF ID:** AF-Q15349-F1-model-v4 | **Chain:** A
- **b-phipsi:** 0.0031808232338456
- **w-rdist:** 0.4826721997624241
- **t-alpha:** 0.0007283954918235

---

---

12

- **AF ID:** AF-P40123-F1-model-v4 | **Chain:** A
- **b-phipsi:** 0.0042100057616756
- **w-rdist:** 0.3365787471877647
- **t-alpha:** 0.0036549441753579

---

---

13

- **AF ID:** AF-Q01518-F1-model-v4 | **Chain:** A
- **b-phipsi:** 0.0019179877394586
- **w-rdist:** 0.232862702747337
- **t-alpha:** 0.0147818972725322

---

---

14

- **AF ID:** AF-Q6ZN11-F1-model-v4 | **Chain:** A
- **b-phipsi:** 0.010119332571359
- **w-rdist:** 0.336461673011481
- **t-alpha:** 0.0007288726764216

---

---

15

- **AF ID:** AF-Q969S9-F1-model-v4 | **Chain:** A
- **b-phipsi:** 0.0042403497796089
- **w-rdist:** 0.3558217782507487
- **t-alpha:** 0.0029220273949486

---

---

16

- **AF ID:** AF-Q9Y2X0-F1-model-v4 | **Chain:** A
- **b-phipsi:** 0.0006540061423279
- **w-rdist:** 0.3409170187502385
- **t-alpha:** 0.0504970964456714

---

---

17

- **AF ID:** AF-O75366-F1-model-v4 | **Chain:** A
- **b-phipsi:** 0.0018895590721252
- **w-rdist:** 0.6103435241245627
- **t-alpha:** 0.0014568440116902

---

---

18

- **AF ID:** AF-Q96PZ0-F1-model-v4 | **Chain:** A
- **b-phipsi:** 0.0001858762358365
- **w-rdist:** 0.4191341047581966
- **t-alpha:** 0.0502551818524676

---

---

19

- **AF ID:** AF-Q9UL01-F1-model-v4 | **Chain:** A
- **b-phipsi:** 0.0063405358476029
- **w-rdist:** 0.3143093020434866
- **t-alpha:** 0.004389354417605

---

---

20

- **AF ID:** AF-O14841-F1-model-v4 | **Chain:** A
- **b-phipsi:** 0.0022705853567763
- **w-rdist:** 0.3169074966484093
- **t-alpha:** 0.0072833804367331

---

---

21

- **AF ID:** AF-Q7Z7A4-F1-model-v4 | **Chain:** A
- **b-phipsi:** 0.0024869909773614
- **w-rdist:** 0.5892044925053923
- **t-alpha:** 0.0014568440116902

---

---

22

- **AF ID:** AF-Q9UBK8-F1-model-v4 | **Chain:** A
- **b-phipsi:** 0.0039205102940396
- **w-rdist:** 0.4525836838142585
- **t-alpha:** 0.0014587541408541

---

---

23

- **AF ID:** AF-Q9UJT9-F1-model-v4 | **Chain:** A
- **b-phipsi:** 0.0024712597230505
- **w-rdist:** 0.5103150504933838
- **t-alpha:** 0.0021896458253802

---

---

24

- **AF ID:** AF-Q9NZI8-F1-model-v4 | **Chain:** A
- **b-phipsi:** 0.0033699174017986
- **w-rdist:** 0.2326820128783328
- **t-alpha:** 0.0196648853517085

---

---

25

- **AF ID:** AF-P34931-F1-model-v4 | **Chain:** A
- **b-phipsi:** 0.0038482057661098
- **w-rdist:** 0.6465642499995109
- **t-alpha:** 0.0

---

---

26

- **AF ID:** AF-Q3KNW1-F1-model-v4 | **Chain:** A
- **b-phipsi:** 0.0068505572928429
- **w-rdist:** 0.3710780999405203
- **t-alpha:** 0.0021848670241468

---

---

27

- **AF ID:** AF-Q96RQ3-F1-model-v4 | **Chain:** A
- **b-phipsi:** 0.0074347470562844
- **w-rdist:** 0.4424766538404435
- **t-alpha:** 0.0

---

---

28

- **AF ID:** AF-Q9HAU4-F1-model-v4 | **Chain:** A
- **b-phipsi:** 0.0025582991345086
- **w-rdist:** 0.1905597379585445
- **t-alpha:** 0.0429718680332931

---

---

29

- **AF ID:** AF-Q8NC26-F1-model-v4 | **Chain:** A
- **b-phipsi:** 0.000356967053683
- **w-rdist:** 0.2795029796716288
- **t-alpha:** 0.2367084387255091

---

---

30

- **AF ID:** AF-O00425-F1-model-v4 | **Chain:** A
- **b-phipsi:** 0.004126510224361
- **w-rdist:** 0.2475192168276953
- **t-alpha:** 0.0131099992438439

---

---

31

- **AF ID:** AF-O75676-F1-model-v4 | **Chain:** A
- **b-phipsi:** 0.0055462909778492
- **w-rdist:** 0.2879867832640781
- **t-alpha:** 0.0065550134155867

---

---

32

- **AF ID:** AF-Q9BQ52-F1-model-v4 | **Chain:** A
- **b-phipsi:** 0.0005140182652912
- **w-rdist:** 0.3290255873397046
- **t-alpha:** 0.1347419169217441

---

---

33

- **AF ID:** AF-Q96Q07-F1-model-v4 | **Chain:** A
- **b-phipsi:** 0.0051682106218869
- **w-rdist:** 0.5969901155099623
- **t-alpha:** 0.0

---

---

34

- **AF ID:** AF-P51178-F1-model-v4 | **Chain:** A
- **b-phipsi:** 0.0004648621423469
- **w-rdist:** 0.6664643217119542
- **t-alpha:** 0.0140326350426887

---

---

35

- **AF ID:** AF-Q9NZ08-F1-model-v4 | **Chain:** A
- **b-phipsi:** 0.0057167306902904
- **w-rdist:** 0.4471017878761965
- **t-alpha:** 0.0014587541408541

---

---

36

- **AF ID:** AF-Q9H091-F1-model-v4 | **Chain:** A
- **b-phipsi:** 0.0006017139735541
- **w-rdist:** 0.7112768607270422
- **t-alpha:** 0.0065550134155867

---

---

37

- **AF ID:** AF-Q460N3-F1-model-v4 | **Chain:** A
- **b-phipsi:** 0.0043972934414173
- **w-rdist:** 0.2148140264615601
- **t-alpha:** 0.0262200978972946

---

---

38

- **AF ID:** AF-O75879-F1-model-v4 | **Chain:** A
- **b-phipsi:** 0.0006677332420321
- **w-rdist:** 0.4302073861233585
- **t-alpha:** 0.036416391876046

---

---

39

- **AF ID:** AF-Q9HA65-F1-model-v4 | **Chain:** A
- **b-phipsi:** 0.0054616787489352
- **w-rdist:** 0.4302324415165067
- **t-alpha:** 0.0021896458253802

---

---

40

- **AF ID:** AF-Q96JY0-F1-model-v4 | **Chain:** A
- **b-phipsi:** 0.0078197713547741
- **w-rdist:** 0.4469270525686398
- **t-alpha:** 0.0007288726764216

---

---

41

- **AF ID:** AF-Q6ZTN6-F1-model-v4 | **Chain:** A
- **b-phipsi:** 0.0011798042362088
- **w-rdist:** 0.7003385349343969
- **t-alpha:** 0.0021896458253802

---

---

42

- **AF ID:** AF-P26639-F1-model-v4 | **Chain:** A
- **b-phipsi:** 0.0039036435431503
- **w-rdist:** 0.6812127095093964
- **t-alpha:** 0.0007288726764216

---

---

43

- **AF ID:** AF-Q68DD2-F1-model-v4 | **Chain:** A
- **b-phipsi:** 0.0010669735979758
- **w-rdist:** 0.283534365650539
- **t-alpha:** 0.0597231737029493

---

---

44

- **AF ID:** AF-Q96J94-F1-model-v4 | **Chain:** A
- **b-phipsi:** 0.0042312013965369
- **w-rdist:** 0.161129481858615
- **t-alpha:** 0.0433130906697325

---

---

45

- **AF ID:** AF-Q9UL63-F1-model-v4 | **Chain:** A
- **b-phipsi:** 0.002588793135836
- **w-rdist:** 0.220002540272522
- **t-alpha:** 0.0594134606378968

---

---

46

- **AF ID:** AF-O43300-F1-model-v4 | **Chain:** A
- **b-phipsi:** 0.0033656760644481
- **w-rdist:** 0.7353118763821469
- **t-alpha:** 0.0007288726764216

---

---

47

- **AF ID:** AF-Q3V5L5-F1-model-v4 | **Chain:** A
- **b-phipsi:** 0.0065632410055385
- **w-rdist:** 0.1784341883601119
- **t-alpha:** 0.0218498381101377

---

---

48

- **AF ID:** AF-Q9Y2L1-F1-model-v4 | **Chain:** A
- **b-phipsi:** 0.0028120019946062
- **w-rdist:** 0.3429913086807502
- **t-alpha:** 0.011653569134763

---

---

49

- **AF ID:** AF-Q8N806-F1-model-v4 | **Chain:** A
- **b-phipsi:** 0.0028946051857684
- **w-rdist:** 0.3486717173004471
- **t-alpha:** 0.0080762040238695

---

---

50

- **AF ID:** AF-O43143-F1-model-v4 | **Chain:** A
- **b-phipsi:** 0.0070687491050394
- **w-rdist:** 0.3288788143183344
- **t-alpha:** 0.0058607467412064

---

---

51

- **AF ID:** AF-Q15937-F1-model-v4 | **Chain:** A
- **b-phipsi:** 0.005700844600732
- **w-rdist:** 0.6467686143441221
- **t-alpha:** 0.0007288726764216

---

---

52

- **AF ID:** AF-Q8NC60-F1-model-v4 | **Chain:** A
- **b-phipsi:** 0.0038158934084664
- **w-rdist:** 0.2935365331803353
- **t-alpha:** 0.036416391876046

---

---

53

- **AF ID:** AF-O15033-F1-model-v4 | **Chain:** A
- **b-phipsi:** 0.0001790186060663
- **w-rdist:** 0.6607314685895532
- **t-alpha:** 0.0569667321740288

---

---

54

- **AF ID:** AF-Q9BW92-F1-model-v4 | **Chain:** A
- **b-phipsi:** 0.0038171275749441
- **w-rdist:** 0.4778945967650733
- **t-alpha:** 0.0043701973936818

---

---

55

- **AF ID:** AF-Q6PI48-F1-model-v4 | **Chain:** A
- **b-phipsi:** 0.0008214877266426
- **w-rdist:** 0.4981159655023905
- **t-alpha:** 0.0167517699463162

---

---

56

- **AF ID:** AF-Q8NI99-F1-model-v4 | **Chain:** A
- **b-phipsi:** 0.0049043390449329
- **w-rdist:** 0.5801165434274829
- **t-alpha:** 0.0021896458253802

---

---

57

- **AF ID:** AF-P19525-F1-model-v4 | **Chain:** A
- **b-phipsi:** 0.0050308277013414
- **w-rdist:** 0.4843364547647412
- **t-alpha:** 0.0029220273949486

---

---

58

- **AF ID:** AF-A8K7I4-F1-model-v4 | **Chain:** A
- **b-phipsi:** 0.0092153687135678
- **w-rdist:** 0.3443020268967584
- **t-alpha:** 0.0050984212340619

---

---

59

- **AF ID:** AF-Q9BZQ2-F1-model-v4 | **Chain:** A
- **b-phipsi:** 0.0007562863597093
- **w-rdist:** 0.6112300151029483
- **t-alpha:** 0.011653569134763

---

---

60

- **AF ID:** AF-Q06124-F1-model-v4 | **Chain:** A
- **b-phipsi:** 0.0014725198006469
- **w-rdist:** 0.6792013901033762
- **t-alpha:** 0.0036549441753579

---

---

61

- **AF ID:** AF-Q86UX7-F1-model-v4 | **Chain:** A
- **b-phipsi:** 0.0005937517805737
- **w-rdist:** 0.6000422467498301
- **t-alpha:** 0.0385778104072074

---

---

62

- **AF ID:** AF-Q96IC2-F1-model-v4 | **Chain:** A
- **b-phipsi:** 0.0011488324023699
- **w-rdist:** 0.347294735122389
- **t-alpha:** 0.0401513644150786

---

---

63

- **AF ID:** AF-Q9N2K0-F1-model-v4 | **Chain:** A
- **b-phipsi:** 0.0005356855461661
- **w-rdist:** 0.7275527246016726
- **t-alpha:** 0.0223381852496014

---

---

64

- **AF ID:** AF-Q9NXP7-F1-model-v4 | **Chain:** A
- **b-phipsi:** 0.0009282682336035
- **w-rdist:** 0.7580636669368799
- **t-alpha:** 0.0050984212340619

---

---

65

- **AF ID:** AF-Q86TM3-F1-model-v4 | **Chain:** A
- **b-phipsi:** 0.0022229814935704
- **w-rdist:** 0.3168191759715351
- **t-alpha:** 0.0496939342883562

---

---

66

- **AF ID:** AF-Q9H4B4-F1-model-v4 | **Chain:** A
- **b-phipsi:** 0.0055116885652779
- **w-rdist:** 0.2497847911477329
- **t-alpha:** 0.0393298454728356

---

---

67

- **AF ID:** AF-Q8N3E9-F1-model-v4 | **Chain:** A
- **b-phipsi:** 0.0006990186097381
- **w-rdist:** 0.4610178658390951
- **t-alpha:** 0.0480914507615701

---

---

68

- **AF ID:** AF-Q9Y6W3-F1-model-v4 | **Chain:** A
- **b-phipsi:** 0.0003718395327692
- **w-rdist:** 0.5366244752050346
- **t-alpha:** 0.0853753468743623

---

---

69

- **AF ID:** AF-Q9BXC9-F1-model-v4 | **Chain:** A
- **b-phipsi:** 0.005201582138507
- **w-rdist:** 0.2877726865368891
- **t-alpha:** 0.0307805727451215

---

---

70

- **AF ID:** AF-P47989-F1-model-v4 | **Chain:** A
- **b-phipsi:** 0.0022801674041496
- **w-rdist:** 0.2820338587600785
- **t-alpha:** 0.0706484408514722

---

---

71

- **AF ID:** AF-Q13724-F1-model-v4 | **Chain:** A
- **b-phipsi:** 0.0058577936741673
- **w-rdist:** 0.550983490103152
- **t-alpha:** 0.0029134192830806

---

---

72

- **AF ID:** AF-Q86XP0-F1-model-v4 | **Chain:** A
- **b-phipsi:** 0.0024402545807624
- **w-rdist:** 0.3670877089562501
- **t-alpha:** 0.0152949457801805

---

---

73

- **AF ID:** AF-O14638-F1-model-v4 | **Chain:** A
- **b-phipsi:** 0.0006726220772263
- **w-rdist:** 0.4713324155635693
- **t-alpha:** 0.0582668457273456

---

---

74

- **AF ID:** AF-Q16394-F1-model-v4 | **Chain:** A
- **b-phipsi:** 0.0030875247654264
- **w-rdist:** 0.3224269055254203
- **t-alpha:** 0.0441063918932529

---

---

75

- **AF ID:** AF-Q5T2T1-F1-model-v4 | **Chain:** A
- **b-phipsi:** 0.0006668754822545
- **w-rdist:** 0.7541804982952017
- **t-alpha:** 0.0073366962737499

---

---

76

- **AF ID:** AF-Q04759-F1-model-v4 | **Chain:** A
- **b-phipsi:** 0.0038502741964588
- **w-rdist:** 0.3432908775635467
- **t-alpha:** 0.0240350781259224

---

---

77

- **AF ID:** AF-Q86VW2-F1-model-v4 | **Chain:** A
- **b-phipsi:** 0.0109622102440941
- **w-rdist:** 0.3804506943663119
- **t-alpha:** 0.0029134192830806

---

---

78

- **AF ID:** AF-Q8NHY0-F1-model-v4 | **Chain:** A
- **b-phipsi:** 0.0008155775641725
- **w-rdist:** 0.594488439764107
- **t-alpha:** 0.0254917294284022

---

---

79

- **AF ID:** AF-Q9NTJ4-F1-model-v4 | **Chain:** A
- **b-phipsi:** 0.0040094948776726
- **w-rdist:** 0.3498729971703247
- **t-alpha:** 0.0177907956813867

---

---

80

- **AF ID:** AF-Q5SY16-F1-model-v4 | **Chain:** A
- **b-phipsi:** 0.0027391727755097
- **w-rdist:** 0.3419605918737526
- **t-alpha:** 0.0451564268098936

---

---

81

- **AF ID:** AF-O43374-F1-model-v4 | **Chain:** A
- **b-phipsi:** 0.0015941302092716
- **w-rdist:** 0.281857134440261
- **t-alpha:** 0.0992795788492999

---

---

82

- **AF ID:** AF-Q14CN2-F1-model-v4 | **Chain:** A
- **b-phipsi:** 0.0067515005016211
- **w-rdist:** 0.3025317400716028
- **t-alpha:** 0.0247633892798655

---

---

83

- **AF ID:** AF-O15067-F1-model-v4 | **Chain:** A
- **b-phipsi:** 0.0020240751194128
- **w-rdist:** 0.3481920953462453
- **t-alpha:** 0.0502551818524676

---

---

84

- **AF ID:** AF-Q96K75-F1-model-v4 | **Chain:** A
- **b-phipsi:** 0.0134297143720729
- **w-rdist:** 0.357478014322832
- **t-alpha:** 0.0036415440256147

---

---

85

- **AF ID:** AF-Q9UIF7-F1-model-v4 | **Chain:** A
- **b-phipsi:** 0.0077515402109007
- **w-rdist:** 0.3142194774777452
- **t-alpha:** 0.0087397720309874

---

---

86

- **AF ID:** AF-Q8N653-F1-model-v4 | **Chain:** A
- **b-phipsi:** 0.0029837745014629
- **w-rdist:** 0.8785268036146492
- **t-alpha:** 0.0014568440116902

---

---

87

- **AF ID:** AF-Q8NAM6-F1-model-v4 | **Chain:** A
- **b-phipsi:** 0.0124778807331227
- **w-rdist:** 0.2806169138594115
- **t-alpha:** 0.0065982109973949

---

---

88

- **AF ID:** AF-Q9BXB7-F1-model-v4 | **Chain:** A
- **b-phipsi:** 0.0105049420779871
- **w-rdist:** 0.5195828102537532
- **t-alpha:** 0.0014587541408541

---

---

89

- **AF ID:** AF-P49641-F1-model-v4 | **Chain:** A
- **b-phipsi:** 0.0008378236730446
- **w-rdist:** 0.3891243083991874
- **t-alpha:** 0.0619083860998908

---

---

90

- **AF ID:** AF-Q9BTE3-F1-model-v4 | **Chain:** A
- **b-phipsi:** 0.0007687198393503
- **w-rdist:** 0.5746857737484894
- **t-alpha:** 0.0466131781632361

---

---

91

- **AF ID:** AF-A6NFN9-F1-model-v4 | **Chain:** A
- **b-phipsi:** 0.0052218789764719
- **w-rdist:** 0.615837825135609
- **t-alpha:** 0.0036415440256147

---

---

92

- **AF ID:** AF-Q8IYK4-F1-model-v4 | **Chain:** A
- **b-phipsi:** 0.000126657664718
- **w-rdist:** 0.6837187327758599
- **t-alpha:** 0.0983998372896508

---

---

93

- **AF ID:** AF-Q9UHY1-F1-model-v4 | **Chain:** A
- **b-phipsi:** 0.0010469760437791
- **w-rdist:** 0.4131229866030809
- **t-alpha:** 0.0323309912234826

---

---

94

- **AF ID:** AF-P37173-F1-model-v4 | **Chain:** A
- **b-phipsi:** 0.0006678049967396
- **w-rdist:** 0.4981645968350738
- **t-alpha:** 0.0718186926297554

---

---

95

- **AF ID:** AF-Q3SY69-F1-model-v4 | **Chain:** A
- **b-phipsi:** 0.0039434315632253
- **w-rdist:** 0.8603669333884739
- **t-alpha:** 0.0014568440116902

---

---

96

- **AF ID:** AF-Q9NZW5-F1-model-v4 | **Chain:** A
- **b-phipsi:** 0.0009935617215847
- **w-rdist:** 0.4491236973794088
- **t-alpha:** 0.0393640418205993

---

---

97

- **AF ID:** AF-Q9H4Z3-F1-model-v4 | **Chain:** A
- **b-phipsi:** 0.0043897278722179
- **w-rdist:** 0.4824532503792904
- **t-alpha:** 0.0058266934612722

---

---

98

- **AF ID:** AF-P48443-F1-model-v4 | **Chain:** A
- **b-phipsi:** 0.0077681238914425
- **w-rdist:** 0.4628940679633859
- **t-alpha:** 0.0036549441753579

---

---

99

- **AF ID:** AF-Q6ZQR2-F1-model-v4 | **Chain:** A
- **b-phipsi:** 0.0062560505946834
- **w-rdist:** 0.7417069042512422
- **t-alpha:** 0.0014587541408541

---

---

100

- **AF ID:** AF-Q9BYG8-F1-model-v4 | **Chain:** A
- **b-phipsi:** 0.0029950272427551
- **w-rdist:** 0.7832353614791551
- **t-alpha:** 0.0029220273949486

---

---

101

- **AF ID:** AF-O95294-F1-model-v4 | **Chain:** A
- **b-phipsi:** 0.0020597790941192
- **w-rdist:** 0.3071952770108285
- **t-alpha:** 0.0983998372896508

---

---

102

- **AF ID:** AF-Q9NXH9-F1-model-v4 | **Chain:** A
- **b-phipsi:** 0.0010043165317791
- **w-rdist:** 0.5232902773990337
- **t-alpha:** 0.0246270663199015

---

---

103

- **AF ID:** AF-Q9Y2E5-F1-model-v4 | **Chain:** A
- **b-phipsi:** 0.0008884216162606
- **w-rdist:** 0.4273546844619379
- **t-alpha:** 0.0618714226546988

---

---

104

- **AF ID:** AF-Q53H47-F1-model-v4 | **Chain:** A
- **b-phipsi:** 0.0004628941738695
- **w-rdist:** 0.8612814372511362
- **t-alpha:** 0.0370091093109781

---

---

105

- **AF ID:** AF-Q5JTZ5-F1-model-v4 | **Chain:** A
- **b-phipsi:** 0.0113891210624455
- **w-rdist:** 0.5359682123755116
- **t-alpha:** 0.0021848670241468

---

---

106

- **AF ID:** AF-Q96JB8-F1-model-v4 | **Chain:** A
- **b-phipsi:** 0.0032429518311675
- **w-rdist:** 0.9004176631043326
- **t-alpha:** 0.0014587541408541

---

---

107

- **AF ID:** AF-Q9H6W3-F1-model-v4 | **Chain:** A
- **b-phipsi:** 0.0028343760052904
- **w-rdist:** 0.3250819344203794
- **t-alpha:** 0.077931746040353

---

---

108

- **AF ID:** AF-Q6NUM6-F1-model-v4 | **Chain:** A
- **b-phipsi:** 0.0071696606896772
- **w-rdist:** 0.417714599733502
- **t-alpha:** 0.0058266934612722

---

---

109

- **AF ID:** AF-Q09013-F1-model-v4 | **Chain:** A
- **b-phipsi:** 0.00673114318516
- **w-rdist:** 0.3122734161616992
- **t-alpha:** 0.0458847982169747

---

---

110

- **AF ID:** AF-Q8NA19-F1-model-v4 | **Chain:** A
- **b-phipsi:** 0.0063791606537925
- **w-rdist:** 0.3592782377117982
- **t-alpha:** 0.0160233389937762

---

---

111

- **AF ID:** AF-O15259-F1-model-v4 | **Chain:** A
- **b-phipsi:** 0.0023579591307223
- **w-rdist:** 0.1720848408264949
- **t-alpha:** 0.1893660442320035

---

---

112

- **AF ID:** AF-A0A087X1G2-F1-model-v4 | **Chain:** A
- **b-phipsi:** 0.0080424083104011
- **w-rdist:** 0.5609837492119722
- **t-alpha:** 0.0036549441753579

---

---

113

- **AF ID:** AF-Q8IY47-F1-model-v4 | **Chain:** A
- **b-phipsi:** 0.0044407059730991
- **w-rdist:** 0.3372460929795613
- **t-alpha:** 0.0545316885212077

---

---

114

- **AF ID:** AF-Q15046-F1-model-v4 | **Chain:** A
- **b-phipsi:** 0.0009114007633423
- **w-rdist:** 0.5772297390597452
- **t-alpha:** 0.0472921254005003

---

---

115

- **AF ID:** AF-Q9BQS7-F1-model-v4 | **Chain:** A
- **b-phipsi:** 0.0076970520360819
- **w-rdist:** 0.2692708188389689
- **t-alpha:** 0.0582668457273456

---

---

116

- **AF ID:** AF-P0C7X1-F1-model-v4 | **Chain:** A
- **b-phipsi:** 0.0094691812807584
- **w-rdist:** 0.50920055388851
- **t-alpha:** 0.0036549441753579

---

---

117

- **AF ID:** AF-C9J798-F1-model-v4 | **Chain:** A
- **b-phipsi:** 0.0029370363098615
- **w-rdist:** 0.3287344208525272
- **t-alpha:** 0.0896826187952655

---

---

118

- **AF ID:** AF-Q9UJX5-F1-model-v4 | **Chain:** A
- **b-phipsi:** 0.0018342075040473
- **w-rdist:** 0.6848330801471858
- **t-alpha:** 0.0065982109973949

---

---

119

- **AF ID:** AF-Q658Y4-F1-model-v4 | **Chain:** A
- **b-phipsi:** 0.0076142851566125
- **w-rdist:** 0.1809325273307173
- **t-alpha:** 0.0786598128498576

---

---

120

- **AF ID:** AF-P17028-F1-model-v4 | **Chain:** A
- **b-phipsi:** 0.0148055914583024
- **w-rdist:** 0.2368202066699873
- **t-alpha:** 0.0230999264545257

---

---

121

- **AF ID:** AF-Q92562-F1-model-v4 | **Chain:** A
- **b-phipsi:** 0.0011704336034589
- **w-rdist:** 0.2993597449417604
- **t-alpha:** 0.1507652002007882

---

---

122

- **AF ID:** AF-Q96M94-F1-model-v4 | **Chain:** A
- **b-phipsi:** 0.0030293439558428
- **w-rdist:** 0.7109888925068348
- **t-alpha:** 0.0058266934612722

---

---

123

- **AF ID:** AF-Q96NY9-F1-model-v4 | **Chain:** A
- **b-phipsi:** 0.0062497877206543
- **w-rdist:** 0.296695775513759
- **t-alpha:** 0.0651667209447612

---

---

124

- **AF ID:** AF-Q8WZA1-F1-model-v4 | **Chain:** A
- **b-phipsi:** 0.0009259634641494
- **w-rdist:** 0.3857122208357022
- **t-alpha:** 0.0954111582921788

---

---

125

- **AF ID:** AF-Q9BSQ5-F1-model-v4 | **Chain:** A
- **b-phipsi:** 0.0135926152283923
- **w-rdist:** 0.7109856222425024
- **t-alpha:** 0.0

---

---

126

- **AF ID:** AF-P35858-F1-model-v4 | **Chain:** A
- **b-phipsi:** 0.0131446652442243
- **w-rdist:** 0.6377010139207502
- **t-alpha:** 0.0014568440116902

---

---

127

- **AF ID:** AF-Q9UBT2-F1-model-v4 | **Chain:** A
- **b-phipsi:** 0.0030811237314507
- **w-rdist:** 0.3506266636387898
- **t-alpha:** 0.0699196593590019

---

---

128

- **AF ID:** AF-P50747-F1-model-v4 | **Chain:** A
- **b-phipsi:** 0.0064986879068654
- **w-rdist:** 0.3172814914589946
- **t-alpha:** 0.0560815732405199

---

---

129

- **AF ID:** AF-Q7Z4K8-F1-model-v4 | **Chain:** A
- **b-phipsi:** 0.000462315557246
- **w-rdist:** 1.4537677052906823
- **t-alpha:** 0.0036549441753579

---

---

130

- **AF ID:** AF-Q8NFF5-F1-model-v4 | **Chain:** A
- **b-phipsi:** 0.0024719891117185
- **w-rdist:** 0.2222047513206378
- **t-alpha:** 0.2053902107995835

---

---

131

- **AF ID:** AF-Q9H0J9-F1-model-v4 | **Chain:** A
- **b-phipsi:** 0.0004652080392326
- **w-rdist:** 0.4807038764037619
- **t-alpha:** 0.2012245600058226

---

---

132

- **AF ID:** AF-Q2NL67-F1-model-v4 | **Chain:** A
- **b-phipsi:** 0.000484484409048
- **w-rdist:** 0.7139439124022491
- **t-alpha:** 0.0975966865124746

---

---

133

- **AF ID:** AF-P29350-F1-model-v4 | **Chain:** A
- **b-phipsi:** 0.0042591278331811
- **w-rdist:** 0.5504215305057711
- **t-alpha:** 0.0065982109973949

---

---

134

- **AF ID:** AF-P0CF97-F1-model-v4 | **Chain:** A
- **b-phipsi:** 0.0324240392418705
- **w-rdist:** 0.1334478098667424
- **t-alpha:** 0.0065550134155867

---

---

135

- **AF ID:** AF-Q8IVL6-F1-model-v4 | **Chain:** A
- **b-phipsi:** 0.0139602974334313
- **w-rdist:** 0.2852311170796359
- **t-alpha:** 0.0182082568765367

---

---

136

- **AF ID:** AF-Q2TBF2-F1-model-v4 | **Chain:** A
- **b-phipsi:** 0.0079974622940905
- **w-rdist:** 0.3345703202600886
- **t-alpha:** 0.0407864162082982

---

---

137

- **AF ID:** AF-Q13822-F1-model-v4 | **Chain:** A
- **b-phipsi:** 0.0008158690196115
- **w-rdist:** 0.450699000955563
- **t-alpha:** 0.1108414573859111

---

---

138

- **AF ID:** AF-Q9Y4C4-F1-model-v4 | **Chain:** A
- **b-phipsi:** 0.0061302645820028
- **w-rdist:** 0.8132194259922555
- **t-alpha:** 0.0029134192830806

---

---

139

- **AF ID:** AF-P60608-F1-model-v4 | **Chain:** A
- **b-phipsi:** 0.0028895151978894
- **w-rdist:** 0.3650968334886395
- **t-alpha:** 0.0662781018568299

---

---

140

- **AF ID:** AF-Q86VX9-F1-model-v4 | **Chain:** A
- **b-phipsi:** 0.0009508774876915
- **w-rdist:** 0.4997650752281402
- **t-alpha:** 0.0706484408514722

---

---

141

- **AF ID:** AF-Q9NRJ5-F1-model-v4 | **Chain:** A
- **b-phipsi:** 0.0006560784794131
- **w-rdist:** 0.4170041153328041
- **t-alpha:** 0.1806258887665859

---

---

142

- **AF ID:** AF-Q14644-F1-model-v4 | **Chain:** A
- **b-phipsi:** 0.0015708078011562
- **w-rdist:** 0.3669429172606573
- **t-alpha:** 0.0896826187952655

---

---

143

- **AF ID:** AF-P57075-F1-model-v4 | **Chain:** A
- **b-phipsi:** 0.0021089599507186
- **w-rdist:** 0.3459944437666244
- **t-alpha:** 0.1201749287180127

---

---

144

- **AF ID:** AF-Q9UQF0-F1-model-v4 | **Chain:** A
- **b-phipsi:** 0.0011163376996854
- **w-rdist:** 1.0891829235199946
- **t-alpha:** 0.0021848670241468

---

---

145

- **AF ID:** AF-Q7Z3Z3-F1-model-v4 | **Chain:** A
- **b-phipsi:** 0.0037795281495148
- **w-rdist:** 0.3797007034518374
- **t-alpha:** 0.0466131781632361

---

---

146

- **AF ID:** AF-Q92784-F1-model-v4 | **Chain:** A
- **b-phipsi:** 0.0076674587628302
- **w-rdist:** 0.3578809093848499
- **t-alpha:** 0.0276945584914771

---

---

147

- **AF ID:** AF-P98170-F1-model-v4 | **Chain:** A
- **b-phipsi:** 0.0094358197041599
- **w-rdist:** 0.3623675587146288
- **t-alpha:** 0.0095587741674756

---

---

148

- **AF ID:** AF-Q9HB09-F1-model-v4 | **Chain:** A
- **b-phipsi:** 0.0071080085314798
- **w-rdist:** 0.2625334403642033
- **t-alpha:** 0.0946832683923559

---

---

149

- **AF ID:** AF-Q8WXF8-F1-model-v4 | **Chain:** A
- **b-phipsi:** 0.0008142147202946
- **w-rdist:** 0.4792248841800336
- **t-alpha:** 0.1199020159026713

---

---

150

- **AF ID:** AF-Q13490-F1-model-v4 | **Chain:** A
- **b-phipsi:** 0.0107827105989036
- **w-rdist:** 0.3471403324255783
- **t-alpha:** 0.0167517699463162

---

---

151

- **AF ID:** AF-Q9NXZ2-F1-model-v4 | **Chain:** A
- **b-phipsi:** 0.0050956371594539
- **w-rdist:** 0.2503889479931973
- **t-alpha:** 0.1412960222621002

---

---

152

- **AF ID:** AF-Q06278-F1-model-v4 | **Chain:** A
- **b-phipsi:** 0.003858773970025
- **w-rdist:** 0.2879809327676016
- **t-alpha:** 0.1478518582637831

---

---

153

- **AF ID:** AF-P10075-F1-model-v4 | **Chain:** A
- **b-phipsi:** 0.0094889680017092
- **w-rdist:** 0.4493987551791102
- **t-alpha:** 0.0058607467412064

---

---

154

- **AF ID:** AF-Q9H8H0-F1-model-v4 | **Chain:** A
- **b-phipsi:** 0.0029536574058474
- **w-rdist:** 1.179597535666648
- **t-alpha:** 0.0

---

---

155

- **AF ID:** AF-P19878-F1-model-v4 | **Chain:** A
- **b-phipsi:** 0.0007994965761571
- **w-rdist:** 0.3846761875444723
- **t-alpha:** 0.1762563687134875

---

---

156

- **AF ID:** AF-Q8N6K7-F1-model-v4 | **Chain:** A
- **b-phipsi:** 0.0201639954090353
- **w-rdist:** 0.2490157942196151
- **t-alpha:** 0.0155326816144203

---

---

157

- **AF ID:** AF-A5YM72-F1-model-v4 | **Chain:** A
- **b-phipsi:** 0.0060775857674449
- **w-rdist:** 0.386528536286097
- **t-alpha:** 0.0200592812463542

---

---

158

- **AF ID:** AF-O15091-F1-model-v4 | **Chain:** A
- **b-phipsi:** 0.0102548750417591
- **w-rdist:** 0.338416570863013
- **t-alpha:** 0.0327750996298437

---

---

159

- **AF ID:** AF-Q53T94-F1-model-v4 | **Chain:** A
- **b-phipsi:** 0.0091540819694272
- **w-rdist:** 0.5630361518319429
- **t-alpha:** 0.00512430205411

---

---

160

- **AF ID:** AF-Q9Y6M1-F1-model-v4 | **Chain:** A
- **b-phipsi:** 0.0136470490759857
- **w-rdist:** 0.1680837302286414
- **t-alpha:** 0.0604515833997143

---

---

161

- **AF ID:** AF-P82933-F1-model-v4 | **Chain:** A
- **b-phipsi:** 0.0032418741970928
- **w-rdist:** 0.808778158842713
- **t-alpha:** 0.0058266934612722

---

---

162

- **AF ID:** AF-Q9NPH2-F1-model-v4 | **Chain:** A
- **b-phipsi:** 3.981630393709759e-05
- **w-rdist:** 0.8494310341031235
- **t-alpha:** 0.1337737842211765

---

---

163

- **AF ID:** AF-Q96PZ2-F1-model-v4 | **Chain:** A
- **b-phipsi:** 0.0010614427845567
- **w-rdist:** 0.4041066529385497
- **t-alpha:** 0.0879557950251648

---

---

164

- **AF ID:** AF-Q96JF0-F1-model-v4 | **Chain:** A
- **b-phipsi:** 0.0145412706197889
- **w-rdist:** 0.6550829037816979
- **t-alpha:** 0.0021848670241468

---

---

165

- **AF ID:** AF-P55055-F1-model-v4 | **Chain:** A
- **b-phipsi:** 0.0174400111555667
- **w-rdist:** 0.4564343533940381
- **t-alpha:** 0.0029220273949486

---

---

166

- **AF ID:** AF-P06241-F1-model-v4 | **Chain:** A
- **b-phipsi:** 0.0100938138204664
- **w-rdist:** 0.754768302450633
- **t-alpha:** 0.0021896458253802

---

---

167

- **AF ID:** AF-Q9NV66-F1-model-v4 | **Chain:** A
- **b-phipsi:** 0.0058944895406606
- **w-rdist:** 0.3856915224136211
- **t-alpha:** 0.0261584429252026

---

---

168

- **AF ID:** AF-A0A1W2PPF3-F1-model-v4 | **Chain:** A
- **b-phipsi:** 0.0140542301364198
- **w-rdist:** 0.6902624269980466
- **t-alpha:** 0.0014587541408541

---

---

169

- **AF ID:** AF-Q6ZW61-F1-model-v4 | **Chain:** A
- **b-phipsi:** 0.0050016568448761
- **w-rdist:** 0.3276489438453355
- **t-alpha:** 0.1056082980484882

---

---

170

- **AF ID:** AF-Q9P2G3-F1-model-v4 | **Chain:** A
- **b-phipsi:** 0.0058043898139466
- **w-rdist:** 0.3470644663925947
- **t-alpha:** 0.0718186926297554

---

---

171

- **AF ID:** AF-Q9NVM9-F1-model-v4 | **Chain:** A
- **b-phipsi:** 0.0032871730733774
- **w-rdist:** 0.989039621902734
- **t-alpha:** 0.0029220273949486

---

---

172

- **AF ID:** AF-P23743-F1-model-v4 | **Chain:** A
- **b-phipsi:** 0.0074480352916007
- **w-rdist:** 0.3395627258952075
- **t-alpha:** 0.0626935301476931

---

---

173

- **AF ID:** AF-Q96LI5-F1-model-v4 | **Chain:** A
- **b-phipsi:** 0.0005690064528616
- **w-rdist:** 0.635563997244491
- **t-alpha:** 0.167516791817055

---

---

174

- **AF ID:** AF-P35626-F1-model-v4 | **Chain:** A
- **b-phipsi:** 0.0115076399202463
- **w-rdist:** 0.3672642929552895
- **t-alpha:** 0.0073366962737499

---

---

175

- **AF ID:** AF-Q9H1B5-F1-model-v4 | **Chain:** A
- **b-phipsi:** 0.0052422037708676
- **w-rdist:** 0.2217247573734986
- **t-alpha:** 0.1922801396614761

---

---

176

- **AF ID:** AF-Q86UR1-F1-model-v4 | **Chain:** A
- **b-phipsi:** 0.0072412954531341
- **w-rdist:** 0.6975145087617888
- **t-alpha:** 0.00512430205411

---

---

177

- **AF ID:** AF-Q96LW9-F1-model-v4 | **Chain:** A
- **b-phipsi:** 0.009590788977138
- **w-rdist:** 0.3168802853344089
- **t-alpha:** 0.0610509630466473

---

---

178

- **AF ID:** AF-Q8NBJ5-F1-model-v4 | **Chain:** A
- **b-phipsi:** 0.0007117617398835
- **w-rdist:** 0.5623472081470287
- **t-alpha:** 0.1499160832876762

---

---

179

- **AF ID:** AF-P0DPD6-F1-model-v4 | **Chain:** A
- **b-phipsi:** 0.0255751736483944
- **w-rdist:** 0.5849142648993597
- **t-alpha:** 0.0007288726764216

---

---

180

- **AF ID:** AF-Q6DHY5-F1-model-v4 | **Chain:** A
- **b-phipsi:** 0.0112688928996026
- **w-rdist:** 0.5400508613606334
- **t-alpha:** 0.00512430205411

---

---

181

- **AF ID:** AF-P00747-F1-model-v4 | **Chain:** A
- **b-phipsi:** 0.0170870898624695
- **w-rdist:** 0.2468638535541327
- **t-alpha:** 0.0449007945645392

---

---

182

- **AF ID:** AF-P49759-F1-model-v4 | **Chain:** A
- **b-phipsi:** 0.0177630619242392
- **w-rdist:** 0.7130649239938054
- **t-alpha:** 0.0007288726764216

---

---

183

- **AF ID:** AF-O43462-F1-model-v4 | **Chain:** A
- **b-phipsi:** 0.0188739430833399
- **w-rdist:** 0.5427384806850134
- **t-alpha:** 0.0029134192830806

---

---

184

- **AF ID:** AF-Q9NXT0-F1-model-v4 | **Chain:** A
- **b-phipsi:** 0.0179216878373015
- **w-rdist:** 0.6863964696216153
- **t-alpha:** 0.0014568440116902

---

---

185

- **AF ID:** AF-Q59H18-F1-model-v4 | **Chain:** A
- **b-phipsi:** 0.0169316596001556
- **w-rdist:** 0.3167857245129189
- **t-alpha:** 0.0247633892798655

---

---

186

- **AF ID:** AF-P58317-F1-model-v4 | **Chain:** A
- **b-phipsi:** 0.0234746584861469
- **w-rdist:** 0.2936894787936283
- **t-alpha:** 0.0088172167453031

---

---

187

- **AF ID:** AF-P26599-F1-model-v4 | **Chain:** A
- **b-phipsi:** 0.029452813531033
- **w-rdist:** 0.4874255477628775
- **t-alpha:** 0.0014587541408541

---

---

188

- **AF ID:** AF-P14735-F1-model-v4 | **Chain:** A
- **b-phipsi:** 0.0041709594731243
- **w-rdist:** 0.3826834536981023
- **t-alpha:** 0.0640930305320786

---

---

189

- **AF ID:** AF-Q07075-F1-model-v4 | **Chain:** A
- **b-phipsi:** 0.0099923175539561
- **w-rdist:** 0.3303110088827435
- **t-alpha:** 0.0597231737029493

---

---

190

- **AF ID:** AF-P0CB48-F1-model-v4 | **Chain:** A
- **b-phipsi:** 0.0211915848272602
- **w-rdist:** 0.2804717909226824
- **t-alpha:** 0.0269259686475575

---

---

191

- **AF ID:** AF-P61565-F1-model-v4 | **Chain:** A
- **b-phipsi:** 0.0012099556115467
- **w-rdist:** 1.0422136649842335
- **t-alpha:** 0.004389354417605

---

---

192

- **AF ID:** AF-Q9NZ20-F1-model-v4 | **Chain:** A
- **b-phipsi:** 0.0039579865645511
- **w-rdist:** 0.3741050973943929
- **t-alpha:** 0.0785543772618266

---

---

193

- **AF ID:** AF-Q6IPX1-F1-model-v4 | **Chain:** A
- **b-phipsi:** 0.0137154756089536
- **w-rdist:** 0.5458997420745172
- **t-alpha:** 0.0050984212340619

---

---

194

- **AF ID:** AF-O43610-F1-model-v4 | **Chain:** A
- **b-phipsi:** 0.0066680120166681
- **w-rdist:** 0.3560618763039662
- **t-alpha:** 0.0684824353053381

---

---

195

- **AF ID:** AF-Q9Y6K8-F1-model-v4 | **Chain:** A
- **b-phipsi:** 0.0211840871537855
- **w-rdist:** 0.2149209991383703
- **t-alpha:** 0.0458847982169747

---

---

196

- **AF ID:** AF-P15144-F1-model-v4 | **Chain:** A
- **b-phipsi:** 0.0121494941918525
- **w-rdist:** 0.3828999998600786
- **t-alpha:** 0.0072833804367331

---

---

197

- **AF ID:** AF-A2RUS2-F1-model-v4 | **Chain:** A
- **b-phipsi:** 0.0007718333656717
- **w-rdist:** 0.8649797841543752
- **t-alpha:** 0.0597231737029493

---

---

198

- **AF ID:** AF-Q9H9S5-F1-model-v4 | **Chain:** A
- **b-phipsi:** 0.0002350438521717
- **w-rdist:** 0.6394213345584349
- **t-alpha:** 0.3126193326963351

---

---

199

- **AF ID:** AF-Q9NXG6-F1-model-v4 | **Chain:** A
- **b-phipsi:** 0.0007891923617567
- **w-rdist:** 0.624751400694471
- **t-alpha:** 0.1403652546715101

---

---

200

- **AF ID:** AF-Q9UL40-F1-model-v4 | **Chain:** A
- **b-phipsi:** 0.0072854119915906
- **w-rdist:** 0.2676687558933344
- **t-alpha:** 0.1479930802623299

---

---

201

- **AF ID:** AF-Q96BT7-F1-model-v4 | **Chain:** A
- **b-phipsi:** 0.0054919026432885
- **w-rdist:** 0.3775016427102492
- **t-alpha:** 0.0619083860998908

---

---

202

- **AF ID:** AF-Q08J23-F1-model-v4 | **Chain:** A
- **b-phipsi:** 0.0044057271545451
- **w-rdist:** 0.3559402542329443
- **t-alpha:** 0.1070653702560531

---

---

203

- **AF ID:** AF-P63252-F1-model-v4 | **Chain:** A
- **b-phipsi:** 0.0002364367315247
- **w-rdist:** 0.938486535034007
- **t-alpha:** 0.1180782532791766

---

---

204

- **AF ID:** AF-P54578-F1-model-v4 | **Chain:** A
- **b-phipsi:** 0.0001718393533977
- **w-rdist:** 0.9331803905644516
- **t-alpha:** 0.1309719463952541

---

---

205

- **AF ID:** AF-Q8NCR0-F1-model-v4 | **Chain:** A
- **b-phipsi:** 0.0005745722169182
- **w-rdist:** 0.5992439215785053
- **t-alpha:** 0.2414102545444916

---

---

206

- **AF ID:** AF-Q9UFP1-F1-model-v4 | **Chain:** A
- **b-phipsi:** 0.0068823033142212
- **w-rdist:** 0.6364247528528346
- **t-alpha:** 0.0072833804367331

---

---

207

- **AF ID:** AF-P38435-F1-model-v4 | **Chain:** A
- **b-phipsi:** 0.0198900691971443
- **w-rdist:** 0.3096766166381665
- **t-alpha:** 0.0300077103542844

---

---

208

- **AF ID:** AF-P53671-F1-model-v4 | **Chain:** A
- **b-phipsi:** 0.0095972444331817
- **w-rdist:** 0.5885753563384579
- **t-alpha:** 0.0065550134155867

---

---

209

- **AF ID:** AF-Q9H489-F1-model-v4 | **Chain:** A
- **b-phipsi:** 0.0220329876607045
- **w-rdist:** 0.7682337064050373
- **t-alpha:** 0.0

---

---

210

- **AF ID:** AF-Q8N427-F1-model-v4 | **Chain:** A
- **b-phipsi:** 0.0026684638644661
- **w-rdist:** 0.3679275925098015
- **t-alpha:** 0.1403652546715101

---

---

211

- **AF ID:** AF-P49619-F1-model-v4 | **Chain:** A
- **b-phipsi:** 0.0121346584721191
- **w-rdist:** 0.1270939400849973
- **t-alpha:** 0.1230876410749617

---

---

212

- **AF ID:** AF-D6RBQ6-F1-model-v4 | **Chain:** A
- **b-phipsi:** 0.0152462628448476
- **w-rdist:** 0.6925468522320191
- **t-alpha:** 0.0029220273949486

---

---

213

- **AF ID:** AF-Q9Y6X5-F1-model-v4 | **Chain:** A
- **b-phipsi:** 0.0009472779458055
- **w-rdist:** 0.8965512595100545
- **t-alpha:** 0.0370091093109781

---

---

214

- **AF ID:** AF-Q8N428-F1-model-v4 | **Chain:** A
- **b-phipsi:** 0.0008080623150027
- **w-rdist:** 0.5646267737679016
- **t-alpha:** 0.1755134337987387

---

---

215

- **AF ID:** AF-Q8N323-F1-model-v4 | **Chain:** A
- **b-phipsi:** 0.0006316544751415
- **w-rdist:** 0.8705671429580509
- **t-alpha:** 0.0966450803523832

---

---

216

- **AF ID:** AF-Q8IUH3-F1-model-v4 | **Chain:** A
- **b-phipsi:** 0.0048911502469206
- **w-rdist:** 0.3793855675723064
- **t-alpha:** 0.0757463891855267

---

---

217

- **AF ID:** AF-Q13237-F1-model-v4 | **Chain:** A
- **b-phipsi:** 0.0038153182239671
- **w-rdist:** 0.3890965101151216
- **t-alpha:** 0.0823013369141532

---

---

218

- **AF ID:** AF-P51795-F1-model-v4 | **Chain:** A
- **b-phipsi:** 0.0259562757549254
- **w-rdist:** 0.5809193206240013
- **t-alpha:** 0.0021848670241468

---

---

219

- **AF ID:** AF-P63136-F1-model-v4 | **Chain:** A
- **b-phipsi:** 0.0001600993715179
- **w-rdist:** 1.2436157909344512
- **t-alpha:** 0.0407864162082982

---

---

220

- **AF ID:** AF-O00255-F1-model-v4 | **Chain:** A
- **b-phipsi:** 0.0143114446452935
- **w-rdist:** 0.525195533964344
- **t-alpha:** 0.0058266934612722

---

---

221

- **AF ID:** AF-Q6P179-F1-model-v4 | **Chain:** A
- **b-phipsi:** 0.0067844858092569
- **w-rdist:** 0.3713992514025492
- **t-alpha:** 0.0640930305320786

---

---

222

- **AF ID:** AF-Q16581-F1-model-v4 | **Chain:** A
- **b-phipsi:** 0.0174429259872644
- **w-rdist:** 0.8509061936931689
- **t-alpha:** 0.0007283954918235

---

---

223

- **AF ID:** AF-O95567-F1-model-v4 | **Chain:** A
- **b-phipsi:** 0.0120001764116078
- **w-rdist:** 0.7844253268118814
- **t-alpha:** 0.0029220273949486

---

---

224

- **AF ID:** AF-Q9Y6S9-F1-model-v4 | **Chain:** A
- **b-phipsi:** 0.0087364671331537
- **w-rdist:** 0.3630998367823632
- **t-alpha:** 0.0604515833997143

---

---

225

- **AF ID:** AF-Q9ULM6-F1-model-v4 | **Chain:** A
- **b-phipsi:** 0.0006368297730471
- **w-rdist:** 0.6518864016207632
- **t-alpha:** 0.2054430912450047

---

---

226

- **AF ID:** AF-Q96KR4-F1-model-v4 | **Chain:** A
- **b-phipsi:** 0.0099341821693515
- **w-rdist:** 0.5472790211135828
- **t-alpha:** 0.0072833804367331

---

---

227

- **AF ID:** AF-Q15916-F1-model-v4 | **Chain:** A
- **b-phipsi:** 0.0125615221010701
- **w-rdist:** 0.3602274755772214
- **t-alpha:** 0.0385778104072074

---

---

228

- **AF ID:** AF-Q96HU1-F1-model-v4 | **Chain:** A
- **b-phipsi:** 0.0394025409557985
- **w-rdist:** 0.2934907803591357
- **t-alpha:** 0.0073366962737499

---

---

229

- **AF ID:** AF-Q14493-F1-model-v4 | **Chain:** A
- **b-phipsi:** 0.0002207093287502
- **w-rdist:** 0.9133487133296476
- **t-alpha:** 0.1547520117601062

---

---

230

- **AF ID:** AF-Q9BWW7-F1-model-v4 | **Chain:** A
- **b-phipsi:** 0.0292157374257912
- **w-rdist:** 0.2553647258257979
- **t-alpha:** 0.034664446127588

---

---

231

- **AF ID:** AF-A8K5M9-F1-model-v4 | **Chain:** A
- **b-phipsi:** 0.0021497062974897
- **w-rdist:** 0.3285843358352993
- **t-alpha:** 0.3051330241412824

---

---

232

- **AF ID:** AF-Q9H5U6-F1-model-v4 | **Chain:** A
- **b-phipsi:** 0.0004082879448305
- **w-rdist:** 0.7898708471474833
- **t-alpha:** 0.2086265064684453

---

---

233

- **AF ID:** AF-Q01432-F1-model-v4 | **Chain:** A
- **b-phipsi:** 0.0152520603625432
- **w-rdist:** 0.662142529314279
- **t-alpha:** 0.0043701973936818

---

---

234

- **AF ID:** AF-P0C860-F1-model-v4 | **Chain:** A
- **b-phipsi:** 0.0048998565084514
- **w-rdist:** 0.87695673945548
- **t-alpha:** 0.0058607467412064

---

---

235

- **AF ID:** AF-Q53QZ3-F1-model-v4 | **Chain:** A
- **b-phipsi:** 0.0010968448808832
- **w-rdist:** 0.6156223155127968
- **t-alpha:** 0.087399807391695

---

---

236

- **AF ID:** AF-P31751-F1-model-v4 | **Chain:** A
- **b-phipsi:** 0.0002575757271148
- **w-rdist:** 0.8845925216454777
- **t-alpha:** 0.1836207206904445

---

---

237

- **AF ID:** AF-B1APH4-F1-model-v4 | **Chain:** A
- **b-phipsi:** 0.0090385789429338
- **w-rdist:** 0.374137813790806
- **t-alpha:** 0.0553531458501084

---

---

238

- **AF ID:** AF-Q5RL73-F1-model-v4 | **Chain:** A
- **b-phipsi:** 0.015968691413419
- **w-rdist:** 0.9234378166087776
- **t-alpha:** 0.0

---

---

239

- **AF ID:** AF-O95786-F1-model-v4 | **Chain:** A
- **b-phipsi:** 0.0206569046700866
- **w-rdist:** 0.3060544504829142
- **t-alpha:** 0.0444280646304331

---

---

240

- **AF ID:** AF-Q14147-F1-model-v4 | **Chain:** A
- **b-phipsi:** 0.0089031056171326
- **w-rdist:** 0.985349639108765
- **t-alpha:** 0.0021896458253802

---

---

241

- **AF ID:** AF-P58335-F1-model-v4 | **Chain:** A
- **b-phipsi:** 0.0034737243228714
- **w-rdist:** 1.369196400586786
- **t-alpha:** 0.0007288726764216

---

---

242

- **AF ID:** AF-Q9BRP7-F1-model-v4 | **Chain:** A
- **b-phipsi:** 0.0009975278061812
- **w-rdist:** 0.5197026968831318
- **t-alpha:** 0.1537816613453546

---

---

243

- **AF ID:** AF-P10253-F1-model-v4 | **Chain:** A
- **b-phipsi:** 0.0120819302408245
- **w-rdist:** 0.3783443248652706
- **t-alpha:** 0.0276945584914771

---

---

244

- **AF ID:** AF-Q96FL9-F1-model-v4 | **Chain:** A
- **b-phipsi:** 0.0004311284257682
- **w-rdist:** 0.7637980008248744
- **t-alpha:** 0.2584782099371299

---

---

245

- **AF ID:** AF-O43155-F1-model-v4 | **Chain:** A
- **b-phipsi:** 0.0088345445860484
- **w-rdist:** 0.893884544571828
- **t-alpha:** 0.0043701973936818

---

---

246

- **AF ID:** AF-O15499-F1-model-v4 | **Chain:** A
- **b-phipsi:** 0.0065600265353868
- **w-rdist:** 0.2417100414372007
- **t-alpha:** 0.2807832558099548

---

---

247

- **AF ID:** AF-Q96LR2-F1-model-v4 | **Chain:** A
- **b-phipsi:** 0.0096611885484151
- **w-rdist:** 0.2807947211752024
- **t-alpha:** 0.1537816613453546

---

---

248

- **AF ID:** AF-Q96LQ0-F1-model-v4 | **Chain:** A
- **b-phipsi:** 0.0366082173254729
- **w-rdist:** 0.7459778093528502
- **t-alpha:** 0.0

---

---

249

- **AF ID:** AF-Q96K49-F1-model-v4 | **Chain:** A
- **b-phipsi:** 0.021693217859689
- **w-rdist:** 0.8701314787760128
- **t-alpha:** 0.0

---

---

250

- **AF ID:** AF-Q6ZV50-F1-model-v4 | **Chain:** A
- **b-phipsi:** 0.0151456486751385
- **w-rdist:** 0.3783349106625225
- **t-alpha:** 0.0170368870295003

---

---

251

- **AF ID:** AF-Q96EX3-F1-model-v4 | **Chain:** A
- **b-phipsi:** 0.0185675831741942
- **w-rdist:** 0.702555587515691
- **t-alpha:** 0.0029220273949486

---

---

252

- **AF ID:** AF-Q13087-F1-model-v4 | **Chain:** A
- **b-phipsi:** 0.0003827175793844
- **w-rdist:** 0.845109396460867
- **t-alpha:** 0.222618037107644

---

---

253

- **AF ID:** AF-Q8IUK5-F1-model-v4 | **Chain:** A
- **b-phipsi:** 0.0084457065407007
- **w-rdist:** 0.7518890729732086
- **t-alpha:** 0.0065550134155867

---

---

254

- **AF ID:** AF-O94923-F1-model-v4 | **Chain:** A
- **b-phipsi:** 0.0109876678070464
- **w-rdist:** 0.3819235168833608
- **t-alpha:** 0.0422430403347799

---

---

255

- **AF ID:** AF-Q16819-F1-model-v4 | **Chain:** A
- **b-phipsi:** 0.010121866211703
- **w-rdist:** 0.3611029939016067
- **t-alpha:** 0.0659939212028208

---

---

256

- **AF ID:** AF-Q6R6M4-F1-model-v4 | **Chain:** A
- **b-phipsi:** 0.0150077279935169
- **w-rdist:** 0.6523277075687883
- **t-alpha:** 0.00512430205411

---

---

257

- **AF ID:** AF-Q9Y5X1-F1-model-v4 | **Chain:** A
- **b-phipsi:** 0.0110785201948137
- **w-rdist:** 0.28946739316761
- **t-alpha:** 0.1332851641169676

---

---

258

- **AF ID:** AF-Q8N4A0-F1-model-v4 | **Chain:** A
- **b-phipsi:** 0.0007763488953606
- **w-rdist:** 0.7142205421645128
- **t-alpha:** 0.1596281837125226

---

---

259

- **AF ID:** AF-Q15036-F1-model-v4 | **Chain:** A
- **b-phipsi:** 0.0002546804897874
- **w-rdist:** 1.0398015686319686
- **t-alpha:** 0.1144481357306117

---

---

260

- **AF ID:** AF-P42336-F1-model-v4 | **Chain:** A
- **b-phipsi:** 0.0092381773541685
- **w-rdist:** 0.3840889015150408
- **t-alpha:** 0.0553531458501084

---

---

261

- **AF ID:** AF-O00329-F1-model-v4 | **Chain:** A
- **b-phipsi:** 0.0100335240841902
- **w-rdist:** 0.3631810265744986
- **t-alpha:** 0.0699196593590019

---

---

262

- **AF ID:** AF-Q68CQ4-F1-model-v4 | **Chain:** A
- **b-phipsi:** 0.0048908273490057
- **w-rdist:** 0.3843161325552537
- **t-alpha:** 0.1143477037129587

---

---

263

- **AF ID:** AF-O43781-F1-model-v4 | **Chain:** A
- **b-phipsi:** 0.021478819301069
- **w-rdist:** 0.6404878459473814
- **t-alpha:** 0.0036549441753579

---

---

264

- **AF ID:** AF-Q9Y4K3-F1-model-v4 | **Chain:** A
- **b-phipsi:** 0.0005332724078942
- **w-rdist:** 1.2610756881929357
- **t-alpha:** 0.0385778104072074

---

---

265

- **AF ID:** AF-A4D0V7-F1-model-v4 | **Chain:** A
- **b-phipsi:** 0.0003787943078209
- **w-rdist:** 0.978720148579054
- **t-alpha:** 0.1456667763786574

---

---

266

- **AF ID:** AF-Q96HY7-F1-model-v4 | **Chain:** A
- **b-phipsi:** 0.0095241990182733
- **w-rdist:** 0.3524013185433681
- **t-alpha:** 0.0975218464555562

---

---

267

- **AF ID:** AF-Q9NYK6-F1-model-v4 | **Chain:** A
- **b-phipsi:** 0.0342332764368122
- **w-rdist:** 0.2731365081665022
- **t-alpha:** 0.0433130906697325

---

---

268

- **AF ID:** AF-Q9NSD9-F1-model-v4 | **Chain:** A
- **b-phipsi:** 0.0010350964140166
- **w-rdist:** 0.6866932511759242
- **t-alpha:** 0.1081517725047538

---

---

269

- **AF ID:** AF-A8MPY1-F1-model-v4 | **Chain:** A
- **b-phipsi:** 0.0043154717433203
- **w-rdist:** 1.3237165428903654
- **t-alpha:** 0.0014568440116902

---

---

270

- **AF ID:** AF-P27694-F1-model-v4 | **Chain:** A
- **b-phipsi:** 0.0166386082079155
- **w-rdist:** 0.6802401405619731
- **t-alpha:** 0.0050984212340619

---

---

271

- **AF ID:** AF-P0CH99-F1-model-v4 | **Chain:** A
- **b-phipsi:** 0.0117882346716223
- **w-rdist:** 0.3111432732030536
- **t-alpha:** 0.1226491277029568

---

---

272

- **AF ID:** AF-Q8WTU2-F1-model-v4 | **Chain:** A
- **b-phipsi:** 0.0425861079720018
- **w-rdist:** 0.4125034565741186
- **t-alpha:** 0.004389354417605

---

---

273

- **AF ID:** AF-Q9H0K6-F1-model-v4 | **Chain:** A
- **b-phipsi:** 0.0005917121136602
- **w-rdist:** 0.7854416325848377
- **t-alpha:** 0.2061180954314494

---

---

274

- **AF ID:** AF-Q6ZNX1-F1-model-v4 | **Chain:** A
- **b-phipsi:** 0.0008371914281085
- **w-rdist:** 0.8071730148446888
- **t-alpha:** 0.1217319626680046

---

---

275

- **AF ID:** AF-Q9BW62-F1-model-v4 | **Chain:** A
- **b-phipsi:** 0.0086098983077988
- **w-rdist:** 0.3620296600793498
- **t-alpha:** 0.1056082980484882

---

---

276

- **AF ID:** AF-P42338-F1-model-v4 | **Chain:** A
- **b-phipsi:** 0.0110634918968404
- **w-rdist:** 0.3736814253267493
- **t-alpha:** 0.0575384238750913

---

---

277

- **AF ID:** AF-Q8N3Y3-F1-model-v4 | **Chain:** A
- **b-phipsi:** 0.0093391648258036
- **w-rdist:** 0.3724698209968327
- **t-alpha:** 0.0718186926297554

---

---

278

- **AF ID:** AF-Q8NFW8-F1-model-v4 | **Chain:** A
- **b-phipsi:** 0.0006555095638663
- **w-rdist:** 0.7481890693583438
- **t-alpha:** 0.2313901212502505

---

---

279

- **AF ID:** AF-Q96GV9-F1-model-v4 | **Chain:** A
- **b-phipsi:** 0.006437660144995
- **w-rdist:** 0.386359155997419
- **t-alpha:** 0.0905480982534083

---

---

280

- **AF ID:** AF-O14772-F1-model-v4 | **Chain:** A
- **b-phipsi:** 0.0001294277195516
- **w-rdist:** 0.9161884573429762
- **t-alpha:** 0.2527370901804473

---

---

281

- **AF ID:** AF-Q5TFG8-F1-model-v4 | **Chain:** A
- **b-phipsi:** 0.0043751819402655
- **w-rdist:** 0.3886482270617329
- **t-alpha:** 0.1309719463952541

---

---

282

- **AF ID:** AF-Q8IUF8-F1-model-v4 | **Chain:** A
- **b-phipsi:** 0.0004839017024336
- **w-rdist:** 0.8123481492057824
- **t-alpha:** 0.2689462558288373

---

---

283

- **AF ID:** AF-Q9H2W6-F1-model-v4 | **Chain:** A
- **b-phipsi:** 0.0003551580032185
- **w-rdist:** 0.7684271321409855
- **t-alpha:** 0.4010201385071092

---

---

284

- **AF ID:** AF-C9JVI0-F1-model-v4 | **Chain:** A
- **b-phipsi:** 0.0114515568619637
- **w-rdist:** 0.6465712941609049
- **t-alpha:** 0.0072833804367331

---

---

285

- **AF ID:** AF-O43847-F1-model-v4 | **Chain:** A
- **b-phipsi:** 0.0063821158038643
- **w-rdist:** 0.3630886481794448
- **t-alpha:** 0.1463946744398983

---

---

286

- **AF ID:** AF-Q9UK28-F1-model-v4 | **Chain:** A
- **b-phipsi:** 0.0091932884760405
- **w-rdist:** 1.011839918715995
- **t-alpha:** 0.0029220273949486

---

---

287

- **AF ID:** AF-Q6UX15-F1-model-v4 | **Chain:** A
- **b-phipsi:** 0.0064250415627869
- **w-rdist:** 0.8498142081700608
- **t-alpha:** 0.0072833804367331

---

---

288

- **AF ID:** AF-Q6P050-F1-model-v4 | **Chain:** A
- **b-phipsi:** 0.0005323240631689
- **w-rdist:** 0.8273060595287087
- **t-alpha:** 0.2459166461742072

---

---

289

- **AF ID:** AF-Q07869-F1-model-v4 | **Chain:** A
- **b-phipsi:** 0.0170796009151354
- **w-rdist:** 0.6848405444263151
- **t-alpha:** 0.00512430205411

---

---

290

- **AF ID:** AF-Q92670-F1-model-v4 | **Chain:** A
- **b-phipsi:** 0.02677265218668
- **w-rdist:** 0.1977831091690551
- **t-alpha:** 0.0866720592869298

---

---

291

- **AF ID:** AF-Q13505-F1-model-v4 | **Chain:** A
- **b-phipsi:** 0.0116171118265345
- **w-rdist:** 0.8153337441402437
- **t-alpha:** 0.00512430205411

---

---

292

- **AF ID:** AF-Q7L190-F1-model-v4 | **Chain:** A
- **b-phipsi:** 0.0180406548935233
- **w-rdist:** 0.7528443727028664
- **t-alpha:** 0.0036549441753579

---

---

293

- **AF ID:** AF-Q9BVP2-F1-model-v4 | **Chain:** A
- **b-phipsi:** 0.0089032046115684
- **w-rdist:** 0.3524310636378391
- **t-alpha:** 0.1340127658618555

---

---

294

- **AF ID:** AF-Q8NHX9-F1-model-v4 | **Chain:** A
- **b-phipsi:** 0.0766141061247681
- **w-rdist:** 0.6951184363664187
- **t-alpha:** 0.0007288726764216

---

---

295

- **AF ID:** AF-Q3SY52-F1-model-v4 | **Chain:** A
- **b-phipsi:** 0.0106324450309902
- **w-rdist:** 0.7453449424919754
- **t-alpha:** 0.0065982109973949

---

---

296

- **AF ID:** AF-Q9BYG3-F1-model-v4 | **Chain:** A
- **b-phipsi:** 0.0008809777801687
- **w-rdist:** 0.6378323603125371
- **t-alpha:** 0.2447871814631508

---

---

297

- **AF ID:** AF-Q9Y6F1-F1-model-v4 | **Chain:** A
- **b-phipsi:** 0.0005896710816439
- **w-rdist:** 0.7965620018737113
- **t-alpha:** 0.2493177459002025

---

---

298

- **AF ID:** AF-Q96BR6-F1-model-v4 | **Chain:** A
- **b-phipsi:** 0.0057961438448748
- **w-rdist:** 0.9946301528297232
- **t-alpha:** 0.0058266934612722

---

---

299

- **AF ID:** AF-Q6ZV89-F1-model-v4 | **Chain:** A
- **b-phipsi:** 0.0009820317763146
- **w-rdist:** 0.7505604585759833
- **t-alpha:** 0.1394189465197122

---

---

300

- **AF ID:** AF-P51449-F1-model-v4 | **Chain:** A
- **b-phipsi:** 0.0231989323510456
- **w-rdist:** 0.3372080261731116
- **t-alpha:** 0.0480914507615701

---

---

301

- **AF ID:** AF-O43791-F1-model-v4 | **Chain:** A
- **b-phipsi:** 0.0010732379687126
- **w-rdist:** 0.4563763623379918
- **t-alpha:** 0.2459166461742072

---

---

302

- **AF ID:** AF-Q96DL1-F1-model-v4 | **Chain:** A
- **b-phipsi:** 0.0005369754035797
- **w-rdist:** 0.89686813412969
- **t-alpha:** 0.2096914347494454

---

---

303

- **AF ID:** AF-Q96GY3-F1-model-v4 | **Chain:** A
- **b-phipsi:** 0.0109209748594852
- **w-rdist:** 0.3642264590738177
- **t-alpha:** 0.0966450803523832

---

---

304

- **AF ID:** AF-Q3MJ62-F1-model-v4 | **Chain:** A
- **b-phipsi:** 0.0113903707288877
- **w-rdist:** 0.3486445962645892
- **t-alpha:** 0.1180782532791766

---

---

305

- **AF ID:** AF-P58215-F1-model-v4 | **Chain:** A
- **b-phipsi:** 0.026210004411101
- **w-rdist:** 0.3529590980660269
- **t-alpha:** 0.0292354274430095

---

---

306

- **AF ID:** AF-Q14654-F1-model-v4 | **Chain:** A
- **b-phipsi:** 0.0004336012709773
- **w-rdist:** 0.9901797236778164
- **t-alpha:** 0.1826012993691217

---

---

307

- **AF ID:** AF-Q53FZ2-F1-model-v4 | **Chain:** A
- **b-phipsi:** 0.0004514067306201
- **w-rdist:** 0.917018895067969
- **t-alpha:** 0.2447871814631508

---

---

308

- **AF ID:** AF-O95460-F1-model-v4 | **Chain:** A
- **b-phipsi:** 0.0022246858571013
- **w-rdist:** 1.3338861012623835
- **t-alpha:** 0.004389354417605

---

---

309

- **AF ID:** AF-Q9UKJ1-F1-model-v4 | **Chain:** A
- **b-phipsi:** 0.0208562371342903
- **w-rdist:** 0.8066632522504877
- **t-alpha:** 0.0029220273949486

---

---

310

- **AF ID:** AF-Q6GPH6-F1-model-v4 | **Chain:** A
- **b-phipsi:** 0.0259506029571624
- **w-rdist:** 0.321797955169068
- **t-alpha:** 0.0569667321740288

---

---

311

- **AF ID:** AF-Q14566-F1-model-v4 | **Chain:** A
- **b-phipsi:** 0.0010065579646908
- **w-rdist:** 0.9878077213082744
- **t-alpha:** 0.0472921254005003

---

---

312

- **AF ID:** AF-Q969Y0-F1-model-v4 | **Chain:** A
- **b-phipsi:** 0.0002745736064844
- **w-rdist:** 0.8927140465472272
- **t-alpha:** 0.3189241522520512

---

---

313

- **AF ID:** AF-A0A494C086-F1-model-v4 | **Chain:** A
- **b-phipsi:** 0.0087477333723804
- **w-rdist:** 1.1194341871620437
- **t-alpha:** 0.0029134192830806

---

---

314

- **AF ID:** AF-P78508-F1-model-v4 | **Chain:** A
- **b-phipsi:** 0.000530789341703
- **w-rdist:** 0.7875292933530408
- **t-alpha:** 0.3868684636625106

---

---

315

- **AF ID:** AF-P10266-F1-model-v4 | **Chain:** A
- **b-phipsi:** 0.0002108412659632
- **w-rdist:** 1.2344953669636003
- **t-alpha:** 0.1077930415888199

---

---

316

- **AF ID:** AF-Q96DV4-F1-model-v4 | **Chain:** A
- **b-phipsi:** 0.0004827855885384
- **w-rdist:** 0.8553929758009393
- **t-alpha:** 0.313875500163689

---

---

317

- **AF ID:** AF-P19113-F1-model-v4 | **Chain:** A
- **b-phipsi:** 0.0086278687156296
- **w-rdist:** 0.3500612532889257
- **t-alpha:** 0.1835400150513015

---

---

318

- **AF ID:** AF-Q49A17-F1-model-v4 | **Chain:** A
- **b-phipsi:** 0.0010828207262928
- **w-rdist:** 0.7702380278797324
- **t-alpha:** 0.1092498194584445

---

---

319

- **AF ID:** AF-Q6J4K2-F1-model-v4 | **Chain:** A
- **b-phipsi:** 0.0759955496792755
- **w-rdist:** 0.7780180021282012
- **t-alpha:** 0.0007288726764216

---

---

320

- **AF ID:** AF-P07237-F1-model-v4 | **Chain:** A
- **b-phipsi:** 0.0027090564576518
- **w-rdist:** 0.3830783518674703
- **t-alpha:** 0.2879923131027375

---

---

321

- **AF ID:** AF-Q86X45-F1-model-v4 | **Chain:** A
- **b-phipsi:** 0.0006364822234046
- **w-rdist:** 1.1360651814476794
- **t-alpha:** 0.0772031433552034

---

---

322

- **AF ID:** AF-Q8IYR2-F1-model-v4 | **Chain:** A
- **b-phipsi:** 0.0277038593472584
- **w-rdist:** 0.2458377498980847
- **t-alpha:** 0.1036978139084012

---

---

323

- **AF ID:** AF-Q9BZG8-F1-model-v4 | **Chain:** A
- **b-phipsi:** 0.0009477536497923
- **w-rdist:** 0.821585301123462
- **t-alpha:** 0.1432138034259678

---

---

324

- **AF ID:** AF-Q9UH90-F1-model-v4 | **Chain:** A
- **b-phipsi:** 0.0005596889180414
- **w-rdist:** 0.963321325345868
- **t-alpha:** 0.1922801396614761

---

---

325

- **AF ID:** AF-Q9NTG7-F1-model-v4 | **Chain:** A
- **b-phipsi:** 0.001077257485396
- **w-rdist:** 0.8839273035527456
- **t-alpha:** 0.0768626469088202

---

---

326

- **AF ID:** AF-P31749-F1-model-v4 | **Chain:** A
- **b-phipsi:** 0.0009356619530009
- **w-rdist:** 0.9022124573277236
- **t-alpha:** 0.1081517725047538

---

---

327

- **AF ID:** AF-O60304-F1-model-v4 | **Chain:** A
- **b-phipsi:** 0.0162172612866993
- **w-rdist:** 0.8568382555782694
- **t-alpha:** 0.004389354417605

---

---

328

- **AF ID:** AF-Q8N7R0-F1-model-v4 | **Chain:** A
- **b-phipsi:** 0.0108434731243497
- **w-rdist:** 0.3719451460043952
- **t-alpha:** 0.1081517725047538

---

---

329

- **AF ID:** AF-B2RU33-F1-model-v4 | **Chain:** A
- **b-phipsi:** 0.0227065681246375
- **w-rdist:** 0.3424184009986506
- **t-alpha:** 0.0640930305320786

---

---

330

- **AF ID:** AF-Q96JB6-F1-model-v4 | **Chain:** A
- **b-phipsi:** 0.0223399326995598
- **w-rdist:** 0.2777022147665202
- **t-alpha:** 0.1272580390591406

---

---

331

- **AF ID:** AF-Q9UBS4-F1-model-v4 | **Chain:** A
- **b-phipsi:** 0.0009061514140885
- **w-rdist:** 0.7399635865580287
- **t-alpha:** 0.2369366769879393

---

---

332

- **AF ID:** AF-Q8WW27-F1-model-v4 | **Chain:** A
- **b-phipsi:** 0.0003960256773135
- **w-rdist:** 0.9469673282772608
- **t-alpha:** 0.2940622142691544

---

---

333

- **AF ID:** AF-Q8TA94-F1-model-v4 | **Chain:** A
- **b-phipsi:** 0.0126774370787668
- **w-rdist:** 0.7188752734607529
- **t-alpha:** 0.0073366962737499

---

---

334

- **AF ID:** AF-Q6EMK4-F1-model-v4 | **Chain:** A
- **b-phipsi:** 0.0120805140842388
- **w-rdist:** 1.018761039202812
- **t-alpha:** 0.0036415440256147

---

---

335

- **AF ID:** AF-Q8N987-F1-model-v4 | **Chain:** A
- **b-phipsi:** 0.0238279316583477
- **w-rdist:** 0.3105518754056445
- **t-alpha:** 0.0975218464555562

---

---

336

- **AF ID:** AF-Q96PH1-F1-model-v4 | **Chain:** A
- **b-phipsi:** 0.0221923956281096
- **w-rdist:** 0.5521833703138233
- **t-alpha:** 0.0072833804367331

---

---

337

- **AF ID:** AF-P59910-F1-model-v4 | **Chain:** A
- **b-phipsi:** 0.0004099644242606
- **w-rdist:** 0.8651916718248995
- **t-alpha:** 0.4684490442190427

---

---

338

- **AF ID:** AF-Q9Y615-F1-model-v4 | **Chain:** A
- **b-phipsi:** 0.0006320367009935
- **w-rdist:** 0.9926570931706852
- **t-alpha:** 0.1635591445426425

---

---

339

- **AF ID:** AF-E9PJI5-F1-model-v4 | **Chain:** A
- **b-phipsi:** 0.0106050750912351
- **w-rdist:** 1.13923424674157
- **t-alpha:** 0.0029134192830806

---

---

340

- **AF ID:** AF-Q5T8I9-F1-model-v4 | **Chain:** A
- **b-phipsi:** 0.0007738751351111
- **w-rdist:** 0.7720094035959715
- **t-alpha:** 0.2952827110519931

---

---

341

- **AF ID:** AF-Q6UXV4-F1-model-v4 | **Chain:** A
- **b-phipsi:** 0.0175911973557736
- **w-rdist:** 0.921721542396868
- **t-alpha:** 0.0036415440256147

---

---

342

- **AF ID:** AF-Q6GPH4-F1-model-v4 | **Chain:** A
- **b-phipsi:** 0.0006913059555457
- **w-rdist:** 1.0250641023482785
- **t-alpha:** 0.1356491413888314

---

---

343

- **AF ID:** AF-Q9BSL1-F1-model-v4 | **Chain:** A
- **b-phipsi:** 0.0301800369107336
- **w-rdist:** 0.336743891304395
- **t-alpha:** 0.0626935301476931

---

---

344

- **AF ID:** AF-A8MX76-F1-model-v4 | **Chain:** A
- **b-phipsi:** 0.0010583570955238
- **w-rdist:** 0.768321015753217
- **t-alpha:** 0.1615906165511234

---

---

345

- **AF ID:** AF-Q8IYM1-F1-model-v4 | **Chain:** A
- **b-phipsi:** 0.0003821685337091
- **w-rdist:** 0.9256265898410516
- **t-alpha:** 0.4110998156919059

---

---

346

- **AF ID:** AF-Q92519-F1-model-v4 | **Chain:** A
- **b-phipsi:** 0.0003339577981493
- **w-rdist:** 1.0456876965551023
- **t-alpha:** 0.247048324988869

---

---

347

- **AF ID:** AF-Q5H8A4-F1-model-v4 | **Chain:** A
- **b-phipsi:** 0.0581099322163253
- **w-rdist:** 0.6088352767376606
- **t-alpha:** 0.0050984212340619

---

---

348

- **AF ID:** AF-Q6STE5-F1-model-v4 | **Chain:** A
- **b-phipsi:** 0.0006994627199166
- **w-rdist:** 1.188445231532925
- **t-alpha:** 0.0768626469088202

---

---

349

- **AF ID:** AF-Q9UHR4-F1-model-v4 | **Chain:** A
- **b-phipsi:** 0.0080946208590419
- **w-rdist:** 1.1586221321478325
- **t-alpha:** 0.0043701973936818

---

---

350

- **AF ID:** AF-P07992-F1-model-v4 | **Chain:** A
- **b-phipsi:** 0.0009824585291641
- **w-rdist:** 0.6683746081925614
- **t-alpha:** 0.2977314906825847

---

---

351

- **AF ID:** AF-Q99689-F1-model-v4 | **Chain:** A
- **b-phipsi:** 0.0157851276230259
- **w-rdist:** 1.1816226930225633
- **t-alpha:** 0.0007288726764216

---

---

352

- **AF ID:** AF-P34903-F1-model-v4 | **Chain:** A
- **b-phipsi:** 0.0040368695964422
- **w-rdist:** 1.2009701312475196
- **t-alpha:** 0.0058607467412064

---

---

353

- **AF ID:** AF-Q6NSI4-F1-model-v4 | **Chain:** A
- **b-phipsi:** 0.0096810685955499
- **w-rdist:** 0.3691109827048018
- **t-alpha:** 0.1595043286742896

---

---

354

- **AF ID:** AF-Q13946-F1-model-v4 | **Chain:** A
- **b-phipsi:** 0.0461031044983175
- **w-rdist:** 0.7474202755311686
- **t-alpha:** 0.0036415440256147

---

---

355

- **AF ID:** AF-Q9UBP9-F1-model-v4 | **Chain:** A
- **b-phipsi:** 0.0006827884082567
- **w-rdist:** 0.8252259148142825
- **t-alpha:** 0.346078343986026

---

---

356

- **AF ID:** AF-Q9Y6H8-F1-model-v4 | **Chain:** A
- **b-phipsi:** 0.0158566087549068
- **w-rdist:** 1.1881249297112526
- **t-alpha:** 0.0007288726764216

---

---

357

- **AF ID:** AF-Q9BYE2-F1-model-v4 | **Chain:** A
- **b-phipsi:** 0.0236923922786828
- **w-rdist:** 0.7436869860112411
- **t-alpha:** 0.0058266934612722

---

---

358

- **AF ID:** AF-P28288-F1-model-v4 | **Chain:** A
- **b-phipsi:** 0.0456635409945025
- **w-rdist:** 0.78055378099984
- **t-alpha:** 0.0029220273949486

---

---

359

- **AF ID:** AF-P63133-F1-model-v4 | **Chain:** A
- **b-phipsi:** 0.0007359587649904
- **w-rdist:** 1.30791296002388
- **t-alpha:** 0.0553531458501084

---

---

360

- **AF ID:** AF-Q8N9L1-F1-model-v4 | **Chain:** A
- **b-phipsi:** 0.0138027122618661
- **w-rdist:** 0.3534138274683455
- **t-alpha:** 0.1422629527700643

---

---

361

- **AF ID:** AF-P31152-F1-model-v4 | **Chain:** A
- **b-phipsi:** 0.0099170421323852
- **w-rdist:** 0.3616003636189393
- **t-alpha:** 0.200291277483134

---

---

362

- **AF ID:** AF-Q09161-F1-model-v4 | **Chain:** A
- **b-phipsi:** 0.1133467745848225
- **w-rdist:** 0.3662854057961
- **t-alpha:** 0.0087397720309874

---

---

363

- **AF ID:** AF-O75541-F1-model-v4 | **Chain:** A
- **b-phipsi:** 0.0123565955019646
- **w-rdist:** 0.3681611064056664
- **t-alpha:** 0.1391114704263809

---

---

364

- **AF ID:** AF-Q9BXS4-F1-model-v4 | **Chain:** A
- **b-phipsi:** 0.017338202900123
- **w-rdist:** 1.081619137597251
- **t-alpha:** 0.0021848670241468

---

---

365

- **AF ID:** AF-P08833-F1-model-v4 | **Chain:** A
- **b-phipsi:** 0.0006839614943642
- **w-rdist:** 0.8183105932168729
- **t-alpha:** 0.4110998156919059

---

---

366

- **AF ID:** AF-Q5TAQ9-F1-model-v4 | **Chain:** A
- **b-phipsi:** 0.0248001998951011
- **w-rdist:** 0.620613469812032
- **t-alpha:** 0.0072833804367331

---

---

367

- **AF ID:** AF-O00330-F1-model-v4 | **Chain:** A
- **b-phipsi:** 0.020282666207896
- **w-rdist:** 0.8980219617954455
- **t-alpha:** 0.004389354417605

---

---

368

- **AF ID:** AF-O95704-F1-model-v4 | **Chain:** A
- **b-phipsi:** 0.0183531499971693
- **w-rdist:** 0.3385147467860121
- **t-alpha:** 0.1394189465197122

---

---

369

- **AF ID:** AF-P31314-F1-model-v4 | **Chain:** A
- **b-phipsi:** 0.0194742822237529
- **w-rdist:** 1.0120004421584865
- **t-alpha:** 0.0029134192830806

---

---

370

- **AF ID:** AF-Q9Y625-F1-model-v4 | **Chain:** A
- **b-phipsi:** 0.0347999930138775
- **w-rdist:** 0.9564480726404884
- **t-alpha:** 0.0014587541408541

---

---

371

- **AF ID:** AF-P14868-F1-model-v4 | **Chain:** A
- **b-phipsi:** 0.0009537372700033
- **w-rdist:** 0.7041711925355375
- **t-alpha:** 0.3594060058461437

---

---

372

- **AF ID:** AF-Q7Z7E8-F1-model-v4 | **Chain:** A
- **b-phipsi:** 0.0009590930985912
- **w-rdist:** 0.9214119023448866
- **t-alpha:** 0.1537816613453546

---

---

373

- **AF ID:** AF-Q9NQ86-F1-model-v4 | **Chain:** A
- **b-phipsi:** 0.0007202711128196
- **w-rdist:** 1.4418969123977483
- **t-alpha:** 0.0546247180047534

---

---

374

- **AF ID:** AF-Q9HAH1-F1-model-v4 | **Chain:** A
- **b-phipsi:** 0.0067593576117885
- **w-rdist:** 1.223011386796644
- **t-alpha:** 0.00512430205411

---

---

375

- **AF ID:** AF-P0C2L3-F1-model-v4 | **Chain:** A
- **b-phipsi:** 0.0109513422307413
- **w-rdist:** 0.3083614917022754
- **t-alpha:** 0.4198552688658383

---

---

376

- **AF ID:** AF-Q8NFJ9-F1-model-v4 | **Chain:** A
- **b-phipsi:** 0.0126711568919239
- **w-rdist:** 0.9078421435425578
- **t-alpha:** 0.0065982109973949

---

---

377

- **AF ID:** AF-Q04741-F1-model-v4 | **Chain:** A
- **b-phipsi:** 0.0441149071942628
- **w-rdist:** 0.9918553625535816
- **t-alpha:** 0.0007288726764216

---

---

378

- **AF ID:** AF-Q9BXL8-F1-model-v4 | **Chain:** A
- **b-phipsi:** 0.0208283525756243
- **w-rdist:** 0.3644254103675913
- **t-alpha:** 0.0940236773146412

---

---

379

- **AF ID:** AF-Q7RTS3-F1-model-v4 | **Chain:** A
- **b-phipsi:** 0.0355131869515116
- **w-rdist:** 1.0210124433015282
- **t-alpha:** 0.0007288726764216

---

---

380

- **AF ID:** AF-Q8N1L9-F1-model-v4 | **Chain:** A
- **b-phipsi:** 0.0156025623947439
- **w-rdist:** 1.1622608861558898
- **t-alpha:** 0.0021896458253802

---

---

381

- **AF ID:** AF-Q13554-F1-model-v4 | **Chain:** A
- **b-phipsi:** 0.0146685057911347
- **w-rdist:** 0.31134351118801
- **t-alpha:** 0.2825928899009824

---

---

382

- **AF ID:** AF-Q9UJV3-F1-model-v4 | **Chain:** A
- **b-phipsi:** 0.0005431783360962
- **w-rdist:** 1.4723658551144507
- **t-alpha:** 0.0888183453868016

---

---

383

- **AF ID:** AF-Q6ZMU1-F1-model-v4 | **Chain:** A
- **b-phipsi:** 0.0008026645300554
- **w-rdist:** 0.8981550543071795
- **t-alpha:** 0.2807832558099548

---

---

384

- **AF ID:** AF-A6NLC5-F1-model-v4 | **Chain:** A
- **b-phipsi:** 0.0244064961177631
- **w-rdist:** 0.3723322997833454
- **t-alpha:** 0.0651667209447612

---

---

385

- **AF ID:** AF-P0C7M4-F1-model-v4 | **Chain:** A
- **b-phipsi:** 0.0235198542178941
- **w-rdist:** 0.9140764895792248
- **t-alpha:** 0.0043701973936818

---

---

386

- **AF ID:** AF-P56192-F1-model-v4 | **Chain:** A
- **b-phipsi:** 0.0160947822062811
- **w-rdist:** 0.3300893586694921
- **t-alpha:** 0.2053902107995835

---

---

387

- **AF ID:** AF-B7ZC32-F1-model-v4 | **Chain:** A
- **b-phipsi:** 0.000913229473514
- **w-rdist:** 0.8993819378971263
- **t-alpha:** 0.2119443043382731

---

---

388

- **AF ID:** AF-O95872-F1-model-v4 | **Chain:** A
- **b-phipsi:** 0.0220599542864814
- **w-rdist:** 0.8712278657414065
- **t-alpha:** 0.00512430205411

---

---

389

- **AF ID:** AF-Q9P2I0-F1-model-v4 | **Chain:** A
- **b-phipsi:** 0.0010112024470758
- **w-rdist:** 0.9273426305997616
- **t-alpha:** 0.1456667763786574

---

---

390

- **AF ID:** AF-Q99748-F1-model-v4 | **Chain:** A
- **b-phipsi:** 0.0038809207804278
- **w-rdist:** 0.3829574039790581
- **t-alpha:** 0.4684490442190427

---

---

391

- **AF ID:** AF-O94886-F1-model-v4 | **Chain:** A
- **b-phipsi:** 0.0761939693577914
- **w-rdist:** 0.5064898881328116
- **t-alpha:** 0.0065982109973949

---

---

392

- **AF ID:** AF-Q7L5Y6-F1-model-v4 | **Chain:** A
- **b-phipsi:** 0.0010685812388412
- **w-rdist:** 0.9027417372228852
- **t-alpha:** 0.1422629527700643

---

---

393

- **AF ID:** AF-Q96AY2-F1-model-v4 | **Chain:** A
- **b-phipsi:** 0.0004999574743037
- **w-rdist:** 1.154840034769327
- **t-alpha:** 0.2068464196023396

---

---

394

- **AF ID:** AF-Q86X67-F1-model-v4 | **Chain:** A
- **b-phipsi:** 0.0005303606449945
- **w-rdist:** 0.9628102030773468
- **t-alpha:** 0.4452630955285339

---

---

395

- **AF ID:** AF-Q96MR7-F1-model-v4 | **Chain:** A
- **b-phipsi:** 0.0152425149316361
- **w-rdist:** 0.2822389729560417
- **t-alpha:** 0.3356030792065776

---

---

396

- **AF ID:** AF-Q6ICH7-F1-model-v4 | **Chain:** A
- **b-phipsi:** 0.0006827688555879
- **w-rdist:** 0.8788696648407774
- **t-alpha:** 0.4331942862850146

---

---

397

- **AF ID:** AF-Q13049-F1-model-v4 | **Chain:** A
- **b-phipsi:** 0.0004497652580305
- **w-rdist:** 1.399755516375773
- **t-alpha:** 0.1319042225962123

---

---

398

- **AF ID:** AF-Q9Y6Y0-F1-model-v4 | **Chain:** A
- **b-phipsi:** 0.0154746594358681
- **w-rdist:** 0.3886755851337689
- **t-alpha:** 0.1048799544056959

---

---

399

- **AF ID:** AF-O94776-F1-model-v4 | **Chain:** A
- **b-phipsi:** 8.142956996077073e-05
- **w-rdist:** 1.077779041150841
- **t-alpha:** 0.4297159062814497

---

---

400

- **AF ID:** AF-Q9HB96-F1-model-v4 | **Chain:** A
- **b-phipsi:** 0.0224778609314053
- **w-rdist:** 0.3785776165083168
- **t-alpha:** 0.0706484408514722

---

---

401

- **AF ID:** AF-P0C6A0-F1-model-v4 | **Chain:** A
- **b-phipsi:** 0.0714619253454017
- **w-rdist:** 0.1814316413620896
- **t-alpha:** 0.1499160832876762

---

---

402

- **AF ID:** AF-P63132-F1-model-v4 | **Chain:** A
- **b-phipsi:** 0.0002027592204408
- **w-rdist:** 1.23917965078773
- **t-alpha:** 0.2352515380442414

---

---

403

- **AF ID:** AF-A8MXV6-F1-model-v4 | **Chain:** A
- **b-phipsi:** 0.0167551448206844
- **w-rdist:** 1.0474748740705309
- **t-alpha:** 0.0036549441753579

---

---

404

- **AF ID:** AF-Q9NZM6-F1-model-v4 | **Chain:** A
- **b-phipsi:** 0.0579625288466359
- **w-rdist:** 0.837466033896149
- **t-alpha:** 0.0036415440256147

---

---

405

- **AF ID:** AF-P20592-F1-model-v4 | **Chain:** A
- **b-phipsi:** 0.0156836523884983
- **w-rdist:** 1.2548330333304134
- **t-alpha:** 0.0014587541408541

---

---

406

- **AF ID:** AF-A6NNY8-F1-model-v4 | **Chain:** A
- **b-phipsi:** 0.0008162381169493
- **w-rdist:** 1.004283670861004
- **t-alpha:** 0.1949523474570458

---

---

407

- **AF ID:** AF-Q8IV36-F1-model-v4 | **Chain:** A
- **b-phipsi:** 0.035975574837334
- **w-rdist:** 0.3916147541863886
- **t-alpha:** 0.0409402761272381

---

---

408

- **AF ID:** AF-Q9H5I1-F1-model-v4 | **Chain:** A
- **b-phipsi:** 0.0008731600210539
- **w-rdist:** 0.8245180810848936
- **t-alpha:** 0.4096512089211832

---

---

409

- **AF ID:** AF-Q9H9P8-F1-model-v4 | **Chain:** A
- **b-phipsi:** 0.0007329467563386
- **w-rdist:** 1.0302493612187589
- **t-alpha:** 0.2193605569322374

---

---

410

- **AF ID:** AF-Q68CJ6-F1-model-v4 | **Chain:** A
- **b-phipsi:** 0.052667647232322
- **w-rdist:** 0.2862442683280552
- **t-alpha:** 0.1403652546715101

---

---

411

- **AF ID:** AF-P26196-F1-model-v4 | **Chain:** A
- **b-phipsi:** 0.000992639077888
- **w-rdist:** 0.9415116976246312
- **t-alpha:** 0.1908063014656433

---

---

412

- **AF ID:** AF-P0DMS9-F1-model-v4 | **Chain:** A
- **b-phipsi:** 0.000466657435559
- **w-rdist:** 1.0113135814944625
- **t-alpha:** 0.4637525526241222

---

---

413

- **AF ID:** AF-P29144-F1-model-v4 | **Chain:** A
- **b-phipsi:** 0.0008668013192426
- **w-rdist:** 0.9704541921390863
- **t-alpha:** 0.2345225541085771

---

---

414

- **AF ID:** AF-Q8TBX8-F1-model-v4 | **Chain:** A
- **b-phipsi:** 0.0006892823057585
- **w-rdist:** 0.9470626455072236
- **t-alpha:** 0.3607530159404146

---

---

415

- **AF ID:** AF-Q7Z4G4-F1-model-v4 | **Chain:** A
- **b-phipsi:** 0.0009953497488959
- **w-rdist:** 0.91633607209118
- **t-alpha:** 0.2065023380700259

---

---

416

- **AF ID:** AF-Q7Z4H8-F1-model-v4 | **Chain:** A
- **b-phipsi:** 0.0007334845943222
- **w-rdist:** 0.9232498817127688
- **t-alpha:** 0.3910840986011614

---

---

417

- **AF ID:** AF-Q9UIW0-F1-model-v4 | **Chain:** A
- **b-phipsi:** 0.019911215932936
- **w-rdist:** 1.0371396633997505
- **t-alpha:** 0.0036549441753579

---

---

418

- **AF ID:** AF-Q9H5J8-F1-model-v4 | **Chain:** A
- **b-phipsi:** 0.0077991273001032
- **w-rdist:** 1.2096832191150924
- **t-alpha:** 0.0058607467412064

---

---

419

- **AF ID:** AF-Q9UHY8-F1-model-v4 | **Chain:** A
- **b-phipsi:** 0.0174532457440844
- **w-rdist:** 1.0984675165539357
- **t-alpha:** 0.0036415440256147

---

---

420

- **AF ID:** AF-Q9Y4K4-F1-model-v4 | **Chain:** A
- **b-phipsi:** 0.012272182339631
- **w-rdist:** 0.3846377700712211
- **t-alpha:** 0.1697011570161091

---

---

421

- **AF ID:** AF-Q9GZY0-F1-model-v4 | **Chain:** A
- **b-phipsi:** 0.0007381677723383
- **w-rdist:** 0.9674747267114516
- **t-alpha:** 0.3058993445495244

---

---

422

- **AF ID:** AF-A6NFK2-F1-model-v4 | **Chain:** A
- **b-phipsi:** 0.0246418122231984
- **w-rdist:** 0.323741782610417
- **t-alpha:** 0.1775302618623218

---

---

423

- **AF ID:** AF-Q03154-F1-model-v4 | **Chain:** A
- **b-phipsi:** 0.0005512489337888
- **w-rdist:** 1.023151982138574
- **t-alpha:** 0.3981670975043241

---

---

424

- **AF ID:** AF-Q9Y471-F1-model-v4 | **Chain:** A
- **b-phipsi:** 0.0009719050751169
- **w-rdist:** 0.9771269442409654
- **t-alpha:** 0.185665027205609

---

---

425

- **AF ID:** AF-Q8NI77-F1-model-v4 | **Chain:** A
- **b-phipsi:** 0.0003229287314775
- **w-rdist:** 1.1949168569870023
- **t-alpha:** 0.2986166858779318

---

---

426

- **AF ID:** AF-Q9Y6R7-F6-model-v4 | **Chain:** A
- **b-phipsi:** 0.0243119001258341
- **w-rdist:** 1.2253550382265153
- **t-alpha:** 0.0007288726764216

---

---

427

- **AF ID:** AF-O94832-F1-model-v4 | **Chain:** A
- **b-phipsi:** 0.0148157163932768
- **w-rdist:** 1.585513994116993
- **t-alpha:** 0.0007288726764216

---

---

428

- **AF ID:** AF-Q75V66-F1-model-v4 | **Chain:** A
- **b-phipsi:** 0.0630139744689156
- **w-rdist:** 0.7948908311096035
- **t-alpha:** 0.0050984212340619

---

---

429

- **AF ID:** AF-P22735-F1-model-v4 | **Chain:** A
- **b-phipsi:** 0.0201100436004473
- **w-rdist:** 0.3781249354945216
- **t-alpha:** 0.1201749287180127

---

---

430

- **AF ID:** AF-Q13426-F1-model-v4 | **Chain:** A
- **b-phipsi:** 0.0082296662397272
- **w-rdist:** 1.2250446348554624
- **t-alpha:** 0.0058607467412064

---

---

431

- **AF ID:** AF-Q07866-F1-model-v4 | **Chain:** A
- **b-phipsi:** 0.090455680264757
- **w-rdist:** 1.01758988534155
- **t-alpha:** 0.0007288726764216

---

---

432

- **AF ID:** AF-A6NCN8-F1-model-v4 | **Chain:** A
- **b-phipsi:** 0.0325016225619356
- **w-rdist:** 0.2376013748630809
- **t-alpha:** 0.2493177459002025

---

---

433

- **AF ID:** AF-Q9UK80-F1-model-v4 | **Chain:** A
- **b-phipsi:** 0.0155720525136663
- **w-rdist:** 0.3752735333689553
- **t-alpha:** 0.1660602202799717

---

---

434

- **AF ID:** AF-Q96C57-F1-model-v4 | **Chain:** A
- **b-phipsi:** 0.0487932664346796
- **w-rdist:** 1.032069131645535
- **t-alpha:** 0.0014587541408541

---

---

435

- **AF ID:** AF-Q9ULA0-F1-model-v4 | **Chain:** A
- **b-phipsi:** 0.0005791083769807
- **w-rdist:** 0.984678661143282
- **t-alpha:** 0.4700211588074106

---

---

436

- **AF ID:** AF-P49748-F1-model-v4 | **Chain:** A
- **b-phipsi:** 0.0303348170698882
- **w-rdist:** 0.8274206109807366
- **t-alpha:** 0.0058607467412064

---

---

437

- **AF ID:** AF-O95279-F1-model-v4 | **Chain:** A
- **b-phipsi:** 0.0271732523300751
- **w-rdist:** 0.974272563766068
- **t-alpha:** 0.0043701973936818

---

---

438

- **AF ID:** AF-Q32M78-F1-model-v4 | **Chain:** A
- **b-phipsi:** 0.0133334399423691
- **w-rdist:** 1.170906743965817
- **t-alpha:** 0.0050984212340619

---

---

439

- **AF ID:** AF-P48547-F1-model-v4 | **Chain:** A
- **b-phipsi:** 0.0392804891663654
- **w-rdist:** 0.8926933948021034
- **t-alpha:** 0.0050984212340619

---

---

440

- **AF ID:** AF-P0C7M7-F1-model-v4 | **Chain:** A
- **b-phipsi:** 0.0009343847014158
- **w-rdist:** 0.9331481081416624
- **t-alpha:** 0.2724745369855814

---

---

441

- **AF ID:** AF-P13056-F1-model-v4 | **Chain:** A
- **b-phipsi:** 0.0122193777491991
- **w-rdist:** 1.09024190857087
- **t-alpha:** 0.0058607467412064

---

---

442

- **AF ID:** AF-Q9Y4L5-F1-model-v4 | **Chain:** A
- **b-phipsi:** 0.0181911576848363
- **w-rdist:** 0.3691197113823051
- **t-alpha:** 0.1528126760301813

---

---

443

- **AF ID:** AF-P53794-F1-model-v4 | **Chain:** A
- **b-phipsi:** 0.0733508873002607
- **w-rdist:** 0.7192774728878673
- **t-alpha:** 0.0058607467412064

---

---

444

- **AF ID:** AF-Q9HAV7-F1-model-v4 | **Chain:** A
- **b-phipsi:** 0.0005036758526236
- **w-rdist:** 1.011287422750007
- **t-alpha:** 0.6562123706107199

---

---

445

- **AF ID:** AF-Q8TDJ6-F4-model-v4 | **Chain:** A
- **b-phipsi:** 0.0005233942865872
- **w-rdist:** 1.2101096097411814
- **t-alpha:** 0.2636562010650927

---

---

446

- **AF ID:** AF-Q9UQG0-F1-model-v4 | **Chain:** A
- **b-phipsi:** 0.0010196952856067
- **w-rdist:** 1.2342372621741509
- **t-alpha:** 0.0713766970124236

---

---

447

- **AF ID:** AF-Q9H7Z6-F1-model-v4 | **Chain:** A
- **b-phipsi:** 0.0010130390317369
- **w-rdist:** 0.8894698521920887
- **t-alpha:** 0.2831777012648411

---

---

448

- **AF ID:** AF-Q5VY09-F1-model-v4 | **Chain:** A
- **b-phipsi:** 0.0418577217692672
- **w-rdist:** 1.0200596836837783
- **t-alpha:** 0.0029134192830806

---

---

449

- **AF ID:** AF-Q86XR7-F1-model-v4 | **Chain:** A
- **b-phipsi:** 0.000903320849565
- **w-rdist:** 0.827985406611928
- **t-alpha:** 0.5289533786135319

---

---

450

- **AF ID:** AF-P17024-F1-model-v4 | **Chain:** A
- **b-phipsi:** 0.0216867206791953
- **w-rdist:** 1.1795129863432865
- **t-alpha:** 0.0029134192830806

---

---

451

- **AF ID:** AF-Q3ZCT1-F1-model-v4 | **Chain:** A
- **b-phipsi:** 0.0277035872825448
- **w-rdist:** 0.8060858252721834
- **t-alpha:** 0.0072833804367331

---

---

452

- **AF ID:** AF-Q96JP0-F1-model-v4 | **Chain:** A
- **b-phipsi:** 0.0529965518477877
- **w-rdist:** 0.3657789791061433
- **t-alpha:** 0.0819540697927871

---

---

453

- **AF ID:** AF-Q6ZNG1-F1-model-v4 | **Chain:** A
- **b-phipsi:** 0.0045956662760383
- **w-rdist:** 1.3988986474281977
- **t-alpha:** 0.0072833804367331

---

---

454

- **AF ID:** AF-Q9H2S6-F1-model-v4 | **Chain:** A
- **b-phipsi:** 0.0006998261143005
- **w-rdist:** 0.9776314904027356
- **t-alpha:** 0.4590863880066733

---

---

455

- **AF ID:** AF-Q5VZT2-F1-model-v4 | **Chain:** A
- **b-phipsi:** 0.0163111764865125
- **w-rdist:** 0.327008621465065
- **t-alpha:** 0.4096512089211832

---

---

456

- **AF ID:** AF-Q9Y485-F4-model-v4 | **Chain:** A
- **b-phipsi:** 0.0004998750571918
- **w-rdist:** 1.2035897701000668
- **t-alpha:** 0.3088130963638931

---

---

457

- **AF ID:** AF-Q8NCU7-F1-model-v4 | **Chain:** A
- **b-phipsi:** 0.0284035202523032
- **w-rdist:** 1.0725552647679215
- **t-alpha:** 0.0036415440256147

---

---

458

- **AF ID:** AF-Q6ZNH5-F1-model-v4 | **Chain:** A
- **b-phipsi:** 0.0102009858501347
- **w-rdist:** 1.1964891404565694
- **t-alpha:** 0.0065550134155867

---

---

459

- **AF ID:** AF-Q8NDG6-F1-model-v4 | **Chain:** A
- **b-phipsi:** 0.0010965981413398
- **w-rdist:** 0.9692255951204176
- **t-alpha:** 0.1966498397026195

---

---

460

- **AF ID:** AF-Q9Y251-F1-model-v4 | **Chain:** A
- **b-phipsi:** 0.0010835346077914
- **w-rdist:** 0.9416495980766182
- **t-alpha:** 0.2139698011127386

---

---

461

- **AF ID:** AF-Q8IYN6-F1-model-v4 | **Chain:** A
- **b-phipsi:** 0.0158991191435697
- **w-rdist:** 0.3127481056647898
- **t-alpha:** 0.5426963486396443

---

---

462

- **AF ID:** AF-Q9ULY5-F1-model-v4 | **Chain:** A
- **b-phipsi:** 0.0004623178622026
- **w-rdist:** 1.0724371841655105
- **t-alpha:** 0.7761965965812012

---

---

463

- **AF ID:** AF-Q96GL9-F1-model-v4 | **Chain:** A
- **b-phipsi:** 0.0150343420040723
- **w-rdist:** 0.3506699490373669
- **t-alpha:** 0.3953251891745846

---

---

464

- **AF ID:** AF-P18509-F1-model-v4 | **Chain:** A
- **b-phipsi:** 0.0444067610258895
- **w-rdist:** 0.2413538152047985
- **t-alpha:** 0.3038932978199846

---

---

465

- **AF ID:** AF-Q9GZR2-F1-model-v4 | **Chain:** A
- **b-phipsi:** 0.0180767789522683
- **w-rdist:** 1.0762467542324448
- **t-alpha:** 0.0058266934612722

---

---

466

- **AF ID:** AF-P78356-F1-model-v4 | **Chain:** A
- **b-phipsi:** 0.0008942584059071
- **w-rdist:** 0.9393469537403688
- **t-alpha:** 0.4272351578282072

---

---

467

- **AF ID:** AF-O95800-F1-model-v4 | **Chain:** A
- **b-phipsi:** 0.0131780041545809
- **w-rdist:** 1.0355306395030903
- **t-alpha:** 0.0073366962737499

---

---

468

- **AF ID:** AF-Q99523-F1-model-v4 | **Chain:** A
- **b-phipsi:** 0.0492799455165221
- **w-rdist:** 0.7692989543335191
- **t-alpha:** 0.0072833804367331

---

---

469

- **AF ID:** AF-Q8TAQ5-F1-model-v4 | **Chain:** A
- **b-phipsi:** 0.0239594129443445
- **w-rdist:** 1.5517476219791482
- **t-alpha:** 0.0007283954918235

---

---

470

- **AF ID:** AF-Q02241-F1-model-v4 | **Chain:** A
- **b-phipsi:** 0.000549368163566
- **w-rdist:** 1.377255259640782
- **t-alpha:** 0.2214124262080892

---

---

471

- **AF ID:** AF-Q9UET6-F1-model-v4 | **Chain:** A
- **b-phipsi:** 0.0008098104901924
- **w-rdist:** 0.9624751509135528
- **t-alpha:** 0.5238620870942268

---

---

472

- **AF ID:** AF-Q9UI47-F1-model-v4 | **Chain:** A
- **b-phipsi:** 0.1905507189573614
- **w-rdist:** 0.8945297919814079
- **t-alpha:** 0.0043701973936818

---

---

473

- **AF ID:** AF-Q99435-F1-model-v4 | **Chain:** A
- **b-phipsi:** 0.0340114062348794
- **w-rdist:** 1.3930554548255272
- **t-alpha:** 0.0

---

---

474

- **AF ID:** AF-Q92730-F1-model-v4 | **Chain:** A
- **b-phipsi:** 0.0006296338390971
- **w-rdist:** 1.0116932316299876
- **t-alpha:** 0.7270435870604988

---

---

475

- **AF ID:** AF-Q9C0B6-F1-model-v4 | **Chain:** A
- **b-phipsi:** 0.000776202316993
- **w-rdist:** 1.4894854424313757
- **t-alpha:** 0.1300409768952239

---

---

476

- **AF ID:** AF-Q9NP66-F1-model-v4 | **Chain:** A
- **b-phipsi:** 0.019625555465384
- **w-rdist:** 1.103782458953677
- **t-alpha:** 0.00512430205411

---

---

477

- **AF ID:** AF-Q9BZ23-F1-model-v4 | **Chain:** A
- **b-phipsi:** 0.0188166024764356
- **w-rdist:** 0.3599794282697021
- **t-alpha:** 0.2651128206331343

---

---

478

- **AF ID:** AF-Q96QS6-F1-model-v4 | **Chain:** A
- **b-phipsi:** 0.0008205490490191
- **w-rdist:** 1.0864408341557663
- **t-alpha:** 0.2928434839907625

---

---

479

- **AF ID:** AF-Q8N8U2-F1-model-v4 | **Chain:** A
- **b-phipsi:** 0.0297855861009524
- **w-rdist:** 0.8502000192405936
- **t-alpha:** 0.0073366962737499

---

---

480

- **AF ID:** AF-Q9Y234-F1-model-v4 | **Chain:** A
- **b-phipsi:** 0.0008900384283807
- **w-rdist:** 0.9814171324490444
- **t-alpha:** 0.4227980207919202

---

---

481

- **AF ID:** AF-Q15822-F1-model-v4 | **Chain:** A
- **b-phipsi:** 0.0010619378803657
- **w-rdist:** 1.30325094868864
- **t-alpha:** 0.0870944463336922

---

---

482

- **AF ID:** AF-Q99797-F1-model-v4 | **Chain:** A
- **b-phipsi:** 0.0405505127430707
- **w-rdist:** 0.8740910197709011
- **t-alpha:** 0.0065982109973949

---

---

483

- **AF ID:** AF-Q8TAM6-F1-model-v4 | **Chain:** A
- **b-phipsi:** 0.0599215073225381
- **w-rdist:** 1.1066984539906894
- **t-alpha:** 0.0021896458253802

---

---

484

- **AF ID:** AF-P43004-F1-model-v4 | **Chain:** A
- **b-phipsi:** 0.0918914936241162
- **w-rdist:** 0.7470699896923683
- **t-alpha:** 0.0072833804367331

---

---

485

- **AF ID:** AF-Q9Y4E6-F1-model-v4 | **Chain:** A
- **b-phipsi:** 0.0007057471394095
- **w-rdist:** 1.1274163079650952
- **t-alpha:** 0.3175532080847634

---

---

486

- **AF ID:** AF-Q9NXC5-F1-model-v4 | **Chain:** A
- **b-phipsi:** 0.0009988337264455
- **w-rdist:** 1.2640038159585514
- **t-alpha:** 0.1217319626680046

---

---

487

- **AF ID:** AF-P35638-F1-model-v4 | **Chain:** A
- **b-phipsi:** 0.0207835613941511
- **w-rdist:** 0.3433644832258138
- **t-alpha:** 0.3176581314030211

---

---

488

- **AF ID:** AF-Q8IV77-F1-model-v4 | **Chain:** A
- **b-phipsi:** 0.0340547468878599
- **w-rdist:** 1.2358929773815692
- **t-alpha:** 0.0021848670241468

---

---

489

- **AF ID:** AF-Q96IR2-F1-model-v4 | **Chain:** A
- **b-phipsi:** 0.0094366766167364
- **w-rdist:** 1.566429123180765
- **t-alpha:** 0.0058266934612722

---

---

490

- **AF ID:** AF-P0C866-F1-model-v4 | **Chain:** A
- **b-phipsi:** 0.0008228684509185
- **w-rdist:** 1.0089053666998886
- **t-alpha:** 0.4513743136001449

---

---

491

- **AF ID:** AF-Q969E3-F1-model-v4 | **Chain:** A
- **b-phipsi:** 0.0172705066112217
- **w-rdist:** 0.3674084757297893
- **t-alpha:** 0.2940622142691544

---

---

492

- **AF ID:** AF-Q96MI6-F1-model-v4 | **Chain:** A
- **b-phipsi:** 0.0006462588744333
- **w-rdist:** 1.0950703983806038
- **t-alpha:** 0.4859308238291024

---

---

493

- **AF ID:** AF-P04818-F1-model-v4 | **Chain:** A
- **b-phipsi:** 0.0005405118402384
- **w-rdist:** 1.130397603211029
- **t-alpha:** 0.6191034313350332

---

---

494

- **AF ID:** AF-Q8TF45-F1-model-v4 | **Chain:** A
- **b-phipsi:** 0.0119937067848554
- **w-rdist:** 1.301699844630393
- **t-alpha:** 0.0058607467412064

---

---

495

- **AF ID:** AF-Q7RTV5-F1-model-v4 | **Chain:** A
- **b-phipsi:** 0.0001328296863177
- **w-rdist:** 1.2812749874082532
- **t-alpha:** 0.6971570131404303

---

---

496

- **AF ID:** AF-Q9NSG2-F1-model-v4 | **Chain:** A
- **b-phipsi:** 0.2035597322035471
- **w-rdist:** 1.0553849351707096
- **t-alpha:** 0.0021896458253802

---

---

497

- **AF ID:** AF-Q9BXK1-F1-model-v4 | **Chain:** A
- **b-phipsi:** 0.0205556174652347
- **w-rdist:** 0.3847057238713109
- **t-alpha:** 0.2001745250914763

---

---

498

- **AF ID:** AF-Q08462-F1-model-v4 | **Chain:** A
- **b-phipsi:** 0.0520168385011786
- **w-rdist:** 1.2301728954786957
- **t-alpha:** 0.0014587541408541

---

---

499

- **AF ID:** AF-Q14689-F1-model-v4 | **Chain:** A
- **b-phipsi:** 0.0005737517331962
- **w-rdist:** 1.2402173046143534
- **t-alpha:** 0.3750912177807666

---

---

500

- **AF ID:** AF-Q15040-F1-model-v4 | **Chain:** A
- **b-phipsi:** 0.0004081534526463
- **w-rdist:** 1.2587288007047095
- **t-alpha:** 0.5426963486396443

---

---

501

- **AF ID:** AF-Q8TEA1-F1-model-v4 | **Chain:** A
- **b-phipsi:** 0.0009873439265071
- **w-rdist:** 1.0775239283196103
- **t-alpha:** 0.2584782099371299

---

---

502

- **AF ID:** AF-P16422-F1-model-v4 | **Chain:** A
- **b-phipsi:** 0.0007703995887902
- **w-rdist:** 1.0084874472028045
- **t-alpha:** 0.7670527563021641

---

---

503

- **AF ID:** AF-P82930-F1-model-v4 | **Chain:** A
- **b-phipsi:** 0.000926768125363
- **w-rdist:** 0.9515305243311272
- **t-alpha:** 0.5655645269348346

---

---

504

- **AF ID:** AF-P0C024-F1-model-v4 | **Chain:** A
- **b-phipsi:** 0.0002388959274154
- **w-rdist:** 1.2851409271647252
- **t-alpha:** 0.6723507164561369

---

---

505

- **AF ID:** AF-P45381-F1-model-v4 | **Chain:** A
- **b-phipsi:** 0.0005509784908983
- **w-rdist:** 1.195846456626206
- **t-alpha:** 0.5238620870942268

---

---

506

- **AF ID:** AF-Q9BQ24-F1-model-v4 | **Chain:** A
- **b-phipsi:** 0.0005627482839742
- **w-rdist:** 1.1560529559401482
- **t-alpha:** 0.6482590666191546

---

---

507

- **AF ID:** AF-O75629-F1-model-v4 | **Chain:** A
- **b-phipsi:** 0.0006267200947288
- **w-rdist:** 1.1234293813336067
- **t-alpha:** 0.5745409296631607

---

---

508

- **AF ID:** AF-P07911-F1-model-v4 | **Chain:** A
- **b-phipsi:** 0.0439917999472915
- **w-rdist:** 1.3940364190374332
- **t-alpha:** 0.0007288726764216

---

---

509

- **AF ID:** AF-Q8TB05-F1-model-v4 | **Chain:** A
- **b-phipsi:** 0.0226365806625142
- **w-rdist:** 0.3525512877247173
- **t-alpha:** 0.307619131659183

---

---

510

- **AF ID:** AF-Q63ZY6-F1-model-v4 | **Chain:** A
- **b-phipsi:** 0.0010020446722776
- **w-rdist:** 1.0103143026496435
- **t-alpha:** 0.3291386707808175

---

---

511

- **AF ID:** AF-Q8N8L6-F1-model-v4 | **Chain:** A
- **b-phipsi:** 0.0006454033708921
- **w-rdist:** 1.1253284253481173
- **t-alpha:** 0.54617088683965

---

---

512

- **AF ID:** AF-P11230-F1-model-v4 | **Chain:** A
- **b-phipsi:** 0.0009251586256177
- **w-rdist:** 1.3168943538604585
- **t-alpha:** 0.1566973677436389

---

---

513

- **AF ID:** AF-P40855-F1-model-v4 | **Chain:** A
- **b-phipsi:** 0.0730191907880134
- **w-rdist:** 0.307085023044832
- **t-alpha:** 0.2783987647377453

---

---

514

- **AF ID:** AF-P62995-F1-model-v4 | **Chain:** A
- **b-phipsi:** 0.0998145249972169
- **w-rdist:** 1.1353164836841731
- **t-alpha:** 0.0021896458253802

---

---

515

- **AF ID:** AF-Q8WUH2-F1-model-v4 | **Chain:** A
- **b-phipsi:** 0.0075605939024178
- **w-rdist:** 1.4715920246742562
- **t-alpha:** 0.0073366962737499

---

---

516

- **AF ID:** AF-Q9UMW8-F1-model-v4 | **Chain:** A
- **b-phipsi:** 0.0009687212283403
- **w-rdist:** 1.0880873130766806
- **t-alpha:** 0.2928434839907625

---

---

517

- **AF ID:** AF-Q6DHV5-F1-model-v4 | **Chain:** A
- **b-phipsi:** 0.000569213935581
- **w-rdist:** 1.5011613748591537
- **t-alpha:** 0.264384301088878

---

---

518

- **AF ID:** AF-P26715-F1-model-v4 | **Chain:** A
- **b-phipsi:** 0.0008904196780595
- **w-rdist:** 1.050597646415497
- **t-alpha:** 0.4272351578282072

---

---

519

- **AF ID:** AF-P13010-F1-model-v4 | **Chain:** A
- **b-phipsi:** 0.0010159510399597
- **w-rdist:** 1.0969451897800102
- **t-alpha:** 0.2410776229138844

---

---

520

- **AF ID:** AF-Q8TC94-F1-model-v4 | **Chain:** A
- **b-phipsi:** 0.0010475734537222
- **w-rdist:** 0.9451803049761396
- **t-alpha:** 0.4376960654520414

---

---

521

- **AF ID:** AF-Q13137-F1-model-v4 | **Chain:** A
- **b-phipsi:** 0.0177799132143805
- **w-rdist:** 1.929167495106405
- **t-alpha:** 0.0029134192830806

---

---

522

- **AF ID:** AF-P20336-F1-model-v4 | **Chain:** A
- **b-phipsi:** 0.00066917590695
- **w-rdist:** 1.0613096760552392
- **t-alpha:** 1.0370915016213322

---

---

523

- **AF ID:** AF-Q96E17-F1-model-v4 | **Chain:** A
- **b-phipsi:** 0.0007522956348411
- **w-rdist:** 1.027389086299345
- **t-alpha:** 1.0043792954684103

---

---

524

- **AF ID:** AF-Q63HN8-F21-model-v4 | **Chain:** A
- **b-phipsi:** 0.052732028958741
- **w-rdist:** 1.0474726750025558
- **t-alpha:** 0.0050984212340619

---

---

525

- **AF ID:** AF-Q9NXX6-F1-model-v4 | **Chain:** A
- **b-phipsi:** 0.0310943327933709
- **w-rdist:** 1.272091906580028
- **t-alpha:** 0.0029220273949486

---

---

526

- **AF ID:** AF-Q96KB5-F1-model-v4 | **Chain:** A
- **b-phipsi:** 0.0007983376680812
- **w-rdist:** 1.096813078339111
- **t-alpha:** 0.4940152935476167

---

---

527

- **AF ID:** AF-O43511-F1-model-v4 | **Chain:** A
- **b-phipsi:** 0.0500537826488355
- **w-rdist:** 0.9672654708732789
- **t-alpha:** 0.0065550134155867

---

---

528

- **AF ID:** AF-P19235-F1-model-v4 | **Chain:** A
- **b-phipsi:** 0.0284562258960879
- **w-rdist:** 1.3873809441440412
- **t-alpha:** 0.0029134192830806

---

---

529

- **AF ID:** AF-Q9H0M5-F1-model-v4 | **Chain:** A
- **b-phipsi:** 0.0119844404196025
- **w-rdist:** 1.429449694168285
- **t-alpha:** 0.0065550134155867

---

---

530

- **AF ID:** AF-Q04760-F1-model-v4 | **Chain:** A
- **b-phipsi:** 0.0005571720411259
- **w-rdist:** 1.201620124811215
- **t-alpha:** 0.7401773376357526

---

---

531

- **AF ID:** AF-Q9NVU7-F1-model-v4 | **Chain:** A
- **b-phipsi:** 0.0585801092036907
- **w-rdist:** 0.9853808418733068
- **t-alpha:** 0.0058607467412064

---

---

532

- **AF ID:** AF-Q9Y2L9-F1-model-v4 | **Chain:** A
- **b-phipsi:** 0.0006921822393505
- **w-rdist:** 1.2742356812822373
- **t-alpha:** 0.3597956390671519

---

---

533

- **AF ID:** AF-A6NNF4-F1-model-v4 | **Chain:** A
- **b-phipsi:** 0.0137878645775635
- **w-rdist:** 1.5531667954338964
- **t-alpha:** 0.0058266934612722

---

---

534

- **AF ID:** AF-O43264-F1-model-v4 | **Chain:** A
- **b-phipsi:** 0.1199726838036604
- **w-rdist:** 1.4204950878780496
- **t-alpha:** 0.0007283954918235

---

---

535

- **AF ID:** AF-Q96DA2-F1-model-v4 | **Chain:** A
- **b-phipsi:** 0.0006219212009011
- **w-rdist:** 1.188889664061598
- **t-alpha:** 0.9042991059388596

---

---

536

- **AF ID:** AF-Q5T011-F11-model-v4 | **Chain:** A
- **b-phipsi:** 0.0008624685002177
- **w-rdist:** 1.5984254900497648
- **t-alpha:** 0.1755285681652634

---

---

537

- **AF ID:** AF-P55160-F1-model-v4 | **Chain:** A
- **b-phipsi:** 0.1617910377441415
- **w-rdist:** 1.2699708944160442
- **t-alpha:** 0.0014587541408541

---

---

538

- **AF ID:** AF-A6NFU8-F1-model-v4 | **Chain:** A
- **b-phipsi:** 0.0005494089635062
- **w-rdist:** 1.238406762597509
- **t-alpha:** 0.9898548854088384

---

---

539

- **AF ID:** AF-Q14532-F1-model-v4 | **Chain:** A
- **b-phipsi:** 0.0718505583114304
- **w-rdist:** 1.655877890781794
- **t-alpha:** 0.0007283954918235

---

---

540

- **AF ID:** AF-Q2WGJ8-F1-model-v4 | **Chain:** A
- **b-phipsi:** 0.000925900834903
- **w-rdist:** 1.0342538925074591
- **t-alpha:** 0.6992575519326867

---

---

541

- **AF ID:** AF-Q8WXI8-F1-model-v4 | **Chain:** A
- **b-phipsi:** 0.0008619920005595
- **w-rdist:** 1.0765678013905804
- **t-alpha:** 0.7248741558575302

---

---

542

- **AF ID:** AF-P78509-F6-model-v4 | **Chain:** A
- **b-phipsi:** 0.0431849513553879
- **w-rdist:** 1.6028662018843731
- **t-alpha:** 0.0014587541408541

---

---

543

- **AF ID:** AF-Q02083-F1-model-v4 | **Chain:** A
- **b-phipsi:** 0.0008864141792909
- **w-rdist:** 1.110778902014867
- **t-alpha:** 0.5673512199485278

---

---

544

- **AF ID:** AF-A6NP61-F1-model-v4 | **Chain:** A
- **b-phipsi:** 0.035368269706328
- **w-rdist:** 0.391251163661398
- **t-alpha:** 0.202476181715995

---

---

545

- **AF ID:** AF-P55259-F1-model-v4 | **Chain:** A
- **b-phipsi:** 0.0318342716498891
- **w-rdist:** 1.0570496095692248
- **t-alpha:** 0.0072833804367331

---

---

546

- **AF ID:** AF-Q99445-F1-model-v4 | **Chain:** A
- **b-phipsi:** 0.0010145553924193
- **w-rdist:** 0.9656715670713082
- **t-alpha:** 1.3390119211824625

---

---

547

- **AF ID:** AF-Q8WZ82-F1-model-v4 | **Chain:** A
- **b-phipsi:** 0.0006080885504118
- **w-rdist:** 1.2860070585892809
- **t-alpha:** 0.7423859560666775

---

---

548

- **AF ID:** AF-Q8N8Q3-F1-model-v4 | **Chain:** A
- **b-phipsi:** 0.0008998218023613
- **w-rdist:** 1.1150483054644975
- **t-alpha:** 0.5928071625401776

---

---

549

- **AF ID:** AF-Q8IVF4-F14-model-v4 | **Chain:** A
- **b-phipsi:** 0.0785397127557206
- **w-rdist:** 1.5770616349945266
- **t-alpha:** 0.0014568440116902

---

---

550

- **AF ID:** AF-Q9Y264-F1-model-v4 | **Chain:** A
- **b-phipsi:** 0.0119981269126123
- **w-rdist:** 1.58158291813309
- **t-alpha:** 0.0072833804367331

---

---

551

- **AF ID:** AF-Q8IWA4-F1-model-v4 | **Chain:** A
- **b-phipsi:** 0.0721890622129476
- **w-rdist:** 1.620442091842729
- **t-alpha:** 0.0014568440116902

---

---

552

- **AF ID:** AF-P62910-F1-model-v4 | **Chain:** A
- **b-phipsi:** 0.000903079467158
- **w-rdist:** 1.112244239676817
- **t-alpha:** 0.6602172562591797

---

---

553

- **AF ID:** AF-Q8NFV4-F1-model-v4 | **Chain:** A
- **b-phipsi:** 0.0009455000902153
- **w-rdist:** 1.1618107344793556
- **t-alpha:** 0.4559915152873357

---

---

554

- **AF ID:** AF-Q6ZRQ5-F1-model-v4 | **Chain:** A
- **b-phipsi:** 0.1584891342354302
- **w-rdist:** 1.493054672352055
- **t-alpha:** 0.0014568440116902

---

---

555

- **AF ID:** AF-Q96PP4-F1-model-v4 | **Chain:** A
- **b-phipsi:** 0.0256989904446644
- **w-rdist:** 1.1068514853689269
- **t-alpha:** 0.0073366962737499

---

---

556

- **AF ID:** AF-Q6P9F7-F1-model-v4 | **Chain:** A
- **b-phipsi:** 0.0175518080969384
- **w-rdist:** 1.3941372569318875
- **t-alpha:** 0.0065550134155867

---

---

557

- **AF ID:** AF-Q99572-F1-model-v4 | **Chain:** A
- **b-phipsi:** 0.0010891483023973
- **w-rdist:** 1.4075458659780502
- **t-alpha:** 0.1557237293482958

---

---

558

- **AF ID:** AF-Q86U42-F1-model-v4 | **Chain:** A
- **b-phipsi:** 0.0670491811790029
- **w-rdist:** 1.0908536326804763
- **t-alpha:** 0.0058607467412064

---

---

559

- **AF ID:** AF-O95071-F2-model-v4 | **Chain:** A
- **b-phipsi:** 0.0009782276766542
- **w-rdist:** 1.2670100660114167
- **t-alpha:** 0.307356032104314

---

---

560

- **AF ID:** AF-O75594-F1-model-v4 | **Chain:** A
- **b-phipsi:** 0.0007895993406634
- **w-rdist:** 1.1849047184457742
- **t-alpha:** 1.239804036071395

---

---

561

- **AF ID:** AF-P63135-F1-model-v4 | **Chain:** A
- **b-phipsi:** 0.000926215865881
- **w-rdist:** 1.2864497765803051
- **t-alpha:** 0.3590683276233792

---

---

562

- **AF ID:** AF-O94812-F1-model-v4 | **Chain:** A
- **b-phipsi:** 0.0159693596912808
- **w-rdist:** 1.5504668809969278
- **t-alpha:** 0.0065982109973949

---

---

563

- **AF ID:** AF-P55290-F1-model-v4 | **Chain:** A
- **b-phipsi:** 0.0272290340198293
- **w-rdist:** 1.601562995038173
- **t-alpha:** 0.0043701973936818

---

---

564

- **AF ID:** AF-P62070-F1-model-v4 | **Chain:** A
- **b-phipsi:** 0.0008528083536273
- **w-rdist:** 1.1874412411506865
- **t-alpha:** 0.8680268214080953

---

---

565

- **AF ID:** AF-Q4W4Y0-F1-model-v4 | **Chain:** A
- **b-phipsi:** 0.0010323154411592
- **w-rdist:** 1.1235582342019648
- **t-alpha:** 0.471597061629184

---

---

566

- **AF ID:** AF-O95873-F1-model-v4 | **Chain:** A
- **b-phipsi:** 0.0321887980829361
- **w-rdist:** 1.3584917751127166
- **t-alpha:** 0.00512430205411

---

---

567

- **AF ID:** AF-Q6NSJ5-F1-model-v4 | **Chain:** A
- **b-phipsi:** 0.0161276041513417
- **w-rdist:** 1.390412933319282
- **t-alpha:** 0.0073366962737499

---

---

568

- **AF ID:** AF-Q9NUL5-F1-model-v4 | **Chain:** A
- **b-phipsi:** 0.0010632040696501
- **w-rdist:** 1.072675291077266
- **t-alpha:** 0.6522258120583067

---

---

569

- **AF ID:** AF-Q9HBG7-F1-model-v4 | **Chain:** A
- **b-phipsi:** 0.0284366627086675
- **w-rdist:** 1.505022737771871
- **t-alpha:** 0.0050984212340619

---

---

570

- **AF ID:** AF-Q9P1W9-F1-model-v4 | **Chain:** A
- **b-phipsi:** 0.001066402089961
- **w-rdist:** 1.173343874790623
- **t-alpha:** 0.4242737205531668

---

---

571

- **AF ID:** AF-Q8IYX3-F1-model-v4 | **Chain:** A
- **b-phipsi:** 0.0010001778691399
- **w-rdist:** 1.34870922058532
- **t-alpha:** 0.3051709871846573

---

---

572

- **AF ID:** AF-Q9H9E3-F1-model-v4 | **Chain:** A
- **b-phipsi:** 0.1861348713717644
- **w-rdist:** 1.443504153750211
- **t-alpha:** 0.0029134192830806

---

---

573

- **AF ID:** AF-Q8N5S3-F1-model-v4 | **Chain:** A
- **b-phipsi:** 0.0645498829932179
- **w-rdist:** 1.229288736087537
- **t-alpha:** 0.0058266934612722

---

---

574

- **AF ID:** AF-Q14CZ8-F1-model-v4 | **Chain:** A
- **b-phipsi:** 0.0512692143860034
- **w-rdist:** 1.3658713078308131
- **t-alpha:** 0.0050984212340619

---

---

575

- **AF ID:** AF-O75608-F1-model-v4 | **Chain:** A
- **b-phipsi:** 0.0008050582849222
- **w-rdist:** 1.2970192531799891
- **t-alpha:** 0.8380189876649526

---

---

576

- **AF ID:** AF-Q3KRA6-F1-model-v4 | **Chain:** A
- **b-phipsi:** 0.0007074627579279
- **w-rdist:** 1.3640948035856988
- **t-alpha:** 1.1058285810634971

---

---

577

- **AF ID:** AF-Q99487-F1-model-v4 | **Chain:** A
- **b-phipsi:** 0.0010985439045555
- **w-rdist:** 1.185819666603115
- **t-alpha:** 0.4529101351111235

---

---

578

- **AF ID:** AF-P09758-F1-model-v4 | **Chain:** A
- **b-phipsi:** 0.0010705435399987
- **w-rdist:** 1.1033617895980978
- **t-alpha:** 0.9530580997962405

---

---

579

- **AF ID:** AF-Q8N8D1-F1-model-v4 | **Chain:** A
- **b-phipsi:** 0.0377666884921889
- **w-rdist:** 1.4546071442468484
- **t-alpha:** 0.0058266934612722

---

---

580

- **AF ID:** AF-Q9Y6W8-F1-model-v4 | **Chain:** A
- **b-phipsi:** 0.0010014632206026
- **w-rdist:** 1.175339560613221
- **t-alpha:** 1.1353035574607393

---

---

581

- **AF ID:** AF-P41219-F1-model-v4 | **Chain:** A
- **b-phipsi:** 0.104603072550183
- **w-rdist:** 1.6692991098076593
- **t-alpha:** 0.0029220273949486

---

---

582

- **AF ID:** AF-P0CAP1-F1-model-v4 | **Chain:** A
- **b-phipsi:** 0.0784761020506889
- **w-rdist:** 1.9932742047081269
- **t-alpha:** 0.0029220273949486

---

---

583

- **AF ID:** AF-Q9Y487-F1-model-v4 | **Chain:** A
- **b-phipsi:** 0.037474104571008
- **w-rdist:** 1.2385518291347275
- **t-alpha:** 0.0072833804367331

---

---

584

- **AF ID:** AF-Q12756-F1-model-v4 | **Chain:** A
- **b-phipsi:** 0.0009581746579332
- **w-rdist:** 1.3531694493018258
- **t-alpha:** 0.531681772119877

---

---

585

- **AF ID:** AF-Q96DT5-F16-model-v4 | **Chain:** A
- **b-phipsi:** 0.0874231444993692
- **w-rdist:** 1.5875837499649872
- **t-alpha:** 0.0036549441753579

---

---

586

- **AF ID:** AF-P51160-F1-model-v4 | **Chain:** A
- **b-phipsi:** 0.0373997416237076
- **w-rdist:** 1.347579482103516
- **t-alpha:** 0.0065982109973949

---

---

587

- **AF ID:** AF-Q96A22-F1-model-v4 | **Chain:** A
- **b-phipsi:** 0.0532384327572825
- **w-rdist:** 0.3768028208510821
- **t-alpha:** 0.525555357896593

---

---

588

- **AF ID:** AF-H3BQW9-F1-model-v4 | **Chain:** A
- **b-phipsi:** 0.1078339631110849
- **w-rdist:** 0.3508908471244742
- **t-alpha:** 0.7248741558575302

---

---

589

- **AF ID:** AF-Q8NCM8-F12-model-v4 | **Chain:** A
- **b-phipsi:** 0.0629899640222277
- **w-rdist:** 1.5835150935347364
- **t-alpha:** 0.0050984212340619

---

---

590

- **AF ID:** AF-O60667-F1-model-v4 | **Chain:** A
- **b-phipsi:** 0.0258826750119587
- **w-rdist:** 1.4590906297007202
- **t-alpha:** 0.0072833804367331

---

---

591

- **AF ID:** AF-A6NEM1-F1-model-v4 | **Chain:** A
- **b-phipsi:** 0.026556825636141
- **w-rdist:** 1.6662839721032232
- **t-alpha:** 0.0065550134155867

---

---

592

- **AF ID:** AF-Q9Y5E1-F1-model-v4 | **Chain:** A
- **b-phipsi:** 0.0243537401354999
- **w-rdist:** 1.8052329404677547
- **t-alpha:** 0.0065982109973949

---

---

593

- **AF ID:** AF-Q5VST9-F16-model-v4 | **Chain:** A
- **b-phipsi:** 0.0574664182343403
- **w-rdist:** 1.9050106583121893
- **t-alpha:** 0.0050984212340619

---

---

594

- **AF ID:** AF-Q13519-F1-model-v4 | **Chain:** A
- **b-phipsi:** 0.0900716866778576
- **w-rdist:** 0.3732104901695553
- **t-alpha:** 0.532366274953326

---

---

595

- **AF ID:** AF-Q13404-F1-model-v4 | **Chain:** A
- **b-phipsi:** 0.0009671190996117
- **w-rdist:** 1.3212280464792294
- **t-alpha:** 1.225283491262589

---

---

596

- **AF ID:** AF-Q9P225-F11-model-v4 | **Chain:** A
- **b-phipsi:** 0.0487905718551149
- **w-rdist:** 1.5864317029121977
- **t-alpha:** 0.0058607467412064

---

---

597

- **AF ID:** AF-P60604-F1-model-v4 | **Chain:** A
- **b-phipsi:** 0.001011074107885
- **w-rdist:** 1.3138255971832031
- **t-alpha:** 0.8834016474385145

---

---

598

- **AF ID:** AF-Q16352-F1-model-v4 | **Chain:** A
- **b-phipsi:** 0.1329549401354309
- **w-rdist:** 1.671298092322386
- **t-alpha:** 0.0050984212340619

---

---

599

- **AF ID:** AF-Q96CV9-F1-model-v4 | **Chain:** A
- **b-phipsi:** 0.1365028766435943
- **w-rdist:** 1.7792249336592325
- **t-alpha:** 0.0050984212340619

---

---

600

- **AF ID:** AF-Q9Y2D4-F1-model-v4 | **Chain:** A
- **b-phipsi:** 0.1587684943894436
- **w-rdist:** 1.7189085532589163
- **t-alpha:** 0.0050984212340619

---

---

601

- **AF ID:** AF-A0A3B3IRV3-F1-model-v4 | **Chain:** A
- **b-phipsi:** 0.0010770061535918
- **w-rdist:** 1.30766096855276
- **t-alpha:** 1.0280652739481408

---

---

602

- **AF ID:** AF-Q17RH7-F1-model-v4 | **Chain:** A
- **b-phipsi:** 0.1608221933569198
- **w-rdist:** 1.3045707455879747
- **t-alpha:** 0.0072833804367331

---

---

603

- **AF ID:** AF-Q5JSS6-F1-model-v4 | **Chain:** A
- **b-phipsi:** 0.0010385108854258
- **w-rdist:** 1.391939866908111
- **t-alpha:** 1.51005527606545

---

---

604

- **AF ID:** AF-Q0VFZ6-F1-model-v4 | **Chain:** A
- **b-phipsi:** 0.2355546310649429
- **w-rdist:** 1.667910665820791
- **t-alpha:** 0.0058607467412064

---

---

605

- **AF ID:** AF-P60371-F1-model-v4 | **Chain:** A
- **b-phipsi:** 0.0968696691662063
- **w-rdist:** 1.5542812355915991
- **t-alpha:** 0.0072833804367331

---

---

606

- **AF ID:** AF-Q03001-F21-model-v4 | **Chain:** A
- **b-phipsi:** 0.3929505750349089
- **w-rdist:** 1.9716604357916567
- **t-alpha:** 0.0058607467412064

---

---

607

- **AF ID:** AF-O95995-F1-model-v4 | **Chain:** A
- **b-phipsi:** 0.2370452602834016
- **w-rdist:** 1.817273887704167
- **t-alpha:** 0.0065550134155867

---

---
