# Supplementary material for: Comparative structural insights and functional analysis for the distinct unbound states of Human AGO proteins
Source: Sci Rep. 2025 Mar 19;15:9432. doi: 10.1038/s41598-025-91849-5 (PMC11923369; doi:10.1038/s41598-025-91849-5)
Supplement: Supplementary file 24 — Supplementary Information 12. [file 41598_2025_91849_MOESM24_ESM.zip › 4Z4Dp_A_mdwhole_AF4REF/go/4Z4Dp_A_mitot_mitosis_48b968e8b0064751aea82538ef7ddf99-pres_report.html]

 

# Structural Comparison Report for 4Z4Dp\_A - whole structures (total: 1)

---

1

- **Protein name:** Serine/threonine-protein kinase PLK3
- **Organism:** Homo sapiens
- **Uniprot Accession Number:** Q9H4B4
- **Protein sequence length:** 646 aa
- **1D identity (%):** 12.26
- **1D identity (%) [Gaps excluded]:** 21.23
- **1D identity - Alignment Gaps:** 403
- **1D aligned content (<aminoacid>:%):** {'A': 5.98, 'F': 5.13, 'P': 12.82, 'L': 10.26, 'E': 4.27, 'D': 4.27, 'R': 9.4, 'T': 3.42, 'G': 8.55, 'K': 5.13, 'Y': 3.42, 'V': 7.69, 'S': 4.27, 'Q': 4.27, 'H': 4.27, 'C': 3.42, 'N': 1.71, 'I': 0.85, 'W': 0.85}
- **Common reported functions (%):** 0.0
- **Common reported locations (%):** 28.57
- **Common reported processes (%):** 0.0

- **AF ID:** Q9H4B4
- **Chain:** A
- **Protein length:** 646 aa
- **Resolution:** N/A
- **b-phipsi ():** 0.005512
- **w-rdist ():** 0.249785
- **t-alpha ():** 0.03933
- **Chemical similarity (Tanimoto Index) (%):** 97.33
- **1D identity (%) [PDB]:** 2.44
- **1D identity (%) [Gaps excluded][PDB]:** 74.47
- **1D identity - Alignment Gaps [PDB]:** 1390
- **1D aligned content [PDB] (<aminoacid>:%):** {'E': 8.57, 'L': 14.29, 'T': 5.71, 'P': 5.71, 'G': 5.71, 'K': 8.57, 'R': 2.86, 'I': 5.71, 'F': 2.86, 'V': 11.43, 'S': 11.43, 'C': 2.86, 'A': 8.57, 'H': 2.86, 'Q': 2.86}
- **2D identity (%) [PDB]:** 41.97
- **2D identity (%) [Gaps excluded][PDB]:** 87.53
- **2D identity - Alignment Gaps [PDB]:** 522
- **2D aligned content [PDB] (<2D-fold>:%):** {'.': 18.29, 'T': 18.53, 'H': 40.14, 'E': 22.33, 'G': 0.71}
- **3D similarity (TM-Score) (%) [PDB]:** 21.37

- **Gene name:** PLK3
- **Entrez ID:** 126300
- **RefSeq ID:** NM\_004073
- **Transcript sequence length:** 2340
- **5-UTR|CDS|3-UTR identity (%):** 40.43 | 43.66 | 1.73
- **5-UTR|CDS|3-UTR identity (%) [Gaps excluded]:** 78.08 | 75.24 | 76.3
- **5-UTR|CDS|3-UTR identity [Alignment Gaps]:** 68 | 1201 | 11660
- **5-UTR aligned content (<base>:%):** {'G': 54.39, 'C': 45.61}
- **CDS aligned content (<base>:%):** {'A': 19.78, 'T': 17.45, 'G': 29.38, 'C': 33.39}
- **3-UTR aligned content (<base>:%):** {'G': 21.36, 'C': 30.58, 'A': 16.02, 'T': 32.04}

**Uniprot Description:**  
  
 Serine/threonine-protein kinase involved in cell cycle regulation, response to stress and Golgi disassembly. Polo-like kinases act by binding and phosphorylating proteins are that already phosphorylated on a specific motif recognized by the POLO box domains. Phosphorylates ATF2, BCL2L1, CDC25A, CDC25C, CHEK2, HIF1A, JUN, p53/TP53, p73/TP73, PTEN, TOP2A and VRK1. Involved in cell cycle regulation: required for entry into S phase and cytokinesis. Phosphorylates BCL2L1, leading to regulate the G2 checkpoint and progression to cytokinesis during mitosis. Plays a key role in response to stress: rapidly activated upon stress stimulation, such as ionizing radiation, reactive oxygen species (ROS), hyperosmotic stress, UV irradiation and hypoxia. Involved in DNA damage response and G1/S transition checkpoint by phosphorylating CDC25A, p53/TP53 and p73/TP73. Phosphorylates p53/TP53 in response to reactive oxygen species (ROS), thereby promoting p53/TP53-mediated apoptosis. Phosphorylates CHEK2 in response to DNA damage, promoting the G2/M transition checkpoint. Phosphorylates the transcription factor p73/TP73 in response to DNA damage, leading to inhibit p73/TP73-mediated transcriptional activation and pro-apoptotic functions. Phosphorylates HIF1A and JUN is response to hypoxia. Phosphorylates ATF2 following hyperosmotic stress in corneal epithelium. Also involved in Golgi disassembly during the cell cycle: part of a MEK1/MAP2K1-dependent pathway that induces Golgi fragmentation during mitosis by mediating phosphorylation of VRK1. May participate in endomitotic cell cycle, a form of mitosis in which both karyokinesis and cytokinesis are interrupted and is a hallmark of megakaryocyte differentiation, via its interaction with CIB1.   
  
Interacts (via the POLO-box domain) with CIB1; leading to inhibit PLK3 kinase activity. Interacts with GOLGB1.   
  
 **Gene Ontology Information:**

Molecular Function

- ATP binding
- p53 binding
- protein serine kinase activity
- protein serine/threonine kinase activity

Location

- centrosome
- cytoplasm
- dendrite
- Golgi stack
- kinetochore
- neuronal cell body
- nucleolus
- nucleoplasm
- nucleus
- spindle pole

Biological process

- apoptotic process
- cytoplasmic microtubule organization
- cellular response to DNA damage stimulus
- DNA damage response, signal transduction by p53 class mediator resulting in cell cycle arrest
- endomitotic cell cycle
- G1/S transition of mitotic cell cycle
- G2/M transition of mitotic cell cycle
- Golgi disassembly
- mitotic G1/S transition checkpoint
- mitotic spindle organization
- negative regulation of apoptotic process
- negative regulation of transcription by RNA polymerase II
- positive regulation of chaperone-mediated autophagy
- positive regulation of intracellular protein transport
- positive regulation of proteasomal ubiquitin-dependent protein catabolic process involved in cellular response to hypoxia
- protein kinase B signaling
- protein phosphorylation
- regulation of cell division
- regulation of cytokinesis
- regulation of signal transduction by p53 class mediator
- response to osmotic stress
- response to radiation
- response to reactive oxygen species

---
