# Supplementary figures and images for: Comparative structural insights and functional analysis for the distinct unbound states of Human AGO proteins
Source: Sci Rep. 2025 Mar 19;15:9432. doi: 10.1038/s41598-025-91849-5 (PMC11923369; doi:10.1038/s41598-025-91849-5)

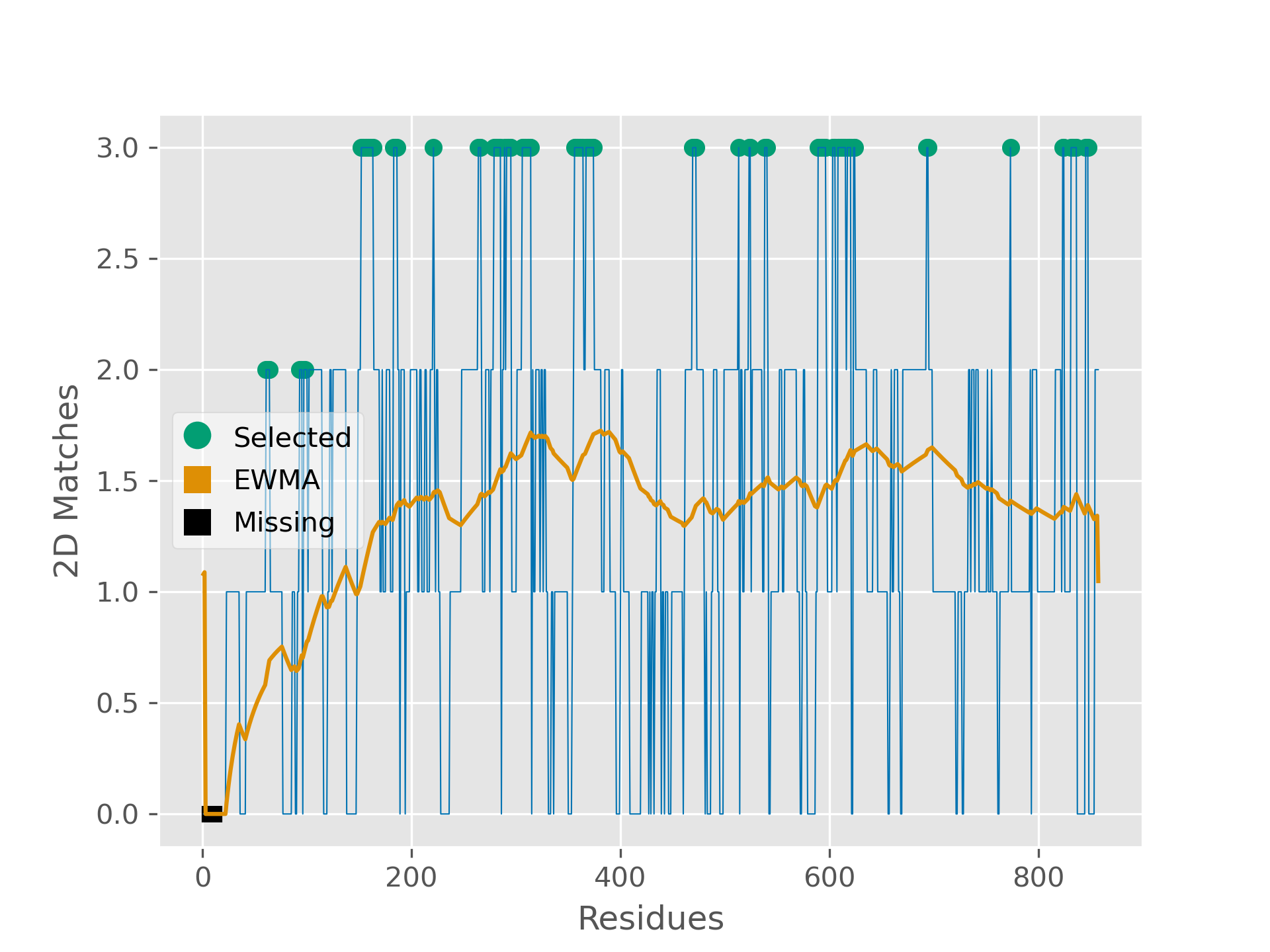

Supplement: Supplementary file 24 — Supplementary Information 12. [file 41598_2025_91849_MOESM24_ESM.zip › 4KREp_A_mdwhole_AF4REF/go/4KREp_A_mitot_mitosis_7bedd80c00a949cdb7afa7a9f8f82984.png]

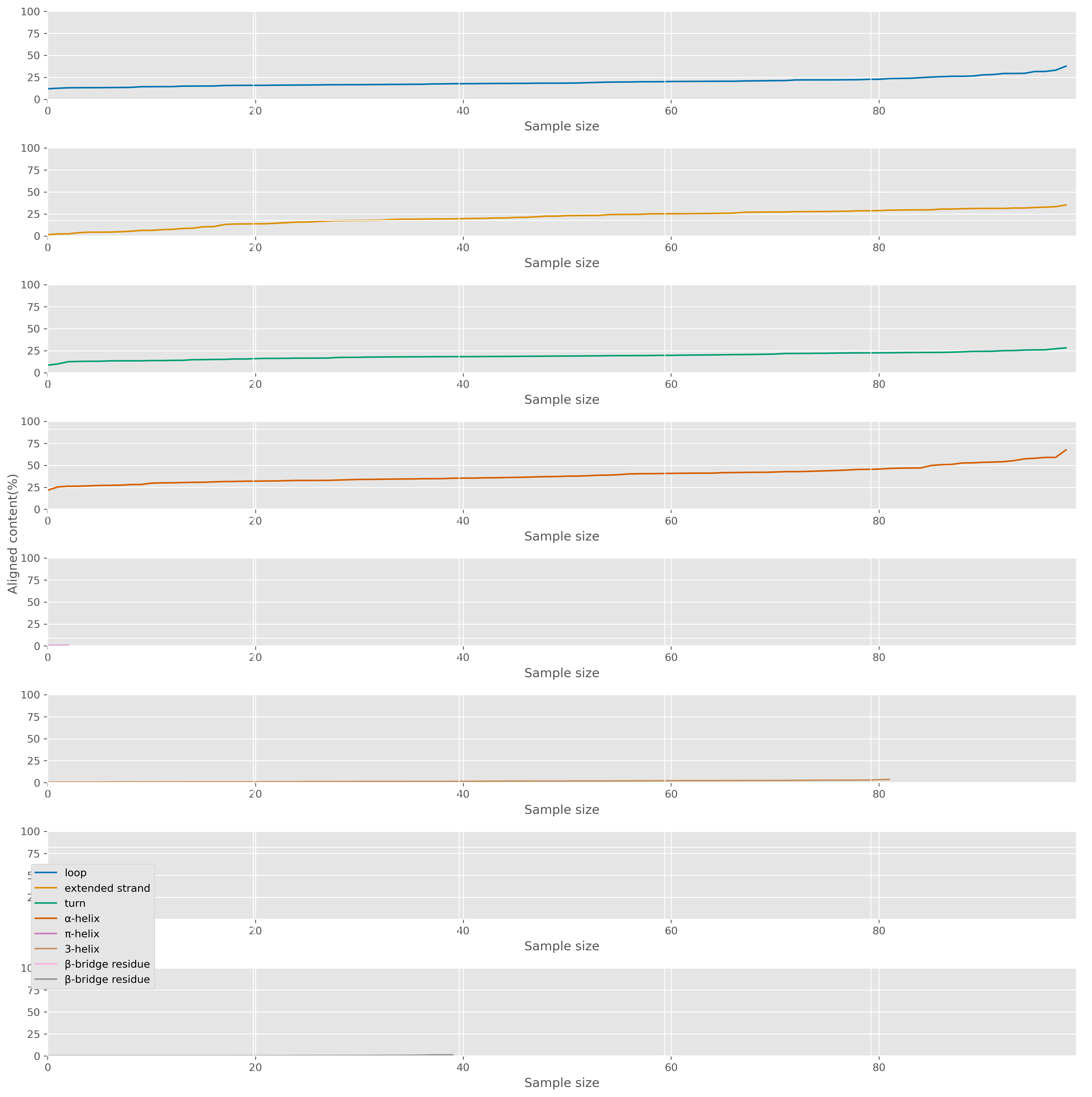

Supplement: Supplementary file 24 — Supplementary Information 12. [file 41598_2025_91849_MOESM24_ESM.zip › 4KREp_A_mdwhole_AF4REF/plots/4KREp_A-2Dfold_coverage.png]

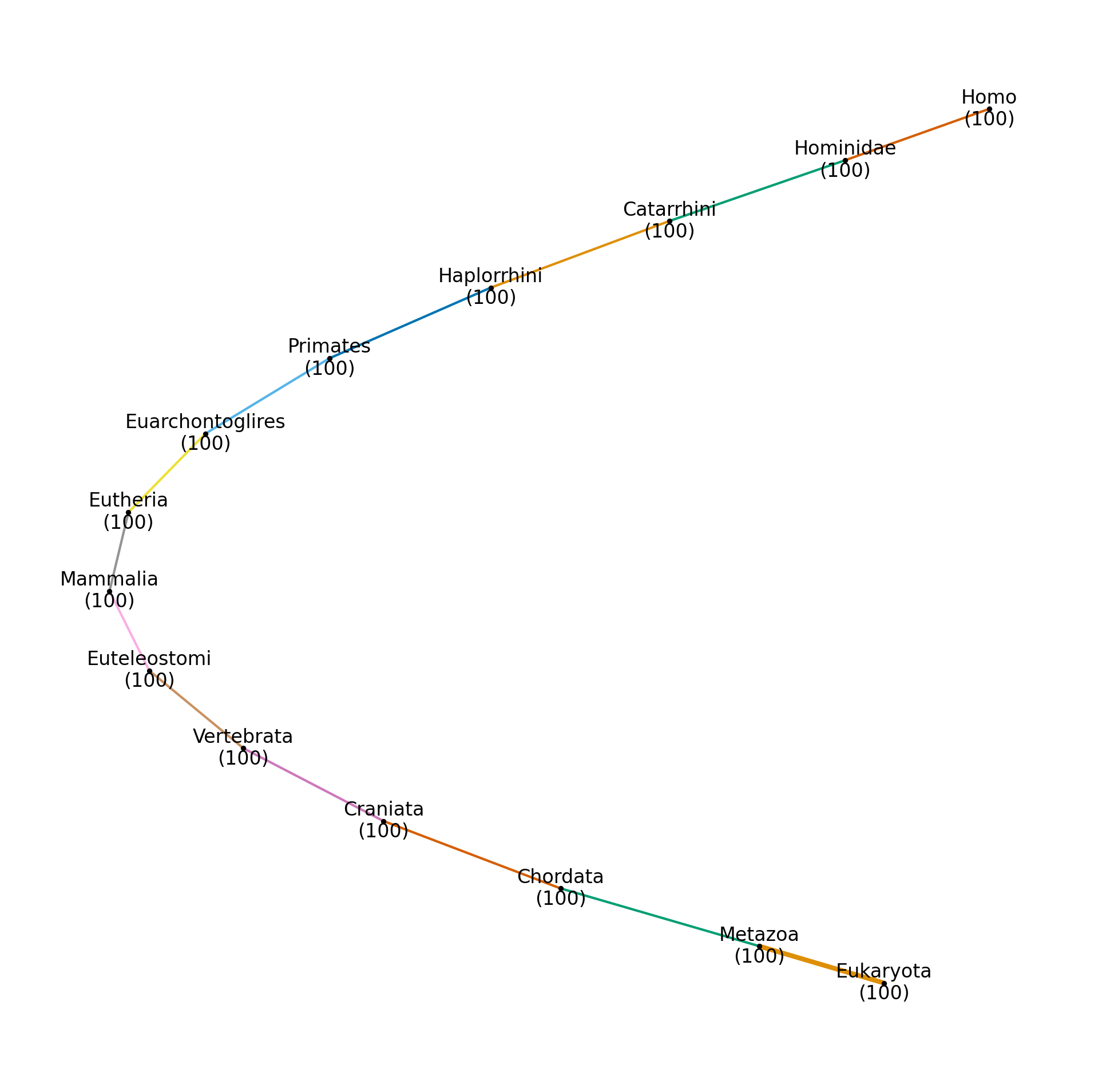

Supplement: Supplementary file 24 — Supplementary Information 12. [file 41598_2025_91849_MOESM24_ESM.zip › 4KREp_A_mdwhole_AF4REF/plots/4KREp_A-Eukaryota-tree.png]

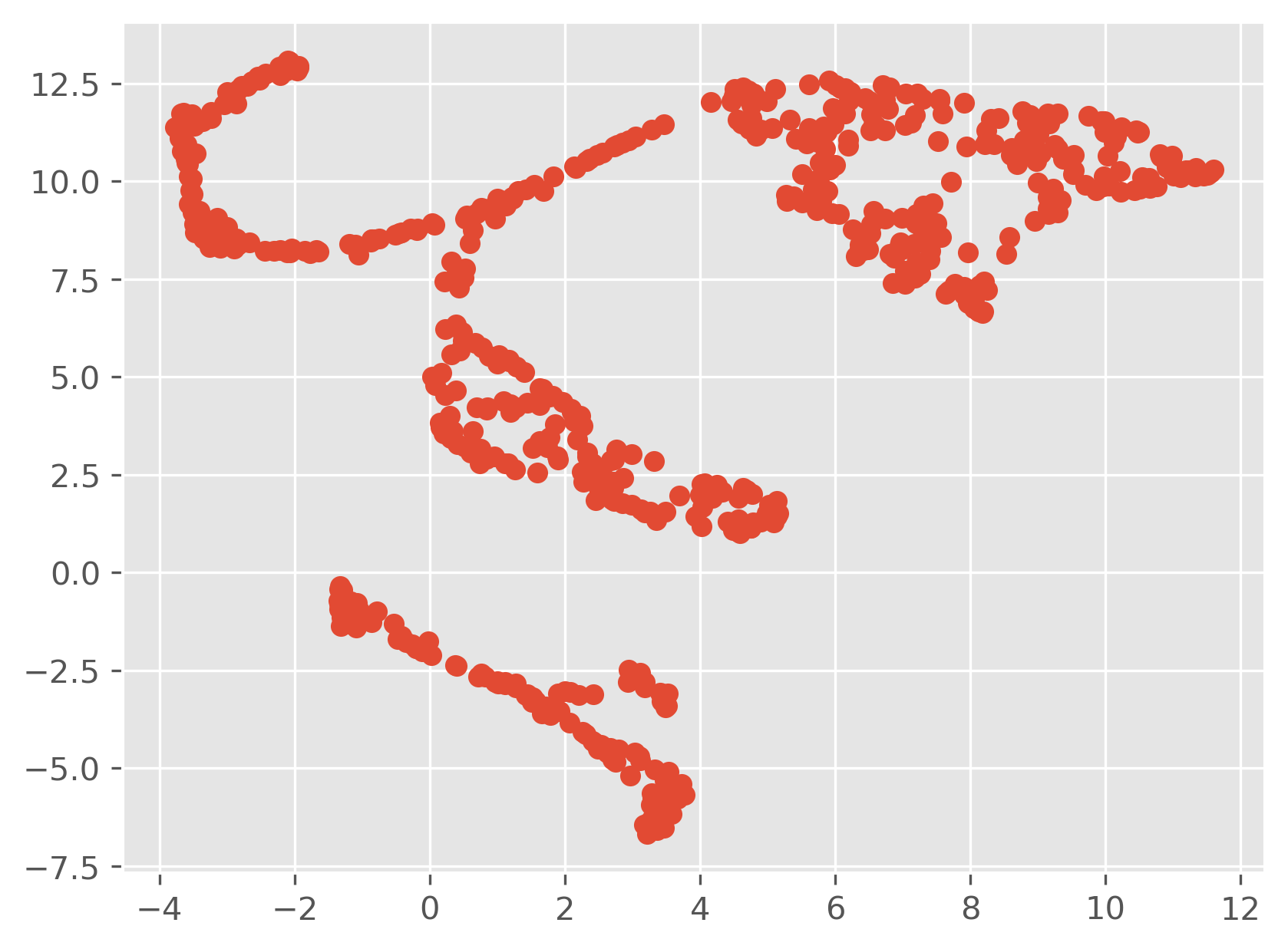

Supplement: Supplementary file 24 — Supplementary Information 12. [file 41598_2025_91849_MOESM24_ESM.zip › 4KREp_A_mdwhole_AF4REF/plots/4KREp_A-UMAP.png]

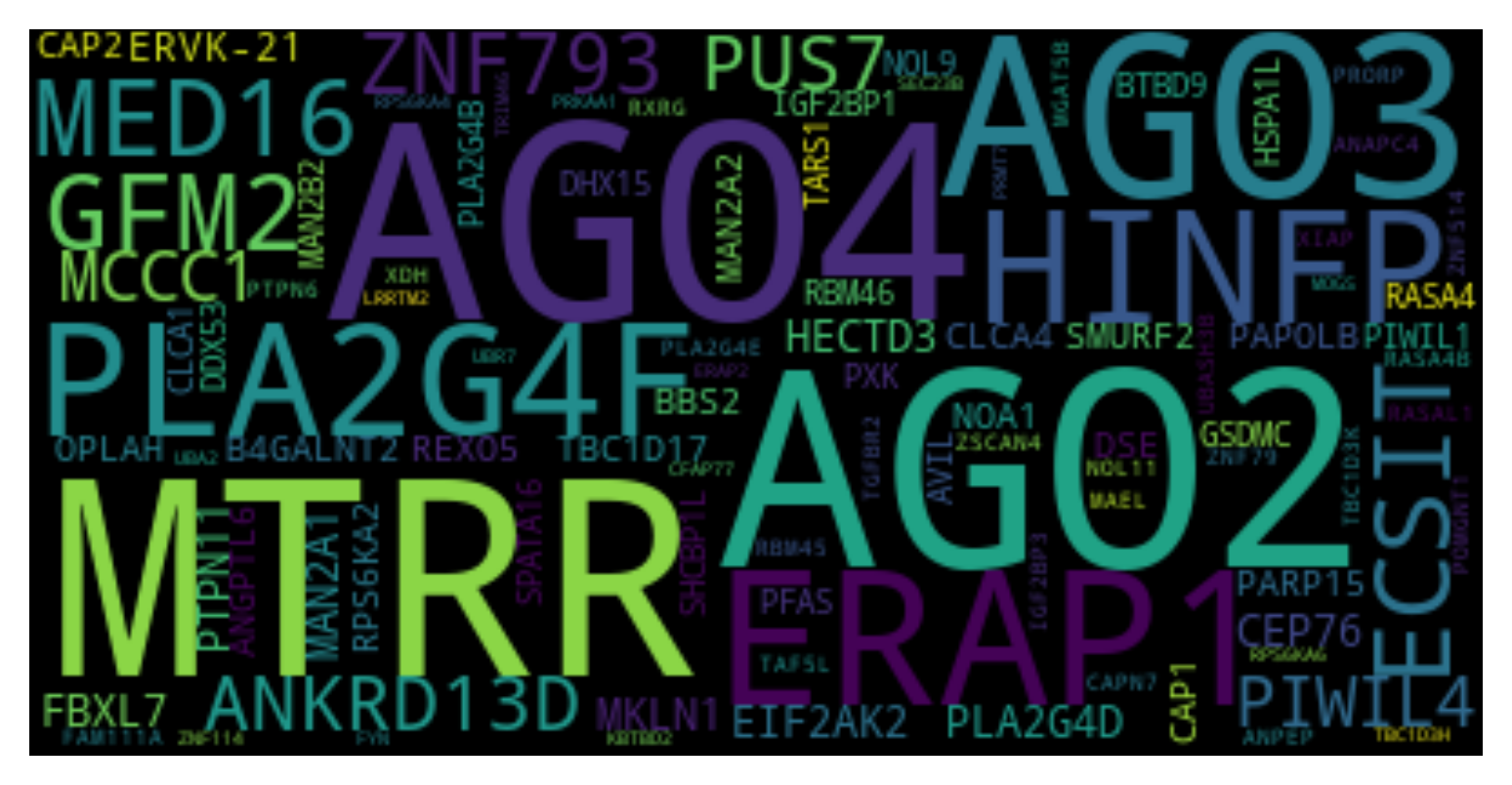

Supplement: Supplementary file 24 — Supplementary Information 12. [file 41598_2025_91849_MOESM24_ESM.zip › 4KREp_A_mdwhole_AF4REF/plots/4KREp_A-wordcloud.png]

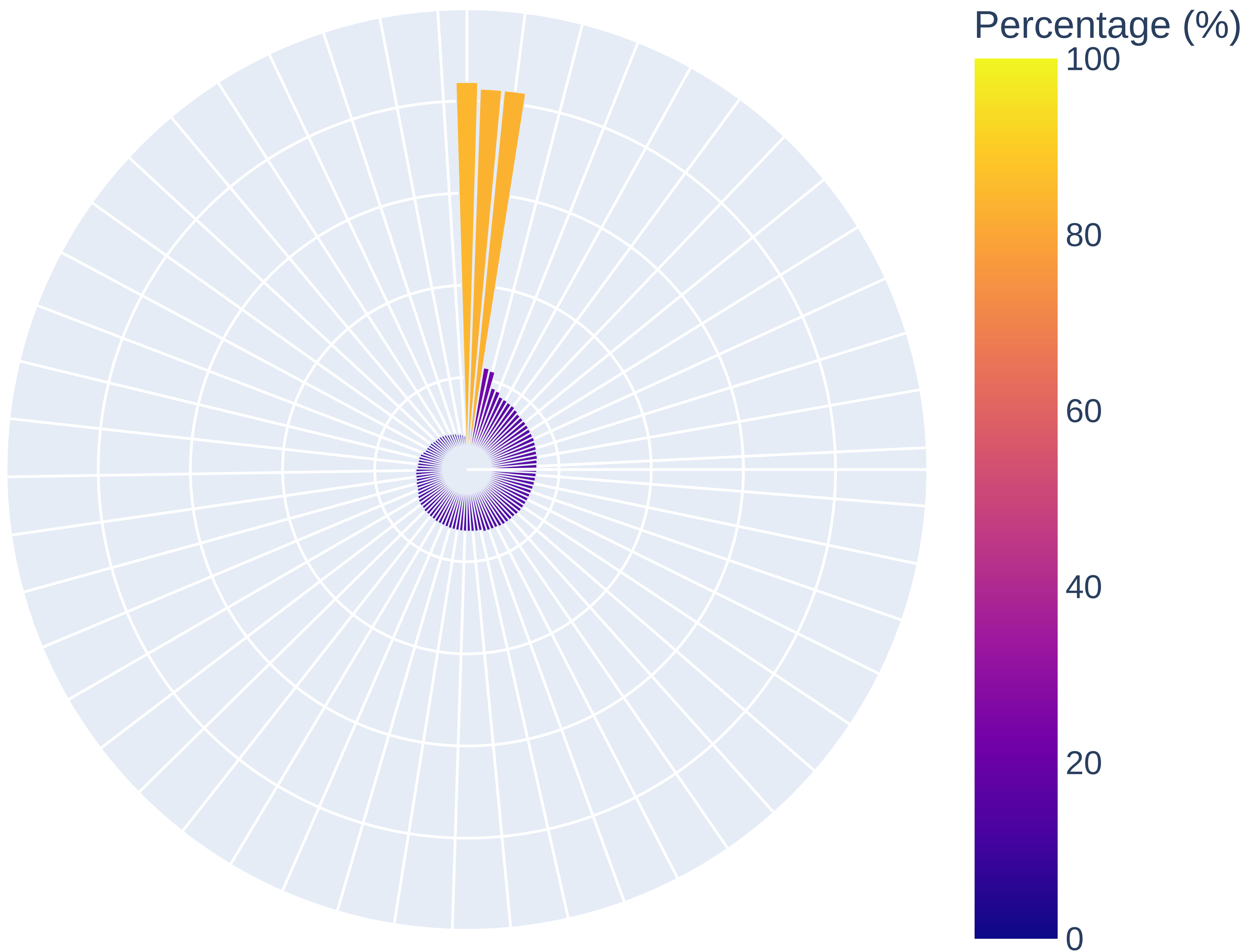

1D identity for 99 proteins in the final set (total: 100)

Supplement: Supplementary file 24 — Supplementary Information 12. [file 41598_2025_91849_MOESM24_ESM.zip › 4KREp_A_mdwhole_AF4REF/plots/4KREp_A_1D-identity.pdf]

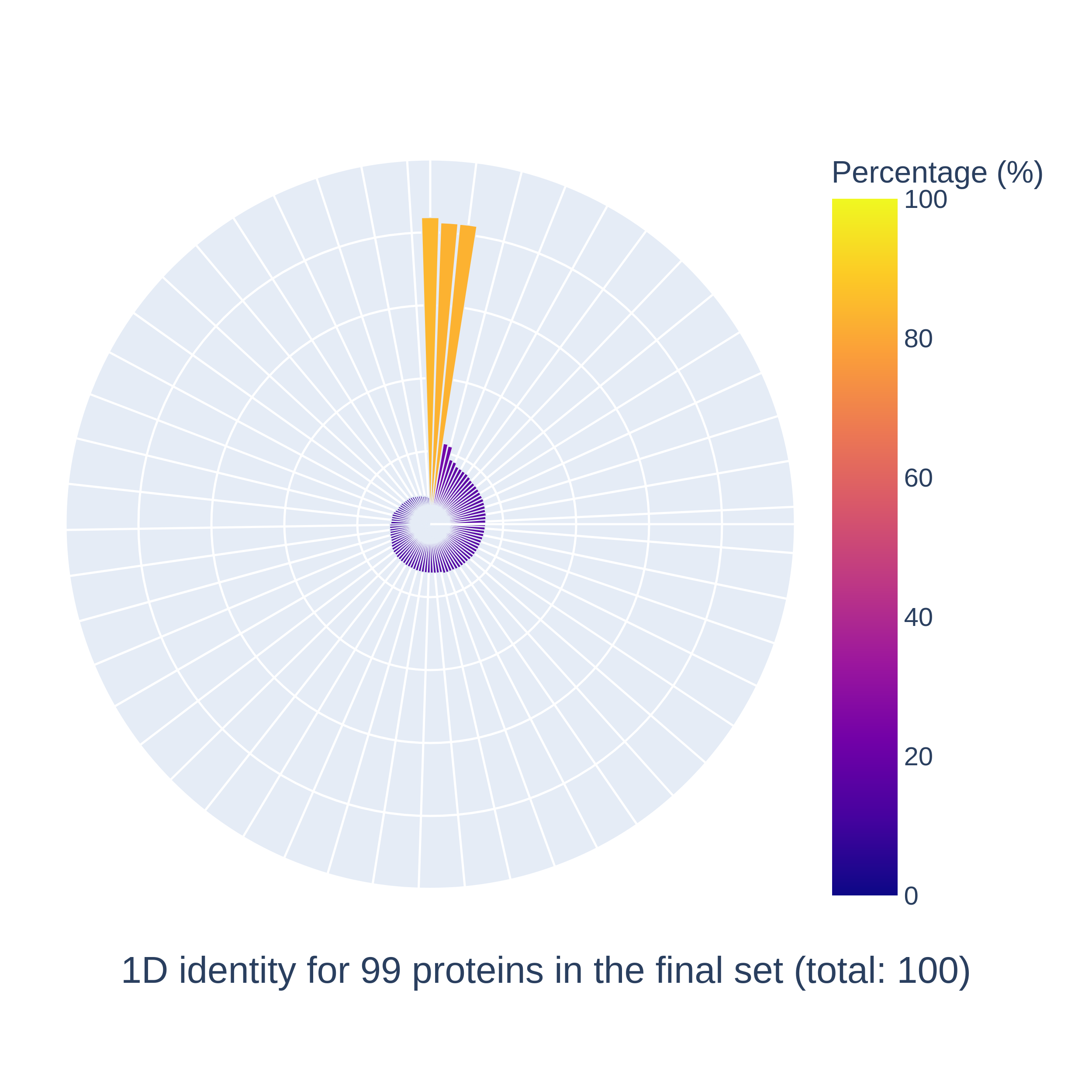

Supplement: Supplementary file 24 — Supplementary Information 12. [file 41598_2025_91849_MOESM24_ESM.zip › 4KREp_A_mdwhole_AF4REF/plots/4KREp_A_1D-identity.png]

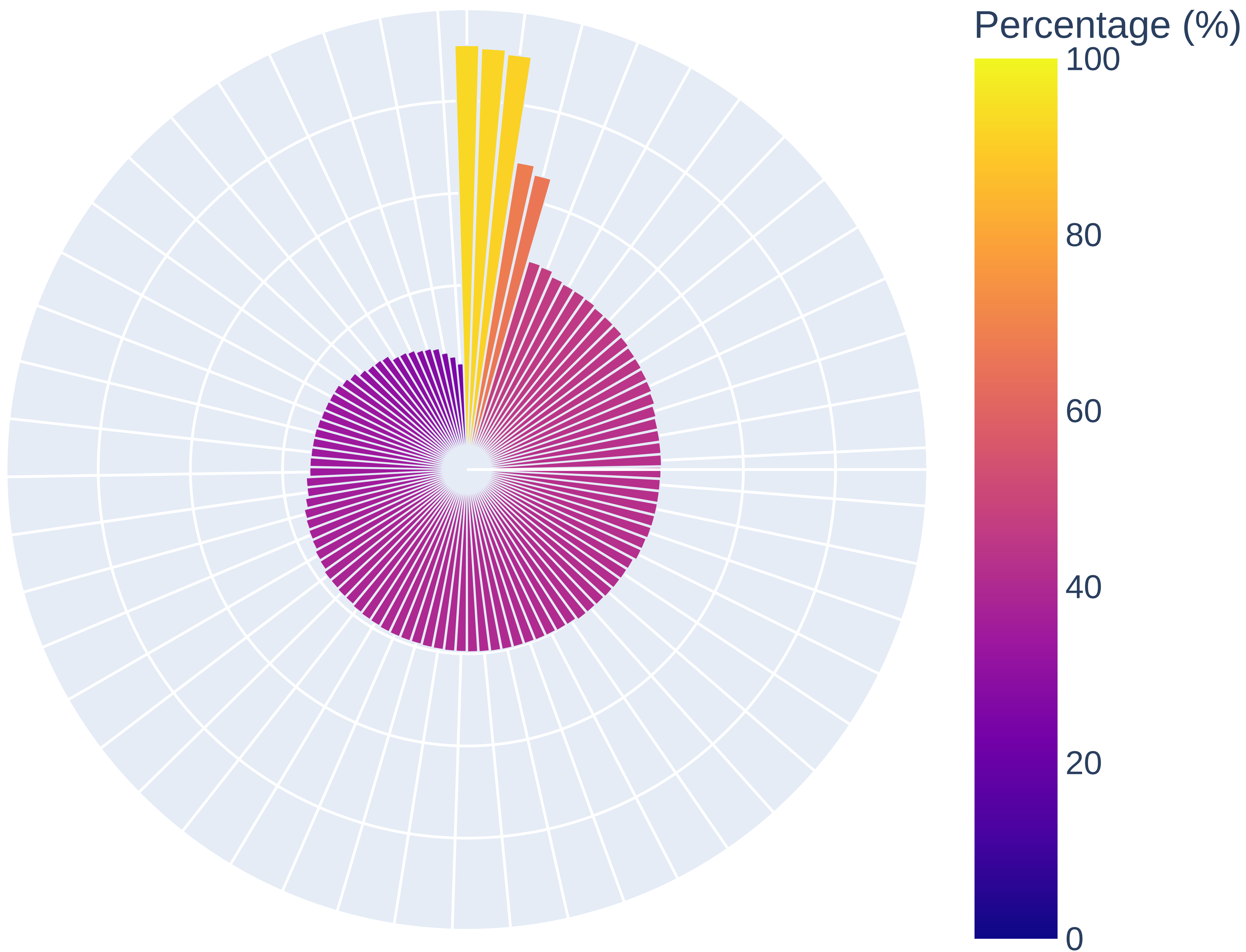

2D identity for 99 proteins in the final set (total: 100)

Supplement: Supplementary file 24 — Supplementary Information 12. [file 41598_2025_91849_MOESM24_ESM.zip › 4KREp_A_mdwhole_AF4REF/plots/4KREp_A_2D-identity.pdf]

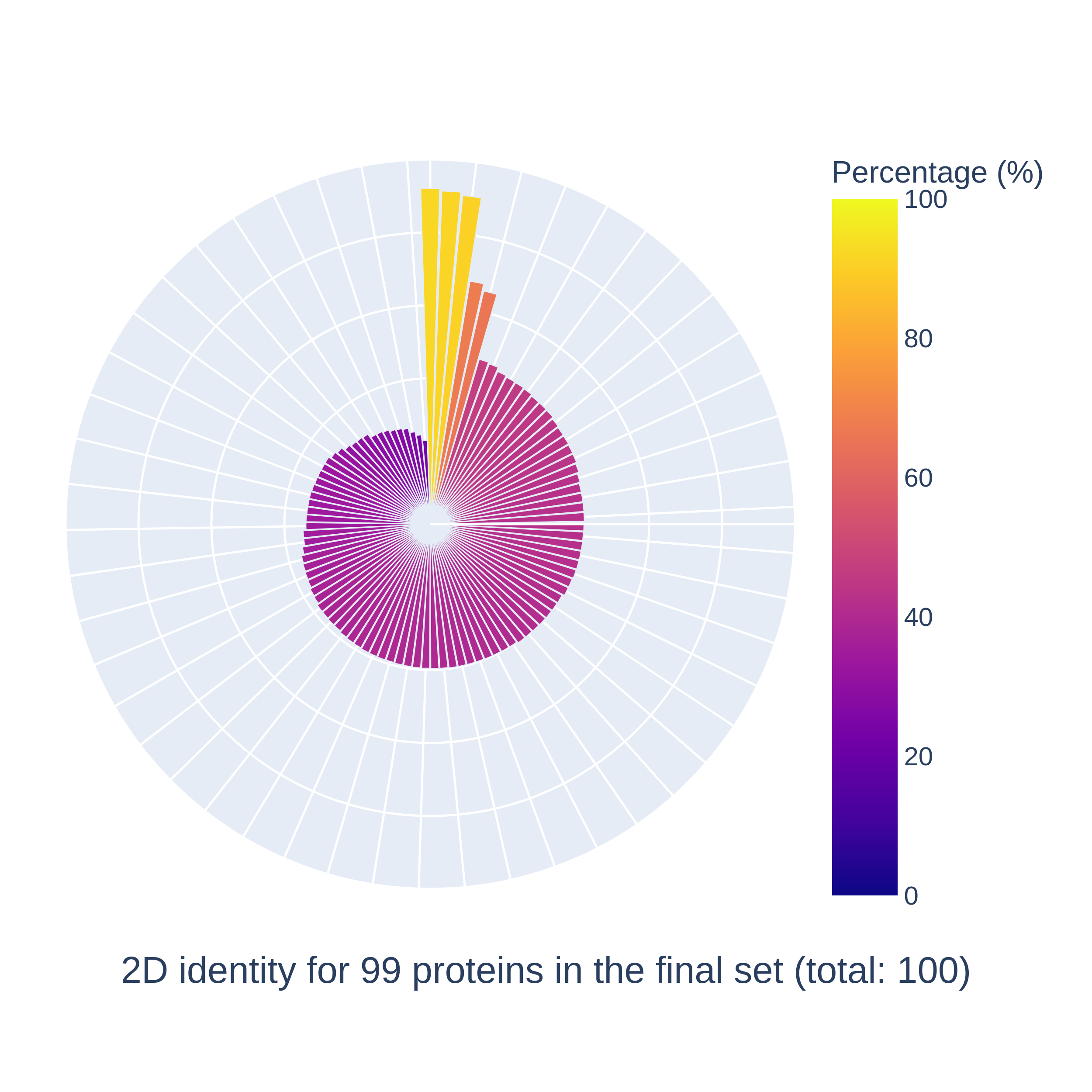

Supplement: Supplementary file 24 — Supplementary Information 12. [file 41598_2025_91849_MOESM24_ESM.zip › 4KREp_A_mdwhole_AF4REF/plots/4KREp_A_2D-identity.png]

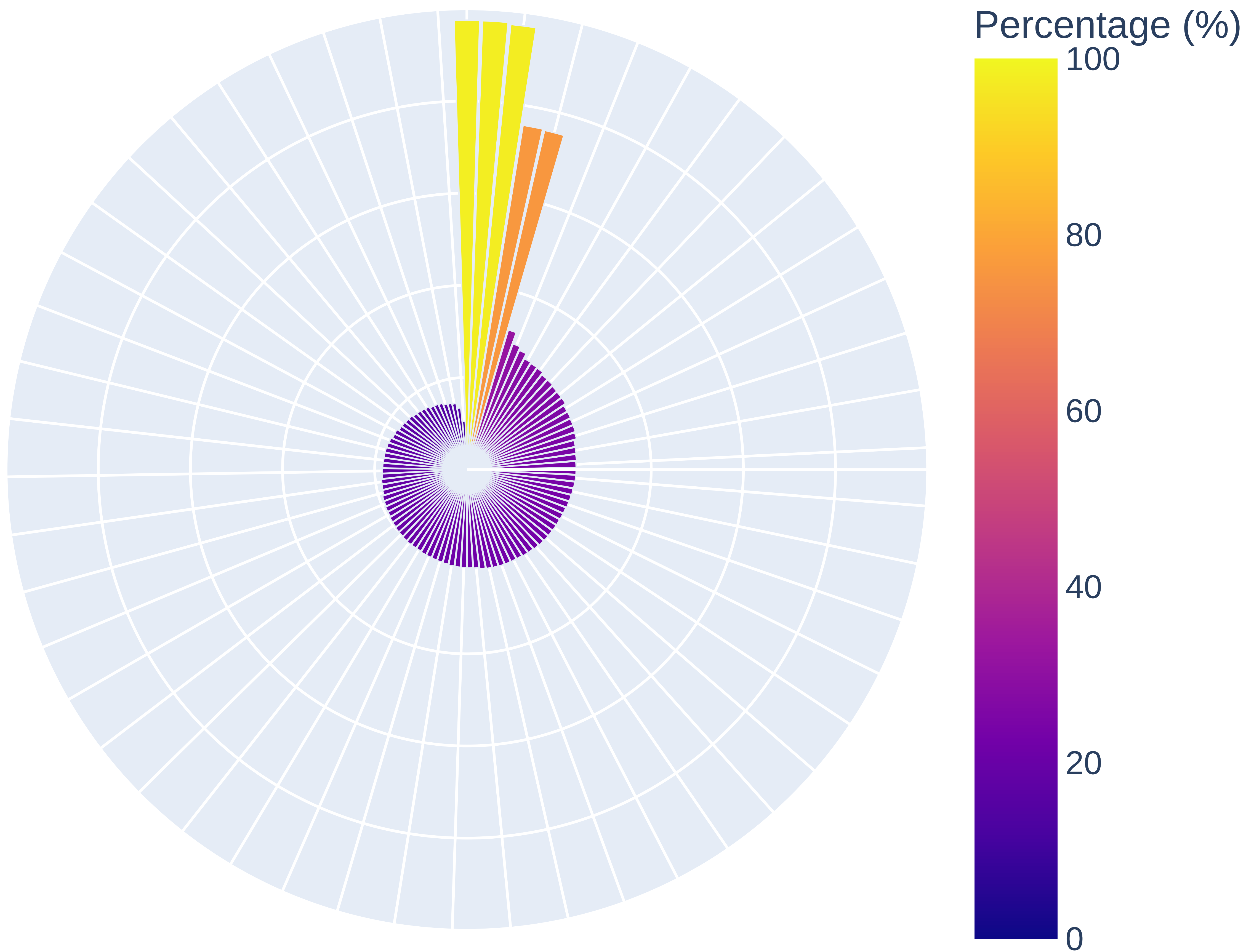

3D Similarity for 99 proteins in the final set (total: 100)

Supplement: Supplementary file 24 — Supplementary Information 12. [file 41598_2025_91849_MOESM24_ESM.zip › 4KREp_A_mdwhole_AF4REF/plots/4KREp_A_3D-score.pdf]

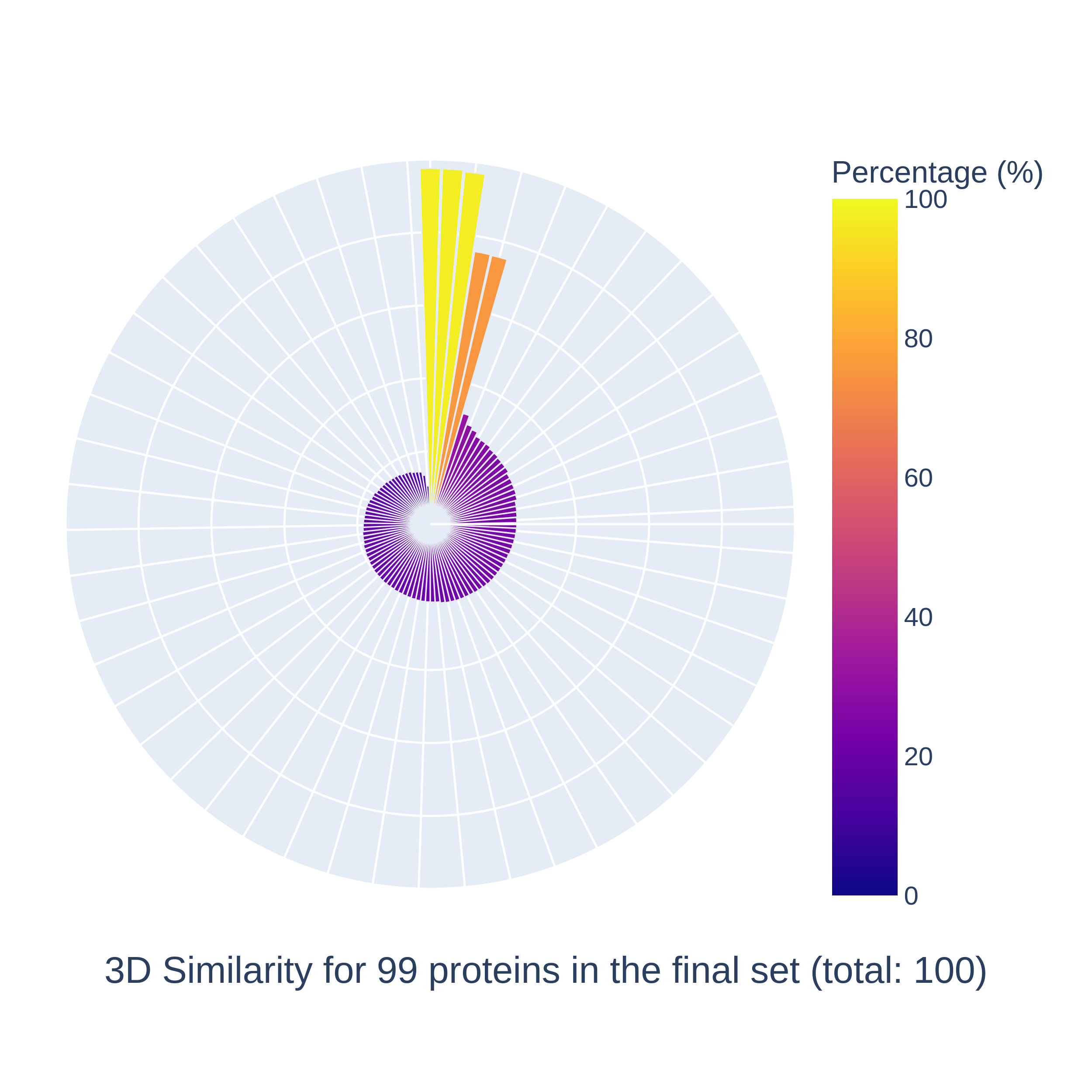

Supplement: Supplementary file 24 — Supplementary Information 12. [file 41598_2025_91849_MOESM24_ESM.zip › 4KREp_A_mdwhole_AF4REF/plots/4KREp_A_3D-score.png]

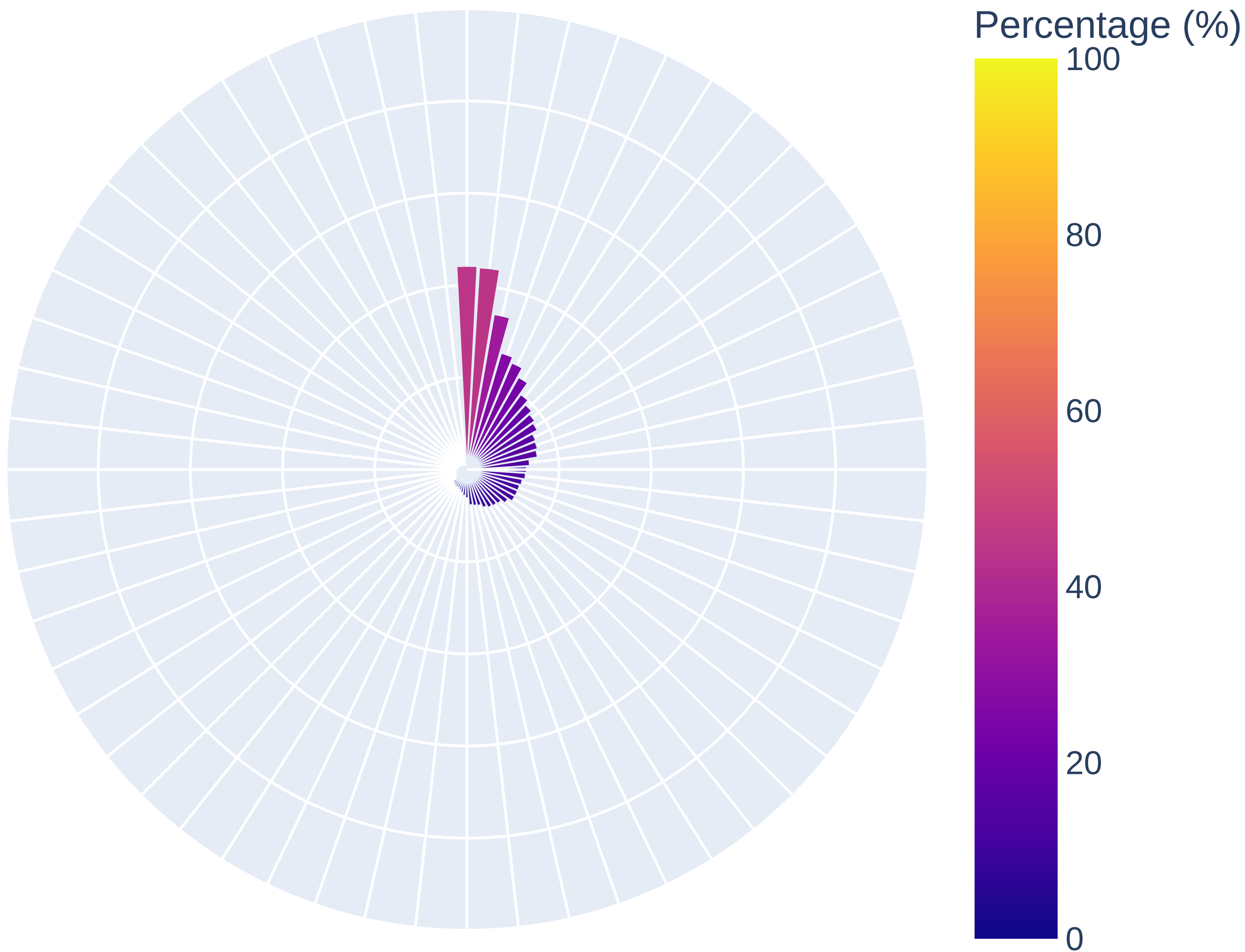

3'-UTR identity for 56 proteins in the final set (total: 100)

Supplement: Supplementary file 24 — Supplementary Information 12. [file 41598_2025_91849_MOESM24_ESM.zip › 4KREp_A_mdwhole_AF4REF/plots/4KREp_A_3UTR-identity.pdf]

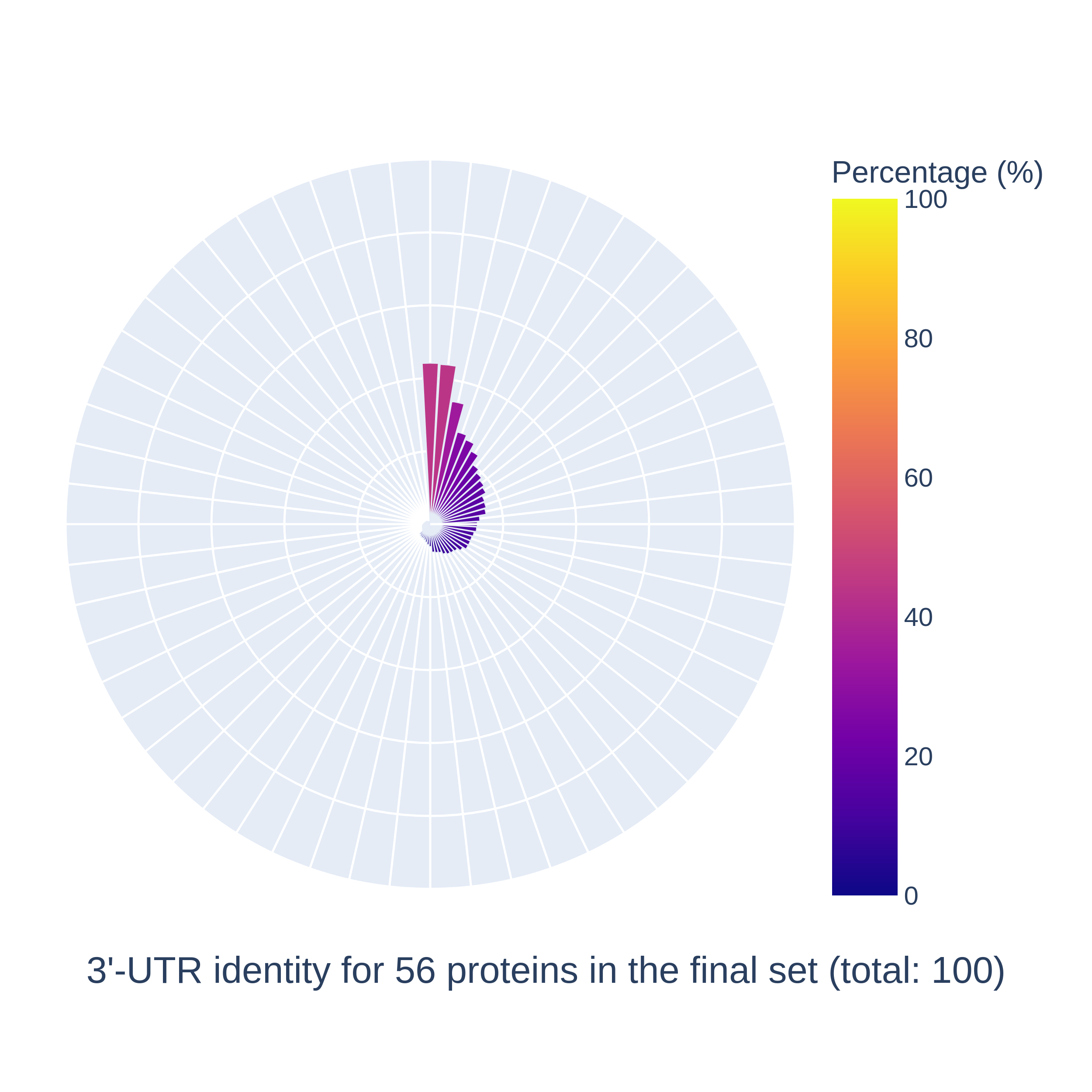

Supplement: Supplementary file 24 — Supplementary Information 12. [file 41598_2025_91849_MOESM24_ESM.zip › 4KREp_A_mdwhole_AF4REF/plots/4KREp_A_3UTR-identity.png]

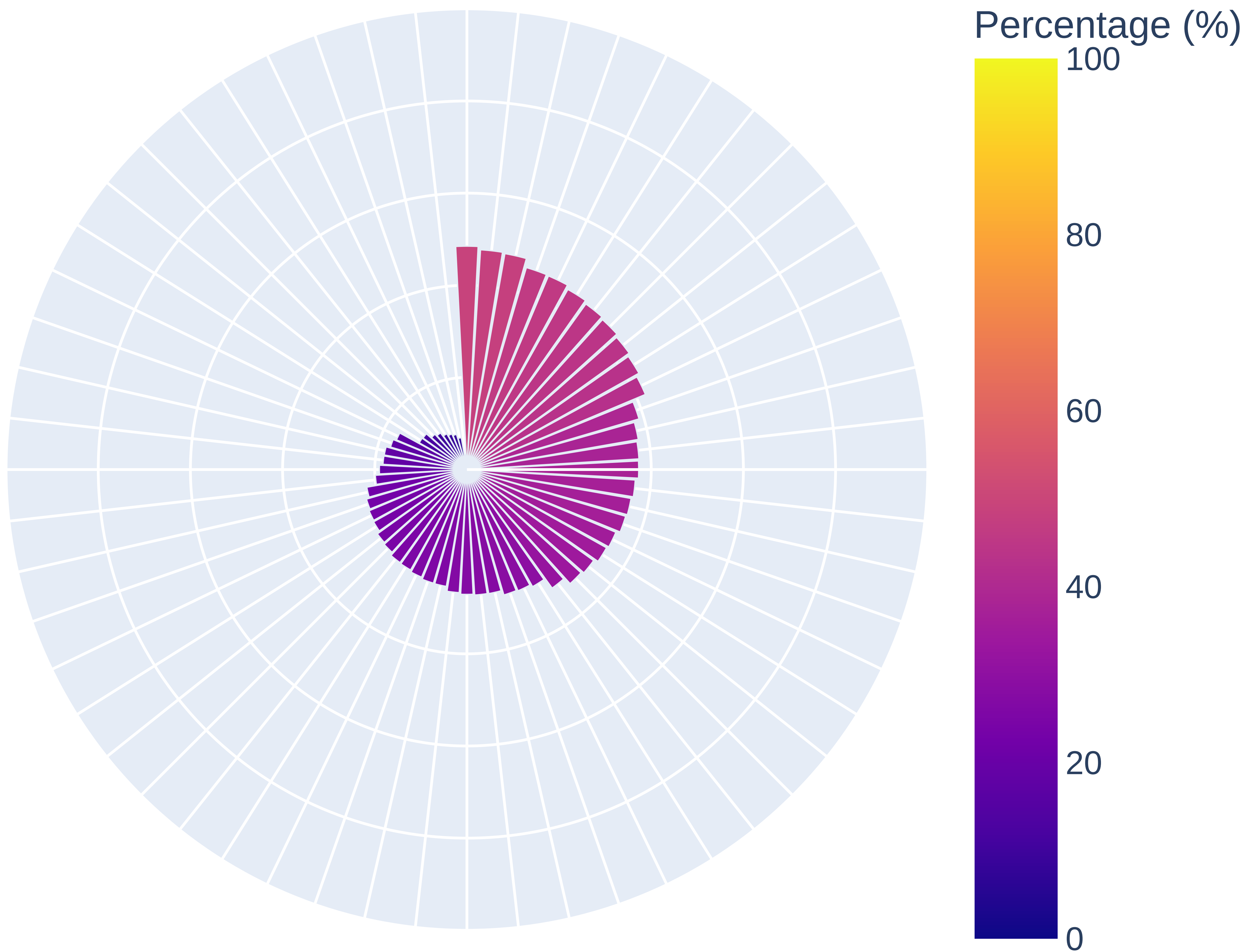

5'-UTR identity for 56 proteins in the final set (total: 100)

Supplement: Supplementary file 24 — Supplementary Information 12. [file 41598_2025_91849_MOESM24_ESM.zip › 4KREp_A_mdwhole_AF4REF/plots/4KREp_A_5UTR-identity.pdf]

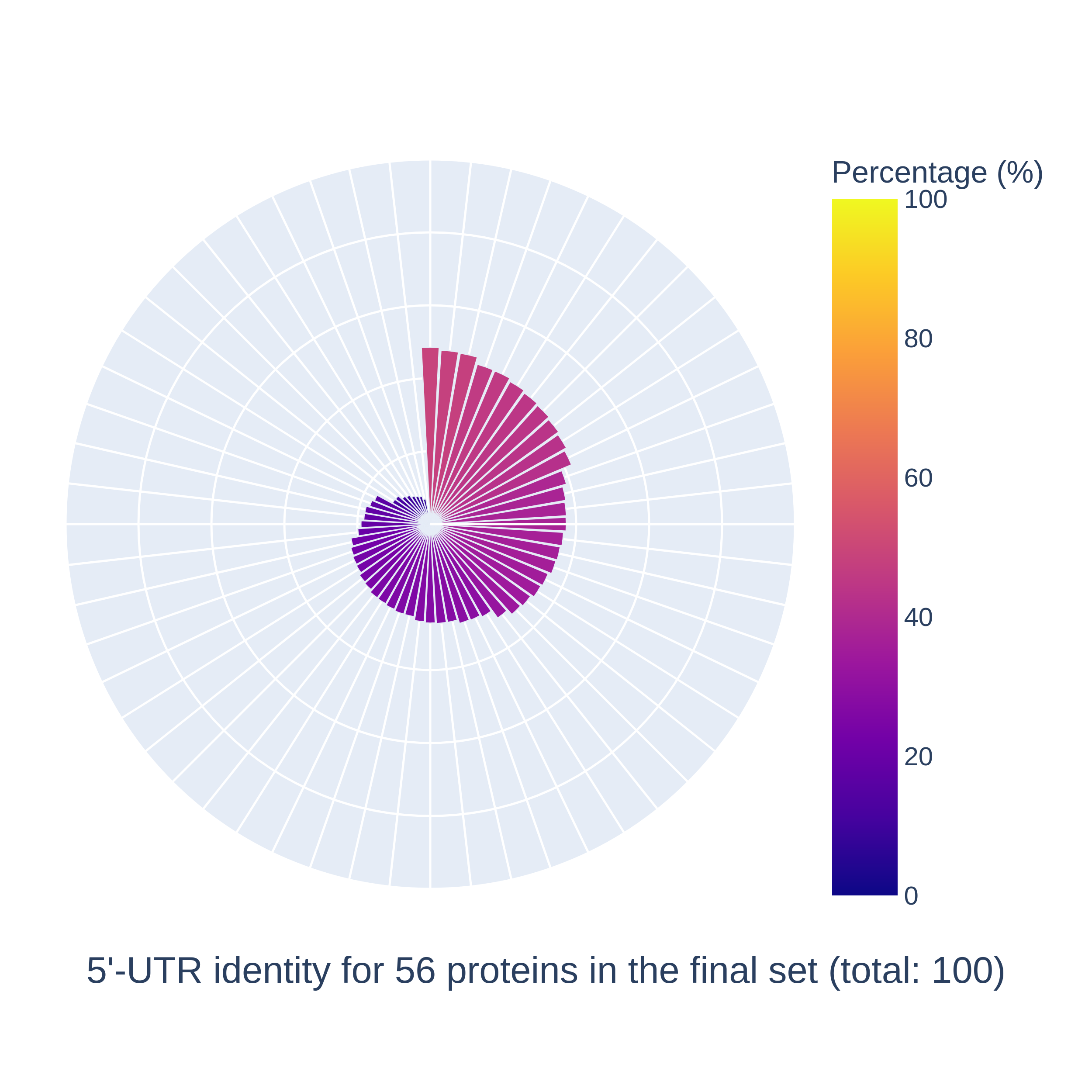

Supplement: Supplementary file 24 — Supplementary Information 12. [file 41598_2025_91849_MOESM24_ESM.zip › 4KREp_A_mdwhole_AF4REF/plots/4KREp_A_5UTR-identity.png]

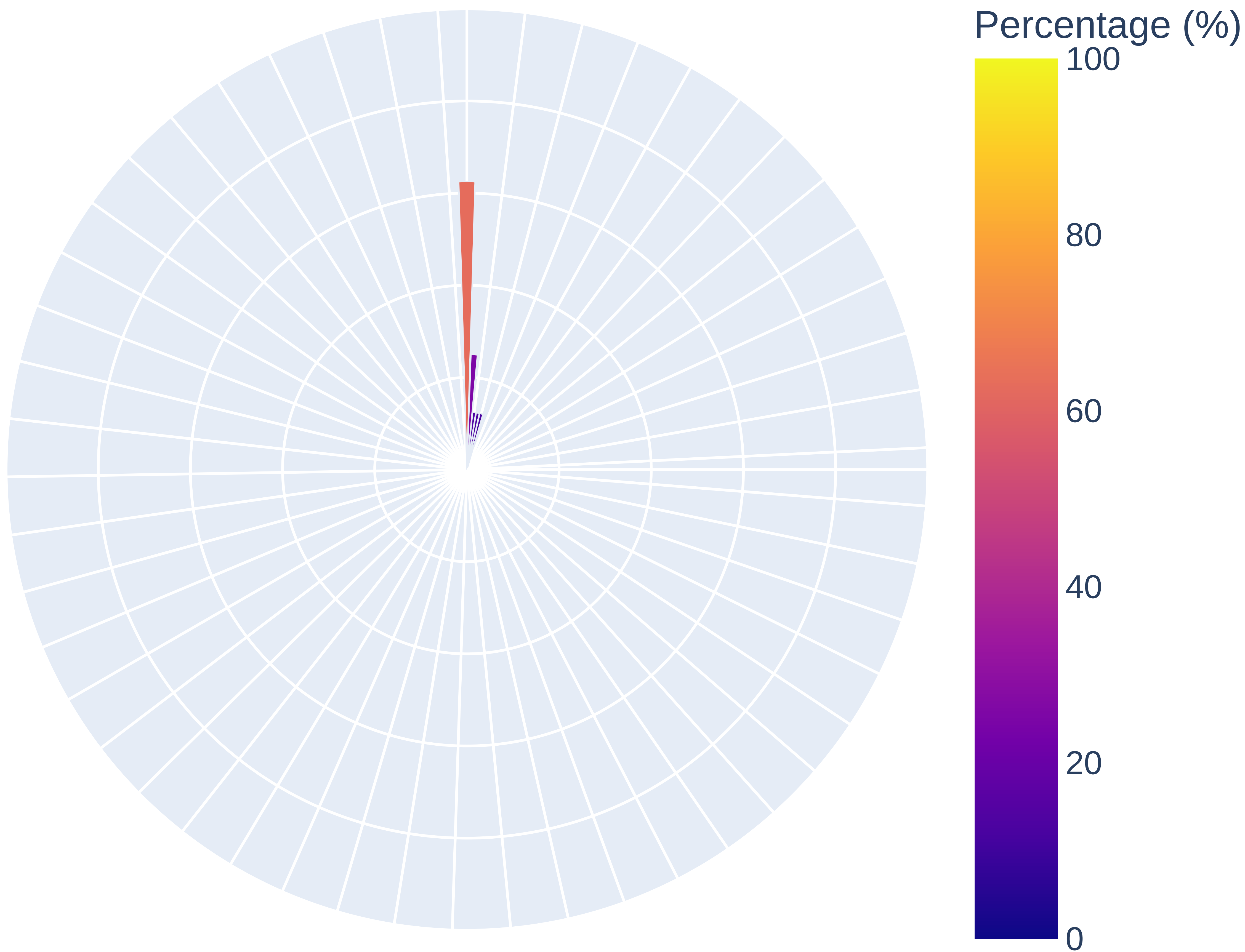

Common pathways for 99 proteins in the final set (total: 100)

Supplement: Supplementary file 24 — Supplementary Information 12. [file 41598_2025_91849_MOESM24_ESM.zip › 4KREp_A_mdwhole_AF4REF/plots/4KREp_A_biologicalProcessSim.pdf]

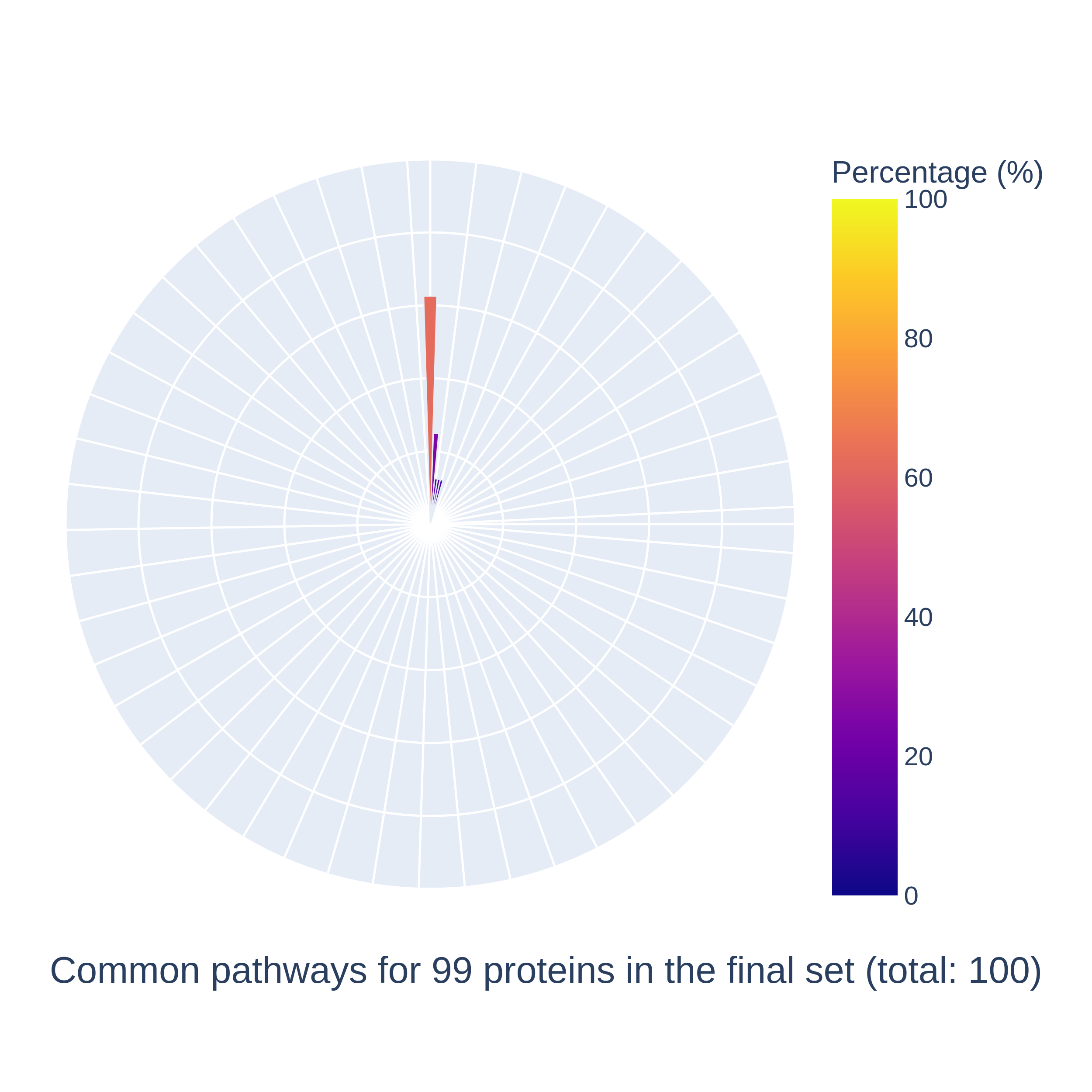

Supplement: Supplementary file 24 — Supplementary Information 12. [file 41598_2025_91849_MOESM24_ESM.zip › 4KREp_A_mdwhole_AF4REF/plots/4KREp_A_biologicalProcessSim.png]

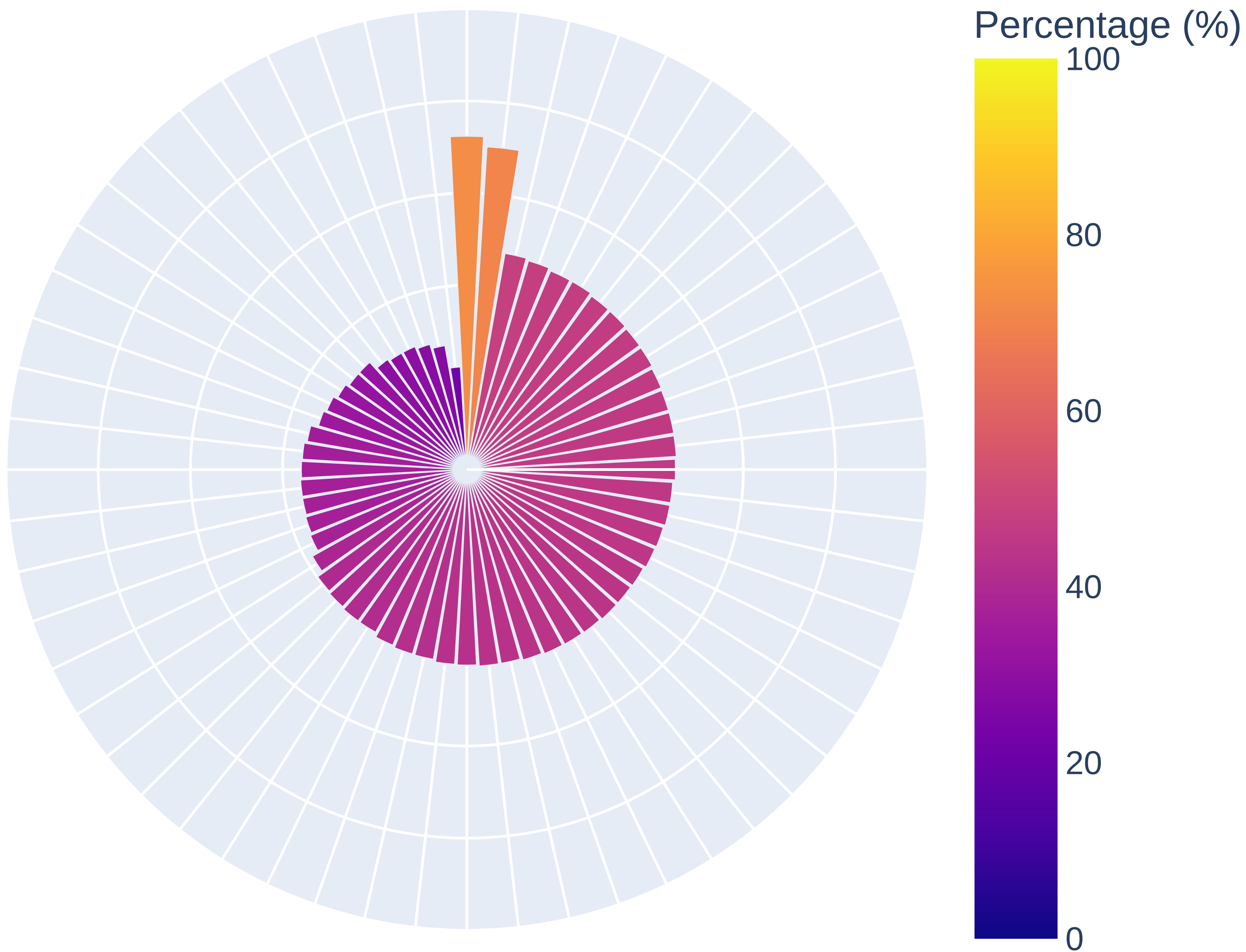

CDS identity for 56 proteins in the final set (total: 100)

Supplement: Supplementary file 24 — Supplementary Information 12. [file 41598_2025_91849_MOESM24_ESM.zip › 4KREp_A_mdwhole_AF4REF/plots/4KREp_A_CDS-identity.pdf]

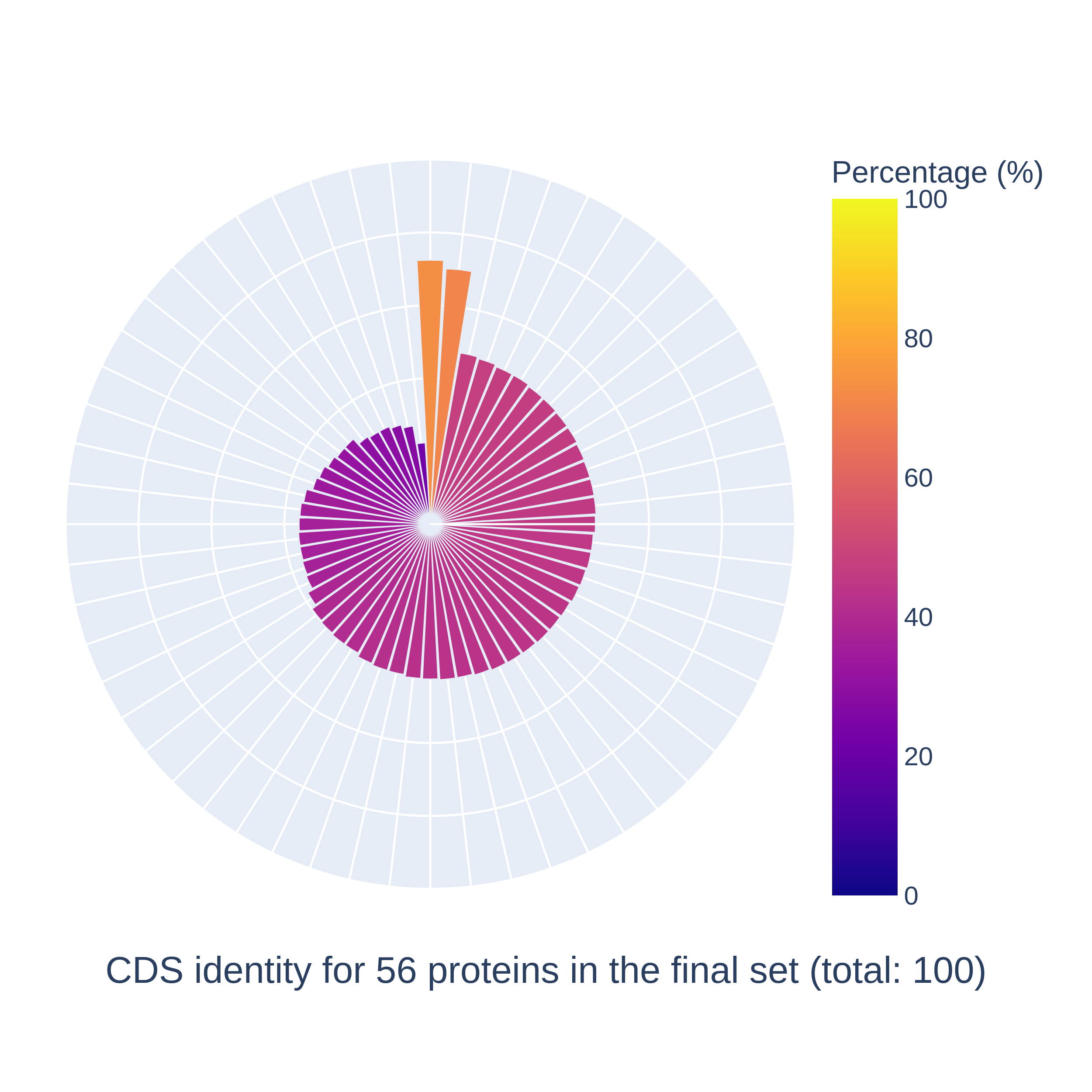

Supplement: Supplementary file 24 — Supplementary Information 12. [file 41598_2025_91849_MOESM24_ESM.zip › 4KREp_A_mdwhole_AF4REF/plots/4KREp_A_CDS-identity.png]

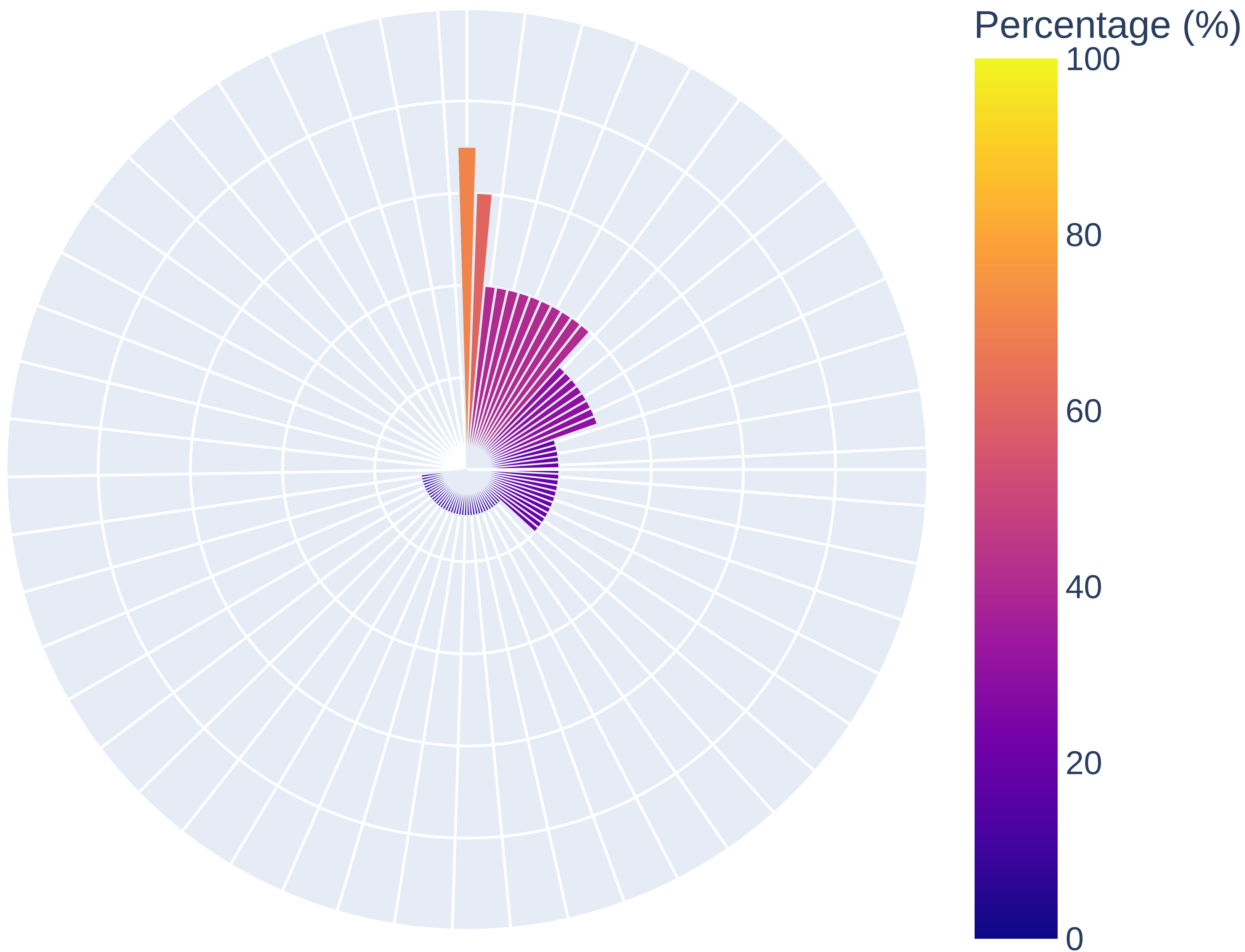

Common locations for 99 proteins in the final set (total: 100)

Supplement: Supplementary file 24 — Supplementary Information 12. [file 41598_2025_91849_MOESM24_ESM.zip › 4KREp_A_mdwhole_AF4REF/plots/4KREp_A_cellularComponentSim.pdf]

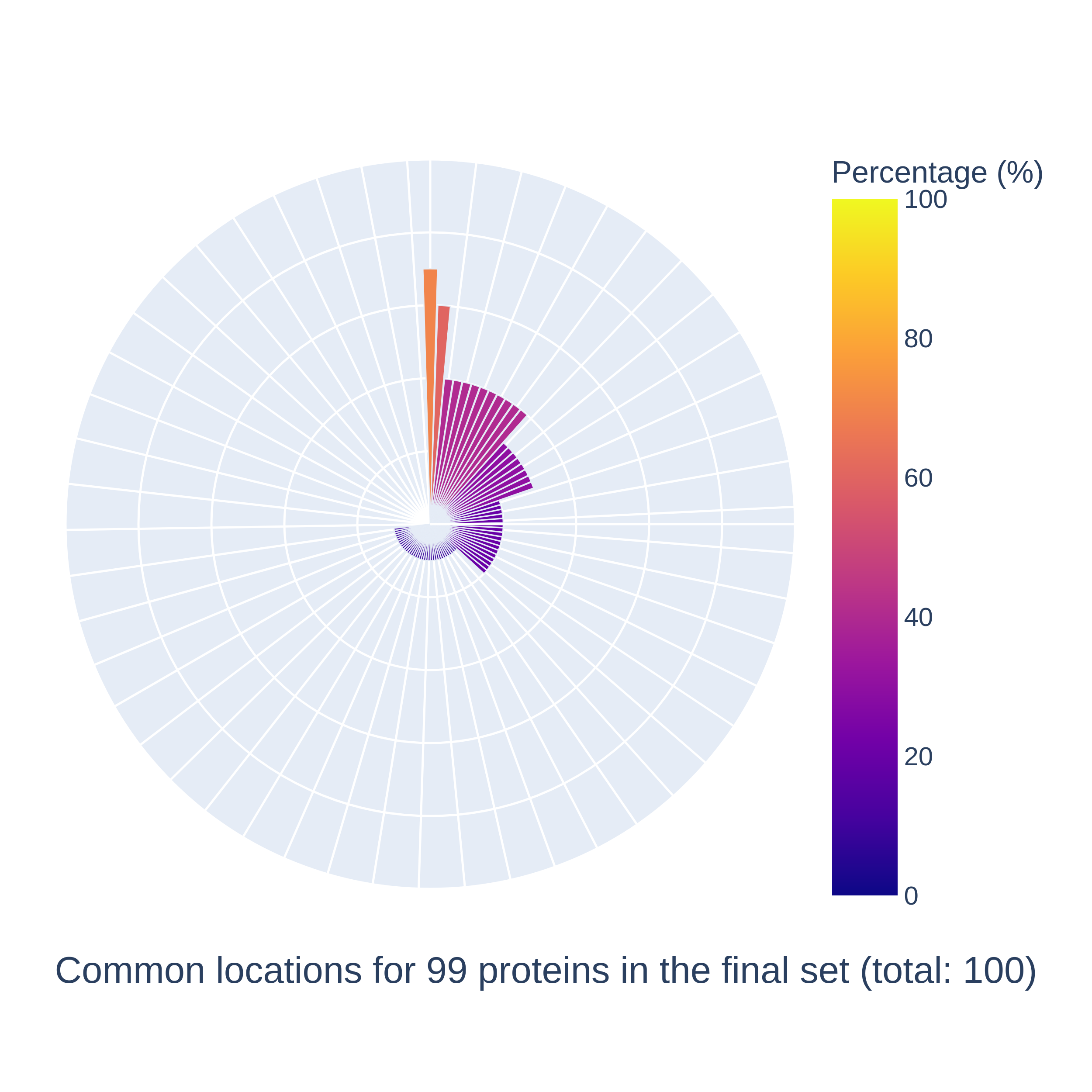

Supplement: Supplementary file 24 — Supplementary Information 12. [file 41598_2025_91849_MOESM24_ESM.zip › 4KREp_A_mdwhole_AF4REF/plots/4KREp_A_cellularComponentSim.png]

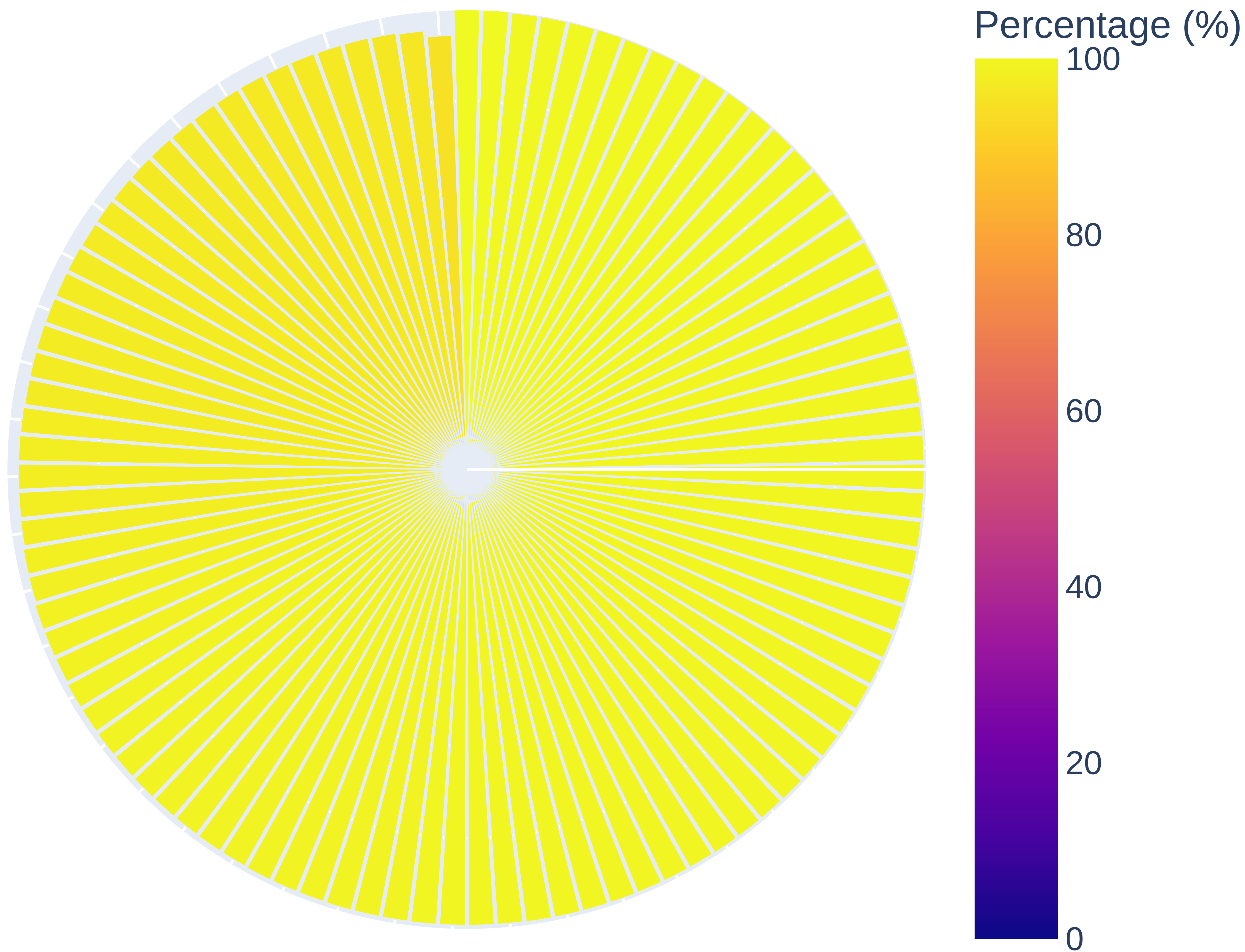

Chemical similarity for 99 proteins in the final set (total: 100)

Supplement: Supplementary file 24 — Supplementary Information 12. [file 41598_2025_91849_MOESM24_ESM.zip › 4KREp_A_mdwhole_AF4REF/plots/4KREp_A_chemSim.pdf]

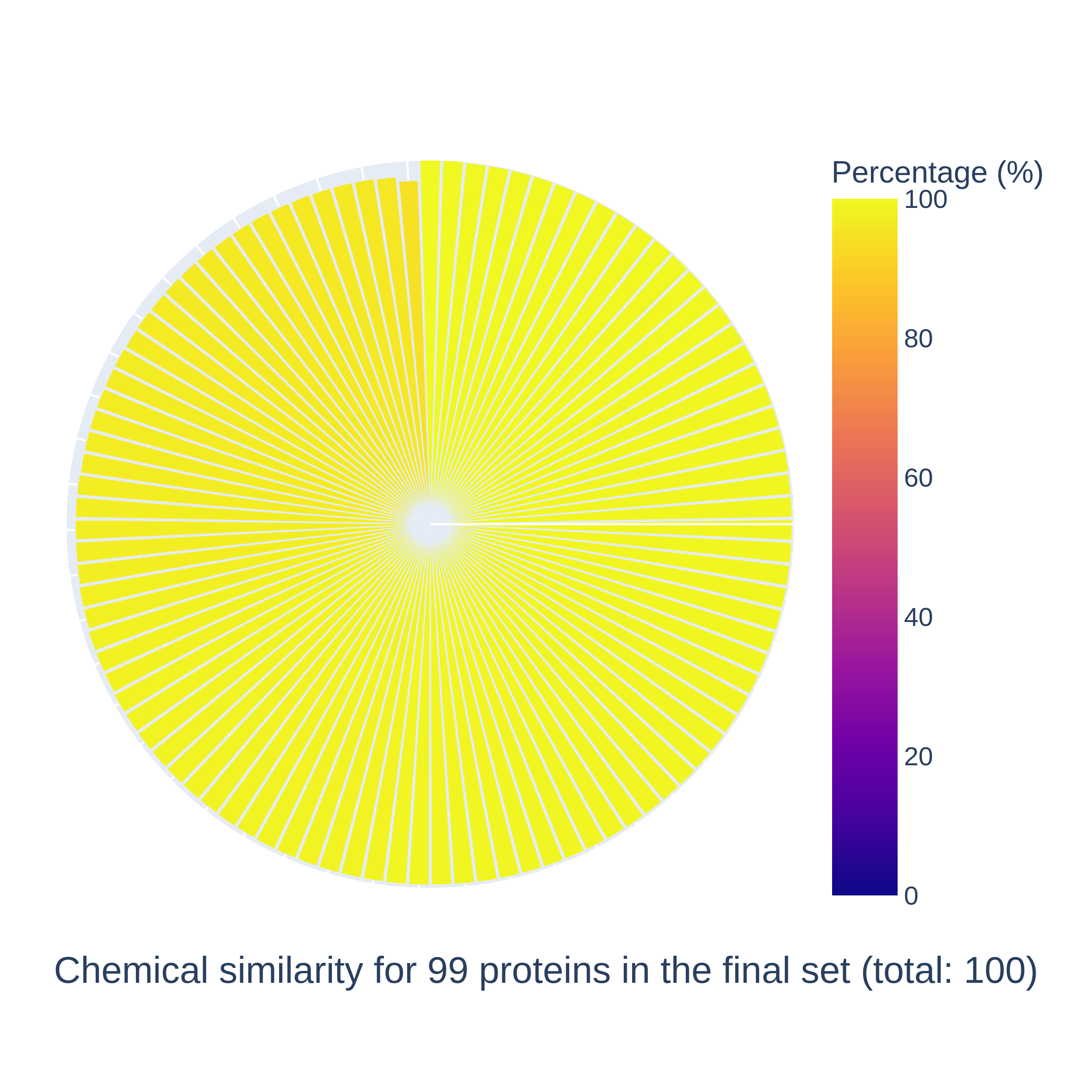

Supplement: Supplementary file 24 — Supplementary Information 12. [file 41598_2025_91849_MOESM24_ESM.zip › 4KREp_A_mdwhole_AF4REF/plots/4KREp_A_chemSim.png]

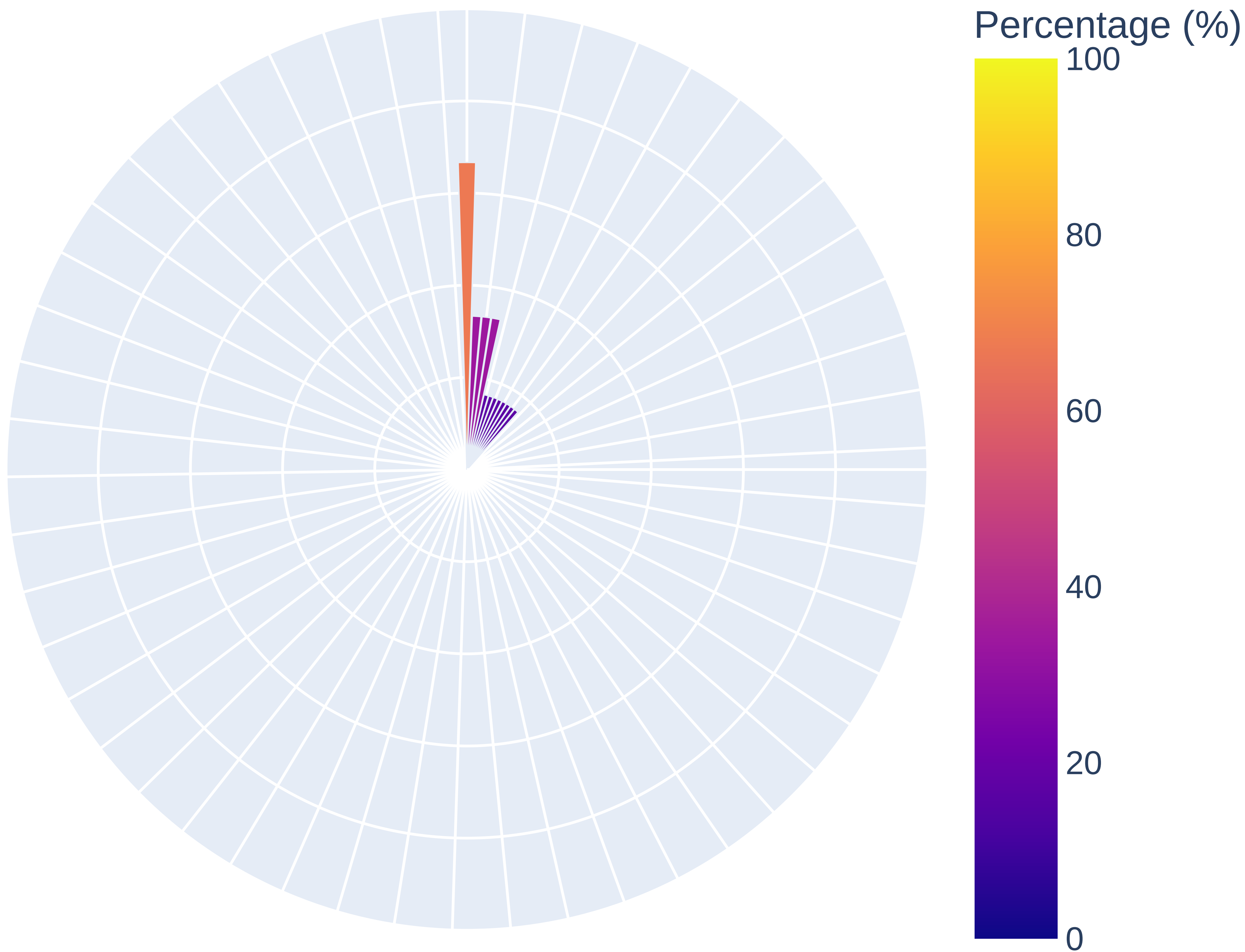

Common functions for 99 proteins in the final set (total: 100)

Supplement: Supplementary file 24 — Supplementary Information 12. [file 41598_2025_91849_MOESM24_ESM.zip › 4KREp_A_mdwhole_AF4REF/plots/4KREp_A_molecularFunctionSim.pdf]

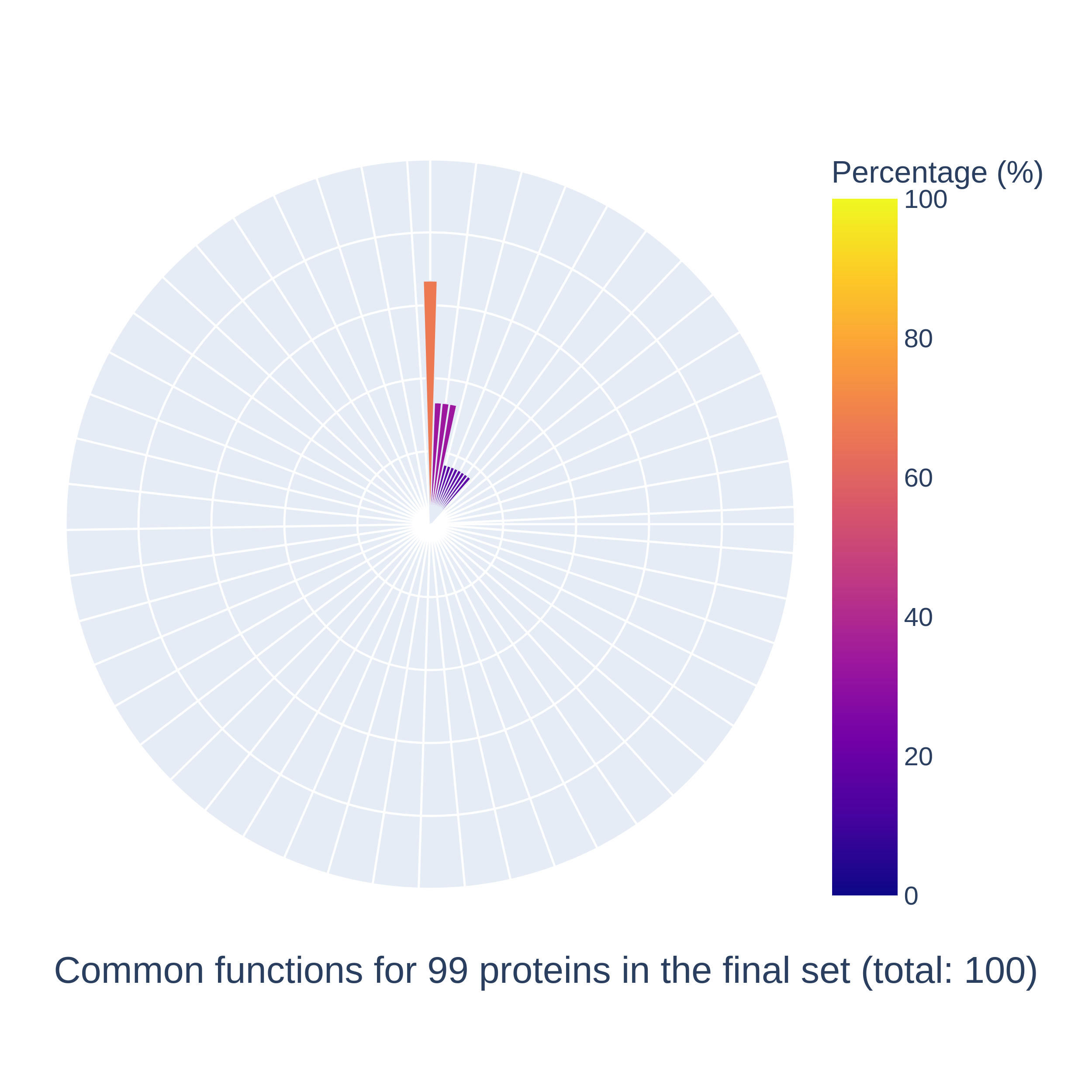

Supplement: Supplementary file 24 — Supplementary Information 12. [file 41598_2025_91849_MOESM24_ESM.zip › 4KREp_A_mdwhole_AF4REF/plots/4KREp_A_molecularFunctionSim.png]

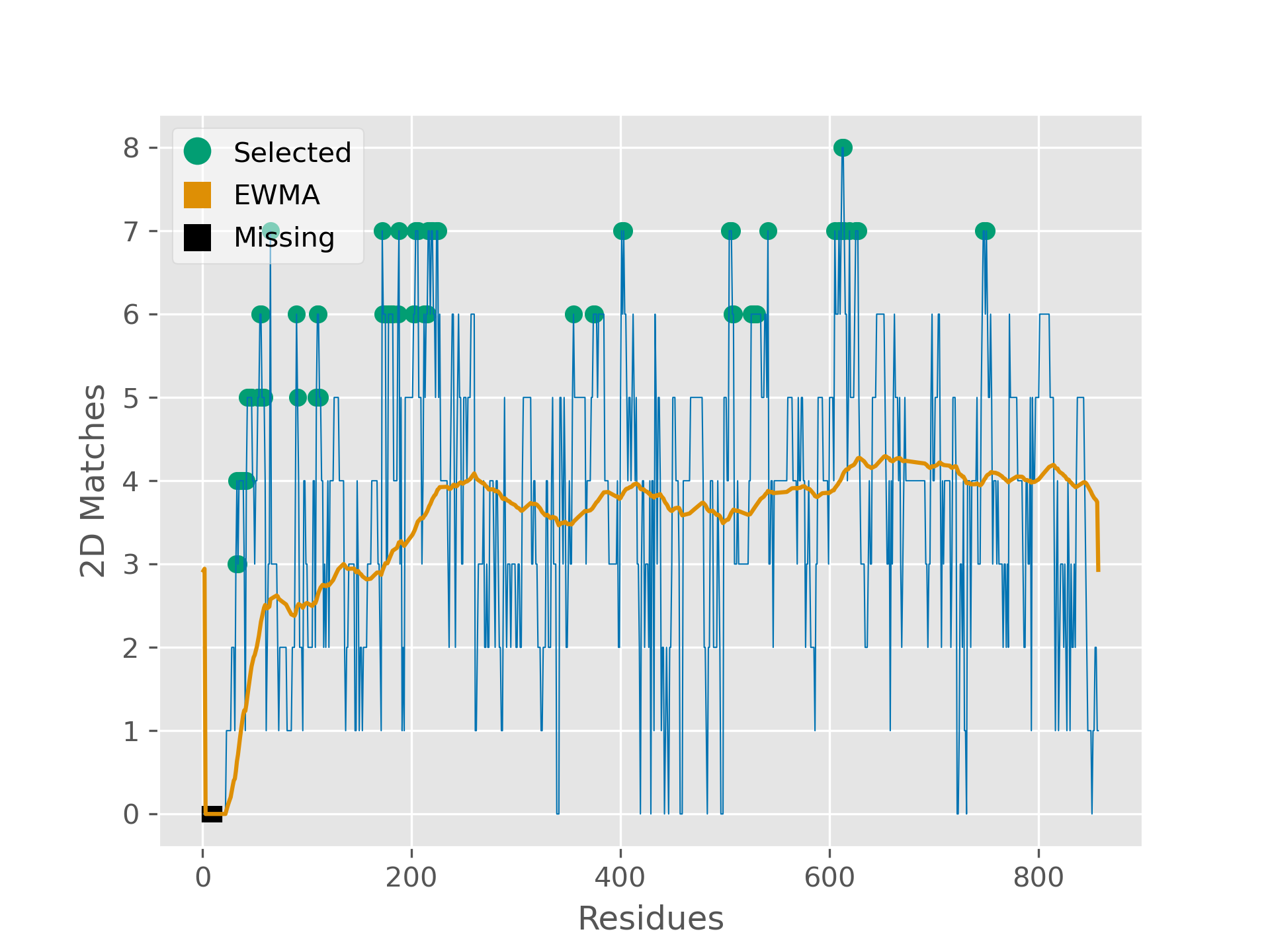

Supplement: Supplementary file 24 — Supplementary Information 12. [file 41598_2025_91849_MOESM24_ESM.zip › 4KREp_A_mdwhole_HL2REF/go/4KREp_A_mitot_mitosis_de7538da1f7844b2a2310efe4413d0e5.png]

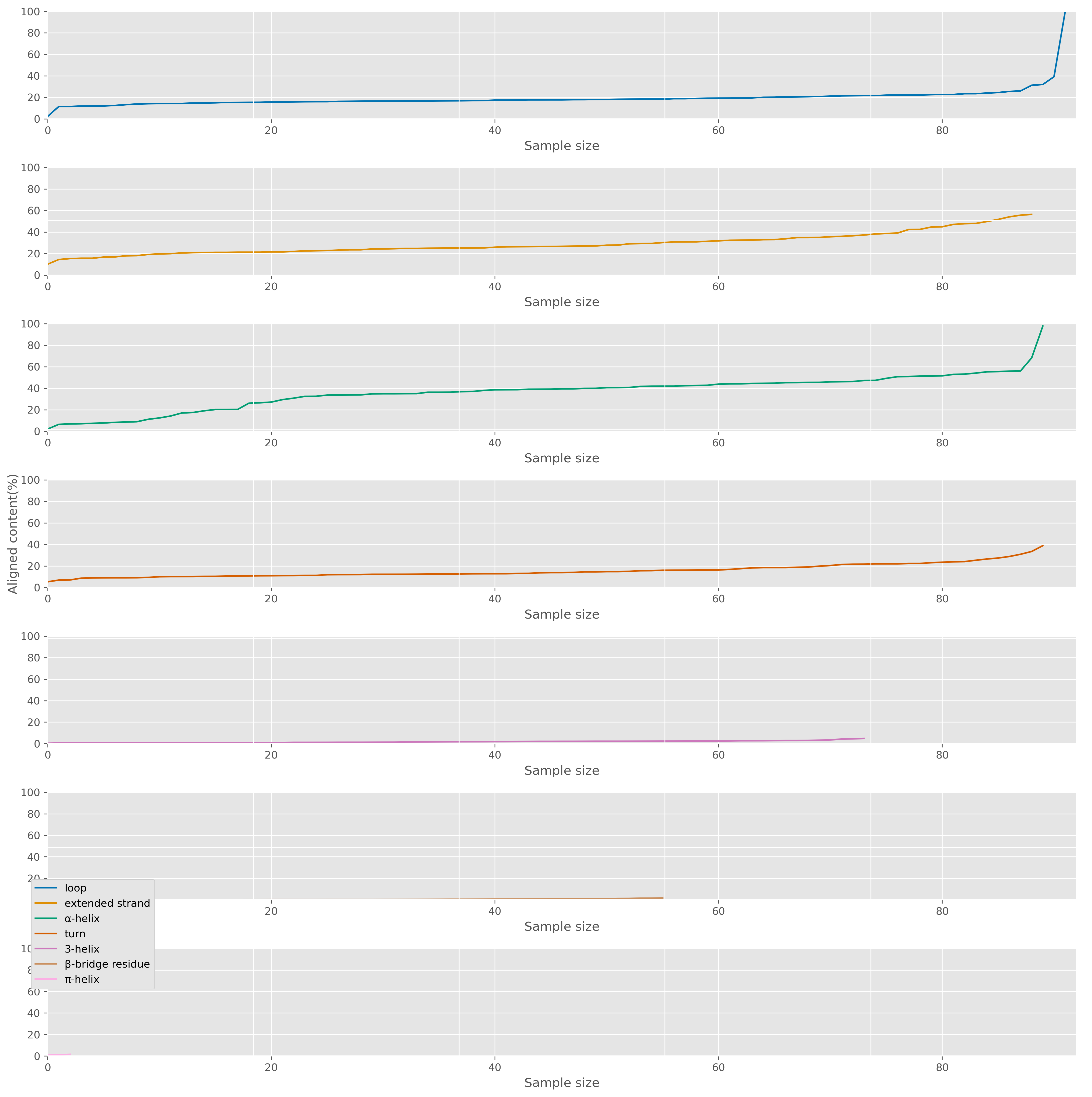

Supplement: Supplementary file 24 — Supplementary Information 12. [file 41598_2025_91849_MOESM24_ESM.zip › 4KREp_A_mdwhole_HL2REF/plots/4KREp_A-2Dfold_coverage.png]

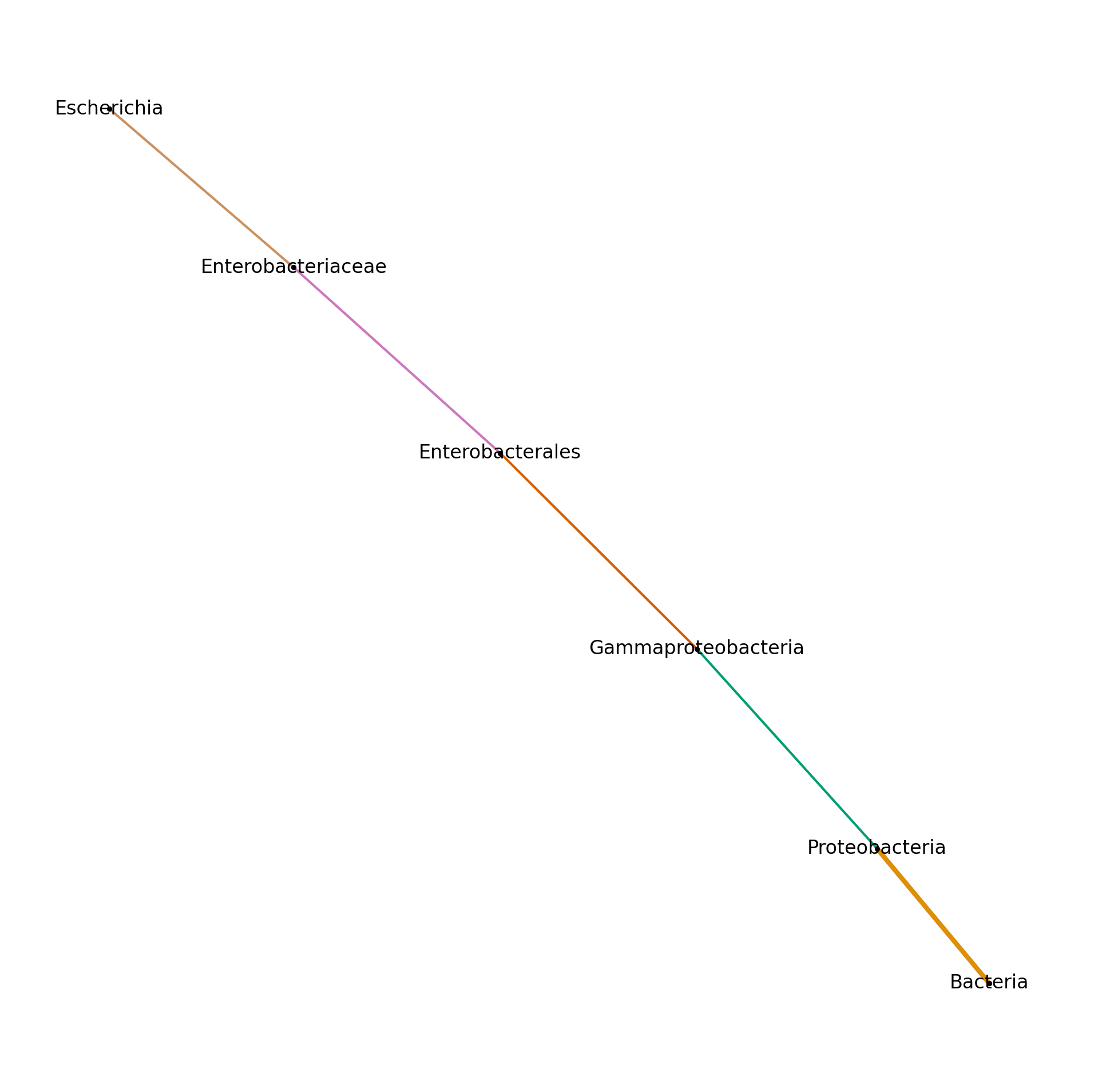

Supplement: Supplementary file 24 — Supplementary Information 12. [file 41598_2025_91849_MOESM24_ESM.zip › 4KREp_A_mdwhole_HL2REF/plots/4KREp_A-Bacteria-tree.png]

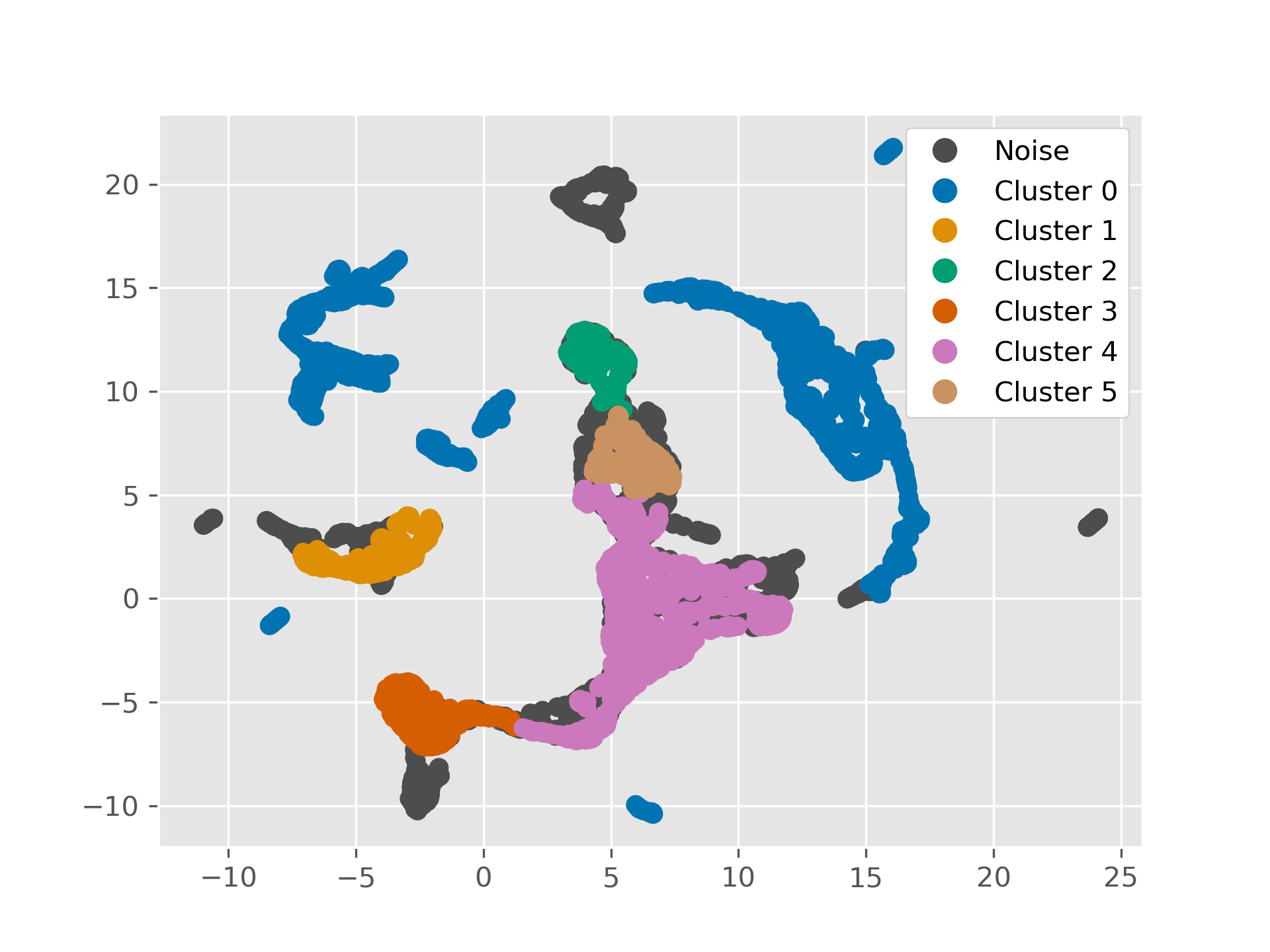

Supplement: Supplementary file 24 — Supplementary Information 12. [file 41598_2025_91849_MOESM24_ESM.zip › 4KREp_A_mdwhole_HL2REF/plots/4KREp_A-clusters-initial.png]

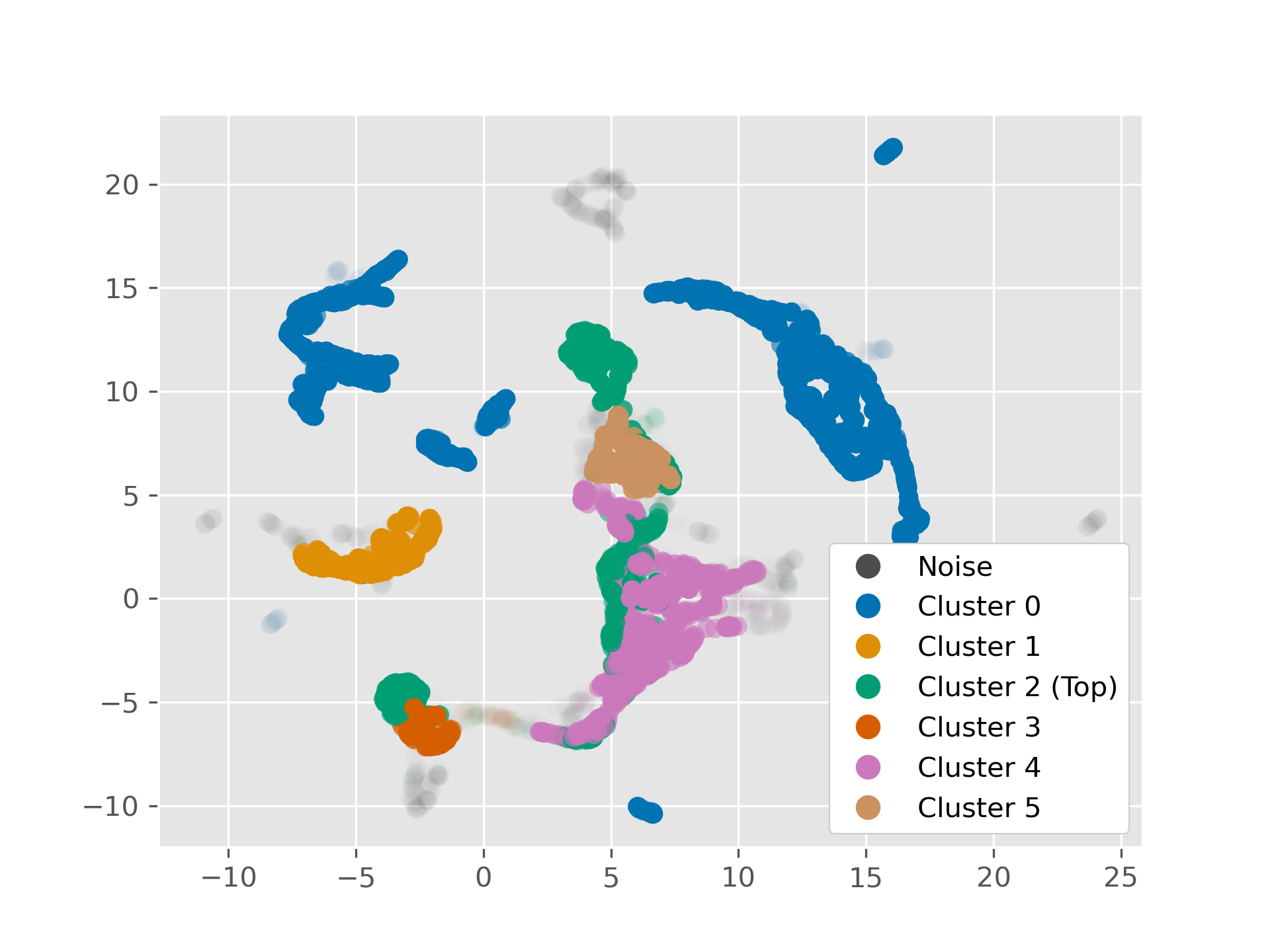

Supplement: Supplementary file 24 — Supplementary Information 12. [file 41598_2025_91849_MOESM24_ESM.zip › 4KREp_A_mdwhole_HL2REF/plots/4KREp_A-clusters.png]

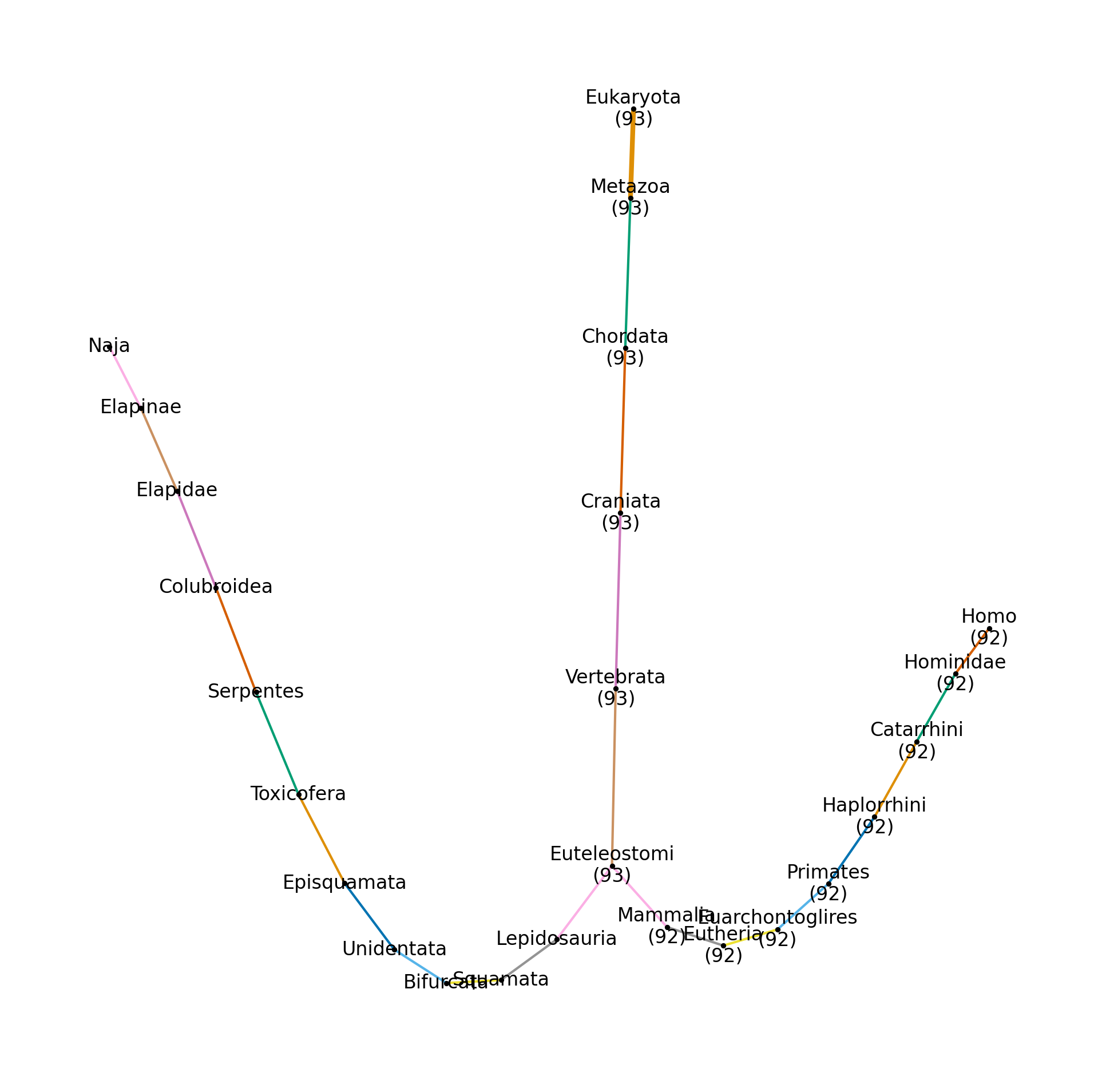

Supplement: Supplementary file 24 — Supplementary Information 12. [file 41598_2025_91849_MOESM24_ESM.zip › 4KREp_A_mdwhole_HL2REF/plots/4KREp_A-Eukaryota-tree.png]

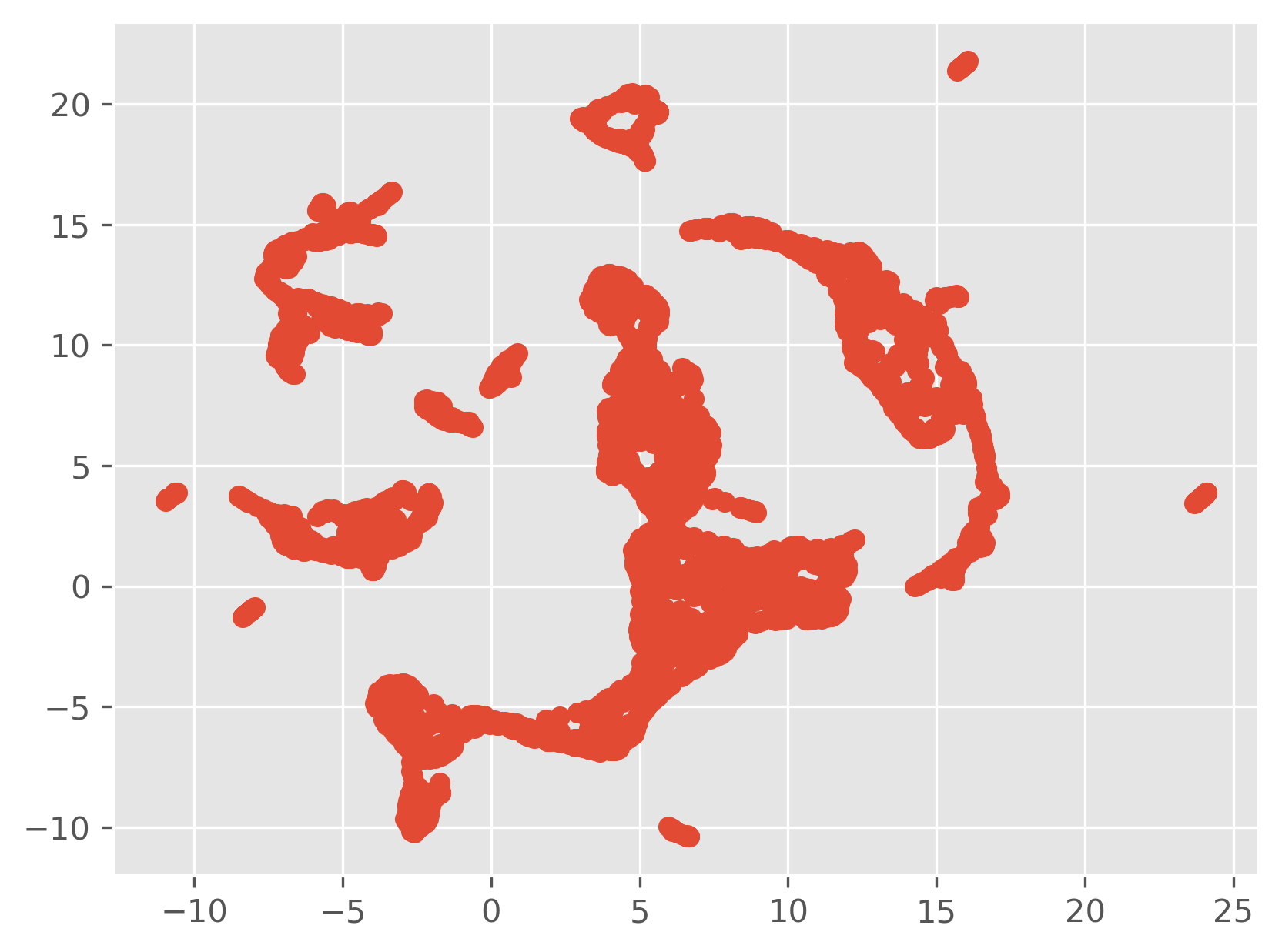

Supplement: Supplementary file 24 — Supplementary Information 12. [file 41598_2025_91849_MOESM24_ESM.zip › 4KREp_A_mdwhole_HL2REF/plots/4KREp_A-UMAP.png]

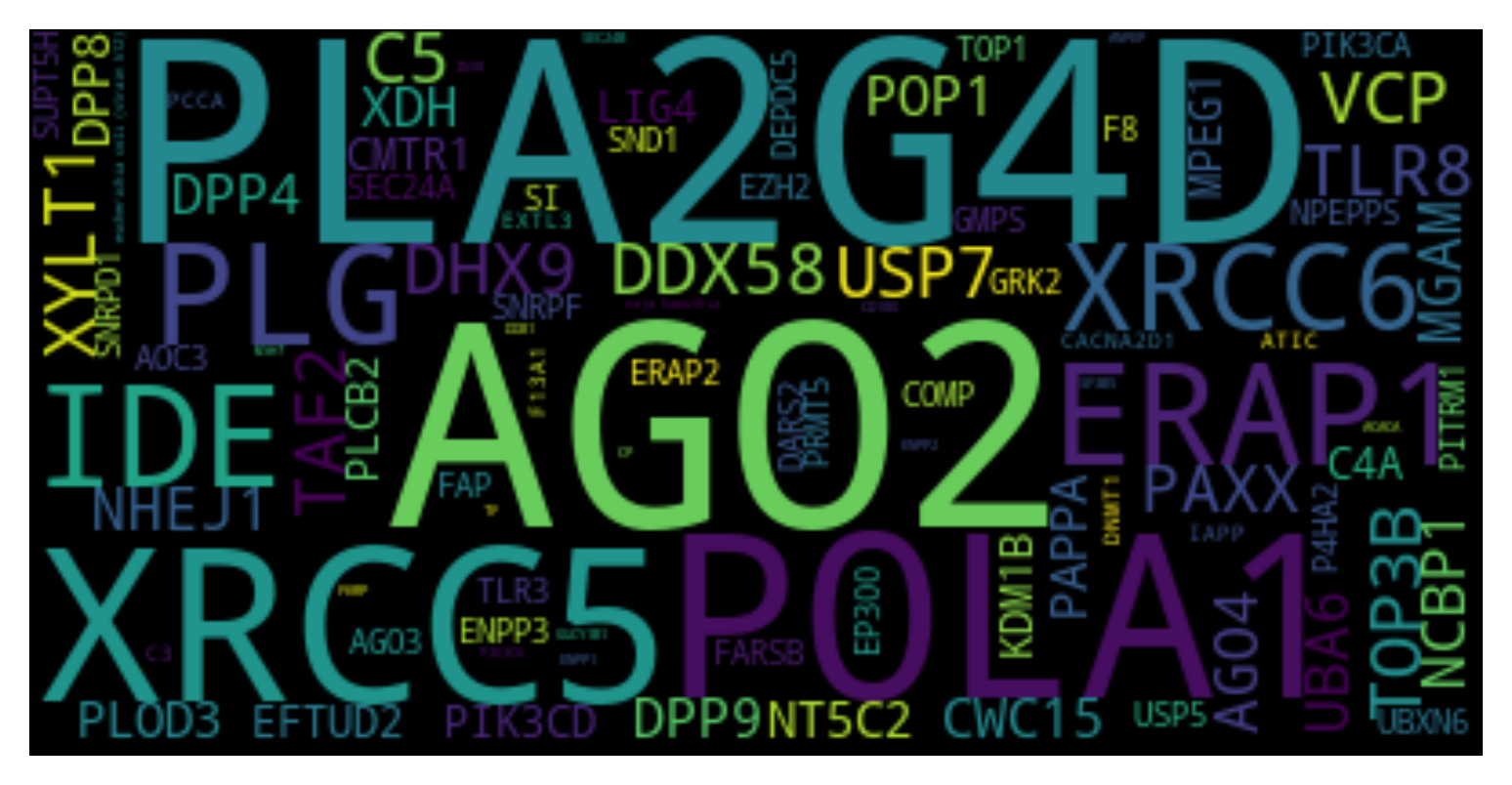

Supplement: Supplementary file 24 — Supplementary Information 12. [file 41598_2025_91849_MOESM24_ESM.zip › 4KREp_A_mdwhole_HL2REF/plots/4KREp_A-wordcloud.png]

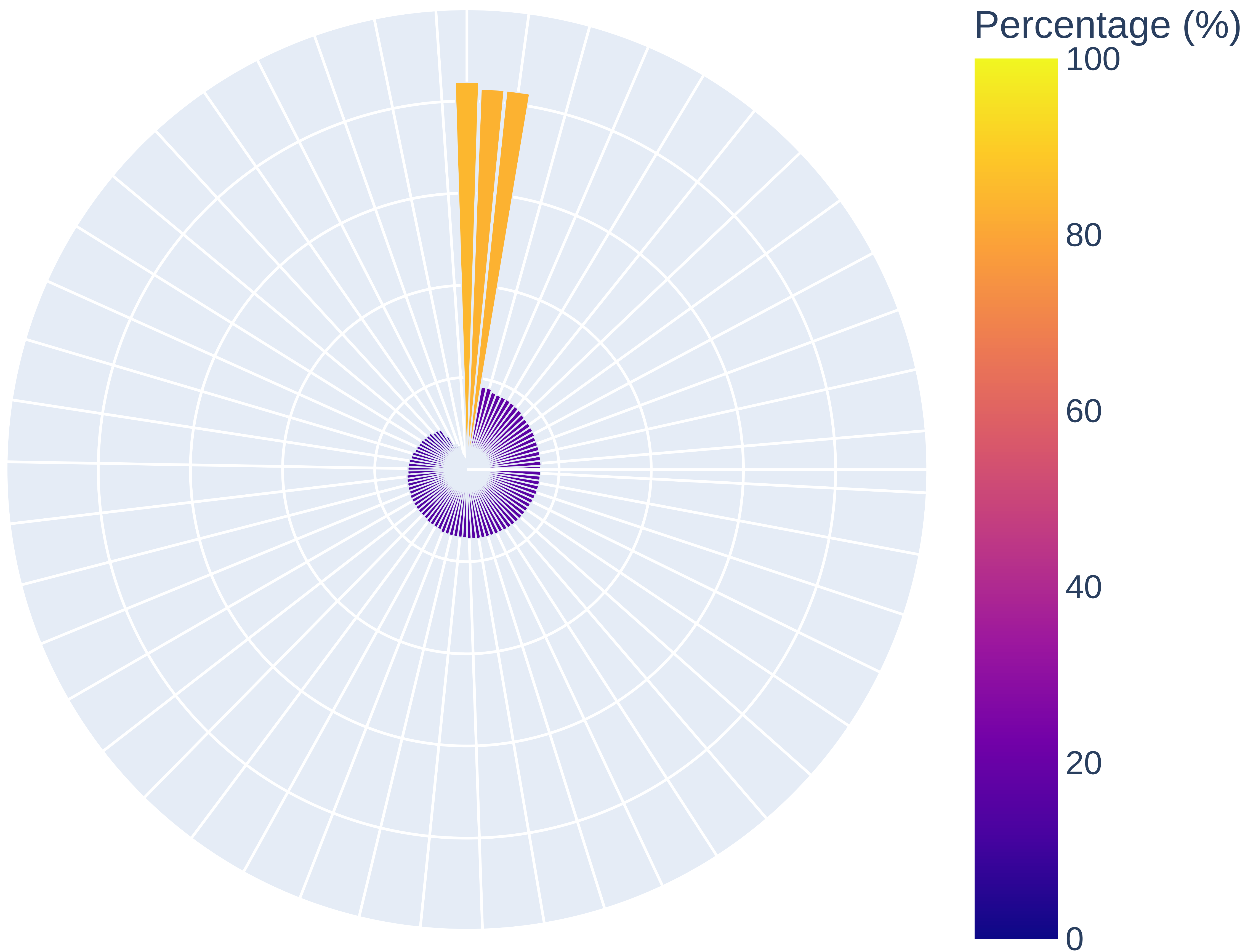

1D identity for 93 proteins in the final set (total: 94)

Supplement: Supplementary file 24 — Supplementary Information 12. [file 41598_2025_91849_MOESM24_ESM.zip › 4KREp_A_mdwhole_HL2REF/plots/4KREp_A_1D-identity.pdf]

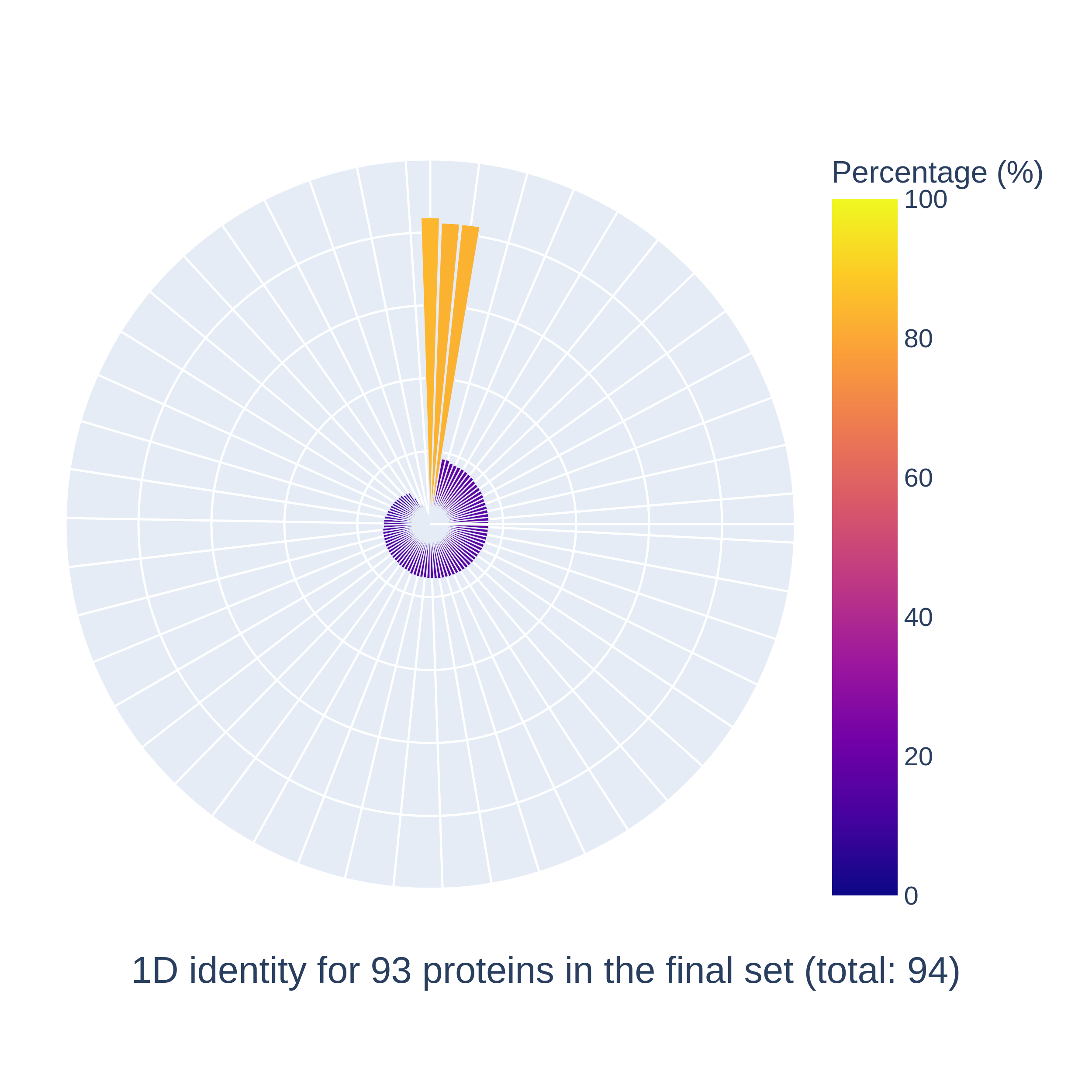

Supplement: Supplementary file 24 — Supplementary Information 12. [file 41598_2025_91849_MOESM24_ESM.zip › 4KREp_A_mdwhole_HL2REF/plots/4KREp_A_1D-identity.png]

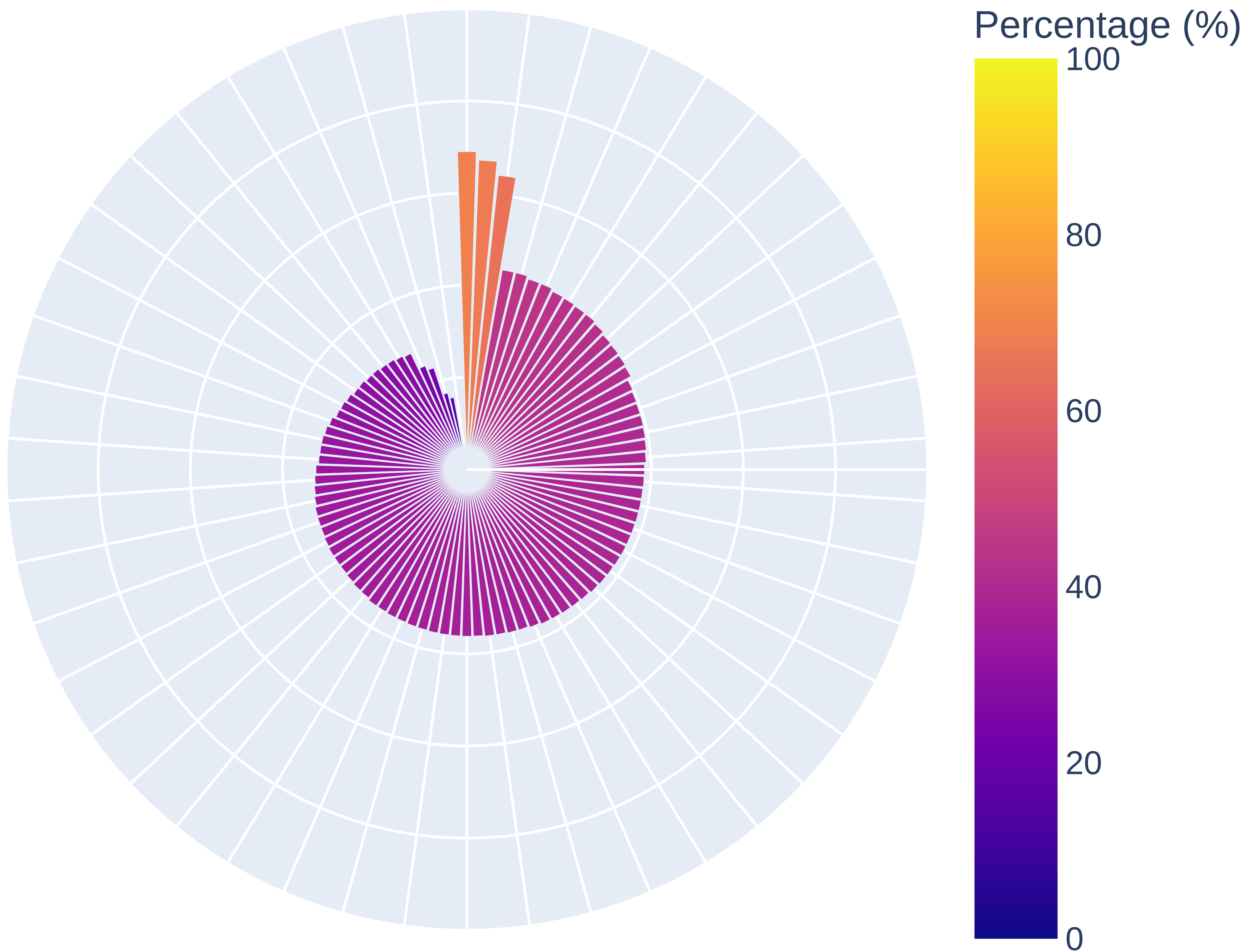

2D identity for 92 proteins in the final set (total: 94)

Supplement: Supplementary file 24 — Supplementary Information 12. [file 41598_2025_91849_MOESM24_ESM.zip › 4KREp_A_mdwhole_HL2REF/plots/4KREp_A_2D-identity.pdf]

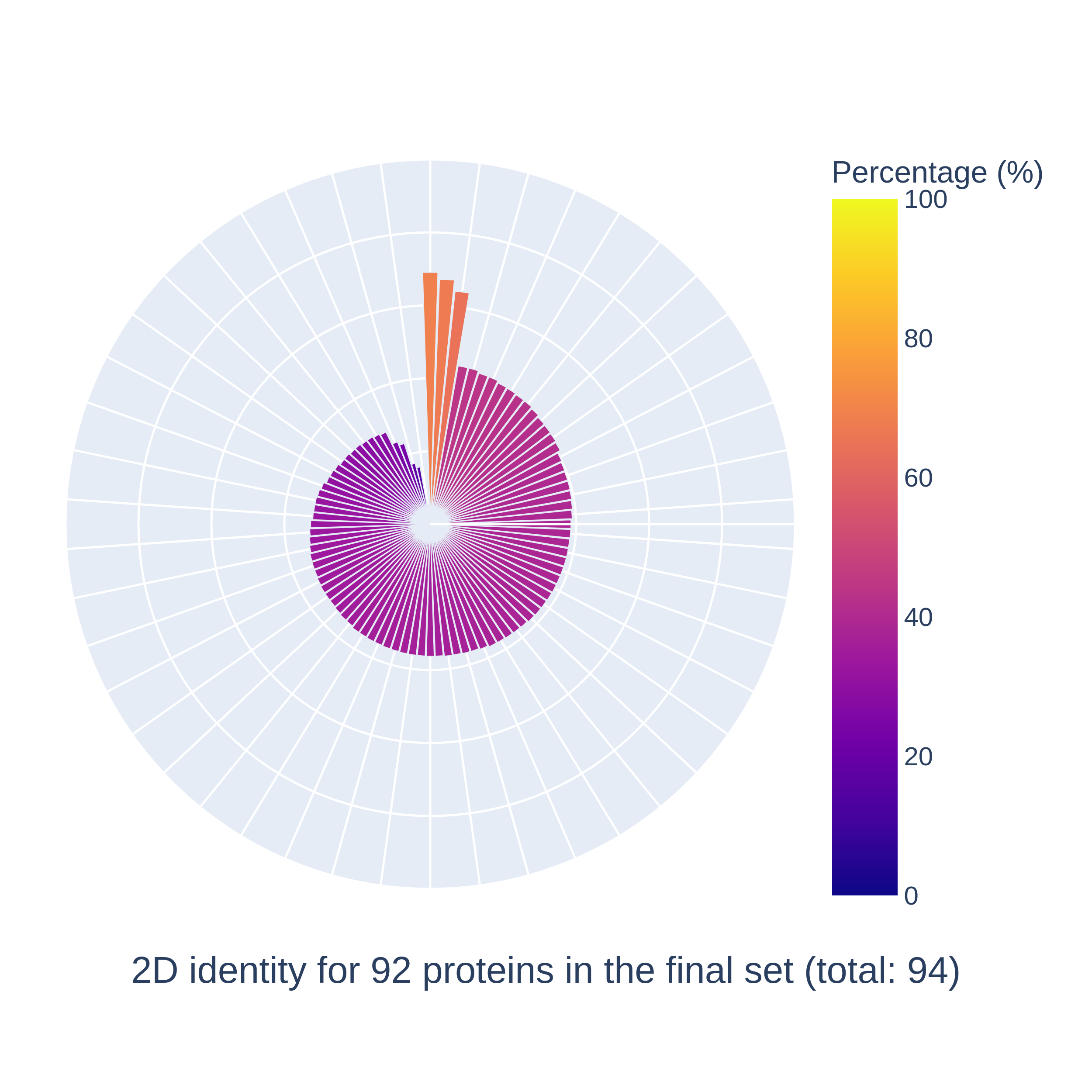

Supplement: Supplementary file 24 — Supplementary Information 12. [file 41598_2025_91849_MOESM24_ESM.zip › 4KREp_A_mdwhole_HL2REF/plots/4KREp_A_2D-identity.png]

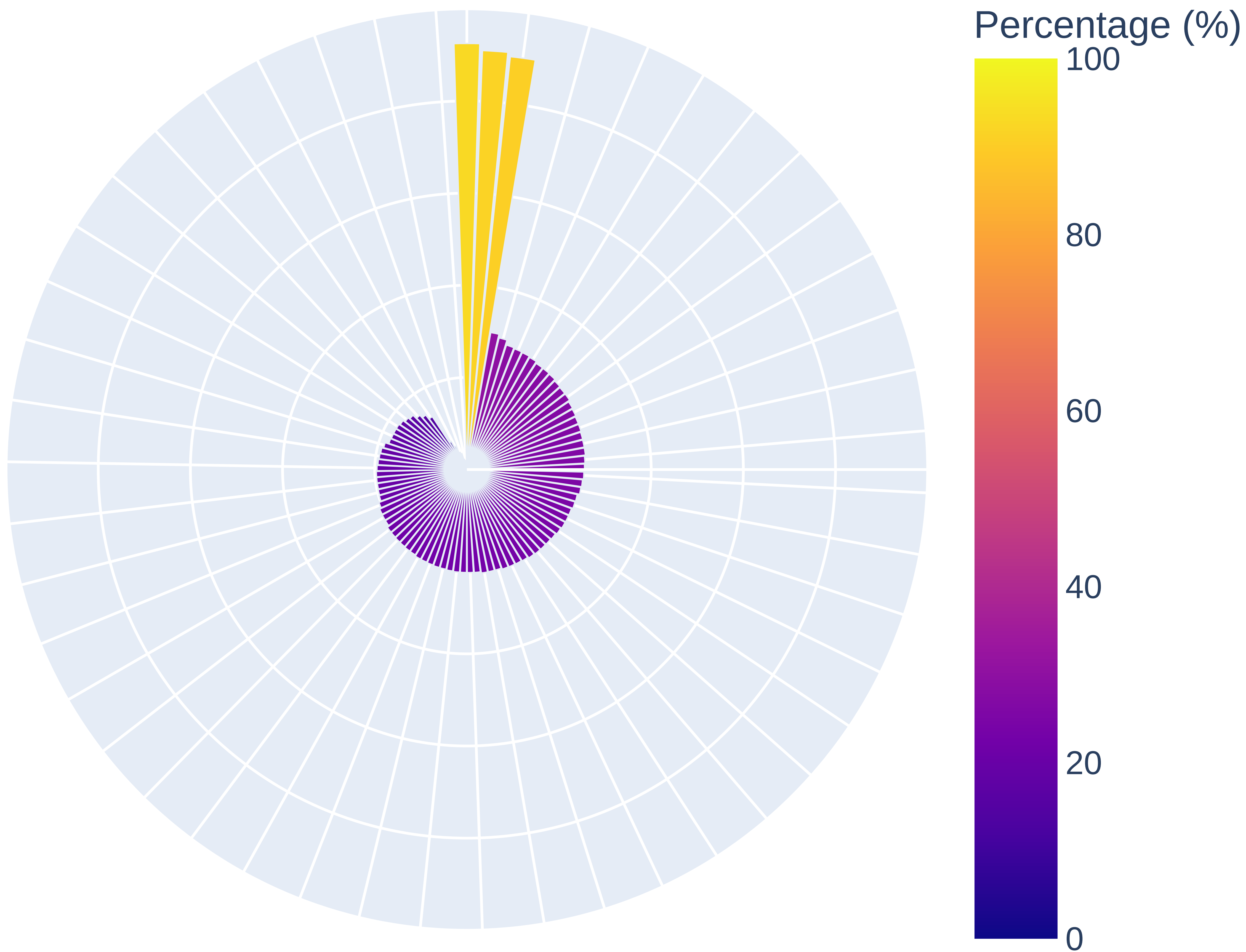

3D Similarity for 93 proteins in the final set (total: 94)

Supplement: Supplementary file 24 — Supplementary Information 12. [file 41598_2025_91849_MOESM24_ESM.zip › 4KREp_A_mdwhole_HL2REF/plots/4KREp_A_3D-score.pdf]

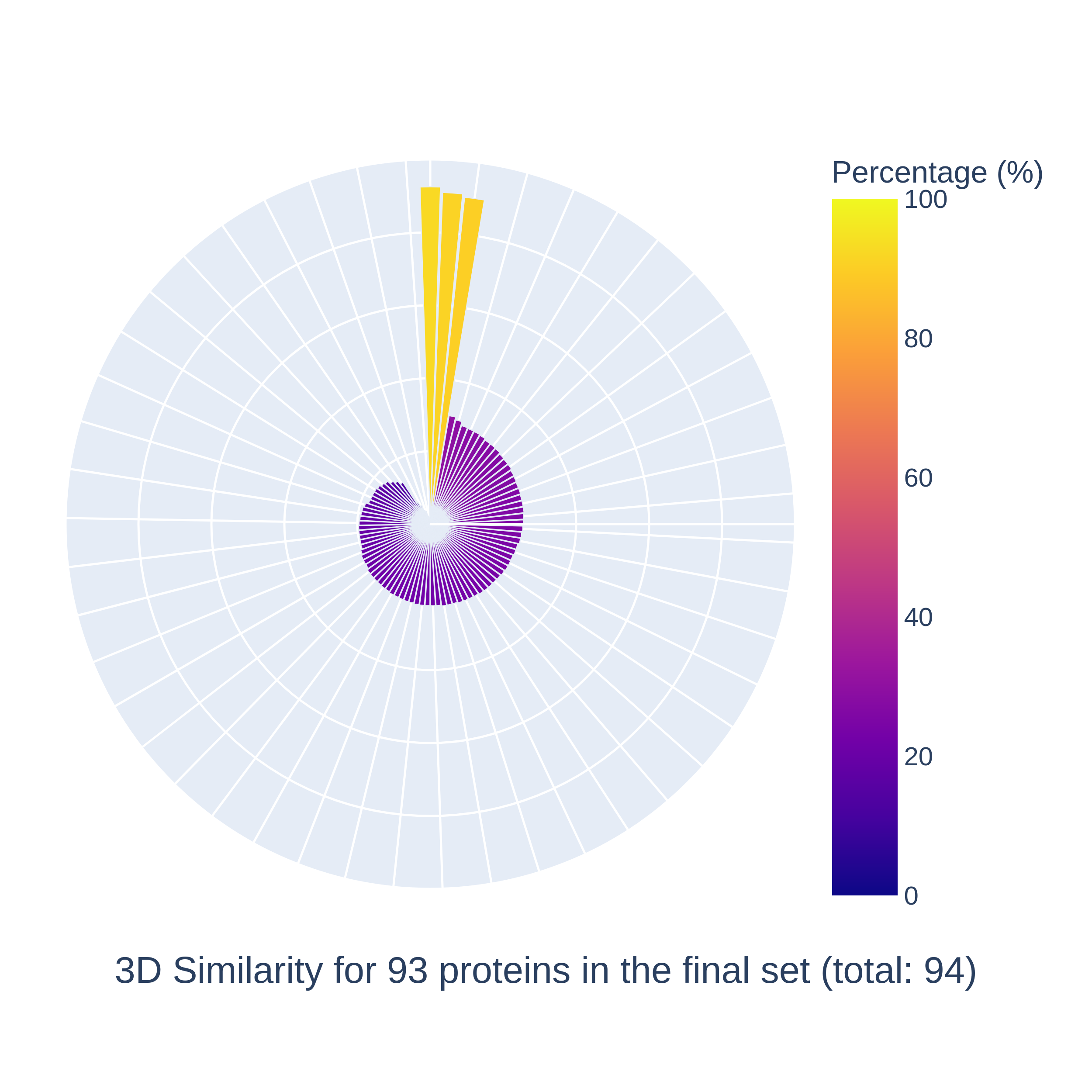

Supplement: Supplementary file 24 — Supplementary Information 12. [file 41598_2025_91849_MOESM24_ESM.zip › 4KREp_A_mdwhole_HL2REF/plots/4KREp_A_3D-score.png]

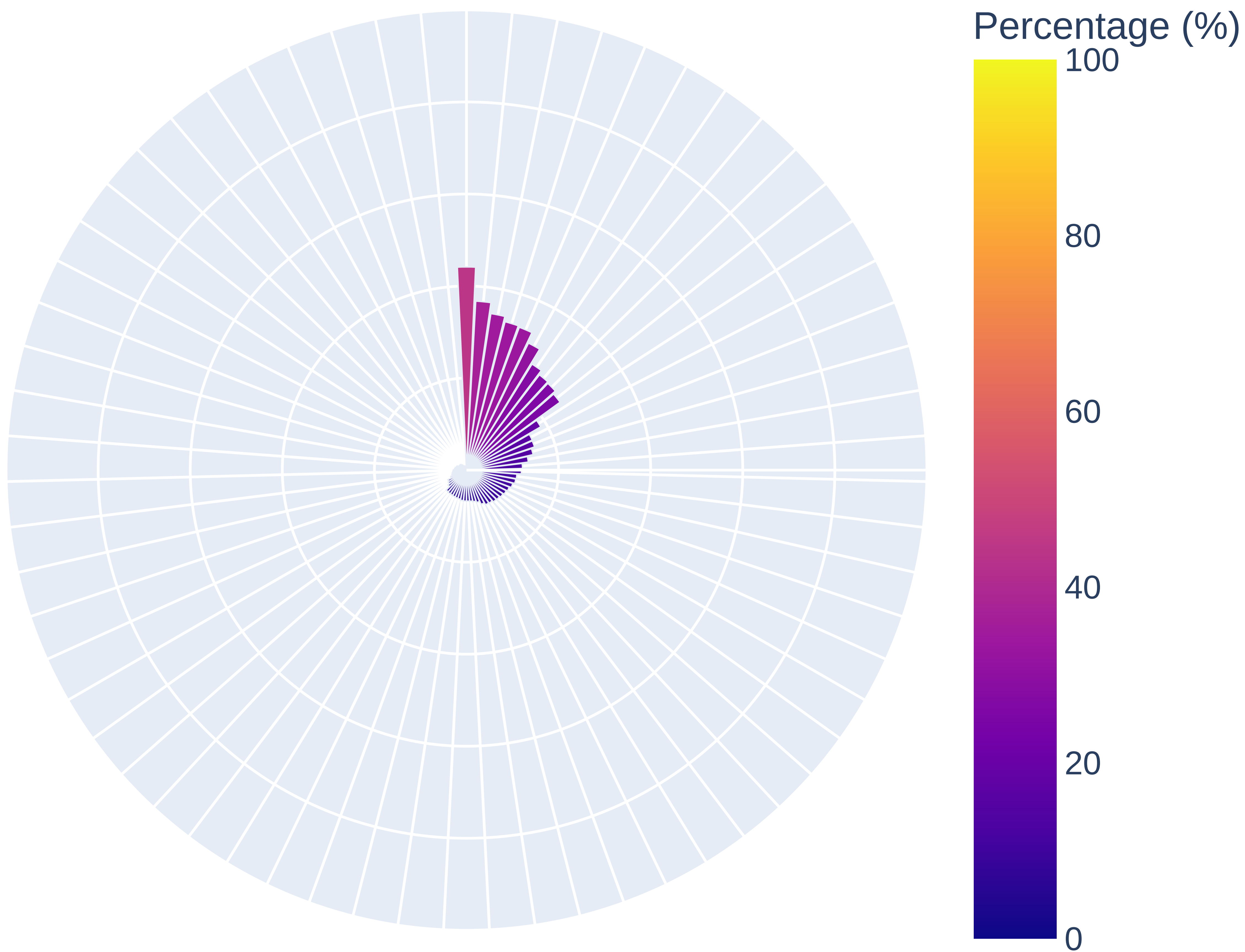

3'-UTR identity for 63 proteins in the final set (total: 94)

Supplement: Supplementary file 24 — Supplementary Information 12. [file 41598_2025_91849_MOESM24_ESM.zip › 4KREp_A_mdwhole_HL2REF/plots/4KREp_A_3UTR-identity.pdf]

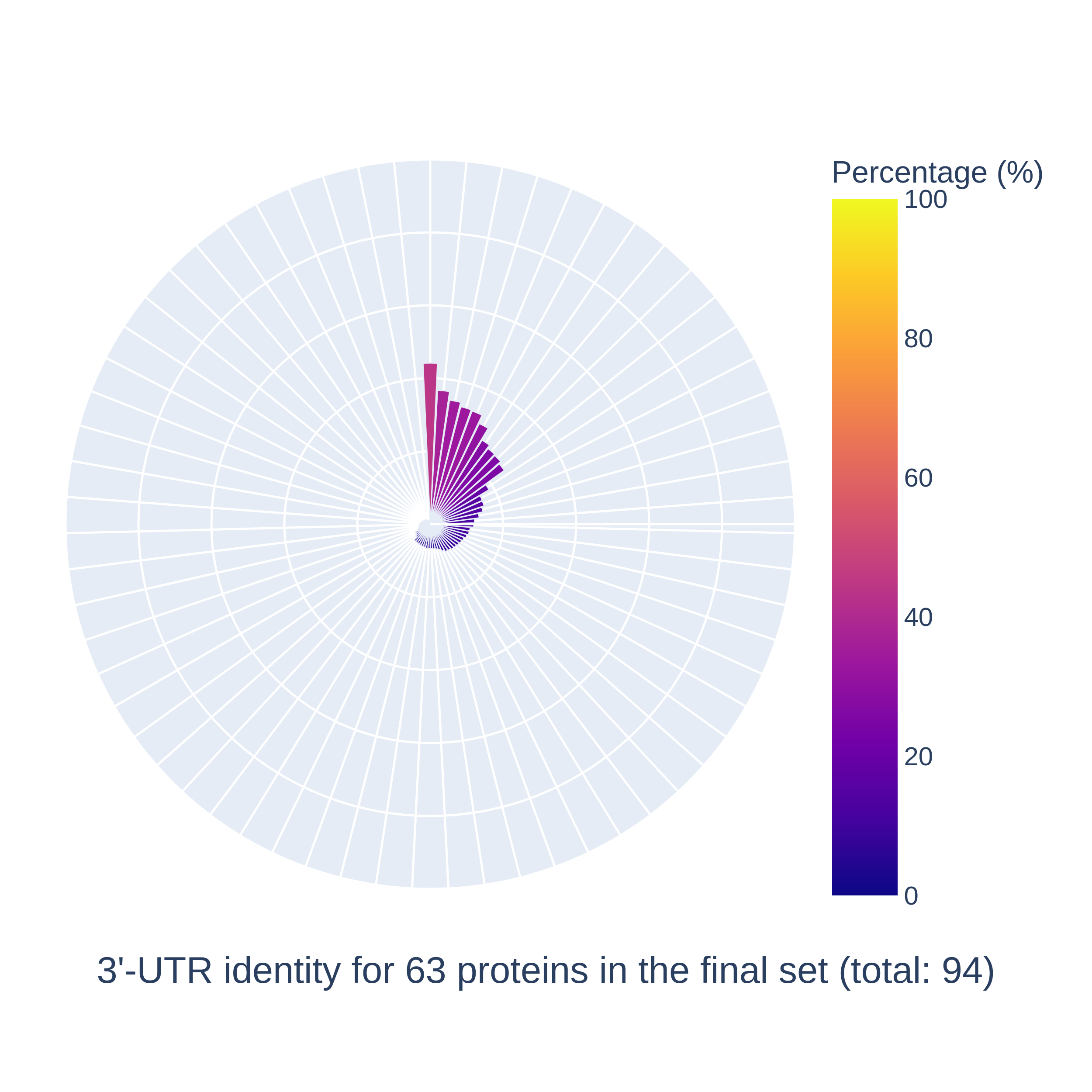

Supplement: Supplementary file 24 — Supplementary Information 12. [file 41598_2025_91849_MOESM24_ESM.zip › 4KREp_A_mdwhole_HL2REF/plots/4KREp_A_3UTR-identity.png]

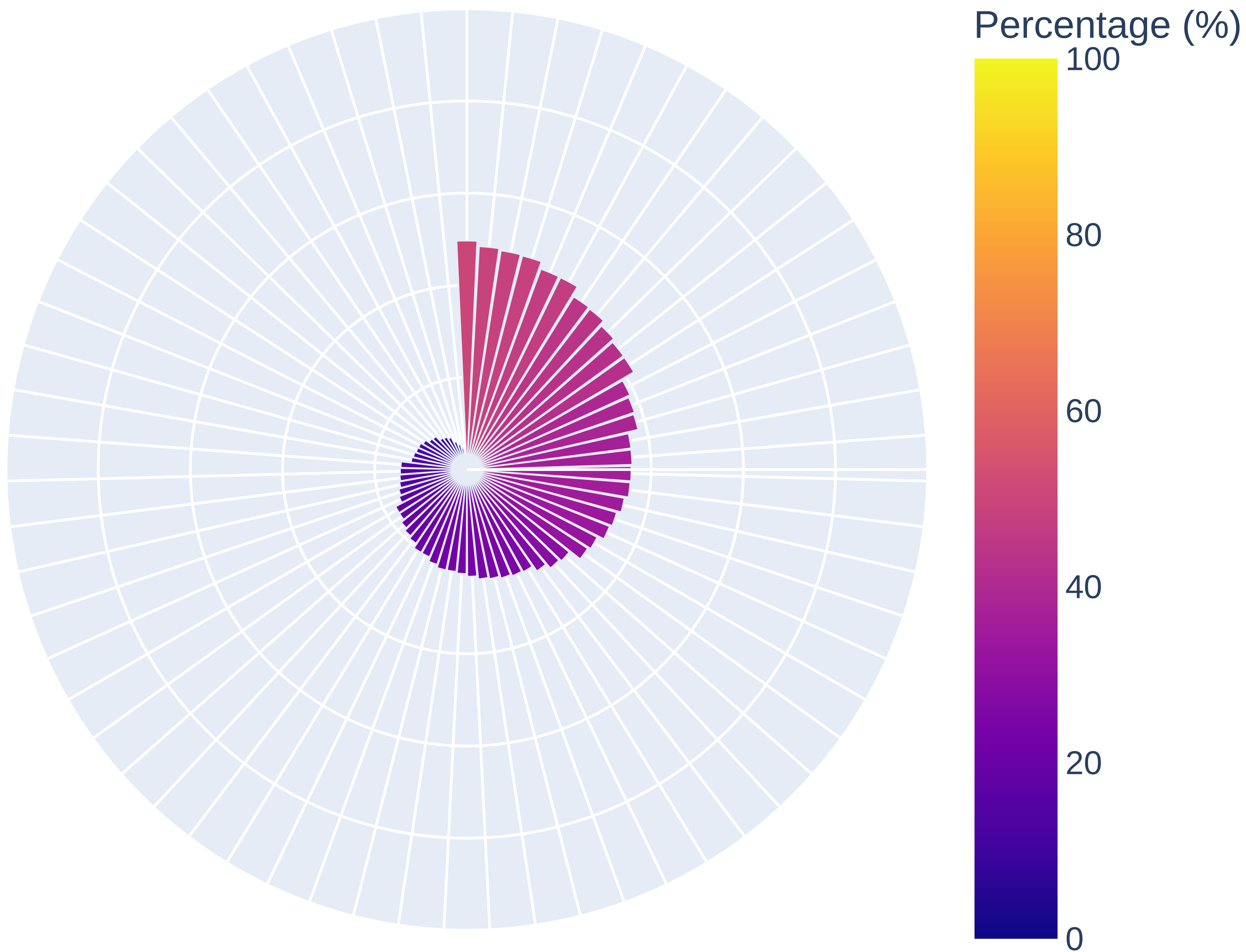

5'-UTR identity for 63 proteins in the final set (total: 94)

Supplement: Supplementary file 24 — Supplementary Information 12. [file 41598_2025_91849_MOESM24_ESM.zip › 4KREp_A_mdwhole_HL2REF/plots/4KREp_A_5UTR-identity.pdf]

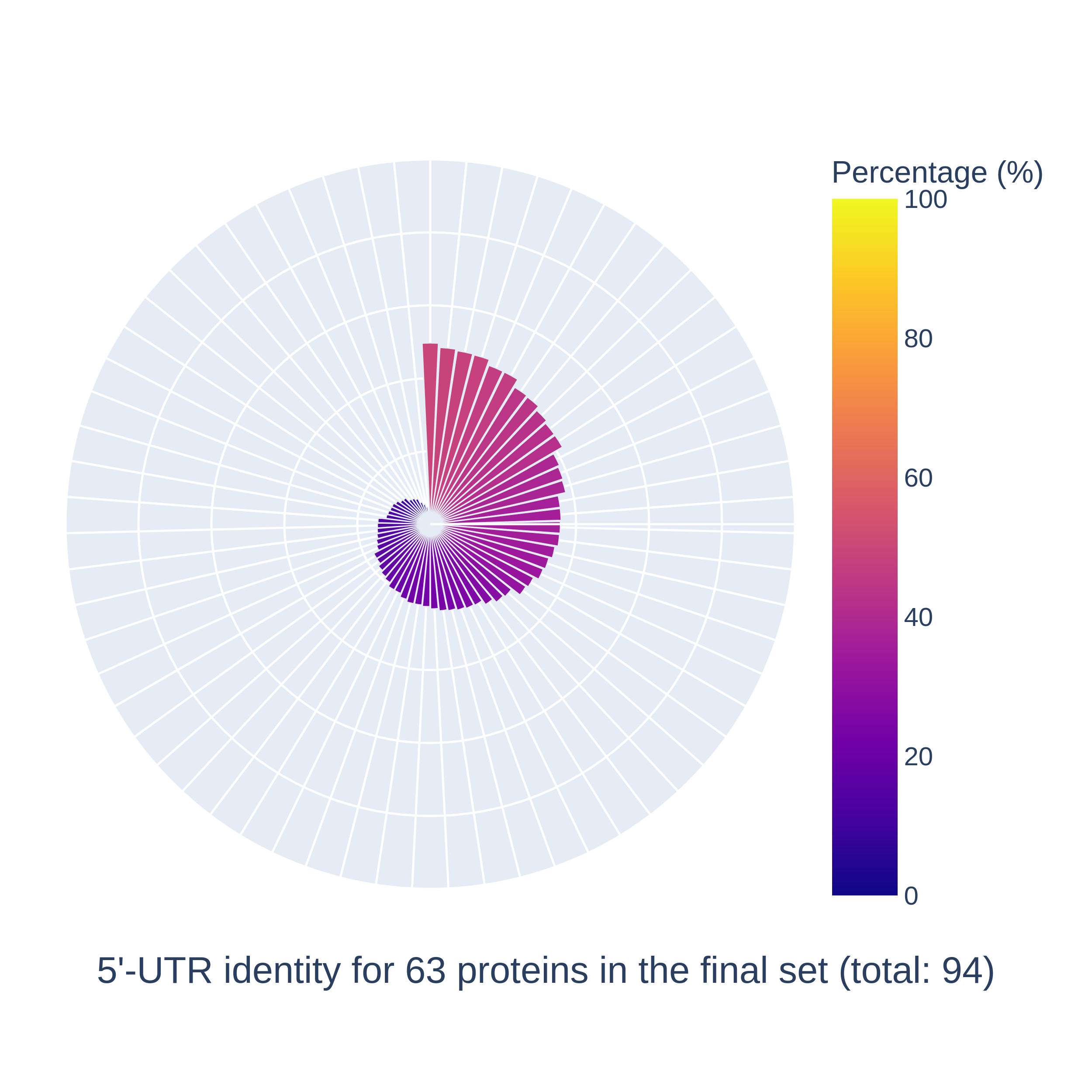

Supplement: Supplementary file 24 — Supplementary Information 12. [file 41598_2025_91849_MOESM24_ESM.zip › 4KREp_A_mdwhole_HL2REF/plots/4KREp_A_5UTR-identity.png]

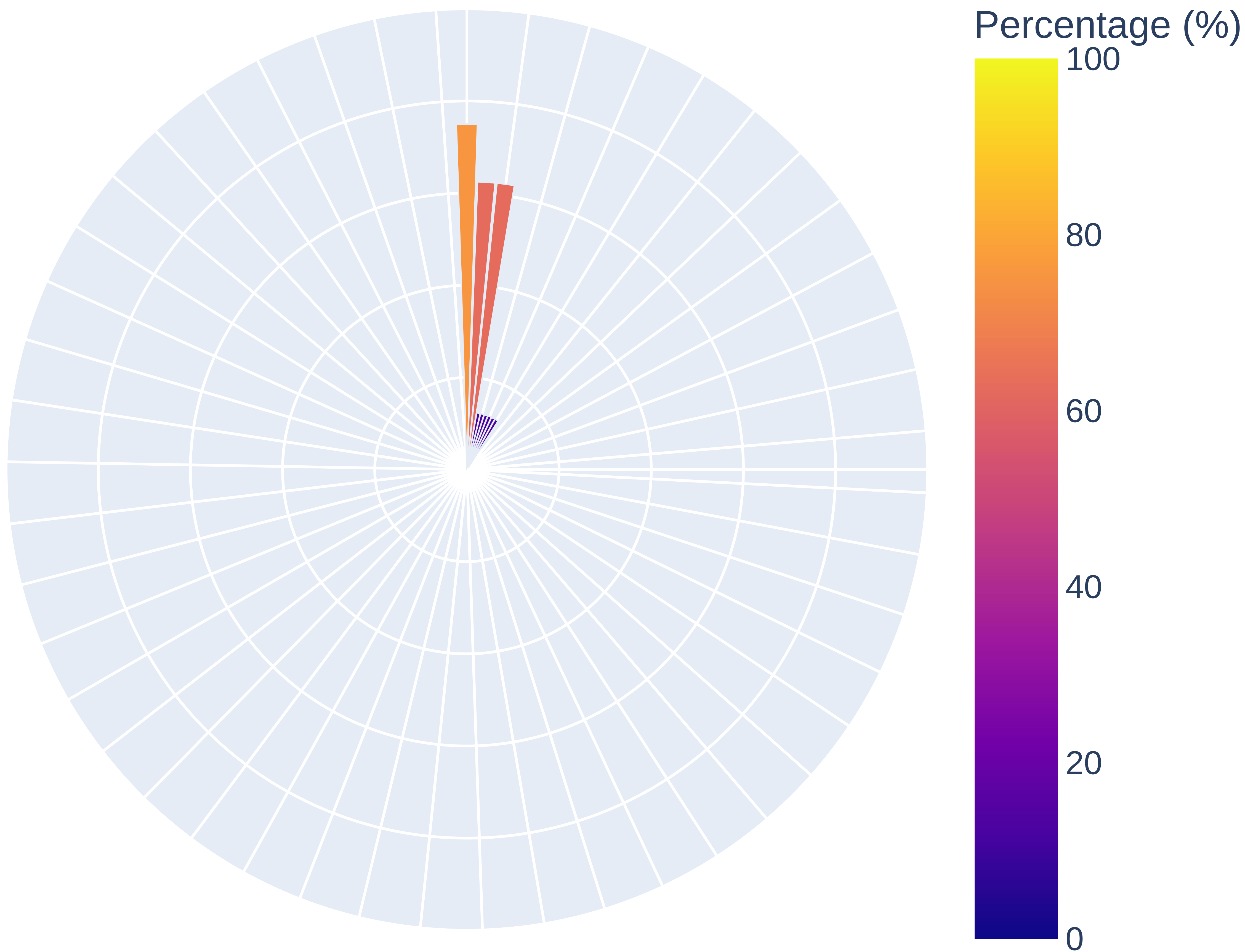

Common pathways for 93 proteins in the final set (total: 94)

Supplement: Supplementary file 24 — Supplementary Information 12. [file 41598_2025_91849_MOESM24_ESM.zip › 4KREp_A_mdwhole_HL2REF/plots/4KREp_A_biologicalProcessSim.pdf]

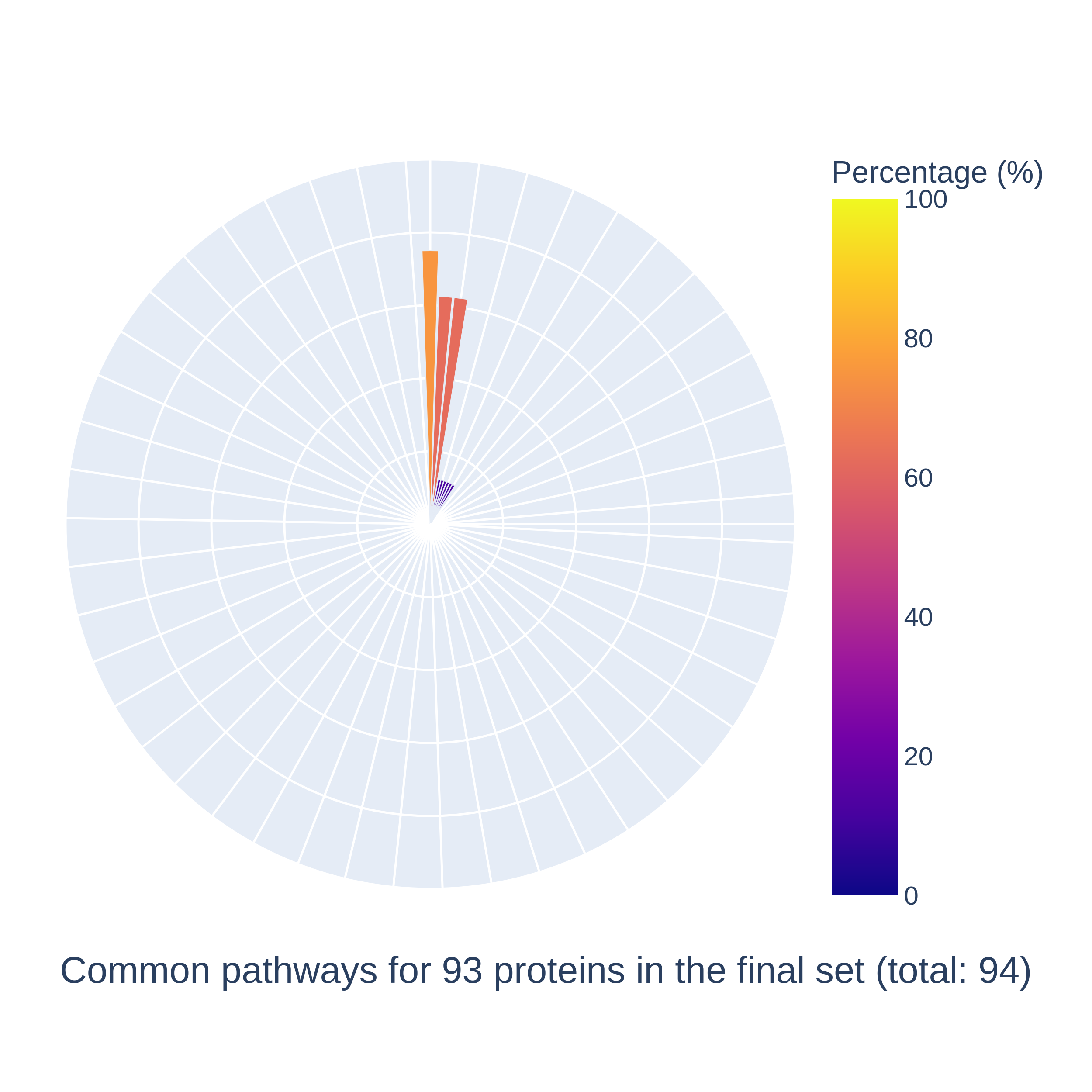

Supplement: Supplementary file 24 — Supplementary Information 12. [file 41598_2025_91849_MOESM24_ESM.zip › 4KREp_A_mdwhole_HL2REF/plots/4KREp_A_biologicalProcessSim.png]

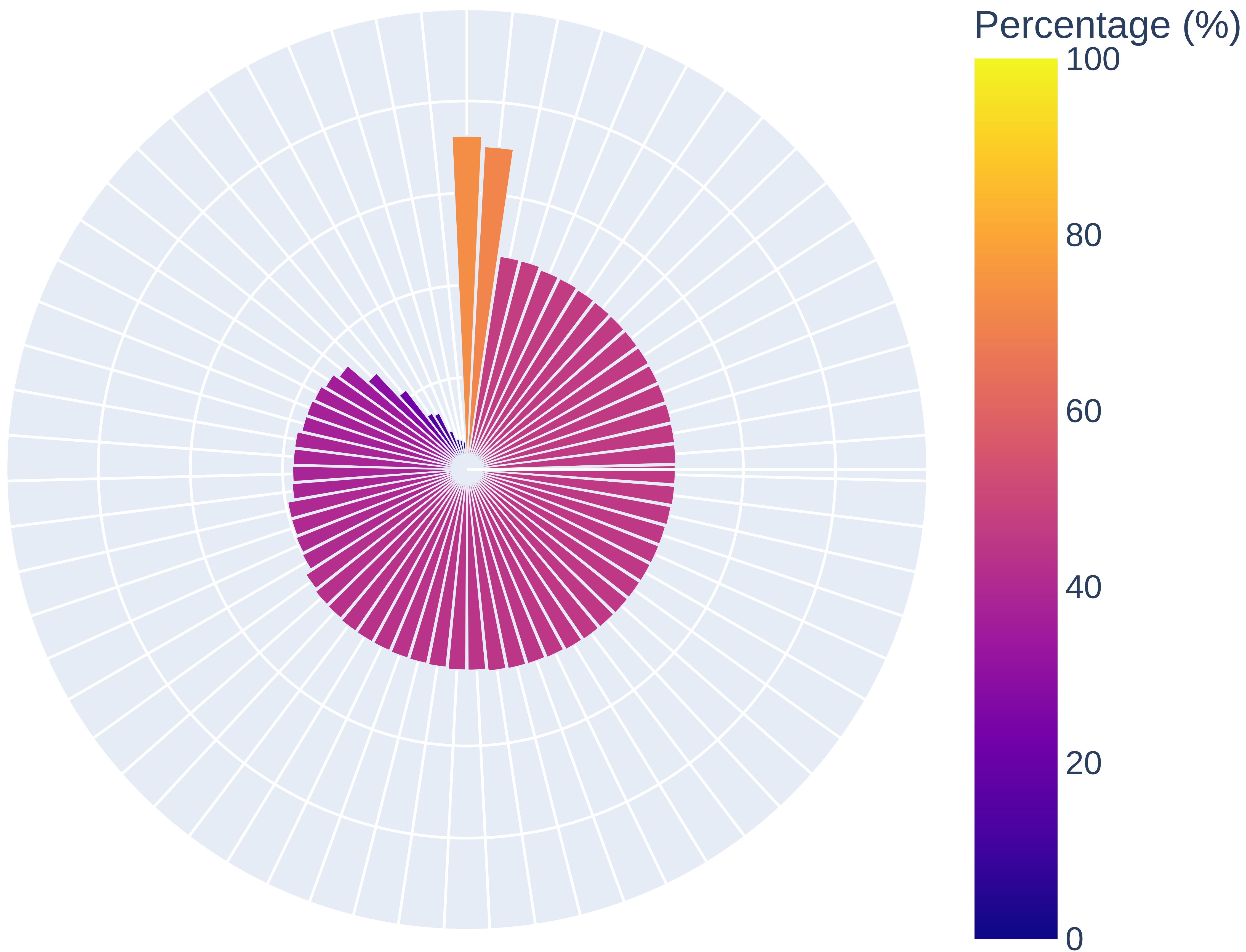

CDS identity for 63 proteins in the final set (total: 94)

Supplement: Supplementary file 24 — Supplementary Information 12. [file 41598_2025_91849_MOESM24_ESM.zip › 4KREp_A_mdwhole_HL2REF/plots/4KREp_A_CDS-identity.pdf]

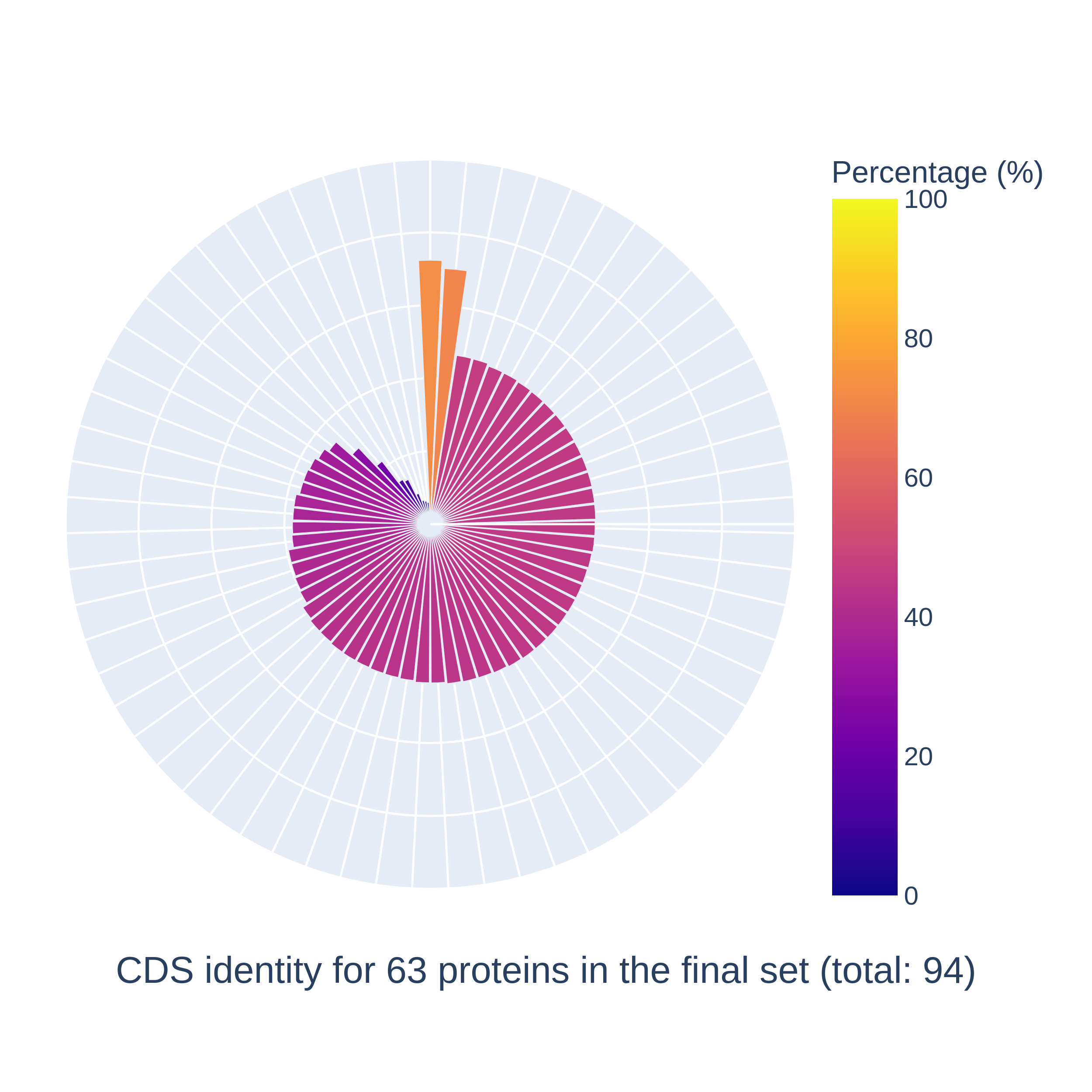

Supplement: Supplementary file 24 — Supplementary Information 12. [file 41598_2025_91849_MOESM24_ESM.zip › 4KREp_A_mdwhole_HL2REF/plots/4KREp_A_CDS-identity.png]

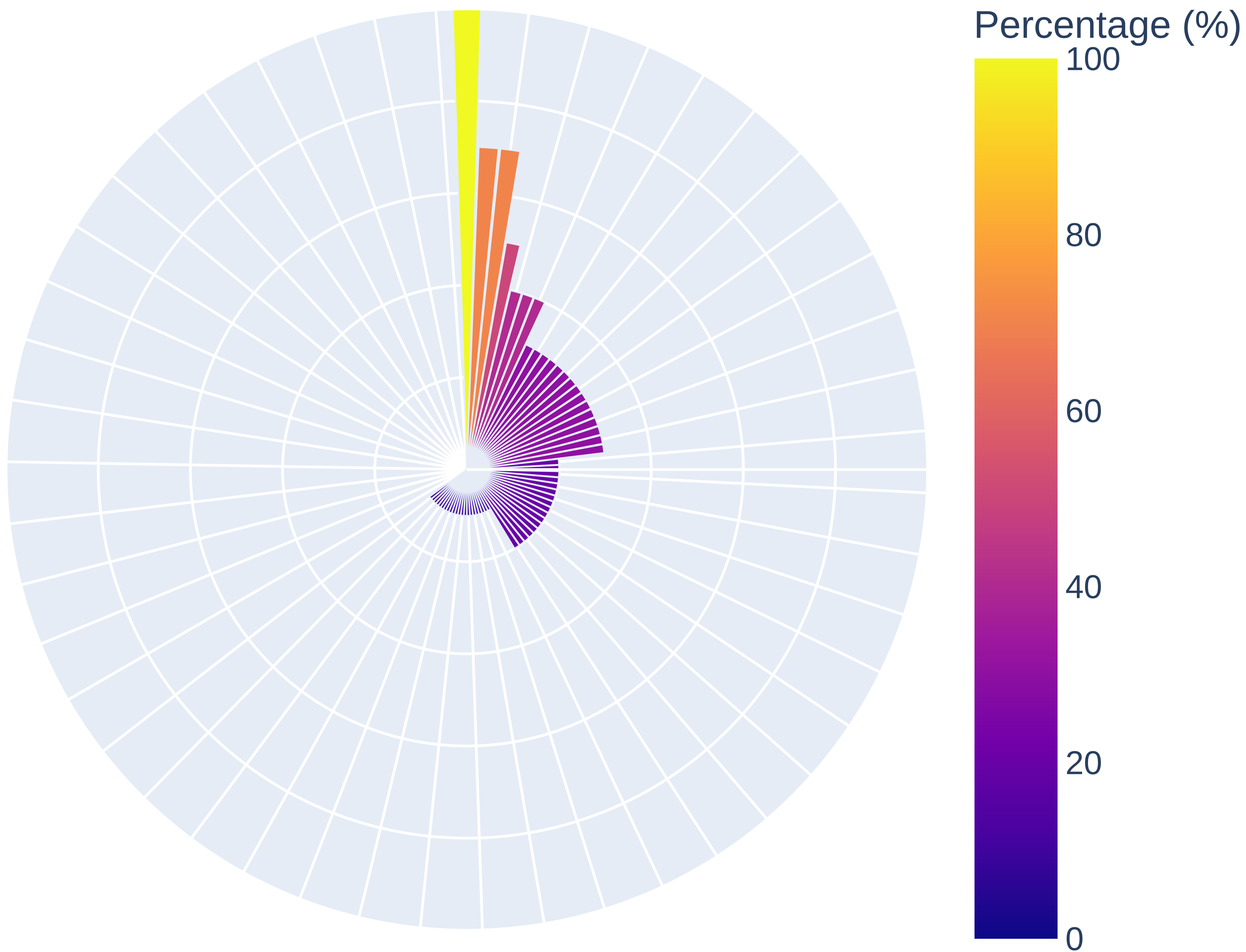

Common locations for 93 proteins in the final set (total: 94)

Supplement: Supplementary file 24 — Supplementary Information 12. [file 41598_2025_91849_MOESM24_ESM.zip › 4KREp_A_mdwhole_HL2REF/plots/4KREp_A_cellularComponentSim.pdf]

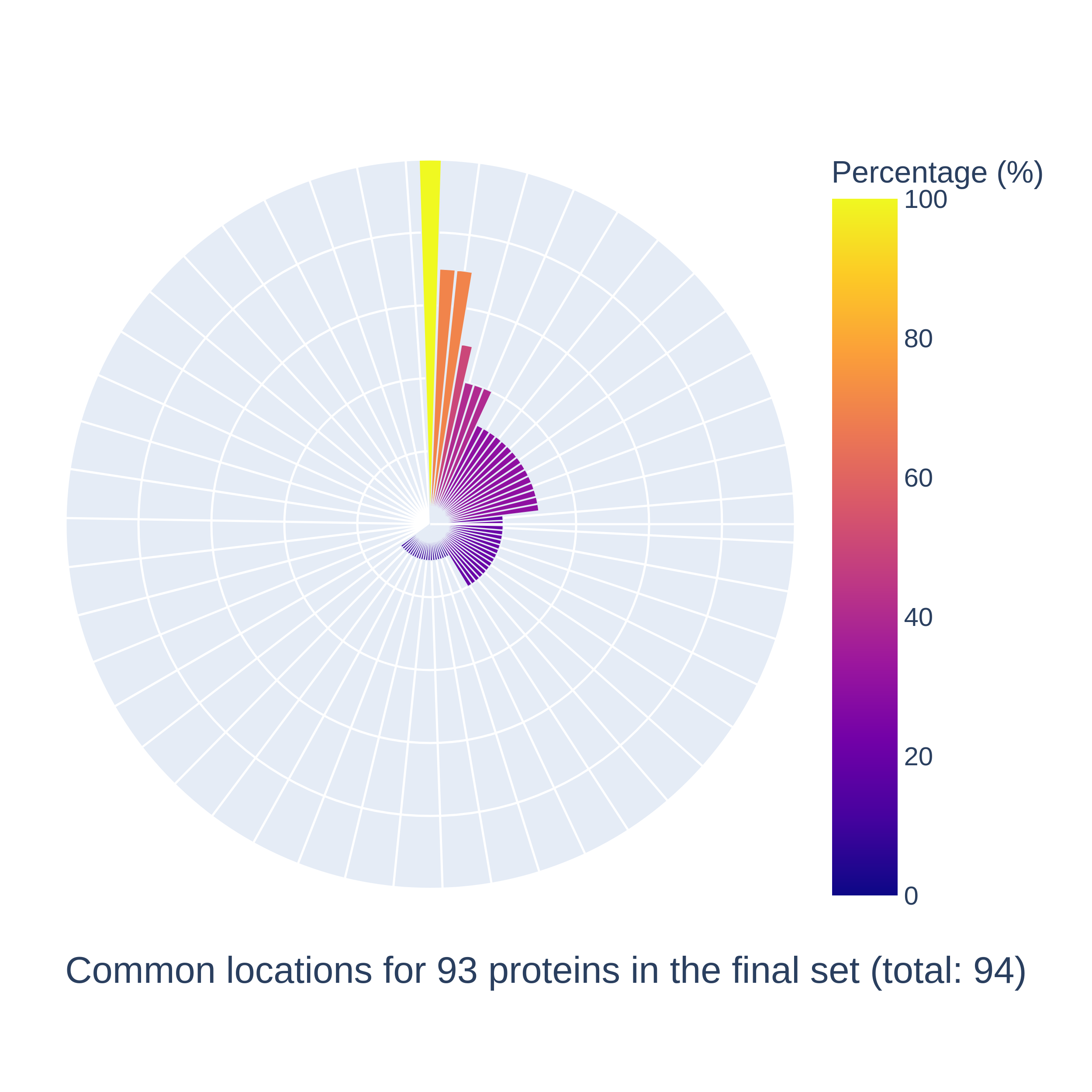

Supplement: Supplementary file 24 — Supplementary Information 12. [file 41598_2025_91849_MOESM24_ESM.zip › 4KREp_A_mdwhole_HL2REF/plots/4KREp_A_cellularComponentSim.png]

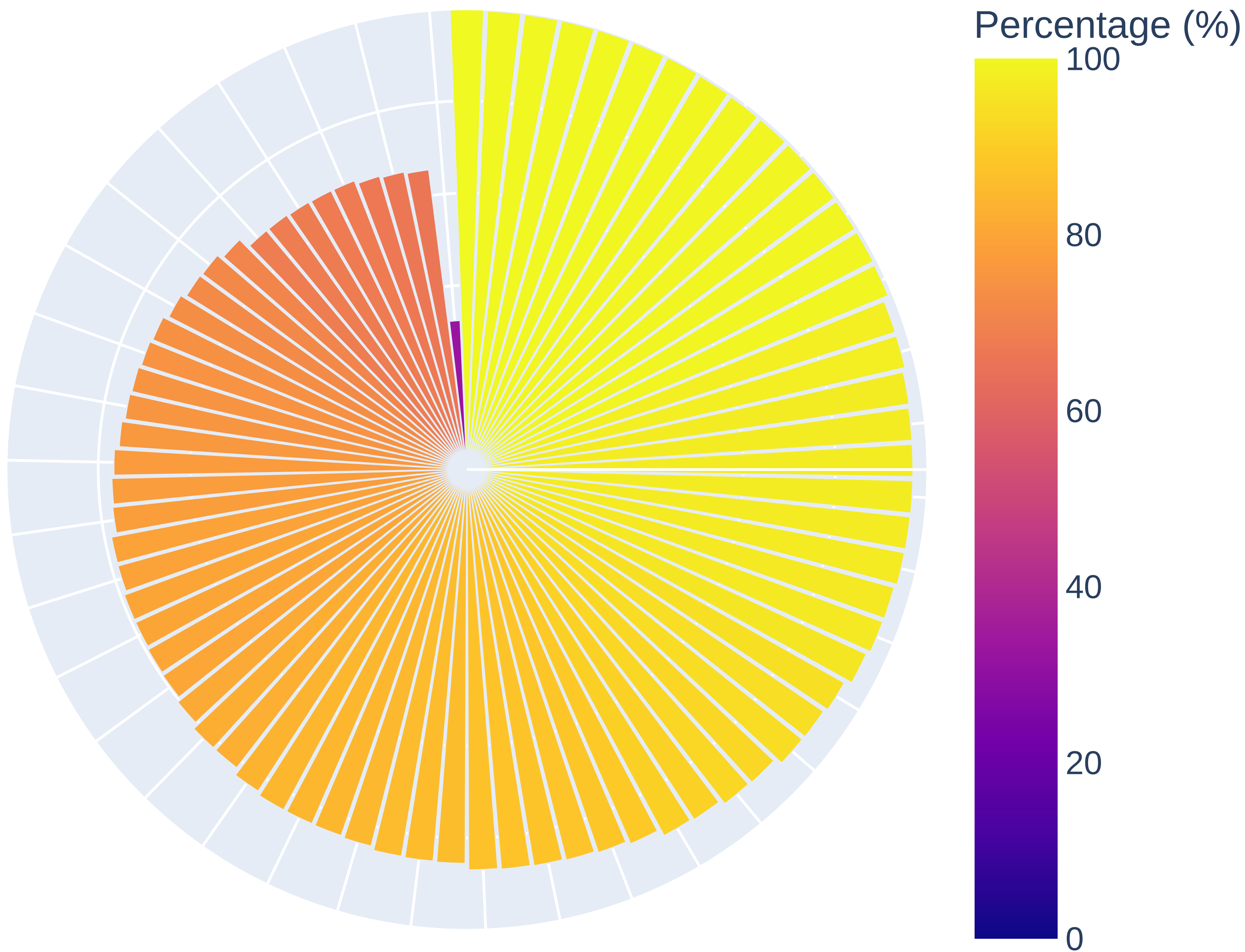

Chemical similarity for 77 proteins in the final set (total: 94)

Supplement: Supplementary file 24 — Supplementary Information 12. [file 41598_2025_91849_MOESM24_ESM.zip › 4KREp_A_mdwhole_HL2REF/plots/4KREp_A_chemSim.pdf]

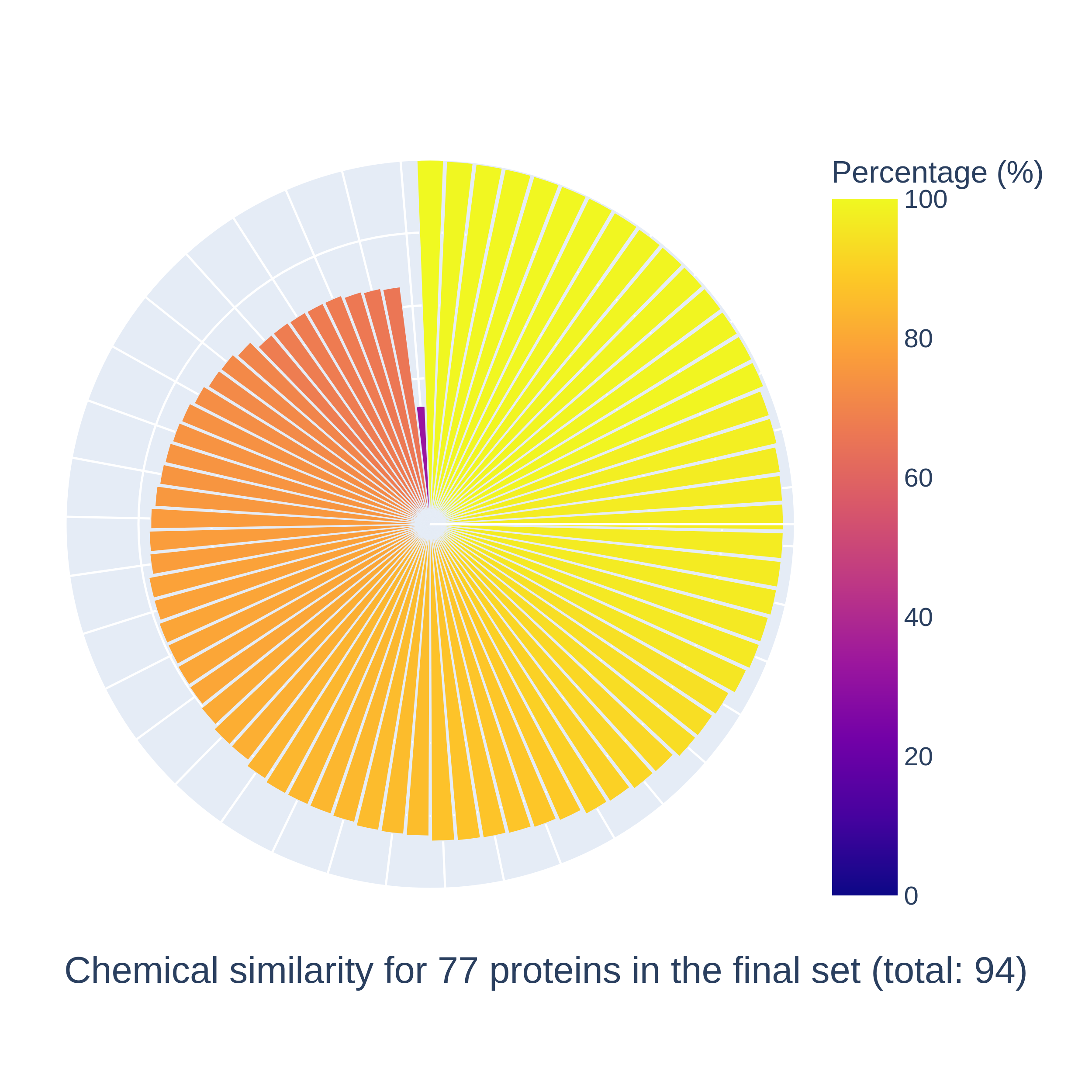

Supplement: Supplementary file 24 — Supplementary Information 12. [file 41598_2025_91849_MOESM24_ESM.zip › 4KREp_A_mdwhole_HL2REF/plots/4KREp_A_chemSim.png]

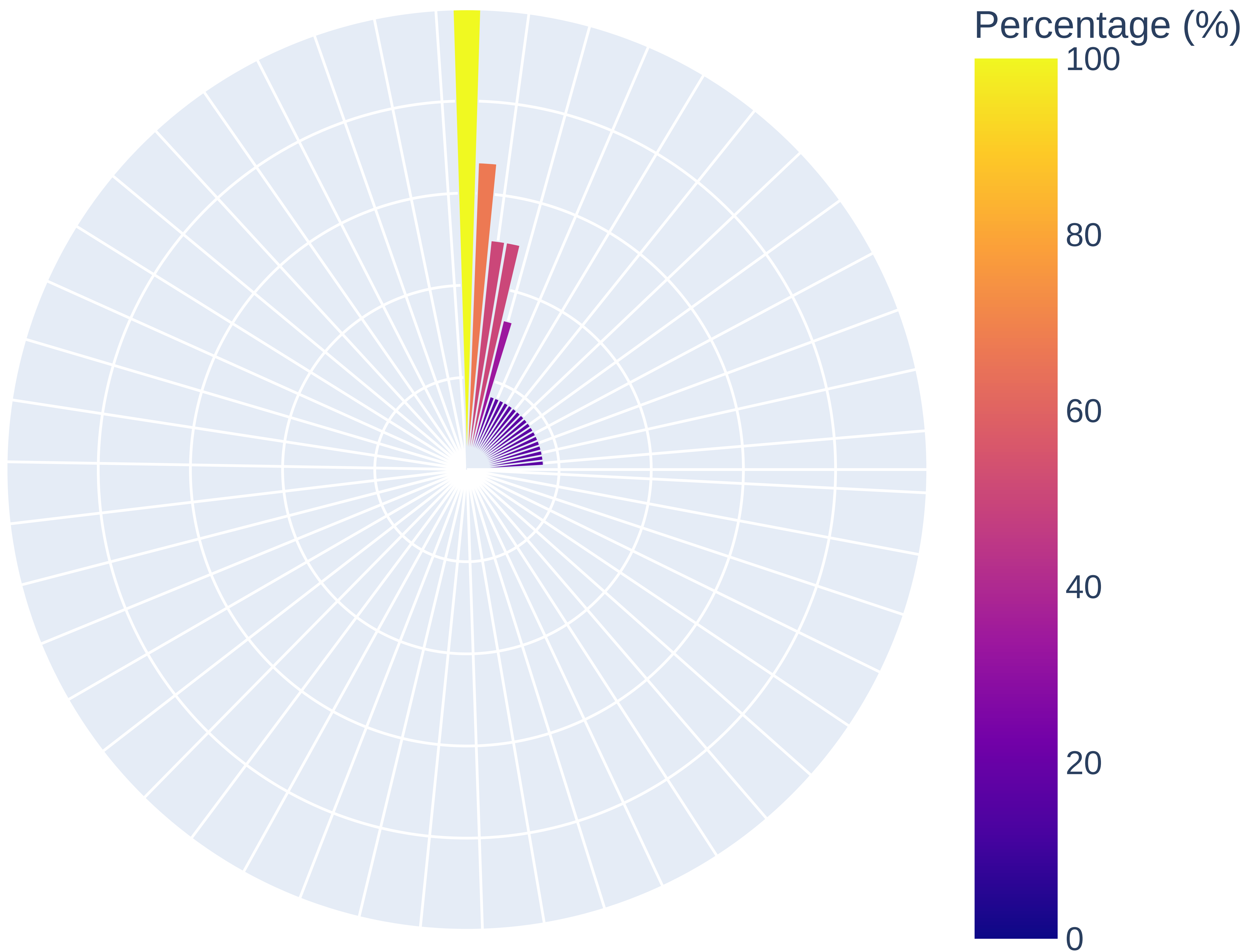

Common functions for 93 proteins in the final set (total: 94)

Supplement: Supplementary file 24 — Supplementary Information 12. [file 41598_2025_91849_MOESM24_ESM.zip › 4KREp_A_mdwhole_HL2REF/plots/4KREp_A_molecularFunctionSim.pdf]

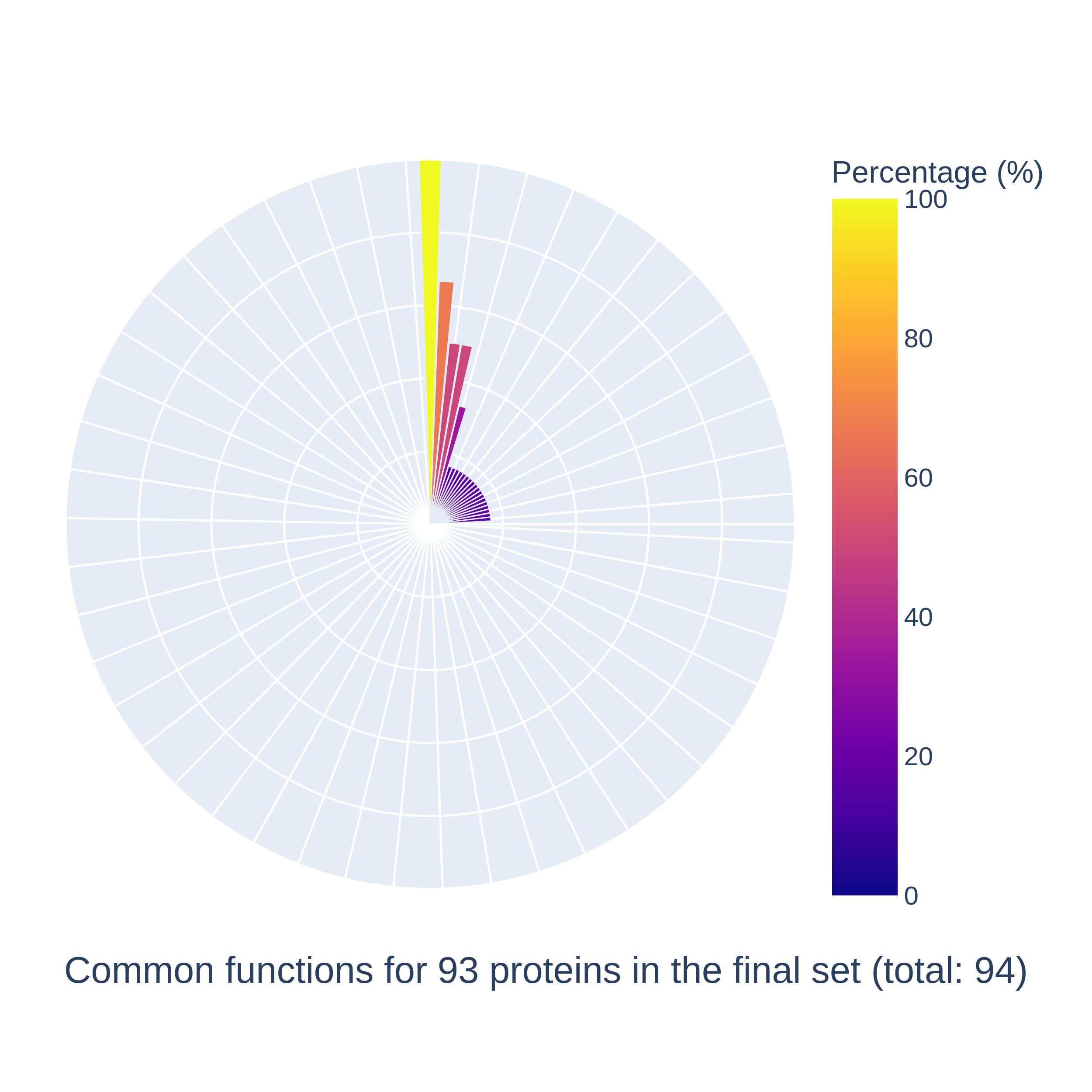

Supplement: Supplementary file 24 — Supplementary Information 12. [file 41598_2025_91849_MOESM24_ESM.zip › 4KREp_A_mdwhole_HL2REF/plots/4KREp_A_molecularFunctionSim.png]

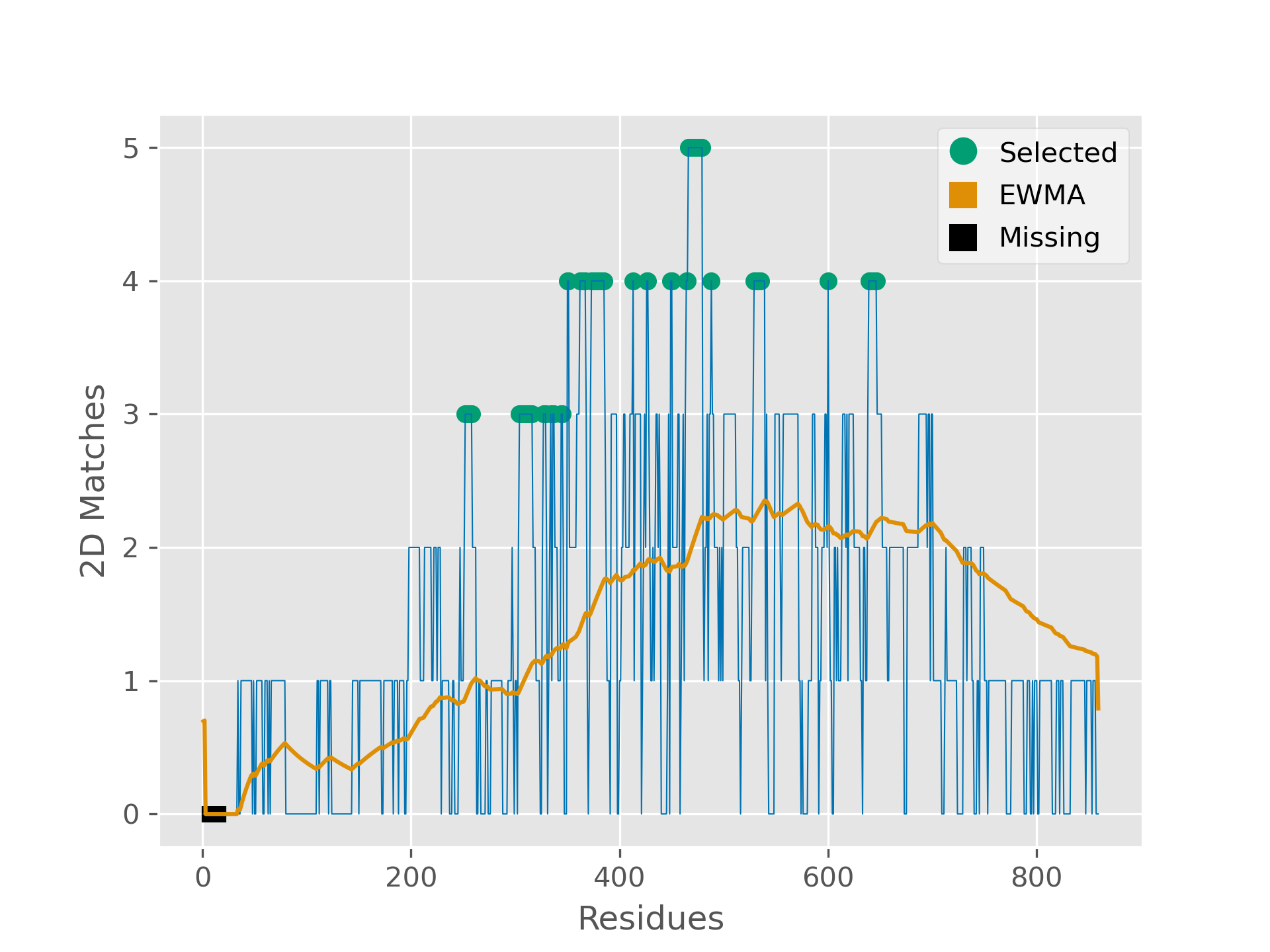

Supplement: Supplementary file 24 — Supplementary Information 12. [file 41598_2025_91849_MOESM24_ESM.zip › 4Z4Dp_A_mddomain_HL2REF/go/4Z4Dp_A_Piwi_mitot_mitosis_e8649561789a4b21afe18d44787a2120.png]

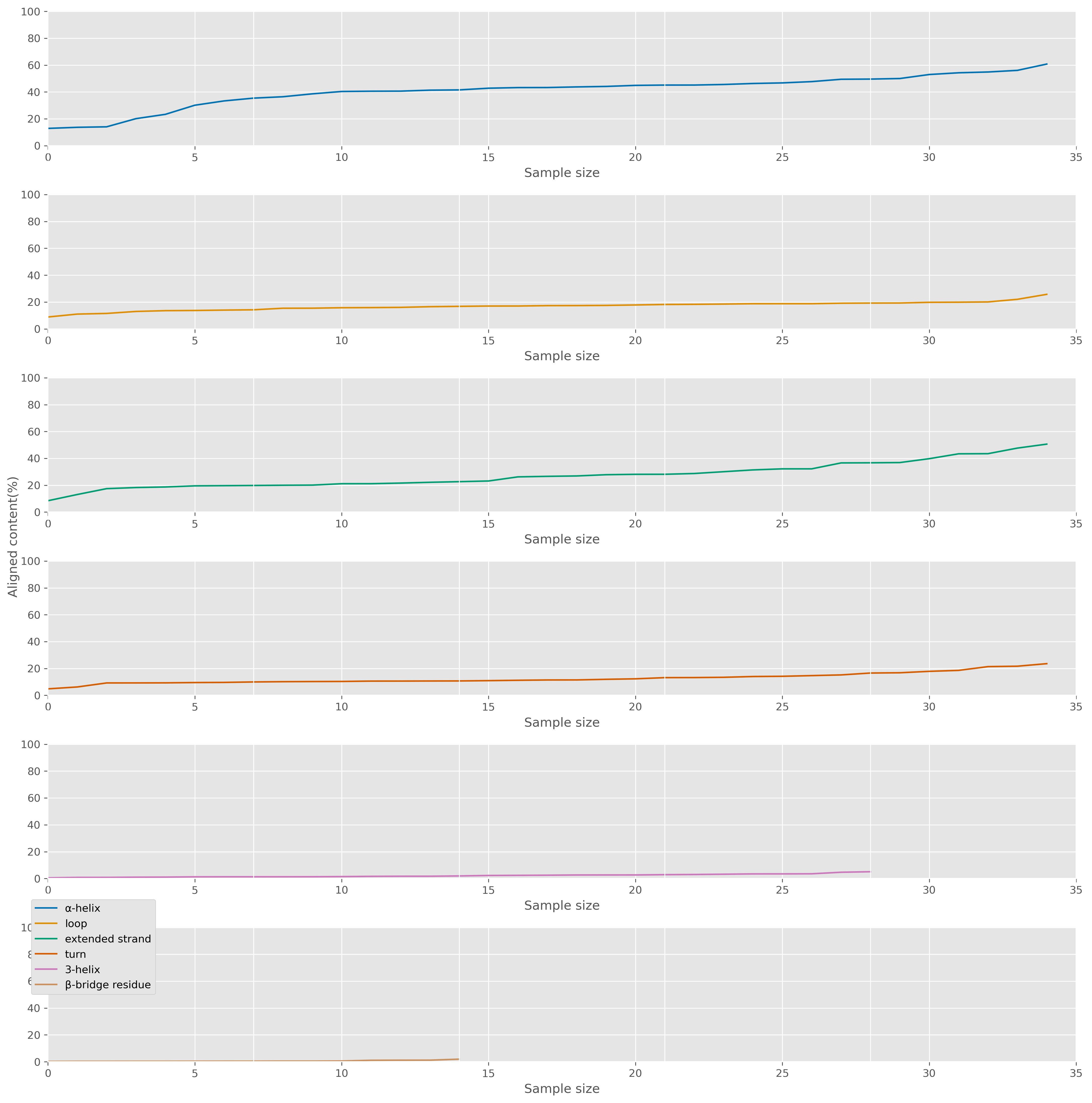

Supplement: Supplementary file 24 — Supplementary Information 12. [file 41598_2025_91849_MOESM24_ESM.zip › 4Z4Dp_A_mddomain_HL2REF/plots/4Z4Dp_A_Piwi-2Dfold_coverage.png]

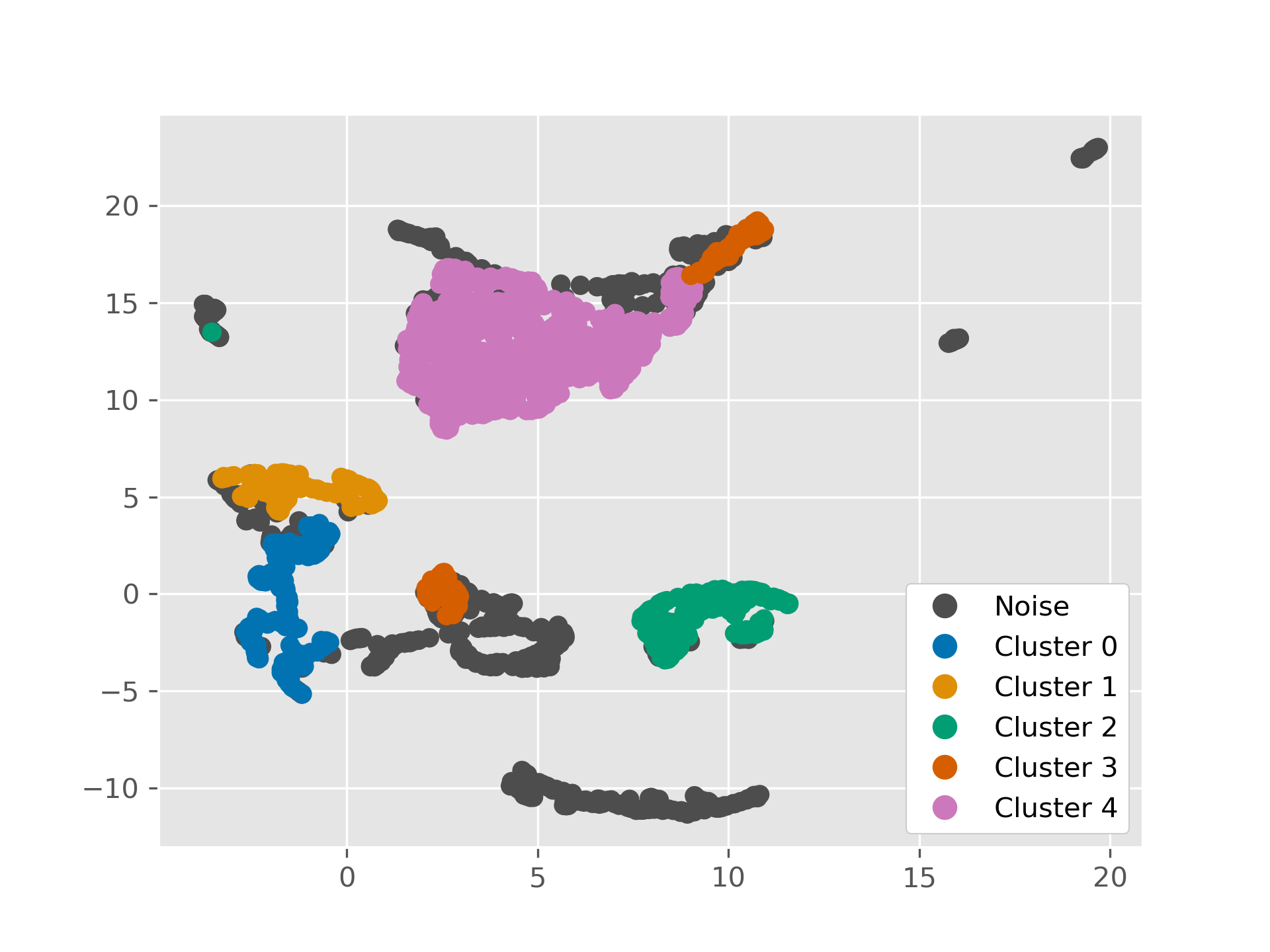

Supplement: Supplementary file 24 — Supplementary Information 12. [file 41598_2025_91849_MOESM24_ESM.zip › 4Z4Dp_A_mddomain_HL2REF/plots/4Z4Dp_A_Piwi-clusters-initial.png]

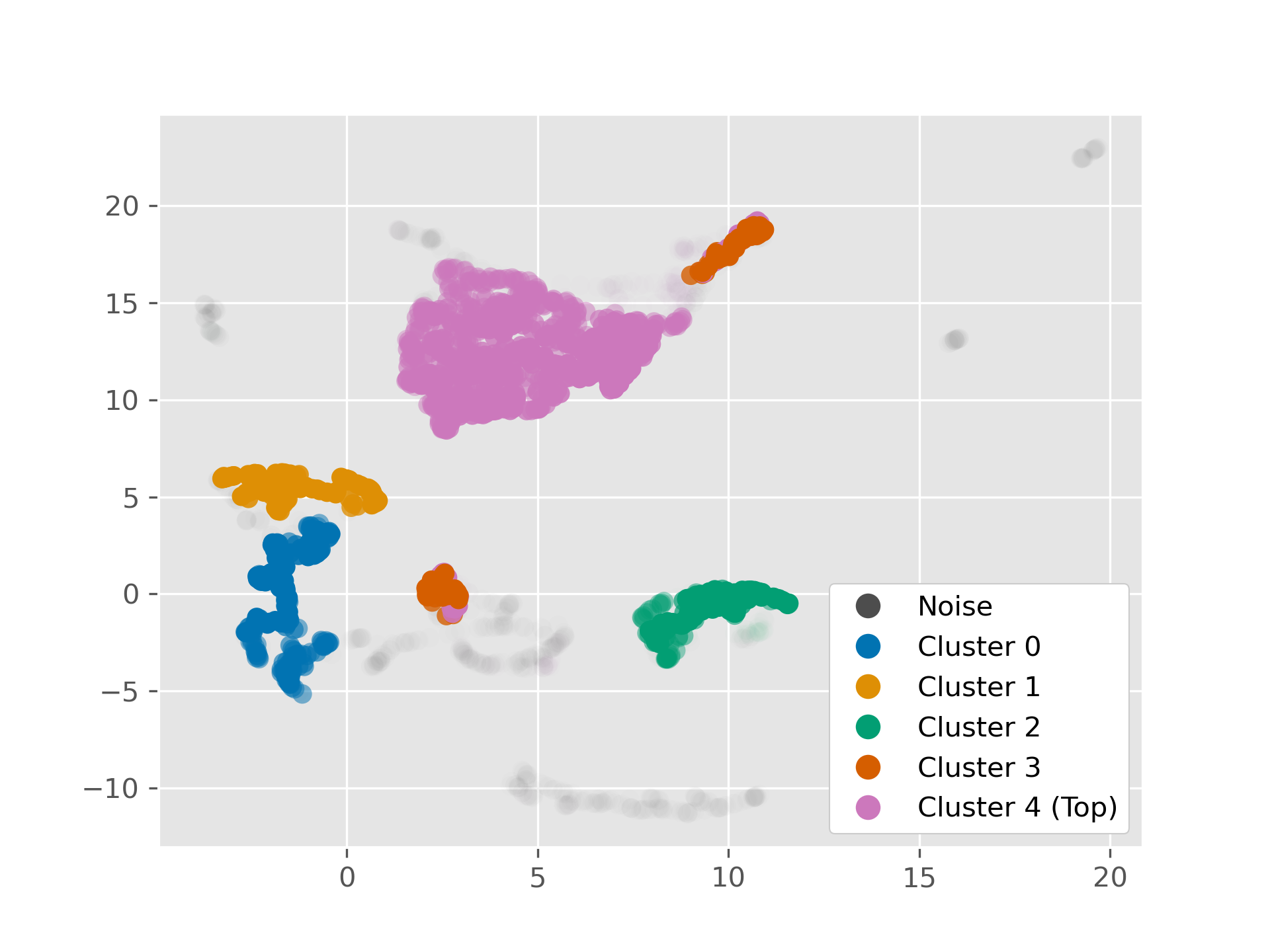

Supplement: Supplementary file 24 — Supplementary Information 12. [file 41598_2025_91849_MOESM24_ESM.zip › 4Z4Dp_A_mddomain_HL2REF/plots/4Z4Dp_A_Piwi-clusters.png]

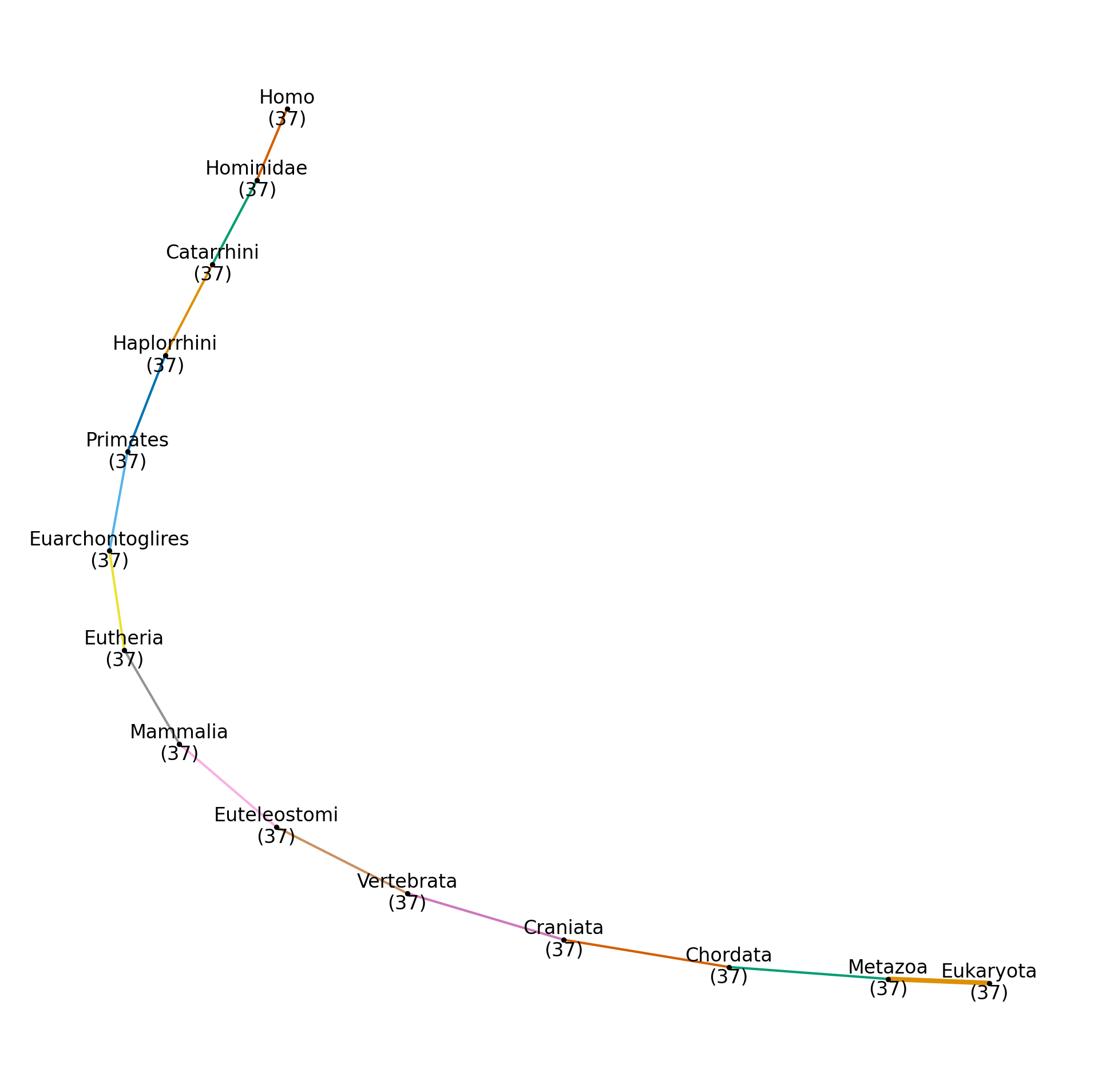

Supplement: Supplementary file 24 — Supplementary Information 12. [file 41598_2025_91849_MOESM24_ESM.zip › 4Z4Dp_A_mddomain_HL2REF/plots/4Z4Dp_A_Piwi-Eukaryota-tree.png]

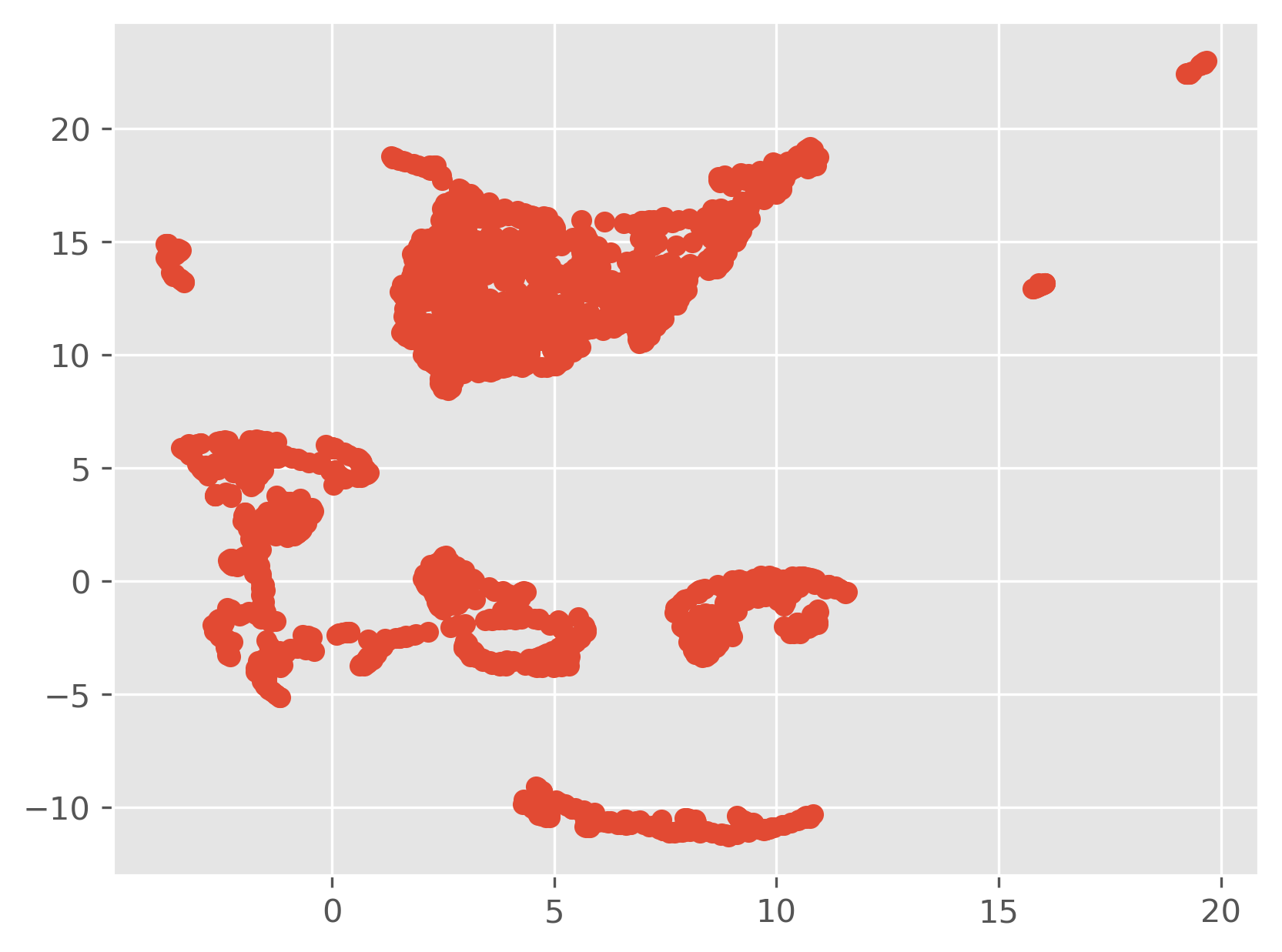

Supplement: Supplementary file 24 — Supplementary Information 12. [file 41598_2025_91849_MOESM24_ESM.zip › 4Z4Dp_A_mddomain_HL2REF/plots/4Z4Dp_A_Piwi-UMAP.png]

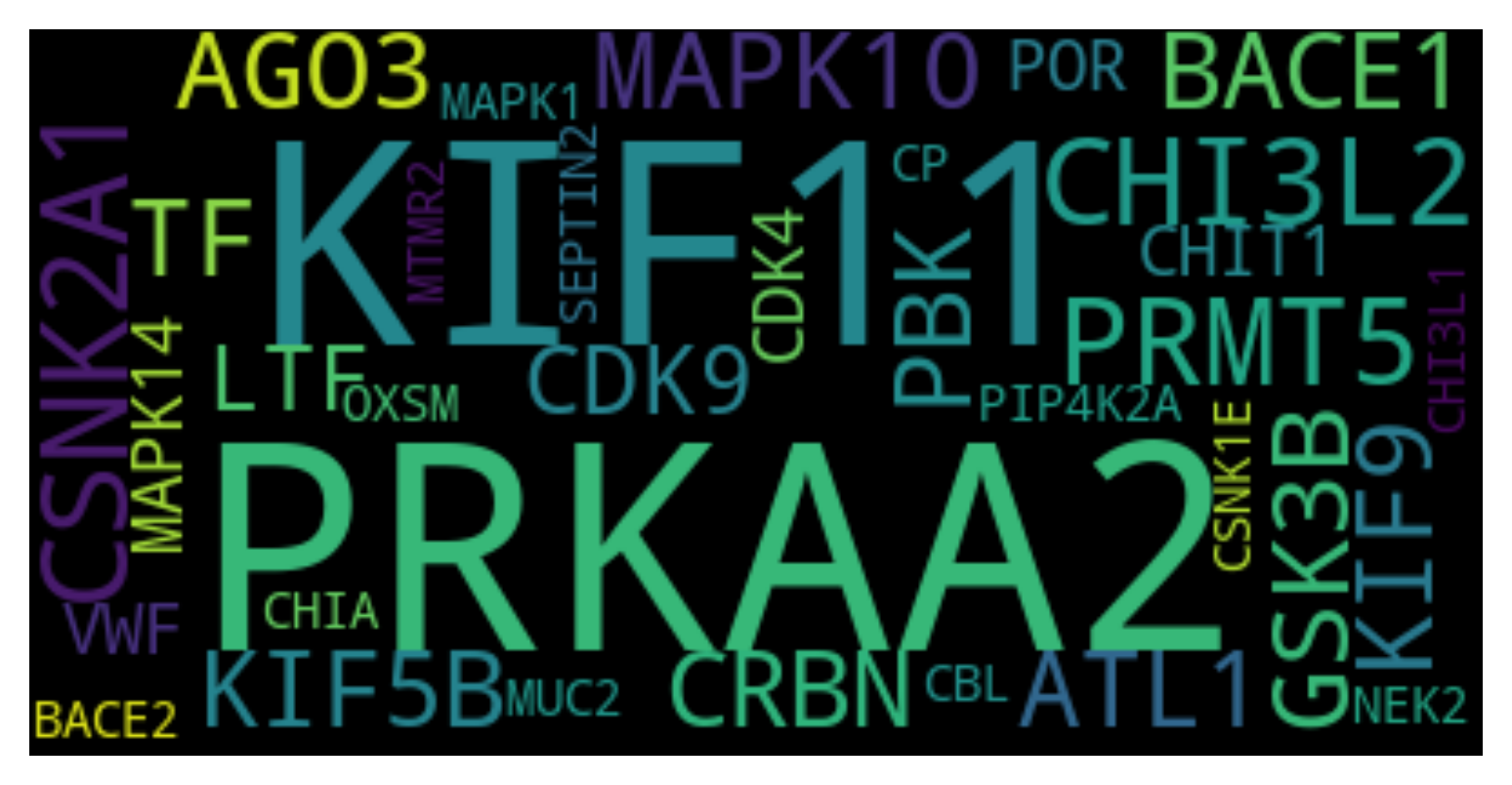

Supplement: Supplementary file 24 — Supplementary Information 12. [file 41598_2025_91849_MOESM24_ESM.zip › 4Z4Dp_A_mddomain_HL2REF/plots/4Z4Dp_A_Piwi-wordcloud.png]

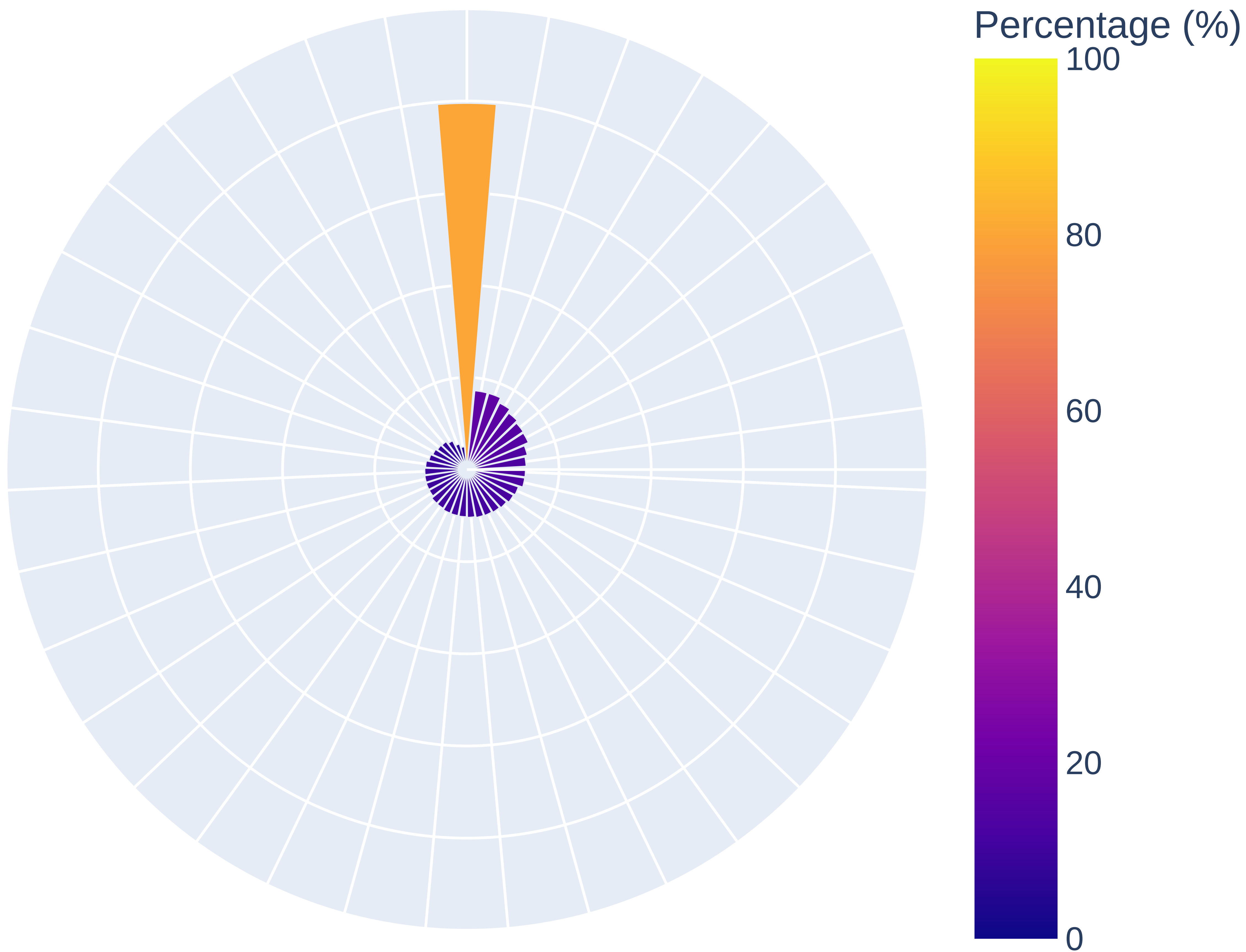

1D identity for 35 proteins in the final set (total: 37)

Supplement: Supplementary file 24 — Supplementary Information 12. [file 41598_2025_91849_MOESM24_ESM.zip › 4Z4Dp_A_mddomain_HL2REF/plots/4Z4Dp_A_Piwi_1D-identity.pdf]

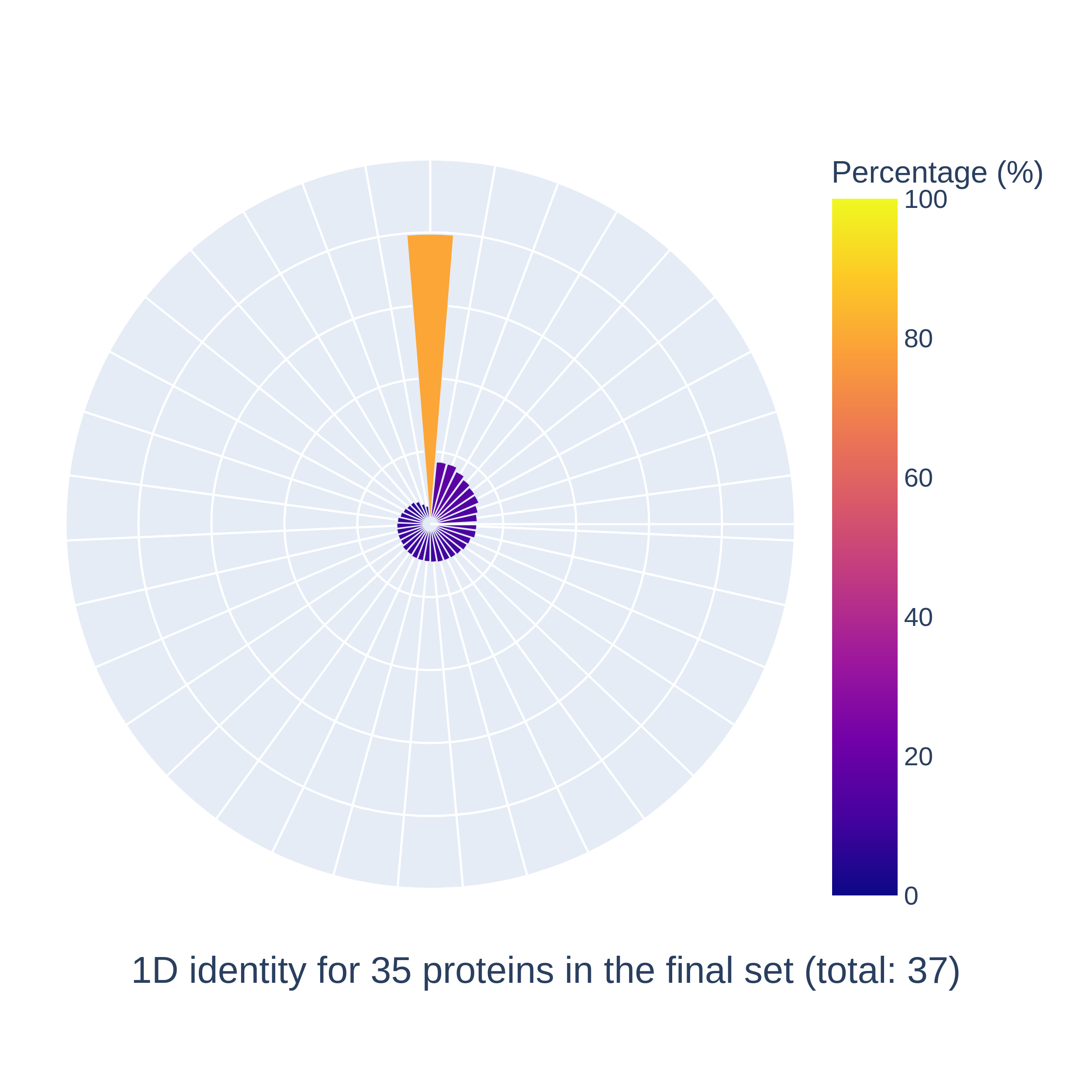

Supplement: Supplementary file 24 — Supplementary Information 12. [file 41598_2025_91849_MOESM24_ESM.zip › 4Z4Dp_A_mddomain_HL2REF/plots/4Z4Dp_A_Piwi_1D-identity.png]

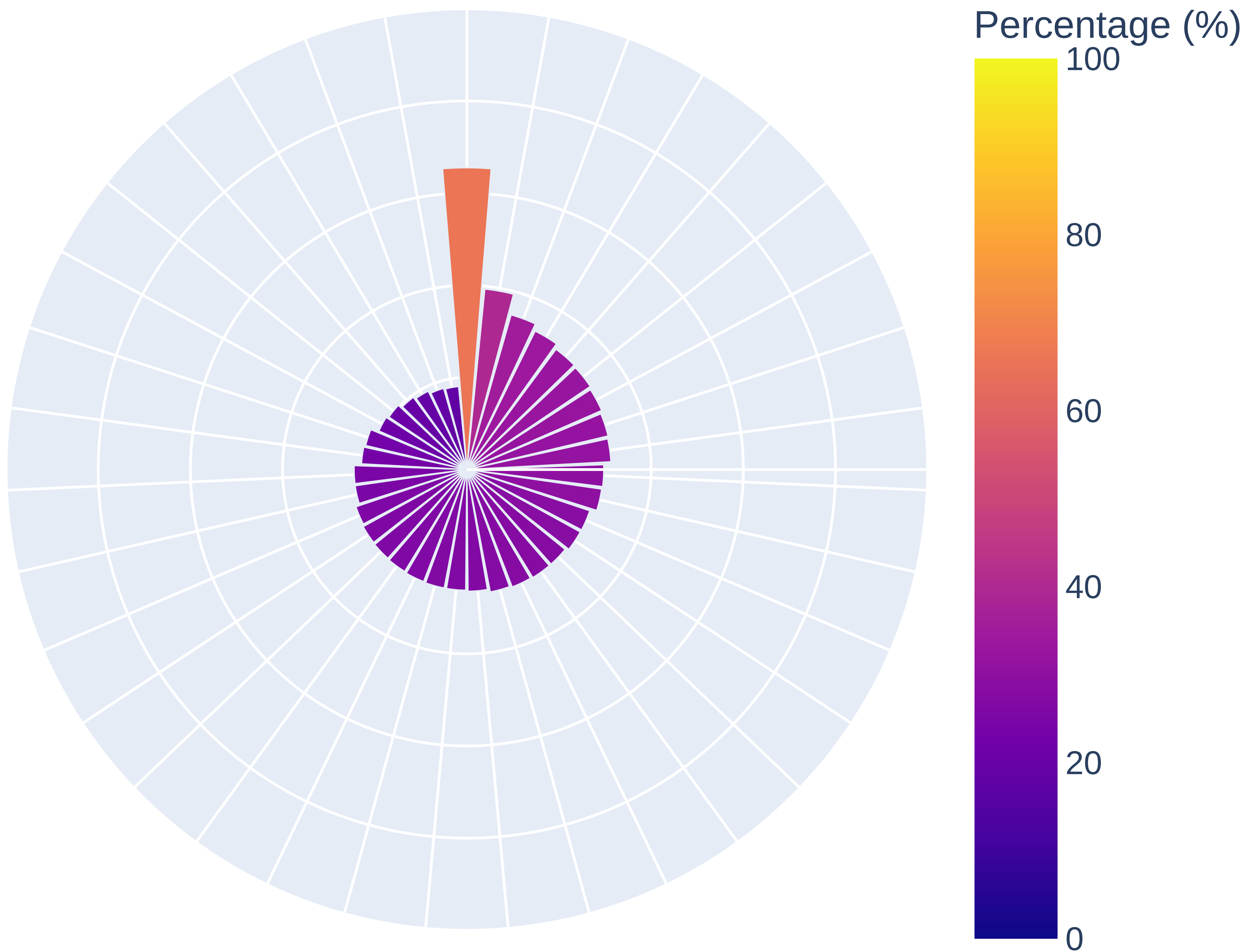

2D identity for 35 proteins in the final set (total: 37)

Supplement: Supplementary file 24 — Supplementary Information 12. [file 41598_2025_91849_MOESM24_ESM.zip › 4Z4Dp_A_mddomain_HL2REF/plots/4Z4Dp_A_Piwi_2D-identity.pdf]

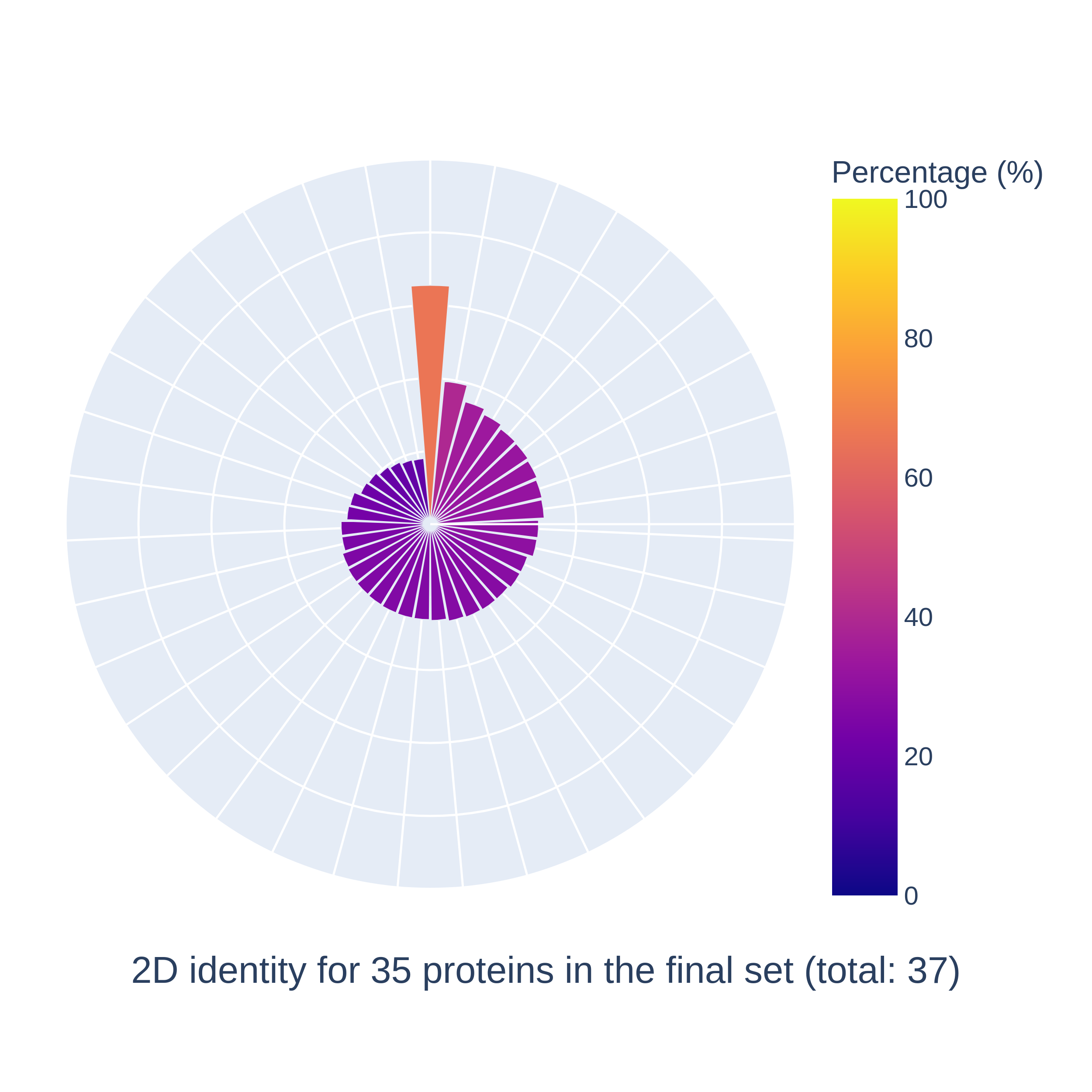

Supplement: Supplementary file 24 — Supplementary Information 12. [file 41598_2025_91849_MOESM24_ESM.zip › 4Z4Dp_A_mddomain_HL2REF/plots/4Z4Dp_A_Piwi_2D-identity.png]

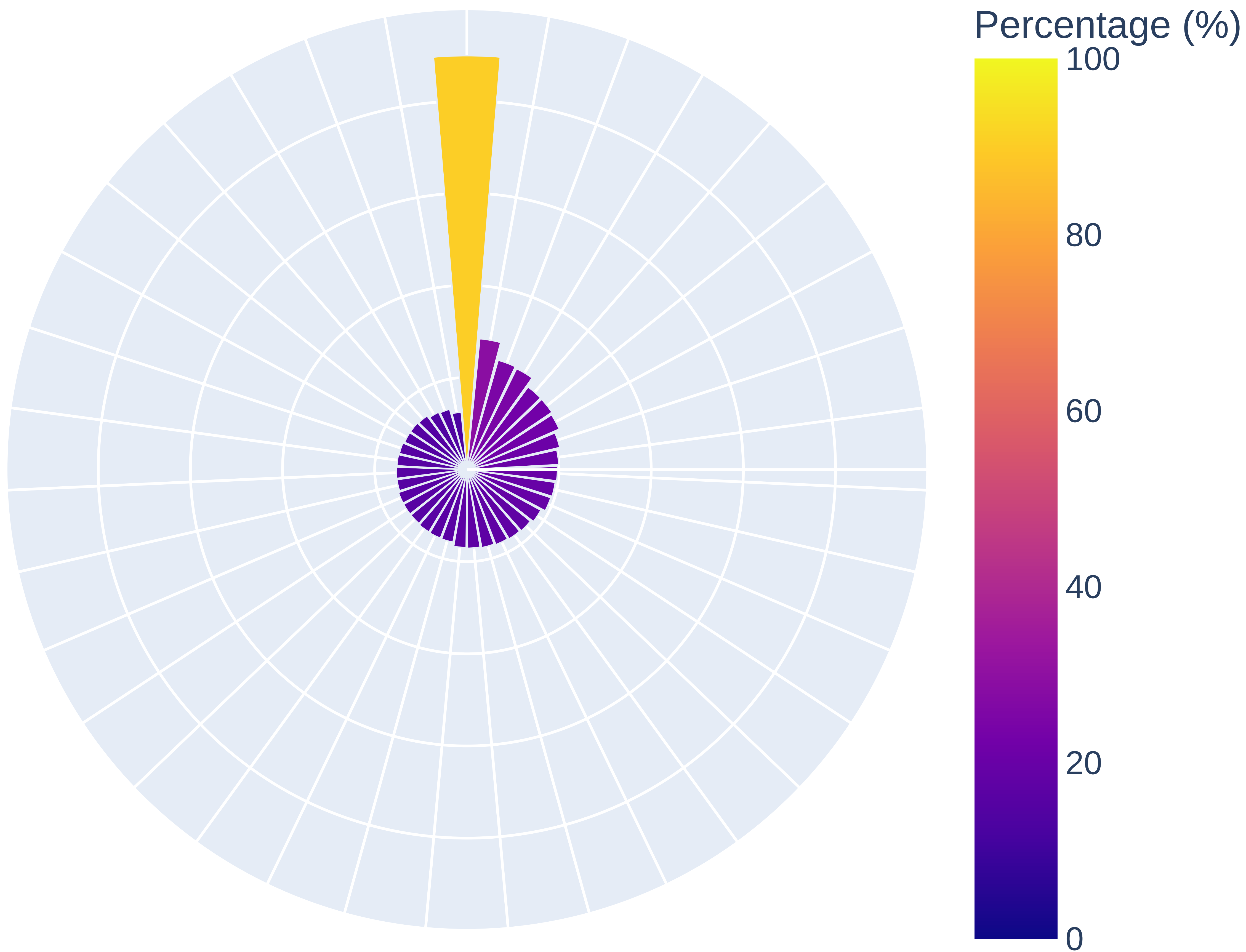

3D Similarity for 35 proteins in the final set (total: 37)

Supplement: Supplementary file 24 — Supplementary Information 12. [file 41598_2025_91849_MOESM24_ESM.zip › 4Z4Dp_A_mddomain_HL2REF/plots/4Z4Dp_A_Piwi_3D-score.pdf]

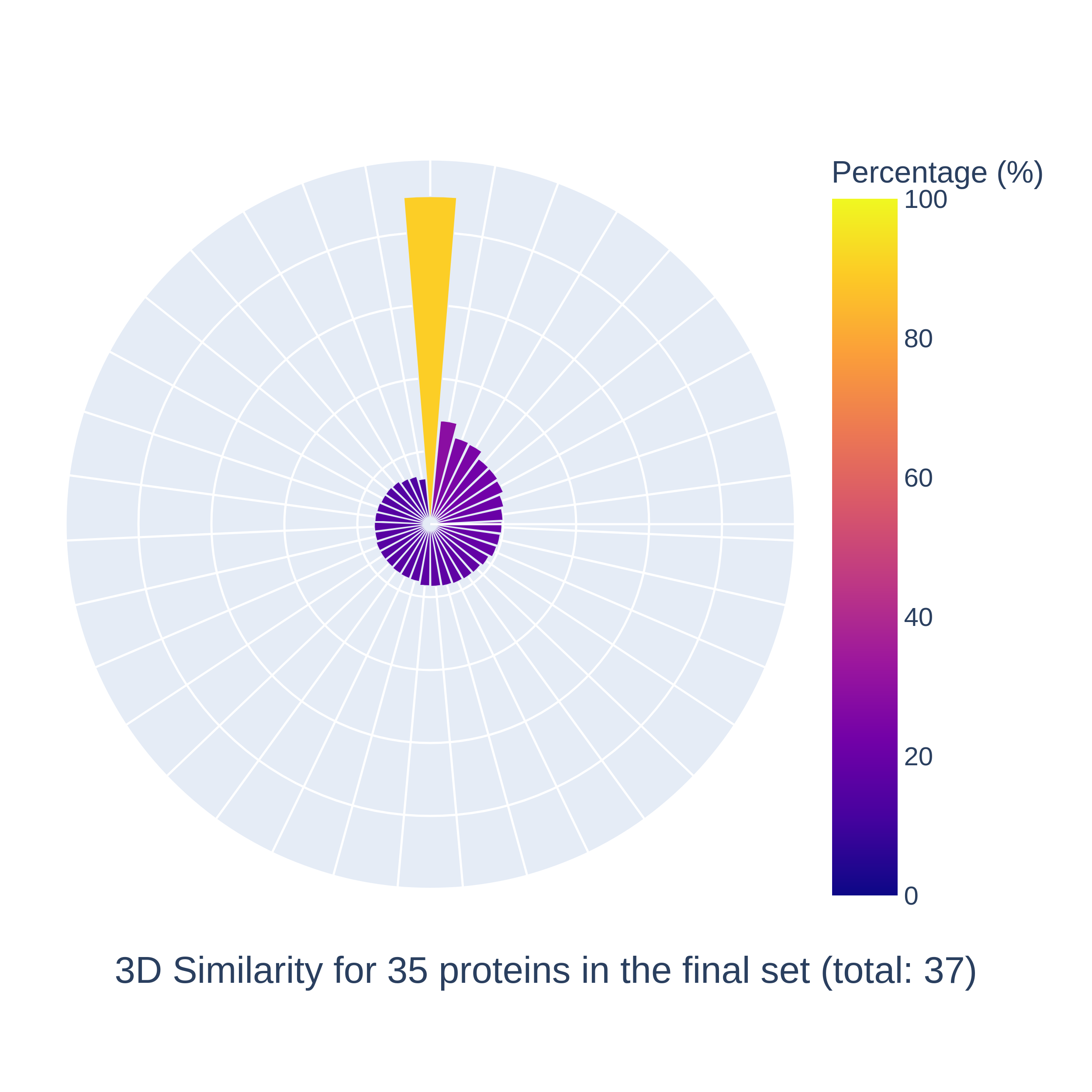

Supplement: Supplementary file 24 — Supplementary Information 12. [file 41598_2025_91849_MOESM24_ESM.zip › 4Z4Dp_A_mddomain_HL2REF/plots/4Z4Dp_A_Piwi_3D-score.png]

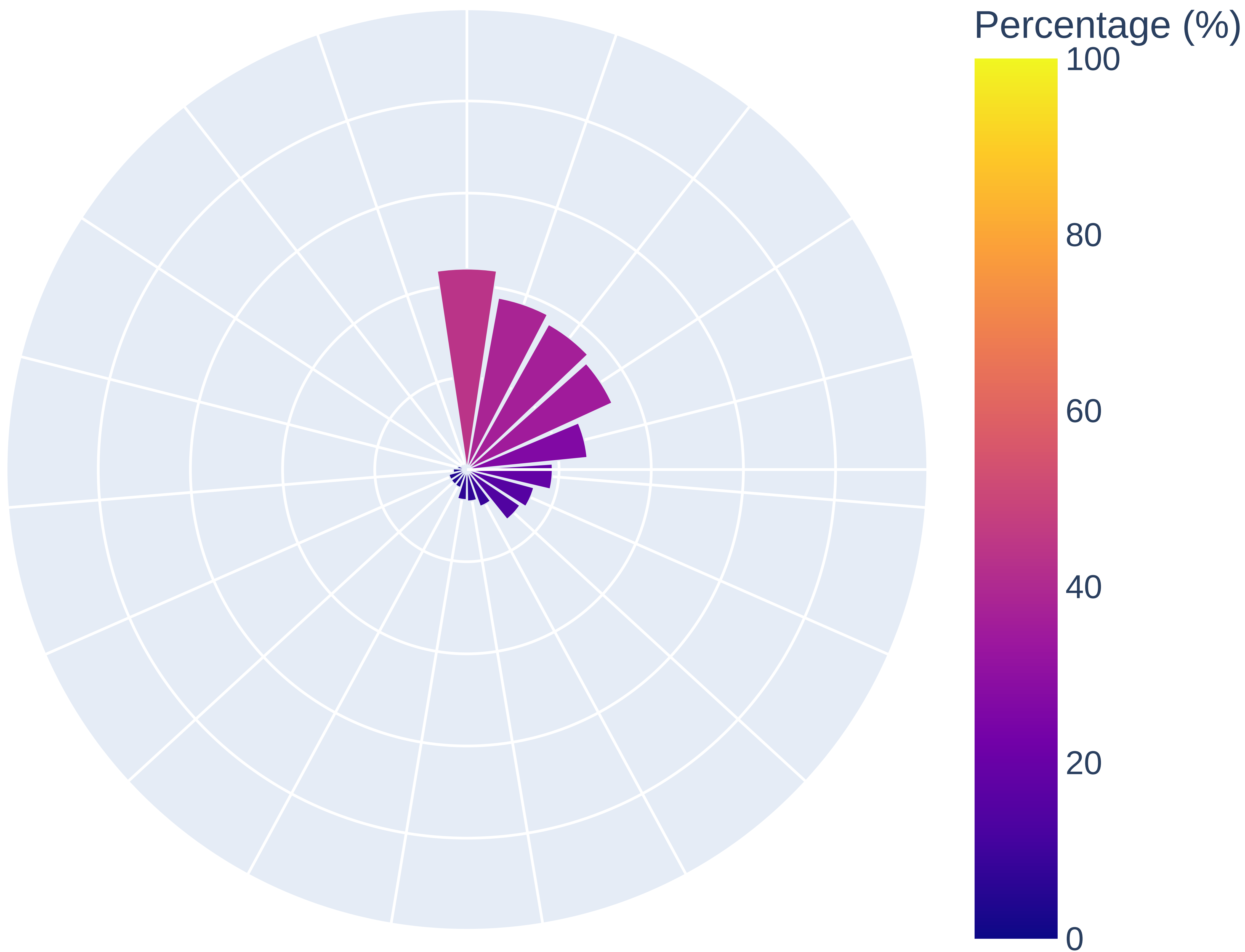

3'-UTR identity for 19 proteins in the final set (total: 37)

Supplement: Supplementary file 24 — Supplementary Information 12. [file 41598_2025_91849_MOESM24_ESM.zip › 4Z4Dp_A_mddomain_HL2REF/plots/4Z4Dp_A_Piwi_3UTR-identity.pdf]

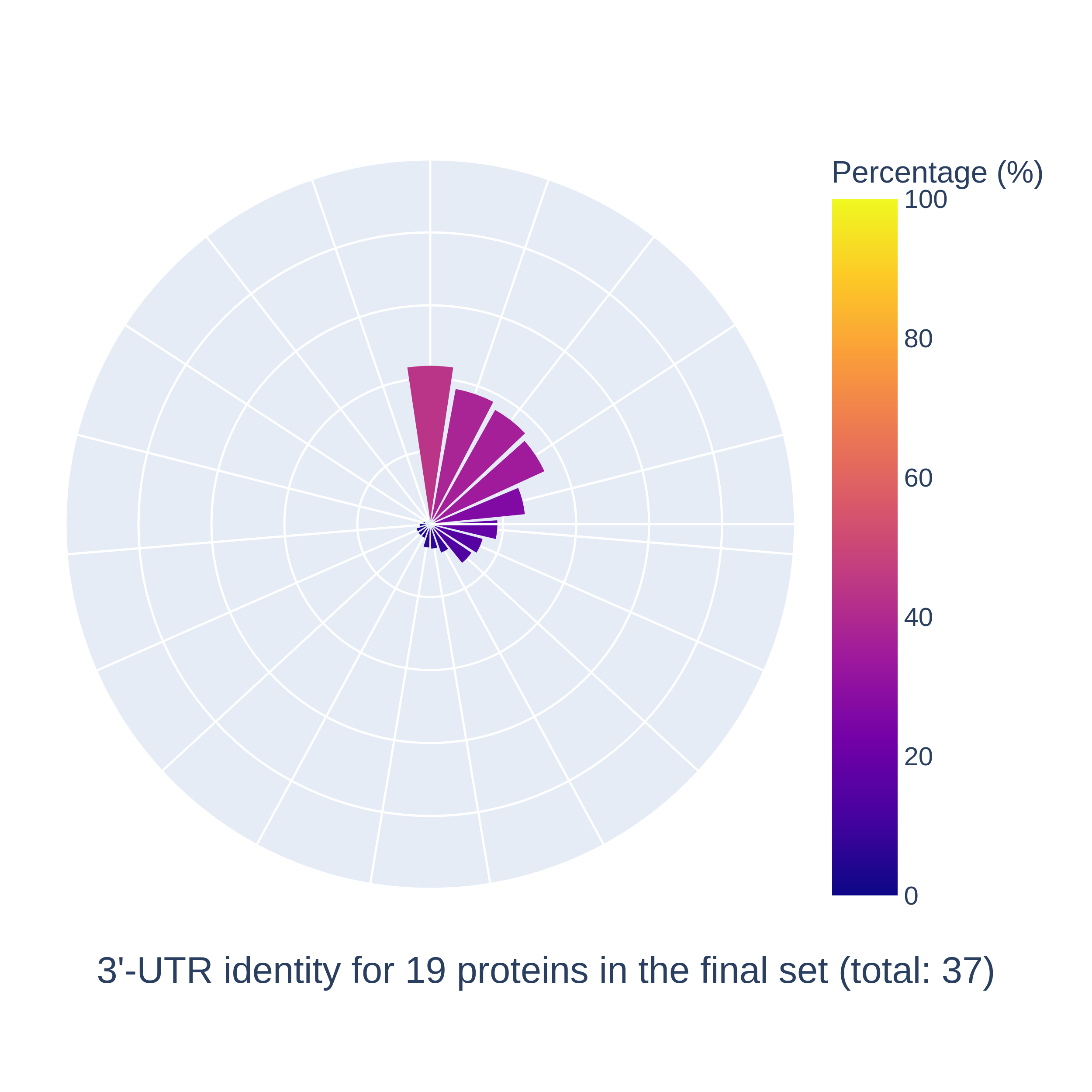

Supplement: Supplementary file 24 — Supplementary Information 12. [file 41598_2025_91849_MOESM24_ESM.zip › 4Z4Dp_A_mddomain_HL2REF/plots/4Z4Dp_A_Piwi_3UTR-identity.png]

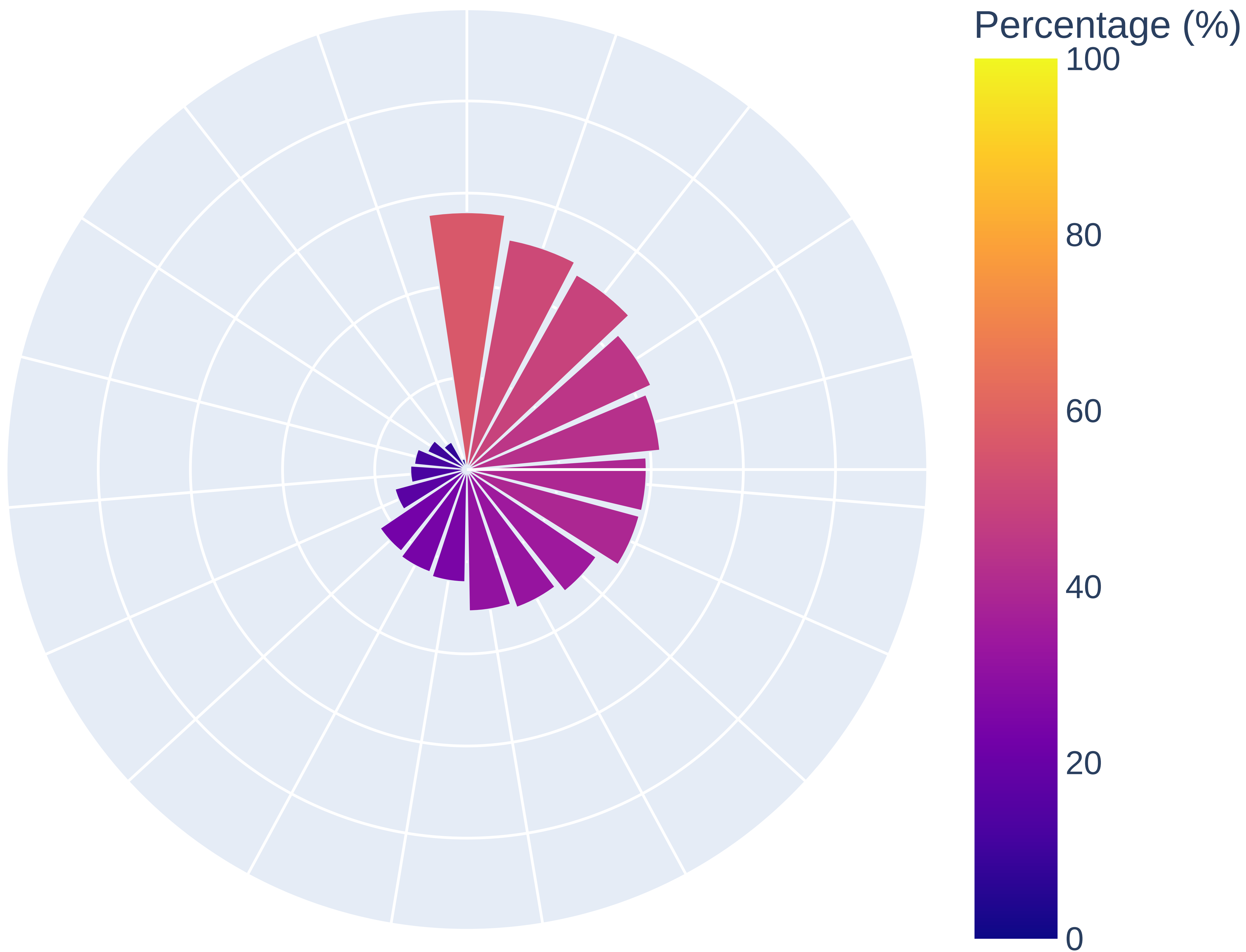

5'-UTR identity for 19 proteins in the final set (total: 37)

Supplement: Supplementary file 24 — Supplementary Information 12. [file 41598_2025_91849_MOESM24_ESM.zip › 4Z4Dp_A_mddomain_HL2REF/plots/4Z4Dp_A_Piwi_5UTR-identity.pdf]

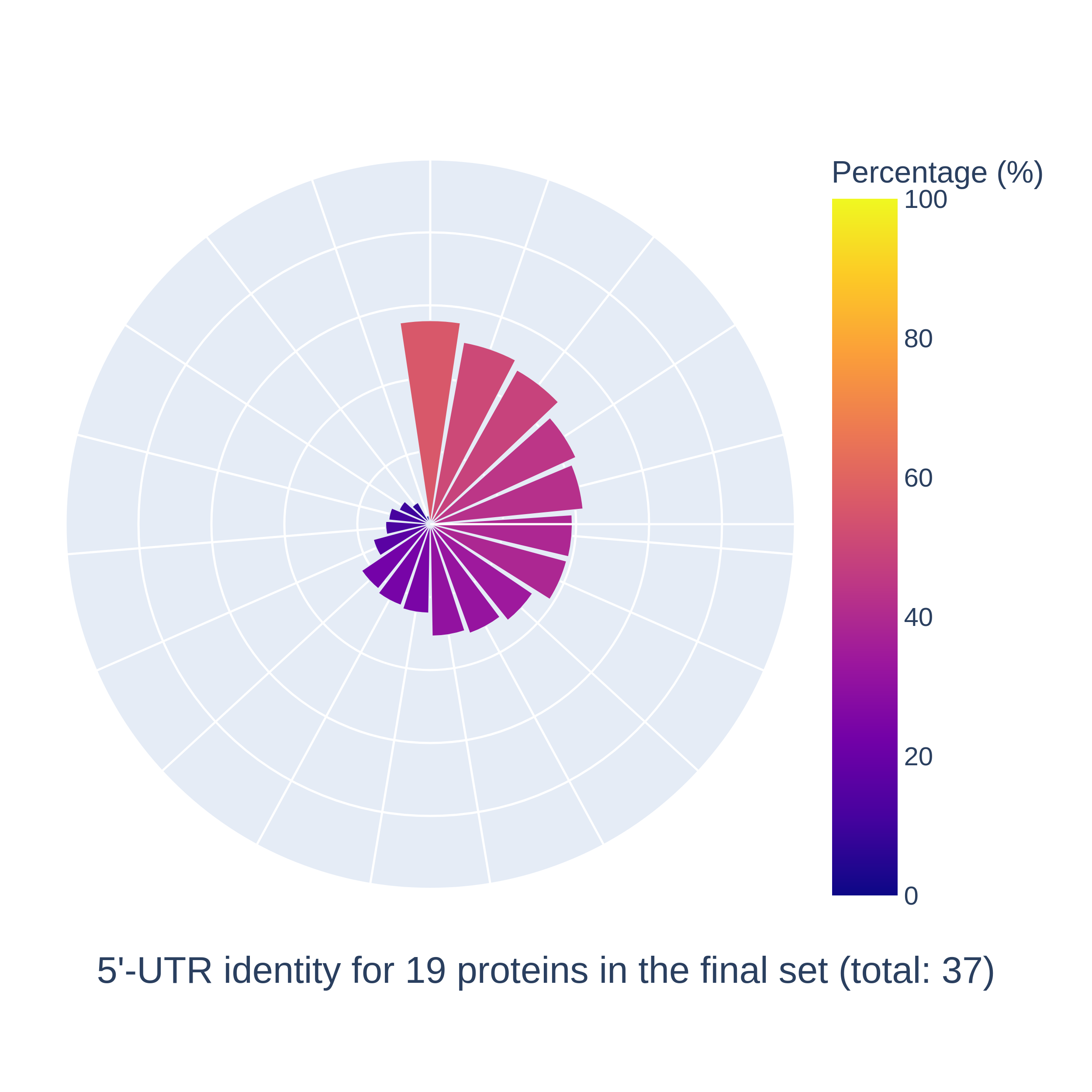

Supplement: Supplementary file 24 — Supplementary Information 12. [file 41598_2025_91849_MOESM24_ESM.zip › 4Z4Dp_A_mddomain_HL2REF/plots/4Z4Dp_A_Piwi_5UTR-identity.png]

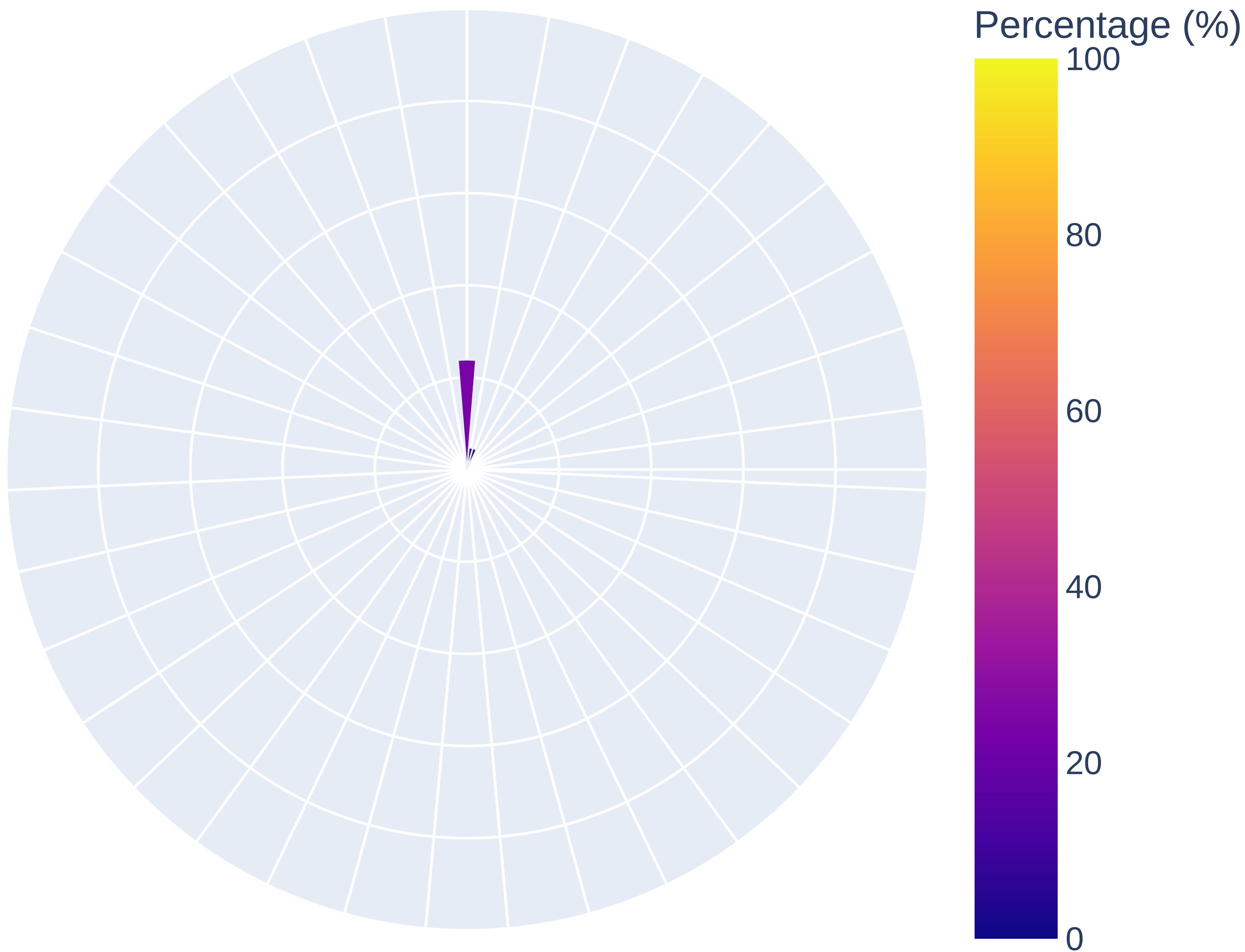

Common pathways for 35 proteins in the final set (total: 37)

Supplement: Supplementary file 24 — Supplementary Information 12. [file 41598_2025_91849_MOESM24_ESM.zip › 4Z4Dp_A_mddomain_HL2REF/plots/4Z4Dp_A_Piwi_biologicalProcessSim.pdf]

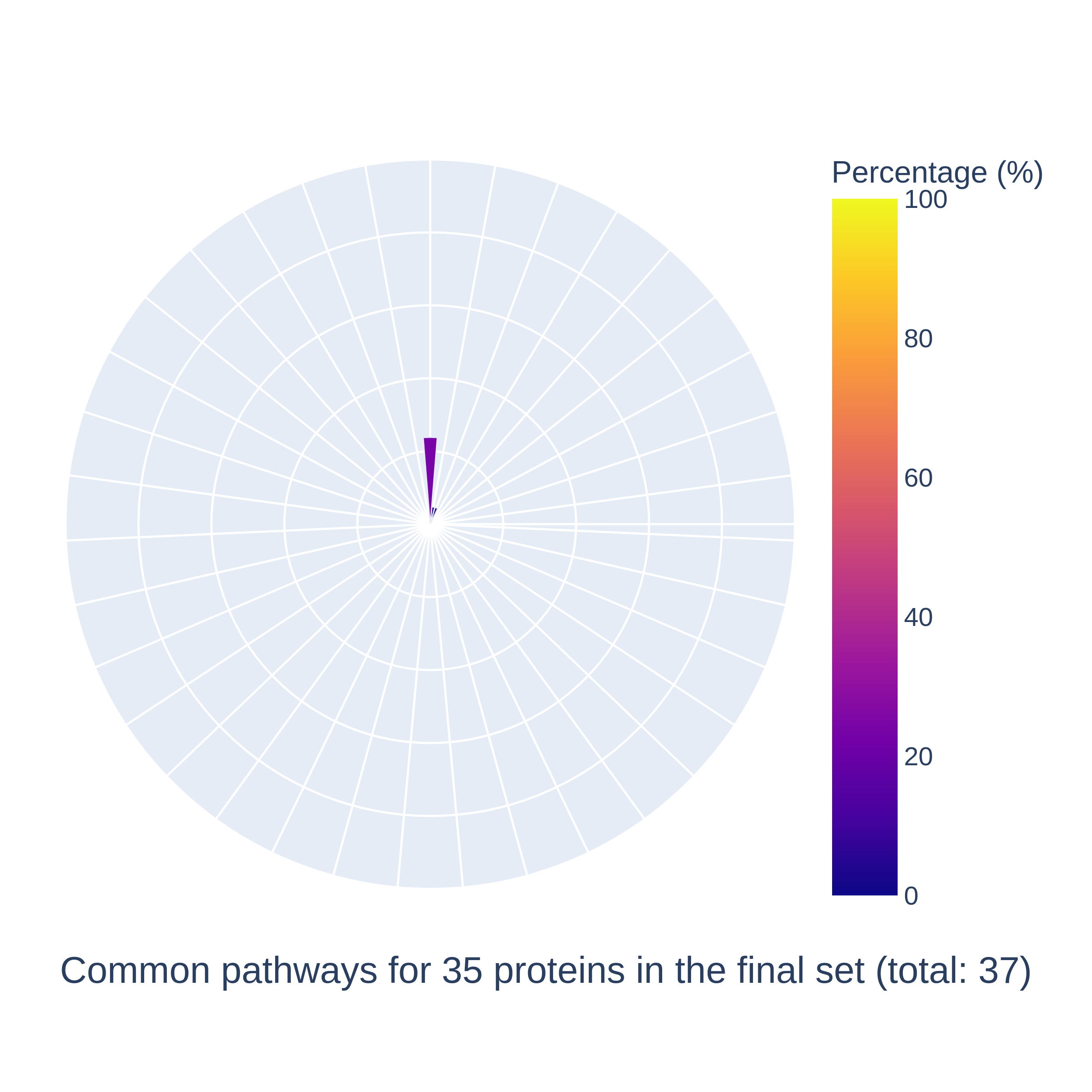

Supplement: Supplementary file 24 — Supplementary Information 12. [file 41598_2025_91849_MOESM24_ESM.zip › 4Z4Dp_A_mddomain_HL2REF/plots/4Z4Dp_A_Piwi_biologicalProcessSim.png]

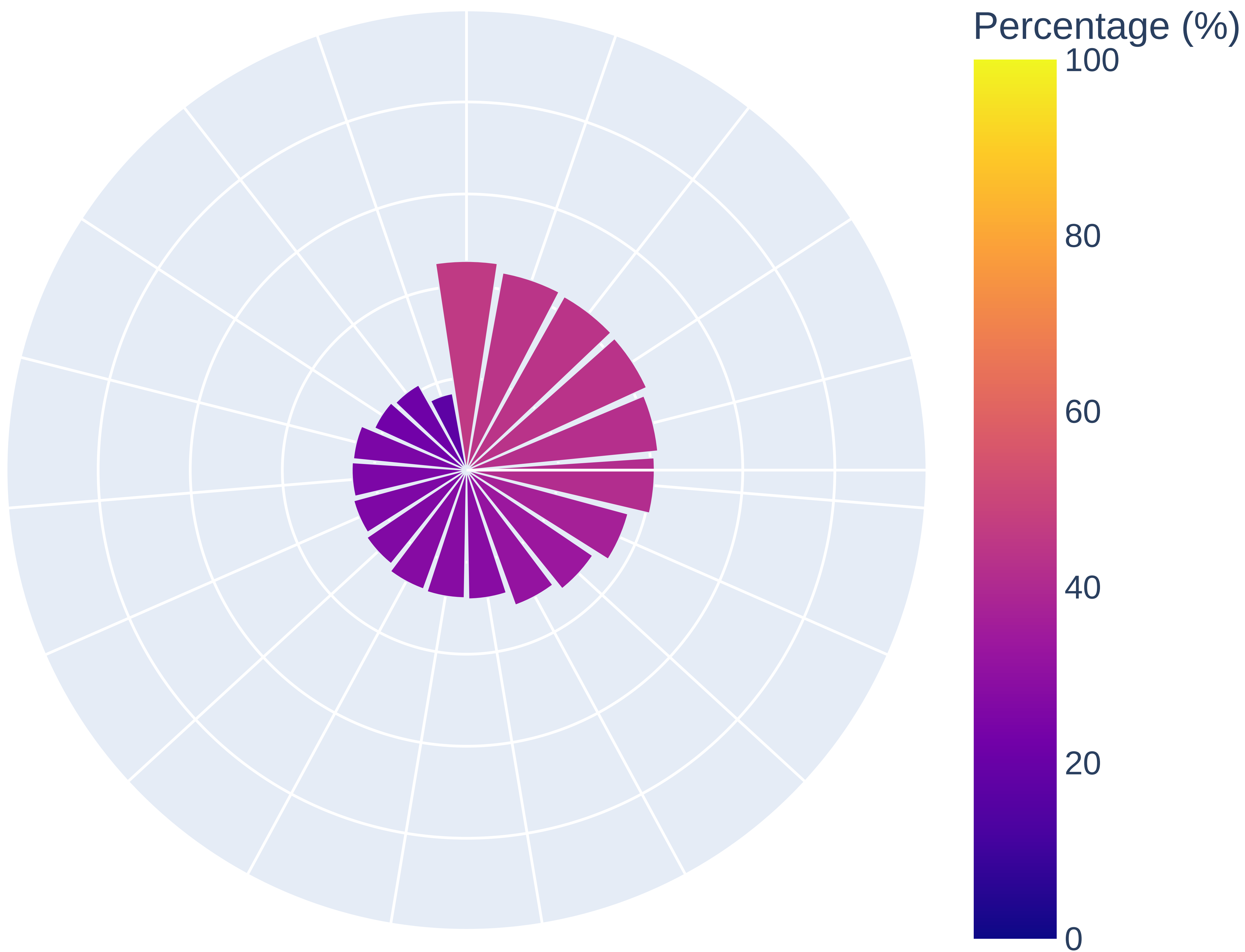

Supplement: Supplementary file 24 — Supplementary Information 12. [file 41598_2025_91849_MOESM24_ESM.zip › 4Z4Dp_A_mddomain_HL2REF/plots/4Z4Dp_A_Piwi_CDS-identity.pdf]

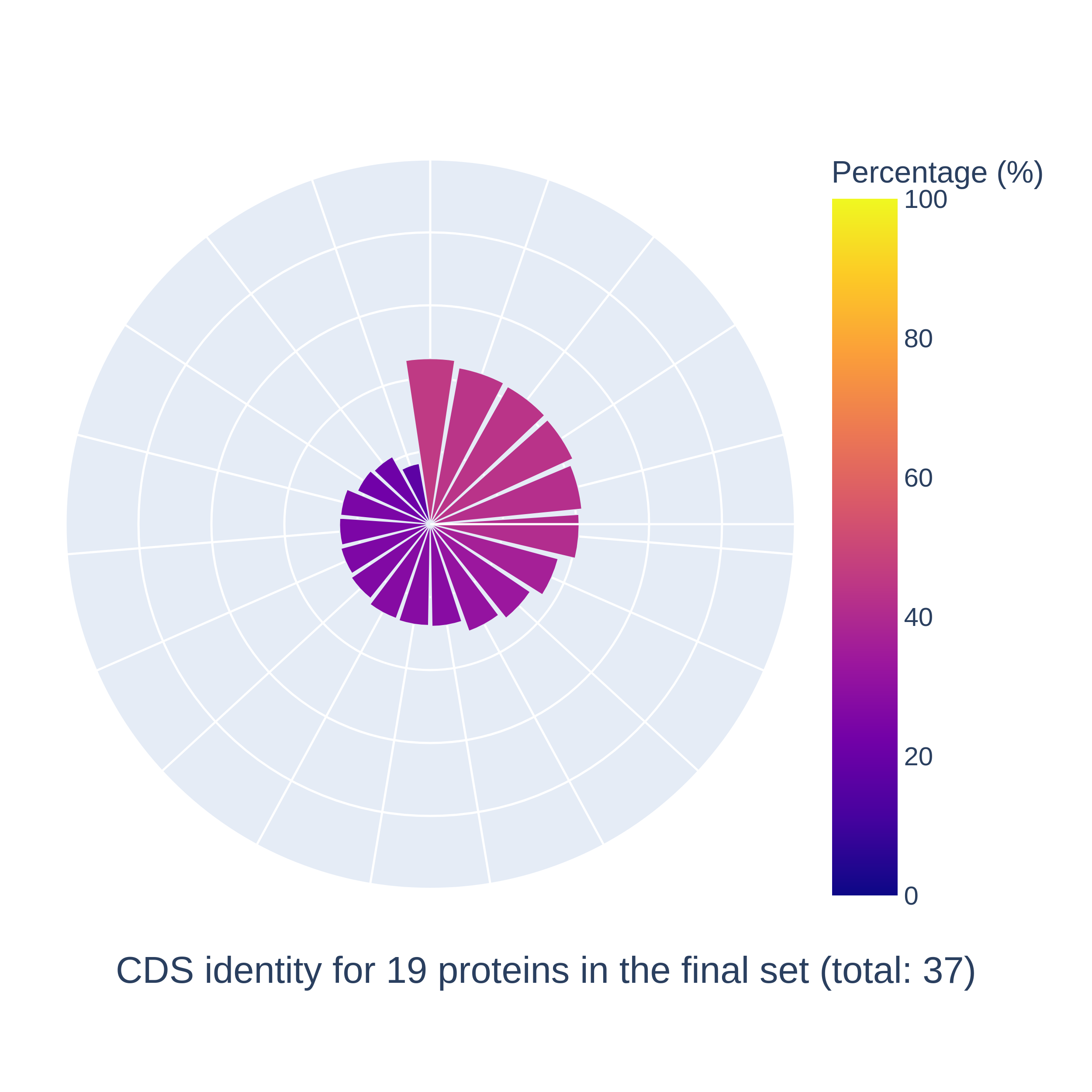

Supplement: Supplementary file 24 — Supplementary Information 12. [file 41598_2025_91849_MOESM24_ESM.zip › 4Z4Dp_A_mddomain_HL2REF/plots/4Z4Dp_A_Piwi_CDS-identity.png]

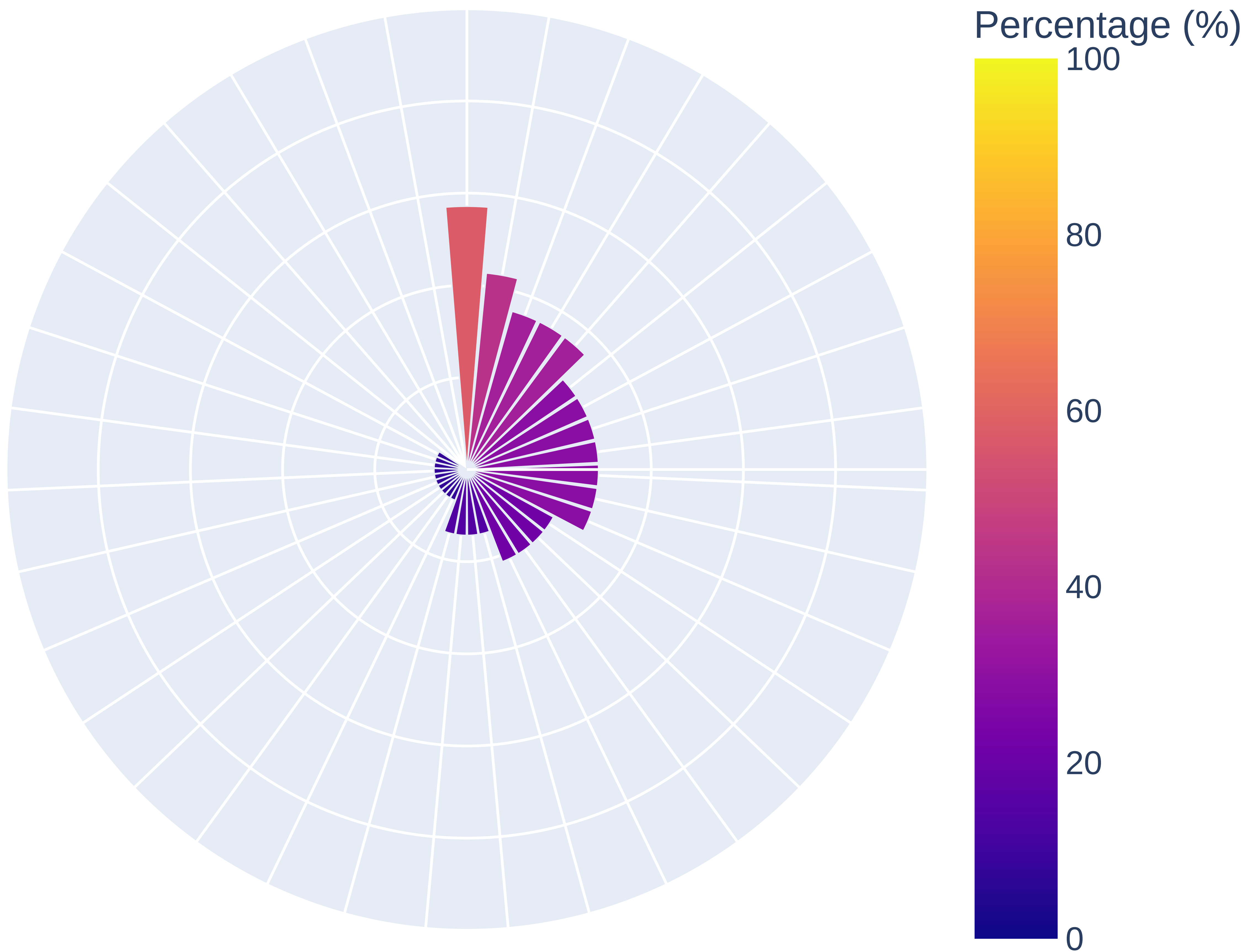

Common locations for 35 proteins in the final set (total: 37)

Supplement: Supplementary file 24 — Supplementary Information 12. [file 41598_2025_91849_MOESM24_ESM.zip › 4Z4Dp_A_mddomain_HL2REF/plots/4Z4Dp_A_Piwi_cellularComponentSim.pdf]

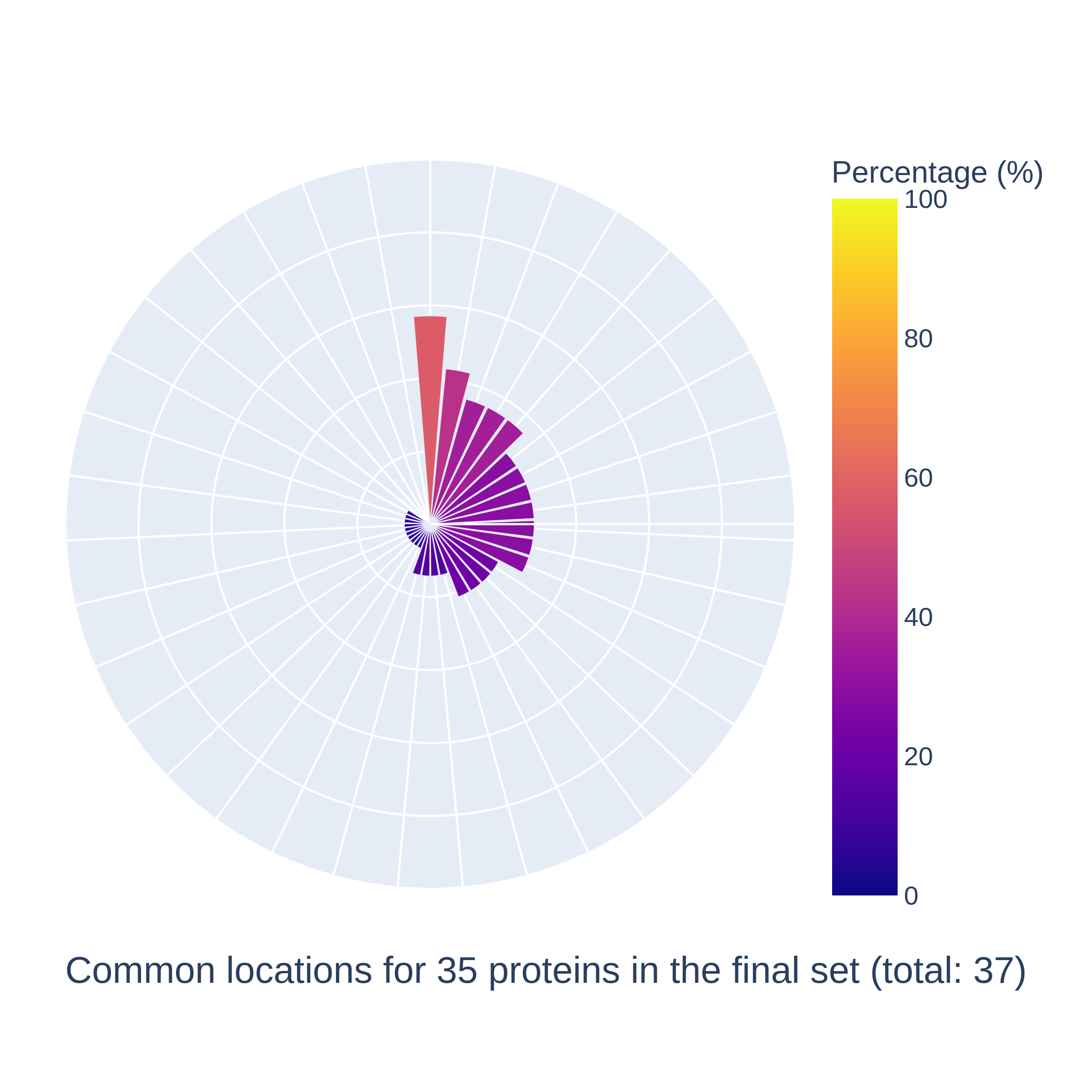

Supplement: Supplementary file 24 — Supplementary Information 12. [file 41598_2025_91849_MOESM24_ESM.zip › 4Z4Dp_A_mddomain_HL2REF/plots/4Z4Dp_A_Piwi_cellularComponentSim.png]

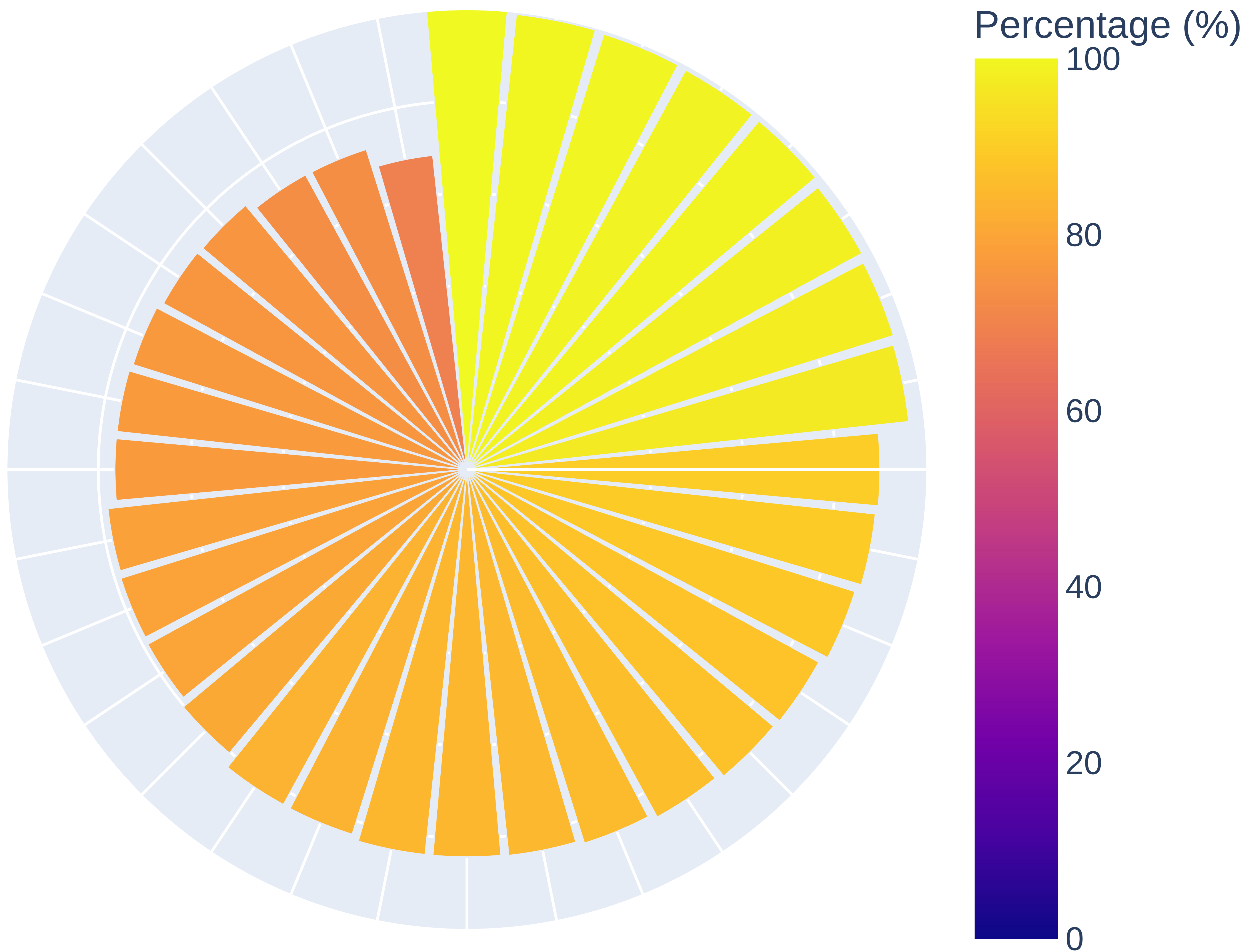

Chemical similarity for 32 proteins in the final set (total: 37)

Supplement: Supplementary file 24 — Supplementary Information 12. [file 41598_2025_91849_MOESM24_ESM.zip › 4Z4Dp_A_mddomain_HL2REF/plots/4Z4Dp_A_Piwi_chemSim.pdf]

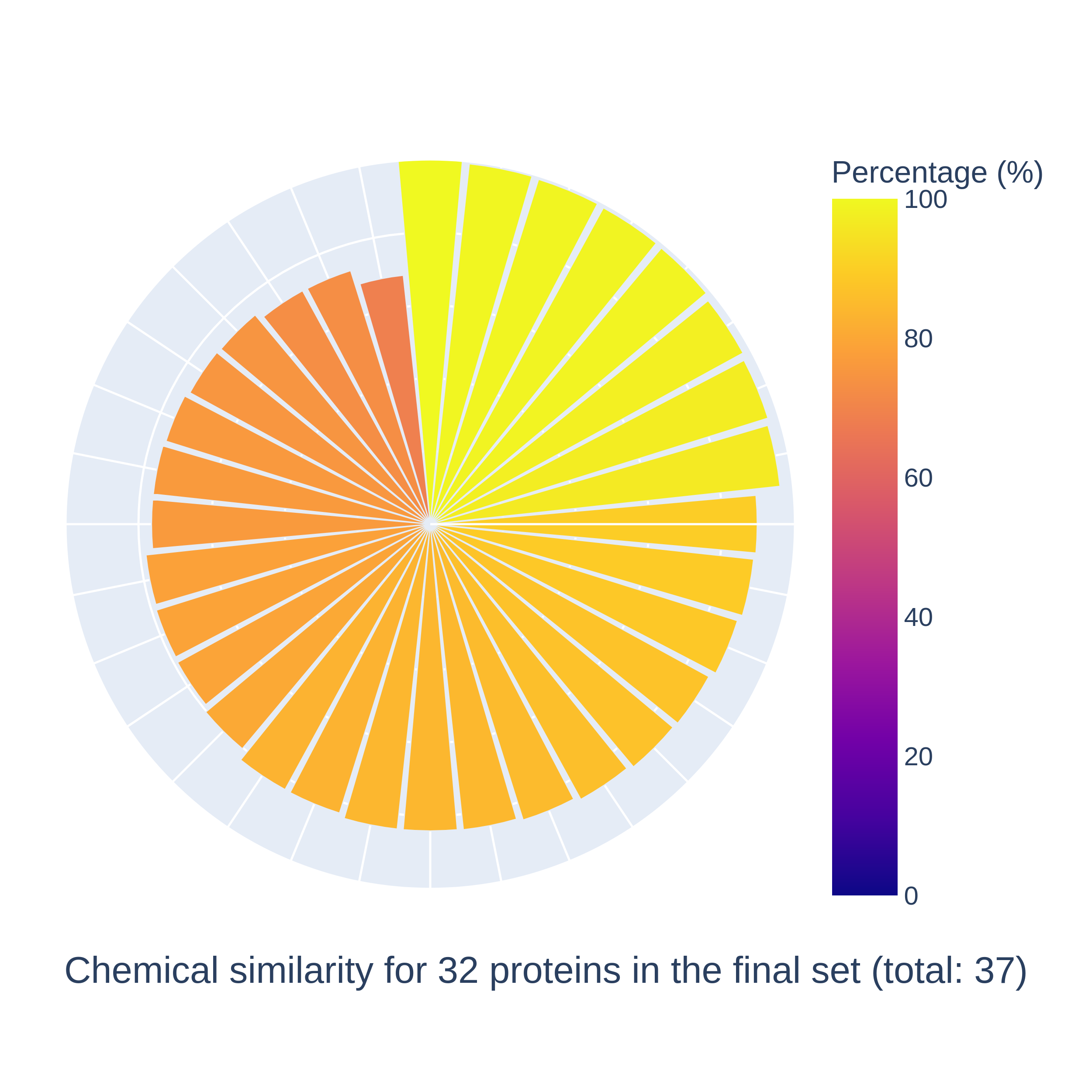

Supplement: Supplementary file 24 — Supplementary Information 12. [file 41598_2025_91849_MOESM24_ESM.zip › 4Z4Dp_A_mddomain_HL2REF/plots/4Z4Dp_A_Piwi_chemSim.png]

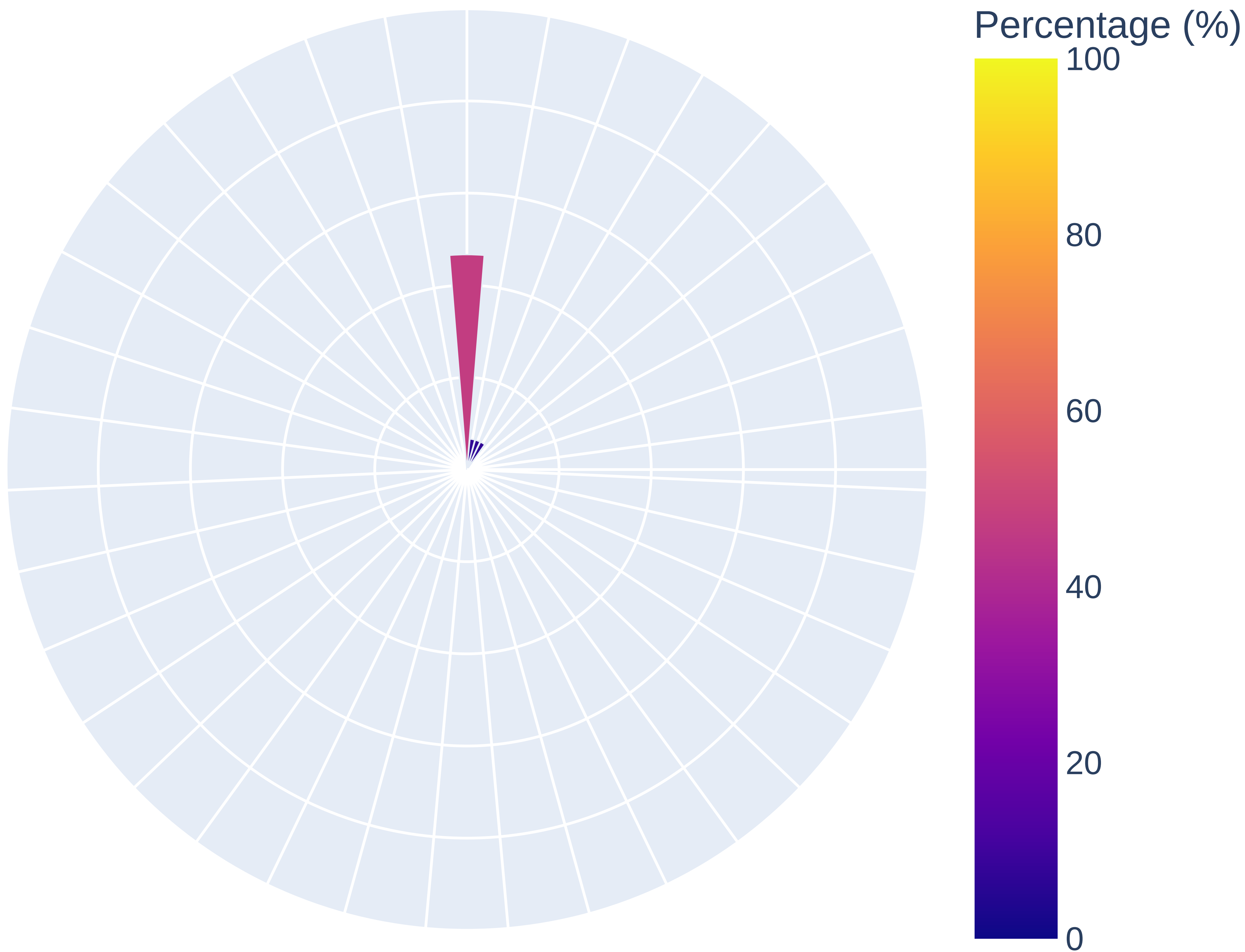

Common functions for 35 proteins in the final set (total: 37)

Supplement: Supplementary file 24 — Supplementary Information 12. [file 41598_2025_91849_MOESM24_ESM.zip › 4Z4Dp_A_mddomain_HL2REF/plots/4Z4Dp_A_Piwi_molecularFunctionSim.pdf]

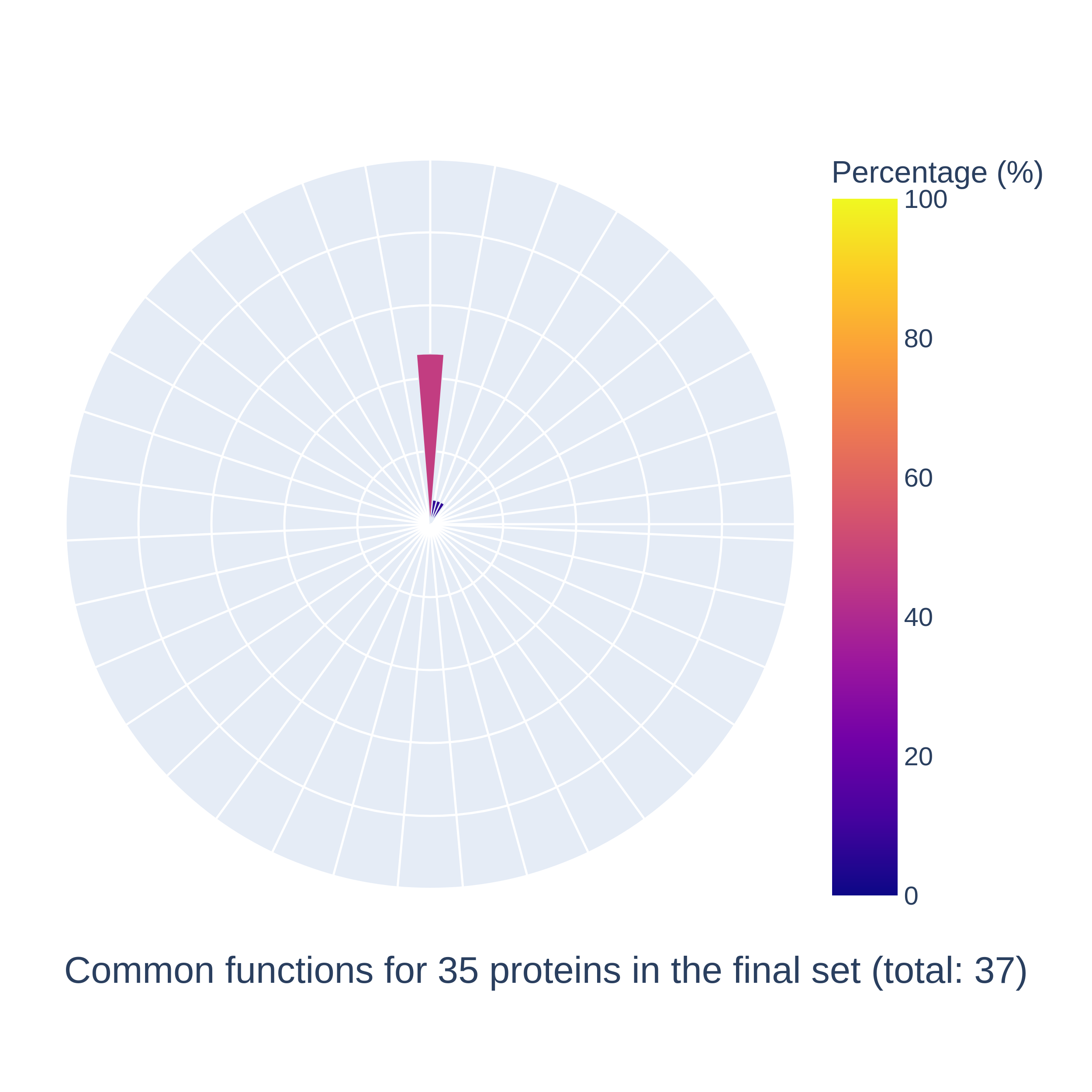

Supplement: Supplementary file 24 — Supplementary Information 12. [file 41598_2025_91849_MOESM24_ESM.zip › 4Z4Dp_A_mddomain_HL2REF/plots/4Z4Dp_A_Piwi_molecularFunctionSim.png]

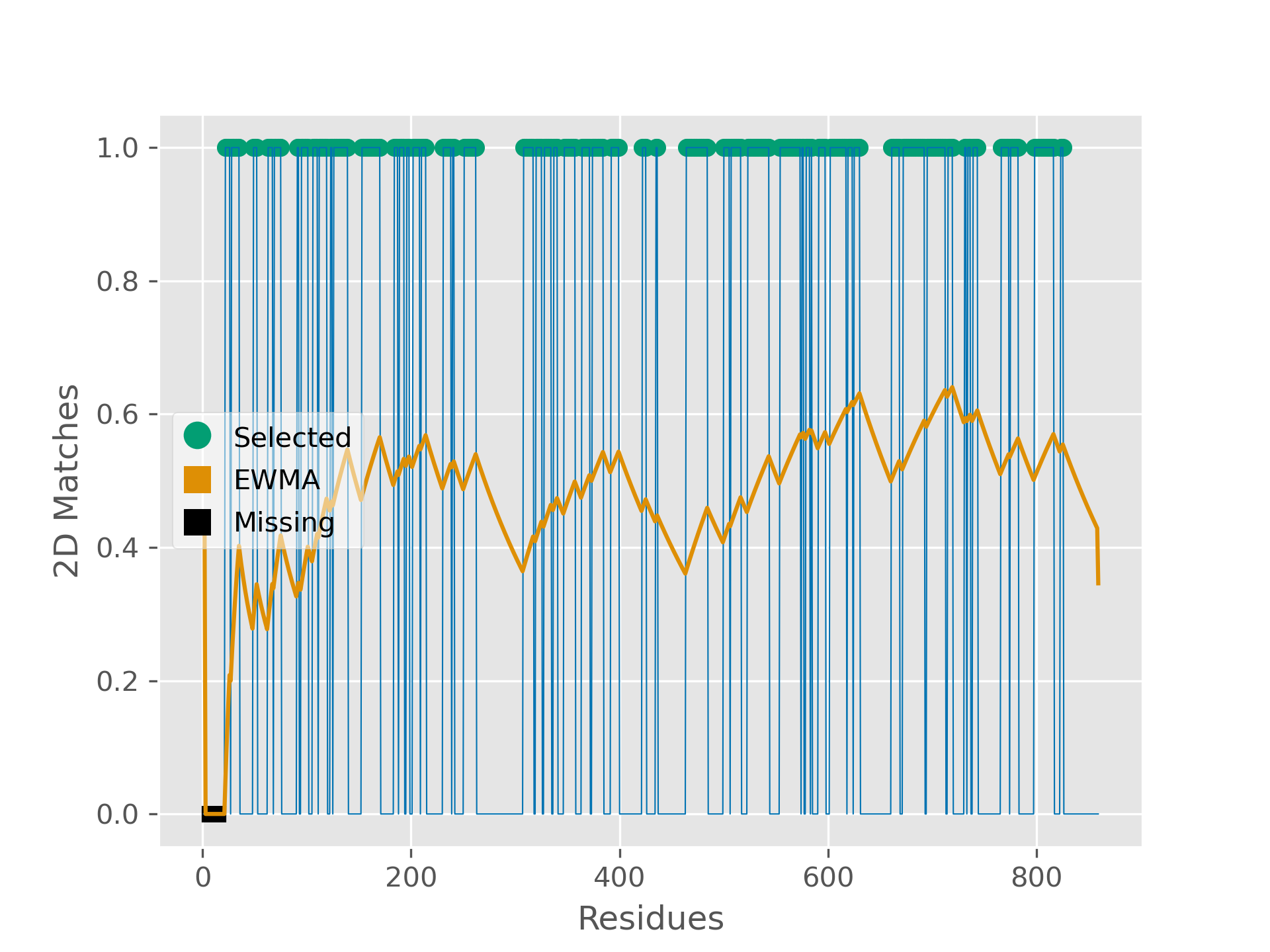

Supplement: Supplementary file 24 — Supplementary Information 12. [file 41598_2025_91849_MOESM24_ESM.zip › 4Z4Dp_A_mdwhole_AF4REF/go/4Z4Dp_A_mitot_mitosis_48b968e8b0064751aea82538ef7ddf99.png]

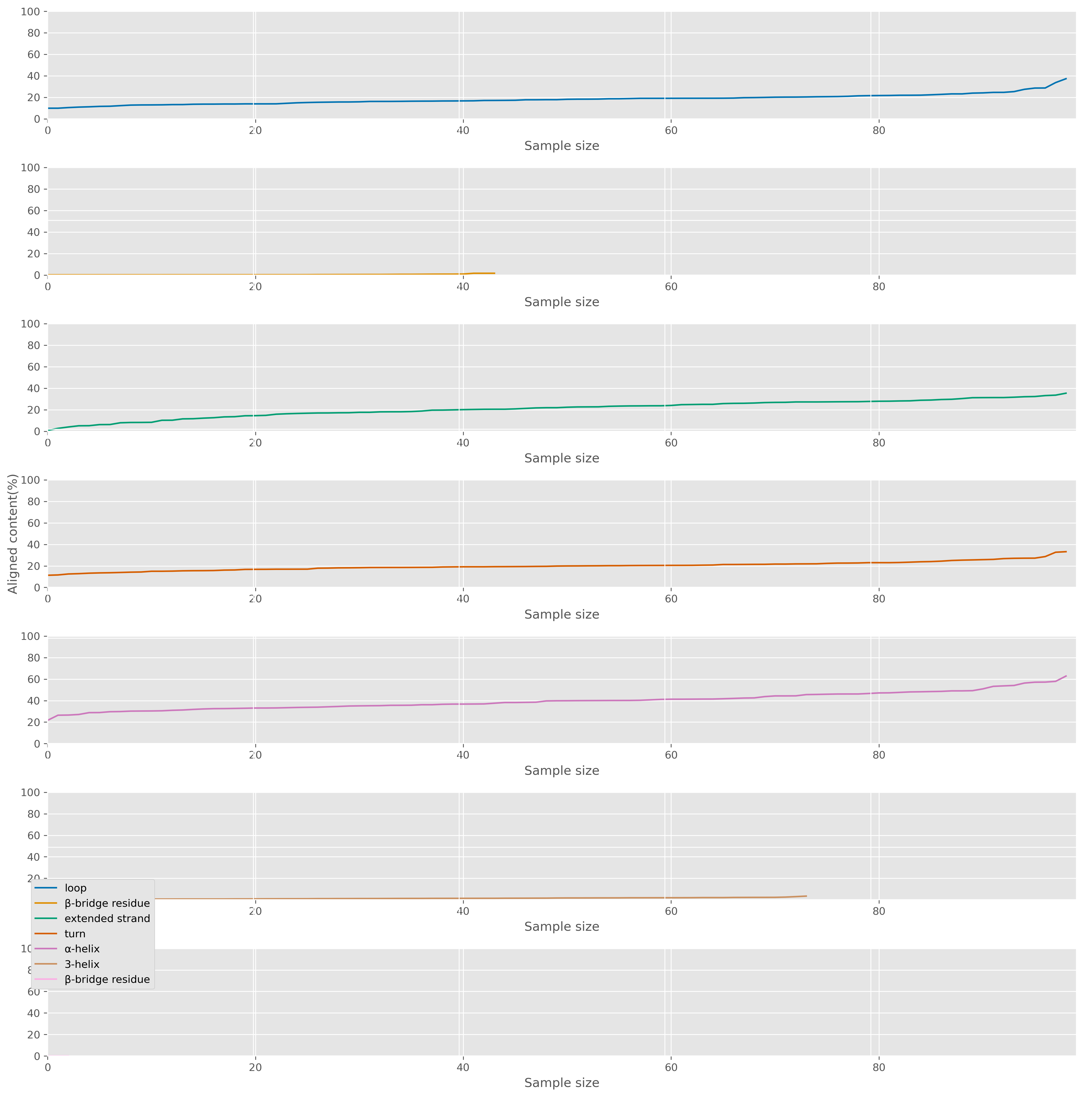

Supplement: Supplementary file 24 — Supplementary Information 12. [file 41598_2025_91849_MOESM24_ESM.zip › 4Z4Dp_A_mdwhole_AF4REF/plots/4Z4Dp_A-2Dfold_coverage.png]

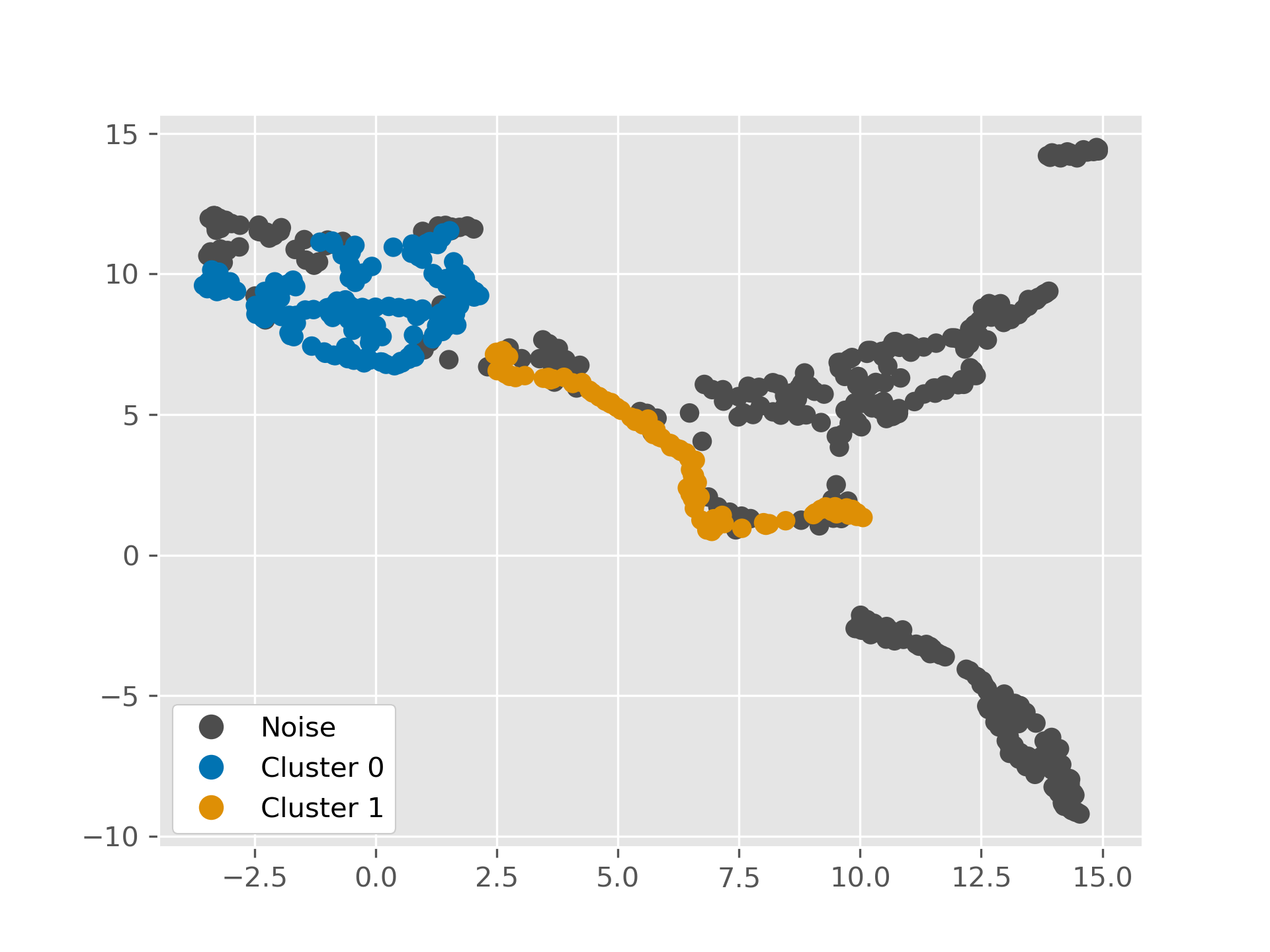

Supplement: Supplementary file 24 — Supplementary Information 12. [file 41598_2025_91849_MOESM24_ESM.zip › 4Z4Dp_A_mdwhole_AF4REF/plots/4Z4Dp_A-clusters-initial.png]

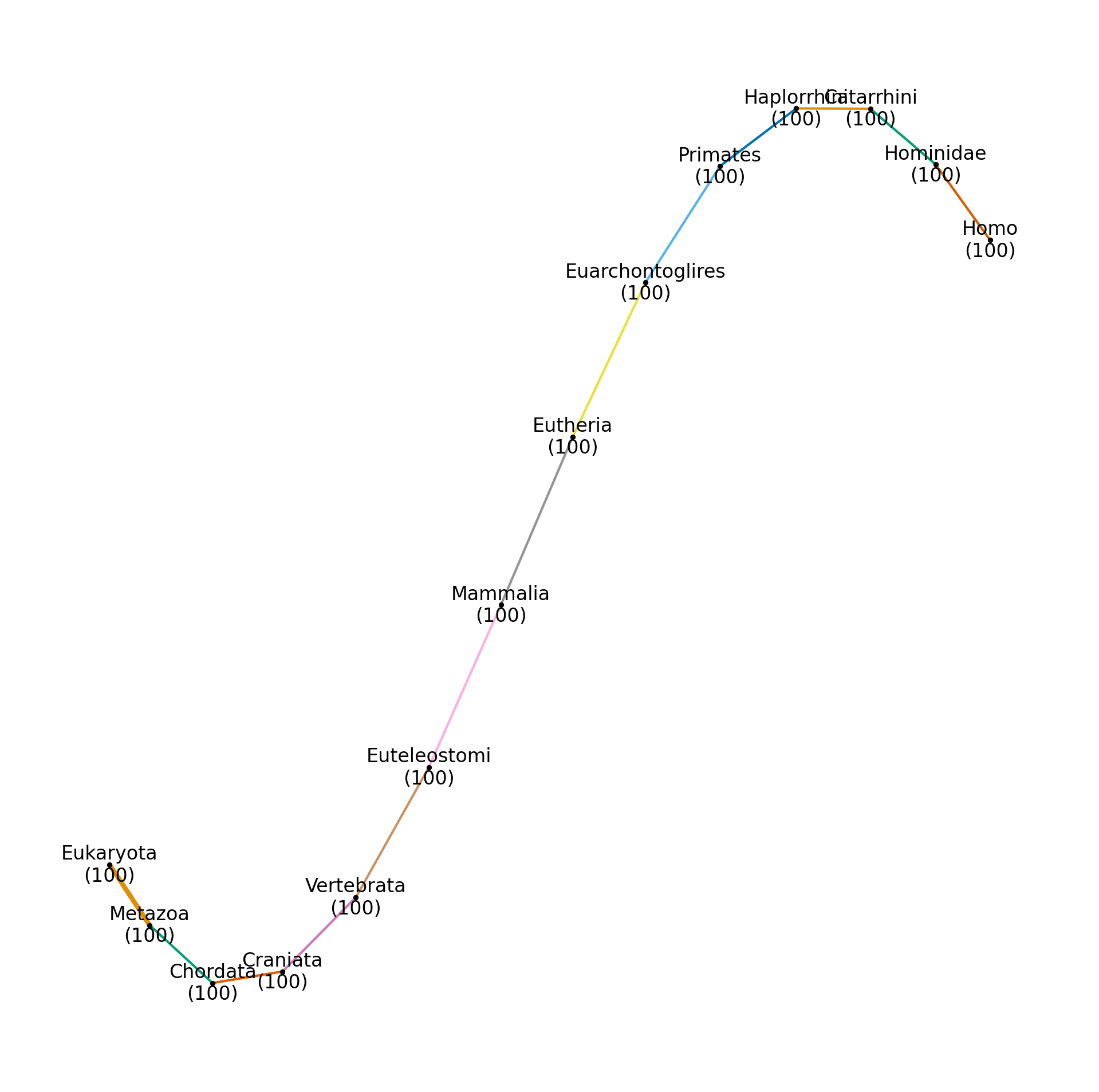

Supplement: Supplementary file 24 — Supplementary Information 12. [file 41598_2025_91849_MOESM24_ESM.zip › 4Z4Dp_A_mdwhole_AF4REF/plots/4Z4Dp_A-Eukaryota-tree.png]

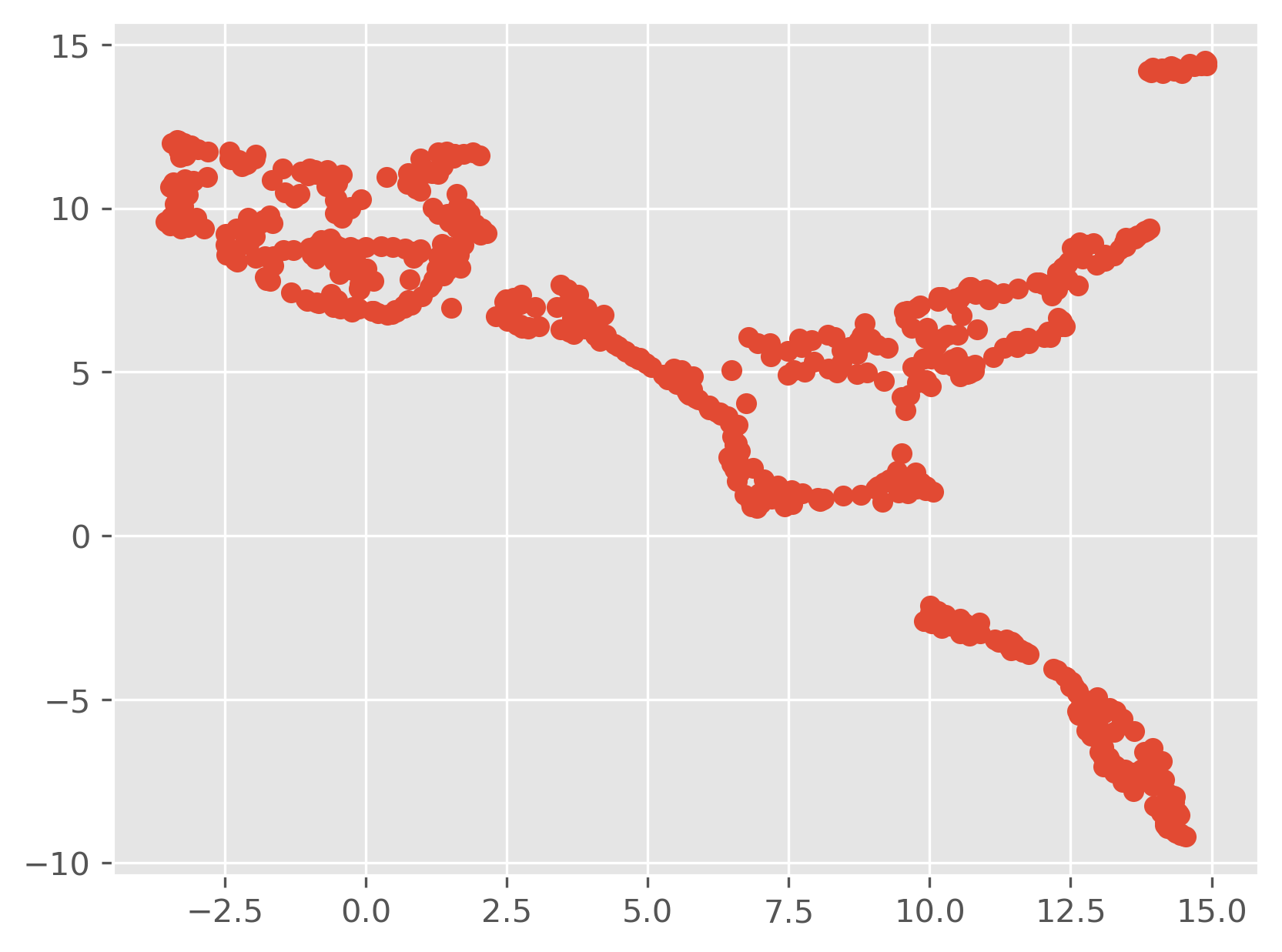

Supplement: Supplementary file 24 — Supplementary Information 12. [file 41598_2025_91849_MOESM24_ESM.zip › 4Z4Dp_A_mdwhole_AF4REF/plots/4Z4Dp_A-UMAP.png]

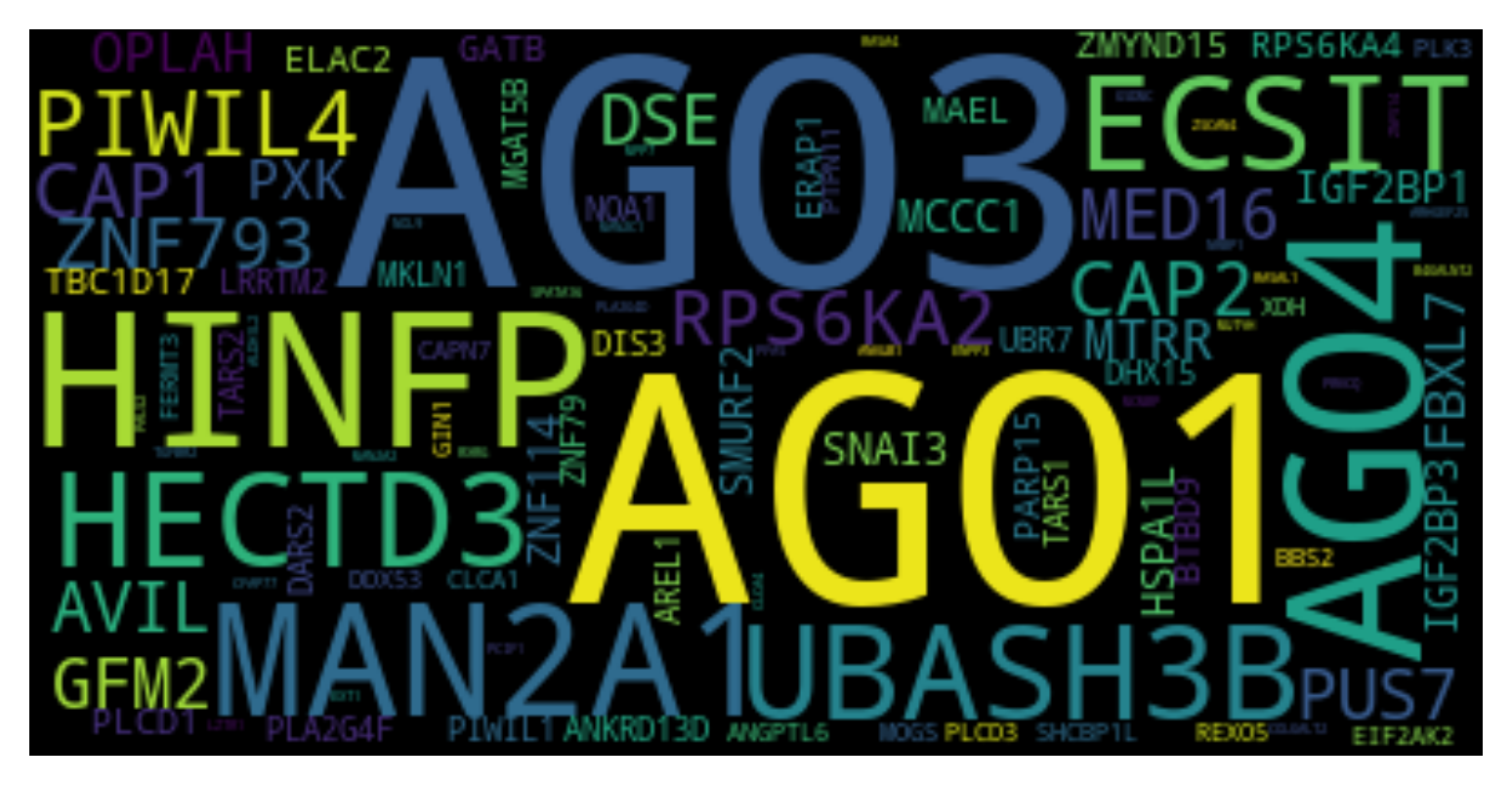

Supplement: Supplementary file 24 — Supplementary Information 12. [file 41598_2025_91849_MOESM24_ESM.zip › 4Z4Dp_A_mdwhole_AF4REF/plots/4Z4Dp_A-wordcloud.png]

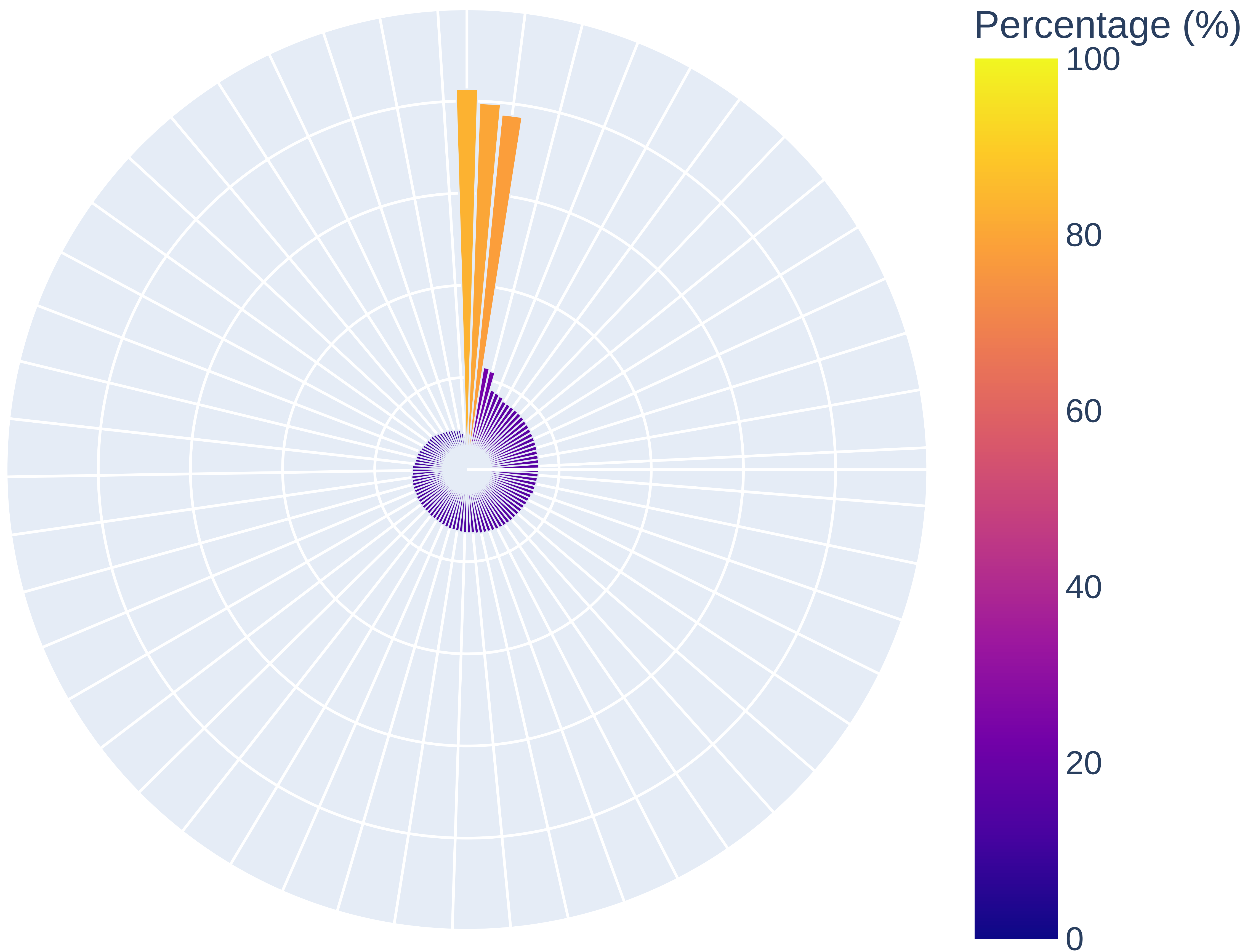

1D identity for 99 proteins in the final set (total: 100)

Supplement: Supplementary file 24 — Supplementary Information 12. [file 41598_2025_91849_MOESM24_ESM.zip › 4Z4Dp_A_mdwhole_AF4REF/plots/4Z4Dp_A_1D-identity.pdf]

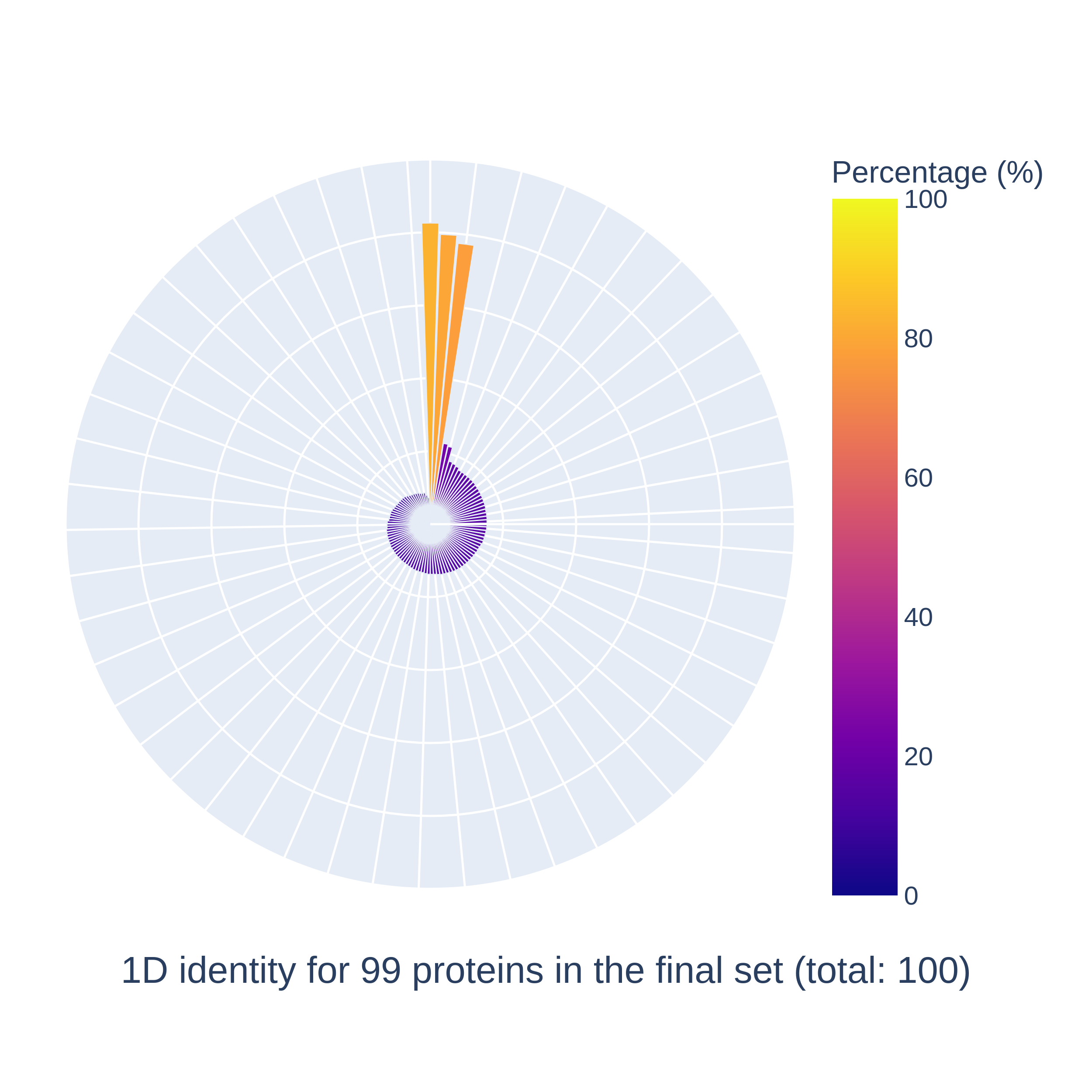

Supplement: Supplementary file 24 — Supplementary Information 12. [file 41598_2025_91849_MOESM24_ESM.zip › 4Z4Dp_A_mdwhole_AF4REF/plots/4Z4Dp_A_1D-identity.png]

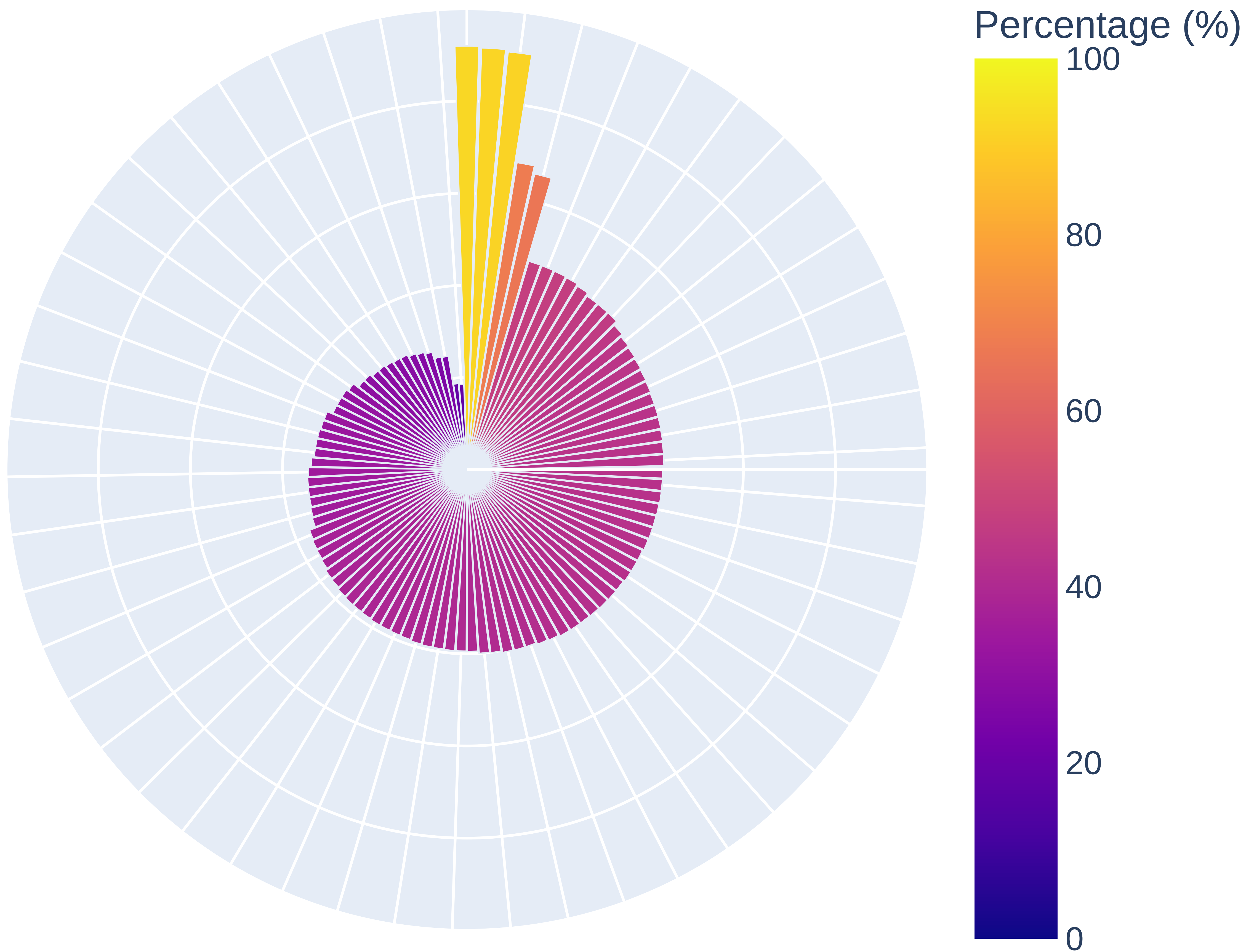

2D identity for 99 proteins in the final set (total: 100)

Supplement: Supplementary file 24 — Supplementary Information 12. [file 41598_2025_91849_MOESM24_ESM.zip › 4Z4Dp_A_mdwhole_AF4REF/plots/4Z4Dp_A_2D-identity.pdf]

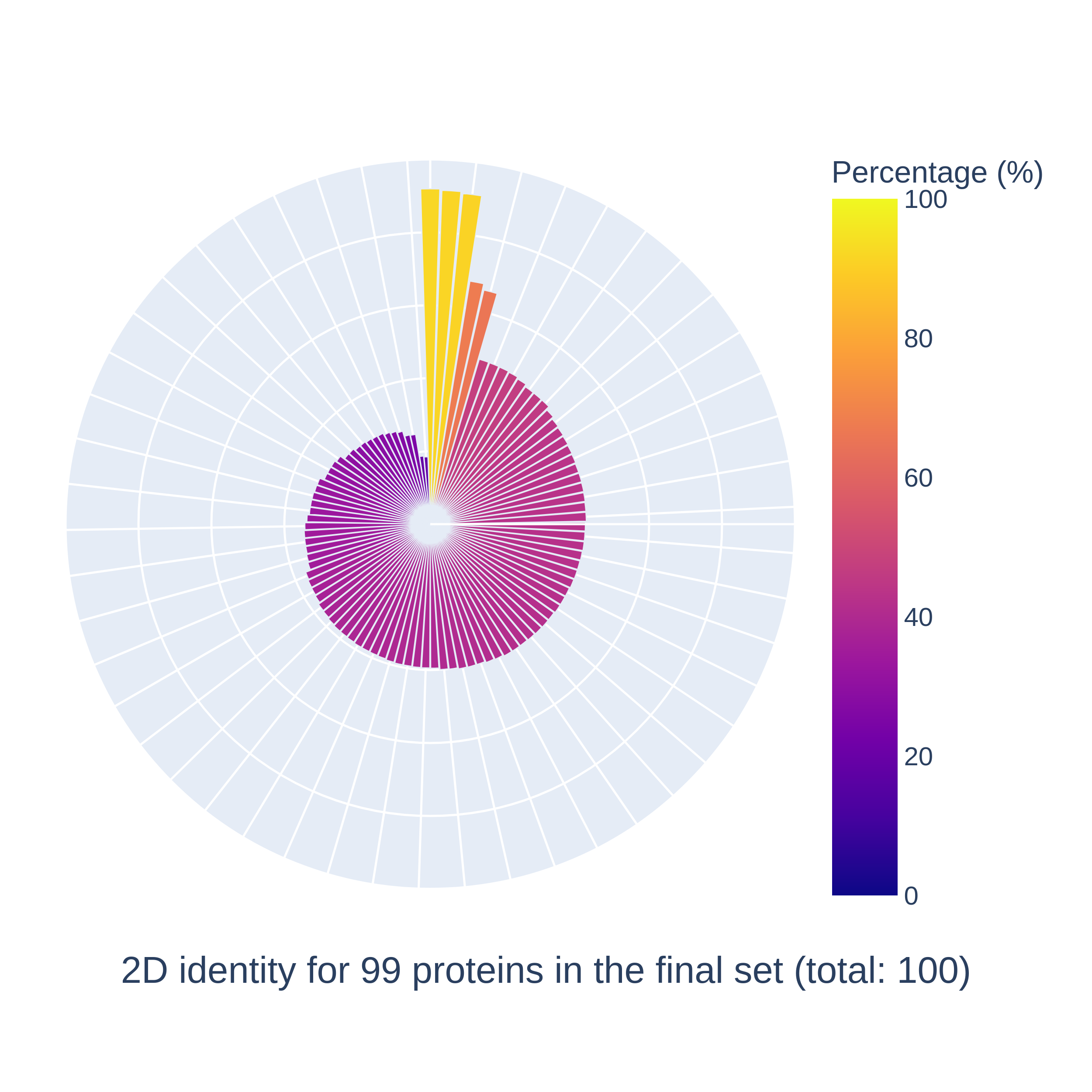

Supplement: Supplementary file 24 — Supplementary Information 12. [file 41598_2025_91849_MOESM24_ESM.zip › 4Z4Dp_A_mdwhole_AF4REF/plots/4Z4Dp_A_2D-identity.png]

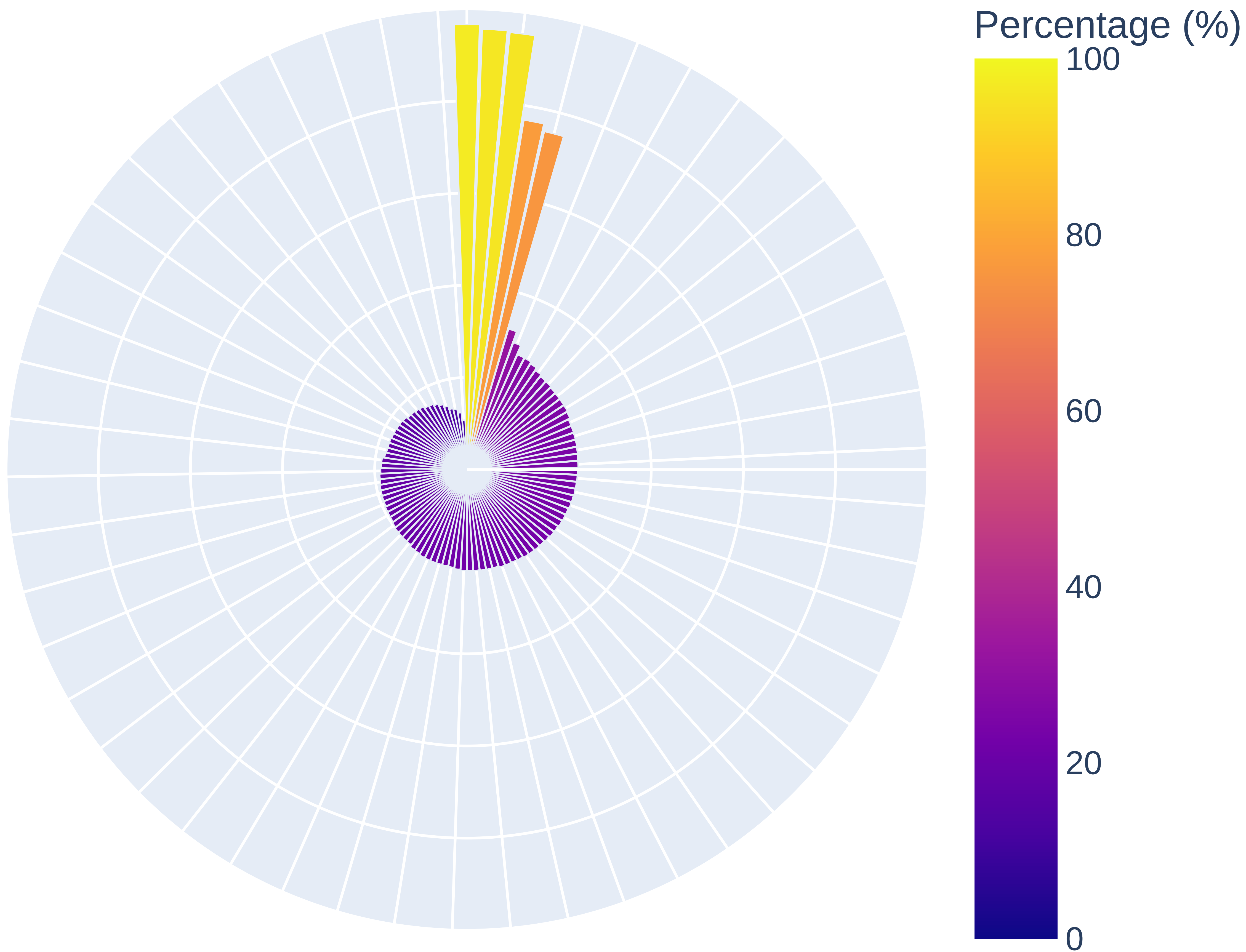

3D Similarity for 99 proteins in the final set (total: 100)

Supplement: Supplementary file 24 — Supplementary Information 12. [file 41598_2025_91849_MOESM24_ESM.zip › 4Z4Dp_A_mdwhole_AF4REF/plots/4Z4Dp_A_3D-score.pdf]

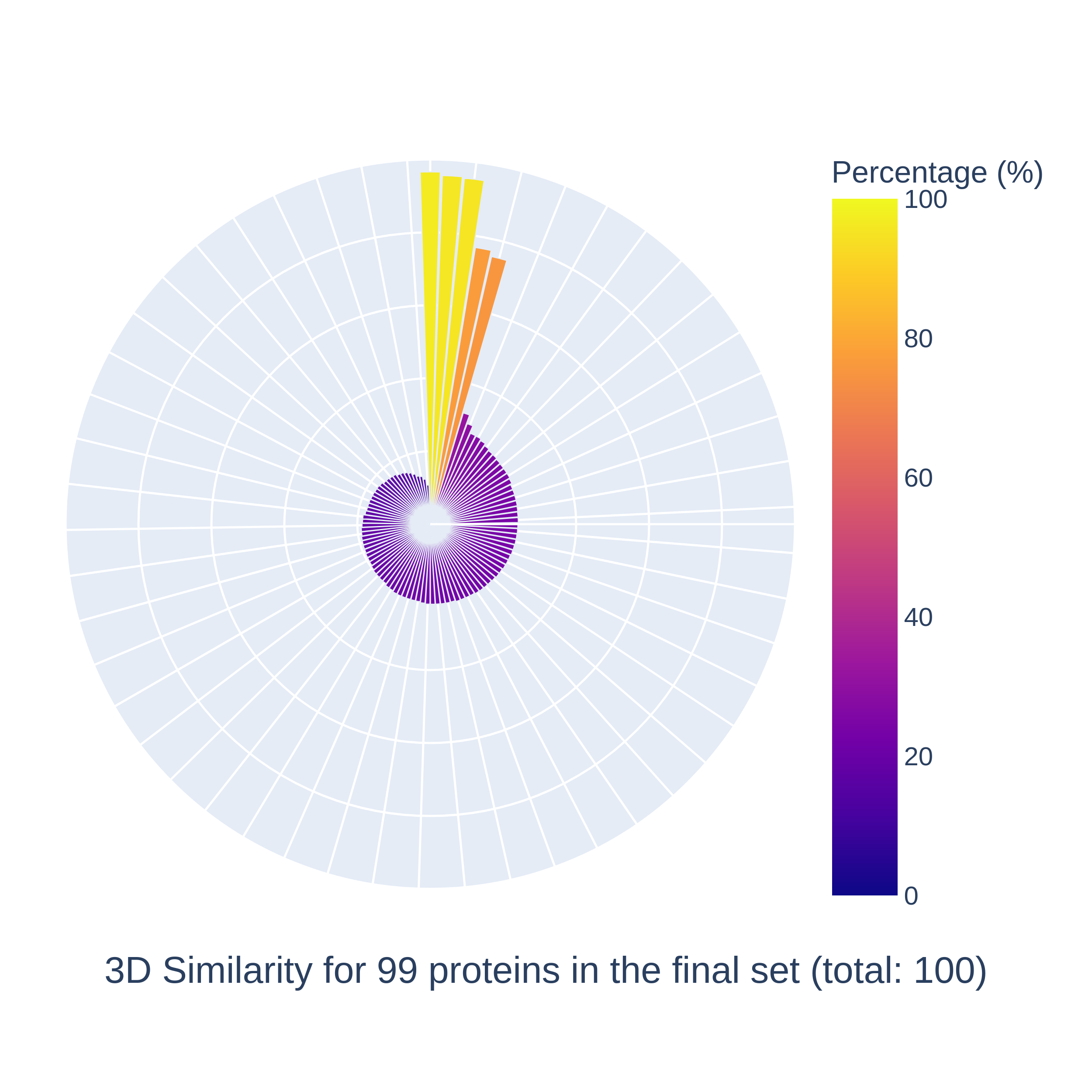

Supplement: Supplementary file 24 — Supplementary Information 12. [file 41598_2025_91849_MOESM24_ESM.zip › 4Z4Dp_A_mdwhole_AF4REF/plots/4Z4Dp_A_3D-score.png]

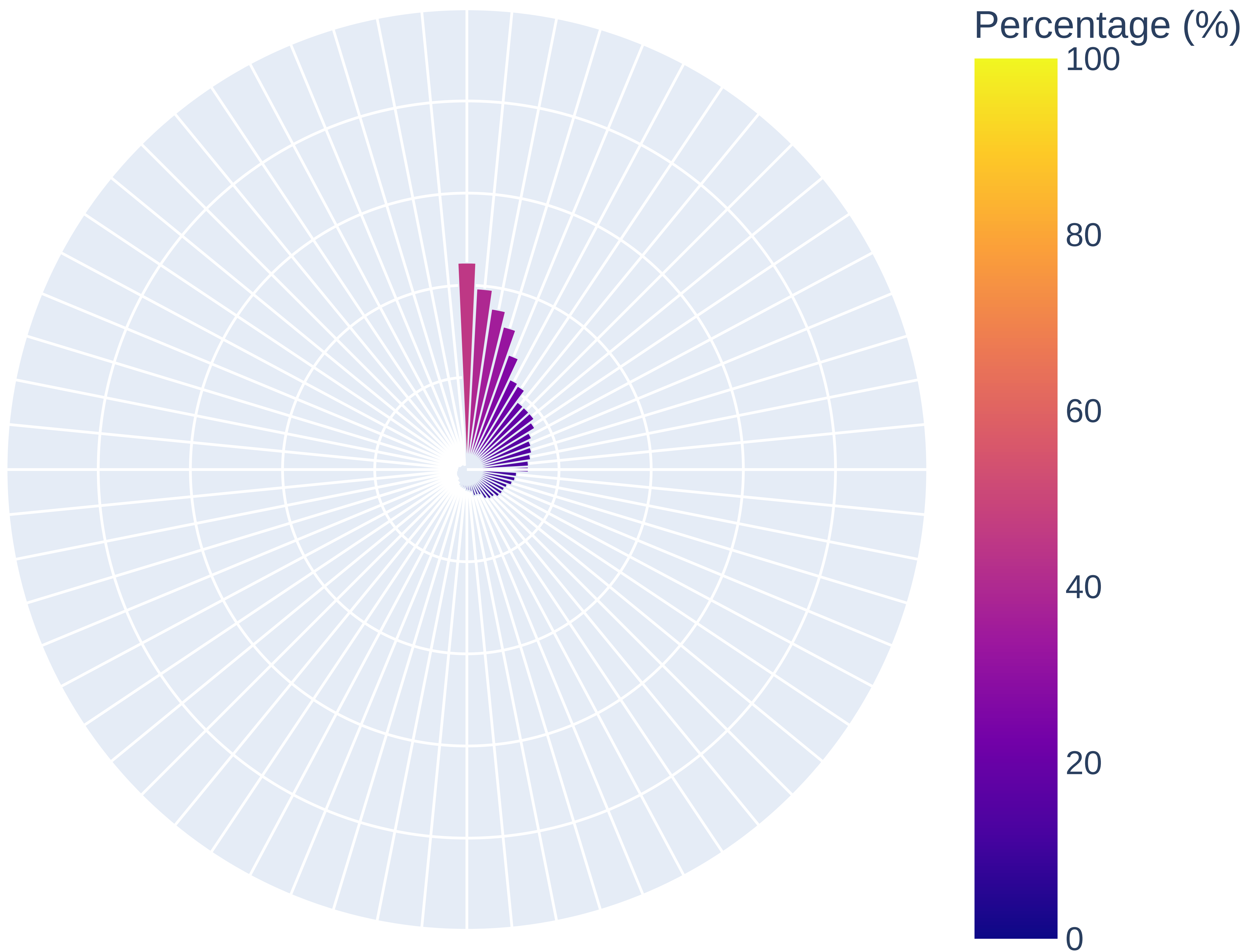

Supplement: Supplementary file 24 — Supplementary Information 12. [file 41598_2025_91849_MOESM24_ESM.zip › 4Z4Dp_A_mdwhole_AF4REF/plots/4Z4Dp_A_3UTR-identity.pdf]

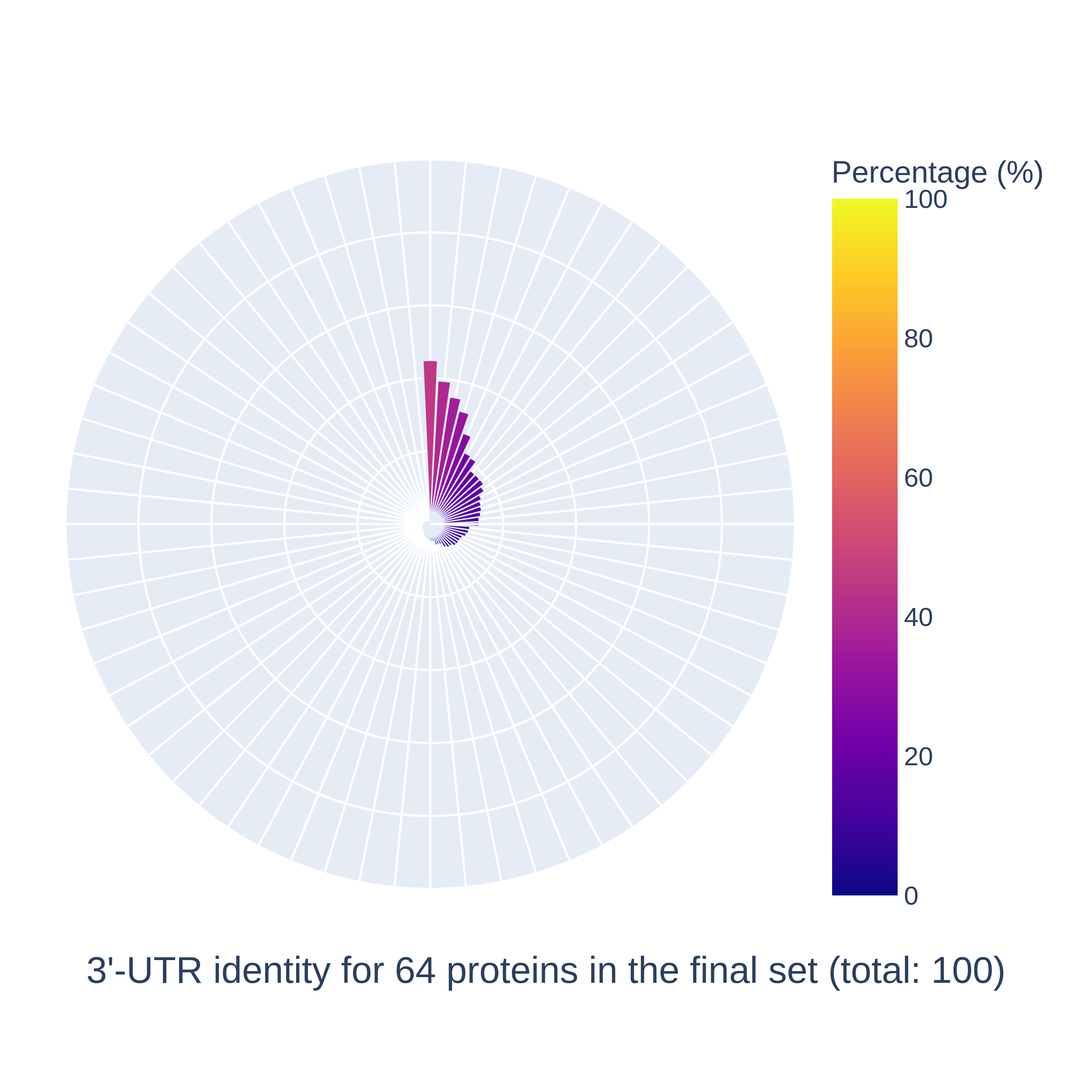

Supplement: Supplementary file 24 — Supplementary Information 12. [file 41598_2025_91849_MOESM24_ESM.zip › 4Z4Dp_A_mdwhole_AF4REF/plots/4Z4Dp_A_3UTR-identity.png]
