# Supplementary material for: Comparative structural insights and functional analysis for the distinct unbound states of Human AGO proteins
Source: Sci Rep. 2025 Mar 19;15:9432. doi: 10.1038/s41598-025-91849-5 (PMC11923369; doi:10.1038/s41598-025-91849-5)
Supplement: Supplementary file 24 — Supplementary Information 12. [file 41598_2025_91849_MOESM24_ESM.zip › 4Z4Dp_A_mdwhole_HL2REF/candidates/4Z4Dp_A-merged-enriched_eval_report.html]

 

# Structural Comparison Report for 4Z4Dp\_A - whole structures (total: 100)

---

1

- **Protein name:** DNA polymerase alpha catalytic subunit
- **Organism:** Homo sapiens
- **Uniprot Accession Number:** P09884
- **Protein sequence length:** 1462 aa
- **1D identity (%):** 14.22
- **1D identity (%) [Gaps excluded]:** 26.23
- **1D identity - Alignment Gaps:** 689
- **1D aligned content (<aminoacid>:%):** {'P': 8.88, 'G': 10.75, 'R': 4.67, 'L': 8.88, 'K': 8.41, 'Y': 3.27, 'E': 7.48, 'V': 6.07, 'D': 7.01, 'F': 4.67, 'T': 5.14, 'Q': 3.27, 'S': 4.67, 'A': 4.67, 'H': 2.34, 'C': 1.87, 'M': 0.93, 'N': 3.74, 'I': 2.8, 'W': 0.47}
- **Common reported functions (%):** 0.0
- **Common reported locations (%):** 21.43
- **Common reported processes (%):** 0.0

- **PDB ID:** 4QCL
- **Chain:** A
- **Crystallized protein length:** 865 aa
- **Resolution:** 2.2 Å
- **b-phipsi:** 0.00526
- **w-rdist:** 0.153242
- **t-alpha:** 0.002185
- **Chemical similarity (Tanimoto Index) (%):** 67.7
- **1D identity (%) [PDB]:** 2.99
- **1D identity (%) [Gaps excluded][PDB]:** 75.38
- **1D identity - Alignment Gaps [PDB]:** 1576
- **1D aligned content [PDB] (<aminoacid>:%):** {'E': 10.2, 'F': 10.2, 'H': 2.04, 'S': 6.12, 'V': 12.24, 'M': 2.04, 'N': 2.04, 'I': 6.12, 'D': 6.12, 'A': 6.12, 'T': 6.12, 'Y': 2.04, 'Q': 6.12, 'P': 4.08, 'L': 6.12, 'K': 10.2, 'G': 2.04}
- **2D identity (%) [PDB]:** 37.94
- **2D identity (%) [Gaps excluded][PDB]:** 88.48
- **2D identity - Alignment Gaps [PDB]:** 682
- **2D aligned content [PDB] (<2D-fold>:%):** {'.': 17.66, 'E': 27.81, 'T': 6.84, 'H': 47.02, 'G': 0.66}
- **3D similarity (TM-Score) (%) [PDB]:** 24.28

- **Gene name:** POLA1
- **Entrez ID:** 5422
- **RefSeq ID:** NM\_016937
- **Transcript sequence length:** 5469
- **5-UTR|CDS|3-UTR identity (%):** 19.85 | 38.99 | 5.48
- **5-UTR|CDS|3-UTR identity (%) [Gaps excluded]:** 77.14 | 76.14 | 75.43
- **5-UTR|CDS|3-UTR identity [Alignment Gaps]:** 101 | 2249 | 11174
- **5-UTR aligned content (<base>:%):** {'G': 51.85, 'C': 33.33, 'T': 14.81}
- **CDS aligned content (<base>:%):** {'T': 20.48, 'G': 26.54, 'C': 24.71, 'A': 28.27}
- **3-UTR aligned content (<base>:%):** {'G': 19.55, 'A': 27.58, 'T': 32.88, 'C': 20.0}

**Uniprot Description:**  
  
 Catalytic subunit of the DNA polymerase alpha complex (also known as the alpha DNA polymerase-primase complex) which plays an essential role in the initiation of DNA synthesis. During the S phase of the cell cycle, the DNA polymerase alpha complex (composed of a catalytic subunit POLA1, a regulatory subunit POLA2 and two primase subunits PRIM1 and PRIM2) is recruited to DNA at the replicative forks via direct interactions with MCM10 and WDHD1. The primase subunit of the polymerase alpha complex initiates DNA synthesis by oligomerising short RNA primers on both leading and lagging strands. These primers are initially extended by the polymerase alpha catalytic subunit and subsequently transferred to polymerase delta and polymerase epsilon for processive synthesis on the lagging and leading strand, respectively. The reason this transfer occurs is because the polymerase alpha has limited processivity and lacks intrinsic 3' exonuclease activity for proofreading error, and therefore is not well suited for replicating long complexes. In the cytosol, responsible for a substantial proportion of the physiological concentration of cytosolic RNA:DNA hybrids, which are necessary to prevent spontaneous activation of type I interferon responses (PubMed:27019227).   
  
Component of the alpha DNA polymerase complex (also known as the alpha DNA polymerase-primase complex) consisting of four subunits: the catalytic subunit POLA1, the regulatory subunit POLA2, and the primase complex subunits PRIM1 and PRIM2 respectively (PubMed:9705292). Interacts with PARP1; this interaction functions as part of the control of replication fork progression (PubMed:9518481). Interacts with MCM10 and WDHD1; these interactions recruit the polymerase alpha complex to the pre-replicative complex bound to DNA (PubMed:19608746). Interacts with RPA1; this interaction stabilizes the replicative complex and reduces the misincorporation rate of DNA polymerase alpha by acting as a fidelity clamp (PubMed:9214288).   
  
 **Gene Ontology Information:**

Molecular Function

- chromatin binding
- DNA binding
- DNA replication origin binding
- DNA-directed DNA polymerase activity
- nucleotide binding
- protein kinase binding
- single-stranded DNA binding
- zinc ion binding

Location

- alpha DNA polymerase:primase complex
- cytosol
- nuclear envelope
- nuclear matrix
- nucleolus
- nucleoplasm
- nucleus

Biological process

- DNA repair
- DNA replication
- DNA replication initiation
- DNA replication, synthesis of RNA primer
- DNA strand elongation involved in DNA replication
- DNA synthesis involved in DNA repair
- double-strand break repair via nonhomologous end joining
- lagging strand elongation
- leading strand elongation
- mitotic DNA replication initiation
- regulation of type I interferon production

---

2

- **Protein name:** Cytosolic phospholipase A2 delta
- **Organism:** Homo sapiens
- **Uniprot Accession Number:** Q86XP0
- **Protein sequence length:** 818 aa
- **1D identity (%):** 14.55
- **1D identity (%) [Gaps excluded]:** 24.05
- **1D identity - Alignment Gaps:** 413
- **1D aligned content (<aminoacid>:%):** {'L': 15.79, 'P': 13.16, 'Q': 5.26, 'G': 7.89, 'A': 5.26, 'F': 3.29, 'T': 3.29, 'K': 5.26, 'D': 5.26, 'I': 1.97, 'Y': 3.29, 'R': 5.92, 'E': 5.92, 'V': 5.26, 'S': 6.58, 'C': 1.32, 'N': 1.32, 'H': 2.63, 'W': 1.32}
- **Common reported functions (%):** 0.0
- **Common reported locations (%):** 14.29
- **Common reported processes (%):** 0.0

- **PDB ID:** 5IZ5
- **Chain:** B
- **Crystallized protein length:** 746 aa
- **Resolution:** 2.2 Å
- **b-phipsi:** 0.003032
- **w-rdist:** 0.347231
- **t-alpha:** 0.002922
- **Chemical similarity (Tanimoto Index) (%):** 99.47
- **1D identity (%) [PDB]:** 2.53
- **1D identity (%) [Gaps excluded][PDB]:** 82.98
- **1D identity - Alignment Gaps [PDB]:** 1492
- **1D aligned content [PDB] (<aminoacid>:%):** {'G': 10.26, 'Q': 7.69, 'L': 10.26, 'V': 20.51, 'I': 2.56, 'P': 7.69, 'K': 5.13, 'T': 12.82, 'E': 2.56, 'D': 2.56, 'M': 2.56, 'A': 2.56, 'C': 2.56, 'N': 5.13, 'R': 2.56, 'S': 2.56}
- **2D identity (%) [PDB]:** 39.5
- **2D identity (%) [Gaps excluded][PDB]:** 82.14
- **2D identity - Alignment Gaps [PDB]:** 556
- **2D aligned content [PDB] (<2D-fold>:%):** {'.': 15.37, 'E': 37.59, 'T': 12.77, 'H': 32.86, 'G': 1.42}
- **3D similarity (TM-Score) (%) [PDB]:** 23.32

- **Gene name:** PLA2G4D
- **Entrez ID:** 283748
- **RefSeq ID:** NM\_178034
- **Transcript sequence length:** 4266
- **5-UTR|CDS|3-UTR identity (%):** 39.44 | 46.31 | 9.08
- **5-UTR|CDS|3-UTR identity (%) [Gaps excluded]:** 63.64 | 75.12 | 76.6
- **5-UTR|CDS|3-UTR identity [Alignment Gaps]:** 54 | 1195 | 10714
- **5-UTR aligned content (<base>:%):** {'G': 41.07, 'A': 5.36, 'T': 12.5, 'C': 41.07}
- **CDS aligned content (<base>:%):** {'A': 21.34, 'T': 16.22, 'G': 29.87, 'C': 32.57}
- **3-UTR aligned content (<base>:%):** {'G': 21.58, 'T': 35.9, 'C': 23.12, 'A': 19.4}

**Uniprot Description:**  
  
 Calcium-dependent phospholipase A2 that selectively hydrolyzes glycerophospholipids in the sn-2 position (PubMed:14709560). Has a preference for linoleic acid at the sn-2 position (PubMed:14709560). N/A   
  
 **Gene Ontology Information:**

Molecular Function

- calcium ion binding
- calcium-dependent phospholipase A2 activity
- calcium-dependent phospholipid binding
- phospholipase A1 activity

Location

- cytosol
- membrane

Biological process

- fatty acid metabolic process
- glycerophospholipid catabolic process
- phosphatidylglycerol acyl-chain remodeling
- phosphatidylinositol acyl-chain remodeling

---

3

- **Protein name:** X-ray repair cross-complementing protein 5
- **Organism:** Homo sapiens
- **Uniprot Accession Number:** P13010
- **Protein sequence length:** 732 aa
- **1D identity (%):** 16.08
- **1D identity (%) [Gaps excluded]:** 24.72
- **1D identity - Alignment Gaps:** 337
- **1D aligned content (<aminoacid>:%):** {'S': 3.87, 'V': 7.74, 'P': 9.03, 'F': 6.45, 'E': 3.87, 'Q': 9.03, 'A': 9.03, 'R': 1.94, 'G': 6.45, 'N': 2.58, 'L': 10.32, 'M': 0.65, 'D': 6.45, 'K': 7.74, 'C': 1.94, 'H': 3.87, 'I': 2.58, 'T': 3.87, 'Y': 2.58}
- **Common reported functions (%):** 6.67
- **Common reported locations (%):** 28.57
- **Common reported processes (%):** 4.76

- **PDB ID:** 8ASC
- **Chain:** O
- **Crystallized protein length:** 498 aa
- **Resolution:** 2.95 Å
- **b-phipsi:** 0.000598
- **w-rdist:** 0.306069
- **t-alpha:** 0.009468
- **Chemical similarity (Tanimoto Index) (%):** N/A
- **1D identity (%) [PDB]:** 1.77
- **1D identity (%) [Gaps excluded][PDB]:** 69.7
- **1D identity - Alignment Gaps [PDB]:** 1267
- **1D aligned content [PDB] (<aminoacid>:%):** {'S': 17.39, 'G': 8.7, 'R': 13.04, 'V': 8.7, 'F': 4.35, 'I': 4.35, 'Q': 4.35, 'L': 8.7, 'D': 4.35, 'M': 4.35, 'H': 4.35, 'P': 8.7, 'Y': 4.35, 'T': 4.35}
- **2D identity (%) [PDB]:** 32.45
- **2D identity (%) [Gaps excluded][PDB]:** 89.04
- **2D identity - Alignment Gaps [PDB]:** 621
- **2D aligned content [PDB] (<2D-fold>:%):** {'.': 25.87, 'E': 31.86, 'T': 40.69, 'G': 1.58}
- **3D similarity (TM-Score) (%) [PDB]:** 17.69

- **Gene name:** XRCC5
- **Entrez ID:** N/A
- **RefSeq ID:** NM\_021141
- **Transcript sequence length:** 3379
- **5-UTR|CDS|3-UTR identity (%):** 38.13 | 45.53 | 5.65
- **5-UTR|CDS|3-UTR identity (%) [Gaps excluded]:** 68.83 | 72.98 | 75.97
- **5-UTR|CDS|3-UTR identity [Alignment Gaps]:** 62 | 1107 | 11181
- **5-UTR aligned content (<base>:%):** {'G': 47.17, 'C': 45.28, 'A': 1.89, 'T': 5.66}
- **CDS aligned content (<base>:%):** {'A': 28.13, 'T': 20.9, 'G': 27.24, 'C': 23.73}
- **3-UTR aligned content (<base>:%):** {'T': 36.6, 'C': 16.84, 'G': 17.72, 'A': 28.84}

**Uniprot Description:**  
  
 Single-stranded DNA-dependent ATP-dependent helicase. Has a role in chromosome translocation. The DNA helicase II complex binds preferentially to fork-like ends of double-stranded DNA in a cell cycle-dependent manner. It works in the 3'-5' direction. Binding to DNA may be mediated by XRCC6. Involved in DNA non-homologous end joining (NHEJ) required for double-strand break repair and V(D)J recombination. The XRCC5/6 dimer acts as regulatory subunit of the DNA-dependent protein kinase complex DNA-PK by increasing the affinity of the catalytic subunit PRKDC to DNA by 100-fold. The XRCC5/6 dimer is probably involved in stabilizing broken DNA ends and bringing them together (PubMed:12145306, PubMed:20383123, PubMed:7957065, PubMed:8621488). The assembly of the DNA-PK complex to DNA ends is required for the NHEJ ligation step. In association with NAA15, the XRCC5/6 dimer binds to the osteocalcin promoter and activates osteocalcin expression (PubMed:20383123). The XRCC5/6 dimer probably also acts as a 5'-deoxyribose-5-phosphate lyase (5'-dRP lyase), by catalyzing the beta-elimination of the 5' deoxyribose-5-phosphate at an abasic site near double-strand breaks. XRCC5 probably acts as the catalytic subunit of 5'-dRP activity, and allows to 'clean' the termini of abasic sites, a class of nucleotide damage commonly associated with strand breaks, before such broken ends can be joined. The XRCC5/6 dimer together with APEX1 acts as a negative regulator of transcription (PubMed:8621488). As part of the DNA-PK complex, involved in the early steps of ribosome assembly by promoting the processing of precursor rRNA into mature 18S rRNA in the small-subunit processome (PubMed:32103174). Binding to U3 small nucleolar RNA, recruits PRKDC and XRCC5/Ku86 to the small-subunit processome (PubMed:32103174). Plays a role in the regulation of DNA virus-mediated innate immune response by assembling into the HDP-RNP complex, a complex that serves as a platform for IRF3 phosphorylation and subsequent innate immune response activation through the cGAS-STING pathway.   
  
Heterodimer composed of XRCC5/Ku80 and XRCC6/Ku70. The dimer associates in a DNA-dependent manner with PRKDC to form the DNA-dependent protein kinase complex DNA-PK, and with the LIG4-XRCC4 complex to form the core of the non-homologous end joining (NHEJ) complex (PubMed:25941166, PubMed:25670504, PubMed:11493912, PubMed:22442688). Additional components of the NHEJ complex include NHEJ1/XLF and PAXX (PubMed:25574025, PubMed:25941166, PubMed:25670504). The dimer also associates with NAA15, and this complex displays DNA binding activity towards the osteocalcin FGF response element (OCFRE) (PubMed:12145306). In addition, XRCC5 binds to the osteoblast-specific transcription factors MSX2 and RUNX2 (PubMed:12145306). Interacts with ELF3 (PubMed:15075319). May interact with APLF (PubMed:17353262, PubMed:17396150). The XRCC5/XRCC6 dimer associates in a DNA-dependent manner with APEX1 (PubMed:8621488). Identified in a complex with DEAF1 and XRCC6. Interacts with NR4A3; the DNA-dependent protein kinase complex DNA-PK phosphorylates and activates NR4A3 and prevents NR4A3 ubiquitinylation and degradation (PubMed:25852083). Interacts with RNF138 (PubMed:26502055). Interacts with CYREN isoform 1 (CYREN-1) and isoform 4 (CYREN-2) (PubMed:24610814, PubMed:28959974). Interacts (via N-terminus) with HSF1 (via N-terminus); this interaction is direct and prevents XRCC5/XRCC6 heterodimeric binding and non-homologous end joining (NHEJ) repair activities induced by ionizing radiation (IR) (PubMed:26359349). Interacts with DHX9; this interaction occurs in a RNA-dependent manner (PubMed:14704337). Part of the HDP-RNP complex composed of at least HEXIM1, PRKDC, XRCC5, XRCC6, paraspeckle proteins (SFPQ, NONO, PSPC1, RBM14, and MATR3) and NEAT1 RNA (PubMed:28712728). Interacts with ERCC6 (PubMed:26030138). Interats with ATF7 (PubMed:29490055).   
  
 **Gene Ontology Information:**

Molecular Function

- 5'-deoxyribose-5-phosphate lyase activity
- ATP binding
- DNA-dependent ATPase activity
- cyclin binding
- damaged DNA binding
- DNA binding
- DNA helicase activity
- double-stranded DNA binding
- hydrolase activity
- protein-containing complex binding
- RNA binding
- scaffold protein binding
- telomeric DNA binding
- transcription regulatory region sequence-specific DNA binding

Location

- chromosome, telomeric region
- cytosol
- DNA-dependent protein kinase complex
- extracellular region
- ficolin-1-rich granule lumen
- Ku70:Ku80 complex
- membrane
- nonhomologous end joining complex
- nuclear telomere cap complex
- nucleolus
- nucleoplasm
- nucleus
- protein-containing complex
- protein-DNA complex
- secretory granule lumen
- transcription regulator complex

Biological process

- activation of innate immune response
- cellular response to gamma radiation
- cellular response to X-ray
- DNA ligation
- double-strand break repair via classical nonhomologous end joining
- double-strand break repair via nonhomologous end joining
- innate immune response
- negative regulation of transcription, DNA-templated
- positive regulation of lymphocyte differentiation
- positive regulation of protein kinase activity
- positive regulation of transcription by RNA polymerase II
- positive regulation of transcription, DNA-templated
- recombinational repair
- regulation of smooth muscle cell proliferation
- telomere maintenance

---

4

- **Protein name:** X-ray repair cross-complementing protein 6
- **Organism:** Homo sapiens
- **Uniprot Accession Number:** P12956
- **Protein sequence length:** 609 aa
- **1D identity (%):** 13.77
- **1D identity (%) [Gaps excluded]:** 26.49
- **1D identity - Alignment Gaps:** 464
- **1D aligned content (<aminoacid>:%):** {'W': 0.75, 'N': 2.26, 'S': 4.51, 'Y': 3.01, 'F': 5.26, 'E': 3.76, 'P': 10.53, 'Q': 5.26, 'V': 6.77, 'K': 11.28, 'I': 4.51, 'G': 8.27, 'L': 9.77, 'A': 4.51, 'D': 4.51, 'H': 2.26, 'R': 4.51, 'T': 6.02, 'C': 1.5, 'M': 0.75}
- **Common reported functions (%):** 6.67
- **Common reported locations (%):** 28.57
- **Common reported processes (%):** 4.76

- **PDB ID:** 7ZYG
- **Chain:** A
- **Crystallized protein length:** 505 aa
- **Resolution:** 2.68 Å
- **b-phipsi:** 0.003222
- **w-rdist:** 0.362111
- **t-alpha:** 0.002913
- **Chemical similarity (Tanimoto Index) (%):** 99.09
- **1D identity (%) [PDB]:** 1.44
- **1D identity (%) [Gaps excluded][PDB]:** 79.17
- **1D identity - Alignment Gaps [PDB]:** 1295
- **1D aligned content [PDB] (<aminoacid>:%):** {'G': 21.05, 'R': 10.53, 'V': 5.26, 'L': 10.53, 'Q': 10.53, 'P': 15.79, 'Y': 5.26, 'N': 5.26, 'K': 5.26, 'A': 5.26, 'T': 5.26}
- **2D identity (%) [PDB]:** 41.89
- **2D identity (%) [Gaps excluded][PDB]:** 90.8
- **2D identity - Alignment Gaps [PDB]:** 495
- **2D aligned content [PDB] (<2D-fold>:%):** {'E': 25.19, '.': 22.08, 'T': 20.78, 'H': 31.95}
- **3D similarity (TM-Score) (%) [PDB]:** 19.62

- **Gene name:** XRCC6
- **Entrez ID:** N/A
- **RefSeq ID:** NM\_001469
- **Transcript sequence length:** 2122
- **5-UTR|CDS|3-UTR identity (%):** 30.3 | 40.25 | 1.31
- **5-UTR|CDS|3-UTR identity (%) [Gaps excluded]:** 65.57 | 75.16 | 80.0
- **5-UTR|CDS|3-UTR identity [Alignment Gaps]:** 71 | 1334 | 11724
- **5-UTR aligned content (<base>:%):** {'T': 15.0, 'C': 42.5, 'G': 37.5, 'A': 5.0}
- **CDS aligned content (<base>:%):** {'A': 27.08, 'T': 20.5, 'G': 28.55, 'C': 23.88}
- **3-UTR aligned content (<base>:%):** {'C': 30.77, 'A': 16.03, 'G': 27.56, 'T': 25.64}

**Uniprot Description:**  
  
 Single-stranded DNA-dependent ATP-dependent helicase. Has a role in chromosome translocation. The DNA helicase II complex binds preferentially to fork-like ends of double-stranded DNA in a cell cycle-dependent manner. It works in the 3'-5' direction. Binding to DNA may be mediated by XRCC6. Involved in DNA non-homologous end joining (NHEJ) required for double-strand break repair and V(D)J recombination. The XRCC5/6 dimer acts as regulatory subunit of the DNA-dependent protein kinase complex DNA-PK by increasing the affinity of the catalytic subunit PRKDC to DNA by 100-fold. The XRCC5/6 dimer is probably involved in stabilizing broken DNA ends and bringing them together. The assembly of the DNA-PK complex to DNA ends is required for the NHEJ ligation step. Required for osteocalcin gene expression. Probably also acts as a 5'-deoxyribose-5-phosphate lyase (5'-dRP lyase), by catalyzing the beta-elimination of the 5' deoxyribose-5-phosphate at an abasic site near double-strand breaks. 5'-dRP lyase activity allows to 'clean' the termini of abasic sites, a class of nucleotide damage commonly associated with strand breaks, before such broken ends can be joined. The XRCC5/6 dimer together with APEX1 acts as a negative regulator of transcription. Plays a role in the regulation of DNA virus-mediated innate immune response by assembling into the HDP-RNP complex, a complex that serves as a platform for IRF3 phosphorylation and subsequent innate immune response activation through the cGAS-STING pathway.   
  
Heterodimer composed of XRCC5/Ku80 and XRCC6/Ku70. The dimer associates in a DNA-dependent manner with PRKDC to form the DNA-dependent protein kinase complex DNA-PK, and with the LIG4-XRCC4 complex to form the core of the non-homologous end joining (NHEJ) complex. Additional components of the NHEJ complex include NHEJ1/XLF and PAXX (PubMed:25574025, PubMed:25941166, PubMed:25670504). The dimer also associates with NAA15, and this complex binds to the osteocalcin promoter and activates osteocalcin expression. In addition, XRCC6 interacts with the osteoblast-specific transcription factors MSX2, RUNX2 and DLX5. Interacts with ELF3. Interacts with ATP23. The XRCC5/6 dimer associates in a DNA-dependent manner with APEX1. Binds to CDK9 isoform 2. Identified in a complex with DEAF1 and XRCC5. Interacts with DEAF1 (via the SAND domain); the interaction is direct and may be inhibited by DNA-binding (PubMed:10219089, PubMed:11493912, PubMed:12145306, PubMed:12509254, PubMed:12547193, PubMed:15075319, PubMed:20493174, PubMed:22442688, PubMed:8621488, PubMed:9742108). Interacts with CLU (By similarity). Interacts with NR4A3; the DNA-dependent protein kinase complex DNA-PK phosphorylates and activates NR4A3 and prevents NR4A3 ubiquitinylation and degradation (PubMed:25852083). Interacts with CYREN isoform 1 (CYREN-1) and isoform 4 (CYREN-2) (PubMed:24610814, PubMed:28959974). Interacts (via N-terminus) with HSF1 (via N-terminus); this interaction is direct and prevents XRCC5/XRCC6 heterodimeric binding and non-homologous end joining (NHEJ) repair activities induced by ionizing radiation (IR) (PubMed:26359349). Part of the HDP-RNP complex composed of at least HEXIM1, PRKDC, XRCC5, XRCC6, paraspeckle proteins (SFPQ, NONO, PSPC1, RBM14, and MATR3) and NEAT1 RNA (PubMed:28712728). Interacts with HMBOX1 (PubMed:23685356). Interacts with ATF7 (PubMed:29490055).   
  
 **Gene Ontology Information:**

Molecular Function

- 5'-deoxyribose-5-phosphate lyase activity
- ATP binding
- DNA-dependent ATPase activity
- cyclin binding
- damaged DNA binding
- DNA binding
- DNA helicase activity
- double-stranded DNA binding
- hydrolase activity
- protein-containing complex binding
- RNA binding
- scaffold protein binding
- telomeric DNA binding
- transcription regulatory region sequence-specific DNA binding

Location

- chromosome, telomeric region
- cytosol
- DNA-dependent protein kinase complex
- extracellular region
- ficolin-1-rich granule lumen
- Ku70:Ku80 complex
- membrane
- nonhomologous end joining complex
- nuclear telomere cap complex
- nucleolus
- nucleoplasm
- nucleus
- protein-containing complex
- protein-DNA complex
- secretory granule lumen
- transcription regulator complex

Biological process

- activation of innate immune response
- cellular response to gamma radiation
- cellular response to X-ray
- DNA ligation
- double-strand break repair via classical nonhomologous end joining
- double-strand break repair via nonhomologous end joining
- innate immune response
- negative regulation of transcription, DNA-templated
- positive regulation of lymphocyte differentiation
- positive regulation of protein kinase activity
- positive regulation of transcription by RNA polymerase II
- positive regulation of transcription, DNA-templated
- recombinational repair
- regulation of smooth muscle cell proliferation
- telomere maintenance

---

5

- **Protein name:** Protein argonaute-1
- **Organism:** Homo sapiens
- **Uniprot Accession Number:** Q9UL18
- **Protein sequence length:** 857 aa
- **1D identity (%):** 82.58
- **1D identity (%) [Gaps excluded]:** 83.16
- **1D identity - Alignment Gaps:** 6
- **1D aligned content (<aminoacid>:%):** {'A': 6.19, 'G': 7.03, 'P': 6.61, 'Q': 6.61, 'F': 4.36, 'R': 6.33, 'T': 6.05, 'I': 5.91, 'K': 6.33, 'L': 7.17, 'N': 2.67, 'E': 4.92, 'D': 4.78, 'Y': 3.66, 'H': 3.23, 'C': 2.81, 'V': 8.02, 'M': 2.25, 'S': 4.22, 'W': 0.84}
- **Common reported functions (%):** 40.0
- **Common reported locations (%):** 71.43
- **Common reported processes (%):** 28.57

- **PDB ID:** 4KXT
- **Chain:** A
- **Crystallized protein length:** 806 aa
- **Resolution:** 2.29 Å
- **b-phipsi:** 0.001056
- **w-rdist:** 0.260581
- **t-alpha:** 0.026948
- **Chemical similarity (Tanimoto Index) (%):** 100.0
- **1D identity (%) [PDB]:** 80.71
- **1D identity (%) [Gaps excluded][PDB]:** 84.12
- **1D identity - Alignment Gaps [PDB]:** 34
- **1D aligned content [PDB] (<aminoacid>:%):** {'P': 6.34, 'R': 6.49, 'G': 6.49, 'T': 6.19, 'I': 6.19, 'K': 6.49, 'L': 7.67, 'A': 6.05, 'N': 2.65, 'F': 4.28, 'E': 4.57, 'D': 4.72, 'Y': 3.83, 'H': 3.1, 'C': 2.95, 'V': 8.26, 'M': 2.36, 'Q': 6.64, 'S': 3.83, 'W': 0.88}
- **2D identity (%) [PDB]:** 70.83
- **2D identity (%) [Gaps excluded][PDB]:** 78.26
- **2D identity - Alignment Gaps [PDB]:** 82
- **2D aligned content [PDB] (<2D-fold>:%):** {'.': 17.32, 'B': 1.96, 'E': 32.52, 'H': 36.44, 'T': 9.31, 'G': 2.45}
- **3D similarity (TM-Score) (%) [PDB]:** 88.14

- **Gene name:** AGO1
- **Entrez ID:** 26523
- **RefSeq ID:** NM\_012199
- **Transcript sequence length:** 13712
- **5-UTR|CDS|3-UTR identity (%):** 42.22 | 75.21 | 44.87
- **5-UTR|CDS|3-UTR identity (%) [Gaps excluded]:** 82.61 | 77.64 | 73.98
- **5-UTR|CDS|3-UTR identity [Alignment Gaps]:** 110 | 82 | 5587
- **5-UTR aligned content (<base>:%):** {'T': 3.16, 'G': 49.47, 'C': 44.21, 'A': 3.16}
- **CDS aligned content (<base>:%):** {'A': 24.94, 'T': 19.3, 'G': 27.78, 'C': 27.98}
- **3-UTR aligned content (<base>:%):** {'A': 25.3, 'G': 22.05, 'C': 18.97, 'T': 33.68}

**Uniprot Description:**  
  
 Required for RNA-mediated gene silencing (RNAi). Binds to short RNAs such as microRNAs (miRNAs) or short interfering RNAs (siRNAs), and represses the translation of mRNAs which are complementary to them. Lacks endonuclease activity and does not appear to cleave target mRNAs. Also required for transcriptional gene silencing (TGS) of promoter regions which are complementary to bound short antigene RNAs (agRNAs).   
  
Interacts with DDB1, DDX5, DDX6, DHX30, DHX36, DDX47, DICER1, AGO2, ELAVL1, HNRNPF, IGF2BP1, ILF3, IMP8, MATR3, MOV10, PABPC1, PRMT5, RBM4, SART3, TNRC6B, UPF1 and YBX1. Associates with polysomes and messenger ribonucleoproteins (mNRPs). Interacts with LIMD1, WTIP and AJUBA. Interacts with APOBEC3F, APOBEC3G and APOBEC3H.   
  
 **Gene Ontology Information:**

Molecular Function

- core promoter sequence-specific DNA binding
- double-stranded RNA binding
- miRNA binding
- RNA binding
- RNA polymerase II complex binding
- single-stranded RNA binding

Location

- cytoplasm
- cytoplasmic ribonucleoprotein granule
- cytosol
- nucleoplasm
- nucleus
- P-body
- polysome
- ribonucleoprotein complex
- RISC complex
- RISC-loading complex

Biological process

- production of miRNAs involved in gene silencing by miRNA
- miRNA mediated inhibition of translation
- negative regulation of angiogenesis
- nuclear-transcribed mRNA catabolic process
- positive regulation of transcription by RNA polymerase II
- pre-miRNA processing
- small RNA loading onto RISC
- RNA secondary structure unwinding

---

6

- **Protein name:** Non-homologous end-joining factor 1
- **Organism:** Homo sapiens
- **Uniprot Accession Number:** Q9H9Q4
- **Protein sequence length:** 299 aa
- **1D identity (%):** 6.83
- **1D identity (%) [Gaps excluded]:** 23.02
- **1D identity - Alignment Gaps:** 628
- **1D aligned content (<aminoacid>:%):** {'E': 4.92, 'G': 4.92, 'P': 8.2, 'W': 1.64, 'A': 9.84, 'F': 3.28, 'Q': 13.11, 'V': 3.28, 'D': 4.92, 'S': 4.92, 'R': 8.2, 'K': 6.56, 'H': 4.92, 'C': 3.28, 'L': 8.2, 'Y': 1.64, 'T': 4.92, 'I': 3.28}
- **Common reported functions (%):** 0.0
- **Common reported locations (%):** 0.0
- **Common reported processes (%):** 0.0

- **PDB ID:** 8EZA
- **Chain:** J
- **Crystallized protein length:** 497 aa
- **Resolution:** 4.39 Å
- **b-phipsi:** 0.002943
- **w-rdist:** 0.330907
- **t-alpha:** 0.016023
- **Chemical similarity (Tanimoto Index) (%):** 99.09
- **1D identity (%) [PDB]:** 1.45
- **1D identity (%) [Gaps excluded][PDB]:** 86.36
- **1D identity - Alignment Gaps [PDB]:** 1285
- **1D aligned content [PDB] (<aminoacid>:%):** {'Y': 10.53, 'E': 21.05, 'L': 5.26, 'D': 5.26, 'I': 10.53, 'K': 10.53, 'P': 10.53, 'C': 5.26, 'R': 10.53, 'V': 10.53}
- **2D identity (%) [PDB]:** 40.41
- **2D identity (%) [Gaps excluded][PDB]:** 90.27
- **2D identity - Alignment Gaps [PDB]:** 507
- **2D aligned content [PDB] (<2D-fold>:%):** {'T': 26.68, 'E': 23.72, 'H': 27.76, '.': 19.95, 'G': 1.62, 'B': 0.27}
- **3D similarity (TM-Score) (%) [PDB]:** 31.31

- **Gene name:** NHEJ1
- **Entrez ID:** 79840
- **RefSeq ID:** NM\_024782
- **Transcript sequence length:** 8020
- **5-UTR|CDS|3-UTR identity (%):** 28.14 | 19.48 | 33.31
- **5-UTR|CDS|3-UTR identity (%) [Gaps excluded]:** 83.93 | 76.96 | 74.79
- **5-UTR|CDS|3-UTR identity [Alignment Gaps]:** 111 | 2074 | 7258
- **5-UTR aligned content (<base>:%):** {'G': 51.06, 'A': 4.26, 'C': 40.43, 'T': 4.26}
- **CDS aligned content (<base>:%):** {'T': 18.11, 'G': 26.25, 'A': 26.06, 'C': 29.57}
- **3-UTR aligned content (<base>:%):** {'T': 31.46, 'C': 22.69, 'A': 23.54, 'G': 22.3}

**Uniprot Description:**  
  
 DNA repair protein involved in DNA non-homologous end joining (NHEJ); required for double-strand break (DSB) repair and V(D)J recombination (PubMed:16439204, PubMed:16439205, PubMed:17717001, PubMed:17317666, PubMed:17470781, PubMed:18644470, PubMed:20558749, PubMed:26100018, PubMed:18158905). Plays a key role in NHEJ by promoting the ligation of various mismatched and non-cohesive ends (PubMed:17717001, PubMed:17470781, PubMed:19056826). Together with PAXX, collaborates with DNA polymerase lambda (POLL) to promote joining of non-cohesive DNA ends (PubMed:30250067, PubMed:25670504). May act in concert with XRCC5-XRCC6 (Ku) to stimulate XRCC4-mediated joining of blunt ends and several types of mismatched ends that are non-complementary or partially complementary (PubMed:16439204, PubMed:16439205, PubMed:17317666, PubMed:17470781). Associates with XRCC4 to form alternating helical filaments that bridge DNA and act like a bandage, holding together the broken DNA until it is repaired (PubMed:22228831, PubMed:26100018, PubMed:28500754, PubMed:27437582, PubMed:21775435, PubMed:22287571, PubMed:21768349). The XRCC4-NHEJ1/XLF subcomplex binds to the DNA fragments of a DSB in a highly diffusive manner and robustly bridges two independent DNA molecules, holding the broken DNA fragments in close proximity to one other (PubMed:28500754, PubMed:27437582). The mobility of the bridges ensures that the ends remain accessible for further processing by other repair factors (PubMed:27437582). Binds DNA in a length-dependent manner (PubMed:17317666, PubMed:18158905).   
  
Homodimer; mainly exists as a homodimer when not associated with XRCC4 (PubMed:18046455, PubMed:25574025, PubMed:25670504, PubMed:25941166, PubMed:18158905). Interacts with XRCC4; the interaction is direct and is mediated via a head-to-head interaction between N-terminal head regions (PubMed:16439205, PubMed:20558749, PubMed:22228831, PubMed:26100018, PubMed:18158905, PubMed:21936820, PubMed:21775435, PubMed:22287571, PubMed:21768349, PubMed:27437582). Component of the core long-range non-homologous end joining (NHEJ) complex (also named DNA-PK complex) composed of PRKDC, LIG4, XRCC4, XRCC6/Ku70, XRCC5/Ku86 and NHEJ1/XLF (PubMed:16571728, PubMed:17317666, PubMed:33854234). Additional component of the NHEJ complex includes PAXX (PubMed:25574025, PubMed:25941166). Following autophosphorylation, PRKDC dissociates from DNA, leading to formation of the short-range NHEJ complex, composed of LIG4, XRCC4, XRCC6/Ku70, XRCC5/Ku86 and NHEJ1/XLF (PubMed:33854234). Interacts with POLL (DNA polymerase lambda); promoting POLL recruitment to double-strand breaks (DSBs) and stimulation of the end-filling activity of POLL (PubMed:30250067).   
  
 **Gene Ontology Information:**

Molecular Function   
  
N/A

Location   
  
N/A

Biological process   
  
N/A

---

7

- **Protein name:** Protein PAXX
- **Organism:** Homo sapiens
- **Uniprot Accession Number:** Q9BUH6
- **Protein sequence length:** 204 aa
- **1D identity (%):** 5.27
- **1D identity (%) [Gaps excluded]:** 24.21
- **1D identity - Alignment Gaps:** 683
- **1D aligned content (<aminoacid>:%):** {'P': 13.04, 'L': 15.22, 'G': 15.22, 'Y': 2.17, 'E': 4.35, 'T': 6.52, 'K': 8.7, 'I': 2.17, 'R': 6.52, 'Q': 4.35, 'V': 4.35, 'D': 2.17, 'S': 4.35, 'A': 8.7, 'F': 2.17}
- **Common reported functions (%):** 0.0
- **Common reported locations (%):** 0.0
- **Common reported processes (%):** 0.0

- **PDB ID:** 8EZA
- **Chain:** A
- **Crystallized protein length:** 497 aa
- **Resolution:** 4.39 Å
- **b-phipsi:** 0.003629
- **w-rdist:** 0.327156
- **t-alpha:** 0.011654
- **Chemical similarity (Tanimoto Index) (%):** 99.09
- **1D identity (%) [PDB]:** 1.45
- **1D identity (%) [Gaps excluded][PDB]:** 86.36
- **1D identity - Alignment Gaps [PDB]:** 1285
- **1D aligned content [PDB] (<aminoacid>:%):** {'Y': 10.53, 'E': 21.05, 'L': 5.26, 'D': 5.26, 'I': 10.53, 'K': 10.53, 'P': 10.53, 'C': 5.26, 'R': 10.53, 'V': 10.53}
- **2D identity (%) [PDB]:** 40.46
- **2D identity (%) [Gaps excluded][PDB]:** 90.05
- **2D identity - Alignment Gaps [PDB]:** 505
- **2D aligned content [PDB] (<2D-fold>:%):** {'T': 26.68, 'E': 23.72, 'H': 27.76, '.': 19.95, 'G': 1.62, 'B': 0.27}
- **3D similarity (TM-Score) (%) [PDB]:** 2.04

- **Gene name:** PAXX
- **Entrez ID:** 286257
- **RefSeq ID:** NM\_183241
- **Transcript sequence length:** 808
- **5-UTR|CDS|3-UTR identity (%):** 14.17 | 15.69 | 1.06
- **5-UTR|CDS|3-UTR identity (%) [Gaps excluded]:** 81.82 | 77.51 | 84.56
- **5-UTR|CDS|3-UTR identity [Alignment Gaps]:** 105 | 2119 | 11761
- **5-UTR aligned content (<base>:%):** {'G': 44.44, 'C': 55.56}
- **CDS aligned content (<base>:%):** {'A': 17.75, 'G': 33.09, 'T': 12.71, 'C': 36.45}
- **3-UTR aligned content (<base>:%):** {'A': 16.67, 'G': 29.37, 'C': 35.71, 'T': 18.25}

**Uniprot Description:**  
  
 Non-essential DNA repair protein involved in DNA non-homologous end joining (NHEJ); participates in double-strand break (DSB) repair and V(D)J recombination (PubMed:25574025, PubMed:25670504, PubMed:25941166, PubMed:27705800). May act as a scaffold required for accumulation of the Ku heterodimer, composed of XRCC5/Ku80 and XRCC6/Ku70, at double-strand break sites and promote the assembly and/or stability of the NHEJ machinery (PubMed:25574025, PubMed:25670504, PubMed:25941166). Involved in NHEJ by promoting the ligation of blunt-ended DNA ends (PubMed:27703001). Together with NHEJ1/XLF, collaborates with DNA polymerase lambda (POLL) to promote joining of non-cohesive DNA ends (PubMed:30250067, PubMed:25670504). Constitutes a non-essential component of classical NHEJ: has a complementary but distinct function with NHEJ1/XLF in DNA repair (PubMed:27705800). Able to restrict infection by herpesvirus 1 (HSV-1) via an unknown mechanism (PubMed:29144403).   
  
Homodimer (PubMed:25574025). Interacts with the DNA-bound XRCC5/Ku80 and XRCC6/Ku70 heterodimer (Ku complex); the interaction is direct (PubMed:27601299, PubMed:27705800, PubMed:25574025). Associated component of the non-homologous end joining (NHEJ) complex, composed of the core proteins PRKDC, LIG4, XRCC4, XRCC6/Ku70, XRCC5/Ku86 and NHEJ1/XLF (PubMed:25670504, PubMed:25941166). Interacts with POLL (DNA polymerase lambda); promoting POLL recruitment to double-strand breaks (DSBs) and stimulation of the end-filling activity of POLL (PubMed:30250067).   
  
 **Gene Ontology Information:**

Molecular Function   
  
N/A

Location   
  
N/A

Biological process   
  
N/A

---

8

- **Protein name:** Transitional endoplasmic reticulum ATPase
- **Organism:** Homo sapiens
- **Uniprot Accession Number:** P55072
- **Protein sequence length:** 806 aa
- **1D identity (%):** 15.04
- **1D identity (%) [Gaps excluded]:** 22.11
- **1D identity - Alignment Gaps:** 317
- **1D aligned content (<aminoacid>:%):** {'D': 9.4, 'L': 8.72, 'K': 4.03, 'R': 10.07, 'N': 2.01, 'I': 6.71, 'V': 8.05, 'P': 7.38, 'G': 11.41, 'E': 4.03, 'S': 3.36, 'Q': 4.7, 'T': 4.7, 'Y': 1.34, 'F': 3.36, 'C': 1.34, 'M': 1.34, 'A': 6.71, 'H': 1.34}
- **Common reported functions (%):** 6.67
- **Common reported locations (%):** 35.71
- **Common reported processes (%):** 0.0

- **PDB ID:** 5C1B
- **Chain:** C
- **Crystallized protein length:** 724 aa
- **Resolution:** 3.08 Å
- **b-phipsi:** 0.005744
- **w-rdist:** 0.278455
- **t-alpha:** 0.00874
- **Chemical similarity (Tanimoto Index) (%):** 74.99
- **1D identity (%) [PDB]:** 2.52
- **1D identity (%) [Gaps excluded][PDB]:** 66.67
- **1D identity - Alignment Gaps [PDB]:** 1450
- **1D aligned content [PDB] (<aminoacid>:%):** {'L': 21.05, 'P': 10.53, 'I': 7.89, 'D': 5.26, 'V': 13.16, 'E': 10.53, 'G': 2.63, 'R': 7.89, 'S': 10.53, 'Q': 2.63, 'A': 5.26, 'M': 2.63}
- **2D identity (%) [PDB]:** 39.46
- **2D identity (%) [Gaps excluded][PDB]:** 84.71
- **2D identity - Alignment Gaps [PDB]:** 570
- **2D aligned content [PDB] (<2D-fold>:%):** {'.': 16.39, 'E': 24.94, 'H': 47.51, 'T': 9.26, 'G': 1.43, 'B': 0.48}
- **3D similarity (TM-Score) (%) [PDB]:** 22.22

- **Gene name:** VCP
- **Entrez ID:** N/A
- **RefSeq ID:** NM\_007126
- **Transcript sequence length:** 3746
- **5-UTR|CDS|3-UTR identity (%):** 37.82 | 46.17 | 5.09
- **5-UTR|CDS|3-UTR identity (%) [Gaps excluded]:** 83.87 | 74.97 | 74.79
- **5-UTR|CDS|3-UTR identity [Alignment Gaps]:** 151 | 1189 | 11291
- **5-UTR aligned content (<base>:%):** {'G': 40.38, 'T': 10.58, 'C': 45.19, 'A': 3.85}
- **CDS aligned content (<base>:%):** {'A': 24.63, 'T': 18.96, 'G': 30.23, 'C': 26.17}
- **3-UTR aligned content (<base>:%):** {'G': 22.37, 'T': 32.25, 'C': 20.91, 'A': 24.47}

**Uniprot Description:**  
  
 Necessary for the fragmentation of Golgi stacks during mitosis and for their reassembly after mitosis. Involved in the formation of the transitional endoplasmic reticulum (tER). The transfer of membranes from the endoplasmic reticulum to the Golgi apparatus occurs via 50-70 nm transition vesicles which derive from part-rough, part-smooth transitional elements of the endoplasmic reticulum (tER). Vesicle budding from the tER is an ATP-dependent process. The ternary complex containing UFD1, VCP and NPLOC4 binds ubiquitinated proteins and is necessary for the export of misfolded proteins from the ER to the cytoplasm, where they are degraded by the proteasome. The NPLOC4-UFD1-VCP complex regulates spindle disassembly at the end of mitosis and is necessary for the formation of a closed nuclear envelope. Regulates E3 ubiquitin-protein ligase activity of RNF19A. Component of the VCP/p97-AMFR/gp78 complex that participates in the final step of the sterol-mediated ubiquitination and endoplasmic reticulum-associated degradation (ERAD) of HMGCR. Involved in endoplasmic reticulum stress-induced pre-emptive quality control, a mechanism that selectively attenuates the translocation of newly synthesized proteins into the endoplasmic reticulum and reroutes them to the cytosol for proteasomal degradation (PubMed:26565908). Plays a role in the regulation of stress granules (SGs) clearance process upon arsenite-induced response (PubMed:29804830). Also involved in DNA damage response: recruited to double-strand breaks (DSBs) sites in a RNF8- and RNF168-dependent manner and promotes the recruitment of TP53BP1 at DNA damage sites (PubMed:22020440, PubMed:22120668). Recruited to stalled replication forks by SPRTN: may act by mediating extraction of DNA polymerase eta (POLH) to prevent excessive translesion DNA synthesis and limit the incidence of mutations induced by DNA damage (PubMed:23042607, PubMed:23042605). Together with SPRTN metalloprotease, involved in the repair of covalent DNA-protein cross-links (DPCs) during DNA synthesis (PubMed:32152270). Involved in interstrand cross-link repair in response to replication stress by mediating unloading of the ubiquitinated CMG helicase complex (By similarity). Required for cytoplasmic retrotranslocation of stressed/damaged mitochondrial outer-membrane proteins and their subsequent proteasomal degradation (PubMed:16186510, PubMed:21118995). Essential for the maturation of ubiquitin-containing autophagosomes and the clearance of ubiquitinated protein by autophagy (PubMed:20104022, PubMed:27753622). Acts as a negative regulator of type I interferon production by interacting with DDX58/RIG-I: interaction takes place when DDX58/RIG-I is ubiquitinated via 'Lys-63'-linked ubiquitin on its CARD domains, leading to recruit RNF125 and promote ubiquitination and degradation of DDX58/RIG-I (PubMed:26471729). May play a role in the ubiquitin-dependent sorting of membrane proteins to lysosomes where they undergo degradation (PubMed:21822278). May more particularly play a role in caveolins sorting in cells (PubMed:21822278, PubMed:23335559). By controlling the steady-state expression of the IGF1R receptor, indirectly regulates the insulin-like growth factor receptor signaling pathway (PubMed:26692333).   
  
Homohexamer. Forms a ring-shaped particle of 12.5 nm diameter, that displays 6-fold radial symmetry. Part of a ternary complex containing STX5A, NSFL1C and VCP. NSFL1C forms a homotrimer that binds to one end of a VCP homohexamer. The complex binds to membranes enriched in phosphatidylethanolamine-containing lipids and promotes Golgi membrane fusion. Binds to a heterodimer of NPLOC4 and UFD1, binding to this heterodimer inhibits Golgi-membrane fusion (PubMed:26471729). Interaction with VCIP135 leads to dissociation of the complex via ATP hydrolysis by VCP. Part of a ternary complex containing NPLOC4, UFD1 and VCP. Interacts with NSFL1C-like protein p37; the complex has membrane fusion activity and is required for Golgi and endoplasmic reticulum biogenesis. Interacts with SELENOS and SYVN1, as well as with DERL1, DERL2 and DERL3; which probably transfer misfolded proteins from the ER to VCP. Interacts with SVIP. Component of a complex required to couple retrotranslocation, ubiquitination and deglycosylation composed of NGLY1, SAKS1, AMFR, VCP and RAD23B. Directly interacts with UBXN4 and RNF19A. Interacts with CASR. Interacts with UBE4B and YOD1. Interacts with clathrin. Interacts with RNF103. Interacts with TRIM13 and TRIM21. Component of a VCP/p97-AMFR/gp78 complex that participates in the final step of the endoplasmic reticulum-associated degradation (ERAD) of HMGCR. Interacts directly with AMFR/gp78 (via its VIM). Interacts with RHBDD1 (via C-terminal domain). Interacts with SPRTN; leading to recruitment to stalled replication forks (PubMed:23042607, PubMed:23042605). Interacts with WASHC5. Interacts with UBOX5. Interacts (via N-terminus) with UBXN7, UBXN8, and probably several other UBX domain-containing proteins (via UBX domains); the interactions are mutually exclusive with VIM-dependent interactions such as those with AMFR and SELENOS. Forms a complex with UBQLN1 and UBXN4. Interacts (via the PIM motif) with RNF31 (via the PUB domain) (PubMed:24726327). Interacts with DDX58/RIG-I and RNF125; interaction takes place when DDX58/RIG-I is ubiquitinated via 'Lys-63'-linked ubiquitin on its CARD domains, leading to recruit RNF125 and promote ubiquitination and degradation of DDX58/RIG-I (PubMed:26471729). Interacts with BAG6 (PubMed:21636303). Interacts with UBXN10 (PubMed:26389662). Interacts with UBXN6; the interaction with UBXN6 is direct and competitive with UFD1 (PubMed:19174149, PubMed:19275885). Forms a ternary complex with CAV1 and UBXN6 (PubMed:21822278, PubMed:18656546, PubMed:19174149). Interacts with PLAA, UBXN6 and YOD1; may form a complex involved in macroautophagy (PubMed:27753622). Interacts with ANKZF1 (PubMed:28302725). Interacts with ubiquitin-binding protein FAF1 (PubMed:26842564). Interacts with ZFAND2B (via VIM motif); the interaction is direct (PubMed:24160817, PubMed:26337389). Interacts with ZFAND1 (via its ubiquitin-like region); this interaction occurs in an arsenite-dependent manner (PubMed:29804830). Interacts with CCDC47 (By similarity). Interacts with UBAC2 (By similarity). Interacts with LMBR1L (PubMed:31073040). Interacts with ATXN3 (PubMed:30455355). Interacts with TEX264; bridging VCP to covalent DNA-protein cross-links (DPCs) (PubMed:32152270).   
  
 **Gene Ontology Information:**

Molecular Function

- ADP binding
- ATP binding
- ATPase activity
- BAT3 complex binding
- deubiquitinase activator activity
- identical protein binding
- K48-linked polyubiquitin modification-dependent protein binding
- lipid binding
- MHC class I protein binding
- polyubiquitin modification-dependent protein binding
- protein domain specific binding
- protein phosphatase binding
- RNA binding
- ubiquitin protein ligase binding
- ubiquitin-dependent protein binding
- ubiquitin-like protein ligase binding
- ubiquitin-specific protease binding

Location

- ATPase complex
- azurophil granule lumen
- cytoplasm
- cytoplasmic stress granule
- cytosol
- Derlin-1 retrotranslocation complex
- endoplasmic reticulum
- endoplasmic reticulum membrane
- extracellular exosome
- extracellular region
- ficolin-1-rich granule lumen
- glutamatergic synapse
- intracellular membrane-bounded organelle
- lipid droplet
- nucleoplasm
- nucleus
- perinuclear region of cytoplasm
- proteasome complex
- protein-containing complex
- secretory granule lumen
- site of double-strand break
- VCP-NPL4-UFD1 AAA ATPase complex
- VCP-NSFL1C complex

Biological process

- activation of cysteine-type endopeptidase activity involved in apoptotic process
- aggresome assembly
- ATP metabolic process
- autophagosome maturation
- autophagy
- cellular response to arsenite ion
- cellular response to heat
- cellular response to DNA damage stimulus
- DNA repair
- double-strand break repair
- endoplasmic reticulum stress-induced pre-emptive quality control
- endoplasmic reticulum to Golgi vesicle-mediated transport
- endoplasmic reticulum unfolded protein response
- endosome to lysosome transport via multivesicular body sorting pathway
- ER-associated misfolded protein catabolic process
- ERAD pathway
- establishment of protein localization
- flavin adenine dinucleotide catabolic process
- interstrand cross-link repair
- macroautophagy
- mitotic spindle disassembly
- NADH metabolic process
- negative regulation of protein localization to chromatin
- negative regulation of smoothened signaling pathway
- positive regulation of ATP biosynthetic process
- positive regulation of canonical Wnt signaling pathway
- positive regulation of Lys63-specific deubiquitinase activity
- positive regulation of mitochondrial membrane potential
- positive regulation of oxidative phosphorylation
- positive regulation of proteasomal ubiquitin-dependent protein catabolic process
- positive regulation of protein catabolic process
- positive regulation of protein K63-linked deubiquitination
- positive regulation of protein-containing complex assembly
- proteasomal protein catabolic process
- proteasome-mediated ubiquitin-dependent protein catabolic process
- protein ubiquitination
- protein-DNA covalent cross-linking repair
- regulation of aerobic respiration
- regulation of apoptotic process
- regulation of protein localization to chromatin
- regulation of synapse organization
- retrograde protein transport, ER to cytosol
- stress granule disassembly
- translesion synthesis
- ubiquitin-dependent ERAD pathway
- viral genome replication

---

9

- **Protein name:** Toll-like receptor 8
- **Organism:** Homo sapiens
- **Uniprot Accession Number:** Q9NR97
- **Protein sequence length:** 1041 aa
- **1D identity (%):** 14.15
- **1D identity (%) [Gaps excluded]:** 23.94
- **1D identity - Alignment Gaps:** 488
- **1D aligned content (<aminoacid>:%):** {'L': 13.61, 'Q': 5.33, 'E': 4.73, 'Y': 4.14, 'D': 5.92, 'K': 6.51, 'C': 2.37, 'P': 8.28, 'V': 3.55, 'H': 3.55, 'F': 7.1, 'G': 7.69, 'N': 6.51, 'I': 5.33, 'S': 4.14, 'A': 2.96, 'T': 4.73, 'R': 2.37, 'M': 0.59, 'W': 0.59}
- **Common reported functions (%):** 20.0
- **Common reported locations (%):** 0.0
- **Common reported processes (%):** 0.0

- **PDB ID:** 4R08
- **Chain:** D
- **Crystallized protein length:** 743 aa
- **Resolution:** 2.4 Å
- **b-phipsi:** 0.007914
- **w-rdist:** 0.359604
- **t-alpha:** 0.001457
- **Chemical similarity (Tanimoto Index) (%):** 71.63
- **1D identity (%) [PDB]:** 2.55
- **1D identity (%) [Gaps excluded][PDB]:** 69.64
- **1D identity - Alignment Gaps [PDB]:** 1472
- **1D aligned content [PDB] (<aminoacid>:%):** {'R': 5.13, 'P': 10.26, 'Q': 10.26, 'L': 12.82, 'N': 7.69, 'K': 7.69, 'I': 7.69, 'G': 12.82, 'V': 7.69, 'F': 2.56, 'A': 5.13, 'T': 2.56, 'H': 2.56, 'D': 2.56, 'S': 2.56}
- **2D identity (%) [PDB]:** 29.87
- **2D identity (%) [Gaps excluded][PDB]:** 77.9
- **2D identity - Alignment Gaps [PDB]:** 706
- **2D aligned content [PDB] (<2D-fold>:%):** {'.': 28.95, 'E': 26.02, 'T': 31.29, 'H': 9.94, 'G': 3.51, 'B': 0.29}
- **3D similarity (TM-Score) (%) [PDB]:** 23.04

- **Gene name:** TLR8
- **Entrez ID:** 51311
- **RefSeq ID:** N/A
- **Sequence length:** N/A
- **5-UTR|CDS|3-UTR identity (%):** N/A | N/A | N/A
- **5-UTR|CDS|3-UTR identity (%) [Gaps excluded]:** N/A | N/A | N/A
- **5-UTR|CDS|3-UTR identity [Alignment Gaps]:** N/A | N/A | N/A
- **5-UTR aligned content (<base>:%):** N/A
- **CDS aligned content (<base>:%):** N/A
- **3-UTR aligned content (<base>:%):** N/A

**Uniprot Description:**  
  
 Endosomal receptor that plays a key role in innate and adaptive immunity (PubMed:25297876, PubMed:32433612). Controls host immune response against pathogens through recognition of RNA degradation products specific to microorganisms that are initially processed by RNASET2 (PubMed:31778653). Upon binding to agonists, undergoes dimerization that brings TIR domains from the two molecules into direct contact, leading to the recruitment of TIR-containing downstream adapter MYD88 through homotypic interaction (PubMed:23520111, PubMed:25599397, PubMed:26929371). In turn, the Myddosome signaling complex is formed involving IRAK4, IRAK1, TRAF6, TRAF3 leading to activation of downstream transcription factors NF-kappa-B and IRF7 to induce proinflammatory cytokines and interferons, respectively (PubMed:16737960, PubMed:17932028, PubMed:29155428).   
  
Homodimer (PubMed:23520111, PubMed:25599397, PubMed:26929371, PubMed:29155428). Interacts with MYD88 via their respective TIR domains (Probable). Interacts with UNC93B1 (By similarity). Interacts with BTK (PubMed:17932028). Interacts with SMPDL3B (By similarity).   
  
 **Gene Ontology Information:**

Molecular Function

- DNA binding
- double-stranded RNA binding
- identical protein binding
- pattern recognition receptor activity
- RNA binding
- signaling receptor activity
- single-stranded RNA binding

Location

- endolysosome membrane
- endoplasmic reticulum membrane
- endosome membrane
- external side of plasma membrane
- Golgi membrane
- plasma membrane

Biological process

- cellular response to mechanical stimulus
- defense response to virus
- I-kappaB kinase/NF-kappaB signaling
- immunoglobulin mediated immune response
- inflammatory response
- innate immune response
- negative regulation of interleukin-12 production
- positive regulation of innate immune response
- positive regulation of interferon-alpha production
- positive regulation of interferon-beta production
- positive regulation of interleukin-1 beta production
- positive regulation of interleukin-6 production
- positive regulation of interleukin-8 production
- positive regulation of interferon-gamma production
- regulation of protein phosphorylation
- response to virus
- toll-like receptor 8 signaling pathway
- toll-like receptor signaling pathway

---

10

- **Protein name:** Endoplasmic reticulum aminopeptidase 1
- **Organism:** Homo sapiens
- **Uniprot Accession Number:** Q9NZ08
- **Protein sequence length:** 941 aa
- **1D identity (%):** 15.27
- **1D identity (%) [Gaps excluded]:** 21.28
- **1D identity - Alignment Gaps:** 296
- **1D aligned content (<aminoacid>:%):** {'F': 5.0, 'P': 9.38, 'T': 11.88, 'N': 4.38, 'E': 5.0, 'H': 2.5, 'I': 5.62, 'L': 11.25, 'G': 9.38, 'K': 4.38, 'V': 6.25, 'S': 5.0, 'R': 3.12, 'A': 3.75, 'Y': 3.75, 'Q': 3.12, 'D': 3.12, 'C': 2.5, 'M': 0.62}
- **Common reported functions (%):** 0.0
- **Common reported locations (%):** 28.57
- **Common reported processes (%):** 4.76

- **PDB ID:** 6MGQ
- **Chain:** A
- **Crystallized protein length:** 861 aa
- **Resolution:** 2.92 Å
- **b-phipsi:** 0.006574
- **w-rdist:** 0.080563
- **t-alpha:** 0.009559
- **Chemical similarity (Tanimoto Index) (%):** N/A
- **1D identity (%) [PDB]:** 2.5
- **1D identity (%) [Gaps excluded][PDB]:** 68.33
- **1D identity - Alignment Gaps [PDB]:** 1582
- **1D aligned content [PDB] (<aminoacid>:%):** {'A': 7.32, 'T': 7.32, 'R': 7.32, 'V': 4.88, 'H': 4.88, 'Q': 4.88, 'E': 12.2, 'I': 9.76, 'L': 12.2, 'F': 9.76, 'Y': 4.88, 'K': 2.44, 'S': 4.88, 'P': 2.44, 'D': 2.44, 'G': 2.44}
- **2D identity (%) [PDB]:** 34.59
- **2D identity (%) [Gaps excluded][PDB]:** 88.31
- **2D identity - Alignment Gaps [PDB]:** 744
- **2D aligned content [PDB] (<2D-fold>:%):** {'.': 13.71, 'E': 27.9, 'T': 10.64, 'H': 47.04, 'G': 0.71}
- **3D similarity (TM-Score) (%) [PDB]:** 25.39

- **Gene name:** ERAP1
- **Entrez ID:** 51752
- **RefSeq ID:** N/A
- **Sequence length:** N/A
- **5-UTR|CDS|3-UTR identity (%):** N/A | N/A | N/A
- **5-UTR|CDS|3-UTR identity (%) [Gaps excluded]:** N/A | N/A | N/A
- **5-UTR|CDS|3-UTR identity [Alignment Gaps]:** N/A | N/A | N/A
- **5-UTR aligned content (<base>:%):** N/A
- **CDS aligned content (<base>:%):** N/A
- **3-UTR aligned content (<base>:%):** N/A

**Uniprot Description:**  
  
 Aminopeptidase that plays a central role in peptide trimming, a step required for the generation of most HLA class I-binding peptides. Peptide trimming is essential to customize longer precursor peptides to fit them to the correct length required for presentation on MHC class I molecules. Strongly prefers substrates 9-16 residues long. Rapidly degrades 13-mer to a 9-mer and then stops. Preferentially hydrolyzes the residue Leu and peptides with a hydrophobic C-terminus, while it has weak activity toward peptides with charged C-terminus. May play a role in the inactivation of peptide hormones. May be involved in the regulation of blood pressure through the inactivation of angiotensin II and/or the generation of bradykinin in the kidney.   
  
Monomer. May also exist as a heterodimer; with ERAP2. Interacts with RBMX.   
  
 **Gene Ontology Information:**

Molecular Function

- aminopeptidase activity
- endopeptidase activity
- interleukin-1, type II receptor binding
- interleukin-6 receptor binding
- metalloaminopeptidase activity
- metalloexopeptidase activity
- peptide binding
- zinc ion binding

Location

- cytoplasm
- cytosol
- endoplasmic reticulum
- endoplasmic reticulum lumen
- endoplasmic reticulum membrane
- extracellular exosome
- extracellular region
- extracellular space
- membrane

Biological process

- adaptive immune response
- angiogenesis
- antigen processing and presentation of endogenous peptide antigen via MHC class I
- antigen processing and presentation of peptide antigen via MHC class I
- fat cell differentiation
- membrane protein ectodomain proteolysis
- peptide catabolic process
- positive regulation of angiogenesis
- proteolysis
- regulation of blood pressure
- regulation of innate immune response
- response to bacterium

---

11

- **Protein name:** Xanthine dehydrogenase/oxidase
- **Organism:** Homo sapiens
- **Uniprot Accession Number:** P47989
- **Protein sequence length:** 1333 aa
- **1D identity (%):** 14.43
- **1D identity (%) [Gaps excluded]:** 23.82
- **1D identity - Alignment Gaps:** 538
- **1D aligned content (<aminoacid>:%):** {'M': 0.51, 'A': 7.61, 'L': 8.12, 'P': 9.14, 'Q': 2.03, 'K': 6.09, 'F': 7.11, 'T': 10.15, 'G': 10.15, 'N': 3.55, 'Y': 4.06, 'C': 2.54, 'V': 5.58, 'E': 4.57, 'W': 0.51, 'D': 4.57, 'R': 4.06, 'S': 2.54, 'I': 3.55, 'H': 3.55}
- **Common reported functions (%):** 0.0
- **Common reported locations (%):** 7.14
- **Common reported processes (%):** 0.0

- **PDB ID:** 2E1Q
- **Chain:** A
- **Crystallized protein length:** 1307 aa
- **Resolution:** 2.6 Å
- **b-phipsi:** 0.004788
- **w-rdist:** 0.268808
- **t-alpha:** 0.02622
- **Chemical similarity (Tanimoto Index) (%):** 65.62
- **1D identity (%) [PDB]:** 3.63
- **1D identity (%) [Gaps excluded][PDB]:** 68.52
- **1D identity - Alignment Gaps [PDB]:** 1930
- **1D aligned content [PDB] (<aminoacid>:%):** {'L': 9.46, 'V': 17.57, 'I': 2.7, 'G': 8.11, 'K': 5.41, 'T': 10.81, 'A': 9.46, 'E': 1.35, 'R': 6.76, 'M': 4.05, 'Q': 4.05, 'N': 4.05, 'S': 2.7, 'P': 5.41, 'F': 2.7, 'D': 2.7, 'H': 1.35, 'C': 1.35}
- **2D identity (%) [PDB]:** 37.63
- **2D identity (%) [Gaps excluded][PDB]:** 87.17
- **2D identity - Alignment Gaps [PDB]:** 852
- **2D aligned content [PDB] (<2D-fold>:%):** {'.': 15.43, 'E': 32.8, 'T': 12.41, 'H': 38.48, 'B': 0.89}
- **3D similarity (TM-Score) (%) [PDB]:** 30.1

- **Gene name:** XDH
- **Entrez ID:** 7498
- **RefSeq ID:** NM\_000379
- **Transcript sequence length:** 5715
- **5-UTR|CDS|3-UTR identity (%):** 24.0 | 41.79 | 8.15
- **5-UTR|CDS|3-UTR identity (%) [Gaps excluded]:** 67.92 | 76.77 | 74.27
- **5-UTR|CDS|3-UTR identity [Alignment Gaps]:** 97 | 1942 | 10851
- **5-UTR aligned content (<base>:%):** {'C': 33.33, 'G': 50.0, 'A': 2.78, 'T': 13.89}
- **CDS aligned content (<base>:%):** {'A': 23.98, 'T': 18.75, 'G': 28.47, 'C': 28.8}
- **3-UTR aligned content (<base>:%):** {'A': 32.33, 'G': 18.03, 'C': 16.62, 'T': 33.03}

**Uniprot Description:**  
  
 Key enzyme in purine degradation. Catalyzes the oxidation of hypoxanthine to xanthine. Catalyzes the oxidation of xanthine to uric acid. Contributes to the generation of reactive oxygen species. Has also low oxidase activity towards aldehydes (in vitro).   
  
Homodimer. Interacts with BTN1A1 (By similarity).   
  
 **Gene Ontology Information:**

Molecular Function

- 2 iron, 2 sulfur cluster binding
- FAD binding
- flavin adenine dinucleotide binding
- hypoxanthine dehydrogenase activity
- hypoxanthine oxidase activity
- iron ion binding
- molybdopterin cofactor binding
- protein homodimerization activity
- xanthine dehydrogenase activity
- xanthine oxidase activity

Location

- cytosol
- extracellular space
- peroxisome
- sarcoplasmic reticulum

Biological process

- activation of cysteine-type endopeptidase activity involved in apoptotic process
- adenosine catabolic process
- allantoin metabolic process
- cellular amide catabolic process
- AMP catabolic process
- dAMP catabolic process
- deoxyadenosine catabolic process
- deoxyguanosine catabolic process
- deoxyinosine catabolic process
- dGMP catabolic process
- GMP catabolic process
- guanine catabolic process
- hypoxanthine catabolic process
- IMP catabolic process
- inosine catabolic process
- iron-sulfur cluster assembly
- lactation
- negative regulation of endothelial cell differentiation
- negative regulation of endothelial cell proliferation
- negative regulation of gene expression
- negative regulation of protein kinase B signaling
- negative regulation of protein phosphorylation
- negative regulation of vascular endothelial growth factor signaling pathway
- negative regulation of vasculogenesis
- positive regulation of p38MAPK cascade
- positive regulation of reactive oxygen species metabolic process
- xanthine catabolic process

---

12

- **Protein name:** Ubiquitin carboxyl-terminal hydrolase 7
- **Organism:** Homo sapiens
- **Uniprot Accession Number:** Q93009
- **Protein sequence length:** 1102 aa
- **1D identity (%):** 16.15
- **1D identity (%) [Gaps excluded]:** 22.1
- **1D identity - Alignment Gaps:** 305
- **1D aligned content (<aminoacid>:%):** {'M': 1.64, 'A': 4.37, 'G': 6.56, 'L': 8.74, 'P': 10.93, 'T': 5.46, 'S': 6.56, 'R': 5.46, 'E': 4.37, 'V': 7.65, 'Q': 4.92, 'H': 4.37, 'D': 6.01, 'F': 4.92, 'I': 3.83, 'K': 7.65, 'C': 2.73, 'Y': 2.19, 'N': 1.64}
- **Common reported functions (%):** 0.0
- **Common reported locations (%):** 21.43
- **Common reported processes (%):** 0.0

- **PDB ID:** 5JTV
- **Chain:** E
- **Crystallized protein length:** 565 aa
- **Resolution:** 3.31 Å
- **b-phipsi:** 0.002134
- **w-rdist:** 0.617131
- **t-alpha:** 0.003642
- **Chemical similarity (Tanimoto Index) (%):** N/A
- **1D identity (%) [PDB]:** 2.73
- **1D identity (%) [Gaps excluded][PDB]:** 74.0
- **1D identity - Alignment Gaps [PDB]:** 1304
- **1D aligned content [PDB] (<aminoacid>:%):** {'K': 2.7, 'T': 8.11, 'P': 8.11, 'E': 2.7, 'D': 10.81, 'F': 5.41, 'Y': 5.41, 'L': 13.51, 'C': 5.41, 'V': 8.11, 'N': 2.7, 'R': 8.11, 'Q': 2.7, 'I': 5.41, 'H': 2.7, 'S': 5.41, 'A': 2.7}
- **2D identity (%) [PDB]:** 34.43
- **2D identity (%) [Gaps excluded][PDB]:** 84.94
- **2D identity - Alignment Gaps [PDB]:** 594
- **2D aligned content [PDB] (<2D-fold>:%):** {'T': 16.57, '.': 17.44, 'H': 29.07, 'E': 33.72, 'B': 0.58, 'G': 2.62}
- **3D similarity (TM-Score) (%) [PDB]:** 19.1

- **Gene name:** USP7
- **Entrez ID:** 7874
- **RefSeq ID:** NM\_003470
- **Transcript sequence length:** 5831
- **5-UTR|CDS|3-UTR identity (%):** 18.72 | 42.86 | 9.79
- **5-UTR|CDS|3-UTR identity (%) [Gaps excluded]:** 95.12 | 74.21 | 73.52
- **5-UTR|CDS|3-UTR identity [Alignment Gaps]:** 502 | 1577 | 10549
- **5-UTR aligned content (<base>:%):** {'G': 46.15, 'T': 6.84, 'C': 43.59, 'A': 3.42}
- **CDS aligned content (<base>:%):** {'T': 19.19, 'G': 27.94, 'A': 28.5, 'C': 24.38}
- **3-UTR aligned content (<base>:%):** {'T': 35.94, 'C': 18.64, 'A': 21.24, 'G': 24.18}

**Uniprot Description:**  
  
 Hydrolase that deubiquitinates target proteins such as FOXO4, p53/TP53, MDM2, ERCC6, DNMT1, UHRF1, PTEN, KMT2E/MLL5 and DAXX (PubMed:11923872, PubMed:15053880, PubMed:16964248, PubMed:18716620, PubMed:25283148, PubMed:26678539, PubMed:28655758). Together with DAXX, prevents MDM2 self-ubiquitination and enhances the E3 ligase activity of MDM2 towards p53/TP53, thereby promoting p53/TP53 ubiquitination and proteasomal degradation (PubMed:15053880, PubMed:16845383, PubMed:18566590, PubMed:20153724). Deubiquitinates p53/TP53, preventing degradation of p53/TP53, and enhances p53/TP53-dependent transcription regulation, cell growth repression and apoptosis (PubMed:25283148). Deubiquitinates p53/TP53 and MDM2 and strongly stabilizes p53/TP53 even in the presence of excess MDM2, and also induces p53/TP53-dependent cell growth repression and apoptosis (PubMed:11923872). Deubiquitination of FOXO4 in presence of hydrogen peroxide is not dependent on p53/TP53 and inhibits FOXO4-induced transcriptional activity (PubMed:16964248). In association with DAXX, is involved in the deubiquitination and translocation of PTEN from the nucleus to the cytoplasm, both processes that are counteracted by PML (PubMed:18716620). Deubiquitinates KMT2E/MLL5 preventing KMT2E/MLL5 proteasomal-mediated degradation (PubMed:26678539). Involved in cell proliferation during early embryonic development. Involved in transcription-coupled nucleotide excision repair (TC-NER) in response to UV damage: recruited to DNA damage sites following interaction with KIAA1530/UVSSA and promotes deubiquitination of ERCC6, preventing UV-induced degradation of ERCC6 (PubMed:22466611, PubMed:22466612). Involved in maintenance of DNA methylation via its interaction with UHRF1 and DNMT1: acts by mediating deubiquitination of UHRF1 and DNMT1, preventing their degradation and promoting DNA methylation by DNMT1 (PubMed:21745816, PubMed:22411829). Deubiquitinates alkylation repair enzyme ALKBH3. OTUD4 recruits USP7 and USP9X to stabilize ALKBH3, thereby promoting the repair of alkylated DNA lesions (PubMed:25944111). Acts as a chromatin regulator via its association with the Polycomb group (PcG) multiprotein PRC1-like complex; may act by deubiquitinating components of the PRC1-like complex (PubMed:20601937). Able to mediate deubiquitination of histone H2B; it is however unsure whether this activity takes place in vivo (PubMed:20601937). Exhibits a preference towards 'Lys-48'-linked ubiquitin chains (PubMed:22689415). Increases regulatory T-cells (Treg) suppressive capacity by deubiquitinating and stabilizing the transcription factor FOXP3 which is crucial for Treg cell function (PubMed:23973222). Plays a role in the maintenance of the circadian clock periodicity via deubiquitination and stabilization of the CRY1 and CRY2 proteins (PubMed:27123980). Deubiquitinates REST, thereby stabilizing REST and promoting the maintenance of neural progenitor cells (PubMed:21258371). Deubiquitinates SIRT7, inhibiting SIRT7 histone deacetylase activity and regulating gluconeogenesis (PubMed:28655758).   
  
Monomer. Homodimer. Part of a complex with DAXX, MDM2, RASSF1 and USP7 (PubMed:18566590). Part of a complex with DAXX, MDM2 and USP7 (PubMed:16845383). Interacts with MDM2; the interaction is independent of p53/TP53. Interacts with DAXX; the interaction is direct and independent of MDM2 and p53/TP53 (PubMed:16845383). Component of a complex composed of KMT2E/MLL5 (isoform 3), OGT (isoform 1) and USP7; the complex stabilizes KMT2E/MLL5, preventing KMT2E/MLL5 ubiquitination and proteosomal-mediated degradation (PubMed:26678539). Interacts (via MATH domain) with KMT2E/MLL5 isoform 3 (PubMed:26678539). Interacts with OGT isoform 1 (PubMed:26678539). Interacts with FOXO4; the interaction is enhanced in presence of hydrogen peroxide and occurs independently of p53/TP53 (PubMed:16964248). Interacts with p53/TP53; the interaction is enhanced in response to DNA damage (PubMed:25283148). Interacts with TSPYL5; this impairs interaction with p53/TP53 (PubMed:21170034). Interacts with PTEN; the interaction is direct (PubMed:18716620). Interacts with ATXN1 and the strength of interaction is influenced by the length of the poly-Gln region in ATXN1 (PubMed:12093161). A weaker interaction seen with mutants having longer poly-Gln regions (PubMed:12093161). Interacts with KIAA1530/UVSSA (PubMed:22466611, PubMed:22466612). Interacts with ABRAXAS2; the interaction is direct (PubMed:25283148). Identified in a complex with TP53/p53 and ABRAXAS2 (PubMed:25283148). Interacts with MEX3C and antagonizes its ability to degrade mRNA (PubMed:22863774). Interacts with DNMT1 and UHRF1 (PubMed:21745816, PubMed:22411829). Interacts with FOXP3 (PubMed:23973222). Interacts (via MATH domain) with RNF220. Associated component of the Polycomb group (PcG) multiprotein PRC1-like complex (PubMed:20601937). Interacts with EPOP (By similarity). Interacts with OTUD4 and USP9X; the interaction is direct (PubMed:25944111). Interacts with CRY2 (PubMed:27123980). Interacts with REST (PubMed:21258371). Interacts with ERCC6 (PubMed:26030138).   
  
 **Gene Ontology Information:**

Molecular Function

- thiol-dependent ubiquitin-specific protease activity
- cysteine-type endopeptidase activity
- ubiquitinyl hydrolase activity
- Lys48-specific deubiquitinase activity
- p53 binding

Location

- chromosome
- cytosol
- nuclear body
- nucleoplasm
- nucleus
- PML body
- protein-containing complex

Biological process

- symbiont-mediated disruption of host cell PML body
- monoubiquitinated protein deubiquitination
- hypermethylation of CpG island
- negative regulation of gluconeogenesis
- negative regulation of NF-kappaB transcription factor activity
- negative regulation of proteasomal ubiquitin-dependent protein catabolic process
- positive regulation of DNA demethylation
- protein deubiquitination
- protein stabilization
- protein ubiquitination
- regulation of circadian rhythm
- regulation of DNA-binding transcription factor activity
- regulation of protein stability
- regulation of retrograde transport, endosome to Golgi
- regulation of signal transduction by p53 class mediator
- regulation of telomere capping
- rhythmic process
- transcription-coupled nucleotide-excision repair
- ubiquitin-dependent protein catabolic process

---

13

- **Protein name:** Phosphatidylinositol 4,5-bisphosphate 3-kinase catalytic subunit delta isoform
- **Organism:** Homo sapiens
- **Uniprot Accession Number:** O00329
- **Protein sequence length:** 1044 aa
- **1D identity (%):** 15.86
- **1D identity (%) [Gaps excluded]:** 23.44
- **1D identity - Alignment Gaps:** 367
- **1D aligned content (<aminoacid>:%):** {'P': 8.89, 'D': 6.67, 'F': 7.78, 'T': 3.89, 'Y': 2.22, 'L': 13.89, 'R': 7.78, 'E': 5.0, 'H': 3.89, 'M': 1.67, 'G': 8.89, 'K': 4.44, 'S': 5.0, 'N': 1.67, 'A': 2.22, 'C': 3.33, 'I': 3.33, 'Q': 3.89, 'V': 4.44, 'W': 1.11}
- **Common reported functions (%):** 0.0
- **Common reported locations (%):** 14.29
- **Common reported processes (%):** 4.76

- **PDB ID:** 6G6W
- **Chain:** A
- **Crystallized protein length:** 925 aa
- **Resolution:** 2.72 Å
- **b-phipsi:** 0.010914
- **w-rdist:** 0.311374
- **t-alpha:** 0.002185
- **Chemical similarity (Tanimoto Index) (%):** 78.1
- **1D identity (%) [PDB]:** 2.69
- **1D identity (%) [Gaps excluded][PDB]:** 73.02
- **1D identity - Alignment Gaps [PDB]:** 1645
- **1D aligned content [PDB] (<aminoacid>:%):** {'Q': 8.7, 'V': 19.57, 'L': 17.39, 'P': 6.52, 'T': 6.52, 'Y': 2.17, 'A': 4.35, 'E': 2.17, 'R': 2.17, 'G': 6.52, 'D': 2.17, 'M': 4.35, 'C': 4.35, 'K': 2.17, 'S': 2.17, 'I': 4.35, 'N': 4.35}
- **2D identity (%) [PDB]:** 41.2
- **2D identity (%) [Gaps excluded][PDB]:** 82.68
- **2D identity - Alignment Gaps [PDB]:** 593
- **2D aligned content [PDB] (<2D-fold>:%):** {'.': 15.2, 'E': 26.28, 'T': 12.32, 'H': 44.35, 'G': 1.85}
- **3D similarity (TM-Score) (%) [PDB]:** 21.3

- **Gene name:** PIK3CD
- **Entrez ID:** 5293
- **RefSeq ID:** N/A
- **Sequence length:** N/A
- **5-UTR|CDS|3-UTR identity (%):** N/A | N/A | N/A
- **5-UTR|CDS|3-UTR identity (%) [Gaps excluded]:** N/A | N/A | N/A
- **5-UTR|CDS|3-UTR identity [Alignment Gaps]:** N/A | N/A | N/A
- **5-UTR aligned content (<base>:%):** N/A
- **CDS aligned content (<base>:%):** N/A
- **3-UTR aligned content (<base>:%):** N/A

**Uniprot Description:**  
  
 Phosphoinositide-3-kinase (PI3K) phosphorylates phosphatidylinositol (PI) and its phosphorylated derivatives at position 3 of the inositol ring to produce 3-phosphoinositides (PubMed:9235916). Uses ATP and PtdIns(4,5)P2 (phosphatidylinositol 4,5-bisphosphate) to generate phosphatidylinositol 3,4,5-trisphosphate (PIP3) (PubMed:15135396). PIP3 plays a key role by recruiting PH domain-containing proteins to the membrane, including AKT1 and PDPK1, activating signaling cascades involved in cell growth, survival, proliferation, motility and morphology. Mediates immune responses. Plays a role in B-cell development, proliferation, migration, and function. Required for B-cell receptor (BCR) signaling. Mediates B-cell proliferation response to anti-IgM, anti-CD40 and IL4 stimulation. Promotes cytokine production in response to TLR4 and TLR9. Required for antibody class switch mediated by TLR9. Involved in the antigen presentation function of B-cells. Involved in B-cell chemotaxis in response to CXCL13 and sphingosine 1-phosphate (S1P). Required for proliferation, signaling and cytokine production of naive, effector and memory T-cells. Required for T-cell receptor (TCR) signaling. Mediates TCR signaling events at the immune synapse. Activation by TCR leads to antigen-dependent memory T-cell migration and retention to antigenic tissues. Together with PIK3CG participates in T-cell development. Contributes to T-helper cell expansion and differentiation. Required for T-cell migration mediated by homing receptors SELL/CD62L, CCR7 and S1PR1 and antigen dependent recruitment of T-cells. Together with PIK3CG is involved in natural killer (NK) cell development and migration towards the sites of inflammation. Participates in NK cell receptor activation. Plays a role in NK cell maturation and cytokine production. Together with PIK3CG is involved in neutrophil chemotaxis and extravasation. Together with PIK3CG participates in neutrophil respiratory burst. Plays important roles in mast-cell development and mast cell mediated allergic response. Involved in stem cell factor (SCF)-mediated proliferation, adhesion and migration. Required for allergen-IgE-induced degranulation and cytokine release. The lipid kinase activity is required for its biological function. Isoform 2 may be involved in stabilizing total RAS levels, resulting in increased ERK phosphorylation and increased PI3K activity.   
  
Heterodimer of a catalytic subunit PIK3CD and a p85 regulatory subunit (PIK3R1, PIK3R2 or PIK3R3). Interacts with ERAS (By similarity). Interacts with HRAS.   
  
 **Gene Ontology Information:**

Molecular Function

- phosphatidylinositol-3,4-bisphosphate 5-kinase activity
- 1-phosphatidylinositol-3-kinase activity
- phosphatidylinositol-4,5-bisphosphate 3-kinase activity
- 1-phosphatidylinositol-4-phosphate 3-kinase activity
- ATP binding
- kinase activity

Location

- cytoplasm
- cytosol
- phosphatidylinositol 3-kinase complex
- phosphatidylinositol 3-kinase complex, class IA
- plasma membrane

Biological process

- adaptive immune response
- B cell activation
- B cell chemotaxis
- B cell differentiation
- B cell receptor signaling pathway
- immune response
- inflammatory response
- innate immune response
- mast cell chemotaxis
- mast cell degranulation
- mast cell differentiation
- natural killer cell activation
- natural killer cell chemotaxis
- natural killer cell differentiation
- neutrophil chemotaxis
- neutrophil extravasation
- phosphatidylinositol 3-kinase signaling
- phosphatidylinositol-3-phosphate biosynthetic process
- phosphorylation
- positive regulation of angiogenesis
- positive regulation of cell migration
- positive regulation of cell migration by vascular endothelial growth factor signaling pathway
- positive regulation of cytokine production
- positive regulation of endothelial cell migration
- positive regulation of endothelial cell proliferation
- positive regulation of epithelial tube formation
- positive regulation of gene expression
- positive regulation of neutrophil apoptotic process
- positive regulation of protein kinase B signaling
- protein phosphorylation
- respiratory burst involved in defense response
- signal transduction
- T cell activation
- T cell chemotaxis
- T cell differentiation
- T cell receptor signaling pathway

---

14

- **Protein name:** Ubiquitin-like modifier-activating enzyme 6
- **Organism:** Homo sapiens
- **Uniprot Accession Number:** A0AVT1
- **Protein sequence length:** 1052 aa
- **1D identity (%):** 17.03
- **1D identity (%) [Gaps excluded]:** 23.6
- **1D identity - Alignment Gaps:** 309
- **1D aligned content (<aminoacid>:%):** {'P': 8.47, 'G': 5.29, 'T': 11.11, 'K': 9.52, 'E': 6.88, 'D': 7.94, 'Y': 3.17, 'L': 8.47, 'I': 5.29, 'V': 4.76, 'F': 8.99, 'R': 3.7, 'N': 2.12, 'S': 2.12, 'Q': 3.17, 'A': 2.65, 'C': 3.7, 'M': 0.53, 'H': 1.59, 'W': 0.53}
- **Common reported functions (%):** 0.0
- **Common reported locations (%):** 14.29
- **Common reported processes (%):** 0.0

- **PDB ID:** 7SOL
- **Chain:** C
- **Crystallized protein length:** 970 aa
- **Resolution:** 2.25 Å
- **b-phipsi:** 0.003149
- **w-rdist:** 0.196677
- **t-alpha:** 0.064821
- **Chemical similarity (Tanimoto Index) (%):** 99.55
- **1D identity (%) [PDB]:** 2.43
- **1D identity (%) [Gaps excluded][PDB]:** 68.85
- **1D identity - Alignment Gaps [PDB]:** 1668
- **1D aligned content [PDB] (<aminoacid>:%):** {'K': 7.14, 'N': 2.38, 'L': 19.05, 'T': 4.76, 'M': 2.38, 'P': 7.14, 'I': 9.52, 'G': 4.76, 'D': 7.14, 'E': 9.52, 'R': 2.38, 'F': 4.76, 'V': 4.76, 'W': 2.38, 'C': 2.38, 'Q': 2.38, 'H': 2.38, 'A': 2.38, 'S': 2.38}
- **2D identity (%) [PDB]:** 38.06
- **2D identity (%) [Gaps excluded][PDB]:** 89.51
- **2D identity - Alignment Gaps [PDB]:** 722
- **2D aligned content [PDB] (<2D-fold>:%):** {'.': 14.85, 'E': 23.85, 'T': 22.18, 'H': 36.4, 'G': 2.51, 'B': 0.21}
- **3D similarity (TM-Score) (%) [PDB]:** 21.27

- **Gene name:** UBA6
- **Entrez ID:** 55236
- **RefSeq ID:** NM\_018227
- **Transcript sequence length:** 9540
- **5-UTR|CDS|3-UTR identity (%):** 15.67 | 43.6 | 29.44
- **5-UTR|CDS|3-UTR identity (%) [Gaps excluded]:** 72.41 | 73.05 | 74.95
- **5-UTR|CDS|3-UTR identity [Alignment Gaps]:** 105 | 1449 | 7949
- **5-UTR aligned content (<base>:%):** {'A': 4.76, 'T': 9.52, 'C': 42.86, 'G': 42.86}
- **CDS aligned content (<base>:%):** {'A': 29.48, 'T': 22.53, 'G': 25.02, 'C': 22.97}
- **3-UTR aligned content (<base>:%):** {'T': 39.31, 'C': 15.44, 'A': 28.18, 'G': 17.07}

**Uniprot Description:**  
  
 Activates ubiquitin by first adenylating its C-terminal glycine residue with ATP, and thereafter linking this residue to the side chain of a cysteine residue in E1, yielding a ubiquitin-E1 thioester and free AMP. Specific for ubiquitin, does not activate ubiquitin-like peptides. Differs from UBE1 in its specificity for substrate E2 charging. Does not charge cell cycle E2s, such as CDC34. Essential for embryonic development. Required for UBD/FAT10 conjugation. Isoform 2 may play a key role in ubiquitin system and may influence spermatogenesis and male fertility.   
  
Forms a thioester with UBD in cells stimulated with tumor necrosis factor-alpha (TNFa) and interferon-gamma (IFNg) (PubMed:17889673, PubMed:25422469).   
  
 **Gene Ontology Information:**

Molecular Function

- ATP binding
- FAT10 activating enzyme activity
- nucleotidyltransferase activity
- thiosulfate sulfurtransferase activity
- ubiquitin activating enzyme activity

Location

- cytoplasm
- cytosol

Biological process

- amygdala development
- dendritic spine development
- hippocampus development
- learning
- locomotory behavior
- protein modification by small protein conjugation
- protein ubiquitination
- ubiquitin-dependent protein catabolic process

---

15

- **Protein name:** DNA topoisomerase 3-beta-1
- **Organism:** Homo sapiens
- **Uniprot Accession Number:** O95985
- **Protein sequence length:** 862 aa
- **1D identity (%):** 16.8
- **1D identity (%) [Gaps excluded]:** 22.65
- **1D identity - Alignment Gaps:** 255
- **1D aligned content (<aminoacid>:%):** {'T': 7.83, 'A': 4.82, 'I': 3.61, 'K': 6.63, 'L': 7.83, 'N': 1.2, 'H': 3.01, 'V': 5.42, 'G': 8.43, 'F': 6.63, 'D': 6.02, 'E': 9.64, 'P': 7.23, 'C': 4.22, 'M': 0.6, 'S': 1.2, 'Q': 4.82, 'R': 6.63, 'Y': 4.22}
- **Common reported functions (%):** 6.67
- **Common reported locations (%):** 7.14
- **Common reported processes (%):** 0.0

- **PDB ID:** 5GVC
- **Chain:** A
- **Crystallized protein length:** 612 aa
- **Resolution:** 2.44 Å
- **b-phipsi:** 0.00497
- **w-rdist:** 0.48229
- **t-alpha:** 0.004389
- **Chemical similarity (Tanimoto Index) (%):** 96.82
- **1D identity (%) [PDB]:** 2.72
- **1D identity (%) [Gaps excluded][PDB]:** 76.0
- **1D identity - Alignment Gaps [PDB]:** 1348
- **1D aligned content [PDB] (<aminoacid>:%):** {'D': 10.53, 'K': 7.89, 'V': 13.16, 'E': 10.53, 'L': 15.79, 'P': 5.26, 'G': 7.89, 'I': 5.26, 'F': 2.63, 'W': 2.63, 'Q': 2.63, 'A': 5.26, 'H': 2.63, 'R': 2.63, 'S': 2.63, 'T': 2.63}
- **2D identity (%) [PDB]:** 36.07
- **2D identity (%) [Gaps excluded][PDB]:** 86.82
- **2D identity - Alignment Gaps [PDB]:** 598
- **2D aligned content [PDB] (<2D-fold>:%):** {'E': 24.39, 'H': 50.68, 'T': 9.76, '.': 13.28, 'G': 1.63, 'B': 0.27}
- **3D similarity (TM-Score) (%) [PDB]:** 21.08

- **Gene name:** TOP3B
- **Entrez ID:** 8940
- **RefSeq ID:** N/A
- **Sequence length:** N/A
- **5-UTR|CDS|3-UTR identity (%):** N/A | N/A | N/A
- **5-UTR|CDS|3-UTR identity (%) [Gaps excluded]:** N/A | N/A | N/A
- **5-UTR|CDS|3-UTR identity [Alignment Gaps]:** N/A | N/A | N/A
- **5-UTR aligned content (<base>:%):** N/A
- **CDS aligned content (<base>:%):** N/A
- **3-UTR aligned content (<base>:%):** N/A

**Uniprot Description:**  
  
 Releases the supercoiling and torsional tension of DNA introduced during the DNA replication and transcription by transiently cleaving and rejoining one strand of the DNA duplex. Introduces a single-strand break via transesterification at a target site in duplex DNA. The scissile phosphodiester is attacked by the catalytic tyrosine of the enzyme, resulting in the formation of a DNA-(5'-phosphotyrosyl)-enzyme intermediate and the expulsion of a 3'-OH DNA strand. The free DNA strand than undergoes passage around the unbroken strand thus removing DNA supercoils. Finally, in the religation step, the DNA 3'-OH attacks the covalent intermediate to expel the active-site tyrosine and restore the DNA phosphodiester backbone (By similarity). Possesses negatively supercoiled DNA relaxing activity. N/A   
  
 **Gene Ontology Information:**

Molecular Function

- DNA binding
- DNA topoisomerase activity
- DNA topoisomerase type I (single strand cut, ATP-independent) activity
- RNA binding

Location

- condensed chromosome
- DNA topoisomerase III-beta-TDRD3 complex
- nucleus

Biological process

- chromosome segregation
- DNA topological change

---

16

- **Protein name:** Spliceosome-associated protein CWC15 homolog
- **Organism:** Homo sapiens
- **Uniprot Accession Number:** Q9P013
- **Protein sequence length:** 229 aa
- **1D identity (%):** 4.95
- **1D identity (%) [Gaps excluded]:** 22.11
- **1D identity - Alignment Gaps:** 690
- **1D aligned content (<aminoacid>:%):** {'T': 6.82, 'A': 9.09, 'P': 6.82, 'G': 9.09, 'R': 9.09, 'L': 4.55, 'K': 11.36, 'D': 6.82, 'E': 15.91, 'F': 4.55, 'Q': 6.82, 'N': 2.27, 'I': 2.27, 'S': 2.27, 'Y': 2.27}
- **Common reported functions (%):** 6.67
- **Common reported locations (%):** 14.29
- **Common reported processes (%):** 0.0

- **PDB ID:** 7ABI
- **Chain:** r
- **Crystallized protein length:** 895 aa
- **Resolution:** 8.0 Å
- **b-phipsi:** 0.010289
- **w-rdist:** 0.261249
- **t-alpha:** 0.005827
- **Chemical similarity (Tanimoto Index) (%):** N/A
- **1D identity (%) [PDB]:** 3.19
- **1D identity (%) [Gaps excluded][PDB]:** 72.6
- **1D identity - Alignment Gaps [PDB]:** 1587
- **1D aligned content [PDB] (<aminoacid>:%):** {'E': 9.43, 'M': 1.89, 'D': 11.32, 'I': 13.21, 'K': 13.21, 'L': 5.66, 'P': 5.66, 'V': 9.43, 'N': 3.77, 'R': 5.66, 'T': 5.66, 'Q': 1.89, 'F': 3.77, 'G': 5.66, 'A': 1.89, 'S': 1.89}
- **2D identity (%) [PDB]:** 32.3
- **2D identity (%) [Gaps excluded][PDB]:** 85.29
- **2D identity - Alignment Gaps [PDB]:** 781
- **2D aligned content [PDB] (<2D-fold>:%):** {'.': 23.89, 'B': 0.99, 'E': 21.18, 'T': 10.59, 'H': 41.87, 'G': 1.48}
- **3D similarity (TM-Score) (%) [PDB]:** 14.94

- **Gene name:** CWC15
- **Entrez ID:** 51503
- **RefSeq ID:** N/A
- **Sequence length:** N/A
- **5-UTR|CDS|3-UTR identity (%):** N/A | N/A | N/A
- **5-UTR|CDS|3-UTR identity (%) [Gaps excluded]:** N/A | N/A | N/A
- **5-UTR|CDS|3-UTR identity [Alignment Gaps]:** N/A | N/A | N/A
- **5-UTR aligned content (<base>:%):** N/A
- **CDS aligned content (<base>:%):** N/A
- **3-UTR aligned content (<base>:%):** N/A

**Uniprot Description:**  
  
 Involved in pre-mRNA splicing as component of the spliceosome (PubMed:28502770, PubMed:28076346). Component of the PRP19-CDC5L complex that forms an integral part of the spliceosome and is required for activating pre-mRNA splicing.   
  
Identified in the spliceosome C complex (PubMed:28502770, PubMed:28076346). Component of the PRP19-CDC5L splicing complex composed of a core complex comprising a homotetramer of PRPF19, CDC5L, PLRG1 and BCAS2, and at least three less stably associated proteins CTNNBL1, CWC15 and HSPA8 (PubMed:20176811). Interacts directly with CTNNBL1 in the complex (PubMed:20176811).   
  
 **Gene Ontology Information:**

Molecular Function

- RNA binding

Location

- catalytic step 2 spliceosome
- mitochondrion
- nuclear speck
- nucleoplasm
- nucleus
- Prp19 complex
- spliceosomal complex
- U2-type catalytic step 2 spliceosome

Biological process

- mRNA cis splicing, via spliceosome
- mRNA splicing, via spliceosome

---

17

- **Protein name:** Xylosyltransferase 1
- **Organism:** Homo sapiens
- **Uniprot Accession Number:** Q86Y38
- **Protein sequence length:** 959 aa
- **1D identity (%):** 14.56
- **1D identity (%) [Gaps excluded]:** 24.48
- **1D identity - Alignment Gaps:** 462
- **1D aligned content (<aminoacid>:%):** {'C': 4.22, 'R': 11.45, 'V': 6.02, 'T': 6.63, 'G': 11.45, 'P': 15.06, 'L': 6.02, 'A': 2.41, 'D': 6.02, 'H': 3.61, 'S': 3.61, 'F': 2.41, 'Q': 4.22, 'K': 4.82, 'E': 4.22, 'Y': 1.2, 'I': 3.01, 'M': 1.2, 'W': 1.2, 'N': 1.2}
- **Common reported functions (%):** 6.67
- **Common reported locations (%):** 0.0
- **Common reported processes (%):** 0.0

- **PDB ID:** 6EJA
- **Chain:** A
- **Crystallized protein length:** 696 aa
- **Resolution:** 1.94 Å
- **b-phipsi:** 0.001679
- **w-rdist:** 0.644525
- **t-alpha:** 0.005861
- **Chemical similarity (Tanimoto Index) (%):** 90.59
- **1D identity (%) [PDB]:** 2.36
- **1D identity (%) [Gaps excluded][PDB]:** 68.63
- **1D identity - Alignment Gaps [PDB]:** 1434
- **1D aligned content [PDB] (<aminoacid>:%):** {'P': 2.86, 'A': 14.29, 'Y': 8.57, 'H': 8.57, 'L': 5.71, 'V': 8.57, 'F': 2.86, 'R': 8.57, 'D': 8.57, 'E': 5.71, 'S': 8.57, 'T': 2.86, 'G': 5.71, 'K': 2.86, 'Q': 5.71}
- **2D identity (%) [PDB]:** 35.39
- **2D identity (%) [Gaps excluded][PDB]:** 80.77
- **2D identity - Alignment Gaps [PDB]:** 600
- **2D aligned content [PDB] (<2D-fold>:%):** {'H': 37.3, '.': 18.25, 'T': 14.81, 'E': 28.31, 'G': 0.79, 'B': 0.53}
- **3D similarity (TM-Score) (%) [PDB]:** 22.05

- **Gene name:** XYLT1
- **Entrez ID:** 64131
- **RefSeq ID:** NM\_022166
- **Transcript sequence length:** 9970
- **5-UTR|CDS|3-UTR identity (%):** 53.76 | 45.08 | 32.62
- **5-UTR|CDS|3-UTR identity (%) [Gaps excluded]:** 78.81 | 75.87 | 75.33
- **5-UTR|CDS|3-UTR identity [Alignment Gaps]:** 55 | 1390 | 7442
- **5-UTR aligned content (<base>:%):** {'C': 40.86, 'T': 4.3, 'G': 53.76, 'A': 1.08}
- **CDS aligned content (<base>:%):** {'A': 21.89, 'T': 15.22, 'G': 29.34, 'C': 33.55}
- **3-UTR aligned content (<base>:%):** {'C': 22.53, 'T': 31.73, 'G': 23.37, 'A': 22.37}

**Uniprot Description:**  
  
 Catalyzes the first step in the biosynthesis of chondroitin sulfate and dermatan sulfate proteoglycans, such as DCN. Transfers D-xylose from UDP-D-xylose to specific serine residues of the core protein (PubMed:15461586, PubMed:17189265, PubMed:24581741, PubMed:23982343). Required for normal embryonic and postnatal skeleton development, especially of the long bones (PubMed:24581741, PubMed:23982343). Required for normal maturation of chondrocytes during bone development, and normal onset of ossification (By similarity).   
  
Monomer.   
  
 **Gene Ontology Information:**

Molecular Function

- metal ion binding
- protein xylosyltransferase activity

Location

- extracellular space
- Golgi cis cisterna
- Golgi membrane

Biological process

- chondroitin sulfate biosynthetic process
- chondroitin sulfate proteoglycan biosynthetic process
- embryonic skeletal system development
- glycosaminoglycan biosynthetic process
- glycosaminoglycan metabolic process
- heparan sulfate proteoglycan biosynthetic process
- ossification involved in bone maturation
- proteoglycan biosynthetic process

---

18

- **Protein name:** Lysine-specific histone demethylase 1B
- **Organism:** Homo sapiens
- **Uniprot Accession Number:** Q8NB78
- **Protein sequence length:** 822 aa
- **1D identity (%):** 16.37
- **1D identity (%) [Gaps excluded]:** 24.15
- **1D identity - Alignment Gaps:** 323
- **1D aligned content (<aminoacid>:%):** {'A': 7.32, 'P': 9.15, 'S': 4.88, 'G': 7.32, 'R': 7.93, 'K': 6.1, 'E': 5.49, 'D': 6.1, 'C': 3.05, 'H': 2.44, 'T': 4.27, 'I': 3.05, 'L': 7.32, 'V': 9.15, 'M': 1.22, 'F': 4.27, 'Y': 1.83, 'Q': 7.93, 'N': 0.61, 'W': 0.61}
- **Common reported functions (%):** 0.0
- **Common reported locations (%):** 14.29
- **Common reported processes (%):** 4.76

- **PDB ID:** 6R25
- **Chain:** K
- **Crystallized protein length:** 737 aa
- **Resolution:** 4.61 Å
- **b-phipsi:** 0.003209
- **w-rdist:** 0.46922
- **t-alpha:** 0.011791
- **Chemical similarity (Tanimoto Index) (%):** N/A
- **1D identity (%) [PDB]:** 2.08
- **1D identity (%) [Gaps excluded][PDB]:** 78.05
- **1D identity - Alignment Gaps [PDB]:** 1495
- **1D aligned content [PDB] (<aminoacid>:%):** {'G': 9.38, 'R': 9.38, 'K': 9.38, 'P': 9.38, 'V': 6.25, 'F': 6.25, 'D': 9.38, 'N': 3.12, 'L': 9.38, 'Y': 3.12, 'T': 6.25, 'A': 3.12, 'I': 6.25, 'E': 9.38}
- **2D identity (%) [PDB]:** 34.17
- **2D identity (%) [Gaps excluded][PDB]:** 82.47
- **2D identity - Alignment Gaps [PDB]:** 653
- **2D aligned content [PDB] (<2D-fold>:%):** {'.': 15.49, 'T': 11.81, 'E': 16.01, 'H': 53.54, 'G': 3.15}
- **3D similarity (TM-Score) (%) [PDB]:** 20.85

- **Gene name:** KDM1B
- **Entrez ID:** 221656
- **RefSeq ID:** N/A
- **Sequence length:** N/A
- **5-UTR|CDS|3-UTR identity (%):** N/A | N/A | N/A
- **5-UTR|CDS|3-UTR identity (%) [Gaps excluded]:** N/A | N/A | N/A
- **5-UTR|CDS|3-UTR identity [Alignment Gaps]:** N/A | N/A | N/A
- **5-UTR aligned content (<base>:%):** N/A
- **CDS aligned content (<base>:%):** N/A
- **3-UTR aligned content (<base>:%):** N/A

**Uniprot Description:**  
  
 Histone demethylase that demethylates 'Lys-4' of histone H3, a specific tag for epigenetic transcriptional activation, thereby acting as a corepressor. Required for de novo DNA methylation of a subset of imprinted genes during oogenesis. Acts by oxidizing the substrate by FAD to generate the corresponding imine that is subsequently hydrolyzed. Demethylates both mono- and di-methylated 'Lys-4' of histone H3. Has no effect on tri-methylated 'Lys-4', mono-, di- or tri-methylated 'Lys-9', mono-, di- or tri-methylated 'Lys-27', mono-, di- or tri-methylated 'Lys-36' of histone H3, or on mono-, di- or tri-methylated 'Lys-20' of histone H4.   
  
Does not form a complex with RCOR1/CoREST (By similarity). Interacts with its cofactor GLYR1 at nucleosomes; this interaction stimulates H3K4me1 and H3K4me2 demethylation (PubMed:23260659).   
  
 **Gene Ontology Information:**

Molecular Function

- chromatin binding
- FAD binding
- FAD-dependent H3K4me/H3K4me3 demethylase activity
- flavin adenine dinucleotide binding
- histone binding
- histone demethylase activity
- oxidoreductase activity
- zinc ion binding

Location

- chromatin
- nucleoplasm
- nucleosome
- nucleus

Biological process

- genetic imprinting
- negative regulation of transcription by RNA polymerase II
- positive regulation of transcription by RNA polymerase II
- regulation of gene expression by genetic imprinting
- positive regulation of gene expression, epigenetic

---

19

- **Protein name:** 1-phosphatidylinositol 4,5-bisphosphate phosphodiesterase beta-2
- **Organism:** Homo sapiens
- **Uniprot Accession Number:** Q00722
- **Protein sequence length:** 1185 aa
- **1D identity (%):** 16.3
- **1D identity (%) [Gaps excluded]:** 24.18
- **1D identity - Alignment Gaps:** 398
- **1D aligned content (<aminoacid>:%):** {'S': 6.53, 'P': 12.56, 'L': 10.05, 'Q': 5.03, 'G': 7.04, 'F': 4.02, 'K': 11.06, 'M': 1.01, 'D': 5.53, 'H': 2.01, 'N': 2.51, 'V': 4.02, 'R': 4.02, 'E': 4.52, 'C': 2.01, 'T': 6.03, 'A': 7.04, 'W': 0.5, 'Y': 1.51, 'I': 3.02}
- **Common reported functions (%):** 0.0
- **Common reported locations (%):** 7.14
- **Common reported processes (%):** 0.0

- **PDB ID:** 2FJU
- **Chain:** B
- **Crystallized protein length:** 696 aa
- **Resolution:** 2.2 Å
- **b-phipsi:** 0.00179
- **w-rdist:** 0.804138
- **t-alpha:** 0.002185
- **Chemical similarity (Tanimoto Index) (%):** 87.24
- **1D identity (%) [PDB]:** 3.05
- **1D identity (%) [Gaps excluded][PDB]:** 70.31
- **1D identity - Alignment Gaps [PDB]:** 1411
- **1D aligned content [PDB] (<aminoacid>:%):** {'P': 15.56, 'K': 11.11, 'D': 8.89, 'I': 11.11, 'Y': 2.22, 'E': 15.56, 'L': 8.89, 'C': 2.22, 'R': 2.22, 'M': 2.22, 'V': 6.67, 'F': 4.44, 'T': 4.44, 'G': 4.44}
- **2D identity (%) [PDB]:** 32.89
- **2D identity (%) [Gaps excluded][PDB]:** 87.62
- **2D identity - Alignment Gaps [PDB]:** 699
- **2D aligned content [PDB] (<2D-fold>:%):** {'.': 20.65, 'E': 25.27, 'H': 42.66, 'T': 11.14, 'B': 0.27}
- **3D similarity (TM-Score) (%) [PDB]:** 22.68

- **Gene name:** PLCB2
- **Entrez ID:** N/A
- **RefSeq ID:** NM\_004573
- **Transcript sequence length:** 4627
- **5-UTR|CDS|3-UTR identity (%):** 34.83 | 43.27 | 4.16
- **5-UTR|CDS|3-UTR identity (%) [Gaps excluded]:** 75.61 | 74.98 | 71.8
- **5-UTR|CDS|3-UTR identity [Alignment Gaps]:** 144 | 1646 | 11304
- **5-UTR aligned content (<base>:%):** {'C': 44.09, 'G': 41.94, 'T': 10.75, 'A': 3.23}
- **CDS aligned content (<base>:%):** {'A': 24.47, 'T': 16.57, 'G': 30.4, 'C': 28.56}
- **3-UTR aligned content (<base>:%):** {'T': 26.45, 'C': 32.46, 'A': 15.63, 'G': 25.45}

**Uniprot Description:**  
  
 The production of the second messenger molecules diacylglycerol (DAG) and inositol 1,4,5-trisphosphate (IP3) is mediated by activated phosphatidylinositol-specific phospholipase C enzymes.   
  
Interacts with RAC1 (PubMed:1644792). Forms a complex composed of at least WDR26, a G-beta:gamma unit, and PLCB2 (PubMed:23625927).   
  
 **Gene Ontology Information:**

Molecular Function

- calcium ion binding
- G-protein beta/gamma-subunit complex binding
- phosphatidylinositol phospholipase C activity
- phospholipase C activity
- phospholipid binding

Location

- cytosol
- G-protein beta/gamma-subunit complex
- neuronal dense core vesicle

Biological process

- activation of phospholipase C activity
- detection of chemical stimulus involved in sensory perception of bitter taste
- G protein-coupled receptor signaling pathway
- lipid catabolic process
- phosphatidylinositol metabolic process
- phosphatidylinositol-mediated signaling
- phospholipase C-activating G protein-coupled receptor signaling pathway
- phospholipid metabolic process
- release of sequestered calcium ion into cytosol

---

20

- **Protein name:** Pappalysin-1
- **Organism:** Homo sapiens
- **Uniprot Accession Number:** Q13219
- **Protein sequence length:** 1627 aa
- **1D identity (%):** 14.14
- **1D identity (%) [Gaps excluded]:** 28.52
- **1D identity - Alignment Gaps:** 838
- **1D aligned content (<aminoacid>:%):** {'M': 1.7, 'A': 5.11, 'G': 7.23, 'P': 11.91, 'F': 5.53, 'L': 9.36, 'D': 7.66, 'E': 2.55, 'K': 2.98, 'C': 5.96, 'R': 4.68, 'Y': 2.13, 'I': 2.55, 'W': 0.85, 'S': 5.11, 'H': 5.11, 'V': 5.53, 'N': 3.4, 'Q': 5.96, 'T': 4.68}
- **Common reported functions (%):** 0.0
- **Common reported locations (%):** 0.0
- **Common reported processes (%):** 0.0

- **PDB ID:** 8A7D
- **Chain:** C
- **Crystallized protein length:** 921 aa
- **Resolution:** 3.06 Å
- **b-phipsi:** 0.019922
- **w-rdist:** 0.148137
- **t-alpha:** 0.00437
- **Chemical similarity (Tanimoto Index) (%):** 72.71
- **1D identity (%) [PDB]:** 3.6
- **1D identity (%) [Gaps excluded][PDB]:** 63.83
- **1D identity - Alignment Gaps [PDB]:** 1571
- **1D aligned content [PDB] (<aminoacid>:%):** {'S': 6.67, 'E': 3.33, 'Q': 8.33, 'L': 11.67, 'R': 5.0, 'I': 5.0, 'D': 3.33, 'G': 8.33, 'P': 5.0, 'F': 3.33, 'K': 8.33, 'A': 5.0, 'M': 1.67, 'N': 5.0, 'Y': 3.33, 'V': 10.0, 'T': 5.0, 'C': 1.67}
- **2D identity (%) [PDB]:** 38.13
- **2D identity (%) [Gaps excluded][PDB]:** 85.61
- **2D identity - Alignment Gaps [PDB]:** 675
- **2D aligned content [PDB] (<2D-fold>:%):** {'.': 20.26, 'E': 35.56, 'T': 31.03, 'H': 10.78, 'G': 1.94, 'B': 0.43}
- **3D similarity (TM-Score) (%) [PDB]:** 24.51

- **Gene name:** PAPPA
- **Entrez ID:** 5069
- **RefSeq ID:** NM\_002581
- **Transcript sequence length:** 10971
- **5-UTR|CDS|3-UTR identity (%):** 23.58 | 37.24 | 28.06
- **5-UTR|CDS|3-UTR identity (%) [Gaps excluded]:** 73.98 | 77.81 | 74.67
- **5-UTR|CDS|3-UTR identity [Alignment Gaps]:** 263 | 2632 | 7983
- **5-UTR aligned content (<base>:%):** {'G': 52.75, 'T': 8.79, 'C': 36.26, 'A': 2.2}
- **CDS aligned content (<base>:%):** {'A': 23.67, 'T': 17.87, 'G': 27.87, 'C': 30.59}
- **3-UTR aligned content (<base>:%):** {'G': 18.76, 'A': 28.79, 'T': 33.14, 'C': 19.31}

**Uniprot Description:**  
  
 Metalloproteinase which specifically cleaves IGFBP-4 and IGFBP-5, resulting in release of bound IGF. Cleavage of IGFBP-4 is dramatically enhanced by the presence of IGF, whereas cleavage of IGFBP-5 is slightly inhibited by the presence of IGF.   
  
Homodimer; disulfide-linked. In pregnancy serum, predominantly found as a disulfide-linked 2:2 heterotetramer with the proform of PRG2.   
  
 **Gene Ontology Information:**

Molecular Function

- metalloendopeptidase activity
- metallopeptidase activity
- zinc ion binding

Location

- extracellular region
- extracellular space

Biological process

- cell surface receptor signaling pathway
- female pregnancy
- protein catabolic process
- protein metabolic process
- proteolysis
- response to dexamethasone
- response to follicle-stimulating hormone

---

21

- **Protein name:** Complement C4-A
- **Organism:** Homo sapiens
- **Uniprot Accession Number:** P0C0L4
- **Protein sequence length:** 1744 aa
- **1D identity (%):** 13.89
- **1D identity (%) [Gaps excluded]:** 29.2
- **1D identity - Alignment Gaps:** 925
- **1D aligned content (<aminoacid>:%):** {'M': 0.82, 'S': 6.94, 'P': 10.61, 'L': 10.2, 'Q': 5.71, 'G': 11.84, 'F': 4.9, 'R': 6.12, 'D': 4.9, 'T': 4.08, 'K': 5.31, 'A': 5.31, 'E': 3.27, 'I': 3.27, 'V': 8.16, 'H': 2.45, 'Y': 2.45, 'C': 2.04, 'N': 1.63}
- **Common reported functions (%):** 0.0
- **Common reported locations (%):** 14.29
- **Common reported processes (%):** 0.0

- **PDB ID:** 5JPN
- **Chain:** B
- **Crystallized protein length:** 697 aa
- **Resolution:** 3.6 Å
- **b-phipsi:** 0.003676
- **w-rdist:** 0.232694
- **t-alpha:** 0.064093
- **Chemical similarity (Tanimoto Index) (%):** 76.29
- **1D identity (%) [PDB]:** 2.84
- **1D identity (%) [Gaps excluded][PDB]:** 71.19
- **1D identity - Alignment Gaps [PDB]:** 1418
- **1D aligned content [PDB] (<aminoacid>:%):** {'P': 9.52, 'V': 7.14, 'F': 2.38, 'D': 9.52, 'G': 7.14, 'N': 2.38, 'Y': 2.38, 'T': 4.76, 'A': 7.14, 'L': 9.52, 'K': 9.52, 'E': 4.76, 'R': 4.76, 'I': 4.76, 'S': 9.52, 'Q': 2.38, 'H': 2.38}
- **2D identity (%) [PDB]:** 34.63
- **2D identity (%) [Gaps excluded][PDB]:** 82.78
- **2D identity - Alignment Gaps [PDB]:** 630
- **2D aligned content [PDB] (<2D-fold>:%):** {'.': 16.8, 'E': 30.67, 'T': 6.13, 'H': 43.47, 'G': 2.4, 'B': 0.53}
- **3D similarity (TM-Score) (%) [PDB]:** 18.76

- **Gene name:** C4A
- **Entrez ID:** 720; 721
- **RefSeq ID:** NM\_007293
- **Transcript sequence length:** 5427
- **5-UTR|CDS|3-UTR identity (%):** 14.29 | 35.3 | 0.76
- **5-UTR|CDS|3-UTR identity (%) [Gaps excluded]:** 67.74 | 78.89 | 80.36
- **5-UTR|CDS|3-UTR identity [Alignment Gaps]:** 116 | 2983 | 11805
- **5-UTR aligned content (<base>:%):** {'A': 9.52, 'G': 47.62, 'C': 33.33, 'T': 9.52}
- **CDS aligned content (<base>:%):** {'A': 22.82, 'T': 18.31, 'G': 28.44, 'C': 30.43}
- **3-UTR aligned content (<base>:%):** {'G': 27.78, 'C': 21.11, 'T': 27.78, 'A': 23.33}

**Uniprot Description:**  
  
 Non-enzymatic component of C3 and C5 convertases and thus essential for the propagation of the classical complement pathway. Covalently binds to immunoglobulins and immune complexes and enhances the solubilization of immune aggregates and the clearance of IC through CR1 on erythrocytes. C4A isotype is responsible for effective binding to form amide bonds with immune aggregates or protein antigens, while C4B isotype catalyzes the transacylation of the thioester carbonyl group to form ester bonds with carbohydrate antigens.   
  
Circulates in blood as a disulfide-linked trimer of an alpha, beta and gamma chain.   
  
 **Gene Ontology Information:**

Molecular Function

- complement component C1q complex binding
- endopeptidase inhibitor activity

Location

- axon
- blood microparticle
- classical-complement-pathway C3/C5 convertase complex
- dendrite
- endoplasmic reticulum lumen
- extracellular exosome
- extracellular region
- extracellular space
- neuronal cell body
- plasma membrane
- synapse

Biological process

- complement activation
- complement activation, classical pathway
- inflammatory response
- innate immune response
- positive regulation of apoptotic cell clearance
- positive regulation of immune response
- positive regulation of opsonization

---

22

- **Protein name:** Ubiquitin carboxyl-terminal hydrolase 5
- **Organism:** Homo sapiens
- **Uniprot Accession Number:** P45974
- **Protein sequence length:** 858 aa
- **1D identity (%):** 15.08
- **1D identity (%) [Gaps excluded]:** 23.22
- **1D identity - Alignment Gaps:** 365
- **1D aligned content (<aminoacid>:%):** {'M': 3.82, 'A': 4.46, 'L': 8.92, 'P': 13.38, 'G': 7.01, 'F': 4.46, 'K': 8.28, 'R': 8.92, 'E': 5.73, 'I': 6.37, 'D': 3.82, 'V': 5.1, 'S': 5.1, 'T': 4.46, 'Y': 1.91, 'Q': 4.46, 'H': 1.91, 'C': 0.64, 'N': 1.27}
- **Common reported functions (%):** 0.0
- **Common reported locations (%):** 14.29
- **Common reported processes (%):** 0.0

- **PDB ID:** 3IHP
- **Chain:** B
- **Crystallized protein length:** 681 aa
- **Resolution:** 2.8 Å
- **b-phipsi:** 0.006614
- **w-rdist:** 0.270184
- **t-alpha:** 0.016284
- **Chemical similarity (Tanimoto Index) (%):** 84.51
- **1D identity (%) [PDB]:** 1.95
- **1D identity (%) [Gaps excluded][PDB]:** 74.36
- **1D identity - Alignment Gaps [PDB]:** 1448
- **1D aligned content [PDB] (<aminoacid>:%):** {'R': 6.9, 'D': 6.9, 'G': 6.9, 'V': 10.34, 'S': 3.45, 'E': 10.34, 'F': 3.45, 'Q': 10.34, 'L': 10.34, 'H': 3.45, 'A': 3.45, 'I': 6.9, 'K': 6.9, 'Y': 3.45, 'P': 3.45, 'T': 3.45}
- **2D identity (%) [PDB]:** 33.27
- **2D identity (%) [Gaps excluded][PDB]:** 86.76
- **2D identity - Alignment Gaps [PDB]:** 680
- **2D aligned content [PDB] (<2D-fold>:%):** {'.': 22.07, 'H': 40.6, 'T': 14.17, 'G': 1.63, 'E': 20.98, 'B': 0.54}
- **3D similarity (TM-Score) (%) [PDB]:** 21.91

- **Gene name:** USP5
- **Entrez ID:** 8078
- **RefSeq ID:** NM\_003481
- **Transcript sequence length:** 3093
- **5-UTR|CDS|3-UTR identity (%):** 16.54 | 42.76 | 2.74
- **5-UTR|CDS|3-UTR identity (%) [Gaps excluded]:** 70.0 | 75.35 | 75.29
- **5-UTR|CDS|3-UTR identity [Alignment Gaps]:** 97 | 1404 | 11569
- **5-UTR aligned content (<base>:%):** {'G': 57.14, 'C': 33.33, 'A': 4.76, 'T': 4.76}
- **CDS aligned content (<base>:%):** {'A': 22.98, 'G': 30.26, 'C': 29.39, 'T': 17.36}
- **3-UTR aligned content (<base>:%):** {'G': 32.52, 'C': 29.79, 'T': 24.32, 'A': 13.37}

**Uniprot Description:**  
  
 Cleaves linear and branched multiubiquitin polymers with a marked preference for branched polymers. Involved in unanchored 'Lys-48'-linked polyubiquitin disassembly. Binds linear and 'Lys-63'-linked polyubiquitin with a lower affinity. Knock-down of USP5 causes the accumulation of p53/TP53 and an increase in p53/TP53 transcriptional activity because the unanchored polyubiquitin that accumulates is able to compete with ubiquitinated p53/TP53 but not with MDM2 for proteasomal recognition.   
  
Interacts with TRIML1.   
  
 **Gene Ontology Information:**

Molecular Function

- thiol-dependent ubiquitin-specific protease activity
- cysteine-type endopeptidase activity
- ubiquitin binding
- zinc ion binding

Location

- cytosol
- lysosome
- nucleus

Biological process

- positive regulation of proteasomal ubiquitin-dependent protein catabolic process
- protein deubiquitination
- protein K48-linked deubiquitination
- protein ubiquitination
- ubiquitin-dependent protein catabolic process

---

23

- **Protein name:** 116 kDa U5 small nuclear ribonucleoprotein component
- **Organism:** Homo sapiens
- **Uniprot Accession Number:** Q15029
- **Protein sequence length:** 972 aa
- **1D identity (%):** 16.92
- **1D identity (%) [Gaps excluded]:** 23.78
- **1D identity - Alignment Gaps:** 309
- **1D aligned content (<aminoacid>:%):** {'F': 4.42, 'G': 8.29, 'R': 4.42, 'K': 7.18, 'E': 4.97, 'M': 1.66, 'D': 4.97, 'P': 9.39, 'V': 9.94, 'Q': 6.63, 'T': 6.63, 'L': 11.05, 'C': 1.66, 'H': 3.31, 'S': 5.52, 'A': 4.42, 'Y': 1.66, 'I': 2.21, 'N': 1.1, 'W': 0.55}
- **Common reported functions (%):** 6.67
- **Common reported locations (%):** 28.57
- **Common reported processes (%):** 0.0

- **PDB ID:** 6ZYM
- **Chain:** B
- **Crystallized protein length:** 895 aa
- **Resolution:** 3.4 Å
- **b-phipsi:** 0.00313
- **w-rdist:** 0.337567
- **t-alpha:** 0.042521
- **Chemical similarity (Tanimoto Index) (%):** N/A
- **1D identity (%) [PDB]:** 2.7
- **1D identity (%) [Gaps excluded][PDB]:** 70.31
- **1D identity - Alignment Gaps [PDB]:** 1605
- **1D aligned content [PDB] (<aminoacid>:%):** {'V': 15.56, 'E': 8.89, 'Q': 2.22, 'H': 2.22, 'K': 15.56, 'T': 4.44, 'F': 4.44, 'D': 6.67, 'R': 4.44, 'P': 8.89, 'G': 6.67, 'L': 6.67, 'A': 2.22, 'M': 2.22, 'I': 6.67, 'S': 2.22}
- **2D identity (%) [PDB]:** 37.09
- **2D identity (%) [Gaps excluded][PDB]:** 85.33
- **2D identity - Alignment Gaps [PDB]:** 683
- **2D aligned content [PDB] (<2D-fold>:%):** {'.': 20.98, 'B': 0.22, 'E': 22.54, 'T': 16.07, 'H': 38.17, 'G': 2.01}
- **3D similarity (TM-Score) (%) [PDB]:** 1.28

- **Gene name:** EFTUD2
- **Entrez ID:** 9343
- **RefSeq ID:** NM\_004247
- **Transcript sequence length:** 4326
- **5-UTR|CDS|3-UTR identity (%):** 37.96 | 44.28 | 7.34
- **5-UTR|CDS|3-UTR identity (%) [Gaps excluded]:** 73.24 | 74.73 | 77.11
- **5-UTR|CDS|3-UTR identity [Alignment Gaps]:** 66 | 1407 | 10916
- **5-UTR aligned content (<base>:%):** {'G': 46.15, 'C': 44.23, 'T': 5.77, 'A': 3.85}
- **CDS aligned content (<base>:%):** {'A': 24.72, 'G': 28.91, 'T': 19.49, 'C': 26.88}
- **3-UTR aligned content (<base>:%):** {'G': 24.6, 'T': 26.75, 'A': 20.43, 'C': 28.22}

**Uniprot Description:**  
  
 Required for pre-mRNA splicing as component of the spliceosome, including pre-catalytic, catalytic and post-catalytic spliceosomal complexes (PubMed:28502770, PubMed:28781166, PubMed:28076346, PubMed:29361316, PubMed:30315277, PubMed:29360106, PubMed:29301961, PubMed:30705154). Component of the U5 snRNP and the U4/U6-U5 tri-snRNP complex, a building block of the spliceosome (PubMed:16723661).   
  
Component of the U5 snRNP and the U4/U6-U5 tri-snRNP complex, a building block of the spliceosome (PubMed:26912367, PubMed:16723661). The U4/U6-U5 tri-snRNP complex is composed of the U4, U6 and U5 snRNAs and at least PRPF3, PRPF4, PRPF6, PRPF8, PRPF31, SNRNP200, TXNL4A, SNRNP40, DDX23, CD2BP2, PPIH, SNU13, EFTUD2, SART1 and USP39 (PubMed:16723661, PubMed:26912367). Component of the pre-catalytic, catalytic and post-catalytic spliceosome complexes (PubMed:28502770, PubMed:28781166, PubMed:28076346, PubMed:29361316, PubMed:30315277, PubMed:29360106, PubMed:29301961, PubMed:30705154). Interacts with ERBB4 and PRPF8. Interacts with PIH1D1 (PubMed:24656813). Interacts with RPAP3 and URI1 in a ZNHIT2-dependent manner (PubMed:28561026). Interacts with NRDE2 (PubMed:30538148). Interacts with FAM50A (PubMed:32703943).   
  
 **Gene Ontology Information:**

Molecular Function

- GTP binding
- GTPase activity
- RNA binding
- U5 snRNA binding

Location

- Cajal body
- catalytic step 2 spliceosome
- cytosol
- intracellular membrane-bounded organelle
- membrane
- nuclear speck
- nucleoplasm
- nucleus
- U2-type catalytic step 2 spliceosome
- U2-type precatalytic spliceosome
- U4/U6 x U5 tri-snRNP complex

Biological process

- cellular response to xenobiotic stimulus
- mRNA splicing, via spliceosome
- response to cocaine

---

24

- **Protein name:** Insulin-degrading enzyme
- **Organism:** Homo sapiens
- **Uniprot Accession Number:** P14735
- **Protein sequence length:** 1019 aa
- **1D identity (%):** 16.86
- **1D identity (%) [Gaps excluded]:** 23.69
- **1D identity - Alignment Gaps:** 316
- **1D aligned content (<aminoacid>:%):** {'M': 1.08, 'L': 11.35, 'P': 12.97, 'G': 7.57, 'R': 3.24, 'A': 3.24, 'N': 3.78, 'D': 6.49, 'H': 3.78, 'K': 5.95, 'Y': 2.16, 'E': 6.49, 'F': 6.49, 'V': 7.57, 'S': 3.24, 'I': 4.32, 'C': 2.16, 'Q': 4.86, 'T': 2.7, 'W': 0.54}
- **Common reported functions (%):** 0.0
- **Common reported locations (%):** 28.57
- **Common reported processes (%):** 0.0

- **PDB ID:** 7RZI
- **Chain:** B
- **Crystallized protein length:** 938 aa
- **Resolution:** 3.0 Å
- **b-phipsi:** 0.006237
- **w-rdist:** 0.489864
- **t-alpha:** 0.00219
- **Chemical similarity (Tanimoto Index) (%):** 97.65
- **1D identity (%) [PDB]:** 0.36
- **1D identity (%) [Gaps excluded][PDB]:** 50.0
- **1D identity - Alignment Gaps [PDB]:** 832
- **1D aligned content [PDB] (<aminoacid>:%):** {'K': 33.33, 'R': 33.33, 'G': 33.33}
- **2D identity (%) [PDB]:** 0.6
- **2D identity (%) [Gaps excluded][PDB]:** 83.33
- **2D identity - Alignment Gaps [PDB]:** 832
- **2D aligned content [PDB] (<2D-fold>:%):** {'.': 100.0}
- **3D similarity (TM-Score) (%) [PDB]:** 24.52

- **Gene name:** IDE
- **Entrez ID:** 3416
- **RefSeq ID:** NM\_004969
- **Transcript sequence length:** 5894
- **5-UTR|CDS|3-UTR identity (%):** 35.04 | 43.77 | 14.18
- **5-UTR|CDS|3-UTR identity (%) [Gaps excluded]:** 75.0 | 75.27 | 75.34
- **5-UTR|CDS|3-UTR identity [Alignment Gaps]:** 73 | 1492 | 10008
- **5-UTR aligned content (<base>:%):** {'A': 2.08, 'G': 54.17, 'C': 33.33, 'T': 10.42}
- **CDS aligned content (<base>:%):** {'A': 28.06, 'T': 20.76, 'G': 25.5, 'C': 25.69}
- **3-UTR aligned content (<base>:%):** {'A': 30.78, 'T': 39.7, 'C': 15.27, 'G': 14.24}

**Uniprot Description:**  
  
 Plays a role in the cellular breakdown of insulin, APP peptides, IAPP peptides, natriuretic peptides, glucagon, bradykinin, kallidin, and other peptides, and thereby plays a role in intercellular peptide signaling (PubMed:2293021, PubMed:10684867, PubMed:26968463, PubMed:17051221, PubMed:17613531, PubMed:18986166, PubMed:19321446, PubMed:23922390, PubMed:24847884, PubMed:26394692, PubMed:29596046, PubMed:21098034). Substrate binding induces important conformation changes, making it possible to bind and degrade larger substrates, such as insulin (PubMed:23922390, PubMed:26394692, PubMed:29596046). Contributes to the regulation of peptide hormone signaling cascades and regulation of blood glucose homeostasis via its role in the degradation of insulin, glucagon and IAPP (By similarity). Plays a role in the degradation and clearance of APP-derived amyloidogenic peptides that are secreted by neurons and microglia (PubMed:9830016, PubMed:26394692) (Probable). Degrades the natriuretic peptides ANP, BNP and CNP, inactivating their ability to raise intracellular cGMP (PubMed:21098034). Also degrades an aberrant frameshifted 40-residue form of NPPA (fsNPPA) which is associated with familial atrial fibrillation in heterozygous patients (PubMed:21098034). Involved in antigen processing. Produces both the N terminus and the C terminus of MAGEA3-derived antigenic peptide (EVDPIGHLY) that is presented to cytotoxic T lymphocytes by MHC class I.   
  
Homodimer (PubMed:17051221, PubMed:19321446, PubMed:23922390, PubMed:26394692, PubMed:29596046) (Probable). Can also form homotetramers (By similarity).   
  
 **Gene Ontology Information:**

Molecular Function

- ATP binding
- endopeptidase activity
- identical protein binding
- insulin binding
- metalloendopeptidase activity
- peptide binding
- protein homodimerization activity
- ubiquitin-dependent protein binding
- virus receptor activity
- zinc ion binding

Location

- basolateral plasma membrane
- cell surface
- cytoplasm
- cytosol
- external side of plasma membrane
- extracellular exosome
- extracellular space
- mitochondrion
- nucleus
- peroxisomal matrix
- peroxisome

Biological process

- amyloid-beta clearance
- amyloid-beta clearance by cellular catabolic process
- amyloid-beta metabolic process
- antigen processing and presentation of endogenous peptide antigen via MHC class I
- bradykinin catabolic process
- hormone catabolic process
- insulin catabolic process
- insulin metabolic process
- insulin receptor signaling pathway
- peptide catabolic process
- positive regulation of protein binding
- positive regulation of protein catabolic process
- protein catabolic process
- proteolysis
- proteolysis involved in cellular protein catabolic process
- regulation of aerobic respiration
- ubiquitin recycling

---

25

- **Protein name:** Antiviral innate immune response receptor RIG-I
- **Organism:** Homo sapiens
- **Uniprot Accession Number:** O95786
- **Protein sequence length:** 925 aa
- **1D identity (%):** 16.23
- **1D identity (%) [Gaps excluded]:** 22.12
- **1D identity - Alignment Gaps:** 274
- **1D aligned content (<aminoacid>:%):** {'T': 5.99, 'L': 10.18, 'Q': 4.79, 'A': 7.78, 'I': 6.59, 'Y': 2.4, 'E': 7.19, 'K': 8.98, 'P': 7.19, 'F': 3.59, 'D': 5.99, 'G': 4.79, 'R': 2.99, 'C': 5.39, 'S': 4.79, 'V': 2.99, 'N': 2.99, 'W': 1.2, 'M': 0.6, 'H': 3.59}
- **Common reported functions (%):** 13.33
- **Common reported locations (%):** 21.43
- **Common reported processes (%):** 4.76

- **PDB ID:** 3ZD6
- **Chain:** A
- **Crystallized protein length:** 632 aa
- **Resolution:** 2.8 Å
- **b-phipsi:** 0.018931
- **w-rdist:** 0.294361
- **t-alpha:** 0.00219
- **Chemical similarity (Tanimoto Index) (%):** 93.43
- **1D identity (%) [PDB]:** 2.96
- **1D identity (%) [Gaps excluded][PDB]:** 77.78
- **1D identity - Alignment Gaps [PDB]:** 1366
- **1D aligned content [PDB] (<aminoacid>:%):** {'N': 2.38, 'L': 19.05, 'Y': 2.38, 'T': 4.76, 'A': 9.52, 'P': 9.52, 'I': 4.76, 'G': 4.76, 'R': 2.38, 'K': 4.76, 'V': 11.9, 'E': 7.14, 'D': 4.76, 'S': 4.76, 'C': 2.38, 'Q': 2.38, 'F': 2.38}
- **2D identity (%) [PDB]:** 35.72
- **2D identity (%) [Gaps excluded][PDB]:** 83.67
- **2D identity - Alignment Gaps [PDB]:** 592
- **2D aligned content [PDB] (<2D-fold>:%):** {'.': 15.45, 'H': 53.12, 'T': 11.92, 'E': 17.62, 'G': 1.63, 'B': 0.27}
- **3D similarity (TM-Score) (%) [PDB]:** 21.72

- **Gene name:** DDX58
- **Entrez ID:** 23586
- **RefSeq ID:** NM\_014314
- **Transcript sequence length:** 4628
- **5-UTR|CDS|3-UTR identity (%):** 9.15 | 45.05 | 10.06
- **5-UTR|CDS|3-UTR identity (%) [Gaps excluded]:** 86.67 | 73.04 | 75.15
- **5-UTR|CDS|3-UTR identity [Alignment Gaps]:** 127 | 1270 | 10472
- **5-UTR aligned content (<base>:%):** {'G': 53.85, 'A': 7.69, 'C': 30.77, 'T': 7.69}
- **CDS aligned content (<base>:%):** {'T': 20.83, 'G': 25.18, 'A': 30.88, 'C': 23.11}
- **3-UTR aligned content (<base>:%):** {'A': 26.15, 'T': 35.61, 'C': 18.67, 'G': 19.57}

**Uniprot Description:**  
  
 Innate immune receptor that senses cytoplasmic viral nucleic acids and activates a downstream signaling cascade leading to the production of type I interferons and proinflammatory cytokines. Forms a ribonucleoprotein complex with viral RNAs on which it homooligomerizes to form filaments. The homooligomerization allows the recruitment of RNF135 an E3 ubiquitin-protein ligase that activates and amplifies the RIG-I-mediated antiviral signaling in an RNA length-dependent manner through ubiquitination-dependent and -independent mechanisms (PubMed:28469175, PubMed:31006531). Upon activation, associates with mitochondria antiviral signaling protein (MAVS/IPS1) that activates the IKK-related kinases TBK1 and IKBKE which in turn phosphorylate the interferon regulatory factors IRF3 and IRF7, activating transcription of antiviral immunological genes including the IFN-alpha and IFN-beta interferons (PubMed:28469175, PubMed:31006531). Ligands include 5'-triphosphorylated ssRNAs and dsRNAs but also short dsRNAs (<1 kb in length). In addition to the 5'-triphosphate moiety, blunt-end base pairing at the 5'-end of the RNA is very essential. Overhangs at the non-triphosphorylated end of the dsRNA RNA have no major impact on its activity. A 3'overhang at the 5'triphosphate end decreases and any 5'overhang at the 5' triphosphate end abolishes its activity. Detects both positive and negative strand RNA viruses including members of the families Paramyxoviridae: Human respiratory syncytial virus and measles virus (MeV), Rhabdoviridae: vesicular stomatitis virus (VSV), Orthomyxoviridae: influenza A and B virus, Flaviviridae: Japanese encephalitis virus (JEV), hepatitis C virus (HCV), dengue virus (DENV) and west Nile virus (WNV). It also detects rotaviruses and reoviruses. Also involved in antiviral signaling in response to viruses containing a dsDNA genome such as Epstein-Barr virus (EBV). Detects dsRNA produced from non-self dsDNA by RNA polymerase III, such as Epstein-Barr virus-encoded RNAs (EBERs). May play important roles in granulocyte production and differentiation, bacterial phagocytosis and in the regulation of cell migration.   
  
Monomer; maintained as a monomer in an autoinhibited state. Upon binding of viral RNAs and conformational shift, homooligomerizes and forms filaments on these molecules (PubMed:26471729). Interacts (via tandem CARD domain) with MAVS/IPS1 promoting its filamentation. Interacts with DHX58/LGP2, IKBKE, TBK1 and STING1. Interacts (via CARD domain) with TRIM25 (via SPRY domain). Interacts (double-stranded RNA-bound oligomeric form) with RNF135 (homodimer); involved in RNA length-dependent activation of the RIG-I signaling pathway (PubMed:19017631, PubMed:19484123, PubMed:23950712, PubMed:28469175, PubMed:31006531). Interacts with CYLD. Interacts with NLRC5; blocks the interaction of MAVS/IPS1 to DDX58. Interacts with SRC. Interacts with DDX60. Interacts with isoform 2 of ZC3HAV1 (via zinc-fingers) in an RNA-dependent manner. Interacts (via tandem CARD domain) with SEC14L1; the interaction is direct and impairs the interaction of DDX58 with MAVS/IPS1. Interacts with VCP/p97; interaction is direct and allows the recruitment of RNF125 and subsequent ubiquitination and degradation (PubMed:26471729). Interacts with NOP53; may regulate DDX58 through USP15-mediated 'Lys-63'-linked deubiquitination (PubMed:27824081). Interacts with SIGLEC10, CBL and PTPN11; within a negative feedback loop leading to DDX58 degradation (By similarity). Interacts with LRRC25 (PubMed:29288164). Interacts with ZCCHC3; leading to activation of DDX58/RIG-I (PubMed:30193849). Interacts with RNF123 (PubMed:27312109). Interacts with UBE2D3 and UBE2N; E2 ubiquitin ligases involved in RNF135-mediated ubiquitination of DDX58 and activation of the RIG-I signaling pathway (PubMed:28469175). Interacts with IFIT3 (PubMed:21813773). Interacts with DDX3X (PubMed:20127681).   
  
 **Gene Ontology Information:**

Molecular Function

- ATP binding
- ATPase activity
- double-stranded DNA binding
- double-stranded RNA binding
- GTP binding
- identical protein binding
- pattern recognition receptor activity
- RNA helicase activity
- single-stranded RNA binding
- ubiquitin protein ligase binding
- zinc ion binding

Location

- actin cytoskeleton
- bicellular tight junction
- cytoplasm
- cytosol
- ribonucleoprotein complex
- ruffle membrane

Biological process

- antiviral innate immune response
- cellular response to exogenous dsRNA
- cytoplasmic pattern recognition receptor signaling pathway
- defense response to virus
- detection of virus
- gene expression
- innate immune response
- positive regulation of defense response to virus by host
- positive regulation of DNA-binding transcription factor activity
- positive regulation of gene expression
- positive regulation of granulocyte macrophage colony-stimulating factor production
- positive regulation of interferon-alpha production
- positive regulation of interferon-beta production
- positive regulation of interleukin-6 production
- positive regulation of interleukin-8 production
- positive regulation of myeloid dendritic cell cytokine production
- positive regulation of response to cytokine stimulus
- positive regulation of transcription by RNA polymerase II
- positive regulation of tumor necrosis factor production
- regulation of cell migration
- regulation of type III interferon production
- response to exogenous dsRNA
- response to virus
- RIG-I signaling pathway

---

26

- **Protein name:** Phosphatidylinositol 4,5-bisphosphate 3-kinase catalytic subunit alpha isoform
- **Organism:** Homo sapiens
- **Uniprot Accession Number:** P42336
- **Protein sequence length:** 1068 aa
- **1D identity (%):** 15.68
- **1D identity (%) [Gaps excluded]:** 23.42
- **1D identity - Alignment Gaps:** 381
- **1D aligned content (<aminoacid>:%):** {'P': 8.84, 'G': 8.84, 'R': 6.63, 'L': 8.29, 'F': 4.97, 'D': 6.63, 'K': 6.63, 'E': 5.52, 'N': 3.87, 'I': 4.42, 'V': 6.63, 'Q': 7.73, 'W': 1.66, 'C': 2.21, 'A': 3.31, 'S': 2.76, 'Y': 2.21, 'T': 3.87, 'H': 3.87, 'M': 1.1}
- **Common reported functions (%):** 0.0
- **Common reported locations (%):** 21.43
- **Common reported processes (%):** 0.0

- **PDB ID:** 8SBJ
- **Chain:** A
- **Crystallized protein length:** 986 aa
- **Resolution:** 3.1 Å
- **b-phipsi:** 0.008478
- **w-rdist:** 0.450119
- **t-alpha:** 0.001457
- **Chemical similarity (Tanimoto Index) (%):** 87.09
- **1D identity (%) [PDB]:** 2.17
- **1D identity (%) [Gaps excluded][PDB]:** 68.52
- **1D identity - Alignment Gaps [PDB]:** 1654
- **1D aligned content [PDB] (<aminoacid>:%):** {'R': 5.41, 'P': 5.41, 'S': 10.81, 'L': 8.11, 'W': 2.7, 'I': 8.11, 'V': 13.51, 'T': 8.11, 'F': 5.41, 'K': 13.51, 'E': 10.81, 'D': 2.7, 'Q': 5.41}
- **2D identity (%) [PDB]:** 45.13
- **2D identity (%) [Gaps excluded][PDB]:** 89.19
- **2D identity - Alignment Gaps [PDB]:** 578
- **2D aligned content [PDB] (<2D-fold>:%):** {'.': 11.74, 'E': 21.78, 'T': 25.19, 'H': 41.29}
- **3D similarity (TM-Score) (%) [PDB]:** 23.72

- **Gene name:** PIK3CA
- **Entrez ID:** N/A
- **RefSeq ID:** N/A
- **Sequence length:** N/A
- **5-UTR|CDS|3-UTR identity (%):** N/A | N/A | N/A
- **5-UTR|CDS|3-UTR identity (%) [Gaps excluded]:** N/A | N/A | N/A
- **5-UTR|CDS|3-UTR identity [Alignment Gaps]:** N/A | N/A | N/A
- **5-UTR aligned content (<base>:%):** N/A
- **CDS aligned content (<base>:%):** N/A
- **3-UTR aligned content (<base>:%):** N/A

**Uniprot Description:**  
  
 Phosphoinositide-3-kinase (PI3K) phosphorylates phosphatidylinositol (PI) and its phosphorylated derivatives at position 3 of the inositol ring to produce 3-phosphoinositides (PubMed:15135396, PubMed:23936502, PubMed:28676499). Uses ATP and PtdIns(4,5)P2 (phosphatidylinositol 4,5-bisphosphate) to generate phosphatidylinositol 3,4,5-trisphosphate (PIP3) (PubMed:15135396, PubMed:28676499). PIP3 plays a key role by recruiting PH domain-containing proteins to the membrane, including AKT1 and PDPK1, activating signaling cascades involved in cell growth, survival, proliferation, motility and morphology. Participates in cellular signaling in response to various growth factors. Involved in the activation of AKT1 upon stimulation by receptor tyrosine kinases ligands such as EGF, insulin, IGF1, VEGFA and PDGF. Involved in signaling via insulin-receptor substrate (IRS) proteins. Essential in endothelial cell migration during vascular development through VEGFA signaling, possibly by regulating RhoA activity. Required for lymphatic vasculature development, possibly by binding to RAS and by activation by EGF and FGF2, but not by PDGF. Regulates invadopodia formation through the PDPK1-AKT1 pathway. Participates in cardiomyogenesis in embryonic stem cells through a AKT1 pathway. Participates in vasculogenesis in embryonic stem cells through PDK1 and protein kinase C pathway. In addition to its lipid kinase activity, it displays a serine-protein kinase activity that results in the autophosphorylation of the p85alpha regulatory subunit as well as phosphorylation of other proteins such as 4EBP1, H-Ras, the IL-3 beta c receptor and possibly others (PubMed:23936502, PubMed:28676499). Plays a role in the positive regulation of phagocytosis and pinocytosis (By similarity).   
  
Heterodimer of a catalytic subunit PIK3CA and a p85 regulatory subunit (PIK3R1, PIK3R2 or PIK3R3) (PubMed:26593112). Interacts with IRS1 in nuclear extracts (By similarity). Interacts with RUFY3 (By similarity). Interacts with RASD2 (By similarity). Interacts with APPL1. Interacts with HRAS and KRAS (By similarity). Interaction with HRAS/KRAS is required for PI3K pathway signaling and cell proliferation stimulated by EGF and FGF2 (By similarity). Interacts with FAM83B; activates the PI3K/AKT signaling cascade (PubMed:23676467).   
  
 **Gene Ontology Information:**

Molecular Function

- 1-phosphatidylinositol-3-kinase activity
- 1-phosphatidylinositol-4-phosphate 3-kinase activity
- ATP binding
- insulin receptor substrate binding
- kinase activity
- phosphatidylinositol kinase activity
- phosphatidylinositol-4,5-bisphosphate 3-kinase activity
- protein kinase activator activity
- protein serine kinase activity
- protein serine/threonine kinase activity

Location

- cytoplasm
- cytosol
- intercalated disc
- lamellipodium
- membrane
- perinuclear region of cytoplasm
- phosphatidylinositol 3-kinase complex
- phosphatidylinositol 3-kinase complex, class IA
- phosphatidylinositol 3-kinase complex, class IB
- plasma membrane

Biological process

- actin cytoskeleton organization
- adipose tissue development
- angiogenesis
- anoikis
- cardiac muscle cell contraction
- cardiac muscle contraction
- cell migration
- cellular response to glucose stimulus
- cellular response to hydrostatic pressure
- endothelial cell migration
- energy homeostasis
- epidermal growth factor receptor signaling pathway
- glucose metabolic process
- hypomethylation of CpG island
- liver development
- negative regulation of actin filament depolymerization
- negative regulation of anoikis
- negative regulation of fibroblast apoptotic process
- negative regulation of gene expression
- negative regulation of macroautophagy
- negative regulation of neuron apoptotic process
- phagocytosis
- phosphatidylinositol phosphorylation
- phosphatidylinositol-3-phosphate biosynthetic process
- phosphatidylinositol-mediated signaling
- phosphorylation
- platelet activation
- positive regulation of lamellipodium assembly
- positive regulation of peptidyl-serine phosphorylation
- positive regulation of protein kinase B signaling
- positive regulation of smooth muscle cell proliferation
- positive regulation of TOR signaling
- protein kinase B signaling
- regulation of actin filament organization
- regulation of cellular respiration
- regulation of multicellular organism growth
- relaxation of cardiac muscle
- response to activity
- response to butyrate
- response to dexamethasone
- response to leucine
- response to muscle inactivity
- response to muscle stretch
- T cell costimulation
- T cell receptor signaling pathway
- vascular endothelial growth factor signaling pathway
- vasculature development

---

27

- **Protein name:** Plasminogen
- **Organism:** Homo sapiens
- **Uniprot Accession Number:** P00747
- **Protein sequence length:** 810 aa
- **1D identity (%):** 15.08
- **1D identity (%) [Gaps excluded]:** 28.7
- **1D identity - Alignment Gaps:** 519
- **1D aligned content (<aminoacid>:%):** {'E': 6.06, 'L': 7.88, 'S': 4.85, 'P': 11.52, 'G': 9.7, 'F': 3.64, 'I': 1.82, 'A': 3.64, 'K': 7.27, 'C': 4.85, 'Q': 6.06, 'V': 7.27, 'M': 0.61, 'R': 6.67, 'H': 4.24, 'Y': 2.42, 'D': 3.64, 'T': 5.45, 'N': 1.21, 'W': 1.21}
- **Common reported functions (%):** 0.0
- **Common reported locations (%):** 7.14
- **Common reported processes (%):** 0.0

- **PDB ID:** 4DUR
- **Chain:** A
- **Crystallized protein length:** 756 aa
- **Resolution:** 2.45 Å
- **b-phipsi:** 0.018188
- **w-rdist:** 0.27563
- **t-alpha:** 0.003655
- **Chemical similarity (Tanimoto Index) (%):** 76.64
- **1D identity (%) [PDB]:** 3.42
- **1D identity (%) [Gaps excluded][PDB]:** 66.67
- **1D identity - Alignment Gaps [PDB]:** 1441
- **1D aligned content [PDB] (<aminoacid>:%):** {'L': 1.92, 'D': 7.69, 'N': 3.85, 'T': 11.54, 'S': 9.62, 'I': 5.77, 'R': 7.69, 'A': 5.77, 'P': 7.69, 'E': 5.77, 'K': 3.85, 'M': 5.77, 'F': 1.92, 'V': 9.62, 'G': 7.69, 'Q': 3.85}
- **2D identity (%) [PDB]:** 25.82
- **2D identity (%) [Gaps excluded][PDB]:** 84.72
- **2D identity - Alignment Gaps [PDB]:** 851
- **2D aligned content [PDB] (<2D-fold>:%):** {'T': 25.4, 'E': 32.38, '.': 30.79, 'H': 7.3, 'G': 2.86, 'B': 1.27}
- **3D similarity (TM-Score) (%) [PDB]:** 23.29

- **Gene name:** PLG
- **Entrez ID:** 5340
- **RefSeq ID:** NM\_000301
- **Transcript sequence length:** 3530
- **5-UTR|CDS|3-UTR identity (%):** 25.76 | 44.17 | 5.01
- **5-UTR|CDS|3-UTR identity (%) [Gaps excluded]:** 60.71 | 76.1 | 72.84
- **5-UTR|CDS|3-UTR identity [Alignment Gaps]:** 76 | 1331 | 11260
- **5-UTR aligned content (<base>:%):** {'G': 38.24, 'C': 47.06, 'T': 11.76, 'A': 2.94}
- **CDS aligned content (<base>:%):** {'A': 26.55, 'T': 17.7, 'G': 27.27, 'C': 28.48}
- **3-UTR aligned content (<base>:%):** {'T': 33.33, 'A': 31.68, 'G': 18.15, 'C': 16.83}

**Uniprot Description:**  
  
 Plasmin dissolves the fibrin of blood clots and acts as a proteolytic factor in a variety of other processes including embryonic development, tissue remodeling, tumor invasion, and inflammation. In ovulation, weakens the walls of the Graafian follicle. It activates the urokinase-type plasminogen activator, collagenases and several complement zymogens, such as C1 and C5. Cleavage of fibronectin and laminin leads to cell detachment and apoptosis. Also cleaves fibrin, thrombospondin and von Willebrand factor. Its role in tissue remodeling and tumor invasion may be modulated by CSPG4. Binds to cells.   
  
Interacts (both mature PLG and the angiostatin peptide) with CSPG4 and AMOT (PubMed:10889192, PubMed:16043488). Interacts (via the Kringle domains) with HRG; the interaction tethers PLG to the cell surface and enhances its activation (PubMed:9102401, PubMed:19712047). Interacts (via Kringle 4 domain) with ADA; the interaction stimulates PLG activation when in complex with DPP4 (PubMed:15016824). Angiostatin: Interacts with ATP5F1A; the interaction inhibits most of the angiogenic effects of angiostatin (PubMed:10077593).   
  
 **Gene Ontology Information:**

Molecular Function

- apolipoprotein binding
- endopeptidase activity
- enzyme binding
- kinase binding
- protease binding
- protein antigen binding
- protein domain specific binding
- chaperone binding
- serine-type endopeptidase activity
- serine-type peptidase activity
- signaling receptor binding

Location

- blood microparticle
- cell surface
- collagen-containing extracellular matrix
- external side of plasma membrane
- extracellular exosome
- extracellular region
- extracellular space
- plasma membrane
- platelet alpha granule lumen

Biological process

- biological process involved in interaction with symbiont
- blood coagulation
- extracellular matrix disassembly
- fibrinolysis
- negative regulation of cell population proliferation
- negative regulation of cell-cell adhesion mediated by cadherin
- negative regulation of cell-substrate adhesion
- negative regulation of fibrinolysis
- positive regulation of blood vessel endothelial cell migration
- positive regulation of fibrinolysis
- proteolysis
- tissue remodeling

---

28

- **Protein name:** Multifunctional procollagen lysine hydroxylase and glycosyltransferase LH3
- **Organism:** Homo sapiens
- **Uniprot Accession Number:** O60568
- **Protein sequence length:** 738 aa
- **1D identity (%):** 15.72
- **1D identity (%) [Gaps excluded]:** 22.96
- **1D identity - Alignment Gaps:** 299
- **1D aligned content (<aminoacid>:%):** {'G': 10.07, 'R': 10.07, 'L': 10.74, 'D': 7.38, 'P': 7.38, 'E': 3.36, 'K': 6.04, 'V': 6.04, 'F': 4.7, 'Y': 3.36, 'T': 4.7, 'W': 2.68, 'A': 3.36, 'C': 2.01, 'H': 5.37, 'Q': 5.37, 'S': 2.01, 'I': 2.01, 'N': 2.68, 'M': 0.67}
- **Common reported functions (%):** 0.0
- **Common reported locations (%):** 0.0
- **Common reported processes (%):** 0.0

- **PDB ID:** 8ONE
- **Chain:** A
- **Crystallized protein length:** 696 aa
- **Resolution:** 2.3 Å
- **b-phipsi:** 0.004756
- **w-rdist:** 0.763534
- **t-alpha:** 0.000729
- **Chemical similarity (Tanimoto Index) (%):** 66.05
- **1D identity (%) [PDB]:** 3.43
- **1D identity (%) [Gaps excluded][PDB]:** 72.46
- **1D identity - Alignment Gaps [PDB]:** 1389
- **1D aligned content [PDB] (<aminoacid>:%):** {'P': 4.0, 'L': 14.0, 'I': 6.0, 'G': 10.0, 'K': 4.0, 'V': 18.0, 'E': 6.0, 'R': 8.0, 'F': 4.0, 'S': 10.0, 'A': 6.0, 'D': 4.0, 'Q': 2.0, 'M': 2.0, 'Y': 2.0}
- **2D identity (%) [PDB]:** 42.4
- **2D identity (%) [Gaps excluded][PDB]:** 88.66
- **2D identity - Alignment Gaps [PDB]:** 539
- **2D aligned content [PDB] (<2D-fold>:%):** {'.': 12.56, 'E': 29.91, 'T': 24.66, 'H': 31.51, 'G': 1.14, 'B': 0.23}
- **3D similarity (TM-Score) (%) [PDB]:** 21.42

- **Gene name:** PLOD3
- **Entrez ID:** N/A
- **RefSeq ID:** NM\_001084
- **Transcript sequence length:** 5126
- **5-UTR|CDS|3-UTR identity (%):** 32.14 | 45.01 | 11.51
- **5-UTR|CDS|3-UTR identity (%) [Gaps excluded]:** 78.57 | 74.94 | 74.83
- **5-UTR|CDS|3-UTR identity [Alignment Gaps]:** 182 | 1197 | 10628
- **5-UTR aligned content (<base>:%):** {'C': 47.47, 'T': 10.1, 'G': 38.38, 'A': 4.04}
- **CDS aligned content (<base>:%):** {'A': 20.98, 'T': 16.68, 'G': 30.47, 'C': 31.88}
- **3-UTR aligned content (<base>:%):** {'C': 25.71, 'T': 27.16, 'A': 21.98, 'G': 25.02, '-': 0.14}

**Uniprot Description:**  
  
 Multifunctional enzyme that catalyzes a series of essential post-translational modifications on Lys residues in procollagen (PubMed:11956192, PubMed:12475640, PubMed:18298658, PubMed:30089812, PubMed:18834968). Plays a redundant role in catalyzing the formation of hydroxylysine residues in -Xaa-Lys-Gly- sequences in collagens (PubMed:9582318, PubMed:9724729, PubMed:11956192, PubMed:12475640, PubMed:18298658, PubMed:30089812, PubMed:18834968). Plays a redundant role in catalyzing the transfer of galactose onto hydroxylysine groups, giving rise to galactosyl 5-hydroxylysine (PubMed:12475640, PubMed:18298658, PubMed:30089812, PubMed:18834968). Has an essential role by catalyzing the subsequent transfer of glucose moieties, giving rise to 1,2-glucosylgalactosyl-5-hydroxylysine residues (PubMed:10934207, PubMed:11896059, PubMed:11956192, PubMed:12475640, PubMed:18298658, PubMed:30089812, PubMed:18834968). Catalyzes hydroxylation and glycosylation of Lys residues in the MBL1 collagen-like domain, giving rise to hydroxylysine and 1,2-glucosylgalactosyl-5-hydroxylysine residues (PubMed:25419660). Essential for normal biosynthesis and secretion of type IV collagens (PubMed:18834968) (Probable). Essential for normal formation of basement membranes (By similarity).   
  
Homodimer.   
  
 **Gene Ontology Information:**

Molecular Function   
  
N/A

Location   
  
N/A

Biological process   
  
N/A

---

29

- **Protein name:** ATP-dependent RNA helicase A
- **Organism:** Homo sapiens
- **Uniprot Accession Number:** Q08211
- **Protein sequence length:** 1270 aa
- **1D identity (%):** N/A
- **1D identity (%) [Gaps excluded]:** N/A
- **1D identity - Alignment Gaps:** N/A
- **1D aligned content (<aminoacid>:%):** N/A
- **Common reported functions (%):** N/A
- **Common reported locations (%):** N/A
- **Common reported processes (%):** N/A

- **PDB ID:** 8SZP
- **Chain:** B
- **Crystallized protein length:** 860 aa
- **Resolution:** 2.62 Å
- **b-phipsi:** 0.009558
- **w-rdist:** 0.483256
- **t-alpha:** 0.0
- **Chemical similarity (Tanimoto Index) (%):** N/A
- **1D identity (%) [PDB]:** N/A
- **1D identity (%) [Gaps excluded][PDB]:** N/A
- **1D identity - Alignment Gaps [PDB]:** N/A
- **1D aligned content [PDB] (<aminoacid>:%):** N/A
- **2D identity (%) [PDB]:** N/A
- **2D identity (%) [Gaps excluded][PDB]:** N/A
- **2D identity - Alignment Gaps [PDB]:** N/A
- **2D aligned content [PDB] (<2D-fold>:%):** N/A
- **3D similarity (TM-Score) (%) [PDB]:** N/A

- **Gene name:** DHX9
- **Entrez ID:** N/A
- **RefSeq ID:** N/A
- **Sequence length:** N/A
- **5-UTR|CDS|3-UTR identity (%):** N/A | N/A | N/A
- **5-UTR|CDS|3-UTR identity (%) [Gaps excluded]:** N/A | N/A | N/A
- **5-UTR|CDS|3-UTR identity [Alignment Gaps]:** N/A | N/A | N/A
- **5-UTR aligned content (<base>:%):** N/A
- **CDS aligned content (<base>:%):** N/A
- **3-UTR aligned content (<base>:%):** N/A

**Uniprot Description:**  
  
 Multifunctional ATP-dependent nucleic acid helicase that unwinds DNA and RNA in a 3' to 5' direction and that plays important roles in many processes, such as DNA replication, transcriptional activation, post-transcriptional RNA regulation, mRNA translation and RNA-mediated gene silencing (PubMed:9111062, PubMed:11416126, PubMed:12711669, PubMed:15355351, PubMed:16680162, PubMed:17531811, PubMed:20669935, PubMed:21561811, PubMed:24049074, PubMed:25062910, PubMed:24990949, PubMed:28221134). Requires a 3'-single-stranded tail as entry site for acid nuclei unwinding activities as well as the binding and hydrolyzing of any of the four ribo- or deoxyribo-nucleotide triphosphates (NTPs) (PubMed:1537828). Unwinds numerous nucleic acid substrates such as double-stranded (ds) DNA and RNA, DNA:RNA hybrids, DNA and RNA forks composed of either partially complementary DNA duplexes or DNA:RNA hybrids, respectively, and also DNA and RNA displacement loops (D- and R-loops), triplex-helical DNA (H-DNA) structure and DNA and RNA-based G-quadruplexes (PubMed:20669935, PubMed:21561811, PubMed:24049074). Binds dsDNA, single-stranded DNA (ssDNA), dsRNA, ssRNA and poly(A)-containing RNA (PubMed:9111062, PubMed:10198287). Binds also to circular dsDNA or dsRNA of either linear and/or circular forms and stimulates the relaxation of supercoiled DNAs catalyzed by topoisomerase TOP2A (PubMed:12711669). Plays a role in DNA replication at origins of replication and cell cycle progression (PubMed:24990949). Plays a role as a transcriptional coactivator acting as a bridging factor between polymerase II holoenzyme and transcription factors or cofactors, such as BRCA1, CREBBP, RELA and SMN1 (PubMed:11149922, PubMed:9323138, PubMed:9662397, PubMed:11038348, PubMed:11416126, PubMed:15355351, PubMed:28221134). Binds to the CDKN2A promoter (PubMed:11038348). Plays several roles in post-transcriptional regulation of gene expression (PubMed:28221134, PubMed:28355180). In cooperation with NUP98, promotes pre-mRNA alternative splicing activities of a subset of genes (PubMed:11402034, PubMed:16680162, PubMed:28221134, PubMed:28355180). As component of a large PER complex, is involved in the negative regulation of 3' transcriptional termination of circadian target genes such as PER1 and NR1D1 and the control of the circadian rhythms (By similarity). Acts also as a nuclear resolvase that is able to bind and neutralize harmful massive secondary double-stranded RNA structures formed by inverted-repeat Alu retrotransposon elements that are inserted and transcribed as parts of genes during the process of gene transposition (PubMed:28355180). Involved in the positive regulation of nuclear export of constitutive transport element (CTE)-containing unspliced mRNA (PubMed:9162007, PubMed:10924507, PubMed:11402034). Component of the coding region determinant (CRD)-mediated complex that promotes cytoplasmic MYC mRNA stability (PubMed:19029303). Plays a role in mRNA translation (PubMed:28355180). Positively regulates translation of selected mRNAs through its binding to post-transcriptional control element (PCE) in the 5'-untranslated region (UTR) (PubMed:16680162). Involved with LARP6 in the translation stimulation of type I collagen mRNAs for CO1A1 and CO1A2 through binding of a specific stem-loop structure in their 5'-UTRs (PubMed:22190748). Stimulates LIN28A-dependent mRNA translation probably by facilitating ribonucleoprotein remodeling during the process of translation (PubMed:21247876). Plays also a role as a small interfering (siRNA)-loading factor involved in the RNA-induced silencing complex (RISC) loading complex (RLC) assembly, and hence functions in the RISC-mediated gene silencing process (PubMed:17531811). Binds preferentially to short double-stranded RNA, such as those produced during rotavirus intestinal infection (PubMed:28636595). This interaction may mediate NLRP9 inflammasome activation and trigger inflammatory response, including IL18 release and pyroptosis (PubMed:28636595). Finally, mediates the attachment of heterogeneous nuclear ribonucleoproteins (hnRNPs) to actin filaments in the nucleus (PubMed:11687588).   
  
Component of the coding region determinant (CRD)-mediated complex, composed of DHX9, HNRNPU, IGF2BP1, SYNCRIP and YBX1 (PubMed:19029303). Identified in a mRNP complex, at least composed of DHX9, DDX3X, ELAVL1, HNRNPU, IGF2BP1, ILF3, PABPC1, PCBP2, PTBP2, STAU1, STAU2, SYNCRIP and YBX1 (PubMed:19029303). Identified in a IGF2BP1-dependent mRNP granule complex containing untranslated mRNAs (PubMed:17289661). The large PER complex involved in the repression of transcriptional termination is composed of at least PER2, CDK9, DDX5, DHX9, NCBP1 and POLR2A (active) (By similarity). Associates (via DRBM domains) with the RISC complex; this association occurs in a small interfering (siRNA)-dependent manner (PubMed:17531811, PubMed:23361462). Associates with the SMN complex; this association induces recruitment of DHX9 to the RNA polymerase II (ref.8). Associates with polysomes in a LIN28A-dependent manner (PubMed:16680162, PubMed:21247876). Interacts (via C-terminus) with ACTB; this interaction is direct and mediates the attachment to nuclear ribonucleoprotein complexes (PubMed:11687588). Interacts with ADAR isoform 1; this interaction occurs in a RNA-independent manner (PubMed:28355180). Interacts (via DRBM domains) with AGO2 (via middle region); this interaction promotes active RISC assembly by promoting the association of siRNA with AGO2 (PubMed:17531811, PubMed:23361462). Interacts (via RGG region) with AKAP8L (via N-terminus) (PubMed:11402034). Interacts with BRCA1 (via C-terminus); this interaction is direct and links BRCA1 to the RNA polymerase II holoenzyme (PubMed:9662397). Interacts (via N-terminus) with CREBBP; this interaction mediates association with RNA polymerase II holoenzyme and stimulates CREB-dependent transcriptional activation (PubMed:9323138). Interacts (via N-terminus) with EIF2AK2/PKR; this interaction is dependent upon the activation of the kinase (PubMed:19229320). Interacts (via DRBM domains) with DICER1 (PubMed:17531811). Interacts with H2AX; this interaction is direct, requires phosphorylation of histone H2AX on 'Ser-140' by PRKDC and promotes binding of DHX9 to transcriptionally stalled sites on chromosomal DNA in response to genotoxic stress (PubMed:15613478, PubMed:17498979). Interacts with HNRNPC; this interaction is direct, enhanced probably by their concomitant binding to RNA and mediates the attachment to actin filaments (PubMed:11687588). Interacts (via RGG region) with PRMT1 (PubMed:15084609). Interacts with IGF2BP1 (PubMed:17289661, PubMed:23640942). Interacts with IGF2BP2, IGF2BP3 (PubMed:23640942). Interacts (via DRBM domains) with ILF3; this interaction occurs in a RNA-independent manner (PubMed:12946349). Interacts with Importin alpha/Importin beta receptor (PubMed:16375861). Interacts with LARP6 (via C-terminus); this interaction occurs in a mRNA-independent manner (PubMed:22190748). Interacts (via N- and C-terminus) with LIN28A (via C-terminus); this interaction occurs in a RNA-independent manner (PubMed:21247876). Interacts with LMX1B (PubMed:23308148). Interacts (via helicase C-terminal domain, HA2 and OB-fold regions) with MAVS (via CARD domain); this interaction occurs in both resting and double-stranded RNA poly(I:C)-induced cells (PubMed:21957149). Interacts with MBD2; this interaction stimulates transcriptional activation in a CREB-dependent manner (PubMed:12665568). Interacts (via H2A and OB-fold regions) with MYD88 (via TIR domain); this interaction is direct (PubMed:20696886). Interacts with NLRP9 upon rotavirus infection; this interaction may trigger NLRP9 inflammasome activation and inflammatory response (PubMed:28636595). Interacts (via DRBM, OB-fold and RGG regions) with NUP98 (via N-terminus); this interaction occurs in a RNA-dependent manner and stimulates DHX9-mediated ATPase activity and regulates transcription and splicing of a subset of genes (PubMed:28221134). Interacts (via N-terminus) with NXF1 (via N-terminus); this interaction is direct and negatively regulates NXF1-mediated nuclear export of constitutive transport element (CTE)-containing cellular mRNAs (PubMed:10924507). Interacts with RELA; this interaction is direct and activates NF-kappa-B-mediated transcription (PubMed:15355351). Interacts (via MTAD region) with RNA polymerase II holoenzyme; this interaction stimulates transcription activation in a CREB-dependent manner (PubMed:11149922, PubMed:9323138, PubMed:11416126). Interacts (via RGG region) with SMN1; this interaction links SMN1 to the RNA polymerase II holoenzyme (PubMed:11149922). Interacts with SP7 (PubMed:17303075). Interacts (via DRBM domains) with TARBP2 (via DRBM first and second domains); this interaction occurs in a small interfering (siRNA)-dependent manner (PubMed:17531811, PubMed:23361462). Interacts with TOP2A; this interaction occurs in a E2 enzyme UBE2I- and RNA-dependent manner, negatively regulates DHX9-mediated double-stranded DNA and RNA duplex helicase activity and stimulates TOP2A-mediated supercoiled DNA relaxation activity (PubMed:12711669). Interacts (via DRBM domains and C-terminus) with WRN (via 3'-5' exonuclease domain); this interaction inhibits the DNA-dependent NTPase and DNA helicase activities of DHX9 and stimulates the 3'-5' exonuclease activity of WRN (PubMed:15995249). Interacts with XRCC5; this interaction occurs in a RNA-dependent manner (PubMed:14704337). Interacts with ZIC2 (via C2H2-type domain 3) (PubMed:17251188). Interacts with MCM3AP isoform GANP (PubMed:23652018).   
  
 **Gene Ontology Information:**

Molecular Function   
  
N/A

Location   
  
N/A

Biological process   
  
N/A

---

30

- **Protein name:** Transcription initiation factor TFIID subunit 2
- **Organism:** Homo sapiens
- **Uniprot Accession Number:** Q6P1X5
- **Protein sequence length:** 1199 aa
- **1D identity (%):** 15.75
- **1D identity (%) [Gaps excluded]:** 23.78
- **1D identity - Alignment Gaps:** 418
- **1D aligned content (<aminoacid>:%):** {'P': 9.23, 'A': 5.13, 'G': 4.1, 'R': 5.64, 'K': 7.18, 'F': 6.15, 'E': 6.67, 'I': 4.1, 'C': 4.1, 'D': 5.13, 'N': 4.1, 'Y': 2.56, 'L': 7.69, 'S': 8.21, 'V': 5.64, 'H': 5.13, 'M': 1.03, 'T': 4.62, 'Q': 3.59}
- **Common reported functions (%):** 0.0
- **Common reported locations (%):** 14.29
- **Common reported processes (%):** 0.0

- **PDB ID:** 6MZC
- **Chain:** B
- **Crystallized protein length:** 968 aa
- **Resolution:** 4.5 Å
- **b-phipsi:** 0.011575
- **w-rdist:** 0.311744
- **t-alpha:** 0.007337
- **Chemical similarity (Tanimoto Index) (%):** 99.55
- **1D identity (%) [PDB]:** 2.89
- **1D identity (%) [Gaps excluded][PDB]:** 66.67
- **1D identity - Alignment Gaps [PDB]:** 1656
- **1D aligned content [PDB] (<aminoacid>:%):** {'V': 12.0, 'E': 6.0, 'L': 14.0, 'T': 6.0, 'P': 6.0, 'G': 6.0, 'K': 2.0, 'R': 6.0, 'I': 6.0, 'F': 4.0, 'S': 16.0, 'C': 2.0, 'A': 6.0, 'D': 4.0, 'H': 2.0, 'Y': 2.0}
- **2D identity (%) [PDB]:** 37.28
- **2D identity (%) [Gaps excluded][PDB]:** 85.58
- **2D identity - Alignment Gaps [PDB]:** 710
- **2D aligned content [PDB] (<2D-fold>:%):** {'.': 20.9, 'E': 20.26, 'T': 12.37, 'H': 45.2, 'G': 0.64, 'B': 0.64}
- **3D similarity (TM-Score) (%) [PDB]:** 27.03

- **Gene name:** TAF2
- **Entrez ID:** 6873
- **RefSeq ID:** NM\_003184
- **Transcript sequence length:** 5027
- **5-UTR|CDS|3-UTR identity (%):** 35.36 | 41.57 | 6.24
- **5-UTR|CDS|3-UTR identity (%) [Gaps excluded]:** 79.84 | 75.26 | 75.02
- **5-UTR|CDS|3-UTR identity [Alignment Gaps]:** 156 | 1782 | 11036
- **5-UTR aligned content (<base>:%):** {'G': 50.51, 'T': 11.11, 'C': 35.35, 'A': 3.03}
- **CDS aligned content (<base>:%):** {'A': 28.64, 'T': 20.48, 'G': 25.5, 'C': 25.38}
- **3-UTR aligned content (<base>:%):** {'G': 14.65, 'A': 31.82, 'C': 13.72, 'T': 39.81}

**Uniprot Description:**  
  
 Transcription factor TFIID is one of the general factors required for accurate and regulated initiation by RNA polymerase II. TFIID is a multimeric protein complex that plays a central role in mediating promoter responses to various activators and repressors. It requires core promoter-specific cofactors for productive transcription stimulation. TAF2 stabilizes TFIID binding to core promoter.   
  
Component of transcription factor TFIID which is composed of TBP and a number of TBP-associated factors. Interacts with TAF2C1. Component of the TFTC-HAT complex.   
  
 **Gene Ontology Information:**

Molecular Function

- chromatin binding
- RNA polymerase II general transcription initiation factor activity
- transcription regulatory region sequence-specific DNA binding

Location

- nucleoplasm
- nucleus
- transcription factor TFIID complex
- transcription factor TFTC complex

Biological process

- G2/M transition of mitotic cell cycle
- histone H3 acetylation
- monoubiquitinated histone deubiquitination
- monoubiquitinated histone H2A deubiquitination
- mRNA transcription by RNA polymerase II
- positive regulation of transcription, DNA-templated
- positive regulation of transcription initiation from RNA polymerase II promoter
- protein phosphorylation
- regulation of DNA repair
- regulation of transcription by RNA polymerase II
- response to organic cyclic compound
- RNA polymerase II preinitiation complex assembly
- transcription initiation from RNA polymerase II promoter

---

31

- **Protein name:** Ectonucleotide pyrophosphatase/phosphodiesterase family member 3
- **Organism:** Homo sapiens
- **Uniprot Accession Number:** O14638
- **Protein sequence length:** 875 aa
- **1D identity (%):** 15.95
- **1D identity (%) [Gaps excluded]:** 24.67
- **1D identity - Alignment Gaps:** 372
- **1D aligned content (<aminoacid>:%):** {'E': 5.95, 'T': 7.14, 'L': 10.71, 'K': 4.17, 'C': 1.79, 'A': 5.36, 'S': 6.55, 'R': 7.74, 'F': 5.95, 'G': 7.14, 'W': 0.6, 'M': 1.19, 'N': 2.98, 'D': 3.57, 'Q': 5.95, 'P': 8.33, 'H': 4.17, 'Y': 3.57, 'I': 1.19, 'V': 5.95}
- **Common reported functions (%):** 0.0
- **Common reported locations (%):** 7.14
- **Common reported processes (%):** 0.0

- **PDB ID:** 6C02
- **Chain:** A
- **Crystallized protein length:** 815 aa
- **Resolution:** 1.94 Å
- **b-phipsi:** 0.000738
- **w-rdist:** 0.510511
- **t-alpha:** 0.029235
- **Chemical similarity (Tanimoto Index) (%):** 66.48
- **1D identity (%) [PDB]:** 3.17
- **1D identity (%) [Gaps excluded][PDB]:** 64.94
- **1D identity - Alignment Gaps [PDB]:** 1500
- **1D aligned content [PDB] (<aminoacid>:%):** {'S': 12.0, 'R': 8.0, 'L': 10.0, 'P': 10.0, 'V': 12.0, 'F': 10.0, 'E': 6.0, 'I': 2.0, 'A': 2.0, 'T': 4.0, 'G': 8.0, 'N': 4.0, 'W': 2.0, 'H': 2.0, 'D': 2.0, 'Y': 2.0, 'K': 2.0, 'Q': 2.0}
- **2D identity (%) [PDB]:** 27.64
- **2D identity (%) [Gaps excluded][PDB]:** 83.05
- **2D identity - Alignment Gaps [PDB]:** 828
- **2D aligned content [PDB] (<2D-fold>:%):** {'.': 20.12, 'T': 18.66, 'H': 39.07, 'E': 19.53, 'B': 0.87, 'G': 1.75}
- **3D similarity (TM-Score) (%) [PDB]:** 23.01

- **Gene name:** ENPP3
- **Entrez ID:** 5169
- **RefSeq ID:** NM\_005021
- **Transcript sequence length:** 3165
- **5-UTR|CDS|3-UTR identity (%):** 20.0 | 43.29 | 2.26
- **5-UTR|CDS|3-UTR identity (%) [Gaps excluded]:** 64.0 | 74.63 | 73.44
- **5-UTR|CDS|3-UTR identity [Alignment Gaps]:** 110 | 1384 | 11604
- **5-UTR aligned content (<base>:%):** {'C': 34.38, 'T': 21.88, 'G': 37.5, 'A': 6.25}
- **CDS aligned content (<base>:%):** {'A': 28.17, 'T': 23.13, 'G': 24.18, 'C': 24.53}
- **3-UTR aligned content (<base>:%):** {'T': 45.39, 'A': 36.16, 'C': 11.81, 'G': 6.64}

**Uniprot Description:**  
  
 Hydrolase that metabolizes extracellular nucleotides, including ATP, GTP, UTP and CTP (PubMed:29717535). Limits mast cell and basophil responses during inflammation and during the chronic phases of allergic responses by eliminating the extracellular ATP that functions as signaling molecule and activates basophils and mast cells and induces the release of inflammatory cytokines. Metabolizes extracellular ATP in the lumen of the small intestine, and thereby prevents ATP-induced apoptosis of intestinal plasmacytoid dendritic cells (By similarity). Has also alkaline phosphodiesterase activity (PubMed:11342463).   
  
Monomer and homodimer.   
  
 **Gene Ontology Information:**

Molecular Function

- calcium ion binding
- nucleic acid binding
- nucleoside-triphosphate diphosphatase activity
- phosphodiesterase I activity
- zinc ion binding

Location

- apical plasma membrane
- external side of plasma membrane
- extracellular exosome
- perinuclear region of cytoplasm

Biological process

- ATP metabolic process
- basophil activation involved in immune response
- negative regulation of inflammatory response
- negative regulation of mast cell activation involved in immune response
- negative regulation of mast cell proliferation
- nucleoside triphosphate catabolic process
- phosphate ion homeostasis
- phosphate-containing compound metabolic process
- pyrimidine nucleotide metabolic process

---

32

- **Protein name:** Small nuclear ribonucleoprotein Sm D1
- **Organism:** Homo sapiens
- **Uniprot Accession Number:** P62314
- **Protein sequence length:** 119 aa
- **1D identity (%):** 2.78
- **1D identity (%) [Gaps excluded]:** 20.87
- **1D identity - Alignment Gaps:** 748
- **1D aligned content (<aminoacid>:%):** {'R': 8.33, 'L': 20.83, 'V': 20.83, 'T': 4.17, 'D': 4.17, 'M': 4.17, 'P': 20.83, 'N': 4.17, 'G': 12.5}
- **Common reported functions (%):** 6.67
- **Common reported locations (%):** 21.43
- **Common reported processes (%):** 0.0

- **PDB ID:** 7DVQ
- **Chain:** C
- **Crystallized protein length:** 902 aa
- **Resolution:** 2.89 Å
- **b-phipsi:** 0.003937
- **w-rdist:** 0.468306
- **t-alpha:** 0.0231
- **Chemical similarity (Tanimoto Index) (%):** 75.65
- **1D identity (%) [PDB]:** 3.18
- **1D identity (%) [Gaps excluded][PDB]:** 72.6
- **1D identity - Alignment Gaps [PDB]:** 1594
- **1D aligned content [PDB] (<aminoacid>:%):** {'E': 9.43, 'M': 1.89, 'D': 11.32, 'I': 13.21, 'K': 13.21, 'L': 5.66, 'P': 5.66, 'V': 9.43, 'N': 3.77, 'R': 5.66, 'T': 5.66, 'Q': 1.89, 'F': 3.77, 'G': 5.66, 'A': 1.89, 'S': 1.89}
- **2D identity (%) [PDB]:** 39.21
- **2D identity (%) [Gaps excluded][PDB]:** 83.94
- **2D identity - Alignment Gaps [PDB]:** 632
- **2D aligned content [PDB] (<2D-fold>:%):** {'.': 16.34, 'E': 25.81, 'T': 15.91, 'H': 41.29, 'G': 0.65}
- **3D similarity (TM-Score) (%) [PDB]:** 7.07

- **Gene name:** SNRPD1
- **Entrez ID:** 6632
- **RefSeq ID:** NM\_006938
- **Transcript sequence length:** 4858
- **5-UTR|CDS|3-UTR identity (%):** 36.48 | 9.57 | 21.08
- **5-UTR|CDS|3-UTR identity (%) [Gaps excluded]:** 69.05 | 76.22 | 73.39
- **5-UTR|CDS|3-UTR identity [Alignment Gaps]:** 75 | 2284 | 9010
- **5-UTR aligned content (<base>:%):** {'C': 37.93, 'T': 10.34, 'G': 48.28, 'A': 3.45}
- **CDS aligned content (<base>:%):** {'A': 36.8, 'T': 21.6, 'G': 28.0, 'C': 13.6}
- **3-UTR aligned content (<base>:%):** {'G': 19.52, 'T': 36.86, 'A': 27.03, 'C': 16.59}

**Uniprot Description:**  
  
 Plays role in pre-mRNA splicing as core component of the SMN-Sm complex that mediates spliceosomal snRNP assembly and as component of the spliceosomal U1, U2, U4 and U5 small nuclear ribonucleoproteins (snRNPs), the building blocks of the spliceosome (PubMed:11991638, PubMed:18984161, PubMed:19325628, PubMed:23333303, PubMed:25555158, PubMed:26912367, PubMed:28502770, PubMed:28781166, PubMed:28076346). Component of both the pre-catalytic spliceosome B complex and activated spliceosome C complexes (PubMed:11991638, PubMed:26912367, PubMed:28502770, PubMed:28781166, PubMed:28076346). Is also a component of the minor U12 spliceosome (PubMed:15146077). May act as a charged protein scaffold to promote snRNP assembly or strengthen snRNP-snRNP interactions through non-specific electrostatic contacts with RNA (Probable).   
  
Core component of the spliceosomal U1, U2, U4 and U5 small nuclear ribonucleoproteins (snRNPs), the building blocks of the spliceosome (PubMed:11991638, PubMed:19325628, PubMed:25555158, PubMed:26912367, PubMed:28502770, PubMed:28781166, PubMed:28076346). Most spliceosomal snRNPs contain a common set of Sm proteins, SNRPB, SNRPD1, SNRPD2, SNRPD3, SNRPE, SNRPF and SNRPG that assemble in a heptameric protein ring on the Sm site of the small nuclear RNA to form the core snRNP (PubMed:10025403, PubMed:19325628, PubMed:21113136, PubMed:25555158, PubMed:26912367, PubMed:28502770, PubMed:28781166, PubMed:28076346). Component of the U1 snRNP (PubMed:19325628, PubMed:21113136, PubMed:25555158). The U1 snRNP is composed of the U1 snRNA and the 7 core Sm proteins SNRPB, SNRPD1, SNRPD2, SNRPD3, SNRPE, SNRPF and SNRPG, and at least three U1 snRNP-specific proteins SNRNP70/U1-70K, SNRPA/U1-A and SNRPC/U1-C (PubMed:19325628, PubMed:21113136, PubMed:25555158). Component of the U4/U6-U5 tri-snRNP complex composed of the U4, U6 and U5 snRNAs and at least PRPF3, PRPF4, PRPF6, PRPF8, PRPF31, SNRNP200, TXNL4A, SNRNP40, SNRPB, SNRPD1, SNRPD2, SNRPD3, SNRPE, SNRPF, SNRPG, DDX23, CD2BP2, PPIH, SNU13, EFTUD2, SART1 and USP39, plus LSM2, LSM3, LSM4, LSM5, LSM6, LSM7 and LSM8 (PubMed:26912367). Component of the U11/U12 snRNPs that are part of the U12-type spliceosome (PubMed:15146077). Part of the SMN-Sm complex that contains SMN1, GEMIN2/SIP1, DDX20/GEMIN3, GEMIN4, GEMIN5, GEMIN6, GEMIN7, GEMIN8, STRAP/UNRIP and the Sm proteins SNRPB, SNRPD1, SNRPD2, SNRPD3, SNRPE, SNRPF and SNRPG; catalyzes core snRNPs assembly. Forms a 6S pICln-Sm complex composed of CLNS1A/pICln, SNRPD1, SNRPD2, SNRPE, SNRPF and SNRPG; ring-like structure where CLNS1A/pICln mimics additional Sm proteins and which is unable to assemble into the core snRNP.   
  
 **Gene Ontology Information:**

Molecular Function

- RNA binding
- U1 snRNP binding

Location

- catalytic step 2 spliceosome
- commitment complex
- cytosol
- methylosome
- nucleoplasm
- nucleus
- pICln-Sm protein complex
- precatalytic spliceosome
- small nuclear ribonucleoprotein complex
- SMN-Sm protein complex
- spliceosomal complex
- spliceosomal tri-snRNP complex
- U1 snRNP
- U12-type spliceosomal complex
- U2 snRNP
- U2-type catalytic step 2 spliceosome
- U2-type precatalytic spliceosome
- U4 snRNP
- U4/U6 x U5 tri-snRNP complex
- U5 snRNP

Biological process

- 7-methylguanosine cap hypermethylation
- mRNA splicing, via spliceosome
- RNA splicing
- spliceosomal complex assembly
- spliceosomal snRNP assembly
- U2-type prespliceosome assembly

---

33

- **Protein name:** Nuclear cap-binding protein subunit 1
- **Organism:** Homo sapiens
- **Uniprot Accession Number:** Q09161
- **Protein sequence length:** 790 aa
- **1D identity (%):** 14.97
- **1D identity (%) [Gaps excluded]:** 22.78
- **1D identity - Alignment Gaps:** 341
- **1D aligned content (<aminoacid>:%):** {'D': 4.03, 'K': 8.72, 'T': 6.04, 'E': 3.36, 'L': 13.42, 'I': 4.03, 'V': 6.04, 'P': 7.38, 'R': 8.05, 'G': 3.36, 'N': 2.68, 'F': 4.7, 'A': 5.37, 'Q': 7.38, 'Y': 2.68, 'H': 6.04, 'C': 2.68, 'M': 1.34, 'S': 2.01, 'W': 0.67}
- **Common reported functions (%):** 13.33
- **Common reported locations (%):** 42.86
- **Common reported processes (%):** 0.0

- **PDB ID:** 1N52
- **Chain:** A
- **Crystallized protein length:** 732 aa
- **Resolution:** 2.11 Å
- **b-phipsi:** 0.080155
- **w-rdist:** 0.286562
- **t-alpha:** 0.004389
- **Chemical similarity (Tanimoto Index) (%):** 97.63
- **1D identity (%) [PDB]:** 1.43
- **1D identity (%) [Gaps excluded][PDB]:** 75.86
- **1D identity - Alignment Gaps [PDB]:** 1514
- **1D aligned content [PDB] (<aminoacid>:%):** {'E': 9.09, 'V': 9.09, 'T': 4.55, 'L': 9.09, 'P': 4.55, 'G': 4.55, 'K': 4.55, 'D': 4.55, 'R': 4.55, 'I': 9.09, 'F': 4.55, 'S': 13.64, 'W': 4.55, 'C': 4.55, 'Q': 4.55, 'A': 4.55}
- **2D identity (%) [PDB]:** 29.53
- **2D identity (%) [Gaps excluded][PDB]:** 89.49
- **2D identity - Alignment Gaps [PDB]:** 792
- **2D aligned content [PDB] (<2D-fold>:%):** {'.': 12.32, 'H': 68.48, 'T': 16.05, 'G': 3.15}
- **3D similarity (TM-Score) (%) [PDB]:** 27.28

- **Gene name:** NCBP1
- **Entrez ID:** 4686
- **RefSeq ID:** NM\_002486
- **Transcript sequence length:** 4983
- **5-UTR|CDS|3-UTR identity (%):** 35.43 | 44.03 | 12.83
- **5-UTR|CDS|3-UTR identity (%) [Gaps excluded]:** 73.77 | 74.24 | 74.13
- **5-UTR|CDS|3-UTR identity [Alignment Gaps]:** 66 | 1265 | 10177
- **5-UTR aligned content (<base>:%):** {'G': 42.22, 'C': 42.22, 'T': 13.33, 'A': 2.22}
- **CDS aligned content (<base>:%):** {'A': 29.51, 'T': 21.69, 'G': 25.27, 'C': 23.52}
- **3-UTR aligned content (<base>:%):** {'G': 17.35, 'T': 38.06, 'C': 14.0, 'A': 30.59}

**Uniprot Description:**  
  
 Component of the cap-binding complex (CBC), which binds cotranscriptionally to the 5'-cap of pre-mRNAs and is involved in various processes such as pre-mRNA splicing, translation regulation, nonsense-mediated mRNA decay, RNA-mediated gene silencing (RNAi) by microRNAs (miRNAs) and mRNA export. The CBC complex is involved in mRNA export from the nucleus via its interaction with ALYREF/THOC4/ALY, leading to the recruitment of the mRNA export machinery to the 5'-end of mRNA and to mRNA export in a 5' to 3' direction through the nuclear pore. The CBC complex is also involved in mediating U snRNA and intronless mRNAs export from the nucleus. The CBC complex is essential for a pioneer round of mRNA translation, before steady state translation when the CBC complex is replaced by cytoplasmic cap-binding protein eIF4E. The pioneer round of mRNA translation mediated by the CBC complex plays a central role in nonsense-mediated mRNA decay (NMD), NMD only taking place in mRNAs bound to the CBC complex, but not on eIF4E-bound mRNAs. The CBC complex enhances NMD in mRNAs containing at least one exon-junction complex (EJC) via its interaction with UPF1, promoting the interaction between UPF1 and UPF2. The CBC complex is also involved in 'failsafe' NMD, which is independent of the EJC complex, while it does not participate in Staufen-mediated mRNA decay (SMD). During cell proliferation, the CBC complex is also involved in microRNAs (miRNAs) biogenesis via its interaction with SRRT/ARS2 and is required for miRNA-mediated RNA interference. The CBC complex also acts as a negative regulator of PARN, thereby acting as an inhibitor of mRNA deadenylation. In the CBC complex, NCBP1/CBP80 does not bind directly capped RNAs (m7GpppG-capped RNA) but is required to stabilize the movement of the N-terminal loop of NCBP2/CBP20 and lock the CBC into a high affinity cap-binding state with the cap structure. Associates with NCBP3 to form an alternative cap-binding complex (CBC) which plays a key role in mRNA export and is particularly important in cellular stress situations such as virus infections. The conventional CBC with NCBP2 binds both small nuclear RNA (snRNA) and messenger (mRNA) and is involved in their export from the nucleus whereas the alternative CBC with NCBP3 does not bind snRNA and associates only with mRNA thereby playing a role only in mRNA export. NCBP1/CBP80 is required for cell growth and viability (PubMed:26382858).   
  
Component of the nuclear cap-binding complex (CBC), a heterodimer composed of NCBP1/CBP80 and NCBP2/CBP20 that interacts with m7GpppG-capped RNA. Found in a U snRNA export complex containing PHAX/RNUXA, NCBP1/CBP80, NCBP2/CBP20, RAN, XPO1 and m7G-capped RNA. Identified in a IGF2BP1-dependent mRNP granule complex containing untranslated mRNAs. Interacts with PHAX/RNUXA, SRRT/ARS2, EIF4G2, IGF2BP1, HNRNPF, HNRNPH1, KIAA0427/CTIF, PARN, DROSHA, UPF1 and ALYREF/THOC4. May interact with EIF4G1; the interaction is however controversial since it is reported by PubMed:11340157, PubMed:15059963 and PubMed:15361857, but is not observed by PubMed:19648179. The large PER complex involved in the repression of transcriptional termination is composed of at least PER2, CDK9, DDX5, DHX9, NCBP1/CBP80 and POLR2A. Component of an alternative nuclear cap-binding complex (CBC) composed of NCBP1/CBP80 and NCBP3 (PubMed:26382858). Interacts with METTL3 (PubMed:27117702). Interacts with ZFC3H1 in a RNase-insensitive manner (PubMed:27871484). Interacts with MTREX (PubMed:30842217). Interacts with TASOR (By similarity).   
  
 **Gene Ontology Information:**

Molecular Function

- molecular adaptor activity
- mRNA binding
- RNA 7-methylguanosine cap binding
- RNA binding
- RNA cap binding

Location

- cytoplasm
- cytosol
- mitochondrion
- mRNA cap binding complex
- nuclear cap binding complex
- nucleoplasm
- nucleus
- ribonucleoprotein complex
- RNA cap binding complex

Biological process

- 7-methylguanosine mRNA capping
- alternative mRNA splicing, via spliceosome
- cap-dependent translational initiation
- defense response to virus
- histone mRNA metabolic process
- gene silencing by miRNA
- mRNA 3'-end processing
- mRNA export from nucleus
- mRNA metabolic process
- mRNA splicing, via spliceosome
- mRNA transcription by RNA polymerase II
- nuclear-transcribed mRNA catabolic process, nonsense-mediated decay
- positive regulation of cell growth
- positive regulation of mRNA 3'-end processing
- positive regulation of mRNA splicing, via spliceosome
- positive regulation of RNA binding
- positive regulation of transcription elongation from RNA polymerase II promoter
- primary miRNA processing
- regulation of mRNA processing
- regulation of translational initiation
- RNA catabolic process
- RNA splicing
- snRNA export from nucleus
- spliceosomal complex assembly

---

34

- **Protein name:** Complement C5
- **Organism:** Homo sapiens
- **Uniprot Accession Number:** P01031
- **Protein sequence length:** 1676 aa
- **1D identity (%):** 14.23
- **1D identity (%) [Gaps excluded]:** 29.02
- **1D identity - Alignment Gaps:** 867
- **1D aligned content (<aminoacid>:%):** {'M': 2.07, 'Y': 5.37, 'P': 8.68, 'A': 2.89, 'G': 9.09, 'K': 7.02, 'F': 6.2, 'S': 4.96, 'L': 10.74, 'D': 5.79, 'V': 7.44, 'E': 3.72, 'H': 4.96, 'R': 4.13, 'T': 4.96, 'I': 3.72, 'N': 2.07, 'Q': 4.13, 'C': 2.07}
- **Common reported functions (%):** 0.0
- **Common reported locations (%):** 0.0
- **Common reported processes (%):** 0.0

- **PDB ID:** 6I2X
- **Chain:** A
- **Crystallized protein length:** 815 aa
- **Resolution:** 3.35 Å
- **b-phipsi:** 0.002937
- **w-rdist:** 0.379372
- **t-alpha:** 0.085215
- **Chemical similarity (Tanimoto Index) (%):** 86.91
- **1D identity (%) [PDB]:** 2.44
- **1D identity (%) [Gaps excluded][PDB]:** 72.22
- **1D identity - Alignment Gaps [PDB]:** 1547
- **1D aligned content [PDB] (<aminoacid>:%):** {'T': 5.13, 'L': 17.95, 'S': 7.69, 'N': 7.69, 'I': 5.13, 'V': 15.38, 'K': 5.13, 'G': 15.38, 'P': 12.82, 'Q': 2.56, 'F': 2.56, 'A': 2.56}
- **2D identity (%) [PDB]:** 32.11
- **2D identity (%) [Gaps excluded][PDB]:** 84.43
- **2D identity - Alignment Gaps [PDB]:** 743
- **2D aligned content [PDB] (<2D-fold>:%):** {'.': 17.14, 'H': 44.42, 'T': 14.55, 'E': 22.86, 'B': 1.04}
- **3D similarity (TM-Score) (%) [PDB]:** 23.78

- **Gene name:** C5
- **Entrez ID:** N/A
- **RefSeq ID:** NM\_001735
- **Transcript sequence length:** 5464
- **5-UTR|CDS|3-UTR identity (%):** 12.03 | 36.0 | 2.37
- **5-UTR|CDS|3-UTR identity (%) [Gaps excluded]:** 69.57 | 77.13 | 77.75
- **5-UTR|CDS|3-UTR identity [Alignment Gaps]:** 110 | 2767 | 11564
- **5-UTR aligned content (<base>:%):** {'A': 6.25, 'T': 25.0, 'C': 43.75, 'G': 25.0}
- **CDS aligned content (<base>:%):** {'A': 27.94, 'T': 21.04, 'G': 25.21, 'C': 25.8}
- **3-UTR aligned content (<base>:%):** {'A': 32.16, 'T': 36.4, 'G': 16.25, 'C': 15.19}

**Uniprot Description:**  
  
 Activation of C5 by a C5 convertase initiates the spontaneous assembly of the late complement components, C5-C9, into the membrane attack complex. C5b has a transient binding site for C6. The C5b-C6 complex is the foundation upon which the lytic complex is assembled.   
  
C5 precursor is first processed by the removal of 4 basic residues, forming two chains, beta and alpha, linked by a disulfide bond. C5 convertase activates C5 by cleaving the alpha chain, releasing C5a anaphylatoxin and generating C5b (beta chain + alpha' chain). The C5a anaphylatoxin interacts with C5AR1. Interacts with tick complement inhibitor.   
  
 **Gene Ontology Information:**

Molecular Function   
  
N/A

Location   
  
N/A

Biological process   
  
N/A

---

35

- **Protein name:** Insulin receptor
- **Organism:** Homo sapiens
- **Uniprot Accession Number:** P06213
- **Protein sequence length:** 1382 aa
- **1D identity (%):** 14.87
- **1D identity (%) [Gaps excluded]:** 25.67
- **1D identity - Alignment Gaps:** 597
- **1D aligned content (<aminoacid>:%):** {'M': 1.42, 'G': 9.48, 'A': 3.79, 'P': 10.9, 'L': 10.43, 'F': 4.27, 'K': 4.74, 'Y': 2.84, 'E': 5.69, 'D': 5.21, 'R': 6.16, 'V': 6.16, 'I': 4.27, 'W': 1.42, 'Q': 3.79, 'H': 3.79, 'S': 5.69, 'C': 2.37, 'T': 6.16, 'N': 1.42}
- **Common reported functions (%):** 0.0
- **Common reported locations (%):** 14.29
- **Common reported processes (%):** 0.0

- **PDB ID:** 7BW8
- **Chain:** A
- **Crystallized protein length:** 551 aa
- **Resolution:** 3.8 Å
- **b-phipsi:** 0.022729
- **w-rdist:** 0.418381
- **t-alpha:** 0.001457
- **Chemical similarity (Tanimoto Index) (%):** 97.19
- **1D identity (%) [PDB]:** 2.38
- **1D identity (%) [Gaps excluded][PDB]:** 66.67
- **1D identity - Alignment Gaps [PDB]:** 1296
- **1D aligned content [PDB] (<aminoacid>:%):** {'T': 12.5, 'I': 9.38, 'R': 6.25, 'A': 3.12, 'S': 12.5, 'P': 6.25, 'D': 12.5, 'E': 12.5, 'K': 6.25, 'F': 6.25, 'N': 3.12, 'Y': 3.12, 'V': 6.25}
- **2D identity (%) [PDB]:** 23.67
- **2D identity (%) [Gaps excluded][PDB]:** 78.33
- **2D identity - Alignment Gaps [PDB]:** 746
- **2D aligned content [PDB] (<2D-fold>:%):** {'E': 32.81, '.': 35.18, 'T': 17.0, 'G': 2.37, 'H': 11.46, 'B': 1.19}
- **3D similarity (TM-Score) (%) [PDB]:** 14.71

- **Gene name:** INSR
- **Entrez ID:** 3643
- **RefSeq ID:** NM\_001079817
- **Transcript sequence length:** 9427
- **5-UTR|CDS|3-UTR identity (%):** 20.99 | 41.46 | 23.88
- **5-UTR|CDS|3-UTR identity (%) [Gaps excluded]:** 87.3 | 77.03 | 74.67
- **5-UTR|CDS|3-UTR identity [Alignment Gaps]:** 398 | 2009 | 8595
- **5-UTR aligned content (<base>:%):** {'T': 9.09, 'G': 42.73, 'C': 45.45, 'A': 2.73}
- **CDS aligned content (<base>:%):** {'A': 24.06, 'T': 18.02, 'G': 28.16, 'C': 29.77}
- **3-UTR aligned content (<base>:%):** {'C': 18.95, 'A': 26.04, 'T': 34.13, 'G': 20.87}

**Uniprot Description:**  
  
 Receptor tyrosine kinase which mediates the pleiotropic actions of insulin. Binding of insulin leads to phosphorylation of several intracellular substrates, including, insulin receptor substrates (IRS1, 2, 3, 4), SHC, GAB1, CBL and other signaling intermediates. Each of these phosphorylated proteins serve as docking proteins for other signaling proteins that contain Src-homology-2 domains (SH2 domain) that specifically recognize different phosphotyrosine residues, including the p85 regulatory subunit of PI3K and SHP2. Phosphorylation of IRSs proteins lead to the activation of two main signaling pathways: the PI3K-AKT/PKB pathway, which is responsible for most of the metabolic actions of insulin, and the Ras-MAPK pathway, which regulates expression of some genes and cooperates with the PI3K pathway to control cell growth and differentiation. Binding of the SH2 domains of PI3K to phosphotyrosines on IRS1 leads to the activation of PI3K and the generation of phosphatidylinositol-(3, 4, 5)-triphosphate (PIP3), a lipid second messenger, which activates several PIP3-dependent serine/threonine kinases, such as PDPK1 and subsequently AKT/PKB. The net effect of this pathway is to produce a translocation of the glucose transporter SLC2A4/GLUT4 from cytoplasmic vesicles to the cell membrane to facilitate glucose transport. Moreover, upon insulin stimulation, activated AKT/PKB is responsible for: anti-apoptotic effect of insulin by inducing phosphorylation of BAD; regulates the expression of gluconeogenic and lipogenic enzymes by controlling the activity of the winged helix or forkhead (FOX) class of transcription factors. Another pathway regulated by PI3K-AKT/PKB activation is mTORC1 signaling pathway which regulates cell growth and metabolism and integrates signals from insulin. AKT mediates insulin-stimulated protein synthesis by phosphorylating TSC2 thereby activating mTORC1 pathway. The Ras/RAF/MAP2K/MAPK pathway is mainly involved in mediating cell growth, survival and cellular differentiation of insulin. Phosphorylated IRS1 recruits GRB2/SOS complex, which triggers the activation of the Ras/RAF/MAP2K/MAPK pathway. In addition to binding insulin, the insulin receptor can bind insulin-like growth factors (IGFI and IGFII). Isoform Short has a higher affinity for IGFII binding. When present in a hybrid receptor with IGF1R, binds IGF1. PubMed:12138094 shows that hybrid receptors composed of IGF1R and INSR isoform Long are activated with a high affinity by IGF1, with low affinity by IGF2 and not significantly activated by insulin, and that hybrid receptors composed of IGF1R and INSR isoform Short are activated by IGF1, IGF2 and insulin. In contrast, PubMed:16831875 shows that hybrid receptors composed of IGF1R and INSR isoform Long and hybrid receptors composed of IGF1R and INSR isoform Short have similar binding characteristics, both bind IGF1 and have a low affinity for insulin. In adipocytes, inhibits lipolysis (By similarity).   
  
Tetramer of 2 alpha and 2 beta chains linked by disulfide bonds. The alpha chains carry the insulin-binding regions, while the beta chains carry the kinase domain. Forms a hybrid receptor with IGF1R, the hybrid is a tetramer consisting of 1 alpha chain and 1 beta chain of INSR and 1 alpha chain and 1 beta chain of IGF1R. Interacts with SORBS1 but dissociates from it following insulin stimulation. Binds SH2B2. Activated form of INSR interacts (via Tyr-999) with the PTB/PID domains of IRS1 and SHC1. The sequences surrounding the phosphorylated NPXY motif contribute differentially to either IRS1 or SHC1 recognition. Interacts (via tyrosines in the C-terminus) with IRS2 (via PTB domain and 591-786 AA); the 591-786 would be the primary anchor of IRS2 to INSR while the PTB domain would have a stabilizing action on the interaction with INSR. Interacts with the SH2 domains of the 85 kDa regulatory subunit of PI3K (PIK3R1) in vitro, when autophosphorylated on tyrosine residues. Interacts with SOCS7. Interacts (via the phosphorylated Tyr-999), with SOCS3. Interacts (via the phosphorylated Tyr-1185, Tyr-1189, Tyr-1190) with SOCS1. Interacts with CAV2 (tyrosine-phosphorylated form); the interaction is increased with 'Tyr-27'phosphorylation of CAV2 (By similarity). Interacts with ARRB2 (By similarity). Interacts with GRB10; this interaction blocks the association between IRS1/IRS2 and INSR, significantly reduces insulin-stimulated tyrosine phosphorylation of IRS1 and IRS2 and thus decreases insulin signaling. Interacts with GRB7. Interacts with PDPK1. Interacts (via Tyr-1190) with GRB14 (via BPS domain); this interaction protects the tyrosines in the activation loop from dephosphorylation, but promotes dephosphorylation of Tyr-999, this results in decreased interaction with, and phosphorylation of, IRS1. Interacts (via subunit alpha) with ENPP1 (via 485-599 AA); this interaction blocks autophosphorylation. Interacts with PTPRE; this interaction is dependent of Tyr-1185, Tyr-1189 and Tyr-1190 of the INSR. Interacts with STAT5B (via SH2 domain). Interacts with PTPRF. Interacts with ATIC; ATIC together with PRKAA2/AMPK2 and HACD3/PTPLAD1 is proposed to be part of a signaling netwok regulating INSR autophosphorylation and endocytosis (By similarity). Interacts with the cone snail venom insulin Con-Ins G1 (PubMed:27617429). Interacts with the insulin receptor SORL1; this interaction strongly increases its surface exposure, hence strengthens insulin signal reception (PubMed:27322061). Interacts (tyrosine phosphorylated) with CCDC88A/GIV (via SH2-like region); binding requires autophosphorylation of the INSR C-terminal region (PubMed:25187647). Interacts with GNAI3; the interaction is probably mediated by CCDC88A/GIV (PubMed:25187647). Interacts with LMBRD1 (By similarity).   
  
 **Gene Ontology Information:**

Molecular Function

- amyloid-beta binding
- ATP binding
- cargo receptor activity
- GTP binding
- identical protein binding
- insulin binding
- insulin-activated receptor activity
- insulin receptor substrate binding
- insulin-like growth factor I binding
- insulin-like growth factor II binding
- insulin-like growth factor receptor binding
- phosphatidylinositol 3-kinase binding
- protein domain specific binding
- protein tyrosine kinase activity
- protein-containing complex binding
- PTB domain binding
- structural molecule activity

Location

- axon
- caveola
- dendrite membrane
- endosome membrane
- external side of plasma membrane
- extracellular exosome
- insulin receptor complex
- late endosome
- lysosome
- membrane
- neuronal cell body membrane
- plasma membrane
- receptor complex

Biological process

- activation of protein kinase activity
- activation of protein kinase B activity
- adrenal gland development
- amyloid-beta clearance
- carbohydrate metabolic process
- cellular response to growth factor stimulus
- cellular response to insulin stimulus
- dendritic spine maintenance
- epidermis development
- exocrine pancreas development
- G protein-coupled receptor signaling pathway
- glucose homeostasis
- heart morphogenesis
- insulin receptor signaling pathway
- learning
- male gonad development
- male sex determination
- memory
- neuron projection maintenance
- peptidyl-tyrosine autophosphorylation
- peptidyl-tyrosine phosphorylation
- positive regulation of cell migration
- positive regulation of cell population proliferation
- positive regulation of developmental growth
- positive regulation of transcription, DNA-templated
- positive regulation of glucose import
- positive regulation of glycogen biosynthetic process
- positive regulation of glycolytic process
- positive regulation of kinase activity
- positive regulation of MAP kinase activity
- positive regulation of MAPK cascade
- positive regulation of meiotic cell cycle
- positive regulation of mitotic nuclear division
- positive regulation of nitric oxide biosynthetic process
- positive regulation of phosphatidylinositol 3-kinase signaling
- positive regulation of protein kinase B signaling
- positive regulation of protein phosphorylation
- positive regulation of protein-containing complex disassembly
- positive regulation of receptor internalization
- positive regulation of respiratory burst
- protein autophosphorylation
- protein phosphorylation
- receptor internalization
- receptor-mediated endocytosis
- regulation of transcription, DNA-templated
- regulation of embryonic development
- regulation of female gonad development
- transmembrane receptor protein tyrosine kinase signaling pathway
- transport across blood-brain barrier
- viral entry into host cell

---

36

- **Protein name:** Dipeptidyl peptidase 4
- **Organism:** Homo sapiens
- **Uniprot Accession Number:** P27487
- **Protein sequence length:** 766 aa
- **1D identity (%):** 14.16
- **1D identity (%) [Gaps excluded]:** 22.83
- **1D identity - Alignment Gaps:** 381
- **1D aligned content (<aminoacid>:%):** {'K': 4.93, 'P': 8.45, 'G': 9.86, 'D': 5.63, 'R': 6.34, 'N': 3.52, 'V': 7.75, 'L': 9.86, 'E': 2.82, 'I': 7.04, 'S': 7.04, 'W': 2.11, 'T': 7.75, 'F': 4.23, 'Q': 2.11, 'C': 4.23, 'Y': 3.52, 'M': 0.7, 'A': 2.11}
- **Common reported functions (%):** 0.0
- **Common reported locations (%):** 14.29
- **Common reported processes (%):** 0.0

- **PDB ID:** 2P8S
- **Chain:** A
- **Crystallized protein length:** 728 aa
- **Resolution:** 2.2 Å
- **b-phipsi:** 0.005292
- **w-rdist:** 0.833767
- **t-alpha:** 0.0
- **Chemical similarity (Tanimoto Index) (%):** 73.71
- **1D identity (%) [PDB]:** 1.96
- **1D identity (%) [Gaps excluded][PDB]:** 81.08
- **1D identity - Alignment Gaps [PDB]:** 1492
- **1D aligned content [PDB] (<aminoacid>:%):** {'R': 10.0, 'E': 6.67, 'F': 3.33, 'G': 13.33, 'I': 10.0, 'V': 6.67, 'K': 6.67, 'T': 6.67, 'D': 3.33, 'L': 6.67, 'Q': 3.33, 'P': 6.67, 'S': 3.33, 'Y': 3.33, 'N': 3.33, 'A': 6.67}
- **2D identity (%) [PDB]:** 36.33
- **2D identity (%) [Gaps excluded][PDB]:** 83.19
- **2D identity - Alignment Gaps [PDB]:** 614
- **2D aligned content [PDB] (<2D-fold>:%):** {'.': 17.93, 'E': 46.72, 'T': 17.93, 'G': 0.76, 'H': 16.16, 'B': 0.51}
- **3D similarity (TM-Score) (%) [PDB]:** 25.59

- **Gene name:** DPP4
- **Entrez ID:** 1803
- **RefSeq ID:** NM\_001935
- **Transcript sequence length:** 3573
- **5-UTR|CDS|3-UTR identity (%):** 36.0 | 43.68 | 5.56
- **5-UTR|CDS|3-UTR identity (%) [Gaps excluded]:** 81.08 | 74.5 | 76.57
- **5-UTR|CDS|3-UTR identity [Alignment Gaps]:** 139 | 1273 | 11176
- **5-UTR aligned content (<base>:%):** {'A': 2.22, 'G': 42.22, 'C': 47.78, 'T': 7.78}
- **CDS aligned content (<base>:%):** {'A': 29.99, 'T': 22.99, 'C': 22.32, 'G': 24.7}
- **3-UTR aligned content (<base>:%):** {'A': 33.73, 'C': 15.37, 'T': 35.22, 'G': 15.67}

**Uniprot Description:**  
  
 Cell surface glycoprotein receptor involved in the costimulatory signal essential for T-cell receptor (TCR)-mediated T-cell activation (PubMed:10951221, PubMed:10900005, PubMed:11772392, PubMed:17287217). Acts as a positive regulator of T-cell coactivation, by binding at least ADA, CAV1, IGF2R, and PTPRC (PubMed:10951221, PubMed:10900005, PubMed:11772392, PubMed:14691230). Its binding to CAV1 and CARD11 induces T-cell proliferation and NF-kappa-B activation in a T-cell receptor/CD3-dependent manner (PubMed:17287217). Its interaction with ADA also regulates lymphocyte-epithelial cell adhesion (PubMed:11772392). In association with FAP is involved in the pericellular proteolysis of the extracellular matrix (ECM), the migration and invasion of endothelial cells into the ECM (PubMed:16651416, PubMed:10593948). May be involved in the promotion of lymphatic endothelial cells adhesion, migration and tube formation (PubMed:18708048). When overexpressed, enhanced cell proliferation, a process inhibited by GPC3 (PubMed:17549790). Acts also as a serine exopeptidase with a dipeptidyl peptidase activity that regulates various physiological processes by cleaving peptides in the circulation, including many chemokines, mitogenic growth factors, neuropeptides and peptide hormones such as brain natriuretic peptide 32 (PubMed:16254193, PubMed:10570924). Removes N-terminal dipeptides sequentially from polypeptides having unsubstituted N-termini provided that the penultimate residue is proline (PubMed:10593948).   
  
Monomer. Homodimer (PubMed:12832764, PubMed:15448155, PubMed:17287217, PubMed:12646248, PubMed:12483204, PubMed:12906826). Heterodimer with Seprase (FAP) (PubMed:16651416). Requires homodimerization for optimal dipeptidyl peptidase activity and T-cell costimulation. Found in a membrane raft complex, at least composed of BCL10, CARD11, DPP4 and IKBKB (PubMed:17287217). Associates with collagen (PubMed:8526932). Interacts with PTPRC; the interaction is enhanced in an interleukin-12-dependent manner in activated lymphocytes (PubMed:12676959). Interacts (via extracellular domain) with ADA; does not inhibit its dipeptidyl peptidase activity (PubMed:15016824, PubMed:10951221, PubMed:14691230, PubMed:7907293, PubMed:8101391). Interacts with CAV1 (via the N-terminus); the interaction is direct (PubMed:17287217). Interacts (via cytoplasmic tail) with CARD11 (via PDZ domain); its homodimerization is necessary for interaction with CARD11 (PubMed:17287217). Interacts with IGF2R; the interaction is direct (PubMed:10900005). Interacts with GPC3 (PubMed:17549790). Interacts with human coronavirus-EMC spike protein and acts as a receptor for this virus (PubMed:23486063).   
  
 **Gene Ontology Information:**

Molecular Function

- aminopeptidase activity
- chemorepellent activity
- dipeptidyl-peptidase activity
- identical protein binding
- protease binding
- protein homodimerization activity
- serine-type endopeptidase activity
- serine-type peptidase activity
- signaling receptor binding
- virus receptor activity

Location

- apical plasma membrane
- cell surface
- endocytic vesicle
- extracellular exosome
- extracellular region
- focal adhesion
- intercellular canaliculus
- lamellipodium
- lamellipodium membrane
- lysosomal membrane
- membrane
- membrane raft
- plasma membrane

Biological process

- behavioral fear response
- cell adhesion
- endothelial cell migration
- glucagon processing
- locomotory exploration behavior
- membrane fusion
- negative regulation of extracellular matrix disassembly
- negative regulation of neutrophil chemotaxis
- peptide hormone processing
- positive regulation of cell population proliferation
- proteolysis
- psychomotor behavior
- receptor-mediated endocytosis of virus by host cell
- receptor-mediated virion attachment to host cell
- regulation of cell-cell adhesion mediated by integrin
- response to hypoxia
- T cell activation
- T cell costimulation
- viral entry into host cell

---

37

- **Protein name:** Cap-specific mRNA (nucleoside-2'-O-)-methyltransferase 1
- **Organism:** Homo sapiens
- **Uniprot Accession Number:** Q8N1G2
- **Protein sequence length:** 835 aa
- **1D identity (%):** 16.68
- **1D identity (%) [Gaps excluded]:** 22.41
- **1D identity - Alignment Gaps:** 248
- **1D aligned content (<aminoacid>:%):** {'M': 1.85, 'P': 10.49, 'A': 3.09, 'G': 7.41, 'T': 4.94, 'L': 11.11, 'E': 6.17, 'D': 6.17, 'R': 9.26, 'V': 5.56, 'F': 8.02, 'K': 8.02, 'S': 3.09, 'I': 4.32, 'W': 0.62, 'N': 0.62, 'Q': 2.47, 'H': 3.09, 'C': 1.23, 'Y': 2.47}
- **Common reported functions (%):** 0.0
- **Common reported locations (%):** 21.43
- **Common reported processes (%):** 0.0

- **PDB ID:** 8P4E
- **Chain:** O
- **Crystallized protein length:** 695 aa
- **Resolution:** 3.9 Å
- **b-phipsi:** 0.001562
- **w-rdist:** 0.534916
- **t-alpha:** 0.028464
- **Chemical similarity (Tanimoto Index) (%):** N/A
- **1D identity (%) [PDB]:** 2.56
- **1D identity (%) [Gaps excluded][PDB]:** 74.51
- **1D identity - Alignment Gaps [PDB]:** 1431
- **1D aligned content [PDB] (<aminoacid>:%):** {'V': 13.16, 'C': 5.26, 'E': 7.89, 'L': 7.89, 'K': 21.05, 'S': 2.63, 'P': 5.26, 'F': 2.63, 'T': 5.26, 'I': 5.26, 'G': 2.63, 'H': 2.63, 'Q': 2.63, 'R': 7.89, 'Y': 2.63, 'N': 2.63, 'A': 2.63}
- **2D identity (%) [PDB]:** 45.31
- **2D identity (%) [Gaps excluded][PDB]:** 88.27
- **2D identity - Alignment Gaps [PDB]:** 493
- **2D aligned content [PDB] (<2D-fold>:%):** {'.': 17.21, 'T': 23.53, 'E': 21.35, 'H': 35.29, 'G': 2.61}
- **3D similarity (TM-Score) (%) [PDB]:** 5.37

- **Gene name:** CMTR1
- **Entrez ID:** 23070
- **RefSeq ID:** NM\_015050
- **Transcript sequence length:** 4034
- **5-UTR|CDS|3-UTR identity (%):** 44.32 | 45.93 | 6.74
- **5-UTR|CDS|3-UTR identity (%) [Gaps excluded]:** 78.1 | 77.32 | 75.09
- **5-UTR|CDS|3-UTR identity [Alignment Gaps]:** 80 | 1296 | 11067
- **5-UTR aligned content (<base>:%):** {'G': 43.9, 'C': 46.34, 'A': 2.44, 'T': 7.32}
- **CDS aligned content (<base>:%):** {'A': 25.03, 'T': 19.71, 'G': 28.92, 'C': 26.33}
- **3-UTR aligned content (<base>:%):** {'G': 25.37, 'A': 17.44, 'C': 27.07, 'T': 30.12}

**Uniprot Description:**  
  
 S-adenosyl-L-methionine-dependent methyltransferase that mediates mRNA cap1 2'-O-ribose methylation to the 5'-cap structure of mRNAs. Methylates the ribose of the first nucleotide of a m(7)GpppG-capped mRNA and small nuclear RNA (snRNA) to produce m(7)GpppRm (cap1). Displays a preference for cap0 transcripts. Cap1 modification is linked to higher levels of translation. May be involved in the interferon response pathway.   
  
Interacts with POLR2A (via C-terminus).   
  
 **Gene Ontology Information:**

Molecular Function

- mRNA (nucleoside-2'-O-)-methyltransferase activity
- nucleic acid binding

Location

- cytoplasm
- intracellular membrane-bounded organelle
- nucleoplasm
- nucleus

Biological process

- 7-methylguanosine mRNA capping
- cap1 mRNA methylation
- mRNA methylation

---

38

- **Protein name:** Toll-like receptor 3
- **Organism:** Homo sapiens
- **Uniprot Accession Number:** O15455
- **Protein sequence length:** 904 aa
- **1D identity (%):** 16.25
- **1D identity (%) [Gaps excluded]:** 22.72
- **1D identity - Alignment Gaps:** 293
- **1D aligned content (<aminoacid>:%):** {'P': 5.99, 'I': 4.79, 'L': 13.77, 'C': 2.99, 'V': 6.59, 'H': 5.99, 'K': 5.39, 'T': 4.19, 'Q': 2.99, 'D': 4.19, 'N': 5.99, 'G': 4.79, 'E': 7.19, 'F': 6.59, 'S': 5.39, 'R': 6.59, 'Y': 2.99, 'A': 3.59}
- **Common reported functions (%):** 6.67
- **Common reported locations (%):** 14.29
- **Common reported processes (%):** 9.52

- **PDB ID:** 7WVJ
- **Chain:** B
- **Crystallized protein length:** 659 aa
- **Resolution:** 3.26 Å
- **b-phipsi:** 0.008361
- **w-rdist:** 0.626898
- **t-alpha:** 0.0
- **Chemical similarity (Tanimoto Index) (%):** 96.75
- **1D identity (%) [PDB]:** 1.43
- **1D identity (%) [Gaps excluded][PDB]:** 77.78
- **1D identity - Alignment Gaps [PDB]:** 1443
- **1D aligned content [PDB] (<aminoacid>:%):** {'T': 9.52, 'E': 4.76, 'V': 4.76, 'H': 4.76, 'L': 9.52, 'K': 9.52, 'S': 4.76, 'F': 4.76, 'Q': 4.76, 'R': 4.76, 'I': 9.52, 'D': 4.76, 'A': 4.76, 'G': 9.52, 'P': 9.52}
- **2D identity (%) [PDB]:** N/A
- **2D identity (%) [Gaps excluded][PDB]:** N/A
- **2D identity - Alignment Gaps [PDB]:** N/A
- **2D aligned content [PDB] (<2D-fold>:%):** N/A
- **3D similarity (TM-Score) (%) [PDB]:** 21.67

- **Gene name:** TLR3
- **Entrez ID:** 7098
- **RefSeq ID:** N/A
- **Sequence length:** N/A
- **5-UTR|CDS|3-UTR identity (%):** N/A | N/A | N/A
- **5-UTR|CDS|3-UTR identity (%) [Gaps excluded]:** N/A | N/A | N/A
- **5-UTR|CDS|3-UTR identity [Alignment Gaps]:** N/A | N/A | N/A
- **5-UTR aligned content (<base>:%):** N/A
- **CDS aligned content (<base>:%):** N/A
- **3-UTR aligned content (<base>:%):** N/A

**Uniprot Description:**  
  
 Key component of innate and adaptive immunity. TLRs (Toll-like receptors) control host immune response against pathogens through recognition of molecular patterns specific to microorganisms. TLR3 is a nucleotide-sensing TLR which is activated by double-stranded RNA, a sign of viral infection. Acts via the adapter TRIF/TICAM1, leading to NF-kappa-B activation, IRF3 nuclear translocation, cytokine secretion and the inflammatory response.   
  
Monomer and homodimer; dimerization is triggered by ligand-binding, the signaling unit is composed of one ds-RNA of around 40 bp and two TLR3 molecules, and lateral clustering of signaling units along the length of the ds-RNA ligand is required for TLR3 signal transduction. Interacts (via transmembrane domain) with UNC93B1; the interaction is required for transport from the ER to the endosomes (PubMed:33432245). Interacts with SRC; upon binding of double-stranded RNA. Interacts with TICAM1 (via the TIR domain) in response to poly(I:C) and this interaction is enhanced in the presence of WDFY1 (PubMed:25736436). The tyrosine-phosphorylated form (via TIR domain) interacts with WDFY1 (via WD repeat 2) in response to poly(I:C) (PubMed:25736436).   
  
 **Gene Ontology Information:**

Molecular Function

- double-stranded RNA binding
- identical protein binding
- pattern recognition receptor activity
- signaling receptor activity
- transmembrane signaling receptor activity

Location

- cytoplasm
- early endosome
- endolysosome membrane
- endoplasmic reticulum membrane
- endosome membrane
- extracellular matrix
- extracellular space
- Golgi membrane
- lysosomal membrane
- membrane
- plasma membrane

Biological process

- activation of NF-kappaB-inducing kinase activity
- cellular response to exogenous dsRNA
- cellular response to interferon-beta
- cellular response to mechanical stimulus
- cellular response to interferon-gamma
- cellular response to virus
- cellular response to xenobiotic stimulus
- defense response to bacterium
- defense response to virus
- detection of virus
- extrinsic apoptotic signaling pathway
- hyperosmotic response
- I-kappaB phosphorylation
- inflammatory response to wounding
- innate immune response
- JNK cascade
- male gonad development
- microglial cell activation
- necroptotic signaling pathway
- negative regulation of osteoclast differentiation
- positive regulation of angiogenesis
- positive regulation of apoptotic process
- positive regulation of chemokine production
- positive regulation of cytokine production involved in inflammatory response
- positive regulation of gene expression
- positive regulation of I-kappaB kinase/NF-kappaB signaling
- positive regulation of inflammatory response
- positive regulation of interferon-alpha production
- positive regulation of interferon-beta production
- positive regulation of interleukin-12 production
- positive regulation of interleukin-6 production
- positive regulation of interleukin-8 production
- positive regulation of JNK cascade
- positive regulation of macrophage cytokine production
- positive regulation of NF-kappaB transcription factor activity
- positive regulation of NIK/NF-kappaB signaling
- positive regulation of transcription by RNA polymerase II
- positive regulation of tumor necrosis factor production
- positive regulation of interferon-gamma production
- positive regulation of type III interferon production
- regulation of dendritic cell cytokine production
- response to dsRNA
- response to exogenous dsRNA
- signal transduction
- toll-like receptor 3 signaling pathway
- toll-like receptor signaling pathway
- type III interferon production

---

39

- **Protein name:** Macrophage-expressed gene 1 protein
- **Organism:** Homo sapiens
- **Uniprot Accession Number:** Q2M385
- **Protein sequence length:** 716 aa
- **1D identity (%):** 14.24
- **1D identity (%) [Gaps excluded]:** 23.65
- **1D identity - Alignment Gaps:** 391
- **1D aligned content (<aminoacid>:%):** {'F': 5.0, 'I': 2.86, 'G': 10.71, 'V': 8.57, 'A': 4.29, 'L': 8.57, 'P': 10.71, 'E': 5.0, 'D': 4.29, 'R': 6.43, 'T': 10.0, 'S': 4.29, 'N': 3.57, 'W': 0.71, 'K': 5.0, 'H': 2.14, 'Q': 4.29, 'C': 2.14, 'M': 0.71, 'Y': 0.71}
- **Common reported functions (%):** 0.0
- **Common reported locations (%):** 0.0
- **Common reported processes (%):** 0.0

- **PDB ID:** 6U23
- **Chain:** O
- **Crystallized protein length:** 583 aa
- **Resolution:** 3.49 Å
- **b-phipsi:** 0.012095
- **w-rdist:** 0.296664
- **t-alpha:** 0.011045
- **Chemical similarity (Tanimoto Index) (%):** N/A
- **1D identity (%) [PDB]:** 2.38
- **1D identity (%) [Gaps excluded][PDB]:** 86.84
- **1D identity - Alignment Gaps [PDB]:** 1349
- **1D aligned content [PDB] (<aminoacid>:%):** {'E': 6.06, 'L': 15.15, 'V': 12.12, 'P': 9.09, 'G': 9.09, 'D': 3.03, 'I': 6.06, 'F': 6.06, 'K': 6.06, 'S': 12.12, 'C': 3.03, 'Q': 3.03, 'H': 3.03, 'A': 3.03, 'R': 3.03}
- **2D identity (%) [PDB]:** 23.64
- **2D identity (%) [Gaps excluded][PDB]:** 81.31
- **2D identity - Alignment Gaps [PDB]:** 783
- **2D aligned content [PDB] (<2D-fold>:%):** {'.': 22.61, 'H': 31.03, 'E': 26.44, 'T': 18.39, 'B': 1.53}
- **3D similarity (TM-Score) (%) [PDB]:** 20.91

- **Gene name:** MPEG1
- **Entrez ID:** 219972
- **RefSeq ID:** NM\_001039396
- **Transcript sequence length:** 4418
- **5-UTR|CDS|3-UTR identity (%):** 28.89 | 44.83 | 10.81
- **5-UTR|CDS|3-UTR identity (%) [Gaps excluded]:** 72.22 | 74.33 | 75.08
- **5-UTR|CDS|3-UTR identity [Alignment Gaps]:** 81 | 1171 | 10545
- **5-UTR aligned content (<base>:%):** {'G': 58.97, 'T': 15.38, 'C': 25.64}
- **CDS aligned content (<base>:%):** {'A': 25.02, 'T': 18.9, 'G': 26.76, 'C': 29.33}
- **3-UTR aligned content (<base>:%):** {'T': 33.71, 'C': 20.8, 'A': 27.18, 'G': 18.32}

**Uniprot Description:**  
  
 Plays a key role in the innate immune response following bacterial infection by inserting into the bacterial surface to form pores (By similarity). By breaching the surface of phagocytosed bacteria, allows antimicrobial effectors to enter the bacterial periplasmic space and degrade bacterial proteins such as superoxide dismutase sodC which contributes to bacterial virulence (By similarity). Shows antibacterial activity against a wide spectrum of Gram-positive, Gram-negative and acid-fast bacteria (PubMed:23753625, PubMed:26402460, PubMed:30609079). Reduces the viability of the intracytosolic pathogen L.monocytogenes by inhibiting acidification of the phagocytic vacuole of host cells which restricts bacterial translocation from the vacuole to the cytosol (By similarity). Required for the antibacterial activity of reactive oxygen species and nitric oxide (By similarity). N/A   
  
 **Gene Ontology Information:**

Molecular Function   
  
N/A

Location

- cytoplasmic vesicle
- cytoplasmic vesicle membrane
- extracellular region
- phagocytic vesicle

Biological process

- antibacterial innate immune response
- defense response to bacterium
- defense response to Gram-negative bacterium
- defense response to Gram-positive bacterium

---

40

- **Protein name:** Voltage-dependent calcium channel subunit alpha-2/delta-1
- **Organism:** Homo sapiens
- **Uniprot Accession Number:** P54289
- **Protein sequence length:** 1103 aa
- **1D identity (%):** 16.1
- **1D identity (%) [Gaps excluded]:** 23.68
- **1D identity - Alignment Gaps:** 374
- **1D aligned content (<aminoacid>:%):** {'A': 4.26, 'G': 8.51, 'L': 6.91, 'Q': 4.26, 'P': 9.04, 'F': 3.72, 'S': 5.32, 'T': 5.85, 'I': 7.45, 'K': 6.38, 'D': 9.04, 'Y': 5.85, 'E': 4.26, 'V': 6.91, 'R': 4.26, 'N': 3.19, 'W': 0.53, 'M': 1.06, 'C': 1.6, 'H': 1.6}
- **Common reported functions (%):** 6.67
- **Common reported locations (%):** 7.14
- **Common reported processes (%):** 0.0

- **PDB ID:** 8IF3
- **Chain:** A
- **Crystallized protein length:** 917 aa
- **Resolution:** 3.2 Å
- **b-phipsi:** 0.00555
- **w-rdist:** 0.429452
- **t-alpha:** 0.024627
- **Chemical similarity (Tanimoto Index) (%):** 81.79
- **1D identity (%) [PDB]:** 3.02
- **1D identity (%) [Gaps excluded][PDB]:** 67.61
- **1D identity - Alignment Gaps [PDB]:** 1521
- **1D aligned content [PDB] (<aminoacid>:%):** {'T': 6.25, 'A': 10.42, 'R': 12.5, 'S': 6.25, 'D': 8.33, 'Q': 6.25, 'I': 6.25, 'K': 6.25, 'L': 4.17, 'F': 4.17, 'N': 4.17, 'Y': 2.08, 'V': 10.42, 'E': 2.08, 'G': 6.25, 'P': 4.17}
- **2D identity (%) [PDB]:** 39.55
- **2D identity (%) [Gaps excluded][PDB]:** 89.41
- **2D identity - Alignment Gaps [PDB]:** 643
- **2D aligned content [PDB] (<2D-fold>:%):** {'.': 16.45, 'H': 37.28, 'T': 23.46, 'E': 22.81}
- **3D similarity (TM-Score) (%) [PDB]:** 23.56

- **Gene name:** CACNA2D1
- **Entrez ID:** 781
- **RefSeq ID:** N/A
- **Sequence length:** N/A
- **5-UTR|CDS|3-UTR identity (%):** N/A | N/A | N/A
- **5-UTR|CDS|3-UTR identity (%) [Gaps excluded]:** N/A | N/A | N/A
- **5-UTR|CDS|3-UTR identity [Alignment Gaps]:** N/A | N/A | N/A
- **5-UTR aligned content (<base>:%):** N/A
- **CDS aligned content (<base>:%):** N/A
- **3-UTR aligned content (<base>:%):** N/A

**Uniprot Description:**  
  
 The alpha-2/delta subunit of voltage-dependent calcium channels regulates calcium current density and activation/inactivation kinetics of the calcium channel. Plays an important role in excitation-contraction coupling (By similarity).   
  
Dimer formed of alpha-2-1 and delta-1 chains; disulfide-linked. Voltage-dependent calcium channels are multisubunit complexes, consisting of alpha-1 (CACNA1), alpha-2 (CACNA2D), beta (CACNB) and delta (CACNA2D) subunits in a 1:1:1:1 ratio (By similarity).   
  
 **Gene Ontology Information:**

Molecular Function

- metal ion binding
- voltage-gated calcium channel activity

Location

- extracellular exosome
- L-type voltage-gated calcium channel complex
- neuronal dense core vesicle
- plasma membrane
- sarcoplasmic reticulum
- voltage-gated calcium channel complex

Biological process

- calcium ion import across plasma membrane
- calcium ion transmembrane transport
- calcium ion transmembrane transport via high voltage-gated calcium channel
- calcium ion transport
- calcium ion transport into cytosol
- cardiac muscle cell action potential involved in contraction
- cellular response to amyloid-beta
- membrane depolarization during bundle of His cell action potential
- positive regulation of high voltage-gated calcium channel activity
- positive regulation of muscle contraction
- regulation of calcium ion transmembrane transport via high voltage-gated calcium channel
- regulation of calcium ion transport
- regulation of heart rate by cardiac conduction
- regulation of membrane repolarization during action potential
- regulation of ventricular cardiac muscle cell membrane repolarization

---

41

- **Protein name:** GATOR complex protein DEPDC5
- **Organism:** Homo sapiens
- **Uniprot Accession Number:** O75140
- **Protein sequence length:** 1603 aa
- **1D identity (%):** 14.47
- **1D identity (%) [Gaps excluded]:** 27.69
- **1D identity - Alignment Gaps:** 772
- **1D aligned content (<aminoacid>:%):** {'M': 2.14, 'G': 9.4, 'L': 8.12, 'P': 7.26, 'Y': 4.27, 'F': 5.13, 'D': 5.56, 'R': 6.84, 'I': 2.56, 'Q': 5.56, 'A': 2.99, 'E': 5.56, 'K': 5.56, 'C': 2.14, 'V': 7.26, 'N': 1.71, 'H': 5.13, 'S': 5.13, 'T': 7.26, 'W': 0.43}
- **Common reported functions (%):** 0.0
- **Common reported locations (%):** 7.14
- **Common reported processes (%):** 0.0

- **PDB ID:** 7T3B
- **Chain:** A
- **Crystallized protein length:** 989 aa
- **Resolution:** 3.9 Å
- **b-phipsi:** 0.003456
- **w-rdist:** 0.443784
- **t-alpha:** 0.03496
- **Chemical similarity (Tanimoto Index) (%):** 99.47
- **1D identity (%) [PDB]:** 2.78
- **1D identity (%) [Gaps excluded][PDB]:** 69.01
- **1D identity - Alignment Gaps [PDB]:** 1692
- **1D aligned content [PDB] (<aminoacid>:%):** {'K': 6.12, 'L': 8.16, 'D': 6.12, 'Q': 4.08, 'T': 10.2, 'I': 6.12, 'R': 6.12, 'A': 10.2, 'S': 8.16, 'E': 4.08, 'F': 4.08, 'N': 4.08, 'P': 4.08, 'V': 8.16, 'G': 6.12, 'M': 2.04, 'Y': 2.04}
- **2D identity (%) [PDB]:** 37.45
- **2D identity (%) [Gaps excluded][PDB]:** 86.31
- **2D identity - Alignment Gaps [PDB]:** 724
- **2D aligned content [PDB] (<2D-fold>:%):** {'.': 16.91, 'E': 30.27, 'H': 42.59, 'T': 8.98, 'G': 1.25}
- **3D similarity (TM-Score) (%) [PDB]:** 14.62

- **Gene name:** DEPDC5
- **Entrez ID:** 9681
- **RefSeq ID:** N/A
- **Sequence length:** N/A
- **5-UTR|CDS|3-UTR identity (%):** N/A | N/A | N/A
- **5-UTR|CDS|3-UTR identity (%) [Gaps excluded]:** N/A | N/A | N/A
- **5-UTR|CDS|3-UTR identity [Alignment Gaps]:** N/A | N/A | N/A
- **5-UTR aligned content (<base>:%):** N/A
- **CDS aligned content (<base>:%):** N/A
- **3-UTR aligned content (<base>:%):** N/A

**Uniprot Description:**  
  
 As a component of the GATOR1 complex functions as an inhibitor of the amino acid-sensing branch of the TORC1 pathway. The GATOR1 complex strongly increases GTP hydrolysis by RRAGA and RRAGB within RRAGC-containing heterodimers, thereby deactivating RRAGs, releasing mTORC1 from lysosomal surface and inhibiting mTORC1 signaling. The GATOR1 complex is negatively regulated by GATOR2 the other GATOR subcomplex in this amino acid-sensing branch of the TORC1 pathway.   
  
Within the GATOR complex, component of the GATOR1 subcomplex, made of DEPDC5, NPRL2 and NPRL3. GATOR1 mediates the strong interaction of the GATOR complex with RRAGA/RRAGC and RRAGB/RRAGC heterodimers. Interacts (via DEP domain) with KLHL22; the interaction depends on amino acid availability (PubMed:29769719).   
  
 **Gene Ontology Information:**

Molecular Function

- GTPase activator activity
- protein-containing complex binding

Location

- cytosol
- GATOR1 complex
- lysosomal membrane
- lysosome
- perinuclear region of cytoplasm

Biological process

- cellular response to amino acid starvation
- intracellular signal transduction
- negative regulation of TOR signaling
- negative regulation of TORC1 signaling
- positive regulation of autophagy

---

42

- **Protein name:** Phenylalanine--tRNA ligase beta subunit
- **Organism:** Homo sapiens
- **Uniprot Accession Number:** Q9NSD9
- **Protein sequence length:** 589 aa
- **1D identity (%):** 14.35
- **1D identity (%) [Gaps excluded]:** 26.63
- **1D identity - Alignment Gaps:** 434
- **1D aligned content (<aminoacid>:%):** {'M': 0.74, 'P': 9.63, 'R': 4.44, 'D': 5.93, 'L': 11.85, 'T': 7.41, 'E': 3.7, 'F': 5.19, 'S': 2.22, 'G': 4.44, 'A': 5.19, 'V': 8.89, 'Y': 3.7, 'C': 4.44, 'K': 8.15, 'I': 8.15, 'Q': 2.22, 'H': 2.22, 'N': 1.48}
- **Common reported functions (%):** 6.67
- **Common reported locations (%):** 21.43
- **Common reported processes (%):** 4.76

- **PDB ID:** 3L4G
- **Chain:** H
- **Crystallized protein length:** 589 aa
- **Resolution:** 3.3 Å
- **b-phipsi:** 0.002196
- **w-rdist:** 0.682222
- **t-alpha:** 0.014033
- **Chemical similarity (Tanimoto Index) (%):** 87.49
- **1D identity (%) [PDB]:** 2.93
- **1D identity (%) [Gaps excluded][PDB]:** 64.52
- **1D identity - Alignment Gaps [PDB]:** 1303
- **1D aligned content [PDB] (<aminoacid>:%):** {'M': 2.5, 'F': 2.5, 'L': 15.0, 'T': 10.0, 'Y': 5.0, 'A': 7.5, 'G': 10.0, 'V': 20.0, 'K': 5.0, 'P': 5.0, 'E': 2.5, 'D': 2.5, 'N': 5.0, 'R': 2.5, 'S': 2.5, 'C': 2.5}
- **2D identity (%) [PDB]:** 35.69
- **2D identity (%) [Gaps excluded][PDB]:** 85.27
- **2D identity - Alignment Gaps [PDB]:** 585
- **2D aligned content [PDB] (<2D-fold>:%):** {'.': 16.43, 'E': 29.53, 'H': 34.54, 'T': 16.71, 'B': 0.56, 'G': 2.23}
- **3D similarity (TM-Score) (%) [PDB]:** 18.3

- **Gene name:** FARSB
- **Entrez ID:** 10056
- **RefSeq ID:** N/A
- **Sequence length:** N/A
- **5-UTR|CDS|3-UTR identity (%):** N/A | N/A | N/A
- **5-UTR|CDS|3-UTR identity (%) [Gaps excluded]:** N/A | N/A | N/A
- **5-UTR|CDS|3-UTR identity [Alignment Gaps]:** N/A | N/A | N/A
- **5-UTR aligned content (<base>:%):** N/A
- **CDS aligned content (<base>:%):** N/A
- **3-UTR aligned content (<base>:%):** N/A

**Uniprot Description:**  
  
 N/A   
  
Heterotetramer; dimer of two heterodimers formed by FARSA and FARSB.   
  
 **Gene Ontology Information:**

Molecular Function

- ATP binding
- magnesium ion binding
- phenylalanine-tRNA ligase activity
- RNA binding

Location

- cytoplasm
- cytosol
- membrane
- phenylalanine-tRNA ligase complex

Biological process

- phenylalanyl-tRNA aminoacylation
- protein heterotetramerization
- translation

---

43

- **Protein name:** Histone-lysine N-methyltransferase EZH2
- **Organism:** Homo sapiens
- **Uniprot Accession Number:** Q15910
- **Protein sequence length:** 746 aa
- **1D identity (%):** 12.62
- **1D identity (%) [Gaps excluded]:** 22.13
- **1D identity - Alignment Gaps:** 439
- **1D aligned content (<aminoacid>:%):** {'K': 8.53, 'E': 10.85, 'P': 10.08, 'R': 6.2, 'V': 6.98, 'F': 2.33, 'D': 4.65, 'L': 9.3, 'T': 3.88, 'S': 3.1, 'A': 5.43, 'M': 0.78, 'G': 5.43, 'N': 4.65, 'Q': 3.1, 'I': 3.88, 'Y': 3.88, 'H': 2.33, 'C': 4.65}
- **Common reported functions (%):** 0.0
- **Common reported locations (%):** 14.29
- **Common reported processes (%):** 0.0

- **PDB ID:** 5LS6
- **Chain:** J
- **Crystallized protein length:** 570 aa
- **Resolution:** 3.47 Å
- **b-phipsi:** 0.00571
- **w-rdist:** 0.688466
- **t-alpha:** 0.00219
- **Chemical similarity (Tanimoto Index) (%):** N/A
- **1D identity (%) [PDB]:** 1.37
- **1D identity (%) [Gaps excluded][PDB]:** 79.17
- **1D identity - Alignment Gaps [PDB]:** 1365
- **1D aligned content [PDB] (<aminoacid>:%):** {'R': 15.79, 'T': 5.26, 'S': 10.53, 'V': 10.53, 'I': 5.26, 'A': 21.05, 'P': 5.26, 'Y': 10.53, 'H': 5.26, 'L': 5.26, 'F': 5.26}
- **2D identity (%) [PDB]:** 30.12
- **2D identity (%) [Gaps excluded][PDB]:** 86.81
- **2D identity - Alignment Gaps [PDB]:** 685
- **2D aligned content [PDB] (<2D-fold>:%):** {'.': 20.57, 'H': 49.05, 'E': 18.99, 'T': 10.44, 'G': 0.95}
- **3D similarity (TM-Score) (%) [PDB]:** 19.6

- **Gene name:** EZH2
- **Entrez ID:** 2146
- **RefSeq ID:** N/A
- **Sequence length:** N/A
- **5-UTR|CDS|3-UTR identity (%):** N/A | N/A | N/A
- **5-UTR|CDS|3-UTR identity (%) [Gaps excluded]:** N/A | N/A | N/A
- **5-UTR|CDS|3-UTR identity [Alignment Gaps]:** N/A | N/A | N/A
- **5-UTR aligned content (<base>:%):** N/A
- **CDS aligned content (<base>:%):** N/A
- **3-UTR aligned content (<base>:%):** N/A

**Uniprot Description:**  
  
 Polycomb group (PcG) protein. Catalytic subunit of the PRC2/EED-EZH2 complex, which methylates 'Lys-9' (H3K9me) and 'Lys-27' (H3K27me) of histone H3, leading to transcriptional repression of the affected target gene. Able to mono-, di- and trimethylate 'Lys-27' of histone H3 to form H3K27me1, H3K27me2 and H3K27me3, respectively. Displays a preference for substrates with less methylation, loses activity when progressively more methyl groups are incorporated into H3K27, H3K27me0 > H3K27me1 > H3K27me2 (PubMed:22323599, PubMed:30923826). Compared to EZH1-containing complexes, it is more abundant in embryonic stem cells and plays a major role in forming H3K27me3, which is required for embryonic stem cell identity and proper differentiation. The PRC2/EED-EZH2 complex may also serve as a recruiting platform for DNA methyltransferases, thereby linking two epigenetic repression systems. Genes repressed by the PRC2/EED-EZH2 complex include HOXC8, HOXA9, MYT1, CDKN2A and retinoic acid target genes. EZH2 can also methylate non-histone proteins such as the transcription factor GATA4 and the nuclear receptor RORA. Regulates the circadian clock via histone methylation at the promoter of the circadian genes. Essential for the CRY1/2-mediated repression of the transcriptional activation of PER1/2 by the CLOCK-ARNTL/BMAL1 heterodimer; involved in the di and trimethylation of 'Lys-27' of histone H3 on PER1/2 promoters which is necessary for the CRY1/2 proteins to inhibit transcription.   
  
Component of the PRC2/EED-EZH2 complex, which includes EED, EZH2, SUZ12, RBBP4 and RBBP7 and possibly AEBP2. The minimum components required for methyltransferase activity of the PRC2/EED-EZH2 complex are EED, EZH2 and SUZ12. The PRC2 complex may also interact with DNMT1, DNMT3A, DNMT3B and PHF1 via the EZH2 subunit and with SIRT1 via the SUZ12 subunit. Interacts with HDAC1 and HDAC2. Binds ATRX via the SET domain (Probable). Interacts with PRAME. Interacts with CDYL. Interacts with CLOCK, ARNTL/BMAL1 and CRY1 (By similarity). Interacts with DNMT3L; the interaction is direct (By similarity). Interacts with EZHIP; the interaction blocks EZH2 methyltransferase activity (PubMed:30923826, PubMed:31086175, PubMed:31451685). Interacts with ZNF263; recruited to the SIX3 promoter along with other proteins involved in chromatin modification and transcriptional corepression where it contributes to transcriptional repression (PubMed:32051553).   
  
 **Gene Ontology Information:**

Molecular Function

- chromatin binding
- chromatin DNA binding
- histone methyltransferase activity (H3-K27 specific)
- histone H3K27 trimethyltransferase activity
- histone methyltransferase activity
- lncRNA binding
- primary miRNA binding
- promoter-specific chromatin binding
- protein-lysine N-methyltransferase activity
- ribonucleoprotein complex binding
- RNA polymerase II cis-regulatory region sequence-specific DNA binding
- RNA polymerase II core promoter sequence-specific DNA binding
- transcription corepressor activity
- transcription corepressor binding

Location

- chromatin
- chromatin silencing complex
- chromosome, telomeric region
- ESC/E(Z) complex
- nucleoplasm
- nucleus
- pericentric heterochromatin
- pronucleus
- synapse

Biological process

- B cell differentiation
- cardiac muscle hypertrophy in response to stress
- cellular response to hydrogen peroxide
- cellular response to trichostatin A
- cerebellar cortex development
- chromatin organization
- DNA methylation
- facultative heterochromatin formation
- G1 to G0 transition
- G1/S transition of mitotic cell cycle
- hepatocyte homeostasis
- heterochromatin assembly
- hippocampus development
- histone H3-K27 methylation
- keratinocyte differentiation
- liver regeneration
- negative regulation of cytokine production involved in inflammatory response
- negative regulation of DNA-binding transcription factor activity
- negative regulation of transcription, DNA-templated
- negative regulation of G1/S transition of mitotic cell cycle
- negative regulation of gene expression, epigenetic
- negative regulation of keratinocyte differentiation
- negative regulation of retinoic acid receptor signaling pathway
- negative regulation of stem cell differentiation
- negative regulation of striated muscle cell differentiation
- negative regulation of transcription by RNA polymerase II
- negative regulation of transcription elongation from RNA polymerase II promoter
- positive regulation of cell cycle G1/S phase transition
- positive regulation of cell population proliferation
- positive regulation of dendrite development
- positive regulation of epithelial to mesenchymal transition
- positive regulation of GTPase activity
- positive regulation of MAP kinase activity
- positive regulation of protein serine/threonine kinase activity
- protein localization to chromatin
- regulation of circadian rhythm
- regulation of transcription, DNA-templated
- regulation of gliogenesis
- regulation of kidney development
- response to estradiol
- response to tetrachloromethane
- rhythmic process
- skeletal muscle satellite cell maintenance involved in skeletal muscle regeneration
- stem cell differentiation
- subtelomeric heterochromatin assembly
- synaptic transmission, GABAergic

---

44

- **Protein name:** Protein transport protein Sec24A
- **Organism:** Homo sapiens
- **Uniprot Accession Number:** O95486
- **Protein sequence length:** 1093 aa
- **1D identity (%):** 16.1
- **1D identity (%) [Gaps excluded]:** 24.29
- **1D identity - Alignment Gaps:** 396
- **1D aligned content (<aminoacid>:%):** {'M': 1.06, 'S': 6.88, 'G': 9.52, 'P': 14.29, 'A': 4.23, 'L': 11.64, 'Q': 5.82, 'Y': 3.7, 'F': 4.76, 'T': 6.35, 'C': 1.59, 'R': 3.7, 'V': 8.99, 'D': 5.29, 'H': 2.65, 'I': 2.65, 'K': 3.17, 'N': 2.12, 'E': 1.59}
- **Common reported functions (%):** 0.0
- **Common reported locations (%):** 7.14
- **Common reported processes (%):** 0.0

- **PDB ID:** 5VNE
- **Chain:** B
- **Crystallized protein length:** 729 aa
- **Resolution:** 2.7 Å
- **b-phipsi:** 0.003322
- **w-rdist:** 0.735875
- **t-alpha:** 0.009559
- **Chemical similarity (Tanimoto Index) (%):** 94.1
- **1D identity (%) [PDB]:** 2.36
- **1D identity (%) [Gaps excluded][PDB]:** 80.0
- **1D identity - Alignment Gaps [PDB]:** 1480
- **1D aligned content [PDB] (<aminoacid>:%):** {'P': 8.33, 'Q': 11.11, 'T': 5.56, 'L': 19.44, 'N': 5.56, 'G': 13.89, 'V': 11.11, 'R': 2.78, 'F': 5.56, 'A': 5.56, 'D': 2.78, 'H': 2.78, 'K': 5.56}
- **2D identity (%) [PDB]:** 38.08
- **2D identity (%) [Gaps excluded][PDB]:** 84.43
- **2D identity - Alignment Gaps [PDB]:** 594
- **2D aligned content [PDB] (<2D-fold>:%):** {'.': 17.23, 'E': 23.79, 'T': 12.38, 'H': 44.42, 'G': 2.18}
- **3D similarity (TM-Score) (%) [PDB]:** 22.81

- **Gene name:** SEC24A
- **Entrez ID:** 10802
- **RefSeq ID:** NM\_021982
- **Transcript sequence length:** 6389
- **5-UTR|CDS|3-UTR identity (%):** 33.33 | 44.49 | 15.46
- **5-UTR|CDS|3-UTR identity (%) [Gaps excluded]:** 78.4 | 74.76 | 75.44
- **5-UTR|CDS|3-UTR identity [Alignment Gaps]:** 169 | 1488 | 9703
- **5-UTR aligned content (<base>:%):** {'T': 8.16, 'G': 37.76, 'C': 51.02, 'A': 3.06}
- **CDS aligned content (<base>:%):** {'A': 26.61, 'T': 21.9, 'G': 23.43, 'C': 28.07}
- **3-UTR aligned content (<base>:%):** {'A': 33.09, 'T': 40.19, 'G': 15.16, 'C': 11.56}

**Uniprot Description:**  
  
 Component of the coat protein complex II (COPII) which promotes the formation of transport vesicles from the endoplasmic reticulum (ER). The coat has two main functions, the physical deformation of the endoplasmic reticulum membrane into vesicles and the selection of cargo molecules for their transport to the Golgi complex (PubMed:20427317, PubMed:17499046, PubMed:18843296). Plays a central role in cargo selection within the COPII complex and together with SEC24B may have a different specificity compared to SEC24C and SEC24D. May package preferentially cargos with cytoplasmic DxE or LxxLE motifs and may also recognize conformational epitopes (PubMed:17499046, PubMed:18843296).   
  
COPII is composed of at least five proteins: the Sec23/24 complex, the Sec13/31 complex and Sar1 (PubMed:17499046). Interacts with TMED2 (PubMed:20427317). Interacts (as part of the Sec23/24 complex) with SEC22B; recruits SEC22B into COPII-coated vesicles for its transport from the endoplasmic reticulum to the Golgi (PubMed:17499046). Interacts with TMEM39A (PubMed:31806350). Interacts with SACM1L; this interaction is reduced in the absence of TMEM39A (PubMed:31806350).   
  
 **Gene Ontology Information:**

Molecular Function

- SNARE binding
- zinc ion binding

Location

- COPII vesicle coat
- cytosol
- endoplasmic reticulum exit site
- endoplasmic reticulum membrane
- ER to Golgi transport vesicle membrane

Biological process

- cholesterol homeostasis
- COPII-coated vesicle cargo loading
- endoplasmic reticulum to Golgi vesicle-mediated transport
- intracellular protein transport
- positive regulation of protein secretion
- regulation of cholesterol transport

---

45

- **Protein name:** Dipeptidyl peptidase 8
- **Organism:** Homo sapiens
- **Uniprot Accession Number:** Q6V1X1
- **Protein sequence length:** 898 aa
- **1D identity (%):** 15.51
- **1D identity (%) [Gaps excluded]:** 24.16
- **1D identity - Alignment Gaps:** 383
- **1D aligned content (<aminoacid>:%):** {'G': 7.83, 'N': 4.22, 'A': 4.22, 'M': 1.2, 'E': 5.42, 'S': 4.22, 'L': 10.24, 'H': 4.82, 'P': 9.04, 'F': 6.02, 'D': 4.22, 'Y': 4.22, 'I': 5.42, 'R': 8.43, 'V': 6.02, 'Q': 4.22, 'K': 3.01, 'T': 5.42, 'C': 1.2, 'W': 0.6}
- **Common reported functions (%):** 0.0
- **Common reported locations (%):** 14.29
- **Common reported processes (%):** 0.0

- **PDB ID:** 7SVO
- **Chain:** A
- **Crystallized protein length:** 848 aa
- **Resolution:** 2.58 Å
- **b-phipsi:** 0.005603
- **w-rdist:** 0.670833
- **t-alpha:** 0.005098
- **Chemical similarity (Tanimoto Index) (%):** 85.5
- **1D identity (%) [PDB]:** 2.66
- **1D identity (%) [Gaps excluded][PDB]:** 63.24
- **1D identity - Alignment Gaps [PDB]:** 1548
- **1D aligned content [PDB] (<aminoacid>:%):** {'L': 16.28, 'Y': 2.33, 'A': 6.98, 'M': 2.33, 'P': 6.98, 'I': 9.3, 'R': 6.98, 'V': 13.95, 'E': 6.98, 'T': 2.33, 'G': 2.33, 'K': 4.65, 'D': 4.65, 'F': 2.33, 'S': 6.98, 'C': 2.33, 'H': 2.33}
- **2D identity (%) [PDB]:** 43.11
- **2D identity (%) [Gaps excluded][PDB]:** 86.76
- **2D identity - Alignment Gaps [PDB]:** 566
- **2D aligned content [PDB] (<2D-fold>:%):** {'E': 36.36, '.': 14.26, 'H': 22.93, 'T': 24.79, 'G': 1.24, 'B': 0.41}
- **3D similarity (TM-Score) (%) [PDB]:** 27.05

- **Gene name:** DPP8
- **Entrez ID:** 54878
- **RefSeq ID:** N/A
- **Sequence length:** N/A
- **5-UTR|CDS|3-UTR identity (%):** N/A | N/A | N/A
- **5-UTR|CDS|3-UTR identity (%) [Gaps excluded]:** N/A | N/A | N/A
- **5-UTR|CDS|3-UTR identity [Alignment Gaps]:** N/A | N/A | N/A
- **5-UTR aligned content (<base>:%):** N/A
- **CDS aligned content (<base>:%):** N/A
- **3-UTR aligned content (<base>:%):** N/A

**Uniprot Description:**  
  
 Dipeptidyl peptidase that cleaves off N-terminal dipeptides from proteins having a Pro or Ala residue at position 2.   
  
Homodimer.   
  
 **Gene Ontology Information:**

Molecular Function

- aminopeptidase activity
- dipeptidyl-peptidase activity
- serine-type peptidase activity

Location

- cytoplasm
- cytosol

Biological process

- apoptotic process
- immune response
- negative regulation of programmed cell death
- proteolysis

---

46

- **Protein name:** Maltase-glucoamylase, intestinal
- **Organism:** Homo sapiens
- **Uniprot Accession Number:** O43451
- **Protein sequence length:** 1857 aa
- **1D identity (%):** 13.63
- **1D identity (%) [Gaps excluded]:** 30.55
- **1D identity - Alignment Gaps:** 1040
- **1D aligned content (<aminoacid>:%):** {'M': 1.17, 'A': 4.3, 'L': 10.16, 'P': 11.33, 'G': 10.94, 'D': 3.91, 'T': 6.64, 'F': 4.3, 'H': 4.3, 'Y': 5.86, 'I': 3.52, 'K': 2.34, 'R': 5.47, 'N': 2.34, 'E': 5.47, 'Q': 3.52, 'V': 8.2, 'S': 3.91, 'W': 1.17, 'C': 1.17}
- **Common reported functions (%):** 0.0
- **Common reported locations (%):** 7.14
- **Common reported processes (%):** 0.0

- **PDB ID:** 3TON
- **Chain:** A
- **Crystallized protein length:** 890 aa
- **Resolution:** 2.95 Å
- **b-phipsi:** 0.005234
- **w-rdist:** 0.680082
- **t-alpha:** 0.007337
- **Chemical similarity (Tanimoto Index) (%):** 96.82
- **1D identity (%) [PDB]:** 2.95
- **1D identity (%) [Gaps excluded][PDB]:** 74.24
- **1D identity - Alignment Gaps [PDB]:** 1596
- **1D aligned content [PDB] (<aminoacid>:%):** {'D': 10.2, 'K': 14.29, 'I': 12.24, 'Y': 4.08, 'E': 8.16, 'L': 10.2, 'P': 10.2, 'R': 8.16, 'T': 6.12, 'F': 4.08, 'G': 4.08, 'V': 6.12, 'S': 2.04}
- **2D identity (%) [PDB]:** 36.95
- **2D identity (%) [Gaps excluded][PDB]:** 83.74
- **2D identity - Alignment Gaps [PDB]:** 670
- **2D aligned content [PDB] (<2D-fold>:%):** {'.': 21.67, 'B': 0.45, 'E': 36.57, 'H': 27.77, 'T': 12.19, 'G': 1.35}
- **3D similarity (TM-Score) (%) [PDB]:** 27.16

- **Gene name:** MGAM
- **Entrez ID:** 8972
- **RefSeq ID:** NM\_004668
- **Transcript sequence length:** 6484
- **5-UTR|CDS|3-UTR identity (%):** 17.57 | 33.88 | 4.23
- **5-UTR|CDS|3-UTR identity (%) [Gaps excluded]:** 78.79 | 79.24 | 75.11
- **5-UTR|CDS|3-UTR identity [Alignment Gaps]:** 115 | 3270 | 11386
- **5-UTR aligned content (<base>:%):** {'A': 3.85, 'T': 23.08, 'G': 38.46, 'C': 34.62}
- **CDS aligned content (<base>:%):** {'A': 25.01, 'T': 19.02, 'G': 27.55, 'C': 28.42}
- **3-UTR aligned content (<base>:%):** {'A': 35.88, 'T': 34.12, 'G': 18.82, 'C': 11.18}

**Uniprot Description:**  
  
 May serve as an alternate pathway for starch digestion when luminal alpha-amylase activity is reduced because of immaturity or malnutrition. May play a unique role in the digestion of malted dietary oligosaccharides used in food manufacturing.   
  
Monomer.   
  
 **Gene Ontology Information:**

Molecular Function

- alpha-1,4-glucosidase activity
- amylase activity
- carbohydrate binding
- catalytic activity
- glucan 1,4-alpha-glucosidase activity
- maltose alpha-glucosidase activity

Location

- apical plasma membrane
- extracellular exosome
- ficolin-1-rich granule membrane
- plasma membrane
- tertiary granule membrane

Biological process

- dextrin catabolic process
- maltose catabolic process
- starch catabolic process

---

47

- **Protein name:** Protein argonaute-4
- **Organism:** Homo sapiens
- **Uniprot Accession Number:** Q9HCK5
- **Protein sequence length:** 861 aa
- **1D identity (%):** 77.38
- **1D identity (%) [Gaps excluded]:** 79.39
- **1D identity - Alignment Gaps:** 22
- **1D aligned content (<aminoacid>:%):** {'P': 7.42, 'F': 4.45, 'R': 6.23, 'G': 7.27, 'T': 6.08, 'I': 5.19, 'L': 7.57, 'A': 6.08, 'N': 2.37, 'K': 5.49, 'D': 4.75, 'Y': 3.86, 'H': 3.12, 'E': 4.75, 'V': 8.16, 'M': 2.08, 'Q': 6.68, 'S': 4.75, 'W': 0.89, 'C': 2.82}
- **Common reported functions (%):** 26.67
- **Common reported locations (%):** 57.14
- **Common reported processes (%):** 28.57

- **PDB ID:** 6OON
- **Chain:** A
- **Crystallized protein length:** 785 aa
- **Resolution:** 1.9 Å
- **b-phipsi:** 0.007896
- **w-rdist:** 0.237756
- **t-alpha:** 0.043313
- **Chemical similarity (Tanimoto Index) (%):** 99.62
- **1D identity (%) [PDB]:** 74.49
- **1D identity (%) [Gaps excluded][PDB]:** 78.81
- **1D identity - Alignment Gaps [PDB]:** 46
- **1D aligned content [PDB] (<aminoacid>:%):** {'F': 4.8, 'P': 7.36, 'R': 6.56, 'G': 6.24, 'T': 6.4, 'I': 5.44, 'L': 8.16, 'A': 6.24, 'N': 2.4, 'K': 5.44, 'D': 4.64, 'Y': 4.16, 'H': 2.72, 'E': 4.32, 'V': 8.8, 'M': 2.08, 'Q': 6.08, 'S': 4.32, 'W': 0.96, 'C': 2.88}
- **2D identity (%) [PDB]:** 71.03
- **2D identity (%) [Gaps excluded][PDB]:** 78.35
- **2D identity - Alignment Gaps [PDB]:** 80
- **2D aligned content [PDB] (<2D-fold>:%):** {'.': 17.96, 'B': 2.14, 'E': 32.45, 'H': 35.75, 'T': 10.21, 'G': 1.48}
- **3D similarity (TM-Score) (%) [PDB]:** 90.43

- **Gene name:** AGO4
- **Entrez ID:** 192670
- **RefSeq ID:** NM\_017629
- **Transcript sequence length:** 7272
- **5-UTR|CDS|3-UTR identity (%):** 25.74 | 69.33 | 21.24
- **5-UTR|CDS|3-UTR identity (%) [Gaps excluded]:** 87.5 | 74.74 | 75.77
- **5-UTR|CDS|3-UTR identity [Alignment Gaps]:** 288 | 194 | 9091
- **5-UTR aligned content (<base>:%):** {'G': 46.67, 'T': 5.71, 'C': 45.71, 'A': 1.9}
- **CDS aligned content (<base>:%):** {'A': 26.26, 'T': 20.99, 'G': 27.02, 'C': 25.73}
- **3-UTR aligned content (<base>:%):** {'G': 22.74, 'T': 33.92, 'C': 17.7, 'A': 25.64}

**Uniprot Description:**  
  
 Required for RNA-mediated gene silencing (RNAi). Binds to short RNAs such as microRNAs (miRNAs) and represses the translation of mRNAs which are complementary to them. Lacks endonuclease activity and does not appear to cleave target mRNAs. Also required for RNA-directed transcription and replication of the human hapatitis delta virus (HDV).   
  
Interacts with EIF4B, IMP8, PRMT5, TNRC6A and TNRC6B (PubMed:19167051). Interacts with ZFP36 (PubMed:15766526).   
  
 **Gene Ontology Information:**

Molecular Function

- double-stranded RNA binding
- miRNA binding
- endoribonuclease activity
- single-stranded RNA binding

Location

- cytoplasm
- cytoplasmic ribonucleoprotein granule
- cytosol
- membrane
- nucleus
- P-body
- RISC complex
- RISC-loading complex

Biological process

- male gonad development
- male meiotic nuclear division
- miRNA metabolic process
- production of miRNAs involved in gene silencing by miRNA
- miRNA mediated inhibition of translation
- mRNA catabolic process
- negative regulation of apoptotic process
- pre-miRNA processing
- regulation of cell morphogenesis
- small RNA loading onto RISC
- RNA secondary structure unwinding
- synaptonemal complex assembly

---

48

- **Protein name:** Coagulation factor VIII
- **Organism:** Homo sapiens
- **Uniprot Accession Number:** P00451
- **Protein sequence length:** 2351 aa
- **1D identity (%):** 11.22
- **1D identity (%) [Gaps excluded]:** 31.21
- **1D identity - Alignment Gaps:** 1512
- **1D aligned content (<aminoacid>:%):** {'M': 0.38, 'Y': 3.77, 'G': 8.68, 'A': 3.02, 'P': 11.7, 'K': 4.53, 'R': 6.42, 'T': 8.3, 'I': 3.02, 'Q': 3.77, 'E': 7.17, 'V': 5.66, 'H': 5.28, 'F': 6.04, 'D': 5.28, 'L': 7.17, 'S': 4.15, 'C': 2.64, 'W': 0.75, 'N': 2.26}
- **Common reported functions (%):** 0.0
- **Common reported locations (%):** 0.0
- **Common reported processes (%):** 0.0

- **PDB ID:** 3CDZ
- **Chain:** A
- **Crystallized protein length:** 630 aa
- **Resolution:** 3.98 Å
- **b-phipsi:** 0.040926
- **w-rdist:** 0.362502
- **t-alpha:** 0.005098
- **Chemical similarity (Tanimoto Index) (%):** 73.79
- **1D identity (%) [PDB]:** 3.28
- **1D identity (%) [Gaps excluded][PDB]:** 66.67
- **1D identity - Alignment Gaps [PDB]:** 1333
- **1D aligned content [PDB] (<aminoacid>:%):** {'T': 8.7, 'R': 13.04, 'I': 6.52, 'D': 6.52, 'V': 8.7, 'S': 4.35, 'E': 10.87, 'G': 8.7, 'Q': 2.17, 'L': 10.87, 'F': 4.35, 'H': 2.17, 'C': 2.17, 'K': 4.35, 'N': 2.17, 'P': 2.17, 'A': 2.17}
- **2D identity (%) [PDB]:** 19.48
- **2D identity (%) [Gaps excluded][PDB]:** 86.67
- **2D identity - Alignment Gaps [PDB]:** 931
- **2D aligned content [PDB] (<2D-fold>:%):** {'.': 24.36, 'B': 1.28, 'E': 46.15, 'T': 16.24, 'H': 10.68, 'G': 1.28}
- **3D similarity (TM-Score) (%) [PDB]:** 23.5

- **Gene name:** F8
- **Entrez ID:** 2157
- **RefSeq ID:** NM\_019863
- **Transcript sequence length:** 2616
- **5-UTR|CDS|3-UTR identity (%):** 37.88 | 14.29 | 9.21
- **5-UTR|CDS|3-UTR identity (%) [Gaps excluded]:** 84.27 | 75.19 | 76.37
- **5-UTR|CDS|3-UTR identity [Alignment Gaps]:** 109 | 2199 | 10747
- **5-UTR aligned content (<base>:%):** {'G': 42.67, 'C': 48.0, 'T': 8.0, 'A': 1.33}
- **CDS aligned content (<base>:%):** {'A': 27.58, 'C': 23.97, 'G': 27.32, 'T': 21.13}
- **3-UTR aligned content (<base>:%):** {'G': 16.8, 'T': 35.02, 'C': 18.67, 'A': 29.51}

**Uniprot Description:**  
  
 Factor VIII, along with calcium and phospholipid, acts as a cofactor for F9/factor IXa when it converts F10/factor X to the activated form, factor Xa.   
  
Interacts with VWF/vWF. vWF binding is essential for the stabilization of F8 in circulation.   
  
 **Gene Ontology Information:**

Molecular Function

- copper ion binding
- oxidoreductase activity

Location

- COPII-coated ER to Golgi transport vesicle
- endoplasmic reticulum lumen
- endoplasmic reticulum-Golgi intermediate compartment membrane
- extracellular region
- extracellular space
- Golgi lumen
- plasma membrane
- platelet alpha granule lumen
- protein-containing complex
- serine-type endopeptidase complex

Biological process

- acute-phase response
- blood coagulation
- blood coagulation, intrinsic pathway
- proteolysis
- zymogen activation

---

49

- **Protein name:** Bifunctional purine biosynthesis protein ATIC
- **Organism:** Homo sapiens
- **Uniprot Accession Number:** P31939
- **Protein sequence length:** 592 aa
- **1D identity (%):** 14.36
- **1D identity (%) [Gaps excluded]:** 24.3
- **1D identity - Alignment Gaps:** 373
- **1D aligned content (<aminoacid>:%):** {'G': 11.45, 'F': 4.58, 'V': 10.69, 'S': 4.58, 'K': 5.34, 'T': 3.82, 'L': 8.4, 'E': 6.11, 'H': 3.82, 'M': 0.76, 'D': 3.82, 'A': 5.34, 'Y': 4.58, 'P': 7.63, 'R': 7.63, 'Q': 3.82, 'N': 2.29, 'I': 4.58, 'C': 0.76}
- **Common reported functions (%):** 0.0
- **Common reported locations (%):** 21.43
- **Common reported processes (%):** 0.0

- **PDB ID:** 5UY8
- **Chain:** A
- **Crystallized protein length:** 589 aa
- **Resolution:** 2.39 Å
- **b-phipsi:** 0.005773
- **w-rdist:** 0.733345
- **t-alpha:** 0.003642
- **Chemical similarity (Tanimoto Index) (%):** 71.02
- **1D identity (%) [PDB]:** 2.85
- **1D identity (%) [Gaps excluded][PDB]:** 66.1
- **1D identity - Alignment Gaps [PDB]:** 1309
- **1D aligned content [PDB] (<aminoacid>:%):** {'G': 7.69, 'Q': 10.26, 'T': 5.13, 'V': 12.82, 'E': 7.69, 'A': 5.13, 'Y': 7.69, 'K': 12.82, 'L': 10.26, 'R': 2.56, 'P': 5.13, 'H': 2.56, 'I': 5.13, 'D': 2.56, 'N': 2.56}
- **2D identity (%) [PDB]:** 37.33
- **2D identity (%) [Gaps excluded][PDB]:** 88.0
- **2D identity - Alignment Gaps [PDB]:** 577
- **2D aligned content [PDB] (<2D-fold>:%):** {'.': 13.14, 'E': 17.96, 'T': 10.99, 'H': 57.64, 'B': 0.27}
- **3D similarity (TM-Score) (%) [PDB]:** 20.24

- **Gene name:** ATIC
- **Entrez ID:** 471
- **RefSeq ID:** NM\_004044
- **Transcript sequence length:** 1972
- **5-UTR|CDS|3-UTR identity (%):** 45.11 | 37.0 | 0.61
- **5-UTR|CDS|3-UTR identity (%) [Gaps excluded]:** 76.92 | 73.32 | 73.74
- **5-UTR|CDS|3-UTR identity [Alignment Gaps]:** 55 | 1435 | 11799
- **5-UTR aligned content (<base>:%):** {'G': 30.0, 'C': 58.33, 'A': 3.33, 'T': 8.33}
- **CDS aligned content (<base>:%):** {'A': 26.77, 'G': 26.87, 'C': 25.47, 'T': 20.9}
- **3-UTR aligned content (<base>:%):** {'T': 38.36, 'C': 16.44, 'A': 30.14, 'G': 15.07}

**Uniprot Description:**  
  
 Bifunctional enzyme that catalyzes the last two steps of purine biosynthesis (PubMed:11948179, PubMed:14756554). Acts as a transformylase that incorporates a formyl group to the AMP analog AICAR (5-amino-1-(5-phospho-beta-D-ribosyl)imidazole-4-carboxamide) to produce the intermediate formyl-AICAR (FAICAR) (PubMed:9378707, PubMed:11948179, PubMed:10985775). Can use both 10-formyldihydrofolate and 10-formyltetrahydrofolate as the formyl donor in this reaction (PubMed:10985775). Also catalyzes the cyclization of FAICAR to IMP (PubMed:11948179, PubMed:14756554). Is able to convert thio-AICAR to 6-mercaptopurine ribonucleotide, an inhibitor of purine biosynthesis used in the treatment of human leukemias (PubMed:10985775). Promotes insulin receptor/INSR autophosphorylation and is involved in INSR internalization (PubMed:25687571).   
  
Homodimer (PubMed:14756553, PubMed:14966129). Associates with internalized INSR complexes on Golgi/endosomal membranes (PubMed:25687571). Interacts with INSR; ATIC together with PRKAA2/AMPK2 and HACD3/PTPLAD1 is proposed to be part of a signaling network regulating INSR autophosphorylation and endocytosis (PubMed:25687571).   
  
 **Gene Ontology Information:**

Molecular Function

- cadherin binding
- IMP cyclohydrolase activity
- phosphoribosylaminoimidazolecarboxamide formyltransferase activity
- protein homodimerization activity

Location

- cytosol
- extracellular exosome
- membrane
- plasma membrane

Biological process

- 'de novo' AMP biosynthetic process
- 'de novo' IMP biosynthetic process
- 'de novo' XMP biosynthetic process
- animal organ regeneration
- brainstem development
- cellular response to interleukin-7
- cerebellum development
- cerebral cortex development
- dihydrofolate metabolic process
- GMP biosynthetic process
- nucleobase-containing compound metabolic process
- response to inorganic substance
- tetrahydrofolate biosynthetic process

---

50

- **Protein name:** Ribonucleases P/MRP protein subunit POP1
- **Organism:** Homo sapiens
- **Uniprot Accession Number:** Q99575
- **Protein sequence length:** 1024 aa
- **1D identity (%):** 15.57
- **1D identity (%) [Gaps excluded]:** 24.42
- **1D identity - Alignment Gaps:** 417
- **1D aligned content (<aminoacid>:%):** {'A': 5.03, 'L': 8.94, 'F': 4.47, 'H': 3.35, 'K': 9.5, 'P': 10.61, 'E': 6.15, 'R': 10.06, 'T': 7.82, 'S': 5.03, 'V': 5.03, 'N': 1.68, 'W': 0.56, 'Y': 1.12, 'Q': 4.47, 'C': 2.79, 'G': 7.26, 'I': 1.68, 'D': 3.35, 'M': 1.12}
- **Common reported functions (%):** 6.67
- **Common reported locations (%):** 7.14
- **Common reported processes (%):** 0.0

- **PDB ID:** 6AHU
- **Chain:** B
- **Crystallized protein length:** 774 aa
- **Resolution:** 3.66 Å
- **b-phipsi:** 0.015209
- **w-rdist:** 0.402253
- **t-alpha:** 0.008817
- **Chemical similarity (Tanimoto Index) (%):** 99.55
- **1D identity (%) [PDB]:** 2.03
- **1D identity (%) [Gaps excluded][PDB]:** 76.19
- **1D identity - Alignment Gaps [PDB]:** 1532
- **1D aligned content [PDB] (<aminoacid>:%):** {'K': 9.38, 'N': 12.5, 'V': 12.5, 'Q': 9.38, 'R': 6.25, 'T': 6.25, 'P': 15.62, 'L': 15.62, 'S': 3.12, 'G': 6.25, 'I': 3.12}
- **2D identity (%) [PDB]:** 35.14
- **2D identity (%) [Gaps excluded][PDB]:** 80.98
- **2D identity - Alignment Gaps [PDB]:** 638
- **2D aligned content [PDB] (<2D-fold>:%):** {'.': 21.21, 'H': 41.16, 'T': 13.64, 'E': 22.98, 'G': 0.76, 'B': 0.25}
- **3D similarity (TM-Score) (%) [PDB]:** 21.59

- **Gene name:** POP1
- **Entrez ID:** 10940
- **RefSeq ID:** NM\_015029
- **Transcript sequence length:** 4667
- **5-UTR|CDS|3-UTR identity (%):** 13.43 | 44.0 | 7.54
- **5-UTR|CDS|3-UTR identity (%) [Gaps excluded]:** 85.71 | 74.99 | 75.14
- **5-UTR|CDS|3-UTR identity [Alignment Gaps]:** 113 | 1473 | 10998
- **5-UTR aligned content (<base>:%):** {'G': 33.33, 'A': 5.56, 'T': 22.22, 'C': 38.89}
- **CDS aligned content (<base>:%):** {'A': 26.79, 'T': 17.35, 'G': 28.19, 'C': 27.68}
- **3-UTR aligned content (<base>:%):** {'A': 31.02, 'T': 36.12, 'G': 16.81, 'C': 16.05}

**Uniprot Description:**  
  
 Component of ribonuclease P, a ribonucleoprotein complex that generates mature tRNA molecules by cleaving their 5'-ends (PubMed:8918471, PubMed:30454648). Also a component of the MRP ribonuclease complex, which cleaves pre-rRNA sequences (PubMed:28115465).   
  
Component of nuclear RNase P and RNase MRP ribonucleoproteins (PubMed:8918471, PubMed:16723659). RNase P consists of a catalytic RNA moiety and 10 different protein chains; POP1, POP4, POP5, POP7, RPP14, RPP21, RPP25, RPP30, RPP38 and RPP40 (PubMed:16723659, PubMed:30454648). Within the RNase P complex, POP1, POP7 and RPP25 form the 'finger' subcomplex, POP5, RPP14, RPP40 and homodimeric RPP30 form the 'palm' subcomplex, and RPP21, POP4 and RPP38 form the 'wrist' subcomplex. All subunits of the RNase P complex interact with the catalytic RNA (PubMed:30454648). Several subunits of RNase P are also part of the RNase MRP complex. RNase MRP consists of a catalytic RNA moiety and about 8 protein subunits; POP1, POP7, RPP25, RPP30, RPP38, RPP40 and possibly also POP4 and POP5 (PubMed:16723659, PubMed:28115465).   
  
 **Gene Ontology Information:**

Molecular Function

- ribonuclease P activity
- ribonuclease P RNA binding
- RNA binding

Location

- extracellular space
- multimeric ribonuclease P complex
- nucleolar ribonuclease P complex
- nucleolus
- nucleoplasm
- ribonuclease MRP complex

Biological process

- RNA phosphodiester bond hydrolysis, endonucleolytic
- tRNA 5'-leader removal
- tRNA catabolic process
- tRNA processing

---

51

- **Protein name:** UBX domain-containing protein 6
- **Organism:** Homo sapiens
- **Uniprot Accession Number:** Q9BZV1
- **Protein sequence length:** 441 aa
- **1D identity (%):** 10.8
- **1D identity (%) [Gaps excluded]:** 21.86
- **1D identity - Alignment Gaps:** 440
- **1D aligned content (<aminoacid>:%):** {'K': 9.57, 'F': 5.32, 'E': 8.51, 'G': 7.45, 'Q': 11.7, 'V': 3.19, 'P': 8.51, 'T': 5.32, 'A': 5.32, 'I': 4.26, 'R': 8.51, 'L': 12.77, 'S': 2.13, 'D': 5.32, 'H': 1.06, 'N': 1.06}
- **Common reported functions (%):** 6.67
- **Common reported locations (%):** 35.71
- **Common reported processes (%):** 0.0

- **PDB ID:** 8FCN
- **Chain:** A
- **Crystallized protein length:** 721 aa
- **Resolution:** 2.95 Å
- **b-phipsi:** 0.006365
- **w-rdist:** 0.280749
- **t-alpha:** 0.07866
- **Chemical similarity (Tanimoto Index) (%):** 79.7
- **1D identity (%) [PDB]:** 2.49
- **1D identity (%) [Gaps excluded][PDB]:** 66.07
- **1D identity - Alignment Gaps [PDB]:** 1429
- **1D aligned content [PDB] (<aminoacid>:%):** {'P': 13.51, 'L': 21.62, 'I': 8.11, 'D': 5.41, 'V': 13.51, 'E': 10.81, 'G': 2.7, 'R': 5.41, 'S': 10.81, 'Q': 2.7, 'A': 5.41}
- **2D identity (%) [PDB]:** 44.6
- **2D identity (%) [Gaps excluded][PDB]:** 89.11
- **2D identity - Alignment Gaps [PDB]:** 513
- **2D aligned content [PDB] (<2D-fold>:%):** {'.': 15.28, 'E': 25.55, 'H': 37.55, 'T': 18.56, 'B': 0.44, 'G': 2.62}
- **3D similarity (TM-Score) (%) [PDB]:** 2.26

- **Gene name:** UBXN6
- **Entrez ID:** 80700
- **RefSeq ID:** N/A
- **Sequence length:** N/A
- **5-UTR|CDS|3-UTR identity (%):** N/A | N/A | N/A
- **5-UTR|CDS|3-UTR identity (%) [Gaps excluded]:** N/A | N/A | N/A
- **5-UTR|CDS|3-UTR identity [Alignment Gaps]:** N/A | N/A | N/A
- **5-UTR aligned content (<base>:%):** N/A
- **CDS aligned content (<base>:%):** N/A
- **3-UTR aligned content (<base>:%):** N/A

**Uniprot Description:**  
  
 May negatively regulate the ATPase activity of VCP, an ATP-driven segregase that associates with different cofactors to control a wide variety of cellular processes (PubMed:26475856). As a cofactor of VCP, it may play a role in the transport of CAV1 to lysosomes for degradation (PubMed:21822278, PubMed:23335559). It may also play a role in endoplasmic reticulum-associated degradation (ERAD) of misfolded proteins (PubMed:19275885). Together with VCP and other cofactors, it may play a role in macroautophagy, regulating for instance the clearance of damaged lysosomes (PubMed:27753622).   
  
Interacts with VCP through the PUB domain (via C-terminus) and VIM motif (via N-terminus); the interaction is direct (PubMed:18656546, PubMed:19174149, PubMed:21896481, PubMed:21822278, PubMed:26475856). Forms a ternary complex with CAV1 and VCP (PubMed:21822278). Interacts with SYVN1 (PubMed:18656546). Interacts with HERPUD1 (PubMed:18656546). Interacts with VCPKMT (PubMed:23349634). May interact with DERL1 (PubMed:19275885). Interacts with PLAA, VCP and YOD1; may form a complex involved in macroautophagy (PubMed:27753622). Interacts with LMAN1 (PubMed:22337587).   
  
 **Gene Ontology Information:**

Molecular Function

- ADP binding
- ATP binding
- ATPase activity
- BAT3 complex binding
- deubiquitinase activator activity
- identical protein binding
- K48-linked polyubiquitin modification-dependent protein binding
- lipid binding
- MHC class I protein binding
- polyubiquitin modification-dependent protein binding
- protein domain specific binding
- protein phosphatase binding
- RNA binding
- ubiquitin protein ligase binding
- ubiquitin-like protein ligase binding
- ubiquitin-specific protease binding

Location

- ATPase complex
- azurophil granule lumen
- cytoplasm
- cytoplasmic stress granule
- cytosol
- Derlin-1 retrotranslocation complex
- endoplasmic reticulum
- endoplasmic reticulum membrane
- extracellular exosome
- extracellular region
- ficolin-1-rich granule lumen
- glutamatergic synapse
- intracellular membrane-bounded organelle
- lipid droplet
- nucleoplasm
- nucleus
- perinuclear region of cytoplasm
- proteasome complex
- protein-containing complex
- secretory granule lumen
- site of double-strand break
- VCP-NPL4-UFD1 AAA ATPase complex
- VCP-NSFL1C complex

Biological process

- activation of cysteine-type endopeptidase activity involved in apoptotic process
- aggresome assembly
- ATP metabolic process
- autophagosome maturation
- autophagy
- cellular response to arsenite ion
- cellular response to DNA damage stimulus
- cellular response to heat
- DNA repair
- double-strand break repair
- endoplasmic reticulum stress-induced pre-emptive quality control
- endoplasmic reticulum to Golgi vesicle-mediated transport
- endoplasmic reticulum unfolded protein response
- endosome to lysosome transport via multivesicular body sorting pathway
- ER-associated misfolded protein catabolic process
- ERAD pathway
- establishment of protein localization
- flavin adenine dinucleotide catabolic process
- interstrand cross-link repair
- macroautophagy
- mitotic spindle disassembly
- NADH metabolic process
- negative regulation of smoothened signaling pathway
- positive regulation of ATP biosynthetic process
- positive regulation of canonical Wnt signaling pathway
- positive regulation of Lys63-specific deubiquitinase activity
- positive regulation of mitochondrial membrane potential
- positive regulation of oxidative phosphorylation
- positive regulation of proteasomal ubiquitin-dependent protein catabolic process
- positive regulation of protein catabolic process
- positive regulation of protein K63-linked deubiquitination
- positive regulation of protein-containing complex assembly
- proteasomal protein catabolic process
- proteasome-mediated ubiquitin-dependent protein catabolic process
- protein ubiquitination
- protein-DNA covalent cross-linking repair
- regulation of aerobic respiration
- regulation of apoptotic process
- regulation of protein localization to chromatin
- regulation of synapse organization
- retrograde protein transport, ER to cytosol
- stress granule disassembly
- translesion synthesis
- ubiquitin-dependent ERAD pathway
- viral genome replication

---

52

- **Protein name:** Aspartate--tRNA ligase, mitochondrial
- **Organism:** Homo sapiens
- **Uniprot Accession Number:** Q6PI48
- **Protein sequence length:** 645 aa
- **1D identity (%):** 14.6
- **1D identity (%) [Gaps excluded]:** 25.69
- **1D identity - Alignment Gaps:** 414
- **1D aligned content (<aminoacid>:%):** {'P': 6.43, 'S': 7.14, 'W': 0.71, 'L': 12.14, 'Q': 7.86, 'R': 9.29, 'E': 7.86, 'V': 7.14, 'C': 3.57, 'G': 6.43, 'H': 3.57, 'Y': 3.57, 'T': 3.57, 'I': 3.57, 'A': 5.0, 'F': 3.57, 'K': 2.14, 'M': 1.43, 'D': 3.57, 'N': 1.43}
- **Common reported functions (%):** 0.0
- **Common reported locations (%):** 7.14
- **Common reported processes (%):** 0.0

- **PDB ID:** 4AH6
- **Chain:** B
- **Crystallized protein length:** 589 aa
- **Resolution:** 3.7 Å
- **b-phipsi:** 0.004115
- **w-rdist:** 0.398513
- **t-alpha:** 0.129112
- **Chemical similarity (Tanimoto Index) (%):** 99.17
- **1D identity (%) [PDB]:** 2.02
- **1D identity (%) [Gaps excluded][PDB]:** 63.64
- **1D identity - Alignment Gaps [PDB]:** 1339
- **1D aligned content [PDB] (<aminoacid>:%):** {'E': 10.71, 'L': 14.29, 'V': 14.29, 'T': 3.57, 'G': 7.14, 'D': 7.14, 'R': 3.57, 'F': 3.57, 'S': 14.29, 'I': 3.57, 'W': 3.57, 'Q': 3.57, 'A': 3.57, 'H': 3.57, 'P': 3.57}
- **2D identity (%) [PDB]:** 32.32
- **2D identity (%) [Gaps excluded][PDB]:** 82.13
- **2D identity - Alignment Gaps [PDB]:** 621
- **2D aligned content [PDB] (<2D-fold>:%):** {'.': 19.94, 'T': 12.69, 'E': 27.49, 'H': 39.88}
- **3D similarity (TM-Score) (%) [PDB]:** 19.77

- **Gene name:** DARS2
- **Entrez ID:** 55157
- **RefSeq ID:** NM\_018122
- **Transcript sequence length:** 3336
- **5-UTR|CDS|3-UTR identity (%):** 18.89 | 39.93 | 4.6
- **5-UTR|CDS|3-UTR identity (%) [Gaps excluded]:** 86.18 | 76.08 | 76.03
- **5-UTR|CDS|3-UTR identity [Alignment Gaps]:** 438 | 1408 | 11277
- **5-UTR aligned content (<base>:%):** {'G': 41.51, 'T': 10.38, 'C': 44.34, 'A': 3.77}
- **CDS aligned content (<base>:%):** {'A': 27.3, 'T': 21.05, 'G': 26.12, 'C': 25.53}
- **3-UTR aligned content (<base>:%):** {'A': 34.42, 'T': 37.32, 'G': 14.31, 'C': 13.95}

**Uniprot Description:**  
  
 N/A   
  
Homodimer.   
  
 **Gene Ontology Information:**

Molecular Function

- aspartate-tRNA ligase activity
- aspartate-tRNA(Asn) ligase activity
- ATP binding
- protein homodimerization activity
- tRNA binding

Location

- mitochondrial matrix
- mitochondrion
- nucleoplasm

Biological process

- aspartyl-tRNA aminoacylation
- mitochondrial asparaginyl-tRNA aminoacylation
- tRNA aminoacylation

---

53

- **Protein name:** GMP synthase [glutamine-hydrolyzing]
- **Organism:** Homo sapiens
- **Uniprot Accession Number:** P49915
- **Protein sequence length:** 693 aa
- **1D identity (%):** 11.26
- **1D identity (%) [Gaps excluded]:** 26.74
- **1D identity - Alignment Gaps:** 632
- **1D aligned content (<aminoacid>:%):** {'K': 8.13, 'L': 11.38, 'H': 4.07, 'Y': 2.44, 'E': 6.5, 'D': 4.88, 'R': 4.07, 'V': 9.76, 'T': 7.32, 'F': 4.88, 'G': 8.94, 'N': 1.63, 'A': 3.25, 'P': 5.69, 'I': 4.88, 'S': 5.69, 'C': 3.25, 'Q': 3.25}
- **Common reported functions (%):** 0.0
- **Common reported locations (%):** 7.14
- **Common reported processes (%):** 0.0

- **PDB ID:** 2VXO
- **Chain:** A
- **Crystallized protein length:** 643 aa
- **Resolution:** 2.5 Å
- **b-phipsi:** 0.004225
- **w-rdist:** 0.48458
- **t-alpha:** 0.032331
- **Chemical similarity (Tanimoto Index) (%):** 78.58
- **1D identity (%) [PDB]:** 5.32
- **1D identity (%) [Gaps excluded][PDB]:** 65.18
- **1D identity - Alignment Gaps [PDB]:** 1261
- **1D aligned content [PDB] (<aminoacid>:%):** {'P': 5.48, 'L': 8.22, 'E': 8.22, 'N': 2.74, 'I': 10.96, 'V': 9.59, 'A': 4.11, 'G': 5.48, 'K': 9.59, 'T': 12.33, 'R': 4.11, 'Q': 4.11, 'S': 5.48, 'F': 4.11, 'D': 4.11, 'Y': 1.37}
- **2D identity (%) [PDB]:** 37.74
- **2D identity (%) [Gaps excluded][PDB]:** 89.34
- **2D identity - Alignment Gaps [PDB]:** 603
- **2D aligned content [PDB] (<2D-fold>:%):** {'.': 18.02, 'E': 27.16, 'H': 43.4, 'T': 9.9, 'B': 0.76, 'G': 0.76}
- **3D similarity (TM-Score) (%) [PDB]:** 20.85

- **Gene name:** GMPS
- **Entrez ID:** 8833
- **RefSeq ID:** NM\_003875
- **Transcript sequence length:** 8631
- **5-UTR|CDS|3-UTR identity (%):** 43.56 | 42.73 | 29.73
- **5-UTR|CDS|3-UTR identity (%) [Gaps excluded]:** 79.67 | 74.18 | 74.53
- **5-UTR|CDS|3-UTR identity [Alignment Gaps]:** 102 | 1254 | 7828
- **5-UTR aligned content (<base>:%):** {'C': 50.0, 'T': 10.2, 'G': 39.8}
- **CDS aligned content (<base>:%):** {'A': 30.14, 'G': 27.14, 'T': 21.68, 'C': 21.04}
- **3-UTR aligned content (<base>:%):** {'T': 34.31, 'A': 27.33, 'C': 18.91, 'G': 19.45}

**Uniprot Description:**  
  
 Involved in the de novo synthesis of guanine nucleotides which are not only essential for DNA and RNA synthesis, but also provide GTP, which is involved in a number of cellular processes important for cell division.   
  
Homodimer.   
  
 **Gene Ontology Information:**

Molecular Function

- ATP binding
- GMP synthase (glutamine-hydrolyzing) activity
- GMP synthase activity

Location

- cytosol

Biological process

- glutamine metabolic process
- GMP biosynthetic process
- purine nucleobase biosynthetic process
- purine ribonucleoside monophosphate biosynthetic process

---

54

- **Protein name:** DNA ligase 4
- **Organism:** Homo sapiens
- **Uniprot Accession Number:** P49917
- **Protein sequence length:** 911 aa
- **1D identity (%):** 14.88
- **1D identity (%) [Gaps excluded]:** 22.32
- **1D identity - Alignment Gaps:** 354
- **1D aligned content (<aminoacid>:%):** {'T': 6.96, 'A': 3.16, 'P': 6.96, 'D': 6.33, 'I': 4.43, 'K': 10.76, 'R': 6.96, 'E': 6.96, 'F': 5.06, 'V': 3.8, 'Y': 1.9, 'M': 1.27, 'L': 11.39, 'G': 10.76, 'Q': 3.16, 'S': 2.53, 'H': 1.27, 'C': 4.43, 'N': 0.63, 'W': 1.27}
- **Common reported functions (%):** 6.67
- **Common reported locations (%):** 21.43
- **Common reported processes (%):** 0.0

- **PDB ID:** 3W1B
- **Chain:** A
- **Crystallized protein length:** 589 aa
- **Resolution:** 2.4 Å
- **b-phipsi:** 0.002072
- **w-rdist:** 0.514834
- **t-alpha:** 0.049694
- **Chemical similarity (Tanimoto Index) (%):** 87.93
- **1D identity (%) [PDB]:** 2.85
- **1D identity (%) [Gaps excluded][PDB]:** 65.0
- **1D identity - Alignment Gaps [PDB]:** 1309
- **1D aligned content [PDB] (<aminoacid>:%):** {'T': 10.26, 'S': 10.26, 'M': 7.69, 'I': 10.26, 'A': 2.56, 'R': 10.26, 'Q': 2.56, 'E': 7.69, 'K': 7.69, 'F': 2.56, 'N': 2.56, 'D': 7.69, 'G': 7.69, 'V': 5.13, 'P': 2.56, 'L': 2.56}
- **2D identity (%) [PDB]:** 31.92
- **2D identity (%) [Gaps excluded][PDB]:** 84.44
- **2D identity - Alignment Gaps [PDB]:** 645
- **2D aligned content [PDB] (<2D-fold>:%):** {'H': 47.73, '.': 19.34, 'T': 12.08, 'G': 1.81, 'E': 19.03}
- **3D similarity (TM-Score) (%) [PDB]:** 18.76

- **Gene name:** LIG4
- **Entrez ID:** 3981
- **RefSeq ID:** N/A
- **Sequence length:** N/A
- **5-UTR|CDS|3-UTR identity (%):** N/A | N/A | N/A
- **5-UTR|CDS|3-UTR identity (%) [Gaps excluded]:** N/A | N/A | N/A
- **5-UTR|CDS|3-UTR identity [Alignment Gaps]:** N/A | N/A | N/A
- **5-UTR aligned content (<base>:%):** N/A
- **CDS aligned content (<base>:%):** N/A
- **3-UTR aligned content (<base>:%):** N/A

**Uniprot Description:**  
  
 Efficiently joins single-strand breaks in a double-stranded polydeoxynucleotide in an ATP-dependent reaction. Involved in DNA non-homologous end joining (NHEJ) required for double-strand break repair and V(D)J recombination. The LIG4-XRCC4 complex is responsible for the NHEJ ligation step, and XRCC4 enhances the joining activity of LIG4. Binding of the LIG4-XRCC4 complex to DNA ends is dependent on the assembly of the DNA-dependent protein kinase complex DNA-PK to these DNA ends.   
  
Interacts with XRCC4. The LIG4-XRCC4 complex has probably a 1:2 stoichiometry. The LIG4-XRCC4 complex associates in a DNA-dependent manner with the DNA-PK complex composed of PRKDC, XRCC6/Ku70 and XRCC5/Ku86 to form the core non-homologous end joining (NHEJ) complex. Additional components of the NHEJ complex include NHEJ1/XLF and PAXX. Interacts with APLF.   
  
 **Gene Ontology Information:**

Molecular Function

- ATP binding
- DNA binding
- DNA ligase (ATP) activity
- DNA ligase activity
- ligase activity
- metal ion binding

Location

- chromosome, telomeric region
- condensed chromosome
- cytoplasmic ribonucleoprotein granule
- DNA ligase IV complex
- DNA-dependent protein kinase-DNA ligase 4 complex
- nonhomologous end joining complex
- nucleoplasm
- nucleus

Biological process

- cell cycle
- cell division
- cell population proliferation
- cellular response to ionizing radiation
- cellular response to lithium ion
- central nervous system development
- chromosome organization
- DN2 thymocyte differentiation
- DNA biosynthetic process
- DNA ligation
- DNA ligation involved in DNA recombination
- DNA ligation involved in DNA repair
- DNA replication
- double-strand break repair
- double-strand break repair via classical nonhomologous end joining
- double-strand break repair via nonhomologous end joining
- establishment of integrated proviral latency
- fibroblast proliferation
- immunoglobulin V(D)J recombination
- in utero embryonic development
- isotype switching
- negative regulation of neuron apoptotic process
- neurogenesis
- neuron apoptotic process
- nucleotide-excision repair, DNA gap filling
- positive regulation of chromosome organization
- positive regulation of fibroblast proliferation
- positive regulation of neurogenesis
- pro-B cell differentiation
- response to gamma radiation
- response to X-ray
- single strand break repair
- somatic stem cell population maintenance
- stem cell proliferation
- T cell differentiation in thymus
- T cell receptor V(D)J recombination
- V(D)J recombination

---

55

- **Protein name:** Cytosolic purine 5'-nucleotidase
- **Organism:** Homo sapiens
- **Uniprot Accession Number:** P49902
- **Protein sequence length:** 561 aa
- **1D identity (%):** 12.1
- **1D identity (%) [Gaps excluded]:** 22.67
- **1D identity - Alignment Gaps:** 432
- **1D aligned content (<aminoacid>:%):** {'M': 0.89, 'R': 6.25, 'N': 3.57, 'P': 7.14, 'A': 6.25, 'L': 10.71, 'F': 8.04, 'C': 1.79, 'G': 8.04, 'T': 8.04, 'E': 2.68, 'V': 6.25, 'K': 6.25, 'D': 7.14, 'I': 6.25, 'S': 5.36, 'Q': 1.79, 'H': 3.57}
- **Common reported functions (%):** 6.67
- **Common reported locations (%):** 14.29
- **Common reported processes (%):** 0.0

- **PDB ID:** 6DDQ
- **Chain:** A
- **Crystallized protein length:** 507 aa
- **Resolution:** 2.31 Å
- **b-phipsi:** 0.006552
- **w-rdist:** 0.713402
- **t-alpha:** 0.002922
- **Chemical similarity (Tanimoto Index) (%):** 99.17
- **1D identity (%) [PDB]:** 3.3
- **1D identity (%) [Gaps excluded][PDB]:** 57.53
- **1D identity - Alignment Gaps [PDB]:** 1200
- **1D aligned content [PDB] (<aminoacid>:%):** {'K': 14.29, 'Y': 4.76, 'R': 9.52, 'A': 2.38, 'H': 7.14, 'F': 4.76, 'Q': 11.9, 'E': 9.52, 'S': 2.38, 'T': 7.14, 'V': 4.76, 'D': 4.76, 'P': 4.76, 'L': 4.76, 'G': 2.38, 'I': 4.76}
- **2D identity (%) [PDB]:** 27.69
- **2D identity (%) [Gaps excluded][PDB]:** 81.29
- **2D identity - Alignment Gaps [PDB]:** 662
- **2D aligned content [PDB] (<2D-fold>:%):** {'.': 18.05, 'H': 49.82, 'G': 2.17, 'E': 15.52, 'T': 14.08, 'B': 0.36}
- **3D similarity (TM-Score) (%) [PDB]:** 19.12

- **Gene name:** NT5C2
- **Entrez ID:** 22978
- **RefSeq ID:** N/A
- **Sequence length:** N/A
- **5-UTR|CDS|3-UTR identity (%):** N/A | N/A | N/A
- **5-UTR|CDS|3-UTR identity (%) [Gaps excluded]:** N/A | N/A | N/A
- **5-UTR|CDS|3-UTR identity [Alignment Gaps]:** N/A | N/A | N/A
- **5-UTR aligned content (<base>:%):** N/A
- **CDS aligned content (<base>:%):** N/A
- **3-UTR aligned content (<base>:%):** N/A

**Uniprot Description:**  
  
 May have a critical role in the maintenance of a constant composition of intracellular purine/pyrimidine nucleotides in cooperation with other nucleotidases. Preferentially hydrolyzes inosine 5'-monophosphate (IMP) and other purine nucleotides.   
  
Homotetramer.   
  
 **Gene Ontology Information:**

Molecular Function

- 5'-nucleotidase activity
- ATP binding
- GMP 5'-nucleotidase activity
- identical protein binding
- IMP 5'-nucleotidase activity
- metal ion binding
- nucleoside phosphotransferase activity
- ubiquitin protein ligase activity
- XMP 5'-nucleosidase activity

Location

- cytoplasm
- cytosol

Biological process

- adenosine metabolic process
- allantoin metabolic process
- dGMP metabolic process
- GMP metabolic process
- IMP catabolic process
- IMP metabolic process
- negative regulation of defense response to virus by host
- protein K48-linked ubiquitination

---

56

- **Protein name:** DNA (cytosine-5)-methyltransferase 1
- **Organism:** Homo sapiens
- **Uniprot Accession Number:** P26358
- **Protein sequence length:** 1616 aa
- **1D identity (%):** 14.64
- **1D identity (%) [Gaps excluded]:** 29.82
- **1D identity - Alignment Gaps:** 845
- **1D aligned content (<aminoacid>:%):** {'P': 11.52, 'A': 8.64, 'D': 6.58, 'T': 5.76, 'K': 7.41, 'N': 2.47, 'F': 4.53, 'I': 4.53, 'Y': 2.47, 'E': 5.76, 'R': 7.82, 'H': 1.65, 'Q': 5.35, 'L': 8.23, 'V': 4.12, 'M': 0.41, 'S': 3.7, 'C': 2.06, 'G': 6.17, 'W': 0.82}
- **Common reported functions (%):** 6.67
- **Common reported locations (%):** 14.29
- **Common reported processes (%):** 0.0

- **PDB ID:** 3SWR
- **Chain:** A
- **Crystallized protein length:** 965 aa
- **Resolution:** 2.49 Å
- **b-phipsi:** 0.006745
- **w-rdist:** 0.447636
- **t-alpha:** 0.018937
- **Chemical similarity (Tanimoto Index) (%):** 73.73
- **1D identity (%) [PDB]:** 5.01
- **1D identity (%) [Gaps excluded][PDB]:** 64.12
- **1D identity - Alignment Gaps [PDB]:** 1544
- **1D aligned content [PDB] (<aminoacid>:%):** {'Q': 2.38, 'I': 3.57, 'F': 4.76, 'G': 10.71, 'D': 2.38, 'K': 7.14, 'P': 7.14, 'V': 7.14, 'R': 10.71, 'N': 2.38, 'L': 13.1, 'E': 2.38, 'T': 3.57, 'S': 8.33, 'A': 5.95, 'H': 2.38, 'M': 2.38, 'Y': 1.19, 'C': 1.19, 'W': 1.19}
- **2D identity (%) [PDB]:** 35.98
- **2D identity (%) [Gaps excluded][PDB]:** 82.82
- **2D identity - Alignment Gaps [PDB]:** 712
- **2D aligned content [PDB] (<2D-fold>:%):** {'.': 24.28, 'H': 30.91, 'T': 15.67, 'E': 27.15, 'G': 1.99}
- **3D similarity (TM-Score) (%) [PDB]:** 27.88

- **Gene name:** DNMT1
- **Entrez ID:** 1786
- **RefSeq ID:** N/A
- **Sequence length:** N/A
- **5-UTR|CDS|3-UTR identity (%):** N/A | N/A | N/A
- **5-UTR|CDS|3-UTR identity (%) [Gaps excluded]:** N/A | N/A | N/A
- **5-UTR|CDS|3-UTR identity [Alignment Gaps]:** N/A | N/A | N/A
- **5-UTR aligned content (<base>:%):** N/A
- **CDS aligned content (<base>:%):** N/A
- **3-UTR aligned content (<base>:%):** N/A

**Uniprot Description:**  
  
 Methylates CpG residues. Preferentially methylates hemimethylated DNA. Associates with DNA replication sites in S phase maintaining the methylation pattern in the newly synthesized strand, that is essential for epigenetic inheritance. Associates with chromatin during G2 and M phases to maintain DNA methylation independently of replication. It is responsible for maintaining methylation patterns established in development. DNA methylation is coordinated with methylation of histones. Mediates transcriptional repression by direct binding to HDAC2. In association with DNMT3B and via the recruitment of CTCFL/BORIS, involved in activation of BAG1 gene expression by modulating dimethylation of promoter histone H3 at H3K4 and H3K9. Probably forms a corepressor complex required for activated KRAS-mediated promoter hypermethylation and transcriptional silencing of tumor suppressor genes (TSGs) or other tumor-related genes in colorectal cancer (CRC) cells (PubMed:24623306). Also required to maintain a transcriptionally repressive state of genes in undifferentiated embryonic stem cells (ESCs) (PubMed:24623306). Associates at promoter regions of tumor suppressor genes (TSGs) leading to their gene silencing (PubMed:24623306). Promotes tumor growth (PubMed:24623306).   
  
Homodimer (PubMed:19173286). Forms a stable complex with E2F1, BB1 and HDAC1 (PubMed:10888886). Forms a complex with DMAP1 and HDAC2, with direct interaction (PubMed:10888872). Interacts with the PRC2/EED-EZH2 complex (PubMed:16357870). Probably part of a corepressor complex containing ZNF304, TRIM28, SETDB1 and DNMT1 (PubMed:24623306). Interacts with UHRF1; promoting its recruitment to hemimethylated DNA (PubMed:21745816). Interacts with USP7, promoting its deubiquitination (PubMed:21745816). Interacts with PCNA (PubMed:9302295). Interacts with MBD2 and MBD3 (PubMed:10947852). Interacts with DNMT3A and DNMT3B (PubMed:12145218). Interacts with UBC9 (PubMed:19450230). Interacts with CSNK1D (By similarity). Interacts with HDAC1 (By similarity). Interacts with BAZ2A/TIP5 (By similarity). Interacts with SIRT7 (By similarity). Interacts with ZNF263; recruited to the SIX3 promoter along with other proteins involved in chromatin modification and transcriptional corepression where it contributes to transcriptional repression (PubMed:32051553).   
  
 **Gene Ontology Information:**

Molecular Function

- DNA (cytosine-5-)-methyltransferase activity
- DNA binding
- DNA-methyltransferase activity
- methyl-CpG binding
- promoter-specific chromatin binding
- RNA binding
- zinc ion binding

Location

- female germ cell nucleus
- nucleoplasm
- nucleus
- pericentric heterochromatin
- replication fork

Biological process

- cellular response to amino acid stimulus
- cellular response to bisphenol A
- DNA methylation on cytosine within a CG sequence
- DNA methylation-dependent heterochromatin assembly
- transcription, DNA-templated
- DNA methylation involved in embryo development
- negative regulation of gene expression
- hypermethylation of CpG island
- negative regulation of transcription by RNA polymerase II
- negative regulation of vascular associated smooth muscle cell apoptotic process
- negative regulation of vascular associated smooth muscle cell differentiation involved in phenotypic switching
- positive regulation of gene expression
- positive regulation of vascular associated smooth muscle cell proliferation

---

57

- **Protein name:** 1-phosphatidylinositol 4,5-bisphosphate phosphodiesterase beta-3
- **Organism:** Homo sapiens
- **Uniprot Accession Number:** Q01970
- **Protein sequence length:** 1234 aa
- **1D identity (%):** 16.51
- **1D identity (%) [Gaps excluded]:** 25.89
- **1D identity - Alignment Gaps:** 463
- **1D aligned content (<aminoacid>:%):** {'G': 8.53, 'A': 7.58, 'P': 10.9, 'F': 4.27, 'D': 5.69, 'T': 6.64, 'S': 5.21, 'R': 5.21, 'N': 2.37, 'M': 1.9, 'I': 2.84, 'L': 11.85, 'E': 8.06, 'K': 4.74, 'V': 4.74, 'Q': 4.74, 'Y': 1.9, 'H': 2.37, 'C': 0.47}
- **Common reported functions (%):** 0.0
- **Common reported locations (%):** 21.43
- **Common reported processes (%):** 0.0

- **PDB ID:** 4QJ3
- **Chain:** B
- **Crystallized protein length:** 756 aa
- **Resolution:** 3.0 Å
- **b-phipsi:** 0.000902
- **w-rdist:** 0.654091
- **t-alpha:** 0.04173
- **Chemical similarity (Tanimoto Index) (%):** 87.15
- **1D identity (%) [PDB]:** 4.43
- **1D identity (%) [Gaps excluded][PDB]:** 62.26
- **1D identity - Alignment Gaps [PDB]:** 1384
- **1D aligned content [PDB] (<aminoacid>:%):** {'D': 7.58, 'T': 3.03, 'S': 4.55, 'R': 7.58, 'K': 10.61, 'L': 10.61, 'Q': 1.52, 'N': 3.03, 'F': 4.55, 'E': 9.09, 'I': 10.61, 'Y': 1.52, 'P': 4.55, 'V': 9.09, 'G': 7.58, 'A': 3.03, 'M': 1.52}
- **2D identity (%) [PDB]:** 37.35
- **2D identity (%) [Gaps excluded][PDB]:** 85.57
- **2D identity - Alignment Gaps [PDB]:** 626
- **2D aligned content [PDB] (<2D-fold>:%):** {'.': 17.59, 'E': 24.82, 'T': 10.84, 'H': 45.3, 'G': 1.45}
- **3D similarity (TM-Score) (%) [PDB]:** 23.53

- **Gene name:** PLCB3
- **Entrez ID:** 5331
- **RefSeq ID:** NM\_001316314
- **Transcript sequence length:** 4734
- **5-UTR|CDS|3-UTR identity (%):** 57.75 | 42.11 | 5.2
- **5-UTR|CDS|3-UTR identity (%) [Gaps excluded]:** 78.1 | 75.99 | 78.89
- **5-UTR|CDS|3-UTR identity [Alignment Gaps]:** 37 | 1803 | 11215
- **5-UTR aligned content (<base>:%):** {'G': 51.22, 'T': 4.88, 'C': 42.68, 'A': 1.22}
- **CDS aligned content (<base>:%):** {'A': 23.19, 'T': 15.38, 'G': 30.42, 'C': 31.0}
- **3-UTR aligned content (<base>:%):** {'A': 15.22, 'C': 32.85, 'T': 22.92, 'G': 29.01}

**Uniprot Description:**  
  
 The production of the second messenger molecules diacylglycerol (DAG) and inositol 1,4,5-trisphosphate (IP3) is mediated by activated phosphatidylinositol-specific phospholipase C enzymes.   
  
Interacts with SHANK2 (By similarity). Interacts with LPAR2 (PubMed:15143197).   
  
 **Gene Ontology Information:**

Molecular Function

- cadherin binding
- calcium ion binding
- calmodulin binding
- molecular adaptor activity
- molecular function activator activity
- phosphatidylinositol phospholipase C activity
- phospholipase C activity

Location

- cytosol
- membrane
- nucleus
- postsynaptic cytosol
- protein-containing complex

Biological process

- G protein-coupled receptor signaling pathway
- lipid catabolic process
- phosphatidylinositol metabolic process
- phosphatidylinositol-mediated signaling
- regulation of systemic arterial blood pressure
- release of sequestered calcium ion into cytosol

---

58

- **Protein name:** Astrotactin-2
- **Organism:** Homo sapiens
- **Uniprot Accession Number:** O75129
- **Protein sequence length:** 1339 aa
- **1D identity (%):** 14.45
- **1D identity (%) [Gaps excluded]:** 24.57
- **1D identity - Alignment Gaps:** 570
- **1D aligned content (<aminoacid>:%):** {'A': 4.5, 'G': 12.5, 'L': 8.5, 'P': 10.5, 'D': 5.0, 'S': 6.0, 'R': 6.5, 'N': 2.5, 'F': 4.0, 'I': 2.5, 'H': 2.5, 'K': 5.0, 'T': 6.5, 'V': 7.0, 'E': 4.5, 'Q': 3.0, 'C': 4.5, 'W': 1.0, 'Y': 3.0, 'M': 0.5}
- **Common reported functions (%):** 0.0
- **Common reported locations (%):** 7.14
- **Common reported processes (%):** 0.0

- **PDB ID:** 5J68
- **Chain:** A
- **Crystallized protein length:** 559 aa
- **Resolution:** 5.22 Å
- **b-phipsi:** 0.004833
- **w-rdist:** 0.407507
- **t-alpha:** 0.093153
- **Chemical similarity (Tanimoto Index) (%):** 77.72
- **1D identity (%) [PDB]:** 1.83
- **1D identity (%) [Gaps excluded][PDB]:** 71.43
- **1D identity - Alignment Gaps [PDB]:** 1329
- **1D aligned content [PDB] (<aminoacid>:%):** {'K': 8.0, 'N': 4.0, 'Y': 4.0, 'A': 8.0, 'G': 16.0, 'V': 16.0, 'I': 4.0, 'L': 8.0, 'P': 8.0, 'T': 8.0, 'E': 4.0, 'R': 4.0, 'D': 4.0, 'M': 4.0}
- **2D identity (%) [PDB]:** 31.85
- **2D identity (%) [Gaps excluded][PDB]:** 89.89
- **2D identity - Alignment Gaps [PDB]:** 667
- **2D aligned content [PDB] (<2D-fold>:%):** {'.': 15.81, 'B': 0.61, 'E': 40.73, 'T': 8.21, 'H': 34.65}
- **3D similarity (TM-Score) (%) [PDB]:** 18.61

- **Gene name:** ASTN2
- **Entrez ID:** 23245
- **RefSeq ID:** NM\_198188
- **Transcript sequence length:** 1799
- **5-UTR|CDS|3-UTR identity (%):** 36.33 | 27.2 | 2.14
- **5-UTR|CDS|3-UTR identity (%) [Gaps excluded]:** 75.42 | 76.44 | 77.98
- **5-UTR|CDS|3-UTR identity [Alignment Gaps]:** 127 | 1790 | 11609
- **5-UTR aligned content (<base>:%):** {'C': 46.07, 'G': 46.07, 'A': 3.37, 'T': 4.49}
- **CDS aligned content (<base>:%):** {'A': 26.06, 'T': 21.03, 'G': 27.65, 'C': 25.26}
- **3-UTR aligned content (<base>:%):** {'A': 18.04, 'C': 25.49, 'T': 35.29, 'G': 21.18}

**Uniprot Description:**  
  
 Mediates recycling of the neuronal cell adhesion molecule ASTN1 to the anterior pole of the cell membrane in migrating neurons. Promotes ASTN1 internalization and intracellular transport of endocytosed ASTN1 (By similarity). Selectively binds inositol-4,5-bisphosphate, inositol-3,4,5-trisphosphate and inositol-1,3,4,5-tetrakisphosphate, suggesting it is recruited to membranes that contain lipids with a phosphoinositide headgroup (Ref.6).   
  
Interacts with ASTN1; the interaction is not calcium-dependent.   
  
 **Gene Ontology Information:**

Molecular Function

- calcium ion binding
- inositol 1,3,4,5 tetrakisphosphate binding

Location

- cell cortex
- cell pole
- clathrin-coated vesicle
- early endosome
- endosome
- late endosome
- membrane
- perikaryon

Biological process

- establishment of body hair planar orientation
- negative regulation of protein localization to cell surface
- neuron cell-cell adhesion
- neuron migration
- protein localization to cell surface
- protein transport

---

59

- **Protein name:** Phosphatidylinositol 4,5-bisphosphate 3-kinase catalytic subunit gamma isoform
- **Organism:** Homo sapiens
- **Uniprot Accession Number:** P48736
- **Protein sequence length:** 1102 aa
- **1D identity (%):** 16.5
- **1D identity (%) [Gaps excluded]:** 25.03
- **1D identity - Alignment Gaps:** 403
- **1D aligned content (<aminoacid>:%):** {'Y': 3.08, 'R': 6.67, 'D': 8.72, 'K': 7.18, 'A': 3.59, 'M': 1.03, 'I': 4.1, 'L': 10.77, 'C': 2.05, 'P': 6.67, 'H': 4.1, 'V': 5.64, 'Q': 6.67, 'G': 9.74, 'E': 4.1, 'T': 4.62, 'W': 1.54, 'S': 4.62, 'F': 2.56, 'N': 2.56}
- **Common reported functions (%):** 0.0
- **Common reported locations (%):** 21.43
- **Common reported processes (%):** 0.0

- **PDB ID:** 8DP0
- **Chain:** A
- **Crystallized protein length:** 979 aa
- **Resolution:** 2.96 Å
- **b-phipsi:** 0.010461
- **w-rdist:** 0.36705
- **t-alpha:** 0.019665
- **Chemical similarity (Tanimoto Index) (%):** 99.17
- **1D identity (%) [PDB]:** 2.36
- **1D identity (%) [Gaps excluded][PDB]:** 71.43
- **1D identity - Alignment Gaps [PDB]:** 1639
- **1D aligned content [PDB] (<aminoacid>:%):** {'I': 5.0, 'F': 7.5, 'Y': 5.0, 'R': 7.5, 'G': 7.5, 'S': 2.5, 'Q': 7.5, 'V': 5.0, 'H': 10.0, 'E': 7.5, 'L': 10.0, 'A': 5.0, 'K': 7.5, 'P': 2.5, 'T': 5.0, 'C': 2.5, 'D': 2.5}
- **2D identity (%) [PDB]:** 43.29
- **2D identity (%) [Gaps excluded][PDB]:** 89.01
- **2D identity - Alignment Gaps [PDB]:** 605
- **2D aligned content [PDB] (<2D-fold>:%):** {'.': 15.29, 'E': 19.61, 'T': 20.78, 'H': 43.73, 'G': 0.59}
- **3D similarity (TM-Score) (%) [PDB]:** 21.96

- **Gene name:** PIK3CG
- **Entrez ID:** N/A
- **RefSeq ID:** N/A
- **Sequence length:** N/A
- **5-UTR|CDS|3-UTR identity (%):** N/A | N/A | N/A
- **5-UTR|CDS|3-UTR identity (%) [Gaps excluded]:** N/A | N/A | N/A
- **5-UTR|CDS|3-UTR identity [Alignment Gaps]:** N/A | N/A | N/A
- **5-UTR aligned content (<base>:%):** N/A
- **CDS aligned content (<base>:%):** N/A
- **3-UTR aligned content (<base>:%):** N/A

**Uniprot Description:**  
  
 Phosphoinositide-3-kinase (PI3K) that phosphorylates PtdIns(4,5)P2 (Phosphatidylinositol 4,5-bisphosphate) to generate phosphatidylinositol 3,4,5-trisphosphate (PIP3). PIP3 plays a key role by recruiting PH domain-containing proteins to the membrane, including AKT1 and PDPK1, activating signaling cascades involved in cell growth, survival, proliferation, motility and morphology. Links G-protein coupled receptor activation to PIP3 production. Involved in immune, inflammatory and allergic responses. Modulates leukocyte chemotaxis to inflammatory sites and in response to chemoattractant agents. May control leukocyte polarization and migration by regulating the spatial accumulation of PIP3 and by regulating the organization of F-actin formation and integrin-based adhesion at the leading edge. Controls motility of dendritic cells. Together with PIK3CD is involved in natural killer (NK) cell development and migration towards the sites of inflammation. Participates in T-lymphocyte migration. Regulates T-lymphocyte proliferation and cytokine production. Together with PIK3CD participates in T-lymphocyte development. Required for B-lymphocyte development and signaling. Together with PIK3CD participates in neutrophil respiratory burst. Together with PIK3CD is involved in neutrophil chemotaxis and extravasation. Together with PIK3CB promotes platelet aggregation and thrombosis. Regulates alpha-IIb/beta-3 integrins (ITGA2B/ ITGB3) adhesive function in platelets downstream of P2Y12 through a lipid kinase activity-independent mechanism. May have also a lipid kinase activity-dependent function in platelet aggregation. Involved in endothelial progenitor cell migration. Negative regulator of cardiac contractility. Modulates cardiac contractility by anchoring protein kinase A (PKA) and PDE3B activation, reducing cAMP levels. Regulates cardiac contractility also by promoting beta-adrenergic receptor internalization by binding to GRK2 and by non-muscle tropomyosin phosphorylation. Also has serine/threonine protein kinase activity: both lipid and protein kinase activities are required for beta-adrenergic receptor endocytosis. May also have a scaffolding role in modulating cardiac contractility. Contributes to cardiac hypertrophy under pathological stress. Through simultaneous binding of PDE3B to RAPGEF3 and PIK3R6 is assembled in a signaling complex in which the PI3K gamma complex is activated by RAPGEF3 and which is involved in angiogenesis.   
  
Heterodimer of a catalytic subunit PIK3CG and a PIK3R5 or PIK3R6 regulatory subunit. Interacts with GRK2 through the PIK helical domain. Interaction with GRK2 is required for targeting to agonist-occupied receptor. Interacts with PDE3B (By similarity). Interacts with TPM2. Interacts with EPHA8; regulates integrin-mediated cell adhesion to substrate. Interacts with HRAS; the interaction is required for membrane recruitment and beta-gamma G protein dimer-dependent activation of the PI3K gamma complex PIK3CG:PIK3R6 (By similarity).   
  
 **Gene Ontology Information:**

Molecular Function

- 1-phosphatidylinositol-3-kinase activity
- 1-phosphatidylinositol-4-phosphate 3-kinase activity
- ATP binding
- ephrin receptor binding
- identical protein binding
- kinase activity
- phosphatidylinositol kinase activity
- phosphatidylinositol-4,5-bisphosphate 3-kinase activity
- protein kinase activity
- protein serine kinase activity
- protein serine/threonine kinase activity

Location

- cytoplasm
- cytosol
- membrane
- phosphatidylinositol 3-kinase complex
- phosphatidylinositol 3-kinase complex, class IA
- phosphatidylinositol 3-kinase complex, class IB
- plasma membrane

Biological process

- adaptive immune response
- angiogenesis
- cell migration
- cellular response to cAMP
- dendritic cell chemotaxis
- endocytosis
- G protein-coupled receptor signaling pathway
- hepatocyte apoptotic process
- immune response
- inflammatory response
- innate immune response
- mast cell degranulation
- natural killer cell chemotaxis
- negative regulation of cardiac muscle contraction
- negative regulation of fibroblast apoptotic process
- negative regulation of triglyceride catabolic process
- neutrophil chemotaxis
- neutrophil extravasation
- phosphatidylinositol phosphorylation
- phosphatidylinositol-3-phosphate biosynthetic process
- phosphatidylinositol-mediated signaling
- phosphorylation
- platelet aggregation
- positive regulation of acute inflammatory response
- positive regulation of cytokine production
- positive regulation of cytosolic calcium ion concentration
- positive regulation of endothelial cell migration
- positive regulation of MAP kinase activity
- positive regulation of protein kinase B signaling
- positive regulation of Rac protein signal transduction
- protein phosphorylation
- regulation of calcium ion transmembrane transport
- regulation of cell adhesion mediated by integrin
- respiratory burst involved in defense response
- secretory granule localization
- sphingosine-1-phosphate receptor signaling pathway
- T cell activation
- T cell chemotaxis
- T cell proliferation

---

60

- **Protein name:** Mucolipin-3
- **Organism:** Homo sapiens
- **Uniprot Accession Number:** Q8TDD5
- **Protein sequence length:** 553 aa
- **1D identity (%):** 8.56
- **1D identity (%) [Gaps excluded]:** 19.49
- **1D identity - Alignment Gaps:** 550
- **1D aligned content (<aminoacid>:%):** {'V': 5.95, 'C': 4.76, 'R': 2.38, 'N': 2.38, 'L': 13.1, 'F': 3.57, 'P': 5.95, 'Q': 7.14, 'K': 8.33, 'T': 7.14, 'D': 7.14, 'S': 4.76, 'Y': 3.57, 'G': 8.33, 'I': 9.52, 'H': 1.19, 'A': 1.19, 'M': 1.19, 'E': 2.38}
- **Common reported functions (%):** 0.0
- **Common reported locations (%):** 0.0
- **Common reported processes (%):** 0.0

- **PDB ID:** 6AYG
- **Chain:** A
- **Crystallized protein length:** 439 aa
- **Resolution:** 4.65 Å
- **b-phipsi:** 0.040348
- **w-rdist:** 0.366821
- **t-alpha:** 0.008817
- **Chemical similarity (Tanimoto Index) (%):** 97.5
- **1D identity (%) [PDB]:** 1.11
- **1D identity (%) [Gaps excluded][PDB]:** 73.68
- **1D identity - Alignment Gaps [PDB]:** 1244
- **1D aligned content [PDB] (<aminoacid>:%):** {'R': 7.14, 'L': 28.57, 'K': 7.14, 'N': 7.14, 'T': 7.14, 'A': 7.14, 'G': 14.29, 'Q': 7.14, 'V': 7.14, 'I': 7.14}
- **2D identity (%) [PDB]:** 31.83
- **2D identity (%) [Gaps excluded][PDB]:** 92.97
- **2D identity - Alignment Gaps [PDB]:** 628
- **2D aligned content [PDB] (<2D-fold>:%):** {'.': 9.87, 'H': 76.32, 'T': 2.96, 'E': 10.86}
- **3D similarity (TM-Score) (%) [PDB]:** 18.71

- **Gene name:** MCOLN3
- **Entrez ID:** 55283
- **RefSeq ID:** N/A
- **Sequence length:** N/A
- **5-UTR|CDS|3-UTR identity (%):** N/A | N/A | N/A
- **5-UTR|CDS|3-UTR identity (%) [Gaps excluded]:** N/A | N/A | N/A
- **5-UTR|CDS|3-UTR identity [Alignment Gaps]:** N/A | N/A | N/A
- **5-UTR aligned content (<base>:%):** N/A
- **CDS aligned content (<base>:%):** N/A
- **3-UTR aligned content (<base>:%):** N/A

**Uniprot Description:**  
  
 Nonselective ligand-gated cation channel probably playing a role in the regulation of membrane trafficking events. Acts as Ca(2+)-permeable cation channel with inwardly rectifying activity (PubMed:18369318, PubMed:19497048, PubMed:19522758, PubMed:19885840, PubMed:29106414). Mediates release of Ca(2+) from endosomes to the cytoplasm, contributes to endosomal acidification and is involved in the regulation of membrane trafficking and fusion in the endosomal pathway (PubMed:21245134). Does not seem to act as mechanosensory transduction channel in inner ear sensory hair cells. Proposed to play a critical role at the cochlear stereocilia ankle-link region during hair-bundle growth (By similarity). Involved in the regulation of autophagy (PubMed:19522758). Through association with GABARAPL2 may be involved in autophagosome formation possibly providing Ca(2+) for the fusion process (By similarity). Through a possible and probably tissue-specific heteromerization with MCOLN1 may be at least in part involved in many lysosome-dependent cellular events (PubMed:19885840). Possible heteromeric ion channel assemblies with TRPV5 show pharmacological similarity with TRPML3 (PubMed:23469151).   
  
Homotetramer (PubMed:29106414). Can heterooligomerize with MCOLN1; heteromeric assemblies have different channel properties as compared to the respective homooligomers and may be tissue-specific (PubMed:19885840). May heterooligomerize with TRPV5 to form a functional distinct ion channel (PubMed:23469151). Interacts with GABARAPL2 (By similarity).   
  
 **Gene Ontology Information:**

Molecular Function

- calcium channel activity
- lipid binding
- NAADP-sensitive calcium-release channel activity

Location

- autophagosome membrane
- early endosome membrane
- late endosome membrane
- lysosomal membrane
- plasma membrane

Biological process

- calcium ion transmembrane transport
- inner ear auditory receptor cell differentiation
- locomotory behavior

---

61

- **Protein name:** Guanylate cyclase soluble subunit beta-1
- **Organism:** Homo sapiens
- **Uniprot Accession Number:** Q02153
- **Protein sequence length:** 619 aa
- **1D identity (%):** 12.62
- **1D identity (%) [Gaps excluded]:** 23.87
- **1D identity - Alignment Gaps:** 456
- **1D aligned content (<aminoacid>:%):** {'V': 7.38, 'N': 2.46, 'D': 8.2, 'K': 9.84, 'L': 11.48, 'G': 9.02, 'E': 8.2, 'I': 2.46, 'S': 4.1, 'Q': 2.46, 'H': 2.46, 'P': 8.2, 'R': 5.74, 'T': 6.56, 'F': 4.1, 'C': 4.92, 'Y': 1.64, 'M': 0.82}
- **Common reported functions (%):** 6.67
- **Common reported locations (%):** 7.14
- **Common reported processes (%):** 0.0

- **PDB ID:** 6JT1
- **Chain:** B
- **Crystallized protein length:** 576 aa
- **Resolution:** 3.9 Å
- **b-phipsi:** 0.012437
- **w-rdist:** 0.356208
- **t-alpha:** 0.018208
- **Chemical similarity (Tanimoto Index) (%):** 90.28
- **1D identity (%) [PDB]:** 2.19
- **1D identity (%) [Gaps excluded][PDB]:** 65.22
- **1D identity - Alignment Gaps [PDB]:** 1324
- **1D aligned content [PDB] (<aminoacid>:%):** {'M': 3.33, 'V': 6.67, 'R': 13.33, 'E': 13.33, 'L': 10.0, 'I': 13.33, 'Y': 3.33, 'S': 3.33, 'F': 10.0, 'P': 3.33, 'D': 3.33, 'G': 3.33, 'Q': 6.67, 'C': 3.33, 'K': 3.33}
- **2D identity (%) [PDB]:** 29.71
- **2D identity (%) [Gaps excluded][PDB]:** 87.47
- **2D identity - Alignment Gaps [PDB]:** 698
- **2D aligned content [PDB] (<2D-fold>:%):** {'.': 11.46, 'H': 48.73, 'T': 11.46, 'E': 27.39, 'G': 0.96}
- **3D similarity (TM-Score) (%) [PDB]:** 22.14

- **Gene name:** GUCY1B1
- **Entrez ID:** 2983
- **RefSeq ID:** NM\_001291954
- **Transcript sequence length:** 3283
- **5-UTR|CDS|3-UTR identity (%):** 52.74 | 38.54 | 7.5
- **5-UTR|CDS|3-UTR identity (%) [Gaps excluded]:** 76.24 | 74.58 | 75.52
- **5-UTR|CDS|3-UTR identity [Alignment Gaps]:** 45 | 1383 | 10888
- **5-UTR aligned content (<base>:%):** {'G': 40.26, 'C': 49.35, 'A': 2.6, 'T': 7.79}
- **CDS aligned content (<base>:%):** {'A': 29.65, 'T': 20.76, 'G': 26.38, 'C': 23.21}
- **3-UTR aligned content (<base>:%):** {'A': 30.1, 'C': 14.44, 'G': 14.11, 'T': 41.35}

**Uniprot Description:**  
  
 Mediates responses to nitric oxide (NO) by catalyzing the biosynthesis of the signaling molecule cGMP.   
  
The active enzyme is formed by a heterodimer of an alpha and a beta subunit. Heterodimer with GUCY1A1 (PubMed:1352257, PubMed:23505436, PubMed:24669844). Can also form inactive homodimers in vitro (PubMed:23505436, PubMed:24669844).   
  
 **Gene Ontology Information:**

Molecular Function

- adenylate cyclase activity
- cytidylate cyclase activity
- GTP binding
- guanylate cyclase activity
- heme binding
- Hsp90 protein binding
- metal ion binding
- protein-containing complex binding
- signaling receptor activity

Location

- cytosol
- glutamatergic synapse
- guanylate cyclase complex, soluble
- presynaptic active zone cytoplasmic component

Biological process

- blood circulation
- cellular response to nitric oxide
- cGMP biosynthetic process
- cGMP-mediated signaling
- nitric oxide mediated signal transduction
- nitric oxide-cGMP-mediated signaling pathway
- response to oxygen levels
- trans-synaptic signaling by nitric oxide, modulating synaptic transmission

---

62

- **Protein name:** Presequence protease, mitochondrial
- **Organism:** Homo sapiens
- **Uniprot Accession Number:** Q5JRX3
- **Protein sequence length:** 1037 aa
- **1D identity (%):** 15.21
- **1D identity (%) [Gaps excluded]:** 24.18
- **1D identity - Alignment Gaps:** 432
- **1D aligned content (<aminoacid>:%):** {'G': 7.91, 'R': 6.21, 'L': 7.91, 'E': 5.08, 'D': 6.21, 'I': 3.39, 'V': 6.78, 'H': 3.39, 'S': 2.26, 'F': 7.34, 'T': 9.6, 'Y': 2.26, 'P': 7.91, 'N': 1.69, 'A': 5.65, 'Q': 6.78, 'C': 2.82, 'K': 5.65, 'M': 0.56, 'W': 0.56}
- **Common reported functions (%):** 0.0
- **Common reported locations (%):** 0.0
- **Common reported processes (%):** 0.0

- **PDB ID:** 6XOV
- **Chain:** A
- **Crystallized protein length:** 966 aa
- **Resolution:** 3.3 Å
- **b-phipsi:** 0.009662
- **w-rdist:** 0.439506
- **t-alpha:** 0.016023
- **Chemical similarity (Tanimoto Index) (%):** 99.17
- **1D identity (%) [PDB]:** 3.81
- **1D identity (%) [Gaps excluded][PDB]:** 65.66
- **1D identity - Alignment Gaps [PDB]:** 1607
- **1D aligned content [PDB] (<aminoacid>:%):** {'A': 3.08, 'Q': 4.62, 'Y': 7.69, 'K': 7.69, 'L': 12.31, 'R': 10.77, 'H': 3.08, 'P': 4.62, 'C': 3.08, 'V': 4.62, 'E': 6.15, 'T': 9.23, 'I': 3.08, 'S': 7.69, 'D': 3.08, 'N': 1.54, 'F': 1.54, 'M': 1.54, 'G': 4.62}
- **2D identity (%) [PDB]:** 38.16
- **2D identity (%) [Gaps excluded][PDB]:** 85.95
- **2D identity - Alignment Gaps [PDB]:** 695
- **2D aligned content [PDB] (<2D-fold>:%):** {'H': 42.23, 'T': 14.92, '.': 17.02, 'E': 23.95, 'G': 1.26, 'B': 0.63}
- **3D similarity (TM-Score) (%) [PDB]:** 24.27

- **Gene name:** PITRM1
- **Entrez ID:** 10531
- **RefSeq ID:** NM\_014889
- **Transcript sequence length:** 3427
- **5-UTR|CDS|3-UTR identity (%):** 4.72 | 45.25 | 1.69
- **5-UTR|CDS|3-UTR identity (%) [Gaps excluded]:** 60.0 | 75.76 | 73.36
- **5-UTR|CDS|3-UTR identity [Alignment Gaps]:** 117 | 1436 | 11643
- **5-UTR aligned content (<base>:%):** {'T': 33.33, 'C': 33.33, 'G': 33.33}
- **CDS aligned content (<base>:%):** {'A': 25.05, 'T': 19.65, 'G': 25.85, 'C': 29.45}
- **3-UTR aligned content (<base>:%):** {'G': 20.4, 'A': 31.84, 'C': 17.41, 'T': 30.35}

**Uniprot Description:**  
  
 Metalloendopeptidase of the mitochondrial matrix that functions in peptide cleavage and degradation rather than in protein processing (PubMed:10360838, PubMed:16849325, PubMed:19196155, PubMed:24931469). Has an ATP-independent activity (PubMed:16849325). Specifically cleaves peptides in the range of 5 to 65 residues (PubMed:19196155). Shows a preference for cleavage after small polar residues and before basic residues, but without any positional preference (PubMed:10360838, PubMed:19196155, PubMed:24931469). Degrades the transit peptides of mitochondrial proteins after their cleavage (PubMed:19196155). Also degrades other unstructured peptides (PubMed:19196155). It is also able to degrade amyloid-beta protein 40, one of the peptides produced by APP processing, when it accumulates in mitochondrion (PubMed:16849325, PubMed:24931469). It is a highly efficient protease, at least toward amyloid-beta protein 40 (PubMed:24931469). Cleaves that peptide at a specific position and is probably not processive, releasing digested peptides intermediates that can be further cleaved subsequently (PubMed:24931469).   
  
Monomer and homodimer; homodimerization is induced by binding of the substrate.   
  
 **Gene Ontology Information:**

Molecular Function

- enzyme activator activity
- metalloendopeptidase activity
- metallopeptidase activity
- zinc ion binding

Location

- mitochondrial matrix
- mitochondrion

Biological process

- protein processing
- protein targeting to mitochondrion
- proteolysis

---

63

- **Protein name:** Dipeptidyl peptidase 9
- **Organism:** Homo sapiens
- **Uniprot Accession Number:** Q86TI2
- **Protein sequence length:** 863 aa
- **1D identity (%):** 17.0
- **1D identity (%) [Gaps excluded]:** 23.55
- **1D identity - Alignment Gaps:** 278
- **1D aligned content (<aminoacid>:%):** {'A': 7.65, 'P': 11.76, 'D': 2.94, 'G': 10.0, 'T': 5.29, 'F': 7.65, 'R': 5.88, 'I': 2.94, 'V': 5.29, 'Q': 4.71, 'Y': 4.12, 'M': 1.18, 'L': 8.24, 'W': 1.18, 'H': 4.12, 'E': 3.53, 'S': 3.53, 'K': 3.53, 'C': 4.71, 'N': 1.76}
- **Common reported functions (%):** 0.0
- **Common reported locations (%):** 14.29
- **Common reported processes (%):** 0.0

- **PDB ID:** 7JN7
- **Chain:** A
- **Crystallized protein length:** 846 aa
- **Resolution:** 3.3 Å
- **b-phipsi:** 0.00846
- **w-rdist:** 0.736336
- **t-alpha:** 0.001459
- **Chemical similarity (Tanimoto Index) (%):** N/A
- **1D identity (%) [PDB]:** 3.37
- **1D identity (%) [Gaps excluded][PDB]:** 65.06
- **1D identity - Alignment Gaps [PDB]:** 1518
- **1D aligned content [PDB] (<aminoacid>:%):** {'D': 3.7, 'P': 5.56, 'Y': 3.7, 'R': 3.7, 'E': 7.41, 'F': 5.56, 'G': 9.26, 'I': 11.11, 'M': 3.7, 'V': 7.41, 'K': 3.7, 'T': 5.56, 'L': 7.41, 'N': 3.7, 'A': 7.41, 'Q': 7.41, 'W': 1.85, 'H': 1.85}
- **2D identity (%) [PDB]:** 36.77
- **2D identity (%) [Gaps excluded][PDB]:** 84.18
- **2D identity - Alignment Gaps [PDB]:** 660
- **2D aligned content [PDB] (<2D-fold>:%):** {'.': 21.35, 'E': 44.78, 'H': 20.65, 'T': 13.23}
- **3D similarity (TM-Score) (%) [PDB]:** 27.21

- **Gene name:** DPP9
- **Entrez ID:** 91039
- **RefSeq ID:** N/A
- **Sequence length:** N/A
- **5-UTR|CDS|3-UTR identity (%):** N/A | N/A | N/A
- **5-UTR|CDS|3-UTR identity (%) [Gaps excluded]:** N/A | N/A | N/A
- **5-UTR|CDS|3-UTR identity [Alignment Gaps]:** N/A | N/A | N/A
- **5-UTR aligned content (<base>:%):** N/A
- **CDS aligned content (<base>:%):** N/A
- **3-UTR aligned content (<base>:%):** N/A

**Uniprot Description:**  
  
 Dipeptidyl peptidase that cleaves off N-terminal dipeptides from proteins having a Pro or Ala residue at position 2 (PubMed:12662155, PubMed:16475979, PubMed:19667070, PubMed:30291141, PubMed:29382749). Acts as an inhibitor of caspase-1-dependent monocyte and macrophage pyroptosis: inhibits pyroptosis by preventing activation of NLRP1 and CARD8 via an unknown mechanism (PubMed:27820798, PubMed:30291141, PubMed:29967349, PubMed:31525884, PubMed:32796818).   
  
Homodimer.   
  
 **Gene Ontology Information:**

Molecular Function

- aminopeptidase activity
- dipeptidyl-peptidase activity
- identical protein binding
- serine-type peptidase activity

Location

- cell leading edge
- cytosol
- microtubule
- nucleus

Biological process

- negative regulation of programmed cell death
- proteolysis
- pyroptosis

---

64

- **Protein name:** Cartilage oligomeric matrix protein
- **Organism:** Homo sapiens
- **Uniprot Accession Number:** P49747
- **Protein sequence length:** 757 aa
- **1D identity (%):** 13.92
- **1D identity (%) [Gaps excluded]:** 23.83
- **1D identity - Alignment Gaps:** 424
- **1D aligned content (<aminoacid>:%):** {'V': 9.15, 'L': 5.63, 'P': 10.56, 'E': 5.63, 'D': 9.86, 'Q': 7.04, 'K': 3.52, 'C': 4.93, 'G': 9.86, 'M': 0.7, 'R': 9.86, 'H': 4.23, 'F': 3.52, 'S': 2.82, 'T': 2.82, 'N': 2.11, 'W': 1.41, 'A': 2.11, 'I': 1.41, 'Y': 2.82}
- **Common reported functions (%):** 0.0
- **Common reported locations (%):** 7.14
- **Common reported processes (%):** 0.0

- **PDB ID:** 3FBY
- **Chain:** C
- **Crystallized protein length:** 535 aa
- **Resolution:** 3.15 Å
- **b-phipsi:** 0.042921
- **w-rdist:** 0.436765
- **t-alpha:** 0.005098
- **Chemical similarity (Tanimoto Index) (%):** 67.73
- **1D identity (%) [PDB]:** 2.64
- **1D identity (%) [Gaps excluded][PDB]:** 71.43
- **1D identity - Alignment Gaps [PDB]:** 1275
- **1D aligned content [PDB] (<aminoacid>:%):** {'N': 5.71, 'E': 5.71, 'R': 5.71, 'V': 8.57, 'G': 11.43, 'I': 5.71, 'A': 5.71, 'T': 8.57, 'D': 8.57, 'K': 2.86, 'H': 2.86, 'P': 5.71, 'Q': 2.86, 'S': 8.57, 'Y': 2.86, 'L': 2.86, 'W': 2.86, 'F': 2.86}
- **2D identity (%) [PDB]:** 16.46
- **2D identity (%) [Gaps excluded][PDB]:** 84.0
- **2D identity - Alignment Gaps [PDB]:** 923
- **2D aligned content [PDB] (<2D-fold>:%):** {'.': 16.4, 'B': 0.53, 'E': 53.44, 'T': 22.22, 'H': 7.41}
- **3D similarity (TM-Score) (%) [PDB]:** 19.76

- **Gene name:** COMP
- **Entrez ID:** 1311
- **RefSeq ID:** NM\_000095
- **Transcript sequence length:** 2452
- **5-UTR|CDS|3-UTR identity (%):** 18.9 | 45.16 | 0.82
- **5-UTR|CDS|3-UTR identity (%) [Gaps excluded]:** 66.67 | 73.66 | 76.56
- **5-UTR|CDS|3-UTR identity [Alignment Gaps]:** 91 | 1164 | 11774
- **5-UTR aligned content (<base>:%):** {'G': 33.33, 'C': 62.5, 'A': 4.17}
- **CDS aligned content (<base>:%):** {'A': 20.53, 'T': 14.57, 'G': 32.52, 'C': 32.38}
- **3-UTR aligned content (<base>:%):** {'G': 40.82, 'A': 19.39, 'C': 23.47, 'T': 16.33}

**Uniprot Description:**  
  
 May play a role in the structural integrity of cartilage via its interaction with other extracellular matrix proteins such as the collagens and fibronectin. Can mediate the interaction of chondrocytes with the cartilage extracellular matrix through interaction with cell surface integrin receptors. Could play a role in the pathogenesis of osteoarthritis. Potent suppressor of apoptosis in both primary chondrocytes and transformed cells. Suppresses apoptosis by blocking the activation of caspase-3 and by inducing the IAP family of survival proteins (BIRC3, BIRC2, BIRC5 and XIAP). Essential for maintaining a vascular smooth muscle cells (VSMCs) contractile/differentiated phenotype under physiological and pathological stimuli. Maintains this phenotype of VSMCs by interacting with ITGA7 (By similarity).   
  
Pentamer; disulfide-linked. Exists in a more compact conformation in the presence of calcium and shows a more extended conformation in the absence of calcium. Interacts with ITGB3, ITGA5 and FN1. Binding to FN1 requires the presence of divalent cations (Ca(2+), Mg(2+) or Mn(2+)). The greatest amount of binding is seen in the presence of Mn(2+). Interacts with MATN1, MATN3, MATN4 and ACAN. Binds heparin, heparan sulfate and chondroitin sulfate. EDTA dimishes significantly its binding to ACAN and abolishes its binding to MATN3, MATN4 and chondroitin sulfate. Interacts with collagen I, II and IX, and interaction with these collagens is dependent on the presence of zinc ions. Interacts with ADAMTS12. Interacts with ITGA7 (By similarity).   
  
 **Gene Ontology Information:**

Molecular Function

- BMP binding
- calcium ion binding
- collagen binding
- extracellular matrix structural constituent
- heparan sulfate proteoglycan binding
- heparin binding
- integrin binding
- protease binding
- proteoglycan binding

Location

- collagen-containing extracellular matrix
- extracellular exosome
- extracellular matrix
- extracellular region
- extracellular space
- protein-containing complex

Biological process

- animal organ morphogenesis
- apoptotic process
- artery morphogenesis
- BMP signaling pathway
- bone mineralization
- cartilage homeostasis
- cellular senescence
- chondrocyte development
- chondrocyte proliferation
- collagen fibril organization
- growth plate cartilage development
- limb development
- multicellular organism growth
- musculoskeletal movement
- negative regulation of apoptotic process
- negative regulation of hemostasis
- platelet aggregation
- positive regulation of chondrocyte proliferation
- protein homooligomerization
- protein processing
- protein secretion
- regulation of bone mineralization
- regulation of gene expression
- response to unfolded protein
- skeletal system development
- skin development
- tendon development
- vascular associated smooth muscle cell development
- vascular associated smooth muscle contraction

---

65

- **Protein name:** Small nuclear ribonucleoprotein F
- **Organism:** Homo sapiens
- **Uniprot Accession Number:** P62306
- **Protein sequence length:** 86 aa
- **1D identity (%):** 2.16
- **1D identity (%) [Gaps excluded]:** 29.23
- **1D identity - Alignment Gaps:** 815
- **1D aligned content (<aminoacid>:%):** {'M': 10.53, 'L': 31.58, 'P': 5.26, 'G': 10.53, 'V': 15.79, 'S': 10.53, 'A': 5.26, 'D': 5.26, 'R': 5.26}
- **Common reported functions (%):** 6.67
- **Common reported locations (%):** 21.43
- **Common reported processes (%):** 0.0

- **PDB ID:** 5O9Z
- **Chain:** B
- **Crystallized protein length:** 844 aa
- **Resolution:** 4.5 Å
- **b-phipsi:** 0.005406
- **w-rdist:** 0.433397
- **t-alpha:** 0.052914
- **Chemical similarity (Tanimoto Index) (%):** N/A
- **1D identity (%) [PDB]:** 3.3
- **1D identity (%) [Gaps excluded][PDB]:** 71.62
- **1D identity - Alignment Gaps [PDB]:** 1534
- **1D aligned content [PDB] (<aminoacid>:%):** {'E': 9.43, 'M': 1.89, 'D': 11.32, 'I': 13.21, 'K': 13.21, 'L': 5.66, 'P': 5.66, 'V': 9.43, 'N': 3.77, 'R': 5.66, 'T': 5.66, 'Q': 1.89, 'F': 3.77, 'G': 5.66, 'A': 1.89, 'S': 1.89}
- **2D identity (%) [PDB]:** 38.44
- **2D identity (%) [Gaps excluded][PDB]:** 87.35
- **2D identity - Alignment Gaps [PDB]:** 654
- **2D aligned content [PDB] (<2D-fold>:%):** {'.': 19.15, 'E': 23.83, 'H': 42.32, 'T': 12.92, 'B': 0.45, 'G': 1.34}
- **3D similarity (TM-Score) (%) [PDB]:** 24.52

- **Gene name:** SNRPF
- **Entrez ID:** 6636
- **RefSeq ID:** NM\_003095
- **Transcript sequence length:** 452
- **5-UTR|CDS|3-UTR identity (%):** 49.35 | 6.47 | 0.41
- **5-UTR|CDS|3-UTR identity (%) [Gaps excluded]:** 80.0 | 73.8 | 74.24
- **5-UTR|CDS|3-UTR identity [Alignment Gaps]:** 59 | 2383 | 11825
- **5-UTR aligned content (<base>:%):** {'C': 36.84, 'T': 14.47, 'G': 47.37, 'A': 1.32}
- **CDS aligned content (<base>:%):** {'A': 33.14, 'T': 20.12, 'G': 28.99, 'C': 17.75}
- **3-UTR aligned content (<base>:%):** {'C': 6.12, 'A': 20.41, 'T': 57.14, 'G': 16.33}

**Uniprot Description:**  
  
 Plays role in pre-mRNA splicing as core component of the SMN-Sm complex that mediates spliceosomal snRNP assembly and as component of the spliceosomal U1, U2, U4 and U5 small nuclear ribonucleoproteins (snRNPs), the building blocks of the spliceosome (PubMed:11991638, PubMed:18984161, PubMed:19325628, PubMed:23333303, PubMed:25555158, PubMed:26912367, PubMed:28502770, PubMed:28781166, PubMed:28076346). Component of both the pre-catalytic spliceosome B complex and activated spliceosome C complexes (PubMed:11991638, PubMed:28502770, PubMed:28781166, PubMed:28076346). Is also a component of the minor U12 spliceosome (PubMed:15146077). As part of the U7 snRNP it is involved in histone 3'-end processing (PubMed:12975319).   
  
Core component of the spliceosomal U1, U2, U4 and U5 small nuclear ribonucleoproteins (snRNPs), the building blocks of the spliceosome (PubMed:11991638, PubMed:19325628, PubMed:21516107, PubMed:25555158, PubMed:26912367, PubMed:28502770, PubMed:28781166, PubMed:28076346). Most spliceosomal snRNPs contain a common set of Sm proteins, SNRPB, SNRPD1, SNRPD2, SNRPD3, SNRPE, SNRPF and SNRPG that assemble in a heptameric protein ring on the Sm site of the small nuclear RNA to form the core snRNP (PubMed:19325628, PubMed:21516107, PubMed:25555158, PubMed:26912367, PubMed:28502770, PubMed:28781166, PubMed:28076346). Component of the U1 snRNP (PubMed:19325628, PubMed:25555158). The U1 snRNP is composed of the U1 snRNA and the 7 core Sm proteins SNRPB, SNRPD1, SNRPD2, SNRPD3, SNRPE, SNRPF and SNRPG, and at least three U1 snRNP-specific proteins SNRNP70/U1-70K, SNRPA/U1-A and SNRPC/U1-C (PubMed:19325628, PubMed:25555158). Component of the U4/U6-U5 tri-snRNP complex composed of the U4, U6 and U5 snRNAs and at least PRPF3, PRPF4, PRPF6, PRPF8, PRPF31, SNRNP200, TXNL4A, SNRNP40, SNRPB, SNRPD1, SNRPD2, SNRPD3, SNRPE, SNRPF, SNRPG, DDX23, CD2BP2, PPIH, SNU13, EFTUD2, SART1 and USP39, plus LSM2, LSM3, LSM4, LSM5, LSM6, LSM7 and LSM8 (PubMed:26912367). Component of the U7 snRNP complex, or U7 Sm protein core complex, that is composed of the U7 snRNA and at least LSM10, LSM11, SNRPB, SNRPD3, SNRPE, SNRPF and SNRPG; the complex does not contain SNRPD1 and SNRPD2 (PubMed:11574479). Component of the U11/U12 snRNPs that are part of the U12-type spliceosome (PubMed:15146077). Part of the SMN-Sm complex that contains SMN1, GEMIN2/SIP1, DDX20/GEMIN3, GEMIN4, GEMIN5, GEMIN6, GEMIN7, GEMIN8, STRAP/UNRIP and the Sm proteins SNRPB, SNRPD1, SNRPD2, SNRPD3, SNRPE, SNRPF and SNRPG; catalyzes core snRNPs assembly (PubMed:18984161). Forms a 6S pICln-Sm complex composed of CLNS1A/pICln, SNRPD1, SNRPD2, SNRPE, SNRPF and SNRPG; ring-like structure where CLNS1A/pICln mimics additional Sm proteins and which is unable to assemble into the core snRNP (PubMed:18984161, PubMed:23333303).   
  
 **Gene Ontology Information:**

Molecular Function

- RNA binding

Location

- catalytic step 2 spliceosome
- cytosol
- methylosome
- nucleoplasm
- nucleus
- pICln-Sm protein complex
- small nuclear ribonucleoprotein complex
- SMN-Sm protein complex
- spliceosomal complex
- U1 snRNP
- U12-type spliceosomal complex
- U2 snRNP
- U2-type catalytic step 2 spliceosome
- U2-type precatalytic spliceosome
- U4 snRNP
- U4/U6 x U5 tri-snRNP complex
- U5 snRNP
- U7 snRNP

Biological process

- 7-methylguanosine cap hypermethylation
- mRNA splicing, via spliceosome
- RNA splicing
- spliceosomal snRNP assembly
- U2-type prespliceosome assembly

---

66

- **Protein name:** Transcription elongation factor SPT5
- **Organism:** Homo sapiens
- **Uniprot Accession Number:** O00267
- **Protein sequence length:** 1087 aa
- **1D identity (%):** 15.48
- **1D identity (%) [Gaps excluded]:** 23.29
- **1D identity - Alignment Gaps:** 392
- **1D aligned content (<aminoacid>:%):** {'N': 0.55, 'F': 2.76, 'E': 6.63, 'D': 6.63, 'P': 7.18, 'R': 5.52, 'K': 9.39, 'L': 8.84, 'I': 4.42, 'S': 6.63, 'G': 11.6, 'V': 5.52, 'W': 0.55, 'A': 4.42, 'T': 7.18, 'Q': 5.52, 'M': 1.1, 'C': 1.1, 'H': 1.66, 'Y': 2.76}
- **Common reported functions (%):** 0.0
- **Common reported locations (%):** 0.0
- **Common reported processes (%):** 0.0

- **PDB ID:** 8P4F
- **Chain:** O
- **Crystallized protein length:** 695 aa
- **Resolution:** 4.0 Å
- **b-phipsi:** 0.002055
- **w-rdist:** 0.533118
- **t-alpha:** 0.077708
- **Chemical similarity (Tanimoto Index) (%):** N/A
- **1D identity (%) [PDB]:** 2.56
- **1D identity (%) [Gaps excluded][PDB]:** 74.51
- **1D identity - Alignment Gaps [PDB]:** 1431
- **1D aligned content [PDB] (<aminoacid>:%):** {'V': 13.16, 'C': 5.26, 'E': 7.89, 'L': 7.89, 'K': 21.05, 'S': 2.63, 'P': 5.26, 'F': 2.63, 'T': 5.26, 'I': 5.26, 'G': 2.63, 'H': 2.63, 'Q': 2.63, 'R': 7.89, 'Y': 2.63, 'N': 2.63, 'A': 2.63}
- **2D identity (%) [PDB]:** 45.31
- **2D identity (%) [Gaps excluded][PDB]:** 88.27
- **2D identity - Alignment Gaps [PDB]:** 493
- **2D aligned content [PDB] (<2D-fold>:%):** {'.': 17.21, 'T': 23.53, 'E': 21.35, 'H': 35.29, 'G': 2.61}
- **3D similarity (TM-Score) (%) [PDB]:** 28.03

- **Gene name:** SUPT5H
- **Entrez ID:** N/A
- **RefSeq ID:** NM\_003169
- **Transcript sequence length:** 3782
- **5-UTR|CDS|3-UTR identity (%):** 36.0 | 45.65 | 1.44
- **5-UTR|CDS|3-UTR identity (%) [Gaps excluded]:** 72.58 | 75.44 | 76.11
- **5-UTR|CDS|3-UTR identity [Alignment Gaps]:** 126 | 1438 | 11707
- **5-UTR aligned content (<base>:%):** {'G': 52.22, 'T': 7.78, 'A': 3.33, 'C': 36.67}
- **CDS aligned content (<base>:%):** {'A': 25.03, 'T': 16.0, 'G': 30.32, 'C': 28.64}
- **3-UTR aligned content (<base>:%):** {'A': 16.28, 'G': 23.84, 'C': 20.35, 'T': 39.53}

**Uniprot Description:**  
  
 Component of the DRB sensitivity-inducing factor complex (DSIF complex), which regulates mRNA processing and transcription elongation by RNA polymerase II. DSIF positively regulates mRNA capping by stimulating the mRNA guanylyltransferase activity of RNGTT/CAP1A. DSIF also acts cooperatively with the negative elongation factor complex (NELF complex) to enhance transcriptional pausing at sites proximal to the promoter. Transcriptional pausing may facilitate the assembly of an elongation competent RNA polymerase II complex. DSIF and NELF promote pausing by inhibition of the transcription elongation factor TFIIS/S-II. TFIIS/S-II binds to RNA polymerase II at transcription pause sites and stimulates the weak intrinsic nuclease activity of the enzyme. Cleavage of blocked transcripts by RNA polymerase II promotes the resumption of transcription from the new 3' terminus and may allow repeated attempts at transcription through natural pause sites. DSIF can also positively regulate transcriptional elongation and is required for the efficient activation of transcriptional elongation by the HIV-1 nuclear transcriptional activator, Tat. DSIF acts to suppress transcriptional pausing in transcripts derived from the HIV-1 LTR and blocks premature release of HIV-1 transcripts at terminator sequences.   
  
Interacts with SUPT4H1 to form DSIF. DSIF interacts with the positive transcription elongation factor b complex (P-TEFb complex), which is composed of CDK9 and cyclin-T (CCNT1 or CCNT2). DSIF interacts with RNA polymerase II, and this interaction is reduced by phosphorylation of the C-terminal domain (CTD) of POLR2A by P-TEFb. DSIF also interacts with the NELF complex, which is composed of NELFA, NELFB, NELFD and NELFE, and this interaction occurs following prior binding of DSIF to RNA polymerase II. DSIF also interacts with PRMT1/HRMT1L2, HTATSF1/TATSF1, RNGTT/CAP1A, PRMT5/SKB1, SUPT6H, and can interact with PIN1. Component of a complex which is at least composed of HTATSF1/Tat-SF1, the P-TEFb complex components CDK9 and CCNT1, RNA polymerase II, SUPT5H, and NCL/nucleolin. Interacts with MCM3AP isoform GANP (PubMed:23652018).   
  
 **Gene Ontology Information:**

Molecular Function   
  
N/A

Location   
  
N/A

Biological process   
  
N/A

---

67

- **Protein name:** Protein argonaute-3
- **Organism:** Homo sapiens
- **Uniprot Accession Number:** Q9H9G7
- **Protein sequence length:** 860 aa
- **1D identity (%):** 79.52
- **1D identity (%) [Gaps excluded]:** 81.29
- **1D identity - Alignment Gaps:** 19
- **1D aligned content (<aminoacid>:%):** {'M': 2.17, 'G': 7.09, 'A': 6.8, 'P': 6.8, 'R': 6.66, 'T': 6.37, 'I': 5.21, 'K': 5.35, 'L': 7.67, 'N': 2.32, 'F': 4.49, 'D': 4.78, 'Y': 3.62, 'E': 4.78, 'C': 2.75, 'V': 8.54, 'Q': 6.08, 'H': 3.47, 'S': 4.34, 'W': 0.72}
- **Common reported functions (%):** 46.67
- **Common reported locations (%):** 57.14
- **Common reported processes (%):** 23.81

- **PDB ID:** 5VM9
- **Chain:** A
- **Crystallized protein length:** 779 aa
- **Resolution:** 3.28 Å
- **b-phipsi:** 0.009833
- **w-rdist:** 0.164679
- **t-alpha:** 0.065167
- **Chemical similarity (Tanimoto Index) (%):** 100.0
- **1D identity (%) [PDB]:** 75.18
- **1D identity (%) [Gaps excluded][PDB]:** 82.35
- **1D identity - Alignment Gaps [PDB]:** 74
- **1D aligned content [PDB] (<aminoacid>:%):** {'P': 6.89, 'R': 7.04, 'G': 5.79, 'T': 6.73, 'I': 5.63, 'K': 5.63, 'L': 7.82, 'A': 6.73, 'N': 2.35, 'F': 4.85, 'D': 4.69, 'Y': 3.91, 'E': 4.07, 'C': 2.97, 'V': 8.92, 'M': 2.03, 'Q': 6.1, 'H': 3.29, 'S': 3.76, 'W': 0.78}
- **2D identity (%) [PDB]:** 65.64
- **2D identity (%) [Gaps excluded][PDB]:** 77.24
- **2D identity - Alignment Gaps [PDB]:** 132
- **2D aligned content [PDB] (<2D-fold>:%):** {'.': 20.31, 'B': 1.74, 'E': 30.56, 'H': 36.28, 'T': 10.07, 'G': 1.04}
- **3D similarity (TM-Score) (%) [PDB]:** 89.19

- **Gene name:** AGO3
- **Entrez ID:** 192669
- **RefSeq ID:** N/A
- **Sequence length:** N/A
- **5-UTR|CDS|3-UTR identity (%):** N/A | N/A | N/A
- **5-UTR|CDS|3-UTR identity (%) [Gaps excluded]:** N/A | N/A | N/A
- **5-UTR|CDS|3-UTR identity [Alignment Gaps]:** N/A | N/A | N/A
- **5-UTR aligned content (<base>:%):** N/A
- **CDS aligned content (<base>:%):** N/A
- **3-UTR aligned content (<base>:%):** N/A

**Uniprot Description:**  
  
 Required for RNA-mediated gene silencing (RNAi). Binds to short RNAs such as microRNAs (miRNAs) and represses the translation of mRNAs which are complementary to them. Proposed to be involved in stabilization of small RNA derivates (siRNA) derived from processed RNA polymerase III-transcribed Alu repeats containing a DR2 retinoic acid response element (RARE) in stem cells and in the subsequent siRNA-dependent degradation of a subset of RNA polymerase II-transcribed coding mRNAs by recruiting a mRNA decapping complex involving EDC4. Possesses RNA slicer activity but only on select RNAs bearing 5'- and 3'-flanking sequences to the region of guide-target complementarity (PubMed:29040713).   
  
Interacts with EIF4B, IMP8, PRMT5 and TNRC6B. Interacts with APOBEC3F, APOBEC3G and APOBEC3H. Interacts with EDC4.   
  
 **Gene Ontology Information:**

Molecular Function

- double-stranded RNA binding
- endoribonuclease activity, cleaving miRNA-paired mRNA
- metal ion binding
- miRNA binding
- RNA binding
- endoribonuclease activity
- single-stranded RNA binding

Location

- condensed nuclear chromosome
- cytoplasm
- cytoplasmic ribonucleoprotein granule
- cytosol
- membrane
- nucleoplasm
- P-body
- RISC complex
- RISC-loading complex

Biological process

- production of miRNAs involved in gene silencing by miRNA
- miRNA mediated inhibition of translation
- mRNA catabolic process
- positive regulation of gene expression
- positive regulation of NIK/NF-kappaB signaling
- pre-miRNA processing
- regulation of stem cell proliferation
- small RNA loading onto RISC
- RNA secondary structure unwinding

---

68

- **Protein name:** Ceruloplasmin
- **Organism:** Homo sapiens
- **Uniprot Accession Number:** P00450
- **Protein sequence length:** 1065 aa
- **1D identity (%):** 17.23
- **1D identity (%) [Gaps excluded]:** 26.21
- **1D identity - Alignment Gaps:** 398
- **1D aligned content (<aminoacid>:%):** {'I': 5.5, 'G': 12.5, 'A': 5.0, 'T': 6.5, 'E': 4.5, 'D': 7.5, 'H': 4.0, 'P': 6.5, 'R': 6.0, 'F': 5.0, 'K': 6.0, 'V': 8.0, 'N': 3.0, 'L': 6.5, 'Y': 3.0, 'C': 3.0, 'S': 4.0, 'M': 0.5, 'Q': 3.0}
- **Common reported functions (%):** 0.0
- **Common reported locations (%):** 7.14
- **Common reported processes (%):** 0.0

- **PDB ID:** 4ENZ
- **Chain:** A
- **Crystallized protein length:** 1029 aa
- **Resolution:** 2.6 Å
- **b-phipsi:** 0.017028
- **w-rdist:** 0.620285
- **t-alpha:** 0.001457
- **Chemical similarity (Tanimoto Index) (%):** N/A
- **1D identity (%) [PDB]:** 1.69
- **1D identity (%) [Gaps excluded][PDB]:** 81.58
- **1D identity - Alignment Gaps [PDB]:** 1793
- **1D aligned content [PDB] (<aminoacid>:%):** {'F': 3.23, 'R': 3.23, 'H': 3.23, 'L': 16.13, 'K': 6.45, 'N': 3.23, 'T': 9.68, 'Y': 6.45, 'A': 6.45, 'G': 9.68, 'Q': 3.23, 'V': 19.35, 'P': 3.23, 'E': 3.23, 'D': 3.23}
- **2D identity (%) [PDB]:** 35.56
- **2D identity (%) [Gaps excluded][PDB]:** 85.27
- **2D identity - Alignment Gaps [PDB]:** 769
- **2D aligned content [PDB] (<2D-fold>:%):** {'.': 18.12, 'E': 44.78, 'T': 16.63, 'H': 17.91, 'G': 2.56}
- **3D similarity (TM-Score) (%) [PDB]:** 28.43

- **Gene name:** CP
- **Entrez ID:** 1356
- **RefSeq ID:** NM\_000096
- **Transcript sequence length:** 4452
- **5-UTR|CDS|3-UTR identity (%):** 12.23 | 43.29 | 6.57
- **5-UTR|CDS|3-UTR identity (%) [Gaps excluded]:** 68.0 | 74.37 | 73.08
- **5-UTR|CDS|3-UTR identity [Alignment Gaps]:** 114 | 1526 | 10943
- **5-UTR aligned content (<base>:%):** {'C': 41.18, 'T': 23.53, 'G': 35.29}
- **CDS aligned content (<base>:%):** {'A': 29.16, 'T': 20.68, 'G': 25.87, 'C': 24.29}
- **3-UTR aligned content (<base>:%):** {'T': 32.03, 'A': 40.0, 'G': 13.92, 'C': 14.05}

**Uniprot Description:**  
  
 Ceruloplasmin is a blue, copper-binding (6-7 atoms per molecule) glycoprotein. It has ferroxidase activity oxidizing Fe(2+) to Fe(3+) without releasing radical oxygen species. It is involved in iron transport across the cell membrane. Provides Cu(2+) ions for the ascorbate-mediated deaminase degradation of the heparan sulfate chains of GPC1. May also play a role in fetal lung development or pulmonary antioxidant defense (By similarity). N/A   
  
 **Gene Ontology Information:**

Molecular Function

- copper ion binding
- ferroxidase activity
- oxidoreductase activity
- chaperone binding

Location

- blood microparticle
- endoplasmic reticulum lumen
- extracellular exosome
- extracellular region
- extracellular space
- lysosomal membrane
- plasma membrane

Biological process

- copper ion transport
- cellular iron ion homeostasis
- iron ion transport

---

69

- **Protein name:** Regulator of nonsense transcripts 1
- **Organism:** Homo sapiens
- **Uniprot Accession Number:** Q92900
- **Protein sequence length:** 1129 aa
- **1D identity (%):** 16.19
- **1D identity (%) [Gaps excluded]:** 25.55
- **1D identity - Alignment Gaps:** 446
- **1D aligned content (<aminoacid>:%):** {'A': 5.08, 'P': 12.18, 'Q': 5.08, 'F': 7.11, 'G': 10.15, 'T': 3.55, 'L': 7.61, 'E': 5.08, 'D': 5.58, 'Y': 3.55, 'C': 2.54, 'V': 9.14, 'I': 3.05, 'H': 1.02, 'K': 5.08, 'N': 3.05, 'W': 1.02, 'S': 3.55, 'R': 6.09, 'M': 0.51}
- **Common reported functions (%):** 6.67
- **Common reported locations (%):** 35.71
- **Common reported processes (%):** 0.0

- **PDB ID:** 2WJY
- **Chain:** A
- **Crystallized protein length:** 773 aa
- **Resolution:** 2.5 Å
- **b-phipsi:** 0.003909
- **w-rdist:** 0.480527
- **t-alpha:** 0.087956
- **Chemical similarity (Tanimoto Index) (%):** 80.8
- **1D identity (%) [PDB]:** 2.64
- **1D identity (%) [Gaps excluded][PDB]:** 68.33
- **1D identity - Alignment Gaps [PDB]:** 1495
- **1D aligned content [PDB] (<aminoacid>:%):** {'K': 7.32, 'P': 7.32, 'T': 9.76, 'D': 2.44, 'S': 7.32, 'R': 7.32, 'V': 12.2, 'I': 2.44, 'G': 4.88, 'E': 7.32, 'H': 4.88, 'C': 2.44, 'Y': 4.88, 'Q': 9.76, 'F': 4.88, 'L': 2.44, 'A': 2.44}
- **2D identity (%) [PDB]:** 37.07
- **2D identity (%) [Gaps excluded][PDB]:** 89.24
- **2D identity - Alignment Gaps [PDB]:** 667
- **2D aligned content [PDB] (<2D-fold>:%):** {'.': 17.73, 'E': 25.3, 'T': 13.24, 'H': 41.61, 'G': 2.13}
- **3D similarity (TM-Score) (%) [PDB]:** 20.37

- **Gene name:** UPF1
- **Entrez ID:** 5976
- **RefSeq ID:** NM\_002911
- **Transcript sequence length:** 5321
- **5-UTR|CDS|3-UTR identity (%):** 42.19 | 45.52 | 8.57
- **5-UTR|CDS|3-UTR identity (%) [Gaps excluded]:** 88.52 | 75.39 | 75.2
- **5-UTR|CDS|3-UTR identity [Alignment Gaps]:** 134 | 1467 | 10819
- **5-UTR aligned content (<base>:%):** {'G': 46.3, 'T': 8.33, 'C': 41.67, 'A': 3.7}
- **CDS aligned content (<base>:%):** {'A': 23.09, 'T': 16.08, 'G': 29.79, 'C': 31.04}
- **3-UTR aligned content (<base>:%):** {'A': 19.5, 'G': 30.31, 'T': 25.81, 'C': 24.38}

**Uniprot Description:**  
  
 RNA-dependent helicase and ATPase required for nonsense-mediated decay (NMD) of mRNAs containing premature stop codons. Is recruited to mRNAs upon translation termination and undergoes a cycle of phosphorylation and dephosphorylation; its phosphorylation appears to be a key step in NMD. Recruited by release factors to stalled ribosomes together with the SMG1C protein kinase complex to form the transient SURF (SMG1-UPF1-eRF1-eRF3) complex. In EJC-dependent NMD, the SURF complex associates with the exon junction complex (EJC) (located 50-55 or more nucleotides downstream from the termination codon) through UPF2 and allows the formation of an UPF1-UPF2-UPF3 surveillance complex which is believed to activate NMD. Phosphorylated UPF1 is recognized by EST1B/SMG5, SMG6 and SMG7 which are thought to provide a link to the mRNA degradation machinery involving exonucleolytic and endonucleolytic pathways, and to serve as adapters to protein phosphatase 2A (PP2A), thereby triggering UPF1 dephosphorylation and allowing the recycling of NMD factors. UPF1 can also activate NMD without UPF2 or UPF3, and in the absence of the NMD-enhancing downstream EJC indicative for alternative NMD pathways. Plays a role in replication-dependent histone mRNA degradation at the end of phase S; the function is independent of UPF2. For the recognition of premature termination codons (PTC) and initiation of NMD a competitive interaction between UPF1 and PABPC1 with the ribosome-bound release factors is proposed. The ATPase activity of UPF1 is required for disassembly of mRNPs undergoing NMD. Essential for embryonic viability. Together with UPF2 and dependent on TDRD6, mediates the degradation of mRNA hardoring long 3'UTR by inducing the NMD machinery (By similarity).   
  
Found in a post-splicing messenger ribonucleoprotein (mRNP) complex (PubMed:21419344). Associates with the exon junction complex (EJC) (PubMed:11546874, PubMed:16452507). Associates with the SGM1C complex; is phosphorylated by the complex kinase component SGM1 (PubMed:19417104). Interacts with UPF2 (PubMed:11163187, PubMed:11073994, PubMed:11113196, PubMed:19556969). Interacts with UPF3A and UPF3B (PubMed:11163187). Interacts with EST1A (PubMed:12554878). Interacts with SLBP (PubMed:16086026). Interacts (when hyperphosphorylated) with PNRC2 (PubMed:19150429). Interacts with AGO1 and AGO2 (PubMed:17932509). Interacts with GSPT2 (PubMed:18447585). Interacts with isoform 1 and isoform 5 of ADAR/ADAR1 (PubMed:18362360). Interacts with SMG7 (PubMed:15721257). Interacts with ZC3H12A; this interaction occurs in a mRNA translationally active- and termination-dependent manner and is essential for ZC3H12A-mediated degradation of target mRNAs (PubMed:26000482). Interacts with CPSF6 (PubMed:19864460). Interacts with MOV10; the interaction is direct and RNA-dependent (PubMed:24726324). Interacts with SHFL; the interaction increases in the presence of RNA (PubMed:27974568). Interacts with UPF2 and DDX4; interactions are mediated by TDRD6 (By similarity).   
  
 **Gene Ontology Information:**

Molecular Function

- ATP binding
- ATPase activity
- chromatin binding
- double-stranded DNA helicase activity
- helicase activity
- protein-containing complex binding
- RNA binding
- RNA helicase activity
- telomeric DNA binding
- zinc ion binding

Location

- chromatin
- chromosome, telomeric region
- cytoplasm
- cytosol
- exon-exon junction complex
- nucleoplasm
- nucleus
- P-body
- perinuclear region of cytoplasm
- supraspliceosomal complex

Biological process

- 3'-UTR-mediated mRNA destabilization
- cell cycle phase transition
- cellular response to interleukin-1
- cellular response to lipopolysaccharide
- DNA duplex unwinding
- DNA repair
- DNA replication
- histone mRNA catabolic process
- mRNA export from nucleus
- nuclear-transcribed mRNA catabolic process
- nuclear-transcribed mRNA catabolic process, nonsense-mediated decay
- positive regulation of mRNA catabolic process
- regulation of telomere maintenance
- regulation of translational termination
- telomere maintenance via semi-conservative replication

---

70

- **Protein name:** Histone acetyltransferase p300
- **Organism:** Homo sapiens
- **Uniprot Accession Number:** Q09472
- **Protein sequence length:** 2414 aa
- **1D identity (%):** 10.75
- **1D identity (%) [Gaps excluded]:** 30.85
- **1D identity - Alignment Gaps:** 1581
- **1D aligned content (<aminoacid>:%):** {'M': 4.21, 'S': 7.66, 'P': 16.09, 'L': 8.43, 'A': 7.66, 'Q': 10.34, 'G': 8.81, 'F': 2.3, 'D': 2.68, 'K': 3.45, 'C': 2.3, 'R': 4.6, 'H': 4.6, 'Y': 1.15, 'V': 4.21, 'T': 3.83, 'N': 2.3, 'I': 1.92, 'E': 3.45}
- **Common reported functions (%):** 0.0
- **Common reported locations (%):** 28.57
- **Common reported processes (%):** 4.76

- **PDB ID:** 6GYR
- **Chain:** B
- **Crystallized protein length:** 583 aa
- **Resolution:** 3.1 Å
- **b-phipsi:** 0.004967
- **w-rdist:** 0.497294
- **t-alpha:** 0.04173
- **Chemical similarity (Tanimoto Index) (%):** N/A
- **1D identity (%) [PDB]:** 2.1
- **1D identity (%) [Gaps excluded][PDB]:** 67.44
- **1D identity - Alignment Gaps [PDB]:** 1336
- **1D aligned content [PDB] (<aminoacid>:%):** {'A': 3.45, 'M': 3.45, 'P': 10.34, 'L': 10.34, 'I': 6.9, 'R': 3.45, 'D': 10.34, 'V': 13.79, 'E': 10.34, 'T': 3.45, 'G': 3.45, 'F': 3.45, 'K': 6.9, 'S': 6.9, 'H': 3.45}
- **2D identity (%) [PDB]:** 32.02
- **2D identity (%) [Gaps excluded][PDB]:** 87.17
- **2D identity - Alignment Gaps [PDB]:** 658
- **2D aligned content [PDB] (<2D-fold>:%):** {'.': 15.02, 'H': 54.95, 'T': 13.21, 'E': 15.92, 'G': 0.9}
- **3D similarity (TM-Score) (%) [PDB]:** 22.9

- **Gene name:** EP300
- **Entrez ID:** 2033
- **RefSeq ID:** NM\_001429
- **Transcript sequence length:** 8779
- **5-UTR|CDS|3-UTR identity (%):** 23.88 | 27.46 | 5.61
- **5-UTR|CDS|3-UTR identity (%) [Gaps excluded]:** 86.32 | 81.05 | 74.62
- **5-UTR|CDS|3-UTR identity [Alignment Gaps]:** 306 | 4853 | 11189
- **5-UTR aligned content (<base>:%):** {'G': 48.51, 'A': 2.97, 'C': 42.57, 'T': 5.94}
- **CDS aligned content (<base>:%):** {'A': 25.96, 'T': 17.52, 'G': 27.05, 'C': 29.48}
- **3-UTR aligned content (<base>:%):** {'G': 14.14, 'A': 33.14, 'C': 11.49, 'T': 41.24}

**Uniprot Description:**  
  
 Functions as histone acetyltransferase and regulates transcription via chromatin remodeling (PubMed:23415232, PubMed:23934153, PubMed:8945521). Acetylates all four core histones in nucleosomes. Histone acetylation gives an epigenetic tag for transcriptional activation (PubMed:23415232, PubMed:23934153, PubMed:8945521). Mediates cAMP-gene regulation by binding specifically to phosphorylated CREB protein. Mediates acetylation of histone H3 at 'Lys-122' (H3K122ac), a modification that localizes at the surface of the histone octamer and stimulates transcription, possibly by promoting nucleosome instability. Mediates acetylation of histone H3 at 'Lys-27' (H3K27ac) (PubMed:23911289). Also functions as acetyltransferase for non-histone targets, such as ALX1, HDAC1, PRMT1 or SIRT2 (PubMed:12929931, PubMed:16762839, PubMed:18722353). Acetylates 'Lys-131' of ALX1 and acts as its coactivator (PubMed:12929931). Acetylates SIRT2 and is proposed to indirectly increase the transcriptional activity of TP53 through acetylation and subsequent attenuation of SIRT2 deacetylase function (PubMed:18722353). Acetylates HDAC1 leading to its inactivation and modulation of transcription (PubMed:16762839). Acetylates 'Lys-247' of EGR2 (By similarity). Acts as a TFAP2A-mediated transcriptional coactivator in presence of CITED2 (PubMed:12586840). Plays a role as a coactivator of NEUROD1-dependent transcription of the secretin and p21 genes and controls terminal differentiation of cells in the intestinal epithelium. Promotes cardiac myocyte enlargement. Can also mediate transcriptional repression. Acetylates FOXO1 and enhances its transcriptional activity (PubMed:15890677). Acetylates BCL6 wich disrupts its ability to recruit histone deacetylases and hinders its transcriptional repressor activity (PubMed:12402037). Participates in CLOCK or NPAS2-regulated rhythmic gene transcription; exhibits a circadian association with CLOCK or NPAS2, correlating with increase in PER1/2 mRNA and histone H3 acetylation on the PER1/2 promoter (PubMed:14645221). Acetylates MTA1 at 'Lys-626' which is essential for its transcriptional coactivator activity (PubMed:16617102). Acetylates XBP1 isoform 2; acetylation increases protein stability of XBP1 isoform 2 and enhances its transcriptional activity (PubMed:20955178). Acetylates PCNA; acetylation promotes removal of chromatin-bound PCNA and its degradation during nucleotide excision repair (NER) (PubMed:24939902). Acetylates MEF2D (PubMed:21030595). Acetylates and stabilizes ZBTB7B protein by antagonizing ubiquitin conjugation and degragation, this mechanism may be involved in CD4/CD8 lineage differentiation (PubMed:20810990). Acetylates GABPB1, impairing GABPB1 heterotetramerization and activity (By similarity). In addition to protein acetyltransferase, can use different acyl-CoA substrates, such as (2E)-butenoyl-CoA (crotonyl-CoA), butanoyl-CoA (butyryl-CoA), 2-hydroxyisobutanoyl-CoA (2-hydroxyisobutyryl-CoA), lactoyl-CoA or propanoyl-CoA (propionyl-CoA), and is able to mediate protein crotonylation, butyrylation, 2-hydroxyisobutyrylation, lactylation or propionylation, respectively (PubMed:17267393, PubMed:25818647, PubMed:29775581, PubMed:31645732). Acts as a histone crotonyltransferase; crotonylation marks active promoters and enhancers and confers resistance to transcriptional repressors (PubMed:25818647). Histone crotonyltransferase activity is dependent on the concentration of (2E)-butenoyl-CoA (crotonyl-CoA) substrate and such activity is weak when (2E)-butenoyl-CoA (crotonyl-CoA) concentration is low (PubMed:25818647). Also acts as a histone butyryltransferase; butyrylation marks active promoters (PubMed:17267393). Catalyzes histone lactylation in macrophages by using lactoyl-CoA directly derived from endogenous or exogenous lactate, leading to stimulates gene transcription (PubMed:31645732). Acts as a protein-lysine 2-hydroxyisobutyryltransferase; regulates glycolysis by mediating 2-hydroxyisobutyrylation of glycolytic enzymes (PubMed:29775581). Functions as a transcriptional coactivator for SMAD4 in the TGF-beta signaling pathway (PubMed:25514493). Acetylates PCK1 and promotes PCK1 anaplerotic activity (PubMed:30193097). Acetylates RXRA and RXRG (PubMed:17761950).   
  
Interacts with HIF1A; the interaction is stimulated in response to hypoxia and inhibited by CITED2 (PubMed:9887100, PubMed:11959990). Probably part of a complex with HIF1A and CREBBP (PubMed:8917528). Interacts (via N-terminus) with TFAP2A (via N-terminus); the interaction requires CITED2 (PubMed:12586840). Interacts (via CH1 domain) with CITED2 (via C-terminus) (PubMed:12586840, PubMed:12778114). Interacts with CITED1 (unphosphorylated form preferentially and via C-terminus) (PubMed:10722728, PubMed:16864582). Interacts with ESR1; the interaction is estrogen-dependent and enhanced by CITED1 (PubMed:11581164). Interacts with DTX1, EID1, ELF3, FEN1, LEF1, NCOA1, NCOA6, NR3C1, PCAF, PELP1, PRDM6, SP1, SP3, SPIB, SRY, TCF7L2, TP53, DDX5, DDX17, SATB1, SRCAP, TTC5, JMY and TRERF1 (PubMed:11073989, PubMed:11073990, PubMed:10823961, PubMed:11349124, PubMed:11430825, PubMed:11481323, PubMed:11564735, PubMed:11581372, PubMed:11864910, PubMed:12446687, PubMed:12527917, PubMed:12837748, PubMed:14605447, PubMed:15075319, PubMed:15186775, PubMed:15297880, PubMed:16478997, PubMed:8684459, PubMed:17226766, PubMed:19217391, PubMed:9590696). Part of a complex containing CARM1 and NCOA2/GRIP1 (PubMed:11701890, PubMed:11997499, PubMed:15731352). Interacts with ING4 and this interaction may be indirect (PubMed:12750254). Interacts with ING5 (PubMed:12750254). Interacts with the C-terminal region of CITED4 (PubMed:11744733). Non-sumoylated EP300 preferentially interacts with SENP3 (PubMed:19680224). Interacts with SS18L1/CREST (PubMed:14716005). Interacts with ALX1 (via homeobox domain) (PubMed:12929931). Interacts with NEUROD1; the interaction is inhibited by NR0B2 (PubMed:14752053). Interacts with TCF3 (PubMed:14752053). Interacts (via CREB-binding domain) with MYOCD (via C-terminus) (By similarity). Interacts with ROCK2 and PPARG (PubMed:11518699, PubMed:16574662). Forms a complex made of CDK9, CCNT1/cyclin-T1, EP300 and GATA4 that stimulates hypertrophy in cardiomyocytes (PubMed:20081228). Interacts with IRF1 and this interaction enhances acetylation of p53/TP53 and stimulation of its activity (PubMed:15509808). Interacts with FOXO1; the interaction acetylates FOXO1 and enhances its transcriptional activity (PubMed:15890677). Interacts with ALKBH4 and DDIT3/CHOP (PubMed:17872950, PubMed:23145062). Interacts with KLF15 (PubMed:23999430). Interacts with CEBPB and RORA (PubMed:9862959). Interacts with NPAS2, ARNTL/BMAL1 and CLOCK (PubMed:14645221). Interacts with SIRT2 isoform 1, isoform 2 and isoform 5 (PubMed:24177535). Interacts with MTA1 (PubMed:16617102). Interacts with HDAC4 and HDAC5 in the presence of TFAP2C (PubMed:24413532). Interacts with TRIP4 (PubMed:25219498). Directly interacts with ZBTB49; this interaction leads to synergistic transactivation of CDKN1A (PubMed:25245946). Interacts with NR4A3 (By similarity). Interacts with ZNF451 (PubMed:24324267). Interacts with ATF5; EP300 is required for ATF5 and CEBPB interaction and DNA binding (By similarity). Interacts with HSF1 (PubMed:27189267). Interacts with ZBTB48/TZAP (PubMed:24382891). Interacts with STAT1; the interaction is enhanced upon IFN-gamma stimulation (PubMed:26479788). Interacts with HNRNPU (via C-terminus); this interaction enhances DNA-binding of HNRNPU to nuclear scaffold/matrix attachment region (S/MAR) elements (PubMed:11909954). Interacts with BCL11B (PubMed:27959755, PubMed:16809611). Interacts with SMAD4; negatively regulated by ZBTB7A (PubMed:25514493). Interacts with DUX4 (via C-terminus) (PubMed:26951377). Interacts with NUPR1; this interaction enhances the effect of EP300 on PAX2 transcription factor activity (PubMed:11940591). Interacts with RXRA; the interaction is decreased by 9-cis retinoic acid (PubMed:17761950). NR4A1 competes with EP300 for interaction with RXRA and thereby attenuates EP300 mediated acetylation of RXRA (PubMed:17761950). Interacts with RB1 (By similarity). Interacts with DDX3X; this interaction may facilitate HNF4A acetylation (PubMed:28128295). Interacts with SOX9 (PubMed:12732631). Interacts with ATF4; EP300/p300 stabilizes ATF4 and increases its transcriptional activity independently of its catalytic activity by preventing its ubiquitination (PubMed:16219772).   
  
 **Gene Ontology Information:**

Molecular Function

- acetyltransferase activity
- transferase activity, transferring acyl groups
- beta-catenin binding
- chromatin binding
- chromatin DNA binding
- damaged DNA binding
- DNA binding
- DNA-binding transcription factor binding
- histone acetyltransferase activity
- histone butyryltransferase activity
- histone crotonyltransferase activity
- histone H2B acetyltransferase activity
- H3 histone acetyltransferase activity
- histone H3K122 acetyltransferase activity
- H4 histone acetyltransferase activity
- histone lactyltransferase activity
- lysine N-acetyltransferase activity, acting on acetyl phosphate as donor
- NF-kappaB binding
- androgen receptor binding
- nuclear receptor binding
- p53 binding
- peptide 2-hydroxyisobutyryltransferase activity
- peptide butyryltransferase activity
- peptide N-acetyltransferase activity
- peptide-lysine-N-acetyltransferase activity
- pre-mRNA intronic binding
- protein propionyltransferase activity
- RNA polymerase II-specific DNA-binding transcription factor binding
- STAT family protein binding
- tau protein binding
- transcription coactivator activity
- transcription coactivator binding
- transcription coregulator binding
- zinc ion binding

Location

- chromatin
- cytoplasm
- cytosol
- histone acetyltransferase complex
- nucleoplasm
- nucleus
- protein-DNA complex
- transcription regulator complex

Biological process

- animal organ morphogenesis
- apoptotic process
- B cell differentiation
- behavioral defense response
- cell cycle
- cellular response to UV
- circadian rhythm
- face morphogenesis
- fat cell differentiation
- heart development
- histone acetylation
- internal peptidyl-lysine acetylation
- internal protein amino acid acetylation
- intrinsic apoptotic signaling pathway in response to DNA damage by p53 class mediator
- learning or memory
- lung development
- macrophage derived foam cell differentiation
- megakaryocyte development
- multicellular organism growth
- N-terminal peptidyl-lysine acetylation
- negative regulation of gluconeogenesis
- negative regulation of protein-containing complex assembly
- negative regulation of transcription by RNA polymerase II
- nervous system development
- peptidyl-lysine acetylation
- peptidyl-lysine butyrylation
- peptidyl-lysine crotonylation
- peptidyl-lysine propionylation
- platelet formation
- positive regulation by host of viral transcription
- positive regulation of DNA-binding transcription factor activity
- positive regulation of transcription, DNA-templated
- positive regulation of neuron projection development
- positive regulation of NF-kappaB transcription factor activity
- positive regulation of NIK/NF-kappaB signaling
- positive regulation of protein binding
- positive regulation of protein import into nucleus
- positive regulation of receptor signaling pathway via JAK-STAT
- positive regulation of RNA polymerase II regulatory region sequence-specific DNA binding
- positive regulation of transcription by RNA polymerase II
- positive regulation of transforming growth factor beta receptor signaling pathway
- protein acetylation
- protein destabilization
- protein stabilization
- regulation of androgen receptor signaling pathway
- regulation of autophagy
- regulation of cellular response to heat
- regulation of glycolytic process
- regulation of mitochondrion organization
- regulation of signal transduction by p53 class mediator
- regulation of tubulin deacetylation
- response to estrogen
- response to hypoxia
- skeletal muscle tissue development
- somitogenesis
- stimulatory C-type lectin receptor signaling pathway
- swimming
- thigmotaxis
- transcription by RNA polymerase II
- positive regulation of gene expression, epigenetic

---

71

- **Protein name:** Cobra venom factor
- **Organism:** Naja kaouthia
- **Uniprot Accession Number:** Q91132
- **Protein sequence length:** 1642 aa
- **1D identity (%):** 13.18
- **1D identity (%) [Gaps excluded]:** 26.07
- **1D identity - Alignment Gaps:** 821
- **1D aligned content (<aminoacid>:%):** {'M': 0.91, 'G': 9.59, 'A': 4.57, 'L': 8.68, 'P': 8.68, 'R': 4.57, 'D': 7.31, 'T': 6.85, 'I': 4.11, 'K': 7.76, 'Y': 4.11, 'N': 2.74, 'E': 3.2, 'H': 3.65, 'F': 5.48, 'V': 7.76, 'S': 3.65, 'Q': 4.11, 'W': 0.46, 'C': 1.83}
- **Common reported functions (%):** 0.0
- **Common reported locations (%):** 0.0
- **Common reported processes (%):** 0.0

- **PDB ID:** 8AYH
- **Chain:** C
- **Crystallized protein length:** 648 aa
- **Resolution:** 3.35 Å
- **b-phipsi:** 0.038292
- **w-rdist:** 0.438306
- **t-alpha:** 0.00874
- **Chemical similarity (Tanimoto Index) (%):** 96.67
- **1D identity (%) [PDB]:** 2.76
- **1D identity (%) [Gaps excluded][PDB]:** 60.0
- **1D identity - Alignment Gaps [PDB]:** 1350
- **1D aligned content [PDB] (<aminoacid>:%):** {'Q': 5.13, 'V': 15.38, 'I': 5.13, 'L': 15.38, 'P': 5.13, 'G': 10.26, 'Y': 2.56, 'E': 2.56, 'K': 7.69, 'R': 2.56, 'T': 12.82, 'M': 2.56, 'S': 2.56, 'N': 10.26}
- **2D identity (%) [PDB]:** 39.64
- **2D identity (%) [Gaps excluded][PDB]:** 84.93
- **2D identity - Alignment Gaps [PDB]:** 538
- **2D aligned content [PDB] (<2D-fold>:%):** {'.': 14.04, 'E': 51.13, 'T': 32.83, 'H': 2.01}
- **3D similarity (TM-Score) (%) [PDB]:** 21.86

- **Gene name:** N/A
- **Entrez ID:** N/A
- **RefSeq ID:** N/A
- **Sequence length:** N/A
- **5-UTR|CDS|3-UTR identity (%):** N/A | N/A | N/A
- **5-UTR|CDS|3-UTR identity (%) [Gaps excluded]:** N/A | N/A | N/A
- **5-UTR|CDS|3-UTR identity [Alignment Gaps]:** N/A | N/A | N/A
- **5-UTR aligned content (<base>:%):** N/A
- **CDS aligned content (<base>:%):** N/A
- **3-UTR aligned content (<base>:%):** N/A

**Uniprot Description:**  
  
 Complement-activating protein in cobra venom. It is a structural and functional analog of complement component C3b, the activated form of C3. It binds factor B (CFB), which is subsequently cleaved by factor D (CFD) to form the bimolecular complex CVF/Bb. CVF/Bb is a C3/C5 convertase that cleaves both complement components C3 and C5. Structurally, it resembles the C3b degradation product C3c, which is not able to form a C3/C5 convertase. Unlike C3b/Bb, CVF/Bb is a stable complex and completely resistant to the actions of complement regulatory factors H (CFH) and I (CFI). Therefore, CVF continuously activates complement resulting in the depletion of complement activity.   
  
Heterotrimer of alpha, beta and gamma chains; disulfide-linked. Is active with factor B in the presence of factor D.   
  
 **Gene Ontology Information:**

Molecular Function   
  
N/A

Location   
  
N/A

Biological process   
  
N/A

---

72

- **Protein name:** Aldehyde oxidase
- **Organism:** Homo sapiens
- **Uniprot Accession Number:** Q06278
- **Protein sequence length:** 1338 aa
- **1D identity (%):** 15.27
- **1D identity (%) [Gaps excluded]:** 24.91
- **1D identity - Alignment Gaps:** 527
- **1D aligned content (<aminoacid>:%):** {'M': 1.92, 'Y': 2.4, 'G': 11.06, 'P': 7.69, 'A': 7.69, 'F': 5.29, 'T': 4.33, 'D': 6.25, 'E': 8.65, 'L': 10.1, 'Q': 4.81, 'V': 8.17, 'R': 4.81, 'I': 2.88, 'S': 3.85, 'H': 3.85, 'N': 1.92, 'C': 1.44, 'K': 2.88}
- **Common reported functions (%):** 0.0
- **Common reported locations (%):** 14.29
- **Common reported processes (%):** 0.0

- **PDB ID:** 7ORC
- **Chain:** A
- **Crystallized protein length:** 1295 aa
- **Resolution:** 2.7 Å
- **b-phipsi:** 0.006455
- **w-rdist:** 0.35236
- **t-alpha:** 0.099054
- **Chemical similarity (Tanimoto Index) (%):** 67.52
- **1D identity (%) [PDB]:** 2.62
- **1D identity (%) [Gaps excluded][PDB]:** 72.0
- **1D identity - Alignment Gaps [PDB]:** 1986
- **1D aligned content [PDB] (<aminoacid>:%):** {'A': 7.41, 'S': 7.41, 'E': 7.41, 'G': 7.41, 'C': 3.7, 'N': 1.85, 'P': 7.41, 'L': 7.41, 'V': 5.56, 'F': 9.26, 'H': 1.85, 'M': 1.85, 'I': 5.56, 'D': 5.56, 'T': 5.56, 'Y': 1.85, 'K': 9.26, 'Q': 3.7}
- **2D identity (%) [PDB]:** 38.55
- **2D identity (%) [Gaps excluded][PDB]:** 89.72
- **2D identity - Alignment Gaps [PDB]:** 852
- **2D aligned content [PDB] (<2D-fold>:%):** {'.': 14.41, 'E': 32.99, 'T': 12.15, 'H': 39.93, 'B': 0.52}
- **3D similarity (TM-Score) (%) [PDB]:** 28.52

- **Gene name:** AOX1
- **Entrez ID:** 316
- **RefSeq ID:** N/A
- **Sequence length:** N/A
- **5-UTR|CDS|3-UTR identity (%):** N/A | N/A | N/A
- **5-UTR|CDS|3-UTR identity (%) [Gaps excluded]:** N/A | N/A | N/A
- **5-UTR|CDS|3-UTR identity [Alignment Gaps]:** N/A | N/A | N/A
- **5-UTR aligned content (<base>:%):** N/A
- **CDS aligned content (<base>:%):** N/A
- **3-UTR aligned content (<base>:%):** N/A

**Uniprot Description:**  
  
 Oxidase with broad substrate specificity, oxidizing aromatic azaheterocycles, such as N1-methylnicotinamide, N-methylphthalazinium and phthalazine, as well as aldehydes, such as benzaldehyde, retinal, pyridoxal, and vanillin. Plays a key role in the metabolism of xenobiotics and drugs containing aromatic azaheterocyclic substituents. Participates in the bioactivation of prodrugs such as famciclovir, catalyzing the oxidation step from 6-deoxypenciclovir to penciclovir, which is a potent antiviral agent. Is probably involved in the regulation of reactive oxygen species homeostasis. May be a prominent source of superoxide generation via the one-electron reduction of molecular oxygen. Also may catalyze nitric oxide (NO) production via the reduction of nitrite to NO with NADH or aldehyde as electron donor. May play a role in adipogenesis.   
  
Homodimer.   
  
 **Gene Ontology Information:**

Molecular Function

- 2 iron, 2 sulfur cluster binding
- aldehyde oxidase activity
- FAD binding
- flavin adenine dinucleotide binding
- identical protein binding
- iron ion binding
- molybdopterin cofactor binding
- NAD binding
- protein homodimerization activity

Location

- cytosol
- extracellular exosome

Biological process

- lipid metabolic process
- xenobiotic metabolic process

---

73

- **Protein name:** Serotransferrin
- **Organism:** Homo sapiens
- **Uniprot Accession Number:** P02787
- **Protein sequence length:** 698 aa
- **1D identity (%):** 11.82
- **1D identity (%) [Gaps excluded]:** 24.41
- **1D identity - Alignment Gaps:** 541
- **1D aligned content (<aminoacid>:%):** {'M': 1.61, 'R': 4.84, 'V': 8.06, 'G': 9.68, 'A': 8.06, 'W': 1.61, 'F': 4.84, 'H': 3.23, 'S': 4.03, 'P': 7.26, 'K': 7.26, 'D': 9.68, 'Q': 3.23, 'C': 5.65, 'E': 4.84, 'L': 6.45, 'N': 4.03, 'I': 2.42, 'Y': 0.81, 'T': 2.42}
- **Common reported functions (%):** 0.0
- **Common reported locations (%):** 7.14
- **Common reported processes (%):** 0.0

- **PDB ID:** 5DYH
- **Chain:** B
- **Crystallized protein length:** 677 aa
- **Resolution:** 2.68 Å
- **b-phipsi:** 0.004049
- **w-rdist:** 0.521934
- **t-alpha:** 0.052107
- **Chemical similarity (Tanimoto Index) (%):** 93.55
- **1D identity (%) [PDB]:** 1.75
- **1D identity (%) [Gaps excluded][PDB]:** 83.87
- **1D identity - Alignment Gaps [PDB]:** 1453
- **1D aligned content [PDB] (<aminoacid>:%):** {'D': 7.69, 'V': 7.69, 'S': 3.85, 'E': 15.38, 'G': 3.85, 'F': 3.85, 'Q': 7.69, 'L': 15.38, 'A': 3.85, 'I': 7.69, 'R': 3.85, 'C': 3.85, 'K': 7.69, 'Y': 3.85, 'P': 3.85}
- **2D identity (%) [PDB]:** 40.08
- **2D identity (%) [Gaps excluded][PDB]:** 79.68
- **2D identity - Alignment Gaps [PDB]:** 501
- **2D aligned content [PDB] (<2D-fold>:%):** {'.': 14.36, 'E': 24.75, 'H': 43.81, 'T': 14.6, 'G': 2.48}
- **3D similarity (TM-Score) (%) [PDB]:** 21.7

- **Gene name:** TF
- **Entrez ID:** 7018
- **RefSeq ID:** N/A
- **Sequence length:** N/A
- **5-UTR|CDS|3-UTR identity (%):** N/A | N/A | N/A
- **5-UTR|CDS|3-UTR identity (%) [Gaps excluded]:** N/A | N/A | N/A
- **5-UTR|CDS|3-UTR identity [Alignment Gaps]:** N/A | N/A | N/A
- **5-UTR aligned content (<base>:%):** N/A
- **CDS aligned content (<base>:%):** N/A
- **3-UTR aligned content (<base>:%):** N/A

**Uniprot Description:**  
  
 Transferrins are iron binding transport proteins which can bind two Fe(3+) ions in association with the binding of an anion, usually bicarbonate. It is responsible for the transport of iron from sites of absorption and heme degradation to those of storage and utilization. Serum transferrin may also have a further role in stimulating cell proliferation.   
  
Monomer.   
  
 **Gene Ontology Information:**

Molecular Function

- ferric iron binding
- ferrous iron binding
- iron chaperone activity
- transferrin receptor binding

Location

- apical plasma membrane
- basal part of cell
- basal plasma membrane
- blood microparticle
- cell surface
- clathrin-coated endocytic vesicle membrane
- clathrin-coated pit
- cytoplasmic vesicle
- early endosome
- endocytic vesicle
- endoplasmic reticulum lumen
- endosome membrane
- extracellular exosome
- extracellular region
- extracellular space
- HFE-transferrin receptor complex
- late endosome
- perinuclear region of cytoplasm
- plasma membrane
- recycling endosome
- secretory granule lumen
- vesicle

Biological process

- actin filament organization
- antibacterial humoral response
- cellular response to iron ion
- ERK1 and ERK2 cascade
- cellular iron ion homeostasis
- iron ion transmembrane transport
- iron ion transport
- osteoclast differentiation
- positive regulation of bone resorption
- positive regulation of cell motility
- positive regulation of transcription, DNA-templated
- positive regulation of phosphorylation
- positive regulation of receptor-mediated endocytosis
- regulation of iron ion transport
- regulation of protein stability
- retina homeostasis
- SMAD protein signal transduction

---

74

- **Protein name:** DNA topoisomerase 1
- **Organism:** Homo sapiens
- **Uniprot Accession Number:** P11387
- **Protein sequence length:** 765 aa
- **1D identity (%):** 13.65
- **1D identity (%) [Gaps excluded]:** 22.1
- **1D identity - Alignment Gaps:** 384
- **1D aligned content (<aminoacid>:%):** {'S': 2.92, 'G': 7.3, 'A': 6.57, 'F': 2.19, 'D': 9.49, 'K': 14.6, 'H': 2.92, 'R': 6.57, 'E': 7.3, 'V': 2.92, 'P': 5.84, 'I': 2.92, 'L': 9.49, 'Y': 3.65, 'W': 1.46, 'T': 4.38, 'C': 1.46, 'Q': 4.38, 'M': 0.73, 'N': 2.92}
- **Common reported functions (%):** 6.67
- **Common reported locations (%):** 21.43
- **Common reported processes (%):** 0.0

- **PDB ID:** 1SC7
- **Chain:** A
- **Crystallized protein length:** 566 aa
- **Resolution:** 3.0 Å
- **b-phipsi:** 0.015378
- **w-rdist:** 0.474997
- **t-alpha:** 0.008817
- **Chemical similarity (Tanimoto Index) (%):** 91.64
- **1D identity (%) [PDB]:** 2.9
- **1D identity (%) [Gaps excluded][PDB]:** 63.93
- **1D identity - Alignment Gaps [PDB]:** 1283
- **1D aligned content [PDB] (<aminoacid>:%):** {'E': 12.82, 'Q': 2.56, 'F': 7.69, 'L': 10.26, 'H': 2.56, 'I': 7.69, 'K': 12.82, 'Y': 2.56, 'P': 5.13, 'G': 7.69, 'C': 2.56, 'D': 2.56, 'N': 5.13, 'R': 2.56, 'V': 5.13, 'A': 2.56, 'T': 7.69}
- **2D identity (%) [PDB]:** 38.23
- **2D identity (%) [Gaps excluded][PDB]:** 86.11
- **2D identity - Alignment Gaps [PDB]:** 541
- **2D aligned content [PDB] (<2D-fold>:%):** {'.': 18.01, 'E': 10.22, 'H': 54.84, 'T': 16.13, 'B': 0.81}
- **3D similarity (TM-Score) (%) [PDB]:** 18.05

- **Gene name:** TOP1
- **Entrez ID:** 7150
- **RefSeq ID:** NM\_003286
- **Transcript sequence length:** 3734
- **5-UTR|CDS|3-UTR identity (%):** 37.89 | 43.08 | 6.92
- **5-UTR|CDS|3-UTR identity (%) [Gaps excluded]:** 82.91 | 73.22 | 77.45
- **5-UTR|CDS|3-UTR identity [Alignment Gaps]:** 139 | 1264 | 10932
- **5-UTR aligned content (<base>:%):** {'C': 47.42, 'T': 11.34, 'G': 39.18, 'A': 2.06}
- **CDS aligned content (<base>:%):** {'A': 34.62, 'T': 16.48, 'G': 26.15, 'C': 22.75}
- **3-UTR aligned content (<base>:%):** {'C': 13.84, 'A': 30.2, 'G': 17.81, 'T': 38.15}

**Uniprot Description:**  
  
 Releases the supercoiling and torsional tension of DNA introduced during the DNA replication and transcription by transiently cleaving and rejoining one strand of the DNA duplex. Introduces a single-strand break via transesterification at a target site in duplex DNA. The scissile phosphodiester is attacked by the catalytic tyrosine of the enzyme, resulting in the formation of a DNA-(3'-phosphotyrosyl)-enzyme intermediate and the expulsion of a 5'-OH DNA strand. The free DNA strand then rotates around the intact phosphodiester bond on the opposing strand, thus removing DNA supercoils. Finally, in the religation step, the DNA 5'-OH attacks the covalent intermediate to expel the active-site tyrosine and restore the DNA phosphodiester backbone (By similarity). Regulates the alternative splicing of tissue factor (F3) pre-mRNA in endothelial cells. Involved in the circadian transcription of the core circadian clock component ARNTL/BMAL1 by altering the chromatin structure around the ROR response elements (ROREs) on the ARNTL/BMAL1 promoter.   
  
Monomer. Interacts with ERCC6 (PubMed:26030138).   
  
 **Gene Ontology Information:**

Molecular Function

- ATP binding
- chromatin binding
- DNA binding
- DNA binding, bending
- DNA topoisomerase type I (single strand cut, ATP-independent) activity
- double-stranded DNA binding
- protein domain specific binding
- protein serine/threonine kinase activity
- RNA binding
- RNA polymerase II cis-regulatory region sequence-specific DNA binding
- single-stranded DNA binding
- supercoiled DNA binding

Location

- chromosome
- fibrillar center
- male germ cell nucleus
- nucleolus
- nucleoplasm
- nucleus
- P-body
- perikaryon
- protein-DNA complex

Biological process

- chromatin remodeling
- chromosome segregation
- circadian regulation of gene expression
- circadian rhythm
- DNA replication
- DNA topological change
- embryonic cleavage
- peptidyl-serine phosphorylation
- phosphorylation
- programmed cell death
- response to xenobiotic stimulus

---

75

- **Protein name:** Protein transport protein Sec24D
- **Organism:** Homo sapiens
- **Uniprot Accession Number:** O94855
- **Protein sequence length:** 1032 aa
- **1D identity (%):** 17.25
- **1D identity (%) [Gaps excluded]:** 23.17
- **1D identity - Alignment Gaps:** 277
- **1D aligned content (<aminoacid>:%):** {'S': 5.35, 'G': 9.09, 'P': 15.51, 'T': 5.35, 'L': 9.09, 'A': 4.81, 'H': 3.74, 'Q': 3.21, 'F': 6.95, 'Y': 4.81, 'R': 4.28, 'D': 3.74, 'V': 5.35, 'C': 3.21, 'K': 6.42, 'N': 2.14, 'E': 2.67, 'I': 3.21, 'M': 1.07}
- **Common reported functions (%):** 0.0
- **Common reported locations (%):** 7.14
- **Common reported processes (%):** 0.0

- **PDB ID:** 5KYU
- **Chain:** B
- **Crystallized protein length:** 764 aa
- **Resolution:** 3.51 Å
- **b-phipsi:** 0.001334
- **w-rdist:** 0.741806
- **t-alpha:** 0.044106
- **Chemical similarity (Tanimoto Index) (%):** 94.12
- **1D identity (%) [PDB]:** 2.66
- **1D identity (%) [Gaps excluded][PDB]:** 68.33
- **1D identity - Alignment Gaps [PDB]:** 1483
- **1D aligned content [PDB] (<aminoacid>:%):** {'P': 7.32, 'V': 4.88, 'C': 2.44, 'N': 4.88, 'I': 4.88, 'A': 12.2, 'G': 2.44, 'R': 9.76, 'K': 4.88, 'L': 4.88, 'T': 12.2, 'D': 7.32, 'S': 7.32, 'Q': 2.44, 'E': 4.88, 'F': 2.44, 'Y': 2.44, 'M': 2.44}
- **2D identity (%) [PDB]:** 36.31
- **2D identity (%) [Gaps excluded][PDB]:** 84.44
- **2D identity - Alignment Gaps [PDB]:** 639
- **2D aligned content [PDB] (<2D-fold>:%):** {'.': 17.69, 'H': 40.05, 'T': 14.74, 'E': 25.06, 'B': 0.25, 'G': 2.21}
- **3D similarity (TM-Score) (%) [PDB]:** 23.16

- **Gene name:** SEC24D
- **Entrez ID:** 9871
- **RefSeq ID:** N/A
- **Sequence length:** N/A
- **5-UTR|CDS|3-UTR identity (%):** N/A | N/A | N/A
- **5-UTR|CDS|3-UTR identity (%) [Gaps excluded]:** N/A | N/A | N/A
- **5-UTR|CDS|3-UTR identity [Alignment Gaps]:** N/A | N/A | N/A
- **5-UTR aligned content (<base>:%):** N/A
- **CDS aligned content (<base>:%):** N/A
- **3-UTR aligned content (<base>:%):** N/A

**Uniprot Description:**  
  
 Component of the coat protein complex II (COPII) which promotes the formation of transport vesicles from the endoplasmic reticulum (ER). The coat has two main functions, the physical deformation of the endoplasmic reticulum membrane into vesicles and the selection of cargo molecules for their transport to the Golgi complex (PubMed:17499046, PubMed:20427317, PubMed:18843296). Plays a central role in cargo selection within the COPII complex and together with SEC24C may have a different specificity compared to SEC24A and SEC24B (PubMed:17499046, PubMed:20427317, PubMed:18843296). May more specifically package GPI-anchored proteins through the cargo receptor TMED10 (PubMed:20427317). May also be specific for IxM motif-containing cargos like the SNAREs GOSR2 and STX5 (PubMed:18843296).   
  
COPII is composed of at least five proteins: the Sec23/24 complex, the Sec13/31 complex and Sar1 (PubMed:17499046, PubMed:27551091). Interacts with TMED2 and TMED10 (PubMed:20427317). Interacts with CNIH4 (PubMed:24405750). Interacts with GOSR2 (via IxM motif) and STX5 (via IxM motif); recruits GOSR2 and STX5 into COPII-coated vesicles (PubMed:18843296).   
  
 **Gene Ontology Information:**

Molecular Function

- SNARE binding
- zinc ion binding

Location

- COPII vesicle coat
- cytosol
- endoplasmic reticulum exit site
- endoplasmic reticulum membrane
- ER to Golgi transport vesicle membrane
- intracellular membrane-bounded organelle

Biological process

- COPII-coated vesicle cargo loading
- endoplasmic reticulum to Golgi vesicle-mediated transport
- in utero embryonic development
- intracellular protein transport

---

76

- **Protein name:** Endoplasmic reticulum aminopeptidase 2
- **Organism:** Homo sapiens
- **Uniprot Accession Number:** Q6P179
- **Protein sequence length:** 960 aa
- **1D identity (%):** 17.98
- **1D identity (%) [Gaps excluded]:** 25.23
- **1D identity - Alignment Gaps:** 305
- **1D aligned content (<aminoacid>:%):** {'M': 1.57, 'P': 7.85, 'I': 3.66, 'Y': 4.71, 'F': 5.76, 'T': 7.85, 'G': 6.81, 'Q': 4.71, 'E': 6.81, 'H': 2.62, 'L': 13.09, 'V': 6.28, 'D': 4.19, 'R': 3.66, 'K': 6.81, 'S': 5.24, 'A': 3.66, 'C': 2.09, 'N': 2.09, 'W': 0.52}
- **Common reported functions (%):** 0.0
- **Common reported locations (%):** 7.14
- **Common reported processes (%):** 0.0

- **PDB ID:** 7P7P
- **Chain:** B
- **Crystallized protein length:** 871 aa
- **Resolution:** 3.0 Å
- **b-phipsi:** 0.00925
- **w-rdist:** 0.664383
- **t-alpha:** 0.005124
- **Chemical similarity (Tanimoto Index) (%):** 69.61
- **1D identity (%) [PDB]:** 1.91
- **1D identity (%) [Gaps excluded][PDB]:** 84.21
- **1D identity - Alignment Gaps [PDB]:** 1638
- **1D aligned content [PDB] (<aminoacid>:%):** {'G': 3.12, 'A': 6.25, 'D': 3.12, 'S': 3.12, 'E': 6.25, 'P': 9.38, 'F': 3.12, 'R': 6.25, 'H': 3.12, 'L': 12.5, 'K': 9.38, 'T': 6.25, 'Y': 6.25, 'Q': 3.12, 'V': 15.62, 'I': 3.12}
- **2D identity (%) [PDB]:** 35.92
- **2D identity (%) [Gaps excluded][PDB]:** 83.85
- **2D identity - Alignment Gaps [PDB]:** 686
- **2D aligned content [PDB] (<2D-fold>:%):** {'.': 16.94, 'B': 0.23, 'E': 26.22, 'T': 11.14, 'H': 44.08, 'G': 1.39}
- **3D similarity (TM-Score) (%) [PDB]:** 26.12

- **Gene name:** ERAP2
- **Entrez ID:** 64167
- **RefSeq ID:** NM\_022350
- **Transcript sequence length:** 5131
- **5-UTR|CDS|3-UTR identity (%):** 23.15 | 44.91 | 10.65
- **5-UTR|CDS|3-UTR identity (%) [Gaps excluded]:** 63.51 | 74.14 | 75.22
- **5-UTR|CDS|3-UTR identity [Alignment Gaps]:** 129 | 1341 | 10516
- **5-UTR aligned content (<base>:%):** {'G': 44.68, 'C': 38.3, 'A': 4.26, 'T': 12.77}
- **CDS aligned content (<base>:%):** {'A': 27.29, 'T': 22.71, 'G': 25.52, 'C': 24.48}
- **3-UTR aligned content (<base>:%):** {'T': 34.02, 'G': 19.08, 'C': 14.79, 'A': 32.11}

**Uniprot Description:**  
  
 Aminopeptidase that plays a central role in peptide trimming, a step required for the generation of most HLA class I-binding peptides. Peptide trimming is essential to customize longer precursor peptides to fit them to the correct length required for presentation on MHC class I molecules. Preferentially hydrolyzes the basic residues Arg and Lys.   
  
Heterodimer with ERAP1.   
  
 **Gene Ontology Information:**

Molecular Function

- aminopeptidase activity
- endopeptidase activity
- metalloaminopeptidase activity
- metallopeptidase activity
- peptide binding
- zinc ion binding

Location

- cytoplasm
- endoplasmic reticulum lumen
- endoplasmic reticulum membrane

Biological process

- adaptive immune response
- antigen processing and presentation of endogenous peptide antigen via MHC class I
- antigen processing and presentation of peptide antigen via MHC class I
- peptide catabolic process
- proteolysis
- regulation of blood pressure

---

77

- **Protein name:** DNA topoisomerase 2-beta
- **Organism:** Homo sapiens
- **Uniprot Accession Number:** Q02880
- **Protein sequence length:** 1626 aa
- **1D identity (%):** 14.46
- **1D identity (%) [Gaps excluded]:** 28.01
- **1D identity - Alignment Gaps:** 793
- **1D aligned content (<aminoacid>:%):** {'M': 0.84, 'G': 7.59, 'A': 7.59, 'P': 6.33, 'D': 6.75, 'R': 5.06, 'L': 9.28, 'F': 4.64, 'E': 5.49, 'I': 4.64, 'V': 8.44, 'H': 2.11, 'K': 10.55, 'N': 1.69, 'Y': 3.8, 'S': 4.22, 'T': 5.49, 'Q': 4.22, 'W': 0.42, 'C': 0.84}
- **Common reported functions (%):** 6.67
- **Common reported locations (%):** 28.57
- **Common reported processes (%):** 0.0

- **PDB ID:** 5ZEN
- **Chain:** A
- **Crystallized protein length:** 708 aa
- **Resolution:** 2.75 Å
- **b-phipsi:** 0.015333
- **w-rdist:** 0.437116
- **t-alpha:** 0.016752
- **Chemical similarity (Tanimoto Index) (%):** N/A
- **1D identity (%) [PDB]:** 3.17
- **1D identity (%) [Gaps excluded][PDB]:** 69.12
- **1D identity - Alignment Gaps [PDB]:** 1414
- **1D aligned content [PDB] (<aminoacid>:%):** {'K': 4.26, 'L': 10.64, 'G': 12.77, 'V': 10.64, 'N': 4.26, 'I': 4.26, 'F': 2.13, 'T': 4.26, 'P': 4.26, 'A': 10.64, 'D': 6.38, 'S': 4.26, 'R': 8.51, 'Y': 2.13, 'Q': 6.38, 'E': 4.26}
- **2D identity (%) [PDB]:** 35.08
- **2D identity (%) [Gaps excluded][PDB]:** 88.21
- **2D identity - Alignment Gaps [PDB]:** 668
- **2D aligned content [PDB] (<2D-fold>:%):** {'.': 16.71, 'E': 17.99, 'H': 53.21, 'T': 10.8, 'G': 1.29}
- **3D similarity (TM-Score) (%) [PDB]:** 21.71

- **Gene name:** TOP2B
- **Entrez ID:** 7155
- **RefSeq ID:** NM\_001330700
- **Transcript sequence length:** 5814
- **5-UTR|CDS|3-UTR identity (%):** 18.66 | 35.0 | 1.77
- **5-UTR|CDS|3-UTR identity (%) [Gaps excluded]:** 90.48 | 76.41 | 75.63
- **5-UTR|CDS|3-UTR identity [Alignment Gaps]:** 485 | 2773 | 11653
- **5-UTR aligned content (<base>:%):** {'C': 45.61, 'T': 9.65, 'G': 42.11, 'A': 2.63}
- **CDS aligned content (<base>:%):** {'A': 29.15, 'T': 22.22, 'G': 26.13, 'C': 22.5}
- **3-UTR aligned content (<base>:%):** {'T': 42.18, 'C': 16.59, 'A': 27.01, 'G': 14.22}

**Uniprot Description:**  
  
 Key decatenating enzyme that alters DNA topology by binding to two double-stranded DNA molecules, generating a double-stranded break in one of the strands, passing the intact strand through the broken strand, and religating the broken strand.   
  
Homodimer (PubMed:21778401). Interacts with KIAA1210 (By similarity). Interacts with PLSCR1 (PubMed:19690332).   
  
 **Gene Ontology Information:**

Molecular Function

- ATP binding
- chromatin binding
- DNA binding
- DNA topoisomerase type II (double strand cut, ATP-hydrolyzing) activity
- metal ion binding
- ribonucleoprotein complex binding

Location

- cytosol
- nucleolus
- nucleoplasm
- nucleus
- ribonucleoprotein complex

Biological process

- axonogenesis
- B cell differentiation
- cellular response to ATP
- cellular response to hydrogen peroxide
- cellular senescence
- DNA topological change
- forebrain development
- neuron migration
- positive regulation of double-strand break repair via nonhomologous end joining
- positive regulation of single stranded viral RNA replication via double stranded DNA intermediate
- resolution of meiotic recombination intermediates
- sister chromatid segregation

---

78

- **Protein name:** Beta-adrenergic receptor kinase 1
- **Organism:** Homo sapiens
- **Uniprot Accession Number:** P25098
- **Protein sequence length:** 689 aa
- **1D identity (%):** 15.45
- **1D identity (%) [Gaps excluded]:** 23.38
- **1D identity - Alignment Gaps:** 316
- **1D aligned content (<aminoacid>:%):** {'A': 6.94, 'L': 11.11, 'M': 2.78, 'T': 4.86, 'P': 7.64, 'R': 4.86, 'S': 3.47, 'E': 10.42, 'V': 6.25, 'F': 6.94, 'Q': 4.86, 'D': 4.86, 'I': 2.78, 'C': 4.17, 'K': 3.47, 'H': 3.47, 'G': 9.03, 'N': 1.39, 'Y': 0.69}
- **Common reported functions (%):** 0.0
- **Common reported locations (%):** 21.43
- **Common reported processes (%):** 0.0

- **PDB ID:** 3KRW
- **Chain:** A
- **Crystallized protein length:** 618 aa
- **Resolution:** 2.9 Å
- **b-phipsi:** 0.011037
- **w-rdist:** 0.414918
- **t-alpha:** 0.028464
- **Chemical similarity (Tanimoto Index) (%):** 83.24
- **1D identity (%) [PDB]:** 3.34
- **1D identity (%) [Gaps excluded][PDB]:** 57.5
- **1D identity - Alignment Gaps [PDB]:** 1299
- **1D aligned content [PDB] (<aminoacid>:%):** {'K': 6.52, 'P': 4.35, 'S': 4.35, 'I': 10.87, 'V': 13.04, 'A': 8.7, 'H': 6.52, 'T': 4.35, 'Q': 6.52, 'E': 8.7, 'L': 8.7, 'R': 8.7, 'F': 2.17, 'Y': 2.17, 'D': 2.17, 'G': 2.17}
- **2D identity (%) [PDB]:** 37.93
- **2D identity (%) [Gaps excluded][PDB]:** 88.99
- **2D identity - Alignment Gaps [PDB]:** 587
- **2D aligned content [PDB] (<2D-fold>:%):** {'.': 17.27, 'T': 11.86, 'H': 51.03, 'E': 19.07, 'G': 0.77}
- **3D similarity (TM-Score) (%) [PDB]:** 20.95

- **Gene name:** GRK2
- **Entrez ID:** 156
- **RefSeq ID:** NM\_001619
- **Transcript sequence length:** 3403
- **5-UTR|CDS|3-UTR identity (%):** 43.04 | 43.75 | 5.99
- **5-UTR|CDS|3-UTR identity (%) [Gaps excluded]:** 79.84 | 75.99 | 73.69
- **5-UTR|CDS|3-UTR identity [Alignment Gaps]:** 106 | 1252 | 11040
- **5-UTR aligned content (<base>:%):** {'G': 53.54, 'T': 3.03, 'C': 40.4, 'A': 3.03}
- **CDS aligned content (<base>:%):** {'A': 22.85, 'T': 15.96, 'G': 31.45, 'C': 29.74}
- **3-UTR aligned content (<base>:%):** {'C': 33.89, 'G': 28.89, 'A': 14.17, 'T': 23.06}

**Uniprot Description:**  
  
 Specifically phosphorylates the agonist-occupied form of the beta-adrenergic and closely related receptors, probably inducing a desensitization of them (PubMed:19715378). Key regulator of LPAR1 signaling (PubMed:19306925). Competes with RALA for binding to LPAR1 thus affecting the signaling properties of the receptor (PubMed:19306925). Desensitizes LPAR1 and LPAR2 in a phosphorylation-independent manner (PubMed:19306925). Positively regulates ciliary smoothened (SMO)-dependent Hedgehog (Hh) signaling pathway by facilitating the trafficking of SMO into the cilium and the stimulation of SMO activity (By similarity). Inhibits relaxation of airway smooth muscle in response to blue light (PubMed:30284927).   
  
Interacts with the heterodimer formed by GNB1 and GNG2 (By similarity). Interacts with GIT1 (By similarity). Interacts with, and phosphorylates chemokine-stimulated CCR5 (PubMed:10085131). Interacts with ARRB1 (PubMed:9501202). Interacts with LPAR1 and LPAR2 (PubMed:19306925). Interacts with RALA in response to LPAR1 activation (PubMed:19306925). ADRBK1 and RALA mutually inhibit each other's binding to LPAR1 (PubMed:19306925). Interacts with ADRB2 (PubMed:19715378).   
  
 **Gene Ontology Information:**

Molecular Function

- alpha-2A adrenergic receptor binding
- ATP binding
- beta-adrenergic receptor kinase activity
- Edg-2 lysophosphatidic acid receptor binding
- G protein-coupled receptor binding
- G protein-coupled receptor kinase activity
- protein kinase activity

Location

- cilium
- cytoplasm
- cytosol
- membrane
- plasma membrane
- postsynapse
- presynapse

Biological process

- cardiac muscle contraction
- desensitization of G protein-coupled receptor signaling pathway
- G protein-coupled acetylcholine receptor signaling pathway
- G protein-coupled receptor signaling pathway
- heart development
- negative regulation of relaxation of smooth muscle
- negative regulation of striated muscle contraction
- negative regulation of the force of heart contraction by chemical signal
- peptidyl-serine phosphorylation
- peptidyl-threonine phosphorylation
- positive regulation of catecholamine secretion
- protein phosphorylation
- receptor internalization
- regulation of the force of heart contraction
- tachykinin receptor signaling pathway
- viral entry into host cell
- viral genome replication

---

79

- **Protein name:** Exostosin-like 3
- **Organism:** Homo sapiens
- **Uniprot Accession Number:** O43909
- **Protein sequence length:** 919 aa
- **1D identity (%):** 17.2
- **1D identity (%) [Gaps excluded]:** 24.29
- **1D identity - Alignment Gaps:** 304
- **1D aligned content (<aminoacid>:%):** {'G': 8.94, 'Y': 3.91, 'T': 2.79, 'L': 11.73, 'N': 2.23, 'H': 4.47, 'D': 6.15, 'E': 8.94, 'R': 5.03, 'V': 9.5, 'I': 1.68, 'K': 2.23, 'C': 1.68, 'S': 3.91, 'A': 7.82, 'M': 1.12, 'P': 10.06, 'F': 3.91, 'Q': 2.79, 'W': 1.12}
- **Common reported functions (%):** 6.67
- **Common reported locations (%):** 0.0
- **Common reported processes (%):** 0.0

- **PDB ID:** 8OG4
- **Chain:** A
- **Crystallized protein length:** 709 aa
- **Resolution:** 2.1 Å
- **b-phipsi:** 0.006319
- **w-rdist:** 0.402804
- **t-alpha:** 0.105475
- **Chemical similarity (Tanimoto Index) (%):** 75.5
- **1D identity (%) [PDB]:** 2.55
- **1D identity (%) [Gaps excluded][PDB]:** 72.55
- **1D identity - Alignment Gaps [PDB]:** 1399
- **1D aligned content [PDB] (<aminoacid>:%):** {'S': 10.81, 'I': 5.41, 'R': 16.22, 'A': 5.41, 'T': 8.11, 'P': 2.7, 'D': 10.81, 'E': 10.81, 'L': 5.41, 'F': 5.41, 'N': 2.7, 'Y': 2.7, 'M': 5.41, 'V': 8.11}
- **2D identity (%) [PDB]:** 41.51
- **2D identity (%) [Gaps excluded][PDB]:** 87.76
- **2D identity - Alignment Gaps [PDB]:** 537
- **2D aligned content [PDB] (<2D-fold>:%):** {'.': 20.09, 'E': 21.99, 'T': 21.51, 'H': 35.22, 'B': 0.47, 'G': 0.71}
- **3D similarity (TM-Score) (%) [PDB]:** 23.65

- **Gene name:** EXTL3
- **Entrez ID:** N/A
- **RefSeq ID:** N/A
- **Sequence length:** N/A
- **5-UTR|CDS|3-UTR identity (%):** N/A | N/A | N/A
- **5-UTR|CDS|3-UTR identity (%) [Gaps excluded]:** N/A | N/A | N/A
- **5-UTR|CDS|3-UTR identity [Alignment Gaps]:** N/A | N/A | N/A
- **5-UTR aligned content (<base>:%):** N/A
- **CDS aligned content (<base>:%):** N/A
- **3-UTR aligned content (<base>:%):** N/A

**Uniprot Description:**  
  
 Glycosyltransferase which regulates the biosynthesis of heparan sulfate (HS). Important for both skeletal development and hematopoiesis, through the formation of HS proteoglycans (HSPGs) (PubMed:28132690, PubMed:28148688). Required for the function of REG3A in regulating keratinocyte proliferation and differentiation (PubMed:22727489).   
  
Interacts with REG3A.   
  
 **Gene Ontology Information:**

Molecular Function

- glucuronyl-galactosyl-proteoglycan 4-alpha-N-acetylglucosaminyltransferase activity
- transferase activity, transferring glycosyl groups
- metal ion binding

Location

- endoplasmic reticulum
- endoplasmic reticulum membrane
- Golgi apparatus

Biological process

- heparan sulfate proteoglycan biosynthetic process
- positive regulation of cell growth
- protein glycosylation

---

80

- **Protein name:** Coagulation factor XIII A chain
- **Organism:** Homo sapiens
- **Uniprot Accession Number:** P00488
- **Protein sequence length:** 732 aa
- **1D identity (%):** 12.49
- **1D identity (%) [Gaps excluded]:** 23.64
- **1D identity - Alignment Gaps:** 491
- **1D aligned content (<aminoacid>:%):** {'S': 4.62, 'T': 9.23, 'A': 6.15, 'F': 5.38, 'P': 8.46, 'E': 3.08, 'Q': 4.62, 'V': 11.54, 'G': 7.69, 'K': 3.85, 'L': 5.38, 'R': 9.23, 'Y': 5.38, 'D': 4.62, 'I': 3.08, 'M': 0.77, 'N': 0.77, 'W': 1.54, 'C': 2.31, 'H': 2.31}
- **Common reported functions (%):** 6.67
- **Common reported locations (%):** 0.0
- **Common reported processes (%):** 0.0

- **PDB ID:** 5MHL
- **Chain:** A
- **Crystallized protein length:** 692 aa
- **Resolution:** 2.4 Å
- **b-phipsi:** 0.017657
- **w-rdist:** 0.301034
- **t-alpha:** 0.035445
- **Chemical similarity (Tanimoto Index) (%):** 84.03
- **1D identity (%) [PDB]:** 2.72
- **1D identity (%) [Gaps excluded][PDB]:** 63.49
- **1D identity - Alignment Gaps [PDB]:** 1406
- **1D aligned content [PDB] (<aminoacid>:%):** {'V': 20.0, 'L': 7.5, 'G': 12.5, 'N': 5.0, 'P': 7.5, 'Q': 7.5, 'F': 5.0, 'I': 10.0, 'D': 5.0, 'T': 5.0, 'K': 2.5, 'S': 5.0, 'M': 2.5, 'H': 2.5, 'R': 2.5}
- **2D identity (%) [PDB]:** 36.28
- **2D identity (%) [Gaps excluded][PDB]:** 82.48
- **2D identity - Alignment Gaps [PDB]:** 596
- **2D aligned content [PDB] (<2D-fold>:%):** {'.': 19.22, 'E': 45.97, 'T': 13.77, 'H': 20.0, 'G': 0.78, 'B': 0.26}
- **3D similarity (TM-Score) (%) [PDB]:** 19.59

- **Gene name:** F13A1
- **Entrez ID:** 2162
- **RefSeq ID:** NM\_000129
- **Transcript sequence length:** 3828
- **5-UTR|CDS|3-UTR identity (%):** 35.62 | 43.33 | 8.28
- **5-UTR|CDS|3-UTR identity (%) [Gaps excluded]:** 69.33 | 75.55 | 75.26
- **5-UTR|CDS|3-UTR identity [Alignment Gaps]:** 71 | 1295 | 10763
- **5-UTR aligned content (<base>:%):** {'G': 46.15, 'C': 44.23, 'T': 5.77, 'A': 3.85}
- **CDS aligned content (<base>:%):** {'A': 26.37, 'T': 19.07, 'G': 26.98, 'C': 27.58}
- **3-UTR aligned content (<base>:%):** {'T': 34.07, 'G': 17.48, 'A': 31.97, 'C': 16.48}

**Uniprot Description:**  
  
 Factor XIII is activated by thrombin and calcium ion to a transglutaminase that catalyzes the formation of gamma-glutamyl-epsilon-lysine cross-links between fibrin chains, thus stabilizing the fibrin clot. Also cross-link alpha-2-plasmin inhibitor, or fibronectin, to the alpha chains of fibrin.   
  
Tetramer of two A chains (F13A1) and two B (F13B) chains.   
  
 **Gene Ontology Information:**

Molecular Function

- metal ion binding
- protein-glutamine gamma-glutamyltransferase activity

Location

- blood microparticle
- collagen-containing extracellular matrix
- extracellular region
- extracellular space
- platelet alpha granule lumen
- transferase complex

Biological process

- blood coagulation
- blood coagulation, fibrin clot formation
- peptide cross-linking

---

81

- **Protein name:** Acetyl-CoA carboxylase 1
- **Organism:** Homo sapiens
- **Uniprot Accession Number:** Q13085
- **Protein sequence length:** 2346 aa
- **1D identity (%):** 11.55
- **1D identity (%) [Gaps excluded]:** 31.96
- **1D identity - Alignment Gaps:** 1503
- **1D aligned content (<aminoacid>:%):** {'M': 1.84, 'A': 5.15, 'P': 11.03, 'Q': 4.04, 'G': 8.46, 'F': 4.41, 'S': 3.68, 'N': 1.47, 'Y': 3.31, 'I': 5.88, 'K': 7.35, 'R': 7.35, 'V': 8.46, 'H': 4.04, 'D': 4.41, 'L': 7.35, 'E': 3.68, 'T': 6.62, 'C': 1.47}
- **Common reported functions (%):** 6.67
- **Common reported locations (%):** 7.14
- **Common reported processes (%):** 0.0

- **PDB ID:** 4ASI
- **Chain:** C
- **Crystallized protein length:** 755 aa
- **Resolution:** 2.8 Å
- **b-phipsi:** 0.008575
- **w-rdist:** 0.671675
- **t-alpha:** 0.008012
- **Chemical similarity (Tanimoto Index) (%):** 96.11
- **1D identity (%) [PDB]:** 3.52
- **1D identity (%) [Gaps excluded][PDB]:** 60.92
- **1D identity - Alignment Gaps [PDB]:** 1420
- **1D aligned content [PDB] (<aminoacid>:%):** {'D': 3.77, 'L': 13.21, 'S': 11.32, 'R': 7.55, 'P': 7.55, 'T': 3.77, 'V': 7.55, 'M': 3.77, 'Y': 3.77, 'G': 11.32, 'F': 7.55, 'A': 3.77, 'E': 3.77, 'N': 1.89, 'W': 1.89, 'Q': 1.89, 'K': 3.77, 'I': 1.89}
- **2D identity (%) [PDB]:** 37.07
- **2D identity (%) [Gaps excluded][PDB]:** 88.91
- **2D identity - Alignment Gaps [PDB]:** 656
- **2D aligned content [PDB] (<2D-fold>:%):** {'T': 7.43, '.': 11.75, 'H': 53.96, 'E': 25.42, 'G': 1.44}
- **3D similarity (TM-Score) (%) [PDB]:** 21.78

- **Gene name:** ACACA
- **Entrez ID:** 31
- **RefSeq ID:** N/A
- **Sequence length:** N/A
- **5-UTR|CDS|3-UTR identity (%):** N/A | N/A | N/A
- **5-UTR|CDS|3-UTR identity (%) [Gaps excluded]:** N/A | N/A | N/A
- **5-UTR|CDS|3-UTR identity [Alignment Gaps]:** N/A | N/A | N/A
- **5-UTR aligned content (<base>:%):** N/A
- **CDS aligned content (<base>:%):** N/A
- **3-UTR aligned content (<base>:%):** N/A

**Uniprot Description:**  
  
 Cytosolic enzyme that catalyzes the carboxylation of acetyl-CoA to malonyl-CoA, the first and rate-limiting step of de novo fatty acid biosynthesis (PubMed:20952656, PubMed:20457939, PubMed:29899443). This is a 2 steps reaction starting with the ATP-dependent carboxylation of the biotin carried by the biotin carboxyl carrier (BCC) domain followed by the transfer of the carboxyl group from carboxylated biotin to acetyl-CoA (PubMed:20952656, PubMed:20457939, PubMed:29899443).   
  
Monomer, homodimer, and homotetramer (PubMed:20952656, PubMed:29899443). Can form filamentous polymers (PubMed:20457939, PubMed:20952656, PubMed:29899443). Interacts in its inactive phosphorylated form with the BRCT domains of BRCA1 which prevents ACACA dephosphorylation and inhibits lipid synthesis (PubMed:12360400, PubMed:16326698, PubMed:18452305, PubMed:29899443). Interacts with MID1IP1; interaction with MID1IP1 promotes oligomerization and increases its activity (PubMed:20457939).   
  
 **Gene Ontology Information:**

Molecular Function

- acetyl-CoA carboxylase activity
- ATP binding
- identical protein binding
- metal ion binding

Location

- actin cytoskeleton
- cytosol
- fibrillar center
- mitochondrion

Biological process

- acetyl-CoA metabolic process
- cellular response to prostaglandin E stimulus
- fatty acid biosynthetic process
- fatty-acyl-CoA biosynthetic process
- lipid homeostasis
- malonyl-CoA biosynthetic process
- protein homotetramerization
- protein metabolic process
- tissue homeostasis

---

82

- **Protein name:** Complement C3
- **Organism:** Homo sapiens
- **Uniprot Accession Number:** P01024
- **Protein sequence length:** 1663 aa
- **1D identity (%):** 14.0
- **1D identity (%) [Gaps excluded]:** 27.84
- **1D identity - Alignment Gaps:** 834
- **1D aligned content (<aminoacid>:%):** {'M': 0.85, 'G': 11.06, 'P': 9.79, 'L': 7.66, 'A': 5.96, 'Q': 6.38, 'F': 5.11, 'R': 5.96, 'D': 4.68, 'I': 2.98, 'E': 5.11, 'V': 9.36, 'K': 7.66, 'Y': 3.4, 'T': 5.11, 'S': 2.13, 'C': 2.55, 'N': 1.28, 'H': 2.98}
- **Common reported functions (%):** 0.0
- **Common reported locations (%):** 0.0
- **Common reported processes (%):** 0.0

- **PDB ID:** 8EOK
- **Chain:** G
- **Crystallized protein length:** 629 aa
- **Resolution:** 3.53 Å
- **b-phipsi:** 0.033799
- **w-rdist:** 0.440551
- **t-alpha:** 0.011654
- **Chemical similarity (Tanimoto Index) (%):** 97.04
- **1D identity (%) [PDB]:** 3.25
- **1D identity (%) [Gaps excluded][PDB]:** 60.81
- **1D identity - Alignment Gaps [PDB]:** 1312
- **1D aligned content [PDB] (<aminoacid>:%):** {'T': 8.89, 'V': 20.0, 'L': 8.89, 'A': 4.44, 'M': 2.22, 'N': 8.89, 'P': 13.33, 'K': 6.67, 'G': 8.89, 'R': 2.22, 'Q': 2.22, 'I': 2.22, 'F': 2.22, 'D': 4.44, 'S': 4.44}
- **2D identity (%) [PDB]:** 42.71
- **2D identity (%) [Gaps excluded][PDB]:** 85.6
- **2D identity - Alignment Gaps [PDB]:** 488
- **2D aligned content [PDB] (<2D-fold>:%):** {'.': 22.36, 'E': 48.32, 'T': 21.88, 'H': 6.97, 'B': 0.48}
- **3D similarity (TM-Score) (%) [PDB]:** 4.52

- **Gene name:** C3
- **Entrez ID:** N/A
- **RefSeq ID:** NM\_000064
- **Transcript sequence length:** 5231
- **5-UTR|CDS|3-UTR identity (%):** 28.79 | 36.79 | 1.01
- **5-UTR|CDS|3-UTR identity (%) [Gaps excluded]:** 67.86 | 79.04 | 71.86
- **5-UTR|CDS|3-UTR identity [Alignment Gaps]:** 76 | 2762 | 11732
- **5-UTR aligned content (<base>:%):** {'C': 68.42, 'T': 15.79, 'G': 13.16, 'A': 2.63}
- **CDS aligned content (<base>:%):** {'A': 23.93, 'T': 17.15, 'G': 28.77, 'C': 30.14}
- **3-UTR aligned content (<base>:%):** {'A': 17.5, 'C': 37.5, 'T': 25.83, 'G': 19.17}

**Uniprot Description:**  
  
 C3 plays a central role in the activation of the complement system. Its processing by C3 convertase is the central reaction in both classical and alternative complement pathways. After activation C3b can bind covalently, via its reactive thioester, to cell surface carbohydrates or immune aggregates.   
  
C3 precursor is first processed by the removal of 4 Arg residues, forming two chains, beta and alpha, linked by a disulfide bond. C3 convertase activates C3 by cleaving the alpha chain, releasing C3a anaphylatoxin and generating C3b (beta chain + alpha' chain). Forms the pro-C3-convertase enzyme complex by interacting with Complement factor B Bb fragment (Bb), which is then stabilized by binding CFP, allowing the complex to become active (PubMed:28264884, PubMed:31507604). The interaction with Bb is dependent on Mg2+ (PubMed:31507604). C3b interacts with CR1 (via Sushi 8 and Sushi 9 domains) (PubMed:8175757, PubMed:2972794). C3b interacts with CFH (PubMed:21285368). C3d interacts with CFH (PubMed:21285368, PubMed:21317894). C3dg interacts with CR2 (via the N-terminal Sushi domains 1 and 2). During pregnancy, C3dg exists as a complex (probably a 2:2:2 heterohexamer) with AGT and the proform of PRG2. Interacts with VSIG4. Interacts (both C3a and ASP) with C5AR2; the interaction occurs with higher affinity for ASP, enhancing the phosphorylation and activation of C5AR2, recruitment of ARRB2 to the cell surface and endocytosis of GRP77.   
  
 **Gene Ontology Information:**

Molecular Function   
  
N/A

Location   
  
N/A

Biological process   
  
N/A

---

83

- **Protein name:** Ectonucleotide pyrophosphatase/phosphodiesterase family member 2
- **Organism:** Homo sapiens
- **Uniprot Accession Number:** Q13822
- **Protein sequence length:** 863 aa
- **1D identity (%):** 13.41
- **1D identity (%) [Gaps excluded]:** 23.06
- **1D identity - Alignment Gaps:** 456
- **1D aligned content (<aminoacid>:%):** {'M': 0.68, 'G': 8.9, 'L': 9.59, 'A': 3.42, 'P': 12.33, 'S': 6.16, 'R': 5.48, 'Q': 4.79, 'E': 7.53, 'K': 7.53, 'C': 3.42, 'N': 2.74, 'H': 4.11, 'V': 5.48, 'D': 3.42, 'T': 4.79, 'W': 2.05, 'F': 3.42, 'Y': 2.74, 'I': 1.37}
- **Common reported functions (%):** 0.0
- **Common reported locations (%):** 0.0
- **Common reported processes (%):** 0.0

- **PDB ID:** 4ZG6
- **Chain:** A
- **Crystallized protein length:** 754 aa
- **Resolution:** 1.8 Å
- **b-phipsi:** 0.006482
- **w-rdist:** 0.534897
- **t-alpha:** 0.026158
- **Chemical similarity (Tanimoto Index) (%):** 67.35
- **1D identity (%) [PDB]:** 2.94
- **1D identity (%) [Gaps excluded][PDB]:** 67.16
- **1D identity - Alignment Gaps [PDB]:** 1462
- **1D aligned content [PDB] (<aminoacid>:%):** {'G': 6.67, 'R': 6.67, 'E': 13.33, 'F': 11.11, 'V': 11.11, 'P': 4.44, 'L': 6.67, 'K': 11.11, 'M': 2.22, 'N': 2.22, 'I': 6.67, 'D': 4.44, 'A': 4.44, 'T': 4.44, 'Y': 2.22, 'Q': 2.22}
- **2D identity (%) [PDB]:** 28.31
- **2D identity (%) [Gaps excluded][PDB]:** 80.29
- **2D identity - Alignment Gaps [PDB]:** 764
- **2D aligned content [PDB] (<2D-fold>:%):** {'T': 14.67, '.': 19.16, 'H': 41.02, 'E': 20.96, 'G': 3.59, 'B': 0.6}
- **3D similarity (TM-Score) (%) [PDB]:** 23.91

- **Gene name:** ENPP2
- **Entrez ID:** 5168
- **RefSeq ID:** NM\_006209
- **Transcript sequence length:** 3242
- **5-UTR|CDS|3-UTR identity (%):** 15.79 | 44.29 | 2.8
- **5-UTR|CDS|3-UTR identity (%) [Gaps excluded]:** 70.59 | 73.7 | 82.27
- **5-UTR|CDS|3-UTR identity [Alignment Gaps]:** 118 | 1328 | 11511
- **5-UTR aligned content (<base>:%):** {'G': 41.67, 'C': 50.0, 'T': 8.33}
- **CDS aligned content (<base>:%):** {'A': 27.88, 'T': 21.03, 'G': 25.37, 'C': 25.71}
- **3-UTR aligned content (<base>:%):** {'T': 43.41, 'C': 12.57, 'A': 29.64, 'G': 14.37}

**Uniprot Description:**  
  
 Hydrolyzes lysophospholipids to produce the signaling molecule lysophosphatidic acid (LPA) in extracellular fluids (PubMed:15769751, PubMed:26371182, PubMed:27754931, PubMed:14500380, PubMed:12354767,). Major substrate is lysophosphatidylcholine (PubMed:12176993, PubMed:27754931, PubMed:14500380). Also can act on sphingosylphosphorylcholine producing sphingosine-1-phosphate, a modulator of cell motility (PubMed:14500380). Can hydrolyze, in vitro, bis-pNPP, to some extent pNP-TMP, and barely ATP (PubMed:15769751, PubMed:12176993). Involved in several motility-related processes such as angiogenesis and neurite outgrowth. Acts as an angiogenic factor by stimulating migration of smooth muscle cells and microtubule formation (PubMed:11559573). Stimulates migration of melanoma cells, probably via a pertussis toxin-sensitive G protein (PubMed:1733949). May have a role in induction of parturition (PubMed:12176993). Possible involvement in cell proliferation and adipose tissue development (Probable). Tumor cell motility-stimulating factor (PubMed:1733949, PubMed:11559573). Required for LPA production in activated platelets, cleaves the sn-1 lysophospholipids to generate sn-1 lysophosphatidic acids containing predominantly 18:2 and 20:4 fatty acids (PubMed:21393252). Shows a preference for the sn-1 to the sn-2 isomer of 1-O-alkyl-sn-glycero-3-phosphocholine (lyso-PAF) (PubMed:21393252). N/A   
  
 **Gene Ontology Information:**

Molecular Function

- alkylglycerophosphoethanolamine phosphodiesterase activity
- calcium ion binding
- hydrolase activity
- lysophospholipase activity
- nucleic acid binding
- phosphodiesterase I activity
- polysaccharide binding
- scavenger receptor activity
- zinc ion binding

Location

- extracellular space
- plasma membrane

Biological process

- cell motility
- chemotaxis
- immune response
- phosphatidylcholine catabolic process
- phospholipid catabolic process
- positive regulation of epithelial cell migration
- positive regulation of lamellipodium morphogenesis
- positive regulation of peptidyl-tyrosine phosphorylation
- regulation of cell migration
- sphingolipid catabolic process

---

84

- **Protein name:** Membrane primary amine oxidase
- **Organism:** Homo sapiens
- **Uniprot Accession Number:** Q16853
- **Protein sequence length:** 763 aa
- **1D identity (%):** 16.12
- **1D identity (%) [Gaps excluded]:** 24.61
- **1D identity - Alignment Gaps:** 338
- **1D aligned content (<aminoacid>:%):** {'K': 3.8, 'L': 8.23, 'V': 8.86, 'G': 8.23, 'D': 5.7, 'S': 5.7, 'A': 9.49, 'H': 4.43, 'E': 5.06, 'M': 1.27, 'R': 5.06, 'P': 8.23, 'C': 3.16, 'F': 5.7, 'Q': 6.96, 'T': 4.43, 'Y': 3.16, 'N': 1.9, 'I': 0.63}
- **Common reported functions (%):** 0.0
- **Common reported locations (%):** 14.29
- **Common reported processes (%):** 0.0

- **PDB ID:** 2C11
- **Chain:** D
- **Crystallized protein length:** 671 aa
- **Resolution:** 2.9 Å
- **b-phipsi:** 0.011402
- **w-rdist:** 0.790089
- **t-alpha:** 0.001459
- **Chemical similarity (Tanimoto Index) (%):** 70.2
- **1D identity (%) [PDB]:** 2.46
- **1D identity (%) [Gaps excluded][PDB]:** 75.0
- **1D identity - Alignment Gaps [PDB]:** 1414
- **1D aligned content [PDB] (<aminoacid>:%):** {'Q': 5.56, 'A': 11.11, 'D': 5.56, 'S': 2.78, 'V': 19.44, 'E': 5.56, 'P': 8.33, 'R': 5.56, 'L': 8.33, 'K': 2.78, 'Y': 5.56, 'G': 8.33, 'I': 2.78, 'T': 5.56, 'M': 2.78}
- **2D identity (%) [PDB]:** 28.8
- **2D identity (%) [Gaps excluded][PDB]:** 84.16
- **2D identity - Alignment Gaps [PDB]:** 740
- **2D aligned content [PDB] (<2D-fold>:%):** {'.': 14.2, 'E': 59.26, 'H': 8.95, 'T': 14.81, 'G': 2.78}
- **3D similarity (TM-Score) (%) [PDB]:** 18.97

- **Gene name:** AOC3
- **Entrez ID:** 8639
- **RefSeq ID:** NM\_003734
- **Transcript sequence length:** 4011
- **5-UTR|CDS|3-UTR identity (%):** 34.62 | 45.75 | 8.13
- **5-UTR|CDS|3-UTR identity (%) [Gaps excluded]:** 70.0 | 75.52 | 74.6
- **5-UTR|CDS|3-UTR identity [Alignment Gaps]:** 92 | 1196 | 10816
- **5-UTR aligned content (<base>:%):** {'G': 36.51, 'T': 12.7, 'C': 47.62, 'A': 3.17}
- **CDS aligned content (<base>:%):** {'A': 18.44, 'T': 18.23, 'G': 28.96, 'C': 34.37}
- **3-UTR aligned content (<base>:%):** {'G': 24.92, 'C': 25.63, 'T': 29.08, 'A': 20.36}

**Uniprot Description:**  
  
 Cell adhesion protein that participates in lymphocyte extravasation and recirculation by mediating the binding of lymphocytes to peripheral lymph node vascular endothelial cells in an L-selectin-independent fashion. Has semicarbazide-sensitive (SSAO) monoamine oxidase activity. May play a role in adipogenesis.   
  
Homodimer; disulfide-linked. Can heterodimerize with isoform 2 leading to reduced surface expression. Forms a heterodimer with AOC2.   
  
 **Gene Ontology Information:**

Molecular Function

- calcium ion binding
- copper ion binding
- identical protein binding
- primary amine oxidase activity
- protein heterodimerization activity
- quinone binding
- tryptamine:oxygen oxidoreductase (deaminating) activity

Location

- cell surface
- cytoplasm
- early endosome
- endoplasmic reticulum
- Golgi apparatus
- membrane
- microvillus
- plasma membrane

Biological process

- amine metabolic process
- cell adhesion
- inflammatory response
- negative regulation of primary amine oxidase activity
- response to antibiotic

---

85

- **Protein name:** Microsomal triglyceride transfer protein large subunit
- **Organism:** Homo sapiens
- **Uniprot Accession Number:** P55157
- **Protein sequence length:** 894 aa
- **1D identity (%):** 17.26
- **1D identity (%) [Gaps excluded]:** 23.68
- **1D identity - Alignment Gaps:** 275
- **1D aligned content (<aminoacid>:%):** {'I': 5.71, 'L': 12.0, 'F': 4.57, 'Y': 2.29, 'K': 5.71, 'N': 2.29, 'T': 8.57, 'D': 2.86, 'R': 4.57, 'G': 10.29, 'V': 7.43, 'P': 5.14, 'S': 6.86, 'E': 5.71, 'A': 6.29, 'H': 3.43, 'Q': 4.57, 'M': 0.57, 'C': 0.57, 'W': 0.57}
- **Common reported functions (%):** 0.0
- **Common reported locations (%):** 0.0
- **Common reported processes (%):** 0.0

- **PDB ID:** 6I7S
- **Chain:** H
- **Crystallized protein length:** 862 aa
- **Resolution:** 2.5 Å
- **b-phipsi:** 0.005095
- **w-rdist:** 0.57495
- **t-alpha:** 0.037009
- **Chemical similarity (Tanimoto Index) (%):** 96.69
- **1D identity (%) [PDB]:** 2.37
- **1D identity (%) [Gaps excluded][PDB]:** 70.91
- **1D identity - Alignment Gaps [PDB]:** 1591
- **1D aligned content [PDB] (<aminoacid>:%):** {'G': 12.82, 'T': 10.26, 'V': 10.26, 'L': 17.95, 'Q': 17.95, 'M': 2.56, 'K': 5.13, 'N': 5.13, 'R': 5.13, 'P': 7.69, 'S': 2.56, 'I': 2.56}
- **2D identity (%) [PDB]:** 33.39
- **2D identity (%) [Gaps excluded][PDB]:** 88.22
- **2D identity - Alignment Gaps [PDB]:** 767
- **2D aligned content [PDB] (<2D-fold>:%):** {'.': 16.02, 'E': 30.83, 'T': 12.86, 'H': 38.11, 'G': 2.18}
- **3D similarity (TM-Score) (%) [PDB]:** 23.39

- **Gene name:** MTTP
- **Entrez ID:** N/A
- **RefSeq ID:** N/A
- **Sequence length:** N/A
- **5-UTR|CDS|3-UTR identity (%):** N/A | N/A | N/A
- **5-UTR|CDS|3-UTR identity (%) [Gaps excluded]:** N/A | N/A | N/A
- **5-UTR|CDS|3-UTR identity [Alignment Gaps]:** N/A | N/A | N/A
- **5-UTR aligned content (<base>:%):** N/A
- **CDS aligned content (<base>:%):** N/A
- **3-UTR aligned content (<base>:%):** N/A

**Uniprot Description:**  
  
 Catalyzes the transport of triglyceride, cholesteryl ester, and phospholipid between phospholipid surfaces (PubMed:23475612, PubMed:8939939, PubMed:26224785, PubMed:25108285, PubMed:22236406, PubMed:16478722, PubMed:15897609, PubMed:8876250). Required for the assembly and secretion of plasma lipoproteins that contain apolipoprotein B (PubMed:23475612, PubMed:8939939, PubMed:26224785, PubMed:8876250, PubMed:16478722). May be involved in regulating cholesteryl ester biosynthesis in cells that produce lipoproteins (By similarity).   
  
Heterodimer; heterodimerizes with the protein disulfide isomerase (P4HB/PDI) (PubMed:23475612, PubMed:26224785, PubMed:25108285, PubMed:16478722). Interacts with APOB (PubMed:26224785, PubMed:25108285, PubMed:27206948). Interacts with PRAP1 (By similarity).   
  
 **Gene Ontology Information:**

Molecular Function   
  
N/A

Location   
  
N/A

Biological process   
  
N/A

---

86

- **Protein name:** Protein zwilch homolog
- **Organism:** Homo sapiens
- **Uniprot Accession Number:** Q9H900
- **Protein sequence length:** 591 aa
- **1D identity (%):** 11.35
- **1D identity (%) [Gaps excluded]:** 22.24
- **1D identity - Alignment Gaps:** 470
- **1D aligned content (<aminoacid>:%):** {'R': 5.5, 'N': 4.59, 'Q': 3.67, 'F': 4.59, 'G': 9.17, 'K': 6.42, 'L': 10.09, 'Y': 2.75, 'A': 5.5, 'V': 9.17, 'P': 9.17, 'E': 4.59, 'H': 2.75, 'S': 5.5, 'C': 3.67, 'I': 2.75, 'T': 3.67, 'D': 5.5, 'M': 0.92}
- **Common reported functions (%):** 0.0
- **Common reported locations (%):** 7.14
- **Common reported processes (%):** 0.0

- **PDB ID:** 7QPG
- **Chain:** B
- **Crystallized protein length:** 591 aa
- **Resolution:** 3.9 Å
- **b-phipsi:** 0.004152
- **w-rdist:** 0.495404
- **t-alpha:** 0.108152
- **Chemical similarity (Tanimoto Index) (%):** 98.72
- **1D identity (%) [PDB]:** 1.95
- **1D identity (%) [Gaps excluded][PDB]:** 64.29
- **1D identity - Alignment Gaps [PDB]:** 1345
- **1D aligned content [PDB] (<aminoacid>:%):** {'G': 11.11, 'R': 11.11, 'K': 14.81, 'P': 3.7, 'V': 7.41, 'N': 3.7, 'L': 11.11, 'T': 7.41, 'A': 3.7, 'M': 3.7, 'I': 7.41, 'D': 3.7, 'E': 7.41, 'F': 3.7}
- **2D identity (%) [PDB]:** 35.42
- **2D identity (%) [Gaps excluded][PDB]:** 84.8
- **2D identity - Alignment Gaps [PDB]:** 587
- **2D aligned content [PDB] (<2D-fold>:%):** {'.': 19.33, 'H': 47.06, 'T': 8.96, 'E': 24.65}
- **3D similarity (TM-Score) (%) [PDB]:** 20.4

- **Gene name:** ZWILCH
- **Entrez ID:** 55055
- **RefSeq ID:** NM\_017975
- **Transcript sequence length:** 3595
- **5-UTR|CDS|3-UTR identity (%):** 6.3 | 37.42 | 8.83
- **5-UTR|CDS|3-UTR identity (%) [Gaps excluded]:** 61.54 | 73.08 | 75.14
- **5-UTR|CDS|3-UTR identity [Alignment Gaps]:** 114 | 1406 | 10814
- **5-UTR aligned content (<base>:%):** {'C': 25.0, 'G': 75.0}
- **CDS aligned content (<base>:%):** {'A': 29.87, 'G': 24.3, 'T': 22.26, 'C': 23.56}
- **3-UTR aligned content (<base>:%):** {'A': 33.83, 'G': 16.45, 'T': 37.15, 'C': 12.57}

**Uniprot Description:**  
  
 Essential component of the mitotic checkpoint, which prevents cells from prematurely exiting mitosis. Required for the assembly of the dynein-dynactin and MAD1-MAD2 complexes onto kinetochores. Its function related to the spindle assembly machinery is proposed to depend on its association in the mitotic RZZ complex (PubMed:15824131).   
  
Component of the RZZ complex composed of KNTC1/ROD, ZW10 and ZWILCH; in the complex interacts directly with KNTC1/ROD.   
  
 **Gene Ontology Information:**

Molecular Function   
  
N/A

Location

- cytosol
- kinetochore
- kinetochore microtubule
- RZZ complex

Biological process

- cell division
- mitotic spindle assembly checkpoint
- protein localization to kinetochore

---

87

- **Protein name:** Propionyl-CoA carboxylase alpha chain, mitochondrial
- **Organism:** Homo sapiens
- **Uniprot Accession Number:** P05165
- **Protein sequence length:** 728 aa
- **1D identity (%):** 13.7
- **1D identity (%) [Gaps excluded]:** 25.27
- **1D identity - Alignment Gaps:** 471
- **1D aligned content (<aminoacid>:%):** {'W': 1.42, 'V': 12.06, 'L': 8.51, 'Q': 4.96, 'A': 9.22, 'R': 5.67, 'S': 4.96, 'Y': 1.42, 'P': 3.55, 'F': 4.96, 'G': 9.22, 'C': 2.84, 'H': 2.13, 'K': 8.51, 'N': 1.42, 'D': 4.96, 'E': 4.96, 'I': 4.96, 'T': 4.26}
- **Common reported functions (%):** 0.0
- **Common reported locations (%):** 0.0
- **Common reported processes (%):** 0.0

- **PDB ID:** 7YBU
- **Chain:** D
- **Crystallized protein length:** 670 aa
- **Resolution:** 2.2 Å
- **b-phipsi:** 0.007869
- **w-rdist:** 0.546676
- **t-alpha:** 0.020818
- **Chemical similarity (Tanimoto Index) (%):** N/A
- **1D identity (%) [PDB]:** 2.04
- **1D identity (%) [Gaps excluded][PDB]:** 78.95
- **1D identity - Alignment Gaps [PDB]:** 1432
- **1D aligned content [PDB] (<aminoacid>:%):** {'N': 6.67, 'R': 3.33, 'E': 13.33, 'I': 6.67, 'V': 13.33, 'H': 6.67, 'Q': 6.67, 'F': 6.67, 'K': 6.67, 'P': 6.67, 'D': 3.33, 'G': 6.67, 'L': 10.0, 'A': 3.33}
- **2D identity (%) [PDB]:** 41.69
- **2D identity (%) [Gaps excluded][PDB]:** 89.56
- **2D identity - Alignment Gaps [PDB]:** 550
- **2D aligned content [PDB] (<2D-fold>:%):** {'.': 15.38, 'E': 29.6, 'H': 34.5, 'T': 19.81, 'G': 0.7}
- **3D similarity (TM-Score) (%) [PDB]:** 20.32

- **Gene name:** PCCA
- **Entrez ID:** N/A
- **RefSeq ID:** NM\_001178004
- **Transcript sequence length:** 2343
- **5-UTR|CDS|3-UTR identity (%):** 14.5 | 41.44 | 1.54
- **5-UTR|CDS|3-UTR identity (%) [Gaps excluded]:** 79.17 | 74.04 | 75.0
- **5-UTR|CDS|3-UTR identity [Alignment Gaps]:** 107 | 1306 | 11669
- **5-UTR aligned content (<base>:%):** {'G': 63.16, 'C': 26.32, 'T': 10.53}
- **CDS aligned content (<base>:%):** {'A': 28.32, 'G': 27.91, 'C': 21.81, 'T': 21.97}
- **3-UTR aligned content (<base>:%):** {'G': 14.75, 'A': 33.33, 'T': 36.61, 'C': 15.3}

**Uniprot Description:**  
  
 This is one of the 2 subunits of the biotin-dependent propionyl-CoA carboxylase (PCC), a mitochondrial enzyme involved in the catabolism of odd chain fatty acids, branched-chain amino acids isoleucine, threonine, methionine, and valine and other metabolites (PubMed:8434582, PubMed:6765947). Propionyl-CoA carboxylase catalyzes the carboxylation of propionyl-CoA/propanoyl-CoA to D-methylmalonyl-CoA/(S)-methylmalonyl-CoA (PubMed:8434582, PubMed:6765947, PubMed:10101253). Within the holoenzyme, the alpha subunit catalyzes the ATP-dependent carboxylation of the biotin carried by the biotin carboxyl carrier (BCC) domain, while the beta subunit then transfers the carboxyl group from carboxylated biotin to propionyl-CoA (By similarity). Propionyl-CoA carboxylase also significantly acts on butyryl-CoA/butanoyl-CoA, which is converted to ethylmalonyl-CoA/(2S)-ethylmalonyl-CoA at a much lower rate (PubMed:6765947). Other alternative minor substrates include (2E)-butenoyl-CoA/crotonoyl-CoA (By similarity).   
  
The holoenzyme is a dodecamer composed of 6 PCCA/alpha subunits and 6 PCCB/beta subunits (PubMed:6765947, PubMed:20725044). Interacts (via the biotin carboxylation domain) with SIRT4 (PubMed:23438705). Interacts with SIRT3 and SIRT5 (PubMed:23438705).   
  
 **Gene Ontology Information:**

Molecular Function   
  
N/A

Location   
  
N/A

Biological process   
  
N/A

---

88

- **Protein name:** Transforming growth factor-beta-induced protein ig-h3
- **Organism:** Homo sapiens
- **Uniprot Accession Number:** Q15582
- **Protein sequence length:** 683 aa
- **1D identity (%):** 13.23
- **1D identity (%) [Gaps excluded]:** 22.75
- **1D identity - Alignment Gaps:** 408
- **1D aligned content (<aminoacid>:%):** {'L': 12.4, 'A': 6.2, 'P': 8.53, 'Y': 2.33, 'C': 2.33, 'N': 3.1, 'R': 6.2, 'Q': 5.43, 'I': 4.65, 'G': 15.5, 'K': 3.1, 'D': 3.88, 'E': 3.1, 'F': 2.33, 'S': 3.88, 'W': 0.78, 'V': 9.3, 'T': 5.43, 'H': 1.55}
- **Common reported functions (%):** 0.0
- **Common reported locations (%):** 7.14
- **Common reported processes (%):** 0.0

- **PDB ID:** 5NV6
- **Chain:** A
- **Crystallized protein length:** 595 aa
- **Resolution:** 2.93 Å
- **b-phipsi:** 0.003734
- **w-rdist:** 0.784228
- **t-alpha:** 0.031555
- **Chemical similarity (Tanimoto Index) (%):** 97.12
- **1D identity (%) [PDB]:** 1.35
- **1D identity (%) [Gaps excluded][PDB]:** 79.17
- **1D identity - Alignment Gaps [PDB]:** 1385
- **1D aligned content [PDB] (<aminoacid>:%):** {'Q': 10.53, 'G': 5.26, 'T': 5.26, 'R': 10.53, 'P': 5.26, 'S': 10.53, 'H': 5.26, 'Y': 5.26, 'V': 5.26, 'L': 10.53, 'D': 10.53, 'N': 5.26, 'F': 5.26, 'E': 5.26}
- **2D identity (%) [PDB]:** 36.17
- **2D identity (%) [Gaps excluded][PDB]:** 86.94
- **2D identity - Alignment Gaps [PDB]:** 591
- **2D aligned content [PDB] (<2D-fold>:%):** {'.': 14.48, 'E': 33.33, 'T': 11.2, 'H': 38.25, 'G': 2.46, 'B': 0.27}
- **3D similarity (TM-Score) (%) [PDB]:** 16.22

- **Gene name:** TGFBI
- **Entrez ID:** 7045
- **RefSeq ID:** NM\_000358
- **Transcript sequence length:** 2712
- **5-UTR|CDS|3-UTR identity (%):** 34.33 | 39.3 | 2.86
- **5-UTR|CDS|3-UTR identity (%) [Gaps excluded]:** 75.41 | 73.83 | 72.67
- **5-UTR|CDS|3-UTR identity [Alignment Gaps]:** 73 | 1414 | 11536
- **5-UTR aligned content (<base>:%):** {'C': 43.48, 'T': 17.39, 'G': 36.96, 'A': 2.17}
- **CDS aligned content (<base>:%):** {'A': 24.24, 'T': 18.86, 'G': 28.28, 'C': 28.62}
- **3-UTR aligned content (<base>:%):** {'T': 34.11, 'G': 18.95, 'A': 33.24, 'C': 13.7}

**Uniprot Description:**  
  
 Plays a role in cell adhesion (PubMed:8024701). May play a role in cell-collagen interactions (By similarity).   
  
Binds to type I, II, and IV collagens.   
  
 **Gene Ontology Information:**

Molecular Function

- cell adhesion molecule binding
- collagen binding
- extracellular matrix binding
- extracellular matrix structural constituent
- identical protein binding
- integrin binding

Location

- basement membrane
- collagen-containing extracellular matrix
- extracellular exosome
- extracellular matrix
- extracellular region
- extracellular space
- plasma membrane
- trans-Golgi network

Biological process

- angiogenesis
- cell adhesion
- cell population proliferation
- chondrocyte differentiation
- extracellular matrix organization
- localization
- negative regulation of cell adhesion
- response to stimulus
- visual perception

---

89

- **Protein name:** Puromycin-sensitive aminopeptidase
- **Organism:** Homo sapiens
- **Uniprot Accession Number:** P55786
- **Protein sequence length:** 919 aa
- **1D identity (%):** 17.74
- **1D identity (%) [Gaps excluded]:** 22.39
- **1D identity - Alignment Gaps:** 206
- **1D aligned content (<aminoacid>:%):** {'M': 1.14, 'A': 8.52, 'P': 10.8, 'L': 9.66, 'F': 4.55, 'R': 5.11, 'G': 9.66, 'E': 6.25, 'Y': 2.84, 'K': 6.25, 'V': 10.23, 'Q': 2.27, 'I': 3.98, 'T': 5.11, 'D': 2.84, 'S': 3.98, 'N': 3.41, 'C': 0.57, 'H': 2.27, 'W': 0.57}
- **Common reported functions (%):** 0.0
- **Common reported locations (%):** 0.0
- **Common reported processes (%):** 0.0

- **PDB ID:** 8SW1
- **Chain:** A
- **Crystallized protein length:** 864 aa
- **Resolution:** 3.65 Å
- **b-phipsi:** 0.008414
- **w-rdist:** 0.411377
- **t-alpha:** 0.062637
- **Chemical similarity (Tanimoto Index) (%):** 87.83
- **1D identity (%) [PDB]:** 2.8
- **1D identity (%) [Gaps excluded][PDB]:** 76.67
- **1D identity - Alignment Gaps [PDB]:** 1582
- **1D aligned content [PDB] (<aminoacid>:%):** {'F': 4.35, 'D': 8.7, 'R': 4.35, 'K': 8.7, 'P': 6.52, 'N': 2.17, 'L': 15.22, 'Y': 2.17, 'T': 4.35, 'A': 6.52, 'I': 6.52, 'G': 8.7, 'V': 6.52, 'E': 8.7, 'S': 4.35, 'Q': 2.17}
- **2D identity (%) [PDB]:** 39.64
- **2D identity (%) [Gaps excluded][PDB]:** 89.12
- **2D identity - Alignment Gaps [PDB]:** 654
- **2D aligned content [PDB] (<2D-fold>:%):** {'.': 12.63, 'E': 28.69, 'T': 21.41, 'H': 35.97, 'G': 1.28}
- **3D similarity (TM-Score) (%) [PDB]:** 25.78

- **Gene name:** NPEPPS
- **Entrez ID:** N/A
- **RefSeq ID:** N/A
- **Sequence length:** N/A
- **5-UTR|CDS|3-UTR identity (%):** N/A | N/A | N/A
- **5-UTR|CDS|3-UTR identity (%) [Gaps excluded]:** N/A | N/A | N/A
- **5-UTR|CDS|3-UTR identity [Alignment Gaps]:** N/A | N/A | N/A
- **5-UTR aligned content (<base>:%):** N/A
- **CDS aligned content (<base>:%):** N/A
- **3-UTR aligned content (<base>:%):** N/A

**Uniprot Description:**  
  
 Aminopeptidase with broad substrate specificity for several peptides. Involved in proteolytic events essential for cell growth and viability. May act as regulator of neuropeptide activity. Plays a role in the antigen-processing pathway for MHC class I molecules. Involved in the N-terminal trimming of cytotoxic T-cell epitope precursors. Digests the poly-Q peptides found in many cellular proteins. Digests tau from normal brain more efficiently than tau from Alzheimer disease brain.   
  
Monomer.   
  
 **Gene Ontology Information:**

Molecular Function   
  
N/A

Location   
  
N/A

Biological process   
  
N/A

---

90

- **Protein name:** Sucrase-isomaltase, intestinal
- **Organism:** Homo sapiens
- **Uniprot Accession Number:** P14410
- **Protein sequence length:** 1827 aa
- **1D identity (%):** 13.07
- **1D identity (%) [Gaps excluded]:** 28.98
- **1D identity - Alignment Gaps:** 1016
- **1D aligned content (<aminoacid>:%):** {'M': 2.07, 'S': 3.72, 'G': 9.5, 'P': 11.98, 'A': 3.31, 'F': 6.61, 'N': 2.89, 'I': 4.13, 'Y': 5.79, 'E': 4.13, 'D': 4.55, 'K': 3.72, 'R': 6.2, 'L': 8.26, 'V': 6.61, 'W': 2.48, 'T': 5.79, 'Q': 4.55, 'H': 2.07, 'C': 1.65}
- **Common reported functions (%):** 0.0
- **Common reported locations (%):** 7.14
- **Common reported processes (%):** 0.0

- **PDB ID:** 3LPP
- **Chain:** C
- **Crystallized protein length:** 871 aa
- **Resolution:** 2.15 Å
- **b-phipsi:** 0.007215
- **w-rdist:** 0.694276
- **t-alpha:** 0.011791
- **Chemical similarity (Tanimoto Index) (%):** 79.28
- **1D identity (%) [PDB]:** 2.1
- **1D identity (%) [Gaps excluded][PDB]:** 83.33
- **1D identity - Alignment Gaps [PDB]:** 1625
- **1D aligned content [PDB] (<aminoacid>:%):** {'R': 8.57, 'K': 11.43, 'P': 11.43, 'V': 5.71, 'F': 5.71, 'G': 8.57, 'N': 2.86, 'L': 11.43, 'Y': 2.86, 'A': 2.86, 'M': 2.86, 'I': 5.71, 'D': 5.71, 'E': 8.57, 'T': 2.86, 'S': 2.86}
- **2D identity (%) [PDB]:** 37.85
- **2D identity (%) [Gaps excluded][PDB]:** 82.84
- **2D identity - Alignment Gaps [PDB]:** 637
- **2D aligned content [PDB] (<2D-fold>:%):** {'.': 18.69, 'E': 35.36, 'T': 15.77, 'H': 27.7, 'G': 2.03, 'B': 0.45}
- **3D similarity (TM-Score) (%) [PDB]:** 27.43

- **Gene name:** SI
- **Entrez ID:** 6476
- **RefSeq ID:** NM\_001041
- **Transcript sequence length:** 6012
- **5-UTR|CDS|3-UTR identity (%):** 13.12 | 33.83 | 2.74
- **5-UTR|CDS|3-UTR identity (%) [Gaps excluded]:** 67.74 | 77.1 | 74.43
- **5-UTR|CDS|3-UTR identity [Alignment Gaps]:** 129 | 3146 | 11476
- **5-UTR aligned content (<base>:%):** {'A': 4.76, 'T': 19.05, 'G': 38.1, 'C': 38.1}
- **CDS aligned content (<base>:%):** {'A': 28.11, 'T': 20.73, 'G': 25.74, 'C': 25.42}
- **3-UTR aligned content (<base>:%):** {'A': 35.28, 'T': 43.25, 'C': 10.74, 'G': 10.74}

**Uniprot Description:**  
  
 Plays an important role in the final stage of carbohydrate digestion. Isomaltase activity is specific for both alpha-1,4- and alpha-1,6-oligosaccharides.   
  
The resulting sucrase and isomaltase subunits stay associated with one another in a complex by non-covalent linkages.   
  
 **Gene Ontology Information:**

Molecular Function

- alpha-1,4-glucosidase activity
- carbohydrate binding
- oligo-1,6-glucosidase activity
- sucrose alpha-glucosidase activity

Location

- apical plasma membrane
- brush border
- extracellular exosome
- Golgi apparatus
- plasma membrane

Biological process

- polysaccharide digestion
- sucrose catabolic process

---

91

- **Protein name:** Toll-like receptor 4
- **Organism:** Homo sapiens
- **Uniprot Accession Number:** O00206
- **Protein sequence length:** 839 aa
- **1D identity (%):** 15.1
- **1D identity (%) [Gaps excluded]:** 23.86
- **1D identity - Alignment Gaps:** 382
- **1D aligned content (<aminoacid>:%):** {'M': 0.64, 'S': 5.73, 'A': 5.1, 'G': 6.37, 'P': 9.55, 'K': 6.37, 'F': 4.46, 'T': 3.18, 'L': 15.29, 'D': 2.55, 'C': 7.01, 'H': 3.82, 'I': 4.46, 'N': 3.18, 'E': 5.73, 'V': 7.01, 'Q': 3.82, 'R': 5.73}
- **Common reported functions (%):** 0.0
- **Common reported locations (%):** 7.14
- **Common reported processes (%):** 4.76

- **PDB ID:** 3FXI
- **Chain:** A
- **Crystallized protein length:** 601 aa
- **Resolution:** 3.1 Å
- **b-phipsi:** 0.010502
- **w-rdist:** 0.493777
- **t-alpha:** 0.018546
- **Chemical similarity (Tanimoto Index) (%):** 74.59
- **1D identity (%) [PDB]:** 1.85
- **1D identity (%) [Gaps excluded][PDB]:** 72.22
- **1D identity - Alignment Gaps [PDB]:** 1367
- **1D aligned content [PDB] (<aminoacid>:%):** {'E': 19.23, 'V': 11.54, 'L': 11.54, 'D': 3.85, 'F': 7.69, 'K': 11.54, 'I': 11.54, 'T': 11.54, 'S': 3.85, 'Q': 3.85, 'R': 3.85}
- **2D identity (%) [PDB]:** 24.52
- **2D identity (%) [Gaps excluded][PDB]:** 81.14
- **2D identity - Alignment Gaps [PDB]:** 771
- **2D aligned content [PDB] (<2D-fold>:%):** {'.': 33.21, 'E': 29.52, 'T': 26.94, 'H': 8.49, 'G': 1.11, 'B': 0.74}
- **3D similarity (TM-Score) (%) [PDB]:** 21.25

- **Gene name:** TLR4
- **Entrez ID:** 7099
- **RefSeq ID:** NM\_138557
- **Transcript sequence length:** 12510
- **5-UTR|CDS|3-UTR identity (%):** 18.06 | 39.08 | 44.61
- **5-UTR|CDS|3-UTR identity (%) [Gaps excluded]:** 80.87 | 74.02 | 74.63
- **5-UTR|CDS|3-UTR identity [Alignment Gaps]:** 400 | 1390 | 5533
- **5-UTR aligned content (<base>:%):** {'A': 4.3, 'G': 44.09, 'C': 41.94, 'T': 9.68}
- **CDS aligned content (<base>:%):** {'A': 27.98, 'T': 24.33, 'G': 23.46, 'C': 24.24}
- **3-UTR aligned content (<base>:%):** {'A': 27.29, 'G': 18.74, 'T': 35.99, 'C': 17.98}

**Uniprot Description:**  
  
 Cooperates with LY96 and CD14 to mediate the innate immune response to bacterial lipopolysaccharide (LPS) (PubMed:27022195). Acts via MYD88, TIRAP and TRAF6, leading to NF-kappa-B activation, cytokine secretion and the inflammatory response (PubMed:9237759, PubMed:10835634, PubMed:27022195). Also involved in LPS-independent inflammatory responses triggered by free fatty acids, such as palmitate, and Ni(2+). Responses triggered by Ni(2+) require non-conserved histidines and are, therefore, species-specific (PubMed:20711192). Both M.tuberculosis HSP70 (dnaK) and HSP65 (groEL-2) act via this protein to stimulate NF-kappa-B expression (PubMed:15809303). In complex with TLR6, promotes sterile inflammation in monocytes/macrophages in response to oxidized low-density lipoprotein (oxLDL) or amyloid-beta 42. In this context, the initial signal is provided by oxLDL- or amyloid-beta 42-binding to CD36. This event induces the formation of a heterodimer of TLR4 and TLR6, which is rapidly internalized and triggers inflammatory response, leading to the NF-kappa-B-dependent production of CXCL1, CXCL2 and CCL9 cytokines, via MYD88 signaling pathway, and CCL5 cytokine, via TICAM1 signaling pathway, as well as IL1B secretion. Binds electronegative LDL (LDL(-)) and mediates the cytokine release induced by LDL(-) (PubMed:23880187). Stimulation of monocytes in vitro with M.tuberculosis PstS1 induces p38 MAPK and ERK1/2 activation primarily via TLR2, but also partially via this receptor (PubMed:16622205).   
  
Belongs to the lipopolysaccharide (LPS) receptor, a multi-protein complex containing at least CD14, LY96 and TLR4 (PubMed:11274165). Binding to bacterial LPS leads to homodimerization. Interacts with LY96 via the extracellular domain (PubMed:17803912, PubMed:19252480). Interacts with MYD88 and TIRAP via their respective TIR domains (By similarity). Interacts with TICAM2 (PubMed:14519765, PubMed:25736436). Interacts with NOX4 (PubMed:15356101). Interacts with CNPY3 (By similarity). Interacts with HSP90B1. The interaction with both CNPY3 and HSP90B1 is required for proper folding in the endoplasmic reticulum. Interacts with MAP3K21; this interaction leads to negative regulation of TLR4 signaling (PubMed:21602844). Interacts with CD36, following CD36 stimulation by oxLDL or amyloid-beta 42, and forms a heterodimer with TLR6 (PubMed:20037584). The trimeric complex is internalized and triggers inflammatory response. LYN kinase activity facilitates TLR4-TLR6 heterodimerization and signal initiation. Interacts with TICAM1 in response to LPS in a WDFY1-dependent manner (PubMed:25736436). Interacts with WDFY1 in response to LPS (By similarity). Interacts with SMPDL3B (By similarity). Interacts with CEACAM1; upon lipopolysaccharide stimulation, forms a complex including TLR4 and the phosphorylated form of SYK and CEACAM1, which in turn, recruits PTPN6 that dephosphorylates SYK, reducing the production of reactive oxygen species (ROS) and lysosome disruption, which in turn, reduces the activity of the inflammasome (By similarity). Interacts with RFTN1; the interaction occurs in response to lipopolysaccharide stimulation (PubMed:27022195). Interacts with SCIMP; the interaction occurs in response to lipopolysaccharide stimulation and is enhanced by phosphorylation of SCIMP by LYN (By similarity). This interaction facilitates the phosphorylation of TLR4 by LYN which elicits a selective cytokine response in macrophages (By similarity). Interacts with TRAF3IP3 (PubMed:30573680).   
  
 **Gene Ontology Information:**

Molecular Function

- amyloid-beta binding
- identical protein binding
- lipopolysaccharide binding
- lipopolysaccharide immune receptor activity
- NAD(P)+ nucleosidase activity
- NAD+ nucleotidase, cyclic ADP-ribose generating
- protein heterodimerization activity
- signaling receptor activity
- signaling receptor binding
- transmembrane signaling receptor activity

Location

- cell surface
- cytoplasm
- early endosome
- endosome membrane
- external side of plasma membrane
- Golgi apparatus
- lipopolysaccharide receptor complex
- perinuclear region of cytoplasm
- phagocytic cup
- plasma membrane
- receptor complex
- ruffle

Biological process

- astrocyte development
- B cell proliferation involved in immune response
- cellular response to amyloid-beta
- cellular response to lipopolysaccharide
- cellular response to lipoteichoic acid
- cellular response to mechanical stimulus
- cellular response to oxidised low-density lipoprotein particle stimulus
- cellular response to platelet-derived growth factor stimulus
- cellular response to interferon-gamma
- defense response to bacterium
- defense response to Gram-negative bacterium
- detection of fungus
- detection of lipopolysaccharide
- ERK1 and ERK2 cascade
- gene expression
- I-kappaB phosphorylation
- immune response
- inflammatory response
- innate immune response
- intestinal epithelial structure maintenance
- JNK cascade
- lipopolysaccharide-mediated signaling pathway
- macrophage activation
- MHC class II biosynthetic process
- microglia differentiation
- MyD88-dependent toll-like receptor signaling pathway
- negative regulation of cold-induced thermogenesis
- negative regulation of ERK1 and ERK2 cascade
- negative regulation of interleukin-17 production
- negative regulation of interleukin-23 production
- negative regulation of interleukin-6 production
- negative regulation of osteoclast differentiation
- negative regulation of tumor necrosis factor production
- negative regulation of interferon-gamma production
- nitric oxide biosynthetic process
- nitric oxide production involved in inflammatory response
- nucleotide-binding oligomerization domain containing 1 signaling pathway
- nucleotide-binding oligomerization domain containing 2 signaling pathway
- phagocytosis
- positive regulation of B cell proliferation
- positive regulation of cellular response to macrophage colony-stimulating factor stimulus
- positive regulation of chemokine (C-X-C motif) ligand 2 production
- positive regulation of chemokine production
- positive regulation of cytokine production involved in inflammatory response
- positive regulation of ERK1 and ERK2 cascade
- positive regulation of extrinsic apoptotic signaling pathway
- positive regulation of gene expression
- positive regulation of I-kappaB kinase/NF-kappaB signaling
- positive regulation of inflammatory response
- positive regulation of interferon-alpha production
- positive regulation of interferon-beta production
- positive regulation of interleukin-1 beta production
- positive regulation of interleukin-1 production
- positive regulation of interleukin-10 production
- positive regulation of interleukin-12 production
- positive regulation of interleukin-6 production
- positive regulation of interleukin-8 production
- positive regulation of JNK cascade
- positive regulation of macrophage activation
- positive regulation of macrophage cytokine production
- positive regulation of MAP kinase activity
- positive regulation of matrix metallopeptidase secretion
- positive regulation of MHC class II biosynthetic process
- positive regulation of NF-kappaB transcription factor activity
- positive regulation of NIK/NF-kappaB signaling
- positive regulation of nitric oxide biosynthetic process
- positive regulation of nitric-oxide synthase biosynthetic process
- positive regulation of NLRP3 inflammasome complex assembly
- positive regulation of nucleotide-binding oligomerization domain containing 1 signaling pathway
- positive regulation of nucleotide-binding oligomerization domain containing 2 signaling pathway
- positive regulation of platelet activation
- positive regulation of reactive oxygen species biosynthetic process
- positive regulation of smooth muscle cell migration
- positive regulation of smooth muscle cell proliferation
- positive regulation of transcription by RNA polymerase II
- positive regulation of tumor necrosis factor production
- positive regulation of interferon-gamma production
- regulation of dendritic cell cytokine production
- response to lipopolysaccharide
- T-helper 1 type immune response
- toll-like receptor 4 signaling pathway
- toll-like receptor signaling pathway
- TRIF-dependent toll-like receptor signaling pathway
- wound healing involved in inflammatory response

---

92

- **Protein name:** Ubiquitin-like modifier-activating enzyme 1
- **Organism:** Homo sapiens
- **Uniprot Accession Number:** P22314
- **Protein sequence length:** 1058 aa
- **1D identity (%):** 16.67
- **1D identity (%) [Gaps excluded]:** 24.14
- **1D identity - Alignment Gaps:** 351
- **1D aligned content (<aminoacid>:%):** {'M': 0.53, 'S': 5.82, 'G': 8.47, 'P': 10.58, 'Q': 6.88, 'T': 6.88, 'N': 2.12, 'E': 5.82, 'D': 5.82, 'I': 5.82, 'Y': 3.17, 'L': 7.41, 'R': 5.82, 'V': 6.88, 'K': 4.76, 'F': 3.7, 'C': 3.17, 'A': 4.23, 'H': 1.59, 'W': 0.53}
- **Common reported functions (%):** 6.67
- **Common reported locations (%):** 35.71
- **Common reported processes (%):** 0.0

- **PDB ID:** 6DC6
- **Chain:** C
- **Crystallized protein length:** 996 aa
- **Resolution:** 3.14 Å
- **b-phipsi:** 0.005246
- **w-rdist:** 0.649624
- **t-alpha:** 0.037145
- **Chemical similarity (Tanimoto Index) (%):** 90.12
- **1D identity (%) [PDB]:** 2.24
- **1D identity (%) [Gaps excluded][PDB]:** 78.43
- **1D identity - Alignment Gaps [PDB]:** 1733
- **1D aligned content [PDB] (<aminoacid>:%):** {'D': 7.5, 'I': 5.0, 'K': 5.0, 'V': 7.5, 'S': 15.0, 'L': 15.0, 'Q': 5.0, 'A': 5.0, 'H': 5.0, 'G': 2.5, 'P': 5.0, 'F': 2.5, 'E': 2.5, 'T': 5.0, 'M': 5.0, 'R': 5.0, 'Y': 2.5}
- **2D identity (%) [PDB]:** 31.86
- **2D identity (%) [Gaps excluded][PDB]:** 85.54
- **2D identity - Alignment Gaps [PDB]:** 839
- **2D aligned content [PDB] (<2D-fold>:%):** {'.': 17.61, 'H': 44.37, 'T': 13.15, 'E': 24.88}
- **3D similarity (TM-Score) (%) [PDB]:** 24.6

- **Gene name:** UBA1
- **Entrez ID:** 7317
- **RefSeq ID:** N/A
- **Sequence length:** N/A
- **5-UTR|CDS|3-UTR identity (%):** N/A | N/A | N/A
- **5-UTR|CDS|3-UTR identity (%) [Gaps excluded]:** N/A | N/A | N/A
- **5-UTR|CDS|3-UTR identity [Alignment Gaps]:** N/A | N/A | N/A
- **5-UTR aligned content (<base>:%):** N/A
- **CDS aligned content (<base>:%):** N/A
- **3-UTR aligned content (<base>:%):** N/A

**Uniprot Description:**  
  
 Catalyzes the first step in ubiquitin conjugation to mark cellular proteins for degradation through the ubiquitin-proteasome system (PubMed:1606621, PubMed:1447181). Activates ubiquitin by first adenylating its C-terminal glycine residue with ATP, and thereafter linking this residue to the side chain of a cysteine residue in E1, yielding a ubiquitin-E1 thioester and free AMP (PubMed:1447181). Essential for the formation of radiation-induced foci, timely DNA repair and for response to replication stress. Promotes the recruitment of TP53BP1 and BRCA1 at DNA damage sites (PubMed:22456334).   
  
Monomer (By similarity). Interacts with GAN (via BTB domain).   
  
 **Gene Ontology Information:**

Molecular Function

- ATP binding
- RNA binding
- ubiquitin activating enzyme activity

Location

- cytoplasm
- cytosol
- extracellular exosome
- mitochondrion
- nucleoplasm
- nucleus

Biological process

- cellular response to DNA damage stimulus
- protein ubiquitination
- ubiquitin-dependent protein catabolic process

---

93

- **Protein name:** Schlafen family member 5
- **Organism:** Homo sapiens
- **Uniprot Accession Number:** Q08AF3
- **Protein sequence length:** 891 aa
- **1D identity (%):** 17.97
- **1D identity (%) [Gaps excluded]:** 26.7
- **1D identity - Alignment Gaps:** 342
- **1D aligned content (<aminoacid>:%):** {'I': 7.98, 'N': 3.19, 'F': 3.19, 'E': 6.91, 'D': 4.79, 'K': 6.38, 'R': 6.38, 'V': 6.38, 'G': 6.38, 'Y': 3.72, 'P': 6.91, 'S': 4.79, 'L': 12.77, 'T': 2.66, 'Q': 4.26, 'A': 5.85, 'H': 2.66, 'C': 4.26, 'W': 0.53}
- **Common reported functions (%):** 0.0
- **Common reported locations (%):** 7.14
- **Common reported processes (%):** 0.0

- **PDB ID:** 7PPJ
- **Chain:** A
- **Crystallized protein length:** 643 aa
- **Resolution:** 3.44 Å
- **b-phipsi:** 0.008375
- **w-rdist:** 0.491848
- **t-alpha:** 0.030781
- **Chemical similarity (Tanimoto Index) (%):** 87.78
- **1D identity (%) [PDB]:** 2.07
- **1D identity (%) [Gaps excluded][PDB]:** 83.33
- **1D identity - Alignment Gaps [PDB]:** 1411
- **1D aligned content [PDB] (<aminoacid>:%):** {'L': 23.33, 'S': 3.33, 'N': 13.33, 'C': 3.33, 'K': 3.33, 'I': 10.0, 'V': 13.33, 'G': 10.0, 'Q': 6.67, 'R': 3.33, 'P': 6.67, 'F': 3.33}
- **2D identity (%) [PDB]:** 35.65
- **2D identity (%) [Gaps excluded][PDB]:** 86.18
- **2D identity - Alignment Gaps [PDB]:** 615
- **2D aligned content [PDB] (<2D-fold>:%):** {'.': 19.52, 'B': 0.27, 'E': 27.01, 'H': 42.78, 'T': 8.82, 'G': 1.6}
- **3D similarity (TM-Score) (%) [PDB]:** 21.42

- **Gene name:** SLFN5
- **Entrez ID:** 162394
- **RefSeq ID:** NM\_144975
- **Transcript sequence length:** 10555
- **5-UTR|CDS|3-UTR identity (%):** 34.57 | 45.08 | 34.51
- **5-UTR|CDS|3-UTR identity (%) [Gaps excluded]:** 72.73 | 73.56 | 74.71
- **5-UTR|CDS|3-UTR identity [Alignment Gaps]:** 85 | 1262 | 7233
- **5-UTR aligned content (<base>:%):** {'T': 10.71, 'C': 41.07, 'G': 46.43, 'A': 1.79}
- **CDS aligned content (<base>:%):** {'A': 28.25, 'T': 21.72, 'G': 25.8, 'C': 24.23}
- **3-UTR aligned content (<base>:%):** {'A': 31.4, 'G': 19.4, 'C': 16.75, 'T': 32.46}

**Uniprot Description:**  
  
 May have a role in hematopoietic cell differentiation. N/A   
  
 **Gene Ontology Information:**

Molecular Function

- ATP binding

Location

- nucleus

Biological process

- cell differentiation

---

94

- **Protein name:** Prolyl endopeptidase FAP
- **Organism:** Homo sapiens
- **Uniprot Accession Number:** Q12884
- **Protein sequence length:** 760 aa
- **1D identity (%):** 14.69
- **1D identity (%) [Gaps excluded]:** 22.94
- **1D identity - Alignment Gaps:** 355
- **1D aligned content (<aminoacid>:%):** {'K': 5.52, 'T': 4.14, 'V': 6.9, 'F': 7.59, 'A': 3.45, 'P': 8.97, 'L': 11.03, 'G': 6.21, 'W': 2.76, 'S': 5.52, 'H': 2.76, 'E': 4.14, 'Q': 4.14, 'Y': 5.52, 'R': 2.76, 'D': 6.9, 'I': 5.52, 'C': 3.45, 'N': 2.76}
- **Common reported functions (%):** 0.0
- **Common reported locations (%):** 14.29
- **Common reported processes (%):** 0.0

- **PDB ID:** 6Y0F
- **Chain:** C
- **Crystallized protein length:** 722 aa
- **Resolution:** 2.92 Å
- **b-phipsi:** 0.007317
- **w-rdist:** 0.835257
- **t-alpha:** 0.008076
- **Chemical similarity (Tanimoto Index) (%):** N/A
- **1D identity (%) [PDB]:** 2.86
- **1D identity (%) [Gaps excluded][PDB]:** 72.88
- **1D identity - Alignment Gaps [PDB]:** 1442
- **1D aligned content [PDB] (<aminoacid>:%):** {'T': 6.98, 'R': 13.95, 'S': 6.98, 'A': 6.98, 'P': 9.3, 'D': 6.98, 'Q': 4.65, 'K': 4.65, 'L': 4.65, 'F': 4.65, 'N': 4.65, 'V': 9.3, 'I': 4.65, 'E': 2.33, 'G': 6.98, 'Y': 2.33}
- **2D identity (%) [PDB]:** 36.87
- **2D identity (%) [Gaps excluded][PDB]:** 84.21
- **2D identity - Alignment Gaps [PDB]:** 610
- **2D aligned content [PDB] (<2D-fold>:%):** {'.': 15.75, 'B': 1.0, 'E': 48.5, 'H': 17.5, 'T': 16.5, 'G': 0.75}
- **3D similarity (TM-Score) (%) [PDB]:** 25.56

- **Gene name:** FAP
- **Entrez ID:** 2191
- **RefSeq ID:** NM\_004460
- **Transcript sequence length:** 2696
- **5-UTR|CDS|3-UTR identity (%):** 22.47 | 43.73 | 1.86
- **5-UTR|CDS|3-UTR identity (%) [Gaps excluded]:** 59.7 | 71.85 | 79.5
- **5-UTR|CDS|3-UTR identity [Alignment Gaps]:** 111 | 1183 | 11627
- **5-UTR aligned content (<base>:%):** {'G': 35.0, 'T': 15.0, 'C': 50.0}
- **CDS aligned content (<base>:%):** {'A': 30.48, 'T': 23.3, 'G': 24.66, 'C': 21.56}
- **3-UTR aligned content (<base>:%):** {'A': 40.27, 'C': 12.67, 'G': 14.48, 'T': 32.58}

**Uniprot Description:**  
  
 Cell surface glycoprotein serine protease that participates in extracellular matrix degradation and involved in many cellular processes including tissue remodeling, fibrosis, wound healing, inflammation and tumor growth. Both plasma membrane and soluble forms exhibit post-proline cleaving endopeptidase activity, with a marked preference for Ala/Ser-Gly-Pro-Ser/Asn/Ala consensus sequences, on substrate such as alpha-2-antiplasmin SERPINF2 and SPRY2 (PubMed:14751930, PubMed:16223769, PubMed:16480718, PubMed:16410248, PubMed:17381073, PubMed:18095711, PubMed:21288888, PubMed:24371721). Degrade also gelatin, heat-denatured type I collagen, but not native collagen type I and IV, vitronectin, tenascin, laminin, fibronectin, fibrin or casein (PubMed:9065413, PubMed:2172980, PubMed:7923219, PubMed:10347120, PubMed:10455171, PubMed:12376466, PubMed:16223769, PubMed:16651416, PubMed:18095711). Also has dipeptidyl peptidase activity, exhibiting the ability to hydrolyze the prolyl bond two residues from the N-terminus of synthetic dipeptide substrates provided that the penultimate residue is proline, with a preference for Ala-Pro, Ile-Pro, Gly-Pro, Arg-Pro and Pro-Pro (PubMed:10347120, PubMed:10593948, PubMed:16175601, PubMed:16223769, PubMed:16651416, PubMed:16410248, PubMed:17381073, PubMed:21314817, PubMed:24371721, PubMed:24717288). Natural neuropeptide hormones for dipeptidyl peptidase are the neuropeptide Y (NPY), peptide YY (PYY), substance P (TAC1) and brain natriuretic peptide 32 (NPPB) (PubMed:21314817). The plasma membrane form, in association with either DPP4, PLAUR or integrins, is involved in the pericellular proteolysis of the extracellular matrix (ECM), and hence promotes cell adhesion, migration and invasion through the ECM. Plays a role in tissue remodeling during development and wound healing. Participates in the cell invasiveness towards the ECM in malignant melanoma cancers. Enhances tumor growth progression by increasing angiogenesis, collagen fiber degradation and apoptosis and by reducing antitumor response of the immune system. Promotes glioma cell invasion through the brain parenchyma by degrading the proteoglycan brevican. Acts as a tumor suppressor in melanocytic cells through regulation of cell proliferation and survival in a serine protease activity-independent manner.   
  
Homodimer; homodimerization is required for activity of both plasma membrane and soluble forms. The monomer is inactive. Heterodimer with DPP4. Interacts with PLAUR; the interaction occurs at the cell surface of invadopodia membranes. Interacts with ITGB1. Interacts with ITGA3. Associates with integrin alpha-3/beta-1; the association occurs in a collagen-dependent manner at the cell surface of invadopodia membranes.   
  
 **Gene Ontology Information:**

Molecular Function

- dipeptidyl-peptidase activity
- endopeptidase activity
- identical protein binding
- integrin binding
- peptidase activity
- protease binding
- protein homodimerization activity
- serine-type endopeptidase activity
- serine-type peptidase activity

Location

- apical part of cell
- basal part of cell
- cell surface
- cytoplasm
- extracellular space
- focal adhesion
- lamellipodium
- lamellipodium membrane
- membrane
- peptidase complex
- plasma membrane
- ruffle membrane

Biological process

- angiogenesis
- cell adhesion
- endothelial cell migration
- melanocyte apoptotic process
- melanocyte proliferation
- negative regulation of cell proliferation involved in contact inhibition
- negative regulation of extracellular matrix disassembly
- negative regulation of extracellular matrix organization
- positive regulation of execution phase of apoptosis
- proteolysis
- proteolysis involved in cellular protein catabolic process
- regulation of cell cycle
- regulation of collagen catabolic process
- regulation of fibrinolysis

---

95

- **Protein name:** Maltose/maltodextrin-binding periplasmic protein
- **Organism:** Escherichia coli (strain K12)
- **Uniprot Accession Number:** P0AEX9
- **Protein sequence length:** 396 aa
- **1D identity (%):** 8.13
- **1D identity (%) [Gaps excluded]:** 22.52
- **1D identity - Alignment Gaps:** 589
- **1D aligned content (<aminoacid>:%):** {'G': 12.0, 'L': 5.33, 'M': 2.67, 'S': 5.33, 'A': 6.67, 'E': 6.67, 'K': 13.33, 'F': 5.33, 'V': 4.0, 'H': 2.67, 'P': 6.67, 'T': 6.67, 'D': 2.67, 'Y': 5.33, 'N': 6.67, 'I': 4.0, 'W': 1.33, 'Q': 1.33, 'R': 1.33}
- **Common reported functions (%):** 0.0
- **Common reported locations (%):** 0.0
- **Common reported processes (%):** 0.0

- **PDB ID:** 2NVU
- **Chain:** B
- **Crystallized protein length:** 789 aa
- **Resolution:** 2.8 Å
- **b-phipsi:** 0.005824
- **w-rdist:** 0.553399
- **t-alpha:** 0.034664
- **Chemical similarity (Tanimoto Index) (%):** N/A
- **1D identity (%) [PDB]:** 2.28
- **1D identity (%) [Gaps excluded][PDB]:** 66.67
- **1D identity - Alignment Gaps [PDB]:** 1522
- **1D aligned content [PDB] (<aminoacid>:%):** {'K': 8.33, 'E': 5.56, 'L': 16.67, 'V': 13.89, 'G': 8.33, 'D': 8.33, 'I': 5.56, 'F': 2.78, 'S': 11.11, 'Q': 2.78, 'A': 2.78, 'R': 5.56, 'P': 5.56, 'H': 2.78}
- **2D identity (%) [PDB]:** 38.72
- **2D identity (%) [Gaps excluded][PDB]:** 86.51
- **2D identity - Alignment Gaps [PDB]:** 622
- **2D aligned content [PDB] (<2D-fold>:%):** {'.': 15.6, 'E': 19.04, 'T': 14.68, 'H': 49.77, 'B': 0.23, 'G': 0.69}
- **3D similarity (TM-Score) (%) [PDB]:** 18.97

- **Gene name:** malE
- **Entrez ID:** 75204178; 948538
- **RefSeq ID:** N/A
- **Sequence length:** N/A
- **5-UTR|CDS|3-UTR identity (%):** N/A | N/A | N/A
- **5-UTR|CDS|3-UTR identity (%) [Gaps excluded]:** N/A | N/A | N/A
- **5-UTR|CDS|3-UTR identity [Alignment Gaps]:** N/A | N/A | N/A
- **5-UTR aligned content (<base>:%):** N/A
- **CDS aligned content (<base>:%):** N/A
- **3-UTR aligned content (<base>:%):** N/A

**Uniprot Description:**  
  
 Part of the ABC transporter complex MalEFGK involved in maltose/maltodextrin import. Binds maltose and higher maltodextrins such as maltotriose.   
  
The complex is composed of two ATP-binding proteins (MalK), two transmembrane proteins (MalG and MalF) and a solute-binding protein (MalE).   
  
 **Gene Ontology Information:**

Molecular Function

- carbohydrate transmembrane transporter activity
- maltose binding

Location

- ATP-binding cassette (ABC) transporter complex
- ATP-binding cassette (ABC) transporter complex, substrate-binding subunit-containing
- maltose transport complex
- outer membrane-bounded periplasmic space
- periplasmic space

Biological process

- carbohydrate transport
- cell chemotaxis
- detection of maltose stimulus
- cellular response to DNA damage stimulus
- maltodextrin transport
- maltose transport

---

96

- **Protein name:** Islet amyloid polypeptide
- **Organism:** Homo sapiens
- **Uniprot Accession Number:** P10997
- **Protein sequence length:** 89 aa
- **1D identity (%):** 2.33
- **1D identity (%) [Gaps excluded]:** 22.73
- **1D identity - Alignment Gaps:** 772
- **1D aligned content (<aminoacid>:%):** {'G': 5.0, 'L': 20.0, 'F': 10.0, 'V': 15.0, 'H': 10.0, 'T': 5.0, 'P': 5.0, 'S': 10.0, 'C': 5.0, 'R': 5.0, 'K': 5.0, 'E': 5.0}
- **Common reported functions (%):** 0.0
- **Common reported locations (%):** 0.0
- **Common reported processes (%):** 0.0

- **PDB ID:** 7BG0
- **Chain:** A
- **Crystallized protein length:** 403 aa
- **Resolution:** 2.89 Å
- **b-phipsi:** 0.011985
- **w-rdist:** 0.478256
- **t-alpha:** 0.022338
- **Chemical similarity (Tanimoto Index) (%):** 96.82
- **1D identity (%) [PDB]:** 1.56
- **1D identity (%) [Gaps excluded][PDB]:** 86.36
- **1D identity - Alignment Gaps [PDB]:** 1197
- **1D aligned content [PDB] (<aminoacid>:%):** {'K': 10.53, 'I': 5.26, 'N': 15.79, 'L': 15.79, 'G': 15.79, 'V': 10.53, 'P': 15.79, 'Q': 5.26, 'R': 5.26}
- **2D identity (%) [PDB]:** 28.54
- **2D identity (%) [Gaps excluded][PDB]:** 86.08
- **2D identity - Alignment Gaps [PDB]:** 623
- **2D aligned content [PDB] (<2D-fold>:%):** {'.': 13.96, 'E': 20.75, 'H': 53.58, 'T': 11.32, 'B': 0.38}
- **3D similarity (TM-Score) (%) [PDB]:** 16.23

- **Gene name:** IAPP
- **Entrez ID:** 3375
- **RefSeq ID:** NM\_001329201
- **Transcript sequence length:** 1894
- **5-UTR|CDS|3-UTR identity (%):** 21.43 | 6.6 | 8.91
- **5-UTR|CDS|3-UTR identity (%) [Gaps excluded]:** 52.63 | 76.21 | 77.32
- **5-UTR|CDS|3-UTR identity [Alignment Gaps]:** 83 | 2396 | 10664
- **5-UTR aligned content (<base>:%):** {'G': 26.67, 'T': 23.33, 'C': 46.67, 'A': 3.33}
- **CDS aligned content (<base>:%):** {'A': 27.17, 'G': 25.43, 'T': 19.65, 'C': 27.75}
- **3-UTR aligned content (<base>:%):** {'A': 31.1, 'G': 18.62, 'C': 13.87, 'T': 36.41}

**Uniprot Description:**  
  
 Selectively inhibits insulin-stimulated glucose utilization and glycogen deposition in muscle, while not affecting adipocyte glucose metabolism.   
  
Interacts with IDE and INS. Can form homodimers. Interaction with INS inhibits homodimerization and fibril formation.   
  
 **Gene Ontology Information:**

Molecular Function

- amyloid-beta binding
- hormone activity
- identical protein binding
- lipid binding
- signaling receptor binding

Location

- extracellular region
- extracellular space
- inclusion body

Biological process

- adenylate cyclase-activating G protein-coupled receptor signaling pathway
- amylin receptor signaling pathway
- amyloid fibril formation
- apoptotic process
- bone resorption
- cell-cell signaling
- eating behavior
- negative regulation of amyloid fibril formation
- negative regulation of bone resorption
- negative regulation of cell population proliferation
- negative regulation of mitochondrion organization
- negative regulation of osteoclast differentiation
- negative regulation of protein-containing complex assembly
- osteoclast differentiation
- positive regulation of apoptotic process
- positive regulation of calcium ion import across plasma membrane
- positive regulation of cytosolic calcium ion concentration
- positive regulation of ERK1 and ERK2 cascade
- positive regulation of gene expression
- positive regulation of MAPK cascade
- positive regulation of peptidyl-serine phosphorylation
- positive regulation of protein kinase A signaling
- positive regulation of protein kinase B signaling
- protein destabilization
- protein homooligomerization
- sensory perception of pain
- signal transduction

---

97

- **Protein name:** CD180 antigen
- **Organism:** Homo sapiens
- **Uniprot Accession Number:** Q99467
- **Protein sequence length:** 661 aa
- **1D identity (%):** 11.95
- **1D identity (%) [Gaps excluded]:** 22.71
- **1D identity - Alignment Gaps:** 472
- **1D aligned content (<aminoacid>:%):** {'F': 5.88, 'S': 6.72, 'K': 5.88, 'M': 0.84, 'I': 4.2, 'Y': 3.36, 'E': 5.88, 'L': 21.01, 'P': 6.72, 'T': 5.88, 'N': 3.36, 'D': 4.2, 'G': 7.56, 'H': 5.04, 'R': 0.84, 'W': 0.84, 'A': 0.84, 'C': 3.36, 'Q': 4.2, 'V': 3.36}
- **Common reported functions (%):** 0.0
- **Common reported locations (%):** 7.14
- **Common reported processes (%):** 0.0

- **PDB ID:** 3B2D
- **Chain:** A
- **Crystallized protein length:** 601 aa
- **Resolution:** 2.8 Å
- **b-phipsi:** 0.010238
- **w-rdist:** 0.448186
- **t-alpha:** 0.031555
- **Chemical similarity (Tanimoto Index) (%):** 79.22
- **1D identity (%) [PDB]:** 1.27
- **1D identity (%) [Gaps excluded][PDB]:** 90.0
- **1D identity - Alignment Gaps [PDB]:** 1399
- **1D aligned content [PDB] (<aminoacid>:%):** {'Q': 11.11, 'R': 5.56, 'T': 16.67, 'P': 5.56, 'L': 22.22, 'S': 5.56, 'N': 11.11, 'K': 11.11, 'V': 5.56, 'G': 5.56}
- **2D identity (%) [PDB]:** 29.02
- **2D identity (%) [Gaps excluded][PDB]:** 80.58
- **2D identity - Alignment Gaps [PDB]:** 677
- **2D aligned content [PDB] (<2D-fold>:%):** {'.': 28.99, 'E': 30.62, 'T': 30.29, 'G': 2.93, 'H': 6.84, 'B': 0.33}
- **3D similarity (TM-Score) (%) [PDB]:** 20.03

- **Gene name:** CD180
- **Entrez ID:** 4064
- **RefSeq ID:** NM\_005582
- **Transcript sequence length:** 5388
- **5-UTR|CDS|3-UTR identity (%):** 32.98 | 42.01 | 16.24
- **5-UTR|CDS|3-UTR identity (%) [Gaps excluded]:** 67.02 | 74.24 | 75.13
- **5-UTR|CDS|3-UTR identity [Alignment Gaps]:** 97 | 1266 | 9752
- **5-UTR aligned content (<base>:%):** {'C': 52.38, 'G': 34.92, 'A': 1.59, 'T': 11.11}
- **CDS aligned content (<base>:%):** {'A': 26.94, 'G': 21.71, 'T': 23.1, 'C': 28.24}
- **3-UTR aligned content (<base>:%):** {'T': 31.17, 'C': 22.17, 'G': 20.63, 'A': 26.03}

**Uniprot Description:**  
  
 May cooperate with MD-1 and TLR4 to mediate the innate immune response to bacterial lipopolysaccharide (LPS) in B-cells. Leads to NF-kappa-B activation. Also involved in the life/death decision of B-cells (By similarity).   
  
M-shaped tetramer of two CD180-LY86 heterodimers.   
  
 **Gene Ontology Information:**

Molecular Function   
  
N/A

Location

- extracellular matrix
- extracellular space
- mitotic spindle
- nucleolus
- nucleoplasm
- plasma membrane

Biological process

- B cell proliferation involved in immune response
- cellular response to lipopolysaccharide
- inflammatory response
- innate immune response
- lipopolysaccharide-mediated signaling pathway
- positive regulation of lipopolysaccharide-mediated signaling pathway

---

98

- **Protein name:** Myosin-7
- **Organism:** Homo sapiens
- **Uniprot Accession Number:** P12883
- **Protein sequence length:** 1935 aa
- **1D identity (%):** 11.79
- **1D identity (%) [Gaps excluded]:** 26.91
- **1D identity - Alignment Gaps:** 1092
- **1D aligned content (<aminoacid>:%):** {'M': 2.18, 'A': 6.55, 'P': 5.68, 'L': 10.92, 'F': 3.93, 'G': 6.99, 'T': 6.11, 'K': 9.61, 'D': 5.24, 'I': 2.62, 'Y': 2.62, 'E': 9.61, 'V': 3.93, 'Q': 6.55, 'R': 6.55, 'H': 3.93, 'S': 3.06, 'N': 2.18, 'C': 1.75}
- **Common reported functions (%):** 0.0
- **Common reported locations (%):** 7.14
- **Common reported processes (%):** 0.0

- **PDB ID:** 4DB1
- **Chain:** A
- **Crystallized protein length:** 716 aa
- **Resolution:** 2.6 Å
- **b-phipsi:** 0.012226
- **w-rdist:** 0.49825
- **t-alpha:** 0.017791
- **Chemical similarity (Tanimoto Index) (%):** 74.21
- **1D identity (%) [PDB]:** 3.66
- **1D identity (%) [Gaps excluded][PDB]:** 65.85
- **1D identity - Alignment Gaps [PDB]:** 1395
- **1D aligned content [PDB] (<aminoacid>:%):** {'G': 7.41, 'D': 3.7, 'S': 3.7, 'E': 3.7, 'M': 5.56, 'F': 1.85, 'H': 1.85, 'L': 16.67, 'K': 5.56, 'T': 9.26, 'Y': 1.85, 'V': 14.81, 'P': 3.7, 'R': 5.56, 'A': 1.85, 'Q': 3.7, 'N': 5.56, 'I': 3.7}
- **2D identity (%) [PDB]:** 40.35
- **2D identity (%) [Gaps excluded][PDB]:** 89.09
- **2D identity - Alignment Gaps [PDB]:** 587
- **2D aligned content [PDB] (<2D-fold>:%):** {'.': 19.4, 'E': 21.02, 'T': 8.55, 'H': 50.12, 'B': 0.23, 'G': 0.69}
- **3D similarity (TM-Score) (%) [PDB]:** 25.12

- **Gene name:** MYH7
- **Entrez ID:** 4625
- **RefSeq ID:** N/A
- **Sequence length:** N/A
- **5-UTR|CDS|3-UTR identity (%):** N/A | N/A | N/A
- **5-UTR|CDS|3-UTR identity (%) [Gaps excluded]:** N/A | N/A | N/A
- **5-UTR|CDS|3-UTR identity [Alignment Gaps]:** N/A | N/A | N/A
- **5-UTR aligned content (<base>:%):** N/A
- **CDS aligned content (<base>:%):** N/A
- **3-UTR aligned content (<base>:%):** N/A

**Uniprot Description:**  
  
 Myosins are actin-based motor molecules with ATPase activity essential for muscle contraction. Forms regular bipolar thick filaments that, together with actin thin filaments, constitute the fundamental contractile unit of skeletal and cardiac muscle.   
  
Muscle myosin is a hexameric protein that consists of 2 heavy chain subunits (MHC), 2 alkali light chain subunits (MLC) and 2 regulatory light chain subunits (MLC-2). Interacts with ECPAS (PubMed:20682791). Interacts (via C-terminus) with LRRC39 (PubMed:20847312).   
  
 **Gene Ontology Information:**

Molecular Function

- actin filament binding
- ATP binding
- calmodulin binding
- microfilament motor activity

Location

- cytoplasm
- muscle myosin complex
- myofibril
- myosin complex
- myosin filament
- myosin II complex
- sarcomere
- stress fiber
- Z disc

Biological process

- adult heart development
- ATP metabolic process
- cardiac muscle contraction
- cardiac muscle hypertrophy in response to stress
- muscle contraction
- muscle filament sliding
- regulation of heart rate
- regulation of slow-twitch skeletal muscle fiber contraction
- regulation of the force of heart contraction
- regulation of the force of skeletal muscle contraction
- sarcomere organization
- skeletal muscle contraction
- striated muscle contraction
- transition between fast and slow fiber
- ventricular cardiac muscle tissue morphogenesis

---

99

- **Protein name:** DNA ligase 1
- **Organism:** Homo sapiens
- **Uniprot Accession Number:** P18858
- **Protein sequence length:** 919 aa
- **1D identity (%):** 16.84
- **1D identity (%) [Gaps excluded]:** 25.75
- **1D identity - Alignment Gaps:** 372
- **1D aligned content (<aminoacid>:%):** {'R': 9.39, 'P': 10.5, 'G': 9.39, 'K': 8.29, 'E': 9.94, 'W': 1.1, 'V': 5.52, 'S': 6.63, 'L': 9.94, 'A': 3.87, 'Q': 4.97, 'D': 3.87, 'T': 5.52, 'F': 2.21, 'Y': 2.21, 'I': 3.31, 'C': 1.66, 'N': 0.55, 'H': 1.1}
- **Common reported functions (%):** 6.67
- **Common reported locations (%):** 14.29
- **Common reported processes (%):** 0.0

- **PDB ID:** 7QO1
- **Chain:** A
- **Crystallized protein length:** 640 aa
- **Resolution:** 4.4 Å
- **b-phipsi:** 0.005512
- **w-rdist:** 0.758206
- **t-alpha:** 0.029133
- **Chemical similarity (Tanimoto Index) (%):** 99.17
- **1D identity (%) [PDB]:** 2.31
- **1D identity (%) [Gaps excluded][PDB]:** 64.71
- **1D identity - Alignment Gaps [PDB]:** 1376
- **1D aligned content [PDB] (<aminoacid>:%):** {'A': 9.09, 'R': 12.12, 'S': 12.12, 'P': 6.06, 'D': 6.06, 'Q': 6.06, 'E': 6.06, 'I': 3.03, 'L': 6.06, 'N': 3.03, 'T': 6.06, 'Y': 3.03, 'V': 9.09, 'G': 6.06, 'M': 3.03, 'K': 3.03}
- **2D identity (%) [PDB]:** 38.95
- **2D identity (%) [Gaps excluded][PDB]:** 88.69
- **2D identity - Alignment Gaps [PDB]:** 576
- **2D aligned content [PDB] (<2D-fold>:%):** {'T': 25.25, '.': 17.5, 'H': 44.25, 'G': 0.75, 'E': 12.25}
- **3D similarity (TM-Score) (%) [PDB]:** 23.3

- **Gene name:** LIG1
- **Entrez ID:** 3978
- **RefSeq ID:** NM\_001320971
- **Transcript sequence length:** 3035
- **5-UTR|CDS|3-UTR identity (%):** 46.63 | 41.54 | 1.07
- **5-UTR|CDS|3-UTR identity (%) [Gaps excluded]:** 75.45 | 75.52 | 74.85
- **5-UTR|CDS|3-UTR identity [Alignment Gaps]:** 68 | 1524 | 11750
- **5-UTR aligned content (<base>:%):** {'A': 4.82, 'G': 48.19, 'C': 40.96, 'T': 6.02}
- **CDS aligned content (<base>:%):** {'G': 31.34, 'C': 30.92, 'A': 24.52, 'T': 13.22}
- **3-UTR aligned content (<base>:%):** {'G': 33.59, 'C': 20.31, 'T': 32.03, 'A': 14.06}

**Uniprot Description:**  
  
 DNA ligase that seals nicks in double-stranded DNA during DNA replication, DNA recombination and DNA repair.   
  
Interacts with PCNA.   
  
 **Gene Ontology Information:**

Molecular Function

- ATP binding
- DNA binding
- DNA ligase (ATP) activity
- DNA ligase activity
- metal ion binding

Location

- intracellular membrane-bounded organelle
- mitochondrion
- nucleoplasm
- nucleus

Biological process

- anatomical structure morphogenesis
- base-excision repair
- base-excision repair, gap-filling
- cell division
- DNA biosynthetic process
- DNA ligation
- DNA recombination
- DNA repair
- lagging strand elongation
- mismatch repair
- Okazaki fragment processing involved in mitotic DNA replication

---

100

- **Protein name:** Anthrax toxin receptor 2
- **Organism:** Homo sapiens
- **Uniprot Accession Number:** P58335
- **Protein sequence length:** 489 aa
- **1D identity (%):** 10.02
- **1D identity (%) [Gaps excluded]:** 24.62
- **1D identity - Alignment Gaps:** 568
- **1D aligned content (<aminoacid>:%):** {'M': 1.04, 'P': 17.71, 'A': 3.12, 'G': 12.5, 'R': 5.21, 'L': 7.29, 'F': 4.17, 'D': 2.08, 'I': 5.21, 'Y': 2.08, 'V': 7.29, 'E': 6.25, 'Q': 4.17, 'K': 5.21, 'S': 6.25, 'T': 5.21, 'C': 3.12, 'W': 2.08}
- **Common reported functions (%):** 6.67
- **Common reported locations (%):** 0.0
- **Common reported processes (%):** 0.0

- **PDB ID:** 1TZN
- **Chain:** e
- **Crystallized protein length:** 181 aa
- **Resolution:** 4.3 Å
- **b-phipsi:** 0.006101
- **w-rdist:** 0.815846
- **t-alpha:** 0.021577
- **Chemical similarity (Tanimoto Index) (%):** 96.19
- **1D identity (%) [PDB]:** 1.6
- **1D identity (%) [Gaps excluded][PDB]:** 84.21
- **1D identity - Alignment Gaps [PDB]:** 981
- **1D aligned content [PDB] (<aminoacid>:%):** {'E': 18.75, 'V': 6.25, 'L': 12.5, 'D': 12.5, 'K': 12.5, 'S': 12.5, 'I': 6.25, 'Q': 12.5, 'T': 6.25}
- **2D identity (%) [PDB]:** 14.19
- **2D identity (%) [Gaps excluded][PDB]:** 85.52
- **2D identity - Alignment Gaps [PDB]:** 729
- **2D aligned content [PDB] (<2D-fold>:%):** {'.': 18.55, 'B': 0.81, 'E': 16.94, 'H': 54.84, 'T': 7.26, 'G': 1.61}
- **3D similarity (TM-Score) (%) [PDB]:** 17.57

- **Gene name:** ANTXR2
- **Entrez ID:** 118429
- **RefSeq ID:** NM\_058172
- **Transcript sequence length:** 8419
- **5-UTR|CDS|3-UTR identity (%):** 16.29 | 29.39 | 29.86
- **5-UTR|CDS|3-UTR identity (%) [Gaps excluded]:** 85.83 | 73.94 | 74.0
- **5-UTR|CDS|3-UTR identity [Alignment Gaps]:** 542 | 1745 | 7723
- **5-UTR aligned content (<base>:%):** {'C': 42.2, 'T': 9.17, 'G': 45.87, 'A': 2.75}
- **CDS aligned content (<base>:%):** {'A': 26.91, 'G': 29.73, 'C': 22.33, 'T': 21.03}
- **3-UTR aligned content (<base>:%):** {'A': 29.93, 'G': 18.65, 'C': 16.43, 'T': 35.0}

**Uniprot Description:**  
  
 Necessary for cellular interactions with laminin and the extracellular matrix.   
  
Binds laminin, and possibly also collagen type IV.   
  
 **Gene Ontology Information:**

Molecular Function

- metal ion binding
- transmembrane signaling receptor activity

Location

- cell surface
- endoplasmic reticulum membrane
- endosome membrane
- extracellular region
- plasma membrane

Biological process

- toxin transport

---
