# Supplementary material for: Comparative structural insights and functional analysis for the distinct unbound states of Human AGO proteins
Source: Sci Rep. 2025 Mar 19;15:9432. doi: 10.1038/s41598-025-91849-5 (PMC11923369; doi:10.1038/s41598-025-91849-5)
Supplement: Supplementary file 24 — Supplementary Information 12. [file 41598_2025_91849_MOESM24_ESM.zip › 4Z4Dp_A_mdwhole_HL2REF/candidates/4Z4Dp_A-merged-h-enriched_report.html]

 

# Structural Comparison Report for 4Z4Dp\_A - whole structures (total: 91)

---

0

- **PDB ID:** 4QCL | **Chain:** A
- **b-phipsi:** 0.0052602819231083
- **w-rdist:** 0.1532424937614729
- **t-alpha:** 0.0021848670241468

---

---

1

- **PDB ID:** 5IZ5 | **Chain:** B
- **b-phipsi:** 0.0030320553184234
- **w-rdist:** 0.3472313369506615
- **t-alpha:** 0.0029220273949486

---

---

2

- **PDB ID:** 8ASC | **Chain:** O
- **b-phipsi:** 0.0005980702607657
- **w-rdist:** 0.3060688185576871
- **t-alpha:** 0.009468275938171

---

---

3

- **PDB ID:** 7ZYG | **Chain:** A
- **b-phipsi:** 0.0032219258560405
- **w-rdist:** 0.3621111493603766
- **t-alpha:** 0.0029134192830806

---

---

4

- **PDB ID:** 4KXT | **Chain:** A
- **b-phipsi:** 0.0010555604683578
- **w-rdist:** 0.2605813557766685
- **t-alpha:** 0.0269484940117843

---

---

5

- **PDB ID:** 5C1B | **Chain:** C
- **b-phipsi:** 0.0057443587069979
- **w-rdist:** 0.2784551364116782
- **t-alpha:** 0.0087397720309874

---

---

6

- **PDB ID:** 4R08 | **Chain:** D
- **b-phipsi:** 0.0079139092159989
- **w-rdist:** 0.3596043897192201
- **t-alpha:** 0.0014568440116902

---

---

7

- **PDB ID:** 6MGQ | **Chain:** A
- **b-phipsi:** 0.0065742716102258
- **w-rdist:** 0.0805625123287563
- **t-alpha:** 0.0095587741674756

---

---

8

- **PDB ID:** 2E1Q | **Chain:** A
- **b-phipsi:** 0.0047876699429319
- **w-rdist:** 0.2688079670531421
- **t-alpha:** 0.0262200978972946

---

---

9

- **PDB ID:** 5JTV | **Chain:** E
- **b-phipsi:** 0.0021339208690932
- **w-rdist:** 0.6171310414770145
- **t-alpha:** 0.0036415440256147

---

---

10

- **PDB ID:** 6G6W | **Chain:** A
- **b-phipsi:** 0.0109141552535157
- **w-rdist:** 0.311373719308453
- **t-alpha:** 0.0021848670241468

---

---

11

- **PDB ID:** 7SOL | **Chain:** C
- **b-phipsi:** 0.003149308714924
- **w-rdist:** 0.1966770386493286
- **t-alpha:** 0.0648213974041245

---

---

12

- **PDB ID:** 5GVC | **Chain:** A
- **b-phipsi:** 0.0049697781005776
- **w-rdist:** 0.4822897873102144
- **t-alpha:** 0.004389354417605

---

---

13

- **PDB ID:** 7ABI | **Chain:** r
- **b-phipsi:** 0.0102888822125315
- **w-rdist:** 0.261249088681681
- **t-alpha:** 0.0058266934612722

---

---

14

- **PDB ID:** 6EJA | **Chain:** A
- **b-phipsi:** 0.0016791650121734
- **w-rdist:** 0.6445247037656157
- **t-alpha:** 0.0058607467412064

---

---

15

- **PDB ID:** 6R25 | **Chain:** K
- **b-phipsi:** 0.0032088551061344
- **w-rdist:** 0.4692199154627208
- **t-alpha:** 0.0117905783112548

---

---

16

- **PDB ID:** 2FJU | **Chain:** B
- **b-phipsi:** 0.0017902354555213
- **w-rdist:** 0.8041375136537998
- **t-alpha:** 0.0021848670241468

---

---

17

- **PDB ID:** 8A7D | **Chain:** C
- **b-phipsi:** 0.0199224340908551
- **w-rdist:** 0.1481366941676064
- **t-alpha:** 0.0043701973936818

---

---

18

- **PDB ID:** 5JPN | **Chain:** B
- **b-phipsi:** 0.0036755762352329
- **w-rdist:** 0.2326944077278183
- **t-alpha:** 0.0640930305320786

---

---

19

- **PDB ID:** 3IHP | **Chain:** B
- **b-phipsi:** 0.0066138707333892
- **w-rdist:** 0.2701840924377264
- **t-alpha:** 0.016284021422591

---

---

20

- **PDB ID:** 6ZYM | **Chain:** B
- **b-phipsi:** 0.0031300074509888
- **w-rdist:** 0.3375668956945657
- **t-alpha:** 0.0425208893008459

---

---

21

- **PDB ID:** 7RZI | **Chain:** B
- **b-phipsi:** 0.006237183781244
- **w-rdist:** 0.4898643332494637
- **t-alpha:** 0.0021896458253802

---

---

22

- **PDB ID:** 3ZD6 | **Chain:** A
- **b-phipsi:** 0.0189314617237864
- **w-rdist:** 0.2943614231531589
- **t-alpha:** 0.0021896458253802

---

---

23

- **PDB ID:** 8SBJ | **Chain:** A
- **b-phipsi:** 0.0084775377750693
- **w-rdist:** 0.4501191889213313
- **t-alpha:** 0.0014568440116902

---

---

24

- **PDB ID:** 4DUR | **Chain:** A
- **b-phipsi:** 0.018188432613707
- **w-rdist:** 0.2756299205476912
- **t-alpha:** 0.0036549441753579

---

---

25

- **PDB ID:** 6MZC | **Chain:** B
- **b-phipsi:** 0.0115753394578801
- **w-rdist:** 0.3117436352494714
- **t-alpha:** 0.0073366962737499

---

---

26

- **PDB ID:** 6C02 | **Chain:** A
- **b-phipsi:** 0.0007376179573695
- **w-rdist:** 0.5105109469458755
- **t-alpha:** 0.0292354274430095

---

---

27

- **PDB ID:** 7DVQ | **Chain:** C
- **b-phipsi:** 0.0039369480106831
- **w-rdist:** 0.468305861563199
- **t-alpha:** 0.0230999264545257

---

---

28

- **PDB ID:** 1N52 | **Chain:** A
- **b-phipsi:** 0.0801549999786274
- **w-rdist:** 0.2865617244247226
- **t-alpha:** 0.004389354417605

---

---

29

- **PDB ID:** 7BW8 | **Chain:** A
- **b-phipsi:** 0.0227288800861709
- **w-rdist:** 0.4183810893771259
- **t-alpha:** 0.0014568440116902

---

---

30

- **PDB ID:** 2P8S | **Chain:** A
- **b-phipsi:** 0.0052919240944148
- **w-rdist:** 0.8337673629196358
- **t-alpha:** 0.0

---

---

31

- **PDB ID:** 8P4E | **Chain:** O
- **b-phipsi:** 0.0015623217896402
- **w-rdist:** 0.5349155238952266
- **t-alpha:** 0.0284642139866488

---

---

32

- **PDB ID:** 7WVJ | **Chain:** B
- **b-phipsi:** 0.0083610224946656
- **w-rdist:** 0.62689800431722
- **t-alpha:** 0.0

---

---

33

- **PDB ID:** 6U23 | **Chain:** O
- **b-phipsi:** 0.0120946829329232
- **w-rdist:** 0.2966643935402481
- **t-alpha:** 0.0110454531302417

---

---

34

- **PDB ID:** 8IF3 | **Chain:** A
- **b-phipsi:** 0.0055503717212547
- **w-rdist:** 0.4294520455628693
- **t-alpha:** 0.0246270663199015

---

---

35

- **PDB ID:** 7T3B | **Chain:** A
- **b-phipsi:** 0.0034564606988133
- **w-rdist:** 0.4437840218775543
- **t-alpha:** 0.0349600148426103

---

---

36

- **PDB ID:** 3L4G | **Chain:** H
- **b-phipsi:** 0.0021963697476728
- **w-rdist:** 0.682222061702094
- **t-alpha:** 0.0140326350426887

---

---

37

- **PDB ID:** 5LS6 | **Chain:** J
- **b-phipsi:** 0.0057099855316525
- **w-rdist:** 0.6884664584779117
- **t-alpha:** 0.0021896458253802

---

---

38

- **PDB ID:** 5VNE | **Chain:** B
- **b-phipsi:** 0.0033215267946985
- **w-rdist:** 0.7358747829081145
- **t-alpha:** 0.0095587741674756

---

---

39

- **PDB ID:** 7SVO | **Chain:** A
- **b-phipsi:** 0.0056033376209954
- **w-rdist:** 0.6708326118129316
- **t-alpha:** 0.0050984212340619

---

---

40

- **PDB ID:** 3TON | **Chain:** A
- **b-phipsi:** 0.0052340752051919
- **w-rdist:** 0.6800815560577769
- **t-alpha:** 0.0073366962737499

---

---

41

- **PDB ID:** 6OON | **Chain:** A
- **b-phipsi:** 0.0078963214233278
- **w-rdist:** 0.2377556069108367
- **t-alpha:** 0.0433130906697325

---

---

42

- **PDB ID:** 3CDZ | **Chain:** A
- **b-phipsi:** 0.0409261798605325
- **w-rdist:** 0.3625017793524294
- **t-alpha:** 0.0050984212340619

---

---

43

- **PDB ID:** 5UY8 | **Chain:** A
- **b-phipsi:** 0.0057728200153513
- **w-rdist:** 0.7333446883418808
- **t-alpha:** 0.0036415440256147

---

---

44

- **PDB ID:** 6AHU | **Chain:** B
- **b-phipsi:** 0.0152091642492406
- **w-rdist:** 0.4022534939194734
- **t-alpha:** 0.0088172167453031

---

---

45

- **PDB ID:** 8FCN | **Chain:** A
- **b-phipsi:** 0.0063648125063553
- **w-rdist:** 0.280749056069955
- **t-alpha:** 0.0786598128498576

---

---

46

- **PDB ID:** 4AH6 | **Chain:** B
- **b-phipsi:** 0.0041149947226531
- **w-rdist:** 0.3985134643298203
- **t-alpha:** 0.1291118505353805

---

---

47

- **PDB ID:** 2VXO | **Chain:** A
- **b-phipsi:** 0.0042252717997938
- **w-rdist:** 0.4845802859761593
- **t-alpha:** 0.0323309912234826

---

---

48

- **PDB ID:** 3W1B | **Chain:** A
- **b-phipsi:** 0.0020721104241361
- **w-rdist:** 0.5148342345425531
- **t-alpha:** 0.0496939342883562

---

---

49

- **PDB ID:** 6DDQ | **Chain:** A
- **b-phipsi:** 0.006551661874528
- **w-rdist:** 0.713402310661824
- **t-alpha:** 0.0029220273949486

---

---

50

- **PDB ID:** 3SWR | **Chain:** A
- **b-phipsi:** 0.0067446315950706
- **w-rdist:** 0.4476356608627592
- **t-alpha:** 0.0189367966891196

---

---

51

- **PDB ID:** 4QJ3 | **Chain:** B
- **b-phipsi:** 0.0009017506954001
- **w-rdist:** 0.6540910365774196
- **t-alpha:** 0.0417297861962435

---

---

52

- **PDB ID:** 5J68 | **Chain:** A
- **b-phipsi:** 0.0048332320493505
- **w-rdist:** 0.4075067092592313
- **t-alpha:** 0.0931528332891025

---

---

53

- **PDB ID:** 8DP0 | **Chain:** A
- **b-phipsi:** 0.0104611811792592
- **w-rdist:** 0.3670495494254605
- **t-alpha:** 0.0196648853517085

---

---

54

- **PDB ID:** 6AYG | **Chain:** A
- **b-phipsi:** 0.0403482701696468
- **w-rdist:** 0.3668206771691688
- **t-alpha:** 0.0088172167453031

---

---

55

- **PDB ID:** 6JT1 | **Chain:** B
- **b-phipsi:** 0.012437387277431
- **w-rdist:** 0.356207525438995
- **t-alpha:** 0.0182082568765367

---

---

56

- **PDB ID:** 6XOV | **Chain:** A
- **b-phipsi:** 0.0096622648150704
- **w-rdist:** 0.43950642934174
- **t-alpha:** 0.0160233389937762

---

---

57

- **PDB ID:** 7JN7 | **Chain:** A
- **b-phipsi:** 0.0084599501797507
- **w-rdist:** 0.7363360062143126
- **t-alpha:** 0.0014587541408541

---

---

58

- **PDB ID:** 3FBY | **Chain:** C
- **b-phipsi:** 0.0429211591362305
- **w-rdist:** 0.4367648143683592
- **t-alpha:** 0.0050984212340619

---

---

59

- **PDB ID:** 5O9Z | **Chain:** B
- **b-phipsi:** 0.0054056860766018
- **w-rdist:** 0.4333974385966259
- **t-alpha:** 0.0529142885263107

---

---

60

- **PDB ID:** 5VM9 | **Chain:** A
- **b-phipsi:** 0.0098333585689871
- **w-rdist:** 0.1646792217983402
- **t-alpha:** 0.0651667209447612

---

---

61

- **PDB ID:** 4ENZ | **Chain:** A
- **b-phipsi:** 0.0170276843173366
- **w-rdist:** 0.62028526769934
- **t-alpha:** 0.0014568440116902

---

---

62

- **PDB ID:** 2WJY | **Chain:** A
- **b-phipsi:** 0.0039087567947213
- **w-rdist:** 0.4805266332420173
- **t-alpha:** 0.0879557950251648

---

---

63

- **PDB ID:** 6GYR | **Chain:** B
- **b-phipsi:** 0.0049669726530023
- **w-rdist:** 0.4972936216569463
- **t-alpha:** 0.0417297861962435

---

---

64

- **PDB ID:** 7ORC | **Chain:** A
- **b-phipsi:** 0.0064546585505066
- **w-rdist:** 0.3523603262817474
- **t-alpha:** 0.0990536816248905

---

---

65

- **PDB ID:** 5DYH | **Chain:** B
- **b-phipsi:** 0.0040487073325827
- **w-rdist:** 0.5219337500575548
- **t-alpha:** 0.0521072715569208

---

---

66

- **PDB ID:** 1SC7 | **Chain:** A
- **b-phipsi:** 0.0153778411858484
- **w-rdist:** 0.4749967934077385
- **t-alpha:** 0.0088172167453031

---

---

67

- **PDB ID:** 5KYU | **Chain:** B
- **b-phipsi:** 0.001333696970895
- **w-rdist:** 0.7418063394045575
- **t-alpha:** 0.0441063918932529

---

---

68

- **PDB ID:** 7P7P | **Chain:** B
- **b-phipsi:** 0.0092503221063408
- **w-rdist:** 0.664382914778899
- **t-alpha:** 0.00512430205411

---

---

69

- **PDB ID:** 5ZEN | **Chain:** A
- **b-phipsi:** 0.0153328163897711
- **w-rdist:** 0.4371163882566072
- **t-alpha:** 0.0167517699463162

---

---

70

- **PDB ID:** 3KRW | **Chain:** A
- **b-phipsi:** 0.0110372202154018
- **w-rdist:** 0.4149177348015097
- **t-alpha:** 0.0284642139866488

---

---

71

- **PDB ID:** 8OG4 | **Chain:** A
- **b-phipsi:** 0.0063192544644425
- **w-rdist:** 0.4028041860919502
- **t-alpha:** 0.1054749257285665

---

---

72

- **PDB ID:** 5MHL | **Chain:** A
- **b-phipsi:** 0.0176565554138629
- **w-rdist:** 0.3010339024123524
- **t-alpha:** 0.0354447525832375

---

---

73

- **PDB ID:** 4ASI | **Chain:** C
- **b-phipsi:** 0.008575235384088
- **w-rdist:** 0.671675210599459
- **t-alpha:** 0.0080117938638943

---

---

74

- **PDB ID:** 4ZG6 | **Chain:** A
- **b-phipsi:** 0.00648172526878
- **w-rdist:** 0.5348965119551472
- **t-alpha:** 0.0261584429252026

---

---

75

- **PDB ID:** 2C11 | **Chain:** D
- **b-phipsi:** 0.0114024099478992
- **w-rdist:** 0.790088959484816
- **t-alpha:** 0.0014587541408541

---

---

76

- **PDB ID:** 7QPG | **Chain:** B
- **b-phipsi:** 0.0041519022491283
- **w-rdist:** 0.4954040309124952
- **t-alpha:** 0.1081517725047538

---

---

77

- **PDB ID:** 5NV6 | **Chain:** A
- **b-phipsi:** 0.0037337485851924
- **w-rdist:** 0.7842280617749704
- **t-alpha:** 0.0315549988173708

---

---

78

- **PDB ID:** 3LPP | **Chain:** C
- **b-phipsi:** 0.0072146550713031
- **w-rdist:** 0.6942759785383008
- **t-alpha:** 0.0117905783112548

---

---

79

- **PDB ID:** 3FXI | **Chain:** A
- **b-phipsi:** 0.0105015405884777
- **w-rdist:** 0.493777358377487
- **t-alpha:** 0.0185457488706886

---

---

80

- **PDB ID:** 6DC6 | **Chain:** C
- **b-phipsi:** 0.0052463124615591
- **w-rdist:** 0.6496240675807382
- **t-alpha:** 0.0371451014582198

---

---

81

- **PDB ID:** 7PPJ | **Chain:** A
- **b-phipsi:** 0.0083746234839353
- **w-rdist:** 0.4918478431173556
- **t-alpha:** 0.0307805727451215

---

---

82

- **PDB ID:** 6Y0F | **Chain:** C
- **b-phipsi:** 0.0073173359976774
- **w-rdist:** 0.8352570999389719
- **t-alpha:** 0.0080762040238695

---

---

83

- **PDB ID:** 7BG0 | **Chain:** A
- **b-phipsi:** 0.0119853114610755
- **w-rdist:** 0.4782555612941439
- **t-alpha:** 0.0223381852496014

---

---

84

- **PDB ID:** 3B2D | **Chain:** A
- **b-phipsi:** 0.0102377638555675
- **w-rdist:** 0.448185700277658
- **t-alpha:** 0.0315549988173708

---

---

85

- **PDB ID:** 4DB1 | **Chain:** A
- **b-phipsi:** 0.012225842401641
- **w-rdist:** 0.4982495550087842
- **t-alpha:** 0.0177907956813867

---

---

86

- **PDB ID:** 7QO1 | **Chain:** A
- **b-phipsi:** 0.005512119943936
- **w-rdist:** 0.7582056725245028
- **t-alpha:** 0.0291333507443458

---

---

87

- **PDB ID:** 1TZN | **Chain:** e
- **b-phipsi:** 0.0061012918609379
- **w-rdist:** 0.815846481811475
- **t-alpha:** 0.0215774983192449

---

---

88

- **PDB ID:** 7ZSC | **Chain:** D
- **b-phipsi:** 0.0063464243387128
- **w-rdist:** 0.545919563815706
- **t-alpha:** 0.0464939086507718

---

---

89

- **PDB ID:** 6TNY | **Chain:** A
- **b-phipsi:** 0.0060604704440685
- **w-rdist:** 0.7579842308793633
- **t-alpha:** 0.0291333507443458

---

---

90

- **PDB ID:** 7SCH | **Chain:** B
- **b-phipsi:** 0.0091820463586081
- **w-rdist:** 0.5279334007471008
- **t-alpha:** 0.0313184298622246

---

---
