# Supplementary material for: Comparative structural insights and functional analysis for the distinct unbound states of Human AGO proteins
Source: Sci Rep. 2025 Mar 19;15:9432. doi: 10.1038/s41598-025-91849-5 (PMC11923369; doi:10.1038/s41598-025-91849-5)
Supplement: Supplementary file 24 — Supplementary Information 12. [file 41598_2025_91849_MOESM24_ESM.zip › 4Z4Dp_A_mdwhole_HL2REF/candidates/4Z4Dp_A-merged-notenriched_report.html]

 

# Structural Comparison Report for 4Z4Dp\_A - whole structures (total: 800)

---

1

- **PDB ID:** 4W5Q | **Chain:** A
- **b-phipsi:** 0.0024376811210827
- **w-rdist:** 0.0712594206321134
- **t-alpha:** 0.0029134192830806

---

---

2

- **PDB ID:** 4Z4E | **Chain:** A
- **b-phipsi:** 0.0009583645252032
- **w-rdist:** 0.112675871592887
- **t-alpha:** 0.0095587741674756

---

---

3

- **PDB ID:** 4QCL | **Chain:** A
- **b-phipsi:** 0.0052602819231083
- **w-rdist:** 0.1532424937614729
- **t-alpha:** 0.0021848670241468

---

---

4

- **PDB ID:** 4W5T | **Chain:** A
- **b-phipsi:** 0.0023490492760846
- **w-rdist:** 0.1569973617400714
- **t-alpha:** 0.0087397720309874

---

---

5

- **PDB ID:** 8ASC | **Chain:** O
- **b-phipsi:** 0.0005980702607657
- **w-rdist:** 0.3060688185576871
- **t-alpha:** 0.009468275938171

---

---

6

- **PDB ID:** 5IZ5 | **Chain:** B
- **b-phipsi:** 0.0030320553184234
- **w-rdist:** 0.3472313369506615
- **t-alpha:** 0.0029220273949486

---

---

7

- **PDB ID:** 7NFC | **Chain:** G
- **b-phipsi:** 0.0014581401456665
- **w-rdist:** 0.3040006103502842
- **t-alpha:** 0.0110454531302417

---

---

8

- **PDB ID:** 4Z4G | **Chain:** A
- **b-phipsi:** 0.0019927414636392
- **w-rdist:** 0.0957410921426041
- **t-alpha:** 0.0218498381101377

---

---

9

- **PDB ID:** 7ZYG | **Chain:** A
- **b-phipsi:** 0.0032219258560405
- **w-rdist:** 0.3621111493603766
- **t-alpha:** 0.0029134192830806

---

---

10

- **PDB ID:** 4W5R | **Chain:** A
- **b-phipsi:** 0.0027408691800642
- **w-rdist:** 0.1458696441128715
- **t-alpha:** 0.0200592812463542

---

---

11

- **PDB ID:** 4W5N | **Chain:** A
- **b-phipsi:** 0.0028605666759252
- **w-rdist:** 0.3237548512958296
- **t-alpha:** 0.0080762040238695

---

---

12

- **PDB ID:** 6MGQ | **Chain:** A
- **b-phipsi:** 0.0065742716102258
- **w-rdist:** 0.0805625123287563
- **t-alpha:** 0.0095587741674756

---

---

13

- **PDB ID:** 6CBD | **Chain:** A
- **b-phipsi:** 0.0012616759437286
- **w-rdist:** 0.0896910076364217
- **t-alpha:** 0.0269259686475575

---

---

14

- **PDB ID:** 8D71 | **Chain:** A
- **b-phipsi:** 0.0029815522646256
- **w-rdist:** 0.3745972108237999
- **t-alpha:** 0.004389354417605

---

---

15

- **PDB ID:** 5C1B | **Chain:** C
- **b-phipsi:** 0.0057443587069979
- **w-rdist:** 0.2784551364116782
- **t-alpha:** 0.0087397720309874

---

---

16

- **PDB ID:** 8ASC | **Chain:** E
- **b-phipsi:** 0.0011673809414168
- **w-rdist:** 0.3096656591432542
- **t-alpha:** 0.016284021422591

---

---

17

- **PDB ID:** 7ZWA | **Chain:** A
- **b-phipsi:** 0.0024490735410098
- **w-rdist:** 0.3248848116093839
- **t-alpha:** 0.0110454531302417

---

---

18

- **PDB ID:** 6MGQ | **Chain:** C
- **b-phipsi:** 0.0072875138723614
- **w-rdist:** 0.1337321339385553
- **t-alpha:** 0.0072833804367331

---

---

19

- **PDB ID:** 8EZA | **Chain:** A
- **b-phipsi:** 0.0036290260231332
- **w-rdist:** 0.3271555546725032
- **t-alpha:** 0.011653569134763

---

---

20

- **PDB ID:** 7NFC | **Chain:** C
- **b-phipsi:** 0.0062597509313172
- **w-rdist:** 0.3224618638309195
- **t-alpha:** 0.004389354417605

---

---

21

- **PDB ID:** 3ZIM | **Chain:** A
- **b-phipsi:** 0.008216405752952
- **w-rdist:** 0.2302620379289736
- **t-alpha:** 0.0

---

---

22

- **PDB ID:** 4Z4D | **Chain:** A
- **b-phipsi:** 0.0036071618041506
- **w-rdist:** 0.0980057887240716
- **t-alpha:** 0.0284642139866488

---

---

23

- **PDB ID:** 4Z4H | **Chain:** A
- **b-phipsi:** 0.0017159400225969
- **w-rdist:** 0.1105202956093712
- **t-alpha:** 0.032046507746164

---

---

24

- **PDB ID:** 4OLA | **Chain:** A
- **b-phipsi:** 0.0012724423327829
- **w-rdist:** 0.3696826710439034
- **t-alpha:** 0.0145665909728742

---

---

25

- **PDB ID:** 4Z4C | **Chain:** A
- **b-phipsi:** 0.0048726330163714
- **w-rdist:** 0.0993660126596962
- **t-alpha:** 0.0269484940117843

---

---

26

- **PDB ID:** 8EZA | **Chain:** J
- **b-phipsi:** 0.0029427060470615
- **w-rdist:** 0.3309073760090678
- **t-alpha:** 0.0160233389937762

---

---

27

- **PDB ID:** 4KXT | **Chain:** A
- **b-phipsi:** 0.0010555604683578
- **w-rdist:** 0.2605813557766685
- **t-alpha:** 0.0269484940117843

---

---

28

- **PDB ID:** 6N4O | **Chain:** A
- **b-phipsi:** 0.005079801077739
- **w-rdist:** 0.1423170914320043
- **t-alpha:** 0.0261584429252026

---

---

29

- **PDB ID:** 4W5O | **Chain:** A
- **b-phipsi:** 0.0029983179932889
- **w-rdist:** 0.0972587178478639
- **t-alpha:** 0.0327750996298437

---

---

30

- **PDB ID:** 4TUU | **Chain:** A
- **b-phipsi:** 0.0074568917585154
- **w-rdist:** 0.2197998138205177
- **t-alpha:** 0.0087397720309874

---

---

31

- **PDB ID:** 7NFC | **Chain:** B
- **b-phipsi:** 0.0029249474160694
- **w-rdist:** 0.3206311659029561
- **t-alpha:** 0.0200592812463542

---

---

32

- **PDB ID:** 4Z4I | **Chain:** A
- **b-phipsi:** 0.0016404003286257
- **w-rdist:** 0.0954670779927621
- **t-alpha:** 0.037792668599949

---

---

33

- **PDB ID:** 5GVC | **Chain:** A
- **b-phipsi:** 0.0049697781005776
- **w-rdist:** 0.4822897873102144
- **t-alpha:** 0.004389354417605

---

---

34

- **PDB ID:** 3IHP | **Chain:** B
- **b-phipsi:** 0.0066138707333892
- **w-rdist:** 0.2701840924377264
- **t-alpha:** 0.016284021422591

---

---

35

- **PDB ID:** 2E1Q | **Chain:** A
- **b-phipsi:** 0.0047876699429319
- **w-rdist:** 0.2688079670531421
- **t-alpha:** 0.0262200978972946

---

---

36

- **PDB ID:** 7ZT6 | **Chain:** B
- **b-phipsi:** 0.0012205176415049
- **w-rdist:** 0.522562625946333
- **t-alpha:** 0.0072833804367331

---

---

37

- **PDB ID:** 6ZHA | **Chain:** C
- **b-phipsi:** 0.00480741322004
- **w-rdist:** 0.5086370414515695
- **t-alpha:** 0.0036549441753579

---

---

38

- **PDB ID:** 6MFR | **Chain:** A
- **b-phipsi:** 0.0039837716683323
- **w-rdist:** 0.3173691589306231
- **t-alpha:** 0.0240350781259224

---

---

39

- **PDB ID:** 6ERH | **Chain:** B
- **b-phipsi:** 0.0041489526684541
- **w-rdist:** 0.2302158489207952
- **t-alpha:** 0.0313184298622246

---

---

40

- **PDB ID:** 7N2M | **Chain:** A
- **b-phipsi:** 0.0069751283791954
- **w-rdist:** 0.1795674262659495
- **t-alpha:** 0.020393494275321

---

---

41

- **PDB ID:** 6PYS | **Chain:** A
- **b-phipsi:** 0.0097735924986866
- **w-rdist:** 0.2229841332546117
- **t-alpha:** 0.0050984212340619

---

---

42

- **PDB ID:** 7R9Y | **Chain:** A
- **b-phipsi:** 0.0115002412189747
- **w-rdist:** 0.2014133069381798
- **t-alpha:** 0.0014568440116902

---

---

43

- **PDB ID:** 6R25 | **Chain:** K
- **b-phipsi:** 0.0032088551061344
- **w-rdist:** 0.4692199154627208
- **t-alpha:** 0.0117905783112548

---

---

44

- **PDB ID:** 4R08 | **Chain:** D
- **b-phipsi:** 0.0079139092159989
- **w-rdist:** 0.3596043897192201
- **t-alpha:** 0.0014568440116902

---

---

45

- **PDB ID:** 6MFN | **Chain:** A
- **b-phipsi:** 0.001451180693035
- **w-rdist:** 0.1606493689005715
- **t-alpha:** 0.0451564268098936

---

---

46

- **PDB ID:** 4L2Y | **Chain:** A
- **b-phipsi:** 0.0058712508673961
- **w-rdist:** 0.404470999835619
- **t-alpha:** 0.010924934303695

---

---

47

- **PDB ID:** 7KI3 | **Chain:** A
- **b-phipsi:** 0.0034928604436058
- **w-rdist:** 0.3118819787629631
- **t-alpha:** 0.0276764270621712

---

---

48

- **PDB ID:** 7SGL | **Chain:** B
- **b-phipsi:** 0.0031807793472151
- **w-rdist:** 0.3306217496770306
- **t-alpha:** 0.0261584429252026

---

---

49

- **PDB ID:** 3IHP | **Chain:** A
- **b-phipsi:** 0.0066644436075326
- **w-rdist:** 0.261517237046786
- **t-alpha:** 0.0200592812463542

---

---

50

- **PDB ID:** 7RZI | **Chain:** B
- **b-phipsi:** 0.006237183781244
- **w-rdist:** 0.4898643332494637
- **t-alpha:** 0.0021896458253802

---

---

51

- **PDB ID:** 4FWE | **Chain:** A
- **b-phipsi:** 0.0043818452664217
- **w-rdist:** 0.4394865189768218
- **t-alpha:** 0.0152949457801805

---

---

52

- **PDB ID:** 3HIZ | **Chain:** A
- **b-phipsi:** 0.0108498973289656
- **w-rdist:** 0.2459380130338126
- **t-alpha:** 0.0036415440256147

---

---

53

- **PDB ID:** 4FWF | **Chain:** A
- **b-phipsi:** 0.005397724296864
- **w-rdist:** 0.448598122198495
- **t-alpha:** 0.0131099992438439

---

---

54

- **PDB ID:** 6B3Q | **Chain:** B
- **b-phipsi:** 0.0061307400127199
- **w-rdist:** 0.5041164759055488
- **t-alpha:** 0.0029220273949486

---

---

55

- **PDB ID:** 7WUB | **Chain:** G
- **b-phipsi:** 0.0069378990657235
- **w-rdist:** 0.1278491433257975
- **t-alpha:** 0.0284048760941568

---

---

56

- **PDB ID:** 7ABI | **Chain:** r
- **b-phipsi:** 0.0102888822125315
- **w-rdist:** 0.261249088681681
- **t-alpha:** 0.0058266934612722

---

---

57

- **PDB ID:** 7K71 | **Chain:** A
- **b-phipsi:** 0.013487903122695
- **w-rdist:** 0.162970501088405
- **t-alpha:** 0.0043701973936818

---

---

58

- **PDB ID:** 5C1B | **Chain:** A
- **b-phipsi:** 0.0071458747952924
- **w-rdist:** 0.2181593000811512
- **t-alpha:** 0.0233067966402911

---

---

59

- **PDB ID:** 3MDJ | **Chain:** C
- **b-phipsi:** 0.0102769379722238
- **w-rdist:** 0.3043619332099617
- **t-alpha:** 0.0036549441753579

---

---

60

- **PDB ID:** 5UL1 | **Chain:** A
- **b-phipsi:** 0.0065623495672057
- **w-rdist:** 0.4373304169226378
- **t-alpha:** 0.0088172167453031

---

---

61

- **PDB ID:** 4TV3 | **Chain:** A
- **b-phipsi:** 0.0090803017655319
- **w-rdist:** 0.2458509093610573
- **t-alpha:** 0.011653569134763

---

---

62

- **PDB ID:** 6ERF | **Chain:** E
- **b-phipsi:** 0.0015958641478748
- **w-rdist:** 0.5302005954703963
- **t-alpha:** 0.0123817636541396

---

---

63

- **PDB ID:** 6G6W | **Chain:** A
- **b-phipsi:** 0.0109141552535157
- **w-rdist:** 0.311373719308453
- **t-alpha:** 0.0021848670241468

---

---

64

- **PDB ID:** 7ABI | **Chain:** R
- **b-phipsi:** 0.0092657233445036
- **w-rdist:** 0.2622934035974124
- **t-alpha:** 0.011653569134763

---

---

65

- **PDB ID:** 3N56 | **Chain:** B
- **b-phipsi:** 0.0060807077395947
- **w-rdist:** 0.5060977809860514
- **t-alpha:** 0.0072833804367331

---

---

66

- **PDB ID:** 7KI3 | **Chain:** D
- **b-phipsi:** 0.0079177860289213
- **w-rdist:** 0.1694741482852863
- **t-alpha:** 0.0238627234462787

---

---

67

- **PDB ID:** 7LT3 | **Chain:** A
- **b-phipsi:** 0.0067438833500739
- **w-rdist:** 0.3491179592886733
- **t-alpha:** 0.0177907956813867

---

---

68

- **PDB ID:** 7ZWA | **Chain:** B
- **b-phipsi:** 0.0011944803844036
- **w-rdist:** 0.5367033348466393
- **t-alpha:** 0.0138382752386696

---

---

69

- **PDB ID:** 4F3T | **Chain:** A
- **b-phipsi:** 0.0032482207979658
- **w-rdist:** 0.2810733070203272
- **t-alpha:** 0.0386015818388498

---

---

70

- **PDB ID:** 5C1B | **Chain:** D
- **b-phipsi:** 0.0076639235043833
- **w-rdist:** 0.1980612349967487
- **t-alpha:** 0.0247633892798655

---

---

71

- **PDB ID:** 8AG5 | **Chain:** B
- **b-phipsi:** 0.0039451055119231
- **w-rdist:** 0.5456095515594173
- **t-alpha:** 0.0080762040238695

---

---

72

- **PDB ID:** 6ERG | **Chain:** B
- **b-phipsi:** 0.0012590566484775
- **w-rdist:** 0.2608727732656647
- **t-alpha:** 0.0560815732405199

---

---

73

- **PDB ID:** 8FCO | **Chain:** B
- **b-phipsi:** 0.0073774659053547
- **w-rdist:** 0.3364925868307021
- **t-alpha:** 0.0160233389937762

---

---

74

- **PDB ID:** 6ERG | **Chain:** E
- **b-phipsi:** 0.0011537584547189
- **w-rdist:** 0.2550614255728027
- **t-alpha:** 0.0582668457273456

---

---

75

- **PDB ID:** 8D96 | **Chain:** C
- **b-phipsi:** 0.0030706846042551
- **w-rdist:** 0.2799923983402735
- **t-alpha:** 0.0449007945645392

---

---

76

- **PDB ID:** 4Z4F | **Chain:** A
- **b-phipsi:** 0.0046893563479259
- **w-rdist:** 0.0763567016827712
- **t-alpha:** 0.0626935301476931

---

---

77

- **PDB ID:** 5JTV | **Chain:** E
- **b-phipsi:** 0.0021339208690932
- **w-rdist:** 0.6171310414770145
- **t-alpha:** 0.0036415440256147

---

---

78

- **PDB ID:** 8SBJ | **Chain:** A
- **b-phipsi:** 0.0084775377750693
- **w-rdist:** 0.4501191889213313
- **t-alpha:** 0.0014568440116902

---

---

79

- **PDB ID:** 6MGQ | **Chain:** B
- **b-phipsi:** 0.0115018187758333
- **w-rdist:** 0.2337249187959184
- **t-alpha:** 0.0101968242656327

---

---

80

- **PDB ID:** 7AAV | **Chain:** r
- **b-phipsi:** 0.0113727100541058
- **w-rdist:** 0.2449235838679932
- **t-alpha:** 0.0101968242656327

---

---

81

- **PDB ID:** 6PYR | **Chain:** A
- **b-phipsi:** 0.0102810447373386
- **w-rdist:** 0.3490673668959869
- **t-alpha:** 0.004389354417605

---

---

82

- **PDB ID:** 6PYU | **Chain:** A
- **b-phipsi:** 0.0092786421897528
- **w-rdist:** 0.3206012351035331
- **t-alpha:** 0.0095587741674756

---

---

83

- **PDB ID:** 7DVQ | **Chain:** C
- **b-phipsi:** 0.0039369480106831
- **w-rdist:** 0.468305861563199
- **t-alpha:** 0.0230999264545257

---

---

84

- **PDB ID:** 5UKJ | **Chain:** A
- **b-phipsi:** 0.0076750380363397
- **w-rdist:** 0.4022758124053051
- **t-alpha:** 0.010924934303695

---

---

85

- **PDB ID:** 7SOL | **Chain:** C
- **b-phipsi:** 0.003149308714924
- **w-rdist:** 0.1966770386493286
- **t-alpha:** 0.0648213974041245

---

---

86

- **PDB ID:** 5JS1 | **Chain:** A
- **b-phipsi:** 0.0013910228866128
- **w-rdist:** 0.2844116068983141
- **t-alpha:** 0.058994759372676

---

---

87

- **PDB ID:** 8D6J | **Chain:** A
- **b-phipsi:** 0.000918217136535
- **w-rdist:** 0.3324121528718834
- **t-alpha:** 0.0495267695540877

---

---

88

- **PDB ID:** 8IF3 | **Chain:** A
- **b-phipsi:** 0.0055503717212547
- **w-rdist:** 0.4294520455628693
- **t-alpha:** 0.0246270663199015

---

---

89

- **PDB ID:** 2E1Q | **Chain:** B
- **b-phipsi:** 0.0046957591516663
- **w-rdist:** 0.2726009702392985
- **t-alpha:** 0.0480699609170811

---

---

90

- **PDB ID:** 8ASC | **Chain:** F
- **b-phipsi:** 0.0015410447544579
- **w-rdist:** 0.5751319656219812
- **t-alpha:** 0.0110454531302417

---

---

91

- **PDB ID:** 6EJA | **Chain:** A
- **b-phipsi:** 0.0016791650121734
- **w-rdist:** 0.6445247037656157
- **t-alpha:** 0.0058607467412064

---

---

92

- **PDB ID:** 7ZYG | **Chain:** B
- **b-phipsi:** 0.0024536218805701
- **w-rdist:** 0.3301269492084282
- **t-alpha:** 0.0451564268098936

---

---

93

- **PDB ID:** 6ERF | **Chain:** D
- **b-phipsi:** 0.0042653838934358
- **w-rdist:** 0.5194499096378797
- **t-alpha:** 0.0189367966891196

---

---

94

- **PDB ID:** 6MZC | **Chain:** B
- **b-phipsi:** 0.0115753394578801
- **w-rdist:** 0.3117436352494714
- **t-alpha:** 0.0073366962737499

---

---

95

- **PDB ID:** 4R07 | **Chain:** D
- **b-phipsi:** 0.0088760127144803
- **w-rdist:** 0.3614459228721989
- **t-alpha:** 0.010924934303695

---

---

96

- **PDB ID:** 5JPN | **Chain:** B
- **b-phipsi:** 0.0036755762352329
- **w-rdist:** 0.2326944077278183
- **t-alpha:** 0.0640930305320786

---

---

97

- **PDB ID:** 5C1B | **Chain:** F
- **b-phipsi:** 0.0058657915147247
- **w-rdist:** 0.2407221954034222
- **t-alpha:** 0.0466131781632361

---

---

98

- **PDB ID:** 6BFC | **Chain:** b
- **b-phipsi:** 0.007038252914101
- **w-rdist:** 0.5130780365201655
- **t-alpha:** 0.0065550134155867

---

---

99

- **PDB ID:** 6ZYM | **Chain:** B
- **b-phipsi:** 0.0031300074509888
- **w-rdist:** 0.3375668956945657
- **t-alpha:** 0.0425208893008459

---

---

100

- **PDB ID:** 8A7D | **Chain:** C
- **b-phipsi:** 0.0199224340908551
- **w-rdist:** 0.1481366941676064
- **t-alpha:** 0.0043701973936818

---

---

101

- **PDB ID:** 6B70 | **Chain:** B
- **b-phipsi:** 0.0087497575226754
- **w-rdist:** 0.4886502439049781
- **t-alpha:** 0.0007288726764216

---

---

102

- **PDB ID:** 6B3Q | **Chain:** b
- **b-phipsi:** 0.0079122357238896
- **w-rdist:** 0.5103035367163821
- **t-alpha:** 0.0029134192830806

---

---

103

- **PDB ID:** 7LM2 | **Chain:** A
- **b-phipsi:** 0.0109307395515948
- **w-rdist:** 0.2782134950775536
- **t-alpha:** 0.0138382752386696

---

---

104

- **PDB ID:** 5UBT | **Chain:** A
- **b-phipsi:** 0.0118522240440913
- **w-rdist:** 0.326747139557329
- **t-alpha:** 0.0058607467412064

---

---

105

- **PDB ID:** 5C19 | **Chain:** C
- **b-phipsi:** 0.006249185287178
- **w-rdist:** 0.1352305861022304
- **t-alpha:** 0.0568099994807522

---

---

106

- **PDB ID:** 8BCY | **Chain:** A
- **b-phipsi:** 0.0121674815237005
- **w-rdist:** 0.2909976435065112
- **t-alpha:** 0.0095587741674756

---

---

107

- **PDB ID:** 2E1Q | **Chain:** D
- **b-phipsi:** 0.0058277216938588
- **w-rdist:** 0.2778858480521926
- **t-alpha:** 0.0429718680332931

---

---

108

- **PDB ID:** 7PG6 | **Chain:** A
- **b-phipsi:** 0.006290717196378
- **w-rdist:** 0.4513500298309255
- **t-alpha:** 0.020393494275321

---

---

109

- **PDB ID:** 3E4Z | **Chain:** B
- **b-phipsi:** 0.0060793349751283
- **w-rdist:** 0.5240192018193991
- **t-alpha:** 0.0131099992438439

---

---

110

- **PDB ID:** 5M6U | **Chain:** A
- **b-phipsi:** 0.013289341062301
- **w-rdist:** 0.3241792409798686
- **t-alpha:** 0.0043701973936818

---

---

111

- **PDB ID:** 5JTV | **Chain:** G
- **b-phipsi:** 0.002786475035475
- **w-rdist:** 0.6153799436759539
- **t-alpha:** 0.0088172167453031

---

---

112

- **PDB ID:** 8EZB | **Chain:** A
- **b-phipsi:** 0.0038706666035117
- **w-rdist:** 0.3196540437947688
- **t-alpha:** 0.0495267695540877

---

---

113

- **PDB ID:** 6C02 | **Chain:** A
- **b-phipsi:** 0.0007376179573695
- **w-rdist:** 0.5105109469458755
- **t-alpha:** 0.0292354274430095

---

---

114

- **PDB ID:** 6OAC | **Chain:** A
- **b-phipsi:** 0.020137343764026
- **w-rdist:** 0.1788715759727634
- **t-alpha:** 0.0043701973936818

---

---

115

- **PDB ID:** 8SZP | **Chain:** B
- **b-phipsi:** 0.0095580525467291
- **w-rdist:** 0.483256420856626
- **t-alpha:** 0.0

---

---

116

- **PDB ID:** 5JS2 | **Chain:** A
- **b-phipsi:** 0.0035250080122701
- **w-rdist:** 0.2913153001712234
- **t-alpha:** 0.0604515833997143

---

---

117

- **PDB ID:** 6U23 | **Chain:** O
- **b-phipsi:** 0.0120946829329232
- **w-rdist:** 0.2966643935402481
- **t-alpha:** 0.0110454531302417

---

---

118

- **PDB ID:** 3SWR | **Chain:** A
- **b-phipsi:** 0.0067446315950706
- **w-rdist:** 0.4476356608627592
- **t-alpha:** 0.0189367966891196

---

---

119

- **PDB ID:** 2FJU | **Chain:** B
- **b-phipsi:** 0.0017902354555213
- **w-rdist:** 0.8041375136537998
- **t-alpha:** 0.0021848670241468

---

---

120

- **PDB ID:** 7Z6O | **Chain:** B
- **b-phipsi:** 0.0009837908758786
- **w-rdist:** 0.5147050521875489
- **t-alpha:** 0.0298618503359113

---

---

121

- **PDB ID:** 8IF4 | **Chain:** A
- **b-phipsi:** 0.0055985688693108
- **w-rdist:** 0.3918820646095302
- **t-alpha:** 0.0323309912234826

---

---

122

- **PDB ID:** 7JIS | **Chain:** A
- **b-phipsi:** 0.0100404676389758
- **w-rdist:** 0.2971297476827228
- **t-alpha:** 0.0182082568765367

---

---

123

- **PDB ID:** 8EZB | **Chain:** K
- **b-phipsi:** 0.0046014137127632
- **w-rdist:** 0.2739838285364382
- **t-alpha:** 0.0604515833997143

---

---

124

- **PDB ID:** 6ZHA | **Chain:** B
- **b-phipsi:** 0.0107159587579968
- **w-rdist:** 0.3220808459030894
- **t-alpha:** 0.0125367354532617

---

---

125

- **PDB ID:** 8EZB | **Chain:** J
- **b-phipsi:** 0.0043496728103966
- **w-rdist:** 0.3202068262630198
- **t-alpha:** 0.0509830974011791

---

---

126

- **PDB ID:** 4FXK | **Chain:** B
- **b-phipsi:** 0.004688006395303
- **w-rdist:** 0.2196207152211521
- **t-alpha:** 0.0728331610517756

---

---

127

- **PDB ID:** 7TZ7 | **Chain:** A
- **b-phipsi:** 0.0073110321694285
- **w-rdist:** 0.4342327271345467
- **t-alpha:** 0.0174797527976524

---

---

128

- **PDB ID:** 5T8F | **Chain:** A
- **b-phipsi:** 0.0110625480301829
- **w-rdist:** 0.3424525974579193
- **t-alpha:** 0.0101968242656327

---

---

129

- **PDB ID:** 4OVU | **Chain:** A
- **b-phipsi:** 0.0076877894013438
- **w-rdist:** 0.287817128772296
- **t-alpha:** 0.0292354274430095

---

---

130

- **PDB ID:** 1JEY | **Chain:** A
- **b-phipsi:** 0.0003094079036895
- **w-rdist:** 0.626511227985237
- **t-alpha:** 0.0147818972725322

---

---

131

- **PDB ID:** 5C18 | **Chain:** A
- **b-phipsi:** 0.0063277705687845
- **w-rdist:** 0.281750350664689
- **t-alpha:** 0.041514721940334

---

---

132

- **PDB ID:** 4FLH | **Chain:** A
- **b-phipsi:** 0.0254627802175565
- **w-rdist:** 0.1962131085773414
- **t-alpha:** 0.0014587541408541

---

---

133

- **PDB ID:** 4DUR | **Chain:** A
- **b-phipsi:** 0.018188432613707
- **w-rdist:** 0.2756299205476912
- **t-alpha:** 0.0036549441753579

---

---

134

- **PDB ID:** 7NFE | **Chain:** B
- **b-phipsi:** 0.0055779533886985
- **w-rdist:** 0.3358763129142361
- **t-alpha:** 0.0393640418205993

---

---

135

- **PDB ID:** 7MEZ | **Chain:** A
- **b-phipsi:** 0.0080247077493062
- **w-rdist:** 0.3321800173826441
- **t-alpha:** 0.0225785454961677

---

---

136

- **PDB ID:** 5DXU | **Chain:** A
- **b-phipsi:** 0.0106612766219886
- **w-rdist:** 0.3279291831936556
- **t-alpha:** 0.0138382752386696

---

---

137

- **PDB ID:** 6BFC | **Chain:** B
- **b-phipsi:** 0.0086375282790545
- **w-rdist:** 0.5092406047633499
- **t-alpha:** 0.0043701973936818

---

---

138

- **PDB ID:** 8ONE | **Chain:** A
- **b-phipsi:** 0.0047560336952411
- **w-rdist:** 0.7635335761337426
- **t-alpha:** 0.0007288726764216

---

---

139

- **PDB ID:** 7K6N | **Chain:** A
- **b-phipsi:** 0.0103358856547213
- **w-rdist:** 0.2172131533820649
- **t-alpha:** 0.0247633892798655

---

---

140

- **PDB ID:** 7T3B | **Chain:** A
- **b-phipsi:** 0.0034564606988133
- **w-rdist:** 0.4437840218775543
- **t-alpha:** 0.0349600148426103

---

---

141

- **PDB ID:** 8FCQ | **Chain:** A
- **b-phipsi:** 0.0061241924788788
- **w-rdist:** 0.2150304761482985
- **t-alpha:** 0.0604515833997143

---

---

142

- **PDB ID:** 6OCO | **Chain:** A
- **b-phipsi:** 0.0125217289889727
- **w-rdist:** 0.3332097950191453
- **t-alpha:** 0.0087397720309874

---

---

143

- **PDB ID:** 3ZD6 | **Chain:** A
- **b-phipsi:** 0.0189314617237864
- **w-rdist:** 0.2943614231531589
- **t-alpha:** 0.0021896458253802

---

---

144

- **PDB ID:** 6MFR | **Chain:** B
- **b-phipsi:** 0.00470917130767
- **w-rdist:** 0.3076385508379488
- **t-alpha:** 0.0597231737029493

---

---

145

- **PDB ID:** 2E1Q | **Chain:** C
- **b-phipsi:** 0.005838019366086
- **w-rdist:** 0.2764363228693054
- **t-alpha:** 0.0560815732405199

---

---

146

- **PDB ID:** 5KI6 | **Chain:** A
- **b-phipsi:** 0.0045196251346696
- **w-rdist:** 0.3888142477840122
- **t-alpha:** 0.0407864162082982

---

---

147

- **PDB ID:** 6ZHE | **Chain:** G
- **b-phipsi:** 0.0087355627344137
- **w-rdist:** 0.3079528150411869
- **t-alpha:** 0.0246270663199015

---

---

148

- **PDB ID:** 6TY5 | **Chain:** A
- **b-phipsi:** 0.0179088184026167
- **w-rdist:** 0.3133553357236293
- **t-alpha:** 0.0029134192830806

---

---

149

- **PDB ID:** 6GVF | **Chain:** A
- **b-phipsi:** 0.0104069945961098
- **w-rdist:** 0.2212728749174677
- **t-alpha:** 0.0254917294284022

---

---

150

- **PDB ID:** 7RC9 | **Chain:** A
- **b-phipsi:** 0.0142662052148757
- **w-rdist:** 0.3568158970491892
- **t-alpha:** 0.0050984212340619

---

---

151

- **PDB ID:** 7SFC | **Chain:** A
- **b-phipsi:** 0.0046909294205221
- **w-rdist:** 0.3088650808363311
- **t-alpha:** 0.0611799877648819

---

---

152

- **PDB ID:** 6ERF | **Chain:** G
- **b-phipsi:** 0.0012015178376238
- **w-rdist:** 0.5614838731442946
- **t-alpha:** 0.0225785454961677

---

---

153

- **PDB ID:** 5LS6 | **Chain:** J
- **b-phipsi:** 0.0057099855316525
- **w-rdist:** 0.6884664584779117
- **t-alpha:** 0.0021896458253802

---

---

154

- **PDB ID:** 6ICZ | **Chain:** C
- **b-phipsi:** 0.0044817800170768
- **w-rdist:** 0.3592318912615262
- **t-alpha:** 0.0504970964456714

---

---

155

- **PDB ID:** 6ICZ | **Chain:** c
- **b-phipsi:** 0.0044817800170768
- **w-rdist:** 0.3592318912615262
- **t-alpha:** 0.0504970964456714

---

---

156

- **PDB ID:** 7AAV | **Chain:** R
- **b-phipsi:** 0.0108625853905735
- **w-rdist:** 0.2469754060277252
- **t-alpha:** 0.0225785454961677

---

---

157

- **PDB ID:** 5VLR | **Chain:** A
- **b-phipsi:** 0.0115941361398753
- **w-rdist:** 0.3280304330606561
- **t-alpha:** 0.0131099992438439

---

---

158

- **PDB ID:** 6V9U | **Chain:** A
- **b-phipsi:** 0.0134610736347513
- **w-rdist:** 0.3498004198055033
- **t-alpha:** 0.0072833804367331

---

---

159

- **PDB ID:** 4L1B | **Chain:** A
- **b-phipsi:** 0.0074310545421945
- **w-rdist:** 0.463320390206238
- **t-alpha:** 0.0167517699463162

---

---

160

- **PDB ID:** 8AG4 | **Chain:** B
- **b-phipsi:** 0.0021434095507181
- **w-rdist:** 0.501223262453105
- **t-alpha:** 0.0327750996298437

---

---

161

- **PDB ID:** 6C01 | **Chain:** A
- **b-phipsi:** 0.0005090493418481
- **w-rdist:** 0.5154145616229626
- **t-alpha:** 0.0338857136971153

---

---

162

- **PDB ID:** 8AG4 | **Chain:** A
- **b-phipsi:** 0.0022517072591146
- **w-rdist:** 0.6329178327229386
- **t-alpha:** 0.0140326350426887

---

---

163

- **PDB ID:** 7R7T | **Chain:** D
- **b-phipsi:** 0.0126446603629269
- **w-rdist:** 0.4463557642635223
- **t-alpha:** 0.0007288726764216

---

---

164

- **PDB ID:** 7DVQ | **Chain:** c
- **b-phipsi:** 0.0041708339605496
- **w-rdist:** 0.47205120116296
- **t-alpha:** 0.0331075673737624

---

---

165

- **PDB ID:** 7SVO | **Chain:** A
- **b-phipsi:** 0.0056033376209954
- **w-rdist:** 0.6708326118129316
- **t-alpha:** 0.0050984212340619

---

---

166

- **PDB ID:** 8P4E | **Chain:** O
- **b-phipsi:** 0.0015623217896402
- **w-rdist:** 0.5349155238952266
- **t-alpha:** 0.0284642139866488

---

---

167

- **PDB ID:** 3W3G | **Chain:** A
- **b-phipsi:** 0.0096267928428435
- **w-rdist:** 0.3587543484505893
- **t-alpha:** 0.0174797527976524

---

---

168

- **PDB ID:** 4AY2 | **Chain:** A
- **b-phipsi:** 0.0114467297885832
- **w-rdist:** 0.4331588446012683
- **t-alpha:** 0.0058266934612722

---

---

169

- **PDB ID:** 2VXO | **Chain:** A
- **b-phipsi:** 0.0042252717997938
- **w-rdist:** 0.4845802859761593
- **t-alpha:** 0.0323309912234826

---

---

170

- **PDB ID:** 5IUD | **Chain:** J
- **b-phipsi:** 0.0036320735362093
- **w-rdist:** 0.2512497789783475
- **t-alpha:** 0.088856766054028

---

---

171

- **PDB ID:** 7K6O | **Chain:** A
- **b-phipsi:** 0.0108227593008055
- **w-rdist:** 0.2018119509527951
- **t-alpha:** 0.0276764270621712

---

---

172

- **PDB ID:** 8EZB | **Chain:** B
- **b-phipsi:** 0.0055185729804895
- **w-rdist:** 0.2868433312821713
- **t-alpha:** 0.0648213974041245

---

---

173

- **PDB ID:** 6WKR | **Chain:** C
- **b-phipsi:** 0.0046017145896792
- **w-rdist:** 0.8066430632083119
- **t-alpha:** 0.0029134192830806

---

---

174

- **PDB ID:** 3HHM | **Chain:** A
- **b-phipsi:** 0.0079806740576213
- **w-rdist:** 0.3091159469262522
- **t-alpha:** 0.0300077103542844

---

---

175

- **PDB ID:** 5C18 | **Chain:** C
- **b-phipsi:** 0.0070152982038033
- **w-rdist:** 0.2609798439584397
- **t-alpha:** 0.0436997123665408

---

---

176

- **PDB ID:** 7WUB | **Chain:** J
- **b-phipsi:** 0.0080053386422382
- **w-rdist:** 0.1255985315854946
- **t-alpha:** 0.0495267695540877

---

---

177

- **PDB ID:** 5W6V | **Chain:** A
- **b-phipsi:** 0.0061132948898572
- **w-rdist:** 0.240153653867428
- **t-alpha:** 0.0677347625762181

---

---

178

- **PDB ID:** 1JEQ | **Chain:** B
- **b-phipsi:** 0.0073908374271097
- **w-rdist:** 0.5281442158946571
- **t-alpha:** 0.010924934303695

---

---

179

- **PDB ID:** 5C18 | **Chain:** D
- **b-phipsi:** 0.0066340758596687
- **w-rdist:** 0.2471707802455769
- **t-alpha:** 0.0560815732405199

---

---

180

- **PDB ID:** 3TON | **Chain:** A
- **b-phipsi:** 0.0052340752051919
- **w-rdist:** 0.6800815560577769
- **t-alpha:** 0.0073366962737499

---

---

181

- **PDB ID:** 5UK8 | **Chain:** A
- **b-phipsi:** 0.0054456374100252
- **w-rdist:** 0.4484366831092689
- **t-alpha:** 0.0342318542055553

---

---

182

- **PDB ID:** 6ID0 | **Chain:** c
- **b-phipsi:** 0.004914440392516
- **w-rdist:** 0.3755193818607135
- **t-alpha:** 0.0513018757330945

---

---

183

- **PDB ID:** 7AXZ | **Chain:** B
- **b-phipsi:** 0.0031298481290204
- **w-rdist:** 0.5666980532777812
- **t-alpha:** 0.0215774983192449

---

---

184

- **PDB ID:** 5UY8 | **Chain:** A
- **b-phipsi:** 0.0057728200153513
- **w-rdist:** 0.7333446883418808
- **t-alpha:** 0.0036415440256147

---

---

185

- **PDB ID:** 6GVG | **Chain:** A
- **b-phipsi:** 0.0097105548810472
- **w-rdist:** 0.2044694246989093
- **t-alpha:** 0.0327750996298437

---

---

186

- **PDB ID:** 2P8S | **Chain:** A
- **b-phipsi:** 0.0052919240944148
- **w-rdist:** 0.8337673629196358
- **t-alpha:** 0.0

---

---

187

- **PDB ID:** 4FAD | **Chain:** A
- **b-phipsi:** 0.0208689078326068
- **w-rdist:** 0.2407388167981405
- **t-alpha:** 0.0087397720309874

---

---

188

- **PDB ID:** 1NU8 | **Chain:** A
- **b-phipsi:** 0.0049563860212418
- **w-rdist:** 0.8357328422068755
- **t-alpha:** 0.0

---

---

189

- **PDB ID:** 5WOB | **Chain:** H
- **b-phipsi:** 0.0057557446684995
- **w-rdist:** 0.4520394083045846
- **t-alpha:** 0.0335032202501839

---

---

190

- **PDB ID:** 7UHG | **Chain:** D
- **b-phipsi:** 0.0058489454116795
- **w-rdist:** 0.4600320323619355
- **t-alpha:** 0.0323309912234826

---

---

191

- **PDB ID:** 5VNE | **Chain:** B
- **b-phipsi:** 0.0033215267946985
- **w-rdist:** 0.7358747829081145
- **t-alpha:** 0.0095587741674756

---

---

192

- **PDB ID:** 6TES | **Chain:** A
- **b-phipsi:** 0.0052752238724672
- **w-rdist:** 0.7709252080131307
- **t-alpha:** 0.0050984212340619

---

---

193

- **PDB ID:** 4RAL | **Chain:** B
- **b-phipsi:** 0.0061092729876289
- **w-rdist:** 0.5136606938972195
- **t-alpha:** 0.0247633892798655

---

---

194

- **PDB ID:** 5C1B | **Chain:** E
- **b-phipsi:** 0.0060020015961019
- **w-rdist:** 0.2039811088386118
- **t-alpha:** 0.0837581824319544

---

---

195

- **PDB ID:** 6TEU | **Chain:** A
- **b-phipsi:** 0.0056556532128208
- **w-rdist:** 0.7736415328568055
- **t-alpha:** 0.0029220273949486

---

---

196

- **PDB ID:** 5UBR | **Chain:** A
- **b-phipsi:** 0.0132647279154244
- **w-rdist:** 0.2127218483694809
- **t-alpha:** 0.0233067966402911

---

---

197

- **PDB ID:** 3L4G | **Chain:** H
- **b-phipsi:** 0.0021963697476728
- **w-rdist:** 0.682222061702094
- **t-alpha:** 0.0140326350426887

---

---

198

- **PDB ID:** 5C18 | **Chain:** F
- **b-phipsi:** 0.0078388145309169
- **w-rdist:** 0.2457913072870368
- **t-alpha:** 0.0393298454728356

---

---

199

- **PDB ID:** 5XGJ | **Chain:** A
- **b-phipsi:** 0.0089066924666288
- **w-rdist:** 0.3796208485523922
- **t-alpha:** 0.0211216500137569

---

---

200

- **PDB ID:** 7MDM | **Chain:** A
- **b-phipsi:** 0.0070366492579885
- **w-rdist:** 0.140555007856246
- **t-alpha:** 0.0670064380342809

---

---

201

- **PDB ID:** 6ERF | **Chain:** C
- **b-phipsi:** 0.0020892734685424
- **w-rdist:** 0.5599236497898274
- **t-alpha:** 0.0261584429252026

---

---

202

- **PDB ID:** 4Q5V | **Chain:** E
- **b-phipsi:** 0.004849004887158
- **w-rdist:** 0.1719078562663324
- **t-alpha:** 0.1092498194584445

---

---

203

- **PDB ID:** 4OLB | **Chain:** A
- **b-phipsi:** 0.0051876061141668
- **w-rdist:** 0.3022221473576613
- **t-alpha:** 0.0757463891855267

---

---

204

- **PDB ID:** 8ASC | **Chain:** B
- **b-phipsi:** 0.0015892206895191
- **w-rdist:** 0.585992543557017
- **t-alpha:** 0.0230999264545257

---

---

205

- **PDB ID:** 5C1B | **Chain:** B
- **b-phipsi:** 0.0056181526333601
- **w-rdist:** 0.209902028965572
- **t-alpha:** 0.0946832683923559

---

---

206

- **PDB ID:** 7LT3 | **Chain:** J
- **b-phipsi:** 0.0107743530139059
- **w-rdist:** 0.3621235867822751
- **t-alpha:** 0.0174797527976524

---

---

207

- **PDB ID:** 6TEX | **Chain:** A
- **b-phipsi:** 0.005841499060539
- **w-rdist:** 0.7711005663333409
- **t-alpha:** 0.0029220273949486

---

---

208

- **PDB ID:** 6TY5 | **Chain:** B
- **b-phipsi:** 0.0175148396493227
- **w-rdist:** 0.3105473221308794
- **t-alpha:** 0.0088172167453031

---

---

209

- **PDB ID:** 6MDZ | **Chain:** B
- **b-phipsi:** 0.0043367591863432
- **w-rdist:** 0.2912208466103346
- **t-alpha:** 0.0881280428719286

---

---

210

- **PDB ID:** 6OON | **Chain:** A
- **b-phipsi:** 0.0078963214233278
- **w-rdist:** 0.2377556069108367
- **t-alpha:** 0.0433130906697325

---

---

211

- **PDB ID:** 6NIT | **Chain:** A
- **b-phipsi:** 0.006244522327525
- **w-rdist:** 0.2628762431677279
- **t-alpha:** 0.0684630747798777

---

---

212

- **PDB ID:** 8FCQ | **Chain:** D
- **b-phipsi:** 0.0071224816140039
- **w-rdist:** 0.1549314234917417
- **t-alpha:** 0.0684630747798777

---

---

213

- **PDB ID:** 8OW2 | **Chain:** A
- **b-phipsi:** 0.0104488469271441
- **w-rdist:** 0.1591103446742745
- **t-alpha:** 0.0349600148426103

---

---

214

- **PDB ID:** 8ASC | **Chain:** P
- **b-phipsi:** 0.0024413917153298
- **w-rdist:** 0.5713108199894098
- **t-alpha:** 0.0246270663199015

---

---

215

- **PDB ID:** 7SVM | **Chain:** B
- **b-phipsi:** 0.0061376027111988
- **w-rdist:** 0.6730354597215581
- **t-alpha:** 0.0065550134155867

---

---

216

- **PDB ID:** 4KRF | **Chain:** A
- **b-phipsi:** 0.0020625722844762
- **w-rdist:** 0.2756179612447421
- **t-alpha:** 0.1107063945241015

---

---

217

- **PDB ID:** 7LQ1 | **Chain:** A
- **b-phipsi:** 0.0077425895599699
- **w-rdist:** 0.3529812389352127
- **t-alpha:** 0.032046507746164

---

---

218

- **PDB ID:** 8DP0 | **Chain:** A
- **b-phipsi:** 0.0104611811792592
- **w-rdist:** 0.3670495494254605
- **t-alpha:** 0.0196648853517085

---

---

219

- **PDB ID:** 6XOV | **Chain:** A
- **b-phipsi:** 0.0096622648150704
- **w-rdist:** 0.43950642934174
- **t-alpha:** 0.0160233389937762

---

---

220

- **PDB ID:** 5UZ0 | **Chain:** A
- **b-phipsi:** 0.0060491376782685
- **w-rdist:** 0.7334207626350788
- **t-alpha:** 0.0050984212340619

---

---

221

- **PDB ID:** 5C18 | **Chain:** E
- **b-phipsi:** 0.0068217673468853
- **w-rdist:** 0.2047984828714695
- **t-alpha:** 0.0721049373143647

---

---

222

- **PDB ID:** 7BP8 | **Chain:** D
- **b-phipsi:** 0.008065846276167
- **w-rdist:** 0.2222918154637818
- **t-alpha:** 0.0495267695540877

---

---

223

- **PDB ID:** 6X9K | **Chain:** A
- **b-phipsi:** 0.0048945799181802
- **w-rdist:** 0.277350835522565
- **t-alpha:** 0.0954111582921788

---

---

224

- **PDB ID:** 6V9U | **Chain:** B
- **b-phipsi:** 0.0191724099834631
- **w-rdist:** 0.3148958135528327
- **t-alpha:** 0.0080117938638943

---

---

225

- **PDB ID:** 5IFW | **Chain:** B
- **b-phipsi:** 0.0046989392420944
- **w-rdist:** 0.7116646186159664
- **t-alpha:** 0.011653569134763

---

---

226

- **PDB ID:** 1JEY | **Chain:** B
- **b-phipsi:** 0.0020356965649493
- **w-rdist:** 0.5636701334964744
- **t-alpha:** 0.0284048760941568

---

---

227

- **PDB ID:** 4OVV | **Chain:** A
- **b-phipsi:** 0.0068229612278703
- **w-rdist:** 0.2644246380456521
- **t-alpha:** 0.0610509630466473

---

---

228

- **PDB ID:** 6BF7 | **Chain:** B
- **b-phipsi:** 0.0097757522561881
- **w-rdist:** 0.4248840723098412
- **t-alpha:** 0.0182082568765367

---

---

229

- **PDB ID:** 8FCN | **Chain:** D
- **b-phipsi:** 0.0053172720348255
- **w-rdist:** 0.3056940751458327
- **t-alpha:** 0.0852149190700788

---

---

230

- **PDB ID:** 7MYO | **Chain:** A
- **b-phipsi:** 0.0080419599283484
- **w-rdist:** 0.1767091244323365
- **t-alpha:** 0.0560815732405199

---

---

231

- **PDB ID:** 7BP9 | **Chain:** F
- **b-phipsi:** 0.0073879490325403
- **w-rdist:** 0.1653088489496375
- **t-alpha:** 0.0684630747798777

---

---

232

- **PDB ID:** 3VJK | **Chain:** B
- **b-phipsi:** 0.0061608204840994
- **w-rdist:** 0.8280968264231303
- **t-alpha:** 0.0014568440116902

---

---

233

- **PDB ID:** 4FWJ | **Chain:** A
- **b-phipsi:** 0.0045328048973331
- **w-rdist:** 0.4522159304520046
- **t-alpha:** 0.0488923864934398

---

---

234

- **PDB ID:** 5T4B | **Chain:** B
- **b-phipsi:** 0.0058287011958196
- **w-rdist:** 0.8338787027278964
- **t-alpha:** 0.0021848670241468

---

---

235

- **PDB ID:** 6DDQ | **Chain:** A
- **b-phipsi:** 0.006551661874528
- **w-rdist:** 0.713402310661824
- **t-alpha:** 0.0029220273949486

---

---

236

- **PDB ID:** 6U23 | **Chain:** K
- **b-phipsi:** 0.009636175819997
- **w-rdist:** 0.3011500939800377
- **t-alpha:** 0.0307805727451215

---

---

237

- **PDB ID:** 7A3L | **Chain:** C
- **b-phipsi:** 0.0062812413228874
- **w-rdist:** 0.6166165483760283
- **t-alpha:** 0.011653569134763

---

---

238

- **PDB ID:** 5IUD | **Chain:** D
- **b-phipsi:** 0.006362923317383
- **w-rdist:** 0.267750855355971
- **t-alpha:** 0.0728331610517756

---

---

239

- **PDB ID:** 4DTT | **Chain:** A
- **b-phipsi:** 0.0057635032640341
- **w-rdist:** 0.53384537447108
- **t-alpha:** 0.0276764270621712

---

---

240

- **PDB ID:** 7R9V | **Chain:** A
- **b-phipsi:** 0.012368216912395
- **w-rdist:** 0.1885012276547164
- **t-alpha:** 0.0307805727451215

---

---

241

- **PDB ID:** 4RE9 | **Chain:** B
- **b-phipsi:** 0.0059334484358001
- **w-rdist:** 0.5334016161457362
- **t-alpha:** 0.0262200978972946

---

---

242

- **PDB ID:** 6AS7 | **Chain:** A
- **b-phipsi:** 0.0034250666239505
- **w-rdist:** 0.3150513237063371
- **t-alpha:** 0.0968683904054403

---

---

243

- **PDB ID:** 6GVH | **Chain:** A
- **b-phipsi:** 0.0091592006696796
- **w-rdist:** 0.2487833274315384
- **t-alpha:** 0.036416391876046

---

---

244

- **PDB ID:** 4R08 | **Chain:** C
- **b-phipsi:** 0.0098598667690227
- **w-rdist:** 0.3575202261770501
- **t-alpha:** 0.0247633892798655

---

---

245

- **PDB ID:** 7AD7 | **Chain:** A
- **b-phipsi:** 0.0038042209516197
- **w-rdist:** 0.3725254090302493
- **t-alpha:** 0.0734948207675663

---

---

246

- **PDB ID:** 4Q5V | **Chain:** A
- **b-phipsi:** 0.005606858474608
- **w-rdist:** 0.1709111459709628
- **t-alpha:** 0.1165326797387364

---

---

247

- **PDB ID:** 6EJD | **Chain:** A
- **b-phipsi:** 0.0018226173723416
- **w-rdist:** 0.6106762640811003
- **t-alpha:** 0.0253919797973902

---

---

248

- **PDB ID:** 5IXC | **Chain:** A
- **b-phipsi:** 0.0149661654868982
- **w-rdist:** 0.3370211482215693
- **t-alpha:** 0.0140326350426887

---

---

249

- **PDB ID:** 7PVN | **Chain:** A
- **b-phipsi:** 0.0046353665316864
- **w-rdist:** 0.7219134103380728
- **t-alpha:** 0.0131099992438439

---

---

250

- **PDB ID:** 3VJM | **Chain:** B
- **b-phipsi:** 0.0063038846472704
- **w-rdist:** 0.8317910865825251
- **t-alpha:** 0.0007283954918235

---

---

251

- **PDB ID:** 1JEQ | **Chain:** A
- **b-phipsi:** 0.0031437846413906
- **w-rdist:** 0.7022643554274355
- **t-alpha:** 0.0174797527976524

---

---

252

- **PDB ID:** 5XGH | **Chain:** A
- **b-phipsi:** 0.0062133426241836
- **w-rdist:** 0.3987843290368205
- **t-alpha:** 0.047341565959746

---

---

253

- **PDB ID:** 7LN1 | **Chain:** E
- **b-phipsi:** 0.0050660590119266
- **w-rdist:** 0.7100682397986087
- **t-alpha:** 0.0131099992438439

---

---

254

- **PDB ID:** 5O9Z | **Chain:** B
- **b-phipsi:** 0.0054056860766018
- **w-rdist:** 0.4333974385966259
- **t-alpha:** 0.0529142885263107

---

---

255

- **PDB ID:** 6JT1 | **Chain:** B
- **b-phipsi:** 0.012437387277431
- **w-rdist:** 0.356207525438995
- **t-alpha:** 0.0182082568765367

---

---

256

- **PDB ID:** 6MDZ | **Chain:** A
- **b-phipsi:** 0.0031247756916694
- **w-rdist:** 0.3404716869184425
- **t-alpha:** 0.0932268541315344

---

---

257

- **PDB ID:** 5ITD | **Chain:** A
- **b-phipsi:** 0.0069482756426544
- **w-rdist:** 0.4649904829724564
- **t-alpha:** 0.0298618503359113

---

---

258

- **PDB ID:** 6AHU | **Chain:** B
- **b-phipsi:** 0.0152091642492406
- **w-rdist:** 0.4022534939194734
- **t-alpha:** 0.0088172167453031

---

---

259

- **PDB ID:** 6C01 | **Chain:** B
- **b-phipsi:** 0.0011033072375484
- **w-rdist:** 0.5145671572163238
- **t-alpha:** 0.0529142885263107

---

---

260

- **PDB ID:** 6GVI | **Chain:** A
- **b-phipsi:** 0.0100937088889697
- **w-rdist:** 0.1983446257583245
- **t-alpha:** 0.0378733336061549

---

---

261

- **PDB ID:** 2QJR | **Chain:** A
- **b-phipsi:** 0.0061696770736193
- **w-rdist:** 0.8287347375270612
- **t-alpha:** 0.0029220273949486

---

---

262

- **PDB ID:** 7PVN | **Chain:** B
- **b-phipsi:** 0.0050747968855712
- **w-rdist:** 0.7105121653618739
- **t-alpha:** 0.0132839260291477

---

---

263

- **PDB ID:** 3LJ3 | **Chain:** A
- **b-phipsi:** 0.0191648872183312
- **w-rdist:** 0.2264906587539924
- **t-alpha:** 0.0182082568765367

---

---

264

- **PDB ID:** 7ZT6 | **Chain:** A
- **b-phipsi:** 0.0016534253466755
- **w-rdist:** 0.6479485486802155
- **t-alpha:** 0.0246270663199015

---

---

265

- **PDB ID:** 7BP8 | **Chain:** A
- **b-phipsi:** 0.0087560753123932
- **w-rdist:** 0.2312639446723182
- **t-alpha:** 0.0466131781632361

---

---

266

- **PDB ID:** 7VCX | **Chain:** E
- **b-phipsi:** 0.006175194544004
- **w-rdist:** 0.3818659351208801
- **t-alpha:** 0.0531683659174533

---

---

267

- **PDB ID:** 7WVJ | **Chain:** B
- **b-phipsi:** 0.0083610224946656
- **w-rdist:** 0.62689800431722
- **t-alpha:** 0.0

---

---

268

- **PDB ID:** 7LN5 | **Chain:** D
- **b-phipsi:** 0.0070708219232788
- **w-rdist:** 0.7410455966700153
- **t-alpha:** 0.0007283954918235

---

---

269

- **PDB ID:** 6FXR | **Chain:** A
- **b-phipsi:** 0.0054205661695056
- **w-rdist:** 0.7692088635518509
- **t-alpha:** 0.0103018401892411

---

---

270

- **PDB ID:** 6MZM | **Chain:** B
- **b-phipsi:** 0.020103466915403
- **w-rdist:** 0.3537448472974312
- **t-alpha:** 0.0058607467412064

---

---

271

- **PDB ID:** 8FCQ | **Chain:** C
- **b-phipsi:** 0.0059798261061507
- **w-rdist:** 0.1567081456700226
- **t-alpha:** 0.1187176724156662

---

---

272

- **PDB ID:** 2IIT | **Chain:** A
- **b-phipsi:** 0.0052758001602877
- **w-rdist:** 0.8366135010865512
- **t-alpha:** 0.00512430205411

---

---

273

- **PDB ID:** 3L4G | **Chain:** F
- **b-phipsi:** 0.0030961316255374
- **w-rdist:** 0.6659903490342455
- **t-alpha:** 0.0215774983192449

---

---

274

- **PDB ID:** 6TEC | **Chain:** A
- **b-phipsi:** 0.0058891358881107
- **w-rdist:** 0.763467013095103
- **t-alpha:** 0.0087397720309874

---

---

275

- **PDB ID:** 7A3J | **Chain:** C
- **b-phipsi:** 0.0087053494230663
- **w-rdist:** 0.5859620050443402
- **t-alpha:** 0.0021848670241468

---

---

276

- **PDB ID:** 7VCT | **Chain:** C
- **b-phipsi:** 0.0050384211540932
- **w-rdist:** 0.3654372276303581
- **t-alpha:** 0.0728331610517756

---

---

277

- **PDB ID:** 6JT0 | **Chain:** B
- **b-phipsi:** 0.017426013261133
- **w-rdist:** 0.3598592454954067
- **t-alpha:** 0.0095587741674756

---

---

278

- **PDB ID:** 6I2X | **Chain:** A
- **b-phipsi:** 0.0029366648989769
- **w-rdist:** 0.3793722278978494
- **t-alpha:** 0.0852149190700788

---

---

279

- **PDB ID:** 4L23 | **Chain:** A
- **b-phipsi:** 0.0056582377771757
- **w-rdist:** 0.3871225538176427
- **t-alpha:** 0.0619083860998908

---

---

280

- **PDB ID:** 6U23 | **Chain:** G
- **b-phipsi:** 0.012899052251155
- **w-rdist:** 0.3027212539847144
- **t-alpha:** 0.0246270663199015

---

---

281

- **PDB ID:** 3W1B | **Chain:** A
- **b-phipsi:** 0.0020721104241361
- **w-rdist:** 0.5148342345425531
- **t-alpha:** 0.0496939342883562

---

---

282

- **PDB ID:** 8FCN | **Chain:** A
- **b-phipsi:** 0.0063648125063553
- **w-rdist:** 0.280749056069955
- **t-alpha:** 0.0786598128498576

---

---

283

- **PDB ID:** 4GU1 | **Chain:** B
- **b-phipsi:** 0.003981968315734
- **w-rdist:** 0.510176900530464
- **t-alpha:** 0.0433130906697325

---

---

284

- **PDB ID:** 2BGR | **Chain:** B
- **b-phipsi:** 0.0062498158420921
- **w-rdist:** 0.8251433257526264
- **t-alpha:** 0.0043701973936818

---

---

285

- **PDB ID:** 7K6M | **Chain:** A
- **b-phipsi:** 0.0111566353962055
- **w-rdist:** 0.1842675028930985
- **t-alpha:** 0.036416391876046

---

---

286

- **PDB ID:** 2RD0 | **Chain:** A
- **b-phipsi:** 0.0102961496090321
- **w-rdist:** 0.120053699199055
- **t-alpha:** 0.0504970964456714

---

---

287

- **PDB ID:** 7VCU | **Chain:** J
- **b-phipsi:** 0.0061957208389997
- **w-rdist:** 0.4453284014079819
- **t-alpha:** 0.0407864162082982

---

---

288

- **PDB ID:** 7RLF | **Chain:** D
- **b-phipsi:** 0.0076865735744051
- **w-rdist:** 0.1389805087462033
- **t-alpha:** 0.0772031433552034

---

---

289

- **PDB ID:** 6GYR | **Chain:** B
- **b-phipsi:** 0.0049669726530023
- **w-rdist:** 0.4972936216569463
- **t-alpha:** 0.0417297861962435

---

---

290

- **PDB ID:** 2QOE | **Chain:** A
- **b-phipsi:** 0.0053407482549475
- **w-rdist:** 0.8361092547151352
- **t-alpha:** 0.0058607467412064

---

---

291

- **PDB ID:** 7MDM | **Chain:** C
- **b-phipsi:** 0.0199912422247431
- **w-rdist:** 0.3107841727545569
- **t-alpha:** 0.0110454531302417

---

---

292

- **PDB ID:** 8FCR | **Chain:** D
- **b-phipsi:** 0.0067016383352983
- **w-rdist:** 0.1573335972042622
- **t-alpha:** 0.1005094622253814

---

---

293

- **PDB ID:** 3D4L | **Chain:** A
- **b-phipsi:** 0.0061187292784675
- **w-rdist:** 0.8352916381857813
- **t-alpha:** 0.0029134192830806

---

---

294

- **PDB ID:** 3HAC | **Chain:** B
- **b-phipsi:** 0.0062572376061614
- **w-rdist:** 0.8343602720588488
- **t-alpha:** 0.0021896458253802

---

---

295

- **PDB ID:** 6EOP | **Chain:** B
- **b-phipsi:** 0.0067526298675212
- **w-rdist:** 0.6769398272574791
- **t-alpha:** 0.0065982109973949

---

---

296

- **PDB ID:** 6ERF | **Chain:** B
- **b-phipsi:** 0.0028482952302509
- **w-rdist:** 0.5306190011781888
- **t-alpha:** 0.0386015818388498

---

---

297

- **PDB ID:** 7ZVT | **Chain:** B
- **b-phipsi:** 0.0007518664416953
- **w-rdist:** 0.50704488396041
- **t-alpha:** 0.0604515833997143

---

---

298

- **PDB ID:** 6B1E | **Chain:** B
- **b-phipsi:** 0.0060564310628659
- **w-rdist:** 0.837057370040956
- **t-alpha:** 0.0021896458253802

---

---

299

- **PDB ID:** 5AWC | **Chain:** C
- **b-phipsi:** 0.0160977235220908
- **w-rdist:** 0.3018722547108743
- **t-alpha:** 0.0196648853517085

---

---

300

- **PDB ID:** 5AZ5 | **Chain:** C
- **b-phipsi:** 0.01654163927869
- **w-rdist:** 0.3619480684369305
- **t-alpha:** 0.0123817636541396

---

---

301

- **PDB ID:** 3IBE | **Chain:** A
- **b-phipsi:** 0.0178984761038052
- **w-rdist:** 0.2469689827806713
- **t-alpha:** 0.0211216500137569

---

---

302

- **PDB ID:** 6ID0 | **Chain:** C
- **b-phipsi:** 0.0055958573194046
- **w-rdist:** 0.3767543973799023
- **t-alpha:** 0.0684824353053381

---

---

303

- **PDB ID:** 5SWG | **Chain:** A
- **b-phipsi:** 0.0069179381461006
- **w-rdist:** 0.4638468860438145
- **t-alpha:** 0.0327750996298437

---

---

304

- **PDB ID:** 5IUD | **Chain:** A
- **b-phipsi:** 0.0051292952101887
- **w-rdist:** 0.2752862782638984
- **t-alpha:** 0.1128916601201213

---

---

305

- **PDB ID:** 4ZG6 | **Chain:** A
- **b-phipsi:** 0.00648172526878
- **w-rdist:** 0.5348965119551472
- **t-alpha:** 0.0261584429252026

---

---

306

- **PDB ID:** 3F8S | **Chain:** B
- **b-phipsi:** 0.0064075305863962
- **w-rdist:** 0.8324535124763073
- **t-alpha:** 0.0029220273949486

---

---

307

- **PDB ID:** 4DSZ | **Chain:** B
- **b-phipsi:** 0.0061077142657585
- **w-rdist:** 0.8420564772923343
- **t-alpha:** 0.0014568440116902

---

---

308

- **PDB ID:** 6EJ7 | **Chain:** A
- **b-phipsi:** 0.0012576631609101
- **w-rdist:** 0.6305091121661791
- **t-alpha:** 0.0292354274430095

---

---

309

- **PDB ID:** 7SVO | **Chain:** C
- **b-phipsi:** 0.0075756716209226
- **w-rdist:** 0.6815940512825598
- **t-alpha:** 0.0029134192830806

---

---

310

- **PDB ID:** 1RWQ | **Chain:** A
- **b-phipsi:** 0.0060979492947062
- **w-rdist:** 0.8288653291780789
- **t-alpha:** 0.0065550134155867

---

---

311

- **PDB ID:** 2BGR | **Chain:** A
- **b-phipsi:** 0.0061354911005432
- **w-rdist:** 0.8279423552973738
- **t-alpha:** 0.0065550134155867

---

---

312

- **PDB ID:** 7BW8 | **Chain:** A
- **b-phipsi:** 0.0227288800861709
- **w-rdist:** 0.4183810893771259
- **t-alpha:** 0.0014568440116902

---

---

313

- **PDB ID:** 3VJL | **Chain:** A
- **b-phipsi:** 0.0059842812747628
- **w-rdist:** 0.8297831776156246
- **t-alpha:** 0.0073366962737499

---

---

314

- **PDB ID:** 5WOB | **Chain:** c
- **b-phipsi:** 0.0083686609521178
- **w-rdist:** 0.5043376035149436
- **t-alpha:** 0.0211216500137569

---

---

315

- **PDB ID:** 8EXV | **Chain:** A
- **b-phipsi:** 0.0089283033408927
- **w-rdist:** 0.2164596044945312
- **t-alpha:** 0.0553531458501084

---

---

316

- **PDB ID:** 7WVJ | **Chain:** A
- **b-phipsi:** 0.0082634814099854
- **w-rdist:** 0.6284891314504832
- **t-alpha:** 0.0036549441753579

---

---

317

- **PDB ID:** 7BP9 | **Chain:** C
- **b-phipsi:** 0.0078106464991659
- **w-rdist:** 0.1685547790100163
- **t-alpha:** 0.0786598128498576

---

---

318

- **PDB ID:** 8BFU | **Chain:** A
- **b-phipsi:** 0.0102095729953207
- **w-rdist:** 0.2274916809897911
- **t-alpha:** 0.0436997123665408

---

---

319

- **PDB ID:** 7JIU | **Chain:** A
- **b-phipsi:** 0.0089463658209402
- **w-rdist:** 0.2223083198306074
- **t-alpha:** 0.0546247180047534

---

---

320

- **PDB ID:** 7AUA | **Chain:** A
- **b-phipsi:** 0.0045601543030294
- **w-rdist:** 0.4284324906792025
- **t-alpha:** 0.070148243271072

---

---

321

- **PDB ID:** 7WUB | **Chain:** K
- **b-phipsi:** 0.0072448769761361
- **w-rdist:** 0.127073410715423
- **t-alpha:** 0.0975966865124746

---

---

322

- **PDB ID:** 7LMZ | **Chain:** E
- **b-phipsi:** 0.0074933452104573
- **w-rdist:** 0.7064259010739397
- **t-alpha:** 0.0029134192830806

---

---

323

- **PDB ID:** 7XE1 | **Chain:** A
- **b-phipsi:** 0.0032295356573046
- **w-rdist:** 0.4472635646623589
- **t-alpha:** 0.0726561735687285

---

---

324

- **PDB ID:** 7RC9 | **Chain:** B
- **b-phipsi:** 0.0144301674022056
- **w-rdist:** 0.3602583766856866
- **t-alpha:** 0.0182082568765367

---

---

325

- **PDB ID:** 8EXL | **Chain:** A
- **b-phipsi:** 0.0085525246280597
- **w-rdist:** 0.2212173822927725
- **t-alpha:** 0.0604515833997143

---

---

326

- **PDB ID:** 6EOT | **Chain:** G
- **b-phipsi:** 0.0080363849507621
- **w-rdist:** 0.6585257766290904
- **t-alpha:** 0.0029220273949486

---

---

327

- **PDB ID:** 6EOT | **Chain:** A
- **b-phipsi:** 0.0067187270267479
- **w-rdist:** 0.6750868242982929
- **t-alpha:** 0.0101968242656327

---

---

328

- **PDB ID:** 7WVE | **Chain:** B
- **b-phipsi:** 0.0079319212388943
- **w-rdist:** 0.6342813812888098
- **t-alpha:** 0.00512430205411

---

---

329

- **PDB ID:** 6TRX | **Chain:** B
- **b-phipsi:** 0.0057495407695643
- **w-rdist:** 0.6758548330884496
- **t-alpha:** 0.0177907956813867

---

---

330

- **PDB ID:** 2QT9 | **Chain:** A
- **b-phipsi:** 0.006195969360955
- **w-rdist:** 0.8308952448070424
- **t-alpha:** 0.0065982109973949

---

---

331

- **PDB ID:** 1R9N | **Chain:** A
- **b-phipsi:** 0.0068679024717205
- **w-rdist:** 0.8332325118598387
- **t-alpha:** 0.0014568440116902

---

---

332

- **PDB ID:** 5LS6 | **Chain:** D
- **b-phipsi:** 0.0045594711611636
- **w-rdist:** 0.6900902984140727
- **t-alpha:** 0.020817864153829

---

---

333

- **PDB ID:** 7A3I | **Chain:** C
- **b-phipsi:** 0.0114453624602521
- **w-rdist:** 0.5604551856011843
- **t-alpha:** 0.0014568440116902

---

---

334

- **PDB ID:** 8AYH | **Chain:** A
- **b-phipsi:** 0.0035520322204025
- **w-rdist:** 0.3791946877159445
- **t-alpha:** 0.0961395321892653

---

---

335

- **PDB ID:** 7XE2 | **Chain:** A
- **b-phipsi:** 0.004044213069757
- **w-rdist:** 0.4461294992800468
- **t-alpha:** 0.0726561735687285

---

---

336

- **PDB ID:** 4LKO | **Chain:** A
- **b-phipsi:** 0.0061167996257879
- **w-rdist:** 0.8357700371477895
- **t-alpha:** 0.0058266934612722

---

---

337

- **PDB ID:** 7SFF | **Chain:** A
- **b-phipsi:** 0.0077887138992431
- **w-rdist:** 0.2487315380277398
- **t-alpha:** 0.0684630747798777

---

---

338

- **PDB ID:** 7VCU | **Chain:** F
- **b-phipsi:** 0.0059330739786813
- **w-rdist:** 0.4367344897342314
- **t-alpha:** 0.0582668457273456

---

---

339

- **PDB ID:** 5LS6 | **Chain:** G
- **b-phipsi:** 0.0042006189179406
- **w-rdist:** 0.6762218156985639
- **t-alpha:** 0.0225785454961677

---

---

340

- **PDB ID:** 7R53 | **Chain:** A
- **b-phipsi:** 0.0185205611891506
- **w-rdist:** 0.3132249266944709
- **t-alpha:** 0.0174797527976524

---

---

341

- **PDB ID:** 5SWR | **Chain:** A
- **b-phipsi:** 0.0064297084866942
- **w-rdist:** 0.4831485385191188
- **t-alpha:** 0.0371451014582198

---

---

342

- **PDB ID:** 6ERF | **Chain:** F
- **b-phipsi:** 0.0020328309490282
- **w-rdist:** 0.5447011203355737
- **t-alpha:** 0.0429718680332931

---

---

343

- **PDB ID:** 2QTB | **Chain:** A
- **b-phipsi:** 0.0067533717425353
- **w-rdist:** 0.8368717962251782
- **t-alpha:** 0.0007283954918235

---

---

344

- **PDB ID:** 5WOB | **Chain:** B
- **b-phipsi:** 0.0054485431722776
- **w-rdist:** 0.5015709208460464
- **t-alpha:** 0.0480699609170811

---

---

345

- **PDB ID:** 7SFE | **Chain:** A
- **b-phipsi:** 0.0072398520164495
- **w-rdist:** 0.2610944757870186
- **t-alpha:** 0.0772031433552034

---

---

346

- **PDB ID:** 4WAF | **Chain:** A
- **b-phipsi:** 0.0061566895239796
- **w-rdist:** 0.452569939331619
- **t-alpha:** 0.0509830974011791

---

---

347

- **PDB ID:** 8FCL | **Chain:** A
- **b-phipsi:** 0.0101248624507002
- **w-rdist:** 0.2723963056415363
- **t-alpha:** 0.041514721940334

---

---

348

- **PDB ID:** 4BPB | **Chain:** A
- **b-phipsi:** 0.0095629738066711
- **w-rdist:** 0.4475019900812547
- **t-alpha:** 0.0246270663199015

---

---

349

- **PDB ID:** 3MDJ | **Chain:** A
- **b-phipsi:** 0.007406241442214
- **w-rdist:** 0.1846140580234407
- **t-alpha:** 0.0924983319030738

---

---

350

- **PDB ID:** 5FTM | **Chain:** B
- **b-phipsi:** 0.014401753211407
- **w-rdist:** 0.3545679082327921
- **t-alpha:** 0.0211216500137569

---

---

351

- **PDB ID:** 5WOB | **Chain:** f
- **b-phipsi:** 0.007213220339973
- **w-rdist:** 0.4578452469052847
- **t-alpha:** 0.0342318542055553

---

---

352

- **PDB ID:** 7VCU | **Chain:** C
- **b-phipsi:** 0.0063021472884611
- **w-rdist:** 0.4414316187697646
- **t-alpha:** 0.0524399405265496

---

---

353

- **PDB ID:** 7BP9 | **Chain:** B
- **b-phipsi:** 0.0082457955377051
- **w-rdist:** 0.1663858885410689
- **t-alpha:** 0.0808449036036103

---

---

354

- **PDB ID:** 6C02 | **Chain:** B
- **b-phipsi:** 0.0013478160693033
- **w-rdist:** 0.5090872083069813
- **t-alpha:** 0.0684824353053381

---

---

355

- **PDB ID:** 5C19 | **Chain:** E
- **b-phipsi:** 0.0091842970866781
- **w-rdist:** 0.1895129101481312
- **t-alpha:** 0.0626367777057093

---

---

356

- **PDB ID:** 8EXU | **Chain:** A
- **b-phipsi:** 0.0086890247958129
- **w-rdist:** 0.2292122571692917
- **t-alpha:** 0.0633651618828348

---

---

357

- **PDB ID:** 5T4F | **Chain:** B
- **b-phipsi:** 0.0053712125973635
- **w-rdist:** 0.8356216531377415
- **t-alpha:** 0.0110454531302417

---

---

358

- **PDB ID:** 5I7U | **Chain:** B
- **b-phipsi:** 0.0058928122801527
- **w-rdist:** 0.8299562481660467
- **t-alpha:** 0.0103018401892411

---

---

359

- **PDB ID:** 3MDJ | **Chain:** B
- **b-phipsi:** 0.0102552719526871
- **w-rdist:** 0.2453705537690258
- **t-alpha:** 0.0472921254005003

---

---

360

- **PDB ID:** 3NOX | **Chain:** A
- **b-phipsi:** 0.0066167450314615
- **w-rdist:** 0.8272253053737959
- **t-alpha:** 0.0058607467412064

---

---

361

- **PDB ID:** 1X70 | **Chain:** B
- **b-phipsi:** 0.0062007674885785
- **w-rdist:** 0.8335752852537924
- **t-alpha:** 0.0073366962737499

---

---

362

- **PDB ID:** 5DYH | **Chain:** B
- **b-phipsi:** 0.0040487073325827
- **w-rdist:** 0.5219337500575548
- **t-alpha:** 0.0521072715569208

---

---

363

- **PDB ID:** 7BP8 | **Chain:** C
- **b-phipsi:** 0.0074878811389244
- **w-rdist:** 0.2276544065686682
- **t-alpha:** 0.0852149190700788

---

---

364

- **PDB ID:** 3ML9 | **Chain:** A
- **b-phipsi:** 0.0179057506550048
- **w-rdist:** 0.2740876547845607
- **t-alpha:** 0.0233067966402911

---

---

365

- **PDB ID:** 5C18 | **Chain:** B
- **b-phipsi:** 0.006130848265462
- **w-rdist:** 0.2525388786880022
- **t-alpha:** 0.1143477037129587

---

---

366

- **PDB ID:** 5UZ0 | **Chain:** D
- **b-phipsi:** 0.0059518636163564
- **w-rdist:** 0.7380656538528533
- **t-alpha:** 0.0152949457801805

---

---

367

- **PDB ID:** 1J2E | **Chain:** B
- **b-phipsi:** 0.0067453292079235
- **w-rdist:** 0.8305757746061926
- **t-alpha:** 0.0050984212340619

---

---

368

- **PDB ID:** 6R1U | **Chain:** K
- **b-phipsi:** 0.0023389001884641
- **w-rdist:** 0.5079797896223106
- **t-alpha:** 0.0659939212028208

---

---

369

- **PDB ID:** 7BP9 | **Chain:** E
- **b-phipsi:** 0.0084635398237106
- **w-rdist:** 0.1658735391902618
- **t-alpha:** 0.0801163919944811

---

---

370

- **PDB ID:** 3EIO | **Chain:** A
- **b-phipsi:** 0.006742903689088
- **w-rdist:** 0.8405224149082628
- **t-alpha:** 0.0007288726764216

---

---

371

- **PDB ID:** 5ZID | **Chain:** B
- **b-phipsi:** 0.0075800170486169
- **w-rdist:** 0.8215754076246711
- **t-alpha:** 0.0007283954918235

---

---

372

- **PDB ID:** 2G48 | **Chain:** A
- **b-phipsi:** 0.0053241151351496
- **w-rdist:** 0.5275562233311251
- **t-alpha:** 0.0422430403347799

---

---

373

- **PDB ID:** 7MYN | **Chain:** A
- **b-phipsi:** 0.0101313523560761
- **w-rdist:** 0.4503393700603847
- **t-alpha:** 0.0240350781259224

---

---

374

- **PDB ID:** 1SC7 | **Chain:** A
- **b-phipsi:** 0.0153778411858484
- **w-rdist:** 0.4749967934077385
- **t-alpha:** 0.0088172167453031

---

---

375

- **PDB ID:** 7WVF | **Chain:** B
- **b-phipsi:** 0.0095385916281533
- **w-rdist:** 0.6279979077131099
- **t-alpha:** 0.0021896458253802

---

---

376

- **PDB ID:** 3W2T | **Chain:** A
- **b-phipsi:** 0.0063089869372373
- **w-rdist:** 0.8350191016053862
- **t-alpha:** 0.0065982109973949

---

---

377

- **PDB ID:** 4LTE | **Chain:** A
- **b-phipsi:** 0.0065439208242297
- **w-rdist:** 0.5369656344540223
- **t-alpha:** 0.0298618503359113

---

---

378

- **PDB ID:** 5VM9 | **Chain:** A
- **b-phipsi:** 0.0098333585689871
- **w-rdist:** 0.1646792217983402
- **t-alpha:** 0.0651667209447612

---

---

379

- **PDB ID:** 8FCN | **Chain:** E
- **b-phipsi:** 0.0050175083413363
- **w-rdist:** 0.3013560806413254
- **t-alpha:** 0.1289142741113467

---

---

380

- **PDB ID:** 3FXI | **Chain:** A
- **b-phipsi:** 0.0105015405884777
- **w-rdist:** 0.493777358377487
- **t-alpha:** 0.0185457488706886

---

---

381

- **PDB ID:** 3EIO | **Chain:** B
- **b-phipsi:** 0.0072498853128378
- **w-rdist:** 0.835032771660134
- **t-alpha:** 0.0

---

---

382

- **PDB ID:** 4KZ0 | **Chain:** A
- **b-phipsi:** 0.0172233815203304
- **w-rdist:** 0.2461958781120976
- **t-alpha:** 0.0276764270621712

---

---

383

- **PDB ID:** 5T4F | **Chain:** A
- **b-phipsi:** 0.0068132210334138
- **w-rdist:** 0.8395007058842943
- **t-alpha:** 0.0014568440116902

---

---

384

- **PDB ID:** 8ASC | **Chain:** L
- **b-phipsi:** 0.0013463622686616
- **w-rdist:** 0.5602551720050237
- **t-alpha:** 0.0456967989064831

---

---

385

- **PDB ID:** 6ID1 | **Chain:** c
- **b-phipsi:** 0.0067726579063313
- **w-rdist:** 0.3698274729348545
- **t-alpha:** 0.0610509630466473

---

---

386

- **PDB ID:** 3QZ2 | **Chain:** B
- **b-phipsi:** 0.0059820804794121
- **w-rdist:** 0.5165256753491432
- **t-alpha:** 0.0407864162082982

---

---

387

- **PDB ID:** 5WOB | **Chain:** e
- **b-phipsi:** 0.0074514639712633
- **w-rdist:** 0.4801879774202365
- **t-alpha:** 0.0327750996298437

---

---

388

- **PDB ID:** 5SXE | **Chain:** A
- **b-phipsi:** 0.0067646450674256
- **w-rdist:** 0.4908496508325541
- **t-alpha:** 0.0356881942932585

---

---

389

- **PDB ID:** 1J2E | **Chain:** A
- **b-phipsi:** 0.005445844116986
- **w-rdist:** 0.8311111848780248
- **t-alpha:** 0.0140326350426887

---

---

390

- **PDB ID:** 2QKY | **Chain:** D
- **b-phipsi:** 0.006049827821468
- **w-rdist:** 0.8422104532506669
- **t-alpha:** 0.0058607467412064

---

---

391

- **PDB ID:** 7AU2 | **Chain:** B
- **b-phipsi:** 0.0047648951117408
- **w-rdist:** 0.4186260938206498
- **t-alpha:** 0.0862342975398848

---

---

392

- **PDB ID:** 3N57 | **Chain:** B
- **b-phipsi:** 0.0059842931855483
- **w-rdist:** 0.5203377417222508
- **t-alpha:** 0.0378733336061549

---

---

393

- **PDB ID:** 7VCU | **Chain:** D
- **b-phipsi:** 0.0053201111256017
- **w-rdist:** 0.4401187651991424
- **t-alpha:** 0.0735613675186812

---

---

394

- **PDB ID:** 5XGI | **Chain:** A
- **b-phipsi:** 0.0066335212785154
- **w-rdist:** 0.4157734420053657
- **t-alpha:** 0.0568099994807522

---

---

395

- **PDB ID:** 7VCX | **Chain:** A
- **b-phipsi:** 0.0061314937692771
- **w-rdist:** 0.3974511728766586
- **t-alpha:** 0.0721049373143647

---

---

396

- **PDB ID:** 5I7U | **Chain:** A
- **b-phipsi:** 0.0065874074503651
- **w-rdist:** 0.8318004746280545
- **t-alpha:** 0.0065982109973949

---

---

397

- **PDB ID:** 6ERH | **Chain:** C
- **b-phipsi:** 0.0050550482614247
- **w-rdist:** 0.6145567741456939
- **t-alpha:** 0.0284642139866488

---

---

398

- **PDB ID:** 4NGE | **Chain:** A
- **b-phipsi:** 0.0106759335551365
- **w-rdist:** 0.5748722121303552
- **t-alpha:** 0.0036415440256147

---

---

399

- **PDB ID:** 7BP9 | **Chain:** A
- **b-phipsi:** 0.0074867397658661
- **w-rdist:** 0.1744377153455746
- **t-alpha:** 0.1005094622253814

---

---

400

- **PDB ID:** 4PNZ | **Chain:** B
- **b-phipsi:** 0.0068053917924875
- **w-rdist:** 0.8353625883879618
- **t-alpha:** 0.0043701973936818

---

---

401

- **PDB ID:** 5FTK | **Chain:** E
- **b-phipsi:** 0.0102341059200769
- **w-rdist:** 0.1783662581499498
- **t-alpha:** 0.0619083860998908

---

---

402

- **PDB ID:** 5SWT | **Chain:** A
- **b-phipsi:** 0.0075349297064593
- **w-rdist:** 0.4481307805382551
- **t-alpha:** 0.0356881942932585

---

---

403

- **PDB ID:** 3CWW | **Chain:** A
- **b-phipsi:** 0.0059263611307153
- **w-rdist:** 0.5366425494259085
- **t-alpha:** 0.0349600148426103

---

---

404

- **PDB ID:** 7VCT | **Chain:** F
- **b-phipsi:** 0.0054459389581575
- **w-rdist:** 0.3608995403687575
- **t-alpha:** 0.0990536816248905

---

---

405

- **PDB ID:** 6EOT | **Chain:** I
- **b-phipsi:** 0.0062866907540755
- **w-rdist:** 0.6746381304813205
- **t-alpha:** 0.0177907956813867

---

---

406

- **PDB ID:** 6MQ3 | **Chain:** B
- **b-phipsi:** 0.0054066474695023
- **w-rdist:** 0.5271648948057903
- **t-alpha:** 0.0458847982169747

---

---

407

- **PDB ID:** 5UY8 | **Chain:** D
- **b-phipsi:** 0.0055322434855558
- **w-rdist:** 0.7377122481099441
- **t-alpha:** 0.0196648853517085

---

---

408

- **PDB ID:** 4PNZ | **Chain:** A
- **b-phipsi:** 0.0068310235381521
- **w-rdist:** 0.8325046792449845
- **t-alpha:** 0.00512430205411

---

---

409

- **PDB ID:** 8FCL | **Chain:** B
- **b-phipsi:** 0.0064869215335799
- **w-rdist:** 0.1877168895882541
- **t-alpha:** 0.1318281226615005

---

---

410

- **PDB ID:** 7WVE | **Chain:** A
- **b-phipsi:** 0.0077080505970438
- **w-rdist:** 0.6366437336588301
- **t-alpha:** 0.0103018401892411

---

---

411

- **PDB ID:** 5J68 | **Chain:** A
- **b-phipsi:** 0.0048332320493505
- **w-rdist:** 0.4075067092592313
- **t-alpha:** 0.0931528332891025

---

---

412

- **PDB ID:** 7RL6 | **Chain:** C
- **b-phipsi:** 0.0074547406398715
- **w-rdist:** 0.1141939083741413
- **t-alpha:** 0.1136199749619588

---

---

413

- **PDB ID:** 7JN7 | **Chain:** A
- **b-phipsi:** 0.0084599501797507
- **w-rdist:** 0.7363360062143126
- **t-alpha:** 0.0014587541408541

---

---

414

- **PDB ID:** 7VCX | **Chain:** B
- **b-phipsi:** 0.0068653741808011
- **w-rdist:** 0.3941572139909713
- **t-alpha:** 0.0597231737029493

---

---

415

- **PDB ID:** 7AU2 | **Chain:** A
- **b-phipsi:** 0.0053731723551906
- **w-rdist:** 0.4229793674401012
- **t-alpha:** 0.0845181097092675

---

---

416

- **PDB ID:** 3O95 | **Chain:** D
- **b-phipsi:** 0.0064987896682234
- **w-rdist:** 0.8365888048148156
- **t-alpha:** 0.0058607467412064

---

---

417

- **PDB ID:** 8FCN | **Chain:** B
- **b-phipsi:** 0.0058844708366768
- **w-rdist:** 0.2933318325255037
- **t-alpha:** 0.1216319439163229

---

---

418

- **PDB ID:** 5WOB | **Chain:** F
- **b-phipsi:** 0.0065192318935638
- **w-rdist:** 0.4594740476772143
- **t-alpha:** 0.0517115174568478

---

---

419

- **PDB ID:** 5FUR | **Chain:** I
- **b-phipsi:** 0.0114330156586556
- **w-rdist:** 0.2207340877664292
- **t-alpha:** 0.0504970964456714

---

---

420

- **PDB ID:** 7YBU | **Chain:** D
- **b-phipsi:** 0.007869342391232
- **w-rdist:** 0.5466761801550414
- **t-alpha:** 0.020817864153829

---

---

421

- **PDB ID:** 6EOS | **Chain:** E
- **b-phipsi:** 0.0133289903977715
- **w-rdist:** 0.5210164585412516
- **t-alpha:** 0.0088172167453031

---

---

422

- **PDB ID:** 3W3L | **Chain:** C
- **b-phipsi:** 0.0154341963046071
- **w-rdist:** 0.3141576181795834
- **t-alpha:** 0.0262200978972946

---

---

423

- **PDB ID:** 4R09 | **Chain:** D
- **b-phipsi:** 0.0082274571964557
- **w-rdist:** 0.3616130592364319
- **t-alpha:** 0.0466131781632361

---

---

424

- **PDB ID:** 7U6E | **Chain:** E
- **b-phipsi:** 0.0156204345514541
- **w-rdist:** 0.4479579449888254
- **t-alpha:** 0.0132839260291477

---

---

425

- **PDB ID:** 5F98 | **Chain:** A
- **b-phipsi:** 0.0106408942044209
- **w-rdist:** 0.6134367463784651
- **t-alpha:** 0.0021896458253802

---

---

426

- **PDB ID:** 8AG5 | **Chain:** A
- **b-phipsi:** 0.0009059004702824
- **w-rdist:** 0.607662913484923
- **t-alpha:** 0.037792668599949

---

---

427

- **PDB ID:** 7A3K | **Chain:** C
- **b-phipsi:** 0.0105600743913417
- **w-rdist:** 0.6037538218737565
- **t-alpha:** 0.0029220273949486

---

---

428

- **PDB ID:** 3OC0 | **Chain:** A
- **b-phipsi:** 0.00622952659959
- **w-rdist:** 0.8322237509609309
- **t-alpha:** 0.010924934303695

---

---

429

- **PDB ID:** 8FCL | **Chain:** D
- **b-phipsi:** 0.0086329496165302
- **w-rdist:** 0.1471567636066133
- **t-alpha:** 0.0910417030770296

---

---

430

- **PDB ID:** 4DUR | **Chain:** B
- **b-phipsi:** 0.0197698363613219
- **w-rdist:** 0.1693286374561248
- **t-alpha:** 0.0300077103542844

---

---

431

- **PDB ID:** 7RLF | **Chain:** B
- **b-phipsi:** 0.0087533937304575
- **w-rdist:** 0.139935622198157
- **t-alpha:** 0.088856766054028

---

---

432

- **PDB ID:** 6ERF | **Chain:** H
- **b-phipsi:** 0.0016469330497396
- **w-rdist:** 0.5437360161055642
- **t-alpha:** 0.0575384238750913

---

---

433

- **PDB ID:** 6U23 | **Chain:** C
- **b-phipsi:** 0.0126348233708036
- **w-rdist:** 0.3076358376346602
- **t-alpha:** 0.0331075673737624

---

---

434

- **PDB ID:** 6BKF | **Chain:** A
- **b-phipsi:** 0.0082750522404403
- **w-rdist:** 0.5645614175920278
- **t-alpha:** 0.0155326816144203

---

---

435

- **PDB ID:** 2ONC | **Chain:** D
- **b-phipsi:** 0.0063094165900434
- **w-rdist:** 0.835844107190471
- **t-alpha:** 0.0088172167453031

---

---

436

- **PDB ID:** 6TRW | **Chain:** B
- **b-phipsi:** 0.0080559069473065
- **w-rdist:** 0.6895662735089275
- **t-alpha:** 0.0058607467412064

---

---

437

- **PDB ID:** 6TRW | **Chain:** C
- **b-phipsi:** 0.0079672657081281
- **w-rdist:** 0.6780413396747547
- **t-alpha:** 0.0073366962737499

---

---

438

- **PDB ID:** 5Y7J | **Chain:** D
- **b-phipsi:** 0.0062500688224665
- **w-rdist:** 0.8405682792699818
- **t-alpha:** 0.0073366962737499

---

---

439

- **PDB ID:** 8BH3 | **Chain:** T
- **b-phipsi:** 0.0224406737089957
- **w-rdist:** 0.3715559786212553
- **t-alpha:** 0.0110454531302417

---

---

440

- **PDB ID:** 3F8S | **Chain:** A
- **b-phipsi:** 0.0059569798561355
- **w-rdist:** 0.8348161863708585
- **t-alpha:** 0.0125367354532617

---

---

441

- **PDB ID:** 2NVU | **Chain:** B
- **b-phipsi:** 0.0058244145677929
- **w-rdist:** 0.5533986581450695
- **t-alpha:** 0.034664446127588

---

---

442

- **PDB ID:** 7RLI | **Chain:** K
- **b-phipsi:** 0.0086756231897052
- **w-rdist:** 0.1076609299499696
- **t-alpha:** 0.0983244187475649

---

---

443

- **PDB ID:** 4N8D | **Chain:** B
- **b-phipsi:** 0.0052327999430394
- **w-rdist:** 0.827193292248943
- **t-alpha:** 0.0193022341335096

---

---

444

- **PDB ID:** 7RLH | **Chain:** E
- **b-phipsi:** 0.0093759579110486
- **w-rdist:** 0.1667440722991206
- **t-alpha:** 0.0786598128498576

---

---

445

- **PDB ID:** 1N52 | **Chain:** A
- **b-phipsi:** 0.0801549999786274
- **w-rdist:** 0.2865617244247226
- **t-alpha:** 0.004389354417605

---

---

446

- **PDB ID:** 3VJM | **Chain:** A
- **b-phipsi:** 0.0060508092387603
- **w-rdist:** 0.8290812505106179
- **t-alpha:** 0.0140326350426887

---

---

447

- **PDB ID:** 7VCT | **Chain:** E
- **b-phipsi:** 0.0049786108072296
- **w-rdist:** 0.3658127160362059
- **t-alpha:** 0.1107063945241015

---

---

448

- **PDB ID:** 7YBU | **Chain:** F
- **b-phipsi:** 0.0080032852964627
- **w-rdist:** 0.5453178808076127
- **t-alpha:** 0.0215774983192449

---

---

449

- **PDB ID:** 5ZEN | **Chain:** A
- **b-phipsi:** 0.0153328163897711
- **w-rdist:** 0.4371163882566072
- **t-alpha:** 0.0167517699463162

---

---

450

- **PDB ID:** 5O9Z | **Chain:** b
- **b-phipsi:** 0.0092155502761985
- **w-rdist:** 0.4796407564173965
- **t-alpha:** 0.0276945584914771

---

---

451

- **PDB ID:** 3W5O | **Chain:** B
- **b-phipsi:** 0.0026998335998134
- **w-rdist:** 0.5582426021011069
- **t-alpha:** 0.0496939342883562

---

---

452

- **PDB ID:** 3KRW | **Chain:** A
- **b-phipsi:** 0.0110372202154018
- **w-rdist:** 0.4149177348015097
- **t-alpha:** 0.0284642139866488

---

---

453

- **PDB ID:** 5J67 | **Chain:** D
- **b-phipsi:** 0.0022554803674006
- **w-rdist:** 0.4244400860112994
- **t-alpha:** 0.1126417435120965

---

---

454

- **PDB ID:** 7VCU | **Chain:** A
- **b-phipsi:** 0.005464051675922
- **w-rdist:** 0.4402720977600145
- **t-alpha:** 0.0830297729265951

---

---

455

- **PDB ID:** 5SX9 | **Chain:** A
- **b-phipsi:** 0.0063386294421677
- **w-rdist:** 0.4421420642051606
- **t-alpha:** 0.0640930305320786

---

---

456

- **PDB ID:** 6NIT | **Chain:** B
- **b-phipsi:** 0.0063880394512338
- **w-rdist:** 0.2556285119768484
- **t-alpha:** 0.1267297821689152

---

---

457

- **PDB ID:** 6EOP | **Chain:** C
- **b-phipsi:** 0.0084426774181426
- **w-rdist:** 0.6882749381222442
- **t-alpha:** 0.0058266934612722

---

---

458

- **PDB ID:** 3LPP | **Chain:** C
- **b-phipsi:** 0.0072146550713031
- **w-rdist:** 0.6942759785383008
- **t-alpha:** 0.0117905783112548

---

---

459

- **PDB ID:** 4GU1 | **Chain:** A
- **b-phipsi:** 0.0094687126890336
- **w-rdist:** 0.5138390096573515
- **t-alpha:** 0.0230999264545257

---

---

460

- **PDB ID:** 3QBJ | **Chain:** B
- **b-phipsi:** 0.0077882455528179
- **w-rdist:** 0.8310652668896127
- **t-alpha:** 0.0029134192830806

---

---

461

- **PDB ID:** 3KWF | **Chain:** B
- **b-phipsi:** 0.0062723824160342
- **w-rdist:** 0.8284044810459403
- **t-alpha:** 0.0132839260291477

---

---

462

- **PDB ID:** 5CJO | **Chain:** A
- **b-phipsi:** 0.0058203039390689
- **w-rdist:** 0.5323344261223661
- **t-alpha:** 0.0436997123665408

---

---

463

- **PDB ID:** 7K1N | **Chain:** B
- **b-phipsi:** 0.011080281410138
- **w-rdist:** 0.5864464439896921
- **t-alpha:** 0.004389354417605

---

---

464

- **PDB ID:** 3C43 | **Chain:** A
- **b-phipsi:** 0.0068232449015159
- **w-rdist:** 0.8361877505096157
- **t-alpha:** 0.0058607467412064

---

---

465

- **PDB ID:** 7VCU | **Chain:** G
- **b-phipsi:** 0.0064926340546344
- **w-rdist:** 0.4458952615616751
- **t-alpha:** 0.0604515833997143

---

---

466

- **PDB ID:** 3KWF | **Chain:** A
- **b-phipsi:** 0.0066055088626531
- **w-rdist:** 0.8417770386620782
- **t-alpha:** 0.0058266934612722

---

---

467

- **PDB ID:** 7LN0 | **Chain:** E
- **b-phipsi:** 0.0066052261581587
- **w-rdist:** 0.70856021539853
- **t-alpha:** 0.016284021422591

---

---

468

- **PDB ID:** 7OP0 | **Chain:** A
- **b-phipsi:** 0.0025923541457971
- **w-rdist:** 0.5528355846796706
- **t-alpha:** 0.0538967929362443

---

---

469

- **PDB ID:** 7WUB | **Chain:** H
- **b-phipsi:** 0.0084793235213679
- **w-rdist:** 0.1254074045288601
- **t-alpha:** 0.1019661202012538

---

---

470

- **PDB ID:** 8FCO | **Chain:** F
- **b-phipsi:** 0.0091622225438427
- **w-rdist:** 0.181173578806251
- **t-alpha:** 0.0830297729265951

---

---

471

- **PDB ID:** 7BP8 | **Chain:** F
- **b-phipsi:** 0.0075026319196013
- **w-rdist:** 0.2296470973301
- **t-alpha:** 0.1026946462477864

---

---

472

- **PDB ID:** 5SWP | **Chain:** A
- **b-phipsi:** 0.0074215595477922
- **w-rdist:** 0.4513180852869304
- **t-alpha:** 0.0422430403347799

---

---

473

- **PDB ID:** 2FJP | **Chain:** A
- **b-phipsi:** 0.0062744187891595
- **w-rdist:** 0.8399545810230007
- **t-alpha:** 0.0088172167453031

---

---

474

- **PDB ID:** 5FTK | **Chain:** C
- **b-phipsi:** 0.0093553283183787
- **w-rdist:** 0.1820720309735247
- **t-alpha:** 0.0801163919944811

---

---

475

- **PDB ID:** 3SWW | **Chain:** A
- **b-phipsi:** 0.0066803978998718
- **w-rdist:** 0.8298620079079608
- **t-alpha:** 0.0103018401892411

---

---

476

- **PDB ID:** 2HHA | **Chain:** A
- **b-phipsi:** 0.0064923230222781
- **w-rdist:** 0.8377723997215767
- **t-alpha:** 0.0080762040238695

---

---

477

- **PDB ID:** 8FCM | **Chain:** F
- **b-phipsi:** 0.008598053389091
- **w-rdist:** 0.3303408487424421
- **t-alpha:** 0.0553531458501084

---

---

478

- **PDB ID:** 7RLI | **Chain:** B
- **b-phipsi:** 0.0094719364400502
- **w-rdist:** 0.1222555326374342
- **t-alpha:** 0.0895849381549418

---

---

479

- **PDB ID:** 3G0D | **Chain:** A
- **b-phipsi:** 0.0074481833683222
- **w-rdist:** 0.8340494924942343
- **t-alpha:** 0.0036549441753579

---

---

480

- **PDB ID:** 7NFE | **Chain:** C
- **b-phipsi:** 0.0093315912899041
- **w-rdist:** 0.2707235986393398
- **t-alpha:** 0.0633651618828348

---

---

481

- **PDB ID:** 8B5R | **Chain:** B
- **b-phipsi:** 0.0082739295165088
- **w-rdist:** 0.8173174659562362
- **t-alpha:** 0.0021896458253802

---

---

482

- **PDB ID:** 4PES | **Chain:** B
- **b-phipsi:** 0.0067448015297789
- **w-rdist:** 0.4867402215133749
- **t-alpha:** 0.0458847982169747

---

---

483

- **PDB ID:** 3CCB | **Chain:** D
- **b-phipsi:** 0.0060640405513182
- **w-rdist:** 0.8431049524202077
- **t-alpha:** 0.0095587741674756

---

---

484

- **PDB ID:** 7PPJ | **Chain:** A
- **b-phipsi:** 0.0083746234839353
- **w-rdist:** 0.4918478431173556
- **t-alpha:** 0.0307805727451215

---

---

485

- **PDB ID:** 6HP8 | **Chain:** B
- **b-phipsi:** 0.0086327310354277
- **w-rdist:** 0.6764958543776378
- **t-alpha:** 0.0065550134155867

---

---

486

- **PDB ID:** 7P7P | **Chain:** B
- **b-phipsi:** 0.0092503221063408
- **w-rdist:** 0.664382914778899
- **t-alpha:** 0.00512430205411

---

---

487

- **PDB ID:** 7PG5 | **Chain:** A
- **b-phipsi:** 0.0073302796535874
- **w-rdist:** 0.4525155273034325
- **t-alpha:** 0.0444280646304331

---

---

488

- **PDB ID:** 5WOB | **Chain:** b
- **b-phipsi:** 0.0066983921114299
- **w-rdist:** 0.5033848349413471
- **t-alpha:** 0.0429718680332931

---

---

489

- **PDB ID:** 7A3L | **Chain:** A
- **b-phipsi:** 0.0074436328840825
- **w-rdist:** 0.6195055754306558
- **t-alpha:** 0.0170368870295003

---

---

490

- **PDB ID:** 3C45 | **Chain:** A
- **b-phipsi:** 0.0055716251814377
- **w-rdist:** 0.8361692921903541
- **t-alpha:** 0.0160233389937762

---

---

491

- **PDB ID:** 8FCT | **Chain:** C
- **b-phipsi:** 0.0079231666546294
- **w-rdist:** 0.1613716585186319
- **t-alpha:** 0.1099778680663861

---

---

492

- **PDB ID:** 1R9N | **Chain:** C
- **b-phipsi:** 0.0073209989991527
- **w-rdist:** 0.8301412916344165
- **t-alpha:** 0.0058607467412064

---

---

493

- **PDB ID:** 3CDZ | **Chain:** A
- **b-phipsi:** 0.0409261798605325
- **w-rdist:** 0.3625017793524294
- **t-alpha:** 0.0050984212340619

---

---

494

- **PDB ID:** 2G5P | **Chain:** B
- **b-phipsi:** 0.0064745715481948
- **w-rdist:** 0.8384793308078936
- **t-alpha:** 0.0087397720309874

---

---

495

- **PDB ID:** 7WV3 | **Chain:** A
- **b-phipsi:** 0.0106508777825215
- **w-rdist:** 0.6540397452493968
- **t-alpha:** 0.0021896458253802

---

---

496

- **PDB ID:** 4DB1 | **Chain:** A
- **b-phipsi:** 0.012225842401641
- **w-rdist:** 0.4982495550087842
- **t-alpha:** 0.0177907956813867

---

---

497

- **PDB ID:** 6GYR | **Chain:** A
- **b-phipsi:** 0.0044948358679864
- **w-rdist:** 0.5034786553050707
- **t-alpha:** 0.0726561735687285

---

---

498

- **PDB ID:** 2WJY | **Chain:** A
- **b-phipsi:** 0.0039087567947213
- **w-rdist:** 0.4805266332420173
- **t-alpha:** 0.0879557950251648

---

---

499

- **PDB ID:** 6TEZ | **Chain:** A
- **b-phipsi:** 0.0044316419751439
- **w-rdist:** 0.7676722690892475
- **t-alpha:** 0.0253919797973902

---

---

500

- **PDB ID:** 2YKG | **Chain:** A
- **b-phipsi:** 0.0129681837230373
- **w-rdist:** 0.3524578416201601
- **t-alpha:** 0.0305898827678434

---

---

501

- **PDB ID:** 2G47 | **Chain:** A
- **b-phipsi:** 0.00579678419108
- **w-rdist:** 0.534309218350353
- **t-alpha:** 0.0458847982169747

---

---

502

- **PDB ID:** 5FTJ | **Chain:** A
- **b-phipsi:** 0.0151293948847133
- **w-rdist:** 0.1325651860466532
- **t-alpha:** 0.0495267695540877

---

---

503

- **PDB ID:** 7RLB | **Chain:** A
- **b-phipsi:** 0.0091834811051424
- **w-rdist:** 0.172097850679289
- **t-alpha:** 0.087399807391695

---

---

504

- **PDB ID:** 4ASI | **Chain:** C
- **b-phipsi:** 0.008575235384088
- **w-rdist:** 0.671675210599459
- **t-alpha:** 0.0080117938638943

---

---

505

- **PDB ID:** 6FXM | **Chain:** A
- **b-phipsi:** 0.0049971007729912
- **w-rdist:** 0.7679979627654423
- **t-alpha:** 0.0246270663199015

---

---

506

- **PDB ID:** 7W5A | **Chain:** C
- **b-phipsi:** 0.0045612438426427
- **w-rdist:** 0.4033867229703399
- **t-alpha:** 0.1108414573859111

---

---

507

- **PDB ID:** 5FTL | **Chain:** B
- **b-phipsi:** 0.0079200696592689
- **w-rdist:** 0.2210048010326547
- **t-alpha:** 0.1005094622253814

---

---

508

- **PDB ID:** 3L4G | **Chain:** J
- **b-phipsi:** 0.0020155449804922
- **w-rdist:** 0.6623381220070855
- **t-alpha:** 0.0349600148426103

---

---

509

- **PDB ID:** 7RLF | **Chain:** A
- **b-phipsi:** 0.0091829045720899
- **w-rdist:** 0.1397434960015005
- **t-alpha:** 0.0946832683923559

---

---

510

- **PDB ID:** 3CST | **Chain:** A
- **b-phipsi:** 0.0119620886253142
- **w-rdist:** 0.2863826215793612
- **t-alpha:** 0.0429718680332931

---

---

511

- **PDB ID:** 3D4L | **Chain:** B
- **b-phipsi:** 0.0060513870690997
- **w-rdist:** 0.8351513965764414
- **t-alpha:** 0.0140326350426887

---

---

512

- **PDB ID:** 6EJ9 | **Chain:** A
- **b-phipsi:** 0.0015265241730693
- **w-rdist:** 0.6270744054611924
- **t-alpha:** 0.0401513644150786

---

---

513

- **PDB ID:** 3NOX | **Chain:** B
- **b-phipsi:** 0.0068678284871243
- **w-rdist:** 0.8311461867130381
- **t-alpha:** 0.0095587741674756

---

---

514

- **PDB ID:** 4E36 | **Chain:** A
- **b-phipsi:** 0.0069783311152778
- **w-rdist:** 0.5144643027102777
- **t-alpha:** 0.0370091093109781

---

---

515

- **PDB ID:** 5Y3R | **Chain:** A
- **b-phipsi:** 0.0103876007121792
- **w-rdist:** 0.4930723424547392
- **t-alpha:** 0.0246270663199015

---

---

516

- **PDB ID:** 4ZG7 | **Chain:** A
- **b-phipsi:** 0.0054207901088054
- **w-rdist:** 0.4950687899109667
- **t-alpha:** 0.070148243271072

---

---

517

- **PDB ID:** 6ERH | **Chain:** D
- **b-phipsi:** 0.0089495627520817
- **w-rdist:** 0.2433633176666967
- **t-alpha:** 0.0772031433552034

---

---

518

- **PDB ID:** 4KR0 | **Chain:** A
- **b-phipsi:** 0.0059244465904915
- **w-rdist:** 0.8356640160780053
- **t-alpha:** 0.0152949457801805

---

---

519

- **PDB ID:** 5Y7H | **Chain:** A
- **b-phipsi:** 0.0065131542278136
- **w-rdist:** 0.8362271282404501
- **t-alpha:** 0.0101968242656327

---

---

520

- **PDB ID:** 5CJO | **Chain:** a
- **b-phipsi:** 0.0060411910409346
- **w-rdist:** 0.5308432778315667
- **t-alpha:** 0.0458847982169747

---

---

521

- **PDB ID:** 7K0Y | **Chain:** B
- **b-phipsi:** 0.0121180460818242
- **w-rdist:** 0.5695126047936082
- **t-alpha:** 0.0050984212340619

---

---

522

- **PDB ID:** 3LPO | **Chain:** B
- **b-phipsi:** 0.0089087947067363
- **w-rdist:** 0.6886160218295454
- **t-alpha:** 0.00512430205411

---

---

523

- **PDB ID:** 2QJR | **Chain:** B
- **b-phipsi:** 0.0079792944423798
- **w-rdist:** 0.835320858222619
- **t-alpha:** 0.0014587541408541

---

---

524

- **PDB ID:** 5CU5 | **Chain:** A
- **b-phipsi:** 0.0088925251087587
- **w-rdist:** 0.500779119081794
- **t-alpha:** 0.0284642139866488

---

---

525

- **PDB ID:** 4GS8 | **Chain:** B
- **b-phipsi:** 0.005864189068707
- **w-rdist:** 0.5266432175416389
- **t-alpha:** 0.0524399405265496

---

---

526

- **PDB ID:** 3W1G | **Chain:** A
- **b-phipsi:** 0.0030733901025659
- **w-rdist:** 0.5295267910194582
- **t-alpha:** 0.0693147599636081

---

---

527

- **PDB ID:** 3LPP | **Chain:** A
- **b-phipsi:** 0.0069153518230039
- **w-rdist:** 0.6969459810961887
- **t-alpha:** 0.016284021422591

---

---

528

- **PDB ID:** 7RL6 | **Chain:** F
- **b-phipsi:** 0.0075415292284821
- **w-rdist:** 0.1161225148482957
- **t-alpha:** 0.1340127658618555

---

---

529

- **PDB ID:** 5J67 | **Chain:** B
- **b-phipsi:** 0.003859796608997
- **w-rdist:** 0.4238535510724849
- **t-alpha:** 0.1126417435120965

---

---

530

- **PDB ID:** 3SWW | **Chain:** B
- **b-phipsi:** 0.0065862667224651
- **w-rdist:** 0.8306491530161223
- **t-alpha:** 0.0125367354532617

---

---

531

- **PDB ID:** 7BP9 | **Chain:** D
- **b-phipsi:** 0.007801344368494
- **w-rdist:** 0.1680857619263692
- **t-alpha:** 0.1158045881950033

---

---

532

- **PDB ID:** 8FCN | **Chain:** F
- **b-phipsi:** 0.0052387774972491
- **w-rdist:** 0.2819010567769653
- **t-alpha:** 0.1777133218901017

---

---

533

- **PDB ID:** 8EFD | **Chain:** A
- **b-phipsi:** 0.0132855234127314
- **w-rdist:** 0.4533903113667908
- **t-alpha:** 0.0215774983192449

---

---

534

- **PDB ID:** 7Z6O | **Chain:** A
- **b-phipsi:** 0.0017825697853594
- **w-rdist:** 0.6371287667205328
- **t-alpha:** 0.037792668599949

---

---

535

- **PDB ID:** 5SXC | **Chain:** A
- **b-phipsi:** 0.0068311757994863
- **w-rdist:** 0.4862179589905256
- **t-alpha:** 0.0495267695540877

---

---

536

- **PDB ID:** 8EXO | **Chain:** A
- **b-phipsi:** 0.0094484100510017
- **w-rdist:** 0.2368792028950328
- **t-alpha:** 0.0757463891855267

---

---

537

- **PDB ID:** 5WOB | **Chain:** C
- **b-phipsi:** 0.0063353836898465
- **w-rdist:** 0.5038720911901852
- **t-alpha:** 0.0538967929362443

---

---

538

- **PDB ID:** 7WUB | **Chain:** L
- **b-phipsi:** 0.0071775616149972
- **w-rdist:** 0.1250990834968448
- **t-alpha:** 0.1456667763786574

---

---

539

- **PDB ID:** 7XE3 | **Chain:** A
- **b-phipsi:** 0.0056300746235395
- **w-rdist:** 0.4615645524605923
- **t-alpha:** 0.081102112321876

---

---

540

- **PDB ID:** 7BG0 | **Chain:** A
- **b-phipsi:** 0.0119853114610755
- **w-rdist:** 0.4782555612941439
- **t-alpha:** 0.0223381852496014

---

---

541

- **PDB ID:** 7RLH | **Chain:** A
- **b-phipsi:** 0.0116144372524805
- **w-rdist:** 0.1357734846452183
- **t-alpha:** 0.0706484408514722

---

---

542

- **PDB ID:** 8FCL | **Chain:** F
- **b-phipsi:** 0.0074126081752507
- **w-rdist:** 0.1729312237663616
- **t-alpha:** 0.1267297821689152

---

---

543

- **PDB ID:** 7VCU | **Chain:** K
- **b-phipsi:** 0.0063303626644702
- **w-rdist:** 0.432545541719877
- **t-alpha:** 0.0764750331519523

---

---

544

- **PDB ID:** 6ZJZ | **Chain:** A
- **b-phipsi:** 0.0100755163314637
- **w-rdist:** 0.3137084959592618
- **t-alpha:** 0.0531683659174533

---

---

545

- **PDB ID:** 1R9M | **Chain:** C
- **b-phipsi:** 0.0061005932717089
- **w-rdist:** 0.8393870081024686
- **t-alpha:** 0.0125367354532617

---

---

546

- **PDB ID:** 8FCM | **Chain:** A
- **b-phipsi:** 0.0073839315839351
- **w-rdist:** 0.3923507706227253
- **t-alpha:** 0.0633651618828348

---

---

547

- **PDB ID:** 7ORC | **Chain:** A
- **b-phipsi:** 0.0064546585505066
- **w-rdist:** 0.3523603262817474
- **t-alpha:** 0.0990536816248905

---

---

548

- **PDB ID:** 5WOB | **Chain:** G
- **b-phipsi:** 0.0072999470584438
- **w-rdist:** 0.4694212866436098
- **t-alpha:** 0.0458847982169747

---

---

549

- **PDB ID:** 5WOB | **Chain:** a
- **b-phipsi:** 0.0060694056771624
- **w-rdist:** 0.5284582755122296
- **t-alpha:** 0.0495267695540877

---

---

550

- **PDB ID:** 3N57 | **Chain:** A
- **b-phipsi:** 0.0057175061405615
- **w-rdist:** 0.5403616756349413
- **t-alpha:** 0.0466131781632361

---

---

551

- **PDB ID:** 8FCO | **Chain:** C
- **b-phipsi:** 0.0087507158579926
- **w-rdist:** 0.3547397126353415
- **t-alpha:** 0.0538967929362443

---

---

552

- **PDB ID:** 7ZVT | **Chain:** A
- **b-phipsi:** 0.0011521029367331
- **w-rdist:** 0.6294071354867666
- **t-alpha:** 0.0449007945645392

---

---

553

- **PDB ID:** 3G0C | **Chain:** A
- **b-phipsi:** 0.0063461511071863
- **w-rdist:** 0.8342156323527384
- **t-alpha:** 0.0132839260291477

---

---

554

- **PDB ID:** 2CKJ | **Chain:** A
- **b-phipsi:** 0.0124964504156097
- **w-rdist:** 0.3883356137565408
- **t-alpha:** 0.0298618503359113

---

---

555

- **PDB ID:** 6MQ3 | **Chain:** A
- **b-phipsi:** 0.0053329332111137
- **w-rdist:** 0.5268072641155086
- **t-alpha:** 0.0604515833997143

---

---

556

- **PDB ID:** 5J6S | **Chain:** A
- **b-phipsi:** 0.009626748132018
- **w-rdist:** 0.6003780110317014
- **t-alpha:** 0.0101968242656327

---

---

557

- **PDB ID:** 4QJ3 | **Chain:** B
- **b-phipsi:** 0.0009017506954001
- **w-rdist:** 0.6540910365774196
- **t-alpha:** 0.0417297861962435

---

---

558

- **PDB ID:** 6I7S | **Chain:** H
- **b-phipsi:** 0.0050948950968702
- **w-rdist:** 0.5749504858284491
- **t-alpha:** 0.0370091093109781

---

---

559

- **PDB ID:** 5T4B | **Chain:** A
- **b-phipsi:** 0.0062628693051235
- **w-rdist:** 0.8403868789161768
- **t-alpha:** 0.0110454531302417

---

---

560

- **PDB ID:** 5SWO | **Chain:** A
- **b-phipsi:** 0.0080845340720331
- **w-rdist:** 0.4427891173941178
- **t-alpha:** 0.041514721940334

---

---

561

- **PDB ID:** 3TOP | **Chain:** A
- **b-phipsi:** 0.0069328584165542
- **w-rdist:** 0.6841650174982744
- **t-alpha:** 0.0177907956813867

---

---

562

- **PDB ID:** 3OPM | **Chain:** C
- **b-phipsi:** 0.0076837294061446
- **w-rdist:** 0.8440108146322158
- **t-alpha:** 0.0007288726764216

---

---

563

- **PDB ID:** 7WUB | **Chain:** I
- **b-phipsi:** 0.0072071270428968
- **w-rdist:** 0.1201478288371626
- **t-alpha:** 0.1522213463235975

---

---

564

- **PDB ID:** 1RWQ | **Chain:** B
- **b-phipsi:** 0.0071508969997549
- **w-rdist:** 0.8381946694799531
- **t-alpha:** 0.0058266934612722

---

---

565

- **PDB ID:** 5T4H | **Chain:** B
- **b-phipsi:** 0.0061319206274263
- **w-rdist:** 0.8329861076534322
- **t-alpha:** 0.016284021422591

---

---

566

- **PDB ID:** 5C19 | **Chain:** B
- **b-phipsi:** 0.0085022713411162
- **w-rdist:** 0.1669796020957567
- **t-alpha:** 0.1070653702560531

---

---

567

- **PDB ID:** 5FTL | **Chain:** C
- **b-phipsi:** 0.0075212084006746
- **w-rdist:** 0.2133903855561534
- **t-alpha:** 0.1172612463811577

---

---

568

- **PDB ID:** 5HE1 | **Chain:** A
- **b-phipsi:** 0.0107991039828527
- **w-rdist:** 0.3704681847355447
- **t-alpha:** 0.0354447525832375

---

---

569

- **PDB ID:** 8ASC | **Chain:** A
- **b-phipsi:** 0.0024728567249914
- **w-rdist:** 0.657466802272676
- **t-alpha:** 0.0362266357400147

---

---

570

- **PDB ID:** 2JID | **Chain:** B
- **b-phipsi:** 0.0067027725856965
- **w-rdist:** 0.8385814508146281
- **t-alpha:** 0.0088172167453031

---

---

571

- **PDB ID:** 5VM9 | **Chain:** C
- **b-phipsi:** 0.0104394650186182
- **w-rdist:** 0.1393800099812611
- **t-alpha:** 0.0862342975398848

---

---

572

- **PDB ID:** 6ERG | **Chain:** D
- **b-phipsi:** 0.0006639502644265
- **w-rdist:** 0.6672692698066793
- **t-alpha:** 0.0409402761272381

---

---

573

- **PDB ID:** 3B2D | **Chain:** A
- **b-phipsi:** 0.0102377638555675
- **w-rdist:** 0.448185700277658
- **t-alpha:** 0.0315549988173708

---

---

574

- **PDB ID:** 5WOB | **Chain:** g
- **b-phipsi:** 0.0076016404961959
- **w-rdist:** 0.4694687651594582
- **t-alpha:** 0.0429718680332931

---

---

575

- **PDB ID:** 5IXC | **Chain:** B
- **b-phipsi:** 0.0261509890416955
- **w-rdist:** 0.4632171972560033
- **t-alpha:** 0.00512430205411

---

---

576

- **PDB ID:** 6XRM | **Chain:** A
- **b-phipsi:** 0.0141369224116838
- **w-rdist:** 0.2564714545371404
- **t-alpha:** 0.041514721940334

---

---

577

- **PDB ID:** 7P7P | **Chain:** A
- **b-phipsi:** 0.0147821077934201
- **w-rdist:** 0.5781440221586258
- **t-alpha:** 0.0007283954918235

---

---

578

- **PDB ID:** 8FCT | **Chain:** F
- **b-phipsi:** 0.0090308953659715
- **w-rdist:** 0.1692644592767367
- **t-alpha:** 0.0990536816248905

---

---

579

- **PDB ID:** 6OCU | **Chain:** A
- **b-phipsi:** 0.0106696826269251
- **w-rdist:** 0.3237989365259934
- **t-alpha:** 0.0466131781632361

---

---

580

- **PDB ID:** 2RGU | **Chain:** A
- **b-phipsi:** 0.0076878455004271
- **w-rdist:** 0.8384729044343351
- **t-alpha:** 0.0029220273949486

---

---

581

- **PDB ID:** 4AH6 | **Chain:** B
- **b-phipsi:** 0.0041149947226531
- **w-rdist:** 0.3985134643298203
- **t-alpha:** 0.1291118505353805

---

---

582

- **PDB ID:** 8FCM | **Chain:** D
- **b-phipsi:** 0.0085220016910049
- **w-rdist:** 0.1647738092967602
- **t-alpha:** 0.1099778680663861

---

---

583

- **PDB ID:** 7LN4 | **Chain:** E
- **b-phipsi:** 0.0054400319286185
- **w-rdist:** 0.7160234903000043
- **t-alpha:** 0.0269484940117843

---

---

584

- **PDB ID:** 7RLF | **Chain:** E
- **b-phipsi:** 0.0095221264073715
- **w-rdist:** 0.1374731913262661
- **t-alpha:** 0.0990536816248905

---

---

585

- **PDB ID:** 8HBE | **Chain:** B
- **b-phipsi:** 0.0138211599847339
- **w-rdist:** 0.3491555802435268
- **t-alpha:** 0.032046507746164

---

---

586

- **PDB ID:** 8P4F | **Chain:** O
- **b-phipsi:** 0.0020546222812976
- **w-rdist:** 0.5331184138217339
- **t-alpha:** 0.0777081801365642

---

---

587

- **PDB ID:** 7BPA | **Chain:** D
- **b-phipsi:** 0.007027003956997
- **w-rdist:** 0.3299760870876081
- **t-alpha:** 0.0946832683923559

---

---

588

- **PDB ID:** 2BUB | **Chain:** A
- **b-phipsi:** 0.0069457843963221
- **w-rdist:** 0.8380741501542538
- **t-alpha:** 0.0080762040238695

---

---

589

- **PDB ID:** 2BUB | **Chain:** B
- **b-phipsi:** 0.0077219873414569
- **w-rdist:** 0.8335215989491369
- **t-alpha:** 0.0058266934612722

---

---

590

- **PDB ID:** 2WK3 | **Chain:** A
- **b-phipsi:** 0.0058381548936652
- **w-rdist:** 0.5426180633897233
- **t-alpha:** 0.047341565959746

---

---

591

- **PDB ID:** 3G0D | **Chain:** D
- **b-phipsi:** 0.0069508071100741
- **w-rdist:** 0.8391377453751581
- **t-alpha:** 0.0080117938638943

---

---

592

- **PDB ID:** 2OPH | **Chain:** B
- **b-phipsi:** 0.0072304859963193
- **w-rdist:** 0.8306737493158932
- **t-alpha:** 0.0095587741674756

---

---

593

- **PDB ID:** 4R07 | **Chain:** C
- **b-phipsi:** 0.0099418816185498
- **w-rdist:** 0.3587698704431464
- **t-alpha:** 0.0466131781632361

---

---

594

- **PDB ID:** 7KKE | **Chain:** A
- **b-phipsi:** 0.012023393976148
- **w-rdist:** 0.2239098177133595
- **t-alpha:** 0.0604515833997143

---

---

595

- **PDB ID:** 2JBU | **Chain:** B
- **b-phipsi:** 0.0063681438587086
- **w-rdist:** 0.5245932444788571
- **t-alpha:** 0.0495267695540877

---

---

596

- **PDB ID:** 5Z58 | **Chain:** C
- **b-phipsi:** 0.0058162488863928
- **w-rdist:** 0.3979129843718317
- **t-alpha:** 0.1090467283444061

---

---

597

- **PDB ID:** 5WEA | **Chain:** A
- **b-phipsi:** 0.0114491118195398
- **w-rdist:** 0.3305772856624838
- **t-alpha:** 0.0407864162082982

---

---

598

- **PDB ID:** 3BJM | **Chain:** A
- **b-phipsi:** 0.0066963384982031
- **w-rdist:** 0.8326264946065958
- **t-alpha:** 0.0132839260291477

---

---

599

- **PDB ID:** 6EOT | **Chain:** B
- **b-phipsi:** 0.0085119932861698
- **w-rdist:** 0.6764793462979249
- **t-alpha:** 0.0103018401892411

---

---

600

- **PDB ID:** 7VCX | **Chain:** D
- **b-phipsi:** 0.0072337564615803
- **w-rdist:** 0.403641256416955
- **t-alpha:** 0.0677347625762181

---

---

601

- **PDB ID:** 6ERG | **Chain:** A
- **b-phipsi:** 0.0003023577009538
- **w-rdist:** 0.6452289270169057
- **t-alpha:** 0.0504970964456714

---

---

602

- **PDB ID:** 7VCU | **Chain:** H
- **b-phipsi:** 0.0064545432935763
- **w-rdist:** 0.4390028849415364
- **t-alpha:** 0.077931746040353

---

---

603

- **PDB ID:** 3G0D | **Chain:** C
- **b-phipsi:** 0.007650065882296
- **w-rdist:** 0.8316467262917968
- **t-alpha:** 0.0073366962737499

---

---

604

- **PDB ID:** 2CKJ | **Chain:** C
- **b-phipsi:** 0.0151416764380709
- **w-rdist:** 0.4566709236804596
- **t-alpha:** 0.020393494275321

---

---

605

- **PDB ID:** 6U23 | **Chain:** N
- **b-phipsi:** 0.0116029305585195
- **w-rdist:** 0.2982209826921706
- **t-alpha:** 0.0521072715569208

---

---

606

- **PDB ID:** 7BPA | **Chain:** C
- **b-phipsi:** 0.0065234707650438
- **w-rdist:** 0.3145958479230658
- **t-alpha:** 0.1209026650898825

---

---

607

- **PDB ID:** 2YPU | **Chain:** A
- **b-phipsi:** 0.0067025411018171
- **w-rdist:** 0.5330491258928327
- **t-alpha:** 0.0378733336061549

---

---

608

- **PDB ID:** 6Y0F | **Chain:** C
- **b-phipsi:** 0.0073173359976774
- **w-rdist:** 0.8352570999389719
- **t-alpha:** 0.0080762040238695

---

---

609

- **PDB ID:** 2WBY | **Chain:** B
- **b-phipsi:** 0.0058842607805081
- **w-rdist:** 0.5244134998537207
- **t-alpha:** 0.0597231737029493

---

---

610

- **PDB ID:** 6BF6 | **Chain:** B
- **b-phipsi:** 0.0084290074117916
- **w-rdist:** 0.4137290072641987
- **t-alpha:** 0.0517115174568478

---

---

611

- **PDB ID:** 2IIV | **Chain:** B
- **b-phipsi:** 0.0064780958235588
- **w-rdist:** 0.8367838737201035
- **t-alpha:** 0.0132839260291477

---

---

612

- **PDB ID:** 5WOB | **Chain:** E
- **b-phipsi:** 0.0064114799602
- **w-rdist:** 0.4850023089055826
- **t-alpha:** 0.0648213974041245

---

---

613

- **PDB ID:** 7A3G | **Chain:** A
- **b-phipsi:** 0.0100200804101961
- **w-rdist:** 0.5910801298751502
- **t-alpha:** 0.0123817636541396

---

---

614

- **PDB ID:** 7VCT | **Chain:** B
- **b-phipsi:** 0.0055784805080917
- **w-rdist:** 0.3688779174086707
- **t-alpha:** 0.1260014841031005

---

---

615

- **PDB ID:** 5GVE | **Chain:** A
- **b-phipsi:** 0.0058666775832087
- **w-rdist:** 0.4946388802035264
- **t-alpha:** 0.0734948207675663

---

---

616

- **PDB ID:** 6AYG | **Chain:** A
- **b-phipsi:** 0.0403482701696468
- **w-rdist:** 0.3668206771691688
- **t-alpha:** 0.0088172167453031

---

---

617

- **PDB ID:** 4DWK | **Chain:** A
- **b-phipsi:** 0.0065948678718955
- **w-rdist:** 0.533498205912675
- **t-alpha:** 0.041514721940334

---

---

618

- **PDB ID:** 8FCR | **Chain:** A
- **b-phipsi:** 0.0087109943949126
- **w-rdist:** 0.3621003985035769
- **t-alpha:** 0.058994759372676

---

---

619

- **PDB ID:** 3H44 | **Chain:** B
- **b-phipsi:** 0.0071796241615085
- **w-rdist:** 0.5225738835368164
- **t-alpha:** 0.0371451014582198

---

---

620

- **PDB ID:** 3KRX | **Chain:** A
- **b-phipsi:** 0.0101746317611145
- **w-rdist:** 0.4029437413139499
- **t-alpha:** 0.037792668599949

---

---

621

- **PDB ID:** 7BPA | **Chain:** F
- **b-phipsi:** 0.0067165319532988
- **w-rdist:** 0.3233094559214855
- **t-alpha:** 0.1114343391983352

---

---

622

- **PDB ID:** 3L4G | **Chain:** N
- **b-phipsi:** 0.0016757543638192
- **w-rdist:** 0.677190312226006
- **t-alpha:** 0.0378733336061549

---

---

623

- **PDB ID:** 5SXB | **Chain:** A
- **b-phipsi:** 0.0068858059844441
- **w-rdist:** 0.4738154801972181
- **t-alpha:** 0.0597231737029493

---

---

624

- **PDB ID:** 7NSK | **Chain:** A
- **b-phipsi:** 0.0108786996040978
- **w-rdist:** 0.5915676105796042
- **t-alpha:** 0.0095587741674756

---

---

625

- **PDB ID:** 2HHA | **Chain:** B
- **b-phipsi:** 0.0055543437110529
- **w-rdist:** 0.8350254097445172
- **t-alpha:** 0.0215774983192449

---

---

626

- **PDB ID:** 7RLI | **Chain:** E
- **b-phipsi:** 0.0100491587493172
- **w-rdist:** 0.1360123699683423
- **t-alpha:** 0.1005094622253814

---

---

627

- **PDB ID:** 4GSC | **Chain:** B
- **b-phipsi:** 0.0061812179518746
- **w-rdist:** 0.5205470781513815
- **t-alpha:** 0.058994759372676

---

---

628

- **PDB ID:** 5MQF | **Chain:** b
- **b-phipsi:** 0.0110096384731155
- **w-rdist:** 0.3580718998781949
- **t-alpha:** 0.0417297861962435

---

---

629

- **PDB ID:** 1W1I | **Chain:** C
- **b-phipsi:** 0.0072312512836885
- **w-rdist:** 0.8395767931066922
- **t-alpha:** 0.0072833804367331

---

---

630

- **PDB ID:** 1R9M | **Chain:** D
- **b-phipsi:** 0.0070043260120636
- **w-rdist:** 0.8381701424574357
- **t-alpha:** 0.009468275938171

---

---

631

- **PDB ID:** 1TZN | **Chain:** e
- **b-phipsi:** 0.0061012918609379
- **w-rdist:** 0.815846481811475
- **t-alpha:** 0.0215774983192449

---

---

632

- **PDB ID:** 7AD6 | **Chain:** B
- **b-phipsi:** 0.0373881104734794
- **w-rdist:** 0.3603168367888424
- **t-alpha:** 0.011653569134763

---

---

633

- **PDB ID:** 5EPG | **Chain:** A
- **b-phipsi:** 0.0071894436403644
- **w-rdist:** 0.3575426831706388
- **t-alpha:** 0.088856766054028

---

---

634

- **PDB ID:** 3HAB | **Chain:** A
- **b-phipsi:** 0.0057669559506721
- **w-rdist:** 0.8388304705079799
- **t-alpha:** 0.0185457488706886

---

---

635

- **PDB ID:** 4DB1 | **Chain:** B
- **b-phipsi:** 0.0140863122157367
- **w-rdist:** 0.5415519664743849
- **t-alpha:** 0.0103018401892411

---

---

636

- **PDB ID:** 5SXK | **Chain:** A
- **b-phipsi:** 0.0091704030006973
- **w-rdist:** 0.4239425617435377
- **t-alpha:** 0.0429718680332931

---

---

637

- **PDB ID:** 3H0C | **Chain:** B
- **b-phipsi:** 0.0064507638548385
- **w-rdist:** 0.829627657792382
- **t-alpha:** 0.0182082568765367

---

---

638

- **PDB ID:** 4EZJ | **Chain:** A
- **b-phipsi:** 0.0188872110181597
- **w-rdist:** 0.267966249300769
- **t-alpha:** 0.0327750996298437

---

---

639

- **PDB ID:** 3G0B | **Chain:** C
- **b-phipsi:** 0.0060167670539989
- **w-rdist:** 0.8456894358314424
- **t-alpha:** 0.0155326816144203

---

---

640

- **PDB ID:** 3TON | **Chain:** B
- **b-phipsi:** 0.0047556597367352
- **w-rdist:** 0.6821211238762253
- **t-alpha:** 0.0331075673737624

---

---

641

- **PDB ID:** 6Q6Q | **Chain:** A
- **b-phipsi:** 0.0063507313335068
- **w-rdist:** 0.3454594936318673
- **t-alpha:** 0.1179902884322814

---

---

642

- **PDB ID:** 3ZD7 | **Chain:** A
- **b-phipsi:** 0.0237247167680928
- **w-rdist:** 0.3304729660150665
- **t-alpha:** 0.0218498381101377

---

---

643

- **PDB ID:** 2WBY | **Chain:** A
- **b-phipsi:** 0.0070894201016194
- **w-rdist:** 0.537975103315155
- **t-alpha:** 0.0349600148426103

---

---

644

- **PDB ID:** 4JPS | **Chain:** A
- **b-phipsi:** 0.0081229212970318
- **w-rdist:** 0.483361953457715
- **t-alpha:** 0.0386015818388498

---

---

645

- **PDB ID:** 6BYZ | **Chain:** B
- **b-phipsi:** 0.0071435834803945
- **w-rdist:** 0.5221651723867193
- **t-alpha:** 0.0407864162082982

---

---

646

- **PDB ID:** 5NV6 | **Chain:** A
- **b-phipsi:** 0.0037337485851924
- **w-rdist:** 0.7842280617749704
- **t-alpha:** 0.0315549988173708

---

---

647

- **PDB ID:** 7R7T | **Chain:** B
- **b-phipsi:** 0.0145697619870867
- **w-rdist:** 0.5586855677628402
- **t-alpha:** 0.0072833804367331

---

---

648

- **PDB ID:** 7NSK | **Chain:** B
- **b-phipsi:** 0.0117195420653397
- **w-rdist:** 0.6658898286449041
- **t-alpha:** 0.0036549441753579

---

---

649

- **PDB ID:** 6EOT | **Chain:** D
- **b-phipsi:** 0.0105426310488732
- **w-rdist:** 0.679722106459782
- **t-alpha:** 0.0058266934612722

---

---

650

- **PDB ID:** 2A0Z | **Chain:** A
- **b-phipsi:** 0.0084328464165318
- **w-rdist:** 0.7407050321590479
- **t-alpha:** 0.0101968242656327

---

---

651

- **PDB ID:** 3HGZ | **Chain:** A
- **b-phipsi:** 0.0064107580419136
- **w-rdist:** 0.5341256533544277
- **t-alpha:** 0.0495267695540877

---

---

652

- **PDB ID:** 7W5A | **Chain:** c
- **b-phipsi:** 0.0049997527890134
- **w-rdist:** 0.4048539168118812
- **t-alpha:** 0.1309719463952541

---

---

653

- **PDB ID:** 7BP8 | **Chain:** E
- **b-phipsi:** 0.0080053340694052
- **w-rdist:** 0.2085047344731822
- **t-alpha:** 0.1230876410749617

---

---

654

- **PDB ID:** 7A3L | **Chain:** B
- **b-phipsi:** 0.007646956031772
- **w-rdist:** 0.6555983975772363
- **t-alpha:** 0.0196648853517085

---

---

655

- **PDB ID:** 7RLH | **Chain:** D
- **b-phipsi:** 0.0124358950329833
- **w-rdist:** 0.1458172884764043
- **t-alpha:** 0.0772031433552034

---

---

656

- **PDB ID:** 5FTK | **Chain:** F
- **b-phipsi:** 0.0105998198823913
- **w-rdist:** 0.1742086237521845
- **t-alpha:** 0.0924983319030738

---

---

657

- **PDB ID:** 7SVO | **Chain:** B
- **b-phipsi:** 0.0057253963364327
- **w-rdist:** 0.6666949738072196
- **t-alpha:** 0.0323309912234826

---

---

658

- **PDB ID:** 2OGZ | **Chain:** B
- **b-phipsi:** 0.0067494071847995
- **w-rdist:** 0.83582866487489
- **t-alpha:** 0.0138382752386696

---

---

659

- **PDB ID:** 7QO1 | **Chain:** A
- **b-phipsi:** 0.005512119943936
- **w-rdist:** 0.7582056725245028
- **t-alpha:** 0.0291333507443458

---

---

660

- **PDB ID:** 5WOB | **Chain:** A
- **b-phipsi:** 0.0063963059004418
- **w-rdist:** 0.5283299665173403
- **t-alpha:** 0.0538967929362443

---

---

661

- **PDB ID:** 3O95 | **Chain:** C
- **b-phipsi:** 0.0071494600942063
- **w-rdist:** 0.8393182400611524
- **t-alpha:** 0.009468275938171

---

---

662

- **PDB ID:** 5DXH | **Chain:** A
- **b-phipsi:** 0.0068206430849693
- **w-rdist:** 0.4807516290964201
- **t-alpha:** 0.0635167812456465

---

---

663

- **PDB ID:** 6EDS | **Chain:** A
- **b-phipsi:** 0.0070151892902764
- **w-rdist:** 0.5311739018409636
- **t-alpha:** 0.0393298454728356

---

---

664

- **PDB ID:** 4KRE | **Chain:** A
- **b-phipsi:** 0.0078337418924007
- **w-rdist:** 0.2250044633102681
- **t-alpha:** 0.1260014841031005

---

---

665

- **PDB ID:** 3OFI | **Chain:** A
- **b-phipsi:** 0.0057076014932762
- **w-rdist:** 0.5436930883109666
- **t-alpha:** 0.0575384238750913

---

---

666

- **PDB ID:** 5J67 | **Chain:** A
- **b-phipsi:** 0.004136912999082
- **w-rdist:** 0.4110730535505711
- **t-alpha:** 0.1403652546715101

---

---

667

- **PDB ID:** 5F9H | **Chain:** I
- **b-phipsi:** 0.0147389103056319
- **w-rdist:** 0.6065791339802652
- **t-alpha:** 0.0021896458253802

---

---

668

- **PDB ID:** 7MDM | **Chain:** D
- **b-phipsi:** 0.0213661318596178
- **w-rdist:** 0.3307010705053738
- **t-alpha:** 0.0246270663199015

---

---

669

- **PDB ID:** 7RLI | **Chain:** I
- **b-phipsi:** 0.0117518199330654
- **w-rdist:** 0.1231293402411713
- **t-alpha:** 0.088856766054028

---

---

670

- **PDB ID:** 7MLK | **Chain:** A
- **b-phipsi:** 0.0136191894411123
- **w-rdist:** 0.2274715791704719
- **t-alpha:** 0.0597231737029493

---

---

671

- **PDB ID:** 6TE3 | **Chain:** A
- **b-phipsi:** 0.0067660672233602
- **w-rdist:** 0.7683678551419406
- **t-alpha:** 0.0200592812463542

---

---

672

- **PDB ID:** 7VCT | **Chain:** A
- **b-phipsi:** 0.0048756423081904
- **w-rdist:** 0.3521297048627784
- **t-alpha:** 0.1689729531736603

---

---

673

- **PDB ID:** 5ISM | **Chain:** A
- **b-phipsi:** 0.0058181251891354
- **w-rdist:** 0.8321743314299344
- **t-alpha:** 0.0230999264545257

---

---

674

- **PDB ID:** 5FTK | **Chain:** D
- **b-phipsi:** 0.0074038315071905
- **w-rdist:** 0.1827907459001606
- **t-alpha:** 0.1529501102018282

---

---

675

- **PDB ID:** 2QTB | **Chain:** B
- **b-phipsi:** 0.0066453755292249
- **w-rdist:** 0.8341203263126009
- **t-alpha:** 0.016284021422591

---

---

676

- **PDB ID:** 7AUA | **Chain:** B
- **b-phipsi:** 0.0083661163393502
- **w-rdist:** 0.4145101789222455
- **t-alpha:** 0.057781007302982

---

---

677

- **PDB ID:** 7OR4 | **Chain:** A
- **b-phipsi:** 0.013248893934876
- **w-rdist:** 0.6590589574866184
- **t-alpha:** 0.0021848670241468

---

---

678

- **PDB ID:** 8OG4 | **Chain:** A
- **b-phipsi:** 0.0063192544644425
- **w-rdist:** 0.4028041860919502
- **t-alpha:** 0.1054749257285665

---

---

679

- **PDB ID:** 3EGX | **Chain:** B
- **b-phipsi:** 0.0034989793697344
- **w-rdist:** 0.7459593335123555
- **t-alpha:** 0.0338857136971153

---

---

680

- **PDB ID:** 7SVM | **Chain:** A
- **b-phipsi:** 0.0069336005959831
- **w-rdist:** 0.6706570404891168
- **t-alpha:** 0.0238627234462787

---

---

681

- **PDB ID:** 5ISM | **Chain:** B
- **b-phipsi:** 0.0062256228164385
- **w-rdist:** 0.8372193336447569
- **t-alpha:** 0.0177907956813867

---

---

682

- **PDB ID:** 5C19 | **Chain:** F
- **b-phipsi:** 0.0084290103507877
- **w-rdist:** 0.2023427200263268
- **t-alpha:** 0.1201749287180127

---

---

683

- **PDB ID:** 5FTK | **Chain:** B
- **b-phipsi:** 0.0113543701224201
- **w-rdist:** 0.1715072523380607
- **t-alpha:** 0.087399807391695

---

---

684

- **PDB ID:** 6EJB | **Chain:** A
- **b-phipsi:** 0.0015286809382371
- **w-rdist:** 0.6186135546901624
- **t-alpha:** 0.057781007302982

---

---

685

- **PDB ID:** 4FWJ | **Chain:** B
- **b-phipsi:** 0.0041333404694299
- **w-rdist:** 0.4391659161354027
- **t-alpha:** 0.1300409768952239

---

---

686

- **PDB ID:** 5WOB | **Chain:** D
- **b-phipsi:** 0.0058518116875543
- **w-rdist:** 0.5045433497996757
- **t-alpha:** 0.0793883714171022

---

---

687

- **PDB ID:** 3FBY | **Chain:** C
- **b-phipsi:** 0.0429211591362305
- **w-rdist:** 0.4367648143683592
- **t-alpha:** 0.0050984212340619

---

---

688

- **PDB ID:** 1NU6 | **Chain:** A
- **b-phipsi:** 0.0058774928910767
- **w-rdist:** 0.8256354243079146
- **t-alpha:** 0.0246270663199015

---

---

689

- **PDB ID:** 7SVM | **Chain:** C
- **b-phipsi:** 0.007511667239716
- **w-rdist:** 0.6797277019669224
- **t-alpha:** 0.0193022341335096

---

---

690

- **PDB ID:** 4ANV | **Chain:** A
- **b-phipsi:** 0.0168088729339897
- **w-rdist:** 0.2756681395039456
- **t-alpha:** 0.0371451014582198

---

---

691

- **PDB ID:** 1WCY | **Chain:** B
- **b-phipsi:** 0.0060165134863197
- **w-rdist:** 0.8321578929716043
- **t-alpha:** 0.0223381852496014

---

---

692

- **PDB ID:** 5FTL | **Chain:** E
- **b-phipsi:** 0.0096473966436037
- **w-rdist:** 0.2032832003014399
- **t-alpha:** 0.1005094622253814

---

---

693

- **PDB ID:** 8ASC | **Chain:** K
- **b-phipsi:** 0.0005134979703846
- **w-rdist:** 0.6455674600841353
- **t-alpha:** 0.057781007302982

---

---

694

- **PDB ID:** 5AWC | **Chain:** D
- **b-phipsi:** 0.0254475520920112
- **w-rdist:** 0.3134697514942035
- **t-alpha:** 0.0246270663199015

---

---

695

- **PDB ID:** 2P8S | **Chain:** B
- **b-phipsi:** 0.0054844843618892
- **w-rdist:** 0.8380332283438219
- **t-alpha:** 0.0223381852496014

---

---

696

- **PDB ID:** 4JH0 | **Chain:** B
- **b-phipsi:** 0.0063914037989195
- **w-rdist:** 0.8283255426633364
- **t-alpha:** 0.020817864153829

---

---

697

- **PDB ID:** 8FCP | **Chain:** E
- **b-phipsi:** 0.0083067282640412
- **w-rdist:** 0.1805204438796885
- **t-alpha:** 0.1296433788581392

---

---

698

- **PDB ID:** 7ZSC | **Chain:** D
- **b-phipsi:** 0.0063464243387128
- **w-rdist:** 0.545919563815706
- **t-alpha:** 0.0464939086507718

---

---

699

- **PDB ID:** 6DDO | **Chain:** A
- **b-phipsi:** 0.0096410078971505
- **w-rdist:** 0.7185925699449872
- **t-alpha:** 0.0073366962737499

---

---

700

- **PDB ID:** 8FCP | **Chain:** A
- **b-phipsi:** 0.0074616255010232
- **w-rdist:** 0.3987080511244236
- **t-alpha:** 0.0772031433552034

---

---

701

- **PDB ID:** 6QZW | **Chain:** B
- **b-phipsi:** 0.0087940487712423
- **w-rdist:** 0.6674015687640844
- **t-alpha:** 0.0138382752386696

---

---

702

- **PDB ID:** 4PF7 | **Chain:** B
- **b-phipsi:** 0.0070367272689755
- **w-rdist:** 0.4830211237890882
- **t-alpha:** 0.0604515833997143

---

---

703

- **PDB ID:** 3L4G | **Chain:** P
- **b-phipsi:** 0.0016675503553014
- **w-rdist:** 0.6724250098302165
- **t-alpha:** 0.0487983623453289

---

---

704

- **PDB ID:** 7RLI | **Chain:** C
- **b-phipsi:** 0.0099183954528341
- **w-rdist:** 0.1234128034353769
- **t-alpha:** 0.1128916601201213

---

---

705

- **PDB ID:** 7ZXS | **Chain:** A
- **b-phipsi:** 0.0071169563056837
- **w-rdist:** 0.7187274817595405
- **t-alpha:** 0.0200592812463542

---

---

706

- **PDB ID:** 8SBC | **Chain:** A
- **b-phipsi:** 0.0100520396259793
- **w-rdist:** 0.4555131657663593
- **t-alpha:** 0.0356881942932585

---

---

707

- **PDB ID:** 8FCL | **Chain:** C
- **b-phipsi:** 0.0079645366142189
- **w-rdist:** 0.1597915437354221
- **t-alpha:** 0.1449382485942429

---

---

708

- **PDB ID:** 4EZL | **Chain:** A
- **b-phipsi:** 0.0145675762382264
- **w-rdist:** 0.2570847271834434
- **t-alpha:** 0.0524399405265496

---

---

709

- **PDB ID:** 7LT3 | **Chain:** B
- **b-phipsi:** 0.0024316947790682
- **w-rdist:** 0.4746076370202249
- **t-alpha:** 0.1300409768952239

---

---

710

- **PDB ID:** 4L3T | **Chain:** B
- **b-phipsi:** 0.0090436817959774
- **w-rdist:** 0.5758212036758215
- **t-alpha:** 0.0200592812463542

---

---

711

- **PDB ID:** 4GSC | **Chain:** A
- **b-phipsi:** 0.006570988445677
- **w-rdist:** 0.5289688408827632
- **t-alpha:** 0.0538967929362443

---

---

712

- **PDB ID:** 5ZID | **Chain:** A
- **b-phipsi:** 0.0054129937516187
- **w-rdist:** 0.8223357469694795
- **t-alpha:** 0.0284048760941568

---

---

713

- **PDB ID:** 6RQJ | **Chain:** A
- **b-phipsi:** 0.0015482616876153
- **w-rdist:** 0.5234163407151553
- **t-alpha:** 0.1077930415888199

---

---

714

- **PDB ID:** 1W1I | **Chain:** A
- **b-phipsi:** 0.0076204921439238
- **w-rdist:** 0.8332055961934044
- **t-alpha:** 0.010924934303695

---

---

715

- **PDB ID:** 7BPA | **Chain:** A
- **b-phipsi:** 0.0079157138957416
- **w-rdist:** 0.3274940371900417
- **t-alpha:** 0.0932268541315344

---

---

716

- **PDB ID:** 6ZHE | **Chain:** B
- **b-phipsi:** 0.0150318869353087
- **w-rdist:** 0.3079112225272087
- **t-alpha:** 0.0401513644150786

---

---

717

- **PDB ID:** 6EOO | **Chain:** A
- **b-phipsi:** 0.0089496615185815
- **w-rdist:** 0.6115036171852278
- **t-alpha:** 0.0177907956813867

---

---

718

- **PDB ID:** 6DC6 | **Chain:** C
- **b-phipsi:** 0.0052463124615591
- **w-rdist:** 0.6496240675807382
- **t-alpha:** 0.0371451014582198

---

---

719

- **PDB ID:** 3QZ2 | **Chain:** A
- **b-phipsi:** 0.007028421032507
- **w-rdist:** 0.5325090070162478
- **t-alpha:** 0.0429718680332931

---

---

720

- **PDB ID:** 7AXZ | **Chain:** A
- **b-phipsi:** 0.0004996564781416
- **w-rdist:** 0.6511572776398554
- **t-alpha:** 0.0594134606378968

---

---

721

- **PDB ID:** 8SZP | **Chain:** A
- **b-phipsi:** 0.009114595053025
- **w-rdist:** 0.5719901237551182
- **t-alpha:** 0.020817864153829

---

---

722

- **PDB ID:** 7QPG | **Chain:** B
- **b-phipsi:** 0.0041519022491283
- **w-rdist:** 0.4954040309124952
- **t-alpha:** 0.1081517725047538

---

---

723

- **PDB ID:** 6BYZ | **Chain:** A
- **b-phipsi:** 0.0064699653774203
- **w-rdist:** 0.5292827902506536
- **t-alpha:** 0.0568099994807522

---

---

724

- **PDB ID:** 6KYV | **Chain:** L
- **b-phipsi:** 0.0100927831719323
- **w-rdist:** 0.7132087070269421
- **t-alpha:** 0.0073366962737499

---

---

725

- **PDB ID:** 8FCR | **Chain:** C
- **b-phipsi:** 0.0077834552148691
- **w-rdist:** 0.1659201386484041
- **t-alpha:** 0.1565909222786323

---

---

726

- **PDB ID:** 6B1O | **Chain:** B
- **b-phipsi:** 0.0064498410777323
- **w-rdist:** 0.8331464093076181
- **t-alpha:** 0.0200592812463542

---

---

727

- **PDB ID:** 8FCO | **Chain:** A
- **b-phipsi:** 0.0083396196800519
- **w-rdist:** 0.1732844054025994
- **t-alpha:** 0.1347419169217441

---

---

728

- **PDB ID:** 5ZQF | **Chain:** A
- **b-phipsi:** 0.0129820306544885
- **w-rdist:** 0.4343381792023089
- **t-alpha:** 0.0313184298622246

---

---

729

- **PDB ID:** 6EOP | **Chain:** A
- **b-phipsi:** 0.0066040319536886
- **w-rdist:** 0.6806915253705129
- **t-alpha:** 0.0269259686475575

---

---

730

- **PDB ID:** 3C45 | **Chain:** B
- **b-phipsi:** 0.0070371949196118
- **w-rdist:** 0.8337617549283783
- **t-alpha:** 0.0147818972725322

---

---

731

- **PDB ID:** 7SQ2 | **Chain:** B
- **b-phipsi:** 0.0008173396550844
- **w-rdist:** 0.6665179707817391
- **t-alpha:** 0.0561535874836141

---

---

732

- **PDB ID:** 2OQI | **Chain:** B
- **b-phipsi:** 0.0070229866962436
- **w-rdist:** 0.8409034227527934
- **t-alpha:** 0.011653569134763

---

---

733

- **PDB ID:** 7W59 | **Chain:** C
- **b-phipsi:** 0.0044069545282756
- **w-rdist:** 0.406916008463174
- **t-alpha:** 0.1499160832876762

---

---

734

- **PDB ID:** 7K1E | **Chain:** B
- **b-phipsi:** 0.0057897611764798
- **w-rdist:** 0.5236109478470868
- **t-alpha:** 0.0735613675186812

---

---

735

- **PDB ID:** 2WJV | **Chain:** B
- **b-phipsi:** 0.0032892764535223
- **w-rdist:** 0.4717558876960187
- **t-alpha:** 0.1281840260686497

---

---

736

- **PDB ID:** 8FCO | **Chain:** E
- **b-phipsi:** 0.0086936789883066
- **w-rdist:** 0.2011437291528312
- **t-alpha:** 0.1223595564912818

---

---

737

- **PDB ID:** 6Y0F | **Chain:** B
- **b-phipsi:** 0.0068815400295391
- **w-rdist:** 0.8419851095123984
- **t-alpha:** 0.0125367354532617

---

---

738

- **PDB ID:** 5T4H | **Chain:** A
- **b-phipsi:** 0.0062570983378417
- **w-rdist:** 0.8335468346534656
- **t-alpha:** 0.0215774983192449

---

---

739

- **PDB ID:** 7BP8 | **Chain:** B
- **b-phipsi:** 0.0082305235011983
- **w-rdist:** 0.2262651576414563
- **t-alpha:** 0.1267297821689152

---

---

740

- **PDB ID:** 4AOF | **Chain:** A
- **b-phipsi:** 0.0242450420569811
- **w-rdist:** 0.2141575020441098
- **t-alpha:** 0.0342318542055553

---

---

741

- **PDB ID:** 2VXO | **Chain:** B
- **b-phipsi:** 0.0063689295010219
- **w-rdist:** 0.416205148772471
- **t-alpha:** 0.1054749257285665

---

---

742

- **PDB ID:** 7W59 | **Chain:** c
- **b-phipsi:** 0.0044241916643776
- **w-rdist:** 0.4067448753949365
- **t-alpha:** 0.1528126760301813

---

---

743

- **PDB ID:** 7AD6 | **Chain:** A
- **b-phipsi:** 0.0034468862473437
- **w-rdist:** 0.5501248810012992
- **t-alpha:** 0.0772031433552034

---

---

744

- **PDB ID:** 5DXT | **Chain:** A
- **b-phipsi:** 0.0124377264736515
- **w-rdist:** 0.2373603937276341
- **t-alpha:** 0.0691913739420344

---

---

745

- **PDB ID:** 8SW1 | **Chain:** A
- **b-phipsi:** 0.0084142429976159
- **w-rdist:** 0.4113767365323159
- **t-alpha:** 0.0626367777057093

---

---

746

- **PDB ID:** 5MHL | **Chain:** A
- **b-phipsi:** 0.0176565554138629
- **w-rdist:** 0.3010339024123524
- **t-alpha:** 0.0354447525832375

---

---

747

- **PDB ID:** 2C11 | **Chain:** D
- **b-phipsi:** 0.0114024099478992
- **w-rdist:** 0.790088959484816
- **t-alpha:** 0.0014587541408541

---

---

748

- **PDB ID:** 3DPD | **Chain:** A
- **b-phipsi:** 0.0187272036641525
- **w-rdist:** 0.3016452308792359
- **t-alpha:** 0.0335032202501839

---

---

749

- **PDB ID:** 7RL6 | **Chain:** D
- **b-phipsi:** 0.0087665418740209
- **w-rdist:** 0.1121642573513557
- **t-alpha:** 0.1427532640502136

---

---

750

- **PDB ID:** 5F98 | **Chain:** G
- **b-phipsi:** 0.0112907615276731
- **w-rdist:** 0.6329984033911568
- **t-alpha:** 0.0095587741674756

---

---

751

- **PDB ID:** 2I78 | **Chain:** D
- **b-phipsi:** 0.0066817420465575
- **w-rdist:** 0.8365519830788745
- **t-alpha:** 0.0167517699463162

---

---

752

- **PDB ID:** 8FCM | **Chain:** C
- **b-phipsi:** 0.0073521987428001
- **w-rdist:** 0.1699044645657665
- **t-alpha:** 0.1842682580627777

---

---

753

- **PDB ID:** 6I2X | **Chain:** C
- **b-phipsi:** 0.0361894871651838
- **w-rdist:** 0.4425384867361662
- **t-alpha:** 0.0088172167453031

---

---

754

- **PDB ID:** 4R0A | **Chain:** A
- **b-phipsi:** 0.0138553899723012
- **w-rdist:** 0.3116793954201526
- **t-alpha:** 0.047341565959746

---

---

755

- **PDB ID:** 4IFH | **Chain:** A
- **b-phipsi:** 0.0056603487591906
- **w-rdist:** 0.532694846906263
- **t-alpha:** 0.0713766970124236

---

---

756

- **PDB ID:** 5KYU | **Chain:** B
- **b-phipsi:** 0.001333696970895
- **w-rdist:** 0.7418063394045575
- **t-alpha:** 0.0441063918932529

---

---

757

- **PDB ID:** 5J67 | **Chain:** C
- **b-phipsi:** 0.0044647451177137
- **w-rdist:** 0.4263541778178981
- **t-alpha:** 0.1441665367574605

---

---

758

- **PDB ID:** 7RZE | **Chain:** A
- **b-phipsi:** 0.0063512246368466
- **w-rdist:** 0.5181180833917167
- **t-alpha:** 0.0670064380342809

---

---

759

- **PDB ID:** 8AYH | **Chain:** C
- **b-phipsi:** 0.0382924227563515
- **w-rdist:** 0.4383055051264222
- **t-alpha:** 0.0087397720309874

---

---

760

- **PDB ID:** 8FCT | **Chain:** B
- **b-phipsi:** 0.0079189689918771
- **w-rdist:** 0.1291165665391084
- **t-alpha:** 0.1660602202799717

---

---

761

- **PDB ID:** 7QPG | **Chain:** C
- **b-phipsi:** 0.004025263473759
- **w-rdist:** 0.4965053665298635
- **t-alpha:** 0.1117407059204289

---

---

762

- **PDB ID:** 7Z61 | **Chain:** A
- **b-phipsi:** 0.0180200805282652
- **w-rdist:** 0.3140839873839124
- **t-alpha:** 0.0335032202501839

---

---

763

- **PDB ID:** 7SCH | **Chain:** B
- **b-phipsi:** 0.0091820463586081
- **w-rdist:** 0.5279334007471008
- **t-alpha:** 0.0313184298622246

---

---

764

- **PDB ID:** 5DXH | **Chain:** D
- **b-phipsi:** 0.0091241765703123
- **w-rdist:** 0.4819598614317164
- **t-alpha:** 0.037792668599949

---

---

765

- **PDB ID:** 4R09 | **Chain:** C
- **b-phipsi:** 0.0110270516919833
- **w-rdist:** 0.3609151465130412
- **t-alpha:** 0.0524399405265496

---

---

766

- **PDB ID:** 3PRZ | **Chain:** A
- **b-phipsi:** 0.0150494742435441
- **w-rdist:** 0.2417890722286385
- **t-alpha:** 0.0568099994807522

---

---

767

- **PDB ID:** 7RLI | **Chain:** L
- **b-phipsi:** 0.0103452355596932
- **w-rdist:** 0.1246339084981129
- **t-alpha:** 0.1136199749619588

---

---

768

- **PDB ID:** 7RLB | **Chain:** F
- **b-phipsi:** 0.0083017061791971
- **w-rdist:** 0.1915563287313385
- **t-alpha:** 0.1369267629961306

---

---

769

- **PDB ID:** 7VCT | **Chain:** D
- **b-phipsi:** 0.0053079620778355
- **w-rdist:** 0.3691557144490021
- **t-alpha:** 0.1631458709176767

---

---

770

- **PDB ID:** 7R54 | **Chain:** A
- **b-phipsi:** 0.0141445483738923
- **w-rdist:** 0.3174731840625183
- **t-alpha:** 0.0436997123665408

---

---

771

- **PDB ID:** 5IUD | **Chain:** G
- **b-phipsi:** 0.011423609519278
- **w-rdist:** 0.3021616256887723
- **t-alpha:** 0.0662781018568299

---

---

772

- **PDB ID:** 6U23 | **Chain:** H
- **b-phipsi:** 0.0096196153771353
- **w-rdist:** 0.3001783522368423
- **t-alpha:** 0.0853753468743623

---

---

773

- **PDB ID:** 5FTL | **Chain:** F
- **b-phipsi:** 0.0084605257511348
- **w-rdist:** 0.2206942721341174
- **t-alpha:** 0.1274585512981167

---

---

774

- **PDB ID:** 3O9V | **Chain:** C
- **b-phipsi:** 0.00755184303206
- **w-rdist:** 0.8320398132802989
- **t-alpha:** 0.0132839260291477

---

---

775

- **PDB ID:** 3HAB | **Chain:** B
- **b-phipsi:** 0.0062224958044519
- **w-rdist:** 0.83418639947707
- **t-alpha:** 0.0223381852496014

---

---

776

- **PDB ID:** 5GS0 | **Chain:** B
- **b-phipsi:** 0.0096473055271457
- **w-rdist:** 0.6499435171737802
- **t-alpha:** 0.0140326350426887

---

---

777

- **PDB ID:** 7WVF | **Chain:** A
- **b-phipsi:** 0.0087020886418004
- **w-rdist:** 0.628613878537297
- **t-alpha:** 0.0196648853517085

---

---

778

- **PDB ID:** 6TNY | **Chain:** A
- **b-phipsi:** 0.0060604704440685
- **w-rdist:** 0.7579842308793633
- **t-alpha:** 0.0291333507443458

---

---

779

- **PDB ID:** 4G1F | **Chain:** C
- **b-phipsi:** 0.0063450723277815
- **w-rdist:** 0.8439529391319234
- **t-alpha:** 0.0174797527976524

---

---

780

- **PDB ID:** 4N8E | **Chain:** B
- **b-phipsi:** 0.0067280711367044
- **w-rdist:** 0.8276675096607985
- **t-alpha:** 0.020817864153829

---

---

781

- **PDB ID:** 6U23 | **Chain:** P
- **b-phipsi:** 0.0119232777768123
- **w-rdist:** 0.2915487743304258
- **t-alpha:** 0.0651667209447612

---

---

782

- **PDB ID:** 4LTE | **Chain:** B
- **b-phipsi:** 0.0055405045951329
- **w-rdist:** 0.5326517287269947
- **t-alpha:** 0.0764750331519523

---

---

783

- **PDB ID:** 4R09 | **Chain:** A
- **b-phipsi:** 0.0072307567335693
- **w-rdist:** 0.3690933588611668
- **t-alpha:** 0.0990536816248905

---

---

784

- **PDB ID:** 5AWC | **Chain:** A
- **b-phipsi:** 0.0087919772611702
- **w-rdist:** 0.3142738371534879
- **t-alpha:** 0.0910417030770296

---

---

785

- **PDB ID:** 4GSF | **Chain:** A
- **b-phipsi:** 0.0064652218097223
- **w-rdist:** 0.5349626062700343
- **t-alpha:** 0.0568099994807522

---

---

786

- **PDB ID:** 6X9J | **Chain:** A
- **b-phipsi:** 0.0083181138953006
- **w-rdist:** 0.2472203523069417
- **t-alpha:** 0.1223595564912818

---

---

787

- **PDB ID:** 7RLJ | **Chain:** L
- **b-phipsi:** 0.0126111593454122
- **w-rdist:** 0.1207427151289687
- **t-alpha:** 0.0946832683923559

---

---

788

- **PDB ID:** 5SXJ | **Chain:** A
- **b-phipsi:** 0.0088058433540731
- **w-rdist:** 0.436410271139128
- **t-alpha:** 0.0560815732405199

---

---

789

- **PDB ID:** 7WV3 | **Chain:** D
- **b-phipsi:** 0.010537914737373
- **w-rdist:** 0.6558215229155206
- **t-alpha:** 0.0117905783112548

---

---

790

- **PDB ID:** 3SE6 | **Chain:** A
- **b-phipsi:** 0.0096789315930941
- **w-rdist:** 0.5347702478725997
- **t-alpha:** 0.0284642139866488

---

---

791

- **PDB ID:** 8EOK | **Chain:** G
- **b-phipsi:** 0.0337986386806919
- **w-rdist:** 0.440550735134674
- **t-alpha:** 0.011653569134763

---

---

792

- **PDB ID:** 7RLG | **Chain:** A
- **b-phipsi:** 0.0103804061782364
- **w-rdist:** 0.1910882392445212
- **t-alpha:** 0.1070653702560531

---

---

793

- **PDB ID:** 8FCQ | **Chain:** B
- **b-phipsi:** 0.0078973111438065
- **w-rdist:** 0.1700523106097195
- **t-alpha:** 0.1653309490792112

---

---

794

- **PDB ID:** 4FA6 | **Chain:** A
- **b-phipsi:** 0.0159517509927607
- **w-rdist:** 0.2521904695653206
- **t-alpha:** 0.0531683659174533

---

---

795

- **PDB ID:** 3LPO | **Chain:** A
- **b-phipsi:** 0.0081953896617705
- **w-rdist:** 0.6892922612159544
- **t-alpha:** 0.0182082568765367

---

---

796

- **PDB ID:** 3R7R | **Chain:** A
- **b-phipsi:** 0.0220682048504916
- **w-rdist:** 0.2644385353711629
- **t-alpha:** 0.0342318542055553

---

---

797

- **PDB ID:** 4ENZ | **Chain:** A
- **b-phipsi:** 0.0170276843173366
- **w-rdist:** 0.62028526769934
- **t-alpha:** 0.0014568440116902

---

---

798

- **PDB ID:** 4AH6 | **Chain:** A
- **b-phipsi:** 0.0053014618277592
- **w-rdist:** 0.3913596230036079
- **t-alpha:** 0.1625741867166441

---

---

799

- **PDB ID:** 4L72 | **Chain:** A
- **b-phipsi:** 0.00772606334658
- **w-rdist:** 0.8342388727671135
- **t-alpha:** 0.0125367354532617

---

---

800

- **PDB ID:** 2QOE | **Chain:** B
- **b-phipsi:** 0.0064322794547385
- **w-rdist:** 0.8362188107294688
- **t-alpha:** 0.020817864153829

---

---
