# Supplementary material for: Comparative structural insights and functional analysis for the distinct unbound states of Human AGO proteins
Source: Sci Rep. 2025 Mar 19;15:9432. doi: 10.1038/s41598-025-91849-5 (PMC11923369; doi:10.1038/s41598-025-91849-5)
Supplement: Supplementary file 24 — Supplementary Information 12. [file 41598_2025_91849_MOESM24_ESM.zip › 5VM9p_A_mdwhole_AF4REF/candidates/5VM9p_A-merged-enriched_eval_report.html]

 

# Structural Comparison Report for 5VM9p\_A - whole structures (total: 100)

---

1

- **Protein name:** Zinc finger protein DPF3
- **Organism:** Homo sapiens
- **Uniprot Accession Number:** Q92784
- **Protein sequence length:** 378 aa
- **1D identity (%):** 8.67
- **1D identity (%) [Gaps excluded]:** 22.0
- **1D identity - Alignment Gaps:** 538
- **1D aligned content (<aminoacid>:%):** {'A': 10.39, 'T': 3.9, 'Q': 3.9, 'F': 2.6, 'E': 3.9, 'H': 1.3, 'R': 12.99, 'S': 2.6, 'G': 15.58, 'V': 3.9, 'C': 5.19, 'M': 1.3, 'P': 10.39, 'K': 5.19, 'L': 10.39, 'N': 3.9, 'D': 1.3, 'I': 1.3}
- **Common reported functions (%):** 0.0
- **Common reported locations (%):** 11.11
- **Common reported processes (%):** 0.0

- **AF ID:** Q92784
- **Chain:** A
- **Protein length:** 378 aa
- **Resolution:** N/A
- **b-phipsi:** 0.00028
- **w-rdist:** 0.371153
- **t-alpha:** 0.058637
- **Chemical similarity (Tanimoto Index) (%):** 99.62
- **1D identity (%) [PDB]:** 1.5
- **1D identity (%) [Gaps excluded][PDB]:** 72.0
- **1D identity - Alignment Gaps [PDB]:** 1173
- **1D aligned content [PDB] (<aminoacid>:%):** {'Y': 5.56, 'I': 5.56, 'K': 5.56, 'R': 11.11, 'H': 11.11, 'T': 5.56, 'L': 5.56, 'C': 5.56, 'A': 11.11, 'D': 5.56, 'G': 11.11, 'S': 5.56, 'N': 5.56, 'P': 5.56}
- **2D identity (%) [PDB]:** 26.01
- **2D identity (%) [Gaps excluded][PDB]:** 85.61
- **2D identity - Alignment Gaps [PDB]:** 653
- **2D aligned content [PDB] (<2D-fold>:%):** {'.': 24.18, 'H': 34.43, 'E': 8.2, 'T': 30.33, 'G': 2.46, 'B': 0.41}
- **3D similarity (TM-Score) (%) [PDB]:** 14.18

- **Gene name:** DPF3
- **Entrez ID:** 8110
- **RefSeq ID:** N/A
- **Sequence length:** N/A
- **5-UTR|CDS|3-UTR identity (%):** N/A | N/A | N/A
- **5-UTR|CDS|3-UTR identity (%) [Gaps excluded]:** N/A | N/A | N/A
- **5-UTR|CDS|3-UTR identity [Alignment Gaps]:** N/A | N/A | N/A
- **5-UTR aligned content (<base>:%):** N/A
- **CDS aligned content (<base>:%):** N/A
- **3-UTR aligned content (<base>:%):** N/A

**Uniprot Description:**  
  
 Belongs to the neuron-specific chromatin remodeling complex (nBAF complex). During neural development a switch from a stem/progenitor to a post-mitotic chromatin remodeling mechanism occurs as neurons exit the cell cycle and become committed to their adult state. The transition from proliferating neural stem/progenitor cells to post-mitotic neurons requires a switch in subunit composition of the npBAF and nBAF complexes. As neural progenitors exit mitosis and differentiate into neurons, npBAF complexes which contain ACTL6A/BAF53A and PHF10/BAF45A, are exchanged for homologous alternative ACTL6B/BAF53B and DPF1/BAF45B or DPF3/BAF45C subunits in neuron-specific complexes (nBAF). The npBAF complex is essential for the self-renewal/proliferative capacity of the multipotent neural stem cells. The nBAF complex along with CREST plays a role regulating the activity of genes essential for dendrite growth (By similarity). Muscle-specific component of the BAF complex, a multiprotein complex involved in transcriptional activation and repression of select genes by chromatin remodeling (alteration of DNA-nucleosome topology). Specifically binds acetylated lysines on histone 3 and 4 (H3K14ac, H3K9ac, H4K5ac, H4K8ac, H4K12ac, H4K16ac). In the complex, it acts as a tissue-specific anchor between histone acetylations and methylations and chromatin remodeling. It thereby probably plays an essential role in heart and skeletal muscle development.   
  
Component of the BAF complex, which includes at least actin (ACTB), ARID1A, ARID1B/BAF250, SMARCA2, SMARCA4/BRG1/BAF190A, ACTL6A/BAF53, ACTL6B/BAF53B, SMARCE1/BAF57, SMARCC1/BAF155, SMARCC2/BAF170, SMARCB1/SNF5/INI1, and one or more of SMARCD1/BAF60A, SMARCD2/BAF60B, or SMARCD3/BAF60C (PubMed:18765789). In muscle cells, the BAF complex also contains DPF3 (PubMed:18765789). Interacts with acetylated histones H3 and H4 (PubMed:18765789). Component of neuron-specific chromatin remodeling complex (nBAF complex) composed of at least, ARID1A/BAF250A or ARID1B/BAF250B, SMARCD1/BAF60A, SMARCD3/BAF60C, SMARCA2/BRM/BAF190B, SMARCA4/BRG1/BAF190A, SMARCB1/BAF47, SMARCC1/BAF155, SMARCE1/BAF57, SMARCC2/BAF170, DPF1/BAF45B, DPF3/BAF45C, ACTL6B/BAF53B and actin (By similarity).   
  
 **Gene Ontology Information:**

Molecular Function

- zinc ion binding

Location

- brahma complex
- chromatin
- nBAF complex
- nucleoplasm
- SWI/SNF complex

Biological process

- chromatin remodeling
- muscle organ development
- nervous system development
- positive regulation of cell differentiation
- positive regulation of double-strand break repair
- positive regulation of myoblast differentiation
- regulation of G0 to G1 transition
- regulation of G1/S transition of mitotic cell cycle
- regulation of mitotic metaphase/anaphase transition
- regulation of nucleotide-excision repair
- regulation of transcription by RNA polymerase II

---

2

- **Protein name:** Serine/threonine-protein kinase PLK3
- **Organism:** Homo sapiens
- **Uniprot Accession Number:** Q9H4B4
- **Protein sequence length:** 646 aa
- **1D identity (%):** 12.26
- **1D identity (%) [Gaps excluded]:** 22.24
- **1D identity - Alignment Gaps:** 436
- **1D aligned content (<aminoacid>:%):** {'P': 13.45, 'F': 5.04, 'R': 13.45, 'G': 7.56, 'L': 12.61, 'T': 4.2, 'K': 5.04, 'V': 5.88, 'E': 4.2, 'H': 3.36, 'Y': 2.52, 'C': 4.2, 'N': 0.84, 'D': 5.04, 'Q': 3.36, 'W': 0.84, 'A': 4.2, 'I': 0.84, 'S': 3.36}
- **Common reported functions (%):** 0.0
- **Common reported locations (%):** 22.22
- **Common reported processes (%):** 0.0

- **AF ID:** Q9H4B4
- **Chain:** A
- **Protein length:** 646 aa
- **Resolution:** N/A
- **b-phipsi:** 0.000435
- **w-rdist:** 0.271844
- **t-alpha:** 0.130745
- **Chemical similarity (Tanimoto Index) (%):** 97.33
- **1D identity (%) [PDB]:** 1.79
- **1D identity (%) [Gaps excluded][PDB]:** 74.29
- **1D identity - Alignment Gaps [PDB]:** 1421
- **1D aligned content [PDB] (<aminoacid>:%):** {'P': 19.23, 'A': 15.38, 'G': 11.54, 'K': 7.69, 'S': 7.69, 'I': 3.85, 'V': 15.38, 'D': 3.85, 'C': 3.85, 'T': 3.85, 'Q': 3.85, 'R': 3.85}
- **2D identity (%) [PDB]:** 40.23
- **2D identity (%) [Gaps excluded][PDB]:** 92.48
- **2D identity - Alignment Gaps [PDB]:** 587
- **2D aligned content [PDB] (<2D-fold>:%):** {'.': 19.14, 'T': 22.01, 'H': 34.45, 'E': 24.4}
- **3D similarity (TM-Score) (%) [PDB]:** 20.74

- **Gene name:** PLK3
- **Entrez ID:** 1263
- **RefSeq ID:** NM\_004073
- **Transcript sequence length:** 2340
- **5-UTR|CDS|3-UTR identity (%):** 15.87 | 39.07 | 1.22
- **5-UTR|CDS|3-UTR identity (%) [Gaps excluded]:** 70.67 | 72.94 | 74.28
- **5-UTR|CDS|3-UTR identity [Alignment Gaps]:** 259 | 1368 | 16515
- **5-UTR aligned content (<base>:%):** {'C': 47.17, 'A': 7.55, 'G': 45.28}
- **CDS aligned content (<base>:%):** {'A': 22.5, 'T': 20.59, 'G': 28.32, 'C': 28.58}
- **3-UTR aligned content (<base>:%):** {'G': 23.41, 'C': 30.24, 'T': 29.76, 'A': 16.59}

**Uniprot Description:**  
  
 Serine/threonine-protein kinase involved in cell cycle regulation, response to stress and Golgi disassembly. Polo-like kinases act by binding and phosphorylating proteins are that already phosphorylated on a specific motif recognized by the POLO box domains. Phosphorylates ATF2, BCL2L1, CDC25A, CDC25C, CHEK2, HIF1A, JUN, p53/TP53, p73/TP73, PTEN, TOP2A and VRK1. Involved in cell cycle regulation: required for entry into S phase and cytokinesis. Phosphorylates BCL2L1, leading to regulate the G2 checkpoint and progression to cytokinesis during mitosis. Plays a key role in response to stress: rapidly activated upon stress stimulation, such as ionizing radiation, reactive oxygen species (ROS), hyperosmotic stress, UV irradiation and hypoxia. Involved in DNA damage response and G1/S transition checkpoint by phosphorylating CDC25A, p53/TP53 and p73/TP73. Phosphorylates p53/TP53 in response to reactive oxygen species (ROS), thereby promoting p53/TP53-mediated apoptosis. Phosphorylates CHEK2 in response to DNA damage, promoting the G2/M transition checkpoint. Phosphorylates the transcription factor p73/TP73 in response to DNA damage, leading to inhibit p73/TP73-mediated transcriptional activation and pro-apoptotic functions. Phosphorylates HIF1A and JUN is response to hypoxia. Phosphorylates ATF2 following hyperosmotic stress in corneal epithelium. Also involved in Golgi disassembly during the cell cycle: part of a MEK1/MAP2K1-dependent pathway that induces Golgi fragmentation during mitosis by mediating phosphorylation of VRK1. May participate in endomitotic cell cycle, a form of mitosis in which both karyokinesis and cytokinesis are interrupted and is a hallmark of megakaryocyte differentiation, via its interaction with CIB1.   
  
Interacts (via the POLO-box domain) with CIB1; leading to inhibit PLK3 kinase activity. Interacts with GOLGB1.   
  
 **Gene Ontology Information:**

Molecular Function

- ATP binding
- p53 binding
- protein serine kinase activity
- protein serine/threonine kinase activity

Location

- centrosome
- cytoplasm
- dendrite
- Golgi stack
- kinetochore
- neuronal cell body
- nucleolus
- nucleoplasm
- nucleus
- spindle pole

Biological process

- apoptotic process
- cytoplasmic microtubule organization
- cellular response to DNA damage stimulus
- DNA damage response, signal transduction by p53 class mediator resulting in cell cycle arrest
- endomitotic cell cycle
- G1/S transition of mitotic cell cycle
- G2/M transition of mitotic cell cycle
- Golgi disassembly
- mitotic G1/S transition checkpoint
- mitotic spindle organization
- negative regulation of apoptotic process
- negative regulation of transcription by RNA polymerase II
- positive regulation of chaperone-mediated autophagy
- positive regulation of intracellular protein transport
- positive regulation of proteasomal ubiquitin-dependent protein catabolic process involved in cellular response to hypoxia
- protein kinase B signaling
- protein phosphorylation
- regulation of cell division
- regulation of cytokinesis
- regulation of signal transduction by p53 class mediator
- response to osmotic stress
- response to radiation
- response to reactive oxygen species

---

3

- **Protein name:** Calpain-1 catalytic subunit
- **Organism:** Homo sapiens
- **Uniprot Accession Number:** P07384
- **Protein sequence length:** 714 aa
- **1D identity (%):** 14.47
- **1D identity (%) [Gaps excluded]:** 24.87
- **1D identity - Alignment Gaps:** 416
- **1D aligned content (<aminoacid>:%):** {'P': 8.33, 'V': 9.03, 'T': 4.86, 'G': 8.33, 'E': 4.17, 'R': 9.03, 'I': 2.78, 'K': 6.25, 'L': 13.19, 'N': 2.78, 'D': 6.94, 'S': 4.17, 'F': 6.25, 'H': 1.39, 'W': 0.69, 'Y': 4.17, 'A': 3.47, 'C': 1.39, 'Q': 2.08, 'M': 0.69}
- **Common reported functions (%):** 0.0
- **Common reported locations (%):** 11.11
- **Common reported processes (%):** 0.0

- **AF ID:** P07384
- **Chain:** A
- **Protein length:** 714 aa
- **Resolution:** N/A
- **b-phipsi:** 0.000401
- **w-rdist:** 0.490586
- **t-alpha:** 0.014263
- **Chemical similarity (Tanimoto Index) (%):** 99.62
- **1D identity (%) [PDB]:** 2.95
- **1D identity (%) [Gaps excluded][PDB]:** 67.69
- **1D identity - Alignment Gaps [PDB]:** 1429
- **1D aligned content [PDB] (<aminoacid>:%):** {'P': 11.36, 'V': 20.45, 'T': 4.55, 'G': 4.55, 'D': 9.09, 'L': 15.91, 'K': 9.09, 'F': 2.27, 'S': 6.82, 'I': 2.27, 'R': 2.27, 'H': 2.27, 'E': 6.82, 'A': 2.27}
- **2D identity (%) [PDB]:** 39.3
- **2D identity (%) [Gaps excluded][PDB]:** 91.06
- **2D identity - Alignment Gaps [PDB]:** 619
- **2D aligned content [PDB] (<2D-fold>:%):** {'.': 18.69, 'H': 35.28, 'T': 24.77, 'E': 21.26}
- **3D similarity (TM-Score) (%) [PDB]:** 22.63

- **Gene name:** CAPN1
- **Entrez ID:** 408218
- **RefSeq ID:** N/A
- **Sequence length:** N/A
- **5-UTR|CDS|3-UTR identity (%):** N/A | N/A | N/A
- **5-UTR|CDS|3-UTR identity (%) [Gaps excluded]:** N/A | N/A | N/A
- **5-UTR|CDS|3-UTR identity [Alignment Gaps]:** N/A | N/A | N/A
- **5-UTR aligned content (<base>:%):** N/A
- **CDS aligned content (<base>:%):** N/A
- **3-UTR aligned content (<base>:%):** N/A

**Uniprot Description:**  
  
 Calcium-regulated non-lysosomal thiol-protease which catalyzes limited proteolysis of substrates involved in cytoskeletal remodeling and signal transduction (PubMed:19617626, PubMed:21531719, PubMed:2400579). Proteolytically cleaves CTBP1 at 'Asn-375', 'Gly-387' and 'His-409' (PubMed:23707407). Cleaves and activates caspase-7 (CASP7) (PubMed:19617626).   
  
Forms a heterodimer with a small (regulatory) subunit CAPNS1.   
  
 **Gene Ontology Information:**

Molecular Function

- calcium ion binding
- calcium-dependent cysteine-type endopeptidase activity

Location

- acrosomal vesicle
- cytoplasm

Biological process

- proteolysis

---

4

- **Protein name:** Ras GTPase-activating protein 3
- **Organism:** Homo sapiens
- **Uniprot Accession Number:** Q14644
- **Protein sequence length:** 834 aa
- **1D identity (%):** 13.98
- **1D identity (%) [Gaps excluded]:** 21.31
- **1D identity - Alignment Gaps:** 352
- **1D aligned content (<aminoacid>:%):** {'M': 0.7, 'G': 6.99, 'Q': 5.59, 'P': 12.59, 'Y': 4.2, 'T': 4.2, 'K': 8.39, 'C': 2.1, 'F': 6.29, 'E': 5.59, 'I': 3.5, 'D': 6.99, 'V': 5.59, 'H': 2.1, 'L': 8.39, 'S': 4.2, 'W': 0.7, 'N': 3.5, 'R': 4.2, 'A': 4.2}
- **Common reported functions (%):** 14.29
- **Common reported locations (%):** 11.11
- **Common reported processes (%):** 0.0

- **AF ID:** Q14644
- **Chain:** A
- **Protein length:** 834 aa
- **Resolution:** N/A
- **b-phipsi:** 0.003956
- **w-rdist:** 0.37244
- **t-alpha:** 0.001587
- **Chemical similarity (Tanimoto Index) (%):** 98.95
- **1D identity (%) [PDB]:** 2.53
- **1D identity (%) [Gaps excluded][PDB]:** 69.49
- **1D identity - Alignment Gaps [PDB]:** 1561
- **1D aligned content [PDB] (<aminoacid>:%):** {'M': 4.88, 'F': 2.44, 'R': 4.88, 'H': 2.44, 'L': 14.63, 'T': 14.63, 'Q': 9.76, 'V': 9.76, 'I': 4.88, 'P': 2.44, 'K': 9.76, 'A': 4.88, 'E': 2.44, 'D': 2.44, 'G': 2.44, 'C': 2.44, 'S': 4.88}
- **2D identity (%) [PDB]:** 42.14
- **2D identity (%) [Gaps excluded][PDB]:** 88.89
- **2D identity - Alignment Gaps [PDB]:** 599
- **2D aligned content [PDB] (<2D-fold>:%):** {'E': 24.17, '.': 18.12, 'T': 26.25, 'H': 31.46}
- **3D similarity (TM-Score) (%) [PDB]:** 21.81

- **Gene name:** RASA3
- **Entrez ID:** 22821
- **RefSeq ID:** N/A
- **Sequence length:** N/A
- **5-UTR|CDS|3-UTR identity (%):** N/A | N/A | N/A
- **5-UTR|CDS|3-UTR identity (%) [Gaps excluded]:** N/A | N/A | N/A
- **5-UTR|CDS|3-UTR identity [Alignment Gaps]:** N/A | N/A | N/A
- **5-UTR aligned content (<base>:%):** N/A
- **CDS aligned content (<base>:%):** N/A
- **3-UTR aligned content (<base>:%):** N/A

**Uniprot Description:**  
  
 Inhibitory regulator of the Ras-cyclic AMP pathway. Binds inositol tetrakisphosphate (IP4) with high affinity. Might be a specific IP4 receptor. N/A   
  
 **Gene Ontology Information:**

Molecular Function

- calcium-release channel activity
- GTPase activator activity
- metal ion binding

Location

- cytoplasmic side of plasma membrane
- cytosol

Biological process

- negative regulation of Ras protein signal transduction
- regulation of GTPase activity
- signal transduction

---

5

- **Protein name:** G protein-activated inward rectifier potassium channel 4
- **Organism:** Homo sapiens
- **Uniprot Accession Number:** P48544
- **Protein sequence length:** 419 aa
- **1D identity (%):** 8.26
- **1D identity (%) [Gaps excluded]:** 21.17
- **1D identity - Alignment Gaps:** 561
- **1D aligned content (<aminoacid>:%):** {'M': 2.63, 'G': 15.79, 'E': 6.58, 'I': 6.58, 'Q': 2.63, 'R': 7.89, 'T': 3.95, 'D': 6.58, 'K': 2.63, 'C': 5.26, 'V': 6.58, 'L': 14.47, 'N': 2.63, 'P': 5.26, 'F': 3.95, 'S': 2.63, 'A': 1.32, 'H': 1.32, 'Y': 1.32}
- **Common reported functions (%):** 14.29
- **Common reported locations (%):** 0.0
- **Common reported processes (%):** 0.0

- **AF ID:** P48544
- **Chain:** A
- **Protein length:** 419 aa
- **Resolution:** N/A
- **b-phipsi:** 0.000314
- **w-rdist:** 0.914135
- **t-alpha:** 0.002383
- **Chemical similarity (Tanimoto Index) (%):** 96.62
- **1D identity (%) [PDB]:** 2.71
- **1D identity (%) [Gaps excluded][PDB]:** 70.21
- **1D identity - Alignment Gaps [PDB]:** 1170
- **1D aligned content [PDB] (<aminoacid>:%):** {'A': 3.03, 'T': 12.12, 'V': 12.12, 'D': 9.09, 'L': 15.15, 'G': 12.12, 'E': 9.09, 'R': 6.06, 'F': 3.03, 'S': 6.06, 'H': 3.03, 'P': 9.09}
- **2D identity (%) [PDB]:** 33.12
- **2D identity (%) [Gaps excluded][PDB]:** 91.1
- **2D identity - Alignment Gaps [PDB]:** 590
- **2D aligned content [PDB] (<2D-fold>:%):** {'.': 18.57, 'T': 31.6, 'E': 14.66, 'H': 34.2, 'B': 0.98}
- **3D similarity (TM-Score) (%) [PDB]:** 15.82

- **Gene name:** KCNJ5
- **Entrez ID:** 4512
- **RefSeq ID:** N/A
- **Sequence length:** N/A
- **5-UTR|CDS|3-UTR identity (%):** N/A | N/A | N/A
- **5-UTR|CDS|3-UTR identity (%) [Gaps excluded]:** N/A | N/A | N/A
- **5-UTR|CDS|3-UTR identity [Alignment Gaps]:** N/A | N/A | N/A
- **5-UTR aligned content (<base>:%):** N/A
- **CDS aligned content (<base>:%):** N/A
- **3-UTR aligned content (<base>:%):** N/A

**Uniprot Description:**  
  
 This potassium channel is controlled by G proteins. Inward rectifier potassium channels are characterized by a greater tendency to allow potassium to flow into the cell rather than out of it. Their voltage dependence is regulated by the concentration of extracellular potassium; as external potassium is raised, the voltage range of the channel opening shifts to more positive voltages. The inward rectification is mainly due to the blockage of outward current by internal magnesium. Can be blocked by external barium.   
  
May associate with GIRK1 and GIRK2 to form a G-protein-activated heteromultimer pore-forming unit. The resulting inward current is much larger.   
  
 **Gene Ontology Information:**

Molecular Function

- cytochrome-c oxidase activity
- heme binding
- metal ion binding

Location

- mitochondrial inner membrane
- mitochondrial respiratory chain complex III
- mitochondrial respiratory chain complex IV
- respiratory chain complex IV

Biological process

- cellular respiration
- cerebellum development
- electron transport coupled proton transport
- mitochondrial electron transport, cytochrome c to oxygen
- response to copper ion
- response to electrical stimulus
- response to oxidative stress

---

6

- **Protein name:** Piwi-like protein 1
- **Organism:** Homo sapiens
- **Uniprot Accession Number:** Q96J94
- **Protein sequence length:** 861 aa
- **1D identity (%):** 22.7
- **1D identity (%) [Gaps excluded]:** 27.78
- **1D identity - Alignment Gaps:** 173
- **1D aligned content (<aminoacid>:%):** {'G': 11.16, 'A': 3.72, 'Q': 5.58, 'P': 6.98, 'Y': 5.58, 'T': 6.05, 'K': 3.72, 'L': 8.84, 'E': 5.58, 'D': 5.12, 'R': 9.77, 'N': 3.26, 'V': 6.51, 'H': 2.79, 'F': 4.19, 'I': 2.33, 'S': 3.72, 'M': 2.33, 'W': 1.4, 'C': 1.4}
- **Common reported functions (%):** 42.86
- **Common reported locations (%):** 11.11
- **Common reported processes (%):** 0.0

- **AF ID:** Q96J94
- **Chain:** A
- **Protein length:** 861 aa
- **Resolution:** N/A
- **b-phipsi:** 0.001347
- **w-rdist:** 0.145832
- **t-alpha:** 0.042789
- **Chemical similarity (Tanimoto Index) (%):** 99.4
- **1D identity (%) [PDB]:** 5.08
- **1D identity (%) [Gaps excluded][PDB]:** 52.32
- **1D identity - Alignment Gaps [PDB]:** 1404
- **1D aligned content [PDB] (<aminoacid>:%):** {'M': 1.27, 'V': 7.59, 'R': 10.13, 'E': 2.53, 'L': 5.06, 'I': 6.33, 'Q': 3.8, 'F': 6.33, 'Y': 11.39, 'K': 3.8, 'S': 2.53, 'T': 7.59, 'P': 7.59, 'D': 6.33, 'G': 6.33, 'N': 1.27, 'A': 5.06, 'H': 3.8, 'C': 1.27}
- **2D identity (%) [PDB]:** 59.02
- **2D identity (%) [Gaps excluded][PDB]:** 87.76
- **2D identity - Alignment Gaps [PDB]:** 334
- **2D aligned content [PDB] (<2D-fold>:%):** {'.': 23.83, 'T': 19.67, 'E': 23.67, 'H': 32.0, 'B': 0.33, 'G': 0.5}
- **3D similarity (TM-Score) (%) [PDB]:** 75.98

- **Gene name:** PIWIL1
- **Entrez ID:** 9271
- **RefSeq ID:** N/A
- **Sequence length:** N/A
- **5-UTR|CDS|3-UTR identity (%):** N/A | N/A | N/A
- **5-UTR|CDS|3-UTR identity (%) [Gaps excluded]:** N/A | N/A | N/A
- **5-UTR|CDS|3-UTR identity [Alignment Gaps]:** N/A | N/A | N/A
- **5-UTR aligned content (<base>:%):** N/A
- **CDS aligned content (<base>:%):** N/A
- **3-UTR aligned content (<base>:%):** N/A

**Uniprot Description:**  
  
 Endoribonuclease that plays a central role in postnatal germ cells by repressing transposable elements and preventing their mobilization, which is essential for the germline integrity. Acts via the piRNA metabolic process, which mediates the repression of transposable elements during meiosis by forming complexes composed of piRNAs and Piwi proteins and governs the methylation and subsequent repression of transposons. Directly binds methylated piRNAs, a class of 24 to 30 nucleotide RNAs that are generated by a Dicer-independent mechanism and are primarily derived from transposons and other repeated sequence elements. Strongly prefers a uridine in the first position of their guide (g1U preference, also named 1U-bias). Not involved in the piRNA amplification loop, also named ping-pong amplification cycle. Acts as an endoribonuclease that cleaves transposon messenger RNAs. Besides their function in transposable elements repression, piRNAs are probably involved in other processes during meiosis such as translation regulation. Probable component of some RISC complex, which mediates RNA cleavage and translational silencing. Also plays a role in the formation of chromatoid bodies and is required for some miRNAs stability. Required to sequester RNF8 in the cytoplasm until late spermatogenesis; RNF8 being released upon ubiquitination and degradation of PIWIL1.   
  
Interacts (via Piwi domain) with DICER1, suggesting that it forms ribonucleoprotein RISC complexes; this interaction is regulated by HSP90AB1 activity. Interacts with MAEL, KIF17, PABPC1, PRMT5 and WDR77. Interacts (when methylated on arginine residues) with TDRD1, TDRKH/TDRD2, RNF17/TDRD4, TDRD6, TDRD7 and TDRD9. Interacts with CLOCK. Interacts with MOV10L1. Interacts with ANAPC10; interaction oly takes place following piRNA-binding. Interacts with RNF8; leading to sequester RNF8 in the cytoplasm. Interacts with TEX19 (By similarity).   
  
 **Gene Ontology Information:**

Molecular Function

- metal ion binding
- mRNA binding
- mRNA cap binding complex binding
- piRNA binding
- polysome binding
- protein kinase binding
- endoribonuclease activity
- single-stranded RNA binding

Location

- chromatoid body
- cytoplasm
- dense body
- nucleus
- P granule

Biological process

- meiotic cell cycle
- piRNA-mediated retrotransposon silencing by heterochromatin formation
- primary piRNA processing
- regulation of translation
- gene silencing by RNA
- sperm chromatin condensation
- spermatid development
- spermatogenesis

---

7

- **Protein name:** Carnosine synthase 1
- **Organism:** Homo sapiens
- **Uniprot Accession Number:** A5YM72
- **Protein sequence length:** 827 aa
- **1D identity (%):** 15.54
- **1D identity (%) [Gaps excluded]:** 23.95
- **1D identity - Alignment Gaps:** 359
- **1D aligned content (<aminoacid>:%):** {'L': 18.87, 'P': 9.43, 'G': 13.84, 'C': 5.66, 'E': 5.66, 'V': 9.43, 'T': 3.77, 'A': 7.55, 'K': 1.89, 'R': 5.66, 'W': 1.26, 'Y': 1.89, 'Q': 4.4, 'D': 3.14, 'M': 1.26, 'S': 1.89, 'N': 0.63, 'F': 1.26, 'I': 1.89, 'H': 0.63}
- **Common reported functions (%):** 14.29
- **Common reported locations (%):** 11.11
- **Common reported processes (%):** 0.0

- **AF ID:** A5YM72
- **Chain:** A
- **Protein length:** 827 aa
- **Resolution:** N/A
- **b-phipsi:** 0.000373
- **w-rdist:** 0.392751
- **t-alpha:** 0.066561
- **Chemical similarity (Tanimoto Index) (%):** 99.47
- **1D identity (%) [PDB]:** 2.81
- **1D identity (%) [Gaps excluded][PDB]:** 65.22
- **1D identity - Alignment Gaps [PDB]:** 1534
- **1D aligned content [PDB] (<aminoacid>:%):** {'K': 6.67, 'V': 17.78, 'T': 2.22, 'F': 4.44, 'G': 15.56, 'D': 4.44, 'R': 4.44, 'P': 6.67, 'L': 20.0, 'N': 2.22, 'A': 2.22, 'S': 4.44, 'H': 4.44, 'E': 4.44}
- **2D identity (%) [PDB]:** 45.89
- **2D identity (%) [Gaps excluded][PDB]:** 86.85
- **2D identity - Alignment Gaps [PDB]:** 516
- **2D aligned content [PDB] (<2D-fold>:%):** {'.': 17.13, 'E': 27.29, 'T': 18.33, 'H': 36.06, 'G': 1.2}
- **3D similarity (TM-Score) (%) [PDB]:** 26.06

- **Gene name:** CARNS1
- **Entrez ID:** 57571
- **RefSeq ID:** N/A
- **Sequence length:** N/A
- **5-UTR|CDS|3-UTR identity (%):** N/A | N/A | N/A
- **5-UTR|CDS|3-UTR identity (%) [Gaps excluded]:** N/A | N/A | N/A
- **5-UTR|CDS|3-UTR identity [Alignment Gaps]:** N/A | N/A | N/A
- **5-UTR aligned content (<base>:%):** N/A
- **CDS aligned content (<base>:%):** N/A
- **3-UTR aligned content (<base>:%):** N/A

**Uniprot Description:**  
  
 Catalyzes the synthesis of carnosine and homocarnosine. Carnosine is synthesized more efficiently than homocarnosine.   
  
Homotetramer.   
  
 **Gene Ontology Information:**

Molecular Function

- ATP binding
- ATPase activity
- carnosine synthase activity
- homocarnosine synthase activity
- metal ion binding

Location

- cytosol

Biological process

- carnosine biosynthetic process
- histidine catabolic process

---

8

- **Protein name:** Serine protease FAM111A
- **Organism:** Homo sapiens
- **Uniprot Accession Number:** Q96PZ2
- **Protein sequence length:** 611 aa
- **1D identity (%):** 13.92
- **1D identity (%) [Gaps excluded]:** 24.21
- **1D identity - Alignment Gaps:** 397
- **1D aligned content (<aminoacid>:%):** {'K': 12.31, 'E': 6.92, 'H': 4.62, 'P': 6.92, 'A': 6.15, 'T': 8.46, 'D': 3.85, 'R': 5.38, 'V': 6.15, 'L': 6.15, 'I': 3.08, 'Y': 3.08, 'G': 10.0, 'F': 3.85, 'Q': 6.15, 'S': 3.08, 'C': 3.08, 'W': 0.77}
- **Common reported functions (%):** 0.0
- **Common reported locations (%):** 22.22
- **Common reported processes (%):** 0.0

- **AF ID:** Q96PZ2
- **Chain:** A
- **Protein length:** 611 aa
- **Resolution:** N/A
- **b-phipsi:** 0.005212
- **w-rdist:** 0.40049
- **t-alpha:** 0.0
- **Chemical similarity (Tanimoto Index) (%):** 98.94
- **1D identity (%) [PDB]:** 3.26
- **1D identity (%) [Gaps excluded][PDB]:** 59.21
- **1D identity - Alignment Gaps [PDB]:** 1304
- **1D aligned content [PDB] (<aminoacid>:%):** {'M': 2.22, 'P': 4.44, 'R': 11.11, 'Y': 6.67, 'G': 6.67, 'T': 6.67, 'I': 6.67, 'K': 8.89, 'N': 2.22, 'C': 4.44, 'F': 4.44, 'Q': 4.44, 'V': 15.56, 'D': 6.67, 'E': 2.22, 'S': 4.44, 'L': 2.22}
- **2D identity (%) [PDB]:** 41.03
- **2D identity (%) [Gaps excluded][PDB]:** 87.72
- **2D identity - Alignment Gaps [PDB]:** 528
- **2D aligned content [PDB] (<2D-fold>:%):** {'.': 25.06, 'T': 22.6, 'E': 22.11, 'H': 29.73, 'B': 0.49}
- **3D similarity (TM-Score) (%) [PDB]:** 20.7

- **Gene name:** FAM111A
- **Entrez ID:** 63901
- **RefSeq ID:** NM\_198847
- **Transcript sequence length:** 3473
- **5-UTR|CDS|3-UTR identity (%):** 17.25 | 38.15 | 5.82
- **5-UTR|CDS|3-UTR identity (%) [Gaps excluded]:** 71.95 | 74.53 | 77.89
- **5-UTR|CDS|3-UTR identity [Alignment Gaps]:** 260 | 1427 | 15748
- **5-UTR aligned content (<base>:%):** {'G': 33.9, 'C': 40.68, 'T': 18.64, 'A': 6.78}
- **CDS aligned content (<base>:%):** {'A': 36.14, 'T': 24.66, 'G': 21.97, 'C': 17.22}
- **3-UTR aligned content (<base>:%):** {'G': 18.59, 'A': 27.68, 'T': 35.56, 'C': 18.18}

**Uniprot Description:**  
  
 Single-stranded DNA-binding serine protease that mediates the proteolytic cleavage of covalent DNA-protein cross-links (DPCs) during DNA synthesis, thereby playing a key role in maintaining genomic integrity (PubMed:32165630). DPCs are highly toxic DNA lesions that interfere with essential chromatin transactions, such as replication and transcription, and which are induced by reactive agents, such as UV light or formaldehyde (PubMed:32165630). Protects replication fork from stalling by removing DPCs, such as covalently trapped topoisomerase 1 (TOP1) adducts on DNA lesion, or poly(ADP-ribose) polymerase 1 (PARP1)-DNA complexes trapped by PARP inhibitors (PubMed:32165630). Required for PCNA loading on replication sites (PubMed:24561620). Promotes S-phase entry and DNA synthesis (PubMed:24561620).   
  
Interacts (via PIP-box) with PCNA; then interaction is direct.   
  
 **Gene Ontology Information:**

Molecular Function

- peptidase activity
- single-stranded DNA binding

Location

- chromatin
- cytoplasm
- fibrillar center
- nucleoplasm
- nucleus

Biological process

- cellular response to DNA damage stimulus
- DNA replication
- negative regulation of viral genome replication
- protein autoprocessing
- protein-DNA covalent cross-linking repair
- proteolysis
- replication fork processing

---

9

- **Protein name:** Group 3 secretory phospholipase A2
- **Organism:** Homo sapiens
- **Uniprot Accession Number:** Q9NZ20
- **Protein sequence length:** 509 aa
- **1D identity (%):** 10.85
- **1D identity (%) [Gaps excluded]:** 26.18
- **1D identity - Alignment Gaps:** 567
- **1D aligned content (<aminoacid>:%):** {'G': 10.48, 'Q': 8.57, 'L': 11.43, 'R': 12.38, 'T': 3.81, 'A': 4.76, 'D': 1.9, 'S': 4.76, 'E': 4.76, 'P': 11.43, 'H': 3.81, 'W': 1.9, 'K': 3.81, 'C': 4.76, 'N': 1.9, 'I': 2.86, 'V': 4.76, 'F': 1.9}
- **Common reported functions (%):** 14.29
- **Common reported locations (%):** 0.0
- **Common reported processes (%):** 0.0

- **AF ID:** Q9NZ20
- **Chain:** A
- **Protein length:** 509 aa
- **Resolution:** N/A
- **b-phipsi:** 0.00077
- **w-rdist:** 0.382102
- **t-alpha:** 0.008717
- **Chemical similarity (Tanimoto Index) (%):** 97.2
- **1D identity (%) [PDB]:** 1.59
- **1D identity (%) [Gaps excluded][PDB]:** 61.76
- **1D identity - Alignment Gaps [PDB]:** 1286
- **1D aligned content [PDB] (<aminoacid>:%):** {'M': 4.76, 'A': 4.76, 'H': 9.52, 'R': 14.29, 'L': 9.52, 'P': 9.52, 'Q': 9.52, 'Y': 4.76, 'G': 9.52, 'N': 4.76, 'T': 4.76, 'S': 4.76, 'D': 4.76, 'K': 4.76}
- **2D identity (%) [PDB]:** 38.53
- **2D identity (%) [Gaps excluded][PDB]:** 86.57
- **2D identity - Alignment Gaps [PDB]:** 520
- **2D aligned content [PDB] (<2D-fold>:%):** {'T': 32.41, '.': 17.17, 'E': 18.28, 'H': 31.86, 'B': 0.28}
- **3D similarity (TM-Score) (%) [PDB]:** 20.33

- **Gene name:** PLA2G3
- **Entrez ID:** 50487
- **RefSeq ID:** NM\_015715
- **Transcript sequence length:** 2600
- **5-UTR|CDS|3-UTR identity (%):** 26.29 | 31.41 | 3.63
- **5-UTR|CDS|3-UTR identity (%) [Gaps excluded]:** 73.02 | 74.84 | 73.35
- **5-UTR|CDS|3-UTR identity [Alignment Gaps]:** 224 | 1681 | 16005
- **5-UTR aligned content (<base>:%):** {'C': 44.57, 'T': 18.48, 'A': 8.7, 'G': 28.26}
- **CDS aligned content (<base>:%):** {'A': 25.82, 'G': 29.23, 'T': 17.14, 'C': 27.8}
- **3-UTR aligned content (<base>:%):** {'C': 19.8, 'T': 28.64, 'A': 26.84, 'G': 24.71}

**Uniprot Description:**  
  
 Secretory calcium-dependent phospholipase A2 that primarily targets extracellular phospholipids. Hydrolyzes the ester bond of the fatty acyl group attached at sn-2 position of phospholipids without apparent head group selectivity (PubMed:12522102, PubMed:15863501, PubMed:18801741, PubMed:28947740). Contributes to phospholipid remodeling of low-density lipoprotein (LDL) and high-density lipoprotein (HDL) particles. Hydrolyzes LDL phospholipids releasing unsaturated fatty acids that regulate macrophage differentiation toward foam cells (PubMed:18801741). May act in an autocrine and paracrine manner (PubMed:23624557). Secreted by immature mast cells, acts on nearby fibroblasts upstream to PTDGS to synthesize prostaglandin D2 (PGD2), which in turn promotes mast cell maturation and degranulation via PTGDR (PubMed:23624557). Secreted by epididymal epithelium, acts on immature sperm cells within the duct, modulating the degree of unsaturation of the fatty acyl components of phosphatidylcholines required for acrosome assembly and sperm cell motility. Facilitates the replacement of fatty acyl chains in phosphatidylcholines in sperm membranes from omega-6 and omega-9 to omega-3 polyunsaturated fatty acids (PUFAs). Coupled to lipoxygenase pathway, may process omega-6 PUFAs to generate oxygenated lipid mediators in the male reproductive tract (By similarity). At pericentrosomal preciliary compartment, negatively regulates ciliogenesis likely by regulating endocytotic recycling of ciliary membrane protein (PubMed:20393563). Coupled to cyclooxygenase pathway provides arachidonate to generate prostaglandin E2 (PGE2), a potent immunomodulatory lipid in inflammation and tumorigenesis (PubMed:12522102, PubMed:15863501). At colonic epithelial barrier, preferentially hydrolyzes phospholipids having arachidonate and docosahexaenoate at sn-2 position, contributing to the generation of oxygenated metabolites involved in colonic stem cell homeostasis (PubMed:28947740). Releases C16:0 and C18:0 lysophosphatidylcholine subclasses from neuron plasma membranes and promotes neurite outgrowth and neuron survival (PubMed:17868035). N/A   
  
 **Gene Ontology Information:**

Molecular Function

- calcium-dependent phospholipase A2 activity
- metal ion binding

Location

- centriole
- extracellular region
- extracellular space
- plasma membrane
- recycling endosome

Biological process

- acrosome assembly
- arachidonic acid secretion
- cell maturation
- cilium assembly
- high-density lipoprotein particle remodeling
- lipoxygenase pathway
- low-density lipoprotein particle remodeling
- macrophage activation
- mast cell degranulation
- negative regulation of amyloid-beta clearance
- negative regulation of gene expression
- negative regulation of neuron apoptotic process
- phosphatidic acid metabolic process
- phosphatidylcholine metabolic process
- phosphatidylethanolamine metabolic process
- phosphatidylglycerol metabolic process
- phosphatidylinositol metabolic process
- phosphatidylserine metabolic process
- phospholipid metabolic process
- positive regulation of cytokine production involved in inflammatory response
- positive regulation of histamine secretion by mast cell
- positive regulation of macrophage derived foam cell differentiation
- positive regulation of mast cell differentiation
- positive regulation of neuron projection development
- positive regulation of prostaglandin biosynthetic process
- positive regulation of prostaglandin secretion
- production of molecular mediator involved in inflammatory response
- regulation of endocytic recycling
- sperm axoneme assembly

---

10

- **Protein name:** EF-hand calcium-binding domain-containing protein 7
- **Organism:** Homo sapiens
- **Uniprot Accession Number:** A8K855
- **Protein sequence length:** 629 aa
- **1D identity (%):** 13.43
- **1D identity (%) [Gaps excluded]:** 22.4
- **1D identity - Alignment Gaps:** 373
- **1D aligned content (<aminoacid>:%):** {'M': 1.6, 'P': 8.8, 'F': 3.2, 'S': 6.4, 'L': 8.8, 'E': 8.0, 'K': 8.8, 'Y': 4.0, 'A': 1.6, 'Q': 3.2, 'I': 8.8, 'T': 5.6, 'D': 4.8, 'V': 4.8, 'G': 6.4, 'C': 4.8, 'R': 6.4, 'N': 1.6, 'W': 0.8, 'H': 1.6}
- **Common reported functions (%):** 0.0
- **Common reported locations (%):** 0.0
- **Common reported processes (%):** 0.0

- **AF ID:** A8K855
- **Chain:** A
- **Protein length:** 629 aa
- **Resolution:** N/A
- **b-phipsi:** 0.003087
- **w-rdist:** 0.610132
- **t-alpha:** 0.000792
- **Chemical similarity (Tanimoto Index) (%):** 99.47
- **1D identity (%) [PDB]:** 1.96
- **1D identity (%) [Gaps excluded][PDB]:** 66.67
- **1D identity - Alignment Gaps [PDB]:** 1390
- **1D aligned content [PDB] (<aminoacid>:%):** {'M': 7.14, 'R': 3.57, 'K': 14.29, 'F': 7.14, 'T': 7.14, 'E': 10.71, 'I': 14.29, 'A': 7.14, 'Q': 7.14, 'C': 3.57, 'L': 7.14, 'G': 3.57, 'D': 3.57, 'S': 3.57}
- **2D identity (%) [PDB]:** 37.91
- **2D identity (%) [Gaps excluded][PDB]:** 86.83
- **2D identity - Alignment Gaps [PDB]:** 578
- **2D aligned content [PDB] (<2D-fold>:%):** {'.': 20.82, 'H': 27.25, 'T': 23.65, 'E': 28.28}
- **3D similarity (TM-Score) (%) [PDB]:** 22.87

- **Gene name:** EFCAB7
- **Entrez ID:** 84455
- **RefSeq ID:** N/A
- **Sequence length:** N/A
- **5-UTR|CDS|3-UTR identity (%):** N/A | N/A | N/A
- **5-UTR|CDS|3-UTR identity (%) [Gaps excluded]:** N/A | N/A | N/A
- **5-UTR|CDS|3-UTR identity [Alignment Gaps]:** N/A | N/A | N/A
- **5-UTR aligned content (<base>:%):** N/A
- **CDS aligned content (<base>:%):** N/A
- **3-UTR aligned content (<base>:%):** N/A

**Uniprot Description:**  
  
 Component of the EvC complex that positively regulates ciliary Hedgehog (Hh) signaling. Required for the localization of the EVC2:EVC subcomplex at the base of primary cilia.   
  
Component of the EvC complex composed of EFCAB7, IQCE, EVC2 and EVC; built from two subcomplexes, EVC2:EVC and EFCAB7:IQCE. Interacts (via EF-hand 1 and 2) with IQCE (via N-terminus); this interaction anchors the EVC-EVC2 complex in a signaling microdomain at the base of cilia and stimulates the Hedgehog (Hh) pathway. Interacts with EVC2 (via N-terminal end). Interacts with EVC.   
  
 **Gene Ontology Information:**

Molecular Function

- calcium ion binding

Location

- ciliary membrane
- cilium
- cytoplasmic side of plasma membrane
- plasma membrane protein complex

Biological process

- positive regulation of protein import into nucleus
- positive regulation of protein localization to ciliary membrane
- positive regulation of transcription by RNA polymerase II

---

11

- **Protein name:** Serine/threonine-protein kinase OSR1
- **Organism:** Homo sapiens
- **Uniprot Accession Number:** O95747
- **Protein sequence length:** 527 aa
- **1D identity (%):** 10.46
- **1D identity (%) [Gaps excluded]:** 21.78
- **1D identity - Alignment Gaps:** 487
- **1D aligned content (<aminoacid>:%):** {'E': 5.1, 'P': 11.22, 'R': 5.1, 'L': 12.24, 'G': 10.2, 'T': 6.12, 'D': 3.06, 'M': 2.04, 'S': 4.08, 'H': 2.04, 'A': 4.08, 'I': 8.16, 'N': 4.08, 'F': 4.08, 'K': 7.14, 'V': 3.06, 'Y': 2.04, 'Q': 5.1, 'W': 1.02}
- **Common reported functions (%):** 0.0
- **Common reported locations (%):** 22.22
- **Common reported processes (%):** 0.0

- **AF ID:** O95747
- **Chain:** A
- **Protein length:** 527 aa
- **Resolution:** N/A
- **b-phipsi:** 0.000434
- **w-rdist:** 0.609901
- **t-alpha:** 0.024351
- **Chemical similarity (Tanimoto Index) (%):** 99.09
- **1D identity (%) [PDB]:** 2.57
- **1D identity (%) [Gaps excluded][PDB]:** 70.83
- **1D identity - Alignment Gaps [PDB]:** 1276
- **1D aligned content [PDB] (<aminoacid>:%):** {'K': 11.76, 'V': 14.71, 'I': 5.88, 'F': 2.94, 'S': 11.76, 'L': 17.65, 'E': 5.88, 'G': 5.88, 'T': 5.88, 'P': 8.82, 'D': 2.94, 'R': 5.88}
- **2D identity (%) [PDB]:** 36.6
- **2D identity (%) [Gaps excluded][PDB]:** 88.31
- **2D identity - Alignment Gaps [PDB]:** 568
- **2D aligned content [PDB] (<2D-fold>:%):** {'.': 16.06, 'T': 30.7, 'E': 16.9, 'H': 36.06, 'B': 0.28}
- **3D similarity (TM-Score) (%) [PDB]:** 17.31

- **Gene name:** OXSR1
- **Entrez ID:** 9943
- **RefSeq ID:** NM\_005109
- **Transcript sequence length:** 4517
- **5-UTR|CDS|3-UTR identity (%):** 44.79 | 35.3 | 9.3
- **5-UTR|CDS|3-UTR identity (%) [Gaps excluded]:** 74.3 | 76.64 | 76.13
- **5-UTR|CDS|3-UTR identity [Alignment Gaps]:** 164 | 1539 | 15134
- **5-UTR aligned content (<base>:%):** {'G': 44.86, 'C': 38.38, 'A': 4.86, 'T': 11.89}
- **CDS aligned content (<base>:%):** {'A': 32.87, 'T': 23.73, 'G': 24.03, 'C': 19.36}
- **3-UTR aligned content (<base>:%):** {'C': 16.46, 'T': 35.6, 'G': 18.52, 'A': 29.43}

**Uniprot Description:**  
  
 Phosphorylates RELL1, RELL2 and RELT (PubMed:16389068, PubMed:28688764). Phosphorylates PAK1 (PubMed:14707132). Phosphorylates PLSCR1 in the presence of RELT (PubMed:22052202).   
  
Interacts with PAK1 (PubMed:14707132). Interacts with chloride channel proteins SLC12A6 isoform 2, SLC12A1 and SLC12A2 but not with SLC12A4 and SLC12A7, possibly establishing sensor/signaling modules that initiate the cellular response to environmental stress (By similarity). Interacts with RELL1, RELL2 and RELT (PubMed:16389068). Interacts with PLSCR1 in the presence of RELT (PubMed:22052202).   
  
 **Gene Ontology Information:**

Molecular Function

- ATP binding
- identical protein binding
- magnesium ion binding
- protein kinase binding
- protein serine kinase activity
- protein serine/threonine kinase activity

Location

- cytoplasm
- cytosol
- extracellular exosome

Biological process

- cellular hypotonic response
- cellular response to chemokine
- chemokine (C-C motif) ligand 21 signaling pathway
- chemokine (C-X-C motif) ligand 12 signaling pathway
- intracellular signal transduction
- negative regulation of potassium ion transmembrane transporter activity
- osmosensory signaling pathway
- peptidyl-threonine phosphorylation
- positive regulation of T cell chemotaxis
- protein autophosphorylation
- protein phosphorylation
- response to oxidative stress
- response to xenobiotic stimulus
- signal transduction

---

12

- **Protein name:** Ras GTPase-activating protein 4B
- **Organism:** Homo sapiens
- **Uniprot Accession Number:** C9J798
- **Protein sequence length:** 803 aa
- **1D identity (%):** 16.52
- **1D identity (%) [Gaps excluded]:** 27.23
- **1D identity - Alignment Gaps:** 407
- **1D aligned content (<aminoacid>:%):** {'R': 7.02, 'S': 5.26, 'G': 8.19, 'L': 10.53, 'P': 9.36, 'D': 5.85, 'V': 11.11, 'A': 5.85, 'F': 4.68, 'Y': 2.92, 'I': 2.34, 'C': 2.92, 'H': 3.51, 'E': 6.43, 'T': 4.09, 'Q': 4.68, 'K': 3.51, 'N': 0.58, 'M': 1.17}
- **Common reported functions (%):** 14.29
- **Common reported locations (%):** 11.11
- **Common reported processes (%):** 0.0

- **AF ID:** C9J798
- **Chain:** A
- **Protein length:** 803 aa
- **Resolution:** N/A
- **b-phipsi:** 0.011922
- **w-rdist:** 0.331238
- **t-alpha:** 0.001587
- **Chemical similarity (Tanimoto Index) (%):** 98.95
- **1D identity (%) [PDB]:** 3.04
- **1D identity (%) [Gaps excluded][PDB]:** 71.64
- **1D identity - Alignment Gaps [PDB]:** 1514
- **1D aligned content [PDB] (<aminoacid>:%):** {'K': 10.42, 'R': 8.33, 'S': 6.25, 'L': 14.58, 'Y': 2.08, 'T': 10.42, 'A': 4.17, 'P': 6.25, 'V': 10.42, 'D': 8.33, 'G': 2.08, 'E': 8.33, 'F': 2.08, 'I': 2.08, 'H': 4.17}
- **2D identity (%) [PDB]:** 41.27
- **2D identity (%) [Gaps excluded][PDB]:** 86.82
- **2D identity - Alignment Gaps [PDB]:** 586
- **2D aligned content [PDB] (<2D-fold>:%):** {'.': 18.87, 'E': 22.56, 'T': 21.26, 'H': 37.31}
- **3D similarity (TM-Score) (%) [PDB]:** 22.16

- **Gene name:** RASA4B
- **Entrez ID:** N/A
- **RefSeq ID:** N/A
- **Sequence length:** N/A
- **5-UTR|CDS|3-UTR identity (%):** N/A | N/A | N/A
- **5-UTR|CDS|3-UTR identity (%) [Gaps excluded]:** N/A | N/A | N/A
- **5-UTR|CDS|3-UTR identity [Alignment Gaps]:** N/A | N/A | N/A
- **5-UTR aligned content (<base>:%):** N/A
- **CDS aligned content (<base>:%):** N/A
- **3-UTR aligned content (<base>:%):** N/A

**Uniprot Description:**  
  
 Ca(2+)-dependent Ras GTPase-activating protein, that may play a role in the Ras-MAPK pathway. N/A   
  
 **Gene Ontology Information:**

Molecular Function

- GTPase activator activity
- metal ion binding
- phospholipid binding

Location

- cytosol
- plasma membrane

Biological process

- cellular response to calcium ion
- intracellular signal transduction
- negative regulation of Ras protein signal transduction
- regulation of GTPase activity

---

13

- **Protein name:** tRNA-dihydrouridine(47) synthase [NAD(P)(+)]-like
- **Organism:** Homo sapiens
- **Uniprot Accession Number:** Q96G46
- **Protein sequence length:** 650 aa
- **1D identity (%):** 12.12
- **1D identity (%) [Gaps excluded]:** 23.63
- **1D identity - Alignment Gaps:** 486
- **1D aligned content (<aminoacid>:%):** {'T': 11.57, 'L': 11.57, 'E': 6.61, 'N': 4.13, 'G': 10.74, 'R': 10.74, 'V': 6.61, 'A': 5.79, 'H': 4.13, 'Q': 9.09, 'D': 1.65, 'Y': 2.48, 'K': 4.13, 'P': 4.96, 'C': 1.65, 'F': 1.65, 'M': 0.83, 'S': 0.83, 'I': 0.83}
- **Common reported functions (%):** 28.57
- **Common reported locations (%):** 0.0
- **Common reported processes (%):** 0.0

- **AF ID:** Q96G46
- **Chain:** A
- **Protein length:** 650 aa
- **Resolution:** N/A
- **b-phipsi:** 0.002755
- **w-rdist:** 0.569915
- **t-alpha:** 0.001587
- **Chemical similarity (Tanimoto Index) (%):** 99.47
- **1D identity (%) [PDB]:** 1.99
- **1D identity (%) [Gaps excluded][PDB]:** 72.5
- **1D identity - Alignment Gaps [PDB]:** 1415
- **1D aligned content [PDB] (<aminoacid>:%):** {'M': 3.45, 'E': 10.34, 'V': 10.34, 'D': 10.34, 'I': 6.9, 'Q': 3.45, 'P': 6.9, 'R': 10.34, 'L': 6.9, 'T': 10.34, 'K': 13.79, 'F': 3.45, 'G': 3.45}
- **2D identity (%) [PDB]:** 43.13
- **2D identity (%) [Gaps excluded][PDB]:** 86.35
- **2D identity - Alignment Gaps [PDB]:** 499
- **2D aligned content [PDB] (<2D-fold>:%):** {'.': 31.86, 'H': 40.7, 'T': 20.0, 'E': 7.44}
- **3D similarity (TM-Score) (%) [PDB]:** 22.54

- **Gene name:** DUS3L
- **Entrez ID:** 56931
- **RefSeq ID:** NM\_020175
- **Transcript sequence length:** 2038
- **5-UTR|CDS|3-UTR identity (%):** 5.9 | 40.46 | 0.27
- **5-UTR|CDS|3-UTR identity (%) [Gaps excluded]:** 86.36 | 73.54 | 78.95
- **5-UTR|CDS|3-UTR identity [Alignment Gaps]:** 300 | 1316 | 16704
- **5-UTR aligned content (<base>:%):** {'A': 10.53, 'G': 57.89, 'C': 21.05, 'T': 10.53}
- **CDS aligned content (<base>:%):** {'T': 18.92, 'G': 30.49, 'A': 21.88, 'C': 28.72}
- **3-UTR aligned content (<base>:%):** {'C': 17.78, 'T': 28.89, 'A': 35.56, 'G': 17.78}

**Uniprot Description:**  
  
 Catalyzes the synthesis of dihydrouridine, a modified base, in various RNAs, such as tRNAs, mRNAs and some long non-coding RNAs (lncRNAs) (PubMed:34556860). Mainly modifies the uridine in position 47 (U47) in the D-loop of most cytoplasmic tRNAs (PubMed:34556860). Also able to mediate the formation of dihydrouridine in some mRNAs, thereby regulating their translation (PubMed:34556860). N/A   
  
 **Gene Ontology Information:**

Molecular Function

- flavin adenine dinucleotide binding
- metal ion binding
- mRNA dihydrouridine synthase activity
- RNA binding
- tRNA dihydrouridine synthase activity

Location   
  
N/A

Biological process

- mRNA processing
- regulation of translation
- tRNA dihydrouridine synthesis

---

14

- **Protein name:** Apoptosis-inducing factor 1, mitochondrial
- **Organism:** Homo sapiens
- **Uniprot Accession Number:** O95831
- **Protein sequence length:** 613 aa
- **1D identity (%):** 13.96
- **1D identity (%) [Gaps excluded]:** 23.02
- **1D identity - Alignment Gaps:** 361
- **1D aligned content (<aminoacid>:%):** {'F': 4.69, 'C': 1.56, 'L': 7.03, 'P': 6.25, 'V': 10.94, 'G': 11.72, 'T': 7.03, 'R': 5.47, 'A': 9.38, 'S': 5.47, 'N': 2.34, 'Y': 3.12, 'K': 5.47, 'E': 3.91, 'Q': 4.69, 'M': 0.78, 'I': 3.91, 'D': 4.69, 'H': 1.56}
- **Common reported functions (%):** 0.0
- **Common reported locations (%):** 22.22
- **Common reported processes (%):** 0.0

- **AF ID:** O95831
- **Chain:** A
- **Protein length:** 613 aa
- **Resolution:** N/A
- **b-phipsi:** 0.000546
- **w-rdist:** 0.617962
- **t-alpha:** 0.015056
- **Chemical similarity (Tanimoto Index) (%):** 98.72
- **1D identity (%) [PDB]:** 2.12
- **1D identity (%) [Gaps excluded][PDB]:** 68.18
- **1D identity - Alignment Gaps [PDB]:** 1370
- **1D aligned content [PDB] (<aminoacid>:%):** {'T': 10.0, 'V': 13.33, 'P': 6.67, 'R': 6.67, 'Q': 6.67, 'E': 3.33, 'D': 6.67, 'A': 3.33, 'S': 6.67, 'L': 6.67, 'I': 10.0, 'F': 10.0, 'Y': 6.67, 'K': 3.33}
- **2D identity (%) [PDB]:** 42.86
- **2D identity (%) [Gaps excluded][PDB]:** 87.87
- **2D identity - Alignment Gaps [PDB]:** 502
- **2D aligned content [PDB] (<2D-fold>:%):** {'.': 28.57, 'H': 36.43, 'T': 17.14, 'E': 17.62, 'B': 0.24}
- **3D similarity (TM-Score) (%) [PDB]:** 18.79

- **Gene name:** AIFM1
- **Entrez ID:** N/A
- **RefSeq ID:** NM\_145812
- **Transcript sequence length:** 2210
- **5-UTR|CDS|3-UTR identity (%):** 26.4 | 38.57 | 0.79
- **5-UTR|CDS|3-UTR identity (%) [Gaps excluded]:** 75.0 | 74.22 | 81.1
- **5-UTR|CDS|3-UTR identity [Alignment Gaps]:** 243 | 1395 | 16622
- **5-UTR aligned content (<base>:%):** {'T': 20.2, 'C': 38.38, 'G': 35.35, 'A': 6.06}
- **CDS aligned content (<base>:%):** {'A': 30.98, 'G': 26.16, 'C': 19.73, 'T': 23.12}
- **3-UTR aligned content (<base>:%):** {'A': 29.32, 'C': 16.54, 'G': 19.55, 'T': 34.59}

**Uniprot Description:**  
  
 Functions both as NADH oxidoreductase and as regulator of apoptosis (PubMed:20362274, PubMed:23217327, PubMed:17094969). In response to apoptotic stimuli, it is released from the mitochondrion intermembrane space into the cytosol and to the nucleus, where it functions as a proapoptotic factor in a caspase-independent pathway. The soluble form (AIFsol) found in the nucleus induces 'parthanatos' i.e. caspase-independent fragmentation of chromosomal DNA (By similarity). Binds to DNA in a sequence-independent manner (PubMed:27178839). Interacts with EIF3G, and thereby inhibits the EIF3 machinery and protein synthesis, and activates caspase-7 to amplify apoptosis (PubMed:17094969). Plays a critical role in caspase-independent, pyknotic cell death in hydrogen peroxide-exposed cells (PubMed:19418225). In contrast, participates in normal mitochondrial metabolism. Plays an important role in the regulation of respiratory chain biogenesis by interacting with CHCHD4 and controlling CHCHD4 mitochondrial import (PubMed:26004228).   
  
Monomer (oxidized form). Homodimer (reduced form). Upon reduction with NADH, undergoes dimerization and forms tight, long-lived FADH2-NAD charge transfer complexes (CTC) resistant to oxidation (PubMed:24914854, PubMed:20111043, PubMed:23217327, PubMed:27818101). Also dimerizes with isoform 3 preventing its release from mitochondria (PubMed:20111043). Interacts with XIAP/BIRC4 (PubMed:17967870). Interacts (via N-terminus) with EIF3G (via C-terminus) (PubMed:17094969). Interacts with PRELID1 (PubMed:21364629). Interacts with CHCHD4; the interaction increases in presence of NADH (PubMed:26004228).   
  
 **Gene Ontology Information:**

Molecular Function

- DNA binding
- FAD binding
- NAD(P)H oxidase H2O2-forming activity
- NADH dehydrogenase activity
- oxidoreductase activity, acting on NAD(P)H
- poly-ADP-D-ribose binding
- protein dimerization activity

Location

- cytoplasm
- cytosol
- mitochondrial inner membrane
- mitochondrial intermembrane space
- mitochondrion
- nucleus
- perinuclear region of cytoplasm

Biological process

- activation of cysteine-type endopeptidase activity involved in apoptotic process
- apoptotic process
- cellular response to aldosterone
- cellular response to estradiol stimulus
- cellular response to hydrogen peroxide
- cellular response to nitric oxide
- cellular response to oxygen-glucose deprivation
- chromosome condensation
- intrinsic apoptotic signaling pathway in response to endoplasmic reticulum stress
- mitochondrial respiratory chain complex assembly
- mitochondrial respiratory chain complex I assembly
- neuron apoptotic process
- neuron differentiation
- positive regulation of apoptotic process
- positive regulation of necroptotic process
- positive regulation of neuron apoptotic process
- protein import into mitochondrial intermembrane space
- regulation of apoptotic DNA fragmentation
- response to ischemia
- response to L-glutamate
- response to toxic substance

---

15

- **Protein name:** Protein arginine N-methyltransferase 3
- **Organism:** Homo sapiens
- **Uniprot Accession Number:** O60678
- **Protein sequence length:** 531 aa
- **1D identity (%):** 8.68
- **1D identity (%) [Gaps excluded]:** 23.34
- **1D identity - Alignment Gaps:** 637
- **1D aligned content (<aminoacid>:%):** {'M': 2.27, 'A': 7.95, 'T': 7.95, 'E': 5.68, 'L': 10.23, 'D': 9.09, 'P': 9.09, 'Q': 2.27, 'C': 5.68, 'F': 3.41, 'S': 3.41, 'H': 3.41, 'K': 4.55, 'Y': 3.41, 'V': 3.41, 'N': 2.27, 'I': 5.68, 'G': 4.55, 'R': 4.55, 'W': 1.14}
- **Common reported functions (%):** 14.29
- **Common reported locations (%):** 22.22
- **Common reported processes (%):** 0.0

- **AF ID:** O60678
- **Chain:** A
- **Protein length:** 531 aa
- **Resolution:** N/A
- **b-phipsi:** 0.000399
- **w-rdist:** 0.654193
- **t-alpha:** 0.030204
- **Chemical similarity (Tanimoto Index) (%):** 99.17
- **1D identity (%) [PDB]:** 1.18
- **1D identity (%) [Gaps excluded][PDB]:** 64.0
- **1D identity - Alignment Gaps [PDB]:** 1326
- **1D aligned content [PDB] (<aminoacid>:%):** {'C': 6.25, 'A': 12.5, 'G': 12.5, 'S': 6.25, 'E': 6.25, 'F': 6.25, 'H': 6.25, 'L': 12.5, 'K': 6.25, 'N': 6.25, 'T': 6.25, 'Y': 6.25, 'Q': 6.25}
- **2D identity (%) [PDB]:** 37.86
- **2D identity (%) [Gaps excluded][PDB]:** 91.09
- **2D identity - Alignment Gaps [PDB]:** 568
- **2D aligned content [PDB] (<2D-fold>:%):** {'.': 20.38, 'T': 27.99, 'E': 14.4, 'H': 37.23}
- **3D similarity (TM-Score) (%) [PDB]:** 17.45

- **Gene name:** PRMT3
- **Entrez ID:** 10196
- **RefSeq ID:** NM\_005788
- **Transcript sequence length:** 2552
- **5-UTR|CDS|3-UTR identity (%):** 6.77 | 37.78 | 3.66
- **5-UTR|CDS|3-UTR identity (%) [Gaps excluded]:** 78.57 | 75.88 | 74.97
- **5-UTR|CDS|3-UTR identity [Alignment Gaps]:** 297 | 1401 | 16034
- **5-UTR aligned content (<base>:%):** {'A': 13.64, 'G': 36.36, 'C': 40.91, 'T': 9.09}
- **CDS aligned content (<base>:%):** {'A': 31.78, 'T': 26.0, 'G': 23.15, 'C': 19.07}
- **3-UTR aligned content (<base>:%):** {'A': 36.63, 'C': 10.7, 'T': 39.87, 'G': 12.8}

**Uniprot Description:**  
  
 Protein-arginine N-methyltransferase that catalyzes both the monomethylation and asymmetric dimethylation of the guanidino nitrogens of arginine residues in target proteins, and therefore falls into the group of type I methyltransferases (Probable). May regulate retinoic acid synthesis and signaling by inhibiting ALDH1A1 retinal dehydrogenase activity (PubMed:33495566).   
  
Monomer and homodimer (By similarity). Interacts with EPB41L3 (via FERM domain); the interaction is direct and inhibits the protein-arginine N-methyltransferase activity of PRMT3 (PubMed:15334060). Interacts with the 40S ribosomal protein RPS2 (PubMed:21059412, PubMed:30530495). Interacts with ALDH1A1; the interaction is direct, inhibits ALDH1A1 aldehyde dehydrogenase activity and is independent of the methyltransferase activity of PRMT3 (PubMed:33495566).   
  
 **Gene Ontology Information:**

Molecular Function

- metal ion binding
- methyltransferase activity
- protein-arginine N-methyltransferase activity
- protein-arginine omega-N asymmetric methyltransferase activity
- protein-arginine omega-N monomethyltransferase activity
- ribosome binding

Location

- cytoplasm
- cytosol
- nucleus

Biological process

- negative regulation of protein ubiquitination
- negative regulation of retinoic acid biosynthetic process
- peptidyl-arginine methylation
- protein methylation

---

16

- **Protein name:** Proto-oncogene tyrosine-protein kinase Src
- **Organism:** Homo sapiens
- **Uniprot Accession Number:** P12931
- **Protein sequence length:** 536 aa
- **1D identity (%):** 12.28
- **1D identity (%) [Gaps excluded]:** 23.14
- **1D identity - Alignment Gaps:** 428
- **1D aligned content (<aminoacid>:%):** {'N': 5.36, 'D': 5.36, 'R': 8.04, 'P': 9.82, 'G': 10.71, 'F': 3.57, 'A': 6.25, 'L': 7.14, 'T': 5.36, 'Y': 4.46, 'S': 2.68, 'Q': 7.14, 'E': 7.14, 'C': 1.79, 'K': 4.46, 'I': 2.68, 'H': 1.79, 'V': 4.46, 'W': 0.89, 'M': 0.89}
- **Common reported functions (%):** 0.0
- **Common reported locations (%):** 0.0
- **Common reported processes (%):** 0.0

- **AF ID:** P12931
- **Chain:** A
- **Protein length:** 536 aa
- **Resolution:** N/A
- **b-phipsi:** 0.001645
- **w-rdist:** 0.720502
- **t-alpha:** 0.000792
- **Chemical similarity (Tanimoto Index) (%):** 99.25
- **1D identity (%) [PDB]:** 2.09
- **1D identity (%) [Gaps excluded][PDB]:** 68.29
- **1D identity - Alignment Gaps [PDB]:** 1299
- **1D aligned content [PDB] (<aminoacid>:%):** {'M': 3.57, 'G': 3.57, 'K': 14.29, 'P': 10.71, 'L': 10.71, 'N': 7.14, 'F': 3.57, 'V': 17.86, 'E': 7.14, 'I': 7.14, 'Y': 3.57, 'D': 7.14, 'R': 3.57}
- **2D identity (%) [PDB]:** 43.81
- **2D identity (%) [Gaps excluded][PDB]:** 85.47
- **2D identity - Alignment Gaps [PDB]:** 445
- **2D aligned content [PDB] (<2D-fold>:%):** {'.': 26.0, 'T': 30.25, 'E': 18.25, 'H': 25.0, 'G': 0.5}
- **3D similarity (TM-Score) (%) [PDB]:** 17.84

- **Gene name:** SRC
- **Entrez ID:** 929038
- **RefSeq ID:** N/A
- **Sequence length:** N/A
- **5-UTR|CDS|3-UTR identity (%):** N/A | N/A | N/A
- **5-UTR|CDS|3-UTR identity (%) [Gaps excluded]:** N/A | N/A | N/A
- **5-UTR|CDS|3-UTR identity [Alignment Gaps]:** N/A | N/A | N/A
- **5-UTR aligned content (<base>:%):** N/A
- **CDS aligned content (<base>:%):** N/A
- **3-UTR aligned content (<base>:%):** N/A

**Uniprot Description:**  
  
 Non-receptor protein tyrosine kinase which is activated following engagement of many different classes of cellular receptors including immune response receptors, integrins and other adhesion receptors, receptor protein tyrosine kinases, G protein-coupled receptors as well as cytokine receptors. Participates in signaling pathways that control a diverse spectrum of biological activities including gene transcription, immune response, cell adhesion, cell cycle progression, apoptosis, migration, and transformation. Due to functional redundancy between members of the SRC kinase family, identification of the specific role of each SRC kinase is very difficult. SRC appears to be one of the primary kinases activated following engagement of receptors and plays a role in the activation of other protein tyrosine kinase (PTK) families. Receptor clustering or dimerization leads to recruitment of SRC to the receptor complexes where it phosphorylates the tyrosine residues within the receptor cytoplasmic domains. Plays an important role in the regulation of cytoskeletal organization through phosphorylation of specific substrates such as AFAP1. Phosphorylation of AFAP1 allows the SRC SH2 domain to bind AFAP1 and to localize to actin filaments. Cytoskeletal reorganization is also controlled through the phosphorylation of cortactin (CTTN) (Probable). When cells adhere via focal adhesions to the extracellular matrix, signals are transmitted by integrins into the cell resulting in tyrosine phosphorylation of a number of focal adhesion proteins, including PTK2/FAK1 and paxillin (PXN) (PubMed:21411625). In addition to phosphorylating focal adhesion proteins, SRC is also active at the sites of cell-cell contact adherens junctions and phosphorylates substrates such as beta-catenin (CTNNB1), delta-catenin (CTNND1), and plakoglobin (JUP). Another type of cell-cell junction, the gap junction, is also a target for SRC, which phosphorylates connexin-43 (GJA1). SRC is implicated in regulation of pre-mRNA-processing and phosphorylates RNA-binding proteins such as KHDRBS1 (Probable). Also plays a role in PDGF-mediated tyrosine phosphorylation of both STAT1 and STAT3, leading to increased DNA binding activity of these transcription factors (By similarity). Involved in the RAS pathway through phosphorylation of RASA1 and RASGRF1 (PubMed:11389730). Plays a role in EGF-mediated calcium-activated chloride channel activation (PubMed:18586953). Required for epidermal growth factor receptor (EGFR) internalization through phosphorylation of clathrin heavy chain (CLTC and CLTCL1) at 'Tyr-1477'. Involved in beta-arrestin (ARRB1 and ARRB2) desensitization through phosphorylation and activation of GRK2, leading to beta-arrestin phosphorylation and internalization. Has a critical role in the stimulation of the CDK20/MAPK3 mitogen-activated protein kinase cascade by epidermal growth factor (Probable). Might be involved not only in mediating the transduction of mitogenic signals at the level of the plasma membrane but also in controlling progression through the cell cycle via interaction with regulatory proteins in the nucleus (PubMed:7853507). Plays an important role in osteoclastic bone resorption in conjunction with PTK2B/PYK2. Both the formation of a SRC-PTK2B/PYK2 complex and SRC kinase activity are necessary for this function. Recruited to activated integrins by PTK2B/PYK2, thereby phosphorylating CBL, which in turn induces the activation and recruitment of phosphatidylinositol 3-kinase to the cell membrane in a signaling pathway that is critical for osteoclast function (PubMed:8755529, PubMed:14585963). Promotes energy production in osteoclasts by activating mitochondrial cytochrome C oxidase (PubMed:12615910). Phosphorylates DDR2 on tyrosine residues, thereby promoting its subsequent autophosphorylation (PubMed:16186108). Phosphorylates RUNX3 and COX2 on tyrosine residues, TNK2 on 'Tyr-284' and CBL on 'Tyr-731' (PubMed:20100835, PubMed:21309750). Enhances DDX58/RIG-I-elicited antiviral signaling (PubMed:19419966). Phosphorylates PDPK1 at 'Tyr-9', 'Tyr-373' and 'Tyr-376' (PubMed:14585963). Phosphorylates BCAR1 at 'Tyr-128' (PubMed:22710723). Phosphorylates CBLC at multiple tyrosine residues, phosphorylation at 'Tyr-341' activates CBLC E3 activity (PubMed:20525694). Involved in anchorage-independent cell growth (PubMed:19307596). Required for podosome formation (By similarity). Mediates IL6 signaling by activating YAP1-NOTCH pathway to induce inflammation-induced epithelial regeneration (PubMed:25731159).   
  
Part of a complex comprised of PTPRA, BCAR1, BCAR3 (via SH2 domain) and SRC; the formation of the complex is dependent on integrin mediated-tyrosine phosphorylation of PTPRA (PubMed:22801373). Interacts with DDEF1/ASAP1; via the SH3 domain (By similarity). Interacts with CCPG1 (By similarity). Identified in a complex containing FGFR4, NCAM1, CDH2, PLCG1, FRS2, SRC, SHC1, GAP43 and CTTN (By similarity). Interacts with ERBB2, STAT1 and PNN (By similarity). Interacts with DDR1, DDR2 and DAB2 (By similarity). Interacts with CDCP1, TGFB1I1 and TOM1L2 (PubMed:15851033, PubMed:16479011, PubMed:17202804). Interacts with the cytoplasmic domain of MUC1, phosphorylates it and increases binding of MUC1 with beta-catenin (PubMed:11152665). Interacts with RALGPS1; via the SH3 domain (PubMed:10747847). Interacts with CAV2 (tyrosine phosphorylated form) (PubMed:12091389, PubMed:15504032). Interacts (via the SH3 domain and the protein kinase domain) with ARRB1; the interaction is independent of the phosphorylation state of SRC C-terminus (By similarity). Interacts with ARRB1 and ARRB2 (PubMed:10753943, PubMed:9924018). Interacts with SRCIN1 (PubMed:17525734). Interacts with NDFIP2 and more weakly with NDFIP1 (PubMed:20534535). Interacts with PIK3CA and/or PIK3C2B, PTK2/FAK1 and ESR1 (dimethylated on arginine) (PubMed:18657504, PubMed:21411625). Interacts with FASLG (PubMed:19807924). Interacts (via SH2 domain) with the 'Tyr-402' phosphorylated form of PTK2B/PYK2 (PubMed:14585963). Interacts (via SH2 domain) with FLT3 (tyrosine phosphorylated) (By similarity). Interacts with PDGFRA (tyrosine phosphorylated) (By similarity). Interacts with CSF1R (By similarity). Interacts (via SH2 and SH3 domain) with TNK2 (PubMed:21309750). Interacts (via protein kinase domain) with the tyrosine phosphorylated form of RUNX3 (via runt domain) (PubMed:20100835). Interacts with TRAF3 (via RING-type zinc finger domain) (PubMed:19419966). Interacts with DDX58, MAVS and TBK1 (PubMed:19419966). Interacts (via SH2 domain) with RACK1; the interaction is enhanced by tyrosine phosphorylation of RACK1 and inhibits SRC activity (PubMed:9584165, PubMed:11279199). Interacts with EPHB1; activates the MAPK/ERK cascade to regulate cell migration (PubMed:12925710). Interacts with FCAMR (PubMed:8759729). Interacts (via SH2 domain) with the 'Tyr-9' phosphorylated form of PDPK1 (PubMed:18024423). Interacts with AMOTL2; this interaction regulates the translocation of phosphorylated SRC to peripheral cell-matrix adhesion sites (PubMed:17293535). Interacts with TRAP1 (PubMed:23564345). Interacts with CBLC; the interaction is enhanced when SRC is phosphorylated at Tyr-419 (PubMed:14661060, PubMed:22888118). Interacts with ARHGEF5 (By similarity). Interacts (via cytoplasmic domain) with CEACAM1 (via SH2 domain); this interaction is regulated by trans-homophilic cell adhesion (PubMed:7478590). Interacts with MPP2 (PubMed:19665017). Interacts with PRR7 (PubMed:21460222). Interacts (via kinase domain and to a lesser extent the SH2 domain) directly with PDLIM4; this interaction results in PTPN13-mediated dephosphorylation of this protein leading to its inactivation (PubMed:19307596). Interacts with P85 (PIK3R1 or PIK3R2) (PubMed:28903391). Interacts with HNRNPA2B1 (PubMed:31320558). Interacts with IL6ST/gp130 (PubMed:25731159). Interacts (via SH3 domain) with PELP1 in the presence of 17-beta-estradiol.   
  
 **Gene Ontology Information:**

Molecular Function   
  
N/A

Location

- virion component

Biological process   
  
N/A

---

17

- **Protein name:** Twinkle mtDNA helicase
- **Organism:** Homo sapiens
- **Uniprot Accession Number:** Q96RR1
- **Protein sequence length:** 684 aa
- **1D identity (%):** 16.52
- **1D identity (%) [Gaps excluded]:** 23.58
- **1D identity - Alignment Gaps:** 272
- **1D aligned content (<aminoacid>:%):** {'V': 8.67, 'R': 10.67, 'G': 9.33, 'Y': 1.33, 'P': 7.33, 'L': 14.67, 'E': 4.0, 'D': 6.0, 'F': 4.67, 'H': 3.33, 'T': 4.67, 'S': 4.0, 'A': 8.0, 'W': 1.33, 'K': 4.67, 'C': 1.33, 'Q': 2.0, 'N': 1.33, 'I': 2.0, 'M': 0.67}
- **Common reported functions (%):** 0.0
- **Common reported locations (%):** 0.0
- **Common reported processes (%):** 0.0

- **AF ID:** Q96RR1
- **Chain:** A
- **Protein length:** 684 aa
- **Resolution:** N/A
- **b-phipsi:** 0.000717
- **w-rdist:** 0.500227
- **t-alpha:** 0.011094
- **Chemical similarity (Tanimoto Index) (%):** 99.02
- **1D identity (%) [PDB]:** 1.53
- **1D identity (%) [Gaps excluded][PDB]:** 76.67
- **1D identity - Alignment Gaps [PDB]:** 1469
- **1D aligned content [PDB] (<aminoacid>:%):** {'M': 4.35, 'W': 4.35, 'A': 4.35, 'I': 8.7, 'C': 4.35, 'F': 8.7, 'R': 8.7, 'Q': 8.7, 'E': 8.7, 'L': 8.7, 'K': 13.04, 'G': 4.35, 'T': 4.35, 'D': 4.35, 'S': 4.35}
- **2D identity (%) [PDB]:** 41.37
- **2D identity (%) [Gaps excluded][PDB]:** 87.2
- **2D identity - Alignment Gaps [PDB]:** 545
- **2D aligned content [PDB] (<2D-fold>:%):** {'.': 26.11, 'H': 33.33, 'T': 20.51, 'E': 19.81, 'B': 0.23}
- **3D similarity (TM-Score) (%) [PDB]:** 20.36

- **Gene name:** TWNK
- **Entrez ID:** 425626
- **RefSeq ID:** NM\_021830
- **Transcript sequence length:** 3616
- **5-UTR|CDS|3-UTR identity (%):** 34.06 | 43.86 | 3.51
- **5-UTR|CDS|3-UTR identity (%) [Gaps excluded]:** 76.14 | 76.66 | 76.09
- **5-UTR|CDS|3-UTR identity [Alignment Gaps]:** 378 | 1262 | 16092
- **5-UTR aligned content (<base>:%):** {'A': 12.02, 'G': 39.06, 'T': 16.74, 'C': 32.19}
- **CDS aligned content (<base>:%):** {'A': 26.89, 'T': 23.11, 'G': 25.04, 'C': 24.96}
- **3-UTR aligned content (<base>:%):** {'A': 28.38, 'G': 28.04, 'T': 21.11, 'C': 22.47}

**Uniprot Description:**  
  
 Isoform 1
Mitochondrial helicase involved in mtDNA replication and repair (PubMed:12975372, PubMed:15167897, PubMed:17324440, PubMed:18039713, PubMed:18971204, PubMed:25824949, PubMed:26887820, PubMed:27226550). Might have a role in mtDNA repair (PubMed:27226550). Has DNA strand separation activity needed to form a processive replication fork for leading strand synthesis which is catalyzed by the formation of a replisome complex with POLG and mtSDB (PubMed:12975372, PubMed:15167897, PubMed:18039713, PubMed:22383523, PubMed:26887820, PubMed:27226550). Preferentially unwinds DNA substrates with pre-existing 5'-and 3'- single-stranded tails but is also active on a 5'- flap substrate (PubMed:12975372, PubMed:15167897, PubMed:18039713, PubMed:22383523, PubMed:26887820, PubMed:27226550). Can dissociate the invading strand of immobile or mobile D-loop DNA structures irrespective of the single strand polarity of the third strand (PubMed:27226550). In addition to its DNA strand separation activity, also has DNA strand annealing, DNA strand-exchange and DNA branch migration activities (PubMed:22383523, PubMed:26887820, PubMed:27226550).   
  
Interacts with LONP1.   
  
 **Gene Ontology Information:**

Molecular Function

- 5'-3' DNA helicase activity
- ATP binding
- ATPase activity
- DNA helicase activity
- single-stranded DNA binding

Location

- mitochondrial nucleoid
- mitochondrion

Biological process

- mitochondrial DNA replication

---

18

- **Protein name:** ATP-dependent DNA helicase PIF1
- **Organism:** Homo sapiens
- **Uniprot Accession Number:** Q9H611
- **Protein sequence length:** 641 aa
- **1D identity (%):** 12.4
- **1D identity (%) [Gaps excluded]:** 24.17
- **1D identity - Alignment Gaps:** 483
- **1D aligned content (<aminoacid>:%):** {'M': 2.44, 'I': 4.07, 'A': 4.88, 'E': 4.07, 'L': 15.45, 'G': 10.57, 'R': 13.01, 'T': 9.76, 'Q': 7.32, 'K': 2.44, 'P': 7.32, 'V': 6.5, 'S': 2.44, 'H': 1.63, 'C': 2.44, 'F': 1.63, 'D': 3.25, 'Y': 0.81}
- **Common reported functions (%):** 0.0
- **Common reported locations (%):** 11.11
- **Common reported processes (%):** 0.0

- **AF ID:** Q9H611
- **Chain:** A
- **Protein length:** 641 aa
- **Resolution:** N/A
- **b-phipsi:** 0.000537
- **w-rdist:** 0.523804
- **t-alpha:** 0.052544
- **Chemical similarity (Tanimoto Index) (%):** 99.4
- **1D identity (%) [PDB]:** 2.44
- **1D identity (%) [Gaps excluded][PDB]:** 64.81
- **1D identity - Alignment Gaps [PDB]:** 1378
- **1D aligned content [PDB] (<aminoacid>:%):** {'L': 20.0, 'G': 11.43, 'K': 11.43, 'P': 5.71, 'V': 11.43, 'A': 5.71, 'E': 2.86, 'R': 2.86, 'T': 5.71, 'Q': 5.71, 'C': 2.86, 'S': 5.71, 'N': 5.71, 'I': 2.86}
- **2D identity (%) [PDB]:** 41.9
- **2D identity (%) [Gaps excluded][PDB]:** 86.21
- **2D identity - Alignment Gaps [PDB]:** 514
- **2D aligned content [PDB] (<2D-fold>:%):** {'.': 19.38, 'T': 26.79, 'E': 21.05, 'H': 31.34, 'G': 1.44}
- **3D similarity (TM-Score) (%) [PDB]:** 21.06

- **Gene name:** PIF1
- **Entrez ID:** 80119
- **RefSeq ID:** N/A
- **Sequence length:** N/A
- **5-UTR|CDS|3-UTR identity (%):** N/A | N/A | N/A
- **5-UTR|CDS|3-UTR identity (%) [Gaps excluded]:** N/A | N/A | N/A
- **5-UTR|CDS|3-UTR identity [Alignment Gaps]:** N/A | N/A | N/A
- **5-UTR aligned content (<base>:%):** N/A
- **CDS aligned content (<base>:%):** N/A
- **3-UTR aligned content (<base>:%):** N/A

**Uniprot Description:**  
  
 DNA-dependent ATPase and 5'-3' DNA helicase required for the maintenance of both mitochondrial and nuclear genome stability. Efficiently unwinds G-quadruplex (G4) DNA structures and forked RNA-DNA hybrids. Resolves G4 structures, preventing replication pausing and double-strand breaks (DSBs) at G4 motifs. Involved in the maintenance of telomeric DNA. Inhibits telomere elongation, de novo telomere formation and telomere addition to DSBs via catalytic inhibition of telomerase. Reduces the processivity of telomerase by displacing active telomerase from DNA ends. Releases telomerase by unwinding the short telomerase RNA/telomeric DNA hybrid that is the intermediate in the telomerase reaction. Possesses an intrinsic strand annealing activity.   
  
Monomer. Interacts with telomerase.   
  
 **Gene Ontology Information:**

Molecular Function

- 5'-3' DNA helicase activity
- 5'-3' DNA/RNA helicase activity
- ATP binding
- ATPase activity
- G-quadruplex DNA binding
- magnesium ion binding
- single-stranded DNA helicase activity
- telomerase inhibitor activity
- telomeric DNA binding

Location

- chromosome, telomeric region
- mitochondrion
- nucleoplasm
- replication fork

Biological process

- DNA duplex unwinding
- DNA recombination
- DNA repair
- DNA replication
- mitochondrial genome maintenance
- negative regulation of telomerase activity
- negative regulation of telomere maintenance via telomerase
- regulation of telomere maintenance
- telomere maintenance via telomerase

---

19

- **Protein name:** Neutral alpha-glucosidase AB
- **Organism:** Homo sapiens
- **Uniprot Accession Number:** Q14697
- **Protein sequence length:** 944 aa
- **1D identity (%):** 17.07
- **1D identity (%) [Gaps excluded]:** 23.39
- **1D identity - Alignment Gaps:** 282
- **1D aligned content (<aminoacid>:%):** {'A': 6.18, 'R': 7.87, 'L': 10.67, 'C': 1.12, 'P': 11.8, 'Y': 3.37, 'D': 7.3, 'E': 3.93, 'V': 7.3, 'F': 4.49, 'T': 5.06, 'G': 11.24, 'K': 2.81, 'S': 5.06, 'N': 2.25, 'W': 1.69, 'H': 2.81, 'Q': 1.69, 'M': 1.12, 'I': 2.25}
- **Common reported functions (%):** 14.29
- **Common reported locations (%):** 11.11
- **Common reported processes (%):** 0.0

- **AF ID:** Q14697
- **Chain:** A
- **Protein length:** 944 aa
- **Resolution:** N/A
- **b-phipsi:** 0.002992
- **w-rdist:** 0.43271
- **t-alpha:** 0.003962
- **Chemical similarity (Tanimoto Index) (%):** 96.83
- **1D identity (%) [PDB]:** 1.89
- **1D identity (%) [Gaps excluded][PDB]:** 82.5
- **1D identity - Alignment Gaps [PDB]:** 1709
- **1D aligned content [PDB] (<aminoacid>:%):** {'R': 9.09, 'S': 6.06, 'W': 3.03, 'H': 12.12, 'L': 18.18, 'E': 3.03, 'V': 15.15, 'T': 6.06, 'P': 9.09, 'D': 6.06, 'K': 3.03, 'I': 3.03, 'N': 3.03, 'A': 3.03}
- **2D identity (%) [PDB]:** 37.06
- **2D identity (%) [Gaps excluded][PDB]:** 90.93
- **2D identity - Alignment Gaps [PDB]:** 753
- **2D aligned content [PDB] (<2D-fold>:%):** {'.': 16.35, 'H': 27.39, 'T': 28.03, 'E': 27.6, 'G': 0.64}
- **3D similarity (TM-Score) (%) [PDB]:** 27.38

- **Gene name:** GANAB
- **Entrez ID:** 23193
- **RefSeq ID:** NM\_198335
- **Transcript sequence length:** 3901
- **5-UTR|CDS|3-UTR identity (%):** 3.42 | 44.37 | 3.64
- **5-UTR|CDS|3-UTR identity (%) [Gaps excluded]:** 78.57 | 75.15 | 73.33
- **5-UTR|CDS|3-UTR identity [Alignment Gaps]:** 308 | 1412 | 16061
- **5-UTR aligned content (<base>:%):** {'A': 27.27, 'C': 36.36, 'T': 18.18, 'G': 18.18}
- **CDS aligned content (<base>:%):** {'A': 24.9, 'T': 23.59, 'G': 27.25, 'C': 24.25}
- **3-UTR aligned content (<base>:%):** {'C': 21.27, 'A': 23.38, 'G': 22.89, 'T': 32.47}

**Uniprot Description:**  
  
 Catalytic subunit of glucosidase II that cleaves sequentially the 2 innermost alpha-1,3-linked glucose residues from the Glc(2)Man(9)GlcNAc(2) oligosaccharide precursor of immature glycoproteins (PubMed:10929008). Required for PKD1/Polycystin-1 and PKD2/Polycystin-2 maturation and localization to the cell surface and cilia (PubMed:27259053).   
  
Heterodimer of a catalytic alpha subunit (GANAB) and a beta subunit (PRKCSH) (PubMed:10929008). Binds glycosylated PTPRC (By similarity).   
  
 **Gene Ontology Information:**

Molecular Function

- alpha-glucosidase activity
- carbohydrate binding
- glucan 1,3-alpha-glucosidase activity
- RNA binding

Location

- endoplasmic reticulum lumen
- extracellular exosome
- glucosidase II complex
- Golgi apparatus
- intracellular membrane-bounded organelle
- melanosome
- membrane

Biological process

- carbohydrate metabolic process
- N-glycan processing

---

20

- **Protein name:** Ras-specific guanine nucleotide-releasing factor RalGPS1
- **Organism:** Homo sapiens
- **Uniprot Accession Number:** Q5JS13
- **Protein sequence length:** 557 aa
- **1D identity (%):** 11.28
- **1D identity (%) [Gaps excluded]:** 22.86
- **1D identity - Alignment Gaps:** 481
- **1D aligned content (<aminoacid>:%):** {'Y': 4.67, 'N': 1.87, 'L': 12.15, 'T': 5.61, 'G': 6.54, 'D': 1.87, 'S': 6.54, 'V': 4.67, 'P': 12.15, 'E': 5.61, 'A': 3.74, 'K': 9.35, 'W': 0.93, 'F': 4.67, 'H': 2.8, 'R': 8.41, 'I': 1.87, 'Q': 3.74, 'C': 2.8}
- **Common reported functions (%):** 0.0
- **Common reported locations (%):** 11.11
- **Common reported processes (%):** 0.0

- **AF ID:** Q5JS13
- **Chain:** A
- **Protein length:** 557 aa
- **Resolution:** N/A
- **b-phipsi:** 0.000308
- **w-rdist:** 0.713449
- **t-alpha:** 0.040412
- **Chemical similarity (Tanimoto Index) (%):** 99.4
- **1D identity (%) [PDB]:** 2.96
- **1D identity (%) [Gaps excluded][PDB]:** 78.43
- **1D identity - Alignment Gaps [PDB]:** 1300
- **1D aligned content [PDB] (<aminoacid>:%):** {'K': 10.0, 'V': 12.5, 'S': 12.5, 'F': 5.0, 'W': 2.5, 'L': 15.0, 'H': 5.0, 'T': 10.0, 'P': 12.5, 'E': 5.0, 'A': 2.5, 'R': 2.5, 'M': 2.5, 'G': 2.5}
- **2D identity (%) [PDB]:** 32.94
- **2D identity (%) [Gaps excluded][PDB]:** 88.92
- **2D identity - Alignment Gaps [PDB]:** 644
- **2D aligned content [PDB] (<2D-fold>:%):** {'.': 23.15, 'T': 25.52, 'H': 43.62, 'B': 0.89, 'E': 6.82}
- **3D similarity (TM-Score) (%) [PDB]:** 17.06

- **Gene name:** RALGPS1
- **Entrez ID:** 100101642
- **RefSeq ID:** N/A
- **Sequence length:** N/A
- **5-UTR|CDS|3-UTR identity (%):** N/A | N/A | N/A
- **5-UTR|CDS|3-UTR identity (%) [Gaps excluded]:** N/A | N/A | N/A
- **5-UTR|CDS|3-UTR identity [Alignment Gaps]:** N/A | N/A | N/A
- **5-UTR aligned content (<base>:%):** N/A
- **CDS aligned content (<base>:%):** N/A
- **3-UTR aligned content (<base>:%):** N/A

**Uniprot Description:**  
  
 Guanine nucleotide exchange factor (GEF) for the small GTPase RALA. May be involved in cytoskeletal organization (By similarity). Guanine nucleotide exchange factor for.   
  
Interacts with the SH3 domains of GRB2, NCK1, PLCG1 and SRC.   
  
 **Gene Ontology Information:**

Molecular Function

- guanyl-nucleotide exchange factor activity

Location

- cytoplasm
- plasma membrane

Biological process

- positive regulation of GTPase activity
- Ras protein signal transduction

---

21

- **Protein name:** Histone H4 transcription factor
- **Organism:** Homo sapiens
- **Uniprot Accession Number:** Q9BQA5
- **Protein sequence length:** 517 aa
- **1D identity (%):** 11.38
- **1D identity (%) [Gaps excluded]:** 23.13
- **1D identity - Alignment Gaps:** 469
- **1D aligned content (<aminoacid>:%):** {'P': 12.38, 'G': 12.38, 'R': 4.76, 'K': 4.76, 'F': 5.71, 'V': 6.67, 'H': 5.71, 'L': 5.71, 'E': 6.67, 'D': 4.76, 'S': 4.76, 'I': 3.81, 'C': 4.76, 'N': 3.81, 'T': 4.76, 'A': 3.81, 'Q': 3.81, 'Y': 0.95}
- **Common reported functions (%):** 14.29
- **Common reported locations (%):** 0.0
- **Common reported processes (%):** 0.0

- **AF ID:** Q9BQA5
- **Chain:** A
- **Protein length:** 517 aa
- **Resolution:** N/A
- **b-phipsi:** 0.001389
- **w-rdist:** 0.247886
- **t-alpha:** 0.091125
- **Chemical similarity (Tanimoto Index) (%):** 95.71
- **1D identity (%) [PDB]:** 2.13
- **1D identity (%) [Gaps excluded][PDB]:** 62.22
- **1D identity - Alignment Gaps [PDB]:** 1272
- **1D aligned content [PDB] (<aminoacid>:%):** {'P': 14.29, 'G': 7.14, 'K': 3.57, 'S': 10.71, 'A': 14.29, 'V': 7.14, 'M': 7.14, 'H': 3.57, 'T': 3.57, 'R': 10.71, 'Q': 7.14, 'I': 7.14, 'D': 3.57}
- **2D identity (%) [PDB]:** 38.53
- **2D identity (%) [Gaps excluded][PDB]:** 88.83
- **2D identity - Alignment Gaps [PDB]:** 538
- **2D aligned content [PDB] (<2D-fold>:%):** {'.': 30.6, 'E': 13.11, 'H': 37.98, 'T': 18.31}
- **3D similarity (TM-Score) (%) [PDB]:** 19.25

- **Gene name:** HINFP
- **Entrez ID:** 511965
- **RefSeq ID:** N/A
- **Sequence length:** N/A
- **5-UTR|CDS|3-UTR identity (%):** N/A | N/A | N/A
- **5-UTR|CDS|3-UTR identity (%) [Gaps excluded]:** N/A | N/A | N/A
- **5-UTR|CDS|3-UTR identity [Alignment Gaps]:** N/A | N/A | N/A
- **5-UTR aligned content (<base>:%):** N/A
- **CDS aligned content (<base>:%):** N/A
- **3-UTR aligned content (<base>:%):** N/A

**Uniprot Description:**  
  
 Transcriptional repressor that binds to the consensus sequence 5'-CGGACGTT-3' and to the RB1 promoter. Transcriptional activator that promotes histone H4 gene transcription at the G1/S phase transition in conjunction with NPAT. Also activates transcription of the ATM and PRKDC genes. Autoregulates its expression by associating with its own promoter.   
  
Binds MBD2 and a histone deacetylase complex. Interacts with NPAT.   
  
 **Gene Ontology Information:**

Molecular Function

- DNA-binding transcription factor activity, RNA polymerase II-specific
- metal ion binding
- RNA polymerase II cis-regulatory region sequence-specific DNA binding

Location

- nucleus

Biological process

- anatomical structure development
- regulation of transcription by RNA polymerase II

---

22

- **Protein name:** Disintegrin and metalloproteinase domain-containing protein 10
- **Organism:** Homo sapiens
- **Uniprot Accession Number:** O14672
- **Protein sequence length:** 748 aa
- **1D identity (%):** N/A
- **1D identity (%) [Gaps excluded]:** N/A
- **1D identity - Alignment Gaps:** N/A
- **1D aligned content (<aminoacid>:%):** N/A
- **Common reported functions (%):** N/A
- **Common reported locations (%):** N/A
- **Common reported processes (%):** N/A

- **AF ID:** O14672
- **Chain:** A
- **Protein length:** 748 aa
- **Resolution:** N/A
- **b-phipsi:** 0.001747
- **w-rdist:** 0.669721
- **t-alpha:** 0.002377
- **Chemical similarity (Tanimoto Index) (%):** N/A
- **1D identity (%) [PDB]:** N/A
- **1D identity (%) [Gaps excluded][PDB]:** N/A
- **1D identity - Alignment Gaps [PDB]:** N/A
- **1D aligned content [PDB] (<aminoacid>:%):** N/A
- **2D identity (%) [PDB]:** N/A
- **2D identity (%) [Gaps excluded][PDB]:** N/A
- **2D identity - Alignment Gaps [PDB]:** N/A
- **2D aligned content [PDB] (<2D-fold>:%):** N/A
- **3D similarity (TM-Score) (%) [PDB]:** N/A

- **Gene name:** ADAM10
- **Entrez ID:** 102
- **RefSeq ID:** N/A
- **Sequence length:** N/A
- **5-UTR|CDS|3-UTR identity (%):** N/A | N/A | N/A
- **5-UTR|CDS|3-UTR identity (%) [Gaps excluded]:** N/A | N/A | N/A
- **5-UTR|CDS|3-UTR identity [Alignment Gaps]:** N/A | N/A | N/A
- **5-UTR aligned content (<base>:%):** N/A
- **CDS aligned content (<base>:%):** N/A
- **3-UTR aligned content (<base>:%):** N/A

**Uniprot Description:**  
  
 Cleaves the membrane-bound precursor of TNF-alpha at '76-Ala-|-Val-77' to its mature soluble form. Responsible for the proteolytical release of soluble JAM3 from endothelial cells surface (PubMed:20592283). Responsible for the proteolytic release of several other cell-surface proteins, including heparin-binding epidermal growth-like factor, ephrin-A2, CD44, CDH2 and for constitutive and regulated alpha-secretase cleavage of amyloid precursor protein (APP) (PubMed:26686862, PubMed:11786905, PubMed:29224781). Contributes to the normal cleavage of the cellular prion protein (PubMed:11477090). Involved in the cleavage of the adhesion molecule L1 at the cell surface and in released membrane vesicles, suggesting a vesicle-based protease activity (PubMed:12475894). Controls also the proteolytic processing of Notch and mediates lateral inhibition during neurogenesis (By similarity). Responsible for the FasL ectodomain shedding and for the generation of the remnant ADAM10-processed FasL (FasL APL) transmembrane form (PubMed:17557115). Also cleaves the ectodomain of the integral membrane proteins CORIN and ITM2B (PubMed:19114711, PubMed:21288900). Mediates the proteolytic cleavage of LAG3, leading to release the secreted form of LAG3 (By similarity). Mediates the proteolytic cleavage of IL6R and IL11RA, leading to the release of secreted forms of IL6R and IL11RA (PubMed:26876177). Enhances the cleavage of CHL1 by BACE1 (By similarity). Cleaves NRCAM (By similarity). Cleaves TREM2, resulting in shedding of the TREM2 ectodomain (PubMed:24990881). Involved in the development and maturation of glomerular and coronary vasculature (By similarity). During development of the cochlear organ of Corti, promotes pillar cell separation by forming a ternary complex with CADH1 and EPHA4 and cleaving CADH1 at adherens junctions (By similarity). May regulate the EFNA5-EPHA3 signaling (PubMed:16239146).   
  
Forms a ternary EFNA5-EPHA3-ADAM10 complex mediating EFNA5 extracellular domain shedding by ADAM10 which regulates the EFNA5-EPHA3 complex internalization and function, the cleavage occurs in trans, with ADAM10 and its substrate being on the membranes of opposing cells (PubMed:16239146). Interacts with the clathrin adapter AP2 complex subunits AP2A1, AP2A2, AP2B1, and AP2M1; this interaction facilitates ADAM10 endocytosis from the plasma membrane during long-term potentiation in hippocampal neurons (PubMed:23676497). Interacts (via extracellular domain) with TSPAN33 (via extracellular domain) and (via cytoplasmic domain) with AFDN; interaction with TSPAN33 allows the docking of ADAM10 to zonula adherens through a PDZ11-dependent interaction between TSPAN33 and PLEKHA7 while interaction with AFDN locks ADAM10 at zonula adherens (PubMed:30463011). Forms a ternary complex composed of ADAM10, EPHA4 and CADH1; within the complex, ADAM10 cleaves CADH1 which disrupts adherens junctions (By similarity). Interacts with EPHA2 (By similarity). Interacts with NGF in a divalent cation-dependent manner (PubMed:20164177). Interacts with TSPAN14; the interaction promotes ADAM10 maturation and cell surface expression (PubMed:26686862, PubMed:26668317). Interacts with TSPAN5, TSPAN10, TSPAN15, TSPAN17 and TSPAN33; these interactions regulate ADAM10 substrate specificity (PubMed:26686862). Interacts with DLG1; this interaction recruits ADAM10 to the cell membrane during long-term depression in hippocampal neurons (PubMed:23676497). Interacts (via extracellular domain) with BACE1 (via extracellular domain) (By similarity). Interacts with FAM171A1 (PubMed:30312582).   
  
 **Gene Ontology Information:**

Molecular Function

- endopeptidase activity
- integrin binding
- metal ion binding
- metallodipeptidase activity
- metalloendopeptidase activity
- metalloendopeptidase activity involved in amyloid precursor protein catabolic process
- metallopeptidase activity
- molecular adaptor activity
- protein homodimerization activity
- protein kinase binding
- SH3 domain binding
- signaling receptor binding

Location

- adherens junction
- axon
- cell surface
- clathrin-coated vesicle
- cytoplasm
- dendrite
- endoplasmic reticulum lumen
- extracellular exosome
- focal adhesion
- glutamatergic synapse
- Golgi apparatus
- Golgi membrane
- Golgi-associated vesicle
- intracellular membrane-bounded organelle
- membrane
- nucleus
- perinuclear endoplasmic reticulum
- plasma membrane
- pore complex
- postsynaptic density
- specific granule membrane
- synaptic membrane
- tertiary granule membrane
- tetraspanin-enriched microdomain

Biological process

- adherens junction organization
- amyloid precursor protein catabolic process
- cell-cell signaling
- cochlea development
- constitutive protein ectodomain proteolysis
- epidermal growth factor receptor ligand maturation
- extracellular matrix disassembly
- in utero embryonic development
- integrin-mediated signaling pathway
- membrane protein ectodomain proteolysis
- monocyte activation
- negative regulation of cell adhesion
- negative regulation of gene expression
- Notch signaling pathway
- pore complex assembly
- positive regulation of cell growth
- positive regulation of cell migration
- positive regulation of cell population proliferation
- positive regulation of T cell chemotaxis
- postsynapse organization
- protein catabolic process at postsynapse
- protein phosphorylation
- protein processing
- regulation of neurotransmitter receptor localization to postsynaptic specialization membrane
- regulation of Notch signaling pathway
- regulation of postsynapse organization
- regulation of vasculature development
- response to tumor necrosis factor
- toxin transport

---

23

- **Protein name:** Biotin--protein ligase
- **Organism:** Homo sapiens
- **Uniprot Accession Number:** P50747
- **Protein sequence length:** 726 aa
- **1D identity (%):** 13.36
- **1D identity (%) [Gaps excluded]:** 23.94
- **1D identity - Alignment Gaps:** 450
- **1D aligned content (<aminoacid>:%):** {'M': 0.74, 'L': 13.24, 'N': 3.68, 'P': 9.56, 'H': 4.41, 'T': 5.15, 'K': 5.15, 'V': 8.82, 'S': 6.62, 'Q': 5.15, 'E': 3.68, 'G': 10.29, 'C': 3.68, 'A': 5.15, 'D': 2.94, 'W': 1.47, 'F': 2.21, 'Y': 3.68, 'I': 1.47, 'R': 2.94}
- **Common reported functions (%):** 0.0
- **Common reported locations (%):** 22.22
- **Common reported processes (%):** 0.0

- **AF ID:** P50747
- **Chain:** A
- **Protein length:** 726 aa
- **Resolution:** N/A
- **b-phipsi:** 0.000722
- **w-rdist:** 0.324547
- **t-alpha:** 0.14897
- **Chemical similarity (Tanimoto Index) (%):** 97.34
- **1D identity (%) [PDB]:** 2.85
- **1D identity (%) [Gaps excluded][PDB]:** 68.25
- **1D identity - Alignment Gaps [PDB]:** 1445
- **1D aligned content [PDB] (<aminoacid>:%):** {'P': 4.65, 'S': 9.3, 'H': 6.98, 'Y': 9.3, 'V': 9.3, 'L': 13.95, 'W': 2.33, 'D': 4.65, 'N': 2.33, 'C': 2.33, 'F': 2.33, 'T': 6.98, 'A': 9.3, 'E': 4.65, 'Q': 2.33, 'R': 6.98, 'G': 2.33}
- **2D identity (%) [PDB]:** 39.67
- **2D identity (%) [Gaps excluded][PDB]:** 86.99
- **2D identity - Alignment Gaps [PDB]:** 587
- **2D aligned content [PDB] (<2D-fold>:%):** {'.': 21.96, 'H': 24.07, 'T': 27.1, 'E': 26.64, 'B': 0.23}
- **3D similarity (TM-Score) (%) [PDB]:** 21.93

- **Gene name:** HLCS
- **Entrez ID:** 3141
- **RefSeq ID:** N/A
- **Sequence length:** N/A
- **5-UTR|CDS|3-UTR identity (%):** N/A | N/A | N/A
- **5-UTR|CDS|3-UTR identity (%) [Gaps excluded]:** N/A | N/A | N/A
- **5-UTR|CDS|3-UTR identity [Alignment Gaps]:** N/A | N/A | N/A
- **5-UTR aligned content (<base>:%):** N/A
- **CDS aligned content (<base>:%):** N/A
- **3-UTR aligned content (<base>:%):** N/A

**Uniprot Description:**  
  
 Biotin--protein ligase catalyzing the biotinylation of the 4 biotin-dependent carboxylases acetyl-CoA-carboxylase, pyruvate carboxylase, propionyl-CoA carboxylase, and methylcrotonyl-CoA carboxylase.   
  
Monomer.   
  
 **Gene Ontology Information:**

Molecular Function

- ATP binding
- biotin binding
- biotin-[acetyl-CoA-carboxylase] ligase activity
- biotin-[methylcrotonoyl-CoA-carboxylase] ligase activity
- biotin-[methylmalonyl-CoA-carboxytransferase] ligase activity
- biotin-[propionyl-CoA-carboxylase (ATP-hydrolyzing)] ligase activity
- biotin-protein ligase activity
- enzyme binding
- identical protein binding

Location

- chromatin
- cytoplasm
- cytosol
- mitochondrion
- nuclear lamina
- nuclear matrix

Biological process

- biotin metabolic process
- post-translational protein modification
- response to biotin

---

24

- **Protein name:** RNA cytosine C(5)-methyltransferase NSUN2
- **Organism:** Homo sapiens
- **Uniprot Accession Number:** Q08J23
- **Protein sequence length:** 767 aa
- **1D identity (%):** 12.87
- **1D identity (%) [Gaps excluded]:** 21.96
- **1D identity - Alignment Gaps:** 425
- **1D aligned content (<aminoacid>:%):** {'G': 10.61, 'L': 9.09, 'R': 6.82, 'P': 13.64, 'K': 6.06, 'E': 6.82, 'I': 3.79, 'Y': 2.27, 'T': 6.82, 'V': 5.3, 'S': 4.55, 'H': 5.3, 'Q': 3.79, 'M': 1.52, 'N': 0.76, 'D': 3.79, 'A': 6.06, 'C': 2.27, 'F': 0.76}
- **Common reported functions (%):** 0.0
- **Common reported locations (%):** 11.11
- **Common reported processes (%):** 0.0

- **AF ID:** Q08J23
- **Chain:** A
- **Protein length:** 767 aa
- **Resolution:** N/A
- **b-phipsi:** 0.000339
- **w-rdist:** 0.361615
- **t-alpha:** 0.204438
- **Chemical similarity (Tanimoto Index) (%):** 99.62
- **1D identity (%) [PDB]:** 2.98
- **1D identity (%) [Gaps excluded][PDB]:** 66.67
- **1D identity - Alignment Gaps [PDB]:** 1474
- **1D aligned content [PDB] (<aminoacid>:%):** {'M': 2.17, 'A': 10.87, 'T': 4.35, 'Q': 4.35, 'V': 15.22, 'P': 10.87, 'L': 6.52, 'S': 6.52, 'N': 4.35, 'K': 6.52, 'I': 8.7, 'H': 4.35, 'R': 4.35, 'F': 2.17, 'G': 4.35, 'D': 4.35}
- **2D identity (%) [PDB]:** 37.15
- **2D identity (%) [Gaps excluded][PDB]:** 88.66
- **2D identity - Alignment Gaps [PDB]:** 660
- **2D aligned content [PDB] (<2D-fold>:%):** {'.': 26.3, 'T': 24.17, 'E': 18.48, 'H': 29.15, 'G': 1.42, 'B': 0.47}
- **3D similarity (TM-Score) (%) [PDB]:** 23.39

- **Gene name:** NSUN2
- **Entrez ID:** 420938
- **RefSeq ID:** NM\_017755
- **Transcript sequence length:** 3056
- **5-UTR|CDS|3-UTR identity (%):** 10.45 | 46.77 | 2.67
- **5-UTR|CDS|3-UTR identity (%) [Gaps excluded]:** 67.31 | 75.59 | 74.5
- **5-UTR|CDS|3-UTR identity [Alignment Gaps]:** 283 | 1151 | 16234
- **5-UTR aligned content (<base>:%):** {'G': 60.0, 'C': 22.86, 'A': 2.86, 'T': 14.29}
- **CDS aligned content (<base>:%):** {'A': 31.59, 'T': 22.73, 'G': 25.28, 'C': 20.4}
- **3-UTR aligned content (<base>:%):** {'C': 14.0, 'A': 29.56, 'G': 19.56, 'T': 36.89}

**Uniprot Description:**  
  
 RNA cytosine C(5)-methyltransferase that methylates cytosine to 5-methylcytosine (m5C) in various RNAs, such as tRNAs, mRNAs and some long non-coding RNAs (lncRNAs) (PubMed:17071714, PubMed:22995836, PubMed:31358969, PubMed:31199786). Involved in various processes, such as epidermal stem cell differentiation, testis differentiation and maternal to zygotic transition during early development: acts by increasing protein synthesis; cytosine C(5)-methylation promoting tRNA stability and preventing mRNA decay (PubMed:31199786). Methylates cytosine to 5-methylcytosine (m5C) at positions 34 and 48 of intron-containing tRNA(Leu)(CAA) precursors, and at positions 48, 49 and 50 of tRNA(Gly)(GCC) precursors (PubMed:17071714, PubMed:22995836, PubMed:31199786). tRNA methylation is required generation of RNA fragments derived from tRNAs (tRFs) (PubMed:31199786). Also mediates C(5)-methylation of mitochondrial tRNAs (PubMed:31276587). Catalyzes cytosine C(5)-methylation of mRNAs, leading to stabilize them and prevent mRNA decay: mRNA stabilization involves YBX1 that specifically recognizes and binds m5C-modified transcripts (PubMed:22395603, PubMed:31358969, PubMed:34556860). Cytosine C(5)-methylation of mRNAs also regulates mRNA export: methylated transcripts are specifically recognized by THOC4/ALYREF, which mediates mRNA nucleo-cytoplasmic shuttling (PubMed:28418038). Also mediates cytosine C(5)-methylation of non-coding RNAs, such as vault RNAs (vtRNAs), promoting their processing into regulatory small RNAs (PubMed:23871666). Cytosine C(5)-methylation of vtRNA VTRNA1.1 promotes its processing into small-vault RNA4 (svRNA4) and regulates epidermal differentiation (PubMed:31186410). May act downstream of Myc to regulate epidermal cell growth and proliferation (By similarity). Required for proper spindle assembly and chromosome segregation, independently of its methyltransferase activity (PubMed:19596847).   
  
Interacts with NPM1 and NCL during interphase; interaction is disrupted following phosphorylation at Ser-139.   
  
 **Gene Ontology Information:**

Molecular Function

- mRNA (cytidine-5-)-methyltransferase activity
- tRNA (cytosine-5-)-methyltransferase activity
- tRNA binding

Location

- cytoplasm
- extracellular exosome
- mitochondrion
- nucleolus
- nucleus
- spindle

Biological process

- mRNA methylation
- regulation of mRNA export from nucleus
- regulation of stem cell differentiation
- tRNA methylation
- tRNA stabilization

---

25

- **Protein name:** PHD finger protein 6
- **Organism:** Homo sapiens
- **Uniprot Accession Number:** Q8IWS0
- **Protein sequence length:** 365 aa
- **1D identity (%):** 8.55
- **1D identity (%) [Gaps excluded]:** 24.92
- **1D identity - Alignment Gaps:** 599
- **1D aligned content (<aminoacid>:%):** {'G': 14.1, 'R': 6.41, 'F': 2.56, 'S': 5.13, 'A': 5.13, 'K': 12.82, 'H': 5.13, 'E': 8.97, 'L': 7.69, 'I': 3.85, 'C': 6.41, 'T': 8.97, 'Q': 2.56, 'D': 5.13, 'P': 1.28, 'V': 1.28, 'M': 2.56}
- **Common reported functions (%):** 14.29
- **Common reported locations (%):** 11.11
- **Common reported processes (%):** 0.0

- **AF ID:** Q8IWS0
- **Chain:** A
- **Protein length:** 365 aa
- **Resolution:** N/A
- **b-phipsi:** 0.001017
- **w-rdist:** 0.668095
- **t-alpha:** 0.00317
- **Chemical similarity (Tanimoto Index) (%):** 85.94
- **1D identity (%) [PDB]:** 1.52
- **1D identity (%) [Gaps excluded][PDB]:** 64.29
- **1D identity - Alignment Gaps [PDB]:** 1154
- **1D aligned content [PDB] (<aminoacid>:%):** {'G': 22.22, 'R': 16.67, 'T': 16.67, 'S': 5.56, 'H': 5.56, 'M': 5.56, 'K': 11.11, 'Q': 5.56, 'E': 5.56, 'I': 5.56}
- **2D identity (%) [PDB]:** 25.79
- **2D identity (%) [Gaps excluded][PDB]:** 82.93
- **2D identity - Alignment Gaps [PDB]:** 636
- **2D aligned content [PDB] (<2D-fold>:%):** {'T': 44.54, '.': 21.85, 'E': 7.14, 'H': 26.05, 'B': 0.42}
- **3D similarity (TM-Score) (%) [PDB]:** 16.28

- **Gene name:** PHF6
- **Entrez ID:** 518186
- **RefSeq ID:** NM\_032458
- **Transcript sequence length:** 4760
- **5-UTR|CDS|3-UTR identity (%):** 32.87 | 24.61 | 12.53
- **5-UTR|CDS|3-UTR identity (%) [Gaps excluded]:** 73.46 | 76.73 | 75.64
- **5-UTR|CDS|3-UTR identity [Alignment Gaps]:** 200 | 1893 | 14469
- **5-UTR aligned content (<base>:%):** {'A': 11.76, 'G': 28.57, 'C': 40.34, 'T': 19.33}
- **CDS aligned content (<base>:%):** {'T': 23.62, 'C': 16.18, 'G': 22.59, 'A': 37.61}
- **3-UTR aligned content (<base>:%):** {'G': 14.54, 'A': 32.54, 'T': 40.22, 'C': 12.7}

**Uniprot Description:**  
  
 Transcriptional regulator that associates with ribosomal RNA promoters and suppresses ribosomal RNA (rRNA) transcription.   
  
Interacts with UBTF. Interacts with the NuRD complex component RBBP4 (via the nucleolar localization motif), the interaction mediates transcriptional repression activity.   
  
 **Gene Ontology Information:**

Molecular Function

- DNA binding
- histone binding
- histone deacetylase binding
- metal ion binding
- phosphoprotein binding
- ribonucleoprotein complex binding
- scaffold protein binding
- tubulin binding

Location

- kinetochore
- nucleolus
- nucleoplasm
- nucleus

Biological process

- blastocyst hatching
- negative regulation of transcription by RNA polymerase II

---

26

- **Protein name:** Diacylglycerol kinase alpha
- **Organism:** Homo sapiens
- **Uniprot Accession Number:** P23743
- **Protein sequence length:** 735 aa
- **1D identity (%):** 11.71
- **1D identity (%) [Gaps excluded]:** 23.13
- **1D identity - Alignment Gaps:** 523
- **1D aligned content (<aminoacid>:%):** {'K': 7.26, 'E': 6.45, 'G': 12.9, 'L': 9.68, 'Y': 3.23, 'F': 4.84, 'V': 5.65, 'P': 8.87, 'T': 7.26, 'C': 4.03, 'R': 5.65, 'A': 3.23, 'Q': 4.84, 'W': 0.81, 'D': 4.84, 'H': 2.42, 'I': 5.65, 'N': 0.81, 'S': 1.61}
- **Common reported functions (%):** 0.0
- **Common reported locations (%):** 22.22
- **Common reported processes (%):** 0.0

- **AF ID:** P23743
- **Chain:** A
- **Protein length:** 735 aa
- **Resolution:** N/A
- **b-phipsi:** 0.002469
- **w-rdist:** 0.346361
- **t-alpha:** 0.023772
- **Chemical similarity (Tanimoto Index) (%):** 99.17
- **1D identity (%) [PDB]:** 3.68
- **1D identity (%) [Gaps excluded][PDB]:** 64.71
- **1D identity - Alignment Gaps [PDB]:** 1410
- **1D aligned content [PDB] (<aminoacid>:%):** {'M': 1.82, 'V': 5.45, 'Q': 1.82, 'F': 5.45, 'K': 9.09, 'T': 10.91, 'D': 5.45, 'R': 7.27, 'P': 10.91, 'Y': 3.64, 'G': 9.09, 'L': 16.36, 'A': 1.82, 'N': 3.64, 'S': 3.64, 'E': 3.64}
- **2D identity (%) [PDB]:** 36.94
- **2D identity (%) [Gaps excluded][PDB]:** 87.23
- **2D identity - Alignment Gaps [PDB]:** 640
- **2D aligned content [PDB] (<2D-fold>:%):** {'T': 22.68, '.': 17.56, 'H': 43.41, 'E': 16.1, 'B': 0.24}
- **3D similarity (TM-Score) (%) [PDB]:** 23.59

- **Gene name:** DGKA
- **Entrez ID:** 1606
- **RefSeq ID:** N/A
- **Sequence length:** N/A
- **5-UTR|CDS|3-UTR identity (%):** N/A | N/A | N/A
- **5-UTR|CDS|3-UTR identity (%) [Gaps excluded]:** N/A | N/A | N/A
- **5-UTR|CDS|3-UTR identity [Alignment Gaps]:** N/A | N/A | N/A
- **5-UTR aligned content (<base>:%):** N/A
- **CDS aligned content (<base>:%):** N/A
- **3-UTR aligned content (<base>:%):** N/A

**Uniprot Description:**  
  
 Diacylglycerol kinase that converts diacylglycerol/DAG into phosphatidic acid/phosphatidate/PA and regulates the respective levels of these two bioactive lipids (PubMed:2175712, PubMed:15544348). Thereby, acts as a central switch between the signaling pathways activated by these second messengers with different cellular targets and opposite effects in numerous biological processes (PubMed:2175712, PubMed:15544348). Also plays an important role in the biosynthesis of complex lipids (Probable). Can also phosphorylate 1-alkyl-2-acylglycerol in vitro as efficiently as diacylglycerol provided it contains an arachidonoyl group (PubMed:15544348). Also involved in the production of alkyl-lysophosphatidic acid, another bioactive lipid, through the phosphorylation of 1-alkyl-2-acetyl glycerol (PubMed:22627129).   
  
Monomer.   
  
 **Gene Ontology Information:**

Molecular Function

- ATP binding
- diacylglycerol kinase activity
- calcium ion binding
- kinase activity
- lipid binding
- phospholipid binding

Location

- cytosol
- membrane
- plasma membrane

Biological process

- diacylglycerol metabolic process
- glycerolipid metabolic process
- intracellular signal transduction
- lipid phosphorylation
- phosphatidic acid biosynthetic process
- platelet activation
- protein kinase C-activating G protein-coupled receptor signaling pathway

---

27

- **Protein name:** Insulin-like growth factor 2 mRNA-binding protein 3
- **Organism:** Homo sapiens
- **Uniprot Accession Number:** O00425
- **Protein sequence length:** 579 aa
- **1D identity (%):** 13.54
- **1D identity (%) [Gaps excluded]:** 26.54
- **1D identity - Alignment Gaps:** 467
- **1D aligned content (<aminoacid>:%):** {'Y': 1.55, 'G': 7.75, 'S': 6.2, 'N': 2.33, 'P': 11.63, 'D': 3.88, 'F': 3.1, 'K': 9.3, 'V': 8.53, 'W': 0.78, 'L': 6.2, 'E': 5.43, 'I': 7.75, 'H': 2.33, 'R': 7.75, 'A': 6.98, 'Q': 4.65, 'T': 3.1, 'M': 0.78}
- **Common reported functions (%):** 14.29
- **Common reported locations (%):** 33.33
- **Common reported processes (%):** 0.0

- **AF ID:** O00425
- **Chain:** A
- **Protein length:** 579 aa
- **Resolution:** N/A
- **b-phipsi:** 0.001069
- **w-rdist:** 0.257559
- **t-alpha:** 0.102219
- **Chemical similarity (Tanimoto Index) (%):** 99.4
- **1D identity (%) [PDB]:** 2.25
- **1D identity (%) [Gaps excluded][PDB]:** 68.89
- **1D identity - Alignment Gaps [PDB]:** 1334
- **1D aligned content [PDB] (<aminoacid>:%):** {'M': 3.23, 'E': 9.68, 'V': 9.68, 'L': 6.45, 'I': 9.68, 'N': 3.23, 'D': 6.45, 'Q': 3.23, 'P': 6.45, 'R': 12.9, 'H': 3.23, 'K': 12.9, 'F': 3.23, 'T': 3.23, 'G': 6.45}
- **2D identity (%) [PDB]:** 41.37
- **2D identity (%) [Gaps excluded][PDB]:** 87.53
- **2D identity - Alignment Gaps [PDB]:** 510
- **2D aligned content [PDB] (<2D-fold>:%):** {'.': 18.0, 'E': 25.75, 'H': 41.5, 'T': 14.75}
- **3D similarity (TM-Score) (%) [PDB]:** 17.98

- **Gene name:** IGF2BP3
- **Entrez ID:** 10643
- **RefSeq ID:** NM\_006547
- **Transcript sequence length:** 4274
- **5-UTR|CDS|3-UTR identity (%):** 36.5 | 39.13 | 7.94
- **5-UTR|CDS|3-UTR identity (%) [Gaps excluded]:** 70.42 | 75.87 | 73.88
- **5-UTR|CDS|3-UTR identity [Alignment Gaps]:** 223 | 1381 | 15240
- **5-UTR aligned content (<base>:%):** {'A': 10.65, 'G': 31.95, 'T': 20.12, 'C': 37.28}
- **CDS aligned content (<base>:%):** {'A': 31.09, 'T': 23.21, 'C': 21.59, 'G': 24.1}
- **3-UTR aligned content (<base>:%):** {'A': 34.02, 'G': 13.73, 'C': 14.1, 'T': 38.15}

**Uniprot Description:**  
  
 RNA-binding factor that may recruit target transcripts to cytoplasmic protein-RNA complexes (mRNPs). This transcript 'caging' into mRNPs allows mRNA transport and transient storage. It also modulates the rate and location at which target transcripts encounter the translational apparatus and shields them from endonuclease attacks or microRNA-mediated degradation. Preferentially binds to N6-methyladenosine (m6A)-containing mRNAs and increases their stability (PubMed:29476152). Binds to the 3'-UTR of CD44 mRNA and stabilizes it, hence promotes cell adhesion and invadopodia formation in cancer cells. Binds to beta-actin/ACTB and MYC transcripts. Increases MYC mRNA stability by binding to the coding region instability determinant (CRD) and binding is enhanced by m6A-modification of the CRD (PubMed:29476152). Binds to the 5'-UTR of the insulin-like growth factor 2 (IGF2) mRNAs.   
  
Can form homooligomers and heterooligomers with IGF2BP1 and IGF2BP3 in an RNA-dependent manner (PubMed:23640942). Interacts with IGF2BP1 (PubMed:17289661). Interacts with ELAVL1, DHX9, HNRNPU, MATR3 and PABPC1 (PubMed:23640942, PubMed:29476152).   
  
 **Gene Ontology Information:**

Molecular Function

- mRNA 3'-UTR binding
- mRNA 5'-UTR binding
- N6-methyladenosine-containing RNA binding
- RNA binding
- translation regulator activity

Location

- cytoplasm
- cytoplasmic stress granule
- cytosol
- nucleus
- P-body

Biological process

- anatomical structure morphogenesis
- CRD-mediated mRNA stabilization
- mRNA transport
- negative regulation of translation
- nervous system development
- regulation of cytokine production
- regulation of gene expression
- translation

---

28

- **Protein name:** NudC domain-containing protein 3
- **Organism:** Homo sapiens
- **Uniprot Accession Number:** Q8IVD9
- **Protein sequence length:** 361 aa
- **1D identity (%):** 8.0
- **1D identity (%) [Gaps excluded]:** 21.26
- **1D identity - Alignment Gaps:** 553
- **1D aligned content (<aminoacid>:%):** {'L': 11.27, 'D': 2.82, 'V': 7.04, 'G': 14.08, 'R': 4.23, 'K': 9.86, 'P': 12.68, 'F': 2.82, 'T': 2.82, 'Q': 8.45, 'E': 7.04, 'I': 5.63, 'A': 4.23, 'Y': 1.41, 'H': 1.41, 'S': 1.41, 'N': 1.41, 'M': 1.41}
- **Common reported functions (%):** 0.0
- **Common reported locations (%):** 11.11
- **Common reported processes (%):** 0.0

- **AF ID:** Q8IVD9
- **Chain:** A
- **Protein length:** 361 aa
- **Resolution:** N/A
- **b-phipsi:** 0.000287
- **w-rdist:** 0.800511
- **t-alpha:** 0.019386
- **Chemical similarity (Tanimoto Index) (%):** 98.2
- **1D identity (%) [PDB]:** 1.61
- **1D identity (%) [Gaps excluded][PDB]:** 67.86
- **1D identity - Alignment Gaps [PDB]:** 1150
- **1D aligned content [PDB] (<aminoacid>:%):** {'E': 10.53, 'T': 5.26, 'V': 15.79, 'Q': 5.26, 'F': 10.53, 'K': 5.26, 'D': 5.26, 'M': 5.26, 'A': 10.53, 'H': 5.26, 'G': 5.26, 'R': 5.26, 'L': 5.26, 'P': 5.26}
- **2D identity (%) [PDB]:** 27.2
- **2D identity (%) [Gaps excluded][PDB]:** 87.11
- **2D identity - Alignment Gaps [PDB]:** 632
- **2D aligned content [PDB] (<2D-fold>:%):** {'T': 24.4, 'H': 46.0, '.': 18.4, 'E': 9.6, 'B': 0.4, 'G': 1.2}
- **3D similarity (TM-Score) (%) [PDB]:** 14.86

- **Gene name:** NUDCD3
- **Entrez ID:** 23386
- **RefSeq ID:** NM\_015332
- **Transcript sequence length:** 8036
- **5-UTR|CDS|3-UTR identity (%):** 12.2 | 24.85 | 25.08
- **5-UTR|CDS|3-UTR identity (%) [Gaps excluded]:** 76.92 | 74.67 | 74.3
- **5-UTR|CDS|3-UTR identity [Alignment Gaps]:** 276 | 1837 | 11711
- **5-UTR aligned content (<base>:%):** {'G': 60.0, 'T': 10.0, 'C': 22.5, 'A': 7.5}
- **CDS aligned content (<base>:%):** {'A': 29.24, 'T': 20.18, 'G': 27.92, 'C': 22.66}
- **3-UTR aligned content (<base>:%):** {'T': 27.63, 'G': 24.09, 'A': 24.94, 'C': 23.34}

**Uniprot Description:**  
  
 N/A N/A   
  
 **Gene Ontology Information:**

Molecular Function

- unfolded protein binding

Location

- cytoplasm
- cytoplasmic dynein complex

Biological process

- cilium assembly
- protein folding
- protein localization to pericentriolar material

---

29

- **Protein name:** Golgi-associated kinase 1A
- **Organism:** Homo sapiens
- **Uniprot Accession Number:** Q9UFP1
- **Protein sequence length:** 575 aa
- **1D identity (%):** 11.29
- **1D identity (%) [Gaps excluded]:** 22.59
- **1D identity - Alignment Gaps:** 479
- **1D aligned content (<aminoacid>:%):** {'R': 12.96, 'G': 11.11, 'K': 1.85, 'A': 7.41, 'C': 1.85, 'V': 9.26, 'P': 15.74, 'D': 5.56, 'H': 3.7, 'L': 12.04, 'S': 2.78, 'E': 6.48, 'T': 0.93, 'W': 1.85, 'F': 2.78, 'Y': 1.85, 'N': 0.93, 'Q': 0.93}
- **Common reported functions (%):** 0.0
- **Common reported locations (%):** 0.0
- **Common reported processes (%):** 0.0

- **AF ID:** Q9UFP1
- **Chain:** A
- **Protein length:** 575 aa
- **Resolution:** N/A
- **b-phipsi:** 0.00024
- **w-rdist:** 0.640363
- **t-alpha:** 0.09588
- **Chemical similarity (Tanimoto Index) (%):** 96.56
- **1D identity (%) [PDB]:** 3.08
- **1D identity (%) [Gaps excluded][PDB]:** 73.68
- **1D identity - Alignment Gaps [PDB]:** 1306
- **1D aligned content [PDB] (<aminoacid>:%):** {'A': 7.14, 'L': 21.43, 'P': 11.9, 'V': 11.9, 'T': 9.52, 'G': 9.52, 'D': 9.52, 'K': 4.76, 'H': 2.38, 'E': 7.14, 'R': 2.38, 'S': 2.38}
- **2D identity (%) [PDB]:** 30.42
- **2D identity (%) [Gaps excluded][PDB]:** 86.96
- **2D identity - Alignment Gaps [PDB]:** 684
- **2D aligned content [PDB] (<2D-fold>:%):** {'.': 26.25, 'T': 35.94, 'H': 31.25, 'E': 5.94, 'B': 0.62}
- **3D similarity (TM-Score) (%) [PDB]:** 17.35

- **Gene name:** GASK1A
- **Entrez ID:** 729085
- **RefSeq ID:** NM\_001129908
- **Transcript sequence length:** 3389
- **5-UTR|CDS|3-UTR identity (%):** 43.33 | 37.12 | 5.14
- **5-UTR|CDS|3-UTR identity (%) [Gaps excluded]:** 77.78 | 74.08 | 74.4
- **5-UTR|CDS|3-UTR identity [Alignment Gaps]:** 186 | 1433 | 15748
- **5-UTR aligned content (<base>:%):** {'C': 41.76, 'G': 35.71, 'A': 9.34, 'T': 13.19}
- **CDS aligned content (<base>:%):** {'G': 29.46, 'C': 24.95, 'T': 21.48, 'A': 24.11}
- **3-UTR aligned content (<base>:%):** {'G': 19.56, 'C': 16.8, 'A': 30.03, 'T': 33.6}

**Uniprot Description:**  
  
 N/A N/A   
  
 **Gene Ontology Information:**

Molecular Function   
  
N/A

Location

- caveola
- endoplasmic reticulum
- extracellular region
- Golgi apparatus
- intracellular membrane-bounded organelle

Biological process   
  
N/A

---

30

- **Protein name:** Long-chain-fatty-acid--CoA ligase ACSBG1
- **Organism:** Homo sapiens
- **Uniprot Accession Number:** Q96GR2
- **Protein sequence length:** 724 aa
- **1D identity (%):** 12.94
- **1D identity (%) [Gaps excluded]:** 23.4
- **1D identity - Alignment Gaps:** 456
- **1D aligned content (<aminoacid>:%):** {'P': 8.33, 'G': 11.36, 'R': 5.3, 'L': 9.85, 'E': 5.3, 'N': 2.27, 'A': 2.27, 'V': 7.58, 'Y': 3.79, 'T': 3.79, 'F': 5.3, 'W': 2.27, 'I': 7.58, 'S': 3.03, 'H': 2.27, 'C': 4.55, 'Q': 6.06, 'K': 6.06, 'D': 2.27, 'M': 0.76}
- **Common reported functions (%):** 0.0
- **Common reported locations (%):** 11.11
- **Common reported processes (%):** 0.0

- **AF ID:** Q96GR2
- **Chain:** A
- **Protein length:** 724 aa
- **Resolution:** N/A
- **b-phipsi:** 0.003261
- **w-rdist:** 0.723198
- **t-alpha:** 0.000793
- **Chemical similarity (Tanimoto Index) (%):** 98.8
- **1D identity (%) [PDB]:** 2.04
- **1D identity (%) [Gaps excluded][PDB]:** 67.39
- **1D identity - Alignment Gaps [PDB]:** 1477
- **1D aligned content [PDB] (<aminoacid>:%):** {'E': 9.68, 'K': 9.68, 'Y': 6.45, 'T': 9.68, 'L': 22.58, 'H': 3.23, 'P': 6.45, 'V': 9.68, 'Q': 3.23, 'N': 6.45, 'I': 3.23, 'G': 3.23, 'C': 3.23, 'D': 3.23}
- **2D identity (%) [PDB]:** 44.28
- **2D identity (%) [Gaps excluded][PDB]:** 87.31
- **2D identity - Alignment Gaps [PDB]:** 513
- **2D aligned content [PDB] (<2D-fold>:%):** {'T': 23.21, '.': 21.04, 'H': 36.01, 'E': 19.52, 'B': 0.22}
- **3D similarity (TM-Score) (%) [PDB]:** 22.88

- **Gene name:** ACSBG1
- **Entrez ID:** 515577
- **RefSeq ID:** NM\_015162
- **Transcript sequence length:** 6215
- **5-UTR|CDS|3-UTR identity (%):** 13.62 | 41.35 | 14.66
- **5-UTR|CDS|3-UTR identity (%) [Gaps excluded]:** 69.84 | 73.97 | 75.01
- **5-UTR|CDS|3-UTR identity [Alignment Gaps]:** 260 | 1346 | 13951
- **5-UTR aligned content (<base>:%):** {'C': 31.82, 'T': 22.73, 'G': 31.82, 'A': 13.64}
- **CDS aligned content (<base>:%):** {'A': 26.7, 'T': 20.6, 'G': 26.78, 'C': 25.91}
- **3-UTR aligned content (<base>:%):** {'T': 31.77, 'C': 16.87, 'A': 33.35, 'G': 18.01}

**Uniprot Description:**  
  
 Catalyzes the conversion of fatty acids such as long-chain and very long-chain fatty acids to their active form acyl-CoAs for both synthesis of cellular lipids, and degradation via beta-oxidation (PubMed:10954726, PubMed:12975357, PubMed:24269233). Can activate diverse saturated, monosaturated and polyunsaturated fatty acids (PubMed:10954726). N/A   
  
 **Gene Ontology Information:**

Molecular Function

- ATP binding
- long-chain fatty acid-CoA ligase activity

Location

- cytoplasm
- cytoplasmic vesicle
- endoplasmic reticulum
- plasma membrane

Biological process

- long-chain fatty acid biosynthetic process

---

31

- **Protein name:** Protein mono-ADP-ribosyltransferase PARP15
- **Organism:** Homo sapiens
- **Uniprot Accession Number:** Q460N3
- **Protein sequence length:** 678 aa
- **1D identity (%):** 12.21
- **1D identity (%) [Gaps excluded]:** 22.12
- **1D identity - Alignment Gaps:** 444
- **1D aligned content (<aminoacid>:%):** {'P': 10.74, 'L': 10.74, 'A': 7.44, 'G': 9.09, 'D': 4.96, 'S': 3.31, 'R': 8.26, 'E': 3.31, 'K': 7.44, 'I': 2.48, 'V': 6.61, 'F': 7.44, 'N': 2.48, 'Y': 1.65, 'Q': 6.61, 'M': 0.83, 'T': 4.96, 'C': 0.83, 'H': 0.83}
- **Common reported functions (%):** 0.0
- **Common reported locations (%):** 11.11
- **Common reported processes (%):** 0.0

- **AF ID:** Q460N3
- **Chain:** A
- **Protein length:** 678 aa
- **Resolution:** N/A
- **b-phipsi:** 0.001862
- **w-rdist:** 0.217791
- **t-alpha:** 0.116482
- **Chemical similarity (Tanimoto Index) (%):** 96.34
- **1D identity (%) [PDB]:** 1.67
- **1D identity (%) [Gaps excluded][PDB]:** 86.21
- **1D identity - Alignment Gaps [PDB]:** 1465
- **1D aligned content [PDB] (<aminoacid>:%):** {'M': 8.0, 'A': 12.0, 'V': 12.0, 'T': 12.0, 'G': 16.0, 'L': 8.0, 'P': 12.0, 'Q': 4.0, 'R': 8.0, 'S': 4.0, 'H': 4.0}
- **2D identity (%) [PDB]:** 42.63
- **2D identity (%) [Gaps excluded][PDB]:** 87.75
- **2D identity - Alignment Gaps [PDB]:** 527
- **2D aligned content [PDB] (<2D-fold>:%):** {'.': 24.26, 'T': 24.71, 'E': 21.74, 'H': 29.29}
- **3D similarity (TM-Score) (%) [PDB]:** 23.2

- **Gene name:** PARP15
- **Entrez ID:** 165631
- **RefSeq ID:** NM\_152615
- **Transcript sequence length:** 4377
- **5-UTR|CDS|3-UTR identity (%):** 12.19 | 31.73 | 10.6
- **5-UTR|CDS|3-UTR identity (%) [Gaps excluded]:** 78.57 | 73.71 | 75.12
- **5-UTR|CDS|3-UTR identity [Alignment Gaps]:** 305 | 1560 | 14830
- **5-UTR aligned content (<base>:%):** {'T': 40.91, 'C': 29.55, 'G': 20.45, 'A': 9.09}
- **CDS aligned content (<base>:%):** {'T': 25.43, 'C': 19.79, 'A': 35.33, 'G': 19.45}
- **3-UTR aligned content (<base>:%):** {'A': 29.51, 'T': 39.51, 'C': 15.63, 'G': 15.36}

**Uniprot Description:**  
  
 Mono-ADP-ribosyltransferase that mediates mono-ADP-ribosylation of target proteins (PubMed:16061477, PubMed:25043379, PubMed:25635049). Acts as a negative regulator of transcription (PubMed:16061477). N/A   
  
 **Gene Ontology Information:**

Molecular Function

- NAD+ ADP-ribosyltransferase activity
- NAD+ binding
- protein ADP-ribosylase activity
- nucleotidyltransferase activity
- transcription corepressor activity

Location

- cytoplasm
- nucleus

Biological process

- negative regulation of gene expression
- negative regulation of transcription by RNA polymerase II
- protein poly-ADP-ribosylation

---

32

- **Protein name:** Heparan-sulfate 6-O-sulfotransferase 3
- **Organism:** Homo sapiens
- **Uniprot Accession Number:** Q8IZP7
- **Protein sequence length:** 471 aa
- **1D identity (%):** 8.27
- **1D identity (%) [Gaps excluded]:** 23.08
- **1D identity - Alignment Gaps:** 629
- **1D aligned content (<aminoacid>:%):** {'E': 7.41, 'L': 6.17, 'P': 7.41, 'C': 4.94, 'G': 11.11, 'V': 7.41, 'R': 12.35, 'A': 1.23, 'D': 6.17, 'F': 7.41, 'T': 6.17, 'H': 2.47, 'Q': 7.41, 'W': 1.23, 'K': 1.23, 'S': 1.23, 'N': 3.7, 'I': 3.7, 'Y': 1.23}
- **Common reported functions (%):** 0.0
- **Common reported locations (%):** 11.11
- **Common reported processes (%):** 0.0

- **AF ID:** Q8IZP7
- **Chain:** A
- **Protein length:** 471 aa
- **Resolution:** N/A
- **b-phipsi:** 0.003892
- **w-rdist:** 0.661693
- **t-alpha:** 0.001587
- **Chemical similarity (Tanimoto Index) (%):** 95.86
- **1D identity (%) [PDB]:** 1.63
- **1D identity (%) [Gaps excluded][PDB]:** 84.0
- **1D identity - Alignment Gaps [PDB]:** 1266
- **1D aligned content [PDB] (<aminoacid>:%):** {'M': 9.52, 'V': 9.52, 'P': 14.29, 'R': 9.52, 'G': 4.76, 'Y': 4.76, 'T': 4.76, 'L': 9.52, 'A': 4.76, 'N': 4.76, 'Q': 4.76, 'E': 4.76, 'I': 4.76, 'K': 4.76, 'D': 4.76}
- **2D identity (%) [PDB]:** 37.88
- **2D identity (%) [Gaps excluded][PDB]:** 89.29
- **2D identity - Alignment Gaps [PDB]:** 532
- **2D aligned content [PDB] (<2D-fold>:%):** {'.': 20.86, 'H': 40.29, 'T': 33.43, 'E': 4.57, 'B': 0.86}
- **3D similarity (TM-Score) (%) [PDB]:** 17.69

- **Gene name:** HS6ST3
- **Entrez ID:** 266722
- **RefSeq ID:** NM\_153456
- **Transcript sequence length:** 8536
- **5-UTR|CDS|3-UTR identity (%):** 33.73 | 31.02 | 22.5
- **5-UTR|CDS|3-UTR identity (%) [Gaps excluded]:** 81.33 | 72.54 | 75.33
- **5-UTR|CDS|3-UTR identity [Alignment Gaps]:** 446 | 1603 | 12483
- **5-UTR aligned content (<base>:%):** {'A': 7.78, 'G': 38.91, 'C': 43.58, 'T': 9.73}
- **CDS aligned content (<base>:%):** {'T': 18.87, 'G': 28.54, 'A': 25.89, 'C': 26.7}
- **3-UTR aligned content (<base>:%):** {'C': 15.55, 'T': 33.65, 'G': 18.3, 'A': 32.5}

**Uniprot Description:**  
  
 6-O-sulfation enzyme which catalyzes the transfer of sulfate from 3'-phosphoadenosine 5'-phosphosulfate (PAPS) to position 6 of the N-sulfoglucosamine residue (GlcNS) of heparan sulfate. N/A   
  
 **Gene Ontology Information:**

Molecular Function

- heparan sulfate 6-O-sulfotransferase activity

Location

- Golgi apparatus
- membrane

Biological process

- heparan sulfate proteoglycan biosynthetic process, enzymatic modification

---

33

- **Protein name:** Protein odd-skipped-related 1
- **Organism:** Homo sapiens
- **Uniprot Accession Number:** Q8TAX0
- **Protein sequence length:** 266 aa
- **1D identity (%):** 5.97
- **1D identity (%) [Gaps excluded]:** 26.96
- **1D identity - Alignment Gaps:** 718
- **1D aligned content (<aminoacid>:%):** {'G': 5.45, 'K': 7.27, 'P': 9.09, 'V': 12.73, 'I': 1.82, 'L': 9.09, 'T': 12.73, 'H': 9.09, 'A': 5.45, 'S': 3.64, 'E': 9.09, 'D': 1.82, 'F': 3.64, 'R': 3.64, 'Y': 1.82, 'C': 1.82, 'Q': 1.82}
- **Common reported functions (%):** 14.29
- **Common reported locations (%):** 11.11
- **Common reported processes (%):** 11.11

- **AF ID:** Q8TAX0
- **Chain:** A
- **Protein length:** 266 aa
- **Resolution:** N/A
- **b-phipsi:** 0.005411
- **w-rdist:** 0.574098
- **t-alpha:** 0.001587
- **Chemical similarity (Tanimoto Index) (%):** 99.02
- **1D identity (%) [PDB]:** 3.02
- **1D identity (%) [Gaps excluded][PDB]:** 62.75
- **1D identity - Alignment Gaps [PDB]:** 1009
- **1D aligned content [PDB] (<aminoacid>:%):** {'G': 18.75, 'T': 6.25, 'L': 15.62, 'P': 12.5, 'N': 3.12, 'H': 6.25, 'A': 3.12, 'V': 3.12, 'R': 6.25, 'S': 9.38, 'F': 6.25, 'E': 6.25, 'Q': 3.12}
- **2D identity (%) [PDB]:** 22.0
- **2D identity (%) [Gaps excluded][PDB]:** 89.09
- **2D identity - Alignment Gaps [PDB]:** 671
- **2D aligned content [PDB] (<2D-fold>:%):** {'.': 38.27, 'T': 29.08, 'H': 28.57, 'E': 4.08}
- **3D similarity (TM-Score) (%) [PDB]:** 13.06

- **Gene name:** OSR1
- **Entrez ID:** 130497
- **RefSeq ID:** N/A
- **Sequence length:** N/A
- **5-UTR|CDS|3-UTR identity (%):** N/A | N/A | N/A
- **5-UTR|CDS|3-UTR identity (%) [Gaps excluded]:** N/A | N/A | N/A
- **5-UTR|CDS|3-UTR identity [Alignment Gaps]:** N/A | N/A | N/A
- **5-UTR aligned content (<base>:%):** N/A
- **CDS aligned content (<base>:%):** N/A
- **3-UTR aligned content (<base>:%):** N/A

**Uniprot Description:**  
  
 Transcription factor that plays a role in the regulation of embryonic heart and urogenital development. N/A   
  
 **Gene Ontology Information:**

Molecular Function

- DNA-binding transcription factor activity, RNA polymerase II-specific
- metal ion binding
- RNA polymerase II transcription regulatory region sequence-specific DNA binding
- sequence-specific double-stranded DNA binding

Location

- cell cortex
- chromatin
- cytosol
- nucleus

Biological process

- cell differentiation
- cell proliferation involved in kidney development
- cellular response to retinoic acid
- chondrocyte differentiation
- embryonic digit morphogenesis
- embryonic forelimb morphogenesis
- embryonic hindlimb morphogenesis
- embryonic skeletal joint development
- embryonic skeletal joint morphogenesis
- embryonic skeletal limb joint morphogenesis
- gonad development
- heart development
- intermediate mesoderm development
- mesangial cell development
- mesonephric duct morphogenesis
- mesonephros development
- metanephric cap mesenchymal cell proliferation involved in metanephros development
- metanephric epithelium development
- metanephric glomerulus vasculature development
- metanephric interstitial fibroblast development
- metanephric mesenchymal cell differentiation
- metanephric mesenchyme development
- metanephric mesenchyme morphogenesis
- metanephric nephron tubule development
- metanephric smooth muscle tissue development
- middle ear morphogenesis
- negative regulation of apoptotic process
- negative regulation of creatine transmembrane transporter activity
- negative regulation of epithelial cell differentiation
- negative regulation of nephron tubule epithelial cell differentiation
- negative regulation of sodium ion transmembrane transporter activity
- negative regulation of transcription by RNA polymerase II
- odontogenesis
- pattern specification involved in metanephros development
- positive regulation of bone mineralization
- positive regulation of epithelial cell proliferation
- positive regulation of gastrulation
- positive regulation of gene expression
- positive regulation of transcription by RNA polymerase II
- posterior mesonephric tubule development
- pronephros development
- regulation of transcription by RNA polymerase II
- renal vesicle progenitor cell differentiation
- roof of mouth development
- sodium ion transmembrane transport
- specification of anterior mesonephric tubule identity
- specification of posterior mesonephric tubule identity
- stem cell differentiation
- ureter urothelium development
- ureteric bud development
- urogenital system development

---

34

- **Protein name:** Calcium/calmodulin-dependent protein kinase type IV
- **Organism:** Homo sapiens
- **Uniprot Accession Number:** Q16566
- **Protein sequence length:** 473 aa
- **1D identity (%):** 9.46
- **1D identity (%) [Gaps excluded]:** 21.84
- **1D identity - Alignment Gaps:** 527
- **1D aligned content (<aminoacid>:%):** {'M': 3.41, 'S': 2.27, 'A': 11.36, 'P': 12.5, 'Y': 3.41, 'G': 12.5, 'L': 9.09, 'F': 2.27, 'V': 6.82, 'E': 7.95, 'K': 7.95, 'D': 2.27, 'T': 4.55, 'R': 4.55, 'H': 2.27, 'W': 1.14, 'N': 2.27, 'I': 1.14, 'Q': 2.27}
- **Common reported functions (%):** 0.0
- **Common reported locations (%):** 22.22
- **Common reported processes (%):** 0.0

- **AF ID:** Q16566
- **Chain:** A
- **Protein length:** 473 aa
- **Resolution:** N/A
- **b-phipsi:** 0.000674
- **w-rdist:** 0.668138
- **t-alpha:** 0.009509
- **Chemical similarity (Tanimoto Index) (%):** 98.2
- **1D identity (%) [PDB]:** 1.71
- **1D identity (%) [Gaps excluded][PDB]:** 75.86
- **1D identity - Alignment Gaps [PDB]:** 1260
- **1D aligned content [PDB] (<aminoacid>:%):** {'L': 9.09, 'V': 9.09, 'D': 9.09, 'K': 9.09, 'E': 4.55, 'H': 9.09, 'S': 18.18, 'A': 13.64, 'Q': 9.09, 'R': 4.55, 'P': 4.55}
- **2D identity (%) [PDB]:** 36.58
- **2D identity (%) [Gaps excluded][PDB]:** 93.01
- **2D identity - Alignment Gaps [PDB]:** 574
- **2D aligned content [PDB] (<2D-fold>:%):** {'.': 28.03, 'T': 21.68, 'E': 10.12, 'H': 39.31, 'G': 0.87}
- **3D similarity (TM-Score) (%) [PDB]:** 14.85

- **Gene name:** CAMK4
- **Entrez ID:** 814
- **RefSeq ID:** NM\_001744
- **Transcript sequence length:** 11942
- **5-UTR|CDS|3-UTR identity (%):** 22.12 | 33.08 | 35.45
- **5-UTR|CDS|3-UTR identity (%) [Gaps excluded]:** 79.35 | 75.97 | 75.96
- **5-UTR|CDS|3-UTR identity [Alignment Gaps]:** 238 | 1575 | 9881
- **5-UTR aligned content (<base>:%):** {'C': 42.47, 'T': 17.81, 'G': 36.99, 'A': 2.74}
- **CDS aligned content (<base>:%):** {'A': 32.18, 'T': 22.54, 'G': 24.7, 'C': 20.59}
- **3-UTR aligned content (<base>:%):** {'A': 34.61, 'C': 15.09, 'G': 16.34, 'T': 33.97}

**Uniprot Description:**  
  
 Calcium/calmodulin-dependent protein kinase that operates in the calcium-triggered CaMKK-CaMK4 signaling cascade and regulates, mainly by phosphorylation, the activity of several transcription activators, such as CREB1, MEF2D, JUN and RORA, which play pivotal roles in immune response, inflammation, and memory consolidation. In the thymus, regulates the CD4(+)/CD8(+) double positive thymocytes selection threshold during T-cell ontogeny. In CD4 memory T-cells, is required to link T-cell antigen receptor (TCR) signaling to the production of IL2, IFNG and IL4 (through the regulation of CREB and MEF2). Regulates the differentiation and survival phases of osteoclasts and dendritic cells (DCs). Mediates DCs survival by linking TLR4 and the regulation of temporal expression of BCL2. Phosphorylates the transcription activator CREB1 on 'Ser-133' in hippocampal neuron nuclei and contribute to memory consolidation and long term potentiation (LTP) in the hippocampus. Can activate the MAP kinases MAPK1/ERK2, MAPK8/JNK1 and MAPK14/p38 and stimulate transcription through the phosphorylation of ELK1 and ATF2. Can also phosphorylate in vitro CREBBP, PRM2, MEF2A and STMN1/OP18.   
  
Monomer (By similarity). Interacts with protein phosphatase 2A (PPP2CA/PPP2CB); the interaction is mutually exclusive with binding to Ca(2+)/calmodulin.   
  
 **Gene Ontology Information:**

Molecular Function

- ATP binding
- calcium-dependent protein serine/threonine kinase activity
- calmodulin binding
- calmodulin-dependent protein kinase activity
- protein serine kinase activity

Location

- cytoplasm
- extracellular exosome
- fibrillar center
- nucleoplasm
- nucleus

Biological process

- adaptive immune response
- inflammatory response
- intracellular signal transduction
- long-term memory
- myeloid dendritic cell differentiation
- peptidyl-serine phosphorylation
- positive regulation of transcription, DNA-templated
- protein autophosphorylation
- protein phosphorylation
- regulation of osteoclast differentiation
- regulation of T cell differentiation in thymus
- signal transduction

---

35

- **Protein name:** Dihydropyrimidinase-related protein 3
- **Organism:** Homo sapiens
- **Uniprot Accession Number:** Q14195
- **Protein sequence length:** 570 aa
- **1D identity (%):** 11.74
- **1D identity (%) [Gaps excluded]:** 24.84
- **1D identity - Alignment Gaps:** 512
- **1D aligned content (<aminoacid>:%):** {'G': 14.04, 'I': 6.14, 'T': 7.02, 'K': 4.39, 'R': 4.39, 'D': 7.02, 'Q': 3.51, 'V': 6.14, 'M': 0.88, 'H': 3.51, 'F': 4.39, 'A': 8.77, 'E': 0.88, 'C': 2.63, 'Y': 3.51, 'S': 7.02, 'L': 7.02, 'P': 6.14, 'N': 2.63}
- **Common reported functions (%):** 0.0
- **Common reported locations (%):** 11.11
- **Common reported processes (%):** 0.0

- **AF ID:** Q14195
- **Chain:** A
- **Protein length:** 570 aa
- **Resolution:** N/A
- **b-phipsi:** 0.000242
- **w-rdist:** 0.784709
- **t-alpha:** 0.03962
- **Chemical similarity (Tanimoto Index) (%):** 99.55
- **1D identity (%) [PDB]:** 4.67
- **1D identity (%) [Gaps excluded][PDB]:** 70.45
- **1D identity - Alignment Gaps [PDB]:** 1239
- **1D aligned content [PDB] (<aminoacid>:%):** {'G': 9.68, 'R': 8.06, 'N': 3.23, 'I': 6.45, 'P': 3.23, 'T': 8.06, 'V': 4.84, 'D': 9.68, 'H': 4.84, 'Y': 6.45, 'E': 3.23, 'L': 4.84, 'S': 9.68, 'A': 11.29, 'W': 1.61, 'F': 1.61, 'Q': 1.61, 'K': 1.61}
- **2D identity (%) [PDB]:** 33.59
- **2D identity (%) [Gaps excluded][PDB]:** 92.82
- **2D identity - Alignment Gaps [PDB]:** 663
- **2D aligned content [PDB] (<2D-fold>:%):** {'.': 24.07, 'E': 20.34, 'T': 24.07, 'H': 30.66, 'G': 0.86}
- **3D similarity (TM-Score) (%) [PDB]:** 16.91

- **Gene name:** DPYSL3
- **Entrez ID:** 1809
- **RefSeq ID:** NM\_001387
- **Transcript sequence length:** 4640498
- **5-UTR|CDS|3-UTR identity (%):** 16.4 | 38.71 | N/A
- **5-UTR|CDS|3-UTR identity (%) [Gaps excluded]:** 86.79 | 79.08 | N/A
- **5-UTR|CDS|3-UTR identity [Alignment Gaps]:** 1365 | 2473 | N/A
- **5-UTR aligned content (<base>:%):** {'A': 6.52, 'G': 34.06, 'T': 17.39, 'C': 42.03}
- **CDS aligned content (<base>:%):** {'A': 31.04, 'T': 25.07, 'G': 22.03, 'C': 21.87}
- **3-UTR aligned content (<base>:%):** N/A

**Uniprot Description:**  
  
 Necessary for signaling by class 3 semaphorins and subsequent remodeling of the cytoskeleton. Plays a role in axon guidance, neuronal growth cone collapse and cell migration (By similarity).   
  
Homotetramer, and heterotetramer with CRMP1, DPYSL2, DPYSL4 or DPYSL5 (By similarity). Interacts with synaptic vesicle protein 2 and SH3A domain of intersectin (By similarity). Interacts with FLNA (PubMed:25358863).   
  
 **Gene Ontology Information:**

Molecular Function

- chondroitin sulfate binding
- filamin binding
- hydrolase activity, acting on carbon-nitrogen (but not peptide) bonds, in cyclic amides
- identical protein binding
- SH3 domain binding

Location

- cell body
- cytosol
- exocytic vesicle
- extracellular space
- filamentous actin
- growth cone
- lamellipodium
- synapse

Biological process

- actin crosslink formation
- actin filament bundle assembly
- cellular response to cytokine stimulus
- negative regulation of cell migration
- negative regulation of neuron projection development
- neuron development
- positive regulation of filopodium assembly
- positive regulation of neuron projection development
- response to axon injury

---

36

- **Protein name:** Insulin-like growth factor 2 mRNA-binding protein 1
- **Organism:** Homo sapiens
- **Uniprot Accession Number:** Q9NZI8
- **Protein sequence length:** 577 aa
- **1D identity (%):** 11.9
- **1D identity (%) [Gaps excluded]:** 22.02
- **1D identity - Alignment Gaps:** 429
- **1D aligned content (<aminoacid>:%):** {'N': 2.7, 'V': 10.81, 'T': 3.6, 'F': 1.8, 'Y': 4.5, 'G': 7.21, 'S': 2.7, 'P': 9.91, 'L': 6.31, 'K': 6.31, 'R': 9.01, 'I': 7.21, 'W': 0.9, 'E': 7.21, 'H': 4.5, 'A': 6.31, 'D': 2.7, 'Q': 6.31}
- **Common reported functions (%):** 0.0
- **Common reported locations (%):** 33.33
- **Common reported processes (%):** 0.0

- **AF ID:** Q9NZI8
- **Chain:** A
- **Protein length:** 577 aa
- **Resolution:** N/A
- **b-phipsi:** 0.002139
- **w-rdist:** 0.240254
- **t-alpha:** 0.10935
- **Chemical similarity (Tanimoto Index) (%):** 99.4
- **1D identity (%) [PDB]:** 1.72
- **1D identity (%) [Gaps excluded][PDB]:** 82.76
- **1D identity - Alignment Gaps [PDB]:** 1364
- **1D aligned content [PDB] (<aminoacid>:%):** {'M': 4.17, 'K': 16.67, 'P': 8.33, 'L': 12.5, 'A': 4.17, 'N': 4.17, 'C': 4.17, 'Q': 4.17, 'V': 12.5, 'I': 12.5, 'D': 8.33, 'Y': 4.17, 'E': 4.17}
- **2D identity (%) [PDB]:** 41.62
- **2D identity (%) [Gaps excluded][PDB]:** 90.2
- **2D identity - Alignment Gaps [PDB]:** 524
- **2D aligned content [PDB] (<2D-fold>:%):** {'.': 16.54, 'E': 25.93, 'H': 43.21, 'T': 13.58, 'G': 0.74}
- **3D similarity (TM-Score) (%) [PDB]:** 17.95

- **Gene name:** IGF2BP1
- **Entrez ID:** 395953
- **RefSeq ID:** NM\_006546
- **Transcript sequence length:** 8796
- **5-UTR|CDS|3-UTR identity (%):** 44.5 | 37.77 | 23.62
- **5-UTR|CDS|3-UTR identity (%) [Gaps excluded]:** 78.54 | 75.14 | 74.49
- **5-UTR|CDS|3-UTR identity [Alignment Gaps]:** 189 | 1429 | 12160
- **5-UTR aligned content (<base>:%):** {'C': 45.88, 'G': 34.54, 'A': 6.7, 'T': 12.89}
- **CDS aligned content (<base>:%):** {'A': 29.4, 'T': 18.8, 'G': 26.27, 'C': 25.53}
- **3-UTR aligned content (<base>:%):** {'C': 19.42, 'A': 28.6, 'G': 20.94, 'T': 31.04}

**Uniprot Description:**  
  
 RNA-binding factor that recruits target transcripts to cytoplasmic protein-RNA complexes (mRNPs). This transcript 'caging' into mRNPs allows mRNA transport and transient storage. It also modulates the rate and location at which target transcripts encounter the translational apparatus and shields them from endonuclease attacks or microRNA-mediated degradation. Plays a direct role in the transport and translation of transcripts required for axonal regeneration in adult sensory neurons (By similarity). Regulates localized beta-actin/ACTB mRNA translation, a crucial process for cell polarity, cell migration and neurite outgrowth. Co-transcriptionally associates with the ACTB mRNA in the nucleus. This binding involves a conserved 54-nucleotide element in the ACTB mRNA 3'-UTR, known as the 'zipcode'. The RNP thus formed is exported to the cytoplasm, binds to a motor protein and is transported along the cytoskeleton to the cell periphery. During transport, prevents ACTB mRNA from being translated into protein. When the RNP complex reaches its destination near the plasma membrane, IGF2BP1 is phosphorylated. This releases the mRNA, allowing ribosomal 40S and 60S subunits to assemble and initiate ACTB protein synthesis. Monomeric ACTB then assembles into the subcortical actin cytoskeleton (By similarity). During neuronal development, key regulator of neurite outgrowth, growth cone guidance and neuronal cell migration, presumably through the spatiotemporal fine tuning of protein synthesis, such as that of ACTB (By similarity). May regulate mRNA transport to activated synapses (By similarity). Binds to and stabilizes ABCB1/MDR-1 mRNA (By similarity). During interstinal wound repair, interacts with and stabilizes PTGS2 transcript. PTGS2 mRNA stabilization may be crucial for colonic mucosal wound healing (By similarity). Binds to the 3'-UTR of IGF2 mRNA by a mechanism of cooperative and sequential dimerization and regulates IGF2 mRNA subcellular localization and translation. Binds to MYC mRNA, in the coding region instability determinant (CRD) of the open reading frame (ORF), hence prevents MYC cleavage by endonucleases and possibly microRNA targeting to MYC-CRD. Binds to the 3'-UTR of CD44 mRNA and stabilizes it, hence promotes cell adhesion and invadopodia formation in cancer cells. Binds to the oncofetal H19 transcript and to the neuron-specific TAU mRNA and regulates their localizations. Binds to and stabilizes BTRC/FBW1A mRNA. Binds to the adenine-rich autoregulatory sequence (ARS) located in PABPC1 mRNA and represses its translation. PABPC1 mRNA-binding is stimulated by PABPC1 protein. Prevents BTRC/FBW1A mRNA degradation by disrupting microRNA-dependent interaction with AGO2. Promotes the directed movement of tumor-derived cells by fine-tuning intracellular signaling networks. Binds to MAPK4 3'-UTR and inhibits its translation. Interacts with PTEN transcript open reading frame (ORF) and prevents mRNA decay. This combined action on MAPK4 (down-regulation) and PTEN (up-regulation) antagonizes HSPB1 phosphorylation, consequently it prevents G-actin sequestration by phosphorylated HSPB1, allowing F-actin polymerization. Hence enhances the velocity of cell migration and stimulates directed cell migration by PTEN-modulated polarization. Interacts with Hepatitis C virus (HCV) 5'-UTR and 3'-UTR and specifically enhances translation at the HCV IRES, but not 5'-cap-dependent translation, possibly by recruiting eIF3. Interacts with HIV-1 GAG protein and blocks the formation of infectious HIV-1 particles. Reduces HIV-1 assembly by inhibiting viral RNA packaging, as well as assembly and processing of GAG protein on cellular membranes. During cellular stress, such as oxidative stress or heat shock, stabilizes target mRNAs that are recruited to stress granules, including CD44, IGF2, MAPK4, MYC, PTEN, RAPGEF2 and RPS6KA5 transcripts.   
  
Can form homodimers and heterodimers with IGF2BP1 and IGF2BP3. Component of the coding region determinant (CRD)-mediated complex, composed of DHX9, HNRNPU, IGF2BP1, SYNCRIP and YBX1. During HCV infection, identified in a HCV IRES-mediated translation complex, at least composed of EIF3C, IGF2BP1, RPS3 and HCV RNA-replicon. Interacts (via the KH domains) with HIV-1 GAG (via the second zinc finger motif of NC). Associates (via the RRM domains and KH domains) with HIV-1 particles. Identified in a mRNP complex, composed of at least DHX9, DDX3X, ELAVL1, HNRNPU, IGF2BP1, ILF3, PABPC1, PCBP2, PTBP2, STAU1, STAU2, SYNCRIP and YBX1. Identified in a IGF2BP1-dependent mRNP granule complex containing untranslated mRNAs. Interacts with DHX9, ELAVL2, HNRNPA2B1, HNRNPC, HNRNPH1, HNRNPU, IGF2BP2, ILF2, and YBX1. Interacts with FMR1. Component of a multisubunit autoregulatory RNP complex (ARC), at least composed of IGF2BP1, PABPC1 and CSDE1/UNR. Directly interacts with PABPC1. Component of a TAU mRNP complex, at least composed of IGF2BP1, ELAVL4 and G3BP. Interacts with ELAVL4 in an RNA-dependent manner. Associates with microtubules and polysomes. Interacts with AGO1 and AGO2.   
  
 **Gene Ontology Information:**

Molecular Function

- mRNA 3'-UTR binding
- mRNA binding
- N6-methyladenosine-containing RNA binding

Location

- CRD-mediated mRNA stability complex
- cytoplasm
- cytoplasmic stress granule
- cytosol
- filopodium
- growth cone
- lamellipodium
- nucleus
- P-body
- perinuclear region of cytoplasm

Biological process

- CRD-mediated mRNA stabilization
- mRNA transport
- negative regulation of translation
- nervous system development
- positive regulation of neuron projection development
- regulation of gene expression

---

37

- **Protein name:** Nuclear protein localization protein 4 homolog
- **Organism:** Homo sapiens
- **Uniprot Accession Number:** Q8TAT6
- **Protein sequence length:** 608 aa
- **1D identity (%):** 12.59
- **1D identity (%) [Gaps excluded]:** 22.75
- **1D identity - Alignment Gaps:** 422
- **1D aligned content (<aminoacid>:%):** {'E': 5.88, 'S': 3.36, 'V': 7.56, 'R': 9.24, 'F': 3.36, 'G': 11.76, 'L': 6.72, 'I': 3.36, 'A': 5.88, 'P': 10.08, 'D': 5.88, 'K': 6.72, 'Y': 2.52, 'H': 1.68, 'C': 4.2, 'N': 1.68, 'T': 4.2, 'Q': 5.04, 'M': 0.84}
- **Common reported functions (%):** 14.29
- **Common reported locations (%):** 22.22
- **Common reported processes (%):** 0.0

- **AF ID:** Q8TAT6
- **Chain:** A
- **Protein length:** 608 aa
- **Resolution:** N/A
- **b-phipsi:** 0.00397
- **w-rdist:** 0.508245
- **t-alpha:** 0.003962
- **Chemical similarity (Tanimoto Index) (%):** 98.57
- **1D identity (%) [PDB]:** 2.13
- **1D identity (%) [Gaps excluded][PDB]:** 71.43
- **1D identity - Alignment Gaps [PDB]:** 1369
- **1D aligned content [PDB] (<aminoacid>:%):** {'M': 3.33, 'V': 20.0, 'Q': 3.33, 'F': 6.67, 'K': 6.67, 'G': 10.0, 'D': 6.67, 'R': 3.33, 'S': 3.33, 'L': 13.33, 'Y': 3.33, 'A': 3.33, 'P': 10.0, 'T': 6.67}
- **2D identity (%) [PDB]:** 36.9
- **2D identity (%) [Gaps excluded][PDB]:** 86.64
- **2D identity - Alignment Gaps [PDB]:** 585
- **2D aligned content [PDB] (<2D-fold>:%):** {'.': 20.48, 'E': 21.28, 'T': 29.26, 'H': 28.99}
- **3D similarity (TM-Score) (%) [PDB]:** 20.25

- **Gene name:** NPLOC4
- **Entrez ID:** 55666
- **RefSeq ID:** N/A
- **Sequence length:** N/A
- **5-UTR|CDS|3-UTR identity (%):** N/A | N/A | N/A
- **5-UTR|CDS|3-UTR identity (%) [Gaps excluded]:** N/A | N/A | N/A
- **5-UTR|CDS|3-UTR identity [Alignment Gaps]:** N/A | N/A | N/A
- **5-UTR aligned content (<base>:%):** N/A
- **CDS aligned content (<base>:%):** N/A
- **3-UTR aligned content (<base>:%):** N/A

**Uniprot Description:**  
  
 The ternary complex containing UFD1, VCP and NPLOC4 binds ubiquitinated proteins and is necessary for the export of misfolded proteins from the ER to the cytoplasm, where they are degraded by the proteasome. The NPLOC4-UFD1-VCP complex regulates spindle disassembly at the end of mitosis and is necessary for the formation of a closed nuclear envelope (By similarity). Acts as a negative regulator of type I interferon production via the complex formed with VCP and UFD1, which binds to RIGI and recruits RNF125 to promote ubiquitination and degradation of RIGI (PubMed:26471729).   
  
Heterodimer with UFD1 (PubMed:11574150, PubMed:26471729). The heterodimer binds ubiquitinated proteins (By similarity). The heterodimer binds to VCP and inhibits Golgi membrane fusion (PubMed:11574150, PubMed:26471729). Interacts with ZFAND2B; probably through VCP (PubMed:24160817).   
  
 **Gene Ontology Information:**

Molecular Function

- ATPase binding
- K48-linked polyubiquitin modification-dependent protein binding
- K63-linked polyubiquitin modification-dependent protein binding
- metal ion binding
- protein-containing complex binding
- ubiquitin binding
- ubiquitin protein ligase binding

Location

- cytosol
- endoplasmic reticulum
- nuclear outer membrane-endoplasmic reticulum membrane network
- nucleoplasm
- nucleus
- UFD1-NPL4 complex
- VCP-NPL4-UFD1 AAA ATPase complex

Biological process

- Golgi organization
- negative regulation of RIG-I signaling pathway
- negative regulation of type I interferon production
- retrograde protein transport, ER to cytosol
- ubiquitin-dependent ERAD pathway
- ubiquitin-dependent protein catabolic process

---

38

- **Protein name:** cAMP-dependent protein kinase type II-beta regulatory subunit
- **Organism:** Homo sapiens
- **Uniprot Accession Number:** P31323
- **Protein sequence length:** 418 aa
- **1D identity (%):** 9.46
- **1D identity (%) [Gaps excluded]:** 25.29
- **1D identity - Alignment Gaps:** 582
- **1D aligned content (<aminoacid>:%):** {'M': 1.14, 'I': 4.55, 'T': 5.68, 'F': 7.95, 'E': 7.95, 'V': 6.82, 'L': 9.09, 'Q': 5.68, 'P': 7.95, 'G': 12.5, 'R': 6.82, 'A': 6.82, 'C': 2.27, 'Y': 1.14, 'D': 5.68, 'S': 4.55, 'W': 1.14, 'K': 2.27}
- **Common reported functions (%):** 0.0
- **Common reported locations (%):** 22.22
- **Common reported processes (%):** 0.0

- **AF ID:** P31323
- **Chain:** A
- **Protein length:** 418 aa
- **Resolution:** N/A
- **b-phipsi:** 0.000453
- **w-rdist:** 0.772712
- **t-alpha:** 0.01981
- **Chemical similarity (Tanimoto Index) (%):** 99.55
- **1D identity (%) [PDB]:** 2.12
- **1D identity (%) [Gaps excluded][PDB]:** 76.47
- **1D identity - Alignment Gaps [PDB]:** 1195
- **1D aligned content [PDB] (<aminoacid>:%):** {'E': 7.69, 'L': 7.69, 'G': 11.54, 'F': 7.69, 'T': 3.85, 'D': 11.54, 'R': 3.85, 'K': 11.54, 'S': 7.69, 'A': 11.54, 'C': 3.85, 'Q': 3.85, 'V': 3.85, 'P': 3.85}
- **2D identity (%) [PDB]:** 30.14
- **2D identity (%) [Gaps excluded][PDB]:** 87.35
- **2D identity - Alignment Gaps [PDB]:** 615
- **2D aligned content [PDB] (<2D-fold>:%):** {'.': 12.06, 'T': 26.24, 'H': 43.26, 'E': 17.38, 'G': 1.06}
- **3D similarity (TM-Score) (%) [PDB]:** 15.71

- **Gene name:** PRKAR2B
- **Entrez ID:** 5577
- **RefSeq ID:** NM\_002736
- **Transcript sequence length:** 3689
- **5-UTR|CDS|3-UTR identity (%):** 44.55 | 26.81 | 8.55
- **5-UTR|CDS|3-UTR identity (%) [Gaps excluded]:** 75.38 | 74.83 | 76.85
- **5-UTR|CDS|3-UTR identity [Alignment Gaps]:** 135 | 1814 | 15182
- **5-UTR aligned content (<base>:%):** {'C': 45.58, 'G': 46.26, 'A': 4.76, 'T': 3.4}
- **CDS aligned content (<base>:%):** {'A': 32.32, 'T': 21.37, 'G': 27.04, 'C': 19.26}
- **3-UTR aligned content (<base>:%):** {'A': 32.85, 'G': 13.55, 'T': 40.73, 'C': 12.87}

**Uniprot Description:**  
  
 Regulatory subunit of the cAMP-dependent protein kinases involved in cAMP signaling in cells. Type II regulatory chains mediate membrane association by binding to anchoring proteins, including the MAP2 kinase.   
  
The inactive form of the enzyme is composed of two regulatory chains and two catalytic chains. Activation by cAMP produces two active catalytic monomers and a regulatory dimer that binds four cAMP molecules. Interacts with PRKACA and PRKACB (PubMed:33058759). Interacts with the phosphorylated form of PJA2. Forms a complex composed of PRKAR2B, GSK3B and GSKIP through GSKIP interaction; facilitates PKA-induced phosphorylation and regulates GSK3B activity (PubMed:25920809).   
  
 **Gene Ontology Information:**

Molecular Function

- cAMP binding
- cAMP-dependent protein kinase inhibitor activity
- cAMP-dependent protein kinase regulator activity
- protein domain specific binding
- protein kinase A catalytic subunit binding
- ubiquitin protein ligase binding

Location

- cAMP-dependent protein kinase complex
- centrosome
- ciliary base
- cytoplasm
- cytosol
- dendritic shaft
- dendritic spine
- extracellular exosome
- glutamatergic synapse
- neuronal cell body
- perinuclear region of cytoplasm
- plasma membrane

Biological process

- fatty acid metabolic process
- intracellular signal transduction
- learning
- modulation of chemical synaptic transmission
- negative regulation of cAMP-dependent protein kinase activity
- response to antipsychotic drug

---

39

- **Protein name:** Adenosylhomocysteinase 3
- **Organism:** Homo sapiens
- **Uniprot Accession Number:** Q96HN2
- **Protein sequence length:** 611 aa
- **1D identity (%):** 12.88
- **1D identity (%) [Gaps excluded]:** 23.28
- **1D identity - Alignment Gaps:** 423
- **1D aligned content (<aminoacid>:%):** {'S': 3.28, 'V': 9.02, 'E': 5.74, 'P': 13.11, 'T': 6.56, 'A': 7.38, 'G': 9.84, 'K': 6.56, 'Q': 2.46, 'R': 4.92, 'F': 1.64, 'Y': 3.28, 'N': 4.1, 'L': 8.2, 'I': 3.28, 'C': 5.74, 'H': 2.46, 'D': 2.46}
- **Common reported functions (%):** 0.0
- **Common reported locations (%):** 11.11
- **Common reported processes (%):** 0.0

- **AF ID:** Q96HN2
- **Chain:** A
- **Protein length:** 611 aa
- **Resolution:** N/A
- **b-phipsi:** 0.000285
- **w-rdist:** 0.532334
- **t-alpha:** 0.142631
- **Chemical similarity (Tanimoto Index) (%):** 98.28
- **1D identity (%) [PDB]:** 1.62
- **1D identity (%) [Gaps excluded][PDB]:** 67.65
- **1D identity - Alignment Gaps [PDB]:** 1388
- **1D aligned content [PDB] (<aminoacid>:%):** {'M': 4.35, 'V': 13.04, 'P': 8.7, 'R': 8.7, 'G': 13.04, 'Y': 13.04, 'K': 13.04, 'I': 8.7, 'L': 8.7, 'Q': 4.35, 'D': 4.35}
- **2D identity (%) [PDB]:** 34.32
- **2D identity (%) [Gaps excluded][PDB]:** 87.56
- **2D identity - Alignment Gaps [PDB]:** 636
- **2D aligned content [PDB] (<2D-fold>:%):** {'.': 29.81, 'T': 16.99, 'H': 41.23, 'E': 11.98}
- **3D similarity (TM-Score) (%) [PDB]:** 22.24

- **Gene name:** AHCYL2
- **Entrez ID:** 532836
- **RefSeq ID:** NM\_015328
- **Transcript sequence length:** 5049
- **5-UTR|CDS|3-UTR identity (%):** 8.76 | 39.6 | 11.88
- **5-UTR|CDS|3-UTR identity (%) [Gaps excluded]:** 76.32 | 75.53 | 73.76
- **5-UTR|CDS|3-UTR identity [Alignment Gaps]:** 293 | 1379 | 14395
- **5-UTR aligned content (<base>:%):** {'G': 65.52, 'C': 17.24, 'A': 10.34, 'T': 6.9}
- **CDS aligned content (<base>:%):** {'T': 23.08, 'G': 25.52, 'C': 22.47, 'A': 28.92}
- **3-UTR aligned content (<base>:%):** {'G': 16.88, 'C': 16.73, 'T': 36.56, 'A': 29.83}

**Uniprot Description:**  
  
 May regulate the electrogenic sodium/bicarbonate cotransporter SLC4A4 activity and Mg(2+)-sensitivity. On the contrary of its homolog AHCYL1, does not regulate ITPR1 sensitivity to inositol 1,4,5-trisphosphate (PubMed:19220705).   
  
Homotetramer (Probable). Forms heteromultimers with AHCYL1 (via the C-terminal region) (PubMed:19220705). Interacts with ITPR1; with lower affinity than AHCYL1 and maybe via ITPR1 (PubMed:19220705). Interacts with SLC4A4 (By similarity). Interacts with ZCCHC4 (PubMed:31799605).   
  
 **Gene Ontology Information:**

Molecular Function

- hydrolase activity

Location

- cytosol
- endoplasmic reticulum
- neuron projection

Biological process

- one-carbon metabolic process
- S-adenosylmethionine cycle

---

40

- **Protein name:** E3 ubiquitin-protein ligase SMURF2
- **Organism:** Homo sapiens
- **Uniprot Accession Number:** Q9HAU4
- **Protein sequence length:** 748 aa
- **1D identity (%):** 15.08
- **1D identity (%) [Gaps excluded]:** 24.88
- **1D identity - Alignment Gaps:** 394
- **1D aligned content (<aminoacid>:%):** {'P': 10.6, 'G': 9.93, 'K': 5.3, 'L': 13.25, 'V': 8.61, 'D': 3.97, 'Q': 4.64, 'H': 1.99, 'T': 7.28, 'Y': 3.97, 'F': 4.64, 'S': 1.32, 'W': 1.99, 'R': 7.95, 'A': 1.99, 'E': 3.31, 'C': 1.32, 'N': 2.65, 'I': 5.3}
- **Common reported functions (%):** 0.0
- **Common reported locations (%):** 33.33
- **Common reported processes (%):** 0.0

- **AF ID:** Q9HAU4
- **Chain:** A
- **Protein length:** 748 aa
- **Resolution:** N/A
- **b-phipsi:** 0.002037
- **w-rdist:** 0.185394
- **t-alpha:** 0.134707
- **Chemical similarity (Tanimoto Index) (%):** 99.02
- **1D identity (%) [PDB]:** 3.16
- **1D identity (%) [Gaps excluded][PDB]:** 66.67
- **1D identity - Alignment Gaps [PDB]:** 1449
- **1D aligned content [PDB] (<aminoacid>:%):** {'M': 2.08, 'R': 16.67, 'K': 2.08, 'V': 8.33, 'N': 6.25, 'T': 10.42, 'A': 4.17, 'S': 2.08, 'H': 4.17, 'Q': 6.25, 'E': 6.25, 'G': 6.25, 'Y': 6.25, 'L': 12.5, 'P': 2.08, 'C': 2.08, 'I': 2.08}
- **2D identity (%) [PDB]:** 40.75
- **2D identity (%) [Gaps excluded][PDB]:** 90.12
- **2D identity - Alignment Gaps [PDB]:** 601
- **2D aligned content [PDB] (<2D-fold>:%):** {'.': 17.67, 'E': 25.06, 'T': 22.82, 'H': 34.45}
- **3D similarity (TM-Score) (%) [PDB]:** 22.81

- **Gene name:** SMURF2
- **Entrez ID:** 64750
- **RefSeq ID:** NM\_022739
- **Transcript sequence length:** 6240
- **5-UTR|CDS|3-UTR identity (%):** 40.73 | 45.79 | 13.41
- **5-UTR|CDS|3-UTR identity (%) [Gaps excluded]:** 79.84 | 74.67 | 77.12
- **5-UTR|CDS|3-UTR identity [Alignment Gaps]:** 243 | 1158 | 14299
- **5-UTR aligned content (<base>:%):** {'A': 3.96, 'G': 39.11, 'C': 46.53, 'T': 10.4}
- **CDS aligned content (<base>:%):** {'A': 32.9, 'T': 25.97, 'G': 22.1, 'C': 19.04}
- **3-UTR aligned content (<base>:%):** {'C': 14.13, 'A': 33.29, 'T': 34.75, 'G': 17.83}

**Uniprot Description:**  
  
 E3 ubiquitin-protein ligase which accepts ubiquitin from an E2 ubiquitin-conjugating enzyme in the form of a thioester and then directly transfers the ubiquitin to targeted substrates (PubMed:11016919). Interacts with SMAD7 to trigger SMAD7-mediated transforming growth factor beta/TGF-beta receptor ubiquitin-dependent degradation, thereby downregulating TGF-beta signaling (PubMed:11163210, PubMed:12717440). In addition, interaction with SMAD7 activates autocatalytic degradation, which is prevented by interaction with AIMP1 (PubMed:18448069). Also forms a stable complex with TGF-beta receptor-mediated phosphorylated SMAD1, SMAD2 and SMAD3, and targets SMAD1 and SMAD2 for ubiquitination and proteasome-mediated degradation (PubMed:11016919, PubMed:11158580, PubMed:11389444). SMAD2 may recruit substrates, such as SNON, for ubiquitin-dependent degradation (PubMed:11389444). Negatively regulates TGFB1-induced epithelial-mesenchymal transition and myofibroblast differentiation (PubMed:30696809).   
  
Interacts (via WW domains) with SMAD1 (PubMed:11158580). Interacts (via WW domains) with SMAD2 (via PY-motif) (PubMed:11158580, PubMed:11389444). Interacts (via WW domains) with SMAD3 (via PY-motif) (PubMed:11158580, PubMed:11389444). Interacts with SMAD6 (PubMed:11158580). Interacts with SMAD7 (via PY-motif) and TGFBR1; SMAD7 recruits SMURF2 to the TGF-beta receptor and regulates its degradation (PubMed:11163210, PubMed:11158580, PubMed:33673144, PubMed:16061177, PubMed:16641086). Does not interact with SMAD4; SMAD4 lacks a PY-motif (PubMed:11158580). Interacts with AIMP1 (PubMed:18448069). Interacts with SNON (PubMed:11389444). Interacts with STAMBP and RNF11 (PubMed:14562029, PubMed:14755250). May interact with NDFIP1 and NDFIP2; this interaction induces the E3 ubiquitin-protein ligase activity. Interacts with TTC3 (Probable).   
  
 **Gene Ontology Information:**

Molecular Function

- identical protein binding
- SMAD binding
- ubiquitin protein ligase activity
- ubiquitin-protein transferase activity

Location

- cytoplasm
- cytosol
- membrane raft
- nuclear speck
- nucleoplasm
- nucleus
- plasma membrane
- ubiquitin ligase complex

Biological process

- negative regulation of BMP signaling pathway
- negative regulation of transcription, DNA-templated
- negative regulation of transcription by RNA polymerase II
- negative regulation of transforming growth factor beta receptor signaling pathway
- positive regulation of canonical Wnt signaling pathway
- positive regulation of trophoblast cell migration
- proteasome-mediated ubiquitin-dependent protein catabolic process
- protein ubiquitination
- regulation of transforming growth factor beta receptor signaling pathway
- ubiquitin-dependent protein catabolic process
- ubiquitin-dependent SMAD protein catabolic process
- Wnt signaling pathway, planar cell polarity pathway

---

41

- **Protein name:** Protein phosphatase 1H
- **Organism:** Homo sapiens
- **Uniprot Accession Number:** Q9ULR3
- **Protein sequence length:** 514 aa
- **1D identity (%):** 11.06
- **1D identity (%) [Gaps excluded]:** 21.28
- **1D identity - Alignment Gaps:** 434
- **1D aligned content (<aminoacid>:%):** {'M': 2.0, 'K': 7.0, 'G': 11.0, 'D': 4.0, 'L': 10.0, 'P': 10.0, 'S': 6.0, 'V': 8.0, 'H': 3.0, 'R': 6.0, 'E': 8.0, 'T': 7.0, 'W': 1.0, 'A': 4.0, 'Q': 2.0, 'I': 5.0, 'F': 2.0, 'C': 2.0, 'N': 2.0}
- **Common reported functions (%):** 0.0
- **Common reported locations (%):** 22.22
- **Common reported processes (%):** 0.0

- **AF ID:** Q9ULR3
- **Chain:** A
- **Protein length:** 514 aa
- **Resolution:** N/A
- **b-phipsi:** 0.000585
- **w-rdist:** 0.746822
- **t-alpha:** 0.017433
- **Chemical similarity (Tanimoto Index) (%):** 99.17
- **1D identity (%) [PDB]:** 2.52
- **1D identity (%) [Gaps excluded][PDB]:** 70.21
- **1D identity - Alignment Gaps [PDB]:** 1265
- **1D aligned content [PDB] (<aminoacid>:%):** {'F': 3.03, 'L': 6.06, 'G': 6.06, 'A': 18.18, 'D': 9.09, 'V': 12.12, 'T': 3.03, 'P': 6.06, 'S': 12.12, 'M': 3.03, 'R': 9.09, 'Y': 3.03, 'Q': 6.06, 'I': 3.03}
- **2D identity (%) [PDB]:** 36.43
- **2D identity (%) [Gaps excluded][PDB]:** 90.51
- **2D identity - Alignment Gaps [PDB]:** 579
- **2D aligned content [PDB] (<2D-fold>:%):** {'H': 27.2, 'T': 28.61, '.': 22.95, 'E': 20.11, 'B': 0.28, 'G': 0.85}
- **3D similarity (TM-Score) (%) [PDB]:** 19.39

- **Gene name:** PPM1H
- **Entrez ID:** 57460
- **RefSeq ID:** NM\_020700
- **Transcript sequence length:** 6454
- **5-UTR|CDS|3-UTR identity (%):** 49.66 | 32.94 | 16.49
- **5-UTR|CDS|3-UTR identity (%) [Gaps excluded]:** 76.82 | 74.04 | 74.71
- **5-UTR|CDS|3-UTR identity [Alignment Gaps]:** 158 | 1586 | 13564
- **5-UTR aligned content (<base>:%):** {'A': 6.76, 'T': 11.26, 'C': 43.69, 'G': 38.29}
- **CDS aligned content (<base>:%):** {'A': 27.52, 'T': 20.83, 'C': 24.23, 'G': 27.42}
- **3-UTR aligned content (<base>:%):** {'A': 28.67, 'T': 35.81, 'C': 16.79, 'G': 18.74}

**Uniprot Description:**  
  
 Dephosphorylates CDKN1B at 'Thr-187', thus removing a signal for proteasomal degradation. N/A   
  
 **Gene Ontology Information:**

Molecular Function

- [pyruvate dehydrogenase (lipoamide)] phosphatase activity
- identical protein binding
- myosin phosphatase activity
- phosphoprotein phosphatase activity

Location

- cytoplasm
- glutamatergic synapse
- mitochondrion
- nucleoplasm
- nucleus

Biological process

- positive regulation of pyruvate dehydrogenase activity
- protein dephosphorylation

---

42

- **Protein name:** RasGAP-activating-like protein 1
- **Organism:** Homo sapiens
- **Uniprot Accession Number:** O95294
- **Protein sequence length:** 804 aa
- **1D identity (%):** 14.39
- **1D identity (%) [Gaps excluded]:** 26.9
- **1D identity - Alignment Gaps:** 504
- **1D aligned content (<aminoacid>:%):** {'V': 10.26, 'E': 8.33, 'P': 9.62, 'K': 4.49, 'C': 2.56, 'G': 11.54, 'S': 3.21, 'L': 14.74, 'T': 5.77, 'A': 4.49, 'D': 6.41, 'R': 7.69, 'W': 0.64, 'Y': 0.64, 'Q': 5.77, 'F': 2.56, 'M': 0.64, 'N': 0.64}
- **Common reported functions (%):** 14.29
- **Common reported locations (%):** 11.11
- **Common reported processes (%):** 0.0

- **AF ID:** O95294
- **Chain:** A
- **Protein length:** 804 aa
- **Resolution:** N/A
- **b-phipsi:** 0.007121
- **w-rdist:** 0.308366
- **t-alpha:** 0.0096
- **Chemical similarity (Tanimoto Index) (%):** 98.88
- **1D identity (%) [PDB]:** 2.66
- **1D identity (%) [Gaps excluded][PDB]:** 59.15
- **1D identity - Alignment Gaps [PDB]:** 1507
- **1D aligned content [PDB] (<aminoacid>:%):** {'M': 7.14, 'A': 11.9, 'V': 4.76, 'G': 11.9, 'R': 11.9, 'L': 9.52, 'P': 4.76, 'S': 2.38, 'W': 2.38, 'D': 4.76, 'K': 4.76, 'Q': 9.52, 'H': 2.38, 'E': 2.38, 'I': 2.38, 'F': 2.38, 'C': 2.38, 'T': 2.38}
- **2D identity (%) [PDB]:** 38.08
- **2D identity (%) [Gaps excluded][PDB]:** 91.93
- **2D identity - Alignment Gaps [PDB]:** 683
- **2D aligned content [PDB] (<2D-fold>:%):** {'.': 18.92, 'E': 23.2, 'T': 19.82, 'H': 36.71, 'G': 1.35}
- **3D similarity (TM-Score) (%) [PDB]:** 22.39

- **Gene name:** RASAL1
- **Entrez ID:** 8437
- **RefSeq ID:** N/A
- **Sequence length:** N/A
- **5-UTR|CDS|3-UTR identity (%):** N/A | N/A | N/A
- **5-UTR|CDS|3-UTR identity (%) [Gaps excluded]:** N/A | N/A | N/A
- **5-UTR|CDS|3-UTR identity [Alignment Gaps]:** N/A | N/A | N/A
- **5-UTR aligned content (<base>:%):** N/A
- **CDS aligned content (<base>:%):** N/A
- **3-UTR aligned content (<base>:%):** N/A

**Uniprot Description:**  
  
 Probable inhibitory regulator of the Ras-cyclic AMP pathway (PubMed:9751798). Plays a role in dendrite formation by melanocytes (PubMed:23999003). N/A   
  
 **Gene Ontology Information:**

Molecular Function

- GTPase activator activity
- metal ion binding
- phospholipid binding

Location

- cytosol

Biological process

- cell differentiation
- cellular response to calcium ion
- intracellular signal transduction
- negative regulation of Ras protein signal transduction
- positive regulation of dendrite extension
- regulation of GTPase activity
- signal transduction

---

43

- **Protein name:** Muskelin
- **Organism:** Homo sapiens
- **Uniprot Accession Number:** Q9UL63
- **Protein sequence length:** 735 aa
- **1D identity (%):** 13.27
- **1D identity (%) [Gaps excluded]:** 23.36
- **1D identity - Alignment Gaps:** 439
- **1D aligned content (<aminoacid>:%):** {'G': 7.41, 'A': 5.19, 'P': 7.41, 'E': 5.93, 'W': 2.22, 'F': 4.44, 'N': 2.96, 'I': 3.7, 'V': 5.19, 'S': 2.22, 'Y': 5.19, 'Q': 5.93, 'T': 7.41, 'K': 6.67, 'R': 7.41, 'L': 8.89, 'C': 3.7, 'D': 4.44, 'H': 2.96, 'M': 0.74}
- **Common reported functions (%):** 0.0
- **Common reported locations (%):** 33.33
- **Common reported processes (%):** 0.0

- **AF ID:** Q9UL63
- **Chain:** A
- **Protein length:** 735 aa
- **Resolution:** N/A
- **b-phipsi:** 0.008399
- **w-rdist:** 0.2182
- **t-alpha:** 0.026942
- **Chemical similarity (Tanimoto Index) (%):** 99.4
- **1D identity (%) [PDB]:** 2.69
- **1D identity (%) [Gaps excluded][PDB]:** 75.93
- **1D identity - Alignment Gaps [PDB]:** 1472
- **1D aligned content [PDB] (<aminoacid>:%):** {'T': 7.32, 'Y': 4.88, 'L': 9.76, 'P': 2.44, 'E': 7.32, 'V': 7.32, 'C': 4.88, 'N': 4.88, 'I': 7.32, 'Q': 9.76, 'K': 7.32, 'D': 7.32, 'S': 7.32, 'R': 4.88, 'F': 7.32}
- **2D identity (%) [PDB]:** 41.84
- **2D identity (%) [Gaps excluded][PDB]:** 89.84
- **2D identity - Alignment Gaps [PDB]:** 576
- **2D aligned content [PDB] (<2D-fold>:%):** {'.': 24.83, 'E': 28.82, 'T': 24.17, 'H': 21.06, 'B': 0.44, 'G': 0.67}
- **3D similarity (TM-Score) (%) [PDB]:** 22.56

- **Gene name:** MKLN1
- **Entrez ID:** 4289
- **RefSeq ID:** NM\_013255
- **Transcript sequence length:** 11136
- **5-UTR|CDS|3-UTR identity (%):** 5.23 | 45.25 | 32.57
- **5-UTR|CDS|3-UTR identity (%) [Gaps excluded]:** 80.95 | 76.33 | 76.08
- **5-UTR|CDS|3-UTR identity [Alignment Gaps]:** 304 | 1225 | 10275
- **5-UTR aligned content (<base>:%):** {'G': 52.94, 'C': 29.41, 'T': 5.88, 'A': 11.76}
- **CDS aligned content (<base>:%):** {'A': 32.4, 'T': 27.26, 'G': 21.75, 'C': 18.59}
- **3-UTR aligned content (<base>:%):** {'C': 15.14, 'T': 35.65, 'A': 32.3, 'G': 16.92}

**Uniprot Description:**  
  
 Component of the CTLH E3 ubiquitin-protein ligase complex that selectively accepts ubiquitin from UBE2H and mediates ubiquitination and subsequent proteasomal degradation of the transcription factor HBP1 (PubMed:29911972). Required for internalization of the GABA receptor GABRA1 from the cell membrane via endosomes and subsequent GABRA1 degradation (By similarity). Acts as a mediator of cell spreading and cytoskeletal responses to the extracellular matrix component THBS1 (PubMed:18710924).   
  
Homodimer; may form higher oligomers (By similarity). Identified in the CTLH complex that contains GID4, RANBP9 and/or RANBP10, MKLN1, MAEA, RMND5A (or alternatively its paralog RMND5B), GID8, ARMC8, WDR26 and YPEL5 (PubMed:17467196, PubMed:29911972). Within this complex, MAEA, RMND5A (or alternatively its paralog RMND5B), GID8, WDR26, and RANBP9 and/or RANBP10 form the catalytic core, while GID4, MKLN1, ARMC8 and YPEL5 have ancillary roles (PubMed:29911972). Interacts with RANBP9 (PubMed:18710924). Part of a complex consisting of RANBP9, MKLN1 and GID8 (PubMed:12559565). Interacts with GABRA1. Interacts with the C-terminal tail of PTGER3 (By similarity).   
  
 **Gene Ontology Information:**

Molecular Function

- identical protein binding
- protein homodimerization activity

Location

- cell cortex
- cytoplasm
- cytosol
- nucleoplasm
- postsynapse
- ruffle
- ubiquitin ligase complex

Biological process

- actin cytoskeleton reorganization
- cell-matrix adhesion
- regulation of cell shape
- regulation of receptor internalization
- signal transduction

---

44

- **Protein name:** Ras GTPase-activating protein 4
- **Organism:** Homo sapiens
- **Uniprot Accession Number:** O43374
- **Protein sequence length:** 803 aa
- **1D identity (%):** 16.43
- **1D identity (%) [Gaps excluded]:** 27.07
- **1D identity - Alignment Gaps:** 407
- **1D aligned content (<aminoacid>:%):** {'R': 7.06, 'S': 5.29, 'G': 8.24, 'L': 10.59, 'P': 9.41, 'D': 5.88, 'V': 10.59, 'A': 5.88, 'F': 4.71, 'Y': 2.94, 'I': 2.35, 'C': 2.94, 'H': 3.53, 'E': 6.47, 'T': 4.12, 'Q': 4.71, 'K': 3.53, 'N': 0.59, 'M': 1.18}
- **Common reported functions (%):** 14.29
- **Common reported locations (%):** 11.11
- **Common reported processes (%):** 0.0

- **AF ID:** O43374
- **Chain:** A
- **Protein length:** 803 aa
- **Resolution:** N/A
- **b-phipsi:** 0.008349
- **w-rdist:** 0.285865
- **t-alpha:** 0.010408
- **Chemical similarity (Tanimoto Index) (%):** 98.95
- **1D identity (%) [PDB]:** 3.04
- **1D identity (%) [Gaps excluded][PDB]:** 71.64
- **1D identity - Alignment Gaps [PDB]:** 1514
- **1D aligned content [PDB] (<aminoacid>:%):** {'K': 10.42, 'R': 8.33, 'S': 6.25, 'L': 14.58, 'Y': 2.08, 'T': 10.42, 'A': 4.17, 'P': 6.25, 'V': 10.42, 'D': 8.33, 'G': 2.08, 'E': 8.33, 'F': 2.08, 'I': 2.08, 'H': 4.17}
- **2D identity (%) [PDB]:** 41.37
- **2D identity (%) [Gaps excluded][PDB]:** 88.74
- **2D identity - Alignment Gaps [PDB]:** 600
- **2D aligned content [PDB] (<2D-fold>:%):** {'.': 18.06, 'E': 22.15, 'T': 22.8, 'H': 36.99}
- **3D similarity (TM-Score) (%) [PDB]:** 20.5

- **Gene name:** RASA4
- **Entrez ID:** 10156
- **RefSeq ID:** NM\_006989
- **Transcript sequence length:** 5604
- **5-UTR|CDS|3-UTR identity (%):** 14.89 | 45.65 | 11.24
- **5-UTR|CDS|3-UTR identity (%) [Gaps excluded]:** 81.67 | 73.68 | 74.34
- **5-UTR|CDS|3-UTR identity [Alignment Gaps]:** 269 | 1173 | 14658
- **5-UTR aligned content (<base>:%):** {'A': 8.16, 'G': 28.57, 'C': 57.14, 'T': 6.12}
- **CDS aligned content (<base>:%):** {'A': 23.93, 'T': 18.96, 'G': 29.19, 'C': 27.91}
- **3-UTR aligned content (<base>:%):** {'G': 25.55, 'C': 24.37, 'T': 26.07, 'A': 24.01}

**Uniprot Description:**  
  
 Ca(2+)-dependent Ras GTPase-activating protein, that switches off the Ras-MAPK pathway following a stimulus that elevates intracellular calcium. Functions as an adaptor for Cdc42 and Rac1 during FcR-mediated phagocytosis. N/A   
  
 **Gene Ontology Information:**

Molecular Function

- GTPase activator activity
- metal ion binding
- phospholipid binding

Location

- cytosol
- plasma membrane

Biological process

- cellular response to calcium ion
- intracellular signal transduction
- negative regulation of GTPase activity
- negative regulation of Ras protein signal transduction

---

45

- **Protein name:** Ribosomal protein S6 kinase alpha-4
- **Organism:** Homo sapiens
- **Uniprot Accession Number:** O75676
- **Protein sequence length:** 772 aa
- **1D identity (%):** 14.68
- **1D identity (%) [Gaps excluded]:** 26.42
- **1D identity - Alignment Gaps:** 466
- **1D aligned content (<aminoacid>:%):** {'M': 0.65, 'E': 7.14, 'G': 9.74, 'A': 7.14, 'R': 7.14, 'K': 5.84, 'L': 13.64, 'V': 7.79, 'Y': 3.25, 'D': 3.25, 'Q': 4.55, 'F': 4.55, 'I': 0.65, 'W': 1.95, 'T': 1.3, 'P': 11.04, 'H': 1.95, 'N': 2.6, 'S': 4.55, 'C': 1.3}
- **Common reported functions (%):** 0.0
- **Common reported locations (%):** 33.33
- **Common reported processes (%):** 0.0

- **AF ID:** O75676
- **Chain:** A
- **Protein length:** 772 aa
- **Resolution:** N/A
- **b-phipsi:** 0.002498
- **w-rdist:** 0.295796
- **t-alpha:** 0.095087
- **Chemical similarity (Tanimoto Index) (%):** 99.17
- **1D identity (%) [PDB]:** 1.85
- **1D identity (%) [Gaps excluded][PDB]:** 61.7
- **1D identity - Alignment Gaps [PDB]:** 1523
- **1D aligned content [PDB] (<aminoacid>:%):** {'L': 24.14, 'E': 13.79, 'N': 3.45, 'G': 6.9, 'V': 6.9, 'R': 3.45, 'T': 6.9, 'A': 3.45, 'Q': 13.79, 'F': 3.45, 'Y': 6.9, 'P': 3.45, 'H': 3.45}
- **2D identity (%) [PDB]:** 42.18
- **2D identity (%) [Gaps excluded][PDB]:** 87.98
- **2D identity - Alignment Gaps [PDB]:** 569
- **2D aligned content [PDB] (<2D-fold>:%):** {'.': 21.26, 'E': 12.58, 'T': 24.95, 'H': 39.05, 'B': 0.22, 'G': 1.95}
- **3D similarity (TM-Score) (%) [PDB]:** 23.23

- **Gene name:** RPS6KA4
- **Entrez ID:** 8986
- **RefSeq ID:** NM\_003942
- **Transcript sequence length:** 3128
- **5-UTR|CDS|3-UTR identity (%):** 18.21 | 42.36 | 2.59
- **5-UTR|CDS|3-UTR identity (%) [Gaps excluded]:** 81.94 | 73.0 | 72.4
- **5-UTR|CDS|3-UTR identity [Alignment Gaps]:** 252 | 1302 | 16280
- **5-UTR aligned content (<base>:%):** {'G': 33.9, 'C': 61.02, 'A': 3.39, 'T': 1.69}
- **CDS aligned content (<base>:%):** {'A': 21.39, 'T': 20.32, 'G': 29.76, 'C': 28.54}
- **3-UTR aligned content (<base>:%):** {'T': 22.37, 'C': 29.22, 'A': 21.69, 'G': 26.71}

**Uniprot Description:**  
  
 Serine/threonine-protein kinase that is required for the mitogen or stress-induced phosphorylation of the transcription factors CREB1 and ATF1 and for the regulation of the transcription factor RELA, and that contributes to gene activation by histone phosphorylation and functions in the regulation of inflammatory genes. Phosphorylates CREB1 and ATF1 in response to mitogenic or stress stimuli such as UV-C irradiation, epidermal growth factor (EGF) and anisomycin. Plays an essential role in the control of RELA transcriptional activity in response to TNF. Phosphorylates 'Ser-10' of histone H3 in response to mitogenics, stress stimuli and EGF, which results in the transcriptional activation of several immediate early genes, including proto-oncogenes c-fos/FOS and c-jun/JUN. May also phosphorylate 'Ser-28' of histone H3. Mediates the mitogen- and stress-induced phosphorylation of high mobility group protein 1 (HMGN1/HMG14). In lipopolysaccharide-stimulated primary macrophages, acts downstream of the Toll-like receptor TLR4 to limit the production of pro-inflammatory cytokines. Functions probably by inducing transcription of the MAP kinase phosphatase DUSP1 and the anti-inflammatory cytokine interleukin 10 (IL10), via CREB1 and ATF1 transcription factors.   
  
Forms a complex with either MAPK1/ERK2 or MAPK3/ERK1 in quiescent cells which transiently dissociates following mitogenic stimulation. Also associates with MAPK14/p38-alpha. Activated RPS6KA4 associates with and phosphorylates the NF-kappa-B p65 subunit RELA.   
  
 **Gene Ontology Information:**

Molecular Function

- ATP binding
- histone kinase activity (H3-S10 specific)
- histone H3S28 kinase activity
- magnesium ion binding
- protein serine kinase activity
- protein serine/threonine kinase activity
- ribosomal protein S6 kinase activity

Location

- cytoplasm
- cytosol
- nucleoplasm
- nucleus
- synapse

Biological process

- inflammatory response
- interleukin-1-mediated signaling pathway
- intracellular signal transduction
- negative regulation of cytokine production
- peptidyl-serine phosphorylation
- positive regulation of CREB transcription factor activity
- positive regulation of NF-kappaB transcription factor activity
- positive regulation of transcription by RNA polymerase II
- post-translational protein modification
- protein phosphorylation
- regulation of transcription, DNA-templated

---

46

- **Protein name:** Prolyl endopeptidase FAP
- **Organism:** Homo sapiens
- **Uniprot Accession Number:** Q12884
- **Protein sequence length:** 760 aa
- **1D identity (%):** 13.38
- **1D identity (%) [Gaps excluded]:** 22.09
- **1D identity - Alignment Gaps:** 398
- **1D aligned content (<aminoacid>:%):** {'V': 9.63, 'A': 3.7, 'T': 5.93, 'L': 8.15, 'R': 4.44, 'P': 7.41, 'G': 6.67, 'I': 5.19, 'S': 5.93, 'H': 2.96, 'Y': 9.63, 'F': 5.93, 'W': 1.48, 'D': 5.19, 'Q': 3.7, 'C': 3.7, 'N': 2.22, 'K': 4.44, 'E': 3.7}
- **Common reported functions (%):** 0.0
- **Common reported locations (%):** 0.0
- **Common reported processes (%):** 0.0

- **AF ID:** Q12884
- **Chain:** A
- **Protein length:** 760 aa
- **Resolution:** N/A
- **b-phipsi:** 0.004588
- **w-rdist:** 0.690311
- **t-alpha:** 0.001587
- **Chemical similarity (Tanimoto Index) (%):** 96.77
- **1D identity (%) [PDB]:** 2.59
- **1D identity (%) [Gaps excluded][PDB]:** 65.57
- **1D identity - Alignment Gaps [PDB]:** 1483
- **1D aligned content [PDB] (<aminoacid>:%):** {'V': 10.0, 'L': 7.5, 'R': 10.0, 'P': 7.5, 'S': 5.0, 'M': 5.0, 'K': 5.0, 'Y': 7.5, 'T': 2.5, 'F': 7.5, 'G': 12.5, 'H': 2.5, 'E': 2.5, 'A': 10.0, 'W': 2.5, 'N': 2.5}
- **2D identity (%) [PDB]:** 38.68
- **2D identity (%) [Gaps excluded][PDB]:** 86.49
- **2D identity - Alignment Gaps [PDB]:** 613
- **2D aligned content [PDB] (<2D-fold>:%):** {'.': 13.75, 'H': 21.45, 'T': 32.63, 'E': 31.24, 'G': 0.7, 'B': 0.23}
- **3D similarity (TM-Score) (%) [PDB]:** 26.45

- **Gene name:** FAP
- **Entrez ID:** 508882
- **RefSeq ID:** NM\_004460
- **Transcript sequence length:** 2696
- **5-UTR|CDS|3-UTR identity (%):** 16.62 | 45.8 | 1.16
- **5-UTR|CDS|3-UTR identity (%) [Gaps excluded]:** 69.41 | 74.61 | 76.17
- **5-UTR|CDS|3-UTR identity [Alignment Gaps]:** 270 | 1164 | 16538
- **5-UTR aligned content (<base>:%):** {'G': 16.95, 'T': 32.2, 'C': 40.68, 'A': 10.17}
- **CDS aligned content (<base>:%):** {'A': 33.24, 'G': 20.93, 'C': 18.61, 'T': 27.23}
- **3-UTR aligned content (<base>:%):** {'A': 33.85, 'C': 14.36, 'G': 16.41, 'T': 35.38}

**Uniprot Description:**  
  
 Cell surface glycoprotein serine protease that participates in extracellular matrix degradation and involved in many cellular processes including tissue remodeling, fibrosis, wound healing, inflammation and tumor growth. Both plasma membrane and soluble forms exhibit post-proline cleaving endopeptidase activity, with a marked preference for Ala/Ser-Gly-Pro-Ser/Asn/Ala consensus sequences, on substrate such as alpha-2-antiplasmin SERPINF2 and SPRY2 (PubMed:14751930, PubMed:16223769, PubMed:16480718, PubMed:16410248, PubMed:17381073, PubMed:18095711, PubMed:21288888, PubMed:24371721). Degrade also gelatin, heat-denatured type I collagen, but not native collagen type I and IV, vitronectin, tenascin, laminin, fibronectin, fibrin or casein (PubMed:9065413, PubMed:2172980, PubMed:7923219, PubMed:10347120, PubMed:10455171, PubMed:12376466, PubMed:16223769, PubMed:16651416, PubMed:18095711). Also has dipeptidyl peptidase activity, exhibiting the ability to hydrolyze the prolyl bond two residues from the N-terminus of synthetic dipeptide substrates provided that the penultimate residue is proline, with a preference for Ala-Pro, Ile-Pro, Gly-Pro, Arg-Pro and Pro-Pro (PubMed:10347120, PubMed:10593948, PubMed:16175601, PubMed:16223769, PubMed:16651416, PubMed:16410248, PubMed:17381073, PubMed:21314817, PubMed:24371721, PubMed:24717288). Natural neuropeptide hormones for dipeptidyl peptidase are the neuropeptide Y (NPY), peptide YY (PYY), substance P (TAC1) and brain natriuretic peptide 32 (NPPB) (PubMed:21314817). The plasma membrane form, in association with either DPP4, PLAUR or integrins, is involved in the pericellular proteolysis of the extracellular matrix (ECM), and hence promotes cell adhesion, migration and invasion through the ECM. Plays a role in tissue remodeling during development and wound healing. Participates in the cell invasiveness towards the ECM in malignant melanoma cancers. Enhances tumor growth progression by increasing angiogenesis, collagen fiber degradation and apoptosis and by reducing antitumor response of the immune system. Promotes glioma cell invasion through the brain parenchyma by degrading the proteoglycan brevican. Acts as a tumor suppressor in melanocytic cells through regulation of cell proliferation and survival in a serine protease activity-independent manner.   
  
Homodimer; homodimerization is required for activity of both plasma membrane and soluble forms. The monomer is inactive. Heterodimer with DPP4. Interacts with PLAUR; the interaction occurs at the cell surface of invadopodia membranes. Interacts with ITGB1. Interacts with ITGA3. Associates with integrin alpha-3/beta-1; the association occurs in a collagen-dependent manner at the cell surface of invadopodia membranes.   
  
 **Gene Ontology Information:**

Molecular Function

- dipeptidyl-peptidase activity
- integrin binding
- protease binding
- protein homodimerization activity
- serine-type endopeptidase activity

Location

- anchoring junction
- apical part of cell
- basal part of cell
- cell surface
- extracellular space
- lamellipodium membrane
- peptidase complex
- plasma membrane
- ruffle membrane

Biological process

- angiogenesis
- cell adhesion
- endothelial cell migration
- melanocyte apoptotic process
- melanocyte proliferation
- negative regulation of cell proliferation involved in contact inhibition
- negative regulation of extracellular matrix disassembly
- positive regulation of execution phase of apoptosis
- proteolysis
- proteolysis involved in cellular protein catabolic process
- regulation of cell cycle
- regulation of collagen catabolic process

---

47

- **Protein name:** Crossover junction endonuclease MUS81
- **Organism:** Homo sapiens
- **Uniprot Accession Number:** Q96NY9
- **Protein sequence length:** 551 aa
- **1D identity (%):** 12.57
- **1D identity (%) [Gaps excluded]:** 25.0
- **1D identity - Alignment Gaps:** 467
- **1D aligned content (<aminoacid>:%):** {'M': 0.85, 'R': 11.86, 'P': 13.56, 'L': 12.71, 'T': 4.24, 'K': 2.54, 'Y': 3.39, 'G': 11.86, 'E': 7.63, 'C': 3.39, 'D': 5.93, 'A': 5.93, 'S': 3.39, 'Q': 2.54, 'V': 3.39, 'W': 0.85, 'H': 1.69, 'F': 2.54, 'I': 0.85, 'N': 0.85}
- **Common reported functions (%):** 14.29
- **Common reported locations (%):** 11.11
- **Common reported processes (%):** 0.0

- **AF ID:** Q96NY9
- **Chain:** A
- **Protein length:** 551 aa
- **Resolution:** N/A
- **b-phipsi:** 0.007125
- **w-rdist:** 0.293173
- **t-alpha:** 0.021395
- **Chemical similarity (Tanimoto Index) (%):** 98.95
- **1D identity (%) [PDB]:** 2.98
- **1D identity (%) [Gaps excluded][PDB]:** 72.73
- **1D identity - Alignment Gaps [PDB]:** 1286
- **1D aligned content [PDB] (<aminoacid>:%):** {'A': 5.0, 'N': 2.5, 'P': 12.5, 'L': 20.0, 'V': 12.5, 'T': 7.5, 'G': 12.5, 'D': 5.0, 'E': 5.0, 'R': 2.5, 'F': 5.0, 'S': 2.5, 'I': 2.5, 'K': 2.5, 'H': 2.5}
- **2D identity (%) [PDB]:** 41.57
- **2D identity (%) [Gaps excluded][PDB]:** 90.02
- **2D identity - Alignment Gaps [PDB]:** 514
- **2D aligned content [PDB] (<2D-fold>:%):** {'.': 30.48, 'H': 38.04, 'T': 20.65, 'E': 10.83}
- **3D similarity (TM-Score) (%) [PDB]:** 19.06

- **Gene name:** MUS81
- **Entrez ID:** 80198
- **RefSeq ID:** N/A
- **Sequence length:** N/A
- **5-UTR|CDS|3-UTR identity (%):** N/A | N/A | N/A
- **5-UTR|CDS|3-UTR identity (%) [Gaps excluded]:** N/A | N/A | N/A
- **5-UTR|CDS|3-UTR identity [Alignment Gaps]:** N/A | N/A | N/A
- **5-UTR aligned content (<base>:%):** N/A
- **CDS aligned content (<base>:%):** N/A
- **3-UTR aligned content (<base>:%):** N/A

**Uniprot Description:**  
  
 Interacts with EME1 and EME2 to form a DNA structure-specific endonuclease with substrate preference for branched DNA structures with a 5'-end at the branch nick. Typical substrates include 3'-flap structures, replication forks and nicked Holliday junctions. May be required in mitosis for the processing of stalled or collapsed replication forks.   
  
May self-associate. Interacts with EME1, EME2 and CHEK2. Interacts with BLM, and this interaction may stimulate the endonuclease activity of MUS81. Interacts with SLX4/BTBD12; this interaction is direct and links the MUS81-EME1 complex to SLX4, which may coordinate the action of the structure-specific endonuclease during DNA repair. Interacts with DCLRE1B/Apollo.   
  
 **Gene Ontology Information:**

Molecular Function

- 3'-flap endonuclease activity
- crossover junction endodeoxyribonuclease activity
- DNA binding
- endonuclease activity
- metal ion binding

Location

- endodeoxyribonuclease complex
- Holliday junction resolvase complex
- nuclear replication fork
- nucleolus
- nucleoplasm
- nucleus
- replication fork

Biological process

- DNA catabolic process
- DNA repair
- double-strand break repair
- double-strand break repair via break-induced replication
- intra-S DNA damage checkpoint
- osteoblast proliferation
- replication fork processing
- resolution of meiotic recombination intermediates
- response to intra-S DNA damage checkpoint signaling

---

48

- **Protein name:** Zinc finger and BTB domain-containing protein 6
- **Organism:** Homo sapiens
- **Uniprot Accession Number:** Q15916
- **Protein sequence length:** 424 aa
- **1D identity (%):** 8.9
- **1D identity (%) [Gaps excluded]:** 21.66
- **1D identity - Alignment Gaps:** 536
- **1D aligned content (<aminoacid>:%):** {'V': 13.58, 'F': 3.7, 'E': 6.17, 'L': 16.05, 'N': 3.7, 'Q': 9.88, 'D': 4.94, 'G': 6.17, 'K': 6.17, 'C': 7.41, 'T': 2.47, 'R': 3.7, 'Y': 2.47, 'I': 1.23, 'P': 3.7, 'H': 3.7, 'S': 2.47, 'A': 2.47}
- **Common reported functions (%):** 14.29
- **Common reported locations (%):** 11.11
- **Common reported processes (%):** 0.0

- **AF ID:** Q15916
- **Chain:** A
- **Protein length:** 424 aa
- **Resolution:** N/A
- **b-phipsi:** 0.001951
- **w-rdist:** 0.365356
- **t-alpha:** 0.047544
- **Chemical similarity (Tanimoto Index) (%):** 87.42
- **1D identity (%) [PDB]:** 1.37
- **1D identity (%) [Gaps excluded][PDB]:** 68.0
- **1D identity - Alignment Gaps [PDB]:** 1219
- **1D aligned content [PDB] (<aminoacid>:%):** {'L': 17.65, 'Q': 17.65, 'E': 11.76, 'N': 5.88, 'G': 5.88, 'T': 11.76, 'V': 11.76, 'A': 5.88, 'F': 5.88, 'K': 5.88}
- **2D identity (%) [PDB]:** 34.24
- **2D identity (%) [Gaps excluded][PDB]:** 91.33
- **2D identity - Alignment Gaps [PDB]:** 577
- **2D aligned content [PDB] (<2D-fold>:%):** {'.': 24.37, 'E': 10.13, 'T': 34.49, 'H': 31.01}
- **3D similarity (TM-Score) (%) [PDB]:** 15.8

- **Gene name:** ZBTB6
- **Entrez ID:** 540218
- **RefSeq ID:** NM\_006626
- **Transcript sequence length:** 4099
- **5-UTR|CDS|3-UTR identity (%):** 14.58 | 30.38 | 10.65
- **5-UTR|CDS|3-UTR identity (%) [Gaps excluded]:** 81.97 | 76.18 | 76.74
- **5-UTR|CDS|3-UTR identity [Alignment Gaps]:** 282 | 1658 | 14743
- **5-UTR aligned content (<base>:%):** {'C': 34.0, 'T': 38.0, 'G': 20.0, 'A': 8.0}
- **CDS aligned content (<base>:%):** {'A': 33.65, 'T': 25.54, 'G': 22.32, 'C': 18.5}
- **3-UTR aligned content (<base>:%):** {'T': 42.6, 'A': 31.74, 'C': 11.51, 'G': 14.14}

**Uniprot Description:**  
  
 May be involved in transcriptional regulation. N/A   
  
 **Gene Ontology Information:**

Molecular Function

- DNA-binding transcription repressor activity, RNA polymerase II-specific
- metal ion binding
- RNA polymerase II cis-regulatory region sequence-specific DNA binding

Location

- nucleoplasm

Biological process

- negative regulation of transcription by RNA polymerase II
- regulation of cytokine production
- regulation of immune system process

---

49

- **Protein name:** 5-oxoprolinase
- **Organism:** Homo sapiens
- **Uniprot Accession Number:** O14841
- **Protein sequence length:** 1288 aa
- **1D identity (%):** 15.1
- **1D identity (%) [Gaps excluded]:** 26.28
- **1D identity - Alignment Gaps:** 580
- **1D aligned content (<aminoacid>:%):** {'M': 0.97, 'G': 12.62, 'P': 10.68, 'F': 4.37, 'I': 2.43, 'D': 3.88, 'V': 10.68, 'C': 3.4, 'R': 8.25, 'T': 5.34, 'L': 9.22, 'H': 3.88, 'E': 4.37, 'Y': 1.94, 'S': 3.4, 'A': 7.28, 'Q': 5.34, 'N': 1.46, 'K': 0.49}
- **Common reported functions (%):** 0.0
- **Common reported locations (%):** 11.11
- **Common reported processes (%):** 0.0

- **AF ID:** O14841
- **Chain:** A
- **Protein length:** 1288 aa
- **Resolution:** N/A
- **b-phipsi:** 0.002108
- **w-rdist:** 0.309569
- **t-alpha:** 0.09588
- **Chemical similarity (Tanimoto Index) (%):** 99.25
- **1D identity (%) [PDB]:** 3.91
- **1D identity (%) [Gaps excluded][PDB]:** 71.17
- **1D identity - Alignment Gaps [PDB]:** 1911
- **1D aligned content [PDB] (<aminoacid>:%):** {'G': 8.86, 'I': 7.59, 'L': 8.86, 'V': 12.66, 'P': 8.86, 'Q': 6.33, 'A': 10.13, 'S': 5.06, 'H': 1.27, 'R': 12.66, 'T': 2.53, 'D': 2.53, 'M': 1.27, 'E': 3.8, 'F': 3.8, 'Y': 3.8}
- **2D identity (%) [PDB]:** 40.97
- **2D identity (%) [Gaps excluded][PDB]:** 90.24
- **2D identity - Alignment Gaps [PDB]:** 801
- **2D aligned content [PDB] (<2D-fold>:%):** {'.': 18.8, 'E': 23.96, 'T': 24.63, 'H': 32.11, 'B': 0.5}
- **3D similarity (TM-Score) (%) [PDB]:** 28.34

- **Gene name:** OPLAH
- **Entrez ID:** 26873
- **RefSeq ID:** NM\_017570
- **Transcript sequence length:** 4020
- **5-UTR|CDS|3-UTR identity (%):** 18.37 | 40.03 | 0.24
- **5-UTR|CDS|3-UTR identity (%) [Gaps excluded]:** 73.49 | 73.31 | 69.49
- **5-UTR|CDS|3-UTR identity [Alignment Gaps]:** 249 | 1894 | 16697
- **5-UTR aligned content (<base>:%):** {'G': 37.7, 'C': 47.54, 'A': 6.56, 'T': 8.2}
- **CDS aligned content (<base>:%):** {'A': 22.46, 'T': 21.44, 'G': 28.62, 'C': 27.49}
- **3-UTR aligned content (<base>:%):** {'G': 29.27, 'T': 17.07, 'C': 36.59, 'A': 17.07}

**Uniprot Description:**  
  
 Catalyzes the cleavage of 5-oxo-L-proline to form L-glutamate coupled to the hydrolysis of ATP to ADP and inorganic phosphate.   
  
Homodimer.   
  
 **Gene Ontology Information:**

Molecular Function

- 5-oxoprolinase (ATP-hydrolyzing) activity
- ATP binding
- identical protein binding

Location

- cytosol

Biological process

- glutathione metabolic process

---

50

- **Protein name:** Zinc finger and SCAN domain-containing protein 4
- **Organism:** Homo sapiens
- **Uniprot Accession Number:** Q8NAM6
- **Protein sequence length:** 433 aa
- **1D identity (%):** 8.41
- **1D identity (%) [Gaps excluded]:** 21.37
- **1D identity - Alignment Gaps:** 563
- **1D aligned content (<aminoacid>:%):** {'M': 1.28, 'R': 10.26, 'C': 3.85, 'Q': 8.97, 'G': 8.97, 'V': 6.41, 'L': 6.41, 'Y': 1.28, 'H': 6.41, 'K': 7.69, 'S': 3.85, 'E': 6.41, 'F': 2.56, 'D': 3.85, 'T': 7.69, 'P': 8.97, 'W': 1.28, 'I': 1.28, 'A': 2.56}
- **Common reported functions (%):** 14.29
- **Common reported locations (%):** 0.0
- **Common reported processes (%):** 0.0

- **AF ID:** Q8NAM6
- **Chain:** A
- **Protein length:** 433 aa
- **Resolution:** N/A
- **b-phipsi:** 0.003916
- **w-rdist:** 0.282112
- **t-alpha:** 0.080824
- **Chemical similarity (Tanimoto Index) (%):** 99.09
- **1D identity (%) [PDB]:** 1.35
- **1D identity (%) [Gaps excluded][PDB]:** 73.91
- **1D identity - Alignment Gaps [PDB]:** 1232
- **1D aligned content [PDB] (<aminoacid>:%):** {'A': 11.76, 'D': 5.88, 'R': 11.76, 'Q': 5.88, 'E': 11.76, 'I': 5.88, 'S': 5.88, 'L': 5.88, 'V': 5.88, 'N': 5.88, 'Y': 5.88, 'T': 5.88, 'P': 5.88, 'F': 5.88}
- **2D identity (%) [PDB]:** 28.19
- **2D identity (%) [Gaps excluded][PDB]:** 89.54
- **2D identity - Alignment Gaps [PDB]:** 666
- **2D aligned content [PDB] (<2D-fold>:%):** {'.': 31.39, 'T': 25.91, 'H': 39.05, 'E': 3.65}
- **3D similarity (TM-Score) (%) [PDB]:** 17.06

- **Gene name:** ZSCAN4
- **Entrez ID:** N/A
- **RefSeq ID:** N/A
- **Sequence length:** N/A
- **5-UTR|CDS|3-UTR identity (%):** N/A | N/A | N/A
- **5-UTR|CDS|3-UTR identity (%) [Gaps excluded]:** N/A | N/A | N/A
- **5-UTR|CDS|3-UTR identity [Alignment Gaps]:** N/A | N/A | N/A
- **5-UTR aligned content (<base>:%):** N/A
- **CDS aligned content (<base>:%):** N/A
- **3-UTR aligned content (<base>:%):** N/A

**Uniprot Description:**  
  
 Embryonic stem (ES) cell-specific transcription factor required to regulate ES cell pluripotency. Binds telomeres and plays a key role in genomic stability in ES cells by regulating telomere elongation. Acts as an activator of spontaneous telomere sister chromatid exchange (T-SCE) and telomere elongation in undifferentiated ES cells (By similarity). N/A   
  
 **Gene Ontology Information:**

Molecular Function

- DNA binding
- metal ion binding

Location

- chromosome, telomeric region
- nucleus

Biological process

- telomere maintenance via telomere lengthening

---

51

- **Protein name:** Putative homeobox protein NANOG2
- **Organism:** Homo sapiens
- **Uniprot Accession Number:** Q8N7R0
- **Protein sequence length:** 232 aa
- **1D identity (%):** 4.84
- **1D identity (%) [Gaps excluded]:** 18.67
- **1D identity - Alignment Gaps:** 642
- **1D aligned content (<aminoacid>:%):** {'L': 16.67, 'H': 2.38, 'K': 2.38, 'A': 2.38, 'T': 16.67, 'R': 4.76, 'V': 2.38, 'Q': 14.29, 'C': 4.76, 'Y': 2.38, 'I': 2.38, 'S': 4.76, 'G': 4.76, 'P': 4.76, 'M': 2.38, 'N': 4.76, 'W': 2.38, 'E': 4.76}
- **Common reported functions (%):** 0.0
- **Common reported locations (%):** 0.0
- **Common reported processes (%):** 0.0

- **AF ID:** Q8N7R0
- **Chain:** A
- **Protein length:** 232 aa
- **Resolution:** N/A
- **b-phipsi:** 0.002422
- **w-rdist:** 0.382156
- **t-alpha:** 0.018563
- **Chemical similarity (Tanimoto Index) (%):** 98.42
- **1D identity (%) [PDB]:** 1.42
- **1D identity (%) [Gaps excluded][PDB]:** 88.24
- **1D identity - Alignment Gaps [PDB]:** 1043
- **1D aligned content [PDB] (<aminoacid>:%):** {'M': 6.67, 'A': 6.67, 'T': 20.0, 'C': 6.67, 'V': 20.0, 'Q': 13.33, 'K': 6.67, 'N': 6.67, 'I': 6.67, 'P': 6.67}
- **2D identity (%) [PDB]:** 16.52
- **2D identity (%) [Gaps excluded][PDB]:** 88.76
- **2D identity - Alignment Gaps [PDB]:** 739
- **2D aligned content [PDB] (<2D-fold>:%):** {'.': 36.0, 'T': 44.0, 'H': 20.0}
- **3D similarity (TM-Score) (%) [PDB]:** 10.6

- **Gene name:** NANOGP1
- **Entrez ID:** N/A
- **RefSeq ID:** N/A
- **Sequence length:** N/A
- **5-UTR|CDS|3-UTR identity (%):** N/A | N/A | N/A
- **5-UTR|CDS|3-UTR identity (%) [Gaps excluded]:** N/A | N/A | N/A
- **5-UTR|CDS|3-UTR identity [Alignment Gaps]:** N/A | N/A | N/A
- **5-UTR aligned content (<base>:%):** N/A
- **CDS aligned content (<base>:%):** N/A
- **3-UTR aligned content (<base>:%):** N/A

**Uniprot Description:**  
  
 Probable transcriptional regulator. N/A   
  
 **Gene Ontology Information:**

Molecular Function

- DNA-binding transcription factor activity, RNA polymerase II-specific
- RNA polymerase II cis-regulatory region sequence-specific DNA binding

Location

- nucleus

Biological process

- regulation of transcription by RNA polymerase II

---

52

- **Protein name:** LIM domain kinase 1
- **Organism:** Homo sapiens
- **Uniprot Accession Number:** P53667
- **Protein sequence length:** 647 aa
- **1D identity (%):** 12.86
- **1D identity (%) [Gaps excluded]:** 23.91
- **1D identity - Alignment Gaps:** 453
- **1D aligned content (<aminoacid>:%):** {'M': 0.79, 'R': 10.32, 'C': 6.35, 'P': 10.32, 'G': 14.29, 'Q': 2.38, 'Y': 2.38, 'L': 9.52, 'H': 7.14, 'E': 2.38, 'V': 8.73, 'A': 1.59, 'T': 7.14, 'D': 7.14, 'S': 3.17, 'I': 2.38, 'K': 1.59, 'F': 1.59, 'N': 0.79}
- **Common reported functions (%):** 14.29
- **Common reported locations (%):** 11.11
- **Common reported processes (%):** 0.0

- **AF ID:** P53667
- **Chain:** A
- **Protein length:** 647 aa
- **Resolution:** N/A
- **b-phipsi:** 0.000351
- **w-rdist:** 0.620545
- **t-alpha:** 0.129953
- **Chemical similarity (Tanimoto Index) (%):** 99.17
- **1D identity (%) [PDB]:** 4.37
- **1D identity (%) [Gaps excluded][PDB]:** 64.21
- **1D identity - Alignment Gaps [PDB]:** 1302
- **1D aligned content [PDB] (<aminoacid>:%):** {'R': 8.2, 'P': 8.2, 'V': 11.48, 'Y': 3.28, 'D': 8.2, 'G': 8.2, 'K': 6.56, 'L': 16.39, 'T': 8.2, 'F': 4.92, 'S': 4.92, 'H': 1.64, 'E': 6.56, 'N': 1.64, 'A': 1.64}
- **2D identity (%) [PDB]:** 42.61
- **2D identity (%) [Gaps excluded][PDB]:** 81.8
- **2D identity - Alignment Gaps [PDB]:** 470
- **2D aligned content [PDB] (<2D-fold>:%):** {'.': 18.18, 'E': 15.55, 'T': 33.49, 'H': 32.54, 'B': 0.24}
- **3D similarity (TM-Score) (%) [PDB]:** 20.17

- **Gene name:** LIMK1
- **Entrez ID:** 373922
- **RefSeq ID:** NM\_002314
- **Transcript sequence length:** 3355
- **5-UTR|CDS|3-UTR identity (%):** 35.65 | 37.31 | 4.71
- **5-UTR|CDS|3-UTR identity (%) [Gaps excluded]:** 75.0 | 72.86 | 76.36
- **5-UTR|CDS|3-UTR identity [Alignment Gaps]:** 181 | 1461 | 15889
- **5-UTR aligned content (<base>:%):** {'C': 57.72, 'T': 7.32, 'G': 33.33, 'A': 1.63}
- **CDS aligned content (<base>:%):** {'T': 20.68, 'G': 27.39, 'C': 27.93, 'A': 23.99}
- **3-UTR aligned content (<base>:%):** {'G': 26.69, 'A': 23.56, 'C': 25.94, 'T': 23.81}

**Uniprot Description:**  
  
 Serine/threonine-protein kinase that plays an essential role in the regulation of actin filament dynamics. Acts downstream of several Rho family GTPase signal transduction pathways (PubMed:10436159, PubMed:11832213, PubMed:12807904, PubMed:15660133, PubMed:16230460, PubMed:18028908, PubMed:22328514, PubMed:23633677). Activated by upstream kinases including ROCK1, PAK1 and PAK4, which phosphorylate LIMK1 on a threonine residue located in its activation loop (PubMed:10436159). LIMK1 subsequently phosphorylates and inactivates the actin binding/depolymerizing factors cofilin-1/CFL1, cofilin-2/CFL2 and destrin/DSTN, thereby preventing the cleavage of filamentous actin (F-actin), and stabilizing the actin cytoskeleton (PubMed:11832213, PubMed:15660133, PubMed:16230460, PubMed:23633677). In this way LIMK1 regulates several actin-dependent biological processes including cell motility, cell cycle progression, and differentiation (PubMed:11832213, PubMed:15660133, PubMed:16230460, PubMed:23633677). Phosphorylates TPPP on serine residues, thereby promoting microtubule disassembly (PubMed:18028908). Stimulates axonal outgrowth and may be involved in brain development (PubMed:18028908).   
  
Interacts (via LIM domain) with the cytoplasmic domain of NRG1. Interacts with NISCH. Interacts with RLIM and RNF6 (By similarity). Self-associates to form homodimers (PubMed:10196227). Interacts with HSP90AA1; this interaction promotes LIMK1 dimerization and subsequent transphosphorylation (PubMed:16641196). Interacts with CDKN1C (PubMed:14530263). Interacts with SSH1 (PubMed:15660133). Interacts with ROCK1 (PubMed:10436159, PubMed:10652353). Interacts (via LIM zinc-binding domains) with FAM89B/LRAP25 (via LRR repeat). Forms a tripartite complex with CDC42BPA, CDC42BPB and FAM89B/LRAP25 (By similarity).   
  
 **Gene Ontology Information:**

Molecular Function

- ATP binding
- metal ion binding
- protein serine kinase activity
- protein serine/threonine kinase activity

Location

- cytoplasm
- cytoskeleton
- growth cone
- neuron projection
- nucleus

Biological process

- actin cytoskeleton organization
- positive regulation of stress fiber assembly
- protein phosphorylation

---

53

- **Protein name:** BTB/POZ domain-containing protein 2
- **Organism:** Homo sapiens
- **Uniprot Accession Number:** Q9BX70
- **Protein sequence length:** 525 aa
- **1D identity (%):** 11.41
- **1D identity (%) [Gaps excluded]:** 26.13
- **1D identity - Alignment Gaps:** 543
- **1D aligned content (<aminoacid>:%):** {'G': 8.18, 'P': 10.0, 'V': 6.36, 'N': 1.82, 'T': 8.18, 'A': 5.45, 'R': 4.55, 'E': 8.18, 'L': 7.27, 'Y': 5.45, 'Q': 3.64, 'F': 6.36, 'H': 3.64, 'D': 3.64, 'I': 7.27, 'K': 4.55, 'C': 4.55, 'S': 0.91}
- **Common reported functions (%):** 0.0
- **Common reported locations (%):** 22.22
- **Common reported processes (%):** 0.0

- **AF ID:** Q9BX70
- **Chain:** A
- **Protein length:** 525 aa
- **Resolution:** N/A
- **b-phipsi:** 0.000401
- **w-rdist:** 0.729203
- **t-alpha:** 0.066779
- **Chemical similarity (Tanimoto Index) (%):** 98.87
- **1D identity (%) [PDB]:** 2.17
- **1D identity (%) [Gaps excluded][PDB]:** 80.56
- **1D identity - Alignment Gaps [PDB]:** 1298
- **1D aligned content [PDB] (<aminoacid>:%):** {'M': 3.45, 'E': 13.79, 'V': 13.79, 'L': 10.34, 'D': 10.34, 'H': 3.45, 'N': 3.45, 'I': 3.45, 'R': 6.9, 'P': 3.45, 'T': 6.9, 'S': 3.45, 'F': 3.45, 'K': 10.34, 'G': 3.45}
- **2D identity (%) [PDB]:** 38.41
- **2D identity (%) [Gaps excluded][PDB]:** 85.41
- **2D identity - Alignment Gaps [PDB]:** 520
- **2D aligned content [PDB] (<2D-fold>:%):** {'.': 26.45, 'H': 36.91, 'T': 19.01, 'E': 17.63}
- **3D similarity (TM-Score) (%) [PDB]:** 20.67

- **Gene name:** BTBD2
- **Entrez ID:** 55643
- **RefSeq ID:** N/A
- **Sequence length:** N/A
- **5-UTR|CDS|3-UTR identity (%):** N/A | N/A | N/A
- **5-UTR|CDS|3-UTR identity (%) [Gaps excluded]:** N/A | N/A | N/A
- **5-UTR|CDS|3-UTR identity [Alignment Gaps]:** N/A | N/A | N/A
- **5-UTR aligned content (<base>:%):** N/A
- **CDS aligned content (<base>:%):** N/A
- **3-UTR aligned content (<base>:%):** N/A

**Uniprot Description:**  
  
 N/A   
  
Interacts with topoisomerase 1 and with TRIM5 isoform Delta.   
  
 **Gene Ontology Information:**

Molecular Function   
  
N/A

Location

- cytosol
- P-body

Biological process

- neurogenesis

---

54

- **Protein name:** D-glucuronyl C5-epimerase
- **Organism:** Homo sapiens
- **Uniprot Accession Number:** O94923
- **Protein sequence length:** 617 aa
- **1D identity (%):** 13.08
- **1D identity (%) [Gaps excluded]:** 23.44
- **1D identity - Alignment Gaps:** 419
- **1D aligned content (<aminoacid>:%):** {'A': 5.65, 'Y': 8.87, 'K': 6.45, 'I': 2.42, 'C': 2.42, 'D': 8.06, 'F': 5.65, 'G': 11.29, 'V': 7.26, 'E': 4.84, 'N': 3.23, 'S': 3.23, 'P': 4.84, 'L': 9.68, 'H': 0.81, 'T': 4.03, 'R': 5.65, 'Q': 4.03, 'W': 1.61}
- **Common reported functions (%):** 0.0
- **Common reported locations (%):** 0.0
- **Common reported processes (%):** 0.0

- **AF ID:** O94923
- **Chain:** A
- **Protein length:** 617 aa
- **Resolution:** N/A
- **b-phipsi:** 0.000696
- **w-rdist:** 0.381889
- **t-alpha:** 0.133914
- **Chemical similarity (Tanimoto Index) (%):** 99.17
- **1D identity (%) [PDB]:** 1.83
- **1D identity (%) [Gaps excluded][PDB]:** 66.67
- **1D identity - Alignment Gaps [PDB]:** 1384
- **1D aligned content [PDB] (<aminoacid>:%):** {'N': 11.54, 'V': 7.69, 'A': 15.38, 'Q': 3.85, 'C': 3.85, 'I': 7.69, 'K': 3.85, 'L': 7.69, 'T': 15.38, 'D': 3.85, 'S': 15.38, 'R': 3.85}
- **2D identity (%) [PDB]:** 40.3
- **2D identity (%) [Gaps excluded][PDB]:** 84.53
- **2D identity - Alignment Gaps [PDB]:** 518
- **2D aligned content [PDB] (<2D-fold>:%):** {'.': 25.06, 'H': 36.59, 'E': 15.04, 'T': 21.3, 'B': 0.5, 'G': 1.5}
- **3D similarity (TM-Score) (%) [PDB]:** 17.17

- **Gene name:** GLCE
- **Entrez ID:** 26035
- **RefSeq ID:** N/A
- **Sequence length:** N/A
- **5-UTR|CDS|3-UTR identity (%):** N/A | N/A | N/A
- **5-UTR|CDS|3-UTR identity (%) [Gaps excluded]:** N/A | N/A | N/A
- **5-UTR|CDS|3-UTR identity [Alignment Gaps]:** N/A | N/A | N/A
- **5-UTR aligned content (<base>:%):** N/A
- **CDS aligned content (<base>:%):** N/A
- **3-UTR aligned content (<base>:%):** N/A

**Uniprot Description:**  
  
 Converts D-glucuronic acid residues adjacent to N-sulfate sugar residues to L-iduronic acid residues, both in maturing heparan sulfate (HS) and heparin chains. This is important for further modifications that determine the specificity of interactions between these glycosaminoglycans and proteins.   
  
Homodimer (PubMed:30872481). Interacts with HS2ST1.   
  
 **Gene Ontology Information:**

Molecular Function

- calcium ion binding
- heparosan-N-sulfate-glucuronate 5-epimerase activity
- protein homodimerization activity
- racemase and epimerase activity, acting on carbohydrates and derivatives

Location

- Golgi apparatus
- Golgi membrane

Biological process

- heparan sulfate proteoglycan biosynthetic process
- heparin biosynthetic process

---

55

- **Protein name:** Piwi-like protein 4
- **Organism:** Homo sapiens
- **Uniprot Accession Number:** Q7Z3Z4
- **Protein sequence length:** 852 aa
- **1D identity (%):** 21.09
- **1D identity (%) [Gaps excluded]:** 26.79
- **1D identity - Alignment Gaps:** 204
- **1D aligned content (<aminoacid>:%):** {'M': 0.99, 'E': 4.46, 'G': 9.41, 'S': 3.96, 'A': 3.47, 'L': 10.4, 'R': 9.41, 'P': 7.92, 'K': 6.44, 'N': 1.49, 'F': 4.95, 'Y': 6.44, 'V': 7.92, 'D': 5.45, 'H': 1.98, 'T': 5.94, 'I': 2.97, 'W': 1.49, 'Q': 3.47, 'C': 1.49}
- **Common reported functions (%):** 14.29
- **Common reported locations (%):** 22.22
- **Common reported processes (%):** 0.0

- **AF ID:** Q7Z3Z4
- **Chain:** A
- **Protein length:** 852 aa
- **Resolution:** N/A
- **b-phipsi:** 0.005956
- **w-rdist:** 0.25506
- **t-alpha:** 0.062599
- **Chemical similarity (Tanimoto Index) (%):** 99.4
- **1D identity (%) [PDB]:** 5.52
- **1D identity (%) [Gaps excluded][PDB]:** 53.8
- **1D identity - Alignment Gaps [PDB]:** 1381
- **1D aligned content [PDB] (<aminoacid>:%):** {'I': 7.06, 'D': 9.41, 'L': 9.41, 'A': 5.88, 'S': 5.88, 'V': 8.24, 'E': 5.88, 'Q': 3.53, 'Y': 8.24, 'K': 4.71, 'R': 3.53, 'P': 7.06, 'T': 8.24, 'G': 3.53, 'C': 2.35, 'F': 2.35, 'N': 2.35, 'H': 2.35}
- **2D identity (%) [PDB]:** 62.13
- **2D identity (%) [Gaps excluded][PDB]:** 87.64
- **2D identity - Alignment Gaps [PDB]:** 289
- **2D aligned content [PDB] (<2D-fold>:%):** {'.': 18.48, 'H': 32.9, 'T': 21.72, 'E': 25.77, 'B': 0.65, 'G': 0.49}
- **3D similarity (TM-Score) (%) [PDB]:** 75.82

- **Gene name:** PIWIL4
- **Entrez ID:** 143689
- **RefSeq ID:** NM\_152431
- **Transcript sequence length:** 3139
- **5-UTR|CDS|3-UTR identity (%):** 25.21 | 47.25 | 1.82
- **5-UTR|CDS|3-UTR identity (%) [Gaps excluded]:** 74.17 | 75.2 | 78.01
- **5-UTR|CDS|3-UTR identity [Alignment Gaps]:** 233 | 1174 | 16402
- **5-UTR aligned content (<base>:%):** {'C': 44.94, 'T': 19.1, 'G': 31.46, 'A': 4.49}
- **CDS aligned content (<base>:%):** {'A': 29.69, 'T': 26.27, 'G': 22.52, 'C': 21.51}
- **3-UTR aligned content (<base>:%):** {'T': 37.05, 'G': 18.03, 'C': 11.15, 'A': 33.77}

**Uniprot Description:**  
  
 Plays a central role during spermatogenesis by repressing transposable elements and preventing their mobilization, which is essential for the germline integrity (By similarity). Acts via the piRNA metabolic process, which mediates the repression of transposable elements during meiosis by forming complexes composed of piRNAs and Piwi proteins and governs the methylation and subsequent repression of transposons (By similarity). Directly binds piRNAs, a class of 24 to 30 nucleotide RNAs that are generated by a Dicer-independent mechanism and are primarily derived from transposons and other repeated sequence elements (By similarity). Associates with secondary piRNAs antisense and PIWIL2/MILI is required for such association (By similarity). The piRNA process acts upstream of known mediators of DNA methylation (By similarity). Does not show endonuclease activity (By similarity). Plays a key role in the piRNA amplification loop, also named ping-pong amplification cycle, by acting as a 'slicer-incompetent' component that loads cleaved piRNAs from the 'slicer-competent' component PIWIL2 and target them on genomic transposon loci in the nucleus (By similarity). May be involved in the chromatin-modifying pathway by inducing 'Lys-9' methylation of histone H3 at some loci (PubMed:17544373). In addition to its role in germline, PIWIL4 also plays a role in the regulation of somatic cells activities. Plays a role in pancreatic beta cell function and insulin secretion (By similarity). Involved in maintaining cell morphology and functional integrity of retinal epithelial through Akt/GSK3alpha/beta signaling pathway (PubMed:28025795). When overexpressed, acts as an oncogene by inhibition of apoptosis and promotion of cells proliferation in tumors (PubMed:22483988).   
  
Interacts with PRMT5 and WDR77. Interacts (when methylated on arginine residues) with TDRD1, TDRKH/TDRD2 and TDRD9. Interacts with MOV10L1 (By similarity). Interacts with TEX15 and SPOCD1 (By similarity).   
  
 **Gene Ontology Information:**

Molecular Function

- piRNA binding
- endoribonuclease activity

Location

- cytoplasm
- mitochondrion
- nucleoplasm
- nucleus
- P granule
- piP-body

Biological process

- epithelial structure maintenance
- genetic imprinting
- meiotic cell cycle
- regulation of translation
- retrotransposon silencing by heterochromatin formation
- gene silencing by RNA
- secondary piRNA processing
- spermatogenesis

---

56

- **Protein name:** Adenine DNA glycosylase
- **Organism:** Homo sapiens
- **Uniprot Accession Number:** Q9UIF7
- **Protein sequence length:** 546 aa
- **1D identity (%):** 12.54
- **1D identity (%) [Gaps excluded]:** 22.94
- **1D identity - Alignment Gaps:** 412
- **1D aligned content (<aminoacid>:%):** {'R': 7.02, 'P': 10.53, 'H': 2.63, 'E': 4.39, 'Q': 7.89, 'G': 11.4, 'V': 7.02, 'A': 11.4, 'D': 5.26, 'T': 8.77, 'S': 3.51, 'M': 0.88, 'L': 13.16, 'W': 0.88, 'F': 1.75, 'C': 0.88, 'K': 0.88, 'Y': 1.75}
- **Common reported functions (%):** 14.29
- **Common reported locations (%):** 11.11
- **Common reported processes (%):** 0.0

- **AF ID:** Q9UIF7
- **Chain:** A
- **Protein length:** 546 aa
- **Resolution:** N/A
- **b-phipsi:** 0.002109
- **w-rdist:** 0.315745
- **t-alpha:** 0.097464
- **Chemical similarity (Tanimoto Index) (%):** 99.62
- **1D identity (%) [PDB]:** 1.92
- **1D identity (%) [Gaps excluded][PDB]:** 74.29
- **1D identity - Alignment Gaps [PDB]:** 1321
- **1D aligned content [PDB] (<aminoacid>:%):** {'M': 3.85, 'R': 15.38, 'Y': 3.85, 'V': 11.54, 'N': 3.85, 'P': 7.69, 'A': 7.69, 'S': 3.85, 'Q': 11.54, 'T': 11.54, 'F': 3.85, 'L': 7.69, 'E': 3.85, 'G': 3.85}
- **2D identity (%) [PDB]:** 38.62
- **2D identity (%) [Gaps excluded][PDB]:** 89.29
- **2D identity - Alignment Gaps [PDB]:** 551
- **2D aligned content [PDB] (<2D-fold>:%):** {'.': 28.27, 'T': 18.4, 'H': 45.07, 'G': 0.8, 'E': 7.47}
- **3D similarity (TM-Score) (%) [PDB]:** 18.45

- **Gene name:** MUTYH
- **Entrez ID:** 4595
- **RefSeq ID:** N/A
- **Sequence length:** N/A
- **5-UTR|CDS|3-UTR identity (%):** N/A | N/A | N/A
- **5-UTR|CDS|3-UTR identity (%) [Gaps excluded]:** N/A | N/A | N/A
- **5-UTR|CDS|3-UTR identity [Alignment Gaps]:** N/A | N/A | N/A
- **5-UTR aligned content (<base>:%):** N/A
- **CDS aligned content (<base>:%):** N/A
- **3-UTR aligned content (<base>:%):** N/A

**Uniprot Description:**  
  
 Involved in oxidative DNA damage repair. Initiates repair of A\*oxoG to C\*G by removing the inappropriately paired adenine base from the DNA backbone. Possesses both adenine and 2-OH-A DNA glycosylase activities. N/A   
  
 **Gene Ontology Information:**

Molecular Function

- 4 iron, 4 sulfur cluster binding
- 8-oxo-7,8-dihydroguanine DNA N-glycosylase activity
- adenine/guanine mispair binding
- DNA N-glycosylase activity
- metal ion binding
- MutSalpha complex binding
- oxidized purine DNA binding
- purine-specific mismatch base pair DNA N-glycosylase activity

Location

- mitochondrion
- nucleoplasm
- nucleus

Biological process

- base-excision repair
- depurination
- DNA repair
- mismatch repair
- negative regulation of necroptotic process

---

57

- **Protein name:** Kelch-like protein 14
- **Organism:** Homo sapiens
- **Uniprot Accession Number:** Q9P2G3
- **Protein sequence length:** 628 aa
- **1D identity (%):** 14.24
- **1D identity (%) [Gaps excluded]:** 22.61
- **1D identity - Alignment Gaps:** 338
- **1D aligned content (<aminoacid>:%):** {'G': 8.46, 'D': 6.15, 'R': 5.38, 'S': 6.15, 'L': 16.15, 'N': 4.62, 'V': 6.92, 'T': 5.38, 'K': 2.31, 'H': 0.77, 'P': 13.85, 'Y': 4.62, 'C': 3.85, 'E': 3.85, 'F': 2.31, 'Q': 4.62, 'M': 1.54, 'W': 0.77, 'I': 1.54, 'A': 0.77}
- **Common reported functions (%):** 0.0
- **Common reported locations (%):** 22.22
- **Common reported processes (%):** 0.0

- **AF ID:** Q9P2G3
- **Chain:** A
- **Protein length:** 628 aa
- **Resolution:** N/A
- **b-phipsi:** 0.005679
- **w-rdist:** 0.353527
- **t-alpha:** 0.015056
- **Chemical similarity (Tanimoto Index) (%):** 99.47
- **1D identity (%) [PDB]:** 3.44
- **1D identity (%) [Gaps excluded][PDB]:** 63.16
- **1D identity - Alignment Gaps [PDB]:** 1321
- **1D aligned content [PDB] (<aminoacid>:%):** {'R': 6.25, 'S': 12.5, 'L': 16.67, 'Y': 2.08, 'T': 2.08, 'A': 4.17, 'N': 2.08, 'V': 14.58, 'D': 6.25, 'P': 12.5, 'G': 8.33, 'K': 4.17, 'F': 2.08, 'W': 2.08, 'H': 2.08, 'I': 2.08}
- **2D identity (%) [PDB]:** 34.77
- **2D identity (%) [Gaps excluded][PDB]:** 85.45
- **2D identity - Alignment Gaps [PDB]:** 621
- **2D aligned content [PDB] (<2D-fold>:%):** {'.': 28.57, 'E': 21.7, 'T': 24.45, 'H': 23.63, 'B': 0.82, 'G': 0.82}
- **3D similarity (TM-Score) (%) [PDB]:** 21.05

- **Gene name:** KLHL14
- **Entrez ID:** 421100
- **RefSeq ID:** NM\_020805
- **Transcript sequence length:** 4271
- **5-UTR|CDS|3-UTR identity (%):** 34.85 | 36.31 | 7.76
- **5-UTR|CDS|3-UTR identity (%) [Gaps excluded]:** 71.31 | 75.09 | 75.98
- **5-UTR|CDS|3-UTR identity [Alignment Gaps]:** 248 | 1556 | 15267
- **5-UTR aligned content (<base>:%):** {'A': 8.88, 'G': 30.77, 'T': 24.26, 'C': 36.09}
- **CDS aligned content (<base>:%):** {'A': 26.33, 'T': 22.3, 'C': 24.77, 'G': 26.6}
- **3-UTR aligned content (<base>:%):** {'C': 15.47, 'A': 34.65, 'G': 15.62, 'T': 34.27}

**Uniprot Description:**  
  
 N/A   
  
Interacts with TOR1A, preferentially with the ATP-free form.   
  
 **Gene Ontology Information:**

Molecular Function   
  
N/A

Location

- cytoplasm
- cytosol
- endoplasmic reticulum
- endoplasmic reticulum membrane
- neuron projection
- neuronal cell body

Biological process   
  
N/A

---

58

- **Protein name:** Amyloid-beta A4 precursor protein-binding family B member 3
- **Organism:** Homo sapiens
- **Uniprot Accession Number:** O95704
- **Protein sequence length:** 486 aa
- **1D identity (%):** 9.81
- **1D identity (%) [Gaps excluded]:** 23.37
- **1D identity - Alignment Gaps:** 550
- **1D aligned content (<aminoacid>:%):** {'G': 6.45, 'K': 5.38, 'D': 4.3, 'W': 3.23, 'H': 5.38, 'L': 10.75, 'E': 4.3, 'P': 9.68, 'A': 8.6, 'S': 3.23, 'T': 6.45, 'R': 7.53, 'Y': 2.15, 'I': 2.15, 'Q': 7.53, 'M': 4.3, 'C': 4.3, 'V': 4.3}
- **Common reported functions (%):** 0.0
- **Common reported locations (%):** 33.33
- **Common reported processes (%):** 0.0

- **AF ID:** O95704
- **Chain:** A
- **Protein length:** 486 aa
- **Resolution:** N/A
- **b-phipsi:** 0.003739
- **w-rdist:** 0.345514
- **t-alpha:** 0.047303
- **Chemical similarity (Tanimoto Index) (%):** 98.88
- **1D identity (%) [PDB]:** 2.58
- **1D identity (%) [Gaps excluded][PDB]:** 63.46
- **1D identity - Alignment Gaps [PDB]:** 1227
- **1D aligned content [PDB] (<aminoacid>:%):** {'P': 12.12, 'G': 6.06, 'R': 6.06, 'S': 12.12, 'F': 9.09, 'A': 15.15, 'E': 3.03, 'D': 3.03, 'H': 3.03, 'L': 6.06, 'V': 9.09, 'W': 3.03, 'M': 3.03, 'N': 3.03, 'I': 3.03, 'K': 3.03}
- **2D identity (%) [PDB]:** 38.62
- **2D identity (%) [Gaps excluded][PDB]:** 84.65
- **2D identity - Alignment Gaps [PDB]:** 497
- **2D aligned content [PDB] (<2D-fold>:%):** {'.': 28.05, 'E': 23.51, 'T': 19.55, 'H': 28.05, 'G': 0.85}
- **3D similarity (TM-Score) (%) [PDB]:** 17.01

- **Gene name:** APBB3
- **Entrez ID:** 10307
- **RefSeq ID:** NM\_133174
- **Transcript sequence length:** 2125
- **5-UTR|CDS|3-UTR identity (%):** 45.15 | 31.05 | 1.28
- **5-UTR|CDS|3-UTR identity (%) [Gaps excluded]:** 75.79 | 75.66 | 76.79
- **5-UTR|CDS|3-UTR identity [Alignment Gaps]:** 171 | 1688 | 16512
- **5-UTR aligned content (<base>:%):** {'G': 40.31, 'T': 14.66, 'C': 38.74, 'A': 6.28}
- **CDS aligned content (<base>:%):** {'A': 21.93, 'T': 21.93, 'G': 29.92, 'C': 26.21}
- **3-UTR aligned content (<base>:%):** {'A': 22.33, 'C': 24.19, 'T': 37.21, 'G': 16.28}

**Uniprot Description:**  
  
 May modulate the internalization of amyloid-beta precursor protein.   
  
Interacts with APP (via intracellular domain) (PubMed:10081969). Interacts with APLP1 and APLP2 (via intracellular domain) (PubMed:10081969).   
  
 **Gene Ontology Information:**

Molecular Function

- amyloid-beta binding
- low-density lipoprotein particle receptor binding

Location

- actin cytoskeleton
- cytoplasm
- cytosol
- membrane
- nucleus

Biological process

- positive regulation of protein secretion
- regulation of transcription, DNA-templated

---

59

- **Protein name:** Ribosomal protein S6 kinase-like 1
- **Organism:** Homo sapiens
- **Uniprot Accession Number:** Q9Y6S9
- **Protein sequence length:** 549 aa
- **1D identity (%):** 14.57
- **1D identity (%) [Gaps excluded]:** 25.15
- **1D identity - Alignment Gaps:** 375
- **1D aligned content (<aminoacid>:%):** {'L': 9.23, 'P': 10.0, 'G': 13.08, 'C': 3.08, 'V': 6.92, 'Y': 3.08, 'D': 3.85, 'R': 10.0, 'Q': 5.38, 'N': 1.54, 'K': 3.08, 'E': 6.15, 'S': 4.62, 'A': 6.92, 'T': 6.15, 'H': 3.08, 'W': 1.54, 'I': 0.77, 'F': 1.54}
- **Common reported functions (%):** 0.0
- **Common reported locations (%):** 0.0
- **Common reported processes (%):** 0.0

- **AF ID:** Q9Y6S9
- **Chain:** A
- **Protein length:** 549 aa
- **Resolution:** N/A
- **b-phipsi:** 0.000686
- **w-rdist:** 0.373331
- **t-alpha:** 0.153724
- **Chemical similarity (Tanimoto Index) (%):** 97.33
- **1D identity (%) [PDB]:** 1.54
- **1D identity (%) [Gaps excluded][PDB]:** 80.77
- **1D identity - Alignment Gaps [PDB]:** 1342
- **1D aligned content [PDB] (<aminoacid>:%):** {'L': 9.52, 'V': 9.52, 'A': 14.29, 'R': 9.52, 'Y': 4.76, 'D': 9.52, 'K': 4.76, 'E': 9.52, 'S': 14.29, 'G': 9.52, 'H': 4.76}
- **2D identity (%) [PDB]:** 26.25
- **2D identity (%) [Gaps excluded][PDB]:** 89.56
- **2D identity - Alignment Gaps [PDB]:** 762
- **2D aligned content [PDB] (<2D-fold>:%):** {'.': 17.67, 'H': 47.7, 'T': 21.91, 'G': 2.83, 'E': 9.89}
- **3D similarity (TM-Score) (%) [PDB]:** 21.33

- **Gene name:** RPS6KL1
- **Entrez ID:** 83694
- **RefSeq ID:** NM\_031464
- **Transcript sequence length:** 5322
- **5-UTR|CDS|3-UTR identity (%):** 35.59 | 37.6 | 11.1
- **5-UTR|CDS|3-UTR identity (%) [Gaps excluded]:** 76.35 | 73.31 | 74.03
- **5-UTR|CDS|3-UTR identity [Alignment Gaps]:** 339 | 1363 | 14650
- **5-UTR aligned content (<base>:%):** {'G': 30.97, 'C': 46.46, 'T': 15.93, 'A': 6.64}
- **CDS aligned content (<base>:%):** {'T': 19.2, 'G': 29.75, 'A': 23.0, 'C': 28.04}
- **3-UTR aligned content (<base>:%):** {'G': 23.16, 'A': 28.33, 'C': 23.16, 'T': 25.35}

**Uniprot Description:**  
  
 N/A N/A   
  
 **Gene Ontology Information:**

Molecular Function

- ATP binding
- protein serine kinase activity
- protein serine/threonine kinase activity

Location

- ribonucleoprotein complex
- ribosome

Biological process

- protein phosphorylation

---

60

- **Protein name:** Insulin-like growth factor 2 mRNA-binding protein 2
- **Organism:** Homo sapiens
- **Uniprot Accession Number:** Q9Y6M1
- **Protein sequence length:** 599 aa
- **1D identity (%):** 14.15
- **1D identity (%) [Gaps excluded]:** 25.63
- **1D identity - Alignment Gaps:** 421
- **1D aligned content (<aminoacid>:%):** {'N': 3.01, 'F': 1.5, 'G': 12.03, 'D': 3.76, 'R': 6.77, 'S': 3.76, 'Y': 1.5, 'P': 8.27, 'T': 6.77, 'L': 6.77, 'K': 6.77, 'V': 8.27, 'H': 4.51, 'E': 5.26, 'Q': 6.02, 'I': 9.02, 'A': 5.26, 'M': 0.75}
- **Common reported functions (%):** 14.29
- **Common reported locations (%):** 33.33
- **Common reported processes (%):** 0.0

- **AF ID:** Q9Y6M1
- **Chain:** A
- **Protein length:** 599 aa
- **Resolution:** N/A
- **b-phipsi:** 0.002415
- **w-rdist:** 0.189135
- **t-alpha:** 0.153724
- **Chemical similarity (Tanimoto Index) (%):** 98.94
- **1D identity (%) [PDB]:** 2.14
- **1D identity (%) [Gaps excluded][PDB]:** 71.43
- **1D identity - Alignment Gaps [PDB]:** 1360
- **1D aligned content [PDB] (<aminoacid>:%):** {'L': 10.0, 'P': 6.67, 'A': 10.0, 'G': 3.33, 'Q': 6.67, 'R': 13.33, 'I': 10.0, 'K': 10.0, 'T': 6.67, 'D': 6.67, 'N': 3.33, 'S': 6.67, 'E': 6.67}
- **2D identity (%) [PDB]:** 36.96
- **2D identity (%) [Gaps excluded][PDB]:** 91.35
- **2D identity - Alignment Gaps [PDB]:** 612
- **2D aligned content [PDB] (<2D-fold>:%):** {'.': 13.68, 'E': 28.68, 'H': 43.95, 'T': 12.89, 'G': 0.79}
- **3D similarity (TM-Score) (%) [PDB]:** 20.94

- **Gene name:** IGF2BP2
- **Entrez ID:** 10644
- **RefSeq ID:** NM\_006548
- **Transcript sequence length:** 4283
- **5-UTR|CDS|3-UTR identity (%):** 14.84 | 41.06 | 8.62
- **5-UTR|CDS|3-UTR identity (%) [Gaps excluded]:** 74.63 | 73.93 | 74.8
- **5-UTR|CDS|3-UTR identity [Alignment Gaps]:** 270 | 1253 | 15196
- **5-UTR aligned content (<base>:%):** {'G': 66.0, 'C': 20.0, 'A': 12.0, 'T': 2.0}
- **CDS aligned content (<base>:%):** {'A': 29.04, 'T': 20.57, 'G': 26.27, 'C': 24.11}
- **3-UTR aligned content (<base>:%):** {'G': 19.99, 'C': 20.93, 'A': 32.55, 'T': 26.54}

**Uniprot Description:**  
  
 RNA-binding factor that recruits target transcripts to cytoplasmic protein-RNA complexes (mRNPs). This transcript 'caging' into mRNPs allows mRNA transport and transient storage. It also modulates the rate and location at which target transcripts encounter the translational apparatus and shields them from endonuclease attacks or microRNA-mediated degradation (By similarity). Binds to the 5'-UTR of the insulin-like growth factor 2 (IGF2) mRNAs. Binding is isoform-specific. Binds to beta-actin/ACTB and MYC transcripts.   
  
Can form homooligomers and heterooligomers with IGF2BP1 and IGF2BP3 in an RNA-dependent manner. Interacts with HNRPD and IGF2BP1. Interacts with ELAVL1, DHX9 and HNRNPU.   
  
 **Gene Ontology Information:**

Molecular Function

- mRNA 3'-UTR binding
- mRNA 5'-UTR binding
- N6-methyladenosine-containing RNA binding
- RNA binding
- translation regulator activity
- translation regulator activity, nucleic acid binding

Location

- cytoplasm
- cytoplasmic stress granule
- cytoskeleton
- cytosol
- nucleus
- P-body

Biological process

- anatomical structure morphogenesis
- cold-induced thermogenesis
- CRD-mediated mRNA stabilization
- energy homeostasis
- mRNA transport
- negative regulation of translation
- nervous system development
- regulation of cytokine production
- regulation of gene expression

---

61

- **Protein name:** Uncharacterized protein ZSWIM9
- **Organism:** Homo sapiens
- **Uniprot Accession Number:** Q86XI8
- **Protein sequence length:** 627 aa
- **1D identity (%):** 13.38
- **1D identity (%) [Gaps excluded]:** 22.6
- **1D identity - Alignment Gaps:** 381
- **1D aligned content (<aminoacid>:%):** {'P': 14.4, 'E': 4.0, 'F': 4.0, 'D': 5.6, 'R': 13.6, 'K': 3.2, 'S': 5.6, 'A': 5.6, 'T': 2.4, 'V': 8.0, 'G': 12.0, 'L': 10.4, 'N': 0.8, 'H': 0.8, 'Q': 3.2, 'C': 3.2, 'M': 0.8, 'W': 0.8, 'I': 0.8, 'Y': 0.8}
- **Common reported functions (%):** 0.0
- **Common reported locations (%):** 0.0
- **Common reported processes (%):** 0.0

- **AF ID:** Q86XI8
- **Chain:** A
- **Protein length:** 627 aa
- **Resolution:** N/A
- **b-phipsi:** 0.000626
- **w-rdist:** 0.51889
- **t-alpha:** 0.098257
- **Chemical similarity (Tanimoto Index) (%):** 99.55
- **1D identity (%) [PDB]:** 1.73
- **1D identity (%) [Gaps excluded][PDB]:** 89.29
- **1D identity - Alignment Gaps [PDB]:** 1416
- **1D aligned content [PDB] (<aminoacid>:%):** {'L': 16.0, 'Y': 4.0, 'T': 16.0, 'A': 4.0, 'N': 4.0, 'P': 8.0, 'V': 12.0, 'G': 16.0, 'D': 12.0, 'E': 4.0, 'R': 4.0}
- **2D identity (%) [PDB]:** 39.47
- **2D identity (%) [Gaps excluded][PDB]:** 90.81
- **2D identity - Alignment Gaps [PDB]:** 580
- **2D aligned content [PDB] (<2D-fold>:%):** {'.': 19.51, 'H': 42.47, 'E': 10.37, 'T': 26.17, 'G': 1.48}
- **3D similarity (TM-Score) (%) [PDB]:** 18.51

- **Gene name:** ZSWIM9
- **Entrez ID:** 374920
- **RefSeq ID:** N/A
- **Sequence length:** N/A
- **5-UTR|CDS|3-UTR identity (%):** N/A | N/A | N/A
- **5-UTR|CDS|3-UTR identity (%) [Gaps excluded]:** N/A | N/A | N/A
- **5-UTR|CDS|3-UTR identity [Alignment Gaps]:** N/A | N/A | N/A
- **5-UTR aligned content (<base>:%):** N/A
- **CDS aligned content (<base>:%):** N/A
- **3-UTR aligned content (<base>:%):** N/A

**Uniprot Description:**  
  
 N/A N/A   
  
 **Gene Ontology Information:**

Molecular Function   
  
N/A

Location   
  
N/A

Biological process   
  
N/A

---

62

- **Protein name:** Putative E3 ubiquitin-protein ligase UBR7
- **Organism:** Homo sapiens
- **Uniprot Accession Number:** Q8N806
- **Protein sequence length:** 425 aa
- **1D identity (%):** 10.08
- **1D identity (%) [Gaps excluded]:** 24.73
- **1D identity - Alignment Gaps:** 541
- **1D aligned content (<aminoacid>:%):** {'G': 10.87, 'V': 5.43, 'E': 7.61, 'Y': 4.35, 'Q': 8.7, 'C': 4.35, 'P': 4.35, 'K': 8.7, 'L': 7.61, 'T': 4.35, 'S': 4.35, 'N': 1.09, 'D': 7.61, 'F': 5.43, 'R': 4.35, 'M': 2.17, 'A': 3.26, 'I': 5.43}
- **Common reported functions (%):** 0.0
- **Common reported locations (%):** 0.0
- **Common reported processes (%):** 0.0

- **AF ID:** Q8N806
- **Chain:** A
- **Protein length:** 425 aa
- **Resolution:** N/A
- **b-phipsi:** 0.000965
- **w-rdist:** 0.359292
- **t-alpha:** 0.07924
- **Chemical similarity (Tanimoto Index) (%):** 99.17
- **1D identity (%) [PDB]:** 1.78
- **1D identity (%) [Gaps excluded][PDB]:** 59.46
- **1D identity - Alignment Gaps [PDB]:** 1196
- **1D aligned content [PDB] (<aminoacid>:%):** {'A': 13.64, 'R': 13.64, 'Q': 9.09, 'E': 13.64, 'I': 4.55, 'S': 9.09, 'L': 4.55, 'V': 4.55, 'N': 4.55, 'T': 4.55, 'D': 4.55, 'F': 9.09, 'K': 4.55}
- **2D identity (%) [PDB]:** 29.26
- **2D identity (%) [Gaps excluded][PDB]:** 89.46
- **2D identity - Alignment Gaps [PDB]:** 644
- **2D aligned content [PDB] (<2D-fold>:%):** {'.': 28.93, 'H': 42.86, 'T': 20.0, 'E': 8.21}
- **3D similarity (TM-Score) (%) [PDB]:** 15.3

- **Gene name:** UBR7
- **Entrez ID:** N/A
- **RefSeq ID:** NM\_175748
- **Transcript sequence length:** 3494
- **5-UTR|CDS|3-UTR identity (%):** 7.72 | 28.8 | 8.04
- **5-UTR|CDS|3-UTR identity (%) [Gaps excluded]:** 73.53 | 75.89 | 75.69
- **5-UTR|CDS|3-UTR identity [Alignment Gaps]:** 290 | 1737 | 15299
- **5-UTR aligned content (<base>:%):** {'G': 48.0, 'C': 40.0, 'T': 8.0, 'A': 4.0}
- **CDS aligned content (<base>:%):** {'A': 32.88, 'T': 23.33, 'G': 25.68, 'C': 18.11}
- **3-UTR aligned content (<base>:%):** {'A': 26.6, 'G': 17.73, 'T': 37.72, 'C': 17.95}

**Uniprot Description:**  
  
 E3 ubiquitin-protein ligase which is a component of the N-end rule pathway. Recognizes and binds to proteins bearing specific N-terminal residues that are destabilizing according to the N-end rule, leading to their ubiquitination and subsequent degradation. N/A   
  
 **Gene Ontology Information:**

Molecular Function

- ubiquitin protein ligase activity
- zinc ion binding

Location   
  
N/A

Biological process

- protein ubiquitination

---

63

- **Protein name:** E3 ubiquitin-protein ligase rififylin
- **Organism:** Homo sapiens
- **Uniprot Accession Number:** Q8WZ73
- **Protein sequence length:** 363 aa
- **1D identity (%):** 7.44
- **1D identity (%) [Gaps excluded]:** 24.82
- **1D identity - Alignment Gaps:** 659
- **1D aligned content (<aminoacid>:%):** {'C': 8.57, 'F': 4.29, 'Q': 7.14, 'P': 12.86, 'Y': 1.43, 'S': 5.71, 'T': 5.71, 'K': 7.14, 'G': 7.14, 'A': 5.71, 'N': 2.86, 'L': 10.0, 'V': 7.14, 'I': 4.29, 'D': 4.29, 'E': 4.29, 'R': 1.43}
- **Common reported functions (%):** 14.29
- **Common reported locations (%):** 44.44
- **Common reported processes (%):** 0.0

- **AF ID:** Q8WZ73
- **Chain:** A
- **Protein length:** 363 aa
- **Resolution:** N/A
- **b-phipsi:** 0.000328
- **w-rdist:** 0.46211
- **t-alpha:** 0.179873
- **Chemical similarity (Tanimoto Index) (%):** 99.47
- **1D identity (%) [PDB]:** 1.87
- **1D identity (%) [Gaps excluded][PDB]:** 75.86
- **1D identity - Alignment Gaps [PDB]:** 1150
- **1D aligned content [PDB] (<aminoacid>:%):** {'M': 4.55, 'W': 4.55, 'A': 13.64, 'F': 4.55, 'T': 4.55, 'R': 9.09, 'C': 4.55, 'E': 9.09, 'I': 9.09, 'L': 9.09, 'K': 9.09, 'G': 4.55, 'D': 4.55, 'Q': 4.55, 'S': 4.55}
- **2D identity (%) [PDB]:** 25.51
- **2D identity (%) [Gaps excluded][PDB]:** 88.19
- **2D identity - Alignment Gaps [PDB]:** 666
- **2D aligned content [PDB] (<2D-fold>:%):** {'.': 20.92, 'T': 39.33, 'E': 9.62, 'H': 30.13}
- **3D similarity (TM-Score) (%) [PDB]:** 14.28

- **Gene name:** RFFL
- **Entrez ID:** N/A
- **RefSeq ID:** NM\_001017368
- **Transcript sequence length:** 7210
- **5-UTR|CDS|3-UTR identity (%):** 21.35 | 23.94 | 21.72
- **5-UTR|CDS|3-UTR identity (%) [Gaps excluded]:** 71.7 | 76.02 | 76.13
- **5-UTR|CDS|3-UTR identity [Alignment Gaps]:** 250 | 1915 | 12643
- **5-UTR aligned content (<base>:%):** {'C': 35.53, 'T': 31.58, 'G': 23.68, 'A': 9.21}
- **CDS aligned content (<base>:%):** {'A': 26.91, 'G': 26.16, 'T': 20.63, 'C': 26.31}
- **3-UTR aligned content (<base>:%):** {'A': 31.11, 'C': 18.38, 'T': 27.83, 'G': 22.68}

**Uniprot Description:**  
  
 E3 ubiquitin-protein ligase that regulates several biological processes through the ubiquitin-mediated proteasomal degradation of various target proteins. Mediates 'Lys-48'-linked polyubiquitination of PRR5L and its subsequent proteasomal degradation thereby indirectly regulating cell migration through the mTORC2 complex. Ubiquitinates the caspases CASP8 and CASP10, promoting their proteasomal degradation, to negatively regulate cell death downstream of death domain receptors in the extrinsic pathway of apoptosis. Negatively regulates the tumor necrosis factor-mediated signaling pathway through targeting of RIPK1 to ubiquitin-mediated proteasomal degradation. Negatively regulates p53/TP53 through its direct ubiquitination and targeting to proteasomal degradation. Indirectly, may also negatively regulate p53/TP53 through ubiquitination and degradation of SFN. May also play a role in endocytic recycling.   
  
Interacts with CASP8 and CASP10. Interacts with RIPK1 (via protein kinase domain); involved in RIPK1 ubiquitination. Interacts with PRR5L. Interacts (via RING-type zinc finger) with p53/TP53; involved in p53/TP53 ubiquitination. Interacts (via RING-type zinc finger) with MDM2; the interaction stabilizes MDM2.   
  
 **Gene Ontology Information:**

Molecular Function

- metal ion binding
- p53 binding
- protease binding
- protein kinase binding
- ubiquitin protein ligase activity
- ubiquitin protein ligase binding

Location

- cytoplasm
- cytosol
- endosome membrane
- membrane
- nucleoplasm
- plasma membrane
- recycling endosome membrane

Biological process

- apoptotic process
- negative regulation of cysteine-type endopeptidase activity involved in execution phase of apoptosis
- negative regulation of extrinsic apoptotic signaling pathway via death domain receptors
- negative regulation of signal transduction by p53 class mediator
- negative regulation of tumor necrosis factor-mediated signaling pathway
- proteasome-mediated ubiquitin-dependent protein catabolic process
- protein K48-linked ubiquitination
- regulation of fibroblast migration
- regulation of signal transduction by p53 class mediator
- regulation of TOR signaling
- ubiquitin-dependent protein catabolic process

---

64

- **Protein name:** Calpain-7
- **Organism:** Homo sapiens
- **Uniprot Accession Number:** Q9Y6W3
- **Protein sequence length:** 813 aa
- **1D identity (%):** 16.06
- **1D identity (%) [Gaps excluded]:** 22.93
- **1D identity - Alignment Gaps:** 295
- **1D aligned content (<aminoacid>:%):** {'M': 1.27, 'Q': 5.06, 'R': 5.06, 'G': 8.23, 'I': 6.96, 'K': 7.59, 'Y': 2.53, 'L': 6.96, 'V': 7.59, 'D': 6.33, 'H': 1.9, 'F': 7.59, 'T': 7.59, 'A': 1.9, 'E': 3.8, 'P': 10.76, 'N': 3.16, 'S': 3.16, 'C': 1.9, 'W': 0.63}
- **Common reported functions (%):** 0.0
- **Common reported locations (%):** 11.11
- **Common reported processes (%):** 0.0

- **AF ID:** Q9Y6W3
- **Chain:** A
- **Protein length:** 813 aa
- **Resolution:** N/A
- **b-phipsi:** 0.007587
- **w-rdist:** 0.544838
- **t-alpha:** 0.002377
- **Chemical similarity (Tanimoto Index) (%):** 99.62
- **1D identity (%) [PDB]:** 3.29
- **1D identity (%) [Gaps excluded][PDB]:** 66.67
- **1D identity - Alignment Gaps [PDB]:** 1502
- **1D aligned content [PDB] (<aminoacid>:%):** {'M': 1.92, 'V': 13.46, 'Q': 1.92, 'H': 1.92, 'F': 3.85, 'T': 9.62, 'D': 7.69, 'K': 5.77, 'L': 13.46, 'Y': 1.92, 'A': 3.85, 'N': 1.92, 'P': 9.62, 'G': 7.69, 'E': 5.77, 'S': 5.77, 'I': 1.92, 'R': 1.92}
- **2D identity (%) [PDB]:** 36.32
- **2D identity (%) [Gaps excluded][PDB]:** 87.84
- **2D identity - Alignment Gaps [PDB]:** 688
- **2D aligned content [PDB] (<2D-fold>:%):** {'.': 26.76, 'H': 21.13, 'T': 24.41, 'E': 27.23, 'B': 0.47}
- **3D similarity (TM-Score) (%) [PDB]:** 21.96

- **Gene name:** CAPN7
- **Entrez ID:** 23473
- **RefSeq ID:** NM\_014296
- **Transcript sequence length:** 4348
- **5-UTR|CDS|3-UTR identity (%):** 47.09 | 47.46 | 6.17
- **5-UTR|CDS|3-UTR identity (%) [Gaps excluded]:** 80.57 | 75.72 | 75.87
- **5-UTR|CDS|3-UTR identity [Alignment Gaps]:** 150 | 1153 | 15643
- **5-UTR aligned content (<base>:%):** {'G': 37.65, 'C': 43.53, 'A': 6.47, 'T': 12.35}
- **CDS aligned content (<base>:%):** {'A': 32.67, 'T': 25.58, 'G': 21.69, 'C': 20.05}
- **3-UTR aligned content (<base>:%):** {'T': 38.48, 'A': 35.81, 'G': 14.19, 'C': 11.52}

**Uniprot Description:**  
  
 Calcium-regulated non-lysosomal thiol-protease. N/A   
  
 **Gene Ontology Information:**

Molecular Function

- calcium-dependent cysteine-type endopeptidase activity
- cysteine-type endopeptidase activity
- endopeptidase activity
- MIT domain binding

Location

- centrosome
- cytosol
- extracellular exosome
- nucleus

Biological process

- positive regulation of epithelial cell migration
- proteolysis
- self proteolysis

---

65

- **Protein name:** RNA exonuclease 5
- **Organism:** Homo sapiens
- **Uniprot Accession Number:** Q96IC2
- **Protein sequence length:** 774 aa
- **1D identity (%):** 15.52
- **1D identity (%) [Gaps excluded]:** 24.41
- **1D identity - Alignment Gaps:** 364
- **1D aligned content (<aminoacid>:%):** {'E': 7.74, 'G': 7.74, 'P': 9.68, 'K': 7.74, 'V': 8.39, 'W': 1.29, 'L': 12.9, 'T': 2.58, 'Y': 2.58, 'S': 3.87, 'F': 4.52, 'H': 4.52, 'Q': 4.52, 'C': 5.16, 'D': 4.52, 'R': 5.16, 'N': 1.29, 'A': 3.87, 'I': 1.94}
- **Common reported functions (%):** 14.29
- **Common reported locations (%):** 0.0
- **Common reported processes (%):** 0.0

- **AF ID:** Q96IC2
- **Chain:** A
- **Protein length:** 774 aa
- **Resolution:** N/A
- **b-phipsi:** 0.003908
- **w-rdist:** 0.349171
- **t-alpha:** 0.045959
- **Chemical similarity (Tanimoto Index) (%):** 99.55
- **1D identity (%) [PDB]:** 2.56
- **1D identity (%) [Gaps excluded][PDB]:** 70.18
- **1D identity - Alignment Gaps [PDB]:** 1505
- **1D aligned content [PDB] (<aminoacid>:%):** {'E': 7.5, 'V': 15.0, 'L': 15.0, 'G': 5.0, 'R': 2.5, 'T': 5.0, 'P': 17.5, 'K': 5.0, 'I': 2.5, 'N': 2.5, 'A': 5.0, 'H': 5.0, 'S': 7.5, 'F': 2.5, 'D': 2.5}
- **2D identity (%) [PDB]:** 43.77
- **2D identity (%) [Gaps excluded][PDB]:** 90.7
- **2D identity - Alignment Gaps [PDB]:** 565
- **2D aligned content [PDB] (<2D-fold>:%):** {'.': 23.22, 'T': 19.87, 'E': 18.2, 'H': 38.7}
- **3D similarity (TM-Score) (%) [PDB]:** 24.69

- **Gene name:** REXO5
- **Entrez ID:** 532864
- **RefSeq ID:** N/A
- **Sequence length:** N/A
- **5-UTR|CDS|3-UTR identity (%):** N/A | N/A | N/A
- **5-UTR|CDS|3-UTR identity (%) [Gaps excluded]:** N/A | N/A | N/A
- **5-UTR|CDS|3-UTR identity [Alignment Gaps]:** N/A | N/A | N/A
- **5-UTR aligned content (<base>:%):** N/A
- **CDS aligned content (<base>:%):** N/A
- **3-UTR aligned content (<base>:%):** N/A

**Uniprot Description:**  
  
 N/A N/A   
  
 **Gene Ontology Information:**

Molecular Function

- exonuclease activity
- RNA binding

Location

- nucleolus
- nucleus

Biological process   
  
N/A

---

66

- **Protein name:** DNA topoisomerase I, mitochondrial
- **Organism:** Homo sapiens
- **Uniprot Accession Number:** Q969P6
- **Protein sequence length:** 601 aa
- **1D identity (%):** 13.21
- **1D identity (%) [Gaps excluded]:** 23.75
- **1D identity - Alignment Gaps:** 417
- **1D aligned content (<aminoacid>:%):** {'V': 7.26, 'R': 9.68, 'L': 14.52, 'E': 8.06, 'P': 8.06, 'K': 4.84, 'H': 4.84, 'G': 6.45, 'F': 4.84, 'D': 4.84, 'I': 4.84, 'C': 2.42, 'N': 2.42, 'T': 3.23, 'Q': 4.03, 'S': 3.23, 'A': 5.65, 'Y': 0.81}
- **Common reported functions (%):** 0.0
- **Common reported locations (%):** 11.11
- **Common reported processes (%):** 0.0

- **AF ID:** Q969P6
- **Chain:** A
- **Protein length:** 601 aa
- **Resolution:** N/A
- **b-phipsi:** 0.017694
- **w-rdist:** 0.390474
- **t-alpha:** 0.000792
- **Chemical similarity (Tanimoto Index) (%):** 98.28
- **1D identity (%) [PDB]:** 2.6
- **1D identity (%) [Gaps excluded][PDB]:** 59.02
- **1D identity - Alignment Gaps [PDB]:** 1324
- **1D aligned content [PDB] (<aminoacid>:%):** {'R': 11.11, 'V': 8.33, 'P': 5.56, 'A': 5.56, 'S': 2.78, 'T': 5.56, 'F': 5.56, 'L': 16.67, 'Q': 11.11, 'E': 5.56, 'G': 5.56, 'Y': 5.56, 'K': 8.33, 'H': 2.78}
- **2D identity (%) [PDB]:** 37.78
- **2D identity (%) [Gaps excluded][PDB]:** 87.82
- **2D identity - Alignment Gaps [PDB]:** 576
- **2D aligned content [PDB] (<2D-fold>:%):** {'T': 18.59, '.': 21.47, 'E': 13.09, 'H': 45.03, 'G': 1.57, 'B': 0.26}
- **3D similarity (TM-Score) (%) [PDB]:** 19.05

- **Gene name:** TOP1MT
- **Entrez ID:** 116447
- **RefSeq ID:** NM\_052963
- **Transcript sequence length:** 1942
- **5-UTR|CDS|3-UTR identity (%):** 4.6 | 40.46 | 0.45
- **5-UTR|CDS|3-UTR identity (%) [Gaps excluded]:** 100.0 | 74.34 | 81.52
- **5-UTR|CDS|3-UTR identity [Alignment Gaps]:** 311 | 1295 | 16688
- **5-UTR aligned content (<base>:%):** {'C': 26.67, 'T': 26.67, 'G': 33.33, 'A': 13.33}
- **CDS aligned content (<base>:%):** {'A': 28.61, 'T': 18.35, 'C': 23.13, 'G': 29.91}
- **3-UTR aligned content (<base>:%):** {'G': 16.0, 'A': 36.0, 'C': 13.33, 'T': 34.67}

**Uniprot Description:**  
  
 Releases the supercoiling and torsional tension of DNA introduced during duplication of mitochondrial DNA by transiently cleaving and rejoining one strand of the DNA duplex. Introduces a single-strand break via transesterification at a target site in duplex DNA. The scissile phosphodiester is attacked by the catalytic tyrosine of the enzyme, resulting in the formation of a DNA-(3'-phosphotyrosyl)-enzyme intermediate and the expulsion of a 5'-OH DNA strand. The free DNA strand then rotates around the intact phosphodiester bond on the opposing strand, thus removing DNA supercoils. Finally, in the religation step, the DNA 5'-OH attacks the covalent intermediate to expel the active-site tyrosine and restore the DNA phosphodiester backbone (By similarity). N/A   
  
 **Gene Ontology Information:**

Molecular Function

- DNA binding
- DNA topoisomerase type I (single strand cut, ATP-independent) activity

Location

- chromosome
- mitochondrial nucleoid
- mitochondrion
- nucleoplasm

Biological process

- DNA replication
- DNA topological change

---

67

- **Protein name:** Protein argonaute-1
- **Organism:** Homo sapiens
- **Uniprot Accession Number:** Q9UL18
- **Protein sequence length:** 857 aa
- **1D identity (%):** 84.06
- **1D identity (%) [Gaps excluded]:** 85.55
- **1D identity - Alignment Gaps:** 15
- **1D aligned content (<aminoacid>:%):** {'M': 2.34, 'E': 4.67, 'G': 7.01, 'A': 6.18, 'Q': 6.32, 'P': 6.73, 'R': 6.59, 'T': 5.91, 'K': 6.04, 'I': 5.49, 'L': 7.55, 'N': 2.34, 'F': 4.12, 'V': 8.52, 'D': 5.08, 'Y': 4.26, 'C': 2.61, 'H': 3.16, 'S': 4.26, 'W': 0.82}
- **Common reported functions (%):** 57.14
- **Common reported locations (%):** 77.78
- **Common reported processes (%):** 55.56

- **AF ID:** Q9UL18
- **Chain:** A
- **Protein length:** 857 aa
- **Resolution:** N/A
- **b-phipsi:** 0.007862
- **w-rdist:** 0.231244
- **t-alpha:** 0.052298
- **Chemical similarity (Tanimoto Index) (%):** 100.0
- **1D identity (%) [PDB]:** 83.26
- **1D identity (%) [Gaps excluded][PDB]:** 86.24
- **1D identity - Alignment Gaps [PDB]:** 30
- **1D aligned content [PDB] (<aminoacid>:%):** {'M': 2.36, 'P': 6.8, 'R': 6.66, 'G': 6.66, 'T': 5.96, 'K': 6.1, 'I': 5.55, 'L': 7.63, 'A': 5.96, 'N': 2.36, 'F': 4.16, 'V': 8.6, 'D': 5.13, 'Y': 4.3, 'E': 4.58, 'C': 2.64, 'Q': 6.24, 'H': 3.19, 'S': 4.3, 'W': 0.83}
- **2D identity (%) [PDB]:** 75.06
- **2D identity (%) [Gaps excluded][PDB]:** 85.43
- **2D identity - Alignment Gaps [PDB]:** 110
- **2D aligned content [PDB] (<2D-fold>:%):** {'.': 20.18, 'B': 1.47, 'E': 26.07, 'H': 32.25, 'T': 19.29, 'G': 0.74}
- **3D similarity (TM-Score) (%) [PDB]:** 94.06

- **Gene name:** AGO1
- **Entrez ID:** 26523
- **RefSeq ID:** NM\_012199
- **Transcript sequence length:** 13712
- **5-UTR|CDS|3-UTR identity (%):** 40.56 | 73.37 | 37.87
- **5-UTR|CDS|3-UTR identity (%) [Gaps excluded]:** 80.0 | 76.48 | 74.74
- **5-UTR|CDS|3-UTR identity [Alignment Gaps]:** 175 | 107 | 9062
- **5-UTR aligned content (<base>:%):** {'A': 9.72, 'G': 46.53, 'C': 36.81, 'T': 6.94}
- **CDS aligned content (<base>:%):** {'A': 26.88, 'T': 22.68, 'G': 26.41, 'C': 24.03}
- **3-UTR aligned content (<base>:%):** {'A': 29.76, 'G': 20.62, 'C': 17.03, 'T': 32.58}

**Uniprot Description:**  
  
 Required for RNA-mediated gene silencing (RNAi). Binds to short RNAs such as microRNAs (miRNAs) or short interfering RNAs (siRNAs), and represses the translation of mRNAs which are complementary to them. Lacks endonuclease activity and does not appear to cleave target mRNAs. Also required for transcriptional gene silencing (TGS) of promoter regions which are complementary to bound short antigene RNAs (agRNAs).   
  
Interacts with DDB1, DDX5, DDX6, DHX30, DHX36, DDX47, DICER1, AGO2, ELAVL1, HNRNPF, IGF2BP1, ILF3, IMP8, MATR3, MOV10, PABPC1, PRMT5, RBM4, SART3, TNRC6B, UPF1 and YBX1. Associates with polysomes and messenger ribonucleoproteins (mNRPs). Interacts with LIMD1, WTIP and AJUBA. Interacts with APOBEC3F, APOBEC3G and APOBEC3H.   
  
 **Gene Ontology Information:**

Molecular Function

- core promoter sequence-specific DNA binding
- double-stranded RNA binding
- miRNA binding
- RNA binding
- RNA polymerase II complex binding
- single-stranded RNA binding

Location

- cytoplasm
- cytoplasmic ribonucleoprotein granule
- cytosol
- nucleoplasm
- nucleus
- P-body
- polysome
- ribonucleoprotein complex
- RISC complex
- RISC-loading complex

Biological process

- production of miRNAs involved in gene silencing by miRNA
- miRNA mediated inhibition of translation
- negative regulation of angiogenesis
- nuclear-transcribed mRNA catabolic process
- positive regulation of transcription by RNA polymerase II
- pre-miRNA processing
- small RNA loading onto RISC
- RNA secondary structure unwinding

---

68

- **Protein name:** Zinc finger protein 391
- **Organism:** Homo sapiens
- **Uniprot Accession Number:** Q9UJN7
- **Protein sequence length:** 358 aa
- **1D identity (%):** 7.17
- **1D identity (%) [Gaps excluded]:** 20.83
- **1D identity - Alignment Gaps:** 594
- **1D aligned content (<aminoacid>:%):** {'P': 13.85, 'E': 7.69, 'S': 6.15, 'R': 6.15, 'F': 4.62, 'K': 7.69, 'V': 1.54, 'T': 4.62, 'G': 13.85, 'L': 9.23, 'H': 6.15, 'I': 4.62, 'C': 4.62, 'Q': 6.15, 'Y': 1.54, 'D': 1.54}
- **Common reported functions (%):** 14.29
- **Common reported locations (%):** 0.0
- **Common reported processes (%):** 0.0

- **AF ID:** Q9UJN7
- **Chain:** A
- **Protein length:** 358 aa
- **Resolution:** N/A
- **b-phipsi:** 0.00635
- **w-rdist:** 0.464304
- **t-alpha:** 0.003978
- **Chemical similarity (Tanimoto Index) (%):** 98.49
- **1D identity (%) [PDB]:** 1.37
- **1D identity (%) [Gaps excluded][PDB]:** 51.61
- **1D identity - Alignment Gaps [PDB]:** 1141
- **1D aligned content [PDB] (<aminoacid>:%):** {'E': 6.25, 'L': 6.25, 'G': 12.5, 'T': 6.25, 'D': 6.25, 'K': 18.75, 'I': 12.5, 'P': 12.5, 'Q': 6.25, 'C': 12.5}
- **2D identity (%) [PDB]:** 31.5
- **2D identity (%) [Gaps excluded][PDB]:** 90.35
- **2D identity - Alignment Gaps [PDB]:** 581
- **2D aligned content [PDB] (<2D-fold>:%):** {'T': 34.16, '.': 19.57, 'E': 11.74, 'H': 34.52}
- **3D similarity (TM-Score) (%) [PDB]:** 14.94

- **Gene name:** ZNF391
- **Entrez ID:** 346157
- **RefSeq ID:** N/A
- **Sequence length:** N/A
- **5-UTR|CDS|3-UTR identity (%):** N/A | N/A | N/A
- **5-UTR|CDS|3-UTR identity (%) [Gaps excluded]:** N/A | N/A | N/A
- **5-UTR|CDS|3-UTR identity [Alignment Gaps]:** N/A | N/A | N/A
- **5-UTR aligned content (<base>:%):** N/A
- **CDS aligned content (<base>:%):** N/A
- **3-UTR aligned content (<base>:%):** N/A

**Uniprot Description:**  
  
 May be involved in transcriptional regulation. N/A   
  
 **Gene Ontology Information:**

Molecular Function

- DNA-binding transcription factor activity, RNA polymerase II-specific
- metal ion binding
- RNA polymerase II transcription regulatory region sequence-specific DNA binding

Location

- nucleus

Biological process

- regulation of transcription by RNA polymerase II

---

69

- **Protein name:** BTB/POZ domain-containing protein 1
- **Organism:** Homo sapiens
- **Uniprot Accession Number:** Q9H0C5
- **Protein sequence length:** 482 aa
- **1D identity (%):** 10.82
- **1D identity (%) [Gaps excluded]:** 23.19
- **1D identity - Alignment Gaps:** 488
- **1D aligned content (<aminoacid>:%):** {'A': 8.08, 'P': 13.13, 'G': 13.13, 'L': 8.08, 'R': 8.08, 'F': 5.05, 'M': 1.01, 'N': 4.04, 'T': 6.06, 'I': 5.05, 'D': 3.03, 'E': 3.03, 'K': 3.03, 'Y': 5.05, 'V': 4.04, 'Q': 3.03, 'H': 3.03, 'C': 3.03, 'S': 1.01}
- **Common reported functions (%):** 0.0
- **Common reported locations (%):** 44.44
- **Common reported processes (%):** 0.0

- **AF ID:** Q9H0C5
- **Chain:** A
- **Protein length:** 482 aa
- **Resolution:** N/A
- **b-phipsi:** 0.000368
- **w-rdist:** 0.761902
- **t-alpha:** 0.074042
- **Chemical similarity (Tanimoto Index) (%):** 96.62
- **1D identity (%) [PDB]:** 1.46
- **1D identity (%) [Gaps excluded][PDB]:** 73.08
- **1D identity - Alignment Gaps [PDB]:** 1275
- **1D aligned content [PDB] (<aminoacid>:%):** {'L': 15.79, 'S': 10.53, 'V': 5.26, 'R': 15.79, 'E': 5.26, 'K': 10.53, 'T': 5.26, 'F': 10.53, 'P': 5.26, 'I': 10.53, 'Y': 5.26}
- **2D identity (%) [PDB]:** 34.42
- **2D identity (%) [Gaps excluded][PDB]:** 87.7
- **2D identity - Alignment Gaps [PDB]:** 579
- **2D aligned content [PDB] (<2D-fold>:%):** {'.': 25.3, 'T': 22.87, 'H': 33.23, 'E': 18.6}
- **3D similarity (TM-Score) (%) [PDB]:** 16.03

- **Gene name:** BTBD1
- **Entrez ID:** 53339
- **RefSeq ID:** NM\_025238
- **Transcript sequence length:** 3194
- **5-UTR|CDS|3-UTR identity (%):** 19.64 | 32.4 | 6.54
- **5-UTR|CDS|3-UTR identity (%) [Gaps excluded]:** 75.86 | 75.75 | 78.17
- **5-UTR|CDS|3-UTR identity [Alignment Gaps]:** 249 | 1616 | 15559
- **5-UTR aligned content (<base>:%):** {'G': 40.91, 'C': 36.36, 'T': 12.12, 'A': 10.61}
- **CDS aligned content (<base>:%):** {'A': 29.4, 'T': 24.04, 'G': 24.48, 'C': 22.08}
- **3-UTR aligned content (<base>:%):** {'T': 38.38, 'A': 36.67, 'C': 12.07, 'G': 12.88}

**Uniprot Description:**  
  
 Probable substrate-specific adapter of an E3 ubiquitin-protein ligase complex which mediates the ubiquitination and subsequent proteasomal degradation of target proteins (PubMed:14528312). Seems to regulate expression levels and/or subnuclear distribution of TOP1, via an unknown mechanism (By similarity). May play a role in mesenchymal differentiation where it promotes myogenic differentiation and suppresses adipogenesis (By similarity).   
  
Interacts (via C-terminus) with TOP1 (PubMed:11818025). Interacts with TRIM5 isoform Delta (PubMed:12878161). Interacts with CUL3 (PubMed:14528312).   
  
 **Gene Ontology Information:**

Molecular Function

- cullin family protein binding
- identical protein binding

Location

- cytoplasmic ribonucleoprotein granule
- cytosol
- nucleoplasm
- P-body
- protein-containing complex

Biological process

- muscle organ development
- neurogenesis
- protein ubiquitination
- regulation of protein binding

---

70

- **Protein name:** Meprin A subunit alpha
- **Organism:** Homo sapiens
- **Uniprot Accession Number:** Q16819
- **Protein sequence length:** 746 aa
- **1D identity (%):** 12.8
- **1D identity (%) [Gaps excluded]:** 21.57
- **1D identity - Alignment Gaps:** 410
- **1D aligned content (<aminoacid>:%):** {'S': 4.65, 'F': 6.98, 'V': 7.75, 'Y': 3.88, 'A': 4.65, 'G': 13.18, 'D': 6.2, 'I': 8.53, 'L': 6.2, 'T': 6.98, 'P': 4.65, 'N': 1.55, 'R': 5.43, 'K': 5.43, 'Q': 6.2, 'H': 3.1, 'C': 2.33, 'W': 0.78, 'E': 0.78, 'M': 0.78}
- **Common reported functions (%):** 0.0
- **Common reported locations (%):** 0.0
- **Common reported processes (%):** 0.0

- **AF ID:** Q16819
- **Chain:** A
- **Protein length:** 746 aa
- **Resolution:** N/A
- **b-phipsi:** 0.004977
- **w-rdist:** 0.360687
- **t-alpha:** 0.020602
- **Chemical similarity (Tanimoto Index) (%):** 96.98
- **1D identity (%) [PDB]:** 2.27
- **1D identity (%) [Gaps excluded][PDB]:** 70.0
- **1D identity - Alignment Gaps [PDB]:** 1491
- **1D aligned content [PDB] (<aminoacid>:%):** {'A': 2.86, 'E': 2.86, 'K': 8.57, 'V': 11.43, 'G': 8.57, 'D': 2.86, 'T': 8.57, 'L': 14.29, 'Q': 11.43, 'C': 2.86, 'I': 11.43, 'S': 2.86, 'P': 5.71, 'N': 2.86, 'R': 2.86}
- **2D identity (%) [PDB]:** 37.16
- **2D identity (%) [Gaps excluded][PDB]:** 89.7
- **2D identity - Alignment Gaps [PDB]:** 659
- **2D aligned content [PDB] (<2D-fold>:%):** {'.': 22.01, 'T': 32.78, 'H': 19.86, 'E': 24.64, 'B': 0.24, 'G': 0.48}
- **3D similarity (TM-Score) (%) [PDB]:** 23.25

- **Gene name:** MEP1A
- **Entrez ID:** 4224
- **RefSeq ID:** NM\_005588
- **Transcript sequence length:** 2893
- **5-UTR|CDS|3-UTR identity (%):** 2.17 | 43.61 | 2.47
- **5-UTR|CDS|3-UTR identity (%) [Gaps excluded]:** 70.0 | 75.25 | 77.76
- **5-UTR|CDS|3-UTR identity [Alignment Gaps]:** 312 | 1284 | 16327
- **5-UTR aligned content (<base>:%):** {'C': 42.86, 'T': 28.57, 'G': 28.57}
- **CDS aligned content (<base>:%):** {'A': 29.2, 'T': 23.2, 'G': 25.53, 'C': 22.07}
- **3-UTR aligned content (<base>:%):** {'C': 17.79, 'G': 13.94, 'T': 37.5, 'A': 30.77}

**Uniprot Description:**  
  
 N/A   
  
Homotetramer consisting of disulfide-linked alpha subunits, homooligomer consisting of disulfide-linked alpha subunit homodimers, or heterotetramer of two alpha and two beta subunits formed by non-covalent association of two disulfide-linked heterodimers (By similarity). Interacts with MBL2 through its carbohydrate moiety. This interaction may inhibit its catalytic activity (By similarity).   
  
 **Gene Ontology Information:**

Molecular Function

- metallodipeptidase activity
- metalloendopeptidase activity
- metallopeptidase activity
- zinc ion binding

Location

- extracellular exosome
- extracellular space
- meprin A complex
- plasma membrane

Biological process

- epidermal growth factor receptor ligand maturation
- signaling receptor ligand precursor processing

---

71

- **Protein name:** Kelch-like protein 36
- **Organism:** Homo sapiens
- **Uniprot Accession Number:** Q8N4N3
- **Protein sequence length:** 616 aa
- **1D identity (%):** 12.88
- **1D identity (%) [Gaps excluded]:** 23.06
- **1D identity - Alignment Gaps:** 418
- **1D aligned content (<aminoacid>:%):** {'E': 4.92, 'G': 12.3, 'R': 12.3, 'P': 4.92, 'K': 3.28, 'S': 3.28, 'V': 11.48, 'L': 9.84, 'D': 4.1, 'H': 5.74, 'A': 5.74, 'N': 1.64, 'I': 2.46, 'T': 5.74, 'F': 3.28, 'C': 4.92, 'Q': 2.46, 'W': 0.82, 'Y': 0.82}
- **Common reported functions (%):** 0.0
- **Common reported locations (%):** 0.0
- **Common reported processes (%):** 0.0

- **AF ID:** Q8N4N3
- **Chain:** A
- **Protein length:** 616 aa
- **Resolution:** N/A
- **b-phipsi:** 0.002662
- **w-rdist:** 0.82964
- **t-alpha:** 0.001585
- **Chemical similarity (Tanimoto Index) (%):** 99.17
- **1D identity (%) [PDB]:** 1.82
- **1D identity (%) [Gaps excluded][PDB]:** 86.67
- **1D identity - Alignment Gaps [PDB]:** 1401
- **1D aligned content [PDB] (<aminoacid>:%):** {'R': 19.23, 'V': 19.23, 'D': 11.54, 'S': 3.85, 'Q': 3.85, 'H': 3.85, 'F': 7.69, 'K': 7.69, 'T': 3.85, 'I': 3.85, 'G': 7.69, 'P': 3.85, 'Y': 3.85}
- **2D identity (%) [PDB]:** 35.34
- **2D identity (%) [Gaps excluded][PDB]:** 93.75
- **2D identity - Alignment Gaps [PDB]:** 661
- **2D aligned content [PDB] (<2D-fold>:%):** {'.': 21.33, 'T': 25.6, 'H': 35.47, 'E': 17.33, 'B': 0.27}
- **3D similarity (TM-Score) (%) [PDB]:** 18.47

- **Gene name:** KLHL36
- **Entrez ID:** 768061
- **RefSeq ID:** N/A
- **Sequence length:** N/A
- **5-UTR|CDS|3-UTR identity (%):** N/A | N/A | N/A
- **5-UTR|CDS|3-UTR identity (%) [Gaps excluded]:** N/A | N/A | N/A
- **5-UTR|CDS|3-UTR identity [Alignment Gaps]:** N/A | N/A | N/A
- **5-UTR aligned content (<base>:%):** N/A
- **CDS aligned content (<base>:%):** N/A
- **3-UTR aligned content (<base>:%):** N/A

**Uniprot Description:**  
  
 Probable substrate-specific adapter of an E3 ubiquitin-protein ligase complex which mediates the ubiquitination and subsequent proteasomal degradation of target proteins.   
  
Interacts with CUL3.   
  
 **Gene Ontology Information:**

Molecular Function

- cullin family protein binding

Location   
  
N/A

Biological process

- protein ubiquitination

---

72

- **Protein name:** Calpain-2 catalytic subunit
- **Organism:** Homo sapiens
- **Uniprot Accession Number:** P17655
- **Protein sequence length:** 700 aa
- **1D identity (%):** 12.59
- **1D identity (%) [Gaps excluded]:** 21.57
- **1D identity - Alignment Gaps:** 410
- **1D aligned content (<aminoacid>:%):** {'K': 8.06, 'R': 8.06, 'E': 9.68, 'H': 2.42, 'T': 7.26, 'F': 5.65, 'D': 8.06, 'P': 8.06, 'G': 11.29, 'L': 8.87, 'N': 1.61, 'V': 4.84, 'S': 2.42, 'A': 3.23, 'W': 0.81, 'C': 2.42, 'I': 3.23, 'Y': 1.61, 'Q': 2.42}
- **Common reported functions (%):** 0.0
- **Common reported locations (%):** 11.11
- **Common reported processes (%):** 0.0

- **AF ID:** P17655
- **Chain:** A
- **Protein length:** 700 aa
- **Resolution:** N/A
- **b-phipsi:** 0.000908
- **w-rdist:** 0.517633
- **t-alpha:** 0.02352
- **Chemical similarity (Tanimoto Index) (%):** 99.4
- **1D identity (%) [PDB]:** 2.35
- **1D identity (%) [Gaps excluded][PDB]:** 64.81
- **1D identity - Alignment Gaps [PDB]:** 1437
- **1D aligned content [PDB] (<aminoacid>:%):** {'A': 14.29, 'G': 2.86, 'R': 5.71, 'I': 5.71, 'K': 8.57, 'L': 5.71, 'N': 5.71, 'Q': 5.71, 'T': 5.71, 'S': 8.57, 'D': 5.71, 'E': 11.43, 'V': 8.57, 'F': 5.71}
- **2D identity (%) [PDB]:** 38.27
- **2D identity (%) [Gaps excluded][PDB]:** 90.83
- **2D identity - Alignment Gaps [PDB]:** 629
- **2D aligned content [PDB] (<2D-fold>:%):** {'.': 16.83, 'H': 35.82, 'T': 25.0, 'E': 22.36}
- **3D similarity (TM-Score) (%) [PDB]:** 21.29

- **Gene name:** CAPN2
- **Entrez ID:** 281662
- **RefSeq ID:** NM\_001748
- **Transcript sequence length:** 3366
- **5-UTR|CDS|3-UTR identity (%):** 18.37 | 45.38 | 4.38
- **5-UTR|CDS|3-UTR identity (%) [Gaps excluded]:** 77.78 | 74.56 | 73.22
- **5-UTR|CDS|3-UTR identity [Alignment Gaps]:** 262 | 1140 | 15892
- **5-UTR aligned content (<base>:%):** {'A': 7.94, 'G': 36.51, 'T': 7.94, 'C': 47.62}
- **CDS aligned content (<base>:%):** {'A': 27.46, 'T': 21.63, 'G': 28.29, 'C': 22.62}
- **3-UTR aligned content (<base>:%):** {'G': 14.3, 'T': 35.49, 'A': 35.63, 'C': 14.57}

**Uniprot Description:**  
  
 Calcium-regulated non-lysosomal thiol-protease which catalyzes limited proteolysis of substrates involved in cytoskeletal remodeling and signal transduction. Proteolytically cleaves MYOC at 'Arg-226' (PubMed:17650508). Proteolytically cleaves CPEB3 following neuronal stimulation which abolishes CPEB3 translational repressor activity, leading to translation of CPEB3 target mRNAs (By similarity).   
  
Forms a heterodimer with a small (regulatory) subunit (CAPNS1). Interacts with CPEB3; this leads to cleavage of CPEB3.   
  
 **Gene Ontology Information:**

Molecular Function

- calcium ion binding
- calcium-dependent cysteine-type endopeptidase activity

Location

- cytoplasm
- dendrite
- plasma membrane

Biological process

- cellular response to amino acid stimulus
- proteolysis

---

73

- **Protein name:** 1,4-alpha-glucan-branching enzyme
- **Organism:** Homo sapiens
- **Uniprot Accession Number:** Q04446
- **Protein sequence length:** 702 aa
- **1D identity (%):** 13.72
- **1D identity (%) [Gaps excluded]:** 20.74
- **1D identity - Alignment Gaps:** 318
- **1D aligned content (<aminoacid>:%):** {'T': 2.33, 'P': 6.2, 'E': 8.53, 'L': 10.08, 'A': 6.98, 'V': 6.2, 'D': 4.65, 'R': 5.43, 'G': 10.85, 'F': 6.98, 'H': 3.88, 'W': 1.55, 'Q': 3.1, 'K': 4.65, 'I': 4.65, 'Y': 4.65, 'C': 2.33, 'N': 1.55, 'S': 3.1, 'M': 2.33}
- **Common reported functions (%):** 0.0
- **Common reported locations (%):** 11.11
- **Common reported processes (%):** 0.0

- **AF ID:** Q04446
- **Chain:** A
- **Protein length:** 702 aa
- **Resolution:** N/A
- **b-phipsi:** 0.005383
- **w-rdist:** 0.759755
- **t-alpha:** 0.000793
- **Chemical similarity (Tanimoto Index) (%):** 99.62
- **1D identity (%) [PDB]:** 2.63
- **1D identity (%) [Gaps excluded][PDB]:** 62.9
- **1D identity - Alignment Gaps [PDB]:** 1423
- **1D aligned content [PDB] (<aminoacid>:%):** {'M': 2.56, 'V': 10.26, 'E': 10.26, 'L': 10.26, 'Q': 5.13, 'F': 7.69, 'Y': 7.69, 'K': 7.69, 'S': 7.69, 'R': 2.56, 'I': 10.26, 'D': 5.13, 'G': 5.13, 'A': 5.13, 'P': 2.56}
- **2D identity (%) [PDB]:** 39.89
- **2D identity (%) [Gaps excluded][PDB]:** 87.6
- **2D identity - Alignment Gaps [PDB]:** 579
- **2D aligned content [PDB] (<2D-fold>:%):** {'.': 14.39, 'H': 34.67, 'T': 26.65, 'E': 23.82, 'B': 0.47}
- **3D similarity (TM-Score) (%) [PDB]:** 25.13

- **Gene name:** GBE1
- **Entrez ID:** 100034152
- **RefSeq ID:** NM\_000158
- **Transcript sequence length:** 2941
- **5-UTR|CDS|3-UTR identity (%):** 22.9 | 46.68 | 2.88
- **5-UTR|CDS|3-UTR identity (%) [Gaps excluded]:** 75.24 | 75.74 | 79.51
- **5-UTR|CDS|3-UTR identity [Alignment Gaps]:** 240 | 1114 | 16239
- **5-UTR aligned content (<base>:%):** {'C': 46.84, 'G': 37.97, 'T': 10.13, 'A': 5.06}
- **CDS aligned content (<base>:%):** {'A': 29.74, 'T': 26.57, 'G': 23.47, 'C': 20.22}
- **3-UTR aligned content (<base>:%):** {'A': 31.75, 'G': 13.2, 'C': 9.48, 'T': 45.57}

**Uniprot Description:**  
  
 Required for normal glycogen accumulation (PubMed:8463281, PubMed:26199317, PubMed:8613547). The alpha 1-6 branches of glycogen play an important role in increasing the solubility of the molecule (Probable).   
  
Monomer.   
  
 **Gene Ontology Information:**

Molecular Function

- 1,4-alpha-glucan branching enzyme activity
- 1,4-alpha-glucan branching enzyme activity (using a glucosylated glycogenin as primer for glycogen synthesis)
- cation binding
- hydrolase activity, hydrolyzing O-glycosyl compounds

Location

- cytoplasm

Biological process

- glycogen biosynthetic process

---

74

- **Protein name:** STE20/SPS1-related proline-alanine-rich protein kinase
- **Organism:** Homo sapiens
- **Uniprot Accession Number:** Q9UEW8
- **Protein sequence length:** 545 aa
- **1D identity (%):** 10.43
- **1D identity (%) [Gaps excluded]:** 20.42
- **1D identity - Alignment Gaps:** 455
- **1D aligned content (<aminoacid>:%):** {'P': 12.37, 'V': 13.4, 'A': 5.15, 'T': 5.15, 'W': 3.09, 'L': 10.31, 'E': 8.25, 'G': 10.31, 'K': 7.22, 'D': 6.19, 'M': 2.06, 'N': 2.06, 'R': 3.09, 'F': 3.09, 'H': 1.03, 'Y': 2.06, 'Q': 2.06, 'S': 1.03, 'I': 1.03, 'C': 1.03}
- **Common reported functions (%):** 0.0
- **Common reported locations (%):** 22.22
- **Common reported processes (%):** 0.0

- **AF ID:** Q9UEW8
- **Chain:** A
- **Protein length:** 545 aa
- **Resolution:** N/A
- **b-phipsi:** 0.000747
- **w-rdist:** 0.656757
- **t-alpha:** 0.027734
- **Chemical similarity (Tanimoto Index) (%):** 99.17
- **1D identity (%) [PDB]:** 3.18
- **1D identity (%) [Gaps excluded][PDB]:** 60.0
- **1D identity - Alignment Gaps [PDB]:** 1250
- **1D aligned content [PDB] (<aminoacid>:%):** {'V': 19.05, 'L': 19.05, 'P': 9.52, 'G': 9.52, 'K': 9.52, 'R': 9.52, 'I': 2.38, 'F': 2.38, 'E': 4.76, 'S': 7.14, 'H': 2.38, 'A': 2.38, 'T': 2.38}
- **2D identity (%) [PDB]:** 40.46
- **2D identity (%) [Gaps excluded][PDB]:** 88.53
- **2D identity - Alignment Gaps [PDB]:** 518
- **2D aligned content [PDB] (<2D-fold>:%):** {'.': 28.76, 'T': 22.28, 'H': 33.94, 'E': 15.03}
- **3D similarity (TM-Score) (%) [PDB]:** 16.91

- **Gene name:** STK39
- **Entrez ID:** 106505565; 397618
- **RefSeq ID:** NM\_013233
- **Transcript sequence length:** 3272
- **5-UTR|CDS|3-UTR identity (%):** 30.92 | 36.97 | 5.7
- **5-UTR|CDS|3-UTR identity (%) [Gaps excluded]:** 78.68 | 73.86 | 77.01
- **5-UTR|CDS|3-UTR identity [Alignment Gaps]:** 210 | 1405 | 15715
- **5-UTR aligned content (<base>:%):** {'C': 44.86, 'T': 11.21, 'A': 5.61, 'G': 38.32}
- **CDS aligned content (<base>:%):** {'A': 32.4, 'G': 24.81, 'T': 22.21, 'C': 20.58}
- **3-UTR aligned content (<base>:%):** {'T': 38.33, 'A': 32.54, 'G': 14.57, 'C': 14.57}

**Uniprot Description:**  
  
 May act as a mediator of stress-activated signals. Mediates the inhibition of SLC4A4, SLC26A6 as well as CFTR activities by the WNK scaffolds, probably through phosphorylation. Phosphorylates RELT.   
  
The phosphorylated form forms a complex with WNK2 (By similarity). Interacts with RELT (By similarity). Interacts with SORL1 (via cytosolic C-terminus) (PubMed:20385770).   
  
 **Gene Ontology Information:**

Molecular Function

- ATP binding
- magnesium ion binding
- protein serine kinase activity
- protein serine/threonine kinase activity

Location

- cytoplasm
- cytosol

Biological process

- intracellular signal transduction
- negative regulation of potassium ion transmembrane transporter activity
- peptidyl-threonine phosphorylation
- positive regulation of T cell chemotaxis
- protein autophosphorylation
- protein phosphorylation

---

75

- **Protein name:** Polypeptide N-acetylgalactosaminyltransferase 2
- **Organism:** Homo sapiens
- **Uniprot Accession Number:** Q10471
- **Protein sequence length:** 571 aa
- **1D identity (%):** 11.28
- **1D identity (%) [Gaps excluded]:** 21.0
- **1D identity - Alignment Gaps:** 431
- **1D aligned content (<aminoacid>:%):** {'R': 8.57, 'M': 0.95, 'F': 3.81, 'G': 11.43, 'Y': 0.95, 'S': 1.9, 'A': 3.81, 'D': 3.81, 'P': 11.43, 'V': 10.48, 'W': 1.9, 'E': 3.81, 'T': 3.81, 'N': 4.76, 'H': 2.86, 'K': 7.62, 'I': 1.9, 'L': 6.67, 'C': 2.86, 'Q': 6.67}
- **Common reported functions (%):** 0.0
- **Common reported locations (%):** 11.11
- **Common reported processes (%):** 0.0

- **AF ID:** Q10471
- **Chain:** A
- **Protein length:** 571 aa
- **Resolution:** N/A
- **b-phipsi:** 0.000878
- **w-rdist:** 0.613932
- **t-alpha:** 0.012028
- **Chemical similarity (Tanimoto Index) (%):** 96.83
- **1D identity (%) [PDB]:** 2.86
- **1D identity (%) [Gaps excluded][PDB]:** 73.58
- **1D identity - Alignment Gaps [PDB]:** 1310
- **1D aligned content [PDB] (<aminoacid>:%):** {'M': 2.56, 'L': 10.26, 'I': 10.26, 'A': 5.13, 'Y': 2.56, 'K': 10.26, 'Q': 5.13, 'V': 12.82, 'F': 5.13, 'E': 10.26, 'H': 5.13, 'T': 5.13, 'D': 2.56, 'S': 2.56, 'R': 2.56, 'G': 5.13, 'C': 2.56}
- **2D identity (%) [PDB]:** 39.03
- **2D identity (%) [Gaps excluded][PDB]:** 85.17
- **2D identity - Alignment Gaps [PDB]:** 526
- **2D aligned content [PDB] (<2D-fold>:%):** {'H': 32.19, '.': 19.26, 'T': 27.97, 'E': 20.58}
- **3D similarity (TM-Score) (%) [PDB]:** 19.62

- **Gene name:** GALNT2
- **Entrez ID:** 2590
- **RefSeq ID:** NM\_004481
- **Transcript sequence length:** 4423
- **5-UTR|CDS|3-UTR identity (%):** 9.23 | 39.38 | 9.18
- **5-UTR|CDS|3-UTR identity (%) [Gaps excluded]:** 75.0 | 76.98 | 74.37
- **5-UTR|CDS|3-UTR identity [Alignment Gaps]:** 285 | 1389 | 15151
- **5-UTR aligned content (<base>:%):** {'A': 13.33, 'T': 3.33, 'C': 23.33, 'G': 60.0}
- **CDS aligned content (<base>:%):** {'A': 26.96, 'T': 22.14, 'C': 21.79, 'G': 29.11}
- **3-UTR aligned content (<base>:%):** {'G': 26.21, 'T': 25.46, 'C': 24.57, 'A': 23.76}

**Uniprot Description:**  
  
 Catalyzes the initial reaction in O-linked oligosaccharide biosynthesis, the transfer of an N-acetyl-D-galactosamine residue to a serine or threonine residue on the protein receptor. Has a broad spectrum of substrates for peptides such as EA2, Muc5AC, Muc1a, Muc1b. Probably involved in O-linked glycosylation of the immunoglobulin A1 (IgA1) hinge region. Involved in O-linked glycosylation of APOC-III, ANGPTL3 and PLTP. It participates to the regulation of HDL-C metabolism (PubMed:27508872, PubMed:32293671). N/A   
  
 **Gene Ontology Information:**

Molecular Function

- carbohydrate binding
- manganese ion binding
- polypeptide N-acetylgalactosaminyltransferase activity

Location

- endoplasmic reticulum membrane
- extracellular region
- Golgi apparatus
- Golgi cisterna membrane
- Golgi membrane
- Golgi stack
- membrane
- perinuclear region of cytoplasm

Biological process

- O-glycan processing
- protein maturation
- protein O-linked glycosylation
- protein O-linked glycosylation via serine
- protein O-linked glycosylation via threonine

---

76

- **Protein name:** Protein kinase C gamma type
- **Organism:** Homo sapiens
- **Uniprot Accession Number:** P05129
- **Protein sequence length:** 697 aa
- **1D identity (%):** 13.61
- **1D identity (%) [Gaps excluded]:** 25.41
- **1D identity - Alignment Gaps:** 471
- **1D aligned content (<aminoacid>:%):** {'M': 0.72, 'P': 10.87, 'R': 10.14, 'L': 7.25, 'K': 7.25, 'E': 2.9, 'H': 4.35, 'C': 4.35, 'G': 9.42, 'V': 7.97, 'T': 7.25, 'F': 5.07, 'Q': 2.17, 'Y': 4.35, 'A': 2.9, 'N': 0.72, 'D': 5.8, 'W': 1.45, 'S': 2.17, 'I': 2.9}
- **Common reported functions (%):** 0.0
- **Common reported locations (%):** 11.11
- **Common reported processes (%):** 0.0

- **AF ID:** P05129
- **Chain:** A
- **Protein length:** 697 aa
- **Resolution:** N/A
- **b-phipsi:** 0.001858
- **w-rdist:** 0.590441
- **t-alpha:** 0.00634
- **Chemical similarity (Tanimoto Index) (%):** 99.17
- **1D identity (%) [PDB]:** 2.69
- **1D identity (%) [Gaps excluded][PDB]:** 75.47
- **1D identity - Alignment Gaps [PDB]:** 1436
- **1D aligned content [PDB] (<aminoacid>:%):** {'G': 10.0, 'S': 7.5, 'E': 7.5, 'R': 17.5, 'Q': 2.5, 'V': 7.5, 'L': 12.5, 'Y': 2.5, 'I': 7.5, 'A': 5.0, 'C': 2.5, 'K': 5.0, 'D': 2.5, 'H': 2.5, 'T': 5.0, 'F': 2.5}
- **2D identity (%) [PDB]:** 42.44
- **2D identity (%) [Gaps excluded][PDB]:** 87.67
- **2D identity - Alignment Gaps [PDB]:** 536
- **2D aligned content [PDB] (<2D-fold>:%):** {'.': 26.3, 'E': 18.59, 'T': 24.72, 'H': 29.71, 'B': 0.23, 'G': 0.45}
- **3D similarity (TM-Score) (%) [PDB]:** 19.74

- **Gene name:** PRKCG
- **Entrez ID:** 282002
- **RefSeq ID:** NM\_002739
- **Transcript sequence length:** 3149
- **5-UTR|CDS|3-UTR identity (%):** 46.7 | 44.03 | 2.56
- **5-UTR|CDS|3-UTR identity (%) [Gaps excluded]:** 73.44 | 73.52 | 71.69
- **5-UTR|CDS|3-UTR identity [Alignment Gaps]:** 138 | 1173 | 16304
- **5-UTR aligned content (<base>:%):** {'C': 51.41, 'G': 26.55, 'A': 5.65, 'T': 16.38}
- **CDS aligned content (<base>:%):** {'A': 22.9, 'T': 22.52, 'G': 27.25, 'C': 27.33}
- **3-UTR aligned content (<base>:%):** {'T': 23.09, 'C': 35.8, 'A': 18.48, 'G': 22.63}

**Uniprot Description:**  
  
 Calcium-activated, phospholipid- and diacylglycerol (DAG)-dependent serine/threonine-protein kinase that plays diverse roles in neuronal cells and eye tissues, such as regulation of the neuronal receptors GRIA4/GLUR4 and GRIN1/NMDAR1, modulation of receptors and neuronal functions related to sensitivity to opiates, pain and alcohol, mediation of synaptic function and cell survival after ischemia, and inhibition of gap junction activity after oxidative stress. Binds and phosphorylates GRIA4/GLUR4 glutamate receptor and regulates its function by increasing plasma membrane-associated GRIA4 expression. In primary cerebellar neurons treated with the agonist 3,5-dihyidroxyphenylglycine, functions downstream of the metabotropic glutamate receptor GRM5/MGLUR5 and phosphorylates GRIN1/NMDAR1 receptor which plays a key role in synaptic plasticity, synaptogenesis, excitotoxicity, memory acquisition and learning. May be involved in the regulation of hippocampal long-term potentiation (LTP), but may be not necessary for the process of synaptic plasticity. May be involved in desensitization of mu-type opioid receptor-mediated G-protein activation in the spinal cord, and may be critical for the development and/or maintenance of morphine-induced reinforcing effects in the limbic forebrain. May modulate the functionality of mu-type-opioid receptors by participating in a signaling pathway which leads to the phosphorylation and degradation of opioid receptors. May also contributes to chronic morphine-induced changes in nociceptive processing. Plays a role in neuropathic pain mechanisms and contributes to the maintenance of the allodynia pain produced by peripheral inflammation. Plays an important role in initial sensitivity and tolerance to ethanol, by mediating the behavioral effects of ethanol as well as the effects of this drug on the GABA(A) receptors. During and after cerebral ischemia modulate neurotransmission and cell survival in synaptic membranes, and is involved in insulin-induced inhibition of necrosis, an important mechanism for minimizing ischemic injury. Required for the elimination of multiple climbing fibers during innervation of Purkinje cells in developing cerebellum. Is activated in lens epithelial cells upon hydrogen peroxide treatment, and phosphorylates connexin-43 (GJA1/CX43), resulting in disassembly of GJA1 gap junction plaques and inhibition of gap junction activity which could provide a protective effect against oxidative stress (By similarity). Phosphorylates p53/TP53 and promotes p53/TP53-dependent apoptosis in response to DNA damage. Involved in the phase resetting of the cerebral cortex circadian clock during temporally restricted feeding. Stabilizes the core clock component BMAL1 by interfering with its ubiquitination, thus suppressing its degradation, resulting in phase resetting of the cerebral cortex clock (By similarity).   
  
Interacts with GRIA4 (By similarity). Interacts with CDCP1. Interacts with TP53INP1 and p53/TP53. Interacts with BMAL1 (By similarity).   
  
 **Gene Ontology Information:**

Molecular Function

- ATP binding
- calcium-dependent protein kinase C activity
- protein serine kinase activity
- protein serine/threonine kinase activity
- zinc ion binding

Location

- cytosol
- dendrite
- perinuclear region of cytoplasm
- plasma membrane
- synapse

Biological process

- intracellular signal transduction
- negative regulation of neuron apoptotic process
- negative regulation of proteasomal protein catabolic process
- negative regulation of protein ubiquitination
- peptidyl-serine phosphorylation
- regulation of circadian rhythm
- regulation of response to food
- response to morphine
- response to pain
- rhythmic process

---

77

- **Protein name:** N-acetylglutamate synthase, mitochondrial
- **Organism:** Homo sapiens
- **Uniprot Accession Number:** Q8N159
- **Protein sequence length:** 534 aa
- **1D identity (%):** 11.76
- **1D identity (%) [Gaps excluded]:** 24.67
- **1D identity - Alignment Gaps:** 494
- **1D aligned content (<aminoacid>:%):** {'T': 3.6, 'L': 14.41, 'V': 8.11, 'A': 9.01, 'R': 9.91, 'P': 11.71, 'E': 4.5, 'Q': 3.6, 'G': 5.41, 'N': 1.8, 'W': 1.8, 'H': 4.5, 'K': 3.6, 'F': 3.6, 'C': 2.7, 'S': 1.8, 'Y': 4.5, 'I': 1.8, 'D': 3.6}
- **Common reported functions (%):** 0.0
- **Common reported locations (%):** 0.0
- **Common reported processes (%):** 0.0

- **AF ID:** Q8N159
- **Chain:** A
- **Protein length:** 534 aa
- **Resolution:** N/A
- **b-phipsi:** 0.000733
- **w-rdist:** 0.540199
- **t-alpha:** 0.074485
- **Chemical similarity (Tanimoto Index) (%):** 99.17
- **1D identity (%) [PDB]:** 1.86
- **1D identity (%) [Gaps excluded][PDB]:** 75.76
- **1D identity - Alignment Gaps [PDB]:** 1313
- **1D aligned content [PDB] (<aminoacid>:%):** {'A': 4.0, 'T': 8.0, 'G': 16.0, 'V': 16.0, 'L': 8.0, 'D': 4.0, 'P': 8.0, 'K': 8.0, 'R': 8.0, 'S': 12.0, 'I': 4.0, 'F': 4.0}
- **2D identity (%) [PDB]:** 37.8
- **2D identity (%) [Gaps excluded][PDB]:** 89.95
- **2D identity - Alignment Gaps [PDB]:** 563
- **2D aligned content [PDB] (<2D-fold>:%):** {'.': 25.89, 'H': 37.87, 'T': 17.71, 'E': 17.44, 'B': 0.27, 'G': 0.82}
- **3D similarity (TM-Score) (%) [PDB]:** 21.25

- **Gene name:** NAGS
- **Entrez ID:** 162417
- **RefSeq ID:** NM\_153006
- **Transcript sequence length:** 2114
- **5-UTR|CDS|3-UTR identity (%):** 7.65 | 30.78 | 1.83
- **5-UTR|CDS|3-UTR identity (%) [Gaps excluded]:** 67.57 | 73.34 | 77.19
- **5-UTR|CDS|3-UTR identity [Alignment Gaps]:** 290 | 1712 | 16424
- **5-UTR aligned content (<base>:%):** {'G': 52.0, 'C': 24.0, 'A': 8.0, 'T': 16.0}
- **CDS aligned content (<base>:%):** {'A': 21.59, 'G': 30.51, 'C': 29.41, 'T': 18.5}
- **3-UTR aligned content (<base>:%):** {'T': 25.32, 'A': 28.25, 'C': 23.7, 'G': 22.73}

**Uniprot Description:**  
  
 Plays a role in the regulation of ureagenesis by producing the essential cofactor N-acetylglutamate (NAG), thus modulating carbamoylphosphate synthase I (CPS1) activity.   
  
Homodimer. Homotetramer.   
  
 **Gene Ontology Information:**

Molecular Function

- acetyl-CoA:L-glutamate N-acetyltransferase activity
- methione N-acyltransferase activity

Location

- mitochondrial matrix
- mitochondrion

Biological process

- arginine biosynthetic process
- glutamate metabolic process
- urea cycle

---

78

- **Protein name:** Ribosome-releasing factor 2, mitochondrial
- **Organism:** Homo sapiens
- **Uniprot Accession Number:** Q969S9
- **Protein sequence length:** 779 aa
- **1D identity (%):** 14.86
- **1D identity (%) [Gaps excluded]:** 23.02
- **1D identity - Alignment Gaps:** 353
- **1D aligned content (<aminoacid>:%):** {'M': 1.35, 'A': 4.05, 'Q': 6.76, 'I': 4.05, 'P': 8.78, 'Y': 2.7, 'K': 6.08, 'S': 4.73, 'G': 10.14, 'R': 6.08, 'L': 11.49, 'V': 6.76, 'D': 4.73, 'T': 4.73, 'W': 0.68, 'E': 9.46, 'F': 2.7, 'N': 2.03, 'H': 1.35, 'C': 1.35}
- **Common reported functions (%):** 0.0
- **Common reported locations (%):** 0.0
- **Common reported processes (%):** 0.0

- **AF ID:** Q969S9
- **Chain:** A
- **Protein length:** 779 aa
- **Resolution:** N/A
- **b-phipsi:** 0.001232
- **w-rdist:** 0.362466
- **t-alpha:** 0.084786
- **Chemical similarity (Tanimoto Index) (%):** 98.57
- **1D identity (%) [PDB]:** 3.5
- **1D identity (%) [Gaps excluded][PDB]:** 66.67
- **1D identity - Alignment Gaps [PDB]:** 1462
- **1D aligned content [PDB] (<aminoacid>:%):** {'K': 7.41, 'R': 5.56, 'S': 7.41, 'Y': 1.85, 'T': 7.41, 'A': 3.7, 'P': 9.26, 'L': 18.52, 'G': 3.7, 'V': 14.81, 'D': 9.26, 'E': 5.56, 'F': 1.85, 'I': 3.7}
- **2D identity (%) [PDB]:** 45.99
- **2D identity (%) [Gaps excluded][PDB]:** 86.68
- **2D identity - Alignment Gaps [PDB]:** 498
- **2D aligned content [PDB] (<2D-fold>:%):** {'.': 20.9, 'T': 18.65, 'H': 35.45, 'E': 24.39, 'G': 0.61}
- **3D similarity (TM-Score) (%) [PDB]:** 22.81

- **Gene name:** GFM2
- **Entrez ID:** 527467
- **RefSeq ID:** N/A
- **Sequence length:** N/A
- **5-UTR|CDS|3-UTR identity (%):** N/A | N/A | N/A
- **5-UTR|CDS|3-UTR identity (%) [Gaps excluded]:** N/A | N/A | N/A
- **5-UTR|CDS|3-UTR identity [Alignment Gaps]:** N/A | N/A | N/A
- **5-UTR aligned content (<base>:%):** N/A
- **CDS aligned content (<base>:%):** N/A
- **3-UTR aligned content (<base>:%):** N/A

**Uniprot Description:**  
  
 Mitochondrial GTPase that mediates the disassembly of ribosomes from messenger RNA at the termination of mitochondrial protein biosynthesis. Acts in collaboration with MRRF. GTP hydrolysis follows the ribosome disassembly and probably occurs on the ribosome large subunit. Not involved in the GTP-dependent ribosomal translocation step during translation elongation. N/A   
  
 **Gene Ontology Information:**

Molecular Function

- GTP binding
- GTPase activity

Location

- mitochondrion

Biological process

- mitochondrial translation
- ribosome disassembly

---

79

- **Protein name:** Zinc finger protein ZIC 4
- **Organism:** Homo sapiens
- **Uniprot Accession Number:** Q8N9L1
- **Protein sequence length:** 334 aa
- **1D identity (%):** 7.57
- **1D identity (%) [Gaps excluded]:** 24.47
- **1D identity - Alignment Gaps:** 630
- **1D aligned content (<aminoacid>:%):** {'K': 2.9, 'V': 7.25, 'N': 2.9, 'G': 7.25, 'L': 7.25, 'P': 10.14, 'S': 7.25, 'F': 2.9, 'D': 4.35, 'A': 11.59, 'M': 1.45, 'R': 8.7, 'Q': 4.35, 'E': 4.35, 'I': 1.45, 'T': 5.8, 'C': 4.35, 'Y': 1.45, 'H': 4.35}
- **Common reported functions (%):** 14.29
- **Common reported locations (%):** 0.0
- **Common reported processes (%):** 0.0

- **AF ID:** Q8N9L1
- **Chain:** A
- **Protein length:** 334 aa
- **Resolution:** N/A
- **b-phipsi:** 0.00291
- **w-rdist:** 0.370088
- **t-alpha:** 0.049917
- **Chemical similarity (Tanimoto Index) (%):** 99.17
- **1D identity (%) [PDB]:** 2.09
- **1D identity (%) [Gaps excluded][PDB]:** 75.0
- **1D identity - Alignment Gaps [PDB]:** 1115
- **1D aligned content [PDB] (<aminoacid>:%):** {'R': 4.17, 'P': 16.67, 'S': 8.33, 'V': 12.5, 'F': 4.17, 'Q': 4.17, 'I': 4.17, 'L': 4.17, 'A': 12.5, 'D': 4.17, 'T': 4.17, 'H': 4.17, 'G': 8.33, 'K': 8.33}
- **2D identity (%) [PDB]:** 32.83
- **2D identity (%) [Gaps excluded][PDB]:** 89.27
- **2D identity - Alignment Gaps [PDB]:** 545
- **2D aligned content [PDB] (<2D-fold>:%):** {'.': 32.16, 'H': 22.26, 'T': 43.46, 'B': 0.71, 'E': 1.41}
- **3D similarity (TM-Score) (%) [PDB]:** 12.78

- **Gene name:** ZIC4
- **Entrez ID:** 84107
- **RefSeq ID:** NM\_032153
- **Transcript sequence length:** 4014
- **5-UTR|CDS|3-UTR identity (%):** 29.28 | 23.87 | 10.02
- **5-UTR|CDS|3-UTR identity (%) [Gaps excluded]:** 67.95 | 73.77 | 74.99
- **5-UTR|CDS|3-UTR identity [Alignment Gaps]:** 206 | 1834 | 14954
- **5-UTR aligned content (<base>:%):** {'G': 40.57, 'T': 16.98, 'C': 28.3, 'A': 14.15}
- **CDS aligned content (<base>:%):** {'A': 25.35, 'G': 28.59, 'T': 16.85, 'C': 29.21}
- **3-UTR aligned content (<base>:%):** {'G': 19.42, 'C': 17.86, 'A': 30.17, 'T': 32.54}

**Uniprot Description:**  
  
 Binds to DNA. N/A   
  
 **Gene Ontology Information:**

Molecular Function

- DNA binding
- DNA-binding transcription factor activity, RNA polymerase II-specific
- metal ion binding
- RNA polymerase II cis-regulatory region sequence-specific DNA binding
- sequence-specific double-stranded DNA binding

Location

- nucleus

Biological process

- central nervous system development
- regulation of transcription by RNA polymerase II

---

80

- **Protein name:** Protein argonaute-2
- **Organism:** Homo sapiens
- **Uniprot Accession Number:** Q9UKV8
- **Protein sequence length:** 859 aa
- **1D identity (%):** 79.29
- **1D identity (%) [Gaps excluded]:** 81.06
- **1D identity - Alignment Gaps:** 19
- **1D aligned content (<aminoacid>:%):** {'A': 6.82, 'G': 6.97, 'P': 6.82, 'R': 6.68, 'T': 6.39, 'I': 5.22, 'K': 5.37, 'L': 7.69, 'N': 2.32, 'F': 4.5, 'D': 4.79, 'Y': 3.63, 'E': 4.79, 'C': 2.76, 'V': 8.56, 'M': 2.03, 'Q': 6.1, 'H': 3.48, 'S': 4.35, 'W': 0.73}
- **Common reported functions (%):** 57.14
- **Common reported locations (%):** 33.33
- **Common reported processes (%):** 22.22

- **AF ID:** Q9UKV8
- **Chain:** A
- **Protein length:** 859 aa
- **Resolution:** N/A
- **b-phipsi:** 0.006973
- **w-rdist:** 0.248198
- **t-alpha:** 0.071315
- **Chemical similarity (Tanimoto Index) (%):** 100.0
- **1D identity (%) [PDB]:** 78.99
- **1D identity (%) [Gaps excluded][PDB]:** 82.59
- **1D identity - Alignment Gaps [PDB]:** 38
- **1D aligned content [PDB] (<aminoacid>:%):** {'M': 2.18, 'P': 6.69, 'R': 6.83, 'G': 6.83, 'T': 6.4, 'I': 5.23, 'K': 5.52, 'L': 7.85, 'A': 6.4, 'N': 2.33, 'F': 4.51, 'D': 4.8, 'Y': 3.63, 'E': 4.8, 'C': 2.76, 'V': 8.58, 'Q': 6.1, 'H': 3.49, 'S': 4.36, 'W': 0.73}
- **2D identity (%) [PDB]:** 76.52
- **2D identity (%) [Gaps excluded][PDB]:** 86.27
- **2D identity - Alignment Gaps [PDB]:** 102
- **2D aligned content [PDB] (<2D-fold>:%):** {'.': 18.7, 'B': 1.45, 'E': 25.36, 'H': 31.88, 'T': 21.88, 'G': 0.72}
- **3D similarity (TM-Score) (%) [PDB]:** 93.49

- **Gene name:** AGO2
- **Entrez ID:** 404130
- **RefSeq ID:** NM\_012154
- **Transcript sequence length:** 14595
- **5-UTR|CDS|3-UTR identity (%):** 27.69 | 70.93 | 37.29
- **5-UTR|CDS|3-UTR identity (%) [Gaps excluded]:** 72.58 | 74.91 | 74.74
- **5-UTR|CDS|3-UTR identity [Alignment Gaps]:** 201 | 141 | 9575
- **5-UTR aligned content (<base>:%):** {'C': 47.78, 'G': 38.89, 'T': 10.0, 'A': 3.33}
- **CDS aligned content (<base>:%):** {'A': 27.38, 'T': 21.8, 'G': 25.78, 'C': 25.04}
- **3-UTR aligned content (<base>:%):** {'C': 17.51, 'A': 27.67, 'T': 34.55, 'G': 20.26}

**Uniprot Description:**  
  
 Required for RNA-mediated gene silencing (RNAi) by the RNA-induced silencing complex (RISC). The 'minimal RISC' appears to include AGO2 bound to a short guide RNA such as a microRNA (miRNA) or short interfering RNA (siRNA). These guide RNAs direct RISC to complementary mRNAs that are targets for RISC-mediated gene silencing. The precise mechanism of gene silencing depends on the degree of complementarity between the miRNA or siRNA and its target. Binding of RISC to a perfectly complementary mRNA generally results in silencing due to endonucleolytic cleavage of the mRNA specifically by AGO2. Binding of RISC to a partially complementary mRNA results in silencing through inhibition of translation, and this is independent of endonuclease activity. May inhibit translation initiation by binding to the 7-methylguanosine cap, thereby preventing the recruitment of the translation initiation factor eIF4-E. May also inhibit translation initiation via interaction with EIF6, which itself binds to the 60S ribosomal subunit and prevents its association with the 40S ribosomal subunit. The inhibition of translational initiation leads to the accumulation of the affected mRNA in cytoplasmic processing bodies (P-bodies), where mRNA degradation may subsequently occur. In some cases RISC-mediated translational repression is also observed for miRNAs that perfectly match the 3' untranslated region (3'-UTR). Can also up-regulate the translation of specific mRNAs under certain growth conditions. Binds to the AU element of the 3'-UTR of the TNF (TNF-alpha) mRNA and up-regulates translation under conditions of serum starvation. Also required for transcriptional gene silencing (TGS), in which short RNAs known as antigene RNAs or agRNAs direct the transcriptional repression of complementary promoter regions.   
  
Interacts with DICER1 through its Piwi domain and with TARBP2 during assembly of the RNA-induced silencing complex (RISC). Together, DICER1, AGO2 and TARBP2 constitute the trimeric RISC loading complex (RLC), or micro-RNA (miRNA) loading complex (miRLC). Within the RLC/miRLC, DICER1 and TARBP2 are required to process precursor miRNAs (pre-miRNAs) to mature miRNAs and then load them onto AGO2. AGO2 bound to the mature miRNA constitutes the minimal RISC and may subsequently dissociate from DICER1 and TARBP2. Note however that the term RISC has also been used to describe the trimeric RLC/miRLC. The formation of RISC complexes containing siRNAs rather than miRNAs appears to occur independently of DICER1. Interacts with AGO1. Also interacts with DDB1, DDX5, DDX6, DDX20, DHX30, DHX36, DDX47, DHX9, ELAVL, FXR1, GEMIN4, HNRNPF, IGF2BP1, ILF3, IMP8, MATR3, PABPC1, PRMT5, P4HA1, P4HB, RBM4, SART3, TNRC6A, TNRC6B, UPF1 and YBX1. Interacts with the P-body components DCP1A and XRN1. Associates with polysomes and messenger ribonucleoproteins (mNRPs). Interacts with RBM4; the interaction is modulated under stress-induced conditions, occurs under both cell proliferation and differentiation conditions and in an RNA- and phosphorylation-independent manner. Interacts with LIMD1, WTIP and AJUBA. Interacts with TRIM71; the interaction increases in presence of RNA (PubMed:23125361). Interacts with APOBEC3G in an RNA-dependent manner. Interacts with APOBEC3A, APOBEC3C, APOBEC3F and APOBEC3H. Interacts with DICER1, TARBP2, EIF6, MOV10 and RPL7A (60S ribosome subunit); they form a large RNA-induced silencing complex (RISC) (PubMed:17507929, PubMed:24726324). Interacts with FMR1 (PubMed:14703574). Interacts with ZFP36 (PubMed:15766526). Found in a complex, composed of AGO2, CHD7 and FAM172A (By similarity). Interacts with RC3H1; the interaction is RNA independent (PubMed:25697406). Interacts with SND1 (PubMed:14508492, PubMed:28546213). Interacts with SYT11 (By similarity). Interacts with CLNK (PubMed:26009488). Interacts with GARRE1 (PubMed:29395067).   
  
 **Gene Ontology Information:**

Molecular Function

- endoribonuclease activity, cleaving siRNA-paired mRNA
- metal ion binding
- miRNA binding
- RNA 7-methylguanosine cap binding
- endoribonuclease activity
- single-stranded RNA binding
- siRNA binding

Location

- mRNA cap binding complex
- nucleus
- P-body
- polysome
- ribonucleoprotein complex
- RISC complex
- RISC-loading complex

Biological process

- miRNA mediated inhibition of translation
- mRNA cleavage involved in gene silencing by miRNA
- negative regulation of translational initiation
- pre-miRNA processing
- regulation of transcription, DNA-templated

---

81

- **Protein name:** Inactive ubiquitin carboxyl-terminal hydrolase 17-like protein 8
- **Organism:** Homo sapiens
- **Uniprot Accession Number:** P0C7I0
- **Protein sequence length:** 530 aa
- **1D identity (%):** 11.61
- **1D identity (%) [Gaps excluded]:** 23.48
- **1D identity - Alignment Gaps:** 470
- **1D aligned content (<aminoacid>:%):** {'M': 1.85, 'L': 14.81, 'Q': 9.26, 'P': 9.26, 'D': 6.48, 'E': 5.56, 'K': 7.41, 'V': 7.41, 'R': 2.78, 'T': 6.48, 'H': 7.41, 'G': 2.78, 'F': 1.85, 'A': 4.63, 'C': 4.63, 'I': 0.93, 'Y': 1.85, 'N': 1.85, 'S': 1.85, 'W': 0.93}
- **Common reported functions (%):** 0.0
- **Common reported locations (%):** 11.11
- **Common reported processes (%):** 0.0

- **AF ID:** P0C7I0
- **Chain:** A
- **Protein length:** 530 aa
- **Resolution:** N/A
- **b-phipsi:** 0.003463
- **w-rdist:** 0.717471
- **t-alpha:** 0.003179
- **Chemical similarity (Tanimoto Index) (%):** 97.34
- **1D identity (%) [PDB]:** 2.89
- **1D identity (%) [Gaps excluded][PDB]:** 64.41
- **1D identity - Alignment Gaps [PDB]:** 1257
- **1D aligned content [PDB] (<aminoacid>:%):** {'R': 10.53, 'P': 7.89, 'A': 5.26, 'S': 2.63, 'Q': 15.79, 'T': 7.89, 'L': 13.16, 'E': 7.89, 'V': 7.89, 'Y': 2.63, 'F': 2.63, 'K': 5.26, 'H': 5.26, 'C': 5.26}
- **2D identity (%) [PDB]:** 37.6
- **2D identity (%) [Gaps excluded][PDB]:** 89.43
- **2D identity - Alignment Gaps [PDB]:** 561
- **2D aligned content [PDB] (<2D-fold>:%):** {'.': 29.67, 'T': 27.47, 'E': 17.31, 'H': 25.55}
- **3D similarity (TM-Score) (%) [PDB]:** 16.38

- **Gene name:** USP17L8
- **Entrez ID:** 392188
- **RefSeq ID:** N/A
- **Sequence length:** N/A
- **5-UTR|CDS|3-UTR identity (%):** N/A | N/A | N/A
- **5-UTR|CDS|3-UTR identity (%) [Gaps excluded]:** N/A | N/A | N/A
- **5-UTR|CDS|3-UTR identity [Alignment Gaps]:** N/A | N/A | N/A
- **5-UTR aligned content (<base>:%):** N/A
- **CDS aligned content (<base>:%):** N/A
- **3-UTR aligned content (<base>:%):** N/A

**Uniprot Description:**  
  
 N/A N/A   
  
 **Gene Ontology Information:**

Molecular Function

- thiol-dependent ubiquitin-specific protease activity
- cysteine-type endopeptidase activity

Location

- cytosol
- endoplasmic reticulum
- nucleus

Biological process

- protein deubiquitination
- regulation of apoptotic process
- ubiquitin-dependent protein catabolic process

---

82

- **Protein name:** Zinc finger protein 597
- **Organism:** Homo sapiens
- **Uniprot Accession Number:** Q96LX8
- **Protein sequence length:** 424 aa
- **1D identity (%):** 9.96
- **1D identity (%) [Gaps excluded]:** 25.56
- **1D identity - Alignment Gaps:** 564
- **1D aligned content (<aminoacid>:%):** {'M': 3.26, 'G': 6.52, 'I': 4.35, 'A': 4.35, 'Q': 3.26, 'C': 4.35, 'K': 6.52, 'D': 5.43, 'P': 8.7, 'S': 7.61, 'E': 3.26, 'L': 11.96, 'T': 8.7, 'Y': 2.17, 'R': 3.26, 'V': 4.35, 'N': 1.09, 'H': 8.7, 'F': 2.17}
- **Common reported functions (%):** 14.29
- **Common reported locations (%):** 0.0
- **Common reported processes (%):** 0.0

- **AF ID:** Q96LX8
- **Chain:** A
- **Protein length:** 424 aa
- **Resolution:** N/A
- **b-phipsi:** 0.003621
- **w-rdist:** 0.464222
- **t-alpha:** 0.006379
- **Chemical similarity (Tanimoto Index) (%):** 98.87
- **1D identity (%) [PDB]:** 2.38
- **1D identity (%) [Gaps excluded][PDB]:** 55.77
- **1D identity - Alignment Gaps [PDB]:** 1165
- **1D aligned content [PDB] (<aminoacid>:%):** {'D': 10.34, 'L': 24.14, 'G': 6.9, 'E': 13.79, 'K': 10.34, 'P': 6.9, 'S': 6.9, 'V': 6.9, 'R': 3.45, 'T': 6.9, 'I': 3.45}
- **2D identity (%) [PDB]:** 29.68
- **2D identity (%) [Gaps excluded][PDB]:** 84.8
- **2D identity - Alignment Gaps [PDB]:** 611
- **2D aligned content [PDB] (<2D-fold>:%):** {'.': 35.84, 'H': 40.86, 'T': 16.85, 'E': 6.45}
- **3D similarity (TM-Score) (%) [PDB]:** 12.63

- **Gene name:** ZNF597
- **Entrez ID:** 146434
- **RefSeq ID:** NM\_152457
- **Transcript sequence length:** 5483
- **5-UTR|CDS|3-UTR identity (%):** 34.37 | 30.15 | 14.53
- **5-UTR|CDS|3-UTR identity (%) [Gaps excluded]:** 73.94 | 76.07 | 76.67
- **5-UTR|CDS|3-UTR identity [Alignment Gaps]:** 190 | 1668 | 14147
- **5-UTR aligned content (<base>:%):** {'G': 33.61, 'C': 38.52, 'A': 7.38, 'T': 20.49}
- **CDS aligned content (<base>:%):** {'A': 31.69, 'T': 24.61, 'G': 20.77, 'C': 22.93}
- **3-UTR aligned content (<base>:%):** {'A': 31.93, 'G': 17.42, 'T': 37.13, 'C': 13.52}

**Uniprot Description:**  
  
 May be involved in transcriptional regulation. N/A   
  
 **Gene Ontology Information:**

Molecular Function

- DNA-binding transcription activator activity, RNA polymerase II-specific
- metal ion binding
- RNA polymerase II cis-regulatory region sequence-specific DNA binding
- sequence-specific double-stranded DNA binding

Location

- nucleus

Biological process

- regulation of transcription by RNA polymerase II

---

83

- **Protein name:** Protein kinase C alpha type
- **Organism:** Homo sapiens
- **Uniprot Accession Number:** P17252
- **Protein sequence length:** 672 aa
- **1D identity (%):** 12.11
- **1D identity (%) [Gaps excluded]:** 21.68
- **1D identity - Alignment Gaps:** 434
- **1D aligned content (<aminoacid>:%):** {'M': 1.68, 'V': 10.08, 'D': 5.88, 'N': 5.04, 'L': 8.4, 'H': 5.04, 'K': 5.88, 'I': 3.36, 'T': 5.04, 'C': 5.04, 'G': 9.24, 'F': 5.04, 'R': 7.56, 'Y': 4.2, 'A': 2.52, 'E': 3.36, 'P': 6.72, 'W': 1.68, 'Q': 2.52, 'S': 1.68}
- **Common reported functions (%):** 0.0
- **Common reported locations (%):** 33.33
- **Common reported processes (%):** 0.0

- **AF ID:** P17252
- **Chain:** A
- **Protein length:** 672 aa
- **Resolution:** N/A
- **b-phipsi:** 0.000369
- **w-rdist:** 0.684324
- **t-alpha:** 0.131838
- **Chemical similarity (Tanimoto Index) (%):** 99.25
- **1D identity (%) [PDB]:** 1.89
- **1D identity (%) [Gaps excluded][PDB]:** 73.68
- **1D identity - Alignment Gaps [PDB]:** 1441
- **1D aligned content [PDB] (<aminoacid>:%):** {'M': 7.14, 'G': 3.57, 'K': 10.71, 'Q': 10.71, 'F': 10.71, 'T': 7.14, 'V': 3.57, 'E': 10.71, 'I': 10.71, 'A': 10.71, 'R': 7.14, 'D': 3.57, 'L': 3.57}
- **2D identity (%) [PDB]:** 42.29
- **2D identity (%) [Gaps excluded][PDB]:** 89.71
- **2D identity - Alignment Gaps [PDB]:** 545
- **2D aligned content [PDB] (<2D-fold>:%):** {'.': 16.97, 'H': 26.83, 'T': 34.63, 'E': 20.18, 'G': 1.38}
- **3D similarity (TM-Score) (%) [PDB]:** 21.27

- **Gene name:** PRKCA
- **Entrez ID:** 5578
- **RefSeq ID:** NM\_002737
- **Transcript sequence length:** 8964
- **5-UTR|CDS|3-UTR identity (%):** 42.35 | 43.11 | 24.63
- **5-UTR|CDS|3-UTR identity (%) [Gaps excluded]:** 79.49 | 75.04 | 75.09
- **5-UTR|CDS|3-UTR identity [Alignment Gaps]:** 171 | 1244 | 11873
- **5-UTR aligned content (<base>:%):** {'G': 29.68, 'T': 7.1, 'A': 5.16, 'C': 58.06}
- **CDS aligned content (<base>:%):** {'T': 23.97, 'G': 24.21, 'C': 21.43, 'A': 30.4}
- **3-UTR aligned content (<base>:%):** {'A': 28.22, 'C': 20.68, 'G': 21.14, 'T': 29.95}

**Uniprot Description:**  
  
 Calcium-activated, phospholipid- and diacylglycerol (DAG)-dependent serine/threonine-protein kinase that is involved in positive and negative regulation of cell proliferation, apoptosis, differentiation, migration and adhesion, tumorigenesis, cardiac hypertrophy, angiogenesis, platelet function and inflammation, by directly phosphorylating targets such as RAF1, BCL2, CSPG4, TNNT2/CTNT, or activating signaling cascade involving MAPK1/3 (ERK1/2) and RAP1GAP. Involved in cell proliferation and cell growth arrest by positive and negative regulation of the cell cycle. Can promote cell growth by phosphorylating and activating RAF1, which mediates the activation of the MAPK/ERK signaling cascade, and/or by up-regulating CDKN1A, which facilitates active cyclin-dependent kinase (CDK) complex formation in glioma cells. In intestinal cells stimulated by the phorbol ester PMA, can trigger a cell cycle arrest program which is associated with the accumulation of the hyper-phosphorylated growth-suppressive form of RB1 and induction of the CDK inhibitors CDKN1A and CDKN1B. Exhibits anti-apoptotic function in glioma cells and protects them from apoptosis by suppressing the p53/TP53-mediated activation of IGFBP3, and in leukemia cells mediates anti-apoptotic action by phosphorylating BCL2. During macrophage differentiation induced by macrophage colony-stimulating factor (CSF1), is translocated to the nucleus and is associated with macrophage development. After wounding, translocates from focal contacts to lamellipodia and participates in the modulation of desmosomal adhesion. Plays a role in cell motility by phosphorylating CSPG4, which induces association of CSPG4 with extensive lamellipodia at the cell periphery and polarization of the cell accompanied by increases in cell motility. During chemokine-induced CD4(+) T cell migration, phosphorylates CDC42-guanine exchange factor DOCK8 resulting in its dissociation from LRCH1 and the activation of GTPase CDC42 (PubMed:28028151). Is highly expressed in a number of cancer cells where it can act as a tumor promoter and is implicated in malignant phenotypes of several tumors such as gliomas and breast cancers. Negatively regulates myocardial contractility and positively regulates angiogenesis, platelet aggregation and thrombus formation in arteries. Mediates hypertrophic growth of neonatal cardiomyocytes, in part through a MAPK1/3 (ERK1/2)-dependent signaling pathway, and upon PMA treatment, is required to induce cardiomyocyte hypertrophy up to heart failure and death, by increasing protein synthesis, protein-DNA ratio and cell surface area. Regulates cardiomyocyte function by phosphorylating cardiac troponin T (TNNT2/CTNT), which induces significant reduction in actomyosin ATPase activity, myofilament calcium sensitivity and myocardial contractility. In angiogenesis, is required for full endothelial cell migration, adhesion to vitronectin (VTN), and vascular endothelial growth factor A (VEGFA)-dependent regulation of kinase activation and vascular tube formation. Involved in the stabilization of VEGFA mRNA at post-transcriptional level and mediates VEGFA-induced cell proliferation. In the regulation of calcium-induced platelet aggregation, mediates signals from the CD36/GP4 receptor for granule release, and activates the integrin heterodimer ITGA2B-ITGB3 through the RAP1GAP pathway for adhesion. During response to lipopolysaccharides (LPS), may regulate selective LPS-induced macrophage functions involved in host defense and inflammation. But in some inflammatory responses, may negatively regulate NF-kappa-B-induced genes, through IL1A-dependent induction of NF-kappa-B inhibitor alpha (NFKBIA/IKBA). Upon stimulation with 12-O-tetradecanoylphorbol-13-acetate (TPA), phosphorylates EIF4G1, which modulates EIF4G1 binding to MKNK1 and may be involved in the regulation of EIF4E phosphorylation. Phosphorylates KIT, leading to inhibition of KIT activity. Phosphorylates ATF2 which promotes cooperation between ATF2 and JUN, activating transcription. Phosphorylates SOCS2 at 'Ser-52' facilitating its ubiquitination and proteosomal degradation (By similarity).   
  
Recruited in a circadian manner into a nuclear complex which also includes BMAL1 and RACK1 (By similarity). Interacts with ADAP1/CENTA1 (PubMed:12893243). Interacts with CSPG4 (PubMed:15504744). Binds to CAVIN2 in the presence of phosphatidylserine (By similarity). Interacts with PRKCABP/PICK1 (via PDZ domain) (PubMed:15247289). Interacts with TRIM41 (PubMed:17893151). Interacts with PARD3 (PubMed:27925688). Interacts with SOCS2 (By similarity).   
  
 **Gene Ontology Information:**

Molecular Function

- ATP binding
- calcium-dependent protein kinase C activity
- enzyme binding
- histone kinase activity (H3-T6 specific)
- integrin binding
- protein kinase activity
- protein kinase C activity
- protein serine kinase activity
- protein serine/threonine kinase activity
- zinc ion binding

Location

- alphav-beta3 integrin-PKCalpha complex
- ciliary basal body
- cytoplasm
- cytosol
- endoplasmic reticulum
- extracellular exosome
- mitochondrial membrane
- mitochondrion
- nucleoplasm
- perinuclear region of cytoplasm
- plasma membrane

Biological process

- angiogenesis
- apoptotic signaling pathway
- cell adhesion
- desmosome assembly
- intracellular signal transduction
- mitotic nuclear envelope disassembly
- negative regulation of glial cell apoptotic process
- peptidyl-serine phosphorylation
- peptidyl-threonine phosphorylation
- positive regulation of adenylate cyclase-activating G protein-coupled receptor signaling pathway
- positive regulation of angiogenesis
- positive regulation of blood vessel endothelial cell migration
- positive regulation of bone resorption
- positive regulation of cardiac muscle hypertrophy
- positive regulation of cell adhesion
- positive regulation of cell migration
- positive regulation of dense core granule biogenesis
- positive regulation of endothelial cell migration
- positive regulation of endothelial cell proliferation
- positive regulation of ERK1 and ERK2 cascade
- positive regulation of lipopolysaccharide-mediated signaling pathway
- positive regulation of macrophage differentiation
- positive regulation of mitotic cell cycle
- post-translational protein modification
- protein phosphorylation
- regulation of mRNA stability
- regulation of platelet aggregation
- response to interleukin-1

---

84

- **Protein name:** Adenylyl cyclase-associated protein 1
- **Organism:** Homo sapiens
- **Uniprot Accession Number:** Q01518
- **Protein sequence length:** 475 aa
- **1D identity (%):** 8.89
- **1D identity (%) [Gaps excluded]:** 23.37
- **1D identity - Alignment Gaps:** 599
- **1D aligned content (<aminoacid>:%):** {'D': 8.14, 'Q': 6.98, 'R': 3.49, 'L': 9.3, 'P': 11.63, 'V': 8.14, 'F': 4.65, 'Y': 4.65, 'G': 9.3, 'H': 4.65, 'T': 5.81, 'A': 3.49, 'C': 2.33, 'I': 6.98, 'S': 2.33, 'K': 4.65, 'E': 2.33, 'W': 1.16}
- **Common reported functions (%):** 0.0
- **Common reported locations (%):** 11.11
- **Common reported processes (%):** 0.0

- **AF ID:** Q01518
- **Chain:** A
- **Protein length:** 475 aa
- **Resolution:** N/A
- **b-phipsi:** 0.007307
- **w-rdist:** 0.241708
- **t-alpha:** 0.072108
- **Chemical similarity (Tanimoto Index) (%):** 99.62
- **1D identity (%) [PDB]:** 2.58
- **1D identity (%) [Gaps excluded][PDB]:** 76.74
- **1D identity - Alignment Gaps [PDB]:** 1234
- **1D aligned content [PDB] (<aminoacid>:%):** {'L': 15.15, 'E': 9.09, 'P': 12.12, 'I': 3.03, 'S': 12.12, 'T': 6.06, 'N': 3.03, 'V': 12.12, 'H': 3.03, 'D': 6.06, 'M': 3.03, 'K': 3.03, 'R': 3.03, 'F': 3.03, 'A': 3.03, 'G': 3.03}
- **2D identity (%) [PDB]:** 31.73
- **2D identity (%) [Gaps excluded][PDB]:** 90.38
- **2D identity - Alignment Gaps [PDB]:** 634
- **2D aligned content [PDB] (<2D-fold>:%):** {'H': 47.74, 'T': 20.32, '.': 16.77, 'E': 15.16}
- **3D similarity (TM-Score) (%) [PDB]:** 18.23

- **Gene name:** CAP1
- **Entrez ID:** 10487
- **RefSeq ID:** N/A
- **Sequence length:** N/A
- **5-UTR|CDS|3-UTR identity (%):** N/A | N/A | N/A
- **5-UTR|CDS|3-UTR identity (%) [Gaps excluded]:** N/A | N/A | N/A
- **5-UTR|CDS|3-UTR identity [Alignment Gaps]:** N/A | N/A | N/A
- **5-UTR aligned content (<base>:%):** N/A
- **CDS aligned content (<base>:%):** N/A
- **3-UTR aligned content (<base>:%):** N/A

**Uniprot Description:**  
  
 Directly regulates filament dynamics and has been implicated in a number of complex developmental and morphological processes, including mRNA localization and the establishment of cell polarity.   
  
Homodimer. Binds actin monomers.   
  
 **Gene Ontology Information:**

Molecular Function

- actin binding
- adenylate cyclase binding

Location

- azurophil granule lumen
- cortical actin cytoskeleton
- cytoplasm
- extracellular exosome
- extracellular region
- focal adhesion
- plasma membrane

Biological process

- actin filament organization
- activation of adenylate cyclase activity
- ameboidal-type cell migration
- cAMP-mediated signaling
- cell morphogenesis
- establishment or maintenance of cell polarity
- receptor-mediated endocytosis
- signal transduction

---

85

- **Protein name:** Plasminogen
- **Organism:** Homo sapiens
- **Uniprot Accession Number:** P00747
- **Protein sequence length:** 810 aa
- **1D identity (%):** 13.53
- **1D identity (%) [Gaps excluded]:** 23.33
- **1D identity - Alignment Gaps:** 444
- **1D aligned content (<aminoacid>:%):** {'V': 6.99, 'L': 7.69, 'S': 4.9, 'P': 13.29, 'G': 8.39, 'F': 3.5, 'I': 2.8, 'A': 2.8, 'K': 8.39, 'C': 5.59, 'H': 2.8, 'Q': 4.2, 'T': 6.99, 'N': 1.4, 'R': 5.59, 'Y': 4.9, 'E': 6.99, 'W': 1.4, 'D': 1.4}
- **Common reported functions (%):** 0.0
- **Common reported locations (%):** 0.0
- **Common reported processes (%):** 0.0

- **AF ID:** P00747
- **Chain:** A
- **Protein length:** 810 aa
- **Resolution:** N/A
- **b-phipsi:** 0.009472
- **w-rdist:** 0.248733
- **t-alpha:** 0.041205
- **Chemical similarity (Tanimoto Index) (%):** 97.2
- **1D identity (%) [PDB]:** 3.17
- **1D identity (%) [Gaps excluded][PDB]:** 63.29
- **1D identity - Alignment Gaps [PDB]:** 1497
- **1D aligned content [PDB] (<aminoacid>:%):** {'M': 2.0, 'V': 10.0, 'L': 12.0, 'F': 4.0, 'K': 8.0, 'S': 8.0, 'R': 12.0, 'I': 6.0, 'D': 4.0, 'E': 6.0, 'G': 8.0, 'Y': 6.0, 'C': 2.0, 'T': 6.0, 'Q': 2.0, 'A': 2.0, 'N': 2.0}
- **2D identity (%) [PDB]:** 36.55
- **2D identity (%) [Gaps excluded][PDB]:** 88.43
- **2D identity - Alignment Gaps [PDB]:** 687
- **2D aligned content [PDB] (<2D-fold>:%):** {'.': 26.17, 'E': 23.13, 'T': 42.29, 'H': 7.01, 'B': 0.7, 'G': 0.7}
- **3D similarity (TM-Score) (%) [PDB]:** 24.09

- **Gene name:** PLG
- **Entrez ID:** N/A
- **RefSeq ID:** NM\_000301
- **Transcript sequence length:** 3530
- **5-UTR|CDS|3-UTR identity (%):** 12.01 | 44.35 | 4.11
- **5-UTR|CDS|3-UTR identity (%) [Gaps excluded]:** 80.0 | 75.0 | 78.18
- **5-UTR|CDS|3-UTR identity [Alignment Gaps]:** 283 | 1288 | 16013
- **5-UTR aligned content (<base>:%):** {'G': 27.5, 'C': 42.5, 'A': 15.0, 'T': 15.0}
- **CDS aligned content (<base>:%):** {'T': 22.96, 'G': 24.25, 'A': 29.97, 'C': 22.82}
- **3-UTR aligned content (<base>:%):** {'T': 30.65, 'A': 32.37, 'C': 17.12, 'G': 19.86}

**Uniprot Description:**  
  
 Plasmin dissolves the fibrin of blood clots and acts as a proteolytic factor in a variety of other processes including embryonic development, tissue remodeling, tumor invasion, and inflammation. In ovulation, weakens the walls of the Graafian follicle. It activates the urokinase-type plasminogen activator, collagenases and several complement zymogens, such as C1 and C5. Cleavage of fibronectin and laminin leads to cell detachment and apoptosis. Also cleaves fibrin, thrombospondin and von Willebrand factor. Its role in tissue remodeling and tumor invasion may be modulated by CSPG4. Binds to cells.   
  
Interacts (both mature PLG and the angiostatin peptide) with CSPG4 and AMOT (PubMed:10889192, PubMed:16043488). Interacts (via the Kringle domains) with HRG; the interaction tethers PLG to the cell surface and enhances its activation (PubMed:9102401, PubMed:19712047). Interacts (via Kringle 4 domain) with ADA; the interaction stimulates PLG activation when in complex with DPP4 (PubMed:15016824). Angiostatin: Interacts with ATP5F1A; the interaction inhibits most of the angiogenic effects of angiostatin (PubMed:10077593).   
  
 **Gene Ontology Information:**

Molecular Function

- endopeptidase activity
- serine-type endopeptidase activity
- signaling receptor binding

Location

- extracellular space

Biological process

- blood coagulation
- fibrinolysis
- proteolysis
- tissue remodeling

---

86

- **Protein name:** CWF19-like protein 1
- **Organism:** Homo sapiens
- **Uniprot Accession Number:** Q69YN2
- **Protein sequence length:** 538 aa
- **1D identity (%):** 11.56
- **1D identity (%) [Gaps excluded]:** 23.28
- **1D identity - Alignment Gaps:** 470
- **1D aligned content (<aminoacid>:%):** {'C': 2.78, 'G': 13.89, 'K': 6.48, 'R': 6.48, 'V': 6.48, 'F': 6.48, 'L': 6.48, 'T': 6.48, 'A': 5.56, 'E': 4.63, 'Y': 3.7, 'N': 2.78, 'Q': 4.63, 'S': 3.7, 'P': 9.26, 'D': 4.63, 'I': 1.85, 'M': 0.93, 'H': 2.78}
- **Common reported functions (%):** 0.0
- **Common reported locations (%):** 0.0
- **Common reported processes (%):** 0.0

- **AF ID:** Q69YN2
- **Chain:** A
- **Protein length:** 538 aa
- **Resolution:** N/A
- **b-phipsi:** 0.00068
- **w-rdist:** 0.776441
- **t-alpha:** 0.019386
- **Chemical similarity (Tanimoto Index) (%):** 98.72
- **1D identity (%) [PDB]:** 2.24
- **1D identity (%) [Gaps excluded][PDB]:** 73.17
- **1D identity - Alignment Gaps [PDB]:** 1301
- **1D aligned content [PDB] (<aminoacid>:%):** {'M': 3.33, 'K': 13.33, 'P': 10.0, 'L': 10.0, 'A': 3.33, 'C': 3.33, 'V': 16.67, 'E': 10.0, 'I': 6.67, 'Y': 3.33, 'D': 10.0, 'R': 6.67, 'N': 3.33}
- **2D identity (%) [PDB]:** 41.67
- **2D identity (%) [Gaps excluded][PDB]:** 87.25
- **2D identity - Alignment Gaps [PDB]:** 489
- **2D aligned content [PDB] (<2D-fold>:%):** {'.': 25.64, 'E': 21.28, 'H': 24.1, 'T': 27.44, 'G': 1.54}
- **3D similarity (TM-Score) (%) [PDB]:** 20.75

- **Gene name:** CWF19L1
- **Entrez ID:** 55280
- **RefSeq ID:** NM\_018294
- **Transcript sequence length:** 2591
- **5-UTR|CDS|3-UTR identity (%):** 9.12 | 37.02 | 3.75
- **5-UTR|CDS|3-UTR identity (%) [Gaps excluded]:** 78.95 | 74.77 | 82.36
- **5-UTR|CDS|3-UTR identity [Alignment Gaps]:** 291 | 1418 | 16142
- **5-UTR aligned content (<base>:%):** {'A': 16.67, 'T': 30.0, 'C': 36.67, 'G': 16.67}
- **CDS aligned content (<base>:%):** {'A': 30.87, 'T': 24.9, 'G': 23.75, 'C': 20.48}
- **3-UTR aligned content (<base>:%):** {'A': 28.82, 'G': 23.31, 'T': 25.67, 'C': 22.2}

**Uniprot Description:**  
  
 N/A N/A   
  
 **Gene Ontology Information:**

Molecular Function

- RNA lariat debranching enzyme activator activity

Location

- post-mRNA release spliceosomal complex

Biological process

- mRNA splicing, via spliceosome

---

87

- **Protein name:** Protein FAM43A
- **Organism:** Homo sapiens
- **Uniprot Accession Number:** Q8N2R8
- **Protein sequence length:** 423 aa
- **1D identity (%):** 8.11
- **1D identity (%) [Gaps excluded]:** 21.97
- **1D identity - Alignment Gaps:** 591
- **1D aligned content (<aminoacid>:%):** {'L': 10.53, 'P': 14.47, 'S': 5.26, 'Y': 2.63, 'E': 6.58, 'G': 14.47, 'R': 5.26, 'D': 3.95, 'T': 7.89, 'I': 2.63, 'Q': 3.95, 'C': 5.26, 'K': 2.63, 'V': 3.95, 'A': 7.89, 'H': 1.32, 'F': 1.32}
- **Common reported functions (%):** 0.0
- **Common reported locations (%):** 0.0
- **Common reported processes (%):** 0.0

- **AF ID:** Q8N2R8
- **Chain:** A
- **Protein length:** 423 aa
- **Resolution:** N/A
- **b-phipsi:** 0.000501
- **w-rdist:** 0.664469
- **t-alpha:** 0.105388
- **Chemical similarity (Tanimoto Index) (%):** 98.94
- **1D identity (%) [PDB]:** 2.81
- **1D identity (%) [Gaps excluded][PDB]:** 57.63
- **1D identity - Alignment Gaps [PDB]:** 1150
- **1D aligned content [PDB] (<aminoacid>:%):** {'L': 14.71, 'P': 5.88, 'V': 11.76, 'G': 2.94, 'Q': 8.82, 'K': 5.88, 'H': 2.94, 'Y': 2.94, 'A': 11.76, 'R': 8.82, 'T': 2.94, 'S': 11.76, 'E': 5.88, 'I': 2.94}
- **2D identity (%) [PDB]:** 35.45
- **2D identity (%) [Gaps excluded][PDB]:** 85.71
- **2D identity - Alignment Gaps [PDB]:** 526
- **2D aligned content [PDB] (<2D-fold>:%):** {'.': 21.7, 'T': 37.42, 'H': 30.19, 'E': 9.75, 'G': 0.94}
- **3D similarity (TM-Score) (%) [PDB]:** 13.03

- **Gene name:** FAM43A
- **Entrez ID:** 131583
- **RefSeq ID:** NM\_153690
- **Transcript sequence length:** 3155
- **5-UTR|CDS|3-UTR identity (%):** 27.68 | 24.97 | 3.49
- **5-UTR|CDS|3-UTR identity (%) [Gaps excluded]:** 83.23 | 72.94 | 73.84
- **5-UTR|CDS|3-UTR identity [Alignment Gaps]:** 634 | 1889 | 16096
- **5-UTR aligned content (<base>:%):** {'A': 8.37, 'G': 33.46, 'C': 41.83, 'T': 16.35}
- **CDS aligned content (<base>:%):** {'T': 16.04, 'G': 31.24, 'C': 29.43, 'A': 23.29}
- **3-UTR aligned content (<base>:%):** {'G': 18.47, 'T': 35.42, 'C': 20.0, 'A': 26.1}

**Uniprot Description:**  
  
 N/A N/A   
  
 **Gene Ontology Information:**

Molecular Function   
  
N/A

Location   
  
N/A

Biological process   
  
N/A

---

88

- **Protein name:** Deoxynucleotidyltransferase terminal-interacting protein 1
- **Organism:** Homo sapiens
- **Uniprot Accession Number:** Q9H147
- **Protein sequence length:** 329 aa
- **1D identity (%):** 6.95
- **1D identity (%) [Gaps excluded]:** 22.34
- **1D identity - Alignment Gaps:** 625
- **1D aligned content (<aminoacid>:%):** {'G': 9.52, 'D': 6.35, 'P': 11.11, 'R': 11.11, 'V': 4.76, 'W': 1.59, 'H': 7.94, 'T': 4.76, 'L': 7.94, 'S': 3.17, 'E': 11.11, 'F': 1.59, 'K': 7.94, 'N': 1.59, 'A': 3.17, 'I': 1.59, 'Q': 3.17, 'C': 1.59}
- **Common reported functions (%):** 0.0
- **Common reported locations (%):** 11.11
- **Common reported processes (%):** 0.0

- **AF ID:** Q9H147
- **Chain:** A
- **Protein length:** 329 aa
- **Resolution:** N/A
- **b-phipsi:** 0.002604
- **w-rdist:** 0.662842
- **t-alpha:** 0.005547
- **Chemical similarity (Tanimoto Index) (%):** 98.57
- **1D identity (%) [PDB]:** 1.04
- **1D identity (%) [Gaps excluded][PDB]:** 70.59
- **1D identity - Alignment Gaps [PDB]:** 1140
- **1D aligned content [PDB] (<aminoacid>:%):** {'A': 8.33, 'T': 16.67, 'Q': 16.67, 'R': 8.33, 'E': 16.67, 'I': 8.33, 'L': 8.33, 'K': 8.33, 'F': 8.33}
- **2D identity (%) [PDB]:** 26.31
- **2D identity (%) [Gaps excluded][PDB]:** 85.2
- **2D identity - Alignment Gaps [PDB]:** 620
- **2D aligned content [PDB] (<2D-fold>:%):** {'.': 25.0, 'T': 33.9, 'H': 36.86, 'E': 4.24}
- **3D similarity (TM-Score) (%) [PDB]:** 13.35

- **Gene name:** DNTTIP1
- **Entrez ID:** 116092
- **RefSeq ID:** NM\_052951
- **Transcript sequence length:** 1264
- **5-UTR|CDS|3-UTR identity (%):** 11.28 | 24.55 | 0.92
- **5-UTR|CDS|3-UTR identity (%) [Gaps excluded]:** 82.22 | 74.13 | 73.33
- **5-UTR|CDS|3-UTR identity [Alignment Gaps]:** 283 | 1795 | 16558
- **5-UTR aligned content (<base>:%):** {'A': 10.81, 'C': 35.14, 'T': 13.51, 'G': 40.54}
- **CDS aligned content (<base>:%):** {'A': 30.05, 'T': 17.6, 'G': 28.68, 'C': 23.67}
- **3-UTR aligned content (<base>:%):** {'G': 25.97, 'C': 24.68, 'T': 24.68, 'A': 24.68}

**Uniprot Description:**  
  
 Increases DNTT terminal deoxynucleotidyltransferase activity (in vitro) (PubMed:11473582). Also acts as a transcriptional regulator, binding to the consensus sequence 5'-GNTGCATG-3' following an AT-tract. Associates with RAB20 promoter and positively regulates its transcription. Binds DNA and nucleosomes; may recruit HDAC1 complexes to nucleosomes or naked DNA.   
  
Monomer and homodimer (PubMed:11473582, PubMed:25653165). A minor proportion may form homotrimers (PubMed:11473582). Interacts with ZNF541 (PubMed:21573134). Interacts with the terminal deoxynucleotidyltransferase DNTT (PubMed:11473582, PubMed:16371131). Interacts with TRERF1 (PubMed:16371131, PubMed:21573134). Identified in a histone deacetylase complex that contains DNTTIP1, HDAC1 and MIDEAS; this complex assembles into a tetramer that contains four copies of each protein chain (PubMed:25653165). Component of a histone deacetylase complex containing DNTTIP1, ZNF541, HDAC1 and HDAC2 (PubMed:21573134). Identified in a complex with KCTD19, HDAC1, HDAC2 and ZNF541 (By similarity).   
  
 **Gene Ontology Information:**

Molecular Function

- DNA binding
- nucleosome binding
- protein homodimerization activity

Location

- chromosome
- histone deacetylase complex
- nucleolus
- nucleoplasm
- nucleus

Biological process   
  
N/A

---

89

- **Protein name:** Zinc finger protein 121
- **Organism:** Homo sapiens
- **Uniprot Accession Number:** P58317
- **Protein sequence length:** 390 aa
- **1D identity (%):** 8.23
- **1D identity (%) [Gaps excluded]:** 23.24
- **1D identity - Alignment Gaps:** 596
- **1D aligned content (<aminoacid>:%):** {'E': 9.21, 'C': 5.26, 'N': 2.63, 'H': 10.53, 'G': 11.84, 'D': 3.95, 'Y': 5.26, 'P': 7.89, 'T': 6.58, 'L': 6.58, 'F': 6.58, 'S': 1.32, 'V': 5.26, 'A': 1.32, 'K': 6.58, 'I': 3.95, 'R': 3.95, 'Q': 1.32}
- **Common reported functions (%):** 14.29
- **Common reported locations (%):** 0.0
- **Common reported processes (%):** 0.0

- **AF ID:** P58317
- **Chain:** A
- **Protein length:** 390 aa
- **Resolution:** N/A
- **b-phipsi:** 0.006281
- **w-rdist:** 0.293712
- **t-alpha:** 0.078447
- **Chemical similarity (Tanimoto Index) (%):** 97.03
- **1D identity (%) [PDB]:** 2.08
- **1D identity (%) [Gaps excluded][PDB]:** 75.76
- **1D identity - Alignment Gaps [PDB]:** 1169
- **1D aligned content [PDB] (<aminoacid>:%):** {'P': 8.0, 'A': 8.0, 'G': 12.0, 'T': 12.0, 'V': 4.0, 'D': 4.0, 'I': 4.0, 'H': 12.0, 'Y': 8.0, 'E': 4.0, 'F': 8.0, 'L': 4.0, 'S': 8.0, 'R': 4.0}
- **2D identity (%) [PDB]:** 32.14
- **2D identity (%) [Gaps excluded][PDB]:** 84.96
- **2D identity - Alignment Gaps [PDB]:** 557
- **2D aligned content [PDB] (<2D-fold>:%):** {'.': 33.33, 'T': 21.18, 'H': 36.11, 'E': 9.38}
- **3D similarity (TM-Score) (%) [PDB]:** 12.67

- **Gene name:** ZNF121
- **Entrez ID:** 7675
- **RefSeq ID:** N/A
- **Sequence length:** N/A
- **5-UTR|CDS|3-UTR identity (%):** N/A | N/A | N/A
- **5-UTR|CDS|3-UTR identity (%) [Gaps excluded]:** N/A | N/A | N/A
- **5-UTR|CDS|3-UTR identity [Alignment Gaps]:** N/A | N/A | N/A
- **5-UTR aligned content (<base>:%):** N/A
- **CDS aligned content (<base>:%):** N/A
- **3-UTR aligned content (<base>:%):** N/A

**Uniprot Description:**  
  
 May be involved in transcriptional regulation. N/A   
  
 **Gene Ontology Information:**

Molecular Function

- DNA-binding transcription factor activity, RNA polymerase II-specific
- metal ion binding
- RNA polymerase II cis-regulatory region sequence-specific DNA binding

Location

- nucleus

Biological process

- regulation of transcription by RNA polymerase II

---

90

- **Protein name:** Lysyl oxidase homolog 4
- **Organism:** Homo sapiens
- **Uniprot Accession Number:** Q96JB6
- **Protein sequence length:** 756 aa
- **1D identity (%):** 15.25
- **1D identity (%) [Gaps excluded]:** 26.31
- **1D identity - Alignment Gaps:** 430
- **1D aligned content (<aminoacid>:%):** {'P': 12.18, 'L': 10.26, 'G': 12.82, 'E': 3.85, 'V': 10.9, 'H': 3.85, 'Q': 6.41, 'W': 1.92, 'A': 6.41, 'C': 6.41, 'T': 4.49, 'K': 3.21, 'R': 5.13, 'D': 3.21, 'M': 1.28, 'I': 1.28, 'S': 1.92, 'N': 0.64, 'Y': 2.56, 'F': 1.28}
- **Common reported functions (%):** 0.0
- **Common reported locations (%):** 11.11
- **Common reported processes (%):** 0.0

- **AF ID:** Q96JB6
- **Chain:** A
- **Protein length:** 756 aa
- **Resolution:** N/A
- **b-phipsi:** 0.009448
- **w-rdist:** 0.285598
- **t-alpha:** 0.036125
- **Chemical similarity (Tanimoto Index) (%):** 96.7
- **1D identity (%) [PDB]:** 2.25
- **1D identity (%) [Gaps excluded][PDB]:** 72.92
- **1D identity - Alignment Gaps [PDB]:** 1505
- **1D aligned content [PDB] (<aminoacid>:%):** {'T': 2.86, 'L': 11.43, 'P': 14.29, 'E': 8.57, 'D': 5.71, 'K': 2.86, 'I': 2.86, 'S': 5.71, 'V': 11.43, 'H': 2.86, 'A': 5.71, 'R': 5.71, 'F': 5.71, 'G': 8.57, 'Y': 2.86, 'W': 2.86}
- **2D identity (%) [PDB]:** 41.58
- **2D identity (%) [Gaps excluded][PDB]:** 87.94
- **2D identity - Alignment Gaps [PDB]:** 573
- **2D aligned content [PDB] (<2D-fold>:%):** {'.': 22.35, 'E': 28.54, 'T': 30.75, 'H': 18.36}
- **3D similarity (TM-Score) (%) [PDB]:** 24.06

- **Gene name:** LOXL4
- **Entrez ID:** 281904
- **RefSeq ID:** NM\_032211
- **Transcript sequence length:** 3597
- **5-UTR|CDS|3-UTR identity (%):** 17.8 | 44.09 | 4.79
- **5-UTR|CDS|3-UTR identity (%) [Gaps excluded]:** 75.0 | 73.11 | 75.7
- **5-UTR|CDS|3-UTR identity [Alignment Gaps]:** 257 | 1202 | 15846
- **5-UTR aligned content (<base>:%):** {'G': 28.33, 'C': 51.67, 'T': 15.0, 'A': 5.0}
- **CDS aligned content (<base>:%):** {'A': 23.22, 'T': 20.52, 'G': 29.29, 'C': 26.97}
- **3-UTR aligned content (<base>:%):** {'A': 31.23, 'G': 20.25, 'C': 23.46, 'T': 25.06}

**Uniprot Description:**  
  
 May modulate the formation of a collagenous extracellular matrix. N/A   
  
 **Gene Ontology Information:**

Molecular Function

- copper ion binding
- protein-lysine 6-oxidase activity
- scavenger receptor activity

Location

- collagen-containing extracellular matrix
- extracellular space
- membrane

Biological process

- collagen fibril organization
- peptidyl-lysine oxidation

---

91

- **Protein name:** Kelch repeat and BTB domain-containing protein 2
- **Organism:** Homo sapiens
- **Uniprot Accession Number:** Q8IY47
- **Protein sequence length:** 623 aa
- **1D identity (%):** 13.25
- **1D identity (%) [Gaps excluded]:** 26.52
- **1D identity - Alignment Gaps:** 495
- **1D aligned content (<aminoacid>:%):** {'D': 8.4, 'E': 3.05, 'R': 6.11, 'V': 9.16, 'K': 6.11, 'L': 8.4, 'T': 8.4, 'C': 3.05, 'F': 3.82, 'G': 4.58, 'Q': 6.11, 'Y': 6.87, 'I': 3.05, 'N': 3.82, 'A': 6.11, 'S': 4.58, 'M': 1.53, 'P': 5.34, 'H': 0.76, 'W': 0.76}
- **Common reported functions (%):** 0.0
- **Common reported locations (%):** 0.0
- **Common reported processes (%):** 0.0

- **AF ID:** Q8IY47
- **Chain:** A
- **Protein length:** 623 aa
- **Resolution:** N/A
- **b-phipsi:** 0.006707
- **w-rdist:** 0.343872
- **t-alpha:** 0.031696
- **Chemical similarity (Tanimoto Index) (%):** 98.87
- **1D identity (%) [PDB]:** 3.22
- **1D identity (%) [Gaps excluded][PDB]:** 65.22
- **1D identity - Alignment Gaps [PDB]:** 1330
- **1D aligned content [PDB] (<aminoacid>:%):** {'T': 6.67, 'Q': 8.89, 'R': 6.67, 'C': 4.44, 'E': 6.67, 'L': 8.89, 'K': 2.22, 'F': 4.44, 'D': 6.67, 'I': 4.44, 'S': 6.67, 'A': 4.44, 'M': 2.22, 'G': 4.44, 'P': 6.67, 'Y': 4.44, 'V': 6.67, 'H': 2.22, 'N': 2.22}
- **2D identity (%) [PDB]:** 34.14
- **2D identity (%) [Gaps excluded][PDB]:** 91.48
- **2D identity - Alignment Gaps [PDB]:** 670
- **2D aligned content [PDB] (<2D-fold>:%):** {'.': 20.82, 'H': 35.34, 'T': 24.38, 'E': 17.53, 'G': 1.64, 'B': 0.27}
- **3D similarity (TM-Score) (%) [PDB]:** 21.08

- **Gene name:** KBTBD2
- **Entrez ID:** 25948
- **RefSeq ID:** N/A
- **Sequence length:** N/A
- **5-UTR|CDS|3-UTR identity (%):** N/A | N/A | N/A
- **5-UTR|CDS|3-UTR identity (%) [Gaps excluded]:** N/A | N/A | N/A
- **5-UTR|CDS|3-UTR identity [Alignment Gaps]:** N/A | N/A | N/A
- **5-UTR aligned content (<base>:%):** N/A
- **CDS aligned content (<base>:%):** N/A
- **3-UTR aligned content (<base>:%):** N/A

**Uniprot Description:**  
  
 N/A N/A   
  
 **Gene Ontology Information:**

Molecular Function   
  
N/A

Location   
  
N/A

Biological process

- gene expression
- glucose metabolic process
- lipid metabolic process
- phosphatidylinositol 3-kinase signaling
- protein ubiquitination
- response to insulin

---

92

- **Protein name:** Adenylyl cyclase-associated protein 2
- **Organism:** Homo sapiens
- **Uniprot Accession Number:** P40123
- **Protein sequence length:** 477 aa
- **1D identity (%):** 8.71
- **1D identity (%) [Gaps excluded]:** 21.61
- **1D identity - Alignment Gaps:** 569
- **1D aligned content (<aminoacid>:%):** {'A': 7.23, 'S': 8.43, 'P': 15.66, 'E': 6.02, 'V': 7.23, 'F': 3.61, 'K': 8.43, 'D': 6.02, 'M': 2.41, 'R': 2.41, 'G': 7.23, 'H': 3.61, 'Q': 6.02, 'I': 4.82, 'L': 3.61, 'Y': 3.61, 'C': 1.2, 'T': 1.2, 'W': 1.2}
- **Common reported functions (%):** 0.0
- **Common reported locations (%):** 11.11
- **Common reported processes (%):** 0.0

- **AF ID:** P40123
- **Chain:** A
- **Protein length:** 477 aa
- **Resolution:** N/A
- **b-phipsi:** 0.00287
- **w-rdist:** 0.354924
- **t-alpha:** 0.083994
- **Chemical similarity (Tanimoto Index) (%):** 99.47
- **1D identity (%) [PDB]:** 2.26
- **1D identity (%) [Gaps excluded][PDB]:** 72.5
- **1D identity - Alignment Gaps [PDB]:** 1242
- **1D aligned content [PDB] (<aminoacid>:%):** {'L': 13.79, 'E': 6.9, 'K': 6.9, 'P': 10.34, 'I': 3.45, 'S': 13.79, 'T': 3.45, 'N': 3.45, 'V': 13.79, 'H': 6.9, 'D': 3.45, 'R': 6.9, 'G': 3.45, 'A': 3.45}
- **2D identity (%) [PDB]:** 30.91
- **2D identity (%) [Gaps excluded][PDB]:** 92.17
- **2D identity - Alignment Gaps [PDB]:** 658
- **2D aligned content [PDB] (<2D-fold>:%):** {'H': 49.35, '.': 19.61, 'T': 15.36, 'G': 0.98, 'E': 14.71}
- **3D similarity (TM-Score) (%) [PDB]:** 16.9

- **Gene name:** CAP2
- **Entrez ID:** 10486
- **RefSeq ID:** NM\_006366
- **Transcript sequence length:** 2925
- **5-UTR|CDS|3-UTR identity (%):** 29.33 | 33.24 | 5.18
- **5-UTR|CDS|3-UTR identity (%) [Gaps excluded]:** 74.63 | 74.18 | 74.7
- **5-UTR|CDS|3-UTR identity [Alignment Gaps]:** 207 | 1531 | 15745
- **5-UTR aligned content (<base>:%):** {'G': 46.0, 'A': 12.0, 'C': 32.0, 'T': 10.0}
- **CDS aligned content (<base>:%):** {'A': 31.89, 'T': 22.34, 'G': 24.08, 'C': 21.69}
- **3-UTR aligned content (<base>:%):** {'T': 40.48, 'C': 14.25, 'G': 12.43, 'A': 32.84}

**Uniprot Description:**  
  
 May have a regulatory bifunctional role. N/A   
  
 **Gene Ontology Information:**

Molecular Function

- actin binding
- adenylate cyclase binding
- identical protein binding

Location

- cytoplasm
- plasma membrane
- postsynaptic density

Biological process

- actin filament organization
- activation of adenylate cyclase activity
- cAMP-mediated signaling
- cell morphogenesis
- establishment or maintenance of cell polarity
- presynaptic actin cytoskeleton organization
- signal transduction

---

93

- **Protein name:** Diacylglycerol kinase gamma
- **Organism:** Homo sapiens
- **Uniprot Accession Number:** P49619
- **Protein sequence length:** 791 aa
- **1D identity (%):** N/A
- **1D identity (%) [Gaps excluded]:** N/A
- **1D identity - Alignment Gaps:** N/A
- **1D aligned content (<aminoacid>:%):** N/A
- **Common reported functions (%):** N/A
- **Common reported locations (%):** N/A
- **Common reported processes (%):** N/A

- **AF ID:** P49619
- **Chain:** A
- **Protein length:** 791 aa
- **Resolution:** N/A
- **b-phipsi:** 0.001365
- **w-rdist:** 0.136383
- **t-alpha:** 0.22187
- **Chemical similarity (Tanimoto Index) (%):** N/A
- **1D identity (%) [PDB]:** N/A
- **1D identity (%) [Gaps excluded][PDB]:** N/A
- **1D identity - Alignment Gaps [PDB]:** N/A
- **1D aligned content [PDB] (<aminoacid>:%):** N/A
- **2D identity (%) [PDB]:** N/A
- **2D identity (%) [Gaps excluded][PDB]:** N/A
- **2D identity - Alignment Gaps [PDB]:** N/A
- **2D aligned content [PDB] (<2D-fold>:%):** N/A
- **3D similarity (TM-Score) (%) [PDB]:** N/A

- **Gene name:** DGKG
- **Entrez ID:** 1608
- **RefSeq ID:** N/A
- **Sequence length:** N/A
- **5-UTR|CDS|3-UTR identity (%):** N/A | N/A | N/A
- **5-UTR|CDS|3-UTR identity (%) [Gaps excluded]:** N/A | N/A | N/A
- **5-UTR|CDS|3-UTR identity [Alignment Gaps]:** N/A | N/A | N/A
- **5-UTR aligned content (<base>:%):** N/A
- **CDS aligned content (<base>:%):** N/A
- **3-UTR aligned content (<base>:%):** N/A

**Uniprot Description:**  
  
 Diacylglycerol kinase that converts diacylglycerol/DAG into phosphatidic acid/phosphatidate/PA and regulates the respective levels of these two bioactive lipids (PubMed:8034597). Thereby, acts as a central switch between the signaling pathways activated by these second messengers with different cellular targets and opposite effects in numerous biological processes (By similarity). Has no apparent specificity with regard to the acyl compositions of diacylglycerol (PubMed:8034597). Specifically expressed in the cerebellum where it controls the level of diacylglycerol which in turn regulates the activity of protein kinase C gamma. Through protein kinase C gamma, indirectly regulates the dendritic development of Purkinje cells, cerebellar long term depression and ultimately cerebellar motor coordination (By similarity). N/A   
  
 **Gene Ontology Information:**

Molecular Function

- ATP binding
- diacylglycerol kinase activity
- calcium ion binding
- lipid binding

Location

- cytoskeleton
- cytosol
- membrane
- plasma membrane

Biological process

- diacylglycerol metabolic process
- glycerolipid metabolic process
- intracellular signal transduction
- lipid phosphorylation
- negative regulation of protein kinase C signaling
- neuron development
- phosphatidic acid biosynthetic process
- platelet activation
- protein kinase C-activating G protein-coupled receptor signaling pathway
- regulation of dendrite development

---

94

- **Protein name:** Long-chain fatty acid transport protein 5
- **Organism:** Homo sapiens
- **Uniprot Accession Number:** Q9Y2P5
- **Protein sequence length:** 690 aa
- **1D identity (%):** 11.66
- **1D identity (%) [Gaps excluded]:** 23.63
- **1D identity - Alignment Gaps:** 526
- **1D aligned content (<aminoacid>:%):** {'G': 13.22, 'L': 12.4, 'P': 16.53, 'A': 7.44, 'R': 5.79, 'V': 9.09, 'H': 4.13, 'I': 2.48, 'T': 5.79, 'D': 3.31, 'E': 1.65, 'K': 2.48, 'W': 1.65, 'M': 1.65, 'Y': 0.83, 'F': 3.31, 'C': 3.31, 'N': 2.48, 'Q': 2.48}
- **Common reported functions (%):** 0.0
- **Common reported locations (%):** 0.0
- **Common reported processes (%):** 0.0

- **AF ID:** Q9Y2P5
- **Chain:** A
- **Protein length:** 690 aa
- **Resolution:** N/A
- **b-phipsi:** 0.009049
- **w-rdist:** 0.703234
- **t-alpha:** 0.000792
- **Chemical similarity (Tanimoto Index) (%):** 99.4
- **1D identity (%) [PDB]:** 2.85
- **1D identity (%) [Gaps excluded][PDB]:** 71.19
- **1D identity - Alignment Gaps [PDB]:** 1417
- **1D aligned content [PDB] (<aminoacid>:%):** {'V': 19.05, 'Q': 9.52, 'P': 11.9, 'L': 7.14, 'A': 9.52, 'D': 9.52, 'T': 4.76, 'G': 4.76, 'S': 4.76, 'R': 9.52, 'Y': 2.38, 'E': 4.76, 'I': 2.38}
- **2D identity (%) [PDB]:** 37.73
- **2D identity (%) [Gaps excluded][PDB]:** 90.69
- **2D identity - Alignment Gaps [PDB]:** 633
- **2D aligned content [PDB] (<2D-fold>:%):** {'.': 14.91, 'H': 46.94, 'T': 23.47, 'E': 14.18, 'B': 0.49}
- **3D similarity (TM-Score) (%) [PDB]:** 22.58

- **Gene name:** SLC27A5
- **Entrez ID:** 10998
- **RefSeq ID:** NM\_012254
- **Transcript sequence length:** 2292
- **5-UTR|CDS|3-UTR identity (%):** 7.76 | 42.47 | 0.78
- **5-UTR|CDS|3-UTR identity (%) [Gaps excluded]:** 67.57 | 72.83 | 79.88
- **5-UTR|CDS|3-UTR identity [Alignment Gaps]:** 285 | 1226 | 16609
- **5-UTR aligned content (<base>:%):** {'T': 8.0, 'C': 32.0, 'G': 44.0, 'A': 16.0}
- **CDS aligned content (<base>:%):** {'A': 19.3, 'T': 22.1, 'G': 30.9, 'C': 27.7}
- **3-UTR aligned content (<base>:%):** {'C': 33.59, 'A': 23.66, 'T': 16.79, 'G': 25.95}

**Uniprot Description:**  
  
 May mediate the import of long-chain fatty acids (LCFA) by facilitating their transport across cell membranes (PubMed:20448275, PubMed:20530735). Also catalyzes the ATP-dependent formation of fatty acyl-CoA using LCFA and very-long-chain fatty acids (VLCFA) as substrates (PubMed:10479480). Mainly functions as a bile acyl-CoA synthetase catalyzing the activation of bile acids via ATP-dependent formation of bile acid CoA thioesters which is necessary for their subsequent conjugation with glycine or taurine (PubMed:10749848, PubMed:11980911). Both primary bile acids (cholic acid and chenodeoxycholic acid) and secondary bile acids (deoxycholic acid and lithocholic acid) are the principal substrates (PubMed:10749848, PubMed:11980911). In vitro, activates 3-alpha,7-alpha,12-alpha-trihydroxy-5-beta-cholestanate ((25R)-3alpha,7alpha,12alpha-trihydroxy-5beta-cholestan-26-oate or THCA), the C27 precursor of cholic acid deriving from the de novo synthesis from cholesterol (PubMed:11980911). Plays an important role in hepatic fatty acid uptake and bile acid reconjugation and recycling but not in de novo synthesis of bile acids (By similarity). N/A   
  
 **Gene Ontology Information:**

Molecular Function

- ATP binding
- cholate-CoA ligase activity
- fatty acid transmembrane transporter activity
- long-chain fatty acid transporter activity
- long-chain fatty acid-CoA ligase activity
- oxidoreductase activity
- protein-containing complex binding
- very long-chain fatty acid-CoA ligase activity

Location

- basal plasma membrane
- endoplasmic reticulum
- endoplasmic reticulum membrane
- protein-containing complex

Biological process

- bile acid and bile salt transport
- bile acid biosynthetic process
- establishment of localization in cell
- ketone body biosynthetic process
- long-chain fatty acid import across plasma membrane
- triglyceride mobilization
- very long-chain fatty acid metabolic process

---

95

- **Protein name:** Protein kinase C theta type
- **Organism:** Homo sapiens
- **Uniprot Accession Number:** Q04759
- **Protein sequence length:** 706 aa
- **1D identity (%):** 12.79
- **1D identity (%) [Gaps excluded]:** 21.22
- **1D identity - Alignment Gaps:** 388
- **1D aligned content (<aminoacid>:%):** {'P': 9.6, 'L': 11.2, 'D': 4.0, 'G': 8.8, 'V': 4.8, 'K': 10.4, 'I': 3.2, 'T': 4.0, 'S': 1.6, 'E': 6.4, 'W': 0.8, 'M': 1.6, 'N': 3.2, 'F': 4.0, 'H': 6.4, 'Q': 4.0, 'R': 7.2, 'C': 4.8, 'Y': 1.6, 'A': 2.4}
- **Common reported functions (%):** 14.29
- **Common reported locations (%):** 11.11
- **Common reported processes (%):** 0.0

- **AF ID:** Q04759
- **Chain:** A
- **Protein length:** 706 aa
- **Resolution:** N/A
- **b-phipsi:** 0.00127
- **w-rdist:** 0.356015
- **t-alpha:** 0.114105
- **Chemical similarity (Tanimoto Index) (%):** 99.02
- **1D identity (%) [PDB]:** 2.32
- **1D identity (%) [Gaps excluded][PDB]:** 79.55
- **1D identity - Alignment Gaps [PDB]:** 1463
- **1D aligned content [PDB] (<aminoacid>:%):** {'G': 8.57, 'E': 11.43, 'K': 11.43, 'D': 5.71, 'P': 8.57, 'F': 5.71, 'V': 8.57, 'S': 8.57, 'I': 5.71, 'R': 5.71, 'W': 2.86, 'L': 17.14}
- **2D identity (%) [PDB]:** 41.08
- **2D identity (%) [Gaps excluded][PDB]:** 88.41
- **2D identity - Alignment Gaps [PDB]:** 567
- **2D aligned content [PDB] (<2D-fold>:%):** {'.': 25.98, 'E': 22.53, 'T': 24.6, 'H': 25.98, 'B': 0.23, 'G': 0.69}
- **3D similarity (TM-Score) (%) [PDB]:** 25.16

- **Gene name:** PRKCQ
- **Entrez ID:** 5588
- **RefSeq ID:** N/A
- **Sequence length:** N/A
- **5-UTR|CDS|3-UTR identity (%):** N/A | N/A | N/A
- **5-UTR|CDS|3-UTR identity (%) [Gaps excluded]:** N/A | N/A | N/A
- **5-UTR|CDS|3-UTR identity [Alignment Gaps]:** N/A | N/A | N/A
- **5-UTR aligned content (<base>:%):** N/A
- **CDS aligned content (<base>:%):** N/A
- **3-UTR aligned content (<base>:%):** N/A

**Uniprot Description:**  
  
 Calcium-independent, phospholipid- and diacylglycerol (DAG)-dependent serine/threonine-protein kinase that mediates non-redundant functions in T-cell receptor (TCR) signaling, including T-cells activation, proliferation, differentiation and survival, by mediating activation of multiple transcription factors such as NF-kappa-B, JUN, NFATC1 and NFATC2. In TCR-CD3/CD28-co-stimulated T-cells, is required for the activation of NF-kappa-B and JUN, which in turn are essential for IL2 production, and participates in the calcium-dependent NFATC1 and NFATC2 transactivation (PubMed:21964608). Mediates the activation of the canonical NF-kappa-B pathway (NFKB1) by direct phosphorylation of CARD11 on several serine residues, inducing CARD11 association with lipid rafts and recruitment of the BCL10-MALT1 complex, which then activates IKK complex, resulting in nuclear translocation and activation of NFKB1. May also play an indirect role in activation of the non-canonical NF-kappa-B (NFKB2) pathway. In the signaling pathway leading to JUN activation, acts by phosphorylating the mediator STK39/SPAK and may not act through MAP kinases signaling. Plays a critical role in TCR/CD28-induced NFATC1 and NFATC2 transactivation by participating in the regulation of reduced inositol 1,4,5-trisphosphate generation and intracellular calcium mobilization. After costimulation of T-cells through CD28 can phosphorylate CBLB and is required for the ubiquitination and subsequent degradation of CBLB, which is a prerequisite for the activation of TCR. During T-cells differentiation, plays an important role in the development of T-helper 2 (Th2) cells following immune and inflammatory responses, and, in the development of inflammatory autoimmune diseases, is necessary for the activation of IL17-producing Th17 cells. May play a minor role in Th1 response. Upon TCR stimulation, mediates T-cell protective survival signal by phosphorylating BAD, thus protecting T-cells from BAD-induced apoptosis, and by up-regulating BCL-X(L)/BCL2L1 levels through NF-kappa-B and JUN pathways. In platelets, regulates signal transduction downstream of the ITGA2B, CD36/GP4, F2R/PAR1 and F2RL3/PAR4 receptors, playing a positive role in 'outside-in' signaling and granule secretion signal transduction. May relay signals from the activated ITGA2B receptor by regulating the uncoupling of WASP and WIPF1, thereby permitting the regulation of actin filament nucleation and branching activity of the Arp2/3 complex. May mediate inhibitory effects of free fatty acids on insulin signaling by phosphorylating IRS1, which in turn blocks IRS1 tyrosine phosphorylation and downstream activation of the PI3K/AKT pathway. Phosphorylates MSN (moesin) in the presence of phosphatidylglycerol or phosphatidylinositol. Phosphorylates PDPK1 at 'Ser-504' and 'Ser-532' and negatively regulates its ability to phosphorylate PKB/AKT1. Phosphorylates CCDC88A/GIV and inhibits its guanine nucleotide exchange factor activity (PubMed:23509302).   
  
Part of a lipid raft complex composed at least of BCL10, CARD11, MALT1 and IKBKB (PubMed:16356855). Interacts with GLRX3 (via N-terminus) (PubMed:10636891). Interacts with ECT2 (PubMed:15254234). Interacts with CCDC88A/GIV; the interaction leads to phosphorylation of CCDC88A and inhibition of its guanine nucleotide exchange factor activity (PubMed:23509302). Interacts with PRKCH upstream open reading frame 2; the interaction leads to inhibition of kinase activity (PubMed:34593629). Interacts with CD28 (PubMed:21964608).   
  
 **Gene Ontology Information:**

Molecular Function

- ATP binding
- calcium-dependent protein kinase C activity
- metal ion binding
- protein kinase activity
- protein kinase C activity
- protein serine kinase activity
- protein serine/threonine kinase activity

Location

- aggresome
- centriolar satellite
- cytosol
- immunological synapse
- plasma membrane

Biological process

- axon guidance
- CD4-positive, alpha-beta T cell proliferation
- cell chemotaxis
- Fc-epsilon receptor signaling pathway
- inflammatory response
- intracellular signal transduction
- membrane protein ectodomain proteolysis
- negative regulation of insulin receptor signaling pathway
- negative regulation of T cell apoptotic process
- peptidyl-serine phosphorylation
- positive regulation of CD4-positive, alpha-beta T cell proliferation
- positive regulation of interleukin-17 production
- positive regulation of interleukin-2 production
- positive regulation of interleukin-4 production
- positive regulation of NF-kappaB transcription factor activity
- positive regulation of T cell activation
- positive regulation of T-helper 17 type immune response
- positive regulation of T-helper 2 cell activation
- positive regulation of telomerase activity
- positive regulation of telomere capping
- positive regulation of telomere maintenance via telomerase
- protein phosphorylation
- regulation of cell growth
- regulation of transcription, DNA-templated
- regulation of platelet aggregation

---

96

- **Protein name:** E3 ubiquitin-protein ligase HECTD3
- **Organism:** Homo sapiens
- **Uniprot Accession Number:** Q5T447
- **Protein sequence length:** 861 aa
- **1D identity (%):** 14.58
- **1D identity (%) [Gaps excluded]:** 23.96
- **1D identity - Alignment Gaps:** 419
- **1D aligned content (<aminoacid>:%):** {'A': 5.13, 'G': 10.26, 'P': 14.1, 'L': 8.33, 'R': 8.33, 'Y': 3.85, 'K': 3.85, 'V': 11.54, 'S': 4.49, 'T': 3.21, 'E': 5.13, 'D': 8.33, 'F': 3.21, 'W': 1.28, 'H': 1.28, 'I': 1.28, 'Q': 4.49, 'C': 1.28, 'M': 0.64}
- **Common reported functions (%):** 0.0
- **Common reported locations (%):** 0.0
- **Common reported processes (%):** 0.0

- **AF ID:** Q5T447
- **Chain:** A
- **Protein length:** 861 aa
- **Resolution:** N/A
- **b-phipsi:** 0.007232
- **w-rdist:** 0.134316
- **t-alpha:** 0.102219
- **Chemical similarity (Tanimoto Index) (%):** 99.4
- **1D identity (%) [PDB]:** 2.49
- **1D identity (%) [Gaps excluded][PDB]:** 67.21
- **1D identity - Alignment Gaps [PDB]:** 1584
- **1D aligned content [PDB] (<aminoacid>:%):** {'R': 7.32, 'S': 9.76, 'L': 14.63, 'P': 14.63, 'V': 14.63, 'T': 7.32, 'G': 9.76, 'E': 9.76, 'D': 2.44, 'F': 2.44, 'I': 2.44, 'K': 2.44, 'W': 2.44}
- **2D identity (%) [PDB]:** 39.4
- **2D identity (%) [Gaps excluded][PDB]:** 91.62
- **2D identity - Alignment Gaps [PDB]:** 680
- **2D aligned content [PDB] (<2D-fold>:%):** {'.': 22.13, 'H': 38.3, 'T': 22.98, 'E': 16.6}
- **3D similarity (TM-Score) (%) [PDB]:** 25.14

- **Gene name:** HECTD3
- **Entrez ID:** 79654
- **RefSeq ID:** NM\_024602
- **Transcript sequence length:** 3597
- **5-UTR|CDS|3-UTR identity (%):** 14.68 | 46.54 | 3.54
- **5-UTR|CDS|3-UTR identity (%) [Gaps excluded]:** 77.42 | 72.73 | 76.79
- **5-UTR|CDS|3-UTR identity [Alignment Gaps]:** 265 | 1135 | 16139
- **5-UTR aligned content (<base>:%):** {'G': 31.25, 'T': 22.92, 'C': 45.83}
- **CDS aligned content (<base>:%):** {'A': 24.88, 'T': 21.4, 'G': 28.29, 'C': 25.43}
- **3-UTR aligned content (<base>:%):** {'G': 22.54, 'T': 22.37, 'C': 30.88, 'A': 24.21}

**Uniprot Description:**  
  
 E3 ubiquitin ligases accepts ubiquitin from an E2 ubiquitin-conjugating enzyme in the form of a thioester and then directly transfers the ubiquitin to targeted substrates. Mediates ubiquitination of TRIOBP and its subsequent proteasomal degradation, thus facilitating cell cycle progression by regulating the turn-over of TRIOBP. Mediates also ubiquitination of STX8 (By similarity).   
  
Interacts with TRIOBP. Interacts with STX8 (By similarity).   
  
 **Gene Ontology Information:**

Molecular Function

- syntaxin binding
- ubiquitin-protein transferase activity

Location

- perinuclear region of cytoplasm

Biological process

- proteasome-mediated ubiquitin-dependent protein catabolic process
- protein ubiquitination

---

97

- **Protein name:** BTB/POZ domain-containing protein 3
- **Organism:** Homo sapiens
- **Uniprot Accession Number:** Q9Y2F9
- **Protein sequence length:** 522 aa
- **1D identity (%):** 9.97
- **1D identity (%) [Gaps excluded]:** 22.14
- **1D identity - Alignment Gaps:** 524
- **1D aligned content (<aminoacid>:%):** {'D': 9.47, 'K': 7.37, 'T': 6.32, 'L': 8.42, 'S': 3.16, 'E': 6.32, 'P': 9.47, 'A': 3.16, 'W': 2.11, 'G': 5.26, 'R': 5.26, 'M': 2.11, 'N': 3.16, 'V': 7.37, 'I': 4.21, 'Y': 2.11, 'F': 4.21, 'Q': 7.37, 'C': 3.16}
- **Common reported functions (%):** 0.0
- **Common reported locations (%):** 11.11
- **Common reported processes (%):** 0.0

- **AF ID:** Q9Y2F9
- **Chain:** A
- **Protein length:** 522 aa
- **Resolution:** N/A
- **b-phipsi:** 0.004808
- **w-rdist:** 0.477145
- **t-alpha:** 0.00634
- **Chemical similarity (Tanimoto Index) (%):** 99.17
- **1D identity (%) [PDB]:** 2.2
- **1D identity (%) [Gaps excluded][PDB]:** 59.18
- **1D identity - Alignment Gaps [PDB]:** 1269
- **1D aligned content [PDB] (<aminoacid>:%):** {'V': 17.24, 'K': 10.34, 'N': 10.34, 'S': 6.9, 'Q': 13.79, 'L': 17.24, 'I': 10.34, 'P': 3.45, 'R': 3.45, 'F': 6.9}
- **2D identity (%) [PDB]:** 35.92
- **2D identity (%) [Gaps excluded][PDB]:** 83.25
- **2D identity - Alignment Gaps [PDB]:** 543
- **2D aligned content [PDB] (<2D-fold>:%):** {'.': 21.28, 'H': 39.36, 'T': 21.28, 'E': 17.78, 'B': 0.29}
- **3D similarity (TM-Score) (%) [PDB]:** 19.55

- **Gene name:** BTBD3
- **Entrez ID:** 100385566
- **RefSeq ID:** N/A
- **Sequence length:** N/A
- **5-UTR|CDS|3-UTR identity (%):** N/A | N/A | N/A
- **5-UTR|CDS|3-UTR identity (%) [Gaps excluded]:** N/A | N/A | N/A
- **5-UTR|CDS|3-UTR identity [Alignment Gaps]:** N/A | N/A | N/A
- **5-UTR aligned content (<base>:%):** N/A
- **CDS aligned content (<base>:%):** N/A
- **3-UTR aligned content (<base>:%):** N/A

**Uniprot Description:**  
  
 Acts as a key regulator of dendritic field orientation during development of sensory cortex. Also directs dendrites toward active axon terminals when ectopically expressed (By similarity). N/A   
  
 **Gene Ontology Information:**

Molecular Function   
  
N/A

Location

- cytosol
- nucleus

Biological process

- cerebral cortex development
- dendrite morphogenesis

---

98

- **Protein name:** Protein kinase C iota type
- **Organism:** Homo sapiens
- **Uniprot Accession Number:** P41743
- **Protein sequence length:** 596 aa
- **1D identity (%):** 11.9
- **1D identity (%) [Gaps excluded]:** 24.74
- **1D identity - Alignment Gaps:** 510
- **1D aligned content (<aminoacid>:%):** {'P': 7.69, 'D': 8.55, 'H': 5.13, 'G': 5.98, 'V': 6.84, 'R': 5.98, 'K': 4.27, 'T': 7.69, 'F': 7.69, 'I': 2.56, 'C': 5.98, 'E': 5.13, 'S': 4.27, 'L': 7.69, 'A': 3.42, 'N': 2.56, 'Y': 3.42, 'Q': 4.27, 'M': 0.85}
- **Common reported functions (%):** 14.29
- **Common reported locations (%):** 22.22
- **Common reported processes (%):** 0.0

- **AF ID:** P41743
- **Chain:** A
- **Protein length:** 596 aa
- **Resolution:** N/A
- **b-phipsi:** 0.000881
- **w-rdist:** 0.533928
- **t-alpha:** 0.049921
- **Chemical similarity (Tanimoto Index) (%):** 99.25
- **1D identity (%) [PDB]:** 1.56
- **1D identity (%) [Gaps excluded][PDB]:** 70.97
- **1D identity - Alignment Gaps [PDB]:** 1379
- **1D aligned content [PDB] (<aminoacid>:%):** {'M': 4.55, 'V': 9.09, 'P': 13.64, 'R': 4.55, 'G': 9.09, 'K': 13.64, 'I': 13.64, 'L': 9.09, 'C': 4.55, 'F': 4.55, 'Q': 4.55, 'E': 4.55, 'D': 4.55}
- **2D identity (%) [PDB]:** 40.02
- **2D identity (%) [Gaps excluded][PDB]:** 89.86
- **2D identity - Alignment Gaps [PDB]:** 553
- **2D aligned content [PDB] (<2D-fold>:%):** {'.': 23.31, 'E': 18.55, 'T': 23.31, 'H': 32.33, 'B': 0.5, 'G': 2.01}
- **3D similarity (TM-Score) (%) [PDB]:** 20.13

- **Gene name:** PRKCI
- **Entrez ID:** 5584
- **RefSeq ID:** NM\_002740
- **Transcript sequence length:** 4887
- **5-UTR|CDS|3-UTR identity (%):** 44.67 | 42.43 | 11.42
- **5-UTR|CDS|3-UTR identity (%) [Gaps excluded]:** 70.14 | 76.54 | 76.3
- **5-UTR|CDS|3-UTR identity [Alignment Gaps]:** 126 | 1254 | 14499
- **5-UTR aligned content (<base>:%):** {'G': 46.45, 'C': 38.71, 'T': 10.32, 'A': 4.52}
- **CDS aligned content (<base>:%):** {'A': 30.65, 'T': 27.81, 'G': 22.78, 'C': 18.76}
- **3-UTR aligned content (<base>:%):** {'T': 38.24, 'C': 12.63, 'A': 33.52, 'G': 15.61}

**Uniprot Description:**  
  
 Calcium- and diacylglycerol-independent serine/ threonine-protein kinase that plays a general protective role against apoptotic stimuli, is involved in NF-kappa-B activation, cell survival, differentiation and polarity, and contributes to the regulation of microtubule dynamics in the early secretory pathway. Is necessary for BCR-ABL oncogene-mediated resistance to apoptotic drug in leukemia cells, protecting leukemia cells against drug-induced apoptosis. In cultured neurons, prevents amyloid beta protein-induced apoptosis by interrupting cell death process at a very early step. In glioblastoma cells, may function downstream of phosphatidylinositol 3-kinase (PI(3)K) and PDPK1 in the promotion of cell survival by phosphorylating and inhibiting the pro-apoptotic factor BAD. Can form a protein complex in non-small cell lung cancer (NSCLC) cells with PARD6A and ECT2 and regulate ECT2 oncogenic activity by phosphorylation, which in turn promotes transformed growth and invasion. In response to nerve growth factor (NGF), acts downstream of SRC to phosphorylate and activate IRAK1, allowing the subsequent activation of NF-kappa-B and neuronal cell survival. Functions in the organization of the apical domain in epithelial cells by phosphorylating EZR. This step is crucial for activation and normal distribution of EZR at the early stages of intestinal epithelial cell differentiation. Forms a protein complex with LLGL1 and PARD6B independently of PARD3 to regulate epithelial cell polarity. Plays a role in microtubule dynamics in the early secretory pathway through interaction with RAB2A and GAPDH and recruitment to vesicular tubular clusters (VTCs). In human coronary artery endothelial cells (HCAEC), is activated by saturated fatty acids and mediates lipid-induced apoptosis. Involved in early synaptic long term potentiation phase in CA1 hippocampal cells and short term memory formation (By similarity).   
  
Forms a complex with SQSTM1 and MP2K5 (By similarity). Interacts directly with SQSTM1 (Probable). Interacts with IKBKB. Interacts with PARD6A, PARD6B and PARD6G. Part of a quaternary complex containing aPKC, PARD3, a PARD6 protein (PARD6A, PARD6B or PARD6G) and a GTPase protein (CDC42 or RAC1). Part of a complex with LLGL1 and PARD6B. Interacts with ADAP1/CENTA1. Interaction with SMG1, through the ZN-finger domain, activates the kinase activity. Interacts with CDK7. Forms a complex with RAB2A and GAPDH involved in recruitment onto the membrane of vesicular tubular clusters (VTCs). Interacts with ECT2 ('Thr-359' phosphorylated form). Interacts with VAMP2 (PubMed:17313651). Interacts with WDFY2 (via WD repeats 1-3) (PubMed:16792529).   
  
 **Gene Ontology Information:**

Molecular Function

- ATP binding
- calcium-dependent protein kinase C activity
- metal ion binding
- phospholipid binding
- protein kinase activity
- protein kinase C activity
- protein serine kinase activity
- protein serine/threonine kinase activity

Location

- apical plasma membrane
- bicellular tight junction
- brush border
- cell leading edge
- cytosol
- endosome
- extracellular exosome
- glutamatergic synapse
- Golgi membrane
- intercellular bridge
- microtubule cytoskeleton
- nucleoplasm
- nucleus
- PAR polarity complex
- plasma membrane
- Schaffer collateral - CA1 synapse
- Schmidt-Lanterman incisure
- tight junction

Biological process

- actin filament organization
- cell migration
- cell-cell junction organization
- cellular response to insulin stimulus
- cytoskeleton organization
- establishment of apical/basal cell polarity
- establishment or maintenance of epithelial cell apical/basal polarity
- eye photoreceptor cell development
- Golgi vesicle budding
- intracellular signal transduction
- membrane organization
- negative regulation of apoptotic process
- negative regulation of glial cell apoptotic process
- negative regulation of neuron apoptotic process
- peptidyl-serine phosphorylation
- positive regulation of endothelial cell apoptotic process
- positive regulation of glial cell proliferation
- positive regulation of glucose import
- positive regulation of neuron projection development
- positive regulation of NF-kappaB transcription factor activity
- positive regulation of Notch signaling pathway
- positive regulation of protein localization to plasma membrane
- protein phosphorylation
- protein targeting to membrane
- regulation of postsynaptic membrane neurotransmitter receptor levels
- response to interleukin-1
- secretion
- vesicle-mediated transport

---

99

- **Protein name:** Voltage-dependent L-type calcium channel subunit beta-3
- **Organism:** Homo sapiens
- **Uniprot Accession Number:** P54284
- **Protein sequence length:** 484 aa
- **1D identity (%):** 10.62
- **1D identity (%) [Gaps excluded]:** 22.51
- **1D identity - Alignment Gaps:** 482
- **1D aligned content (<aminoacid>:%):** {'D': 11.34, 'T': 2.06, 'R': 5.15, 'S': 8.25, 'A': 8.25, 'E': 5.15, 'Q': 9.28, 'F': 3.09, 'V': 7.22, 'P': 10.31, 'G': 5.15, 'N': 2.06, 'H': 5.15, 'K': 6.19, 'I': 3.09, 'L': 5.15, 'C': 1.03, 'Y': 2.06}
- **Common reported functions (%):** 0.0
- **Common reported locations (%):** 22.22
- **Common reported processes (%):** 0.0

- **AF ID:** P54284
- **Chain:** A
- **Protein length:** 484 aa
- **Resolution:** N/A
- **b-phipsi:** 0.000448
- **w-rdist:** 0.90284
- **t-alpha:** 0.030903
- **Chemical similarity (Tanimoto Index) (%):** 96.63
- **1D identity (%) [PDB]:** 1.46
- **1D identity (%) [Gaps excluded][PDB]:** 76.0
- **1D identity - Alignment Gaps [PDB]:** 1279
- **1D aligned content [PDB] (<aminoacid>:%):** {'M': 5.26, 'I': 5.26, 'K': 5.26, 'T': 5.26, 'A': 15.79, 'R': 15.79, 'S': 15.79, 'P': 5.26, 'E': 10.53, 'L': 5.26, 'N': 5.26, 'Y': 5.26}
- **2D identity (%) [PDB]:** 37.5
- **2D identity (%) [Gaps excluded][PDB]:** 86.78
- **2D identity - Alignment Gaps [PDB]:** 527
- **2D aligned content [PDB] (<2D-fold>:%):** {'.': 22.99, 'T': 27.01, 'H': 37.36, 'E': 11.78, 'G': 0.86}
- **3D similarity (TM-Score) (%) [PDB]:** 14.7

- **Gene name:** CACNB3
- **Entrez ID:** 784
- **RefSeq ID:** NM\_001206917
- **Transcript sequence length:** 2526
- **5-UTR|CDS|3-UTR identity (%):** 14.98 | 32.18 | 4.2
- **5-UTR|CDS|3-UTR identity (%) [Gaps excluded]:** 77.78 | 75.42 | 75.08
- **5-UTR|CDS|3-UTR identity [Alignment Gaps]:** 264 | 1607 | 15911
- **5-UTR aligned content (<base>:%):** {'G': 32.65, 'T': 16.33, 'C': 46.94, 'A': 4.08}
- **CDS aligned content (<base>:%):** {'A': 28.27, 'T': 19.18, 'G': 25.94, 'C': 26.61}
- **3-UTR aligned content (<base>:%):** {'C': 26.27, 'A': 22.32, 'T': 22.74, 'G': 28.67}

**Uniprot Description:**  
  
 Regulatory subunit of the voltage-gated calcium channel that gives rise to L-type calcium currents (PubMed:8119293). Increases CACNA1B peak calcium current and shifts the voltage dependencies of channel activation and inactivation (By similarity). Increases CACNA1C peak calcium current and shifts the voltage dependencies of channel activation and inactivation (By similarity).   
  
Component of a calcium channel complex consisting of a pore-forming alpha subunit (CACNA1C) and the ancillary subunits CACNB3 and CACNA2D1 (PubMed:29742403). The channel complex contains alpha, beta, gamma and delta subunits in a 1:1:1:1 ratio (Probable). Interacts with CACNA2D4 (PubMed:12181424). Interacts with FASLG (PubMed:19807924). Interacts with CBARP; prevents the interaction of CACNB3 with the alpha subunit CACNA1C thereby negatively regulating the activity of the corresponding calcium channel (By similarity).   
  
 **Gene Ontology Information:**

Molecular Function

- calcium channel regulator activity
- high voltage-gated calcium channel activity
- voltage-gated calcium channel activity

Location

- cytosol
- L-type voltage-gated calcium channel complex
- membrane
- synapse
- voltage-gated calcium channel complex

Biological process

- calcium ion transmembrane transport via high voltage-gated calcium channel
- calcium ion transport
- calcium ion transport into cytosol
- chemical synaptic transmission
- neuromuscular junction development
- positive regulation of high voltage-gated calcium channel activity
- protein localization to plasma membrane
- regulation of membrane repolarization during action potential
- regulation of voltage-gated calcium channel activity
- T cell receptor signaling pathway

---

100

- **Protein name:** Beta-secretase 1
- **Organism:** Homo sapiens
- **Uniprot Accession Number:** P56817
- **Protein sequence length:** 501 aa
- **1D identity (%):** 11.4
- **1D identity (%) [Gaps excluded]:** 23.16
- **1D identity - Alignment Gaps:** 463
- **1D aligned content (<aminoacid>:%):** {'L': 10.58, 'P': 11.54, 'H': 2.88, 'R': 9.62, 'G': 9.62, 'E': 4.81, 'F': 6.73, 'N': 4.81, 'I': 4.81, 'D': 1.92, 'V': 5.77, 'T': 4.81, 'S': 6.73, 'A': 4.81, 'K': 2.88, 'Q': 1.92, 'C': 2.88, 'Y': 2.88}
- **Common reported functions (%):** 0.0
- **Common reported locations (%):** 0.0
- **Common reported processes (%):** 0.0

- **AF ID:** P56817
- **Chain:** A
- **Protein length:** 501 aa
- **Resolution:** N/A
- **b-phipsi:** 0.005092
- **w-rdist:** 0.785923
- **t-alpha:** 0.001587
- **Chemical similarity (Tanimoto Index) (%):** 96.54
- **1D identity (%) [PDB]:** 1.67
- **1D identity (%) [Gaps excluded][PDB]:** 70.97
- **1D identity - Alignment Gaps [PDB]:** 1284
- **1D aligned content [PDB] (<aminoacid>:%):** {'A': 9.09, 'Q': 9.09, 'G': 4.55, 'D': 4.55, 'S': 9.09, 'E': 4.55, 'P': 9.09, 'F': 4.55, 'H': 4.55, 'N': 4.55, 'L': 13.64, 'I': 13.64, 'V': 4.55, 'K': 4.55}
- **2D identity (%) [PDB]:** 38.57
- **2D identity (%) [Gaps excluded][PDB]:** 88.05
- **2D identity - Alignment Gaps [PDB]:** 526
- **2D aligned content [PDB] (<2D-fold>:%):** {'.': 21.05, 'T': 27.7, 'E': 30.47, 'H': 19.94, 'G': 0.55, 'B': 0.28}
- **3D similarity (TM-Score) (%) [PDB]:** 20.0

- **Gene name:** BACE1
- **Entrez ID:** 614333
- **RefSeq ID:** NM\_138973
- **Transcript sequence length:** 5628
- **5-UTR|CDS|3-UTR identity (%):** 44.11 | 29.52 | 13.59
- **5-UTR|CDS|3-UTR identity (%) [Gaps excluded]:** 74.57 | 75.23 | 74.38
- **5-UTR|CDS|3-UTR identity [Alignment Gaps]:** 201 | 1694 | 14253
- **5-UTR aligned content (<base>:%):** {'A': 6.45, 'T': 11.06, 'C': 51.15, 'G': 31.34}
- **CDS aligned content (<base>:%):** {'A': 23.45, 'T': 20.17, 'G': 30.13, 'C': 26.25}
- **3-UTR aligned content (<base>:%):** {'G': 20.56, 'C': 18.28, 'A': 32.46, 'T': 28.7}

**Uniprot Description:**  
  
 Responsible for the proteolytic processing of the amyloid precursor protein (APP). Cleaves at the N-terminus of the A-beta peptide sequence, between residues 671 and 672 of APP, leads to the generation and extracellular release of beta-cleaved soluble APP, and a corresponding cell-associated C-terminal fragment which is later released by gamma-secretase (PubMed:10656250, PubMed:10677483, PubMed:20354142). Cleaves CHL1 (By similarity).   
  
Monomer. Interacts (via DXXLL motif) with GGA1, GGA2 and GGA3 (via their VHS domain); the interaction highly increases when BACE1 is phosphorylated at Ser-498 (PubMed:14567678, PubMed:15886016). Interacts with RTN1; RTN2; RTN3 and RTN4; the interaction leads to inhibition of amyloid precursor protein processing (PubMed:15286784, PubMed:16965550, PubMed:16979658). Interacts with SNX6 (PubMed:20354142). Interacts with PCSK9 (PubMed:18660751). Interacts with NAT8 and NAT8B (PubMed:19011241). Interacts with BIN1 (PubMed:27179792). Interacts (via extracellular domain) with ADAM10 (via extracellular domain) (By similarity). Interacts with SORL1; this interaction may affect binding with APP and hence reduce APP cleavage (PubMed:16407538).   
  
 **Gene Ontology Information:**

Molecular Function

- amyloid-beta binding
- aspartic-type endopeptidase activity
- endopeptidase activity
- enzyme binding

Location

- axon
- cell surface
- cytoplasmic vesicle membrane
- dendrite
- early endosome
- endoplasmic reticulum
- endosome
- Golgi apparatus
- hippocampal mossy fiber to CA3 synapse
- late endosome
- lysosome
- membrane raft
- multivesicular body
- plasma membrane
- presynapse
- recycling endosome
- trans-Golgi network

Biological process

- amyloid-beta formation
- amyloid-beta metabolic process
- membrane protein ectodomain proteolysis
- positive regulation of neuron apoptotic process
- presynaptic modulation of chemical synaptic transmission
- proteolysis
- signaling receptor ligand precursor processing

---
