# Supplementary material for: Comparative structural insights and functional analysis for the distinct unbound states of Human AGO proteins
Source: Sci Rep. 2025 Mar 19;15:9432. doi: 10.1038/s41598-025-91849-5 (PMC11923369; doi:10.1038/s41598-025-91849-5)
Supplement: Supplementary file 24 — Supplementary Information 12. [file 41598_2025_91849_MOESM24_ESM.zip › 5VM9p_A_mdwhole_AF4REF/candidates/5VM9p_A-merged-h-enriched_report.html]

 

# Structural Comparison Report for 5VM9p\_A - whole structures (total: 100)

---

0

- **AF ID:** AF-Q92784-F1-model-v4 | **Chain:** A
- **b-phipsi:** 0.0002796752659364
- **w-rdist:** 0.3711528281702805
- **t-alpha:** 0.0586373023344051

---

---

1

- **AF ID:** AF-Q9H4B4-F1-model-v4 | **Chain:** A
- **b-phipsi:** 0.000434730762734
- **w-rdist:** 0.271843637242495
- **t-alpha:** 0.1307449283247805

---

---

2

- **AF ID:** AF-Q14644-F1-model-v4 | **Chain:** A
- **b-phipsi:** 0.0039562666924833
- **w-rdist:** 0.3724400231082415
- **t-alpha:** 0.0015872186884768

---

---

3

- **AF ID:** AF-P48544-F1-model-v4 | **Chain:** A
- **b-phipsi:** 0.0003135316616275
- **w-rdist:** 0.9141345241045958
- **t-alpha:** 0.0023827284528445

---

---

4

- **AF ID:** AF-Q96J94-F1-model-v4 | **Chain:** A
- **b-phipsi:** 0.0013470011998628
- **w-rdist:** 0.1458324040394728
- **t-alpha:** 0.0427893647215475

---

---

5

- **AF ID:** AF-A5YM72-F1-model-v4 | **Chain:** A
- **b-phipsi:** 0.0003726303114721
- **w-rdist:** 0.3927505138851829
- **t-alpha:** 0.0665613411172061

---

---

6

- **AF ID:** AF-Q96PZ2-F1-model-v4 | **Chain:** A
- **b-phipsi:** 0.0052119535509232
- **w-rdist:** 0.4004903777354442
- **t-alpha:** 0.0

---

---

7

- **AF ID:** AF-Q9NZ20-F1-model-v4 | **Chain:** A
- **b-phipsi:** 0.0007698248360263
- **w-rdist:** 0.3821023291170035
- **t-alpha:** 0.0087166840741086

---

---

8

- **AF ID:** AF-A8K855-F1-model-v4 | **Chain:** A
- **b-phipsi:** 0.0030872659860307
- **w-rdist:** 0.6101321619234064
- **t-alpha:** 0.0007923402556029

---

---

9

- **AF ID:** AF-O95747-F1-model-v4 | **Chain:** A
- **b-phipsi:** 0.0004336709907287
- **w-rdist:** 0.6099009176295259
- **t-alpha:** 0.0243505672074058

---

---

10

- **AF ID:** AF-C9J798-F1-model-v4 | **Chain:** A
- **b-phipsi:** 0.0119219552455604
- **w-rdist:** 0.3312378275721747
- **t-alpha:** 0.0015872186884768

---

---

11

- **AF ID:** AF-Q96G46-F1-model-v4 | **Chain:** A
- **b-phipsi:** 0.0027552857290858
- **w-rdist:** 0.5699145481147629
- **t-alpha:** 0.0015872186884768

---

---

12

- **AF ID:** AF-O95831-F1-model-v4 | **Chain:** A
- **b-phipsi:** 0.0005463823270872
- **w-rdist:** 0.617961551772173
- **t-alpha:** 0.0150558135497862

---

---

13

- **AF ID:** AF-O60678-F1-model-v4 | **Chain:** A
- **b-phipsi:** 0.0003994756528204
- **w-rdist:** 0.6541928400943612
- **t-alpha:** 0.0302036962314997

---

---

14

- **AF ID:** AF-Q9H611-F1-model-v4 | **Chain:** A
- **b-phipsi:** 0.0005368391369652
- **w-rdist:** 0.5238042168260176
- **t-alpha:** 0.052543827044917

---

---

15

- **AF ID:** AF-Q14697-F1-model-v4 | **Chain:** A
- **b-phipsi:** 0.0029921373382371
- **w-rdist:** 0.4327097239853683
- **t-alpha:** 0.0039622395506779

---

---

16

- **AF ID:** AF-O14672-F1-model-v4 | **Chain:** A
- **b-phipsi:** 0.0017469001798336
- **w-rdist:** 0.6697206683398877
- **t-alpha:** 0.002377470759985

---

---

17

- **AF ID:** AF-P50747-F1-model-v4 | **Chain:** A
- **b-phipsi:** 0.0007224711581059
- **w-rdist:** 0.3245466898021097
- **t-alpha:** 0.1489700676263168

---

---

18

- **AF ID:** AF-P23743-F1-model-v4 | **Chain:** A
- **b-phipsi:** 0.0024690786105629
- **w-rdist:** 0.3463612325489945
- **t-alpha:** 0.0237719193359167

---

---

19

- **AF ID:** AF-O00425-F1-model-v4 | **Chain:** A
- **b-phipsi:** 0.0010693023259971
- **w-rdist:** 0.2575585229483806
- **t-alpha:** 0.1022188946752804

---

---

20

- **AF ID:** AF-Q8IVD9-F1-model-v4 | **Chain:** A
- **b-phipsi:** 0.000286818409079
- **w-rdist:** 0.8005109369948294
- **t-alpha:** 0.0193858366449102

---

---

21

- **AF ID:** AF-Q9UFP1-F1-model-v4 | **Chain:** A
- **b-phipsi:** 0.0002396345402754
- **w-rdist:** 0.6403626287406284
- **t-alpha:** 0.0958797909786817

---

---

22

- **AF ID:** AF-Q460N3-F1-model-v4 | **Chain:** A
- **b-phipsi:** 0.0018623046297258
- **w-rdist:** 0.2177910502718875
- **t-alpha:** 0.1164821024786537

---

---

23

- **AF ID:** AF-Q8IZP7-F1-model-v4 | **Chain:** A
- **b-phipsi:** 0.003891657223553
- **w-rdist:** 0.6616925104227238
- **t-alpha:** 0.0015872186884768

---

---

24

- **AF ID:** AF-Q8TAX0-F1-model-v4 | **Chain:** A
- **b-phipsi:** 0.0054112984281105
- **w-rdist:** 0.5740984810902323
- **t-alpha:** 0.0015872186884768

---

---

25

- **AF ID:** AF-Q16566-F1-model-v4 | **Chain:** A
- **b-phipsi:** 0.0006741898516719
- **w-rdist:** 0.6681375712948441
- **t-alpha:** 0.0095087103146067

---

---

26

- **AF ID:** AF-Q14195-F1-model-v4 | **Chain:** A
- **b-phipsi:** 0.0002416405159575
- **w-rdist:** 0.7847090660212412
- **t-alpha:** 0.039619806700881

---

---

27

- **AF ID:** AF-Q8TAT6-F1-model-v4 | **Chain:** A
- **b-phipsi:** 0.0039697155790612
- **w-rdist:** 0.5082446141517756
- **t-alpha:** 0.0039622395506779

---

---

28

- **AF ID:** AF-P31323-F1-model-v4 | **Chain:** A
- **b-phipsi:** 0.0004528703220395
- **w-rdist:** 0.7727123374429885
- **t-alpha:** 0.0198097874502385

---

---

29

- **AF ID:** AF-Q9HAU4-F1-model-v4 | **Chain:** A
- **b-phipsi:** 0.0020373037221957
- **w-rdist:** 0.1853944095027655
- **t-alpha:** 0.1347072878750428

---

---

30

- **AF ID:** AF-Q9ULR3-F1-model-v4 | **Chain:** A
- **b-phipsi:** 0.0005850289433206
- **w-rdist:** 0.7468221715056953
- **t-alpha:** 0.017432692187089

---

---

31

- **AF ID:** AF-O95294-F1-model-v4 | **Chain:** A
- **b-phipsi:** 0.0071208782699365
- **w-rdist:** 0.3083659702076993
- **t-alpha:** 0.0095996935833633

---

---

32

- **AF ID:** AF-Q9UL63-F1-model-v4 | **Chain:** A
- **b-phipsi:** 0.008398666854891
- **w-rdist:** 0.2182003223275578
- **t-alpha:** 0.0269416384138463

---

---

33

- **AF ID:** AF-O43374-F1-model-v4 | **Chain:** A
- **b-phipsi:** 0.0083489193960566
- **w-rdist:** 0.285864684938989
- **t-alpha:** 0.0104083124295257

---

---

34

- **AF ID:** AF-O75676-F1-model-v4 | **Chain:** A
- **b-phipsi:** 0.0024977156187094
- **w-rdist:** 0.2957960604580699
- **t-alpha:** 0.09508735985712

---

---

35

- **AF ID:** AF-Q96NY9-F1-model-v4 | **Chain:** A
- **b-phipsi:** 0.0071250526344601
- **w-rdist:** 0.2931728625874575
- **t-alpha:** 0.0213948423587535

---

---

36

- **AF ID:** AF-O14841-F1-model-v4 | **Chain:** A
- **b-phipsi:** 0.0021083937260303
- **w-rdist:** 0.3095689807404926
- **t-alpha:** 0.0958797909786817

---

---

37

- **AF ID:** AF-Q8N7R0-F1-model-v4 | **Chain:** A
- **b-phipsi:** 0.0024215248398512
- **w-rdist:** 0.382156382305193
- **t-alpha:** 0.0185632335173342

---

---

38

- **AF ID:** AF-Q9BX70-F1-model-v4 | **Chain:** A
- **b-phipsi:** 0.0004013456896593
- **w-rdist:** 0.7292031020572817
- **t-alpha:** 0.0667790338922971

---

---

39

- **AF ID:** AF-O94923-F1-model-v4 | **Chain:** A
- **b-phipsi:** 0.0006957154411794
- **w-rdist:** 0.3818885157162966
- **t-alpha:** 0.1339143555568707

---

---

40

- **AF ID:** AF-Q7Z3Z4-F1-model-v4 | **Chain:** A
- **b-phipsi:** 0.0059563289424277
- **w-rdist:** 0.2550597483934069
- **t-alpha:** 0.0625992822193501

---

---

41

- **AF ID:** AF-Q9UIF7-F1-model-v4 | **Chain:** A
- **b-phipsi:** 0.0021094571496286
- **w-rdist:** 0.3157450298986002
- **t-alpha:** 0.0974642806534764

---

---

42

- **AF ID:** AF-O95704-F1-model-v4 | **Chain:** A
- **b-phipsi:** 0.0037387062602943
- **w-rdist:** 0.3455141325060059
- **t-alpha:** 0.0473026125968261

---

---

43

- **AF ID:** AF-Q9Y6S9-F1-model-v4 | **Chain:** A
- **b-phipsi:** 0.0006860137592574
- **w-rdist:** 0.3733305033133314
- **t-alpha:** 0.1537244455033311

---

---

44

- **AF ID:** AF-Q9Y6M1-F1-model-v4 | **Chain:** A
- **b-phipsi:** 0.0024152272202774
- **w-rdist:** 0.1891352029918035
- **t-alpha:** 0.1537244455033311

---

---

45

- **AF ID:** AF-Q86XI8-F1-model-v4 | **Chain:** A
- **b-phipsi:** 0.0006258257386891
- **w-rdist:** 0.5188900820689564
- **t-alpha:** 0.0982568607009952

---

---

46

- **AF ID:** AF-Q8N806-F1-model-v4 | **Chain:** A
- **b-phipsi:** 0.0009650789479177
- **w-rdist:** 0.3592923904444868
- **t-alpha:** 0.0792396355379139

---

---

47

- **AF ID:** AF-Q8WZ73-F1-model-v4 | **Chain:** A
- **b-phipsi:** 0.0003280992552344
- **w-rdist:** 0.4621097933267063
- **t-alpha:** 0.1798734427113426

---

---

48

- **AF ID:** AF-Q9Y6W3-F1-model-v4 | **Chain:** A
- **b-phipsi:** 0.0075872190215815
- **w-rdist:** 0.5448376441089542
- **t-alpha:** 0.002377470759985

---

---

49

- **AF ID:** AF-Q969P6-F1-model-v4 | **Chain:** A
- **b-phipsi:** 0.0176938181536997
- **w-rdist:** 0.3904740440552063
- **t-alpha:** 0.0007923402556029

---

---

50

- **AF ID:** AF-Q9UL18-F1-model-v4 | **Chain:** A
- **b-phipsi:** 0.0078623873301343
- **w-rdist:** 0.2312440037475
- **t-alpha:** 0.0522979286895239

---

---

51

- **AF ID:** AF-Q9UJN7-F1-model-v4 | **Chain:** A
- **b-phipsi:** 0.0063495031078038
- **w-rdist:** 0.4643044705215828
- **t-alpha:** 0.0039775589155952

---

---

52

- **AF ID:** AF-Q9H0C5-F1-model-v4 | **Chain:** A
- **b-phipsi:** 0.0003679076857376
- **w-rdist:** 0.7619018696849581
- **t-alpha:** 0.0740421623492073

---

---

53

- **AF ID:** AF-Q16819-F1-model-v4 | **Chain:** A
- **b-phipsi:** 0.0049766498599598
- **w-rdist:** 0.3606874141717947
- **t-alpha:** 0.0206022505246215

---

---

54

- **AF ID:** AF-Q10471-F1-model-v4 | **Chain:** A
- **b-phipsi:** 0.0008778166858717
- **w-rdist:** 0.6139315417530243
- **t-alpha:** 0.0120284587273298

---

---

55

- **AF ID:** AF-Q8N159-F1-model-v4 | **Chain:** A
- **b-phipsi:** 0.0007334733328778
- **w-rdist:** 0.5401988041771149
- **t-alpha:** 0.0744852576021766

---

---

56

- **AF ID:** AF-Q8N9L1-F1-model-v4 | **Chain:** A
- **b-phipsi:** 0.0029101670598967
- **w-rdist:** 0.3700883839857528
- **t-alpha:** 0.0499166951389262

---

---

57

- **AF ID:** AF-P0C7I0-F1-model-v4 | **Chain:** A
- **b-phipsi:** 0.0034632664070093
- **w-rdist:** 0.7174709878449959
- **t-alpha:** 0.003179348403216

---

---

58

- **AF ID:** AF-Q96LX8-F1-model-v4 | **Chain:** A
- **b-phipsi:** 0.0036212188798847
- **w-rdist:** 0.4642224518818655
- **t-alpha:** 0.0063793623469805

---

---

59

- **AF ID:** AF-P17252-F1-model-v4 | **Chain:** A
- **b-phipsi:** 0.0003688463412913
- **w-rdist:** 0.6843237997096245
- **t-alpha:** 0.1318383769643583

---

---

60

- **AF ID:** AF-Q01518-F1-model-v4 | **Chain:** A
- **b-phipsi:** 0.0073069163706713
- **w-rdist:** 0.2417078937825318
- **t-alpha:** 0.0721080046355822

---

---

61

- **AF ID:** AF-Q69YN2-F1-model-v4 | **Chain:** A
- **b-phipsi:** 0.0006803205402667
- **w-rdist:** 0.7764411922467991
- **t-alpha:** 0.0193858366449102

---

---

62

- **AF ID:** AF-Q8N2R8-F1-model-v4 | **Chain:** A
- **b-phipsi:** 0.0005010665774407
- **w-rdist:** 0.6644685082563686
- **t-alpha:** 0.1053884795390964

---

---

63

- **AF ID:** AF-Q9H147-F1-model-v4 | **Chain:** A
- **b-phipsi:** 0.0026043365121717
- **w-rdist:** 0.6628418832357855
- **t-alpha:** 0.0055471164626488

---

---

64

- **AF ID:** AF-P58317-F1-model-v4 | **Chain:** A
- **b-phipsi:** 0.006281476488101
- **w-rdist:** 0.2937115851462523
- **t-alpha:** 0.0784468950036187

---

---

65

- **AF ID:** AF-Q8IY47-F1-model-v4 | **Chain:** A
- **b-phipsi:** 0.0067073910314758
- **w-rdist:** 0.3438718202987501
- **t-alpha:** 0.031695687164061

---

---

66

- **AF ID:** AF-P40123-F1-model-v4 | **Chain:** A
- **b-phipsi:** 0.0028697333241984
- **w-rdist:** 0.3549237450822869
- **t-alpha:** 0.0839938579877885

---

---

67

- **AF ID:** AF-P49619-F1-model-v4 | **Chain:** A
- **b-phipsi:** 0.001364773940395
- **w-rdist:** 0.1363827990077107
- **t-alpha:** 0.2218697074286468

---

---

68

- **AF ID:** AF-Q9Y2P5-F1-model-v4 | **Chain:** A
- **b-phipsi:** 0.0090486911493354
- **w-rdist:** 0.7032340513822388
- **t-alpha:** 0.0007923402556029

---

---

69

- **AF ID:** AF-Q04759-F1-model-v4 | **Chain:** A
- **b-phipsi:** 0.0012704961187832
- **w-rdist:** 0.3560153942590944
- **t-alpha:** 0.1141048975561447

---

---

70

- **AF ID:** AF-Q5T447-F1-model-v4 | **Chain:** A
- **b-phipsi:** 0.0072317669598775
- **w-rdist:** 0.1343161132616565
- **t-alpha:** 0.1022188946752804

---

---

71

- **AF ID:** AF-P41743-F1-model-v4 | **Chain:** A
- **b-phipsi:** 0.000881405997902
- **w-rdist:** 0.5339280140080234
- **t-alpha:** 0.0499207002609116

---

---

72

- **AF ID:** AF-P54284-F1-model-v4 | **Chain:** A
- **b-phipsi:** 0.0004478363812177
- **w-rdist:** 0.9028396483238222
- **t-alpha:** 0.0309034586893088

---

---

73

- **AF ID:** AF-Q86Y07-F1-model-v4 | **Chain:** A
- **b-phipsi:** 0.0025610050315748
- **w-rdist:** 0.6475228781080116
- **t-alpha:** 0.006339533171912

---

---

74

- **AF ID:** AF-Q6IQ19-F1-model-v4 | **Chain:** A
- **b-phipsi:** 0.0013130974899187
- **w-rdist:** 0.8061231653078419
- **t-alpha:** 0.0039775589155952

---

---

75

- **AF ID:** AF-Q9UFC0-F1-model-v4 | **Chain:** A
- **b-phipsi:** 0.0057363482331006
- **w-rdist:** 0.6041782639761237
- **t-alpha:** 0.0047543655130677

---

---

76

- **AF ID:** AF-O15371-F1-model-v4 | **Chain:** A
- **b-phipsi:** 0.0079495910123268
- **w-rdist:** 0.6128661915151095
- **t-alpha:** 0.0031697813850419

---

---

77

- **AF ID:** AF-O15067-F1-model-v4 | **Chain:** A
- **b-phipsi:** 0.0020609071258239
- **w-rdist:** 0.3333983204590733
- **t-alpha:** 0.1426312113516006

---

---

78

- **AF ID:** AF-Q2TBF2-F1-model-v4 | **Chain:** A
- **b-phipsi:** 0.0025815477158426
- **w-rdist:** 0.3415709921893254
- **t-alpha:** 0.1323296128972912

---

---

79

- **AF ID:** AF-Q9UL40-F1-model-v4 | **Chain:** A
- **b-phipsi:** 0.009469244873131
- **w-rdist:** 0.2804963761728593
- **t-alpha:** 0.0551835704278558

---

---

80

- **AF ID:** AF-Q04771-F1-model-v4 | **Chain:** A
- **b-phipsi:** 0.0008190093951515
- **w-rdist:** 0.7049727040073831
- **t-alpha:** 0.0276872078734735

---

---

81

- **AF ID:** AF-Q9UI17-F1-model-v4 | **Chain:** A
- **b-phipsi:** 0.0008404425280736
- **w-rdist:** 0.5639229319383507
- **t-alpha:** 0.0721080046355822

---

---

82

- **AF ID:** AF-Q9HCG7-F1-model-v4 | **Chain:** A
- **b-phipsi:** 0.0006588019920632
- **w-rdist:** 0.4248060792862148
- **t-alpha:** 0.1703645180917901

---

---

83

- **AF ID:** AF-Q9BXL8-F1-model-v4 | **Chain:** A
- **b-phipsi:** 0.0100818369841815
- **w-rdist:** 0.3774445283139138
- **t-alpha:** 0.0055773242968351

---

---

84

- **AF ID:** AF-Q15418-F1-model-v4 | **Chain:** A
- **b-phipsi:** 0.0071738374258311
- **w-rdist:** 0.5044824577294555
- **t-alpha:** 0.0055471164626488

---

---

85

- **AF ID:** AF-Q9BWW7-F1-model-v4 | **Chain:** A
- **b-phipsi:** 0.0110714066811571
- **w-rdist:** 0.270788168966612
- **t-alpha:** 0.0515059245507361

---

---

86

- **AF ID:** AF-Q9NTJ4-F1-model-v4 | **Chain:** A
- **b-phipsi:** 0.0063202706699071
- **w-rdist:** 0.3463140862272972
- **t-alpha:** 0.0689385280761989

---

---

87

- **AF ID:** AF-Q96LR2-F1-model-v4 | **Chain:** A
- **b-phipsi:** 0.009138885395925
- **w-rdist:** 0.2925374084383938
- **t-alpha:** 0.0605041736265279

---

---

88

- **AF ID:** AF-Q9H4B8-F1-model-v4 | **Chain:** A
- **b-phipsi:** 0.0043847762233046
- **w-rdist:** 0.7322506785075235
- **t-alpha:** 0.0039775589155952

---

---

89

- **AF ID:** AF-Q9Y2X0-F1-model-v4 | **Chain:** A
- **b-phipsi:** 0.0089997721627276
- **w-rdist:** 0.3406156613949861
- **t-alpha:** 0.0356580695998436

---

---

90

- **AF ID:** AF-Q86TM3-F1-model-v4 | **Chain:** A
- **b-phipsi:** 0.0099600019055251
- **w-rdist:** 0.3211187445250222
- **t-alpha:** 0.0364504923644704

---

---

91

- **AF ID:** AF-Q9Y4L5-F1-model-v4 | **Chain:** A
- **b-phipsi:** 0.0043554124737749
- **w-rdist:** 0.3756319309318319
- **t-alpha:** 0.0596135259369763

---

---

92

- **AF ID:** AF-P10253-F1-model-v4 | **Chain:** A
- **b-phipsi:** 0.0045502133006232
- **w-rdist:** 0.3759570795327699
- **t-alpha:** 0.0586373023344051

---

---

93

- **AF ID:** AF-Q6UY01-F1-model-v4 | **Chain:** A
- **b-phipsi:** 0.0134374353500463
- **w-rdist:** 0.6969356271750485
- **t-alpha:** 0.0007923402556029

---

---

94

- **AF ID:** AF-P51449-F1-model-v4 | **Chain:** A
- **b-phipsi:** 0.008932783394432
- **w-rdist:** 0.3532876004468009
- **t-alpha:** 0.0380351774023424

---

---

95

- **AF ID:** AF-Q9BPU6-F1-model-v4 | **Chain:** A
- **b-phipsi:** 0.0011101000340979
- **w-rdist:** 0.8207377089875205
- **t-alpha:** 0.0055471164626488

---

---

96

- **AF ID:** AF-Q8NA19-F1-model-v4 | **Chain:** A
- **b-phipsi:** 0.0033445167006746
- **w-rdist:** 0.3657531865077554
- **t-alpha:** 0.1053884795390964

---

---

97

- **AF ID:** AF-Q9Y6T7-F1-model-v4 | **Chain:** A
- **b-phipsi:** 0.0004606029354383
- **w-rdist:** 0.5578738259221658
- **t-alpha:** 0.2202859732691042

---

---

98

- **AF ID:** AF-Q9UG63-F1-model-v4 | **Chain:** A
- **b-phipsi:** 0.0145104162370848
- **w-rdist:** 0.5545245618295742
- **t-alpha:** 0.002377470759985

---

---

99

- **AF ID:** AF-Q96LW9-F1-model-v4 | **Chain:** A
- **b-phipsi:** 0.0116003831956703
- **w-rdist:** 0.3316076503516659
- **t-alpha:** 0.0253567763618671

---

---
