# Supplementary material for: Comparative structural insights and functional analysis for the distinct unbound states of Human AGO proteins
Source: Sci Rep. 2025 Mar 19;15:9432. doi: 10.1038/s41598-025-91849-5 (PMC11923369; doi:10.1038/s41598-025-91849-5)
Supplement: Supplementary file 24 — Supplementary Information 12. [file 41598_2025_91849_MOESM24_ESM.zip › 5VM9p_A_mdwhole_AF4REF/candidates/5VM9p_A-merged-notenriched_report.html]

 

# Structural Comparison Report for 5VM9p\_A - whole structures (total: 644)

---

1

- **AF ID:** AF-Q92784-F1-model-v4 | **Chain:** A
- **b-phipsi:** 0.0002796752659364
- **w-rdist:** 0.3711528281702805
- **t-alpha:** 0.0586373023344051

---

---

2

- **AF ID:** AF-Q9H4B4-F1-model-v4 | **Chain:** A
- **b-phipsi:** 0.000434730762734
- **w-rdist:** 0.271843637242495
- **t-alpha:** 0.1307449283247805

---

---

3

- **AF ID:** AF-P07384-F1-model-v4 | **Chain:** A
- **b-phipsi:** 0.0004009495293778
- **w-rdist:** 0.4905863071046543
- **t-alpha:** 0.0142633043406019

---

---

4

- **AF ID:** AF-Q14644-F1-model-v4 | **Chain:** A
- **b-phipsi:** 0.0039562666924833
- **w-rdist:** 0.3724400231082415
- **t-alpha:** 0.0015872186884768

---

---

5

- **AF ID:** AF-P48544-F1-model-v4 | **Chain:** A
- **b-phipsi:** 0.0003135316616275
- **w-rdist:** 0.9141345241045958
- **t-alpha:** 0.0023827284528445

---

---

6

- **AF ID:** AF-Q96J94-F1-model-v4 | **Chain:** A
- **b-phipsi:** 0.0013470011998628
- **w-rdist:** 0.1458324040394728
- **t-alpha:** 0.0427893647215475

---

---

7

- **AF ID:** AF-A5YM72-F1-model-v4 | **Chain:** A
- **b-phipsi:** 0.0003726303114721
- **w-rdist:** 0.3927505138851829
- **t-alpha:** 0.0665613411172061

---

---

8

- **AF ID:** AF-Q96PZ2-F1-model-v4 | **Chain:** A
- **b-phipsi:** 0.0052119535509232
- **w-rdist:** 0.4004903777354442
- **t-alpha:** 0.0

---

---

9

- **AF ID:** AF-Q9NZ20-F1-model-v4 | **Chain:** A
- **b-phipsi:** 0.0007698248360263
- **w-rdist:** 0.3821023291170035
- **t-alpha:** 0.0087166840741086

---

---

10

- **AF ID:** AF-A8K855-F1-model-v4 | **Chain:** A
- **b-phipsi:** 0.0030872659860307
- **w-rdist:** 0.6101321619234064
- **t-alpha:** 0.0007923402556029

---

---

11

- **AF ID:** AF-O95747-F1-model-v4 | **Chain:** A
- **b-phipsi:** 0.0004336709907287
- **w-rdist:** 0.6099009176295259
- **t-alpha:** 0.0243505672074058

---

---

12

- **AF ID:** AF-O95831-F1-model-v4 | **Chain:** A
- **b-phipsi:** 0.0005463823270872
- **w-rdist:** 0.617961551772173
- **t-alpha:** 0.0150558135497862

---

---

13

- **AF ID:** AF-C9J798-F1-model-v4 | **Chain:** A
- **b-phipsi:** 0.0119219552455604
- **w-rdist:** 0.3312378275721747
- **t-alpha:** 0.0015872186884768

---

---

14

- **AF ID:** AF-Q96G46-F1-model-v4 | **Chain:** A
- **b-phipsi:** 0.0027552857290858
- **w-rdist:** 0.5699145481147629
- **t-alpha:** 0.0015872186884768

---

---

15

- **AF ID:** AF-O60678-F1-model-v4 | **Chain:** A
- **b-phipsi:** 0.0003994756528204
- **w-rdist:** 0.6541928400943612
- **t-alpha:** 0.0302036962314997

---

---

16

- **AF ID:** AF-P12931-F1-model-v4 | **Chain:** A
- **b-phipsi:** 0.0016454868407142
- **w-rdist:** 0.7205018272426769
- **t-alpha:** 0.0007923402556029

---

---

17

- **AF ID:** AF-Q96RR1-F1-model-v4 | **Chain:** A
- **b-phipsi:** 0.0007172223893036
- **w-rdist:** 0.5002267731093648
- **t-alpha:** 0.011093664683754

---

---

18

- **AF ID:** AF-Q9H611-F1-model-v4 | **Chain:** A
- **b-phipsi:** 0.0005368391369652
- **w-rdist:** 0.5238042168260176
- **t-alpha:** 0.052543827044917

---

---

19

- **AF ID:** AF-Q14697-F1-model-v4 | **Chain:** A
- **b-phipsi:** 0.0029921373382371
- **w-rdist:** 0.4327097239853683
- **t-alpha:** 0.0039622395506779

---

---

20

- **AF ID:** AF-Q5JS13-F1-model-v4 | **Chain:** A
- **b-phipsi:** 0.0003078622564663
- **w-rdist:** 0.7134493293969004
- **t-alpha:** 0.0404122840988643

---

---

21

- **AF ID:** AF-Q9BQA5-F1-model-v4 | **Chain:** A
- **b-phipsi:** 0.0013893601067271
- **w-rdist:** 0.2478859493921505
- **t-alpha:** 0.0911254664175305

---

---

22

- **AF ID:** AF-O14672-F1-model-v4 | **Chain:** A
- **b-phipsi:** 0.0017469001798336
- **w-rdist:** 0.6697206683398877
- **t-alpha:** 0.002377470759985

---

---

23

- **AF ID:** AF-P50747-F1-model-v4 | **Chain:** A
- **b-phipsi:** 0.0007224711581059
- **w-rdist:** 0.3245466898021097
- **t-alpha:** 0.1489700676263168

---

---

24

- **AF ID:** AF-Q08J23-F1-model-v4 | **Chain:** A
- **b-phipsi:** 0.0003388829332563
- **w-rdist:** 0.3616146314674822
- **t-alpha:** 0.2044381850417527

---

---

25

- **AF ID:** AF-Q9H9G7-F1-model-v4 | **Chain:** A
- **b-phipsi:** 0.0051519983581889
- **w-rdist:** 0.2165348259397449
- **t-alpha:** 0.0427893647215475

---

---

26

- **AF ID:** AF-Q8IWS0-F1-model-v4 | **Chain:** A
- **b-phipsi:** 0.0010168519132977
- **w-rdist:** 0.6680952697902183
- **t-alpha:** 0.0031697813850419

---

---

27

- **AF ID:** AF-P23743-F1-model-v4 | **Chain:** A
- **b-phipsi:** 0.0024690786105629
- **w-rdist:** 0.3463612325489945
- **t-alpha:** 0.0237719193359167

---

---

28

- **AF ID:** AF-Q8IVD9-F1-model-v4 | **Chain:** A
- **b-phipsi:** 0.000286818409079
- **w-rdist:** 0.8005109369948294
- **t-alpha:** 0.0193858366449102

---

---

29

- **AF ID:** AF-O00425-F1-model-v4 | **Chain:** A
- **b-phipsi:** 0.0010693023259971
- **w-rdist:** 0.2575585229483806
- **t-alpha:** 0.1022188946752804

---

---

30

- **AF ID:** AF-Q9UFP1-F1-model-v4 | **Chain:** A
- **b-phipsi:** 0.0002396345402754
- **w-rdist:** 0.6403626287406284
- **t-alpha:** 0.0958797909786817

---

---

31

- **AF ID:** AF-Q96GR2-F1-model-v4 | **Chain:** A
- **b-phipsi:** 0.0032610627247137
- **w-rdist:** 0.723197750361408
- **t-alpha:** 0.0007928174706921

---

---

32

- **AF ID:** AF-Q460N3-F1-model-v4 | **Chain:** A
- **b-phipsi:** 0.0018623046297258
- **w-rdist:** 0.2177910502718875
- **t-alpha:** 0.1164821024786537

---

---

33

- **AF ID:** AF-Q8IZP7-F1-model-v4 | **Chain:** A
- **b-phipsi:** 0.003891657223553
- **w-rdist:** 0.6616925104227238
- **t-alpha:** 0.0015872186884768

---

---

34

- **AF ID:** AF-Q16566-F1-model-v4 | **Chain:** A
- **b-phipsi:** 0.0006741898516719
- **w-rdist:** 0.6681375712948441
- **t-alpha:** 0.0095087103146067

---

---

35

- **AF ID:** AF-Q8TAX0-F1-model-v4 | **Chain:** A
- **b-phipsi:** 0.0054112984281105
- **w-rdist:** 0.5740984810902323
- **t-alpha:** 0.0015872186884768

---

---

36

- **AF ID:** AF-Q14195-F1-model-v4 | **Chain:** A
- **b-phipsi:** 0.0002416405159575
- **w-rdist:** 0.7847090660212412
- **t-alpha:** 0.039619806700881

---

---

37

- **AF ID:** AF-P31323-F1-model-v4 | **Chain:** A
- **b-phipsi:** 0.0004528703220395
- **w-rdist:** 0.7727123374429885
- **t-alpha:** 0.0198097874502385

---

---

38

- **AF ID:** AF-Q8TAT6-F1-model-v4 | **Chain:** A
- **b-phipsi:** 0.0039697155790612
- **w-rdist:** 0.5082446141517756
- **t-alpha:** 0.0039622395506779

---

---

39

- **AF ID:** AF-Q9NZI8-F1-model-v4 | **Chain:** A
- **b-phipsi:** 0.0021394887158891
- **w-rdist:** 0.2402541638192585
- **t-alpha:** 0.1093503210020616

---

---

40

- **AF ID:** AF-Q96HN2-F1-model-v4 | **Chain:** A
- **b-phipsi:** 0.0002849004918139
- **w-rdist:** 0.5323342057198779
- **t-alpha:** 0.1426312113516006

---

---

41

- **AF ID:** AF-Q9HAU4-F1-model-v4 | **Chain:** A
- **b-phipsi:** 0.0020373037221957
- **w-rdist:** 0.1853944095027655
- **t-alpha:** 0.1347072878750428

---

---

42

- **AF ID:** AF-Q9ULR3-F1-model-v4 | **Chain:** A
- **b-phipsi:** 0.0005850289433206
- **w-rdist:** 0.7468221715056953
- **t-alpha:** 0.017432692187089

---

---

43

- **AF ID:** AF-O95294-F1-model-v4 | **Chain:** A
- **b-phipsi:** 0.0071208782699365
- **w-rdist:** 0.3083659702076993
- **t-alpha:** 0.0095996935833633

---

---

44

- **AF ID:** AF-Q9UL63-F1-model-v4 | **Chain:** A
- **b-phipsi:** 0.008398666854891
- **w-rdist:** 0.2182003223275578
- **t-alpha:** 0.0269416384138463

---

---

45

- **AF ID:** AF-O43374-F1-model-v4 | **Chain:** A
- **b-phipsi:** 0.0083489193960566
- **w-rdist:** 0.285864684938989
- **t-alpha:** 0.0104083124295257

---

---

46

- **AF ID:** AF-Q12884-F1-model-v4 | **Chain:** A
- **b-phipsi:** 0.0045875637067317
- **w-rdist:** 0.6903113030314112
- **t-alpha:** 0.0015872186884768

---

---

47

- **AF ID:** AF-O75676-F1-model-v4 | **Chain:** A
- **b-phipsi:** 0.0024977156187094
- **w-rdist:** 0.2957960604580699
- **t-alpha:** 0.09508735985712

---

---

48

- **AF ID:** AF-Q96NY9-F1-model-v4 | **Chain:** A
- **b-phipsi:** 0.0071250526344601
- **w-rdist:** 0.2931728625874575
- **t-alpha:** 0.0213948423587535

---

---

49

- **AF ID:** AF-Q15916-F1-model-v4 | **Chain:** A
- **b-phipsi:** 0.0019510888424897
- **w-rdist:** 0.3653563424890857
- **t-alpha:** 0.0475438470985598

---

---

50

- **AF ID:** AF-P53667-F1-model-v4 | **Chain:** A
- **b-phipsi:** 0.0003514652066718
- **w-rdist:** 0.6205453731389481
- **t-alpha:** 0.1299526096838796

---

---

51

- **AF ID:** AF-O14841-F1-model-v4 | **Chain:** A
- **b-phipsi:** 0.0021083937260303
- **w-rdist:** 0.3095689807404926
- **t-alpha:** 0.0958797909786817

---

---

52

- **AF ID:** AF-Q9BX70-F1-model-v4 | **Chain:** A
- **b-phipsi:** 0.0004013456896593
- **w-rdist:** 0.7292031020572817
- **t-alpha:** 0.0667790338922971

---

---

53

- **AF ID:** AF-Q8N7R0-F1-model-v4 | **Chain:** A
- **b-phipsi:** 0.0024215248398512
- **w-rdist:** 0.382156382305193
- **t-alpha:** 0.0185632335173342

---

---

54

- **AF ID:** AF-Q8NAM6-F1-model-v4 | **Chain:** A
- **b-phipsi:** 0.003916168948067
- **w-rdist:** 0.2821116135854754
- **t-alpha:** 0.0808242883197218

---

---

55

- **AF ID:** AF-O94923-F1-model-v4 | **Chain:** A
- **b-phipsi:** 0.0006957154411794
- **w-rdist:** 0.3818885157162966
- **t-alpha:** 0.1339143555568707

---

---

56

- **AF ID:** AF-Q9Y6S9-F1-model-v4 | **Chain:** A
- **b-phipsi:** 0.0006860137592574
- **w-rdist:** 0.3733305033133314
- **t-alpha:** 0.1537244455033311

---

---

57

- **AF ID:** AF-Q86XI8-F1-model-v4 | **Chain:** A
- **b-phipsi:** 0.0006258257386891
- **w-rdist:** 0.5188900820689564
- **t-alpha:** 0.0982568607009952

---

---

58

- **AF ID:** AF-Q7Z3Z4-F1-model-v4 | **Chain:** A
- **b-phipsi:** 0.0059563289424277
- **w-rdist:** 0.2550597483934069
- **t-alpha:** 0.0625992822193501

---

---

59

- **AF ID:** AF-Q9UIF7-F1-model-v4 | **Chain:** A
- **b-phipsi:** 0.0021094571496286
- **w-rdist:** 0.3157450298986002
- **t-alpha:** 0.0974642806534764

---

---

60

- **AF ID:** AF-Q9Y6M1-F1-model-v4 | **Chain:** A
- **b-phipsi:** 0.0024152272202774
- **w-rdist:** 0.1891352029918035
- **t-alpha:** 0.1537244455033311

---

---

61

- **AF ID:** AF-Q8WZ73-F1-model-v4 | **Chain:** A
- **b-phipsi:** 0.0003280992552344
- **w-rdist:** 0.4621097933267063
- **t-alpha:** 0.1798734427113426

---

---

62

- **AF ID:** AF-O95704-F1-model-v4 | **Chain:** A
- **b-phipsi:** 0.0037387062602943
- **w-rdist:** 0.3455141325060059
- **t-alpha:** 0.0473026125968261

---

---

63

- **AF ID:** AF-Q9P2G3-F1-model-v4 | **Chain:** A
- **b-phipsi:** 0.0056794686736286
- **w-rdist:** 0.3535267984666272
- **t-alpha:** 0.0150558135497862

---

---

64

- **AF ID:** AF-Q8N806-F1-model-v4 | **Chain:** A
- **b-phipsi:** 0.0009650789479177
- **w-rdist:** 0.3592923904444868
- **t-alpha:** 0.0792396355379139

---

---

65

- **AF ID:** AF-Q9Y6W3-F1-model-v4 | **Chain:** A
- **b-phipsi:** 0.0075872190215815
- **w-rdist:** 0.5448376441089542
- **t-alpha:** 0.002377470759985

---

---

66

- **AF ID:** AF-Q96IC2-F1-model-v4 | **Chain:** A
- **b-phipsi:** 0.0039077049634843
- **w-rdist:** 0.3491706850981996
- **t-alpha:** 0.0459591096503235

---

---

67

- **AF ID:** AF-Q9H0C5-F1-model-v4 | **Chain:** A
- **b-phipsi:** 0.0003679076857376
- **w-rdist:** 0.7619018696849581
- **t-alpha:** 0.0740421623492073

---

---

68

- **AF ID:** AF-Q969P6-F1-model-v4 | **Chain:** A
- **b-phipsi:** 0.0176938181536997
- **w-rdist:** 0.3904740440552063
- **t-alpha:** 0.0007923402556029

---

---

69

- **AF ID:** AF-Q9UJN7-F1-model-v4 | **Chain:** A
- **b-phipsi:** 0.0063495031078038
- **w-rdist:** 0.4643044705215828
- **t-alpha:** 0.0039775589155952

---

---

70

- **AF ID:** AF-Q9UL18-F1-model-v4 | **Chain:** A
- **b-phipsi:** 0.0078623873301343
- **w-rdist:** 0.2312440037475
- **t-alpha:** 0.0522979286895239

---

---

71

- **AF ID:** AF-Q16819-F1-model-v4 | **Chain:** A
- **b-phipsi:** 0.0049766498599598
- **w-rdist:** 0.3606874141717947
- **t-alpha:** 0.0206022505246215

---

---

72

- **AF ID:** AF-Q8N4N3-F1-model-v4 | **Chain:** A
- **b-phipsi:** 0.0026623596365557
- **w-rdist:** 0.8296399601328954
- **t-alpha:** 0.0015848307213084

---

---

73

- **AF ID:** AF-P17655-F1-model-v4 | **Chain:** A
- **b-phipsi:** 0.000907749782848
- **w-rdist:** 0.5176329164580684
- **t-alpha:** 0.0235195652642572

---

---

74

- **AF ID:** AF-Q9UEW8-F1-model-v4 | **Chain:** A
- **b-phipsi:** 0.0007465590619797
- **w-rdist:** 0.6567566827061276
- **t-alpha:** 0.0277337626754545

---

---

75

- **AF ID:** AF-Q10471-F1-model-v4 | **Chain:** A
- **b-phipsi:** 0.0008778166858717
- **w-rdist:** 0.6139315417530243
- **t-alpha:** 0.0120284587273298

---

---

76

- **AF ID:** AF-Q04446-F1-model-v4 | **Chain:** A
- **b-phipsi:** 0.0053831461677842
- **w-rdist:** 0.7597548220387331
- **t-alpha:** 0.0007928174706921

---

---

77

- **AF ID:** AF-P05129-F1-model-v4 | **Chain:** A
- **b-phipsi:** 0.0018578408160412
- **w-rdist:** 0.5904413510300182
- **t-alpha:** 0.006339533171912

---

---

78

- **AF ID:** AF-Q8N159-F1-model-v4 | **Chain:** A
- **b-phipsi:** 0.0007334733328778
- **w-rdist:** 0.5401988041771149
- **t-alpha:** 0.0744852576021766

---

---

79

- **AF ID:** AF-Q969S9-F1-model-v4 | **Chain:** A
- **b-phipsi:** 0.0012319666964088
- **w-rdist:** 0.3624656654299981
- **t-alpha:** 0.0847860205554447

---

---

80

- **AF ID:** AF-Q8N9L1-F1-model-v4 | **Chain:** A
- **b-phipsi:** 0.0029101670598967
- **w-rdist:** 0.3700883839857528
- **t-alpha:** 0.0499166951389262

---

---

81

- **AF ID:** AF-P0C7I0-F1-model-v4 | **Chain:** A
- **b-phipsi:** 0.0034632664070093
- **w-rdist:** 0.7174709878449959
- **t-alpha:** 0.003179348403216

---

---

82

- **AF ID:** AF-Q9UKV8-F1-model-v4 | **Chain:** A
- **b-phipsi:** 0.00697319306268
- **w-rdist:** 0.2481978163641483
- **t-alpha:** 0.0713153940999824

---

---

83

- **AF ID:** AF-P17252-F1-model-v4 | **Chain:** A
- **b-phipsi:** 0.0003688463412913
- **w-rdist:** 0.6843237997096245
- **t-alpha:** 0.1318383769643583

---

---

84

- **AF ID:** AF-Q96LX8-F1-model-v4 | **Chain:** A
- **b-phipsi:** 0.0036212188798847
- **w-rdist:** 0.4642224518818655
- **t-alpha:** 0.0063793623469805

---

---

85

- **AF ID:** AF-Q69YN2-F1-model-v4 | **Chain:** A
- **b-phipsi:** 0.0006803205402667
- **w-rdist:** 0.7764411922467991
- **t-alpha:** 0.0193858366449102

---

---

86

- **AF ID:** AF-Q8N2R8-F1-model-v4 | **Chain:** A
- **b-phipsi:** 0.0005010665774407
- **w-rdist:** 0.6644685082563686
- **t-alpha:** 0.1053884795390964

---

---

87

- **AF ID:** AF-P00747-F1-model-v4 | **Chain:** A
- **b-phipsi:** 0.0094722542507961
- **w-rdist:** 0.2487328339141407
- **t-alpha:** 0.0412048690981889

---

---

88

- **AF ID:** AF-Q01518-F1-model-v4 | **Chain:** A
- **b-phipsi:** 0.0073069163706713
- **w-rdist:** 0.2417078937825318
- **t-alpha:** 0.0721080046355822

---

---

89

- **AF ID:** AF-Q9H147-F1-model-v4 | **Chain:** A
- **b-phipsi:** 0.0026043365121717
- **w-rdist:** 0.6628418832357855
- **t-alpha:** 0.0055471164626488

---

---

90

- **AF ID:** AF-P58317-F1-model-v4 | **Chain:** A
- **b-phipsi:** 0.006281476488101
- **w-rdist:** 0.2937115851462523
- **t-alpha:** 0.0784468950036187

---

---

91

- **AF ID:** AF-Q96JB6-F1-model-v4 | **Chain:** A
- **b-phipsi:** 0.0094476889870072
- **w-rdist:** 0.2855982071683806
- **t-alpha:** 0.0361248537980045

---

---

92

- **AF ID:** AF-P49619-F1-model-v4 | **Chain:** A
- **b-phipsi:** 0.001364773940395
- **w-rdist:** 0.1363827990077107
- **t-alpha:** 0.2218697074286468

---

---

93

- **AF ID:** AF-P40123-F1-model-v4 | **Chain:** A
- **b-phipsi:** 0.0028697333241984
- **w-rdist:** 0.3549237450822869
- **t-alpha:** 0.0839938579877885

---

---

94

- **AF ID:** AF-Q8IY47-F1-model-v4 | **Chain:** A
- **b-phipsi:** 0.0067073910314758
- **w-rdist:** 0.3438718202987501
- **t-alpha:** 0.031695687164061

---

---

95

- **AF ID:** AF-Q9Y2P5-F1-model-v4 | **Chain:** A
- **b-phipsi:** 0.0090486911493354
- **w-rdist:** 0.7032340513822388
- **t-alpha:** 0.0007923402556029

---

---

96

- **AF ID:** AF-Q04759-F1-model-v4 | **Chain:** A
- **b-phipsi:** 0.0012704961187832
- **w-rdist:** 0.3560153942590944
- **t-alpha:** 0.1141048975561447

---

---

97

- **AF ID:** AF-Q9Y2F9-F1-model-v4 | **Chain:** A
- **b-phipsi:** 0.0048081735522913
- **w-rdist:** 0.4771453287847057
- **t-alpha:** 0.006339533171912

---

---

98

- **AF ID:** AF-P54284-F1-model-v4 | **Chain:** A
- **b-phipsi:** 0.0004478363812177
- **w-rdist:** 0.9028396483238222
- **t-alpha:** 0.0309034586893088

---

---

99

- **AF ID:** AF-Q5T447-F1-model-v4 | **Chain:** A
- **b-phipsi:** 0.0072317669598775
- **w-rdist:** 0.1343161132616565
- **t-alpha:** 0.1022188946752804

---

---

100

- **AF ID:** AF-P41743-F1-model-v4 | **Chain:** A
- **b-phipsi:** 0.000881405997902
- **w-rdist:** 0.5339280140080234
- **t-alpha:** 0.0499207002609116

---

---

101

- **AF ID:** AF-P56817-F1-model-v4 | **Chain:** A
- **b-phipsi:** 0.0050922251501322
- **w-rdist:** 0.7859228058427133
- **t-alpha:** 0.0015872186884768

---

---

102

- **AF ID:** AF-Q86Y07-F1-model-v4 | **Chain:** A
- **b-phipsi:** 0.0025610050315748
- **w-rdist:** 0.6475228781080116
- **t-alpha:** 0.006339533171912

---

---

103

- **AF ID:** AF-Q6IQ19-F1-model-v4 | **Chain:** A
- **b-phipsi:** 0.0013130974899187
- **w-rdist:** 0.8061231653078419
- **t-alpha:** 0.0039775589155952

---

---

104

- **AF ID:** AF-Q9BXC9-F1-model-v4 | **Chain:** A
- **b-phipsi:** 0.0079609811831271
- **w-rdist:** 0.2955781342834534
- **t-alpha:** 0.0554678888910149

---

---

105

- **AF ID:** AF-Q9UFC0-F1-model-v4 | **Chain:** A
- **b-phipsi:** 0.0057363482331006
- **w-rdist:** 0.6041782639761237
- **t-alpha:** 0.0047543655130677

---

---

106

- **AF ID:** AF-O15371-F1-model-v4 | **Chain:** A
- **b-phipsi:** 0.0079495910123268
- **w-rdist:** 0.6128661915151095
- **t-alpha:** 0.0031697813850419

---

---

107

- **AF ID:** AF-O15067-F1-model-v4 | **Chain:** A
- **b-phipsi:** 0.0020609071258239
- **w-rdist:** 0.3333983204590733
- **t-alpha:** 0.1426312113516006

---

---

108

- **AF ID:** AF-P05771-F1-model-v4 | **Chain:** A
- **b-phipsi:** 0.0005922247009228
- **w-rdist:** 0.6989306293194273
- **t-alpha:** 0.0993031670831436

---

---

109

- **AF ID:** AF-Q04771-F1-model-v4 | **Chain:** A
- **b-phipsi:** 0.0008190093951515
- **w-rdist:** 0.7049727040073831
- **t-alpha:** 0.0276872078734735

---

---

110

- **AF ID:** AF-O43610-F1-model-v4 | **Chain:** A
- **b-phipsi:** 0.0065464347966418
- **w-rdist:** 0.3733997253251752
- **t-alpha:** 0.0182252502019479

---

---

111

- **AF ID:** AF-Q2TBF2-F1-model-v4 | **Chain:** A
- **b-phipsi:** 0.0025815477158426
- **w-rdist:** 0.3415709921893254
- **t-alpha:** 0.1323296128972912

---

---

112

- **AF ID:** AF-Q8NAT1-F1-model-v4 | **Chain:** A
- **b-phipsi:** 0.0019556410484188
- **w-rdist:** 0.4069165631803854
- **t-alpha:** 0.0451663942458306

---

---

113

- **AF ID:** AF-Q9UL40-F1-model-v4 | **Chain:** A
- **b-phipsi:** 0.009469244873131
- **w-rdist:** 0.2804963761728593
- **t-alpha:** 0.0551835704278558

---

---

114

- **AF ID:** AF-Q9UI17-F1-model-v4 | **Chain:** A
- **b-phipsi:** 0.0008404425280736
- **w-rdist:** 0.5639229319383507
- **t-alpha:** 0.0721080046355822

---

---

115

- **AF ID:** AF-Q9HCG7-F1-model-v4 | **Chain:** A
- **b-phipsi:** 0.0006588019920632
- **w-rdist:** 0.4248060792862148
- **t-alpha:** 0.1703645180917901

---

---

116

- **AF ID:** AF-Q9BQ95-F1-model-v4 | **Chain:** A
- **b-phipsi:** 0.0066510498232194
- **w-rdist:** 0.3070657541251649
- **t-alpha:** 0.0816167133968897

---

---

117

- **AF ID:** AF-Q99965-F1-model-v4 | **Chain:** A
- **b-phipsi:** 0.002507454129778
- **w-rdist:** 0.9445832650845428
- **t-alpha:** 0.0015872186884768

---

---

118

- **AF ID:** AF-Q96GV9-F1-model-v4 | **Chain:** A
- **b-phipsi:** 0.0168176866295972
- **w-rdist:** 0.3973719930394581
- **t-alpha:** 0.0023827284528445

---

---

119

- **AF ID:** AF-Q9BXL8-F1-model-v4 | **Chain:** A
- **b-phipsi:** 0.0100818369841815
- **w-rdist:** 0.3774445283139138
- **t-alpha:** 0.0055773242968351

---

---

120

- **AF ID:** AF-Q15418-F1-model-v4 | **Chain:** A
- **b-phipsi:** 0.0071738374258311
- **w-rdist:** 0.5044824577294555
- **t-alpha:** 0.0055471164626488

---

---

121

- **AF ID:** AF-Q9BWW7-F1-model-v4 | **Chain:** A
- **b-phipsi:** 0.0110714066811571
- **w-rdist:** 0.270788168966612
- **t-alpha:** 0.0515059245507361

---

---

122

- **AF ID:** AF-P47989-F1-model-v4 | **Chain:** A
- **b-phipsi:** 0.0038554974148349
- **w-rdist:** 0.2744741871475388
- **t-alpha:** 0.1648181756590165

---

---

123

- **AF ID:** AF-Q9H4B8-F1-model-v4 | **Chain:** A
- **b-phipsi:** 0.0043847762233046
- **w-rdist:** 0.7322506785075235
- **t-alpha:** 0.0039775589155952

---

---

124

- **AF ID:** AF-Q9NTJ4-F1-model-v4 | **Chain:** A
- **b-phipsi:** 0.0063202706699071
- **w-rdist:** 0.3463140862272972
- **t-alpha:** 0.0689385280761989

---

---

125

- **AF ID:** AF-Q96LR2-F1-model-v4 | **Chain:** A
- **b-phipsi:** 0.009138885395925
- **w-rdist:** 0.2925374084383938
- **t-alpha:** 0.0605041736265279

---

---

126

- **AF ID:** AF-Q9Y2X0-F1-model-v4 | **Chain:** A
- **b-phipsi:** 0.0089997721627276
- **w-rdist:** 0.3406156613949861
- **t-alpha:** 0.0356580695998436

---

---

127

- **AF ID:** AF-Q86TM3-F1-model-v4 | **Chain:** A
- **b-phipsi:** 0.0099600019055251
- **w-rdist:** 0.3211187445250222
- **t-alpha:** 0.0364504923644704

---

---

128

- **AF ID:** AF-Q9H5Q4-F1-model-v4 | **Chain:** A
- **b-phipsi:** 0.0034847050681856
- **w-rdist:** 0.901214479004488
- **t-alpha:** 0.002377470759985

---

---

129

- **AF ID:** AF-Q9Y4L5-F1-model-v4 | **Chain:** A
- **b-phipsi:** 0.0043554124737749
- **w-rdist:** 0.3756319309318319
- **t-alpha:** 0.0596135259369763

---

---

130

- **AF ID:** AF-P10253-F1-model-v4 | **Chain:** A
- **b-phipsi:** 0.0045502133006232
- **w-rdist:** 0.3759570795327699
- **t-alpha:** 0.0586373023344051

---

---

131

- **AF ID:** AF-Q9H1B5-F1-model-v4 | **Chain:** A
- **b-phipsi:** 0.0009770052332637
- **w-rdist:** 0.2242182747295841
- **t-alpha:** 0.2971480872381158

---

---

132

- **AF ID:** AF-Q9BPU6-F1-model-v4 | **Chain:** A
- **b-phipsi:** 0.0011101000340979
- **w-rdist:** 0.8207377089875205
- **t-alpha:** 0.0055471164626488

---

---

133

- **AF ID:** AF-Q9Y6T7-F1-model-v4 | **Chain:** A
- **b-phipsi:** 0.0004606029354383
- **w-rdist:** 0.5578738259221658
- **t-alpha:** 0.2202859732691042

---

---

134

- **AF ID:** AF-Q6UY01-F1-model-v4 | **Chain:** A
- **b-phipsi:** 0.0134374353500463
- **w-rdist:** 0.6969356271750485
- **t-alpha:** 0.0007923402556029

---

---

135

- **AF ID:** AF-P51449-F1-model-v4 | **Chain:** A
- **b-phipsi:** 0.008932783394432
- **w-rdist:** 0.3532876004468009
- **t-alpha:** 0.0380351774023424

---

---

136

- **AF ID:** AF-Q8NA19-F1-model-v4 | **Chain:** A
- **b-phipsi:** 0.0033445167006746
- **w-rdist:** 0.3657531865077554
- **t-alpha:** 0.1053884795390964

---

---

137

- **AF ID:** AF-P15586-F1-model-v4 | **Chain:** A
- **b-phipsi:** 0.0005168125100829
- **w-rdist:** 0.9170035883036504
- **t-alpha:** 0.0499166951389262

---

---

138

- **AF ID:** AF-Q9UG63-F1-model-v4 | **Chain:** A
- **b-phipsi:** 0.0145104162370848
- **w-rdist:** 0.5545245618295742
- **t-alpha:** 0.002377470759985

---

---

139

- **AF ID:** AF-Q96LW9-F1-model-v4 | **Chain:** A
- **b-phipsi:** 0.0116003831956703
- **w-rdist:** 0.3316076503516659
- **t-alpha:** 0.0253567763618671

---

---

140

- **AF ID:** AF-Q14435-F1-model-v4 | **Chain:** A
- **b-phipsi:** 0.0069533699043947
- **w-rdist:** 0.5519866654930417
- **t-alpha:** 0.0055773242968351

---

---

141

- **AF ID:** AF-Q8N6R0-F1-model-v4 | **Chain:** A
- **b-phipsi:** 0.0081243032171089
- **w-rdist:** 0.7737386711833272
- **t-alpha:** 0.0015872186884768

---

---

142

- **AF ID:** AF-Q5MJ70-F1-model-v4 | **Chain:** A
- **b-phipsi:** 0.0009128727269005
- **w-rdist:** 0.7429148240478809
- **t-alpha:** 0.0193858366449102

---

---

143

- **AF ID:** AF-Q9Y2Y9-F1-model-v4 | **Chain:** A
- **b-phipsi:** 0.0053449311159708
- **w-rdist:** 0.5534517294066341
- **t-alpha:** 0.0071316138654509

---

---

144

- **AF ID:** AF-Q9BQS7-F1-model-v4 | **Chain:** A
- **b-phipsi:** 0.0063320419026674
- **w-rdist:** 0.2718376225958623
- **t-alpha:** 0.1513475474920678

---

---

145

- **AF ID:** AF-P78545-F1-model-v4 | **Chain:** A
- **b-phipsi:** 0.0098479532414332
- **w-rdist:** 0.812694088725354
- **t-alpha:** 0.0

---

---

146

- **AF ID:** AF-P31321-F1-model-v4 | **Chain:** A
- **b-phipsi:** 0.0053734951031155
- **w-rdist:** 0.4050472141870076
- **t-alpha:** 0.0268510508819761

---

---

147

- **AF ID:** AF-Q02556-F1-model-v4 | **Chain:** A
- **b-phipsi:** 0.000424600916264
- **w-rdist:** 0.8215739785361322
- **t-alpha:** 0.1277924591090977

---

---

148

- **AF ID:** AF-P29350-F1-model-v4 | **Chain:** A
- **b-phipsi:** 0.0009232767204733
- **w-rdist:** 0.5543289769113705
- **t-alpha:** 0.0808242883197218

---

---

149

- **AF ID:** AF-O75582-F1-model-v4 | **Chain:** A
- **b-phipsi:** 0.0006544176092592
- **w-rdist:** 0.651780523411155
- **t-alpha:** 0.1553093824431966

---

---

150

- **AF ID:** AF-P07948-F1-model-v4 | **Chain:** A
- **b-phipsi:** 0.0011724354936137
- **w-rdist:** 0.8259416053486698
- **t-alpha:** 0.0055773242968351

---

---

151

- **AF ID:** AF-P10276-F1-model-v4 | **Chain:** A
- **b-phipsi:** 0.0056318942569241
- **w-rdist:** 0.7774930105722114
- **t-alpha:** 0.0039622395506779

---

---

152

- **AF ID:** AF-Q86UE6-F1-model-v4 | **Chain:** A
- **b-phipsi:** 0.0029505697810432
- **w-rdist:** 0.8979432354891015
- **t-alpha:** 0.0039622395506779

---

---

153

- **AF ID:** AF-O15522-F1-model-v4 | **Chain:** A
- **b-phipsi:** 0.0083648309658797
- **w-rdist:** 0.8409102238801401
- **t-alpha:** 0.0007928174706921

---

---

154

- **AF ID:** AF-Q6DD87-F1-model-v4 | **Chain:** A
- **b-phipsi:** 0.0082921465869128
- **w-rdist:** 0.6518666271608359
- **t-alpha:** 0.0047543655130677

---

---

155

- **AF ID:** AF-O15499-F1-model-v4 | **Chain:** A
- **b-phipsi:** 0.0057988067029841
- **w-rdist:** 0.2544810948427307
- **t-alpha:** 0.177238323162137

---

---

156

- **AF ID:** AF-P98170-F1-model-v4 | **Chain:** A
- **b-phipsi:** 0.0064576601578707
- **w-rdist:** 0.36309514741037
- **t-alpha:** 0.077654736765909

---

---

157

- **AF ID:** AF-P09769-F1-model-v4 | **Chain:** A
- **b-phipsi:** 0.000559479474875
- **w-rdist:** 0.8598090708293629
- **t-alpha:** 0.0888697463155465

---

---

158

- **AF ID:** AF-Q8N205-F1-model-v4 | **Chain:** A
- **b-phipsi:** 0.000354299291276
- **w-rdist:** 1.1005145313013238
- **t-alpha:** 0.0435817701022656

---

---

159

- **AF ID:** AF-O15259-F1-model-v4 | **Chain:** A
- **b-phipsi:** 0.0034537293380872
- **w-rdist:** 0.164709600500772
- **t-alpha:** 0.2939776802283649

---

---

160

- **AF ID:** AF-Q3MJ62-F1-model-v4 | **Chain:** A
- **b-phipsi:** 0.011878370277352
- **w-rdist:** 0.3551505230286828
- **t-alpha:** 0.0276872078734735

---

---

161

- **AF ID:** AF-O95727-F1-model-v4 | **Chain:** A
- **b-phipsi:** 0.0046377365300218
- **w-rdist:** 1.0605898904983655
- **t-alpha:** 0.0

---

---

162

- **AF ID:** AF-P20807-F1-model-v4 | **Chain:** A
- **b-phipsi:** 0.0006211352172443
- **w-rdist:** 0.7323235619430459
- **t-alpha:** 0.1481775708256634

---

---

163

- **AF ID:** AF-Q86VW2-F1-model-v4 | **Chain:** A
- **b-phipsi:** 0.003685253556323
- **w-rdist:** 0.3901223671418962
- **t-alpha:** 0.0911254664175305

---

---

164

- **AF ID:** AF-A6NM28-F1-model-v4 | **Chain:** A
- **b-phipsi:** 0.000668368342486
- **w-rdist:** 0.8553251646306546
- **t-alpha:** 0.0705233695421561

---

---

165

- **AF ID:** AF-Q3V5L5-F1-model-v4 | **Chain:** A
- **b-phipsi:** 0.0126941176310428
- **w-rdist:** 0.1804947640199163
- **t-alpha:** 0.1117274530174508

---

---

166

- **AF ID:** AF-P14635-F1-model-v4 | **Chain:** A
- **b-phipsi:** 0.0129469520539085
- **w-rdist:** 0.7253559289393694
- **t-alpha:** 0.0015872186884768

---

---

167

- **AF ID:** AF-Q14CN2-F1-model-v4 | **Chain:** A
- **b-phipsi:** 0.0089882273383735
- **w-rdist:** 0.2953175820743751
- **t-alpha:** 0.1148972678966586

---

---

168

- **AF ID:** AF-O14757-F1-model-v4 | **Chain:** A
- **b-phipsi:** 0.0006576938607539
- **w-rdist:** 0.9673853419645124
- **t-alpha:** 0.0310455330972876

---

---

169

- **AF ID:** AF-P22413-F1-model-v4 | **Chain:** A
- **b-phipsi:** 0.0001966010781664
- **w-rdist:** 0.7962556247407109
- **t-alpha:** 0.190174484708248

---

---

170

- **AF ID:** AF-Q96GY3-F1-model-v4 | **Chain:** A
- **b-phipsi:** 0.0135958393999682
- **w-rdist:** 0.3683180304671389
- **t-alpha:** 0.0079867999848444

---

---

171

- **AF ID:** AF-Q92993-F1-model-v4 | **Chain:** A
- **b-phipsi:** 0.013982721515531
- **w-rdist:** 0.7795407655238176
- **t-alpha:** 0.0

---

---

172

- **AF ID:** AF-Q02156-F1-model-v4 | **Chain:** A
- **b-phipsi:** 0.0009791604980882
- **w-rdist:** 0.3999284477334657
- **t-alpha:** 0.1378764271488828

---

---

173

- **AF ID:** AF-Q06278-F1-model-v4 | **Chain:** A
- **b-phipsi:** 0.0031211196569167
- **w-rdist:** 0.2809692672058776
- **t-alpha:** 0.248812081028487

---

---

174

- **AF ID:** AF-Q5SY16-F1-model-v4 | **Chain:** A
- **b-phipsi:** 0.0054748864824403
- **w-rdist:** 0.3484950133956866
- **t-alpha:** 0.1370839912556183

---

---

175

- **AF ID:** AF-Q8NFI3-F1-model-v4 | **Chain:** A
- **b-phipsi:** 0.0067761026381273
- **w-rdist:** 0.5930634496545002
- **t-alpha:** 0.0063793623469805

---

---

176

- **AF ID:** AF-Q9BQS2-F1-model-v4 | **Chain:** A
- **b-phipsi:** 0.0175248064565424
- **w-rdist:** 0.5599837764275831
- **t-alpha:** 0.0031697813850419

---

---

177

- **AF ID:** AF-Q9HCK5-F1-model-v4 | **Chain:** A
- **b-phipsi:** 0.0072915425523741
- **w-rdist:** 0.3187101745532025
- **t-alpha:** 0.1315372629774758

---

---

178

- **AF ID:** AF-Q14512-F1-model-v4 | **Chain:** A
- **b-phipsi:** 0.0004802724856833
- **w-rdist:** 0.7059736695448183
- **t-alpha:** 0.2019046524118539

---

---

179

- **AF ID:** AF-Q7Z6G3-F1-model-v4 | **Chain:** A
- **b-phipsi:** 0.0288373145347269
- **w-rdist:** 0.5683335335261415
- **t-alpha:** 0.0007928174706921

---

---

180

- **AF ID:** AF-Q96MR7-F1-model-v4 | **Chain:** A
- **b-phipsi:** 0.0041487380088407
- **w-rdist:** 0.2808439228609501
- **t-alpha:** 0.2276262374940376

---

---

181

- **AF ID:** AF-Q4G0N4-F1-model-v4 | **Chain:** A
- **b-phipsi:** 0.0025818943924703
- **w-rdist:** 0.935728232108527
- **t-alpha:** 0.0039775589155952

---

---

182

- **AF ID:** AF-P49448-F1-model-v4 | **Chain:** A
- **b-phipsi:** 0.0082330074490449
- **w-rdist:** 0.7392159738251274
- **t-alpha:** 0.0039622395506779

---

---

183

- **AF ID:** AF-Q86UR1-F1-model-v4 | **Chain:** A
- **b-phipsi:** 0.0008609713699586
- **w-rdist:** 0.7008723781126516
- **t-alpha:** 0.0824092033211982

---

---

184

- **AF ID:** AF-Q8IYR2-F1-model-v4 | **Chain:** A
- **b-phipsi:** 0.0423931672866998
- **w-rdist:** 0.2385823114801336
- **t-alpha:** 0.0144693552396331

---

---

185

- **AF ID:** AF-Q16394-F1-model-v4 | **Chain:** A
- **b-phipsi:** 0.0142577767019004
- **w-rdist:** 0.3336576030898412
- **t-alpha:** 0.0419970641616329

---

---

186

- **AF ID:** AF-P50579-F1-model-v4 | **Chain:** A
- **b-phipsi:** 0.0041604124479794
- **w-rdist:** 0.8215335915728956
- **t-alpha:** 0.0047768836635659

---

---

187

- **AF ID:** AF-Q8N8B7-F1-model-v4 | **Chain:** A
- **b-phipsi:** 0.0240772238797949
- **w-rdist:** 0.6463794048568176
- **t-alpha:** 0.0007928174706921

---

---

188

- **AF ID:** AF-A6NJG2-F1-model-v4 | **Chain:** A
- **b-phipsi:** 0.0167382817277153
- **w-rdist:** 0.6550242849118189
- **t-alpha:** 0.002377470759985

---

---

189

- **AF ID:** AF-Q9UL01-F1-model-v4 | **Chain:** A
- **b-phipsi:** 0.0110654465351122
- **w-rdist:** 0.3112776484893109
- **t-alpha:** 0.0832012408733819

---

---

190

- **AF ID:** AF-P31152-F1-model-v4 | **Chain:** A
- **b-phipsi:** 0.00078162536024
- **w-rdist:** 0.369349464654264
- **t-alpha:** 0.3058638510559339

---

---

191

- **AF ID:** AF-Q8IXN7-F1-model-v4 | **Chain:** A
- **b-phipsi:** 0.0007331236502165
- **w-rdist:** 0.963554852445984
- **t-alpha:** 0.0210354708025768

---

---

192

- **AF ID:** AF-P0CH99-F1-model-v4 | **Chain:** A
- **b-phipsi:** 0.0198761048277963
- **w-rdist:** 0.3077338900725569
- **t-alpha:** 0.0318885499175907

---

---

193

- **AF ID:** AF-P51513-F1-model-v4 | **Chain:** A
- **b-phipsi:** 0.0069219415225272
- **w-rdist:** 0.8193611204786074
- **t-alpha:** 0.003179348403216

---

---

194

- **AF ID:** AF-Q9H6W3-F1-model-v4 | **Chain:** A
- **b-phipsi:** 0.0049116157051473
- **w-rdist:** 0.3336652850965926
- **t-alpha:** 0.1727420897461962

---

---

195

- **AF ID:** AF-Q16706-F1-model-v4 | **Chain:** A
- **b-phipsi:** 0.0090934305531891
- **w-rdist:** 0.2928076438219772
- **t-alpha:** 0.1386688723323981

---

---

196

- **AF ID:** AF-Q96HY7-F1-model-v4 | **Chain:** A
- **b-phipsi:** 0.018761897189532
- **w-rdist:** 0.356924557381925
- **t-alpha:** 0.0087926839253336

---

---

197

- **AF ID:** AF-P60608-F1-model-v4 | **Chain:** A
- **b-phipsi:** 0.0024520957150697
- **w-rdist:** 0.3773839906549256
- **t-alpha:** 0.1600634400235712

---

---

198

- **AF ID:** AF-A8K7I4-F1-model-v4 | **Chain:** A
- **b-phipsi:** 0.0090460713981082
- **w-rdist:** 0.3445788024701506
- **t-alpha:** 0.0935026519522419

---

---

199

- **AF ID:** AF-D6RA61-F1-model-v4 | **Chain:** A
- **b-phipsi:** 0.0061661187803536
- **w-rdist:** 0.7799964915291433
- **t-alpha:** 0.0047768836635659

---

---

200

- **AF ID:** AF-Q7Z7J5-F1-model-v4 | **Chain:** A
- **b-phipsi:** 0.0084896141253707
- **w-rdist:** 0.4017262447507781
- **t-alpha:** 0.0218620195820675

---

---

201

- **AF ID:** AF-B2RXF5-F1-model-v4 | **Chain:** A
- **b-phipsi:** 0.0007629597509064
- **w-rdist:** 0.4593114994129537
- **t-alpha:** 0.2202859732691042

---

---

202

- **AF ID:** AF-Q9BUB4-F1-model-v4 | **Chain:** A
- **b-phipsi:** 0.00076582416793
- **w-rdist:** 0.8581131478247306
- **t-alpha:** 0.0534220023088101

---

---

203

- **AF ID:** AF-Q7Z3Z3-F1-model-v4 | **Chain:** A
- **b-phipsi:** 0.0032372573353496
- **w-rdist:** 0.3846456221026981
- **t-alpha:** 0.1386688723323981

---

---

204

- **AF ID:** AF-Q96CM8-F1-model-v4 | **Chain:** A
- **b-phipsi:** 0.0009119497054788
- **w-rdist:** 0.8884088688359967
- **t-alpha:** 0.0120284587273298

---

---

205

- **AF ID:** AF-Q8TCG5-F1-model-v4 | **Chain:** A
- **b-phipsi:** 0.0461609769275035
- **w-rdist:** 0.5439154116039563
- **t-alpha:** 0.0007928174706921

---

---

206

- **AF ID:** AF-A6NNA5-F1-model-v4 | **Chain:** A
- **b-phipsi:** 0.0031557207656733
- **w-rdist:** 0.8564991911194573
- **t-alpha:** 0.0055773242968351

---

---

207

- **AF ID:** AF-A6NLC5-F1-model-v4 | **Chain:** A
- **b-phipsi:** 0.0113743481334607
- **w-rdist:** 0.3815250008705116
- **t-alpha:** 0.0213948423587535

---

---

208

- **AF ID:** AF-Q9BWT1-F1-model-v4 | **Chain:** A
- **b-phipsi:** 0.0004499771699097
- **w-rdist:** 0.6425870857256576
- **t-alpha:** 0.2987324868058021

---

---

209

- **AF ID:** AF-Q8WVV9-F1-model-v4 | **Chain:** A
- **b-phipsi:** 0.0105913173570211
- **w-rdist:** 0.4983588613188738
- **t-alpha:** 0.0063793623469805

---

---

210

- **AF ID:** AF-Q9UBX1-F1-model-v4 | **Chain:** A
- **b-phipsi:** 0.0002926459862322
- **w-rdist:** 0.9079970259440964
- **t-alpha:** 0.1588609377563099

---

---

211

- **AF ID:** AF-Q9NV66-F1-model-v4 | **Chain:** A
- **b-phipsi:** 0.0066989574265834
- **w-rdist:** 0.3930542403729762
- **t-alpha:** 0.0602220373725139

---

---

212

- **AF ID:** AF-Q8N6K7-F1-model-v4 | **Chain:** A
- **b-phipsi:** 0.0198105077333439
- **w-rdist:** 0.2569839441316825
- **t-alpha:** 0.0713153940999824

---

---

213

- **AF ID:** AF-A6NFK2-F1-model-v4 | **Chain:** A
- **b-phipsi:** 0.0110245494282615
- **w-rdist:** 0.3384400744710491
- **t-alpha:** 0.0823328183431248

---

---

214

- **AF ID:** AF-P00488-F1-model-v4 | **Chain:** A
- **b-phipsi:** 0.0185859367363133
- **w-rdist:** 0.5744915393556392
- **t-alpha:** 0.003179348403216

---

---

215

- **AF ID:** AF-Q9ULW8-F1-model-v4 | **Chain:** A
- **b-phipsi:** 0.0052700651277616
- **w-rdist:** 0.7798067920921813
- **t-alpha:** 0.0055773242968351

---

---

216

- **AF ID:** AF-Q8NC60-F1-model-v4 | **Chain:** A
- **b-phipsi:** 0.0108258642347243
- **w-rdist:** 0.3012110134275447
- **t-alpha:** 0.1275752196006165

---

---

217

- **AF ID:** AF-P0CB48-F1-model-v4 | **Chain:** A
- **b-phipsi:** 0.0198626080462088
- **w-rdist:** 0.2891567092717849
- **t-alpha:** 0.0594296261277553

---

---

218

- **AF ID:** AF-P27037-F1-model-v4 | **Chain:** A
- **b-phipsi:** 0.0078597823416774
- **w-rdist:** 0.6654608167183194
- **t-alpha:** 0.006339533171912

---

---

219

- **AF ID:** AF-P58215-F1-model-v4 | **Chain:** A
- **b-phipsi:** 0.0112337238027408
- **w-rdist:** 0.3597500975639901
- **t-alpha:** 0.0570524158190295

---

---

220

- **AF ID:** AF-A8K5M9-F1-model-v4 | **Chain:** A
- **b-phipsi:** 0.0049386516380352
- **w-rdist:** 0.3287147013858528
- **t-alpha:** 0.1996195342762932

---

---

221

- **AF ID:** AF-Q8N9I0-F1-model-v4 | **Chain:** A
- **b-phipsi:** 0.0099989807691384
- **w-rdist:** 0.5703153713306984
- **t-alpha:** 0.006339533171912

---

---

222

- **AF ID:** AF-P49959-F1-model-v4 | **Chain:** A
- **b-phipsi:** 0.0002597331854233
- **w-rdist:** 0.7274171107859017
- **t-alpha:** 0.3193342666536947

---

---

223

- **AF ID:** AF-Q9BQ52-F1-model-v4 | **Chain:** A
- **b-phipsi:** 0.0036499316962661
- **w-rdist:** 0.33554213311678
- **t-alpha:** 0.2345490443729756

---

---

224

- **AF ID:** AF-Q09013-F1-model-v4 | **Chain:** A
- **b-phipsi:** 0.0090888424899449
- **w-rdist:** 0.3193234631580854
- **t-alpha:** 0.1378764271488828

---

---

225

- **AF ID:** AF-Q8IUH3-F1-model-v4 | **Chain:** A
- **b-phipsi:** 0.0033856624213911
- **w-rdist:** 0.3746083689925162
- **t-alpha:** 0.1703645180917901

---

---

226

- **AF ID:** AF-Q92544-F1-model-v4 | **Chain:** A
- **b-phipsi:** 0.0393944296766384
- **w-rdist:** 0.5288961526289174
- **t-alpha:** 0.002377470759985

---

---

227

- **AF ID:** AF-P17023-F1-model-v4 | **Chain:** A
- **b-phipsi:** 0.0080806404662261
- **w-rdist:** 0.9148152672983896
- **t-alpha:** 0.002377470759985

---

---

228

- **AF ID:** AF-Q16822-F1-model-v4 | **Chain:** A
- **b-phipsi:** 0.0008655947338971
- **w-rdist:** 0.9355759007058831
- **t-alpha:** 0.0202100770637112

---

---

229

- **AF ID:** AF-Q9NX74-F1-model-v4 | **Chain:** A
- **b-phipsi:** 0.0075719547132714
- **w-rdist:** 0.7249945737972502
- **t-alpha:** 0.0055773242968351

---

---

230

- **AF ID:** AF-Q6ZT52-F1-model-v4 | **Chain:** A
- **b-phipsi:** 0.0006659595743122
- **w-rdist:** 0.6523934297294803
- **t-alpha:** 0.228821575168636

---

---

231

- **AF ID:** AF-P06576-F1-model-v4 | **Chain:** A
- **b-phipsi:** 0.000853321397212
- **w-rdist:** 0.7151183654418817
- **t-alpha:** 0.128801235033463

---

---

232

- **AF ID:** AF-Q8IUD6-F1-model-v4 | **Chain:** A
- **b-phipsi:** 0.012169608545318
- **w-rdist:** 0.5130574170069551
- **t-alpha:** 0.0063793623469805

---

---

233

- **AF ID:** AF-P17028-F1-model-v4 | **Chain:** A
- **b-phipsi:** 0.0266135787053446
- **w-rdist:** 0.2521171389656892
- **t-alpha:** 0.0633915289148658

---

---

234

- **AF ID:** AF-Q8N987-F1-model-v4 | **Chain:** A
- **b-phipsi:** 0.0461993715850781
- **w-rdist:** 0.3105969431291565
- **t-alpha:** 0.0087926839253336

---

---

235

- **AF ID:** AF-Q9UBT2-F1-model-v4 | **Chain:** A
- **b-phipsi:** 0.0061998634233481
- **w-rdist:** 0.3519976604941481
- **t-alpha:** 0.1640252936109762

---

---

236

- **AF ID:** AF-Q5TGY1-F1-model-v4 | **Chain:** A
- **b-phipsi:** 0.0135811627437786
- **w-rdist:** 0.5848459150265719
- **t-alpha:** 0.0055471164626488

---

---

237

- **AF ID:** AF-Q96BT7-F1-model-v4 | **Chain:** A
- **b-phipsi:** 0.0030776167532512
- **w-rdist:** 0.3928002178716098
- **t-alpha:** 0.1553093824431966

---

---

238

- **AF ID:** AF-O95073-F1-model-v4 | **Chain:** A
- **b-phipsi:** 0.0280308533824474
- **w-rdist:** 0.4755526733755343
- **t-alpha:** 0.0039622395506779

---

---

239

- **AF ID:** AF-Q92562-F1-model-v4 | **Chain:** A
- **b-phipsi:** 0.0047329220523767
- **w-rdist:** 0.3052959825013325
- **t-alpha:** 0.2519816682717417

---

---

240

- **AF ID:** AF-P54829-F1-model-v4 | **Chain:** A
- **b-phipsi:** 0.0001628924501026
- **w-rdist:** 0.9350953771869372
- **t-alpha:** 0.1838352099719058

---

---

241

- **AF ID:** AF-Q9Y6K8-F1-model-v4 | **Chain:** A
- **b-phipsi:** 0.0156607654978725
- **w-rdist:** 0.2261787171851782
- **t-alpha:** 0.1378764271488828

---

---

242

- **AF ID:** AF-P19113-F1-model-v4 | **Chain:** A
- **b-phipsi:** 0.0015479408976973
- **w-rdist:** 0.3567032785576652
- **t-alpha:** 0.2876392180192344

---

---

243

- **AF ID:** AF-P20226-F1-model-v4 | **Chain:** A
- **b-phipsi:** 0.0069622949428465
- **w-rdist:** 0.7813883581710033
- **t-alpha:** 0.0055471164626488

---

---

244

- **AF ID:** AF-Q8TE96-F1-model-v4 | **Chain:** A
- **b-phipsi:** 0.0135782231382253
- **w-rdist:** 0.7509250780928263
- **t-alpha:** 0.0031697813850419

---

---

245

- **AF ID:** AF-A0A1W2PR82-F1-model-v4 | **Chain:** A
- **b-phipsi:** 0.0009451430351636
- **w-rdist:** 0.7390538059143053
- **t-alpha:** 0.0786320385731313

---

---

246

- **AF ID:** AF-Q8NCE2-F1-model-v4 | **Chain:** A
- **b-phipsi:** 0.0005171003577135
- **w-rdist:** 0.6718781064936283
- **t-alpha:** 0.2963553761383701

---

---

247

- **AF ID:** AF-P53779-F1-model-v4 | **Chain:** A
- **b-phipsi:** 0.0006498237986226
- **w-rdist:** 0.7438029883417164
- **t-alpha:** 0.1916901583156363

---

---

248

- **AF ID:** AF-O43143-F1-model-v4 | **Chain:** A
- **b-phipsi:** 0.0154706180513551
- **w-rdist:** 0.3285505112256376
- **t-alpha:** 0.0816167133968897

---

---

249

- **AF ID:** AF-Q8NC26-F1-model-v4 | **Chain:** A
- **b-phipsi:** 0.0040070490482097
- **w-rdist:** 0.277632424872278
- **t-alpha:** 0.3454841126679415

---

---

250

- **AF ID:** AF-Q96MH2-F1-model-v4 | **Chain:** A
- **b-phipsi:** 0.0028267943813394
- **w-rdist:** 1.0916660279180337
- **t-alpha:** 0.0039622395506779

---

---

251

- **AF ID:** AF-Q8NCG7-F1-model-v4 | **Chain:** A
- **b-phipsi:** 0.0438451990444516
- **w-rdist:** 0.474034756121667
- **t-alpha:** 0.0031697813850419

---

---

252

- **AF ID:** AF-Q9UKA2-F1-model-v4 | **Chain:** A
- **b-phipsi:** 0.0189567067599155
- **w-rdist:** 0.813938749335871
- **t-alpha:** 0.0007923402556029

---

---

253

- **AF ID:** AF-B7U540-F1-model-v4 | **Chain:** A
- **b-phipsi:** 0.0004009298872463
- **w-rdist:** 1.0276450964876267
- **t-alpha:** 0.1308241907388465

---

---

254

- **AF ID:** AF-P43365-F1-model-v4 | **Chain:** A
- **b-phipsi:** 0.0005356271777915
- **w-rdist:** 0.7560532628876393
- **t-alpha:** 0.2348334090064667

---

---

255

- **AF ID:** AF-P09016-F1-model-v4 | **Chain:** A
- **b-phipsi:** 0.0110512305680336
- **w-rdist:** 0.8177244641052823
- **t-alpha:** 0.0031697813850419

---

---

256

- **AF ID:** AF-P78396-F1-model-v4 | **Chain:** A
- **b-phipsi:** 0.0008855344512371
- **w-rdist:** 0.4824390844518487
- **t-alpha:** 0.211569305215642

---

---

257

- **AF ID:** AF-Q9UJW2-F1-model-v4 | **Chain:** A
- **b-phipsi:** 0.0007296814804032
- **w-rdist:** 0.8131866534581298
- **t-alpha:** 0.1369365213586904

---

---

258

- **AF ID:** AF-Q13490-F1-model-v4 | **Chain:** A
- **b-phipsi:** 0.0102037548459413
- **w-rdist:** 0.3539541178577495
- **t-alpha:** 0.1061809802151878

---

---

259

- **AF ID:** AF-Q68DD2-F1-model-v4 | **Chain:** A
- **b-phipsi:** 0.0119748407506944
- **w-rdist:** 0.2853171230864522
- **t-alpha:** 0.1529319679525831

---

---

260

- **AF ID:** AF-Q9Y616-F1-model-v4 | **Chain:** A
- **b-phipsi:** 0.0007792626919984
- **w-rdist:** 0.5882249431331962
- **t-alpha:** 0.2187008051406629

---

---

261

- **AF ID:** AF-P0C6A0-F1-model-v4 | **Chain:** A
- **b-phipsi:** 0.0464218784786128
- **w-rdist:** 0.1974030660640569
- **t-alpha:** 0.0569511082581055

---

---

262

- **AF ID:** AF-P0CG32-F1-model-v4 | **Chain:** A
- **b-phipsi:** 0.005847530403018
- **w-rdist:** 0.9595182200309328
- **t-alpha:** 0.0039622395506779

---

---

263

- **AF ID:** AF-Q969E3-F1-model-v4 | **Chain:** A
- **b-phipsi:** 0.0044809419219197
- **w-rdist:** 0.3664526741550536
- **t-alpha:** 0.1894437441175835

---

---

264

- **AF ID:** AF-P18846-F1-model-v4 | **Chain:** A
- **b-phipsi:** 0.0064373750768321
- **w-rdist:** 0.8300342156902322
- **t-alpha:** 0.0055471164626488

---

---

265

- **AF ID:** AF-A2RRH5-F1-model-v4 | **Chain:** A
- **b-phipsi:** 0.0270742115366148
- **w-rdist:** 0.763103170222639
- **t-alpha:** 0.0007923402556029

---

---

266

- **AF ID:** AF-P17483-F1-model-v4 | **Chain:** A
- **b-phipsi:** 0.009114291175125
- **w-rdist:** 0.891436929720079
- **t-alpha:** 0.0031697813850419

---

---

267

- **AF ID:** AF-Q9HB09-F1-model-v4 | **Chain:** A
- **b-phipsi:** 0.0099474952760438
- **w-rdist:** 0.253441087197324
- **t-alpha:** 0.1909670055645516

---

---

268

- **AF ID:** AF-Q6NVV9-F1-model-v4 | **Chain:** A
- **b-phipsi:** 0.0139736452006971
- **w-rdist:** 0.5665710763928575
- **t-alpha:** 0.006339533171912

---

---

269

- **AF ID:** AF-Q5TFG8-F1-model-v4 | **Chain:** A
- **b-phipsi:** 0.0113245131534183
- **w-rdist:** 0.3973815245452332
- **t-alpha:** 0.0395385102655705

---

---

270

- **AF ID:** AF-Q6ZW61-F1-model-v4 | **Chain:** A
- **b-phipsi:** 0.0059846022025401
- **w-rdist:** 0.3418569667321432
- **t-alpha:** 0.2028529548897624

---

---

271

- **AF ID:** AF-Q15043-F1-model-v4 | **Chain:** A
- **b-phipsi:** 0.0673862277356649
- **w-rdist:** 0.6424082181409594
- **t-alpha:** 0.0007928174706921

---

---

272

- **AF ID:** AF-Q8NBF6-F1-model-v4 | **Chain:** A
- **b-phipsi:** 0.0009036696544985
- **w-rdist:** 0.4550875829277042
- **t-alpha:** 0.2282088266060671

---

---

273

- **AF ID:** AF-Q96JP0-F1-model-v4 | **Chain:** A
- **b-phipsi:** 0.0603607097541736
- **w-rdist:** 0.3728724140620887
- **t-alpha:** 0.0055471164626488

---

---

274

- **AF ID:** AF-O43422-F1-model-v4 | **Chain:** A
- **b-phipsi:** 0.0770597838856524
- **w-rdist:** 0.589791913314151
- **t-alpha:** 0.0015848307213084

---

---

275

- **AF ID:** AF-Q9Y5X1-F1-model-v4 | **Chain:** A
- **b-phipsi:** 0.0067546039164436
- **w-rdist:** 0.3094921039701327
- **t-alpha:** 0.2329641617170998

---

---

276

- **AF ID:** AF-Q8TF42-F1-model-v4 | **Chain:** A
- **b-phipsi:** 0.0088765053038106
- **w-rdist:** 0.3593830159940195
- **t-alpha:** 0.1410462561265033

---

---

277

- **AF ID:** AF-Q8NBB4-F1-model-v4 | **Chain:** A
- **b-phipsi:** 0.0015969648958941
- **w-rdist:** 0.9890463155763376
- **t-alpha:** 0.0063793623469805

---

---

278

- **AF ID:** AF-Q8NFF5-F1-model-v4 | **Chain:** A
- **b-phipsi:** 0.006847471492799
- **w-rdist:** 0.2268349055633142
- **t-alpha:** 0.3114112651060119

---

---

279

- **AF ID:** AF-P41214-F1-model-v4 | **Chain:** A
- **b-phipsi:** 0.0009253502190525
- **w-rdist:** 0.5597475896181519
- **t-alpha:** 0.1782885176402775

---

---

280

- **AF ID:** AF-Q8N427-F1-model-v4 | **Chain:** A
- **b-phipsi:** 0.0128074521705304
- **w-rdist:** 0.3847588876378172
- **t-alpha:** 0.0481724164584582

---

---

281

- **AF ID:** AF-O43847-F1-model-v4 | **Chain:** A
- **b-phipsi:** 0.0041296903653066
- **w-rdist:** 0.3541961913986474
- **t-alpha:** 0.2472267294428747

---

---

282

- **AF ID:** AF-Q9NYK6-F1-model-v4 | **Chain:** A
- **b-phipsi:** 0.0619081541471269
- **w-rdist:** 0.277866164200968
- **t-alpha:** 0.0427893647215475

---

---

283

- **AF ID:** AF-Q9BVP2-F1-model-v4 | **Chain:** A
- **b-phipsi:** 0.004018081876621
- **w-rdist:** 0.362998264091959
- **t-alpha:** 0.2337557602519211

---

---

284

- **AF ID:** AF-Q5TC79-F1-model-v4 | **Chain:** A
- **b-phipsi:** 0.0005754650965084
- **w-rdist:** 0.7606442286605181
- **t-alpha:** 0.248812081028487

---

---

285

- **AF ID:** AF-Q96GW9-F1-model-v4 | **Chain:** A
- **b-phipsi:** 0.0167185826184571
- **w-rdist:** 0.750122513282132
- **t-alpha:** 0.003179348403216

---

---

286

- **AF ID:** AF-Q8IYS2-F1-model-v4 | **Chain:** A
- **b-phipsi:** 0.0120079556411614
- **w-rdist:** 0.6857335903636966
- **t-alpha:** 0.0055773242968351

---

---

287

- **AF ID:** AF-Q6ZN11-F1-model-v4 | **Chain:** A
- **b-phipsi:** 0.0176466892052716
- **w-rdist:** 0.3370673985192336
- **t-alpha:** 0.0871633913344154

---

---

288

- **AF ID:** AF-Q96K75-F1-model-v4 | **Chain:** A
- **b-phipsi:** 0.0146383306921349
- **w-rdist:** 0.353607771197881
- **t-alpha:** 0.0919176339506717

---

---

289

- **AF ID:** AF-Q16594-F1-model-v4 | **Chain:** A
- **b-phipsi:** 0.0132106356373932
- **w-rdist:** 0.7973828820569951
- **t-alpha:** 0.003179348403216

---

---

290

- **AF ID:** AF-Q92988-F1-model-v4 | **Chain:** A
- **b-phipsi:** 0.0419664725013619
- **w-rdist:** 0.672482486862321
- **t-alpha:** 0.0015872186884768

---

---

291

- **AF ID:** AF-Q9UJD0-F1-model-v4 | **Chain:** A
- **b-phipsi:** 0.055011449020191
- **w-rdist:** 0.6919976228023185
- **t-alpha:** 0.0007928174706921

---

---

292

- **AF ID:** AF-Q6ZVZ8-F1-model-v4 | **Chain:** A
- **b-phipsi:** 0.0355115687205389
- **w-rdist:** 0.5397508372914488
- **t-alpha:** 0.0039775589155952

---

---

293

- **AF ID:** AF-Q92503-F1-model-v4 | **Chain:** A
- **b-phipsi:** 0.0038579186089451
- **w-rdist:** 1.004924995689508
- **t-alpha:** 0.0055471164626488

---

---

294

- **AF ID:** AF-Q8WZA1-F1-model-v4 | **Chain:** A
- **b-phipsi:** 0.003917488936704
- **w-rdist:** 0.3819477087359084
- **t-alpha:** 0.191758917599204

---

---

295

- **AF ID:** AF-Q5T2E6-F1-model-v4 | **Chain:** A
- **b-phipsi:** 0.1108663413206938
- **w-rdist:** 0.6405319849479554
- **t-alpha:** 0.0007928174706921

---

---

296

- **AF ID:** AF-Q9BSL1-F1-model-v4 | **Chain:** A
- **b-phipsi:** 0.0441902453725926
- **w-rdist:** 0.3358928154963123
- **t-alpha:** 0.0237719193359167

---

---

297

- **AF ID:** AF-P05107-F1-model-v4 | **Chain:** A
- **b-phipsi:** 0.0006567529751353
- **w-rdist:** 1.2600810685778578
- **t-alpha:** 0.0302036962314997

---

---

298

- **AF ID:** AF-P38435-F1-model-v4 | **Chain:** A
- **b-phipsi:** 0.0345687065686922
- **w-rdist:** 0.3116502202471426
- **t-alpha:** 0.0562598552305482

---

---

299

- **AF ID:** AF-Q96GL9-F1-model-v4 | **Chain:** A
- **b-phipsi:** 0.0037011684530823
- **w-rdist:** 0.3585126030872555
- **t-alpha:** 0.2825201129999128

---

---

300

- **AF ID:** AF-Q400G9-F1-model-v4 | **Chain:** A
- **b-phipsi:** 0.0194001511189142
- **w-rdist:** 0.7763716780488906
- **t-alpha:** 0.0023827284528445

---

---

301

- **AF ID:** AF-Q96QD5-F1-model-v4 | **Chain:** A
- **b-phipsi:** 0.0723888681107818
- **w-rdist:** 0.6588413954404382
- **t-alpha:** 0.0015848307213084

---

---

302

- **AF ID:** AF-Q8IVL6-F1-model-v4 | **Chain:** A
- **b-phipsi:** 0.0207779685150598
- **w-rdist:** 0.2935621856225727
- **t-alpha:** 0.1077655736112999

---

---

303

- **AF ID:** AF-Q6GPH6-F1-model-v4 | **Chain:** A
- **b-phipsi:** 0.0475950936253082
- **w-rdist:** 0.329948808228294
- **t-alpha:** 0.0293188630330833

---

---

304

- **AF ID:** AF-Q86U70-F1-model-v4 | **Chain:** A
- **b-phipsi:** 0.0086787189550779
- **w-rdist:** 1.010660348384461
- **t-alpha:** 0.002377470759985

---

---

305

- **AF ID:** AF-Q8WYA6-F1-model-v4 | **Chain:** A
- **b-phipsi:** 0.1081405735106161
- **w-rdist:** 0.6252278473702092
- **t-alpha:** 0.0015848307213084

---

---

306

- **AF ID:** AF-Q9NYL9-F1-model-v4 | **Chain:** A
- **b-phipsi:** 0.033810654880378
- **w-rdist:** 0.738887355178418
- **t-alpha:** 0.0015872186884768

---

---

307

- **AF ID:** AF-P48730-F1-model-v4 | **Chain:** A
- **b-phipsi:** 0.0008181168491638
- **w-rdist:** 0.8596833011679577
- **t-alpha:** 0.1070173333169104

---

---

308

- **AF ID:** AF-Q6IA86-F1-model-v4 | **Chain:** A
- **b-phipsi:** 0.0375162015184376
- **w-rdist:** 0.5153409394608683
- **t-alpha:** 0.0047543655130677

---

---

309

- **AF ID:** AF-Q9BZE2-F1-model-v4 | **Chain:** A
- **b-phipsi:** 0.0092867741594535
- **w-rdist:** 0.7112768857432431
- **t-alpha:** 0.0071316138654509

---

---

310

- **AF ID:** AF-Q8N3Y3-F1-model-v4 | **Chain:** A
- **b-phipsi:** 0.0276065808545298
- **w-rdist:** 0.3760463672940229
- **t-alpha:** 0.0150558135497862

---

---

311

- **AF ID:** AF-P0C2L3-F1-model-v4 | **Chain:** A
- **b-phipsi:** 0.0056269490003355
- **w-rdist:** 0.3181442443138567
- **t-alpha:** 0.3050670582006474

---

---

312

- **AF ID:** AF-Q658Y4-F1-model-v4 | **Chain:** A
- **b-phipsi:** 0.0183909546226262
- **w-rdist:** 0.1749461004497355
- **t-alpha:** 0.1735341942507624

---

---

313

- **AF ID:** AF-P35790-F1-model-v4 | **Chain:** A
- **b-phipsi:** 0.0086903285985853
- **w-rdist:** 0.8279857815099958
- **t-alpha:** 0.0055471164626488

---

---

314

- **AF ID:** AF-Q6ZR03-F1-model-v4 | **Chain:** A
- **b-phipsi:** 0.0360461958003699
- **w-rdist:** 0.6595043551306355
- **t-alpha:** 0.0031697813850419

---

---

315

- **AF ID:** AF-Q93063-F1-model-v4 | **Chain:** A
- **b-phipsi:** 0.0199713549211122
- **w-rdist:** 0.7928711951010904
- **t-alpha:** 0.002377470759985

---

---

316

- **AF ID:** AF-Q3KNW1-F1-model-v4 | **Chain:** A
- **b-phipsi:** 0.0111209806995787
- **w-rdist:** 0.381765937096586
- **t-alpha:** 0.0903328337654447

---

---

317

- **AF ID:** AF-P0CF97-F1-model-v4 | **Chain:** A
- **b-phipsi:** 0.0504050680853041
- **w-rdist:** 0.1394748252432735
- **t-alpha:** 0.09508735985712

---

---

318

- **AF ID:** AF-P24347-F1-model-v4 | **Chain:** A
- **b-phipsi:** 0.018387906885764
- **w-rdist:** 0.5164203191915623
- **t-alpha:** 0.0071316138654509

---

---

319

- **AF ID:** AF-Q9Y2L1-F1-model-v4 | **Chain:** A
- **b-phipsi:** 0.0167600559555125
- **w-rdist:** 0.3483637021822886
- **t-alpha:** 0.1006343630980566

---

---

320

- **AF ID:** AF-P29120-F1-model-v4 | **Chain:** A
- **b-phipsi:** 0.0007559568427954
- **w-rdist:** 0.749638181483046
- **t-alpha:** 0.1965134723742219

---

---

321

- **AF ID:** AF-Q7Z6A9-F1-model-v4 | **Chain:** A
- **b-phipsi:** 0.0063706555085185
- **w-rdist:** 1.0426985022425397
- **t-alpha:** 0.0039622395506779

---

---

322

- **AF ID:** AF-Q13554-F1-model-v4 | **Chain:** A
- **b-phipsi:** 0.0042825927137546
- **w-rdist:** 0.3188136008240041
- **t-alpha:** 0.3954043672258471

---

---

323

- **AF ID:** AF-P04062-F1-model-v4 | **Chain:** A
- **b-phipsi:** 0.0007009386790708
- **w-rdist:** 0.9215401849189264
- **t-alpha:** 0.1410484325015955

---

---

324

- **AF ID:** AF-Q68CJ6-F1-model-v4 | **Chain:** A
- **b-phipsi:** 0.073614390847602
- **w-rdist:** 0.293740589492308
- **t-alpha:** 0.0481724164584582

---

---

325

- **AF ID:** AF-P36578-F1-model-v4 | **Chain:** A
- **b-phipsi:** 0.0005053432270339
- **w-rdist:** 1.4135941766241895
- **t-alpha:** 0.038827437577757

---

---

326

- **AF ID:** AF-Q13237-F1-model-v4 | **Chain:** A
- **b-phipsi:** 0.0050078294843423
- **w-rdist:** 0.3952133168722301
- **t-alpha:** 0.1774960114592363

---

---

327

- **AF ID:** AF-P23921-F1-model-v4 | **Chain:** A
- **b-phipsi:** 0.033575514935731
- **w-rdist:** 0.7015749747890433
- **t-alpha:** 0.0031697813850419

---

---

328

- **AF ID:** AF-Q9NR56-F1-model-v4 | **Chain:** A
- **b-phipsi:** 0.0006021072076731
- **w-rdist:** 0.937167283200322
- **t-alpha:** 0.1679873111070899

---

---

329

- **AF ID:** AF-P43304-F1-model-v4 | **Chain:** A
- **b-phipsi:** 0.0174891622217181
- **w-rdist:** 0.5676880799619815
- **t-alpha:** 0.0071316138654509

---

---

330

- **AF ID:** AF-Q8IYQ7-F1-model-v4 | **Chain:** A
- **b-phipsi:** 0.0221124514433426
- **w-rdist:** 0.6268800652538017
- **t-alpha:** 0.0055471164626488

---

---

331

- **AF ID:** AF-Q6NUI2-F1-model-v4 | **Chain:** A
- **b-phipsi:** 0.0421199061489417
- **w-rdist:** 0.5791117201536232
- **t-alpha:** 0.0039775589155952

---

---

332

- **AF ID:** AF-Q9UKN8-F1-model-v4 | **Chain:** A
- **b-phipsi:** 0.0026131643971368
- **w-rdist:** 0.3993651222623502
- **t-alpha:** 0.2385101300086731

---

---

333

- **AF ID:** AF-Q9P1W3-F1-model-v4 | **Chain:** A
- **b-phipsi:** 0.097808780031512
- **w-rdist:** 0.4358071841487194
- **t-alpha:** 0.0039775589155952

---

---

334

- **AF ID:** AF-Q9UNE2-F1-model-v4 | **Chain:** A
- **b-phipsi:** 0.0159815063853522
- **w-rdist:** 0.7497491662814456
- **t-alpha:** 0.0047768836635659

---

---

335

- **AF ID:** AF-Q9H4K7-F1-model-v4 | **Chain:** A
- **b-phipsi:** 0.0164579723083836
- **w-rdist:** 0.6885316764943855
- **t-alpha:** 0.0055773242968351

---

---

336

- **AF ID:** AF-Q8NFG4-F1-model-v4 | **Chain:** A
- **b-phipsi:** 0.0095939844721858
- **w-rdist:** 1.00980797241428
- **t-alpha:** 0.0023827284528445

---

---

337

- **AF ID:** AF-Q9H5H4-F1-model-v4 | **Chain:** A
- **b-phipsi:** 0.0004973463801487
- **w-rdist:** 0.7736814698835595
- **t-alpha:** 0.3668783508365418

---

---

338

- **AF ID:** AF-O75439-F1-model-v4 | **Chain:** A
- **b-phipsi:** 0.0237293373944538
- **w-rdist:** 0.8598220555807203
- **t-alpha:** 0.0015848307213084

---

---

339

- **AF ID:** AF-Q6ZRI8-F1-model-v4 | **Chain:** A
- **b-phipsi:** 0.0333290829112999
- **w-rdist:** 0.8248512166251574
- **t-alpha:** 0.0007928174706921

---

---

340

- **AF ID:** AF-Q9UJV3-F1-model-v4 | **Chain:** A
- **b-phipsi:** 0.0043630444841687
- **w-rdist:** 1.4728051789826269
- **t-alpha:** 0.0007928174706921

---

---

341

- **AF ID:** AF-Q86WA9-F1-model-v4 | **Chain:** A
- **b-phipsi:** 0.076126392392116
- **w-rdist:** 0.6625988851737769
- **t-alpha:** 0.002377470759985

---

---

342

- **AF ID:** AF-Q59H18-F1-model-v4 | **Chain:** A
- **b-phipsi:** 0.0206548737439362
- **w-rdist:** 0.3316027077091218
- **t-alpha:** 0.1148972678966586

---

---

343

- **AF ID:** AF-Q9BSJ6-F1-model-v4 | **Chain:** A
- **b-phipsi:** 0.0022800530752817
- **w-rdist:** 1.2404006189434174
- **t-alpha:** 0.0055471164626488

---

---

344

- **AF ID:** AF-Q9H825-F1-model-v4 | **Chain:** A
- **b-phipsi:** 0.0069139410355889
- **w-rdist:** 0.8922909740504383
- **t-alpha:** 0.0063793623469805

---

---

345

- **AF ID:** AF-B2RXH8-F1-model-v4 | **Chain:** A
- **b-phipsi:** 0.0063993045086234
- **w-rdist:** 1.0461216554507502
- **t-alpha:** 0.0047543655130677

---

---

346

- **AF ID:** AF-Q9H6E5-F1-model-v4 | **Chain:** A
- **b-phipsi:** 0.0004522448790068
- **w-rdist:** 0.9208436508251348
- **t-alpha:** 0.2456422025040694

---

---

347

- **AF ID:** AF-B1AL88-F1-model-v4 | **Chain:** A
- **b-phipsi:** 0.0093233357004292
- **w-rdist:** 1.0542740461720677
- **t-alpha:** 0.0023827284528445

---

---

348

- **AF ID:** AF-Q6ZNG9-F1-model-v4 | **Chain:** A
- **b-phipsi:** 0.0202302352448827
- **w-rdist:** 0.596043592672553
- **t-alpha:** 0.0063793623469805

---

---

349

- **AF ID:** AF-Q8IYL2-F1-model-v4 | **Chain:** A
- **b-phipsi:** 0.0008739922183238
- **w-rdist:** 0.7966389033871075
- **t-alpha:** 0.1576861790132646

---

---

350

- **AF ID:** AF-Q13508-F1-model-v4 | **Chain:** A
- **b-phipsi:** 0.0185406308052102
- **w-rdist:** 0.7551359406124252
- **t-alpha:** 0.0047768836635659

---

---

351

- **AF ID:** AF-Q96HU1-F1-model-v4 | **Chain:** A
- **b-phipsi:** 0.0579975132528669
- **w-rdist:** 0.3026356528602495
- **t-alpha:** 0.0800319287976243

---

---

352

- **AF ID:** AF-P35626-F1-model-v4 | **Chain:** A
- **b-phipsi:** 0.0195994355485548
- **w-rdist:** 0.3736549222603546
- **t-alpha:** 0.0800319287976243

---

---

353

- **AF ID:** AF-Q86XP0-F1-model-v4 | **Chain:** A
- **b-phipsi:** 0.0160594025679608
- **w-rdist:** 0.3725485312546861
- **t-alpha:** 0.1045960199213078

---

---

354

- **AF ID:** AF-Q8NEG4-F1-model-v4 | **Chain:** A
- **b-phipsi:** 0.0007306291578282
- **w-rdist:** 0.7084159878872853
- **t-alpha:** 0.3201271804060723

---

---

355

- **AF ID:** AF-A8K0S8-F1-model-v4 | **Chain:** A
- **b-phipsi:** 0.0168671889770714
- **w-rdist:** 0.9354574793900698
- **t-alpha:** 0.002377470759985

---

---

356

- **AF ID:** AF-Q13487-F1-model-v4 | **Chain:** A
- **b-phipsi:** 0.0074187905517082
- **w-rdist:** 0.965402364215388
- **t-alpha:** 0.0055471164626488

---

---

357

- **AF ID:** AF-O15091-F1-model-v4 | **Chain:** A
- **b-phipsi:** 0.0253137106422393
- **w-rdist:** 0.3329954004672937
- **t-alpha:** 0.1236136545999804

---

---

358

- **AF ID:** AF-Q8TBH0-F1-model-v4 | **Chain:** A
- **b-phipsi:** 0.0715143729260352
- **w-rdist:** 0.4064441231793303
- **t-alpha:** 0.0055773242968351

---

---

359

- **AF ID:** AF-Q8TD20-F1-model-v4 | **Chain:** A
- **b-phipsi:** 0.0706097246732188
- **w-rdist:** 0.7526735229585226
- **t-alpha:** 0.0015872186884768

---

---

360

- **AF ID:** AF-Q9Y620-F1-model-v4 | **Chain:** A
- **b-phipsi:** 0.0002668772642969
- **w-rdist:** 0.8785180150499816
- **t-alpha:** 0.466719142209856

---

---

361

- **AF ID:** AF-Q9BXK1-F1-model-v4 | **Chain:** A
- **b-phipsi:** 0.013506675109615
- **w-rdist:** 0.388682761262226
- **t-alpha:** 0.1031464059288511

---

---

362

- **AF ID:** AF-Q6NYC1-F1-model-v4 | **Chain:** A
- **b-phipsi:** 0.0007003571388908
- **w-rdist:** 1.0211933267445177
- **t-alpha:** 0.1410484325015955

---

---

363

- **AF ID:** AF-Q96EF0-F1-model-v4 | **Chain:** A
- **b-phipsi:** 0.0085761926881006
- **w-rdist:** 0.402821067489053
- **t-alpha:** 0.1473850735300519

---

---

364

- **AF ID:** AF-Q8N0W4-F1-model-v4 | **Chain:** A
- **b-phipsi:** 0.0006939611738031
- **w-rdist:** 0.8560318689527726
- **t-alpha:** 0.2290020603405205

---

---

365

- **AF ID:** AF-B1APH4-F1-model-v4 | **Chain:** A
- **b-phipsi:** 0.0132445046905499
- **w-rdist:** 0.3715845798748716
- **t-alpha:** 0.1481775708256634

---

---

366

- **AF ID:** AF-A0A1B0GVN3-F1-model-v4 | **Chain:** A
- **b-phipsi:** 0.0131887961095818
- **w-rdist:** 1.039093348466398
- **t-alpha:** 0.002377470759985

---

---

367

- **AF ID:** AF-Q6ZV50-F1-model-v4 | **Chain:** A
- **b-phipsi:** 0.0254952308775309
- **w-rdist:** 0.3739152975146119
- **t-alpha:** 0.0697309103535077

---

---

368

- **AF ID:** AF-P49641-F1-model-v4 | **Chain:** A
- **b-phipsi:** 0.0089710198367585
- **w-rdist:** 0.3939583848257573
- **t-alpha:** 0.1553093824431966

---

---

369

- **AF ID:** AF-Q9Y6A9-F1-model-v4 | **Chain:** A
- **b-phipsi:** 0.0007440853222348
- **w-rdist:** 0.6183154291490754
- **t-alpha:** 0.4882070013677189

---

---

370

- **AF ID:** AF-Q9UNX9-F1-model-v4 | **Chain:** A
- **b-phipsi:** 0.0007482304055033
- **w-rdist:** 0.861052786761433
- **t-alpha:** 0.1860902610364538

---

---

371

- **AF ID:** AF-B2RU33-F1-model-v4 | **Chain:** A
- **b-phipsi:** 0.01486086509001
- **w-rdist:** 0.3543814828957181
- **t-alpha:** 0.1576861790132646

---

---

372

- **AF ID:** AF-Q9Y5A6-F1-model-v4 | **Chain:** A
- **b-phipsi:** 0.0098890490903032
- **w-rdist:** 0.9964384378625478
- **t-alpha:** 0.0039775589155952

---

---

373

- **AF ID:** AF-Q07075-F1-model-v4 | **Chain:** A
- **b-phipsi:** 0.0238185926936121
- **w-rdist:** 0.3142283413268437
- **t-alpha:** 0.1529319679525831

---

---

374

- **AF ID:** AF-P22415-F1-model-v4 | **Chain:** A
- **b-phipsi:** 0.0008679916853999
- **w-rdist:** 1.1389380938749578
- **t-alpha:** 0.0546755105374459

---

---

375

- **AF ID:** AF-Q9UJY4-F1-model-v4 | **Chain:** A
- **b-phipsi:** 0.0003232285932075
- **w-rdist:** 1.0228059561194622
- **t-alpha:** 0.2717908986329644

---

---

376

- **AF ID:** AF-Q7L592-F1-model-v4 | **Chain:** A
- **b-phipsi:** 0.0004037205405899
- **w-rdist:** 0.9643371930728898
- **t-alpha:** 0.2956879455645322

---

---

377

- **AF ID:** AF-Q92670-F1-model-v4 | **Chain:** A
- **b-phipsi:** 0.0312131505195188
- **w-rdist:** 0.2123527340014319
- **t-alpha:** 0.1822511641931448

---

---

378

- **AF ID:** AF-Q8TB05-F1-model-v4 | **Chain:** A
- **b-phipsi:** 0.0098004694607298
- **w-rdist:** 0.3681378258842299
- **t-alpha:** 0.2019046524118539

---

---

379

- **AF ID:** AF-O95786-F1-model-v4 | **Chain:** A
- **b-phipsi:** 0.0380364424253871
- **w-rdist:** 0.2986419251738792
- **t-alpha:** 0.136291565401597

---

---

380

- **AF ID:** AF-Q6NW40-F1-model-v4 | **Chain:** A
- **b-phipsi:** 0.0006829305466464
- **w-rdist:** 0.98340140622956
- **t-alpha:** 0.1706860124558415

---

---

381

- **AF ID:** AF-Q8IV48-F1-model-v4 | **Chain:** A
- **b-phipsi:** 0.0005124432478859
- **w-rdist:** 0.9307196898757388
- **t-alpha:** 0.2877546541077096

---

---

382

- **AF ID:** AF-Q9Y6Y0-F1-model-v4 | **Chain:** A
- **b-phipsi:** 0.0073402572768133
- **w-rdist:** 0.3853008946151801
- **t-alpha:** 0.2020605492028166

---

---

383

- **AF ID:** AF-Q9NXZ2-F1-model-v4 | **Chain:** A
- **b-phipsi:** 0.0176743300473875
- **w-rdist:** 0.2647611783252495
- **t-alpha:** 0.2416796212592216

---

---

384

- **AF ID:** AF-Q15822-F1-model-v4 | **Chain:** A
- **b-phipsi:** 0.01038461493925
- **w-rdist:** 1.3048437612153956
- **t-alpha:** 0.0007923402556029

---

---

385

- **AF ID:** AF-Q8NFI4-F1-model-v4 | **Chain:** A
- **b-phipsi:** 0.0355017634259184
- **w-rdist:** 0.675424650687138
- **t-alpha:** 0.0055471164626488

---

---

386

- **AF ID:** AF-P15144-F1-model-v4 | **Chain:** A
- **b-phipsi:** 0.0262227933007256
- **w-rdist:** 0.3671990141948062
- **t-alpha:** 0.0958797909786817

---

---

387

- **AF ID:** AF-Q7Z7C8-F1-model-v4 | **Chain:** A
- **b-phipsi:** 0.0247214732597763
- **w-rdist:** 0.8407727695229479
- **t-alpha:** 0.003179348403216

---

---

388

- **AF ID:** AF-Q9BZ23-F1-model-v4 | **Chain:** A
- **b-phipsi:** 0.005309051494484
- **w-rdist:** 0.3672971753089151
- **t-alpha:** 0.3763868245684505

---

---

389

- **AF ID:** AF-Q8IVJ1-F1-model-v4 | **Chain:** A
- **b-phipsi:** 0.0888274170136072
- **w-rdist:** 0.8472403852380523
- **t-alpha:** 0.0007923402556029

---

---

390

- **AF ID:** AF-Q96EZ8-F1-model-v4 | **Chain:** A
- **b-phipsi:** 0.0006217058113973
- **w-rdist:** 0.8490513482594724
- **t-alpha:** 0.3335971879649484

---

---

391

- **AF ID:** AF-Q9BW62-F1-model-v4 | **Chain:** A
- **b-phipsi:** 0.0095889674733512
- **w-rdist:** 0.3726430689319434
- **t-alpha:** 0.2028529548897624

---

---

392

- **AF ID:** AF-A6NCN8-F1-model-v4 | **Chain:** A
- **b-phipsi:** 0.0554498816688117
- **w-rdist:** 0.250761671350924
- **t-alpha:** 0.1483166426548656

---

---

393

- **AF ID:** AF-Q9Y6N9-F1-model-v4 | **Chain:** A
- **b-phipsi:** 0.0008590939723736
- **w-rdist:** 0.9175931091364544
- **t-alpha:** 0.1553093824431966

---

---

394

- **AF ID:** AF-O43314-F1-model-v4 | **Chain:** A
- **b-phipsi:** 0.0001896820759626
- **w-rdist:** 0.9425223412650386
- **t-alpha:** 0.523772713724916

---

---

395

- **AF ID:** AF-P14735-F1-model-v4 | **Chain:** A
- **b-phipsi:** 0.0127329425046788
- **w-rdist:** 0.3775298858030264
- **t-alpha:** 0.1576861790132646

---

---

396

- **AF ID:** AF-O60602-F1-model-v4 | **Chain:** A
- **b-phipsi:** 0.0061567897829964
- **w-rdist:** 1.422501764747134
- **t-alpha:** 0.002377470759985

---

---

397

- **AF ID:** AF-Q9Y5Q3-F1-model-v4 | **Chain:** A
- **b-phipsi:** 0.0005816728674173
- **w-rdist:** 1.1399495047449475
- **t-alpha:** 0.1719494022326169

---

---

398

- **AF ID:** AF-P18509-F1-model-v4 | **Chain:** A
- **b-phipsi:** 0.0287285749871685
- **w-rdist:** 0.2482597718629781
- **t-alpha:** 0.1984800336394414

---

---

399

- **AF ID:** AF-Q9Y4K4-F1-model-v4 | **Chain:** A
- **b-phipsi:** 0.005984137657691
- **w-rdist:** 0.3895385255780892
- **t-alpha:** 0.2725831522233162

---

---

400

- **AF ID:** AF-P19878-F1-model-v4 | **Chain:** A
- **b-phipsi:** 0.0051112557718237
- **w-rdist:** 0.3974247048760773
- **t-alpha:** 0.2797149327770958

---

---

401

- **AF ID:** AF-Q8NC42-F1-model-v4 | **Chain:** A
- **b-phipsi:** 0.0036684113155029
- **w-rdist:** 1.3494644856071392
- **t-alpha:** 0.0047768836635659

---

---

402

- **AF ID:** AF-Q96GM5-F1-model-v4 | **Chain:** A
- **b-phipsi:** 0.0002679352851412
- **w-rdist:** 1.251221119147251
- **t-alpha:** 0.2179084126827131

---

---

403

- **AF ID:** AF-P37088-F1-model-v4 | **Chain:** A
- **b-phipsi:** 0.0005662108471713
- **w-rdist:** 1.1899139636683196
- **t-alpha:** 0.1703645180917901

---

---

404

- **AF ID:** AF-O75342-F1-model-v4 | **Chain:** A
- **b-phipsi:** 0.0177618261708232
- **w-rdist:** 0.7818467176859911
- **t-alpha:** 0.006339533171912

---

---

405

- **AF ID:** AF-Q6P179-F1-model-v4 | **Chain:** A
- **b-phipsi:** 0.0197740131192228
- **w-rdist:** 0.3559359533054861
- **t-alpha:** 0.1576861790132646

---

---

406

- **AF ID:** AF-Q04900-F1-model-v4 | **Chain:** A
- **b-phipsi:** 0.0141240578705632
- **w-rdist:** 0.4016703469791747
- **t-alpha:** 0.128801235033463

---

---

407

- **AF ID:** AF-Q9H2H9-F1-model-v4 | **Chain:** A
- **b-phipsi:** 0.1229313660657215
- **w-rdist:** 0.8946940104466727
- **t-alpha:** 0.0

---

---

408

- **AF ID:** AF-Q9Y5K3-F1-model-v4 | **Chain:** A
- **b-phipsi:** 0.0004097018340962
- **w-rdist:** 1.3714082681302473
- **t-alpha:** 0.1504102281981949

---

---

409

- **AF ID:** AF-Q5SRH9-F1-model-v4 | **Chain:** A
- **b-phipsi:** 0.1598120479240671
- **w-rdist:** 0.7846975663802738
- **t-alpha:** 0.0015872186884768

---

---

410

- **AF ID:** AF-O95149-F1-model-v4 | **Chain:** A
- **b-phipsi:** 0.0004408951552603
- **w-rdist:** 0.979181417432944
- **t-alpha:** 0.3298207959494841

---

---

411

- **AF ID:** AF-P48050-F1-model-v4 | **Chain:** A
- **b-phipsi:** 0.0007452535452847
- **w-rdist:** 0.8854777062847022
- **t-alpha:** 0.2445758917588425

---

---

412

- **AF ID:** AF-Q8N8U3-F1-model-v4 | **Chain:** A
- **b-phipsi:** 0.0004134711252119
- **w-rdist:** 1.199966014160445
- **t-alpha:** 0.216324013081756

---

---

413

- **AF ID:** AF-Q6P1Q9-F1-model-v4 | **Chain:** A
- **b-phipsi:** 0.0006247217249318
- **w-rdist:** 0.973587661551188
- **t-alpha:** 0.2458043664560305

---

---

414

- **AF ID:** AF-Q6UXD7-F1-model-v4 | **Chain:** A
- **b-phipsi:** 0.0442625061549074
- **w-rdist:** 0.7264088583319637
- **t-alpha:** 0.0047768836635659

---

---

415

- **AF ID:** AF-Q8NBA8-F1-model-v4 | **Chain:** A
- **b-phipsi:** 0.0007457960459493
- **w-rdist:** 0.8751742397346488
- **t-alpha:** 0.2495048163374138

---

---

416

- **AF ID:** AF-Q76MJ5-F1-model-v4 | **Chain:** A
- **b-phipsi:** 0.0005789984800001
- **w-rdist:** 0.9145231425745778
- **t-alpha:** 0.3652928425047437

---

---

417

- **AF ID:** AF-Q8IXM2-F1-model-v4 | **Chain:** A
- **b-phipsi:** 0.0007304292415727
- **w-rdist:** 0.8225999449234103
- **t-alpha:** 0.3242390766415903

---

---

418

- **AF ID:** AF-Q9UK80-F1-model-v4 | **Chain:** A
- **b-phipsi:** 0.0073603433990051
- **w-rdist:** 0.3839306209321547
- **t-alpha:** 0.2686219740019155

---

---

419

- **AF ID:** AF-Q6NXE6-F1-model-v4 | **Chain:** A
- **b-phipsi:** 0.1311231135672431
- **w-rdist:** 0.7672927263321806
- **t-alpha:** 0.0023827284528445

---

---

420

- **AF ID:** AF-O15228-F1-model-v4 | **Chain:** A
- **b-phipsi:** 0.0426221140742788
- **w-rdist:** 0.8616313372536633
- **t-alpha:** 0.0023827284528445

---

---

421

- **AF ID:** AF-A0A2U3TZM8-F1-model-v4 | **Chain:** A
- **b-phipsi:** 0.0048617224889493
- **w-rdist:** 1.1603383811098942
- **t-alpha:** 0.0071316138654509

---

---

422

- **AF ID:** AF-Q6UWV6-F1-model-v4 | **Chain:** A
- **b-phipsi:** 0.0008172722569814
- **w-rdist:** 0.8741157617716284
- **t-alpha:** 0.2312192658877143

---

---

423

- **AF ID:** AF-O75781-F1-model-v4 | **Chain:** A
- **b-phipsi:** 0.000710484963376
- **w-rdist:** 0.9727803212846444
- **t-alpha:** 0.2218697074286468

---

---

424

- **AF ID:** AF-P35638-F1-model-v4 | **Chain:** A
- **b-phipsi:** 0.0147290716535168
- **w-rdist:** 0.3590022673053157
- **t-alpha:** 0.2111320491404185

---

---

425

- **AF ID:** AF-P53701-F1-model-v4 | **Chain:** A
- **b-phipsi:** 0.0005745922289798
- **w-rdist:** 0.9573628448443892
- **t-alpha:** 0.3284207889118367

---

---

426

- **AF ID:** AF-Q15526-F1-model-v4 | **Chain:** A
- **b-phipsi:** 0.000937836653088
- **w-rdist:** 0.8956924211341573
- **t-alpha:** 0.1588609377563099

---

---

427

- **AF ID:** AF-P57075-F1-model-v4 | **Chain:** A
- **b-phipsi:** 0.0151860990039431
- **w-rdist:** 0.3556108329103055
- **t-alpha:** 0.2187008051406629

---

---

428

- **AF ID:** AF-Q8IYN6-F1-model-v4 | **Chain:** A
- **b-phipsi:** 0.010294563350639
- **w-rdist:** 0.3102032429782818
- **t-alpha:** 0.4179770498892017

---

---

429

- **AF ID:** AF-Q8IYX4-F1-model-v4 | **Chain:** A
- **b-phipsi:** 0.0008080832310071
- **w-rdist:** 0.9554005532630102
- **t-alpha:** 0.1838645641599305

---

---

430

- **AF ID:** AF-P18505-F1-model-v4 | **Chain:** A
- **b-phipsi:** 0.0006648373754386
- **w-rdist:** 1.4218529782634273
- **t-alpha:** 0.083261674299526

---

---

431

- **AF ID:** AF-Q5GH72-F1-model-v4 | **Chain:** A
- **b-phipsi:** 0.0416066717842148
- **w-rdist:** 0.7124712576327488
- **t-alpha:** 0.0055773242968351

---

---

432

- **AF ID:** AF-Q9UL41-F1-model-v4 | **Chain:** A
- **b-phipsi:** 0.0135698175634701
- **w-rdist:** 1.2101862947565265
- **t-alpha:** 0.002377470759985

---

---

433

- **AF ID:** AF-Q8TDQ1-F1-model-v4 | **Chain:** A
- **b-phipsi:** 0.0113490786040768
- **w-rdist:** 1.2697539924525278
- **t-alpha:** 0.002377470759985

---

---

434

- **AF ID:** AF-Q14CW9-F1-model-v4 | **Chain:** A
- **b-phipsi:** 0.0007508926716655
- **w-rdist:** 1.0103652095772004
- **t-alpha:** 0.1806658600500577

---

---

435

- **AF ID:** AF-Q8IV36-F1-model-v4 | **Chain:** A
- **b-phipsi:** 0.0535295960678565
- **w-rdist:** 0.3977180100290308
- **t-alpha:** 0.0451663942458306

---

---

436

- **AF ID:** AF-Q9H972-F1-model-v4 | **Chain:** A
- **b-phipsi:** 0.0006124942011082
- **w-rdist:** 0.910809317736026
- **t-alpha:** 0.3993663345655274

---

---

437

- **AF ID:** AF-O60235-F1-model-v4 | **Chain:** A
- **b-phipsi:** 0.0006577003166098
- **w-rdist:** 0.9230197061278352
- **t-alpha:** 0.3214655154429202

---

---

438

- **AF ID:** AF-Q8N9P0-F1-model-v4 | **Chain:** A
- **b-phipsi:** 0.0433487236503243
- **w-rdist:** 1.04632089705773
- **t-alpha:** 0.0

---

---

439

- **AF ID:** AF-P07237-F1-model-v4 | **Chain:** A
- **b-phipsi:** 0.0133005191807135
- **w-rdist:** 0.3830688851504105
- **t-alpha:** 0.1838645641599305

---

---

440

- **AF ID:** AF-O60812-F1-model-v4 | **Chain:** A
- **b-phipsi:** 0.0119018861784026
- **w-rdist:** 1.0761029636086794
- **t-alpha:** 0.0047543655130677

---

---

441

- **AF ID:** AF-P22570-F1-model-v4 | **Chain:** A
- **b-phipsi:** 0.0008981533382868
- **w-rdist:** 0.9139362978080317
- **t-alpha:** 0.1827552563400034

---

---

442

- **AF ID:** AF-P22735-F1-model-v4 | **Chain:** A
- **b-phipsi:** 0.0108949688381858
- **w-rdist:** 0.3841769539659235
- **t-alpha:** 0.2187008051406629

---

---

443

- **AF ID:** AF-P17980-F1-model-v4 | **Chain:** A
- **b-phipsi:** 0.0269705300800101
- **w-rdist:** 0.9000918726736002
- **t-alpha:** 0.0039775589155952

---

---

444

- **AF ID:** AF-O75382-F1-model-v4 | **Chain:** A
- **b-phipsi:** 0.0008785442159809
- **w-rdist:** 1.258046254270469
- **t-alpha:** 0.0729001783846572

---

---

445

- **AF ID:** AF-Q8TF50-F1-model-v4 | **Chain:** A
- **b-phipsi:** 0.0002790973594427
- **w-rdist:** 1.207935597905467
- **t-alpha:** 0.3011089930050006

---

---

446

- **AF ID:** AF-A6PVC2-F1-model-v4 | **Chain:** A
- **b-phipsi:** 0.0005425706763756
- **w-rdist:** 0.927748796132861
- **t-alpha:** 0.4587955949061209

---

---

447

- **AF ID:** AF-O75190-F1-model-v4 | **Chain:** A
- **b-phipsi:** 0.015387149528427
- **w-rdist:** 0.922410126618158
- **t-alpha:** 0.0055773242968351

---

---

448

- **AF ID:** AF-Q6IN97-F1-model-v4 | **Chain:** A
- **b-phipsi:** 0.025880383577865
- **w-rdist:** 0.9712838839115392
- **t-alpha:** 0.0031697813850419

---

---

449

- **AF ID:** AF-Q6UWJ1-F1-model-v4 | **Chain:** A
- **b-phipsi:** 0.1426403573820158
- **w-rdist:** 0.7801417008156668
- **t-alpha:** 0.0031697813850419

---

---

450

- **AF ID:** AF-Q9UKU9-F1-model-v4 | **Chain:** A
- **b-phipsi:** 0.0006378419474764
- **w-rdist:** 1.2093955149325653
- **t-alpha:** 0.1782885176402775

---

---

451

- **AF ID:** AF-Q495C1-F1-model-v4 | **Chain:** A
- **b-phipsi:** 0.0009357345791044
- **w-rdist:** 0.9416800895821488
- **t-alpha:** 0.1556774385457986

---

---

452

- **AF ID:** AF-P55347-F1-model-v4 | **Chain:** A
- **b-phipsi:** 0.0008944038836235
- **w-rdist:** 0.9692641976583876
- **t-alpha:** 0.1600634400235712

---

---

453

- **AF ID:** AF-Q9ULD6-F1-model-v4 | **Chain:** A
- **b-phipsi:** 0.000750746418895
- **w-rdist:** 0.9066019685192518
- **t-alpha:** 0.2844687774403429

---

---

454

- **AF ID:** AF-Q86WT1-F1-model-v4 | **Chain:** A
- **b-phipsi:** 0.1861619797859199
- **w-rdist:** 0.4050477396165341
- **t-alpha:** 0.0169214728656399

---

---

455

- **AF ID:** AF-Q9HC73-F1-model-v4 | **Chain:** A
- **b-phipsi:** 0.0181411732223179
- **w-rdist:** 1.3270722417005996
- **t-alpha:** 0.0

---

---

456

- **AF ID:** AF-P01854-F1-model-v4 | **Chain:** A
- **b-phipsi:** 0.0415274211849837
- **w-rdist:** 0.7154855125518081
- **t-alpha:** 0.0063793623469805

---

---

457

- **AF ID:** AF-O00329-F1-model-v4 | **Chain:** A
- **b-phipsi:** 0.0243746355235131
- **w-rdist:** 0.3682277896021275
- **t-alpha:** 0.1640252936109762

---

---

458

- **AF ID:** AF-P21589-F1-model-v4 | **Chain:** A
- **b-phipsi:** 0.0007290070878223
- **w-rdist:** 0.8978847893907371
- **t-alpha:** 0.3511772863652134

---

---

459

- **AF ID:** AF-Q8IZK6-F1-model-v4 | **Chain:** A
- **b-phipsi:** 0.0656367668538298
- **w-rdist:** 0.9492838919021884
- **t-alpha:** 0.0015872186884768

---

---

460

- **AF ID:** AF-Q6NSZ9-F1-model-v4 | **Chain:** A
- **b-phipsi:** 0.0008655408287199
- **w-rdist:** 0.9804648237580166
- **t-alpha:** 0.1782885176402775

---

---

461

- **AF ID:** AF-Q9H4S2-F1-model-v4 | **Chain:** A
- **b-phipsi:** 0.0227207330959079
- **w-rdist:** 0.9653246111875216
- **t-alpha:** 0.0039775589155952

---

---

462

- **AF ID:** AF-P42336-F1-model-v4 | **Chain:** A
- **b-phipsi:** 0.0214308948451339
- **w-rdist:** 0.3889789901910279
- **t-alpha:** 0.1481775708256634

---

---

463

- **AF ID:** AF-Q6ZQN5-F1-model-v4 | **Chain:** A
- **b-phipsi:** 0.0220287404126441
- **w-rdist:** 1.0212825086211976
- **t-alpha:** 0.003179348403216

---

---

464

- **AF ID:** AF-Q5HYA8-F1-model-v4 | **Chain:** A
- **b-phipsi:** 0.0125740331800462
- **w-rdist:** 1.27067223897139
- **t-alpha:** 0.0031697813850419

---

---

465

- **AF ID:** AF-Q9Y3A6-F1-model-v4 | **Chain:** A
- **b-phipsi:** 0.0005600189511206
- **w-rdist:** 0.9001563252441275
- **t-alpha:** 0.6871653810492291

---

---

466

- **AF ID:** AF-Q92934-F1-model-v4 | **Chain:** A
- **b-phipsi:** 0.0100944311325393
- **w-rdist:** 0.3970705899491832
- **t-alpha:** 0.2409042399915573

---

---

467

- **AF ID:** AF-P42338-F1-model-v4 | **Chain:** A
- **b-phipsi:** 0.0261913652848064
- **w-rdist:** 0.3785985830321431
- **t-alpha:** 0.1505550567166846

---

---

468

- **AF ID:** AF-P12525-F1-model-v4 | **Chain:** A
- **b-phipsi:** 0.0006640472452515
- **w-rdist:** 1.0539013950592828
- **t-alpha:** 0.2765457422035273

---

---

469

- **AF ID:** AF-Q14651-F1-model-v4 | **Chain:** A
- **b-phipsi:** 0.0602905499637108
- **w-rdist:** 0.8246484850657524
- **t-alpha:** 0.0047543655130677

---

---

470

- **AF ID:** AF-O75541-F1-model-v4 | **Chain:** A
- **b-phipsi:** 0.014726089705119
- **w-rdist:** 0.3757403008271854
- **t-alpha:** 0.2393029254300178

---

---

471

- **AF ID:** AF-O94887-F1-model-v4 | **Chain:** A
- **b-phipsi:** 0.0006529985650451
- **w-rdist:** 0.9539217067180352
- **t-alpha:** 0.4453258100075898

---

---

472

- **AF ID:** AF-Q9HB96-F1-model-v4 | **Chain:** A
- **b-phipsi:** 0.021632020793772
- **w-rdist:** 0.3833963705823922
- **t-alpha:** 0.1648181756590165

---

---

473

- **AF ID:** AF-Q9UPI3-F1-model-v4 | **Chain:** A
- **b-phipsi:** 0.1101433662135243
- **w-rdist:** 0.7871158593036285
- **t-alpha:** 0.0047543655130677

---

---

474

- **AF ID:** AF-Q8WWR8-F1-model-v4 | **Chain:** A
- **b-phipsi:** 0.0340828928780161
- **w-rdist:** 0.7905919010788346
- **t-alpha:** 0.0063793623469805

---

---

475

- **AF ID:** AF-Q86V86-F1-model-v4 | **Chain:** A
- **b-phipsi:** 0.0006804326849537
- **w-rdist:** 1.0808126783629717
- **t-alpha:** 0.2670682661396322

---

---

476

- **AF ID:** AF-Q9NR09-F4-model-v4 | **Chain:** A
- **b-phipsi:** 0.0002970322153566
- **w-rdist:** 1.2640721427672996
- **t-alpha:** 0.3502375919252167

---

---

477

- **AF ID:** AF-Q99575-F1-model-v4 | **Chain:** A
- **b-phipsi:** 0.000405979081913
- **w-rdist:** 1.1362865784247398
- **t-alpha:** 0.4350235484624076

---

---

478

- **AF ID:** AF-O75460-F1-model-v4 | **Chain:** A
- **b-phipsi:** 0.0006101442423695
- **w-rdist:** 0.9952392704278908
- **t-alpha:** 0.4199683390062632

---

---

479

- **AF ID:** AF-Q68CQ4-F1-model-v4 | **Chain:** A
- **b-phipsi:** 0.0155167963545024
- **w-rdist:** 0.3896616443950702
- **t-alpha:** 0.212361041927499

---

---

480

- **AF ID:** AF-P21953-F1-model-v4 | **Chain:** A
- **b-phipsi:** 0.0008654277434872
- **w-rdist:** 0.9794391423198684
- **t-alpha:** 0.2264332322029261

---

---

481

- **AF ID:** AF-Q15256-F1-model-v4 | **Chain:** A
- **b-phipsi:** 0.0006889930401789
- **w-rdist:** 0.9619056397772892
- **t-alpha:** 0.3866885988935387

---

---

482

- **AF ID:** AF-Q86YW5-F1-model-v4 | **Chain:** A
- **b-phipsi:** 0.022573309565677
- **w-rdist:** 1.09534918669582
- **t-alpha:** 0.003179348403216

---

---

483

- **AF ID:** AF-Q9BSA9-F1-model-v4 | **Chain:** A
- **b-phipsi:** 0.1965411527573321
- **w-rdist:** 0.7942415623021339
- **t-alpha:** 0.0039775589155952

---

---

484

- **AF ID:** AF-Q9UL25-F1-model-v4 | **Chain:** A
- **b-phipsi:** 0.000204910551614
- **w-rdist:** 1.0815627452253072
- **t-alpha:** 0.7359009297238215

---

---

485

- **AF ID:** AF-P40855-F1-model-v4 | **Chain:** A
- **b-phipsi:** 0.0898554179061821
- **w-rdist:** 0.3264687269496178
- **t-alpha:** 0.1750466062898956

---

---

486

- **AF ID:** AF-P17098-F1-model-v4 | **Chain:** A
- **b-phipsi:** 0.0008856425541313
- **w-rdist:** 0.9096088924126204
- **t-alpha:** 0.2757534549854332

---

---

487

- **AF ID:** AF-Q8IZQ1-F11-model-v4 | **Chain:** A
- **b-phipsi:** 0.000322085911076
- **w-rdist:** 1.3147887332311954
- **t-alpha:** 0.3375596870792907

---

---

488

- **AF ID:** AF-Q96BY9-F1-model-v4 | **Chain:** A
- **b-phipsi:** 0.0007846138144452
- **w-rdist:** 1.1656519565821246
- **t-alpha:** 0.1849760424819018

---

---

489

- **AF ID:** AF-Q5VZT2-F1-model-v4 | **Chain:** A
- **b-phipsi:** 0.0243794483286518
- **w-rdist:** 0.3392604438908915
- **t-alpha:** 0.2956879455645322

---

---

490

- **AF ID:** AF-Q9H0Z9-F1-model-v4 | **Chain:** A
- **b-phipsi:** 0.0361228380021865
- **w-rdist:** 1.0091559577207274
- **t-alpha:** 0.0039622395506779

---

---

491

- **AF ID:** AF-Q9NPD8-F1-model-v4 | **Chain:** A
- **b-phipsi:** 0.0003707481084546
- **w-rdist:** 1.037518760167728
- **t-alpha:** 0.8779761180303607

---

---

492

- **AF ID:** AF-O75683-F1-model-v4 | **Chain:** A
- **b-phipsi:** 0.0410483435227572
- **w-rdist:** 1.2026876120851624
- **t-alpha:** 0.0015848307213084

---

---

493

- **AF ID:** AF-Q9Y226-F1-model-v4 | **Chain:** A
- **b-phipsi:** 0.1236284506166336
- **w-rdist:** 0.7670010185406659
- **t-alpha:** 0.0055773242968351

---

---

494

- **AF ID:** AF-Q96LD4-F1-model-v4 | **Chain:** A
- **b-phipsi:** 0.0064139544981465
- **w-rdist:** 1.3887811104927883
- **t-alpha:** 0.006339533171912

---

---

495

- **AF ID:** AF-Q09161-F1-model-v4 | **Chain:** A
- **b-phipsi:** 0.1453838494038171
- **w-rdist:** 0.3785598355201523
- **t-alpha:** 0.0974642806534764

---

---

496

- **AF ID:** AF-Q13467-F1-model-v4 | **Chain:** A
- **b-phipsi:** 0.029034945006415
- **w-rdist:** 0.9728476981006602
- **t-alpha:** 0.0047768836635659

---

---

497

- **AF ID:** AF-Q8TEY7-F1-model-v4 | **Chain:** A
- **b-phipsi:** 0.0007761006176444
- **w-rdist:** 0.922726699029841
- **t-alpha:** 0.384310232103257

---

---

498

- **AF ID:** AF-Q05823-F1-model-v4 | **Chain:** A
- **b-phipsi:** 0.0244892346258906
- **w-rdist:** 1.05185233677606
- **t-alpha:** 0.0047543655130677

---

---

499

- **AF ID:** AF-Q8WZA2-F1-model-v4 | **Chain:** A
- **b-phipsi:** 0.0324496932801473
- **w-rdist:** 0.4015828656367373
- **t-alpha:** 0.1481775708256634

---

---

500

- **AF ID:** AF-P56192-F1-model-v4 | **Chain:** A
- **b-phipsi:** 0.0258653571043394
- **w-rdist:** 0.3357945295351754
- **t-alpha:** 0.3114112651060119

---

---

501

- **AF ID:** AF-O75346-F1-model-v4 | **Chain:** A
- **b-phipsi:** 0.0223393472808594
- **w-rdist:** 1.358309894975141
- **t-alpha:** 0.0015848307213084

---

---

502

- **AF ID:** AF-Q8NHK3-F1-model-v4 | **Chain:** A
- **b-phipsi:** 0.0163757319900057
- **w-rdist:** 1.0217725907943238
- **t-alpha:** 0.0063793623469805

---

---

503

- **AF ID:** AF-Q8TEQ6-F1-model-v4 | **Chain:** A
- **b-phipsi:** 0.0004251930623058
- **w-rdist:** 1.279854681308739
- **t-alpha:** 0.3621234439593428

---

---

504

- **AF ID:** AF-A8MYP8-F1-model-v4 | **Chain:** A
- **b-phipsi:** 0.0199528964817011
- **w-rdist:** 1.323760643563621
- **t-alpha:** 0.002377470759985

---

---

505

- **AF ID:** AF-Q14155-F1-model-v4 | **Chain:** A
- **b-phipsi:** 0.00060083529399
- **w-rdist:** 1.0647415480370817
- **t-alpha:** 0.4754355125016942

---

---

506

- **AF ID:** AF-Q2M3M2-F1-model-v4 | **Chain:** A
- **b-phipsi:** 0.0850699644036468
- **w-rdist:** 0.8108227667551069
- **t-alpha:** 0.0055773242968351

---

---

507

- **AF ID:** AF-Q8NI60-F1-model-v4 | **Chain:** A
- **b-phipsi:** 0.0140921261845362
- **w-rdist:** 0.4019102894776923
- **t-alpha:** 0.2456422025040694

---

---

508

- **AF ID:** AF-Q9NS73-F1-model-v4 | **Chain:** A
- **b-phipsi:** 0.0188594757043262
- **w-rdist:** 0.992431065520733
- **t-alpha:** 0.0063793623469805

---

---

509

- **AF ID:** AF-Q8N3J6-F1-model-v4 | **Chain:** A
- **b-phipsi:** 0.0350897869973515
- **w-rdist:** 1.2843727297992953
- **t-alpha:** 0.0015848307213084

---

---

510

- **AF ID:** AF-P35612-F1-model-v4 | **Chain:** A
- **b-phipsi:** 0.000559922288357
- **w-rdist:** 1.2137339291805147
- **t-alpha:** 0.3629160779989906

---

---

511

- **AF ID:** AF-Q6NSI4-F1-model-v4 | **Chain:** A
- **b-phipsi:** 0.0204870889261758
- **w-rdist:** 0.3741708799370609
- **t-alpha:** 0.2614894537379568

---

---

512

- **AF ID:** AF-Q15007-F1-model-v4 | **Chain:** A
- **b-phipsi:** 0.0065577138413876
- **w-rdist:** 1.5796587547921288
- **t-alpha:** 0.0055773242968351

---

---

513

- **AF ID:** AF-Q8TCY9-F1-model-v4 | **Chain:** A
- **b-phipsi:** 0.027625220877143
- **w-rdist:** 0.392068091826229
- **t-alpha:** 0.1806658600500577

---

---

514

- **AF ID:** AF-Q9NVC6-F1-model-v4 | **Chain:** A
- **b-phipsi:** 0.0161202168453083
- **w-rdist:** 1.3379623073218605
- **t-alpha:** 0.003179348403216

---

---

515

- **AF ID:** AF-Q9NRM0-F1-model-v4 | **Chain:** A
- **b-phipsi:** 0.1616095142318154
- **w-rdist:** 0.8196089770076169
- **t-alpha:** 0.0055471164626488

---

---

516

- **AF ID:** AF-Q96LJ8-F1-model-v4 | **Chain:** A
- **b-phipsi:** 0.0215231729126728
- **w-rdist:** 1.0650991537794876
- **t-alpha:** 0.0055471164626488

---

---

517

- **AF ID:** AF-Q4V9L6-F1-model-v4 | **Chain:** A
- **b-phipsi:** 0.0282276846029933
- **w-rdist:** 1.3780150524892454
- **t-alpha:** 0.0007928174706921

---

---

518

- **AF ID:** AF-Q9Y2E4-F1-model-v4 | **Chain:** A
- **b-phipsi:** 0.0004824525984514
- **w-rdist:** 1.2156226890202917
- **t-alpha:** 0.4580028173420525

---

---

519

- **AF ID:** AF-P40394-F1-model-v4 | **Chain:** A
- **b-phipsi:** 0.0005879448653651
- **w-rdist:** 1.1525796583875776
- **t-alpha:** 0.465737532622738

---

---

520

- **AF ID:** AF-Q99748-F1-model-v4 | **Chain:** A
- **b-phipsi:** 0.0144630935688327
- **w-rdist:** 0.383265759417719
- **t-alpha:** 0.3497322693934241

---

---

521

- **AF ID:** AF-Q92806-F1-model-v4 | **Chain:** A
- **b-phipsi:** 0.0007863264829799
- **w-rdist:** 1.0200128398914892
- **t-alpha:** 0.3540771284344679

---

---

522

- **AF ID:** AF-P25791-F1-model-v4 | **Chain:** A
- **b-phipsi:** 0.0004544924413584
- **w-rdist:** 1.1055564766840036
- **t-alpha:** 0.76998573804687

---

---

523

- **AF ID:** AF-Q8IZQ1-F12-model-v4 | **Chain:** A
- **b-phipsi:** 0.0008486929081116
- **w-rdist:** 1.0395512655879455
- **t-alpha:** 0.2995254121653938

---

---

524

- **AF ID:** AF-Q8N5W9-F1-model-v4 | **Chain:** A
- **b-phipsi:** 0.078512519915768
- **w-rdist:** 0.4066131396919545
- **t-alpha:** 0.1207813428706752

---

---

525

- **AF ID:** AF-Q9NP74-F1-model-v4 | **Chain:** A
- **b-phipsi:** 0.0003570844099483
- **w-rdist:** 1.2814213797721945
- **t-alpha:** 0.5689386926056668

---

---

526

- **AF ID:** AF-Q96EG1-F1-model-v4 | **Chain:** A
- **b-phipsi:** 0.0007179173055016
- **w-rdist:** 1.0214575066417328
- **t-alpha:** 0.5259969787842145

---

---

527

- **AF ID:** AF-Q8TD35-F1-model-v4 | **Chain:** A
- **b-phipsi:** 0.0195934812522328
- **w-rdist:** 0.4016024240148816
- **t-alpha:** 0.2360428369658773

---

---

528

- **AF ID:** AF-Q8TEV9-F1-model-v4 | **Chain:** A
- **b-phipsi:** 0.0006506157892462
- **w-rdist:** 1.1383477090522738
- **t-alpha:** 0.4881140424270673

---

---

529

- **AF ID:** AF-O95071-F3-model-v4 | **Chain:** A
- **b-phipsi:** 0.0004687627663912
- **w-rdist:** 1.2506623057558564
- **t-alpha:** 0.5293194114364446

---

---

530

- **AF ID:** AF-Q9UKN7-F12-model-v4 | **Chain:** A
- **b-phipsi:** 0.0002986871549368
- **w-rdist:** 1.2129925851251282
- **t-alpha:** 0.8423132729407334

---

---

531

- **AF ID:** AF-Q8IZ40-F1-model-v4 | **Chain:** A
- **b-phipsi:** 0.0005702342347884
- **w-rdist:** 1.2407783391768956
- **t-alpha:** 0.41283754891727

---

---

532

- **AF ID:** AF-Q14833-F1-model-v4 | **Chain:** A
- **b-phipsi:** 0.0100156375144336
- **w-rdist:** 1.5096852206994948
- **t-alpha:** 0.0047768836635659

---

---

533

- **AF ID:** AF-Q14149-F1-model-v4 | **Chain:** A
- **b-phipsi:** 0.0007524099628046
- **w-rdist:** 1.125708895627283
- **t-alpha:** 0.3209193109685022

---

---

534

- **AF ID:** AF-Q9H7L9-F1-model-v4 | **Chain:** A
- **b-phipsi:** 0.0184627598375558
- **w-rdist:** 1.232173605933622
- **t-alpha:** 0.0055471164626488

---

---

535

- **AF ID:** AF-O00499-F1-model-v4 | **Chain:** A
- **b-phipsi:** 0.0006713264062821
- **w-rdist:** 1.1483387469184565
- **t-alpha:** 0.4191763603580545

---

---

536

- **AF ID:** AF-Q8ND04-F1-model-v4 | **Chain:** A
- **b-phipsi:** 0.0004338535333943
- **w-rdist:** 1.3818319187621255
- **t-alpha:** 0.370840376735208

---

---

537

- **AF ID:** AF-P31785-F1-model-v4 | **Chain:** A
- **b-phipsi:** 0.0129476656931566
- **w-rdist:** 1.409429573529425
- **t-alpha:** 0.0047768836635659

---

---

538

- **AF ID:** AF-Q8N6K0-F1-model-v4 | **Chain:** A
- **b-phipsi:** 0.0007673892472039
- **w-rdist:** 0.9874139199804794
- **t-alpha:** 0.5296964730906049

---

---

539

- **AF ID:** AF-P29475-F1-model-v4 | **Chain:** A
- **b-phipsi:** 0.0004910339001593
- **w-rdist:** 1.3094572440547032
- **t-alpha:** 0.4286848031268502

---

---

540

- **AF ID:** AF-P00540-F1-model-v4 | **Chain:** A
- **b-phipsi:** 0.0008617043610217
- **w-rdist:** 1.060873794610817
- **t-alpha:** 0.302373396636866

---

---

541

- **AF ID:** AF-Q8NFA0-F1-model-v4 | **Chain:** A
- **b-phipsi:** 0.0001149141830945
- **w-rdist:** 1.331777815666818
- **t-alpha:** 0.7076075674057383

---

---

542

- **AF ID:** AF-P28332-F1-model-v4 | **Chain:** A
- **b-phipsi:** 0.0007311657254066
- **w-rdist:** 1.1734876709088948
- **t-alpha:** 0.33403794550915

---

---

543

- **AF ID:** AF-Q9UNI6-F1-model-v4 | **Chain:** A
- **b-phipsi:** 0.0009202654560189
- **w-rdist:** 0.8577879677504308
- **t-alpha:** 0.5874207343328242

---

---

544

- **AF ID:** AF-P29074-F1-model-v4 | **Chain:** A
- **b-phipsi:** 0.0008414104565918
- **w-rdist:** 0.9789228546827958
- **t-alpha:** 0.4619654412063634

---

---

545

- **AF ID:** AF-Q6IQ22-F1-model-v4 | **Chain:** A
- **b-phipsi:** 0.0007417600196032
- **w-rdist:** 1.0439132205154382
- **t-alpha:** 0.5315532336637465

---

---

546

- **AF ID:** AF-Q38SD2-F1-model-v4 | **Chain:** A
- **b-phipsi:** 0.0003582726194237
- **w-rdist:** 1.322223546888606
- **t-alpha:** 0.611727146769057

---

---

547

- **AF ID:** AF-P42262-F1-model-v4 | **Chain:** A
- **b-phipsi:** 0.0080940790668355
- **w-rdist:** 1.4355007555928918
- **t-alpha:** 0.0071316138654509

---

---

548

- **AF ID:** AF-P49585-F1-model-v4 | **Chain:** A
- **b-phipsi:** 0.0110868562713526
- **w-rdist:** 1.302430485092544
- **t-alpha:** 0.0071316138654509

---

---

549

- **AF ID:** AF-Q13255-F1-model-v4 | **Chain:** A
- **b-phipsi:** 0.0001354677877957
- **w-rdist:** 1.494306996995625
- **t-alpha:** 0.5673534620640364

---

---

550

- **AF ID:** AF-P61006-F1-model-v4 | **Chain:** A
- **b-phipsi:** 0.0006301682565359
- **w-rdist:** 1.1136379402522003
- **t-alpha:** 0.7123473729467027

---

---

551

- **AF ID:** AF-Q9H321-F1-model-v4 | **Chain:** A
- **b-phipsi:** 0.0915030846394993
- **w-rdist:** 1.3010360674443324
- **t-alpha:** 0.0015848307213084

---

---

552

- **AF ID:** AF-Q92560-F1-model-v4 | **Chain:** A
- **b-phipsi:** 0.0006988251974816
- **w-rdist:** 1.142105760873665
- **t-alpha:** 0.5372433323173718

---

---

553

- **AF ID:** AF-Q8IZF5-F1-model-v4 | **Chain:** A
- **b-phipsi:** 0.0007105884247832
- **w-rdist:** 1.3495269561173262
- **t-alpha:** 0.2717908986329644

---

---

554

- **AF ID:** AF-E9PJ23-F1-model-v4 | **Chain:** A
- **b-phipsi:** 0.0008359665243495
- **w-rdist:** 1.215663051808587
- **t-alpha:** 0.2646598588873914

---

---

555

- **AF ID:** AF-P09237-F1-model-v4 | **Chain:** A
- **b-phipsi:** 0.0006332641839063
- **w-rdist:** 1.1252378839996893
- **t-alpha:** 0.7406893879673939

---

---

556

- **AF ID:** AF-Q00688-F1-model-v4 | **Chain:** A
- **b-phipsi:** 0.0008801578352116
- **w-rdist:** 1.0521591593554944
- **t-alpha:** 0.3540771284344679

---

---

557

- **AF ID:** AF-P01266-F3-model-v4 | **Chain:** A
- **b-phipsi:** 0.0006682980922872
- **w-rdist:** 1.3006755152554426
- **t-alpha:** 0.3645014287824946

---

---

558

- **AF ID:** AF-P61599-F1-model-v4 | **Chain:** A
- **b-phipsi:** 0.0004376770073943
- **w-rdist:** 1.3090841080675453
- **t-alpha:** 0.6432288110939464

---

---

559

- **AF ID:** AF-Q5SVQ8-F1-model-v4 | **Chain:** A
- **b-phipsi:** 0.0005404091367517
- **w-rdist:** 1.311995425608781
- **t-alpha:** 0.5475442964191188

---

---

560

- **AF ID:** AF-Q96JB1-F17-model-v4 | **Chain:** A
- **b-phipsi:** 0.077966453164319
- **w-rdist:** 1.4353100446006943
- **t-alpha:** 0.0

---

---

561

- **AF ID:** AF-P15104-F1-model-v4 | **Chain:** A
- **b-phipsi:** 0.0009280070345941
- **w-rdist:** 1.1549998904775165
- **t-alpha:** 0.2445758917588425

---

---

562

- **AF ID:** AF-Q8TDB6-F1-model-v4 | **Chain:** A
- **b-phipsi:** 0.0008987842940777
- **w-rdist:** 1.1674807713860784
- **t-alpha:** 0.2741679251567999

---

---

563

- **AF ID:** AF-A3KN83-F1-model-v4 | **Chain:** A
- **b-phipsi:** 0.0007131235595602
- **w-rdist:** 1.0935974577516097
- **t-alpha:** 0.689381632564039

---

---

564

- **AF ID:** AF-Q9BVT8-F1-model-v4 | **Chain:** A
- **b-phipsi:** 0.0009357951684974
- **w-rdist:** 1.0053421231149529
- **t-alpha:** 0.3687632888590515

---

---

565

- **AF ID:** AF-Q13477-F1-model-v4 | **Chain:** A
- **b-phipsi:** 0.0277786882136079
- **w-rdist:** 1.1880597454697477
- **t-alpha:** 0.0055773242968351

---

---

566

- **AF ID:** AF-A1KZ92-F1-model-v4 | **Chain:** A
- **b-phipsi:** 0.0007220802645113
- **w-rdist:** 1.2588462379207142
- **t-alpha:** 0.384310232103257

---

---

567

- **AF ID:** AF-I0J062-F1-model-v4 | **Chain:** A
- **b-phipsi:** 0.0517320161513676
- **w-rdist:** 1.3015278326547337
- **t-alpha:** 0.0031697813850419

---

---

568

- **AF ID:** AF-Q6ZUJ8-F1-model-v4 | **Chain:** A
- **b-phipsi:** 0.0004917091655171
- **w-rdist:** 1.3305940752366667
- **t-alpha:** 0.623613834092273

---

---

569

- **AF ID:** AF-Q92626-F1-model-v4 | **Chain:** A
- **b-phipsi:** 0.0007301135258575
- **w-rdist:** 1.254482873963545
- **t-alpha:** 0.4041213528673766

---

---

570

- **AF ID:** AF-P43234-F1-model-v4 | **Chain:** A
- **b-phipsi:** 0.000813742198652
- **w-rdist:** 1.190611353215313
- **t-alpha:** 0.3822562354456378

---

---

571

- **AF ID:** AF-Q9Y5E3-F1-model-v4 | **Chain:** A
- **b-phipsi:** 0.0212127145939801
- **w-rdist:** 1.8156875949673088
- **t-alpha:** 0.003179348403216

---

---

572

- **AF ID:** AF-Q8TDX9-F6-model-v4 | **Chain:** A
- **b-phipsi:** 0.0003540645736308
- **w-rdist:** 1.615779213254414
- **t-alpha:** 0.6315376830417603

---

---

573

- **AF ID:** AF-O95714-F10-model-v4 | **Chain:** A
- **b-phipsi:** 0.0006655591872003
- **w-rdist:** 1.2485952505964355
- **t-alpha:** 0.6497624904960149

---

---

574

- **AF ID:** AF-Q4V339-F1-model-v4 | **Chain:** A
- **b-phipsi:** 0.0009253372039356
- **w-rdist:** 1.0738985757240165
- **t-alpha:** 0.3777289810409012

---

---

575

- **AF ID:** AF-Q8N394-F1-model-v4 | **Chain:** A
- **b-phipsi:** 0.1677139580521455
- **w-rdist:** 1.0624620807522074
- **t-alpha:** 0.0047768836635659

---

---

576

- **AF ID:** AF-Q92567-F1-model-v4 | **Chain:** A
- **b-phipsi:** 0.0901751760956098
- **w-rdist:** 1.1656299501794822
- **t-alpha:** 0.0047543655130677

---

---

577

- **AF ID:** AF-Q6ZTK2-F1-model-v4 | **Chain:** A
- **b-phipsi:** 0.1113095974980883
- **w-rdist:** 1.4264378749247704
- **t-alpha:** 0.0015848307213084

---

---

578

- **AF ID:** AF-P60673-F1-model-v4 | **Chain:** A
- **b-phipsi:** 0.0004189730226851
- **w-rdist:** 1.3789026803288866
- **t-alpha:** 0.8779761180303607

---

---

579

- **AF ID:** AF-P83731-F1-model-v4 | **Chain:** A
- **b-phipsi:** 0.000728829197183
- **w-rdist:** 1.258634626946572
- **t-alpha:** 0.542786831931056

---

---

580

- **AF ID:** AF-A6NP61-F1-model-v4 | **Chain:** A
- **b-phipsi:** 0.0340757690566426
- **w-rdist:** 0.397978431120488
- **t-alpha:** 0.3082409302776501

---

---

581

- **AF ID:** AF-Q9UGR2-F1-model-v4 | **Chain:** A
- **b-phipsi:** 0.000866570390962
- **w-rdist:** 1.055416853484905
- **t-alpha:** 0.5839939808367314

---

---

582

- **AF ID:** AF-P36776-F1-model-v4 | **Chain:** A
- **b-phipsi:** 0.0007683170745623
- **w-rdist:** 1.1961555077525554
- **t-alpha:** 0.5522979264727486

---

---

583

- **AF ID:** AF-Q12770-F1-model-v4 | **Chain:** A
- **b-phipsi:** 0.0007612072525826
- **w-rdist:** 1.4170707610624316
- **t-alpha:** 0.29080886705517

---

---

584

- **AF ID:** AF-Q9BSJ2-F1-model-v4 | **Chain:** A
- **b-phipsi:** 0.1307970669033412
- **w-rdist:** 1.2816253962157107
- **t-alpha:** 0.0031697813850419

---

---

585

- **AF ID:** AF-Q9NRR4-F1-model-v4 | **Chain:** A
- **b-phipsi:** 0.0007321151612147
- **w-rdist:** 1.115104549073217
- **t-alpha:** 0.8977823782472802

---

---

586

- **AF ID:** AF-Q96DY7-F1-model-v4 | **Chain:** A
- **b-phipsi:** 0.0009252087071276
- **w-rdist:** 1.1056102837593906
- **t-alpha:** 0.3946114588493568

---

---

587

- **AF ID:** AF-A0A1B0GWB2-F1-model-v4 | **Chain:** A
- **b-phipsi:** 0.0319089178064659
- **w-rdist:** 1.2040529112579543
- **t-alpha:** 0.0063793623469805

---

---

588

- **AF ID:** AF-O60244-F1-model-v4 | **Chain:** A
- **b-phipsi:** 0.0003808721154163
- **w-rdist:** 1.4800324700164675
- **t-alpha:** 0.8367678634210749

---

---

589

- **AF ID:** AF-Q9H2S1-F1-model-v4 | **Chain:** A
- **b-phipsi:** 0.0402169469585442
- **w-rdist:** 1.3579394228841826
- **t-alpha:** 0.0047543655130677

---

---

590

- **AF ID:** AF-Q16557-F1-model-v4 | **Chain:** A
- **b-phipsi:** 0.0490202866053399
- **w-rdist:** 1.3429097410552646
- **t-alpha:** 0.0039775589155952

---

---

591

- **AF ID:** AF-Q9BZY9-F1-model-v4 | **Chain:** A
- **b-phipsi:** 0.0367276108274112
- **w-rdist:** 1.3811300650057718
- **t-alpha:** 0.0047543655130677

---

---

592

- **AF ID:** AF-Q9UPW6-F1-model-v4 | **Chain:** A
- **b-phipsi:** 0.0008538861645809
- **w-rdist:** 1.161747097867725
- **t-alpha:** 0.5594304816052347

---

---

593

- **AF ID:** AF-A2IDD5-F1-model-v4 | **Chain:** A
- **b-phipsi:** 0.0367469470598192
- **w-rdist:** 1.2061525896772685
- **t-alpha:** 0.0063793623469805

---

---

594

- **AF ID:** AF-Q15751-F14-model-v4 | **Chain:** A
- **b-phipsi:** 0.0005944197188885
- **w-rdist:** 1.3165546950572338
- **t-alpha:** 0.9841529708914638

---

---

595

- **AF ID:** AF-Q9NZ63-F1-model-v4 | **Chain:** A
- **b-phipsi:** 0.0484549908910799
- **w-rdist:** 1.1086031601694435
- **t-alpha:** 0.0071316138654509

---

---

596

- **AF ID:** AF-P42701-F1-model-v4 | **Chain:** A
- **b-phipsi:** 0.0277476320561604
- **w-rdist:** 1.5284572068463402
- **t-alpha:** 0.0047543655130677

---

---

597

- **AF ID:** AF-Q12967-F1-model-v4 | **Chain:** A
- **b-phipsi:** 0.0008148625073013
- **w-rdist:** 1.143566074732577
- **t-alpha:** 0.7115694148772245

---

---

598

- **AF ID:** AF-Q96A22-F1-model-v4 | **Chain:** A
- **b-phipsi:** 0.0745743320029304
- **w-rdist:** 0.370509601056224
- **t-alpha:** 0.4022218226810459

---

---

599

- **AF ID:** AF-Q9P2K1-F1-model-v4 | **Chain:** A
- **b-phipsi:** 0.0005604714737488
- **w-rdist:** 1.491705992696636
- **t-alpha:** 0.6458008743418924

---

---

600

- **AF ID:** AF-O95625-F1-model-v4 | **Chain:** A
- **b-phipsi:** 0.0006569486937914
- **w-rdist:** 1.3311352250652344
- **t-alpha:** 0.7226622705839278

---

---

601

- **AF ID:** AF-Q502W6-F1-model-v4 | **Chain:** A
- **b-phipsi:** 0.000667275416331
- **w-rdist:** 1.3580313651414602
- **t-alpha:** 0.6347071271748359

---

---

602

- **AF ID:** AF-Q8IYM9-F1-model-v4 | **Chain:** A
- **b-phipsi:** 0.0207709088246905
- **w-rdist:** 1.411194441701582
- **t-alpha:** 0.0063793623469805

---

---

603

- **AF ID:** AF-Q7Z3Y7-F1-model-v4 | **Chain:** A
- **b-phipsi:** 0.0645273645203272
- **w-rdist:** 1.6478789003718086
- **t-alpha:** 0.0031697813850419

---

---

604

- **AF ID:** AF-O95789-F1-model-v4 | **Chain:** A
- **b-phipsi:** 0.0008680456380523
- **w-rdist:** 1.2106693165570108
- **t-alpha:** 0.5570527940205876

---

---

605

- **AF ID:** AF-O95965-F1-model-v4 | **Chain:** A
- **b-phipsi:** 0.0377264118722854
- **w-rdist:** 1.2388804277507186
- **t-alpha:** 0.0071316138654509

---

---

606

- **AF ID:** AF-Q99973-F1-model-v4 | **Chain:** A
- **b-phipsi:** 0.0006201060560536
- **w-rdist:** 1.4277080004804883
- **t-alpha:** 0.7147389688095571

---

---

607

- **AF ID:** AF-Q9UH65-F1-model-v4 | **Chain:** A
- **b-phipsi:** 0.0792196698838467
- **w-rdist:** 1.95731006799676
- **t-alpha:** 0.0023827284528445

---

---

608

- **AF ID:** AF-Q6ZN55-F1-model-v4 | **Chain:** A
- **b-phipsi:** 0.0007780439809997
- **w-rdist:** 1.2601687722413557
- **t-alpha:** 0.6426302859697797

---

---

609

- **AF ID:** AF-P50851-F9-model-v4 | **Chain:** A
- **b-phipsi:** 0.0007979933630302
- **w-rdist:** 1.317176709077704
- **t-alpha:** 0.5808248651240295

---

---

610

- **AF ID:** AF-Q96EK2-F1-model-v4 | **Chain:** A
- **b-phipsi:** 0.0007358655054829
- **w-rdist:** 1.393940208480576
- **t-alpha:** 0.5736935050151362

---

---

611

- **AF ID:** AF-P57729-F1-model-v4 | **Chain:** A
- **b-phipsi:** 0.0009191643154343
- **w-rdist:** 1.1420620733231803
- **t-alpha:** 0.7008083039838859

---

---

612

- **AF ID:** AF-A6PVI3-F1-model-v4 | **Chain:** A
- **b-phipsi:** 0.0007337579105395
- **w-rdist:** 1.311078315803606
- **t-alpha:** 0.8132181751916234

---

---

613

- **AF ID:** AF-Q8N1N0-F1-model-v4 | **Chain:** A
- **b-phipsi:** 0.0779902285172943
- **w-rdist:** 1.810686997605226
- **t-alpha:** 0.003179348403216

---

---

614

- **AF ID:** AF-Q13519-F1-model-v4 | **Chain:** A
- **b-phipsi:** 0.1203737190361784
- **w-rdist:** 0.3780058301325257
- **t-alpha:** 0.4084821110933847

---

---

615

- **AF ID:** AF-O00443-F1-model-v4 | **Chain:** A
- **b-phipsi:** 0.0009142933512977
- **w-rdist:** 1.1690715060421313
- **t-alpha:** 0.6497624904960149

---

---

616

- **AF ID:** AF-A8TX70-F1-model-v4 | **Chain:** A
- **b-phipsi:** 0.0006233363328073
- **w-rdist:** 1.4778239475526698
- **t-alpha:** 1.022187333219076

---

---

617

- **AF ID:** AF-Q9NZ52-F1-model-v4 | **Chain:** A
- **b-phipsi:** 0.00091486905545
- **w-rdist:** 1.1602953457552283
- **t-alpha:** 0.7329641047358588

---

---

618

- **AF ID:** AF-H3BQW9-F1-model-v4 | **Chain:** A
- **b-phipsi:** 0.1309303467561117
- **w-rdist:** 0.366955838309289
- **t-alpha:** 0.5854266908129602

---

---

619

- **AF ID:** AF-Q9Y6X9-F1-model-v4 | **Chain:** A
- **b-phipsi:** 0.0008922763134702
- **w-rdist:** 1.1622934529312012
- **t-alpha:** 0.8827264154398471

---

---

620

- **AF ID:** AF-O43933-F1-model-v4 | **Chain:** A
- **b-phipsi:** 0.0008104149227971
- **w-rdist:** 1.3457943001137294
- **t-alpha:** 0.6030110610207624

---

---

621

- **AF ID:** AF-Q13617-F1-model-v4 | **Chain:** A
- **b-phipsi:** 0.1295338691912587
- **w-rdist:** 1.2787529657504055
- **t-alpha:** 0.0055773242968351

---

---

622

- **AF ID:** AF-Q86XQ3-F1-model-v4 | **Chain:** A
- **b-phipsi:** 0.1972434714186215
- **w-rdist:** 1.1982161166690446
- **t-alpha:** 0.0063793623469805

---

---

623

- **AF ID:** AF-Q8IX01-F1-model-v4 | **Chain:** A
- **b-phipsi:** 0.000651740572776
- **w-rdist:** 1.5586909962813429
- **t-alpha:** 1.0990495394830595

---

---

624

- **AF ID:** AF-Q86XL3-F1-model-v4 | **Chain:** A
- **b-phipsi:** 0.0009073438383822
- **w-rdist:** 1.2507820576854478
- **t-alpha:** 0.6703654752684229

---

---

625

- **AF ID:** AF-P21817-F1-model-v4 | **Chain:** A
- **b-phipsi:** 0.0008916683830028
- **w-rdist:** 1.4121427309673356
- **t-alpha:** 0.4033288445166683

---

---

626

- **AF ID:** AF-Q9P203-F1-model-v4 | **Chain:** A
- **b-phipsi:** 0.0009212178635505
- **w-rdist:** 1.1994779865957117
- **t-alpha:** 0.8034863986423078

---

---

627

- **AF ID:** AF-P41567-F1-model-v4 | **Chain:** A
- **b-phipsi:** 0.0008311106430549
- **w-rdist:** 1.267970655393684
- **t-alpha:** 1.3413720293711875

---

---

628

- **AF ID:** AF-Q9GZZ8-F1-model-v4 | **Chain:** A
- **b-phipsi:** 0.060241631607658
- **w-rdist:** 0.3964933299439543
- **t-alpha:** 0.591424695782957

---

---

629

- **AF ID:** AF-Q14690-F1-model-v4 | **Chain:** A
- **b-phipsi:** 0.000820404812846
- **w-rdist:** 1.3844462536388589
- **t-alpha:** 0.6877970342305582

---

---

630

- **AF ID:** AF-Q16787-F5-model-v4 | **Chain:** A
- **b-phipsi:** 0.0008820302005016
- **w-rdist:** 1.4109127963080923
- **t-alpha:** 0.5586380114023168

---

---

631

- **AF ID:** AF-P27708-F1-model-v4 | **Chain:** A
- **b-phipsi:** 0.0009273402054803
- **w-rdist:** 1.3210044512247694
- **t-alpha:** 0.6022193638398652

---

---

632

- **AF ID:** AF-Q53HC0-F1-model-v4 | **Chain:** A
- **b-phipsi:** 0.0373457517005085
- **w-rdist:** 1.5897930553714337
- **t-alpha:** 0.0071316138654509

---

---

633

- **AF ID:** AF-P14174-F1-model-v4 | **Chain:** A
- **b-phipsi:** 0.000818554501319
- **w-rdist:** 1.358622770047154
- **t-alpha:** 1.1833906677903665

---

---

634

- **AF ID:** AF-Q12805-F1-model-v4 | **Chain:** A
- **b-phipsi:** 0.0466560062780717
- **w-rdist:** 1.436690947421856
- **t-alpha:** 0.0071316138654509

---

---

635

- **AF ID:** AF-P10163-F1-model-v4 | **Chain:** A
- **b-phipsi:** 0.1163040615418305
- **w-rdist:** 1.4709275987880577
- **t-alpha:** 0.0055773242968351

---

---

636

- **AF ID:** AF-Q13616-F1-model-v4 | **Chain:** A
- **b-phipsi:** 0.10744926345611
- **w-rdist:** 1.346936506775755
- **t-alpha:** 0.0071316138654509

---

---

637

- **AF ID:** AF-P35080-F1-model-v4 | **Chain:** A
- **b-phipsi:** 0.0008602958571572
- **w-rdist:** 1.3763468362457258
- **t-alpha:** 0.929662843081562

---

---

638

- **AF ID:** AF-Q2M1K9-F1-model-v4 | **Chain:** A
- **b-phipsi:** 0.0008335871457919
- **w-rdist:** 1.4204698948545675
- **t-alpha:** 0.896989822343039

---

---

639

- **AF ID:** AF-Q8NCM8-F16-model-v4 | **Chain:** A
- **b-phipsi:** 0.0830646263294135
- **w-rdist:** 1.560439462284413
- **t-alpha:** 0.006339533171912

---

---

640

- **AF ID:** AF-P50851-F8-model-v4 | **Chain:** A
- **b-phipsi:** 0.0009163740701394
- **w-rdist:** 1.4896517565781258
- **t-alpha:** 0.5935030253306877

---

---

641

- **AF ID:** AF-Q9Y4D8-F10-model-v4 | **Chain:** A
- **b-phipsi:** 0.0009088034351493
- **w-rdist:** 1.433932979901669
- **t-alpha:** 0.700475012110326

---

---

642

- **AF ID:** AF-A4UGR9-F4-model-v4 | **Chain:** A
- **b-phipsi:** 0.0008495096920881
- **w-rdist:** 1.6126019419088236
- **t-alpha:** 0.9595880264431966

---

---

643

- **AF ID:** AF-P08727-F1-model-v4 | **Chain:** A
- **b-phipsi:** 0.1364096958156361
- **w-rdist:** 1.6321365228740798
- **t-alpha:** 0.0063793623469805

---

---

644

- **AF ID:** AF-Q8NFP9-F8-model-v4 | **Chain:** A
- **b-phipsi:** 0.0009498441647088
- **w-rdist:** 1.537166967046231
- **t-alpha:** 0.7480189598651505

---

---
