# Supplementary material for: Comparative structural insights and functional analysis for the distinct unbound states of Human AGO proteins
Source: Sci Rep. 2025 Mar 19;15:9432. doi: 10.1038/s41598-025-91849-5 (PMC11923369; doi:10.1038/s41598-025-91849-5)
Supplement: Supplementary file 24 — Supplementary Information 12. [file 41598_2025_91849_MOESM24_ESM.zip › 5VM9p_A_mdwhole_AF4REF/go/5VM9p_A_mitot_mitosis_81f45c35dd7b4ca39e19a5166202d846-pres_report.html]

 

# Structural Comparison Report for 5VM9p\_A - whole structures (total: 3)

---

1

- **Protein name:** Zinc finger protein DPF3
- **Organism:** Homo sapiens
- **Uniprot Accession Number:** Q92784
- **Protein sequence length:** 378 aa
- **1D identity (%):** 8.67
- **1D identity (%) [Gaps excluded]:** 22.0
- **1D identity - Alignment Gaps:** 538
- **1D aligned content (<aminoacid>:%):** {'A': 10.39, 'T': 3.9, 'Q': 3.9, 'F': 2.6, 'E': 3.9, 'H': 1.3, 'R': 12.99, 'S': 2.6, 'G': 15.58, 'V': 3.9, 'C': 5.19, 'M': 1.3, 'P': 10.39, 'K': 5.19, 'L': 10.39, 'N': 3.9, 'D': 1.3, 'I': 1.3}
- **Common reported functions (%):** 0.0
- **Common reported locations (%):** 11.11
- **Common reported processes (%):** 0.0

- **AF ID:** Q92784
- **Chain:** A
- **Protein length:** 378 aa
- **Resolution:** N/A
- **b-phipsi ():** 0.00028
- **w-rdist ():** 0.371153
- **t-alpha ():** 0.058637
- **Chemical similarity (Tanimoto Index) (%):** 99.62
- **1D identity (%) [PDB]:** 1.5
- **1D identity (%) [Gaps excluded][PDB]:** 72.0
- **1D identity - Alignment Gaps [PDB]:** 1173
- **1D aligned content [PDB] (<aminoacid>:%):** {'Y': 5.56, 'I': 5.56, 'K': 5.56, 'R': 11.11, 'H': 11.11, 'T': 5.56, 'L': 5.56, 'C': 5.56, 'A': 11.11, 'D': 5.56, 'G': 11.11, 'S': 5.56, 'N': 5.56, 'P': 5.56}
- **2D identity (%) [PDB]:** 26.01
- **2D identity (%) [Gaps excluded][PDB]:** 85.61
- **2D identity - Alignment Gaps [PDB]:** 653
- **2D aligned content [PDB] (<2D-fold>:%):** {'.': 24.18, 'H': 34.43, 'E': 8.2, 'T': 30.33, 'G': 2.46, 'B': 0.41}
- **3D similarity (TM-Score) (%) [PDB]:** 14.18

- **Gene name:** DPF3
- **Entrez ID:** 811000
- **RefSeq ID:** N/A
- **Sequence length:** N/A
- **5-UTR|CDS|3-UTR identity (%):** N/A | N/A | N/A
- **5-UTR|CDS|3-UTR identity (%) [Gaps excluded]:** N/A | N/A | N/A
- **5-UTR|CDS|3-UTR identity [Alignment Gaps]:** N/A | N/A | N/A
- **5-UTR aligned content (<base>:%):** N/A
- **CDS aligned content (<base>:%):** N/A
- **3-UTR aligned content (<base>:%):** N/A

**Uniprot Description:**  
  
 Belongs to the neuron-specific chromatin remodeling complex (nBAF complex). During neural development a switch from a stem/progenitor to a post-mitotic chromatin remodeling mechanism occurs as neurons exit the cell cycle and become committed to their adult state. The transition from proliferating neural stem/progenitor cells to post-mitotic neurons requires a switch in subunit composition of the npBAF and nBAF complexes. As neural progenitors exit mitosis and differentiate into neurons, npBAF complexes which contain ACTL6A/BAF53A and PHF10/BAF45A, are exchanged for homologous alternative ACTL6B/BAF53B and DPF1/BAF45B or DPF3/BAF45C subunits in neuron-specific complexes (nBAF). The npBAF complex is essential for the self-renewal/proliferative capacity of the multipotent neural stem cells. The nBAF complex along with CREST plays a role regulating the activity of genes essential for dendrite growth (By similarity). Muscle-specific component of the BAF complex, a multiprotein complex involved in transcriptional activation and repression of select genes by chromatin remodeling (alteration of DNA-nucleosome topology). Specifically binds acetylated lysines on histone 3 and 4 (H3K14ac, H3K9ac, H4K5ac, H4K8ac, H4K12ac, H4K16ac). In the complex, it acts as a tissue-specific anchor between histone acetylations and methylations and chromatin remodeling. It thereby probably plays an essential role in heart and skeletal muscle development.   
  
Component of the BAF complex, which includes at least actin (ACTB), ARID1A, ARID1B/BAF250, SMARCA2, SMARCA4/BRG1/BAF190A, ACTL6A/BAF53, ACTL6B/BAF53B, SMARCE1/BAF57, SMARCC1/BAF155, SMARCC2/BAF170, SMARCB1/SNF5/INI1, and one or more of SMARCD1/BAF60A, SMARCD2/BAF60B, or SMARCD3/BAF60C (PubMed:18765789). In muscle cells, the BAF complex also contains DPF3 (PubMed:18765789). Interacts with acetylated histones H3 and H4 (PubMed:18765789). Component of neuron-specific chromatin remodeling complex (nBAF complex) composed of at least, ARID1A/BAF250A or ARID1B/BAF250B, SMARCD1/BAF60A, SMARCD3/BAF60C, SMARCA2/BRM/BAF190B, SMARCA4/BRG1/BAF190A, SMARCB1/BAF47, SMARCC1/BAF155, SMARCE1/BAF57, SMARCC2/BAF170, DPF1/BAF45B, DPF3/BAF45C, ACTL6B/BAF53B and actin (By similarity).   
  
 **Gene Ontology Information:**

Molecular Function

- zinc ion binding

Location

- brahma complex
- chromatin
- nBAF complex
- nucleoplasm
- SWI/SNF complex

Biological process

- chromatin remodeling
- muscle organ development
- nervous system development
- positive regulation of cell differentiation
- positive regulation of double-strand break repair
- positive regulation of myoblast differentiation
- regulation of G0 to G1 transition
- regulation of G1/S transition of mitotic cell cycle
- regulation of mitotic metaphase/anaphase transition
- regulation of nucleotide-excision repair
- regulation of transcription by RNA polymerase II

---

2

- **Protein name:** Serine/threonine-protein kinase PLK3
- **Organism:** Homo sapiens
- **Uniprot Accession Number:** Q9H4B4
- **Protein sequence length:** 646 aa
- **1D identity (%):** 12.26
- **1D identity (%) [Gaps excluded]:** 22.24
- **1D identity - Alignment Gaps:** 436
- **1D aligned content (<aminoacid>:%):** {'P': 13.45, 'F': 5.04, 'R': 13.45, 'G': 7.56, 'L': 12.61, 'T': 4.2, 'K': 5.04, 'V': 5.88, 'E': 4.2, 'H': 3.36, 'Y': 2.52, 'C': 4.2, 'N': 0.84, 'D': 5.04, 'Q': 3.36, 'W': 0.84, 'A': 4.2, 'I': 0.84, 'S': 3.36}
- **Common reported functions (%):** 0.0
- **Common reported locations (%):** 22.22
- **Common reported processes (%):** 0.0

- **AF ID:** Q9H4B4
- **Chain:** A
- **Protein length:** 646 aa
- **Resolution:** N/A
- **b-phipsi ():** 0.000435
- **w-rdist ():** 0.271844
- **t-alpha ():** 0.130745
- **Chemical similarity (Tanimoto Index) (%):** 97.33
- **1D identity (%) [PDB]:** 1.79
- **1D identity (%) [Gaps excluded][PDB]:** 74.29
- **1D identity - Alignment Gaps [PDB]:** 1421
- **1D aligned content [PDB] (<aminoacid>:%):** {'P': 19.23, 'A': 15.38, 'G': 11.54, 'K': 7.69, 'S': 7.69, 'I': 3.85, 'V': 15.38, 'D': 3.85, 'C': 3.85, 'T': 3.85, 'Q': 3.85, 'R': 3.85}
- **2D identity (%) [PDB]:** 40.23
- **2D identity (%) [Gaps excluded][PDB]:** 92.48
- **2D identity - Alignment Gaps [PDB]:** 587
- **2D aligned content [PDB] (<2D-fold>:%):** {'.': 19.14, 'T': 22.01, 'H': 34.45, 'E': 24.4}
- **3D similarity (TM-Score) (%) [PDB]:** 20.74

- **Gene name:** PLK3
- **Entrez ID:** 126300
- **RefSeq ID:** NM\_004073
- **Transcript sequence length:** 2340
- **5-UTR|CDS|3-UTR identity (%):** 15.87 | 39.07 | 1.22
- **5-UTR|CDS|3-UTR identity (%) [Gaps excluded]:** 70.67 | 72.94 | 74.28
- **5-UTR|CDS|3-UTR identity [Alignment Gaps]:** 259 | 1368 | 16515
- **5-UTR aligned content (<base>:%):** {'C': 47.17, 'A': 7.55, 'G': 45.28}
- **CDS aligned content (<base>:%):** {'A': 22.5, 'T': 20.59, 'G': 28.32, 'C': 28.58}
- **3-UTR aligned content (<base>:%):** {'G': 23.41, 'C': 30.24, 'T': 29.76, 'A': 16.59}

**Uniprot Description:**  
  
 Serine/threonine-protein kinase involved in cell cycle regulation, response to stress and Golgi disassembly. Polo-like kinases act by binding and phosphorylating proteins are that already phosphorylated on a specific motif recognized by the POLO box domains. Phosphorylates ATF2, BCL2L1, CDC25A, CDC25C, CHEK2, HIF1A, JUN, p53/TP53, p73/TP73, PTEN, TOP2A and VRK1. Involved in cell cycle regulation: required for entry into S phase and cytokinesis. Phosphorylates BCL2L1, leading to regulate the G2 checkpoint and progression to cytokinesis during mitosis. Plays a key role in response to stress: rapidly activated upon stress stimulation, such as ionizing radiation, reactive oxygen species (ROS), hyperosmotic stress, UV irradiation and hypoxia. Involved in DNA damage response and G1/S transition checkpoint by phosphorylating CDC25A, p53/TP53 and p73/TP73. Phosphorylates p53/TP53 in response to reactive oxygen species (ROS), thereby promoting p53/TP53-mediated apoptosis. Phosphorylates CHEK2 in response to DNA damage, promoting the G2/M transition checkpoint. Phosphorylates the transcription factor p73/TP73 in response to DNA damage, leading to inhibit p73/TP73-mediated transcriptional activation and pro-apoptotic functions. Phosphorylates HIF1A and JUN is response to hypoxia. Phosphorylates ATF2 following hyperosmotic stress in corneal epithelium. Also involved in Golgi disassembly during the cell cycle: part of a MEK1/MAP2K1-dependent pathway that induces Golgi fragmentation during mitosis by mediating phosphorylation of VRK1. May participate in endomitotic cell cycle, a form of mitosis in which both karyokinesis and cytokinesis are interrupted and is a hallmark of megakaryocyte differentiation, via its interaction with CIB1.   
  
Interacts (via the POLO-box domain) with CIB1; leading to inhibit PLK3 kinase activity. Interacts with GOLGB1.   
  
 **Gene Ontology Information:**

Molecular Function

- ATP binding
- p53 binding
- protein serine kinase activity
- protein serine/threonine kinase activity

Location

- centrosome
- cytoplasm
- dendrite
- Golgi stack
- kinetochore
- neuronal cell body
- nucleolus
- nucleoplasm
- nucleus
- spindle pole

Biological process

- apoptotic process
- cytoplasmic microtubule organization
- cellular response to DNA damage stimulus
- DNA damage response, signal transduction by p53 class mediator resulting in cell cycle arrest
- endomitotic cell cycle
- G1/S transition of mitotic cell cycle
- G2/M transition of mitotic cell cycle
- Golgi disassembly
- mitotic G1/S transition checkpoint
- mitotic spindle organization
- negative regulation of apoptotic process
- negative regulation of transcription by RNA polymerase II
- positive regulation of chaperone-mediated autophagy
- positive regulation of intracellular protein transport
- positive regulation of proteasomal ubiquitin-dependent protein catabolic process involved in cellular response to hypoxia
- protein kinase B signaling
- protein phosphorylation
- regulation of cell division
- regulation of cytokinesis
- regulation of signal transduction by p53 class mediator
- response to osmotic stress
- response to radiation
- response to reactive oxygen species

---

3

- **Protein name:** Protein kinase C alpha type
- **Organism:** Homo sapiens
- **Uniprot Accession Number:** P17252
- **Protein sequence length:** 672 aa
- **1D identity (%):** 12.11
- **1D identity (%) [Gaps excluded]:** 21.68
- **1D identity - Alignment Gaps:** 434
- **1D aligned content (<aminoacid>:%):** {'M': 1.68, 'V': 10.08, 'D': 5.88, 'N': 5.04, 'L': 8.4, 'H': 5.04, 'K': 5.88, 'I': 3.36, 'T': 5.04, 'C': 5.04, 'G': 9.24, 'F': 5.04, 'R': 7.56, 'Y': 4.2, 'A': 2.52, 'E': 3.36, 'P': 6.72, 'W': 1.68, 'Q': 2.52, 'S': 1.68}
- **Common reported functions (%):** 0.0
- **Common reported locations (%):** 33.33
- **Common reported processes (%):** 0.0

- **AF ID:** P17252
- **Chain:** A
- **Protein length:** 672 aa
- **Resolution:** N/A
- **b-phipsi ():** 0.000369
- **w-rdist ():** 0.684324
- **t-alpha ():** 0.131838
- **Chemical similarity (Tanimoto Index) (%):** 99.25
- **1D identity (%) [PDB]:** 1.89
- **1D identity (%) [Gaps excluded][PDB]:** 73.68
- **1D identity - Alignment Gaps [PDB]:** 1441
- **1D aligned content [PDB] (<aminoacid>:%):** {'M': 7.14, 'G': 3.57, 'K': 10.71, 'Q': 10.71, 'F': 10.71, 'T': 7.14, 'V': 3.57, 'E': 10.71, 'I': 10.71, 'A': 10.71, 'R': 7.14, 'D': 3.57, 'L': 3.57}
- **2D identity (%) [PDB]:** 42.29
- **2D identity (%) [Gaps excluded][PDB]:** 89.71
- **2D identity - Alignment Gaps [PDB]:** 545
- **2D aligned content [PDB] (<2D-fold>:%):** {'.': 16.97, 'H': 26.83, 'T': 34.63, 'E': 20.18, 'G': 1.38}
- **3D similarity (TM-Score) (%) [PDB]:** 21.27

- **Gene name:** PRKCA
- **Entrez ID:** 557800
- **RefSeq ID:** NM\_002737
- **Transcript sequence length:** 8964
- **5-UTR|CDS|3-UTR identity (%):** 42.35 | 43.11 | 24.63
- **5-UTR|CDS|3-UTR identity (%) [Gaps excluded]:** 79.49 | 75.04 | 75.09
- **5-UTR|CDS|3-UTR identity [Alignment Gaps]:** 171 | 1244 | 11873
- **5-UTR aligned content (<base>:%):** {'G': 29.68, 'T': 7.1, 'A': 5.16, 'C': 58.06}
- **CDS aligned content (<base>:%):** {'T': 23.97, 'G': 24.21, 'C': 21.43, 'A': 30.4}
- **3-UTR aligned content (<base>:%):** {'A': 28.22, 'C': 20.68, 'G': 21.14, 'T': 29.95}

**Uniprot Description:**  
  
 Calcium-activated, phospholipid- and diacylglycerol (DAG)-dependent serine/threonine-protein kinase that is involved in positive and negative regulation of cell proliferation, apoptosis, differentiation, migration and adhesion, tumorigenesis, cardiac hypertrophy, angiogenesis, platelet function and inflammation, by directly phosphorylating targets such as RAF1, BCL2, CSPG4, TNNT2/CTNT, or activating signaling cascade involving MAPK1/3 (ERK1/2) and RAP1GAP. Involved in cell proliferation and cell growth arrest by positive and negative regulation of the cell cycle. Can promote cell growth by phosphorylating and activating RAF1, which mediates the activation of the MAPK/ERK signaling cascade, and/or by up-regulating CDKN1A, which facilitates active cyclin-dependent kinase (CDK) complex formation in glioma cells. In intestinal cells stimulated by the phorbol ester PMA, can trigger a cell cycle arrest program which is associated with the accumulation of the hyper-phosphorylated growth-suppressive form of RB1 and induction of the CDK inhibitors CDKN1A and CDKN1B. Exhibits anti-apoptotic function in glioma cells and protects them from apoptosis by suppressing the p53/TP53-mediated activation of IGFBP3, and in leukemia cells mediates anti-apoptotic action by phosphorylating BCL2. During macrophage differentiation induced by macrophage colony-stimulating factor (CSF1), is translocated to the nucleus and is associated with macrophage development. After wounding, translocates from focal contacts to lamellipodia and participates in the modulation of desmosomal adhesion. Plays a role in cell motility by phosphorylating CSPG4, which induces association of CSPG4 with extensive lamellipodia at the cell periphery and polarization of the cell accompanied by increases in cell motility. During chemokine-induced CD4(+) T cell migration, phosphorylates CDC42-guanine exchange factor DOCK8 resulting in its dissociation from LRCH1 and the activation of GTPase CDC42 (PubMed:28028151). Is highly expressed in a number of cancer cells where it can act as a tumor promoter and is implicated in malignant phenotypes of several tumors such as gliomas and breast cancers. Negatively regulates myocardial contractility and positively regulates angiogenesis, platelet aggregation and thrombus formation in arteries. Mediates hypertrophic growth of neonatal cardiomyocytes, in part through a MAPK1/3 (ERK1/2)-dependent signaling pathway, and upon PMA treatment, is required to induce cardiomyocyte hypertrophy up to heart failure and death, by increasing protein synthesis, protein-DNA ratio and cell surface area. Regulates cardiomyocyte function by phosphorylating cardiac troponin T (TNNT2/CTNT), which induces significant reduction in actomyosin ATPase activity, myofilament calcium sensitivity and myocardial contractility. In angiogenesis, is required for full endothelial cell migration, adhesion to vitronectin (VTN), and vascular endothelial growth factor A (VEGFA)-dependent regulation of kinase activation and vascular tube formation. Involved in the stabilization of VEGFA mRNA at post-transcriptional level and mediates VEGFA-induced cell proliferation. In the regulation of calcium-induced platelet aggregation, mediates signals from the CD36/GP4 receptor for granule release, and activates the integrin heterodimer ITGA2B-ITGB3 through the RAP1GAP pathway for adhesion. During response to lipopolysaccharides (LPS), may regulate selective LPS-induced macrophage functions involved in host defense and inflammation. But in some inflammatory responses, may negatively regulate NF-kappa-B-induced genes, through IL1A-dependent induction of NF-kappa-B inhibitor alpha (NFKBIA/IKBA). Upon stimulation with 12-O-tetradecanoylphorbol-13-acetate (TPA), phosphorylates EIF4G1, which modulates EIF4G1 binding to MKNK1 and may be involved in the regulation of EIF4E phosphorylation. Phosphorylates KIT, leading to inhibition of KIT activity. Phosphorylates ATF2 which promotes cooperation between ATF2 and JUN, activating transcription. Phosphorylates SOCS2 at 'Ser-52' facilitating its ubiquitination and proteosomal degradation (By similarity).   
  
Recruited in a circadian manner into a nuclear complex which also includes BMAL1 and RACK1 (By similarity). Interacts with ADAP1/CENTA1 (PubMed:12893243). Interacts with CSPG4 (PubMed:15504744). Binds to CAVIN2 in the presence of phosphatidylserine (By similarity). Interacts with PRKCABP/PICK1 (via PDZ domain) (PubMed:15247289). Interacts with TRIM41 (PubMed:17893151). Interacts with PARD3 (PubMed:27925688). Interacts with SOCS2 (By similarity).   
  
 **Gene Ontology Information:**

Molecular Function

- ATP binding
- calcium-dependent protein kinase C activity
- enzyme binding
- histone kinase activity (H3-T6 specific)
- integrin binding
- protein kinase activity
- protein kinase C activity
- protein serine kinase activity
- protein serine/threonine kinase activity
- zinc ion binding

Location

- alphav-beta3 integrin-PKCalpha complex
- ciliary basal body
- cytoplasm
- cytosol
- endoplasmic reticulum
- extracellular exosome
- mitochondrial membrane
- mitochondrion
- nucleoplasm
- perinuclear region of cytoplasm
- plasma membrane

Biological process

- angiogenesis
- apoptotic signaling pathway
- cell adhesion
- desmosome assembly
- intracellular signal transduction
- mitotic nuclear envelope disassembly
- negative regulation of glial cell apoptotic process
- peptidyl-serine phosphorylation
- peptidyl-threonine phosphorylation
- positive regulation of adenylate cyclase-activating G protein-coupled receptor signaling pathway
- positive regulation of angiogenesis
- positive regulation of blood vessel endothelial cell migration
- positive regulation of bone resorption
- positive regulation of cardiac muscle hypertrophy
- positive regulation of cell adhesion
- positive regulation of cell migration
- positive regulation of dense core granule biogenesis
- positive regulation of endothelial cell migration
- positive regulation of endothelial cell proliferation
- positive regulation of ERK1 and ERK2 cascade
- positive regulation of lipopolysaccharide-mediated signaling pathway
- positive regulation of macrophage differentiation
- positive regulation of mitotic cell cycle
- post-translational protein modification
- protein phosphorylation
- regulation of mRNA stability
- regulation of platelet aggregation
- response to interleukin-1

---
