# Supplementary material for: Comparative structural insights and functional analysis for the distinct unbound states of Human AGO proteins
Source: Sci Rep. 2025 Mar 19;15:9432. doi: 10.1038/s41598-025-91849-5 (PMC11923369; doi:10.1038/s41598-025-91849-5)
Supplement: Supplementary file 24 — Supplementary Information 12. [file 41598_2025_91849_MOESM24_ESM.zip › 5VM9p_A_mdwhole_HL2REF/candidates/5VM9p_A-merged-enriched_eval_report.html]

 

# Structural Comparison Report for 5VM9p\_A - whole structures (total: 100)

---

1

- **Protein name:** Macrophage-expressed gene 1 protein
- **Organism:** Homo sapiens
- **Uniprot Accession Number:** Q2M385
- **Protein sequence length:** 716 aa
- **1D identity (%):** 13.07
- **1D identity (%) [Gaps excluded]:** 21.9
- **1D identity - Alignment Gaps:** 398
- **1D aligned content (<aminoacid>:%):** {'F': 5.43, 'I': 2.33, 'W': 0.78, 'G': 8.53, 'E': 7.75, 'N': 3.88, 'L': 11.63, 'P': 9.3, 'D': 4.65, 'Q': 6.2, 'K': 3.88, 'V': 6.2, 'T': 8.53, 'A': 4.65, 'Y': 2.33, 'H': 2.33, 'S': 3.1, 'R': 3.88, 'M': 0.78, 'C': 3.88}
- **Common reported functions (%):** 0.0
- **Common reported locations (%):** 0.0
- **Common reported processes (%):** 0.0

- **PDB ID:** 6U23
- **Chain:** H
- **Crystallized protein length:** 583 aa
- **Resolution:** 3.49 Å
- **b-phipsi:** 0.000889
- **w-rdist:** 0.300809
- **t-alpha:** 0.002377
- **Chemical similarity (Tanimoto Index) (%):** N/A
- **1D identity (%) [PDB]:** 2.45
- **1D identity (%) [Gaps excluded][PDB]:** 73.91
- **1D identity - Alignment Gaps [PDB]:** 1340
- **1D aligned content [PDB] (<aminoacid>:%):** {'G': 14.71, 'V': 14.71, 'L': 17.65, 'P': 11.76, 'K': 8.82, 'D': 2.94, 'R': 2.94, 'F': 2.94, 'S': 5.88, 'H': 5.88, 'E': 8.82, 'T': 2.94}
- **2D identity (%) [PDB]:** 29.44
- **2D identity (%) [Gaps excluded][PDB]:** 81.79
- **2D identity - Alignment Gaps [PDB]:** 674
- **2D aligned content [PDB] (<2D-fold>:%):** {'.': 23.55, 'H': 24.52, 'T': 20.65, 'E': 30.97, 'B': 0.32}
- **3D similarity (TM-Score) (%) [PDB]:** 20.47

- **Gene name:** MPEG1
- **Entrez ID:** 219972
- **RefSeq ID:** NM\_001039396
- **Transcript sequence length:** 4418
- **5-UTR|CDS|3-UTR identity (%):** 13.5 | 44.83 | 8.51
- **5-UTR|CDS|3-UTR identity (%) [Gaps excluded]:** 75.86 | 75.41 | 75.08
- **5-UTR|CDS|3-UTR identity [Alignment Gaps]:** 268 | 1204 | 15100
- **5-UTR aligned content (<base>:%):** {'A': 11.36, 'G': 45.45, 'T': 13.64, 'C': 29.55}
- **CDS aligned content (<base>:%):** {'A': 27.87, 'G': 23.82, 'C': 25.54, 'T': 22.76}
- **3-UTR aligned content (<base>:%):** {'A': 31.4, 'T': 35.4, 'C': 17.05, 'G': 16.15}

**Uniprot Description:**  
  
 Plays a key role in the innate immune response following bacterial infection by inserting into the bacterial surface to form pores (By similarity). By breaching the surface of phagocytosed bacteria, allows antimicrobial effectors to enter the bacterial periplasmic space and degrade bacterial proteins such as superoxide dismutase sodC which contributes to bacterial virulence (By similarity). Shows antibacterial activity against a wide spectrum of Gram-positive, Gram-negative and acid-fast bacteria (PubMed:23753625, PubMed:26402460, PubMed:30609079). Reduces the viability of the intracytosolic pathogen L.monocytogenes by inhibiting acidification of the phagocytic vacuole of host cells which restricts bacterial translocation from the vacuole to the cytosol (By similarity). Required for the antibacterial activity of reactive oxygen species and nitric oxide (By similarity). N/A   
  
 **Gene Ontology Information:**

Molecular Function   
  
N/A

Location

- cytoplasmic vesicle
- cytoplasmic vesicle membrane
- extracellular region
- phagocytic vesicle

Biological process

- antibacterial innate immune response
- defense response to bacterium
- defense response to Gram-negative bacterium
- defense response to Gram-positive bacterium

---

2

- **Protein name:** Protein argonaute-2
- **Organism:** Homo sapiens
- **Uniprot Accession Number:** Q9UKV8
- **Protein sequence length:** 859 aa
- **1D identity (%):** 79.29
- **1D identity (%) [Gaps excluded]:** 81.06
- **1D identity - Alignment Gaps:** 19
- **1D aligned content (<aminoacid>:%):** {'A': 6.82, 'G': 6.97, 'P': 6.82, 'R': 6.68, 'T': 6.39, 'I': 5.22, 'K': 5.37, 'L': 7.69, 'N': 2.32, 'F': 4.5, 'D': 4.79, 'Y': 3.63, 'E': 4.79, 'C': 2.76, 'V': 8.56, 'M': 2.03, 'Q': 6.1, 'H': 3.48, 'S': 4.35, 'W': 0.73}
- **Common reported functions (%):** 100.0
- **Common reported locations (%):** 88.89
- **Common reported processes (%):** 55.56

- **PDB ID:** 4Z4F
- **Chain:** A
- **Crystallized protein length:** 801 aa
- **Resolution:** 2.8 Å
- **b-phipsi:** 0.000194
- **w-rdist:** 0.087913
- **t-alpha:** 0.023772
- **Chemical similarity (Tanimoto Index) (%):** 91.98
- **1D identity (%) [PDB]:** 76.88
- **1D identity (%) [Gaps excluded][PDB]:** 81.98
- **1D identity - Alignment Gaps [PDB]:** 53
- **1D aligned content [PDB] (<aminoacid>:%):** {'P': 7.02, 'R': 7.02, 'G': 6.26, 'T': 6.41, 'I': 5.5, 'K': 5.5, 'L': 8.09, 'A': 6.56, 'N': 2.29, 'F': 4.73, 'D': 4.73, 'Y': 3.82, 'E': 4.58, 'C': 2.75, 'V': 8.85, 'M': 1.98, 'Q': 5.95, 'H': 3.21, 'S': 3.97, 'W': 0.76}
- **2D identity (%) [PDB]:** 62.8
- **2D identity (%) [Gaps excluded][PDB]:** 79.42
- **2D identity - Alignment Gaps [PDB]:** 193
- **2D aligned content [PDB] (<2D-fold>:%):** {'.': 19.86, 'B': 1.38, 'E': 29.53, 'H': 35.92, 'T': 12.26, 'G': 1.04}
- **3D similarity (TM-Score) (%) [PDB]:** 90.09

- **Gene name:** AGO2
- **Entrez ID:** N/A
- **RefSeq ID:** NM\_012154
- **Transcript sequence length:** 14595
- **5-UTR|CDS|3-UTR identity (%):** 27.69 | 70.93 | 37.29
- **5-UTR|CDS|3-UTR identity (%) [Gaps excluded]:** 72.58 | 74.91 | 74.74
- **5-UTR|CDS|3-UTR identity [Alignment Gaps]:** 201 | 141 | 9575
- **5-UTR aligned content (<base>:%):** {'C': 47.78, 'G': 38.89, 'T': 10.0, 'A': 3.33}
- **CDS aligned content (<base>:%):** {'A': 27.38, 'T': 21.8, 'G': 25.78, 'C': 25.04}
- **3-UTR aligned content (<base>:%):** {'C': 17.51, 'A': 27.67, 'T': 34.55, 'G': 20.26}

**Uniprot Description:**  
  
 Required for RNA-mediated gene silencing (RNAi) by the RNA-induced silencing complex (RISC). The 'minimal RISC' appears to include AGO2 bound to a short guide RNA such as a microRNA (miRNA) or short interfering RNA (siRNA). These guide RNAs direct RISC to complementary mRNAs that are targets for RISC-mediated gene silencing. The precise mechanism of gene silencing depends on the degree of complementarity between the miRNA or siRNA and its target. Binding of RISC to a perfectly complementary mRNA generally results in silencing due to endonucleolytic cleavage of the mRNA specifically by AGO2. Binding of RISC to a partially complementary mRNA results in silencing through inhibition of translation, and this is independent of endonuclease activity. May inhibit translation initiation by binding to the 7-methylguanosine cap, thereby preventing the recruitment of the translation initiation factor eIF4-E. May also inhibit translation initiation via interaction with EIF6, which itself binds to the 60S ribosomal subunit and prevents its association with the 40S ribosomal subunit. The inhibition of translational initiation leads to the accumulation of the affected mRNA in cytoplasmic processing bodies (P-bodies), where mRNA degradation may subsequently occur. In some cases RISC-mediated translational repression is also observed for miRNAs that perfectly match the 3' untranslated region (3'-UTR). Can also up-regulate the translation of specific mRNAs under certain growth conditions. Binds to the AU element of the 3'-UTR of the TNF (TNF-alpha) mRNA and up-regulates translation under conditions of serum starvation. Also required for transcriptional gene silencing (TGS), in which short RNAs known as antigene RNAs or agRNAs direct the transcriptional repression of complementary promoter regions.   
  
Interacts with DICER1 through its Piwi domain and with TARBP2 during assembly of the RNA-induced silencing complex (RISC). Together, DICER1, AGO2 and TARBP2 constitute the trimeric RISC loading complex (RLC), or micro-RNA (miRNA) loading complex (miRLC). Within the RLC/miRLC, DICER1 and TARBP2 are required to process precursor miRNAs (pre-miRNAs) to mature miRNAs and then load them onto AGO2. AGO2 bound to the mature miRNA constitutes the minimal RISC and may subsequently dissociate from DICER1 and TARBP2. Note however that the term RISC has also been used to describe the trimeric RLC/miRLC. The formation of RISC complexes containing siRNAs rather than miRNAs appears to occur independently of DICER1. Interacts with AGO1. Also interacts with DDB1, DDX5, DDX6, DDX20, DHX30, DHX36, DDX47, DHX9, ELAVL, FXR1, GEMIN4, HNRNPF, IGF2BP1, ILF3, IMP8, MATR3, PABPC1, PRMT5, P4HA1, P4HB, RBM4, SART3, TNRC6A, TNRC6B, UPF1 and YBX1. Interacts with the P-body components DCP1A and XRN1. Associates with polysomes and messenger ribonucleoproteins (mNRPs). Interacts with RBM4; the interaction is modulated under stress-induced conditions, occurs under both cell proliferation and differentiation conditions and in an RNA- and phosphorylation-independent manner. Interacts with LIMD1, WTIP and AJUBA. Interacts with TRIM71; the interaction increases in presence of RNA (PubMed:23125361). Interacts with APOBEC3G in an RNA-dependent manner. Interacts with APOBEC3A, APOBEC3C, APOBEC3F and APOBEC3H. Interacts with DICER1, TARBP2, EIF6, MOV10 and RPL7A (60S ribosome subunit); they form a large RNA-induced silencing complex (RISC) (PubMed:17507929, PubMed:24726324). Interacts with FMR1 (PubMed:14703574). Interacts with ZFP36 (PubMed:15766526). Found in a complex, composed of AGO2, CHD7 and FAM172A (By similarity). Interacts with RC3H1; the interaction is RNA independent (PubMed:25697406). Interacts with SND1 (PubMed:14508492, PubMed:28546213). Interacts with SYT11 (By similarity). Interacts with CLNK (PubMed:26009488). Interacts with GARRE1 (PubMed:29395067).   
  
 **Gene Ontology Information:**

Molecular Function

- core promoter sequence-specific DNA binding
- double-stranded RNA binding
- endoribonuclease activity, cleaving miRNA-paired mRNA
- endoribonuclease activity, cleaving siRNA-paired mRNA
- metal ion binding
- miRNA binding
- mRNA 3'-UTR AU-rich region binding
- mRNA cap binding
- RNA 7-methylguanosine cap binding
- RNA binding
- endoribonuclease activity
- RNA polymerase II complex binding
- single-stranded RNA binding
- siRNA binding
- translation initiation factor activity

Location

- cytoplasm
- cytoplasmic ribonucleoprotein granule
- cytosol
- dendrite
- extracellular exosome
- membrane
- mRNA cap binding complex
- nucleoplasm
- nucleus
- P-body
- polysome
- ribonucleoprotein complex
- RISC complex
- RISC-loading complex

Biological process

- miRNA metabolic process
- production of miRNAs involved in gene silencing by miRNA
- miRNA mediated inhibition of translation
- mRNA cleavage involved in gene silencing by miRNA
- post-transcriptional gene silencing by RNA
- negative regulation of amyloid precursor protein biosynthetic process
- negative regulation of translational initiation
- positive regulation of angiogenesis
- positive regulation of nuclear-transcribed mRNA catabolic process, deadenylation-dependent decay
- positive regulation of nuclear-transcribed mRNA poly(A) tail shortening
- positive regulation of transcription by RNA polymerase II
- positive regulation of translation
- positive regulation of trophoblast cell migration
- post-embryonic development
- pre-miRNA processing
- small RNA loading onto RISC
- RNA secondary structure unwinding
- gene silencing by RNA
- production of siRNA involved in RNA interference
- mRNA cleavage involved in gene silencing by siRNA
- translation

---

3

- **Protein name:** Small nuclear ribonucleoprotein F
- **Organism:** Homo sapiens
- **Uniprot Accession Number:** P62306
- **Protein sequence length:** 86 aa
- **1D identity (%):** 2.53
- **1D identity (%) [Gaps excluded]:** 28.95
- **1D identity - Alignment Gaps:** 794
- **1D aligned content (<aminoacid>:%):** {'S': 4.55, 'P': 9.09, 'F': 4.55, 'L': 13.64, 'T': 4.55, 'G': 18.18, 'K': 4.55, 'V': 9.09, 'M': 4.55, 'I': 9.09, 'E': 9.09, 'Y': 4.55, 'R': 4.55}
- **Common reported functions (%):** 14.29
- **Common reported locations (%):** 22.22
- **Common reported processes (%):** 0.0

- **PDB ID:** 6FF7
- **Chain:** b
- **Crystallized protein length:** 73 aa
- **Resolution:** 4.5 Å
- **b-phipsi:** 0.003465
- **w-rdist:** 0.372703
- **t-alpha:** 0.003962
- **Chemical similarity (Tanimoto Index) (%):** N/A
- **1D identity (%) [PDB]:** 0.88
- **1D identity (%) [Gaps excluded][PDB]:** 88.89
- **1D identity - Alignment Gaps [PDB]:** 900
- **1D aligned content [PDB] (<aminoacid>:%):** {'E': 37.5, 'V': 12.5, 'L': 25.0, 'T': 12.5, 'P': 12.5}
- **2D identity (%) [PDB]:** 6.76
- **2D identity (%) [Gaps excluded][PDB]:** 96.67
- **2D identity - Alignment Gaps [PDB]:** 798
- **2D aligned content [PDB] (<2D-fold>:%):** {'.': 50.0, 'T': 50.0}
- **3D similarity (TM-Score) (%) [PDB]:** 7.86

- **Gene name:** SNRPF
- **Entrez ID:** 6636
- **RefSeq ID:** NM\_003095
- **Transcript sequence length:** 452
- **5-UTR|CDS|3-UTR identity (%):** 20.11 | 6.23 | 0.27
- **5-UTR|CDS|3-UTR identity (%) [Gaps excluded]:** 78.02 | 77.73 | 80.7
- **5-UTR|CDS|3-UTR identity [Alignment Gaps]:** 262 | 2422 | 16710
- **5-UTR aligned content (<base>:%):** {'C': 32.39, 'G': 42.25, 'T': 21.13, 'A': 4.23}
- **CDS aligned content (<base>:%):** {'A': 39.63, 'G': 23.78, 'C': 15.85, 'T': 20.73}
- **3-UTR aligned content (<base>:%):** {'C': 4.35, 'A': 23.91, 'T': 60.87, 'G': 10.87}

**Uniprot Description:**  
  
 Plays role in pre-mRNA splicing as core component of the SMN-Sm complex that mediates spliceosomal snRNP assembly and as component of the spliceosomal U1, U2, U4 and U5 small nuclear ribonucleoproteins (snRNPs), the building blocks of the spliceosome (PubMed:11991638, PubMed:18984161, PubMed:19325628, PubMed:23333303, PubMed:25555158, PubMed:26912367, PubMed:28502770, PubMed:28781166, PubMed:28076346). Component of both the pre-catalytic spliceosome B complex and activated spliceosome C complexes (PubMed:11991638, PubMed:28502770, PubMed:28781166, PubMed:28076346). Is also a component of the minor U12 spliceosome (PubMed:15146077). As part of the U7 snRNP it is involved in histone 3'-end processing (PubMed:12975319).   
  
Core component of the spliceosomal U1, U2, U4 and U5 small nuclear ribonucleoproteins (snRNPs), the building blocks of the spliceosome (PubMed:11991638, PubMed:19325628, PubMed:21516107, PubMed:25555158, PubMed:26912367, PubMed:28502770, PubMed:28781166, PubMed:28076346). Most spliceosomal snRNPs contain a common set of Sm proteins, SNRPB, SNRPD1, SNRPD2, SNRPD3, SNRPE, SNRPF and SNRPG that assemble in a heptameric protein ring on the Sm site of the small nuclear RNA to form the core snRNP (PubMed:19325628, PubMed:21516107, PubMed:25555158, PubMed:26912367, PubMed:28502770, PubMed:28781166, PubMed:28076346). Component of the U1 snRNP (PubMed:19325628, PubMed:25555158). The U1 snRNP is composed of the U1 snRNA and the 7 core Sm proteins SNRPB, SNRPD1, SNRPD2, SNRPD3, SNRPE, SNRPF and SNRPG, and at least three U1 snRNP-specific proteins SNRNP70/U1-70K, SNRPA/U1-A and SNRPC/U1-C (PubMed:19325628, PubMed:25555158). Component of the U4/U6-U5 tri-snRNP complex composed of the U4, U6 and U5 snRNAs and at least PRPF3, PRPF4, PRPF6, PRPF8, PRPF31, SNRNP200, TXNL4A, SNRNP40, SNRPB, SNRPD1, SNRPD2, SNRPD3, SNRPE, SNRPF, SNRPG, DDX23, CD2BP2, PPIH, SNU13, EFTUD2, SART1 and USP39, plus LSM2, LSM3, LSM4, LSM5, LSM6, LSM7 and LSM8 (PubMed:26912367). Component of the U7 snRNP complex, or U7 Sm protein core complex, that is composed of the U7 snRNA and at least LSM10, LSM11, SNRPB, SNRPD3, SNRPE, SNRPF and SNRPG; the complex does not contain SNRPD1 and SNRPD2 (PubMed:11574479). Component of the U11/U12 snRNPs that are part of the U12-type spliceosome (PubMed:15146077). Part of the SMN-Sm complex that contains SMN1, GEMIN2/SIP1, DDX20/GEMIN3, GEMIN4, GEMIN5, GEMIN6, GEMIN7, GEMIN8, STRAP/UNRIP and the Sm proteins SNRPB, SNRPD1, SNRPD2, SNRPD3, SNRPE, SNRPF and SNRPG; catalyzes core snRNPs assembly (PubMed:18984161). Forms a 6S pICln-Sm complex composed of CLNS1A/pICln, SNRPD1, SNRPD2, SNRPE, SNRPF and SNRPG; ring-like structure where CLNS1A/pICln mimics additional Sm proteins and which is unable to assemble into the core snRNP (PubMed:18984161, PubMed:23333303).   
  
 **Gene Ontology Information:**

Molecular Function

- RNA binding

Location

- catalytic step 2 spliceosome
- cytosol
- methylosome
- nucleoplasm
- nucleus
- pICln-Sm protein complex
- small nuclear ribonucleoprotein complex
- SMN-Sm protein complex
- spliceosomal complex
- U1 snRNP
- U12-type spliceosomal complex
- U2 snRNP
- U2-type catalytic step 2 spliceosome
- U2-type precatalytic spliceosome
- U4 snRNP
- U4/U6 x U5 tri-snRNP complex
- U5 snRNP
- U7 snRNP

Biological process

- 7-methylguanosine cap hypermethylation
- mRNA splicing, via spliceosome
- RNA splicing
- spliceosomal snRNP assembly
- U2-type prespliceosome assembly

---

4

- **Protein name:** Astrotactin-2
- **Organism:** Homo sapiens
- **Uniprot Accession Number:** O75129
- **Protein sequence length:** 1339 aa
- **1D identity (%):** 16.15
- **1D identity (%) [Gaps excluded]:** 28.25
- **1D identity - Alignment Gaps:** 599
- **1D aligned content (<aminoacid>:%):** {'A': 6.19, 'G': 10.18, 'P': 9.29, 'L': 10.18, 'C': 2.65, 'F': 3.1, 'R': 6.19, 'E': 5.31, 'D': 3.98, 'S': 6.64, 'V': 7.08, 'T': 7.08, 'H': 1.77, 'Y': 6.19, 'K': 3.1, 'Q': 5.75, 'I': 3.54, 'N': 1.33, 'W': 0.44}
- **Common reported functions (%):** 0.0
- **Common reported locations (%):** 11.11
- **Common reported processes (%):** 0.0

- **PDB ID:** 5J68
- **Chain:** A
- **Crystallized protein length:** 559 aa
- **Resolution:** 5.22 Å
- **b-phipsi:** 0.002574
- **w-rdist:** 0.416856
- **t-alpha:** 0.004777
- **Chemical similarity (Tanimoto Index) (%):** 77.72
- **1D identity (%) [PDB]:** 3.81
- **1D identity (%) [Gaps excluded][PDB]:** 73.91
- **1D identity - Alignment Gaps [PDB]:** 1268
- **1D aligned content [PDB] (<aminoacid>:%):** {'V': 15.69, 'N': 3.92, 'R': 9.8, 'E': 5.88, 'S': 7.84, 'M': 1.96, 'Q': 1.96, 'H': 5.88, 'F': 3.92, 'K': 5.88, 'T': 5.88, 'P': 3.92, 'G': 5.88, 'L': 11.76, 'Y': 1.96, 'A': 1.96, 'D': 3.92, 'I': 1.96}
- **2D identity (%) [PDB]:** 25.7
- **2D identity (%) [Gaps excluded][PDB]:** 81.85
- **2D identity - Alignment Gaps [PDB]:** 734
- **2D aligned content [PDB] (<2D-fold>:%):** {'.': 18.91, 'T': 16.36, 'E': 31.27, 'H': 33.45}
- **3D similarity (TM-Score) (%) [PDB]:** 19.8

- **Gene name:** ASTN2
- **Entrez ID:** 23245
- **RefSeq ID:** NM\_198188
- **Transcript sequence length:** 1799
- **5-UTR|CDS|3-UTR identity (%):** 41.85 | 29.7 | 1.57
- **5-UTR|CDS|3-UTR identity (%) [Gaps excluded]:** 73.76 | 76.25 | 80.92
- **5-UTR|CDS|3-UTR identity [Alignment Gaps]:** 154 | 1657 | 16480
- **5-UTR aligned content (<base>:%):** {'C': 39.6, 'G': 36.24, 'T': 17.45, 'A': 6.71}
- **CDS aligned content (<base>:%):** {'A': 26.92, 'T': 21.71, 'G': 27.17, 'C': 24.19}
- **3-UTR aligned content (<base>:%):** {'A': 23.57, 'C': 24.71, 'T': 30.42, 'G': 21.29}

**Uniprot Description:**  
  
 Mediates recycling of the neuronal cell adhesion molecule ASTN1 to the anterior pole of the cell membrane in migrating neurons. Promotes ASTN1 internalization and intracellular transport of endocytosed ASTN1 (By similarity). Selectively binds inositol-4,5-bisphosphate, inositol-3,4,5-trisphosphate and inositol-1,3,4,5-tetrakisphosphate, suggesting it is recruited to membranes that contain lipids with a phosphoinositide headgroup (Ref.6).   
  
Interacts with ASTN1; the interaction is not calcium-dependent.   
  
 **Gene Ontology Information:**

Molecular Function

- calcium ion binding
- inositol 1,3,4,5 tetrakisphosphate binding

Location

- cell cortex
- cell pole
- clathrin-coated vesicle
- early endosome
- endosome
- late endosome
- membrane
- perikaryon

Biological process

- establishment of body hair planar orientation
- negative regulation of protein localization to cell surface
- neuron cell-cell adhesion
- neuron migration
- protein localization to cell surface
- protein transport

---

5

- **Protein name:** Exostosin-like 3
- **Organism:** Homo sapiens
- **Uniprot Accession Number:** O43909
- **Protein sequence length:** 919 aa
- **1D identity (%):** 16.99
- **1D identity (%) [Gaps excluded]:** 23.36
- **1D identity - Alignment Gaps:** 281
- **1D aligned content (<aminoacid>:%):** {'M': 1.14, 'G': 8.0, 'A': 8.57, 'L': 12.0, 'R': 5.14, 'P': 13.71, 'E': 7.43, 'V': 7.43, 'D': 5.14, 'C': 1.14, 'Q': 2.86, 'K': 4.57, 'H': 3.43, 'I': 1.71, 'T': 4.0, 'F': 3.43, 'N': 2.86, 'Y': 2.86, 'W': 1.14, 'S': 3.43}
- **Common reported functions (%):** 14.29
- **Common reported locations (%):** 0.0
- **Common reported processes (%):** 0.0

- **PDB ID:** 8OG4
- **Chain:** A
- **Crystallized protein length:** 709 aa
- **Resolution:** 2.1 Å
- **b-phipsi:** 0.000572
- **w-rdist:** 0.408782
- **t-alpha:** 0.016103
- **Chemical similarity (Tanimoto Index) (%):** 75.5
- **1D identity (%) [PDB]:** 3.33
- **1D identity (%) [Gaps excluded][PDB]:** 72.73
- **1D identity - Alignment Gaps [PDB]:** 1376
- **1D aligned content [PDB] (<aminoacid>:%):** {'L': 10.42, 'R': 8.33, 'P': 16.67, 'E': 4.17, 'S': 8.33, 'V': 12.5, 'D': 2.08, 'Y': 2.08, 'T': 2.08, 'G': 12.5, 'F': 8.33, 'A': 4.17, 'W': 2.08, 'H': 2.08, 'Q': 2.08, 'M': 2.08}
- **2D identity (%) [PDB]:** 39.21
- **2D identity (%) [Gaps excluded][PDB]:** 87.96
- **2D identity - Alignment Gaps [PDB]:** 578
- **2D aligned content [PDB] (<2D-fold>:%):** {'.': 18.09, 'H': 34.96, 'T': 27.63, 'E': 18.09, 'B': 0.49, 'G': 0.73}
- **3D similarity (TM-Score) (%) [PDB]:** 23.79

- **Gene name:** EXTL3
- **Entrez ID:** N/A
- **RefSeq ID:** N/A
- **Sequence length:** N/A
- **5-UTR|CDS|3-UTR identity (%):** N/A | N/A | N/A
- **5-UTR|CDS|3-UTR identity (%) [Gaps excluded]:** N/A | N/A | N/A
- **5-UTR|CDS|3-UTR identity [Alignment Gaps]:** N/A | N/A | N/A
- **5-UTR aligned content (<base>:%):** N/A
- **CDS aligned content (<base>:%):** N/A
- **3-UTR aligned content (<base>:%):** N/A

**Uniprot Description:**  
  
 Glycosyltransferase which regulates the biosynthesis of heparan sulfate (HS). Important for both skeletal development and hematopoiesis, through the formation of HS proteoglycans (HSPGs) (PubMed:28132690, PubMed:28148688). Required for the function of REG3A in regulating keratinocyte proliferation and differentiation (PubMed:22727489).   
  
Interacts with REG3A.   
  
 **Gene Ontology Information:**

Molecular Function

- glucuronyl-galactosyl-proteoglycan 4-alpha-N-acetylglucosaminyltransferase activity
- transferase activity, transferring glycosyl groups
- metal ion binding

Location

- endoplasmic reticulum
- endoplasmic reticulum membrane
- Golgi apparatus

Biological process

- heparan sulfate proteoglycan biosynthetic process
- positive regulation of cell growth
- protein glycosylation

---

6

- **Protein name:** Ubiquitin carboxyl-terminal hydrolase 5
- **Organism:** Homo sapiens
- **Uniprot Accession Number:** P45974
- **Protein sequence length:** 858 aa
- **1D identity (%):** 15.0
- **1D identity (%) [Gaps excluded]:** 22.29
- **1D identity - Alignment Gaps:** 336
- **1D aligned content (<aminoacid>:%):** {'E': 5.84, 'S': 5.19, 'V': 9.09, 'G': 7.79, 'D': 5.19, 'R': 9.74, 'P': 10.39, 'L': 12.34, 'F': 3.9, 'K': 5.19, 'H': 3.9, 'T': 3.25, 'A': 3.25, 'Q': 5.19, 'C': 1.95, 'I': 2.6, 'N': 0.65, 'M': 0.65, 'Y': 3.9}
- **Common reported functions (%):** 0.0
- **Common reported locations (%):** 11.11
- **Common reported processes (%):** 0.0

- **PDB ID:** 3IHP
- **Chain:** A
- **Crystallized protein length:** 673 aa
- **Resolution:** 2.8 Å
- **b-phipsi:** 0.00053
- **w-rdist:** 0.27827
- **t-alpha:** 0.066561
- **Chemical similarity (Tanimoto Index) (%):** 84.36
- **1D identity (%) [PDB]:** 2.88
- **1D identity (%) [Gaps excluded][PDB]:** 63.64
- **1D identity - Alignment Gaps [PDB]:** 1394
- **1D aligned content [PDB] (<aminoacid>:%):** {'A': 4.76, 'E': 2.38, 'V': 19.05, 'G': 11.9, 'D': 2.38, 'T': 4.76, 'L': 16.67, 'Q': 7.14, 'K': 4.76, 'I': 7.14, 'S': 7.14, 'N': 7.14, 'P': 2.38, 'F': 2.38}
- **2D identity (%) [PDB]:** 32.76
- **2D identity (%) [Gaps excluded][PDB]:** 86.84
- **2D identity - Alignment Gaps [PDB]:** 690
- **2D aligned content [PDB] (<2D-fold>:%):** {'.': 19.28, 'H': 40.77, 'T': 19.28, 'E': 18.18, 'B': 0.83, 'G': 1.65}
- **3D similarity (TM-Score) (%) [PDB]:** 21.21

- **Gene name:** USP5
- **Entrez ID:** 8078
- **RefSeq ID:** NM\_003481
- **Transcript sequence length:** 3093
- **5-UTR|CDS|3-UTR identity (%):** 6.17 | 44.37 | 2.11
- **5-UTR|CDS|3-UTR identity (%) [Gaps excluded]:** 71.43 | 74.26 | 72.9
- **5-UTR|CDS|3-UTR identity [Alignment Gaps]:** 296 | 1283 | 16336
- **5-UTR aligned content (<base>:%):** {'G': 55.0, 'C': 15.0, 'T': 20.0, 'A': 10.0}
- **CDS aligned content (<base>:%):** {'T': 21.71, 'G': 27.23, 'C': 24.12, 'A': 26.94}
- **3-UTR aligned content (<base>:%):** {'G': 29.58, 'C': 28.17, 'T': 23.1, 'A': 19.15}

**Uniprot Description:**  
  
 Cleaves linear and branched multiubiquitin polymers with a marked preference for branched polymers. Involved in unanchored 'Lys-48'-linked polyubiquitin disassembly. Binds linear and 'Lys-63'-linked polyubiquitin with a lower affinity. Knock-down of USP5 causes the accumulation of p53/TP53 and an increase in p53/TP53 transcriptional activity because the unanchored polyubiquitin that accumulates is able to compete with ubiquitinated p53/TP53 but not with MDM2 for proteasomal recognition.   
  
Interacts with TRIML1.   
  
 **Gene Ontology Information:**

Molecular Function

- thiol-dependent ubiquitin-specific protease activity
- cysteine-type endopeptidase activity
- ubiquitin binding
- zinc ion binding

Location

- cytosol
- lysosome
- nucleus

Biological process

- positive regulation of proteasomal ubiquitin-dependent protein catabolic process
- protein deubiquitination
- protein K48-linked deubiquitination
- protein ubiquitination
- ubiquitin-dependent protein catabolic process

---

7

- **Protein name:** Protein PAXX
- **Organism:** Homo sapiens
- **Uniprot Accession Number:** Q9BUH6
- **Protein sequence length:** 204 aa
- **1D identity (%):** 6.06
- **1D identity (%) [Gaps excluded]:** 27.89
- **1D identity - Alignment Gaps:** 684
- **1D aligned content (<aminoacid>:%):** {'P': 15.09, 'L': 11.32, 'G': 16.98, 'Y': 1.89, 'E': 3.77, 'T': 7.55, 'K': 3.77, 'I': 3.77, 'R': 7.55, 'Q': 3.77, 'V': 5.66, 'D': 5.66, 'S': 3.77, 'A': 7.55, 'C': 1.89}
- **Common reported functions (%):** 0.0
- **Common reported locations (%):** 0.0
- **Common reported processes (%):** 0.0

- **PDB ID:** 8EZA
- **Chain:** B
- **Crystallized protein length:** 531 aa
- **Resolution:** 4.39 Å
- **b-phipsi:** 0.003208
- **w-rdist:** 0.565128
- **t-alpha:** 0.000793
- **Chemical similarity (Tanimoto Index) (%):** 99.01
- **1D identity (%) [PDB]:** 2.37
- **1D identity (%) [Gaps excluded][PDB]:** 70.45
- **1D identity - Alignment Gaps [PDB]:** 1265
- **1D aligned content [PDB] (<aminoacid>:%):** {'K': 12.9, 'A': 3.23, 'Q': 6.45, 'P': 6.45, 'V': 12.9, 'I': 12.9, 'F': 3.23, 'L': 9.68, 'D': 6.45, 'E': 9.68, 'T': 9.68, 'R': 3.23, 'G': 3.23}
- **2D identity (%) [PDB]:** 36.39
- **2D identity (%) [Gaps excluded][PDB]:** 92.17
- **2D identity - Alignment Gaps [PDB]:** 587
- **2D aligned content [PDB] (<2D-fold>:%):** {'.': 24.08, 'E': 22.38, 'T': 22.66, 'H': 30.88}
- **3D similarity (TM-Score) (%) [PDB]:** 19.53

- **Gene name:** PAXX
- **Entrez ID:** N/A
- **RefSeq ID:** NM\_183241
- **Transcript sequence length:** 808
- **5-UTR|CDS|3-UTR identity (%):** 4.95 | 13.11 | 0.64
- **5-UTR|CDS|3-UTR identity (%) [Gaps excluded]:** 76.19 | 73.71 | 75.52
- **5-UTR|CDS|3-UTR identity [Alignment Gaps]:** 302 | 2232 | 16640
- **5-UTR aligned content (<base>:%):** {'G': 37.5, 'T': 12.5, 'C': 50.0}
- **CDS aligned content (<base>:%):** {'A': 19.94, 'T': 17.7, 'G': 31.46, 'C': 30.9}
- **3-UTR aligned content (<base>:%):** {'G': 29.63, 'T': 14.81, 'C': 32.41, 'A': 23.15}

**Uniprot Description:**  
  
 Non-essential DNA repair protein involved in DNA non-homologous end joining (NHEJ); participates in double-strand break (DSB) repair and V(D)J recombination (PubMed:25574025, PubMed:25670504, PubMed:25941166, PubMed:27705800). May act as a scaffold required for accumulation of the Ku heterodimer, composed of XRCC5/Ku80 and XRCC6/Ku70, at double-strand break sites and promote the assembly and/or stability of the NHEJ machinery (PubMed:25574025, PubMed:25670504, PubMed:25941166). Involved in NHEJ by promoting the ligation of blunt-ended DNA ends (PubMed:27703001). Together with NHEJ1/XLF, collaborates with DNA polymerase lambda (POLL) to promote joining of non-cohesive DNA ends (PubMed:30250067, PubMed:25670504). Constitutes a non-essential component of classical NHEJ: has a complementary but distinct function with NHEJ1/XLF in DNA repair (PubMed:27705800). Able to restrict infection by herpesvirus 1 (HSV-1) via an unknown mechanism (PubMed:29144403).   
  
Homodimer (PubMed:25574025). Interacts with the DNA-bound XRCC5/Ku80 and XRCC6/Ku70 heterodimer (Ku complex); the interaction is direct (PubMed:27601299, PubMed:27705800, PubMed:25574025). Associated component of the non-homologous end joining (NHEJ) complex, composed of the core proteins PRKDC, LIG4, XRCC4, XRCC6/Ku70, XRCC5/Ku86 and NHEJ1/XLF (PubMed:25670504, PubMed:25941166). Interacts with POLL (DNA polymerase lambda); promoting POLL recruitment to double-strand breaks (DSBs) and stimulation of the end-filling activity of POLL (PubMed:30250067).   
  
 **Gene Ontology Information:**

Molecular Function   
  
N/A

Location   
  
N/A

Biological process   
  
N/A

---

8

- **Protein name:** Epidermal growth factor receptor
- **Organism:** Homo sapiens
- **Uniprot Accession Number:** P00533
- **Protein sequence length:** 1210 aa
- **1D identity (%):** 16.44
- **1D identity (%) [Gaps excluded]:** 25.52
- **1D identity - Alignment Gaps:** 448
- **1D aligned content (<aminoacid>:%):** {'G': 11.11, 'A': 4.83, 'L': 9.18, 'T': 4.83, 'K': 7.25, 'N': 1.93, 'C': 3.38, 'V': 7.25, 'Y': 5.8, 'D': 2.42, 'P': 9.66, 'S': 4.83, 'F': 2.9, 'H': 3.38, 'R': 5.8, 'M': 0.97, 'I': 6.28, 'E': 3.86, 'Q': 4.35}
- **Common reported functions (%):** 0.0
- **Common reported locations (%):** 22.22
- **Common reported processes (%):** 11.11

- **PDB ID:** 7OM4
- **Chain:** A
- **Crystallized protein length:** 614 aa
- **Resolution:** 6.05 Å
- **b-phipsi:** 0.00191
- **w-rdist:** 0.748419
- **t-alpha:** 0.0
- **Chemical similarity (Tanimoto Index) (%):** 83.93
- **1D identity (%) [PDB]:** 1.89
- **1D identity (%) [Gaps excluded][PDB]:** 79.41
- **1D identity - Alignment Gaps [PDB]:** 1391
- **1D aligned content [PDB] (<aminoacid>:%):** {'L': 3.7, 'E': 7.41, 'K': 7.41, 'Q': 7.41, 'G': 7.41, 'I': 11.11, 'T': 11.11, 'Y': 3.7, 'V': 7.41, 'C': 3.7, 'A': 7.41, 'D': 3.7, 'R': 7.41, 'S': 3.7, 'N': 3.7, 'P': 3.7}
- **2D identity (%) [PDB]:** 19.52
- **2D identity (%) [Gaps excluded][PDB]:** 78.35
- **2D identity - Alignment Gaps [PDB]:** 877
- **2D aligned content [PDB] (<2D-fold>:%):** {'.': 35.53, 'E': 30.26, 'H': 11.84, 'T': 22.37}
- **3D similarity (TM-Score) (%) [PDB]:** 21.07

- **Gene name:** EGFR
- **Entrez ID:** 1956
- **RefSeq ID:** NM\_201284
- **Transcript sequence length:** 2872
- **5-UTR|CDS|3-UTR identity (%):** 43.05 | 43.1 | 1.76
- **5-UTR|CDS|3-UTR identity (%) [Gaps excluded]:** 77.03 | 73.95 | 75.19
- **5-UTR|CDS|3-UTR identity [Alignment Gaps]:** 165 | 1239 | 16458
- **5-UTR aligned content (<base>:%):** {'A': 4.97, 'G': 36.65, 'C': 50.93, 'T': 7.45}
- **CDS aligned content (<base>:%):** {'A': 28.28, 'T': 22.27, 'G': 24.45, 'C': 25.0}
- **3-UTR aligned content (<base>:%):** {'G': 16.5, 'C': 25.25, 'A': 30.64, 'T': 27.61}

**Uniprot Description:**  
  
 Receptor tyrosine kinase binding ligands of the EGF family and activating several signaling cascades to convert extracellular cues into appropriate cellular responses (PubMed:2790960, PubMed:10805725, PubMed:27153536). Known ligands include EGF, TGFA/TGF-alpha, AREG, epigen/EPGN, BTC/betacellulin, epiregulin/EREG and HBEGF/heparin-binding EGF (PubMed:2790960, PubMed:7679104, PubMed:8144591, PubMed:9419975, PubMed:15611079, PubMed:12297049, PubMed:27153536, PubMed:20837704, PubMed:17909029). Ligand binding triggers receptor homo- and/or heterodimerization and autophosphorylation on key cytoplasmic residues. The phosphorylated receptor recruits adapter proteins like GRB2 which in turn activates complex downstream signaling cascades. Activates at least 4 major downstream signaling cascades including the RAS-RAF-MEK-ERK, PI3 kinase-AKT, PLCgamma-PKC and STATs modules (PubMed:27153536). May also activate the NF-kappa-B signaling cascade (PubMed:11116146). Also directly phosphorylates other proteins like RGS16, activating its GTPase activity and probably coupling the EGF receptor signaling to the G protein-coupled receptor signaling (PubMed:11602604). Also phosphorylates MUC1 and increases its interaction with SRC and CTNNB1/beta-catenin (PubMed:11483589). Positively regulates cell migration via interaction with CCDC88A/GIV which retains EGFR at the cell membrane following ligand stimulation, promoting EGFR signaling which triggers cell migration (PubMed:20462955). Plays a role in enhancing learning and memory performance (By similarity).   
  
Binding of the ligand triggers homo- and/or heterodimerization of the receptor triggering its autophosphorylation. Heterodimer with ERBB2 (PubMed:10805725). Forms a complex with CCDC88A/GIV (via SH2-like regions) and GNAI3 which leads to enhanced EGFR signaling and triggering of cell migration; binding to CCDC88A requires autophosphorylation of the EGFR C-terminal region, and ligand stimulation is required for recruitment of GNAI3 to the complex (PubMed:20462955, PubMed:25187647). Interacts with ERRFI1; inhibits dimerization of the kinase domain and autophosphorylation (PubMed:18046415). Part of a complex with ERBB2 and either PIK3C2A or PIK3C2B (PubMed:10805725). Interacts with GRB2; an adapter protein coupling the receptor to downstream signaling pathways. Interacts with GAB2; involved in signaling downstream of EGFR. Interacts with STAT3; mediates EGFR downstream signaling in cell proliferation. Interacts with RIPK1; involved in NF-kappa-B activation. Interacts (autophosphorylated) with CBL, CBLB and CBLC; involved in EGFR ubiquitination and regulation. Interacts with SOCS5; regulates EGFR degradation through ELOC- and ELOB-mediated ubiquitination and proteasomal degradation. Interacts with PRMT5; methylates EGFR and enhances interaction with PTPN6. Interacts (phosphorylated) with PTPN6; inhibits EGFR-dependent activation of MAPK/ERK. Interacts with COPG1; essential for regulation of EGF-dependent nuclear transport of EGFR by retrograde trafficking from the Golgi to the ER. Interacts with TNK2; this interaction is dependent on EGF stimulation and kinase activity of EGFR. Interacts with PCNA; positively regulates PCNA (PubMed:17115032). Interacts with PELP1. Interacts with MUC1. Interacts with AP2M1. Interacts with FER. May interact with EPS8; mediates EPS8 phosphorylation. Interacts (via SH2 domains) with GRB2, NCK1 and NCK2 (PubMed:10026169). Interacts with ATXN2. Interacts with GAREM1. Interacts (ubiquitinated) with ANKRD13A/B/D; the interaction is direct and may regulate EGFR internalization after EGF stimulation. Interacts with GPER1; the interaction occurs in an estrogen-dependent manner. Interacts (via C-terminal cytoplasmic kinase domain) with ZPR1 (via zinc fingers). Interacts with RNF115 and RNF126 (PubMed:23418353). Interacts with GPRC5A (via its transmembrane domain) (PubMed:25311788). Interacts with FAM83B; positively regulates EGFR inducing its autophosphorylation in absence of stimulation by EGF (PubMed:23912460). Interacts with LAPTM4B; positively correlates with EGFR activation (PubMed:28479384). Interacts with STX19 (PubMed:16420529). Interacts with CD44 (PubMed:23589287). Interacts with PGRMC1; the interaction requires PGRMC1 homodimerization (PubMed:26988023). Interacts with PIKFYVE (PubMed:17909029). Interacts with NEU3.   
  
 **Gene Ontology Information:**

Molecular Function

- actin filament binding
- ATP binding
- ATPase binding
- cadherin binding
- chromatin binding
- double-stranded DNA binding
- enzyme binding
- epidermal growth factor binding
- epidermal growth factor-activated receptor activity
- identical protein binding
- kinase binding
- MAP kinase kinase kinase activity
- protein phosphatase binding
- protein tyrosine kinase activator activity
- protein tyrosine kinase activity
- transmembrane receptor protein tyrosine kinase activator activity
- transmembrane receptor protein tyrosine kinase activity
- transmembrane signaling receptor activity
- ubiquitin protein ligase binding
- virus receptor activity

Location

- basal plasma membrane
- basolateral plasma membrane
- cell junction
- cell surface
- clathrin-coated endocytic vesicle membrane
- cytoplasm
- early endosome membrane
- endoplasmic reticulum membrane
- endosome
- endosome membrane
- extracellular space
- focal adhesion
- Golgi membrane
- intracellular vesicle
- membrane
- membrane raft
- multivesicular body, internal vesicle lumen
- nuclear membrane
- nucleus
- perinuclear region of cytoplasm
- plasma membrane
- protein-containing complex
- receptor complex
- ruffle membrane
- Shc-EGFR complex

Biological process

- activation of phospholipase C activity
- cell morphogenesis
- cell surface receptor signaling pathway
- cell-cell adhesion
- cellular response to amino acid stimulus
- cellular response to cadmium ion
- cellular response to epidermal growth factor stimulus
- cellular response to estradiol stimulus
- cellular response to reactive oxygen species
- cerebral cortex cell migration
- digestive tract morphogenesis
- embryonic placenta development
- epidermal growth factor receptor signaling pathway
- epithelial cell proliferation
- ERBB2-EGFR signaling pathway
- eyelid development in camera-type eye
- hair follicle development
- learning or memory
- morphogenesis of an epithelial fold
- negative regulation of apoptotic process
- negative regulation of cardiocyte differentiation
- negative regulation of epidermal growth factor receptor signaling pathway
- negative regulation of protein catabolic process
- neurogenesis
- ossification
- peptidyl-tyrosine autophosphorylation
- peptidyl-tyrosine phosphorylation
- positive regulation of canonical Wnt signaling pathway
- positive regulation of cell growth
- positive regulation of cell migration
- positive regulation of cell population proliferation
- positive regulation of cyclin-dependent protein serine/threonine kinase activity
- positive regulation of DNA repair
- positive regulation of DNA replication
- positive regulation of transcription, DNA-templated
- positive regulation of epithelial cell proliferation
- positive regulation of ERK1 and ERK2 cascade
- positive regulation of fibroblast proliferation
- positive regulation of G1/S transition of mitotic cell cycle
- positive regulation of kinase activity
- positive regulation of MAP kinase activity
- positive regulation of pri-miRNA transcription by RNA polymerase II
- positive regulation of NIK/NF-kappaB signaling
- positive regulation of nitric oxide mediated signal transduction
- positive regulation of peptidyl-serine phosphorylation
- positive regulation of phosphorylation
- positive regulation of protein kinase B signaling
- positive regulation of protein kinase C activity
- positive regulation of protein localization to plasma membrane
- positive regulation of protein phosphorylation
- positive regulation of transcription by RNA polymerase II
- protein autophosphorylation
- protein insertion into membrane
- regulation of ERK1 and ERK2 cascade
- regulation of JNK cascade
- regulation of nitric-oxide synthase activity
- regulation of peptidyl-tyrosine phosphorylation
- regulation of phosphatidylinositol 3-kinase signaling
- response to UV-A
- salivary gland morphogenesis
- signal transduction
- transmembrane receptor protein tyrosine kinase signaling pathway

---

9

- **Protein name:** Small nuclear ribonucleoprotein Sm D2
- **Organism:** Homo sapiens
- **Uniprot Accession Number:** P62316
- **Protein sequence length:** 118 aa
- **1D identity (%):** 2.86
- **1D identity (%) [Gaps excluded]:** 24.04
- **1D identity - Alignment Gaps:** 770
- **1D aligned content (<aminoacid>:%):** {'S': 8.0, 'K': 12.0, 'P': 8.0, 'L': 12.0, 'V': 28.0, 'T': 4.0, 'D': 8.0, 'R': 8.0, 'W': 4.0, 'G': 4.0, 'I': 4.0}
- **Common reported functions (%):** 14.29
- **Common reported locations (%):** 22.22
- **Common reported processes (%):** 0.0

- **PDB ID:** 5Z58
- **Chain:** c
- **Crystallized protein length:** 82 aa
- **Resolution:** 4.9 Å
- **b-phipsi:** 0.002824
- **w-rdist:** 0.402982
- **t-alpha:** 0.007183
- **Chemical similarity (Tanimoto Index) (%):** 32.41
- **1D identity (%) [PDB]:** 0.98
- **1D identity (%) [Gaps excluded][PDB]:** 75.0
- **1D identity - Alignment Gaps [PDB]:** 903
- **1D aligned content [PDB] (<aminoacid>:%):** {'K': 11.11, 'L': 22.22, 'G': 11.11, 'I': 22.22, 'N': 22.22, 'V': 11.11}
- **2D identity (%) [PDB]:** 6.25
- **2D identity (%) [Gaps excluded][PDB]:** 85.71
- **2D identity - Alignment Gaps [PDB]:** 801
- **2D aligned content [PDB] (<2D-fold>:%):** {'H': 18.52, '.': 9.26, 'E': 64.81, 'T': 7.41}
- **3D similarity (TM-Score) (%) [PDB]:** 23.36

- **Gene name:** SNRPD2
- **Entrez ID:** 6632
- **RefSeq ID:** NM\_177542
- **Transcript sequence length:** 689
- **5-UTR|CDS|3-UTR identity (%):** 34.04 | 8.3 | 0.52
- **5-UTR|CDS|3-UTR identity (%) [Gaps excluded]:** 67.19 | 77.03 | 79.09
- **5-UTR|CDS|3-UTR identity [Alignment Gaps]:** 187 | 2344 | 16648
- **5-UTR aligned content (<base>:%):** {'G': 36.43, 'C': 37.21, 'A': 13.18, 'T': 13.18}
- **CDS aligned content (<base>:%):** {'A': 29.82, 'G': 29.36, 'C': 24.31, 'T': 16.51}
- **3-UTR aligned content (<base>:%):** {'G': 14.94, 'C': 17.24, 'T': 35.63, 'A': 32.18}

**Uniprot Description:**  
  
 Plays role in pre-mRNA splicing as core component of the SMN-Sm complex that mediates spliceosomal snRNP assembly and as component of the spliceosomal U1, U2, U4 and U5 small nuclear ribonucleoproteins (snRNPs), the building blocks of the spliceosome (PubMed:11991638, PubMed:18984161, PubMed:19325628, PubMed:23333303, PubMed:25555158, PubMed:26912367, PubMed:28502770, PubMed:28781166, PubMed:28076346). Component of both the pre-catalytic spliceosome B complex and activated spliceosome C complexes (PubMed:11991638, PubMed:28502770, PubMed:28781166, PubMed:28076346). Is also a component of the minor U12 spliceosome (PubMed:15146077).   
  
Core component of the spliceosomal U1, U2, U4 and U5 small nuclear ribonucleoproteins (snRNPs), the building blocks of the spliceosome (PubMed:11991638, PubMed:19325628, PubMed:21516107, PubMed:25555158, PubMed:26912367, PubMed:28502770, PubMed:28781166, PubMed:28076346). Most spliceosomal snRNPs contain a common set of Sm proteins, SNRPB, SNRPD1, SNRPD2, SNRPD3, SNRPE, SNRPF and SNRPG that assemble in a heptameric protein ring on the Sm site of the small nuclear RNA to form the core snRNP (PubMed:10025403, PubMed:19325628, PubMed:21516107, PubMed:25555158, PubMed:26912367, PubMed:28502770, PubMed:28781166, PubMed:28076346). Component of the U1 snRNP (PubMed:19325628, PubMed:25555158). The U1 snRNP is composed of the U1 snRNA and the 7 core Sm proteins SNRPB, SNRPD1, SNRPD2, SNRPD3, SNRPE, SNRPF and SNRPG, and at least three U1 snRNP-specific proteins SNRNP70/U1-70K, SNRPA/U1-A and SNRPC/U1-C (PubMed:19325628, PubMed:25555158). Component of the U4/U6-U5 tri-snRNP complex composed of the U4, U6 and U5 snRNAs and at least PRPF3, PRPF4, PRPF6, PRPF8, PRPF31, SNRNP200, TXNL4A, SNRNP40, SNRPB, SNRPD1, SNRPD2, SNRPD3, SNRPE, SNRPF, SNRPG, DDX23, CD2BP2, PPIH, SNU13, EFTUD2, SART1 and USP39, plus LSM2, LSM3, LSM4, LSM5, LSM6, LSM7 and LSM8 (PubMed:26912367). Component of the U11/U12 snRNPs that are part of the U12-type spliceosome (PubMed:15146077). Part of the SMN-Sm complex that contains SMN1, GEMIN2/SIP1, DDX20/GEMIN3, GEMIN4, GEMIN5, GEMIN6, GEMIN7, GEMIN8, STRAP/UNRIP and the Sm proteins SNRPB, SNRPD1, SNRPD2, SNRPD3, SNRPE, SNRPF and SNRPG; catalyzes core snRNPs assembly. Forms a 6S pICln-Sm complex composed of CLNS1A/pICln, SNRPD1, SNRPD2, SNRPE, SNRPF and SNRPG; ring-like structure where CLNS1A/pICln mimics additional Sm proteins and which is unable to assemble into the core snRNP.   
  
 **Gene Ontology Information:**

Molecular Function

- RNA binding
- U1 snRNP binding

Location

- catalytic step 2 spliceosome
- commitment complex
- cytosol
- methylosome
- nucleoplasm
- nucleus
- pICln-Sm protein complex
- precatalytic spliceosome
- small nuclear ribonucleoprotein complex
- SMN-Sm protein complex
- spliceosomal complex
- spliceosomal tri-snRNP complex
- U1 snRNP
- U12-type spliceosomal complex
- U2 snRNP
- U2-type catalytic step 2 spliceosome
- U2-type precatalytic spliceosome
- U4 snRNP
- U4/U6 x U5 tri-snRNP complex
- U5 snRNP

Biological process

- 7-methylguanosine cap hypermethylation
- mRNA splicing, via spliceosome
- RNA splicing
- spliceosomal complex assembly
- spliceosomal snRNP assembly
- U2-type prespliceosome assembly

---

10

- **Protein name:** Complement C3
- **Organism:** Homo sapiens
- **Uniprot Accession Number:** P01024
- **Protein sequence length:** 1663 aa
- **1D identity (%):** 14.45
- **1D identity (%) [Gaps excluded]:** 29.59
- **1D identity - Alignment Gaps:** 867
- **1D aligned content (<aminoacid>:%):** {'G': 9.8, 'P': 11.02, 'L': 9.39, 'V': 9.8, 'T': 6.94, 'K': 4.9, 'Q': 6.53, 'I': 3.27, 'E': 2.86, 'F': 3.67, 'R': 6.53, 'D': 4.49, 'S': 2.86, 'Y': 4.9, 'H': 3.67, 'A': 6.12, 'C': 2.45, 'N': 0.82}
- **Common reported functions (%):** 0.0
- **Common reported locations (%):** 0.0
- **Common reported processes (%):** 0.0

- **PDB ID:** 8ENU
- **Chain:** D
- **Crystallized protein length:** 706 aa
- **Resolution:** 3.22 Å
- **b-phipsi:** 0.001464
- **w-rdist:** 0.762512
- **t-alpha:** 0.000793
- **Chemical similarity (Tanimoto Index) (%):** 83.93
- **1D identity (%) [PDB]:** 2.28
- **1D identity (%) [Gaps excluded][PDB]:** 85.0
- **1D identity - Alignment Gaps [PDB]:** 1453
- **1D aligned content [PDB] (<aminoacid>:%):** {'P': 8.82, 'V': 17.65, 'Y': 5.88, 'D': 8.82, 'G': 11.76, 'T': 5.88, 'A': 5.88, 'N': 2.94, 'L': 2.94, 'E': 2.94, 'K': 8.82, 'R': 2.94, 'F': 5.88, 'S': 5.88, 'I': 2.94}
- **2D identity (%) [PDB]:** 40.97
- **2D identity (%) [Gaps excluded][PDB]:** 85.14
- **2D identity - Alignment Gaps [PDB]:** 537
- **2D aligned content [PDB] (<2D-fold>:%):** {'.': 16.51, 'T': 29.25, 'E': 30.42, 'H': 21.93, 'G': 1.42, 'B': 0.47}
- **3D similarity (TM-Score) (%) [PDB]:** 13.44

- **Gene name:** C3
- **Entrez ID:** N/A
- **RefSeq ID:** NM\_000064
- **Transcript sequence length:** 5231
- **5-UTR|CDS|3-UTR identity (%):** 13.54 | 35.73 | 0.64
- **5-UTR|CDS|3-UTR identity (%) [Gaps excluded]:** 75.86 | 77.15 | 76.6
- **5-UTR|CDS|3-UTR identity [Alignment Gaps]:** 267 | 2779 | 16651
- **5-UTR aligned content (<base>:%):** {'A': 9.09, 'C': 56.82, 'T': 25.0, 'G': 9.09}
- **CDS aligned content (<base>:%):** {'A': 28.32, 'T': 22.54, 'G': 24.65, 'C': 24.49}
- **3-UTR aligned content (<base>:%):** {'C': 36.11, 'A': 15.74, 'T': 29.63, 'G': 18.52}

**Uniprot Description:**  
  
 C3 plays a central role in the activation of the complement system. Its processing by C3 convertase is the central reaction in both classical and alternative complement pathways. After activation C3b can bind covalently, via its reactive thioester, to cell surface carbohydrates or immune aggregates.   
  
C3 precursor is first processed by the removal of 4 Arg residues, forming two chains, beta and alpha, linked by a disulfide bond. C3 convertase activates C3 by cleaving the alpha chain, releasing C3a anaphylatoxin and generating C3b (beta chain + alpha' chain). Forms the pro-C3-convertase enzyme complex by interacting with Complement factor B Bb fragment (Bb), which is then stabilized by binding CFP, allowing the complex to become active (PubMed:28264884, PubMed:31507604). The interaction with Bb is dependent on Mg2+ (PubMed:31507604). C3b interacts with CR1 (via Sushi 8 and Sushi 9 domains) (PubMed:8175757, PubMed:2972794). C3b interacts with CFH (PubMed:21285368). C3d interacts with CFH (PubMed:21285368, PubMed:21317894). C3dg interacts with CR2 (via the N-terminal Sushi domains 1 and 2). During pregnancy, C3dg exists as a complex (probably a 2:2:2 heterohexamer) with AGT and the proform of PRG2. Interacts with VSIG4. Interacts (both C3a and ASP) with C5AR2; the interaction occurs with higher affinity for ASP, enhancing the phosphorylation and activation of C5AR2, recruitment of ARRB2 to the cell surface and endocytosis of GRP77.   
  
 **Gene Ontology Information:**

Molecular Function   
  
N/A

Location   
  
N/A

Biological process   
  
N/A

---

11

- **Protein name:** Protein argonaute-4
- **Organism:** Homo sapiens
- **Uniprot Accession Number:** Q9HCK5
- **Protein sequence length:** 861 aa
- **1D identity (%):** 80.5
- **1D identity (%) [Gaps excluded]:** 82.69
- **1D identity - Alignment Gaps:** 23
- **1D aligned content (<aminoacid>:%):** {'G': 7.12, 'P': 6.98, 'L': 7.83, 'R': 6.27, 'T': 6.13, 'K': 5.41, 'I': 5.13, 'A': 6.27, 'N': 2.42, 'F': 4.13, 'Q': 6.13, 'V': 8.97, 'D': 4.84, 'Y': 4.13, 'E': 4.27, 'M': 2.42, 'H': 3.13, 'S': 4.99, 'W': 0.71, 'C': 2.71}
- **Common reported functions (%):** 57.14
- **Common reported locations (%):** 77.78
- **Common reported processes (%):** 66.67

- **PDB ID:** 6OON
- **Chain:** A
- **Crystallized protein length:** 785 aa
- **Resolution:** 1.9 Å
- **b-phipsi:** 0.001793
- **w-rdist:** 0.230722
- **t-alpha:** 0.042789
- **Chemical similarity (Tanimoto Index) (%):** 99.62
- **1D identity (%) [PDB]:** 76.91
- **1D identity (%) [Gaps excluded][PDB]:** 83.46
- **1D identity - Alignment Gaps [PDB]:** 67
- **1D aligned content [PDB] (<aminoacid>:%):** {'P': 7.32, 'R': 6.55, 'G': 5.95, 'T': 6.4, 'K': 5.49, 'I': 5.34, 'L': 8.23, 'A': 6.4, 'N': 2.29, 'F': 4.42, 'Q': 5.64, 'V': 9.45, 'D': 4.73, 'Y': 4.42, 'E': 3.96, 'M': 2.44, 'H': 2.74, 'S': 4.73, 'W': 0.76, 'C': 2.74}
- **2D identity (%) [PDB]:** 62.02
- **2D identity (%) [Gaps excluded][PDB]:** 76.09
- **2D identity - Alignment Gaps [PDB]:** 167
- **2D aligned content [PDB] (<2D-fold>:%):** {'.': 19.29, 'B': 1.96, 'E': 30.54, 'H': 36.07, 'T': 11.07, 'G': 1.07}
- **3D similarity (TM-Score) (%) [PDB]:** 89.8

- **Gene name:** AGO4
- **Entrez ID:** 192670
- **RefSeq ID:** NM\_017629
- **Transcript sequence length:** 7272
- **5-UTR|CDS|3-UTR identity (%):** 49.77 | 73.5 | 16.25
- **5-UTR|CDS|3-UTR identity (%) [Gaps excluded]:** 71.38 | 78.65 | 75.62
- **5-UTR|CDS|3-UTR identity [Alignment Gaps]:** 129 | 175 | 13598
- **5-UTR aligned content (<base>:%):** {'G': 36.32, 'T': 11.32, 'C': 48.58, 'A': 3.77}
- **CDS aligned content (<base>:%):** {'A': 28.46, 'T': 24.59, 'G': 24.19, 'C': 22.76}
- **3-UTR aligned content (<base>:%):** {'G': 19.37, 'A': 30.74, 'T': 34.12, 'C': 15.78}

**Uniprot Description:**  
  
 Required for RNA-mediated gene silencing (RNAi). Binds to short RNAs such as microRNAs (miRNAs) and represses the translation of mRNAs which are complementary to them. Lacks endonuclease activity and does not appear to cleave target mRNAs. Also required for RNA-directed transcription and replication of the human hapatitis delta virus (HDV).   
  
Interacts with EIF4B, IMP8, PRMT5, TNRC6A and TNRC6B (PubMed:19167051). Interacts with ZFP36 (PubMed:15766526).   
  
 **Gene Ontology Information:**

Molecular Function

- double-stranded RNA binding
- miRNA binding
- endoribonuclease activity
- single-stranded RNA binding

Location

- cytoplasm
- cytoplasmic ribonucleoprotein granule
- cytosol
- membrane
- nucleus
- P-body
- RISC complex
- RISC-loading complex

Biological process

- male gonad development
- male meiotic nuclear division
- miRNA metabolic process
- production of miRNAs involved in gene silencing by miRNA
- miRNA mediated inhibition of translation
- mRNA catabolic process
- negative regulation of apoptotic process
- pre-miRNA processing
- regulation of cell morphogenesis
- small RNA loading onto RISC
- RNA secondary structure unwinding
- synaptonemal complex assembly

---

12

- **Protein name:** Regulator of nonsense transcripts 1
- **Organism:** Homo sapiens
- **Uniprot Accession Number:** Q92900
- **Protein sequence length:** 1129 aa
- **1D identity (%):** 15.47
- **1D identity (%) [Gaps excluded]:** 24.29
- **1D identity - Alignment Gaps:** 441
- **1D aligned content (<aminoacid>:%):** {'M': 0.53, 'G': 11.7, 'P': 10.64, 'Q': 5.85, 'L': 9.57, 'N': 3.72, 'K': 5.32, 'E': 4.79, 'D': 4.79, 'C': 2.13, 'R': 6.38, 'V': 9.04, 'T': 5.85, 'H': 1.6, 'Y': 3.72, 'W': 0.53, 'I': 3.72, 'F': 3.19, 'A': 4.79, 'S': 2.13}
- **Common reported functions (%):** 14.29
- **Common reported locations (%):** 44.44
- **Common reported processes (%):** 0.0

- **PDB ID:** 2WJY
- **Chain:** A
- **Crystallized protein length:** 773 aa
- **Resolution:** 2.5 Å
- **b-phipsi:** 0.004913
- **w-rdist:** 0.485287
- **t-alpha:** 0.0
- **Chemical similarity (Tanimoto Index) (%):** 80.8
- **1D identity (%) [PDB]:** 2.69
- **1D identity (%) [Gaps excluded][PDB]:** 66.67
- **1D identity - Alignment Gaps [PDB]:** 1496
- **1D aligned content [PDB] (<aminoacid>:%):** {'D': 4.76, 'E': 4.76, 'Q': 9.52, 'R': 11.9, 'P': 7.14, 'T': 11.9, 'S': 4.76, 'V': 11.9, 'I': 2.38, 'K': 2.38, 'G': 4.76, 'H': 4.76, 'C': 2.38, 'Y': 4.76, 'F': 4.76, 'L': 2.38, 'N': 2.38, 'A': 2.38}
- **2D identity (%) [PDB]:** 38.16
- **2D identity (%) [Gaps excluded][PDB]:** 82.97
- **2D identity - Alignment Gaps [PDB]:** 600
- **2D aligned content [PDB] (<2D-fold>:%):** {'.': 20.05, 'E': 23.11, 'T': 17.45, 'H': 37.97, 'G': 1.42}
- **3D similarity (TM-Score) (%) [PDB]:** 20.16

- **Gene name:** UPF1
- **Entrez ID:** 5976
- **RefSeq ID:** NM\_002911
- **Transcript sequence length:** 5321
- **5-UTR|CDS|3-UTR identity (%):** 45.94 | 42.74 | 6.1
- **5-UTR|CDS|3-UTR identity (%) [Gaps excluded]:** 75.93 | 76.91 | 74.43
- **5-UTR|CDS|3-UTR identity [Alignment Gaps]:** 141 | 1696 | 15668
- **5-UTR aligned content (<base>:%):** {'A': 3.05, 'G': 46.95, 'T': 10.98, 'C': 39.02}
- **CDS aligned content (<base>:%):** {'A': 27.33, 'T': 19.42, 'G': 27.14, 'C': 26.1}
- **3-UTR aligned content (<base>:%):** {'A': 22.46, 'G': 29.75, 'T': 23.03, 'C': 24.76}

**Uniprot Description:**  
  
 RNA-dependent helicase and ATPase required for nonsense-mediated decay (NMD) of mRNAs containing premature stop codons. Is recruited to mRNAs upon translation termination and undergoes a cycle of phosphorylation and dephosphorylation; its phosphorylation appears to be a key step in NMD. Recruited by release factors to stalled ribosomes together with the SMG1C protein kinase complex to form the transient SURF (SMG1-UPF1-eRF1-eRF3) complex. In EJC-dependent NMD, the SURF complex associates with the exon junction complex (EJC) (located 50-55 or more nucleotides downstream from the termination codon) through UPF2 and allows the formation of an UPF1-UPF2-UPF3 surveillance complex which is believed to activate NMD. Phosphorylated UPF1 is recognized by EST1B/SMG5, SMG6 and SMG7 which are thought to provide a link to the mRNA degradation machinery involving exonucleolytic and endonucleolytic pathways, and to serve as adapters to protein phosphatase 2A (PP2A), thereby triggering UPF1 dephosphorylation and allowing the recycling of NMD factors. UPF1 can also activate NMD without UPF2 or UPF3, and in the absence of the NMD-enhancing downstream EJC indicative for alternative NMD pathways. Plays a role in replication-dependent histone mRNA degradation at the end of phase S; the function is independent of UPF2. For the recognition of premature termination codons (PTC) and initiation of NMD a competitive interaction between UPF1 and PABPC1 with the ribosome-bound release factors is proposed. The ATPase activity of UPF1 is required for disassembly of mRNPs undergoing NMD. Essential for embryonic viability. Together with UPF2 and dependent on TDRD6, mediates the degradation of mRNA hardoring long 3'UTR by inducing the NMD machinery (By similarity).   
  
Found in a post-splicing messenger ribonucleoprotein (mRNP) complex (PubMed:21419344). Associates with the exon junction complex (EJC) (PubMed:11546874, PubMed:16452507). Associates with the SGM1C complex; is phosphorylated by the complex kinase component SGM1 (PubMed:19417104). Interacts with UPF2 (PubMed:11163187, PubMed:11073994, PubMed:11113196, PubMed:19556969). Interacts with UPF3A and UPF3B (PubMed:11163187). Interacts with EST1A (PubMed:12554878). Interacts with SLBP (PubMed:16086026). Interacts (when hyperphosphorylated) with PNRC2 (PubMed:19150429). Interacts with AGO1 and AGO2 (PubMed:17932509). Interacts with GSPT2 (PubMed:18447585). Interacts with isoform 1 and isoform 5 of ADAR/ADAR1 (PubMed:18362360). Interacts with SMG7 (PubMed:15721257). Interacts with ZC3H12A; this interaction occurs in a mRNA translationally active- and termination-dependent manner and is essential for ZC3H12A-mediated degradation of target mRNAs (PubMed:26000482). Interacts with CPSF6 (PubMed:19864460). Interacts with MOV10; the interaction is direct and RNA-dependent (PubMed:24726324). Interacts with SHFL; the interaction increases in the presence of RNA (PubMed:27974568). Interacts with UPF2 and DDX4; interactions are mediated by TDRD6 (By similarity).   
  
 **Gene Ontology Information:**

Molecular Function

- ATP binding
- ATPase activity
- chromatin binding
- double-stranded DNA helicase activity
- helicase activity
- protein-containing complex binding
- RNA binding
- RNA helicase activity
- telomeric DNA binding
- zinc ion binding

Location

- chromatin
- chromosome, telomeric region
- cytoplasm
- cytosol
- exon-exon junction complex
- nucleoplasm
- nucleus
- P-body
- perinuclear region of cytoplasm
- supraspliceosomal complex

Biological process

- 3'-UTR-mediated mRNA destabilization
- cell cycle phase transition
- cellular response to interleukin-1
- cellular response to lipopolysaccharide
- DNA duplex unwinding
- DNA repair
- DNA replication
- histone mRNA catabolic process
- mRNA export from nucleus
- nuclear-transcribed mRNA catabolic process
- nuclear-transcribed mRNA catabolic process, nonsense-mediated decay
- positive regulation of mRNA catabolic process
- regulation of telomere maintenance
- regulation of translational termination
- telomere maintenance via semi-conservative replication

---

13

- **Protein name:** Integrin beta-3
- **Organism:** Homo sapiens
- **Uniprot Accession Number:** P05106
- **Protein sequence length:** 788 aa
- **1D identity (%):** 15.98
- **1D identity (%) [Gaps excluded]:** 24.73
- **1D identity - Alignment Gaps:** 354
- **1D aligned content (<aminoacid>:%):** {'A': 5.0, 'P': 10.62, 'L': 8.75, 'G': 7.5, 'I': 3.12, 'C': 5.0, 'Q': 5.62, 'D': 5.0, 'E': 5.62, 'K': 5.62, 'V': 7.5, 'R': 4.38, 'S': 6.25, 'T': 7.5, 'Y': 3.75, 'F': 3.12, 'H': 2.5, 'M': 0.62, 'N': 2.5}
- **Common reported functions (%):** 14.29
- **Common reported locations (%):** 11.11
- **Common reported processes (%):** 11.11

- **PDB ID:** 4G1M
- **Chain:** B
- **Crystallized protein length:** 692 aa
- **Resolution:** 2.9 Å
- **b-phipsi:** 0.00246
- **w-rdist:** 0.659858
- **t-alpha:** 0.001587
- **Chemical similarity (Tanimoto Index) (%):** 73.79
- **1D identity (%) [PDB]:** 1.8
- **1D identity (%) [Gaps excluded][PDB]:** 72.97
- **1D identity - Alignment Gaps [PDB]:** 1463
- **1D aligned content [PDB] (<aminoacid>:%):** {'G': 7.41, 'V': 11.11, 'S': 7.41, 'E': 11.11, 'Q': 11.11, 'R': 7.41, 'Y': 11.11, 'L': 7.41, 'A': 7.41, 'I': 11.11, 'D': 3.7, 'P': 3.7}
- **2D identity (%) [PDB]:** 31.95
- **2D identity (%) [Gaps excluded][PDB]:** 83.33
- **2D identity - Alignment Gaps [PDB]:** 685
- **2D aligned content [PDB] (<2D-fold>:%):** {'.': 20.0, 'H': 30.42, 'T': 17.75, 'E': 31.55, 'B': 0.28}
- **3D similarity (TM-Score) (%) [PDB]:** 20.49

- **Gene name:** ITGB3
- **Entrez ID:** 3690
- **RefSeq ID:** NM\_000212
- **Transcript sequence length:** 5941
- **5-UTR|CDS|3-UTR identity (%):** 8.7 | 45.05 | 12.82
- **5-UTR|CDS|3-UTR identity (%) [Gaps excluded]:** 80.0 | 75.69 | 75.36
- **5-UTR|CDS|3-UTR identity [Alignment Gaps]:** 287 | 1256 | 14394
- **5-UTR aligned content (<base>:%):** {'G': 67.86, 'C': 25.0, 'A': 7.14}
- **CDS aligned content (<base>:%):** {'A': 26.97, 'T': 22.32, 'G': 26.25, 'C': 24.46}
- **3-UTR aligned content (<base>:%):** {'T': 35.94, 'A': 26.41, 'G': 19.66, 'C': 17.99}

**Uniprot Description:**  
  
 Integrin alpha-V/beta-3 (ITGAV:ITGB3) is a receptor for cytotactin, fibronectin, laminin, matrix metalloproteinase-2, osteopontin, osteomodulin, prothrombin, thrombospondin, vitronectin and von Willebrand factor. Integrin alpha-IIb/beta-3 (ITGA2B:ITGB3) is a receptor for fibronectin, fibrinogen, plasminogen, prothrombin, thrombospondin and vitronectin. Integrins alpha-IIb/beta-3 and alpha-V/beta-3 recognize the sequence R-G-D in a wide array of ligands. Integrin alpha-IIb/beta-3 recognizes the sequence H-H-L-G-G-G-A-K-Q-A-G-D-V in fibrinogen gamma chain. Following activation integrin alpha-IIb/beta-3 brings about platelet/platelet interaction through binding of soluble fibrinogen. This step leads to rapid platelet aggregation which physically plugs ruptured endothelial surface. Fibrinogen binding enhances SELP expression in activated platelets (By similarity). ITGAV:ITGB3 binds to fractalkine (CX3CL1) and acts as its coreceptor in CX3CR1-dependent fractalkine signaling (PubMed:23125415, PubMed:24789099). ITGAV:ITGB3 binds to NRG1 (via EGF domain) and this binding is essential for NRG1-ERBB signaling (PubMed:20682778). ITGAV:ITGB3 binds to FGF1 and this binding is essential for FGF1 signaling (PubMed:18441324). ITGAV:ITGB3 binds to FGF2 and this binding is essential for FGF2 signaling (PubMed:28302677). ITGAV:ITGB3 binds to IGF1 and this binding is essential for IGF1 signaling (PubMed:19578119). ITGAV:ITGB3 binds to IGF2 and this binding is essential for IGF2 signaling (PubMed:28873464). ITGAV:ITGB3 binds to IL1B and this binding is essential for IL1B signaling (PubMed:29030430). ITGAV:ITGB3 binds to PLA2G2A via a site (site 2) which is distinct from the classical ligand-binding site (site 1) and this induces integrin conformational changes and enhanced ligand binding to site 1 (PubMed:18635536, PubMed:25398877). ITGAV:ITGB3 acts as a receptor for fibrillin-1 (FBN1) and mediates R-G-D-dependent cell adhesion to FBN1 (PubMed:12807887). In brain, plays a role in synaptic transmission and plasticity. Involved in the regulation of the serotonin neurotransmission, is required to localize to specific compartments within the synapse the serotonin receptor SLC6A4 and for an appropriate reuptake of serotonin. Controls excitatory synaptic strength by regulating GRIA2-containing AMPAR endocytosis, which affects AMPAR abundance and composition (By similarity). ITGAV:ITGB3 act as a receptor for CD40LG (PubMed:31331973).   
  
Heterodimer of an alpha and a beta subunit. Beta-3 (ITGB3) associates with either alpha-IIb (ITGA2B) or alpha-V (ITGAV). Isoform Beta-3C interacts with FLNB. Interacts with COMP. Interacts with PDIA6 following platelet stimulation. Interacts with SYK; upon activation by ITGB3 promotes platelet adhesion. Interacts with MYO10. Interacts with DAB2. Interacts with FERMT2. Interacts with EMP2; regulates the levels of the heterodimer ITGA5:ITGB3 integrin expression on the plasma membrane (PubMed:16216233). Integrin ITGAV:ITGB3 interacts with FBLN5 (via N-terminus) (By similarity). ITGAV:ITGB3 interacts with CCN3 (PubMed:12695522). ITGAV:ITGB3 is found in a ternary complex with CX3CR1 and CX3CL1 (PubMed:23125415). ITGAV:ITGB3 is found in a ternary complex with NRG1 and ERBB3 (PubMed:20682778). ITGAV:ITGB3 is found in a ternary complex with FGF1 and FGFR1 (PubMed:18441324). ITGAV:ITGB3 interacts with FGF2; it is likely that FGF2 can simultaneously bind ITGAV:ITGB3 and FGF receptors (PubMed:28302677). ITGAV:ITGB3 binds to IL1B (PubMed:29030430). ITGAV:ITGB3 is found in a ternary complex with IGF1 and IGF1R (PubMed:19578119). ITGAV:ITGB3 interacts with IGF2 (PubMed:28873464). ITGAV:ITGB3 interacts with FBN1 (PubMed:12807887). ITGAV:ITGB3 interacts with CD9, CD81 and CD151 (via second extracellular domain) (PubMed:27993971). Interacts (via the allosteric site (site 2)) with CXCL12 in a CXCR4-independent manner (PubMed:29301984). Interacts with MXRA8/DICAM; the interaction inhibits ITGAV:ITGB3 heterodimer formation (PubMed:22492581). ITGAV:ITGB3 interacts with PTN (PubMed:19141530). Forms a complex with PTPRZ1 and PTN that stimulates endothelial cell migration through ITGB3 Tyr-773 phosphorylation (PubMed:19141530). ITGAV:ITGB3 interacts with SLC6A4 (By similarity). ITGA2B:ITGB3 interacts with PPIA/CYPA; the interaction is ROS and PPIase activity-dependent and is increased in the presence of thrombin (By similarity).   
  
 **Gene Ontology Information:**

Molecular Function

- cell adhesion molecule binding
- coreceptor activity
- enzyme binding
- extracellular matrix binding
- fibrinogen binding
- fibronectin binding
- identical protein binding
- integrin binding
- metal ion binding
- platelet-derived growth factor receptor binding
- protease binding
- protein disulfide isomerase activity
- protein kinase C binding
- vascular endothelial growth factor receptor 2 binding
- virus receptor activity

Location

- alpha9-beta1 integrin-ADAM8 complex
- alphav-beta3 integrin-HMGB1 complex
- alphav-beta3 integrin-IGF-1-IGF1R complex
- alphav-beta3 integrin-PKCalpha complex
- alphav-beta3 integrin-vitronectin complex
- apical plasma membrane
- cell surface
- cell-cell junction
- external side of plasma membrane
- extracellular exosome
- filopodium membrane
- focal adhesion
- glutamatergic synapse
- glycinergic synapse
- integrin alphav-beta3 complex
- integrin complex
- lamellipodium membrane
- melanosome
- microvillus membrane
- nucleoplasm
- nucleus
- plasma membrane
- platelet alpha granule membrane
- postsynaptic membrane
- protein-containing complex
- receptor complex
- ruffle membrane
- synapse

Biological process

- activation of protein kinase activity
- angiogenesis involved in wound healing
- apolipoprotein A-I-mediated signaling pathway
- apoptotic cell clearance
- blood coagulation
- blood coagulation, fibrin clot formation
- cell adhesion
- cell adhesion mediated by integrin
- cell-matrix adhesion
- cell-substrate adhesion
- cell-substrate junction assembly
- cellular response to insulin-like growth factor stimulus
- cellular response to mechanical stimulus
- cellular response to platelet-derived growth factor stimulus
- cellular response to xenobiotic stimulus
- embryo implantation
- heterotypic cell-cell adhesion
- integrin-mediated signaling pathway
- maintenance of postsynaptic specialization structure
- mesodermal cell differentiation
- negative chemotaxis
- negative regulation of endothelial cell apoptotic process
- negative regulation of lipid storage
- negative regulation of lipid transport
- negative regulation of lipoprotein metabolic process
- negative regulation of low-density lipoprotein receptor activity
- negative regulation of macrophage derived foam cell differentiation
- platelet activation
- platelet aggregation
- platelet-derived growth factor receptor signaling pathway
- positive regulation of adenylate cyclase-inhibiting opioid receptor signaling pathway
- positive regulation of angiogenesis
- positive regulation of bone resorption
- positive regulation of cell adhesion mediated by integrin
- positive regulation of cell-matrix adhesion
- positive regulation of endothelial cell migration
- positive regulation of endothelial cell proliferation
- positive regulation of ERK1 and ERK2 cascade
- positive regulation of fibroblast migration
- positive regulation of fibroblast proliferation
- positive regulation of gene expression
- positive regulation of glomerular mesangial cell proliferation
- positive regulation of osteoblast proliferation
- positive regulation of peptidyl-tyrosine phosphorylation
- positive regulation of protein phosphorylation
- positive regulation of smooth muscle cell migration
- positive regulation of smooth muscle cell proliferation
- positive regulation of substrate adhesion-dependent cell spreading
- positive regulation of T cell migration
- positive regulation of vascular endothelial growth factor receptor signaling pathway
- regulation of actin cytoskeleton organization
- regulation of bone resorption
- regulation of extracellular matrix organization
- regulation of postsynaptic neurotransmitter receptor diffusion trapping
- regulation of postsynaptic neurotransmitter receptor internalization
- regulation of protein localization
- regulation of protein tyrosine kinase activity
- regulation of release of sequestered calcium ion into cytosol
- regulation of serotonin uptake
- regulation of trophoblast cell migration
- response to activity
- smooth muscle cell migration
- substrate adhesion-dependent cell spreading
- tube development
- viral entry into host cell
- wound healing

---

14

- **Protein name:** DNA replication licensing factor MCM7
- **Organism:** Homo sapiens
- **Uniprot Accession Number:** P33993
- **Protein sequence length:** 719 aa
- **1D identity (%):** 15.3
- **1D identity (%) [Gaps excluded]:** 25.51
- **1D identity - Alignment Gaps:** 395
- **1D aligned content (<aminoacid>:%):** {'K': 2.65, 'E': 5.3, 'V': 11.26, 'Q': 6.62, 'F': 2.65, 'G': 6.62, 'Y': 2.65, 'A': 6.62, 'D': 5.96, 'L': 12.58, 'P': 8.61, 'H': 0.66, 'M': 1.99, 'S': 3.97, 'I': 5.3, 'C': 0.66, 'T': 7.28, 'R': 8.61}
- **Common reported functions (%):** 0.0
- **Common reported locations (%):** 33.33
- **Common reported processes (%):** 0.0

- **PDB ID:** 6XTY
- **Chain:** 7
- **Crystallized protein length:** 598 aa
- **Resolution:** 6.77 Å
- **b-phipsi:** 0.00423
- **w-rdist:** 0.462394
- **t-alpha:** 0.003962
- **Chemical similarity (Tanimoto Index) (%):** N/A
- **1D identity (%) [PDB]:** 2.8
- **1D identity (%) [Gaps excluded][PDB]:** 75.0
- **1D identity - Alignment Gaps [PDB]:** 1341
- **1D aligned content [PDB] (<aminoacid>:%):** {'Q': 10.26, 'E': 12.82, 'D': 5.13, 'A': 7.69, 'V': 5.13, 'L': 12.82, 'Y': 2.56, 'K': 5.13, 'R': 7.69, 'F': 7.69, 'P': 2.56, 'T': 2.56, 'I': 10.26, 'S': 5.13, 'G': 2.56}
- **2D identity (%) [PDB]:** 29.98
- **2D identity (%) [Gaps excluded][PDB]:** 86.79
- **2D identity - Alignment Gaps [PDB]:** 703
- **2D aligned content [PDB] (<2D-fold>:%):** {'T': 19.25, 'H': 36.34, '.': 19.88, 'E': 23.6, 'G': 0.93}
- **3D similarity (TM-Score) (%) [PDB]:** 20.73

- **Gene name:** MCM7
- **Entrez ID:** 4176
- **RefSeq ID:** NM\_182776
- **Transcript sequence length:** 2725
- **5-UTR|CDS|3-UTR identity (%):** 26.97 | 32.46 | 0.86
- **5-UTR|CDS|3-UTR identity (%) [Gaps excluded]:** 78.39 | 76.63 | 79.56
- **5-UTR|CDS|3-UTR identity [Alignment Gaps]:** 591 | 1707 | 16597
- **5-UTR aligned content (<base>:%):** {'A': 9.05, 'G': 35.39, 'T': 19.34, 'C': 36.21}
- **CDS aligned content (<base>:%):** {'A': 27.16, 'T': 20.5, 'G': 28.82, 'C': 23.52}
- **3-UTR aligned content (<base>:%):** {'T': 28.47, 'C': 25.0, 'A': 19.44, 'G': 27.08}

**Uniprot Description:**  
  
 Acts as component of the MCM2-7 complex (MCM complex) which is the putative replicative helicase essential for 'once per cell cycle' DNA replication initiation and elongation in eukaryotic cells. The active ATPase sites in the MCM2-7 ring are formed through the interaction surfaces of two neighboring subunits such that a critical structure of a conserved arginine finger motif is provided in trans relative to the ATP-binding site of the Walker A box of the adjacent subunit. The six ATPase active sites, however, are likely to contribute differentially to the complex helicase activity. Required for S-phase checkpoint activation upon UV-induced damage.   
  
Component of the MCM2-7 complex (PubMed:9305914, PubMed:16899510, PubMed:17296731). The complex forms a toroidal hexameric ring with the proposed subunit order MCM2-MCM6-MCM4-MCM7-MCM3-MCM5 (PubMed:9305914, PubMed:16899510, PubMed:17296731). Interacts with the ATR-ATRIP complex and with RAD17 (PubMed:15210935, PubMed:15538388). Interacts with TIPIN (PubMed:17116885). Interacts with MCMBP (PubMed:17296731). Interacts with ANKRD17 (PubMed:23711367). Component of the replisome complex composed of at least DONSON, MCM2, MCM7, PCNA and TICRR (PubMed:28191891). Component of the CMG helicase complex, composed of the MCM2-7 complex, the GINS complex and CDC45 (By similarity).   
  
 **Gene Ontology Information:**

Molecular Function

- ATP binding
- ATPase activity
- DNA helicase activity
- single-stranded DNA binding

Location

- chromatin
- chromosome, telomeric region
- CMG complex
- cytosol
- MCM complex
- membrane
- nucleoplasm
- nucleus

Biological process

- cell cycle
- cell population proliferation
- cellular response to epidermal growth factor stimulus
- cellular response to organic substance
- cellular response to xenobiotic stimulus
- cellular response to DNA damage stimulus
- DNA replication
- DNA replication initiation
- DNA strand elongation involved in DNA replication
- DNA unwinding involved in DNA replication
- double-strand break repair via break-induced replication
- regulation of DNA-dependent DNA replication initiation
- regulation of phosphorylation

---

15

- **Protein name:** DNA polymerase alpha catalytic subunit
- **Organism:** Homo sapiens
- **Uniprot Accession Number:** P09884
- **Protein sequence length:** 1462 aa
- **1D identity (%):** 15.31
- **1D identity (%) [Gaps excluded]:** 28.41
- **1D identity - Alignment Gaps:** 696
- **1D aligned content (<aminoacid>:%):** {'M': 0.43, 'P': 10.82, 'R': 6.93, 'G': 8.23, 'L': 11.69, 'E': 2.6, 'K': 9.52, 'V': 7.36, 'Y': 3.9, 'D': 6.93, 'T': 3.9, 'S': 3.03, 'N': 3.9, 'H': 3.9, 'F': 4.33, 'W': 0.43, 'Q': 3.46, 'C': 2.16, 'I': 3.46, 'A': 3.03}
- **Common reported functions (%):** 0.0
- **Common reported locations (%):** 22.22
- **Common reported processes (%):** 0.0

- **PDB ID:** 4QCL
- **Chain:** A
- **Crystallized protein length:** 865 aa
- **Resolution:** 2.2 Å
- **b-phipsi:** 0.001961
- **w-rdist:** 0.135904
- **t-alpha:** 0.090333
- **Chemical similarity (Tanimoto Index) (%):** 67.7
- **1D identity (%) [PDB]:** 2.47
- **1D identity (%) [Gaps excluded][PDB]:** 74.55
- **1D identity - Alignment Gaps [PDB]:** 1603
- **1D aligned content [PDB] (<aminoacid>:%):** {'Q': 12.2, 'C': 2.44, 'V': 17.07, 'K': 9.76, 'N': 9.76, 'S': 7.32, 'P': 7.32, 'L': 14.63, 'G': 7.32, 'I': 2.44, 'R': 2.44, 'F': 2.44, 'D': 2.44, 'T': 2.44}
- **2D identity (%) [PDB]:** 37.71
- **2D identity (%) [Gaps excluded][PDB]:** 87.23
- **2D identity - Alignment Gaps [PDB]:** 679
- **2D aligned content [PDB] (<2D-fold>:%):** {'.': 19.51, 'E': 27.27, 'T': 9.53, 'H': 43.02, 'G': 0.67}
- **3D similarity (TM-Score) (%) [PDB]:** 22.63

- **Gene name:** POLA1
- **Entrez ID:** 5422
- **RefSeq ID:** NM\_016937
- **Transcript sequence length:** 5469
- **5-UTR|CDS|3-UTR identity (%):** 10.22 | 40.36 | 3.8
- **5-UTR|CDS|3-UTR identity (%) [Gaps excluded]:** 76.74 | 76.01 | 74.94
- **5-UTR|CDS|3-UTR identity [Alignment Gaps]:** 280 | 2136 | 16075
- **5-UTR aligned content (<base>:%):** {'G': 54.55, 'C': 21.21, 'A': 9.09, 'T': 15.15}
- **CDS aligned content (<base>:%):** {'A': 31.45, 'T': 25.03, 'G': 23.72, 'C': 19.8}
- **3-UTR aligned content (<base>:%):** {'G': 19.28, 'A': 30.79, 'T': 32.81, 'C': 17.11}

**Uniprot Description:**  
  
 Catalytic subunit of the DNA polymerase alpha complex (also known as the alpha DNA polymerase-primase complex) which plays an essential role in the initiation of DNA synthesis. During the S phase of the cell cycle, the DNA polymerase alpha complex (composed of a catalytic subunit POLA1, a regulatory subunit POLA2 and two primase subunits PRIM1 and PRIM2) is recruited to DNA at the replicative forks via direct interactions with MCM10 and WDHD1. The primase subunit of the polymerase alpha complex initiates DNA synthesis by oligomerising short RNA primers on both leading and lagging strands. These primers are initially extended by the polymerase alpha catalytic subunit and subsequently transferred to polymerase delta and polymerase epsilon for processive synthesis on the lagging and leading strand, respectively. The reason this transfer occurs is because the polymerase alpha has limited processivity and lacks intrinsic 3' exonuclease activity for proofreading error, and therefore is not well suited for replicating long complexes. In the cytosol, responsible for a substantial proportion of the physiological concentration of cytosolic RNA:DNA hybrids, which are necessary to prevent spontaneous activation of type I interferon responses (PubMed:27019227).   
  
Component of the alpha DNA polymerase complex (also known as the alpha DNA polymerase-primase complex) consisting of four subunits: the catalytic subunit POLA1, the regulatory subunit POLA2, and the primase complex subunits PRIM1 and PRIM2 respectively (PubMed:9705292). Interacts with PARP1; this interaction functions as part of the control of replication fork progression (PubMed:9518481). Interacts with MCM10 and WDHD1; these interactions recruit the polymerase alpha complex to the pre-replicative complex bound to DNA (PubMed:19608746). Interacts with RPA1; this interaction stabilizes the replicative complex and reduces the misincorporation rate of DNA polymerase alpha by acting as a fidelity clamp (PubMed:9214288).   
  
 **Gene Ontology Information:**

Molecular Function

- chromatin binding
- DNA binding
- DNA replication origin binding
- DNA-directed DNA polymerase activity
- nucleotide binding
- protein kinase binding
- single-stranded DNA binding
- zinc ion binding

Location

- alpha DNA polymerase:primase complex
- cytosol
- nuclear envelope
- nuclear matrix
- nucleolus
- nucleoplasm
- nucleus

Biological process

- DNA repair
- DNA replication
- DNA replication initiation
- DNA replication, synthesis of RNA primer
- DNA strand elongation involved in DNA replication
- DNA synthesis involved in DNA repair
- double-strand break repair via nonhomologous end joining
- lagging strand elongation
- leading strand elongation
- mitotic DNA replication initiation
- regulation of type I interferon production

---

16

- **Protein name:** Lysine-specific histone demethylase 1B
- **Organism:** Homo sapiens
- **Uniprot Accession Number:** Q8NB78
- **Protein sequence length:** 822 aa
- **1D identity (%):** 16.23
- **1D identity (%) [Gaps excluded]:** 23.33
- **1D identity - Alignment Gaps:** 302
- **1D aligned content (<aminoacid>:%):** {'A': 5.59, 'P': 9.94, 'L': 7.45, 'Q': 9.32, 'D': 6.83, 'K': 4.97, 'C': 2.48, 'R': 8.7, 'S': 3.11, 'H': 3.11, 'F': 4.35, 'Y': 3.73, 'T': 3.11, 'N': 3.11, 'E': 4.97, 'I': 3.73, 'G': 7.45, 'V': 6.21, 'M': 1.24, 'W': 0.62}
- **Common reported functions (%):** 0.0
- **Common reported locations (%):** 11.11
- **Common reported processes (%):** 0.0

- **PDB ID:** 7XE3
- **Chain:** A
- **Crystallized protein length:** 747 aa
- **Resolution:** 2.82 Å
- **b-phipsi:** 0.003064
- **w-rdist:** 0.467304
- **t-alpha:** 0.00634
- **Chemical similarity (Tanimoto Index) (%):** 69.99
- **1D identity (%) [PDB]:** 4.51
- **1D identity (%) [Gaps excluded][PDB]:** 66.0
- **1D identity - Alignment Gaps [PDB]:** 1365
- **1D aligned content [PDB] (<aminoacid>:%):** {'E': 4.55, 'F': 4.55, 'D': 4.55, 'Y': 3.03, 'S': 10.61, 'H': 4.55, 'A': 12.12, 'Q': 7.58, 'G': 4.55, 'P': 6.06, 'V': 9.09, 'L': 6.06, 'W': 1.52, 'N': 3.03, 'C': 3.03, 'T': 6.06, 'R': 4.55, 'I': 1.52, 'K': 3.03}
- **2D identity (%) [PDB]:** 41.87
- **2D identity (%) [Gaps excluded][PDB]:** 87.38
- **2D identity - Alignment Gaps [PDB]:** 551
- **2D aligned content [PDB] (<2D-fold>:%):** {'.': 20.09, 'T': 27.99, 'H': 36.79, 'E': 13.09, 'G': 2.03}
- **3D similarity (TM-Score) (%) [PDB]:** 22.22

- **Gene name:** KDM1B
- **Entrez ID:** 221656
- **RefSeq ID:** N/A
- **Sequence length:** N/A
- **5-UTR|CDS|3-UTR identity (%):** N/A | N/A | N/A
- **5-UTR|CDS|3-UTR identity (%) [Gaps excluded]:** N/A | N/A | N/A
- **5-UTR|CDS|3-UTR identity [Alignment Gaps]:** N/A | N/A | N/A
- **5-UTR aligned content (<base>:%):** N/A
- **CDS aligned content (<base>:%):** N/A
- **3-UTR aligned content (<base>:%):** N/A

**Uniprot Description:**  
  
 Histone demethylase that demethylates 'Lys-4' of histone H3, a specific tag for epigenetic transcriptional activation, thereby acting as a corepressor. Required for de novo DNA methylation of a subset of imprinted genes during oogenesis. Acts by oxidizing the substrate by FAD to generate the corresponding imine that is subsequently hydrolyzed. Demethylates both mono- and di-methylated 'Lys-4' of histone H3. Has no effect on tri-methylated 'Lys-4', mono-, di- or tri-methylated 'Lys-9', mono-, di- or tri-methylated 'Lys-27', mono-, di- or tri-methylated 'Lys-36' of histone H3, or on mono-, di- or tri-methylated 'Lys-20' of histone H4.   
  
Does not form a complex with RCOR1/CoREST (By similarity). Interacts with its cofactor GLYR1 at nucleosomes; this interaction stimulates H3K4me1 and H3K4me2 demethylation (PubMed:23260659).   
  
 **Gene Ontology Information:**

Molecular Function

- chromatin binding
- FAD binding
- FAD-dependent H3K4me/H3K4me3 demethylase activity
- flavin adenine dinucleotide binding
- histone binding
- histone demethylase activity
- oxidoreductase activity
- zinc ion binding

Location

- chromatin
- nucleoplasm
- nucleosome
- nucleus

Biological process

- genetic imprinting
- negative regulation of transcription by RNA polymerase II
- positive regulation of transcription by RNA polymerase II
- regulation of gene expression by genetic imprinting
- positive regulation of gene expression, epigenetic

---

17

- **Protein name:** Xanthine dehydrogenase/oxidase
- **Organism:** Homo sapiens
- **Uniprot Accession Number:** P47989
- **Protein sequence length:** 1333 aa
- **1D identity (%):** 15.49
- **1D identity (%) [Gaps excluded]:** 25.39
- **1D identity - Alignment Gaps:** 531
- **1D aligned content (<aminoacid>:%):** {'M': 1.42, 'A': 6.64, 'P': 9.48, 'L': 11.85, 'R': 7.58, 'G': 9.48, 'K': 6.64, 'N': 2.37, 'C': 3.79, 'V': 7.58, 'E': 5.21, 'Q': 2.37, 'D': 3.79, 'F': 4.74, 'W': 0.47, 'T': 6.16, 'S': 2.37, 'I': 2.84, 'H': 2.84, 'Y': 2.37}
- **Common reported functions (%):** 0.0
- **Common reported locations (%):** 11.11
- **Common reported processes (%):** 0.0

- **PDB ID:** 2E1Q
- **Chain:** A
- **Crystallized protein length:** 1307 aa
- **Resolution:** 2.6 Å
- **b-phipsi:** 0.001487
- **w-rdist:** 0.262199
- **t-alpha:** 0.116482
- **Chemical similarity (Tanimoto Index) (%):** 65.62
- **1D identity (%) [PDB]:** 3.72
- **1D identity (%) [Gaps excluded][PDB]:** 67.86
- **1D identity - Alignment Gaps [PDB]:** 1929
- **1D aligned content [PDB] (<aminoacid>:%):** {'A': 7.89, 'D': 5.26, 'V': 11.84, 'E': 2.63, 'K': 5.26, 'N': 5.26, 'Y': 2.63, 'L': 10.53, 'Q': 3.95, 'I': 6.58, 'G': 9.21, 'T': 7.89, 'R': 3.95, 'M': 2.63, 'S': 6.58, 'P': 5.26, 'F': 1.32, 'H': 1.32}
- **2D identity (%) [PDB]:** 36.99
- **2D identity (%) [Gaps excluded][PDB]:** 86.09
- **2D identity - Alignment Gaps [PDB]:** 859
- **2D aligned content [PDB] (<2D-fold>:%):** {'.': 19.57, 'E': 28.73, 'T': 15.62, 'H': 35.55, 'B': 0.54}
- **3D similarity (TM-Score) (%) [PDB]:** 27.63

- **Gene name:** XDH
- **Entrez ID:** 7498
- **RefSeq ID:** NM\_000379
- **Transcript sequence length:** 5715
- **5-UTR|CDS|3-UTR identity (%):** 16.46 | 40.54 | 6.15
- **5-UTR|CDS|3-UTR identity (%) [Gaps excluded]:** 77.14 | 76.65 | 76.78
- **5-UTR|CDS|3-UTR identity [Alignment Gaps]:** 258 | 2029 | 15662
- **5-UTR aligned content (<base>:%):** {'C': 29.63, 'G': 37.04, 'T': 22.22, 'A': 11.11}
- **CDS aligned content (<base>:%):** {'A': 29.04, 'T': 22.97, 'G': 25.89, 'C': 22.11}
- **3-UTR aligned content (<base>:%):** {'A': 33.11, 'G': 20.9, 'T': 30.06, 'C': 15.94}

**Uniprot Description:**  
  
 Key enzyme in purine degradation. Catalyzes the oxidation of hypoxanthine to xanthine. Catalyzes the oxidation of xanthine to uric acid. Contributes to the generation of reactive oxygen species. Has also low oxidase activity towards aldehydes (in vitro).   
  
Homodimer. Interacts with BTN1A1 (By similarity).   
  
 **Gene Ontology Information:**

Molecular Function

- 2 iron, 2 sulfur cluster binding
- FAD binding
- flavin adenine dinucleotide binding
- hypoxanthine dehydrogenase activity
- hypoxanthine oxidase activity
- iron ion binding
- molybdopterin cofactor binding
- protein homodimerization activity
- xanthine dehydrogenase activity
- xanthine oxidase activity

Location

- cytosol
- extracellular space
- peroxisome
- sarcoplasmic reticulum

Biological process

- activation of cysteine-type endopeptidase activity involved in apoptotic process
- adenosine catabolic process
- allantoin metabolic process
- cellular amide catabolic process
- AMP catabolic process
- dAMP catabolic process
- deoxyadenosine catabolic process
- deoxyguanosine catabolic process
- deoxyinosine catabolic process
- dGMP catabolic process
- GMP catabolic process
- guanine catabolic process
- hypoxanthine catabolic process
- IMP catabolic process
- inosine catabolic process
- iron-sulfur cluster assembly
- lactation
- negative regulation of endothelial cell differentiation
- negative regulation of endothelial cell proliferation
- negative regulation of gene expression
- negative regulation of protein kinase B signaling
- negative regulation of protein phosphorylation
- negative regulation of vascular endothelial growth factor signaling pathway
- negative regulation of vasculogenesis
- positive regulation of p38MAPK cascade
- positive regulation of reactive oxygen species metabolic process
- xanthine catabolic process

---

18

- **Protein name:** Cytosolic phospholipase A2 delta
- **Organism:** Homo sapiens
- **Uniprot Accession Number:** Q86XP0
- **Protein sequence length:** 818 aa
- **1D identity (%):** 15.29
- **1D identity (%) [Gaps excluded]:** 22.22
- **1D identity - Alignment Gaps:** 310
- **1D aligned content (<aminoacid>:%):** {'P': 9.87, 'G': 9.21, 'W': 1.32, 'L': 17.76, 'V': 5.26, 'R': 3.95, 'D': 5.92, 'M': 1.32, 'K': 2.63, 'F': 4.61, 'S': 5.92, 'Y': 5.92, 'E': 4.61, 'T': 3.95, 'A': 8.55, 'Q': 3.95, 'N': 1.97, 'I': 1.32, 'C': 1.32, 'H': 0.66}
- **Common reported functions (%):** 0.0
- **Common reported locations (%):** 22.22
- **Common reported processes (%):** 0.0

- **PDB ID:** 5IZ5
- **Chain:** A
- **Crystallized protein length:** 757 aa
- **Resolution:** 2.2 Å
- **b-phipsi:** 0.000724
- **w-rdist:** 0.453158
- **t-alpha:** 0.032733
- **Chemical similarity (Tanimoto Index) (%):** 97.12
- **1D identity (%) [PDB]:** 2.37
- **1D identity (%) [Gaps excluded][PDB]:** 77.08
- **1D identity - Alignment Gaps [PDB]:** 1510
- **1D aligned content [PDB] (<aminoacid>:%):** {'G': 8.11, 'A': 2.7, 'C': 5.41, 'Q': 13.51, 'V': 8.11, 'K': 8.11, 'N': 10.81, 'T': 2.7, 'S': 8.11, 'P': 8.11, 'L': 13.51, 'I': 2.7, 'H': 2.7, 'R': 2.7, 'F': 2.7}
- **2D identity (%) [PDB]:** 36.38
- **2D identity (%) [Gaps excluded][PDB]:** 82.86
- **2D identity - Alignment Gaps [PDB]:** 626
- **2D aligned content [PDB] (<2D-fold>:%):** {'.': 20.94, 'E': 32.51, 'T': 12.07, 'H': 33.0, 'G': 1.48}
- **3D similarity (TM-Score) (%) [PDB]:** 23.28

- **Gene name:** PLA2G4D
- **Entrez ID:** 283748
- **RefSeq ID:** NM\_178034
- **Transcript sequence length:** 4266
- **5-UTR|CDS|3-UTR identity (%):** 20.41 | 40.69 | 6.78
- **5-UTR|CDS|3-UTR identity (%) [Gaps excluded]:** 79.31 | 76.04 | 75.43
- **5-UTR|CDS|3-UTR identity [Alignment Gaps]:** 251 | 1526 | 15417
- **5-UTR aligned content (<base>:%):** {'A': 11.59, 'G': 39.13, 'T': 15.94, 'C': 33.33}
- **CDS aligned content (<base>:%):** {'A': 25.37, 'T': 20.51, 'G': 26.95, 'C': 27.17}
- **3-UTR aligned content (<base>:%):** {'G': 18.55, 'T': 37.89, 'C': 19.86, 'A': 23.69}

**Uniprot Description:**  
  
 Calcium-dependent phospholipase A2 that selectively hydrolyzes glycerophospholipids in the sn-2 position (PubMed:14709560). Has a preference for linoleic acid at the sn-2 position (PubMed:14709560). N/A   
  
 **Gene Ontology Information:**

Molecular Function

- calcium ion binding
- calcium-dependent phospholipase A2 activity
- calcium-dependent phospholipid binding
- phospholipase A1 activity

Location

- cytosol
- membrane

Biological process

- fatty acid metabolic process
- glycerophospholipid catabolic process
- phosphatidylglycerol acyl-chain remodeling
- phosphatidylinositol acyl-chain remodeling

---

19

- **Protein name:** 116 kDa U5 small nuclear ribonucleoprotein component
- **Organism:** Homo sapiens
- **Uniprot Accession Number:** Q15029
- **Protein sequence length:** 972 aa
- **1D identity (%):** 18.82
- **1D identity (%) [Gaps excluded]:** 28.61
- **1D identity - Alignment Gaps:** 378
- **1D aligned content (<aminoacid>:%):** {'M': 0.96, 'E': 5.77, 'G': 8.17, 'P': 7.69, 'L': 11.54, 'Y': 3.85, 'K': 7.21, 'F': 4.81, 'V': 7.69, 'D': 8.65, 'N': 1.44, 'H': 3.85, 'T': 6.73, 'R': 4.81, 'I': 4.33, 'S': 4.33, 'Q': 3.37, 'W': 1.44, 'A': 3.37}
- **Common reported functions (%):** 14.29
- **Common reported locations (%):** 33.33
- **Common reported processes (%):** 0.0

- **PDB ID:** 6FF4
- **Chain:** B
- **Crystallized protein length:** 902 aa
- **Resolution:** 3.4 Å
- **b-phipsi:** 0.003131
- **w-rdist:** 0.376743
- **t-alpha:** 0.011218
- **Chemical similarity (Tanimoto Index) (%):** 78.39
- **1D identity (%) [PDB]:** 3.06
- **1D identity (%) [Gaps excluded][PDB]:** 65.38
- **1D identity - Alignment Gaps [PDB]:** 1591
- **1D aligned content [PDB] (<aminoacid>:%):** {'E': 5.88, 'V': 13.73, 'I': 5.88, 'K': 11.76, 'P': 11.76, 'N': 1.96, 'D': 5.88, 'Q': 1.96, 'H': 1.96, 'T': 7.84, 'R': 7.84, 'G': 5.88, 'S': 3.92, 'L': 7.84, 'A': 3.92, 'F': 1.96}
- **2D identity (%) [PDB]:** 36.69
- **2D identity (%) [Gaps excluded][PDB]:** 85.17
- **2D identity - Alignment Gaps [PDB]:** 695
- **2D aligned content [PDB] (<2D-fold>:%):** {'.': 30.8, 'E': 19.2, 'H': 34.38, 'T': 14.96, 'B': 0.67}
- **3D similarity (TM-Score) (%) [PDB]:** 26.36

- **Gene name:** EFTUD2
- **Entrez ID:** 9343
- **RefSeq ID:** NM\_004247
- **Transcript sequence length:** 4326
- **5-UTR|CDS|3-UTR identity (%):** 13.91 | 46.1 | 4.91
- **5-UTR|CDS|3-UTR identity (%) [Gaps excluded]:** 82.76 | 73.54 | 75.54
- **5-UTR|CDS|3-UTR identity [Alignment Gaps]:** 287 | 1262 | 15873
- **5-UTR aligned content (<base>:%):** {'G': 43.75, 'A': 12.5, 'C': 31.25, 'T': 12.5}
- **CDS aligned content (<base>:%):** {'A': 27.52, 'T': 24.12, 'G': 25.27, 'C': 23.09}
- **3-UTR aligned content (<base>:%):** {'T': 27.46, 'C': 26.62, 'G': 20.74, 'A': 25.18}

**Uniprot Description:**  
  
 Required for pre-mRNA splicing as component of the spliceosome, including pre-catalytic, catalytic and post-catalytic spliceosomal complexes (PubMed:28502770, PubMed:28781166, PubMed:28076346, PubMed:29361316, PubMed:30315277, PubMed:29360106, PubMed:29301961, PubMed:30705154). Component of the U5 snRNP and the U4/U6-U5 tri-snRNP complex, a building block of the spliceosome (PubMed:16723661).   
  
Component of the U5 snRNP and the U4/U6-U5 tri-snRNP complex, a building block of the spliceosome (PubMed:26912367, PubMed:16723661). The U4/U6-U5 tri-snRNP complex is composed of the U4, U6 and U5 snRNAs and at least PRPF3, PRPF4, PRPF6, PRPF8, PRPF31, SNRNP200, TXNL4A, SNRNP40, DDX23, CD2BP2, PPIH, SNU13, EFTUD2, SART1 and USP39 (PubMed:16723661, PubMed:26912367). Component of the pre-catalytic, catalytic and post-catalytic spliceosome complexes (PubMed:28502770, PubMed:28781166, PubMed:28076346, PubMed:29361316, PubMed:30315277, PubMed:29360106, PubMed:29301961, PubMed:30705154). Interacts with ERBB4 and PRPF8. Interacts with PIH1D1 (PubMed:24656813). Interacts with RPAP3 and URI1 in a ZNHIT2-dependent manner (PubMed:28561026). Interacts with NRDE2 (PubMed:30538148). Interacts with FAM50A (PubMed:32703943).   
  
 **Gene Ontology Information:**

Molecular Function

- GTP binding
- GTPase activity
- RNA binding
- U5 snRNA binding

Location

- Cajal body
- catalytic step 2 spliceosome
- cytosol
- intracellular membrane-bounded organelle
- membrane
- nuclear speck
- nucleoplasm
- nucleus
- U2-type catalytic step 2 spliceosome
- U2-type precatalytic spliceosome
- U4/U6 x U5 tri-snRNP complex

Biological process

- cellular response to xenobiotic stimulus
- mRNA splicing, via spliceosome
- response to cocaine

---

20

- **Protein name:** Mediator of RNA polymerase II transcription subunit 1
- **Organism:** Homo sapiens
- **Uniprot Accession Number:** Q15648
- **Protein sequence length:** 1581 aa
- **1D identity (%):** 12.99
- **1D identity (%) [Gaps excluded]:** 24.76
- **1D identity - Alignment Gaps:** 761
- **1D aligned content (<aminoacid>:%):** {'M': 0.48, 'S': 9.62, 'G': 13.94, 'P': 15.87, 'A': 4.33, 'L': 7.21, 'R': 5.29, 'F': 3.85, 'V': 5.29, 'E': 2.4, 'D': 6.25, 'K': 6.73, 'C': 0.96, 'H': 3.85, 'Y': 1.44, 'T': 5.77, 'N': 1.92, 'Q': 3.37, 'I': 1.44}
- **Common reported functions (%):** 0.0
- **Common reported locations (%):** 22.22
- **Common reported processes (%):** 11.11

- **PDB ID:** 7ENJ
- **Chain:** A
- **Crystallized protein length:** 467 aa
- **Resolution:** 4.4 Å
- **b-phipsi:** 0.001339
- **w-rdist:** 0.611115
- **t-alpha:** 0.006379
- **Chemical similarity (Tanimoto Index) (%):** 99.47
- **1D identity (%) [PDB]:** 1.79
- **1D identity (%) [Gaps excluded][PDB]:** 69.7
- **1D identity - Alignment Gaps [PDB]:** 1251
- **1D aligned content [PDB] (<aminoacid>:%):** {'H': 8.7, 'L': 8.7, 'V': 13.04, 'D': 4.35, 'K': 8.7, 'E': 4.35, 'S': 13.04, 'A': 8.7, 'G': 8.7, 'Q': 8.7, 'R': 4.35, 'P': 4.35, 'I': 4.35}
- **2D identity (%) [PDB]:** 26.8
- **2D identity (%) [Gaps excluded][PDB]:** 84.54
- **2D identity - Alignment Gaps [PDB]:** 683
- **2D aligned content [PDB] (<2D-fold>:%):** {'.': 20.9, 'H': 52.99, 'E': 21.64, 'T': 4.48}
- **3D similarity (TM-Score) (%) [PDB]:** 10.17

- **Gene name:** MED1
- **Entrez ID:** 5469
- **RefSeq ID:** NM\_004774
- **Transcript sequence length:** 8137
- **5-UTR|CDS|3-UTR identity (%):** 36.6 | 37.59 | 11.54
- **5-UTR|CDS|3-UTR identity (%) [Gaps excluded]:** 72.16 | 77.21 | 75.67
- **5-UTR|CDS|3-UTR identity [Alignment Gaps]:** 171 | 2529 | 14667
- **5-UTR aligned content (<base>:%):** {'A': 9.45, 'T': 22.83, 'C': 40.16, 'G': 27.56}
- **CDS aligned content (<base>:%):** {'A': 30.33, 'T': 25.2, 'G': 21.91, 'C': 22.56}
- **3-UTR aligned content (<base>:%):** {'G': 20.33, 'A': 31.1, 'C': 15.52, 'T': 33.05}

**Uniprot Description:**  
  
 Component of the Mediator complex, a coactivator involved in the regulated transcription of nearly all RNA polymerase II-dependent genes. Mediator functions as a bridge to convey information from gene-specific regulatory proteins to the basal RNA polymerase II transcription machinery. Mediator is recruited to promoters by direct interactions with regulatory proteins and serves as a scaffold for the assembly of a functional preinitiation complex with RNA polymerase II and the general transcription factors (PubMed:10406464, PubMed:11867769, PubMed:12037571, PubMed:12218053, PubMed:12556447, PubMed:14636573, PubMed:15340084, PubMed:15471764, PubMed:15989967, PubMed:16574658, PubMed:9653119). Acts as a coactivator for GATA1-mediated transcriptional activation during erythroid differentiation of K562 erythroleukemia cells (PubMed:24245781).   
  
Component of the Mediator complex, which is composed of MED1, MED4, MED6, MED7, MED8, MED9, MED10, MED11, MED12, MED13, MED13L, MED14, MED15, MED16, MED17, MED18, MED19, MED20, MED21, MED22, MED23, MED24, MED25, MED26, MED27, MED29, MED30, MED31, CCNC, CDK8 and CDC2L6/CDK11. The MED12, MED13, CCNC and CDK8 subunits form a distinct module termed the CDK8 module. Mediator containing the CDK8 module is less active than Mediator lacking this module in supporting transcriptional activation. Individual preparations of the Mediator complex lacking one or more distinct subunits have been variously termed ARC, CRSP, DRIP, PC2, SMCC and TRAP. This subunit specifically interacts with a number of nuclear receptors in a ligand-dependent fashion including AR, ESR1, ESR2, PPARA, PPARG, RORA, RXRA, RXRG, THRA, THRB and VDR. Interacts with CTNNB1, GABPA, GLI3, PPARGC1A and TP53. Interacts with YWHAH. Interacts with CLOCK; this interaction requires the presence of THRAP3 (By similarity). Interacts with GATA1 and CCAR1. Interacts with NR4A3 (By similarity). Interacts (via IBM motif) with PSIP1 (via IBD domain); phosphorylation increases its affinity for PSIP1 (PubMed:29997176).   
  
 **Gene Ontology Information:**

Molecular Function

- chromatin binding
- chromatin DNA binding
- DNA-binding transcription factor binding
- general transcription initiation factor binding
- histone acetyltransferase binding
- LBD domain binding
- estrogen receptor binding
- nuclear receptor binding
- nuclear receptor coactivator activity
- retinoic acid receptor binding
- thyroid hormone receptor binding
- vitamin D receptor binding
- peroxisome proliferator activated receptor binding
- promoter-specific chromatin binding
- RNA polymerase II cis-regulatory region sequence-specific DNA binding
- transcription coactivator activity
- transcription coactivator binding
- transcription coregulator activity
- transcription corepressor activity
- ubiquitin protein ligase activity

Location

- chromatin
- core mediator complex
- mediator complex
- membrane
- nucleolus
- nucleoplasm
- nucleus
- ubiquitin ligase complex

Biological process

- androgen biosynthetic process
- angiogenesis
- animal organ regeneration
- brain development
- cell morphogenesis
- cellular response to epidermal growth factor stimulus
- cellular response to hepatocyte growth factor stimulus
- cellular response to steroid hormone stimulus
- cellular response to thyroid hormone stimulus
- embryonic heart tube development
- embryonic hemopoiesis
- embryonic hindlimb morphogenesis
- embryonic placenta development
- enucleate erythrocyte development
- epithelial cell proliferation involved in mammary gland duct elongation
- erythrocyte development
- fat cell differentiation
- G0 to G1 transition
- hematopoietic stem cell differentiation
- intracellular steroid hormone receptor signaling pathway
- keratinocyte differentiation
- lactation
- lens development in camera-type eye
- liver development
- mammary gland branching involved in pregnancy
- mammary gland branching involved in thelarche
- megakaryocyte development
- monocyte differentiation
- mRNA transcription by RNA polymerase II
- negative regulation of apoptotic process
- negative regulation of keratinocyte proliferation
- negative regulation of neuron differentiation
- negative regulation of transcription by RNA polymerase II
- peroxisome proliferator activated receptor signaling pathway
- positive regulation of transcription, DNA-templated
- positive regulation of erythrocyte differentiation
- positive regulation of G0 to G1 transition
- positive regulation of gene expression
- positive regulation of hepatocyte proliferation
- positive regulation of intracellular estrogen receptor signaling pathway
- positive regulation of keratinocyte differentiation
- positive regulation of transcription by RNA polymerase II
- positive regulation of transcription elongation from RNA polymerase II promoter
- positive regulation of transcription initiation from RNA polymerase II promoter
- positive regulation of interferon-gamma-mediated signaling pathway
- protein import into nucleus
- protein ubiquitination
- regulation of RNA biosynthetic process
- regulation of transcription by RNA polymerase II
- regulation of vitamin D receptor signaling pathway
- retinal pigment epithelium development
- RNA polymerase II preinitiation complex assembly
- thyroid hormone generation
- thyroid hormone mediated signaling pathway
- ventricular trabecula myocardium morphogenesis

---

21

- **Protein name:** DNA (cytosine-5)-methyltransferase 1
- **Organism:** Homo sapiens
- **Uniprot Accession Number:** P26358
- **Protein sequence length:** 1616 aa
- **1D identity (%):** 14.19
- **1D identity (%) [Gaps excluded]:** 28.3
- **1D identity - Alignment Gaps:** 822
- **1D aligned content (<aminoacid>:%):** {'M': 1.28, 'A': 5.56, 'P': 12.82, 'R': 8.12, 'L': 6.41, 'Q': 3.85, 'E': 5.56, 'I': 2.99, 'K': 6.41, 'Y': 2.99, 'V': 7.69, 'H': 1.71, 'F': 5.13, 'D': 6.84, 'T': 5.98, 'G': 9.4, 'S': 3.85, 'C': 3.42}
- **Common reported functions (%):** 14.29
- **Common reported locations (%):** 11.11
- **Common reported processes (%):** 11.11

- **PDB ID:** 7SFF
- **Chain:** A
- **Crystallized protein length:** 842 aa
- **Resolution:** 2.05 Å
- **b-phipsi:** 0.001128
- **w-rdist:** 0.263594
- **t-alpha:** 0.162441
- **Chemical similarity (Tanimoto Index) (%):** 88.84
- **1D identity (%) [PDB]:** 3.76
- **1D identity (%) [Gaps excluded][PDB]:** 65.22
- **1D identity - Alignment Gaps [PDB]:** 1504
- **1D aligned content [PDB] (<aminoacid>:%):** {'R': 11.67, 'C': 1.67, 'I': 5.0, 'K': 6.67, 'L': 5.0, 'T': 11.67, 'D': 6.67, 'A': 11.67, 'Q': 3.33, 'E': 6.67, 'S': 1.67, 'N': 3.33, 'V': 5.0, 'G': 8.33, 'P': 3.33, 'Y': 1.67, 'H': 1.67, 'M': 3.33, 'F': 1.67}
- **2D identity (%) [PDB]:** 31.9
- **2D identity (%) [Gaps excluded][PDB]:** 80.04
- **2D identity - Alignment Gaps [PDB]:** 726
- **2D aligned content [PDB] (<2D-fold>:%):** {'.': 19.22, 'E': 29.09, 'T': 18.18, 'H': 32.73, 'B': 0.78}
- **3D similarity (TM-Score) (%) [PDB]:** 24.02

- **Gene name:** DNMT1
- **Entrez ID:** 1786
- **RefSeq ID:** N/A
- **Sequence length:** N/A
- **5-UTR|CDS|3-UTR identity (%):** N/A | N/A | N/A
- **5-UTR|CDS|3-UTR identity (%) [Gaps excluded]:** N/A | N/A | N/A
- **5-UTR|CDS|3-UTR identity [Alignment Gaps]:** N/A | N/A | N/A
- **5-UTR aligned content (<base>:%):** N/A
- **CDS aligned content (<base>:%):** N/A
- **3-UTR aligned content (<base>:%):** N/A

**Uniprot Description:**  
  
 Methylates CpG residues. Preferentially methylates hemimethylated DNA. Associates with DNA replication sites in S phase maintaining the methylation pattern in the newly synthesized strand, that is essential for epigenetic inheritance. Associates with chromatin during G2 and M phases to maintain DNA methylation independently of replication. It is responsible for maintaining methylation patterns established in development. DNA methylation is coordinated with methylation of histones. Mediates transcriptional repression by direct binding to HDAC2. In association with DNMT3B and via the recruitment of CTCFL/BORIS, involved in activation of BAG1 gene expression by modulating dimethylation of promoter histone H3 at H3K4 and H3K9. Probably forms a corepressor complex required for activated KRAS-mediated promoter hypermethylation and transcriptional silencing of tumor suppressor genes (TSGs) or other tumor-related genes in colorectal cancer (CRC) cells (PubMed:24623306). Also required to maintain a transcriptionally repressive state of genes in undifferentiated embryonic stem cells (ESCs) (PubMed:24623306). Associates at promoter regions of tumor suppressor genes (TSGs) leading to their gene silencing (PubMed:24623306). Promotes tumor growth (PubMed:24623306).   
  
Homodimer (PubMed:19173286). Forms a stable complex with E2F1, BB1 and HDAC1 (PubMed:10888886). Forms a complex with DMAP1 and HDAC2, with direct interaction (PubMed:10888872). Interacts with the PRC2/EED-EZH2 complex (PubMed:16357870). Probably part of a corepressor complex containing ZNF304, TRIM28, SETDB1 and DNMT1 (PubMed:24623306). Interacts with UHRF1; promoting its recruitment to hemimethylated DNA (PubMed:21745816). Interacts with USP7, promoting its deubiquitination (PubMed:21745816). Interacts with PCNA (PubMed:9302295). Interacts with MBD2 and MBD3 (PubMed:10947852). Interacts with DNMT3A and DNMT3B (PubMed:12145218). Interacts with UBC9 (PubMed:19450230). Interacts with CSNK1D (By similarity). Interacts with HDAC1 (By similarity). Interacts with BAZ2A/TIP5 (By similarity). Interacts with SIRT7 (By similarity). Interacts with ZNF263; recruited to the SIX3 promoter along with other proteins involved in chromatin modification and transcriptional corepression where it contributes to transcriptional repression (PubMed:32051553).   
  
 **Gene Ontology Information:**

Molecular Function

- DNA (cytosine-5-)-methyltransferase activity
- DNA binding
- DNA-methyltransferase activity
- methyl-CpG binding
- promoter-specific chromatin binding
- RNA binding
- zinc ion binding

Location

- female germ cell nucleus
- nucleoplasm
- nucleus
- pericentric heterochromatin
- replication fork

Biological process

- cellular response to amino acid stimulus
- cellular response to bisphenol A
- DNA methylation on cytosine within a CG sequence
- DNA methylation-dependent heterochromatin assembly
- transcription, DNA-templated
- DNA methylation involved in embryo development
- negative regulation of gene expression
- hypermethylation of CpG island
- negative regulation of transcription by RNA polymerase II
- negative regulation of vascular associated smooth muscle cell apoptotic process
- negative regulation of vascular associated smooth muscle cell differentiation involved in phenotypic switching
- positive regulation of gene expression
- positive regulation of vascular associated smooth muscle cell proliferation

---

22

- **Protein name:** Aspartate--tRNA ligase, mitochondrial
- **Organism:** Homo sapiens
- **Uniprot Accession Number:** Q6PI48
- **Protein sequence length:** 645 aa
- **1D identity (%):** 12.81
- **1D identity (%) [Gaps excluded]:** 25.3
- **1D identity - Alignment Gaps:** 493
- **1D aligned content (<aminoacid>:%):** {'Y': 3.12, 'R': 9.38, 'P': 6.25, 'T': 6.25, 'L': 9.38, 'Q': 7.81, 'H': 7.81, 'C': 2.34, 'K': 5.47, 'S': 3.91, 'E': 7.03, 'I': 2.34, 'V': 6.25, 'A': 3.91, 'F': 5.47, 'D': 5.47, 'G': 5.47, 'M': 0.78, 'N': 1.56}
- **Common reported functions (%):** 0.0
- **Common reported locations (%):** 11.11
- **Common reported processes (%):** 0.0

- **PDB ID:** 4AH6
- **Chain:** C
- **Crystallized protein length:** 589 aa
- **Resolution:** 3.7 Å
- **b-phipsi:** 0.002324
- **w-rdist:** 0.394138
- **t-alpha:** 0.024351
- **Chemical similarity (Tanimoto Index) (%):** 99.17
- **1D identity (%) [PDB]:** 3.69
- **1D identity (%) [Gaps excluded][PDB]:** 63.29
- **1D identity - Alignment Gaps [PDB]:** 1276
- **1D aligned content [PDB] (<aminoacid>:%):** {'P': 4.0, 'H': 4.0, 'L': 8.0, 'G': 6.0, 'Q': 10.0, 'E': 10.0, 'T': 10.0, 'V': 6.0, 'A': 6.0, 'C': 2.0, 'K': 6.0, 'D': 8.0, 'R': 6.0, 'S': 4.0, 'I': 2.0, 'N': 2.0, 'F': 6.0}
- **2D identity (%) [PDB]:** 29.77
- **2D identity (%) [Gaps excluded][PDB]:** 85.91
- **2D identity - Alignment Gaps [PDB]:** 696
- **2D aligned content [PDB] (<2D-fold>:%):** {'T': 14.2, 'E': 24.61, '.': 16.09, 'H': 44.48, 'B': 0.63}
- **3D similarity (TM-Score) (%) [PDB]:** 19.88

- **Gene name:** DARS2
- **Entrez ID:** 55157
- **RefSeq ID:** NM\_018122
- **Transcript sequence length:** 3336
- **5-UTR|CDS|3-UTR identity (%):** 38.42 | 39.04 | 3.52
- **5-UTR|CDS|3-UTR identity (%) [Gaps excluded]:** 75.68 | 75.42 | 78.31
- **5-UTR|CDS|3-UTR identity [Alignment Gaps]:** 287 | 1437 | 16084
- **5-UTR aligned content (<base>:%):** {'G': 31.25, 'C': 39.29, 'T': 18.3, 'A': 11.16}
- **CDS aligned content (<base>:%):** {'A': 30.01, 'T': 25.97, 'C': 20.64, 'G': 23.39}
- **3-UTR aligned content (<base>:%):** {'A': 36.32, 'T': 35.3, 'C': 12.84, 'G': 15.54}

**Uniprot Description:**  
  
 N/A   
  
Homodimer.   
  
 **Gene Ontology Information:**

Molecular Function

- aspartate-tRNA ligase activity
- aspartate-tRNA(Asn) ligase activity
- ATP binding
- protein homodimerization activity
- tRNA binding

Location

- mitochondrial matrix
- mitochondrion
- nucleoplasm

Biological process

- aspartyl-tRNA aminoacylation
- mitochondrial asparaginyl-tRNA aminoacylation
- tRNA aminoacylation

---

23

- **Protein name:** Tapasin
- **Organism:** Homo sapiens
- **Uniprot Accession Number:** O15533
- **Protein sequence length:** 448 aa
- **1D identity (%):** 10.64
- **1D identity (%) [Gaps excluded]:** 23.65
- **1D identity - Alignment Gaps:** 496
- **1D aligned content (<aminoacid>:%):** {'M': 1.04, 'G': 9.38, 'S': 6.25, 'A': 9.38, 'P': 16.67, 'V': 8.33, 'L': 14.58, 'E': 6.25, 'D': 2.08, 'Y': 4.17, 'R': 3.12, 'H': 2.08, 'F': 2.08, 'W': 2.08, 'T': 7.29, 'Q': 4.17, 'K': 1.04}
- **Common reported functions (%):** 14.29
- **Common reported locations (%):** 0.0
- **Common reported processes (%):** 0.0

- **PDB ID:** 7QNG
- **Chain:** B
- **Crystallized protein length:** 464 aa
- **Resolution:** 2.7 Å
- **b-phipsi:** 0.005869
- **w-rdist:** 0.469769
- **t-alpha:** 0.002377
- **Chemical similarity (Tanimoto Index) (%):** 97.19
- **1D identity (%) [PDB]:** 2.87
- **1D identity (%) [Gaps excluded][PDB]:** 67.92
- **1D identity - Alignment Gaps [PDB]:** 1202
- **1D aligned content [PDB] (<aminoacid>:%):** {'S': 5.56, 'V': 8.33, 'E': 11.11, 'L': 13.89, 'F': 8.33, 'T': 5.56, 'R': 11.11, 'K': 5.56, 'P': 2.78, 'I': 5.56, 'D': 5.56, 'G': 5.56, 'Y': 2.78, 'A': 5.56, 'C': 2.78}
- **2D identity (%) [PDB]:** 34.67
- **2D identity (%) [Gaps excluded][PDB]:** 90.61
- **2D identity - Alignment Gaps [PDB]:** 584
- **2D aligned content [PDB] (<2D-fold>:%):** {'.': 15.24, 'E': 18.29, 'H': 38.11, 'T': 27.44, 'G': 0.91}
- **3D similarity (TM-Score) (%) [PDB]:** 17.9

- **Gene name:** TAPBP
- **Entrez ID:** 2923
- **RefSeq ID:** NM\_172209
- **Transcript sequence length:** 3189
- **5-UTR|CDS|3-UTR identity (%):** 7.69 | 24.44 | 7.23
- **5-UTR|CDS|3-UTR identity (%) [Gaps excluded]:** 73.53 | 73.98 | 75.69
- **5-UTR|CDS|3-UTR identity [Alignment Gaps]:** 291 | 1847 | 15539
- **5-UTR aligned content (<base>:%):** {'G': 60.0, 'C': 16.0, 'T': 12.0, 'A': 12.0}
- **CDS aligned content (<base>:%):** {'A': 19.88, 'G': 28.78, 'T': 21.66, 'C': 29.67}
- **3-UTR aligned content (<base>:%):** {'G': 21.01, 'C': 23.19, 'A': 23.51, 'T': 32.29}

**Uniprot Description:**  
  
 Involved in the association of MHC class I with transporter associated with antigen processing (TAP) and in the assembly of MHC class I with peptide (peptide loading).   
  
Heterodimer with PDIA3; disulfide-linked. Obligatory mediator for the interaction between newly assembled MHC class I molecules, calreticulin, PDIA3 and TAP. Up to 4 MHC class I/tapasin complexes bind to 1 TAP (PubMed:19119025, PubMed:21263072, PubMed:26611325). Interacts with HLA-G-B2M complex; this interaction is required for loading of high affinity peptides.   
  
 **Gene Ontology Information:**

Molecular Function

- cysteine-type endopeptidase activity
- disulfide oxidoreductase activity
- identical protein binding
- phospholipase C activity
- protein disulfide isomerase activity
- protein disulfide oxidoreductase activity
- RNA binding

Location

- cell surface
- endoplasmic reticulum
- endoplasmic reticulum lumen
- extracellular exosome
- extracellular space
- focal adhesion
- melanosome
- MHC class I peptide loading complex
- nucleus
- phagocytic vesicle
- recycling endosome membrane
- Tapasin-ERp57 complex

Biological process

- cellular response to interleukin-7
- extrinsic apoptotic signaling pathway
- peptide antigen assembly with MHC class I protein complex
- platelet aggregation
- positive regulation of extrinsic apoptotic signaling pathway
- protein folding
- protein folding in endoplasmic reticulum
- response to endoplasmic reticulum stress

---

24

- **Protein name:** Protein transport protein Sec23A
- **Organism:** Homo sapiens
- **Uniprot Accession Number:** Q15436
- **Protein sequence length:** 765 aa
- **1D identity (%):** 15.04
- **1D identity (%) [Gaps excluded]:** 23.5
- **1D identity - Alignment Gaps:** 357
- **1D aligned content (<aminoacid>:%):** {'F': 3.36, 'Q': 9.4, 'V': 6.71, 'R': 8.05, 'W': 0.67, 'A': 6.04, 'T': 6.71, 'P': 9.4, 'S': 5.37, 'K': 4.7, 'C': 2.01, 'N': 2.01, 'E': 4.03, 'Y': 4.03, 'L': 8.72, 'D': 5.37, 'G': 6.71, 'I': 4.03, 'M': 0.67, 'H': 2.01}
- **Common reported functions (%):** 0.0
- **Common reported locations (%):** 11.11
- **Common reported processes (%):** 0.0

- **PDB ID:** 5KYN
- **Chain:** B
- **Crystallized protein length:** 719 aa
- **Resolution:** 2.55 Å
- **b-phipsi:** 0.002188
- **w-rdist:** 0.693687
- **t-alpha:** 0.003978
- **Chemical similarity (Tanimoto Index) (%):** N/A
- **1D identity (%) [PDB]:** 2.66
- **1D identity (%) [Gaps excluded][PDB]:** 62.5
- **1D identity - Alignment Gaps [PDB]:** 1439
- **1D aligned content [PDB] (<aminoacid>:%):** {'V': 20.0, 'D': 10.0, 'M': 2.5, 'Q': 2.5, 'G': 12.5, 'P': 5.0, 'K': 7.5, 'R': 7.5, 'L': 5.0, 'T': 10.0, 'A': 2.5, 'E': 2.5, 'S': 7.5, 'I': 2.5, 'F': 2.5}
- **2D identity (%) [PDB]:** 34.82
- **2D identity (%) [Gaps excluded][PDB]:** 82.01
- **2D identity - Alignment Gaps [PDB]:** 633
- **2D aligned content [PDB] (<2D-fold>:%):** {'.': 15.14, 'H': 44.65, 'E': 26.11, 'T': 12.79, 'B': 0.52, 'G': 0.78}
- **3D similarity (TM-Score) (%) [PDB]:** 24.42

- **Gene name:** SEC23A
- **Entrez ID:** 10484
- **RefSeq ID:** NM\_006364
- **Transcript sequence length:** 3843
- **5-UTR|CDS|3-UTR identity (%):** 40.91 | 44.4 | 4.88
- **5-UTR|CDS|3-UTR identity (%) [Gaps excluded]:** 73.85 | 73.77 | 78.17
- **5-UTR|CDS|3-UTR identity [Alignment Gaps]:** 157 | 1213 | 15949
- **5-UTR aligned content (<base>:%):** {'G': 45.14, 'T': 9.72, 'C': 36.11, 'A': 9.03}
- **CDS aligned content (<base>:%):** {'A': 31.04, 'T': 27.27, 'G': 21.73, 'C': 19.96}
- **3-UTR aligned content (<base>:%):** {'A': 36.22, 'G': 13.0, 'T': 40.43, 'C': 10.35}

**Uniprot Description:**  
  
 Component of the coat protein complex II (COPII) which promotes the formation of transport vesicles from the endoplasmic reticulum (ER). The coat has two main functions, the physical deformation of the endoplasmic reticulum membrane into vesicles and the selection of cargo molecules for their transport to the Golgi complex. Required for the translocation of insulin-induced glucose transporter SLC2A4/GLUT4 to the cell membrane (By similarity).   
  
COPII is composed of at least five proteins: the Sec23/24 complex, the Sec13/31 complex and Sar1 (PubMed:17499046). Interacts with SEC23IP. Interacts with HTR4 (By similarity). Interacts with SEC16A (PubMed:17192411). Interacts with SLC6A4 (By similarity). Interacts (as part of the Sec23/24 complex) with SEC22B; recruits SEC22B into COPII-coated vesicles and allows the transport of this cargo from the endoplasmic reticulum to the Golgi (PubMed:17499046). Interacts (via Gelsolin-like repeat) with MIA2 and MIA3; specifically involved in the transport of large cargos like the collagen COL7A1 (PubMed:27551091, PubMed:28442536). Interacts with DDHD1 (By similarity). Interacts with TMEM39A (PubMed:31806350). Interacts with SACM1L; this interaction is reduced in the absence of TMEM39A (PubMed:31806350).   
  
 **Gene Ontology Information:**

Molecular Function

- GTPase activator activity
- zinc ion binding

Location

- COPII vesicle coat
- cytosol
- endoplasmic reticulum exit site
- endoplasmic reticulum membrane
- ER to Golgi transport vesicle membrane
- Golgi membrane
- perinuclear region of cytoplasm

Biological process

- COPII-coated vesicle cargo loading
- intracellular protein transport
- protein localization to plasma membrane

---

25

- **Protein name:** Complement C4-A
- **Organism:** Homo sapiens
- **Uniprot Accession Number:** P0C0L4
- **Protein sequence length:** 1744 aa
- **1D identity (%):** 14.35
- **1D identity (%) [Gaps excluded]:** 30.08
- **1D identity - Alignment Gaps:** 922
- **1D aligned content (<aminoacid>:%):** {'M': 1.19, 'G': 9.09, 'L': 11.07, 'P': 10.28, 'R': 8.7, 'N': 1.58, 'K': 3.95, 'D': 5.93, 'V': 7.51, 'C': 2.77, 'S': 5.14, 'F': 4.74, 'Y': 3.95, 'T': 7.51, 'I': 1.98, 'H': 2.77, 'E': 2.77, 'A': 5.53, 'Q': 3.16, 'W': 0.4}
- **Common reported functions (%):** 0.0
- **Common reported locations (%):** 0.0
- **Common reported processes (%):** 0.0

- **PDB ID:** 4FXK
- **Chain:** B
- **Crystallized protein length:** 697 aa
- **Resolution:** 3.6 Å
- **b-phipsi:** 0.001919
- **w-rdist:** 0.235603
- **t-alpha:** 0.167195
- **Chemical similarity (Tanimoto Index) (%):** 78.24
- **1D identity (%) [PDB]:** 3.77
- **1D identity (%) [Gaps excluded][PDB]:** 66.27
- **1D identity - Alignment Gaps [PDB]:** 1377
- **1D aligned content [PDB] (<aminoacid>:%):** {'P': 10.91, 'L': 16.36, 'V': 16.36, 'T': 5.45, 'D': 7.27, 'E': 5.45, 'G': 7.27, 'R': 7.27, 'S': 10.91, 'K': 3.64, 'H': 1.82, 'A': 3.64, 'M': 1.82, 'F': 1.82}
- **2D identity (%) [PDB]:** 35.1
- **2D identity (%) [Gaps excluded][PDB]:** 81.12
- **2D identity - Alignment Gaps [PDB]:** 611
- **2D aligned content [PDB] (<2D-fold>:%):** {'.': 25.4, 'E': 24.07, 'T': 11.9, 'H': 37.57, 'B': 0.26, 'G': 0.79}
- **3D similarity (TM-Score) (%) [PDB]:** 20.17

- **Gene name:** C4A
- **Entrez ID:** 721
- **RefSeq ID:** NM\_007293
- **Transcript sequence length:** 5427
- **5-UTR|CDS|3-UTR identity (%):** 8.87 | 34.96 | 0.56
- **5-UTR|CDS|3-UTR identity (%) [Gaps excluded]:** 63.04 | 77.02 | 70.68
- **5-UTR|CDS|3-UTR identity [Alignment Gaps]:** 281 | 2936 | 16630
- **5-UTR aligned content (<base>:%):** {'G': 20.69, 'A': 17.24, 'C': 44.83, 'T': 17.24}
- **CDS aligned content (<base>:%):** {'A': 26.33, 'T': 23.19, 'G': 26.33, 'C': 24.15}
- **3-UTR aligned content (<base>:%):** {'G': 31.91, 'C': 23.4, 'T': 22.34, 'A': 22.34}

**Uniprot Description:**  
  
 Non-enzymatic component of C3 and C5 convertases and thus essential for the propagation of the classical complement pathway. Covalently binds to immunoglobulins and immune complexes and enhances the solubilization of immune aggregates and the clearance of IC through CR1 on erythrocytes. C4A isotype is responsible for effective binding to form amide bonds with immune aggregates or protein antigens, while C4B isotype catalyzes the transacylation of the thioester carbonyl group to form ester bonds with carbohydrate antigens.   
  
Circulates in blood as a disulfide-linked trimer of an alpha, beta and gamma chain.   
  
 **Gene Ontology Information:**

Molecular Function   
  
N/A

Location   
  
N/A

Biological process   
  
N/A

---

26

- **Protein name:** Dipeptidyl peptidase 8
- **Organism:** Homo sapiens
- **Uniprot Accession Number:** Q6V1X1
- **Protein sequence length:** 898 aa
- **1D identity (%):** 15.59
- **1D identity (%) [Gaps excluded]:** 23.23
- **1D identity - Alignment Gaps:** 346
- **1D aligned content (<aminoacid>:%):** {'G': 7.93, 'K': 6.71, 'A': 6.1, 'V': 7.32, 'T': 8.54, 'E': 4.27, 'D': 3.66, 'R': 9.15, 'P': 7.32, 'S': 4.27, 'W': 1.83, 'L': 9.15, 'H': 3.05, 'Y': 4.88, 'I': 3.66, 'F': 5.49, 'C': 3.05, 'N': 1.22, 'M': 0.61, 'Q': 1.83}
- **Common reported functions (%):** 0.0
- **Common reported locations (%):** 22.22
- **Common reported processes (%):** 0.0

- **PDB ID:** 6EOS
- **Chain:** C
- **Crystallized protein length:** 829 aa
- **Resolution:** 3.1 Å
- **b-phipsi:** 0.005727
- **w-rdist:** 0.514916
- **t-alpha:** 0.001585
- **Chemical similarity (Tanimoto Index) (%):** 99.55
- **1D identity (%) [PDB]:** 3.79
- **1D identity (%) [Gaps excluded][PDB]:** 65.22
- **1D identity - Alignment Gaps [PDB]:** 1493
- **1D aligned content [PDB] (<aminoacid>:%):** {'K': 6.67, 'L': 15.0, 'A': 3.33, 'F': 3.33, 'E': 5.0, 'I': 6.67, 'P': 11.67, 'D': 6.67, 'V': 16.67, 'C': 1.67, 'R': 5.0, 'S': 5.0, 'T': 6.67, 'G': 3.33, 'H': 3.33}
- **2D identity (%) [PDB]:** 34.0
- **2D identity (%) [Gaps excluded][PDB]:** 84.06
- **2D identity - Alignment Gaps [PDB]:** 711
- **2D aligned content [PDB] (<2D-fold>:%):** {'.': 23.4, 'E': 33.0, 'H': 25.37, 'T': 18.23}
- **3D similarity (TM-Score) (%) [PDB]:** 27.18

- **Gene name:** DPP8
- **Entrez ID:** 54878
- **RefSeq ID:** N/A
- **Sequence length:** N/A
- **5-UTR|CDS|3-UTR identity (%):** N/A | N/A | N/A
- **5-UTR|CDS|3-UTR identity (%) [Gaps excluded]:** N/A | N/A | N/A
- **5-UTR|CDS|3-UTR identity [Alignment Gaps]:** N/A | N/A | N/A
- **5-UTR aligned content (<base>:%):** N/A
- **CDS aligned content (<base>:%):** N/A
- **3-UTR aligned content (<base>:%):** N/A

**Uniprot Description:**  
  
 Dipeptidyl peptidase that cleaves off N-terminal dipeptides from proteins having a Pro or Ala residue at position 2.   
  
Homodimer.   
  
 **Gene Ontology Information:**

Molecular Function

- aminopeptidase activity
- dipeptidyl-peptidase activity
- serine-type peptidase activity

Location

- cytoplasm
- cytosol

Biological process

- apoptotic process
- immune response
- negative regulation of programmed cell death
- proteolysis

---

27

- **Protein name:** D-glucuronyl C5-epimerase
- **Organism:** Homo sapiens
- **Uniprot Accession Number:** O94923
- **Protein sequence length:** 617 aa
- **1D identity (%):** 13.08
- **1D identity (%) [Gaps excluded]:** 23.44
- **1D identity - Alignment Gaps:** 419
- **1D aligned content (<aminoacid>:%):** {'A': 5.65, 'Y': 8.87, 'K': 6.45, 'I': 2.42, 'C': 2.42, 'D': 8.06, 'F': 5.65, 'G': 11.29, 'V': 7.26, 'E': 4.84, 'N': 3.23, 'S': 3.23, 'P': 4.84, 'L': 9.68, 'H': 0.81, 'T': 4.03, 'R': 5.65, 'Q': 4.03, 'W': 1.61}
- **Common reported functions (%):** 0.0
- **Common reported locations (%):** 0.0
- **Common reported processes (%):** 0.0

- **PDB ID:** 6I01
- **Chain:** A
- **Crystallized protein length:** 512 aa
- **Resolution:** 2.1 Å
- **b-phipsi:** 0.002092
- **w-rdist:** 0.838067
- **t-alpha:** 0.002377
- **Chemical similarity (Tanimoto Index) (%):** 72.22
- **1D identity (%) [PDB]:** 2.69
- **1D identity (%) [Gaps excluded][PDB]:** 60.34
- **1D identity - Alignment Gaps [PDB]:** 1242
- **1D aligned content [PDB] (<aminoacid>:%):** {'L': 11.43, 'K': 5.71, 'Y': 5.71, 'C': 5.71, 'V': 8.57, 'Q': 5.71, 'E': 2.86, 'H': 2.86, 'R': 5.71, 'I': 5.71, 'T': 11.43, 'D': 2.86, 'N': 5.71, 'S': 11.43, 'A': 8.57}
- **2D identity (%) [PDB]:** 35.82
- **2D identity (%) [Gaps excluded][PDB]:** 91.86
- **2D identity - Alignment Gaps [PDB]:** 596
- **2D aligned content [PDB] (<2D-fold>:%):** {'.': 17.43, 'E': 31.71, 'T': 10.57, 'H': 38.29, 'G': 1.71, 'B': 0.29}
- **3D similarity (TM-Score) (%) [PDB]:** 18.55

- **Gene name:** GLCE
- **Entrez ID:** 26035
- **RefSeq ID:** N/A
- **Sequence length:** N/A
- **5-UTR|CDS|3-UTR identity (%):** N/A | N/A | N/A
- **5-UTR|CDS|3-UTR identity (%) [Gaps excluded]:** N/A | N/A | N/A
- **5-UTR|CDS|3-UTR identity [Alignment Gaps]:** N/A | N/A | N/A
- **5-UTR aligned content (<base>:%):** N/A
- **CDS aligned content (<base>:%):** N/A
- **3-UTR aligned content (<base>:%):** N/A

**Uniprot Description:**  
  
 Converts D-glucuronic acid residues adjacent to N-sulfate sugar residues to L-iduronic acid residues, both in maturing heparan sulfate (HS) and heparin chains. This is important for further modifications that determine the specificity of interactions between these glycosaminoglycans and proteins.   
  
Homodimer (PubMed:30872481). Interacts with HS2ST1.   
  
 **Gene Ontology Information:**

Molecular Function

- calcium ion binding
- heparosan-N-sulfate-glucuronate 5-epimerase activity
- protein homodimerization activity
- racemase and epimerase activity, acting on carbohydrates and derivatives

Location

- Golgi apparatus
- Golgi membrane

Biological process

- heparan sulfate proteoglycan biosynthetic process
- heparin biosynthetic process

---

28

- **Protein name:** Beta-adrenergic receptor kinase 1
- **Organism:** Homo sapiens
- **Uniprot Accession Number:** P25098
- **Protein sequence length:** 689 aa
- **1D identity (%):** 15.56
- **1D identity (%) [Gaps excluded]:** 22.7
- **1D identity - Alignment Gaps:** 289
- **1D aligned content (<aminoacid>:%):** {'A': 3.5, 'V': 7.69, 'L': 9.79, 'M': 0.7, 'K': 3.5, 'T': 4.2, 'P': 7.69, 'R': 6.29, 'S': 6.29, 'Y': 1.4, 'G': 11.19, 'E': 10.49, 'F': 8.39, 'Q': 4.9, 'D': 3.5, 'I': 4.9, 'C': 3.5, 'H': 1.4, 'N': 0.7}
- **Common reported functions (%):** 0.0
- **Common reported locations (%):** 33.33
- **Common reported processes (%):** 0.0

- **PDB ID:** 7K7Z
- **Chain:** A
- **Crystallized protein length:** 625 aa
- **Resolution:** 2.61 Å
- **b-phipsi:** 0.013259
- **w-rdist:** 0.427897
- **t-alpha:** 0.0
- **Chemical similarity (Tanimoto Index) (%):** 90.12
- **1D identity (%) [PDB]:** 2.18
- **1D identity (%) [Gaps excluded][PDB]:** 63.27
- **1D identity - Alignment Gaps [PDB]:** 1373
- **1D aligned content [PDB] (<aminoacid>:%):** {'K': 12.9, 'L': 6.45, 'T': 6.45, 'N': 6.45, 'S': 6.45, 'I': 6.45, 'A': 9.68, 'P': 6.45, 'R': 12.9, 'Q': 6.45, 'E': 9.68, 'D': 3.23, 'F': 3.23, 'V': 3.23}
- **2D identity (%) [PDB]:** 36.9
- **2D identity (%) [Gaps excluded][PDB]:** 83.19
- **2D identity - Alignment Gaps [PDB]:** 567
- **2D aligned content [PDB] (<2D-fold>:%):** {'.': 20.74, 'H': 48.14, 'T': 14.89, 'E': 16.22}
- **3D similarity (TM-Score) (%) [PDB]:** 21.66

- **Gene name:** GRK2
- **Entrez ID:** 156
- **RefSeq ID:** NM\_001619
- **Transcript sequence length:** 3403
- **5-UTR|CDS|3-UTR identity (%):** 42.05 | 42.21 | 4.21
- **5-UTR|CDS|3-UTR identity (%) [Gaps excluded]:** 75.13 | 72.92 | 75.03
- **5-UTR|CDS|3-UTR identity [Alignment Gaps]:** 155 | 1241 | 15963
- **5-UTR aligned content (<base>:%):** {'A': 8.11, 'G': 49.32, 'T': 3.38, 'C': 39.19}
- **CDS aligned content (<base>:%):** {'A': 26.85, 'G': 29.9, 'C': 24.04, 'T': 19.21}
- **3-UTR aligned content (<base>:%):** {'C': 30.06, 'G': 26.4, 'A': 17.28, 'T': 26.26}

**Uniprot Description:**  
  
 Specifically phosphorylates the agonist-occupied form of the beta-adrenergic and closely related receptors, probably inducing a desensitization of them (PubMed:19715378). Key regulator of LPAR1 signaling (PubMed:19306925). Competes with RALA for binding to LPAR1 thus affecting the signaling properties of the receptor (PubMed:19306925). Desensitizes LPAR1 and LPAR2 in a phosphorylation-independent manner (PubMed:19306925). Positively regulates ciliary smoothened (SMO)-dependent Hedgehog (Hh) signaling pathway by facilitating the trafficking of SMO into the cilium and the stimulation of SMO activity (By similarity). Inhibits relaxation of airway smooth muscle in response to blue light (PubMed:30284927).   
  
Interacts with the heterodimer formed by GNB1 and GNG2 (By similarity). Interacts with GIT1 (By similarity). Interacts with, and phosphorylates chemokine-stimulated CCR5 (PubMed:10085131). Interacts with ARRB1 (PubMed:9501202). Interacts with LPAR1 and LPAR2 (PubMed:19306925). Interacts with RALA in response to LPAR1 activation (PubMed:19306925). ADRBK1 and RALA mutually inhibit each other's binding to LPAR1 (PubMed:19306925). Interacts with ADRB2 (PubMed:19715378).   
  
 **Gene Ontology Information:**

Molecular Function

- alpha-2A adrenergic receptor binding
- ATP binding
- beta-adrenergic receptor kinase activity
- Edg-2 lysophosphatidic acid receptor binding
- G protein-coupled receptor binding
- G protein-coupled receptor kinase activity
- protein kinase activity

Location

- cilium
- cytoplasm
- cytosol
- membrane
- plasma membrane
- postsynapse
- presynapse

Biological process

- cardiac muscle contraction
- desensitization of G protein-coupled receptor signaling pathway
- G protein-coupled acetylcholine receptor signaling pathway
- G protein-coupled receptor signaling pathway
- heart development
- negative regulation of relaxation of smooth muscle
- negative regulation of striated muscle contraction
- negative regulation of the force of heart contraction by chemical signal
- peptidyl-serine phosphorylation
- peptidyl-threonine phosphorylation
- positive regulation of catecholamine secretion
- protein phosphorylation
- receptor internalization
- regulation of the force of heart contraction
- tachykinin receptor signaling pathway
- viral entry into host cell
- viral genome replication

---

29

- **Protein name:** Lysine--tRNA ligase
- **Organism:** Homo sapiens
- **Uniprot Accession Number:** Q15046
- **Protein sequence length:** 597 aa
- **1D identity (%):** 13.14
- **1D identity (%) [Gaps excluded]:** 23.61
- **1D identity - Alignment Gaps:** 415
- **1D aligned content (<aminoacid>:%):** {'V': 6.5, 'L': 13.01, 'K': 5.69, 'A': 4.88, 'G': 10.57, 'E': 8.94, 'S': 1.63, 'P': 7.32, 'N': 2.44, 'D': 4.07, 'T': 8.13, 'F': 5.69, 'Q': 4.07, 'I': 4.88, 'R': 4.88, 'Y': 1.63, 'H': 2.44, 'M': 1.63, 'C': 1.63}
- **Common reported functions (%):** 0.0
- **Common reported locations (%):** 11.11
- **Common reported processes (%):** 0.0

- **PDB ID:** 4DPG
- **Chain:** A
- **Crystallized protein length:** 501 aa
- **Resolution:** 2.84 Å
- **b-phipsi:** 0.003016
- **w-rdist:** 0.655854
- **t-alpha:** 0.003179
- **Chemical similarity (Tanimoto Index) (%):** 77.35
- **1D identity (%) [PDB]:** 2.29
- **1D identity (%) [Gaps excluded][PDB]:** 83.33
- **1D identity - Alignment Gaps [PDB]:** 1275
- **1D aligned content [PDB] (<aminoacid>:%):** {'V': 10.0, 'Y': 6.67, 'D': 6.67, 'G': 16.67, 'K': 3.33, 'R': 6.67, 'S': 3.33, 'L': 13.33, 'T': 13.33, 'N': 3.33, 'P': 13.33, 'A': 3.33}
- **2D identity (%) [PDB]:** 29.49
- **2D identity (%) [Gaps excluded][PDB]:** 87.35
- **2D identity - Alignment Gaps [PDB]:** 667
- **2D aligned content [PDB] (<2D-fold>:%):** {'.': 13.13, 'H': 48.82, 'T': 9.76, 'E': 27.27, 'G': 1.01}
- **3D similarity (TM-Score) (%) [PDB]:** 17.42

- **Gene name:** KARS1
- **Entrez ID:** 3735
- **RefSeq ID:** NM\_005548
- **Transcript sequence length:** 1991
- **5-UTR|CDS|3-UTR identity (%):** 6.21 | 42.81 | 0.74
- **5-UTR|CDS|3-UTR identity (%) [Gaps excluded]:** 76.92 | 77.68 | 75.61
- **5-UTR|CDS|3-UTR identity [Alignment Gaps]:** 296 | 1267 | 16598
- **5-UTR aligned content (<base>:%):** {'C': 20.0, 'T': 25.0, 'G': 45.0, 'A': 10.0}
- **CDS aligned content (<base>:%):** {'A': 31.87, 'T': 21.69, 'G': 24.92, 'C': 21.52}
- **3-UTR aligned content (<base>:%):** {'A': 32.26, 'T': 35.48, 'C': 16.13, 'G': 16.13}

**Uniprot Description:**  
  
 Catalyzes the specific attachment of an amino acid to its cognate tRNA in a 2 step reaction: the amino acid (AA) is first activated by ATP to form AA-AMP and then transferred to the acceptor end of the tRNA (PubMed:9278442, PubMed:18029264, PubMed:18272479). When secreted, acts as a signaling molecule that induces immune response through the activation of monocyte/macrophages (PubMed:15851690). Catalyzes the synthesis of the signaling molecule diadenosine tetraphosphate (Ap4A), and thereby mediates disruption of the complex between HINT1 and MITF and the concomitant activation of MITF transcriptional activity (PubMed:5338216, PubMed:14975237, PubMed:19524539, PubMed:23159739).   
  
Homodimer and tetradimer (PubMed:18272479, PubMed:23159739, PubMed:26074468, PubMed:28887846). Part of the multisynthetase complex (MSC), a multisubunit complex that groups tRNA ligases for Arg (RARS), Asp (DARS), Gln (QARS), Ile (IARS), Leu (LARS), Lys (KARS), Met (MARS) the bifunctional ligase for Glu and Pro (EPRS) and the auxiliary subunits AIMP1/p43, AIMP2/p38 and EEF1E1/p18 (PubMed:19131329, PubMed:19289464, PubMed:24312579, PubMed:23159739). Interacts with AIMP2 (via N-terminus) and MITF (PubMed:9878398, PubMed:14975237, PubMed:15220430, PubMed:23159739, PubMed:26074468). Interacts with TARSL2 (PubMed:24312579).   
  
 **Gene Ontology Information:**

Molecular Function

- amino acid binding
- ATP adenylyltransferase activity
- ATP binding
- identical protein binding
- lysine-tRNA ligase activity
- protein homodimerization activity
- tRNA binding

Location

- aminoacyl-tRNA synthetase multienzyme complex
- cytosol
- extracellular space
- mitochondrial matrix
- mitochondrion
- nucleus
- plasma membrane

Biological process

- basophil activation involved in immune response
- diadenosine tetraphosphate biosynthetic process
- ERK1 and ERK2 cascade
- lysyl-tRNA aminoacylation
- positive regulation of transcription, DNA-templated
- positive regulation of inflammatory response to antigenic stimulus
- positive regulation of macrophage activation
- response to X-ray
- tRNA processing

---

30

- **Protein name:** Serotransferrin
- **Organism:** Homo sapiens
- **Uniprot Accession Number:** P02787
- **Protein sequence length:** 698 aa
- **1D identity (%):** 13.37
- **1D identity (%) [Gaps excluded]:** 24.1
- **1D identity - Alignment Gaps:** 446
- **1D aligned content (<aminoacid>:%):** {'M': 0.75, 'L': 11.19, 'C': 5.22, 'P': 10.45, 'V': 6.72, 'K': 8.96, 'S': 4.48, 'H': 2.99, 'I': 2.24, 'D': 5.97, 'Y': 3.73, 'A': 4.48, 'N': 2.99, 'T': 2.99, 'G': 13.43, 'F': 5.97, 'Q': 1.49, 'R': 2.24, 'E': 2.24, 'W': 1.49}
- **Common reported functions (%):** 0.0
- **Common reported locations (%):** 0.0
- **Common reported processes (%):** 0.0

- **PDB ID:** 2HAV
- **Chain:** B
- **Crystallized protein length:** 676 aa
- **Resolution:** 2.7 Å
- **b-phipsi:** 0.004259
- **w-rdist:** 0.523593
- **t-alpha:** 0.004754
- **Chemical similarity (Tanimoto Index) (%):** 94.13
- **1D identity (%) [PDB]:** 3.31
- **1D identity (%) [Gaps excluded][PDB]:** 65.75
- **1D identity - Alignment Gaps [PDB]:** 1375
- **1D aligned content [PDB] (<aminoacid>:%):** {'K': 6.25, 'V': 20.83, 'R': 8.33, 'S': 6.25, 'H': 4.17, 'E': 6.25, 'T': 4.17, 'G': 8.33, 'L': 6.25, 'P': 10.42, 'D': 2.08, 'N': 2.08, 'A': 4.17, 'F': 6.25, 'Y': 2.08, 'Q': 2.08}
- **2D identity (%) [PDB]:** 34.62
- **2D identity (%) [Gaps excluded][PDB]:** 80.35
- **2D identity - Alignment Gaps [PDB]:** 605
- **2D aligned content [PDB] (<2D-fold>:%):** {'.': 15.49, 'H': 42.93, 'E': 22.55, 'T': 18.21, 'G': 0.82}
- **3D similarity (TM-Score) (%) [PDB]:** 21.0

- **Gene name:** TF
- **Entrez ID:** 7018
- **RefSeq ID:** N/A
- **Sequence length:** N/A
- **5-UTR|CDS|3-UTR identity (%):** N/A | N/A | N/A
- **5-UTR|CDS|3-UTR identity (%) [Gaps excluded]:** N/A | N/A | N/A
- **5-UTR|CDS|3-UTR identity [Alignment Gaps]:** N/A | N/A | N/A
- **5-UTR aligned content (<base>:%):** N/A
- **CDS aligned content (<base>:%):** N/A
- **3-UTR aligned content (<base>:%):** N/A

**Uniprot Description:**  
  
 Transferrins are iron binding transport proteins which can bind two Fe(3+) ions in association with the binding of an anion, usually bicarbonate. It is responsible for the transport of iron from sites of absorption and heme degradation to those of storage and utilization. Serum transferrin may also have a further role in stimulating cell proliferation.   
  
Monomer.   
  
 **Gene Ontology Information:**

Molecular Function

- ferric iron binding
- ferrous iron binding
- iron chaperone activity
- transferrin receptor binding

Location

- apical plasma membrane
- basal part of cell
- basal plasma membrane
- blood microparticle
- cell surface
- clathrin-coated endocytic vesicle membrane
- clathrin-coated pit
- cytoplasmic vesicle
- early endosome
- endocytic vesicle
- endoplasmic reticulum lumen
- endosome membrane
- extracellular exosome
- extracellular region
- extracellular space
- HFE-transferrin receptor complex
- late endosome
- perinuclear region of cytoplasm
- plasma membrane
- recycling endosome
- secretory granule lumen
- vesicle

Biological process

- actin filament organization
- antibacterial humoral response
- cellular response to iron ion
- ERK1 and ERK2 cascade
- cellular iron ion homeostasis
- iron ion transmembrane transport
- iron ion transport
- osteoclast differentiation
- positive regulation of bone resorption
- positive regulation of cell motility
- positive regulation of transcription, DNA-templated
- positive regulation of phosphorylation
- positive regulation of receptor-mediated endocytosis
- regulation of iron ion transport
- regulation of protein stability
- retina homeostasis
- SMAD protein signal transduction

---

31

- **Protein name:** Dipeptidyl aminopeptidase-like protein 6
- **Organism:** Homo sapiens
- **Uniprot Accession Number:** P42658
- **Protein sequence length:** 865 aa
- **1D identity (%):** 18.31
- **1D identity (%) [Gaps excluded]:** 26.23
- **1D identity - Alignment Gaps:** 307
- **1D aligned content (<aminoacid>:%):** {'M': 1.08, 'G': 10.75, 'P': 8.06, 'A': 7.53, 'L': 9.14, 'R': 7.53, 'N': 3.76, 'Q': 2.69, 'I': 4.3, 'C': 1.08, 'V': 8.06, 'S': 3.76, 'K': 3.76, 'T': 7.53, 'F': 2.15, 'D': 8.06, 'H': 3.76, 'Y': 3.23, 'E': 3.76}
- **Common reported functions (%):** 0.0
- **Common reported locations (%):** 11.11
- **Common reported processes (%):** 0.0

- **PDB ID:** 7E8H
- **Chain:** I
- **Crystallized protein length:** 760 aa
- **Resolution:** 4.5 Å
- **b-phipsi:** 0.00238
- **w-rdist:** 0.71315
- **t-alpha:** 0.003978
- **Chemical similarity (Tanimoto Index) (%):** 96.33
- **1D identity (%) [PDB]:** 3.14
- **1D identity (%) [Gaps excluded][PDB]:** 64.0
- **1D identity - Alignment Gaps [PDB]:** 1455
- **1D aligned content [PDB] (<aminoacid>:%):** {'S': 6.25, 'Q': 2.08, 'K': 2.08, 'V': 16.67, 'T': 8.33, 'I': 2.08, 'D': 10.42, 'R': 10.42, 'G': 10.42, 'Y': 2.08, 'A': 4.17, 'N': 2.08, 'P': 2.08, 'L': 10.42, 'E': 4.17, 'F': 2.08, 'H': 4.17}
- **2D identity (%) [PDB]:** 29.52
- **2D identity (%) [Gaps excluded][PDB]:** 87.19
- **2D identity - Alignment Gaps [PDB]:** 793
- **2D aligned content [PDB] (<2D-fold>:%):** {'.': 24.86, 'H': 25.42, 'T': 18.08, 'E': 30.79, 'G': 0.85}
- **3D similarity (TM-Score) (%) [PDB]:** 10.75

- **Gene name:** DPP6
- **Entrez ID:** 1804
- **RefSeq ID:** NM\_130797
- **Transcript sequence length:** 4826
- **5-UTR|CDS|3-UTR identity (%):** 38.93 | 45.13 | 6.31
- **5-UTR|CDS|3-UTR identity (%) [Gaps excluded]:** 73.93 | 73.14 | 73.68
- **5-UTR|CDS|3-UTR identity [Alignment Gaps]:** 231 | 1227 | 15634
- **5-UTR aligned content (<base>:%):** {'G': 33.68, 'A': 9.47, 'T': 20.53, 'C': 36.32}
- **CDS aligned content (<base>:%):** {'A': 29.46, 'T': 20.61, 'G': 24.62, 'C': 25.31}
- **3-UTR aligned content (<base>:%):** {'G': 23.28, 'C': 23.1, 'T': 29.5, 'A': 24.12}

**Uniprot Description:**  
  
 Promotes cell surface expression of the potassium channel KCND2 (PubMed:15454437, PubMed:19441798). Modulates the activity and gating characteristics of the potassium channel KCND2 (PubMed:18364354). Has no dipeptidyl aminopeptidase activity (PubMed:8103397, PubMed:15476821).   
  
Homodimer (in vitro) (PubMed:15476821). Interacts with KCND2 (PubMed:15454437, PubMed:18364354). Identified in a complex with KCND2 and KCNIP2 (PubMed:18364354). Forms an octameric complex composed of four DPP6 subunits bound to the KCND2 tetramer (PubMed:18364354).   
  
 **Gene Ontology Information:**

Molecular Function

- potassium channel regulator activity
- serine-type peptidase activity

Location

- membrane
- plasma membrane
- voltage-gated potassium channel complex

Biological process

- protein localization to plasma membrane
- proteolysis
- regulation of potassium ion transmembrane transport

---

32

- **Protein name:** Spliceosome-associated protein CWC15 homolog
- **Organism:** Homo sapiens
- **Uniprot Accession Number:** Q9P013
- **Protein sequence length:** 229 aa
- **1D identity (%):** 4.93
- **1D identity (%) [Gaps excluded]:** 19.91
- **1D identity - Alignment Gaps:** 657
- **1D aligned content (<aminoacid>:%):** {'A': 6.98, 'P': 6.98, 'G': 9.3, 'R': 11.63, 'K': 13.95, 'D': 9.3, 'Q': 9.3, 'E': 16.28, 'N': 4.65, 'L': 4.65, 'T': 2.33, 'I': 2.33, 'F': 2.33}
- **Common reported functions (%):** 14.29
- **Common reported locations (%):** 11.11
- **Common reported processes (%):** 0.0

- **PDB ID:** 7ABI
- **Chain:** r
- **Crystallized protein length:** 895 aa
- **Resolution:** 8.0 Å
- **b-phipsi:** 0.002706
- **w-rdist:** 0.261168
- **t-alpha:** 0.094295
- **Chemical similarity (Tanimoto Index) (%):** N/A
- **1D identity (%) [PDB]:** 3.26
- **1D identity (%) [Gaps excluded][PDB]:** 63.53
- **1D identity - Alignment Gaps [PDB]:** 1570
- **1D aligned content [PDB] (<aminoacid>:%):** {'K': 12.96, 'V': 12.96, 'Y': 1.85, 'L': 9.26, 'E': 5.56, 'I': 5.56, 'P': 11.11, 'N': 1.85, 'D': 5.56, 'Q': 1.85, 'H': 1.85, 'T': 7.41, 'R': 7.41, 'G': 5.56, 'S': 3.7, 'A': 3.7, 'F': 1.85}
- **2D identity (%) [PDB]:** 33.39
- **2D identity (%) [Gaps excluded][PDB]:** 86.39
- **2D identity - Alignment Gaps [PDB]:** 770
- **2D aligned content [PDB] (<2D-fold>:%):** {'.': 28.64, 'E': 17.9, 'H': 38.66, 'T': 14.08, 'B': 0.72}
- **3D similarity (TM-Score) (%) [PDB]:** 14.74

- **Gene name:** CWC15
- **Entrez ID:** 51503
- **RefSeq ID:** N/A
- **Sequence length:** N/A
- **5-UTR|CDS|3-UTR identity (%):** N/A | N/A | N/A
- **5-UTR|CDS|3-UTR identity (%) [Gaps excluded]:** N/A | N/A | N/A
- **5-UTR|CDS|3-UTR identity [Alignment Gaps]:** N/A | N/A | N/A
- **5-UTR aligned content (<base>:%):** N/A
- **CDS aligned content (<base>:%):** N/A
- **3-UTR aligned content (<base>:%):** N/A

**Uniprot Description:**  
  
 Involved in pre-mRNA splicing as component of the spliceosome (PubMed:28502770, PubMed:28076346). Component of the PRP19-CDC5L complex that forms an integral part of the spliceosome and is required for activating pre-mRNA splicing.   
  
Identified in the spliceosome C complex (PubMed:28502770, PubMed:28076346). Component of the PRP19-CDC5L splicing complex composed of a core complex comprising a homotetramer of PRPF19, CDC5L, PLRG1 and BCAS2, and at least three less stably associated proteins CTNNBL1, CWC15 and HSPA8 (PubMed:20176811). Interacts directly with CTNNBL1 in the complex (PubMed:20176811).   
  
 **Gene Ontology Information:**

Molecular Function

- RNA binding

Location

- catalytic step 2 spliceosome
- mitochondrion
- nuclear speck
- nucleoplasm
- nucleus
- Prp19 complex
- spliceosomal complex
- U2-type catalytic step 2 spliceosome

Biological process

- mRNA cis splicing, via spliceosome
- mRNA splicing, via spliceosome

---

33

- **Protein name:** RuvB-like 2
- **Organism:** Homo sapiens
- **Uniprot Accession Number:** Q9Y230
- **Protein sequence length:** 463 aa
- **1D identity (%):** 10.03
- **1D identity (%) [Gaps excluded]:** 21.88
- **1D identity - Alignment Gaps:** 491
- **1D aligned content (<aminoacid>:%):** {'K': 8.79, 'V': 7.69, 'I': 5.49, 'R': 10.99, 'H': 2.2, 'G': 10.99, 'L': 8.79, 'E': 8.79, 'S': 3.3, 'A': 6.59, 'P': 5.49, 'Q': 5.49, 'T': 7.69, 'F': 3.3, 'Y': 1.1, 'D': 1.1, 'M': 1.1, 'N': 1.1}
- **Common reported functions (%):** 0.0
- **Common reported locations (%):** 44.44
- **Common reported processes (%):** 0.0

- **PDB ID:** 6HTS
- **Chain:** D
- **Crystallized protein length:** 434 aa
- **Resolution:** 4.8 Å
- **b-phipsi:** 0.003084
- **w-rdist:** 0.525244
- **t-alpha:** 0.007132
- **Chemical similarity (Tanimoto Index) (%):** 79.52
- **1D identity (%) [PDB]:** 2.42
- **1D identity (%) [Gaps excluded][PDB]:** 71.43
- **1D identity - Alignment Gaps [PDB]:** 1196
- **1D aligned content [PDB] (<aminoacid>:%):** {'D': 10.0, 'V': 16.67, 'T': 10.0, 'P': 6.67, 'G': 13.33, 'F': 3.33, 'I': 3.33, 'K': 3.33, 'S': 6.67, 'L': 13.33, 'H': 3.33, 'E': 6.67, 'N': 3.33}
- **2D identity (%) [PDB]:** 30.81
- **2D identity (%) [Gaps excluded][PDB]:** 89.06
- **2D identity - Alignment Gaps [PDB]:** 622
- **2D aligned content [PDB] (<2D-fold>:%):** {'.': 16.04, 'H': 47.78, 'E': 25.26, 'T': 10.92}
- **3D similarity (TM-Score) (%) [PDB]:** 15.48

- **Gene name:** RUVBL2
- **Entrez ID:** 10856
- **RefSeq ID:** N/A
- **Sequence length:** N/A
- **5-UTR|CDS|3-UTR identity (%):** N/A | N/A | N/A
- **5-UTR|CDS|3-UTR identity (%) [Gaps excluded]:** N/A | N/A | N/A
- **5-UTR|CDS|3-UTR identity [Alignment Gaps]:** N/A | N/A | N/A
- **5-UTR aligned content (<base>:%):** N/A
- **CDS aligned content (<base>:%):** N/A
- **3-UTR aligned content (<base>:%):** N/A

**Uniprot Description:**  
  
 Possesses single-stranded DNA-stimulated ATPase and ATP-dependent DNA helicase (5' to 3') activity; hexamerization is thought to be critical for ATP hydrolysis and adjacent subunits in the ring-like structure contribute to the ATPase activity (PubMed:10428817, PubMed:17157868). Component of the NuA4 histone acetyltransferase complex which is involved in transcriptional activation of select genes principally by acetylation of nucleosomal histones H4 and H2A (PubMed:14966270). This modification may both alter nucleosome -DNA interactions and promote interaction of the modified histones with other proteins which positively regulate transcription (PubMed:14966270). This complex may be required for the activation of transcriptional programs associated with oncogene and proto-oncogene mediated growth induction, tumor suppressor mediated growth arrest and replicative senescence, apoptosis, and DNA repair (PubMed:14966270). The NuA4 complex ATPase and helicase activities seem to be, at least in part, contributed by the association of RUVBL1 and RUVBL2 with EP400 (PubMed:14966270). NuA4 may also play a direct role in DNA repair when recruited to sites of DNA damage (PubMed:14966270). Component of a SWR1-like complex that specifically mediates the removal of histone H2A.Z/H2AZ1 from the nucleosome (PubMed:24463511). Proposed core component of the chromatin remodeling INO80 complex which exhibits DNA- and nucleosome-activated ATPase activity and catalyzes ATP-dependent nucleosome sliding (PubMed:16230350, PubMed:21303910). Plays an essential role in oncogenic transformation by MYC and also modulates transcriptional activation by the LEF1/TCF1-CTNNB1 complex (PubMed:10882073, PubMed:16014379). May also inhibit the transcriptional activity of ATF2 (PubMed:11713276). Involved in the endoplasmic reticulum (ER)-associated degradation (ERAD) pathway where it negatively regulates expression of ER stress response genes (PubMed:25652260). May play a role in regulating the composition of the U5 snRNP complex (PubMed:28561026).   
  
Forms homohexameric rings (Probable). Can form a dodecamer with RUVBL1 made of two stacked hexameric rings; however, even though RUVBL1 and RUVBL2 are present in equimolar ratio, the oligomeric status of each hexamer is not known. Oligomerization may regulate binding to nucleic acids and conversely, binding to nucleic acids may affect the dodecameric assembly. Interacts with the transcriptional activation domain of MYC. Interacts With ATF2. Component of the RNA polymerase II holoenzyme complex. May also act to bridge the LEF1/TCF1-CTNNB1 complex and TBP. Component of the NuA4 histone acetyltransferase complex which contains the catalytic subunit KAT5/TIP60 and the subunits EP400, TRRAP/PAF400, BRD8/SMAP, EPC1, DMAP1/DNMAP1, RUVBL1/TIP49, RUVBL2, ING3, actin, ACTL6A/BAF53A, MORF4L1/MRG15, MORF4L2/MRGX, MRGBP, YEATS4/GAS41, VPS72/YL1 and MEAF6. The NuA4 complex interacts with MYC and the adenovirus E1A protein. RUVBL2 interacts with EP400. Component of a NuA4-related complex which contains EP400, TRRAP/PAF400, SRCAP, BRD8/SMAP, EPC1, DMAP1/DNMAP1, RUVBL1/TIP49, RUVBL2, actin, ACTL6A/BAF53A, VPS72 and YEATS4/GAS41. Interacts with NPAT. Component of the chromatin-remodeling INO80 complex; specifically part of a complex module associated with the helicase ATP-binding and the helicase C-terminal domain of INO80. Component of some MLL1/MLL complex, at least composed of the core components KMT2A/MLL1, ASH2L, HCFC1/HCF1, WDR5 and RBBP5, as well as the facultative components BAP18, CHD8, E2F6, HSP70, INO80C, KANSL1, LAS1L, MAX, MCRS1, MGA, MYST1/MOF, PELP1, PHF20, PRP31, RING2, RUVB1/TIP49A, RUVB2/TIP49B, SENP3, TAF1, TAF4, TAF6, TAF7, TAF9 and TEX10. Interacts with IGHMBP2. Interacts with TELO2. Interacts with HINT1. Component of a SWR1-like complex. Component of the R2TP complex composed at least of PIHD1, RUVBL1, RUVBL2 and RPAP3 (PubMed:20864032). Interacts with ITFG1 (PubMed:25437307). Interacts with ZMYND10 (PubMed:29601588). Interacts with WAC; WAC positively regulates MTOR activity by promoting the assembly of the TTT complex composed of TELO2, TTI1 and TTI2 and the RUVBL complex composed of RUVBL1 and RUVBL2 into the TTT-RUVBL complex which leads to the dimerization of the mTORC1 complex and its subsequent activation (PubMed:26812014). Forms a complex with APPL1 and APPL2 (PubMed:19433865). Interacts with ZNHIT2 (via HIT-type zinc finger) in the presence of ATP or ADP; shows a stronger interaction in the presence of ADP (PubMed:28561026). The RUVBL1/RUVBL2 complex interacts with ZNHIT1 (via HIT-type zinc finger), ZNHIT3 (via HIT-type zinc finger), ZNHIT6 (via HIT-type zinc finger) and DDX59/ZNHIT5 (via HIT-type zinc finger) in the presence of ADP (PubMed:28561026). Interacts with NOPCHAP1; the interaction is direct and disrupted upon ATP binding (PubMed:33367824).   
  
 **Gene Ontology Information:**

Molecular Function

- ADP binding
- ATP binding
- ATPase activity
- ATPase binding
- beta-catenin binding
- chromatin DNA binding
- DNA helicase activity
- identical protein binding
- promoter-enhancer loop anchoring activity
- protein homodimerization activity
- RNA polymerase II cis-regulatory region sequence-specific DNA binding
- RNA polymerase II core promoter sequence-specific DNA binding
- TBP-class protein binding
- TFIID-class transcription factor complex binding
- transcription corepressor activity
- unfolded protein binding

Location

- centrosome
- cytoplasm
- cytosol
- dynein axonemal particle
- euchromatin
- extracellular exosome
- Ino80 complex
- membrane
- MLL1 complex
- NuA4 histone acetyltransferase complex
- nuclear matrix
- nucleoplasm
- nucleosome
- nucleus
- chaperone complex
- R2TP complex
- ribonucleoprotein complex
- RPAP3/R2TP/prefoldin-like complex
- Swr1 complex

Biological process

- box C/D snoRNP assembly
- cellular response to estradiol stimulus
- cellular response to UV
- chromatin remodeling
- DNA recombination
- DNA repair
- establishment of protein localization to chromatin
- histone acetylation
- histone H2A acetylation
- histone H4 acetylation
- negative regulation of canonical Wnt signaling pathway
- positive regulation of DNA repair
- positive regulation of transcription, DNA-templated
- positive regulation of double-strand break repair via homologous recombination
- positive regulation of telomerase RNA localization to Cajal body
- positive regulation of telomere maintenance in response to DNA damage
- positive regulation of transcription by RNA polymerase II
- protein folding
- protein stabilization
- regulation of apoptotic process
- regulation of cell cycle
- regulation of chromosome organization
- regulation of DNA repair
- regulation of DNA replication
- regulation of DNA strand elongation
- regulation of transcription, DNA-templated
- regulation of double-strand break repair
- regulation of embryonic development
- regulation of transcription by RNA polymerase II
- telomere maintenance

---

34

- **Protein name:** Aldehyde oxidase
- **Organism:** Homo sapiens
- **Uniprot Accession Number:** Q06278
- **Protein sequence length:** 1338 aa
- **1D identity (%):** 13.86
- **1D identity (%) [Gaps excluded]:** 23.62
- **1D identity - Alignment Gaps:** 572
- **1D aligned content (<aminoacid>:%):** {'M': 2.6, 'S': 4.69, 'G': 10.94, 'A': 6.77, 'P': 13.02, 'Y': 4.17, 'T': 5.21, 'C': 4.69, 'D': 4.69, 'E': 3.65, 'Q': 4.17, 'F': 3.65, 'R': 3.65, 'L': 8.85, 'V': 5.21, 'K': 5.73, 'I': 3.12, 'H': 3.65, 'N': 1.56}
- **Common reported functions (%):** 0.0
- **Common reported locations (%):** 11.11
- **Common reported processes (%):** 0.0

- **PDB ID:** 7ORC
- **Chain:** A
- **Crystallized protein length:** 1295 aa
- **Resolution:** 2.7 Å
- **b-phipsi:** 0.001396
- **w-rdist:** 0.357618
- **t-alpha:** 0.195722
- **Chemical similarity (Tanimoto Index) (%):** 67.52
- **1D identity (%) [PDB]:** 4.55
- **1D identity (%) [Gaps excluded][PDB]:** 63.19
- **1D identity - Alignment Gaps [PDB]:** 1855
- **1D aligned content [PDB] (<aminoacid>:%):** {'N': 2.2, 'V': 12.09, 'K': 5.49, 'L': 8.79, 'G': 7.69, 'P': 6.59, 'Q': 7.69, 'R': 6.59, 'F': 4.4, 'I': 7.69, 'A': 7.69, 'D': 4.4, 'H': 3.3, 'S': 3.3, 'M': 1.1, 'E': 4.4, 'T': 4.4, 'C': 2.2}
- **2D identity (%) [PDB]:** 37.59
- **2D identity (%) [Gaps excluded][PDB]:** 85.21
- **2D identity - Alignment Gaps [PDB]:** 831
- **2D aligned content [PDB] (<2D-fold>:%):** {'.': 18.78, 'E': 28.62, 'T': 14.67, 'H': 37.39, 'B': 0.54}
- **3D similarity (TM-Score) (%) [PDB]:** 27.53

- **Gene name:** AOX1
- **Entrez ID:** 316
- **RefSeq ID:** N/A
- **Sequence length:** N/A
- **5-UTR|CDS|3-UTR identity (%):** N/A | N/A | N/A
- **5-UTR|CDS|3-UTR identity (%) [Gaps excluded]:** N/A | N/A | N/A
- **5-UTR|CDS|3-UTR identity [Alignment Gaps]:** N/A | N/A | N/A
- **5-UTR aligned content (<base>:%):** N/A
- **CDS aligned content (<base>:%):** N/A
- **3-UTR aligned content (<base>:%):** N/A

**Uniprot Description:**  
  
 Oxidase with broad substrate specificity, oxidizing aromatic azaheterocycles, such as N1-methylnicotinamide, N-methylphthalazinium and phthalazine, as well as aldehydes, such as benzaldehyde, retinal, pyridoxal, and vanillin. Plays a key role in the metabolism of xenobiotics and drugs containing aromatic azaheterocyclic substituents. Participates in the bioactivation of prodrugs such as famciclovir, catalyzing the oxidation step from 6-deoxypenciclovir to penciclovir, which is a potent antiviral agent. Is probably involved in the regulation of reactive oxygen species homeostasis. May be a prominent source of superoxide generation via the one-electron reduction of molecular oxygen. Also may catalyze nitric oxide (NO) production via the reduction of nitrite to NO with NADH or aldehyde as electron donor. May play a role in adipogenesis.   
  
Homodimer.   
  
 **Gene Ontology Information:**

Molecular Function

- 2 iron, 2 sulfur cluster binding
- aldehyde oxidase activity
- FAD binding
- flavin adenine dinucleotide binding
- identical protein binding
- iron ion binding
- molybdopterin cofactor binding
- NAD binding
- protein homodimerization activity

Location

- cytosol
- extracellular exosome

Biological process

- lipid metabolic process
- xenobiotic metabolic process

---

35

- **Protein name:** DNA replication licensing factor MCM6
- **Organism:** Homo sapiens
- **Uniprot Accession Number:** Q14566
- **Protein sequence length:** 821 aa
- **1D identity (%):** 14.96
- **1D identity (%) [Gaps excluded]:** 22.86
- **1D identity - Alignment Gaps:** 351
- **1D aligned content (<aminoacid>:%):** {'D': 7.89, 'P': 8.55, 'R': 9.21, 'K': 5.26, 'F': 4.61, 'G': 9.87, 'L': 8.55, 'E': 5.26, 'N': 1.97, 'S': 7.24, 'T': 6.58, 'Y': 3.95, 'C': 4.61, 'H': 1.97, 'A': 3.29, 'V': 5.92, 'Q': 1.32, 'I': 3.29, 'W': 0.66}
- **Common reported functions (%):** 0.0
- **Common reported locations (%):** 11.11
- **Common reported processes (%):** 0.0

- **PDB ID:** 6XTY
- **Chain:** 6
- **Crystallized protein length:** 609 aa
- **Resolution:** 6.77 Å
- **b-phipsi:** 0.002389
- **w-rdist:** 0.467083
- **t-alpha:** 0.020602
- **Chemical similarity (Tanimoto Index) (%):** N/A
- **1D identity (%) [PDB]:** 3.51
- **1D identity (%) [Gaps excluded][PDB]:** 80.33
- **1D identity - Alignment Gaps [PDB]:** 1334
- **1D aligned content [PDB] (<aminoacid>:%):** {'K': 6.12, 'Y': 4.08, 'L': 16.33, 'Q': 6.12, 'P': 8.16, 'V': 8.16, 'T': 10.2, 'E': 8.16, 'C': 2.04, 'I': 6.12, 'A': 8.16, 'G': 2.04, 'D': 2.04, 'N': 2.04, 'R': 6.12, 'S': 4.08}
- **2D identity (%) [PDB]:** 35.23
- **2D identity (%) [Gaps excluded][PDB]:** 82.95
- **2D identity - Alignment Gaps [PDB]:** 588
- **2D aligned content [PDB] (<2D-fold>:%):** {'.': 19.44, 'H': 41.39, 'E': 24.72, 'T': 13.61, 'G': 0.83}
- **3D similarity (TM-Score) (%) [PDB]:** 20.73

- **Gene name:** MCM6
- **Entrez ID:** 4175
- **RefSeq ID:** NM\_005915
- **Transcript sequence length:** 3753
- **5-UTR|CDS|3-UTR identity (%):** 14.2 | 47.18 | 4.59
- **5-UTR|CDS|3-UTR identity (%) [Gaps excluded]:** 77.42 | 76.7 | 75.15
- **5-UTR|CDS|3-UTR identity [Alignment Gaps]:** 276 | 1203 | 15896
- **5-UTR aligned content (<base>:%):** {'G': 45.83, 'C': 27.08, 'T': 12.5, 'A': 14.58}
- **CDS aligned content (<base>:%):** {'A': 30.51, 'T': 24.61, 'G': 24.14, 'C': 20.75}
- **3-UTR aligned content (<base>:%):** {'G': 14.29, 'A': 29.6, 'T': 42.08, 'C': 14.03}

**Uniprot Description:**  
  
 Acts as component of the MCM2-7 complex (MCM complex) which is the putative replicative helicase essential for 'once per cell cycle' DNA replication initiation and elongation in eukaryotic cells. The active ATPase sites in the MCM2-7 ring are formed through the interaction surfaces of two neighboring subunits such that a critical structure of a conserved arginine finger motif is provided in trans relative to the ATP-binding site of the Walker A box of the adjacent subunit. The six ATPase active sites, however, are likely to contribute differentially to the complex helicase activity.   
  
Component of the MCM2-7 complex (PubMed:16899510, PubMed:17296731, PubMed:9305914). The complex forms a toroidal hexameric ring with the proposed subunit order MCM2-MCM6-MCM4-MCM7-MCM3-MCM5 (PubMed:16899510, PubMed:17296731, PubMed:9305914). May interact with MCM10 (PubMed:11095689). Interacts with TIPIN (PubMed:17116885). Interacts with CDT1 (PubMed:20202939). Interacts with MCMBP (PubMed:17296731). Interacts with DDI2 (PubMed:29290612). Component of the CMG helicase complex, composed of the MCM2-7 complex, the GINS complex and CDC45 (By similarity).   
  
 **Gene Ontology Information:**

Molecular Function

- ATP binding
- ATPase activity
- DNA helicase activity
- identical protein binding
- single-stranded DNA binding

Location

- chromosome, telomeric region
- CMG complex
- MCM complex
- nucleoplasm
- nucleus

Biological process

- DNA replication
- DNA replication initiation
- DNA unwinding involved in DNA replication
- double-strand break repair via break-induced replication
- mitotic DNA replication
- regulation of DNA-dependent DNA replication initiation

---

36

- **Protein name:** Complement factor B
- **Organism:** Homo sapiens
- **Uniprot Accession Number:** P00751
- **Protein sequence length:** 764 aa
- **1D identity (%):** 13.92
- **1D identity (%) [Gaps excluded]:** 23.08
- **1D identity - Alignment Gaps:** 402
- **1D aligned content (<aminoacid>:%):** {'S': 4.96, 'L': 11.35, 'G': 10.64, 'P': 7.09, 'V': 7.09, 'Y': 3.55, 'K': 4.96, 'A': 5.67, 'I': 5.67, 'F': 3.55, 'C': 4.26, 'D': 2.13, 'T': 7.8, 'H': 2.84, 'R': 7.09, 'Q': 2.13, 'N': 2.84, 'E': 4.26, 'M': 1.42, 'W': 0.71}
- **Common reported functions (%):** 0.0
- **Common reported locations (%):** 0.0
- **Common reported processes (%):** 0.0

- **PDB ID:** 2XWB
- **Chain:** H
- **Crystallized protein length:** 711 aa
- **Resolution:** 3.49 Å
- **b-phipsi:** 0.002217
- **w-rdist:** 0.710831
- **t-alpha:** 0.004777
- **Chemical similarity (Tanimoto Index) (%):** 78.15
- **1D identity (%) [PDB]:** 2.11
- **1D identity (%) [Gaps excluded][PDB]:** 76.19
- **1D identity - Alignment Gaps [PDB]:** 1474
- **1D aligned content [PDB] (<aminoacid>:%):** {'G': 12.5, 'S': 9.38, 'L': 9.38, 'Y': 3.12, 'T': 3.12, 'A': 6.25, 'N': 3.12, 'P': 6.25, 'V': 15.62, 'D': 9.38, 'K': 9.38, 'R': 3.12, 'F': 6.25, 'I': 3.12}
- **2D identity (%) [PDB]:** 33.82
- **2D identity (%) [Gaps excluded][PDB]:** 81.98
- **2D identity - Alignment Gaps [PDB]:** 648
- **2D aligned content [PDB] (<2D-fold>:%):** {'.': 22.79, 'E': 35.39, 'T': 13.67, 'H': 27.88, 'B': 0.27}
- **3D similarity (TM-Score) (%) [PDB]:** 21.94

- **Gene name:** CFB
- **Entrez ID:** 629
- **RefSeq ID:** NM\_001710
- **Transcript sequence length:** 2476
- **5-UTR|CDS|3-UTR identity (%):** 21.11 | 45.15 | 0.19
- **5-UTR|CDS|3-UTR identity (%) [Gaps excluded]:** 66.67 | 75.47 | 88.89
- **5-UTR|CDS|3-UTR identity [Alignment Gaps]:** 233 | 1226 | 16737
- **5-UTR aligned content (<base>:%):** {'G': 36.11, 'C': 41.67, 'T': 12.5, 'A': 9.72}
- **CDS aligned content (<base>:%):** {'T': 21.92, 'G': 25.76, 'C': 23.95, 'A': 28.37}
- **3-UTR aligned content (<base>:%):** {'G': 28.12, 'T': 18.75, 'C': 18.75, 'A': 34.38}

**Uniprot Description:**  
  
 Factor B which is part of the alternate pathway of the complement system is cleaved by factor D into 2 fragments: Ba and Bb. Bb, a serine protease, then combines with complement factor 3b to generate the C3 or C5 convertase. It has also been implicated in proliferation and differentiation of preactivated B-lymphocytes, rapid spreading of peripheral blood monocytes, stimulation of lymphocyte blastogenesis and lysis of erythrocytes. Ba inhibits the proliferation of preactivated B-lymphocytes.   
  
Monomer (PubMed:19574954). Part of the C3-convertase enzyme complex comprised of Complement C3 beta chain (C3b) and Complement factor B Bb fragment (Bb) and CFP (PubMed:28264884, PubMed:31507604). Interacts to C3b; this interaction is dependent on the presence of Mg2+ (PubMed:28264884, PubMed:31507604). Interacts to CFP; this interaction contributes to the stabilization of the active C3-convertase enzyme complex (PubMed:31507604).   
  
 **Gene Ontology Information:**

Molecular Function

- complement binding
- serine-type endopeptidase activity

Location

- blood microparticle
- classical-complement-pathway C3/C5 convertase complex
- extracellular exosome
- extracellular region
- extracellular space
- plasma membrane

Biological process

- complement activation
- complement activation, alternative pathway
- positive regulation of immune response
- positive regulation of opsonization
- proteolysis
- response to bacterium

---

37

- **Protein name:** DNA replication licensing factor MCM3
- **Organism:** Homo sapiens
- **Uniprot Accession Number:** P25205
- **Protein sequence length:** 808 aa
- **1D identity (%):** 14.98
- **1D identity (%) [Gaps excluded]:** 24.06
- **1D identity - Alignment Gaps:** 388
- **1D aligned content (<aminoacid>:%):** {'M': 1.3, 'T': 8.44, 'V': 6.49, 'A': 6.49, 'Y': 3.25, 'L': 10.39, 'G': 7.14, 'Q': 5.84, 'S': 4.55, 'N': 0.65, 'D': 9.09, 'F': 3.25, 'I': 2.6, 'P': 7.79, 'R': 9.09, 'C': 3.25, 'E': 4.55, 'K': 3.25, 'H': 2.6}
- **Common reported functions (%):** 0.0
- **Common reported locations (%):** 22.22
- **Common reported processes (%):** 0.0

- **PDB ID:** 6XTY
- **Chain:** 4
- **Crystallized protein length:** 598 aa
- **Resolution:** 6.77 Å
- **b-phipsi:** 0.002561
- **w-rdist:** 0.411996
- **t-alpha:** 0.027734
- **Chemical similarity (Tanimoto Index) (%):** N/A
- **1D identity (%) [PDB]:** 3.67
- **1D identity (%) [Gaps excluded][PDB]:** 60.98
- **1D identity - Alignment Gaps [PDB]:** 1281
- **1D aligned content [PDB] (<aminoacid>:%):** {'N': 4.0, 'V': 12.0, 'L': 8.0, 'G': 2.0, 'I': 10.0, 'P': 10.0, 'H': 4.0, 'Q': 8.0, 'S': 8.0, 'A': 6.0, 'D': 6.0, 'K': 4.0, 'Y': 2.0, 'T': 2.0, 'R': 8.0, 'E': 4.0, 'M': 2.0}
- **2D identity (%) [PDB]:** 32.08
- **2D identity (%) [Gaps excluded][PDB]:** 82.67
- **2D identity - Alignment Gaps [PDB]:** 637
- **2D aligned content [PDB] (<2D-fold>:%):** {'.': 29.64, 'H': 37.72, 'T': 15.57, 'E': 16.47, 'B': 0.6}
- **3D similarity (TM-Score) (%) [PDB]:** 20.73

- **Gene name:** MCM3
- **Entrez ID:** 4173
- **RefSeq ID:** NM\_002388
- **Transcript sequence length:** 3068
- **5-UTR|CDS|3-UTR identity (%):** 10.29 | 45.76 | 2.34
- **5-UTR|CDS|3-UTR identity (%) [Gaps excluded]:** 70.0 | 74.46 | 78.13
- **5-UTR|CDS|3-UTR identity [Alignment Gaps]:** 290 | 1196 | 16322
- **5-UTR aligned content (<base>:%):** {'G': 60.0, 'C': 20.0, 'A': 11.43, 'T': 8.57}
- **CDS aligned content (<base>:%):** {'A': 29.51, 'T': 21.69, 'G': 26.9, 'C': 21.9}
- **3-UTR aligned content (<base>:%):** {'G': 20.87, 'A': 25.7, 'C': 19.85, 'T': 33.59}

**Uniprot Description:**  
  
 Acts as component of the MCM2-7 complex (MCM complex) which is the putative replicative helicase essential for 'once per cell cycle' DNA replication initiation and elongation in eukaryotic cells. The active ATPase sites in the MCM2-7 ring are formed through the interaction surfaces of two neighboring subunits such that a critical structure of a conserved arginine finger motif is provided in trans relative to the ATP-binding site of the Walker A box of the adjacent subunit. The six ATPase active sites, however, are likely to contribute differentially to the complex helicase activity. Required for DNA replication and cell proliferation.   
  
Component of the MCM2-7 complex (PubMed:16899510, PubMed:17296731). The complex forms a toroidal hexameric ring with the proposed subunit order MCM2-MCM6-MCM4-MCM7-MCM3-MCM5 (PubMed:16899510, PubMed:17296731). Associated with the replication-specific DNA polymerase alpha (By similarity). Interacts with MCMBP (PubMed:17296731). Interacts with ANKRD17 (PubMed:23711367). Interacts with MCM3AP isoform MCM3AP; this interaction leads to MCM3 acetylation (PubMed:9712829, PubMed:11258703, PubMed:12226073). Component of the CMG helicase complex, composed of the MCM2-7 complex, the GINS complex and CDC45 (By similarity).   
  
 **Gene Ontology Information:**

Molecular Function

- ATP binding
- ATPase activity
- DNA helicase activity
- single-stranded DNA binding

Location

- chromosome, telomeric region
- CMG complex
- MCM complex
- membrane
- nucleoplasm
- nucleus

Biological process

- DNA replication
- DNA strand elongation involved in DNA replication
- DNA unwinding involved in DNA replication
- double-strand break repair via break-induced replication
- mitotic DNA replication initiation
- regulation of DNA-dependent DNA replication initiation

---

38

- **Protein name:** Ectonucleotide pyrophosphatase/phosphodiesterase family member 2
- **Organism:** Homo sapiens
- **Uniprot Accession Number:** Q13822
- **Protein sequence length:** 863 aa
- **1D identity (%):** 15.81
- **1D identity (%) [Gaps excluded]:** 22.18
- **1D identity - Alignment Gaps:** 289
- **1D aligned content (<aminoacid>:%):** {'M': 1.89, 'S': 5.03, 'G': 8.81, 'L': 6.92, 'R': 3.14, 'P': 10.06, 'C': 5.03, 'F': 4.4, 'D': 7.55, 'N': 2.52, 'E': 5.03, 'V': 10.06, 'A': 3.14, 'K': 7.55, 'I': 1.89, 'H': 2.52, 'T': 5.03, 'Y': 6.29, 'W': 1.26, 'Q': 1.89}
- **Common reported functions (%):** 0.0
- **Common reported locations (%):** 0.0
- **Common reported processes (%):** 0.0

- **PDB ID:** 4ZG9
- **Chain:** B
- **Crystallized protein length:** 764 aa
- **Resolution:** 2.95 Å
- **b-phipsi:** 0.003296
- **w-rdist:** 0.48495
- **t-alpha:** 0.009509
- **Chemical similarity (Tanimoto Index) (%):** 66.67
- **1D identity (%) [PDB]:** 2.3
- **1D identity (%) [Gaps excluded][PDB]:** 75.0
- **1D identity - Alignment Gaps [PDB]:** 1517
- **1D aligned content [PDB] (<aminoacid>:%):** {'S': 8.33, 'L': 13.89, 'Q': 5.56, 'I': 8.33, 'V': 19.44, 'P': 5.56, 'K': 8.33, 'T': 11.11, 'Y': 2.78, 'A': 2.78, 'G': 5.56, 'D': 2.78, 'M': 2.78, 'N': 2.78}
- **2D identity (%) [PDB]:** 26.39
- **2D identity (%) [Gaps excluded][PDB]:** 83.03
- **2D identity - Alignment Gaps [PDB]:** 835
- **2D aligned content [PDB] (<2D-fold>:%):** {'.': 18.58, 'T': 19.81, 'H': 40.56, 'E': 19.5, 'B': 0.62, 'G': 0.93}
- **3D similarity (TM-Score) (%) [PDB]:** 25.29

- **Gene name:** ENPP2
- **Entrez ID:** 5168
- **RefSeq ID:** NM\_006209
- **Transcript sequence length:** 3242
- **5-UTR|CDS|3-UTR identity (%):** 10.54 | 47.14 | 1.99
- **5-UTR|CDS|3-UTR identity (%) [Gaps excluded]:** 71.43 | 75.75 | 81.66
- **5-UTR|CDS|3-UTR identity [Alignment Gaps]:** 283 | 1241 | 16372
- **5-UTR aligned content (<base>:%):** {'A': 17.14, 'G': 31.43, 'T': 17.14, 'C': 34.29}
- **CDS aligned content (<base>:%):** {'A': 30.34, 'T': 26.21, 'G': 21.43, 'C': 22.01}
- **3-UTR aligned content (<base>:%):** {'C': 12.28, 'T': 40.42, 'A': 33.23, 'G': 14.07}

**Uniprot Description:**  
  
 Hydrolyzes lysophospholipids to produce the signaling molecule lysophosphatidic acid (LPA) in extracellular fluids (PubMed:15769751, PubMed:26371182, PubMed:27754931, PubMed:14500380, PubMed:12354767,). Major substrate is lysophosphatidylcholine (PubMed:12176993, PubMed:27754931, PubMed:14500380). Also can act on sphingosylphosphorylcholine producing sphingosine-1-phosphate, a modulator of cell motility (PubMed:14500380). Can hydrolyze, in vitro, bis-pNPP, to some extent pNP-TMP, and barely ATP (PubMed:15769751, PubMed:12176993). Involved in several motility-related processes such as angiogenesis and neurite outgrowth. Acts as an angiogenic factor by stimulating migration of smooth muscle cells and microtubule formation (PubMed:11559573). Stimulates migration of melanoma cells, probably via a pertussis toxin-sensitive G protein (PubMed:1733949). May have a role in induction of parturition (PubMed:12176993). Possible involvement in cell proliferation and adipose tissue development (Probable). Tumor cell motility-stimulating factor (PubMed:1733949, PubMed:11559573). Required for LPA production in activated platelets, cleaves the sn-1 lysophospholipids to generate sn-1 lysophosphatidic acids containing predominantly 18:2 and 20:4 fatty acids (PubMed:21393252). Shows a preference for the sn-1 to the sn-2 isomer of 1-O-alkyl-sn-glycero-3-phosphocholine (lyso-PAF) (PubMed:21393252). N/A   
  
 **Gene Ontology Information:**

Molecular Function

- alkylglycerophosphoethanolamine phosphodiesterase activity
- calcium ion binding
- hydrolase activity
- lysophospholipase activity
- nucleic acid binding
- phosphodiesterase I activity
- polysaccharide binding
- scavenger receptor activity
- zinc ion binding

Location

- extracellular space
- plasma membrane

Biological process

- cell motility
- chemotaxis
- immune response
- phosphatidylcholine catabolic process
- phospholipid catabolic process
- positive regulation of epithelial cell migration
- positive regulation of lamellipodium morphogenesis
- positive regulation of peptidyl-tyrosine phosphorylation
- regulation of cell migration
- sphingolipid catabolic process

---

39

- **Protein name:** Maltose/maltodextrin-binding periplasmic protein
- **Organism:** Escherichia coli (strain K12)
- **Uniprot Accession Number:** P0AEX9
- **Protein sequence length:** 396 aa
- **1D identity (%):** 7.52
- **1D identity (%) [Gaps excluded]:** 20.41
- **1D identity - Alignment Gaps:** 580
- **1D aligned content (<aminoacid>:%):** {'L': 10.14, 'T': 7.25, 'A': 5.8, 'I': 5.8, 'N': 7.25, 'D': 5.8, 'K': 13.04, 'F': 5.8, 'G': 10.14, 'V': 11.59, 'H': 2.9, 'P': 7.25, 'E': 4.35, 'Y': 2.9}
- **Common reported functions (%):** 0.0
- **Common reported locations (%):** 0.0
- **Common reported processes (%):** 0.0

- **PDB ID:** 4BLB
- **Chain:** A
- **Crystallized protein length:** 734 aa
- **Resolution:** 2.8 Å
- **b-phipsi:** 0.00519
- **w-rdist:** 0.599792
- **t-alpha:** 0.000792
- **Chemical similarity (Tanimoto Index) (%):** 84.36
- **1D identity (%) [PDB]:** 2.55
- **1D identity (%) [Gaps excluded][PDB]:** 70.91
- **1D identity - Alignment Gaps [PDB]:** 1472
- **1D aligned content [PDB] (<aminoacid>:%):** {'K': 10.26, 'T': 5.13, 'P': 12.82, 'Q': 10.26, 'L': 10.26, 'S': 5.13, 'I': 10.26, 'N': 7.69, 'V': 7.69, 'G': 7.69, 'F': 5.13, 'A': 5.13, 'H': 2.56}
- **2D identity (%) [PDB]:** 36.53
- **2D identity (%) [Gaps excluded][PDB]:** 84.87
- **2D identity - Alignment Gaps [PDB]:** 630
- **2D aligned content [PDB] (<2D-fold>:%):** {'.': 17.82, 'E': 25.74, 'H': 43.56, 'T': 11.39, 'G': 1.49}
- **3D similarity (TM-Score) (%) [PDB]:** 20.89

- **Gene name:** malE
- **Entrez ID:** 75204178; 948538
- **RefSeq ID:** N/A
- **Sequence length:** N/A
- **5-UTR|CDS|3-UTR identity (%):** N/A | N/A | N/A
- **5-UTR|CDS|3-UTR identity (%) [Gaps excluded]:** N/A | N/A | N/A
- **5-UTR|CDS|3-UTR identity [Alignment Gaps]:** N/A | N/A | N/A
- **5-UTR aligned content (<base>:%):** N/A
- **CDS aligned content (<base>:%):** N/A
- **3-UTR aligned content (<base>:%):** N/A

**Uniprot Description:**  
  
 Part of the ABC transporter complex MalEFGK involved in maltose/maltodextrin import. Binds maltose and higher maltodextrins such as maltotriose.   
  
The complex is composed of two ATP-binding proteins (MalK), two transmembrane proteins (MalG and MalF) and a solute-binding protein (MalE).   
  
 **Gene Ontology Information:**

Molecular Function

- carbohydrate transmembrane transporter activity
- maltose binding

Location

- ATP-binding cassette (ABC) transporter complex
- ATP-binding cassette (ABC) transporter complex, substrate-binding subunit-containing
- maltose transport complex
- outer membrane-bounded periplasmic space
- periplasmic space

Biological process

- carbohydrate transport
- cell chemotaxis
- detection of maltose stimulus
- cellular response to DNA damage stimulus
- maltodextrin transport
- maltose transport

---

40

- **Protein name:** Voltage-dependent calcium channel subunit alpha-2/delta-1
- **Organism:** Homo sapiens
- **Uniprot Accession Number:** P54289
- **Protein sequence length:** 1103 aa
- **1D identity (%):** 16.58
- **1D identity (%) [Gaps excluded]:** 23.26
- **1D identity - Alignment Gaps:** 329
- **1D aligned content (<aminoacid>:%):** {'M': 2.63, 'G': 8.95, 'P': 6.32, 'V': 6.32, 'L': 8.95, 'A': 5.26, 'D': 8.42, 'Y': 7.37, 'R': 6.84, 'K': 7.37, 'N': 3.68, 'E': 3.68, 'I': 4.74, 'F': 3.68, 'S': 4.21, 'H': 1.58, 'T': 6.84, 'W': 0.53, 'Q': 2.11, 'C': 0.53}
- **Common reported functions (%):** 14.29
- **Common reported locations (%):** 0.0
- **Common reported processes (%):** 0.0

- **PDB ID:** 7UHG
- **Chain:** D
- **Crystallized protein length:** 948 aa
- **Resolution:** 3.0 Å
- **b-phipsi:** 0.001717
- **w-rdist:** 0.465892
- **t-alpha:** 0.053883
- **Chemical similarity (Tanimoto Index) (%):** 81.99
- **1D identity (%) [PDB]:** 2.72
- **1D identity (%) [Gaps excluded][PDB]:** 66.2
- **1D identity - Alignment Gaps [PDB]:** 1656
- **1D aligned content [PDB] (<aminoacid>:%):** {'P': 10.64, 'A': 4.26, 'T': 8.51, 'D': 6.38, 'L': 12.77, 'V': 17.02, 'G': 6.38, 'E': 4.26, 'K': 6.38, 'R': 6.38, 'S': 6.38, 'F': 2.13, 'W': 2.13, 'I': 2.13, 'N': 2.13, 'H': 2.13}
- **2D identity (%) [PDB]:** 34.94
- **2D identity (%) [Gaps excluded][PDB]:** 82.28
- **2D identity - Alignment Gaps [PDB]:** 726
- **2D aligned content [PDB] (<2D-fold>:%):** {'.': 18.59, 'H': 40.59, 'T': 13.61, 'E': 25.62, 'G': 1.36, 'B': 0.23}
- **3D similarity (TM-Score) (%) [PDB]:** N/A

- **Gene name:** CACNA2D1
- **Entrez ID:** 781
- **RefSeq ID:** N/A
- **Sequence length:** N/A
- **5-UTR|CDS|3-UTR identity (%):** N/A | N/A | N/A
- **5-UTR|CDS|3-UTR identity (%) [Gaps excluded]:** N/A | N/A | N/A
- **5-UTR|CDS|3-UTR identity [Alignment Gaps]:** N/A | N/A | N/A
- **5-UTR aligned content (<base>:%):** N/A
- **CDS aligned content (<base>:%):** N/A
- **3-UTR aligned content (<base>:%):** N/A

**Uniprot Description:**  
  
 The alpha-2/delta subunit of voltage-dependent calcium channels regulates calcium current density and activation/inactivation kinetics of the calcium channel. Plays an important role in excitation-contraction coupling (By similarity).   
  
Dimer formed of alpha-2-1 and delta-1 chains; disulfide-linked. Voltage-dependent calcium channels are multisubunit complexes, consisting of alpha-1 (CACNA1), alpha-2 (CACNA2D), beta (CACNB) and delta (CACNA2D) subunits in a 1:1:1:1 ratio (By similarity).   
  
 **Gene Ontology Information:**

Molecular Function

- metal ion binding
- voltage-gated calcium channel activity

Location

- extracellular exosome
- L-type voltage-gated calcium channel complex
- neuronal dense core vesicle
- plasma membrane
- sarcoplasmic reticulum
- voltage-gated calcium channel complex

Biological process

- calcium ion import across plasma membrane
- calcium ion transmembrane transport
- calcium ion transmembrane transport via high voltage-gated calcium channel
- calcium ion transport
- calcium ion transport into cytosol
- cardiac muscle cell action potential involved in contraction
- cellular response to amyloid-beta
- membrane depolarization during bundle of His cell action potential
- positive regulation of high voltage-gated calcium channel activity
- positive regulation of muscle contraction
- regulation of calcium ion transmembrane transport via high voltage-gated calcium channel
- regulation of calcium ion transport
- regulation of heart rate by cardiac conduction
- regulation of membrane repolarization during action potential
- regulation of ventricular cardiac muscle cell membrane repolarization

---

41

- **Protein name:** Neutral and basic amino acid transport protein rBAT
- **Organism:** Homo sapiens
- **Uniprot Accession Number:** Q07837
- **Protein sequence length:** 685 aa
- **1D identity (%):** 15.93
- **1D identity (%) [Gaps excluded]:** 23.63
- **1D identity - Alignment Gaps:** 301
- **1D aligned content (<aminoacid>:%):** {'D': 10.2, 'K': 5.44, 'R': 6.8, 'S': 2.72, 'P': 8.84, 'T': 6.12, 'L': 11.56, 'G': 10.88, 'F': 4.76, 'V': 8.16, 'E': 6.12, 'A': 2.04, 'N': 3.4, 'I': 3.4, 'Y': 2.04, 'H': 2.04, 'Q': 4.08, 'C': 1.36}
- **Common reported functions (%):** 0.0
- **Common reported locations (%):** 11.11
- **Common reported processes (%):** 0.0

- **PDB ID:** 6LI9
- **Chain:** C
- **Crystallized protein length:** 623 aa
- **Resolution:** 2.3 Å
- **b-phipsi:** 0.00267
- **w-rdist:** 0.77038
- **t-alpha:** 0.00317
- **Chemical similarity (Tanimoto Index) (%):** 88.16
- **1D identity (%) [PDB]:** 3.3
- **1D identity (%) [Gaps excluded][PDB]:** 63.89
- **1D identity - Alignment Gaps [PDB]:** 1324
- **1D aligned content [PDB] (<aminoacid>:%):** {'Q': 8.7, 'A': 4.35, 'M': 2.17, 'E': 6.52, 'L': 4.35, 'F': 6.52, 'Y': 10.87, 'K': 6.52, 'S': 4.35, 'T': 8.7, 'R': 8.7, 'D': 4.35, 'G': 4.35, 'V': 8.7, 'I': 6.52, 'P': 2.17, 'C': 2.17}
- **2D identity (%) [PDB]:** 30.27
- **2D identity (%) [Gaps excluded][PDB]:** 86.35
- **2D identity - Alignment Gaps [PDB]:** 706
- **2D aligned content [PDB] (<2D-fold>:%):** {'.': 20.97, 'H': 41.95, 'T': 19.15, 'E': 16.41, 'B': 0.61, 'G': 0.91}
- **3D similarity (TM-Score) (%) [PDB]:** 20.58

- **Gene name:** SLC3A1
- **Entrez ID:** 6519
- **RefSeq ID:** NM\_000341
- **Transcript sequence length:** 2969
- **5-UTR|CDS|3-UTR identity (%):** 11.48 | 43.46 | 3.56
- **5-UTR|CDS|3-UTR identity (%) [Gaps excluded]:** 80.85 | 76.46 | 75.92
- **5-UTR|CDS|3-UTR identity [Alignment Gaps]:** 284 | 1277 | 16032
- **5-UTR aligned content (<base>:%):** {'A': 15.79, 'C': 36.84, 'T': 21.05, 'G': 26.32}
- **CDS aligned content (<base>:%):** {'A': 29.94, 'T': 25.97, 'G': 22.24, 'C': 21.85}
- **3-UTR aligned content (<base>:%):** {'G': 16.03, 'C': 12.69, 'T': 35.06, 'A': 36.23}

**Uniprot Description:**  
  
 Involved in the high-affinity, sodium-independent transport of cystine and neutral and dibasic amino acids (system B(0,+)-like activity). May function as an activator of SLC7A9 and be involved in the high-affinity reabsorption of cystine in the kidney tubule.   
  
Disulfide-linked heterodimer with the amino acid transport protein SLC7A9.   
  
 **Gene Ontology Information:**

Molecular Function

- amino acid transmembrane transporter activity
- basic amino acid transmembrane transporter activity
- L-cystine transmembrane transporter activity
- protein heterodimerization activity
- protein-containing complex binding

Location

- apical plasma membrane
- brush border membrane
- extracellular exosome
- membrane
- plasma membrane
- vacuolar membrane

Biological process

- amino acid transport
- aspartate transmembrane transport
- basic amino acid transport
- carbohydrate metabolic process
- gene expression
- L-cystine transport
- L-glutamate transmembrane transport

---

42

- **Protein name:** Protein arginine N-methyltransferase 9
- **Organism:** Homo sapiens
- **Uniprot Accession Number:** Q6P2P2
- **Protein sequence length:** 845 aa
- **1D identity (%):** 14.24
- **1D identity (%) [Gaps excluded]:** 21.84
- **1D identity - Alignment Gaps:** 359
- **1D aligned content (<aminoacid>:%):** {'P': 4.08, 'S': 6.12, 'V': 7.48, 'R': 5.44, 'L': 12.93, 'F': 3.4, 'A': 5.44, 'Y': 4.08, 'E': 7.48, 'D': 6.12, 'Q': 5.44, 'H': 4.08, 'K': 5.44, 'N': 2.04, 'T': 5.44, 'C': 2.72, 'G': 7.48, 'I': 3.4, 'M': 1.36}
- **Common reported functions (%):** 0.0
- **Common reported locations (%):** 11.11
- **Common reported processes (%):** 0.0

- **PDB ID:** 7T39
- **Chain:** A
- **Crystallized protein length:** 613 aa
- **Resolution:** 2.81 Å
- **b-phipsi:** 0.004462
- **w-rdist:** 0.477973
- **t-alpha:** 0.007132
- **Chemical similarity (Tanimoto Index) (%):** 81.5
- **1D identity (%) [PDB]:** 2.41
- **1D identity (%) [Gaps excluded][PDB]:** 64.15
- **1D identity - Alignment Gaps [PDB]:** 1357
- **1D aligned content [PDB] (<aminoacid>:%):** {'A': 2.94, 'T': 11.76, 'G': 11.76, 'V': 8.82, 'L': 20.59, 'E': 8.82, 'K': 8.82, 'P': 2.94, 'F': 5.88, 'S': 8.82, 'R': 5.88, 'D': 2.94}
- **2D identity (%) [PDB]:** 29.8
- **2D identity (%) [Gaps excluded][PDB]:** 82.26
- **2D identity - Alignment Gaps [PDB]:** 685
- **2D aligned content [PDB] (<2D-fold>:%):** {'.': 21.56, 'H': 32.5, 'T': 13.75, 'E': 31.25, 'G': 0.94}
- **3D similarity (TM-Score) (%) [PDB]:** 23.38

- **Gene name:** PRMT9
- **Entrez ID:** 90826
- **RefSeq ID:** NM\_138364
- **Transcript sequence length:** 3461
- **5-UTR|CDS|3-UTR identity (%):** 30.11 | 43.47 | 2.91
- **5-UTR|CDS|3-UTR identity (%) [Gaps excluded]:** 72.6 | 76.41 | 76.48
- **5-UTR|CDS|3-UTR identity [Alignment Gaps]:** 206 | 1407 | 16218
- **5-UTR aligned content (<base>:%):** {'A': 13.21, 'G': 35.85, 'C': 28.3, 'T': 22.64}
- **CDS aligned content (<base>:%):** {'A': 32.28, 'T': 26.78, 'G': 23.47, 'C': 17.48}
- **3-UTR aligned content (<base>:%):** {'A': 35.64, 'G': 12.83, 'T': 41.14, 'C': 10.39}

**Uniprot Description:**  
  
 Arginine methyltransferase that can both catalyze the formation of omega-N monomethylarginine (MMA) and symmetrical dimethylarginine (sDMA). Specifically mediates the symmetrical dimethylation of SF3B2. Involved in the regulation of alternative splicing of pre-mRNA (PubMed:25737013, PubMed:25979344).   
  
Found in a complex with PRMT9, SF3B2 and SF3B4 (PubMed:25737013). Interacts with SF3B2 (PubMed:25737013).   
  
 **Gene Ontology Information:**

Molecular Function

- protein-arginine N-methyltransferase activity
- protein-arginine omega-N monomethyltransferase activity
- protein-arginine omega-N symmetric methyltransferase activity

Location

- cytoplasm

Biological process

- mRNA processing
- peptidyl-arginine methylation

---

43

- **Protein name:** Mediator of RNA polymerase II transcription subunit 16
- **Organism:** Homo sapiens
- **Uniprot Accession Number:** Q9Y2X0
- **Protein sequence length:** 877 aa
- **1D identity (%):** 15.23
- **1D identity (%) [Gaps excluded]:** 22.24
- **1D identity - Alignment Gaps:** 325
- **1D aligned content (<aminoacid>:%):** {'D': 8.92, 'P': 10.83, 'G': 9.55, 'S': 7.64, 'H': 4.46, 'L': 12.1, 'R': 7.64, 'T': 7.01, 'E': 3.18, 'W': 0.64, 'M': 0.64, 'N': 1.27, 'F': 3.82, 'K': 3.82, 'V': 6.37, 'Q': 3.82, 'C': 3.82, 'A': 3.18, 'Y': 0.64, 'I': 0.64}
- **Common reported functions (%):** 0.0
- **Common reported locations (%):** 22.22
- **Common reported processes (%):** 0.0

- **PDB ID:** 7EMF
- **Chain:** P
- **Crystallized protein length:** 766 aa
- **Resolution:** 3.5 Å
- **b-phipsi:** 0.00084
- **w-rdist:** 0.542664
- **t-alpha:** 0.029319
- **Chemical similarity (Tanimoto Index) (%):** 95.91
- **1D identity (%) [PDB]:** 2.7
- **1D identity (%) [Gaps excluded][PDB]:** 70.0
- **1D identity - Alignment Gaps [PDB]:** 1495
- **1D aligned content [PDB] (<aminoacid>:%):** {'S': 16.67, 'I': 4.76, 'K': 7.14, 'F': 4.76, 'R': 7.14, 'V': 14.29, 'L': 14.29, 'T': 7.14, 'G': 4.76, 'P': 9.52, 'E': 2.38, 'N': 2.38, 'H': 2.38, 'M': 2.38}
- **2D identity (%) [PDB]:** 30.65
- **2D identity (%) [Gaps excluded][PDB]:** 82.61
- **2D identity - Alignment Gaps [PDB]:** 741
- **2D aligned content [PDB] (<2D-fold>:%):** {'.': 21.61, 'E': 26.32, 'T': 11.91, 'H': 39.34, 'G': 0.83}
- **3D similarity (TM-Score) (%) [PDB]:** 7.5

- **Gene name:** MED16
- **Entrez ID:** 10025
- **RefSeq ID:** NM\_005481
- **Transcript sequence length:** 2892
- **5-UTR|CDS|3-UTR identity (%):** 23.37 | 45.03 | 0.6
- **5-UTR|CDS|3-UTR identity (%) [Gaps excluded]:** 75.96 | 72.49 | 77.1
- **5-UTR|CDS|3-UTR identity [Alignment Gaps]:** 234 | 1219 | 16631
- **5-UTR aligned content (<base>:%):** {'A': 8.86, 'G': 39.24, 'C': 43.04, 'T': 8.86}
- **CDS aligned content (<base>:%):** {'A': 21.33, 'T': 19.32, 'G': 29.95, 'C': 29.4}
- **3-UTR aligned content (<base>:%):** {'G': 35.64, 'C': 34.65, 'A': 15.84, 'T': 13.86}

**Uniprot Description:**  
  
 Component of the Mediator complex, a coactivator involved in the regulated transcription of nearly all RNA polymerase II-dependent genes. Mediator functions as a bridge to convey information from gene-specific regulatory proteins to the basal RNA polymerase II transcription machinery. Mediator is recruited to promoters by direct interactions with regulatory proteins and serves as a scaffold for the assembly of a functional preinitiation complex with RNA polymerase II and the general transcription factors.   
  
Component of the Mediator complex, which is composed of MED1, MED4, MED6, MED7, MED8, MED9, MED10, MED11, MED12, MED13, MED13L, MED14, MED15, MED16, MED17, MED18, MED19, MED20, MED21, MED22, MED23, MED24, MED25, MED26, MED27, MED29, MED30, MED31, CCNC, CDK8 and CDC2L6/CDK11. The MED12, MED13, CCNC and CDK8 subunits form a distinct module termed the CDK8 module. Mediator containing the CDK8 module is less active than Mediator lacking this module in supporting transcriptional activation. Individual preparations of the Mediator complex lacking one or more distinct subunits have been variously termed ARC, CRSP, DRIP, PC2, SMCC and TRAP.   
  
 **Gene Ontology Information:**

Molecular Function

- thyroid hormone receptor binding
- vitamin D receptor binding
- transcription coactivator activity

Location

- core mediator complex
- mediator complex
- membrane
- nucleoplasm
- nucleus

Biological process

- positive regulation of transcription, DNA-templated
- positive regulation of transcription elongation from RNA polymerase II promoter
- positive regulation of transcription initiation from RNA polymerase II promoter
- regulation of transcription by RNA polymerase II
- RNA polymerase II preinitiation complex assembly
- transcription by RNA polymerase II

---

44

- **Protein name:** Protein argonaute-1
- **Organism:** Homo sapiens
- **Uniprot Accession Number:** Q9UL18
- **Protein sequence length:** 857 aa
- **1D identity (%):** 84.06
- **1D identity (%) [Gaps excluded]:** 85.55
- **1D identity - Alignment Gaps:** 15
- **1D aligned content (<aminoacid>:%):** {'M': 2.34, 'E': 4.67, 'G': 7.01, 'A': 6.18, 'Q': 6.32, 'P': 6.73, 'R': 6.59, 'T': 5.91, 'K': 6.04, 'I': 5.49, 'L': 7.55, 'N': 2.34, 'F': 4.12, 'V': 8.52, 'D': 5.08, 'Y': 4.26, 'C': 2.61, 'H': 3.16, 'S': 4.26, 'W': 0.82}
- **Common reported functions (%):** 57.14
- **Common reported locations (%):** 77.78
- **Common reported processes (%):** 55.56

- **PDB ID:** 4KXT
- **Chain:** A
- **Crystallized protein length:** 806 aa
- **Resolution:** 2.29 Å
- **b-phipsi:** 0.003142
- **w-rdist:** 0.254084
- **t-alpha:** 0.117275
- **Chemical similarity (Tanimoto Index) (%):** 100.0
- **1D identity (%) [PDB]:** 82.37
- **1D identity (%) [Gaps excluded][PDB]:** 86.14
- **1D identity - Alignment Gaps [PDB]:** 37
- **1D aligned content [PDB] (<aminoacid>:%):** {'P': 6.9, 'R': 6.75, 'G': 6.18, 'T': 6.03, 'K': 6.18, 'I': 5.75, 'L': 7.9, 'A': 6.18, 'N': 2.3, 'F': 4.17, 'V': 8.76, 'D': 5.03, 'Y': 4.45, 'E': 4.31, 'C': 2.73, 'M': 2.3, 'Q': 6.32, 'H': 3.02, 'S': 3.88, 'W': 0.86}
- **2D identity (%) [PDB]:** 61.12
- **2D identity (%) [Gaps excluded][PDB]:** 77.85
- **2D identity - Alignment Gaps [PDB]:** 199
- **2D aligned content [PDB] (<2D-fold>:%):** {'.': 19.79, 'B': 1.41, 'E': 29.68, 'H': 37.63, 'T': 10.42, 'G': 1.06}
- **3D similarity (TM-Score) (%) [PDB]:** 90.03

- **Gene name:** AGO1
- **Entrez ID:** 26523
- **RefSeq ID:** NM\_012199
- **Transcript sequence length:** 13712
- **5-UTR|CDS|3-UTR identity (%):** 40.56 | 73.37 | 37.87
- **5-UTR|CDS|3-UTR identity (%) [Gaps excluded]:** 80.0 | 76.48 | 74.74
- **5-UTR|CDS|3-UTR identity [Alignment Gaps]:** 175 | 107 | 9062
- **5-UTR aligned content (<base>:%):** {'A': 9.72, 'G': 46.53, 'C': 36.81, 'T': 6.94}
- **CDS aligned content (<base>:%):** {'A': 26.88, 'T': 22.68, 'G': 26.41, 'C': 24.03}
- **3-UTR aligned content (<base>:%):** {'A': 29.76, 'G': 20.62, 'C': 17.03, 'T': 32.58}

**Uniprot Description:**  
  
 Required for RNA-mediated gene silencing (RNAi). Binds to short RNAs such as microRNAs (miRNAs) or short interfering RNAs (siRNAs), and represses the translation of mRNAs which are complementary to them. Lacks endonuclease activity and does not appear to cleave target mRNAs. Also required for transcriptional gene silencing (TGS) of promoter regions which are complementary to bound short antigene RNAs (agRNAs).   
  
Interacts with DDB1, DDX5, DDX6, DHX30, DHX36, DDX47, DICER1, AGO2, ELAVL1, HNRNPF, IGF2BP1, ILF3, IMP8, MATR3, MOV10, PABPC1, PRMT5, RBM4, SART3, TNRC6B, UPF1 and YBX1. Associates with polysomes and messenger ribonucleoproteins (mNRPs). Interacts with LIMD1, WTIP and AJUBA. Interacts with APOBEC3F, APOBEC3G and APOBEC3H.   
  
 **Gene Ontology Information:**

Molecular Function

- core promoter sequence-specific DNA binding
- double-stranded RNA binding
- miRNA binding
- RNA binding
- RNA polymerase II complex binding
- single-stranded RNA binding

Location

- cytoplasm
- cytoplasmic ribonucleoprotein granule
- cytosol
- nucleoplasm
- nucleus
- P-body
- polysome
- ribonucleoprotein complex
- RISC complex
- RISC-loading complex

Biological process

- production of miRNAs involved in gene silencing by miRNA
- miRNA mediated inhibition of translation
- negative regulation of angiogenesis
- nuclear-transcribed mRNA catabolic process
- positive regulation of transcription by RNA polymerase II
- pre-miRNA processing
- small RNA loading onto RISC
- RNA secondary structure unwinding

---

45

- **Protein name:** GMP synthase [glutamine-hydrolyzing]
- **Organism:** Homo sapiens
- **Uniprot Accession Number:** P49915
- **Protein sequence length:** 693 aa
- **1D identity (%):** 13.93
- **1D identity (%) [Gaps excluded]:** 22.67
- **1D identity - Alignment Gaps:** 371
- **1D aligned content (<aminoacid>:%):** {'D': 5.97, 'L': 8.96, 'K': 9.7, 'Q': 3.73, 'V': 13.43, 'R': 5.97, 'P': 9.7, 'T': 6.72, 'G': 8.96, 'F': 2.24, 'I': 5.22, 'S': 5.22, 'N': 2.24, 'E': 8.21, 'C': 1.49, 'Y': 0.75, 'A': 0.75, 'M': 0.75}
- **Common reported functions (%):** 0.0
- **Common reported locations (%):** 11.11
- **Common reported processes (%):** 0.0

- **PDB ID:** 2VXO
- **Chain:** B
- **Crystallized protein length:** 658 aa
- **Resolution:** 2.5 Å
- **b-phipsi:** 0.003893
- **w-rdist:** 0.411455
- **t-alpha:** 0.016103
- **Chemical similarity (Tanimoto Index) (%):** 78.58
- **1D identity (%) [PDB]:** 3.49
- **1D identity (%) [Gaps excluded][PDB]:** 69.44
- **1D identity - Alignment Gaps [PDB]:** 1361
- **1D aligned content [PDB] (<aminoacid>:%):** {'V': 18.0, 'I': 8.0, 'L': 14.0, 'P': 10.0, 'T': 8.0, 'Y': 2.0, 'A': 4.0, 'E': 2.0, 'R': 4.0, 'G': 4.0, 'Q': 8.0, 'K': 8.0, 'S': 2.0, 'N': 6.0, 'H': 2.0}
- **2D identity (%) [PDB]:** 37.24
- **2D identity (%) [Gaps excluded][PDB]:** 85.93
- **2D identity - Alignment Gaps [PDB]:** 595
- **2D aligned content [PDB] (<2D-fold>:%):** {'.': 17.9, 'E': 25.83, 'H': 43.22, 'T': 11.51, 'B': 0.77, 'G': 0.77}
- **3D similarity (TM-Score) (%) [PDB]:** 21.05

- **Gene name:** GMPS
- **Entrez ID:** 8833
- **RefSeq ID:** NM\_003875
- **Transcript sequence length:** 8631
- **5-UTR|CDS|3-UTR identity (%):** 39.72 | 45.58 | 24.12
- **5-UTR|CDS|3-UTR identity (%) [Gaps excluded]:** 75.0 | 75.99 | 76.27
- **5-UTR|CDS|3-UTR identity [Alignment Gaps]:** 167 | 1167 | 11991
- **5-UTR aligned content (<base>:%):** {'A': 2.84, 'G': 31.91, 'T': 15.6, 'C': 49.65}
- **CDS aligned content (<base>:%):** {'A': 33.26, 'T': 26.64, 'G': 22.57, 'C': 17.53}
- **3-UTR aligned content (<base>:%):** {'A': 32.53, 'C': 16.19, 'T': 33.07, 'G': 18.2}

**Uniprot Description:**  
  
 Involved in the de novo synthesis of guanine nucleotides which are not only essential for DNA and RNA synthesis, but also provide GTP, which is involved in a number of cellular processes important for cell division.   
  
Homodimer.   
  
 **Gene Ontology Information:**

Molecular Function

- ATP binding
- GMP synthase (glutamine-hydrolyzing) activity
- GMP synthase activity

Location

- cytosol

Biological process

- glutamine metabolic process
- GMP biosynthetic process
- purine nucleobase biosynthetic process
- purine ribonucleoside monophosphate biosynthetic process

---

46

- **Protein name:** Propionyl-CoA carboxylase alpha chain, mitochondrial
- **Organism:** Homo sapiens
- **Uniprot Accession Number:** P05165
- **Protein sequence length:** 728 aa
- **1D identity (%):** 15.18
- **1D identity (%) [Gaps excluded]:** 25.89
- **1D identity - Alignment Gaps:** 414
- **1D aligned content (<aminoacid>:%):** {'A': 7.24, 'V': 9.21, 'G': 9.21, 'P': 7.24, 'R': 8.55, 'H': 3.95, 'L': 6.58, 'D': 5.26, 'K': 8.55, 'N': 1.97, 'M': 1.32, 'S': 3.95, 'F': 5.26, 'E': 5.26, 'T': 3.29, 'C': 1.97, 'I': 3.95, 'Q': 4.61, 'W': 0.66, 'Y': 1.97}
- **Common reported functions (%):** 0.0
- **Common reported locations (%):** 0.0
- **Common reported processes (%):** 0.0

- **PDB ID:** 7YBU
- **Chain:** A
- **Crystallized protein length:** 670 aa
- **Resolution:** 2.2 Å
- **b-phipsi:** 0.00177
- **w-rdist:** 0.543502
- **t-alpha:** 0.018225
- **Chemical similarity (Tanimoto Index) (%):** N/A
- **1D identity (%) [PDB]:** 1.62
- **1D identity (%) [Gaps excluded][PDB]:** 80.0
- **1D identity - Alignment Gaps [PDB]:** 1455
- **1D aligned content [PDB] (<aminoacid>:%):** {'P': 4.17, 'T': 4.17, 'R': 16.67, 'I': 8.33, 'Y': 12.5, 'D': 4.17, 'G': 4.17, 'F': 4.17, 'Q': 4.17, 'V': 4.17, 'L': 16.67, 'A': 8.33, 'E': 8.33}
- **2D identity (%) [PDB]:** 40.17
- **2D identity (%) [Gaps excluded][PDB]:** 87.42
- **2D identity - Alignment Gaps [PDB]:** 561
- **2D aligned content [PDB] (<2D-fold>:%):** {'.': 19.66, 'E': 26.62, 'H': 31.65, 'T': 22.06}
- **3D similarity (TM-Score) (%) [PDB]:** 20.69

- **Gene name:** PCCA
- **Entrez ID:** N/A
- **RefSeq ID:** NM\_001178004
- **Transcript sequence length:** 2343
- **5-UTR|CDS|3-UTR identity (%):** 7.12 | 40.53 | 1.13
- **5-UTR|CDS|3-UTR identity (%) [Gaps excluded]:** 85.19 | 74.65 | 78.42
- **5-UTR|CDS|3-UTR identity [Alignment Gaps]:** 296 | 1371 | 16542
- **5-UTR aligned content (<base>:%):** {'A': 17.39, 'G': 56.52, 'C': 26.09}
- **CDS aligned content (<base>:%):** {'A': 30.84, 'T': 25.99, 'G': 24.18, 'C': 19.0}
- **3-UTR aligned content (<base>:%):** {'A': 38.62, 'G': 10.05, 'T': 37.04, 'C': 14.29}

**Uniprot Description:**  
  
 This is one of the 2 subunits of the biotin-dependent propionyl-CoA carboxylase (PCC), a mitochondrial enzyme involved in the catabolism of odd chain fatty acids, branched-chain amino acids isoleucine, threonine, methionine, and valine and other metabolites (PubMed:8434582, PubMed:6765947). Propionyl-CoA carboxylase catalyzes the carboxylation of propionyl-CoA/propanoyl-CoA to D-methylmalonyl-CoA/(S)-methylmalonyl-CoA (PubMed:8434582, PubMed:6765947, PubMed:10101253). Within the holoenzyme, the alpha subunit catalyzes the ATP-dependent carboxylation of the biotin carried by the biotin carboxyl carrier (BCC) domain, while the beta subunit then transfers the carboxyl group from carboxylated biotin to propionyl-CoA (By similarity). Propionyl-CoA carboxylase also significantly acts on butyryl-CoA/butanoyl-CoA, which is converted to ethylmalonyl-CoA/(2S)-ethylmalonyl-CoA at a much lower rate (PubMed:6765947). Other alternative minor substrates include (2E)-butenoyl-CoA/crotonoyl-CoA (By similarity).   
  
The holoenzyme is a dodecamer composed of 6 PCCA/alpha subunits and 6 PCCB/beta subunits (PubMed:6765947, PubMed:20725044). Interacts (via the biotin carboxylation domain) with SIRT4 (PubMed:23438705). Interacts with SIRT3 and SIRT5 (PubMed:23438705).   
  
 **Gene Ontology Information:**

Molecular Function   
  
N/A

Location   
  
N/A

Biological process   
  
N/A

---

47

- **Protein name:** Protein O-linked-mannose beta-1,2-N-acetylglucosaminyltransferase 1
- **Organism:** Homo sapiens
- **Uniprot Accession Number:** Q8WZA1
- **Protein sequence length:** 660 aa
- **1D identity (%):** 14.19
- **1D identity (%) [Gaps excluded]:** 26.81
- **1D identity - Alignment Gaps:** 468
- **1D aligned content (<aminoacid>:%):** {'D': 4.96, 'I': 4.26, 'K': 4.26, 'P': 9.93, 'R': 9.93, 'V': 9.22, 'T': 4.96, 'A': 5.67, 'N': 2.84, 'L': 9.22, 'G': 7.09, 'S': 4.26, 'E': 6.38, 'H': 2.13, 'W': 2.84, 'F': 2.84, 'C': 1.42, 'Y': 2.13, 'Q': 4.96, 'M': 0.71}
- **Common reported functions (%):** 0.0
- **Common reported locations (%):** 11.11
- **Common reported processes (%):** 0.0

- **PDB ID:** 5GGF
- **Chain:** C
- **Crystallized protein length:** 536 aa
- **Resolution:** 2.49 Å
- **b-phipsi:** 0.002457
- **w-rdist:** 0.728667
- **t-alpha:** 0.004777
- **Chemical similarity (Tanimoto Index) (%):** 96.82
- **1D identity (%) [PDB]:** 3.78
- **1D identity (%) [Gaps excluded][PDB]:** 57.65
- **1D identity - Alignment Gaps [PDB]:** 1212
- **1D aligned content [PDB] (<aminoacid>:%):** {'R': 10.2, 'V': 10.2, 'E': 4.08, 'D': 8.16, 'H': 4.08, 'K': 6.12, 'T': 8.16, 'I': 4.08, 'G': 8.16, 'P': 10.2, 'Y': 2.04, 'S': 6.12, 'L': 12.24, 'A': 4.08, 'N': 2.04}
- **2D identity (%) [PDB]:** 32.46
- **2D identity (%) [Gaps excluded][PDB]:** 83.46
- **2D identity - Alignment Gaps [PDB]:** 608
- **2D aligned content [PDB] (<2D-fold>:%):** {'E': 30.34, 'T': 17.96, '.': 19.2, 'H': 31.89, 'B': 0.62}
- **3D similarity (TM-Score) (%) [PDB]:** 16.56

- **Gene name:** POMGNT1
- **Entrez ID:** 55624
- **RefSeq ID:** N/A
- **Sequence length:** N/A
- **5-UTR|CDS|3-UTR identity (%):** N/A | N/A | N/A
- **5-UTR|CDS|3-UTR identity (%) [Gaps excluded]:** N/A | N/A | N/A
- **5-UTR|CDS|3-UTR identity [Alignment Gaps]:** N/A | N/A | N/A
- **5-UTR aligned content (<base>:%):** N/A
- **CDS aligned content (<base>:%):** N/A
- **3-UTR aligned content (<base>:%):** N/A

**Uniprot Description:**  
  
 Participates in O-mannosyl glycosylation by catalyzing the addition of N-acetylglucosamine to O-linked mannose on glycoproteins (PubMed:11709191, PubMed:27493216, PubMed:28512129). Catalyzes the synthesis of the GlcNAc(beta1-2)Man(alpha1-)O-Ser/Thr moiety on alpha-dystroglycan and other O-mannosylated proteins, providing the necessary basis for the addition of further carbohydrate moieties (PubMed:11709191, PubMed:27493216). Is specific for alpha linked terminal mannose and does not have MGAT3, MGAT4, MGAT5, MGAT7 or MGAT8 activity.   
  
Interacts with DAG1 (via O-linked mannose moiety) (PubMed:27493216). Interacts (via transmembrane domain) with FKTN; the interaction is direct and is required for normal location in Golgi membranes (PubMed:17034757).   
  
 **Gene Ontology Information:**

Molecular Function

- acetylglucosaminyltransferase activity
- beta-1,3-galactosyl-O-glycosyl-glycoprotein beta-1,3-N-acetylglucosaminyltransferase activity
- manganese ion binding

Location

- Golgi membrane
- membrane

Biological process

- basement membrane organization
- dentate gyrus development
- gene expression
- localization of cell
- myelination
- O-glycan processing
- protein O-linked glycosylation
- protein O-linked mannosylation
- reactive gliosis
- sensory perception of sound

---

48

- **Protein name:** Schlafen family member 5
- **Organism:** Homo sapiens
- **Uniprot Accession Number:** Q08AF3
- **Protein sequence length:** 891 aa
- **1D identity (%):** 17.52
- **1D identity (%) [Gaps excluded]:** 23.89
- **1D identity - Alignment Gaps:** 269
- **1D aligned content (<aminoacid>:%):** {'M': 1.13, 'P': 8.47, 'A': 5.65, 'G': 7.34, 'L': 14.12, 'R': 6.78, 'I': 4.52, 'C': 3.95, 'K': 5.08, 'Y': 3.39, 'E': 7.34, 'D': 5.65, 'H': 3.39, 'F': 3.39, 'V': 5.65, 'T': 4.52, 'S': 2.26, 'N': 1.69, 'Q': 5.65}
- **Common reported functions (%):** 0.0
- **Common reported locations (%):** 0.0
- **Common reported processes (%):** 0.0

- **PDB ID:** 7PPJ
- **Chain:** A
- **Crystallized protein length:** 643 aa
- **Resolution:** 3.44 Å
- **b-phipsi:** 0.001502
- **w-rdist:** 0.497101
- **t-alpha:** 0.055468
- **Chemical similarity (Tanimoto Index) (%):** 87.78
- **1D identity (%) [PDB]:** 2.3
- **1D identity (%) [Gaps excluded][PDB]:** 60.0
- **1D identity - Alignment Gaps [PDB]:** 1380
- **1D aligned content [PDB] (<aminoacid>:%):** {'L': 21.21, 'V': 15.15, 'T': 9.09, 'G': 9.09, 'D': 9.09, 'E': 9.09, 'K': 3.03, 'R': 3.03, 'P': 9.09, 'F': 6.06, 'S': 6.06}
- **2D identity (%) [PDB]:** 34.31
- **2D identity (%) [Gaps excluded][PDB]:** 84.03
- **2D identity - Alignment Gaps [PDB]:** 626
- **2D aligned content [PDB] (<2D-fold>:%):** {'.': 22.59, 'E': 22.59, 'H': 39.94, 'T': 14.05, 'G': 0.83}
- **3D similarity (TM-Score) (%) [PDB]:** 21.69

- **Gene name:** SLFN5
- **Entrez ID:** 162394
- **RefSeq ID:** NM\_144975
- **Transcript sequence length:** 10555
- **5-UTR|CDS|3-UTR identity (%):** 19.76 | 46.13 | 28.09
- **5-UTR|CDS|3-UTR identity (%) [Gaps excluded]:** 70.53 | 75.65 | 75.9
- **5-UTR|CDS|3-UTR identity [Alignment Gaps]:** 244 | 1275 | 11276
- **5-UTR aligned content (<base>:%):** {'C': 31.34, 'T': 14.93, 'G': 41.79, 'A': 11.94}
- **CDS aligned content (<base>:%):** {'A': 29.4, 'T': 26.68, 'G': 23.62, 'C': 20.31}
- **3-UTR aligned content (<base>:%):** {'C': 15.64, 'A': 35.99, 'T': 29.76, 'G': 18.62}

**Uniprot Description:**  
  
 May have a role in hematopoietic cell differentiation. N/A   
  
 **Gene Ontology Information:**

Molecular Function

- ATP binding

Location

- nucleus

Biological process

- cell differentiation

---

49

- **Protein name:** X-ray repair cross-complementing protein 5
- **Organism:** Homo sapiens
- **Uniprot Accession Number:** P13010
- **Protein sequence length:** 732 aa
- **1D identity (%):** 14.39
- **1D identity (%) [Gaps excluded]:** 21.02
- **1D identity - Alignment Gaps:** 298
- **1D aligned content (<aminoacid>:%):** {'V': 7.35, 'G': 6.62, 'L': 13.24, 'D': 8.09, 'N': 1.47, 'R': 3.68, 'F': 4.41, 'P': 8.82, 'Q': 8.09, 'M': 2.21, 'K': 9.56, 'I': 4.41, 'E': 3.68, 'C': 2.21, 'S': 2.94, 'A': 5.15, 'Y': 2.94, 'H': 2.21, 'T': 2.94}
- **Common reported functions (%):** 14.29
- **Common reported locations (%):** 33.33
- **Common reported processes (%):** 0.0

- **PDB ID:** 8EZB
- **Chain:** B
- **Crystallized protein length:** 524 aa
- **Resolution:** 8.9 Å
- **b-phipsi:** 0.003006
- **w-rdist:** 0.300547
- **t-alpha:** 0.158479
- **Chemical similarity (Tanimoto Index) (%):** N/A
- **1D identity (%) [PDB]:** 2.38
- **1D identity (%) [Gaps excluded][PDB]:** 70.45
- **1D identity - Alignment Gaps [PDB]:** 1258
- **1D aligned content [PDB] (<aminoacid>:%):** {'K': 12.9, 'A': 3.23, 'Q': 6.45, 'P': 6.45, 'V': 12.9, 'I': 12.9, 'F': 3.23, 'L': 9.68, 'D': 6.45, 'E': 9.68, 'T': 9.68, 'R': 3.23, 'G': 3.23}
- **2D identity (%) [PDB]:** 36.16
- **2D identity (%) [Gaps excluded][PDB]:** 92.59
- **2D identity - Alignment Gaps [PDB]:** 590
- **2D aligned content [PDB] (<2D-fold>:%):** {'.': 25.14, 'E': 22.57, 'T': 21.14, 'H': 31.14}
- **3D similarity (TM-Score) (%) [PDB]:** 19.58

- **Gene name:** XRCC5
- **Entrez ID:** N/A
- **RefSeq ID:** NM\_021141
- **Transcript sequence length:** 3379
- **5-UTR|CDS|3-UTR identity (%):** 16.06 | 46.17 | 4.0
- **5-UTR|CDS|3-UTR identity (%) [Gaps excluded]:** 65.43 | 74.34 | 74.67
- **5-UTR|CDS|3-UTR identity [Alignment Gaps]:** 249 | 1118 | 16030
- **5-UTR aligned content (<base>:%):** {'G': 39.62, 'C': 52.83, 'T': 5.66, 'A': 1.89}
- **CDS aligned content (<base>:%):** {'A': 33.19, 'T': 25.55, 'G': 23.35, 'C': 17.91}
- **3-UTR aligned content (<base>:%):** {'T': 34.07, 'C': 18.44, 'G': 19.76, 'A': 27.73}

**Uniprot Description:**  
  
 Single-stranded DNA-dependent ATP-dependent helicase. Has a role in chromosome translocation. The DNA helicase II complex binds preferentially to fork-like ends of double-stranded DNA in a cell cycle-dependent manner. It works in the 3'-5' direction. Binding to DNA may be mediated by XRCC6. Involved in DNA non-homologous end joining (NHEJ) required for double-strand break repair and V(D)J recombination. The XRCC5/6 dimer acts as regulatory subunit of the DNA-dependent protein kinase complex DNA-PK by increasing the affinity of the catalytic subunit PRKDC to DNA by 100-fold. The XRCC5/6 dimer is probably involved in stabilizing broken DNA ends and bringing them together (PubMed:12145306, PubMed:20383123, PubMed:7957065, PubMed:8621488). The assembly of the DNA-PK complex to DNA ends is required for the NHEJ ligation step. In association with NAA15, the XRCC5/6 dimer binds to the osteocalcin promoter and activates osteocalcin expression (PubMed:20383123). The XRCC5/6 dimer probably also acts as a 5'-deoxyribose-5-phosphate lyase (5'-dRP lyase), by catalyzing the beta-elimination of the 5' deoxyribose-5-phosphate at an abasic site near double-strand breaks. XRCC5 probably acts as the catalytic subunit of 5'-dRP activity, and allows to 'clean' the termini of abasic sites, a class of nucleotide damage commonly associated with strand breaks, before such broken ends can be joined. The XRCC5/6 dimer together with APEX1 acts as a negative regulator of transcription (PubMed:8621488). As part of the DNA-PK complex, involved in the early steps of ribosome assembly by promoting the processing of precursor rRNA into mature 18S rRNA in the small-subunit processome (PubMed:32103174). Binding to U3 small nucleolar RNA, recruits PRKDC and XRCC5/Ku86 to the small-subunit processome (PubMed:32103174). Plays a role in the regulation of DNA virus-mediated innate immune response by assembling into the HDP-RNP complex, a complex that serves as a platform for IRF3 phosphorylation and subsequent innate immune response activation through the cGAS-STING pathway.   
  
Heterodimer composed of XRCC5/Ku80 and XRCC6/Ku70. The dimer associates in a DNA-dependent manner with PRKDC to form the DNA-dependent protein kinase complex DNA-PK, and with the LIG4-XRCC4 complex to form the core of the non-homologous end joining (NHEJ) complex (PubMed:25941166, PubMed:25670504, PubMed:11493912, PubMed:22442688). Additional components of the NHEJ complex include NHEJ1/XLF and PAXX (PubMed:25574025, PubMed:25941166, PubMed:25670504). The dimer also associates with NAA15, and this complex displays DNA binding activity towards the osteocalcin FGF response element (OCFRE) (PubMed:12145306). In addition, XRCC5 binds to the osteoblast-specific transcription factors MSX2 and RUNX2 (PubMed:12145306). Interacts with ELF3 (PubMed:15075319). May interact with APLF (PubMed:17353262, PubMed:17396150). The XRCC5/XRCC6 dimer associates in a DNA-dependent manner with APEX1 (PubMed:8621488). Identified in a complex with DEAF1 and XRCC6. Interacts with NR4A3; the DNA-dependent protein kinase complex DNA-PK phosphorylates and activates NR4A3 and prevents NR4A3 ubiquitinylation and degradation (PubMed:25852083). Interacts with RNF138 (PubMed:26502055). Interacts with CYREN isoform 1 (CYREN-1) and isoform 4 (CYREN-2) (PubMed:24610814, PubMed:28959974). Interacts (via N-terminus) with HSF1 (via N-terminus); this interaction is direct and prevents XRCC5/XRCC6 heterodimeric binding and non-homologous end joining (NHEJ) repair activities induced by ionizing radiation (IR) (PubMed:26359349). Interacts with DHX9; this interaction occurs in a RNA-dependent manner (PubMed:14704337). Part of the HDP-RNP complex composed of at least HEXIM1, PRKDC, XRCC5, XRCC6, paraspeckle proteins (SFPQ, NONO, PSPC1, RBM14, and MATR3) and NEAT1 RNA (PubMed:28712728). Interacts with ERCC6 (PubMed:26030138). Interats with ATF7 (PubMed:29490055).   
  
 **Gene Ontology Information:**

Molecular Function

- ATP binding
- DNA-dependent ATPase activity
- damaged DNA binding
- DNA binding
- DNA end binding
- DNA helicase activity
- double-stranded DNA binding
- enzyme activator activity
- hydrolase activity
- protein-containing complex binding
- RNA binding
- telomeric DNA binding
- transcription regulatory region sequence-specific DNA binding
- U3 snoRNA binding
- ubiquitin protein ligase binding

Location

- chromosome, telomeric region
- cytosol
- DNA-dependent protein kinase complex
- extracellular region
- Ku70:Ku80 complex
- membrane
- nonhomologous end joining complex
- nuclear telomere cap complex
- nucleolus
- nucleoplasm
- nucleus
- plasma membrane
- protein-containing complex
- protein-DNA complex
- ribonucleoprotein complex
- secretory granule lumen
- site of DNA damage
- small-subunit processome

Biological process

- activation of innate immune response
- brain development
- cellular hyperosmotic salinity response
- cellular response to DNA damage stimulus
- cellular response to fatty acid
- cellular response to gamma radiation
- cellular response to leukemia inhibitory factor
- cellular response to X-ray
- DNA recombination
- double-strand break repair
- double-strand break repair via nonhomologous end joining
- hematopoietic stem cell differentiation
- hematopoietic stem cell proliferation
- innate immune response
- negative regulation of t-circle formation
- negative regulation of transcription, DNA-templated
- neurogenesis
- positive regulation of catalytic activity
- positive regulation of neurogenesis
- positive regulation of protein kinase activity
- positive regulation of telomerase activity
- positive regulation of telomere maintenance via telomerase
- protein localization to chromosome, telomeric region
- recombinational repair
- regulation of smooth muscle cell proliferation
- regulation of telomere maintenance
- response to xenobiotic stimulus
- small-subunit processome assembly
- telomere maintenance

---

50

- **Protein name:** X-ray repair cross-complementing protein 6
- **Organism:** Homo sapiens
- **Uniprot Accession Number:** P12956
- **Protein sequence length:** 609 aa
- **1D identity (%):** 13.57
- **1D identity (%) [Gaps excluded]:** 22.81
- **1D identity - Alignment Gaps:** 373
- **1D aligned content (<aminoacid>:%):** {'K': 9.6, 'G': 8.0, 'D': 4.8, 'L': 13.6, 'V': 6.4, 'P': 8.0, 'F': 4.8, 'S': 4.8, 'I': 1.6, 'R': 8.0, 'N': 3.2, 'W': 0.8, 'H': 3.2, 'T': 5.6, 'Y': 2.4, 'A': 4.0, 'E': 6.4, 'Q': 4.0, 'C': 0.8}
- **Common reported functions (%):** 14.29
- **Common reported locations (%):** 33.33
- **Common reported processes (%):** 0.0

- **PDB ID:** 8ASC
- **Chain:** E
- **Crystallized protein length:** 497 aa
- **Resolution:** 2.95 Å
- **b-phipsi:** 0.003521
- **w-rdist:** 0.314119
- **t-alpha:** 0.070523
- **Chemical similarity (Tanimoto Index) (%):** N/A
- **1D identity (%) [PDB]:** 2.07
- **1D identity (%) [Gaps excluded][PDB]:** 79.41
- **1D identity - Alignment Gaps [PDB]:** 1270
- **1D aligned content [PDB] (<aminoacid>:%):** {'S': 7.41, 'R': 7.41, 'L': 22.22, 'E': 11.11, 'V': 11.11, 'T': 7.41, 'G': 3.7, 'P': 7.41, 'D': 7.41, 'K': 3.7, 'I': 3.7, 'N': 3.7, 'A': 3.7}
- **2D identity (%) [PDB]:** 37.07
- **2D identity (%) [Gaps excluded][PDB]:** 86.32
- **2D identity - Alignment Gaps [PDB]:** 534
- **2D aligned content [PDB] (<2D-fold>:%):** {'E': 23.34, '.': 17.87, 'H': 35.73, 'T': 22.19, 'G': 0.86}
- **3D similarity (TM-Score) (%) [PDB]:** 18.31

- **Gene name:** XRCC6
- **Entrez ID:** N/A
- **RefSeq ID:** NM\_001469
- **Transcript sequence length:** 2122
- **5-UTR|CDS|3-UTR identity (%):** 12.73 | 39.81 | 0.9
- **5-UTR|CDS|3-UTR identity (%) [Gaps excluded]:** 72.41 | 74.12 | 74.38
- **5-UTR|CDS|3-UTR identity [Alignment Gaps]:** 272 | 1329 | 16575
- **5-UTR aligned content (<base>:%):** {'A': 7.14, 'C': 38.1, 'T': 23.81, 'G': 30.95}
- **CDS aligned content (<base>:%):** {'A': 31.23, 'T': 22.31, 'G': 26.42, 'C': 20.03}
- **3-UTR aligned content (<base>:%):** {'C': 25.83, 'A': 19.87, 'G': 21.85, 'T': 32.45}

**Uniprot Description:**  
  
 Single-stranded DNA-dependent ATP-dependent helicase. Has a role in chromosome translocation. The DNA helicase II complex binds preferentially to fork-like ends of double-stranded DNA in a cell cycle-dependent manner. It works in the 3'-5' direction. Binding to DNA may be mediated by XRCC6. Involved in DNA non-homologous end joining (NHEJ) required for double-strand break repair and V(D)J recombination. The XRCC5/6 dimer acts as regulatory subunit of the DNA-dependent protein kinase complex DNA-PK by increasing the affinity of the catalytic subunit PRKDC to DNA by 100-fold. The XRCC5/6 dimer is probably involved in stabilizing broken DNA ends and bringing them together. The assembly of the DNA-PK complex to DNA ends is required for the NHEJ ligation step. Required for osteocalcin gene expression. Probably also acts as a 5'-deoxyribose-5-phosphate lyase (5'-dRP lyase), by catalyzing the beta-elimination of the 5' deoxyribose-5-phosphate at an abasic site near double-strand breaks. 5'-dRP lyase activity allows to 'clean' the termini of abasic sites, a class of nucleotide damage commonly associated with strand breaks, before such broken ends can be joined. The XRCC5/6 dimer together with APEX1 acts as a negative regulator of transcription. Plays a role in the regulation of DNA virus-mediated innate immune response by assembling into the HDP-RNP complex, a complex that serves as a platform for IRF3 phosphorylation and subsequent innate immune response activation through the cGAS-STING pathway.   
  
Heterodimer composed of XRCC5/Ku80 and XRCC6/Ku70. The dimer associates in a DNA-dependent manner with PRKDC to form the DNA-dependent protein kinase complex DNA-PK, and with the LIG4-XRCC4 complex to form the core of the non-homologous end joining (NHEJ) complex. Additional components of the NHEJ complex include NHEJ1/XLF and PAXX (PubMed:25574025, PubMed:25941166, PubMed:25670504). The dimer also associates with NAA15, and this complex binds to the osteocalcin promoter and activates osteocalcin expression. In addition, XRCC6 interacts with the osteoblast-specific transcription factors MSX2, RUNX2 and DLX5. Interacts with ELF3. Interacts with ATP23. The XRCC5/6 dimer associates in a DNA-dependent manner with APEX1. Binds to CDK9 isoform 2. Identified in a complex with DEAF1 and XRCC5. Interacts with DEAF1 (via the SAND domain); the interaction is direct and may be inhibited by DNA-binding (PubMed:10219089, PubMed:11493912, PubMed:12145306, PubMed:12509254, PubMed:12547193, PubMed:15075319, PubMed:20493174, PubMed:22442688, PubMed:8621488, PubMed:9742108). Interacts with CLU (By similarity). Interacts with NR4A3; the DNA-dependent protein kinase complex DNA-PK phosphorylates and activates NR4A3 and prevents NR4A3 ubiquitinylation and degradation (PubMed:25852083). Interacts with CYREN isoform 1 (CYREN-1) and isoform 4 (CYREN-2) (PubMed:24610814, PubMed:28959974). Interacts (via N-terminus) with HSF1 (via N-terminus); this interaction is direct and prevents XRCC5/XRCC6 heterodimeric binding and non-homologous end joining (NHEJ) repair activities induced by ionizing radiation (IR) (PubMed:26359349). Part of the HDP-RNP complex composed of at least HEXIM1, PRKDC, XRCC5, XRCC6, paraspeckle proteins (SFPQ, NONO, PSPC1, RBM14, and MATR3) and NEAT1 RNA (PubMed:28712728). Interacts with HMBOX1 (PubMed:23685356). Interacts with ATF7 (PubMed:29490055).   
  
 **Gene Ontology Information:**

Molecular Function

- 5'-deoxyribose-5-phosphate lyase activity
- ATP binding
- DNA-dependent ATPase activity
- cyclin binding
- damaged DNA binding
- DNA binding
- DNA helicase activity
- double-stranded DNA binding
- hydrolase activity
- protein-containing complex binding
- RNA binding
- scaffold protein binding
- telomeric DNA binding
- transcription regulatory region sequence-specific DNA binding

Location

- chromosome, telomeric region
- cytosol
- DNA-dependent protein kinase complex
- extracellular region
- ficolin-1-rich granule lumen
- Ku70:Ku80 complex
- membrane
- nonhomologous end joining complex
- nuclear telomere cap complex
- nucleolus
- nucleoplasm
- nucleus
- protein-containing complex
- protein-DNA complex
- secretory granule lumen
- transcription regulator complex

Biological process

- activation of innate immune response
- cellular response to gamma radiation
- cellular response to X-ray
- DNA ligation
- double-strand break repair via classical nonhomologous end joining
- double-strand break repair via nonhomologous end joining
- innate immune response
- negative regulation of transcription, DNA-templated
- positive regulation of lymphocyte differentiation
- positive regulation of protein kinase activity
- positive regulation of transcription by RNA polymerase II
- positive regulation of transcription, DNA-templated
- recombinational repair
- regulation of smooth muscle cell proliferation
- telomere maintenance

---

51

- **Protein name:** 1-phosphatidylinositol 4,5-bisphosphate phosphodiesterase beta-3
- **Organism:** Homo sapiens
- **Uniprot Accession Number:** Q01970
- **Protein sequence length:** 1234 aa
- **1D identity (%):** 15.79
- **1D identity (%) [Gaps excluded]:** 24.48
- **1D identity - Alignment Gaps:** 452
- **1D aligned content (<aminoacid>:%):** {'A': 6.97, 'G': 6.47, 'Q': 4.98, 'P': 11.44, 'L': 13.43, 'T': 5.47, 'K': 4.98, 'N': 2.49, 'Y': 2.49, 'E': 8.96, 'V': 5.97, 'D': 4.98, 'R': 8.96, 'F': 3.98, 'S': 3.98, 'H': 1.99, 'M': 1.0, 'I': 1.49}
- **Common reported functions (%):** 0.0
- **Common reported locations (%):** 22.22
- **Common reported processes (%):** 0.0

- **PDB ID:** 4QJ4
- **Chain:** B
- **Crystallized protein length:** 749 aa
- **Resolution:** 3.3 Å
- **b-phipsi:** 0.003946
- **w-rdist:** 0.660178
- **t-alpha:** 0.003962
- **Chemical similarity (Tanimoto Index) (%):** 78.34
- **1D identity (%) [PDB]:** 3.49
- **1D identity (%) [Gaps excluded][PDB]:** 67.95
- **1D identity - Alignment Gaps [PDB]:** 1440
- **1D aligned content [PDB] (<aminoacid>:%):** {'Q': 3.77, 'E': 7.55, 'P': 7.55, 'K': 7.55, 'D': 13.21, 'V': 16.98, 'L': 7.55, 'I': 5.66, 'C': 1.89, 'R': 5.66, 'N': 1.89, 'S': 5.66, 'F': 1.89, 'G': 5.66, 'A': 1.89, 'T': 5.66}
- **2D identity (%) [PDB]:** 33.1
- **2D identity (%) [Gaps excluded][PDB]:** 84.04
- **2D identity - Alignment Gaps [PDB]:** 694
- **2D aligned content [PDB] (<2D-fold>:%):** {'H': 41.95, '.': 20.05, 'E': 22.69, 'T': 14.25, 'B': 0.26, 'G': 0.79}
- **3D similarity (TM-Score) (%) [PDB]:** 22.57

- **Gene name:** PLCB3
- **Entrez ID:** 5331
- **RefSeq ID:** NM\_001316314
- **Transcript sequence length:** 4734
- **5-UTR|CDS|3-UTR identity (%):** 27.44 | 42.74 | 3.43
- **5-UTR|CDS|3-UTR identity (%) [Gaps excluded]:** 78.95 | 74.52 | 75.69
- **5-UTR|CDS|3-UTR identity [Alignment Gaps]:** 214 | 1704 | 16134
- **5-UTR aligned content (<base>:%):** {'A': 6.67, 'G': 43.33, 'C': 45.56, 'T': 4.44}
- **CDS aligned content (<base>:%):** {'A': 26.93, 'T': 19.44, 'G': 27.81, 'C': 25.82}
- **3-UTR aligned content (<base>:%):** {'T': 22.97, 'G': 29.19, 'C': 30.57, 'A': 17.27}

**Uniprot Description:**  
  
 The production of the second messenger molecules diacylglycerol (DAG) and inositol 1,4,5-trisphosphate (IP3) is mediated by activated phosphatidylinositol-specific phospholipase C enzymes.   
  
Interacts with SHANK2 (By similarity). Interacts with LPAR2 (PubMed:15143197).   
  
 **Gene Ontology Information:**

Molecular Function

- cadherin binding
- calcium ion binding
- calmodulin binding
- molecular adaptor activity
- molecular function activator activity
- phosphatidylinositol phospholipase C activity
- phospholipase C activity

Location

- cytosol
- membrane
- nucleus
- postsynaptic cytosol
- protein-containing complex

Biological process

- G protein-coupled receptor signaling pathway
- lipid catabolic process
- phosphatidylinositol metabolic process
- phosphatidylinositol-mediated signaling
- regulation of systemic arterial blood pressure
- release of sequestered calcium ion into cytosol

---

52

- **Protein name:** Lactotransferrin
- **Organism:** Homo sapiens
- **Uniprot Accession Number:** P02788
- **Protein sequence length:** 710 aa
- **1D identity (%):** 13.66
- **1D identity (%) [Gaps excluded]:** 25.69
- **1D identity - Alignment Gaps:** 480
- **1D aligned content (<aminoacid>:%):** {'F': 5.0, 'L': 10.71, 'G': 10.71, 'R': 8.57, 'P': 10.0, 'T': 6.43, 'V': 7.86, 'A': 5.71, 'Y': 1.43, 'K': 7.14, 'Q': 2.14, 'E': 5.0, 'C': 5.0, 'N': 2.86, 'D': 4.29, 'S': 3.57, 'I': 2.14, 'W': 1.43}
- **Common reported functions (%):** 0.0
- **Common reported locations (%):** 11.11
- **Common reported processes (%):** 0.0

- **PDB ID:** 1BKA
- **Chain:** A
- **Crystallized protein length:** 688 aa
- **Resolution:** 2.4 Å
- **b-phipsi:** 0.004518
- **w-rdist:** 0.618643
- **t-alpha:** 0.003962
- **Chemical similarity (Tanimoto Index) (%):** 76.72
- **1D identity (%) [PDB]:** 2.58
- **1D identity (%) [Gaps excluded][PDB]:** 63.33
- **1D identity - Alignment Gaps [PDB]:** 1413
- **1D aligned content [PDB] (<aminoacid>:%):** {'R': 7.89, 'S': 15.79, 'V': 13.16, 'P': 7.89, 'A': 15.79, 'Y': 7.89, 'L': 7.89, 'D': 10.53, 'K': 2.63, 'E': 2.63, 'G': 2.63, 'Q': 2.63, 'T': 2.63}
- **2D identity (%) [PDB]:** 39.37
- **2D identity (%) [Gaps excluded][PDB]:** 78.52
- **2D identity - Alignment Gaps [PDB]:** 509
- **2D aligned content [PDB] (<2D-fold>:%):** {'.': 16.17, 'E': 22.39, 'H': 45.52, 'T': 15.42, 'G': 0.5}
- **3D similarity (TM-Score) (%) [PDB]:** 21.74

- **Gene name:** LTF
- **Entrez ID:** 4057
- **RefSeq ID:** NM\_002343
- **Transcript sequence length:** 2721
- **5-UTR|CDS|3-UTR identity (%):** 9.26 | 45.37 | 2.26
- **5-UTR|CDS|3-UTR identity (%) [Gaps excluded]:** 83.33 | 75.86 | 77.39
- **5-UTR|CDS|3-UTR identity [Alignment Gaps]:** 288 | 1186 | 16323
- **5-UTR aligned content (<base>:%):** {'A': 13.33, 'C': 46.67, 'T': 20.0, 'G': 20.0}
- **CDS aligned content (<base>:%):** {'A': 25.99, 'T': 23.45, 'G': 28.45, 'C': 22.11}
- **3-UTR aligned content (<base>:%):** {'A': 25.53, 'G': 23.95, 'T': 26.84, 'C': 23.68}

**Uniprot Description:**  
  
 Transferrins are iron binding transport proteins which can bind two Fe(3+) ions in association with the binding of an anion, usually bicarbonate.   
  
Monomer. Found in a complex with LTF, CLU, EPPIN and SEMG1.   
  
 **Gene Ontology Information:**

Molecular Function

- cysteine-type endopeptidase inhibitor activity
- DNA binding
- heparin binding
- iron ion binding
- lipopolysaccharide binding
- membrane destabilizing activity
- protein serine/threonine kinase activator activity
- serine-type endopeptidase activity

Location

- cell surface
- cytoplasm
- early endosome
- extracellular exosome
- extracellular region
- extracellular space
- nucleus
- phagocytic vesicle lumen
- plasma membrane
- protein-containing complex
- recycling endosome
- secretory granule
- specific granule
- specific granule lumen
- tertiary granule lumen

Biological process

- antibacterial humoral response
- antifungal humoral response
- antimicrobial humoral immune response mediated by antimicrobial peptide
- bone morphogenesis
- cytolysis by host of symbiont cells
- defense response to Gram-negative bacterium
- defense response to Gram-positive bacterium
- humoral immune response
- innate immune response
- innate immune response in mucosa
- iron ion transmembrane transport
- iron ion transport
- killing of cells of other organism
- negative regulation by host of viral process
- negative regulation of apoptotic process
- negative regulation of ATPase activity
- negative regulation of cysteine-type endopeptidase activity
- negative regulation of lipopolysaccharide-mediated signaling pathway
- negative regulation of membrane potential
- negative regulation of osteoclast development
- negative regulation of single-species biofilm formation in or on host organism
- negative regulation of tumor necrosis factor (ligand) superfamily member 11 production
- negative regulation of viral genome replication
- negative regulation of viral process
- ossification
- positive regulation of bone mineralization involved in bone maturation
- positive regulation of chondrocyte proliferation
- positive regulation of I-kappaB kinase/NF-kappaB signaling
- positive regulation of NF-kappaB transcription factor activity
- positive regulation of osteoblast differentiation
- positive regulation of osteoblast proliferation
- positive regulation of protein serine/threonine kinase activity
- positive regulation of toll-like receptor 4 signaling pathway
- proteolysis
- regulation of cytokine production
- regulation of tumor necrosis factor production
- retina homeostasis

---

53

- **Protein name:** Ribonuclease SLFN12
- **Organism:** Homo sapiens
- **Uniprot Accession Number:** Q8IYM2
- **Protein sequence length:** 578 aa
- **1D identity (%):** 12.02
- **1D identity (%) [Gaps excluded]:** 25.16
- **1D identity - Alignment Gaps:** 508
- **1D aligned content (<aminoacid>:%):** {'N': 5.13, 'I': 7.69, 'V': 7.69, 'Y': 6.84, 'L': 11.11, 'D': 5.13, 'R': 4.27, 'K': 9.4, 'G': 5.98, 'E': 5.13, 'F': 3.42, 'A': 4.27, 'Q': 7.69, 'S': 2.56, 'T': 3.42, 'P': 4.27, 'C': 4.27, 'H': 1.71}
- **Common reported functions (%):** 0.0
- **Common reported locations (%):** 11.11
- **Common reported processes (%):** 0.0

- **PDB ID:** 7LRD
- **Chain:** C
- **Crystallized protein length:** 512 aa
- **Resolution:** 3.22 Å
- **b-phipsi:** 0.005615
- **w-rdist:** 0.558765
- **t-alpha:** 0.00317
- **Chemical similarity (Tanimoto Index) (%):** 85.69
- **1D identity (%) [PDB]:** 2.51
- **1D identity (%) [Gaps excluded][PDB]:** 73.33
- **1D identity - Alignment Gaps [PDB]:** 1269
- **1D aligned content [PDB] (<aminoacid>:%):** {'V': 9.09, 'D': 6.06, 'L': 15.15, 'T': 9.09, 'G': 9.09, 'E': 9.09, 'K': 9.09, 'R': 6.06, 'P': 6.06, 'F': 6.06, 'S': 6.06, 'W': 3.03, 'H': 6.06}
- **2D identity (%) [PDB]:** 32.28
- **2D identity (%) [Gaps excluded][PDB]:** 83.16
- **2D identity - Alignment Gaps [PDB]:** 599
- **2D aligned content [PDB] (<2D-fold>:%):** {'E': 24.05, '.': 18.99, 'H': 47.15, 'T': 9.81}
- **3D similarity (TM-Score) (%) [PDB]:** 18.66

- **Gene name:** SLFN12
- **Entrez ID:** 55106
- **RefSeq ID:** N/A
- **Sequence length:** N/A
- **5-UTR|CDS|3-UTR identity (%):** N/A | N/A | N/A
- **5-UTR|CDS|3-UTR identity (%) [Gaps excluded]:** N/A | N/A | N/A
- **5-UTR|CDS|3-UTR identity [Alignment Gaps]:** N/A | N/A | N/A
- **5-UTR aligned content (<base>:%):** N/A
- **CDS aligned content (<base>:%):** N/A
- **3-UTR aligned content (<base>:%):** N/A

**Uniprot Description:**  
  
 Ribonuclease which is part of an E2/17beta-estradiol-induced pro-apoptotic signaling pathway. E2 stabilizes the PDE3A/SLFN12 complex in the cytosol, promoting the dephosphorylation of SLFN12 and activating its pro-apoptotic ribosomal RNA/rRNA ribonuclease activity. This apoptotic pathway might be relevant in tissues with high concentration of E2 and be for instance involved in placenta remodeling (PubMed:31420216, PubMed:35104454, PubMed:34272366, PubMed:34707099). May play a role in cell differentiation (PubMed:30045019).   
  
Homodimer (PubMed:34272366). Interacts with PDE3A; direct low affinity interaction which is stimulated by binding of 17beta-estradiol/E2 to PDE3A and that positively regulates the ribonuclease activity of SLFN12 (PubMed:31420216, PubMed:35104454, PubMed:34272366, PubMed:34707099). Interacts with SERPINB12; as part of a pathway regulating cell differentiation (PubMed:30045019).   
  
 **Gene Ontology Information:**

Molecular Function

- catalytic activity, acting on a rRNA
- ribosome binding
- ribonuclease activity

Location

- cytosol
- nucleus

Biological process

- apoptotic signaling pathway
- rRNA catabolic process

---

54

- **Protein name:** Dipeptidyl peptidase 4
- **Organism:** Homo sapiens
- **Uniprot Accession Number:** P27487
- **Protein sequence length:** 766 aa
- **1D identity (%):** 13.43
- **1D identity (%) [Gaps excluded]:** 21.34
- **1D identity - Alignment Gaps:** 370
- **1D aligned content (<aminoacid>:%):** {'M': 2.24, 'P': 9.7, 'K': 5.22, 'L': 9.7, 'V': 10.45, 'I': 8.21, 'R': 2.99, 'D': 5.22, 'Y': 3.73, 'N': 3.73, 'E': 2.99, 'G': 9.7, 'S': 5.97, 'T': 5.22, 'H': 1.49, 'W': 1.49, 'A': 4.48, 'F': 2.99, 'C': 2.99, 'Q': 1.49}
- **Common reported functions (%):** 0.0
- **Common reported locations (%):** 11.11
- **Common reported processes (%):** 0.0

- **PDB ID:** 1W1I
- **Chain:** B
- **Crystallized protein length:** 728 aa
- **Resolution:** 3.03 Å
- **b-phipsi:** 0.003779
- **w-rdist:** 0.841856
- **t-alpha:** 0.001587
- **Chemical similarity (Tanimoto Index) (%):** 79.31
- **1D identity (%) [PDB]:** 2.84
- **1D identity (%) [Gaps excluded][PDB]:** 75.44
- **1D identity - Alignment Gaps [PDB]:** 1459
- **1D aligned content [PDB] (<aminoacid>:%):** {'R': 6.98, 'K': 9.3, 'I': 9.3, 'S': 6.98, 'D': 4.65, 'G': 9.3, 'Q': 4.65, 'F': 4.65, 'Y': 6.98, 'A': 6.98, 'E': 4.65, 'P': 6.98, 'H': 2.33, 'T': 2.33, 'L': 6.98, 'V': 6.98}
- **2D identity (%) [PDB]:** 31.18
- **2D identity (%) [Gaps excluded][PDB]:** 80.05
- **2D identity - Alignment Gaps [PDB]:** 691
- **2D aligned content [PDB] (<2D-fold>:%):** {'.': 17.28, 'E': 37.96, 'T': 18.98, 'H': 24.93, 'G': 0.85}
- **3D similarity (TM-Score) (%) [PDB]:** 25.79

- **Gene name:** DPP4
- **Entrez ID:** 1803
- **RefSeq ID:** NM\_001935
- **Transcript sequence length:** 3573
- **5-UTR|CDS|3-UTR identity (%):** 41.46 | 45.87 | 3.99
- **5-UTR|CDS|3-UTR identity (%) [Gaps excluded]:** 74.37 | 74.84 | 74.97
- **5-UTR|CDS|3-UTR identity [Alignment Gaps]:** 158 | 1172 | 15995
- **5-UTR aligned content (<base>:%):** {'A': 6.08, 'T': 13.51, 'C': 52.03, 'G': 28.38}
- **CDS aligned content (<base>:%):** {'A': 33.55, 'T': 26.21, 'G': 21.53, 'C': 18.72}
- **3-UTR aligned content (<base>:%):** {'C': 14.84, 'A': 33.98, 'T': 36.2, 'G': 14.99}

**Uniprot Description:**  
  
 Cell surface glycoprotein receptor involved in the costimulatory signal essential for T-cell receptor (TCR)-mediated T-cell activation (PubMed:10951221, PubMed:10900005, PubMed:11772392, PubMed:17287217). Acts as a positive regulator of T-cell coactivation, by binding at least ADA, CAV1, IGF2R, and PTPRC (PubMed:10951221, PubMed:10900005, PubMed:11772392, PubMed:14691230). Its binding to CAV1 and CARD11 induces T-cell proliferation and NF-kappa-B activation in a T-cell receptor/CD3-dependent manner (PubMed:17287217). Its interaction with ADA also regulates lymphocyte-epithelial cell adhesion (PubMed:11772392). In association with FAP is involved in the pericellular proteolysis of the extracellular matrix (ECM), the migration and invasion of endothelial cells into the ECM (PubMed:16651416, PubMed:10593948). May be involved in the promotion of lymphatic endothelial cells adhesion, migration and tube formation (PubMed:18708048). When overexpressed, enhanced cell proliferation, a process inhibited by GPC3 (PubMed:17549790). Acts also as a serine exopeptidase with a dipeptidyl peptidase activity that regulates various physiological processes by cleaving peptides in the circulation, including many chemokines, mitogenic growth factors, neuropeptides and peptide hormones such as brain natriuretic peptide 32 (PubMed:16254193, PubMed:10570924). Removes N-terminal dipeptides sequentially from polypeptides having unsubstituted N-termini provided that the penultimate residue is proline (PubMed:10593948).   
  
Monomer. Homodimer (PubMed:12832764, PubMed:15448155, PubMed:17287217, PubMed:12646248, PubMed:12483204, PubMed:12906826). Heterodimer with Seprase (FAP) (PubMed:16651416). Requires homodimerization for optimal dipeptidyl peptidase activity and T-cell costimulation. Found in a membrane raft complex, at least composed of BCL10, CARD11, DPP4 and IKBKB (PubMed:17287217). Associates with collagen (PubMed:8526932). Interacts with PTPRC; the interaction is enhanced in an interleukin-12-dependent manner in activated lymphocytes (PubMed:12676959). Interacts (via extracellular domain) with ADA; does not inhibit its dipeptidyl peptidase activity (PubMed:15016824, PubMed:10951221, PubMed:14691230, PubMed:7907293, PubMed:8101391). Interacts with CAV1 (via the N-terminus); the interaction is direct (PubMed:17287217). Interacts (via cytoplasmic tail) with CARD11 (via PDZ domain); its homodimerization is necessary for interaction with CARD11 (PubMed:17287217). Interacts with IGF2R; the interaction is direct (PubMed:10900005). Interacts with GPC3 (PubMed:17549790). Interacts with human coronavirus-EMC spike protein and acts as a receptor for this virus (PubMed:23486063).   
  
 **Gene Ontology Information:**

Molecular Function

- aminopeptidase activity
- chemorepellent activity
- dipeptidyl-peptidase activity
- identical protein binding
- protease binding
- protein homodimerization activity
- serine-type endopeptidase activity
- serine-type peptidase activity
- signaling receptor binding
- virus receptor activity

Location

- apical plasma membrane
- cell surface
- endocytic vesicle
- extracellular exosome
- extracellular region
- focal adhesion
- intercellular canaliculus
- lamellipodium
- lamellipodium membrane
- lysosomal membrane
- membrane
- membrane raft
- plasma membrane

Biological process

- behavioral fear response
- cell adhesion
- endothelial cell migration
- glucagon processing
- locomotory exploration behavior
- membrane fusion
- negative regulation of extracellular matrix disassembly
- negative regulation of neutrophil chemotaxis
- peptide hormone processing
- positive regulation of cell population proliferation
- proteolysis
- psychomotor behavior
- receptor-mediated endocytosis of virus by host cell
- receptor-mediated virion attachment to host cell
- regulation of cell-cell adhesion mediated by integrin
- response to hypoxia
- T cell activation
- T cell costimulation
- viral entry into host cell

---

55

- **Protein name:** Non-homologous end-joining factor 1
- **Organism:** Homo sapiens
- **Uniprot Accession Number:** Q9H9Q4
- **Protein sequence length:** 299 aa
- **1D identity (%):** 7.72
- **1D identity (%) [Gaps excluded]:** 27.78
- **1D identity - Alignment Gaps:** 655
- **1D aligned content (<aminoacid>:%):** {'L': 12.86, 'E': 5.71, 'G': 10.0, 'Q': 8.57, 'A': 7.14, 'F': 2.86, 'K': 5.71, 'V': 7.14, 'T': 5.71, 'R': 4.29, 'D': 5.71, 'N': 1.43, 'P': 10.0, 'Y': 2.86, 'S': 4.29, 'H': 1.43, 'I': 1.43, 'C': 2.86}
- **Common reported functions (%):** 14.29
- **Common reported locations (%):** 33.33
- **Common reported processes (%):** 0.0

- **PDB ID:** 8BHY
- **Chain:** T
- **Crystallized protein length:** 502 aa
- **Resolution:** 5.33 Å
- **b-phipsi:** 0.00701
- **w-rdist:** 0.391852
- **t-alpha:** 0.007183
- **Chemical similarity (Tanimoto Index) (%):** 99.09
- **1D identity (%) [PDB]:** 2.38
- **1D identity (%) [Gaps excluded][PDB]:** 70.45
- **1D identity - Alignment Gaps [PDB]:** 1256
- **1D aligned content [PDB] (<aminoacid>:%):** {'V': 16.13, 'D': 12.9, 'S': 6.45, 'Q': 3.23, 'F': 3.23, 'K': 6.45, 'T': 9.68, 'G': 6.45, 'R': 6.45, 'P': 9.68, 'Y': 3.23, 'A': 6.45, 'L': 6.45, 'E': 3.23}
- **2D identity (%) [PDB]:** 42.57
- **2D identity (%) [Gaps excluded][PDB]:** 88.97
- **2D identity - Alignment Gaps [PDB]:** 474
- **2D aligned content [PDB] (<2D-fold>:%):** {'.': 24.81, 'E': 21.19, 'T': 22.74, 'H': 31.27}
- **3D similarity (TM-Score) (%) [PDB]:** 19.59

- **Gene name:** NHEJ1
- **Entrez ID:** 79840
- **RefSeq ID:** NM\_024782
- **Transcript sequence length:** 8020
- **5-UTR|CDS|3-UTR identity (%):** 19.7 | 21.64 | 26.15
- **5-UTR|CDS|3-UTR identity (%) [Gaps excluded]:** 73.86 | 75.61 | 76.08
- **5-UTR|CDS|3-UTR identity [Alignment Gaps]:** 242 | 1933 | 11615
- **5-UTR aligned content (<base>:%):** {'G': 44.62, 'C': 29.23, 'T': 16.92, 'A': 9.23}
- **CDS aligned content (<base>:%):** {'A': 27.82, 'G': 23.38, 'C': 23.55, 'T': 25.26}
- **3-UTR aligned content (<base>:%):** {'T': 32.07, 'C': 19.49, 'G': 19.84, 'A': 28.59}

**Uniprot Description:**  
  
 DNA repair protein involved in DNA non-homologous end joining (NHEJ); required for double-strand break (DSB) repair and V(D)J recombination (PubMed:16439204, PubMed:16439205, PubMed:17717001, PubMed:17317666, PubMed:17470781, PubMed:18644470, PubMed:20558749, PubMed:26100018, PubMed:18158905). Plays a key role in NHEJ by promoting the ligation of various mismatched and non-cohesive ends (PubMed:17717001, PubMed:17470781, PubMed:19056826). Together with PAXX, collaborates with DNA polymerase lambda (POLL) to promote joining of non-cohesive DNA ends (PubMed:30250067, PubMed:25670504). May act in concert with XRCC5-XRCC6 (Ku) to stimulate XRCC4-mediated joining of blunt ends and several types of mismatched ends that are non-complementary or partially complementary (PubMed:16439204, PubMed:16439205, PubMed:17317666, PubMed:17470781). Associates with XRCC4 to form alternating helical filaments that bridge DNA and act like a bandage, holding together the broken DNA until it is repaired (PubMed:22228831, PubMed:26100018, PubMed:28500754, PubMed:27437582, PubMed:21775435, PubMed:22287571, PubMed:21768349). The XRCC4-NHEJ1/XLF subcomplex binds to the DNA fragments of a DSB in a highly diffusive manner and robustly bridges two independent DNA molecules, holding the broken DNA fragments in close proximity to one other (PubMed:28500754, PubMed:27437582). The mobility of the bridges ensures that the ends remain accessible for further processing by other repair factors (PubMed:27437582). Binds DNA in a length-dependent manner (PubMed:17317666, PubMed:18158905).   
  
Homodimer; mainly exists as a homodimer when not associated with XRCC4 (PubMed:18046455, PubMed:25574025, PubMed:25670504, PubMed:25941166, PubMed:18158905). Interacts with XRCC4; the interaction is direct and is mediated via a head-to-head interaction between N-terminal head regions (PubMed:16439205, PubMed:20558749, PubMed:22228831, PubMed:26100018, PubMed:18158905, PubMed:21936820, PubMed:21775435, PubMed:22287571, PubMed:21768349, PubMed:27437582). Component of the core long-range non-homologous end joining (NHEJ) complex (also named DNA-PK complex) composed of PRKDC, LIG4, XRCC4, XRCC6/Ku70, XRCC5/Ku86 and NHEJ1/XLF (PubMed:16571728, PubMed:17317666, PubMed:33854234). Additional component of the NHEJ complex includes PAXX (PubMed:25574025, PubMed:25941166). Following autophosphorylation, PRKDC dissociates from DNA, leading to formation of the short-range NHEJ complex, composed of LIG4, XRCC4, XRCC6/Ku70, XRCC5/Ku86 and NHEJ1/XLF (PubMed:33854234). Interacts with POLL (DNA polymerase lambda); promoting POLL recruitment to double-strand breaks (DSBs) and stimulation of the end-filling activity of POLL (PubMed:30250067).   
  
 **Gene Ontology Information:**

Molecular Function

- 5'-deoxyribose-5-phosphate lyase activity
- ATP binding
- DNA-dependent ATPase activity
- cyclin binding
- damaged DNA binding
- DNA binding
- DNA helicase activity
- double-stranded DNA binding
- hydrolase activity
- protein-containing complex binding
- RNA binding
- scaffold protein binding
- telomeric DNA binding
- transcription regulatory region sequence-specific DNA binding

Location

- chromosome, telomeric region
- cytosol
- DNA-dependent protein kinase complex
- extracellular region
- ficolin-1-rich granule lumen
- Ku70:Ku80 complex
- membrane
- nonhomologous end joining complex
- nuclear telomere cap complex
- nucleolus
- nucleoplasm
- nucleus
- protein-containing complex
- protein-DNA complex
- secretory granule lumen
- transcription regulator complex

Biological process

- activation of innate immune response
- cellular response to gamma radiation
- cellular response to X-ray
- DNA ligation
- double-strand break repair via classical nonhomologous end joining
- double-strand break repair via nonhomologous end joining
- innate immune response
- negative regulation of transcription, DNA-templated
- positive regulation of lymphocyte differentiation
- positive regulation of protein kinase activity
- positive regulation of transcription by RNA polymerase II
- positive regulation of transcription, DNA-templated
- recombinational repair
- regulation of smooth muscle cell proliferation
- telomere maintenance

---

56

- **Protein name:** Dimethylglycine dehydrogenase, mitochondrial
- **Organism:** Homo sapiens
- **Uniprot Accession Number:** Q9UI17
- **Protein sequence length:** 866 aa
- **1D identity (%):** 16.98
- **1D identity (%) [Gaps excluded]:** 23.78
- **1D identity - Alignment Gaps:** 288
- **1D aligned content (<aminoacid>:%):** {'R': 7.6, 'P': 9.94, 'L': 11.11, 'G': 11.7, 'V': 4.68, 'H': 3.51, 'E': 7.02, 'A': 7.02, 'F': 5.26, 'Q': 4.09, 'S': 5.26, 'M': 1.75, 'T': 2.92, 'Y': 3.51, 'I': 2.92, 'D': 4.09, 'C': 0.58, 'K': 4.09, 'N': 1.75, 'W': 1.17}
- **Common reported functions (%):** 14.29
- **Common reported locations (%):** 11.11
- **Common reported processes (%):** 0.0

- **PDB ID:** 5L46
- **Chain:** A
- **Crystallized protein length:** 809 aa
- **Resolution:** 3.09 Å
- **b-phipsi:** 0.001236
- **w-rdist:** 0.77932
- **t-alpha:** 0.008793
- **Chemical similarity (Tanimoto Index) (%):** 74.43
- **1D identity (%) [PDB]:** 4.12
- **1D identity (%) [Gaps excluded][PDB]:** 64.65
- **1D identity - Alignment Gaps [PDB]:** 1456
- **1D aligned content [PDB] (<aminoacid>:%):** {'K': 6.25, 'D': 7.81, 'V': 20.31, 'L': 12.5, 'E': 7.81, 'I': 4.69, 'P': 10.94, 'R': 6.25, 'N': 1.56, 'M': 1.56, 'Q': 1.56, 'H': 1.56, 'T': 4.69, 'G': 7.81, 'S': 3.12, 'W': 1.56}
- **2D identity (%) [PDB]:** 40.04
- **2D identity (%) [Gaps excluded][PDB]:** 82.59
- **2D identity - Alignment Gaps [PDB]:** 574
- **2D aligned content [PDB] (<2D-fold>:%):** {'.': 15.02, 'E': 27.8, 'H': 33.41, 'T': 23.32, 'G': 0.45}
- **3D similarity (TM-Score) (%) [PDB]:** 24.36

- **Gene name:** DMGDH
- **Entrez ID:** 29958
- **RefSeq ID:** NM\_013391
- **Transcript sequence length:** 3173
- **5-UTR|CDS|3-UTR identity (%):** 11.96 | 47.42 | 2.01
- **5-UTR|CDS|3-UTR identity (%) [Gaps excluded]:** 78.0 | 73.61 | 80.09
- **5-UTR|CDS|3-UTR identity [Alignment Gaps]:** 276 | 1122 | 16429
- **5-UTR aligned content (<base>:%):** {'A': 2.56, 'G': 38.46, 'C': 46.15, 'T': 12.82}
- **CDS aligned content (<base>:%):** {'A': 31.37, 'T': 25.35, 'G': 23.41, 'C': 19.87}
- **3-UTR aligned content (<base>:%):** {'A': 40.24, 'C': 11.83, 'T': 34.32, 'G': 13.61}

**Uniprot Description:**  
  
 Catalyzes the demethylation of N,N-dimethylglycine to sarcosine. Also has activity with sarcosine in vitro.   
  
Monomer.   
  
 **Gene Ontology Information:**

Molecular Function

- dimethylglycine dehydrogenase activity
- electron transfer activity
- RNA binding

Location

- cytoplasm
- mitochondrial matrix
- mitochondrion

Biological process

- amino-acid betaine catabolic process
- choline catabolic process
- choline metabolic process

---

57

- **Protein name:** Lysosomal alpha-glucosidase
- **Organism:** Homo sapiens
- **Uniprot Accession Number:** P10253
- **Protein sequence length:** 952 aa
- **1D identity (%):** 15.0
- **1D identity (%) [Gaps excluded]:** 25.86
- **1D identity - Alignment Gaps:** 482
- **1D aligned content (<aminoacid>:%):** {'G': 11.05, 'P': 16.86, 'L': 11.63, 'V': 8.14, 'R': 4.07, 'C': 1.74, 'D': 5.23, 'K': 1.74, 'M': 1.16, 'H': 2.33, 'F': 5.23, 'Y': 2.33, 'T': 5.81, 'A': 4.65, 'S': 4.65, 'W': 1.74, 'E': 3.49, 'Q': 4.65, 'N': 1.74, 'I': 1.74}
- **Common reported functions (%):** 0.0
- **Common reported locations (%):** 11.11
- **Common reported processes (%):** 0.0

- **PDB ID:** 5KZW
- **Chain:** A
- **Crystallized protein length:** 850 aa
- **Resolution:** 2.0 Å
- **b-phipsi:** 0.004711
- **w-rdist:** 0.686457
- **t-alpha:** 0.002377
- **Chemical similarity (Tanimoto Index) (%):** N/A
- **1D identity (%) [PDB]:** 2.18
- **1D identity (%) [Gaps excluded][PDB]:** 72.0
- **1D identity - Alignment Gaps [PDB]:** 1598
- **1D aligned content [PDB] (<aminoacid>:%):** {'P': 11.11, 'Q': 13.89, 'L': 16.67, 'E': 8.33, 'N': 2.78, 'G': 5.56, 'T': 8.33, 'V': 8.33, 'A': 2.78, 'Y': 8.33, 'F': 2.78, 'R': 2.78, 'H': 2.78, 'K': 2.78, 'C': 2.78}
- **2D identity (%) [PDB]:** 36.03
- **2D identity (%) [Gaps excluded][PDB]:** 83.24
- **2D identity - Alignment Gaps [PDB]:** 672
- **2D aligned content [PDB] (<2D-fold>:%):** {'.': 25.29, 'H': 29.51, 'T': 17.56, 'E': 26.93, 'G': 0.7}
- **3D similarity (TM-Score) (%) [PDB]:** 24.56

- **Gene name:** GAA
- **Entrez ID:** 2548
- **RefSeq ID:** N/A
- **Sequence length:** N/A
- **5-UTR|CDS|3-UTR identity (%):** N/A | N/A | N/A
- **5-UTR|CDS|3-UTR identity (%) [Gaps excluded]:** N/A | N/A | N/A
- **5-UTR|CDS|3-UTR identity [Alignment Gaps]:** N/A | N/A | N/A
- **5-UTR aligned content (<base>:%):** N/A
- **CDS aligned content (<base>:%):** N/A
- **3-UTR aligned content (<base>:%):** N/A

**Uniprot Description:**  
  
 Essential for the degradation of glycogen in lysosomes (PubMed:1856189, PubMed:7717400, PubMed:14695532, PubMed:18429042). Has highest activity on alpha-1,4-linked glycosidic linkages, but can also hydrolyze alpha-1,6-linked glucans (PubMed:29061980). N/A   
  
 **Gene Ontology Information:**

Molecular Function

- alpha-1,4-glucosidase activity
- alpha-glucosidase activity
- carbohydrate binding
- maltose alpha-glucosidase activity

Location

- autolysosome lumen
- azurophil granule membrane
- extracellular exosome
- ficolin-1-rich granule membrane
- intracellular membrane-bounded organelle
- lysosomal lumen
- lysosomal membrane
- lysosome
- membrane
- plasma membrane
- tertiary granule membrane

Biological process

- aorta development
- cardiac muscle contraction
- diaphragm contraction
- glucose metabolic process
- glycogen catabolic process
- glycophagy
- heart morphogenesis
- locomotory behavior
- lysosome organization
- maltose metabolic process
- muscle cell cellular homeostasis
- neuromuscular process controlling balance
- neuromuscular process controlling posture
- regulation of the force of heart contraction
- sucrose metabolic process
- tissue development
- vacuolar sequestering

---

58

- **Protein name:** DNA helicase MCM8
- **Organism:** Homo sapiens
- **Uniprot Accession Number:** Q9UJA3
- **Protein sequence length:** 840 aa
- **1D identity (%):** 14.6
- **1D identity (%) [Gaps excluded]:** 24.29
- **1D identity - Alignment Gaps:** 424
- **1D aligned content (<aminoacid>:%):** {'M': 1.94, 'G': 10.97, 'R': 9.03, 'F': 2.58, 'P': 5.16, 'W': 0.65, 'K': 7.1, 'S': 4.52, 'Q': 7.1, 'D': 7.1, 'E': 8.39, 'L': 8.39, 'T': 5.16, 'C': 0.65, 'A': 5.81, 'H': 1.94, 'Y': 2.58, 'V': 4.52, 'N': 3.87, 'I': 2.58}
- **Common reported functions (%):** 0.0
- **Common reported locations (%):** 0.0
- **Common reported processes (%):** 0.0

- **PDB ID:** 8S91
- **Chain:** B
- **Crystallized protein length:** 602 aa
- **Resolution:** 4.3 Å
- **b-phipsi:** 0.003879
- **w-rdist:** 0.628009
- **t-alpha:** 0.005547
- **Chemical similarity (Tanimoto Index) (%):** 78.04
- **1D identity (%) [PDB]:** 3.54
- **1D identity (%) [Gaps excluded][PDB]:** 63.89
- **1D identity - Alignment Gaps [PDB]:** 1229
- **1D aligned content [PDB] (<aminoacid>:%):** {'I': 8.7, 'P': 13.04, 'D': 10.87, 'L': 8.7, 'Y': 4.35, 'E': 4.35, 'V': 17.39, 'K': 4.35, 'C': 2.17, 'R': 6.52, 'S': 2.17, 'T': 6.52, 'G': 6.52, 'A': 4.35}
- **2D identity (%) [PDB]:** 40.36
- **2D identity (%) [Gaps excluded][PDB]:** 88.81
- **2D identity - Alignment Gaps [PDB]:** 515
- **2D aligned content [PDB] (<2D-fold>:%):** {'.': 25.72, 'H': 38.58, 'T': 23.88, 'E': 11.81}
- **3D similarity (TM-Score) (%) [PDB]:** 18.05

- **Gene name:** MCM8
- **Entrez ID:** N/A
- **RefSeq ID:** N/A
- **Sequence length:** N/A
- **5-UTR|CDS|3-UTR identity (%):** N/A | N/A | N/A
- **5-UTR|CDS|3-UTR identity (%) [Gaps excluded]:** N/A | N/A | N/A
- **5-UTR|CDS|3-UTR identity [Alignment Gaps]:** N/A | N/A | N/A
- **5-UTR aligned content (<base>:%):** N/A
- **CDS aligned content (<base>:%):** N/A
- **3-UTR aligned content (<base>:%):** N/A

**Uniprot Description:**  
  
 Component of the MCM8-MCM9 complex, a complex involved in the repair of double-stranded DNA breaks (DBSs) and DNA interstrand cross-links (ICLs) by homologous recombination (HR) (PubMed:23401855). Required for DNA resection by the MRE11-RAD50-NBN/NBS1 (MRN) complex by recruiting the MRN complex to the repair site and by promoting the complex nuclease activity (PubMed:26215093). Probably by regulating the localization of the MNR complex, indirectly regulates the recruitment of downstream effector RAD51 to DNA damage sites including DBSs and ICLs (PubMed:23401855). The MCM8-MCM9 complex is dispensable for DNA replication and S phase progression (PubMed:23401855). However, may play a non-essential for DNA replication: may be involved in the activation of the prereplicative complex (pre-RC) during G(1) phase by recruiting CDC6 to the origin recognition complex (ORC) (PubMed:15684404). Probably by regulating HR, plays a key role during gametogenesis (By similarity). Stabilizes MCM9 protein (PubMed:23401855, PubMed:26215093).   
  
Component of the MCM8-MCM9 complex, which forms a hexamer composed of MCM8 and MCM9 (PubMed:23401855, PubMed:26300262). Interacts with the DNA mismatch repair (MMR) complex composed at least of MSH2, MSH3, MSH6, PMS1 and MLH1 (PubMed:26300262). Interacts with RAD51; the interaction recruits RAD51 to DNA damage sites (PubMed:23401855). Interacts with the MRN complex composed of MRE11, RAD50 and NBN/NBS1 (PubMed:26215093). Interacts with CDC6 and ORC2 (PubMed:15684404). Interacts with HROB; the interaction recruits the MCM8-MCM9 complex to DNA damage sites (PubMed:31467087).   
  
 **Gene Ontology Information:**

Molecular Function   
  
N/A

Location   
  
N/A

Biological process   
  
N/A

---

59

- **Protein name:** Bifunctional 3'-phosphoadenosine 5'-phosphosulfate synthase 1
- **Organism:** Homo sapiens
- **Uniprot Accession Number:** O43252
- **Protein sequence length:** 624 aa
- **1D identity (%):** 13.13
- **1D identity (%) [Gaps excluded]:** 22.49
- **1D identity - Alignment Gaps:** 390
- **1D aligned content (<aminoacid>:%):** {'E': 5.69, 'I': 2.44, 'G': 10.57, 'K': 5.69, 'V': 11.38, 'R': 6.5, 'N': 3.25, 'T': 4.88, 'A': 8.94, 'H': 4.07, 'Q': 4.07, 'L': 8.94, 'Y': 5.69, 'C': 4.07, 'D': 4.88, 'S': 1.63, 'P': 6.5, 'F': 0.81}
- **Common reported functions (%):** 0.0
- **Common reported locations (%):** 11.11
- **Common reported processes (%):** 0.0

- **PDB ID:** 1X6V
- **Chain:** B
- **Crystallized protein length:** 586 aa
- **Resolution:** 1.75 Å
- **b-phipsi:** 0.005114
- **w-rdist:** 0.564868
- **t-alpha:** 0.004754
- **Chemical similarity (Tanimoto Index) (%):** 79.58
- **1D identity (%) [PDB]:** 2.31
- **1D identity (%) [Gaps excluded][PDB]:** 69.57
- **1D identity - Alignment Gaps [PDB]:** 1340
- **1D aligned content [PDB] (<aminoacid>:%):** {'V': 15.62, 'T': 6.25, 'L': 18.75, 'G': 6.25, 'E': 12.5, 'K': 12.5, 'D': 3.12, 'R': 6.25, 'P': 6.25, 'F': 3.12, 'I': 3.12, 'W': 3.12, 'H': 3.12}
- **2D identity (%) [PDB]:** 38.41
- **2D identity (%) [Gaps excluded][PDB]:** 84.38
- **2D identity - Alignment Gaps [PDB]:** 536
- **2D aligned content [PDB] (<2D-fold>:%):** {'.': 18.78, 'E': 18.52, 'H': 47.62, 'T': 13.23, 'G': 1.59, 'B': 0.26}
- **3D similarity (TM-Score) (%) [PDB]:** 17.98

- **Gene name:** PAPSS1
- **Entrez ID:** 9061
- **RefSeq ID:** N/A
- **Sequence length:** N/A
- **5-UTR|CDS|3-UTR identity (%):** N/A | N/A | N/A
- **5-UTR|CDS|3-UTR identity (%) [Gaps excluded]:** N/A | N/A | N/A
- **5-UTR|CDS|3-UTR identity [Alignment Gaps]:** N/A | N/A | N/A
- **5-UTR aligned content (<base>:%):** N/A
- **CDS aligned content (<base>:%):** N/A
- **3-UTR aligned content (<base>:%):** N/A

**Uniprot Description:**  
  
 Bifunctional enzyme with both ATP sulfurylase and APS kinase activity, which mediates two steps in the sulfate activation pathway. The first step is the transfer of a sulfate group to ATP to yield adenosine 5'-phosphosulfate (APS), and the second step is the transfer of a phosphate group from ATP to APS yielding 3'-phosphoadenylylsulfate (PAPS: activated sulfate donor used by sulfotransferase). In mammals, PAPS is the sole source of sulfate; APS appears to be only an intermediate in the sulfate-activation pathway (PubMed:9576487, PubMed:9668121, PubMed:9648242, PubMed:14747722). Required for normal biosynthesis of sulfated L-selectin ligands in endothelial cells (PubMed:9576487).   
  
Homodimer.   
  
 **Gene Ontology Information:**

Molecular Function

- adenylylsulfate kinase activity
- ATP binding
- nucleotidyltransferase activity
- protein homodimerization activity
- sulfate adenylyltransferase (ATP) activity

Location

- cytosol

Biological process

- 3'-phosphoadenosine 5'-phosphosulfate biosynthetic process
- phosphorylation
- skeletal system development
- sulfate assimilation

---

60

- **Protein name:** GATOR complex protein DEPDC5
- **Organism:** Homo sapiens
- **Uniprot Accession Number:** O75140
- **Protein sequence length:** 1603 aa
- **1D identity (%):** 13.64
- **1D identity (%) [Gaps excluded]:** 26.56
- **1D identity - Alignment Gaps:** 791
- **1D aligned content (<aminoacid>:%):** {'M': 3.15, 'I': 2.25, 'G': 7.66, 'L': 7.66, 'V': 8.56, 'P': 10.36, 'Y': 4.05, 'K': 4.95, 'Q': 5.41, 'E': 4.05, 'D': 8.56, 'C': 3.15, 'R': 7.66, 'F': 3.6, 'S': 5.41, 'H': 4.05, 'A': 3.15, 'T': 4.05, 'N': 1.35, 'W': 0.9}
- **Common reported functions (%):** 0.0
- **Common reported locations (%):** 11.11
- **Common reported processes (%):** 0.0

- **PDB ID:** 7T3B
- **Chain:** A
- **Crystallized protein length:** 989 aa
- **Resolution:** 3.9 Å
- **b-phipsi:** 0.002418
- **w-rdist:** 0.448049
- **t-alpha:** 0.125991
- **Chemical similarity (Tanimoto Index) (%):** 99.47
- **1D identity (%) [PDB]:** 3.67
- **1D identity (%) [Gaps excluded][PDB]:** 64.65
- **1D identity - Alignment Gaps [PDB]:** 1643
- **1D aligned content [PDB] (<aminoacid>:%):** {'V': 17.19, 'H': 3.12, 'K': 7.81, 'T': 7.81, 'I': 1.56, 'G': 6.25, 'D': 9.38, 'R': 6.25, 'Y': 1.56, 'S': 6.25, 'L': 12.5, 'A': 3.12, 'P': 4.69, 'E': 6.25, 'F': 3.12, 'N': 1.56, 'M': 1.56}
- **2D identity (%) [PDB]:** 36.8
- **2D identity (%) [Gaps excluded][PDB]:** 83.96
- **2D identity - Alignment Gaps [PDB]:** 719
- **2D aligned content [PDB] (<2D-fold>:%):** {'E': 28.03, '.': 22.08, 'T': 15.07, 'H': 34.18, 'G': 0.64}
- **3D similarity (TM-Score) (%) [PDB]:** 17.01

- **Gene name:** DEPDC5
- **Entrez ID:** 9681
- **RefSeq ID:** N/A
- **Sequence length:** N/A
- **5-UTR|CDS|3-UTR identity (%):** N/A | N/A | N/A
- **5-UTR|CDS|3-UTR identity (%) [Gaps excluded]:** N/A | N/A | N/A
- **5-UTR|CDS|3-UTR identity [Alignment Gaps]:** N/A | N/A | N/A
- **5-UTR aligned content (<base>:%):** N/A
- **CDS aligned content (<base>:%):** N/A
- **3-UTR aligned content (<base>:%):** N/A

**Uniprot Description:**  
  
 As a component of the GATOR1 complex functions as an inhibitor of the amino acid-sensing branch of the TORC1 pathway. The GATOR1 complex strongly increases GTP hydrolysis by RRAGA and RRAGB within RRAGC-containing heterodimers, thereby deactivating RRAGs, releasing mTORC1 from lysosomal surface and inhibiting mTORC1 signaling. The GATOR1 complex is negatively regulated by GATOR2 the other GATOR subcomplex in this amino acid-sensing branch of the TORC1 pathway.   
  
Within the GATOR complex, component of the GATOR1 subcomplex, made of DEPDC5, NPRL2 and NPRL3. GATOR1 mediates the strong interaction of the GATOR complex with RRAGA/RRAGC and RRAGB/RRAGC heterodimers. Interacts (via DEP domain) with KLHL22; the interaction depends on amino acid availability (PubMed:29769719).   
  
 **Gene Ontology Information:**

Molecular Function

- GTPase activator activity
- protein-containing complex binding

Location

- cytosol
- GATOR1 complex
- lysosomal membrane
- lysosome
- perinuclear region of cytoplasm

Biological process

- cellular response to amino acid starvation
- intracellular signal transduction
- negative regulation of TOR signaling
- negative regulation of TORC1 signaling
- positive regulation of autophagy

---

61

- **Protein name:** Macrophage mannose receptor 1
- **Organism:** Homo sapiens
- **Uniprot Accession Number:** P22897
- **Protein sequence length:** 1456 aa
- **1D identity (%):** 14.62
- **1D identity (%) [Gaps excluded]:** 26.08
- **1D identity - Alignment Gaps:** 652
- **1D aligned content (<aminoacid>:%):** {'P': 5.99, 'L': 9.68, 'V': 3.23, 'G': 9.68, 'Y': 4.61, 'K': 6.45, 'A': 6.45, 'C': 5.99, 'Q': 3.23, 'D': 5.07, 'N': 2.3, 'R': 4.15, 'M': 0.92, 'T': 7.37, 'E': 5.99, 'S': 5.07, 'I': 4.61, 'W': 1.38, 'H': 4.61, 'F': 3.23}
- **Common reported functions (%):** 0.0
- **Common reported locations (%):** 0.0
- **Common reported processes (%):** 0.0

- **PDB ID:** 6INN
- **Chain:** B
- **Crystallized protein length:** 587 aa
- **Resolution:** 3.0 Å
- **b-phipsi:** 0.008116
- **w-rdist:** 0.596121
- **t-alpha:** 0.001585
- **Chemical similarity (Tanimoto Index) (%):** 88.82
- **1D identity (%) [PDB]:** 2.89
- **1D identity (%) [Gaps excluded][PDB]:** 68.97
- **1D identity - Alignment Gaps [PDB]:** 1327
- **1D aligned content [PDB] (<aminoacid>:%):** {'T': 12.5, 'V': 10.0, 'D': 7.5, 'L': 17.5, 'G': 7.5, 'P': 10.0, 'F': 2.5, 'K': 2.5, 'S': 7.5, 'I': 5.0, 'W': 2.5, 'H': 7.5, 'E': 7.5}
- **2D identity (%) [PDB]:** 32.65
- **2D identity (%) [Gaps excluded][PDB]:** 81.16
- **2D identity - Alignment Gaps [PDB]:** 615
- **2D aligned content [PDB] (<2D-fold>:%):** {'.': 26.49, 'E': 35.71, 'T': 17.86, 'H': 18.75, 'G': 0.89, 'B': 0.3}
- **3D similarity (TM-Score) (%) [PDB]:** 23.0

- **Gene name:** MRC1
- **Entrez ID:** 4360
- **RefSeq ID:** NM\_002438
- **Transcript sequence length:** 5188
- **5-UTR|CDS|3-UTR identity (%):** 19.65 | 39.87 | 2.94
- **5-UTR|CDS|3-UTR identity (%) [Gaps excluded]:** 72.34 | 77.52 | 78.57
- **5-UTR|CDS|3-UTR identity [Alignment Gaps]:** 252 | 2230 | 16194
- **5-UTR aligned content (<base>:%):** {'C': 30.88, 'A': 10.29, 'G': 38.24, 'T': 20.59}
- **CDS aligned content (<base>:%):** {'T': 24.74, 'G': 23.48, 'C': 20.48, 'A': 31.29}
- **3-UTR aligned content (<base>:%):** {'A': 36.36, 'C': 11.31, 'T': 37.37, 'G': 14.95}

**Uniprot Description:**  
  
 Mediates the endocytosis of glycoproteins by macrophages. Binds both sulfated and non-sulfated polysaccharide chains.   
  
(Microbial infection) Interacts with Dengue virus.   
  
 **Gene Ontology Information:**

Molecular Function

- cargo receptor activity
- mannose binding
- signaling receptor activity
- transmembrane signaling receptor activity
- virus receptor activity

Location

- cell surface
- endosome membrane
- plasma membrane

Biological process

- cellular response to interleukin-4
- cellular response to lipopolysaccharide
- cellular response to interferon-gamma
- receptor-mediated endocytosis

---

62

- **Protein name:** Protein O-linked-mannose beta-1,4-N-acetylglucosaminyltransferase 2
- **Organism:** Homo sapiens
- **Uniprot Accession Number:** Q8NAT1
- **Protein sequence length:** 580 aa
- **1D identity (%):** 12.36
- **1D identity (%) [Gaps excluded]:** 21.48
- **1D identity - Alignment Gaps:** 388
- **1D aligned content (<aminoacid>:%):** {'H': 2.65, 'S': 1.77, 'A': 7.08, 'L': 13.27, 'V': 8.85, 'K': 2.65, 'Q': 4.42, 'R': 12.39, 'T': 7.96, 'C': 2.65, 'F': 2.65, 'E': 4.42, 'Y': 4.42, 'P': 10.62, 'N': 1.77, 'D': 1.77, 'G': 8.85, 'I': 1.77}
- **Common reported functions (%):** 0.0
- **Common reported locations (%):** 0.0
- **Common reported processes (%):** 0.0

- **PDB ID:** 6XI2
- **Chain:** D
- **Crystallized protein length:** 521 aa
- **Resolution:** 2.57 Å
- **b-phipsi:** 0.001518
- **w-rdist:** 0.626153
- **t-alpha:** 0.02021
- **Chemical similarity (Tanimoto Index) (%):** 83.63
- **1D identity (%) [PDB]:** 1.96
- **1D identity (%) [Gaps excluded][PDB]:** 65.0
- **1D identity - Alignment Gaps [PDB]:** 1288
- **1D aligned content [PDB] (<aminoacid>:%):** {'L': 15.38, 'H': 7.69, 'V': 15.38, 'T': 15.38, 'G': 3.85, 'R': 7.69, 'P': 15.38, 'D': 7.69, 'S': 7.69, 'K': 3.85}
- **2D identity (%) [PDB]:** 29.08
- **2D identity (%) [Gaps excluded][PDB]:** 82.35
- **2D identity - Alignment Gaps [PDB]:** 654
- **2D aligned content [PDB] (<2D-fold>:%):** {'H': 27.21, '.': 23.13, 'E': 34.01, 'T': 15.31, 'B': 0.34}
- **3D similarity (TM-Score) (%) [PDB]:** 19.99

- **Gene name:** POMGNT2
- **Entrez ID:** N/A
- **RefSeq ID:** N/A
- **Sequence length:** N/A
- **5-UTR|CDS|3-UTR identity (%):** N/A | N/A | N/A
- **5-UTR|CDS|3-UTR identity (%) [Gaps excluded]:** N/A | N/A | N/A
- **5-UTR|CDS|3-UTR identity [Alignment Gaps]:** N/A | N/A | N/A
- **5-UTR aligned content (<base>:%):** N/A
- **CDS aligned content (<base>:%):** N/A
- **3-UTR aligned content (<base>:%):** N/A

**Uniprot Description:**  
  
 O-linked mannose beta-1,4-N-acetylglucosaminyltransferase that transfers UDP-N-acetyl-D-glucosamine to the 4-position of the mannose to generate N-acetyl-D-glucosamine-beta-1,4-O-D-mannosylprotein. Involved in the biosynthesis of the phosphorylated O-mannosyl trisaccharide (N-acetylgalactosamine-beta-3-N-acetylglucosamine-beta-4-(phosphate-6-)mannose), a carbohydrate structure present in alpha-dystroglycan (DAG1), which is required for binding laminin G-like domain-containing extracellular proteins with high affinity. N/A   
  
 **Gene Ontology Information:**

Molecular Function   
  
N/A

Location   
  
N/A

Biological process   
  
N/A

---

63

- **Protein name:** Toll-like receptor 3
- **Organism:** Homo sapiens
- **Uniprot Accession Number:** O15455
- **Protein sequence length:** 904 aa
- **1D identity (%):** 16.47
- **1D identity (%) [Gaps excluded]:** 22.27
- **1D identity - Alignment Gaps:** 264
- **1D aligned content (<aminoacid>:%):** {'P': 4.79, 'I': 4.79, 'L': 15.57, 'K': 5.99, 'C': 2.99, 'V': 5.99, 'E': 6.59, 'D': 4.19, 'H': 2.99, 'T': 7.19, 'R': 5.39, 'A': 2.4, 'N': 7.19, 'G': 5.99, 'S': 7.19, 'Q': 2.4, 'Y': 2.99, 'F': 5.39}
- **Common reported functions (%):** 14.29
- **Common reported locations (%):** 22.22
- **Common reported processes (%):** 22.22

- **PDB ID:** 3ULU
- **Chain:** A
- **Crystallized protein length:** 659 aa
- **Resolution:** 3.52 Å
- **b-phipsi:** 0.006302
- **w-rdist:** 0.638853
- **t-alpha:** 0.001587
- **Chemical similarity (Tanimoto Index) (%):** 79.4
- **1D identity (%) [PDB]:** 2.33
- **1D identity (%) [Gaps excluded][PDB]:** 79.07
- **1D identity - Alignment Gaps [PDB]:** 1418
- **1D aligned content [PDB] (<aminoacid>:%):** {'T': 5.88, 'L': 17.65, 'S': 5.88, 'N': 8.82, 'C': 2.94, 'I': 8.82, 'K': 2.94, 'G': 8.82, 'V': 8.82, 'P': 11.76, 'Q': 5.88, 'R': 2.94, 'F': 2.94, 'A': 2.94, 'H': 2.94}
- **2D identity (%) [PDB]:** 36.17
- **2D identity (%) [Gaps excluded][PDB]:** 76.92
- **2D identity - Alignment Gaps [PDB]:** 542
- **2D aligned content [PDB] (<2D-fold>:%):** {'.': 36.76, 'E': 24.32, 'T': 28.11, 'H': 9.19, 'G': 1.62}
- **3D similarity (TM-Score) (%) [PDB]:** 17.16

- **Gene name:** TLR3
- **Entrez ID:** 7098
- **RefSeq ID:** N/A
- **Sequence length:** N/A
- **5-UTR|CDS|3-UTR identity (%):** N/A | N/A | N/A
- **5-UTR|CDS|3-UTR identity (%) [Gaps excluded]:** N/A | N/A | N/A
- **5-UTR|CDS|3-UTR identity [Alignment Gaps]:** N/A | N/A | N/A
- **5-UTR aligned content (<base>:%):** N/A
- **CDS aligned content (<base>:%):** N/A
- **3-UTR aligned content (<base>:%):** N/A

**Uniprot Description:**  
  
 Key component of innate and adaptive immunity. TLRs (Toll-like receptors) control host immune response against pathogens through recognition of molecular patterns specific to microorganisms. TLR3 is a nucleotide-sensing TLR which is activated by double-stranded RNA, a sign of viral infection. Acts via the adapter TRIF/TICAM1, leading to NF-kappa-B activation, IRF3 nuclear translocation, cytokine secretion and the inflammatory response.   
  
Monomer and homodimer; dimerization is triggered by ligand-binding, the signaling unit is composed of one ds-RNA of around 40 bp and two TLR3 molecules, and lateral clustering of signaling units along the length of the ds-RNA ligand is required for TLR3 signal transduction. Interacts (via transmembrane domain) with UNC93B1; the interaction is required for transport from the ER to the endosomes (PubMed:33432245). Interacts with SRC; upon binding of double-stranded RNA. Interacts with TICAM1 (via the TIR domain) in response to poly(I:C) and this interaction is enhanced in the presence of WDFY1 (PubMed:25736436). The tyrosine-phosphorylated form (via TIR domain) interacts with WDFY1 (via WD repeat 2) in response to poly(I:C) (PubMed:25736436).   
  
 **Gene Ontology Information:**

Molecular Function

- double-stranded RNA binding
- identical protein binding
- pattern recognition receptor activity
- signaling receptor activity
- transmembrane signaling receptor activity

Location

- cytoplasm
- early endosome
- endolysosome membrane
- endoplasmic reticulum membrane
- endosome membrane
- extracellular matrix
- extracellular space
- Golgi membrane
- lysosomal membrane
- membrane
- plasma membrane

Biological process

- activation of NF-kappaB-inducing kinase activity
- cellular response to exogenous dsRNA
- cellular response to interferon-beta
- cellular response to mechanical stimulus
- cellular response to interferon-gamma
- cellular response to virus
- cellular response to xenobiotic stimulus
- defense response to bacterium
- defense response to virus
- detection of virus
- extrinsic apoptotic signaling pathway
- hyperosmotic response
- I-kappaB phosphorylation
- inflammatory response to wounding
- innate immune response
- JNK cascade
- male gonad development
- microglial cell activation
- necroptotic signaling pathway
- negative regulation of osteoclast differentiation
- positive regulation of angiogenesis
- positive regulation of apoptotic process
- positive regulation of chemokine production
- positive regulation of cytokine production involved in inflammatory response
- positive regulation of gene expression
- positive regulation of I-kappaB kinase/NF-kappaB signaling
- positive regulation of inflammatory response
- positive regulation of interferon-alpha production
- positive regulation of interferon-beta production
- positive regulation of interleukin-12 production
- positive regulation of interleukin-6 production
- positive regulation of interleukin-8 production
- positive regulation of JNK cascade
- positive regulation of macrophage cytokine production
- positive regulation of NF-kappaB transcription factor activity
- positive regulation of NIK/NF-kappaB signaling
- positive regulation of transcription by RNA polymerase II
- positive regulation of tumor necrosis factor production
- positive regulation of interferon-gamma production
- positive regulation of type III interferon production
- regulation of dendritic cell cytokine production
- response to dsRNA
- response to exogenous dsRNA
- signal transduction
- toll-like receptor 3 signaling pathway
- toll-like receptor signaling pathway
- type III interferon production

---

64

- **Protein name:** Integrin beta-2
- **Organism:** Homo sapiens
- **Uniprot Accession Number:** P05107
- **Protein sequence length:** 769 aa
- **1D identity (%):** 13.64
- **1D identity (%) [Gaps excluded]:** 26.12
- **1D identity - Alignment Gaps:** 511
- **1D aligned content (<aminoacid>:%):** {'G': 12.33, 'L': 11.64, 'A': 3.42, 'V': 5.48, 'K': 5.48, 'C': 4.79, 'R': 6.85, 'E': 5.48, 'S': 2.74, 'Q': 6.85, 'T': 7.53, 'I': 3.42, 'D': 4.79, 'P': 8.9, 'H': 3.42, 'F': 1.37, 'Y': 3.42, 'N': 2.05}
- **Common reported functions (%):** 14.29
- **Common reported locations (%):** 11.11
- **Common reported processes (%):** 0.0

- **PDB ID:** 3K6S
- **Chain:** F
- **Crystallized protein length:** 674 aa
- **Resolution:** 3.5 Å
- **b-phipsi:** 0.004809
- **w-rdist:** 0.747349
- **t-alpha:** 0.001587
- **Chemical similarity (Tanimoto Index) (%):** 77.18
- **1D identity (%) [PDB]:** 2.74
- **1D identity (%) [Gaps excluded][PDB]:** 70.18
- **1D identity - Alignment Gaps [PDB]:** 1405
- **1D aligned content [PDB] (<aminoacid>:%):** {'S': 10.0, 'P': 15.0, 'Q': 7.5, 'T': 5.0, 'L': 12.5, 'N': 7.5, 'V': 10.0, 'K': 5.0, 'G': 12.5, 'I': 5.0, 'F': 2.5, 'H': 2.5, 'A': 5.0}
- **2D identity (%) [PDB]:** 25.72
- **2D identity (%) [Gaps excluded][PDB]:** 77.11
- **2D identity - Alignment Gaps [PDB]:** 759
- **2D aligned content [PDB] (<2D-fold>:%):** {'.': 31.74, 'T': 21.16, 'E': 25.26, 'H': 21.16, 'B': 0.68}
- **3D similarity (TM-Score) (%) [PDB]:** 19.74

- **Gene name:** ITGB2
- **Entrez ID:** 3689
- **RefSeq ID:** NM\_001303238
- **Transcript sequence length:** 2850
- **5-UTR|CDS|3-UTR identity (%):** 45.59 | 41.35 | 1.49
- **5-UTR|CDS|3-UTR identity (%) [Gaps excluded]:** 75.61 | 73.99 | 74.63
- **5-UTR|CDS|3-UTR identity [Alignment Gaps]:** 162 | 1326 | 16500
- **5-UTR aligned content (<base>:%):** {'A': 8.06, 'G': 32.8, 'C': 44.62, 'T': 14.52}
- **CDS aligned content (<base>:%):** {'A': 26.15, 'G': 28.0, 'C': 26.15, 'T': 19.71}
- **3-UTR aligned content (<base>:%):** {'A': 30.8, 'G': 25.6, 'C': 19.6, 'T': 24.0}

**Uniprot Description:**  
  
 Integrin ITGAL/ITGB2 is a receptor for ICAM1, ICAM2, ICAM3 and ICAM4. Integrin ITGAL/ITGB2 is also a receptor for the secreted form of ubiquitin-like protein ISG15; the interaction is mediated by ITGAL (PubMed:29100055). Integrins ITGAM/ITGB2 and ITGAX/ITGB2 are receptors for the iC3b fragment of the third complement component and for fibrinogen. Integrin ITGAX/ITGB2 recognizes the sequence G-P-R in fibrinogen alpha-chain. Integrin ITGAM/ITGB2 recognizes P1 and P2 peptides of fibrinogen gamma chain. Integrin ITGAM/ITGB2 is also a receptor for factor X. Integrin ITGAD/ITGB2 is a receptor for ICAM3 and VCAM1. Contributes to natural killer cell cytotoxicity (PubMed:15356110). Involved in leukocyte adhesion and transmigration of leukocytes including T-cells and neutrophils (PubMed:11812992, PubMed:28807980). Triggers neutrophil transmigration during lung injury through PTK2B/PYK2-mediated activation (PubMed:18587400). Integrin ITGAL/ITGB2 in association with ICAM3, contributes to apoptotic neutrophil phagocytosis by macrophages (PubMed:23775590). In association with alpha subunit ITGAM/CD11b, required for CD177-PRTN3-mediated activation of TNF primed neutrophils (PubMed:21193407).   
  
Heterodimer of an alpha and a beta subunit (PubMed:20033057). The ITGB2 beta subunit associates with the ITGAL, ITGAM, ITGAX or ITGAD alpha subunits. Found in a complex with CD177 and ITGAM/CD11b (PubMed:21193407, PubMed:28807980). Interacts with FGR (By similarity). Interacts with COPS5 and RANBP9 (PubMed:10766246, PubMed:14722085). Interacts with FLNA (via filamin repeats 4, 9, 12, 17, 19, 21, and 23) (PubMed:19828450). Interacts with THBD (PubMed:27055590).   
  
 **Gene Ontology Information:**

Molecular Function

- amyloid-beta binding
- cell adhesion molecule binding
- complement component C3b binding
- heat shock protein binding
- ICAM-3 receptor activity
- integrin binding
- metal ion binding
- protein kinase binding

Location

- cell surface
- external side of plasma membrane
- extracellular exosome
- extracellular vesicle
- ficolin-1-rich granule membrane
- focal adhesion
- integrin alphaL-beta2 complex
- integrin alphaM-beta2 complex
- integrin alphaX-beta2 complex
- integrin complex
- membrane
- plasma membrane
- plasma membrane raft
- receptor complex
- specific granule membrane
- tertiary granule membrane

Biological process

- amyloid-beta clearance
- apoptotic process
- cell adhesion
- cell-cell adhesion
- cell-cell adhesion via plasma-membrane adhesion molecules
- cell-cell signaling
- cell-matrix adhesion
- cellular response to low-density lipoprotein particle stimulus
- endodermal cell differentiation
- heterotypic cell-cell adhesion
- inflammatory response
- integrin-mediated signaling pathway
- leukocyte cell-cell adhesion
- microglial cell activation
- negative regulation of dopamine metabolic process
- neutrophil chemotaxis
- neutrophil migration
- phagocytosis, engulfment
- positive regulation of leukocyte adhesion to vascular endothelial cell
- positive regulation of neutrophil degranulation
- positive regulation of prostaglandin-E synthase activity
- positive regulation of protein targeting to membrane
- positive regulation of superoxide anion generation
- receptor clustering
- receptor internalization
- receptor-mediated endocytosis
- regulation of cell shape
- regulation of peptidyl-tyrosine phosphorylation

---

65

- **Protein name:** Plasminogen
- **Organism:** Homo sapiens
- **Uniprot Accession Number:** P00747
- **Protein sequence length:** 810 aa
- **1D identity (%):** 13.53
- **1D identity (%) [Gaps excluded]:** 23.33
- **1D identity - Alignment Gaps:** 444
- **1D aligned content (<aminoacid>:%):** {'V': 6.99, 'L': 7.69, 'S': 4.9, 'P': 13.29, 'G': 8.39, 'F': 3.5, 'I': 2.8, 'A': 2.8, 'K': 8.39, 'C': 5.59, 'H': 2.8, 'Q': 4.2, 'T': 6.99, 'N': 1.4, 'R': 5.59, 'Y': 4.9, 'E': 6.99, 'W': 1.4, 'D': 1.4}
- **Common reported functions (%):** 0.0
- **Common reported locations (%):** 0.0
- **Common reported processes (%):** 0.0

- **PDB ID:** 4A5T
- **Chain:** S
- **Crystallized protein length:** 767 aa
- **Resolution:** 3.49 Å
- **b-phipsi:** 0.009513
- **w-rdist:** 0.207673
- **t-alpha:** 0.014263
- **Chemical similarity (Tanimoto Index) (%):** 80.97
- **1D identity (%) [PDB]:** 1.64
- **1D identity (%) [Gaps excluded][PDB]:** 83.87
- **1D identity - Alignment Gaps [PDB]:** 1551
- **1D aligned content [PDB] (<aminoacid>:%):** {'P': 3.85, 'G': 11.54, 'I': 7.69, 'T': 11.54, 'Y': 3.85, 'V': 11.54, 'Q': 3.85, 'K': 3.85, 'R': 15.38, 'L': 3.85, 'F': 3.85, 'A': 3.85, 'D': 3.85, 'E': 3.85, 'S': 3.85, 'N': 3.85}
- **2D identity (%) [PDB]:** 24.8
- **2D identity (%) [Gaps excluded][PDB]:** 83.02
- **2D identity - Alignment Gaps [PDB]:** 871
- **2D aligned content [PDB] (<2D-fold>:%):** {'.': 31.82, 'T': 29.55, 'E': 32.79, 'H': 4.55, 'B': 1.3}
- **3D similarity (TM-Score) (%) [PDB]:** 23.96

- **Gene name:** PLG
- **Entrez ID:** 5340
- **RefSeq ID:** NM\_000301
- **Transcript sequence length:** 3530
- **5-UTR|CDS|3-UTR identity (%):** 12.01 | 44.35 | 4.11
- **5-UTR|CDS|3-UTR identity (%) [Gaps excluded]:** 80.0 | 75.0 | 78.18
- **5-UTR|CDS|3-UTR identity [Alignment Gaps]:** 283 | 1288 | 16013
- **5-UTR aligned content (<base>:%):** {'G': 27.5, 'C': 42.5, 'A': 15.0, 'T': 15.0}
- **CDS aligned content (<base>:%):** {'T': 22.96, 'G': 24.25, 'A': 29.97, 'C': 22.82}
- **3-UTR aligned content (<base>:%):** {'T': 30.65, 'A': 32.37, 'C': 17.12, 'G': 19.86}

**Uniprot Description:**  
  
 Plasmin dissolves the fibrin of blood clots and acts as a proteolytic factor in a variety of other processes including embryonic development, tissue remodeling, tumor invasion, and inflammation. In ovulation, weakens the walls of the Graafian follicle. It activates the urokinase-type plasminogen activator, collagenases and several complement zymogens, such as C1 and C5. Cleavage of fibronectin and laminin leads to cell detachment and apoptosis. Also cleaves fibrin, thrombospondin and von Willebrand factor. Its role in tissue remodeling and tumor invasion may be modulated by CSPG4. Binds to cells.   
  
Interacts (both mature PLG and the angiostatin peptide) with CSPG4 and AMOT (PubMed:10889192, PubMed:16043488). Interacts (via the Kringle domains) with HRG; the interaction tethers PLG to the cell surface and enhances its activation (PubMed:9102401, PubMed:19712047). Interacts (via Kringle 4 domain) with ADA; the interaction stimulates PLG activation when in complex with DPP4 (PubMed:15016824). Angiostatin: Interacts with ATP5F1A; the interaction inhibits most of the angiogenic effects of angiostatin (PubMed:10077593).   
  
 **Gene Ontology Information:**

Molecular Function

- apolipoprotein binding
- endopeptidase activity
- enzyme binding
- kinase binding
- protease binding
- protein antigen binding
- protein domain specific binding
- chaperone binding
- serine-type endopeptidase activity
- serine-type peptidase activity
- signaling receptor binding

Location

- blood microparticle
- cell surface
- collagen-containing extracellular matrix
- external side of plasma membrane
- extracellular exosome
- extracellular region
- extracellular space
- plasma membrane
- platelet alpha granule lumen

Biological process

- biological process involved in interaction with symbiont
- blood coagulation
- extracellular matrix disassembly
- fibrinolysis
- negative regulation of cell population proliferation
- negative regulation of cell-cell adhesion mediated by cadherin
- negative regulation of cell-substrate adhesion
- negative regulation of fibrinolysis
- positive regulation of blood vessel endothelial cell migration
- positive regulation of fibrinolysis
- proteolysis
- tissue remodeling

---

66

- **Protein name:** Protein 4.2
- **Organism:** Homo sapiens
- **Uniprot Accession Number:** P16452
- **Protein sequence length:** 691 aa
- **1D identity (%):** 12.91
- **1D identity (%) [Gaps excluded]:** 23.37
- **1D identity - Alignment Gaps:** 447
- **1D aligned content (<aminoacid>:%):** {'M': 0.78, 'L': 10.85, 'I': 3.1, 'D': 1.55, 'Q': 9.3, 'H': 1.55, 'K': 3.1, 'F': 3.1, 'R': 5.43, 'T': 7.75, 'P': 12.4, 'V': 10.08, 'G': 6.98, 'N': 2.33, 'A': 6.98, 'S': 3.1, 'W': 1.55, 'C': 4.65, 'Y': 1.55, 'E': 3.88}
- **Common reported functions (%):** 0.0
- **Common reported locations (%):** 0.0
- **Common reported processes (%):** 0.0

- **PDB ID:** 7TW1
- **Chain:** E
- **Crystallized protein length:** 665 aa
- **Resolution:** 4.6 Å
- **b-phipsi:** 0.00898
- **w-rdist:** 0.582166
- **t-alpha:** 0.001585
- **Chemical similarity (Tanimoto Index) (%):** 99.09
- **1D identity (%) [PDB]:** 2.53
- **1D identity (%) [Gaps excluded][PDB]:** 71.15
- **1D identity - Alignment Gaps [PDB]:** 1408
- **1D aligned content [PDB] (<aminoacid>:%):** {'A': 13.51, 'G': 2.7, 'D': 8.11, 'K': 5.41, 'S': 10.81, 'V': 13.51, 'H': 2.7, 'R': 10.81, 'T': 2.7, 'Q': 8.11, 'I': 5.41, 'L': 8.11, 'E': 2.7, 'F': 2.7, 'Y': 2.7}
- **2D identity (%) [PDB]:** 32.5
- **2D identity (%) [Gaps excluded][PDB]:** 79.68
- **2D identity - Alignment Gaps [PDB]:** 636
- **2D aligned content [PDB] (<2D-fold>:%):** {'.': 24.07, 'E': 40.4, 'H': 23.21, 'T': 11.75, 'B': 0.57}
- **3D similarity (TM-Score) (%) [PDB]:** 20.03

- **Gene name:** EPB42
- **Entrez ID:** 2038
- **RefSeq ID:** N/A
- **Sequence length:** N/A
- **5-UTR|CDS|3-UTR identity (%):** N/A | N/A | N/A
- **5-UTR|CDS|3-UTR identity (%) [Gaps excluded]:** N/A | N/A | N/A
- **5-UTR|CDS|3-UTR identity [Alignment Gaps]:** N/A | N/A | N/A
- **5-UTR aligned content (<base>:%):** N/A
- **CDS aligned content (<base>:%):** N/A
- **3-UTR aligned content (<base>:%):** N/A

**Uniprot Description:**  
  
 Probably plays an important role in the regulation of erythrocyte shape and mechanical properties.   
  
Oligomer. Interacts with the cytoplasmic domain of SLC4A1/band 3 anion transport protein.   
  
 **Gene Ontology Information:**

Molecular Function

- ATP binding
- protein-glutamine gamma-glutamyltransferase activity
- structural constituent of cytoskeleton

Location

- ankyrin-1 complex
- cortical cytoskeleton
- cytoskeleton
- plasma membrane

Biological process

- cell morphogenesis
- erythrocyte maturation
- hemoglobin metabolic process
- multicellular organismal iron ion homeostasis
- peptide cross-linking
- regulation of cell shape
- spleen development

---

67

- **Protein name:** Melanotransferrin
- **Organism:** Homo sapiens
- **Uniprot Accession Number:** P08582
- **Protein sequence length:** 738 aa
- **1D identity (%):** 16.56
- **1D identity (%) [Gaps excluded]:** 24.92
- **1D identity - Alignment Gaps:** 322
- **1D aligned content (<aminoacid>:%):** {'G': 12.58, 'P': 8.81, 'A': 8.18, 'L': 10.69, 'R': 5.66, 'M': 1.26, 'Q': 4.4, 'E': 4.4, 'I': 1.26, 'D': 5.03, 'C': 3.77, 'H': 1.26, 'V': 7.55, 'Y': 3.14, 'T': 3.14, 'K': 5.03, 'W': 0.63, 'S': 5.03, 'F': 5.66, 'N': 2.52}
- **Common reported functions (%):** 0.0
- **Common reported locations (%):** 0.0
- **Common reported processes (%):** 0.0

- **PDB ID:** 6XR0
- **Chain:** M
- **Crystallized protein length:** 683 aa
- **Resolution:** 3.06 Å
- **b-phipsi:** 0.002512
- **w-rdist:** 0.501188
- **t-alpha:** 0.042789
- **Chemical similarity (Tanimoto Index) (%):** N/A
- **1D identity (%) [PDB]:** 2.79
- **1D identity (%) [Gaps excluded][PDB]:** 69.49
- **1D identity - Alignment Gaps [PDB]:** 1411
- **1D aligned content [PDB] (<aminoacid>:%):** {'A': 9.76, 'G': 4.88, 'I': 2.44, 'Q': 2.44, 'T': 4.88, 'S': 12.2, 'R': 9.76, 'H': 2.44, 'Y': 9.76, 'V': 7.32, 'L': 7.32, 'D': 9.76, 'N': 2.44, 'F': 4.88, 'E': 2.44, 'P': 4.88, 'K': 2.44}
- **2D identity (%) [PDB]:** 30.69
- **2D identity (%) [Gaps excluded][PDB]:** 84.31
- **2D identity - Alignment Gaps [PDB]:** 713
- **2D aligned content [PDB] (<2D-fold>:%):** {'.': 16.86, 'H': 40.12, 'E': 23.55, 'T': 18.6, 'G': 0.87}
- **3D similarity (TM-Score) (%) [PDB]:** 11.62

- **Gene name:** MELTF
- **Entrez ID:** 4241
- **RefSeq ID:** NM\_033316
- **Transcript sequence length:** 1676
- **5-UTR|CDS|3-UTR identity (%):** 22.29 | 20.63 | 2.61
- **5-UTR|CDS|3-UTR identity (%) [Gaps excluded]:** 79.17 | 75.03 | 76.39
- **5-UTR|CDS|3-UTR identity [Alignment Gaps]:** 245 | 1986 | 16255
- **5-UTR aligned content (<base>:%):** {'G': 42.11, 'C': 47.37, 'A': 9.21, 'T': 1.32}
- **CDS aligned content (<base>:%):** {'A': 24.78, 'T': 17.7, 'G': 33.81, 'C': 23.72}
- **3-UTR aligned content (<base>:%):** {'G': 20.23, 'A': 30.91, 'T': 33.18, 'C': 15.68}

**Uniprot Description:**  
  
 Involved in iron cellular uptake. Seems to be internalized and then recycled back to the cell membrane. Binds a single atom of iron per subunit. Could also bind zinc. N/A   
  
 **Gene Ontology Information:**

Molecular Function

- iron ion binding

Location

- cell surface
- early endosome
- endoplasmic reticulum lumen
- extracellular exosome
- extracellular region
- extracellular space
- plasma membrane
- recycling endosome
- side of membrane

Biological process

- iron ion transmembrane transport
- iron ion transport
- negative regulation of substrate adhesion-dependent cell spreading
- positive regulation of extracellular matrix disassembly
- positive regulation of plasminogen activation

---

68

- **Protein name:** Probable 2-oxoglutarate dehydrogenase E1 component DHKTD1, mitochondrial
- **Organism:** Homo sapiens
- **Uniprot Accession Number:** Q96HY7
- **Protein sequence length:** 919 aa
- **1D identity (%):** 16.26
- **1D identity (%) [Gaps excluded]:** 21.9
- **1D identity - Alignment Gaps:** 263
- **1D aligned content (<aminoacid>:%):** {'S': 6.02, 'A': 7.83, 'R': 6.02, 'G': 10.84, 'L': 9.04, 'Q': 6.02, 'E': 4.22, 'V': 8.43, 'Y': 1.81, 'P': 9.64, 'D': 4.82, 'T': 8.43, 'K': 4.82, 'H': 1.81, 'F': 4.22, 'M': 1.2, 'C': 2.41, 'N': 0.6, 'I': 1.81}
- **Common reported functions (%):** 0.0
- **Common reported locations (%):** 0.0
- **Common reported processes (%):** 0.0

- **PDB ID:** 6U3J
- **Chain:** A
- **Crystallized protein length:** 874 aa
- **Resolution:** 2.25 Å
- **b-phipsi:** 0.014906
- **w-rdist:** 0.381968
- **t-alpha:** 0.005577
- **Chemical similarity (Tanimoto Index) (%):** 77.84
- **1D identity (%) [PDB]:** 2.71
- **1D identity (%) [Gaps excluded][PDB]:** 71.43
- **1D identity - Alignment Gaps [PDB]:** 1596
- **1D aligned content [PDB] (<aminoacid>:%):** {'G': 13.33, 'F': 6.67, 'T': 6.67, 'Q': 8.89, 'L': 8.89, 'K': 4.44, 'S': 6.67, 'A': 2.22, 'M': 2.22, 'P': 11.11, 'C': 2.22, 'R': 4.44, 'H': 2.22, 'N': 2.22, 'Y': 4.44, 'I': 4.44, 'V': 8.89}
- **2D identity (%) [PDB]:** 37.83
- **2D identity (%) [Gaps excluded][PDB]:** 82.14
- **2D identity - Alignment Gaps [PDB]:** 636
- **2D aligned content [PDB] (<2D-fold>:%):** {'.': 22.87, 'H': 41.26, 'T': 17.26, 'E': 18.61}
- **3D similarity (TM-Score) (%) [PDB]:** 22.1

- **Gene name:** DHTKD1
- **Entrez ID:** 55526
- **RefSeq ID:** NM\_018706
- **Transcript sequence length:** 5173
- **5-UTR|CDS|3-UTR identity (%):** 15.09 | 47.21 | 9.38
- **5-UTR|CDS|3-UTR identity (%) [Gaps excluded]:** 79.69 | 75.0 | 80.36
- **5-UTR|CDS|3-UTR identity [Alignment Gaps]:** 274 | 1215 | 15096
- **5-UTR aligned content (<base>:%):** {'G': 43.14, 'C': 39.22, 'A': 5.88, 'T': 11.76}
- **CDS aligned content (<base>:%):** {'A': 28.04, 'T': 23.26, 'G': 25.06, 'C': 23.64}
- **3-UTR aligned content (<base>:%):** {'T': 27.49, 'G': 23.44, 'A': 29.24, 'C': 19.83}

**Uniprot Description:**  
  
 The 2-oxoglutarate dehydrogenase complex catalyzes the overall conversion of 2-oxoglutarate to succinyl-CoA and CO(2). It contains multiple copies of three enzymatic components: 2-oxoglutarate dehydrogenase (E1), dihydrolipoamide succinyltransferase (E2) and lipoamide dehydrogenase (E3) (By similarity). N/A   
  
 **Gene Ontology Information:**

Molecular Function

- oxoglutarate dehydrogenase (succinyl-transferring) activity
- thiamine pyrophosphate binding

Location

- mitochondrial matrix
- mitochondrion

Biological process

- generation of precursor metabolites and energy
- glycolytic process
- hematopoietic progenitor cell differentiation
- tricarboxylic acid cycle

---

69

- **Protein name:** DNA polymerase theta
- **Organism:** Homo sapiens
- **Uniprot Accession Number:** O75417
- **Protein sequence length:** 2590 aa
- **1D identity (%):** 10.98
- **1D identity (%) [Gaps excluded]:** 33.85
- **1D identity - Alignment Gaps:** 1760
- **1D aligned content (<aminoacid>:%):** {'M': 1.05, 'G': 8.74, 'S': 5.24, 'A': 5.59, 'P': 9.79, 'K': 6.29, 'L': 9.09, 'N': 2.1, 'Q': 6.99, 'E': 4.2, 'V': 7.69, 'Y': 2.1, 'I': 2.45, 'D': 5.24, 'R': 4.9, 'T': 7.34, 'F': 3.5, 'H': 4.55, 'C': 3.15}
- **Common reported functions (%):** 0.0
- **Common reported locations (%):** 22.22
- **Common reported processes (%):** 0.0

- **PDB ID:** 4X0P
- **Chain:** A
- **Crystallized protein length:** 625 aa
- **Resolution:** 3.91 Å
- **b-phipsi:** 0.027322
- **w-rdist:** 0.274937
- **t-alpha:** 0.007132
- **Chemical similarity (Tanimoto Index) (%):** 72.62
- **1D identity (%) [PDB]:** 2.68
- **1D identity (%) [Gaps excluded][PDB]:** 69.09
- **1D identity - Alignment Gaps [PDB]:** 1365
- **1D aligned content [PDB] (<aminoacid>:%):** {'L': 18.42, 'D': 7.89, 'V': 13.16, 'T': 10.53, 'K': 10.53, 'R': 5.26, 'S': 10.53, 'H': 5.26, 'E': 7.89, 'G': 2.63, 'P': 2.63, 'I': 2.63, 'A': 2.63}
- **2D identity (%) [PDB]:** 36.08
- **2D identity (%) [Gaps excluded][PDB]:** 86.84
- **2D identity - Alignment Gaps [PDB]:** 609
- **2D aligned content [PDB] (<2D-fold>:%):** {'.': 23.14, 'E': 15.43, 'H': 50.53, 'T': 10.64, 'B': 0.27}
- **3D similarity (TM-Score) (%) [PDB]:** 23.56

- **Gene name:** POLQ
- **Entrez ID:** 10721
- **RefSeq ID:** NM\_199420
- **Transcript sequence length:** 8757
- **5-UTR|CDS|3-UTR identity (%):** 20.65 | 26.67 | 3.4
- **5-UTR|CDS|3-UTR identity (%) [Gaps excluded]:** 74.47 | 83.45 | 77.25
- **5-UTR|CDS|3-UTR identity [Alignment Gaps]:** 245 | 5340 | 16142
- **5-UTR aligned content (<base>:%):** {'A': 10.0, 'G': 40.0, 'T': 20.0, 'C': 30.0}
- **CDS aligned content (<base>:%):** {'A': 31.87, 'T': 24.75, 'G': 23.36, 'C': 20.02}
- **3-UTR aligned content (<base>:%):** {'C': 13.94, 'T': 38.33, 'G': 15.68, 'A': 32.06}

**Uniprot Description:**  
  
 DNA polymerase that promotes microhomology-mediated end-joining (MMEJ), an alternative non-homologous end-joining (NHEJ) machinery triggered in response to double-strand breaks in DNA (PubMed:25642963, PubMed:25643323). MMEJ is an error-prone repair pathway that produces deletions of sequences from the strand being repaired and promotes genomic rearrangements, such as telomere fusions, some of them leading to cellular transformation (PubMed:25642963, PubMed:25643323). POLQ acts as an inhibitor of homology-recombination repair (HR) pathway by limiting RAD51 accumulation at resected ends (PubMed:25642963). POLQ-mediated MMEJ may be required to promote the survival of cells with a compromised HR repair pathway, thereby preventing genomic havoc by resolving unrepaired lesions (By similarity). The polymerase acts by binding directly the 2 ends of resected double-strand breaks, allowing microhomologous sequences in the overhangs to form base pairs. It then extends each strand from the base-paired region using the opposing overhang as a template. Requires partially resected DNA containing 2 to 6 base pairs of microhomology to perform MMEJ (PubMed:25643323). The polymerase activity is highly promiscuous: unlike most polymerases, promotes extension of ssDNA and partial ssDNA (pssDNA) substrates (PubMed:18503084, PubMed:21050863, PubMed:22135286). Also exhibits low-fidelity DNA synthesis, translesion synthesis and lyase activity, and it is implicated in interstrand-cross-link repair, base excision repair and DNA end-joining (PubMed:14576298, PubMed:18503084, PubMed:19188258, PubMed:24648516). Involved in somatic hypermutation of immunoglobulin genes, a process that requires the activity of DNA polymerases to ultimately introduce mutations at both A/T and C/G base pairs (By similarity).   
  
Homomultimer; forms dimers and multimers (PubMed:25643323). Interacts with RAD51 (PubMed:25642963). Interacts with ORC2 and ORC4 (PubMed:24989122).   
  
 **Gene Ontology Information:**

Molecular Function

- 5'-deoxyribose-5-phosphate lyase activity
- ATP binding
- chromatin binding
- damaged DNA binding
- DNA-directed DNA polymerase activity
- identical protein binding
- single-stranded DNA helicase activity

Location

- chromosome
- cytosol
- Golgi apparatus
- nucleoplasm

Biological process

- base-excision repair
- cellular response to DNA damage stimulus
- DNA repair
- DNA-dependent DNA replication
- double-strand break repair
- double-strand break repair via alternative nonhomologous end joining
- negative regulation of double-strand break repair via homologous recombination
- protein homooligomerization
- somatic hypermutation of immunoglobulin genes

---

70

- **Protein name:** Ectonucleotide pyrophosphatase/phosphodiesterase family member 3
- **Organism:** Homo sapiens
- **Uniprot Accession Number:** O14638
- **Protein sequence length:** 875 aa
- **1D identity (%):** 17.58
- **1D identity (%) [Gaps excluded]:** 24.97
- **1D identity - Alignment Gaps:** 301
- **1D aligned content (<aminoacid>:%):** {'M': 1.68, 'E': 4.47, 'A': 5.03, 'Q': 4.47, 'P': 12.85, 'L': 10.06, 'V': 8.94, 'G': 6.7, 'C': 4.47, 'F': 3.91, 'K': 2.23, 'D': 3.91, 'R': 5.59, 'S': 3.91, 'N': 2.23, 'T': 6.7, 'H': 6.7, 'W': 1.12, 'I': 1.68, 'Y': 3.35}
- **Common reported functions (%):** 0.0
- **Common reported locations (%):** 0.0
- **Common reported processes (%):** 0.0

- **PDB ID:** 6C02
- **Chain:** B
- **Crystallized protein length:** 815 aa
- **Resolution:** 1.94 Å
- **b-phipsi:** 0.003379
- **w-rdist:** 0.513774
- **t-alpha:** 0.018225
- **Chemical similarity (Tanimoto Index) (%):** 66.48
- **1D identity (%) [PDB]:** 1.73
- **1D identity (%) [Gaps excluded][PDB]:** 66.67
- **1D identity - Alignment Gaps [PDB]:** 1577
- **1D aligned content [PDB] (<aminoacid>:%):** {'G': 7.14, 'D': 14.29, 'P': 17.86, 'V': 10.71, 'R': 7.14, 'L': 10.71, 'Y': 3.57, 'T': 7.14, 'A': 7.14, 'N': 3.57, 'E': 3.57, 'K': 3.57, 'F': 3.57}
- **2D identity (%) [PDB]:** 30.17
- **2D identity (%) [Gaps excluded][PDB]:** 79.26
- **2D identity - Alignment Gaps [PDB]:** 745
- **2D aligned content [PDB] (<2D-fold>:%):** {'.': 19.28, 'T': 21.76, 'H': 36.91, 'E': 18.46, 'B': 1.93, 'G': 1.65}
- **3D similarity (TM-Score) (%) [PDB]:** 22.67

- **Gene name:** ENPP3
- **Entrez ID:** 5169
- **RefSeq ID:** NM\_005021
- **Transcript sequence length:** 3165
- **5-UTR|CDS|3-UTR identity (%):** 10.11 | 46.97 | 1.72
- **5-UTR|CDS|3-UTR identity (%) [Gaps excluded]:** 73.47 | 74.74 | 75.52
- **5-UTR|CDS|3-UTR identity [Alignment Gaps]:** 307 | 1189 | 16441
- **5-UTR aligned content (<base>:%):** {'T': 33.33, 'C': 30.56, 'G': 22.22, 'A': 13.89}
- **CDS aligned content (<base>:%):** {'A': 32.27, 'T': 24.88, 'G': 22.02, 'C': 20.83}
- **3-UTR aligned content (<base>:%):** {'C': 12.41, 'T': 43.79, 'A': 32.07, 'G': 11.72}

**Uniprot Description:**  
  
 Hydrolase that metabolizes extracellular nucleotides, including ATP, GTP, UTP and CTP (PubMed:29717535). Limits mast cell and basophil responses during inflammation and during the chronic phases of allergic responses by eliminating the extracellular ATP that functions as signaling molecule and activates basophils and mast cells and induces the release of inflammatory cytokines. Metabolizes extracellular ATP in the lumen of the small intestine, and thereby prevents ATP-induced apoptosis of intestinal plasmacytoid dendritic cells (By similarity). Has also alkaline phosphodiesterase activity (PubMed:11342463).   
  
Monomer and homodimer.   
  
 **Gene Ontology Information:**

Molecular Function

- calcium ion binding
- nucleic acid binding
- nucleoside-triphosphate diphosphatase activity
- phosphodiesterase I activity
- zinc ion binding

Location

- apical plasma membrane
- external side of plasma membrane
- extracellular exosome
- perinuclear region of cytoplasm

Biological process

- ATP metabolic process
- basophil activation involved in immune response
- negative regulation of inflammatory response
- negative regulation of mast cell activation involved in immune response
- negative regulation of mast cell proliferation
- nucleoside triphosphate catabolic process
- phosphate ion homeostasis
- phosphate-containing compound metabolic process
- pyrimidine nucleotide metabolic process

---

71

- **Protein name:** Calpain-2 catalytic subunit
- **Organism:** Homo sapiens
- **Uniprot Accession Number:** P17655
- **Protein sequence length:** 700 aa
- **1D identity (%):** 12.59
- **1D identity (%) [Gaps excluded]:** 21.57
- **1D identity - Alignment Gaps:** 410
- **1D aligned content (<aminoacid>:%):** {'K': 8.06, 'R': 8.06, 'E': 9.68, 'H': 2.42, 'T': 7.26, 'F': 5.65, 'D': 8.06, 'P': 8.06, 'G': 11.29, 'L': 8.87, 'N': 1.61, 'V': 4.84, 'S': 2.42, 'A': 3.23, 'W': 0.81, 'C': 2.42, 'I': 3.23, 'Y': 1.61, 'Q': 2.42}
- **Common reported functions (%):** 0.0
- **Common reported locations (%):** 22.22
- **Common reported processes (%):** 0.0

- **PDB ID:** 1KFU
- **Chain:** L
- **Crystallized protein length:** 699 aa
- **Resolution:** 2.5 Å
- **b-phipsi:** 0.002243
- **w-rdist:** 0.510143
- **t-alpha:** 0.08887
- **Chemical similarity (Tanimoto Index) (%):** 99.4
- **1D identity (%) [PDB]:** 2.35
- **1D identity (%) [Gaps excluded][PDB]:** 66.04
- **1D identity - Alignment Gaps [PDB]:** 1438
- **1D aligned content [PDB] (<aminoacid>:%):** {'A': 14.29, 'G': 2.86, 'R': 5.71, 'I': 5.71, 'K': 8.57, 'L': 5.71, 'N': 5.71, 'Q': 5.71, 'T': 5.71, 'S': 8.57, 'D': 5.71, 'E': 11.43, 'V': 8.57, 'F': 5.71}
- **2D identity (%) [PDB]:** 35.71
- **2D identity (%) [Gaps excluded][PDB]:** 83.37
- **2D identity - Alignment Gaps [PDB]:** 618
- **2D aligned content [PDB] (<2D-fold>:%):** {'.': 25.65, 'H': 33.68, 'T': 17.62, 'E': 22.8, 'B': 0.26}
- **3D similarity (TM-Score) (%) [PDB]:** 21.56

- **Gene name:** CAPN2
- **Entrez ID:** 824
- **RefSeq ID:** NM\_001748
- **Transcript sequence length:** 3366
- **5-UTR|CDS|3-UTR identity (%):** 18.37 | 45.38 | 4.38
- **5-UTR|CDS|3-UTR identity (%) [Gaps excluded]:** 77.78 | 74.56 | 73.22
- **5-UTR|CDS|3-UTR identity [Alignment Gaps]:** 262 | 1140 | 15892
- **5-UTR aligned content (<base>:%):** {'A': 7.94, 'G': 36.51, 'T': 7.94, 'C': 47.62}
- **CDS aligned content (<base>:%):** {'A': 27.46, 'T': 21.63, 'G': 28.29, 'C': 22.62}
- **3-UTR aligned content (<base>:%):** {'G': 14.3, 'T': 35.49, 'A': 35.63, 'C': 14.57}

**Uniprot Description:**  
  
 Calcium-regulated non-lysosomal thiol-protease which catalyzes limited proteolysis of substrates involved in cytoskeletal remodeling and signal transduction. Proteolytically cleaves MYOC at 'Arg-226' (PubMed:17650508). Proteolytically cleaves CPEB3 following neuronal stimulation which abolishes CPEB3 translational repressor activity, leading to translation of CPEB3 target mRNAs (By similarity).   
  
Forms a heterodimer with a small (regulatory) subunit (CAPNS1). Interacts with CPEB3; this leads to cleavage of CPEB3.   
  
 **Gene Ontology Information:**

Molecular Function

- calcium ion binding
- calcium-dependent cysteine-type endopeptidase activity
- cysteine-type peptidase activity
- cytoskeletal protein binding
- enzyme binding
- protein-containing complex binding

Location

- calpain complex
- chromatin
- cortical actin cytoskeleton
- cytoplasm
- cytosol
- dendrite
- endoplasmic reticulum
- external side of plasma membrane
- extracellular exosome
- focal adhesion
- Golgi apparatus
- lysosome
- membrane raft
- neuronal cell body
- nucleus
- perinuclear endoplasmic reticulum
- plasma membrane
- pseudopodium

Biological process

- behavioral response to pain
- blastocyst development
- cellular response to amino acid stimulus
- cellular response to interferon-beta
- cellular response to lipopolysaccharide
- female pregnancy
- myoblast fusion
- positive regulation of cardiac muscle cell apoptotic process
- positive regulation of myoblast fusion
- positive regulation of neuron death
- positive regulation of phosphatidylcholine biosynthetic process
- protein autoprocessing
- proteolysis
- proteolysis involved in cellular protein catabolic process
- regulation of cytoskeleton organization
- regulation of interleukin-6 production
- response to hydrogen peroxide
- response to hypoxia
- response to mechanical stimulus

---

72

- **Protein name:** Phosphatidylinositol 4,5-bisphosphate 3-kinase catalytic subunit alpha isoform
- **Organism:** Homo sapiens
- **Uniprot Accession Number:** P42336
- **Protein sequence length:** 1068 aa
- **1D identity (%):** 13.41
- **1D identity (%) [Gaps excluded]:** 23.64
- **1D identity - Alignment Gaps:** 532
- **1D aligned content (<aminoacid>:%):** {'P': 10.91, 'R': 4.24, 'G': 6.06, 'L': 12.73, 'C': 3.64, 'E': 4.85, 'I': 4.24, 'K': 8.48, 'D': 7.27, 'F': 4.85, 'V': 5.45, 'T': 3.03, 'N': 3.64, 'W': 2.42, 'H': 4.85, 'S': 1.21, 'Y': 4.24, 'A': 3.03, 'Q': 4.85}
- **Common reported functions (%):** 0.0
- **Common reported locations (%):** 22.22
- **Common reported processes (%):** 0.0

- **PDB ID:** 2RD0
- **Chain:** A
- **Crystallized protein length:** 997 aa
- **Resolution:** 3.05 Å
- **b-phipsi:** 0.006789
- **w-rdist:** 0.11047
- **t-alpha:** 0.035658
- **Chemical similarity (Tanimoto Index) (%):** 99.17
- **1D identity (%) [PDB]:** 3.99
- **1D identity (%) [Gaps excluded][PDB]:** 67.65
- **1D identity - Alignment Gaps [PDB]:** 1629
- **1D aligned content [PDB] (<aminoacid>:%):** {'L': 8.7, 'N': 5.8, 'G': 4.35, 'V': 10.14, 'E': 11.59, 'T': 7.25, 'Q': 7.25, 'Y': 4.35, 'F': 4.35, 'K': 4.35, 'P': 7.25, 'C': 4.35, 'H': 2.9, 'R': 7.25, 'D': 2.9, 'S': 1.45, 'M': 2.9, 'I': 1.45, 'A': 1.45}
- **2D identity (%) [PDB]:** 37.76
- **2D identity (%) [Gaps excluded][PDB]:** 86.05
- **2D identity - Alignment Gaps [PDB]:** 715
- **2D aligned content [PDB] (<2D-fold>:%):** {'.': 19.96, 'B': 0.21, 'E': 22.04, 'H': 41.58, 'T': 16.22}
- **3D similarity (TM-Score) (%) [PDB]:** 23.0

- **Gene name:** PIK3CA
- **Entrez ID:** 5290
- **RefSeq ID:** N/A
- **Sequence length:** N/A
- **5-UTR|CDS|3-UTR identity (%):** N/A | N/A | N/A
- **5-UTR|CDS|3-UTR identity (%) [Gaps excluded]:** N/A | N/A | N/A
- **5-UTR|CDS|3-UTR identity [Alignment Gaps]:** N/A | N/A | N/A
- **5-UTR aligned content (<base>:%):** N/A
- **CDS aligned content (<base>:%):** N/A
- **3-UTR aligned content (<base>:%):** N/A

**Uniprot Description:**  
  
 Phosphoinositide-3-kinase (PI3K) phosphorylates phosphatidylinositol (PI) and its phosphorylated derivatives at position 3 of the inositol ring to produce 3-phosphoinositides (PubMed:15135396, PubMed:23936502, PubMed:28676499). Uses ATP and PtdIns(4,5)P2 (phosphatidylinositol 4,5-bisphosphate) to generate phosphatidylinositol 3,4,5-trisphosphate (PIP3) (PubMed:15135396, PubMed:28676499). PIP3 plays a key role by recruiting PH domain-containing proteins to the membrane, including AKT1 and PDPK1, activating signaling cascades involved in cell growth, survival, proliferation, motility and morphology. Participates in cellular signaling in response to various growth factors. Involved in the activation of AKT1 upon stimulation by receptor tyrosine kinases ligands such as EGF, insulin, IGF1, VEGFA and PDGF. Involved in signaling via insulin-receptor substrate (IRS) proteins. Essential in endothelial cell migration during vascular development through VEGFA signaling, possibly by regulating RhoA activity. Required for lymphatic vasculature development, possibly by binding to RAS and by activation by EGF and FGF2, but not by PDGF. Regulates invadopodia formation through the PDPK1-AKT1 pathway. Participates in cardiomyogenesis in embryonic stem cells through a AKT1 pathway. Participates in vasculogenesis in embryonic stem cells through PDK1 and protein kinase C pathway. In addition to its lipid kinase activity, it displays a serine-protein kinase activity that results in the autophosphorylation of the p85alpha regulatory subunit as well as phosphorylation of other proteins such as 4EBP1, H-Ras, the IL-3 beta c receptor and possibly others (PubMed:23936502, PubMed:28676499). Plays a role in the positive regulation of phagocytosis and pinocytosis (By similarity).   
  
Heterodimer of a catalytic subunit PIK3CA and a p85 regulatory subunit (PIK3R1, PIK3R2 or PIK3R3) (PubMed:26593112). Interacts with IRS1 in nuclear extracts (By similarity). Interacts with RUFY3 (By similarity). Interacts with RASD2 (By similarity). Interacts with APPL1. Interacts with HRAS and KRAS (By similarity). Interaction with HRAS/KRAS is required for PI3K pathway signaling and cell proliferation stimulated by EGF and FGF2 (By similarity). Interacts with FAM83B; activates the PI3K/AKT signaling cascade (PubMed:23676467).   
  
 **Gene Ontology Information:**

Molecular Function

- phosphatidylinositol-3,4-bisphosphate 5-kinase activity
- 1-phosphatidylinositol-3-kinase activity
- phosphatidylinositol-4,5-bisphosphate 3-kinase activity
- 1-phosphatidylinositol-4-phosphate 3-kinase activity
- ATP binding
- insulin receptor substrate binding
- kinase activity
- protein kinase activator activity
- protein serine kinase activity
- protein serine/threonine kinase activity

Location

- cytoplasm
- cytosol
- intercalated disc
- lamellipodium
- perinuclear region of cytoplasm
- phosphatidylinositol 3-kinase complex
- phosphatidylinositol 3-kinase complex, class IA
- phosphatidylinositol 3-kinase complex, class IB
- plasma membrane

Biological process

- actin cytoskeleton organization
- adipose tissue development
- angiogenesis
- anoikis
- cardiac muscle cell contraction
- cardiac muscle contraction
- cellular response to glucose stimulus
- cellular response to hydrostatic pressure
- endothelial cell migration
- energy homeostasis
- epidermal growth factor receptor signaling pathway
- genetic imprinting
- glucose metabolic process
- insulin receptor signaling pathway via phosphatidylinositol 3-kinase
- insulin-like growth factor receptor signaling pathway
- liver development
- negative regulation of actin filament depolymerization
- negative regulation of anoikis
- negative regulation of fibroblast apoptotic process
- negative regulation of gene expression
- negative regulation of macroautophagy
- negative regulation of neuron apoptotic process
- phagocytosis
- phosphatidylinositol 3-kinase signaling
- phosphatidylinositol phosphorylation
- phosphatidylinositol-3-phosphate biosynthetic process
- phosphorylation
- platelet activation
- hypomethylation of CpG island
- positive regulation of lamellipodium assembly
- positive regulation of peptidyl-serine phosphorylation
- positive regulation of protein kinase B signaling
- positive regulation of smooth muscle cell proliferation
- positive regulation of TOR signaling
- protein kinase B signaling
- regulation of actin filament organization
- regulation of cellular respiration
- regulation of multicellular organism growth
- relaxation of cardiac muscle
- response to activity
- response to butyrate
- response to dexamethasone
- response to leucine
- response to muscle inactivity
- response to muscle stretch
- T cell costimulation
- T cell receptor signaling pathway
- vascular endothelial growth factor signaling pathway
- vasculature development

---

73

- **Protein name:** 1-phosphatidylinositol 4,5-bisphosphate phosphodiesterase beta-2
- **Organism:** Homo sapiens
- **Uniprot Accession Number:** Q00722
- **Protein sequence length:** 1185 aa
- **1D identity (%):** 14.87
- **1D identity (%) [Gaps excluded]:** 21.86
- **1D identity - Alignment Gaps:** 389
- **1D aligned content (<aminoacid>:%):** {'P': 12.15, 'L': 11.6, 'Y': 2.21, 'G': 7.18, 'I': 2.21, 'K': 7.18, 'A': 6.63, 'E': 8.29, 'D': 5.52, 'T': 7.18, 'F': 3.31, 'R': 4.97, 'N': 3.87, 'V': 4.42, 'H': 3.31, 'S': 2.21, 'M': 2.21, 'C': 1.66, 'Q': 3.87}
- **Common reported functions (%):** 0.0
- **Common reported locations (%):** 11.11
- **Common reported processes (%):** 0.0

- **PDB ID:** 2ZKM
- **Chain:** X
- **Crystallized protein length:** 708 aa
- **Resolution:** 1.62 Å
- **b-phipsi:** 0.000856
- **w-rdist:** 0.615441
- **t-alpha:** 0.047303
- **Chemical similarity (Tanimoto Index) (%):** 87.24
- **1D identity (%) [PDB]:** 3.23
- **1D identity (%) [Gaps excluded][PDB]:** 66.67
- **1D identity - Alignment Gaps [PDB]:** 1413
- **1D aligned content [PDB] (<aminoacid>:%):** {'P': 10.42, 'K': 12.5, 'Y': 6.25, 'L': 8.33, 'E': 6.25, 'I': 4.17, 'D': 10.42, 'R': 6.25, 'V': 8.33, 'N': 2.08, 'S': 4.17, 'Q': 2.08, 'T': 8.33, 'F': 2.08, 'G': 6.25, 'A': 2.08}
- **2D identity (%) [PDB]:** 30.45
- **2D identity (%) [Gaps excluded][PDB]:** 84.91
- **2D identity - Alignment Gaps [PDB]:** 735
- **2D aligned content [PDB] (<2D-fold>:%):** {'H': 33.52, '.': 17.19, 'E': 34.1, 'T': 13.47, 'G': 1.43, 'B': 0.29}
- **3D similarity (TM-Score) (%) [PDB]:** 22.17

- **Gene name:** PLCB2
- **Entrez ID:** N/A
- **RefSeq ID:** NM\_004573
- **Transcript sequence length:** 4627
- **5-UTR|CDS|3-UTR identity (%):** 45.58 | 42.88 | 3.07
- **5-UTR|CDS|3-UTR identity (%) [Gaps excluded]:** 73.99 | 73.99 | 76.32
- **5-UTR|CDS|3-UTR identity [Alignment Gaps]:** 139 | 1635 | 16201
- **5-UTR aligned content (<base>:%):** {'A': 9.7, 'G': 38.79, 'C': 36.36, 'T': 15.15}
- **CDS aligned content (<base>:%):** {'A': 29.09, 'T': 20.76, 'G': 25.91, 'C': 24.24}
- **3-UTR aligned content (<base>:%):** {'T': 21.97, 'C': 30.83, 'A': 19.85, 'G': 27.36}

**Uniprot Description:**  
  
 The production of the second messenger molecules diacylglycerol (DAG) and inositol 1,4,5-trisphosphate (IP3) is mediated by activated phosphatidylinositol-specific phospholipase C enzymes.   
  
Interacts with RAC1 (PubMed:1644792). Forms a complex composed of at least WDR26, a G-beta:gamma unit, and PLCB2 (PubMed:23625927).   
  
 **Gene Ontology Information:**

Molecular Function

- calcium ion binding
- G-protein beta/gamma-subunit complex binding
- phosphatidylinositol phospholipase C activity
- phospholipase C activity
- phospholipid binding

Location

- cytosol
- G-protein beta/gamma-subunit complex
- neuronal dense core vesicle

Biological process

- activation of phospholipase C activity
- detection of chemical stimulus involved in sensory perception of bitter taste
- G protein-coupled receptor signaling pathway
- lipid catabolic process
- phosphatidylinositol metabolic process
- phosphatidylinositol-mediated signaling
- phospholipase C-activating G protein-coupled receptor signaling pathway
- phospholipid metabolic process
- release of sequestered calcium ion into cytosol

---

74

- **Protein name:** Microsomal triglyceride transfer protein large subunit
- **Organism:** Homo sapiens
- **Uniprot Accession Number:** P55157
- **Protein sequence length:** 894 aa
- **1D identity (%):** 16.05
- **1D identity (%) [Gaps excluded]:** 20.23
- **1D identity - Alignment Gaps:** 202
- **1D aligned content (<aminoacid>:%):** {'L': 10.19, 'A': 6.37, 'F': 4.46, 'I': 5.1, 'Y': 4.46, 'K': 8.28, 'N': 3.82, 'V': 5.73, 'D': 5.73, 'R': 4.46, 'G': 10.19, 'E': 5.73, 'P': 4.46, 'H': 3.82, 'S': 5.1, 'T': 5.1, 'Q': 5.1, 'M': 0.64, 'C': 1.27}
- **Common reported functions (%):** 0.0
- **Common reported locations (%):** 11.11
- **Common reported processes (%):** 0.0

- **PDB ID:** 8EOJ
- **Chain:** B
- **Crystallized protein length:** 781 aa
- **Resolution:** 3.07 Å
- **b-phipsi:** 0.004262
- **w-rdist:** 0.455454
- **t-alpha:** 0.021862
- **Chemical similarity (Tanimoto Index) (%):** 97.04
- **1D identity (%) [PDB]:** 3.28
- **1D identity (%) [Gaps excluded][PDB]:** 68.06
- **1D identity - Alignment Gaps [PDB]:** 1422
- **1D aligned content [PDB] (<aminoacid>:%):** {'D': 6.12, 'I': 6.12, 'S': 10.2, 'N': 2.04, 'V': 12.24, 'L': 6.12, 'R': 6.12, 'T': 2.04, 'G': 4.08, 'F': 6.12, 'A': 8.16, 'P': 4.08, 'E': 2.04, 'Y': 4.08, 'H': 2.04, 'Q': 6.12, 'M': 6.12, 'K': 4.08, 'C': 2.04}
- **2D identity (%) [PDB]:** 38.08
- **2D identity (%) [Gaps excluded][PDB]:** 85.12
- **2D identity - Alignment Gaps [PDB]:** 598
- **2D aligned content [PDB] (<2D-fold>:%):** {'.': 12.62, 'E': 27.18, 'T': 27.67, 'H': 32.52}
- **3D similarity (TM-Score) (%) [PDB]:** 24.87

- **Gene name:** MTTP
- **Entrez ID:** 4547
- **RefSeq ID:** N/A
- **Sequence length:** N/A
- **5-UTR|CDS|3-UTR identity (%):** N/A | N/A | N/A
- **5-UTR|CDS|3-UTR identity (%) [Gaps excluded]:** N/A | N/A | N/A
- **5-UTR|CDS|3-UTR identity [Alignment Gaps]:** N/A | N/A | N/A
- **5-UTR aligned content (<base>:%):** N/A
- **CDS aligned content (<base>:%):** N/A
- **3-UTR aligned content (<base>:%):** N/A

**Uniprot Description:**  
  
 Catalyzes the transport of triglyceride, cholesteryl ester, and phospholipid between phospholipid surfaces (PubMed:23475612, PubMed:8939939, PubMed:26224785, PubMed:25108285, PubMed:22236406, PubMed:16478722, PubMed:15897609, PubMed:8876250). Required for the assembly and secretion of plasma lipoproteins that contain apolipoprotein B (PubMed:23475612, PubMed:8939939, PubMed:26224785, PubMed:8876250, PubMed:16478722). May be involved in regulating cholesteryl ester biosynthesis in cells that produce lipoproteins (By similarity).   
  
Heterodimer; heterodimerizes with the protein disulfide isomerase (P4HB/PDI) (PubMed:23475612, PubMed:26224785, PubMed:25108285, PubMed:16478722). Interacts with APOB (PubMed:26224785, PubMed:25108285, PubMed:27206948). Interacts with PRAP1 (By similarity).   
  
 **Gene Ontology Information:**

Molecular Function

- apolipoprotein binding
- ceramide 1-phosphate transfer activity
- cholesterol transfer activity
- lipid binding
- lipid transporter activity
- phosphatidylcholine transfer activity
- phosphatidylethanolamine transfer activity
- phospholipid transfer activity
- phospholipid transporter activity
- protein heterodimerization activity
- protein-containing complex binding
- triglyceride transfer activity

Location

- basolateral plasma membrane
- brush border membrane
- cytosol
- endoplasmic reticulum
- endoplasmic reticulum lumen
- Golgi apparatus
- microvillus membrane
- receptor complex
- vesicle

Biological process

- cholesterol homeostasis
- chylomicron assembly
- circadian rhythm
- establishment of localization in cell
- lipid metabolic process
- lipoprotein metabolic process
- lipoprotein transport
- low-density lipoprotein particle remodeling
- phospholipid transport
- plasma lipoprotein particle assembly
- protein lipidation
- protein secretion
- response to calcium ion
- triglyceride metabolic process
- triglyceride transport
- very-low-density lipoprotein particle assembly

---

75

- **Protein name:** Transforming growth factor-beta-induced protein ig-h3
- **Organism:** Homo sapiens
- **Uniprot Accession Number:** Q15582
- **Protein sequence length:** 683 aa
- **1D identity (%):** 13.29
- **1D identity (%) [Gaps excluded]:** 22.07
- **1D identity - Alignment Gaps:** 383
- **1D aligned content (<aminoacid>:%):** {'V': 7.03, 'R': 9.38, 'L': 10.94, 'A': 9.38, 'T': 7.81, 'Q': 3.91, 'P': 7.03, 'F': 4.69, 'K': 4.69, 'C': 2.34, 'G': 5.47, 'Y': 3.12, 'E': 4.69, 'N': 2.34, 'H': 2.34, 'I': 6.25, 'D': 5.47, 'S': 2.34, 'M': 0.78}
- **Common reported functions (%):** 0.0
- **Common reported locations (%):** 0.0
- **Common reported processes (%):** 0.0

- **PDB ID:** 7AS7
- **Chain:** A
- **Crystallized protein length:** 584 aa
- **Resolution:** 2.65 Å
- **b-phipsi:** 0.001312
- **w-rdist:** 0.857136
- **t-alpha:** 0.008793
- **Chemical similarity (Tanimoto Index) (%):** N/A
- **1D identity (%) [PDB]:** 2.47
- **1D identity (%) [Gaps excluded][PDB]:** 60.71
- **1D identity - Alignment Gaps [PDB]:** 1318
- **1D aligned content [PDB] (<aminoacid>:%):** {'P': 11.76, 'G': 8.82, 'E': 8.82, 'K': 2.94, 'R': 2.94, 'V': 23.53, 'S': 5.88, 'L': 20.59, 'T': 8.82, 'N': 2.94, 'H': 2.94}
- **2D identity (%) [PDB]:** 35.2
- **2D identity (%) [Gaps excluded][PDB]:** 87.56
- **2D identity - Alignment Gaps [PDB]:** 610
- **2D aligned content [PDB] (<2D-fold>:%):** {'.': 15.04, 'E': 35.38, 'T': 11.42, 'H': 37.05, 'B': 0.28, 'G': 0.84}
- **3D similarity (TM-Score) (%) [PDB]:** 16.69

- **Gene name:** TGFBI
- **Entrez ID:** 7045
- **RefSeq ID:** NM\_000358
- **Transcript sequence length:** 2712
- **5-UTR|CDS|3-UTR identity (%):** 14.11 | 45.22 | 2.24
- **5-UTR|CDS|3-UTR identity (%) [Gaps excluded]:** 71.88 | 74.61 | 74.65
- **5-UTR|CDS|3-UTR identity [Alignment Gaps]:** 262 | 1137 | 16337
- **5-UTR aligned content (<base>:%):** {'T': 17.39, 'C': 58.7, 'G': 23.91}
- **CDS aligned content (<base>:%):** {'A': 28.05, 'T': 21.76, 'G': 24.37, 'C': 25.82}
- **3-UTR aligned content (<base>:%):** {'C': 21.22, 'T': 24.93, 'G': 21.75, 'A': 32.1}

**Uniprot Description:**  
  
 Plays a role in cell adhesion (PubMed:8024701). May play a role in cell-collagen interactions (By similarity).   
  
Binds to type I, II, and IV collagens.   
  
 **Gene Ontology Information:**

Molecular Function

- cell adhesion molecule binding
- collagen binding
- extracellular matrix binding
- extracellular matrix structural constituent
- identical protein binding
- integrin binding

Location

- basement membrane
- collagen-containing extracellular matrix
- extracellular exosome
- extracellular matrix
- extracellular region
- extracellular space
- plasma membrane
- trans-Golgi network

Biological process

- angiogenesis
- cell adhesion
- cell population proliferation
- chondrocyte differentiation
- extracellular matrix organization
- localization
- negative regulation of cell adhesion
- response to stimulus
- visual perception

---

76

- **Protein name:** DNA ligase 1
- **Organism:** Homo sapiens
- **Uniprot Accession Number:** P18858
- **Protein sequence length:** 919 aa
- **1D identity (%):** 17.04
- **1D identity (%) [Gaps excluded]:** 22.64
- **1D identity - Alignment Gaps:** 251
- **1D aligned content (<aminoacid>:%):** {'Q': 2.89, 'M': 1.16, 'P': 16.76, 'G': 12.72, 'K': 6.36, 'N': 0.58, 'L': 7.51, 'E': 6.36, 'R': 9.83, 'V': 2.89, 'S': 3.47, 'D': 5.2, 'T': 6.36, 'F': 3.47, 'A': 6.36, 'H': 1.73, 'C': 1.73, 'I': 2.89, 'Y': 1.16, 'W': 0.58}
- **Common reported functions (%):** 14.29
- **Common reported locations (%):** 11.11
- **Common reported processes (%):** 0.0

- **PDB ID:** 7L34
- **Chain:** A
- **Crystallized protein length:** 637 aa
- **Resolution:** 1.9 Å
- **b-phipsi:** 0.003334
- **w-rdist:** 0.789276
- **t-alpha:** 0.004777
- **Chemical similarity (Tanimoto Index) (%):** 99.17
- **1D identity (%) [PDB]:** 2.66
- **1D identity (%) [Gaps excluded][PDB]:** 73.08
- **1D identity - Alignment Gaps [PDB]:** 1379
- **1D aligned content [PDB] (<aminoacid>:%):** {'N': 2.63, 'P': 7.89, 'L': 18.42, 'V': 10.53, 'A': 2.63, 'T': 10.53, 'G': 10.53, 'D': 5.26, 'E': 10.53, 'K': 7.89, 'I': 5.26, 'S': 7.89}
- **2D identity (%) [PDB]:** 32.62
- **2D identity (%) [Gaps excluded][PDB]:** 88.03
- **2D identity - Alignment Gaps [PDB]:** 681
- **2D aligned content [PDB] (<2D-fold>:%):** {'.': 17.56, 'T': 17.0, 'H': 48.44, 'E': 15.58, 'G': 1.42}
- **3D similarity (TM-Score) (%) [PDB]:** 24.26

- **Gene name:** LIG1
- **Entrez ID:** 3978
- **RefSeq ID:** NM\_001320971
- **Transcript sequence length:** 3035
- **5-UTR|CDS|3-UTR identity (%):** 26.04 | 44.45 | 0.82
- **5-UTR|CDS|3-UTR identity (%) [Gaps excluded]:** 77.05 | 73.24 | 76.11
- **5-UTR|CDS|3-UTR identity [Alignment Gaps]:** 239 | 1285 | 16599
- **5-UTR aligned content (<base>:%):** {'A': 11.7, 'G': 38.3, 'C': 35.11, 'T': 14.89}
- **CDS aligned content (<base>:%):** {'A': 26.91, 'T': 18.51, 'G': 28.49, 'C': 26.08}
- **3-UTR aligned content (<base>:%):** {'G': 24.82, 'C': 18.98, 'T': 35.77, 'A': 20.44}

**Uniprot Description:**  
  
 DNA ligase that seals nicks in double-stranded DNA during DNA replication, DNA recombination and DNA repair.   
  
Interacts with PCNA.   
  
 **Gene Ontology Information:**

Molecular Function

- ATP binding
- DNA binding
- DNA ligase (ATP) activity
- DNA ligase activity
- metal ion binding

Location

- intracellular membrane-bounded organelle
- mitochondrion
- nucleoplasm
- nucleus

Biological process

- anatomical structure morphogenesis
- base-excision repair
- base-excision repair, gap-filling
- cell division
- DNA biosynthetic process
- DNA ligation
- DNA recombination
- DNA repair
- lagging strand elongation
- mismatch repair
- Okazaki fragment processing involved in mitotic DNA replication

---

77

- **Protein name:** Dipeptidyl peptidase 9
- **Organism:** Homo sapiens
- **Uniprot Accession Number:** Q86TI2
- **Protein sequence length:** 863 aa
- **1D identity (%):** 16.37
- **1D identity (%) [Gaps excluded]:** 23.08
- **1D identity - Alignment Gaps:** 293
- **1D aligned content (<aminoacid>:%):** {'P': 9.09, 'R': 8.48, 'D': 5.45, 'F': 6.06, 'V': 6.06, 'G': 10.91, 'S': 4.85, 'L': 9.09, 'E': 4.24, 'H': 6.06, 'A': 3.64, 'K': 3.03, 'N': 2.42, 'I': 1.82, 'T': 6.06, 'Y': 4.85, 'Q': 3.64, 'W': 0.61, 'C': 3.64}
- **Common reported functions (%):** 0.0
- **Common reported locations (%):** 11.11
- **Common reported processes (%):** 0.0

- **PDB ID:** 6EOQ
- **Chain:** C
- **Crystallized protein length:** 766 aa
- **Resolution:** 3.0 Å
- **b-phipsi:** 0.006109
- **w-rdist:** 0.549073
- **t-alpha:** 0.004777
- **Chemical similarity (Tanimoto Index) (%):** 99.55
- **1D identity (%) [PDB]:** 2.62
- **1D identity (%) [Gaps excluded][PDB]:** 71.93
- **1D identity - Alignment Gaps [PDB]:** 1508
- **1D aligned content [PDB] (<aminoacid>:%):** {'P': 9.76, 'D': 9.76, 'G': 9.76, 'R': 4.88, 'S': 4.88, 'L': 17.07, 'Y': 2.44, 'V': 12.2, 'A': 2.44, 'T': 2.44, 'E': 4.88, 'K': 7.32, 'F': 4.88, 'I': 2.44, 'H': 4.88}
- **2D identity (%) [PDB]:** 35.18
- **2D identity (%) [Gaps excluded][PDB]:** 82.47
- **2D identity - Alignment Gaps [PDB]:** 652
- **2D aligned content [PDB] (<2D-fold>:%):** {'.': 27.5, 'B': 0.5, 'E': 32.5, 'T': 14.5, 'H': 24.25, 'G': 0.75}
- **3D similarity (TM-Score) (%) [PDB]:** 26.08

- **Gene name:** DPP9
- **Entrez ID:** 91039
- **RefSeq ID:** N/A
- **Sequence length:** N/A
- **5-UTR|CDS|3-UTR identity (%):** N/A | N/A | N/A
- **5-UTR|CDS|3-UTR identity (%) [Gaps excluded]:** N/A | N/A | N/A
- **5-UTR|CDS|3-UTR identity [Alignment Gaps]:** N/A | N/A | N/A
- **5-UTR aligned content (<base>:%):** N/A
- **CDS aligned content (<base>:%):** N/A
- **3-UTR aligned content (<base>:%):** N/A

**Uniprot Description:**  
  
 Dipeptidyl peptidase that cleaves off N-terminal dipeptides from proteins having a Pro or Ala residue at position 2 (PubMed:12662155, PubMed:16475979, PubMed:19667070, PubMed:30291141, PubMed:29382749). Acts as an inhibitor of caspase-1-dependent monocyte and macrophage pyroptosis: inhibits pyroptosis by preventing activation of NLRP1 and CARD8 via an unknown mechanism (PubMed:27820798, PubMed:30291141, PubMed:29967349, PubMed:31525884, PubMed:32796818).   
  
Homodimer.   
  
 **Gene Ontology Information:**

Molecular Function

- aminopeptidase activity
- dipeptidyl-peptidase activity
- identical protein binding
- serine-type peptidase activity

Location

- cell leading edge
- cytosol
- microtubule
- nucleus

Biological process

- negative regulation of programmed cell death
- proteolysis
- pyroptosis

---

78

- **Protein name:** Insulin receptor
- **Organism:** Homo sapiens
- **Uniprot Accession Number:** P06213
- **Protein sequence length:** 1382 aa
- **1D identity (%):** 16.09
- **1D identity (%) [Gaps excluded]:** 27.96
- **1D identity - Alignment Gaps:** 604
- **1D aligned content (<aminoacid>:%):** {'M': 0.87, 'G': 10.04, 'A': 3.49, 'P': 10.48, 'L': 10.04, 'I': 3.93, 'N': 4.37, 'C': 1.75, 'E': 4.8, 'K': 3.93, 'V': 8.3, 'D': 5.24, 'R': 7.42, 'H': 3.93, 'T': 5.24, 'F': 3.49, 'Y': 3.06, 'S': 5.24, 'Q': 3.49, 'W': 0.87}
- **Common reported functions (%):** 0.0
- **Common reported locations (%):** 0.0
- **Common reported processes (%):** 0.0

- **PDB ID:** 6CEB
- **Chain:** B
- **Crystallized protein length:** 562 aa
- **Resolution:** 4.7 Å
- **b-phipsi:** 0.009087
- **w-rdist:** 0.397733
- **t-alpha:** 0.008717
- **Chemical similarity (Tanimoto Index) (%):** 79.52
- **1D identity (%) [PDB]:** 2.8
- **1D identity (%) [Gaps excluded][PDB]:** 71.7
- **1D identity - Alignment Gaps [PDB]:** 1305
- **1D aligned content [PDB] (<aminoacid>:%):** {'T': 7.89, 'R': 13.16, 'F': 5.26, 'K': 2.63, 'D': 5.26, 'V': 7.89, 'G': 5.26, 'Q': 10.53, 'L': 10.53, 'Y': 7.89, 'A': 5.26, 'I': 7.89, 'E': 5.26, 'S': 2.63, 'P': 2.63}
- **2D identity (%) [PDB]:** 33.13
- **2D identity (%) [Gaps excluded][PDB]:** 77.12
- **2D identity - Alignment Gaps [PDB]:** 563
- **2D aligned content [PDB] (<2D-fold>:%):** {'.': 39.76, 'E': 36.7, 'T': 13.76, 'H': 8.87, 'B': 0.92}
- **3D similarity (TM-Score) (%) [PDB]:** 17.64

- **Gene name:** INSR
- **Entrez ID:** N/A
- **RefSeq ID:** NM\_001079817
- **Transcript sequence length:** 9427
- **5-UTR|CDS|3-UTR identity (%):** 42.05 | 40.62 | 17.31
- **5-UTR|CDS|3-UTR identity (%) [Gaps excluded]:** 77.18 | 77.03 | 75.12
- **5-UTR|CDS|3-UTR identity [Alignment Gaps]:** 249 | 2072 | 13476
- **5-UTR aligned content (<base>:%):** {'A': 6.96, 'G': 36.09, 'T': 10.43, 'C': 46.52}
- **CDS aligned content (<base>:%):** {'A': 27.68, 'T': 23.19, 'G': 25.04, 'C': 24.09}
- **3-UTR aligned content (<base>:%):** {'C': 16.2, 'G': 19.1, 'T': 34.58, 'A': 30.12}

**Uniprot Description:**  
  
 Receptor tyrosine kinase which mediates the pleiotropic actions of insulin. Binding of insulin leads to phosphorylation of several intracellular substrates, including, insulin receptor substrates (IRS1, 2, 3, 4), SHC, GAB1, CBL and other signaling intermediates. Each of these phosphorylated proteins serve as docking proteins for other signaling proteins that contain Src-homology-2 domains (SH2 domain) that specifically recognize different phosphotyrosine residues, including the p85 regulatory subunit of PI3K and SHP2. Phosphorylation of IRSs proteins lead to the activation of two main signaling pathways: the PI3K-AKT/PKB pathway, which is responsible for most of the metabolic actions of insulin, and the Ras-MAPK pathway, which regulates expression of some genes and cooperates with the PI3K pathway to control cell growth and differentiation. Binding of the SH2 domains of PI3K to phosphotyrosines on IRS1 leads to the activation of PI3K and the generation of phosphatidylinositol-(3, 4, 5)-triphosphate (PIP3), a lipid second messenger, which activates several PIP3-dependent serine/threonine kinases, such as PDPK1 and subsequently AKT/PKB. The net effect of this pathway is to produce a translocation of the glucose transporter SLC2A4/GLUT4 from cytoplasmic vesicles to the cell membrane to facilitate glucose transport. Moreover, upon insulin stimulation, activated AKT/PKB is responsible for: anti-apoptotic effect of insulin by inducing phosphorylation of BAD; regulates the expression of gluconeogenic and lipogenic enzymes by controlling the activity of the winged helix or forkhead (FOX) class of transcription factors. Another pathway regulated by PI3K-AKT/PKB activation is mTORC1 signaling pathway which regulates cell growth and metabolism and integrates signals from insulin. AKT mediates insulin-stimulated protein synthesis by phosphorylating TSC2 thereby activating mTORC1 pathway. The Ras/RAF/MAP2K/MAPK pathway is mainly involved in mediating cell growth, survival and cellular differentiation of insulin. Phosphorylated IRS1 recruits GRB2/SOS complex, which triggers the activation of the Ras/RAF/MAP2K/MAPK pathway. In addition to binding insulin, the insulin receptor can bind insulin-like growth factors (IGFI and IGFII). Isoform Short has a higher affinity for IGFII binding. When present in a hybrid receptor with IGF1R, binds IGF1. PubMed:12138094 shows that hybrid receptors composed of IGF1R and INSR isoform Long are activated with a high affinity by IGF1, with low affinity by IGF2 and not significantly activated by insulin, and that hybrid receptors composed of IGF1R and INSR isoform Short are activated by IGF1, IGF2 and insulin. In contrast, PubMed:16831875 shows that hybrid receptors composed of IGF1R and INSR isoform Long and hybrid receptors composed of IGF1R and INSR isoform Short have similar binding characteristics, both bind IGF1 and have a low affinity for insulin. In adipocytes, inhibits lipolysis (By similarity).   
  
Tetramer of 2 alpha and 2 beta chains linked by disulfide bonds. The alpha chains carry the insulin-binding regions, while the beta chains carry the kinase domain. Forms a hybrid receptor with IGF1R, the hybrid is a tetramer consisting of 1 alpha chain and 1 beta chain of INSR and 1 alpha chain and 1 beta chain of IGF1R. Interacts with SORBS1 but dissociates from it following insulin stimulation. Binds SH2B2. Activated form of INSR interacts (via Tyr-999) with the PTB/PID domains of IRS1 and SHC1. The sequences surrounding the phosphorylated NPXY motif contribute differentially to either IRS1 or SHC1 recognition. Interacts (via tyrosines in the C-terminus) with IRS2 (via PTB domain and 591-786 AA); the 591-786 would be the primary anchor of IRS2 to INSR while the PTB domain would have a stabilizing action on the interaction with INSR. Interacts with the SH2 domains of the 85 kDa regulatory subunit of PI3K (PIK3R1) in vitro, when autophosphorylated on tyrosine residues. Interacts with SOCS7. Interacts (via the phosphorylated Tyr-999), with SOCS3. Interacts (via the phosphorylated Tyr-1185, Tyr-1189, Tyr-1190) with SOCS1. Interacts with CAV2 (tyrosine-phosphorylated form); the interaction is increased with 'Tyr-27'phosphorylation of CAV2 (By similarity). Interacts with ARRB2 (By similarity). Interacts with GRB10; this interaction blocks the association between IRS1/IRS2 and INSR, significantly reduces insulin-stimulated tyrosine phosphorylation of IRS1 and IRS2 and thus decreases insulin signaling. Interacts with GRB7. Interacts with PDPK1. Interacts (via Tyr-1190) with GRB14 (via BPS domain); this interaction protects the tyrosines in the activation loop from dephosphorylation, but promotes dephosphorylation of Tyr-999, this results in decreased interaction with, and phosphorylation of, IRS1. Interacts (via subunit alpha) with ENPP1 (via 485-599 AA); this interaction blocks autophosphorylation. Interacts with PTPRE; this interaction is dependent of Tyr-1185, Tyr-1189 and Tyr-1190 of the INSR. Interacts with STAT5B (via SH2 domain). Interacts with PTPRF. Interacts with ATIC; ATIC together with PRKAA2/AMPK2 and HACD3/PTPLAD1 is proposed to be part of a signaling netwok regulating INSR autophosphorylation and endocytosis (By similarity). Interacts with the cone snail venom insulin Con-Ins G1 (PubMed:27617429). Interacts with the insulin receptor SORL1; this interaction strongly increases its surface exposure, hence strengthens insulin signal reception (PubMed:27322061). Interacts (tyrosine phosphorylated) with CCDC88A/GIV (via SH2-like region); binding requires autophosphorylation of the INSR C-terminal region (PubMed:25187647). Interacts with GNAI3; the interaction is probably mediated by CCDC88A/GIV (PubMed:25187647). Interacts with LMBRD1 (By similarity).   
  
 **Gene Ontology Information:**

Molecular Function   
  
N/A

Location   
  
N/A

Biological process   
  
N/A

---

79

- **Protein name:** Complement C5
- **Organism:** Homo sapiens
- **Uniprot Accession Number:** P01031
- **Protein sequence length:** 1676 aa
- **1D identity (%):** 14.57
- **1D identity (%) [Gaps excluded]:** 29.74
- **1D identity - Alignment Gaps:** 868
- **1D aligned content (<aminoacid>:%):** {'G': 10.08, 'P': 7.66, 'R': 6.05, 'Y': 5.65, 'K': 6.85, 'N': 2.42, 'F': 4.03, 'Q': 4.44, 'V': 7.26, 'D': 6.05, 'E': 5.24, 'H': 2.02, 'I': 6.05, 'T': 4.44, 'A': 4.03, 'L': 9.68, 'S': 5.65, 'C': 2.02, 'M': 0.4}
- **Common reported functions (%):** 0.0
- **Common reported locations (%):** 0.0
- **Common reported processes (%):** 0.0

- **PDB ID:** 7AD7
- **Chain:** A
- **Crystallized protein length:** 782 aa
- **Resolution:** 2.3 Å
- **b-phipsi:** 0.007379
- **w-rdist:** 0.367445
- **t-alpha:** 0.013471
- **Chemical similarity (Tanimoto Index) (%):** N/A
- **1D identity (%) [PDB]:** 3.19
- **1D identity (%) [Gaps excluded][PDB]:** 75.76
- **1D identity - Alignment Gaps [PDB]:** 1499
- **1D aligned content [PDB] (<aminoacid>:%):** {'I': 12.0, 'R': 6.0, 'A': 8.0, 'S': 10.0, 'L': 4.0, 'E': 4.0, 'D': 8.0, 'Y': 6.0, 'P': 6.0, 'G': 6.0, 'T': 12.0, 'V': 6.0, 'N': 2.0, 'H': 4.0, 'F': 4.0, 'Q': 2.0}
- **2D identity (%) [PDB]:** 30.39
- **2D identity (%) [Gaps excluded][PDB]:** 87.62
- **2D identity - Alignment Gaps [PDB]:** 791
- **2D aligned content [PDB] (<2D-fold>:%):** {'.': 17.93, 'H': 44.84, 'T': 13.86, 'E': 23.37}
- **3D similarity (TM-Score) (%) [PDB]:** 23.64

- **Gene name:** C5
- **Entrez ID:** 727
- **RefSeq ID:** NM\_001735
- **Transcript sequence length:** 5464
- **5-UTR|CDS|3-UTR identity (%):** 5.26 | 36.87 | 1.48
- **5-UTR|CDS|3-UTR identity (%) [Gaps excluded]:** 60.71 | 79.52 | 74.33
- **5-UTR|CDS|3-UTR identity [Alignment Gaps]:** 295 | 2790 | 16489
- **5-UTR aligned content (<base>:%):** {'A': 17.65, 'C': 41.18, 'G': 17.65, 'T': 23.53}
- **CDS aligned content (<base>:%):** {'A': 31.54, 'T': 26.02, 'G': 22.11, 'C': 20.33}
- **3-UTR aligned content (<base>:%):** {'A': 30.12, 'T': 30.12, 'C': 17.67, 'G': 22.09}

**Uniprot Description:**  
  
 Activation of C5 by a C5 convertase initiates the spontaneous assembly of the late complement components, C5-C9, into the membrane attack complex. C5b has a transient binding site for C6. The C5b-C6 complex is the foundation upon which the lytic complex is assembled.   
  
C5 precursor is first processed by the removal of 4 basic residues, forming two chains, beta and alpha, linked by a disulfide bond. C5 convertase activates C5 by cleaving the alpha chain, releasing C5a anaphylatoxin and generating C5b (beta chain + alpha' chain). The C5a anaphylatoxin interacts with C5AR1. Interacts with tick complement inhibitor.   
  
 **Gene Ontology Information:**

Molecular Function

- chemokine activity
- endopeptidase inhibitor activity
- signaling receptor binding

Location

- extracellular exosome
- extracellular region
- extracellular space
- membrane attack complex

Biological process

- cell surface receptor signaling pathway
- chemotaxis
- complement activation
- complement activation, alternative pathway
- complement activation, classical pathway
- G protein-coupled receptor signaling pathway
- inflammatory response
- killing of cells of other organism
- negative regulation of macrophage chemotaxis
- positive regulation of chemokine production
- positive regulation of immune response
- positive regulation of vascular endothelial growth factor production

---

80

- **Protein name:** Endoplasmic reticulum aminopeptidase 1
- **Organism:** Homo sapiens
- **Uniprot Accession Number:** Q9NZ08
- **Protein sequence length:** 941 aa
- **1D identity (%):** 17.61
- **1D identity (%) [Gaps excluded]:** 24.02
- **1D identity - Alignment Gaps:** 277
- **1D aligned content (<aminoacid>:%):** {'P': 9.29, 'T': 10.38, 'M': 1.09, 'L': 13.11, 'V': 8.2, 'D': 4.37, 'N': 3.83, 'Y': 4.92, 'A': 4.92, 'S': 5.46, 'H': 3.28, 'E': 2.19, 'G': 7.65, 'F': 4.37, 'I': 4.37, 'R': 2.73, 'K': 5.46, 'Q': 2.73, 'C': 1.09, 'W': 0.55}
- **Common reported functions (%):** 0.0
- **Common reported locations (%):** 33.33
- **Common reported processes (%):** 0.0

- **PDB ID:** 3MDJ
- **Chain:** B
- **Crystallized protein length:** 821 aa
- **Resolution:** 2.95 Å
- **b-phipsi:** 0.006353
- **w-rdist:** 0.252218
- **t-alpha:** 0.038827
- **Chemical similarity (Tanimoto Index) (%):** 69.93
- **1D identity (%) [PDB]:** 2.29
- **1D identity (%) [Gaps excluded][PDB]:** 68.52
- **1D identity - Alignment Gaps [PDB]:** 1563
- **1D aligned content [PDB] (<aminoacid>:%):** {'P': 5.41, 'S': 8.11, 'R': 16.22, 'A': 5.41, 'T': 5.41, 'Q': 8.11, 'E': 8.11, 'I': 8.11, 'D': 5.41, 'L': 5.41, 'M': 2.7, 'V': 5.41, 'Y': 2.7, 'K': 5.41, 'F': 5.41, 'G': 2.7}
- **2D identity (%) [PDB]:** 33.92
- **2D identity (%) [Gaps excluded][PDB]:** 85.65
- **2D identity - Alignment Gaps [PDB]:** 723
- **2D aligned content [PDB] (<2D-fold>:%):** {'.': 14.53, 'E': 24.38, 'T': 16.01, 'H': 45.07}
- **3D similarity (TM-Score) (%) [PDB]:** 25.79

- **Gene name:** ERAP1
- **Entrez ID:** 51752
- **RefSeq ID:** N/A
- **Sequence length:** N/A
- **5-UTR|CDS|3-UTR identity (%):** N/A | N/A | N/A
- **5-UTR|CDS|3-UTR identity (%) [Gaps excluded]:** N/A | N/A | N/A
- **5-UTR|CDS|3-UTR identity [Alignment Gaps]:** N/A | N/A | N/A
- **5-UTR aligned content (<base>:%):** N/A
- **CDS aligned content (<base>:%):** N/A
- **3-UTR aligned content (<base>:%):** N/A

**Uniprot Description:**  
  
 Aminopeptidase that plays a central role in peptide trimming, a step required for the generation of most HLA class I-binding peptides. Peptide trimming is essential to customize longer precursor peptides to fit them to the correct length required for presentation on MHC class I molecules. Strongly prefers substrates 9-16 residues long. Rapidly degrades 13-mer to a 9-mer and then stops. Preferentially hydrolyzes the residue Leu and peptides with a hydrophobic C-terminus, while it has weak activity toward peptides with charged C-terminus. May play a role in the inactivation of peptide hormones. May be involved in the regulation of blood pressure through the inactivation of angiotensin II and/or the generation of bradykinin in the kidney.   
  
Monomer. May also exist as a heterodimer; with ERAP2. Interacts with RBMX.   
  
 **Gene Ontology Information:**

Molecular Function

- aminopeptidase activity
- endopeptidase activity
- interleukin-1, type II receptor binding
- interleukin-6 receptor binding
- metalloaminopeptidase activity
- metalloexopeptidase activity
- peptide binding
- zinc ion binding

Location

- cytoplasm
- cytosol
- endoplasmic reticulum
- endoplasmic reticulum lumen
- endoplasmic reticulum membrane
- extracellular exosome
- extracellular region
- extracellular space
- membrane

Biological process

- adaptive immune response
- angiogenesis
- antigen processing and presentation of endogenous peptide antigen via MHC class I
- antigen processing and presentation of peptide antigen via MHC class I
- fat cell differentiation
- membrane protein ectodomain proteolysis
- peptide catabolic process
- positive regulation of angiogenesis
- proteolysis
- regulation of blood pressure
- regulation of innate immune response
- response to bacterium

---

81

- **Protein name:** Toll-like receptor 8
- **Organism:** Homo sapiens
- **Uniprot Accession Number:** Q9NR97
- **Protein sequence length:** 1041 aa
- **1D identity (%):** 14.52
- **1D identity (%) [Gaps excluded]:** 21.9
- **1D identity - Alignment Gaps:** 385
- **1D aligned content (<aminoacid>:%):** {'M': 1.2, 'E': 6.02, 'G': 7.83, 'S': 6.02, 'R': 5.42, 'A': 3.01, 'C': 2.41, 'P': 6.63, 'Y': 3.61, 'D': 6.02, 'V': 1.81, 'Q': 5.42, 'H': 3.01, 'I': 4.82, 'L': 14.46, 'N': 5.42, 'T': 6.02, 'W': 1.2, 'F': 4.82, 'K': 4.82}
- **Common reported functions (%):** 0.0
- **Common reported locations (%):** 0.0
- **Common reported processes (%):** 0.0

- **PDB ID:** 8PFI
- **Chain:** A
- **Crystallized protein length:** 733 aa
- **Resolution:** 2.79 Å
- **b-phipsi:** 0.00423
- **w-rdist:** 0.315249
- **t-alpha:** 0.131537
- **Chemical similarity (Tanimoto Index) (%):** 76.7
- **1D identity (%) [PDB]:** 2.62
- **1D identity (%) [Gaps excluded][PDB]:** 75.47
- **1D identity - Alignment Gaps [PDB]:** 1472
- **1D aligned content [PDB] (<aminoacid>:%):** {'S': 5.0, 'P': 7.5, 'Q': 7.5, 'T': 5.0, 'L': 12.5, 'N': 10.0, 'K': 7.5, 'I': 10.0, 'G': 10.0, 'V': 7.5, 'H': 5.0, 'R': 2.5, 'F': 2.5, 'A': 5.0, 'D': 2.5}
- **2D identity (%) [PDB]:** N/A
- **2D identity (%) [Gaps excluded][PDB]:** N/A
- **2D identity - Alignment Gaps [PDB]:** N/A
- **2D aligned content [PDB] (<2D-fold>:%):** N/A
- **3D similarity (TM-Score) (%) [PDB]:** 22.86

- **Gene name:** TLR8
- **Entrez ID:** N/A
- **RefSeq ID:** N/A
- **Sequence length:** N/A
- **5-UTR|CDS|3-UTR identity (%):** N/A | N/A | N/A
- **5-UTR|CDS|3-UTR identity (%) [Gaps excluded]:** N/A | N/A | N/A
- **5-UTR|CDS|3-UTR identity [Alignment Gaps]:** N/A | N/A | N/A
- **5-UTR aligned content (<base>:%):** N/A
- **CDS aligned content (<base>:%):** N/A
- **3-UTR aligned content (<base>:%):** N/A

**Uniprot Description:**  
  
 Endosomal receptor that plays a key role in innate and adaptive immunity (PubMed:25297876, PubMed:32433612). Controls host immune response against pathogens through recognition of RNA degradation products specific to microorganisms that are initially processed by RNASET2 (PubMed:31778653). Upon binding to agonists, undergoes dimerization that brings TIR domains from the two molecules into direct contact, leading to the recruitment of TIR-containing downstream adapter MYD88 through homotypic interaction (PubMed:23520111, PubMed:25599397, PubMed:26929371). In turn, the Myddosome signaling complex is formed involving IRAK4, IRAK1, TRAF6, TRAF3 leading to activation of downstream transcription factors NF-kappa-B and IRF7 to induce proinflammatory cytokines and interferons, respectively (PubMed:16737960, PubMed:17932028, PubMed:29155428).   
  
Homodimer (PubMed:23520111, PubMed:25599397, PubMed:26929371, PubMed:29155428). Interacts with MYD88 via their respective TIR domains (Probable). Interacts with UNC93B1 (By similarity). Interacts with BTK (PubMed:17932028). Interacts with SMPDL3B (By similarity).   
  
 **Gene Ontology Information:**

Molecular Function   
  
N/A

Location   
  
N/A

Biological process   
  
N/A

---

82

- **Protein name:** Xylosyltransferase 1
- **Organism:** Homo sapiens
- **Uniprot Accession Number:** Q86Y38
- **Protein sequence length:** 959 aa
- **1D identity (%):** 14.12
- **1D identity (%) [Gaps excluded]:** 22.21
- **1D identity - Alignment Gaps:** 405
- **1D aligned content (<aminoacid>:%):** {'C': 2.55, 'R': 12.74, 'V': 6.37, 'G': 11.46, 'P': 12.1, 'A': 3.18, 'E': 4.46, 'H': 3.18, 'S': 4.46, 'N': 2.55, 'K': 4.46, 'D': 5.1, 'I': 4.46, 'L': 5.1, 'T': 5.1, 'F': 3.82, 'Y': 3.18, 'Q': 3.82, 'M': 1.27, 'W': 0.64}
- **Common reported functions (%):** 14.29
- **Common reported locations (%):** 0.0
- **Common reported processes (%):** 0.0

- **PDB ID:** 6EJC
- **Chain:** A
- **Crystallized protein length:** 701 aa
- **Resolution:** 2.06 Å
- **b-phipsi:** 0.002513
- **w-rdist:** 0.628424
- **t-alpha:** 0.01664
- **Chemical similarity (Tanimoto Index) (%):** 90.53
- **1D identity (%) [PDB]:** 2.33
- **1D identity (%) [Gaps excluded][PDB]:** 71.43
- **1D identity - Alignment Gaps [PDB]:** 1450
- **1D aligned content [PDB] (<aminoacid>:%):** {'P': 14.29, 'K': 5.71, 'V': 17.14, 'I': 2.86, 'F': 2.86, 'S': 8.57, 'R': 8.57, 'W': 2.86, 'L': 20.0, 'H': 2.86, 'T': 5.71, 'N': 2.86, 'A': 2.86, 'D': 2.86}
- **2D identity (%) [PDB]:** 37.25
- **2D identity (%) [Gaps excluded][PDB]:** 81.65
- **2D identity - Alignment Gaps [PDB]:** 578
- **2D aligned content [PDB] (<2D-fold>:%):** {'.': 22.22, 'H': 35.86, 'T': 16.92, 'E': 25.0}
- **3D similarity (TM-Score) (%) [PDB]:** 25.26

- **Gene name:** XYLT1
- **Entrez ID:** 64131
- **RefSeq ID:** NM\_022166
- **Transcript sequence length:** 9970
- **5-UTR|CDS|3-UTR identity (%):** 33.93 | 44.84 | 21.92
- **5-UTR|CDS|3-UTR identity (%) [Gaps excluded]:** 73.86 | 74.48 | 74.44
- **5-UTR|CDS|3-UTR identity [Alignment Gaps]:** 180 | 1357 | 12907
- **5-UTR aligned content (<base>:%):** {'A': 7.08, 'C': 41.59, 'T': 7.96, 'G': 43.36}
- **CDS aligned content (<base>:%):** {'A': 25.57, 'T': 19.29, 'G': 28.58, 'C': 26.55}
- **3-UTR aligned content (<base>:%):** {'A': 27.53, 'C': 20.07, 'T': 30.8, 'G': 21.6}

**Uniprot Description:**  
  
 Catalyzes the first step in the biosynthesis of chondroitin sulfate and dermatan sulfate proteoglycans, such as DCN. Transfers D-xylose from UDP-D-xylose to specific serine residues of the core protein (PubMed:15461586, PubMed:17189265, PubMed:24581741, PubMed:23982343). Required for normal embryonic and postnatal skeleton development, especially of the long bones (PubMed:24581741, PubMed:23982343). Required for normal maturation of chondrocytes during bone development, and normal onset of ossification (By similarity).   
  
Monomer.   
  
 **Gene Ontology Information:**

Molecular Function

- metal ion binding
- protein xylosyltransferase activity

Location

- extracellular space
- Golgi cis cisterna
- Golgi membrane

Biological process

- chondroitin sulfate biosynthetic process
- chondroitin sulfate proteoglycan biosynthetic process
- embryonic skeletal system development
- glycosaminoglycan biosynthetic process
- glycosaminoglycan metabolic process
- heparan sulfate proteoglycan biosynthetic process
- ossification involved in bone maturation
- proteoglycan biosynthetic process

---

83

- **Protein name:** Receptor tyrosine-protein kinase erbB-3
- **Organism:** Homo sapiens
- **Uniprot Accession Number:** P21860
- **Protein sequence length:** 1342 aa
- **1D identity (%):** 15.58
- **1D identity (%) [Gaps excluded]:** 26.47
- **1D identity - Alignment Gaps:** 570
- **1D aligned content (<aminoacid>:%):** {'M': 0.93, 'A': 3.7, 'G': 14.81, 'K': 4.63, 'L': 9.26, 'C': 3.7, 'V': 9.72, 'D': 4.17, 'R': 6.94, 'Y': 2.78, 'S': 4.63, 'H': 3.24, 'E': 3.24, 'P': 10.19, 'T': 7.41, 'F': 2.78, 'N': 3.24, 'Q': 3.24, 'I': 1.39}
- **Common reported functions (%):** 0.0
- **Common reported locations (%):** 0.0
- **Common reported processes (%):** 11.11

- **PDB ID:** 7MN8
- **Chain:** A
- **Crystallized protein length:** 599 aa
- **Resolution:** 3.45 Å
- **b-phipsi:** 0.002448
- **w-rdist:** 0.699442
- **t-alpha:** 0.012841
- **Chemical similarity (Tanimoto Index) (%):** 83.74
- **1D identity (%) [PDB]:** 2.23
- **1D identity (%) [Gaps excluded][PDB]:** 59.62
- **1D identity - Alignment Gaps [PDB]:** 1341
- **1D aligned content [PDB] (<aminoacid>:%):** {'L': 19.35, 'V': 12.9, 'T': 9.68, 'G': 6.45, 'P': 16.13, 'D': 6.45, 'S': 12.9, 'H': 3.23, 'R': 6.45, 'F': 3.23, 'E': 3.23}
- **2D identity (%) [PDB]:** 24.44
- **2D identity (%) [Gaps excluded][PDB]:** 80.65
- **2D identity - Alignment Gaps [PDB]:** 773
- **2D aligned content [PDB] (<2D-fold>:%):** {'.': 29.52, 'E': 38.75, 'H': 6.64, 'T': 23.99, 'B': 1.11}
- **3D similarity (TM-Score) (%) [PDB]:** 21.23

- **Gene name:** ERBB3
- **Entrez ID:** 2065
- **RefSeq ID:** NM\_001982
- **Transcript sequence length:** 5615
- **5-UTR|CDS|3-UTR identity (%):** 27.57 | 41.87 | 5.75
- **5-UTR|CDS|3-UTR identity (%) [Gaps excluded]:** 80.34 | 77.09 | 76.37
- **5-UTR|CDS|3-UTR identity [Alignment Gaps]:** 224 | 1958 | 15657
- **5-UTR aligned content (<base>:%):** {'G': 32.98, 'C': 42.55, 'T': 19.15, 'A': 5.32}
- **CDS aligned content (<base>:%):** {'A': 28.04, 'T': 23.13, 'G': 26.42, 'C': 22.41}
- **3-UTR aligned content (<base>:%):** {'C': 21.07, 'T': 28.88, 'G': 19.22, 'A': 30.83}

**Uniprot Description:**  
  
 Tyrosine-protein kinase that plays an essential role as cell surface receptor for neuregulins. Binds to neuregulin-1 (NRG1) and is activated by it; ligand-binding increases phosphorylation on tyrosine residues and promotes its association with the p85 subunit of phosphatidylinositol 3-kinase (PubMed:20682778). May also be activated by CSPG5 (PubMed:15358134). Involved in the regulation of myeloid cell differentiation (PubMed:27416908).   
  
Monomer and homodimer. Heterodimer with each of the other ERBB receptors (Potential). Interacts with CSPG5 (PubMed:15358134). Interacts with GRB7 (PubMed:9516479). Interacts with MUC1 (PubMed:12939402). Interacts with MYOC (By similarity). Interacts with isoform 2 of PA2G4 (PubMed:11325528, PubMed:16832058). Found in a ternary complex with NRG1 and ITGAV:ITGB3 or ITGA6:ITGB4 (PubMed:20682778).   
  
 **Gene Ontology Information:**

Molecular Function

- ATP binding
- ErbB-3 class receptor binding
- growth factor binding
- identical protein binding
- neuregulin binding
- neuregulin receptor activity
- protein heterodimerization activity
- protein kinase activity
- protein tyrosine kinase activator activity
- transmembrane signaling receptor activity
- ubiquitin protein ligase binding

Location

- apical plasma membrane
- basal plasma membrane
- basolateral plasma membrane
- ERBB3:ERBB2 complex
- extracellular space
- lateral plasma membrane
- plasma membrane
- receptor complex

Biological process

- cranial nerve development
- endocardial cushion development
- ERBB2-ERBB3 signaling pathway
- extrinsic apoptotic signaling pathway in absence of ligand
- heart development
- motor neuron apoptotic process
- negative regulation of apoptotic process
- negative regulation of cell adhesion
- negative regulation of motor neuron apoptotic process
- negative regulation of neuron apoptotic process
- negative regulation of secretion
- negative regulation of signal transduction
- neurogenesis
- neuron apoptotic process
- peripheral nervous system development
- phosphatidylinositol 3-kinase signaling
- positive regulation of calcineurin-NFAT signaling cascade
- positive regulation of cardiac muscle tissue development
- positive regulation of cell population proliferation
- positive regulation of gene expression
- positive regulation of kinase activity
- positive regulation of phosphatidylinositol 3-kinase signaling
- positive regulation of protein tyrosine kinase activity
- protein phosphorylation
- regulation of cell population proliferation
- Schwann cell differentiation
- signal transduction
- transmembrane receptor protein tyrosine kinase signaling pathway
- wound healing

---

84

- **Protein name:** Anthrax toxin receptor 2
- **Organism:** Homo sapiens
- **Uniprot Accession Number:** P58335
- **Protein sequence length:** 489 aa
- **1D identity (%):** 9.7
- **1D identity (%) [Gaps excluded]:** 21.38
- **1D identity - Alignment Gaps:** 507
- **1D aligned content (<aminoacid>:%):** {'R': 6.67, 'P': 8.89, 'W': 1.11, 'L': 12.22, 'A': 6.67, 'Q': 3.33, 'C': 3.33, 'D': 4.44, 'F': 4.44, 'E': 3.33, 'M': 1.11, 'V': 12.22, 'S': 4.44, 'T': 3.33, 'Y': 2.22, 'H': 1.11, 'I': 6.67, 'G': 7.78, 'N': 1.11, 'K': 5.56}
- **Common reported functions (%):** 14.29
- **Common reported locations (%):** 0.0
- **Common reported processes (%):** 0.0

- **PDB ID:** 1TZN
- **Chain:** O
- **Crystallized protein length:** 552 aa
- **Resolution:** 4.3 Å
- **b-phipsi:** 0.003963
- **w-rdist:** 0.811211
- **t-alpha:** 0.004754
- **Chemical similarity (Tanimoto Index) (%):** 75.3
- **1D identity (%) [PDB]:** 1.81
- **1D identity (%) [Gaps excluded][PDB]:** 66.67
- **1D identity - Alignment Gaps [PDB]:** 967
- **1D aligned content [PDB] (<aminoacid>:%):** {'V': 16.67, 'R': 11.11, 'A': 11.11, 'L': 5.56, 'D': 5.56, 'E': 11.11, 'H': 5.56, 'G': 11.11, 'S': 16.67, 'N': 5.56}
- **2D identity (%) [PDB]:** 14.27
- **2D identity (%) [Gaps excluded][PDB]:** 86.21
- **2D identity - Alignment Gaps [PDB]:** 731
- **2D aligned content [PDB] (<2D-fold>:%):** {'.': 16.0, 'T': 42.4, 'E': 29.6, 'H': 12.0}
- **3D similarity (TM-Score) (%) [PDB]:** 12.61

- **Gene name:** ANTXR2
- **Entrez ID:** 118429
- **RefSeq ID:** NM\_058172
- **Transcript sequence length:** 8419
- **5-UTR|CDS|3-UTR identity (%):** 36.75 | 32.43 | 22.71
- **5-UTR|CDS|3-UTR identity (%) [Gaps excluded]:** 81.49 | 75.31 | 76.15
- **5-UTR|CDS|3-UTR identity [Alignment Gaps]:** 375 | 1612 | 12454
- **5-UTR aligned content (<base>:%):** {'A': 8.76, 'G': 37.45, 'C': 38.25, 'T': 15.54}
- **CDS aligned content (<base>:%):** {'A': 28.98, 'T': 26.47, 'G': 24.62, 'C': 19.93}
- **3-UTR aligned content (<base>:%):** {'A': 33.75, 'G': 16.33, 'C': 14.04, 'T': 35.88}

**Uniprot Description:**  
  
 Necessary for cellular interactions with laminin and the extracellular matrix.   
  
Binds laminin, and possibly also collagen type IV.   
  
 **Gene Ontology Information:**

Molecular Function

- metal ion binding
- transmembrane signaling receptor activity

Location

- cell surface
- endoplasmic reticulum membrane
- endosome membrane
- extracellular region
- plasma membrane

Biological process

- toxin transport

---

85

- **Protein name:** Endoplasmic reticulum aminopeptidase 2
- **Organism:** Homo sapiens
- **Uniprot Accession Number:** Q6P179
- **Protein sequence length:** 960 aa
- **1D identity (%):** 17.49
- **1D identity (%) [Gaps excluded]:** 28.78
- **1D identity - Alignment Gaps:** 444
- **1D aligned content (<aminoacid>:%):** {'H': 6.06, 'R': 5.56, 'P': 7.07, 'Y': 5.05, 'T': 8.08, 'V': 5.05, 'A': 7.07, 'G': 8.59, 'L': 11.62, 'D': 5.05, 'K': 4.55, 'E': 4.55, 'F': 3.54, 'S': 6.06, 'Q': 3.54, 'C': 1.52, 'N': 1.52, 'W': 1.01, 'I': 3.54, 'M': 1.01}
- **Common reported functions (%):** 0.0
- **Common reported locations (%):** 11.11
- **Common reported processes (%):** 0.0

- **PDB ID:** 6EA4
- **Chain:** B
- **Crystallized protein length:** 876 aa
- **Resolution:** 2.45 Å
- **b-phipsi:** 0.018397
- **w-rdist:** 0.531259
- **t-alpha:** 0.002377
- **Chemical similarity (Tanimoto Index) (%):** N/A
- **1D identity (%) [PDB]:** 2.84
- **1D identity (%) [Gaps excluded][PDB]:** 69.12
- **1D identity - Alignment Gaps [PDB]:** 1586
- **1D aligned content [PDB] (<aminoacid>:%):** {'P': 8.51, 'V': 14.89, 'A': 4.26, 'T': 8.51, 'G': 8.51, 'D': 8.51, 'L': 14.89, 'E': 4.26, 'F': 2.13, 'K': 4.26, 'S': 4.26, 'I': 4.26, 'R': 4.26, 'H': 6.38, 'N': 2.13}
- **2D identity (%) [PDB]:** 31.35
- **2D identity (%) [Gaps excluded][PDB]:** 90.52
- **2D identity - Alignment Gaps [PDB]:** 836
- **2D aligned content [PDB] (<2D-fold>:%):** {'.': 15.46, 'E': 24.19, 'T': 14.21, 'H': 45.39, 'G': 0.75}
- **3D similarity (TM-Score) (%) [PDB]:** 26.15

- **Gene name:** ERAP2
- **Entrez ID:** 64167
- **RefSeq ID:** NM\_022350
- **Transcript sequence length:** 5131
- **5-UTR|CDS|3-UTR identity (%):** 17.91 | 46.64 | 8.34
- **5-UTR|CDS|3-UTR identity (%) [Gaps excluded]:** 68.37 | 73.78 | 77.64
- **5-UTR|CDS|3-UTR identity [Alignment Gaps]:** 276 | 1232 | 15195
- **5-UTR aligned content (<base>:%):** {'A': 16.42, 'G': 25.37, 'T': 26.87, 'C': 31.34}
- **CDS aligned content (<base>:%):** {'A': 30.99, 'G': 21.45, 'C': 20.93, 'T': 26.63}
- **3-UTR aligned content (<base>:%):** {'A': 33.87, 'T': 33.94, 'C': 14.44, 'G': 17.75}

**Uniprot Description:**  
  
 Aminopeptidase that plays a central role in peptide trimming, a step required for the generation of most HLA class I-binding peptides. Peptide trimming is essential to customize longer precursor peptides to fit them to the correct length required for presentation on MHC class I molecules. Preferentially hydrolyzes the basic residues Arg and Lys.   
  
Heterodimer with ERAP1.   
  
 **Gene Ontology Information:**

Molecular Function

- aminopeptidase activity
- endopeptidase activity
- metalloaminopeptidase activity
- metallopeptidase activity
- peptide binding
- zinc ion binding

Location

- cytoplasm
- endoplasmic reticulum lumen
- endoplasmic reticulum membrane

Biological process

- adaptive immune response
- antigen processing and presentation of endogenous peptide antigen via MHC class I
- antigen processing and presentation of peptide antigen via MHC class I
- peptide catabolic process
- proteolysis
- regulation of blood pressure

---

86

- **Protein name:** Transitional endoplasmic reticulum ATPase
- **Organism:** Homo sapiens
- **Uniprot Accession Number:** P55072
- **Protein sequence length:** 806 aa
- **1D identity (%):** 16.77
- **1D identity (%) [Gaps excluded]:** 24.19
- **1D identity - Alignment Gaps:** 302
- **1D aligned content (<aminoacid>:%):** {'M': 0.61, 'G': 10.3, 'S': 4.24, 'L': 3.03, 'R': 9.7, 'P': 10.3, 'I': 3.64, 'K': 8.48, 'D': 7.88, 'E': 6.06, 'V': 6.67, 'T': 7.27, 'F': 4.85, 'A': 7.27, 'Y': 1.82, 'N': 2.42, 'H': 1.21, 'C': 1.82, 'Q': 2.42}
- **Common reported functions (%):** 14.29
- **Common reported locations (%):** 33.33
- **Common reported processes (%):** 0.0

- **PDB ID:** 5FTJ
- **Chain:** A
- **Crystallized protein length:** 723 aa
- **Resolution:** 2.3 Å
- **b-phipsi:** 0.005297
- **w-rdist:** 0.123707
- **t-alpha:** 0.141839
- **Chemical similarity (Tanimoto Index) (%):** 77.76
- **1D identity (%) [PDB]:** 3.36
- **1D identity (%) [Gaps excluded][PDB]:** 60.24
- **1D identity - Alignment Gaps [PDB]:** 1403
- **1D aligned content [PDB] (<aminoacid>:%):** {'G': 10.0, 'D': 6.0, 'K': 6.0, 'I': 8.0, 'V': 6.0, 'M': 2.0, 'A': 8.0, 'P': 4.0, 'S': 6.0, 'Y': 4.0, 'C': 2.0, 'R': 10.0, 'E': 6.0, 'Q': 6.0, 'L': 8.0, 'F': 6.0, 'T': 2.0}
- **2D identity (%) [PDB]:** 36.56
- **2D identity (%) [Gaps excluded][PDB]:** 87.07
- **2D identity - Alignment Gaps [PDB]:** 641
- **2D aligned content [PDB] (<2D-fold>:%):** {'.': 25.99, 'E': 16.09, 'H': 45.54, 'T': 12.13, 'B': 0.25}
- **3D similarity (TM-Score) (%) [PDB]:** 22.38

- **Gene name:** VCP
- **Entrez ID:** N/A
- **RefSeq ID:** NM\_007126
- **Transcript sequence length:** 3746
- **5-UTR|CDS|3-UTR identity (%):** 43.98 | 44.69 | 3.86
- **5-UTR|CDS|3-UTR identity (%) [Gaps excluded]:** 79.25 | 74.83 | 76.4
- **5-UTR|CDS|3-UTR identity [Alignment Gaps]:** 170 | 1262 | 16096
- **5-UTR aligned content (<base>:%):** {'A': 7.74, 'G': 38.69, 'T': 10.71, 'C': 42.86}
- **CDS aligned content (<base>:%):** {'A': 27.29, 'T': 24.07, 'G': 27.71, 'C': 20.93}
- **3-UTR aligned content (<base>:%):** {'G': 23.09, 'T': 27.52, 'C': 23.09, 'A': 26.3}

**Uniprot Description:**  
  
 Necessary for the fragmentation of Golgi stacks during mitosis and for their reassembly after mitosis. Involved in the formation of the transitional endoplasmic reticulum (tER). The transfer of membranes from the endoplasmic reticulum to the Golgi apparatus occurs via 50-70 nm transition vesicles which derive from part-rough, part-smooth transitional elements of the endoplasmic reticulum (tER). Vesicle budding from the tER is an ATP-dependent process. The ternary complex containing UFD1, VCP and NPLOC4 binds ubiquitinated proteins and is necessary for the export of misfolded proteins from the ER to the cytoplasm, where they are degraded by the proteasome. The NPLOC4-UFD1-VCP complex regulates spindle disassembly at the end of mitosis and is necessary for the formation of a closed nuclear envelope. Regulates E3 ubiquitin-protein ligase activity of RNF19A. Component of the VCP/p97-AMFR/gp78 complex that participates in the final step of the sterol-mediated ubiquitination and endoplasmic reticulum-associated degradation (ERAD) of HMGCR. Involved in endoplasmic reticulum stress-induced pre-emptive quality control, a mechanism that selectively attenuates the translocation of newly synthesized proteins into the endoplasmic reticulum and reroutes them to the cytosol for proteasomal degradation (PubMed:26565908). Plays a role in the regulation of stress granules (SGs) clearance process upon arsenite-induced response (PubMed:29804830). Also involved in DNA damage response: recruited to double-strand breaks (DSBs) sites in a RNF8- and RNF168-dependent manner and promotes the recruitment of TP53BP1 at DNA damage sites (PubMed:22020440, PubMed:22120668). Recruited to stalled replication forks by SPRTN: may act by mediating extraction of DNA polymerase eta (POLH) to prevent excessive translesion DNA synthesis and limit the incidence of mutations induced by DNA damage (PubMed:23042607, PubMed:23042605). Together with SPRTN metalloprotease, involved in the repair of covalent DNA-protein cross-links (DPCs) during DNA synthesis (PubMed:32152270). Involved in interstrand cross-link repair in response to replication stress by mediating unloading of the ubiquitinated CMG helicase complex (By similarity). Required for cytoplasmic retrotranslocation of stressed/damaged mitochondrial outer-membrane proteins and their subsequent proteasomal degradation (PubMed:16186510, PubMed:21118995). Essential for the maturation of ubiquitin-containing autophagosomes and the clearance of ubiquitinated protein by autophagy (PubMed:20104022, PubMed:27753622). Acts as a negative regulator of type I interferon production by interacting with DDX58/RIG-I: interaction takes place when DDX58/RIG-I is ubiquitinated via 'Lys-63'-linked ubiquitin on its CARD domains, leading to recruit RNF125 and promote ubiquitination and degradation of DDX58/RIG-I (PubMed:26471729). May play a role in the ubiquitin-dependent sorting of membrane proteins to lysosomes where they undergo degradation (PubMed:21822278). May more particularly play a role in caveolins sorting in cells (PubMed:21822278, PubMed:23335559). By controlling the steady-state expression of the IGF1R receptor, indirectly regulates the insulin-like growth factor receptor signaling pathway (PubMed:26692333).   
  
Homohexamer. Forms a ring-shaped particle of 12.5 nm diameter, that displays 6-fold radial symmetry. Part of a ternary complex containing STX5A, NSFL1C and VCP. NSFL1C forms a homotrimer that binds to one end of a VCP homohexamer. The complex binds to membranes enriched in phosphatidylethanolamine-containing lipids and promotes Golgi membrane fusion. Binds to a heterodimer of NPLOC4 and UFD1, binding to this heterodimer inhibits Golgi-membrane fusion (PubMed:26471729). Interaction with VCIP135 leads to dissociation of the complex via ATP hydrolysis by VCP. Part of a ternary complex containing NPLOC4, UFD1 and VCP. Interacts with NSFL1C-like protein p37; the complex has membrane fusion activity and is required for Golgi and endoplasmic reticulum biogenesis. Interacts with SELENOS and SYVN1, as well as with DERL1, DERL2 and DERL3; which probably transfer misfolded proteins from the ER to VCP. Interacts with SVIP. Component of a complex required to couple retrotranslocation, ubiquitination and deglycosylation composed of NGLY1, SAKS1, AMFR, VCP and RAD23B. Directly interacts with UBXN4 and RNF19A. Interacts with CASR. Interacts with UBE4B and YOD1. Interacts with clathrin. Interacts with RNF103. Interacts with TRIM13 and TRIM21. Component of a VCP/p97-AMFR/gp78 complex that participates in the final step of the endoplasmic reticulum-associated degradation (ERAD) of HMGCR. Interacts directly with AMFR/gp78 (via its VIM). Interacts with RHBDD1 (via C-terminal domain). Interacts with SPRTN; leading to recruitment to stalled replication forks (PubMed:23042607, PubMed:23042605). Interacts with WASHC5. Interacts with UBOX5. Interacts (via N-terminus) with UBXN7, UBXN8, and probably several other UBX domain-containing proteins (via UBX domains); the interactions are mutually exclusive with VIM-dependent interactions such as those with AMFR and SELENOS. Forms a complex with UBQLN1 and UBXN4. Interacts (via the PIM motif) with RNF31 (via the PUB domain) (PubMed:24726327). Interacts with DDX58/RIG-I and RNF125; interaction takes place when DDX58/RIG-I is ubiquitinated via 'Lys-63'-linked ubiquitin on its CARD domains, leading to recruit RNF125 and promote ubiquitination and degradation of DDX58/RIG-I (PubMed:26471729). Interacts with BAG6 (PubMed:21636303). Interacts with UBXN10 (PubMed:26389662). Interacts with UBXN6; the interaction with UBXN6 is direct and competitive with UFD1 (PubMed:19174149, PubMed:19275885). Forms a ternary complex with CAV1 and UBXN6 (PubMed:21822278, PubMed:18656546, PubMed:19174149). Interacts with PLAA, UBXN6 and YOD1; may form a complex involved in macroautophagy (PubMed:27753622). Interacts with ANKZF1 (PubMed:28302725). Interacts with ubiquitin-binding protein FAF1 (PubMed:26842564). Interacts with ZFAND2B (via VIM motif); the interaction is direct (PubMed:24160817, PubMed:26337389). Interacts with ZFAND1 (via its ubiquitin-like region); this interaction occurs in an arsenite-dependent manner (PubMed:29804830). Interacts with CCDC47 (By similarity). Interacts with UBAC2 (By similarity). Interacts with LMBR1L (PubMed:31073040). Interacts with ATXN3 (PubMed:30455355). Interacts with TEX264; bridging VCP to covalent DNA-protein cross-links (DPCs) (PubMed:32152270).   
  
 **Gene Ontology Information:**

Molecular Function

- ADP binding
- ATP binding
- ATPase activity
- BAT3 complex binding
- deubiquitinase activator activity
- identical protein binding
- K48-linked polyubiquitin modification-dependent protein binding
- lipid binding
- MHC class I protein binding
- polyubiquitin modification-dependent protein binding
- protein domain specific binding
- protein phosphatase binding
- RNA binding
- ubiquitin protein ligase binding
- ubiquitin-dependent protein binding
- ubiquitin-like protein ligase binding
- ubiquitin-specific protease binding

Location

- ATPase complex
- azurophil granule lumen
- cytoplasm
- cytoplasmic stress granule
- cytosol
- Derlin-1 retrotranslocation complex
- endoplasmic reticulum
- endoplasmic reticulum membrane
- extracellular exosome
- extracellular region
- ficolin-1-rich granule lumen
- glutamatergic synapse
- intracellular membrane-bounded organelle
- lipid droplet
- nucleoplasm
- nucleus
- perinuclear region of cytoplasm
- proteasome complex
- protein-containing complex
- secretory granule lumen
- site of double-strand break
- VCP-NPL4-UFD1 AAA ATPase complex
- VCP-NSFL1C complex

Biological process

- activation of cysteine-type endopeptidase activity involved in apoptotic process
- aggresome assembly
- ATP metabolic process
- autophagosome maturation
- autophagy
- cellular response to arsenite ion
- cellular response to heat
- cellular response to DNA damage stimulus
- DNA repair
- double-strand break repair
- endoplasmic reticulum stress-induced pre-emptive quality control
- endoplasmic reticulum to Golgi vesicle-mediated transport
- endoplasmic reticulum unfolded protein response
- endosome to lysosome transport via multivesicular body sorting pathway
- ER-associated misfolded protein catabolic process
- ERAD pathway
- establishment of protein localization
- flavin adenine dinucleotide catabolic process
- interstrand cross-link repair
- macroautophagy
- mitotic spindle disassembly
- NADH metabolic process
- negative regulation of protein localization to chromatin
- negative regulation of smoothened signaling pathway
- positive regulation of ATP biosynthetic process
- positive regulation of canonical Wnt signaling pathway
- positive regulation of Lys63-specific deubiquitinase activity
- positive regulation of mitochondrial membrane potential
- positive regulation of oxidative phosphorylation
- positive regulation of proteasomal ubiquitin-dependent protein catabolic process
- positive regulation of protein catabolic process
- positive regulation of protein K63-linked deubiquitination
- positive regulation of protein-containing complex assembly
- proteasomal protein catabolic process
- proteasome-mediated ubiquitin-dependent protein catabolic process
- protein ubiquitination
- protein-DNA covalent cross-linking repair
- regulation of aerobic respiration
- regulation of apoptotic process
- regulation of protein localization to chromatin
- regulation of synapse organization
- retrograde protein transport, ER to cytosol
- stress granule disassembly
- translesion synthesis
- ubiquitin-dependent ERAD pathway
- viral genome replication

---

87

- **Protein name:** DNA replication licensing factor MCM2
- **Organism:** Homo sapiens
- **Uniprot Accession Number:** P49736
- **Protein sequence length:** 904 aa
- **1D identity (%):** 15.36
- **1D identity (%) [Gaps excluded]:** 21.81
- **1D identity - Alignment Gaps:** 306
- **1D aligned content (<aminoacid>:%):** {'S': 6.29, 'A': 7.55, 'G': 8.81, 'D': 6.92, 'P': 7.55, 'F': 3.14, 'L': 11.32, 'E': 5.03, 'R': 7.55, 'V': 7.55, 'M': 0.63, 'I': 3.14, 'H': 4.4, 'K': 5.66, 'N': 2.52, 'Q': 5.66, 'Y': 2.52, 'C': 1.26, 'T': 2.52}
- **Common reported functions (%):** 14.29
- **Common reported locations (%):** 22.22
- **Common reported processes (%):** 0.0

- **PDB ID:** 6XTY
- **Chain:** 2
- **Crystallized protein length:** 611 aa
- **Resolution:** 6.77 Å
- **b-phipsi:** 0.003504
- **w-rdist:** 0.41116
- **t-alpha:** 0.146593
- **Chemical similarity (Tanimoto Index) (%):** N/A
- **1D identity (%) [PDB]:** 5.4
- **1D identity (%) [Gaps excluded][PDB]:** 67.59
- **1D identity - Alignment Gaps [PDB]:** 1243
- **1D aligned content [PDB] (<aminoacid>:%):** {'N': 4.11, 'R': 8.22, 'E': 2.74, 'V': 17.81, 'S': 10.96, 'M': 1.37, 'Q': 1.37, 'K': 6.85, 'T': 4.11, 'I': 2.74, 'G': 6.85, 'P': 8.22, 'Y': 2.74, 'D': 5.48, 'L': 8.22, 'A': 4.11, 'W': 1.37, 'H': 1.37, 'F': 1.37}
- **2D identity (%) [PDB]:** 30.05
- **2D identity (%) [Gaps excluded][PDB]:** 84.11
- **2D identity - Alignment Gaps [PDB]:** 691
- **2D aligned content [PDB] (<2D-fold>:%):** {'.': 26.63, 'E': 16.41, 'H': 42.72, 'T': 13.93, 'B': 0.31}
- **3D similarity (TM-Score) (%) [PDB]:** 20.73

- **Gene name:** MCM2
- **Entrez ID:** 4171
- **RefSeq ID:** NM\_004526
- **Transcript sequence length:** 3434
- **5-UTR|CDS|3-UTR identity (%):** 11.62 | 46.12 | 2.52
- **5-UTR|CDS|3-UTR identity (%) [Gaps excluded]:** 74.51 | 73.96 | 75.71
- **5-UTR|CDS|3-UTR identity [Alignment Gaps]:** 276 | 1228 | 16298
- **5-UTR aligned content (<base>:%):** {'C': 23.68, 'T': 28.95, 'G': 34.21, 'A': 13.16}
- **CDS aligned content (<base>:%):** {'A': 26.38, 'T': 19.0, 'G': 29.37, 'C': 25.25}
- **3-UTR aligned content (<base>:%):** {'G': 26.89, 'C': 20.52, 'A': 19.81, 'T': 32.78}

**Uniprot Description:**  
  
 Acts as component of the MCM2-7 complex (MCM complex) which is the putative replicative helicase essential for 'once per cell cycle' DNA replication initiation and elongation in eukaryotic cells. The active ATPase sites in the MCM2-7 ring are formed through the interaction surfaces of two neighboring subunits such that a critical structure of a conserved arginine finger motif is provided in trans relative to the ATP-binding site of the Walker A box of the adjacent subunit. The six ATPase active sites, however, are likely to contribute differentially to the complex helicase activity. Required for the entry in S phase and for cell division. Plays a role in terminally differentiated hair cells development of the cochlea and induces cells apoptosis.   
  
Component of the MCM2-7 complex (PubMed:9305914, PubMed:16899510, PubMed:17296731). The complex forms a toroidal hexameric ring with the proposed subunit order MCM2-MCM6-MCM4-MCM7-MCM3-MCM5 (PubMed:9305914, PubMed:16899510, PubMed:17296731). Interacts with DBF4 (By similarity). Interacts with KAT7 (PubMed:16387653). May interact with MCM10 (PubMed:11095689). Component of the replisome complex composed of at least DONSON, MCM2, MCM7, PCNA and TICRR (PubMed:28191891). Component of the CMG helicase complex, composed of the MCM2-7 complex, the GINS complex and CDC45 (By similarity).   
  
 **Gene Ontology Information:**

Molecular Function

- ATP binding
- ATPase activity
- DNA binding
- DNA replication origin binding
- enzyme binding
- helicase activity
- histone binding
- metal ion binding
- single-stranded DNA binding

Location

- chromatin
- chromosome, telomeric region
- CMG complex
- cytoplasm
- MCM complex
- nuclear origin of replication recognition complex
- nucleoplasm
- nucleus

Biological process

- apoptotic process
- cellular response to interleukin-4
- cochlea development
- DNA replication
- DNA replication initiation
- DNA unwinding involved in DNA replication
- double-strand break repair via break-induced replication
- mitotic DNA replication initiation
- negative regulation of DNA helicase activity
- nucleosome assembly
- regulation of DNA-dependent DNA replication initiation

---

88

- **Protein name:** DNA topoisomerase 3-alpha
- **Organism:** Homo sapiens
- **Uniprot Accession Number:** Q13472
- **Protein sequence length:** 1001 aa
- **1D identity (%):** 14.65
- **1D identity (%) [Gaps excluded]:** 25.04
- **1D identity - Alignment Gaps:** 487
- **1D aligned content (<aminoacid>:%):** {'F': 5.81, 'V': 6.98, 'R': 7.56, 'P': 7.56, 'A': 4.65, 'G': 11.05, 'K': 4.65, 'H': 4.65, 'L': 8.72, 'T': 8.14, 'S': 1.74, 'N': 2.33, 'Y': 2.91, 'W': 0.58, 'C': 4.07, 'E': 4.07, 'D': 5.23, 'Q': 6.4, 'I': 2.33, 'M': 0.58}
- **Common reported functions (%):** 0.0
- **Common reported locations (%):** 11.11
- **Common reported processes (%):** 0.0

- **PDB ID:** 4CGY
- **Chain:** A
- **Crystallized protein length:** 619 aa
- **Resolution:** 2.85 Å
- **b-phipsi:** 0.014617
- **w-rdist:** 0.488642
- **t-alpha:** 0.004777
- **Chemical similarity (Tanimoto Index) (%):** 97.19
- **1D identity (%) [PDB]:** 2.47
- **1D identity (%) [Gaps excluded][PDB]:** 76.09
- **1D identity - Alignment Gaps [PDB]:** 1372
- **1D aligned content [PDB] (<aminoacid>:%):** {'S': 11.43, 'R': 8.57, 'V': 11.43, 'H': 8.57, 'L': 17.14, 'T': 8.57, 'E': 5.71, 'P': 11.43, 'D': 2.86, 'K': 2.86, 'N': 2.86, 'A': 2.86, 'M': 2.86, 'Y': 2.86}
- **2D identity (%) [PDB]:** 35.1
- **2D identity (%) [Gaps excluded][PDB]:** 85.25
- **2D identity - Alignment Gaps [PDB]:** 610
- **2D aligned content [PDB] (<2D-fold>:%):** {'.': 13.74, 'H': 50.55, 'T': 13.46, 'E': 21.7, 'B': 0.55}
- **3D similarity (TM-Score) (%) [PDB]:** 21.2

- **Gene name:** TOP3A
- **Entrez ID:** 7156
- **RefSeq ID:** NM\_004618
- **Transcript sequence length:** 6596
- **5-UTR|CDS|3-UTR identity (%):** 34.68 | 44.44 | 13.72
- **5-UTR|CDS|3-UTR identity (%) [Gaps excluded]:** 77.71 | 75.04 | 77.11
- **5-UTR|CDS|3-UTR identity [Alignment Gaps]:** 206 | 1431 | 14047
- **5-UTR aligned content (<base>:%):** {'G': 42.64, 'C': 34.11, 'A': 7.75, 'T': 15.5}
- **CDS aligned content (<base>:%):** {'A': 28.85, 'T': 21.67, 'G': 26.03, 'C': 23.46}
- **3-UTR aligned content (<base>:%):** {'G': 23.8, 'C': 21.83, 'A': 26.06, 'T': 28.32}

**Uniprot Description:**  
  
 Releases the supercoiling and torsional tension of DNA introduced during the DNA replication and transcription by transiently cleaving and rejoining one strand of the DNA duplex. Introduces a single-strand break via transesterification at a target site in duplex DNA. The scissile phosphodiester is attacked by the catalytic tyrosine of the enzyme, resulting in the formation of a DNA-(5'-phosphotyrosyl)-enzyme intermediate and the expulsion of a 3'-OH DNA strand. The free DNA strand then undergoes passage around the unbroken strand thus removing DNA supercoils. Finally, in the religation step, the DNA 3'-OH attacks the covalent intermediate to expel the active-site tyrosine and restore the DNA phosphodiester backbone. As an essential component of the RMI complex it is involved in chromosome separation and the processing of homologous recombination intermediates to limit DNA crossover formation in cells. Has DNA decatenation activity (PubMed:30057030). It is required for mtDNA decatenation and segregation after completion of replication, in a process that does not require BLM, RMI1 and RMI2 (PubMed:29290614).   
  
Binds ssDNA (PubMed:29290614). Interacts (via N-terminal region) with BLM; the interaction is direct. Directly interacts with RMI1. Component of the RMI complex, containing at least TOP3A, RMI1 and RMI2. The RMI complex interacts with BLM.   
  
 **Gene Ontology Information:**

Molecular Function

- DNA binding
- DNA topoisomerase activity
- DNA topoisomerase type I (single strand cut, ATP-independent) activity
- single-stranded DNA binding
- zinc ion binding

Location

- mitochondrial matrix
- nucleoplasm
- nucleus
- PML body
- RecQ family helicase-topoisomerase III complex

Biological process

- chromosome separation
- DNA topological change
- double-strand break repair via homologous recombination
- meiotic cell cycle
- mitochondrial DNA metabolic process
- resolution of recombination intermediates

---

89

- **Protein name:** Plexin-C1
- **Organism:** Homo sapiens
- **Uniprot Accession Number:** O60486
- **Protein sequence length:** 1568 aa
- **1D identity (%):** 14.0
- **1D identity (%) [Gaps excluded]:** 26.03
- **1D identity - Alignment Gaps:** 730
- **1D aligned content (<aminoacid>:%):** {'M': 0.9, 'E': 6.79, 'P': 8.14, 'A': 4.52, 'L': 9.95, 'G': 6.33, 'I': 2.71, 'C': 4.07, 'Y': 5.43, 'V': 8.14, 'R': 6.33, 'Q': 4.07, 'H': 2.71, 'D': 6.79, 'S': 4.98, 'N': 1.36, 'T': 6.79, 'F': 3.17, 'W': 1.81, 'K': 4.98}
- **Common reported functions (%):** 0.0
- **Common reported locations (%):** 11.11
- **Common reported processes (%):** 0.0

- **PDB ID:** 6VXK
- **Chain:** B
- **Crystallized protein length:** 747 aa
- **Resolution:** 3.1 Å
- **b-phipsi:** 0.019323
- **w-rdist:** 0.305439
- **t-alpha:** 0.009509
- **Chemical similarity (Tanimoto Index) (%):** 83.93
- **1D identity (%) [PDB]:** 2.19
- **1D identity (%) [Gaps excluded][PDB]:** 70.83
- **1D identity - Alignment Gaps [PDB]:** 1508
- **1D aligned content [PDB] (<aminoacid>:%):** {'E': 8.82, 'L': 17.65, 'T': 8.82, 'G': 5.88, 'P': 11.76, 'S': 5.88, 'V': 8.82, 'A': 5.88, 'D': 2.94, 'R': 5.88, 'H': 2.94, 'M': 2.94, 'K': 2.94, 'F': 5.88, 'Y': 2.94}
- **2D identity (%) [PDB]:** 30.72
- **2D identity (%) [Gaps excluded][PDB]:** 80.77
- **2D identity - Alignment Gaps [PDB]:** 720
- **2D aligned content [PDB] (<2D-fold>:%):** {'.': 32.21, 'E': 47.62, 'T': 15.97, 'H': 3.36, 'G': 0.84}
- **3D similarity (TM-Score) (%) [PDB]:** 21.03

- **Gene name:** PLXNC1
- **Entrez ID:** 10154
- **RefSeq ID:** NM\_005761
- **Transcript sequence length:** 7492
- **5-UTR|CDS|3-UTR identity (%):** 44.84 | 36.93 | 9.06
- **5-UTR|CDS|3-UTR identity (%) [Gaps excluded]:** 77.86 | 78.26 | 76.67
- **5-UTR|CDS|3-UTR identity [Alignment Gaps]:** 193 | 2616 | 15099
- **5-UTR aligned content (<base>:%):** {'C': 53.43, 'T': 10.78, 'G': 29.41, 'A': 6.37}
- **CDS aligned content (<base>:%):** {'A': 29.41, 'T': 24.77, 'G': 23.67, 'C': 22.14}
- **3-UTR aligned content (<base>:%):** {'G': 16.83, 'C': 14.89, 'T': 36.11, 'A': 32.17}

**Uniprot Description:**  
  
 Receptor for SEMA7A, for smallpox semaphorin A39R, vaccinia virus semaphorin A39R and for herpesvirus Sema protein. Binding of semaphorins triggers cellular responses leading to the rearrangement of the cytoskeleton and to secretion of IL6 and IL8 (By similarity).   
  
Monomer. Homodimer. Interacts with SEMA7A.   
  
 **Gene Ontology Information:**

Molecular Function

- semaphorin receptor activity
- signaling receptor binding

Location

- cerebellar climbing fiber to Purkinje cell synapse
- membrane
- plasma membrane
- semaphorin receptor complex

Biological process

- cell adhesion
- negative regulation of cell adhesion
- positive regulation of axonogenesis
- regulation of cell migration
- regulation of cell shape
- regulation of GTPase activity
- regulation of synapse pruning
- semaphorin-plexin signaling pathway involved in axon guidance

---

90

- **Protein name:** Toll-like receptor 7
- **Organism:** Homo sapiens
- **Uniprot Accession Number:** Q9NYK1
- **Protein sequence length:** 1049 aa
- **1D identity (%):** 15.33
- **1D identity (%) [Gaps excluded]:** 23.13
- **1D identity - Alignment Gaps:** 387
- **1D aligned content (<aminoacid>:%):** {'P': 7.95, 'T': 6.82, 'I': 3.98, 'L': 16.48, 'N': 5.68, 'K': 6.25, 'D': 4.55, 'V': 7.95, 'H': 3.98, 'G': 5.11, 'S': 3.98, 'E': 5.68, 'Y': 3.98, 'A': 2.84, 'R': 2.84, 'Q': 3.98, 'F': 5.68, 'C': 2.27}
- **Common reported functions (%):** 28.57
- **Common reported locations (%):** 11.11
- **Common reported processes (%):** 11.11

- **PDB ID:** 7CYN
- **Chain:** B
- **Crystallized protein length:** 786 aa
- **Resolution:** 4.2 Å
- **b-phipsi:** 0.003781
- **w-rdist:** 0.532117
- **t-alpha:** 0.01981
- **Chemical similarity (Tanimoto Index) (%):** 97.12
- **1D identity (%) [PDB]:** 2.75
- **1D identity (%) [Gaps excluded][PDB]:** 63.24
- **1D identity - Alignment Gaps [PDB]:** 1497
- **1D aligned content [PDB] (<aminoacid>:%):** {'P': 6.98, 'K': 6.98, 'I': 6.98, 'D': 16.28, 'V': 13.95, 'L': 4.65, 'C': 2.33, 'R': 6.98, 'N': 2.33, 'E': 2.33, 'S': 4.65, 'M': 2.33, 'F': 4.65, 'G': 9.3, 'T': 6.98, 'A': 2.33}
- **2D identity (%) [PDB]:** 27.6
- **2D identity (%) [Gaps excluded][PDB]:** 81.8
- **2D identity - Alignment Gaps [PDB]:** 809
- **2D aligned content [PDB] (<2D-fold>:%):** {'.': 35.31, 'E': 25.22, 'T': 29.08, 'H': 10.09, 'B': 0.3}
- **3D similarity (TM-Score) (%) [PDB]:** 22.42

- **Gene name:** TLR7
- **Entrez ID:** 51284
- **RefSeq ID:** NM\_016562
- **Transcript sequence length:** 5003
- **5-UTR|CDS|3-UTR identity (%):** 19.62 | 44.01 | 6.52
- **5-UTR|CDS|3-UTR identity (%) [Gaps excluded]:** 73.0 | 74.73 | 77.51
- **5-UTR|CDS|3-UTR identity [Alignment Gaps]:** 272 | 1483 | 15594
- **5-UTR aligned content (<base>:%):** {'A': 13.7, 'C': 41.1, 'T': 32.88, 'G': 12.33}
- **CDS aligned content (<base>:%):** {'A': 31.36, 'T': 27.96, 'G': 19.77, 'C': 20.91}
- **3-UTR aligned content (<base>:%):** {'C': 16.4, 'T': 30.9, 'A': 33.06, 'G': 19.64}

**Uniprot Description:**  
  
 Endosomal receptor that plays a key role in innate and adaptive immunity (PubMed:14976261, PubMed:32433612). Controls host immune response against pathogens through recognition of uridine-containing single strand RNAs (ssRNAs) of viral origin or guanosine analogs (PubMed:31608988, PubMed:27742543, PubMed:12738885, PubMed:32706371, PubMed:35477763). Upon binding to agonists, undergoes dimerization that brings TIR domains from the two molecules into direct contact, leading to the recruitment of TIR-containing downstream adapter MYD88 through homotypic interaction (PubMed:27742543). In turn, the Myddosome signaling complex is formed involving IRAK4, IRAK1, TRAF6, TRAF3 leading to activation of downstream transcription factors NF-kappa-B and IRF7 to induce pro-inflammatory cytokines and interferons, respectively (PubMed:27742543, PubMed:32706371).   
  
Homodimer (PubMed:27742543). Interacts with MYD88 via their respective TIR domains (Probable). Interacts with UNC93B1 (PubMed:33432245). Interacts with SMPDL3B (By similarity).   
  
 **Gene Ontology Information:**

Molecular Function

- double-stranded RNA binding
- pattern recognition receptor activity
- single-stranded RNA binding
- siRNA binding

Location

- cytoplasm
- early phagosome
- endolysosome membrane
- endoplasmic reticulum
- endoplasmic reticulum membrane
- endosome
- endosome membrane
- Golgi membrane
- lysosome
- plasma membrane
- receptor complex

Biological process

- cellular response to mechanical stimulus
- cellular response to virus
- defense response to virus
- I-kappaB kinase/NF-kappaB signaling
- I-kappaB phosphorylation
- inflammatory response
- innate immune response
- JNK cascade
- positive regulation of chemokine production
- positive regulation of inflammatory response
- positive regulation of interferon-alpha production
- positive regulation of interferon-beta production
- positive regulation of interleukin-6 production
- positive regulation of interleukin-8 production
- positive regulation of macrophage cytokine production
- positive regulation of NIK/NF-kappaB signaling
- positive regulation of transcription by RNA polymerase II
- positive regulation of interferon-gamma production
- regulation of protein phosphorylation
- response to cGMP
- toll-like receptor 7 signaling pathway
- toll-like receptor signaling pathway

---

91

- **Protein name:** [Histone H3]-lysine(27) N-trimethyltransferase
- **Organism:** Anolis carolinensis
- **Uniprot Accession Number:** G1KPH4
- **Protein sequence length:** 737 aa
- **1D identity (%):** 15.48
- **1D identity (%) [Gaps excluded]:** 23.89
- **1D identity - Alignment Gaps:** 341
- **1D aligned content (<aminoacid>:%):** {'G': 8.67, 'K': 8.0, 'I': 6.0, 'R': 8.0, 'V': 8.0, 'L': 7.33, 'E': 7.33, 'P': 10.67, 'S': 2.0, 'F': 5.33, 'N': 4.0, 'Y': 4.67, 'Q': 3.33, 'M': 1.33, 'H': 0.67, 'D': 2.67, 'C': 2.67, 'T': 5.33, 'A': 4.0}
- **Common reported functions (%):** 0.0
- **Common reported locations (%):** 11.11
- **Common reported processes (%):** 0.0

- **PDB ID:** 5IJ7
- **Chain:** B
- **Crystallized protein length:** 469 aa
- **Resolution:** 2.62 Å
- **b-phipsi:** 0.001281
- **w-rdist:** 0.714462
- **t-alpha:** 0.030111
- **Chemical similarity (Tanimoto Index) (%):** 71.77
- **1D identity (%) [PDB]:** 1.62
- **1D identity (%) [Gaps excluded][PDB]:** 91.3
- **1D identity - Alignment Gaps [PDB]:** 1275
- **1D aligned content [PDB] (<aminoacid>:%):** {'R': 19.05, 'V': 9.52, 'Q': 14.29, 'E': 9.52, 'I': 14.29, 'D': 4.76, 'L': 14.29, 'A': 4.76, 'S': 4.76, 'M': 4.76}
- **2D identity (%) [PDB]:** 23.04
- **2D identity (%) [Gaps excluded][PDB]:** 87.64
- **2D identity - Alignment Gaps [PDB]:** 771
- **2D aligned content [PDB] (<2D-fold>:%):** {'.': 21.99, 'H': 53.53, 'E': 9.96, 'T': 12.86, 'G': 1.24, 'B': 0.41}
- **3D similarity (TM-Score) (%) [PDB]:** 17.19

- **Gene name:** EZH2
- **Entrez ID:** 2146
- **RefSeq ID:** N/A
- **Sequence length:** N/A
- **5-UTR|CDS|3-UTR identity (%):** N/A | N/A | N/A
- **5-UTR|CDS|3-UTR identity (%) [Gaps excluded]:** N/A | N/A | N/A
- **5-UTR|CDS|3-UTR identity [Alignment Gaps]:** N/A | N/A | N/A
- **5-UTR aligned content (<base>:%):** N/A
- **CDS aligned content (<base>:%):** N/A
- **3-UTR aligned content (<base>:%):** N/A

**Uniprot Description:**  
  
 N/A N/A   
  
 **Gene Ontology Information:**

Molecular Function

- chromatin binding
- chromatin DNA binding
- histone methyltransferase activity (H3-K27 specific)
- histone H3K27 trimethyltransferase activity
- histone methyltransferase activity
- lncRNA binding
- primary miRNA binding
- promoter-specific chromatin binding
- protein-lysine N-methyltransferase activity
- ribonucleoprotein complex binding
- RNA polymerase II cis-regulatory region sequence-specific DNA binding
- RNA polymerase II core promoter sequence-specific DNA binding
- transcription corepressor activity
- transcription corepressor binding

Location

- chromatin
- chromatin silencing complex
- chromosome, telomeric region
- ESC/E(Z) complex
- nucleoplasm
- nucleus
- pericentric heterochromatin
- pronucleus
- synapse

Biological process

- B cell differentiation
- cardiac muscle hypertrophy in response to stress
- cellular response to hydrogen peroxide
- cellular response to trichostatin A
- cerebellar cortex development
- chromatin organization
- DNA methylation
- facultative heterochromatin formation
- G1 to G0 transition
- G1/S transition of mitotic cell cycle
- hepatocyte homeostasis
- heterochromatin assembly
- hippocampus development
- histone H3-K27 methylation
- keratinocyte differentiation
- liver regeneration
- negative regulation of cytokine production involved in inflammatory response
- negative regulation of DNA-binding transcription factor activity
- negative regulation of transcription, DNA-templated
- negative regulation of G1/S transition of mitotic cell cycle
- negative regulation of gene expression, epigenetic
- negative regulation of keratinocyte differentiation
- negative regulation of retinoic acid receptor signaling pathway
- negative regulation of stem cell differentiation
- negative regulation of striated muscle cell differentiation
- negative regulation of transcription by RNA polymerase II
- negative regulation of transcription elongation from RNA polymerase II promoter
- positive regulation of cell cycle G1/S phase transition
- positive regulation of cell population proliferation
- positive regulation of dendrite development
- positive regulation of epithelial to mesenchymal transition
- positive regulation of GTPase activity
- positive regulation of MAP kinase activity
- positive regulation of protein serine/threonine kinase activity
- protein localization to chromatin
- regulation of circadian rhythm
- regulation of transcription, DNA-templated
- regulation of gliogenesis
- regulation of kidney development
- response to estradiol
- response to tetrachloromethane
- rhythmic process
- skeletal muscle satellite cell maintenance involved in skeletal muscle regeneration
- stem cell differentiation
- subtelomeric heterochromatin assembly
- synaptic transmission, GABAergic

---

92

- **Protein name:** Guanylate cyclase soluble subunit alpha-1
- **Organism:** Homo sapiens
- **Uniprot Accession Number:** Q02108
- **Protein sequence length:** 690 aa
- **1D identity (%):** 12.33
- **1D identity (%) [Gaps excluded]:** 23.32
- **1D identity - Alignment Gaps:** 478
- **1D aligned content (<aminoacid>:%):** {'F': 6.4, 'D': 8.0, 'E': 7.2, 'P': 6.4, 'L': 8.0, 'V': 7.2, 'G': 12.0, 'K': 7.2, 'C': 6.4, 'R': 4.8, 'S': 5.6, 'T': 6.4, 'A': 4.0, 'N': 1.6, 'H': 4.8, 'Q': 2.4, 'I': 0.8, 'Y': 0.8}
- **Common reported functions (%):** 14.29
- **Common reported locations (%):** 11.11
- **Common reported processes (%):** 0.0

- **PDB ID:** 8HBE
- **Chain:** B
- **Crystallized protein length:** 572 aa
- **Resolution:** 3.2 Å
- **b-phipsi:** 0.004482
- **w-rdist:** 0.356066
- **t-alpha:** 0.122821
- **Chemical similarity (Tanimoto Index) (%):** 82.78
- **1D identity (%) [PDB]:** 2.23
- **1D identity (%) [Gaps excluded][PDB]:** 78.95
- **1D identity - Alignment Gaps [PDB]:** 1309
- **1D aligned content [PDB] (<aminoacid>:%):** {'Y': 3.33, 'S': 3.33, 'G': 6.67, 'L': 16.67, 'Q': 10.0, 'I': 10.0, 'V': 16.67, 'K': 10.0, 'P': 3.33, 'A': 6.67, 'R': 3.33, 'D': 3.33, 'T': 3.33, 'M': 3.33}
- **2D identity (%) [PDB]:** 38.21
- **2D identity (%) [Gaps excluded][PDB]:** 89.61
- **2D identity - Alignment Gaps [PDB]:** 557
- **2D aligned content [PDB] (<2D-fold>:%):** {'.': 8.36, 'H': 39.35, 'T': 24.26, 'E': 28.03}
- **3D similarity (TM-Score) (%) [PDB]:** 19.38

- **Gene name:** GUCY1A1
- **Entrez ID:** 2983
- **RefSeq ID:** N/A
- **Sequence length:** N/A
- **5-UTR|CDS|3-UTR identity (%):** N/A | N/A | N/A
- **5-UTR|CDS|3-UTR identity (%) [Gaps excluded]:** N/A | N/A | N/A
- **5-UTR|CDS|3-UTR identity [Alignment Gaps]:** N/A | N/A | N/A
- **5-UTR aligned content (<base>:%):** N/A
- **CDS aligned content (<base>:%):** N/A
- **3-UTR aligned content (<base>:%):** N/A

**Uniprot Description:**  
  
 N/A   
  
The active enzyme is formed by a heterodimer of an alpha and a beta subunit. Heterodimer with GUCY1B1 (PubMed:9742212, PubMed:23505436, PubMed:24669844).   
  
 **Gene Ontology Information:**

Molecular Function

- adenylate cyclase activity
- cytidylate cyclase activity
- GTP binding
- guanylate cyclase activity
- heme binding
- Hsp90 protein binding
- metal ion binding
- protein-containing complex binding
- signaling receptor activity

Location

- cytosol
- glutamatergic synapse
- guanylate cyclase complex, soluble
- presynaptic active zone cytoplasmic component

Biological process

- blood circulation
- cellular response to nitric oxide
- cGMP biosynthetic process
- cGMP-mediated signaling
- nitric oxide mediated signal transduction
- nitric oxide-cGMP-mediated signaling pathway
- response to oxygen levels
- trans-synaptic signaling by nitric oxide, modulating synaptic transmission

---

93

- **Protein name:** Protein-glutamine gamma-glutamyltransferase E
- **Organism:** Homo sapiens
- **Uniprot Accession Number:** Q08188
- **Protein sequence length:** 693 aa
- **1D identity (%):** 14.24
- **1D identity (%) [Gaps excluded]:** 26.05
- **1D identity - Alignment Gaps:** 455
- **1D aligned content (<aminoacid>:%):** {'V': 11.89, 'W': 1.4, 'N': 4.9, 'T': 6.99, 'Q': 5.59, 'I': 6.29, 'F': 5.59, 'L': 9.09, 'R': 5.59, 'E': 5.59, 'M': 1.4, 'G': 8.39, 'Y': 1.4, 'P': 5.59, 'S': 6.99, 'D': 5.59, 'A': 3.5, 'K': 2.1, 'C': 2.1}
- **Common reported functions (%):** 0.0
- **Common reported locations (%):** 11.11
- **Common reported processes (%):** 0.0

- **PDB ID:** 1NUG
- **Chain:** A
- **Crystallized protein length:** 675 aa
- **Resolution:** 2.4 Å
- **b-phipsi:** 0.01177
- **w-rdist:** 0.650668
- **t-alpha:** 0.000793
- **Chemical similarity (Tanimoto Index) (%):** 77.9
- **1D identity (%) [PDB]:** 3.09
- **1D identity (%) [Gaps excluded][PDB]:** 70.31
- **1D identity - Alignment Gaps [PDB]:** 1393
- **1D aligned content [PDB] (<aminoacid>:%):** {'D': 6.67, 'K': 4.44, 'P': 11.11, 'I': 4.44, 'S': 11.11, 'T': 4.44, 'N': 2.22, 'A': 6.67, 'V': 13.33, 'L': 6.67, 'Y': 2.22, 'R': 6.67, 'F': 4.44, 'G': 8.89, 'E': 2.22, 'W': 2.22, 'M': 2.22}
- **2D identity (%) [PDB]:** 35.43
- **2D identity (%) [Gaps excluded][PDB]:** 83.26
- **2D identity - Alignment Gaps [PDB]:** 613
- **2D aligned content [PDB] (<2D-fold>:%):** {'.': 19.05, 'E': 36.77, 'H': 24.87, 'T': 18.52, 'B': 0.79}
- **3D similarity (TM-Score) (%) [PDB]:** 20.72

- **Gene name:** TGM3
- **Entrez ID:** N/A
- **RefSeq ID:** NM\_003245
- **Transcript sequence length:** 2643
- **5-UTR|CDS|3-UTR identity (%):** 11.85 | 44.17 | 1.91
- **5-UTR|CDS|3-UTR identity (%) [Gaps excluded]:** 69.64 | 75.49 | 74.83
- **5-UTR|CDS|3-UTR identity [Alignment Gaps]:** 273 | 1221 | 16395
- **5-UTR aligned content (<base>:%):** {'T': 10.26, 'C': 33.33, 'G': 38.46, 'A': 17.95}
- **CDS aligned content (<base>:%):** {'T': 22.69, 'G': 26.38, 'C': 22.46, 'A': 28.46}
- **3-UTR aligned content (<base>:%):** {'G': 24.3, 'C': 34.58, 'T': 19.94, 'A': 21.18}

**Uniprot Description:**  
  
 Catalyzes the calcium-dependent formation of isopeptide cross-links between glutamine and lysine residues in various proteins, as well as the conjugation of polyamines to proteins. Involved in the formation of the cornified envelope (CE), a specialized component consisting of covalent cross-links of proteins beneath the plasma membrane of terminally differentiated keratinocytes. Catalyzes small proline-rich proteins (SPRR1 and SPRR2) and LOR cross-linking to form small interchain oligomers, which are further cross-linked by TGM1 onto the growing CE scaffold (By similarity). In hair follicles, involved in cross-linking structural proteins to hardening the inner root sheath.   
  
Consists of two polypeptide chains, which are synthesized as a precursor form of a single polypeptide.   
  
 **Gene Ontology Information:**

Molecular Function

- transferase activity, transferring acyl groups
- calcium ion binding
- catalytic activity
- protein-glutamine gamma-glutamyltransferase activity
- structural molecule activity

Location

- cytoplasm
- extracellular exosome
- extrinsic component of cytoplasmic side of plasma membrane
- protein-containing complex

Biological process

- cell envelope organization
- hair follicle morphogenesis
- keratinization
- keratinocyte differentiation
- peptide cross-linking
- protein modification process

---

94

- **Protein name:** Mucolipin-1
- **Organism:** Homo sapiens
- **Uniprot Accession Number:** Q9GZU1
- **Protein sequence length:** 580 aa
- **1D identity (%):** 12.46
- **1D identity (%) [Gaps excluded]:** 23.35
- **1D identity - Alignment Gaps:** 438
- **1D aligned content (<aminoacid>:%):** {'A': 5.13, 'G': 14.53, 'P': 9.4, 'Y': 4.27, 'T': 3.42, 'E': 1.71, 'L': 11.97, 'D': 5.13, 'K': 4.27, 'V': 7.69, 'F': 5.13, 'N': 2.56, 'H': 0.85, 'R': 5.98, 'W': 1.71, 'C': 5.13, 'Q': 3.42, 'I': 3.42, 'S': 4.27}
- **Common reported functions (%):** 0.0
- **Common reported locations (%):** 22.22
- **Common reported processes (%):** 0.0

- **PDB ID:** 5WJ5
- **Chain:** C
- **Crystallized protein length:** 457 aa
- **Resolution:** 3.7 Å
- **b-phipsi:** 0.048381
- **w-rdist:** 0.344632
- **t-alpha:** 0.007924
- **Chemical similarity (Tanimoto Index) (%):** N/A
- **1D identity (%) [PDB]:** 1.56
- **1D identity (%) [Gaps excluded][PDB]:** 86.96
- **1D identity - Alignment Gaps [PDB]:** 1257
- **1D aligned content [PDB] (<aminoacid>:%):** {'G': 5.0, 'K': 15.0, 'P': 10.0, 'L': 15.0, 'A': 5.0, 'F': 5.0, 'Q': 5.0, 'V': 10.0, 'E': 5.0, 'I': 10.0, 'D': 5.0, 'Y': 10.0}
- **2D identity (%) [PDB]:** 27.01
- **2D identity (%) [Gaps excluded][PDB]:** 91.89
- **2D identity - Alignment Gaps [PDB]:** 711
- **2D aligned content [PDB] (<2D-fold>:%):** {'.': 14.71, 'H': 60.66, 'T': 6.62, 'E': 16.91, 'G': 1.1}
- **3D similarity (TM-Score) (%) [PDB]:** 19.48

- **Gene name:** MCOLN1
- **Entrez ID:** 57192
- **RefSeq ID:** NM\_020533
- **Transcript sequence length:** 2084
- **5-UTR|CDS|3-UTR identity (%):** 27.81 | 34.1 | 0.86
- **5-UTR|CDS|3-UTR identity (%) [Gaps excluded]:** 84.68 | 73.11 | 79.56
- **5-UTR|CDS|3-UTR identity [Alignment Gaps]:** 227 | 1574 | 16607
- **5-UTR aligned content (<base>:%):** {'C': 42.55, 'A': 9.57, 'G': 38.3, 'T': 9.57}
- **CDS aligned content (<base>:%):** {'A': 22.66, 'T': 22.07, 'G': 26.54, 'C': 28.73}
- **3-UTR aligned content (<base>:%):** {'T': 27.78, 'C': 22.92, 'G': 29.86, 'A': 19.44}

**Uniprot Description:**  
  
 Nonselective cation channel probably playing a role in the regulation of membrane trafficking events and of metal homeostasis. Proposed to play a major role in Ca(2+) release from late endosome and lysosome vesicles to the cytoplasm, which is important for many lysosome-dependent cellular events, including the fusion and trafficking of these organelles, exocytosis and autophagy (PubMed:11013137, PubMed:12459486, PubMed:15336987, PubMed:14749347, PubMed:29019983, PubMed:27623384). Required for efficient uptake of large particles in macrophages in which Ca(2+) release from the lysosomes triggers lysosomal exocytosis. May also play a role in phagosome-lysosome fusion (By similarity). Involved in lactosylceramide trafficking indicative for a role in the regulation of late endocytic membrane fusion/fission events (PubMed:16978393). By mediating lysosomal Ca(2+) release is involved in regulation of mTORC1 signaling and in mTOR/TFEB-dependent lysosomal adaptation to environmental cues such as nutrient levels (PubMed:27787197, PubMed:25733853). Seems to act as lysosomal active oxygen species (ROS) sensor involved in ROS-induced TFEB activation and autophagy (PubMed:27357649). Functions as a Fe(2+) permeable channel in late endosomes and lysosomes (PubMed:18794901). Proposed to play a role in zinc homeostasis probably implicating its association with TMEM163 (PubMed:25130899) In adaptive immunity, TRPML2 and TRPML1 may play redundant roles in the function of the specialized lysosomes of B cells (By similarity).   
  
Homotetramer (PubMed:28112729, PubMed:29019983). Homooligomer (PubMed:14749347). Can heterooligomerize with MCOLN2 or MCOLN3; heteromeric assemblies have different channel properties as compared to the respective homooligomers and may be tissue-specific (PubMed:19885840). Interacts with PDCD6 (PubMed:19864416). Interacts with TMEM163 (PubMed:25130899). Interacts with LAPTM4B (PubMed:21224396).   
  
 **Gene Ontology Information:**

Molecular Function

- calcium channel activity
- identical protein binding
- intracellular phosphatidylinositol-3,5-bisphosphate-sensitive cation channel activity
- iron ion transmembrane transporter activity
- ligand-gated calcium channel activity
- lipid binding
- cation channel activity
- NAADP-sensitive calcium-release channel activity

Location

- cell projection
- endosome membrane
- Golgi apparatus
- intracellular membrane-bounded organelle
- late endosome
- late endosome membrane
- lysosomal membrane
- lysosome
- membrane
- nucleoplasm
- phagocytic cup
- phagocytic vesicle membrane
- plasma membrane
- receptor complex

Biological process

- adaptive immune response
- autophagosome maturation
- calcium ion export
- calcium ion transmembrane transport
- cellular response to calcium ion
- cellular response to pH
- cation transport
- phagosome maturation
- positive regulation of lysosome organization
- protein homotetramerization
- transferrin transport

---

95

- **Protein name:** DNA polymerase nu
- **Organism:** Homo sapiens
- **Uniprot Accession Number:** Q7Z5Q5
- **Protein sequence length:** 900 aa
- **1D identity (%):** 15.6
- **1D identity (%) [Gaps excluded]:** 19.92
- **1D identity - Alignment Gaps:** 214
- **1D aligned content (<aminoacid>:%):** {'G': 8.44, 'P': 8.44, 'A': 4.55, 'K': 7.79, 'I': 4.55, 'D': 4.55, 'E': 3.25, 'V': 6.49, 'R': 7.14, 'L': 8.44, 'T': 5.19, 'S': 9.09, 'H': 5.84, 'F': 4.55, 'M': 1.3, 'Q': 5.84, 'N': 1.3, 'Y': 1.95, 'C': 1.3}
- **Common reported functions (%):** 0.0
- **Common reported locations (%):** 11.11
- **Common reported processes (%):** 0.0

- **PDB ID:** 4XVM
- **Chain:** A
- **Crystallized protein length:** 627 aa
- **Resolution:** 3.2 Å
- **b-phipsi:** 0.024547
- **w-rdist:** 0.525071
- **t-alpha:** 0.002383
- **Chemical similarity (Tanimoto Index) (%):** N/A
- **1D identity (%) [PDB]:** 2.23
- **1D identity (%) [Gaps excluded][PDB]:** 71.11
- **1D identity - Alignment Gaps [PDB]:** 1387
- **1D aligned content [PDB] (<aminoacid>:%):** {'Q': 12.5, 'E': 6.25, 'K': 12.5, 'H': 3.12, 'T': 12.5, 'Y': 3.12, 'L': 6.25, 'P': 3.12, 'I': 6.25, 'V': 3.12, 'A': 9.38, 'G': 3.12, 'R': 6.25, 'C': 3.12, 'N': 3.12, 'S': 3.12, 'D': 3.12}
- **2D identity (%) [PDB]:** 35.37
- **2D identity (%) [Gaps excluded][PDB]:** 85.85
- **2D identity - Alignment Gaps [PDB]:** 615
- **2D aligned content [PDB] (<2D-fold>:%):** {'.': 18.11, 'E': 11.35, 'H': 56.22, 'T': 12.43, 'B': 0.54, 'G': 1.35}
- **3D similarity (TM-Score) (%) [PDB]:** 24.02

- **Gene name:** POLN
- **Entrez ID:** 353497
- **RefSeq ID:** N/A
- **Sequence length:** N/A
- **5-UTR|CDS|3-UTR identity (%):** N/A | N/A | N/A
- **5-UTR|CDS|3-UTR identity (%) [Gaps excluded]:** N/A | N/A | N/A
- **5-UTR|CDS|3-UTR identity [Alignment Gaps]:** N/A | N/A | N/A
- **5-UTR aligned content (<base>:%):** N/A
- **CDS aligned content (<base>:%):** N/A
- **3-UTR aligned content (<base>:%):** N/A

**Uniprot Description:**  
  
 DNA polymerase with very low fidelity that catalyzes considerable misincorporation by inserting dTTP opposite a G template, and dGTP opposite a T template (PubMed:16787914, PubMed:17118716). Is the least accurate of the DNA polymerase A family (i.e. POLG, POLN and POLQ) (PubMed:17118716). Can perform accurate translesion DNA synthesis (TLS) past a 5S-thymine glycol. Can perform efficient strand displacement past a nick or a gap and gives rise to an amount of product similar to that on non-damaged template. Has no exonuclease activity (PubMed:16787914). Error-prone DNA polymerase that preferentially misincorporates dT regardless of template sequence (PubMed:25775266). May play a role in TLS during interstrand cross-link (ICL) repair (PubMed:19908865). May be involved in TLS when genomic replication is blocked by extremely large major groove DNA lesions. May function in the bypass of some DNA-protein and DNA-DNA cross-links. May have a role in cellular tolerance to DNA cross-linking agents (PubMed:20102227). Involved in the repair of DNA cross-links and double-strand break (DSB) resistance. Participates in FANCD2-mediated repair. Forms a complex with HELQ helicase that participates in homologous recombination (HR) repair and is essential for cellular protection against DNA cross-links (PubMed:19995904).   
  
Interacts with FANCD2, FANCI, PCNA, RAD51 and HELQ.   
  
 **Gene Ontology Information:**

Molecular Function

- cyclin binding
- DNA binding
- DNA-directed DNA polymerase activity

Location

- nucleoplasm
- nucleus

Biological process

- DNA-dependent DNA replication
- double-strand break repair
- double-strand break repair via homologous recombination
- interstrand cross-link repair
- translesion synthesis

---

96

- **Protein name:** Polycomb protein SUZ12
- **Organism:** Homo sapiens
- **Uniprot Accession Number:** Q15022
- **Protein sequence length:** 739 aa
- **1D identity (%):** 12.27
- **1D identity (%) [Gaps excluded]:** 21.09
- **1D identity - Alignment Gaps:** 423
- **1D aligned content (<aminoacid>:%):** {'G': 8.87, 'S': 4.03, 'A': 4.03, 'K': 8.87, 'P': 12.9, 'H': 4.84, 'Y': 1.61, 'F': 0.81, 'R': 9.68, 'D': 5.65, 'L': 8.06, 'T': 4.84, 'E': 5.65, 'V': 5.65, 'C': 2.42, 'Q': 6.45, 'I': 4.84, 'M': 0.81}
- **Common reported functions (%):** 14.29
- **Common reported locations (%):** 11.11
- **Common reported processes (%):** 0.0

- **PDB ID:** 5WAI
- **Chain:** B
- **Crystallized protein length:** 300 aa
- **Resolution:** 2.9 Å
- **b-phipsi:** 0.016456
- **w-rdist:** 0.460818
- **t-alpha:** 0.005577
- **Chemical similarity (Tanimoto Index) (%):** N/A
- **1D identity (%) [PDB]:** 1.59
- **1D identity (%) [Gaps excluded][PDB]:** 81.82
- **1D identity - Alignment Gaps [PDB]:** 1109
- **1D aligned content [PDB] (<aminoacid>:%):** {'V': 11.11, 'S': 11.11, 'R': 11.11, 'H': 5.56, 'L': 22.22, 'E': 16.67, 'T': 11.11, 'G': 5.56, 'P': 5.56}
- **2D identity (%) [PDB]:** 16.19
- **2D identity (%) [Gaps excluded][PDB]:** 85.79
- **2D identity - Alignment Gaps [PDB]:** 787
- **2D aligned content [PDB] (<2D-fold>:%):** {'H': 29.3, '.': 27.39, 'T': 9.55, 'E': 33.76}
- **3D similarity (TM-Score) (%) [PDB]:** 16.66

- **Gene name:** SUZ12
- **Entrez ID:** 23512
- **RefSeq ID:** NM\_015355
- **Transcript sequence length:** 4495
- **5-UTR|CDS|3-UTR identity (%):** 38.81 | 40.15 | 8.15
- **5-UTR|CDS|3-UTR identity (%) [Gaps excluded]:** 75.39 | 75.33 | 74.61
- **5-UTR|CDS|3-UTR identity [Alignment Gaps]:** 180 | 1463 | 15088
- **5-UTR aligned content (<base>:%):** {'G': 34.72, 'A': 5.56, 'C': 43.75, 'T': 15.97}
- **CDS aligned content (<base>:%):** {'T': 24.96, 'G': 22.58, 'C': 19.16, 'A': 33.31}
- **3-UTR aligned content (<base>:%):** {'A': 33.74, 'T': 40.91, 'C': 10.5, 'G': 14.84}

**Uniprot Description:**  
  
 Polycomb group (PcG) protein. Component of the PRC2 complex, which methylates 'Lys-9' (H3K9me) and 'Lys-27' (H3K27me) of histone H3, leading to transcriptional repression of the affected target gene (PubMed:15225548, PubMed:15231737, PubMed:15385962, PubMed:16618801, PubMed:17344414, PubMed:18285464, PubMed:28229514, PubMed:29499137, PubMed:31959557). The PRC2 complex may also serve as a recruiting platform for DNA methyltransferases, thereby linking two epigenetic repression systems (PubMed:12435631, PubMed:12351676, PubMed:15385962, PubMed:15099518, PubMed:15225548, PubMed:15684044, PubMed:16431907, PubMed:18086877, PubMed:18285464). Genes repressed by the PRC2 complex include HOXC8, HOXA9, MYT1 and CDKN2A (PubMed:15231737, PubMed:16618801, PubMed:17200670, PubMed:31959557).   
  
Component of the PRC2 complex, which consists of the core subunits EED, EZH1 or EZH2, SUZ12, and RBBP4, and various combinations of accessory subunits including AEBP2, JARID2, PHF19, MTF2 and EPOP (PubMed:12435631, PubMed:12351676, PubMed:15385962, PubMed:15099518, PubMed:15225548, PubMed:15684044, PubMed:16224021, PubMed:19026781, PubMed:18285464, PubMed:29499137, PubMed:31959557). Within the complex, interacts (via C2H2 zinc finger domain) with JARID2 and EPOP; JARID2 and EPOP compete for SUZ12 binding (PubMed:29499137). Also interacts with AEBP2 and PHF19 (PubMed:29499137). Forms a monomeric PRC2.2 (class 2) complex consisting of at least SUZ12, RBBP4, AEBP2 and JARID2 (PubMed:29499137). Forms a dimeric PRC2.1 (class 1, PRC-PCL) complex consisting of at least SUZ12, RBBP4, and PHF19 or MTF2; PHF19 and MTF2 stabilize the dimeric structure which enhances PRC2 interaction with chromatin (PubMed:31959557). The minimum components required for methyltransferase activity of the PRC2/EZH2 complex are EED, EZH2 and SUZ12 (PubMed:12435631, PubMed:12351676, PubMed:15385962, PubMed:15099518, PubMed:15225548, PubMed:15684044, PubMed:16431907, PubMed:18086877, PubMed:18285464). The PRC2 complex may also interact with DNMT1, DNMT3A, DNMT3B and PHF1 via the EZH2 subunit and with SIRT1 via the SUZ12 subunit (PubMed:15684044, PubMed:18285464). Interacts with WDR77 (PubMed:16712789). Interacts with histone H1 (PubMed:16431907). Interacts with CDYL (PubMed:22009739). Interacts with ARNTL/BMAL1 (By similarity). Interacts with EZHIP (via C-terminal region) (PubMed:30923826). Interacts with ARMC12 (PubMed:30026490).   
  
 **Gene Ontology Information:**

Molecular Function

- chromatin DNA binding
- enzyme activator activity
- histone methyltransferase activity
- lncRNA binding
- metal ion binding
- methylated histone binding
- promoter-specific chromatin binding
- RNA polymerase II cis-regulatory region sequence-specific DNA binding
- transcription corepressor binding

Location

- chromatin silencing complex
- ESC/E(Z) complex
- nuclear body
- nucleolus
- nucleoplasm
- nucleus
- ribonucleoprotein complex
- RSC-type complex
- sex chromatin

Biological process

- cell population proliferation
- dosage compensation by inactivation of X chromosome
- facultative heterochromatin formation
- negative regulation of cell differentiation
- negative regulation of transcription by RNA polymerase II
- oligodendrocyte differentiation
- positive regulation of cell population proliferation

---

97

- **Protein name:** Exostosin-2
- **Organism:** Homo sapiens
- **Uniprot Accession Number:** Q93063
- **Protein sequence length:** 718 aa
- **1D identity (%):** 13.89
- **1D identity (%) [Gaps excluded]:** 24.09
- **1D identity - Alignment Gaps:** 424
- **1D aligned content (<aminoacid>:%):** {'C': 1.44, 'V': 12.95, 'Y': 3.6, 'P': 12.23, 'R': 7.91, 'F': 3.6, 'G': 6.47, 'D': 8.63, 'A': 4.32, 'L': 10.07, 'K': 5.04, 'I': 3.6, 'E': 2.88, 'T': 4.32, 'S': 5.04, 'W': 0.72, 'H': 0.72, 'Q': 5.04, 'N': 1.44}
- **Common reported functions (%):** 14.29
- **Common reported locations (%):** 11.11
- **Common reported processes (%):** 0.0

- **PDB ID:** 7SCH
- **Chain:** B
- **Crystallized protein length:** 587 aa
- **Resolution:** 3.1 Å
- **b-phipsi:** 0.00229
- **w-rdist:** 0.533745
- **t-alpha:** 0.122029
- **Chemical similarity (Tanimoto Index) (%):** 96.82
- **1D identity (%) [PDB]:** 1.91
- **1D identity (%) [Gaps excluded][PDB]:** 63.41
- **1D identity - Alignment Gaps [PDB]:** 1321
- **1D aligned content [PDB] (<aminoacid>:%):** {'P': 7.69, 'I': 11.54, 'L': 11.54, 'A': 3.85, 'F': 3.85, 'Q': 3.85, 'V': 23.08, 'E': 7.69, 'K': 11.54, 'Y': 3.85, 'D': 3.85, 'C': 3.85, 'R': 3.85}
- **2D identity (%) [PDB]:** 40.79
- **2D identity (%) [Gaps excluded][PDB]:** 90.16
- **2D identity - Alignment Gaps [PDB]:** 529
- **2D aligned content [PDB] (<2D-fold>:%):** {'.': 19.8, 'E': 18.27, 'H': 39.09, 'T': 21.83, 'G': 0.76, 'B': 0.25}
- **3D similarity (TM-Score) (%) [PDB]:** 21.7

- **Gene name:** EXT2
- **Entrez ID:** 2132
- **RefSeq ID:** NM\_207122
- **Transcript sequence length:** 10037
- **5-UTR|CDS|3-UTR identity (%):** 34.81 | 43.84 | 26.13
- **5-UTR|CDS|3-UTR identity (%) [Gaps excluded]:** 76.36 | 73.24 | 75.15
- **5-UTR|CDS|3-UTR identity [Alignment Gaps]:** 197 | 1190 | 11826
- **5-UTR aligned content (<base>:%):** {'G': 38.89, 'C': 43.65, 'T': 12.7, 'A': 4.76}
- **CDS aligned content (<base>:%):** {'A': 26.31, 'T': 26.54, 'G': 23.62, 'C': 23.54}
- **3-UTR aligned content (<base>:%):** {'A': 28.46, 'C': 18.18, 'T': 33.38, 'G': 19.97}

**Uniprot Description:**  
  
 Glycosyltransferase required for the biosynthesis of heparan-sulfate. The EXT1/EXT2 complex possesses substantially higher glycosyltransferase activity than EXT1 or EXT2 alone. Appears to be a tumor suppressor. Required for the exosomal release of SDCBP, CD63 and syndecan (PubMed:22660413).   
  
Forms a homo/heterooligomeric complex with EXT1. Interacts with GALNT5. Interacts with NDST1.   
  
 **Gene Ontology Information:**

Molecular Function

- glucuronosyl-N-acetylglucosaminyl-proteoglycan 4-alpha-N-acetylglucosaminyltransferase activity
- glucuronosyltransferase activity
- transferase activity, transferring glycosyl groups
- heparan sulfate N-acetylglucosaminyltransferase activity
- metal ion binding
- N-acetylglucosaminyl-proteoglycan 4-beta-glucuronosyltransferase activity
- protein heterodimerization activity

Location

- endoplasmic reticulum
- endoplasmic reticulum membrane
- extracellular exosome
- Golgi apparatus
- Golgi membrane
- membrane
- UDP-N-acetylglucosamine transferase complex

Biological process

- cellular response to fibroblast growth factor stimulus
- chondrocyte differentiation
- endochondral bone morphogenesis
- fluid transport
- gene expression
- glycosaminoglycan biosynthetic process
- heart contraction
- heparan sulfate proteoglycan biosynthetic process
- heparan sulfate proteoglycan biosynthetic process, polysaccharide chain biosynthetic process
- heparin biosynthetic process
- mesoderm formation
- multicellular organismal water homeostasis
- ossification
- polysaccharide biosynthetic process
- protein N-linked glycosylation
- regulation of blood pressure
- signal transduction
- sodium ion homeostasis
- sulfation
- vasodilation

---

98

- **Protein name:** Receptor tyrosine-protein kinase erbB-4
- **Organism:** Homo sapiens
- **Uniprot Accession Number:** Q15303
- **Protein sequence length:** 1308 aa
- **1D identity (%):** 15.68
- **1D identity (%) [Gaps excluded]:** 25.67
- **1D identity - Alignment Gaps:** 524
- **1D aligned content (<aminoacid>:%):** {'M': 1.9, 'G': 8.53, 'P': 10.43, 'Q': 3.79, 'T': 8.06, 'K': 3.32, 'N': 3.79, 'C': 3.79, 'V': 8.53, 'E': 4.74, 'I': 6.16, 'D': 7.11, 'R': 6.16, 'F': 3.32, 'L': 6.64, 'S': 2.84, 'H': 3.32, 'A': 4.27, 'Y': 3.32}
- **Common reported functions (%):** 0.0
- **Common reported locations (%):** 22.22
- **Common reported processes (%):** 0.0

- **PDB ID:** 3U7U
- **Chain:** E
- **Crystallized protein length:** 593 aa
- **Resolution:** 3.03 Å
- **b-phipsi:** 0.004678
- **w-rdist:** 0.628635
- **t-alpha:** 0.007132
- **Chemical similarity (Tanimoto Index) (%):** 83.61
- **1D identity (%) [PDB]:** 1.63
- **1D identity (%) [Gaps excluded][PDB]:** 69.7
- **1D identity - Alignment Gaps [PDB]:** 1375
- **1D aligned content [PDB] (<aminoacid>:%):** {'Q': 17.39, 'L': 21.74, 'E': 8.7, 'N': 4.35, 'T': 13.04, 'R': 8.7, 'V': 4.35, 'Y': 13.04, 'K': 4.35, 'H': 4.35}
- **2D identity (%) [PDB]:** 20.63
- **2D identity (%) [Gaps excluded][PDB]:** 79.46
- **2D identity - Alignment Gaps [PDB]:** 847
- **2D aligned content [PDB] (<2D-fold>:%):** {'.': 32.2, 'E': 30.51, 'H': 11.02, 'T': 26.27}
- **3D similarity (TM-Score) (%) [PDB]:** 18.92

- **Gene name:** ERBB4
- **Entrez ID:** 2066
- **RefSeq ID:** N/A
- **Sequence length:** N/A
- **5-UTR|CDS|3-UTR identity (%):** N/A | N/A | N/A
- **5-UTR|CDS|3-UTR identity (%) [Gaps excluded]:** N/A | N/A | N/A
- **5-UTR|CDS|3-UTR identity [Alignment Gaps]:** N/A | N/A | N/A
- **5-UTR aligned content (<base>:%):** N/A
- **CDS aligned content (<base>:%):** N/A
- **3-UTR aligned content (<base>:%):** N/A

**Uniprot Description:**  
  
 Tyrosine-protein kinase that plays an essential role as cell surface receptor for neuregulins and EGF family members and regulates development of the heart, the central nervous system and the mammary gland, gene transcription, cell proliferation, differentiation, migration and apoptosis. Required for normal cardiac muscle differentiation during embryonic development, and for postnatal cardiomyocyte proliferation. Required for normal development of the embryonic central nervous system, especially for normal neural crest cell migration and normal axon guidance. Required for mammary gland differentiation, induction of milk proteins and lactation. Acts as cell-surface receptor for the neuregulins NRG1, NRG2, NRG3 and NRG4 and the EGF family members BTC, EREG and HBEGF. Ligand binding triggers receptor dimerization and autophosphorylation at specific tyrosine residues that then serve as binding sites for scaffold proteins and effectors. Ligand specificity and signaling is modulated by alternative splicing, proteolytic processing, and by the formation of heterodimers with other ERBB family members, thereby creating multiple combinations of intracellular phosphotyrosines that trigger ligand- and context-specific cellular responses. Mediates phosphorylation of SHC1 and activation of the MAP kinases MAPK1/ERK2 and MAPK3/ERK1. Isoform JM-A CYT-1 and isoform JM-B CYT-1 phosphorylate PIK3R1, leading to the activation of phosphatidylinositol 3-kinase and AKT1 and protect cells against apoptosis. Isoform JM-A CYT-1 and isoform JM-B CYT-1 mediate reorganization of the actin cytoskeleton and promote cell migration in response to NRG1. Isoform JM-A CYT-2 and isoform JM-B CYT-2 lack the phosphotyrosine that mediates interaction with PIK3R1, and hence do not phosphorylate PIK3R1, do not protect cells against apoptosis, and do not promote reorganization of the actin cytoskeleton and cell migration. Proteolytic processing of isoform JM-A CYT-1 and isoform JM-A CYT-2 gives rise to the corresponding soluble intracellular domains (4ICD) that translocate to the nucleus, promote nuclear import of STAT5A, activation of STAT5A, mammary epithelium differentiation, cell proliferation and activation of gene expression. The ERBB4 soluble intracellular domains (4ICD) colocalize with STAT5A at the CSN2 promoter to regulate transcription of milk proteins during lactation. The ERBB4 soluble intracellular domains can also translocate to mitochondria and promote apoptosis.   
  
Monomer in the absence of bound ligand. Homodimer or heterodimer with another ERBB family member upon ligand binding, thus forming heterotetramers. Interacts with EGFR and ERBB2. Interacts with CBFA2T3 (By similarity). Interacts with DLG2 (via its PDZ domain), DLG3 (via its PDZ domain), DLG4 (via its PDZ domain) and SNTB2 (via its PDZ domain). Interacts with MUC1. Interacts (via its PPxy motifs) with WWOX. Interacts (via the PPxY motif 3 of isoform JM-A CYT-2) with YAP1 (via the WW domain 1 of isoform 1). Interacts (isoform JM-A CYT-1 and isoform JM-B CYT-1) with WWP1. Interacts (via its intracellular domain) with TRIM28. Interacts (via the intracellular domains of both CYT-1 and CYT-2 isoforms) with KAP1; the interaction does not phosphorylate KAP1 but represses ERBB4-mediated transcriptional activity. Interacts with PRPU, DDX23, MATR3, RBM15, ILF3, KAP1, U5S1, U2SURP, ITCH, HNRNPU, AP2A1, NULC, LEO1, WWP2, IGHG1, HXK1, GRB7 AND SRRT. Interacts (phosphorylated isoform JM-A CYT-1 and isoform JM-B CYT-1) with PIK3R1. Interacts with SHC1. Interacts with GRB2. Interacts (soluble intracellular domain) with STAT5A. Interacts (soluble intracellular domain) with BCL2. Interacts (phosphorylated) with STAT1.   
  
 **Gene Ontology Information:**

Molecular Function

- ATP binding
- epidermal growth factor-activated receptor activity
- epidermal growth factor receptor binding
- GABA receptor binding
- neuregulin receptor activity
- protein homodimerization activity
- protein tyrosine kinase activity
- transcription regulatory region sequence-specific DNA binding
- transmembrane receptor protein tyrosine kinase activity

Location

- basal plasma membrane
- basolateral plasma membrane
- cytosol
- extracellular region
- GABA-ergic synapse
- glutamatergic synapse
- mitochondrial matrix
- mitochondrion
- neuromuscular junction
- nucleoplasm
- nucleus
- plasma membrane
- postsynaptic density membrane
- postsynaptic membrane
- presynaptic membrane
- receptor complex

Biological process

- cardiac muscle tissue regeneration
- cell fate commitment
- cell migration
- cellular response to epidermal growth factor stimulus
- central nervous system morphogenesis
- embryonic pattern specification
- ERBB2-ERBB4 signaling pathway
- ERBB4 signaling pathway
- ERBB4-ERBB4 signaling pathway
- establishment of planar polarity involved in nephron morphogenesis
- heart development
- lactation
- mammary gland alveolus development
- mammary gland epithelial cell differentiation
- mitochondrial fragmentation involved in apoptotic process
- negative regulation of apoptotic process
- negative regulation of cell population proliferation
- nervous system development
- neural crest cell migration
- neurogenesis
- neurotransmitter receptor localization to postsynaptic specialization membrane
- olfactory bulb interneuron differentiation
- peptidyl-tyrosine phosphorylation
- positive regulation of cardiac muscle cell proliferation
- positive regulation of cell population proliferation
- positive regulation of transcription, DNA-templated
- positive regulation of ERK1 and ERK2 cascade
- positive regulation of kinase activity
- positive regulation of phosphatidylinositol 3-kinase activity
- positive regulation of phosphatidylinositol 3-kinase signaling
- positive regulation of protein localization to cell surface
- positive regulation of protein phosphorylation
- positive regulation of receptor signaling pathway via JAK-STAT
- positive regulation of tyrosine phosphorylation of STAT protein
- protein autophosphorylation
- regulation of cell migration
- signal transduction
- synapse assembly
- transmembrane receptor protein tyrosine kinase signaling pathway

---

99

- **Protein name:** Exostosin-1
- **Organism:** Homo sapiens
- **Uniprot Accession Number:** Q16394
- **Protein sequence length:** 746 aa
- **1D identity (%):** 14.61
- **1D identity (%) [Gaps excluded]:** 21.6
- **1D identity - Alignment Gaps:** 310
- **1D aligned content (<aminoacid>:%):** {'F': 5.71, 'G': 6.43, 'R': 12.14, 'L': 6.43, 'P': 10.71, 'D': 7.86, 'V': 7.14, 'W': 2.14, 'T': 7.14, 'Y': 5.71, 'Q': 5.71, 'A': 3.57, 'K': 2.86, 'H': 0.71, 'N': 2.86, 'E': 4.29, 'S': 5.0, 'I': 3.57}
- **Common reported functions (%):** 14.29
- **Common reported locations (%):** 11.11
- **Common reported processes (%):** 0.0

- **PDB ID:** 7UQX
- **Chain:** A
- **Crystallized protein length:** 583 aa
- **Resolution:** 3.3 Å
- **b-phipsi:** 0.002876
- **w-rdist:** 0.691359
- **t-alpha:** 0.013471
- **Chemical similarity (Tanimoto Index) (%):** 77.67
- **1D identity (%) [PDB]:** 2.21
- **1D identity (%) [Gaps excluded][PDB]:** 75.0
- **1D identity - Alignment Gaps [PDB]:** 1319
- **1D aligned content [PDB] (<aminoacid>:%):** {'I': 13.33, 'A': 13.33, 'G': 3.33, 'S': 6.67, 'M': 6.67, 'H': 3.33, 'P': 6.67, 'R': 6.67, 'C': 3.33, 'V': 10.0, 'Q': 10.0, 'E': 3.33, 'D': 3.33, 'L': 10.0}
- **2D identity (%) [PDB]:** 39.42
- **2D identity (%) [Gaps excluded][PDB]:** 88.84
- **2D identity - Alignment Gaps [PDB]:** 539
- **2D aligned content [PDB] (<2D-fold>:%):** {'T': 31.94, '.': 14.14, 'H': 39.01, 'E': 13.87, 'G': 0.79, 'B': 0.26}
- **3D similarity (TM-Score) (%) [PDB]:** 22.57

- **Gene name:** EXT1
- **Entrez ID:** 2131
- **RefSeq ID:** NM\_000127
- **Transcript sequence length:** 8243
- **5-UTR|CDS|3-UTR identity (%):** 30.24 | 44.05 | 19.76
- **5-UTR|CDS|3-UTR identity (%) [Gaps excluded]:** 79.02 | 75.34 | 75.85
- **5-UTR|CDS|3-UTR identity [Alignment Gaps]:** 492 | 1264 | 12893
- **5-UTR aligned content (<base>:%):** {'A': 8.71, 'T': 12.45, 'C': 42.74, 'G': 36.1}
- **CDS aligned content (<base>:%):** {'A': 27.96, 'T': 24.68, 'G': 23.27, 'C': 24.09}
- **3-UTR aligned content (<base>:%):** {'G': 16.72, 'A': 33.24, 'C': 15.07, 'T': 34.98}

**Uniprot Description:**  
  
 Glycosyltransferase forming with EXT2 the heterodimeric heparan sulfate polymerase which catalyzes the elongation of the heparan sulfate glycan backbone (PubMed:10639137, PubMed:22660413, PubMed:36402845, PubMed:36593275, PubMed:9620772). Glycan backbone extension consists in the alternating transfer of (1->4)-beta-D-GlcA and (1->4)-alpha-D-GlcNAc residues from their respective UDP-sugar donors. Both EXT1 and EXT2 are required for the full activity of the polymerase since EXT1 bears the N-acetylglucosaminyl-proteoglycan 4-beta-glucuronosyltransferase activity within the complex while EXT2 carries the glucuronosyl-N-acetylglucosaminyl-proteoglycan 4-alpha-N-acetylglucosaminyltransferase activity (PubMed:36402845, PubMed:36593275). Heparan sulfate proteoglycans are ubiquitous components of the extracellular matrix and play an important role in tissue homeostasis and signaling (PubMed:10639137, PubMed:11391482, PubMed:22660413, PubMed:9620772).   
  
Part of the heparan sulfate polymerase, a dimeric complex composed of EXT1 and EXT2 (PubMed:10639137, PubMed:10679296, PubMed:36402845, PubMed:36593275). Could also form homooligomeric complexes (PubMed:10679296). Interacts with NDST1 (PubMed:35137078).   
  
 **Gene Ontology Information:**

Molecular Function

- acetylglucosaminyltransferase activity
- glucuronosyl-N-acetylglucosaminyl-proteoglycan 4-alpha-N-acetylglucosaminyltransferase activity
- glucuronosyltransferase activity
- transferase activity, transferring glycosyl groups
- heparan sulfate N-acetylglucosaminyltransferase activity
- metal ion binding
- N-acetylglucosaminyl-proteoglycan 4-beta-glucuronosyltransferase activity
- protein heterodimerization activity
- protein homodimerization activity

Location

- endoplasmic reticulum
- endoplasmic reticulum membrane
- Golgi apparatus
- Golgi membrane
- membrane
- synapse

Biological process

- antigen processing and presentation
- axon guidance
- basement membrane organization
- blood vessel remodeling
- BMP signaling pathway
- bone resorption
- canonical Wnt signaling pathway
- cartilage development involved in endochondral bone morphogenesis
- cell adhesion mediated by integrin
- cell fate commitment
- cellular response to virus
- chondrocyte hypertrophy
- chondrocyte proliferation
- chondroitin sulfate metabolic process
- collagen fibril organization
- cranial skeletal system development
- dendrite self-avoidance
- dendritic cell migration
- developmental growth involved in morphogenesis
- embryonic skeletal joint development
- endochondral bone growth
- endochondral ossification
- endoderm development
- epithelial tube branching involved in lung morphogenesis
- fear response
- fibroblast growth factor receptor signaling pathway
- fluid transport
- gastrulation
- gene expression
- glandular epithelial cell differentiation
- glomerular basement membrane development
- glycosaminoglycan biosynthetic process
- hair follicle morphogenesis
- heart contraction
- heart field specification
- hematopoietic stem cell differentiation
- hematopoietic stem cell homeostasis
- hematopoietic stem cell migration to bone marrow
- heparan sulfate proteoglycan biosynthetic process
- heparan sulfate proteoglycan biosynthetic process, polysaccharide chain biosynthetic process
- heparin biosynthetic process
- hypersensitivity
- leukocyte tethering or rolling
- limb joint morphogenesis
- lymphocyte adhesion to endothelial cell of high endothelial venule
- lymphocyte migration into lymphoid organs
- mesenchymal cell differentiation involved in bone development
- mesoderm development
- motor behavior
- multicellular organism growth
- multicellular organismal water homeostasis
- neural crest cell differentiation
- olfactory bulb development
- optic nerve development
- ossification
- ossification involved in bone maturation
- perichondral bone morphogenesis
- glomerular visceral epithelial cell differentiation
- polysaccharide biosynthetic process
- protein catabolic process
- protein glycosylation
- protein-containing complex assembly
- regulation of blood pressure
- response to heparin
- response to leukemia inhibitory factor
- response to light intensity
- sebaceous gland development
- signal transduction
- skeletal system development
- smoothened signaling pathway involved in lung development
- social behavior
- sodium ion homeostasis
- stem cell division
- stomach development
- sulfation
- sweat gland development
- synaptic transmission, glutamatergic
- tight junction organization
- TNFSF11-mediated signaling pathway
- vacuole organization
- vasodilation
- vocalization behavior
- wound healing

---

100

- **Protein name:** ATP-citrate synthase
- **Organism:** Homo sapiens
- **Uniprot Accession Number:** P53396
- **Protein sequence length:** 1101 aa
- **1D identity (%):** 16.9
- **1D identity (%) [Gaps excluded]:** 24.47
- **1D identity - Alignment Gaps:** 359
- **1D aligned content (<aminoacid>:%):** {'M': 2.55, 'L': 12.76, 'R': 7.65, 'Y': 3.06, 'K': 6.12, 'P': 8.16, 'V': 7.14, 'H': 2.55, 'F': 2.04, 'I': 5.1, 'G': 13.78, 'E': 5.1, 'T': 2.55, 'D': 5.61, 'W': 0.51, 'A': 7.65, 'C': 1.53, 'N': 2.04, 'S': 2.04, 'Q': 2.04}
- **Common reported functions (%):** 14.29
- **Common reported locations (%):** 33.33
- **Common reported processes (%):** 0.0

- **PDB ID:** 5TE1
- **Chain:** A
- **Crystallized protein length:** 742 aa
- **Resolution:** 2.25 Å
- **b-phipsi:** 0.003711
- **w-rdist:** 0.496093
- **t-alpha:** 0.054676
- **Chemical similarity (Tanimoto Index) (%):** 86.75
- **1D identity (%) [PDB]:** 3.56
- **1D identity (%) [Gaps excluded][PDB]:** 75.0
- **1D identity - Alignment Gaps [PDB]:** 1445
- **1D aligned content [PDB] (<aminoacid>:%):** {'S': 7.41, 'P': 11.11, 'L': 9.26, 'N': 3.7, 'V': 12.96, 'K': 3.7, 'G': 11.11, 'H': 3.7, 'Q': 3.7, 'D': 7.41, 'T': 3.7, 'A': 7.41, 'M': 1.85, 'R': 3.7, 'Y': 1.85, 'C': 1.85, 'E': 1.85, 'I': 3.7}
- **2D identity (%) [PDB]:** 40.57
- **2D identity (%) [Gaps excluded][PDB]:** 86.59
- **2D identity - Alignment Gaps [PDB]:** 575
- **2D aligned content [PDB] (<2D-fold>:%):** {'.': 15.72, 'E': 29.61, 'H': 40.77, 'T': 13.67, 'B': 0.23}
- **3D similarity (TM-Score) (%) [PDB]:** 28.35

- **Gene name:** ACLY
- **Entrez ID:** 47
- **RefSeq ID:** N/A
- **Sequence length:** N/A
- **5-UTR|CDS|3-UTR identity (%):** N/A | N/A | N/A
- **5-UTR|CDS|3-UTR identity (%) [Gaps excluded]:** N/A | N/A | N/A
- **5-UTR|CDS|3-UTR identity [Alignment Gaps]:** N/A | N/A | N/A
- **5-UTR aligned content (<base>:%):** N/A
- **CDS aligned content (<base>:%):** N/A
- **3-UTR aligned content (<base>:%):** N/A

**Uniprot Description:**  
  
 Catalyzes the cleavage of citrate into oxaloacetate and acetyl-CoA, the latter serving as common substrate for de novo cholesterol and fatty acid synthesis.   
  
Homotetramer.   
  
 **Gene Ontology Information:**

Molecular Function

- ATP binding
- ATP citrate synthase activity
- metal ion binding

Location

- azurophil granule lumen
- cytosol
- extracellular exosome
- extracellular region
- ficolin-1-rich granule lumen
- membrane
- nucleoplasm

Biological process

- acetyl-CoA biosynthetic process
- cholesterol biosynthetic process
- citrate metabolic process
- coenzyme A metabolic process
- fatty acid biosynthetic process
- lipid biosynthetic process
- oxaloacetate metabolic process
- tricarboxylic acid cycle

---
