# Supplementary material for: Comparative structural insights and functional analysis for the distinct unbound states of Human AGO proteins
Source: Sci Rep. 2025 Mar 19;15:9432. doi: 10.1038/s41598-025-91849-5 (PMC11923369; doi:10.1038/s41598-025-91849-5)
Supplement: Supplementary file 24 — Supplementary Information 12. [file 41598_2025_91849_MOESM24_ESM.zip › 5VM9p_A_mdwhole_HL2REF/candidates/5VM9p_A-merged-h-enriched_report.html]

 

# Structural Comparison Report for 5VM9p\_A - whole structures (total: 100)

---

0

- **PDB ID:** 6U23 | **Chain:** H
- **b-phipsi:** 0.0008892132434517
- **w-rdist:** 0.3008086950955793
- **t-alpha:** 0.002377470759985

---

---

1

- **PDB ID:** 4Z4F | **Chain:** A
- **b-phipsi:** 0.000193657588726
- **w-rdist:** 0.0879128194521469
- **t-alpha:** 0.0237719193359167

---

---

2

- **PDB ID:** 6FF7 | **Chain:** b
- **b-phipsi:** 0.0034652779735162
- **w-rdist:** 0.3727032079506256
- **t-alpha:** 0.0039622395506779

---

---

3

- **PDB ID:** 5J68 | **Chain:** A
- **b-phipsi:** 0.0025739163795269
- **w-rdist:** 0.4168561477370409
- **t-alpha:** 0.0047768836635659

---

---

4

- **PDB ID:** 8OG4 | **Chain:** A
- **b-phipsi:** 0.0005722673491763
- **w-rdist:** 0.4087817239635772
- **t-alpha:** 0.0161027964403612

---

---

5

- **PDB ID:** 3IHP | **Chain:** A
- **b-phipsi:** 0.0005304479911781
- **w-rdist:** 0.2782695504083858
- **t-alpha:** 0.0665613411172061

---

---

6

- **PDB ID:** 7OM4 | **Chain:** A
- **b-phipsi:** 0.0019097165438755
- **w-rdist:** 0.748419288246852
- **t-alpha:** 0.0

---

---

7

- **PDB ID:** 5Z58 | **Chain:** c
- **b-phipsi:** 0.0028237479680548
- **w-rdist:** 0.4029819024895402
- **t-alpha:** 0.0071825203562971

---

---

8

- **PDB ID:** 6OON | **Chain:** A
- **b-phipsi:** 0.0017932092649952
- **w-rdist:** 0.2307221282342427
- **t-alpha:** 0.0427893647215475

---

---

9

- **PDB ID:** 2WJY | **Chain:** A
- **b-phipsi:** 0.0049126096741579
- **w-rdist:** 0.4852868301421132
- **t-alpha:** 0.0

---

---

10

- **PDB ID:** 4G1M | **Chain:** B
- **b-phipsi:** 0.0024603570994749
- **w-rdist:** 0.6598583422772034
- **t-alpha:** 0.0015872186884768

---

---

11

- **PDB ID:** 6XTY | **Chain:** 7
- **b-phipsi:** 0.0042304617543708
- **w-rdist:** 0.4623943550765823
- **t-alpha:** 0.0039622395506779

---

---

12

- **PDB ID:** 4QCL | **Chain:** A
- **b-phipsi:** 0.0019611427343597
- **w-rdist:** 0.1359038562507484
- **t-alpha:** 0.0903328337654447

---

---

13

- **PDB ID:** 7XE3 | **Chain:** A
- **b-phipsi:** 0.0030637490531205
- **w-rdist:** 0.4673039317991387
- **t-alpha:** 0.006339533171912

---

---

14

- **PDB ID:** 2E1Q | **Chain:** A
- **b-phipsi:** 0.001486770913253
- **w-rdist:** 0.2621994675146065
- **t-alpha:** 0.1164821024786537

---

---

15

- **PDB ID:** 5IZ5 | **Chain:** A
- **b-phipsi:** 0.0007243579668951
- **w-rdist:** 0.4531582365991532
- **t-alpha:** 0.0327332409069371

---

---

16

- **PDB ID:** 6FF4 | **Chain:** B
- **b-phipsi:** 0.0031312519762633
- **w-rdist:** 0.3767426995259328
- **t-alpha:** 0.0112175789229118

---

---

17

- **PDB ID:** 7ENJ | **Chain:** A
- **b-phipsi:** 0.0013394363990223
- **w-rdist:** 0.6111154032274817
- **t-alpha:** 0.0063793623469805

---

---

18

- **PDB ID:** 7SFF | **Chain:** A
- **b-phipsi:** 0.0011283680145137
- **w-rdist:** 0.2635941437574629
- **t-alpha:** 0.162440593977174

---

---

19

- **PDB ID:** 4AH6 | **Chain:** C
- **b-phipsi:** 0.0023239286750264
- **w-rdist:** 0.3941384754899958
- **t-alpha:** 0.0243505672074058

---

---

20

- **PDB ID:** 7QNG | **Chain:** B
- **b-phipsi:** 0.005868689378779
- **w-rdist:** 0.4697691831298261
- **t-alpha:** 0.002377470759985

---

---

21

- **PDB ID:** 5KYN | **Chain:** B
- **b-phipsi:** 0.0021879737483416
- **w-rdist:** 0.6936865808070732
- **t-alpha:** 0.0039775589155952

---

---

22

- **PDB ID:** 6EOS | **Chain:** C
- **b-phipsi:** 0.0057268797660507
- **w-rdist:** 0.5149163168521069
- **t-alpha:** 0.0015848307213084

---

---

23

- **PDB ID:** 6I01 | **Chain:** A
- **b-phipsi:** 0.0020915188242351
- **w-rdist:** 0.8380665768756527
- **t-alpha:** 0.002377470759985

---

---

24

- **PDB ID:** 7K7Z | **Chain:** A
- **b-phipsi:** 0.0132588085176053
- **w-rdist:** 0.427897264588459
- **t-alpha:** 0.0

---

---

25

- **PDB ID:** 4DPG | **Chain:** A
- **b-phipsi:** 0.0030164248933693
- **w-rdist:** 0.6558542402337948
- **t-alpha:** 0.003179348403216

---

---

26

- **PDB ID:** 2HAV | **Chain:** B
- **b-phipsi:** 0.0042590603423451
- **w-rdist:** 0.523593452898076
- **t-alpha:** 0.0047543655130677

---

---

27

- **PDB ID:** 7E8H | **Chain:** I
- **b-phipsi:** 0.0023803556676839
- **w-rdist:** 0.7131496288894863
- **t-alpha:** 0.0039775589155952

---

---

28

- **PDB ID:** 7ABI | **Chain:** r
- **b-phipsi:** 0.0027057224204415
- **w-rdist:** 0.2611675268583068
- **t-alpha:** 0.0942949799421912

---

---

29

- **PDB ID:** 6HTS | **Chain:** D
- **b-phipsi:** 0.0030835404722346
- **w-rdist:** 0.5252435342511752
- **t-alpha:** 0.0071316138654509

---

---

30

- **PDB ID:** 7ORC | **Chain:** A
- **b-phipsi:** 0.0013961362320437
- **w-rdist:** 0.3576178981425165
- **t-alpha:** 0.1957218219675423

---

---

31

- **PDB ID:** 6XTY | **Chain:** 6
- **b-phipsi:** 0.0023892028908141
- **w-rdist:** 0.4670832800692017
- **t-alpha:** 0.0206022505246215

---

---

32

- **PDB ID:** 2XWB | **Chain:** H
- **b-phipsi:** 0.0022174211095771
- **w-rdist:** 0.7108313963037216
- **t-alpha:** 0.0047768836635659

---

---

33

- **PDB ID:** 6XTY | **Chain:** 4
- **b-phipsi:** 0.0025614044627058
- **w-rdist:** 0.4119955381579739
- **t-alpha:** 0.0277337626754545

---

---

34

- **PDB ID:** 4ZG9 | **Chain:** B
- **b-phipsi:** 0.0032957622901348
- **w-rdist:** 0.4849500923588657
- **t-alpha:** 0.0095087103146067

---

---

35

- **PDB ID:** 7UHG | **Chain:** D
- **b-phipsi:** 0.0017166573901687
- **w-rdist:** 0.4658917768870012
- **t-alpha:** 0.0538827219899284

---

---

36

- **PDB ID:** 6LI9 | **Chain:** C
- **b-phipsi:** 0.0026697371755335
- **w-rdist:** 0.7703803218722591
- **t-alpha:** 0.0031697813850419

---

---

37

- **PDB ID:** 7T39 | **Chain:** A
- **b-phipsi:** 0.0044617718382561
- **w-rdist:** 0.4779729081355099
- **t-alpha:** 0.0071316138654509

---

---

38

- **PDB ID:** 7EMF | **Chain:** P
- **b-phipsi:** 0.000839893034861
- **w-rdist:** 0.5426642155229777
- **t-alpha:** 0.0293188630330833

---

---

39

- **PDB ID:** 4KXT | **Chain:** A
- **b-phipsi:** 0.0031423136838832
- **w-rdist:** 0.2540842900684347
- **t-alpha:** 0.1172745652524867

---

---

40

- **PDB ID:** 2VXO | **Chain:** B
- **b-phipsi:** 0.0038932818286318
- **w-rdist:** 0.4114553066671341
- **t-alpha:** 0.0161027964403612

---

---

41

- **PDB ID:** 5GGF | **Chain:** C
- **b-phipsi:** 0.0024574245743787
- **w-rdist:** 0.728666503068166
- **t-alpha:** 0.0047768836635659

---

---

42

- **PDB ID:** 7PPJ | **Chain:** A
- **b-phipsi:** 0.0015021077583291
- **w-rdist:** 0.4971005658104505
- **t-alpha:** 0.0554678888910149

---

---

43

- **PDB ID:** 8EZB | **Chain:** B
- **b-phipsi:** 0.0030057035936239
- **w-rdist:** 0.3005466786849501
- **t-alpha:** 0.1584786099726112

---

---

44

- **PDB ID:** 8ASC | **Chain:** E
- **b-phipsi:** 0.0035207476681388
- **w-rdist:** 0.3141189959106518
- **t-alpha:** 0.0705233695421561

---

---

45

- **PDB ID:** 4QJ4 | **Chain:** B
- **b-phipsi:** 0.0039457034152635
- **w-rdist:** 0.6601779476579921
- **t-alpha:** 0.0039622395506779

---

---

46

- **PDB ID:** 1BKA | **Chain:** A
- **b-phipsi:** 0.0045177710409041
- **w-rdist:** 0.6186430369019257
- **t-alpha:** 0.0039622395506779

---

---

47

- **PDB ID:** 7LRD | **Chain:** C
- **b-phipsi:** 0.0056148717045177
- **w-rdist:** 0.5587647997133769
- **t-alpha:** 0.0031697813850419

---

---

48

- **PDB ID:** 1W1I | **Chain:** B
- **b-phipsi:** 0.0037786473762071
- **w-rdist:** 0.8418558205341391
- **t-alpha:** 0.0015872186884768

---

---

49

- **PDB ID:** 8BHY | **Chain:** T
- **b-phipsi:** 0.0070102081216301
- **w-rdist:** 0.3918522628110036
- **t-alpha:** 0.0071825203562971

---

---

50

- **PDB ID:** 5L46 | **Chain:** A
- **b-phipsi:** 0.0012357954889878
- **w-rdist:** 0.7793204866760921
- **t-alpha:** 0.0087926839253336

---

---

51

- **PDB ID:** 5KZW | **Chain:** A
- **b-phipsi:** 0.0047106778931313
- **w-rdist:** 0.6864568818774863
- **t-alpha:** 0.002377470759985

---

---

52

- **PDB ID:** 1X6V | **Chain:** B
- **b-phipsi:** 0.0051138979013394
- **w-rdist:** 0.5648680967953266
- **t-alpha:** 0.0047543655130677

---

---

53

- **PDB ID:** 7T3B | **Chain:** A
- **b-phipsi:** 0.0024179909436063
- **w-rdist:** 0.4480488693443627
- **t-alpha:** 0.1259907457673486

---

---

54

- **PDB ID:** 6INN | **Chain:** B
- **b-phipsi:** 0.0081164781482751
- **w-rdist:** 0.5961212738666799
- **t-alpha:** 0.0015848307213084

---

---

55

- **PDB ID:** 3ULU | **Chain:** A
- **b-phipsi:** 0.0063018427487742
- **w-rdist:** 0.6388525747978236
- **t-alpha:** 0.0015872186884768

---

---

56

- **PDB ID:** 3K6S | **Chain:** F
- **b-phipsi:** 0.004809095386698
- **w-rdist:** 0.7473494089765231
- **t-alpha:** 0.0015872186884768

---

---

57

- **PDB ID:** 4A5T | **Chain:** S
- **b-phipsi:** 0.0095125594020864
- **w-rdist:** 0.2076727031388011
- **t-alpha:** 0.0142633043406019

---

---

58

- **PDB ID:** 7TW1 | **Chain:** E
- **b-phipsi:** 0.0089799575227523
- **w-rdist:** 0.5821656661595869
- **t-alpha:** 0.0015848307213084

---

---

59

- **PDB ID:** 6XR0 | **Chain:** M
- **b-phipsi:** 0.0025120001376466
- **w-rdist:** 0.501188132709612
- **t-alpha:** 0.0427893647215475

---

---

60

- **PDB ID:** 6U3J | **Chain:** A
- **b-phipsi:** 0.0149057722895768
- **w-rdist:** 0.381968162074106
- **t-alpha:** 0.0055773242968351

---

---

61

- **PDB ID:** 4X0P | **Chain:** A
- **b-phipsi:** 0.0273219057214977
- **w-rdist:** 0.2749373982683809
- **t-alpha:** 0.0071316138654509

---

---

62

- **PDB ID:** 6C02 | **Chain:** B
- **b-phipsi:** 0.0033788775723232
- **w-rdist:** 0.51377411908464
- **t-alpha:** 0.0182252502019479

---

---

63

- **PDB ID:** 1KFU | **Chain:** L
- **b-phipsi:** 0.0022425258018261
- **w-rdist:** 0.5101426963372342
- **t-alpha:** 0.0888697463155465

---

---

64

- **PDB ID:** 2RD0 | **Chain:** A
- **b-phipsi:** 0.0067889904915279
- **w-rdist:** 0.1104703719168629
- **t-alpha:** 0.0356580695998436

---

---

65

- **PDB ID:** 2ZKM | **Chain:** X
- **b-phipsi:** 0.0008561943995074
- **w-rdist:** 0.6154410933618143
- **t-alpha:** 0.0473026125968261

---

---

66

- **PDB ID:** 8EOJ | **Chain:** B
- **b-phipsi:** 0.004261655248687
- **w-rdist:** 0.4554543699287945
- **t-alpha:** 0.0218620195820675

---

---

67

- **PDB ID:** 7AS7 | **Chain:** A
- **b-phipsi:** 0.0013116649425222
- **w-rdist:** 0.8571363469177009
- **t-alpha:** 0.0087926839253336

---

---

68

- **PDB ID:** 7L34 | **Chain:** A
- **b-phipsi:** 0.0033344944693558
- **w-rdist:** 0.7892757029358745
- **t-alpha:** 0.0047768836635659

---

---

69

- **PDB ID:** 6EOQ | **Chain:** C
- **b-phipsi:** 0.0061089689035382
- **w-rdist:** 0.5490732934426286
- **t-alpha:** 0.0047768836635659

---

---

70

- **PDB ID:** 7AD7 | **Chain:** A
- **b-phipsi:** 0.0073794921379084
- **w-rdist:** 0.3674449090355521
- **t-alpha:** 0.013470930625691

---

---

71

- **PDB ID:** 3MDJ | **Chain:** B
- **b-phipsi:** 0.0063533439696447
- **w-rdist:** 0.252217712081588
- **t-alpha:** 0.038827437577757

---

---

72

- **PDB ID:** 6EJC | **Chain:** A
- **b-phipsi:** 0.0025128653706637
- **w-rdist:** 0.6284241024113788
- **t-alpha:** 0.0166402663052187

---

---

73

- **PDB ID:** 7MN8 | **Chain:** A
- **b-phipsi:** 0.0024479632561553
- **w-rdist:** 0.6994415188085482
- **t-alpha:** 0.0128409546837204

---

---

74

- **PDB ID:** 1TZN | **Chain:** O
- **b-phipsi:** 0.0039629240501071
- **w-rdist:** 0.8112112891741461
- **t-alpha:** 0.0047543655130677

---

---

75

- **PDB ID:** 6EA4 | **Chain:** B
- **b-phipsi:** 0.0183965022187064
- **w-rdist:** 0.5312589829141389
- **t-alpha:** 0.002377470759985

---

---

76

- **PDB ID:** 5FTJ | **Chain:** A
- **b-phipsi:** 0.0052973061150515
- **w-rdist:** 0.1237072422794325
- **t-alpha:** 0.1418387309704105

---

---

77

- **PDB ID:** 6XTY | **Chain:** 2
- **b-phipsi:** 0.0035035724027054
- **w-rdist:** 0.4111598217426045
- **t-alpha:** 0.1465931232334234

---

---

78

- **PDB ID:** 4CGY | **Chain:** A
- **b-phipsi:** 0.0146168215842464
- **w-rdist:** 0.4886421718207977
- **t-alpha:** 0.0047768836635659

---

---

79

- **PDB ID:** 6VXK | **Chain:** B
- **b-phipsi:** 0.0193229018218343
- **w-rdist:** 0.3054392039071469
- **t-alpha:** 0.0095087103146067

---

---

80

- **PDB ID:** 7CYN | **Chain:** B
- **b-phipsi:** 0.003780751945932
- **w-rdist:** 0.5321166194109858
- **t-alpha:** 0.0198097874502385

---

---

81

- **PDB ID:** 5IJ7 | **Chain:** B
- **b-phipsi:** 0.0012808045649414
- **w-rdist:** 0.7144619491087362
- **t-alpha:** 0.0301113473631449

---

---

82

- **PDB ID:** 8HBE | **Chain:** B
- **b-phipsi:** 0.0044819462541632
- **w-rdist:** 0.3560660123882703
- **t-alpha:** 0.1228209788379228

---

---

83

- **PDB ID:** 1NUG | **Chain:** A
- **b-phipsi:** 0.0117703989140633
- **w-rdist:** 0.6506680107380138
- **t-alpha:** 0.0007928174706921

---

---

84

- **PDB ID:** 5WJ5 | **Chain:** C
- **b-phipsi:** 0.0483805143453556
- **w-rdist:** 0.3446315148765989
- **t-alpha:** 0.0079243179984995

---

---

85

- **PDB ID:** 4XVM | **Chain:** A
- **b-phipsi:** 0.0245469780770673
- **w-rdist:** 0.5250705363700692
- **t-alpha:** 0.0023827284528445

---

---

86

- **PDB ID:** 5WAI | **Chain:** B
- **b-phipsi:** 0.0164564090158235
- **w-rdist:** 0.4608184998590733
- **t-alpha:** 0.0055773242968351

---

---

87

- **PDB ID:** 7SCH | **Chain:** B
- **b-phipsi:** 0.0022897177573609
- **w-rdist:** 0.5337454755405613
- **t-alpha:** 0.1220288622848613

---

---

88

- **PDB ID:** 3U7U | **Chain:** E
- **b-phipsi:** 0.0046784057269588
- **w-rdist:** 0.6286353431883684
- **t-alpha:** 0.0071316138654509

---

---

89

- **PDB ID:** 7UQX | **Chain:** A
- **b-phipsi:** 0.0028761025648444
- **w-rdist:** 0.691358940488077
- **t-alpha:** 0.013470930625691

---

---

90

- **PDB ID:** 5TE1 | **Chain:** A
- **b-phipsi:** 0.0037113524303989
- **w-rdist:** 0.4960929892678193
- **t-alpha:** 0.0546755105374459

---

---

91

- **PDB ID:** 8A7D | **Chain:** C
- **b-phipsi:** 0.0072678116716724
- **w-rdist:** 0.1558829267864882
- **t-alpha:** 0.0927103766050247

---

---

92

- **PDB ID:** 5MHL | **Chain:** B
- **b-phipsi:** 0.0098425894667589
- **w-rdist:** 0.3124743639559472
- **t-alpha:** 0.0198097874502385

---

---

93

- **PDB ID:** 6WM3 | **Chain:** B
- **b-phipsi:** 0.0049219657753696
- **w-rdist:** 0.6613525022818125
- **t-alpha:** 0.0055773242968351

---

---

94

- **PDB ID:** 8DP0 | **Chain:** A
- **b-phipsi:** 0.0048625438988401
- **w-rdist:** 0.3721332439081602
- **t-alpha:** 0.1093503210020616

---

---

95

- **PDB ID:** 4ZG4 | **Chain:** B
- **b-phipsi:** 0.0098348792745314
- **w-rdist:** 0.4306672870607264
- **t-alpha:** 0.0095087103146067

---

---

96

- **PDB ID:** 1RRJ | **Chain:** A
- **b-phipsi:** 0.0259582938532902
- **w-rdist:** 0.4730625962517829
- **t-alpha:** 0.0055471164626488

---

---

97

- **PDB ID:** 5ISO | **Chain:** C
- **b-phipsi:** 0.0023613319733114
- **w-rdist:** 0.8454764156348403
- **t-alpha:** 0.0112175789229118

---

---

98

- **PDB ID:** 6AHR | **Chain:** B
- **b-phipsi:** 0.0081159604390969
- **w-rdist:** 0.3596710351074818
- **t-alpha:** 0.026149146068277

---

---

99

- **PDB ID:** 5FUR | **Chain:** I
- **b-phipsi:** 0.0124404652801566
- **w-rdist:** 0.210075684226529
- **t-alpha:** 0.0356580695998436

---

---
