# Supplementary material for: Comparative structural insights and functional analysis for the distinct unbound states of Human AGO proteins
Source: Sci Rep. 2025 Mar 19;15:9432. doi: 10.1038/s41598-025-91849-5 (PMC11923369; doi:10.1038/s41598-025-91849-5)
Supplement: Supplementary file 24 — Supplementary Information 12. [file 41598_2025_91849_MOESM24_ESM.zip › 5VM9p_A_mdwhole_HL2REF/candidates/5VM9p_A-merged-notenriched_report.html]

 

# Structural Comparison Report for 5VM9p\_A - whole structures (total: 800)

---

1

- **PDB ID:** 5VM9 | **Chain:** C
- **b-phipsi:** 0.0017453748302197
- **w-rdist:** 0.1491621826687278
- **t-alpha:** 0.0015848307213084

---

---

2

- **PDB ID:** 6U23 | **Chain:** H
- **b-phipsi:** 0.0008892132434517
- **w-rdist:** 0.3008086950955793
- **t-alpha:** 0.002377470759985

---

---

3

- **PDB ID:** 6U23 | **Chain:** F
- **b-phipsi:** 0.0025860619591025
- **w-rdist:** 0.299255344211237
- **t-alpha:** 0.006339533171912

---

---

4

- **PDB ID:** 7AU2 | **Chain:** B
- **b-phipsi:** 0.0015031585564856
- **w-rdist:** 0.4244525725023511
- **t-alpha:** 0.0015848307213084

---

---

5

- **PDB ID:** 7AU2 | **Chain:** A
- **b-phipsi:** 0.0012293990640331
- **w-rdist:** 0.4287222803318675
- **t-alpha:** 0.0031697813850419

---

---

6

- **PDB ID:** 6U23 | **Chain:** M
- **b-phipsi:** 0.0019414798384253
- **w-rdist:** 0.2959532228816955
- **t-alpha:** 0.0112175789229118

---

---

7

- **PDB ID:** 6U2J | **Chain:** H
- **b-phipsi:** 0.001408671808295
- **w-rdist:** 0.3561568571937746
- **t-alpha:** 0.0087926839253336

---

---

8

- **PDB ID:** 6U23 | **Chain:** E
- **b-phipsi:** 0.000971691276952
- **w-rdist:** 0.2980844474626159
- **t-alpha:** 0.0136545862916419

---

---

9

- **PDB ID:** 6FF7 | **Chain:** b
- **b-phipsi:** 0.0034652779735162
- **w-rdist:** 0.3727032079506256
- **t-alpha:** 0.0039622395506779

---

---

10

- **PDB ID:** 6U2W | **Chain:** P
- **b-phipsi:** 0.0033419718832157
- **w-rdist:** 0.4317441437885599
- **t-alpha:** 0.0015848307213084

---

---

11

- **PDB ID:** 5J68 | **Chain:** A
- **b-phipsi:** 0.0025739163795269
- **w-rdist:** 0.4168561477370409
- **t-alpha:** 0.0047768836635659

---

---

12

- **PDB ID:** 6U23 | **Chain:** B
- **b-phipsi:** 0.0037814742753827
- **w-rdist:** 0.3051842199294587
- **t-alpha:** 0.0087166840741086

---

---

13

- **PDB ID:** 6U2J | **Chain:** K
- **b-phipsi:** 0.0024410359232149
- **w-rdist:** 0.3685774501497869
- **t-alpha:** 0.0079243179984995

---

---

14

- **PDB ID:** 5VM9 | **Chain:** A
- **b-phipsi:** 0.0012282528796794
- **w-rdist:** 0.1563152655077996
- **t-alpha:** 0.0213948423587535

---

---

15

- **PDB ID:** 5Z58 | **Chain:** c
- **b-phipsi:** 0.0028237479680548
- **w-rdist:** 0.4029819024895402
- **t-alpha:** 0.0071825203562971

---

---

16

- **PDB ID:** 4Z4F | **Chain:** A
- **b-phipsi:** 0.000193657588726
- **w-rdist:** 0.0879128194521469
- **t-alpha:** 0.0237719193359167

---

---

17

- **PDB ID:** 6U2W | **Chain:** L
- **b-phipsi:** 0.0027642085508278
- **w-rdist:** 0.462702534419354
- **t-alpha:** 0.0047768836635659

---

---

18

- **PDB ID:** 6FF7 | **Chain:** B
- **b-phipsi:** 0.0016238179651549
- **w-rdist:** 0.3812797993328441
- **t-alpha:** 0.0136545862916419

---

---

19

- **PDB ID:** 7XE3 | **Chain:** A
- **b-phipsi:** 0.0030637490531205
- **w-rdist:** 0.4673039317991387
- **t-alpha:** 0.006339533171912

---

---

20

- **PDB ID:** 6XTY | **Chain:** 7
- **b-phipsi:** 0.0042304617543708
- **w-rdist:** 0.4623943550765823
- **t-alpha:** 0.0039622395506779

---

---

21

- **PDB ID:** 6FF4 | **Chain:** B
- **b-phipsi:** 0.0031312519762633
- **w-rdist:** 0.3767426995259328
- **t-alpha:** 0.0112175789229118

---

---

22

- **PDB ID:** 2WJY | **Chain:** A
- **b-phipsi:** 0.0049126096741579
- **w-rdist:** 0.4852868301421132
- **t-alpha:** 0.0

---

---

23

- **PDB ID:** 6U2W | **Chain:** K
- **b-phipsi:** 0.0039229821489575
- **w-rdist:** 0.4536699658292199
- **t-alpha:** 0.0063793623469805

---

---

24

- **PDB ID:** 6U23 | **Chain:** L
- **b-phipsi:** 0.0034629621734682
- **w-rdist:** 0.3029328205724194
- **t-alpha:** 0.0161027964403612

---

---

25

- **PDB ID:** 8OG4 | **Chain:** A
- **b-phipsi:** 0.0005722673491763
- **w-rdist:** 0.4087817239635772
- **t-alpha:** 0.0161027964403612

---

---

26

- **PDB ID:** 4Z4D | **Chain:** A
- **b-phipsi:** 0.00072703025335
- **w-rdist:** 0.097279327673884
- **t-alpha:** 0.0578450666823961

---

---

27

- **PDB ID:** 6U23 | **Chain:** D
- **b-phipsi:** 0.0024231670069784
- **w-rdist:** 0.3037200490226114
- **t-alpha:** 0.0193858366449102

---

---

28

- **PDB ID:** 6U23 | **Chain:** J
- **b-phipsi:** 0.0029238715148255
- **w-rdist:** 0.3027771072945729
- **t-alpha:** 0.0190174537910852

---

---

29

- **PDB ID:** 4Z4I | **Chain:** A
- **b-phipsi:** 0.0019108488088869
- **w-rdist:** 0.1027560868451121
- **t-alpha:** 0.0483363661576923

---

---

30

- **PDB ID:** 6N4O | **Chain:** A
- **b-phipsi:** 0.0006600173498893
- **w-rdist:** 0.1697120975954549
- **t-alpha:** 0.0602220373725139

---

---

31

- **PDB ID:** 4W5R | **Chain:** A
- **b-phipsi:** 0.0008146049303977
- **w-rdist:** 0.1375492504544532
- **t-alpha:** 0.0665613411172061

---

---

32

- **PDB ID:** 7QNG | **Chain:** B
- **b-phipsi:** 0.005868689378779
- **w-rdist:** 0.4697691831298261
- **t-alpha:** 0.002377470759985

---

---

33

- **PDB ID:** 6U23 | **Chain:** P
- **b-phipsi:** 0.0025631588346875
- **w-rdist:** 0.2949758812737119
- **t-alpha:** 0.0213948423587535

---

---

34

- **PDB ID:** 4ZG9 | **Chain:** B
- **b-phipsi:** 0.0032957622901348
- **w-rdist:** 0.4849500923588657
- **t-alpha:** 0.0095087103146067

---

---

35

- **PDB ID:** 6CBD | **Chain:** A
- **b-phipsi:** 0.0021823403573769
- **w-rdist:** 0.1017100495089108
- **t-alpha:** 0.0594296261277553

---

---

36

- **PDB ID:** 8EZA | **Chain:** B
- **b-phipsi:** 0.0032079874903797
- **w-rdist:** 0.5651277157976015
- **t-alpha:** 0.0007928174706921

---

---

37

- **PDB ID:** 7T39 | **Chain:** A
- **b-phipsi:** 0.0044617718382561
- **w-rdist:** 0.4779729081355099
- **t-alpha:** 0.0071316138654509

---

---

38

- **PDB ID:** 7KI3 | **Chain:** D
- **b-phipsi:** 0.0013136427025656
- **w-rdist:** 0.1613340681673014
- **t-alpha:** 0.0625992822193501

---

---

39

- **PDB ID:** 7AUA | **Chain:** A
- **b-phipsi:** 0.0018010007260808
- **w-rdist:** 0.4339908803828045
- **t-alpha:** 0.0166402663052187

---

---

40

- **PDB ID:** 6U23 | **Chain:** I
- **b-phipsi:** 0.0010374720721193
- **w-rdist:** 0.3063861116412018
- **t-alpha:** 0.0302036962314997

---

---

41

- **PDB ID:** 6OON | **Chain:** A
- **b-phipsi:** 0.0017932092649952
- **w-rdist:** 0.2307221282342427
- **t-alpha:** 0.0427893647215475

---

---

42

- **PDB ID:** 4W5Q | **Chain:** A
- **b-phipsi:** 0.0010240210306663
- **w-rdist:** 0.068137439023606
- **t-alpha:** 0.0911254664175305

---

---

43

- **PDB ID:** 6HTS | **Chain:** D
- **b-phipsi:** 0.0030835404722346
- **w-rdist:** 0.5252435342511752
- **t-alpha:** 0.0071316138654509

---

---

44

- **PDB ID:** 6EOS | **Chain:** C
- **b-phipsi:** 0.0057268797660507
- **w-rdist:** 0.5149163168521069
- **t-alpha:** 0.0015848307213084

---

---

45

- **PDB ID:** 2HAV | **Chain:** B
- **b-phipsi:** 0.0042590603423451
- **w-rdist:** 0.523593452898076
- **t-alpha:** 0.0047543655130677

---

---

46

- **PDB ID:** 6U23 | **Chain:** N
- **b-phipsi:** 0.0016155434546863
- **w-rdist:** 0.2996190491382687
- **t-alpha:** 0.0340730688185386

---

---

47

- **PDB ID:** 6U2J | **Chain:** P
- **b-phipsi:** 0.0013231608048223
- **w-rdist:** 0.3904739920148187
- **t-alpha:** 0.0213948423587535

---

---

48

- **PDB ID:** 8BHY | **Chain:** T
- **b-phipsi:** 0.0070102081216301
- **w-rdist:** 0.3918522628110036
- **t-alpha:** 0.0071825203562971

---

---

49

- **PDB ID:** 4ZG7 | **Chain:** A
- **b-phipsi:** 0.0001512083345543
- **w-rdist:** 0.4984790663462144
- **t-alpha:** 0.0166402663052187

---

---

50

- **PDB ID:** 3V83 | **Chain:** C
- **b-phipsi:** 0.0022305927438704
- **w-rdist:** 0.6520171570643626
- **t-alpha:** 0.002377470759985

---

---

51

- **PDB ID:** 4Z4C | **Chain:** A
- **b-phipsi:** 0.0002489283297071
- **w-rdist:** 0.1102658875296972
- **t-alpha:** 0.1172745652524867

---

---

52

- **PDB ID:** 4W5T | **Chain:** A
- **b-phipsi:** 0.001140151369359
- **w-rdist:** 0.1514035011043696
- **t-alpha:** 0.0974642806534764

---

---

53

- **PDB ID:** 6U23 | **Chain:** A
- **b-phipsi:** 0.002291252281848
- **w-rdist:** 0.3224662054170443
- **t-alpha:** 0.0251827326913391

---

---

54

- **PDB ID:** 4QCL | **Chain:** A
- **b-phipsi:** 0.0019611427343597
- **w-rdist:** 0.1359038562507484
- **t-alpha:** 0.0903328337654447

---

---

55

- **PDB ID:** 6ID0 | **Chain:** C
- **b-phipsi:** 0.00371750858056
- **w-rdist:** 0.3736445584254202
- **t-alpha:** 0.0182252502019479

---

---

56

- **PDB ID:** 4G1M | **Chain:** B
- **b-phipsi:** 0.0024603570994749
- **w-rdist:** 0.6598583422772034
- **t-alpha:** 0.0015872186884768

---

---

57

- **PDB ID:** 2VXO | **Chain:** B
- **b-phipsi:** 0.0038932818286318
- **w-rdist:** 0.4114553066671341
- **t-alpha:** 0.0161027964403612

---

---

58

- **PDB ID:** 5MQF | **Chain:** B
- **b-phipsi:** 0.0030978494874683
- **w-rdist:** 0.3599535906282845
- **t-alpha:** 0.0206022505246215

---

---

59

- **PDB ID:** 7ENJ | **Chain:** A
- **b-phipsi:** 0.0013394363990223
- **w-rdist:** 0.6111154032274817
- **t-alpha:** 0.0063793623469805

---

---

60

- **PDB ID:** 6U23 | **Chain:** K
- **b-phipsi:** 0.0011036726361268
- **w-rdist:** 0.3033151390342956
- **t-alpha:** 0.0554678888910149

---

---

61

- **PDB ID:** 3IHP | **Chain:** A
- **b-phipsi:** 0.0005304479911781
- **w-rdist:** 0.2782695504083858
- **t-alpha:** 0.0665613411172061

---

---

62

- **PDB ID:** 4Z4G | **Chain:** A
- **b-phipsi:** 0.0016152342414359
- **w-rdist:** 0.1038839085695156
- **t-alpha:** 0.1117274530174508

---

---

63

- **PDB ID:** 7Q1L | **Chain:** B
- **b-phipsi:** 0.005429175397311
- **w-rdist:** 0.5450323372755869
- **t-alpha:** 0.0015848307213084

---

---

64

- **PDB ID:** 4ZG6 | **Chain:** B
- **b-phipsi:** 0.0008131963853432
- **w-rdist:** 0.5185163062641392
- **t-alpha:** 0.0161027964403612

---

---

65

- **PDB ID:** 6ID1 | **Chain:** C
- **b-phipsi:** 0.0024927810578489
- **w-rdist:** 0.3690488973094996
- **t-alpha:** 0.0221870748795647

---

---

66

- **PDB ID:** 7XE2 | **Chain:** A
- **b-phipsi:** 0.0043178607619158
- **w-rdist:** 0.4516332382132979
- **t-alpha:** 0.0142633043406019

---

---

67

- **PDB ID:** 5Z58 | **Chain:** C
- **b-phipsi:** 0.0033892884931198
- **w-rdist:** 0.4027944970572931
- **t-alpha:** 0.0193858366449102

---

---

68

- **PDB ID:** 7NFE | **Chain:** B
- **b-phipsi:** 0.001368664540196
- **w-rdist:** 0.3308778249250991
- **t-alpha:** 0.0467514280361767

---

---

69

- **PDB ID:** 6ZHE | **Chain:** G
- **b-phipsi:** 0.0011305771629568
- **w-rdist:** 0.3089129672175936
- **t-alpha:** 0.0618066131443488

---

---

70

- **PDB ID:** 4W5O | **Chain:** A
- **b-phipsi:** 0.0016121110024301
- **w-rdist:** 0.1066269413588796
- **t-alpha:** 0.1236136545999804

---

---

71

- **PDB ID:** 6U23 | **Chain:** C
- **b-phipsi:** 0.00198034767182
- **w-rdist:** 0.3057162977148036
- **t-alpha:** 0.0530905293732681

---

---

72

- **PDB ID:** 7OM4 | **Chain:** A
- **b-phipsi:** 0.0019097165438755
- **w-rdist:** 0.748419288246852
- **t-alpha:** 0.0

---

---

73

- **PDB ID:** 6U2J | **Chain:** F
- **b-phipsi:** 0.0037111793245937
- **w-rdist:** 0.3652408623635142
- **t-alpha:** 0.0210354708025768

---

---

74

- **PDB ID:** 4A5T | **Chain:** S
- **b-phipsi:** 0.0095125594020864
- **w-rdist:** 0.2076727031388011
- **t-alpha:** 0.0142633043406019

---

---

75

- **PDB ID:** 4DPG | **Chain:** A
- **b-phipsi:** 0.0030164248933693
- **w-rdist:** 0.6558542402337948
- **t-alpha:** 0.003179348403216

---

---

76

- **PDB ID:** 6ZHE | **Chain:** B
- **b-phipsi:** 0.0025821312073421
- **w-rdist:** 0.3094041760208569
- **t-alpha:** 0.0459591096503235

---

---

77

- **PDB ID:** 7N2M | **Chain:** A
- **b-phipsi:** 0.0021054996838876
- **w-rdist:** 0.1572018763588685
- **t-alpha:** 0.1101430153028129

---

---

78

- **PDB ID:** 7LT3 | **Chain:** K
- **b-phipsi:** 0.0019009783485804
- **w-rdist:** 0.5085124420882766
- **t-alpha:** 0.0169214728656399

---

---

79

- **PDB ID:** 6U2W | **Chain:** C
- **b-phipsi:** 0.005287729291675
- **w-rdist:** 0.4569116475132843
- **t-alpha:** 0.0120284587273298

---

---

80

- **PDB ID:** 6U2J | **Chain:** D
- **b-phipsi:** 0.0036817427162621
- **w-rdist:** 0.3480964434068668
- **t-alpha:** 0.0226902127823684

---

---

81

- **PDB ID:** 5KYN | **Chain:** B
- **b-phipsi:** 0.0021879737483416
- **w-rdist:** 0.6936865808070732
- **t-alpha:** 0.0039775589155952

---

---

82

- **PDB ID:** 7AUA | **Chain:** B
- **b-phipsi:** 0.0008423328134644
- **w-rdist:** 0.4204088326014552
- **t-alpha:** 0.0285264979365806

---

---

83

- **PDB ID:** 6U23 | **Chain:** G
- **b-phipsi:** 0.0020928888611245
- **w-rdist:** 0.29972922743247
- **t-alpha:** 0.0618066131443488

---

---

84

- **PDB ID:** 8OG1 | **Chain:** A
- **b-phipsi:** 0.0007152772825959
- **w-rdist:** 0.4389587834298315
- **t-alpha:** 0.0251827326913391

---

---

85

- **PDB ID:** 8ENU | **Chain:** D
- **b-phipsi:** 0.0014642389956574
- **w-rdist:** 0.7625116123880467
- **t-alpha:** 0.0007928174706921

---

---

86

- **PDB ID:** 4Z4E | **Chain:** A
- **b-phipsi:** 0.0042113305930335
- **w-rdist:** 0.121492992286762
- **t-alpha:** 0.077654736765909

---

---

87

- **PDB ID:** 4BLB | **Chain:** A
- **b-phipsi:** 0.0051897649361371
- **w-rdist:** 0.5997917448056223
- **t-alpha:** 0.0007923402556029

---

---

88

- **PDB ID:** 4AH6 | **Chain:** C
- **b-phipsi:** 0.0023239286750264
- **w-rdist:** 0.3941384754899958
- **t-alpha:** 0.0243505672074058

---

---

89

- **PDB ID:** 3IHP | **Chain:** B
- **b-phipsi:** 0.0020372113355719
- **w-rdist:** 0.2910987690495987
- **t-alpha:** 0.0705233695421561

---

---

90

- **PDB ID:** 6U23 | **Chain:** O
- **b-phipsi:** 0.0015568361063108
- **w-rdist:** 0.2980418788402889
- **t-alpha:** 0.0760701130268726

---

---

91

- **PDB ID:** 8OG4 | **Chain:** B
- **b-phipsi:** 0.0007398473023873
- **w-rdist:** 0.4121437845642788
- **t-alpha:** 0.0361248537980045

---

---

92

- **PDB ID:** 6U2J | **Chain:** N
- **b-phipsi:** 0.0038774355541831
- **w-rdist:** 0.3885476198410712
- **t-alpha:** 0.0210354708025768

---

---

93

- **PDB ID:** 6U2J | **Chain:** M
- **b-phipsi:** 0.0016061588544695
- **w-rdist:** 0.3800734759926243
- **t-alpha:** 0.0412540222902821

---

---

94

- **PDB ID:** 6XTY | **Chain:** 6
- **b-phipsi:** 0.0023892028908141
- **w-rdist:** 0.4670832800692017
- **t-alpha:** 0.0206022505246215

---

---

95

- **PDB ID:** 7LRD | **Chain:** C
- **b-phipsi:** 0.0056148717045177
- **w-rdist:** 0.5587647997133769
- **t-alpha:** 0.0031697813850419

---

---

96

- **PDB ID:** 6ID1 | **Chain:** c
- **b-phipsi:** 0.0032112222043421
- **w-rdist:** 0.3667371174315659
- **t-alpha:** 0.0253567763618671

---

---

97

- **PDB ID:** 7E8H | **Chain:** I
- **b-phipsi:** 0.0023803556676839
- **w-rdist:** 0.7131496288894863
- **t-alpha:** 0.0039775589155952

---

---

98

- **PDB ID:** 7AD7 | **Chain:** A
- **b-phipsi:** 0.0073794921379084
- **w-rdist:** 0.3674449090355521
- **t-alpha:** 0.013470930625691

---

---

99

- **PDB ID:** 7XE1 | **Chain:** A
- **b-phipsi:** 0.0053965399542508
- **w-rdist:** 0.4528329023245303
- **t-alpha:** 0.0142633043406019

---

---

100

- **PDB ID:** 2XWB | **Chain:** H
- **b-phipsi:** 0.0022174211095771
- **w-rdist:** 0.7108313963037216
- **t-alpha:** 0.0047768836635659

---

---

101

- **PDB ID:** 5VNJ | **Chain:** B
- **b-phipsi:** 0.0022680172388458
- **w-rdist:** 0.7351116418854924
- **t-alpha:** 0.0031697813850419

---

---

102

- **PDB ID:** 6ERF | **Chain:** A
- **b-phipsi:** 0.0042662041292309
- **w-rdist:** 0.5812604569959241
- **t-alpha:** 0.0055471164626488

---

---

103

- **PDB ID:** 7LRE | **Chain:** B
- **b-phipsi:** 0.002887074521279
- **w-rdist:** 0.5753696998967821
- **t-alpha:** 0.0087926839253336

---

---

104

- **PDB ID:** 7K7Z | **Chain:** A
- **b-phipsi:** 0.0132588085176053
- **w-rdist:** 0.427897264588459
- **t-alpha:** 0.0

---

---

105

- **PDB ID:** 5VNJ | **Chain:** A
- **b-phipsi:** 0.0017503462380709
- **w-rdist:** 0.6789235096188909
- **t-alpha:** 0.0079243179984995

---

---

106

- **PDB ID:** 5IZ5 | **Chain:** A
- **b-phipsi:** 0.0007243579668951
- **w-rdist:** 0.4531582365991532
- **t-alpha:** 0.0327332409069371

---

---

107

- **PDB ID:** 4QJ4 | **Chain:** B
- **b-phipsi:** 0.0039457034152635
- **w-rdist:** 0.6601779476579921
- **t-alpha:** 0.0039622395506779

---

---

108

- **PDB ID:** 8S91 | **Chain:** B
- **b-phipsi:** 0.0038794124186887
- **w-rdist:** 0.6280091029687872
- **t-alpha:** 0.0055471164626488

---

---

109

- **PDB ID:** 6U2J | **Chain:** G
- **b-phipsi:** 0.0028012041303866
- **w-rdist:** 0.3910639341614471
- **t-alpha:** 0.0285264979365806

---

---

110

- **PDB ID:** 6U2J | **Chain:** J
- **b-phipsi:** 0.0026912872471107
- **w-rdist:** 0.3986878479664758
- **t-alpha:** 0.0285245361798418

---

---

111

- **PDB ID:** 6CEB | **Chain:** B
- **b-phipsi:** 0.0090871518764292
- **w-rdist:** 0.3977331051400421
- **t-alpha:** 0.0087166840741086

---

---

112

- **PDB ID:** 1X6V | **Chain:** B
- **b-phipsi:** 0.0051138979013394
- **w-rdist:** 0.5648680967953266
- **t-alpha:** 0.0047543655130677

---

---

113

- **PDB ID:** 1BKA | **Chain:** A
- **b-phipsi:** 0.0045177710409041
- **w-rdist:** 0.6186430369019257
- **t-alpha:** 0.0039622395506779

---

---

114

- **PDB ID:** 2RD0 | **Chain:** A
- **b-phipsi:** 0.0067889904915279
- **w-rdist:** 0.1104703719168629
- **t-alpha:** 0.0356580695998436

---

---

115

- **PDB ID:** 6U2J | **Chain:** I
- **b-phipsi:** 0.0031384709332908
- **w-rdist:** 0.3589995270663371
- **t-alpha:** 0.0369759793802602

---

---

116

- **PDB ID:** 5WG5 | **Chain:** A
- **b-phipsi:** 0.0126486801264233
- **w-rdist:** 0.432372040747654
- **t-alpha:** 0.0015848307213084

---

---

117

- **PDB ID:** 6XTY | **Chain:** 4
- **b-phipsi:** 0.0025614044627058
- **w-rdist:** 0.4119955381579739
- **t-alpha:** 0.0277337626754545

---

---

118

- **PDB ID:** 7SGL | **Chain:** B
- **b-phipsi:** 0.0023162157214226
- **w-rdist:** 0.3381450742049339
- **t-alpha:** 0.0602220373725139

---

---

119

- **PDB ID:** 5KYW | **Chain:** B
- **b-phipsi:** 0.0010074785192163
- **w-rdist:** 0.7538921369989533
- **t-alpha:** 0.0055471164626488

---

---

120

- **PDB ID:** 4AH6 | **Chain:** B
- **b-phipsi:** 0.0024510214860529
- **w-rdist:** 0.3973178448351634
- **t-alpha:** 0.0378287938704933

---

---

121

- **PDB ID:** 6U2J | **Chain:** A
- **b-phipsi:** 0.0035518866687499
- **w-rdist:** 0.3624649698611306
- **t-alpha:** 0.0344261775693652

---

---

122

- **PDB ID:** 6U2J | **Chain:** O
- **b-phipsi:** 0.0025269537341041
- **w-rdist:** 0.4049944082447767
- **t-alpha:** 0.0348652657606178

---

---

123

- **PDB ID:** 2E1Q | **Chain:** A
- **b-phipsi:** 0.001486770913253
- **w-rdist:** 0.2621994675146065
- **t-alpha:** 0.1164821024786537

---

---

124

- **PDB ID:** 6ICZ | **Chain:** C
- **b-phipsi:** 0.0036197258186867
- **w-rdist:** 0.3585675058987845
- **t-alpha:** 0.0356580695998436

---

---

125

- **PDB ID:** 6ICZ | **Chain:** c
- **b-phipsi:** 0.0036197258186867
- **w-rdist:** 0.3585675058987845
- **t-alpha:** 0.0356580695998436

---

---

126

- **PDB ID:** 6ZHA | **Chain:** B
- **b-phipsi:** 0.0023009054285717
- **w-rdist:** 0.3200040530369582
- **t-alpha:** 0.0744852576021766

---

---

127

- **PDB ID:** 4W5N | **Chain:** A
- **b-phipsi:** 0.0021542352292043
- **w-rdist:** 0.3180632394957963
- **t-alpha:** 0.0792396355379139

---

---

128

- **PDB ID:** 4Z4H | **Chain:** A
- **b-phipsi:** 0.0037517747589096
- **w-rdist:** 0.1152210708521185
- **t-alpha:** 0.1228209788379228

---

---

129

- **PDB ID:** 6MFN | **Chain:** A
- **b-phipsi:** 0.0021830647279299
- **w-rdist:** 0.187814935487582
- **t-alpha:** 0.1370839912556183

---

---

130

- **PDB ID:** 5J67 | **Chain:** B
- **b-phipsi:** 0.0032196528668641
- **w-rdist:** 0.4342863013007563
- **t-alpha:** 0.0226902127823684

---

---

131

- **PDB ID:** 7LT3 | **Chain:** A
- **b-phipsi:** 0.0018414643909002
- **w-rdist:** 0.344045715649808
- **t-alpha:** 0.0689385280761989

---

---

132

- **PDB ID:** 5MQF | **Chain:** b
- **b-phipsi:** 0.003308705872737
- **w-rdist:** 0.3537596111935985
- **t-alpha:** 0.0443742796274553

---

---

133

- **PDB ID:** 6XTX | **Chain:** 7
- **b-phipsi:** 0.00517226100139
- **w-rdist:** 0.4326889578724915
- **t-alpha:** 0.0177417794941832

---

---

134

- **PDB ID:** 7ABI | **Chain:** r
- **b-phipsi:** 0.0027057224204415
- **w-rdist:** 0.2611675268583068
- **t-alpha:** 0.0942949799421912

---

---

135

- **PDB ID:** 6EOQ | **Chain:** C
- **b-phipsi:** 0.0061089689035382
- **w-rdist:** 0.5490732934426286
- **t-alpha:** 0.0047768836635659

---

---

136

- **PDB ID:** 5GGF | **Chain:** C
- **b-phipsi:** 0.0024574245743787
- **w-rdist:** 0.728666503068166
- **t-alpha:** 0.0047768836635659

---

---

137

- **PDB ID:** 7SZ1 | **Chain:** B
- **b-phipsi:** 0.0037829971617044
- **w-rdist:** 0.6735250303408082
- **t-alpha:** 0.0055471164626488

---

---

138

- **PDB ID:** 7SVL | **Chain:** B
- **b-phipsi:** 0.0043958066091817
- **w-rdist:** 0.7277362837154009
- **t-alpha:** 0.0

---

---

139

- **PDB ID:** 5KZW | **Chain:** A
- **b-phipsi:** 0.0047106778931313
- **w-rdist:** 0.6864568818774863
- **t-alpha:** 0.002377470759985

---

---

140

- **PDB ID:** 6CE9 | **Chain:** A
- **b-phipsi:** 0.0081600165723104
- **w-rdist:** 0.3948762074958005
- **t-alpha:** 0.013470930625691

---

---

141

- **PDB ID:** 7NFC | **Chain:** B
- **b-phipsi:** 0.0034213444515356
- **w-rdist:** 0.3156305911648343
- **t-alpha:** 0.0665613411172061

---

---

142

- **PDB ID:** 6U2J | **Chain:** B
- **b-phipsi:** 0.0037149058351414
- **w-rdist:** 0.3828502120581705
- **t-alpha:** 0.0335786233497044

---

---

143

- **PDB ID:** 6C02 | **Chain:** B
- **b-phipsi:** 0.0033788775723232
- **w-rdist:** 0.51377411908464
- **t-alpha:** 0.0182252502019479

---

---

144

- **PDB ID:** 4F3T | **Chain:** A
- **b-phipsi:** 0.0013680641161216
- **w-rdist:** 0.2747832756510465
- **t-alpha:** 0.1299526096838796

---

---

145

- **PDB ID:** 5NN5 | **Chain:** A
- **b-phipsi:** 0.0037573417837446
- **w-rdist:** 0.6860733255810261
- **t-alpha:** 0.0055773242968351

---

---

146

- **PDB ID:** 7AAV | **Chain:** R
- **b-phipsi:** 0.0027451272799576
- **w-rdist:** 0.2447799516128539
- **t-alpha:** 0.11252025444096

---

---

147

- **PDB ID:** 7AAV | **Chain:** r
- **b-phipsi:** 0.0034741470679703
- **w-rdist:** 0.2433275067147261
- **t-alpha:** 0.0990494890758131

---

---

148

- **PDB ID:** 6LI9 | **Chain:** C
- **b-phipsi:** 0.0026697371755335
- **w-rdist:** 0.7703803218722591
- **t-alpha:** 0.0031697813850419

---

---

149

- **PDB ID:** 8ASC | **Chain:** E
- **b-phipsi:** 0.0035207476681388
- **w-rdist:** 0.3141189959106518
- **t-alpha:** 0.0705233695421561

---

---

150

- **PDB ID:** 4AH6 | **Chain:** D
- **b-phipsi:** 0.0024346838980226
- **w-rdist:** 0.4002846084920614
- **t-alpha:** 0.0516663838635056

---

---

151

- **PDB ID:** 6MFR | **Chain:** A
- **b-phipsi:** 0.0005036955595058
- **w-rdist:** 0.3346698789970271
- **t-alpha:** 0.1141048975561447

---

---

152

- **PDB ID:** 7YBU | **Chain:** A
- **b-phipsi:** 0.0017697885763739
- **w-rdist:** 0.5435022075629583
- **t-alpha:** 0.0182252502019479

---

---

153

- **PDB ID:** 1SQY | **Chain:** A
- **b-phipsi:** 0.0063571641381652
- **w-rdist:** 0.6053452918958947
- **t-alpha:** 0.0015872186884768

---

---

154

- **PDB ID:** 5J67 | **Chain:** A
- **b-phipsi:** 0.0022303366634241
- **w-rdist:** 0.4210203365899908
- **t-alpha:** 0.0481724164584582

---

---

155

- **PDB ID:** 2E1Q | **Chain:** D
- **b-phipsi:** 0.001447231935857
- **w-rdist:** 0.2708726799187758
- **t-alpha:** 0.1347072878750428

---

---

156

- **PDB ID:** 7SYD | **Chain:** A
- **b-phipsi:** 0.0026535844426365
- **w-rdist:** 0.7416749768307233
- **t-alpha:** 0.0055471164626488

---

---

157

- **PDB ID:** 4UV7 | **Chain:** A
- **b-phipsi:** 0.0057382120658367
- **w-rdist:** 0.562940275490065
- **t-alpha:** 0.006339533171912

---

---

158

- **PDB ID:** 4BLD | **Chain:** B
- **b-phipsi:** 0.0041859133960448
- **w-rdist:** 0.5629006971888213
- **t-alpha:** 0.0112175789229118

---

---

159

- **PDB ID:** 6U2K | **Chain:** B
- **b-phipsi:** 0.0010555808735728
- **w-rdist:** 0.5061329019506551
- **t-alpha:** 0.0268510508819761

---

---

160

- **PDB ID:** 8D71 | **Chain:** A
- **b-phipsi:** 0.0014793921220328
- **w-rdist:** 0.3695385503065267
- **t-alpha:** 0.0832012408733819

---

---

161

- **PDB ID:** 4GU0 | **Chain:** D
- **b-phipsi:** 0.0019928510104394
- **w-rdist:** 0.4564483487543253
- **t-alpha:** 0.0403954165895417

---

---

162

- **PDB ID:** 2HAU | **Chain:** B
- **b-phipsi:** 0.0060954354894266
- **w-rdist:** 0.5639237812275855
- **t-alpha:** 0.0055471164626488

---

---

163

- **PDB ID:** 7YBU | **Chain:** H
- **b-phipsi:** 0.0016983242745692
- **w-rdist:** 0.533553621902956
- **t-alpha:** 0.0198097874502385

---

---

164

- **PDB ID:** 2E1Q | **Chain:** B
- **b-phipsi:** 0.0016609491394019
- **w-rdist:** 0.2657829242153136
- **t-alpha:** 0.1402537875715366

---

---

165

- **PDB ID:** 3ULU | **Chain:** A
- **b-phipsi:** 0.0063018427487742
- **w-rdist:** 0.6388525747978236
- **t-alpha:** 0.0015872186884768

---

---

166

- **PDB ID:** 3U7U | **Chain:** E
- **b-phipsi:** 0.0046784057269588
- **w-rdist:** 0.6286353431883684
- **t-alpha:** 0.0071316138654509

---

---

167

- **PDB ID:** 4ZG4 | **Chain:** B
- **b-phipsi:** 0.0098348792745314
- **w-rdist:** 0.4306672870607264
- **t-alpha:** 0.0095087103146067

---

---

168

- **PDB ID:** 3EG9 | **Chain:** B
- **b-phipsi:** 0.0007740389057637
- **w-rdist:** 0.7272945632643407
- **t-alpha:** 0.011093664683754

---

---

169

- **PDB ID:** 4ZGA | **Chain:** A
- **b-phipsi:** 0.0015930707444909
- **w-rdist:** 0.5312607530391835
- **t-alpha:** 0.0206022505246215

---

---

170

- **PDB ID:** 7NFC | **Chain:** G
- **b-phipsi:** 0.0040063466122633
- **w-rdist:** 0.3051443445620465
- **t-alpha:** 0.0760701130268726

---

---

171

- **PDB ID:** 6U2J | **Chain:** C
- **b-phipsi:** 0.0048695988262879
- **w-rdist:** 0.3650695587080571
- **t-alpha:** 0.0285264979365806

---

---

172

- **PDB ID:** 6I01 | **Chain:** A
- **b-phipsi:** 0.0020915188242351
- **w-rdist:** 0.8380665768756527
- **t-alpha:** 0.002377470759985

---

---

173

- **PDB ID:** 4KXT | **Chain:** A
- **b-phipsi:** 0.0031423136838832
- **w-rdist:** 0.2540842900684347
- **t-alpha:** 0.1172745652524867

---

---

174

- **PDB ID:** 6SOY | **Chain:** C
- **b-phipsi:** 0.0042803513945882
- **w-rdist:** 0.724848145696084
- **t-alpha:** 0.003179348403216

---

---

175

- **PDB ID:** 6PDM | **Chain:** A
- **b-phipsi:** 0.0095828469123421
- **w-rdist:** 0.4596057517657905
- **t-alpha:** 0.0079867999848444

---

---

176

- **PDB ID:** 8EOJ | **Chain:** B
- **b-phipsi:** 0.004261655248687
- **w-rdist:** 0.4554543699287945
- **t-alpha:** 0.0218620195820675

---

---

177

- **PDB ID:** 5WTD | **Chain:** A
- **b-phipsi:** 0.0053348951414289
- **w-rdist:** 0.6922241400881842
- **t-alpha:** 0.002377470759985

---

---

178

- **PDB ID:** 3MDJ | **Chain:** B
- **b-phipsi:** 0.0063533439696447
- **w-rdist:** 0.252217712081588
- **t-alpha:** 0.038827437577757

---

---

179

- **PDB ID:** 7SVL | **Chain:** C
- **b-phipsi:** 0.0045339752241492
- **w-rdist:** 0.7170083710468531
- **t-alpha:** 0.003179348403216

---

---

180

- **PDB ID:** 4ZG9 | **Chain:** A
- **b-phipsi:** 0.0011129123240631
- **w-rdist:** 0.5027098221688413
- **t-alpha:** 0.0348652657606178

---

---

181

- **PDB ID:** 7LT3 | **Chain:** J
- **b-phipsi:** 0.001380162625941
- **w-rdist:** 0.35691958688306
- **t-alpha:** 0.1069729933769783

---

---

182

- **PDB ID:** 5KXA | **Chain:** A
- **b-phipsi:** 0.0006094281565432
- **w-rdist:** 0.5271037039851577
- **t-alpha:** 0.022979434091199

---

---

183

- **PDB ID:** 1NQL | **Chain:** A
- **b-phipsi:** 0.0043819147414063
- **w-rdist:** 0.5598081401589354
- **t-alpha:** 0.0126786930545719

---

---

184

- **PDB ID:** 6U2J | **Chain:** E
- **b-phipsi:** 0.0040181686611562
- **w-rdist:** 0.3654347001203641
- **t-alpha:** 0.050790674265883

---

---

185

- **PDB ID:** 5WEA | **Chain:** A
- **b-phipsi:** 0.0011419459752658
- **w-rdist:** 0.3249543117394801
- **t-alpha:** 0.1323296128972912

---

---

186

- **PDB ID:** 7KI3 | **Chain:** A
- **b-phipsi:** 0.001783768717122
- **w-rdist:** 0.3292771712083466
- **t-alpha:** 0.1180665242330454

---

---

187

- **PDB ID:** 6LID | **Chain:** C
- **b-phipsi:** 0.0030041182851912
- **w-rdist:** 0.7706946756758876
- **t-alpha:** 0.0047543655130677

---

---

188

- **PDB ID:** 4X1B | **Chain:** A
- **b-phipsi:** 0.0037057553248059
- **w-rdist:** 0.6905120567896976
- **t-alpha:** 0.0079867999848444

---

---

189

- **PDB ID:** 5J67 | **Chain:** D
- **b-phipsi:** 0.0046529791180396
- **w-rdist:** 0.4340427655840311
- **t-alpha:** 0.0226902127823684

---

---

190

- **PDB ID:** 6U3J | **Chain:** A
- **b-phipsi:** 0.0149057722895768
- **w-rdist:** 0.381968162074106
- **t-alpha:** 0.0055773242968351

---

---

191

- **PDB ID:** 6WM3 | **Chain:** B
- **b-phipsi:** 0.0049219657753696
- **w-rdist:** 0.6613525022818125
- **t-alpha:** 0.0055773242968351

---

---

192

- **PDB ID:** 7ZWA | **Chain:** A
- **b-phipsi:** 0.0036997615495229
- **w-rdist:** 0.3309506017939294
- **t-alpha:** 0.0760701130268726

---

---

193

- **PDB ID:** 7ABI | **Chain:** R
- **b-phipsi:** 0.0040343214590203
- **w-rdist:** 0.2629592145829005
- **t-alpha:** 0.1006343630980566

---

---

194

- **PDB ID:** 7SYE | **Chain:** B
- **b-phipsi:** 0.0017181087545765
- **w-rdist:** 0.6819769738328345
- **t-alpha:** 0.013470930625691

---

---

195

- **PDB ID:** 2E1Q | **Chain:** C
- **b-phipsi:** 0.0017189899801424
- **w-rdist:** 0.2694883883929084
- **t-alpha:** 0.1489700676263168

---

---

196

- **PDB ID:** 5JS2 | **Chain:** A
- **b-phipsi:** 0.0011613922836974
- **w-rdist:** 0.2851796860439171
- **t-alpha:** 0.1537244455033311

---

---

197

- **PDB ID:** 3IJE | **Chain:** B
- **b-phipsi:** 0.0061687273989043
- **w-rdist:** 0.6796541356890494
- **t-alpha:** 0.0015872186884768

---

---

198

- **PDB ID:** 7UHG | **Chain:** D
- **b-phipsi:** 0.0017166573901687
- **w-rdist:** 0.4658917768870012
- **t-alpha:** 0.0538827219899284

---

---

199

- **PDB ID:** 2HAU | **Chain:** A
- **b-phipsi:** 0.0032478660928184
- **w-rdist:** 0.5692324002107494
- **t-alpha:** 0.0144693552396331

---

---

200

- **PDB ID:** 7YBU | **Chain:** J
- **b-phipsi:** 0.0026204094113666
- **w-rdist:** 0.5400546409315345
- **t-alpha:** 0.0193858366449102

---

---

201

- **PDB ID:** 5KYX | **Chain:** B
- **b-phipsi:** 0.0007522325633204
- **w-rdist:** 0.7409404633649208
- **t-alpha:** 0.0118861097710496

---

---

202

- **PDB ID:** 7SFF | **Chain:** A
- **b-phipsi:** 0.0011283680145137
- **w-rdist:** 0.2635941437574629
- **t-alpha:** 0.162440593977174

---

---

203

- **PDB ID:** 6NIT | **Chain:** A
- **b-phipsi:** 0.0006313118557669
- **w-rdist:** 0.2834751347139282
- **t-alpha:** 0.162440593977174

---

---

204

- **PDB ID:** 5KYY | **Chain:** A
- **b-phipsi:** 0.0023357928601579
- **w-rdist:** 0.7233499845600022
- **t-alpha:** 0.0103013584398974

---

---

205

- **PDB ID:** 5J67 | **Chain:** C
- **b-phipsi:** 0.0028271287644033
- **w-rdist:** 0.4359254200226842
- **t-alpha:** 0.0516663838635056

---

---

206

- **PDB ID:** 3K6S | **Chain:** F
- **b-phipsi:** 0.004809095386698
- **w-rdist:** 0.7473494089765231
- **t-alpha:** 0.0015872186884768

---

---

207

- **PDB ID:** 4GU0 | **Chain:** C
- **b-phipsi:** 0.0025213610457662
- **w-rdist:** 0.4743056601080015
- **t-alpha:** 0.0395385102655705

---

---

208

- **PDB ID:** 8EZA | **Chain:** K
- **b-phipsi:** 0.0075650749466934
- **w-rdist:** 0.5770237989144693
- **t-alpha:** 0.0023827284528445

---

---

209

- **PDB ID:** 5W6V | **Chain:** A
- **b-phipsi:** 0.0020442262729785
- **w-rdist:** 0.2332332003876996
- **t-alpha:** 0.1616482224946149

---

---

210

- **PDB ID:** 7RBQ | **Chain:** A
- **b-phipsi:** 0.0033739447368295
- **w-rdist:** 0.468576055091119
- **t-alpha:** 0.031695687164061

---

---

211

- **PDB ID:** 6ID0 | **Chain:** c
- **b-phipsi:** 0.0049915650167773
- **w-rdist:** 0.372256688912746
- **t-alpha:** 0.0348652657606178

---

---

212

- **PDB ID:** 5DYH | **Chain:** A
- **b-phipsi:** 0.0047098375982585
- **w-rdist:** 0.5522093584469109
- **t-alpha:** 0.0136545862916419

---

---

213

- **PDB ID:** 4AH6 | **Chain:** A
- **b-phipsi:** 0.0030058127208909
- **w-rdist:** 0.3924339487060064
- **t-alpha:** 0.0685858672132477

---

---

214

- **PDB ID:** 6INN | **Chain:** B
- **b-phipsi:** 0.0081164781482751
- **w-rdist:** 0.5961212738666799
- **t-alpha:** 0.0015848307213084

---

---

215

- **PDB ID:** 5L46 | **Chain:** A
- **b-phipsi:** 0.0012357954889878
- **w-rdist:** 0.7793204866760921
- **t-alpha:** 0.0087926839253336

---

---

216

- **PDB ID:** 8EZB | **Chain:** J
- **b-phipsi:** 0.0013880020248123
- **w-rdist:** 0.3231418226817978
- **t-alpha:** 0.1434231512911101

---

---

217

- **PDB ID:** 5JS1 | **Chain:** A
- **b-phipsi:** 0.0019971767065898
- **w-rdist:** 0.2781761858416838
- **t-alpha:** 0.1521394853607829

---

---

218

- **PDB ID:** 4X1D | **Chain:** A
- **b-phipsi:** 0.0029603132163977
- **w-rdist:** 0.7088627283604441
- **t-alpha:** 0.0104083124295257

---

---

219

- **PDB ID:** 4FXK | **Chain:** B
- **b-phipsi:** 0.0019191328853484
- **w-rdist:** 0.235602844398045
- **t-alpha:** 0.1671950546614453

---

---

220

- **PDB ID:** 5IZ5 | **Chain:** B
- **b-phipsi:** 0.0034924183180328
- **w-rdist:** 0.3551639171617906
- **t-alpha:** 0.0847860205554447

---

---

221

- **PDB ID:** 4OLA | **Chain:** A
- **b-phipsi:** 0.0023076186844815
- **w-rdist:** 0.3645661690817027
- **t-alpha:** 0.1038036020878645

---

---

222

- **PDB ID:** 7ZYG | **Chain:** A
- **b-phipsi:** 0.0025327019044396
- **w-rdist:** 0.3752093748179806
- **t-alpha:** 0.0911254664175305

---

---

223

- **PDB ID:** 3HS0 | **Chain:** D
- **b-phipsi:** 0.0027500050761375
- **w-rdist:** 0.7042348782265104
- **t-alpha:** 0.0112175789229118

---

---

224

- **PDB ID:** 5ES4 | **Chain:** B
- **b-phipsi:** 0.0050627121951924
- **w-rdist:** 0.6978241266905106
- **t-alpha:** 0.0055471164626488

---

---

225

- **PDB ID:** 7Q1L | **Chain:** A
- **b-phipsi:** 0.0049428763629593
- **w-rdist:** 0.5441649187278825
- **t-alpha:** 0.0142633043406019

---

---

226

- **PDB ID:** 5AWC | **Chain:** C
- **b-phipsi:** 0.0038255356334844
- **w-rdist:** 0.3044527106054994
- **t-alpha:** 0.1093503210020616

---

---

227

- **PDB ID:** 5O9Z | **Chain:** B
- **b-phipsi:** 0.0044253189512811
- **w-rdist:** 0.430283152826686
- **t-alpha:** 0.0332804928954846

---

---

228

- **PDB ID:** 6V9U | **Chain:** A
- **b-phipsi:** 0.0030123482091353
- **w-rdist:** 0.3552585329415224
- **t-alpha:** 0.0958797909786817

---

---

229

- **PDB ID:** 6ERH | **Chain:** B
- **b-phipsi:** 0.0043794694676785
- **w-rdist:** 0.2293882145038853
- **t-alpha:** 0.1220288622848613

---

---

230

- **PDB ID:** 6EOR | **Chain:** D
- **b-phipsi:** 0.0058885275080397
- **w-rdist:** 0.7091039646771963
- **t-alpha:** 0.002377470759985

---

---

231

- **PDB ID:** 7L34 | **Chain:** A
- **b-phipsi:** 0.0033344944693558
- **w-rdist:** 0.7892757029358745
- **t-alpha:** 0.0047768836635659

---

---

232

- **PDB ID:** 4Q5V | **Chain:** E
- **b-phipsi:** 0.0017696237099736
- **w-rdist:** 0.1577347932354648
- **t-alpha:** 0.2068147692104327

---

---

233

- **PDB ID:** 7LRD | **Chain:** A
- **b-phipsi:** 0.0030430704810474
- **w-rdist:** 0.5659909822165465
- **t-alpha:** 0.0169214728656399

---

---

234

- **PDB ID:** 7MN8 | **Chain:** A
- **b-phipsi:** 0.0024479632561553
- **w-rdist:** 0.6994415188085482
- **t-alpha:** 0.0128409546837204

---

---

235

- **PDB ID:** 6XTX | **Chain:** 4
- **b-phipsi:** 0.0024524562795077
- **w-rdist:** 0.4864407315468374
- **t-alpha:** 0.0451663942458306

---

---

236

- **PDB ID:** 6U2W | **Chain:** E
- **b-phipsi:** 0.0038678422394122
- **w-rdist:** 0.4678756920143366
- **t-alpha:** 0.0327332409069371

---

---

237

- **PDB ID:** 1LFI | **Chain:** A
- **b-phipsi:** 0.0055118280168284
- **w-rdist:** 0.6144165743315702
- **t-alpha:** 0.0079867999848444

---

---

238

- **PDB ID:** 4G8A | **Chain:** B
- **b-phipsi:** 0.0103678351744468
- **w-rdist:** 0.4549283050515738
- **t-alpha:** 0.0104083124295257

---

---

239

- **PDB ID:** 8EZA | **Chain:** J
- **b-phipsi:** 0.0031646742622721
- **w-rdist:** 0.3382398998675822
- **t-alpha:** 0.1053884795390964

---

---

240

- **PDB ID:** 5H52 | **Chain:** A
- **b-phipsi:** 0.0027143511110208
- **w-rdist:** 0.6754870703117204
- **t-alpha:** 0.0136545862916419

---

---

241

- **PDB ID:** 7KTR | **Chain:** B
- **b-phipsi:** 0.0091922305951827
- **w-rdist:** 0.5270063162269304
- **t-alpha:** 0.006339533171912

---

---

242

- **PDB ID:** 6P0A | **Chain:** A
- **b-phipsi:** 0.00437515824724
- **w-rdist:** 0.7924669441310855
- **t-alpha:** 0.002377470759985

---

---

243

- **PDB ID:** 8EZB | **Chain:** A
- **b-phipsi:** 0.0020562828925124
- **w-rdist:** 0.3248149472543173
- **t-alpha:** 0.1418387309704105

---

---

244

- **PDB ID:** 4OLB | **Chain:** A
- **b-phipsi:** 0.0011712496200884
- **w-rdist:** 0.2962381812795513
- **t-alpha:** 0.1703645180917901

---

---

245

- **PDB ID:** 6U2W | **Chain:** G
- **b-phipsi:** 0.0056502126123827
- **w-rdist:** 0.4431989764765876
- **t-alpha:** 0.0218620195820675

---

---

246

- **PDB ID:** 7CYN | **Chain:** B
- **b-phipsi:** 0.003780751945932
- **w-rdist:** 0.5321166194109858
- **t-alpha:** 0.0198097874502385

---

---

247

- **PDB ID:** 1W1I | **Chain:** B
- **b-phipsi:** 0.0037786473762071
- **w-rdist:** 0.8418558205341391
- **t-alpha:** 0.0015872186884768

---

---

248

- **PDB ID:** 6VXK | **Chain:** B
- **b-phipsi:** 0.0193229018218343
- **w-rdist:** 0.3054392039071469
- **t-alpha:** 0.0095087103146067

---

---

249

- **PDB ID:** 5IXC | **Chain:** A
- **b-phipsi:** 0.0043401708716119
- **w-rdist:** 0.3495654670567006
- **t-alpha:** 0.0729001783846572

---

---

250

- **PDB ID:** 6MFR | **Chain:** B
- **b-phipsi:** 0.0012465823783414
- **w-rdist:** 0.3266765032124991
- **t-alpha:** 0.1529319679525831

---

---

251

- **PDB ID:** 6LI9 | **Chain:** A
- **b-phipsi:** 0.0027846064509234
- **w-rdist:** 0.7714029131152592
- **t-alpha:** 0.0079243179984995

---

---

252

- **PDB ID:** 3QWQ | **Chain:** A
- **b-phipsi:** 0.0036575784315045
- **w-rdist:** 0.6131907585895444
- **t-alpha:** 0.0144693552396331

---

---

253

- **PDB ID:** 7TW1 | **Chain:** E
- **b-phipsi:** 0.0089799575227523
- **w-rdist:** 0.5821656661595869
- **t-alpha:** 0.0015848307213084

---

---

254

- **PDB ID:** 5MHL | **Chain:** B
- **b-phipsi:** 0.0098425894667589
- **w-rdist:** 0.3124743639559472
- **t-alpha:** 0.0198097874502385

---

---

255

- **PDB ID:** 7SFC | **Chain:** A
- **b-phipsi:** 0.0014922336321939
- **w-rdist:** 0.3162038785958003
- **t-alpha:** 0.1545169172535367

---

---

256

- **PDB ID:** 7LT3 | **Chain:** B
- **b-phipsi:** 0.0031188186589969
- **w-rdist:** 0.4874160631149072
- **t-alpha:** 0.03868280500228

---

---

257

- **PDB ID:** 7PPJ | **Chain:** A
- **b-phipsi:** 0.0015021077583291
- **w-rdist:** 0.4971005658104505
- **t-alpha:** 0.0554678888910149

---

---

258

- **PDB ID:** 8IF4 | **Chain:** A
- **b-phipsi:** 0.0043592458328775
- **w-rdist:** 0.4003907754232746
- **t-alpha:** 0.0538827219899284

---

---

259

- **PDB ID:** 7SFE | **Chain:** A
- **b-phipsi:** 0.0016076116775103
- **w-rdist:** 0.2720738648115386
- **t-alpha:** 0.1719494022326169

---

---

260

- **PDB ID:** 7EMF | **Chain:** P
- **b-phipsi:** 0.000839893034861
- **w-rdist:** 0.5426642155229777
- **t-alpha:** 0.0293188630330833

---

---

261

- **PDB ID:** 7UQX | **Chain:** A
- **b-phipsi:** 0.0028761025648444
- **w-rdist:** 0.691358940488077
- **t-alpha:** 0.013470930625691

---

---

262

- **PDB ID:** 8EZA | **Chain:** A
- **b-phipsi:** 0.0038511824724212
- **w-rdist:** 0.3326874830643792
- **t-alpha:** 0.1006343630980566

---

---

263

- **PDB ID:** 5LST | **Chain:** A
- **b-phipsi:** 0.0083137389955676
- **w-rdist:** 0.5468882726165638
- **t-alpha:** 0.0063793623469805

---

---

264

- **PDB ID:** 7XE2 | **Chain:** B
- **b-phipsi:** 0.0029476377338216
- **w-rdist:** 0.4615753342982902
- **t-alpha:** 0.0551835704278558

---

---

265

- **PDB ID:** 1XNJ | **Chain:** B
- **b-phipsi:** 0.0060084121002991
- **w-rdist:** 0.5634498050091632
- **t-alpha:** 0.0104083124295257

---

---

266

- **PDB ID:** 5IZR | **Chain:** A
- **b-phipsi:** 0.004387257198877
- **w-rdist:** 0.4416198856996758
- **t-alpha:** 0.0380351774023424

---

---

267

- **PDB ID:** 6EJC | **Chain:** A
- **b-phipsi:** 0.0025128653706637
- **w-rdist:** 0.6284241024113788
- **t-alpha:** 0.0166402663052187

---

---

268

- **PDB ID:** 4BLD | **Chain:** D
- **b-phipsi:** 0.0091285559642684
- **w-rdist:** 0.5966390931727107
- **t-alpha:** 0.0007928174706921

---

---

269

- **PDB ID:** 4GUR | **Chain:** A
- **b-phipsi:** 0.0025318880035137
- **w-rdist:** 0.4653854750942745
- **t-alpha:** 0.0605041736265279

---

---

270

- **PDB ID:** 6X9I | **Chain:** A
- **b-phipsi:** 0.0017162492683157
- **w-rdist:** 0.2130918249781314
- **t-alpha:** 0.1980983459162797

---

---

271

- **PDB ID:** 5FTJ | **Chain:** A
- **b-phipsi:** 0.0052973061150515
- **w-rdist:** 0.1237072422794325
- **t-alpha:** 0.1418387309704105

---

---

272

- **PDB ID:** 6TY5 | **Chain:** B
- **b-phipsi:** 0.0049908212978556
- **w-rdist:** 0.315851296824245
- **t-alpha:** 0.0784468950036187

---

---

273

- **PDB ID:** 6XR0 | **Chain:** M
- **b-phipsi:** 0.0025120001376466
- **w-rdist:** 0.501188132709612
- **t-alpha:** 0.0427893647215475

---

---

274

- **PDB ID:** 6TY5 | **Chain:** A
- **b-phipsi:** 0.0046448905999084
- **w-rdist:** 0.3170277266754085
- **t-alpha:** 0.0911254664175305

---

---

275

- **PDB ID:** 4R07 | **Chain:** D
- **b-phipsi:** 0.0031644808157116
- **w-rdist:** 0.3654947713166805
- **t-alpha:** 0.099841640611139

---

---

276

- **PDB ID:** 5KYX | **Chain:** A
- **b-phipsi:** 0.0044731671010096
- **w-rdist:** 0.7260813648322153
- **t-alpha:** 0.0071316138654509

---

---

277

- **PDB ID:** 2VXO | **Chain:** A
- **b-phipsi:** 0.0025486060704308
- **w-rdist:** 0.4806606988284197
- **t-alpha:** 0.0538827219899284

---

---

278

- **PDB ID:** 5X5P | **Chain:** A
- **b-phipsi:** 0.0044710137708678
- **w-rdist:** 0.6818860068566118
- **t-alpha:** 0.0095996935833633

---

---

279

- **PDB ID:** 4X0P | **Chain:** A
- **b-phipsi:** 0.0273219057214977
- **w-rdist:** 0.2749373982683809
- **t-alpha:** 0.0071316138654509

---

---

280

- **PDB ID:** 5KI6 | **Chain:** A
- **b-phipsi:** 0.0012066277540498
- **w-rdist:** 0.3839195317907489
- **t-alpha:** 0.1323296128972912

---

---

281

- **PDB ID:** 2HAV | **Chain:** A
- **b-phipsi:** 0.0030283220290313
- **w-rdist:** 0.5683188671706367
- **t-alpha:** 0.0177417794941832

---

---

282

- **PDB ID:** 7XE3 | **Chain:** B
- **b-phipsi:** 0.002618297389635
- **w-rdist:** 0.4459426653734546
- **t-alpha:** 0.0749572397256044

---

---

283

- **PDB ID:** 4GU0 | **Chain:** A
- **b-phipsi:** 0.0021617387808318
- **w-rdist:** 0.4742435944581216
- **t-alpha:** 0.0649788104250272

---

---

284

- **PDB ID:** 3V83 | **Chain:** A
- **b-phipsi:** 0.0028936472162968
- **w-rdist:** 0.6656030857864844
- **t-alpha:** 0.0150558135497862

---

---

285

- **PDB ID:** 4BLB | **Chain:** B
- **b-phipsi:** 0.0079076208912847
- **w-rdist:** 0.5622515876530458
- **t-alpha:** 0.0063793623469805

---

---

286

- **PDB ID:** 5GS0 | **Chain:** A
- **b-phipsi:** 0.0053241725400976
- **w-rdist:** 0.6607533346210283
- **t-alpha:** 0.0079867999848444

---

---

287

- **PDB ID:** 8IF3 | **Chain:** A
- **b-phipsi:** 0.0035778475844228
- **w-rdist:** 0.4375366628523019
- **t-alpha:** 0.0618066131443488

---

---

288

- **PDB ID:** 6EOR | **Chain:** C
- **b-phipsi:** 0.005999686849144
- **w-rdist:** 0.7135611369185607
- **t-alpha:** 0.0039622395506779

---

---

289

- **PDB ID:** 3SWR | **Chain:** A
- **b-phipsi:** 0.0009070815514748
- **w-rdist:** 0.451862518220019
- **t-alpha:** 0.1085581927223058

---

---

290

- **PDB ID:** 4FWJ | **Chain:** B
- **b-phipsi:** 0.0046733933955384
- **w-rdist:** 0.4443886789878599
- **t-alpha:** 0.03868280500228

---

---

291

- **PDB ID:** 3HIZ | **Chain:** A
- **b-phipsi:** 0.0056608223157812
- **w-rdist:** 0.2526449638492246
- **t-alpha:** 0.0919176339506717

---

---

292

- **PDB ID:** 7YBU | **Chain:** B
- **b-phipsi:** 0.0023154416128179
- **w-rdist:** 0.5451130647602316
- **t-alpha:** 0.0226902127823684

---

---

293

- **PDB ID:** 4R08 | **Chain:** C
- **b-phipsi:** 0.0031369871381339
- **w-rdist:** 0.3609911999956188
- **t-alpha:** 0.1148972678966586

---

---

294

- **PDB ID:** 4YCU | **Chain:** A
- **b-phipsi:** 0.0066178620420096
- **w-rdist:** 0.6677728842052153
- **t-alpha:** 0.0047768836635659

---

---

295

- **PDB ID:** 3EFO | **Chain:** B
- **b-phipsi:** 0.0014987557281979
- **w-rdist:** 0.7492664329202832
- **t-alpha:** 0.013470930625691

---

---

296

- **PDB ID:** 1TZN | **Chain:** O
- **b-phipsi:** 0.0039629240501071
- **w-rdist:** 0.8112112891741461
- **t-alpha:** 0.0047543655130677

---

---

297

- **PDB ID:** 8A7D | **Chain:** C
- **b-phipsi:** 0.0072678116716724
- **w-rdist:** 0.1558829267864882
- **t-alpha:** 0.0927103766050247

---

---

298

- **PDB ID:** 5O9Z | **Chain:** b
- **b-phipsi:** 0.0028192294218035
- **w-rdist:** 0.4764472447163701
- **t-alpha:** 0.0586373023344051

---

---

299

- **PDB ID:** 4BLB | **Chain:** C
- **b-phipsi:** 0.005148735977003
- **w-rdist:** 0.5527366575696941
- **t-alpha:** 0.0152852632192168

---

---

300

- **PDB ID:** 4Q5V | **Chain:** A
- **b-phipsi:** 0.0025094537537844
- **w-rdist:** 0.1611186504972351
- **t-alpha:** 0.2147381992567347

---

---

301

- **PDB ID:** 3HS0 | **Chain:** I
- **b-phipsi:** 0.001245976274389
- **w-rdist:** 0.7280045983238408
- **t-alpha:** 0.0152852632192168

---

---

302

- **PDB ID:** 6GYR | **Chain:** A
- **b-phipsi:** 0.0080282609387165
- **w-rdist:** 0.5003074184420606
- **t-alpha:** 0.0142633043406019

---

---

303

- **PDB ID:** 4FWJ | **Chain:** A
- **b-phipsi:** 0.004473255314091
- **w-rdist:** 0.4582107507761355
- **t-alpha:** 0.0372425322509186

---

---

304

- **PDB ID:** 6MDZ | **Chain:** B
- **b-phipsi:** 0.0011975880561342
- **w-rdist:** 0.310778404960512
- **t-alpha:** 0.1838352099719058

---

---

305

- **PDB ID:** 4BLB | **Chain:** D
- **b-phipsi:** 0.005517108708447
- **w-rdist:** 0.5937492696559242
- **t-alpha:** 0.0112175789229118

---

---

306

- **PDB ID:** 8D6J | **Chain:** A
- **b-phipsi:** 0.0029198544113819
- **w-rdist:** 0.3268330491275267
- **t-alpha:** 0.1418387309704105

---

---

307

- **PDB ID:** 6EOR | **Chain:** B
- **b-phipsi:** 0.0048623224653018
- **w-rdist:** 0.7129201086930453
- **t-alpha:** 0.0079867999848444

---

---

308

- **PDB ID:** 5Y3R | **Chain:** A
- **b-phipsi:** 0.0023804853229767
- **w-rdist:** 0.4892309344009721
- **t-alpha:** 0.0618066131443488

---

---

309

- **PDB ID:** 4PNK | **Chain:** A
- **b-phipsi:** 0.014410656488604
- **w-rdist:** 0.3959492676155842
- **t-alpha:** 0.011093664683754

---

---

310

- **PDB ID:** 7SFG | **Chain:** A
- **b-phipsi:** 0.0009617831423696
- **w-rdist:** 0.2343244095870837
- **t-alpha:** 0.2250398398369042

---

---

311

- **PDB ID:** 6MSU | **Chain:** B
- **b-phipsi:** 0.0040743905766415
- **w-rdist:** 0.6488863845127317
- **t-alpha:** 0.0142633043406019

---

---

312

- **PDB ID:** 6XI2 | **Chain:** D
- **b-phipsi:** 0.0015175694636422
- **w-rdist:** 0.6261531597984161
- **t-alpha:** 0.0202100770637112

---

---

313

- **PDB ID:** 6X9K | **Chain:** A
- **b-phipsi:** 0.0014313636546018
- **w-rdist:** 0.2856688903807108
- **t-alpha:** 0.191758917599204

---

---

314

- **PDB ID:** 4CGY | **Chain:** A
- **b-phipsi:** 0.0146168215842464
- **w-rdist:** 0.4886421718207977
- **t-alpha:** 0.0047768836635659

---

---

315

- **PDB ID:** 4DUR | **Chain:** B
- **b-phipsi:** 0.0092390037095146
- **w-rdist:** 0.1670552264436763
- **t-alpha:** 0.0562598552305482

---

---

316

- **PDB ID:** 7XE1 | **Chain:** B
- **b-phipsi:** 0.0027412487953394
- **w-rdist:** 0.4662877470819876
- **t-alpha:** 0.0713072206433529

---

---

317

- **PDB ID:** 4GU0 | **Chain:** B
- **b-phipsi:** 0.003702093052292
- **w-rdist:** 0.4688828221702578
- **t-alpha:** 0.0551835704278558

---

---

318

- **PDB ID:** 7RC9 | **Chain:** A
- **b-phipsi:** 0.004202120091303
- **w-rdist:** 0.362733464518072
- **t-alpha:** 0.0935026519522419

---

---

319

- **PDB ID:** 4KRE | **Chain:** A
- **b-phipsi:** 0.0014519615636429
- **w-rdist:** 0.2178480407527682
- **t-alpha:** 0.2250398398369042

---

---

320

- **PDB ID:** 7AS7 | **Chain:** A
- **b-phipsi:** 0.0013116649425222
- **w-rdist:** 0.8571363469177009
- **t-alpha:** 0.0087926839253336

---

---

321

- **PDB ID:** 2WJV | **Chain:** A
- **b-phipsi:** 0.0042611079735394
- **w-rdist:** 0.4594906934413348
- **t-alpha:** 0.0464345284809148

---

---

322

- **PDB ID:** 5IUD | **Chain:** G
- **b-phipsi:** 0.0027413365234961
- **w-rdist:** 0.3018509697636231
- **t-alpha:** 0.1600634400235712

---

---

323

- **PDB ID:** 8FCQ | **Chain:** D
- **b-phipsi:** 0.0047929921279396
- **w-rdist:** 0.1511981435295
- **t-alpha:** 0.162440593977174

---

---

324

- **PDB ID:** 5AZ5 | **Chain:** C
- **b-phipsi:** 0.0038293113309985
- **w-rdist:** 0.368973419771661
- **t-alpha:** 0.1014266065453182

---

---

325

- **PDB ID:** 4X0P | **Chain:** B
- **b-phipsi:** 0.03073029393511
- **w-rdist:** 0.3155719540946511
- **t-alpha:** 0.0055773242968351

---

---

326

- **PDB ID:** 5VNM | **Chain:** B
- **b-phipsi:** 0.0015675549463126
- **w-rdist:** 0.7188995387175237
- **t-alpha:** 0.0161027964403612

---

---

327

- **PDB ID:** 6U2W | **Chain:** O
- **b-phipsi:** 0.0052711773218787
- **w-rdist:** 0.4292889665688691
- **t-alpha:** 0.0403954165895417

---

---

328

- **PDB ID:** 8EZB | **Chain:** B
- **b-phipsi:** 0.0030057035936239
- **w-rdist:** 0.3005466786849501
- **t-alpha:** 0.1584786099726112

---

---

329

- **PDB ID:** 6P0C | **Chain:** A
- **b-phipsi:** 0.0043762866982376
- **w-rdist:** 0.8066215807246446
- **t-alpha:** 0.0047543655130677

---

---

330

- **PDB ID:** 5IUD | **Chain:** J
- **b-phipsi:** 0.0027413901985437
- **w-rdist:** 0.2435664994011023
- **t-alpha:** 0.1846280285808399

---

---

331

- **PDB ID:** 6U7C | **Chain:** A
- **b-phipsi:** 0.0139519599701618
- **w-rdist:** 0.4149882916209778
- **t-alpha:** 0.011093664683754

---

---

332

- **PDB ID:** 6V9U | **Chain:** B
- **b-phipsi:** 0.0050314631019117
- **w-rdist:** 0.319801802641154
- **t-alpha:** 0.0966722725879358

---

---

333

- **PDB ID:** 7K71 | **Chain:** A
- **b-phipsi:** 0.0075993965721959
- **w-rdist:** 0.1676634831001402
- **t-alpha:** 0.0927103766050247

---

---

334

- **PDB ID:** 4R08 | **Chain:** D
- **b-phipsi:** 0.0044750533030467
- **w-rdist:** 0.3639289582895145
- **t-alpha:** 0.089540776910131

---

---

335

- **PDB ID:** 4R09 | **Chain:** D
- **b-phipsi:** 0.0024187759181797
- **w-rdist:** 0.3658405082153163
- **t-alpha:** 0.1386688723323981

---

---

336

- **PDB ID:** 7R9V | **Chain:** A
- **b-phipsi:** 0.0094872039500588
- **w-rdist:** 0.1720648509362785
- **t-alpha:** 0.0554678888910149

---

---

337

- **PDB ID:** 7JTN | **Chain:** C
- **b-phipsi:** 0.0057297086425594
- **w-rdist:** 0.7479109707361908
- **t-alpha:** 0.0039622395506779

---

---

338

- **PDB ID:** 6ILH | **Chain:** A
- **b-phipsi:** 0.0063845460379812
- **w-rdist:** 0.6601459800617829
- **t-alpha:** 0.0071825203562971

---

---

339

- **PDB ID:** 5JTW | **Chain:** B
- **b-phipsi:** 0.0022149912120837
- **w-rdist:** 1.1837246189321975
- **t-alpha:** 0.0

---

---

340

- **PDB ID:** 3K6S | **Chain:** B
- **b-phipsi:** 0.0053057336648165
- **w-rdist:** 0.6955309676876291
- **t-alpha:** 0.0087166840741086

---

---

341

- **PDB ID:** 3PTA | **Chain:** A
- **b-phipsi:** 0.0026778088792204
- **w-rdist:** 0.3747373185352739
- **t-alpha:** 0.1291603077989023

---

---

342

- **PDB ID:** 7ASG | **Chain:** A
- **b-phipsi:** 0.0018326039754341
- **w-rdist:** 0.8559377005489619
- **t-alpha:** 0.0087166840741086

---

---

343

- **PDB ID:** 4BLD | **Chain:** C
- **b-phipsi:** 0.0049305351979958
- **w-rdist:** 0.5557233975629047
- **t-alpha:** 0.0169214728656399

---

---

344

- **PDB ID:** 5WG4 | **Chain:** A
- **b-phipsi:** 0.0121330560494599
- **w-rdist:** 0.403190780741818
- **t-alpha:** 0.0142633043406019

---

---

345

- **PDB ID:** 8PFI | **Chain:** A
- **b-phipsi:** 0.0042295064723601
- **w-rdist:** 0.3152491493576407
- **t-alpha:** 0.1315372629774758

---

---

346

- **PDB ID:** 6NIT | **Chain:** B
- **b-phipsi:** 0.0001884663874837
- **w-rdist:** 0.2760042094581763
- **t-alpha:** 0.2258321959381128

---

---

347

- **PDB ID:** 5WAI | **Chain:** B
- **b-phipsi:** 0.0164564090158235
- **w-rdist:** 0.4608184998590733
- **t-alpha:** 0.0055773242968351

---

---

348

- **PDB ID:** 4R0A | **Chain:** A
- **b-phipsi:** 0.0039806783412136
- **w-rdist:** 0.3156560211999481
- **t-alpha:** 0.1394613260566366

---

---

349

- **PDB ID:** 3W3G | **Chain:** A
- **b-phipsi:** 0.0040815990410498
- **w-rdist:** 0.3643962518096275
- **t-alpha:** 0.1069729933769783

---

---

350

- **PDB ID:** 4BLD | **Chain:** A
- **b-phipsi:** 0.0043654739838761
- **w-rdist:** 0.5949426490844825
- **t-alpha:** 0.0161027964403612

---

---

351

- **PDB ID:** 8P4F | **Chain:** O
- **b-phipsi:** 0.0087532377033367
- **w-rdist:** 0.5375888067462644
- **t-alpha:** 0.0095087103146067

---

---

352

- **PDB ID:** 6WM4 | **Chain:** C
- **b-phipsi:** 0.0040306078754938
- **w-rdist:** 0.6359481570722162
- **t-alpha:** 0.0161027964403612

---

---

353

- **PDB ID:** 1R9N | **Chain:** B
- **b-phipsi:** 0.0040357196468625
- **w-rdist:** 0.8240208545419547
- **t-alpha:** 0.0055773242968351

---

---

354

- **PDB ID:** 4YCW | **Chain:** F
- **b-phipsi:** 0.0054592970732287
- **w-rdist:** 0.663567576502073
- **t-alpha:** 0.0104083124295257

---

---

355

- **PDB ID:** 5FTK | **Chain:** E
- **b-phipsi:** 0.0049629683899981
- **w-rdist:** 0.1971951721051294
- **t-alpha:** 0.1553093824431966

---

---

356

- **PDB ID:** 4ZG6 | **Chain:** A
- **b-phipsi:** 0.0002554174200611
- **w-rdist:** 0.5337746237719164
- **t-alpha:** 0.0602220373725139

---

---

357

- **PDB ID:** 8T2V | **Chain:** B
- **b-phipsi:** 0.0029046372167789
- **w-rdist:** 0.8626453024075738
- **t-alpha:** 0.0071316138654509

---

---

358

- **PDB ID:** 7R53 | **Chain:** A
- **b-phipsi:** 0.0050846086700308
- **w-rdist:** 0.3178771269648471
- **t-alpha:** 0.1069729933769783

---

---

359

- **PDB ID:** 2CKJ | **Chain:** A
- **b-phipsi:** 0.0031637983010671
- **w-rdist:** 0.3931774982076785
- **t-alpha:** 0.1204441681482937

---

---

360

- **PDB ID:** 7WUB | **Chain:** J
- **b-phipsi:** 0.0063306054125851
- **w-rdist:** 0.1430843477324042
- **t-alpha:** 0.1418387309704105

---

---

361

- **PDB ID:** 6J71 | **Chain:** A
- **b-phipsi:** 0.00818940980521
- **w-rdist:** 0.5814844037989283
- **t-alpha:** 0.0071316138654509

---

---

362

- **PDB ID:** 1YGR | **Chain:** B
- **b-phipsi:** 0.0049838143858643
- **w-rdist:** 0.7754795715849037
- **t-alpha:** 0.0055471164626488

---

---

363

- **PDB ID:** 7E8B | **Chain:** J
- **b-phipsi:** 0.0040321225967862
- **w-rdist:** 0.7113061197811793
- **t-alpha:** 0.0128409546837204

---

---

364

- **PDB ID:** 6AHR | **Chain:** B
- **b-phipsi:** 0.0081159604390969
- **w-rdist:** 0.3596710351074818
- **t-alpha:** 0.026149146068277

---

---

365

- **PDB ID:** 3BJU | **Chain:** C
- **b-phipsi:** 0.0034358499747109
- **w-rdist:** 0.6684509656905284
- **t-alpha:** 0.0166402663052187

---

---

366

- **PDB ID:** 8FCL | **Chain:** A
- **b-phipsi:** 0.0050910801829136
- **w-rdist:** 0.2659877537131512
- **t-alpha:** 0.1331219773390095

---

---

367

- **PDB ID:** 7R54 | **Chain:** A
- **b-phipsi:** 0.0041483441782687
- **w-rdist:** 0.322349892750758
- **t-alpha:** 0.1354991503352756

---

---

368

- **PDB ID:** 7RC9 | **Chain:** B
- **b-phipsi:** 0.0042322639971984
- **w-rdist:** 0.3659509842782271
- **t-alpha:** 0.1077655736112999

---

---

369

- **PDB ID:** 7UN3 | **Chain:** A
- **b-phipsi:** 0.006434290493909
- **w-rdist:** 0.4190358289879044
- **t-alpha:** 0.0310455330972876

---

---

370

- **PDB ID:** 3HHM | **Chain:** A
- **b-phipsi:** 0.007367917919995
- **w-rdist:** 0.3149205501998283
- **t-alpha:** 0.0562598552305482

---

---

371

- **PDB ID:** 7WUB | **Chain:** G
- **b-phipsi:** 0.0074151578853688
- **w-rdist:** 0.145557411506449
- **t-alpha:** 0.1188590445787747

---

---

372

- **PDB ID:** 7CYN | **Chain:** A
- **b-phipsi:** 0.0029388001369879
- **w-rdist:** 0.540580796503654
- **t-alpha:** 0.0285264979365806

---

---

373

- **PDB ID:** 4MMY | **Chain:** B
- **b-phipsi:** 0.006395324217925
- **w-rdist:** 0.6452882535482507
- **t-alpha:** 0.0095087103146067

---

---

374

- **PDB ID:** 4NEN | **Chain:** B
- **b-phipsi:** 0.0048184309630376
- **w-rdist:** 0.8108344570945248
- **t-alpha:** 0.0047543655130677

---

---

375

- **PDB ID:** 6MGQ | **Chain:** C
- **b-phipsi:** 0.0084704563233276
- **w-rdist:** 0.1432351853871405
- **t-alpha:** 0.0958797909786817

---

---

376

- **PDB ID:** 6XFI | **Chain:** A
- **b-phipsi:** 0.0010232052703907
- **w-rdist:** 0.5991733831200248
- **t-alpha:** 0.0260160634552359

---

---

377

- **PDB ID:** 8BRC | **Chain:** A
- **b-phipsi:** 0.0033732364155054
- **w-rdist:** 0.7039037944823857
- **t-alpha:** 0.0152852632192168

---

---

378

- **PDB ID:** 3W3L | **Chain:** B
- **b-phipsi:** 0.0030024217279152
- **w-rdist:** 0.3108783557442214
- **t-alpha:** 0.1695723222503973

---

---

379

- **PDB ID:** 6C01 | **Chain:** B
- **b-phipsi:** 0.0039586603538507
- **w-rdist:** 0.5192578443879322
- **t-alpha:** 0.0332804928954846

---

---

380

- **PDB ID:** 8D96 | **Chain:** C
- **b-phipsi:** 0.0090615999161294
- **w-rdist:** 0.286449636511551
- **t-alpha:** 0.0412048690981889

---

---

381

- **PDB ID:** 5OAF | **Chain:** D
- **b-phipsi:** 0.0023064372499107
- **w-rdist:** 0.7035175980335373
- **t-alpha:** 0.0177417794941832

---

---

382

- **PDB ID:** 5TE1 | **Chain:** A
- **b-phipsi:** 0.0037113524303989
- **w-rdist:** 0.4960929892678193
- **t-alpha:** 0.0546755105374459

---

---

383

- **PDB ID:** 5C19 | **Chain:** E
- **b-phipsi:** 0.0054283206764313
- **w-rdist:** 0.1817444598144391
- **t-alpha:** 0.1561018403117944

---

---

384

- **PDB ID:** 3QYT | **Chain:** A
- **b-phipsi:** 0.0053194286414656
- **w-rdist:** 0.7250251951180677
- **t-alpha:** 0.0079867999848444

---

---

385

- **PDB ID:** 8FCT | **Chain:** B
- **b-phipsi:** 0.0029114514016869
- **w-rdist:** 0.139866999993196
- **t-alpha:** 0.2686219740019155

---

---

386

- **PDB ID:** 7MQO | **Chain:** E
- **b-phipsi:** 0.0050920416589159
- **w-rdist:** 0.4793963848366344
- **t-alpha:** 0.0340730688185386

---

---

387

- **PDB ID:** 5JPN | **Chain:** A
- **b-phipsi:** 0.0337858716013362
- **w-rdist:** 0.4094489549052818
- **t-alpha:** 0.0015848307213084

---

---

388

- **PDB ID:** 7MQO | **Chain:** F
- **b-phipsi:** 0.0042522854277263
- **w-rdist:** 0.4771957485199761
- **t-alpha:** 0.0554678888910149

---

---

389

- **PDB ID:** 3W5O | **Chain:** A
- **b-phipsi:** 0.0049103539027393
- **w-rdist:** 0.5691673238440652
- **t-alpha:** 0.0166402663052187

---

---

390

- **PDB ID:** 7SVL | **Chain:** A
- **b-phipsi:** 0.0064013652376668
- **w-rdist:** 0.7064587846768329
- **t-alpha:** 0.0071316138654509

---

---

391

- **PDB ID:** 6X9J | **Chain:** A
- **b-phipsi:** 0.0019693415560115
- **w-rdist:** 0.2581212683322209
- **t-alpha:** 0.2210775835865639

---

---

392

- **PDB ID:** 7TW1 | **Chain:** F
- **b-phipsi:** 0.0101149758206712
- **w-rdist:** 0.5933762769577684
- **t-alpha:** 0.003179348403216

---

---

393

- **PDB ID:** 4GUU | **Chain:** A
- **b-phipsi:** 0.0040729093246847
- **w-rdist:** 0.4910347081170166
- **t-alpha:** 0.0516663838635056

---

---

394

- **PDB ID:** 6ZJZ | **Chain:** A
- **b-phipsi:** 0.0041764878708467
- **w-rdist:** 0.3178856801882267
- **t-alpha:** 0.1458006268370766

---

---

395

- **PDB ID:** 3U7U | **Chain:** C
- **b-phipsi:** 0.0068542158581213
- **w-rdist:** 0.6731697144528153
- **t-alpha:** 0.0071825203562971

---

---

396

- **PDB ID:** 6U2J | **Chain:** L
- **b-phipsi:** 0.0073177656962221
- **w-rdist:** 0.2752795996669246
- **t-alpha:** 0.0777112634888108

---

---

397

- **PDB ID:** 6SOZ | **Chain:** C
- **b-phipsi:** 0.0040391597986049
- **w-rdist:** 0.7244301373850806
- **t-alpha:** 0.0128409546837204

---

---

398

- **PDB ID:** 5JPN | **Chain:** B
- **b-phipsi:** 0.0045760421387383
- **w-rdist:** 0.2469165811148407
- **t-alpha:** 0.1576861790132646

---

---

399

- **PDB ID:** 4GU1 | **Chain:** A
- **b-phipsi:** 0.0021278641995168
- **w-rdist:** 0.5208971138448756
- **t-alpha:** 0.0633915289148658

---

---

400

- **PDB ID:** 5KZX | **Chain:** A
- **b-phipsi:** 0.0047881039554228
- **w-rdist:** 0.6871743492475976
- **t-alpha:** 0.0128409546837204

---

---

401

- **PDB ID:** 5C19 | **Chain:** C
- **b-phipsi:** 0.0062537019083586
- **w-rdist:** 0.1496370668898192
- **t-alpha:** 0.1497625631756258

---

---

402

- **PDB ID:** 4FWF | **Chain:** A
- **b-phipsi:** 0.0029498517204496
- **w-rdist:** 0.4541568925281066
- **t-alpha:** 0.1022188946752804

---

---

403

- **PDB ID:** 1LCF | **Chain:** A
- **b-phipsi:** 0.0041114554140098
- **w-rdist:** 0.6047657731508885
- **t-alpha:** 0.0182252502019479

---

---

404

- **PDB ID:** 6NAJ | **Chain:** B
- **b-phipsi:** 0.0066860394762492
- **w-rdist:** 0.6426315821255612
- **t-alpha:** 0.0095087103146067

---

---

405

- **PDB ID:** 5VNI | **Chain:** B
- **b-phipsi:** 0.0026497619432319
- **w-rdist:** 0.6818220694926874
- **t-alpha:** 0.0185632335173342

---

---

406

- **PDB ID:** 6ERG | **Chain:** E
- **b-phipsi:** 0.0048752407622519
- **w-rdist:** 0.2503231288883913
- **t-alpha:** 0.1513475474920678

---

---

407

- **PDB ID:** 6INN | **Chain:** C
- **b-phipsi:** 0.007635752596643
- **w-rdist:** 0.6032305312463486
- **t-alpha:** 0.0087166840741086

---

---

408

- **PDB ID:** 3CIK | **Chain:** A
- **b-phipsi:** 0.0152906853061471
- **w-rdist:** 0.4344588192586121
- **t-alpha:** 0.011093664683754

---

---

409

- **PDB ID:** 7WUB | **Chain:** H
- **b-phipsi:** 0.0046360470069132
- **w-rdist:** 0.1425867656705518
- **t-alpha:** 0.1988904263943514

---

---

410

- **PDB ID:** 8FCT | **Chain:** C
- **b-phipsi:** 0.0040876546279161
- **w-rdist:** 0.1726887968157335
- **t-alpha:** 0.2076068539125026

---

---

411

- **PDB ID:** 6XI2 | **Chain:** A
- **b-phipsi:** 0.0004640602714421
- **w-rdist:** 0.6219201837140392
- **t-alpha:** 0.0293635283929596

---

---

412

- **PDB ID:** 1XFD | **Chain:** C
- **b-phipsi:** 0.0045609516021547
- **w-rdist:** 0.8708408642437471
- **t-alpha:** 0.003179348403216

---

---

413

- **PDB ID:** 5MHM | **Chain:** B
- **b-phipsi:** 0.0114494960559673
- **w-rdist:** 0.3516286110889237
- **t-alpha:** 0.0198097874502385

---

---

414

- **PDB ID:** 5DXH | **Chain:** A
- **b-phipsi:** 0.0060113794782655
- **w-rdist:** 0.4846695862017446
- **t-alpha:** 0.022979434091199

---

---

415

- **PDB ID:** 5AWC | **Chain:** B
- **b-phipsi:** 0.0040262608899372
- **w-rdist:** 0.3310614091537527
- **t-alpha:** 0.1450081322117995

---

---

416

- **PDB ID:** 1LFG | **Chain:** A
- **b-phipsi:** 0.0046025373490663
- **w-rdist:** 0.6074893733589644
- **t-alpha:** 0.0169214728656399

---

---

417

- **PDB ID:** 8HGO | **Chain:** A
- **b-phipsi:** 0.006082783378386
- **w-rdist:** 0.5617090023303905
- **t-alpha:** 0.0152852632192168

---

---

418

- **PDB ID:** 3MDJ | **Chain:** C
- **b-phipsi:** 0.0067294624714348
- **w-rdist:** 0.3103801233843933
- **t-alpha:** 0.0839938579877885

---

---

419

- **PDB ID:** 5MHM | **Chain:** A
- **b-phipsi:** 0.0101324659183264
- **w-rdist:** 0.3034498296572654
- **t-alpha:** 0.0285264979365806

---

---

420

- **PDB ID:** 7MDM | **Chain:** C
- **b-phipsi:** 0.0065344473337576
- **w-rdist:** 0.3263248792777394
- **t-alpha:** 0.0760701130268726

---

---

421

- **PDB ID:** 1B0L | **Chain:** A
- **b-phipsi:** 0.004715673096957
- **w-rdist:** 0.6130517838849859
- **t-alpha:** 0.0166402663052187

---

---

422

- **PDB ID:** 6NIG | **Chain:** D
- **b-phipsi:** 0.0045698728872101
- **w-rdist:** 0.5088562119630797
- **t-alpha:** 0.0361248537980045

---

---

423

- **PDB ID:** 7T3B | **Chain:** A
- **b-phipsi:** 0.0024179909436063
- **w-rdist:** 0.4480488693443627
- **t-alpha:** 0.1259907457673486

---

---

424

- **PDB ID:** 7LM2 | **Chain:** A
- **b-phipsi:** 0.0064353635933952
- **w-rdist:** 0.2844428137042078
- **t-alpha:** 0.1030112267642286

---

---

425

- **PDB ID:** 8BCY | **Chain:** A
- **b-phipsi:** 0.0073357222953753
- **w-rdist:** 0.2970474932044461
- **t-alpha:** 0.077654736765909

---

---

426

- **PDB ID:** 4GUS | **Chain:** A
- **b-phipsi:** 0.0040789437816883
- **w-rdist:** 0.4685463117181496
- **t-alpha:** 0.0703983128434551

---

---

427

- **PDB ID:** 8HBE | **Chain:** B
- **b-phipsi:** 0.0044819462541632
- **w-rdist:** 0.3560660123882703
- **t-alpha:** 0.1228209788379228

---

---

428

- **PDB ID:** 4DPG | **Chain:** C
- **b-phipsi:** 0.0026842681150804
- **w-rdist:** 0.6549334723780801
- **t-alpha:** 0.0202100770637112

---

---

429

- **PDB ID:** 4R6A | **Chain:** A
- **b-phipsi:** 0.0024755386936228
- **w-rdist:** 0.3144559075892796
- **t-alpha:** 0.1925513562014842

---

---

430

- **PDB ID:** 7MDM | **Chain:** A
- **b-phipsi:** 0.0058069677701353
- **w-rdist:** 0.1657527904230575
- **t-alpha:** 0.1608558375885556

---

---

431

- **PDB ID:** 3VE1 | **Chain:** D
- **b-phipsi:** 0.0166411626075183
- **w-rdist:** 0.4733333599799296
- **t-alpha:** 0.0071825203562971

---

---

432

- **PDB ID:** 6U2W | **Chain:** B
- **b-phipsi:** 0.0061958351022738
- **w-rdist:** 0.4566785511811626
- **t-alpha:** 0.0318885499175907

---

---

433

- **PDB ID:** 5AWC | **Chain:** D
- **b-phipsi:** 0.007653197384022
- **w-rdist:** 0.3160995426544567
- **t-alpha:** 0.0618066131443488

---

---

434

- **PDB ID:** 6OAC | **Chain:** A
- **b-phipsi:** 0.00895888200967
- **w-rdist:** 0.166964330925768
- **t-alpha:** 0.0927103766050247

---

---

435

- **PDB ID:** 7JTQ | **Chain:** C
- **b-phipsi:** 0.0054289822979819
- **w-rdist:** 0.76416217998728
- **t-alpha:** 0.0063793623469805

---

---

436

- **PDB ID:** 3W3L | **Chain:** C
- **b-phipsi:** 0.0054455624196883
- **w-rdist:** 0.319659846800779
- **t-alpha:** 0.1164821024786537

---

---

437

- **PDB ID:** 4KTY | **Chain:** A
- **b-phipsi:** 0.0091020822924568
- **w-rdist:** 0.3168529811841061
- **t-alpha:** 0.039619806700881

---

---

438

- **PDB ID:** 4HSU | **Chain:** A
- **b-phipsi:** 0.0039324167441861
- **w-rdist:** 0.4745922577761459
- **t-alpha:** 0.0740421623492073

---

---

439

- **PDB ID:** 3B2D | **Chain:** B
- **b-phipsi:** 0.0059096489565872
- **w-rdist:** 0.4560174006785089
- **t-alpha:** 0.0372425322509186

---

---

440

- **PDB ID:** 5AWC | **Chain:** A
- **b-phipsi:** 0.002687067617657
- **w-rdist:** 0.3198156912522218
- **t-alpha:** 0.1870051434767796

---

---

441

- **PDB ID:** 7SZ0 | **Chain:** B
- **b-phipsi:** 0.0054814922355272
- **w-rdist:** 0.7089960429605342
- **t-alpha:** 0.0104083124295257

---

---

442

- **PDB ID:** 1KFU | **Chain:** L
- **b-phipsi:** 0.0022425258018261
- **w-rdist:** 0.5101426963372342
- **t-alpha:** 0.0888697463155465

---

---

443

- **PDB ID:** 5FTK | **Chain:** C
- **b-phipsi:** 0.0049821063714686
- **w-rdist:** 0.2021096152287547
- **t-alpha:** 0.1751188879720684

---

---

444

- **PDB ID:** 5FTK | **Chain:** F
- **b-phipsi:** 0.004758592944786
- **w-rdist:** 0.1942650721158617
- **t-alpha:** 0.1885898912492751

---

---

445

- **PDB ID:** 6D03 | **Chain:** C
- **b-phipsi:** 0.0042301838803386
- **w-rdist:** 0.5961523715268121
- **t-alpha:** 0.0193858366449102

---

---

446

- **PDB ID:** 5FUR | **Chain:** I
- **b-phipsi:** 0.0124404652801566
- **w-rdist:** 0.210075684226529
- **t-alpha:** 0.0356580695998436

---

---

447

- **PDB ID:** 4R6A | **Chain:** B
- **b-phipsi:** 0.0047344095509346
- **w-rdist:** 0.292586150379575
- **t-alpha:** 0.1529319679525831

---

---

448

- **PDB ID:** 5ISO | **Chain:** C
- **b-phipsi:** 0.0023613319733114
- **w-rdist:** 0.8454764156348403
- **t-alpha:** 0.0112175789229118

---

---

449

- **PDB ID:** 8FCO | **Chain:** B
- **b-phipsi:** 0.0052875448808208
- **w-rdist:** 0.3548396464548444
- **t-alpha:** 0.1053884795390964

---

---

450

- **PDB ID:** 2CKJ | **Chain:** C
- **b-phipsi:** 0.0029643303062006
- **w-rdist:** 0.4608112080213521
- **t-alpha:** 0.1101430153028129

---

---

451

- **PDB ID:** 2WJV | **Chain:** B
- **b-phipsi:** 0.0054814097294283
- **w-rdist:** 0.4810372367952473
- **t-alpha:** 0.0369759793802602

---

---

452

- **PDB ID:** 3W3N | **Chain:** B
- **b-phipsi:** 0.0039824392481814
- **w-rdist:** 0.3294591669070464
- **t-alpha:** 0.1537244455033311

---

---

453

- **PDB ID:** 5MHP | **Chain:** A
- **b-phipsi:** 0.0038295229796312
- **w-rdist:** 0.5934517534384933
- **t-alpha:** 0.0206022505246215

---

---

454

- **PDB ID:** 1JEQ | **Chain:** B
- **b-phipsi:** 0.0003326849138591
- **w-rdist:** 0.5246002587700143
- **t-alpha:** 0.099841640611139

---

---

455

- **PDB ID:** 4X1D | **Chain:** B
- **b-phipsi:** 0.004654356146345
- **w-rdist:** 0.7002671433474502
- **t-alpha:** 0.0142633043406019

---

---

456

- **PDB ID:** 8EZB | **Chain:** K
- **b-phipsi:** 0.0048639928969313
- **w-rdist:** 0.2818770276203171
- **t-alpha:** 0.1537244455033311

---

---

457

- **PDB ID:** 6ERH | **Chain:** D
- **b-phipsi:** 0.0047179278240923
- **w-rdist:** 0.2381427679491354
- **t-alpha:** 0.1719494022326169

---

---

458

- **PDB ID:** 6HTS | **Chain:** B
- **b-phipsi:** 0.0021933625124576
- **w-rdist:** 0.5420797168771524
- **t-alpha:** 0.0560669695523419

---

---

459

- **PDB ID:** 6JT0 | **Chain:** B
- **b-phipsi:** 0.0059387083605288
- **w-rdist:** 0.3679979873816599
- **t-alpha:** 0.077654736765909

---

---

460

- **PDB ID:** 3EGD | **Chain:** B
- **b-phipsi:** 0.003120423821112
- **w-rdist:** 0.7246836479636498
- **t-alpha:** 0.0166402663052187

---

---

461

- **PDB ID:** 6ZYM | **Chain:** B
- **b-phipsi:** 0.0085374594519755
- **w-rdist:** 0.334919092503787
- **t-alpha:** 0.0435817701022656

---

---

462

- **PDB ID:** 7ORC | **Chain:** A
- **b-phipsi:** 0.0013961362320437
- **w-rdist:** 0.3576178981425165
- **t-alpha:** 0.1957218219675423

---

---

463

- **PDB ID:** 8DP0 | **Chain:** A
- **b-phipsi:** 0.0048625438988401
- **w-rdist:** 0.3721332439081602
- **t-alpha:** 0.1093503210020616

---

---

464

- **PDB ID:** 6U2W | **Chain:** A
- **b-phipsi:** 0.0048718222744656
- **w-rdist:** 0.4844865781163828
- **t-alpha:** 0.050790674265883

---

---

465

- **PDB ID:** 5DYH | **Chain:** B
- **b-phipsi:** 0.0046627908192133
- **w-rdist:** 0.5183383581156538
- **t-alpha:** 0.0340730688185386

---

---

466

- **PDB ID:** 8BH3 | **Chain:** T
- **b-phipsi:** 0.0057035542553994
- **w-rdist:** 0.3834456514103797
- **t-alpha:** 0.0760701130268726

---

---

467

- **PDB ID:** 5GGF | **Chain:** A
- **b-phipsi:** 0.0034780371005834
- **w-rdist:** 0.7569951398375452
- **t-alpha:** 0.0136545862916419

---

---

468

- **PDB ID:** 7YBU | **Chain:** F
- **b-phipsi:** 0.0014395754855528
- **w-rdist:** 0.5419911034720765
- **t-alpha:** 0.0649762713207064

---

---

469

- **PDB ID:** 6CWZ | **Chain:** D
- **b-phipsi:** 0.005600880792752
- **w-rdist:** 0.8555247673530268
- **t-alpha:** 0.002377470759985

---

---

470

- **PDB ID:** 7RLI | **Chain:** K
- **b-phipsi:** 0.0053336148874279
- **w-rdist:** 0.1272195729671113
- **t-alpha:** 0.1949284161940592

---

---

471

- **PDB ID:** 8ENC | **Chain:** M
- **b-phipsi:** 0.014686035798334
- **w-rdist:** 0.5196483713821779
- **t-alpha:** 0.006339533171912

---

---

472

- **PDB ID:** 2NUP | **Chain:** B
- **b-phipsi:** 0.0018855387937149
- **w-rdist:** 0.7291684197522679
- **t-alpha:** 0.0190174537910852

---

---

473

- **PDB ID:** 6PYR | **Chain:** A
- **b-phipsi:** 0.0062770231153609
- **w-rdist:** 0.3543646515485637
- **t-alpha:** 0.0832012408733819

---

---

474

- **PDB ID:** 3W3G | **Chain:** B
- **b-phipsi:** 0.0052846371331696
- **w-rdist:** 0.3233028643321431
- **t-alpha:** 0.1267829728204994

---

---

475

- **PDB ID:** 7A3F | **Chain:** A
- **b-phipsi:** 0.0061250862842032
- **w-rdist:** 0.5367922410445598
- **t-alpha:** 0.0190174537910852

---

---

476

- **PDB ID:** 4OVV | **Chain:** A
- **b-phipsi:** 0.0121685505429214
- **w-rdist:** 0.2708536723004089
- **t-alpha:** 0.0253567763618671

---

---

477

- **PDB ID:** 5UBT | **Chain:** A
- **b-phipsi:** 0.0066922632944919
- **w-rdist:** 0.3323219711857286
- **t-alpha:** 0.0816167133968897

---

---

478

- **PDB ID:** 5MHN | **Chain:** B
- **b-phipsi:** 0.0095773415934249
- **w-rdist:** 0.337074244476699
- **t-alpha:** 0.0301113473631449

---

---

479

- **PDB ID:** 3V83 | **Chain:** B
- **b-phipsi:** 0.0040474732087633
- **w-rdist:** 0.6457493908149513
- **t-alpha:** 0.0190174537910852

---

---

480

- **PDB ID:** 4KRF | **Chain:** A
- **b-phipsi:** 0.0032594369729138
- **w-rdist:** 0.2692539108170698
- **t-alpha:** 0.2083994584940032

---

---

481

- **PDB ID:** 7SU3 | **Chain:** B
- **b-phipsi:** 0.0014092978470769
- **w-rdist:** 0.5703933058135313
- **t-alpha:** 0.0435817701022656

---

---

482

- **PDB ID:** 7MD5 | **Chain:** B
- **b-phipsi:** 0.0088398955812261
- **w-rdist:** 0.4044383849216332
- **t-alpha:** 0.022979434091199

---

---

483

- **PDB ID:** 6RYF | **Chain:** A
- **b-phipsi:** 0.0147427526934641
- **w-rdist:** 0.5592404267931756
- **t-alpha:** 0.0015872186884768

---

---

484

- **PDB ID:** 6G6W | **Chain:** A
- **b-phipsi:** 0.0068324940096092
- **w-rdist:** 0.3171469338418045
- **t-alpha:** 0.0903328337654447

---

---

485

- **PDB ID:** 5IUD | **Chain:** A
- **b-phipsi:** 0.0031392058622381
- **w-rdist:** 0.2677879610115289
- **t-alpha:** 0.2107769308628622

---

---

486

- **PDB ID:** 6C02 | **Chain:** A
- **b-phipsi:** 0.0039019115642555
- **w-rdist:** 0.5151749337051007
- **t-alpha:** 0.0570524158190295

---

---

487

- **PDB ID:** 8FCL | **Chain:** D
- **b-phipsi:** 0.0056008459752444
- **w-rdist:** 0.1462322032591005
- **t-alpha:** 0.1870051434767796

---

---

488

- **PDB ID:** 5HMP | **Chain:** A
- **b-phipsi:** 0.0065614804012376
- **w-rdist:** 0.4698699975462003
- **t-alpha:** 0.0285245361798418

---

---

489

- **PDB ID:** 1NUG | **Chain:** A
- **b-phipsi:** 0.0117703989140633
- **w-rdist:** 0.6506680107380138
- **t-alpha:** 0.0007928174706921

---

---

490

- **PDB ID:** 3W3K | **Chain:** B
- **b-phipsi:** 0.0022869171758221
- **w-rdist:** 0.3285575569225269
- **t-alpha:** 0.2020605492028166

---

---

491

- **PDB ID:** 6LID | **Chain:** A
- **b-phipsi:** 0.0032764119095289
- **w-rdist:** 0.7672945561934743
- **t-alpha:** 0.0142633043406019

---

---

492

- **PDB ID:** 6Q1V | **Chain:** A
- **b-phipsi:** 0.0047115982095133
- **w-rdist:** 0.8093304013601499
- **t-alpha:** 0.0079243179984995

---

---

493

- **PDB ID:** 8FCO | **Chain:** A
- **b-phipsi:** 0.0040799405795889
- **w-rdist:** 0.1733534345933256
- **t-alpha:** 0.2345490443729756

---

---

494

- **PDB ID:** 8FCT | **Chain:** E
- **b-phipsi:** 0.0043188488244911
- **w-rdist:** 0.1285355795581998
- **t-alpha:** 0.2448501004961611

---

---

495

- **PDB ID:** 6XRM | **Chain:** A
- **b-phipsi:** 0.0066111133252647
- **w-rdist:** 0.2375186979435282
- **t-alpha:** 0.1331219773390095

---

---

496

- **PDB ID:** 4R07 | **Chain:** C
- **b-phipsi:** 0.0043118412979494
- **w-rdist:** 0.3623394843020088
- **t-alpha:** 0.1386688723323981

---

---

497

- **PDB ID:** 3W3L | **Chain:** A
- **b-phipsi:** 0.0039826702919671
- **w-rdist:** 0.3123429956189129
- **t-alpha:** 0.1751188879720684

---

---

498

- **PDB ID:** 4FWE | **Chain:** A
- **b-phipsi:** 0.0040893155349694
- **w-rdist:** 0.4448001476247625
- **t-alpha:** 0.1045960199213078

---

---

499

- **PDB ID:** 4GU1 | **Chain:** B
- **b-phipsi:** 0.0045044214528629
- **w-rdist:** 0.5169555912831509
- **t-alpha:** 0.0427893647215475

---

---

500

- **PDB ID:** 7DVQ | **Chain:** c
- **b-phipsi:** 0.005206084231468
- **w-rdist:** 0.476011536498263
- **t-alpha:** 0.0530905293732681

---

---

501

- **PDB ID:** 7MDM | **Chain:** D
- **b-phipsi:** 0.0074992574606197
- **w-rdist:** 0.3488396620745931
- **t-alpha:** 0.0618066131443488

---

---

502

- **PDB ID:** 5VNN | **Chain:** B
- **b-phipsi:** 0.0014612736114405
- **w-rdist:** 0.7499535851207474
- **t-alpha:** 0.0190174537910852

---

---

503

- **PDB ID:** 7DVQ | **Chain:** C
- **b-phipsi:** 0.0048630420141398
- **w-rdist:** 0.4707858136763017
- **t-alpha:** 0.0633915289148658

---

---

504

- **PDB ID:** 7SVL | **Chain:** D
- **b-phipsi:** 0.0058043500445958
- **w-rdist:** 0.7058351545347378
- **t-alpha:** 0.0112175789229118

---

---

505

- **PDB ID:** 6PYU | **Chain:** A
- **b-phipsi:** 0.0073361604522708
- **w-rdist:** 0.3262550157888479
- **t-alpha:** 0.077654736765909

---

---

506

- **PDB ID:** 6N2W | **Chain:** A
- **b-phipsi:** 0.003810938509932
- **w-rdist:** 0.5614665180280856
- **t-alpha:** 0.0243505672074058

---

---

507

- **PDB ID:** 7K6M | **Chain:** A
- **b-phipsi:** 0.0081775740583992
- **w-rdist:** 0.1678220065729999
- **t-alpha:** 0.1275752196006165

---

---

508

- **PDB ID:** 3L4G | **Chain:** B
- **b-phipsi:** 0.0065776284214449
- **w-rdist:** 0.6928573081681413
- **t-alpha:** 0.0103013584398974

---

---

509

- **PDB ID:** 3B2D | **Chain:** A
- **b-phipsi:** 0.005900262988172
- **w-rdist:** 0.4495471467266152
- **t-alpha:** 0.0546755105374459

---

---

510

- **PDB ID:** 4WJL | **Chain:** B
- **b-phipsi:** 0.0047076902463112
- **w-rdist:** 0.8761361386864401
- **t-alpha:** 0.0055471164626488

---

---

511

- **PDB ID:** 5WYZ | **Chain:** A
- **b-phipsi:** 0.0043828037319634
- **w-rdist:** 0.3198469872931204
- **t-alpha:** 0.1584786099726112

---

---

512

- **PDB ID:** 6WML | **Chain:** B
- **b-phipsi:** 0.0044374105505716
- **w-rdist:** 0.3394889885882214
- **t-alpha:** 0.1465931232334234

---

---

513

- **PDB ID:** 7OLE | **Chain:** A
- **b-phipsi:** 0.0102282435862807
- **w-rdist:** 0.6779177095274683
- **t-alpha:** 0.0023827284528445

---

---

514

- **PDB ID:** 6ZHA | **Chain:** C
- **b-phipsi:** 0.0033600634695129
- **w-rdist:** 0.5049299375662161
- **t-alpha:** 0.0839938579877885

---

---

515

- **PDB ID:** 7RL6 | **Chain:** C
- **b-phipsi:** 0.005152975489305
- **w-rdist:** 0.1280275316601203
- **t-alpha:** 0.211569305215642

---

---

516

- **PDB ID:** 6ERH | **Chain:** A
- **b-phipsi:** 0.001384142890131
- **w-rdist:** 0.6230474903975929
- **t-alpha:** 0.0372425322509186

---

---

517

- **PDB ID:** 7K6O | **Chain:** A
- **b-phipsi:** 0.0085601148530493
- **w-rdist:** 0.1867645166068549
- **t-alpha:** 0.1180665242330454

---

---

518

- **PDB ID:** 7W5A | **Chain:** C
- **b-phipsi:** 0.0102026456850929
- **w-rdist:** 0.4080866106223542
- **t-alpha:** 0.0210354708025768

---

---

519

- **PDB ID:** 5WYX | **Chain:** A
- **b-phipsi:** 0.0046672924357345
- **w-rdist:** 0.3183543593882315
- **t-alpha:** 0.1545169172535367

---

---

520

- **PDB ID:** 7RLI | **Chain:** C
- **b-phipsi:** 0.0051156966939541
- **w-rdist:** 0.1439132352230228
- **t-alpha:** 0.2107769308628622

---

---

521

- **PDB ID:** 8ASC | **Chain:** O
- **b-phipsi:** 0.0071582007095562
- **w-rdist:** 0.3111211899204086
- **t-alpha:** 0.0982568607009952

---

---

522

- **PDB ID:** 3U1K | **Chain:** C
- **b-phipsi:** 0.0043676419039318
- **w-rdist:** 0.8580924114516114
- **t-alpha:** 0.0071825203562971

---

---

523

- **PDB ID:** 8FCL | **Chain:** C
- **b-phipsi:** 0.0042908003019383
- **w-rdist:** 0.1539179717781618
- **t-alpha:** 0.2456422025040694

---

---

524

- **PDB ID:** 8FCQ | **Chain:** F
- **b-phipsi:** 0.0044875863288284
- **w-rdist:** 0.1423868024456601
- **t-alpha:** 0.2432650359073522

---

---

525

- **PDB ID:** 8FCT | **Chain:** F
- **b-phipsi:** 0.0053832173978633
- **w-rdist:** 0.166528177578259
- **t-alpha:** 0.1957218219675423

---

---

526

- **PDB ID:** 7RLI | **Chain:** B
- **b-phipsi:** 0.0060291027306767
- **w-rdist:** 0.1427520949847269
- **t-alpha:** 0.1854202476378048

---

---

527

- **PDB ID:** 3ALA | **Chain:** E
- **b-phipsi:** 0.0095630045678512
- **w-rdist:** 0.7001924671400884
- **t-alpha:** 0.0031697813850419

---

---

528

- **PDB ID:** 4GUT | **Chain:** A
- **b-phipsi:** 0.0042608389070782
- **w-rdist:** 0.4892558302044369
- **t-alpha:** 0.0731288873644446

---

---

529

- **PDB ID:** 2ZKM | **Chain:** X
- **b-phipsi:** 0.0008561943995074
- **w-rdist:** 0.6154410933618143
- **t-alpha:** 0.0473026125968261

---

---

530

- **PDB ID:** 6R1U | **Chain:** K
- **b-phipsi:** 0.0072274079639776
- **w-rdist:** 0.5124338832547961
- **t-alpha:** 0.0206022505246215

---

---

531

- **PDB ID:** 7R7T | **Chain:** D
- **b-phipsi:** 0.0047331557094417
- **w-rdist:** 0.4530023996794652
- **t-alpha:** 0.0871633913344154

---

---

532

- **PDB ID:** 7WUB | **Chain:** K
- **b-phipsi:** 0.0056988672559853
- **w-rdist:** 0.1443868213626293
- **t-alpha:** 0.194136675691666

---

---

533

- **PDB ID:** 8FCO | **Chain:** F
- **b-phipsi:** 0.0059733799415823
- **w-rdist:** 0.1732527347655139
- **t-alpha:** 0.1782885176402775

---

---

534

- **PDB ID:** 8FCQ | **Chain:** A
- **b-phipsi:** 0.0064543287553088
- **w-rdist:** 0.2078160360182305
- **t-alpha:** 0.1537244455033311

---

---

535

- **PDB ID:** 5FTM | **Chain:** B
- **b-phipsi:** 0.0053967476577616
- **w-rdist:** 0.3669876814193206
- **t-alpha:** 0.1109352165581252

---

---

536

- **PDB ID:** 6EA4 | **Chain:** B
- **b-phipsi:** 0.0183965022187064
- **w-rdist:** 0.5312589829141389
- **t-alpha:** 0.002377470759985

---

---

537

- **PDB ID:** 3LJ3 | **Chain:** A
- **b-phipsi:** 0.0087221498817851
- **w-rdist:** 0.2106856053336882
- **t-alpha:** 0.1077655736112999

---

---

538

- **PDB ID:** 6MDZ | **Chain:** A
- **b-phipsi:** 0.00261568681198
- **w-rdist:** 0.3561453282133622
- **t-alpha:** 0.1893824912295334

---

---

539

- **PDB ID:** 7YQ4 | **Chain:** E
- **b-phipsi:** 0.0075042553581002
- **w-rdist:** 0.4207049562227618
- **t-alpha:** 0.0412048690981889

---

---

540

- **PDB ID:** 5AWD | **Chain:** A
- **b-phipsi:** 0.0035877984529749
- **w-rdist:** 0.3250921031939899
- **t-alpha:** 0.1870051434767796

---

---

541

- **PDB ID:** 5MHO | **Chain:** A
- **b-phipsi:** 0.0099107248760263
- **w-rdist:** 0.3367717321877765
- **t-alpha:** 0.0356580695998436

---

---

542

- **PDB ID:** 7RLH | **Chain:** A
- **b-phipsi:** 0.0070170054630529
- **w-rdist:** 0.1279121652225929
- **t-alpha:** 0.1648181756590165

---

---

543

- **PDB ID:** 3CST | **Chain:** A
- **b-phipsi:** 0.0065034725748472
- **w-rdist:** 0.2685532693106791
- **t-alpha:** 0.1347072878750428

---

---

544

- **PDB ID:** 8FCR | **Chain:** D
- **b-phipsi:** 0.0054924210351985
- **w-rdist:** 0.1617329340220955
- **t-alpha:** 0.1973056469081315

---

---

545

- **PDB ID:** 6ERF | **Chain:** C
- **b-phipsi:** 0.0021529172035259
- **w-rdist:** 0.556630709656811
- **t-alpha:** 0.0602220373725139

---

---

546

- **PDB ID:** 5IUD | **Chain:** D
- **b-phipsi:** 0.0051775191434179
- **w-rdist:** 0.2602611917771255
- **t-alpha:** 0.1671950546614453

---

---

547

- **PDB ID:** 7RLI | **Chain:** I
- **b-phipsi:** 0.0062316222646502
- **w-rdist:** 0.1439342567999992
- **t-alpha:** 0.1846280285808399

---

---

548

- **PDB ID:** 6R25 | **Chain:** K
- **b-phipsi:** 0.0046894979990287
- **w-rdist:** 0.4754761479053577
- **t-alpha:** 0.075277649690151

---

---

549

- **PDB ID:** 5FTK | **Chain:** B
- **b-phipsi:** 0.0057533950682563
- **w-rdist:** 0.1898704535402048
- **t-alpha:** 0.1830429219610427

---

---

550

- **PDB ID:** 6XTY | **Chain:** 2
- **b-phipsi:** 0.0035035724027054
- **w-rdist:** 0.4111598217426045
- **t-alpha:** 0.1465931232334234

---

---

551

- **PDB ID:** 4R09 | **Chain:** C
- **b-phipsi:** 0.0043904719274437
- **w-rdist:** 0.3653114739823342
- **t-alpha:** 0.1450081322117995

---

---

552

- **PDB ID:** 3W3K | **Chain:** A
- **b-phipsi:** 0.0035988137079307
- **w-rdist:** 0.3256924231907485
- **t-alpha:** 0.1885898912492751

---

---

553

- **PDB ID:** 8FCL | **Chain:** B
- **b-phipsi:** 0.0045498196316159
- **w-rdist:** 0.1798891462767684
- **t-alpha:** 0.2313789650220328

---

---

554

- **PDB ID:** 5F9H | **Chain:** C
- **b-phipsi:** 0.0095257594238282
- **w-rdist:** 0.6802433718542518
- **t-alpha:** 0.0047768836635659

---

---

555

- **PDB ID:** 6U2W | **Chain:** N
- **b-phipsi:** 0.0058557124710217
- **w-rdist:** 0.4840470339695297
- **t-alpha:** 0.0455671638991892

---

---

556

- **PDB ID:** 3G0B | **Chain:** B
- **b-phipsi:** 0.004363892377946
- **w-rdist:** 0.8283115632856728
- **t-alpha:** 0.0095996935833633

---

---

557

- **PDB ID:** 6GVF | **Chain:** A
- **b-phipsi:** 0.0089145125593334
- **w-rdist:** 0.1978301664604562
- **t-alpha:** 0.1156896697820086

---

---

558

- **PDB ID:** 3ZIM | **Chain:** A
- **b-phipsi:** 0.0100014436668843
- **w-rdist:** 0.2085292502927414
- **t-alpha:** 0.0879557950251648

---

---

559

- **PDB ID:** 2BJJ | **Chain:** X
- **b-phipsi:** 0.0050364986510946
- **w-rdist:** 0.6103086225076487
- **t-alpha:** 0.0190174537910852

---

---

560

- **PDB ID:** 7W5A | **Chain:** c
- **b-phipsi:** 0.008154747910842
- **w-rdist:** 0.409580183769491
- **t-alpha:** 0.0395385102655705

---

---

561

- **PDB ID:** 8EFH | **Chain:** A
- **b-phipsi:** 0.0131227453321098
- **w-rdist:** 0.5059088580018104
- **t-alpha:** 0.0120284587273298

---

---

562

- **PDB ID:** 5FWI | **Chain:** C
- **b-phipsi:** 0.0031947906596662
- **w-rdist:** 0.9387976066518308
- **t-alpha:** 0.0079243179984995

---

---

563

- **PDB ID:** 7YBU | **Chain:** D
- **b-phipsi:** 0.0024957903093801
- **w-rdist:** 0.5433600554075988
- **t-alpha:** 0.0657687656426226

---

---

564

- **PDB ID:** 5EPG | **Chain:** A
- **b-phipsi:** 0.0029345818131265
- **w-rdist:** 0.3627382758731929
- **t-alpha:** 0.1846280285808399

---

---

565

- **PDB ID:** 7RLI | **Chain:** L
- **b-phipsi:** 0.0054048806705754
- **w-rdist:** 0.1445797948395348
- **t-alpha:** 0.211569305215642

---

---

566

- **PDB ID:** 1U8C | **Chain:** B
- **b-phipsi:** 0.0035599283684343
- **w-rdist:** 0.5289732420936407
- **t-alpha:** 0.0613960760541711

---

---

567

- **PDB ID:** 6AHU | **Chain:** B
- **b-phipsi:** 0.0063330333778469
- **w-rdist:** 0.3981497053298969
- **t-alpha:** 0.0784468950036187

---

---

568

- **PDB ID:** 5FTK | **Chain:** A
- **b-phipsi:** 0.0042891851086779
- **w-rdist:** 0.1969926371689319
- **t-alpha:** 0.2416796212592216

---

---

569

- **PDB ID:** 6OCO | **Chain:** A
- **b-phipsi:** 0.0067100401707447
- **w-rdist:** 0.3387028243561252
- **t-alpha:** 0.0974642806534764

---

---

570

- **PDB ID:** 7RLF | **Chain:** B
- **b-phipsi:** 0.0063438104682725
- **w-rdist:** 0.1521896757620768
- **t-alpha:** 0.1846280285808399

---

---

571

- **PDB ID:** 4FLH | **Chain:** A
- **b-phipsi:** 0.0108164218183897
- **w-rdist:** 0.1836379959260708
- **t-alpha:** 0.0863710467621965

---

---

572

- **PDB ID:** 4DUR | **Chain:** A
- **b-phipsi:** 0.0090514647129524
- **w-rdist:** 0.2730525602654038
- **t-alpha:** 0.0839938579877885

---

---

573

- **PDB ID:** 8AG5 | **Chain:** B
- **b-phipsi:** 0.0021153363614831
- **w-rdist:** 0.5422060853737465
- **t-alpha:** 0.0792396355379139

---

---

574

- **PDB ID:** 5XGJ | **Chain:** A
- **b-phipsi:** 0.0054148436522299
- **w-rdist:** 0.3845603057525299
- **t-alpha:** 0.1109352165581252

---

---

575

- **PDB ID:** 7BP9 | **Chain:** F
- **b-phipsi:** 0.0068093339131996
- **w-rdist:** 0.1833571372726528
- **t-alpha:** 0.162440593977174

---

---

576

- **PDB ID:** 7SCJ | **Chain:** A
- **b-phipsi:** 0.0047882982535052
- **w-rdist:** 0.7029351900382457
- **t-alpha:** 0.0161027964403612

---

---

577

- **PDB ID:** 3O95 | **Chain:** B
- **b-phipsi:** 0.0043125184461872
- **w-rdist:** 0.8278488706565669
- **t-alpha:** 0.0104083124295257

---

---

578

- **PDB ID:** 6WML | **Chain:** D
- **b-phipsi:** 0.006585990574169
- **w-rdist:** 0.3196387646513759
- **t-alpha:** 0.1204441681482937

---

---

579

- **PDB ID:** 4QBZ | **Chain:** B
- **b-phipsi:** 0.0037637780026278
- **w-rdist:** 0.3127915726668098
- **t-alpha:** 0.2028529548897624

---

---

580

- **PDB ID:** 7RLJ | **Chain:** K
- **b-phipsi:** 0.0060788258418781
- **w-rdist:** 0.120879973917709
- **t-alpha:** 0.2036447350497798

---

---

581

- **PDB ID:** 7E8H | **Chain:** K
- **b-phipsi:** 0.002228583961413
- **w-rdist:** 0.7126207396454525
- **t-alpha:** 0.0218620195820675

---

---

582

- **PDB ID:** 6ERH | **Chain:** C
- **b-phipsi:** 0.0008899674019777
- **w-rdist:** 0.6116543848340839
- **t-alpha:** 0.0578450666823961

---

---

583

- **PDB ID:** 6EOS | **Chain:** D
- **b-phipsi:** 0.0064230742658843
- **w-rdist:** 0.5778223887839189
- **t-alpha:** 0.0166402663052187

---

---

584

- **PDB ID:** 1RRJ | **Chain:** A
- **b-phipsi:** 0.0259582938532902
- **w-rdist:** 0.4730625962517829
- **t-alpha:** 0.0055471164626488

---

---

585

- **PDB ID:** 4TUU | **Chain:** A
- **b-phipsi:** 0.0099445514100033
- **w-rdist:** 0.1982509856670857
- **t-alpha:** 0.0974642806534764

---

---

586

- **PDB ID:** 6HTS | **Chain:** A
- **b-phipsi:** 0.0021354812655054
- **w-rdist:** 0.5319152023721814
- **t-alpha:** 0.0926405588175569

---

---

587

- **PDB ID:** 6U2W | **Chain:** M
- **b-phipsi:** 0.0062151041000967
- **w-rdist:** 0.4586068115670456
- **t-alpha:** 0.0560669695523419

---

---

588

- **PDB ID:** 8FCO | **Chain:** E
- **b-phipsi:** 0.0045731361656663
- **w-rdist:** 0.2154294910689212
- **t-alpha:** 0.2210775835865639

---

---

589

- **PDB ID:** 5Z14 | **Chain:** A
- **b-phipsi:** 0.0051057324203502
- **w-rdist:** 0.3144431717595846
- **t-alpha:** 0.1600634400235712

---

---

590

- **PDB ID:** 7RLJ | **Chain:** L
- **b-phipsi:** 0.0065780383417946
- **w-rdist:** 0.1211136213277399
- **t-alpha:** 0.1909670055645516

---

---

591

- **PDB ID:** 2BGN | **Chain:** D
- **b-phipsi:** 0.0041208085109835
- **w-rdist:** 0.8345192468809643
- **t-alpha:** 0.011093664683754

---

---

592

- **PDB ID:** 7Z61 | **Chain:** A
- **b-phipsi:** 0.0071449622118396
- **w-rdist:** 0.294380216417313
- **t-alpha:** 0.1244058176483571

---

---

593

- **PDB ID:** 6MGQ | **Chain:** A
- **b-phipsi:** 0.0133919687968288
- **w-rdist:** 0.0665799148874441
- **t-alpha:** 0.077654736765909

---

---

594

- **PDB ID:** 6ERF | **Chain:** E
- **b-phipsi:** 0.00213710056684
- **w-rdist:** 0.5266734507330572
- **t-alpha:** 0.1014266065453182

---

---

595

- **PDB ID:** 6J1X | **Chain:** B
- **b-phipsi:** 0.0079057372012765
- **w-rdist:** 0.761907199358904
- **t-alpha:** 0.0047543655130677

---

---

596

- **PDB ID:** 5C18 | **Chain:** F
- **b-phipsi:** 0.0077804919239625
- **w-rdist:** 0.2389751044070249
- **t-alpha:** 0.1307449283247805

---

---

597

- **PDB ID:** 6INN | **Chain:** D
- **b-phipsi:** 0.006325781425436
- **w-rdist:** 0.6003190413670845
- **t-alpha:** 0.0166402663052187

---

---

598

- **PDB ID:** 5FTK | **Chain:** D
- **b-phipsi:** 0.004275719795048
- **w-rdist:** 0.2039982231416629
- **t-alpha:** 0.2543587537689815

---

---

599

- **PDB ID:** 8FCM | **Chain:** A
- **b-phipsi:** 0.0039310986708493
- **w-rdist:** 0.387497446296923
- **t-alpha:** 0.1568942900983025

---

---

600

- **PDB ID:** 3MJW | **Chain:** A
- **b-phipsi:** 0.0071960967682422
- **w-rdist:** 0.1923294431012397
- **t-alpha:** 0.1592710305637339

---

---

601

- **PDB ID:** 7RLG | **Chain:** A
- **b-phipsi:** 0.0052665837135631
- **w-rdist:** 0.2099039869141457
- **t-alpha:** 0.2044381850417527

---

---

602

- **PDB ID:** 6BKF | **Chain:** A
- **b-phipsi:** 0.0018317839772522
- **w-rdist:** 0.5613035910739743
- **t-alpha:** 0.0713153940999824

---

---

603

- **PDB ID:** 6X6C | **Chain:** A
- **b-phipsi:** 0.0069980284680502
- **w-rdist:** 0.7300292964943838
- **t-alpha:** 0.0087166840741086

---

---

604

- **PDB ID:** 5AWA | **Chain:** A
- **b-phipsi:** 0.0037535781350837
- **w-rdist:** 0.3095199496081762
- **t-alpha:** 0.2107769308628622

---

---

605

- **PDB ID:** 6NIG | **Chain:** B
- **b-phipsi:** 0.0056429842523575
- **w-rdist:** 0.4944337632243799
- **t-alpha:** 0.050790674265883

---

---

606

- **PDB ID:** 7BP8 | **Chain:** A
- **b-phipsi:** 0.0073132543367608
- **w-rdist:** 0.2477373168511932
- **t-alpha:** 0.1386688723323981

---

---

607

- **PDB ID:** 7ORC | **Chain:** B
- **b-phipsi:** 0.0022186352900608
- **w-rdist:** 0.4275831202308734
- **t-alpha:** 0.1798734427113426

---

---

608

- **PDB ID:** 4KZ0 | **Chain:** A
- **b-phipsi:** 0.0087484024573416
- **w-rdist:** 0.2274734500065257
- **t-alpha:** 0.1180665242330454

---

---

609

- **PDB ID:** 8FCM | **Chain:** F
- **b-phipsi:** 0.0050683048210894
- **w-rdist:** 0.348583085443889
- **t-alpha:** 0.1481775708256634

---

---

610

- **PDB ID:** 6ILD | **Chain:** A
- **b-phipsi:** 0.0066682430936351
- **w-rdist:** 0.6604660408137287
- **t-alpha:** 0.0136545862916419

---

---

611

- **PDB ID:** 7SZ7 | **Chain:** B
- **b-phipsi:** 0.0041349700326115
- **w-rdist:** 0.7136599060155467
- **t-alpha:** 0.0182252502019479

---

---

612

- **PDB ID:** 5M6U | **Chain:** A
- **b-phipsi:** 0.0076574587115281
- **w-rdist:** 0.3297869186474108
- **t-alpha:** 0.0927103766050247

---

---

613

- **PDB ID:** 5RVY | **Chain:** A
- **b-phipsi:** 0.0165284425005418
- **w-rdist:** 0.4067967159480271
- **t-alpha:** 0.0158479732088472

---

---

614

- **PDB ID:** 5Z14 | **Chain:** B
- **b-phipsi:** 0.0053847844446116
- **w-rdist:** 0.3068190474334369
- **t-alpha:** 0.1608558375885556

---

---

615

- **PDB ID:** 7K1K | **Chain:** C
- **b-phipsi:** 0.0065220539534333
- **w-rdist:** 0.2401161297058358
- **t-alpha:** 0.1561018403117944

---

---

616

- **PDB ID:** 5GGI | **Chain:** B
- **b-phipsi:** 0.0039043712275787
- **w-rdist:** 0.8258705347675024
- **t-alpha:** 0.0128409546837204

---

---

617

- **PDB ID:** 7L35 | **Chain:** A
- **b-phipsi:** 0.0048198135984753
- **w-rdist:** 0.8112879615684571
- **t-alpha:** 0.0104083124295257

---

---

618

- **PDB ID:** 8FCL | **Chain:** F
- **b-phipsi:** 0.0053085070812731
- **w-rdist:** 0.1648572384667536
- **t-alpha:** 0.2258321959381128

---

---

619

- **PDB ID:** 5WJ5 | **Chain:** C
- **b-phipsi:** 0.0483805143453556
- **w-rdist:** 0.3446315148765989
- **t-alpha:** 0.0079243179984995

---

---

620

- **PDB ID:** 1E8Y | **Chain:** A
- **b-phipsi:** 0.0067866130442681
- **w-rdist:** 0.2695169375001215
- **t-alpha:** 0.1450081322117995

---

---

621

- **PDB ID:** 8FCL | **Chain:** E
- **b-phipsi:** 0.0043985428091583
- **w-rdist:** 0.1930105752423764
- **t-alpha:** 0.2662444254286378

---

---

622

- **PDB ID:** 8HGP | **Chain:** A
- **b-phipsi:** 0.0049886482897283
- **w-rdist:** 0.5666978223226458
- **t-alpha:** 0.0218620195820675

---

---

623

- **PDB ID:** 7JIS | **Chain:** A
- **b-phipsi:** 0.0080105008090178
- **w-rdist:** 0.3030953664746602
- **t-alpha:** 0.1077655736112999

---

---

624

- **PDB ID:** 5C19 | **Chain:** F
- **b-phipsi:** 0.005152592586892
- **w-rdist:** 0.194801634775036
- **t-alpha:** 0.2187008051406629

---

---

625

- **PDB ID:** 6MCK | **Chain:** C
- **b-phipsi:** 0.0163786976322782
- **w-rdist:** 0.5931862806383259
- **t-alpha:** 0.0007928174706921

---

---

626

- **PDB ID:** 4FJY | **Chain:** A
- **b-phipsi:** 0.0060648223979318
- **w-rdist:** 0.2196721349610133
- **t-alpha:** 0.1806658600500577

---

---

627

- **PDB ID:** 7ZXS | **Chain:** B
- **b-phipsi:** 0.0074817643079952
- **w-rdist:** 0.721067889905262
- **t-alpha:** 0.0087166840741086

---

---

628

- **PDB ID:** 7RLH | **Chain:** D
- **b-phipsi:** 0.0075093479204434
- **w-rdist:** 0.1376941878884913
- **t-alpha:** 0.1719494022326169

---

---

629

- **PDB ID:** 5C18 | **Chain:** E
- **b-phipsi:** 0.0066344370068683
- **w-rdist:** 0.2108957056625012
- **t-alpha:** 0.1664027794262541

---

---

630

- **PDB ID:** 4JBS | **Chain:** B
- **b-phipsi:** 0.0137778456476462
- **w-rdist:** 0.5653622468030974
- **t-alpha:** 0.0055471164626488

---

---

631

- **PDB ID:** 7R53 | **Chain:** B
- **b-phipsi:** 0.0050579018122371
- **w-rdist:** 0.331489294779703
- **t-alpha:** 0.1561018403117944

---

---

632

- **PDB ID:** 3IBE | **Chain:** A
- **b-phipsi:** 0.0091937831127129
- **w-rdist:** 0.2282837316832923
- **t-alpha:** 0.1109352165581252

---

---

633

- **PDB ID:** 7RLF | **Chain:** E
- **b-phipsi:** 0.0064507637238866
- **w-rdist:** 0.1461133664968953
- **t-alpha:** 0.1957218219675423

---

---

634

- **PDB ID:** 7RLI | **Chain:** F
- **b-phipsi:** 0.0061994680057947
- **w-rdist:** 0.1330951338516256
- **t-alpha:** 0.2083994584940032

---

---

635

- **PDB ID:** 6QZV | **Chain:** A
- **b-phipsi:** 0.0046240874706512
- **w-rdist:** 0.7102228432435751
- **t-alpha:** 0.017432692187089

---

---

636

- **PDB ID:** 3TL5 | **Chain:** A
- **b-phipsi:** 0.007857796284439
- **w-rdist:** 0.2416074126257208
- **t-alpha:** 0.1339143555568707

---

---

637

- **PDB ID:** 3CDZ | **Chain:** B
- **b-phipsi:** 0.0287573878931527
- **w-rdist:** 0.3459466236681297
- **t-alpha:** 0.0126786930545719

---

---

638

- **PDB ID:** 5WYX | **Chain:** B
- **b-phipsi:** 0.0035269857682866
- **w-rdist:** 0.3600658147043796
- **t-alpha:** 0.1893824912295334

---

---

639

- **PDB ID:** 7TZ7 | **Chain:** A
- **b-phipsi:** 0.005132775271478
- **w-rdist:** 0.4385914068382494
- **t-alpha:** 0.1069729933769783

---

---

640

- **PDB ID:** 6CE7 | **Chain:** A
- **b-phipsi:** 0.0078992462596226
- **w-rdist:** 0.4838780195245012
- **t-alpha:** 0.0243505672074058

---

---

641

- **PDB ID:** 2Z7X | **Chain:** A
- **b-phipsi:** 0.0058384961447911
- **w-rdist:** 0.515915610290616
- **t-alpha:** 0.0378287938704933

---

---

642

- **PDB ID:** 6U2W | **Chain:** D
- **b-phipsi:** 0.0111503354046251
- **w-rdist:** 0.3467967064361228
- **t-alpha:** 0.0327332409069371

---

---

643

- **PDB ID:** 8FCQ | **Chain:** C
- **b-phipsi:** 0.0056195343425746
- **w-rdist:** 0.1614104091677216
- **t-alpha:** 0.217115374701688

---

---

644

- **PDB ID:** 7NFE | **Chain:** C
- **b-phipsi:** 0.0061702105772054
- **w-rdist:** 0.2755618060658182
- **t-alpha:** 0.1568942900983025

---

---

645

- **PDB ID:** 7W59 | **Chain:** C
- **b-phipsi:** 0.0077228319794933
- **w-rdist:** 0.4116277688575344
- **t-alpha:** 0.0569511082581055

---

---

646

- **PDB ID:** 4FAD | **Chain:** A
- **b-phipsi:** 0.0099891130599817
- **w-rdist:** 0.2238210477972264
- **t-alpha:** 0.0974642806534764

---

---

647

- **PDB ID:** 5OQ4 | **Chain:** A
- **b-phipsi:** 0.0054790082257907
- **w-rdist:** 0.2697715886997234
- **t-alpha:** 0.1782885176402775

---

---

648

- **PDB ID:** 7RLI | **Chain:** H
- **b-phipsi:** 0.0061878855930252
- **w-rdist:** 0.1607390782101913
- **t-alpha:** 0.2036447350497798

---

---

649

- **PDB ID:** 8EOK | **Chain:** D
- **b-phipsi:** 0.0027066044709254
- **w-rdist:** 0.6986374570862134
- **t-alpha:** 0.0226902127823684

---

---

650

- **PDB ID:** 7RLI | **Chain:** E
- **b-phipsi:** 0.0063834272705549
- **w-rdist:** 0.1566481322950943
- **t-alpha:** 0.1973056469081315

---

---

651

- **PDB ID:** 7K6N | **Chain:** A
- **b-phipsi:** 0.0098415132329015
- **w-rdist:** 0.1934049698607437
- **t-alpha:** 0.1148972678966586

---

---

652

- **PDB ID:** 5VNL | **Chain:** A
- **b-phipsi:** 0.0012113167862394
- **w-rdist:** 0.7203991244489669
- **t-alpha:** 0.0285245361798418

---

---

653

- **PDB ID:** 6CE9 | **Chain:** B
- **b-phipsi:** 0.0129726101058594
- **w-rdist:** 0.3968251893978859
- **t-alpha:** 0.0206022505246215

---

---

654

- **PDB ID:** 6JT1 | **Chain:** B
- **b-phipsi:** 0.0064912506247629
- **w-rdist:** 0.3647672628449012
- **t-alpha:** 0.1077655736112999

---

---

655

- **PDB ID:** 7R9Y | **Chain:** A
- **b-phipsi:** 0.011702562608342
- **w-rdist:** 0.1860447863890637
- **t-alpha:** 0.089540776910131

---

---

656

- **PDB ID:** 5IJ7 | **Chain:** B
- **b-phipsi:** 0.0012808045649414
- **w-rdist:** 0.7144619491087362
- **t-alpha:** 0.0301113473631449

---

---

657

- **PDB ID:** 5K1B | **Chain:** B
- **b-phipsi:** 0.0132920294435212
- **w-rdist:** 0.6583920211406814
- **t-alpha:** 0.002377470759985

---

---

658

- **PDB ID:** 5MHN | **Chain:** A
- **b-phipsi:** 0.0105012164292768
- **w-rdist:** 0.3354453793515955
- **t-alpha:** 0.0443742796274553

---

---

659

- **PDB ID:** 7EG4 | **Chain:** D
- **b-phipsi:** 0.0040670214949745
- **w-rdist:** 0.5121111608516985
- **t-alpha:** 0.0898099285166125

---

---

660

- **PDB ID:** 5HMP | **Chain:** B
- **b-phipsi:** 0.00919526903547
- **w-rdist:** 0.3970393330278663
- **t-alpha:** 0.0412540222902821

---

---

661

- **PDB ID:** 1CJY | **Chain:** A
- **b-phipsi:** 0.0033178747428648
- **w-rdist:** 0.5356715728953185
- **t-alpha:** 0.0729001783846572

---

---

662

- **PDB ID:** 5VLR | **Chain:** A
- **b-phipsi:** 0.0074363429135117
- **w-rdist:** 0.3335889738031826
- **t-alpha:** 0.1022188946752804

---

---

663

- **PDB ID:** 7T8C | **Chain:** B
- **b-phipsi:** 0.0068135950887838
- **w-rdist:** 0.8849892386256686
- **t-alpha:** 0.0015872186884768

---

---

664

- **PDB ID:** 6U3J | **Chain:** B
- **b-phipsi:** 0.0155610655420353
- **w-rdist:** 0.381260510910012
- **t-alpha:** 0.0185632335173342

---

---

665

- **PDB ID:** 4WJL | **Chain:** A
- **b-phipsi:** 0.0063865504701924
- **w-rdist:** 0.8862206405743112
- **t-alpha:** 0.0031697813850419

---

---

666

- **PDB ID:** 4EZL | **Chain:** A
- **b-phipsi:** 0.0075910826685028
- **w-rdist:** 0.2388105475859817
- **t-alpha:** 0.1450081322117995

---

---

667

- **PDB ID:** 5WYZ | **Chain:** B
- **b-phipsi:** 0.0034420367038402
- **w-rdist:** 0.443869146744247
- **t-alpha:** 0.1537244455033311

---

---

668

- **PDB ID:** 4QC0 | **Chain:** A
- **b-phipsi:** 0.0022760037018807
- **w-rdist:** 0.3233587407532192
- **t-alpha:** 0.2805072378344706

---

---

669

- **PDB ID:** 3ALA | **Chain:** A
- **b-phipsi:** 0.012923760451611
- **w-rdist:** 0.7029230447165402
- **t-alpha:** 0.0007928174706921

---

---

670

- **PDB ID:** 8ASC | **Chain:** P
- **b-phipsi:** 0.0023000535866984
- **w-rdist:** 0.5681036657270282
- **t-alpha:** 0.0618066131443488

---

---

671

- **PDB ID:** 5UBR | **Chain:** A
- **b-phipsi:** 0.0102283632163988
- **w-rdist:** 0.189755140451818
- **t-alpha:** 0.1133125594934425

---

---

672

- **PDB ID:** 7T8C | **Chain:** F
- **b-phipsi:** 0.0076406007059862
- **w-rdist:** 0.8884143559781884
- **t-alpha:** 0.0

---

---

673

- **PDB ID:** 5C19 | **Chain:** B
- **b-phipsi:** 0.0060786829898222
- **w-rdist:** 0.1831144748759178
- **t-alpha:** 0.2044381850417527

---

---

674

- **PDB ID:** 7MDM | **Chain:** B
- **b-phipsi:** 0.0065553250864416
- **w-rdist:** 0.1797754440404486
- **t-alpha:** 0.191758917599204

---

---

675

- **PDB ID:** 7CRF | **Chain:** A
- **b-phipsi:** 0.0033829138925883
- **w-rdist:** 0.3419493147034477
- **t-alpha:** 0.2083994584940032

---

---

676

- **PDB ID:** 6C01 | **Chain:** A
- **b-phipsi:** 0.0051209696178029
- **w-rdist:** 0.5200447867989217
- **t-alpha:** 0.0522979286895239

---

---

677

- **PDB ID:** 4KTY | **Chain:** B
- **b-phipsi:** 0.0136739442810916
- **w-rdist:** 0.2763849606969022
- **t-alpha:** 0.0404122840988643

---

---

678

- **PDB ID:** 7W59 | **Chain:** c
- **b-phipsi:** 0.0078220785450946
- **w-rdist:** 0.4114576289834681
- **t-alpha:** 0.0596135259369763

---

---

679

- **PDB ID:** 7S0Q | **Chain:** A
- **b-phipsi:** 0.0052351860777205
- **w-rdist:** 0.4741732097549641
- **t-alpha:** 0.0888697463155465

---

---

680

- **PDB ID:** 6O85 | **Chain:** B
- **b-phipsi:** 0.0051808313080882
- **w-rdist:** 0.892669095980854
- **t-alpha:** 0.0063793623469805

---

---

681

- **PDB ID:** 3W3J | **Chain:** B
- **b-phipsi:** 0.0040048607951195
- **w-rdist:** 0.3142403378600008
- **t-alpha:** 0.2155308475574191

---

---

682

- **PDB ID:** 8FCM | **Chain:** B
- **b-phipsi:** 0.0056912241052541
- **w-rdist:** 0.1789619257979216
- **t-alpha:** 0.2194925501436893

---

---

683

- **PDB ID:** 4UHX | **Chain:** A
- **b-phipsi:** 0.0025135653970838
- **w-rdist:** 0.3525582672500765
- **t-alpha:** 0.2290020603405205

---

---

684

- **PDB ID:** 7RLJ | **Chain:** E
- **b-phipsi:** 0.0062306391774149
- **w-rdist:** 0.1248143826184985
- **t-alpha:** 0.2210775835865639

---

---

685

- **PDB ID:** 6MK0 | **Chain:** B
- **b-phipsi:** 0.0079209106485045
- **w-rdist:** 0.6386680107651623
- **t-alpha:** 0.013470930625691

---

---

686

- **PDB ID:** 8FCO | **Chain:** C
- **b-phipsi:** 0.0055287921628251
- **w-rdist:** 0.3494449631148978
- **t-alpha:** 0.1465931232334234

---

---

687

- **PDB ID:** 5Z15 | **Chain:** A
- **b-phipsi:** 0.0053447132481626
- **w-rdist:** 0.3177543020342782
- **t-alpha:** 0.1664027794262541

---

---

688

- **PDB ID:** 4XVM | **Chain:** A
- **b-phipsi:** 0.0245469780770673
- **w-rdist:** 0.5250705363700692
- **t-alpha:** 0.0023827284528445

---

---

689

- **PDB ID:** 7BP9 | **Chain:** E
- **b-phipsi:** 0.0072428337035711
- **w-rdist:** 0.1837294246445592
- **t-alpha:** 0.1751188879720684

---

---

690

- **PDB ID:** 8FCM | **Chain:** D
- **b-phipsi:** 0.0062826387421953
- **w-rdist:** 0.1666179295760837
- **t-alpha:** 0.2076068539125026

---

---

691

- **PDB ID:** 4UHW | **Chain:** A
- **b-phipsi:** 0.0023519287450896
- **w-rdist:** 0.3386257135329176
- **t-alpha:** 0.2519816682717417

---

---

692

- **PDB ID:** 6EA4 | **Chain:** A
- **b-phipsi:** 0.0156105602708508
- **w-rdist:** 0.5059272275135746
- **t-alpha:** 0.0118861097710496

---

---

693

- **PDB ID:** 8FCR | **Chain:** A
- **b-phipsi:** 0.0052507023325854
- **w-rdist:** 0.3568944295326817
- **t-alpha:** 0.1521394853607829

---

---

694

- **PDB ID:** 7RL6 | **Chain:** D
- **b-phipsi:** 0.0058061161162715
- **w-rdist:** 0.1258014878562918
- **t-alpha:** 0.2432650359073522

---

---

695

- **PDB ID:** 6XRN | **Chain:** A
- **b-phipsi:** 0.0055560329892899
- **w-rdist:** 0.2539349703212035
- **t-alpha:** 0.1909670055645516

---

---

696

- **PDB ID:** 6GPG | **Chain:** A
- **b-phipsi:** 0.0143774247942418
- **w-rdist:** 0.6702857951906641
- **t-alpha:** 0.0015848307213084

---

---

697

- **PDB ID:** 5VNF | **Chain:** B
- **b-phipsi:** 0.0014210604665703
- **w-rdist:** 0.7275664349096106
- **t-alpha:** 0.0269416384138463

---

---

698

- **PDB ID:** 4OVU | **Chain:** A
- **b-phipsi:** 0.0116695570388832
- **w-rdist:** 0.2939117509492767
- **t-alpha:** 0.0570524158190295

---

---

699

- **PDB ID:** 1TZN | **Chain:** o
- **b-phipsi:** 0.0022444195264519
- **w-rdist:** 0.8133544083265246
- **t-alpha:** 0.0182252502019479

---

---

700

- **PDB ID:** 6XI2 | **Chain:** B
- **b-phipsi:** 0.0009449116671005
- **w-rdist:** 0.6457687919252664
- **t-alpha:** 0.0640808530234371

---

---

701

- **PDB ID:** 2JJD | **Chain:** F
- **b-phipsi:** 0.0041977934246522
- **w-rdist:** 0.8477618363614511
- **t-alpha:** 0.0120284587273298

---

---

702

- **PDB ID:** 8FCM | **Chain:** C
- **b-phipsi:** 0.0048967734229821
- **w-rdist:** 0.1753153737987453
- **t-alpha:** 0.2884315142237563

---

---

703

- **PDB ID:** 3DBS | **Chain:** A
- **b-phipsi:** 0.006821690436471
- **w-rdist:** 0.2215326488435048
- **t-alpha:** 0.1703645180917901

---

---

704

- **PDB ID:** 6KYA | **Chain:** A
- **b-phipsi:** 0.005157528232812
- **w-rdist:** 0.3215746096493772
- **t-alpha:** 0.1719494022326169

---

---

705

- **PDB ID:** 3SE6 | **Chain:** B
- **b-phipsi:** 0.0116736020434476
- **w-rdist:** 0.5747855640908743
- **t-alpha:** 0.0079867999848444

---

---

706

- **PDB ID:** 5MHL | **Chain:** A
- **b-phipsi:** 0.0121453715769348
- **w-rdist:** 0.3004354593736235
- **t-alpha:** 0.050713514468949

---

---

707

- **PDB ID:** 7T3C | **Chain:** A
- **b-phipsi:** 0.0017963487893434
- **w-rdist:** 0.4107817913802462
- **t-alpha:** 0.2099839207970202

---

---

708

- **PDB ID:** 3W5O | **Chain:** B
- **b-phipsi:** 0.0047269806378919
- **w-rdist:** 0.5549854099324306
- **t-alpha:** 0.0364504923644704

---

---

709

- **PDB ID:** 3W3M | **Chain:** A
- **b-phipsi:** 0.0040131161721917
- **w-rdist:** 0.3221055525291503
- **t-alpha:** 0.2107769308628622

---

---

710

- **PDB ID:** 1L9N | **Chain:** A
- **b-phipsi:** 0.0135910933954306
- **w-rdist:** 0.6554054102100622
- **t-alpha:** 0.0031697813850419

---

---

711

- **PDB ID:** 4QBZ | **Chain:** A
- **b-phipsi:** 0.0049225433155233
- **w-rdist:** 0.3228621821836415
- **t-alpha:** 0.1798734427113426

---

---

712

- **PDB ID:** 6PYS | **Chain:** A
- **b-phipsi:** 0.0115546508448054
- **w-rdist:** 0.2002013594666463
- **t-alpha:** 0.0935026519522419

---

---

713

- **PDB ID:** 6D03 | **Chain:** D
- **b-phipsi:** 0.0056499545480753
- **w-rdist:** 0.5974096419866413
- **t-alpha:** 0.0202100770637112

---

---

714

- **PDB ID:** 2OK5 | **Chain:** A
- **b-phipsi:** 0.0035639864304846
- **w-rdist:** 0.72991801589809
- **t-alpha:** 0.0202100770637112

---

---

715

- **PDB ID:** 4N2E | **Chain:** A
- **b-phipsi:** 0.0049804913080081
- **w-rdist:** 0.8151287464037242
- **t-alpha:** 0.0112175789229118

---

---

716

- **PDB ID:** 8AG4 | **Chain:** B
- **b-phipsi:** 0.0036395509249753
- **w-rdist:** 0.4974522991644713
- **t-alpha:** 0.1236136545999804

---

---

717

- **PDB ID:** 5C18 | **Chain:** A
- **b-phipsi:** 0.0079551664151487
- **w-rdist:** 0.2754797354194501
- **t-alpha:** 0.1331219773390095

---

---

718

- **PDB ID:** 5IZR | **Chain:** B
- **b-phipsi:** 0.0052654881986505
- **w-rdist:** 0.5273825950779452
- **t-alpha:** 0.0459591096503235

---

---

719

- **PDB ID:** 5AZ5 | **Chain:** D
- **b-phipsi:** 0.0048912155809363
- **w-rdist:** 0.3429291753424216
- **t-alpha:** 0.1695723222503973

---

---

720

- **PDB ID:** 1PKX | **Chain:** C
- **b-phipsi:** 0.0101457356371072
- **w-rdist:** 0.7505706766289318
- **t-alpha:** 0.002377470759985

---

---

721

- **PDB ID:** 7RLF | **Chain:** A
- **b-phipsi:** 0.0072505124699229
- **w-rdist:** 0.1483355304847237
- **t-alpha:** 0.1909670055645516

---

---

722

- **PDB ID:** 6MZC | **Chain:** B
- **b-phipsi:** 0.0102174783789498
- **w-rdist:** 0.2924743767987418
- **t-alpha:** 0.0800319287976243

---

---

723

- **PDB ID:** 7R52 | **Chain:** A
- **b-phipsi:** 0.006990180854514
- **w-rdist:** 0.3225080910782535
- **t-alpha:** 0.1331219773390095

---

---

724

- **PDB ID:** 4TV3 | **Chain:** A
- **b-phipsi:** 0.0105450869351213
- **w-rdist:** 0.2242493017223155
- **t-alpha:** 0.1006343630980566

---

---

725

- **PDB ID:** 3U7U | **Chain:** A
- **b-phipsi:** 0.005569329374494
- **w-rdist:** 0.6133788133322076
- **t-alpha:** 0.0202100770637112

---

---

726

- **PDB ID:** 6AS7 | **Chain:** A
- **b-phipsi:** 0.0048844457428093
- **w-rdist:** 0.3141225742831588
- **t-alpha:** 0.1933443217215238

---

---

727

- **PDB ID:** 8E4Y | **Chain:** A
- **b-phipsi:** 0.0154970926862669
- **w-rdist:** 0.610331123395676
- **t-alpha:** 0.0031697813850419

---

---

728

- **PDB ID:** 3U7U | **Chain:** F
- **b-phipsi:** 0.0048120646821182
- **w-rdist:** 0.6598825737654631
- **t-alpha:** 0.0206022505246215

---

---

729

- **PDB ID:** 8FCO | **Chain:** D
- **b-phipsi:** 0.0052420539541049
- **w-rdist:** 0.1782964439894439
- **t-alpha:** 0.263866511387046

---

---

730

- **PDB ID:** 7WUB | **Chain:** C
- **b-phipsi:** 0.0224587579097708
- **w-rdist:** 0.5541219351391393
- **t-alpha:** 0.0015848307213084

---

---

731

- **PDB ID:** 7JKQ | **Chain:** A
- **b-phipsi:** 0.0057357057462921
- **w-rdist:** 0.7079484557195526
- **t-alpha:** 0.0158479732088472

---

---

732

- **PDB ID:** 7RLB | **Chain:** A
- **b-phipsi:** 0.007175985839491
- **w-rdist:** 0.1924724008840347
- **t-alpha:** 0.1830429219610427

---

---

733

- **PDB ID:** 2B3X | **Chain:** A
- **b-phipsi:** 0.0061970552409317
- **w-rdist:** 0.8275062552399121
- **t-alpha:** 0.0079243179984995

---

---

734

- **PDB ID:** 5DXU | **Chain:** A
- **b-phipsi:** 0.0080852769523494
- **w-rdist:** 0.3334889868582681
- **t-alpha:** 0.1030112267642286

---

---

735

- **PDB ID:** 1YGU | **Chain:** B
- **b-phipsi:** 0.0036919872941728
- **w-rdist:** 0.8071298412666326
- **t-alpha:** 0.0161027964403612

---

---

736

- **PDB ID:** 5C19 | **Chain:** A
- **b-phipsi:** 0.0063772948558228
- **w-rdist:** 0.1006422878399983
- **t-alpha:** 0.2345490443729756

---

---

737

- **PDB ID:** 8FCR | **Chain:** C
- **b-phipsi:** 0.0054021679715946
- **w-rdist:** 0.1719079247911531
- **t-alpha:** 0.2583197963665382

---

---

738

- **PDB ID:** 5T23 | **Chain:** A
- **b-phipsi:** 0.0071620191346057
- **w-rdist:** 0.2267393382229475
- **t-alpha:** 0.1671950546614453

---

---

739

- **PDB ID:** 7FFM | **Chain:** A
- **b-phipsi:** 0.0053158673579927
- **w-rdist:** 0.6801138619357836
- **t-alpha:** 0.0185632335173342

---

---

740

- **PDB ID:** 5VNL | **Chain:** B
- **b-phipsi:** 0.0012686939578866
- **w-rdist:** 0.7467582903459586
- **t-alpha:** 0.026149146068277

---

---

741

- **PDB ID:** 7MDO | **Chain:** F
- **b-phipsi:** 0.0141986737090548
- **w-rdist:** 0.5703425512587188
- **t-alpha:** 0.006339533171912

---

---

742

- **PDB ID:** 5EDS | **Chain:** A
- **b-phipsi:** 0.006608452536111
- **w-rdist:** 0.2163594611127724
- **t-alpha:** 0.187797819454637

---

---

743

- **PDB ID:** 6NIG | **Chain:** C
- **b-phipsi:** 0.0058469053666809
- **w-rdist:** 0.4896620448656599
- **t-alpha:** 0.0667790338922971

---

---

744

- **PDB ID:** 2CKJ | **Chain:** B
- **b-phipsi:** 0.0041465565064094
- **w-rdist:** 0.4908405183086164
- **t-alpha:** 0.1220288622848613

---

---

745

- **PDB ID:** 5FTJ | **Chain:** B
- **b-phipsi:** 0.0060241146926103
- **w-rdist:** 0.2241656900002227
- **t-alpha:** 0.2004761586649326

---

---

746

- **PDB ID:** 7MN5 | **Chain:** A
- **b-phipsi:** 0.0058781966806863
- **w-rdist:** 0.7391898440920197
- **t-alpha:** 0.0136545862916419

---

---

747

- **PDB ID:** 6Q6G | **Chain:** I
- **b-phipsi:** 0.0036942670618176
- **w-rdist:** 0.7219714400736562
- **t-alpha:** 0.0210354708025768

---

---

748

- **PDB ID:** 3QAQ | **Chain:** A
- **b-phipsi:** 0.0078217896249382
- **w-rdist:** 0.1865092560846041
- **t-alpha:** 0.1719494022326169

---

---

749

- **PDB ID:** 8ASC | **Chain:** L
- **b-phipsi:** 0.0047417582718285
- **w-rdist:** 0.5569650550235888
- **t-alpha:** 0.0404122840988643

---

---

750

- **PDB ID:** 7ZWA | **Chain:** B
- **b-phipsi:** 0.0028375215990495
- **w-rdist:** 0.5332293828496882
- **t-alpha:** 0.1030112267642286

---

---

751

- **PDB ID:** 7RL6 | **Chain:** A
- **b-phipsi:** 0.0059151980209926
- **w-rdist:** 0.1365639694697008
- **t-alpha:** 0.2519816682717417

---

---

752

- **PDB ID:** 6Q6Q | **Chain:** A
- **b-phipsi:** 0.0034959150356348
- **w-rdist:** 0.3508007430867805
- **t-alpha:** 0.216324013081756

---

---

753

- **PDB ID:** 7WUB | **Chain:** I
- **b-phipsi:** 0.0059300208517019
- **w-rdist:** 0.1338203747591984
- **t-alpha:** 0.2535658908844553

---

---

754

- **PDB ID:** 7KKE | **Chain:** A
- **b-phipsi:** 0.0083035803437648
- **w-rdist:** 0.2078709089043801
- **t-alpha:** 0.1537244455033311

---

---

755

- **PDB ID:** 5TEQ | **Chain:** A
- **b-phipsi:** 0.0053775498523782
- **w-rdist:** 0.515764381225755
- **t-alpha:** 0.0618066131443488

---

---

756

- **PDB ID:** 3ML9 | **Chain:** A
- **b-phipsi:** 0.0094816448746168
- **w-rdist:** 0.2577863598064769
- **t-alpha:** 0.1133125594934425

---

---

757

- **PDB ID:** 7FFU | **Chain:** A
- **b-phipsi:** 0.0041529750516549
- **w-rdist:** 0.6865846282501769
- **t-alpha:** 0.0218620195820675

---

---

758

- **PDB ID:** 8BHY | **Chain:** B
- **b-phipsi:** 0.0041978478666236
- **w-rdist:** 0.4134725269122666
- **t-alpha:** 0.1608558375885556

---

---

759

- **PDB ID:** 4MMX | **Chain:** B
- **b-phipsi:** 0.0052219995808667
- **w-rdist:** 0.6472113967799014
- **t-alpha:** 0.0206022505246215

---

---

760

- **PDB ID:** 3BDL | **Chain:** A
- **b-phipsi:** 0.0041479975871662
- **w-rdist:** 0.5654310669960936
- **t-alpha:** 0.0464345284809148

---

---

761

- **PDB ID:** 8EFI | **Chain:** M
- **b-phipsi:** 0.0155405748585543
- **w-rdist:** 0.5076644979659267
- **t-alpha:** 0.0128409546837204

---

---

762

- **PDB ID:** 7OPN | **Chain:** B
- **b-phipsi:** 0.0031278150622633
- **w-rdist:** 0.350236610192344
- **t-alpha:** 0.2313789650220328

---

---

763

- **PDB ID:** 4XX5 | **Chain:** A
- **b-phipsi:** 0.0064833355907511
- **w-rdist:** 0.2156718397986675
- **t-alpha:** 0.1933443217215238

---

---

764

- **PDB ID:** 5KYY | **Chain:** B
- **b-phipsi:** 0.0013827309071789
- **w-rdist:** 0.7455118908439599
- **t-alpha:** 0.0285264979365806

---

---

765

- **PDB ID:** 8FCQ | **Chain:** B
- **b-phipsi:** 0.005526012178084
- **w-rdist:** 0.1648591068389543
- **t-alpha:** 0.267828559172903

---

---

766

- **PDB ID:** 7E89 | **Chain:** A
- **b-phipsi:** 0.0035967581966993
- **w-rdist:** 0.835929631730749
- **t-alpha:** 0.0152852632192168

---

---

767

- **PDB ID:** 5GVE | **Chain:** A
- **b-phipsi:** 0.0158460334041609
- **w-rdist:** 0.4992262095713531
- **t-alpha:** 0.013470930625691

---

---

768

- **PDB ID:** 7NDL | **Chain:** B
- **b-phipsi:** 0.0083405486404326
- **w-rdist:** 0.8390960151198957
- **t-alpha:** 0.002377470759985

---

---

769

- **PDB ID:** 5I0I | **Chain:** B
- **b-phipsi:** 0.0173495189219631
- **w-rdist:** 0.521632885134691
- **t-alpha:** 0.0087926839253336

---

---

770

- **PDB ID:** 7RLF | **Chain:** D
- **b-phipsi:** 0.0083767238474467
- **w-rdist:** 0.1506131465858423
- **t-alpha:** 0.1719494022326169

---

---

771

- **PDB ID:** 7RLJ | **Chain:** H
- **b-phipsi:** 0.0072922968106948
- **w-rdist:** 0.1209690369759154
- **t-alpha:** 0.2083994584940032

---

---

772

- **PDB ID:** 7MDM | **Chain:** E
- **b-phipsi:** 0.006425154026884
- **w-rdist:** 0.2861053634166083
- **t-alpha:** 0.1671950546614453

---

---

773

- **PDB ID:** 1A36 | **Chain:** A
- **b-phipsi:** 0.027772145730186
- **w-rdist:** 0.5210250357990122
- **t-alpha:** 0.003179348403216

---

---

774

- **PDB ID:** 7RLJ | **Chain:** J
- **b-phipsi:** 0.0062831211406778
- **w-rdist:** 0.1191538633776332
- **t-alpha:** 0.24960432062362

---

---

775

- **PDB ID:** 4GNK | **Chain:** B
- **b-phipsi:** 0.0079193500833764
- **w-rdist:** 0.6306335260540201
- **t-alpha:** 0.0150558135497862

---

---

776

- **PDB ID:** 7RLB | **Chain:** F
- **b-phipsi:** 0.0053837566539308
- **w-rdist:** 0.2115332378996223
- **t-alpha:** 0.2369260603208425

---

---

777

- **PDB ID:** 7RLJ | **Chain:** G
- **b-phipsi:** 0.0058220775282883
- **w-rdist:** 0.1224686481505879
- **t-alpha:** 0.2884315142237563

---

---

778

- **PDB ID:** 7CRF | **Chain:** B
- **b-phipsi:** 0.0047504608824598
- **w-rdist:** 0.3282575486500937
- **t-alpha:** 0.1933443217215238

---

---

779

- **PDB ID:** 5HDH | **Chain:** A
- **b-phipsi:** 0.0066293543756786
- **w-rdist:** 0.3624764816023775
- **t-alpha:** 0.1275752196006165

---

---

780

- **PDB ID:** 3DPD | **Chain:** A
- **b-phipsi:** 0.008753048801139
- **w-rdist:** 0.2830716552873304
- **t-alpha:** 0.1244058176483571

---

---

781

- **PDB ID:** 8BH3 | **Chain:** B
- **b-phipsi:** 0.0026030124703888
- **w-rdist:** 0.4128847391640905
- **t-alpha:** 0.2044381850417527

---

---

782

- **PDB ID:** 8PFI | **Chain:** B
- **b-phipsi:** 0.005304039811912
- **w-rdist:** 0.4523962783783724
- **t-alpha:** 0.1188590445787747

---

---

783

- **PDB ID:** 8FCM | **Chain:** E
- **b-phipsi:** 0.0049228402349227
- **w-rdist:** 0.2067814133119401
- **t-alpha:** 0.3003175815558698

---

---

784

- **PDB ID:** 8FCR | **Chain:** B
- **b-phipsi:** 0.0057138530126466
- **w-rdist:** 0.1640801272155076
- **t-alpha:** 0.2591132644595053

---

---

785

- **PDB ID:** 4FA6 | **Chain:** A
- **b-phipsi:** 0.0085777394945174
- **w-rdist:** 0.2347860870692421
- **t-alpha:** 0.1458006268370766

---

---

786

- **PDB ID:** 8FCP | **Chain:** B
- **b-phipsi:** 0.005840178875047
- **w-rdist:** 0.1916418378763739
- **t-alpha:** 0.2321718959940186

---

---

787

- **PDB ID:** 6SY1 | **Chain:** A
- **b-phipsi:** 0.0183421156890735
- **w-rdist:** 0.4047353239322317
- **t-alpha:** 0.017432692187089

---

---

788

- **PDB ID:** 7SCH | **Chain:** B
- **b-phipsi:** 0.0022897177573609
- **w-rdist:** 0.5337454755405613
- **t-alpha:** 0.1220288622848613

---

---

789

- **PDB ID:** 8FCP | **Chain:** D
- **b-phipsi:** 0.0049358793332107
- **w-rdist:** 0.3771726832776444
- **t-alpha:** 0.1592710305637339

---

---

790

- **PDB ID:** 8EFE | **Chain:** A
- **b-phipsi:** 0.011781327674682
- **w-rdist:** 0.3625830942406242
- **t-alpha:** 0.0369759793802602

---

---

791

- **PDB ID:** 2CKJ | **Chain:** D
- **b-phipsi:** 0.0045196959722852
- **w-rdist:** 0.4616087452461753
- **t-alpha:** 0.1339143555568707

---

---

792

- **PDB ID:** 4R07 | **Chain:** A
- **b-phipsi:** 0.0040289602373169
- **w-rdist:** 0.3631306485985799
- **t-alpha:** 0.1988904263943514

---

---

793

- **PDB ID:** 7D9T | **Chain:** B
- **b-phipsi:** 0.010876810706273
- **w-rdist:** 0.4172959252006489
- **t-alpha:** 0.0269416384138463

---

---

794

- **PDB ID:** 1YGR | **Chain:** A
- **b-phipsi:** 0.0033046477432286
- **w-rdist:** 0.8156664011058217
- **t-alpha:** 0.017432692187089

---

---

795

- **PDB ID:** 2JBJ | **Chain:** A
- **b-phipsi:** 0.0045176079600584
- **w-rdist:** 0.941149741776323
- **t-alpha:** 0.0079867999848444

---

---

796

- **PDB ID:** 1K4T | **Chain:** A
- **b-phipsi:** 0.0240102211484005
- **w-rdist:** 0.47550326844412
- **t-alpha:** 0.0095996935833633

---

---

797

- **PDB ID:** 8FCR | **Chain:** F
- **b-phipsi:** 0.0051743849261251
- **w-rdist:** 0.3372311547743593
- **t-alpha:** 0.1774960114592363

---

---

798

- **PDB ID:** 7MEZ | **Chain:** A
- **b-phipsi:** 0.0080494007625634
- **w-rdist:** 0.3376859877385733
- **t-alpha:** 0.11252025444096

---

---

799

- **PDB ID:** 4MK0 | **Chain:** A
- **b-phipsi:** 0.0130291811486052
- **w-rdist:** 0.4168671331796613
- **t-alpha:** 0.0218620195820675

---

---

800

- **PDB ID:** 7BP9 | **Chain:** A
- **b-phipsi:** 0.0068941289359391
- **w-rdist:** 0.1933020450831707
- **t-alpha:** 0.1973056469081315

---

---
