# Supplementary material for: Comparative structural insights and functional analysis for the distinct unbound states of Human AGO proteins
Source: Sci Rep. 2025 Mar 19;15:9432. doi: 10.1038/s41598-025-91849-5 (PMC11923369; doi:10.1038/s41598-025-91849-5)
Supplement: Supplementary file 24 — Supplementary Information 12. [file 41598_2025_91849_MOESM24_ESM.zip › 5VM9p_A_mdwhole_HL2REF/go/5VM9p_A_mitot_mitosis_7e57613b126b49008a98724222172435-pres_report.html]

 

# Structural Comparison Report for 5VM9p\_A - whole structures (total: 8)

---

1

- **Protein name:** Epidermal growth factor receptor
- **Organism:** Homo sapiens
- **Uniprot Accession Number:** P00533
- **Protein sequence length:** 1210 aa
- **1D identity (%):** 16.44
- **1D identity (%) [Gaps excluded]:** 25.52
- **1D identity - Alignment Gaps:** 448
- **1D aligned content (<aminoacid>:%):** {'G': 11.11, 'A': 4.83, 'L': 9.18, 'T': 4.83, 'K': 7.25, 'N': 1.93, 'C': 3.38, 'V': 7.25, 'Y': 5.8, 'D': 2.42, 'P': 9.66, 'S': 4.83, 'F': 2.9, 'H': 3.38, 'R': 5.8, 'M': 0.97, 'I': 6.28, 'E': 3.86, 'Q': 4.35}
- **Common reported functions (%):** 0.0
- **Common reported locations (%):** 22.22
- **Common reported processes (%):** 11.11

- **PDB ID:** 7OM4
- **Chain:** A
- **Crystallized protein length:** 614 aa
- **Resolution:** 6.05 Å
- **b-phipsi ():** 0.00191
- **w-rdist ():** 0.748419
- **t-alpha ():** 0.0
- **Chemical similarity (Tanimoto Index) (%):** 83.93
- **1D identity (%) [PDB]:** 1.89
- **1D identity (%) [Gaps excluded][PDB]:** 79.41
- **1D identity - Alignment Gaps [PDB]:** 1391
- **1D aligned content [PDB] (<aminoacid>:%):** {'L': 3.7, 'E': 7.41, 'K': 7.41, 'Q': 7.41, 'G': 7.41, 'I': 11.11, 'T': 11.11, 'Y': 3.7, 'V': 7.41, 'C': 3.7, 'A': 7.41, 'D': 3.7, 'R': 7.41, 'S': 3.7, 'N': 3.7, 'P': 3.7}
- **2D identity (%) [PDB]:** 19.52
- **2D identity (%) [Gaps excluded][PDB]:** 78.35
- **2D identity - Alignment Gaps [PDB]:** 877
- **2D aligned content [PDB] (<2D-fold>:%):** {'.': 35.53, 'E': 30.26, 'H': 11.84, 'T': 22.37}
- **3D similarity (TM-Score) (%) [PDB]:** 21.07

- **Gene name:** EGFR
- **Entrez ID:** 1956
- **RefSeq ID:** NM\_201284
- **Transcript sequence length:** 2872
- **5-UTR|CDS|3-UTR identity (%):** 43.05 | 43.1 | 1.76
- **5-UTR|CDS|3-UTR identity (%) [Gaps excluded]:** 77.03 | 73.95 | 75.19
- **5-UTR|CDS|3-UTR identity [Alignment Gaps]:** 165 | 1239 | 16458
- **5-UTR aligned content (<base>:%):** {'A': 4.97, 'G': 36.65, 'C': 50.93, 'T': 7.45}
- **CDS aligned content (<base>:%):** {'A': 28.28, 'T': 22.27, 'G': 24.45, 'C': 25.0}
- **3-UTR aligned content (<base>:%):** {'G': 16.5, 'C': 25.25, 'A': 30.64, 'T': 27.61}

**Uniprot Description:**  
  
 Receptor tyrosine kinase binding ligands of the EGF family and activating several signaling cascades to convert extracellular cues into appropriate cellular responses (PubMed:2790960, PubMed:10805725, PubMed:27153536). Known ligands include EGF, TGFA/TGF-alpha, AREG, epigen/EPGN, BTC/betacellulin, epiregulin/EREG and HBEGF/heparin-binding EGF (PubMed:2790960, PubMed:7679104, PubMed:8144591, PubMed:9419975, PubMed:15611079, PubMed:12297049, PubMed:27153536, PubMed:20837704, PubMed:17909029). Ligand binding triggers receptor homo- and/or heterodimerization and autophosphorylation on key cytoplasmic residues. The phosphorylated receptor recruits adapter proteins like GRB2 which in turn activates complex downstream signaling cascades. Activates at least 4 major downstream signaling cascades including the RAS-RAF-MEK-ERK, PI3 kinase-AKT, PLCgamma-PKC and STATs modules (PubMed:27153536). May also activate the NF-kappa-B signaling cascade (PubMed:11116146). Also directly phosphorylates other proteins like RGS16, activating its GTPase activity and probably coupling the EGF receptor signaling to the G protein-coupled receptor signaling (PubMed:11602604). Also phosphorylates MUC1 and increases its interaction with SRC and CTNNB1/beta-catenin (PubMed:11483589). Positively regulates cell migration via interaction with CCDC88A/GIV which retains EGFR at the cell membrane following ligand stimulation, promoting EGFR signaling which triggers cell migration (PubMed:20462955). Plays a role in enhancing learning and memory performance (By similarity).   
  
Binding of the ligand triggers homo- and/or heterodimerization of the receptor triggering its autophosphorylation. Heterodimer with ERBB2 (PubMed:10805725). Forms a complex with CCDC88A/GIV (via SH2-like regions) and GNAI3 which leads to enhanced EGFR signaling and triggering of cell migration; binding to CCDC88A requires autophosphorylation of the EGFR C-terminal region, and ligand stimulation is required for recruitment of GNAI3 to the complex (PubMed:20462955, PubMed:25187647). Interacts with ERRFI1; inhibits dimerization of the kinase domain and autophosphorylation (PubMed:18046415). Part of a complex with ERBB2 and either PIK3C2A or PIK3C2B (PubMed:10805725). Interacts with GRB2; an adapter protein coupling the receptor to downstream signaling pathways. Interacts with GAB2; involved in signaling downstream of EGFR. Interacts with STAT3; mediates EGFR downstream signaling in cell proliferation. Interacts with RIPK1; involved in NF-kappa-B activation. Interacts (autophosphorylated) with CBL, CBLB and CBLC; involved in EGFR ubiquitination and regulation. Interacts with SOCS5; regulates EGFR degradation through ELOC- and ELOB-mediated ubiquitination and proteasomal degradation. Interacts with PRMT5; methylates EGFR and enhances interaction with PTPN6. Interacts (phosphorylated) with PTPN6; inhibits EGFR-dependent activation of MAPK/ERK. Interacts with COPG1; essential for regulation of EGF-dependent nuclear transport of EGFR by retrograde trafficking from the Golgi to the ER. Interacts with TNK2; this interaction is dependent on EGF stimulation and kinase activity of EGFR. Interacts with PCNA; positively regulates PCNA (PubMed:17115032). Interacts with PELP1. Interacts with MUC1. Interacts with AP2M1. Interacts with FER. May interact with EPS8; mediates EPS8 phosphorylation. Interacts (via SH2 domains) with GRB2, NCK1 and NCK2 (PubMed:10026169). Interacts with ATXN2. Interacts with GAREM1. Interacts (ubiquitinated) with ANKRD13A/B/D; the interaction is direct and may regulate EGFR internalization after EGF stimulation. Interacts with GPER1; the interaction occurs in an estrogen-dependent manner. Interacts (via C-terminal cytoplasmic kinase domain) with ZPR1 (via zinc fingers). Interacts with RNF115 and RNF126 (PubMed:23418353). Interacts with GPRC5A (via its transmembrane domain) (PubMed:25311788). Interacts with FAM83B; positively regulates EGFR inducing its autophosphorylation in absence of stimulation by EGF (PubMed:23912460). Interacts with LAPTM4B; positively correlates with EGFR activation (PubMed:28479384). Interacts with STX19 (PubMed:16420529). Interacts with CD44 (PubMed:23589287). Interacts with PGRMC1; the interaction requires PGRMC1 homodimerization (PubMed:26988023). Interacts with PIKFYVE (PubMed:17909029). Interacts with NEU3.   
  
 **Gene Ontology Information:**

Molecular Function

- actin filament binding
- ATP binding
- ATPase binding
- cadherin binding
- chromatin binding
- double-stranded DNA binding
- enzyme binding
- epidermal growth factor binding
- epidermal growth factor-activated receptor activity
- identical protein binding
- kinase binding
- MAP kinase kinase kinase activity
- protein phosphatase binding
- protein tyrosine kinase activator activity
- protein tyrosine kinase activity
- transmembrane receptor protein tyrosine kinase activator activity
- transmembrane receptor protein tyrosine kinase activity
- transmembrane signaling receptor activity
- ubiquitin protein ligase binding
- virus receptor activity

Location

- basal plasma membrane
- basolateral plasma membrane
- cell junction
- cell surface
- clathrin-coated endocytic vesicle membrane
- cytoplasm
- early endosome membrane
- endoplasmic reticulum membrane
- endosome
- endosome membrane
- extracellular space
- focal adhesion
- Golgi membrane
- intracellular vesicle
- membrane
- membrane raft
- multivesicular body, internal vesicle lumen
- nuclear membrane
- nucleus
- perinuclear region of cytoplasm
- plasma membrane
- protein-containing complex
- receptor complex
- ruffle membrane
- Shc-EGFR complex

Biological process

- activation of phospholipase C activity
- cell morphogenesis
- cell surface receptor signaling pathway
- cell-cell adhesion
- cellular response to amino acid stimulus
- cellular response to cadmium ion
- cellular response to epidermal growth factor stimulus
- cellular response to estradiol stimulus
- cellular response to reactive oxygen species
- cerebral cortex cell migration
- digestive tract morphogenesis
- embryonic placenta development
- epidermal growth factor receptor signaling pathway
- epithelial cell proliferation
- ERBB2-EGFR signaling pathway
- eyelid development in camera-type eye
- hair follicle development
- learning or memory
- morphogenesis of an epithelial fold
- negative regulation of apoptotic process
- negative regulation of cardiocyte differentiation
- negative regulation of epidermal growth factor receptor signaling pathway
- negative regulation of protein catabolic process
- neurogenesis
- ossification
- peptidyl-tyrosine autophosphorylation
- peptidyl-tyrosine phosphorylation
- positive regulation of canonical Wnt signaling pathway
- positive regulation of cell growth
- positive regulation of cell migration
- positive regulation of cell population proliferation
- positive regulation of cyclin-dependent protein serine/threonine kinase activity
- positive regulation of DNA repair
- positive regulation of DNA replication
- positive regulation of transcription, DNA-templated
- positive regulation of epithelial cell proliferation
- positive regulation of ERK1 and ERK2 cascade
- positive regulation of fibroblast proliferation
- positive regulation of G1/S transition of mitotic cell cycle
- positive regulation of kinase activity
- positive regulation of MAP kinase activity
- positive regulation of pri-miRNA transcription by RNA polymerase II
- positive regulation of NIK/NF-kappaB signaling
- positive regulation of nitric oxide mediated signal transduction
- positive regulation of peptidyl-serine phosphorylation
- positive regulation of phosphorylation
- positive regulation of protein kinase B signaling
- positive regulation of protein kinase C activity
- positive regulation of protein localization to plasma membrane
- positive regulation of protein phosphorylation
- positive regulation of transcription by RNA polymerase II
- protein autophosphorylation
- protein insertion into membrane
- regulation of ERK1 and ERK2 cascade
- regulation of JNK cascade
- regulation of nitric-oxide synthase activity
- regulation of peptidyl-tyrosine phosphorylation
- regulation of phosphatidylinositol 3-kinase signaling
- response to UV-A
- salivary gland morphogenesis
- signal transduction
- transmembrane receptor protein tyrosine kinase signaling pathway

---

2

- **Protein name:** DNA polymerase alpha catalytic subunit
- **Organism:** Homo sapiens
- **Uniprot Accession Number:** P09884
- **Protein sequence length:** 1462 aa
- **1D identity (%):** 15.31
- **1D identity (%) [Gaps excluded]:** 28.41
- **1D identity - Alignment Gaps:** 696
- **1D aligned content (<aminoacid>:%):** {'M': 0.43, 'P': 10.82, 'R': 6.93, 'G': 8.23, 'L': 11.69, 'E': 2.6, 'K': 9.52, 'V': 7.36, 'Y': 3.9, 'D': 6.93, 'T': 3.9, 'S': 3.03, 'N': 3.9, 'H': 3.9, 'F': 4.33, 'W': 0.43, 'Q': 3.46, 'C': 2.16, 'I': 3.46, 'A': 3.03}
- **Common reported functions (%):** 0.0
- **Common reported locations (%):** 22.22
- **Common reported processes (%):** 0.0

- **PDB ID:** 4QCL
- **Chain:** A
- **Crystallized protein length:** 865 aa
- **Resolution:** 2.2 Å
- **b-phipsi ():** 0.001961
- **w-rdist ():** 0.135904
- **t-alpha ():** 0.090333
- **Chemical similarity (Tanimoto Index) (%):** 67.7
- **1D identity (%) [PDB]:** 2.47
- **1D identity (%) [Gaps excluded][PDB]:** 74.55
- **1D identity - Alignment Gaps [PDB]:** 1603
- **1D aligned content [PDB] (<aminoacid>:%):** {'Q': 12.2, 'C': 2.44, 'V': 17.07, 'K': 9.76, 'N': 9.76, 'S': 7.32, 'P': 7.32, 'L': 14.63, 'G': 7.32, 'I': 2.44, 'R': 2.44, 'F': 2.44, 'D': 2.44, 'T': 2.44}
- **2D identity (%) [PDB]:** 37.71
- **2D identity (%) [Gaps excluded][PDB]:** 87.23
- **2D identity - Alignment Gaps [PDB]:** 679
- **2D aligned content [PDB] (<2D-fold>:%):** {'.': 19.51, 'E': 27.27, 'T': 9.53, 'H': 43.02, 'G': 0.67}
- **3D similarity (TM-Score) (%) [PDB]:** 22.63

- **Gene name:** POLA1
- **Entrez ID:** 5422
- **RefSeq ID:** NM\_016937
- **Transcript sequence length:** 5469
- **5-UTR|CDS|3-UTR identity (%):** 10.22 | 40.36 | 3.8
- **5-UTR|CDS|3-UTR identity (%) [Gaps excluded]:** 76.74 | 76.01 | 74.94
- **5-UTR|CDS|3-UTR identity [Alignment Gaps]:** 280 | 2136 | 16075
- **5-UTR aligned content (<base>:%):** {'G': 54.55, 'C': 21.21, 'A': 9.09, 'T': 15.15}
- **CDS aligned content (<base>:%):** {'A': 31.45, 'T': 25.03, 'G': 23.72, 'C': 19.8}
- **3-UTR aligned content (<base>:%):** {'G': 19.28, 'A': 30.79, 'T': 32.81, 'C': 17.11}

**Uniprot Description:**  
  
 Catalytic subunit of the DNA polymerase alpha complex (also known as the alpha DNA polymerase-primase complex) which plays an essential role in the initiation of DNA synthesis. During the S phase of the cell cycle, the DNA polymerase alpha complex (composed of a catalytic subunit POLA1, a regulatory subunit POLA2 and two primase subunits PRIM1 and PRIM2) is recruited to DNA at the replicative forks via direct interactions with MCM10 and WDHD1. The primase subunit of the polymerase alpha complex initiates DNA synthesis by oligomerising short RNA primers on both leading and lagging strands. These primers are initially extended by the polymerase alpha catalytic subunit and subsequently transferred to polymerase delta and polymerase epsilon for processive synthesis on the lagging and leading strand, respectively. The reason this transfer occurs is because the polymerase alpha has limited processivity and lacks intrinsic 3' exonuclease activity for proofreading error, and therefore is not well suited for replicating long complexes. In the cytosol, responsible for a substantial proportion of the physiological concentration of cytosolic RNA:DNA hybrids, which are necessary to prevent spontaneous activation of type I interferon responses (PubMed:27019227).   
  
Component of the alpha DNA polymerase complex (also known as the alpha DNA polymerase-primase complex) consisting of four subunits: the catalytic subunit POLA1, the regulatory subunit POLA2, and the primase complex subunits PRIM1 and PRIM2 respectively (PubMed:9705292). Interacts with PARP1; this interaction functions as part of the control of replication fork progression (PubMed:9518481). Interacts with MCM10 and WDHD1; these interactions recruit the polymerase alpha complex to the pre-replicative complex bound to DNA (PubMed:19608746). Interacts with RPA1; this interaction stabilizes the replicative complex and reduces the misincorporation rate of DNA polymerase alpha by acting as a fidelity clamp (PubMed:9214288).   
  
 **Gene Ontology Information:**

Molecular Function

- chromatin binding
- DNA binding
- DNA replication origin binding
- DNA-directed DNA polymerase activity
- nucleotide binding
- protein kinase binding
- single-stranded DNA binding
- zinc ion binding

Location

- alpha DNA polymerase:primase complex
- cytosol
- nuclear envelope
- nuclear matrix
- nucleolus
- nucleoplasm
- nucleus

Biological process

- DNA repair
- DNA replication
- DNA replication initiation
- DNA replication, synthesis of RNA primer
- DNA strand elongation involved in DNA replication
- DNA synthesis involved in DNA repair
- double-strand break repair via nonhomologous end joining
- lagging strand elongation
- leading strand elongation
- mitotic DNA replication initiation
- regulation of type I interferon production

---

3

- **Protein name:** DNA replication licensing factor MCM6
- **Organism:** Homo sapiens
- **Uniprot Accession Number:** Q14566
- **Protein sequence length:** 821 aa
- **1D identity (%):** 14.96
- **1D identity (%) [Gaps excluded]:** 22.86
- **1D identity - Alignment Gaps:** 351
- **1D aligned content (<aminoacid>:%):** {'D': 7.89, 'P': 8.55, 'R': 9.21, 'K': 5.26, 'F': 4.61, 'G': 9.87, 'L': 8.55, 'E': 5.26, 'N': 1.97, 'S': 7.24, 'T': 6.58, 'Y': 3.95, 'C': 4.61, 'H': 1.97, 'A': 3.29, 'V': 5.92, 'Q': 1.32, 'I': 3.29, 'W': 0.66}
- **Common reported functions (%):** 0.0
- **Common reported locations (%):** 11.11
- **Common reported processes (%):** 0.0

- **PDB ID:** 6XTY
- **Chain:** 6
- **Crystallized protein length:** 609 aa
- **Resolution:** 6.77 Å
- **b-phipsi ():** 0.002389
- **w-rdist ():** 0.467083
- **t-alpha ():** 0.020602
- **Chemical similarity (Tanimoto Index) (%):** N/A
- **1D identity (%) [PDB]:** 3.51
- **1D identity (%) [Gaps excluded][PDB]:** 80.33
- **1D identity - Alignment Gaps [PDB]:** 1334
- **1D aligned content [PDB] (<aminoacid>:%):** {'K': 6.12, 'Y': 4.08, 'L': 16.33, 'Q': 6.12, 'P': 8.16, 'V': 8.16, 'T': 10.2, 'E': 8.16, 'C': 2.04, 'I': 6.12, 'A': 8.16, 'G': 2.04, 'D': 2.04, 'N': 2.04, 'R': 6.12, 'S': 4.08}
- **2D identity (%) [PDB]:** 35.23
- **2D identity (%) [Gaps excluded][PDB]:** 82.95
- **2D identity - Alignment Gaps [PDB]:** 588
- **2D aligned content [PDB] (<2D-fold>:%):** {'.': 19.44, 'H': 41.39, 'E': 24.72, 'T': 13.61, 'G': 0.83}
- **3D similarity (TM-Score) (%) [PDB]:** 20.73

- **Gene name:** MCM6
- **Entrez ID:** 4175
- **RefSeq ID:** NM\_005915
- **Transcript sequence length:** 3753
- **5-UTR|CDS|3-UTR identity (%):** 14.2 | 47.18 | 4.59
- **5-UTR|CDS|3-UTR identity (%) [Gaps excluded]:** 77.42 | 76.7 | 75.15
- **5-UTR|CDS|3-UTR identity [Alignment Gaps]:** 276 | 1203 | 15896
- **5-UTR aligned content (<base>:%):** {'G': 45.83, 'C': 27.08, 'T': 12.5, 'A': 14.58}
- **CDS aligned content (<base>:%):** {'A': 30.51, 'T': 24.61, 'G': 24.14, 'C': 20.75}
- **3-UTR aligned content (<base>:%):** {'G': 14.29, 'A': 29.6, 'T': 42.08, 'C': 14.03}

**Uniprot Description:**  
  
 Acts as component of the MCM2-7 complex (MCM complex) which is the putative replicative helicase essential for 'once per cell cycle' DNA replication initiation and elongation in eukaryotic cells. The active ATPase sites in the MCM2-7 ring are formed through the interaction surfaces of two neighboring subunits such that a critical structure of a conserved arginine finger motif is provided in trans relative to the ATP-binding site of the Walker A box of the adjacent subunit. The six ATPase active sites, however, are likely to contribute differentially to the complex helicase activity.   
  
Component of the MCM2-7 complex (PubMed:16899510, PubMed:17296731, PubMed:9305914). The complex forms a toroidal hexameric ring with the proposed subunit order MCM2-MCM6-MCM4-MCM7-MCM3-MCM5 (PubMed:16899510, PubMed:17296731, PubMed:9305914). May interact with MCM10 (PubMed:11095689). Interacts with TIPIN (PubMed:17116885). Interacts with CDT1 (PubMed:20202939). Interacts with MCMBP (PubMed:17296731). Interacts with DDI2 (PubMed:29290612). Component of the CMG helicase complex, composed of the MCM2-7 complex, the GINS complex and CDC45 (By similarity).   
  
 **Gene Ontology Information:**

Molecular Function

- ATP binding
- ATPase activity
- DNA helicase activity
- identical protein binding
- single-stranded DNA binding

Location

- chromosome, telomeric region
- CMG complex
- MCM complex
- nucleoplasm
- nucleus

Biological process

- DNA replication
- DNA replication initiation
- DNA unwinding involved in DNA replication
- double-strand break repair via break-induced replication
- mitotic DNA replication
- regulation of DNA-dependent DNA replication initiation

---

4

- **Protein name:** DNA replication licensing factor MCM3
- **Organism:** Homo sapiens
- **Uniprot Accession Number:** P25205
- **Protein sequence length:** 808 aa
- **1D identity (%):** 14.98
- **1D identity (%) [Gaps excluded]:** 24.06
- **1D identity - Alignment Gaps:** 388
- **1D aligned content (<aminoacid>:%):** {'M': 1.3, 'T': 8.44, 'V': 6.49, 'A': 6.49, 'Y': 3.25, 'L': 10.39, 'G': 7.14, 'Q': 5.84, 'S': 4.55, 'N': 0.65, 'D': 9.09, 'F': 3.25, 'I': 2.6, 'P': 7.79, 'R': 9.09, 'C': 3.25, 'E': 4.55, 'K': 3.25, 'H': 2.6}
- **Common reported functions (%):** 0.0
- **Common reported locations (%):** 22.22
- **Common reported processes (%):** 0.0

- **PDB ID:** 6XTY
- **Chain:** 4
- **Crystallized protein length:** 598 aa
- **Resolution:** 6.77 Å
- **b-phipsi ():** 0.002561
- **w-rdist ():** 0.411996
- **t-alpha ():** 0.027734
- **Chemical similarity (Tanimoto Index) (%):** N/A
- **1D identity (%) [PDB]:** 3.67
- **1D identity (%) [Gaps excluded][PDB]:** 60.98
- **1D identity - Alignment Gaps [PDB]:** 1281
- **1D aligned content [PDB] (<aminoacid>:%):** {'N': 4.0, 'V': 12.0, 'L': 8.0, 'G': 2.0, 'I': 10.0, 'P': 10.0, 'H': 4.0, 'Q': 8.0, 'S': 8.0, 'A': 6.0, 'D': 6.0, 'K': 4.0, 'Y': 2.0, 'T': 2.0, 'R': 8.0, 'E': 4.0, 'M': 2.0}
- **2D identity (%) [PDB]:** 32.08
- **2D identity (%) [Gaps excluded][PDB]:** 82.67
- **2D identity - Alignment Gaps [PDB]:** 637
- **2D aligned content [PDB] (<2D-fold>:%):** {'.': 29.64, 'H': 37.72, 'T': 15.57, 'E': 16.47, 'B': 0.6}
- **3D similarity (TM-Score) (%) [PDB]:** 20.73

- **Gene name:** MCM3
- **Entrez ID:** 4173
- **RefSeq ID:** NM\_002388
- **Transcript sequence length:** 3068
- **5-UTR|CDS|3-UTR identity (%):** 10.29 | 45.76 | 2.34
- **5-UTR|CDS|3-UTR identity (%) [Gaps excluded]:** 70.0 | 74.46 | 78.13
- **5-UTR|CDS|3-UTR identity [Alignment Gaps]:** 290 | 1196 | 16322
- **5-UTR aligned content (<base>:%):** {'G': 60.0, 'C': 20.0, 'A': 11.43, 'T': 8.57}
- **CDS aligned content (<base>:%):** {'A': 29.51, 'T': 21.69, 'G': 26.9, 'C': 21.9}
- **3-UTR aligned content (<base>:%):** {'G': 20.87, 'A': 25.7, 'C': 19.85, 'T': 33.59}

**Uniprot Description:**  
  
 Acts as component of the MCM2-7 complex (MCM complex) which is the putative replicative helicase essential for 'once per cell cycle' DNA replication initiation and elongation in eukaryotic cells. The active ATPase sites in the MCM2-7 ring are formed through the interaction surfaces of two neighboring subunits such that a critical structure of a conserved arginine finger motif is provided in trans relative to the ATP-binding site of the Walker A box of the adjacent subunit. The six ATPase active sites, however, are likely to contribute differentially to the complex helicase activity. Required for DNA replication and cell proliferation.   
  
Component of the MCM2-7 complex (PubMed:16899510, PubMed:17296731). The complex forms a toroidal hexameric ring with the proposed subunit order MCM2-MCM6-MCM4-MCM7-MCM3-MCM5 (PubMed:16899510, PubMed:17296731). Associated with the replication-specific DNA polymerase alpha (By similarity). Interacts with MCMBP (PubMed:17296731). Interacts with ANKRD17 (PubMed:23711367). Interacts with MCM3AP isoform MCM3AP; this interaction leads to MCM3 acetylation (PubMed:9712829, PubMed:11258703, PubMed:12226073). Component of the CMG helicase complex, composed of the MCM2-7 complex, the GINS complex and CDC45 (By similarity).   
  
 **Gene Ontology Information:**

Molecular Function

- ATP binding
- ATPase activity
- DNA helicase activity
- single-stranded DNA binding

Location

- chromosome, telomeric region
- CMG complex
- MCM complex
- membrane
- nucleoplasm
- nucleus

Biological process

- DNA replication
- DNA strand elongation involved in DNA replication
- DNA unwinding involved in DNA replication
- double-strand break repair via break-induced replication
- mitotic DNA replication initiation
- regulation of DNA-dependent DNA replication initiation

---

5

- **Protein name:** DNA ligase 1
- **Organism:** Homo sapiens
- **Uniprot Accession Number:** P18858
- **Protein sequence length:** 919 aa
- **1D identity (%):** 17.04
- **1D identity (%) [Gaps excluded]:** 22.64
- **1D identity - Alignment Gaps:** 251
- **1D aligned content (<aminoacid>:%):** {'Q': 2.89, 'M': 1.16, 'P': 16.76, 'G': 12.72, 'K': 6.36, 'N': 0.58, 'L': 7.51, 'E': 6.36, 'R': 9.83, 'V': 2.89, 'S': 3.47, 'D': 5.2, 'T': 6.36, 'F': 3.47, 'A': 6.36, 'H': 1.73, 'C': 1.73, 'I': 2.89, 'Y': 1.16, 'W': 0.58}
- **Common reported functions (%):** 14.29
- **Common reported locations (%):** 11.11
- **Common reported processes (%):** 0.0

- **PDB ID:** 7L34
- **Chain:** A
- **Crystallized protein length:** 637 aa
- **Resolution:** 1.9 Å
- **b-phipsi ():** 0.003334
- **w-rdist ():** 0.789276
- **t-alpha ():** 0.004777
- **Chemical similarity (Tanimoto Index) (%):** 99.17
- **1D identity (%) [PDB]:** 2.66
- **1D identity (%) [Gaps excluded][PDB]:** 73.08
- **1D identity - Alignment Gaps [PDB]:** 1379
- **1D aligned content [PDB] (<aminoacid>:%):** {'N': 2.63, 'P': 7.89, 'L': 18.42, 'V': 10.53, 'A': 2.63, 'T': 10.53, 'G': 10.53, 'D': 5.26, 'E': 10.53, 'K': 7.89, 'I': 5.26, 'S': 7.89}
- **2D identity (%) [PDB]:** 32.62
- **2D identity (%) [Gaps excluded][PDB]:** 88.03
- **2D identity - Alignment Gaps [PDB]:** 681
- **2D aligned content [PDB] (<2D-fold>:%):** {'.': 17.56, 'T': 17.0, 'H': 48.44, 'E': 15.58, 'G': 1.42}
- **3D similarity (TM-Score) (%) [PDB]:** 24.26

- **Gene name:** LIG1
- **Entrez ID:** 3978
- **RefSeq ID:** NM\_001320971
- **Transcript sequence length:** 3035
- **5-UTR|CDS|3-UTR identity (%):** 26.04 | 44.45 | 0.82
- **5-UTR|CDS|3-UTR identity (%) [Gaps excluded]:** 77.05 | 73.24 | 76.11
- **5-UTR|CDS|3-UTR identity [Alignment Gaps]:** 239 | 1285 | 16599
- **5-UTR aligned content (<base>:%):** {'A': 11.7, 'G': 38.3, 'C': 35.11, 'T': 14.89}
- **CDS aligned content (<base>:%):** {'A': 26.91, 'T': 18.51, 'G': 28.49, 'C': 26.08}
- **3-UTR aligned content (<base>:%):** {'G': 24.82, 'C': 18.98, 'T': 35.77, 'A': 20.44}

**Uniprot Description:**  
  
 DNA ligase that seals nicks in double-stranded DNA during DNA replication, DNA recombination and DNA repair.   
  
Interacts with PCNA.   
  
 **Gene Ontology Information:**

Molecular Function

- ATP binding
- DNA binding
- DNA ligase (ATP) activity
- DNA ligase activity
- metal ion binding

Location

- intracellular membrane-bounded organelle
- mitochondrion
- nucleoplasm
- nucleus

Biological process

- anatomical structure morphogenesis
- base-excision repair
- base-excision repair, gap-filling
- cell division
- DNA biosynthetic process
- DNA ligation
- DNA recombination
- DNA repair
- lagging strand elongation
- mismatch repair
- Okazaki fragment processing involved in mitotic DNA replication

---

6

- **Protein name:** Transitional endoplasmic reticulum ATPase
- **Organism:** Homo sapiens
- **Uniprot Accession Number:** P55072
- **Protein sequence length:** 806 aa
- **1D identity (%):** 16.77
- **1D identity (%) [Gaps excluded]:** 24.19
- **1D identity - Alignment Gaps:** 302
- **1D aligned content (<aminoacid>:%):** {'M': 0.61, 'G': 10.3, 'S': 4.24, 'L': 3.03, 'R': 9.7, 'P': 10.3, 'I': 3.64, 'K': 8.48, 'D': 7.88, 'E': 6.06, 'V': 6.67, 'T': 7.27, 'F': 4.85, 'A': 7.27, 'Y': 1.82, 'N': 2.42, 'H': 1.21, 'C': 1.82, 'Q': 2.42}
- **Common reported functions (%):** 14.29
- **Common reported locations (%):** 33.33
- **Common reported processes (%):** 0.0

- **PDB ID:** 5FTJ
- **Chain:** A
- **Crystallized protein length:** 723 aa
- **Resolution:** 2.3 Å
- **b-phipsi ():** 0.005297
- **w-rdist ():** 0.123707
- **t-alpha ():** 0.141839
- **Chemical similarity (Tanimoto Index) (%):** 77.76
- **1D identity (%) [PDB]:** 3.36
- **1D identity (%) [Gaps excluded][PDB]:** 60.24
- **1D identity - Alignment Gaps [PDB]:** 1403
- **1D aligned content [PDB] (<aminoacid>:%):** {'G': 10.0, 'D': 6.0, 'K': 6.0, 'I': 8.0, 'V': 6.0, 'M': 2.0, 'A': 8.0, 'P': 4.0, 'S': 6.0, 'Y': 4.0, 'C': 2.0, 'R': 10.0, 'E': 6.0, 'Q': 6.0, 'L': 8.0, 'F': 6.0, 'T': 2.0}
- **2D identity (%) [PDB]:** 36.56
- **2D identity (%) [Gaps excluded][PDB]:** 87.07
- **2D identity - Alignment Gaps [PDB]:** 641
- **2D aligned content [PDB] (<2D-fold>:%):** {'.': 25.99, 'E': 16.09, 'H': 45.54, 'T': 12.13, 'B': 0.25}
- **3D similarity (TM-Score) (%) [PDB]:** 22.38

- **Gene name:** VCP
- **Entrez ID:** N/A
- **RefSeq ID:** NM\_007126
- **Transcript sequence length:** 3746
- **5-UTR|CDS|3-UTR identity (%):** 43.98 | 44.69 | 3.86
- **5-UTR|CDS|3-UTR identity (%) [Gaps excluded]:** 79.25 | 74.83 | 76.4
- **5-UTR|CDS|3-UTR identity [Alignment Gaps]:** 170 | 1262 | 16096
- **5-UTR aligned content (<base>:%):** {'A': 7.74, 'G': 38.69, 'T': 10.71, 'C': 42.86}
- **CDS aligned content (<base>:%):** {'A': 27.29, 'T': 24.07, 'G': 27.71, 'C': 20.93}
- **3-UTR aligned content (<base>:%):** {'G': 23.09, 'T': 27.52, 'C': 23.09, 'A': 26.3}

**Uniprot Description:**  
  
 Necessary for the fragmentation of Golgi stacks during mitosis and for their reassembly after mitosis. Involved in the formation of the transitional endoplasmic reticulum (tER). The transfer of membranes from the endoplasmic reticulum to the Golgi apparatus occurs via 50-70 nm transition vesicles which derive from part-rough, part-smooth transitional elements of the endoplasmic reticulum (tER). Vesicle budding from the tER is an ATP-dependent process. The ternary complex containing UFD1, VCP and NPLOC4 binds ubiquitinated proteins and is necessary for the export of misfolded proteins from the ER to the cytoplasm, where they are degraded by the proteasome. The NPLOC4-UFD1-VCP complex regulates spindle disassembly at the end of mitosis and is necessary for the formation of a closed nuclear envelope. Regulates E3 ubiquitin-protein ligase activity of RNF19A. Component of the VCP/p97-AMFR/gp78 complex that participates in the final step of the sterol-mediated ubiquitination and endoplasmic reticulum-associated degradation (ERAD) of HMGCR. Involved in endoplasmic reticulum stress-induced pre-emptive quality control, a mechanism that selectively attenuates the translocation of newly synthesized proteins into the endoplasmic reticulum and reroutes them to the cytosol for proteasomal degradation (PubMed:26565908). Plays a role in the regulation of stress granules (SGs) clearance process upon arsenite-induced response (PubMed:29804830). Also involved in DNA damage response: recruited to double-strand breaks (DSBs) sites in a RNF8- and RNF168-dependent manner and promotes the recruitment of TP53BP1 at DNA damage sites (PubMed:22020440, PubMed:22120668). Recruited to stalled replication forks by SPRTN: may act by mediating extraction of DNA polymerase eta (POLH) to prevent excessive translesion DNA synthesis and limit the incidence of mutations induced by DNA damage (PubMed:23042607, PubMed:23042605). Together with SPRTN metalloprotease, involved in the repair of covalent DNA-protein cross-links (DPCs) during DNA synthesis (PubMed:32152270). Involved in interstrand cross-link repair in response to replication stress by mediating unloading of the ubiquitinated CMG helicase complex (By similarity). Required for cytoplasmic retrotranslocation of stressed/damaged mitochondrial outer-membrane proteins and their subsequent proteasomal degradation (PubMed:16186510, PubMed:21118995). Essential for the maturation of ubiquitin-containing autophagosomes and the clearance of ubiquitinated protein by autophagy (PubMed:20104022, PubMed:27753622). Acts as a negative regulator of type I interferon production by interacting with DDX58/RIG-I: interaction takes place when DDX58/RIG-I is ubiquitinated via 'Lys-63'-linked ubiquitin on its CARD domains, leading to recruit RNF125 and promote ubiquitination and degradation of DDX58/RIG-I (PubMed:26471729). May play a role in the ubiquitin-dependent sorting of membrane proteins to lysosomes where they undergo degradation (PubMed:21822278). May more particularly play a role in caveolins sorting in cells (PubMed:21822278, PubMed:23335559). By controlling the steady-state expression of the IGF1R receptor, indirectly regulates the insulin-like growth factor receptor signaling pathway (PubMed:26692333).   
  
Homohexamer. Forms a ring-shaped particle of 12.5 nm diameter, that displays 6-fold radial symmetry. Part of a ternary complex containing STX5A, NSFL1C and VCP. NSFL1C forms a homotrimer that binds to one end of a VCP homohexamer. The complex binds to membranes enriched in phosphatidylethanolamine-containing lipids and promotes Golgi membrane fusion. Binds to a heterodimer of NPLOC4 and UFD1, binding to this heterodimer inhibits Golgi-membrane fusion (PubMed:26471729). Interaction with VCIP135 leads to dissociation of the complex via ATP hydrolysis by VCP. Part of a ternary complex containing NPLOC4, UFD1 and VCP. Interacts with NSFL1C-like protein p37; the complex has membrane fusion activity and is required for Golgi and endoplasmic reticulum biogenesis. Interacts with SELENOS and SYVN1, as well as with DERL1, DERL2 and DERL3; which probably transfer misfolded proteins from the ER to VCP. Interacts with SVIP. Component of a complex required to couple retrotranslocation, ubiquitination and deglycosylation composed of NGLY1, SAKS1, AMFR, VCP and RAD23B. Directly interacts with UBXN4 and RNF19A. Interacts with CASR. Interacts with UBE4B and YOD1. Interacts with clathrin. Interacts with RNF103. Interacts with TRIM13 and TRIM21. Component of a VCP/p97-AMFR/gp78 complex that participates in the final step of the endoplasmic reticulum-associated degradation (ERAD) of HMGCR. Interacts directly with AMFR/gp78 (via its VIM). Interacts with RHBDD1 (via C-terminal domain). Interacts with SPRTN; leading to recruitment to stalled replication forks (PubMed:23042607, PubMed:23042605). Interacts with WASHC5. Interacts with UBOX5. Interacts (via N-terminus) with UBXN7, UBXN8, and probably several other UBX domain-containing proteins (via UBX domains); the interactions are mutually exclusive with VIM-dependent interactions such as those with AMFR and SELENOS. Forms a complex with UBQLN1 and UBXN4. Interacts (via the PIM motif) with RNF31 (via the PUB domain) (PubMed:24726327). Interacts with DDX58/RIG-I and RNF125; interaction takes place when DDX58/RIG-I is ubiquitinated via 'Lys-63'-linked ubiquitin on its CARD domains, leading to recruit RNF125 and promote ubiquitination and degradation of DDX58/RIG-I (PubMed:26471729). Interacts with BAG6 (PubMed:21636303). Interacts with UBXN10 (PubMed:26389662). Interacts with UBXN6; the interaction with UBXN6 is direct and competitive with UFD1 (PubMed:19174149, PubMed:19275885). Forms a ternary complex with CAV1 and UBXN6 (PubMed:21822278, PubMed:18656546, PubMed:19174149). Interacts with PLAA, UBXN6 and YOD1; may form a complex involved in macroautophagy (PubMed:27753622). Interacts with ANKZF1 (PubMed:28302725). Interacts with ubiquitin-binding protein FAF1 (PubMed:26842564). Interacts with ZFAND2B (via VIM motif); the interaction is direct (PubMed:24160817, PubMed:26337389). Interacts with ZFAND1 (via its ubiquitin-like region); this interaction occurs in an arsenite-dependent manner (PubMed:29804830). Interacts with CCDC47 (By similarity). Interacts with UBAC2 (By similarity). Interacts with LMBR1L (PubMed:31073040). Interacts with ATXN3 (PubMed:30455355). Interacts with TEX264; bridging VCP to covalent DNA-protein cross-links (DPCs) (PubMed:32152270).   
  
 **Gene Ontology Information:**

Molecular Function

- ADP binding
- ATP binding
- ATPase activity
- BAT3 complex binding
- deubiquitinase activator activity
- identical protein binding
- K48-linked polyubiquitin modification-dependent protein binding
- lipid binding
- MHC class I protein binding
- polyubiquitin modification-dependent protein binding
- protein domain specific binding
- protein phosphatase binding
- RNA binding
- ubiquitin protein ligase binding
- ubiquitin-dependent protein binding
- ubiquitin-like protein ligase binding
- ubiquitin-specific protease binding

Location

- ATPase complex
- azurophil granule lumen
- cytoplasm
- cytoplasmic stress granule
- cytosol
- Derlin-1 retrotranslocation complex
- endoplasmic reticulum
- endoplasmic reticulum membrane
- extracellular exosome
- extracellular region
- ficolin-1-rich granule lumen
- glutamatergic synapse
- intracellular membrane-bounded organelle
- lipid droplet
- nucleoplasm
- nucleus
- perinuclear region of cytoplasm
- proteasome complex
- protein-containing complex
- secretory granule lumen
- site of double-strand break
- VCP-NPL4-UFD1 AAA ATPase complex
- VCP-NSFL1C complex

Biological process

- activation of cysteine-type endopeptidase activity involved in apoptotic process
- aggresome assembly
- ATP metabolic process
- autophagosome maturation
- autophagy
- cellular response to arsenite ion
- cellular response to heat
- cellular response to DNA damage stimulus
- DNA repair
- double-strand break repair
- endoplasmic reticulum stress-induced pre-emptive quality control
- endoplasmic reticulum to Golgi vesicle-mediated transport
- endoplasmic reticulum unfolded protein response
- endosome to lysosome transport via multivesicular body sorting pathway
- ER-associated misfolded protein catabolic process
- ERAD pathway
- establishment of protein localization
- flavin adenine dinucleotide catabolic process
- interstrand cross-link repair
- macroautophagy
- mitotic spindle disassembly
- NADH metabolic process
- negative regulation of protein localization to chromatin
- negative regulation of smoothened signaling pathway
- positive regulation of ATP biosynthetic process
- positive regulation of canonical Wnt signaling pathway
- positive regulation of Lys63-specific deubiquitinase activity
- positive regulation of mitochondrial membrane potential
- positive regulation of oxidative phosphorylation
- positive regulation of proteasomal ubiquitin-dependent protein catabolic process
- positive regulation of protein catabolic process
- positive regulation of protein K63-linked deubiquitination
- positive regulation of protein-containing complex assembly
- proteasomal protein catabolic process
- proteasome-mediated ubiquitin-dependent protein catabolic process
- protein ubiquitination
- protein-DNA covalent cross-linking repair
- regulation of aerobic respiration
- regulation of apoptotic process
- regulation of protein localization to chromatin
- regulation of synapse organization
- retrograde protein transport, ER to cytosol
- stress granule disassembly
- translesion synthesis
- ubiquitin-dependent ERAD pathway
- viral genome replication

---

7

- **Protein name:** DNA replication licensing factor MCM2
- **Organism:** Homo sapiens
- **Uniprot Accession Number:** P49736
- **Protein sequence length:** 904 aa
- **1D identity (%):** 15.36
- **1D identity (%) [Gaps excluded]:** 21.81
- **1D identity - Alignment Gaps:** 306
- **1D aligned content (<aminoacid>:%):** {'S': 6.29, 'A': 7.55, 'G': 8.81, 'D': 6.92, 'P': 7.55, 'F': 3.14, 'L': 11.32, 'E': 5.03, 'R': 7.55, 'V': 7.55, 'M': 0.63, 'I': 3.14, 'H': 4.4, 'K': 5.66, 'N': 2.52, 'Q': 5.66, 'Y': 2.52, 'C': 1.26, 'T': 2.52}
- **Common reported functions (%):** 14.29
- **Common reported locations (%):** 22.22
- **Common reported processes (%):** 0.0

- **PDB ID:** 6XTY
- **Chain:** 2
- **Crystallized protein length:** 611 aa
- **Resolution:** 6.77 Å
- **b-phipsi ():** 0.003504
- **w-rdist ():** 0.41116
- **t-alpha ():** 0.146593
- **Chemical similarity (Tanimoto Index) (%):** N/A
- **1D identity (%) [PDB]:** 5.4
- **1D identity (%) [Gaps excluded][PDB]:** 67.59
- **1D identity - Alignment Gaps [PDB]:** 1243
- **1D aligned content [PDB] (<aminoacid>:%):** {'N': 4.11, 'R': 8.22, 'E': 2.74, 'V': 17.81, 'S': 10.96, 'M': 1.37, 'Q': 1.37, 'K': 6.85, 'T': 4.11, 'I': 2.74, 'G': 6.85, 'P': 8.22, 'Y': 2.74, 'D': 5.48, 'L': 8.22, 'A': 4.11, 'W': 1.37, 'H': 1.37, 'F': 1.37}
- **2D identity (%) [PDB]:** 30.05
- **2D identity (%) [Gaps excluded][PDB]:** 84.11
- **2D identity - Alignment Gaps [PDB]:** 691
- **2D aligned content [PDB] (<2D-fold>:%):** {'.': 26.63, 'E': 16.41, 'H': 42.72, 'T': 13.93, 'B': 0.31}
- **3D similarity (TM-Score) (%) [PDB]:** 20.73

- **Gene name:** MCM2
- **Entrez ID:** 4171
- **RefSeq ID:** NM\_004526
- **Transcript sequence length:** 3434
- **5-UTR|CDS|3-UTR identity (%):** 11.62 | 46.12 | 2.52
- **5-UTR|CDS|3-UTR identity (%) [Gaps excluded]:** 74.51 | 73.96 | 75.71
- **5-UTR|CDS|3-UTR identity [Alignment Gaps]:** 276 | 1228 | 16298
- **5-UTR aligned content (<base>:%):** {'C': 23.68, 'T': 28.95, 'G': 34.21, 'A': 13.16}
- **CDS aligned content (<base>:%):** {'A': 26.38, 'T': 19.0, 'G': 29.37, 'C': 25.25}
- **3-UTR aligned content (<base>:%):** {'G': 26.89, 'C': 20.52, 'A': 19.81, 'T': 32.78}

**Uniprot Description:**  
  
 Acts as component of the MCM2-7 complex (MCM complex) which is the putative replicative helicase essential for 'once per cell cycle' DNA replication initiation and elongation in eukaryotic cells. The active ATPase sites in the MCM2-7 ring are formed through the interaction surfaces of two neighboring subunits such that a critical structure of a conserved arginine finger motif is provided in trans relative to the ATP-binding site of the Walker A box of the adjacent subunit. The six ATPase active sites, however, are likely to contribute differentially to the complex helicase activity. Required for the entry in S phase and for cell division. Plays a role in terminally differentiated hair cells development of the cochlea and induces cells apoptosis.   
  
Component of the MCM2-7 complex (PubMed:9305914, PubMed:16899510, PubMed:17296731). The complex forms a toroidal hexameric ring with the proposed subunit order MCM2-MCM6-MCM4-MCM7-MCM3-MCM5 (PubMed:9305914, PubMed:16899510, PubMed:17296731). Interacts with DBF4 (By similarity). Interacts with KAT7 (PubMed:16387653). May interact with MCM10 (PubMed:11095689). Component of the replisome complex composed of at least DONSON, MCM2, MCM7, PCNA and TICRR (PubMed:28191891). Component of the CMG helicase complex, composed of the MCM2-7 complex, the GINS complex and CDC45 (By similarity).   
  
 **Gene Ontology Information:**

Molecular Function

- ATP binding
- ATPase activity
- DNA binding
- DNA replication origin binding
- enzyme binding
- helicase activity
- histone binding
- metal ion binding
- single-stranded DNA binding

Location

- chromatin
- chromosome, telomeric region
- CMG complex
- cytoplasm
- MCM complex
- nuclear origin of replication recognition complex
- nucleoplasm
- nucleus

Biological process

- apoptotic process
- cellular response to interleukin-4
- cochlea development
- DNA replication
- DNA replication initiation
- DNA unwinding involved in DNA replication
- double-strand break repair via break-induced replication
- mitotic DNA replication initiation
- negative regulation of DNA helicase activity
- nucleosome assembly
- regulation of DNA-dependent DNA replication initiation

---

8

- **Protein name:** [Histone H3]-lysine(27) N-trimethyltransferase
- **Organism:** Anolis carolinensis
- **Uniprot Accession Number:** G1KPH4
- **Protein sequence length:** 737 aa
- **1D identity (%):** 15.48
- **1D identity (%) [Gaps excluded]:** 23.89
- **1D identity - Alignment Gaps:** 341
- **1D aligned content (<aminoacid>:%):** {'G': 8.67, 'K': 8.0, 'I': 6.0, 'R': 8.0, 'V': 8.0, 'L': 7.33, 'E': 7.33, 'P': 10.67, 'S': 2.0, 'F': 5.33, 'N': 4.0, 'Y': 4.67, 'Q': 3.33, 'M': 1.33, 'H': 0.67, 'D': 2.67, 'C': 2.67, 'T': 5.33, 'A': 4.0}
- **Common reported functions (%):** 0.0
- **Common reported locations (%):** 11.11
- **Common reported processes (%):** 0.0

- **PDB ID:** 5IJ7
- **Chain:** B
- **Crystallized protein length:** 469 aa
- **Resolution:** 2.62 Å
- **b-phipsi ():** 0.001281
- **w-rdist ():** 0.714462
- **t-alpha ():** 0.030111
- **Chemical similarity (Tanimoto Index) (%):** 71.77
- **1D identity (%) [PDB]:** 1.62
- **1D identity (%) [Gaps excluded][PDB]:** 91.3
- **1D identity - Alignment Gaps [PDB]:** 1275
- **1D aligned content [PDB] (<aminoacid>:%):** {'R': 19.05, 'V': 9.52, 'Q': 14.29, 'E': 9.52, 'I': 14.29, 'D': 4.76, 'L': 14.29, 'A': 4.76, 'S': 4.76, 'M': 4.76}
- **2D identity (%) [PDB]:** 23.04
- **2D identity (%) [Gaps excluded][PDB]:** 87.64
- **2D identity - Alignment Gaps [PDB]:** 771
- **2D aligned content [PDB] (<2D-fold>:%):** {'.': 21.99, 'H': 53.53, 'E': 9.96, 'T': 12.86, 'G': 1.24, 'B': 0.41}
- **3D similarity (TM-Score) (%) [PDB]:** 17.19

- **Gene name:** EZH2
- **Entrez ID:** 2146
- **RefSeq ID:** N/A
- **Sequence length:** N/A
- **5-UTR|CDS|3-UTR identity (%):** N/A | N/A | N/A
- **5-UTR|CDS|3-UTR identity (%) [Gaps excluded]:** N/A | N/A | N/A
- **5-UTR|CDS|3-UTR identity [Alignment Gaps]:** N/A | N/A | N/A
- **5-UTR aligned content (<base>:%):** N/A
- **CDS aligned content (<base>:%):** N/A
- **3-UTR aligned content (<base>:%):** N/A

**Uniprot Description:**  
  
 N/A N/A   
  
 **Gene Ontology Information:**

Molecular Function

- chromatin binding
- chromatin DNA binding
- histone methyltransferase activity (H3-K27 specific)
- histone H3K27 trimethyltransferase activity
- histone methyltransferase activity
- lncRNA binding
- primary miRNA binding
- promoter-specific chromatin binding
- protein-lysine N-methyltransferase activity
- ribonucleoprotein complex binding
- RNA polymerase II cis-regulatory region sequence-specific DNA binding
- RNA polymerase II core promoter sequence-specific DNA binding
- transcription corepressor activity
- transcription corepressor binding

Location

- chromatin
- chromatin silencing complex
- chromosome, telomeric region
- ESC/E(Z) complex
- nucleoplasm
- nucleus
- pericentric heterochromatin
- pronucleus
- synapse

Biological process

- B cell differentiation
- cardiac muscle hypertrophy in response to stress
- cellular response to hydrogen peroxide
- cellular response to trichostatin A
- cerebellar cortex development
- chromatin organization
- DNA methylation
- facultative heterochromatin formation
- G1 to G0 transition
- G1/S transition of mitotic cell cycle
- hepatocyte homeostasis
- heterochromatin assembly
- hippocampus development
- histone H3-K27 methylation
- keratinocyte differentiation
- liver regeneration
- negative regulation of cytokine production involved in inflammatory response
- negative regulation of DNA-binding transcription factor activity
- negative regulation of transcription, DNA-templated
- negative regulation of G1/S transition of mitotic cell cycle
- negative regulation of gene expression, epigenetic
- negative regulation of keratinocyte differentiation
- negative regulation of retinoic acid receptor signaling pathway
- negative regulation of stem cell differentiation
- negative regulation of striated muscle cell differentiation
- negative regulation of transcription by RNA polymerase II
- negative regulation of transcription elongation from RNA polymerase II promoter
- positive regulation of cell cycle G1/S phase transition
- positive regulation of cell population proliferation
- positive regulation of dendrite development
- positive regulation of epithelial to mesenchymal transition
- positive regulation of GTPase activity
- positive regulation of MAP kinase activity
- positive regulation of protein serine/threonine kinase activity
- protein localization to chromatin
- regulation of circadian rhythm
- regulation of transcription, DNA-templated
- regulation of gliogenesis
- regulation of kidney development
- response to estradiol
- response to tetrachloromethane
- rhythmic process
- skeletal muscle satellite cell maintenance involved in skeletal muscle regeneration
- stem cell differentiation
- subtelomeric heterochromatin assembly
- synaptic transmission, GABAergic

---
