# Supplementary material for: Comparative structural insights and functional analysis for the distinct unbound states of Human AGO proteins
Source: Sci Rep. 2025 Mar 19;15:9432. doi: 10.1038/s41598-025-91849-5 (PMC11923369; doi:10.1038/s41598-025-91849-5)
Supplement: Supplementary file 24 — Supplementary Information 12. [file 41598_2025_91849_MOESM24_ESM.zip › 6OONp_A_mdwhole_AF4REF/candidates/6OONp_A-merged-enriched_eval_report.html]

 

# Structural Comparison Report for 6OONp\_A - whole structures (total: 100)

---

1

- **Protein name:** Protein argonaute-3
- **Organism:** Homo sapiens
- **Uniprot Accession Number:** Q9H9G7
- **Protein sequence length:** 860 aa
- **1D identity (%):** 80.73
- **1D identity (%) [Gaps excluded]:** 82.92
- **1D identity - Alignment Gaps:** 23
- **1D aligned content (<aminoacid>:%):** {'M': 2.56, 'E': 4.4, 'G': 7.1, 'P': 6.96, 'L': 7.81, 'R': 6.25, 'T': 6.11, 'K': 5.4, 'I': 5.11, 'A': 6.25, 'N': 2.41, 'F': 4.12, 'Q': 6.11, 'V': 8.95, 'D': 4.83, 'Y': 4.12, 'H': 3.12, 'S': 4.97, 'W': 0.71, 'C': 2.7}
- **Common reported functions (%):** 100.0
- **Common reported locations (%):** 87.5
- **Common reported processes (%):** 50.0

- **AF ID:** Q9H9G7
- **Chain:** A
- **Protein length:** 860 aa
- **Resolution:** N/A
- **b-phipsi:** 0.000608
- **w-rdist:** 0.080933
- **t-alpha:** 0.029734
- **Chemical similarity (Tanimoto Index) (%):** 99.62
- **1D identity (%) [PDB]:** 80.3
- **1D identity (%) [Gaps excluded][PDB]:** 84.74
- **1D identity - Alignment Gaps [PDB]:** 46
- **1D aligned content [PDB] (<aminoacid>:%):** {'Q': 6.24, 'P': 7.09, 'R': 6.24, 'G': 6.95, 'T': 6.1, 'K': 5.39, 'I': 5.11, 'L': 7.8, 'A': 6.24, 'N': 2.55, 'F': 4.11, 'V': 8.94, 'D': 4.82, 'Y': 4.11, 'E': 4.4, 'M': 2.41, 'H': 3.12, 'S': 4.96, 'W': 0.71, 'C': 2.7}
- **2D identity (%) [PDB]:** 84.93
- **2D identity (%) [Gaps excluded][PDB]:** 91.96
- **2D identity - Alignment Gaps [PDB]:** 68
- **2D aligned content [PDB] (<2D-fold>:%):** {'.': 18.83, 'B': 1.59, 'E': 27.98, 'T': 19.23, 'H': 30.9, 'I': 0.66, 'G': 0.8}
- **3D similarity (TM-Score) (%) [PDB]:** 96.75

- **Gene name:** AGO3
- **Entrez ID:** 192669
- **RefSeq ID:** N/A
- **Sequence length:** N/A
- **5-UTR|CDS|3-UTR identity (%):** N/A | N/A | N/A
- **5-UTR|CDS|3-UTR identity (%) [Gaps excluded]:** N/A | N/A | N/A
- **5-UTR|CDS|3-UTR identity [Alignment Gaps]:** N/A | N/A | N/A
- **5-UTR aligned content (<base>:%):** N/A
- **CDS aligned content (<base>:%):** N/A
- **3-UTR aligned content (<base>:%):** N/A

**Uniprot Description:**  
  
 Required for RNA-mediated gene silencing (RNAi). Binds to short RNAs such as microRNAs (miRNAs) and represses the translation of mRNAs which are complementary to them. Proposed to be involved in stabilization of small RNA derivates (siRNA) derived from processed RNA polymerase III-transcribed Alu repeats containing a DR2 retinoic acid response element (RARE) in stem cells and in the subsequent siRNA-dependent degradation of a subset of RNA polymerase II-transcribed coding mRNAs by recruiting a mRNA decapping complex involving EDC4. Possesses RNA slicer activity but only on select RNAs bearing 5'- and 3'-flanking sequences to the region of guide-target complementarity (PubMed:29040713).   
  
Interacts with EIF4B, IMP8, PRMT5 and TNRC6B. Interacts with APOBEC3F, APOBEC3G and APOBEC3H. Interacts with EDC4.   
  
 **Gene Ontology Information:**

Molecular Function

- double-stranded RNA binding
- endoribonuclease activity, cleaving miRNA-paired mRNA
- metal ion binding
- miRNA binding
- RNA binding
- endoribonuclease activity
- single-stranded RNA binding

Location

- condensed nuclear chromosome
- cytoplasm
- cytoplasmic ribonucleoprotein granule
- cytosol
- membrane
- nucleoplasm
- P-body
- RISC complex
- RISC-loading complex

Biological process

- production of miRNAs involved in gene silencing by miRNA
- miRNA mediated inhibition of translation
- mRNA catabolic process
- positive regulation of gene expression
- positive regulation of NIK/NF-kappaB signaling
- pre-miRNA processing
- regulation of stem cell proliferation
- small RNA loading onto RISC
- RNA secondary structure unwinding

---

2

- **Protein name:** Protein argonaute-1
- **Organism:** Homo sapiens
- **Uniprot Accession Number:** Q9UL18
- **Protein sequence length:** 857 aa
- **1D identity (%):** 82.64
- **1D identity (%) [Gaps excluded]:** 84.79
- **1D identity - Alignment Gaps:** 22
- **1D aligned content (<aminoacid>:%):** {'M': 2.36, 'E': 4.59, 'A': 5.98, 'G': 7.09, 'P': 7.09, 'F': 4.17, 'Q': 6.68, 'R': 6.4, 'T': 5.84, 'V': 8.48, 'K': 6.12, 'I': 5.29, 'L': 7.23, 'N': 2.5, 'D': 4.59, 'Y': 4.45, 'H': 3.2, 'S': 4.45, 'W': 0.83, 'C': 2.64}
- **Common reported functions (%):** 75.0
- **Common reported locations (%):** 87.5
- **Common reported processes (%):** 41.67

- **AF ID:** Q9UL18
- **Chain:** A
- **Protein length:** 857 aa
- **Resolution:** N/A
- **b-phipsi:** 0.000549
- **w-rdist:** 0.047114
- **t-alpha:** 0.039123
- **Chemical similarity (Tanimoto Index) (%):** 99.62
- **1D identity (%) [PDB]:** 81.84
- **1D identity (%) [Gaps excluded][PDB]:** 85.07
- **1D identity - Alignment Gaps [PDB]:** 33
- **1D aligned content [PDB] (<aminoacid>:%):** {'F': 4.21, 'Q': 6.74, 'P': 6.74, 'R': 6.46, 'G': 7.02, 'T': 5.9, 'V': 8.57, 'K': 6.18, 'I': 5.34, 'L': 7.3, 'A': 5.9, 'N': 2.53, 'D': 4.63, 'Y': 4.49, 'H': 3.23, 'E': 4.49, 'M': 2.25, 'S': 4.49, 'W': 0.84, 'C': 2.67}
- **2D identity (%) [PDB]:** 85.91
- **2D identity (%) [Gaps excluded][PDB]:** 91.41
- **2D identity - Alignment Gaps [PDB]:** 53
- **2D aligned content [PDB] (<2D-fold>:%):** {'.': 19.34, 'B': 1.59, 'E': 27.68, 'T': 18.41, 'H': 31.13, 'I': 0.66, 'G': 1.19}
- **3D similarity (TM-Score) (%) [PDB]:** 96.74

- **Gene name:** AGO1
- **Entrez ID:** 26523
- **RefSeq ID:** NM\_012199
- **Transcript sequence length:** 13712
- **5-UTR|CDS|3-UTR identity (%):** 31.54 | 72.91 | 29.89
- **5-UTR|CDS|3-UTR identity (%) [Gaps excluded]:** 72.58 | 77.88 | 80.83
- **5-UTR|CDS|3-UTR identity [Alignment Gaps]:** 242 | 170 | 6998
- **5-UTR aligned content (<base>:%):** {'C': 43.7, 'T': 8.15, 'G': 43.7, 'A': 4.44}
- **CDS aligned content (<base>:%):** {'A': 26.35, 'T': 22.23, 'G': 26.35, 'C': 25.06}
- **3-UTR aligned content (<base>:%):** {'A': 27.87, 'G': 21.93, 'C': 16.6, 'T': 33.59}

**Uniprot Description:**  
  
 Required for RNA-mediated gene silencing (RNAi). Binds to short RNAs such as microRNAs (miRNAs) or short interfering RNAs (siRNAs), and represses the translation of mRNAs which are complementary to them. Lacks endonuclease activity and does not appear to cleave target mRNAs. Also required for transcriptional gene silencing (TGS) of promoter regions which are complementary to bound short antigene RNAs (agRNAs).   
  
Interacts with DDB1, DDX5, DDX6, DHX30, DHX36, DDX47, DICER1, AGO2, ELAVL1, HNRNPF, IGF2BP1, ILF3, IMP8, MATR3, MOV10, PABPC1, PRMT5, RBM4, SART3, TNRC6B, UPF1 and YBX1. Associates with polysomes and messenger ribonucleoproteins (mNRPs). Interacts with LIMD1, WTIP and AJUBA. Interacts with APOBEC3F, APOBEC3G and APOBEC3H.   
  
 **Gene Ontology Information:**

Molecular Function

- core promoter sequence-specific DNA binding
- double-stranded RNA binding
- miRNA binding
- RNA binding
- RNA polymerase II complex binding
- single-stranded RNA binding

Location

- cytoplasm
- cytoplasmic ribonucleoprotein granule
- cytosol
- nucleoplasm
- nucleus
- P-body
- polysome
- ribonucleoprotein complex
- RISC complex
- RISC-loading complex

Biological process

- production of miRNAs involved in gene silencing by miRNA
- miRNA mediated inhibition of translation
- negative regulation of angiogenesis
- nuclear-transcribed mRNA catabolic process
- positive regulation of transcription by RNA polymerase II
- pre-miRNA processing
- small RNA loading onto RISC
- RNA secondary structure unwinding

---

3

- **Protein name:** Kelch-like protein 25
- **Organism:** Homo sapiens
- **Uniprot Accession Number:** Q9H0H3
- **Protein sequence length:** 589 aa
- **1D identity (%):** 13.52
- **1D identity (%) [Gaps excluded]:** 22.78
- **1D identity - Alignment Gaps:** 370
- **1D aligned content (<aminoacid>:%):** {'V': 8.13, 'S': 6.5, 'L': 11.38, 'A': 5.69, 'H': 4.07, 'P': 8.13, 'D': 4.07, 'R': 9.76, 'Y': 3.25, 'F': 4.88, 'E': 3.25, 'G': 6.5, 'Q': 3.25, 'C': 2.44, 'T': 8.94, 'K': 4.88, 'I': 2.44, 'M': 0.81, 'N': 1.63}
- **Common reported functions (%):** 0.0
- **Common reported locations (%):** 25.0
- **Common reported processes (%):** 0.0

- **AF ID:** Q9H0H3
- **Chain:** A
- **Protein length:** 589 aa
- **Resolution:** N/A
- **b-phipsi:** 0.000966
- **w-rdist:** 0.590648
- **t-alpha:** 0.000782
- **Chemical similarity (Tanimoto Index) (%):** 99.55
- **1D identity (%) [PDB]:** 2.08
- **1D identity (%) [Gaps excluded][PDB]:** 65.91
- **1D identity - Alignment Gaps [PDB]:** 1351
- **1D aligned content [PDB] (<aminoacid>:%):** {'V': 20.69, 'S': 13.79, 'Q': 3.45, 'W': 3.45, 'L': 17.24, 'E': 3.45, 'A': 10.34, 'G': 3.45, 'H': 6.9, 'N': 3.45, 'D': 3.45, 'I': 3.45, 'T': 3.45, 'P': 3.45}
- **2D identity (%) [PDB]:** 34.23
- **2D identity (%) [Gaps excluded][PDB]:** 89.22
- **2D identity - Alignment Gaps [PDB]:** 641
- **2D aligned content [PDB] (<2D-fold>:%):** {'T': 28.65, '.': 14.33, 'H': 36.52, 'E': 18.54, 'G': 1.69, 'B': 0.28}
- **3D similarity (TM-Score) (%) [PDB]:** 18.57

- **Gene name:** KLHL25
- **Entrez ID:** 64410
- **RefSeq ID:** NM\_022480
- **Transcript sequence length:** 3650
- **5-UTR|CDS|3-UTR identity (%):** 27.12 | 37.3 | 22.41
- **5-UTR|CDS|3-UTR identity (%) [Gaps excluded]:** 78.23 | 73.15 | 75.22
- **5-UTR|CDS|3-UTR identity [Alignment Gaps]:** 277 | 1414 | 3243
- **5-UTR aligned content (<base>:%):** {'G': 53.04, 'T': 8.7, 'C': 33.04, 'A': 5.22}
- **CDS aligned content (<base>:%):** {'A': 22.58, 'T': 19.42, 'G': 29.18, 'C': 28.81}
- **3-UTR aligned content (<base>:%):** {'G': 30.63, 'A': 21.84, 'T': 22.9, 'C': 24.64}

**Uniprot Description:**  
  
 Substrate-specific adapter of a BCR (BTB-CUL3-RBX1) E3 ubiquitin ligase complex required for translational homeostasis. The BCR(KLHL25) ubiquitin ligase complex acts by mediating ubiquitination of hypophosphorylated EIF4EBP1 (4E-BP1): ubiquitination and subsequent degradation of hypophosphorylated EIF4EBP1 (4E-BP1) probably serves as a homeostatic mechanism to maintain translation and prevent eIF4E inhibition when eIF4E levels are low. The BCR(KLHL25) complex does not target EIF4EBP1 (4E-BP1) when it is hyperphosphorylated or associated with eIF4E.   
  
Component of the BCR(KLHL25) E3 ubiquitin ligase complex, at least composed of CUL3, KLHL25 and RBX1. Interacts with EIF4EBP1 (when hypophosphorylated).   
  
 **Gene Ontology Information:**

Molecular Function

- ubiquitin ligase-substrate adaptor activity

Location

- Cul3-RING ubiquitin ligase complex
- cytoplasm
- cytosol

Biological process

- negative regulation of fatty acid biosynthetic process
- positive regulation of CD4-positive, CD25-positive, alpha-beta regulatory T cell differentiation
- positive regulation of fatty acid oxidation
- protein ubiquitination
- regulation of translational initiation
- ubiquitin-dependent protein catabolic process

---

4

- **Protein name:** Protein argonaute-2
- **Organism:** Homo sapiens
- **Uniprot Accession Number:** Q9UKV8
- **Protein sequence length:** 859 aa
- **1D identity (%):** 77.73
- **1D identity (%) [Gaps excluded]:** 79.74
- **1D identity - Alignment Gaps:** 22
- **1D aligned content (<aminoacid>:%):** {'M': 2.22, 'G': 7.39, 'P': 7.39, 'A': 6.2, 'F': 4.43, 'R': 6.2, 'T': 6.06, 'I': 5.17, 'L': 7.53, 'N': 2.36, 'K': 5.47, 'D': 4.73, 'Y': 3.84, 'H': 3.1, 'E': 4.73, 'V': 8.12, 'Q': 6.65, 'S': 4.73, 'W': 0.89, 'C': 2.81}
- **Common reported functions (%):** 75.0
- **Common reported locations (%):** 50.0
- **Common reported processes (%):** 16.67

- **AF ID:** Q9UKV8
- **Chain:** A
- **Protein length:** 859 aa
- **Resolution:** N/A
- **b-phipsi:** 0.000944
- **w-rdist:** 0.059519
- **t-alpha:** 0.057903
- **Chemical similarity (Tanimoto Index) (%):** 99.62
- **1D identity (%) [PDB]:** 76.95
- **1D identity (%) [Gaps excluded][PDB]:** 80.17
- **1D identity - Alignment Gaps [PDB]:** 35
- **1D aligned content [PDB] (<aminoacid>:%):** {'F': 4.47, 'P': 7.0, 'R': 6.26, 'G': 7.3, 'T': 6.11, 'I': 5.22, 'L': 7.6, 'A': 6.11, 'N': 2.38, 'K': 5.51, 'D': 4.77, 'Y': 3.87, 'H': 3.13, 'E': 4.77, 'V': 8.2, 'M': 2.09, 'Q': 6.71, 'S': 4.77, 'W': 0.89, 'C': 2.83}
- **2D identity (%) [PDB]:** 84.3
- **2D identity (%) [Gaps excluded][PDB]:** 92.04
- **2D identity - Alignment Gaps [PDB]:** 75
- **2D aligned content [PDB] (<2D-fold>:%):** {'.': 17.84, 'B': 1.6, 'E': 27.83, 'T': 19.97, 'H': 31.29, 'I': 0.67, 'G': 0.8}
- **3D similarity (TM-Score) (%) [PDB]:** 96.38

- **Gene name:** AGO2
- **Entrez ID:** 404130
- **RefSeq ID:** NM\_012154
- **Transcript sequence length:** 14595
- **5-UTR|CDS|3-UTR identity (%):** 21.14 | 69.33 | 28.08
- **5-UTR|CDS|3-UTR identity (%) [Gaps excluded]:** 83.18 | 74.74 | 81.16
- **5-UTR|CDS|3-UTR identity [Alignment Gaps]:** 314 | 194 | 7859
- **5-UTR aligned content (<base>:%):** {'C': 44.94, 'G': 43.82, 'T': 8.99, 'A': 2.25}
- **CDS aligned content (<base>:%):** {'A': 26.26, 'T': 20.99, 'G': 27.02, 'C': 25.73}
- **3-UTR aligned content (<base>:%):** {'A': 27.12, 'G': 21.34, 'T': 34.2, 'C': 17.34}

**Uniprot Description:**  
  
 Required for RNA-mediated gene silencing (RNAi) by the RNA-induced silencing complex (RISC). The 'minimal RISC' appears to include AGO2 bound to a short guide RNA such as a microRNA (miRNA) or short interfering RNA (siRNA). These guide RNAs direct RISC to complementary mRNAs that are targets for RISC-mediated gene silencing. The precise mechanism of gene silencing depends on the degree of complementarity between the miRNA or siRNA and its target. Binding of RISC to a perfectly complementary mRNA generally results in silencing due to endonucleolytic cleavage of the mRNA specifically by AGO2. Binding of RISC to a partially complementary mRNA results in silencing through inhibition of translation, and this is independent of endonuclease activity. May inhibit translation initiation by binding to the 7-methylguanosine cap, thereby preventing the recruitment of the translation initiation factor eIF4-E. May also inhibit translation initiation via interaction with EIF6, which itself binds to the 60S ribosomal subunit and prevents its association with the 40S ribosomal subunit. The inhibition of translational initiation leads to the accumulation of the affected mRNA in cytoplasmic processing bodies (P-bodies), where mRNA degradation may subsequently occur. In some cases RISC-mediated translational repression is also observed for miRNAs that perfectly match the 3' untranslated region (3'-UTR). Can also up-regulate the translation of specific mRNAs under certain growth conditions. Binds to the AU element of the 3'-UTR of the TNF (TNF-alpha) mRNA and up-regulates translation under conditions of serum starvation. Also required for transcriptional gene silencing (TGS), in which short RNAs known as antigene RNAs or agRNAs direct the transcriptional repression of complementary promoter regions.   
  
Interacts with DICER1 through its Piwi domain and with TARBP2 during assembly of the RNA-induced silencing complex (RISC). Together, DICER1, AGO2 and TARBP2 constitute the trimeric RISC loading complex (RLC), or micro-RNA (miRNA) loading complex (miRLC). Within the RLC/miRLC, DICER1 and TARBP2 are required to process precursor miRNAs (pre-miRNAs) to mature miRNAs and then load them onto AGO2. AGO2 bound to the mature miRNA constitutes the minimal RISC and may subsequently dissociate from DICER1 and TARBP2. Note however that the term RISC has also been used to describe the trimeric RLC/miRLC. The formation of RISC complexes containing siRNAs rather than miRNAs appears to occur independently of DICER1. Interacts with AGO1. Also interacts with DDB1, DDX5, DDX6, DDX20, DHX30, DHX36, DDX47, DHX9, ELAVL, FXR1, GEMIN4, HNRNPF, IGF2BP1, ILF3, IMP8, MATR3, PABPC1, PRMT5, P4HA1, P4HB, RBM4, SART3, TNRC6A, TNRC6B, UPF1 and YBX1. Interacts with the P-body components DCP1A and XRN1. Associates with polysomes and messenger ribonucleoproteins (mNRPs). Interacts with RBM4; the interaction is modulated under stress-induced conditions, occurs under both cell proliferation and differentiation conditions and in an RNA- and phosphorylation-independent manner. Interacts with LIMD1, WTIP and AJUBA. Interacts with TRIM71; the interaction increases in presence of RNA (PubMed:23125361). Interacts with APOBEC3G in an RNA-dependent manner. Interacts with APOBEC3A, APOBEC3C, APOBEC3F and APOBEC3H. Interacts with DICER1, TARBP2, EIF6, MOV10 and RPL7A (60S ribosome subunit); they form a large RNA-induced silencing complex (RISC) (PubMed:17507929, PubMed:24726324). Interacts with FMR1 (PubMed:14703574). Interacts with ZFP36 (PubMed:15766526). Found in a complex, composed of AGO2, CHD7 and FAM172A (By similarity). Interacts with RC3H1; the interaction is RNA independent (PubMed:25697406). Interacts with SND1 (PubMed:14508492, PubMed:28546213). Interacts with SYT11 (By similarity). Interacts with CLNK (PubMed:26009488). Interacts with GARRE1 (PubMed:29395067).   
  
 **Gene Ontology Information:**

Molecular Function

- endoribonuclease activity, cleaving siRNA-paired mRNA
- metal ion binding
- miRNA binding
- RNA 7-methylguanosine cap binding
- endoribonuclease activity
- single-stranded RNA binding
- siRNA binding

Location

- mRNA cap binding complex
- nucleus
- P-body
- polysome
- ribonucleoprotein complex
- RISC complex
- RISC-loading complex

Biological process

- miRNA mediated inhibition of translation
- mRNA cleavage involved in gene silencing by miRNA
- negative regulation of translational initiation
- pre-miRNA processing
- regulation of transcription, DNA-templated

---

5

- **Protein name:** Protein argonaute-4
- **Organism:** Homo sapiens
- **Uniprot Accession Number:** Q9HCK5
- **Protein sequence length:** 861 aa
- **1D identity (%):** 100.0
- **1D identity (%) [Gaps excluded]:** 100.0
- **1D identity - Alignment Gaps:** N/A
- **1D aligned content (<aminoacid>:%):** {'M': 2.9, 'E': 4.53, 'A': 6.04, 'L': 7.67, 'G': 6.74, 'P': 6.62, 'S': 5.23, 'F': 3.48, 'Q': 6.97, 'R': 6.27, 'T': 5.92, 'V': 8.94, 'K': 5.81, 'I': 4.88, 'N': 2.9, 'H': 3.37, 'D': 4.65, 'Y': 3.95, 'W': 0.81, 'C': 2.32}
- **Common reported functions (%):** 75.0
- **Common reported locations (%):** 25.0
- **Common reported processes (%):** 16.67

- **AF ID:** Q9HCK5
- **Chain:** A
- **Protein length:** 861 aa
- **Resolution:** N/A
- **b-phipsi:** 0.000427
- **w-rdist:** 0.102836
- **t-alpha:** 0.11737
- **Chemical similarity (Tanimoto Index) (%):** 100.0
- **1D identity (%) [PDB]:** 98.72
- **1D identity (%) [Gaps excluded][PDB]:** 100.0
- **1D identity - Alignment Gaps [PDB]:** 11
- **1D aligned content [PDB] (<aminoacid>:%):** {'L': 7.65, 'F': 3.53, 'Q': 7.06, 'P': 6.35, 'R': 6.35, 'G': 6.59, 'T': 6.0, 'V': 9.06, 'K': 5.88, 'I': 4.94, 'A': 5.88, 'N': 2.94, 'H': 3.41, 'D': 4.71, 'Y': 4.0, 'E': 4.47, 'M': 2.82, 'S': 5.18, 'W': 0.82, 'C': 2.35}
- **2D identity (%) [PDB]:** 88.36
- **2D identity (%) [Gaps excluded][PDB]:** 90.98
- **2D identity - Alignment Gaps [PDB]:** 25
- **2D aligned content [PDB] (<2D-fold>:%):** {'.': 18.54, 'B': 1.57, 'E': 27.42, 'T': 19.97, 'H': 30.68, 'I': 0.65, 'G': 1.17}
- **3D similarity (TM-Score) (%) [PDB]:** 97.78

- **Gene name:** AGO4
- **Entrez ID:** 419629
- **RefSeq ID:** NM\_017629
- **Transcript sequence length:** 7272
- **5-UTR|CDS|3-UTR identity (%):** 100.0 | 100.0 | 100.0
- **5-UTR|CDS|3-UTR identity (%) [Gaps excluded]:** 100.0 | 100.0 | 100.0
- **5-UTR|CDS|3-UTR identity [Alignment Gaps]:** N/A | N/A | N/A
- **5-UTR aligned content (<base>:%):** {'G': 38.65, 'T': 10.47, 'C': 44.39, 'A': 6.48}
- **CDS aligned content (<base>:%):** {'A': 28.34, 'T': 24.13, 'G': 24.48, 'C': 23.05}
- **3-UTR aligned content (<base>:%):** {'G': 22.24, 'A': 27.98, 'T': 32.02, 'C': 17.76}

**Uniprot Description:**  
  
 Required for RNA-mediated gene silencing (RNAi). Binds to short RNAs such as microRNAs (miRNAs) and represses the translation of mRNAs which are complementary to them. Lacks endonuclease activity and does not appear to cleave target mRNAs. Also required for RNA-directed transcription and replication of the human hapatitis delta virus (HDV).   
  
Interacts with EIF4B, IMP8, PRMT5, TNRC6A and TNRC6B (PubMed:19167051). Interacts with ZFP36 (PubMed:15766526).   
  
 **Gene Ontology Information:**

Molecular Function

- miRNA binding
- endoribonuclease activity
- single-stranded RNA binding

Location

- P-body
- RISC complex

Biological process

- miRNA mediated inhibition of translation
- mRNA catabolic process

---

6

- **Protein name:** Tyrosine-protein kinase BTK
- **Organism:** Homo sapiens
- **Uniprot Accession Number:** Q06187
- **Protein sequence length:** 659 aa
- **1D identity (%):** 13.86
- **1D identity (%) [Gaps excluded]:** 24.23
- **1D identity - Alignment Gaps:** 414
- **1D aligned content (<aminoacid>:%):** {'A': 4.48, 'I': 5.22, 'E': 7.46, 'L': 10.45, 'T': 4.48, 'P': 10.45, 'R': 3.73, 'F': 1.49, 'G': 8.21, 'K': 4.48, 'V': 6.72, 'M': 1.49, 'Y': 5.97, 'Q': 5.22, 'C': 2.24, 'N': 2.24, 'W': 0.75, 'D': 3.73, 'S': 8.21, 'H': 2.99}
- **Common reported functions (%):** 0.0
- **Common reported locations (%):** 37.5
- **Common reported processes (%):** 0.0

- **AF ID:** Q06187
- **Chain:** A
- **Protein length:** 659 aa
- **Resolution:** N/A
- **b-phipsi:** 0.00258
- **w-rdist:** 0.373166
- **t-alpha:** 0.001565
- **Chemical similarity (Tanimoto Index) (%):** 99.55
- **1D identity (%) [PDB]:** 2.04
- **1D identity (%) [Gaps excluded][PDB]:** 73.17
- **1D identity - Alignment Gaps [PDB]:** 1427
- **1D aligned content [PDB] (<aminoacid>:%):** {'M': 3.33, 'A': 3.33, 'T': 6.67, 'C': 3.33, 'V': 13.33, 'Q': 6.67, 'K': 10.0, 'N': 13.33, 'S': 10.0, 'L': 13.33, 'G': 6.67, 'I': 3.33, 'R': 3.33, 'P': 3.33}
- **2D identity (%) [PDB]:** 39.9
- **2D identity (%) [Gaps excluded][PDB]:** 88.49
- **2D identity - Alignment Gaps [PDB]:** 571
- **2D aligned content [PDB] (<2D-fold>:%):** {'.': 16.39, 'T': 27.47, 'E': 22.89, 'H': 32.05, 'B': 0.48, 'G': 0.72}
- **3D similarity (TM-Score) (%) [PDB]:** 22.45

- **Gene name:** BTK
- **Entrez ID:** 695
- **RefSeq ID:** N/A
- **Sequence length:** N/A
- **5-UTR|CDS|3-UTR identity (%):** N/A | N/A | N/A
- **5-UTR|CDS|3-UTR identity (%) [Gaps excluded]:** N/A | N/A | N/A
- **5-UTR|CDS|3-UTR identity [Alignment Gaps]:** N/A | N/A | N/A
- **5-UTR aligned content (<base>:%):** N/A
- **CDS aligned content (<base>:%):** N/A
- **3-UTR aligned content (<base>:%):** N/A

**Uniprot Description:**  
  
 Non-receptor tyrosine kinase indispensable for B lymphocyte development, differentiation and signaling. Binding of antigen to the B-cell antigen receptor (BCR) triggers signaling that ultimately leads to B-cell activation. After BCR engagement and activation at the plasma membrane, phosphorylates PLCG2 at several sites, igniting the downstream signaling pathway through calcium mobilization, followed by activation of the protein kinase C (PKC) family members. PLCG2 phosphorylation is performed in close cooperation with the adapter protein B-cell linker protein BLNK. BTK acts as a platform to bring together a diverse array of signaling proteins and is implicated in cytokine receptor signaling pathways. Plays an important role in the function of immune cells of innate as well as adaptive immunity, as a component of the Toll-like receptors (TLR) pathway. The TLR pathway acts as a primary surveillance system for the detection of pathogens and are crucial to the activation of host defense. Especially, is a critical molecule in regulating TLR9 activation in splenic B-cells. Within the TLR pathway, induces tyrosine phosphorylation of TIRAP which leads to TIRAP degradation. BTK plays also a critical role in transcription regulation. Induces the activity of NF-kappa-B, which is involved in regulating the expression of hundreds of genes. BTK is involved on the signaling pathway linking TLR8 and TLR9 to NF-kappa-B. Transiently phosphorylates transcription factor GTF2I on tyrosine residues in response to BCR. GTF2I then translocates to the nucleus to bind regulatory enhancer elements to modulate gene expression. ARID3A and NFAT are other transcriptional target of BTK. BTK is required for the formation of functional ARID3A DNA-binding complexes. There is however no evidence that BTK itself binds directly to DNA. BTK has a dual role in the regulation of apoptosis.   
  
Binds GTF2I through the PH domain. Interacts with SH3BP5 via the SH3 domain. Interacts with IBTK via its PH domain. Interacts with ARID3A, CAV1, FASLG, PIN1, TLR8 and TLR9.   
  
 **Gene Ontology Information:**

Molecular Function

- ATP binding
- identical protein binding
- metal ion binding
- non-membrane spanning protein tyrosine kinase activity
- phosphatidylinositol-3,4,5-trisphosphate binding
- phospholipase activator activity
- phospholipase binding
- protein tyrosine kinase activity

Location

- cytoplasm
- cytoplasmic vesicle
- cytosol
- membrane raft
- nucleus
- perinuclear region of cytoplasm
- plasma membrane

Biological process

- adaptive immune response
- apoptotic signaling pathway
- B cell activation
- B cell affinity maturation
- B cell receptor signaling pathway
- calcium-mediated signaling
- cell maturation
- cellular response to interleukin-7
- cellular response to molecule of fungal origin
- cellular response to reactive oxygen species
- eosinophil homeostasis
- Fc-epsilon receptor signaling pathway
- histamine secretion by mast cell
- I-kappaB kinase/NF-kappaB signaling
- innate immune response
- intracellular signal transduction
- mesoderm development
- monocyte proliferation
- MyD88-dependent toll-like receptor signaling pathway
- negative regulation of B cell proliferation
- negative regulation of interleukin-10 production
- neutrophil homeostasis
- peptidyl-tyrosine autophosphorylation
- peptidyl-tyrosine phosphorylation
- positive regulation of B cell differentiation
- positive regulation of B cell proliferation
- positive regulation of immunoglobulin production
- positive regulation of interleukin-17A production
- positive regulation of interleukin-6 production
- positive regulation of NF-kappaB transcription factor activity
- positive regulation of NLRP3 inflammasome complex assembly
- positive regulation of phagocytosis
- positive regulation of synoviocyte proliferation
- positive regulation of tumor necrosis factor production
- positive regulation of type I hypersensitivity
- positive regulation of type III hypersensitivity
- protein phosphorylation
- proteoglycan catabolic process
- regulation of B cell apoptotic process
- regulation of B cell cytokine production
- response to lipopolysaccharide
- T cell receptor signaling pathway

---

7

- **Protein name:** TGF-beta receptor type-2
- **Organism:** Homo sapiens
- **Uniprot Accession Number:** P37173
- **Protein sequence length:** 567 aa
- **1D identity (%):** 11.29
- **1D identity (%) [Gaps excluded]:** 20.51
- **1D identity - Alignment Gaps:** 414
- **1D aligned content (<aminoacid>:%):** {'W': 1.92, 'L': 9.62, 'T': 10.58, 'P': 9.62, 'N': 2.88, 'V': 5.77, 'K': 12.5, 'F': 1.92, 'C': 6.73, 'Q': 1.92, 'E': 1.92, 'A': 4.81, 'H': 3.85, 'I': 4.81, 'S': 4.81, 'D': 3.85, 'G': 6.73, 'Y': 0.96, 'R': 4.81}
- **Common reported functions (%):** 0.0
- **Common reported locations (%):** 25.0
- **Common reported processes (%):** 0.0

- **AF ID:** P37173
- **Chain:** A
- **Protein length:** 567 aa
- **Resolution:** N/A
- **b-phipsi:** 0.001075
- **w-rdist:** 0.577155
- **t-alpha:** 0.002347
- **Chemical similarity (Tanimoto Index) (%):** 97.05
- **1D identity (%) [PDB]:** 1.22
- **1D identity (%) [Gaps excluded][PDB]:** 73.91
- **1D identity - Alignment Gaps [PDB]:** 1371
- **1D aligned content [PDB] (<aminoacid>:%):** {'G': 11.76, 'L': 17.65, 'T': 5.88, 'V': 11.76, 'K': 11.76, 'P': 11.76, 'I': 11.76, 'A': 5.88, 'N': 5.88, 'Q': 5.88}
- **2D identity (%) [PDB]:** 38.69
- **2D identity (%) [Gaps excluded][PDB]:** 91.23
- **2D identity - Alignment Gaps [PDB]:** 573
- **2D aligned content [PDB] (<2D-fold>:%):** {'.': 15.06, 'E': 23.12, 'T': 24.16, 'H': 37.4, 'B': 0.26}
- **3D similarity (TM-Score) (%) [PDB]:** 22.74

- **Gene name:** TGFBR2
- **Entrez ID:** 7048
- **RefSeq ID:** NM\_003242
- **Transcript sequence length:** 4530
- **5-UTR|CDS|3-UTR identity (%):** 38.99 | 37.17 | 31.86
- **5-UTR|CDS|3-UTR identity (%) [Gaps excluded]:** 76.96 | 74.74 | 75.56
- **5-UTR|CDS|3-UTR identity [Alignment Gaps]:** 224 | 1440 | 2778
- **5-UTR aligned content (<base>:%):** {'A': 2.82, 'C': 50.28, 'G': 41.24, 'T': 5.65}
- **CDS aligned content (<base>:%):** {'A': 28.26, 'T': 21.88, 'G': 25.92, 'C': 23.94}
- **3-UTR aligned content (<base>:%):** {'C': 17.06, 'T': 33.99, 'G': 18.69, 'A': 30.26}

**Uniprot Description:**  
  
 Transmembrane serine/threonine kinase forming with the TGF-beta type I serine/threonine kinase receptor, TGFBR1, the non-promiscuous receptor for the TGF-beta cytokines TGFB1, TGFB2 and TGFB3. Transduces the TGFB1, TGFB2 and TGFB3 signal from the cell surface to the cytoplasm and thus regulates a plethora of physiological and pathological processes including cell cycle arrest in epithelial and hematopoietic cells, control of mesenchymal cell proliferation and differentiation, wound healing, extracellular matrix production, immunosuppression and carcinogenesis. The formation of the receptor complex composed of 2 TGFBR1 and 2 TGFBR2 molecules symmetrically bound to the cytokine dimer results in the phosphorylation and activation of TGFBR1 by the constitutively active TGFBR2. Activated TGFBR1 phosphorylates SMAD2 which dissociates from the receptor and interacts with SMAD4. The SMAD2-SMAD4 complex is subsequently translocated to the nucleus where it modulates the transcription of the TGF-beta-regulated genes. This constitutes the canonical SMAD-dependent TGF-beta signaling cascade. Also involved in non-canonical, SMAD-independent TGF-beta signaling pathways.   
  
Homodimer. Heterohexamer; TGFB1, TGFB2 and TGFB3 homodimeric ligands assemble a functional receptor composed of two TGFBR1 and TGFBR2 heterodimers to form a ligand-receptor heterohexamer. The respective affinity of TGFRB1 and TGFRB2 for the ligands may modulate the kinetics of assembly of the receptor and may explain the different biological activities of TGFB1, TGFB2 and TGFB3. Interacts with DAXX. Interacts with DYNLT4. Interacts with ZFYVE9; ZFYVE9 recruits SMAD2 and SMAD3 to the TGF-beta receptor. Interacts with and is activated by SCUBE3; this interaction does not affect TGFB1-binding to TGFBR2. Interacts with VPS39; this interaction is independent of the receptor kinase activity and of the presence of TGF-beta. Interacts with CLU (PubMed:8555189).   
  
 **Gene Ontology Information:**

Molecular Function

- activin binding
- activin-activated receptor activity
- ATP binding
- glycosaminoglycan binding
- kinase activator activity
- metal ion binding
- molecular adaptor activity
- SMAD binding
- transforming growth factor beta binding
- transforming growth factor beta-activated receptor activity
- transforming growth factor beta receptor activity, type II
- transmembrane receptor protein serine/threonine kinase activity
- type I transforming growth factor beta receptor binding
- type III transforming growth factor beta receptor binding

Location

- caveola
- cytosol
- external side of plasma membrane
- extracellular region
- extracellular space
- membrane
- membrane raft
- plasma membrane
- receptor complex
- transforming growth factor beta ligand-receptor complex

Biological process

- activation of protein kinase activity
- aorta morphogenesis
- aortic valve morphogenesis
- apoptotic process
- artery morphogenesis
- atrioventricular valve morphogenesis
- blood vessel development
- brain development
- branching involved in blood vessel morphogenesis
- bronchus morphogenesis
- cardiac left ventricle morphogenesis
- cellular response to growth factor stimulus
- embryonic cranial skeleton morphogenesis
- embryonic hemopoiesis
- endocardial cushion fusion
- gastrulation
- growth plate cartilage chondrocyte growth
- heart development
- heart looping
- in utero embryonic development
- inferior endocardial cushion morphogenesis
- Langerhans cell differentiation
- lens development in camera-type eye
- lens fiber cell apoptotic process
- lung lobe morphogenesis
- mammary gland morphogenesis
- membranous septum morphogenesis
- miRNA transport
- myeloid dendritic cell differentiation
- Notch signaling pathway
- outflow tract morphogenesis
- outflow tract septum morphogenesis
- pathway-restricted SMAD protein phosphorylation
- peptidyl-serine phosphorylation
- peptidyl-threonine phosphorylation
- positive regulation of angiogenesis
- positive regulation of B cell tolerance induction
- positive regulation of CD4-positive, alpha-beta T cell proliferation
- positive regulation of cell population proliferation
- positive regulation of epithelial cell migration
- positive regulation of epithelial to mesenchymal transition
- positive regulation of epithelial to mesenchymal transition involved in endocardial cushion formation
- positive regulation of mesenchymal cell proliferation
- positive regulation of NK T cell differentiation
- positive regulation of pathway-restricted SMAD protein phosphorylation
- positive regulation of reactive oxygen species metabolic process
- positive regulation of SMAD protein signal transduction
- positive regulation of T cell tolerance induction
- positive regulation of tolerance induction to self antigen
- protein phosphorylation
- regulation of cell population proliferation
- regulation of gene expression
- regulation of stem cell proliferation
- response to cholesterol
- response to xenobiotic stimulus
- roof of mouth development
- secondary palate development
- smoothened signaling pathway
- trachea formation
- transforming growth factor beta receptor signaling pathway
- tricuspid valve morphogenesis
- vasculogenesis
- ventricular septum morphogenesis

---

8

- **Protein name:** BTB/POZ domain-containing protein 3
- **Organism:** Homo sapiens
- **Uniprot Accession Number:** Q9Y2F9
- **Protein sequence length:** 522 aa
- **1D identity (%):** 9.11
- **1D identity (%) [Gaps excluded]:** 21.1
- **1D identity - Alignment Gaps:** 549
- **1D aligned content (<aminoacid>:%):** {'V': 6.82, 'D': 6.82, 'L': 12.5, 'P': 10.23, 'K': 5.68, 'S': 5.68, 'T': 2.27, 'R': 5.68, 'W': 2.27, 'G': 9.09, 'M': 2.27, 'N': 4.55, 'A': 3.41, 'E': 4.55, 'C': 5.68, 'Y': 3.41, 'Q': 5.68, 'I': 1.14, 'F': 2.27}
- **Common reported functions (%):** 0.0
- **Common reported locations (%):** 25.0
- **Common reported processes (%):** 0.0

- **AF ID:** Q9Y2F9
- **Chain:** A
- **Protein length:** 522 aa
- **Resolution:** N/A
- **b-phipsi:** 0.001243
- **w-rdist:** 0.339147
- **t-alpha:** 0.006299
- **Chemical similarity (Tanimoto Index) (%):** 99.54
- **1D identity (%) [PDB]:** 2.26
- **1D identity (%) [Gaps excluded][PDB]:** 66.67
- **1D identity - Alignment Gaps [PDB]:** 1282
- **1D aligned content [PDB] (<aminoacid>:%):** {'V': 13.33, 'K': 13.33, 'N': 10.0, 'S': 6.67, 'Q': 13.33, 'L': 16.67, 'A': 3.33, 'G': 6.67, 'P': 3.33, 'R': 3.33, 'F': 6.67, 'I': 3.33}
- **2D identity (%) [PDB]:** 32.34
- **2D identity (%) [Gaps excluded][PDB]:** 87.57
- **2D identity - Alignment Gaps [PDB]:** 632
- **2D aligned content [PDB] (<2D-fold>:%):** {'.': 16.98, 'H': 40.74, 'T': 19.14, 'E': 22.84, 'B': 0.31}
- **3D similarity (TM-Score) (%) [PDB]:** 19.63

- **Gene name:** BTBD3
- **Entrez ID:** 100385566
- **RefSeq ID:** N/A
- **Sequence length:** N/A
- **5-UTR|CDS|3-UTR identity (%):** N/A | N/A | N/A
- **5-UTR|CDS|3-UTR identity (%) [Gaps excluded]:** N/A | N/A | N/A
- **5-UTR|CDS|3-UTR identity [Alignment Gaps]:** N/A | N/A | N/A
- **5-UTR aligned content (<base>:%):** N/A
- **CDS aligned content (<base>:%):** N/A
- **3-UTR aligned content (<base>:%):** N/A

**Uniprot Description:**  
  
 Acts as a key regulator of dendritic field orientation during development of sensory cortex. Also directs dendrites toward active axon terminals when ectopically expressed (By similarity). N/A   
  
 **Gene Ontology Information:**

Molecular Function   
  
N/A

Location

- cytosol
- nucleus

Biological process

- cerebral cortex development
- dendrite morphogenesis

---

9

- **Protein name:** Endogenous retrovirus group V member 1 Env polyprotein
- **Organism:** Homo sapiens
- **Uniprot Accession Number:** B6SEH8
- **Protein sequence length:** 477 aa
- **1D identity (%):** 10.38
- **1D identity (%) [Gaps excluded]:** 23.24
- **1D identity - Alignment Gaps:** 512
- **1D aligned content (<aminoacid>:%):** {'T': 5.21, 'K': 7.29, 'S': 6.25, 'L': 10.42, 'A': 4.17, 'N': 5.21, 'E': 4.17, 'H': 4.17, 'F': 3.12, 'Y': 6.25, 'G': 11.46, 'V': 5.21, 'C': 6.25, 'D': 4.17, 'P': 6.25, 'R': 4.17, 'Q': 3.12, 'I': 2.08, 'W': 1.04}
- **Common reported functions (%):** 0.0
- **Common reported locations (%):** 12.5
- **Common reported processes (%):** 0.0

- **AF ID:** B6SEH8
- **Chain:** A
- **Protein length:** 477 aa
- **Resolution:** N/A
- **b-phipsi:** 0.002878
- **w-rdist:** 0.422257
- **t-alpha:** 0.000783
- **Chemical similarity (Tanimoto Index) (%):** 94.66
- **1D identity (%) [PDB]:** 1.46
- **1D identity (%) [Gaps excluded][PDB]:** 79.17
- **1D identity - Alignment Gaps [PDB]:** 1279
- **1D aligned content [PDB] (<aminoacid>:%):** {'V': 10.53, 'S': 10.53, 'E': 10.53, 'G': 5.26, 'Q': 5.26, 'K': 10.53, 'A': 10.53, 'W': 5.26, 'L': 10.53, 'I': 10.53, 'R': 5.26, 'C': 5.26}
- **2D identity (%) [PDB]:** 32.99
- **2D identity (%) [Gaps excluded][PDB]:** 86.61
- **2D identity - Alignment Gaps [PDB]:** 595
- **2D aligned content [PDB] (<2D-fold>:%):** {'H': 39.75, '.': 21.45, 'E': 18.93, 'T': 17.98, 'G': 1.89}
- **3D similarity (TM-Score) (%) [PDB]:** 18.31

- **Gene name:** ERVV-1
- **Entrez ID:** 147664
- **RefSeq ID:** NM\_152473
- **Transcript sequence length:** 2203
- **5-UTR|CDS|3-UTR identity (%):** 18.2 | 31.32 | 7.96
- **5-UTR|CDS|3-UTR identity (%) [Gaps excluded]:** 64.29 | 77.31 | 72.02
- **5-UTR|CDS|3-UTR identity [Alignment Gaps]:** 319 | 1702 | 3912
- **5-UTR aligned content (<base>:%):** {'G': 25.93, 'C': 40.74, 'T': 20.99, 'A': 12.35}
- **CDS aligned content (<base>:%):** {'A': 29.8, 'T': 25.56, 'G': 20.09, 'C': 24.55}
- **3-UTR aligned content (<base>:%):** {'T': 22.86, 'C': 22.0, 'A': 31.43, 'G': 23.71}

**Uniprot Description:**  
  
 N/A N/A   
  
 **Gene Ontology Information:**

Molecular Function   
  
N/A

Location

- membrane

Biological process   
  
N/A

---

10

- **Protein name:** Muskelin
- **Organism:** Homo sapiens
- **Uniprot Accession Number:** Q9UL63
- **Protein sequence length:** 735 aa
- **1D identity (%):** 13.49
- **1D identity (%) [Gaps excluded]:** 22.22
- **1D identity - Alignment Gaps:** 390
- **1D aligned content (<aminoacid>:%):** {'G': 8.96, 'P': 8.96, 'E': 7.46, 'W': 2.24, 'F': 4.48, 'N': 2.99, 'S': 2.24, 'Y': 5.22, 'Q': 7.46, 'I': 2.24, 'T': 6.72, 'K': 8.96, 'L': 10.45, 'C': 3.73, 'D': 4.48, 'A': 3.73, 'H': 2.99, 'R': 3.73, 'V': 2.24, 'M': 0.75}
- **Common reported functions (%):** 0.0
- **Common reported locations (%):** 25.0
- **Common reported processes (%):** 0.0

- **AF ID:** Q9UL63
- **Chain:** A
- **Protein length:** 735 aa
- **Resolution:** N/A
- **b-phipsi:** 0.001596
- **w-rdist:** 0.271883
- **t-alpha:** 0.014084
- **Chemical similarity (Tanimoto Index) (%):** 99.02
- **1D identity (%) [PDB]:** 3.15
- **1D identity (%) [Gaps excluded][PDB]:** 80.0
- **1D identity - Alignment Gaps [PDB]:** 1465
- **1D aligned content [PDB] (<aminoacid>:%):** {'T': 6.25, 'Y': 4.17, 'L': 10.42, 'P': 4.17, 'E': 8.33, 'V': 6.25, 'C': 4.17, 'N': 4.17, 'I': 6.25, 'Q': 4.17, 'K': 10.42, 'D': 6.25, 'S': 10.42, 'A': 4.17, 'R': 4.17, 'G': 4.17, 'F': 2.08}
- **2D identity (%) [PDB]:** 44.44
- **2D identity (%) [Gaps excluded][PDB]:** 87.97
- **2D identity - Alignment Gaps [PDB]:** 521
- **2D aligned content [PDB] (<2D-fold>:%):** {'.': 22.22, 'E': 32.05, 'T': 24.15, 'H': 20.09, 'B': 0.21, 'G': 1.28}
- **3D similarity (TM-Score) (%) [PDB]:** 23.8

- **Gene name:** MKLN1
- **Entrez ID:** 4289
- **RefSeq ID:** NM\_013255
- **Transcript sequence length:** 11136
- **5-UTR|CDS|3-UTR identity (%):** 3.74 | 45.74 | 34.18
- **5-UTR|CDS|3-UTR identity (%) [Gaps excluded]:** 62.5 | 75.41 | 80.31
- **5-UTR|CDS|3-UTR identity [Alignment Gaps]:** 377 | 1174 | 5315
- **5-UTR aligned content (<base>:%):** {'G': 66.67, 'C': 33.33}
- **CDS aligned content (<base>:%):** {'A': 31.14, 'T': 26.15, 'G': 22.78, 'C': 19.93}
- **3-UTR aligned content (<base>:%):** {'G': 19.45, 'A': 29.16, 'T': 35.26, 'C': 16.13}

**Uniprot Description:**  
  
 Component of the CTLH E3 ubiquitin-protein ligase complex that selectively accepts ubiquitin from UBE2H and mediates ubiquitination and subsequent proteasomal degradation of the transcription factor HBP1 (PubMed:29911972). Required for internalization of the GABA receptor GABRA1 from the cell membrane via endosomes and subsequent GABRA1 degradation (By similarity). Acts as a mediator of cell spreading and cytoskeletal responses to the extracellular matrix component THBS1 (PubMed:18710924).   
  
Homodimer; may form higher oligomers (By similarity). Identified in the CTLH complex that contains GID4, RANBP9 and/or RANBP10, MKLN1, MAEA, RMND5A (or alternatively its paralog RMND5B), GID8, ARMC8, WDR26 and YPEL5 (PubMed:17467196, PubMed:29911972). Within this complex, MAEA, RMND5A (or alternatively its paralog RMND5B), GID8, WDR26, and RANBP9 and/or RANBP10 form the catalytic core, while GID4, MKLN1, ARMC8 and YPEL5 have ancillary roles (PubMed:29911972). Interacts with RANBP9 (PubMed:18710924). Part of a complex consisting of RANBP9, MKLN1 and GID8 (PubMed:12559565). Interacts with GABRA1. Interacts with the C-terminal tail of PTGER3 (By similarity).   
  
 **Gene Ontology Information:**

Molecular Function

- identical protein binding
- protein homodimerization activity

Location

- cell cortex
- cytoplasm
- cytosol
- nucleoplasm
- postsynapse
- ruffle
- ubiquitin ligase complex

Biological process

- actin cytoskeleton reorganization
- cell-matrix adhesion
- regulation of cell shape
- regulation of receptor internalization
- signal transduction

---

11

- **Protein name:** Ras GTPase-activating protein 4
- **Organism:** Homo sapiens
- **Uniprot Accession Number:** O43374
- **Protein sequence length:** 803 aa
- **1D identity (%):** 15.92
- **1D identity (%) [Gaps excluded]:** 23.91
- **1D identity - Alignment Gaps:** 334
- **1D aligned content (<aminoacid>:%):** {'M': 0.63, 'I': 3.14, 'G': 9.43, 'P': 9.43, 'D': 4.4, 'Y': 1.89, 'R': 6.92, 'T': 4.4, 'F': 1.89, 'V': 5.03, 'L': 10.06, 'A': 5.66, 'H': 3.77, 'E': 6.92, 'Q': 6.92, 'S': 6.92, 'W': 1.26, 'N': 2.52, 'C': 4.4, 'K': 4.4}
- **Common reported functions (%):** 0.0
- **Common reported locations (%):** 12.5
- **Common reported processes (%):** 0.0

- **AF ID:** O43374
- **Chain:** A
- **Protein length:** 803 aa
- **Resolution:** N/A
- **b-phipsi:** 0.002197
- **w-rdist:** 0.191802
- **t-alpha:** 0.023219
- **Chemical similarity (Tanimoto Index) (%):** 98.57
- **1D identity (%) [PDB]:** 2.12
- **1D identity (%) [Gaps excluded][PDB]:** 66.67
- **1D identity - Alignment Gaps [PDB]:** 1551
- **1D aligned content [PDB] (<aminoacid>:%):** {'A': 5.88, 'H': 8.82, 'L': 11.76, 'E': 5.88, 'V': 11.76, 'P': 14.71, 'D': 2.94, 'Q': 2.94, 'R': 2.94, 'S': 8.82, 'Y': 5.88, 'G': 11.76, 'F': 5.88}
- **2D identity (%) [PDB]:** 45.09
- **2D identity (%) [Gaps excluded][PDB]:** 87.06
- **2D identity - Alignment Gaps [PDB]:** 525
- **2D aligned content [PDB] (<2D-fold>:%):** {'.': 16.5, 'E': 30.75, 'T': 20.37, 'H': 31.16, 'G': 1.22}
- **3D similarity (TM-Score) (%) [PDB]:** 22.85

- **Gene name:** RASA4
- **Entrez ID:** 10156
- **RefSeq ID:** NM\_006989
- **Transcript sequence length:** 5604
- **5-UTR|CDS|3-UTR identity (%):** 12.78 | 43.45 | 35.63
- **5-UTR|CDS|3-UTR identity (%) [Gaps excluded]:** 85.25 | 74.7 | 74.88
- **5-UTR|CDS|3-UTR identity [Alignment Gaps]:** 346 | 1322 | 2632
- **5-UTR aligned content (<base>:%):** {'G': 30.77, 'C': 63.46, 'A': 3.85, 'T': 1.92}
- **CDS aligned content (<base>:%):** {'A': 23.74, 'T': 18.06, 'G': 29.93, 'C': 28.26}
- **3-UTR aligned content (<base>:%):** {'G': 25.32, 'C': 22.08, 'T': 27.84, 'A': 24.76}

**Uniprot Description:**  
  
 Ca(2+)-dependent Ras GTPase-activating protein, that switches off the Ras-MAPK pathway following a stimulus that elevates intracellular calcium. Functions as an adaptor for Cdc42 and Rac1 during FcR-mediated phagocytosis. N/A   
  
 **Gene Ontology Information:**

Molecular Function

- GTPase activator activity
- metal ion binding
- phospholipid binding

Location

- cytosol
- plasma membrane

Biological process

- cellular response to calcium ion
- intracellular signal transduction
- negative regulation of GTPase activity
- negative regulation of Ras protein signal transduction

---

12

- **Protein name:** Ras GTPase-activating protein 4B
- **Organism:** Homo sapiens
- **Uniprot Accession Number:** C9J798
- **Protein sequence length:** 803 aa
- **1D identity (%):** 15.92
- **1D identity (%) [Gaps excluded]:** 23.91
- **1D identity - Alignment Gaps:** 334
- **1D aligned content (<aminoacid>:%):** {'M': 0.63, 'I': 3.14, 'G': 9.43, 'P': 9.43, 'D': 4.4, 'Y': 1.89, 'R': 6.92, 'T': 4.4, 'F': 1.89, 'V': 5.03, 'L': 10.06, 'A': 5.66, 'H': 3.77, 'E': 6.92, 'Q': 6.92, 'S': 6.92, 'W': 1.26, 'N': 2.52, 'C': 4.4, 'K': 4.4}
- **Common reported functions (%):** 0.0
- **Common reported locations (%):** 12.5
- **Common reported processes (%):** 0.0

- **AF ID:** C9J798
- **Chain:** A
- **Protein length:** 803 aa
- **Resolution:** N/A
- **b-phipsi:** 0.002274
- **w-rdist:** 0.229713
- **t-alpha:** 0.014286
- **Chemical similarity (Tanimoto Index) (%):** 98.57
- **1D identity (%) [PDB]:** 2.12
- **1D identity (%) [Gaps excluded][PDB]:** 66.67
- **1D identity - Alignment Gaps [PDB]:** 1551
- **1D aligned content [PDB] (<aminoacid>:%):** {'A': 5.88, 'H': 8.82, 'L': 11.76, 'E': 5.88, 'V': 11.76, 'P': 14.71, 'D': 2.94, 'Q': 2.94, 'R': 2.94, 'S': 8.82, 'Y': 5.88, 'G': 11.76, 'F': 5.88}
- **2D identity (%) [PDB]:** 45.09
- **2D identity (%) [Gaps excluded][PDB]:** 87.06
- **2D identity - Alignment Gaps [PDB]:** 525
- **2D aligned content [PDB] (<2D-fold>:%):** {'.': 16.29, 'E': 30.75, 'T': 20.37, 'H': 31.36, 'G': 1.22}
- **3D similarity (TM-Score) (%) [PDB]:** 20.39

- **Gene name:** RASA4B
- **Entrez ID:** N/A
- **RefSeq ID:** N/A
- **Sequence length:** N/A
- **5-UTR|CDS|3-UTR identity (%):** N/A | N/A | N/A
- **5-UTR|CDS|3-UTR identity (%) [Gaps excluded]:** N/A | N/A | N/A
- **5-UTR|CDS|3-UTR identity [Alignment Gaps]:** N/A | N/A | N/A
- **5-UTR aligned content (<base>:%):** N/A
- **CDS aligned content (<base>:%):** N/A
- **3-UTR aligned content (<base>:%):** N/A

**Uniprot Description:**  
  
 Ca(2+)-dependent Ras GTPase-activating protein, that may play a role in the Ras-MAPK pathway. N/A   
  
 **Gene Ontology Information:**

Molecular Function

- GTPase activator activity
- metal ion binding
- phospholipid binding

Location

- cytosol
- plasma membrane

Biological process

- cellular response to calcium ion
- intracellular signal transduction
- negative regulation of Ras protein signal transduction
- regulation of GTPase activity

---

13

- **Protein name:** Kelch-like protein 14
- **Organism:** Homo sapiens
- **Uniprot Accession Number:** Q9P2G3
- **Protein sequence length:** 628 aa
- **1D identity (%):** 11.17
- **1D identity (%) [Gaps excluded]:** 23.05
- **1D identity - Alignment Gaps:** 517
- **1D aligned content (<aminoacid>:%):** {'G': 10.71, 'S': 3.57, 'F': 6.25, 'P': 14.29, 'L': 12.5, 'Q': 4.46, 'D': 6.25, 'V': 10.71, 'K': 3.57, 'E': 5.36, 'H': 1.79, 'T': 3.57, 'Y': 0.89, 'A': 3.57, 'N': 2.68, 'R': 4.46, 'C': 4.46, 'I': 0.89}
- **Common reported functions (%):** 0.0
- **Common reported locations (%):** 25.0
- **Common reported processes (%):** 0.0

- **AF ID:** Q9P2G3
- **Chain:** A
- **Protein length:** 628 aa
- **Resolution:** N/A
- **b-phipsi:** 0.002944
- **w-rdist:** 0.395314
- **t-alpha:** 0.002347
- **Chemical similarity (Tanimoto Index) (%):** 99.1
- **1D identity (%) [PDB]:** 2.09
- **1D identity (%) [Gaps excluded][PDB]:** 71.43
- **1D identity - Alignment Gaps [PDB]:** 1394
- **1D aligned content [PDB] (<aminoacid>:%):** {'M': 6.67, 'V': 10.0, 'G': 16.67, 'D': 3.33, 'P': 13.33, 'E': 6.67, 'I': 3.33, 'N': 10.0, 'R': 6.67, 'L': 6.67, 'A': 6.67, 'Q': 3.33, 'Y': 3.33, 'T': 3.33}
- **2D identity (%) [PDB]:** 33.4
- **2D identity (%) [Gaps excluded][PDB]:** 88.18
- **2D identity - Alignment Gaps [PDB]:** 666
- **2D aligned content [PDB] (<2D-fold>:%):** {'.': 20.95, 'H': 36.03, 'T': 25.7, 'E': 17.04, 'B': 0.28}
- **3D similarity (TM-Score) (%) [PDB]:** 21.35

- **Gene name:** KLHL14
- **Entrez ID:** 421100
- **RefSeq ID:** NM\_020805
- **Transcript sequence length:** 4271
- **5-UTR|CDS|3-UTR identity (%):** 36.95 | 38.94 | 27.06
- **5-UTR|CDS|3-UTR identity (%) [Gaps excluded]:** 70.29 | 75.28 | 74.16
- **5-UTR|CDS|3-UTR identity [Alignment Gaps]:** 249 | 1423 | 2917
- **5-UTR aligned content (<base>:%):** {'A': 9.28, 'C': 44.33, 'T': 14.95, 'G': 31.44}
- **CDS aligned content (<base>:%):** {'A': 26.66, 'T': 21.86, 'G': 26.83, 'C': 24.65}
- **3-UTR aligned content (<base>:%):** {'A': 32.02, 'C': 15.37, 'G': 17.14, 'T': 35.48}

**Uniprot Description:**  
  
 N/A   
  
Interacts with TOR1A, preferentially with the ATP-free form.   
  
 **Gene Ontology Information:**

Molecular Function   
  
N/A

Location

- cytoplasm
- cytosol
- endoplasmic reticulum
- endoplasmic reticulum membrane
- neuron projection
- neuronal cell body

Biological process   
  
N/A

---

14

- **Protein name:** Anti-Muellerian hormone type-2 receptor
- **Organism:** Homo sapiens
- **Uniprot Accession Number:** Q16671
- **Protein sequence length:** 573 aa
- **1D identity (%):** 9.61
- **1D identity (%) [Gaps excluded]:** 19.96
- **1D identity - Alignment Gaps:** 502
- **1D aligned content (<aminoacid>:%):** {'S': 4.3, 'L': 19.35, 'P': 19.35, 'T': 3.23, 'E': 4.3, 'D': 5.38, 'N': 1.08, 'F': 2.15, 'G': 5.38, 'A': 6.45, 'W': 2.15, 'K': 2.15, 'V': 4.3, 'R': 7.53, 'C': 5.38, 'M': 1.08, 'Y': 2.15, 'I': 1.08, 'H': 3.23}
- **Common reported functions (%):** 0.0
- **Common reported locations (%):** 0.0
- **Common reported processes (%):** 8.33

- **AF ID:** Q16671
- **Chain:** A
- **Protein length:** 573 aa
- **Resolution:** N/A
- **b-phipsi:** 0.005308
- **w-rdist:** 0.355536
- **t-alpha:** 0.002353
- **Chemical similarity (Tanimoto Index) (%):** 96.55
- **1D identity (%) [PDB]:** 2.24
- **1D identity (%) [Gaps excluded][PDB]:** 73.81
- **1D identity - Alignment Gaps [PDB]:** 1339
- **1D aligned content [PDB] (<aminoacid>:%):** {'L': 6.45, 'G': 16.13, 'I': 9.68, 'N': 3.23, 'V': 22.58, 'P': 9.68, 'R': 3.23, 'S': 3.23, 'F': 3.23, 'Q': 3.23, 'A': 12.9, 'K': 6.45}
- **2D identity (%) [PDB]:** 37.24
- **2D identity (%) [Gaps excluded][PDB]:** 87.74
- **2D identity - Alignment Gaps [PDB]:** 575
- **2D aligned content [PDB] (<2D-fold>:%):** {'.': 23.92, 'E': 17.47, 'T': 25.27, 'H': 32.8, 'B': 0.54}
- **3D similarity (TM-Score) (%) [PDB]:** 18.27

- **Gene name:** AMHR2
- **Entrez ID:** 269
- **RefSeq ID:** NM\_020547
- **Transcript sequence length:** 2001
- **5-UTR|CDS|3-UTR identity (%):** 12.5 | 38.29 | 2.63
- **5-UTR|CDS|3-UTR identity (%) [Gaps excluded]:** 69.86 | 76.43 | 77.03
- **5-UTR|CDS|3-UTR identity [Alignment Gaps]:** 335 | 1432 | 4188
- **5-UTR aligned content (<base>:%):** {'G': 35.29, 'T': 11.76, 'C': 41.18, 'A': 11.76}
- **CDS aligned content (<base>:%):** {'T': 21.47, 'G': 26.57, 'C': 28.57, 'A': 23.38}
- **3-UTR aligned content (<base>:%):** {'A': 27.19, 'T': 31.58, 'G': 23.68, 'C': 17.54}

**Uniprot Description:**  
  
 On ligand binding, forms a receptor complex consisting of two type II and two type I transmembrane serine/threonine kinases. Type II receptors phosphorylate and activate type I receptors which autophosphorylate, then bind and activate SMAD transcriptional regulators. Receptor for anti-Muellerian hormone.   
  
Interacts with type I receptor ACVR1.   
  
 **Gene Ontology Information:**

Molecular Function

- anti-Mullerian hormone receptor activity
- ATP binding
- hormone binding
- metal ion binding
- protein homodimerization activity
- transforming growth factor beta-activated receptor activity
- transforming growth factor beta receptor activity, type II

Location

- plasma membrane
- receptor complex

Biological process

- anti-Mullerian hormone signaling pathway
- blood vessel development
- BMP signaling pathway
- cellular response to growth factor stimulus
- female gonad development
- male gonad development
- Mullerian duct regression
- pathway-restricted SMAD protein phosphorylation
- protein phosphorylation
- sex differentiation
- transforming growth factor beta receptor signaling pathway

---

15

- **Protein name:** Probable polypeptide N-acetylgalactosaminyltransferase 8
- **Organism:** Homo sapiens
- **Uniprot Accession Number:** Q9NY28
- **Protein sequence length:** 637 aa
- **1D identity (%):** 13.51
- **1D identity (%) [Gaps excluded]:** 24.81
- **1D identity - Alignment Gaps:** 442
- **1D aligned content (<aminoacid>:%):** {'F': 2.29, 'R': 7.63, 'P': 9.16, 'L': 12.21, 'I': 4.58, 'Q': 2.29, 'N': 3.05, 'K': 9.92, 'V': 6.11, 'E': 3.05, 'M': 3.05, 'T': 5.34, 'A': 4.58, 'H': 2.29, 'G': 6.11, 'Y': 2.29, 'C': 4.58, 'S': 3.82, 'D': 5.34, 'W': 2.29}
- **Common reported functions (%):** 0.0
- **Common reported locations (%):** 0.0
- **Common reported processes (%):** 0.0

- **AF ID:** Q9NY28
- **Chain:** A
- **Protein length:** 637 aa
- **Resolution:** N/A
- **b-phipsi:** 0.003746
- **w-rdist:** 0.517117
- **t-alpha:** 0.0
- **Chemical similarity (Tanimoto Index) (%):** 97.12
- **1D identity (%) [PDB]:** 3.27
- **1D identity (%) [Gaps excluded][PDB]:** 58.23
- **1D identity - Alignment Gaps [PDB]:** 1329
- **1D aligned content [PDB] (<aminoacid>:%):** {'V': 8.7, 'S': 10.87, 'T': 8.7, 'L': 13.04, 'N': 4.35, 'K': 6.52, 'G': 6.52, 'P': 8.7, 'Q': 8.7, 'F': 2.17, 'I': 4.35, 'D': 4.35, 'A': 4.35, 'R': 4.35, 'Y': 2.17, 'C': 2.17}
- **2D identity (%) [PDB]:** 37.49
- **2D identity (%) [Gaps excluded][PDB]:** 90.37
- **2D identity - Alignment Gaps [PDB]:** 615
- **2D aligned content [PDB] (<2D-fold>:%):** {'.': 15.74, 'H': 41.37, 'T': 23.1, 'G': 1.52, 'E': 18.02, 'B': 0.25}
- **3D similarity (TM-Score) (%) [PDB]:** 22.17

- **Gene name:** GALNT8
- **Entrez ID:** 26290
- **RefSeq ID:** NM\_017417
- **Transcript sequence length:** 2321
- **5-UTR|CDS|3-UTR identity (%):** 25.81 | 45.59 | 2.12
- **5-UTR|CDS|3-UTR identity (%) [Gaps excluded]:** 69.95 | 75.31 | 73.39
- **5-UTR|CDS|3-UTR identity [Alignment Gaps]:** 313 | 1106 | 4166
- **5-UTR aligned content (<base>:%):** {'G': 36.72, 'T': 10.94, 'C': 39.84, 'A': 12.5}
- **CDS aligned content (<base>:%):** {'A': 28.09, 'T': 22.61, 'G': 25.59, 'C': 23.71}
- **3-UTR aligned content (<base>:%):** {'T': 37.36, 'C': 17.58, 'A': 24.18, 'G': 20.88}

**Uniprot Description:**  
  
 Probably catalyzes the initial reaction in O-linked oligosaccharide biosynthesis, the transfer of an N-acetyl-D-galactosamine residue to a serine or threonine residue on the protein receptor. N/A   
  
 **Gene Ontology Information:**

Molecular Function

- carbohydrate binding
- metal ion binding
- polypeptide N-acetylgalactosaminyltransferase activity

Location

- Golgi apparatus
- Golgi membrane

Biological process

- O-glycan processing
- protein O-linked glycosylation

---

16

- **Protein name:** Dual specificity protein phosphatase 10
- **Organism:** Homo sapiens
- **Uniprot Accession Number:** Q9Y6W6
- **Protein sequence length:** 482 aa
- **1D identity (%):** 10.83
- **1D identity (%) [Gaps excluded]:** 23.81
- **1D identity - Alignment Gaps:** 503
- **1D aligned content (<aminoacid>:%):** {'P': 9.0, 'L': 14.0, 'D': 4.0, 'V': 6.0, 'R': 3.0, 'G': 9.0, 'N': 3.0, 'T': 6.0, 'S': 6.0, 'A': 5.0, 'C': 3.0, 'Y': 5.0, 'K': 4.0, 'Q': 6.0, 'I': 10.0, 'E': 4.0, 'F': 2.0, 'M': 1.0}
- **Common reported functions (%):** 0.0
- **Common reported locations (%):** 25.0
- **Common reported processes (%):** 0.0

- **AF ID:** Q9Y6W6
- **Chain:** A
- **Protein length:** 482 aa
- **Resolution:** N/A
- **b-phipsi:** 0.008707
- **w-rdist:** 0.36749
- **t-alpha:** 0.000782
- **Chemical similarity (Tanimoto Index) (%):** 85.67
- **1D identity (%) [PDB]:** 2.09
- **1D identity (%) [Gaps excluded][PDB]:** 69.23
- **1D identity - Alignment Gaps [PDB]:** 1254
- **1D aligned content [PDB] (<aminoacid>:%):** {'M': 3.7, 'C': 3.7, 'T': 7.41, 'V': 7.41, 'A': 3.7, 'Y': 3.7, 'F': 3.7, 'K': 7.41, 'Q': 11.11, 'S': 3.7, 'L': 22.22, 'P': 7.41, 'G': 3.7, 'E': 7.41, 'H': 3.7}
- **2D identity (%) [PDB]:** 30.11
- **2D identity (%) [Gaps excluded][PDB]:** 91.79
- **2D identity - Alignment Gaps [PDB]:** 674
- **2D aligned content [PDB] (<2D-fold>:%):** {'.': 22.85, 'T': 26.16, 'E': 15.23, 'H': 35.76}
- **3D similarity (TM-Score) (%) [PDB]:** 17.01

- **Gene name:** DUSP10
- **Entrez ID:** 541175
- **RefSeq ID:** NM\_007207
- **Transcript sequence length:** 2589
- **5-UTR|CDS|3-UTR identity (%):** 15.1 | 32.94 | 15.25
- **5-UTR|CDS|3-UTR identity (%) [Gaps excluded]:** 71.88 | 75.65 | 76.14
- **5-UTR|CDS|3-UTR identity [Alignment Gaps]:** 361 | 1587 | 3513
- **5-UTR aligned content (<base>:%):** {'C': 27.54, 'T': 23.19, 'G': 42.03, 'A': 7.25}
- **CDS aligned content (<base>:%):** {'A': 27.97, 'G': 24.41, 'C': 25.49, 'T': 22.14}
- **3-UTR aligned content (<base>:%):** {'C': 12.39, 'A': 32.99, 'T': 38.06, 'G': 16.57}

**Uniprot Description:**  
  
 Protein phosphatase involved in the inactivation of MAP kinases. Has a specificity for the MAPK11/MAPK12/MAPK13/MAPK14 subfamily. It preferably dephosphorylates p38.   
  
Monomer. Interacts with MAPK14.   
  
 **Gene Ontology Information:**

Molecular Function

- JUN kinase binding
- MAP kinase phosphatase activity
- MAP kinase tyrosine phosphatase activity
- MAP kinase tyrosine/serine/threonine phosphatase activity
- mitogen-activated protein kinase p38 binding
- myosin phosphatase activity
- protein tyrosine/threonine phosphatase activity

Location

- cytoplasm
- cytosol
- nucleoplasm

Biological process

- negative regulation of epithelial cell migration
- negative regulation of epithelial cell proliferation
- negative regulation of epithelium regeneration
- negative regulation of ERK1 and ERK2 cascade
- negative regulation of JUN kinase activity
- negative regulation of p38MAPK cascade
- negative regulation of protein kinase activity by regulation of protein phosphorylation
- negative regulation of respiratory burst involved in inflammatory response
- oligodendrocyte differentiation
- peptidyl-threonine dephosphorylation
- peptidyl-tyrosine dephosphorylation involved in inactivation of protein kinase activity
- positive regulation of regulatory T cell differentiation
- regulation of adaptive immune response
- regulation of brown fat cell differentiation
- response to lipopolysaccharide
- stress-activated MAPK cascade

---

17

- **Protein name:** Epididymis-specific alpha-mannosidase
- **Organism:** Homo sapiens
- **Uniprot Accession Number:** Q9Y2E5
- **Protein sequence length:** 1009 aa
- **1D identity (%):** 18.63
- **1D identity (%) [Gaps excluded]:** 25.41
- **1D identity - Alignment Gaps:** 288
- **1D aligned content (<aminoacid>:%):** {'M': 1.0, 'L': 12.44, 'P': 11.94, 'G': 9.95, 'I': 2.49, 'R': 7.46, 'F': 3.48, 'V': 9.45, 'D': 3.98, 'Y': 3.48, 'Q': 6.97, 'E': 1.49, 'T': 4.48, 'W': 1.0, 'A': 7.96, 'H': 4.48, 'S': 3.48, 'N': 2.49, 'C': 1.49, 'K': 0.5}
- **Common reported functions (%):** 0.0
- **Common reported locations (%):** 0.0
- **Common reported processes (%):** 0.0

- **AF ID:** Q9Y2E5
- **Chain:** A
- **Protein length:** 1009 aa
- **Resolution:** N/A
- **b-phipsi:** 0.002156
- **w-rdist:** 0.282999
- **t-alpha:** 0.011737
- **Chemical similarity (Tanimoto Index) (%):** 97.19
- **1D identity (%) [PDB]:** 2.85
- **1D identity (%) [Gaps excluded][PDB]:** 73.91
- **1D identity - Alignment Gaps [PDB]:** 1721
- **1D aligned content [PDB] (<aminoacid>:%):** {'V': 15.69, 'W': 1.96, 'S': 5.88, 'L': 15.69, 'Q': 5.88, 'A': 5.88, 'G': 7.84, 'H': 5.88, 'N': 1.96, 'P': 7.84, 'D': 3.92, 'I': 1.96, 'T': 1.96, 'M': 1.96, 'R': 3.92, 'Y': 3.92, 'E': 3.92, 'F': 3.92}
- **2D identity (%) [PDB]:** 41.04
- **2D identity (%) [Gaps excluded][PDB]:** 88.93
- **2D identity - Alignment Gaps [PDB]:** 685
- **2D aligned content [PDB] (<2D-fold>:%):** {'.': 19.54, 'E': 17.62, 'T': 29.5, 'H': 32.95, 'B': 0.38}
- **3D similarity (TM-Score) (%) [PDB]:** 24.61

- **Gene name:** MAN2B2
- **Entrez ID:** 23324
- **RefSeq ID:** NM\_015274
- **Transcript sequence length:** 5129
- **5-UTR|CDS|3-UTR identity (%):** 3.96 | 46.26 | 26.62
- **5-UTR|CDS|3-UTR identity (%) [Gaps excluded]:** 84.21 | 73.8 | 77.6
- **5-UTR|CDS|3-UTR identity [Alignment Gaps]:** 385 | 1288 | 3112
- **5-UTR aligned content (<base>:%):** {'A': 6.25, 'C': 50.0, 'T': 12.5, 'G': 31.25}
- **CDS aligned content (<base>:%):** {'A': 23.98, 'T': 19.47, 'G': 28.37, 'C': 28.18}
- **3-UTR aligned content (<base>:%):** {'G': 27.52, 'C': 20.7, 'A': 28.95, 'T': 22.84}

**Uniprot Description:**  
  
 N/A N/A   
  
 **Gene Ontology Information:**

Molecular Function

- alpha-mannosidase activity
- carbohydrate binding
- metal ion binding

Location

- extracellular exosome
- lysosomal lumen
- lysosome

Biological process

- mannose metabolic process
- oligosaccharide catabolic process

---

18

- **Protein name:** Mediator of RNA polymerase II transcription subunit 16
- **Organism:** Homo sapiens
- **Uniprot Accession Number:** Q9Y2X0
- **Protein sequence length:** 877 aa
- **1D identity (%):** 15.18
- **1D identity (%) [Gaps excluded]:** 23.39
- **1D identity - Alignment Gaps:** 370
- **1D aligned content (<aminoacid>:%):** {'R': 5.62, 'P': 10.62, 'G': 7.5, 'Y': 2.5, 'L': 11.88, 'D': 7.5, 'T': 7.5, 'W': 1.25, 'S': 8.75, 'A': 5.0, 'H': 3.12, 'V': 5.62, 'F': 3.75, 'E': 2.5, 'I': 3.12, 'N': 1.25, 'C': 5.0, 'M': 0.62, 'K': 3.75, 'Q': 3.12}
- **Common reported functions (%):** 0.0
- **Common reported locations (%):** 25.0
- **Common reported processes (%):** 0.0

- **AF ID:** Q9Y2X0
- **Chain:** A
- **Protein length:** 877 aa
- **Resolution:** N/A
- **b-phipsi:** 0.001049
- **w-rdist:** 0.374255
- **t-alpha:** 0.022692
- **Chemical similarity (Tanimoto Index) (%):** 96.12
- **1D identity (%) [PDB]:** 1.9
- **1D identity (%) [Gaps excluded][PDB]:** 80.0
- **1D identity - Alignment Gaps [PDB]:** 1647
- **1D aligned content [PDB] (<aminoacid>:%):** {'R': 12.5, 'P': 15.62, 'G': 3.12, 'L': 9.38, 'T': 3.12, 'V': 6.25, 'K': 6.25, 'A': 3.12, 'N': 3.12, 'F': 3.12, 'Q': 6.25, 'I': 9.38, 'D': 9.38, 'Y': 3.12, 'H': 3.12, 'E': 3.12}
- **2D identity (%) [PDB]:** 37.99
- **2D identity (%) [Gaps excluded][PDB]:** 92.45
- **2D identity - Alignment Gaps [PDB]:** 721
- **2D aligned content [PDB] (<2D-fold>:%):** {'.': 15.05, 'E': 26.24, 'T': 24.3, 'H': 33.76, 'G': 0.65}
- **3D similarity (TM-Score) (%) [PDB]:** 23.96

- **Gene name:** MED16
- **Entrez ID:** 10025
- **RefSeq ID:** NM\_005481
- **Transcript sequence length:** 2892
- **5-UTR|CDS|3-UTR identity (%):** 17.99 | 45.28 | 1.78
- **5-UTR|CDS|3-UTR identity (%) [Gaps excluded]:** 82.8 | 74.34 | 70.64
- **5-UTR|CDS|3-UTR identity [Alignment Gaps]:** 335 | 1268 | 4205
- **5-UTR aligned content (<base>:%):** {'A': 12.99, 'G': 44.16, 'C': 40.26, 'T': 2.6}
- **CDS aligned content (<base>:%):** {'A': 21.38, 'T': 18.72, 'G': 29.88, 'C': 30.02}
- **3-UTR aligned content (<base>:%):** {'G': 27.27, 'C': 33.77, 'T': 19.48, 'A': 19.48}

**Uniprot Description:**  
  
 Component of the Mediator complex, a coactivator involved in the regulated transcription of nearly all RNA polymerase II-dependent genes. Mediator functions as a bridge to convey information from gene-specific regulatory proteins to the basal RNA polymerase II transcription machinery. Mediator is recruited to promoters by direct interactions with regulatory proteins and serves as a scaffold for the assembly of a functional preinitiation complex with RNA polymerase II and the general transcription factors.   
  
Component of the Mediator complex, which is composed of MED1, MED4, MED6, MED7, MED8, MED9, MED10, MED11, MED12, MED13, MED13L, MED14, MED15, MED16, MED17, MED18, MED19, MED20, MED21, MED22, MED23, MED24, MED25, MED26, MED27, MED29, MED30, MED31, CCNC, CDK8 and CDC2L6/CDK11. The MED12, MED13, CCNC and CDK8 subunits form a distinct module termed the CDK8 module. Mediator containing the CDK8 module is less active than Mediator lacking this module in supporting transcriptional activation. Individual preparations of the Mediator complex lacking one or more distinct subunits have been variously termed ARC, CRSP, DRIP, PC2, SMCC and TRAP.   
  
 **Gene Ontology Information:**

Molecular Function

- thyroid hormone receptor binding
- vitamin D receptor binding
- transcription coactivator activity

Location

- core mediator complex
- mediator complex
- membrane
- nucleoplasm
- nucleus

Biological process

- positive regulation of transcription, DNA-templated
- positive regulation of transcription elongation from RNA polymerase II promoter
- positive regulation of transcription initiation from RNA polymerase II promoter
- regulation of transcription by RNA polymerase II
- RNA polymerase II preinitiation complex assembly
- transcription by RNA polymerase II

---

19

- **Protein name:** Serine protease FAM111A
- **Organism:** Homo sapiens
- **Uniprot Accession Number:** Q96PZ2
- **Protein sequence length:** 611 aa
- **1D identity (%):** 11.34
- **1D identity (%) [Gaps excluded]:** 21.91
- **1D identity - Alignment Gaps:** 468
- **1D aligned content (<aminoacid>:%):** {'K': 13.64, 'R': 3.64, 'V': 11.82, 'M': 0.91, 'T': 2.73, 'E': 5.45, 'G': 12.73, 'Q': 6.36, 'P': 7.27, 'Y': 3.64, 'L': 4.55, 'N': 1.82, 'S': 4.55, 'A': 1.82, 'I': 6.36, 'F': 3.64, 'C': 2.73, 'D': 4.55, 'H': 1.82}
- **Common reported functions (%):** 0.0
- **Common reported locations (%):** 25.0
- **Common reported processes (%):** 0.0

- **AF ID:** Q96PZ2
- **Chain:** A
- **Protein length:** 611 aa
- **Resolution:** N/A
- **b-phipsi:** 0.002947
- **w-rdist:** 0.246458
- **t-alpha:** 0.012679
- **Chemical similarity (Tanimoto Index) (%):** 99.32
- **1D identity (%) [PDB]:** 2.26
- **1D identity (%) [Gaps excluded][PDB]:** 66.67
- **1D identity - Alignment Gaps [PDB]:** 1365
- **1D aligned content [PDB] (<aminoacid>:%):** {'M': 6.25, 'R': 9.38, 'K': 12.5, 'V': 9.38, 'C': 3.12, 'N': 6.25, 'T': 3.12, 'Q': 9.38, 'L': 15.62, 'E': 6.25, 'G': 3.12, 'A': 3.12, 'Y': 6.25, 'F': 3.12, 'H': 3.12}
- **2D identity (%) [PDB]:** 39.13
- **2D identity (%) [Gaps excluded][PDB]:** 89.64
- **2D identity - Alignment Gaps [PDB]:** 573
- **2D aligned content [PDB] (<2D-fold>:%):** {'.': 21.86, 'T': 24.12, 'E': 23.87, 'H': 30.15}
- **3D similarity (TM-Score) (%) [PDB]:** 20.74

- **Gene name:** FAM111A
- **Entrez ID:** 63901
- **RefSeq ID:** NM\_198847
- **Transcript sequence length:** 3473
- **5-UTR|CDS|3-UTR identity (%):** 13.32 | 37.73 | 20.97
- **5-UTR|CDS|3-UTR identity (%) [Gaps excluded]:** 61.11 | 73.8 | 76.13
- **5-UTR|CDS|3-UTR identity [Alignment Gaps]:** 323 | 1430 | 3306
- **5-UTR aligned content (<base>:%):** {'G': 38.18, 'C': 40.0, 'T': 14.55, 'A': 7.27}
- **CDS aligned content (<base>:%):** {'T': 23.64, 'G': 24.09, 'A': 33.97, 'C': 18.3}
- **3-UTR aligned content (<base>:%):** {'G': 15.88, 'A': 28.94, 'T': 37.83, 'C': 17.35}

**Uniprot Description:**  
  
 Single-stranded DNA-binding serine protease that mediates the proteolytic cleavage of covalent DNA-protein cross-links (DPCs) during DNA synthesis, thereby playing a key role in maintaining genomic integrity (PubMed:32165630). DPCs are highly toxic DNA lesions that interfere with essential chromatin transactions, such as replication and transcription, and which are induced by reactive agents, such as UV light or formaldehyde (PubMed:32165630). Protects replication fork from stalling by removing DPCs, such as covalently trapped topoisomerase 1 (TOP1) adducts on DNA lesion, or poly(ADP-ribose) polymerase 1 (PARP1)-DNA complexes trapped by PARP inhibitors (PubMed:32165630). Required for PCNA loading on replication sites (PubMed:24561620). Promotes S-phase entry and DNA synthesis (PubMed:24561620).   
  
Interacts (via PIP-box) with PCNA; then interaction is direct.   
  
 **Gene Ontology Information:**

Molecular Function

- peptidase activity
- single-stranded DNA binding

Location

- chromatin
- cytoplasm
- fibrillar center
- nucleoplasm
- nucleus

Biological process

- cellular response to DNA damage stimulus
- DNA replication
- negative regulation of viral genome replication
- protein autoprocessing
- protein-DNA covalent cross-linking repair
- proteolysis
- replication fork processing

---

20

- **Protein name:** Exostosin-like 1
- **Organism:** Homo sapiens
- **Uniprot Accession Number:** Q92935
- **Protein sequence length:** 676 aa
- **1D identity (%):** 15.2
- **1D identity (%) [Gaps excluded]:** 23.99
- **1D identity - Alignment Gaps:** 345
- **1D aligned content (<aminoacid>:%):** {'Q': 6.29, 'W': 2.8, 'L': 18.18, 'G': 9.09, 'P': 13.99, 'A': 5.59, 'R': 5.59, 'S': 4.9, 'E': 5.59, 'F': 5.59, 'V': 6.29, 'Y': 1.4, 'D': 3.5, 'T': 6.29, 'M': 0.7, 'H': 1.4, 'K': 1.4, 'I': 0.7, 'C': 0.7}
- **Common reported functions (%):** 0.0
- **Common reported locations (%):** 0.0
- **Common reported processes (%):** 0.0

- **AF ID:** Q92935
- **Chain:** A
- **Protein length:** 676 aa
- **Resolution:** N/A
- **b-phipsi:** 0.005705
- **w-rdist:** 0.395491
- **t-alpha:** 0.001568
- **Chemical similarity (Tanimoto Index) (%):** 96.19
- **1D identity (%) [PDB]:** 1.74
- **1D identity (%) [Gaps excluded][PDB]:** 83.87
- **1D identity - Alignment Gaps [PDB]:** 1464
- **1D aligned content [PDB] (<aminoacid>:%):** {'M': 3.85, 'Y': 3.85, 'T': 7.69, 'A': 3.85, 'H': 3.85, 'P': 11.54, 'L': 7.69, 'G': 11.54, 'R': 7.69, 'D': 11.54, 'V': 11.54, 'E': 3.85, 'K': 3.85, 'S': 3.85, 'Q': 3.85}
- **2D identity (%) [PDB]:** 38.71
- **2D identity (%) [Gaps excluded][PDB]:** 89.98
- **2D identity - Alignment Gaps [PDB]:** 608
- **2D aligned content [PDB] (<2D-fold>:%):** {'H': 34.62, '.': 17.92, 'T': 28.81, 'E': 16.71, 'B': 0.73, 'G': 1.21}
- **3D similarity (TM-Score) (%) [PDB]:** 20.12

- **Gene name:** EXTL1
- **Entrez ID:** 2134
- **RefSeq ID:** NM\_004455
- **Transcript sequence length:** 4020
- **5-UTR|CDS|3-UTR identity (%):** 33.11 | 38.63 | 14.49
- **5-UTR|CDS|3-UTR identity (%) [Gaps excluded]:** 79.89 | 74.76 | 73.04
- **5-UTR|CDS|3-UTR identity [Alignment Gaps]:** 527 | 1471 | 3614
- **5-UTR aligned content (<base>:%):** {'T': 7.72, 'G': 39.93, 'C': 45.64, 'A': 6.71}
- **CDS aligned content (<base>:%):** {'A': 21.77, 'T': 21.85, 'G': 25.85, 'C': 30.53}
- **3-UTR aligned content (<base>:%):** {'G': 35.07, 'C': 26.34, 'A': 19.91, 'T': 18.68}

**Uniprot Description:**  
  
 Glycosyltransferase required for the biosynthesis of heparan-sulfate (HS) (PubMed:11390981). Transfers N-acetyl-alpha-D-glucosamine to the nascent HS chain (GlcNAcT-II activity) (PubMed:11390981). Appears to lack GlcNAcT I and GlcAT-II activities (PubMed:11390981). N/A   
  
 **Gene Ontology Information:**

Molecular Function

- acetylglucosaminyltransferase activity
- glucuronosyl-N-acetylglucosaminyl-proteoglycan 4-alpha-N-acetylglucosaminyltransferase activity
- glucuronosyltransferase activity

Location

- endoplasmic reticulum membrane
- Golgi apparatus

Biological process

- heparan sulfate proteoglycan biosynthetic process
- protein glycosylation
- skeletal system development

---

21

- **Protein name:** RasGAP-activating-like protein 1
- **Organism:** Homo sapiens
- **Uniprot Accession Number:** O95294
- **Protein sequence length:** 804 aa
- **1D identity (%):** 15.85
- **1D identity (%) [Gaps excluded]:** 26.86
- **1D identity - Alignment Gaps:** 429
- **1D aligned content (<aminoacid>:%):** {'S': 5.42, 'V': 7.83, 'R': 9.64, 'L': 12.65, 'P': 8.43, 'G': 6.63, 'W': 2.41, 'D': 4.82, 'A': 6.63, 'F': 2.41, 'Y': 1.2, 'T': 4.22, 'N': 2.41, 'Q': 6.02, 'C': 4.22, 'I': 1.81, 'K': 3.61, 'M': 1.81, 'E': 4.82, 'H': 3.01}
- **Common reported functions (%):** 0.0
- **Common reported locations (%):** 12.5
- **Common reported processes (%):** 0.0

- **AF ID:** O95294
- **Chain:** A
- **Protein length:** 804 aa
- **Resolution:** N/A
- **b-phipsi:** 0.004172
- **w-rdist:** 0.192397
- **t-alpha:** 0.0224
- **Chemical similarity (Tanimoto Index) (%):** 98.5
- **1D identity (%) [PDB]:** 2.51
- **1D identity (%) [Gaps excluded][PDB]:** 63.49
- **1D identity - Alignment Gaps [PDB]:** 1528
- **1D aligned content [PDB] (<aminoacid>:%):** {'K': 2.5, 'S': 10.0, 'V': 15.0, 'L': 15.0, 'Q': 5.0, 'E': 5.0, 'G': 10.0, 'H': 2.5, 'D': 5.0, 'A': 2.5, 'R': 7.5, 'P': 7.5, 'M': 2.5, 'T': 2.5, 'F': 5.0, 'Y': 2.5}
- **2D identity (%) [PDB]:** 42.67
- **2D identity (%) [Gaps excluded][PDB]:** 88.99
- **2D identity - Alignment Gaps [PDB]:** 582
- **2D aligned content [PDB] (<2D-fold>:%):** {'.': 19.08, 'E': 30.19, 'T': 17.61, 'H': 31.87, 'G': 1.26}
- **3D similarity (TM-Score) (%) [PDB]:** 24.06

- **Gene name:** RASAL1
- **Entrez ID:** 8437
- **RefSeq ID:** N/A
- **Sequence length:** N/A
- **5-UTR|CDS|3-UTR identity (%):** N/A | N/A | N/A
- **5-UTR|CDS|3-UTR identity (%) [Gaps excluded]:** N/A | N/A | N/A
- **5-UTR|CDS|3-UTR identity [Alignment Gaps]:** N/A | N/A | N/A
- **5-UTR aligned content (<base>:%):** N/A
- **CDS aligned content (<base>:%):** N/A
- **3-UTR aligned content (<base>:%):** N/A

**Uniprot Description:**  
  
 Probable inhibitory regulator of the Ras-cyclic AMP pathway (PubMed:9751798). Plays a role in dendrite formation by melanocytes (PubMed:23999003). N/A   
  
 **Gene Ontology Information:**

Molecular Function

- GTPase activator activity
- metal ion binding
- phospholipid binding

Location

- cytosol

Biological process

- cell differentiation
- cellular response to calcium ion
- intracellular signal transduction
- negative regulation of Ras protein signal transduction
- positive regulation of dendrite extension
- regulation of GTPase activity
- signal transduction

---

22

- **Protein name:** tRNA wybutosine-synthesizing protein 2 homolog
- **Organism:** Homo sapiens
- **Uniprot Accession Number:** Q53H54
- **Protein sequence length:** 448 aa
- **1D identity (%):** 9.66
- **1D identity (%) [Gaps excluded]:** 23.87
- **1D identity - Alignment Gaps:** 555
- **1D aligned content (<aminoacid>:%):** {'M': 2.22, 'N': 1.11, 'V': 11.11, 'S': 2.22, 'P': 14.44, 'E': 3.33, 'Q': 11.11, 'L': 10.0, 'D': 4.44, 'R': 5.56, 'G': 7.78, 'H': 2.22, 'C': 4.44, 'T': 3.33, 'K': 5.56, 'A': 5.56, 'I': 3.33, 'Y': 1.11, 'F': 1.11}
- **Common reported functions (%):** 0.0
- **Common reported locations (%):** 12.5
- **Common reported processes (%):** 0.0

- **AF ID:** Q53H54
- **Chain:** A
- **Protein length:** 448 aa
- **Resolution:** N/A
- **b-phipsi:** 0.00226
- **w-rdist:** 0.437018
- **t-alpha:** 0.00314
- **Chemical similarity (Tanimoto Index) (%):** 99.24
- **1D identity (%) [PDB]:** 2.22
- **1D identity (%) [Gaps excluded][PDB]:** 71.79
- **1D identity - Alignment Gaps [PDB]:** 1220
- **1D aligned content [PDB] (<aminoacid>:%):** {'N': 10.71, 'V': 17.86, 'T': 3.57, 'P': 10.71, 'Q': 7.14, 'L': 14.29, 'C': 3.57, 'K': 7.14, 'A': 3.57, 'G': 7.14, 'I': 3.57, 'H': 3.57, 'R': 3.57, 'S': 3.57}
- **2D identity (%) [PDB]:** 31.41
- **2D identity (%) [Gaps excluded][PDB]:** 87.46
- **2D identity - Alignment Gaps [PDB]:** 612
- **2D aligned content [PDB] (<2D-fold>:%):** {'.': 19.0, 'T': 25.33, 'H': 33.33, 'E': 20.33, 'G': 2.0}
- **3D similarity (TM-Score) (%) [PDB]:** 18.25

- **Gene name:** TRMT12
- **Entrez ID:** 55039
- **RefSeq ID:** NM\_017956
- **Transcript sequence length:** 2207
- **5-UTR|CDS|3-UTR identity (%):** 12.39 | 31.6 | 10.14
- **5-UTR|CDS|3-UTR identity (%) [Gaps excluded]:** 73.97 | 75.73 | 72.61
- **5-UTR|CDS|3-UTR identity [Alignment Gaps]:** 363 | 1617 | 3803
- **5-UTR aligned content (<base>:%):** {'T': 24.07, 'C': 20.37, 'G': 48.15, 'A': 7.41}
- **CDS aligned content (<base>:%):** {'A': 27.37, 'T': 23.26, 'G': 27.02, 'C': 22.35}
- **3-UTR aligned content (<base>:%):** {'A': 28.12, 'G': 24.33, 'T': 33.26, 'C': 14.29}

**Uniprot Description:**  
  
 S-adenosyl-L-methionine-dependent transferase that acts as a component of the wybutosine biosynthesis pathway. Wybutosine is a hyper modified guanosine with a tricyclic base found at the 3'-position adjacent to the anticodon of eukaryotic phenylalanine tRNA. Catalyzes the transfer of the alpha-amino-alpha-carboxypropyl (acp) group from S-adenosyl-L-methionine to the C-7 position of 4-demethylwyosine (imG-14) to produce wybutosine-86. N/A   
  
 **Gene Ontology Information:**

Molecular Function

- tRNA 4-demethylwyosine alpha-amino-alpha-carboxypropyltransferase activity
- tRNA methyltransferase activity

Location

- cytoplasm

Biological process

- tRNA methylation
- wybutosine biosynthetic process

---

23

- **Protein name:** Kelch-like protein 7
- **Organism:** Homo sapiens
- **Uniprot Accession Number:** Q8IXQ5
- **Protein sequence length:** 586 aa
- **1D identity (%):** 11.59
- **1D identity (%) [Gaps excluded]:** 20.42
- **1D identity - Alignment Gaps:** 399
- **1D aligned content (<aminoacid>:%):** {'K': 8.41, 'T': 7.48, 'E': 7.48, 'L': 9.35, 'M': 1.87, 'C': 5.61, 'V': 6.54, 'R': 3.74, 'H': 3.74, 'I': 2.8, 'D': 4.67, 'Q': 6.54, 'S': 3.74, 'P': 6.54, 'N': 2.8, 'Y': 4.67, 'A': 2.8, 'G': 7.48, 'F': 2.8, 'W': 0.93}
- **Common reported functions (%):** 0.0
- **Common reported locations (%):** 12.5
- **Common reported processes (%):** 0.0

- **AF ID:** Q8IXQ5
- **Chain:** A
- **Protein length:** 586 aa
- **Resolution:** N/A
- **b-phipsi:** 0.002526
- **w-rdist:** 0.676686
- **t-alpha:** 0.000783
- **Chemical similarity (Tanimoto Index) (%):** 96.19
- **1D identity (%) [PDB]:** 2.22
- **1D identity (%) [Gaps excluded][PDB]:** 79.49
- **1D identity - Alignment Gaps [PDB]:** 1358
- **1D aligned content [PDB] (<aminoacid>:%):** {'I': 3.23, 'P': 3.23, 'A': 19.35, 'Y': 6.45, 'R': 12.9, 'L': 6.45, 'H': 9.68, 'V': 6.45, 'D': 6.45, 'K': 3.23, 'E': 3.23, 'G': 9.68, 'S': 6.45, 'Q': 3.23}
- **2D identity (%) [PDB]:** 35.55
- **2D identity (%) [Gaps excluded][PDB]:** 88.35
- **2D identity - Alignment Gaps [PDB]:** 612
- **2D aligned content [PDB] (<2D-fold>:%):** {'.': 15.66, 'H': 41.48, 'T': 22.8, 'E': 18.96, 'G': 0.82, 'B': 0.27}
- **3D similarity (TM-Score) (%) [PDB]:** 19.63

- **Gene name:** KLHL7
- **Entrez ID:** 55975
- **RefSeq ID:** N/A
- **Sequence length:** N/A
- **5-UTR|CDS|3-UTR identity (%):** N/A | N/A | N/A
- **5-UTR|CDS|3-UTR identity (%) [Gaps excluded]:** N/A | N/A | N/A
- **5-UTR|CDS|3-UTR identity [Alignment Gaps]:** N/A | N/A | N/A
- **5-UTR aligned content (<base>:%):** N/A
- **CDS aligned content (<base>:%):** N/A
- **3-UTR aligned content (<base>:%):** N/A

**Uniprot Description:**  
  
 Substrate-specific adapter of a BCR (BTB-CUL3-RBX1) E3 ubiquitin ligase complex. The BCR(KLHL7) complex acts by mediating ubiquitination and subsequent degradation of substrate proteins. Probably mediates 'Lys-48'-linked ubiquitination.   
  
Homodimer. Component of the BCR(KLHL7) E3 ubiquitin ligase complex, at least composed of CUL3 and KLHL7 and RBX1.   
  
 **Gene Ontology Information:**

Molecular Function

- identical protein binding
- protein homodimerization activity

Location

- Cul3-RING ubiquitin ligase complex
- cytosol
- nucleolus
- nucleoplasm
- perinuclear region of cytoplasm
- plasma membrane

Biological process

- protein ubiquitination

---

24

- **Protein name:** Piwi-like protein 4
- **Organism:** Homo sapiens
- **Uniprot Accession Number:** Q7Z3Z4
- **Protein sequence length:** 852 aa
- **1D identity (%):** 20.89
- **1D identity (%) [Gaps excluded]:** 27.08
- **1D identity - Alignment Gaps:** 221
- **1D aligned content (<aminoacid>:%):** {'M': 0.99, 'G': 8.91, 'S': 4.95, 'F': 5.45, 'R': 8.91, 'P': 7.92, 'L': 9.9, 'N': 1.49, 'Y': 6.44, 'V': 8.91, 'H': 1.98, 'D': 4.95, 'T': 6.93, 'E': 3.96, 'K': 6.44, 'Q': 3.96, 'I': 2.48, 'W': 1.49, 'A': 1.98, 'C': 1.98}
- **Common reported functions (%):** 25.0
- **Common reported locations (%):** 25.0
- **Common reported processes (%):** 0.0

- **AF ID:** Q7Z3Z4
- **Chain:** A
- **Protein length:** 852 aa
- **Resolution:** N/A
- **b-phipsi:** 0.002492
- **w-rdist:** 0.190213
- **t-alpha:** 0.049296
- **Chemical similarity (Tanimoto Index) (%):** 99.02
- **1D identity (%) [PDB]:** 5.96
- **1D identity (%) [Gaps excluded][PDB]:** 52.3
- **1D identity - Alignment Gaps [PDB]:** 1354
- **1D aligned content [PDB] (<aminoacid>:%):** {'M': 1.1, 'G': 5.49, 'P': 6.59, 'S': 7.69, 'A': 4.4, 'T': 9.89, 'R': 6.59, 'Q': 6.59, 'E': 3.3, 'I': 7.69, 'L': 7.69, 'V': 8.79, 'N': 3.3, 'Y': 7.69, 'K': 3.3, 'F': 2.2, 'D': 5.49, 'C': 1.1, 'H': 1.1}
- **2D identity (%) [PDB]:** 65.23
- **2D identity (%) [Gaps excluded][PDB]:** 86.85
- **2D identity - Alignment Gaps [PDB]:** 242
- **2D aligned content [PDB] (<2D-fold>:%):** {'.': 13.72, 'E': 32.33, 'T': 18.61, 'H': 33.44, 'G': 0.95, 'B': 0.95}
- **3D similarity (TM-Score) (%) [PDB]:** 74.72

- **Gene name:** PIWIL4
- **Entrez ID:** 143689
- **RefSeq ID:** NM\_152431
- **Transcript sequence length:** 3139
- **5-UTR|CDS|3-UTR identity (%):** 21.7 | 45.82 | 6.5
- **5-UTR|CDS|3-UTR identity (%) [Gaps excluded]:** 71.88 | 75.5 | 74.8
- **5-UTR|CDS|3-UTR identity [Alignment Gaps]:** 296 | 1259 | 3960
- **5-UTR aligned content (<base>:%):** {'G': 36.96, 'A': 13.04, 'C': 41.3, 'T': 8.7}
- **CDS aligned content (<base>:%):** {'A': 29.79, 'T': 24.27, 'G': 23.93, 'C': 22.02}
- **3-UTR aligned content (<base>:%):** {'T': 37.23, 'G': 15.25, 'C': 12.77, 'A': 34.75}

**Uniprot Description:**  
  
 Plays a central role during spermatogenesis by repressing transposable elements and preventing their mobilization, which is essential for the germline integrity (By similarity). Acts via the piRNA metabolic process, which mediates the repression of transposable elements during meiosis by forming complexes composed of piRNAs and Piwi proteins and governs the methylation and subsequent repression of transposons (By similarity). Directly binds piRNAs, a class of 24 to 30 nucleotide RNAs that are generated by a Dicer-independent mechanism and are primarily derived from transposons and other repeated sequence elements (By similarity). Associates with secondary piRNAs antisense and PIWIL2/MILI is required for such association (By similarity). The piRNA process acts upstream of known mediators of DNA methylation (By similarity). Does not show endonuclease activity (By similarity). Plays a key role in the piRNA amplification loop, also named ping-pong amplification cycle, by acting as a 'slicer-incompetent' component that loads cleaved piRNAs from the 'slicer-competent' component PIWIL2 and target them on genomic transposon loci in the nucleus (By similarity). May be involved in the chromatin-modifying pathway by inducing 'Lys-9' methylation of histone H3 at some loci (PubMed:17544373). In addition to its role in germline, PIWIL4 also plays a role in the regulation of somatic cells activities. Plays a role in pancreatic beta cell function and insulin secretion (By similarity). Involved in maintaining cell morphology and functional integrity of retinal epithelial through Akt/GSK3alpha/beta signaling pathway (PubMed:28025795). When overexpressed, acts as an oncogene by inhibition of apoptosis and promotion of cells proliferation in tumors (PubMed:22483988).   
  
Interacts with PRMT5 and WDR77. Interacts (when methylated on arginine residues) with TDRD1, TDRKH/TDRD2 and TDRD9. Interacts with MOV10L1 (By similarity). Interacts with TEX15 and SPOCD1 (By similarity).   
  
 **Gene Ontology Information:**

Molecular Function

- piRNA binding
- endoribonuclease activity

Location

- cytoplasm
- mitochondrion
- nucleoplasm
- nucleus
- P granule
- piP-body

Biological process

- epithelial structure maintenance
- genetic imprinting
- meiotic cell cycle
- regulation of translation
- retrotransposon silencing by heterochromatin formation
- gene silencing by RNA
- secondary piRNA processing
- spermatogenesis

---

25

- **Protein name:** Kelch repeat and BTB domain-containing protein 12
- **Organism:** Homo sapiens
- **Uniprot Accession Number:** Q3ZCT8
- **Protein sequence length:** 623 aa
- **1D identity (%):** 13.98
- **1D identity (%) [Gaps excluded]:** 23.47
- **1D identity - Alignment Gaps:** 376
- **1D aligned content (<aminoacid>:%):** {'I': 6.92, 'Y': 4.62, 'E': 6.15, 'D': 4.62, 'F': 3.85, 'P': 5.38, 'M': 2.31, 'T': 3.08, 'L': 13.85, 'V': 12.31, 'S': 6.15, 'Q': 4.62, 'G': 9.23, 'H': 0.77, 'R': 4.62, 'C': 3.08, 'K': 3.08, 'N': 3.08, 'W': 0.77, 'A': 1.54}
- **Common reported functions (%):** 0.0
- **Common reported locations (%):** 0.0
- **Common reported processes (%):** 0.0

- **AF ID:** Q3ZCT8
- **Chain:** A
- **Protein length:** 623 aa
- **Resolution:** N/A
- **b-phipsi:** 0.000673
- **w-rdist:** 0.638593
- **t-alpha:** 0.015649
- **Chemical similarity (Tanimoto Index) (%):** 97.91
- **1D identity (%) [PDB]:** 3.2
- **1D identity (%) [Gaps excluded][PDB]:** 68.18
- **1D identity - Alignment Gaps [PDB]:** 1341
- **1D aligned content [PDB] (<aminoacid>:%):** {'M': 4.44, 'L': 6.67, 'N': 6.67, 'I': 6.67, 'D': 4.44, 'V': 11.11, 'T': 6.67, 'A': 2.22, 'E': 8.89, 'F': 2.22, 'C': 6.67, 'Q': 4.44, 'K': 11.11, 'S': 4.44, 'R': 4.44, 'G': 2.22, 'Y': 2.22, 'P': 2.22, 'H': 2.22}
- **2D identity (%) [PDB]:** 33.46
- **2D identity (%) [Gaps excluded][PDB]:** 87.04
- **2D identity - Alignment Gaps [PDB]:** 655
- **2D aligned content [PDB] (<2D-fold>:%):** {'.': 15.45, 'H': 38.76, 'T': 25.28, 'E': 19.66, 'G': 0.84}
- **3D similarity (TM-Score) (%) [PDB]:** 19.94

- **Gene name:** KBTBD12
- **Entrez ID:** 166348
- **RefSeq ID:** N/A
- **Sequence length:** N/A
- **5-UTR|CDS|3-UTR identity (%):** N/A | N/A | N/A
- **5-UTR|CDS|3-UTR identity (%) [Gaps excluded]:** N/A | N/A | N/A
- **5-UTR|CDS|3-UTR identity [Alignment Gaps]:** N/A | N/A | N/A
- **5-UTR aligned content (<base>:%):** N/A
- **CDS aligned content (<base>:%):** N/A
- **3-UTR aligned content (<base>:%):** N/A

**Uniprot Description:**  
  
 N/A N/A   
  
 **Gene Ontology Information:**

Molecular Function   
  
N/A

Location   
  
N/A

Biological process   
  
N/A

---

26

- **Protein name:** Calpain-7
- **Organism:** Homo sapiens
- **Uniprot Accession Number:** Q9Y6W3
- **Protein sequence length:** 813 aa
- **1D identity (%):** 14.95
- **1D identity (%) [Gaps excluded]:** 21.64
- **1D identity - Alignment Gaps:** 306
- **1D aligned content (<aminoacid>:%):** {'M': 1.35, 'A': 4.73, 'G': 11.49, 'L': 8.11, 'H': 2.7, 'V': 6.08, 'K': 6.76, 'P': 10.81, 'D': 6.76, 'F': 5.41, 'Q': 4.73, 'N': 2.7, 'E': 5.41, 'R': 4.05, 'Y': 5.41, 'T': 4.05, 'S': 5.41, 'I': 3.38, 'W': 0.68}
- **Common reported functions (%):** 0.0
- **Common reported locations (%):** 25.0
- **Common reported processes (%):** 0.0

- **AF ID:** Q9Y6W3
- **Chain:** A
- **Protein length:** 813 aa
- **Resolution:** N/A
- **b-phipsi:** 0.000816
- **w-rdist:** 0.612613
- **t-alpha:** 0.010277
- **Chemical similarity (Tanimoto Index) (%):** 99.24
- **1D identity (%) [PDB]:** 3.55
- **1D identity (%) [Gaps excluded][PDB]:** 64.37
- **1D identity - Alignment Gaps [PDB]:** 1489
- **1D aligned content [PDB] (<aminoacid>:%):** {'M': 1.79, 'A': 7.14, 'T': 8.93, 'V': 16.07, 'Q': 7.14, 'K': 8.93, 'N': 5.36, 'S': 7.14, 'L': 7.14, 'I': 7.14, 'G': 3.57, 'P': 5.36, 'R': 3.57, 'F': 1.79, 'D': 5.36, 'H': 1.79, 'Y': 1.79}
- **2D identity (%) [PDB]:** 36.68
- **2D identity (%) [Gaps excluded][PDB]:** 88.32
- **2D identity - Alignment Gaps [PDB]:** 687
- **2D aligned content [PDB] (<2D-fold>:%):** {'.': 19.95, 'H': 36.19, 'T': 25.52, 'E': 17.87, 'B': 0.46}
- **3D similarity (TM-Score) (%) [PDB]:** 22.11

- **Gene name:** CAPN7
- **Entrez ID:** 23473
- **RefSeq ID:** NM\_014296
- **Transcript sequence length:** 4348
- **5-UTR|CDS|3-UTR identity (%):** 33.33 | 45.56 | 23.64
- **5-UTR|CDS|3-UTR identity (%) [Gaps excluded]:** 73.04 | 75.13 | 74.72
- **5-UTR|CDS|3-UTR identity [Alignment Gaps]:** 243 | 1232 | 3085
- **5-UTR aligned content (<base>:%):** {'G': 48.99, 'C': 40.27, 'T': 5.37, 'A': 5.37}
- **CDS aligned content (<base>:%):** {'A': 32.19, 'T': 25.67, 'G': 21.74, 'C': 20.41}
- **3-UTR aligned content (<base>:%):** {'T': 37.49, 'G': 16.49, 'A': 33.46, 'C': 12.56}

**Uniprot Description:**  
  
 Calcium-regulated non-lysosomal thiol-protease. N/A   
  
 **Gene Ontology Information:**

Molecular Function

- calcium-dependent cysteine-type endopeptidase activity
- cysteine-type endopeptidase activity
- endopeptidase activity
- MIT domain binding

Location

- centrosome
- cytosol
- extracellular exosome
- nucleus

Biological process

- positive regulation of epithelial cell migration
- proteolysis
- self proteolysis

---

27

- **Protein name:** Pseudouridylate synthase 7 homolog
- **Organism:** Homo sapiens
- **Uniprot Accession Number:** Q96PZ0
- **Protein sequence length:** 661 aa
- **1D identity (%):** 13.98
- **1D identity (%) [Gaps excluded]:** 24.77
- **1D identity - Alignment Gaps:** 424
- **1D aligned content (<aminoacid>:%):** {'V': 6.62, 'K': 11.76, 'Q': 4.41, 'G': 11.03, 'D': 4.41, 'N': 4.41, 'P': 6.62, 'E': 7.35, 'L': 11.03, 'S': 5.15, 'H': 1.47, 'R': 4.41, 'A': 3.68, 'I': 3.68, 'T': 4.41, 'F': 1.47, 'M': 2.94, 'Y': 3.68, 'W': 0.74, 'C': 0.74}
- **Common reported functions (%):** 0.0
- **Common reported locations (%):** 12.5
- **Common reported processes (%):** 0.0

- **AF ID:** Q96PZ0
- **Chain:** A
- **Protein length:** 661 aa
- **Resolution:** N/A
- **b-phipsi:** 0.001041
- **w-rdist:** 0.266009
- **t-alpha:** 0.128326
- **Chemical similarity (Tanimoto Index) (%):** 99.62
- **1D identity (%) [PDB]:** 1.62
- **1D identity (%) [Gaps excluded][PDB]:** 80.0
- **1D identity - Alignment Gaps [PDB]:** 1451
- **1D aligned content [PDB] (<aminoacid>:%):** {'V': 16.67, 'S': 4.17, 'L': 20.83, 'Q': 8.33, 'E': 8.33, 'A': 8.33, 'G': 4.17, 'H': 4.17, 'N': 4.17, 'D': 12.5, 'I': 4.17, 'R': 4.17}
- **2D identity (%) [PDB]:** 38.67
- **2D identity (%) [Gaps excluded][PDB]:** 93.23
- **2D identity - Alignment Gaps [PDB]:** 625
- **2D aligned content [PDB] (<2D-fold>:%):** {'.': 15.5, 'E': 20.58, 'T': 23.0, 'H': 40.92}
- **3D similarity (TM-Score) (%) [PDB]:** 19.81

- **Gene name:** PUS7
- **Entrez ID:** 615644
- **RefSeq ID:** N/A
- **Sequence length:** N/A
- **5-UTR|CDS|3-UTR identity (%):** N/A | N/A | N/A
- **5-UTR|CDS|3-UTR identity (%) [Gaps excluded]:** N/A | N/A | N/A
- **5-UTR|CDS|3-UTR identity [Alignment Gaps]:** N/A | N/A | N/A
- **5-UTR aligned content (<base>:%):** N/A
- **CDS aligned content (<base>:%):** N/A
- **3-UTR aligned content (<base>:%):** N/A

**Uniprot Description:**  
  
 Pseudouridylate synthase that catalyzes pseudouridylation of RNAs (PubMed:28073919, PubMed:29628141, PubMed:30778726). Acts as a regulator of protein synthesis in embryonic stem cells by mediating pseudouridylation of RNA fragments derived from tRNAs (tRFs): pseudouridylated tRFs inhibit translation by targeting the translation initiation complex (PubMed:29628141). Also catalyzes pseudouridylation of mRNAs: mediates pseudouridylation of mRNAs with the consensus sequence 5'-UGUAG-3' (PubMed:28073919). In addition to mRNAs and tRNAs, binds other types of RNAs, such as snRNAs, Y RNAs and vault RNAs, suggesting that it can catalyze pseudouridylation of many RNA types (PubMed:29628141). N/A   
  
 **Gene Ontology Information:**

Molecular Function

- pseudouridine synthase activity
- RNA binding

Location

- nucleus

Biological process

- mRNA processing
- mRNA pseudouridine synthesis
- negative regulation of translation
- pseudouridine synthesis
- regulation of hematopoietic stem cell differentiation
- regulation of mesoderm development
- RNA splicing
- tRNA pseudouridine synthesis

---

28

- **Protein name:** RNA exonuclease 5
- **Organism:** Homo sapiens
- **Uniprot Accession Number:** Q96IC2
- **Protein sequence length:** 774 aa
- **1D identity (%):** 16.1
- **1D identity (%) [Gaps excluded]:** 23.07
- **1D identity - Alignment Gaps:** 291
- **1D aligned content (<aminoacid>:%):** {'R': 5.81, 'H': 4.52, 'P': 7.74, 'G': 8.39, 'K': 8.39, 'W': 0.65, 'S': 3.87, 'E': 3.87, 'A': 5.81, 'L': 12.9, 'D': 3.23, 'Y': 1.29, 'V': 9.03, 'F': 3.87, 'Q': 7.1, 'T': 5.16, 'C': 5.16, 'N': 1.94, 'I': 0.65, 'M': 0.65}
- **Common reported functions (%):** 0.0
- **Common reported locations (%):** 12.5
- **Common reported processes (%):** 0.0

- **AF ID:** Q96IC2
- **Chain:** A
- **Protein length:** 774 aa
- **Resolution:** N/A
- **b-phipsi:** 0.002983
- **w-rdist:** 0.255921
- **t-alpha:** 0.032864
- **Chemical similarity (Tanimoto Index) (%):** 99.17
- **1D identity (%) [PDB]:** 2.28
- **1D identity (%) [Gaps excluded][PDB]:** 75.0
- **1D identity - Alignment Gaps [PDB]:** 1528
- **1D aligned content [PDB] (<aminoacid>:%):** {'M': 2.78, 'E': 5.56, 'V': 16.67, 'T': 5.56, 'L': 22.22, 'G': 2.78, 'K': 5.56, 'S': 8.33, 'Q': 5.56, 'A': 8.33, 'H': 2.78, 'D': 5.56, 'I': 2.78, 'R': 2.78, 'P': 2.78}
- **2D identity (%) [PDB]:** 42.83
- **2D identity (%) [Gaps excluded][PDB]:** 90.42
- **2D identity - Alignment Gaps [PDB]:** 580
- **2D aligned content [PDB] (<2D-fold>:%):** {'.': 20.76, 'T': 17.8, 'E': 20.55, 'H': 40.89}
- **3D similarity (TM-Score) (%) [PDB]:** 24.0

- **Gene name:** REXO5
- **Entrez ID:** 532864
- **RefSeq ID:** N/A
- **Sequence length:** N/A
- **5-UTR|CDS|3-UTR identity (%):** N/A | N/A | N/A
- **5-UTR|CDS|3-UTR identity (%) [Gaps excluded]:** N/A | N/A | N/A
- **5-UTR|CDS|3-UTR identity [Alignment Gaps]:** N/A | N/A | N/A
- **5-UTR aligned content (<base>:%):** N/A
- **CDS aligned content (<base>:%):** N/A
- **3-UTR aligned content (<base>:%):** N/A

**Uniprot Description:**  
  
 N/A N/A   
  
 **Gene Ontology Information:**

Molecular Function

- exonuclease activity
- RNA binding

Location

- nucleolus
- nucleus

Biological process   
  
N/A

---

29

- **Protein name:** Twinkle mtDNA helicase
- **Organism:** Homo sapiens
- **Uniprot Accession Number:** Q96RR1
- **Protein sequence length:** 684 aa
- **1D identity (%):** 16.27
- **1D identity (%) [Gaps excluded]:** 24.47
- **1D identity - Alignment Gaps:** 311
- **1D aligned content (<aminoacid>:%):** {'M': 0.66, 'P': 9.27, 'L': 11.92, 'I': 5.96, 'G': 11.92, 'R': 5.96, 'K': 4.64, 'Q': 3.31, 'V': 5.96, 'H': 2.65, 'D': 5.3, 'A': 3.97, 'T': 5.96, 'S': 5.3, 'F': 5.3, 'E': 3.97, 'W': 1.32, 'N': 3.31, 'C': 1.99, 'Y': 1.32}
- **Common reported functions (%):** 0.0
- **Common reported locations (%):** 0.0
- **Common reported processes (%):** 0.0

- **AF ID:** Q96RR1
- **Chain:** A
- **Protein length:** 684 aa
- **Resolution:** N/A
- **b-phipsi:** 0.005253
- **w-rdist:** 0.48497
- **t-alpha:** 0.001568
- **Chemical similarity (Tanimoto Index) (%):** 99.39
- **1D identity (%) [PDB]:** 3.0
- **1D identity (%) [Gaps excluded][PDB]:** 66.67
- **1D identity - Alignment Gaps [PDB]:** 1402
- **1D aligned content [PDB] (<aminoacid>:%):** {'M': 2.27, 'V': 15.91, 'L': 15.91, 'P': 9.09, 'G': 4.55, 'E': 6.82, 'D': 6.82, 'Q': 2.27, 'T': 4.55, 'K': 2.27, 'S': 11.36, 'W': 2.27, 'A': 4.55, 'N': 2.27, 'I': 2.27, 'R': 4.55, 'Y': 2.27}
- **2D identity (%) [PDB]:** 41.1
- **2D identity (%) [Gaps excluded][PDB]:** 89.44
- **2D identity - Alignment Gaps [PDB]:** 568
- **2D aligned content [PDB] (<2D-fold>:%):** {'.': 18.06, 'T': 21.76, 'H': 37.73, 'E': 21.99, 'B': 0.46}
- **3D similarity (TM-Score) (%) [PDB]:** 20.19

- **Gene name:** TWNK
- **Entrez ID:** 425626
- **RefSeq ID:** NM\_021830
- **Transcript sequence length:** 3616
- **5-UTR|CDS|3-UTR identity (%):** 34.79 | 44.94 | 12.72
- **5-UTR|CDS|3-UTR identity (%) [Gaps excluded]:** 68.8 | 74.66 | 76.77
- **5-UTR|CDS|3-UTR identity [Alignment Gaps]:** 351 | 1153 | 3706
- **5-UTR aligned content (<base>:%):** {'G': 49.8, 'T': 10.53, 'C': 30.36, 'A': 9.31}
- **CDS aligned content (<base>:%):** {'A': 26.42, 'T': 21.51, 'G': 26.27, 'C': 25.81}
- **3-UTR aligned content (<base>:%):** {'A': 28.67, 'G': 27.79, 'C': 19.12, 'T': 24.42}

**Uniprot Description:**  
  
 Isoform 1
Mitochondrial helicase involved in mtDNA replication and repair (PubMed:12975372, PubMed:15167897, PubMed:17324440, PubMed:18039713, PubMed:18971204, PubMed:25824949, PubMed:26887820, PubMed:27226550). Might have a role in mtDNA repair (PubMed:27226550). Has DNA strand separation activity needed to form a processive replication fork for leading strand synthesis which is catalyzed by the formation of a replisome complex with POLG and mtSDB (PubMed:12975372, PubMed:15167897, PubMed:18039713, PubMed:22383523, PubMed:26887820, PubMed:27226550). Preferentially unwinds DNA substrates with pre-existing 5'-and 3'- single-stranded tails but is also active on a 5'- flap substrate (PubMed:12975372, PubMed:15167897, PubMed:18039713, PubMed:22383523, PubMed:26887820, PubMed:27226550). Can dissociate the invading strand of immobile or mobile D-loop DNA structures irrespective of the single strand polarity of the third strand (PubMed:27226550). In addition to its DNA strand separation activity, also has DNA strand annealing, DNA strand-exchange and DNA branch migration activities (PubMed:22383523, PubMed:26887820, PubMed:27226550).   
  
Interacts with LONP1.   
  
 **Gene Ontology Information:**

Molecular Function

- 5'-3' DNA helicase activity
- ATP binding
- ATPase activity
- DNA helicase activity
- single-stranded DNA binding

Location

- mitochondrial nucleoid
- mitochondrion

Biological process

- mitochondrial DNA replication

---

30

- **Protein name:** Villin-1
- **Organism:** Homo sapiens
- **Uniprot Accession Number:** P09327
- **Protein sequence length:** 827 aa
- **1D identity (%):** 15.52
- **1D identity (%) [Gaps excluded]:** 23.58
- **1D identity - Alignment Gaps:** 348
- **1D aligned content (<aminoacid>:%):** {'M': 1.9, 'L': 10.76, 'S': 3.8, 'Q': 6.33, 'R': 3.8, 'V': 8.86, 'P': 7.59, 'A': 5.7, 'H': 3.16, 'D': 5.06, 'G': 11.39, 'N': 2.53, 'Y': 3.8, 'E': 7.59, 'F': 1.9, 'K': 7.59, 'W': 0.63, 'T': 5.7, 'C': 1.27, 'I': 0.63}
- **Common reported functions (%):** 0.0
- **Common reported locations (%):** 12.5
- **Common reported processes (%):** 0.0

- **AF ID:** P09327
- **Chain:** A
- **Protein length:** 827 aa
- **Resolution:** N/A
- **b-phipsi:** 0.002186
- **w-rdist:** 0.356405
- **t-alpha:** 0.010172
- **Chemical similarity (Tanimoto Index) (%):** 98.65
- **1D identity (%) [PDB]:** 3.37
- **1D identity (%) [Gaps excluded][PDB]:** 73.97
- **1D identity - Alignment Gaps [PDB]:** 1531
- **1D aligned content [PDB] (<aminoacid>:%):** {'M': 5.56, 'Q': 5.56, 'I': 3.7, 'F': 1.85, 'G': 9.26, 'D': 11.11, 'Y': 1.85, 'K': 5.56, 'R': 5.56, 'N': 3.7, 'V': 14.81, 'E': 5.56, 'T': 5.56, 'P': 3.7, 'S': 1.85, 'L': 11.11, 'A': 3.7}
- **2D identity (%) [PDB]:** 45.07
- **2D identity (%) [Gaps excluded][PDB]:** 89.66
- **2D identity - Alignment Gaps [PDB]:** 555
- **2D aligned content [PDB] (<2D-fold>:%):** {'.': 11.73, 'E': 28.43, 'T': 25.25, 'H': 33.8, 'G': 0.6, 'B': 0.2}
- **3D similarity (TM-Score) (%) [PDB]:** 25.2

- **Gene name:** VIL1
- **Entrez ID:** N/A
- **RefSeq ID:** NM\_007127
- **Transcript sequence length:** 6500
- **5-UTR|CDS|3-UTR identity (%):** 7.45 | 44.77 | 44.52
- **5-UTR|CDS|3-UTR identity (%) [Gaps excluded]:** 73.81 | 73.7 | 73.77
- **5-UTR|CDS|3-UTR identity [Alignment Gaps]:** 374 | 1238 | 2038
- **5-UTR aligned content (<base>:%):** {'C': 61.29, 'T': 19.35, 'G': 19.35}
- **CDS aligned content (<base>:%):** {'A': 28.68, 'T': 19.55, 'G': 27.9, 'C': 23.87}
- **3-UTR aligned content (<base>:%):** {'G': 17.21, 'A': 32.68, 'T': 33.86, 'C': 16.25}

**Uniprot Description:**  
  
 Epithelial cell-specific Ca(2+)-regulated actin-modifying protein that modulates the reorganization of microvillar actin filaments. Plays a role in the actin nucleation, actin filament bundle assembly, actin filament capping and severing. Binds phosphatidylinositol 4,5-bisphosphate (PIP2) and lysophosphatidic acid (LPA); binds LPA with higher affinity than PIP2. Binding to LPA increases its phosphorylation by SRC and inhibits all actin-modifying activities. Binding to PIP2 inhibits actin-capping and -severing activities but enhances actin-bundling activity. Regulates the intestinal epithelial cell morphology, cell invasion, cell migration and apoptosis. Protects against apoptosis induced by dextran sodium sulfate (DSS) in the gastrointestinal epithelium. Appears to regulate cell death by maintaining mitochondrial integrity. Enhances hepatocyte growth factor (HGF)-induced epithelial cell motility, chemotaxis and wound repair. Upon S.flexneri cell infection, its actin-severing activity enhances actin-based motility of the bacteria and plays a role during the dissemination.   
  
Monomer. Homodimer; homodimerization is necessary for actin-bundling. Associates with F-actin; phosphorylation at tyrosine residues decreases the association with F-actin. Interacts (phosphorylated at C-terminus tyrosine phosphorylation sites) with PLCG1 (via the SH2 domains). Interacts (phosphorylated form) with PLCG1; the interaction is enhanced by hepatocyte growth factor (HGF) (By similarity).   
  
 **Gene Ontology Information:**

Molecular Function

- actin filament binding
- calcium ion binding
- cysteine-type endopeptidase inhibitor activity involved in apoptotic process
- lysophosphatidic acid binding
- phosphatidylinositol-4,5-bisphosphate binding
- protein homodimerization activity

Location

- actin cytoskeleton
- actin filament bundle
- cytoplasm
- filopodium
- filopodium tip
- lamellipodium
- microvillus
- ruffle

Biological process

- actin filament capping
- actin filament depolymerization
- actin filament polymerization
- actin filament severing
- actin polymerization or depolymerization
- apoptotic process
- barbed-end actin filament capping
- cellular response to epidermal growth factor stimulus
- cellular response to hepatocyte growth factor stimulus
- cytoplasmic actin-based contraction involved in cell motility
- epidermal growth factor receptor signaling pathway
- positive regulation of actin filament bundle assembly
- positive regulation of cell migration
- positive regulation of epithelial cell migration
- regulation of actin nucleation
- regulation of cell shape
- regulation of lamellipodium morphogenesis
- regulation of wound healing
- response to bacterium

---

31

- **Protein name:** cAMP-dependent protein kinase type I-beta regulatory subunit
- **Organism:** Homo sapiens
- **Uniprot Accession Number:** P31321
- **Protein sequence length:** 381 aa
- **1D identity (%):** 7.93
- **1D identity (%) [Gaps excluded]:** 21.56
- **1D identity - Alignment Gaps:** 574
- **1D aligned content (<aminoacid>:%):** {'P': 9.72, 'K': 8.33, 'L': 11.11, 'H': 5.56, 'Q': 2.78, 'E': 8.33, 'R': 8.33, 'I': 2.78, 'D': 2.78, 'V': 11.11, 'A': 2.78, 'T': 4.17, 'G': 11.11, 'F': 5.56, 'W': 1.39, 'Y': 1.39, 'C': 1.39, 'N': 1.39}
- **Common reported functions (%):** 0.0
- **Common reported locations (%):** 12.5
- **Common reported processes (%):** 0.0

- **AF ID:** P31321
- **Chain:** A
- **Protein length:** 381 aa
- **Resolution:** N/A
- **b-phipsi:** 0.00097
- **w-rdist:** 0.403924
- **t-alpha:** 0.03987
- **Chemical similarity (Tanimoto Index) (%):** 98.8
- **1D identity (%) [PDB]:** 1.49
- **1D identity (%) [Gaps excluded][PDB]:** 69.23
- **1D identity - Alignment Gaps [PDB]:** 1179
- **1D aligned content [PDB] (<aminoacid>:%):** {'M': 5.56, 'Y': 11.11, 'V': 33.33, 'L': 11.11, 'I': 11.11, 'G': 5.56, 'T': 5.56, 'P': 5.56, 'E': 5.56, 'R': 5.56}
- **2D identity (%) [PDB]:** 31.61
- **2D identity (%) [Gaps excluded][PDB]:** 88.85
- **2D identity - Alignment Gaps [PDB]:** 585
- **2D aligned content [PDB] (<2D-fold>:%):** {'.': 21.6, 'H': 46.34, 'T': 13.24, 'B': 0.35, 'E': 18.47}
- **3D similarity (TM-Score) (%) [PDB]:** 13.62

- **Gene name:** PRKAR1B
- **Entrez ID:** 5575
- **RefSeq ID:** NM\_002735
- **Transcript sequence length:** 2491
- **5-UTR|CDS|3-UTR identity (%):** 18.1 | 27.39 | 16.6
- **5-UTR|CDS|3-UTR identity (%) [Gaps excluded]:** 80.85 | 74.33 | 72.99
- **5-UTR|CDS|3-UTR identity [Alignment Gaps]:** 326 | 1722 | 3473
- **5-UTR aligned content (<base>:%):** {'G': 31.58, 'T': 3.95, 'C': 60.53, 'A': 3.95}
- **CDS aligned content (<base>:%):** {'T': 19.68, 'C': 26.1, 'G': 28.11, 'A': 26.1}
- **3-UTR aligned content (<base>:%):** {'G': 32.84, 'C': 26.14, 'A': 19.57, 'T': 21.45}

**Uniprot Description:**  
  
 Regulatory subunit of the cAMP-dependent protein kinases involved in cAMP signaling in cells.   
  
The inactive holoenzyme is composed of two regulatory chains and two catalytic chains. Activation by cAMP releases the two active catalytic monomers and the regulatory dimer. Interacts with PRKX; regulates this cAMP-dependent protein kinase. Interacts with C2orf88/smAKAP; this interaction may target PRKAR1B to the plasma membrane.   
  
 **Gene Ontology Information:**

Molecular Function

- cAMP binding
- cAMP-dependent protein kinase inhibitor activity
- cAMP-dependent protein kinase regulator activity
- molecular function inhibitor activity
- protein kinase A catalytic subunit binding

Location

- cAMP-dependent protein kinase complex
- ciliary base
- cytosol
- glutamatergic synapse
- hippocampal mossy fiber to CA3 synapse
- multivesicular body
- plasma membrane
- postsynapse
- Schaffer collateral - CA1 synapse

Biological process

- learning or memory
- negative regulation of cAMP-dependent protein kinase activity
- positive regulation of excitatory postsynaptic potential
- positive regulation of fear response
- positive regulation of long-term synaptic potentiation
- protein phosphorylation
- regulation of synaptic vesicle cycle

---

32

- **Protein name:** Piwi-like protein 1
- **Organism:** Homo sapiens
- **Uniprot Accession Number:** Q96J94
- **Protein sequence length:** 861 aa
- **1D identity (%):** 22.26
- **1D identity (%) [Gaps excluded]:** 29.05
- **1D identity - Alignment Gaps:** 228
- **1D aligned content (<aminoacid>:%):** {'M': 2.76, 'A': 3.23, 'G': 11.52, 'P': 9.22, 'L': 11.06, 'F': 4.61, 'R': 9.22, 'N': 2.76, 'H': 1.84, 'Y': 5.53, 'D': 4.61, 'T': 5.99, 'V': 7.37, 'E': 3.69, 'S': 3.69, 'W': 1.38, 'Q': 4.61, 'K': 3.69, 'C': 1.38, 'I': 1.84}
- **Common reported functions (%):** 50.0
- **Common reported locations (%):** 25.0
- **Common reported processes (%):** 0.0

- **AF ID:** Q96J94
- **Chain:** A
- **Protein length:** 861 aa
- **Resolution:** N/A
- **b-phipsi:** 0.005224
- **w-rdist:** 0.20633
- **t-alpha:** 0.029734
- **Chemical similarity (Tanimoto Index) (%):** 99.02
- **1D identity (%) [PDB]:** 5.95
- **1D identity (%) [Gaps excluded][PDB]:** 56.1
- **1D identity - Alignment Gaps [PDB]:** 1383
- **1D aligned content [PDB] (<aminoacid>:%):** {'T': 8.7, 'R': 7.61, 'Q': 6.52, 'E': 5.43, 'I': 6.52, 'S': 4.35, 'L': 8.7, 'Y': 9.78, 'V': 6.52, 'N': 2.17, 'F': 5.43, 'K': 4.35, 'P': 6.52, 'G': 5.43, 'D': 4.35, 'A': 4.35, 'H': 2.17, 'C': 1.09}
- **2D identity (%) [PDB]:** 61.73
- **2D identity (%) [Gaps excluded][PDB]:** 88.09
- **2D identity - Alignment Gaps [PDB]:** 301
- **2D aligned content [PDB] (<2D-fold>:%):** {'.': 15.67, 'E': 32.15, 'T': 17.45, 'H': 33.12, 'G': 0.97, 'B': 0.65}
- **3D similarity (TM-Score) (%) [PDB]:** 74.92

- **Gene name:** PIWIL1
- **Entrez ID:** 9271
- **RefSeq ID:** N/A
- **Sequence length:** N/A
- **5-UTR|CDS|3-UTR identity (%):** N/A | N/A | N/A
- **5-UTR|CDS|3-UTR identity (%) [Gaps excluded]:** N/A | N/A | N/A
- **5-UTR|CDS|3-UTR identity [Alignment Gaps]:** N/A | N/A | N/A
- **5-UTR aligned content (<base>:%):** N/A
- **CDS aligned content (<base>:%):** N/A
- **3-UTR aligned content (<base>:%):** N/A

**Uniprot Description:**  
  
 Endoribonuclease that plays a central role in postnatal germ cells by repressing transposable elements and preventing their mobilization, which is essential for the germline integrity. Acts via the piRNA metabolic process, which mediates the repression of transposable elements during meiosis by forming complexes composed of piRNAs and Piwi proteins and governs the methylation and subsequent repression of transposons. Directly binds methylated piRNAs, a class of 24 to 30 nucleotide RNAs that are generated by a Dicer-independent mechanism and are primarily derived from transposons and other repeated sequence elements. Strongly prefers a uridine in the first position of their guide (g1U preference, also named 1U-bias). Not involved in the piRNA amplification loop, also named ping-pong amplification cycle. Acts as an endoribonuclease that cleaves transposon messenger RNAs. Besides their function in transposable elements repression, piRNAs are probably involved in other processes during meiosis such as translation regulation. Probable component of some RISC complex, which mediates RNA cleavage and translational silencing. Also plays a role in the formation of chromatoid bodies and is required for some miRNAs stability. Required to sequester RNF8 in the cytoplasm until late spermatogenesis; RNF8 being released upon ubiquitination and degradation of PIWIL1.   
  
Interacts (via Piwi domain) with DICER1, suggesting that it forms ribonucleoprotein RISC complexes; this interaction is regulated by HSP90AB1 activity. Interacts with MAEL, KIF17, PABPC1, PRMT5 and WDR77. Interacts (when methylated on arginine residues) with TDRD1, TDRKH/TDRD2, RNF17/TDRD4, TDRD6, TDRD7 and TDRD9. Interacts with CLOCK. Interacts with MOV10L1. Interacts with ANAPC10; interaction oly takes place following piRNA-binding. Interacts with RNF8; leading to sequester RNF8 in the cytoplasm. Interacts with TEX19 (By similarity).   
  
 **Gene Ontology Information:**

Molecular Function

- metal ion binding
- mRNA binding
- mRNA cap binding complex binding
- piRNA binding
- polysome binding
- protein kinase binding
- endoribonuclease activity
- single-stranded RNA binding

Location

- chromatoid body
- cytoplasm
- dense body
- nucleus
- P granule

Biological process

- meiotic cell cycle
- piRNA-mediated retrotransposon silencing by heterochromatin formation
- primary piRNA processing
- regulation of translation
- gene silencing by RNA
- sperm chromatin condensation
- spermatid development
- spermatogenesis

---

33

- **Protein name:** Probable ATP-dependent RNA helicase DDX53
- **Organism:** Homo sapiens
- **Uniprot Accession Number:** Q86TM3
- **Protein sequence length:** 631 aa
- **1D identity (%):** 12.63
- **1D identity (%) [Gaps excluded]:** 21.64
- **1D identity - Alignment Gaps:** 392
- **1D aligned content (<aminoacid>:%):** {'R': 7.56, 'G': 11.76, 'V': 12.61, 'A': 1.68, 'P': 6.72, 'L': 9.24, 'M': 0.84, 'Y': 3.36, 'K': 4.2, 'Q': 5.88, 'T': 4.2, 'I': 5.88, 'S': 5.88, 'D': 8.4, 'N': 2.52, 'E': 1.68, 'F': 2.52, 'C': 1.68, 'H': 3.36}
- **Common reported functions (%):** 0.0
- **Common reported locations (%):** 12.5
- **Common reported processes (%):** 0.0

- **AF ID:** Q86TM3
- **Chain:** A
- **Protein length:** 631 aa
- **Resolution:** N/A
- **b-phipsi:** 0.003877
- **w-rdist:** 0.298496
- **t-alpha:** 0.023474
- **Chemical similarity (Tanimoto Index) (%):** 99.55
- **1D identity (%) [PDB]:** 3.69
- **1D identity (%) [Gaps excluded][PDB]:** 74.29
- **1D identity - Alignment Gaps [PDB]:** 1341
- **1D aligned content [PDB] (<aminoacid>:%):** {'Y': 5.77, 'S': 5.77, 'I': 15.38, 'D': 3.85, 'L': 7.69, 'T': 7.69, 'N': 1.92, 'V': 5.77, 'R': 9.62, 'E': 7.69, 'F': 3.85, 'K': 7.69, 'P': 3.85, 'Q': 3.85, 'A': 3.85, 'W': 1.92, 'G': 1.92, 'H': 1.92}
- **2D identity (%) [PDB]:** 42.66
- **2D identity (%) [Gaps excluded][PDB]:** 90.91
- **2D identity - Alignment Gaps [PDB]:** 535
- **2D aligned content [PDB] (<2D-fold>:%):** {'.': 20.23, 'T': 13.72, 'E': 17.67, 'H': 48.37}
- **3D similarity (TM-Score) (%) [PDB]:** 22.94

- **Gene name:** DDX53
- **Entrez ID:** 168400
- **RefSeq ID:** NM\_182699
- **Transcript sequence length:** 3630
- **5-UTR|CDS|3-UTR identity (%):** 12.91 | 41.66 | 22.57
- **5-UTR|CDS|3-UTR identity (%) [Gaps excluded]:** 75.34 | 75.11 | 75.55
- **5-UTR|CDS|3-UTR identity [Alignment Gaps]:** 353 | 1284 | 3197
- **5-UTR aligned content (<base>:%):** {'T': 16.36, 'G': 34.55, 'C': 40.0, 'A': 9.09}
- **CDS aligned content (<base>:%):** {'A': 33.64, 'T': 22.9, 'G': 24.23, 'C': 19.23}
- **3-UTR aligned content (<base>:%):** {'A': 30.9, 'T': 38.78, 'G': 16.13, 'C': 14.19}

**Uniprot Description:**  
  
 N/A N/A   
  
 **Gene Ontology Information:**

Molecular Function

- ATP binding
- ATPase activity
- RNA binding
- RNA helicase activity

Location

- cytosol
- intracellular membrane-bounded organelle
- nucleolus
- nucleoplasm

Biological process   
  
N/A

---

34

- **Protein name:** Protein mono-ADP-ribosyltransferase PARP12
- **Organism:** Homo sapiens
- **Uniprot Accession Number:** Q9H0J9
- **Protein sequence length:** 701 aa
- **1D identity (%):** 14.81
- **1D identity (%) [Gaps excluded]:** 23.11
- **1D identity - Alignment Gaps:** 342
- **1D aligned content (<aminoacid>:%):** {'V': 8.51, 'A': 6.38, 'G': 6.38, 'E': 4.26, 'P': 10.64, 'S': 6.38, 'L': 9.22, 'R': 4.26, 'H': 3.55, 'Q': 7.09, 'Y': 5.67, 'C': 4.96, 'T': 6.38, 'N': 2.13, 'K': 4.26, 'I': 1.42, 'F': 2.84, 'D': 4.96, 'M': 0.71}
- **Common reported functions (%):** 0.0
- **Common reported locations (%):** 12.5
- **Common reported processes (%):** 0.0

- **AF ID:** Q9H0J9
- **Chain:** A
- **Protein length:** 701 aa
- **Resolution:** N/A
- **b-phipsi:** 0.000767
- **w-rdist:** 0.367739
- **t-alpha:** 0.11811
- **Chemical similarity (Tanimoto Index) (%):** 99.62
- **1D identity (%) [PDB]:** 2.67
- **1D identity (%) [Gaps excluded][PDB]:** 76.92
- **1D identity - Alignment Gaps [PDB]:** 1447
- **1D aligned content [PDB] (<aminoacid>:%):** {'A': 12.5, 'R': 7.5, 'L': 5.0, 'V': 10.0, 'H': 12.5, 'D': 10.0, 'K': 5.0, 'S': 10.0, 'E': 2.5, 'G': 5.0, 'Q': 10.0, 'P': 2.5, 'T': 5.0, 'M': 2.5}
- **2D identity (%) [PDB]:** 36.08
- **2D identity (%) [Gaps excluded][PDB]:** 88.84
- **2D identity - Alignment Gaps [PDB]:** 655
- **2D aligned content [PDB] (<2D-fold>:%):** {'H': 33.92, 'T': 21.61, 'E': 19.35, '.': 23.12, 'G': 1.51, 'B': 0.5}
- **3D similarity (TM-Score) (%) [PDB]:** 22.14

- **Gene name:** PARP12
- **Entrez ID:** 64761
- **RefSeq ID:** NM\_022750
- **Transcript sequence length:** 3021
- **5-UTR|CDS|3-UTR identity (%):** 16.95 | 42.8 | 10.78
- **5-UTR|CDS|3-UTR identity (%) [Gaps excluded]:** 82.56 | 75.06 | 74.53
- **5-UTR|CDS|3-UTR identity [Alignment Gaps]:** 333 | 1284 | 3808
- **5-UTR aligned content (<base>:%):** {'T': 5.63, 'C': 56.34, 'G': 32.39, 'A': 5.63}
- **CDS aligned content (<base>:%):** {'A': 24.0, 'T': 22.91, 'G': 26.11, 'C': 26.97}
- **3-UTR aligned content (<base>:%):** {'G': 18.75, 'C': 17.5, 'A': 24.17, 'T': 39.58}

**Uniprot Description:**  
  
 Mono-ADP-ribosyltransferase that mediates mono-ADP-ribosylation of target proteins. N/A   
  
 **Gene Ontology Information:**

Molecular Function

- metal ion binding
- NAD+ ADP-ribosyltransferase activity
- NAD+- protein-cysteine ADP-ribosyltransferase activity
- protein ADP-ribosylase activity
- nucleotidyltransferase activity
- RNA binding

Location

- nucleus

Biological process

- protein auto-ADP-ribosylation

---

35

- **Protein name:** Xylosyl- and glucuronyltransferase LARGE2
- **Organism:** Homo sapiens
- **Uniprot Accession Number:** Q8N3Y3
- **Protein sequence length:** 721 aa
- **1D identity (%):** 16.77
- **1D identity (%) [Gaps excluded]:** 25.08
- **1D identity - Alignment Gaps:** 314
- **1D aligned content (<aminoacid>:%):** {'P': 14.47, 'R': 10.69, 'F': 2.52, 'G': 6.92, 'D': 5.03, 'K': 1.89, 'V': 10.69, 'L': 11.32, 'H': 3.14, 'T': 5.03, 'Y': 3.14, 'S': 1.89, 'A': 6.29, 'Q': 7.55, 'I': 0.63, 'C': 2.52, 'N': 2.52, 'E': 2.52, 'M': 0.63, 'W': 0.63}
- **Common reported functions (%):** 0.0
- **Common reported locations (%):** 0.0
- **Common reported processes (%):** 0.0

- **AF ID:** Q8N3Y3
- **Chain:** A
- **Protein length:** 721 aa
- **Resolution:** N/A
- **b-phipsi:** 0.011786
- **w-rdist:** 0.362641
- **t-alpha:** 0.002347
- **Chemical similarity (Tanimoto Index) (%):** 96.89
- **1D identity (%) [PDB]:** 2.93
- **1D identity (%) [Gaps excluded][PDB]:** 63.77
- **1D identity - Alignment Gaps [PDB]:** 1433
- **1D aligned content [PDB] (<aminoacid>:%):** {'L': 18.18, 'G': 11.36, 'V': 11.36, 'P': 11.36, 'D': 4.55, 'A': 4.55, 'I': 2.27, 'T': 2.27, 'S': 9.09, 'M': 2.27, 'R': 4.55, 'F': 9.09, 'Y': 2.27, 'H': 4.55, 'Q': 2.27}
- **2D identity (%) [PDB]:** 44.75
- **2D identity (%) [Gaps excluded][PDB]:** 84.71
- **2D identity - Alignment Gaps [PDB]:** 485
- **2D aligned content [PDB] (<2D-fold>:%):** {'.': 16.52, 'T': 22.61, 'H': 40.87, 'E': 19.35, 'G': 0.65}
- **3D similarity (TM-Score) (%) [PDB]:** 20.53

- **Gene name:** LARGE2
- **Entrez ID:** 120071
- **RefSeq ID:** NM\_152312
- **Transcript sequence length:** 2552
- **5-UTR|CDS|3-UTR identity (%):** 22.55 | 44.46 | 4.13
- **5-UTR|CDS|3-UTR identity (%) [Gaps excluded]:** 71.88 | 74.17 | 79.11
- **5-UTR|CDS|3-UTR identity [Alignment Gaps]:** 280 | 1190 | 4086
- **5-UTR aligned content (<base>:%):** {'G': 40.22, 'C': 44.57, 'A': 4.35, 'T': 10.87}
- **CDS aligned content (<base>:%):** {'A': 20.59, 'T': 21.42, 'G': 27.33, 'C': 30.66}
- **3-UTR aligned content (<base>:%):** {'G': 26.4, 'C': 26.97, 'T': 29.21, 'A': 17.42}

**Uniprot Description:**  
  
 Bifunctional glycosyltransferase with both alpha-1,3-xylosyltransferase and beta-1,3-glucuronyltransferase activities involved in the maturation of alpha-dystroglycan (DAG1) by glycosylation leading to DAG1 binding to laminin G-like domain-containing extracellular proteins with high affinity and in a phosphorylated-O-mannosyl trisaccharide dependent manner (PubMed:15661757, PubMed:15752776, PubMed:25138275). Elongates the glucuronyl-beta-1,4-xylose-beta disaccharide primer structure by adding repeating units [-3-Xylose-alpha-1,3-GlcA-beta-1-] to produce a heteropolysaccharide (By similarity). Supports the maturation of DAG1 more effectively than LARGE1 (PubMed:15752776). In addition, can modify both heparan sulfate (HS)- and chondroitin/dermatan sulfate (CS/DS)-proteoglycans (PGs), namely GPC4, with a glycosaminoglycan (GAG)-like polysaccharide composed of xylose and glucuronic acid to confer laminin binding (By similarity).   
  
Interacts with B4GAT1.   
  
 **Gene Ontology Information:**

Molecular Function

- glucuronosyltransferase activity
- manganese ion binding
- UDP-xylosyltransferase activity
- xylosyltransferase activity

Location

- Golgi apparatus
- Golgi membrane

Biological process

- muscle cell cellular homeostasis
- protein O-linked glycosylation
- protein O-linked mannosylation

---

36

- **Protein name:** Galectin-3-binding protein
- **Organism:** Homo sapiens
- **Uniprot Accession Number:** Q08380
- **Protein sequence length:** 585 aa
- **1D identity (%):** 10.81
- **1D identity (%) [Gaps excluded]:** 23.46
- **1D identity - Alignment Gaps:** 534
- **1D aligned content (<aminoacid>:%):** {'P': 9.35, 'R': 4.67, 'L': 11.21, 'V': 8.41, 'D': 6.54, 'Y': 3.74, 'G': 10.28, 'N': 0.93, 'T': 4.67, 'A': 4.67, 'Q': 6.54, 'M': 0.93, 'E': 4.67, 'W': 0.93, 'C': 4.67, 'S': 9.35, 'I': 1.87, 'K': 3.74, 'F': 0.93, 'H': 1.87}
- **Common reported functions (%):** 0.0
- **Common reported locations (%):** 12.5
- **Common reported processes (%):** 0.0

- **AF ID:** Q08380
- **Chain:** A
- **Protein length:** 585 aa
- **Resolution:** N/A
- **b-phipsi:** 0.001419
- **w-rdist:** 0.3416
- **t-alpha:** 0.043265
- **Chemical similarity (Tanimoto Index) (%):** 96.4
- **1D identity (%) [PDB]:** 1.2
- **1D identity (%) [Gaps excluded][PDB]:** 89.47
- **1D identity - Alignment Gaps [PDB]:** 1397
- **1D aligned content [PDB] (<aminoacid>:%):** {'W': 5.88, 'V': 17.65, 'S': 5.88, 'L': 29.41, 'Q': 5.88, 'E': 5.88, 'A': 11.76, 'G': 5.88, 'H': 5.88, 'N': 5.88}
- **2D identity (%) [PDB]:** 38.21
- **2D identity (%) [Gaps excluded][PDB]:** 86.99
- **2D identity - Alignment Gaps [PDB]:** 559
- **2D aligned content [PDB] (<2D-fold>:%):** {'.': 12.34, 'E': 23.62, 'T': 24.15, 'H': 39.11, 'G': 0.79}
- **3D similarity (TM-Score) (%) [PDB]:** 23.15

- **Gene name:** LGALS3BP
- **Entrez ID:** 3959
- **RefSeq ID:** NM\_005567
- **Transcript sequence length:** 2202
- **5-UTR|CDS|3-UTR identity (%):** 14.98 | 38.76 | 4.45
- **5-UTR|CDS|3-UTR identity (%) [Gaps excluded]:** 72.22 | 74.78 | 77.6
- **5-UTR|CDS|3-UTR identity [Alignment Gaps]:** 344 | 1378 | 4106
- **5-UTR aligned content (<base>:%):** {'A': 16.92, 'T': 4.62, 'G': 47.69, 'C': 30.77}
- **CDS aligned content (<base>:%):** {'T': 19.84, 'G': 28.49, 'A': 21.1, 'C': 30.57}
- **3-UTR aligned content (<base>:%):** {'A': 22.16, 'G': 26.29, 'C': 32.47, 'T': 19.07}

**Uniprot Description:**  
  
 Promotes integrin-mediated cell adhesion. May stimulate host defense against viruses and tumor cells.   
  
Homodimers and homomultimers. The multimers form ring-like structures with a diameter of 30-40 nm. Binds LGALS1 and LGALS3. Binds ITGB1, COL4A1, COL5A1, COL6A1, FN1 and NID. Interacts with the gamma-tubulin ring complex (gamma-TuRC), composed of gamma-tubulin, TUBGCP2, TUBGCP3, TUBGCP4, TUBGCP5 and TUBGCP6 (PubMed:29162697). The unglycosylated form interacts with PDE4DIP isoform 13/MMG8/SMYLE; this interaction may connect a pericentrosomal complex, made of AKAP9, CDK5RAP2, EB1/MAPRE1 and PDE4DIP, to the gamma-tubulin ring complex (gamma-TuRC) to promote microtubule assembly and acetylation (PubMed:29162697).   
  
 **Gene Ontology Information:**

Molecular Function

- scavenger receptor activity

Location

- blood microparticle
- collagen-containing extracellular matrix
- extracellular exosome
- extracellular region
- extracellular space
- membrane
- platelet dense granule lumen

Biological process

- cell adhesion
- cellular defense response
- signal transduction

---

37

- **Protein name:** Kelch repeat and BTB domain-containing protein 2
- **Organism:** Homo sapiens
- **Uniprot Accession Number:** Q8IY47
- **Protein sequence length:** 623 aa
- **1D identity (%):** 11.76
- **1D identity (%) [Gaps excluded]:** 18.18
- **1D identity - Alignment Gaps:** 318
- **1D aligned content (<aminoacid>:%):** {'R': 7.55, 'N': 3.77, 'E': 5.66, 'K': 8.49, 'Q': 5.66, 'F': 2.83, 'D': 6.6, 'G': 6.6, 'H': 2.83, 'V': 9.43, 'L': 10.38, 'A': 8.49, 'S': 2.83, 'T': 3.77, 'Y': 1.89, 'P': 8.49, 'C': 1.89, 'I': 1.89, 'M': 0.94}
- **Common reported functions (%):** 0.0
- **Common reported locations (%):** 0.0
- **Common reported processes (%):** 0.0

- **AF ID:** Q8IY47
- **Chain:** A
- **Protein length:** 623 aa
- **Resolution:** N/A
- **b-phipsi:** 0.002073
- **w-rdist:** 0.368833
- **t-alpha:** 0.018779
- **Chemical similarity (Tanimoto Index) (%):** 99.24
- **1D identity (%) [PDB]:** 2.9
- **1D identity (%) [Gaps excluded][PDB]:** 68.33
- **1D identity - Alignment Gaps [PDB]:** 1353
- **1D aligned content [PDB] (<aminoacid>:%):** {'K': 7.32, 'Q': 4.88, 'C': 2.44, 'R': 2.44, 'E': 4.88, 'L': 9.76, 'S': 4.88, 'F': 4.88, 'D': 7.32, 'I': 7.32, 'A': 4.88, 'G': 9.76, 'P': 9.76, 'Y': 4.88, 'V': 9.76, 'M': 2.44, 'T': 2.44}
- **2D identity (%) [PDB]:** 34.82
- **2D identity (%) [Gaps excluded][PDB]:** 87.59
- **2D identity - Alignment Gaps [PDB]:** 635
- **2D aligned content [PDB] (<2D-fold>:%):** {'.': 19.07, 'H': 36.24, 'T': 25.07, 'E': 18.8, 'G': 0.54, 'B': 0.27}
- **3D similarity (TM-Score) (%) [PDB]:** 20.81

- **Gene name:** KBTBD2
- **Entrez ID:** 25948
- **RefSeq ID:** N/A
- **Sequence length:** N/A
- **5-UTR|CDS|3-UTR identity (%):** N/A | N/A | N/A
- **5-UTR|CDS|3-UTR identity (%) [Gaps excluded]:** N/A | N/A | N/A
- **5-UTR|CDS|3-UTR identity [Alignment Gaps]:** N/A | N/A | N/A
- **5-UTR aligned content (<base>:%):** N/A
- **CDS aligned content (<base>:%):** N/A
- **3-UTR aligned content (<base>:%):** N/A

**Uniprot Description:**  
  
 N/A N/A   
  
 **Gene Ontology Information:**

Molecular Function   
  
N/A

Location   
  
N/A

Biological process

- gene expression
- glucose metabolic process
- lipid metabolic process
- phosphatidylinositol 3-kinase signaling
- protein ubiquitination
- response to insulin

---

38

- **Protein name:** tRNA-splicing endonuclease subunit Sen2
- **Organism:** Homo sapiens
- **Uniprot Accession Number:** Q8NCE0
- **Protein sequence length:** 465 aa
- **1D identity (%):** 11.51
- **1D identity (%) [Gaps excluded]:** 26.17
- **1D identity - Alignment Gaps:** 516
- **1D aligned content (<aminoacid>:%):** {'M': 2.83, 'A': 3.77, 'P': 7.55, 'R': 8.49, 'S': 2.83, 'G': 11.32, 'L': 9.43, 'K': 3.77, 'E': 6.6, 'F': 1.89, 'V': 6.6, 'N': 0.94, 'T': 4.72, 'W': 1.89, 'D': 6.6, 'Q': 7.55, 'I': 4.72, 'C': 2.83, 'H': 1.89, 'Y': 3.77}
- **Common reported functions (%):** 0.0
- **Common reported locations (%):** 12.5
- **Common reported processes (%):** 0.0

- **AF ID:** Q8NCE0
- **Chain:** A
- **Protein length:** 465 aa
- **Resolution:** N/A
- **b-phipsi:** 0.007923
- **w-rdist:** 0.51883
- **t-alpha:** 0.000783
- **Chemical similarity (Tanimoto Index) (%):** 99.32
- **1D identity (%) [PDB]:** 2.77
- **1D identity (%) [Gaps excluded][PDB]:** 68.63
- **1D identity - Alignment Gaps [PDB]:** 1213
- **1D aligned content [PDB] (<aminoacid>:%):** {'L': 11.43, 'K': 8.57, 'S': 8.57, 'F': 5.71, 'T': 2.86, 'I': 11.43, 'D': 2.86, 'M': 5.71, 'P': 5.71, 'Q': 5.71, 'G': 8.57, 'C': 5.71, 'Y': 5.71, 'A': 2.86, 'V': 8.57}
- **2D identity (%) [PDB]:** 31.08
- **2D identity (%) [Gaps excluded][PDB]:** 89.12
- **2D identity - Alignment Gaps [PDB]:** 635
- **2D aligned content [PDB] (<2D-fold>:%):** {'T': 23.76, '.': 26.07, 'E': 18.81, 'H': 31.35}
- **3D similarity (TM-Score) (%) [PDB]:** 15.85

- **Gene name:** TSEN2
- **Entrez ID:** 80746
- **RefSeq ID:** N/A
- **Sequence length:** N/A
- **5-UTR|CDS|3-UTR identity (%):** N/A | N/A | N/A
- **5-UTR|CDS|3-UTR identity (%) [Gaps excluded]:** N/A | N/A | N/A
- **5-UTR|CDS|3-UTR identity [Alignment Gaps]:** N/A | N/A | N/A
- **5-UTR aligned content (<base>:%):** N/A
- **CDS aligned content (<base>:%):** N/A
- **3-UTR aligned content (<base>:%):** N/A

**Uniprot Description:**  
  
 Constitutes one of the two catalytic subunit of the tRNA-splicing endonuclease complex, a complex responsible for identification and cleavage of the splice sites in pre-tRNA. It cleaves pre-tRNA at the 5'- and 3'-splice sites to release the intron. The products are an intron and two tRNA half-molecules bearing 2',3'-cyclic phosphate and 5'-OH termini. There are no conserved sequences at the splice sites, but the intron is invariably located at the same site in the gene, placing the splice sites an invariant distance from the constant structural features of the tRNA body. Isoform 1 probably carries the active site for 5'-splice site cleavage. The tRNA splicing endonuclease is also involved in mRNA processing via its association with pre-mRNA 3'-end processing factors, establishing a link between pre-tRNA splicing and pre-mRNA 3'-end formation, suggesting that the endonuclease subunits function in multiple RNA-processing events. Isoform 2 is responsible for processing a yet unknown RNA substrate. The complex containing isoform 2 is not able to cleave pre-tRNAs properly, although it retains endonucleolytic activity.   
  
tRNA splicing endonuclease is a heterotetramer composed of isoform 1 of TSEN2, TSEN15, TSEN34/LENG5 and TSEN54. tRNA splicing endonuclease complex also contains proteins of the pre-mRNA 3'-end processing machinery such as CLP1, CPSF1, CPSF4 and CSTF2. Isoform 2 belongs to a different complex that contains TSEN54 but low level of TSEN15 and TSEN34/LENG5.   
  
 **Gene Ontology Information:**

Molecular Function

- lyase activity
- nucleic acid binding
- tRNA-intron endonuclease activity

Location

- centrosome
- cytosol
- nucleolus
- nucleoplasm
- tRNA-intron endonuclease complex

Biological process

- mRNA processing
- tRNA splicing, via endonucleolytic cleavage and ligation
- tRNA-type intron splice site recognition and cleavage

---

39

- **Protein name:** Bardet-Biedl syndrome 2 protein
- **Organism:** Homo sapiens
- **Uniprot Accession Number:** Q9BXC9
- **Protein sequence length:** 721 aa
- **1D identity (%):** 14.51
- **1D identity (%) [Gaps excluded]:** 22.69
- **1D identity - Alignment Gaps:** 348
- **1D aligned content (<aminoacid>:%):** {'V': 10.71, 'R': 3.57, 'H': 2.86, 'M': 0.71, 'G': 9.29, 'Y': 4.29, 'D': 2.86, 'P': 6.43, 'Q': 2.86, 'S': 6.43, 'L': 12.86, 'A': 5.71, 'N': 3.57, 'T': 7.14, 'F': 5.0, 'W': 0.71, 'E': 3.57, 'I': 5.0, 'K': 5.0, 'C': 1.43}
- **Common reported functions (%):** 0.0
- **Common reported locations (%):** 25.0
- **Common reported processes (%):** 0.0

- **AF ID:** Q9BXC9
- **Chain:** A
- **Protein length:** 721 aa
- **Resolution:** N/A
- **b-phipsi:** 0.002569
- **w-rdist:** 0.308609
- **t-alpha:** 0.042253
- **Chemical similarity (Tanimoto Index) (%):** 99.17
- **1D identity (%) [PDB]:** 2.16
- **1D identity (%) [Gaps excluded][PDB]:** 73.33
- **1D identity - Alignment Gaps [PDB]:** 1481
- **1D aligned content [PDB] (<aminoacid>:%):** {'Q': 6.06, 'T': 6.06, 'K': 3.03, 'V': 12.12, 'S': 18.18, 'L': 21.21, 'E': 6.06, 'A': 3.03, 'N': 3.03, 'P': 6.06, 'D': 3.03, 'R': 3.03, 'M': 3.03, 'F': 6.06}
- **2D identity (%) [PDB]:** 40.55
- **2D identity (%) [Gaps excluded][PDB]:** 91.89
- **2D identity - Alignment Gaps [PDB]:** 609
- **2D aligned content [PDB] (<2D-fold>:%):** {'.': 13.8, 'E': 33.94, 'T': 18.55, 'H': 33.26, 'B': 0.45}
- **3D similarity (TM-Score) (%) [PDB]:** 20.79

- **Gene name:** BBS2
- **Entrez ID:** 259187
- **RefSeq ID:** NM\_031885
- **Transcript sequence length:** 2704
- **5-UTR|CDS|3-UTR identity (%):** 27.49 | 42.85 | 5.85
- **5-UTR|CDS|3-UTR identity (%) [Gaps excluded]:** 74.34 | 75.33 | 75.07
- **5-UTR|CDS|3-UTR identity [Alignment Gaps]:** 259 | 1306 | 3987
- **5-UTR aligned content (<base>:%):** {'G': 38.05, 'C': 44.25, 'A': 4.42, 'T': 13.27}
- **CDS aligned content (<base>:%):** {'T': 25.35, 'C': 21.73, 'G': 23.11, 'A': 29.82}
- **3-UTR aligned content (<base>:%):** {'G': 15.81, 'T': 39.13, 'A': 32.41, 'C': 12.65}

**Uniprot Description:**  
  
 The BBSome complex is thought to function as a coat complex required for sorting of specific membrane proteins to the primary cilia. The BBSome complex is required for ciliogenesis but is dispensable for centriolar satellite function. This ciliogenic function is mediated in part by the Rab8 GDP/GTP exchange factor, which localizes to the basal body and contacts the BBSome. Rab8(GTP) enters the primary cilium and promotes extension of the ciliary membrane. Firstly the BBSome associates with the ciliary membrane and binds to RAB3IP/Rabin8, the guanosyl exchange factor (GEF) for Rab8 and then the Rab8-GTP localizes to the cilium and promotes docking and fusion of carrier vesicles to the base of the ciliary membrane. The BBSome complex, together with the LTZL1, controls SMO ciliary trafficking and contributes to the sonic hedgehog (SHH) pathway regulation. Required for proper BBSome complex assembly and its ciliary localization.   
  
Part of BBSome complex, that contains BBS1, BBS2, BBS4, BBS5, BBS7, BBS8/TTC8, BBS9 and BBIP10. Interacts (via C-terminus) with BBS7. Interacts (via coiled coil domain) with MKKS. Interacts with CCDC28B and ALDOB. Interacts with DLEC1 (PubMed:33144677).   
  
 **Gene Ontology Information:**

Molecular Function   
  
N/A

Location

- BBSome
- ciliary basal body
- ciliary membrane
- cytoplasm
- membrane
- motile cilium
- neuron projection

Biological process

- cilium assembly
- determination of left/right symmetry
- gastrulation
- intracellular transport
- Kupffer's vesicle development
- melanosome transport
- non-motile cilium assembly
- photoreceptor cell maintenance
- pigment granule aggregation in cell center
- regulation of eye photoreceptor cell development

---

40

- **Protein name:** Calpain-1 catalytic subunit
- **Organism:** Homo sapiens
- **Uniprot Accession Number:** P07384
- **Protein sequence length:** 714 aa
- **1D identity (%):** 15.17
- **1D identity (%) [Gaps excluded]:** 25.6
- **1D identity - Alignment Gaps:** 403
- **1D aligned content (<aminoacid>:%):** {'M': 0.67, 'E': 4.0, 'P': 8.0, 'G': 12.0, 'A': 4.67, 'Q': 4.0, 'R': 8.0, 'L': 12.67, 'I': 3.33, 'D': 6.0, 'K': 6.0, 'N': 3.33, 'V': 9.33, 'F': 5.33, 'W': 1.33, 'S': 3.33, 'Y': 2.0, 'C': 2.0, 'T': 4.0}
- **Common reported functions (%):** 0.0
- **Common reported locations (%):** 12.5
- **Common reported processes (%):** 0.0

- **AF ID:** P07384
- **Chain:** A
- **Protein length:** 714 aa
- **Resolution:** N/A
- **b-phipsi:** 0.00861
- **w-rdist:** 0.486877
- **t-alpha:** 0.001565
- **Chemical similarity (Tanimoto Index) (%):** 100.0
- **1D identity (%) [PDB]:** 1.64
- **1D identity (%) [Gaps excluded][PDB]:** 65.79
- **1D identity - Alignment Gaps [PDB]:** 1488
- **1D aligned content [PDB] (<aminoacid>:%):** {'V': 20.0, 'S': 4.0, 'Q': 8.0, 'W': 4.0, 'L': 20.0, 'E': 8.0, 'A': 8.0, 'G': 4.0, 'D': 12.0, 'I': 4.0, 'T': 4.0, 'R': 4.0}
- **2D identity (%) [PDB]:** 40.78
- **2D identity (%) [Gaps excluded][PDB]:** 89.39
- **2D identity - Alignment Gaps [PDB]:** 584
- **2D aligned content [PDB] (<2D-fold>:%):** {'.': 18.72, 'T': 18.72, 'H': 37.67, 'E': 24.2, 'G': 0.68}
- **3D similarity (TM-Score) (%) [PDB]:** 22.53

- **Gene name:** CAPN1
- **Entrez ID:** 408218
- **RefSeq ID:** N/A
- **Sequence length:** N/A
- **5-UTR|CDS|3-UTR identity (%):** N/A | N/A | N/A
- **5-UTR|CDS|3-UTR identity (%) [Gaps excluded]:** N/A | N/A | N/A
- **5-UTR|CDS|3-UTR identity [Alignment Gaps]:** N/A | N/A | N/A
- **5-UTR aligned content (<base>:%):** N/A
- **CDS aligned content (<base>:%):** N/A
- **3-UTR aligned content (<base>:%):** N/A

**Uniprot Description:**  
  
 Calcium-regulated non-lysosomal thiol-protease which catalyzes limited proteolysis of substrates involved in cytoskeletal remodeling and signal transduction (PubMed:19617626, PubMed:21531719, PubMed:2400579). Proteolytically cleaves CTBP1 at 'Asn-375', 'Gly-387' and 'His-409' (PubMed:23707407). Cleaves and activates caspase-7 (CASP7) (PubMed:19617626).   
  
Forms a heterodimer with a small (regulatory) subunit CAPNS1.   
  
 **Gene Ontology Information:**

Molecular Function

- calcium ion binding
- calcium-dependent cysteine-type endopeptidase activity

Location

- acrosomal vesicle
- cytoplasm

Biological process

- proteolysis

---

41

- **Protein name:** Apoptosis-inducing factor 1, mitochondrial
- **Organism:** Homo sapiens
- **Uniprot Accession Number:** O95831
- **Protein sequence length:** 613 aa
- **1D identity (%):** 15.15
- **1D identity (%) [Gaps excluded]:** 24.04
- **1D identity - Alignment Gaps:** 334
- **1D aligned content (<aminoacid>:%):** {'L': 10.22, 'A': 4.38, 'V': 6.57, 'P': 7.3, 'R': 10.22, 'N': 2.19, 'H': 1.46, 'Q': 3.65, 'G': 13.87, 'T': 5.11, 'K': 7.3, 'S': 5.84, 'E': 7.3, 'D': 5.84, 'F': 2.19, 'M': 1.46, 'C': 1.46, 'I': 2.19, 'W': 0.73, 'Y': 0.73}
- **Common reported functions (%):** 0.0
- **Common reported locations (%):** 37.5
- **Common reported processes (%):** 0.0

- **AF ID:** O95831
- **Chain:** A
- **Protein length:** 613 aa
- **Resolution:** N/A
- **b-phipsi:** 0.006094
- **w-rdist:** 0.550391
- **t-alpha:** 0.002347
- **Chemical similarity (Tanimoto Index) (%):** 99.09
- **1D identity (%) [PDB]:** 1.89
- **1D identity (%) [Gaps excluded][PDB]:** 71.05
- **1D identity - Alignment Gaps [PDB]:** 1387
- **1D aligned content [PDB] (<aminoacid>:%):** {'L': 22.22, 'P': 3.7, 'G': 11.11, 'E': 11.11, 'K': 7.41, 'F': 3.7, 'V': 18.52, 'S': 7.41, 'Q': 3.7, 'A': 3.7, 'N': 3.7, 'D': 3.7}
- **2D identity (%) [PDB]:** 37.52
- **2D identity (%) [Gaps excluded][PDB]:** 90.44
- **2D identity - Alignment Gaps [PDB]:** 605
- **2D aligned content [PDB] (<2D-fold>:%):** {'.': 22.16, 'H': 40.46, 'T': 16.24, 'E': 21.13}
- **3D similarity (TM-Score) (%) [PDB]:** 19.73

- **Gene name:** AIFM1
- **Entrez ID:** N/A
- **RefSeq ID:** NM\_145812
- **Transcript sequence length:** 2210
- **5-UTR|CDS|3-UTR identity (%):** 23.91 | 41.02 | 2.71
- **5-UTR|CDS|3-UTR identity (%) [Gaps excluded]:** 68.87 | 75.92 | 74.52
- **5-UTR|CDS|3-UTR identity [Alignment Gaps]:** 284 | 1318 | 4166
- **5-UTR aligned content (<base>:%):** {'G': 50.0, 'T': 6.73, 'C': 34.62, 'A': 8.65}
- **CDS aligned content (<base>:%):** {'T': 22.62, 'G': 27.3, 'C': 21.09, 'A': 29.0}
- **3-UTR aligned content (<base>:%):** {'A': 27.35, 'G': 19.66, 'C': 13.68, 'T': 39.32}

**Uniprot Description:**  
  
 Functions both as NADH oxidoreductase and as regulator of apoptosis (PubMed:20362274, PubMed:23217327, PubMed:17094969). In response to apoptotic stimuli, it is released from the mitochondrion intermembrane space into the cytosol and to the nucleus, where it functions as a proapoptotic factor in a caspase-independent pathway. The soluble form (AIFsol) found in the nucleus induces 'parthanatos' i.e. caspase-independent fragmentation of chromosomal DNA (By similarity). Binds to DNA in a sequence-independent manner (PubMed:27178839). Interacts with EIF3G, and thereby inhibits the EIF3 machinery and protein synthesis, and activates caspase-7 to amplify apoptosis (PubMed:17094969). Plays a critical role in caspase-independent, pyknotic cell death in hydrogen peroxide-exposed cells (PubMed:19418225). In contrast, participates in normal mitochondrial metabolism. Plays an important role in the regulation of respiratory chain biogenesis by interacting with CHCHD4 and controlling CHCHD4 mitochondrial import (PubMed:26004228).   
  
Monomer (oxidized form). Homodimer (reduced form). Upon reduction with NADH, undergoes dimerization and forms tight, long-lived FADH2-NAD charge transfer complexes (CTC) resistant to oxidation (PubMed:24914854, PubMed:20111043, PubMed:23217327, PubMed:27818101). Also dimerizes with isoform 3 preventing its release from mitochondria (PubMed:20111043). Interacts with XIAP/BIRC4 (PubMed:17967870). Interacts (via N-terminus) with EIF3G (via C-terminus) (PubMed:17094969). Interacts with PRELID1 (PubMed:21364629). Interacts with CHCHD4; the interaction increases in presence of NADH (PubMed:26004228).   
  
 **Gene Ontology Information:**

Molecular Function

- DNA binding
- FAD binding
- NAD(P)H oxidase H2O2-forming activity
- NADH dehydrogenase activity
- oxidoreductase activity, acting on NAD(P)H
- poly-ADP-D-ribose binding
- protein dimerization activity

Location

- cytoplasm
- cytosol
- mitochondrial inner membrane
- mitochondrial intermembrane space
- mitochondrion
- nucleus
- perinuclear region of cytoplasm

Biological process

- activation of cysteine-type endopeptidase activity involved in apoptotic process
- apoptotic process
- cellular response to aldosterone
- cellular response to estradiol stimulus
- cellular response to hydrogen peroxide
- cellular response to nitric oxide
- cellular response to oxygen-glucose deprivation
- chromosome condensation
- intrinsic apoptotic signaling pathway in response to endoplasmic reticulum stress
- mitochondrial respiratory chain complex assembly
- mitochondrial respiratory chain complex I assembly
- neuron apoptotic process
- neuron differentiation
- positive regulation of apoptotic process
- positive regulation of necroptotic process
- positive regulation of neuron apoptotic process
- protein import into mitochondrial intermembrane space
- regulation of apoptotic DNA fragmentation
- response to ischemia
- response to L-glutamate
- response to toxic substance

---

42

- **Protein name:** Extracellular tyrosine-protein kinase PKDCC
- **Organism:** Homo sapiens
- **Uniprot Accession Number:** Q504Y2
- **Protein sequence length:** 493 aa
- **1D identity (%):** 9.61
- **1D identity (%) [Gaps excluded]:** 26.03
- **1D identity - Alignment Gaps:** 624
- **1D aligned content (<aminoacid>:%):** {'M': 2.11, 'A': 5.26, 'G': 9.47, 'S': 5.26, 'L': 10.53, 'F': 3.16, 'P': 12.63, 'R': 11.58, 'Y': 5.26, 'D': 7.37, 'N': 4.21, 'V': 5.26, 'E': 3.16, 'I': 1.05, 'T': 4.21, 'H': 2.11, 'C': 2.11, 'K': 2.11, 'Q': 3.16}
- **Common reported functions (%):** 0.0
- **Common reported locations (%):** 0.0
- **Common reported processes (%):** 0.0

- **AF ID:** Q504Y2
- **Chain:** A
- **Protein length:** 493 aa
- **Resolution:** N/A
- **b-phipsi:** 0.003102
- **w-rdist:** 0.758334
- **t-alpha:** 0.0
- **Chemical similarity (Tanimoto Index) (%):** 97.33
- **1D identity (%) [PDB]:** 2.3
- **1D identity (%) [Gaps excluded][PDB]:** 76.92
- **1D identity - Alignment Gaps [PDB]:** 1265
- **1D aligned content [PDB] (<aminoacid>:%):** {'M': 6.67, 'G': 16.67, 'P': 13.33, 'Y': 6.67, 'K': 6.67, 'E': 10.0, 'F': 3.33, 'V': 3.33, 'T': 6.67, 'L': 10.0, 'R': 6.67, 'A': 3.33, 'Q': 3.33, 'N': 3.33}
- **2D identity (%) [PDB]:** 37.08
- **2D identity (%) [Gaps excluded][PDB]:** 87.72
- **2D identity - Alignment Gaps [PDB]:** 545
- **2D aligned content [PDB] (<2D-fold>:%):** {'T': 22.57, 'H': 39.71, '.': 15.14, 'E': 20.86, 'B': 0.86, 'G': 0.86}
- **3D similarity (TM-Score) (%) [PDB]:** 15.21

- **Gene name:** PKDCC
- **Entrez ID:** 91461
- **RefSeq ID:** NM\_138370
- **Transcript sequence length:** 2490
- **5-UTR|CDS|3-UTR identity (%):** 27.74 | 34.09 | 12.63
- **5-UTR|CDS|3-UTR identity (%) [Gaps excluded]:** 78.81 | 73.16 | 75.34
- **5-UTR|CDS|3-UTR identity [Alignment Gaps]:** 278 | 1482 | 3646
- **5-UTR aligned content (<base>:%):** {'A': 7.56, 'G': 52.94, 'C': 36.97, 'T': 2.52}
- **CDS aligned content (<base>:%):** {'A': 17.12, 'T': 18.08, 'G': 32.24, 'C': 32.56}
- **3-UTR aligned content (<base>:%):** {'C': 20.98, 'A': 24.23, 'T': 25.86, 'G': 28.93}

**Uniprot Description:**  
  
 Secreted tyrosine-protein kinase that mediates phosphorylation of extracellular proteins and endogenous proteins in the secretory pathway, which is essential for patterning at organogenesis stages. Mediates phosphorylation of MMP1, MMP13, MMP14, MMP19 and ERP29 (PubMed:25171405). Probably plays a role in platelets: rapidly and quantitatively secreted from platelets in response to stimulation of platelet degranulation (PubMed:25171405). May also have serine/threonine protein kinase activity. Required for longitudinal bone growth through regulation of chondrocyte differentiation. May be indirectly involved in protein transport from the Golgi apparatus to the plasma membrane (By similarity). N/A   
  
 **Gene Ontology Information:**

Molecular Function

- ATP binding
- non-membrane spanning protein tyrosine kinase activity
- protein kinase activity

Location

- extracellular region
- Golgi apparatus

Biological process

- bone mineralization
- cell differentiation
- embryonic digestive tract development
- limb morphogenesis
- lung alveolus development
- multicellular organism growth
- negative regulation of Golgi to plasma membrane protein transport
- peptidyl-tyrosine phosphorylation
- positive regulation of bone mineralization
- positive regulation of chondrocyte differentiation
- protein transport
- roof of mouth development
- skeletal system development

---

43

- **Protein name:** 5'-AMP-activated protein kinase catalytic subunit alpha-2
- **Organism:** Homo sapiens
- **Uniprot Accession Number:** P54646
- **Protein sequence length:** 552 aa
- **1D identity (%):** 10.43
- **1D identity (%) [Gaps excluded]:** 21.34
- **1D identity - Alignment Gaps:** 485
- **1D aligned content (<aminoacid>:%):** {'Q': 4.04, 'R': 9.09, 'V': 11.11, 'K': 8.08, 'G': 10.1, 'N': 5.05, 'L': 8.08, 'P': 10.1, 'H': 4.04, 'E': 3.03, 'C': 5.05, 'S': 5.05, 'A': 2.02, 'D': 2.02, 'F': 4.04, 'M': 1.01, 'Y': 2.02, 'W': 1.01, 'I': 3.03, 'T': 2.02}
- **Common reported functions (%):** 0.0
- **Common reported locations (%):** 25.0
- **Common reported processes (%):** 8.33

- **AF ID:** P54646
- **Chain:** A
- **Protein length:** 552 aa
- **Resolution:** N/A
- **b-phipsi:** 0.004042
- **w-rdist:** 0.388371
- **t-alpha:** 0.004717
- **Chemical similarity (Tanimoto Index) (%):** 97.26
- **1D identity (%) [PDB]:** 1.68
- **1D identity (%) [Gaps excluded][PDB]:** 76.67
- **1D identity - Alignment Gaps [PDB]:** 1342
- **1D aligned content [PDB] (<aminoacid>:%):** {'V': 8.7, 'G': 17.39, 'K': 4.35, 'S': 13.04, 'A': 8.7, 'D': 8.7, 'I': 4.35, 'T': 4.35, 'P': 4.35, 'E': 4.35, 'F': 8.7, 'Y': 4.35, 'L': 4.35, 'C': 4.35}
- **2D identity (%) [PDB]:** 38.43
- **2D identity (%) [Gaps excluded][PDB]:** 89.55
- **2D identity - Alignment Gaps [PDB]:** 560
- **2D aligned content [PDB] (<2D-fold>:%):** {'T': 22.81, 'E': 19.1, 'H': 41.38, '.': 15.92, 'G': 0.8}
- **3D similarity (TM-Score) (%) [PDB]:** 20.46

- **Gene name:** PRKAA2
- **Entrez ID:** 5563
- **RefSeq ID:** NM\_006252
- **Transcript sequence length:** 9355
- **5-UTR|CDS|3-UTR identity (%):** 14.0 | 37.72 | 39.26
- **5-UTR|CDS|3-UTR identity (%) [Gaps excluded]:** 83.82 | 75.76 | 78.32
- **5-UTR|CDS|3-UTR identity [Alignment Gaps]:** 339 | 1423 | 3955
- **5-UTR aligned content (<base>:%):** {'G': 57.89, 'C': 31.58, 'T': 1.75, 'A': 8.77}
- **CDS aligned content (<base>:%):** {'A': 30.59, 'T': 26.47, 'G': 21.98, 'C': 20.95}
- **3-UTR aligned content (<base>:%):** {'G': 18.53, 'T': 36.1, 'C': 14.74, 'A': 30.64}

**Uniprot Description:**  
  
 Catalytic subunit of AMP-activated protein kinase (AMPK), an energy sensor protein kinase that plays a key role in regulating cellular energy metabolism. In response to reduction of intracellular ATP levels, AMPK activates energy-producing pathways and inhibits energy-consuming processes: inhibits protein, carbohydrate and lipid biosynthesis, as well as cell growth and proliferation. AMPK acts via direct phosphorylation of metabolic enzymes, and by longer-term effects via phosphorylation of transcription regulators. Also acts as a regulator of cellular polarity by remodeling the actin cytoskeleton; probably by indirectly activating myosin. Regulates lipid synthesis by phosphorylating and inactivating lipid metabolic enzymes such as ACACA, ACACB, GYS1, HMGCR and LIPE; regulates fatty acid and cholesterol synthesis by phosphorylating acetyl-CoA carboxylase (ACACA and ACACB) and hormone-sensitive lipase (LIPE) enzymes, respectively. Regulates insulin-signaling and glycolysis by phosphorylating IRS1, PFKFB2 and PFKFB3. Involved in insulin receptor/INSR internalization (PubMed:25687571). AMPK stimulates glucose uptake in muscle by increasing the translocation of the glucose transporter SLC2A4/GLUT4 to the plasma membrane, possibly by mediating phosphorylation of TBC1D4/AS160. Regulates transcription and chromatin structure by phosphorylating transcription regulators involved in energy metabolism such as CRTC2/TORC2, FOXO3, histone H2B, HDAC5, MEF2C, MLXIPL/ChREBP, EP300, HNF4A, p53/TP53, SREBF1, SREBF2 and PPARGC1A. Acts as a key regulator of glucose homeostasis in liver by phosphorylating CRTC2/TORC2, leading to CRTC2/TORC2 sequestration in the cytoplasm. In response to stress, phosphorylates 'Ser-36' of histone H2B (H2BS36ph), leading to promote transcription. Acts as a key regulator of cell growth and proliferation by phosphorylating TSC2, RPTOR and ATG1/ULK1: in response to nutrient limitation, negatively regulates the mTORC1 complex by phosphorylating RPTOR component of the mTORC1 complex and by phosphorylating and activating TSC2. In response to nutrient limitation, promotes autophagy by phosphorylating and activating ATG1/ULK1. In that process also activates WDR45 (PubMed:28561066). AMPK also acts as a regulator of circadian rhythm by mediating phosphorylation of CRY1, leading to destabilize it. May regulate the Wnt signaling pathway by phosphorylating CTNNB1, leading to stabilize it. Also phosphorylates CFTR, EEF2K, KLC1, NOS3 and SLC12A1. Plays an important role in the differential regulation of pro-autophagy (composed of PIK3C3, BECN1, PIK3R4 and UVRAG or ATG14) and non-autophagy (composed of PIK3C3, BECN1 and PIK3R4) complexes, in response to glucose starvation. Can inhibit the non-autophagy complex by phosphorylating PIK3C3 and can activate the pro-autophagy complex by phosphorylating BECN1 (By similarity).   
  
AMPK is a heterotrimer of an alpha catalytic subunit (PRKAA1 or PRKAA2), a beta (PRKAB1 or PRKAB2) and a gamma non-catalytic subunits (PRKAG1, PRKAG2 or PRKAG3). Interacts with FNIP1 and FNIP2. Associates with internalized insulin receptor/INSR complexes on Golgi/endosomal membranes; PRKAA2/AMPK2 together with ATIC and HACD3/PTPLAD1 is proposed to be part of a signaling network regulating INSR autophosphorylation and endocytosis (PubMed:25687571).   
  
 **Gene Ontology Information:**

Molecular Function

- [acetyl-CoA carboxylase] kinase activity
- [hydroxymethylglutaryl-CoA reductase (NADPH)] kinase activity
- AMP-activated protein kinase activity
- ATP binding
- chromatin binding
- histone H2BS36 kinase activity
- metal ion binding
- protein kinase activity
- protein serine kinase activity
- protein serine/threonine kinase activity
- protein serine/threonine/tyrosine kinase activity

Location

- axon
- cytoplasmic stress granule
- cytosol
- dendrite
- Golgi apparatus
- neuronal cell body
- nuclear speck
- nucleoplasm
- nucleotide-activated protein kinase complex
- nucleus

Biological process

- autophagy
- cellular response to calcium ion
- cellular response to glucose starvation
- cellular response to glucose stimulus
- cellular response to nutrient levels
- cellular response to oxidative stress
- cellular response to prostaglandin E stimulus
- cellular response to xenobiotic stimulus
- cholesterol biosynthetic process
- energy homeostasis
- fatty acid biosynthetic process
- fatty acid homeostasis
- glucose homeostasis
- intracellular signal transduction
- lipid biosynthetic process
- lipid droplet disassembly
- negative regulation of apoptotic process
- negative regulation of gene expression
- negative regulation of hepatocyte apoptotic process
- negative regulation of TOR signaling
- negative regulation of tubulin deacetylation
- positive regulation of autophagy
- positive regulation of glycolytic process
- positive regulation of macroautophagy
- positive regulation of peptidyl-lysine acetylation
- positive regulation of cellular protein localization
- protein localization to lipid droplet
- protein phosphorylation
- regulation of circadian rhythm
- regulation of macroautophagy
- regulation of microtubule cytoskeleton organization
- regulation of stress granule assembly
- response to muscle activity
- rhythmic process
- signal transduction
- Wnt signaling pathway

---

44

- **Protein name:** Crossover junction endonuclease MUS81
- **Organism:** Homo sapiens
- **Uniprot Accession Number:** Q96NY9
- **Protein sequence length:** 551 aa
- **1D identity (%):** 12.19
- **1D identity (%) [Gaps excluded]:** 23.9
- **1D identity - Alignment Gaps:** 458
- **1D aligned content (<aminoacid>:%):** {'M': 0.88, 'R': 9.65, 'P': 15.79, 'L': 12.28, 'E': 9.65, 'T': 3.51, 'K': 4.39, 'S': 5.26, 'Y': 4.39, 'G': 10.53, 'C': 3.51, 'D': 4.39, 'A': 3.51, 'W': 0.88, 'V': 5.26, 'Q': 2.63, 'I': 0.88, 'H': 0.88, 'N': 0.88, 'F': 0.88}
- **Common reported functions (%):** 0.0
- **Common reported locations (%):** 12.5
- **Common reported processes (%):** 0.0

- **AF ID:** Q96NY9
- **Chain:** A
- **Protein length:** 551 aa
- **Resolution:** N/A
- **b-phipsi:** 0.010134
- **w-rdist:** 0.178816
- **t-alpha:** 0.008607
- **Chemical similarity (Tanimoto Index) (%):** 98.57
- **1D identity (%) [PDB]:** 3.56
- **1D identity (%) [Gaps excluded][PDB]:** 58.02
- **1D identity - Alignment Gaps [PDB]:** 1239
- **1D aligned content [PDB] (<aminoacid>:%):** {'T': 12.77, 'V': 10.64, 'Q': 6.38, 'K': 4.26, 'R': 8.51, 'H': 2.13, 'L': 14.89, 'C': 6.38, 'A': 6.38, 'G': 6.38, 'S': 4.26, 'N': 2.13, 'P': 4.26, 'Y': 4.26, 'D': 6.38}
- **2D identity (%) [PDB]:** 40.71
- **2D identity (%) [Gaps excluded][PDB]:** 89.5
- **2D identity - Alignment Gaps [PDB]:** 525
- **2D aligned content [PDB] (<2D-fold>:%):** {'.': 25.51, 'T': 16.84, 'H': 46.94, 'E': 9.95, 'G': 0.77}
- **3D similarity (TM-Score) (%) [PDB]:** 18.67

- **Gene name:** MUS81
- **Entrez ID:** 80198
- **RefSeq ID:** N/A
- **Sequence length:** N/A
- **5-UTR|CDS|3-UTR identity (%):** N/A | N/A | N/A
- **5-UTR|CDS|3-UTR identity (%) [Gaps excluded]:** N/A | N/A | N/A
- **5-UTR|CDS|3-UTR identity [Alignment Gaps]:** N/A | N/A | N/A
- **5-UTR aligned content (<base>:%):** N/A
- **CDS aligned content (<base>:%):** N/A
- **3-UTR aligned content (<base>:%):** N/A

**Uniprot Description:**  
  
 Interacts with EME1 and EME2 to form a DNA structure-specific endonuclease with substrate preference for branched DNA structures with a 5'-end at the branch nick. Typical substrates include 3'-flap structures, replication forks and nicked Holliday junctions. May be required in mitosis for the processing of stalled or collapsed replication forks.   
  
May self-associate. Interacts with EME1, EME2 and CHEK2. Interacts with BLM, and this interaction may stimulate the endonuclease activity of MUS81. Interacts with SLX4/BTBD12; this interaction is direct and links the MUS81-EME1 complex to SLX4, which may coordinate the action of the structure-specific endonuclease during DNA repair. Interacts with DCLRE1B/Apollo.   
  
 **Gene Ontology Information:**

Molecular Function

- 3'-flap endonuclease activity
- crossover junction endodeoxyribonuclease activity
- DNA binding
- endonuclease activity
- metal ion binding

Location

- endodeoxyribonuclease complex
- Holliday junction resolvase complex
- nuclear replication fork
- nucleolus
- nucleoplasm
- nucleus
- replication fork

Biological process

- DNA catabolic process
- DNA repair
- double-strand break repair
- double-strand break repair via break-induced replication
- intra-S DNA damage checkpoint
- osteoblast proliferation
- replication fork processing
- resolution of meiotic recombination intermediates
- response to intra-S DNA damage checkpoint signaling

---

45

- **Protein name:** Zinc finger protein 200
- **Organism:** Homo sapiens
- **Uniprot Accession Number:** P98182
- **Protein sequence length:** 395 aa
- **1D identity (%):** 8.45
- **1D identity (%) [Gaps excluded]:** 21.29
- **1D identity - Alignment Gaps:** 542
- **1D aligned content (<aminoacid>:%):** {'K': 7.89, 'M': 2.63, 'P': 6.58, 'F': 3.95, 'G': 6.58, 'D': 3.95, 'L': 9.21, 'V': 7.89, 'T': 5.26, 'N': 3.95, 'S': 9.21, 'A': 2.63, 'R': 7.89, 'Q': 3.95, 'E': 3.95, 'I': 2.63, 'C': 2.63, 'Y': 3.95, 'H': 5.26}
- **Common reported functions (%):** 0.0
- **Common reported locations (%):** 12.5
- **Common reported processes (%):** 0.0

- **AF ID:** P98182
- **Chain:** A
- **Protein length:** 395 aa
- **Resolution:** N/A
- **b-phipsi:** 0.006281
- **w-rdist:** 0.613067
- **t-alpha:** 0.001565
- **Chemical similarity (Tanimoto Index) (%):** 99.1
- **1D identity (%) [PDB]:** 3.45
- **1D identity (%) [Gaps excluded][PDB]:** 71.93
- **1D identity - Alignment Gaps [PDB]:** 1131
- **1D aligned content [PDB] (<aminoacid>:%):** {'K': 14.63, 'P': 4.88, 'L': 9.76, 'T': 12.2, 'S': 4.88, 'Q': 9.76, 'V': 9.76, 'I': 2.44, 'R': 12.2, 'H': 4.88, 'C': 4.88, 'G': 2.44, 'M': 2.44, 'F': 2.44, 'N': 2.44}
- **2D identity (%) [PDB]:** 28.8
- **2D identity (%) [Gaps excluded][PDB]:** 86.5
- **2D identity - Alignment Gaps [PDB]:** 623
- **2D aligned content [PDB] (<2D-fold>:%):** {'.': 24.54, 'T': 26.02, 'H': 43.87, 'B': 0.74, 'E': 3.72, 'G': 1.12}
- **3D similarity (TM-Score) (%) [PDB]:** 14.88

- **Gene name:** ZNF200
- **Entrez ID:** 7752
- **RefSeq ID:** N/A
- **Sequence length:** N/A
- **5-UTR|CDS|3-UTR identity (%):** N/A | N/A | N/A
- **5-UTR|CDS|3-UTR identity (%) [Gaps excluded]:** N/A | N/A | N/A
- **5-UTR|CDS|3-UTR identity [Alignment Gaps]:** N/A | N/A | N/A
- **5-UTR aligned content (<base>:%):** N/A
- **CDS aligned content (<base>:%):** N/A
- **3-UTR aligned content (<base>:%):** N/A

**Uniprot Description:**  
  
 Could have a role in spermatogenesis. N/A   
  
 **Gene Ontology Information:**

Molecular Function

- metal ion binding

Location

- nucleus

Biological process   
  
N/A

---

46

- **Protein name:** Phenylalanine--tRNA ligase beta subunit
- **Organism:** Homo sapiens
- **Uniprot Accession Number:** Q9NSD9
- **Protein sequence length:** 589 aa
- **1D identity (%):** 13.34
- **1D identity (%) [Gaps excluded]:** 24.37
- **1D identity - Alignment Gaps:** 424
- **1D aligned content (<aminoacid>:%):** {'V': 8.0, 'S': 4.8, 'L': 14.4, 'A': 5.6, 'D': 4.0, 'I': 10.4, 'T': 4.8, 'G': 6.4, 'P': 10.4, 'R': 4.0, 'K': 8.0, 'F': 2.4, 'E': 5.6, 'Q': 2.4, 'C': 3.2, 'Y': 4.0, 'H': 0.8, 'N': 0.8}
- **Common reported functions (%):** 0.0
- **Common reported locations (%):** 0.0
- **Common reported processes (%):** 0.0

- **AF ID:** Q9NSD9
- **Chain:** A
- **Protein length:** 589 aa
- **Resolution:** N/A
- **b-phipsi:** 0.00047
- **w-rdist:** 0.736757
- **t-alpha:** 0.031477
- **Chemical similarity (Tanimoto Index) (%):** 87.82
- **1D identity (%) [PDB]:** 3.82
- **1D identity (%) [Gaps excluded][PDB]:** 66.67
- **1D identity - Alignment Gaps [PDB]:** 1283
- **1D aligned content [PDB] (<aminoacid>:%):** {'M': 1.92, 'F': 3.85, 'L': 17.31, 'T': 7.69, 'Y': 1.92, 'G': 9.62, 'Q': 3.85, 'I': 7.69, 'V': 17.31, 'K': 9.62, 'P': 5.77, 'A': 5.77, 'E': 1.92, 'N': 1.92, 'S': 1.92, 'C': 1.92}
- **2D identity (%) [PDB]:** 37.03
- **2D identity (%) [Gaps excluded][PDB]:** 87.18
- **2D identity - Alignment Gaps [PDB]:** 581
- **2D aligned content [PDB] (<2D-fold>:%):** {'E': 29.68, 'H': 34.49, '.': 14.97, 'T': 20.05, 'G': 0.8}
- **3D similarity (TM-Score) (%) [PDB]:** 17.6

- **Gene name:** FARSB
- **Entrez ID:** N/A
- **RefSeq ID:** N/A
- **Sequence length:** N/A
- **5-UTR|CDS|3-UTR identity (%):** N/A | N/A | N/A
- **5-UTR|CDS|3-UTR identity (%) [Gaps excluded]:** N/A | N/A | N/A
- **5-UTR|CDS|3-UTR identity [Alignment Gaps]:** N/A | N/A | N/A
- **5-UTR aligned content (<base>:%):** N/A
- **CDS aligned content (<base>:%):** N/A
- **3-UTR aligned content (<base>:%):** N/A

**Uniprot Description:**  
  
 N/A   
  
Heterotetramer; dimer of two heterodimers formed by FARSA and FARSB.   
  
 **Gene Ontology Information:**

Molecular Function

- ATP binding
- magnesium ion binding
- phenylalanine-tRNA ligase activity
- RNA binding

Location

- phenylalanine-tRNA ligase complex

Biological process

- phenylalanyl-tRNA aminoacylation

---

47

- **Protein name:** Elongation factor 2
- **Organism:** Homo sapiens
- **Uniprot Accession Number:** P13639
- **Protein sequence length:** 858 aa
- **1D identity (%):** 14.57
- **1D identity (%) [Gaps excluded]:** 21.05
- **1D identity - Alignment Gaps:** 313
- **1D aligned content (<aminoacid>:%):** {'V': 8.11, 'N': 2.7, 'R': 4.73, 'D': 6.76, 'M': 3.38, 'G': 8.11, 'I': 8.11, 'E': 8.11, 'K': 6.08, 'L': 10.14, 'S': 2.03, 'T': 5.41, 'P': 8.78, 'Y': 3.38, 'Q': 2.7, 'F': 4.05, 'A': 4.05, 'C': 0.68, 'H': 2.7}
- **Common reported functions (%):** 0.0
- **Common reported locations (%):** 37.5
- **Common reported processes (%):** 0.0

- **AF ID:** P13639
- **Chain:** A
- **Protein length:** 858 aa
- **Resolution:** N/A
- **b-phipsi:** 0.00268
- **w-rdist:** 0.358253
- **t-alpha:** 0.025039
- **Chemical similarity (Tanimoto Index) (%):** 99.02
- **1D identity (%) [PDB]:** 3.24
- **1D identity (%) [Gaps excluded][PDB]:** 72.6
- **1D identity - Alignment Gaps [PDB]:** 1562
- **1D aligned content [PDB] (<aminoacid>:%):** {'D': 9.43, 'K': 13.21, 'S': 3.77, 'F': 3.77, 'T': 1.89, 'Q': 5.66, 'R': 3.77, 'A': 7.55, 'G': 11.32, 'I': 5.66, 'P': 7.55, 'Y': 1.89, 'V': 9.43, 'E': 3.77, 'M': 3.77, 'H': 1.89, 'L': 5.66}
- **2D identity (%) [PDB]:** 42.92
- **2D identity (%) [Gaps excluded][PDB]:** 87.06
- **2D identity - Alignment Gaps [PDB]:** 580
- **2D aligned content [PDB] (<2D-fold>:%):** {'.': 16.5, 'T': 21.79, 'E': 22.2, 'H': 38.9, 'G': 0.61}
- **3D similarity (TM-Score) (%) [PDB]:** 26.68

- **Gene name:** EEF2
- **Entrez ID:** 281138
- **RefSeq ID:** NM\_001961
- **Transcript sequence length:** 3158
- **5-UTR|CDS|3-UTR identity (%):** 13.38 | 45.48 | 6.5
- **5-UTR|CDS|3-UTR identity (%) [Gaps excluded]:** 75.34 | 72.76 | 75.07
- **5-UTR|CDS|3-UTR identity [Alignment Gaps]:** 338 | 1191 | 4021
- **5-UTR aligned content (<base>:%):** {'C': 60.0, 'T': 9.09, 'G': 23.64, 'A': 7.27}
- **CDS aligned content (<base>:%):** {'T': 19.03, 'G': 28.72, 'A': 24.91, 'C': 27.34}
- **3-UTR aligned content (<base>:%):** {'G': 31.12, 'C': 20.98, 'T': 21.33, 'A': 26.57}

**Uniprot Description:**  
  
 Catalyzes the GTP-dependent ribosomal translocation step during translation elongation. During this step, the ribosome changes from the pre-translocational (PRE) to the post-translocational (POST) state as the newly formed A-site-bound peptidyl-tRNA and P-site-bound deacylated tRNA move to the P and E sites, respectively. Catalyzes the coordinated movement of the two tRNA molecules, the mRNA and conformational changes in the ribosome.   
  
Component of the mRNA surveillance SURF complex, at least composed of ERF1, ERF3 (ERF3A or ERF3B), EEF2, UPF1/RENT1, SMG1, SMG8 and SMG9 (PubMed:19417104). Interacts with RBPMS2 (PubMed:25064856).   
  
 **Gene Ontology Information:**

Molecular Function

- GTP binding
- GTPase activity
- ribosome binding
- translation elongation factor activity

Location

- cytoplasm
- cytosol
- nucleus
- ribonucleoprotein complex

Biological process

- translational elongation

---

48

- **Protein name:** NAD-dependent protein deacetylase sirtuin-3, mitochondrial
- **Organism:** Homo sapiens
- **Uniprot Accession Number:** Q9NTG7
- **Protein sequence length:** 399 aa
- **1D identity (%):** 9.11
- **1D identity (%) [Gaps excluded]:** 28.52
- **1D identity - Alignment Gaps:** 650
- **1D aligned content (<aminoacid>:%):** {'M': 1.15, 'G': 13.79, 'L': 9.2, 'V': 9.2, 'A': 6.9, 'C': 2.3, 'S': 8.05, 'P': 14.94, 'Q': 3.45, 'R': 8.05, 'I': 3.45, 'D': 6.9, 'E': 3.45, 'K': 1.15, 'Y': 1.15, 'T': 5.75, 'H': 1.15}
- **Common reported functions (%):** 0.0
- **Common reported locations (%):** 12.5
- **Common reported processes (%):** 0.0

- **AF ID:** Q9NTG7
- **Chain:** A
- **Protein length:** 399 aa
- **Resolution:** N/A
- **b-phipsi:** 0.001232
- **w-rdist:** 0.834692
- **t-alpha:** 0.002353
- **Chemical similarity (Tanimoto Index) (%):** 99.1
- **1D identity (%) [PDB]:** 1.64
- **1D identity (%) [Gaps excluded][PDB]:** 74.07
- **1D identity - Alignment Gaps [PDB]:** 1195
- **1D aligned content [PDB] (<aminoacid>:%):** {'S': 15.0, 'V': 15.0, 'L': 30.0, 'Q': 5.0, 'E': 10.0, 'A': 10.0, 'H': 5.0, 'P': 5.0, 'D': 5.0}
- **2D identity (%) [PDB]:** 25.08
- **2D identity (%) [Gaps excluded][PDB]:** 85.21
- **2D identity - Alignment Gaps [PDB]:** 681
- **2D aligned content [PDB] (<2D-fold>:%):** {'.': 27.69, 'T': 31.4, 'H': 29.75, 'E': 9.92, 'G': 1.24}
- **3D similarity (TM-Score) (%) [PDB]:** 18.51

- **Gene name:** SIRT3
- **Entrez ID:** 23410
- **RefSeq ID:** N/A
- **Sequence length:** N/A
- **5-UTR|CDS|3-UTR identity (%):** N/A | N/A | N/A
- **5-UTR|CDS|3-UTR identity (%) [Gaps excluded]:** N/A | N/A | N/A
- **5-UTR|CDS|3-UTR identity [Alignment Gaps]:** N/A | N/A | N/A
- **5-UTR aligned content (<base>:%):** N/A
- **CDS aligned content (<base>:%):** N/A
- **3-UTR aligned content (<base>:%):** N/A

**Uniprot Description:**  
  
 NAD-dependent protein deacetylase (PubMed:12186850, PubMed:12374852, PubMed:16788062, PubMed:18680753, PubMed:18794531, PubMed:23283301, PubMed:24121500, PubMed:24252090, PubMed:19535340). Activates or deactivates mitochondrial target proteins by deacetylating key lysine residues (PubMed:12186850, PubMed:12374852, PubMed:16788062, PubMed:18680753, PubMed:18794531, PubMed:23283301, PubMed:24121500, PubMed:24252090). Known targets include ACSS1, IDH, GDH, SOD2, PDHA1, LCAD, SDHA and the ATP synthase subunit ATP5PO (PubMed:16788062, PubMed:18680753, PubMed:24121500, PubMed:24252090, PubMed:19535340). Contributes to the regulation of the cellular energy metabolism (PubMed:24252090). Important for regulating tissue-specific ATP levels (PubMed:18794531). In response to metabolic stress, deacetylates transcription factor FOXO3 and recruits FOXO3 and mitochondrial RNA polymerase POLRMT to mtDNA to promote mtDNA transcription (PubMed:23283301). Acts as a regulator of ceramide metabolism by mediating deacetylation of ceramide synthases CERS1, CERS2 and CERS6, thereby increasing their activity and promoting mitochondrial ceramide accumulation (By similarity).   
  
Upon metabolic stress, forms a complex composed of FOXO3, SIRT3 and mitochondrial RNA polymerase POLRMT; the complex is recruited to mtDNA in a SIRT3-dependent manner (PubMed:23283301). Also forms a complex composed of FOXO3, SIRT3, TFAM and POLRMT (PubMed:29445193). Interacts with NDUFA9, ACSS1, IDH2 and GDH (PubMed:16788062, PubMed:18794531, PubMed:19535340, PubMed:18680753). Interacts with PCCA (PubMed:23438705).   
  
 **Gene Ontology Information:**

Molecular Function

- enzyme binding
- NAD+ binding
- NAD-dependent histone deacetylase activity
- NAD-dependent protein deacetylase activity
- sequence-specific DNA binding
- transferase activity
- zinc ion binding

Location

- mitochondrial matrix
- mitochondrion
- nucleoplasm
- nucleus
- protein-containing complex

Biological process

- aerobic respiration
- negative regulation of ERK1 and ERK2 cascade
- negative regulation of reactive oxygen species metabolic process
- peptidyl-lysine deacetylation
- positive regulation of catalase activity
- positive regulation of ceramide biosynthetic process
- positive regulation of insulin secretion
- positive regulation of superoxide dismutase activity
- protein deacetylation

---

49

- **Protein name:** WD repeat-containing protein 48
- **Organism:** Homo sapiens
- **Uniprot Accession Number:** Q8TAF3
- **Protein sequence length:** 677 aa
- **1D identity (%):** 12.07
- **1D identity (%) [Gaps excluded]:** 21.07
- **1D identity - Alignment Gaps:** 418
- **1D aligned content (<aminoacid>:%):** {'H': 7.63, 'Q': 2.54, 'R': 5.08, 'K': 8.47, 'V': 10.17, 'N': 2.54, 'F': 2.54, 'T': 10.17, 'G': 8.47, 'I': 1.69, 'D': 12.71, 'L': 9.32, 'P': 5.93, 'C': 2.54, 'Y': 2.54, 'A': 2.54, 'E': 2.54, 'S': 2.54}
- **Common reported functions (%):** 0.0
- **Common reported locations (%):** 12.5
- **Common reported processes (%):** 0.0

- **AF ID:** Q8TAF3
- **Chain:** A
- **Protein length:** 677 aa
- **Resolution:** N/A
- **b-phipsi:** 0.007026
- **w-rdist:** 0.642314
- **t-alpha:** 0.000782
- **Chemical similarity (Tanimoto Index) (%):** 98.95
- **1D identity (%) [PDB]:** 1.33
- **1D identity (%) [Gaps excluded][PDB]:** 90.91
- **1D identity - Alignment Gaps [PDB]:** 1483
- **1D aligned content [PDB] (<aminoacid>:%):** {'E': 5.0, 'V': 10.0, 'C': 10.0, 'N': 10.0, 'I': 10.0, 'G': 5.0, 'Q': 10.0, 'R': 5.0, 'K': 10.0, 'L': 5.0, 'T': 10.0, 'D': 5.0, 'S': 5.0}
- **2D identity (%) [PDB]:** 44.65
- **2D identity (%) [Gaps excluded][PDB]:** 87.23
- **2D identity - Alignment Gaps [PDB]:** 493
- **2D aligned content [PDB] (<2D-fold>:%):** {'.': 19.29, 'E': 34.81, 'T': 26.61, 'H': 18.63, 'G': 0.67}
- **3D similarity (TM-Score) (%) [PDB]:** 19.06

- **Gene name:** WDR48
- **Entrez ID:** 618860
- **RefSeq ID:** NM\_020839
- **Transcript sequence length:** 3965
- **5-UTR|CDS|3-UTR identity (%):** 1.74 | 43.09 | 25.93
- **5-UTR|CDS|3-UTR identity (%) [Gaps excluded]:** 77.78 | 75.91 | 75.08
- **5-UTR|CDS|3-UTR identity [Alignment Gaps]:** 393 | 1274 | 3020
- **5-UTR aligned content (<base>:%):** {'A': 57.14, 'T': 14.29, 'C': 14.29, 'G': 14.29}
- **CDS aligned content (<base>:%):** {'A': 32.68, 'T': 25.04, 'G': 23.78, 'C': 18.5}
- **3-UTR aligned content (<base>:%):** {'A': 28.51, 'G': 20.4, 'C': 18.06, 'T': 33.03}

**Uniprot Description:**  
  
 Regulator of deubiquitinating complexes, which acts as a strong activator of USP1, USP12 and USP46 (PubMed:18082604, PubMed:19075014, PubMed:31253762, PubMed:26388029). Enhances the USP1-mediated deubiquitination of FANCD2; USP1 being almost inactive by itself (PubMed:18082604, PubMed:31253762). Activates deubiquitination by increasing the catalytic turnover without increasing the affinity of deubiquitinating enzymes for the substrate (PubMed:19075014, PubMed:27373336). Also activates deubiquitinating activity of complexes containing USP12 (PubMed:19075014, PubMed:27650958, PubMed:27373336). In complex with USP12, acts as a potential tumor suppressor by positively regulating PHLPP1 stability (PubMed:24145035). Docks at the distal end of the USP12 fingers domain and induces a cascade of structural changes leading to the activation of the enzyme (PubMed:27650958, PubMed:27373336). Together with RAD51AP1, promotes DNA repair by stimulating RAD51-mediated homologous recombination (PubMed:27463890, PubMed:27239033, PubMed:32350107). Binds single-stranded DNA (ssDNA) and double-stranded DNA (dsDNA) (PubMed:27239033, PubMed:31253762, PubMed:32350107). DNA-binding is required both for USP1-mediated deubiquitination of FANCD2 and stimulation of RAD51-mediated homologous recombination: both WDR48/UAF1 and RAD51AP1 have coordinated role in DNA-binding during these processes (PubMed:31253762, PubMed:32350107).   
  
Interacts with USP46 (PubMed:19075014, PubMed:26388029). Interacts with USP1 (PubMed:18082604, PubMed:26388029). Interacts with USP12 (PubMed:19075014, PubMed:27650958, PubMed:27373336). Component of the USP12-WDR20-WDR48 deubiquitinating complex (PubMed:27373336). Interacts with PHLPP1 (PubMed:24145035). Interacts with RAD51AP1; the interaction is direct and promotes formation of a trimeric complex with RAD51 via RAD51AP1 (PubMed:27463890, PubMed:27239033).   
  
 **Gene Ontology Information:**

Molecular Function

- deubiquitinase activator activity
- DNA binding
- double-stranded DNA binding
- single-stranded DNA binding
- ubiquitin binding

Location

- late endosome
- lysosome
- nucleus

Biological process

- cellular response to DNA damage stimulus
- double-strand break repair via homologous recombination
- positive regulation of double-strand break repair via homologous recombination

---

50

- **Protein name:** Glutamine--fructose-6-phosphate aminotransferase [isomerizing] 1
- **Organism:** Homo sapiens
- **Uniprot Accession Number:** Q06210
- **Protein sequence length:** 699 aa
- **1D identity (%):** 15.86
- **1D identity (%) [Gaps excluded]:** 23.6
- **1D identity - Alignment Gaps:** 306
- **1D aligned content (<aminoacid>:%):** {'I': 8.78, 'Y': 5.41, 'V': 10.81, 'R': 6.08, 'E': 6.08, 'T': 7.43, 'G': 8.11, 'D': 4.73, 'A': 4.73, 'K': 5.41, 'Q': 2.7, 'N': 3.38, 'P': 4.73, 'S': 3.38, 'H': 1.35, 'F': 5.41, 'M': 2.03, 'C': 2.7, 'L': 6.76}
- **Common reported functions (%):** 0.0
- **Common reported locations (%):** 12.5
- **Common reported processes (%):** 0.0

- **AF ID:** Q06210
- **Chain:** A
- **Protein length:** 699 aa
- **Resolution:** N/A
- **b-phipsi:** 0.003008
- **w-rdist:** 0.69994
- **t-alpha:** 0.002347
- **Chemical similarity (Tanimoto Index) (%):** 99.24
- **1D identity (%) [PDB]:** 3.16
- **1D identity (%) [Gaps excluded][PDB]:** 74.6
- **1D identity - Alignment Gaps [PDB]:** 1423
- **1D aligned content [PDB] (<aminoacid>:%):** {'M': 2.13, 'C': 6.38, 'E': 10.64, 'V': 8.51, 'L': 10.64, 'D': 4.26, 'I': 4.26, 'N': 4.26, 'K': 10.64, 'T': 6.38, 'S': 4.26, 'Q': 6.38, 'R': 8.51, 'F': 2.13, 'G': 4.26, 'Y': 2.13, 'P': 4.26}
- **2D identity (%) [PDB]:** 44.76
- **2D identity (%) [Gaps excluded][PDB]:** 91.73
- **2D identity - Alignment Gaps [PDB]:** 533
- **2D aligned content [PDB] (<2D-fold>:%):** {'.': 15.45, 'E': 27.04, 'H': 39.7, 'T': 17.38, 'G': 0.43}
- **3D similarity (TM-Score) (%) [PDB]:** 20.23

- **Gene name:** GFPT1
- **Entrez ID:** 2673
- **RefSeq ID:** NM\_002056
- **Transcript sequence length:** 8611
- **5-UTR|CDS|3-UTR identity (%):** 23.22 | 42.73 | 41.6
- **5-UTR|CDS|3-UTR identity (%) [Gaps excluded]:** 72.59 | 75.01 | 76.75
- **5-UTR|CDS|3-UTR identity [Alignment Gaps]:** 287 | 1270 | 3176
- **5-UTR aligned content (<base>:%):** {'G': 36.73, 'C': 48.98, 'T': 10.2, 'A': 4.08}
- **CDS aligned content (<base>:%):** {'T': 25.3, 'G': 25.14, 'A': 30.06, 'C': 19.51}
- **3-UTR aligned content (<base>:%):** {'G': 20.24, 'A': 28.67, 'T': 36.95, 'C': 14.14}

**Uniprot Description:**  
  
 Controls the flux of glucose into the hexosamine pathway. Most likely involved in regulating the availability of precursors for N- and O-linked glycosylation of proteins. Regulates the circadian expression of clock genes ARNTL/BMAL1 and CRY1.   
  
Homotetramer (Probable), may also exist as homodimers.   
  
 **Gene Ontology Information:**

Molecular Function

- carbohydrate derivative binding
- glutamine-fructose-6-phosphate transaminase (isomerizing) activity

Location

- cytosol
- extracellular exosome

Biological process

- circadian regulation of gene expression
- energy reserve metabolic process
- fructose 6-phosphate metabolic process
- glutamine metabolic process
- protein N-linked glycosylation
- UDP-N-acetylglucosamine biosynthetic process
- UDP-N-acetylglucosamine metabolic process

---

51

- **Protein name:** Activin receptor type-2B
- **Organism:** Homo sapiens
- **Uniprot Accession Number:** Q13705
- **Protein sequence length:** 512 aa
- **1D identity (%):** 9.83
- **1D identity (%) [Gaps excluded]:** 22.54
- **1D identity - Alignment Gaps:** 539
- **1D aligned content (<aminoacid>:%):** {'P': 14.89, 'W': 2.13, 'Y': 2.13, 'A': 4.26, 'E': 5.32, 'C': 5.32, 'S': 4.26, 'K': 6.38, 'G': 9.57, 'T': 6.38, 'N': 2.13, 'Q': 4.26, 'F': 4.26, 'H': 3.19, 'L': 10.64, 'R': 3.19, 'V': 6.38, 'D': 3.19, 'I': 2.13}
- **Common reported functions (%):** 0.0
- **Common reported locations (%):** 12.5
- **Common reported processes (%):** 0.0

- **AF ID:** Q13705
- **Chain:** A
- **Protein length:** 512 aa
- **Resolution:** N/A
- **b-phipsi:** 0.002407
- **w-rdist:** 0.786238
- **t-alpha:** 0.001565
- **Chemical similarity (Tanimoto Index) (%):** 96.62
- **1D identity (%) [PDB]:** 1.97
- **1D identity (%) [Gaps excluded][PDB]:** 66.67
- **1D identity - Alignment Gaps [PDB]:** 1284
- **1D aligned content [PDB] (<aminoacid>:%):** {'T': 3.85, 'A': 3.85, 'P': 11.54, 'L': 11.54, 'I': 3.85, 'G': 3.85, 'R': 7.69, 'D': 11.54, 'M': 3.85, 'E': 7.69, 'K': 7.69, 'V': 11.54, 'S': 11.54}
- **2D identity (%) [PDB]:** 40.0
- **2D identity (%) [Gaps excluded][PDB]:** 90.65
- **2D identity - Alignment Gaps [PDB]:** 528
- **2D aligned content [PDB] (<2D-fold>:%):** {'.': 14.02, 'H': 36.51, 'T': 32.54, 'E': 16.93}
- **3D similarity (TM-Score) (%) [PDB]:** 14.89

- **Gene name:** ACVR2B
- **Entrez ID:** 93
- **RefSeq ID:** NM\_001106
- **Transcript sequence length:** 11782
- **5-UTR|CDS|3-UTR identity (%):** 52.87 | 34.45 | 32.51
- **5-UTR|CDS|3-UTR identity (%) [Gaps excluded]:** 74.57 | 75.54 | 80.18
- **5-UTR|CDS|3-UTR identity [Alignment Gaps]:** 142 | 1541 | 5963
- **5-UTR aligned content (<base>:%):** {'G': 38.76, 'C': 52.71, 'T': 3.49, 'A': 5.04}
- **CDS aligned content (<base>:%):** {'A': 23.36, 'T': 21.0, 'G': 30.12, 'C': 25.51}
- **3-UTR aligned content (<base>:%):** {'G': 21.17, 'A': 27.21, 'T': 34.91, 'C': 16.72}

**Uniprot Description:**  
  
 Transmembrane serine/threonine kinase activin type-2 receptor forming an activin receptor complex with activin type-1 serine/threonine kinase receptors (ACVR1, ACVR1B or ACVR1c). Transduces the activin signal from the cell surface to the cytoplasm and is thus regulating many physiological and pathological processes including neuronal differentiation and neuronal survival, hair follicle development and cycling, FSH production by the pituitary gland, wound healing, extracellular matrix production, immunosuppression and carcinogenesis. Activin is also thought to have a paracrine or autocrine role in follicular development in the ovary. Within the receptor complex, the type-2 receptors act as a primary activin receptors (binds activin-A/INHBA, activin-B/INHBB as well as inhibin-A/INHA-INHBA). The type-1 receptors like ACVR1B act as downstream transducers of activin signals. Activin binds to type-2 receptor at the plasma membrane and activates its serine-threonine kinase. The activated receptor type-2 then phosphorylates and activates the type-1 receptor. Once activated, the type-1 receptor binds and phosphorylates the SMAD proteins SMAD2 and SMAD3, on serine residues of the C-terminal tail. Soon after their association with the activin receptor and subsequent phosphorylation, SMAD2 and SMAD3 are released into the cytoplasm where they interact with the common partner SMAD4. This SMAD complex translocates into the nucleus where it mediates activin-induced transcription. Inhibitory SMAD7, which is recruited to ACVR1B through FKBP1A, can prevent the association of SMAD2 and SMAD3 with the activin receptor complex, thereby blocking the activin signal. Activin signal transduction is also antagonized by the binding to the receptor of inhibin-B via the IGSF1 inhibin coreceptor.   
  
Forms an activin receptor complex with activin type II receptors such as ACVR1B. Interacts with VPS39. Interacts with DYNLT1. Interacts with BMP3 (PubMed:31665064). Interacts with BMP2 (PubMed:17295905). Interacts with BMP6 (PubMed:18070108).   
  
 **Gene Ontology Information:**

Molecular Function

- activin binding
- activin-activated receptor activity
- ATP binding
- growth factor binding
- kinase activator activity
- metal ion binding
- protein serine/threonine kinase activity
- protein serine/threonine/tyrosine kinase activity

Location

- activin receptor complex
- cytoplasm
- plasma membrane
- protein-containing complex
- receptor complex

Biological process

- activin receptor signaling pathway
- anterior/posterior pattern specification
- artery development
- blood vessel remodeling
- BMP signaling pathway
- cellular response to growth factor stimulus
- determination of left/right symmetry
- embryonic foregut morphogenesis
- gastrulation with mouth forming second
- heart development
- insulin secretion
- kidney development
- lung development
- lymphangiogenesis
- lymphatic endothelial cell differentiation
- mesoderm development
- negative regulation of cold-induced thermogenesis
- negative regulation of transcription by RNA polymerase II
- odontogenesis of dentin-containing tooth
- organ growth
- pancreas development
- positive regulation of activin receptor signaling pathway
- positive regulation of bone mineralization
- positive regulation of osteoblast differentiation
- post-embryonic development
- protein phosphorylation
- regulation of transcription, DNA-templated
- response to glucose
- retina vasculature development in camera-type eye
- roof of mouth development
- signal transduction
- skeletal system morphogenesis
- transmembrane receptor protein serine/threonine kinase signaling pathway
- venous blood vessel development

---

52

- **Protein name:** Meprin A subunit alpha
- **Organism:** Homo sapiens
- **Uniprot Accession Number:** Q16819
- **Protein sequence length:** 746 aa
- **1D identity (%):** 14.21
- **1D identity (%) [Gaps excluded]:** 22.51
- **1D identity - Alignment Gaps:** 363
- **1D aligned content (<aminoacid>:%):** {'L': 6.43, 'H': 2.14, 'P': 5.71, 'V': 8.57, 'D': 7.14, 'K': 5.0, 'N': 3.57, 'F': 5.0, 'G': 11.43, 'Q': 7.14, 'R': 7.14, 'T': 7.14, 'I': 5.0, 'S': 2.86, 'E': 2.86, 'Y': 4.29, 'W': 0.71, 'C': 2.86, 'A': 3.57, 'M': 1.43}
- **Common reported functions (%):** 0.0
- **Common reported locations (%):** 0.0
- **Common reported processes (%):** 0.0

- **AF ID:** Q16819
- **Chain:** A
- **Protein length:** 746 aa
- **Resolution:** N/A
- **b-phipsi:** 0.010554
- **w-rdist:** 0.232653
- **t-alpha:** 0.007824
- **Chemical similarity (Tanimoto Index) (%):** 96.62
- **1D identity (%) [PDB]:** 2.06
- **1D identity (%) [Gaps excluded][PDB]:** 78.05
- **1D identity - Alignment Gaps [PDB]:** 1514
- **1D aligned content [PDB] (<aminoacid>:%):** {'T': 6.25, 'F': 6.25, 'P': 6.25, 'L': 18.75, 'E': 9.38, 'G': 6.25, 'Q': 18.75, 'A': 6.25, 'C': 3.12, 'Y': 6.25, 'K': 9.38, 'H': 3.12}
- **2D identity (%) [PDB]:** 37.7
- **2D identity (%) [Gaps excluded][PDB]:** 85.1
- **2D identity - Alignment Gaps [PDB]:** 616
- **2D aligned content [PDB] (<2D-fold>:%):** {'.': 22.3, 'T': 26.86, 'E': 29.74, 'H': 21.1}
- **3D similarity (TM-Score) (%) [PDB]:** 23.55

- **Gene name:** MEP1A
- **Entrez ID:** 4224
- **RefSeq ID:** NM\_005588
- **Transcript sequence length:** 2893
- **5-UTR|CDS|3-UTR identity (%):** 1.5 | 45.23 | 9.92
- **5-UTR|CDS|3-UTR identity (%) [Gaps excluded]:** 60.0 | 77.85 | 77.32
- **5-UTR|CDS|3-UTR identity [Alignment Gaps]:** 391 | 1279 | 3807
- **5-UTR aligned content (<base>:%):** {'C': 50.0, 'T': 16.67, 'G': 33.33}
- **CDS aligned content (<base>:%):** {'T': 23.53, 'G': 24.76, 'C': 23.1, 'A': 28.6}
- **3-UTR aligned content (<base>:%):** {'C': 16.63, 'T': 36.72, 'G': 17.09, 'A': 29.56}

**Uniprot Description:**  
  
 N/A   
  
Homotetramer consisting of disulfide-linked alpha subunits, homooligomer consisting of disulfide-linked alpha subunit homodimers, or heterotetramer of two alpha and two beta subunits formed by non-covalent association of two disulfide-linked heterodimers (By similarity). Interacts with MBL2 through its carbohydrate moiety. This interaction may inhibit its catalytic activity (By similarity).   
  
 **Gene Ontology Information:**

Molecular Function

- metallodipeptidase activity
- metalloendopeptidase activity
- metallopeptidase activity
- zinc ion binding

Location

- extracellular exosome
- extracellular space
- meprin A complex
- plasma membrane

Biological process

- epidermal growth factor receptor ligand maturation
- signaling receptor ligand precursor processing

---

53

- **Protein name:** Serine/threonine-protein kinase STK11
- **Organism:** Homo sapiens
- **Uniprot Accession Number:** Q15831
- **Protein sequence length:** 433 aa
- **1D identity (%):** 8.37
- **1D identity (%) [Gaps excluded]:** 22.57
- **1D identity - Alignment Gaps:** 594
- **1D aligned content (<aminoacid>:%):** {'M': 3.8, 'E': 3.8, 'P': 15.19, 'G': 13.92, 'S': 1.27, 'Q': 1.27, 'R': 8.86, 'K': 6.33, 'L': 10.13, 'V': 6.33, 'I': 5.06, 'N': 2.53, 'D': 3.8, 'A': 3.8, 'T': 5.06, 'F': 2.53, 'W': 1.27, 'Y': 3.8, 'C': 1.27}
- **Common reported functions (%):** 0.0
- **Common reported locations (%):** 50.0
- **Common reported processes (%):** 0.0

- **AF ID:** Q15831
- **Chain:** A
- **Protein length:** 433 aa
- **Resolution:** N/A
- **b-phipsi:** 0.005317
- **w-rdist:** 0.717709
- **t-alpha:** 0.000782
- **Chemical similarity (Tanimoto Index) (%):** 99.62
- **1D identity (%) [PDB]:** 1.92
- **1D identity (%) [Gaps excluded][PDB]:** 68.57
- **1D identity - Alignment Gaps [PDB]:** 1213
- **1D aligned content [PDB] (<aminoacid>:%):** {'A': 12.5, 'K': 4.17, 'L': 8.33, 'G': 8.33, 'I': 8.33, 'N': 4.17, 'V': 16.67, 'P': 16.67, 'R': 4.17, 'F': 4.17, 'Q': 4.17, 'T': 4.17, 'H': 4.17}
- **2D identity (%) [PDB]:** 32.14
- **2D identity (%) [Gaps excluded][PDB]:** 91.32
- **2D identity - Alignment Gaps [PDB]:** 615
- **2D aligned content [PDB] (<2D-fold>:%):** {'.': 24.59, 'T': 36.39, 'H': 24.26, 'E': 14.43, 'B': 0.33}
- **3D similarity (TM-Score) (%) [PDB]:** 15.47

- **Gene name:** STK11
- **Entrez ID:** 6794
- **RefSeq ID:** N/A
- **Sequence length:** N/A
- **5-UTR|CDS|3-UTR identity (%):** N/A | N/A | N/A
- **5-UTR|CDS|3-UTR identity (%) [Gaps excluded]:** N/A | N/A | N/A
- **5-UTR|CDS|3-UTR identity [Alignment Gaps]:** N/A | N/A | N/A
- **5-UTR aligned content (<base>:%):** N/A
- **CDS aligned content (<base>:%):** N/A
- **3-UTR aligned content (<base>:%):** N/A

**Uniprot Description:**  
  
 Tumor suppressor serine/threonine-protein kinase that controls the activity of AMP-activated protein kinase (AMPK) family members, thereby playing a role in various processes such as cell metabolism, cell polarity, apoptosis and DNA damage response. Acts by phosphorylating the T-loop of AMPK family proteins, thus promoting their activity: phosphorylates PRKAA1, PRKAA2, BRSK1, BRSK2, MARK1, MARK2, MARK3, MARK4, NUAK1, NUAK2, SIK1, SIK2, SIK3 and SNRK but not MELK. Also phosphorylates non-AMPK family proteins such as STRADA, PTEN and possibly p53/TP53. Acts as a key upstream regulator of AMPK by mediating phosphorylation and activation of AMPK catalytic subunits PRKAA1 and PRKAA2 and thereby regulates processes including: inhibition of signaling pathways that promote cell growth and proliferation when energy levels are low, glucose homeostasis in liver, activation of autophagy when cells undergo nutrient deprivation, and B-cell differentiation in the germinal center in response to DNA damage. Also acts as a regulator of cellular polarity by remodeling the actin cytoskeleton. Required for cortical neuron polarization by mediating phosphorylation and activation of BRSK1 and BRSK2, leading to axon initiation and specification. Involved in DNA damage response: interacts with p53/TP53 and recruited to the CDKN1A/WAF1 promoter to participate in transcription activation. Able to phosphorylate p53/TP53; the relevance of such result in vivo is however unclear and phosphorylation may be indirect and mediated by downstream STK11/LKB1 kinase NUAK1. Also acts as a mediator of p53/TP53-dependent apoptosis via interaction with p53/TP53: translocates to the mitochondrion during apoptosis and regulates p53/TP53-dependent apoptosis pathways. Regulates UV radiation-induced DNA damage response mediated by CDKN1A. In association with NUAK1, phosphorylates CDKN1A in response to UV radiation and contributes to its degradation which is necessary for optimal DNA repair (PubMed:25329316).   
  
Catalytic component of a trimeric complex composed of STK11/LKB1, STRAD (STRADA or STRADB) and CAB39/MO25 (CAB39/MO25alpha or CAB39L/MO25beta): the complex tethers STK11/LKB1 in the cytoplasm and stimulates its catalytic activity. Found in a ternary complex composed of SMAD4, STK11/LKB1 and STK11IP. Interacts with p53/TP53, SMAD4, STK11IP and WDR6. Interacts with NR4A1. Interacts with NISCH; this interaction may increase STK11 activity. Interacts with PTEN; leading to PTEN phosphorylation. Interacts with SIRT1; the interaction deacetylates STK11. Interacts with CDKN1A.   
  
 **Gene Ontology Information:**

Molecular Function

- ATP binding
- LRR domain binding
- magnesium ion binding
- p53 binding
- protein kinase activator activity
- protein serine kinase activity
- protein serine/threonine kinase activity
- protein-containing complex binding

Location

- cytoplasm
- cytosol
- extracellular exosome
- intracellular protein-containing complex
- membrane
- mitochondrion
- nucleoplasm
- nucleus
- serine/threonine protein kinase complex
- Z disc

Biological process

- activation of protein kinase activity
- anoikis
- autophagy
- axonogenesis
- cellular response to UV-B
- dendrite extension
- cellular response to DNA damage stimulus
- epithelial cell proliferation involved in prostate gland development
- establishment of cell polarity
- G1 to G0 transition
- glucose homeostasis
- Golgi localization
- intrinsic apoptotic signaling pathway by p53 class mediator
- negative regulation of canonical Wnt signaling pathway
- negative regulation of cell growth
- negative regulation of cell population proliferation
- negative regulation of cold-induced thermogenesis
- negative regulation of epithelial cell proliferation involved in prostate gland development
- negative regulation of TORC1 signaling
- peptidyl-threonine phosphorylation
- positive regulation of autophagy
- positive regulation of axonogenesis
- positive regulation of gluconeogenesis
- positive regulation of protein localization to nucleus
- positive regulation of transforming growth factor beta receptor signaling pathway
- positive regulation of vesicle transport along microtubule
- positive thymic T cell selection
- protein autophosphorylation
- protein dephosphorylation
- protein localization to nucleus
- protein phosphorylation
- regulation of cell cycle
- regulation of cell growth
- regulation of dendrite morphogenesis
- regulation of protein kinase B signaling
- regulation of signal transduction by p53 class mediator
- regulation of Wnt signaling pathway
- response to activity
- response to glucagon
- response to ionizing radiation
- response to lipid
- response to thyroid hormone
- signal transduction
- spermatogenesis
- T cell receptor signaling pathway
- tissue homeostasis
- vasculature development

---

54

- **Protein name:** 5'-AMP-activated protein kinase catalytic subunit alpha-1
- **Organism:** Homo sapiens
- **Uniprot Accession Number:** Q13131
- **Protein sequence length:** 559 aa
- **1D identity (%):** 11.86
- **1D identity (%) [Gaps excluded]:** 21.76
- **1D identity - Alignment Gaps:** 418
- **1D aligned content (<aminoacid>:%):** {'K': 7.34, 'T': 1.83, 'R': 7.34, 'V': 10.09, 'I': 2.75, 'G': 8.26, 'N': 4.59, 'Q': 5.5, 'L': 11.01, 'P': 10.09, 'H': 1.83, 'E': 3.67, 'C': 3.67, 'D': 5.5, 'S': 4.59, 'F': 1.83, 'M': 1.83, 'Y': 2.75, 'A': 4.59, 'W': 0.92}
- **Common reported functions (%):** 0.0
- **Common reported locations (%):** 37.5
- **Common reported processes (%):** 8.33

- **AF ID:** Q13131
- **Chain:** A
- **Protein length:** 559 aa
- **Resolution:** N/A
- **b-phipsi:** 0.003276
- **w-rdist:** 0.345026
- **t-alpha:** 0.033646
- **Chemical similarity (Tanimoto Index) (%):** 99.62
- **1D identity (%) [PDB]:** 1.89
- **1D identity (%) [Gaps excluded][PDB]:** 76.47
- **1D identity - Alignment Gaps [PDB]:** 1341
- **1D aligned content [PDB] (<aminoacid>:%):** {'T': 3.85, 'F': 3.85, 'K': 3.85, 'V': 19.23, 'W': 3.85, 'S': 7.69, 'L': 19.23, 'E': 7.69, 'A': 7.69, 'H': 3.85, 'N': 3.85, 'P': 3.85, 'D': 7.69, 'Q': 3.85}
- **2D identity (%) [PDB]:** 40.62
- **2D identity (%) [Gaps excluded][PDB]:** 89.75
- **2D identity - Alignment Gaps [PDB]:** 531
- **2D aligned content [PDB] (<2D-fold>:%):** {'.': 20.81, 'E': 16.24, 'T': 20.3, 'H': 41.88, 'G': 0.76}
- **3D similarity (TM-Score) (%) [PDB]:** 19.74

- **Gene name:** PRKAA1
- **Entrez ID:** 5562
- **RefSeq ID:** NM\_206907
- **Transcript sequence length:** 5299
- **5-UTR|CDS|3-UTR identity (%):** 32.77 | 35.9 | 43.1
- **5-UTR|CDS|3-UTR identity (%) [Gaps excluded]:** 77.59 | 75.52 | 74.39
- **5-UTR|CDS|3-UTR identity [Alignment Gaps]:** 238 | 1533 | 2044
- **5-UTR aligned content (<base>:%):** {'A': 2.96, 'C': 56.3, 'T': 5.19, 'G': 35.56}
- **CDS aligned content (<base>:%):** {'G': 22.12, 'C': 20.11, 'A': 32.7, 'T': 25.07}
- **3-UTR aligned content (<base>:%):** {'C': 13.04, 'G': 16.05, 'A': 30.99, 'T': 39.92}

**Uniprot Description:**  
  
 Catalytic subunit of AMP-activated protein kinase (AMPK), an energy sensor protein kinase that plays a key role in regulating cellular energy metabolism. In response to reduction of intracellular ATP levels, AMPK activates energy-producing pathways and inhibits energy-consuming processes: inhibits protein, carbohydrate and lipid biosynthesis, as well as cell growth and proliferation. AMPK acts via direct phosphorylation of metabolic enzymes, and by longer-term effects via phosphorylation of transcription regulators. Also acts as a regulator of cellular polarity by remodeling the actin cytoskeleton; probably by indirectly activating myosin. Regulates lipid synthesis by phosphorylating and inactivating lipid metabolic enzymes such as ACACA, ACACB, GYS1, HMGCR and LIPE; regulates fatty acid and cholesterol synthesis by phosphorylating acetyl-CoA carboxylase (ACACA and ACACB) and hormone-sensitive lipase (LIPE) enzymes, respectively. Regulates insulin-signaling and glycolysis by phosphorylating IRS1, PFKFB2 and PFKFB3. AMPK stimulates glucose uptake in muscle by increasing the translocation of the glucose transporter SLC2A4/GLUT4 to the plasma membrane, possibly by mediating phosphorylation of TBC1D4/AS160. Regulates transcription and chromatin structure by phosphorylating transcription regulators involved in energy metabolism such as CRTC2/TORC2, FOXO3, histone H2B, HDAC5, MEF2C, MLXIPL/ChREBP, EP300, HNF4A, p53/TP53, SREBF1, SREBF2 and PPARGC1A. Acts as a key regulator of glucose homeostasis in liver by phosphorylating CRTC2/TORC2, leading to CRTC2/TORC2 sequestration in the cytoplasm. In response to stress, phosphorylates 'Ser-36' of histone H2B (H2BS36ph), leading to promote transcription. Acts as a key regulator of cell growth and proliferation by phosphorylating TSC2, RPTOR and ATG1/ULK1: in response to nutrient limitation, negatively regulates the mTORC1 complex by phosphorylating RPTOR component of the mTORC1 complex and by phosphorylating and activating TSC2. In response to nutrient limitation, promotes autophagy by phosphorylating and activating ATG1/ULK1. In that process also activates WDR45 (PubMed:28561066). In response to nutrient limitation, phosphorylates transcription factor FOXO3 promoting FOXO3 mitochondrial import (By similarity). AMPK also acts as a regulator of circadian rhythm by mediating phosphorylation of CRY1, leading to destabilize it. May regulate the Wnt signaling pathway by phosphorylating CTNNB1, leading to stabilize it. Also has tau-protein kinase activity: in response to amyloid beta A4 protein (APP) exposure, activated by CAMKK2, leading to phosphorylation of MAPT/TAU; however the relevance of such data remains unclear in vivo. Also phosphorylates CFTR, EEF2K, KLC1, NOS3 and SLC12A1.   
  
AMPK is a heterotrimer of an alpha catalytic subunit (PRKAA1 or PRKAA2), a beta (PRKAB1 or PRKAB2) and a gamma non-catalytic subunits (PRKAG1, PRKAG2 or PRKAG3). Interacts with FNIP1 and FNIP2.   
  
 **Gene Ontology Information:**

Molecular Function

- [acetyl-CoA carboxylase] kinase activity
- [hydroxymethylglutaryl-CoA reductase (NADPH)] kinase activity
- AMP-activated protein kinase activity
- ATP binding
- cAMP-dependent protein kinase activity
- chromatin binding
- histone H2BS36 kinase activity
- metal ion binding
- protein kinase activity
- protein serine kinase activity
- protein serine/threonine kinase activity
- tau protein binding
- tau-protein kinase activity

Location

- apical plasma membrane
- axon
- chromatin
- cytoplasm
- cytosol
- dendrite
- neuronal cell body
- nuclear speck
- nucleoplasm
- nucleotide-activated protein kinase complex
- nucleus

Biological process

- autophagy
- bile acid and bile salt transport
- bile acid signaling pathway
- CAMKK-AMPK signaling cascade
- cellular response to calcium ion
- cellular response to ethanol
- cellular response to glucose starvation
- cellular response to glucose stimulus
- cellular response to hydrogen peroxide
- cellular response to hypoxia
- cellular response to nutrient levels
- cellular response to organonitrogen compound
- cellular response to oxidative stress
- cellular response to prostaglandin E stimulus
- cellular response to xenobiotic stimulus
- cholesterol biosynthetic process
- cold acclimation
- energy homeostasis
- fatty acid biosynthetic process
- fatty acid homeostasis
- fatty acid oxidation
- glucose homeostasis
- glucose metabolic process
- intracellular signal transduction
- lipid biosynthetic process
- lipid droplet disassembly
- motor behavior
- negative regulation of apoptotic process
- negative regulation of gene expression
- negative regulation of glucosylceramide biosynthetic process
- negative regulation of hepatocyte apoptotic process
- negative regulation of insulin receptor signaling pathway
- negative regulation of lipid catabolic process
- negative regulation of TOR signaling
- negative regulation of tubulin deacetylation
- neuron cellular homeostasis
- positive regulation of adipose tissue development
- positive regulation of autophagy
- positive regulation of cell population proliferation
- positive regulation of cholesterol biosynthetic process
- positive regulation of transcription, DNA-templated
- positive regulation of gene expression
- positive regulation of glycolytic process
- positive regulation of mitochondrial transcription
- positive regulation of peptidyl-lysine acetylation
- positive regulation of cellular protein localization
- positive regulation of protein targeting to mitochondrion
- positive regulation of skeletal muscle tissue development
- protein localization to lipid droplet
- protein phosphorylation
- regulation of bile acid secretion
- regulation of circadian rhythm
- regulation of microtubule cytoskeleton organization
- regulation of peptidyl-serine phosphorylation
- regulation of stress granule assembly
- regulation of vesicle-mediated transport
- response to activity
- response to caffeine
- response to estrogen
- response to gamma radiation
- response to hypoxia
- response to UV
- rhythmic process
- signal transduction
- Wnt signaling pathway

---

55

- **Protein name:** Ferredoxin-fold anticodon-binding domain-containing protein 1
- **Organism:** Homo sapiens
- **Uniprot Accession Number:** Q9BRP7
- **Protein sequence length:** 624 aa
- **1D identity (%):** 13.48
- **1D identity (%) [Gaps excluded]:** 25.54
- **1D identity - Alignment Gaps:** 459
- **1D aligned content (<aminoacid>:%):** {'L': 13.74, 'G': 8.4, 'E': 4.58, 'T': 3.05, 'S': 4.58, 'Q': 8.4, 'A': 3.05, 'D': 6.87, 'V': 5.34, 'H': 5.34, 'P': 9.16, 'R': 3.82, 'F': 5.34, 'W': 0.76, 'Y': 1.53, 'I': 4.58, 'K': 4.58, 'N': 3.05, 'C': 3.05, 'M': 0.76}
- **Common reported functions (%):** 0.0
- **Common reported locations (%):** 12.5
- **Common reported processes (%):** 0.0

- **AF ID:** Q9BRP7
- **Chain:** A
- **Protein length:** 624 aa
- **Resolution:** N/A
- **b-phipsi:** 0.000914
- **w-rdist:** 0.45997
- **t-alpha:** 0.07395
- **Chemical similarity (Tanimoto Index) (%):** 99.62
- **1D identity (%) [PDB]:** 1.38
- **1D identity (%) [Gaps excluded][PDB]:** 76.92
- **1D identity - Alignment Gaps [PDB]:** 1422
- **1D aligned content [PDB] (<aminoacid>:%):** {'L': 30.0, 'E': 10.0, 'A': 5.0, 'G': 5.0, 'N': 5.0, 'V': 10.0, 'P': 5.0, 'D': 10.0, 'S': 5.0, 'Q': 5.0, 'I': 5.0, 'R': 5.0}
- **2D identity (%) [PDB]:** 42.51
- **2D identity (%) [Gaps excluded][PDB]:** 88.31
- **2D identity - Alignment Gaps [PDB]:** 516
- **2D aligned content [PDB] (<2D-fold>:%):** {'.': 15.6, 'E': 27.66, 'T': 28.61, 'H': 26.48, 'G': 1.42, 'B': 0.24}
- **3D similarity (TM-Score) (%) [PDB]:** 21.92

- **Gene name:** FDXACB1
- **Entrez ID:** 91893
- **RefSeq ID:** NM\_138378
- **Transcript sequence length:** 2774
- **5-UTR|CDS|3-UTR identity (%):** 5.65 | 39.69 | 12.43
- **5-UTR|CDS|3-UTR identity (%) [Gaps excluded]:** 85.19 | 74.1 | 75.76
- **5-UTR|CDS|3-UTR identity [Alignment Gaps]:** 380 | 1349 | 3699
- **5-UTR aligned content (<base>:%):** {'A': 17.39, 'G': 43.48, 'C': 30.43, 'T': 8.7}
- **CDS aligned content (<base>:%):** {'A': 27.15, 'G': 23.42, 'C': 22.03, 'T': 27.41}
- **3-UTR aligned content (<base>:%):** {'T': 40.91, 'A': 25.82, 'C': 16.55, 'G': 16.73}

**Uniprot Description:**  
  
 N/A N/A   
  
 **Gene Ontology Information:**

Molecular Function

- rRNA (uridine-N3-)-methyltransferase activity

Location

- cytoplasm

Biological process

- rRNA base methylation

---

56

- **Protein name:** Polypeptide N-acetylgalactosaminyltransferase 12
- **Organism:** Homo sapiens
- **Uniprot Accession Number:** Q8IXK2
- **Protein sequence length:** 581 aa
- **1D identity (%):** 12.55
- **1D identity (%) [Gaps excluded]:** 22.39
- **1D identity - Alignment Gaps:** 406
- **1D aligned content (<aminoacid>:%):** {'R': 12.07, 'P': 12.93, 'E': 5.17, 'G': 9.48, 'V': 7.76, 'Q': 5.17, 'L': 9.48, 'H': 1.72, 'N': 3.45, 'D': 1.72, 'Y': 1.72, 'F': 4.31, 'W': 1.72, 'I': 3.45, 'K': 6.9, 'T': 3.45, 'S': 3.45, 'M': 0.86, 'A': 3.45, 'C': 1.72}
- **Common reported functions (%):** 0.0
- **Common reported locations (%):** 0.0
- **Common reported processes (%):** 0.0

- **AF ID:** Q8IXK2
- **Chain:** A
- **Protein length:** 581 aa
- **Resolution:** N/A
- **b-phipsi:** 0.003224
- **w-rdist:** 0.566377
- **t-alpha:** 0.003928
- **Chemical similarity (Tanimoto Index) (%):** 96.54
- **1D identity (%) [PDB]:** 1.86
- **1D identity (%) [Gaps excluded][PDB]:** 74.29
- **1D identity - Alignment Gaps [PDB]:** 1361
- **1D aligned content [PDB] (<aminoacid>:%):** {'R': 7.69, 'A': 3.85, 'Q': 15.38, 'I': 19.23, 'F': 3.85, 'E': 11.54, 'V': 3.85, 'L': 11.54, 'D': 7.69, 'K': 7.69, 'T': 3.85, 'S': 3.85}
- **2D identity (%) [PDB]:** 33.3
- **2D identity (%) [Gaps excluded][PDB]:** 88.27
- **2D identity - Alignment Gaps [PDB]:** 647
- **2D aligned content [PDB] (<2D-fold>:%):** {'.': 22.54, 'H': 36.13, 'T': 21.68, 'E': 18.5, 'B': 0.29, 'G': 0.87}
- **3D similarity (TM-Score) (%) [PDB]:** 18.55

- **Gene name:** GALNT12
- **Entrez ID:** 79695
- **RefSeq ID:** N/A
- **Sequence length:** N/A
- **5-UTR|CDS|3-UTR identity (%):** N/A | N/A | N/A
- **5-UTR|CDS|3-UTR identity (%) [Gaps excluded]:** N/A | N/A | N/A
- **5-UTR|CDS|3-UTR identity [Alignment Gaps]:** N/A | N/A | N/A
- **5-UTR aligned content (<base>:%):** N/A
- **CDS aligned content (<base>:%):** N/A
- **3-UTR aligned content (<base>:%):** N/A

**Uniprot Description:**  
  
 Catalyzes the initial reaction in O-linked oligosaccharide biosynthesis, the transfer of an N-acetyl-D-galactosamine residue to a serine or threonine residue on the protein receptor. Has activity toward non-glycosylated peptides such as Muc5AC, Muc1a and EA2, and no detectable activity with Muc2 and Muc7. Displays enzymatic activity toward the Gal-NAc-Muc5AC glycopeptide, but no detectable activity to mono-GalNAc-glycosylated Muc1a, Muc2, Muc7 and EA2. May play an important role in the initial step of mucin-type oligosaccharide biosynthesis in digestive organs. N/A   
  
 **Gene Ontology Information:**

Molecular Function

- carbohydrate binding
- metal ion binding
- polypeptide N-acetylgalactosaminyltransferase activity

Location

- Golgi apparatus
- Golgi membrane

Biological process

- O-glycan processing
- protein O-linked glycosylation

---

57

- **Protein name:** Group 3 secretory phospholipase A2
- **Organism:** Homo sapiens
- **Uniprot Accession Number:** Q9NZ20
- **Protein sequence length:** 509 aa
- **1D identity (%):** 11.06
- **1D identity (%) [Gaps excluded]:** 25.73
- **1D identity - Alignment Gaps:** 546
- **1D aligned content (<aminoacid>:%):** {'G': 12.26, 'Q': 8.49, 'L': 8.49, 'R': 10.38, 'T': 2.83, 'A': 3.77, 'D': 3.77, 'S': 5.66, 'P': 12.26, 'H': 3.77, 'E': 4.72, 'K': 2.83, 'W': 1.89, 'M': 0.94, 'V': 6.6, 'C': 4.72, 'I': 2.83, 'N': 1.89, 'F': 0.94, 'Y': 0.94}
- **Common reported functions (%):** 0.0
- **Common reported locations (%):** 0.0
- **Common reported processes (%):** 0.0

- **AF ID:** Q9NZ20
- **Chain:** A
- **Protein length:** 509 aa
- **Resolution:** N/A
- **b-phipsi:** 0.005005
- **w-rdist:** 0.463767
- **t-alpha:** 0.003928
- **Chemical similarity (Tanimoto Index) (%):** 96.83
- **1D identity (%) [PDB]:** 2.92
- **1D identity (%) [Gaps excluded][PDB]:** 64.41
- **1D identity - Alignment Gaps [PDB]:** 1241
- **1D aligned content [PDB] (<aminoacid>:%):** {'F': 2.63, 'L': 13.16, 'G': 10.53, 'A': 5.26, 'T': 10.53, 'P': 2.63, 'K': 5.26, 'S': 7.89, 'I': 5.26, 'V': 7.89, 'M': 5.26, 'R': 5.26, 'Q': 7.89, 'E': 7.89, 'N': 2.63}
- **2D identity (%) [PDB]:** 36.27
- **2D identity (%) [Gaps excluded][PDB]:** 88.83
- **2D identity - Alignment Gaps [PDB]:** 571
- **2D aligned content [PDB] (<2D-fold>:%):** {'T': 30.29, '.': 12.86, 'E': 23.71, 'H': 32.57, 'B': 0.57}
- **3D similarity (TM-Score) (%) [PDB]:** 20.56

- **Gene name:** PLA2G3
- **Entrez ID:** 50487
- **RefSeq ID:** NM\_015715
- **Transcript sequence length:** 2600
- **5-UTR|CDS|3-UTR identity (%):** 23.04 | 35.3 | 13.4
- **5-UTR|CDS|3-UTR identity (%) [Gaps excluded]:** 72.39 | 76.21 | 74.03
- **5-UTR|CDS|3-UTR identity [Alignment Gaps]:** 287 | 1510 | 3607
- **5-UTR aligned content (<base>:%):** {'C': 45.36, 'T': 14.43, 'G': 37.11, 'A': 3.09}
- **CDS aligned content (<base>:%):** {'T': 18.43, 'G': 27.29, 'C': 29.71, 'A': 24.57}
- **3-UTR aligned content (<base>:%):** {'C': 20.0, 'T': 31.36, 'A': 25.76, 'G': 22.88}

**Uniprot Description:**  
  
 Secretory calcium-dependent phospholipase A2 that primarily targets extracellular phospholipids. Hydrolyzes the ester bond of the fatty acyl group attached at sn-2 position of phospholipids without apparent head group selectivity (PubMed:12522102, PubMed:15863501, PubMed:18801741, PubMed:28947740). Contributes to phospholipid remodeling of low-density lipoprotein (LDL) and high-density lipoprotein (HDL) particles. Hydrolyzes LDL phospholipids releasing unsaturated fatty acids that regulate macrophage differentiation toward foam cells (PubMed:18801741). May act in an autocrine and paracrine manner (PubMed:23624557). Secreted by immature mast cells, acts on nearby fibroblasts upstream to PTDGS to synthesize prostaglandin D2 (PGD2), which in turn promotes mast cell maturation and degranulation via PTGDR (PubMed:23624557). Secreted by epididymal epithelium, acts on immature sperm cells within the duct, modulating the degree of unsaturation of the fatty acyl components of phosphatidylcholines required for acrosome assembly and sperm cell motility. Facilitates the replacement of fatty acyl chains in phosphatidylcholines in sperm membranes from omega-6 and omega-9 to omega-3 polyunsaturated fatty acids (PUFAs). Coupled to lipoxygenase pathway, may process omega-6 PUFAs to generate oxygenated lipid mediators in the male reproductive tract (By similarity). At pericentrosomal preciliary compartment, negatively regulates ciliogenesis likely by regulating endocytotic recycling of ciliary membrane protein (PubMed:20393563). Coupled to cyclooxygenase pathway provides arachidonate to generate prostaglandin E2 (PGE2), a potent immunomodulatory lipid in inflammation and tumorigenesis (PubMed:12522102, PubMed:15863501). At colonic epithelial barrier, preferentially hydrolyzes phospholipids having arachidonate and docosahexaenoate at sn-2 position, contributing to the generation of oxygenated metabolites involved in colonic stem cell homeostasis (PubMed:28947740). Releases C16:0 and C18:0 lysophosphatidylcholine subclasses from neuron plasma membranes and promotes neurite outgrowth and neuron survival (PubMed:17868035). N/A   
  
 **Gene Ontology Information:**

Molecular Function

- calcium-dependent phospholipase A2 activity
- metal ion binding

Location

- centriole
- extracellular region
- extracellular space
- plasma membrane
- recycling endosome

Biological process

- acrosome assembly
- arachidonic acid secretion
- cell maturation
- cilium assembly
- high-density lipoprotein particle remodeling
- lipoxygenase pathway
- low-density lipoprotein particle remodeling
- macrophage activation
- mast cell degranulation
- negative regulation of amyloid-beta clearance
- negative regulation of gene expression
- negative regulation of neuron apoptotic process
- phosphatidic acid metabolic process
- phosphatidylcholine metabolic process
- phosphatidylethanolamine metabolic process
- phosphatidylglycerol metabolic process
- phosphatidylinositol metabolic process
- phosphatidylserine metabolic process
- phospholipid metabolic process
- positive regulation of cytokine production involved in inflammatory response
- positive regulation of histamine secretion by mast cell
- positive regulation of macrophage derived foam cell differentiation
- positive regulation of mast cell differentiation
- positive regulation of neuron projection development
- positive regulation of prostaglandin biosynthetic process
- positive regulation of prostaglandin secretion
- production of molecular mediator involved in inflammatory response
- regulation of endocytic recycling
- sperm axoneme assembly

---

58

- **Protein name:** Poly(A) polymerase beta
- **Organism:** Homo sapiens
- **Uniprot Accession Number:** Q9NRJ5
- **Protein sequence length:** 637 aa
- **1D identity (%):** 13.26
- **1D identity (%) [Gaps excluded]:** 24.02
- **1D identity - Alignment Gaps:** 432
- **1D aligned content (<aminoacid>:%):** {'P': 12.5, 'G': 5.47, 'R': 7.03, 'V': 10.94, 'I': 2.34, 'L': 12.5, 'K': 7.81, 'E': 6.25, 'F': 3.91, 'D': 5.47, 'T': 3.12, 'W': 1.56, 'H': 3.91, 'M': 0.78, 'A': 3.91, 'N': 4.69, 'Q': 1.56, 'Y': 2.34, 'S': 3.12, 'C': 0.78}
- **Common reported functions (%):** 0.0
- **Common reported locations (%):** 25.0
- **Common reported processes (%):** 0.0

- **AF ID:** Q9NRJ5
- **Chain:** A
- **Protein length:** 636 aa
- **Resolution:** N/A
- **b-phipsi:** 0.000831
- **w-rdist:** 0.313787
- **t-alpha:** 0.268387
- **Chemical similarity (Tanimoto Index) (%):** 99.39
- **1D identity (%) [PDB]:** 2.01
- **1D identity (%) [Gaps excluded][PDB]:** 65.91
- **1D identity - Alignment Gaps [PDB]:** 1398
- **1D aligned content [PDB] (<aminoacid>:%):** {'F': 6.9, 'P': 10.34, 'L': 27.59, 'E': 3.45, 'N': 3.45, 'G': 3.45, 'C': 3.45, 'V': 3.45, 'A': 3.45, 'Q': 13.79, 'K': 6.9, 'Y': 6.9, 'H': 3.45, 'T': 3.45}
- **2D identity (%) [PDB]:** 37.48
- **2D identity (%) [Gaps excluded][PDB]:** 89.09
- **2D identity - Alignment Gaps [PDB]:** 606
- **2D aligned content [PDB] (<2D-fold>:%):** {'.': 13.52, 'T': 23.98, 'H': 44.64, 'E': 15.31, 'G': 2.3, 'B': 0.26}
- **3D similarity (TM-Score) (%) [PDB]:** 20.69

- **Gene name:** PAPOLB
- **Entrez ID:** 56903
- **RefSeq ID:** NM\_020144
- **Transcript sequence length:** 4293
- **5-UTR|CDS|3-UTR identity (%):** 34.34 | 42.35 | 28.65
- **5-UTR|CDS|3-UTR identity (%) [Gaps excluded]:** 77.89 | 76.91 | 76.02
- **5-UTR|CDS|3-UTR identity [Alignment Gaps]:** 241 | 1304 | 2916
- **5-UTR aligned content (<base>:%):** {'G': 57.43, 'C': 31.08, 'T': 6.08, 'A': 5.41}
- **CDS aligned content (<base>:%):** {'A': 30.76, 'T': 24.98, 'G': 22.46, 'C': 21.81}
- **3-UTR aligned content (<base>:%):** {'T': 35.12, 'G': 17.82, 'A': 32.36, 'C': 14.69}

**Uniprot Description:**  
  
 N/A   
  
Interacts with GSG1.   
  
 **Gene Ontology Information:**

Molecular Function

- ATP binding
- metal ion binding
- RNA adenylyltransferase activity
- RNA binding

Location

- cytoplasm
- nucleus

Biological process

- mRNA polyadenylation
- RNA polyadenylation

---

59

- **Protein name:** Probable RNA-binding protein 46
- **Organism:** Homo sapiens
- **Uniprot Accession Number:** Q8TBY0
- **Protein sequence length:** 533 aa
- **1D identity (%):** 10.36
- **1D identity (%) [Gaps excluded]:** 21.18
- **1D identity - Alignment Gaps:** 478
- **1D aligned content (<aminoacid>:%):** {'N': 3.09, 'E': 8.25, 'D': 1.03, 'Q': 5.15, 'Y': 4.12, 'V': 8.25, 'R': 6.19, 'F': 3.09, 'P': 12.37, 'G': 11.34, 'A': 4.12, 'T': 5.15, 'K': 6.19, 'I': 5.15, 'C': 4.12, 'L': 8.25, 'M': 1.03, 'W': 1.03, 'S': 2.06}
- **Common reported functions (%):** 0.0
- **Common reported locations (%):** 25.0
- **Common reported processes (%):** 8.33

- **AF ID:** Q8TBY0
- **Chain:** A
- **Protein length:** 533 aa
- **Resolution:** N/A
- **b-phipsi:** 0.005273
- **w-rdist:** 0.273671
- **t-alpha:** 0.05399
- **Chemical similarity (Tanimoto Index) (%):** 98.65
- **1D identity (%) [PDB]:** 2.78
- **1D identity (%) [Gaps excluded][PDB]:** 69.81
- **1D identity - Alignment Gaps [PDB]:** 1277
- **1D aligned content [PDB] (<aminoacid>:%):** {'D': 2.7, 'E': 2.7, 'L': 13.51, 'Q': 8.11, 'T': 5.41, 'H': 10.81, 'Y': 10.81, 'V': 8.11, 'R': 2.7, 'C': 2.7, 'S': 10.81, 'I': 2.7, 'P': 5.41, 'A': 5.41, 'G': 5.41, 'N': 2.7}
- **2D identity (%) [PDB]:** 34.6
- **2D identity (%) [Gaps excluded][PDB]:** 91.32
- **2D identity - Alignment Gaps [PDB]:** 623
- **2D aligned content [PDB] (<2D-fold>:%):** {'.': 20.17, 'H': 35.45, 'E': 26.8, 'T': 17.58}
- **3D similarity (TM-Score) (%) [PDB]:** 21.2

- **Gene name:** RBM46
- **Entrez ID:** 166863
- **RefSeq ID:** N/A
- **Sequence length:** N/A
- **5-UTR|CDS|3-UTR identity (%):** N/A | N/A | N/A
- **5-UTR|CDS|3-UTR identity (%) [Gaps excluded]:** N/A | N/A | N/A
- **5-UTR|CDS|3-UTR identity [Alignment Gaps]:** N/A | N/A | N/A
- **5-UTR aligned content (<base>:%):** N/A
- **CDS aligned content (<base>:%):** N/A
- **3-UTR aligned content (<base>:%):** N/A

**Uniprot Description:**  
  
 N/A N/A   
  
 **Gene Ontology Information:**

Molecular Function

- mRNA binding

Location

- cytoplasm
- nucleus

Biological process

- cell cycle switching, mitotic to meiotic cell cycle
- female meiotic nuclear division
- male meiotic nuclear division
- mRNA stabilization
- oogenesis
- spermatid differentiation
- spermatogenesis
- trophectodermal cell differentiation

---

60

- **Protein name:** Centrosomal protein of 76 kDa
- **Organism:** Homo sapiens
- **Uniprot Accession Number:** Q8TAP6
- **Protein sequence length:** 659 aa
- **1D identity (%):** 12.18
- **1D identity (%) [Gaps excluded]:** 22.43
- **1D identity - Alignment Gaps:** 450
- **1D aligned content (<aminoacid>:%):** {'L': 14.17, 'P': 11.67, 'E': 9.17, 'Q': 5.0, 'S': 4.17, 'A': 5.83, 'D': 5.0, 'R': 5.83, 'Y': 1.67, 'G': 7.5, 'C': 3.33, 'T': 7.5, 'I': 4.17, 'V': 7.5, 'K': 4.17, 'N': 0.83, 'F': 1.67, 'H': 0.83}
- **Common reported functions (%):** 0.0
- **Common reported locations (%):** 12.5
- **Common reported processes (%):** 0.0

- **AF ID:** Q8TAP6
- **Chain:** A
- **Protein length:** 659 aa
- **Resolution:** N/A
- **b-phipsi:** 0.000552
- **w-rdist:** 0.544146
- **t-alpha:** 0.11737
- **Chemical similarity (Tanimoto Index) (%):** 99.39
- **1D identity (%) [PDB]:** 2.61
- **1D identity (%) [Gaps excluded][PDB]:** 74.51
- **1D identity - Alignment Gaps [PDB]:** 1407
- **1D aligned content [PDB] (<aminoacid>:%):** {'L': 13.16, 'P': 7.89, 'G': 7.89, 'R': 2.63, 'D': 5.26, 'V': 21.05, 'E': 7.89, 'T': 5.26, 'Q': 7.89, 'F': 2.63, 'K': 2.63, 'S': 7.89, 'A': 5.26, 'N': 2.63}
- **2D identity (%) [PDB]:** 36.59
- **2D identity (%) [Gaps excluded][PDB]:** 88.04
- **2D identity - Alignment Gaps [PDB]:** 623
- **2D aligned content [PDB] (<2D-fold>:%):** {'.': 22.05, 'H': 35.9, 'T': 23.08, 'E': 17.95, 'G': 0.77, 'B': 0.26}
- **3D similarity (TM-Score) (%) [PDB]:** 20.35

- **Gene name:** CEP76
- **Entrez ID:** 79959
- **RefSeq ID:** NM\_024899
- **Transcript sequence length:** 2895
- **5-UTR|CDS|3-UTR identity (%):** 29.52 | 44.6 | 11.06
- **5-UTR|CDS|3-UTR identity (%) [Gaps excluded]:** 79.49 | 76.26 | 77.02
- **5-UTR|CDS|3-UTR identity [Alignment Gaps]:** 264 | 1196 | 3763
- **5-UTR aligned content (<base>:%):** {'A': 6.45, 'C': 41.94, 'T': 9.68, 'G': 41.94}
- **CDS aligned content (<base>:%):** {'A': 30.51, 'T': 26.93, 'G': 21.56, 'C': 21.01}
- **3-UTR aligned content (<base>:%):** {'G': 13.58, 'C': 10.29, 'A': 32.92, 'T': 43.21}

**Uniprot Description:**  
  
 Centrosomal protein involved in regulation of centriole duplication. Required to limit centriole duplication to once per cell cycle by preventing centriole reduplication.   
  
Interacts with CCP110 and CEP97.   
  
 **Gene Ontology Information:**

Molecular Function   
  
N/A

Location

- centriole
- centrosome
- cytosol
- protein-containing complex

Biological process

- regulation of centriole replication

---

61

- **Protein name:** General transcription and DNA repair factor IIH helicase subunit XPB
- **Organism:** Homo sapiens
- **Uniprot Accession Number:** P19447
- **Protein sequence length:** 782 aa
- **1D identity (%):** 12.86
- **1D identity (%) [Gaps excluded]:** 22.3
- **1D identity - Alignment Gaps:** 441
- **1D aligned content (<aminoacid>:%):** {'G': 8.96, 'K': 11.19, 'R': 6.72, 'A': 3.73, 'H': 2.24, 'Y': 4.48, 'D': 5.22, 'E': 5.22, 'P': 6.72, 'V': 11.19, 'T': 4.48, 'L': 6.72, 'Q': 5.22, 'F': 5.22, 'S': 3.73, 'I': 2.99, 'N': 1.49, 'C': 2.24, 'M': 1.49, 'W': 0.75}
- **Common reported functions (%):** 0.0
- **Common reported locations (%):** 0.0
- **Common reported processes (%):** 0.0

- **AF ID:** P19447
- **Chain:** A
- **Protein length:** 782 aa
- **Resolution:** N/A
- **b-phipsi:** 0.003245
- **w-rdist:** 0.36802
- **t-alpha:** 0.027386
- **Chemical similarity (Tanimoto Index) (%):** 99.02
- **1D identity (%) [PDB]:** 3.02
- **1D identity (%) [Gaps excluded][PDB]:** 60.26
- **1D identity - Alignment Gaps [PDB]:** 1476
- **1D aligned content [PDB] (<aminoacid>:%):** {'I': 4.26, 'F': 4.26, 'L': 8.51, 'A': 6.38, 'D': 2.13, 'T': 4.26, 'H': 4.26, 'P': 8.51, 'G': 6.38, 'K': 8.51, 'S': 10.64, 'M': 4.26, 'Y': 4.26, 'C': 2.13, 'V': 4.26, 'R': 6.38, 'Q': 6.38, 'E': 4.26}
- **2D identity (%) [PDB]:** 45.23
- **2D identity (%) [Gaps excluded][PDB]:** 86.12
- **2D identity - Alignment Gaps [PDB]:** 508
- **2D aligned content [PDB] (<2D-fold>:%):** {'.': 20.87, 'E': 21.9, 'T': 18.6, 'H': 37.6, 'B': 0.41, 'G': 0.62}
- **3D similarity (TM-Score) (%) [PDB]:** 23.63

- **Gene name:** ERCC3
- **Entrez ID:** N/A
- **RefSeq ID:** NM\_000122
- **Transcript sequence length:** 2718
- **5-UTR|CDS|3-UTR identity (%):** 9.58 | 41.89 | 5.06
- **5-UTR|CDS|3-UTR identity (%) [Gaps excluded]:** 68.42 | 73.99 | 77.3
- **5-UTR|CDS|3-UTR identity [Alignment Gaps]:** 350 | 1367 | 4027
- **5-UTR aligned content (<base>:%):** {'G': 53.85, 'C': 28.21, 'T': 15.38, 'A': 2.56}
- **CDS aligned content (<base>:%):** {'T': 21.06, 'G': 25.76, 'A': 30.0, 'C': 23.18}
- **3-UTR aligned content (<base>:%):** {'T': 37.61, 'G': 22.48, 'C': 13.76, 'A': 26.15}

**Uniprot Description:**  
  
 ATP-dependent 3'-5' DNA helicase, component of the general transcription and DNA repair factor IIH (TFIIH) core complex, which is involved in general and transcription-coupled nucleotide excision repair (NER) of damaged DNA and, when complexed to CAK, in RNA transcription by RNA polymerase II. In NER, TFIIH acts by opening DNA around the lesion to allow the excision of the damaged oligonucleotide and its replacement by a new DNA fragment. The ATPase activity of XPB/ERCC3, but not its helicase activity, is required for DNA opening. In transcription, TFIIH has an essential role in transcription initiation (PubMed:8157004, PubMed:30894545). When the pre-initiation complex (PIC) has been established, TFIIH is required for promoter opening and promoter escape (PubMed:8157004). The ATP-dependent helicase activity of XPB/ERCC3 is required for promoter opening and promoter escape. Phosphorylation of the C-terminal tail (CTD) of the largest subunit of RNA polymerase II by the kinase module CAK controls the initiation of transcription.   
  
Component of the 7-subunit TFIIH core complex composed of XPB/ERCC3, XPD/ERCC2, GTF2H1, GTF2H2, GTF2H3, GTF2H4 and GTF2H5, which is active in NER. The core complex associates with the 3-subunit CDK-activating kinase (CAK) module composed of CCNH/cyclin H, CDK7 and MNAT1 to form the 10-subunit holoenzyme (holo-TFIIH) active in transcription (PubMed:9852112). Interacts with PUF60 (PubMed:10882074, PubMed:11239393). Interacts with ATF7IP (PubMed:19106100). Interacts with KAT2A; leading to KAT2A recruitment to promoters and acetylation of histones (PubMed:30894545).   
  
 **Gene Ontology Information:**

Molecular Function

- 3'-5' DNA helicase activity
- ATP binding
- ATPase activity
- DNA binding

Location

- nucleoplasm
- nucleotide-excision repair factor 3 complex
- transcription factor TFIIH holo complex
- transcription preinitiation complex

Biological process

- nucleotide-excision repair
- transcription by RNA polymerase II
- transcription initiation from RNA polymerase II promoter

---

62

- **Protein name:** Protein O-linked-mannose beta-1,2-N-acetylglucosaminyltransferase 1
- **Organism:** Homo sapiens
- **Uniprot Accession Number:** Q8WZA1
- **Protein sequence length:** 660 aa
- **1D identity (%):** 14.42
- **1D identity (%) [Gaps excluded]:** 26.49
- **1D identity - Alignment Gaps:** 449
- **1D aligned content (<aminoacid>:%):** {'M': 0.7, 'P': 11.97, 'F': 4.23, 'L': 11.27, 'T': 4.23, 'V': 11.97, 'I': 2.82, 'Y': 4.23, 'D': 4.23, 'R': 7.04, 'A': 5.63, 'E': 4.93, 'Q': 2.82, 'H': 1.41, 'N': 2.11, 'S': 4.23, 'G': 6.34, 'W': 2.82, 'C': 2.11, 'K': 4.93}
- **Common reported functions (%):** 0.0
- **Common reported locations (%):** 12.5
- **Common reported processes (%):** 0.0

- **AF ID:** Q8WZA1
- **Chain:** A
- **Protein length:** 660 aa
- **Resolution:** N/A
- **b-phipsi:** 0.00127
- **w-rdist:** 0.233673
- **t-alpha:** 0.176838
- **Chemical similarity (Tanimoto Index) (%):** 96.55
- **1D identity (%) [PDB]:** 3.7
- **1D identity (%) [Gaps excluded][PDB]:** 67.09
- **1D identity - Alignment Gaps [PDB]:** 1352
- **1D aligned content [PDB] (<aminoacid>:%):** {'D': 3.77, 'L': 11.32, 'T': 7.55, 'N': 1.89, 'R': 13.21, 'I': 7.55, 'Q': 3.77, 'Y': 5.66, 'K': 1.89, 'S': 5.66, 'F': 3.77, 'P': 5.66, 'G': 5.66, 'V': 9.43, 'E': 5.66, 'M': 1.89, 'A': 3.77, 'H': 1.89}
- **2D identity (%) [PDB]:** 40.83
- **2D identity (%) [Gaps excluded][PDB]:** 90.62
- **2D identity - Alignment Gaps [PDB]:** 572
- **2D aligned content [PDB] (<2D-fold>:%):** {'.': 16.24, 'H': 40.0, 'T': 23.29, 'E': 19.53, 'G': 0.71, 'B': 0.24}
- **3D similarity (TM-Score) (%) [PDB]:** 20.08

- **Gene name:** POMGNT1
- **Entrez ID:** N/A
- **RefSeq ID:** N/A
- **Sequence length:** N/A
- **5-UTR|CDS|3-UTR identity (%):** N/A | N/A | N/A
- **5-UTR|CDS|3-UTR identity (%) [Gaps excluded]:** N/A | N/A | N/A
- **5-UTR|CDS|3-UTR identity [Alignment Gaps]:** N/A | N/A | N/A
- **5-UTR aligned content (<base>:%):** N/A
- **CDS aligned content (<base>:%):** N/A
- **3-UTR aligned content (<base>:%):** N/A

**Uniprot Description:**  
  
 Participates in O-mannosyl glycosylation by catalyzing the addition of N-acetylglucosamine to O-linked mannose on glycoproteins (PubMed:11709191, PubMed:27493216, PubMed:28512129). Catalyzes the synthesis of the GlcNAc(beta1-2)Man(alpha1-)O-Ser/Thr moiety on alpha-dystroglycan and other O-mannosylated proteins, providing the necessary basis for the addition of further carbohydrate moieties (PubMed:11709191, PubMed:27493216). Is specific for alpha linked terminal mannose and does not have MGAT3, MGAT4, MGAT5, MGAT7 or MGAT8 activity.   
  
Interacts with DAG1 (via O-linked mannose moiety) (PubMed:27493216). Interacts (via transmembrane domain) with FKTN; the interaction is direct and is required for normal location in Golgi membranes (PubMed:17034757).   
  
 **Gene Ontology Information:**

Molecular Function

- acetylglucosaminyltransferase activity
- beta-1,3-galactosyl-O-glycosyl-glycoprotein beta-1,3-N-acetylglucosaminyltransferase activity
- manganese ion binding

Location

- Golgi membrane
- membrane

Biological process

- O-glycan processing
- protein O-linked glycosylation

---

63

- **Protein name:** E3 ubiquitin-protein ligase SMURF2
- **Organism:** Homo sapiens
- **Uniprot Accession Number:** Q9HAU4
- **Protein sequence length:** 748 aa
- **1D identity (%):** 15.02
- **1D identity (%) [Gaps excluded]:** 22.92
- **1D identity - Alignment Gaps:** 335
- **1D aligned content (<aminoacid>:%):** {'P': 14.38, 'G': 12.33, 'L': 13.7, 'K': 6.16, 'D': 6.16, 'V': 6.85, 'H': 2.74, 'Y': 3.42, 'I': 2.74, 'N': 2.05, 'Q': 6.16, 'T': 4.11, 'S': 2.05, 'W': 0.68, 'R': 5.48, 'E': 4.11, 'C': 2.05, 'A': 2.74, 'M': 0.68, 'F': 1.37}
- **Common reported functions (%):** 0.0
- **Common reported locations (%):** 37.5
- **Common reported processes (%):** 0.0

- **AF ID:** Q9HAU4
- **Chain:** A
- **Protein length:** 748 aa
- **Resolution:** N/A
- **b-phipsi:** 0.002129
- **w-rdist:** 0.251179
- **t-alpha:** 0.120501
- **Chemical similarity (Tanimoto Index) (%):** 98.64
- **1D identity (%) [PDB]:** 2.19
- **1D identity (%) [Gaps excluded][PDB]:** 79.07
- **1D identity - Alignment Gaps [PDB]:** 1512
- **1D aligned content [PDB] (<aminoacid>:%):** {'L': 20.59, 'I': 2.94, 'G': 5.88, 'R': 5.88, 'D': 11.76, 'V': 11.76, 'E': 11.76, 'T': 2.94, 'P': 5.88, 'K': 2.94, 'Q': 8.82, 'S': 2.94, 'A': 2.94, 'N': 2.94}
- **2D identity (%) [PDB]:** 43.12
- **2D identity (%) [Gaps excluded][PDB]:** 88.89
- **2D identity - Alignment Gaps [PDB]:** 554
- **2D aligned content [PDB] (<2D-fold>:%):** {'.': 16.81, 'E': 28.88, 'T': 21.12, 'H': 32.97, 'B': 0.22}
- **3D similarity (TM-Score) (%) [PDB]:** 22.87

- **Gene name:** SMURF2
- **Entrez ID:** 64750
- **RefSeq ID:** NM\_022739
- **Transcript sequence length:** 6240
- **5-UTR|CDS|3-UTR identity (%):** 48.93 | 44.97 | 46.25
- **5-UTR|CDS|3-UTR identity (%) [Gaps excluded]:** 79.68 | 74.9 | 76.59
- **5-UTR|CDS|3-UTR identity [Alignment Gaps]:** 198 | 1207 | 1939
- **5-UTR aligned content (<base>:%):** {'G': 47.41, 'A': 3.98, 'C': 42.63, 'T': 5.98}
- **CDS aligned content (<base>:%):** {'A': 31.81, 'T': 24.3, 'G': 24.3, 'C': 19.59}
- **3-UTR aligned content (<base>:%):** {'C': 15.15, 'A': 29.28, 'G': 18.99, 'T': 36.57}

**Uniprot Description:**  
  
 E3 ubiquitin-protein ligase which accepts ubiquitin from an E2 ubiquitin-conjugating enzyme in the form of a thioester and then directly transfers the ubiquitin to targeted substrates (PubMed:11016919). Interacts with SMAD7 to trigger SMAD7-mediated transforming growth factor beta/TGF-beta receptor ubiquitin-dependent degradation, thereby downregulating TGF-beta signaling (PubMed:11163210, PubMed:12717440). In addition, interaction with SMAD7 activates autocatalytic degradation, which is prevented by interaction with AIMP1 (PubMed:18448069). Also forms a stable complex with TGF-beta receptor-mediated phosphorylated SMAD1, SMAD2 and SMAD3, and targets SMAD1 and SMAD2 for ubiquitination and proteasome-mediated degradation (PubMed:11016919, PubMed:11158580, PubMed:11389444). SMAD2 may recruit substrates, such as SNON, for ubiquitin-dependent degradation (PubMed:11389444). Negatively regulates TGFB1-induced epithelial-mesenchymal transition and myofibroblast differentiation (PubMed:30696809).   
  
Interacts (via WW domains) with SMAD1 (PubMed:11158580). Interacts (via WW domains) with SMAD2 (via PY-motif) (PubMed:11158580, PubMed:11389444). Interacts (via WW domains) with SMAD3 (via PY-motif) (PubMed:11158580, PubMed:11389444). Interacts with SMAD6 (PubMed:11158580). Interacts with SMAD7 (via PY-motif) and TGFBR1; SMAD7 recruits SMURF2 to the TGF-beta receptor and regulates its degradation (PubMed:11163210, PubMed:11158580, PubMed:33673144, PubMed:16061177, PubMed:16641086). Does not interact with SMAD4; SMAD4 lacks a PY-motif (PubMed:11158580). Interacts with AIMP1 (PubMed:18448069). Interacts with SNON (PubMed:11389444). Interacts with STAMBP and RNF11 (PubMed:14562029, PubMed:14755250). May interact with NDFIP1 and NDFIP2; this interaction induces the E3 ubiquitin-protein ligase activity. Interacts with TTC3 (Probable).   
  
 **Gene Ontology Information:**

Molecular Function

- identical protein binding
- SMAD binding
- ubiquitin protein ligase activity
- ubiquitin-protein transferase activity

Location

- cytoplasm
- cytosol
- membrane raft
- nuclear speck
- nucleoplasm
- nucleus
- plasma membrane
- ubiquitin ligase complex

Biological process

- negative regulation of BMP signaling pathway
- negative regulation of transcription, DNA-templated
- negative regulation of transcription by RNA polymerase II
- negative regulation of transforming growth factor beta receptor signaling pathway
- positive regulation of canonical Wnt signaling pathway
- positive regulation of trophoblast cell migration
- proteasome-mediated ubiquitin-dependent protein catabolic process
- protein ubiquitination
- regulation of transforming growth factor beta receptor signaling pathway
- ubiquitin-dependent protein catabolic process
- ubiquitin-dependent SMAD protein catabolic process
- Wnt signaling pathway, planar cell polarity pathway

---

64

- **Protein name:** Cytosolic phospholipase A2 zeta
- **Organism:** Homo sapiens
- **Uniprot Accession Number:** Q68DD2
- **Protein sequence length:** 849 aa
- **1D identity (%):** 16.44
- **1D identity (%) [Gaps excluded]:** 25.52
- **1D identity - Alignment Gaps:** 370
- **1D aligned content (<aminoacid>:%):** {'L': 14.04, 'P': 11.11, 'R': 6.43, 'G': 12.28, 'V': 9.94, 'H': 1.75, 'Y': 5.85, 'D': 2.34, 'K': 2.34, 'F': 5.26, 'Q': 5.85, 'I': 2.92, 'T': 3.51, 'E': 5.26, 'A': 4.68, 'C': 2.34, 'S': 4.09}
- **Common reported functions (%):** 0.0
- **Common reported locations (%):** 12.5
- **Common reported processes (%):** 0.0

- **AF ID:** Q68DD2
- **Chain:** A
- **Protein length:** 849 aa
- **Resolution:** N/A
- **b-phipsi:** 0.001895
- **w-rdist:** 0.202792
- **t-alpha:** 0.138497
- **Chemical similarity (Tanimoto Index) (%):** 100.0
- **1D identity (%) [PDB]:** 2.3
- **1D identity (%) [Gaps excluded][PDB]:** 79.17
- **1D identity - Alignment Gaps [PDB]:** 1603
- **1D aligned content [PDB] (<aminoacid>:%):** {'M': 2.63, 'E': 7.89, 'V': 18.42, 'T': 5.26, 'L': 15.79, 'G': 7.89, 'K': 2.63, 'Q': 10.53, 'F': 2.63, 'S': 7.89, 'A': 7.89, 'P': 2.63, 'D': 7.89}
- **2D identity (%) [PDB]:** 44.9
- **2D identity (%) [Gaps excluded][PDB]:** 91.09
- **2D identity - Alignment Gaps [PDB]:** 577
- **2D aligned content [PDB] (<2D-fold>:%):** {'.': 18.4, 'E': 31.9, 'T': 22.7, 'H': 26.22, 'G': 0.59, 'B': 0.2}
- **3D similarity (TM-Score) (%) [PDB]:** 22.57

- **Gene name:** PLA2G4F
- **Entrez ID:** 255189
- **RefSeq ID:** NM\_213600
- **Transcript sequence length:** 5587
- **5-UTR|CDS|3-UTR identity (%):** 12.06 | 44.43 | 39.38
- **5-UTR|CDS|3-UTR identity (%) [Gaps excluded]:** 78.46 | 74.86 | 73.24
- **5-UTR|CDS|3-UTR identity [Alignment Gaps]:** 358 | 1310 | 2175
- **5-UTR aligned content (<base>:%):** {'G': 39.22, 'T': 15.69, 'C': 35.29, 'A': 9.8}
- **CDS aligned content (<base>:%):** {'A': 23.88, 'T': 21.72, 'G': 28.14, 'C': 26.26}
- **3-UTR aligned content (<base>:%):** {'A': 25.09, 'G': 26.23, 'C': 21.91, 'T': 26.77}

**Uniprot Description:**  
  
 Has calcium-dependent phospholipase and lysophospholipase activities with a potential role in membrane lipid remodeling and biosynthesis of lipid mediators (PubMed:29158256). Preferentially hydrolyzes the ester bond of the fatty acyl group attached at sn-2 position of phospholipids (phospholipase A2 activity) (PubMed:29158256). Selectively hydrolyzes sn-2 arachidonoyl group from membrane phospholipids, providing the precursor for eicosanoid biosynthesis (PubMed:29158256). In myocardial mitochondria, plays a major role in arachidonate release that is metabolically channeled to the formation of cardioprotective eicosanoids, epoxyeicosatrienoates (EETs) (PubMed:29158256). N/A   
  
 **Gene Ontology Information:**

Molecular Function

- calcium ion binding
- calcium-dependent phospholipase A2 activity
- calcium-dependent phospholipid binding
- lysophospholipase activity
- phospholipase A1 activity

Location

- cytosol
- mitochondrion
- ruffle membrane
- vesicle

Biological process

- arachidonic acid secretion
- cellular response to antibiotic
- cellular response to organic cyclic compound
- glycerophospholipid catabolic process
- phosphatidylglycerol acyl-chain remodeling
- prostaglandin biosynthetic process

---

65

- **Protein name:** Apoptosis-resistant E3 ubiquitin protein ligase 1
- **Organism:** Homo sapiens
- **Uniprot Accession Number:** O15033
- **Protein sequence length:** 823 aa
- **1D identity (%):** 16.15
- **1D identity (%) [Gaps excluded]:** 25.3
- **1D identity - Alignment Gaps:** 372
- **1D aligned content (<aminoacid>:%):** {'V': 10.24, 'G': 8.43, 'A': 4.22, 'F': 6.02, 'E': 6.63, 'R': 7.83, 'D': 3.61, 'Y': 3.01, 'H': 3.01, 'P': 9.04, 'K': 6.63, 'L': 10.84, 'N': 3.61, 'I': 2.41, 'T': 4.22, 'S': 3.61, 'Q': 4.22, 'C': 2.41}
- **Common reported functions (%):** 0.0
- **Common reported locations (%):** 25.0
- **Common reported processes (%):** 8.33

- **AF ID:** O15033
- **Chain:** A
- **Protein length:** 823 aa
- **Resolution:** N/A
- **b-phipsi:** 0.000883
- **w-rdist:** 0.734276
- **t-alpha:** 0.016432
- **Chemical similarity (Tanimoto Index) (%):** 99.02
- **1D identity (%) [PDB]:** 3.27
- **1D identity (%) [Gaps excluded][PDB]:** 64.2
- **1D identity - Alignment Gaps [PDB]:** 1511
- **1D aligned content [PDB] (<aminoacid>:%):** {'M': 3.85, 'I': 7.69, 'V': 13.46, 'S': 5.77, 'A': 3.85, 'F': 5.77, 'Y': 1.92, 'Q': 5.77, 'P': 5.77, 'E': 5.77, 'L': 7.69, 'D': 3.85, 'N': 1.92, 'T': 5.77, 'R': 7.69, 'K': 9.62, 'C': 1.92, 'H': 1.92}
- **2D identity (%) [PDB]:** 41.48
- **2D identity (%) [Gaps excluded][PDB]:** 89.96
- **2D identity - Alignment Gaps [PDB]:** 617
- **2D aligned content [PDB] (<2D-fold>:%):** {'H': 38.53, '.': 13.26, 'E': 22.11, 'T': 25.26, 'G': 0.63, 'B': 0.21}
- **3D similarity (TM-Score) (%) [PDB]:** 25.11

- **Gene name:** AREL1
- **Entrez ID:** 9870
- **RefSeq ID:** N/A
- **Sequence length:** N/A
- **5-UTR|CDS|3-UTR identity (%):** N/A | N/A | N/A
- **5-UTR|CDS|3-UTR identity (%) [Gaps excluded]:** N/A | N/A | N/A
- **5-UTR|CDS|3-UTR identity [Alignment Gaps]:** N/A | N/A | N/A
- **5-UTR aligned content (<base>:%):** N/A
- **CDS aligned content (<base>:%):** N/A
- **3-UTR aligned content (<base>:%):** N/A

**Uniprot Description:**  
  
 E3 ubiquitin-protein ligase which accepts ubiquitin from an E2 ubiquitin-conjugating enzyme in the form of a thioester and then directly transfers the ubiquitin to targeted substrates. Inhibits apoptosis by ubiquitinating and targeting for degradation a number of proapoptotic proteins including DIABLO/SMAC, HTRA2 and SEPT4/ARTS which are released from the mitochondrion into the cytosol following apoptotic stimulation (PubMed:23479728). Modulates pulmonary inflammation by targeting SOCS2 for ubiquitination and subsequent degradation by the proteasome (PubMed:31578312).   
  
Interacts with SOCS2.   
  
 **Gene Ontology Information:**

Molecular Function

- ubiquitin protein ligase activity
- ubiquitin-protein transferase activity

Location

- cytoplasm
- cytosol

Biological process

- apoptotic process
- negative regulation of apoptotic process
- protein K11-linked ubiquitination
- protein K33-linked ubiquitination
- protein polyubiquitination
- protein ubiquitination
- regulation of inflammatory response
- ubiquitin-dependent protein catabolic process

---

66

- **Protein name:** Zinc finger protein 410
- **Organism:** Homo sapiens
- **Uniprot Accession Number:** Q86VK4
- **Protein sequence length:** 478 aa
- **1D identity (%):** 11.11
- **1D identity (%) [Gaps excluded]:** 24.23
- **1D identity - Alignment Gaps:** 497
- **1D aligned content (<aminoacid>:%):** {'L': 16.67, 'E': 8.82, 'F': 2.94, 'Q': 8.82, 'P': 4.9, 'T': 5.88, 'S': 3.92, 'V': 12.75, 'R': 3.92, 'G': 9.8, 'H': 2.94, 'I': 0.98, 'A': 1.96, 'N': 2.94, 'D': 1.96, 'K': 6.86, 'Y': 1.96, 'C': 1.96}
- **Common reported functions (%):** 0.0
- **Common reported locations (%):** 12.5
- **Common reported processes (%):** 0.0

- **AF ID:** Q86VK4
- **Chain:** A
- **Protein length:** 478 aa
- **Resolution:** N/A
- **b-phipsi:** 0.010181
- **w-rdist:** 0.569565
- **t-alpha:** 0.001565
- **Chemical similarity (Tanimoto Index) (%):** 98.42
- **1D identity (%) [PDB]:** 2.82
- **1D identity (%) [Gaps excluded][PDB]:** 67.92
- **1D identity - Alignment Gaps [PDB]:** 1222
- **1D aligned content [PDB] (<aminoacid>:%):** {'M': 5.56, 'V': 19.44, 'T': 11.11, 'L': 16.67, 'P': 5.56, 'E': 8.33, 'G': 2.78, 'D': 11.11, 'F': 2.78, 'K': 2.78, 'S': 2.78, 'Q': 2.78, 'A': 2.78, 'H': 2.78, 'R': 2.78}
- **2D identity (%) [PDB]:** 33.61
- **2D identity (%) [Gaps excluded][PDB]:** 84.17
- **2D identity - Alignment Gaps [PDB]:** 570
- **2D aligned content [PDB] (<2D-fold>:%):** {'.': 26.96, 'T': 30.09, 'E': 12.85, 'H': 30.09}
- **3D similarity (TM-Score) (%) [PDB]:** 12.85

- **Gene name:** ZNF410
- **Entrez ID:** 507530
- **RefSeq ID:** NM\_021188
- **Transcript sequence length:** 2623
- **5-UTR|CDS|3-UTR identity (%):** 22.54 | 33.74 | 14.24
- **5-UTR|CDS|3-UTR identity (%) [Gaps excluded]:** 63.52 | 74.68 | 74.91
- **5-UTR|CDS|3-UTR identity [Alignment Gaps]:** 289 | 1519 | 3583
- **5-UTR aligned content (<base>:%):** {'T': 18.81, 'G': 38.61, 'A': 8.91, 'C': 33.66}
- **CDS aligned content (<base>:%):** {'T': 24.81, 'G': 23.64, 'A': 28.66, 'C': 22.89}
- **3-UTR aligned content (<base>:%):** {'G': 18.89, 'C': 17.78, 'T': 34.76, 'A': 28.57}

**Uniprot Description:**  
  
 Transcription factor that binds to the sequence motif 5'-CATCCCATAATA-3', and is specifically required to silence expression of fetal hemoglobin in adult erythroid cells (PubMed:33301730, PubMed:33859416). Prevents expression of fetal hemoglobin genes HBG1 and HBG2 through CHD4: acts as a direct transcriptional activator of CHD4, a central component of the NuRD complex that represses transcription of fetal hemoglobin genes HBG1 and HBG2 in erythroid cells (PubMed:33301730, PubMed:33859416). May also activate transcription of matrix-remodeling genes such as MMP1 during fibroblast senescence (PubMed:12370286). May activate transcription of the gap junction gene GJC1, perhaps in response to increasing glucose (PubMed:30078215). However, recent studies suggest that ZNF410 is dedicated to regulate expression of a single gene: CHD4 (PubMed:33301730, PubMed:33859416).   
  
Interacts with CDKN2A/p14ARF.   
  
 **Gene Ontology Information:**

Molecular Function

- DNA binding
- metal ion binding
- transcription coregulator activity

Location

- chromosome
- nucleus

Biological process

- regulation of transcription by RNA polymerase II

---

67

- **Protein name:** Aspartate--tRNA ligase, mitochondrial
- **Organism:** Homo sapiens
- **Uniprot Accession Number:** Q6PI48
- **Protein sequence length:** 645 aa
- **1D identity (%):** 12.49
- **1D identity (%) [Gaps excluded]:** 22.02
- **1D identity - Alignment Gaps:** 416
- **1D aligned content (<aminoacid>:%):** {'Y': 3.33, 'R': 7.5, 'P': 8.33, 'T': 5.83, 'L': 10.83, 'Q': 5.83, 'H': 4.17, 'C': 1.67, 'K': 6.67, 'S': 5.0, 'E': 8.33, 'I': 3.33, 'V': 10.0, 'F': 2.5, 'G': 6.67, 'M': 1.67, 'A': 4.17, 'D': 3.33, 'W': 0.83}
- **Common reported functions (%):** 0.0
- **Common reported locations (%):** 0.0
- **Common reported processes (%):** 0.0

- **AF ID:** Q6PI48
- **Chain:** A
- **Protein length:** 645 aa
- **Resolution:** N/A
- **b-phipsi:** 0.000628
- **w-rdist:** 0.586866
- **t-alpha:** 0.092332
- **Chemical similarity (Tanimoto Index) (%):** 97.91
- **1D identity (%) [PDB]:** 2.56
- **1D identity (%) [Gaps excluded][PDB]:** 78.72
- **1D identity - Alignment Gaps [PDB]:** 1401
- **1D aligned content [PDB] (<aminoacid>:%):** {'M': 2.7, 'E': 8.11, 'V': 18.92, 'T': 5.41, 'L': 21.62, 'G': 5.41, 'Q': 10.81, 'F': 2.7, 'S': 8.11, 'A': 5.41, 'H': 2.7, 'N': 2.7, 'D': 2.7, 'I': 2.7}
- **2D identity (%) [PDB]:** 46.16
- **2D identity (%) [Gaps excluded][PDB]:** 87.07
- **2D identity - Alignment Gaps [PDB]:** 459
- **2D aligned content [PDB] (<2D-fold>:%):** {'.': 15.96, 'T': 21.06, 'H': 37.69, 'E': 24.61, 'B': 0.67}
- **3D similarity (TM-Score) (%) [PDB]:** 18.82

- **Gene name:** DARS2
- **Entrez ID:** 538772
- **RefSeq ID:** NM\_018122
- **Transcript sequence length:** 3336
- **5-UTR|CDS|3-UTR identity (%):** 37.98 | 41.16 | 12.61
- **5-UTR|CDS|3-UTR identity (%) [Gaps excluded]:** 70.96 | 75.3 | 77.55
- **5-UTR|CDS|3-UTR identity [Alignment Gaps]:** 290 | 1326 | 3692
- **5-UTR aligned content (<base>:%):** {'G': 40.51, 'A': 8.02, 'C': 39.24, 'T': 12.24}
- **CDS aligned content (<base>:%):** {'A': 30.4, 'T': 24.42, 'G': 23.17, 'C': 22.01}
- **3-UTR aligned content (<base>:%):** {'A': 35.25, 'T': 35.43, 'C': 14.93, 'G': 14.39}

**Uniprot Description:**  
  
 N/A   
  
Homodimer.   
  
 **Gene Ontology Information:**

Molecular Function

- aspartate-tRNA ligase activity
- ATP binding
- nucleic acid binding

Location

- mitochondrial matrix
- mitochondrion
- organelle membrane

Biological process

- aspartyl-tRNA aminoacylation
- mitochondrial asparaginyl-tRNA aminoacylation

---

68

- **Protein name:** Mothers against decapentaplegic homolog 2
- **Organism:** Homo sapiens
- **Uniprot Accession Number:** Q15796
- **Protein sequence length:** 467 aa
- **1D identity (%):** 11.3
- **1D identity (%) [Gaps excluded]:** 26.32
- **1D identity - Alignment Gaps:** 530
- **1D aligned content (<aminoacid>:%):** {'I': 2.86, 'T': 7.62, 'P': 11.43, 'Q': 5.71, 'K': 3.81, 'L': 11.43, 'G': 6.67, 'C': 3.81, 'N': 3.81, 'V': 9.52, 'Y': 4.76, 'S': 2.86, 'D': 5.71, 'A': 2.86, 'E': 7.62, 'H': 1.9, 'R': 5.71, 'F': 0.95, 'W': 0.95}
- **Common reported functions (%):** 0.0
- **Common reported locations (%):** 37.5
- **Common reported processes (%):** 0.0

- **AF ID:** Q15796
- **Chain:** A
- **Protein length:** 467 aa
- **Resolution:** N/A
- **b-phipsi:** 0.000531
- **w-rdist:** 0.613332
- **t-alpha:** 0.094178
- **Chemical similarity (Tanimoto Index) (%):** 99.55
- **1D identity (%) [PDB]:** 4.29
- **1D identity (%) [Gaps excluded][PDB]:** 64.63
- **1D identity - Alignment Gaps [PDB]:** 1153
- **1D aligned content [PDB] (<aminoacid>:%):** {'I': 5.66, 'L': 9.43, 'P': 11.32, 'V': 15.09, 'K': 5.66, 'R': 5.66, 'G': 9.43, 'A': 7.55, 'S': 9.43, 'Q': 3.77, 'T': 7.55, 'N': 1.89, 'H': 1.89, 'D': 3.77, 'C': 1.89}
- **2D identity (%) [PDB]:** 32.19
- **2D identity (%) [Gaps excluded][PDB]:** 87.57
- **2D identity - Alignment Gaps [PDB]:** 609
- **2D aligned content [PDB] (<2D-fold>:%):** {'.': 22.9, 'H': 30.97, 'T': 30.0, 'E': 16.13}
- **3D similarity (TM-Score) (%) [PDB]:** 20.05

- **Gene name:** SMAD2
- **Entrez ID:** 4087
- **RefSeq ID:** N/A
- **Sequence length:** N/A
- **5-UTR|CDS|3-UTR identity (%):** N/A | N/A | N/A
- **5-UTR|CDS|3-UTR identity (%) [Gaps excluded]:** N/A | N/A | N/A
- **5-UTR|CDS|3-UTR identity [Alignment Gaps]:** N/A | N/A | N/A
- **5-UTR aligned content (<base>:%):** N/A
- **CDS aligned content (<base>:%):** N/A
- **3-UTR aligned content (<base>:%):** N/A

**Uniprot Description:**  
  
 Receptor-regulated SMAD (R-SMAD) that is an intracellular signal transducer and transcriptional modulator activated by TGF-beta (transforming growth factor) and activin type 1 receptor kinases. Binds the TRE element in the promoter region of many genes that are regulated by TGF-beta and, on formation of the SMAD2/SMAD4 complex, activates transcription. May act as a tumor suppressor in colorectal carcinoma. Positively regulates PDPK1 kinase activity by stimulating its dissociation from the 14-3-3 protein YWHAQ which acts as a negative regulator.   
  
Monomer; in the absence of TGF-beta (PubMed:9670020). Heterodimer; in the presence of TGF-beta (PubMed:9670020). Forms a heterodimer with co-SMAD, SMAD4, in the nucleus to form the transactivation complex SMAD2/SMAD4 (PubMed:9670020, PubMed:24324267, PubMed:15350224). Found in a complex with SMAD3 and TRIM33 upon addition of TGF-beta (PubMed:16751102). Identified in a complex that contains at least ZNF451, SMAD2, SMAD3 and SMAD4 (PubMed:24324267). Interacts (via the MH2 domain) with ZFYVE9; may form trimers with the SMAD4 co-SMAD (PubMed:10615055). Interacts with TAZ/WWRT1 (PubMed:18568018). Interacts with FOXH1 (PubMed:9702198). Interacts with SNW1 (PubMed:11278756). Interacts with CREB-binding protein (CBP) and EP300 (PubMed:16862174). Interacts with SNON (PubMed:11389444). Interacts with ALK4/ACVR1B (PubMed:9892009, PubMed:10615055). Interacts with SKOR1 (PubMed:17292623). Interacts with SKOR2 (PubMed:16200078). Interacts with PRDM16 (PubMed:19049980). Interacts (via MH2 domain) with LEMD3 (PubMed:15601644, PubMed:15647271). Interacts with RBPMS (PubMed:17099224). Interacts with WWP1. Interacts (dephosphorylated form, via the MH1 and MH2 domains) with RANBP3 (via its C-terminal R domain); the interaction results in the export of dephosphorylated SMAD3 out of the nucleus and termination of the TGF-beta signaling (PubMed:19289081). Interacts with PDPK1 (via PH domain) (PubMed:17327236). Interacts with DAB2; the interactions are enhanced upon TGF-beta stimulation (PubMed:11387212). Interacts with USP15 (PubMed:21947082). Interacts with PPP5C (PubMed:22781750). Interacts with LDLRAD4 (via the SMAD interaction motif) (PubMed:24627487). Interacts (via MH2 domain) with PMEPA1 (via the SMAD interaction motif) (PubMed:20129061). Interacts with ZFHX3 (PubMed:25105025). Interacts with ZNF451 (PubMed:24324267). Interacts with SMURF2 when phosphorylated on Ser-465/467 (PubMed:11389444). Interacts with PPM1A (PubMed:16751101). Interacts with TGF-beta (PubMed:8980228). Interacts with TGFBR1 (PubMed:9865696). Interacts with TGIF (PubMed:10835638). Interacts with SMAD3 and TRIM33 (PubMed:16751102). Interacts with ZNF580 (PubMed:21599657). Interacts with NEDD4L in response to TGF-beta (By similarity). Interacts with HGS (By similarity). Interacts with AIP1 (By similarity). Interacts with WWP1 (By similarity). Interacts with PML (By similarity). Interacts weakly with ZNF8 (By similarity). Interacts (when phosphorylated) with RNF111; RNF111 acts as an enhancer of the transcriptional responses by mediating ubiquitination and degradation of SMAD2 inhibitors (By similarity). Interacts with YAP1 (when phosphorylated at 'Ser-127') (By similarity).   
  
 **Gene Ontology Information:**

Molecular Function

- chromatin binding
- cis-regulatory region sequence-specific DNA binding
- co-SMAD binding
- disordered domain specific binding
- DNA-binding transcription activator activity, RNA polymerase II-specific
- DNA-binding transcription factor activity
- DNA-binding transcription factor activity, RNA polymerase II-specific
- DNA-binding transcription factor binding
- double-stranded DNA binding
- I-SMAD binding
- identical protein binding
- metal ion binding
- phosphatase binding
- R-SMAD binding
- RNA polymerase II cis-regulatory region sequence-specific DNA binding
- RNA polymerase II-specific DNA-binding transcription factor binding
- SMAD binding
- tau protein binding
- transforming growth factor beta receptor binding
- type I transforming growth factor beta receptor binding
- ubiquitin protein ligase binding

Location

- activin responsive factor complex
- chromatin
- cytoplasm
- cytosol
- heteromeric SMAD protein complex
- homomeric SMAD protein complex
- nucleoplasm
- nucleus
- protein-containing complex
- SMAD protein complex
- transcription regulator complex

Biological process

- activin receptor signaling pathway
- adrenal gland development
- anatomical structure morphogenesis
- anterior/posterior pattern specification
- aortic valve morphogenesis
- cell differentiation
- cell fate commitment
- common-partner SMAD protein phosphorylation
- transcription, DNA-templated
- embryonic cranial skeleton morphogenesis
- embryonic foregut morphogenesis
- endocardial cushion morphogenesis
- endoderm formation
- gastrulation
- in utero embryonic development
- insulin secretion
- intracellular signal transduction
- lung development
- mesoderm formation
- negative regulation of cell population proliferation
- negative regulation of transcription, DNA-templated
- nodal signaling pathway
- organ growth
- pancreas development
- paraxial mesoderm morphogenesis
- pericardium development
- positive regulation of BMP signaling pathway
- positive regulation of transcription, DNA-templated
- positive regulation of epithelial to mesenchymal transition
- positive regulation of gene expression
- positive regulation of nodal signaling pathway involved in determination of lateral mesoderm left/right asymmetry
- positive regulation of transcription by RNA polymerase II
- post-embryonic development
- primary miRNA processing
- pulmonary valve morphogenesis
- regulation of binding
- regulation of transforming growth factor beta receptor signaling pathway
- response to cholesterol
- response to glucose
- secondary palate development
- signal transduction involved in regulation of gene expression
- SMAD protein complex assembly
- SMAD protein signal transduction
- transforming growth factor beta receptor signaling pathway
- ureteric bud development
- zygotic specification of dorsal/ventral axis

---

69

- **Protein name:** Histone H4 transcription factor
- **Organism:** Homo sapiens
- **Uniprot Accession Number:** Q9BQA5
- **Protein sequence length:** 517 aa
- **1D identity (%):** 10.16
- **1D identity (%) [Gaps excluded]:** 20.75
- **1D identity - Alignment Gaps:** 472
- **1D aligned content (<aminoacid>:%):** {'G': 10.64, 'R': 5.32, 'K': 5.32, 'L': 10.64, 'Q': 5.32, 'E': 11.7, 'C': 6.38, 'F': 4.26, 'H': 4.26, 'P': 8.51, 'S': 5.32, 'D': 4.26, 'V': 4.26, 'N': 2.13, 'W': 2.13, 'T': 2.13, 'Y': 2.13, 'M': 1.06, 'I': 2.13, 'A': 2.13}
- **Common reported functions (%):** 0.0
- **Common reported locations (%):** 12.5
- **Common reported processes (%):** 0.0

- **AF ID:** Q9BQA5
- **Chain:** A
- **Protein length:** 517 aa
- **Resolution:** N/A
- **b-phipsi:** 0.004023
- **w-rdist:** 0.251932
- **t-alpha:** 0.077465
- **Chemical similarity (Tanimoto Index) (%):** 95.34
- **1D identity (%) [PDB]:** 2.27
- **1D identity (%) [Gaps excluded][PDB]:** 68.18
- **1D identity - Alignment Gaps [PDB]:** 1279
- **1D aligned content [PDB] (<aminoacid>:%):** {'P': 13.33, 'D': 10.0, 'S': 13.33, 'Q': 3.33, 'A': 3.33, 'L': 6.67, 'V': 6.67, 'I': 3.33, 'T': 6.67, 'M': 3.33, 'R': 6.67, 'E': 6.67, 'G': 10.0, 'Y': 6.67}
- **2D identity (%) [PDB]:** 36.19
- **2D identity (%) [Gaps excluded][PDB]:** 88.41
- **2D identity - Alignment Gaps [PDB]:** 573
- **2D aligned content [PDB] (<2D-fold>:%):** {'.': 26.78, 'E': 14.25, 'T': 18.8, 'H': 40.17}
- **3D similarity (TM-Score) (%) [PDB]:** 19.02

- **Gene name:** HINFP
- **Entrez ID:** 511965
- **RefSeq ID:** N/A
- **Sequence length:** N/A
- **5-UTR|CDS|3-UTR identity (%):** N/A | N/A | N/A
- **5-UTR|CDS|3-UTR identity (%) [Gaps excluded]:** N/A | N/A | N/A
- **5-UTR|CDS|3-UTR identity [Alignment Gaps]:** N/A | N/A | N/A
- **5-UTR aligned content (<base>:%):** N/A
- **CDS aligned content (<base>:%):** N/A
- **3-UTR aligned content (<base>:%):** N/A

**Uniprot Description:**  
  
 Transcriptional repressor that binds to the consensus sequence 5'-CGGACGTT-3' and to the RB1 promoter. Transcriptional activator that promotes histone H4 gene transcription at the G1/S phase transition in conjunction with NPAT. Also activates transcription of the ATM and PRKDC genes. Autoregulates its expression by associating with its own promoter.   
  
Binds MBD2 and a histone deacetylase complex. Interacts with NPAT.   
  
 **Gene Ontology Information:**

Molecular Function

- DNA-binding transcription factor activity, RNA polymerase II-specific
- metal ion binding
- RNA polymerase II cis-regulatory region sequence-specific DNA binding

Location

- nucleus

Biological process

- anatomical structure development
- regulation of transcription by RNA polymerase II

---

70

- **Protein name:** Lysosomal alpha-mannosidase
- **Organism:** Homo sapiens
- **Uniprot Accession Number:** O00754
- **Protein sequence length:** 1011 aa
- **1D identity (%):** 15.93
- **1D identity (%) [Gaps excluded]:** 23.93
- **1D identity - Alignment Gaps:** 376
- **1D aligned content (<aminoacid>:%):** {'M': 1.12, 'G': 11.17, 'P': 13.41, 'L': 9.5, 'H': 1.12, 'V': 10.06, 'D': 7.82, 'Y': 3.91, 'I': 1.68, 'R': 7.26, 'E': 2.79, 'F': 3.91, 'Q': 5.03, 'W': 1.12, 'A': 3.35, 'T': 7.26, 'N': 3.91, 'K': 1.12, 'S': 3.91, 'C': 0.56}
- **Common reported functions (%):** 0.0
- **Common reported locations (%):** 0.0
- **Common reported processes (%):** 0.0

- **AF ID:** O00754
- **Chain:** A
- **Protein length:** 1011 aa
- **Resolution:** N/A
- **b-phipsi:** 0.00437
- **w-rdist:** 0.353945
- **t-alpha:** 0.031299
- **Chemical similarity (Tanimoto Index) (%):** 96.97
- **1D identity (%) [PDB]:** 2.04
- **1D identity (%) [Gaps excluded][PDB]:** 72.55
- **1D identity - Alignment Gaps [PDB]:** 1759
- **1D aligned content [PDB] (<aminoacid>:%):** {'M': 8.11, 'L': 8.11, 'N': 8.11, 'I': 5.41, 'D': 5.41, 'V': 8.11, 'A': 8.11, 'T': 8.11, 'R': 2.7, 'Q': 8.11, 'P': 5.41, 'E': 8.11, 'C': 2.7, 'K': 5.41, 'S': 2.7, 'F': 2.7, 'G': 2.7}
- **2D identity (%) [PDB]:** 38.15
- **2D identity (%) [Gaps excluded][PDB]:** 88.41
- **2D identity - Alignment Gaps [PDB]:** 739
- **2D aligned content [PDB] (<2D-fold>:%):** {'.': 18.95, 'T': 29.84, 'E': 19.76, 'H': 30.65, 'B': 0.2, 'G': 0.6}
- **3D similarity (TM-Score) (%) [PDB]:** 29.85

- **Gene name:** MAN2B1
- **Entrez ID:** 282272
- **RefSeq ID:** N/A
- **Sequence length:** N/A
- **5-UTR|CDS|3-UTR identity (%):** N/A | N/A | N/A
- **5-UTR|CDS|3-UTR identity (%) [Gaps excluded]:** N/A | N/A | N/A
- **5-UTR|CDS|3-UTR identity [Alignment Gaps]:** N/A | N/A | N/A
- **5-UTR aligned content (<base>:%):** N/A
- **CDS aligned content (<base>:%):** N/A
- **3-UTR aligned content (<base>:%):** N/A

**Uniprot Description:**  
  
 Necessary for the catabolism of N-linked carbohydrates released during glycoprotein turnover. Cleaves all known types of alpha-mannosidic linkages. N/A   
  
 **Gene Ontology Information:**

Molecular Function

- alpha-mannosidase activity
- carbohydrate binding
- zinc ion binding

Location

- extracellular space
- lysosome
- nucleoplasm

Biological process

- mannose metabolic process
- protein modification process

---

71

- **Protein name:** Rho-related BTB domain-containing protein 3
- **Organism:** Homo sapiens
- **Uniprot Accession Number:** O94955
- **Protein sequence length:** 611 aa
- **1D identity (%):** 11.5
- **1D identity (%) [Gaps excluded]:** 22.49
- **1D identity - Alignment Gaps:** 476
- **1D aligned content (<aminoacid>:%):** {'G': 6.25, 'D': 5.36, 'N': 4.46, 'S': 9.82, 'I': 4.46, 'R': 2.68, 'E': 3.57, 'L': 8.04, 'V': 9.82, 'P': 8.04, 'F': 1.79, 'H': 3.57, 'T': 2.68, 'Y': 3.57, 'K': 8.04, 'A': 4.46, 'C': 5.36, 'M': 0.89, 'Q': 6.25, 'W': 0.89}
- **Common reported functions (%):** 0.0
- **Common reported locations (%):** 25.0
- **Common reported processes (%):** 8.33

- **AF ID:** O94955
- **Chain:** A
- **Protein length:** 611 aa
- **Resolution:** N/A
- **b-phipsi:** 0.01879
- **w-rdist:** 0.394584
- **t-alpha:** 0.000783
- **Chemical similarity (Tanimoto Index) (%):** 96.33
- **1D identity (%) [PDB]:** 2.33
- **1D identity (%) [Gaps excluded][PDB]:** 70.21
- **1D identity - Alignment Gaps [PDB]:** 1367
- **1D aligned content [PDB] (<aminoacid>:%):** {'G': 3.03, 'D': 3.03, 'T': 6.06, 'L': 18.18, 'A': 6.06, 'V': 18.18, 'K': 12.12, 'S': 6.06, 'P': 9.09, 'Q': 3.03, 'N': 6.06, 'C': 3.03, 'I': 3.03, 'H': 3.03}
- **2D identity (%) [PDB]:** 42.97
- **2D identity (%) [Gaps excluded][PDB]:** 88.1
- **2D identity - Alignment Gaps [PDB]:** 503
- **2D aligned content [PDB] (<2D-fold>:%):** {'.': 21.8, 'H': 48.58, 'T': 18.01, 'E': 11.61}
- **3D similarity (TM-Score) (%) [PDB]:** 22.85

- **Gene name:** RHOBTB3
- **Entrez ID:** 22836
- **RefSeq ID:** NM\_014899
- **Transcript sequence length:** 5370
- **5-UTR|CDS|3-UTR identity (%):** 44.89 | 38.95 | 38.88
- **5-UTR|CDS|3-UTR identity (%) [Gaps excluded]:** 75.9 | 76.03 | 76.23
- **5-UTR|CDS|3-UTR identity [Alignment Gaps]:** 192 | 1426 | 2424
- **5-UTR aligned content (<base>:%):** {'C': 47.39, 'G': 39.81, 'A': 5.21, 'T': 7.58}
- **CDS aligned content (<base>:%):** {'A': 31.61, 'T': 25.72, 'G': 22.48, 'C': 20.19}
- **3-UTR aligned content (<base>:%):** {'C': 14.92, 'T': 34.15, 'G': 19.23, 'A': 31.7}

**Uniprot Description:**  
  
 Rab9-regulated ATPase required for endosome to Golgi transport. Involved in transport vesicle docking at the Golgi complex, possibly by participating in release M6PRBP1/TIP47 from vesicles to permit their efficient docking and fusion at the Golgi. Specifically binds Rab9, but not other Rab proteins. Has low intrinsic ATPase activity due to autoinhibition, which is relieved by Rab9.   
  
Interacts with RAB9A and RAB9B (at lower level compared to RAB9A-binding). Interacts with M6PRBP1/TIP47.   
  
 **Gene Ontology Information:**

Molecular Function

- ATP binding
- ATPase activity
- GTPase activity
- small GTPase binding
- ubiquitin protein ligase binding

Location

- cytoplasm
- cytosol
- extracellular exosome
- trans-Golgi network membrane

Biological process

- male gonad development
- proteasome-mediated ubiquitin-dependent protein catabolic process
- regulation of proteolysis
- retrograde transport, endosome to Golgi

---

72

- **Protein name:** Adenylyl cyclase-associated protein 1
- **Organism:** Homo sapiens
- **Uniprot Accession Number:** Q01518
- **Protein sequence length:** 475 aa
- **1D identity (%):** 9.35
- **1D identity (%) [Gaps excluded]:** 22.28
- **1D identity - Alignment Gaps:** 546
- **1D aligned content (<aminoacid>:%):** {'D': 6.82, 'N': 2.27, 'A': 6.82, 'R': 3.41, 'G': 11.36, 'P': 10.23, 'Y': 3.41, 'F': 4.55, 'L': 10.23, 'K': 10.23, 'V': 5.68, 'C': 1.14, 'Q': 3.41, 'H': 2.27, 'T': 5.68, 'S': 2.27, 'I': 6.82, 'E': 2.27, 'W': 1.14}
- **Common reported functions (%):** 0.0
- **Common reported locations (%):** 12.5
- **Common reported processes (%):** 0.0

- **AF ID:** Q01518
- **Chain:** A
- **Protein length:** 475 aa
- **Resolution:** N/A
- **b-phipsi:** 0.002043
- **w-rdist:** 0.367564
- **t-alpha:** 0.058685
- **Chemical similarity (Tanimoto Index) (%):** 99.24
- **1D identity (%) [PDB]:** 2.66
- **1D identity (%) [Gaps excluded][PDB]:** 72.34
- **1D identity - Alignment Gaps [PDB]:** 1231
- **1D aligned content [PDB] (<aminoacid>:%):** {'G': 8.82, 'D': 2.94, 'L': 14.71, 'A': 2.94, 'Q': 11.76, 'C': 2.94, 'V': 11.76, 'K': 11.76, 'N': 11.76, 'T': 5.88, 'P': 5.88, 'S': 2.94, 'I': 5.88}
- **2D identity (%) [PDB]:** 28.67
- **2D identity (%) [Gaps excluded][PDB]:** 93.87
- **2D identity - Alignment Gaps [PDB]:** 705
- **2D aligned content [PDB] (<2D-fold>:%):** {'H': 53.95, '.': 12.37, 'T': 14.43, 'G': 1.03, 'E': 18.21}
- **3D similarity (TM-Score) (%) [PDB]:** 16.43

- **Gene name:** CAP1
- **Entrez ID:** 10487
- **RefSeq ID:** N/A
- **Sequence length:** N/A
- **5-UTR|CDS|3-UTR identity (%):** N/A | N/A | N/A
- **5-UTR|CDS|3-UTR identity (%) [Gaps excluded]:** N/A | N/A | N/A
- **5-UTR|CDS|3-UTR identity [Alignment Gaps]:** N/A | N/A | N/A
- **5-UTR aligned content (<base>:%):** N/A
- **CDS aligned content (<base>:%):** N/A
- **3-UTR aligned content (<base>:%):** N/A

**Uniprot Description:**  
  
 Directly regulates filament dynamics and has been implicated in a number of complex developmental and morphological processes, including mRNA localization and the establishment of cell polarity.   
  
Homodimer. Binds actin monomers.   
  
 **Gene Ontology Information:**

Molecular Function

- actin binding
- adenylate cyclase binding

Location

- azurophil granule lumen
- cortical actin cytoskeleton
- cytoplasm
- extracellular exosome
- extracellular region
- focal adhesion
- plasma membrane

Biological process

- actin filament organization
- activation of adenylate cyclase activity
- ameboidal-type cell migration
- cAMP-mediated signaling
- cell morphogenesis
- establishment or maintenance of cell polarity
- receptor-mediated endocytosis
- signal transduction

---

73

- **Protein name:** Kelch repeat and BTB domain-containing protein 4
- **Organism:** Homo sapiens
- **Uniprot Accession Number:** Q9NVX7
- **Protein sequence length:** 534 aa
- **1D identity (%):** 10.86
- **1D identity (%) [Gaps excluded]:** 21.72
- **1D identity - Alignment Gaps:** 465
- **1D aligned content (<aminoacid>:%):** {'G': 7.92, 'A': 4.95, 'Q': 2.97, 'P': 5.94, 'V': 10.89, 'D': 4.95, 'H': 1.98, 'I': 3.96, 'E': 8.91, 'R': 7.92, 'F': 2.97, 'M': 0.99, 'T': 3.96, 'L': 14.85, 'S': 4.95, 'Y': 1.98, 'N': 2.97, 'K': 3.96, 'C': 2.97}
- **Common reported functions (%):** 0.0
- **Common reported locations (%):** 0.0
- **Common reported processes (%):** 0.0

- **AF ID:** Q9NVX7
- **Chain:** A
- **Protein length:** 518 aa
- **Resolution:** N/A
- **b-phipsi:** 0.000587
- **w-rdist:** 0.580332
- **t-alpha:** 0.113241
- **Chemical similarity (Tanimoto Index) (%):** 99.54
- **1D identity (%) [PDB]:** 1.95
- **1D identity (%) [Gaps excluded][PDB]:** 76.47
- **1D identity - Alignment Gaps [PDB]:** 1300
- **1D aligned content [PDB] (<aminoacid>:%):** {'M': 3.85, 'E': 11.54, 'V': 15.38, 'T': 3.85, 'L': 19.23, 'P': 3.85, 'G': 7.69, 'K': 3.85, 'D': 3.85, 'S': 11.54, 'Q': 7.69, 'A': 7.69}
- **2D identity (%) [PDB]:** 34.01
- **2D identity (%) [Gaps excluded][PDB]:** 87.47
- **2D identity - Alignment Gaps [PDB]:** 602
- **2D aligned content [PDB] (<2D-fold>:%):** {'.': 16.12, 'H': 37.31, 'T': 25.07, 'E': 21.49}
- **3D similarity (TM-Score) (%) [PDB]:** 18.84

- **Gene name:** KBTBD4
- **Entrez ID:** 55709
- **RefSeq ID:** NM\_018095
- **Transcript sequence length:** 2357
- **5-UTR|CDS|3-UTR identity (%):** 3.74 | 35.15 | 10.53
- **5-UTR|CDS|3-UTR identity (%) [Gaps excluded]:** 78.95 | 75.7 | 74.68
- **5-UTR|CDS|3-UTR identity [Alignment Gaps]:** 382 | 1533 | 3778
- **5-UTR aligned content (<base>:%):** {'G': 66.67, 'A': 6.67, 'C': 26.67}
- **CDS aligned content (<base>:%):** {'A': 25.05, 'T': 24.06, 'G': 27.34, 'C': 23.56}
- **3-UTR aligned content (<base>:%):** {'G': 23.11, 'C': 19.65, 'T': 30.24, 'A': 27.0}

**Uniprot Description:**  
  
 Substrate-specific adapter of a BCR (BTB-CUL3-RBX1) E3 ubiquitin ligase complex which targets CoREST corepressor complex components RCOR1, KDM1A/LSD1 and HDAC2 for proteasomal degradation (PubMed:33417871). RCOR1 is likely to be the primary target while degradation of KDM1A and HDAC2 is likely due to their association with RCOR1 (PubMed:33417871). Also targets RCOR3, MIER2 and MIER3 for proteasomal degradation as well as associated proteins ZNF217 and RREB1 (PubMed:36997086). Degradation is dependent on the presence of an ELM2 domain in the target proteins (PubMed:36997086).   
  
Component of the BCR(KBTBD4) E3 ubiquitin ligase complex, at least composed of CUL3, KBTBD4 and RBX1.   
  
 **Gene Ontology Information:**

Molecular Function   
  
N/A

Location   
  
N/A

Biological process   
  
N/A

---

74

- **Protein name:** Nicastrin
- **Organism:** Homo sapiens
- **Uniprot Accession Number:** Q92542
- **Protein sequence length:** 709 aa
- **1D identity (%):** 14.99
- **1D identity (%) [Gaps excluded]:** 24.05
- **1D identity - Alignment Gaps:** 364
- **1D aligned content (<aminoacid>:%):** {'M': 0.69, 'G': 9.66, 'A': 6.9, 'P': 11.03, 'L': 12.41, 'R': 5.52, 'F': 4.14, 'V': 7.59, 'N': 3.45, 'Q': 6.21, 'I': 4.83, 'D': 4.14, 'S': 4.14, 'E': 4.83, 'T': 4.14, 'W': 1.38, 'K': 4.14, 'Y': 2.76, 'C': 2.07}
- **Common reported functions (%):** 0.0
- **Common reported locations (%):** 12.5
- **Common reported processes (%):** 0.0

- **AF ID:** Q92542
- **Chain:** A
- **Protein length:** 709 aa
- **Resolution:** N/A
- **b-phipsi:** 0.004687
- **w-rdist:** 0.716275
- **t-alpha:** 0.001568
- **Chemical similarity (Tanimoto Index) (%):** 95.98
- **1D identity (%) [PDB]:** 2.81
- **1D identity (%) [Gaps excluded][PDB]:** 65.62
- **1D identity - Alignment Gaps [PDB]:** 1431
- **1D aligned content [PDB] (<aminoacid>:%):** {'M': 2.38, 'T': 9.52, 'A': 9.52, 'P': 7.14, 'L': 16.67, 'I': 4.76, 'D': 7.14, 'R': 2.38, 'V': 14.29, 'G': 4.76, 'E': 2.38, 'K': 2.38, 'S': 7.14, 'Q': 4.76, 'H': 2.38, 'N': 2.38}
- **2D identity (%) [PDB]:** 41.07
- **2D identity (%) [Gaps excluded][PDB]:** 89.59
- **2D identity - Alignment Gaps [PDB]:** 579
- **2D aligned content [PDB] (<2D-fold>:%):** {'.': 17.08, 'H': 38.27, 'T': 23.23, 'E': 20.5, 'G': 0.68, 'B': 0.23}
- **3D similarity (TM-Score) (%) [PDB]:** 24.0

- **Gene name:** NCSTN
- **Entrez ID:** 23385
- **RefSeq ID:** NM\_015331
- **Transcript sequence length:** 2822
- **5-UTR|CDS|3-UTR identity (%):** 2.48 | 42.25 | 9.8
- **5-UTR|CDS|3-UTR identity (%) [Gaps excluded]:** 90.91 | 76.22 | 73.33
- **5-UTR|CDS|3-UTR identity [Alignment Gaps]:** 393 | 1352 | 3793
- **5-UTR aligned content (<base>:%):** {'A': 20.0, 'G': 60.0, 'C': 20.0}
- **CDS aligned content (<base>:%):** {'T': 24.73, 'G': 25.04, 'A': 25.59, 'C': 24.65}
- **3-UTR aligned content (<base>:%):** {'G': 16.08, 'A': 22.14, 'C': 26.57, 'T': 35.2}

**Uniprot Description:**  
  
 Essential subunit of the gamma-secretase complex, an endoprotease complex that catalyzes the intramembrane cleavage of integral membrane proteins such as Notch receptors and APP (amyloid-beta precursor protein) (PubMed:10993067, PubMed:12679784, PubMed:25043039, PubMed:26280335, PubMed:30598546, PubMed:30630874). The gamma-secretase complex plays a role in Notch and Wnt signaling cascades and regulation of downstream processes via its role in processing key regulatory proteins, and by regulating cytosolic CTNNB1 levels.   
  
Component of the gamma-secretase complex (PubMed:10993067, PubMed:30598546, PubMed:30630874). The functional gamma-secretase complex is composed of at least four polypeptides: a presenilin homodimer (PSEN1 or PSEN2), nicastrin (NCSTN), APH1 (APH1A or APH1B) and PSENEN/PEN2 (PubMed:12740439, PubMed:25043039, PubMed:26623517, PubMed:26280335, PubMed:25918421, PubMed:30598546, PubMed:30630874). Binds to proteolytic processed C-terminal fragments C83 and C99 of the amyloid precursor protein (APP) (PubMed:10993067, PubMed:30630874). Interacts with PSEN1 and PSEN2 (PubMed:10993067).   
  
 **Gene Ontology Information:**

Molecular Function

- aspartic endopeptidase activity, intramembrane cleaving
- ATPase binding
- growth factor receptor binding
- protein-macromolecule adaptor activity

Location

- azurophil granule membrane
- early endosome
- endoplasmic reticulum
- endosome membrane
- extracellular exosome
- focal adhesion
- gamma-secretase complex
- Golgi apparatus
- lysosomal membrane
- melanosome
- membrane
- plasma membrane
- presynaptic membrane
- sarcolemma
- synaptic vesicle

Biological process

- adult behavior
- amyloid precursor protein biosynthetic process
- amyloid precursor protein catabolic process
- amyloid precursor protein metabolic process
- amyloid-beta formation
- cellular response to calcium ion
- central nervous system myelination
- cerebellum development
- dopamine receptor signaling pathway
- epithelial cell proliferation
- glutamate receptor signaling pathway
- learning or memory
- membrane protein ectodomain proteolysis
- membrane protein intracellular domain proteolysis
- myeloid cell homeostasis
- neuron apoptotic process
- Notch receptor processing
- Notch signaling pathway
- positive regulation of amyloid precursor protein biosynthetic process
- positive regulation of catalytic activity
- positive regulation of endopeptidase activity
- protein processing
- proteolysis
- regulation of long-term synaptic potentiation
- short-term synaptic potentiation
- T cell proliferation

---

75

- **Protein name:** Heat shock cognate 71 kDa protein
- **Organism:** Homo sapiens
- **Uniprot Accession Number:** P11142
- **Protein sequence length:** 646 aa
- **1D identity (%):** 12.07
- **1D identity (%) [Gaps excluded]:** 22.84
- **1D identity - Alignment Gaps:** 465
- **1D aligned content (<aminoacid>:%):** {'S': 2.52, 'P': 10.92, 'A': 4.2, 'I': 5.04, 'D': 6.72, 'T': 9.24, 'Y': 4.2, 'E': 6.72, 'N': 2.52, 'R': 2.52, 'V': 8.4, 'K': 6.72, 'G': 11.76, 'F': 2.52, 'Q': 4.2, 'L': 7.56, 'M': 2.52, 'C': 0.84, 'H': 0.84}
- **Common reported functions (%):** 0.0
- **Common reported locations (%):** 25.0
- **Common reported processes (%):** 0.0

- **AF ID:** P11142
- **Chain:** A
- **Protein length:** 646 aa
- **Resolution:** N/A
- **b-phipsi:** 0.003804
- **w-rdist:** 0.584635
- **t-alpha:** 0.003928
- **Chemical similarity (Tanimoto Index) (%):** 99.17
- **1D identity (%) [PDB]:** 2.27
- **1D identity (%) [Gaps excluded][PDB]:** 73.33
- **1D identity - Alignment Gaps [PDB]:** 1406
- **1D aligned content [PDB] (<aminoacid>:%):** {'G': 9.09, 'T': 15.15, 'Y': 3.03, 'V': 15.15, 'Q': 3.03, 'K': 6.06, 'R': 9.09, 'L': 3.03, 'A': 6.06, 'E': 6.06, 'S': 9.09, 'N': 3.03, 'D': 6.06, 'I': 3.03, 'P': 3.03}
- **2D identity (%) [PDB]:** 44.26
- **2D identity (%) [Gaps excluded][PDB]:** 89.49
- **2D identity - Alignment Gaps [PDB]:** 506
- **2D aligned content [PDB] (<2D-fold>:%):** {'.': 10.84, 'E': 26.19, 'T': 17.61, 'H': 45.37}
- **3D similarity (TM-Score) (%) [PDB]:** 21.36

- **Gene name:** HSPA8
- **Entrez ID:** 100009679
- **RefSeq ID:** N/A
- **Sequence length:** N/A
- **5-UTR|CDS|3-UTR identity (%):** N/A | N/A | N/A
- **5-UTR|CDS|3-UTR identity (%) [Gaps excluded]:** N/A | N/A | N/A
- **5-UTR|CDS|3-UTR identity [Alignment Gaps]:** N/A | N/A | N/A
- **5-UTR aligned content (<base>:%):** N/A
- **CDS aligned content (<base>:%):** N/A
- **3-UTR aligned content (<base>:%):** N/A

**Uniprot Description:**  
  
 Molecular chaperone implicated in a wide variety of cellular processes, including protection of the proteome from stress, folding and transport of newly synthesized polypeptides, activation of proteolysis of misfolded proteins and the formation and dissociation of protein complexes. Plays a pivotal role in the protein quality control system, ensuring the correct folding of proteins, the re-folding of misfolded proteins and controlling the targeting of proteins for subsequent degradation (PubMed:21150129, PubMed:21148293, PubMed:24732912, PubMed:27916661, PubMed:23018488). This is achieved through cycles of ATP binding, ATP hydrolysis and ADP release, mediated by co-chaperones (PubMed:21150129, PubMed:21148293, PubMed:24732912, PubMed:27916661, PubMed:23018488, PubMed:12526792). The co-chaperones have been shown to not only regulate different steps of the ATPase cycle of HSP70, but they also have an individual specificity such that one co-chaperone may promote folding of a substrate while another may promote degradation (PubMed:21150129, PubMed:21148293, PubMed:24732912, PubMed:27916661, PubMed:23018488, PubMed:12526792). The affinity of HSP70 for polypeptides is regulated by its nucleotide bound state. In the ATP-bound form, it has a low affinity for substrate proteins. However, upon hydrolysis of the ATP to ADP, it undergoes a conformational change that increases its affinity for substrate proteins. HSP70 goes through repeated cycles of ATP hydrolysis and nucleotide exchange, which permits cycles of substrate binding and release. The HSP70-associated co-chaperones are of three types: J-domain co-chaperones HSP40s (stimulate ATPase hydrolysis by HSP70), the nucleotide exchange factors (NEF) such as BAG1/2/3 (facilitate conversion of HSP70 from the ADP-bound to the ATP-bound state thereby promoting substrate release), and the TPR domain chaperones such as HOPX and STUB1 (PubMed:24318877, PubMed:27474739, PubMed:24121476, PubMed:26865365). Plays a critical role in mitochondrial import, delivers preproteins to the mitochondrial import receptor TOMM70 (PubMed:12526792). Acts as a repressor of transcriptional activation. Inhibits the transcriptional coactivator activity of CITED1 on Smad-mediated transcription. Component of the PRP19-CDC5L complex that forms an integral part of the spliceosome and is required for activating pre-mRNA splicing. May have a scaffolding role in the spliceosome assembly as it contacts all other components of the core complex. Binds bacterial lipopolysaccharide (LPS) and mediates LPS-induced inflammatory response, including TNF secretion by monocytes (PubMed:10722728, PubMed:11276205). Participates in the ER-associated degradation (ERAD) quality control pathway in conjunction with J domain-containing co-chaperones and the E3 ligase STUB1 (PubMed:23990462). Interacts with VGF-derived peptide TLQP-21 (PubMed:28934328).   
  
Identified in a IGF2BP1-dependent mRNP granule complex containing untranslated mRNAs. Interacts with PACRG. Interacts with HSPH1/HSP105. Interacts with IRAK1BP1 and BAG1. Interacts with DNAJC7. Interacts with DNAJB12 (via J domain) (PubMed:21150129, PubMed:21148293, PubMed:24732912, PubMed:27916661). Interacts with DNAJB14 (via J domain) (PubMed:23018488, PubMed:24732912, PubMed:27916661). Interacts (via C-terminus) with the E3 ligase CHIP forming a 210 kDa complex of one CHIP and two HSPA8 molecules. Interacts with CITED1 (via N-terminus); the interaction suppresses the association of CITED1 to p300/CBP and Smad-mediated transcription transactivation. Component of the PRP19-CDC5L splicing complex composed of a core complex comprising a homotetramer of PRPF19, CDC5L, PLRG1 and BCAS2, and at least three less stably associated proteins CTNNBL1, CWC15 and HSPA8. Interacts with TRIM5. Part of a complex composed at least of ASCL2, EMSY, HCFC1, HSPA8, CCAR2, MATR3, MKI67, RBBP5, TUBB2A, WDR5 and ZNF335; this complex may have a histone H3-specific methyltransferase activity. Interacts with METTL21A. Following LPS binding, may form a complex with CXCR4, GDF5 and HSP90AA1. Interacts with PRKN. Interacts with FOXP3. Interacts with DNAJC9 (via J domain) (PubMed:17182002). Interacts with MLLT11 (PubMed:24880125). Interacts with RNF207 (PubMed:25281747). Interacts with DNAJC21 (PubMed:27346687). Interacts with DNAJB2 (PubMed:15936278). Interacts with TTC1 (via TPR repeats) (PubMed:15708368). Interacts with SGTA (via TPR repeats) (By similarity). Interacts with HSF1 (via transactivation domain) (PubMed:9499401). Interacts with HOPX, HSP40 and HSP90 (PubMed:27708256). Interacts with STUB1 (PubMed:27708256). Interacts with BAG2 (PubMed:24318877). Interacts with BAG3 (PubMed:27474739, PubMed:24318877). Interacts with DNAJC12 (PubMed:24122553). Interacts with ZMYND10 (PubMed:29601588). Interacts with HSPC138 (PubMed:25760597). Interacts with BCL2L1, GIMAP5 and MCL1; the interaction with BCL2L1 or MCL1 is impaired in the absence of GIMAP5 (By similarity). Interacts with NLPR12 (PubMed:17947705). Interacts with TTC4 (PubMed:18320024). Interacts with TOMM70; the interaction is required for preprotein mitochondrial import (PubMed:12526792). May interact with DNJC9; the interaction seems to be histone-dependent (PubMed:33857403). Interacts with BAG5 and JPH2; the interaction with JPH2 is increased in the presence of BAG5 (PubMed:35044787).   
  
 **Gene Ontology Information:**

Molecular Function

- ATP binding
- ATPase activity
- ATP-dependent protein disaggregase activity
- ATP-dependent protein folding chaperone
- C3HC4-type RING finger domain binding
- clathrin-uncoating ATPase activity
- G protein-coupled receptor binding
- heat shock protein binding
- phosphatidylserine binding
- protein folding chaperone
- chaperone binding
- protein-macromolecule adaptor activity
- ubiquitin protein ligase binding
- unfolded protein binding

Location

- autophagosome
- cytoplasm
- dendrite
- extracellular exosome
- glutamatergic synapse
- glycinergic synapse
- late endosome
- lysosomal membrane
- lysosome
- melanosome
- nucleolus
- nucleus
- perinuclear region of cytoplasm
- photoreceptor ribbon synapse
- plasma membrane
- postsynaptic cytosol
- postsynaptic specialization membrane
- presynaptic cytosol
- chaperone complex
- Prp19 complex
- ribonucleoprotein complex
- spliceosomal complex
- terminal bouton

Biological process

- ATP metabolic process
- chaperone cofactor-dependent protein refolding
- chaperone-mediated autophagy translocation complex disassembly
- clathrin coat disassembly
- late endosomal microautophagy
- mRNA processing
- negative regulation of transcription, DNA-templated
- negative regulation of NLRP3 inflammasome complex assembly
- negative regulation of supramolecular fiber organization
- positive regulation by host of viral genome replication
- positive regulation of mRNA splicing, via spliceosome
- protein refolding
- protein targeting to lysosome involved in chaperone-mediated autophagy
- regulation of cell cycle
- regulation of postsynapse organization
- regulation of protein stability
- RNA splicing
- slow axonal transport

---

76

- **Protein name:** 5-oxoprolinase
- **Organism:** Homo sapiens
- **Uniprot Accession Number:** O14841
- **Protein sequence length:** 1288 aa
- **1D identity (%):** 15.95
- **1D identity (%) [Gaps excluded]:** 25.85
- **1D identity - Alignment Gaps:** 509
- **1D aligned content (<aminoacid>:%):** {'G': 13.21, 'P': 12.74, 'F': 2.83, 'R': 8.02, 'T': 3.77, 'L': 10.38, 'A': 8.49, 'N': 1.42, 'D': 5.66, 'H': 3.3, 'K': 1.89, 'E': 3.77, 'V': 9.43, 'Q': 5.66, 'S': 2.36, 'I': 0.94, 'C': 1.89, 'Y': 3.3, 'M': 0.47, 'W': 0.47}
- **Common reported functions (%):** 0.0
- **Common reported locations (%):** 12.5
- **Common reported processes (%):** 0.0

- **AF ID:** O14841
- **Chain:** A
- **Protein length:** 1288 aa
- **Resolution:** N/A
- **b-phipsi:** 0.001851
- **w-rdist:** 0.34242
- **t-alpha:** 0.082159
- **Chemical similarity (Tanimoto Index) (%):** 98.87
- **1D identity (%) [PDB]:** 2.75
- **1D identity (%) [Gaps excluded][PDB]:** 82.61
- **1D identity - Alignment Gaps [PDB]:** 2000
- **1D aligned content [PDB] (<aminoacid>:%):** {'V': 12.28, 'F': 3.51, 'Q': 8.77, 'P': 5.26, 'I': 5.26, 'L': 8.77, 'G': 8.77, 'A': 8.77, 'D': 7.02, 'H': 3.51, 'K': 1.75, 'S': 8.77, 'T': 5.26, 'R': 3.51, 'E': 7.02, 'N': 1.75}
- **2D identity (%) [PDB]:** 41.35
- **2D identity (%) [Gaps excluded][PDB]:** 89.63
- **2D identity - Alignment Gaps [PDB]:** 788
- **2D aligned content [PDB] (<2D-fold>:%):** {'.': 13.88, 'E': 28.43, 'T': 23.8, 'H': 32.4, 'G': 0.99, 'B': 0.5}
- **3D similarity (TM-Score) (%) [PDB]:** 27.84

- **Gene name:** OPLAH
- **Entrez ID:** 26873
- **RefSeq ID:** NM\_017570
- **Transcript sequence length:** 4020
- **5-UTR|CDS|3-UTR identity (%):** 13.37 | 40.34 | 0.91
- **5-UTR|CDS|3-UTR identity (%) [Gaps excluded]:** 74.67 | 74.9 | 73.58
- **5-UTR|CDS|3-UTR identity [Alignment Gaps]:** 344 | 1935 | 4239
- **5-UTR aligned content (<base>:%):** {'A': 8.93, 'G': 33.93, 'C': 53.57, 'T': 3.57}
- **CDS aligned content (<base>:%):** {'A': 21.16, 'T': 20.15, 'G': 29.91, 'C': 28.78}
- **3-UTR aligned content (<base>:%):** {'G': 25.64, 'A': 25.64, 'T': 23.08, 'C': 25.64}

**Uniprot Description:**  
  
 Catalyzes the cleavage of 5-oxo-L-proline to form L-glutamate coupled to the hydrolysis of ATP to ADP and inorganic phosphate.   
  
Homodimer.   
  
 **Gene Ontology Information:**

Molecular Function

- 5-oxoprolinase (ATP-hydrolyzing) activity
- ATP binding
- identical protein binding

Location

- cytosol

Biological process

- glutathione metabolic process

---

77

- **Protein name:** DNA polymerase alpha subunit B
- **Organism:** Homo sapiens
- **Uniprot Accession Number:** Q14181
- **Protein sequence length:** 598 aa
- **1D identity (%):** 13.12
- **1D identity (%) [Gaps excluded]:** 24.7
- **1D identity - Alignment Gaps:** 447
- **1D aligned content (<aminoacid>:%):** {'Q': 8.8, 'D': 4.0, 'V': 8.8, 'G': 9.6, 'E': 5.6, 'S': 8.0, 'L': 12.8, 'N': 4.0, 'P': 9.6, 'T': 4.0, 'R': 2.4, 'F': 4.0, 'Y': 4.0, 'I': 4.0, 'K': 3.2, 'A': 4.8, 'H': 1.6, 'C': 0.8}
- **Common reported functions (%):** 0.0
- **Common reported locations (%):** 12.5
- **Common reported processes (%):** 0.0

- **AF ID:** Q14181
- **Chain:** A
- **Protein length:** 598 aa
- **Resolution:** N/A
- **b-phipsi:** 0.000513
- **w-rdist:** 0.81609
- **t-alpha:** 0.035993
- **Chemical similarity (Tanimoto Index) (%):** 98.65
- **1D identity (%) [PDB]:** 3.64
- **1D identity (%) [Gaps excluded][PDB]:** 66.67
- **1D identity - Alignment Gaps [PDB]:** 1298
- **1D aligned content [PDB] (<aminoacid>:%):** {'M': 2.0, 'A': 8.0, 'Q': 8.0, 'C': 2.0, 'V': 18.0, 'K': 6.0, 'T': 4.0, 'S': 4.0, 'P': 10.0, 'L': 10.0, 'N': 6.0, 'G': 6.0, 'I': 6.0, 'H': 2.0, 'R': 2.0, 'F': 4.0, 'D': 2.0}
- **2D identity (%) [PDB]:** 39.96
- **2D identity (%) [Gaps excluded][PDB]:** 90.95
- **2D identity - Alignment Gaps [PDB]:** 564
- **2D aligned content [PDB] (<2D-fold>:%):** {'T': 31.34, '.': 15.42, 'H': 36.07, 'E': 16.92, 'B': 0.25}
- **3D similarity (TM-Score) (%) [PDB]:** 22.47

- **Gene name:** POLA2
- **Entrez ID:** 23649
- **RefSeq ID:** NM\_002689
- **Transcript sequence length:** 3544
- **5-UTR|CDS|3-UTR identity (%):** 42.92 | 40.2 | 19.33
- **5-UTR|CDS|3-UTR identity (%) [Gaps excluded]:** 66.55 | 76.32 | 72.5
- **5-UTR|CDS|3-UTR identity [Alignment Gaps]:** 158 | 1359 | 3301
- **5-UTR aligned content (<base>:%):** {'G': 42.93, 'T': 13.09, 'C': 36.65, 'A': 7.33}
- **CDS aligned content (<base>:%):** {'A': 28.34, 'T': 23.22, 'G': 24.09, 'C': 24.35}
- **3-UTR aligned content (<base>:%):** {'G': 27.59, 'C': 24.71, 'T': 25.4, 'A': 22.3}

**Uniprot Description:**  
  
 Accessory subunit of the DNA polymerase alpha complex (also known as the alpha DNA polymerase-primase complex) which plays an essential role in the initiation of DNA synthesis (PubMed:9705292). During the S phase of the cell cycle, the DNA polymerase alpha complex (composed of a catalytic subunit POLA1, an accessory subunit POLA2 and two primase subunits, the catalytic subunit PRIM1 and the regulatory subunit PRIM2) is recruited to DNA at the replicative forks via direct interactions with MCM10 and WDHD1 (By similarity). The primase subunit of the polymerase alpha complex initiates DNA synthesis by oligomerising short RNA primers on both leading and lagging strands (By similarity). These primers are initially extended by the polymerase alpha catalytic subunit and subsequently transferred to polymerase delta and polymerase epsilon for processive synthesis on the lagging and leading strand, respectively (By similarity).   
  
Component of the alpha DNA polymerase complex (also known as the alpha DNA polymerase-primase complex) consisting of four subunits: the catalytic subunit POLA1, the regulatory subunit POLA2, and primase complex subunits PRIM1 and PRIM2 respectively (PubMed:9705292). Within the complex, POLA1 directly interacts with PRIM2/p58 (By similarity).   
  
 **Gene Ontology Information:**

Molecular Function

- DNA binding

Location

- alpha DNA polymerase:primase complex
- cytosol
- nucleoplasm

Biological process

- DNA replication
- DNA replication initiation
- DNA replication, synthesis of RNA primer
- protein import into nucleus

---

78

- **Protein name:** Forkhead box protein R2
- **Organism:** Homo sapiens
- **Uniprot Accession Number:** Q6PJQ5
- **Protein sequence length:** 311 aa
- **1D identity (%):** 6.75
- **1D identity (%) [Gaps excluded]:** 24.51
- **1D identity - Alignment Gaps:** 666
- **1D aligned content (<aminoacid>:%):** {'D': 9.68, 'L': 16.13, 'K': 6.45, 'E': 11.29, 'F': 3.23, 'H': 3.23, 'P': 11.29, 'Q': 6.45, 'A': 3.23, 'G': 6.45, 'V': 4.84, 'M': 1.61, 'W': 1.61, 'C': 4.84, 'N': 3.23, 'S': 3.23, 'R': 3.23}
- **Common reported functions (%):** 0.0
- **Common reported locations (%):** 12.5
- **Common reported processes (%):** 0.0

- **AF ID:** Q6PJQ5
- **Chain:** A
- **Protein length:** 311 aa
- **Resolution:** N/A
- **b-phipsi:** 0.005068
- **w-rdist:** 0.348309
- **t-alpha:** 0.03231
- **Chemical similarity (Tanimoto Index) (%):** 99.02
- **1D identity (%) [PDB]:** 1.23
- **1D identity (%) [Gaps excluded][PDB]:** 60.87
- **1D identity - Alignment Gaps [PDB]:** 1115
- **1D aligned content [PDB] (<aminoacid>:%):** {'L': 28.57, 'K': 14.29, 'I': 14.29, 'A': 7.14, 'N': 14.29, 'V': 7.14, 'Q': 7.14, 'R': 7.14}
- **2D identity (%) [PDB]:** 23.71
- **2D identity (%) [Gaps excluded][PDB]:** 86.4
- **2D identity - Alignment Gaps [PDB]:** 661
- **2D aligned content [PDB] (<2D-fold>:%):** {'.': 25.46, 'T': 26.39, 'H': 45.37, 'G': 1.39, 'E': 1.39}
- **3D similarity (TM-Score) (%) [PDB]:** 14.63

- **Gene name:** FOXR2
- **Entrez ID:** 139628
- **RefSeq ID:** NM\_198451
- **Transcript sequence length:** 2793
- **5-UTR|CDS|3-UTR identity (%):** 20.97 | 21.87 | 22.83
- **5-UTR|CDS|3-UTR identity (%) [Gaps excluded]:** 75.48 | 78.28 | 73.68
- **5-UTR|CDS|3-UTR identity [Alignment Gaps]:** 403 | 1984 | 3072
- **5-UTR aligned content (<base>:%):** {'T': 22.22, 'C': 41.03, 'G': 27.35, 'A': 9.4}
- **CDS aligned content (<base>:%):** {'A': 28.41, 'T': 21.26, 'G': 23.75, 'C': 26.58}
- **3-UTR aligned content (<base>:%):** {'A': 34.45, 'T': 33.86, 'G': 18.31, 'C': 13.39}

**Uniprot Description:**  
  
 N/A N/A   
  
 **Gene Ontology Information:**

Molecular Function

- DNA-binding transcription factor activity, RNA polymerase II-specific
- sequence-specific double-stranded DNA binding

Location

- chromatin
- nucleoplasm
- nucleus

Biological process   
  
N/A

---

79

- **Protein name:** Asparagine--tRNA ligase, cytoplasmic
- **Organism:** Homo sapiens
- **Uniprot Accession Number:** O43776
- **Protein sequence length:** 548 aa
- **1D identity (%):** 9.37
- **1D identity (%) [Gaps excluded]:** 23.15
- **1D identity - Alignment Gaps:** 597
- **1D aligned content (<aminoacid>:%):** {'E': 5.32, 'A': 3.19, 'T': 5.32, 'F': 4.26, 'K': 12.77, 'P': 9.57, 'V': 9.57, 'N': 1.06, 'I': 4.26, 'R': 5.32, 'G': 13.83, 'L': 6.38, 'Q': 4.26, 'Y': 5.32, 'W': 2.13, 'M': 1.06, 'C': 3.19, 'D': 2.13, 'S': 1.06}
- **Common reported functions (%):** 0.0
- **Common reported locations (%):** 25.0
- **Common reported processes (%):** 0.0

- **AF ID:** O43776
- **Chain:** A
- **Protein length:** 548 aa
- **Resolution:** N/A
- **b-phipsi:** 0.001038
- **w-rdist:** 0.739713
- **t-alpha:** 0.008607
- **Chemical similarity (Tanimoto Index) (%):** 98.5
- **1D identity (%) [PDB]:** 1.99
- **1D identity (%) [Gaps excluded][PDB]:** 65.85
- **1D identity - Alignment Gaps [PDB]:** 1316
- **1D aligned content [PDB] (<aminoacid>:%):** {'V': 14.81, 'S': 7.41, 'Q': 7.41, 'L': 18.52, 'E': 7.41, 'A': 11.11, 'G': 3.7, 'H': 3.7, 'N': 3.7, 'D': 7.41, 'R': 7.41, 'T': 3.7, 'P': 3.7}
- **2D identity (%) [PDB]:** 42.43
- **2D identity (%) [Gaps excluded][PDB]:** 86.52
- **2D identity - Alignment Gaps [PDB]:** 478
- **2D aligned content [PDB] (<2D-fold>:%):** {'.': 13.32, 'T': 22.11, 'E': 22.86, 'H': 41.21, 'B': 0.5}
- **3D similarity (TM-Score) (%) [PDB]:** 20.22

- **Gene name:** NARS1
- **Entrez ID:** 4677
- **RefSeq ID:** NM\_004539
- **Transcript sequence length:** 2762
- **5-UTR|CDS|3-UTR identity (%):** 17.66 | 34.74 | 14.2
- **5-UTR|CDS|3-UTR identity (%) [Gaps excluded]:** 73.27 | 76.17 | 75.3
- **5-UTR|CDS|3-UTR identity [Alignment Gaps]:** 318 | 1581 | 3605
- **5-UTR aligned content (<base>:%):** {'C': 37.84, 'T': 13.51, 'G': 45.95, 'A': 2.7}
- **CDS aligned content (<base>:%):** {'A': 29.21, 'T': 24.55, 'G': 26.14, 'C': 20.1}
- **3-UTR aligned content (<base>:%):** {'C': 14.58, 'A': 33.76, 'T': 38.03, 'G': 13.63}

**Uniprot Description:**  
  
 Catalyzes the attachment of asparagine to tRNA(Asn) in a two-step reaction: asparagine is first activated by ATP to form Asn-AMP and then transferred to the acceptor end of tRNA(Asn) (PubMed:9421509, PubMed:32738225, PubMed:32788587). In addition to its essential role in protein synthesis, acts as a signaling molecule that induced migration of CCR3-expressing cells (PubMed:30171954, PubMed:12235211). Has an essential role in the development of the cerebral cortex, being required for proper proliferation of radial glial cells (PubMed:32788587).   
  
Homodimer.   
  
 **Gene Ontology Information:**

Molecular Function

- asparagine-tRNA ligase activity
- ATP binding
- CCR3 chemokine receptor binding
- nucleic acid binding
- protein dimerization activity

Location

- cytoplasm
- cytosol
- extracellular exosome

Biological process

- asparaginyl-tRNA aminoacylation
- cell migration
- cerebral cortex development
- tRNA aminoacylation for protein translation

---

80

- **Protein name:** DNA topoisomerase I, mitochondrial
- **Organism:** Homo sapiens
- **Uniprot Accession Number:** Q969P6
- **Protein sequence length:** 601 aa
- **1D identity (%):** 14.04
- **1D identity (%) [Gaps excluded]:** 27.59
- **1D identity - Alignment Gaps:** 476
- **1D aligned content (<aminoacid>:%):** {'V': 8.82, 'L': 11.76, 'A': 7.35, 'E': 7.35, 'P': 5.88, 'R': 9.56, 'T': 3.68, 'G': 7.35, 'F': 2.21, 'Y': 3.68, 'M': 0.74, 'I': 4.41, 'C': 2.94, 'K': 8.82, 'N': 2.21, 'H': 1.47, 'Q': 4.41, 'S': 2.94, 'D': 3.68, 'W': 0.74}
- **Common reported functions (%):** 0.0
- **Common reported locations (%):** 0.0
- **Common reported processes (%):** 0.0

- **AF ID:** Q969P6
- **Chain:** A
- **Protein length:** 601 aa
- **Resolution:** N/A
- **b-phipsi:** 0.012789
- **w-rdist:** 0.240309
- **t-alpha:** 0.011877
- **Chemical similarity (Tanimoto Index) (%):** 98.65
- **1D identity (%) [PDB]:** 2.42
- **1D identity (%) [Gaps excluded][PDB]:** 72.34
- **1D identity - Alignment Gaps [PDB]:** 1357
- **1D aligned content [PDB] (<aminoacid>:%):** {'M': 2.94, 'V': 14.71, 'T': 5.88, 'L': 11.76, 'G': 8.82, 'E': 8.82, 'K': 2.94, 'S': 11.76, 'W': 2.94, 'Q': 5.88, 'A': 8.82, 'N': 2.94, 'P': 2.94, 'D': 8.82}
- **2D identity (%) [PDB]:** 36.09
- **2D identity (%) [Gaps excluded][PDB]:** 91.02
- **2D identity - Alignment Gaps [PDB]:** 627
- **2D aligned content [PDB] (<2D-fold>:%):** {'.': 12.53, 'E': 16.0, 'T': 12.53, 'H': 58.67, 'B': 0.27}
- **3D similarity (TM-Score) (%) [PDB]:** 20.2

- **Gene name:** TOP1MT
- **Entrez ID:** 116447
- **RefSeq ID:** NM\_052963
- **Transcript sequence length:** 1942
- **5-UTR|CDS|3-UTR identity (%):** 3.24 | 41.22 | 1.82
- **5-UTR|CDS|3-UTR identity (%) [Gaps excluded]:** 68.42 | 74.6 | 72.9
- **5-UTR|CDS|3-UTR identity [Alignment Gaps]:** 382 | 1266 | 4188
- **5-UTR aligned content (<base>:%):** {'T': 15.38, 'C': 23.08, 'G': 46.15, 'A': 15.38}
- **CDS aligned content (<base>:%):** {'A': 27.36, 'T': 17.24, 'C': 24.61, 'G': 30.79}
- **3-UTR aligned content (<base>:%):** {'C': 7.69, 'A': 26.92, 'G': 20.51, 'T': 44.87}

**Uniprot Description:**  
  
 Releases the supercoiling and torsional tension of DNA introduced during duplication of mitochondrial DNA by transiently cleaving and rejoining one strand of the DNA duplex. Introduces a single-strand break via transesterification at a target site in duplex DNA. The scissile phosphodiester is attacked by the catalytic tyrosine of the enzyme, resulting in the formation of a DNA-(3'-phosphotyrosyl)-enzyme intermediate and the expulsion of a 5'-OH DNA strand. The free DNA strand then rotates around the intact phosphodiester bond on the opposing strand, thus removing DNA supercoils. Finally, in the religation step, the DNA 5'-OH attacks the covalent intermediate to expel the active-site tyrosine and restore the DNA phosphodiester backbone (By similarity). N/A   
  
 **Gene Ontology Information:**

Molecular Function

- DNA binding
- DNA topoisomerase type I (single strand cut, ATP-independent) activity

Location

- chromosome
- mitochondrial nucleoid
- mitochondrion
- nucleoplasm

Biological process

- DNA replication
- DNA topological change

---

81

- **Protein name:** Procollagen galactosyltransferase 1
- **Organism:** Homo sapiens
- **Uniprot Accession Number:** Q8NBJ5
- **Protein sequence length:** 622 aa
- **1D identity (%):** 13.69
- **1D identity (%) [Gaps excluded]:** 21.49
- **1D identity - Alignment Gaps:** 329
- **1D aligned content (<aminoacid>:%):** {'A': 4.84, 'R': 12.1, 'G': 8.87, 'L': 11.29, 'P': 9.68, 'D': 7.26, 'Y': 4.03, 'E': 4.03, 'H': 4.03, 'V': 8.87, 'T': 4.03, 'W': 1.61, 'S': 5.65, 'N': 1.61, 'K': 4.03, 'F': 3.23, 'M': 0.81, 'I': 1.61, 'C': 0.81, 'Q': 1.61}
- **Common reported functions (%):** 0.0
- **Common reported locations (%):** 12.5
- **Common reported processes (%):** 0.0

- **AF ID:** Q8NBJ5
- **Chain:** A
- **Protein length:** 622 aa
- **Resolution:** N/A
- **b-phipsi:** 0.001071
- **w-rdist:** 0.541399
- **t-alpha:** 0.070352
- **Chemical similarity (Tanimoto Index) (%):** 96.97
- **1D identity (%) [PDB]:** 1.88
- **1D identity (%) [Gaps excluded][PDB]:** 81.82
- **1D identity - Alignment Gaps [PDB]:** 1406
- **1D aligned content [PDB] (<aminoacid>:%):** {'M': 7.41, 'A': 7.41, 'T': 3.7, 'R': 11.11, 'S': 11.11, 'P': 3.7, 'D': 7.41, 'Q': 3.7, 'E': 7.41, 'I': 3.7, 'L': 7.41, 'V': 7.41, 'K': 3.7, 'N': 3.7, 'G': 7.41, 'Y': 3.7}
- **2D identity (%) [PDB]:** 42.14
- **2D identity (%) [Gaps excluded][PDB]:** 89.01
- **2D identity - Alignment Gaps [PDB]:** 526
- **2D aligned content [PDB] (<2D-fold>:%):** {'.': 20.9, 'E': 17.34, 'T': 22.57, 'H': 37.77, 'B': 0.71, 'G': 0.71}
- **3D similarity (TM-Score) (%) [PDB]:** 20.46

- **Gene name:** COLGALT1
- **Entrez ID:** 79709
- **RefSeq ID:** NM\_024656
- **Transcript sequence length:** 3647
- **5-UTR|CDS|3-UTR identity (%):** 10.1 | 35.94 | 23.9
- **5-UTR|CDS|3-UTR identity (%) [Gaps excluded]:** 84.0 | 73.65 | 74.97
- **5-UTR|CDS|3-UTR identity [Alignment Gaps]:** 366 | 1533 | 3098
- **5-UTR aligned content (<base>:%):** {'G': 35.71, 'C': 42.86, 'T': 9.52, 'A': 11.9}
- **CDS aligned content (<base>:%):** {'A': 22.58, 'T': 19.89, 'G': 29.18, 'C': 28.35}
- **3-UTR aligned content (<base>:%):** {'G': 28.61, 'T': 27.87, 'A': 23.18, 'C': 20.33}

**Uniprot Description:**  
  
 Beta-galactosyltransferase that transfers beta-galactose to hydroxylysine residues of type I collagen (PubMed:19075007, PubMed:22216269, PubMed:27402836). By acting on collagen glycosylation, facilitates the formation of collagen triple helix (PubMed:27402836). Also involved in the biosynthesis of collagen type IV (PubMed:30412317). N/A   
  
 **Gene Ontology Information:**

Molecular Function

- procollagen galactosyltransferase activity

Location

- endoplasmic reticulum lumen
- membrane

Biological process

- collagen fibril organization
- positive regulation of collagen fibril organization

---

82

- **Protein name:** Ran GTPase-activating protein 1
- **Organism:** Homo sapiens
- **Uniprot Accession Number:** P46060
- **Protein sequence length:** 587 aa
- **1D identity (%):** 12.65
- **1D identity (%) [Gaps excluded]:** 20.36
- **1D identity - Alignment Gaps:** 338
- **1D aligned content (<aminoacid>:%):** {'I': 3.54, 'L': 15.04, 'Q': 3.54, 'G': 8.85, 'K': 7.08, 'V': 7.96, 'E': 7.08, 'N': 2.65, 'A': 12.39, 'H': 3.54, 'T': 5.31, 'P': 7.08, 'D': 4.42, 'S': 4.42, 'R': 2.65, 'M': 0.88, 'C': 0.88, 'F': 0.88, 'Y': 1.77}
- **Common reported functions (%):** 0.0
- **Common reported locations (%):** 0.0
- **Common reported processes (%):** 0.0

- **AF ID:** P46060
- **Chain:** A
- **Protein length:** 587 aa
- **Resolution:** N/A
- **b-phipsi:** 0.027904
- **w-rdist:** 0.352546
- **t-alpha:** 0.001568
- **Chemical similarity (Tanimoto Index) (%):** 99.24
- **1D identity (%) [PDB]:** 1.35
- **1D identity (%) [Gaps excluded][PDB]:** 61.29
- **1D identity - Alignment Gaps [PDB]:** 1375
- **1D aligned content [PDB] (<aminoacid>:%):** {'M': 5.26, 'Q': 5.26, 'V': 10.53, 'A': 15.79, 'L': 10.53, 'I': 21.05, 'S': 5.26, 'E': 5.26, 'P': 5.26, 'G': 5.26, 'T': 5.26, 'Y': 5.26}
- **2D identity (%) [PDB]:** 37.12
- **2D identity (%) [Gaps excluded][PDB]:** 88.68
- **2D identity - Alignment Gaps [PDB]:** 589
- **2D aligned content [PDB] (<2D-fold>:%):** {'.': 17.29, 'H': 42.82, 'T': 31.91, 'E': 7.98}
- **3D similarity (TM-Score) (%) [PDB]:** 20.56

- **Gene name:** RANGAP1
- **Entrez ID:** 8761778
- **RefSeq ID:** N/A
- **Sequence length:** N/A
- **5-UTR|CDS|3-UTR identity (%):** N/A | N/A | N/A
- **5-UTR|CDS|3-UTR identity (%) [Gaps excluded]:** N/A | N/A | N/A
- **5-UTR|CDS|3-UTR identity [Alignment Gaps]:** N/A | N/A | N/A
- **5-UTR aligned content (<base>:%):** N/A
- **CDS aligned content (<base>:%):** N/A
- **3-UTR aligned content (<base>:%):** N/A

**Uniprot Description:**  
  
 GTPase activator for RAN (PubMed:8146159, PubMed:8896452, PubMed:16428860). Converts cytoplasmic GTP-bound RAN to GDP-bound RAN, which is essential for RAN-mediated nuclear import and export (PubMed:8896452, PubMed:27160050). Mediates dissociation of cargo from nuclear export complexes containing XPO1, RAN and RANBP2 after nuclear export (PubMed:27160050).   
  
Homodimer (PubMed:8146159). Interacts with RAN (PubMed:7891706, PubMed:8896452, PubMed:16428860). Forms a complex with RANBP2/NUP358, NXF1 and NXT1 (PubMed:14729961). Forms a tight complex in association with RANBP2/NUP358 and UBE2I/UBC9, the ubiquitin-conjugating enzyme E2 (PubMed:15037602, PubMed:27160050, PubMed:15931224, PubMed:22194619). Interacts with UBE2I; the interaction conjugates SUMO1 to RANGAP1, and subsequently stabilizes interactions of sumoylated RANGAP1 with RANBP2/NUP358 (PubMed:15037602, PubMed:27160050, PubMed:15931224). The complex composed of RANBP2, SUMO1, RANGAP1 and UBE2I associates with nuclear pore complexes (PubMed:15037602, PubMed:15931224). Identified in a complex composed of RAN, RANBP2, sumoylated RANGAP1, UBE2I and XPO1 (PubMed:27160050). Identified in a complex composed of RAN, RANGAP1 and RANBP1 (PubMed:16428860). Interacts with TRAF6 (PubMed:18093978). Interacts with SUMO1 and SENP1 (PubMed:17099698). Interacts (when sumoylated) with MYCBP2; interaction inhibits MYCBP2 E3 ubiquitin-protein ligase activity and promotes MYCBP2 translocation to the nucleus (PubMed:26304119).   
  
 **Gene Ontology Information:**

Molecular Function

- 2,3-bisphosphoglycerate-independent phosphoglycerate mutase activity
- metal ion binding

Location   
  
N/A

Biological process

- glycolytic process

---

83

- **Protein name:** 3-phosphoinositide-dependent protein kinase 1
- **Organism:** Homo sapiens
- **Uniprot Accession Number:** O15530
- **Protein sequence length:** 556 aa
- **1D identity (%):** 10.25
- **1D identity (%) [Gaps excluded]:** 20.0
- **1D identity - Alignment Gaps:** 457
- **1D aligned content (<aminoacid>:%):** {'Y': 4.17, 'D': 5.21, 'V': 7.29, 'I': 4.17, 'P': 10.42, 'G': 12.5, 'R': 3.12, 'Q': 6.25, 'A': 4.17, 'E': 3.12, 'T': 6.25, 'S': 1.04, 'L': 8.33, 'H': 1.04, 'N': 3.12, 'M': 1.04, 'F': 8.33, 'K': 7.29, 'C': 3.12}
- **Common reported functions (%):** 0.0
- **Common reported locations (%):** 37.5
- **Common reported processes (%):** 0.0

- **AF ID:** O15530
- **Chain:** A
- **Protein length:** 556 aa
- **Resolution:** N/A
- **b-phipsi:** 0.005065
- **w-rdist:** 0.60116
- **t-alpha:** 0.003912
- **Chemical similarity (Tanimoto Index) (%):** 99.24
- **1D identity (%) [PDB]:** 2.21
- **1D identity (%) [Gaps excluded][PDB]:** 65.22
- **1D identity - Alignment Gaps [PDB]:** 1314
- **1D aligned content [PDB] (<aminoacid>:%):** {'M': 3.33, 'T': 6.67, 'P': 6.67, 'G': 10.0, 'Q': 10.0, 'K': 3.33, 'V': 16.67, 'S': 10.0, 'W': 3.33, 'L': 16.67, 'E': 3.33, 'A': 3.33, 'H': 3.33, 'D': 3.33}
- **2D identity (%) [PDB]:** 36.09
- **2D identity (%) [Gaps excluded][PDB]:** 89.83
- **2D identity - Alignment Gaps [PDB]:** 600
- **2D aligned content [PDB] (<2D-fold>:%):** {'.': 17.4, 'E': 21.55, 'T': 25.69, 'H': 33.7, 'B': 0.28, 'G': 1.38}
- **3D similarity (TM-Score) (%) [PDB]:** 17.32

- **Gene name:** PDPK1
- **Entrez ID:** 5170
- **RefSeq ID:** NM\_031268
- **Transcript sequence length:** 6803
- **5-UTR|CDS|3-UTR identity (%):** 14.56 | 28.11 | 43.05
- **5-UTR|CDS|3-UTR identity (%) [Gaps excluded]:** 74.07 | 76.56 | 73.66
- **5-UTR|CDS|3-UTR identity [Alignment Gaps]:** 331 | 1794 | 2546
- **5-UTR aligned content (<base>:%):** {'A': 6.67, 'T': 6.67, 'C': 31.67, 'G': 55.0}
- **CDS aligned content (<base>:%):** {'A': 28.48, 'G': 25.6, 'C': 22.96, 'T': 22.96}
- **3-UTR aligned content (<base>:%):** {'T': 32.2, 'G': 23.59, 'C': 20.14, 'A': 24.08}

**Uniprot Description:**  
  
 Serine/threonine kinase which acts as a master kinase, phosphorylating and activating a subgroup of the AGC family of protein kinases. Its targets include: protein kinase B (PKB/AKT1, PKB/AKT2, PKB/AKT3), p70 ribosomal protein S6 kinase (RPS6KB1), p90 ribosomal protein S6 kinase (RPS6KA1, RPS6KA2 and RPS6KA3), cyclic AMP-dependent protein kinase (PRKACA), protein kinase C (PRKCD and PRKCZ), serum and glucocorticoid-inducible kinase (SGK1, SGK2 and SGK3), p21-activated kinase-1 (PAK1), protein kinase PKN (PKN1 and PKN2). Plays a central role in the transduction of signals from insulin by providing the activating phosphorylation to PKB/AKT1, thus propagating the signal to downstream targets controlling cell proliferation and survival, as well as glucose and amino acid uptake and storage. Negatively regulates the TGF-beta-induced signaling by: modulating the association of SMAD3 and SMAD7 with TGF-beta receptor, phosphorylating SMAD2, SMAD3, SMAD4 and SMAD7, preventing the nuclear translocation of SMAD3 and SMAD4 and the translocation of SMAD7 from the nucleus to the cytoplasm in response to TGF-beta. Activates PPARG transcriptional activity and promotes adipocyte differentiation. Activates the NF-kappa-B pathway via phosphorylation of IKKB. The tyrosine phosphorylated form is crucial for the regulation of focal adhesions by angiotensin II. Controls proliferation, survival, and growth of developing pancreatic cells. Participates in the regulation of Ca(2+) entry and Ca(2+)-activated K(+) channels of mast cells. Essential for the motility of vascular endothelial cells (ECs) and is involved in the regulation of their chemotaxis. Plays a critical role in cardiac homeostasis by serving as a dual effector for cell survival and beta-adrenergic response. Plays an important role during thymocyte development by regulating the expression of key nutrient receptors on the surface of pre-T cells and mediating Notch-induced cell growth and proliferative responses. Provides negative feedback inhibition to toll-like receptor-mediated NF-kappa-B activation in macrophages. Isoform 3 is catalytically inactive.   
  
Homodimer in its autoinhibited state. Active as monomer. Interacts with NPRL2, PPARG, PAK1, PTK2B, GRB14, PKN1 (via C-terminus), STRAP and IKKB. The Tyr-9 phosphorylated form interacts with SRC, RASA1 and CRK (via their SH2 domains). Interacts with SGK3 in a phosphorylation-dependent manner. The tyrosine-phosphorylated form interacts with PTPN6. The Ser-241 phosphorylated form interacts with YWHAH and YWHAQ. Binds INSR in response to insulin. Interacts (via PH domain) with SMAD3, SMAD4 and SMAD7. Interacts with PKN2; the interaction stimulates PDPK1 autophosphorylation, its PI(3,4,5)P3-dependent kinase activity toward 'Ser-473' of AKT1 but also activates its kinase activity toward PRKCD and PRKCZ.   
  
 **Gene Ontology Information:**

Molecular Function

- 3-phosphoinositide-dependent protein kinase activity
- ATP binding
- phospholipase activator activity
- phospholipase binding
- protein serine kinase activity
- protein serine/threonine kinase activity

Location

- cell projection
- cytoplasm
- cytoplasmic vesicle
- cytosol
- focal adhesion
- nucleus
- plasma membrane
- postsynaptic density

Biological process

- actin cytoskeleton organization
- activation of protein kinase B activity
- calcium-mediated signaling
- cell migration
- cellular response to epidermal growth factor stimulus
- cellular response to insulin stimulus
- epidermal growth factor receptor signaling pathway
- extrinsic apoptotic signaling pathway
- hyperosmotic response
- insulin receptor signaling pathway
- insulin-like growth factor receptor signaling pathway
- intracellular signal transduction
- negative regulation of cardiac muscle cell apoptotic process
- negative regulation of endothelial cell apoptotic process
- negative regulation of protein kinase activity
- negative regulation of toll-like receptor signaling pathway
- negative regulation of transforming growth factor beta receptor signaling pathway
- peptidyl-serine phosphorylation
- peptidyl-threonine phosphorylation
- positive regulation of angiogenesis
- positive regulation of blood vessel endothelial cell migration
- positive regulation of phospholipase activity
- positive regulation of protein localization to plasma membrane
- positive regulation of release of sequestered calcium ion into cytosol
- positive regulation of sprouting angiogenesis
- positive regulation of vascular endothelial cell proliferation
- protein autophosphorylation
- protein phosphorylation
- regulation of I-kappaB kinase/NF-kappaB signaling
- regulation of mast cell degranulation
- type B pancreatic cell development

---

84

- **Protein name:** 1-phosphatidylinositol 4,5-bisphosphate phosphodiesterase delta-3
- **Organism:** Homo sapiens
- **Uniprot Accession Number:** Q8N3E9
- **Protein sequence length:** 789 aa
- **1D identity (%):** 16.14
- **1D identity (%) [Gaps excluded]:** 25.87
- **1D identity - Alignment Gaps:** 382
- **1D aligned content (<aminoacid>:%):** {'L': 14.63, 'G': 9.15, 'P': 11.59, 'A': 4.88, 'V': 6.71, 'S': 4.88, 'R': 7.93, 'M': 0.61, 'D': 4.27, 'I': 2.44, 'T': 5.49, 'E': 5.49, 'H': 2.44, 'F': 3.05, 'C': 2.44, 'Q': 7.32, 'N': 1.83, 'Y': 3.05, 'K': 1.22, 'W': 0.61}
- **Common reported functions (%):** 0.0
- **Common reported locations (%):** 12.5
- **Common reported processes (%):** 0.0

- **AF ID:** Q8N3E9
- **Chain:** A
- **Protein length:** 789 aa
- **Resolution:** N/A
- **b-phipsi:** 0.003093
- **w-rdist:** 0.394022
- **t-alpha:** 0.025039
- **Chemical similarity (Tanimoto Index) (%):** 99.55
- **1D identity (%) [PDB]:** 2.26
- **1D identity (%) [Gaps excluded][PDB]:** 75.0
- **1D identity - Alignment Gaps [PDB]:** 1543
- **1D aligned content [PDB] (<aminoacid>:%):** {'V': 8.33, 'A': 8.33, 'G': 5.56, 'R': 11.11, 'K': 8.33, 'L': 5.56, 'T': 5.56, 'N': 5.56, 'Q': 2.78, 'S': 13.89, 'M': 2.78, 'I': 5.56, 'D': 5.56, 'E': 5.56, 'P': 5.56}
- **2D identity (%) [PDB]:** 38.34
- **2D identity (%) [Gaps excluded][PDB]:** 88.87
- **2D identity - Alignment Gaps [PDB]:** 651
- **2D aligned content [PDB] (<2D-fold>:%):** {'.': 19.59, 'T': 20.96, 'H': 38.72, 'E': 19.36, 'B': 0.68, 'G': 0.68}
- **3D similarity (TM-Score) (%) [PDB]:** 24.34

- **Gene name:** PLCD3
- **Entrez ID:** 113026
- **RefSeq ID:** NM\_133373
- **Transcript sequence length:** 6132
- **5-UTR|CDS|3-UTR identity (%):** 17.43 | 45.57 | 40.44
- **5-UTR|CDS|3-UTR identity (%) [Gaps excluded]:** 77.42 | 73.48 | 74.22
- **5-UTR|CDS|3-UTR identity [Alignment Gaps]:** 320 | 1162 | 2340
- **5-UTR aligned content (<base>:%):** {'C': 61.11, 'T': 4.17, 'G': 33.33, 'A': 1.39}
- **CDS aligned content (<base>:%):** {'A': 22.81, 'T': 18.58, 'G': 29.7, 'C': 28.91}
- **3-UTR aligned content (<base>:%):** {'G': 27.75, 'C': 22.27, 'A': 23.91, 'T': 26.07}

**Uniprot Description:**  
  
 Hydrolyzes the phosphatidylinositol 4,5-bisphosphate (PIP2) to generate 2 second messenger molecules diacylglycerol (DAG) and inositol 1,4,5-trisphosphate (IP3). DAG mediates the activation of protein kinase C (PKC), while IP3 releases Ca(2+) from intracellular stores. Essential for trophoblast and placental development. May participate in cytokinesis by hydrolyzing PIP2 at the cleavage furrow (PubMed:10336610). Regulates neurite outgrowth through the inhibition of RhoA/Rho kinase signaling (By similarity). N/A   
  
 **Gene Ontology Information:**

Molecular Function

- metal ion binding
- phosphatidylinositol phospholipase C activity

Location

- cleavage furrow
- cytoplasm
- nucleoplasm
- plasma membrane

Biological process

- angiogenesis
- intracellular signal transduction
- labyrinthine layer blood vessel development
- lipid catabolic process
- regulation of cell population proliferation

---

85

- **Protein name:** Evolutionarily conserved signaling intermediate in Toll pathway, mitochondrial
- **Organism:** Homo sapiens
- **Uniprot Accession Number:** Q9BQ95
- **Protein sequence length:** 431 aa
- **1D identity (%):** 9.15
- **1D identity (%) [Gaps excluded]:** 22.46
- **1D identity - Alignment Gaps:** 544
- **1D aligned content (<aminoacid>:%):** {'G': 11.9, 'C': 3.57, 'S': 2.38, 'P': 14.29, 'L': 7.14, 'A': 5.95, 'Q': 5.95, 'E': 3.57, 'R': 4.76, 'K': 5.95, 'I': 3.57, 'V': 13.1, 'H': 3.57, 'M': 1.19, 'N': 4.76, 'W': 1.19, 'F': 1.19, 'D': 3.57, 'T': 2.38}
- **Common reported functions (%):** 0.0
- **Common reported locations (%):** 37.5
- **Common reported processes (%):** 0.0

- **AF ID:** Q9BQ95
- **Chain:** A
- **Protein length:** 431 aa
- **Resolution:** N/A
- **b-phipsi:** 0.005573
- **w-rdist:** 0.288612
- **t-alpha:** 0.068075
- **Chemical similarity (Tanimoto Index) (%):** 99.55
- **1D identity (%) [PDB]:** 1.59
- **1D identity (%) [Gaps excluded][PDB]:** 74.07
- **1D identity - Alignment Gaps [PDB]:** 1227
- **1D aligned content [PDB] (<aminoacid>:%):** {'W': 5.0, 'V': 20.0, 'S': 10.0, 'Q': 5.0, 'L': 20.0, 'E': 10.0, 'A': 10.0, 'H': 5.0, 'N': 5.0, 'P': 5.0, 'D': 5.0}
- **2D identity (%) [PDB]:** 29.03
- **2D identity (%) [Gaps excluded][PDB]:** 92.48
- **2D identity - Alignment Gaps [PDB]:** 669
- **2D aligned content [PDB] (<2D-fold>:%):** {'.': 21.91, 'H': 41.34, 'T': 26.86, 'E': 9.89}
- **3D similarity (TM-Score) (%) [PDB]:** 16.93

- **Gene name:** ECSIT
- **Entrez ID:** 507245
- **RefSeq ID:** NM\_016581
- **Transcript sequence length:** 1647
- **5-UTR|CDS|3-UTR identity (%):** 12.94 | 27.52 | 4.01
- **5-UTR|CDS|3-UTR identity (%) [Gaps excluded]:** 76.39 | 75.19 | 77.58
- **5-UTR|CDS|3-UTR identity [Alignment Gaps]:** 353 | 1802 | 4094
- **5-UTR aligned content (<base>:%):** {'C': 27.27, 'T': 12.73, 'G': 56.36, 'A': 3.64}
- **CDS aligned content (<base>:%):** {'A': 24.17, 'T': 17.9, 'G': 28.26, 'C': 29.67}
- **3-UTR aligned content (<base>:%):** {'T': 19.65, 'G': 37.57, 'C': 22.54, 'A': 20.23}

**Uniprot Description:**  
  
 Adapter protein of the Toll-like and IL-1 receptor signaling pathway that is involved in the activation of NF-kappa-B via MAP3K1. Promotes proteolytic activation of MAP3K1. Involved in the BMP signaling pathway. Required for normal embryonic development (By similarity).   
  
Interacts with MAP3K1, SMAD4 and TRAF6. Interacts with SMAD1 only after BMP4-treatment (By similarity). Part of the mitochondrial complex I assembly/MCIA complex that comprises at least the core subunits TMEM126B, NDUFAF1, ECSIT and ACAD9 and complement subunits such as COA1 and TMEM186 (PubMed:32320651). Interacts with NDUFAF1 (PubMed:17344420). Interacts with ACAD9 (PubMed:20816094). Interacts with TRIM59 (By similarity). Interacts with TMEM70 and TMEM242 (PubMed:33753518).   
  
 **Gene Ontology Information:**

Molecular Function

- molecular adaptor activity

Location

- cytoplasm
- cytosol
- mitochondrion
- nucleoplasm
- nucleus

Biological process

- innate immune response
- regulation of oxidoreductase activity
- regulation of protein complex stability
- toll-like receptor 4 signaling pathway

---

86

- **Protein name:** Gelsolin
- **Organism:** Homo sapiens
- **Uniprot Accession Number:** P06396
- **Protein sequence length:** 782 aa
- **1D identity (%):** 16.02
- **1D identity (%) [Gaps excluded]:** 23.32
- **1D identity - Alignment Gaps:** 305
- **1D aligned content (<aminoacid>:%):** {'P': 10.26, 'A': 4.49, 'L': 9.62, 'R': 5.77, 'Q': 5.77, 'V': 8.33, 'K': 5.13, 'G': 12.82, 'D': 5.13, 'T': 6.41, 'E': 5.77, 'F': 1.92, 'S': 4.49, 'W': 1.92, 'M': 1.28, 'C': 0.64, 'Y': 2.56, 'N': 3.85, 'I': 3.21, 'H': 0.64}
- **Common reported functions (%):** 0.0
- **Common reported locations (%):** 12.5
- **Common reported processes (%):** 0.0

- **AF ID:** P06396
- **Chain:** A
- **Protein length:** 782 aa
- **Resolution:** N/A
- **b-phipsi:** 0.004243
- **w-rdist:** 0.35665
- **t-alpha:** 0.039025
- **Chemical similarity (Tanimoto Index) (%):** 100.0
- **1D identity (%) [PDB]:** 2.08
- **1D identity (%) [Gaps excluded][PDB]:** 71.74
- **1D identity - Alignment Gaps [PDB]:** 1540
- **1D aligned content [PDB] (<aminoacid>:%):** {'P': 6.06, 'A': 24.24, 'Y': 3.03, 'R': 9.09, 'L': 9.09, 'H': 6.06, 'V': 3.03, 'K': 6.06, 'D': 6.06, 'S': 9.09, 'E': 3.03, 'G': 9.09, 'N': 3.03, 'Q': 3.03}
- **2D identity (%) [PDB]:** 43.56
- **2D identity (%) [Gaps excluded][PDB]:** 88.83
- **2D identity - Alignment Gaps [PDB]:** 558
- **2D aligned content [PDB] (<2D-fold>:%):** {'.': 14.88, 'E': 26.83, 'T': 27.25, 'H': 28.93, 'G': 1.68, 'B': 0.42}
- **3D similarity (TM-Score) (%) [PDB]:** 26.56

- **Gene name:** GSN
- **Entrez ID:** 535077
- **RefSeq ID:** N/A
- **Sequence length:** N/A
- **5-UTR|CDS|3-UTR identity (%):** N/A | N/A | N/A
- **5-UTR|CDS|3-UTR identity (%) [Gaps excluded]:** N/A | N/A | N/A
- **5-UTR|CDS|3-UTR identity [Alignment Gaps]:** N/A | N/A | N/A
- **5-UTR aligned content (<base>:%):** N/A
- **CDS aligned content (<base>:%):** N/A
- **3-UTR aligned content (<base>:%):** N/A

**Uniprot Description:**  
  
 Calcium-regulated, actin-modulating protein that binds to the plus (or barbed) ends of actin monomers or filaments, preventing monomer exchange (end-blocking or capping). It can promote the assembly of monomers into filaments (nucleation) as well as sever filaments already formed. Plays a role in ciliogenesis.   
  
Binds to actin and to fibronectin. Identified in a complex composed of ACTA1, COBL, GSN AND TMSB4X. Interacts with the inactive form of EIF2AK2/PKR (By similarity).   
  
 **Gene Ontology Information:**

Molecular Function

- actin filament binding
- metal ion binding
- phosphatidylinositol-4,5-bisphosphate binding

Location

- actin cytoskeleton
- cytoplasm
- extracellular space

Biological process

- actin filament severing
- actin polymerization or depolymerization
- barbed-end actin filament capping
- cell projection assembly
- central nervous system development
- cilium assembly

---

87

- **Protein name:** Phosphoribosylformylglycinamidine synthase
- **Organism:** Homo sapiens
- **Uniprot Accession Number:** O15067
- **Protein sequence length:** 1338 aa
- **1D identity (%):** 15.74
- **1D identity (%) [Gaps excluded]:** 27.8
- **1D identity - Alignment Gaps:** 609
- **1D aligned content (<aminoacid>:%):** {'V': 12.22, 'H': 2.26, 'P': 10.41, 'R': 4.98, 'T': 3.62, 'D': 4.98, 'E': 5.88, 'L': 12.67, 'G': 9.05, 'S': 5.88, 'A': 7.69, 'F': 3.62, 'Q': 3.62, 'W': 0.9, 'N': 3.62, 'K': 4.07, 'C': 2.26, 'Y': 2.26}
- **Common reported functions (%):** 0.0
- **Common reported locations (%):** 25.0
- **Common reported processes (%):** 0.0

- **AF ID:** O15067
- **Chain:** A
- **Protein length:** 1338 aa
- **Resolution:** N/A
- **b-phipsi:** 0.00229
- **w-rdist:** 0.277195
- **t-alpha:** 0.128326
- **Chemical similarity (Tanimoto Index) (%):** 99.1
- **1D identity (%) [PDB]:** 4.17
- **1D identity (%) [Gaps excluded][PDB]:** 67.19
- **1D identity - Alignment Gaps [PDB]:** 1932
- **1D aligned content [PDB] (<aminoacid>:%):** {'R': 11.63, 'P': 9.3, 'V': 13.95, 'E': 5.81, 'D': 8.14, 'H': 3.49, 'F': 3.49, 'K': 2.33, 'Q': 6.98, 'G': 10.47, 'Y': 4.65, 'T': 2.33, 'A': 3.49, 'L': 8.14, 'S': 5.81}
- **2D identity (%) [PDB]:** 43.07
- **2D identity (%) [Gaps excluded][PDB]:** 89.84
- **2D identity - Alignment Gaps [PDB]:** 770
- **2D aligned content [PDB] (<2D-fold>:%):** {'.': 14.44, 'E': 29.51, 'H': 32.34, 'T': 23.08, 'G': 0.47, 'B': 0.16}
- **3D similarity (TM-Score) (%) [PDB]:** 32.67

- **Gene name:** PFAS
- **Entrez ID:** 5198
- **RefSeq ID:** NM\_012393
- **Transcript sequence length:** 5369
- **5-UTR|CDS|3-UTR identity (%):** 14.75 | 40.07 | 16.1
- **5-UTR|CDS|3-UTR identity (%) [Gaps excluded]:** 65.98 | 76.44 | 73.29
- **5-UTR|CDS|3-UTR identity [Alignment Gaps]:** 337 | 2061 | 3523
- **5-UTR aligned content (<base>:%):** {'A': 10.94, 'G': 29.69, 'C': 48.44, 'T': 10.94}
- **CDS aligned content (<base>:%):** {'A': 23.04, 'T': 21.77, 'C': 26.04, 'G': 29.15}
- **3-UTR aligned content (<base>:%):** {'C': 20.91, 'T': 36.31, 'A': 25.03, 'G': 17.74}

**Uniprot Description:**  
  
 Phosphoribosylformylglycinamidine synthase involved in the purines biosynthetic pathway. Catalyzes the ATP-dependent conversion of formylglycinamide ribonucleotide (FGAR) and glutamine to yield formylglycinamidine ribonucleotide (FGAM) and glutamate. N/A   
  
 **Gene Ontology Information:**

Molecular Function

- ATP binding
- metal ion binding
- phosphoribosylformylglycinamidine synthase activity

Location

- cytoplasm
- cytosol
- extracellular exosome

Biological process

- 'de novo' AMP biosynthetic process
- 'de novo' IMP biosynthetic process
- 'de novo' XMP biosynthetic process
- anterior head development
- glutamine metabolic process
- GMP biosynthetic process
- purine nucleotide biosynthetic process
- purine ribonucleoside monophosphate biosynthetic process
- response to xenobiotic stimulus

---

88

- **Protein name:** Threonylcarbamoyladenosine tRNA methylthiotransferase
- **Organism:** Homo sapiens
- **Uniprot Accession Number:** Q5VV42
- **Protein sequence length:** 579 aa
- **1D identity (%):** 11.22
- **1D identity (%) [Gaps excluded]:** 23.91
- **1D identity - Alignment Gaps:** 520
- **1D aligned content (<aminoacid>:%):** {'P': 5.45, 'C': 2.73, 'I': 5.45, 'S': 7.27, 'K': 6.36, 'R': 5.45, 'E': 6.36, 'G': 10.0, 'N': 1.82, 'Y': 5.45, 'T': 3.64, 'V': 8.18, 'A': 6.36, 'Q': 7.27, 'D': 5.45, 'L': 8.18, 'H': 1.82, 'M': 1.82, 'F': 0.91}
- **Common reported functions (%):** 0.0
- **Common reported locations (%):** 12.5
- **Common reported processes (%):** 0.0

- **AF ID:** Q5VV42
- **Chain:** A
- **Protein length:** 579 aa
- **Resolution:** N/A
- **b-phipsi:** 0.00322
- **w-rdist:** 0.74527
- **t-alpha:** 0.002353
- **Chemical similarity (Tanimoto Index) (%):** 99.55
- **1D identity (%) [PDB]:** 2.09
- **1D identity (%) [Gaps excluded][PDB]:** 70.73
- **1D identity - Alignment Gaps [PDB]:** 1347
- **1D aligned content [PDB] (<aminoacid>:%):** {'M': 3.45, 'T': 10.34, 'G': 13.79, 'Q': 6.9, 'I': 6.9, 'V': 24.14, 'L': 6.9, 'P': 6.9, 'A': 6.9, 'K': 3.45, 'R': 3.45, 'C': 3.45, 'N': 3.45}
- **2D identity (%) [PDB]:** 44.07
- **2D identity (%) [Gaps excluded][PDB]:** 90.79
- **2D identity - Alignment Gaps [PDB]:** 495
- **2D aligned content [PDB] (<2D-fold>:%):** {'.': 14.62, 'T': 26.18, 'E': 21.46, 'H': 37.74}
- **3D similarity (TM-Score) (%) [PDB]:** 20.38

- **Gene name:** CDKAL1
- **Entrez ID:** 36954
- **RefSeq ID:** NM\_017774
- **Transcript sequence length:** 3272
- **5-UTR|CDS|3-UTR identity (%):** 15.35 | 41.0 | 19.28
- **5-UTR|CDS|3-UTR identity (%) [Gaps excluded]:** 72.73 | 75.61 | 76.09
- **5-UTR|CDS|3-UTR identity [Alignment Gaps]:** 370 | 1284 | 3366
- **5-UTR aligned content (<base>:%):** {'C': 25.0, 'G': 47.22, 'T': 20.83, 'A': 6.94}
- **CDS aligned content (<base>:%):** {'A': 31.3, 'T': 25.04, 'C': 19.57, 'G': 24.09}
- **3-UTR aligned content (<base>:%):** {'C': 19.91, 'A': 25.89, 'T': 34.75, 'G': 19.45}

**Uniprot Description:**  
  
 Catalyzes the methylthiolation of N6-threonylcarbamoyladenosine (t(6)A), leading to the formation of 2-methylthio-N6-threonylcarbamoyladenosine (ms(2)t(6)A) at position 37 in tRNAs that read codons beginning with adenine. N/A   
  
 **Gene Ontology Information:**

Molecular Function

- 4 iron, 4 sulfur cluster binding
- metal ion binding
- N6-threonylcarbomyladenosine methylthiotransferase activity
- tRNA (N(6)-L-threonylcarbamoyladenosine(37)-C(2))-methylthiotransferase

Location

- endomembrane system
- endoplasmic reticulum
- membrane

Biological process

- tRNA methylthiolation

---

89

- **Protein name:** Cyclic nucleotide-binding domain-containing protein 1
- **Organism:** Homo sapiens
- **Uniprot Accession Number:** Q8NA66
- **Protein sequence length:** 436 aa
- **1D identity (%):** 8.65
- **1D identity (%) [Gaps excluded]:** 23.5
- **1D identity - Alignment Gaps:** 599
- **1D aligned content (<aminoacid>:%):** {'M': 1.22, 'A': 2.44, 'P': 13.41, 'L': 10.98, 'G': 7.32, 'R': 3.66, 'N': 4.88, 'H': 3.66, 'F': 6.1, 'Q': 4.88, 'K': 9.76, 'V': 2.44, 'D': 4.88, 'T': 3.66, 'Y': 3.66, 'S': 3.66, 'E': 8.54, 'C': 2.44, 'I': 2.44}
- **Common reported functions (%):** 0.0
- **Common reported locations (%):** 0.0
- **Common reported processes (%):** 0.0

- **AF ID:** Q8NA66
- **Chain:** A
- **Protein length:** 436 aa
- **Resolution:** N/A
- **b-phipsi:** 0.001125
- **w-rdist:** 0.702176
- **t-alpha:** 0.021909
- **Chemical similarity (Tanimoto Index) (%):** 99.55
- **1D identity (%) [PDB]:** 1.91
- **1D identity (%) [Gaps excluded][PDB]:** 75.0
- **1D identity - Alignment Gaps [PDB]:** 1222
- **1D aligned content [PDB] (<aminoacid>:%):** {'Q': 12.5, 'Y': 8.33, 'K': 12.5, 'P': 12.5, 'L': 16.67, 'E': 8.33, 'H': 4.17, 'V': 8.33, 'C': 4.17, 'N': 4.17, 'I': 4.17, 'A': 4.17}
- **2D identity (%) [PDB]:** 29.06
- **2D identity (%) [Gaps excluded][PDB]:** 85.58
- **2D identity - Alignment Gaps [PDB]:** 634
- **2D aligned content [PDB] (<2D-fold>:%):** {'.': 16.13, 'H': 40.5, 'T': 22.22, 'E': 20.43, 'B': 0.72}
- **3D similarity (TM-Score) (%) [PDB]:** 15.74

- **Gene name:** CNBD1
- **Entrez ID:** 168975
- **RefSeq ID:** NM\_173538
- **Transcript sequence length:** 1624
- **5-UTR|CDS|3-UTR identity (%):** 10.34 | 28.52 | 3.54
- **5-UTR|CDS|3-UTR identity (%) [Gaps excluded]:** 65.15 | 74.95 | 76.5
- **5-UTR|CDS|3-UTR identity [Alignment Gaps]:** 350 | 1749 | 4117
- **5-UTR aligned content (<base>:%):** {'G': 39.53, 'C': 34.88, 'A': 6.98, 'T': 18.6}
- **CDS aligned content (<base>:%):** {'A': 36.15, 'T': 24.72, 'G': 18.63, 'C': 20.5}
- **3-UTR aligned content (<base>:%):** {'T': 40.52, 'C': 15.03, 'A': 34.64, 'G': 9.8}

**Uniprot Description:**  
  
 N/A N/A   
  
 **Gene Ontology Information:**

Molecular Function   
  
N/A

Location   
  
N/A

Biological process   
  
N/A

---

90

- **Protein name:** E3 ubiquitin-protein ligase HECTD3
- **Organism:** Homo sapiens
- **Uniprot Accession Number:** Q5T447
- **Protein sequence length:** 861 aa
- **1D identity (%):** 17.38
- **1D identity (%) [Gaps excluded]:** 25.87
- **1D identity - Alignment Gaps:** 338
- **1D aligned content (<aminoacid>:%):** {'G': 11.17, 'P': 11.73, 'A': 3.35, 'L': 12.29, 'R': 7.82, 'V': 9.5, 'F': 1.68, 'Y': 4.47, 'K': 6.15, 'D': 5.03, 'E': 4.47, 'W': 2.79, 'H': 1.68, 'T': 4.47, 'S': 4.47, 'M': 1.68, 'N': 0.56, 'C': 1.12, 'Q': 4.47, 'I': 1.12}
- **Common reported functions (%):** 0.0
- **Common reported locations (%):** 0.0
- **Common reported processes (%):** 0.0

- **AF ID:** Q5T447
- **Chain:** A
- **Protein length:** 861 aa
- **Resolution:** N/A
- **b-phipsi:** 0.002395
- **w-rdist:** 0.338063
- **t-alpha:** 0.088419
- **Chemical similarity (Tanimoto Index) (%):** 99.02
- **1D identity (%) [PDB]:** 1.92
- **1D identity (%) [Gaps excluded][PDB]:** 68.09
- **1D identity - Alignment Gaps [PDB]:** 1617
- **1D aligned content [PDB] (<aminoacid>:%):** {'Q': 6.25, 'L': 18.75, 'E': 6.25, 'A': 3.12, 'H': 6.25, 'V': 12.5, 'P': 12.5, 'D': 6.25, 'T': 6.25, 'M': 3.12, 'R': 6.25, 'Y': 3.12, 'G': 3.12, 'S': 6.25}
- **2D identity (%) [PDB]:** 36.3
- **2D identity (%) [Gaps excluded][PDB]:** 93.92
- **2D identity - Alignment Gaps [PDB]:** 757
- **2D aligned content [PDB] (<2D-fold>:%):** {'.': 22.32, 'H': 39.51, 'E': 17.63, 'T': 19.64, 'G': 0.67, 'B': 0.22}
- **3D similarity (TM-Score) (%) [PDB]:** 26.48

- **Gene name:** HECTD3
- **Entrez ID:** 79654
- **RefSeq ID:** NM\_024602
- **Transcript sequence length:** 3597
- **5-UTR|CDS|3-UTR identity (%):** 11.69 | 44.67 | 14.88
- **5-UTR|CDS|3-UTR identity (%) [Gaps excluded]:** 71.21 | 75.29 | 74.77
- **5-UTR|CDS|3-UTR identity [Alignment Gaps]:** 336 | 1320 | 3493
- **5-UTR aligned content (<base>:%):** {'G': 31.91, 'T': 8.51, 'C': 53.19, 'A': 6.38}
- **CDS aligned content (<base>:%):** {'A': 24.07, 'T': 20.28, 'G': 30.28, 'C': 25.38}
- **3-UTR aligned content (<base>:%):** {'G': 23.42, 'T': 25.73, 'C': 26.96, 'A': 23.88}

**Uniprot Description:**  
  
 E3 ubiquitin ligases accepts ubiquitin from an E2 ubiquitin-conjugating enzyme in the form of a thioester and then directly transfers the ubiquitin to targeted substrates. Mediates ubiquitination of TRIOBP and its subsequent proteasomal degradation, thus facilitating cell cycle progression by regulating the turn-over of TRIOBP. Mediates also ubiquitination of STX8 (By similarity).   
  
Interacts with TRIOBP. Interacts with STX8 (By similarity).   
  
 **Gene Ontology Information:**

Molecular Function

- syntaxin binding
- ubiquitin-protein transferase activity

Location

- perinuclear region of cytoplasm

Biological process

- proteasome-mediated ubiquitin-dependent protein catabolic process
- protein ubiquitination

---

91

- **Protein name:** Kelch-like ECH-associated protein 1
- **Organism:** Homo sapiens
- **Uniprot Accession Number:** Q14145
- **Protein sequence length:** 624 aa
- **1D identity (%):** 13.04
- **1D identity (%) [Gaps excluded]:** 23.22
- **1D identity - Alignment Gaps:** 417
- **1D aligned content (<aminoacid>:%):** {'P': 9.68, 'R': 8.87, 'Q': 4.84, 'G': 11.29, 'M': 1.61, 'Y': 4.03, 'E': 3.23, 'V': 7.26, 'T': 3.23, 'F': 4.84, 'L': 10.48, 'A': 4.03, 'H': 5.65, 'S': 4.03, 'D': 3.23, 'I': 2.42, 'N': 4.03, 'C': 5.65, 'K': 0.81, 'W': 0.81}
- **Common reported functions (%):** 0.0
- **Common reported locations (%):** 25.0
- **Common reported processes (%):** 0.0

- **AF ID:** Q14145
- **Chain:** A
- **Protein length:** 624 aa
- **Resolution:** N/A
- **b-phipsi:** 0.002544
- **w-rdist:** 0.852992
- **t-alpha:** 0.001568
- **Chemical similarity (Tanimoto Index) (%):** 99.02
- **1D identity (%) [PDB]:** 2.76
- **1D identity (%) [Gaps excluded][PDB]:** 62.9
- **1D identity - Alignment Gaps [PDB]:** 1350
- **1D aligned content [PDB] (<aminoacid>:%):** {'Q': 10.26, 'F': 5.13, 'A': 15.38, 'G': 7.69, 'I': 5.13, 'K': 7.69, 'V': 7.69, 'R': 2.56, 'D': 7.69, 'L': 7.69, 'S': 7.69, 'M': 2.56, 'P': 2.56, 'C': 5.13, 'Y': 2.56, 'E': 2.56}
- **2D identity (%) [PDB]:** 37.57
- **2D identity (%) [Gaps excluded][PDB]:** 89.45
- **2D identity - Alignment Gaps [PDB]:** 602
- **2D aligned content [PDB] (<2D-fold>:%):** {'.': 21.54, 'T': 23.59, 'H': 35.38, 'E': 18.97, 'B': 0.51}
- **3D similarity (TM-Score) (%) [PDB]:** 17.24

- **Gene name:** KEAP1
- **Entrez ID:** 9817
- **RefSeq ID:** N/A
- **Sequence length:** N/A
- **5-UTR|CDS|3-UTR identity (%):** N/A | N/A | N/A
- **5-UTR|CDS|3-UTR identity (%) [Gaps excluded]:** N/A | N/A | N/A
- **5-UTR|CDS|3-UTR identity [Alignment Gaps]:** N/A | N/A | N/A
- **5-UTR aligned content (<base>:%):** N/A
- **CDS aligned content (<base>:%):** N/A
- **3-UTR aligned content (<base>:%):** N/A

**Uniprot Description:**  
  
 Substrate-specific adapter of a BCR (BTB-CUL3-RBX1) E3 ubiquitin ligase complex that regulates the response to oxidative stress by targeting NFE2L2/NRF2 for ubiquitination (PubMed:14585973, PubMed:15379550, PubMed:15572695, PubMed:15983046, PubMed:15601839). KEAP1 acts as a key sensor of oxidative and electrophilic stress: in normal conditions, the BCR(KEAP1) complex mediates ubiquitination and degradation of NFE2L2/NRF2, a transcription factor regulating expression of many cytoprotective genes (PubMed:15601839, PubMed:16006525). In response to oxidative stress, different electrophile metabolites trigger non-enzymatic covalent modifications of highly reactive cysteine residues in KEAP1, leading to inactivate the ubiquitin ligase activity of the BCR(KEAP1) complex, promoting NFE2L2/NRF2 nuclear accumulation and expression of phase II detoxifying enzymes (PubMed:19489739, PubMed:16006525, PubMed:17127771, PubMed:18251510, PubMed:29590092). In response to selective autophagy, KEAP1 is sequestered in inclusion bodies following its interaction with SQSTM1/p62, leading to inactivation of the BCR(KEAP1) complex and activation of NFE2L2/NRF2 (PubMed:20452972). The BCR(KEAP1) complex also mediates ubiquitination of SQSTM1/p62, increasing SQSTM1/p62 sequestering activity and degradation (PubMed:28380357). The BCR(KEAP1) complex also targets BPTF and PGAM5 for ubiquitination and degradation by the proteasome (PubMed:15379550, PubMed:17046835).   
  
Component of the BCR(KEAP1) E3 ubiquitin ligase complex, at least composed of 2 molecules of CUL3, 2 molecules of KEAP1, and RBX1 (PubMed:15572695, PubMed:15983046, PubMed:15601839, PubMed:17127771, PubMed:18251510, PubMed:24896564). Interacts with NFE2L2/NRF2; the interaction is direct (PubMed:15379550, PubMed:15601839, PubMed:16006525, PubMed:18387606, PubMed:16888629). Forms a ternary complex with NFE2L2/NRF2 and PGAM5 (PubMed:17046835). Interacts with (phosphorylated) SQSTM1/p62; the interaction is direct and inactivates the BCR(KEAP1) complex by sequestering it in inclusion bodies, promoting its degradation (PubMed:20495340, PubMed:20452972). Interacts with NFE2L1 (PubMed:16687406). Interacts with BPTF and PTMA (PubMed:15657435). Interacts with MAP1LC3B (PubMed:24089205). Interacts indirectly with ENC1 (PubMed:19424503). Interacts with SESN1 and SESN2 (PubMed:23274085). Interacts with HSP90AA1 and HSP90AB1 (PubMed:26517842).   
  
 **Gene Ontology Information:**

Molecular Function

- disordered domain specific binding
- identical protein binding
- RNA polymerase II-specific DNA-binding transcription factor binding
- ubiquitin ligase-substrate adaptor activity

Location

- actin filament
- centriolar satellite
- Cul3-RING ubiquitin ligase complex
- cytoplasm
- cytosol
- endoplasmic reticulum
- inclusion body
- midbody
- nucleoplasm

Biological process

- cellular response to interleukin-4
- cellular response to oxidative stress
- cytoplasmic sequestering of transcription factor
- in utero embryonic development
- negative regulation of DNA-binding transcription factor activity
- positive regulation of proteasomal ubiquitin-dependent protein catabolic process
- protein ubiquitination
- regulation of autophagy
- regulation of epidermal cell differentiation
- ubiquitin-dependent protein catabolic process

---

92

- **Protein name:** Uncharacterized protein KIAA0930
- **Organism:** Homo sapiens
- **Uniprot Accession Number:** Q6ICG6
- **Protein sequence length:** 404 aa
- **1D identity (%):** 8.17
- **1D identity (%) [Gaps excluded]:** 23.91
- **1D identity - Alignment Gaps:** 621
- **1D aligned content (<aminoacid>:%):** {'L': 9.09, 'R': 9.09, 'I': 2.6, 'G': 14.29, 'C': 5.19, 'F': 6.49, 'K': 5.19, 'A': 2.6, 'D': 5.19, 'Y': 3.9, 'P': 9.09, 'V': 9.09, 'E': 5.19, 'H': 3.9, 'S': 2.6, 'Q': 1.3, 'M': 1.3, 'T': 3.9}
- **Common reported functions (%):** 0.0
- **Common reported locations (%):** 0.0
- **Common reported processes (%):** 0.0

- **AF ID:** Q6ICG6
- **Chain:** A
- **Protein length:** 404 aa
- **Resolution:** N/A
- **b-phipsi:** 0.010241
- **w-rdist:** 0.575514
- **t-alpha:** 0.002347
- **Chemical similarity (Tanimoto Index) (%):** 98.95
- **1D identity (%) [PDB]:** 1.88
- **1D identity (%) [Gaps excluded][PDB]:** 74.19
- **1D identity - Alignment Gaps [PDB]:** 1192
- **1D aligned content [PDB] (<aminoacid>:%):** {'M': 4.35, 'T': 8.7, 'Y': 8.7, 'V': 21.74, 'L': 17.39, 'I': 4.35, 'P': 8.7, 'K': 4.35, 'A': 4.35, 'E': 4.35, 'R': 4.35, 'G': 4.35, 'D': 4.35}
- **2D identity (%) [PDB]:** 21.61
- **2D identity (%) [Gaps excluded][PDB]:** 88.98
- **2D identity - Alignment Gaps [PDB]:** 764
- **2D aligned content [PDB] (<2D-fold>:%):** {'H': 35.32, '.': 16.06, 'T': 21.1, 'E': 27.52}
- **3D similarity (TM-Score) (%) [PDB]:** 15.89

- **Gene name:** KIAA0930
- **Entrez ID:** 23313
- **RefSeq ID:** N/A
- **Sequence length:** N/A
- **5-UTR|CDS|3-UTR identity (%):** N/A | N/A | N/A
- **5-UTR|CDS|3-UTR identity (%) [Gaps excluded]:** N/A | N/A | N/A
- **5-UTR|CDS|3-UTR identity [Alignment Gaps]:** N/A | N/A | N/A
- **5-UTR aligned content (<base>:%):** N/A
- **CDS aligned content (<base>:%):** N/A
- **3-UTR aligned content (<base>:%):** N/A

**Uniprot Description:**  
  
 N/A N/A   
  
 **Gene Ontology Information:**

Molecular Function   
  
N/A

Location   
  
N/A

Biological process   
  
N/A

---

93

- **Protein name:** Cyclic AMP-responsive element-binding protein 1
- **Organism:** Homo sapiens
- **Uniprot Accession Number:** P16220
- **Protein sequence length:** 327 aa
- **1D identity (%):** 6.87
- **1D identity (%) [Gaps excluded]:** 20.33
- **1D identity - Alignment Gaps:** 588
- **1D aligned content (<aminoacid>:%):** {'T': 8.2, 'S': 4.92, 'Q': 9.84, 'V': 11.48, 'M': 3.28, 'P': 11.48, 'H': 1.64, 'G': 6.56, 'A': 4.92, 'E': 9.84, 'D': 4.92, 'R': 3.28, 'K': 3.28, 'I': 3.28, 'Y': 1.64, 'N': 3.28, 'L': 6.56, 'C': 1.64}
- **Common reported functions (%):** 0.0
- **Common reported locations (%):** 12.5
- **Common reported processes (%):** 8.33

- **AF ID:** P16220
- **Chain:** A
- **Protein length:** 327 aa
- **Resolution:** N/A
- **b-phipsi:** 0.004697
- **w-rdist:** 0.857198
- **t-alpha:** 0.0
- **Chemical similarity (Tanimoto Index) (%):** 87.02
- **1D identity (%) [PDB]:** 1.74
- **1D identity (%) [Gaps excluded][PDB]:** 76.92
- **1D identity - Alignment Gaps [PDB]:** 1125
- **1D aligned content [PDB] (<aminoacid>:%):** {'T': 10.0, 'M': 5.0, 'I': 10.0, 'A': 10.0, 'S': 20.0, 'P': 5.0, 'D': 5.0, 'Q': 5.0, 'E': 10.0, 'R': 5.0, 'L': 5.0, 'V': 5.0, 'N': 5.0}
- **2D identity (%) [PDB]:** 26.97
- **2D identity (%) [Gaps excluded][PDB]:** 92.83
- **2D identity - Alignment Gaps [PDB]:** 647
- **2D aligned content [PDB] (<2D-fold>:%):** {'.': 21.14, 'T': 28.46, 'E': 14.23, 'H': 36.18}
- **3D similarity (TM-Score) (%) [PDB]:** 14.03

- **Gene name:** CREB1
- **Entrez ID:** 1385
- **RefSeq ID:** N/A
- **Sequence length:** N/A
- **5-UTR|CDS|3-UTR identity (%):** N/A | N/A | N/A
- **5-UTR|CDS|3-UTR identity (%) [Gaps excluded]:** N/A | N/A | N/A
- **5-UTR|CDS|3-UTR identity [Alignment Gaps]:** N/A | N/A | N/A
- **5-UTR aligned content (<base>:%):** N/A
- **CDS aligned content (<base>:%):** N/A
- **3-UTR aligned content (<base>:%):** N/A

**Uniprot Description:**  
  
 Phosphorylation-dependent transcription factor that stimulates transcription upon binding to the DNA cAMP response element (CRE), a sequence present in many viral and cellular promoters (By similarity). Transcription activation is enhanced by the TORC coactivators which act independently of Ser-119 phosphorylation (PubMed:14536081). Involved in different cellular processes including the synchronization of circadian rhythmicity and the differentiation of adipose cells (By similarity). Regulates the expression of apoptotic and inflammatory response factors in cardiomyocytes in response to ERFE-mediated activation of AKT signaling (By similarity).   
  
Interacts with PPRC1. Binds DNA as a dimer. This dimer is stabilized by magnesium ions. Interacts, through the bZIP domain, with the coactivators CRTC1/TORC1, CRTC2/TORC2 and CRTC3/TORC3. When phosphorylated on Ser-119, binds CREBBP (By similarity). Interacts with CREBL2; regulates CREB1 phosphorylation, stability and transcriptional activity (By similarity). Interacts (phosphorylated form) with TOX3. Interacts with ARRB1. Binds to HIPK2. Interacts with SGK1. Interacts with TSSK4; this interaction facilitates phosphorylation on Ser-119 (PubMed:15964553). Forms a complex with KMT2A and CREBBP (By similarity) (PubMed:14506290, PubMed:14536081, PubMed:15454081, PubMed:15733869, PubMed:15964553, PubMed:16325578, PubMed:16908542, PubMed:20573984, PubMed:21172805, PubMed:23651431). Interacts with TOX4; CREB1 is required for full induction of TOX4-dependent activity and the interaction is increased by cAMP and inhibited by insulin (By similarity).   
  
 **Gene Ontology Information:**

Molecular Function

- cAMP response element binding
- DNA-binding transcription activator activity, RNA polymerase II-specific
- DNA-binding transcription factor activity
- DNA-binding transcription factor activity, RNA polymerase II-specific
- enzyme binding
- identical protein binding
- RNA polymerase II cis-regulatory region sequence-specific DNA binding
- RNA polymerase II-specific DNA-binding transcription factor binding
- sequence-specific double-stranded DNA binding

Location

- ATF4-CREB1 transcription factor complex
- chromatin
- euchromatin
- nucleoplasm
- nucleus
- RNA polymerase II transcription regulator complex

Biological process

- axonogenesis
- cAMP-mediated signaling
- cellular response to forskolin
- cellular response to hepatocyte growth factor stimulus
- cellular response to leukemia inhibitory factor
- cellular response to retinoic acid
- cellular response to zinc ion
- circadian rhythm
- hormone secretion
- lactation
- lung saccule development
- memory
- mRNA transcription by RNA polymerase II
- negative regulation of apoptotic process
- negative regulation of transcription by competitive promoter binding
- osteoclast differentiation
- pituitary gland development
- positive regulation of cardiac muscle tissue development
- positive regulation of transcription, DNA-templated
- positive regulation of fat cell differentiation
- positive regulation of hormone secretion
- positive regulation of lipid biosynthetic process
- positive regulation of multicellular organism growth
- positive regulation of osteoclast differentiation
- positive regulation of transcription by RNA polymerase II
- protein phosphorylation
- protein stabilization
- regulation of cell size
- regulation of transcription by RNA polymerase II
- response to glucagon
- response to organic substance
- response to purine-containing compound
- response to xenobiotic stimulus
- secretory granule organization
- signal transduction
- type I pneumocyte differentiation

---

94

- **Protein name:** Succinate--CoA ligase [ADP-forming] subunit beta, mitochondrial
- **Organism:** Homo sapiens
- **Uniprot Accession Number:** Q9P2R7
- **Protein sequence length:** 463 aa
- **1D identity (%):** 9.69
- **1D identity (%) [Gaps excluded]:** 25.55
- **1D identity - Alignment Gaps:** 596
- **1D aligned content (<aminoacid>:%):** {'G': 9.68, 'L': 12.9, 'T': 6.45, 'A': 7.53, 'Q': 6.45, 'S': 3.23, 'M': 2.15, 'E': 4.3, 'V': 10.75, 'P': 4.3, 'Y': 2.15, 'K': 9.68, 'R': 3.23, 'F': 5.38, 'C': 1.08, 'N': 2.15, 'I': 4.3, 'D': 3.23, 'H': 1.08}
- **Common reported functions (%):** 0.0
- **Common reported locations (%):** 0.0
- **Common reported processes (%):** 0.0

- **AF ID:** Q9P2R7
- **Chain:** A
- **Protein length:** 463 aa
- **Resolution:** N/A
- **b-phipsi:** 0.00102
- **w-rdist:** 0.655546
- **t-alpha:** 0.050123
- **Chemical similarity (Tanimoto Index) (%):** 98.87
- **1D identity (%) [PDB]:** 3.53
- **1D identity (%) [Gaps excluded][PDB]:** 67.69
- **1D identity - Alignment Gaps [PDB]:** 1183
- **1D aligned content [PDB] (<aminoacid>:%):** {'R': 9.09, 'L': 6.82, 'N': 6.82, 'H': 4.55, 'Q': 6.82, 'V': 11.36, 'P': 9.09, 'K': 9.09, 'D': 6.82, 'I': 6.82, 'E': 4.55, 'T': 4.55, 'M': 4.55, 'F': 2.27, 'G': 4.55, 'A': 2.27}
- **2D identity (%) [PDB]:** 29.94
- **2D identity (%) [Gaps excluded][PDB]:** 88.82
- **2D identity - Alignment Gaps [PDB]:** 651
- **2D aligned content [PDB] (<2D-fold>:%):** {'.': 15.99, 'E': 32.31, 'T': 10.88, 'H': 40.82}
- **3D similarity (TM-Score) (%) [PDB]:** 16.7

- **Gene name:** SUCLA2
- **Entrez ID:** 8803
- **RefSeq ID:** NM\_003850
- **Transcript sequence length:** 2111
- **5-UTR|CDS|3-UTR identity (%):** 0.75 | 34.03 | 10.02
- **5-UTR|CDS|3-UTR identity (%) [Gaps excluded]:** 75.0 | 75.49 | 75.3
- **5-UTR|CDS|3-UTR identity [Alignment Gaps]:** 397 | 1506 | 3826
- **5-UTR aligned content (<base>:%):** {'G': 33.33, 'A': 66.67}
- **CDS aligned content (<base>:%):** {'A': 33.23, 'G': 23.58, 'C': 17.9, 'T': 25.29}
- **3-UTR aligned content (<base>:%):** {'T': 38.46, 'C': 13.35, 'G': 14.03, 'A': 34.16}

**Uniprot Description:**  
  
 ATP-specific succinyl-CoA synthetase functions in the citric acid cycle (TCA), coupling the hydrolysis of succinyl-CoA to the synthesis of ATP and thus represents the only step of substrate-level phosphorylation in the TCA (PubMed:15877282). The beta subunit provides nucleotide specificity of the enzyme and binds the substrate succinate, while the binding sites for coenzyme A and phosphate are found in the alpha subunit (By similarity).   
  
Heterodimer of an alpha and a beta subunit. The beta subunit determines specificity for ATP (By similarity). Interacts with ALAS2 (PubMed:14643893).   
  
 **Gene Ontology Information:**

Molecular Function

- ATP binding
- magnesium ion binding
- succinate-CoA ligase (ADP-forming) activity

Location

- extracellular exosome
- mitochondrial matrix
- mitochondrion
- succinate-CoA ligase complex
- succinate-CoA ligase complex (ADP-forming)

Biological process

- succinate metabolic process
- succinyl-CoA catabolic process
- succinyl-CoA metabolic process
- succinyl-CoA pathway
- tricarboxylic acid cycle

---

95

- **Protein name:** Kelch-like protein 41
- **Organism:** Homo sapiens
- **Uniprot Accession Number:** O60662
- **Protein sequence length:** 606 aa
- **1D identity (%):** 12.65
- **1D identity (%) [Gaps excluded]:** 21.59
- **1D identity - Alignment Gaps:** 383
- **1D aligned content (<aminoacid>:%):** {'D': 5.98, 'L': 15.38, 'E': 5.98, 'A': 7.69, 'P': 8.55, 'R': 5.13, 'F': 1.71, 'V': 7.69, 'N': 3.42, 'C': 2.56, 'K': 7.69, 'G': 6.84, 'Q': 4.27, 'I': 6.84, 'Y': 2.56, 'H': 0.85, 'T': 3.42, 'S': 3.42}
- **Common reported functions (%):** 0.0
- **Common reported locations (%):** 25.0
- **Common reported processes (%):** 0.0

- **AF ID:** O60662
- **Chain:** A
- **Protein length:** 606 aa
- **Resolution:** N/A
- **b-phipsi:** 0.00131
- **w-rdist:** 0.52779
- **t-alpha:** 0.046683
- **Chemical similarity (Tanimoto Index) (%):** 96.62
- **1D identity (%) [PDB]:** 2.34
- **1D identity (%) [Gaps excluded][PDB]:** 70.21
- **1D identity - Alignment Gaps [PDB]:** 1362
- **1D aligned content [PDB] (<aminoacid>:%):** {'M': 6.06, 'I': 6.06, 'A': 9.09, 'R': 9.09, 'P': 9.09, 'D': 3.03, 'Q': 3.03, 'E': 9.09, 'S': 6.06, 'L': 9.09, 'K': 3.03, 'N': 3.03, 'V': 6.06, 'G': 9.09, 'F': 3.03, 'H': 3.03, 'T': 3.03}
- **2D identity (%) [PDB]:** 31.48
- **2D identity (%) [Gaps excluded][PDB]:** 90.43
- **2D identity - Alignment Gaps [PDB]:** 704
- **2D aligned content [PDB] (<2D-fold>:%):** {'H': 40.29, 'T': 23.82, '.': 14.12, 'E': 20.29, 'G': 0.88, 'B': 0.59}
- **3D similarity (TM-Score) (%) [PDB]:** 18.94

- **Gene name:** KLHL41
- **Entrez ID:** 10324
- **RefSeq ID:** NM\_006063
- **Transcript sequence length:** 2460
- **5-UTR|CDS|3-UTR identity (%):** 11.76 | 38.65 | 8.38
- **5-UTR|CDS|3-UTR identity (%) [Gaps excluded]:** 68.57 | 76.59 | 78.42
- **5-UTR|CDS|3-UTR identity [Alignment Gaps]:** 338 | 1451 | 3911
- **5-UTR aligned content (<base>:%):** {'T': 27.08, 'G': 25.0, 'C': 39.58, 'A': 8.33}
- **CDS aligned content (<base>:%):** {'A': 30.57, 'T': 25.53, 'G': 24.38, 'C': 19.52}
- **3-UTR aligned content (<base>:%):** {'A': 36.51, 'C': 10.9, 'G': 13.62, 'T': 38.96}

**Uniprot Description:**  
  
 Involved in skeletal muscle development and differentiation. Regulates proliferation and differentiation of myoblasts and plays a role in myofibril assembly by promoting lateral fusion of adjacent thin fibrils into mature, wide myofibrils. Required for pseudopod elongation in transformed cells.   
  
Interacts with NRAP. Interacts with LASP1. Part of a complex that contains CUL3, RBX1 and KLHL41.   
  
 **Gene Ontology Information:**

Molecular Function   
  
N/A

Location

- Cul3-RING ubiquitin ligase complex
- cytoplasm
- cytoskeleton
- cytosol
- endoplasmic reticulum membrane
- M band
- nucleoplasm
- plasma membrane
- pseudopodium
- ruffle
- sarcoplasmic reticulum membrane

Biological process

- myofibril assembly
- protein ubiquitination
- pseudopodium assembly
- regulation of myoblast differentiation
- regulation of myoblast proliferation
- regulation of skeletal muscle cell differentiation
- skeletal muscle cell differentiation
- striated muscle contraction

---

96

- **Protein name:** UDP-GalNAc:beta-1,3-N-acetylgalactosaminyltransferase 2
- **Organism:** Homo sapiens
- **Uniprot Accession Number:** Q8NCR0
- **Protein sequence length:** 500 aa
- **1D identity (%):** 11.79
- **1D identity (%) [Gaps excluded]:** 25.7
- **1D identity - Alignment Gaps:** 505
- **1D aligned content (<aminoacid>:%):** {'M': 0.91, 'L': 12.73, 'P': 13.64, 'G': 8.18, 'A': 6.36, 'H': 2.73, 'Y': 3.64, 'D': 4.55, 'V': 5.45, 'T': 4.55, 'R': 5.45, 'F': 2.73, 'I': 5.45, 'E': 7.27, 'S': 3.64, 'Q': 0.91, 'W': 1.82, 'N': 1.82, 'K': 5.45, 'C': 2.73}
- **Common reported functions (%):** 0.0
- **Common reported locations (%):** 12.5
- **Common reported processes (%):** 0.0

- **AF ID:** Q8NCR0
- **Chain:** A
- **Protein length:** 500 aa
- **Resolution:** N/A
- **b-phipsi:** 0.000656
- **w-rdist:** 0.495664
- **t-alpha:** 0.155515
- **Chemical similarity (Tanimoto Index) (%):** 96.83
- **1D identity (%) [PDB]:** 2.37
- **1D identity (%) [Gaps excluded][PDB]:** 73.81
- **1D identity - Alignment Gaps [PDB]:** 1266
- **1D aligned content [PDB] (<aminoacid>:%):** {'A': 9.68, 'V': 16.13, 'G': 6.45, 'S': 12.9, 'D': 6.45, 'P': 3.23, 'Y': 6.45, 'T': 9.68, 'R': 6.45, 'E': 6.45, 'Q': 6.45, 'L': 6.45, 'I': 3.23}
- **2D identity (%) [PDB]:** 39.29
- **2D identity (%) [Gaps excluded][PDB]:** 86.7
- **2D identity - Alignment Gaps [PDB]:** 508
- **2D aligned content [PDB] (<2D-fold>:%):** {'.': 16.44, 'H': 32.05, 'T': 20.82, 'E': 29.86, 'G': 0.82}
- **3D similarity (TM-Score) (%) [PDB]:** 19.31

- **Gene name:** B3GALNT2
- **Entrez ID:** 148789
- **RefSeq ID:** NM\_152490
- **Transcript sequence length:** 4719
- **5-UTR|CDS|3-UTR identity (%):** 27.64 | 33.42 | 38.52
- **5-UTR|CDS|3-UTR identity (%) [Gaps excluded]:** 78.85 | 75.98 | 75.34
- **5-UTR|CDS|3-UTR identity [Alignment Gaps]:** 289 | 1591 | 2361
- **5-UTR aligned content (<base>:%):** {'G': 38.21, 'C': 50.41, 'T': 8.13, 'A': 3.25}
- **CDS aligned content (<base>:%):** {'A': 27.4, 'T': 25.92, 'G': 25.29, 'C': 21.39}
- **3-UTR aligned content (<base>:%):** {'C': 15.8, 'A': 29.93, 'G': 19.67, 'T': 34.61}

**Uniprot Description:**  
  
 Beta-1,3-N-acetylgalactosaminyltransferase that synthesizes a unique carbohydrate structure, GalNAc-beta-1-3GlcNAc, on N- and O-glycans. Has no galactose nor galactosaminyl transferase activity toward any acceptor substrate. Involved in alpha-dystroglycan (DAG1) glycosylation: acts coordinately with GTDC2/POMGnT2 to synthesize a GalNAc-beta3-GlcNAc-beta-terminus at the 4-position of protein O-mannose in the biosynthesis of the phosphorylated O-mannosyl trisaccharide (N-acetylgalactosamine-beta-3-N-acetylglucosamine-beta-4-(phosphate-6-)mannose), a carbohydrate structure present in alpha-dystroglycan, which is required for binding laminin G-like domain-containing extracellular proteins with high affinity. N/A   
  
 **Gene Ontology Information:**

Molecular Function

- acetylgalactosaminyltransferase activity
- UDP-glycosyltransferase activity

Location

- endoplasmic reticulum
- endoplasmic reticulum membrane
- Golgi membrane
- membrane

Biological process

- protein glycosylation
- protein O-linked glycosylation

---

97

- **Protein name:** Kelch repeat and BTB domain-containing protein 3
- **Organism:** Homo sapiens
- **Uniprot Accession Number:** Q8NAB2
- **Protein sequence length:** 612 aa
- **1D identity (%):** 10.55
- **1D identity (%) [Gaps excluded]:** 21.36
- **1D identity - Alignment Gaps:** 499
- **1D aligned content (<aminoacid>:%):** {'C': 5.77, 'N': 1.92, 'L': 11.54, 'I': 2.88, 'R': 3.85, 'D': 6.73, 'F': 6.73, 'T': 7.69, 'A': 3.85, 'Y': 4.81, 'K': 6.73, 'S': 9.62, 'Q': 2.88, 'G': 7.69, 'E': 2.88, 'V': 6.73, 'P': 4.81, 'H': 1.92, 'W': 0.96}
- **Common reported functions (%):** 0.0
- **Common reported locations (%):** 0.0
- **Common reported processes (%):** 0.0

- **AF ID:** Q8NAB2
- **Chain:** A
- **Protein length:** 612 aa
- **Resolution:** N/A
- **b-phipsi:** 0.000967
- **w-rdist:** 0.754085
- **t-alpha:** 0.028986
- **Chemical similarity (Tanimoto Index) (%):** 99.55
- **1D identity (%) [PDB]:** 3.3
- **1D identity (%) [Gaps excluded][PDB]:** 68.66
- **1D identity - Alignment Gaps [PDB]:** 1328
- **1D aligned content [PDB] (<aminoacid>:%):** {'M': 2.17, 'E': 8.7, 'L': 17.39, 'P': 10.87, 'K': 2.17, 'F': 6.52, 'V': 15.22, 'S': 13.04, 'Q': 4.35, 'G': 2.17, 'H': 2.17, 'D': 4.35, 'A': 2.17, 'I': 2.17, 'T': 2.17, 'R': 2.17, 'Y': 2.17}
- **2D identity (%) [PDB]:** 36.01
- **2D identity (%) [Gaps excluded][PDB]:** 90.84
- **2D identity - Alignment Gaps [PDB]:** 632
- **2D aligned content [PDB] (<2D-fold>:%):** {'.': 18.83, 'T': 21.49, 'H': 40.85, 'E': 17.51, 'G': 0.8, 'B': 0.53}
- **3D similarity (TM-Score) (%) [PDB]:** 20.22

- **Gene name:** KBTBD3
- **Entrez ID:** 143879
- **RefSeq ID:** N/A
- **Sequence length:** N/A
- **5-UTR|CDS|3-UTR identity (%):** N/A | N/A | N/A
- **5-UTR|CDS|3-UTR identity (%) [Gaps excluded]:** N/A | N/A | N/A
- **5-UTR|CDS|3-UTR identity [Alignment Gaps]:** N/A | N/A | N/A
- **5-UTR aligned content (<base>:%):** N/A
- **CDS aligned content (<base>:%):** N/A
- **3-UTR aligned content (<base>:%):** N/A

**Uniprot Description:**  
  
 N/A N/A   
  
 **Gene Ontology Information:**

Molecular Function   
  
N/A

Location   
  
N/A

Biological process   
  
N/A

---

98

- **Protein name:** Cyclic GMP-AMP synthase
- **Organism:** Homo sapiens
- **Uniprot Accession Number:** Q8N884
- **Protein sequence length:** 522 aa
- **1D identity (%):** 12.42
- **1D identity (%) [Gaps excluded]:** 23.89
- **1D identity - Alignment Gaps:** 437
- **1D aligned content (<aminoacid>:%):** {'Q': 6.19, 'G': 8.85, 'A': 7.96, 'M': 1.77, 'K': 9.73, 'S': 4.42, 'P': 9.73, 'R': 7.96, 'D': 6.19, 'T': 3.54, 'E': 6.19, 'W': 1.77, 'I': 2.65, 'V': 5.31, 'L': 7.08, 'F': 3.54, 'N': 1.77, 'C': 2.65, 'H': 0.88, 'Y': 1.77}
- **Common reported functions (%):** 0.0
- **Common reported locations (%):** 37.5
- **Common reported processes (%):** 0.0

- **AF ID:** Q8N884
- **Chain:** A
- **Protein length:** 522 aa
- **Resolution:** N/A
- **b-phipsi:** 0.005934
- **w-rdist:** 0.717033
- **t-alpha:** 0.002347
- **Chemical similarity (Tanimoto Index) (%):** 98.65
- **1D identity (%) [PDB]:** 1.41
- **1D identity (%) [Gaps excluded][PDB]:** 82.61
- **1D identity - Alignment Gaps [PDB]:** 1326
- **1D aligned content [PDB] (<aminoacid>:%):** {'M': 5.26, 'K': 10.53, 'A': 15.79, 'R': 10.53, 'S': 15.79, 'P': 5.26, 'D': 5.26, 'Q': 5.26, 'E': 5.26, 'I': 5.26, 'L': 5.26, 'V': 5.26, 'N': 5.26}
- **2D identity (%) [PDB]:** 29.16
- **2D identity (%) [Gaps excluded][PDB]:** 85.14
- **2D identity - Alignment Gaps [PDB]:** 672
- **2D aligned content [PDB] (<2D-fold>:%):** {'.': 23.15, 'T': 32.89, 'H': 32.21, 'E': 11.74}
- **3D similarity (TM-Score) (%) [PDB]:** 18.79

- **Gene name:** CGAS
- **Entrez ID:** 115004
- **RefSeq ID:** NM\_138441
- **Transcript sequence length:** 3201
- **5-UTR|CDS|3-UTR identity (%):** 18.09 | 36.91 | 18.92
- **5-UTR|CDS|3-UTR identity (%) [Gaps excluded]:** 68.52 | 73.51 | 77.87
- **5-UTR|CDS|3-UTR identity [Alignment Gaps]:** 301 | 1377 | 3533
- **5-UTR aligned content (<base>:%):** {'G': 28.38, 'C': 40.54, 'T': 22.97, 'A': 8.11}
- **CDS aligned content (<base>:%):** {'A': 31.54, 'T': 20.27, 'G': 26.35, 'C': 21.84}
- **3-UTR aligned content (<base>:%):** {'A': 28.09, 'T': 36.81, 'G': 19.82, 'C': 15.29}

**Uniprot Description:**  
  
 Nucleotidyltransferase that catalyzes the formation of cyclic GMP-AMP (cGAMP) from ATP and GTP and plays a key role in innate immunity (PubMed:23258413, PubMed:23707061, PubMed:23722159, PubMed:24077100, PubMed:25131990, PubMed:29976794, PubMed:30799039). Catalysis involves both the formation of a 2',5' phosphodiester linkage at the GpA step and the formation of a 3',5' phosphodiester linkage at the ApG step, producing c[G(2',5')pA(3',5')p] (PubMed:28363908, PubMed:28214358). Acts as a key cytosolic DNA sensor, the presence of double-stranded DNA (dsDNA) in the cytoplasm being a danger signal that triggers the immune responses (PubMed:28363908). Binds cytosolic DNA directly, leading to activation and synthesis of cGAMP, a second messenger that binds to and activates TMEM173/STING, thereby triggering type-I interferon production (PubMed:28363908, PubMed:28314590). Preferentially recognizes and binds curved long DNAs (PubMed:30007416). In contrast to other mammals, human CGAS displays species-specific mechanisms of DNA recognition and produces less cyclic GMP-AMP (cGAMP), allowing a more fine-tuned response to pathogens (PubMed:30007416). Has antiviral activity by sensing the presence of dsDNA from DNA viruses in the cytoplasm (PubMed:28363908). Also acts as an innate immune sensor of infection by retroviruses, such as HIV-1, by detecting the presence of reverse-transcribed DNA in the cytosol (PubMed:23929945). Detection of retroviral reverse-transcribed DNA in the cytosol may be indirect and be mediated via interaction with PQBP1, which directly binds reverse-transcribed retroviral DNA (PubMed:26046437). Also detects the presence of DNA from bacteria, such as M.tuberculosis (PubMed:26048138). cGAMP can be transferred from producing cells to neighboring cells through gap junctions, leading to promote TMEM173/STING activation and convey immune response to connecting cells (PubMed:24077100). cGAMP can also be transferred between cells by virtue of packaging within viral particles contributing to IFN-induction in newly infected cells in a cGAS-independent but TMEM173/STING-dependent manner (PubMed:26229115). In addition to antiviral activity, also involved in the response to cellular stresses, such as senescence, DNA damage or genome instability (PubMed:28738408, PubMed:28759889). Acts as a regulator of cellular senescence by binding to cytosolic chromatin fragments that are present in senescent cells, leading to trigger type-I interferon production via TMEM173/STING and promote cellular senescence (By similarity). Also involved in the inflammatory response to genome instability and double-stranded DNA breaks: acts by localizing to micronuclei arising from genome instability (PubMed:28738408, PubMed:28759889). Micronuclei, which as frequently found in cancer cells, consist of chromatin surrounded by its own nuclear membrane: following breakdown of the micronuclear envelope, a process associated with chromothripsis, CGAS binds self-DNA exposed to the cytosol, leading to cGAMP synthesis and subsequent activation of TMEM173/STING and type-I interferon production (PubMed:28738408, PubMed:28759889). Acts as a suppressor of DNA repair in response to DNA damage: translocates to the nucleus following dephosphorylation at Tyr-215 and inhibits homologous recombination repair by interacting with PARP1, the CGAS-PARP1 interaction leading to impede the formation of the PARP1-TIMELESS complex (PubMed:30356214).   
  
Monomer in the absence of DNA (PubMed:28363908). Homodimer in presence of dsDNA: forms a 2:2 dimer with two enzymes binding to two DNA molecules (PubMed:30007416, PubMed:30799039). Interacts with PQBP1 (via WW domain) (PubMed:26046437). Interacts with TRIM14; this interaction stabilizes CGAS and promotes type I interferon production (PubMed:27666593, PubMed:32404352). Interacts with ZCCHC3; promoting sensing of dsDNA by CGAS (PubMed:30135424). Interacts with PARP1; interaction takes place in the nucleus and prevents the formation of the PARP1-TIMELESS complex (PubMed:30356214).   
  
 **Gene Ontology Information:**

Molecular Function

- cyclic-GMP-AMP synthase activity
- ATP binding
- chromatin binding
- DNA binding
- double-stranded DNA binding
- GTP binding
- metal ion binding
- molecular condensate scaffold activity
- nucleosome binding
- phosphatidylinositol-4,5-bisphosphate binding
- poly-ADP-D-ribose modification-dependent protein binding
- protein homodimerization activity

Location

- cytoplasm
- cytosol
- nuclear body
- nucleoplasm
- nucleus
- plasma membrane
- site of double-strand break

Biological process

- activation of innate immune response
- cAMP-mediated signaling
- cellular response to exogenous dsRNA
- cGMP-mediated signaling
- cytoplasmic pattern recognition receptor signaling pathway
- defense response to virus
- determination of adult lifespan
- cellular response to DNA damage stimulus
- DNA repair
- innate immune response
- negative regulation of double-strand break repair via homologous recombination
- negative regulation of innate immune response
- paracrine signaling
- pattern recognition receptor signaling pathway
- positive regulation of cellular senescence
- positive regulation of defense response to virus by host
- positive regulation of type I interferon production
- regulation of immunoglobulin production
- regulation of innate immune response
- regulation of T cell activation

---

99

- **Protein name:** NXPE family member 1
- **Organism:** Homo sapiens
- **Uniprot Accession Number:** Q8N323
- **Protein sequence length:** 547 aa
- **1D identity (%):** 11.58
- **1D identity (%) [Gaps excluded]:** 25.4
- **1D identity - Alignment Gaps:** 526
- **1D aligned content (<aminoacid>:%):** {'T': 5.36, 'L': 13.39, 'F': 3.57, 'V': 7.14, 'K': 8.93, 'I': 5.36, 'P': 7.14, 'E': 4.46, 'R': 7.14, 'H': 4.46, 'G': 8.04, 'Y': 3.57, 'S': 2.68, 'W': 0.89, 'A': 4.46, 'N': 1.79, 'D': 5.36, 'C': 3.57, 'Q': 2.68}
- **Common reported functions (%):** 0.0
- **Common reported locations (%):** 0.0
- **Common reported processes (%):** 0.0

- **AF ID:** Q8N323
- **Chain:** A
- **Protein length:** 547 aa
- **Resolution:** N/A
- **b-phipsi:** 0.000883
- **w-rdist:** 0.818263
- **t-alpha:** 0.020767
- **Chemical similarity (Tanimoto Index) (%):** 96.62
- **1D identity (%) [PDB]:** 1.98
- **1D identity (%) [Gaps excluded][PDB]:** 81.82
- **1D identity - Alignment Gaps [PDB]:** 1331
- **1D aligned content [PDB] (<aminoacid>:%):** {'S': 7.41, 'V': 11.11, 'L': 25.93, 'Q': 7.41, 'E': 7.41, 'A': 11.11, 'G': 3.7, 'D': 11.11, 'I': 3.7, 'T': 3.7, 'R': 3.7, 'H': 3.7}
- **2D identity (%) [PDB]:** 39.71
- **2D identity (%) [Gaps excluded][PDB]:** 89.3
- **2D identity - Alignment Gaps [PDB]:** 537
- **2D aligned content [PDB] (<2D-fold>:%):** {'.': 11.98, 'H': 33.85, 'E': 29.69, 'T': 24.22, 'B': 0.26}
- **3D similarity (TM-Score) (%) [PDB]:** 20.91

- **Gene name:** NXPE1
- **Entrez ID:** 120400
- **RefSeq ID:** N/A
- **Sequence length:** N/A
- **5-UTR|CDS|3-UTR identity (%):** N/A | N/A | N/A
- **5-UTR|CDS|3-UTR identity (%) [Gaps excluded]:** N/A | N/A | N/A
- **5-UTR|CDS|3-UTR identity [Alignment Gaps]:** N/A | N/A | N/A
- **5-UTR aligned content (<base>:%):** N/A
- **CDS aligned content (<base>:%):** N/A
- **3-UTR aligned content (<base>:%):** N/A

**Uniprot Description:**  
  
 N/A N/A   
  
 **Gene Ontology Information:**

Molecular Function   
  
N/A

Location

- extracellular region

Biological process   
  
N/A

---

100

- **Protein name:** DENN domain-containing protein 11
- **Organism:** Homo sapiens
- **Uniprot Accession Number:** A4D1U4
- **Protein sequence length:** 455 aa
- **1D identity (%):** 8.31
- **1D identity (%) [Gaps excluded]:** 23.75
- **1D identity - Alignment Gaps:** 634
- **1D aligned content (<aminoacid>:%):** {'Q': 6.17, 'L': 12.35, 'S': 6.17, 'P': 11.11, 'G': 11.11, 'E': 4.94, 'V': 8.64, 'A': 4.94, 'W': 1.23, 'D': 2.47, 'F': 3.7, 'K': 8.64, 'C': 2.47, 'Y': 6.17, 'H': 2.47, 'N': 1.23, 'I': 3.7, 'R': 2.47}
- **Common reported functions (%):** 0.0
- **Common reported locations (%):** 12.5
- **Common reported processes (%):** 0.0

- **AF ID:** A4D1U4
- **Chain:** A
- **Protein length:** 455 aa
- **Resolution:** N/A
- **b-phipsi:** 0.004491
- **w-rdist:** 0.754001
- **t-alpha:** 0.002347
- **Chemical similarity (Tanimoto Index) (%):** 98.5
- **1D identity (%) [PDB]:** 2.7
- **1D identity (%) [Gaps excluded][PDB]:** 70.83
- **1D identity - Alignment Gaps [PDB]:** 1209
- **1D aligned content [PDB] (<aminoacid>:%):** {'P': 5.88, 'A': 8.82, 'G': 11.76, 'V': 14.71, 'S': 8.82, 'M': 2.94, 'H': 2.94, 'R': 2.94, 'Y': 5.88, 'C': 2.94, 'T': 5.88, 'Q': 11.76, 'E': 5.88, 'L': 5.88, 'D': 2.94}
- **2D identity (%) [PDB]:** 28.74
- **2D identity (%) [Gaps excluded][PDB]:** 92.26
- **2D identity - Alignment Gaps [PDB]:** 685
- **2D aligned content [PDB] (<2D-fold>:%):** {'.': 17.83, 'E': 18.88, 'T': 17.83, 'H': 44.41, 'G': 1.05}
- **3D similarity (TM-Score) (%) [PDB]:** 18.69

- **Gene name:** DENND11
- **Entrez ID:** 556432
- **RefSeq ID:** NM\_001080392
- **Transcript sequence length:** 7309
- **5-UTR|CDS|3-UTR identity (%):** 2.73 | 29.34 | 43.52
- **5-UTR|CDS|3-UTR identity (%) [Gaps excluded]:** 100.0 | 75.36 | 76.06
- **5-UTR|CDS|3-UTR identity [Alignment Gaps]:** 392 | 1738 | 2779
- **5-UTR aligned content (<base>:%):** {'G': 27.27, 'C': 63.64, 'T': 9.09}
- **CDS aligned content (<base>:%):** {'A': 22.28, 'T': 20.0, 'G': 29.1, 'C': 28.62}
- **3-UTR aligned content (<base>:%):** {'G': 22.39, 'A': 27.91, 'C': 16.34, 'T': 33.36}

**Uniprot Description:**  
  
 Probable guanine nucleotide exchange factor (GEF). May promote the exchange of GDP to GTP, converting inactive GDP-bound small GTPases into their active GTP-bound form (Probable). May play a role in neuritogenesis, as well as in neuronal recovery and/or restructuring in the hippocampus following transient cerebral ischemia (By similarity). N/A   
  
 **Gene Ontology Information:**

Molecular Function

- guanyl-nucleotide exchange factor activity

Location

- cytoplasm

Biological process   
  
N/A

---
