# Supplementary material for: Comparative structural insights and functional analysis for the distinct unbound states of Human AGO proteins
Source: Sci Rep. 2025 Mar 19;15:9432. doi: 10.1038/s41598-025-91849-5 (PMC11923369; doi:10.1038/s41598-025-91849-5)
Supplement: Supplementary file 24 — Supplementary Information 12. [file 41598_2025_91849_MOESM24_ESM.zip › 6OONp_A_mdwhole_AF4REF/candidates/6OONp_A-merged-h-enriched_report.html]

 

# Structural Comparison Report for 6OONp\_A - whole structures (total: 100)

---

0

- **AF ID:** AF-Q9H9G7-F1-model-v4 | **Chain:** A
- **b-phipsi:** 0.0006082477711518
- **w-rdist:** 0.0809331213187695
- **t-alpha:** 0.0297336873714129

---

---

1

- **AF ID:** AF-Q9UL18-F1-model-v4 | **Chain:** A
- **b-phipsi:** 0.0005488876760193
- **w-rdist:** 0.0471138153369363
- **t-alpha:** 0.0391232045333622

---

---

2

- **AF ID:** AF-Q9H0H3-F1-model-v4 | **Chain:** A
- **b-phipsi:** 0.0009658069246138
- **w-rdist:** 0.5906484623943691
- **t-alpha:** 0.0007823187912934

---

---

3

- **AF ID:** AF-Q06187-F1-model-v4 | **Chain:** A
- **b-phipsi:** 0.0025801392520136
- **w-rdist:** 0.3731656749039853
- **t-alpha:** 0.0015647720218643

---

---

4

- **AF ID:** AF-P37173-F1-model-v4 | **Chain:** A
- **b-phipsi:** 0.0010753437965379
- **w-rdist:** 0.5771545397070541
- **t-alpha:** 0.0023473590503262

---

---

5

- **AF ID:** AF-B6SEH8-F1-model-v4 | **Chain:** A
- **b-phipsi:** 0.0028783603695361
- **w-rdist:** 0.422257141719303
- **t-alpha:** 0.0007832732121422

---

---

6

- **AF ID:** AF-Q9UL63-F1-model-v4 | **Chain:** A
- **b-phipsi:** 0.0015964987032425
- **w-rdist:** 0.2718826415725286
- **t-alpha:** 0.0140843739056586

---

---

7

- **AF ID:** AF-O43374-F1-model-v4 | **Chain:** A
- **b-phipsi:** 0.0021965532556254
- **w-rdist:** 0.1918019455109033
- **t-alpha:** 0.0232189692826079

---

---

8

- **AF ID:** AF-C9J798-F1-model-v4 | **Chain:** A
- **b-phipsi:** 0.0022736472330602
- **w-rdist:** 0.2297129503044357
- **t-alpha:** 0.0142860356016105

---

---

9

- **AF ID:** AF-Q16671-F1-model-v4 | **Chain:** A
- **b-phipsi:** 0.0053084586583112
- **w-rdist:** 0.3555357284910318
- **t-alpha:** 0.0023530945443304

---

---

10

- **AF ID:** AF-Q9NY28-F1-model-v4 | **Chain:** A
- **b-phipsi:** 0.0037462662511197
- **w-rdist:** 0.5171167764400909
- **t-alpha:** 0.0

---

---

11

- **AF ID:** AF-Q9Y2E5-F1-model-v4 | **Chain:** A
- **b-phipsi:** 0.0021563529973776
- **w-rdist:** 0.2829987654994442
- **t-alpha:** 0.0117370267412904

---

---

12

- **AF ID:** AF-Q9Y2X0-F1-model-v4 | **Chain:** A
- **b-phipsi:** 0.0010487916794057
- **w-rdist:** 0.374255156718853
- **t-alpha:** 0.0226916757534991

---

---

13

- **AF ID:** AF-Q96PZ2-F1-model-v4 | **Chain:** A
- **b-phipsi:** 0.0029466645158733
- **w-rdist:** 0.2464580378377701
- **t-alpha:** 0.0126786930545719

---

---

14

- **AF ID:** AF-Q92935-F1-model-v4 | **Chain:** A
- **b-phipsi:** 0.0057054008677965
- **w-rdist:** 0.3954906976976218
- **t-alpha:** 0.0015676375257613

---

---

15

- **AF ID:** AF-O95294-F1-model-v4 | **Chain:** A
- **b-phipsi:** 0.0041723581026218
- **w-rdist:** 0.1923965260098805
- **t-alpha:** 0.0224000982062968

---

---

16

- **AF ID:** AF-Q53H54-F1-model-v4 | **Chain:** A
- **b-phipsi:** 0.0022597099387611
- **w-rdist:** 0.4370179899706327
- **t-alpha:** 0.0031401242085145

---

---

17

- **AF ID:** AF-Q8IXQ5-F1-model-v4 | **Chain:** A
- **b-phipsi:** 0.0025263575904233
- **w-rdist:** 0.6766863313231747
- **t-alpha:** 0.0007832732121422

---

---

18

- **AF ID:** AF-Q7Z3Z4-F1-model-v4 | **Chain:** A
- **b-phipsi:** 0.0024924565329652
- **w-rdist:** 0.1902126057300489
- **t-alpha:** 0.0492955855664358

---

---

19

- **AF ID:** AF-Q3ZCT8-F1-model-v4 | **Chain:** A
- **b-phipsi:** 0.000673013286145
- **w-rdist:** 0.6385934583572589
- **t-alpha:** 0.0156493910563146

---

---

20

- **AF ID:** AF-Q9Y6W3-F1-model-v4 | **Chain:** A
- **b-phipsi:** 0.0008155983927349
- **w-rdist:** 0.6126130081976835
- **t-alpha:** 0.0102767895279776

---

---

21

- **AF ID:** AF-P31321-F1-model-v4 | **Chain:** A
- **b-phipsi:** 0.0009698379643694
- **w-rdist:** 0.4039244825430616
- **t-alpha:** 0.0398701801688734

---

---

22

- **AF ID:** AF-Q96J94-F1-model-v4 | **Chain:** A
- **b-phipsi:** 0.0052235119836145
- **w-rdist:** 0.2063300545558366
- **t-alpha:** 0.0297336873714129

---

---

23

- **AF ID:** AF-Q86TM3-F1-model-v4 | **Chain:** A
- **b-phipsi:** 0.0038767851812322
- **w-rdist:** 0.2984959190492869
- **t-alpha:** 0.0234741774196856

---

---

24

- **AF ID:** AF-Q9H0J9-F1-model-v4 | **Chain:** A
- **b-phipsi:** 0.0007668986855758
- **w-rdist:** 0.3677386203406908
- **t-alpha:** 0.1181102422121964

---

---

25

- **AF ID:** AF-Q8N3Y3-F1-model-v4 | **Chain:** A
- **b-phipsi:** 0.0117856045390715
- **w-rdist:** 0.3626405275199351
- **t-alpha:** 0.0023473590503262

---

---

26

- **AF ID:** AF-Q08380-F1-model-v4 | **Chain:** A
- **b-phipsi:** 0.0014185126091855
- **w-rdist:** 0.3415998057158072
- **t-alpha:** 0.0432653326797045

---

---

27

- **AF ID:** AF-Q8IY47-F1-model-v4 | **Chain:** A
- **b-phipsi:** 0.0020730120701645
- **w-rdist:** 0.3688327876779206
- **t-alpha:** 0.0187789021729365

---

---

28

- **AF ID:** AF-Q8NCE0-F1-model-v4 | **Chain:** A
- **b-phipsi:** 0.0079225131894249
- **w-rdist:** 0.5188302430596078
- **t-alpha:** 0.0007832732121422

---

---

29

- **AF ID:** AF-O95831-F1-model-v4 | **Chain:** A
- **b-phipsi:** 0.0060937092110865
- **w-rdist:** 0.5503907173731932
- **t-alpha:** 0.0023473590503262

---

---

30

- **AF ID:** AF-Q504Y2-F1-model-v4 | **Chain:** A
- **b-phipsi:** 0.0031017693826659
- **w-rdist:** 0.7583343456823548
- **t-alpha:** 0.0

---

---

31

- **AF ID:** AF-P54646-F1-model-v4 | **Chain:** A
- **b-phipsi:** 0.004041927583506
- **w-rdist:** 0.3883711526699795
- **t-alpha:** 0.0047169960791435

---

---

32

- **AF ID:** AF-Q96NY9-F1-model-v4 | **Chain:** A
- **b-phipsi:** 0.0101337283249819
- **w-rdist:** 0.1788159191616689
- **t-alpha:** 0.0086070234951727

---

---

33

- **AF ID:** AF-P98182-F1-model-v4 | **Chain:** A
- **b-phipsi:** 0.006280949928769
- **w-rdist:** 0.613066644485438
- **t-alpha:** 0.0015647720218643

---

---

34

- **AF ID:** AF-Q9NTG7-F1-model-v4 | **Chain:** A
- **b-phipsi:** 0.0012315145768263
- **w-rdist:** 0.8346919203560298
- **t-alpha:** 0.0023530945443304

---

---

35

- **AF ID:** AF-Q06210-F1-model-v4 | **Chain:** A
- **b-phipsi:** 0.0030076987088355
- **w-rdist:** 0.6999402583007417
- **t-alpha:** 0.0023473590503262

---

---

36

- **AF ID:** AF-Q13705-F1-model-v4 | **Chain:** A
- **b-phipsi:** 0.0024073390364737
- **w-rdist:** 0.7862377346423994
- **t-alpha:** 0.0015647720218643

---

---

37

- **AF ID:** AF-Q16819-F1-model-v4 | **Chain:** A
- **b-phipsi:** 0.0105537537401441
- **w-rdist:** 0.2326532513698452
- **t-alpha:** 0.0078243548762237

---

---

38

- **AF ID:** AF-Q15831-F1-model-v4 | **Chain:** A
- **b-phipsi:** 0.0053172161938833
- **w-rdist:** 0.7177085104830545
- **t-alpha:** 0.0007823187912934

---

---

39

- **AF ID:** AF-Q13131-F1-model-v4 | **Chain:** A
- **b-phipsi:** 0.003275660300548
- **w-rdist:** 0.3450256391594226
- **t-alpha:** 0.0336461458262049

---

---

40

- **AF ID:** AF-Q9BRP7-F1-model-v4 | **Chain:** A
- **b-phipsi:** 0.0009136839641332
- **w-rdist:** 0.459969568131855
- **t-alpha:** 0.0739499805270313

---

---

41

- **AF ID:** AF-Q8IXK2-F1-model-v4 | **Chain:** A
- **b-phipsi:** 0.0032235779258749
- **w-rdist:** 0.5663772417765246
- **t-alpha:** 0.0039277718342689

---

---

42

- **AF ID:** AF-Q9NZ20-F1-model-v4 | **Chain:** A
- **b-phipsi:** 0.005005401933765
- **w-rdist:** 0.4637674104004329
- **t-alpha:** 0.0039277718342689

---

---

43

- **AF ID:** AF-Q9NRJ5-F1-model-v4 | **Chain:** A
- **b-phipsi:** 0.0008311364239527
- **w-rdist:** 0.3137868107210553
- **t-alpha:** 0.2683872843872745

---

---

44

- **AF ID:** AF-Q8TBY0-F1-model-v4 | **Chain:** A
- **b-phipsi:** 0.0052728005694042
- **w-rdist:** 0.2736711089191508
- **t-alpha:** 0.0539902689916904

---

---

45

- **AF ID:** AF-Q8TAP6-F1-model-v4 | **Chain:** A
- **b-phipsi:** 0.0005521581170716
- **w-rdist:** 0.5441462325083098
- **t-alpha:** 0.1173704658131864

---

---

46

- **AF ID:** AF-Q9HAU4-F1-model-v4 | **Chain:** A
- **b-phipsi:** 0.002129229911346
- **w-rdist:** 0.2511792535055234
- **t-alpha:** 0.1205008021373417

---

---

47

- **AF ID:** AF-Q68DD2-F1-model-v4 | **Chain:** A
- **b-phipsi:** 0.001894887260993
- **w-rdist:** 0.2027922104009692
- **t-alpha:** 0.1384973100154414

---

---

48

- **AF ID:** AF-O15033-F1-model-v4 | **Chain:** A
- **b-phipsi:** 0.0008825173223327
- **w-rdist:** 0.7342760840752007
- **t-alpha:** 0.0164318357763795

---

---

49

- **AF ID:** AF-Q15796-F1-model-v4 | **Chain:** A
- **b-phipsi:** 0.000531006656881
- **w-rdist:** 0.6133324827408306
- **t-alpha:** 0.0941781028703137

---

---

50

- **AF ID:** AF-O94955-F1-model-v4 | **Chain:** A
- **b-phipsi:** 0.0187903813719031
- **w-rdist:** 0.394584095049605
- **t-alpha:** 0.0007832732121422

---

---

51

- **AF ID:** AF-Q01518-F1-model-v4 | **Chain:** A
- **b-phipsi:** 0.0020425492773036
- **w-rdist:** 0.3675642108384912
- **t-alpha:** 0.0586852591928757

---

---

52

- **AF ID:** AF-Q9NVX7-F1-model-v4 | **Chain:** A
- **b-phipsi:** 0.0005870839659491
- **w-rdist:** 0.580331796703812
- **t-alpha:** 0.1132408945125096

---

---

53

- **AF ID:** AF-Q92542-F1-model-v4 | **Chain:** A
- **b-phipsi:** 0.0046871231220074
- **w-rdist:** 0.7162751893874599
- **t-alpha:** 0.0015676375257613

---

---

54

- **AF ID:** AF-O14841-F1-model-v4 | **Chain:** A
- **b-phipsi:** 0.0018508876639965
- **w-rdist:** 0.3424202823833285
- **t-alpha:** 0.0821594238081062

---

---

55

- **AF ID:** AF-Q14181-F1-model-v4 | **Chain:** A
- **b-phipsi:** 0.0005131088081659
- **w-rdist:** 0.8160902169913542
- **t-alpha:** 0.0359934414192668

---

---

56

- **AF ID:** AF-Q6PJQ5-F1-model-v4 | **Chain:** A
- **b-phipsi:** 0.0050675165985821
- **w-rdist:** 0.3483093812479473
- **t-alpha:** 0.0323103167719089

---

---

57

- **AF ID:** AF-O43776-F1-model-v4 | **Chain:** A
- **b-phipsi:** 0.0010380372368401
- **w-rdist:** 0.7397131137007908
- **t-alpha:** 0.0086070234951727

---

---

58

- **AF ID:** AF-Q969P6-F1-model-v4 | **Chain:** A
- **b-phipsi:** 0.0127892371139456
- **w-rdist:** 0.2403093533997815
- **t-alpha:** 0.011876942219535

---

---

59

- **AF ID:** AF-Q8NBJ5-F1-model-v4 | **Chain:** A
- **b-phipsi:** 0.0010710292470885
- **w-rdist:** 0.5413990545686231
- **t-alpha:** 0.0703518669333997

---

---

60

- **AF ID:** AF-O15530-F1-model-v4 | **Chain:** A
- **b-phipsi:** 0.0050652854798716
- **w-rdist:** 0.6011602643885208
- **t-alpha:** 0.0039119745263893

---

---

61

- **AF ID:** AF-Q8N3E9-F1-model-v4 | **Chain:** A
- **b-phipsi:** 0.0030931875106035
- **w-rdist:** 0.3940216381266235
- **t-alpha:** 0.0250390222700223

---

---

62

- **AF ID:** AF-O15067-F1-model-v4 | **Chain:** A
- **b-phipsi:** 0.002289840601717
- **w-rdist:** 0.2771953747719335
- **t-alpha:** 0.1283255184376883

---

---

63

- **AF ID:** AF-Q8NA66-F1-model-v4 | **Chain:** A
- **b-phipsi:** 0.0011252635660009
- **w-rdist:** 0.7021758355182107
- **t-alpha:** 0.0219087977837508

---

---

64

- **AF ID:** AF-Q5T447-F1-model-v4 | **Chain:** A
- **b-phipsi:** 0.0023954232942157
- **w-rdist:** 0.3380629402529312
- **t-alpha:** 0.0884191622029941

---

---

65

- **AF ID:** AF-Q14145-F1-model-v4 | **Chain:** A
- **b-phipsi:** 0.0025441850172837
- **w-rdist:** 0.8529921188325944
- **t-alpha:** 0.0015676375257613

---

---

66

- **AF ID:** AF-Q6ICG6-F1-model-v4 | **Chain:** A
- **b-phipsi:** 0.0102409132295435
- **w-rdist:** 0.5755137558544103
- **t-alpha:** 0.0023473590503262

---

---

67

- **AF ID:** AF-P16220-F1-model-v4 | **Chain:** A
- **b-phipsi:** 0.0046968220705803
- **w-rdist:** 0.8571982504534672
- **t-alpha:** 0.0

---

---

68

- **AF ID:** AF-Q9P2R7-F1-model-v4 | **Chain:** A
- **b-phipsi:** 0.0010202693044081
- **w-rdist:** 0.6555461507486418
- **t-alpha:** 0.0501234795277865

---

---

69

- **AF ID:** AF-O60662-F1-model-v4 | **Chain:** A
- **b-phipsi:** 0.0013099724511646
- **w-rdist:** 0.5277904848872851
- **t-alpha:** 0.0466830494629222

---

---

70

- **AF ID:** AF-Q8NCR0-F1-model-v4 | **Chain:** A
- **b-phipsi:** 0.0006558418936457
- **w-rdist:** 0.4956641970087056
- **t-alpha:** 0.1555154353376837

---

---

71

- **AF ID:** AF-Q8NAB2-F1-model-v4 | **Chain:** A
- **b-phipsi:** 0.0009665576015688
- **w-rdist:** 0.7540849460622907
- **t-alpha:** 0.0289856519083209

---

---

72

- **AF ID:** AF-Q8N884-F1-model-v4 | **Chain:** A
- **b-phipsi:** 0.0059340146424255
- **w-rdist:** 0.7170326865574234
- **t-alpha:** 0.0023473590503262

---

---

73

- **AF ID:** AF-Q8N323-F1-model-v4 | **Chain:** A
- **b-phipsi:** 0.0008833471144211
- **w-rdist:** 0.8182625132781132
- **t-alpha:** 0.0207667552249124

---

---

74

- **AF ID:** AF-Q86XP0-F1-model-v4 | **Chain:** A
- **b-phipsi:** 0.0030958282451962
- **w-rdist:** 0.3163050401995855
- **t-alpha:** 0.0907665259446537

---

---

75

- **AF ID:** AF-Q460N3-F1-model-v4 | **Chain:** A
- **b-phipsi:** 0.003512935041836
- **w-rdist:** 0.2908586107215489
- **t-alpha:** 0.1025037952669631

---

---

76

- **AF ID:** AF-P0C869-F1-model-v4 | **Chain:** A
- **b-phipsi:** 0.0029611258532422
- **w-rdist:** 0.3625778842643337
- **t-alpha:** 0.064162546478931

---

---

77

- **AF ID:** AF-P18031-F1-model-v4 | **Chain:** A
- **b-phipsi:** 0.0013886872279637
- **w-rdist:** 0.6223918477838714
- **t-alpha:** 0.0219087977837508

---

---

78

- **AF ID:** AF-Q8WXF8-F1-model-v4 | **Chain:** A
- **b-phipsi:** 0.0022183421504646
- **w-rdist:** 0.4007357968861972
- **t-alpha:** 0.0424145126110251

---

---

79

- **AF ID:** AF-Q9H1Q7-F1-model-v4 | **Chain:** A
- **b-phipsi:** 0.0075476215223086
- **w-rdist:** 0.5903199475855297
- **t-alpha:** 0.0031401242085145

---

---

80

- **AF ID:** AF-Q16566-F1-model-v4 | **Chain:** A
- **b-phipsi:** 0.0061577706101379
- **w-rdist:** 0.6449894807641368
- **t-alpha:** 0.0031401242085145

---

---

81

- **AF ID:** AF-Q96NJ5-F1-model-v4 | **Chain:** A
- **b-phipsi:** 0.0012361643088472
- **w-rdist:** 0.5715271752374301
- **t-alpha:** 0.0570720736347449

---

---

82

- **AF ID:** AF-Q96ME1-F1-model-v4 | **Chain:** A
- **b-phipsi:** 0.0036992654331515
- **w-rdist:** 0.3754278083567123
- **t-alpha:** 0.0422534769714337

---

---

83

- **AF ID:** AF-Q96RP9-F1-model-v4 | **Chain:** A
- **b-phipsi:** 0.0074185272757875
- **w-rdist:** 0.7571403838754567
- **t-alpha:** 0.0007832732121422

---

---

84

- **AF ID:** AF-Q6UWF7-F1-model-v4 | **Chain:** A
- **b-phipsi:** 0.0014805872145444
- **w-rdist:** 0.8143664274698216
- **t-alpha:** 0.0039119745263893

---

---

85

- **AF ID:** AF-Q96BZ4-F1-model-v4 | **Chain:** A
- **b-phipsi:** 0.0002827056820133
- **w-rdist:** 0.749901178752813
- **t-alpha:** 0.0876596132533018

---

---

86

- **AF ID:** AF-Q9BZQ2-F1-model-v4 | **Chain:** A
- **b-phipsi:** 0.0007509972854688
- **w-rdist:** 0.6834564675775673
- **t-alpha:** 0.0868544688919852

---

---

87

- **AF ID:** AF-A8MX76-F1-model-v4 | **Chain:** A
- **b-phipsi:** 0.0007437942839194
- **w-rdist:** 0.7009440755721825
- **t-alpha:** 0.0812186237826302

---

---

88

- **AF ID:** AF-Q9NZW5-F1-model-v4 | **Chain:** A
- **b-phipsi:** 0.0014487223008964
- **w-rdist:** 0.5508673999762334
- **t-alpha:** 0.0336461458262049

---

---

89

- **AF ID:** AF-Q6ZTN6-F1-model-v4 | **Chain:** A
- **b-phipsi:** 0.0010370312073688
- **w-rdist:** 0.620308973734857
- **t-alpha:** 0.0719873632833352

---

---

90

- **AF ID:** AF-P47972-F1-model-v4 | **Chain:** A
- **b-phipsi:** 0.0023070652183437
- **w-rdist:** 0.8140509108943518
- **t-alpha:** 0.0031401242085145

---

---

91

- **AF ID:** AF-Q9NZ08-F1-model-v4 | **Chain:** A
- **b-phipsi:** 0.0056930420954221
- **w-rdist:** 0.3089415955833576
- **t-alpha:** 0.0727697286543513

---

---

92

- **AF ID:** AF-Q08881-F1-model-v4 | **Chain:** A
- **b-phipsi:** 0.0013653212074157
- **w-rdist:** 0.5823374416879894
- **t-alpha:** 0.0390246061928452

---

---

93

- **AF ID:** AF-Q86TI2-F1-model-v4 | **Chain:** A
- **b-phipsi:** 0.0056444429676834
- **w-rdist:** 0.5529801421221743
- **t-alpha:** 0.0054771144938834

---

---

94

- **AF ID:** AF-Q14697-F1-model-v4 | **Chain:** A
- **b-phipsi:** 0.0093244547030807
- **w-rdist:** 0.3722621180514959
- **t-alpha:** 0.0086820531296027

---

---

95

- **AF ID:** AF-P98170-F1-model-v4 | **Chain:** A
- **b-phipsi:** 0.0097380224802077
- **w-rdist:** 0.262678154058052
- **t-alpha:** 0.064162546478931

---

---

96

- **AF ID:** AF-Q8NBE8-F1-model-v4 | **Chain:** A
- **b-phipsi:** 0.0034845980933919
- **w-rdist:** 0.6019377342628524
- **t-alpha:** 0.0062593997286479

---

---

97

- **AF ID:** AF-Q96RQ3-F1-model-v4 | **Chain:** A
- **b-phipsi:** 0.0048413328532891
- **w-rdist:** 0.3407121828461258
- **t-alpha:** 0.0743346359382088

---

---

98

- **AF ID:** AF-P84022-F1-model-v4 | **Chain:** A
- **b-phipsi:** 0.0005660915298645
- **w-rdist:** 0.6978749259746404
- **t-alpha:** 0.1230228438240716

---

---

99

- **AF ID:** AF-Q13939-F1-model-v4 | **Chain:** A
- **b-phipsi:** 0.0007165498406963
- **w-rdist:** 0.5187326601880891
- **t-alpha:** 0.1844303389458708

---

---
