# Supplementary material for: Comparative structural insights and functional analysis for the distinct unbound states of Human AGO proteins
Source: Sci Rep. 2025 Mar 19;15:9432. doi: 10.1038/s41598-025-91849-5 (PMC11923369; doi:10.1038/s41598-025-91849-5)
Supplement: Supplementary file 24 — Supplementary Information 12. [file 41598_2025_91849_MOESM24_ESM.zip › 6OONp_A_mdwhole_AF4REF/candidates/6OONp_A-merged-notenriched_report.html]

 

# Structural Comparison Report for 6OONp\_A - whole structures (total: 622)

---

1

- **AF ID:** AF-Q9H9G7-F1-model-v4 | **Chain:** A
- **b-phipsi:** 0.0006082477711518
- **w-rdist:** 0.0809331213187695
- **t-alpha:** 0.0297336873714129

---

---

2

- **AF ID:** AF-Q9UL18-F1-model-v4 | **Chain:** A
- **b-phipsi:** 0.0005488876760193
- **w-rdist:** 0.0471138153369363
- **t-alpha:** 0.0391232045333622

---

---

3

- **AF ID:** AF-Q9H0H3-F1-model-v4 | **Chain:** A
- **b-phipsi:** 0.0009658069246138
- **w-rdist:** 0.5906484623943691
- **t-alpha:** 0.0007823187912934

---

---

4

- **AF ID:** AF-Q9UKV8-F1-model-v4 | **Chain:** A
- **b-phipsi:** 0.0009443559990526
- **w-rdist:** 0.0595190221470882
- **t-alpha:** 0.0579025721066006

---

---

5

- **AF ID:** AF-Q9HCK5-F1-model-v4 | **Chain:** A
- **b-phipsi:** 0.0004273091918657
- **w-rdist:** 0.1028356317134269
- **t-alpha:** 0.1173704658131864

---

---

6

- **AF ID:** AF-Q06187-F1-model-v4 | **Chain:** A
- **b-phipsi:** 0.0025801392520136
- **w-rdist:** 0.3731656749039853
- **t-alpha:** 0.0015647720218643

---

---

7

- **AF ID:** AF-P37173-F1-model-v4 | **Chain:** A
- **b-phipsi:** 0.0010753437965379
- **w-rdist:** 0.5771545397070541
- **t-alpha:** 0.0023473590503262

---

---

8

- **AF ID:** AF-Q9Y2F9-F1-model-v4 | **Chain:** A
- **b-phipsi:** 0.001242683873795
- **w-rdist:** 0.3391466513675276
- **t-alpha:** 0.0062992257321734

---

---

9

- **AF ID:** AF-B6SEH8-F1-model-v4 | **Chain:** A
- **b-phipsi:** 0.0028783603695361
- **w-rdist:** 0.422257141719303
- **t-alpha:** 0.0007832732121422

---

---

10

- **AF ID:** AF-Q9UL63-F1-model-v4 | **Chain:** A
- **b-phipsi:** 0.0015964987032425
- **w-rdist:** 0.2718826415725286
- **t-alpha:** 0.0140843739056586

---

---

11

- **AF ID:** AF-O43374-F1-model-v4 | **Chain:** A
- **b-phipsi:** 0.0021965532556254
- **w-rdist:** 0.1918019455109033
- **t-alpha:** 0.0232189692826079

---

---

12

- **AF ID:** AF-C9J798-F1-model-v4 | **Chain:** A
- **b-phipsi:** 0.0022736472330602
- **w-rdist:** 0.2297129503044357
- **t-alpha:** 0.0142860356016105

---

---

13

- **AF ID:** AF-Q9P2G3-F1-model-v4 | **Chain:** A
- **b-phipsi:** 0.0029435683164907
- **w-rdist:** 0.3953144250166574
- **t-alpha:** 0.0023473590503262

---

---

14

- **AF ID:** AF-Q16671-F1-model-v4 | **Chain:** A
- **b-phipsi:** 0.0053084586583112
- **w-rdist:** 0.3555357284910318
- **t-alpha:** 0.0023530945443304

---

---

15

- **AF ID:** AF-Q9NY28-F1-model-v4 | **Chain:** A
- **b-phipsi:** 0.0037462662511197
- **w-rdist:** 0.5171167764400909
- **t-alpha:** 0.0

---

---

16

- **AF ID:** AF-Q9Y6W6-F1-model-v4 | **Chain:** A
- **b-phipsi:** 0.0087066921255892
- **w-rdist:** 0.3674896488133773
- **t-alpha:** 0.0007823187912934

---

---

17

- **AF ID:** AF-Q9Y2E5-F1-model-v4 | **Chain:** A
- **b-phipsi:** 0.0021563529973776
- **w-rdist:** 0.2829987654994442
- **t-alpha:** 0.0117370267412904

---

---

18

- **AF ID:** AF-Q9Y2X0-F1-model-v4 | **Chain:** A
- **b-phipsi:** 0.0010487916794057
- **w-rdist:** 0.374255156718853
- **t-alpha:** 0.0226916757534991

---

---

19

- **AF ID:** AF-Q96PZ2-F1-model-v4 | **Chain:** A
- **b-phipsi:** 0.0029466645158733
- **w-rdist:** 0.2464580378377701
- **t-alpha:** 0.0126786930545719

---

---

20

- **AF ID:** AF-Q92935-F1-model-v4 | **Chain:** A
- **b-phipsi:** 0.0057054008677965
- **w-rdist:** 0.3954906976976218
- **t-alpha:** 0.0015676375257613

---

---

21

- **AF ID:** AF-O95294-F1-model-v4 | **Chain:** A
- **b-phipsi:** 0.0041723581026218
- **w-rdist:** 0.1923965260098805
- **t-alpha:** 0.0224000982062968

---

---

22

- **AF ID:** AF-Q53H54-F1-model-v4 | **Chain:** A
- **b-phipsi:** 0.0022597099387611
- **w-rdist:** 0.4370179899706327
- **t-alpha:** 0.0031401242085145

---

---

23

- **AF ID:** AF-Q8IXQ5-F1-model-v4 | **Chain:** A
- **b-phipsi:** 0.0025263575904233
- **w-rdist:** 0.6766863313231747
- **t-alpha:** 0.0007832732121422

---

---

24

- **AF ID:** AF-Q3ZCT8-F1-model-v4 | **Chain:** A
- **b-phipsi:** 0.000673013286145
- **w-rdist:** 0.6385934583572589
- **t-alpha:** 0.0156493910563146

---

---

25

- **AF ID:** AF-Q9Y6W3-F1-model-v4 | **Chain:** A
- **b-phipsi:** 0.0008155983927349
- **w-rdist:** 0.6126130081976835
- **t-alpha:** 0.0102767895279776

---

---

26

- **AF ID:** AF-Q7Z3Z4-F1-model-v4 | **Chain:** A
- **b-phipsi:** 0.0024924565329652
- **w-rdist:** 0.1902126057300489
- **t-alpha:** 0.0492955855664358

---

---

27

- **AF ID:** AF-Q96PZ0-F1-model-v4 | **Chain:** A
- **b-phipsi:** 0.0010410080880228
- **w-rdist:** 0.2660089288671919
- **t-alpha:** 0.1283255184376883

---

---

28

- **AF ID:** AF-Q96IC2-F1-model-v4 | **Chain:** A
- **b-phipsi:** 0.0029830230296662
- **w-rdist:** 0.2559212825246469
- **t-alpha:** 0.0328637472319743

---

---

29

- **AF ID:** AF-Q96RR1-F1-model-v4 | **Chain:** A
- **b-phipsi:** 0.0052528873151784
- **w-rdist:** 0.4849701860349499
- **t-alpha:** 0.0015676375257613

---

---

30

- **AF ID:** AF-P09327-F1-model-v4 | **Chain:** A
- **b-phipsi:** 0.002186276095447
- **w-rdist:** 0.3564047197256664
- **t-alpha:** 0.0101717762082627

---

---

31

- **AF ID:** AF-P31321-F1-model-v4 | **Chain:** A
- **b-phipsi:** 0.0009698379643694
- **w-rdist:** 0.4039244825430616
- **t-alpha:** 0.0398701801688734

---

---

32

- **AF ID:** AF-Q96J94-F1-model-v4 | **Chain:** A
- **b-phipsi:** 0.0052235119836145
- **w-rdist:** 0.2063300545558366
- **t-alpha:** 0.0297336873714129

---

---

33

- **AF ID:** AF-Q9H0J9-F1-model-v4 | **Chain:** A
- **b-phipsi:** 0.0007668986855758
- **w-rdist:** 0.3677386203406908
- **t-alpha:** 0.1181102422121964

---

---

34

- **AF ID:** AF-Q86TM3-F1-model-v4 | **Chain:** A
- **b-phipsi:** 0.0038767851812322
- **w-rdist:** 0.2984959190492869
- **t-alpha:** 0.0234741774196856

---

---

35

- **AF ID:** AF-Q8N3Y3-F1-model-v4 | **Chain:** A
- **b-phipsi:** 0.0117856045390715
- **w-rdist:** 0.3626405275199351
- **t-alpha:** 0.0023473590503262

---

---

36

- **AF ID:** AF-Q08380-F1-model-v4 | **Chain:** A
- **b-phipsi:** 0.0014185126091855
- **w-rdist:** 0.3415998057158072
- **t-alpha:** 0.0432653326797045

---

---

37

- **AF ID:** AF-Q8IY47-F1-model-v4 | **Chain:** A
- **b-phipsi:** 0.0020730120701645
- **w-rdist:** 0.3688327876779206
- **t-alpha:** 0.0187789021729365

---

---

38

- **AF ID:** AF-Q8NCE0-F1-model-v4 | **Chain:** A
- **b-phipsi:** 0.0079225131894249
- **w-rdist:** 0.5188302430596078
- **t-alpha:** 0.0007832732121422

---

---

39

- **AF ID:** AF-Q9BXC9-F1-model-v4 | **Chain:** A
- **b-phipsi:** 0.0025694704019257
- **w-rdist:** 0.3086092095505957
- **t-alpha:** 0.0422534769714337

---

---

40

- **AF ID:** AF-P07384-F1-model-v4 | **Chain:** A
- **b-phipsi:** 0.0086102698166073
- **w-rdist:** 0.486877079759725
- **t-alpha:** 0.0015647720218643

---

---

41

- **AF ID:** AF-O95831-F1-model-v4 | **Chain:** A
- **b-phipsi:** 0.0060937092110865
- **w-rdist:** 0.5503907173731932
- **t-alpha:** 0.0023473590503262

---

---

42

- **AF ID:** AF-Q504Y2-F1-model-v4 | **Chain:** A
- **b-phipsi:** 0.0031017693826659
- **w-rdist:** 0.7583343456823548
- **t-alpha:** 0.0

---

---

43

- **AF ID:** AF-P54646-F1-model-v4 | **Chain:** A
- **b-phipsi:** 0.004041927583506
- **w-rdist:** 0.3883711526699795
- **t-alpha:** 0.0047169960791435

---

---

44

- **AF ID:** AF-Q96NY9-F1-model-v4 | **Chain:** A
- **b-phipsi:** 0.0101337283249819
- **w-rdist:** 0.1788159191616689
- **t-alpha:** 0.0086070234951727

---

---

45

- **AF ID:** AF-Q9NSD9-F1-model-v4 | **Chain:** A
- **b-phipsi:** 0.0004696338062979
- **w-rdist:** 0.7367570935250812
- **t-alpha:** 0.0314772841117727

---

---

46

- **AF ID:** AF-P98182-F1-model-v4 | **Chain:** A
- **b-phipsi:** 0.006280949928769
- **w-rdist:** 0.613066644485438
- **t-alpha:** 0.0015647720218643

---

---

47

- **AF ID:** AF-Q9NTG7-F1-model-v4 | **Chain:** A
- **b-phipsi:** 0.0012315145768263
- **w-rdist:** 0.8346919203560298
- **t-alpha:** 0.0023530945443304

---

---

48

- **AF ID:** AF-P13639-F1-model-v4 | **Chain:** A
- **b-phipsi:** 0.002679709735741
- **w-rdist:** 0.3582527465743377
- **t-alpha:** 0.0250390222700223

---

---

49

- **AF ID:** AF-Q8TAF3-F1-model-v4 | **Chain:** A
- **b-phipsi:** 0.0070262068596566
- **w-rdist:** 0.6423137192946232
- **t-alpha:** 0.0007823187912934

---

---

50

- **AF ID:** AF-Q06210-F1-model-v4 | **Chain:** A
- **b-phipsi:** 0.0030076987088355
- **w-rdist:** 0.6999402583007417
- **t-alpha:** 0.0023473590503262

---

---

51

- **AF ID:** AF-Q13705-F1-model-v4 | **Chain:** A
- **b-phipsi:** 0.0024073390364737
- **w-rdist:** 0.7862377346423994
- **t-alpha:** 0.0015647720218643

---

---

52

- **AF ID:** AF-Q16819-F1-model-v4 | **Chain:** A
- **b-phipsi:** 0.0105537537401441
- **w-rdist:** 0.2326532513698452
- **t-alpha:** 0.0078243548762237

---

---

53

- **AF ID:** AF-Q15831-F1-model-v4 | **Chain:** A
- **b-phipsi:** 0.0053172161938833
- **w-rdist:** 0.7177085104830545
- **t-alpha:** 0.0007823187912934

---

---

54

- **AF ID:** AF-Q13131-F1-model-v4 | **Chain:** A
- **b-phipsi:** 0.003275660300548
- **w-rdist:** 0.3450256391594226
- **t-alpha:** 0.0336461458262049

---

---

55

- **AF ID:** AF-Q9BRP7-F1-model-v4 | **Chain:** A
- **b-phipsi:** 0.0009136839641332
- **w-rdist:** 0.459969568131855
- **t-alpha:** 0.0739499805270313

---

---

56

- **AF ID:** AF-Q8IXK2-F1-model-v4 | **Chain:** A
- **b-phipsi:** 0.0032235779258749
- **w-rdist:** 0.5663772417765246
- **t-alpha:** 0.0039277718342689

---

---

57

- **AF ID:** AF-Q9NZ20-F1-model-v4 | **Chain:** A
- **b-phipsi:** 0.005005401933765
- **w-rdist:** 0.4637674104004329
- **t-alpha:** 0.0039277718342689

---

---

58

- **AF ID:** AF-Q9NRJ5-F1-model-v4 | **Chain:** A
- **b-phipsi:** 0.0008311364239527
- **w-rdist:** 0.3137868107210553
- **t-alpha:** 0.2683872843872745

---

---

59

- **AF ID:** AF-Q8TAP6-F1-model-v4 | **Chain:** A
- **b-phipsi:** 0.0005521581170716
- **w-rdist:** 0.5441462325083098
- **t-alpha:** 0.1173704658131864

---

---

60

- **AF ID:** AF-Q8TBY0-F1-model-v4 | **Chain:** A
- **b-phipsi:** 0.0052728005694042
- **w-rdist:** 0.2736711089191508
- **t-alpha:** 0.0539902689916904

---

---

61

- **AF ID:** AF-Q8WZA1-F1-model-v4 | **Chain:** A
- **b-phipsi:** 0.001269934633173
- **w-rdist:** 0.2336733595053451
- **t-alpha:** 0.1768381479464797

---

---

62

- **AF ID:** AF-P19447-F1-model-v4 | **Chain:** A
- **b-phipsi:** 0.0032454951814456
- **w-rdist:** 0.368019733639459
- **t-alpha:** 0.027386367694415

---

---

63

- **AF ID:** AF-Q9HAU4-F1-model-v4 | **Chain:** A
- **b-phipsi:** 0.002129229911346
- **w-rdist:** 0.2511792535055234
- **t-alpha:** 0.1205008021373417

---

---

64

- **AF ID:** AF-O15033-F1-model-v4 | **Chain:** A
- **b-phipsi:** 0.0008825173223327
- **w-rdist:** 0.7342760840752007
- **t-alpha:** 0.0164318357763795

---

---

65

- **AF ID:** AF-Q68DD2-F1-model-v4 | **Chain:** A
- **b-phipsi:** 0.001894887260993
- **w-rdist:** 0.2027922104009692
- **t-alpha:** 0.1384973100154414

---

---

66

- **AF ID:** AF-Q6PI48-F1-model-v4 | **Chain:** A
- **b-phipsi:** 0.0006280298927302
- **w-rdist:** 0.5868661858376789
- **t-alpha:** 0.092331642604805

---

---

67

- **AF ID:** AF-Q86VK4-F1-model-v4 | **Chain:** A
- **b-phipsi:** 0.010180704618727
- **w-rdist:** 0.5695645103980478
- **t-alpha:** 0.0015647720218643

---

---

68

- **AF ID:** AF-Q15796-F1-model-v4 | **Chain:** A
- **b-phipsi:** 0.000531006656881
- **w-rdist:** 0.6133324827408306
- **t-alpha:** 0.0941781028703137

---

---

69

- **AF ID:** AF-Q9BQA5-F1-model-v4 | **Chain:** A
- **b-phipsi:** 0.0040227428729013
- **w-rdist:** 0.2519320264949251
- **t-alpha:** 0.0774646231830327

---

---

70

- **AF ID:** AF-O00754-F1-model-v4 | **Chain:** A
- **b-phipsi:** 0.0043700550675103
- **w-rdist:** 0.3539454104531831
- **t-alpha:** 0.0312987592118472

---

---

71

- **AF ID:** AF-O94955-F1-model-v4 | **Chain:** A
- **b-phipsi:** 0.0187903813719031
- **w-rdist:** 0.394584095049605
- **t-alpha:** 0.0007832732121422

---

---

72

- **AF ID:** AF-Q9NVX7-F1-model-v4 | **Chain:** A
- **b-phipsi:** 0.0005870839659491
- **w-rdist:** 0.580331796703812
- **t-alpha:** 0.1132408945125096

---

---

73

- **AF ID:** AF-Q92542-F1-model-v4 | **Chain:** A
- **b-phipsi:** 0.0046871231220074
- **w-rdist:** 0.7162751893874599
- **t-alpha:** 0.0015676375257613

---

---

74

- **AF ID:** AF-Q01518-F1-model-v4 | **Chain:** A
- **b-phipsi:** 0.0020425492773036
- **w-rdist:** 0.3675642108384912
- **t-alpha:** 0.0586852591928757

---

---

75

- **AF ID:** AF-P11142-F1-model-v4 | **Chain:** A
- **b-phipsi:** 0.0038039293808072
- **w-rdist:** 0.5846352698827162
- **t-alpha:** 0.0039277718342689

---

---

76

- **AF ID:** AF-Q14181-F1-model-v4 | **Chain:** A
- **b-phipsi:** 0.0005131088081659
- **w-rdist:** 0.8160902169913542
- **t-alpha:** 0.0359934414192668

---

---

77

- **AF ID:** AF-O14841-F1-model-v4 | **Chain:** A
- **b-phipsi:** 0.0018508876639965
- **w-rdist:** 0.3424202823833285
- **t-alpha:** 0.0821594238081062

---

---

78

- **AF ID:** AF-Q6PJQ5-F1-model-v4 | **Chain:** A
- **b-phipsi:** 0.0050675165985821
- **w-rdist:** 0.3483093812479473
- **t-alpha:** 0.0323103167719089

---

---

79

- **AF ID:** AF-O43776-F1-model-v4 | **Chain:** A
- **b-phipsi:** 0.0010380372368401
- **w-rdist:** 0.7397131137007908
- **t-alpha:** 0.0086070234951727

---

---

80

- **AF ID:** AF-Q969P6-F1-model-v4 | **Chain:** A
- **b-phipsi:** 0.0127892371139456
- **w-rdist:** 0.2403093533997815
- **t-alpha:** 0.011876942219535

---

---

81

- **AF ID:** AF-P46060-F1-model-v4 | **Chain:** A
- **b-phipsi:** 0.0279041626203987
- **w-rdist:** 0.3525461623741237
- **t-alpha:** 0.0015676375257613

---

---

82

- **AF ID:** AF-Q8NBJ5-F1-model-v4 | **Chain:** A
- **b-phipsi:** 0.0010710292470885
- **w-rdist:** 0.5413990545686231
- **t-alpha:** 0.0703518669333997

---

---

83

- **AF ID:** AF-O15530-F1-model-v4 | **Chain:** A
- **b-phipsi:** 0.0050652854798716
- **w-rdist:** 0.6011602643885208
- **t-alpha:** 0.0039119745263893

---

---

84

- **AF ID:** AF-Q8N3E9-F1-model-v4 | **Chain:** A
- **b-phipsi:** 0.0030931875106035
- **w-rdist:** 0.3940216381266235
- **t-alpha:** 0.0250390222700223

---

---

85

- **AF ID:** AF-Q9BQ95-F1-model-v4 | **Chain:** A
- **b-phipsi:** 0.0055733658822594
- **w-rdist:** 0.2886115221891955
- **t-alpha:** 0.06807491933535

---

---

86

- **AF ID:** AF-Q5VV42-F1-model-v4 | **Chain:** A
- **b-phipsi:** 0.0032195858763015
- **w-rdist:** 0.7452695588854322
- **t-alpha:** 0.0023530945443304

---

---

87

- **AF ID:** AF-Q8NA66-F1-model-v4 | **Chain:** A
- **b-phipsi:** 0.0011252635660009
- **w-rdist:** 0.7021758355182107
- **t-alpha:** 0.0219087977837508

---

---

88

- **AF ID:** AF-P06396-F1-model-v4 | **Chain:** A
- **b-phipsi:** 0.0042430191318908
- **w-rdist:** 0.3566503275866743
- **t-alpha:** 0.0390246061928452

---

---

89

- **AF ID:** AF-O15067-F1-model-v4 | **Chain:** A
- **b-phipsi:** 0.002289840601717
- **w-rdist:** 0.2771953747719335
- **t-alpha:** 0.1283255184376883

---

---

90

- **AF ID:** AF-Q14145-F1-model-v4 | **Chain:** A
- **b-phipsi:** 0.0025441850172837
- **w-rdist:** 0.8529921188325944
- **t-alpha:** 0.0015676375257613

---

---

91

- **AF ID:** AF-Q5T447-F1-model-v4 | **Chain:** A
- **b-phipsi:** 0.0023954232942157
- **w-rdist:** 0.3380629402529312
- **t-alpha:** 0.0884191622029941

---

---

92

- **AF ID:** AF-Q6ICG6-F1-model-v4 | **Chain:** A
- **b-phipsi:** 0.0102409132295435
- **w-rdist:** 0.5755137558544103
- **t-alpha:** 0.0023473590503262

---

---

93

- **AF ID:** AF-P16220-F1-model-v4 | **Chain:** A
- **b-phipsi:** 0.0046968220705803
- **w-rdist:** 0.8571982504534672
- **t-alpha:** 0.0

---

---

94

- **AF ID:** AF-Q9P2R7-F1-model-v4 | **Chain:** A
- **b-phipsi:** 0.0010202693044081
- **w-rdist:** 0.6555461507486418
- **t-alpha:** 0.0501234795277865

---

---

95

- **AF ID:** AF-Q8NCR0-F1-model-v4 | **Chain:** A
- **b-phipsi:** 0.0006558418936457
- **w-rdist:** 0.4956641970087056
- **t-alpha:** 0.1555154353376837

---

---

96

- **AF ID:** AF-O60662-F1-model-v4 | **Chain:** A
- **b-phipsi:** 0.0013099724511646
- **w-rdist:** 0.5277904848872851
- **t-alpha:** 0.0466830494629222

---

---

97

- **AF ID:** AF-Q8NAB2-F1-model-v4 | **Chain:** A
- **b-phipsi:** 0.0009665576015688
- **w-rdist:** 0.7540849460622907
- **t-alpha:** 0.0289856519083209

---

---

98

- **AF ID:** AF-Q8N323-F1-model-v4 | **Chain:** A
- **b-phipsi:** 0.0008833471144211
- **w-rdist:** 0.8182625132781132
- **t-alpha:** 0.0207667552249124

---

---

99

- **AF ID:** AF-Q8N884-F1-model-v4 | **Chain:** A
- **b-phipsi:** 0.0059340146424255
- **w-rdist:** 0.7170326865574234
- **t-alpha:** 0.0023473590503262

---

---

100

- **AF ID:** AF-A4D1U4-F1-model-v4 | **Chain:** A
- **b-phipsi:** 0.0044908339402261
- **w-rdist:** 0.7540005584125987
- **t-alpha:** 0.0023473590503262

---

---

101

- **AF ID:** AF-Q86XP0-F1-model-v4 | **Chain:** A
- **b-phipsi:** 0.0030958282451962
- **w-rdist:** 0.3163050401995855
- **t-alpha:** 0.0907665259446537

---

---

102

- **AF ID:** AF-Q460N3-F1-model-v4 | **Chain:** A
- **b-phipsi:** 0.003512935041836
- **w-rdist:** 0.2908586107215489
- **t-alpha:** 0.1025037952669631

---

---

103

- **AF ID:** AF-P0C869-F1-model-v4 | **Chain:** A
- **b-phipsi:** 0.0029611258532422
- **w-rdist:** 0.3625778842643337
- **t-alpha:** 0.064162546478931

---

---

104

- **AF ID:** AF-Q9NZI8-F1-model-v4 | **Chain:** A
- **b-phipsi:** 0.0036196856386589
- **w-rdist:** 0.3023475738062205
- **t-alpha:** 0.0954613033833033

---

---

105

- **AF ID:** AF-P18031-F1-model-v4 | **Chain:** A
- **b-phipsi:** 0.0013886872279637
- **w-rdist:** 0.6223918477838714
- **t-alpha:** 0.0219087977837508

---

---

106

- **AF ID:** AF-Q9H1Q7-F1-model-v4 | **Chain:** A
- **b-phipsi:** 0.0075476215223086
- **w-rdist:** 0.5903199475855297
- **t-alpha:** 0.0031401242085145

---

---

107

- **AF ID:** AF-Q16566-F1-model-v4 | **Chain:** A
- **b-phipsi:** 0.0061577706101379
- **w-rdist:** 0.6449894807641368
- **t-alpha:** 0.0031401242085145

---

---

108

- **AF ID:** AF-Q8WXF8-F1-model-v4 | **Chain:** A
- **b-phipsi:** 0.0022183421504646
- **w-rdist:** 0.4007357968861972
- **t-alpha:** 0.0424145126110251

---

---

109

- **AF ID:** AF-Q96NJ5-F1-model-v4 | **Chain:** A
- **b-phipsi:** 0.0012361643088472
- **w-rdist:** 0.5715271752374301
- **t-alpha:** 0.0570720736347449

---

---

110

- **AF ID:** AF-Q9BZE2-F1-model-v4 | **Chain:** A
- **b-phipsi:** 0.0025774374083502
- **w-rdist:** 0.6376127512094303
- **t-alpha:** 0.0055077996884944

---

---

111

- **AF ID:** AF-Q96BZ4-F1-model-v4 | **Chain:** A
- **b-phipsi:** 0.0002827056820133
- **w-rdist:** 0.749901178752813
- **t-alpha:** 0.0876596132533018

---

---

112

- **AF ID:** AF-Q9BZQ2-F1-model-v4 | **Chain:** A
- **b-phipsi:** 0.0007509972854688
- **w-rdist:** 0.6834564675775673
- **t-alpha:** 0.0868544688919852

---

---

113

- **AF ID:** AF-A8MX76-F1-model-v4 | **Chain:** A
- **b-phipsi:** 0.0007437942839194
- **w-rdist:** 0.7009440755721825
- **t-alpha:** 0.0812186237826302

---

---

114

- **AF ID:** AF-Q96RP9-F1-model-v4 | **Chain:** A
- **b-phipsi:** 0.0074185272757875
- **w-rdist:** 0.7571403838754567
- **t-alpha:** 0.0007832732121422

---

---

115

- **AF ID:** AF-Q6UWF7-F1-model-v4 | **Chain:** A
- **b-phipsi:** 0.0014805872145444
- **w-rdist:** 0.8143664274698216
- **t-alpha:** 0.0039119745263893

---

---

116

- **AF ID:** AF-Q96ME1-F1-model-v4 | **Chain:** A
- **b-phipsi:** 0.0036992654331515
- **w-rdist:** 0.3754278083567123
- **t-alpha:** 0.0422534769714337

---

---

117

- **AF ID:** AF-Q9NZW5-F1-model-v4 | **Chain:** A
- **b-phipsi:** 0.0014487223008964
- **w-rdist:** 0.5508673999762334
- **t-alpha:** 0.0336461458262049

---

---

118

- **AF ID:** AF-Q6ZTN6-F1-model-v4 | **Chain:** A
- **b-phipsi:** 0.0010370312073688
- **w-rdist:** 0.620308973734857
- **t-alpha:** 0.0719873632833352

---

---

119

- **AF ID:** AF-O75529-F1-model-v4 | **Chain:** A
- **b-phipsi:** 0.0023083358093805
- **w-rdist:** 0.343845498214064
- **t-alpha:** 0.1071984065049926

---

---

120

- **AF ID:** AF-P47972-F1-model-v4 | **Chain:** A
- **b-phipsi:** 0.0023070652183437
- **w-rdist:** 0.8140509108943518
- **t-alpha:** 0.0031401242085145

---

---

121

- **AF ID:** AF-Q9NZ08-F1-model-v4 | **Chain:** A
- **b-phipsi:** 0.0056930420954221
- **w-rdist:** 0.3089415955833576
- **t-alpha:** 0.0727697286543513

---

---

122

- **AF ID:** AF-Q14CN2-F1-model-v4 | **Chain:** A
- **b-phipsi:** 0.0064309344961863
- **w-rdist:** 0.1926477373962172
- **t-alpha:** 0.1009388027447895

---

---

123

- **AF ID:** AF-Q08881-F1-model-v4 | **Chain:** A
- **b-phipsi:** 0.0013653212074157
- **w-rdist:** 0.5823374416879894
- **t-alpha:** 0.0390246061928452

---

---

124

- **AF ID:** AF-Q86TI2-F1-model-v4 | **Chain:** A
- **b-phipsi:** 0.0056444429676834
- **w-rdist:** 0.5529801421221743
- **t-alpha:** 0.0054771144938834

---

---

125

- **AF ID:** AF-Q8NC60-F1-model-v4 | **Chain:** A
- **b-phipsi:** 0.0035714122531415
- **w-rdist:** 0.3073819432195452
- **t-alpha:** 0.1134580270465441

---

---

126

- **AF ID:** AF-Q9UBK8-F1-model-v4 | **Chain:** A
- **b-phipsi:** 0.0064603107961626
- **w-rdist:** 0.3083328681979896
- **t-alpha:** 0.0727697286543513

---

---

127

- **AF ID:** AF-Q14697-F1-model-v4 | **Chain:** A
- **b-phipsi:** 0.0093244547030807
- **w-rdist:** 0.3722621180514959
- **t-alpha:** 0.0086820531296027

---

---

128

- **AF ID:** AF-Q5SY16-F1-model-v4 | **Chain:** A
- **b-phipsi:** 0.0032017814478932
- **w-rdist:** 0.3105934936618811
- **t-alpha:** 0.1228477492952864

---

---

129

- **AF ID:** AF-Q8NBE8-F1-model-v4 | **Chain:** A
- **b-phipsi:** 0.0034845980933919
- **w-rdist:** 0.6019377342628524
- **t-alpha:** 0.0062593997286479

---

---

130

- **AF ID:** AF-P98170-F1-model-v4 | **Chain:** A
- **b-phipsi:** 0.0097380224802077
- **w-rdist:** 0.262678154058052
- **t-alpha:** 0.064162546478931

---

---

131

- **AF ID:** AF-O43791-F1-model-v4 | **Chain:** A
- **b-phipsi:** 0.0008584505831841
- **w-rdist:** 0.4727154755295734
- **t-alpha:** 0.1597100237638313

---

---

132

- **AF ID:** AF-Q96RQ3-F1-model-v4 | **Chain:** A
- **b-phipsi:** 0.0048413328532891
- **w-rdist:** 0.3407121828461258
- **t-alpha:** 0.0743346359382088

---

---

133

- **AF ID:** AF-P84022-F1-model-v4 | **Chain:** A
- **b-phipsi:** 0.0005660915298645
- **w-rdist:** 0.6978749259746404
- **t-alpha:** 0.1230228438240716

---

---

134

- **AF ID:** AF-Q13939-F1-model-v4 | **Chain:** A
- **b-phipsi:** 0.0007165498406963
- **w-rdist:** 0.5187326601880891
- **t-alpha:** 0.1844303389458708

---

---

135

- **AF ID:** AF-A8K7I4-F1-model-v4 | **Chain:** A
- **b-phipsi:** 0.0082688798078506
- **w-rdist:** 0.2674953198439244
- **t-alpha:** 0.0798120464585645

---

---

136

- **AF ID:** AF-O43610-F1-model-v4 | **Chain:** A
- **b-phipsi:** 0.0077958266167743
- **w-rdist:** 0.4831206505027958
- **t-alpha:** 0.0054771144938834

---

---

137

- **AF ID:** AF-Q9UK97-F1-model-v4 | **Chain:** A
- **b-phipsi:** 0.0010055291597149
- **w-rdist:** 0.8308747385603896
- **t-alpha:** 0.0266038120788794

---

---

138

- **AF ID:** AF-P55786-F1-model-v4 | **Chain:** A
- **b-phipsi:** 0.0056471155151462
- **w-rdist:** 0.3793368653721571
- **t-alpha:** 0.0383411162518374

---

---

139

- **AF ID:** AF-P19801-F1-model-v4 | **Chain:** A
- **b-phipsi:** 0.0122055378450906
- **w-rdist:** 0.5200312172267842
- **t-alpha:** 0.0031401242085145

---

---

140

- **AF ID:** AF-Q15283-F1-model-v4 | **Chain:** A
- **b-phipsi:** 0.0023703085257179
- **w-rdist:** 0.8995594457666696
- **t-alpha:** 0.0031296009050172

---

---

141

- **AF ID:** AF-Q96M94-F1-model-v4 | **Chain:** A
- **b-phipsi:** 0.0007291854464532
- **w-rdist:** 0.7457419279693508
- **t-alpha:** 0.0805944545366483

---

---

142

- **AF ID:** AF-Q14534-F1-model-v4 | **Chain:** A
- **b-phipsi:** 0.0086505551125082
- **w-rdist:** 0.8008155029534861
- **t-alpha:** 0.0007832732121422

---

---

143

- **AF ID:** AF-Q9H4A9-F1-model-v4 | **Chain:** A
- **b-phipsi:** 0.0044549090362766
- **w-rdist:** 0.7093369782327572
- **t-alpha:** 0.0039277718342689

---

---

144

- **AF ID:** AF-P10253-F1-model-v4 | **Chain:** A
- **b-phipsi:** 0.0118316851149639
- **w-rdist:** 0.2947059395528991
- **t-alpha:** 0.0453832094967918

---

---

145

- **AF ID:** AF-P27037-F1-model-v4 | **Chain:** A
- **b-phipsi:** 0.0015786316374422
- **w-rdist:** 0.7144597223736362
- **t-alpha:** 0.0062992257321734

---

---

146

- **AF ID:** AF-Q9UBT2-F1-model-v4 | **Chain:** A
- **b-phipsi:** 0.0045606980282486
- **w-rdist:** 0.2313658390311102
- **t-alpha:** 0.1494517477205856

---

---

147

- **AF ID:** AF-Q8IUH3-F1-model-v4 | **Chain:** A
- **b-phipsi:** 0.0044323869538332
- **w-rdist:** 0.2227114879254782
- **t-alpha:** 0.1557116053874756

---

---

148

- **AF ID:** AF-P23743-F1-model-v4 | **Chain:** A
- **b-phipsi:** 0.0090374865108467
- **w-rdist:** 0.3857107564864988
- **t-alpha:** 0.0109543395722921

---

---

149

- **AF ID:** AF-P57772-F1-model-v4 | **Chain:** A
- **b-phipsi:** 0.0040317569725066
- **w-rdist:** 0.733965394600764
- **t-alpha:** 0.0039277718342689

---

---

150

- **AF ID:** AF-Q5SRI9-F1-model-v4 | **Chain:** A
- **b-phipsi:** 0.0043496934728762
- **w-rdist:** 0.8172518585104848
- **t-alpha:** 0.0031296009050172

---

---

151

- **AF ID:** AF-Q15916-F1-model-v4 | **Chain:** A
- **b-phipsi:** 0.0129661417446243
- **w-rdist:** 0.3068930787671374
- **t-alpha:** 0.0344286438364997

---

---

152

- **AF ID:** AF-P51843-F1-model-v4 | **Chain:** A
- **b-phipsi:** 0.0077481089422838
- **w-rdist:** 0.551436640809752
- **t-alpha:** 0.0054771144938834

---

---

153

- **AF ID:** AF-Q9Y6U3-F1-model-v4 | **Chain:** A
- **b-phipsi:** 0.0094252914408333
- **w-rdist:** 0.320145495626202
- **t-alpha:** 0.0594675149611743

---

---

154

- **AF ID:** AF-P47989-F1-model-v4 | **Chain:** A
- **b-phipsi:** 0.0025343009688274
- **w-rdist:** 0.3362416352099077
- **t-alpha:** 0.1502347029199773

---

---

155

- **AF ID:** AF-Q00973-F1-model-v4 | **Chain:** A
- **b-phipsi:** 0.0014714920928516
- **w-rdist:** 0.5608574142591904
- **t-alpha:** 0.0544557624307391

---

---

156

- **AF ID:** AF-Q9UL40-F1-model-v4 | **Chain:** A
- **b-phipsi:** 0.0093939567018853
- **w-rdist:** 0.3081507167431301
- **t-alpha:** 0.068561919033538

---

---

157

- **AF ID:** AF-D6RJB6-F1-model-v4 | **Chain:** A
- **b-phipsi:** 0.0118453306811883
- **w-rdist:** 0.7059764311649427
- **t-alpha:** 0.0015676375257613

---

---

158

- **AF ID:** AF-Q9H3Y6-F1-model-v4 | **Chain:** A
- **b-phipsi:** 0.0013151684144775
- **w-rdist:** 0.6366539022930637
- **t-alpha:** 0.0605810409571925

---

---

159

- **AF ID:** AF-Q969S9-F1-model-v4 | **Chain:** A
- **b-phipsi:** 0.0039657131978746
- **w-rdist:** 0.3779302472610634
- **t-alpha:** 0.0712045469065545

---

---

160

- **AF ID:** AF-Q8NC26-F1-model-v4 | **Chain:** A
- **b-phipsi:** 0.0020837644304615
- **w-rdist:** 0.1483160173391156
- **t-alpha:** 0.3286387102798807

---

---

161

- **AF ID:** AF-Q1ZZU3-F1-model-v4 | **Chain:** A
- **b-phipsi:** 0.0230580573752301
- **w-rdist:** 0.5970089408873436
- **t-alpha:** 0.0007832732121422

---

---

162

- **AF ID:** AF-Q8N1C3-F1-model-v4 | **Chain:** A
- **b-phipsi:** 0.000877040507194
- **w-rdist:** 1.1509041787582042
- **t-alpha:** 0.0054771144938834

---

---

163

- **AF ID:** AF-P0DPE3-F1-model-v4 | **Chain:** A
- **b-phipsi:** 0.0055206425136305
- **w-rdist:** 0.8360508338471051
- **t-alpha:** 0.0023530945443304

---

---

164

- **AF ID:** AF-P49641-F1-model-v4 | **Chain:** A
- **b-phipsi:** 0.0011492844725674
- **w-rdist:** 0.5177083575165559
- **t-alpha:** 0.1408449593803573

---

---

165

- **AF ID:** AF-Q9ULR3-F1-model-v4 | **Chain:** A
- **b-phipsi:** 0.0068039344645865
- **w-rdist:** 0.687325338456744
- **t-alpha:** 0.0046944792707916

---

---

166

- **AF ID:** AF-P00747-F1-model-v4 | **Chain:** A
- **b-phipsi:** 0.0169774084857775
- **w-rdist:** 0.3160706455534488
- **t-alpha:** 0.0281690295641283

---

---

167

- **AF ID:** AF-Q9UBI4-F1-model-v4 | **Chain:** A
- **b-phipsi:** 0.0010154431120075
- **w-rdist:** 0.7090519151278263
- **t-alpha:** 0.0848899960610365

---

---

168

- **AF ID:** AF-Q05655-F1-model-v4 | **Chain:** A
- **b-phipsi:** 0.0035454350245458
- **w-rdist:** 0.3252113393176734
- **t-alpha:** 0.1478868884570592

---

---

169

- **AF ID:** AF-Q09013-F1-model-v4 | **Chain:** A
- **b-phipsi:** 0.0066833410693551
- **w-rdist:** 0.2918911133148048
- **t-alpha:** 0.1236302639257407

---

---

170

- **AF ID:** AF-P55010-F1-model-v4 | **Chain:** A
- **b-phipsi:** 0.0152711594602526
- **w-rdist:** 0.535432313006882
- **t-alpha:** 0.0031401242085145

---

---

171

- **AF ID:** AF-P63252-F1-model-v4 | **Chain:** A
- **b-phipsi:** 0.0007598710769659
- **w-rdist:** 0.968373554943161
- **t-alpha:** 0.0407169385382113

---

---

172

- **AF ID:** AF-Q16706-F1-model-v4 | **Chain:** A
- **b-phipsi:** 0.0012784596241349
- **w-rdist:** 0.4439184393209455
- **t-alpha:** 0.1244127877301322

---

---

173

- **AF ID:** AF-C9JJH3-F1-model-v4 | **Chain:** A
- **b-phipsi:** 0.0167965270936868
- **w-rdist:** 0.6705803186474426
- **t-alpha:** 0.0015676375257613

---

---

174

- **AF ID:** AF-Q6P179-F1-model-v4 | **Chain:** A
- **b-phipsi:** 0.0075567124741718
- **w-rdist:** 0.2043767775734961
- **t-alpha:** 0.1431919985610661

---

---

175

- **AF ID:** AF-Q8IZ07-F1-model-v4 | **Chain:** A
- **b-phipsi:** 0.0021436890284231
- **w-rdist:** 0.3713547515434308
- **t-alpha:** 0.1463219731492844

---

---

176

- **AF ID:** AF-P43627-F1-model-v4 | **Chain:** A
- **b-phipsi:** 0.0199220432898435
- **w-rdist:** 0.3974339794722528
- **t-alpha:** 0.0039119745263893

---

---

177

- **AF ID:** AF-O00425-F1-model-v4 | **Chain:** A
- **b-phipsi:** 0.0062381222456728
- **w-rdist:** 0.3497497311595895
- **t-alpha:** 0.0884191622029941

---

---

178

- **AF ID:** AF-Q6ZV50-F1-model-v4 | **Chain:** A
- **b-phipsi:** 0.0178070698975727
- **w-rdist:** 0.2050831427403125
- **t-alpha:** 0.0563379260275018

---

---

179

- **AF ID:** AF-Q6ZN11-F1-model-v4 | **Chain:** A
- **b-phipsi:** 0.0142039486786405
- **w-rdist:** 0.1944805758628961
- **t-alpha:** 0.0735521531070955

---

---

180

- **AF ID:** AF-P15144-F1-model-v4 | **Chain:** A
- **b-phipsi:** 0.0126177734904801
- **w-rdist:** 0.223656895676104
- **t-alpha:** 0.0821594238081062

---

---

181

- **AF ID:** AF-Q96LX8-F1-model-v4 | **Chain:** A
- **b-phipsi:** 0.008997435818818
- **w-rdist:** 0.4039336164167541
- **t-alpha:** 0.0191389373786337

---

---

182

- **AF ID:** AF-P07237-F1-model-v4 | **Chain:** A
- **b-phipsi:** 0.0029052458916495
- **w-rdist:** 0.3117714292558317
- **t-alpha:** 0.1988744195870988

---

---

183

- **AF ID:** AF-Q8IYR2-F1-model-v4 | **Chain:** A
- **b-phipsi:** 0.0309730121982867
- **w-rdist:** 0.0647565527002202
- **t-alpha:** 0.0273315008079859

---

---

184

- **AF ID:** AF-Q8TF42-F1-model-v4 | **Chain:** A
- **b-phipsi:** 0.0024386198866481
- **w-rdist:** 0.3767789064264024
- **t-alpha:** 0.1267604067828589

---

---

185

- **AF ID:** AF-Q6ZNX1-F1-model-v4 | **Chain:** A
- **b-phipsi:** 0.0012437668411683
- **w-rdist:** 0.7458965798624316
- **t-alpha:** 0.0441178429367157

---

---

186

- **AF ID:** AF-Q5JR12-F1-model-v4 | **Chain:** A
- **b-phipsi:** 0.0062875208482292
- **w-rdist:** 0.7971756939312636
- **t-alpha:** 0.0031401242085145

---

---

187

- **AF ID:** AF-A5YM72-F1-model-v4 | **Chain:** A
- **b-phipsi:** 0.0069588305667013
- **w-rdist:** 0.3829667986927294
- **t-alpha:** 0.0532080396597527

---

---

188

- **AF ID:** AF-P0CH99-F1-model-v4 | **Chain:** A
- **b-phipsi:** 0.0171376076848707
- **w-rdist:** 0.2844815921157582
- **t-alpha:** 0.0449715481085233

---

---

189

- **AF ID:** AF-Q8NFW8-F1-model-v4 | **Chain:** A
- **b-phipsi:** 0.0007873024547876
- **w-rdist:** 0.7016771142100864
- **t-alpha:** 0.1461886083332741

---

---

190

- **AF ID:** AF-Q96PF1-F1-model-v4 | **Chain:** A
- **b-phipsi:** 0.0108550945197072
- **w-rdist:** 0.5731365159843504
- **t-alpha:** 0.0047169960791435

---

---

191

- **AF ID:** AF-Q7L1V2-F1-model-v4 | **Chain:** A
- **b-phipsi:** 0.001318456251402
- **w-rdist:** 0.7641964892183886
- **t-alpha:** 0.0314772841117727

---

---

192

- **AF ID:** AF-Q9Y2Y9-F1-model-v4 | **Chain:** A
- **b-phipsi:** 0.0070538126170438
- **w-rdist:** 0.6401724398110779
- **t-alpha:** 0.0055077996884944

---

---

193

- **AF ID:** AF-Q15036-F1-model-v4 | **Chain:** A
- **b-phipsi:** 0.0008074013473642
- **w-rdist:** 1.0049939385207216
- **t-alpha:** 0.0373379936293052

---

---

194

- **AF ID:** AF-Q86Y07-F1-model-v4 | **Chain:** A
- **b-phipsi:** 0.006362837488199
- **w-rdist:** 0.6306479911439353
- **t-alpha:** 0.0062992257321734

---

---

195

- **AF ID:** AF-Q9Y6F1-F1-model-v4 | **Chain:** A
- **b-phipsi:** 0.0004284549296009
- **w-rdist:** 0.7470029912380296
- **t-alpha:** 0.1628757968965435

---

---

196

- **AF ID:** AF-Q9BQ52-F1-model-v4 | **Chain:** A
- **b-phipsi:** 0.001867639277135
- **w-rdist:** 0.3446761000116194
- **t-alpha:** 0.2190925441999474

---

---

197

- **AF ID:** AF-Q9Y4K0-F1-model-v4 | **Chain:** A
- **b-phipsi:** 0.0169518875076771
- **w-rdist:** 0.4916514558807628
- **t-alpha:** 0.0039277718342689

---

---

198

- **AF ID:** AF-Q8N5D0-F1-model-v4 | **Chain:** A
- **b-phipsi:** 0.0013398904888638
- **w-rdist:** 0.7725967222623062
- **t-alpha:** 0.0314772841117727

---

---

199

- **AF ID:** AF-P43304-F1-model-v4 | **Chain:** A
- **b-phipsi:** 0.0109712093947581
- **w-rdist:** 0.5390334729553228
- **t-alpha:** 0.0055077996884944

---

---

200

- **AF ID:** AF-Q96I27-F1-model-v4 | **Chain:** A
- **b-phipsi:** 0.0148087916289042
- **w-rdist:** 0.4276716641562573
- **t-alpha:** 0.0047169960791435

---

---

201

- **AF ID:** AF-Q96K75-F1-model-v4 | **Chain:** A
- **b-phipsi:** 0.0152532088029439
- **w-rdist:** 0.2070174825420308
- **t-alpha:** 0.0782468728132206

---

---

202

- **AF ID:** AF-Q9HCE5-F1-model-v4 | **Chain:** A
- **b-phipsi:** 0.0182094668317147
- **w-rdist:** 0.7027253677493336
- **t-alpha:** 0.0015647720218643

---

---

203

- **AF ID:** AF-O75676-F1-model-v4 | **Chain:** A
- **b-phipsi:** 0.008262369364704
- **w-rdist:** 0.3521393482387077
- **t-alpha:** 0.0813769138896134

---

---

204

- **AF ID:** AF-Q9HBM6-F1-model-v4 | **Chain:** A
- **b-phipsi:** 0.0065229147337254
- **w-rdist:** 0.7347308061897
- **t-alpha:** 0.0046944792707916

---

---

205

- **AF ID:** AF-Q3MJ62-F1-model-v4 | **Chain:** A
- **b-phipsi:** 0.0158586398176331
- **w-rdist:** 0.3296714681049177
- **t-alpha:** 0.0407169385382113

---

---

206

- **AF ID:** AF-Q9H9S5-F1-model-v4 | **Chain:** A
- **b-phipsi:** 0.0006737827654375
- **w-rdist:** 0.6653331422638522
- **t-alpha:** 0.2217974630874986

---

---

207

- **AF ID:** AF-Q99542-F1-model-v4 | **Chain:** A
- **b-phipsi:** 0.0104031401232127
- **w-rdist:** 0.832713666527723
- **t-alpha:** 0.0015676375257613

---

---

208

- **AF ID:** AF-P42681-F1-model-v4 | **Chain:** A
- **b-phipsi:** 0.0055707937685554
- **w-rdist:** 0.7037931086501747
- **t-alpha:** 0.0062593997286479

---

---

209

- **AF ID:** AF-Q8NAM6-F1-model-v4 | **Chain:** A
- **b-phipsi:** 0.0146497169377616
- **w-rdist:** 0.3067497012296835
- **t-alpha:** 0.0672924153855754

---

---

210

- **AF ID:** AF-Q15198-F1-model-v4 | **Chain:** A
- **b-phipsi:** 0.0509293479494086
- **w-rdist:** 0.4720800873097242
- **t-alpha:** 0.0015647720218643

---

---

211

- **AF ID:** AF-Q9Y661-F1-model-v4 | **Chain:** A
- **b-phipsi:** 0.0165905369750442
- **w-rdist:** 0.7424646947687851
- **t-alpha:** 0.0015647720218643

---

---

212

- **AF ID:** AF-Q5T5M9-F1-model-v4 | **Chain:** A
- **b-phipsi:** 0.0074381424656031
- **w-rdist:** 0.6666528166263035
- **t-alpha:** 0.0062593997286479

---

---

213

- **AF ID:** AF-Q9BSL1-F1-model-v4 | **Chain:** A
- **b-phipsi:** 0.0355473721069964
- **w-rdist:** 0.2987315188633505
- **t-alpha:** 0.0109543395722921

---

---

214

- **AF ID:** AF-P29597-F1-model-v4 | **Chain:** A
- **b-phipsi:** 0.0005346602209121
- **w-rdist:** 0.7046528905360911
- **t-alpha:** 0.2245691098208821

---

---

215

- **AF ID:** AF-Q02156-F1-model-v4 | **Chain:** A
- **b-phipsi:** 0.0100863400080858
- **w-rdist:** 0.280987730442124
- **t-alpha:** 0.1236302639257407

---

---

216

- **AF ID:** AF-P05546-F1-model-v4 | **Chain:** A
- **b-phipsi:** 0.0013469060312613
- **w-rdist:** 0.822883519967648
- **t-alpha:** 0.0306454151252038

---

---

217

- **AF ID:** AF-O95096-F1-model-v4 | **Chain:** A
- **b-phipsi:** 0.015108048923192
- **w-rdist:** 0.5890869116414406
- **t-alpha:** 0.0039277718342689

---

---

218

- **AF ID:** AF-Q9NUQ7-F1-model-v4 | **Chain:** A
- **b-phipsi:** 0.0008438634990577
- **w-rdist:** 0.8095959667499328
- **t-alpha:** 0.1142111519453266

---

---

219

- **AF ID:** AF-O14682-F1-model-v4 | **Chain:** A
- **b-phipsi:** 0.0011785561872531
- **w-rdist:** 0.7260666976616157
- **t-alpha:** 0.0848899960610365

---

---

220

- **AF ID:** AF-Q8NHY0-F1-model-v4 | **Chain:** A
- **b-phipsi:** 0.0012626139718598
- **w-rdist:** 0.6691454734294461
- **t-alpha:** 0.1017212837931069

---

---

221

- **AF ID:** AF-Q07075-F1-model-v4 | **Chain:** A
- **b-phipsi:** 0.012161627317115
- **w-rdist:** 0.1870851193659856
- **t-alpha:** 0.1384973100154414

---

---

222

- **AF ID:** AF-Q3V5L5-F1-model-v4 | **Chain:** A
- **b-phipsi:** 0.0084491228714651
- **w-rdist:** 0.3461167300526848
- **t-alpha:** 0.0978086738095724

---

---

223

- **AF ID:** AF-O15091-F1-model-v4 | **Chain:** A
- **b-phipsi:** 0.013619382735624
- **w-rdist:** 0.2139987811400375
- **t-alpha:** 0.1095460606668756

---

---

224

- **AF ID:** AF-Q96DM3-F1-model-v4 | **Chain:** A
- **b-phipsi:** 0.0007228696531036
- **w-rdist:** 0.932050547908876
- **t-alpha:** 0.0821341394260981

---

---

225

- **AF ID:** AF-A8K5M9-F1-model-v4 | **Chain:** A
- **b-phipsi:** 0.0032652318857181
- **w-rdist:** 0.3264179081662291
- **t-alpha:** 0.2148291421336507

---

---

226

- **AF ID:** AF-Q9H079-F1-model-v4 | **Chain:** A
- **b-phipsi:** 0.0085324155245785
- **w-rdist:** 0.8035592436126231
- **t-alpha:** 0.0031401242085145

---

---

227

- **AF ID:** AF-Q8IUF8-F1-model-v4 | **Chain:** A
- **b-phipsi:** 0.0005151051219028
- **w-rdist:** 0.7600670194074615
- **t-alpha:** 0.1811461842339983

---

---

228

- **AF ID:** AF-Q8NBF2-F1-model-v4 | **Chain:** A
- **b-phipsi:** 0.0159974007939685
- **w-rdist:** 0.3592583274331811
- **t-alpha:** 0.0328637472319743

---

---

229

- **AF ID:** AF-Q96HY7-F1-model-v4 | **Chain:** A
- **b-phipsi:** 0.0117162331729945
- **w-rdist:** 0.4069825272535075
- **t-alpha:** 0.0215828567205209

---

---

230

- **AF ID:** AF-Q7L3V2-F1-model-v4 | **Chain:** A
- **b-phipsi:** 0.0098892081173849
- **w-rdist:** 0.9288764934098795
- **t-alpha:** 0.0015647720218643

---

---

231

- **AF ID:** AF-Q6ZNA5-F1-model-v4 | **Chain:** A
- **b-phipsi:** 0.0013821541919931
- **w-rdist:** 0.8117997147802467
- **t-alpha:** 0.0364964556675917

---

---

232

- **AF ID:** AF-Q9Y279-F1-model-v4 | **Chain:** A
- **b-phipsi:** 0.0075004067915636
- **w-rdist:** 0.9631253831315124
- **t-alpha:** 0.0023473590503262

---

---

233

- **AF ID:** AF-O15270-F1-model-v4 | **Chain:** A
- **b-phipsi:** 0.0126392986441664
- **w-rdist:** 0.8776930413432869
- **t-alpha:** 0.0007832732121422

---

---

234

- **AF ID:** AF-O15259-F1-model-v4 | **Chain:** A
- **b-phipsi:** 0.0033090675324196
- **w-rdist:** 0.3047949096220604
- **t-alpha:** 0.2777771361272574

---

---

235

- **AF ID:** AF-Q9GZT8-F1-model-v4 | **Chain:** A
- **b-phipsi:** 0.0009143223636035
- **w-rdist:** 0.6964931794643553
- **t-alpha:** 0.1714024362978017

---

---

236

- **AF ID:** AF-Q86Y33-F1-model-v4 | **Chain:** A
- **b-phipsi:** 0.0118900580027822
- **w-rdist:** 0.6992879651424413
- **t-alpha:** 0.0039277718342689

---

---

237

- **AF ID:** AF-Q7Z7L1-F1-model-v4 | **Chain:** A
- **b-phipsi:** 0.0010819577042532
- **w-rdist:** 0.669755269595566
- **t-alpha:** 0.1525818235009972

---

---

238

- **AF ID:** AF-Q9UBS4-F1-model-v4 | **Chain:** A
- **b-phipsi:** 0.0007216604684733
- **w-rdist:** 0.7721033513877551
- **t-alpha:** 0.1513513905355301

---

---

239

- **AF ID:** AF-P50747-F1-model-v4 | **Chain:** A
- **b-phipsi:** 0.0066461051640196
- **w-rdist:** 0.3467575866013151
- **t-alpha:** 0.1345850125084049

---

---

240

- **AF ID:** AF-Q9NR19-F1-model-v4 | **Chain:** A
- **b-phipsi:** 0.0013119511109631
- **w-rdist:** 0.8621174962818907
- **t-alpha:** 0.0339808661347131

---

---

241

- **AF ID:** AF-Q9UIF7-F1-model-v4 | **Chain:** A
- **b-phipsi:** 0.0099946193231797
- **w-rdist:** 0.3555042319311572
- **t-alpha:** 0.0837240757413027

---

---

242

- **AF ID:** AF-Q9Y263-F1-model-v4 | **Chain:** A
- **b-phipsi:** 0.0012909430503541
- **w-rdist:** 0.5774276156501744
- **t-alpha:** 0.1510170937375228

---

---

243

- **AF ID:** AF-O15321-F1-model-v4 | **Chain:** A
- **b-phipsi:** 0.0298611489076037
- **w-rdist:** 0.5586079623115218
- **t-alpha:** 0.0023530945443304

---

---

244

- **AF ID:** AF-Q96GY3-F1-model-v4 | **Chain:** A
- **b-phipsi:** 0.0165475950735352
- **w-rdist:** 0.3878744882002043
- **t-alpha:** 0.0207667552249124

---

---

245

- **AF ID:** AF-Q6NUN0-F1-model-v4 | **Chain:** A
- **b-phipsi:** 0.000865633292829
- **w-rdist:** 0.9056201671129692
- **t-alpha:** 0.0848899960610365

---

---

246

- **AF ID:** AF-Q8NFF5-F1-model-v4 | **Chain:** A
- **b-phipsi:** 0.0025952982663594
- **w-rdist:** 0.3318376657380487
- **t-alpha:** 0.2949924532828514

---

---

247

- **AF ID:** AF-P49005-F1-model-v4 | **Chain:** A
- **b-phipsi:** 0.0002873733048159
- **w-rdist:** 0.943238333765624
- **t-alpha:** 0.1410718287960064

---

---

248

- **AF ID:** AF-A6NJ46-F1-model-v4 | **Chain:** A
- **b-phipsi:** 0.0130010592033661
- **w-rdist:** 0.737805642027112
- **t-alpha:** 0.0031296009050172

---

---

249

- **AF ID:** AF-Q99467-F1-model-v4 | **Chain:** A
- **b-phipsi:** 0.0128982564561379
- **w-rdist:** 0.8718677811529041
- **t-alpha:** 0.0015647720218643

---

---

250

- **AF ID:** AF-Q86YV9-F1-model-v4 | **Chain:** A
- **b-phipsi:** 0.0007675888277235
- **w-rdist:** 1.1039026530538465
- **t-alpha:** 0.0492615627855226

---

---

251

- **AF ID:** AF-Q7Z7J5-F1-model-v4 | **Chain:** A
- **b-phipsi:** 0.0172236727375582
- **w-rdist:** 0.3597421193813316
- **t-alpha:** 0.0348178944724737

---

---

252

- **AF ID:** AF-Q8IWU9-F1-model-v4 | **Chain:** A
- **b-phipsi:** 0.0068578014126461
- **w-rdist:** 0.786132363054606
- **t-alpha:** 0.0047169960791435

---

---

253

- **AF ID:** AF-Q8N987-F1-model-v4 | **Chain:** A
- **b-phipsi:** 0.0262296846290139
- **w-rdist:** 0.3478983633012674
- **t-alpha:** 0.0215828567205209

---

---

254

- **AF ID:** AF-O94923-F1-model-v4 | **Chain:** A
- **b-phipsi:** 0.0123978979236029
- **w-rdist:** 0.2987441343286697
- **t-alpha:** 0.1197177972971978

---

---

255

- **AF ID:** AF-O43304-F1-model-v4 | **Chain:** A
- **b-phipsi:** 0.0011745829648997
- **w-rdist:** 0.9215924610773348
- **t-alpha:** 0.0461656134308179

---

---

256

- **AF ID:** AF-P17858-F1-model-v4 | **Chain:** A
- **b-phipsi:** 0.0103057592527072
- **w-rdist:** 0.7182611387695954
- **t-alpha:** 0.0046944792707916

---

---

257

- **AF ID:** AF-A8MY62-F1-model-v4 | **Chain:** A
- **b-phipsi:** 0.001437626489409
- **w-rdist:** 0.6916987132862725
- **t-alpha:** 0.085811726945665

---

---

258

- **AF ID:** AF-P05129-F1-model-v4 | **Chain:** A
- **b-phipsi:** 0.0099694484018605
- **w-rdist:** 0.6394165460166938
- **t-alpha:** 0.0062992257321734

---

---

259

- **AF ID:** AF-Q13087-F1-model-v4 | **Chain:** A
- **b-phipsi:** 0.0006411606292397
- **w-rdist:** 0.8922477175305588
- **t-alpha:** 0.1380234763072123

---

---

260

- **AF ID:** AF-P11150-F1-model-v4 | **Chain:** A
- **b-phipsi:** 0.0008008125632743
- **w-rdist:** 0.7279163451056516
- **t-alpha:** 0.2033899315381668

---

---

261

- **AF ID:** AF-Q9HAZ1-F1-model-v4 | **Chain:** A
- **b-phipsi:** 0.0123548084738428
- **w-rdist:** 0.7588141940695486
- **t-alpha:** 0.0031401242085145

---

---

262

- **AF ID:** AF-Q6URK8-F1-model-v4 | **Chain:** A
- **b-phipsi:** 0.0127851438345082
- **w-rdist:** 0.8203812932456582
- **t-alpha:** 0.0023530945443304

---

---

263

- **AF ID:** AF-Q8N6K7-F1-model-v4 | **Chain:** A
- **b-phipsi:** 0.0246949374063499
- **w-rdist:** 0.303556343034199
- **t-alpha:** 0.0579025721066006

---

---

264

- **AF ID:** AF-Q6IEE8-F1-model-v4 | **Chain:** A
- **b-phipsi:** 0.0012533565203484
- **w-rdist:** 0.5812954270721717
- **t-alpha:** 0.1833334788306171

---

---

265

- **AF ID:** AF-Q86VX9-F1-model-v4 | **Chain:** A
- **b-phipsi:** 0.0024457579884461
- **w-rdist:** 0.4101582533121939
- **t-alpha:** 0.1502347029199773

---

---

266

- **AF ID:** AF-Q9UJ14-F1-model-v4 | **Chain:** A
- **b-phipsi:** 0.0026603280353466
- **w-rdist:** 0.8807033017785622
- **t-alpha:** 0.0070418134049574

---

---

267

- **AF ID:** AF-Q9H7Z6-F1-model-v4 | **Chain:** A
- **b-phipsi:** 0.0005027530162076
- **w-rdist:** 0.8394100310835765
- **t-alpha:** 0.1943929371170751

---

---

268

- **AF ID:** AF-O15499-F1-model-v4 | **Chain:** A
- **b-phipsi:** 0.0065334342118462
- **w-rdist:** 0.3264184447862106
- **t-alpha:** 0.1921641665135889

---

---

269

- **AF ID:** AF-Q6STE5-F1-model-v4 | **Chain:** A
- **b-phipsi:** 0.002907019391825
- **w-rdist:** 1.211740472623951
- **t-alpha:** 0.0023530945443304

---

---

270

- **AF ID:** AF-Q8N6U8-F1-model-v4 | **Chain:** A
- **b-phipsi:** 0.0970164748900244
- **w-rdist:** 0.6175246880174623
- **t-alpha:** 0.0007823187912934

---

---

271

- **AF ID:** AF-P31751-F1-model-v4 | **Chain:** A
- **b-phipsi:** 0.0010341475049471
- **w-rdist:** 0.833930723354103
- **t-alpha:** 0.1017244358474924

---

---

272

- **AF ID:** AF-Q96F63-F1-model-v4 | **Chain:** A
- **b-phipsi:** 0.0114787114608796
- **w-rdist:** 0.6563118417812663
- **t-alpha:** 0.0062593997286479

---

---

273

- **AF ID:** AF-Q86SF2-F1-model-v4 | **Chain:** A
- **b-phipsi:** 0.0062815930239242
- **w-rdist:** 0.4053485195857602
- **t-alpha:** 0.0884191622029941

---

---

274

- **AF ID:** AF-O95561-F1-model-v4 | **Chain:** A
- **b-phipsi:** 0.0172064554329041
- **w-rdist:** 0.341875020839247
- **t-alpha:** 0.0676695952069212

---

---

275

- **AF ID:** AF-Q9Y679-F1-model-v4 | **Chain:** A
- **b-phipsi:** 0.0184659842862187
- **w-rdist:** 0.5812381309625084
- **t-alpha:** 0.0047169960791435

---

---

276

- **AF ID:** AF-Q7Z304-F1-model-v4 | **Chain:** A
- **b-phipsi:** 0.0328484126170433
- **w-rdist:** 0.3158427872734938
- **t-alpha:** 0.0323103167719089

---

---

277

- **AF ID:** AF-Q49A17-F1-model-v4 | **Chain:** A
- **b-phipsi:** 0.0009856285480128
- **w-rdist:** 0.7030801821018259
- **t-alpha:** 0.191705500952412

---

---

278

- **AF ID:** AF-Q658Y4-F1-model-v4 | **Chain:** A
- **b-phipsi:** 0.0108951854051328
- **w-rdist:** 0.2917628410796243
- **t-alpha:** 0.1588415973392283

---

---

279

- **AF ID:** AF-O95388-F1-model-v4 | **Chain:** A
- **b-phipsi:** 0.0449384334468778
- **w-rdist:** 0.6252677537761346
- **t-alpha:** 0.0015676375257613

---

---

280

- **AF ID:** AF-Q13572-F1-model-v4 | **Chain:** A
- **b-phipsi:** 0.0010162400813408
- **w-rdist:** 1.0078803585543168
- **t-alpha:** 0.0597019506692302

---

---

281

- **AF ID:** AF-Q5TEA3-F1-model-v4 | **Chain:** A
- **b-phipsi:** 0.003183618624661
- **w-rdist:** 1.1650066312129757
- **t-alpha:** 0.0031296009050172

---

---

282

- **AF ID:** AF-P06681-F1-model-v4 | **Chain:** A
- **b-phipsi:** 0.0046501548613578
- **w-rdist:** 0.392410540083425
- **t-alpha:** 0.142091393527403

---

---

283

- **AF ID:** AF-A0A0G2JN68-F1-model-v4 | **Chain:** A
- **b-phipsi:** 0.0139358079761161
- **w-rdist:** 0.9515097745093009
- **t-alpha:** 0.0007832732121422

---

---

284

- **AF ID:** AF-Q9NZP8-F1-model-v4 | **Chain:** A
- **b-phipsi:** 0.0311827937893894
- **w-rdist:** 0.7247018142605257
- **t-alpha:** 0.0007832732121422

---

---

285

- **AF ID:** AF-Q96C03-F1-model-v4 | **Chain:** A
- **b-phipsi:** 0.0136780433648513
- **w-rdist:** 0.5972326921625425
- **t-alpha:** 0.0062593997286479

---

---

286

- **AF ID:** AF-Q6P988-F1-model-v4 | **Chain:** A
- **b-phipsi:** 0.0125747533059254
- **w-rdist:** 0.9243314961976437
- **t-alpha:** 0.0015676375257613

---

---

287

- **AF ID:** AF-P0CB48-F1-model-v4 | **Chain:** A
- **b-phipsi:** 0.0261818838200809
- **w-rdist:** 0.3235473054399594
- **t-alpha:** 0.0461656134308179

---

---

288

- **AF ID:** AF-Q86YD1-F1-model-v4 | **Chain:** A
- **b-phipsi:** 0.0197275768193769
- **w-rdist:** 0.6932627340504037
- **t-alpha:** 0.0031401242085145

---

---

289

- **AF ID:** AF-O43143-F1-model-v4 | **Chain:** A
- **b-phipsi:** 0.0098437914765592
- **w-rdist:** 0.3981253963904572
- **t-alpha:** 0.06807491933535

---

---

290

- **AF ID:** AF-P23109-F1-model-v4 | **Chain:** A
- **b-phipsi:** 0.0180701448279157
- **w-rdist:** 0.6315858795564099
- **t-alpha:** 0.0046944792707916

---

---

291

- **AF ID:** AF-P14735-F1-model-v4 | **Chain:** A
- **b-phipsi:** 0.006491385168927
- **w-rdist:** 0.3741534846359967
- **t-alpha:** 0.1431919985610661

---

---

292

- **AF ID:** AF-P52790-F1-model-v4 | **Chain:** A
- **b-phipsi:** 0.0123866978416493
- **w-rdist:** 1.0736631654536797
- **t-alpha:** 0.0

---

---

293

- **AF ID:** AF-Q8N6M6-F1-model-v4 | **Chain:** A
- **b-phipsi:** 0.0062754314632262
- **w-rdist:** 0.3889331167076449
- **t-alpha:** 0.1244127877301322

---

---

294

- **AF ID:** AF-P48066-F1-model-v4 | **Chain:** A
- **b-phipsi:** 0.0821276430746645
- **w-rdist:** 0.6784110313249044
- **t-alpha:** 0.0

---

---

295

- **AF ID:** AF-O14733-F1-model-v4 | **Chain:** A
- **b-phipsi:** 0.0087373943853048
- **w-rdist:** 0.8102410979207547
- **t-alpha:** 0.0047169960791435

---

---

296

- **AF ID:** AF-Q9H6W3-F1-model-v4 | **Chain:** A
- **b-phipsi:** 0.0030382315127691
- **w-rdist:** 0.4057837841044918
- **t-alpha:** 0.1580594099485004

---

---

297

- **AF ID:** AF-Q08AH3-F1-model-v4 | **Chain:** A
- **b-phipsi:** 0.0008586912995201
- **w-rdist:** 0.9223171950885553
- **t-alpha:** 0.122036887689374

---

---

298

- **AF ID:** AF-Q06278-F1-model-v4 | **Chain:** A
- **b-phipsi:** 0.002869155455332
- **w-rdist:** 0.3813752527994341
- **t-alpha:** 0.233177008258818

---

---

299

- **AF ID:** AF-Q68CQ4-F1-model-v4 | **Chain:** A
- **b-phipsi:** 0.0075602479271167
- **w-rdist:** 0.3314666042174606
- **t-alpha:** 0.1971823345770407

---

---

300

- **AF ID:** AF-Q14654-F1-model-v4 | **Chain:** A
- **b-phipsi:** 0.000778894002403
- **w-rdist:** 1.018266589602061
- **t-alpha:** 0.1007755496371616

---

---

301

- **AF ID:** AF-P23378-F1-model-v4 | **Chain:** A
- **b-phipsi:** 0.0113979463295013
- **w-rdist:** 0.3439440495491683
- **t-alpha:** 0.1283255184376883

---

---

302

- **AF ID:** AF-Q9Y6Y0-F1-model-v4 | **Chain:** A
- **b-phipsi:** 0.0124934733539138
- **w-rdist:** 0.2355008293389405
- **t-alpha:** 0.1870108035718682

---

---

303

- **AF ID:** AF-Q9P2N7-F1-model-v4 | **Chain:** A
- **b-phipsi:** 0.001201677282436
- **w-rdist:** 0.8478927719819151
- **t-alpha:** 0.0899840291479125

---

---

304

- **AF ID:** AF-Q9NXW2-F1-model-v4 | **Chain:** A
- **b-phipsi:** 0.0270735368169637
- **w-rdist:** 0.6650466762793064
- **t-alpha:** 0.0031401242085145

---

---

305

- **AF ID:** AF-Q9H4T2-F1-model-v4 | **Chain:** A
- **b-phipsi:** 0.015758071522224
- **w-rdist:** 0.3753594170396275
- **t-alpha:** 0.0623444493098053

---

---

306

- **AF ID:** AF-O00151-F1-model-v4 | **Chain:** A
- **b-phipsi:** 0.0178880341871712
- **w-rdist:** 0.6869648468578637
- **t-alpha:** 0.0046944792707916

---

---

307

- **AF ID:** AF-O75342-F1-model-v4 | **Chain:** A
- **b-phipsi:** 0.009745837884596
- **w-rdist:** 0.7190274415454255
- **t-alpha:** 0.0062992257321734

---

---

308

- **AF ID:** AF-P58215-F1-model-v4 | **Chain:** A
- **b-phipsi:** 0.025473006645756
- **w-rdist:** 0.3555217444560142
- **t-alpha:** 0.0438181656914413

---

---

309

- **AF ID:** AF-P61565-F1-model-v4 | **Chain:** A
- **b-phipsi:** 0.0009708783482958
- **w-rdist:** 1.074344593919922
- **t-alpha:** 0.0696396085969683

---

---

310

- **AF ID:** AF-Q969Y0-F1-model-v4 | **Chain:** A
- **b-phipsi:** 0.000560271304684
- **w-rdist:** 0.8481302522791754
- **t-alpha:** 0.2276660438302298

---

---

311

- **AF ID:** AF-Q12887-F1-model-v4 | **Chain:** A
- **b-phipsi:** 0.0480097424366843
- **w-rdist:** 0.715679757844929
- **t-alpha:** 0.0007832732121422

---

---

312

- **AF ID:** AF-Q9HB09-F1-model-v4 | **Chain:** A
- **b-phipsi:** 0.0128530689681626
- **w-rdist:** 0.2766597105224845
- **t-alpha:** 0.1760561506159503

---

---

313

- **AF ID:** AF-P54578-F1-model-v4 | **Chain:** A
- **b-phipsi:** 0.001338960547716
- **w-rdist:** 0.9425609885482954
- **t-alpha:** 0.0527184999556347

---

---

314

- **AF ID:** AF-P07992-F1-model-v4 | **Chain:** A
- **b-phipsi:** 0.0010758205528904
- **w-rdist:** 0.7116676872737253
- **t-alpha:** 0.2079397305749943

---

---

315

- **AF ID:** AF-Q902F9-F1-model-v4 | **Chain:** A
- **b-phipsi:** 0.0008224615104484
- **w-rdist:** 1.0549469140216483
- **t-alpha:** 0.0985915493938487

---

---

316

- **AF ID:** AF-Q13569-F1-model-v4 | **Chain:** A
- **b-phipsi:** 0.0112537646232079
- **w-rdist:** 0.7568097891119377
- **t-alpha:** 0.0054771144938834

---

---

317

- **AF ID:** AF-Q96JB6-F1-model-v4 | **Chain:** A
- **b-phipsi:** 0.0211953072337025
- **w-rdist:** 0.3719614934198416
- **t-alpha:** 0.0492615627855226

---

---

318

- **AF ID:** AF-Q494W8-F1-model-v4 | **Chain:** A
- **b-phipsi:** 0.0058104116580863
- **w-rdist:** 1.2283966462624336
- **t-alpha:** 0.0023473590503262

---

---

319

- **AF ID:** AF-P35626-F1-model-v4 | **Chain:** A
- **b-phipsi:** 0.0126839095266017
- **w-rdist:** 0.3985237947947808
- **t-alpha:** 0.0665099761701244

---

---

320

- **AF ID:** AF-P24347-F1-model-v4 | **Chain:** A
- **b-phipsi:** 0.0272122709796127
- **w-rdist:** 0.500765284485057
- **t-alpha:** 0.0055077996884944

---

---

321

- **AF ID:** AF-O00178-F1-model-v4 | **Chain:** A
- **b-phipsi:** 0.0197085776205423
- **w-rdist:** 0.5494505251260823
- **t-alpha:** 0.0070418134049574

---

---

322

- **AF ID:** AF-O60884-F1-model-v4 | **Chain:** A
- **b-phipsi:** 0.0150445717756684
- **w-rdist:** 0.9795238675469676
- **t-alpha:** 0.0015676375257613

---

---

323

- **AF ID:** AF-Q53EQ6-F1-model-v4 | **Chain:** A
- **b-phipsi:** 0.0116958937485468
- **w-rdist:** 0.9200345785187872
- **t-alpha:** 0.0031401242085145

---

---

324

- **AF ID:** AF-Q9H4B4-F1-model-v4 | **Chain:** A
- **b-phipsi:** 0.0072739074247356
- **w-rdist:** 0.4103183366009051
- **t-alpha:** 0.1165880511557739

---

---

325

- **AF ID:** AF-Q9H172-F1-model-v4 | **Chain:** A
- **b-phipsi:** 0.0240761505242971
- **w-rdist:** 0.6201017566983258
- **t-alpha:** 0.0047169960791435

---

---

326

- **AF ID:** AF-P31268-F1-model-v4 | **Chain:** A
- **b-phipsi:** 0.0272831180594313
- **w-rdist:** 0.756469583350903
- **t-alpha:** 0.0023473590503262

---

---

327

- **AF ID:** AF-O43847-F1-model-v4 | **Chain:** A
- **b-phipsi:** 0.0084604257003765
- **w-rdist:** 0.3275571440255045
- **t-alpha:** 0.2316115052058898

---

---

328

- **AF ID:** AF-Q9NPC6-F1-model-v4 | **Chain:** A
- **b-phipsi:** 0.0120335014048528
- **w-rdist:** 0.8738703037048836
- **t-alpha:** 0.0039119745263893

---

---

329

- **AF ID:** AF-Q9H5U6-F1-model-v4 | **Chain:** A
- **b-phipsi:** 0.0013892830488539
- **w-rdist:** 0.7323989226930814
- **t-alpha:** 0.1250000381984894

---

---

330

- **AF ID:** AF-O60909-F1-model-v4 | **Chain:** A
- **b-phipsi:** 0.0007634446209518
- **w-rdist:** 0.8165313478167533
- **t-alpha:** 0.2492671106308526

---

---

331

- **AF ID:** AF-O76064-F1-model-v4 | **Chain:** A
- **b-phipsi:** 0.0075565791105192
- **w-rdist:** 1.426867246027824
- **t-alpha:** 0.0

---

---

332

- **AF ID:** AF-Q08AH1-F1-model-v4 | **Chain:** A
- **b-phipsi:** 0.0002983397029619
- **w-rdist:** 0.9434132579800958
- **t-alpha:** 0.2407770290234512

---

---

333

- **AF ID:** AF-Q9HCG7-F1-model-v4 | **Chain:** A
- **b-phipsi:** 0.0097091195516985
- **w-rdist:** 0.3638571780222124
- **t-alpha:** 0.1557116053874756

---

---

334

- **AF ID:** AF-Q6NSJ0-F1-model-v4 | **Chain:** A
- **b-phipsi:** 0.0014109835586888
- **w-rdist:** 0.8539263637801492
- **t-alpha:** 0.0721478806100059

---

---

335

- **AF ID:** AF-Q06203-F1-model-v4 | **Chain:** A
- **b-phipsi:** 0.0011280723343217
- **w-rdist:** 1.0077501459130862
- **t-alpha:** 0.0803043978200703

---

---

336

- **AF ID:** AF-Q6GPH6-F1-model-v4 | **Chain:** A
- **b-phipsi:** 0.0286824948413238
- **w-rdist:** 0.3946363994894931
- **t-alpha:** 0.0164318357763795

---

---

337

- **AF ID:** AF-P06737-F1-model-v4 | **Chain:** A
- **b-phipsi:** 0.0286227040821322
- **w-rdist:** 0.6040269829335044
- **t-alpha:** 0.0046944792707916

---

---

338

- **AF ID:** AF-P0CF97-F1-model-v4 | **Chain:** A
- **b-phipsi:** 0.0368276164461136
- **w-rdist:** 0.2761560657871184
- **t-alpha:** 0.0813769138896134

---

---

339

- **AF ID:** AF-P31323-F1-model-v4 | **Chain:** A
- **b-phipsi:** 0.008144565291511
- **w-rdist:** 0.8187163541737892
- **t-alpha:** 0.0070418134049574

---

---

340

- **AF ID:** AF-Q13490-F1-model-v4 | **Chain:** A
- **b-phipsi:** 0.0129366356817275
- **w-rdist:** 0.3804753665791623
- **t-alpha:** 0.092331642604805

---

---

341

- **AF ID:** AF-Q96QE2-F1-model-v4 | **Chain:** A
- **b-phipsi:** 0.0619776591687699
- **w-rdist:** 0.6080820479894149
- **t-alpha:** 0.0031296009050172

---

---

342

- **AF ID:** AF-O95427-F1-model-v4 | **Chain:** A
- **b-phipsi:** 0.0624597293447886
- **w-rdist:** 0.5395615563323191
- **t-alpha:** 0.0039119745263893

---

---

343

- **AF ID:** AF-P38435-F1-model-v4 | **Chain:** A
- **b-phipsi:** 0.0234719168979078
- **w-rdist:** 0.3853542069985564
- **t-alpha:** 0.0430355279269489

---

---

344

- **AF ID:** AF-Q8NGU9-F1-model-v4 | **Chain:** A
- **b-phipsi:** 0.034819652781938
- **w-rdist:** 0.6852932588869302
- **t-alpha:** 0.0031401242085145

---

---

345

- **AF ID:** AF-Q2TBF2-F1-model-v4 | **Chain:** A
- **b-phipsi:** 0.0085202928716423
- **w-rdist:** 0.4074954887943487
- **t-alpha:** 0.1181528955465753

---

---

346

- **AF ID:** AF-P78395-F1-model-v4 | **Chain:** A
- **b-phipsi:** 0.0150553649951798
- **w-rdist:** 0.4099629536559361
- **t-alpha:** 0.0570720736347449

---

---

347

- **AF ID:** AF-Q5JRX3-F1-model-v4 | **Chain:** A
- **b-phipsi:** 0.0089407695706053
- **w-rdist:** 0.3708671106977231
- **t-alpha:** 0.1760561506159503

---

---

348

- **AF ID:** AF-Q3MIR4-F1-model-v4 | **Chain:** A
- **b-phipsi:** 0.0011854159416068
- **w-rdist:** 0.7190071951988584
- **t-alpha:** 0.2148291421336507

---

---

349

- **AF ID:** AF-Q9Y6M1-F1-model-v4 | **Chain:** A
- **b-phipsi:** 0.0141005781736621
- **w-rdist:** 0.3477905673470676
- **t-alpha:** 0.1392798657818292

---

---

350

- **AF ID:** AF-P49619-F1-model-v4 | **Chain:** A
- **b-phipsi:** 0.0119882811330794
- **w-rdist:** 0.3212359252203393
- **t-alpha:** 0.2065719520009707

---

---

351

- **AF ID:** AF-Q8IW41-F1-model-v4 | **Chain:** A
- **b-phipsi:** 0.024084168610941
- **w-rdist:** 0.7011491704184214
- **t-alpha:** 0.0046944792707916

---

---

352

- **AF ID:** AF-Q8N9I0-F1-model-v4 | **Chain:** A
- **b-phipsi:** 0.0194232085644787
- **w-rdist:** 0.6394029680810231
- **t-alpha:** 0.0062992257321734

---

---

353

- **AF ID:** AF-O94941-F1-model-v4 | **Chain:** A
- **b-phipsi:** 0.0179864625465216
- **w-rdist:** 0.9123361059728978
- **t-alpha:** 0.0023530945443304

---

---

354

- **AF ID:** AF-O95361-F1-model-v4 | **Chain:** A
- **b-phipsi:** 0.0100203482629771
- **w-rdist:** 1.327406186192246
- **t-alpha:** 0.0

---

---

355

- **AF ID:** AF-Q96MR7-F1-model-v4 | **Chain:** A
- **b-phipsi:** 0.013452786960544
- **w-rdist:** 0.2710368947502165
- **t-alpha:** 0.2431909337449638

---

---

356

- **AF ID:** AF-Q9NQ55-F1-model-v4 | **Chain:** A
- **b-phipsi:** 0.0171045847508843
- **w-rdist:** 0.9102285736657452
- **t-alpha:** 0.0031296009050172

---

---

357

- **AF ID:** AF-Q96LR2-F1-model-v4 | **Chain:** A
- **b-phipsi:** 0.0138833756133022
- **w-rdist:** 0.4055296005083176
- **t-alpha:** 0.0739499805270313

---

---

358

- **AF ID:** AF-Q8IVL6-F1-model-v4 | **Chain:** A
- **b-phipsi:** 0.0168611470407176
- **w-rdist:** 0.3752321259780959
- **t-alpha:** 0.0938963969607324

---

---

359

- **AF ID:** AF-Q4W5G0-F1-model-v4 | **Chain:** A
- **b-phipsi:** 0.0279242049201855
- **w-rdist:** 0.3984350570586464
- **t-alpha:** 0.0305161718713362

---

---

360

- **AF ID:** AF-O95704-F1-model-v4 | **Chain:** A
- **b-phipsi:** 0.0190573411219344
- **w-rdist:** 0.3965912965011053
- **t-alpha:** 0.0605810409571925

---

---

361

- **AF ID:** AF-Q92904-F1-model-v4 | **Chain:** A
- **b-phipsi:** 0.0538094105606171
- **w-rdist:** 0.8525101142542661
- **t-alpha:** 0.0

---

---

362

- **AF ID:** AF-Q17RR3-F1-model-v4 | **Chain:** A
- **b-phipsi:** 0.0009702563214524
- **w-rdist:** 0.832121875423134
- **t-alpha:** 0.2217974630874986

---

---

363

- **AF ID:** AF-P16885-F1-model-v4 | **Chain:** A
- **b-phipsi:** 0.0006911149537613
- **w-rdist:** 0.9315943633974334
- **t-alpha:** 0.239436486351334

---

---

364

- **AF ID:** AF-Q8N594-F1-model-v4 | **Chain:** A
- **b-phipsi:** 0.0352301312310405
- **w-rdist:** 0.6730686194614053
- **t-alpha:** 0.0039277718342689

---

---

365

- **AF ID:** AF-Q9UNZ2-F1-model-v4 | **Chain:** A
- **b-phipsi:** 0.007326108913486
- **w-rdist:** 1.1085511749821746
- **t-alpha:** 0.0039277718342689

---

---

366

- **AF ID:** AF-Q8N109-F1-model-v4 | **Chain:** A
- **b-phipsi:** 0.0178857604851904
- **w-rdist:** 0.9626638948446852
- **t-alpha:** 0.0023530945443304

---

---

367

- **AF ID:** AF-Q9Y6K8-F1-model-v4 | **Chain:** A
- **b-phipsi:** 0.0270887383125164
- **w-rdist:** 0.30444901173617
- **t-alpha:** 0.1236302639257407

---

---

368

- **AF ID:** AF-O43246-F1-model-v4 | **Chain:** A
- **b-phipsi:** 0.0814504566768533
- **w-rdist:** 0.6933817478229208
- **t-alpha:** 0.0023530945443304

---

---

369

- **AF ID:** AF-Q6XYB7-F1-model-v4 | **Chain:** A
- **b-phipsi:** 0.008264876421916
- **w-rdist:** 0.375207154969081
- **t-alpha:** 0.2125240767486365

---

---

370

- **AF ID:** AF-Q9BWW7-F1-model-v4 | **Chain:** A
- **b-phipsi:** 0.0274090476577077
- **w-rdist:** 0.3953374205092465
- **t-alpha:** 0.0383411162518374

---

---

371

- **AF ID:** AF-Q9Y2P4-F1-model-v4 | **Chain:** A
- **b-phipsi:** 0.0014258516374056
- **w-rdist:** 0.8748709326477633
- **t-alpha:** 0.0923076831090272

---

---

372

- **AF ID:** AF-O14772-F1-model-v4 | **Chain:** A
- **b-phipsi:** 0.0011175836324297
- **w-rdist:** 0.8718907218016254
- **t-alpha:** 0.1660585522186397

---

---

373

- **AF ID:** AF-Q9NXZ2-F1-model-v4 | **Chain:** A
- **b-phipsi:** 0.0074852495781933
- **w-rdist:** 0.3778222738218016
- **t-alpha:** 0.2261338465746794

---

---

374

- **AF ID:** AF-A8MTZ7-F1-model-v4 | **Chain:** A
- **b-phipsi:** 0.0197395251209574
- **w-rdist:** 0.8460081620805153
- **t-alpha:** 0.0031401242085145

---

---

375

- **AF ID:** AF-Q6V1X1-F1-model-v4 | **Chain:** A
- **b-phipsi:** 0.0095344436632468
- **w-rdist:** 0.3846186759006202
- **t-alpha:** 0.1737087976681894

---

---

376

- **AF ID:** AF-Q15084-F1-model-v4 | **Chain:** A
- **b-phipsi:** 0.0010701792426773
- **w-rdist:** 0.9950950615469883
- **t-alpha:** 0.1339841070344893

---

---

377

- **AF ID:** AF-Q8NHU2-F1-model-v4 | **Chain:** A
- **b-phipsi:** 0.0007422199871663
- **w-rdist:** 0.883502834104144
- **t-alpha:** 0.2965569088368105

---

---

378

- **AF ID:** AF-P0DMR1-F1-model-v4 | **Chain:** A
- **b-phipsi:** 0.0246722805317734
- **w-rdist:** 0.9870713302267764
- **t-alpha:** 0.0015647720218643

---

---

379

- **AF ID:** AF-P59910-F1-model-v4 | **Chain:** A
- **b-phipsi:** 0.0005937355573534
- **w-rdist:** 0.8961892253833736
- **t-alpha:** 0.3668451105429142

---

---

380

- **AF ID:** AF-P58317-F1-model-v4 | **Chain:** A
- **b-phipsi:** 0.0255476103960864
- **w-rdist:** 0.3820426139401294
- **t-alpha:** 0.0649447869300658

---

---

381

- **AF ID:** AF-P17028-F1-model-v4 | **Chain:** A
- **b-phipsi:** 0.020162783461958
- **w-rdist:** 0.4100773737433666
- **t-alpha:** 0.050077913367889

---

---

382

- **AF ID:** AF-Q9Y5A7-F1-model-v4 | **Chain:** A
- **b-phipsi:** 0.0576267667377656
- **w-rdist:** 0.5945668068645176
- **t-alpha:** 0.0046944792707916

---

---

383

- **AF ID:** AF-Q8IV36-F1-model-v4 | **Chain:** A
- **b-phipsi:** 0.0412283135912685
- **w-rdist:** 0.3871410415524823
- **t-alpha:** 0.0320809565897601

---

---

384

- **AF ID:** AF-Q96PB1-F1-model-v4 | **Chain:** A
- **b-phipsi:** 0.071433142303887
- **w-rdist:** 0.5053451403309215
- **t-alpha:** 0.0054771144938834

---

---

385

- **AF ID:** AF-Q15822-F1-model-v4 | **Chain:** A
- **b-phipsi:** 0.0013202726057484
- **w-rdist:** 1.3212656860822285
- **t-alpha:** 0.011876942219535

---

---

386

- **AF ID:** AF-Q9H1B5-F1-model-v4 | **Chain:** A
- **b-phipsi:** 0.0050711834235776
- **w-rdist:** 0.3949627338936747
- **t-alpha:** 0.2809078497795689

---

---

387

- **AF ID:** AF-Q9NYK6-F1-model-v4 | **Chain:** A
- **b-phipsi:** 0.036898694957171
- **w-rdist:** 0.3972070969212952
- **t-alpha:** 0.0297336873714129

---

---

388

- **AF ID:** AF-Q9H2B2-F1-model-v4 | **Chain:** A
- **b-phipsi:** 0.0325572908683162
- **w-rdist:** 0.771982450835326
- **t-alpha:** 0.0031296009050172

---

---

389

- **AF ID:** AF-Q53G44-F1-model-v4 | **Chain:** A
- **b-phipsi:** 0.0010642945833599
- **w-rdist:** 0.8198345832932296
- **t-alpha:** 0.2444014267390346

---

---

390

- **AF ID:** AF-Q6PI47-F1-model-v4 | **Chain:** A
- **b-phipsi:** 0.0096691111072386
- **w-rdist:** 1.0868655171822106
- **t-alpha:** 0.0039277718342689

---

---

391

- **AF ID:** AF-Q86YL5-F1-model-v4 | **Chain:** A
- **b-phipsi:** 0.0139828973277044
- **w-rdist:** 0.3772432512114768
- **t-alpha:** 0.1410718287960064

---

---

392

- **AF ID:** AF-Q9HB96-F1-model-v4 | **Chain:** A
- **b-phipsi:** 0.026995024944406
- **w-rdist:** 0.3029224961363935
- **t-alpha:** 0.1502347029199773

---

---

393

- **AF ID:** AF-Q9UK23-F1-model-v4 | **Chain:** A
- **b-phipsi:** 0.0240366259374819
- **w-rdist:** 0.8218608297267626
- **t-alpha:** 0.0039119745263893

---

---

394

- **AF ID:** AF-Q8IVP9-F1-model-v4 | **Chain:** A
- **b-phipsi:** 0.0237611154646705
- **w-rdist:** 0.4112587989008231
- **t-alpha:** 0.0492615627855226

---

---

395

- **AF ID:** AF-Q9C0D3-F1-model-v4 | **Chain:** A
- **b-phipsi:** 0.0602607871189734
- **w-rdist:** 0.7012507623948235
- **t-alpha:** 0.0031401242085145

---

---

396

- **AF ID:** AF-Q03431-F1-model-v4 | **Chain:** A
- **b-phipsi:** 0.0176718538253901
- **w-rdist:** 1.1919081101108528
- **t-alpha:** 0.0007823187912934

---

---

397

- **AF ID:** AF-Q9UKY7-F1-model-v4 | **Chain:** A
- **b-phipsi:** 0.0391288134788828
- **w-rdist:** 0.9815290163149192
- **t-alpha:** 0.0007823187912934

---

---

398

- **AF ID:** AF-Q9UP52-F1-model-v4 | **Chain:** A
- **b-phipsi:** 0.0068618956262147
- **w-rdist:** 0.4085079391038784
- **t-alpha:** 0.204224556621507

---

---

399

- **AF ID:** AF-Q9BV79-F1-model-v4 | **Chain:** A
- **b-phipsi:** 0.0009836722396355
- **w-rdist:** 0.9674321458598092
- **t-alpha:** 0.1977510471938199

---

---

400

- **AF ID:** AF-P51654-F1-model-v4 | **Chain:** A
- **b-phipsi:** 0.0354628399694063
- **w-rdist:** 0.9061214019362408
- **t-alpha:** 0.0015676375257613

---

---

401

- **AF ID:** AF-Q5TA45-F1-model-v4 | **Chain:** A
- **b-phipsi:** 0.0012272859254696
- **w-rdist:** 1.0314036183824555
- **t-alpha:** 0.1171328606038772

---

---

402

- **AF ID:** AF-Q6ZUJ4-F1-model-v4 | **Chain:** A
- **b-phipsi:** 0.0201468505109622
- **w-rdist:** 1.159383116820107
- **t-alpha:** 0.0007823187912934

---

---

403

- **AF ID:** AF-P01127-F1-model-v4 | **Chain:** A
- **b-phipsi:** 0.0139319947280576
- **w-rdist:** 0.3519955374628629
- **t-alpha:** 0.2022577417501936

---

---

404

- **AF ID:** AF-A2A3L6-F1-model-v4 | **Chain:** A
- **b-phipsi:** 0.0269254599181337
- **w-rdist:** 0.8769343094720823
- **t-alpha:** 0.0031296009050172

---

---

405

- **AF ID:** AF-Q8NCK3-F1-model-v4 | **Chain:** A
- **b-phipsi:** 0.0207847422760561
- **w-rdist:** 0.987338858230549
- **t-alpha:** 0.0023530945443304

---

---

406

- **AF ID:** AF-P13196-F1-model-v4 | **Chain:** A
- **b-phipsi:** 0.0092698705202374
- **w-rdist:** 0.3967843629803529
- **t-alpha:** 0.198747516036662

---

---

407

- **AF ID:** AF-Q99983-F1-model-v4 | **Chain:** A
- **b-phipsi:** 0.0204723538159261
- **w-rdist:** 0.7338261473685891
- **t-alpha:** 0.0062593997286479

---

---

408

- **AF ID:** AF-Q9Y4L5-F1-model-v4 | **Chain:** A
- **b-phipsi:** 0.0218897503446114
- **w-rdist:** 0.4035702908291125
- **t-alpha:** 0.0730480405888041

---

---

409

- **AF ID:** AF-Q8N4Q0-F1-model-v4 | **Chain:** A
- **b-phipsi:** 0.0005457760725543
- **w-rdist:** 1.0796247184388232
- **t-alpha:** 0.2591138648516393

---

---

410

- **AF ID:** AF-P06858-F1-model-v4 | **Chain:** A
- **b-phipsi:** 0.0013517918717313
- **w-rdist:** 0.804692609783554
- **t-alpha:** 0.1855287810710499

---

---

411

- **AF ID:** AF-Q9Y334-F1-model-v4 | **Chain:** A
- **b-phipsi:** 0.0122155780480301
- **w-rdist:** 0.3543754651592861
- **t-alpha:** 0.2417838315328293

---

---

412

- **AF ID:** AF-Q96HU1-F1-model-v4 | **Chain:** A
- **b-phipsi:** 0.0431659201379251
- **w-rdist:** 0.3701981779048522
- **t-alpha:** 0.0665099761701244

---

---

413

- **AF ID:** AF-Q5TEU4-F1-model-v4 | **Chain:** A
- **b-phipsi:** 0.0011720432468257
- **w-rdist:** 0.8265321318211654
- **t-alpha:** 0.2395736081101118

---

---

414

- **AF ID:** AF-Q9GZP0-F1-model-v4 | **Chain:** A
- **b-phipsi:** 0.0277937867226961
- **w-rdist:** 0.7020858037705798
- **t-alpha:** 0.0055077996884944

---

---

415

- **AF ID:** AF-Q96LD4-F1-model-v4 | **Chain:** A
- **b-phipsi:** 0.0019317116049202
- **w-rdist:** 1.402916741616657
- **t-alpha:** 0.0062992257321734

---

---

416

- **AF ID:** AF-Q6NVV9-F1-model-v4 | **Chain:** A
- **b-phipsi:** 0.0323568158909545
- **w-rdist:** 0.6324357057522556
- **t-alpha:** 0.0062992257321734

---

---

417

- **AF ID:** AF-Q969Y2-F1-model-v4 | **Chain:** A
- **b-phipsi:** 0.0124446163062836
- **w-rdist:** 0.4105857478099887
- **t-alpha:** 0.125991282681704

---

---

418

- **AF ID:** AF-Q15413-F4-model-v4 | **Chain:** A
- **b-phipsi:** 0.0005796686560451
- **w-rdist:** 1.2350509729300216
- **t-alpha:** 0.1557116053874756

---

---

419

- **AF ID:** AF-Q6ZMI3-F1-model-v4 | **Chain:** A
- **b-phipsi:** 0.0101917950929088
- **w-rdist:** 1.058270706958171
- **t-alpha:** 0.0047169960791435

---

---

420

- **AF ID:** AF-P16442-F1-model-v4 | **Chain:** A
- **b-phipsi:** 0.0011909967386912
- **w-rdist:** 0.960523502460564
- **t-alpha:** 0.1628757968965435

---

---

421

- **AF ID:** AF-O95786-F1-model-v4 | **Chain:** A
- **b-phipsi:** 0.0233533102306484
- **w-rdist:** 0.3715210810222046
- **t-alpha:** 0.1220652445783845

---

---

422

- **AF ID:** AF-Q96P65-F1-model-v4 | **Chain:** A
- **b-phipsi:** 0.084822536961565
- **w-rdist:** 0.6712401910534266
- **t-alpha:** 0.0039277718342689

---

---

423

- **AF ID:** AF-Q53HL2-F1-model-v4 | **Chain:** A
- **b-phipsi:** 0.017965352702677
- **w-rdist:** 0.8369659326224091
- **t-alpha:** 0.0054771144938834

---

---

424

- **AF ID:** AF-Q03154-F1-model-v4 | **Chain:** A
- **b-phipsi:** 0.0007809702006766
- **w-rdist:** 0.9869257055595309
- **t-alpha:** 0.3014260647785169

---

---

425

- **AF ID:** AF-Q86VH5-F1-model-v4 | **Chain:** A
- **b-phipsi:** 0.0133273844059606
- **w-rdist:** 1.0750811130005524
- **t-alpha:** 0.0039119745263893

---

---

426

- **AF ID:** AF-P19113-F1-model-v4 | **Chain:** A
- **b-phipsi:** 0.0096855981855159
- **w-rdist:** 0.3830516892964338
- **t-alpha:** 0.2715180311884424

---

---

427

- **AF ID:** AF-Q9BU76-F1-model-v4 | **Chain:** A
- **b-phipsi:** 0.0081958926037391
- **w-rdist:** 1.081783699986706
- **t-alpha:** 0.0062593997286479

---

---

428

- **AF ID:** AF-A8MV23-F1-model-v4 | **Chain:** A
- **b-phipsi:** 0.0008018983468836
- **w-rdist:** 1.026897236220267
- **t-alpha:** 0.2628460200741693

---

---

429

- **AF ID:** AF-Q8N6Q8-F1-model-v4 | **Chain:** A
- **b-phipsi:** 0.0193068980353102
- **w-rdist:** 0.409149557907111
- **t-alpha:** 0.0899840291479125

---

---

430

- **AF ID:** AF-Q96GL9-F1-model-v4 | **Chain:** A
- **b-phipsi:** 0.0151098587001843
- **w-rdist:** 0.3243755557153112
- **t-alpha:** 0.2987807918489538

---

---

431

- **AF ID:** AF-P52569-F1-model-v4 | **Chain:** A
- **b-phipsi:** 0.102823768314114
- **w-rdist:** 0.6903371106022681
- **t-alpha:** 0.0039277718342689

---

---

432

- **AF ID:** AF-Q96QS6-F1-model-v4 | **Chain:** A
- **b-phipsi:** 0.0009158831403159
- **w-rdist:** 1.055307823054827
- **t-alpha:** 0.2033899315381668

---

---

433

- **AF ID:** AF-Q68CJ6-F1-model-v4 | **Chain:** A
- **b-phipsi:** 0.0559529279452937
- **w-rdist:** 0.3748857076649913
- **t-alpha:** 0.061461872795004

---

---

434

- **AF ID:** AF-Q14449-F1-model-v4 | **Chain:** A
- **b-phipsi:** 0.0136841044044689
- **w-rdist:** 0.3889222811417224
- **t-alpha:** 0.1697965373475096

---

---

435

- **AF ID:** AF-Q96KB5-F1-model-v4 | **Chain:** A
- **b-phipsi:** 0.0005090686207774
- **w-rdist:** 1.0696553288812476
- **t-alpha:** 0.390642397229336

---

---

436

- **AF ID:** AF-O95841-F1-model-v4 | **Chain:** A
- **b-phipsi:** 0.0171658573224942
- **w-rdist:** 1.1523546792226884
- **t-alpha:** 0.0023530945443304

---

---

437

- **AF ID:** AF-P0C6A0-F1-model-v4 | **Chain:** A
- **b-phipsi:** 0.0719889530756275
- **w-rdist:** 0.3612192968664421
- **t-alpha:** 0.0703518669333997

---

---

438

- **AF ID:** AF-Q7Z4Q2-F1-model-v4 | **Chain:** A
- **b-phipsi:** 0.1412494944737222
- **w-rdist:** 0.4041237326376999
- **t-alpha:** 0.0232189692826079

---

---

439

- **AF ID:** AF-Q969K4-F1-model-v4 | **Chain:** A
- **b-phipsi:** 0.0458252587105701
- **w-rdist:** 0.6708516120325846
- **t-alpha:** 0.0062593997286479

---

---

440

- **AF ID:** AF-Q7Z4K8-F1-model-v4 | **Chain:** A
- **b-phipsi:** 0.0010904219517909
- **w-rdist:** 1.466482686996176
- **t-alpha:** 0.0704223021796839

---

---

441

- **AF ID:** AF-Q15413-F3-model-v4 | **Chain:** A
- **b-phipsi:** 0.0004127128177433
- **w-rdist:** 1.093464804702741
- **t-alpha:** 0.4092325066664359

---

---

442

- **AF ID:** AF-P15813-F1-model-v4 | **Chain:** A
- **b-phipsi:** 0.0012227661743043
- **w-rdist:** 0.7590526542698974
- **t-alpha:** 0.409041006968238

---

---

443

- **AF ID:** AF-Q03014-F1-model-v4 | **Chain:** A
- **b-phipsi:** 0.0251617672109919
- **w-rdist:** 1.0925230265913353
- **t-alpha:** 0.0023473590503262

---

---

444

- **AF ID:** AF-Q86X67-F1-model-v4 | **Chain:** A
- **b-phipsi:** 0.000969944162294
- **w-rdist:** 0.9274273631272442
- **t-alpha:** 0.3452634283417621

---

---

445

- **AF ID:** AF-Q9NRN7-F1-model-v4 | **Chain:** A
- **b-phipsi:** 0.0003599684491785
- **w-rdist:** 1.1366423909687955
- **t-alpha:** 0.3756729418935771

---

---

446

- **AF ID:** AF-Q6IPT4-F1-model-v4 | **Chain:** A
- **b-phipsi:** 0.0006111285851241
- **w-rdist:** 1.0640199407561362
- **t-alpha:** 0.390642397229336

---

---

447

- **AF ID:** AF-Q6UWY5-F1-model-v4 | **Chain:** A
- **b-phipsi:** 0.0009024644719627
- **w-rdist:** 1.029464282521794
- **t-alpha:** 0.2691162949046146

---

---

448

- **AF ID:** AF-Q9UBR1-F1-model-v4 | **Chain:** A
- **b-phipsi:** 0.0006351175725317
- **w-rdist:** 1.1358311582365537
- **t-alpha:** 0.2831330357652053

---

---

449

- **AF ID:** AF-Q8N145-F1-model-v4 | **Chain:** A
- **b-phipsi:** 0.0365382580193448
- **w-rdist:** 0.9642545123044208
- **t-alpha:** 0.0023530945443304

---

---

450

- **AF ID:** AF-Q7Z3H0-F1-model-v4 | **Chain:** A
- **b-phipsi:** 0.022194542079937
- **w-rdist:** 0.3232607576669094
- **t-alpha:** 0.2339593290487849

---

---

451

- **AF ID:** AF-O15232-F1-model-v4 | **Chain:** A
- **b-phipsi:** 0.0099425634875639
- **w-rdist:** 1.447611257837129
- **t-alpha:** 0.0031296009050172

---

---

452

- **AF ID:** AF-O43623-F1-model-v4 | **Chain:** A
- **b-phipsi:** 0.0152639056384194
- **w-rdist:** 0.3926912991641308
- **t-alpha:** 0.1778806838678062

---

---

453

- **AF ID:** AF-P29144-F1-model-v4 | **Chain:** A
- **b-phipsi:** 0.0008692266905315
- **w-rdist:** 1.0080219358359273
- **t-alpha:** 0.3262903387257461

---

---

454

- **AF ID:** AF-Q86U38-F1-model-v4 | **Chain:** A
- **b-phipsi:** 0.0742621261361829
- **w-rdist:** 0.3456869399833164
- **t-alpha:** 0.09467909251505

---

---

455

- **AF ID:** AF-Q9BWF2-F1-model-v4 | **Chain:** A
- **b-phipsi:** 0.0165680532300609
- **w-rdist:** 1.7057544559565607
- **t-alpha:** 0.0007823187912934

---

---

456

- **AF ID:** AF-P25391-F9-model-v4 | **Chain:** A
- **b-phipsi:** 0.0039939921939846
- **w-rdist:** 1.6789173448204078
- **t-alpha:** 0.0054771144938834

---

---

457

- **AF ID:** AF-Q86XR2-F1-model-v4 | **Chain:** A
- **b-phipsi:** 0.0269741997024763
- **w-rdist:** 0.3561861058282859
- **t-alpha:** 0.1619717550898061

---

---

458

- **AF ID:** AF-Q6NUT2-F1-model-v4 | **Chain:** A
- **b-phipsi:** 0.080668959416699
- **w-rdist:** 0.4044990097897807
- **t-alpha:** 0.040688156290789

---

---

459

- **AF ID:** AF-Q2WGJ8-F1-model-v4 | **Chain:** A
- **b-phipsi:** 0.0006398598588407
- **w-rdist:** 1.002197362244749
- **t-alpha:** 0.5816836720048004

---

---

460

- **AF ID:** AF-Q9H9P8-F1-model-v4 | **Chain:** A
- **b-phipsi:** 0.0014619316456336
- **w-rdist:** 0.9946350484593264
- **t-alpha:** 0.1349913854982236

---

---

461

- **AF ID:** AF-Q86XR7-F1-model-v4 | **Chain:** A
- **b-phipsi:** 0.001176076876562
- **w-rdist:** 0.8232810911008669
- **t-alpha:** 0.4231630699296105

---

---

462

- **AF ID:** AF-Q969E3-F1-model-v4 | **Chain:** A
- **b-phipsi:** 0.0204480978989642
- **w-rdist:** 0.3570023224081377
- **t-alpha:** 0.2045243362549313

---

---

463

- **AF ID:** AF-Q9NSI5-F1-model-v4 | **Chain:** A
- **b-phipsi:** 0.0479084007070313
- **w-rdist:** 1.1476826637893538
- **t-alpha:** 0.0

---

---

464

- **AF ID:** AF-Q6IEG0-F1-model-v4 | **Chain:** A
- **b-phipsi:** 0.0273975954429869
- **w-rdist:** 1.202220363996782
- **t-alpha:** 0.0007832732121422

---

---

465

- **AF ID:** AF-Q8N8Q3-F1-model-v4 | **Chain:** A
- **b-phipsi:** 0.0005438639963809
- **w-rdist:** 1.088822689269373
- **t-alpha:** 0.4825987260004796

---

---

466

- **AF ID:** AF-Q9BQ24-F1-model-v4 | **Chain:** A
- **b-phipsi:** 0.000293881492194
- **w-rdist:** 1.1296739561769455
- **t-alpha:** 0.5342138394148872

---

---

467

- **AF ID:** AF-P55039-F1-model-v4 | **Chain:** A
- **b-phipsi:** 0.0013024933606043
- **w-rdist:** 0.9908337545934056
- **t-alpha:** 0.1833334788306171

---

---

468

- **AF ID:** AF-Q8IYM1-F1-model-v4 | **Chain:** A
- **b-phipsi:** 0.0011730111467356
- **w-rdist:** 0.9145059199129872
- **t-alpha:** 0.3134639510710761

---

---

469

- **AF ID:** AF-P51148-F1-model-v4 | **Chain:** A
- **b-phipsi:** 0.0004491652975265
- **w-rdist:** 1.0319460425938467
- **t-alpha:** 0.8933335200134038

---

---

470

- **AF ID:** AF-P61247-F1-model-v4 | **Chain:** A
- **b-phipsi:** 0.0010736629083187
- **w-rdist:** 0.9083092074312602
- **t-alpha:** 0.4105966025489225

---

---

471

- **AF ID:** AF-Q00059-F1-model-v4 | **Chain:** A
- **b-phipsi:** 0.043470381538433
- **w-rdist:** 0.3728763636178477
- **t-alpha:** 0.1171328606038772

---

---

472

- **AF ID:** AF-A6NK59-F1-model-v4 | **Chain:** A
- **b-phipsi:** 0.0490391030085721
- **w-rdist:** 0.921629457345001
- **t-alpha:** 0.0031296009050172

---

---

473

- **AF ID:** AF-Q8N8N7-F1-model-v4 | **Chain:** A
- **b-phipsi:** 0.0006333624240089
- **w-rdist:** 1.1572692694156896
- **t-alpha:** 0.3340296759895635

---

---

474

- **AF ID:** AF-Q9NYA1-F1-model-v4 | **Chain:** A
- **b-phipsi:** 0.0006338367611942
- **w-rdist:** 1.1554020208941802
- **t-alpha:** 0.336820683237341

---

---

475

- **AF ID:** AF-P0DMS9-F1-model-v4 | **Chain:** A
- **b-phipsi:** 0.0008675416922403
- **w-rdist:** 1.0396015682300097
- **t-alpha:** 0.3624735754198571

---

---

476

- **AF ID:** AF-A1A4G5-F1-model-v4 | **Chain:** A
- **b-phipsi:** 0.0284809628885495
- **w-rdist:** 0.3388777175658991
- **t-alpha:** 0.2011283353911521

---

---

477

- **AF ID:** AF-Q7Z695-F1-model-v4 | **Chain:** A
- **b-phipsi:** 0.0190426942003978
- **w-rdist:** 0.3929841889863509
- **t-alpha:** 0.1690136447929693

---

---

478

- **AF ID:** AF-Q92519-F1-model-v4 | **Chain:** A
- **b-phipsi:** 0.0013811916825491
- **w-rdist:** 1.0113676570232522
- **t-alpha:** 0.1607634002229021

---

---

479

- **AF ID:** AF-P07225-F1-model-v4 | **Chain:** A
- **b-phipsi:** 0.0190499044260389
- **w-rdist:** 1.5288887388097625
- **t-alpha:** 0.0007832732121422

---

---

480

- **AF ID:** AF-Q7Z404-F1-model-v4 | **Chain:** A
- **b-phipsi:** 0.1140289649168687
- **w-rdist:** 0.7367966110103904
- **t-alpha:** 0.0046944792707916

---

---

481

- **AF ID:** AF-Q9BSK4-F1-model-v4 | **Chain:** A
- **b-phipsi:** 0.0399962018129787
- **w-rdist:** 0.714853275084185
- **t-alpha:** 0.0070418134049574

---

---

482

- **AF ID:** AF-Q63ZY6-F1-model-v4 | **Chain:** A
- **b-phipsi:** 0.0012473183162397
- **w-rdist:** 0.973214144635658
- **t-alpha:** 0.2371738062974113

---

---

483

- **AF ID:** AF-Q9NZM5-F1-model-v4 | **Chain:** A
- **b-phipsi:** 0.0221668390532325
- **w-rdist:** 1.480512797958915
- **t-alpha:** 0.0007823187912934

---

---

484

- **AF ID:** AF-Q92934-F1-model-v4 | **Chain:** A
- **b-phipsi:** 0.0282603283379519
- **w-rdist:** 0.3137405704812956
- **t-alpha:** 0.2566372839605271

---

---

485

- **AF ID:** AF-P38117-F1-model-v4 | **Chain:** A
- **b-phipsi:** 0.0005425039622253
- **w-rdist:** 1.1518132827876786
- **t-alpha:** 0.4605718669272309

---

---

486

- **AF ID:** AF-O43237-F1-model-v4 | **Chain:** A
- **b-phipsi:** 0.0172036961764399
- **w-rdist:** 0.9834066821691574
- **t-alpha:** 0.0062593997286479

---

---

487

- **AF ID:** AF-P51798-F1-model-v4 | **Chain:** A
- **b-phipsi:** 0.0219409194552242
- **w-rdist:** 0.4026148591254749
- **t-alpha:** 0.1463219731492844

---

---

488

- **AF ID:** AF-P38936-F1-model-v4 | **Chain:** A
- **b-phipsi:** 0.0133034337272339
- **w-rdist:** 0.3595067722596445
- **t-alpha:** 0.4295304845720977

---

---

489

- **AF ID:** AF-Q15413-F2-model-v4 | **Chain:** A
- **b-phipsi:** 0.0008786393869704
- **w-rdist:** 1.263265115125683
- **t-alpha:** 0.1815331759031522

---

---

490

- **AF ID:** AF-Q7Z444-F1-model-v4 | **Chain:** A
- **b-phipsi:** 0.0007908388091588
- **w-rdist:** 1.013539808067171
- **t-alpha:** 0.5661767924857357

---

---

491

- **AF ID:** AF-O15524-F1-model-v4 | **Chain:** A
- **b-phipsi:** 0.0011002166233344
- **w-rdist:** 0.9036066280676296
- **t-alpha:** 0.525060050671609

---

---

492

- **AF ID:** AF-Q9BV68-F1-model-v4 | **Chain:** A
- **b-phipsi:** 0.0204020947246979
- **w-rdist:** 1.0203300161150104
- **t-alpha:** 0.0047169960791435

---

---

493

- **AF ID:** AF-O95744-F1-model-v4 | **Chain:** A
- **b-phipsi:** 0.0012621447332932
- **w-rdist:** 0.9835206497603248
- **t-alpha:** 0.2578740651988431

---

---

494

- **AF ID:** AF-Q969V5-F1-model-v4 | **Chain:** A
- **b-phipsi:** 0.0008351369932784
- **w-rdist:** 1.1748762776957486
- **t-alpha:** 0.2767234962758196

---

---

495

- **AF ID:** AF-C9JQI7-F1-model-v4 | **Chain:** A
- **b-phipsi:** 0.0726962523169066
- **w-rdist:** 0.7529217044346119
- **t-alpha:** 0.0054771144938834

---

---

496

- **AF ID:** AF-Q9NUJ1-F1-model-v4 | **Chain:** A
- **b-phipsi:** 0.0010362699014297
- **w-rdist:** 1.0199477195327198
- **t-alpha:** 0.3727176871100233

---

---

497

- **AF ID:** AF-Q6ZS94-F1-model-v4 | **Chain:** A
- **b-phipsi:** 0.0271193855399412
- **w-rdist:** 1.100006871930742
- **t-alpha:** 0.0031401242085145

---

---

498

- **AF ID:** AF-P17540-F1-model-v4 | **Chain:** A
- **b-phipsi:** 0.0012120913191452
- **w-rdist:** 0.987166755833626
- **t-alpha:** 0.3067489854426217

---

---

499

- **AF ID:** AF-O15118-F1-model-v4 | **Chain:** A
- **b-phipsi:** 0.0387689576386326
- **w-rdist:** 1.190320133138952
- **t-alpha:** 0.0015647720218643

---

---

500

- **AF ID:** AF-Q7RTX9-F1-model-v4 | **Chain:** A
- **b-phipsi:** 0.1259618160385643
- **w-rdist:** 0.7558443499354601
- **t-alpha:** 0.0047169960791435

---

---

501

- **AF ID:** AF-Q8IUI8-F1-model-v4 | **Chain:** A
- **b-phipsi:** 0.0008008509333459
- **w-rdist:** 1.2846100926805997
- **t-alpha:** 0.2159847671360162

---

---

502

- **AF ID:** AF-O14508-F1-model-v4 | **Chain:** A
- **b-phipsi:** 0.0003857380527046
- **w-rdist:** 1.186006385819821
- **t-alpha:** 0.5642601548085062

---

---

503

- **AF ID:** AF-Q69YZ2-F1-model-v4 | **Chain:** A
- **b-phipsi:** 0.0469301526933313
- **w-rdist:** 1.2127816755565082
- **t-alpha:** 0.0007823187912934

---

---

504

- **AF ID:** AF-P42262-F1-model-v4 | **Chain:** A
- **b-phipsi:** 0.0074635335232516
- **w-rdist:** 1.448314338418661
- **t-alpha:** 0.0055077996884944

---

---

505

- **AF ID:** AF-Q8IYN6-F1-model-v4 | **Chain:** A
- **b-phipsi:** 0.0186109114989056
- **w-rdist:** 0.3431564549003079
- **t-alpha:** 0.4359551456631745

---

---

506

- **AF ID:** AF-Q08257-F1-model-v4 | **Chain:** A
- **b-phipsi:** 0.0006590484067504
- **w-rdist:** 1.168456923153787
- **t-alpha:** 0.4121552622584874

---

---

507

- **AF ID:** AF-P10266-F1-model-v4 | **Chain:** A
- **b-phipsi:** 0.0010029735555444
- **w-rdist:** 1.255351008811523
- **t-alpha:** 0.1901404340302059

---

---

508

- **AF ID:** AF-O94805-F1-model-v4 | **Chain:** A
- **b-phipsi:** 0.0012272357582504
- **w-rdist:** 1.0137155967877396
- **t-alpha:** 0.2805615818030798

---

---

509

- **AF ID:** AF-Q9Y4Z2-F1-model-v4 | **Chain:** A
- **b-phipsi:** 0.0319649191377556
- **w-rdist:** 0.9939596620439628
- **t-alpha:** 0.0039277718342689

---

---

510

- **AF ID:** AF-Q13401-F1-model-v4 | **Chain:** A
- **b-phipsi:** 0.0005631239564819
- **w-rdist:** 1.1610754727421
- **t-alpha:** 0.5397596300889067

---

---

511

- **AF ID:** AF-Q5R372-F1-model-v4 | **Chain:** A
- **b-phipsi:** 0.0014604573541614
- **w-rdist:** 0.8536343461694438
- **t-alpha:** 0.2965569088368105

---

---

512

- **AF ID:** AF-Q6XUX3-F1-model-v4 | **Chain:** A
- **b-phipsi:** 0.0259784294677508
- **w-rdist:** 1.2792435909544495
- **t-alpha:** 0.0015676375257613

---

---

513

- **AF ID:** AF-Q9NQZ5-F1-model-v4 | **Chain:** A
- **b-phipsi:** 0.0014475354245517
- **w-rdist:** 0.8350084597238415
- **t-alpha:** 0.3552495345324398

---

---

514

- **AF ID:** AF-P63132-F1-model-v4 | **Chain:** A
- **b-phipsi:** 0.0006480331234046
- **w-rdist:** 1.259816804463199
- **t-alpha:** 0.3270735114168728

---

---

515

- **AF ID:** AF-Q15386-F1-model-v4 | **Chain:** A
- **b-phipsi:** 0.0602656652300557
- **w-rdist:** 1.2292060882910596
- **t-alpha:** 0.0

---

---

516

- **AF ID:** AF-P0C866-F1-model-v4 | **Chain:** A
- **b-phipsi:** 0.0012448860313854
- **w-rdist:** 0.9714786055318024
- **t-alpha:** 0.3509518031434124

---

---

517

- **AF ID:** AF-P18509-F1-model-v4 | **Chain:** A
- **b-phipsi:** 0.0466022182913217
- **w-rdist:** 0.3383841539421021
- **t-alpha:** 0.2136751941179888

---

---

518

- **AF ID:** AF-Q7RTM1-F1-model-v4 | **Chain:** A
- **b-phipsi:** 0.0594550682996217
- **w-rdist:** 0.8234853524542133
- **t-alpha:** 0.0054771144938834

---

---

519

- **AF ID:** AF-P00492-F1-model-v4 | **Chain:** A
- **b-phipsi:** 0.00035044360973
- **w-rdist:** 1.2522445786138392
- **t-alpha:** 0.5623473526217455

---

---

520

- **AF ID:** AF-Q9GZU8-F1-model-v4 | **Chain:** A
- **b-phipsi:** 0.0227819092670394
- **w-rdist:** 1.1953917210074194
- **t-alpha:** 0.0031401242085145

---

---

521

- **AF ID:** AF-Q92670-F1-model-v4 | **Chain:** A
- **b-phipsi:** 0.0316014074778262
- **w-rdist:** 0.3846161053079406
- **t-alpha:** 0.1674494311982475

---

---

522

- **AF ID:** AF-A6NI47-F1-model-v4 | **Chain:** A
- **b-phipsi:** 0.0327345979988349
- **w-rdist:** 0.3868305733256831
- **t-alpha:** 0.1604064507642828

---

---

523

- **AF ID:** AF-O94776-F1-model-v4 | **Chain:** A
- **b-phipsi:** 0.0008374820225385
- **w-rdist:** 1.107411323381578
- **t-alpha:** 0.5359933176699478

---

---

524

- **AF ID:** AF-A6NCN8-F1-model-v4 | **Chain:** A
- **b-phipsi:** 0.039844581947509
- **w-rdist:** 0.3801185736760522
- **t-alpha:** 0.1628757968965435

---

---

525

- **AF ID:** AF-Q7L8J4-F1-model-v4 | **Chain:** A
- **b-phipsi:** 0.0378235946737256
- **w-rdist:** 1.206393527005561
- **t-alpha:** 0.0023473590503262

---

---

526

- **AF ID:** AF-P26715-F1-model-v4 | **Chain:** A
- **b-phipsi:** 0.0011782324118262
- **w-rdist:** 1.0760161848215173
- **t-alpha:** 0.3284828675208937

---

---

527

- **AF ID:** AF-P49585-F1-model-v4 | **Chain:** A
- **b-phipsi:** 0.0111978538914664
- **w-rdist:** 1.3177466864869758
- **t-alpha:** 0.0055077996884944

---

---

528

- **AF ID:** AF-P32780-F1-model-v4 | **Chain:** A
- **b-phipsi:** 0.054601473494671
- **w-rdist:** 1.135092051887121
- **t-alpha:** 0.0023530945443304

---

---

529

- **AF ID:** AF-A3KMH1-F1-model-v4 | **Chain:** A
- **b-phipsi:** 0.00041016426999
- **w-rdist:** 1.378985716168542
- **t-alpha:** 0.467136035614391

---

---

530

- **AF ID:** AF-Q8IX30-F1-model-v4 | **Chain:** A
- **b-phipsi:** 0.027971349458349
- **w-rdist:** 1.4209064393070696
- **t-alpha:** 0.0015676375257613

---

---

531

- **AF ID:** AF-Q8WZ82-F1-model-v4 | **Chain:** A
- **b-phipsi:** 0.0003866945951082
- **w-rdist:** 1.2666050685543646
- **t-alpha:** 0.6218279647524012

---

---

532

- **AF ID:** AF-O15355-F1-model-v4 | **Chain:** A
- **b-phipsi:** 0.0398577414047324
- **w-rdist:** 1.0428569619730408
- **t-alpha:** 0.0039277718342689

---

---

533

- **AF ID:** AF-Q9UQ80-F1-model-v4 | **Chain:** A
- **b-phipsi:** 0.0012687554662386
- **w-rdist:** 1.058960280720091
- **t-alpha:** 0.3107692088094504

---

---

534

- **AF ID:** AF-Q9HAV7-F1-model-v4 | **Chain:** A
- **b-phipsi:** 0.0010973207303618
- **w-rdist:** 1.0375848184207697
- **t-alpha:** 0.5416168437726678

---

---

535

- **AF ID:** AF-Q6UWM5-F1-model-v4 | **Chain:** A
- **b-phipsi:** 0.0008284897734415
- **w-rdist:** 1.1261871503007308
- **t-alpha:** 0.7725381294030873

---

---

536

- **AF ID:** AF-Q6ZTR6-F1-model-v4 | **Chain:** A
- **b-phipsi:** 0.0250264344980917
- **w-rdist:** 0.3988118283668444
- **t-alpha:** 0.2159847671360162

---

---

537

- **AF ID:** AF-Q8TDT2-F1-model-v4 | **Chain:** A
- **b-phipsi:** 0.0197986787837262
- **w-rdist:** 1.1073123356861585
- **t-alpha:** 0.0062593997286479

---

---

538

- **AF ID:** AF-Q96DA2-F1-model-v4 | **Chain:** A
- **b-phipsi:** 0.000733078618537
- **w-rdist:** 1.165496318910546
- **t-alpha:** 0.7725381294030873

---

---

539

- **AF ID:** AF-O14832-F1-model-v4 | **Chain:** A
- **b-phipsi:** 0.001253117477903
- **w-rdist:** 1.0468087900132417
- **t-alpha:** 0.3727176871100233

---

---

540

- **AF ID:** AF-Q8N6G2-F1-model-v4 | **Chain:** A
- **b-phipsi:** 0.0283932863167492
- **w-rdist:** 1.1200184211460074
- **t-alpha:** 0.0046944792707916

---

---

541

- **AF ID:** AF-A6NDY0-F1-model-v4 | **Chain:** A
- **b-phipsi:** 0.063515710599961
- **w-rdist:** 0.9808317447474766
- **t-alpha:** 0.0046944792707916

---

---

542

- **AF ID:** AF-Q96AY2-F1-model-v4 | **Chain:** A
- **b-phipsi:** 0.0011352216383206
- **w-rdist:** 1.1798121125201064
- **t-alpha:** 0.2965569088368105

---

---

543

- **AF ID:** AF-Q5VYX0-F1-model-v4 | **Chain:** A
- **b-phipsi:** 0.0012371312927225
- **w-rdist:** 1.1544585730443149
- **t-alpha:** 0.2857145579826488

---

---

544

- **AF ID:** AF-Q96K17-F1-model-v4 | **Chain:** A
- **b-phipsi:** 0.0013228096735708
- **w-rdist:** 0.8915134798343065
- **t-alpha:** 0.8205128238177464

---

---

545

- **AF ID:** AF-P20340-F1-model-v4 | **Chain:** A
- **b-phipsi:** 0.0010071984889391
- **w-rdist:** 1.105496348298991
- **t-alpha:** 0.6532992022825934

---

---

546

- **AF ID:** AF-A6NK75-F1-model-v4 | **Chain:** A
- **b-phipsi:** 0.0252937981765734
- **w-rdist:** 1.4296129885222504
- **t-alpha:** 0.0031296009050172

---

---

547

- **AF ID:** AF-Q8WXX0-F15-model-v4 | **Chain:** A
- **b-phipsi:** 0.0457634961769358
- **w-rdist:** 1.2688916933159304
- **t-alpha:** 0.0023473590503262

---

---

548

- **AF ID:** AF-Q9UHP7-F1-model-v4 | **Chain:** A
- **b-phipsi:** 0.0005048075034143
- **w-rdist:** 1.27893414973457
- **t-alpha:** 1.0285720750669496

---

---

549

- **AF ID:** AF-Q6ZNW5-F1-model-v4 | **Chain:** A
- **b-phipsi:** 0.0011524406906876
- **w-rdist:** 1.1608299843785028
- **t-alpha:** 0.3509518031434124

---

---

550

- **AF ID:** AF-Q9UQB9-F1-model-v4 | **Chain:** A
- **b-phipsi:** 0.0012524759272431
- **w-rdist:** 1.1482171838008737
- **t-alpha:** 0.3067489854426217

---

---

551

- **AF ID:** AF-Q01459-F1-model-v4 | **Chain:** A
- **b-phipsi:** 0.0013507875315924
- **w-rdist:** 0.988370625085112
- **t-alpha:** 0.4440679058137677

---

---

552

- **AF ID:** AF-Q96MI6-F1-model-v4 | **Chain:** A
- **b-phipsi:** 0.0013333484067176
- **w-rdist:** 1.0645719290100644
- **t-alpha:** 0.3831173026749244

---

---

553

- **AF ID:** AF-Q14204-F17-model-v4 | **Chain:** A
- **b-phipsi:** 0.0820907097261841
- **w-rdist:** 1.611407108679829
- **t-alpha:** 0.0

---

---

554

- **AF ID:** AF-A0A2U3TZM8-F1-model-v4 | **Chain:** A
- **b-phipsi:** 0.0208968979426809
- **w-rdist:** 1.1839005836597107
- **t-alpha:** 0.0055077996884944

---

---

555

- **AF ID:** AF-Q9BTV5-F1-model-v4 | **Chain:** A
- **b-phipsi:** 0.0010909019701155
- **w-rdist:** 1.2891620518034277
- **t-alpha:** 0.2792793129760946

---

---

556

- **AF ID:** AF-P59095-F1-model-v4 | **Chain:** A
- **b-phipsi:** 0.0008442825969507
- **w-rdist:** 1.242189209526716
- **t-alpha:** 0.5915324561921709

---

---

557

- **AF ID:** AF-Q9NQT4-F1-model-v4 | **Chain:** A
- **b-phipsi:** 0.0009653105083487
- **w-rdist:** 1.2316144760963248
- **t-alpha:** 0.4947371311033361

---

---

558

- **AF ID:** AF-Q96A22-F1-model-v4 | **Chain:** A
- **b-phipsi:** 0.0595114461157394
- **w-rdist:** 0.3196839142713456
- **t-alpha:** 0.4200001627652414

---

---

559

- **AF ID:** AF-O60486-F1-model-v4 | **Chain:** A
- **b-phipsi:** 0.0011246397321616
- **w-rdist:** 1.254784146557646
- **t-alpha:** 0.3239435926985792

---

---

560

- **AF ID:** AF-Q92736-F3-model-v4 | **Chain:** A
- **b-phipsi:** 0.0013983736915343
- **w-rdist:** 1.122471806211433
- **t-alpha:** 0.3012516967665894

---

---

561

- **AF ID:** AF-O95868-F1-model-v4 | **Chain:** A
- **b-phipsi:** 0.0009144169639168
- **w-rdist:** 1.169412171130941
- **t-alpha:** 0.9813960083631263

---

---

562

- **AF ID:** AF-O43602-F1-model-v4 | **Chain:** A
- **b-phipsi:** 0.0553650839533722
- **w-rdist:** 1.0653097898154482
- **t-alpha:** 0.0047169960791435

---

---

563

- **AF ID:** AF-Q9NZS2-F1-model-v4 | **Chain:** A
- **b-phipsi:** 0.0013341860078649
- **w-rdist:** 1.110868702623774
- **t-alpha:** 0.384615887770632

---

---

564

- **AF ID:** AF-O15031-F1-model-v4 | **Chain:** A
- **b-phipsi:** 0.0006728373230341
- **w-rdist:** 1.536293586036879
- **t-alpha:** 0.5610330067829725

---

---

565

- **AF ID:** AF-Q96JQ0-F7-model-v4 | **Chain:** A
- **b-phipsi:** 0.0249640599925105
- **w-rdist:** 1.857820939975204
- **t-alpha:** 0.0031401242085145

---

---

566

- **AF ID:** AF-Q92736-F4-model-v4 | **Chain:** A
- **b-phipsi:** 0.0012906909112494
- **w-rdist:** 1.203178718221243
- **t-alpha:** 0.3035992457036245

---

---

567

- **AF ID:** AF-Q9NW61-F1-model-v4 | **Chain:** A
- **b-phipsi:** 0.0009072679169667
- **w-rdist:** 1.2595207834734965
- **t-alpha:** 0.686016137645864

---

---

568

- **AF ID:** AF-O75592-F7-model-v4 | **Chain:** A
- **b-phipsi:** 0.0009814796633899
- **w-rdist:** 1.3158476745182124
- **t-alpha:** 0.4616587408252333

---

---

569

- **AF ID:** AF-Q9NZ63-F1-model-v4 | **Chain:** A
- **b-phipsi:** 0.0384003045840268
- **w-rdist:** 1.135059810401306
- **t-alpha:** 0.0055077996884944

---

---

570

- **AF ID:** AF-P14920-F1-model-v4 | **Chain:** A
- **b-phipsi:** 0.0013337957960196
- **w-rdist:** 1.1341783592875083
- **t-alpha:** 0.4791668873833732

---

---

571

- **AF ID:** AF-Q5DX21-F1-model-v4 | **Chain:** A
- **b-phipsi:** 0.0460237257087249
- **w-rdist:** 1.4102346475714629
- **t-alpha:** 0.0031296009050172

---

---

572

- **AF ID:** AF-Q9BSA4-F1-model-v4 | **Chain:** A
- **b-phipsi:** 0.1042216204888739
- **w-rdist:** 1.2766055578559758
- **t-alpha:** 0.0023530945443304

---

---

573

- **AF ID:** AF-Q86U90-F1-model-v4 | **Chain:** A
- **b-phipsi:** 0.0012311451718385
- **w-rdist:** 1.1741205739598095
- **t-alpha:** 0.5070755211397251

---

---

574

- **AF ID:** AF-Q9H867-F1-model-v4 | **Chain:** A
- **b-phipsi:** 0.0010185966761722
- **w-rdist:** 1.2250244616085422
- **t-alpha:** 0.8179234239159088

---

---

575

- **AF ID:** AF-Q14914-F1-model-v4 | **Chain:** A
- **b-phipsi:** 0.001343180065268
- **w-rdist:** 1.150991076221434
- **t-alpha:** 0.4231630699296105

---

---

576

- **AF ID:** AF-P40238-F1-model-v4 | **Chain:** A
- **b-phipsi:** 0.0337900188952736
- **w-rdist:** 1.421072261293679
- **t-alpha:** 0.0039119745263893

---

---

577

- **AF ID:** AF-P21108-F1-model-v4 | **Chain:** A
- **b-phipsi:** 0.0013416054282372
- **w-rdist:** 1.1593110862267784
- **t-alpha:** 0.4184240516116737

---

---

578

- **AF ID:** AF-Q9BRL6-F1-model-v4 | **Chain:** A
- **b-phipsi:** 0.1365052689224035
- **w-rdist:** 1.2999369966050849
- **t-alpha:** 0.0023530945443304

---

---

579

- **AF ID:** AF-Q8WXA3-F1-model-v4 | **Chain:** A
- **b-phipsi:** 0.2201563969293531
- **w-rdist:** 1.772363589182821
- **t-alpha:** 0.0015647720218643

---

---

580

- **AF ID:** AF-P08729-F1-model-v4 | **Chain:** A
- **b-phipsi:** 0.0499159609929086
- **w-rdist:** 1.6747647060846864
- **t-alpha:** 0.0023530945443304

---

---

581

- **AF ID:** AF-Q14964-F1-model-v4 | **Chain:** A
- **b-phipsi:** 0.0011959910209378
- **w-rdist:** 1.1682581702955708
- **t-alpha:** 0.7435204141382297

---

---

582

- **AF ID:** AF-Q86YD3-F1-model-v4 | **Chain:** A
- **b-phipsi:** 0.0468033127183829
- **w-rdist:** 1.2975686326366398
- **t-alpha:** 0.0039119745263893

---

---

583

- **AF ID:** AF-Q8NFR7-F1-model-v4 | **Chain:** A
- **b-phipsi:** 0.2568093082173984
- **w-rdist:** 1.7892414442083424
- **t-alpha:** 0.0015647720218643

---

---

584

- **AF ID:** AF-P0C024-F1-model-v4 | **Chain:** A
- **b-phipsi:** 0.0011165915528108
- **w-rdist:** 1.2656993219395016
- **t-alpha:** 0.5566385561009906

---

---

585

- **AF ID:** AF-Q96G27-F1-model-v4 | **Chain:** A
- **b-phipsi:** 0.0285903796348908
- **w-rdist:** 1.3003990167928507
- **t-alpha:** 0.0047169960791435

---

---

586

- **AF ID:** AF-Q12765-F1-model-v4 | **Chain:** A
- **b-phipsi:** 0.0014620057790139
- **w-rdist:** 1.139201725245571
- **t-alpha:** 0.392157101606611

---

---

587

- **AF ID:** AF-Q8IZP6-F1-model-v4 | **Chain:** A
- **b-phipsi:** 0.0356869954349561
- **w-rdist:** 1.170006530751397
- **t-alpha:** 0.0062593997286479

---

---

588

- **AF ID:** AF-P83436-F1-model-v4 | **Chain:** A
- **b-phipsi:** 0.2262021196598911
- **w-rdist:** 1.3399656557585695
- **t-alpha:** 0.0023530945443304

---

---

589

- **AF ID:** AF-Q96L21-F1-model-v4 | **Chain:** A
- **b-phipsi:** 0.0014013989264893
- **w-rdist:** 1.1014317709936974
- **t-alpha:** 0.5935167025355592

---

---

590

- **AF ID:** AF-Q9ULY5-F1-model-v4 | **Chain:** A
- **b-phipsi:** 0.0014591007322533
- **w-rdist:** 1.0718389066534868
- **t-alpha:** 0.6532992022825934

---

---

591

- **AF ID:** AF-P00736-F1-model-v4 | **Chain:** A
- **b-phipsi:** 0.0439333514540206
- **w-rdist:** 1.6367377971332624
- **t-alpha:** 0.0031401242085145

---

---

592

- **AF ID:** AF-Q96B26-F1-model-v4 | **Chain:** A
- **b-phipsi:** 0.0013980164542043
- **w-rdist:** 1.1563023892813056
- **t-alpha:** 0.5287084334768262

---

---

593

- **AF ID:** AF-Q53HC0-F1-model-v4 | **Chain:** A
- **b-phipsi:** 0.0233419591095619
- **w-rdist:** 1.5987561974974145
- **t-alpha:** 0.0055077996884944

---

---

594

- **AF ID:** AF-Q9BY78-F1-model-v4 | **Chain:** A
- **b-phipsi:** 0.1129159817417254
- **w-rdist:** 1.4343148482397416
- **t-alpha:** 0.0031296009050172

---

---

595

- **AF ID:** AF-P62820-F1-model-v4 | **Chain:** A
- **b-phipsi:** 0.0011872002401379
- **w-rdist:** 1.2573910125052397
- **t-alpha:** 0.6927159089549293

---

---

596

- **AF ID:** AF-Q9BQY9-F1-model-v4 | **Chain:** A
- **b-phipsi:** 0.0544244132587253
- **w-rdist:** 1.2708053053982704
- **t-alpha:** 0.0046944792707916

---

---

597

- **AF ID:** AF-Q96HA8-F1-model-v4 | **Chain:** A
- **b-phipsi:** 0.0011364732011048
- **w-rdist:** 1.2594758173427782
- **t-alpha:** 0.8521741912110206

---

---

598

- **AF ID:** AF-Q66K80-F1-model-v4 | **Chain:** A
- **b-phipsi:** 0.1007320989116041
- **w-rdist:** 1.5079486874301706
- **t-alpha:** 0.0031296009050172

---

---

599

- **AF ID:** AF-O75223-F1-model-v4 | **Chain:** A
- **b-phipsi:** 0.0011212347146359
- **w-rdist:** 1.3050066680228318
- **t-alpha:** 0.7085564330478524

---

---

600

- **AF ID:** AF-Q9UIW2-F1-model-v4 | **Chain:** A
- **b-phipsi:** 0.0011252631936561
- **w-rdist:** 1.506575274620769
- **t-alpha:** 0.5172141227714111

---

---

601

- **AF ID:** AF-O94927-F1-model-v4 | **Chain:** A
- **b-phipsi:** 0.1610226216928684
- **w-rdist:** 1.6469204416509122
- **t-alpha:** 0.0023530945443304

---

---

602

- **AF ID:** AF-Q9Y485-F4-model-v4 | **Chain:** A
- **b-phipsi:** 0.0014226569652914
- **w-rdist:** 1.2259446965560588
- **t-alpha:** 0.406103241393263

---

---

603

- **AF ID:** AF-Q9GZZ8-F1-model-v4 | **Chain:** A
- **b-phipsi:** 0.0359479160864463
- **w-rdist:** 0.3911023254685493
- **t-alpha:** 0.6116018810202546

---

---

604

- **AF ID:** AF-P17677-F1-model-v4 | **Chain:** A
- **b-phipsi:** 0.1186290171694711
- **w-rdist:** 1.1722129518473343
- **t-alpha:** 0.0047169960791435

---

---

605

- **AF ID:** AF-Q8WWY3-F1-model-v4 | **Chain:** A
- **b-phipsi:** 0.0395752196423961
- **w-rdist:** 1.3612178105237165
- **t-alpha:** 0.0047169960791435

---

---

606

- **AF ID:** AF-Q96IU4-F1-model-v4 | **Chain:** A
- **b-phipsi:** 0.0011545525458561
- **w-rdist:** 1.3026651199004573
- **t-alpha:** 0.7506849219513847

---

---

607

- **AF ID:** AF-Q969U7-F1-model-v4 | **Chain:** A
- **b-phipsi:** 0.0014265900568614
- **w-rdist:** 1.206730979942223
- **t-alpha:** 0.4375706158417265

---

---

608

- **AF ID:** AF-M0QZD8-F8-model-v4 | **Chain:** A
- **b-phipsi:** 0.0575203818860786
- **w-rdist:** 1.6620375776634124
- **t-alpha:** 0.0039119745263893

---

---

609

- **AF ID:** AF-Q92979-F1-model-v4 | **Chain:** A
- **b-phipsi:** 0.001383589869894
- **w-rdist:** 1.2028262573862651
- **t-alpha:** 0.6447880364194061

---

---

610

- **AF ID:** AF-Q6P5W5-F1-model-v4 | **Chain:** A
- **b-phipsi:** 0.0474515865356761
- **w-rdist:** 1.1920073627006642
- **t-alpha:** 0.0070418134049574

---

---

611

- **AF ID:** AF-Q6IE38-F1-model-v4 | **Chain:** A
- **b-phipsi:** 0.0013384513785021
- **w-rdist:** 1.228496041139196
- **t-alpha:** 1.438931607578112

---

---

612

- **AF ID:** AF-O95965-F1-model-v4 | **Chain:** A
- **b-phipsi:** 0.0729415697564812
- **w-rdist:** 1.2576285130712863
- **t-alpha:** 0.0055077996884944

---

---

613

- **AF ID:** AF-Q96JQ0-F6-model-v4 | **Chain:** A
- **b-phipsi:** 0.0282477070334577
- **w-rdist:** 1.8629544663884328
- **t-alpha:** 0.0062593997286479

---

---

614

- **AF ID:** AF-A6NFU8-F1-model-v4 | **Chain:** A
- **b-phipsi:** 0.0013943985670133
- **w-rdist:** 1.216700260410608
- **t-alpha:** 0.8521741912110206

---

---

615

- **AF ID:** AF-Q7Z442-F1-model-v4 | **Chain:** A
- **b-phipsi:** 0.001244203757275
- **w-rdist:** 1.535138633179833
- **t-alpha:** 0.5931140339923704

---

---

616

- **AF ID:** AF-Q9Y4D7-F1-model-v4 | **Chain:** A
- **b-phipsi:** 0.0013844704744507
- **w-rdist:** 1.51502809045237
- **t-alpha:** 0.4851325058502518

---

---

617

- **AF ID:** AF-Q9H0U4-F1-model-v4 | **Chain:** A
- **b-phipsi:** 0.001451531842777
- **w-rdist:** 1.2617496561154649
- **t-alpha:** 0.7700836441710404

---

---

618

- **AF ID:** AF-Q8N271-F1-model-v4 | **Chain:** A
- **b-phipsi:** 0.1945947159327848
- **w-rdist:** 1.6221266833425512
- **t-alpha:** 0.0046944792707916

---

---

619

- **AF ID:** AF-Q13616-F1-model-v4 | **Chain:** A
- **b-phipsi:** 0.0829941093911962
- **w-rdist:** 1.362538188059546
- **t-alpha:** 0.0055077996884944

---

---

620

- **AF ID:** AF-Q12805-F1-model-v4 | **Chain:** A
- **b-phipsi:** 0.0640038032059091
- **w-rdist:** 1.4492591721653565
- **t-alpha:** 0.0055077996884944

---

---

621

- **AF ID:** AF-P23528-F1-model-v4 | **Chain:** A
- **b-phipsi:** 0.0014095496974787
- **w-rdist:** 1.3078605208662195
- **t-alpha:** 0.8179234239159088

---

---

622

- **AF ID:** AF-Q8NCM8-F16-model-v4 | **Chain:** A
- **b-phipsi:** 0.0608995963230969
- **w-rdist:** 1.5700904680612264
- **t-alpha:** 0.0062992257321734

---

---
