# Supplementary material for: Comparative structural insights and functional analysis for the distinct unbound states of Human AGO proteins
Source: Sci Rep. 2025 Mar 19;15:9432. doi: 10.1038/s41598-025-91849-5 (PMC11923369; doi:10.1038/s41598-025-91849-5)
Supplement: Supplementary file 24 — Supplementary Information 12. [file 41598_2025_91849_MOESM24_ESM.zip › 6OONp_A_mdwhole_AF4REF/go/6OONp_A_mitot_mitosis_e6368db7ea9243f789b9637b6202452c-pres_report.html]

 

# Structural Comparison Report for 6OONp\_A - whole structures (total: 1)

---

1

- **Protein name:** Probable RNA-binding protein 46
- **Organism:** Homo sapiens
- **Uniprot Accession Number:** Q8TBY0
- **Protein sequence length:** 533 aa
- **1D identity (%):** 10.36
- **1D identity (%) [Gaps excluded]:** 21.18
- **1D identity - Alignment Gaps:** 478
- **1D aligned content (<aminoacid>:%):** {'N': 3.09, 'E': 8.25, 'D': 1.03, 'Q': 5.15, 'Y': 4.12, 'V': 8.25, 'R': 6.19, 'F': 3.09, 'P': 12.37, 'G': 11.34, 'A': 4.12, 'T': 5.15, 'K': 6.19, 'I': 5.15, 'C': 4.12, 'L': 8.25, 'M': 1.03, 'W': 1.03, 'S': 2.06}
- **Common reported functions (%):** 0.0
- **Common reported locations (%):** 25.0
- **Common reported processes (%):** 8.33

- **AF ID:** Q8TBY0
- **Chain:** A
- **Protein length:** 533 aa
- **Resolution:** N/A
- **b-phipsi ():** 0.005273
- **w-rdist ():** 0.273671
- **t-alpha ():** 0.05399
- **Chemical similarity (Tanimoto Index) (%):** 98.65
- **1D identity (%) [PDB]:** 2.78
- **1D identity (%) [Gaps excluded][PDB]:** 69.81
- **1D identity - Alignment Gaps [PDB]:** 1277
- **1D aligned content [PDB] (<aminoacid>:%):** {'D': 2.7, 'E': 2.7, 'L': 13.51, 'Q': 8.11, 'T': 5.41, 'H': 10.81, 'Y': 10.81, 'V': 8.11, 'R': 2.7, 'C': 2.7, 'S': 10.81, 'I': 2.7, 'P': 5.41, 'A': 5.41, 'G': 5.41, 'N': 2.7}
- **2D identity (%) [PDB]:** 34.6
- **2D identity (%) [Gaps excluded][PDB]:** 91.32
- **2D identity - Alignment Gaps [PDB]:** 623
- **2D aligned content [PDB] (<2D-fold>:%):** {'.': 20.17, 'H': 35.45, 'E': 26.8, 'T': 17.58}
- **3D similarity (TM-Score) (%) [PDB]:** 21.2

- **Gene name:** RBM46
- **Entrez ID:** 16686300
- **RefSeq ID:** N/A
- **Sequence length:** N/A
- **5-UTR|CDS|3-UTR identity (%):** N/A | N/A | N/A
- **5-UTR|CDS|3-UTR identity (%) [Gaps excluded]:** N/A | N/A | N/A
- **5-UTR|CDS|3-UTR identity [Alignment Gaps]:** N/A | N/A | N/A
- **5-UTR aligned content (<base>:%):** N/A
- **CDS aligned content (<base>:%):** N/A
- **3-UTR aligned content (<base>:%):** N/A

**Uniprot Description:**  
  
 N/A N/A   
  
 **Gene Ontology Information:**

Molecular Function

- n/a

Location

- cytoplasm
- nucleus

Biological process

- cell cycle switching, mitotic to meiotic cell cycle
- female meiotic nuclear division
- male meiotic nuclear division
- mRNA stabilization
- oogenesis
- spermatid differentiation
- spermatogenesis
- trophectodermal cell differentiation

---
