# Supplementary material for: Comparative structural insights and functional analysis for the distinct unbound states of Human AGO proteins
Source: Sci Rep. 2025 Mar 19;15:9432. doi: 10.1038/s41598-025-91849-5 (PMC11923369; doi:10.1038/s41598-025-91849-5)
Supplement: Supplementary file 24 — Supplementary Information 12. [file 41598_2025_91849_MOESM24_ESM.zip › 6OONp_A_mdwhole_HL2REF/candidates/6OONp_A-merged-enriched_eval_report.html]

 

# Structural Comparison Report for 6OONp\_A - whole structures (total: 100)

---

1

- **Protein name:** DNA ligase 4
- **Organism:** Homo sapiens
- **Uniprot Accession Number:** P49917
- **Protein sequence length:** 911 aa
- **1D identity (%):** 15.21
- **1D identity (%) [Gaps excluded]:** 23.29
- **1D identity - Alignment Gaps:** 372
- **1D aligned content (<aminoacid>:%):** {'A': 6.13, 'H': 3.68, 'V': 6.75, 'P': 7.98, 'D': 6.75, 'L': 7.98, 'I': 6.13, 'R': 4.29, 'F': 2.45, 'M': 1.84, 'E': 4.91, 'N': 1.84, 'G': 9.2, 'T': 4.91, 'Q': 4.29, 'K': 6.75, 'S': 4.91, 'Y': 5.52, 'C': 3.07, 'W': 0.61}
- **Common reported functions (%):** 0.0
- **Common reported locations (%):** 25.0
- **Common reported processes (%):** 0.0

- **PDB ID:** 3W1G
- **Chain:** A
- **Crystallized protein length:** 583 aa
- **Resolution:** 2.55 Å
- **b-phipsi:** 0.002324
- **w-rdist:** 0.410636
- **t-alpha:** 0.004694
- **Chemical similarity (Tanimoto Index) (%):** 79.2
- **1D identity (%) [PDB]:** 1.57
- **1D identity (%) [Gaps excluded][PDB]:** 68.75
- **1D identity - Alignment Gaps [PDB]:** 1373
- **1D aligned content [PDB] (<aminoacid>:%):** {'T': 9.09, 'L': 22.73, 'P': 4.55, 'G': 9.09, 'K': 4.55, 'D': 4.55, 'Q': 9.09, 'F': 4.55, 'V': 13.64, 'S': 13.64, 'E': 4.55}
- **2D identity (%) [PDB]:** 31.02
- **2D identity (%) [Gaps excluded][PDB]:** 85.38
- **2D identity - Alignment Gaps [PDB]:** 671
- **2D aligned content [PDB] (<2D-fold>:%):** {'.': 18.65, 'H': 54.43, 'G': 1.83, 'T': 8.87, 'E': 16.21}
- **3D similarity (TM-Score) (%) [PDB]:** 17.91

- **Gene name:** LIG4
- **Entrez ID:** 3981
- **RefSeq ID:** N/A
- **Sequence length:** N/A
- **5-UTR|CDS|3-UTR identity (%):** N/A | N/A | N/A
- **5-UTR|CDS|3-UTR identity (%) [Gaps excluded]:** N/A | N/A | N/A
- **5-UTR|CDS|3-UTR identity [Alignment Gaps]:** N/A | N/A | N/A
- **5-UTR aligned content (<base>:%):** N/A
- **CDS aligned content (<base>:%):** N/A
- **3-UTR aligned content (<base>:%):** N/A

**Uniprot Description:**  
  
 Efficiently joins single-strand breaks in a double-stranded polydeoxynucleotide in an ATP-dependent reaction. Involved in DNA non-homologous end joining (NHEJ) required for double-strand break repair and V(D)J recombination. The LIG4-XRCC4 complex is responsible for the NHEJ ligation step, and XRCC4 enhances the joining activity of LIG4. Binding of the LIG4-XRCC4 complex to DNA ends is dependent on the assembly of the DNA-dependent protein kinase complex DNA-PK to these DNA ends.   
  
Interacts with XRCC4. The LIG4-XRCC4 complex has probably a 1:2 stoichiometry. The LIG4-XRCC4 complex associates in a DNA-dependent manner with the DNA-PK complex composed of PRKDC, XRCC6/Ku70 and XRCC5/Ku86 to form the core non-homologous end joining (NHEJ) complex. Additional components of the NHEJ complex include NHEJ1/XLF and PAXX. Interacts with APLF.   
  
 **Gene Ontology Information:**

Molecular Function

- ATP binding
- DNA binding
- DNA ligase (ATP) activity
- DNA ligase activity
- ligase activity
- metal ion binding

Location

- chromosome, telomeric region
- condensed chromosome
- cytoplasmic ribonucleoprotein granule
- DNA ligase IV complex
- DNA-dependent protein kinase-DNA ligase 4 complex
- nonhomologous end joining complex
- nucleoplasm
- nucleus

Biological process

- cell cycle
- cell division
- cell population proliferation
- cellular response to ionizing radiation
- cellular response to lithium ion
- central nervous system development
- chromosome organization
- DN2 thymocyte differentiation
- DNA biosynthetic process
- DNA ligation
- DNA ligation involved in DNA recombination
- DNA ligation involved in DNA repair
- DNA replication
- double-strand break repair
- double-strand break repair via classical nonhomologous end joining
- double-strand break repair via nonhomologous end joining
- establishment of integrated proviral latency
- fibroblast proliferation
- immunoglobulin V(D)J recombination
- in utero embryonic development
- isotype switching
- negative regulation of neuron apoptotic process
- neurogenesis
- neuron apoptotic process
- nucleotide-excision repair, DNA gap filling
- positive regulation of chromosome organization
- positive regulation of fibroblast proliferation
- positive regulation of neurogenesis
- pro-B cell differentiation
- response to gamma radiation
- response to X-ray
- single strand break repair
- somatic stem cell population maintenance
- stem cell proliferation
- T cell differentiation in thymus
- T cell receptor V(D)J recombination
- V(D)J recombination

---

2

- **Protein name:** Lysine-specific histone demethylase 1B
- **Organism:** Homo sapiens
- **Uniprot Accession Number:** Q8NB78
- **Protein sequence length:** 822 aa
- **1D identity (%):** 14.7
- **1D identity (%) [Gaps excluded]:** 21.16
- **1D identity - Alignment Gaps:** 303
- **1D aligned content (<aminoacid>:%):** {'P': 13.01, 'R': 6.16, 'L': 7.53, 'Q': 7.53, 'D': 6.85, 'E': 4.11, 'K': 4.79, 'H': 3.42, 'F': 4.79, 'G': 8.22, 'Y': 4.11, 'T': 3.42, 'V': 8.22, 'S': 3.42, 'M': 1.37, 'C': 2.05, 'N': 2.74, 'I': 2.74, 'A': 4.79, 'W': 0.68}
- **Common reported functions (%):** 0.0
- **Common reported locations (%):** 12.5
- **Common reported processes (%):** 0.0

- **PDB ID:** 7XE1
- **Chain:** A
- **Crystallized protein length:** 751 aa
- **Resolution:** 2.07 Å
- **b-phipsi:** 0.004468
- **w-rdist:** 0.487088
- **t-alpha:** 0.001565
- **Chemical similarity (Tanimoto Index) (%):** 70.05
- **1D identity (%) [PDB]:** 4.4
- **1D identity (%) [Gaps excluded][PDB]:** 63.73
- **1D identity - Alignment Gaps [PDB]:** 1375
- **1D aligned content [PDB] (<aminoacid>:%):** {'E': 3.08, 'F': 4.62, 'D': 7.69, 'Y': 3.08, 'S': 10.77, 'H': 4.62, 'I': 4.62, 'Q': 6.15, 'T': 6.15, 'P': 4.62, 'L': 7.69, 'W': 1.54, 'N': 1.54, 'C': 3.08, 'A': 13.85, 'V': 6.15, 'R': 4.62, 'K': 3.08, 'G': 3.08}
- **2D identity (%) [PDB]:** 43.21
- **2D identity (%) [Gaps excluded][PDB]:** 86.5
- **2D identity - Alignment Gaps [PDB]:** 527
- **2D aligned content [PDB] (<2D-fold>:%):** {'.': 18.24, 'B': 0.44, 'T': 25.05, 'E': 14.73, 'H': 39.56, 'G': 1.98}
- **3D similarity (TM-Score) (%) [PDB]:** 22.25

- **Gene name:** KDM1B
- **Entrez ID:** 221656
- **RefSeq ID:** N/A
- **Sequence length:** N/A
- **5-UTR|CDS|3-UTR identity (%):** N/A | N/A | N/A
- **5-UTR|CDS|3-UTR identity (%) [Gaps excluded]:** N/A | N/A | N/A
- **5-UTR|CDS|3-UTR identity [Alignment Gaps]:** N/A | N/A | N/A
- **5-UTR aligned content (<base>:%):** N/A
- **CDS aligned content (<base>:%):** N/A
- **3-UTR aligned content (<base>:%):** N/A

**Uniprot Description:**  
  
 Histone demethylase that demethylates 'Lys-4' of histone H3, a specific tag for epigenetic transcriptional activation, thereby acting as a corepressor. Required for de novo DNA methylation of a subset of imprinted genes during oogenesis. Acts by oxidizing the substrate by FAD to generate the corresponding imine that is subsequently hydrolyzed. Demethylates both mono- and di-methylated 'Lys-4' of histone H3. Has no effect on tri-methylated 'Lys-4', mono-, di- or tri-methylated 'Lys-9', mono-, di- or tri-methylated 'Lys-27', mono-, di- or tri-methylated 'Lys-36' of histone H3, or on mono-, di- or tri-methylated 'Lys-20' of histone H4.   
  
Does not form a complex with RCOR1/CoREST (By similarity). Interacts with its cofactor GLYR1 at nucleosomes; this interaction stimulates H3K4me1 and H3K4me2 demethylation (PubMed:23260659).   
  
 **Gene Ontology Information:**

Molecular Function

- chromatin binding
- FAD binding
- FAD-dependent H3K4me/H3K4me3 demethylase activity
- flavin adenine dinucleotide binding
- histone binding
- histone demethylase activity
- oxidoreductase activity
- zinc ion binding

Location

- chromatin
- nucleoplasm
- nucleosome
- nucleus

Biological process

- genetic imprinting
- negative regulation of transcription by RNA polymerase II
- positive regulation of transcription by RNA polymerase II
- regulation of gene expression by genetic imprinting
- positive regulation of gene expression, epigenetic

---

3

- **Protein name:** Transcription elongation factor SPT5
- **Organism:** Homo sapiens
- **Uniprot Accession Number:** O00267
- **Protein sequence length:** 1087 aa
- **1D identity (%):** 15.28
- **1D identity (%) [Gaps excluded]:** 20.92
- **1D identity - Alignment Gaps:** 304
- **1D aligned content (<aminoacid>:%):** {'R': 5.81, 'G': 12.79, 'V': 11.05, 'A': 4.07, 'P': 10.47, 'Y': 2.91, 'D': 8.72, 'K': 6.98, 'M': 2.33, 'L': 5.81, 'T': 5.23, 'S': 4.65, 'W': 0.58, 'Q': 4.65, 'I': 2.91, 'F': 2.33, 'E': 3.49, 'H': 1.74, 'C': 2.33, 'N': 1.16}
- **Common reported functions (%):** 0.0
- **Common reported locations (%):** 0.0
- **Common reported processes (%):** 0.0

- **PDB ID:** 8P4F
- **Chain:** O
- **Crystallized protein length:** 695 aa
- **Resolution:** 4.0 Å
- **b-phipsi:** 0.000889
- **w-rdist:** 0.509809
- **t-alpha:** 0.00314
- **Chemical similarity (Tanimoto Index) (%):** N/A
- **1D identity (%) [PDB]:** 3.98
- **1D identity (%) [Gaps excluded][PDB]:** 67.44
- **1D identity - Alignment Gaps [PDB]:** 1373
- **1D aligned content [PDB] (<aminoacid>:%):** {'V': 6.9, 'Q': 10.34, 'S': 10.34, 'E': 12.07, 'L': 10.34, 'D': 3.45, 'M': 1.72, 'R': 10.34, 'F': 3.45, 'Y': 3.45, 'P': 5.17, 'T': 1.72, 'I': 6.9, 'G': 5.17, 'K': 5.17, 'A': 3.45}
- **2D identity (%) [PDB]:** 43.57
- **2D identity (%) [Gaps excluded][PDB]:** 88.43
- **2D identity - Alignment Gaps [PDB]:** 525
- **2D aligned content [PDB] (<2D-fold>:%):** {'.': 16.63, 'E': 20.84, 'T': 25.28, 'H': 36.59, 'G': 0.67}
- **3D similarity (TM-Score) (%) [PDB]:** 27.84

- **Gene name:** SUPT5H
- **Entrez ID:** N/A
- **RefSeq ID:** NM\_003169
- **Transcript sequence length:** 3782
- **5-UTR|CDS|3-UTR identity (%):** 31.85 | 45.46 | 4.31
- **5-UTR|CDS|3-UTR identity (%) [Gaps excluded]:** 71.86 | 74.77 | 76.54
- **5-UTR|CDS|3-UTR identity [Alignment Gaps]:** 250 | 1426 | 4070
- **5-UTR aligned content (<base>:%):** {'C': 33.57, 'G': 50.35, 'T': 6.29, 'A': 9.79}
- **CDS aligned content (<base>:%):** {'A': 26.78, 'T': 19.77, 'G': 28.11, 'C': 25.33}
- **3-UTR aligned content (<base>:%):** {'A': 20.43, 'C': 23.66, 'G': 20.43, 'T': 35.48}

**Uniprot Description:**  
  
 Component of the DRB sensitivity-inducing factor complex (DSIF complex), which regulates mRNA processing and transcription elongation by RNA polymerase II. DSIF positively regulates mRNA capping by stimulating the mRNA guanylyltransferase activity of RNGTT/CAP1A. DSIF also acts cooperatively with the negative elongation factor complex (NELF complex) to enhance transcriptional pausing at sites proximal to the promoter. Transcriptional pausing may facilitate the assembly of an elongation competent RNA polymerase II complex. DSIF and NELF promote pausing by inhibition of the transcription elongation factor TFIIS/S-II. TFIIS/S-II binds to RNA polymerase II at transcription pause sites and stimulates the weak intrinsic nuclease activity of the enzyme. Cleavage of blocked transcripts by RNA polymerase II promotes the resumption of transcription from the new 3' terminus and may allow repeated attempts at transcription through natural pause sites. DSIF can also positively regulate transcriptional elongation and is required for the efficient activation of transcriptional elongation by the HIV-1 nuclear transcriptional activator, Tat. DSIF acts to suppress transcriptional pausing in transcripts derived from the HIV-1 LTR and blocks premature release of HIV-1 transcripts at terminator sequences.   
  
Interacts with SUPT4H1 to form DSIF. DSIF interacts with the positive transcription elongation factor b complex (P-TEFb complex), which is composed of CDK9 and cyclin-T (CCNT1 or CCNT2). DSIF interacts with RNA polymerase II, and this interaction is reduced by phosphorylation of the C-terminal domain (CTD) of POLR2A by P-TEFb. DSIF also interacts with the NELF complex, which is composed of NELFA, NELFB, NELFD and NELFE, and this interaction occurs following prior binding of DSIF to RNA polymerase II. DSIF also interacts with PRMT1/HRMT1L2, HTATSF1/TATSF1, RNGTT/CAP1A, PRMT5/SKB1, SUPT6H, and can interact with PIN1. Component of a complex which is at least composed of HTATSF1/Tat-SF1, the P-TEFb complex components CDK9 and CCNT1, RNA polymerase II, SUPT5H, and NCL/nucleolin. Interacts with MCM3AP isoform GANP (PubMed:23652018).   
  
 **Gene Ontology Information:**

Molecular Function   
  
N/A

Location   
  
N/A

Biological process   
  
N/A

---

4

- **Protein name:** Endoplasmic reticulum aminopeptidase 2
- **Organism:** Homo sapiens
- **Uniprot Accession Number:** Q6P179
- **Protein sequence length:** 960 aa
- **1D identity (%):** 14.73
- **1D identity (%) [Gaps excluded]:** 24.31
- **1D identity - Alignment Gaps:** 447
- **1D aligned content (<aminoacid>:%):** {'P': 8.98, 'T': 5.99, 'L': 10.78, 'S': 5.99, 'F': 4.79, 'G': 8.98, 'R': 4.79, 'W': 1.8, 'V': 5.99, 'I': 3.59, 'N': 2.99, 'Q': 5.99, 'D': 3.59, 'K': 8.98, 'E': 3.59, 'H': 3.59, 'Y': 2.99, 'A': 4.79, 'C': 1.8}
- **Common reported functions (%):** 0.0
- **Common reported locations (%):** 12.5
- **Common reported processes (%):** 0.0

- **PDB ID:** 6EA4
- **Chain:** A
- **Crystallized protein length:** 883 aa
- **Resolution:** 2.45 Å
- **b-phipsi:** 0.008472
- **w-rdist:** 0.395226
- **t-alpha:** 0.000783
- **Chemical similarity (Tanimoto Index) (%):** N/A
- **1D identity (%) [PDB]:** 4.02
- **1D identity (%) [Gaps excluded][PDB]:** 70.21
- **1D identity - Alignment Gaps [PDB]:** 1546
- **1D aligned content [PDB] (<aminoacid>:%):** {'Q': 1.52, 'E': 9.09, 'L': 10.61, 'S': 7.58, 'V': 10.61, 'I': 7.58, 'D': 3.03, 'T': 9.09, 'N': 3.03, 'F': 1.52, 'Y': 4.55, 'K': 6.06, 'R': 6.06, 'G': 10.61, 'A': 3.03, 'H': 4.55, 'P': 1.52}
- **2D identity (%) [PDB]:** 34.46
- **2D identity (%) [Gaps excluded][PDB]:** 87.73
- **2D identity - Alignment Gaps [PDB]:** 756
- **2D aligned content [PDB] (<2D-fold>:%):** {'.': 16.32, 'E': 30.07, 'T': 10.26, 'H': 42.66, 'G': 0.7}
- **3D similarity (TM-Score) (%) [PDB]:** 26.92

- **Gene name:** ERAP2
- **Entrez ID:** 64167
- **RefSeq ID:** NM\_022350
- **Transcript sequence length:** 5131
- **5-UTR|CDS|3-UTR identity (%):** 15.23 | 44.11 | 19.63
- **5-UTR|CDS|3-UTR identity (%) [Gaps excluded]:** 70.41 | 76.75 | 78.79
- **5-UTR|CDS|3-UTR identity [Alignment Gaps]:** 355 | 1477 | 3837
- **5-UTR aligned content (<base>:%):** {'G': 31.88, 'C': 31.88, 'A': 10.14, 'T': 26.09}
- **CDS aligned content (<base>:%):** {'T': 25.72, 'G': 23.43, 'C': 21.02, 'A': 29.83}
- **3-UTR aligned content (<base>:%):** {'A': 30.51, 'T': 33.5, 'G': 20.74, 'C': 15.25}

**Uniprot Description:**  
  
 Aminopeptidase that plays a central role in peptide trimming, a step required for the generation of most HLA class I-binding peptides. Peptide trimming is essential to customize longer precursor peptides to fit them to the correct length required for presentation on MHC class I molecules. Preferentially hydrolyzes the basic residues Arg and Lys.   
  
Heterodimer with ERAP1.   
  
 **Gene Ontology Information:**

Molecular Function

- aminopeptidase activity
- endopeptidase activity
- metalloaminopeptidase activity
- metallopeptidase activity
- peptide binding
- zinc ion binding

Location

- cytoplasm
- endoplasmic reticulum lumen
- endoplasmic reticulum membrane

Biological process

- adaptive immune response
- antigen processing and presentation of endogenous peptide antigen via MHC class I
- antigen processing and presentation of peptide antigen via MHC class I
- peptide catabolic process
- proteolysis
- regulation of blood pressure

---

5

- **Protein name:** Serotransferrin
- **Organism:** Homo sapiens
- **Uniprot Accession Number:** P02787
- **Protein sequence length:** 698 aa
- **1D identity (%):** 14.02
- **1D identity (%) [Gaps excluded]:** 23.09
- **1D identity - Alignment Gaps:** 381
- **1D aligned content (<aminoacid>:%):** {'L': 8.82, 'A': 8.09, 'G': 14.71, 'V': 9.56, 'P': 7.35, 'D': 3.68, 'R': 2.94, 'N': 1.47, 'Y': 2.94, 'E': 5.88, 'F': 2.21, 'T': 5.88, 'K': 8.09, 'Q': 3.68, 'C': 5.88, 'S': 4.41, 'M': 1.47, 'H': 2.21, 'I': 0.74}
- **Common reported functions (%):** 0.0
- **Common reported locations (%):** 0.0
- **Common reported processes (%):** 0.0

- **PDB ID:** 7Q1L
- **Chain:** A
- **Crystallized protein length:** 676 aa
- **Resolution:** 3.0 Å
- **b-phipsi:** 0.006562
- **w-rdist:** 0.428836
- **t-alpha:** 0.001565
- **Chemical similarity (Tanimoto Index) (%):** 96.61
- **1D identity (%) [PDB]:** 2.64
- **1D identity (%) [Gaps excluded][PDB]:** 78.0
- **1D identity - Alignment Gaps [PDB]:** 1426
- **1D aligned content [PDB] (<aminoacid>:%):** {'V': 12.82, 'S': 12.82, 'I': 2.56, 'P': 7.69, 'A': 17.95, 'Y': 7.69, 'R': 5.13, 'L': 5.13, 'D': 10.26, 'K': 5.13, 'H': 5.13, 'G': 5.13, 'N': 2.56}
- **2D identity (%) [PDB]:** 33.27
- **2D identity (%) [Gaps excluded][PDB]:** 85.92
- **2D identity - Alignment Gaps [PDB]:** 674
- **2D aligned content [PDB] (<2D-fold>:%):** {'E': 22.68, 'H': 45.08, 'T': 14.75, '.': 16.67, 'G': 0.82}
- **3D similarity (TM-Score) (%) [PDB]:** 21.77

- **Gene name:** TF
- **Entrez ID:** 7018
- **RefSeq ID:** N/A
- **Sequence length:** N/A
- **5-UTR|CDS|3-UTR identity (%):** N/A | N/A | N/A
- **5-UTR|CDS|3-UTR identity (%) [Gaps excluded]:** N/A | N/A | N/A
- **5-UTR|CDS|3-UTR identity [Alignment Gaps]:** N/A | N/A | N/A
- **5-UTR aligned content (<base>:%):** N/A
- **CDS aligned content (<base>:%):** N/A
- **3-UTR aligned content (<base>:%):** N/A

**Uniprot Description:**  
  
 Transferrins are iron binding transport proteins which can bind two Fe(3+) ions in association with the binding of an anion, usually bicarbonate. It is responsible for the transport of iron from sites of absorption and heme degradation to those of storage and utilization. Serum transferrin may also have a further role in stimulating cell proliferation.   
  
Monomer.   
  
 **Gene Ontology Information:**

Molecular Function

- ferric iron binding
- ferrous iron binding
- iron chaperone activity
- transferrin receptor binding

Location

- apical plasma membrane
- basal part of cell
- basal plasma membrane
- blood microparticle
- cell surface
- clathrin-coated endocytic vesicle membrane
- clathrin-coated pit
- cytoplasmic vesicle
- early endosome
- endocytic vesicle
- endoplasmic reticulum lumen
- endosome membrane
- extracellular exosome
- extracellular region
- extracellular space
- HFE-transferrin receptor complex
- late endosome
- perinuclear region of cytoplasm
- plasma membrane
- recycling endosome
- secretory granule lumen
- vesicle

Biological process

- actin filament organization
- antibacterial humoral response
- cellular response to iron ion
- ERK1 and ERK2 cascade
- cellular iron ion homeostasis
- iron ion transmembrane transport
- iron ion transport
- osteoclast differentiation
- positive regulation of bone resorption
- positive regulation of cell motility
- positive regulation of transcription, DNA-templated
- positive regulation of phosphorylation
- positive regulation of receptor-mediated endocytosis
- regulation of iron ion transport
- regulation of protein stability
- retina homeostasis
- SMAD protein signal transduction

---

6

- **Protein name:** Histone acetyltransferase p300
- **Organism:** Homo sapiens
- **Uniprot Accession Number:** Q09472
- **Protein sequence length:** 2414 aa
- **1D identity (%):** 10.88
- **1D identity (%) [Gaps excluded]:** 30.69
- **1D identity - Alignment Gaps:** 1561
- **1D aligned content (<aminoacid>:%):** {'M': 2.66, 'P': 14.07, 'G': 11.41, 'S': 3.42, 'L': 7.98, 'F': 1.9, 'Q': 11.03, 'N': 1.52, 'D': 3.42, 'K': 6.84, 'E': 5.32, 'R': 5.32, 'V': 6.84, 'H': 2.66, 'T': 5.32, 'W': 0.38, 'I': 1.14, 'Y': 2.28, 'A': 4.18, 'C': 2.28}
- **Common reported functions (%):** 0.0
- **Common reported locations (%):** 37.5
- **Common reported processes (%):** 0.0

- **PDB ID:** 6GYR
- **Chain:** A
- **Crystallized protein length:** 588 aa
- **Resolution:** 3.1 Å
- **b-phipsi:** 0.007295
- **w-rdist:** 0.41479
- **t-alpha:** 0.001565
- **Chemical similarity (Tanimoto Index) (%):** N/A
- **1D identity (%) [PDB]:** 2.15
- **1D identity (%) [Gaps excluded][PDB]:** 73.17
- **1D identity - Alignment Gaps [PDB]:** 1357
- **1D aligned content [PDB] (<aminoacid>:%):** {'K': 6.67, 'T': 6.67, 'S': 10.0, 'P': 10.0, 'Q': 6.67, 'L': 16.67, 'C': 3.33, 'A': 6.67, 'G': 6.67, 'V': 13.33, 'R': 3.33, 'F': 3.33, 'I': 3.33, 'D': 3.33}
- **2D identity (%) [PDB]:** 31.32
- **2D identity (%) [Gaps excluded][PDB]:** 86.65
- **2D identity - Alignment Gaps [PDB]:** 675
- **2D aligned content [PDB] (<2D-fold>:%):** {'.': 17.52, 'H': 51.06, 'T': 13.9, 'E': 17.52}
- **3D similarity (TM-Score) (%) [PDB]:** 23.13

- **Gene name:** EP300
- **Entrez ID:** 2033
- **RefSeq ID:** NM\_001429
- **Transcript sequence length:** 8779
- **5-UTR|CDS|3-UTR identity (%):** 39.21 | 28.23 | 16.78
- **5-UTR|CDS|3-UTR identity (%) [Gaps excluded]:** 74.38 | 82.38 | 74.85
- **5-UTR|CDS|3-UTR identity [Alignment Gaps]:** 252 | 4813 | 3426
- **5-UTR aligned content (<base>:%):** {'G': 37.32, 'A': 8.13, 'C': 43.54, 'T': 11.0}
- **CDS aligned content (<base>:%):** {'A': 29.41, 'T': 21.38, 'G': 24.0, 'C': 25.21}
- **3-UTR aligned content (<base>:%):** {'A': 34.28, 'G': 12.96, 'C': 13.36, 'T': 39.41}

**Uniprot Description:**  
  
 Functions as histone acetyltransferase and regulates transcription via chromatin remodeling (PubMed:23415232, PubMed:23934153, PubMed:8945521). Acetylates all four core histones in nucleosomes. Histone acetylation gives an epigenetic tag for transcriptional activation (PubMed:23415232, PubMed:23934153, PubMed:8945521). Mediates cAMP-gene regulation by binding specifically to phosphorylated CREB protein. Mediates acetylation of histone H3 at 'Lys-122' (H3K122ac), a modification that localizes at the surface of the histone octamer and stimulates transcription, possibly by promoting nucleosome instability. Mediates acetylation of histone H3 at 'Lys-27' (H3K27ac) (PubMed:23911289). Also functions as acetyltransferase for non-histone targets, such as ALX1, HDAC1, PRMT1 or SIRT2 (PubMed:12929931, PubMed:16762839, PubMed:18722353). Acetylates 'Lys-131' of ALX1 and acts as its coactivator (PubMed:12929931). Acetylates SIRT2 and is proposed to indirectly increase the transcriptional activity of TP53 through acetylation and subsequent attenuation of SIRT2 deacetylase function (PubMed:18722353). Acetylates HDAC1 leading to its inactivation and modulation of transcription (PubMed:16762839). Acetylates 'Lys-247' of EGR2 (By similarity). Acts as a TFAP2A-mediated transcriptional coactivator in presence of CITED2 (PubMed:12586840). Plays a role as a coactivator of NEUROD1-dependent transcription of the secretin and p21 genes and controls terminal differentiation of cells in the intestinal epithelium. Promotes cardiac myocyte enlargement. Can also mediate transcriptional repression. Acetylates FOXO1 and enhances its transcriptional activity (PubMed:15890677). Acetylates BCL6 wich disrupts its ability to recruit histone deacetylases and hinders its transcriptional repressor activity (PubMed:12402037). Participates in CLOCK or NPAS2-regulated rhythmic gene transcription; exhibits a circadian association with CLOCK or NPAS2, correlating with increase in PER1/2 mRNA and histone H3 acetylation on the PER1/2 promoter (PubMed:14645221). Acetylates MTA1 at 'Lys-626' which is essential for its transcriptional coactivator activity (PubMed:16617102). Acetylates XBP1 isoform 2; acetylation increases protein stability of XBP1 isoform 2 and enhances its transcriptional activity (PubMed:20955178). Acetylates PCNA; acetylation promotes removal of chromatin-bound PCNA and its degradation during nucleotide excision repair (NER) (PubMed:24939902). Acetylates MEF2D (PubMed:21030595). Acetylates and stabilizes ZBTB7B protein by antagonizing ubiquitin conjugation and degragation, this mechanism may be involved in CD4/CD8 lineage differentiation (PubMed:20810990). Acetylates GABPB1, impairing GABPB1 heterotetramerization and activity (By similarity). In addition to protein acetyltransferase, can use different acyl-CoA substrates, such as (2E)-butenoyl-CoA (crotonyl-CoA), butanoyl-CoA (butyryl-CoA), 2-hydroxyisobutanoyl-CoA (2-hydroxyisobutyryl-CoA), lactoyl-CoA or propanoyl-CoA (propionyl-CoA), and is able to mediate protein crotonylation, butyrylation, 2-hydroxyisobutyrylation, lactylation or propionylation, respectively (PubMed:17267393, PubMed:25818647, PubMed:29775581, PubMed:31645732). Acts as a histone crotonyltransferase; crotonylation marks active promoters and enhancers and confers resistance to transcriptional repressors (PubMed:25818647). Histone crotonyltransferase activity is dependent on the concentration of (2E)-butenoyl-CoA (crotonyl-CoA) substrate and such activity is weak when (2E)-butenoyl-CoA (crotonyl-CoA) concentration is low (PubMed:25818647). Also acts as a histone butyryltransferase; butyrylation marks active promoters (PubMed:17267393). Catalyzes histone lactylation in macrophages by using lactoyl-CoA directly derived from endogenous or exogenous lactate, leading to stimulates gene transcription (PubMed:31645732). Acts as a protein-lysine 2-hydroxyisobutyryltransferase; regulates glycolysis by mediating 2-hydroxyisobutyrylation of glycolytic enzymes (PubMed:29775581). Functions as a transcriptional coactivator for SMAD4 in the TGF-beta signaling pathway (PubMed:25514493). Acetylates PCK1 and promotes PCK1 anaplerotic activity (PubMed:30193097). Acetylates RXRA and RXRG (PubMed:17761950).   
  
Interacts with HIF1A; the interaction is stimulated in response to hypoxia and inhibited by CITED2 (PubMed:9887100, PubMed:11959990). Probably part of a complex with HIF1A and CREBBP (PubMed:8917528). Interacts (via N-terminus) with TFAP2A (via N-terminus); the interaction requires CITED2 (PubMed:12586840). Interacts (via CH1 domain) with CITED2 (via C-terminus) (PubMed:12586840, PubMed:12778114). Interacts with CITED1 (unphosphorylated form preferentially and via C-terminus) (PubMed:10722728, PubMed:16864582). Interacts with ESR1; the interaction is estrogen-dependent and enhanced by CITED1 (PubMed:11581164). Interacts with DTX1, EID1, ELF3, FEN1, LEF1, NCOA1, NCOA6, NR3C1, PCAF, PELP1, PRDM6, SP1, SP3, SPIB, SRY, TCF7L2, TP53, DDX5, DDX17, SATB1, SRCAP, TTC5, JMY and TRERF1 (PubMed:11073989, PubMed:11073990, PubMed:10823961, PubMed:11349124, PubMed:11430825, PubMed:11481323, PubMed:11564735, PubMed:11581372, PubMed:11864910, PubMed:12446687, PubMed:12527917, PubMed:12837748, PubMed:14605447, PubMed:15075319, PubMed:15186775, PubMed:15297880, PubMed:16478997, PubMed:8684459, PubMed:17226766, PubMed:19217391, PubMed:9590696). Part of a complex containing CARM1 and NCOA2/GRIP1 (PubMed:11701890, PubMed:11997499, PubMed:15731352). Interacts with ING4 and this interaction may be indirect (PubMed:12750254). Interacts with ING5 (PubMed:12750254). Interacts with the C-terminal region of CITED4 (PubMed:11744733). Non-sumoylated EP300 preferentially interacts with SENP3 (PubMed:19680224). Interacts with SS18L1/CREST (PubMed:14716005). Interacts with ALX1 (via homeobox domain) (PubMed:12929931). Interacts with NEUROD1; the interaction is inhibited by NR0B2 (PubMed:14752053). Interacts with TCF3 (PubMed:14752053). Interacts (via CREB-binding domain) with MYOCD (via C-terminus) (By similarity). Interacts with ROCK2 and PPARG (PubMed:11518699, PubMed:16574662). Forms a complex made of CDK9, CCNT1/cyclin-T1, EP300 and GATA4 that stimulates hypertrophy in cardiomyocytes (PubMed:20081228). Interacts with IRF1 and this interaction enhances acetylation of p53/TP53 and stimulation of its activity (PubMed:15509808). Interacts with FOXO1; the interaction acetylates FOXO1 and enhances its transcriptional activity (PubMed:15890677). Interacts with ALKBH4 and DDIT3/CHOP (PubMed:17872950, PubMed:23145062). Interacts with KLF15 (PubMed:23999430). Interacts with CEBPB and RORA (PubMed:9862959). Interacts with NPAS2, ARNTL/BMAL1 and CLOCK (PubMed:14645221). Interacts with SIRT2 isoform 1, isoform 2 and isoform 5 (PubMed:24177535). Interacts with MTA1 (PubMed:16617102). Interacts with HDAC4 and HDAC5 in the presence of TFAP2C (PubMed:24413532). Interacts with TRIP4 (PubMed:25219498). Directly interacts with ZBTB49; this interaction leads to synergistic transactivation of CDKN1A (PubMed:25245946). Interacts with NR4A3 (By similarity). Interacts with ZNF451 (PubMed:24324267). Interacts with ATF5; EP300 is required for ATF5 and CEBPB interaction and DNA binding (By similarity). Interacts with HSF1 (PubMed:27189267). Interacts with ZBTB48/TZAP (PubMed:24382891). Interacts with STAT1; the interaction is enhanced upon IFN-gamma stimulation (PubMed:26479788). Interacts with HNRNPU (via C-terminus); this interaction enhances DNA-binding of HNRNPU to nuclear scaffold/matrix attachment region (S/MAR) elements (PubMed:11909954). Interacts with BCL11B (PubMed:27959755, PubMed:16809611). Interacts with SMAD4; negatively regulated by ZBTB7A (PubMed:25514493). Interacts with DUX4 (via C-terminus) (PubMed:26951377). Interacts with NUPR1; this interaction enhances the effect of EP300 on PAX2 transcription factor activity (PubMed:11940591). Interacts with RXRA; the interaction is decreased by 9-cis retinoic acid (PubMed:17761950). NR4A1 competes with EP300 for interaction with RXRA and thereby attenuates EP300 mediated acetylation of RXRA (PubMed:17761950). Interacts with RB1 (By similarity). Interacts with DDX3X; this interaction may facilitate HNF4A acetylation (PubMed:28128295). Interacts with SOX9 (PubMed:12732631). Interacts with ATF4; EP300/p300 stabilizes ATF4 and increases its transcriptional activity independently of its catalytic activity by preventing its ubiquitination (PubMed:16219772).   
  
 **Gene Ontology Information:**

Molecular Function

- acetyltransferase activity
- transferase activity, transferring acyl groups
- beta-catenin binding
- chromatin binding
- chromatin DNA binding
- damaged DNA binding
- DNA binding
- DNA-binding transcription factor binding
- histone acetyltransferase activity
- histone butyryltransferase activity
- histone crotonyltransferase activity
- histone H2B acetyltransferase activity
- H3 histone acetyltransferase activity
- histone H3K122 acetyltransferase activity
- H4 histone acetyltransferase activity
- histone lactyltransferase activity
- lysine N-acetyltransferase activity, acting on acetyl phosphate as donor
- NF-kappaB binding
- androgen receptor binding
- nuclear receptor binding
- p53 binding
- peptide 2-hydroxyisobutyryltransferase activity
- peptide butyryltransferase activity
- peptide N-acetyltransferase activity
- peptide-lysine-N-acetyltransferase activity
- pre-mRNA intronic binding
- protein propionyltransferase activity
- RNA polymerase II-specific DNA-binding transcription factor binding
- STAT family protein binding
- tau protein binding
- transcription coactivator activity
- transcription coactivator binding
- transcription coregulator binding
- zinc ion binding

Location

- chromatin
- cytoplasm
- cytosol
- histone acetyltransferase complex
- nucleoplasm
- nucleus
- protein-DNA complex
- transcription regulator complex

Biological process

- animal organ morphogenesis
- apoptotic process
- B cell differentiation
- behavioral defense response
- cell cycle
- cellular response to UV
- circadian rhythm
- face morphogenesis
- fat cell differentiation
- heart development
- histone acetylation
- internal peptidyl-lysine acetylation
- internal protein amino acid acetylation
- intrinsic apoptotic signaling pathway in response to DNA damage by p53 class mediator
- learning or memory
- lung development
- macrophage derived foam cell differentiation
- megakaryocyte development
- multicellular organism growth
- N-terminal peptidyl-lysine acetylation
- negative regulation of gluconeogenesis
- negative regulation of protein-containing complex assembly
- negative regulation of transcription by RNA polymerase II
- nervous system development
- peptidyl-lysine acetylation
- peptidyl-lysine butyrylation
- peptidyl-lysine crotonylation
- peptidyl-lysine propionylation
- platelet formation
- positive regulation by host of viral transcription
- positive regulation of DNA-binding transcription factor activity
- positive regulation of transcription, DNA-templated
- positive regulation of neuron projection development
- positive regulation of NF-kappaB transcription factor activity
- positive regulation of NIK/NF-kappaB signaling
- positive regulation of protein binding
- positive regulation of protein import into nucleus
- positive regulation of receptor signaling pathway via JAK-STAT
- positive regulation of RNA polymerase II regulatory region sequence-specific DNA binding
- positive regulation of transcription by RNA polymerase II
- positive regulation of transforming growth factor beta receptor signaling pathway
- protein acetylation
- protein destabilization
- protein stabilization
- regulation of androgen receptor signaling pathway
- regulation of autophagy
- regulation of cellular response to heat
- regulation of glycolytic process
- regulation of mitochondrion organization
- regulation of signal transduction by p53 class mediator
- regulation of tubulin deacetylation
- response to estrogen
- response to hypoxia
- skeletal muscle tissue development
- somitogenesis
- stimulatory C-type lectin receptor signaling pathway
- swimming
- thigmotaxis
- transcription by RNA polymerase II
- positive regulation of gene expression, epigenetic

---

7

- **Protein name:** Protein argonaute-2
- **Organism:** Homo sapiens
- **Uniprot Accession Number:** Q9UKV8
- **Protein sequence length:** 859 aa
- **1D identity (%):** 77.73
- **1D identity (%) [Gaps excluded]:** 79.74
- **1D identity - Alignment Gaps:** 22
- **1D aligned content (<aminoacid>:%):** {'M': 2.22, 'G': 7.39, 'P': 7.39, 'A': 6.2, 'F': 4.43, 'R': 6.2, 'T': 6.06, 'I': 5.17, 'L': 7.53, 'N': 2.36, 'K': 5.47, 'D': 4.73, 'Y': 3.84, 'H': 3.1, 'E': 4.73, 'V': 8.12, 'Q': 6.65, 'S': 4.73, 'W': 0.89, 'C': 2.81}
- **Common reported functions (%):** 100.0
- **Common reported locations (%):** 100.0
- **Common reported processes (%):** 50.0

- **PDB ID:** 4Z4F
- **Chain:** A
- **Crystallized protein length:** 801 aa
- **Resolution:** 2.8 Å
- **b-phipsi:** 0.005718
- **w-rdist:** 0.232851
- **t-alpha:** 0.010954
- **Chemical similarity (Tanimoto Index) (%):** 91.63
- **1D identity (%) [PDB]:** 75.03
- **1D identity (%) [Gaps excluded][PDB]:** 79.7
- **1D identity - Alignment Gaps [PDB]:** 50
- **1D aligned content [PDB] (<aminoacid>:%):** {'F': 4.69, 'P': 7.34, 'R': 6.56, 'G': 6.72, 'T': 6.25, 'I': 5.47, 'L': 7.81, 'A': 6.25, 'N': 2.5, 'K': 5.47, 'D': 4.69, 'Y': 4.06, 'H': 2.81, 'V': 8.59, 'E': 4.38, 'M': 2.03, 'Q': 6.41, 'S': 4.22, 'W': 0.94, 'C': 2.81}
- **2D identity (%) [PDB]:** 65.03
- **2D identity (%) [Gaps excluded][PDB]:** 76.48
- **2D identity - Alignment Gaps [PDB]:** 134
- **2D aligned content [PDB] (<2D-fold>:%):** {'.': 16.32, 'B': 1.89, 'E': 32.65, 'H': 36.94, 'T': 11.17, 'G': 1.03}
- **3D similarity (TM-Score) (%) [PDB]:** 90.72

- **Gene name:** AGO2
- **Entrez ID:** N/A
- **RefSeq ID:** NM\_012154
- **Transcript sequence length:** 14595
- **5-UTR|CDS|3-UTR identity (%):** 21.14 | 69.33 | 28.08
- **5-UTR|CDS|3-UTR identity (%) [Gaps excluded]:** 83.18 | 74.74 | 81.16
- **5-UTR|CDS|3-UTR identity [Alignment Gaps]:** 314 | 194 | 7859
- **5-UTR aligned content (<base>:%):** {'C': 44.94, 'G': 43.82, 'T': 8.99, 'A': 2.25}
- **CDS aligned content (<base>:%):** {'A': 26.26, 'T': 20.99, 'G': 27.02, 'C': 25.73}
- **3-UTR aligned content (<base>:%):** {'A': 27.12, 'G': 21.34, 'T': 34.2, 'C': 17.34}

**Uniprot Description:**  
  
 Required for RNA-mediated gene silencing (RNAi) by the RNA-induced silencing complex (RISC). The 'minimal RISC' appears to include AGO2 bound to a short guide RNA such as a microRNA (miRNA) or short interfering RNA (siRNA). These guide RNAs direct RISC to complementary mRNAs that are targets for RISC-mediated gene silencing. The precise mechanism of gene silencing depends on the degree of complementarity between the miRNA or siRNA and its target. Binding of RISC to a perfectly complementary mRNA generally results in silencing due to endonucleolytic cleavage of the mRNA specifically by AGO2. Binding of RISC to a partially complementary mRNA results in silencing through inhibition of translation, and this is independent of endonuclease activity. May inhibit translation initiation by binding to the 7-methylguanosine cap, thereby preventing the recruitment of the translation initiation factor eIF4-E. May also inhibit translation initiation via interaction with EIF6, which itself binds to the 60S ribosomal subunit and prevents its association with the 40S ribosomal subunit. The inhibition of translational initiation leads to the accumulation of the affected mRNA in cytoplasmic processing bodies (P-bodies), where mRNA degradation may subsequently occur. In some cases RISC-mediated translational repression is also observed for miRNAs that perfectly match the 3' untranslated region (3'-UTR). Can also up-regulate the translation of specific mRNAs under certain growth conditions. Binds to the AU element of the 3'-UTR of the TNF (TNF-alpha) mRNA and up-regulates translation under conditions of serum starvation. Also required for transcriptional gene silencing (TGS), in which short RNAs known as antigene RNAs or agRNAs direct the transcriptional repression of complementary promoter regions.   
  
Interacts with DICER1 through its Piwi domain and with TARBP2 during assembly of the RNA-induced silencing complex (RISC). Together, DICER1, AGO2 and TARBP2 constitute the trimeric RISC loading complex (RLC), or micro-RNA (miRNA) loading complex (miRLC). Within the RLC/miRLC, DICER1 and TARBP2 are required to process precursor miRNAs (pre-miRNAs) to mature miRNAs and then load them onto AGO2. AGO2 bound to the mature miRNA constitutes the minimal RISC and may subsequently dissociate from DICER1 and TARBP2. Note however that the term RISC has also been used to describe the trimeric RLC/miRLC. The formation of RISC complexes containing siRNAs rather than miRNAs appears to occur independently of DICER1. Interacts with AGO1. Also interacts with DDB1, DDX5, DDX6, DDX20, DHX30, DHX36, DDX47, DHX9, ELAVL, FXR1, GEMIN4, HNRNPF, IGF2BP1, ILF3, IMP8, MATR3, PABPC1, PRMT5, P4HA1, P4HB, RBM4, SART3, TNRC6A, TNRC6B, UPF1 and YBX1. Interacts with the P-body components DCP1A and XRN1. Associates with polysomes and messenger ribonucleoproteins (mNRPs). Interacts with RBM4; the interaction is modulated under stress-induced conditions, occurs under both cell proliferation and differentiation conditions and in an RNA- and phosphorylation-independent manner. Interacts with LIMD1, WTIP and AJUBA. Interacts with TRIM71; the interaction increases in presence of RNA (PubMed:23125361). Interacts with APOBEC3G in an RNA-dependent manner. Interacts with APOBEC3A, APOBEC3C, APOBEC3F and APOBEC3H. Interacts with DICER1, TARBP2, EIF6, MOV10 and RPL7A (60S ribosome subunit); they form a large RNA-induced silencing complex (RISC) (PubMed:17507929, PubMed:24726324). Interacts with FMR1 (PubMed:14703574). Interacts with ZFP36 (PubMed:15766526). Found in a complex, composed of AGO2, CHD7 and FAM172A (By similarity). Interacts with RC3H1; the interaction is RNA independent (PubMed:25697406). Interacts with SND1 (PubMed:14508492, PubMed:28546213). Interacts with SYT11 (By similarity). Interacts with CLNK (PubMed:26009488). Interacts with GARRE1 (PubMed:29395067).   
  
 **Gene Ontology Information:**

Molecular Function

- core promoter sequence-specific DNA binding
- double-stranded RNA binding
- endoribonuclease activity, cleaving miRNA-paired mRNA
- endoribonuclease activity, cleaving siRNA-paired mRNA
- metal ion binding
- miRNA binding
- mRNA 3'-UTR AU-rich region binding
- mRNA cap binding
- RNA 7-methylguanosine cap binding
- RNA binding
- endoribonuclease activity
- RNA polymerase II complex binding
- single-stranded RNA binding
- siRNA binding
- translation initiation factor activity

Location

- cytoplasm
- cytoplasmic ribonucleoprotein granule
- cytosol
- dendrite
- extracellular exosome
- membrane
- mRNA cap binding complex
- nucleoplasm
- nucleus
- P-body
- polysome
- ribonucleoprotein complex
- RISC complex
- RISC-loading complex

Biological process

- miRNA metabolic process
- production of miRNAs involved in gene silencing by miRNA
- miRNA mediated inhibition of translation
- mRNA cleavage involved in gene silencing by miRNA
- post-transcriptional gene silencing by RNA
- negative regulation of amyloid precursor protein biosynthetic process
- negative regulation of translational initiation
- positive regulation of angiogenesis
- positive regulation of nuclear-transcribed mRNA catabolic process, deadenylation-dependent decay
- positive regulation of nuclear-transcribed mRNA poly(A) tail shortening
- positive regulation of transcription by RNA polymerase II
- positive regulation of translation
- positive regulation of trophoblast cell migration
- post-embryonic development
- pre-miRNA processing
- small RNA loading onto RISC
- RNA secondary structure unwinding
- gene silencing by RNA
- production of siRNA involved in RNA interference
- mRNA cleavage involved in gene silencing by siRNA
- translation

---

8

- **Protein name:** Xylosyltransferase 1
- **Organism:** Homo sapiens
- **Uniprot Accession Number:** Q86Y38
- **Protein sequence length:** 959 aa
- **1D identity (%):** 16.9
- **1D identity (%) [Gaps excluded]:** 24.17
- **1D identity - Alignment Gaps:** 322
- **1D aligned content (<aminoacid>:%):** {'V': 8.84, 'P': 13.26, 'L': 8.84, 'A': 4.97, 'D': 6.08, 'R': 8.84, 'G': 11.05, 'E': 4.42, 'Q': 2.21, 'S': 4.42, 'F': 2.76, 'N': 2.21, 'C': 3.87, 'I': 3.31, 'T': 4.97, 'K': 3.87, 'Y': 2.76, 'H': 1.66, 'M': 0.55, 'W': 1.1}
- **Common reported functions (%):** 0.0
- **Common reported locations (%):** 0.0
- **Common reported processes (%):** 0.0

- **PDB ID:** 6EJE
- **Chain:** A
- **Crystallized protein length:** 695 aa
- **Resolution:** 2.43 Å
- **b-phipsi:** 0.001161
- **w-rdist:** 0.530081
- **t-alpha:** 0.004694
- **Chemical similarity (Tanimoto Index) (%):** 90.31
- **1D identity (%) [PDB]:** 2.76
- **1D identity (%) [Gaps excluded][PDB]:** 67.21
- **1D identity - Alignment Gaps [PDB]:** 1425
- **1D aligned content [PDB] (<aminoacid>:%):** {'Q': 7.32, 'P': 12.2, 'K': 9.76, 'D': 12.2, 'Y': 7.32, 'H': 2.44, 'V': 7.32, 'R': 14.63, 'N': 4.88, 'T': 4.88, 'M': 2.44, 'F': 4.88, 'G': 7.32, 'A': 2.44}
- **2D identity (%) [PDB]:** 33.88
- **2D identity (%) [Gaps excluded][PDB]:** 85.23
- **2D identity - Alignment Gaps [PDB]:** 667
- **2D aligned content [PDB] (<2D-fold>:%):** {'.': 18.13, 'H': 41.87, 'T': 14.4, 'E': 24.8, 'G': 0.8}
- **3D similarity (TM-Score) (%) [PDB]:** 25.17

- **Gene name:** XYLT1
- **Entrez ID:** 64131
- **RefSeq ID:** NM\_022166
- **Transcript sequence length:** 9970
- **5-UTR|CDS|3-UTR identity (%):** 27.91 | 45.08 | 39.66
- **5-UTR|CDS|3-UTR identity (%) [Gaps excluded]:** 75.16 | 72.82 | 75.71
- **5-UTR|CDS|3-UTR identity [Alignment Gaps]:** 259 | 1286 | 3503
- **5-UTR aligned content (<base>:%):** {'C': 41.74, 'G': 51.3, 'A': 4.35, 'T': 2.61}
- **CDS aligned content (<base>:%):** {'A': 25.56, 'T': 18.4, 'G': 28.12, 'C': 27.92}
- **3-UTR aligned content (<base>:%):** {'G': 23.17, 'A': 26.32, 'T': 32.69, 'C': 17.82}

**Uniprot Description:**  
  
 Catalyzes the first step in the biosynthesis of chondroitin sulfate and dermatan sulfate proteoglycans, such as DCN. Transfers D-xylose from UDP-D-xylose to specific serine residues of the core protein (PubMed:15461586, PubMed:17189265, PubMed:24581741, PubMed:23982343). Required for normal embryonic and postnatal skeleton development, especially of the long bones (PubMed:24581741, PubMed:23982343). Required for normal maturation of chondrocytes during bone development, and normal onset of ossification (By similarity).   
  
Monomer.   
  
 **Gene Ontology Information:**

Molecular Function

- metal ion binding
- protein xylosyltransferase activity

Location

- extracellular space
- Golgi cis cisterna
- Golgi membrane

Biological process

- chondroitin sulfate biosynthetic process
- chondroitin sulfate proteoglycan biosynthetic process
- embryonic skeletal system development
- glycosaminoglycan biosynthetic process
- glycosaminoglycan metabolic process
- heparan sulfate proteoglycan biosynthetic process
- ossification involved in bone maturation
- proteoglycan biosynthetic process

---

9

- **Protein name:** DNA topoisomerase 3-beta-1
- **Organism:** Homo sapiens
- **Uniprot Accession Number:** O95985
- **Protein sequence length:** 862 aa
- **1D identity (%):** 17.27
- **1D identity (%) [Gaps excluded]:** 23.66
- **1D identity - Alignment Gaps:** 269
- **1D aligned content (<aminoacid>:%):** {'M': 1.74, 'A': 6.4, 'P': 9.3, 'S': 4.07, 'R': 9.3, 'L': 8.14, 'G': 10.47, 'T': 4.65, 'H': 2.91, 'K': 5.23, 'D': 4.65, 'E': 5.81, 'N': 1.74, 'V': 8.14, 'Y': 5.23, 'F': 4.65, 'Q': 2.33, 'I': 2.91, 'C': 2.33}
- **Common reported functions (%):** 0.0
- **Common reported locations (%):** 12.5
- **Common reported processes (%):** 0.0

- **PDB ID:** 5GVE
- **Chain:** A
- **Crystallized protein length:** 611 aa
- **Resolution:** 3.61 Å
- **b-phipsi:** 0.007437
- **w-rdist:** 0.45694
- **t-alpha:** 0.000782
- **Chemical similarity (Tanimoto Index) (%):** 98.94
- **1D identity (%) [PDB]:** 3.3
- **1D identity (%) [Gaps excluded][PDB]:** 68.66
- **1D identity - Alignment Gaps [PDB]:** 1327
- **1D aligned content [PDB] (<aminoacid>:%):** {'M': 2.17, 'T': 6.52, 'A': 6.52, 'L': 21.74, 'G': 6.52, 'D': 4.35, 'V': 13.04, 'E': 6.52, 'P': 6.52, 'K': 4.35, 'Q': 4.35, 'F': 4.35, 'W': 2.17, 'N': 2.17, 'I': 2.17, 'R': 2.17, 'H': 2.17, 'S': 2.17}
- **2D identity (%) [PDB]:** 37.06
- **2D identity (%) [Gaps excluded][PDB]:** 87.99
- **2D identity - Alignment Gaps [PDB]:** 595
- **2D aligned content [PDB] (<2D-fold>:%):** {'E': 22.83, 'H': 55.12, 'T': 9.71, '.': 12.07, 'B': 0.26}
- **3D similarity (TM-Score) (%) [PDB]:** 22.08

- **Gene name:** TOP3B
- **Entrez ID:** 8940
- **RefSeq ID:** N/A
- **Sequence length:** N/A
- **5-UTR|CDS|3-UTR identity (%):** N/A | N/A | N/A
- **5-UTR|CDS|3-UTR identity (%) [Gaps excluded]:** N/A | N/A | N/A
- **5-UTR|CDS|3-UTR identity [Alignment Gaps]:** N/A | N/A | N/A
- **5-UTR aligned content (<base>:%):** N/A
- **CDS aligned content (<base>:%):** N/A
- **3-UTR aligned content (<base>:%):** N/A

**Uniprot Description:**  
  
 Releases the supercoiling and torsional tension of DNA introduced during the DNA replication and transcription by transiently cleaving and rejoining one strand of the DNA duplex. Introduces a single-strand break via transesterification at a target site in duplex DNA. The scissile phosphodiester is attacked by the catalytic tyrosine of the enzyme, resulting in the formation of a DNA-(5'-phosphotyrosyl)-enzyme intermediate and the expulsion of a 3'-OH DNA strand. The free DNA strand than undergoes passage around the unbroken strand thus removing DNA supercoils. Finally, in the religation step, the DNA 3'-OH attacks the covalent intermediate to expel the active-site tyrosine and restore the DNA phosphodiester backbone (By similarity). Possesses negatively supercoiled DNA relaxing activity. N/A   
  
 **Gene Ontology Information:**

Molecular Function

- DNA binding
- DNA topoisomerase activity
- DNA topoisomerase type I (single strand cut, ATP-independent) activity
- RNA binding

Location

- condensed chromosome
- DNA topoisomerase III-beta-TDRD3 complex
- nucleus

Biological process

- chromosome segregation
- DNA topological change

---

10

- **Protein name:** Protein argonaute-1
- **Organism:** Homo sapiens
- **Uniprot Accession Number:** Q9UL18
- **Protein sequence length:** 857 aa
- **1D identity (%):** 82.64
- **1D identity (%) [Gaps excluded]:** 84.79
- **1D identity - Alignment Gaps:** 22
- **1D aligned content (<aminoacid>:%):** {'M': 2.36, 'E': 4.59, 'A': 5.98, 'G': 7.09, 'P': 7.09, 'F': 4.17, 'Q': 6.68, 'R': 6.4, 'T': 5.84, 'V': 8.48, 'K': 6.12, 'I': 5.29, 'L': 7.23, 'N': 2.5, 'D': 4.59, 'Y': 4.45, 'H': 3.2, 'S': 4.45, 'W': 0.83, 'C': 2.64}
- **Common reported functions (%):** 75.0
- **Common reported locations (%):** 87.5
- **Common reported processes (%):** 41.67

- **PDB ID:** 4KXT
- **Chain:** A
- **Crystallized protein length:** 806 aa
- **Resolution:** 2.29 Å
- **b-phipsi:** 0.001797
- **w-rdist:** 0.070243
- **t-alpha:** 0.103286
- **Chemical similarity (Tanimoto Index) (%):** 99.62
- **1D identity (%) [PDB]:** 80.94
- **1D identity (%) [Gaps excluded][PDB]:** 85.15
- **1D identity - Alignment Gaps [PDB]:** 42
- **1D aligned content [PDB] (<aminoacid>:%):** {'Q': 6.83, 'P': 6.83, 'R': 6.69, 'G': 6.54, 'T': 5.96, 'V': 8.72, 'K': 6.25, 'I': 5.52, 'L': 7.56, 'A': 6.1, 'N': 2.47, 'F': 4.07, 'D': 4.51, 'Y': 4.65, 'H': 3.05, 'E': 4.22, 'M': 2.33, 'S': 4.07, 'W': 0.87, 'C': 2.76}
- **2D identity (%) [PDB]:** 66.33
- **2D identity (%) [Gaps excluded][PDB]:** 78.76
- **2D identity - Alignment Gaps [PDB]:** 142
- **2D aligned content [PDB] (<2D-fold>:%):** {'.': 17.25, 'B': 1.84, 'E': 32.33, 'H': 36.68, 'T': 9.88, 'G': 2.01}
- **3D similarity (TM-Score) (%) [PDB]:** 91.23

- **Gene name:** AGO1
- **Entrez ID:** 26523
- **RefSeq ID:** NM\_012199
- **Transcript sequence length:** 13712
- **5-UTR|CDS|3-UTR identity (%):** 31.54 | 72.91 | 29.89
- **5-UTR|CDS|3-UTR identity (%) [Gaps excluded]:** 72.58 | 77.88 | 80.83
- **5-UTR|CDS|3-UTR identity [Alignment Gaps]:** 242 | 170 | 6998
- **5-UTR aligned content (<base>:%):** {'C': 43.7, 'T': 8.15, 'G': 43.7, 'A': 4.44}
- **CDS aligned content (<base>:%):** {'A': 26.35, 'T': 22.23, 'G': 26.35, 'C': 25.06}
- **3-UTR aligned content (<base>:%):** {'A': 27.87, 'G': 21.93, 'C': 16.6, 'T': 33.59}

**Uniprot Description:**  
  
 Required for RNA-mediated gene silencing (RNAi). Binds to short RNAs such as microRNAs (miRNAs) or short interfering RNAs (siRNAs), and represses the translation of mRNAs which are complementary to them. Lacks endonuclease activity and does not appear to cleave target mRNAs. Also required for transcriptional gene silencing (TGS) of promoter regions which are complementary to bound short antigene RNAs (agRNAs).   
  
Interacts with DDB1, DDX5, DDX6, DHX30, DHX36, DDX47, DICER1, AGO2, ELAVL1, HNRNPF, IGF2BP1, ILF3, IMP8, MATR3, MOV10, PABPC1, PRMT5, RBM4, SART3, TNRC6B, UPF1 and YBX1. Associates with polysomes and messenger ribonucleoproteins (mNRPs). Interacts with LIMD1, WTIP and AJUBA. Interacts with APOBEC3F, APOBEC3G and APOBEC3H.   
  
 **Gene Ontology Information:**

Molecular Function

- core promoter sequence-specific DNA binding
- double-stranded RNA binding
- miRNA binding
- RNA binding
- RNA polymerase II complex binding
- single-stranded RNA binding

Location

- cytoplasm
- cytoplasmic ribonucleoprotein granule
- cytosol
- nucleoplasm
- nucleus
- P-body
- polysome
- ribonucleoprotein complex
- RISC complex
- RISC-loading complex

Biological process

- production of miRNAs involved in gene silencing by miRNA
- miRNA mediated inhibition of translation
- negative regulation of angiogenesis
- nuclear-transcribed mRNA catabolic process
- positive regulation of transcription by RNA polymerase II
- pre-miRNA processing
- small RNA loading onto RISC
- RNA secondary structure unwinding

---

11

- **Protein name:** Plasminogen
- **Organism:** Homo sapiens
- **Uniprot Accession Number:** P00747
- **Protein sequence length:** 810 aa
- **1D identity (%):** 14.75
- **1D identity (%) [Gaps excluded]:** 21.44
- **1D identity - Alignment Gaps:** 309
- **1D aligned content (<aminoacid>:%):** {'E': 8.22, 'V': 8.22, 'K': 6.85, 'G': 10.27, 'P': 13.01, 'D': 3.42, 'T': 8.22, 'L': 4.11, 'I': 2.05, 'R': 4.79, 'F': 4.11, 'S': 5.48, 'H': 2.05, 'W': 1.37, 'Y': 3.42, 'C': 6.16, 'Q': 2.74, 'A': 2.74, 'M': 1.37, 'N': 1.37}
- **Common reported functions (%):** 0.0
- **Common reported locations (%):** 0.0
- **Common reported processes (%):** 0.0

- **PDB ID:** 4A5T
- **Chain:** S
- **Crystallized protein length:** 767 aa
- **Resolution:** 3.49 Å
- **b-phipsi:** 0.018146
- **w-rdist:** 0.166866
- **t-alpha:** 0.001565
- **Chemical similarity (Tanimoto Index) (%):** 80.66
- **1D identity (%) [PDB]:** 1.96
- **1D identity (%) [Gaps excluded][PDB]:** 83.78
- **1D identity - Alignment Gaps [PDB]:** 1544
- **1D aligned content [PDB] (<aminoacid>:%):** {'S': 6.45, 'L': 6.45, 'E': 9.68, 'D': 6.45, 'Y': 6.45, 'R': 12.9, 'G': 9.68, 'I': 6.45, 'T': 9.68, 'V': 9.68, 'Q': 3.23, 'K': 3.23, 'F': 3.23, 'A': 3.23, 'N': 3.23}
- **2D identity (%) [PDB]:** 25.97
- **2D identity (%) [Gaps excluded][PDB]:** 82.9
- **2D identity - Alignment Gaps [PDB]:** 846
- **2D aligned content [PDB] (<2D-fold>:%):** {'T': 24.69, 'E': 37.81, 'H': 9.69, '.': 25.31, 'G': 1.88, 'B': 0.62}
- **3D similarity (TM-Score) (%) [PDB]:** 23.65

- **Gene name:** PLG
- **Entrez ID:** 5340
- **RefSeq ID:** NM\_000301
- **Transcript sequence length:** 3530
- **5-UTR|CDS|3-UTR identity (%):** 8.44 | 43.88 | 15.11
- **5-UTR|CDS|3-UTR identity (%) [Gaps excluded]:** 57.63 | 75.09 | 76.16
- **5-UTR|CDS|3-UTR identity [Alignment Gaps]:** 344 | 1317 | 3559
- **5-UTR aligned content (<base>:%):** {'T': 8.82, 'A': 17.65, 'G': 32.35, 'C': 41.18}
- **CDS aligned content (<base>:%):** {'T': 21.73, 'G': 25.04, 'A': 30.0, 'C': 23.24}
- **3-UTR aligned content (<base>:%):** {'T': 32.19, 'G': 18.78, 'C': 16.24, 'A': 32.79}

**Uniprot Description:**  
  
 Plasmin dissolves the fibrin of blood clots and acts as a proteolytic factor in a variety of other processes including embryonic development, tissue remodeling, tumor invasion, and inflammation. In ovulation, weakens the walls of the Graafian follicle. It activates the urokinase-type plasminogen activator, collagenases and several complement zymogens, such as C1 and C5. Cleavage of fibronectin and laminin leads to cell detachment and apoptosis. Also cleaves fibrin, thrombospondin and von Willebrand factor. Its role in tissue remodeling and tumor invasion may be modulated by CSPG4. Binds to cells.   
  
Interacts (both mature PLG and the angiostatin peptide) with CSPG4 and AMOT (PubMed:10889192, PubMed:16043488). Interacts (via the Kringle domains) with HRG; the interaction tethers PLG to the cell surface and enhances its activation (PubMed:9102401, PubMed:19712047). Interacts (via Kringle 4 domain) with ADA; the interaction stimulates PLG activation when in complex with DPP4 (PubMed:15016824). Angiostatin: Interacts with ATP5F1A; the interaction inhibits most of the angiogenic effects of angiostatin (PubMed:10077593).   
  
 **Gene Ontology Information:**

Molecular Function

- apolipoprotein binding
- endopeptidase activity
- enzyme binding
- kinase binding
- protease binding
- protein antigen binding
- protein domain specific binding
- chaperone binding
- serine-type endopeptidase activity
- serine-type peptidase activity
- signaling receptor binding

Location

- blood microparticle
- cell surface
- collagen-containing extracellular matrix
- external side of plasma membrane
- extracellular exosome
- extracellular region
- extracellular space
- plasma membrane
- platelet alpha granule lumen

Biological process

- biological process involved in interaction with symbiont
- blood coagulation
- extracellular matrix disassembly
- fibrinolysis
- negative regulation of cell population proliferation
- negative regulation of cell-cell adhesion mediated by cadherin
- negative regulation of cell-substrate adhesion
- negative regulation of fibrinolysis
- positive regulation of blood vessel endothelial cell migration
- positive regulation of fibrinolysis
- proteolysis
- tissue remodeling

---

12

- **Protein name:** Ectonucleotide pyrophosphatase/phosphodiesterase family member 3
- **Organism:** Homo sapiens
- **Uniprot Accession Number:** O14638
- **Protein sequence length:** 875 aa
- **1D identity (%):** 15.55
- **1D identity (%) [Gaps excluded]:** 21.94
- **1D identity - Alignment Gaps:** 296
- **1D aligned content (<aminoacid>:%):** {'T': 7.59, 'L': 11.39, 'E': 5.7, 'K': 5.06, 'V': 8.86, 'S': 6.33, 'D': 5.7, 'R': 7.59, 'W': 0.63, 'A': 3.16, 'Q': 3.16, 'F': 2.53, 'G': 8.23, 'C': 1.9, 'H': 5.06, 'P': 7.59, 'N': 3.16, 'I': 2.53, 'Y': 3.8}
- **Common reported functions (%):** 0.0
- **Common reported locations (%):** 0.0
- **Common reported processes (%):** 0.0

- **PDB ID:** 6C02
- **Chain:** B
- **Crystallized protein length:** 815 aa
- **Resolution:** 1.94 Å
- **b-phipsi:** 0.00393
- **w-rdist:** 0.506981
- **t-alpha:** 0.005477
- **Chemical similarity (Tanimoto Index) (%):** 66.23
- **1D identity (%) [PDB]:** 3.62
- **1D identity (%) [Gaps excluded][PDB]:** 62.64
- **1D identity - Alignment Gaps [PDB]:** 1484
- **1D aligned content [PDB] (<aminoacid>:%):** {'G': 7.02, 'K': 3.51, 'R': 5.26, 'N': 3.51, 'M': 3.51, 'Y': 3.51, 'T': 5.26, 'A': 7.02, 'L': 15.79, 'P': 7.02, 'V': 8.77, 'D': 7.02, 'E': 3.51, 'Q': 5.26, 'F': 3.51, 'W': 1.75, 'S': 5.26, 'I': 1.75, 'H': 1.75}
- **2D identity (%) [PDB]:** 32.38
- **2D identity (%) [Gaps excluded][PDB]:** 82.17
- **2D identity - Alignment Gaps [PDB]:** 724
- **2D aligned content [PDB] (<2D-fold>:%):** {'H': 36.95, 'I': 1.29, 'T': 20.16, '.': 16.8, 'E': 23.0, 'G': 1.55, 'B': 0.26}
- **3D similarity (TM-Score) (%) [PDB]:** 22.54

- **Gene name:** ENPP3
- **Entrez ID:** 5169
- **RefSeq ID:** NM\_005021
- **Transcript sequence length:** 3165
- **5-UTR|CDS|3-UTR identity (%):** 8.58 | 45.75 | 7.33
- **5-UTR|CDS|3-UTR identity (%) [Gaps excluded]:** 69.81 | 76.14 | 76.76
- **5-UTR|CDS|3-UTR identity [Alignment Gaps]:** 378 | 1300 | 3913
- **5-UTR aligned content (<base>:%):** {'A': 16.22, 'T': 29.73, 'G': 35.14, 'C': 18.92}
- **CDS aligned content (<base>:%):** {'A': 31.41, 'T': 26.31, 'G': 22.01, 'C': 20.27}
- **3-UTR aligned content (<base>:%):** {'T': 44.16, 'A': 34.07, 'C': 11.67, 'G': 10.09}

**Uniprot Description:**  
  
 Hydrolase that metabolizes extracellular nucleotides, including ATP, GTP, UTP and CTP (PubMed:29717535). Limits mast cell and basophil responses during inflammation and during the chronic phases of allergic responses by eliminating the extracellular ATP that functions as signaling molecule and activates basophils and mast cells and induces the release of inflammatory cytokines. Metabolizes extracellular ATP in the lumen of the small intestine, and thereby prevents ATP-induced apoptosis of intestinal plasmacytoid dendritic cells (By similarity). Has also alkaline phosphodiesterase activity (PubMed:11342463).   
  
Monomer and homodimer.   
  
 **Gene Ontology Information:**

Molecular Function

- calcium ion binding
- nucleic acid binding
- nucleoside-triphosphate diphosphatase activity
- phosphodiesterase I activity
- zinc ion binding

Location

- apical plasma membrane
- external side of plasma membrane
- extracellular exosome
- perinuclear region of cytoplasm

Biological process

- ATP metabolic process
- basophil activation involved in immune response
- negative regulation of inflammatory response
- negative regulation of mast cell activation involved in immune response
- negative regulation of mast cell proliferation
- nucleoside triphosphate catabolic process
- phosphate ion homeostasis
- phosphate-containing compound metabolic process
- pyrimidine nucleotide metabolic process

---

13

- **Protein name:** Protein transport protein Sec23A
- **Organism:** Homo sapiens
- **Uniprot Accession Number:** Q15436
- **Protein sequence length:** 765 aa
- **1D identity (%):** 15.14
- **1D identity (%) [Gaps excluded]:** 21.71
- **1D identity - Alignment Gaps:** 290
- **1D aligned content (<aminoacid>:%):** {'N': 3.45, 'P': 12.41, 'A': 6.9, 'T': 6.21, 'Y': 4.14, 'R': 8.28, 'D': 5.52, 'C': 1.38, 'Q': 6.9, 'F': 3.45, 'G': 8.97, 'E': 2.76, 'V': 4.83, 'M': 1.38, 'K': 4.83, 'S': 4.83, 'L': 8.28, 'I': 4.14, 'W': 0.69, 'H': 0.69}
- **Common reported functions (%):** 0.0
- **Common reported locations (%):** 12.5
- **Common reported processes (%):** 0.0

- **PDB ID:** 5KYY
- **Chain:** A
- **Crystallized protein length:** 716 aa
- **Resolution:** 3.4 Å
- **b-phipsi:** 0.00221
- **w-rdist:** 0.650503
- **t-alpha:** 0.002353
- **Chemical similarity (Tanimoto Index) (%):** N/A
- **1D identity (%) [PDB]:** 2.1
- **1D identity (%) [Gaps excluded][PDB]:** 76.19
- **1D identity - Alignment Gaps [PDB]:** 1485
- **1D aligned content [PDB] (<aminoacid>:%):** {'T': 9.38, 'Y': 6.25, 'V': 18.75, 'G': 12.5, 'L': 12.5, 'Q': 9.38, 'I': 3.12, 'P': 6.25, 'K': 6.25, 'A': 6.25, 'E': 3.12, 'R': 3.12, 'N': 3.12}
- **2D identity (%) [PDB]:** 37.82
- **2D identity (%) [Gaps excluded][PDB]:** 84.54
- **2D identity - Alignment Gaps [PDB]:** 599
- **2D aligned content [PDB] (<2D-fold>:%):** {'.': 16.59, 'H': 42.44, 'T': 14.15, 'E': 26.34, 'B': 0.49}
- **3D similarity (TM-Score) (%) [PDB]:** 23.48

- **Gene name:** SEC23A
- **Entrez ID:** 10484
- **RefSeq ID:** NM\_006364
- **Transcript sequence length:** 3843
- **5-UTR|CDS|3-UTR identity (%):** 29.82 | 44.59 | 18.96
- **5-UTR|CDS|3-UTR identity (%) [Gaps excluded]:** 73.89 | 74.6 | 75.33
- **5-UTR|CDS|3-UTR identity [Alignment Gaps]:** 266 | 1230 | 3351
- **5-UTR aligned content (<base>:%):** {'T': 6.77, 'G': 50.38, 'C': 34.59, 'A': 8.27}
- **CDS aligned content (<base>:%):** {'A': 29.57, 'T': 26.71, 'C': 21.06, 'G': 22.67}
- **3-UTR aligned content (<base>:%):** {'A': 30.39, 'G': 15.55, 'C': 11.54, 'T': 42.52}

**Uniprot Description:**  
  
 Component of the coat protein complex II (COPII) which promotes the formation of transport vesicles from the endoplasmic reticulum (ER). The coat has two main functions, the physical deformation of the endoplasmic reticulum membrane into vesicles and the selection of cargo molecules for their transport to the Golgi complex. Required for the translocation of insulin-induced glucose transporter SLC2A4/GLUT4 to the cell membrane (By similarity).   
  
COPII is composed of at least five proteins: the Sec23/24 complex, the Sec13/31 complex and Sar1 (PubMed:17499046). Interacts with SEC23IP. Interacts with HTR4 (By similarity). Interacts with SEC16A (PubMed:17192411). Interacts with SLC6A4 (By similarity). Interacts (as part of the Sec23/24 complex) with SEC22B; recruits SEC22B into COPII-coated vesicles and allows the transport of this cargo from the endoplasmic reticulum to the Golgi (PubMed:17499046). Interacts (via Gelsolin-like repeat) with MIA2 and MIA3; specifically involved in the transport of large cargos like the collagen COL7A1 (PubMed:27551091, PubMed:28442536). Interacts with DDHD1 (By similarity). Interacts with TMEM39A (PubMed:31806350). Interacts with SACM1L; this interaction is reduced in the absence of TMEM39A (PubMed:31806350).   
  
 **Gene Ontology Information:**

Molecular Function

- GTPase activator activity
- zinc ion binding

Location

- COPII vesicle coat
- cytosol
- endoplasmic reticulum exit site
- endoplasmic reticulum membrane
- ER to Golgi transport vesicle membrane
- Golgi membrane
- perinuclear region of cytoplasm

Biological process

- COPII-coated vesicle cargo loading
- intracellular protein transport
- protein localization to plasma membrane

---

14

- **Protein name:** Phenylalanine--tRNA ligase beta subunit
- **Organism:** Homo sapiens
- **Uniprot Accession Number:** Q9NSD9
- **Protein sequence length:** 589 aa
- **1D identity (%):** 13.34
- **1D identity (%) [Gaps excluded]:** 24.37
- **1D identity - Alignment Gaps:** 424
- **1D aligned content (<aminoacid>:%):** {'V': 8.0, 'S': 4.8, 'L': 14.4, 'A': 5.6, 'D': 4.0, 'I': 10.4, 'T': 4.8, 'G': 6.4, 'P': 10.4, 'R': 4.0, 'K': 8.0, 'F': 2.4, 'E': 5.6, 'Q': 2.4, 'C': 3.2, 'Y': 4.0, 'H': 0.8, 'N': 0.8}
- **Common reported functions (%):** 0.0
- **Common reported locations (%):** 37.5
- **Common reported processes (%):** 0.0

- **PDB ID:** 3L4G
- **Chain:** B
- **Crystallized protein length:** 589 aa
- **Resolution:** 3.3 Å
- **b-phipsi:** 0.001127
- **w-rdist:** 0.736726
- **t-alpha:** 0.002353
- **Chemical similarity (Tanimoto Index) (%):** 87.82
- **1D identity (%) [PDB]:** 3.82
- **1D identity (%) [Gaps excluded][PDB]:** 66.67
- **1D identity - Alignment Gaps [PDB]:** 1283
- **1D aligned content [PDB] (<aminoacid>:%):** {'M': 1.92, 'F': 3.85, 'L': 17.31, 'T': 7.69, 'Y': 1.92, 'G': 9.62, 'Q': 3.85, 'I': 7.69, 'V': 17.31, 'K': 9.62, 'P': 5.77, 'A': 5.77, 'E': 1.92, 'N': 1.92, 'S': 1.92, 'C': 1.92}
- **2D identity (%) [PDB]:** 34.28
- **2D identity (%) [Gaps excluded][PDB]:** 84.58
- **2D identity - Alignment Gaps [PDB]:** 609
- **2D aligned content [PDB] (<2D-fold>:%):** {'E': 30.2, 'H': 32.48, '.': 18.52, 'T': 16.81, 'G': 1.71, 'B': 0.28}
- **3D similarity (TM-Score) (%) [PDB]:** 17.65

- **Gene name:** FARSB
- **Entrez ID:** 10056
- **RefSeq ID:** N/A
- **Sequence length:** N/A
- **5-UTR|CDS|3-UTR identity (%):** N/A | N/A | N/A
- **5-UTR|CDS|3-UTR identity (%) [Gaps excluded]:** N/A | N/A | N/A
- **5-UTR|CDS|3-UTR identity [Alignment Gaps]:** N/A | N/A | N/A
- **5-UTR aligned content (<base>:%):** N/A
- **CDS aligned content (<base>:%):** N/A
- **3-UTR aligned content (<base>:%):** N/A

**Uniprot Description:**  
  
 N/A   
  
Heterotetramer; dimer of two heterodimers formed by FARSA and FARSB.   
  
 **Gene Ontology Information:**

Molecular Function

- ATP binding
- magnesium ion binding
- phenylalanine-tRNA ligase activity
- RNA binding

Location

- cytoplasm
- cytosol
- membrane
- phenylalanine-tRNA ligase complex

Biological process

- phenylalanyl-tRNA aminoacylation
- protein heterotetramerization
- translation

---

15

- **Protein name:** Beta-adrenergic receptor kinase 1
- **Organism:** Homo sapiens
- **Uniprot Accession Number:** P25098
- **Protein sequence length:** 689 aa
- **1D identity (%):** 15.13
- **1D identity (%) [Gaps excluded]:** 22.03
- **1D identity - Alignment Gaps:** 288
- **1D aligned content (<aminoacid>:%):** {'D': 4.32, 'V': 8.63, 'L': 12.95, 'M': 0.72, 'T': 3.6, 'P': 7.91, 'R': 3.6, 'S': 6.47, 'Y': 2.16, 'G': 12.95, 'E': 7.19, 'F': 5.76, 'A': 3.6, 'I': 2.88, 'K': 7.19, 'C': 3.6, 'Q': 4.32, 'H': 1.44, 'N': 0.72}
- **Common reported functions (%):** 0.0
- **Common reported locations (%):** 37.5
- **Common reported processes (%):** 0.0

- **PDB ID:** 6U7C
- **Chain:** A
- **Crystallized protein length:** 631 aa
- **Resolution:** 2.44 Å
- **b-phipsi:** 0.013535
- **w-rdist:** 0.341922
- **t-alpha:** 0.001568
- **Chemical similarity (Tanimoto Index) (%):** 80.93
- **1D identity (%) [PDB]:** 2.51
- **1D identity (%) [Gaps excluded][PDB]:** 70.59
- **1D identity - Alignment Gaps [PDB]:** 1381
- **1D aligned content [PDB] (<aminoacid>:%):** {'S': 5.56, 'A': 2.78, 'T': 8.33, 'F': 5.56, 'Q': 11.11, 'P': 5.56, 'I': 8.33, 'E': 11.11, 'C': 2.78, 'V': 2.78, 'L': 5.56, 'D': 2.78, 'N': 2.78, 'K': 11.11, 'R': 8.33, 'G': 2.78, 'M': 2.78}
- **2D identity (%) [PDB]:** 36.96
- **2D identity (%) [Gaps excluded][PDB]:** 83.52
- **2D identity - Alignment Gaps [PDB]:** 573
- **2D aligned content [PDB] (<2D-fold>:%):** {'T': 12.89, '.': 18.42, 'H': 48.42, 'I': 1.32, 'E': 18.95}
- **3D similarity (TM-Score) (%) [PDB]:** 21.66

- **Gene name:** GRK2
- **Entrez ID:** 156
- **RefSeq ID:** NM\_001619
- **Transcript sequence length:** 3403
- **5-UTR|CDS|3-UTR identity (%):** 40.05 | 42.64 | 15.07
- **5-UTR|CDS|3-UTR identity (%) [Gaps excluded]:** 79.15 | 75.88 | 72.18
- **5-UTR|CDS|3-UTR identity [Alignment Gaps]:** 206 | 1306 | 3529
- **5-UTR aligned content (<base>:%):** {'T': 1.2, 'C': 39.52, 'G': 53.29, 'A': 5.99}
- **CDS aligned content (<base>:%):** {'A': 24.15, 'T': 20.06, 'G': 29.43, 'C': 26.36}
- **3-UTR aligned content (<base>:%):** {'C': 28.27, 'G': 28.27, 'A': 18.45, 'T': 25.0}

**Uniprot Description:**  
  
 Specifically phosphorylates the agonist-occupied form of the beta-adrenergic and closely related receptors, probably inducing a desensitization of them (PubMed:19715378). Key regulator of LPAR1 signaling (PubMed:19306925). Competes with RALA for binding to LPAR1 thus affecting the signaling properties of the receptor (PubMed:19306925). Desensitizes LPAR1 and LPAR2 in a phosphorylation-independent manner (PubMed:19306925). Positively regulates ciliary smoothened (SMO)-dependent Hedgehog (Hh) signaling pathway by facilitating the trafficking of SMO into the cilium and the stimulation of SMO activity (By similarity). Inhibits relaxation of airway smooth muscle in response to blue light (PubMed:30284927).   
  
Interacts with the heterodimer formed by GNB1 and GNG2 (By similarity). Interacts with GIT1 (By similarity). Interacts with, and phosphorylates chemokine-stimulated CCR5 (PubMed:10085131). Interacts with ARRB1 (PubMed:9501202). Interacts with LPAR1 and LPAR2 (PubMed:19306925). Interacts with RALA in response to LPAR1 activation (PubMed:19306925). ADRBK1 and RALA mutually inhibit each other's binding to LPAR1 (PubMed:19306925). Interacts with ADRB2 (PubMed:19715378).   
  
 **Gene Ontology Information:**

Molecular Function

- alpha-2A adrenergic receptor binding
- ATP binding
- beta-adrenergic receptor kinase activity
- Edg-2 lysophosphatidic acid receptor binding
- G protein-coupled receptor binding
- G protein-coupled receptor kinase activity
- protein kinase activity

Location

- cilium
- cytoplasm
- cytosol
- membrane
- plasma membrane
- postsynapse
- presynapse

Biological process

- cardiac muscle contraction
- desensitization of G protein-coupled receptor signaling pathway
- G protein-coupled acetylcholine receptor signaling pathway
- G protein-coupled receptor signaling pathway
- heart development
- negative regulation of relaxation of smooth muscle
- negative regulation of striated muscle contraction
- negative regulation of the force of heart contraction by chemical signal
- peptidyl-serine phosphorylation
- peptidyl-threonine phosphorylation
- positive regulation of catecholamine secretion
- protein phosphorylation
- receptor internalization
- regulation of the force of heart contraction
- tachykinin receptor signaling pathway
- viral entry into host cell
- viral genome replication

---

16

- **Protein name:** Protein argonaute-3
- **Organism:** Homo sapiens
- **Uniprot Accession Number:** Q9H9G7
- **Protein sequence length:** 860 aa
- **1D identity (%):** 80.73
- **1D identity (%) [Gaps excluded]:** 82.92
- **1D identity - Alignment Gaps:** 23
- **1D aligned content (<aminoacid>:%):** {'M': 2.56, 'E': 4.4, 'G': 7.1, 'P': 6.96, 'L': 7.81, 'R': 6.25, 'T': 6.11, 'K': 5.4, 'I': 5.11, 'A': 6.25, 'N': 2.41, 'F': 4.12, 'Q': 6.11, 'V': 8.95, 'D': 4.83, 'Y': 4.12, 'H': 3.12, 'S': 4.97, 'W': 0.71, 'C': 2.7}
- **Common reported functions (%):** 100.0
- **Common reported locations (%):** 87.5
- **Common reported processes (%):** 50.0

- **PDB ID:** 5VM9
- **Chain:** A
- **Crystallized protein length:** 779 aa
- **Resolution:** 3.28 Å
- **b-phipsi:** 0.009013
- **w-rdist:** 0.145466
- **t-alpha:** 0.008607
- **Chemical similarity (Tanimoto Index) (%):** 99.62
- **1D identity (%) [PDB]:** 76.99
- **1D identity (%) [Gaps excluded][PDB]:** 84.27
- **1D identity - Alignment Gaps [PDB]:** 74
- **1D aligned content [PDB] (<aminoacid>:%):** {'P': 7.28, 'R': 6.68, 'G': 5.92, 'T': 6.53, 'K': 5.46, 'I': 5.46, 'L': 7.59, 'A': 6.53, 'N': 2.43, 'F': 4.4, 'Q': 6.07, 'V': 9.26, 'D': 4.7, 'Y': 4.4, 'E': 3.79, 'M': 2.43, 'H': 3.03, 'S': 4.4, 'W': 0.76, 'C': 2.88}
- **2D identity (%) [PDB]:** 63.85
- **2D identity (%) [Gaps excluded][PDB]:** 77.67
- **2D identity - Alignment Gaps [PDB]:** 160
- **2D aligned content [PDB] (<2D-fold>:%):** {'.': 20.21, 'B': 1.74, 'E': 31.01, 'H': 36.06, 'T': 10.45, 'G': 0.52}
- **3D similarity (TM-Score) (%) [PDB]:** 88.94

- **Gene name:** AGO3
- **Entrez ID:** 192669
- **RefSeq ID:** N/A
- **Sequence length:** N/A
- **5-UTR|CDS|3-UTR identity (%):** N/A | N/A | N/A
- **5-UTR|CDS|3-UTR identity (%) [Gaps excluded]:** N/A | N/A | N/A
- **5-UTR|CDS|3-UTR identity [Alignment Gaps]:** N/A | N/A | N/A
- **5-UTR aligned content (<base>:%):** N/A
- **CDS aligned content (<base>:%):** N/A
- **3-UTR aligned content (<base>:%):** N/A

**Uniprot Description:**  
  
 Required for RNA-mediated gene silencing (RNAi). Binds to short RNAs such as microRNAs (miRNAs) and represses the translation of mRNAs which are complementary to them. Proposed to be involved in stabilization of small RNA derivates (siRNA) derived from processed RNA polymerase III-transcribed Alu repeats containing a DR2 retinoic acid response element (RARE) in stem cells and in the subsequent siRNA-dependent degradation of a subset of RNA polymerase II-transcribed coding mRNAs by recruiting a mRNA decapping complex involving EDC4. Possesses RNA slicer activity but only on select RNAs bearing 5'- and 3'-flanking sequences to the region of guide-target complementarity (PubMed:29040713).   
  
Interacts with EIF4B, IMP8, PRMT5 and TNRC6B. Interacts with APOBEC3F, APOBEC3G and APOBEC3H. Interacts with EDC4.   
  
 **Gene Ontology Information:**

Molecular Function

- double-stranded RNA binding
- endoribonuclease activity, cleaving miRNA-paired mRNA
- metal ion binding
- miRNA binding
- RNA binding
- endoribonuclease activity
- single-stranded RNA binding

Location

- condensed nuclear chromosome
- cytoplasm
- cytoplasmic ribonucleoprotein granule
- cytosol
- membrane
- nucleoplasm
- P-body
- RISC complex
- RISC-loading complex

Biological process

- production of miRNAs involved in gene silencing by miRNA
- miRNA mediated inhibition of translation
- mRNA catabolic process
- positive regulation of gene expression
- positive regulation of NIK/NF-kappaB signaling
- pre-miRNA processing
- regulation of stem cell proliferation
- small RNA loading onto RISC
- RNA secondary structure unwinding

---

17

- **Protein name:** Propionyl-CoA carboxylase alpha chain, mitochondrial
- **Organism:** Homo sapiens
- **Uniprot Accession Number:** P05165
- **Protein sequence length:** 728 aa
- **1D identity (%):** 15.99
- **1D identity (%) [Gaps excluded]:** 27.66
- **1D identity - Alignment Gaps:** 425
- **1D aligned content (<aminoacid>:%):** {'W': 1.24, 'V': 11.8, 'L': 11.18, 'Q': 6.21, 'A': 8.07, 'S': 6.83, 'R': 6.21, 'Y': 1.86, 'P': 4.35, 'F': 3.73, 'G': 8.07, 'H': 1.24, 'N': 1.86, 'D': 3.73, 'E': 3.73, 'I': 3.11, 'T': 4.97, 'K': 8.07, 'M': 2.48, 'C': 1.24}
- **Common reported functions (%):** 0.0
- **Common reported locations (%):** 0.0
- **Common reported processes (%):** 0.0

- **PDB ID:** 7YBU
- **Chain:** H
- **Crystallized protein length:** 670 aa
- **Resolution:** 2.2 Å
- **b-phipsi:** 0.006223
- **w-rdist:** 0.418566
- **t-alpha:** 0.007042
- **Chemical similarity (Tanimoto Index) (%):** N/A
- **1D identity (%) [PDB]:** 3.41
- **1D identity (%) [Gaps excluded][PDB]:** 59.04
- **1D identity - Alignment Gaps [PDB]:** 1354
- **1D aligned content [PDB] (<aminoacid>:%):** {'E': 4.08, 'K': 8.16, 'R': 10.2, 'V': 16.33, 'D': 10.2, 'H': 4.08, 'M': 4.08, 'Q': 4.08, 'G': 8.16, 'Y': 4.08, 'N': 2.04, 'P': 4.08, 'L': 10.2, 'I': 2.04, 'T': 2.04, 'F': 2.04, 'S': 4.08}
- **2D identity (%) [PDB]:** 41.1
- **2D identity (%) [Gaps excluded][PDB]:** 84.34
- **2D identity - Alignment Gaps [PDB]:** 524
- **2D aligned content [PDB] (<2D-fold>:%):** {'.': 16.67, 'T': 20.24, 'E': 28.33, 'H': 34.76}
- **3D similarity (TM-Score) (%) [PDB]:** 19.92

- **Gene name:** PCCA
- **Entrez ID:** N/A
- **RefSeq ID:** NM\_001178004
- **Transcript sequence length:** 2343
- **5-UTR|CDS|3-UTR identity (%):** 4.73 | 41.76 | 4.5
- **5-UTR|CDS|3-UTR identity (%) [Gaps excluded]:** 70.37 | 73.92 | 79.18
- **5-UTR|CDS|3-UTR identity [Alignment Gaps]:** 375 | 1288 | 4064
- **5-UTR aligned content (<base>:%):** {'G': 68.42, 'C': 26.32, 'A': 5.26}
- **CDS aligned content (<base>:%):** {'A': 29.69, 'T': 24.92, 'G': 26.38, 'C': 19.01}
- **3-UTR aligned content (<base>:%):** {'A': 34.02, 'G': 10.31, 'T': 39.18, 'C': 16.49}

**Uniprot Description:**  
  
 This is one of the 2 subunits of the biotin-dependent propionyl-CoA carboxylase (PCC), a mitochondrial enzyme involved in the catabolism of odd chain fatty acids, branched-chain amino acids isoleucine, threonine, methionine, and valine and other metabolites (PubMed:8434582, PubMed:6765947). Propionyl-CoA carboxylase catalyzes the carboxylation of propionyl-CoA/propanoyl-CoA to D-methylmalonyl-CoA/(S)-methylmalonyl-CoA (PubMed:8434582, PubMed:6765947, PubMed:10101253). Within the holoenzyme, the alpha subunit catalyzes the ATP-dependent carboxylation of the biotin carried by the biotin carboxyl carrier (BCC) domain, while the beta subunit then transfers the carboxyl group from carboxylated biotin to propionyl-CoA (By similarity). Propionyl-CoA carboxylase also significantly acts on butyryl-CoA/butanoyl-CoA, which is converted to ethylmalonyl-CoA/(2S)-ethylmalonyl-CoA at a much lower rate (PubMed:6765947). Other alternative minor substrates include (2E)-butenoyl-CoA/crotonoyl-CoA (By similarity).   
  
The holoenzyme is a dodecamer composed of 6 PCCA/alpha subunits and 6 PCCB/beta subunits (PubMed:6765947, PubMed:20725044). Interacts (via the biotin carboxylation domain) with SIRT4 (PubMed:23438705). Interacts with SIRT3 and SIRT5 (PubMed:23438705).   
  
 **Gene Ontology Information:**

Molecular Function   
  
N/A

Location   
  
N/A

Biological process   
  
N/A

---

18

- **Protein name:** Complement C5
- **Organism:** Homo sapiens
- **Uniprot Accession Number:** P01031
- **Protein sequence length:** 1676 aa
- **1D identity (%):** 14.2
- **1D identity (%) [Gaps excluded]:** 29.42
- **1D identity - Alignment Gaps:** 885
- **1D aligned content (<aminoacid>:%):** {'M': 2.47, 'L': 7.41, 'G': 8.64, 'P': 9.88, 'F': 4.12, 'Q': 6.58, 'H': 1.65, 'V': 7.41, 'K': 4.94, 'Y': 6.17, 'D': 4.12, 'R': 5.35, 'E': 5.35, 'I': 2.88, 'N': 2.88, 'T': 7.41, 'S': 5.76, 'A': 4.53, 'C': 2.06, 'W': 0.41}
- **Common reported functions (%):** 0.0
- **Common reported locations (%):** 0.0
- **Common reported processes (%):** 0.0

- **PDB ID:** 6I2X
- **Chain:** A
- **Crystallized protein length:** 815 aa
- **Resolution:** 3.35 Å
- **b-phipsi:** 0.002239
- **w-rdist:** 0.19062
- **t-alpha:** 0.165884
- **Chemical similarity (Tanimoto Index) (%):** 86.58
- **1D identity (%) [PDB]:** 2.82
- **1D identity (%) [Gaps excluded][PDB]:** 65.22
- **1D identity - Alignment Gaps [PDB]:** 1529
- **1D aligned content [PDB] (<aminoacid>:%):** {'L': 15.56, 'Q': 11.11, 'K': 11.11, 'Y': 2.22, 'H': 2.22, 'P': 4.44, 'V': 6.67, 'E': 8.89, 'T': 6.67, 'N': 6.67, 'I': 4.44, 'G': 2.22, 'S': 8.89, 'A': 6.67, 'R': 2.22}
- **2D identity (%) [PDB]:** 32.97
- **2D identity (%) [Gaps excluded][PDB]:** 82.01
- **2D identity - Alignment Gaps [PDB]:** 711
- **2D aligned content [PDB] (<2D-fold>:%):** {'.': 18.11, 'H': 39.54, 'T': 16.07, 'E': 25.51, 'B': 0.77}
- **3D similarity (TM-Score) (%) [PDB]:** 23.84

- **Gene name:** C5
- **Entrez ID:** N/A
- **RefSeq ID:** NM\_001735
- **Transcript sequence length:** 5464
- **5-UTR|CDS|3-UTR identity (%):** 3.93 | 36.48 | 6.25
- **5-UTR|CDS|3-UTR identity (%) [Gaps excluded]:** 69.57 | 78.73 | 76.12
- **5-UTR|CDS|3-UTR identity [Alignment Gaps]:** 384 | 2793 | 3977
- **5-UTR aligned content (<base>:%):** {'T': 12.5, 'C': 50.0, 'G': 25.0, 'A': 12.5}
- **CDS aligned content (<base>:%):** {'A': 30.75, 'T': 24.91, 'G': 23.49, 'C': 20.85}
- **3-UTR aligned content (<base>:%):** {'A': 33.21, 'T': 35.42, 'C': 13.65, 'G': 17.71}

**Uniprot Description:**  
  
 Activation of C5 by a C5 convertase initiates the spontaneous assembly of the late complement components, C5-C9, into the membrane attack complex. C5b has a transient binding site for C6. The C5b-C6 complex is the foundation upon which the lytic complex is assembled.   
  
C5 precursor is first processed by the removal of 4 basic residues, forming two chains, beta and alpha, linked by a disulfide bond. C5 convertase activates C5 by cleaving the alpha chain, releasing C5a anaphylatoxin and generating C5b (beta chain + alpha' chain). The C5a anaphylatoxin interacts with C5AR1. Interacts with tick complement inhibitor.   
  
 **Gene Ontology Information:**

Molecular Function   
  
N/A

Location   
  
N/A

Biological process   
  
N/A

---

19

- **Protein name:** Ectonucleotide pyrophosphatase/phosphodiesterase family member 2
- **Organism:** Homo sapiens
- **Uniprot Accession Number:** Q13822
- **Protein sequence length:** 863 aa
- **1D identity (%):** 15.73
- **1D identity (%) [Gaps excluded]:** 24.44
- **1D identity - Alignment Gaps:** 374
- **1D aligned content (<aminoacid>:%):** {'F': 3.03, 'Q': 3.64, 'I': 3.03, 'V': 9.09, 'R': 4.85, 'E': 6.06, 'T': 8.48, 'G': 7.27, 'D': 3.64, 'Y': 7.88, 'A': 3.03, 'K': 6.67, 'L': 7.88, 'P': 10.3, 'H': 1.82, 'M': 1.21, 'S': 5.45, 'W': 1.21, 'C': 3.03, 'N': 2.42}
- **Common reported functions (%):** 0.0
- **Common reported locations (%):** 0.0
- **Common reported processes (%):** 0.0

- **PDB ID:** 4ZG7
- **Chain:** A
- **Crystallized protein length:** 776 aa
- **Resolution:** 1.75 Å
- **b-phipsi:** 0.007238
- **w-rdist:** 0.438771
- **t-alpha:** 0.003912
- **Chemical similarity (Tanimoto Index) (%):** 64.57
- **1D identity (%) [PDB]:** 2.89
- **1D identity (%) [Gaps excluded][PDB]:** 65.22
- **1D identity - Alignment Gaps [PDB]:** 1490
- **1D aligned content [PDB] (<aminoacid>:%):** {'I': 6.67, 'N': 2.22, 'A': 6.67, 'K': 4.44, 'L': 4.44, 'G': 8.89, 'V': 13.33, 'H': 6.67, 'R': 6.67, 'P': 11.11, 'S': 11.11, 'Q': 2.22, 'F': 2.22, 'D': 4.44, 'T': 4.44, 'Y': 2.22, 'E': 2.22}
- **2D identity (%) [PDB]:** 34.26
- **2D identity (%) [Gaps excluded][PDB]:** 81.7
- **2D identity - Alignment Gaps [PDB]:** 666
- **2D aligned content [PDB] (<2D-fold>:%):** {'.': 19.08, 'T': 16.79, 'H': 37.91, 'E': 24.17, 'G': 1.53, 'B': 0.51}
- **3D similarity (TM-Score) (%) [PDB]:** 24.29

- **Gene name:** ENPP2
- **Entrez ID:** 5168
- **RefSeq ID:** NM\_006209
- **Transcript sequence length:** 3242
- **5-UTR|CDS|3-UTR identity (%):** 8.45 | 47.57 | 7.21
- **5-UTR|CDS|3-UTR identity (%) [Gaps excluded]:** 76.09 | 75.42 | 79.39
- **5-UTR|CDS|3-UTR identity [Alignment Gaps]:** 368 | 1208 | 3934
- **5-UTR aligned content (<base>:%):** {'A': 22.86, 'G': 25.71, 'C': 40.0, 'T': 11.43}
- **CDS aligned content (<base>:%):** {'A': 30.33, 'T': 24.1, 'G': 23.46, 'C': 22.11}
- **3-UTR aligned content (<base>:%):** {'C': 8.65, 'T': 44.87, 'G': 15.06, 'A': 31.41}

**Uniprot Description:**  
  
 Hydrolyzes lysophospholipids to produce the signaling molecule lysophosphatidic acid (LPA) in extracellular fluids (PubMed:15769751, PubMed:26371182, PubMed:27754931, PubMed:14500380, PubMed:12354767,). Major substrate is lysophosphatidylcholine (PubMed:12176993, PubMed:27754931, PubMed:14500380). Also can act on sphingosylphosphorylcholine producing sphingosine-1-phosphate, a modulator of cell motility (PubMed:14500380). Can hydrolyze, in vitro, bis-pNPP, to some extent pNP-TMP, and barely ATP (PubMed:15769751, PubMed:12176993). Involved in several motility-related processes such as angiogenesis and neurite outgrowth. Acts as an angiogenic factor by stimulating migration of smooth muscle cells and microtubule formation (PubMed:11559573). Stimulates migration of melanoma cells, probably via a pertussis toxin-sensitive G protein (PubMed:1733949). May have a role in induction of parturition (PubMed:12176993). Possible involvement in cell proliferation and adipose tissue development (Probable). Tumor cell motility-stimulating factor (PubMed:1733949, PubMed:11559573). Required for LPA production in activated platelets, cleaves the sn-1 lysophospholipids to generate sn-1 lysophosphatidic acids containing predominantly 18:2 and 20:4 fatty acids (PubMed:21393252). Shows a preference for the sn-1 to the sn-2 isomer of 1-O-alkyl-sn-glycero-3-phosphocholine (lyso-PAF) (PubMed:21393252). N/A   
  
 **Gene Ontology Information:**

Molecular Function

- alkylglycerophosphoethanolamine phosphodiesterase activity
- calcium ion binding
- hydrolase activity
- lysophospholipase activity
- nucleic acid binding
- phosphodiesterase I activity
- polysaccharide binding
- scavenger receptor activity
- zinc ion binding

Location

- extracellular space
- plasma membrane

Biological process

- cell motility
- chemotaxis
- immune response
- phosphatidylcholine catabolic process
- phospholipid catabolic process
- positive regulation of epithelial cell migration
- positive regulation of lamellipodium morphogenesis
- positive regulation of peptidyl-tyrosine phosphorylation
- regulation of cell migration
- sphingolipid catabolic process

---

20

- **Protein name:** Unconventional myosin-Vc
- **Organism:** Homo sapiens
- **Uniprot Accession Number:** Q9NQX4
- **Protein sequence length:** 1742 aa
- **1D identity (%):** 13.22
- **1D identity (%) [Gaps excluded]:** 27.71
- **1D identity - Alignment Gaps:** 921
- **1D aligned content (<aminoacid>:%):** {'A': 4.29, 'L': 10.3, 'R': 6.87, 'P': 3.86, 'V': 6.87, 'K': 9.44, 'D': 6.44, 'E': 5.15, 'N': 3.86, 'H': 3.86, 'I': 4.29, 'G': 7.73, 'Y': 3.43, 'M': 1.29, 'S': 3.86, 'T': 4.72, 'F': 3.86, 'C': 3.43, 'Q': 6.01, 'W': 0.43}
- **Common reported functions (%):** 0.0
- **Common reported locations (%):** 12.5
- **Common reported processes (%):** 0.0

- **PDB ID:** 4ZG4
- **Chain:** B
- **Crystallized protein length:** 705 aa
- **Resolution:** 2.36 Å
- **b-phipsi:** 0.011647
- **w-rdist:** 0.367746
- **t-alpha:** 0.00314
- **Chemical similarity (Tanimoto Index) (%):** 73.64
- **1D identity (%) [PDB]:** 2.72
- **1D identity (%) [Gaps excluded][PDB]:** 73.21
- **1D identity - Alignment Gaps [PDB]:** 1450
- **1D aligned content [PDB] (<aminoacid>:%):** {'E': 9.76, 'D': 7.32, 'Y': 7.32, 'R': 7.32, 'G': 9.76, 'T': 12.2, 'I': 2.44, 'V': 12.2, 'F': 7.32, 'A': 2.44, 'K': 4.88, 'S': 4.88, 'N': 2.44, 'P': 4.88, 'H': 2.44, 'L': 2.44}
- **2D identity (%) [PDB]:** 38.96
- **2D identity (%) [Gaps excluded][PDB]:** 85.48
- **2D identity - Alignment Gaps [PDB]:** 584
- **2D aligned content [PDB] (<2D-fold>:%):** {'.': 15.55, 'E': 21.29, 'T': 11.72, 'H': 50.48, 'I': 0.96}
- **3D similarity (TM-Score) (%) [PDB]:** 23.69

- **Gene name:** MYO5C
- **Entrez ID:** 55930
- **RefSeq ID:** NM\_018728
- **Transcript sequence length:** 6977
- **5-UTR|CDS|3-UTR identity (%):** 24.11 | 36.15 | 21.59
- **5-UTR|CDS|3-UTR identity (%) [Gaps excluded]:** 69.86 | 80.02 | 76.28
- **5-UTR|CDS|3-UTR identity [Alignment Gaps]:** 277 | 2951 | 3277
- **5-UTR aligned content (<base>:%):** {'G': 42.16, 'C': 45.1, 'A': 6.86, 'T': 5.88}
- **CDS aligned content (<base>:%):** {'A': 29.91, 'T': 24.15, 'G': 24.87, 'C': 21.07}
- **3-UTR aligned content (<base>:%):** {'G': 17.53, 'A': 28.77, 'T': 36.07, 'C': 17.63}

**Uniprot Description:**  
  
 May be involved in transferrin trafficking. Likely to power actin-based membrane trafficking in many physiologically crucial tissues. N/A   
  
 **Gene Ontology Information:**

Molecular Function

- actin filament binding
- ATP binding
- calmodulin binding
- microfilament motor activity

Location

- actin cytoskeleton
- cytoplasm
- extracellular exosome
- myosin complex
- vesicle

Biological process

- actin filament organization
- vesicle transport along actin filament

---

21

- **Protein name:** Lysine--tRNA ligase
- **Organism:** Homo sapiens
- **Uniprot Accession Number:** Q15046
- **Protein sequence length:** 597 aa
- **1D identity (%):** 11.99
- **1D identity (%) [Gaps excluded]:** 24.27
- **1D identity - Alignment Gaps:** 494
- **1D aligned content (<aminoacid>:%):** {'M': 2.56, 'V': 5.13, 'P': 10.26, 'K': 11.11, 'R': 2.56, 'L': 10.26, 'E': 8.55, 'T': 5.98, 'H': 3.42, 'N': 4.27, 'G': 8.55, 'Q': 4.27, 'Y': 4.27, 'A': 2.56, 'I': 5.98, 'F': 4.27, 'S': 3.42, 'D': 1.71, 'C': 0.85}
- **Common reported functions (%):** 0.0
- **Common reported locations (%):** 25.0
- **Common reported processes (%):** 0.0

- **PDB ID:** 3BJU
- **Chain:** C
- **Crystallized protein length:** 501 aa
- **Resolution:** 2.31 Å
- **b-phipsi:** 0.003302
- **w-rdist:** 0.629329
- **t-alpha:** 0.003912
- **Chemical similarity (Tanimoto Index) (%):** 68.66
- **1D identity (%) [PDB]:** 2.37
- **1D identity (%) [Gaps excluded][PDB]:** 73.81
- **1D identity - Alignment Gaps [PDB]:** 1268
- **1D aligned content [PDB] (<aminoacid>:%):** {'Q': 9.68, 'Y': 9.68, 'F': 3.23, 'K': 12.9, 'S': 3.23, 'L': 16.13, 'P': 9.68, 'H': 3.23, 'C': 6.45, 'V': 6.45, 'G': 3.23, 'T': 6.45, 'E': 3.23, 'I': 3.23, 'D': 3.23}
- **2D identity (%) [PDB]:** 31.96
- **2D identity (%) [Gaps excluded][PDB]:** 88.06
- **2D identity - Alignment Gaps [PDB]:** 632
- **2D aligned content [PDB] (<2D-fold>:%):** {'H': 42.9, 'T': 13.56, '.': 13.88, 'E': 29.02, 'B': 0.63}
- **3D similarity (TM-Score) (%) [PDB]:** 18.22

- **Gene name:** KARS1
- **Entrez ID:** 3735
- **RefSeq ID:** NM\_005548
- **Transcript sequence length:** 1991
- **5-UTR|CDS|3-UTR identity (%):** 4.43 | 39.82 | 3.02
- **5-UTR|CDS|3-UTR identity (%) [Gaps excluded]:** 85.71 | 74.77 | 83.33
- **5-UTR|CDS|3-UTR identity [Alignment Gaps]:** 385 | 1336 | 4144
- **5-UTR aligned content (<base>:%):** {'T': 27.78, 'G': 44.44, 'C': 16.67, 'A': 11.11}
- **CDS aligned content (<base>:%):** {'T': 22.76, 'G': 24.52, 'C': 21.27, 'A': 31.46}
- **3-UTR aligned content (<base>:%):** {'A': 29.23, 'T': 36.92, 'G': 20.0, 'C': 13.85}

**Uniprot Description:**  
  
 Catalyzes the specific attachment of an amino acid to its cognate tRNA in a 2 step reaction: the amino acid (AA) is first activated by ATP to form AA-AMP and then transferred to the acceptor end of the tRNA (PubMed:9278442, PubMed:18029264, PubMed:18272479). When secreted, acts as a signaling molecule that induces immune response through the activation of monocyte/macrophages (PubMed:15851690). Catalyzes the synthesis of the signaling molecule diadenosine tetraphosphate (Ap4A), and thereby mediates disruption of the complex between HINT1 and MITF and the concomitant activation of MITF transcriptional activity (PubMed:5338216, PubMed:14975237, PubMed:19524539, PubMed:23159739).   
  
Homodimer and tetradimer (PubMed:18272479, PubMed:23159739, PubMed:26074468, PubMed:28887846). Part of the multisynthetase complex (MSC), a multisubunit complex that groups tRNA ligases for Arg (RARS), Asp (DARS), Gln (QARS), Ile (IARS), Leu (LARS), Lys (KARS), Met (MARS) the bifunctional ligase for Glu and Pro (EPRS) and the auxiliary subunits AIMP1/p43, AIMP2/p38 and EEF1E1/p18 (PubMed:19131329, PubMed:19289464, PubMed:24312579, PubMed:23159739). Interacts with AIMP2 (via N-terminus) and MITF (PubMed:9878398, PubMed:14975237, PubMed:15220430, PubMed:23159739, PubMed:26074468). Interacts with TARSL2 (PubMed:24312579).   
  
 **Gene Ontology Information:**

Molecular Function

- amino acid binding
- ATP adenylyltransferase activity
- ATP binding
- identical protein binding
- lysine-tRNA ligase activity
- protein homodimerization activity
- tRNA binding

Location

- aminoacyl-tRNA synthetase multienzyme complex
- cytosol
- extracellular space
- mitochondrial matrix
- mitochondrion
- nucleus
- plasma membrane

Biological process

- basophil activation involved in immune response
- diadenosine tetraphosphate biosynthetic process
- ERK1 and ERK2 cascade
- lysyl-tRNA aminoacylation
- positive regulation of transcription, DNA-templated
- positive regulation of inflammatory response to antigenic stimulus
- positive regulation of macrophage activation
- response to X-ray
- tRNA processing

---

22

- **Protein name:** Aspartate--tRNA ligase, mitochondrial
- **Organism:** Homo sapiens
- **Uniprot Accession Number:** Q6PI48
- **Protein sequence length:** 645 aa
- **1D identity (%):** 12.49
- **1D identity (%) [Gaps excluded]:** 22.02
- **1D identity - Alignment Gaps:** 416
- **1D aligned content (<aminoacid>:%):** {'Y': 3.33, 'R': 7.5, 'P': 8.33, 'T': 5.83, 'L': 10.83, 'Q': 5.83, 'H': 4.17, 'C': 1.67, 'K': 6.67, 'S': 5.0, 'E': 8.33, 'I': 3.33, 'V': 10.0, 'F': 2.5, 'G': 6.67, 'M': 1.67, 'A': 4.17, 'D': 3.33, 'W': 0.83}
- **Common reported functions (%):** 0.0
- **Common reported locations (%):** 0.0
- **Common reported processes (%):** 0.0

- **PDB ID:** 4AH6
- **Chain:** B
- **Crystallized protein length:** 589 aa
- **Resolution:** 3.7 Å
- **b-phipsi:** 0.003408
- **w-rdist:** 0.354202
- **t-alpha:** 0.050987
- **Chemical similarity (Tanimoto Index) (%):** 99.54
- **1D identity (%) [PDB]:** 3.13
- **1D identity (%) [Gaps excluded][PDB]:** 64.18
- **1D identity - Alignment Gaps [PDB]:** 1305
- **1D aligned content [PDB] (<aminoacid>:%):** {'P': 9.3, 'H': 2.33, 'L': 11.63, 'G': 4.65, 'Q': 9.3, 'E': 4.65, 'T': 9.3, 'V': 4.65, 'A': 4.65, 'C': 2.33, 'K': 9.3, 'D': 6.98, 'R': 6.98, 'S': 6.98, 'I': 2.33, 'M': 2.33, 'Y': 2.33}
- **2D identity (%) [PDB]:** 30.67
- **2D identity (%) [Gaps excluded][PDB]:** 82.78
- **2D identity - Alignment Gaps [PDB]:** 661
- **2D aligned content [PDB] (<2D-fold>:%):** {'.': 19.88, 'T': 13.98, 'E': 24.84, 'H': 40.99, 'B': 0.31}
- **3D similarity (TM-Score) (%) [PDB]:** 19.9

- **Gene name:** DARS2
- **Entrez ID:** 55157
- **RefSeq ID:** NM\_018122
- **Transcript sequence length:** 3336
- **5-UTR|CDS|3-UTR identity (%):** 37.98 | 41.16 | 12.61
- **5-UTR|CDS|3-UTR identity (%) [Gaps excluded]:** 70.96 | 75.3 | 77.55
- **5-UTR|CDS|3-UTR identity [Alignment Gaps]:** 290 | 1326 | 3692
- **5-UTR aligned content (<base>:%):** {'G': 40.51, 'A': 8.02, 'C': 39.24, 'T': 12.24}
- **CDS aligned content (<base>:%):** {'A': 30.4, 'T': 24.42, 'G': 23.17, 'C': 22.01}
- **3-UTR aligned content (<base>:%):** {'A': 35.25, 'T': 35.43, 'C': 14.93, 'G': 14.39}

**Uniprot Description:**  
  
 N/A   
  
Homodimer.   
  
 **Gene Ontology Information:**

Molecular Function

- aspartate-tRNA ligase activity
- aspartate-tRNA(Asn) ligase activity
- ATP binding
- protein homodimerization activity
- tRNA binding

Location

- mitochondrial matrix
- mitochondrion
- nucleoplasm

Biological process

- aspartyl-tRNA aminoacylation
- mitochondrial asparaginyl-tRNA aminoacylation
- tRNA aminoacylation

---

23

- **Protein name:** Protein transport protein Sec24D
- **Organism:** Homo sapiens
- **Uniprot Accession Number:** O94855
- **Protein sequence length:** 1032 aa
- **1D identity (%):** 17.64
- **1D identity (%) [Gaps excluded]:** 28.41
- **1D identity - Alignment Gaps:** 443
- **1D aligned content (<aminoacid>:%):** {'M': 3.4, 'G': 7.77, 'P': 13.11, 'S': 6.8, 'Q': 5.83, 'L': 9.22, 'A': 4.37, 'R': 2.91, 'V': 6.31, 'N': 2.91, 'H': 2.43, 'Y': 3.4, 'T': 3.88, 'F': 4.37, 'D': 4.37, 'C': 2.43, 'I': 5.34, 'E': 4.85, 'K': 5.83, 'W': 0.49}
- **Common reported functions (%):** 0.0
- **Common reported locations (%):** 12.5
- **Common reported processes (%):** 0.0

- **PDB ID:** 5KYX
- **Chain:** B
- **Crystallized protein length:** 767 aa
- **Resolution:** 3.52 Å
- **b-phipsi:** 0.005659
- **w-rdist:** 0.671239
- **t-alpha:** 0.000783
- **Chemical similarity (Tanimoto Index) (%):** N/A
- **1D identity (%) [PDB]:** 3.03
- **1D identity (%) [Gaps excluded][PDB]:** 68.12
- **1D identity - Alignment Gaps [PDB]:** 1481
- **1D aligned content [PDB] (<aminoacid>:%):** {'A': 2.13, 'H': 4.26, 'I': 4.26, 'G': 8.51, 'R': 4.26, 'D': 6.38, 'M': 4.26, 'V': 17.02, 'T': 6.38, 'E': 4.26, 'K': 2.13, 'Q': 6.38, 'S': 10.64, 'L': 8.51, 'P': 10.64}
- **2D identity (%) [PDB]:** 34.0
- **2D identity (%) [Gaps excluded][PDB]:** 82.63
- **2D identity - Alignment Gaps [PDB]:** 675
- **2D aligned content [PDB] (<2D-fold>:%):** {'.': 20.0, 'H': 42.31, 'T': 14.62, 'E': 22.56, 'G': 0.51}
- **3D similarity (TM-Score) (%) [PDB]:** 21.67

- **Gene name:** SEC24D
- **Entrez ID:** 9871
- **RefSeq ID:** N/A
- **Sequence length:** N/A
- **5-UTR|CDS|3-UTR identity (%):** N/A | N/A | N/A
- **5-UTR|CDS|3-UTR identity (%) [Gaps excluded]:** N/A | N/A | N/A
- **5-UTR|CDS|3-UTR identity [Alignment Gaps]:** N/A | N/A | N/A
- **5-UTR aligned content (<base>:%):** N/A
- **CDS aligned content (<base>:%):** N/A
- **3-UTR aligned content (<base>:%):** N/A

**Uniprot Description:**  
  
 Component of the coat protein complex II (COPII) which promotes the formation of transport vesicles from the endoplasmic reticulum (ER). The coat has two main functions, the physical deformation of the endoplasmic reticulum membrane into vesicles and the selection of cargo molecules for their transport to the Golgi complex (PubMed:17499046, PubMed:20427317, PubMed:18843296). Plays a central role in cargo selection within the COPII complex and together with SEC24C may have a different specificity compared to SEC24A and SEC24B (PubMed:17499046, PubMed:20427317, PubMed:18843296). May more specifically package GPI-anchored proteins through the cargo receptor TMED10 (PubMed:20427317). May also be specific for IxM motif-containing cargos like the SNAREs GOSR2 and STX5 (PubMed:18843296).   
  
COPII is composed of at least five proteins: the Sec23/24 complex, the Sec13/31 complex and Sar1 (PubMed:17499046, PubMed:27551091). Interacts with TMED2 and TMED10 (PubMed:20427317). Interacts with CNIH4 (PubMed:24405750). Interacts with GOSR2 (via IxM motif) and STX5 (via IxM motif); recruits GOSR2 and STX5 into COPII-coated vesicles (PubMed:18843296).   
  
 **Gene Ontology Information:**

Molecular Function

- SNARE binding
- zinc ion binding

Location

- COPII vesicle coat
- cytosol
- endoplasmic reticulum exit site
- endoplasmic reticulum membrane
- ER to Golgi transport vesicle membrane
- intracellular membrane-bounded organelle

Biological process

- COPII-coated vesicle cargo loading
- endoplasmic reticulum to Golgi vesicle-mediated transport
- in utero embryonic development
- intracellular protein transport

---

24

- **Protein name:** X-ray repair cross-complementing protein 6
- **Organism:** Homo sapiens
- **Uniprot Accession Number:** P12956
- **Protein sequence length:** 609 aa
- **1D identity (%):** 12.76
- **1D identity (%) [Gaps excluded]:** 23.18
- **1D identity - Alignment Gaps:** 426
- **1D aligned content (<aminoacid>:%):** {'K': 7.44, 'G': 9.09, 'D': 7.44, 'N': 4.13, 'R': 7.44, 'V': 10.74, 'F': 4.13, 'S': 4.96, 'Q': 3.31, 'A': 2.48, 'E': 6.61, 'L': 10.74, 'W': 0.83, 'I': 2.48, 'Y': 2.48, 'P': 7.44, 'T': 4.96, 'M': 1.65, 'H': 1.65}
- **Common reported functions (%):** 0.0
- **Common reported locations (%):** 37.5
- **Common reported processes (%):** 0.0

- **PDB ID:** 8ASC
- **Chain:** E
- **Crystallized protein length:** 497 aa
- **Resolution:** 2.95 Å
- **b-phipsi:** 0.001847
- **w-rdist:** 0.396122
- **t-alpha:** 0.05712
- **Chemical similarity (Tanimoto Index) (%):** N/A
- **1D identity (%) [PDB]:** 2.62
- **1D identity (%) [Gaps excluded][PDB]:** 77.27
- **1D identity - Alignment Gaps [PDB]:** 1255
- **1D aligned content [PDB] (<aminoacid>:%):** {'S': 11.76, 'V': 14.71, 'L': 20.59, 'Q': 2.94, 'E': 5.88, 'G': 5.88, 'H': 2.94, 'D': 5.88, 'A': 2.94, 'I': 2.94, 'T': 5.88, 'R': 8.82, 'P': 5.88, 'Y': 2.94}
- **2D identity (%) [PDB]:** 42.62
- **2D identity (%) [Gaps excluded][PDB]:** 88.97
- **2D identity - Alignment Gaps [PDB]:** 473
- **2D aligned content [PDB] (<2D-fold>:%):** {'.': 19.43, 'E': 23.06, 'T': 25.39, 'H': 30.83, 'B': 0.52, 'G': 0.78}
- **3D similarity (TM-Score) (%) [PDB]:** 17.27

- **Gene name:** XRCC6
- **Entrez ID:** N/A
- **RefSeq ID:** NM\_001469
- **Transcript sequence length:** 2122
- **5-UTR|CDS|3-UTR identity (%):** 10.54 | 37.69 | 3.3
- **5-UTR|CDS|3-UTR identity (%) [Gaps excluded]:** 72.88 | 74.85 | 80.79
- **5-UTR|CDS|3-UTR identity [Alignment Gaps]:** 349 | 1458 | 4157
- **5-UTR aligned content (<base>:%):** {'G': 37.21, 'C': 32.56, 'A': 6.98, 'T': 23.26}
- **CDS aligned content (<base>:%):** {'T': 22.04, 'G': 25.29, 'C': 20.14, 'A': 32.52}
- **3-UTR aligned content (<base>:%):** {'C': 23.08, 'A': 20.98, 'G': 20.98, 'T': 34.97}

**Uniprot Description:**  
  
 Single-stranded DNA-dependent ATP-dependent helicase. Has a role in chromosome translocation. The DNA helicase II complex binds preferentially to fork-like ends of double-stranded DNA in a cell cycle-dependent manner. It works in the 3'-5' direction. Binding to DNA may be mediated by XRCC6. Involved in DNA non-homologous end joining (NHEJ) required for double-strand break repair and V(D)J recombination. The XRCC5/6 dimer acts as regulatory subunit of the DNA-dependent protein kinase complex DNA-PK by increasing the affinity of the catalytic subunit PRKDC to DNA by 100-fold. The XRCC5/6 dimer is probably involved in stabilizing broken DNA ends and bringing them together. The assembly of the DNA-PK complex to DNA ends is required for the NHEJ ligation step. Required for osteocalcin gene expression. Probably also acts as a 5'-deoxyribose-5-phosphate lyase (5'-dRP lyase), by catalyzing the beta-elimination of the 5' deoxyribose-5-phosphate at an abasic site near double-strand breaks. 5'-dRP lyase activity allows to 'clean' the termini of abasic sites, a class of nucleotide damage commonly associated with strand breaks, before such broken ends can be joined. The XRCC5/6 dimer together with APEX1 acts as a negative regulator of transcription. Plays a role in the regulation of DNA virus-mediated innate immune response by assembling into the HDP-RNP complex, a complex that serves as a platform for IRF3 phosphorylation and subsequent innate immune response activation through the cGAS-STING pathway.   
  
Heterodimer composed of XRCC5/Ku80 and XRCC6/Ku70. The dimer associates in a DNA-dependent manner with PRKDC to form the DNA-dependent protein kinase complex DNA-PK, and with the LIG4-XRCC4 complex to form the core of the non-homologous end joining (NHEJ) complex. Additional components of the NHEJ complex include NHEJ1/XLF and PAXX (PubMed:25574025, PubMed:25941166, PubMed:25670504). The dimer also associates with NAA15, and this complex binds to the osteocalcin promoter and activates osteocalcin expression. In addition, XRCC6 interacts with the osteoblast-specific transcription factors MSX2, RUNX2 and DLX5. Interacts with ELF3. Interacts with ATP23. The XRCC5/6 dimer associates in a DNA-dependent manner with APEX1. Binds to CDK9 isoform 2. Identified in a complex with DEAF1 and XRCC5. Interacts with DEAF1 (via the SAND domain); the interaction is direct and may be inhibited by DNA-binding (PubMed:10219089, PubMed:11493912, PubMed:12145306, PubMed:12509254, PubMed:12547193, PubMed:15075319, PubMed:20493174, PubMed:22442688, PubMed:8621488, PubMed:9742108). Interacts with CLU (By similarity). Interacts with NR4A3; the DNA-dependent protein kinase complex DNA-PK phosphorylates and activates NR4A3 and prevents NR4A3 ubiquitinylation and degradation (PubMed:25852083). Interacts with CYREN isoform 1 (CYREN-1) and isoform 4 (CYREN-2) (PubMed:24610814, PubMed:28959974). Interacts (via N-terminus) with HSF1 (via N-terminus); this interaction is direct and prevents XRCC5/XRCC6 heterodimeric binding and non-homologous end joining (NHEJ) repair activities induced by ionizing radiation (IR) (PubMed:26359349). Part of the HDP-RNP complex composed of at least HEXIM1, PRKDC, XRCC5, XRCC6, paraspeckle proteins (SFPQ, NONO, PSPC1, RBM14, and MATR3) and NEAT1 RNA (PubMed:28712728). Interacts with HMBOX1 (PubMed:23685356). Interacts with ATF7 (PubMed:29490055).   
  
 **Gene Ontology Information:**

Molecular Function

- 5'-deoxyribose-5-phosphate lyase activity
- ATP binding
- DNA-dependent ATPase activity
- cyclin binding
- damaged DNA binding
- DNA binding
- DNA helicase activity
- double-stranded DNA binding
- hydrolase activity
- protein-containing complex binding
- RNA binding
- scaffold protein binding
- telomeric DNA binding
- transcription regulatory region sequence-specific DNA binding

Location

- chromosome, telomeric region
- cytosol
- DNA-dependent protein kinase complex
- extracellular region
- ficolin-1-rich granule lumen
- Ku70:Ku80 complex
- membrane
- nonhomologous end joining complex
- nuclear telomere cap complex
- nucleolus
- nucleoplasm
- nucleus
- protein-containing complex
- protein-DNA complex
- secretory granule lumen
- transcription regulator complex

Biological process

- activation of innate immune response
- cellular response to gamma radiation
- cellular response to X-ray
- DNA ligation
- double-strand break repair via classical nonhomologous end joining
- double-strand break repair via nonhomologous end joining
- innate immune response
- negative regulation of transcription, DNA-templated
- positive regulation of lymphocyte differentiation
- positive regulation of protein kinase activity
- positive regulation of transcription by RNA polymerase II
- positive regulation of transcription, DNA-templated
- recombinational repair
- regulation of smooth muscle cell proliferation
- telomere maintenance

---

25

- **Protein name:** Small nuclear ribonucleoprotein F
- **Organism:** Homo sapiens
- **Uniprot Accession Number:** P62306
- **Protein sequence length:** 86 aa
- **1D identity (%):** 2.31
- **1D identity (%) [Gaps excluded]:** 25.0
- **1D identity - Alignment Gaps:** 787
- **1D aligned content (<aminoacid>:%):** {'P': 10.0, 'G': 15.0, 'K': 5.0, 'M': 10.0, 'Y': 10.0, 'V': 5.0, 'L': 15.0, 'E': 15.0, 'I': 10.0, 'R': 5.0}
- **Common reported functions (%):** 0.0
- **Common reported locations (%):** 25.0
- **Common reported processes (%):** 0.0

- **PDB ID:** 5O9Z
- **Chain:** B
- **Crystallized protein length:** 844 aa
- **Resolution:** 4.5 Å
- **b-phipsi:** 0.006444
- **w-rdist:** 0.298491
- **t-alpha:** 0.020344
- **Chemical similarity (Tanimoto Index) (%):** N/A
- **1D identity (%) [PDB]:** 3.13
- **1D identity (%) [Gaps excluded][PDB]:** 78.46
- **1D identity - Alignment Gaps [PDB]:** 1564
- **1D aligned content [PDB] (<aminoacid>:%):** {'Y': 1.96, 'V': 25.49, 'L': 13.73, 'I': 3.92, 'P': 5.88, 'G': 7.84, 'K': 9.8, 'T': 5.88, 'A': 3.92, 'E': 1.96, 'R': 1.96, 'D': 1.96, 'M': 1.96, 'C': 1.96, 'Q': 3.92, 'S': 3.92, 'N': 1.96, 'F': 1.96}
- **2D identity (%) [PDB]:** 35.46
- **2D identity (%) [Gaps excluded][PDB]:** 84.43
- **2D identity - Alignment Gaps [PDB]:** 692
- **2D aligned content [PDB] (<2D-fold>:%):** {'H': 45.15, '.': 15.6, 'E': 23.4, 'T': 15.13, 'G': 0.71}
- **3D similarity (TM-Score) (%) [PDB]:** 25.27

- **Gene name:** SNRPF
- **Entrez ID:** 6636
- **RefSeq ID:** NM\_003095
- **Transcript sequence length:** 452
- **5-UTR|CDS|3-UTR identity (%):** 17.07 | 7.01 | 1.21
- **5-UTR|CDS|3-UTR identity (%) [Gaps excluded]:** 66.36 | 77.22 | 76.47
- **5-UTR|CDS|3-UTR identity [Alignment Gaps]:** 309 | 2373 | 4218
- **5-UTR aligned content (<base>:%):** {'C': 40.85, 'T': 7.04, 'G': 43.66, 'A': 8.45}
- **CDS aligned content (<base>:%):** {'T': 20.77, 'G': 23.5, 'A': 40.98, 'C': 14.75}
- **3-UTR aligned content (<base>:%):** {'C': 5.77, 'A': 19.23, 'T': 57.69, 'G': 17.31}

**Uniprot Description:**  
  
 Plays role in pre-mRNA splicing as core component of the SMN-Sm complex that mediates spliceosomal snRNP assembly and as component of the spliceosomal U1, U2, U4 and U5 small nuclear ribonucleoproteins (snRNPs), the building blocks of the spliceosome (PubMed:11991638, PubMed:18984161, PubMed:19325628, PubMed:23333303, PubMed:25555158, PubMed:26912367, PubMed:28502770, PubMed:28781166, PubMed:28076346). Component of both the pre-catalytic spliceosome B complex and activated spliceosome C complexes (PubMed:11991638, PubMed:28502770, PubMed:28781166, PubMed:28076346). Is also a component of the minor U12 spliceosome (PubMed:15146077). As part of the U7 snRNP it is involved in histone 3'-end processing (PubMed:12975319).   
  
Core component of the spliceosomal U1, U2, U4 and U5 small nuclear ribonucleoproteins (snRNPs), the building blocks of the spliceosome (PubMed:11991638, PubMed:19325628, PubMed:21516107, PubMed:25555158, PubMed:26912367, PubMed:28502770, PubMed:28781166, PubMed:28076346). Most spliceosomal snRNPs contain a common set of Sm proteins, SNRPB, SNRPD1, SNRPD2, SNRPD3, SNRPE, SNRPF and SNRPG that assemble in a heptameric protein ring on the Sm site of the small nuclear RNA to form the core snRNP (PubMed:19325628, PubMed:21516107, PubMed:25555158, PubMed:26912367, PubMed:28502770, PubMed:28781166, PubMed:28076346). Component of the U1 snRNP (PubMed:19325628, PubMed:25555158). The U1 snRNP is composed of the U1 snRNA and the 7 core Sm proteins SNRPB, SNRPD1, SNRPD2, SNRPD3, SNRPE, SNRPF and SNRPG, and at least three U1 snRNP-specific proteins SNRNP70/U1-70K, SNRPA/U1-A and SNRPC/U1-C (PubMed:19325628, PubMed:25555158). Component of the U4/U6-U5 tri-snRNP complex composed of the U4, U6 and U5 snRNAs and at least PRPF3, PRPF4, PRPF6, PRPF8, PRPF31, SNRNP200, TXNL4A, SNRNP40, SNRPB, SNRPD1, SNRPD2, SNRPD3, SNRPE, SNRPF, SNRPG, DDX23, CD2BP2, PPIH, SNU13, EFTUD2, SART1 and USP39, plus LSM2, LSM3, LSM4, LSM5, LSM6, LSM7 and LSM8 (PubMed:26912367). Component of the U7 snRNP complex, or U7 Sm protein core complex, that is composed of the U7 snRNA and at least LSM10, LSM11, SNRPB, SNRPD3, SNRPE, SNRPF and SNRPG; the complex does not contain SNRPD1 and SNRPD2 (PubMed:11574479). Component of the U11/U12 snRNPs that are part of the U12-type spliceosome (PubMed:15146077). Part of the SMN-Sm complex that contains SMN1, GEMIN2/SIP1, DDX20/GEMIN3, GEMIN4, GEMIN5, GEMIN6, GEMIN7, GEMIN8, STRAP/UNRIP and the Sm proteins SNRPB, SNRPD1, SNRPD2, SNRPD3, SNRPE, SNRPF and SNRPG; catalyzes core snRNPs assembly (PubMed:18984161). Forms a 6S pICln-Sm complex composed of CLNS1A/pICln, SNRPD1, SNRPD2, SNRPE, SNRPF and SNRPG; ring-like structure where CLNS1A/pICln mimics additional Sm proteins and which is unable to assemble into the core snRNP (PubMed:18984161, PubMed:23333303).   
  
 **Gene Ontology Information:**

Molecular Function

- RNA binding

Location

- catalytic step 2 spliceosome
- cytosol
- methylosome
- nucleoplasm
- nucleus
- pICln-Sm protein complex
- small nuclear ribonucleoprotein complex
- SMN-Sm protein complex
- spliceosomal complex
- U1 snRNP
- U12-type spliceosomal complex
- U2 snRNP
- U2-type catalytic step 2 spliceosome
- U2-type precatalytic spliceosome
- U4 snRNP
- U4/U6 x U5 tri-snRNP complex
- U5 snRNP
- U7 snRNP

Biological process

- 7-methylguanosine cap hypermethylation
- mRNA splicing, via spliceosome
- RNA splicing
- spliceosomal snRNP assembly
- U2-type prespliceosome assembly

---

26

- **Protein name:** Coagulation factor XIII A chain
- **Organism:** Homo sapiens
- **Uniprot Accession Number:** P00488
- **Protein sequence length:** 732 aa
- **1D identity (%):** 12.59
- **1D identity (%) [Gaps excluded]:** 22.71
- **1D identity - Alignment Gaps:** 457
- **1D aligned content (<aminoacid>:%):** {'S': 3.88, 'T': 8.53, 'A': 3.1, 'F': 3.1, 'R': 6.98, 'P': 6.98, 'N': 1.55, 'E': 6.2, 'Q': 3.1, 'V': 15.5, 'G': 9.3, 'K': 6.2, 'L': 5.43, 'Y': 6.98, 'D': 4.65, 'I': 2.33, 'M': 1.55, 'C': 1.55, 'H': 2.33, 'W': 0.78}
- **Common reported functions (%):** 0.0
- **Common reported locations (%):** 0.0
- **Common reported processes (%):** 0.0

- **PDB ID:** 5MHL
- **Chain:** B
- **Crystallized protein length:** 697 aa
- **Resolution:** 2.4 Å
- **b-phipsi:** 0.010555
- **w-rdist:** 0.30315
- **t-alpha:** 0.007042
- **Chemical similarity (Tanimoto Index) (%):** 84.18
- **1D identity (%) [PDB]:** 2.33
- **1D identity (%) [Gaps excluded][PDB]:** 74.47
- **1D identity - Alignment Gaps [PDB]:** 1456
- **1D aligned content [PDB] (<aminoacid>:%):** {'P': 5.71, 'N': 2.86, 'Q': 8.57, 'G': 8.57, 'V': 8.57, 'W': 2.86, 'D': 8.57, 'M': 2.86, 'R': 5.71, 'K': 11.43, 'F': 8.57, 'I': 5.71, 'C': 2.86, 'A': 2.86, 'L': 8.57, 'S': 2.86, 'T': 2.86}
- **2D identity (%) [PDB]:** 33.42
- **2D identity (%) [Gaps excluded][PDB]:** 82.74
- **2D identity - Alignment Gaps [PDB]:** 658
- **2D aligned content [PDB] (<2D-fold>:%):** {'.': 19.78, 'E': 44.44, 'T': 13.82, 'H': 20.87, 'G': 0.81, 'B': 0.27}
- **3D similarity (TM-Score) (%) [PDB]:** 20.67

- **Gene name:** F13A1
- **Entrez ID:** 2162
- **RefSeq ID:** NM\_000129
- **Transcript sequence length:** 3828
- **5-UTR|CDS|3-UTR identity (%):** 14.22 | 43.11 | 22.32
- **5-UTR|CDS|3-UTR identity (%) [Gaps excluded]:** 73.75 | 76.27 | 74.63
- **5-UTR|CDS|3-UTR identity [Alignment Gaps]:** 335 | 1329 | 3140
- **5-UTR aligned content (<base>:%):** {'G': 45.76, 'C': 35.59, 'T': 1.69, 'A': 16.95}
- **CDS aligned content (<base>:%):** {'T': 22.38, 'C': 22.99, 'A': 29.14, 'G': 25.49}
- **3-UTR aligned content (<base>:%):** {'T': 30.6, 'G': 18.6, 'C': 18.5, 'A': 32.3}

**Uniprot Description:**  
  
 Factor XIII is activated by thrombin and calcium ion to a transglutaminase that catalyzes the formation of gamma-glutamyl-epsilon-lysine cross-links between fibrin chains, thus stabilizing the fibrin clot. Also cross-link alpha-2-plasmin inhibitor, or fibronectin, to the alpha chains of fibrin.   
  
Tetramer of two A chains (F13A1) and two B (F13B) chains.   
  
 **Gene Ontology Information:**

Molecular Function

- metal ion binding
- protein-glutamine gamma-glutamyltransferase activity

Location

- blood microparticle
- collagen-containing extracellular matrix
- extracellular region
- extracellular space
- platelet alpha granule lumen
- transferase complex

Biological process

- blood coagulation
- blood coagulation, fibrin clot formation
- peptide cross-linking

---

27

- **Protein name:** Insulin receptor
- **Organism:** Homo sapiens
- **Uniprot Accession Number:** P06213
- **Protein sequence length:** 1382 aa
- **1D identity (%):** 14.87
- **1D identity (%) [Gaps excluded]:** 25.61
- **1D identity - Alignment Gaps:** 595
- **1D aligned content (<aminoacid>:%):** {'M': 1.9, 'A': 0.95, 'L': 9.95, 'G': 9.95, 'P': 9.95, 'R': 5.21, 'H': 4.27, 'Q': 4.74, 'K': 6.16, 'V': 6.64, 'Y': 4.74, 'D': 6.16, 'I': 3.79, 'E': 3.32, 'T': 6.64, 'S': 5.21, 'F': 3.32, 'N': 4.27, 'C': 1.9, 'W': 0.95}
- **Common reported functions (%):** 0.0
- **Common reported locations (%):** 0.0
- **Common reported processes (%):** 0.0

- **PDB ID:** 6CE9
- **Chain:** A
- **Crystallized protein length:** 562 aa
- **Resolution:** 4.3 Å
- **b-phipsi:** 0.018038
- **w-rdist:** 0.404216
- **t-alpha:** 0.000782
- **Chemical similarity (Tanimoto Index) (%):** 79.21
- **1D identity (%) [PDB]:** 2.71
- **1D identity (%) [Gaps excluded][PDB]:** 69.81
- **1D identity - Alignment Gaps [PDB]:** 1310
- **1D aligned content [PDB] (<aminoacid>:%):** {'T': 8.11, 'R': 13.51, 'F': 2.7, 'K': 5.41, 'I': 10.81, 'Y': 5.41, 'G': 8.11, 'V': 8.11, 'S': 5.41, 'E': 10.81, 'A': 5.41, 'P': 5.41, 'L': 5.41, 'D': 2.7, 'Q': 2.7}
- **2D identity (%) [PDB]:** 24.43
- **2D identity (%) [Gaps excluded][PDB]:** 85.4
- **2D identity - Alignment Gaps [PDB]:** 786
- **2D aligned content [PDB] (<2D-fold>:%):** {'.': 30.48, 'E': 40.15, 'T': 17.1, 'H': 8.92, 'G': 2.23, 'B': 1.12}
- **3D similarity (TM-Score) (%) [PDB]:** 17.61

- **Gene name:** INSR
- **Entrez ID:** N/A
- **RefSeq ID:** NM\_001079817
- **Transcript sequence length:** 9427
- **5-UTR|CDS|3-UTR identity (%):** 51.24 | 41.56 | 45.36
- **5-UTR|CDS|3-UTR identity (%) [Gaps excluded]:** 80.28 | 75.77 | 73.0
- **5-UTR|CDS|3-UTR identity [Alignment Gaps]:** 204 | 1953 | 2120
- **5-UTR aligned content (<base>:%):** {'G': 41.18, 'T': 5.19, 'C': 49.83, 'A': 3.81}
- **CDS aligned content (<base>:%):** {'A': 27.25, 'T': 22.47, 'G': 25.47, 'C': 24.81}
- **3-UTR aligned content (<base>:%):** {'A': 28.12, 'T': 33.87, 'G': 21.5, 'C': 16.5}

**Uniprot Description:**  
  
 Receptor tyrosine kinase which mediates the pleiotropic actions of insulin. Binding of insulin leads to phosphorylation of several intracellular substrates, including, insulin receptor substrates (IRS1, 2, 3, 4), SHC, GAB1, CBL and other signaling intermediates. Each of these phosphorylated proteins serve as docking proteins for other signaling proteins that contain Src-homology-2 domains (SH2 domain) that specifically recognize different phosphotyrosine residues, including the p85 regulatory subunit of PI3K and SHP2. Phosphorylation of IRSs proteins lead to the activation of two main signaling pathways: the PI3K-AKT/PKB pathway, which is responsible for most of the metabolic actions of insulin, and the Ras-MAPK pathway, which regulates expression of some genes and cooperates with the PI3K pathway to control cell growth and differentiation. Binding of the SH2 domains of PI3K to phosphotyrosines on IRS1 leads to the activation of PI3K and the generation of phosphatidylinositol-(3, 4, 5)-triphosphate (PIP3), a lipid second messenger, which activates several PIP3-dependent serine/threonine kinases, such as PDPK1 and subsequently AKT/PKB. The net effect of this pathway is to produce a translocation of the glucose transporter SLC2A4/GLUT4 from cytoplasmic vesicles to the cell membrane to facilitate glucose transport. Moreover, upon insulin stimulation, activated AKT/PKB is responsible for: anti-apoptotic effect of insulin by inducing phosphorylation of BAD; regulates the expression of gluconeogenic and lipogenic enzymes by controlling the activity of the winged helix or forkhead (FOX) class of transcription factors. Another pathway regulated by PI3K-AKT/PKB activation is mTORC1 signaling pathway which regulates cell growth and metabolism and integrates signals from insulin. AKT mediates insulin-stimulated protein synthesis by phosphorylating TSC2 thereby activating mTORC1 pathway. The Ras/RAF/MAP2K/MAPK pathway is mainly involved in mediating cell growth, survival and cellular differentiation of insulin. Phosphorylated IRS1 recruits GRB2/SOS complex, which triggers the activation of the Ras/RAF/MAP2K/MAPK pathway. In addition to binding insulin, the insulin receptor can bind insulin-like growth factors (IGFI and IGFII). Isoform Short has a higher affinity for IGFII binding. When present in a hybrid receptor with IGF1R, binds IGF1. PubMed:12138094 shows that hybrid receptors composed of IGF1R and INSR isoform Long are activated with a high affinity by IGF1, with low affinity by IGF2 and not significantly activated by insulin, and that hybrid receptors composed of IGF1R and INSR isoform Short are activated by IGF1, IGF2 and insulin. In contrast, PubMed:16831875 shows that hybrid receptors composed of IGF1R and INSR isoform Long and hybrid receptors composed of IGF1R and INSR isoform Short have similar binding characteristics, both bind IGF1 and have a low affinity for insulin. In adipocytes, inhibits lipolysis (By similarity).   
  
Tetramer of 2 alpha and 2 beta chains linked by disulfide bonds. The alpha chains carry the insulin-binding regions, while the beta chains carry the kinase domain. Forms a hybrid receptor with IGF1R, the hybrid is a tetramer consisting of 1 alpha chain and 1 beta chain of INSR and 1 alpha chain and 1 beta chain of IGF1R. Interacts with SORBS1 but dissociates from it following insulin stimulation. Binds SH2B2. Activated form of INSR interacts (via Tyr-999) with the PTB/PID domains of IRS1 and SHC1. The sequences surrounding the phosphorylated NPXY motif contribute differentially to either IRS1 or SHC1 recognition. Interacts (via tyrosines in the C-terminus) with IRS2 (via PTB domain and 591-786 AA); the 591-786 would be the primary anchor of IRS2 to INSR while the PTB domain would have a stabilizing action on the interaction with INSR. Interacts with the SH2 domains of the 85 kDa regulatory subunit of PI3K (PIK3R1) in vitro, when autophosphorylated on tyrosine residues. Interacts with SOCS7. Interacts (via the phosphorylated Tyr-999), with SOCS3. Interacts (via the phosphorylated Tyr-1185, Tyr-1189, Tyr-1190) with SOCS1. Interacts with CAV2 (tyrosine-phosphorylated form); the interaction is increased with 'Tyr-27'phosphorylation of CAV2 (By similarity). Interacts with ARRB2 (By similarity). Interacts with GRB10; this interaction blocks the association between IRS1/IRS2 and INSR, significantly reduces insulin-stimulated tyrosine phosphorylation of IRS1 and IRS2 and thus decreases insulin signaling. Interacts with GRB7. Interacts with PDPK1. Interacts (via Tyr-1190) with GRB14 (via BPS domain); this interaction protects the tyrosines in the activation loop from dephosphorylation, but promotes dephosphorylation of Tyr-999, this results in decreased interaction with, and phosphorylation of, IRS1. Interacts (via subunit alpha) with ENPP1 (via 485-599 AA); this interaction blocks autophosphorylation. Interacts with PTPRE; this interaction is dependent of Tyr-1185, Tyr-1189 and Tyr-1190 of the INSR. Interacts with STAT5B (via SH2 domain). Interacts with PTPRF. Interacts with ATIC; ATIC together with PRKAA2/AMPK2 and HACD3/PTPLAD1 is proposed to be part of a signaling netwok regulating INSR autophosphorylation and endocytosis (By similarity). Interacts with the cone snail venom insulin Con-Ins G1 (PubMed:27617429). Interacts with the insulin receptor SORL1; this interaction strongly increases its surface exposure, hence strengthens insulin signal reception (PubMed:27322061). Interacts (tyrosine phosphorylated) with CCDC88A/GIV (via SH2-like region); binding requires autophosphorylation of the INSR C-terminal region (PubMed:25187647). Interacts with GNAI3; the interaction is probably mediated by CCDC88A/GIV (PubMed:25187647). Interacts with LMBRD1 (By similarity).   
  
 **Gene Ontology Information:**

Molecular Function   
  
N/A

Location   
  
N/A

Biological process   
  
N/A

---

28

- **Protein name:** Coagulation factor VIII
- **Organism:** Homo sapiens
- **Uniprot Accession Number:** P00451
- **Protein sequence length:** 2351 aa
- **1D identity (%):** 11.59
- **1D identity (%) [Gaps excluded]:** 32.35
- **1D identity - Alignment Gaps:** 1518
- **1D aligned content (<aminoacid>:%):** {'M': 1.46, 'L': 10.22, 'G': 10.22, 'P': 11.68, 'F': 2.55, 'R': 5.84, 'I': 3.28, 'N': 4.38, 'H': 5.47, 'Q': 4.38, 'D': 4.74, 'V': 5.84, 'E': 6.57, 'T': 4.38, 'K': 6.57, 'Y': 2.92, 'S': 4.74, 'W': 1.09, 'A': 3.28, 'C': 0.36}
- **Common reported functions (%):** 0.0
- **Common reported locations (%):** 0.0
- **Common reported processes (%):** 0.0

- **PDB ID:** 3CDZ
- **Chain:** B
- **Crystallized protein length:** 631 aa
- **Resolution:** 3.98 Å
- **b-phipsi:** 0.050917
- **w-rdist:** 0.390877
- **t-alpha:** 0.0
- **Chemical similarity (Tanimoto Index) (%):** 77.35
- **1D identity (%) [PDB]:** 1.8
- **1D identity (%) [Gaps excluded][PDB]:** 74.29
- **1D identity - Alignment Gaps [PDB]:** 1412
- **1D aligned content [PDB] (<aminoacid>:%):** {'F': 7.69, 'Q': 7.69, 'P': 11.54, 'V': 11.54, 'G': 15.38, 'A': 11.54, 'D': 11.54, 'T': 3.85, 'K': 7.69, 'I': 3.85, 'S': 3.85, 'H': 3.85}
- **2D identity (%) [PDB]:** 23.83
- **2D identity (%) [Gaps excluded][PDB]:** 83.84
- **2D identity - Alignment Gaps [PDB]:** 826
- **2D aligned content [PDB] (<2D-fold>:%):** {'.': 26.18, 'E': 58.18, 'T': 10.91, 'H': 2.91, 'B': 0.73, 'G': 1.09}
- **3D similarity (TM-Score) (%) [PDB]:** 19.9

- **Gene name:** F8
- **Entrez ID:** 2157
- **RefSeq ID:** NM\_019863
- **Transcript sequence length:** 2616
- **5-UTR|CDS|3-UTR identity (%):** 25.95 | 16.85 | 24.84
- **5-UTR|CDS|3-UTR identity (%) [Gaps excluded]:** 77.3 | 78.5 | 75.46
- **5-UTR|CDS|3-UTR identity [Alignment Gaps]:** 279 | 2093 | 3074
- **5-UTR aligned content (<base>:%):** {'G': 48.62, 'C': 40.37, 'A': 5.5, 'T': 5.5}
- **CDS aligned content (<base>:%):** {'A': 27.84, 'T': 25.84, 'G': 23.39, 'C': 22.94}
- **3-UTR aligned content (<base>:%):** {'G': 17.4, 'C': 17.14, 'A': 32.07, 'T': 33.39}

**Uniprot Description:**  
  
 Factor VIII, along with calcium and phospholipid, acts as a cofactor for F9/factor IXa when it converts F10/factor X to the activated form, factor Xa.   
  
Interacts with VWF/vWF. vWF binding is essential for the stabilization of F8 in circulation.   
  
 **Gene Ontology Information:**

Molecular Function

- copper ion binding
- oxidoreductase activity

Location

- COPII-coated ER to Golgi transport vesicle
- endoplasmic reticulum lumen
- endoplasmic reticulum-Golgi intermediate compartment membrane
- extracellular region
- extracellular space
- Golgi lumen
- plasma membrane
- platelet alpha granule lumen
- protein-containing complex
- serine-type endopeptidase complex

Biological process

- acute-phase response
- blood coagulation
- blood coagulation, intrinsic pathway
- proteolysis
- zymogen activation

---

29

- **Protein name:** Regulator of nonsense transcripts 1
- **Organism:** Homo sapiens
- **Uniprot Accession Number:** Q92900
- **Protein sequence length:** 1129 aa
- **1D identity (%):** 16.34
- **1D identity (%) [Gaps excluded]:** 25.45
- **1D identity - Alignment Gaps:** 434
- **1D aligned content (<aminoacid>:%):** {'M': 1.01, 'E': 5.56, 'A': 8.08, 'G': 9.09, 'P': 9.09, 'V': 6.57, 'K': 6.57, 'L': 10.61, 'D': 4.04, 'Y': 4.55, 'I': 3.03, 'R': 8.08, 'N': 3.54, 'S': 3.54, 'Q': 6.06, 'W': 1.01, 'T': 5.05, 'H': 1.01, 'F': 2.53, 'C': 1.01}
- **Common reported functions (%):** 0.0
- **Common reported locations (%):** 50.0
- **Common reported processes (%):** 0.0

- **PDB ID:** 2WJY
- **Chain:** A
- **Crystallized protein length:** 773 aa
- **Resolution:** 2.5 Å
- **b-phipsi:** 0.003279
- **w-rdist:** 0.502087
- **t-alpha:** 0.012679
- **Chemical similarity (Tanimoto Index) (%):** 80.83
- **1D identity (%) [PDB]:** 3.71
- **1D identity (%) [Gaps excluded][PDB]:** 63.33
- **1D identity - Alignment Gaps [PDB]:** 1447
- **1D aligned content [PDB] (<aminoacid>:%):** {'K': 3.51, 'L': 10.53, 'G': 5.26, 'I': 7.02, 'V': 14.04, 'R': 7.02, 'P': 8.77, 'S': 7.02, 'Q': 10.53, 'F': 3.51, 'A': 5.26, 'D': 3.51, 'T': 1.75, 'H': 1.75, 'M': 1.75, 'Y': 3.51, 'E': 3.51, 'N': 1.75}
- **2D identity (%) [PDB]:** 37.14
- **2D identity (%) [Gaps excluded][PDB]:** 84.68
- **2D identity - Alignment Gaps [PDB]:** 635
- **2D aligned content [PDB] (<2D-fold>:%):** {'.': 16.9, 'E': 25.71, 'T': 12.86, 'H': 42.38, 'G': 2.14}
- **3D similarity (TM-Score) (%) [PDB]:** 21.8

- **Gene name:** UPF1
- **Entrez ID:** 5976
- **RefSeq ID:** NM\_002911
- **Transcript sequence length:** 5321
- **5-UTR|CDS|3-UTR identity (%):** 38.41 | 43.37 | 22.35
- **5-UTR|CDS|3-UTR identity (%) [Gaps excluded]:** 79.72 | 76.01 | 72.92
- **5-UTR|CDS|3-UTR identity [Alignment Gaps]:** 228 | 1625 | 3184
- **5-UTR aligned content (<base>:%):** {'G': 48.52, 'T': 4.73, 'C': 41.42, 'A': 5.33}
- **CDS aligned content (<base>:%):** {'A': 27.36, 'T': 19.26, 'G': 27.85, 'C': 25.53}
- **3-UTR aligned content (<base>:%):** {'A': 21.83, 'G': 30.21, 'C': 21.15, 'T': 26.8}

**Uniprot Description:**  
  
 RNA-dependent helicase and ATPase required for nonsense-mediated decay (NMD) of mRNAs containing premature stop codons. Is recruited to mRNAs upon translation termination and undergoes a cycle of phosphorylation and dephosphorylation; its phosphorylation appears to be a key step in NMD. Recruited by release factors to stalled ribosomes together with the SMG1C protein kinase complex to form the transient SURF (SMG1-UPF1-eRF1-eRF3) complex. In EJC-dependent NMD, the SURF complex associates with the exon junction complex (EJC) (located 50-55 or more nucleotides downstream from the termination codon) through UPF2 and allows the formation of an UPF1-UPF2-UPF3 surveillance complex which is believed to activate NMD. Phosphorylated UPF1 is recognized by EST1B/SMG5, SMG6 and SMG7 which are thought to provide a link to the mRNA degradation machinery involving exonucleolytic and endonucleolytic pathways, and to serve as adapters to protein phosphatase 2A (PP2A), thereby triggering UPF1 dephosphorylation and allowing the recycling of NMD factors. UPF1 can also activate NMD without UPF2 or UPF3, and in the absence of the NMD-enhancing downstream EJC indicative for alternative NMD pathways. Plays a role in replication-dependent histone mRNA degradation at the end of phase S; the function is independent of UPF2. For the recognition of premature termination codons (PTC) and initiation of NMD a competitive interaction between UPF1 and PABPC1 with the ribosome-bound release factors is proposed. The ATPase activity of UPF1 is required for disassembly of mRNPs undergoing NMD. Essential for embryonic viability. Together with UPF2 and dependent on TDRD6, mediates the degradation of mRNA hardoring long 3'UTR by inducing the NMD machinery (By similarity).   
  
Found in a post-splicing messenger ribonucleoprotein (mRNP) complex (PubMed:21419344). Associates with the exon junction complex (EJC) (PubMed:11546874, PubMed:16452507). Associates with the SGM1C complex; is phosphorylated by the complex kinase component SGM1 (PubMed:19417104). Interacts with UPF2 (PubMed:11163187, PubMed:11073994, PubMed:11113196, PubMed:19556969). Interacts with UPF3A and UPF3B (PubMed:11163187). Interacts with EST1A (PubMed:12554878). Interacts with SLBP (PubMed:16086026). Interacts (when hyperphosphorylated) with PNRC2 (PubMed:19150429). Interacts with AGO1 and AGO2 (PubMed:17932509). Interacts with GSPT2 (PubMed:18447585). Interacts with isoform 1 and isoform 5 of ADAR/ADAR1 (PubMed:18362360). Interacts with SMG7 (PubMed:15721257). Interacts with ZC3H12A; this interaction occurs in a mRNA translationally active- and termination-dependent manner and is essential for ZC3H12A-mediated degradation of target mRNAs (PubMed:26000482). Interacts with CPSF6 (PubMed:19864460). Interacts with MOV10; the interaction is direct and RNA-dependent (PubMed:24726324). Interacts with SHFL; the interaction increases in the presence of RNA (PubMed:27974568). Interacts with UPF2 and DDX4; interactions are mediated by TDRD6 (By similarity).   
  
 **Gene Ontology Information:**

Molecular Function

- ATP binding
- ATPase activity
- chromatin binding
- double-stranded DNA helicase activity
- helicase activity
- protein-containing complex binding
- RNA binding
- RNA helicase activity
- telomeric DNA binding
- zinc ion binding

Location

- chromatin
- chromosome, telomeric region
- cytoplasm
- cytosol
- exon-exon junction complex
- nucleoplasm
- nucleus
- P-body
- perinuclear region of cytoplasm
- supraspliceosomal complex

Biological process

- 3'-UTR-mediated mRNA destabilization
- cell cycle phase transition
- cellular response to interleukin-1
- cellular response to lipopolysaccharide
- DNA duplex unwinding
- DNA repair
- DNA replication
- histone mRNA catabolic process
- mRNA export from nucleus
- nuclear-transcribed mRNA catabolic process
- nuclear-transcribed mRNA catabolic process, nonsense-mediated decay
- positive regulation of mRNA catabolic process
- regulation of telomere maintenance
- regulation of translational termination
- telomere maintenance via semi-conservative replication

---

30

- **Protein name:** X-ray repair cross-complementing protein 5
- **Organism:** Homo sapiens
- **Uniprot Accession Number:** P13010
- **Protein sequence length:** 732 aa
- **1D identity (%):** 14.69
- **1D identity (%) [Gaps excluded]:** 21.48
- **1D identity - Alignment Gaps:** 299
- **1D aligned content (<aminoacid>:%):** {'R': 5.04, 'N': 1.44, 'V': 9.35, 'M': 2.88, 'F': 6.47, 'I': 5.04, 'G': 8.63, 'P': 3.6, 'K': 8.63, 'L': 11.51, 'D': 5.76, 'Q': 6.47, 'S': 5.76, 'A': 5.04, 'E': 8.63, 'H': 0.72, 'Y': 2.88, 'T': 1.44, 'W': 0.72}
- **Common reported functions (%):** 0.0
- **Common reported locations (%):** 0.0
- **Common reported processes (%):** 0.0

- **PDB ID:** 8ASC
- **Chain:** L
- **Crystallized protein length:** 530 aa
- **Resolution:** 2.95 Å
- **b-phipsi:** 0.002374
- **w-rdist:** 0.445417
- **t-alpha:** 0.027386
- **Chemical similarity (Tanimoto Index) (%):** N/A
- **1D identity (%) [PDB]:** 1.78
- **1D identity (%) [Gaps excluded][PDB]:** 85.71
- **1D identity - Alignment Gaps [PDB]:** 1318
- **1D aligned content [PDB] (<aminoacid>:%):** {'N': 4.17, 'E': 12.5, 'Q': 8.33, 'K': 16.67, 'P': 4.17, 'L': 8.33, 'T': 12.5, 'D': 4.17, 'S': 4.17, 'R': 4.17, 'V': 12.5, 'I': 4.17, 'G': 4.17}
- **2D identity (%) [PDB]:** 33.64
- **2D identity (%) [Gaps excluded][PDB]:** 84.87
- **2D identity - Alignment Gaps [PDB]:** 594
- **2D aligned content [PDB] (<2D-fold>:%):** {'.': 24.17, 'E': 25.98, 'T': 45.92, 'G': 3.63, 'B': 0.3}
- **3D similarity (TM-Score) (%) [PDB]:** 17.27

- **Gene name:** XRCC5
- **Entrez ID:** N/A
- **RefSeq ID:** NM\_021141
- **Transcript sequence length:** 3379
- **5-UTR|CDS|3-UTR identity (%):** 12.92 | 41.62 | 15.57
- **5-UTR|CDS|3-UTR identity (%) [Gaps excluded]:** 75.0 | 73.43 | 73.75
- **5-UTR|CDS|3-UTR identity [Alignment Gaps]:** 346 | 1323 | 3502
- **5-UTR aligned content (<base>:%):** {'G': 40.74, 'C': 42.59, 'T': 9.26, 'A': 7.41}
- **CDS aligned content (<base>:%):** {'A': 31.71, 'G': 24.0, 'T': 24.39, 'C': 19.91}
- **3-UTR aligned content (<base>:%):** {'G': 16.79, 'T': 38.93, 'A': 27.79, 'C': 16.5}

**Uniprot Description:**  
  
 Single-stranded DNA-dependent ATP-dependent helicase. Has a role in chromosome translocation. The DNA helicase II complex binds preferentially to fork-like ends of double-stranded DNA in a cell cycle-dependent manner. It works in the 3'-5' direction. Binding to DNA may be mediated by XRCC6. Involved in DNA non-homologous end joining (NHEJ) required for double-strand break repair and V(D)J recombination. The XRCC5/6 dimer acts as regulatory subunit of the DNA-dependent protein kinase complex DNA-PK by increasing the affinity of the catalytic subunit PRKDC to DNA by 100-fold. The XRCC5/6 dimer is probably involved in stabilizing broken DNA ends and bringing them together (PubMed:12145306, PubMed:20383123, PubMed:7957065, PubMed:8621488). The assembly of the DNA-PK complex to DNA ends is required for the NHEJ ligation step. In association with NAA15, the XRCC5/6 dimer binds to the osteocalcin promoter and activates osteocalcin expression (PubMed:20383123). The XRCC5/6 dimer probably also acts as a 5'-deoxyribose-5-phosphate lyase (5'-dRP lyase), by catalyzing the beta-elimination of the 5' deoxyribose-5-phosphate at an abasic site near double-strand breaks. XRCC5 probably acts as the catalytic subunit of 5'-dRP activity, and allows to 'clean' the termini of abasic sites, a class of nucleotide damage commonly associated with strand breaks, before such broken ends can be joined. The XRCC5/6 dimer together with APEX1 acts as a negative regulator of transcription (PubMed:8621488). As part of the DNA-PK complex, involved in the early steps of ribosome assembly by promoting the processing of precursor rRNA into mature 18S rRNA in the small-subunit processome (PubMed:32103174). Binding to U3 small nucleolar RNA, recruits PRKDC and XRCC5/Ku86 to the small-subunit processome (PubMed:32103174). Plays a role in the regulation of DNA virus-mediated innate immune response by assembling into the HDP-RNP complex, a complex that serves as a platform for IRF3 phosphorylation and subsequent innate immune response activation through the cGAS-STING pathway.   
  
Heterodimer composed of XRCC5/Ku80 and XRCC6/Ku70. The dimer associates in a DNA-dependent manner with PRKDC to form the DNA-dependent protein kinase complex DNA-PK, and with the LIG4-XRCC4 complex to form the core of the non-homologous end joining (NHEJ) complex (PubMed:25941166, PubMed:25670504, PubMed:11493912, PubMed:22442688). Additional components of the NHEJ complex include NHEJ1/XLF and PAXX (PubMed:25574025, PubMed:25941166, PubMed:25670504). The dimer also associates with NAA15, and this complex displays DNA binding activity towards the osteocalcin FGF response element (OCFRE) (PubMed:12145306). In addition, XRCC5 binds to the osteoblast-specific transcription factors MSX2 and RUNX2 (PubMed:12145306). Interacts with ELF3 (PubMed:15075319). May interact with APLF (PubMed:17353262, PubMed:17396150). The XRCC5/XRCC6 dimer associates in a DNA-dependent manner with APEX1 (PubMed:8621488). Identified in a complex with DEAF1 and XRCC6. Interacts with NR4A3; the DNA-dependent protein kinase complex DNA-PK phosphorylates and activates NR4A3 and prevents NR4A3 ubiquitinylation and degradation (PubMed:25852083). Interacts with RNF138 (PubMed:26502055). Interacts with CYREN isoform 1 (CYREN-1) and isoform 4 (CYREN-2) (PubMed:24610814, PubMed:28959974). Interacts (via N-terminus) with HSF1 (via N-terminus); this interaction is direct and prevents XRCC5/XRCC6 heterodimeric binding and non-homologous end joining (NHEJ) repair activities induced by ionizing radiation (IR) (PubMed:26359349). Interacts with DHX9; this interaction occurs in a RNA-dependent manner (PubMed:14704337). Part of the HDP-RNP complex composed of at least HEXIM1, PRKDC, XRCC5, XRCC6, paraspeckle proteins (SFPQ, NONO, PSPC1, RBM14, and MATR3) and NEAT1 RNA (PubMed:28712728). Interacts with ERCC6 (PubMed:26030138). Interats with ATF7 (PubMed:29490055).   
  
 **Gene Ontology Information:**

Molecular Function   
  
N/A

Location   
  
N/A

Biological process   
  
N/A

---

31

- **Protein name:** Neutral and basic amino acid transport protein rBAT
- **Organism:** Homo sapiens
- **Uniprot Accession Number:** Q07837
- **Protein sequence length:** 685 aa
- **1D identity (%):** 14.57
- **1D identity (%) [Gaps excluded]:** 23.04
- **1D identity - Alignment Gaps:** 348
- **1D aligned content (<aminoacid>:%):** {'E': 5.07, 'R': 5.8, 'N': 4.35, 'H': 2.17, 'I': 2.9, 'G': 11.59, 'P': 9.42, 'Y': 5.07, 'W': 0.72, 'V': 6.52, 'S': 4.35, 'L': 14.49, 'A': 2.17, 'D': 6.52, 'F': 3.62, 'T': 5.07, 'K': 3.62, 'C': 2.17, 'Q': 3.62, 'M': 0.72}
- **Common reported functions (%):** 0.0
- **Common reported locations (%):** 12.5
- **Common reported processes (%):** 0.0

- **PDB ID:** 6LID
- **Chain:** A
- **Crystallized protein length:** 623 aa
- **Resolution:** 2.7 Å
- **b-phipsi:** 0.005894
- **w-rdist:** 0.729416
- **t-alpha:** 0.001565
- **Chemical similarity (Tanimoto Index) (%):** 88.33
- **1D identity (%) [PDB]:** 2.54
- **1D identity (%) [Gaps excluded][PDB]:** 67.92
- **1D identity - Alignment Gaps [PDB]:** 1367
- **1D aligned content [PDB] (<aminoacid>:%):** {'I': 8.33, 'A': 8.33, 'T': 5.56, 'S': 8.33, 'P': 8.33, 'D': 5.56, 'Q': 2.78, 'E': 11.11, 'L': 8.33, 'V': 8.33, 'N': 5.56, 'M': 5.56, 'G': 11.11, 'Y': 2.78}
- **2D identity (%) [PDB]:** 29.37
- **2D identity (%) [Gaps excluded][PDB]:** 84.47
- **2D identity - Alignment Gaps [PDB]:** 713
- **2D aligned content [PDB] (<2D-fold>:%):** {'.': 20.87, 'H': 39.88, 'T': 16.82, 'E': 20.56, 'B': 0.93, 'G': 0.93}
- **3D similarity (TM-Score) (%) [PDB]:** 20.87

- **Gene name:** SLC3A1
- **Entrez ID:** 6519
- **RefSeq ID:** NM\_000341
- **Transcript sequence length:** 2969
- **5-UTR|CDS|3-UTR identity (%):** 5.88 | 42.08 | 12.0
- **5-UTR|CDS|3-UTR identity (%) [Gaps excluded]:** 78.12 | 74.87 | 76.47
- **5-UTR|CDS|3-UTR identity [Alignment Gaps]:** 393 | 1302 | 3746
- **5-UTR aligned content (<base>:%):** {'A': 8.0, 'C': 48.0, 'T': 16.0, 'G': 28.0}
- **CDS aligned content (<base>:%):** {'A': 31.25, 'T': 24.94, 'G': 23.5, 'C': 20.3}
- **3-UTR aligned content (<base>:%):** {'C': 14.82, 'A': 35.83, 'T': 30.77, 'G': 18.57}

**Uniprot Description:**  
  
 Involved in the high-affinity, sodium-independent transport of cystine and neutral and dibasic amino acids (system B(0,+)-like activity). May function as an activator of SLC7A9 and be involved in the high-affinity reabsorption of cystine in the kidney tubule.   
  
Disulfide-linked heterodimer with the amino acid transport protein SLC7A9.   
  
 **Gene Ontology Information:**

Molecular Function

- amino acid transmembrane transporter activity
- basic amino acid transmembrane transporter activity
- L-cystine transmembrane transporter activity
- protein heterodimerization activity
- protein-containing complex binding

Location

- apical plasma membrane
- brush border membrane
- extracellular exosome
- membrane
- plasma membrane
- vacuolar membrane

Biological process

- amino acid transport
- aspartate transmembrane transport
- basic amino acid transport
- carbohydrate metabolic process
- gene expression
- L-cystine transport
- L-glutamate transmembrane transport

---

32

- **Protein name:** Lactotransferrin
- **Organism:** Homo sapiens
- **Uniprot Accession Number:** P02788
- **Protein sequence length:** 710 aa
- **1D identity (%):** 12.32
- **1D identity (%) [Gaps excluded]:** 23.52
- **1D identity - Alignment Gaps:** 491
- **1D aligned content (<aminoacid>:%):** {'L': 13.39, 'A': 6.3, 'G': 12.6, 'V': 10.24, 'R': 6.3, 'P': 7.87, 'Q': 3.15, 'N': 4.72, 'Y': 1.57, 'T': 3.94, 'K': 5.51, 'E': 4.72, 'C': 6.3, 'F': 2.36, 'D': 5.51, 'S': 3.15, 'I': 1.57, 'W': 0.79}
- **Common reported functions (%):** 0.0
- **Common reported locations (%):** 25.0
- **Common reported processes (%):** 8.33

- **PDB ID:** 2BJJ
- **Chain:** X
- **Crystallized protein length:** 692 aa
- **Resolution:** 2.4 Å
- **b-phipsi:** 0.006021
- **w-rdist:** 0.513218
- **t-alpha:** 0.006259
- **Chemical similarity (Tanimoto Index) (%):** 72.68
- **1D identity (%) [PDB]:** 2.55
- **1D identity (%) [Gaps excluded][PDB]:** 71.7
- **1D identity - Alignment Gaps [PDB]:** 1436
- **1D aligned content [PDB] (<aminoacid>:%):** {'R': 10.53, 'S': 15.79, 'V': 10.53, 'P': 7.89, 'A': 10.53, 'Y': 7.89, 'L': 7.89, 'F': 2.63, 'D': 7.89, 'K': 5.26, 'E': 2.63, 'G': 5.26, 'Q': 2.63, 'N': 2.63}
- **2D identity (%) [PDB]:** 39.48
- **2D identity (%) [Gaps excluded][PDB]:** 83.27
- **2D identity - Alignment Gaps [PDB]:** 550
- **2D aligned content [PDB] (<2D-fold>:%):** {'.': 17.43, 'E': 22.76, 'H': 40.92, 'T': 18.16, 'G': 0.73}
- **3D similarity (TM-Score) (%) [PDB]:** 21.65

- **Gene name:** LTF
- **Entrez ID:** 4057
- **RefSeq ID:** NM\_002343
- **Transcript sequence length:** 2721
- **5-UTR|CDS|3-UTR identity (%):** 5.64 | 41.55 | 8.28
- **5-UTR|CDS|3-UTR identity (%) [Gaps excluded]:** 74.19 | 74.39 | 76.16
- **5-UTR|CDS|3-UTR identity [Alignment Gaps]:** 377 | 1337 | 3887
- **5-UTR aligned content (<base>:%):** {'A': 13.04, 'G': 26.09, 'C': 52.17, 'T': 8.7}
- **CDS aligned content (<base>:%):** {'A': 26.87, 'T': 23.05, 'G': 26.63, 'C': 23.45}
- **3-UTR aligned content (<base>:%):** {'A': 24.1, 'C': 22.44, 'G': 19.11, 'T': 34.35}

**Uniprot Description:**  
  
 Transferrins are iron binding transport proteins which can bind two Fe(3+) ions in association with the binding of an anion, usually bicarbonate.   
  
Monomer. Found in a complex with LTF, CLU, EPPIN and SEMG1.   
  
 **Gene Ontology Information:**

Molecular Function

- cysteine-type endopeptidase inhibitor activity
- DNA binding
- heparin binding
- iron ion binding
- lipopolysaccharide binding
- membrane destabilizing activity
- protein serine/threonine kinase activator activity
- serine-type endopeptidase activity

Location

- cell surface
- cytoplasm
- early endosome
- extracellular exosome
- extracellular region
- extracellular space
- nucleus
- phagocytic vesicle lumen
- plasma membrane
- protein-containing complex
- recycling endosome
- secretory granule
- specific granule
- specific granule lumen
- tertiary granule lumen

Biological process

- antibacterial humoral response
- antifungal humoral response
- antimicrobial humoral immune response mediated by antimicrobial peptide
- bone morphogenesis
- cytolysis by host of symbiont cells
- defense response to Gram-negative bacterium
- defense response to Gram-positive bacterium
- humoral immune response
- innate immune response
- innate immune response in mucosa
- iron ion transmembrane transport
- iron ion transport
- killing of cells of other organism
- negative regulation by host of viral process
- negative regulation of apoptotic process
- negative regulation of ATPase activity
- negative regulation of cysteine-type endopeptidase activity
- negative regulation of lipopolysaccharide-mediated signaling pathway
- negative regulation of membrane potential
- negative regulation of osteoclast development
- negative regulation of single-species biofilm formation in or on host organism
- negative regulation of tumor necrosis factor (ligand) superfamily member 11 production
- negative regulation of viral genome replication
- negative regulation of viral process
- ossification
- positive regulation of bone mineralization involved in bone maturation
- positive regulation of chondrocyte proliferation
- positive regulation of I-kappaB kinase/NF-kappaB signaling
- positive regulation of NF-kappaB transcription factor activity
- positive regulation of osteoblast differentiation
- positive regulation of osteoblast proliferation
- positive regulation of protein serine/threonine kinase activity
- positive regulation of toll-like receptor 4 signaling pathway
- proteolysis
- regulation of cytokine production
- regulation of tumor necrosis factor production
- retina homeostasis

---

33

- **Protein name:** Glutamine--fructose-6-phosphate aminotransferase [isomerizing] 1
- **Organism:** Homo sapiens
- **Uniprot Accession Number:** Q06210
- **Protein sequence length:** 699 aa
- **1D identity (%):** 15.86
- **1D identity (%) [Gaps excluded]:** 23.6
- **1D identity - Alignment Gaps:** 306
- **1D aligned content (<aminoacid>:%):** {'I': 8.78, 'Y': 5.41, 'V': 10.81, 'R': 6.08, 'E': 6.08, 'T': 7.43, 'G': 8.11, 'D': 4.73, 'A': 4.73, 'K': 5.41, 'Q': 2.7, 'N': 3.38, 'P': 4.73, 'S': 3.38, 'H': 1.35, 'F': 5.41, 'M': 2.03, 'C': 2.7, 'L': 6.76}
- **Common reported functions (%):** 0.0
- **Common reported locations (%):** 12.5
- **Common reported processes (%):** 0.0

- **PDB ID:** 6SVP
- **Chain:** B
- **Crystallized protein length:** 652 aa
- **Resolution:** 2.53 Å
- **b-phipsi:** 0.004938
- **w-rdist:** 0.789844
- **t-alpha:** 0.001565
- **Chemical similarity (Tanimoto Index) (%):** 73.28
- **1D identity (%) [PDB]:** 2.4
- **1D identity (%) [Gaps excluded][PDB]:** 76.09
- **1D identity - Alignment Gaps [PDB]:** 1412
- **1D aligned content [PDB] (<aminoacid>:%):** {'T': 11.43, 'H': 5.71, 'P': 2.86, 'S': 8.57, 'E': 2.86, 'F': 8.57, 'D': 11.43, 'Y': 8.57, 'A': 5.71, 'I': 2.86, 'Q': 5.71, 'G': 2.86, 'R': 2.86, 'V': 5.71, 'L': 8.57, 'C': 5.71}
- **2D identity (%) [PDB]:** 36.53
- **2D identity (%) [Gaps excluded][PDB]:** 88.61
- **2D identity - Alignment Gaps [PDB]:** 626
- **2D aligned content [PDB] (<2D-fold>:%):** {'E': 28.02, 'H': 47.3, '.': 11.31, 'T': 12.6, 'G': 0.77}
- **3D similarity (TM-Score) (%) [PDB]:** 25.98

- **Gene name:** GFPT1
- **Entrez ID:** 2673
- **RefSeq ID:** NM\_002056
- **Transcript sequence length:** 8611
- **5-UTR|CDS|3-UTR identity (%):** 23.22 | 42.73 | 41.6
- **5-UTR|CDS|3-UTR identity (%) [Gaps excluded]:** 72.59 | 75.01 | 76.75
- **5-UTR|CDS|3-UTR identity [Alignment Gaps]:** 287 | 1270 | 3176
- **5-UTR aligned content (<base>:%):** {'G': 36.73, 'C': 48.98, 'T': 10.2, 'A': 4.08}
- **CDS aligned content (<base>:%):** {'T': 25.3, 'G': 25.14, 'A': 30.06, 'C': 19.51}
- **3-UTR aligned content (<base>:%):** {'G': 20.24, 'A': 28.67, 'T': 36.95, 'C': 14.14}

**Uniprot Description:**  
  
 Controls the flux of glucose into the hexosamine pathway. Most likely involved in regulating the availability of precursors for N- and O-linked glycosylation of proteins. Regulates the circadian expression of clock genes ARNTL/BMAL1 and CRY1.   
  
Homotetramer (Probable), may also exist as homodimers.   
  
 **Gene Ontology Information:**

Molecular Function

- carbohydrate derivative binding
- glutamine-fructose-6-phosphate transaminase (isomerizing) activity

Location

- cytosol
- extracellular exosome

Biological process

- circadian regulation of gene expression
- energy reserve metabolic process
- fructose 6-phosphate metabolic process
- glutamine metabolic process
- protein N-linked glycosylation
- UDP-N-acetylglucosamine biosynthetic process
- UDP-N-acetylglucosamine metabolic process

---

34

- **Protein name:** Prolyl 4-hydroxylase subunit alpha-2
- **Organism:** Homo sapiens
- **Uniprot Accession Number:** O15460
- **Protein sequence length:** 535 aa
- **1D identity (%):** 9.98
- **1D identity (%) [Gaps excluded]:** 23.67
- **1D identity - Alignment Gaps:** 568
- **1D aligned content (<aminoacid>:%):** {'L': 12.24, 'V': 6.12, 'Q': 4.08, 'H': 3.06, 'I': 2.04, 'E': 7.14, 'K': 5.1, 'T': 7.14, 'D': 6.12, 'P': 9.18, 'W': 3.06, 'A': 7.14, 'G': 10.2, 'R': 6.12, 'Y': 5.1, 'S': 3.06, 'M': 2.04, 'C': 1.02}
- **Common reported functions (%):** 0.0
- **Common reported locations (%):** 12.5
- **Common reported processes (%):** 0.0

- **PDB ID:** 7ZSC
- **Chain:** D
- **Crystallized protein length:** 458 aa
- **Resolution:** 3.85 Å
- **b-phipsi:** 0.00432
- **w-rdist:** 0.426647
- **t-alpha:** 0.026604
- **Chemical similarity (Tanimoto Index) (%):** 98.94
- **1D identity (%) [PDB]:** 2.13
- **1D identity (%) [Gaps excluded][PDB]:** 71.05
- **1D identity - Alignment Gaps [PDB]:** 1232
- **1D aligned content [PDB] (<aminoacid>:%):** {'E': 14.81, 'L': 11.11, 'V': 14.81, 'K': 7.41, 'S': 3.7, 'N': 7.41, 'G': 11.11, 'P': 3.7, 'Y': 3.7, 'F': 3.7, 'I': 3.7, 'H': 3.7, 'M': 3.7, 'T': 7.41}
- **2D identity (%) [PDB]:** 36.75
- **2D identity (%) [Gaps excluded][PDB]:** 92.47
- **2D identity - Alignment Gaps [PDB]:** 564
- **2D aligned content [PDB] (<2D-fold>:%):** {'E': 20.64, 'T': 25.0, '.': 12.79, 'H': 40.41, 'G': 0.87, 'B': 0.29}
- **3D similarity (TM-Score) (%) [PDB]:** 17.04

- **Gene name:** P4HA2
- **Entrez ID:** 5034
- **RefSeq ID:** N/A
- **Sequence length:** N/A
- **5-UTR|CDS|3-UTR identity (%):** N/A | N/A | N/A
- **5-UTR|CDS|3-UTR identity (%) [Gaps excluded]:** N/A | N/A | N/A
- **5-UTR|CDS|3-UTR identity [Alignment Gaps]:** N/A | N/A | N/A
- **5-UTR aligned content (<base>:%):** N/A
- **CDS aligned content (<base>:%):** N/A
- **3-UTR aligned content (<base>:%):** N/A

**Uniprot Description:**  
  
 Catalyzes the post-translational formation of 4-hydroxyproline in -Xaa-Pro-Gly- sequences in collagens and other proteins.   
  
Heterotetramer of two alpha-2 chains and two beta chains (P4HB) (the beta chain is the multi-functional PDI), where P4HB plays the role of a structural subunit; this tetramer catalyzes the formation of 4-hydroxyproline in collagen.   
  
 **Gene Ontology Information:**

Molecular Function

- actin binding
- enzyme binding
- integrin binding
- procollagen-proline 4-dioxygenase activity
- protein disulfide isomerase activity
- protein heterodimerization activity
- protein disulfide oxidoreductase activity
- RNA binding
- thiol oxidase activity

Location

- cytoskeleton
- cytosol
- endoplasmic reticulum
- endoplasmic reticulum chaperone complex
- endoplasmic reticulum lumen
- endoplasmic reticulum-Golgi intermediate compartment
- external side of plasma membrane
- extracellular exosome
- extracellular region
- focal adhesion
- lamellipodium
- melanosome
- procollagen-proline 4-dioxygenase complex
- protein-containing complex

Biological process

- cellular response to hypoxia
- cellular response to interleukin-7
- endoplasmic reticulum to Golgi vesicle-mediated transport
- insulin processing
- interleukin-12-mediated signaling pathway
- interleukin-23-mediated signaling pathway
- peptidyl-proline hydroxylation to 4-hydroxy-L-proline
- positive regulation of cell adhesion
- positive regulation of substrate adhesion-dependent cell spreading
- positive regulation of viral entry into host cell
- protein folding
- protein folding in endoplasmic reticulum
- regulation of oxidative stress-induced intrinsic apoptotic signaling pathway
- response to endoplasmic reticulum stress

---

35

- **Protein name:** Receptor-type tyrosine-protein phosphatase alpha
- **Organism:** Homo sapiens
- **Uniprot Accession Number:** P18433
- **Protein sequence length:** 802 aa
- **1D identity (%):** 14.4
- **1D identity (%) [Gaps excluded]:** 24.15
- **1D identity - Alignment Gaps:** 421
- **1D aligned content (<aminoacid>:%):** {'M': 0.67, 'L': 8.67, 'G': 8.67, 'V': 10.0, 'S': 6.0, 'A': 4.0, 'E': 6.0, 'P': 10.67, 'R': 4.0, 'N': 4.0, 'I': 6.67, 'T': 8.0, 'D': 4.0, 'K': 6.67, 'Y': 2.67, 'C': 2.67, 'F': 2.67, 'Q': 1.33, 'W': 1.33, 'H': 1.33}
- **Common reported functions (%):** 0.0
- **Common reported locations (%):** 12.5
- **Common reported processes (%):** 0.0

- **PDB ID:** 6UZT
- **Chain:** A
- **Crystallized protein length:** 587 aa
- **Resolution:** 1.8 Å
- **b-phipsi:** 0.004142
- **w-rdist:** 0.818659
- **t-alpha:** 0.001568
- **Chemical similarity (Tanimoto Index) (%):** 99.09
- **1D identity (%) [PDB]:** 2.6
- **1D identity (%) [Gaps excluded][PDB]:** 67.92
- **1D identity - Alignment Gaps [PDB]:** 1331
- **1D aligned content [PDB] (<aminoacid>:%):** {'R': 8.33, 'E': 11.11, 'I': 5.56, 'L': 13.89, 'S': 2.78, 'V': 8.33, 'G': 13.89, 'P': 5.56, 'D': 2.78, 'K': 2.78, 'F': 2.78, 'H': 2.78, 'T': 5.56, 'A': 2.78, 'M': 2.78, 'Q': 2.78, 'Y': 2.78, 'N': 2.78}
- **2D identity (%) [PDB]:** 34.7
- **2D identity (%) [Gaps excluded][PDB]:** 85.75
- **2D identity - Alignment Gaps [PDB]:** 609
- **2D aligned content [PDB] (<2D-fold>:%):** {'.': 12.39, 'H': 45.35, 'T': 11.83, 'E': 28.73, 'G': 1.69}
- **3D similarity (TM-Score) (%) [PDB]:** 21.61

- **Gene name:** PTPRA
- **Entrez ID:** 5786
- **RefSeq ID:** NM\_080841
- **Transcript sequence length:** 3416
- **5-UTR|CDS|3-UTR identity (%):** 46.34 | 44.31 | 7.35
- **5-UTR|CDS|3-UTR identity (%) [Gaps excluded]:** 71.67 | 75.33 | 73.18
- **5-UTR|CDS|3-UTR identity [Alignment Gaps]:** 193 | 1288 | 3941
- **5-UTR aligned content (<base>:%):** {'G': 34.78, 'C': 50.99, 'A': 8.7, 'T': 5.53}
- **CDS aligned content (<base>:%):** {'A': 29.73, 'T': 21.72, 'G': 26.05, 'C': 22.51}
- **3-UTR aligned content (<base>:%):** {'A': 32.61, 'G': 19.25, 'C': 16.46, 'T': 31.68}

**Uniprot Description:**  
  
 Tyrosine protein phosphatase which is involved in integrin-mediated focal adhesion formation (By similarity). Following integrin engagement, specifically recruits BCAR3, BCAR1 and CRK to focal adhesions thereby promoting SRC-mediated phosphorylation of BRAC1 and the subsequent activation of PAK and small GTPase RAC1 and CDC42 (By similarity).   
  
Part of a complex comprised of PTPRA, BCAR1, BCAR3 (via SH2 domain), and SRC (PubMed:22801373). Within the complex, interacts (when phosphorylated on Tyr-798) with BCAR3 (via SH2 domain) (PubMed:22801373). Interacts with GRB2 (PubMed:22801373).   
  
 **Gene Ontology Information:**

Molecular Function

- protein tyrosine phosphatase activity
- transmembrane receptor protein tyrosine phosphatase activity

Location

- extracellular exosome
- focal adhesion
- membrane
- plasma membrane
- receptor complex
- Schaffer collateral - CA1 synapse
- synaptic membrane

Biological process

- insulin receptor signaling pathway
- integrin-mediated signaling pathway
- modulation of chemical synaptic transmission
- protein dephosphorylation
- protein phosphorylation
- regulation of focal adhesion assembly

---

36

- **Protein name:** Macrophage-expressed gene 1 protein
- **Organism:** Homo sapiens
- **Uniprot Accession Number:** Q2M385
- **Protein sequence length:** 716 aa
- **1D identity (%):** 13.98
- **1D identity (%) [Gaps excluded]:** 22.95
- **1D identity - Alignment Gaps:** 383
- **1D aligned content (<aminoacid>:%):** {'F': 4.38, 'W': 1.46, 'V': 10.22, 'Q': 5.11, 'A': 4.38, 'L': 10.95, 'E': 5.84, 'P': 10.95, 'D': 4.38, 'R': 7.3, 'T': 10.22, 'G': 6.57, 'S': 5.11, 'N': 2.92, 'H': 2.92, 'K': 3.65, 'I': 1.46, 'C': 2.19}
- **Common reported functions (%):** 0.0
- **Common reported locations (%):** 0.0
- **Common reported processes (%):** 0.0

- **PDB ID:** 6U23
- **Chain:** P
- **Crystallized protein length:** 583 aa
- **Resolution:** 3.49 Å
- **b-phipsi:** 0.010094
- **w-rdist:** 0.351565
- **t-alpha:** 0.008607
- **Chemical similarity (Tanimoto Index) (%):** N/A
- **1D identity (%) [PDB]:** 2.15
- **1D identity (%) [Gaps excluded][PDB]:** 75.0
- **1D identity - Alignment Gaps [PDB]:** 1357
- **1D aligned content [PDB] (<aminoacid>:%):** {'E': 10.0, 'I': 3.33, 'S': 10.0, 'R': 3.33, 'L': 10.0, 'K': 6.67, 'N': 6.67, 'V': 10.0, 'G': 13.33, 'P': 10.0, 'F': 3.33, 'H': 3.33, 'M': 3.33, 'T': 6.67}
- **2D identity (%) [PDB]:** 23.49
- **2D identity (%) [Gaps excluded][PDB]:** 84.35
- **2D identity - Alignment Gaps [PDB]:** 811
- **2D aligned content [PDB] (<2D-fold>:%):** {'.': 24.24, 'H': 31.82, 'E': 25.38, 'B': 1.52, 'T': 17.05}
- **3D similarity (TM-Score) (%) [PDB]:** 21.74

- **Gene name:** MPEG1
- **Entrez ID:** 219972
- **RefSeq ID:** NM\_001039396
- **Transcript sequence length:** 4418
- **5-UTR|CDS|3-UTR identity (%):** 8.76 | 40.49 | 30.56
- **5-UTR|CDS|3-UTR identity (%) [Gaps excluded]:** 69.23 | 75.61 | 74.95
- **5-UTR|CDS|3-UTR identity [Alignment Gaps]:** 359 | 1433 | 2730
- **5-UTR aligned content (<base>:%):** {'G': 61.11, 'T': 11.11, 'C': 22.22, 'A': 5.56}
- **CDS aligned content (<base>:%):** {'A': 27.7, 'T': 21.7, 'G': 25.54, 'C': 25.06}
- **3-UTR aligned content (<base>:%):** {'A': 30.66, 'T': 31.37, 'C': 18.88, 'G': 19.09}

**Uniprot Description:**  
  
 Plays a key role in the innate immune response following bacterial infection by inserting into the bacterial surface to form pores (By similarity). By breaching the surface of phagocytosed bacteria, allows antimicrobial effectors to enter the bacterial periplasmic space and degrade bacterial proteins such as superoxide dismutase sodC which contributes to bacterial virulence (By similarity). Shows antibacterial activity against a wide spectrum of Gram-positive, Gram-negative and acid-fast bacteria (PubMed:23753625, PubMed:26402460, PubMed:30609079). Reduces the viability of the intracytosolic pathogen L.monocytogenes by inhibiting acidification of the phagocytic vacuole of host cells which restricts bacterial translocation from the vacuole to the cytosol (By similarity). Required for the antibacterial activity of reactive oxygen species and nitric oxide (By similarity). N/A   
  
 **Gene Ontology Information:**

Molecular Function   
  
N/A

Location

- cytoplasmic vesicle
- cytoplasmic vesicle membrane
- extracellular region
- phagocytic vesicle

Biological process

- antibacterial innate immune response
- defense response to bacterium
- defense response to Gram-negative bacterium
- defense response to Gram-positive bacterium

---

37

- **Protein name:** Plexin-C1
- **Organism:** Homo sapiens
- **Uniprot Accession Number:** O60486
- **Protein sequence length:** 1568 aa
- **1D identity (%):** 13.99
- **1D identity (%) [Gaps excluded]:** 27.41
- **1D identity - Alignment Gaps:** 787
- **1D aligned content (<aminoacid>:%):** {'M': 1.33, 'E': 4.89, 'P': 11.11, 'G': 8.44, 'A': 5.33, 'F': 4.0, 'D': 5.33, 'Y': 2.67, 'V': 9.33, 'R': 3.56, 'T': 8.0, 'Q': 4.44, 'H': 2.22, 'S': 4.44, 'W': 0.44, 'L': 6.67, 'I': 2.67, 'N': 4.0, 'K': 8.44, 'C': 2.67}
- **Common reported functions (%):** 0.0
- **Common reported locations (%):** 12.5
- **Common reported processes (%):** 0.0

- **PDB ID:** 6VXK
- **Chain:** B
- **Crystallized protein length:** 747 aa
- **Resolution:** 3.1 Å
- **b-phipsi:** 0.026137
- **w-rdist:** 0.344036
- **t-alpha:** 0.00314
- **Chemical similarity (Tanimoto Index) (%):** 83.61
- **1D identity (%) [PDB]:** 2.98
- **1D identity (%) [Gaps excluded][PDB]:** 68.66
- **1D identity - Alignment Gaps [PDB]:** 1475
- **1D aligned content [PDB] (<aminoacid>:%):** {'P': 8.7, 'G': 4.35, 'D': 6.52, 'Q': 4.35, 'T': 6.52, 'V': 15.22, 'S': 10.87, 'L': 17.39, 'E': 4.35, 'A': 4.35, 'H': 4.35, 'I': 2.17, 'R': 4.35, 'F': 4.35, 'Y': 2.17}
- **2D identity (%) [PDB]:** 30.93
- **2D identity (%) [Gaps excluded][PDB]:** 80.9
- **2D identity - Alignment Gaps [PDB]:** 719
- **2D aligned content [PDB] (<2D-fold>:%):** {'.': 25.0, 'E': 54.17, 'T': 15.56, 'H': 4.44, 'G': 0.83}
- **3D similarity (TM-Score) (%) [PDB]:** 21.21

- **Gene name:** PLXNC1
- **Entrez ID:** 10154
- **RefSeq ID:** NM\_005761
- **Transcript sequence length:** 7492
- **5-UTR|CDS|3-UTR identity (%):** 50.85 | 37.45 | 32.08
- **5-UTR|CDS|3-UTR identity (%) [Gaps excluded]:** 74.07 | 77.2 | 75.73
- **5-UTR|CDS|3-UTR identity [Alignment Gaps]:** 148 | 2529 | 2703
- **5-UTR aligned content (<base>:%):** {'C': 53.33, 'T': 7.08, 'G': 37.5, 'A': 2.08}
- **CDS aligned content (<base>:%):** {'A': 28.77, 'T': 22.46, 'G': 25.5, 'C': 23.27}
- **3-UTR aligned content (<base>:%):** {'G': 19.08, 'C': 16.36, 'T': 35.31, 'A': 29.26}

**Uniprot Description:**  
  
 Receptor for SEMA7A, for smallpox semaphorin A39R, vaccinia virus semaphorin A39R and for herpesvirus Sema protein. Binding of semaphorins triggers cellular responses leading to the rearrangement of the cytoskeleton and to secretion of IL6 and IL8 (By similarity).   
  
Monomer. Homodimer. Interacts with SEMA7A.   
  
 **Gene Ontology Information:**

Molecular Function

- semaphorin receptor activity
- signaling receptor binding

Location

- cerebellar climbing fiber to Purkinje cell synapse
- membrane
- plasma membrane
- semaphorin receptor complex

Biological process

- cell adhesion
- negative regulation of cell adhesion
- positive regulation of axonogenesis
- regulation of cell migration
- regulation of cell shape
- regulation of GTPase activity
- regulation of synapse pruning
- semaphorin-plexin signaling pathway involved in axon guidance

---

38

- **Protein name:** 116 kDa U5 small nuclear ribonucleoprotein component
- **Organism:** Homo sapiens
- **Uniprot Accession Number:** Q15029
- **Protein sequence length:** 972 aa
- **1D identity (%):** 16.74
- **1D identity (%) [Gaps excluded]:** 24.73
- **1D identity - Alignment Gaps:** 353
- **1D aligned content (<aminoacid>:%):** {'D': 7.1, 'Y': 4.37, 'R': 5.46, 'E': 5.46, 'M': 1.64, 'G': 8.74, 'P': 9.29, 'K': 4.92, 'T': 6.01, 'A': 6.56, 'V': 8.74, 'F': 4.92, 'Q': 6.01, 'L': 10.93, 'H': 3.28, 'S': 2.73, 'N': 1.64, 'I': 1.64, 'C': 0.55}
- **Common reported functions (%):** 0.0
- **Common reported locations (%):** 37.5
- **Common reported processes (%):** 0.0

- **PDB ID:** 6ZYM
- **Chain:** B
- **Crystallized protein length:** 895 aa
- **Resolution:** 3.4 Å
- **b-phipsi:** 0.005335
- **w-rdist:** 0.405404
- **t-alpha:** 0.030516
- **Chemical similarity (Tanimoto Index) (%):** N/A
- **1D identity (%) [PDB]:** 3.42
- **1D identity (%) [Gaps excluded][PDB]:** 74.03
- **1D identity - Alignment Gaps [PDB]:** 1591
- **1D aligned content [PDB] (<aminoacid>:%):** {'V': 22.81, 'E': 3.51, 'P': 8.77, 'K': 12.28, 'H': 1.75, 'L': 12.28, 'T': 7.02, 'G': 8.77, 'A': 3.51, 'R': 1.75, 'D': 1.75, 'M': 1.75, 'C': 1.75, 'Q': 3.51, 'S': 3.51, 'N': 1.75, 'I': 1.75, 'F': 1.75}
- **2D identity (%) [PDB]:** 35.28
- **2D identity (%) [Gaps excluded][PDB]:** 84.27
- **2D identity - Alignment Gaps [PDB]:** 715
- **2D aligned content [PDB] (<2D-fold>:%):** {'.': 21.43, 'B': 0.23, 'E': 22.12, 'T': 14.75, 'H': 40.09, 'G': 1.38}
- **3D similarity (TM-Score) (%) [PDB]:** 1.02

- **Gene name:** EFTUD2
- **Entrez ID:** 9343
- **RefSeq ID:** NM\_004247
- **Transcript sequence length:** 4326
- **5-UTR|CDS|3-UTR identity (%):** 11.99 | 44.82 | 16.97
- **5-UTR|CDS|3-UTR identity (%) [Gaps excluded]:** 76.92 | 75.48 | 74.76
- **5-UTR|CDS|3-UTR identity [Alignment Gaps]:** 352 | 1403 | 3535
- **5-UTR aligned content (<base>:%):** {'G': 50.0, 'C': 30.0, 'T': 12.0, 'A': 8.0}
- **CDS aligned content (<base>:%):** {'T': 23.26, 'G': 26.42, 'A': 27.07, 'C': 23.26}
- **3-UTR aligned content (<base>:%):** {'T': 26.68, 'G': 25.52, 'A': 26.29, 'C': 21.52}

**Uniprot Description:**  
  
 Required for pre-mRNA splicing as component of the spliceosome, including pre-catalytic, catalytic and post-catalytic spliceosomal complexes (PubMed:28502770, PubMed:28781166, PubMed:28076346, PubMed:29361316, PubMed:30315277, PubMed:29360106, PubMed:29301961, PubMed:30705154). Component of the U5 snRNP and the U4/U6-U5 tri-snRNP complex, a building block of the spliceosome (PubMed:16723661).   
  
Component of the U5 snRNP and the U4/U6-U5 tri-snRNP complex, a building block of the spliceosome (PubMed:26912367, PubMed:16723661). The U4/U6-U5 tri-snRNP complex is composed of the U4, U6 and U5 snRNAs and at least PRPF3, PRPF4, PRPF6, PRPF8, PRPF31, SNRNP200, TXNL4A, SNRNP40, DDX23, CD2BP2, PPIH, SNU13, EFTUD2, SART1 and USP39 (PubMed:16723661, PubMed:26912367). Component of the pre-catalytic, catalytic and post-catalytic spliceosome complexes (PubMed:28502770, PubMed:28781166, PubMed:28076346, PubMed:29361316, PubMed:30315277, PubMed:29360106, PubMed:29301961, PubMed:30705154). Interacts with ERBB4 and PRPF8. Interacts with PIH1D1 (PubMed:24656813). Interacts with RPAP3 and URI1 in a ZNHIT2-dependent manner (PubMed:28561026). Interacts with NRDE2 (PubMed:30538148). Interacts with FAM50A (PubMed:32703943).   
  
 **Gene Ontology Information:**

Molecular Function

- GTP binding
- GTPase activity
- RNA binding
- U5 snRNA binding

Location

- Cajal body
- catalytic step 2 spliceosome
- cytosol
- intracellular membrane-bounded organelle
- membrane
- nuclear speck
- nucleoplasm
- nucleus
- U2-type catalytic step 2 spliceosome
- U2-type precatalytic spliceosome
- U4/U6 x U5 tri-snRNP complex

Biological process

- cellular response to xenobiotic stimulus
- mRNA splicing, via spliceosome
- response to cocaine

---

39

- **Protein name:** GMP synthase [glutamine-hydrolyzing]
- **Organism:** Homo sapiens
- **Uniprot Accession Number:** P49915
- **Protein sequence length:** 693 aa
- **1D identity (%):** 14.38
- **1D identity (%) [Gaps excluded]:** 21.54
- **1D identity - Alignment Gaps:** 310
- **1D aligned content (<aminoacid>:%):** {'L': 9.7, 'D': 4.48, 'H': 2.99, 'Y': 2.24, 'V': 14.18, 'R': 3.73, 'Q': 4.48, 'I': 7.46, 'F': 3.73, 'P': 6.72, 'K': 8.96, 'A': 0.75, 'G': 11.19, 'E': 4.48, 'T': 8.21, 'S': 3.73, 'N': 2.24, 'C': 0.75}
- **Common reported functions (%):** 0.0
- **Common reported locations (%):** 12.5
- **Common reported processes (%):** 0.0

- **PDB ID:** 2VXO
- **Chain:** A
- **Crystallized protein length:** 643 aa
- **Resolution:** 2.5 Å
- **b-phipsi:** 0.006129
- **w-rdist:** 0.369923
- **t-alpha:** 0.040688
- **Chemical similarity (Tanimoto Index) (%):** 78.49
- **1D identity (%) [PDB]:** 3.09
- **1D identity (%) [Gaps excluded][PDB]:** 59.46
- **1D identity - Alignment Gaps [PDB]:** 1349
- **1D aligned content [PDB] (<aminoacid>:%):** {'V': 22.73, 'I': 9.09, 'L': 13.64, 'P': 9.09, 'T': 9.09, 'A': 6.82, 'E': 2.27, 'R': 4.55, 'G': 2.27, 'Q': 6.82, 'K': 4.55, 'S': 4.55, 'N': 4.55}
- **2D identity (%) [PDB]:** 36.68
- **2D identity (%) [Gaps excluded][PDB]:** 90.72
- **2D identity - Alignment Gaps [PDB]:** 635
- **2D aligned content [PDB] (<2D-fold>:%):** {'.': 18.67, 'E': 27.11, 'H': 42.97, 'T': 8.95, 'G': 1.53, 'B': 0.77}
- **3D similarity (TM-Score) (%) [PDB]:** 21.33

- **Gene name:** GMPS
- **Entrez ID:** 8833
- **RefSeq ID:** NM\_003875
- **Transcript sequence length:** 8631
- **5-UTR|CDS|3-UTR identity (%):** 33.64 | 43.28 | 42.44
- **5-UTR|CDS|3-UTR identity (%) [Gaps excluded]:** 79.46 | 75.15 | 78.22
- **5-UTR|CDS|3-UTR identity [Alignment Gaps]:** 252 | 1256 | 3147
- **5-UTR aligned content (<base>:%):** {'G': 34.69, 'T': 12.93, 'C': 51.02, 'A': 1.36}
- **CDS aligned content (<base>:%):** {'A': 32.53, 'T': 25.98, 'G': 22.23, 'C': 19.27}
- **3-UTR aligned content (<base>:%):** {'A': 29.73, 'C': 16.34, 'T': 34.11, 'G': 19.83}

**Uniprot Description:**  
  
 Involved in the de novo synthesis of guanine nucleotides which are not only essential for DNA and RNA synthesis, but also provide GTP, which is involved in a number of cellular processes important for cell division.   
  
Homodimer.   
  
 **Gene Ontology Information:**

Molecular Function

- ATP binding
- GMP synthase (glutamine-hydrolyzing) activity
- GMP synthase activity

Location

- cytosol

Biological process

- glutamine metabolic process
- GMP biosynthetic process
- purine nucleobase biosynthetic process
- purine ribonucleoside monophosphate biosynthetic process

---

40

- **Protein name:** Bifunctional purine biosynthesis protein ATIC
- **Organism:** Homo sapiens
- **Uniprot Accession Number:** P31939
- **Protein sequence length:** 592 aa
- **1D identity (%):** 11.87
- **1D identity (%) [Gaps excluded]:** 23.76
- **1D identity - Alignment Gaps:** 485
- **1D aligned content (<aminoacid>:%):** {'G': 8.7, 'Q': 2.61, 'D': 4.35, 'N': 4.35, 'T': 7.83, 'A': 12.17, 'R': 4.35, 'S': 6.09, 'L': 8.7, 'M': 1.74, 'I': 4.35, 'K': 5.22, 'V': 6.09, 'P': 7.83, 'C': 3.48, 'E': 4.35, 'F': 2.61, 'Y': 2.61, 'W': 0.87, 'H': 1.74}
- **Common reported functions (%):** 0.0
- **Common reported locations (%):** 25.0
- **Common reported processes (%):** 0.0

- **PDB ID:** 1PL0
- **Chain:** C
- **Crystallized protein length:** 585 aa
- **Resolution:** 2.6 Å
- **b-phipsi:** 0.006413
- **w-rdist:** 0.767408
- **t-alpha:** 0.000783
- **Chemical similarity (Tanimoto Index) (%):** 68.69
- **1D identity (%) [PDB]:** 2.91
- **1D identity (%) [Gaps excluded][PDB]:** 65.57
- **1D identity - Alignment Gaps [PDB]:** 1314
- **1D aligned content [PDB] (<aminoacid>:%):** {'Q': 5.0, 'L': 20.0, 'V': 17.5, 'G': 10.0, 'K': 7.5, 'T': 10.0, 'Y': 2.5, 'A': 5.0, 'N': 12.5, 'C': 2.5, 'I': 2.5, 'P': 2.5, 'H': 2.5}
- **2D identity (%) [PDB]:** 37.25
- **2D identity (%) [Gaps excluded][PDB]:** 84.32
- **2D identity - Alignment Gaps [PDB]:** 556
- **2D aligned content [PDB] (<2D-fold>:%):** {'E': 17.52, 'T': 8.36, 'H': 56.33, '.': 16.98, 'G': 0.81}
- **3D similarity (TM-Score) (%) [PDB]:** 18.14

- **Gene name:** ATIC
- **Entrez ID:** 471
- **RefSeq ID:** NM\_004044
- **Transcript sequence length:** 1972
- **5-UTR|CDS|3-UTR identity (%):** 13.46 | 39.12 | 1.49
- **5-UTR|CDS|3-UTR identity (%) [Gaps excluded]:** 81.16 | 75.55 | 73.56
- **5-UTR|CDS|3-UTR identity [Alignment Gaps]:** 347 | 1387 | 4220
- **5-UTR aligned content (<base>:%):** {'G': 26.79, 'C': 58.93, 'A': 1.79, 'T': 12.5}
- **CDS aligned content (<base>:%):** {'T': 25.42, 'G': 23.11, 'C': 21.07, 'A': 30.4}
- **3-UTR aligned content (<base>:%):** {'T': 48.44, 'A': 28.12, 'C': 9.38, 'G': 14.06}

**Uniprot Description:**  
  
 Bifunctional enzyme that catalyzes the last two steps of purine biosynthesis (PubMed:11948179, PubMed:14756554). Acts as a transformylase that incorporates a formyl group to the AMP analog AICAR (5-amino-1-(5-phospho-beta-D-ribosyl)imidazole-4-carboxamide) to produce the intermediate formyl-AICAR (FAICAR) (PubMed:9378707, PubMed:11948179, PubMed:10985775). Can use both 10-formyldihydrofolate and 10-formyltetrahydrofolate as the formyl donor in this reaction (PubMed:10985775). Also catalyzes the cyclization of FAICAR to IMP (PubMed:11948179, PubMed:14756554). Is able to convert thio-AICAR to 6-mercaptopurine ribonucleotide, an inhibitor of purine biosynthesis used in the treatment of human leukemias (PubMed:10985775). Promotes insulin receptor/INSR autophosphorylation and is involved in INSR internalization (PubMed:25687571).   
  
Homodimer (PubMed:14756553, PubMed:14966129). Associates with internalized INSR complexes on Golgi/endosomal membranes (PubMed:25687571). Interacts with INSR; ATIC together with PRKAA2/AMPK2 and HACD3/PTPLAD1 is proposed to be part of a signaling network regulating INSR autophosphorylation and endocytosis (PubMed:25687571).   
  
 **Gene Ontology Information:**

Molecular Function

- cadherin binding
- IMP cyclohydrolase activity
- phosphoribosylaminoimidazolecarboxamide formyltransferase activity
- protein homodimerization activity

Location

- cytosol
- extracellular exosome
- membrane
- plasma membrane

Biological process

- 'de novo' AMP biosynthetic process
- 'de novo' IMP biosynthetic process
- 'de novo' XMP biosynthetic process
- animal organ regeneration
- brainstem development
- cellular response to interleukin-7
- cerebellum development
- cerebral cortex development
- dihydrofolate metabolic process
- GMP biosynthetic process
- nucleobase-containing compound metabolic process
- response to inorganic substance
- tetrahydrofolate biosynthetic process

---

41

- **Protein name:** Cytosolic phospholipase A2 delta
- **Organism:** Homo sapiens
- **Uniprot Accession Number:** Q86XP0
- **Protein sequence length:** 818 aa
- **1D identity (%):** 16.6
- **1D identity (%) [Gaps excluded]:** 22.71
- **1D identity - Alignment Gaps:** 261
- **1D aligned content (<aminoacid>:%):** {'P': 11.18, 'G': 11.18, 'Y': 3.11, 'R': 3.73, 'N': 2.48, 'A': 6.83, 'L': 16.77, 'V': 6.21, 'T': 7.45, 'K': 4.35, 'E': 3.73, 'D': 4.97, 'S': 6.83, 'F': 3.73, 'Q': 4.35, 'C': 0.62, 'M': 1.24, 'I': 0.62, 'H': 0.62}
- **Common reported functions (%):** 0.0
- **Common reported locations (%):** 25.0
- **Common reported processes (%):** 0.0

- **PDB ID:** 5IZ5
- **Chain:** B
- **Crystallized protein length:** 746 aa
- **Resolution:** 2.2 Å
- **b-phipsi:** 0.002765
- **w-rdist:** 0.420166
- **t-alpha:** 0.071205
- **Chemical similarity (Tanimoto Index) (%):** 99.09
- **1D identity (%) [PDB]:** 2.58
- **1D identity (%) [Gaps excluded][PDB]:** 83.33
- **1D identity - Alignment Gaps [PDB]:** 1502
- **1D aligned content [PDB] (<aminoacid>:%):** {'G': 7.5, 'A': 5.0, 'C': 5.0, 'Q': 12.5, 'V': 7.5, 'K': 10.0, 'N': 10.0, 'T': 2.5, 'S': 7.5, 'P': 7.5, 'L': 12.5, 'I': 5.0, 'H': 2.5, 'R': 2.5, 'F': 2.5}
- **2D identity (%) [PDB]:** 37.51
- **2D identity (%) [Gaps excluded][PDB]:** 87.11
- **2D identity - Alignment Gaps [PDB]:** 636
- **2D aligned content [PDB] (<2D-fold>:%):** {'E': 38.9, '.': 15.51, 'T': 10.5, 'H': 33.65, 'G': 1.43}
- **3D similarity (TM-Score) (%) [PDB]:** 24.35

- **Gene name:** PLA2G4D
- **Entrez ID:** 283748
- **RefSeq ID:** NM\_178034
- **Transcript sequence length:** 4266
- **5-UTR|CDS|3-UTR identity (%):** 15.98 | 45.08 | 21.71
- **5-UTR|CDS|3-UTR identity (%) [Gaps excluded]:** 72.53 | 75.72 | 76.09
- **5-UTR|CDS|3-UTR identity [Alignment Gaps]:** 322 | 1279 | 3331
- **5-UTR aligned content (<base>:%):** {'A': 12.12, 'G': 34.85, 'C': 42.42, 'T': 10.61}
- **CDS aligned content (<base>:%):** {'A': 23.51, 'T': 20.42, 'G': 27.86, 'C': 28.21}
- **3-UTR aligned content (<base>:%):** {'G': 18.08, 'T': 38.64, 'C': 19.47, 'A': 23.81}

**Uniprot Description:**  
  
 Calcium-dependent phospholipase A2 that selectively hydrolyzes glycerophospholipids in the sn-2 position (PubMed:14709560). Has a preference for linoleic acid at the sn-2 position (PubMed:14709560). N/A   
  
 **Gene Ontology Information:**

Molecular Function

- calcium ion binding
- calcium-dependent phospholipase A2 activity
- calcium-dependent phospholipid binding
- phospholipase A1 activity

Location

- cytosol
- membrane

Biological process

- fatty acid metabolic process
- glycerophospholipid catabolic process
- phosphatidylglycerol acyl-chain remodeling
- phosphatidylinositol acyl-chain remodeling

---

42

- **Protein name:** Staphylococcal nuclease domain-containing protein 1
- **Organism:** Homo sapiens
- **Uniprot Accession Number:** Q7KZF4
- **Protein sequence length:** 910 aa
- **1D identity (%):** 15.35
- **1D identity (%) [Gaps excluded]:** 21.63
- **1D identity - Alignment Gaps:** 301
- **1D aligned content (<aminoacid>:%):** {'A': 5.66, 'S': 1.89, 'Q': 4.4, 'P': 10.06, 'T': 4.4, 'V': 10.06, 'G': 10.69, 'I': 2.52, 'D': 3.14, 'R': 8.18, 'E': 6.29, 'M': 1.89, 'L': 11.32, 'K': 5.66, 'W': 1.26, 'H': 2.52, 'F': 4.4, 'N': 1.26, 'C': 1.89, 'Y': 2.52}
- **Common reported functions (%):** 25.0
- **Common reported locations (%):** 50.0
- **Common reported processes (%):** 8.33

- **PDB ID:** 3BDL
- **Chain:** A
- **Crystallized protein length:** 537 aa
- **Resolution:** 1.9 Å
- **b-phipsi:** 0.000826
- **w-rdist:** 0.472372
- **t-alpha:** 0.059702
- **Chemical similarity (Tanimoto Index) (%):** 95.82
- **1D identity (%) [PDB]:** 2.53
- **1D identity (%) [Gaps excluded][PDB]:** 80.95
- **1D identity - Alignment Gaps [PDB]:** 1304
- **1D aligned content [PDB] (<aminoacid>:%):** {'P': 14.71, 'R': 17.65, 'G': 5.88, 'L': 8.82, 'T': 2.94, 'V': 17.65, 'K': 2.94, 'I': 2.94, 'A': 2.94, 'H': 2.94, 'F': 2.94, 'Q': 2.94, 'D': 5.88, 'Y': 2.94, 'N': 2.94, 'E': 2.94}
- **2D identity (%) [PDB]:** 37.85
- **2D identity (%) [Gaps excluded][PDB]:** 86.94
- **2D identity - Alignment Gaps [PDB]:** 546
- **2D aligned content [PDB] (<2D-fold>:%):** {'.': 12.57, 'E': 38.52, 'T': 13.93, 'H': 33.33, 'G': 1.64}
- **3D similarity (TM-Score) (%) [PDB]:** 18.75

- **Gene name:** SND1
- **Entrez ID:** 27044
- **RefSeq ID:** NM\_014390
- **Transcript sequence length:** 3448
- **5-UTR|CDS|3-UTR identity (%):** 21.95 | 47.01 | 7.49
- **5-UTR|CDS|3-UTR identity (%) [Gaps excluded]:** 76.15 | 74.49 | 76.87
- **5-UTR|CDS|3-UTR identity [Alignment Gaps]:** 321 | 1203 | 3964
- **5-UTR aligned content (<base>:%):** {'C': 56.57, 'G': 29.29, 'T': 14.14}
- **CDS aligned content (<base>:%):** {'A': 27.59, 'T': 21.85, 'G': 26.35, 'C': 24.2}
- **3-UTR aligned content (<base>:%):** {'G': 30.09, 'T': 30.09, 'C': 20.36, 'A': 19.45}

**Uniprot Description:**  
  
 Endonuclease that mediates miRNA decay of both protein-free and AGO2-loaded miRNAs (PubMed:28546213, PubMed:18453631). As part of its function in miRNA decay, regulates mRNAs involved in G1-to-S phase transition (PubMed:28546213). Functions as a bridging factor between STAT6 and the basal transcription factor (PubMed:12234934). Plays a role in PIM1 regulation of MYB activity (PubMed:9809063). Functions as a transcriptional coactivator for STAT5 (By similarity).   
  
Forms a ternary complex with STAT6 and POLR2A (PubMed:12234934). Associates with the RNA-induced silencing complex (RISC) (PubMed:14508492, PubMed:28546213). Interacts with the RISC components AGO2, FMR1 and TNRC6A (PubMed:14508492, PubMed:28546213). Interacts with GTF2E1 and GTF2E2 (PubMed:7651391). Interacts with PIM1 (PubMed:9809063). Interacts with STAT5 (By similarity). Interacts with SYT11 (via C2 2 domain); the interaction with SYT11 is direct (By similarity).   
  
 **Gene Ontology Information:**

Molecular Function

- cadherin binding
- endonuclease activity
- endonuclease activity, active with either ribo- or deoxyribonucleic acids and producing 3'-phosphomonoesters
- nuclease activity
- RISC complex binding
- RNA binding
- endoribonuclease activity
- transcription coregulator activity

Location

- cytosol
- dense body
- extracellular exosome
- melanosome
- membrane
- nucleus
- RISC complex

Biological process

- miRNA catabolic process
- mRNA catabolic process
- osteoblast differentiation
- regulation of cell cycle process
- gene silencing by RNA

---

43

- **Protein name:** Small nuclear ribonucleoprotein Sm D1
- **Organism:** Homo sapiens
- **Uniprot Accession Number:** P62314
- **Protein sequence length:** 119 aa
- **1D identity (%):** 2.99
- **1D identity (%) [Gaps excluded]:** 23.64
- **1D identity - Alignment Gaps:** 760
- **1D aligned content (<aminoacid>:%):** {'K': 11.54, 'L': 15.38, 'T': 7.69, 'V': 15.38, 'M': 3.85, 'P': 19.23, 'N': 3.85, 'I': 3.85, 'G': 15.38, 'R': 3.85}
- **Common reported functions (%):** 0.0
- **Common reported locations (%):** 25.0
- **Common reported processes (%):** 0.0

- **PDB ID:** 6ID0
- **Chain:** C
- **Crystallized protein length:** 888 aa
- **Resolution:** 2.9 Å
- **b-phipsi:** 0.00773
- **w-rdist:** 0.492456
- **t-alpha:** 0.005477
- **Chemical similarity (Tanimoto Index) (%):** N/A
- **1D identity (%) [PDB]:** 2.68
- **1D identity (%) [Gaps excluded][PDB]:** 80.36
- **1D identity - Alignment Gaps [PDB]:** 1626
- **1D aligned content [PDB] (<aminoacid>:%):** {'V': 24.44, 'L': 17.78, 'I': 4.44, 'P': 4.44, 'G': 8.89, 'K': 11.11, 'T': 6.67, 'A': 4.44, 'E': 2.22, 'R': 2.22, 'D': 2.22, 'M': 2.22, 'C': 2.22, 'Q': 2.22, 'S': 2.22, 'N': 2.22}
- **2D identity (%) [PDB]:** 35.9
- **2D identity (%) [Gaps excluded][PDB]:** 85.24
- **2D identity - Alignment Gaps [PDB]:** 708
- **2D aligned content [PDB] (<2D-fold>:%):** {'.': 19.36, 'E': 25.74, 'T': 12.53, 'H': 41.0, 'B': 0.68, 'G': 0.68}
- **3D similarity (TM-Score) (%) [PDB]:** 26.0

- **Gene name:** SNRPD1
- **Entrez ID:** 6632
- **RefSeq ID:** NM\_006938
- **Transcript sequence length:** 4858
- **5-UTR|CDS|3-UTR identity (%):** 16.43 | 9.55 | 47.57
- **5-UTR|CDS|3-UTR identity (%) [Gaps excluded]:** 71.13 | 76.45 | 75.32
- **5-UTR|CDS|3-UTR identity [Alignment Gaps]:** 323 | 2292 | 1957
- **5-UTR aligned content (<base>:%):** {'C': 30.43, 'T': 20.29, 'G': 46.38, 'A': 2.9}
- **CDS aligned content (<base>:%):** {'A': 34.8, 'T': 20.0, 'G': 28.0, 'C': 17.2}
- **3-UTR aligned content (<base>:%):** {'G': 21.33, 'C': 15.12, 'T': 34.74, 'A': 28.81}

**Uniprot Description:**  
  
 Plays role in pre-mRNA splicing as core component of the SMN-Sm complex that mediates spliceosomal snRNP assembly and as component of the spliceosomal U1, U2, U4 and U5 small nuclear ribonucleoproteins (snRNPs), the building blocks of the spliceosome (PubMed:11991638, PubMed:18984161, PubMed:19325628, PubMed:23333303, PubMed:25555158, PubMed:26912367, PubMed:28502770, PubMed:28781166, PubMed:28076346). Component of both the pre-catalytic spliceosome B complex and activated spliceosome C complexes (PubMed:11991638, PubMed:26912367, PubMed:28502770, PubMed:28781166, PubMed:28076346). Is also a component of the minor U12 spliceosome (PubMed:15146077). May act as a charged protein scaffold to promote snRNP assembly or strengthen snRNP-snRNP interactions through non-specific electrostatic contacts with RNA (Probable).   
  
Core component of the spliceosomal U1, U2, U4 and U5 small nuclear ribonucleoproteins (snRNPs), the building blocks of the spliceosome (PubMed:11991638, PubMed:19325628, PubMed:25555158, PubMed:26912367, PubMed:28502770, PubMed:28781166, PubMed:28076346). Most spliceosomal snRNPs contain a common set of Sm proteins, SNRPB, SNRPD1, SNRPD2, SNRPD3, SNRPE, SNRPF and SNRPG that assemble in a heptameric protein ring on the Sm site of the small nuclear RNA to form the core snRNP (PubMed:10025403, PubMed:19325628, PubMed:21113136, PubMed:25555158, PubMed:26912367, PubMed:28502770, PubMed:28781166, PubMed:28076346). Component of the U1 snRNP (PubMed:19325628, PubMed:21113136, PubMed:25555158). The U1 snRNP is composed of the U1 snRNA and the 7 core Sm proteins SNRPB, SNRPD1, SNRPD2, SNRPD3, SNRPE, SNRPF and SNRPG, and at least three U1 snRNP-specific proteins SNRNP70/U1-70K, SNRPA/U1-A and SNRPC/U1-C (PubMed:19325628, PubMed:21113136, PubMed:25555158). Component of the U4/U6-U5 tri-snRNP complex composed of the U4, U6 and U5 snRNAs and at least PRPF3, PRPF4, PRPF6, PRPF8, PRPF31, SNRNP200, TXNL4A, SNRNP40, SNRPB, SNRPD1, SNRPD2, SNRPD3, SNRPE, SNRPF, SNRPG, DDX23, CD2BP2, PPIH, SNU13, EFTUD2, SART1 and USP39, plus LSM2, LSM3, LSM4, LSM5, LSM6, LSM7 and LSM8 (PubMed:26912367). Component of the U11/U12 snRNPs that are part of the U12-type spliceosome (PubMed:15146077). Part of the SMN-Sm complex that contains SMN1, GEMIN2/SIP1, DDX20/GEMIN3, GEMIN4, GEMIN5, GEMIN6, GEMIN7, GEMIN8, STRAP/UNRIP and the Sm proteins SNRPB, SNRPD1, SNRPD2, SNRPD3, SNRPE, SNRPF and SNRPG; catalyzes core snRNPs assembly. Forms a 6S pICln-Sm complex composed of CLNS1A/pICln, SNRPD1, SNRPD2, SNRPE, SNRPF and SNRPG; ring-like structure where CLNS1A/pICln mimics additional Sm proteins and which is unable to assemble into the core snRNP.   
  
 **Gene Ontology Information:**

Molecular Function

- RNA binding
- U1 snRNP binding

Location

- catalytic step 2 spliceosome
- commitment complex
- cytosol
- methylosome
- nucleoplasm
- nucleus
- pICln-Sm protein complex
- precatalytic spliceosome
- small nuclear ribonucleoprotein complex
- SMN-Sm protein complex
- spliceosomal complex
- spliceosomal tri-snRNP complex
- U1 snRNP
- U12-type spliceosomal complex
- U2 snRNP
- U2-type catalytic step 2 spliceosome
- U2-type precatalytic spliceosome
- U4 snRNP
- U4/U6 x U5 tri-snRNP complex
- U5 snRNP

Biological process

- 7-methylguanosine cap hypermethylation
- mRNA splicing, via spliceosome
- RNA splicing
- spliceosomal complex assembly
- spliceosomal snRNP assembly
- U2-type prespliceosome assembly

---

44

- **Protein name:** Astrotactin-2
- **Organism:** Homo sapiens
- **Uniprot Accession Number:** O75129
- **Protein sequence length:** 1339 aa
- **1D identity (%):** 17.06
- **1D identity (%) [Gaps excluded]:** 29.91
- **1D identity - Alignment Gaps:** 602
- **1D aligned content (<aminoacid>:%):** {'M': 0.84, 'A': 3.35, 'G': 11.3, 'P': 12.55, 'L': 11.72, 'R': 9.21, 'V': 5.44, 'T': 4.6, 'D': 4.6, 'E': 5.86, 'S': 7.53, 'Q': 3.35, 'W': 0.84, 'Y': 4.6, 'N': 1.26, 'F': 2.93, 'K': 3.77, 'C': 3.35, 'I': 2.51, 'H': 0.42}
- **Common reported functions (%):** 0.0
- **Common reported locations (%):** 12.5
- **Common reported processes (%):** 0.0

- **PDB ID:** 5J68
- **Chain:** A
- **Crystallized protein length:** 559 aa
- **Resolution:** 5.22 Å
- **b-phipsi:** 0.004841
- **w-rdist:** 0.491276
- **t-alpha:** 0.017516
- **Chemical similarity (Tanimoto Index) (%):** 77.42
- **1D identity (%) [PDB]:** 2.97
- **1D identity (%) [Gaps excluded][PDB]:** 64.52
- **1D identity - Alignment Gaps [PDB]:** 1287
- **1D aligned content [PDB] (<aminoacid>:%):** {'F': 2.5, 'A': 5.0, 'D': 5.0, 'L': 12.5, 'Q': 2.5, 'T': 5.0, 'Y': 7.5, 'H': 10.0, 'V': 10.0, 'R': 10.0, 'S': 15.0, 'I': 2.5, 'P': 2.5, 'K': 2.5, 'G': 7.5}
- **2D identity (%) [PDB]:** 29.33
- **2D identity (%) [Gaps excluded][PDB]:** 85.32
- **2D identity - Alignment Gaps [PDB]:** 689
- **2D aligned content [PDB] (<2D-fold>:%):** {'.': 16.56, 'T': 12.01, 'E': 32.47, 'H': 37.66, 'G': 0.97, 'B': 0.32}
- **3D similarity (TM-Score) (%) [PDB]:** 18.16

- **Gene name:** ASTN2
- **Entrez ID:** 23245
- **RefSeq ID:** NM\_198188
- **Transcript sequence length:** 1799
- **5-UTR|CDS|3-UTR identity (%):** 30.38 | 29.06 | 6.0
- **5-UTR|CDS|3-UTR identity (%) [Gaps excluded]:** 73.66 | 75.89 | 79.51
- **5-UTR|CDS|3-UTR identity [Alignment Gaps]:** 265 | 1684 | 4006
- **5-UTR aligned content (<base>:%):** {'C': 39.42, 'G': 40.15, 'A': 5.84, 'T': 14.6}
- **CDS aligned content (<base>:%):** {'T': 23.08, 'C': 25.47, 'A': 27.49, 'G': 23.96}
- **3-UTR aligned content (<base>:%):** {'A': 23.46, 'C': 16.92, 'T': 36.92, 'G': 22.69}

**Uniprot Description:**  
  
 Mediates recycling of the neuronal cell adhesion molecule ASTN1 to the anterior pole of the cell membrane in migrating neurons. Promotes ASTN1 internalization and intracellular transport of endocytosed ASTN1 (By similarity). Selectively binds inositol-4,5-bisphosphate, inositol-3,4,5-trisphosphate and inositol-1,3,4,5-tetrakisphosphate, suggesting it is recruited to membranes that contain lipids with a phosphoinositide headgroup (Ref.6).   
  
Interacts with ASTN1; the interaction is not calcium-dependent.   
  
 **Gene Ontology Information:**

Molecular Function

- calcium ion binding
- inositol 1,3,4,5 tetrakisphosphate binding

Location

- cell cortex
- cell pole
- clathrin-coated vesicle
- early endosome
- endosome
- late endosome
- membrane
- perikaryon

Biological process

- establishment of body hair planar orientation
- negative regulation of protein localization to cell surface
- neuron cell-cell adhesion
- neuron migration
- protein localization to cell surface
- protein transport

---

45

- **Protein name:** Probable 2-oxoglutarate dehydrogenase E1 component DHKTD1, mitochondrial
- **Organism:** Homo sapiens
- **Uniprot Accession Number:** Q96HY7
- **Protein sequence length:** 919 aa
- **1D identity (%):** 16.57
- **1D identity (%) [Gaps excluded]:** 23.18
- **1D identity - Alignment Gaps:** 296
- **1D aligned content (<aminoacid>:%):** {'A': 8.14, 'S': 4.07, 'R': 5.23, 'G': 12.21, 'L': 9.3, 'Q': 6.98, 'V': 6.98, 'Y': 2.91, 'K': 4.07, 'P': 8.72, 'E': 6.98, 'D': 2.91, 'H': 5.23, 'F': 3.49, 'T': 5.81, 'W': 0.58, 'M': 1.74, 'N': 1.74, 'C': 1.74, 'I': 1.16}
- **Common reported functions (%):** 0.0
- **Common reported locations (%):** 0.0
- **Common reported processes (%):** 0.0

- **PDB ID:** 6SY1
- **Chain:** A
- **Crystallized protein length:** 859 aa
- **Resolution:** 1.87 Å
- **b-phipsi:** 0.012893
- **w-rdist:** 0.408531
- **t-alpha:** 0.004694
- **Chemical similarity (Tanimoto Index) (%):** 79.85
- **1D identity (%) [PDB]:** 1.98
- **1D identity (%) [Gaps excluded][PDB]:** 75.0
- **1D identity - Alignment Gaps [PDB]:** 1623
- **1D aligned content [PDB] (<aminoacid>:%):** {'Q': 6.06, 'L': 15.15, 'E': 6.06, 'A': 6.06, 'G': 6.06, 'N': 3.03, 'V': 9.09, 'P': 9.09, 'D': 6.06, 'S': 9.09, 'I': 3.03, 'T': 3.03, 'R': 6.06, 'H': 3.03, 'M': 3.03, 'Y': 3.03, 'F': 3.03}
- **2D identity (%) [PDB]:** 34.31
- **2D identity (%) [Gaps excluded][PDB]:** 86.98
- **2D identity - Alignment Gaps [PDB]:** 743
- **2D aligned content [PDB] (<2D-fold>:%):** {'.': 18.53, 'H': 51.31, 'T': 10.69, 'E': 17.34, 'G': 2.14}
- **3D similarity (TM-Score) (%) [PDB]:** 24.24

- **Gene name:** DHTKD1
- **Entrez ID:** 55526
- **RefSeq ID:** NM\_018706
- **Transcript sequence length:** 5173
- **5-UTR|CDS|3-UTR identity (%):** 13.24 | 46.14 | 32.13
- **5-UTR|CDS|3-UTR identity (%) [Gaps excluded]:** 73.97 | 73.71 | 77.13
- **5-UTR|CDS|3-UTR identity [Alignment Gaps]:** 335 | 1230 | 2726
- **5-UTR aligned content (<base>:%):** {'G': 37.04, 'C': 48.15, 'T': 11.11, 'A': 3.7}
- **CDS aligned content (<base>:%):** {'A': 26.1, 'T': 22.87, 'G': 27.29, 'C': 23.73}
- **3-UTR aligned content (<base>:%):** {'T': 29.25, 'G': 23.72, 'A': 28.98, 'C': 18.05}

**Uniprot Description:**  
  
 The 2-oxoglutarate dehydrogenase complex catalyzes the overall conversion of 2-oxoglutarate to succinyl-CoA and CO(2). It contains multiple copies of three enzymatic components: 2-oxoglutarate dehydrogenase (E1), dihydrolipoamide succinyltransferase (E2) and lipoamide dehydrogenase (E3) (By similarity). N/A   
  
 **Gene Ontology Information:**

Molecular Function

- oxoglutarate dehydrogenase (succinyl-transferring) activity
- thiamine pyrophosphate binding

Location

- mitochondrial matrix
- mitochondrion

Biological process

- generation of precursor metabolites and energy
- glycolytic process
- hematopoietic progenitor cell differentiation
- tricarboxylic acid cycle

---

46

- **Protein name:** DNA topoisomerase 1
- **Organism:** Homo sapiens
- **Uniprot Accession Number:** P11387
- **Protein sequence length:** 765 aa
- **1D identity (%):** 14.55
- **1D identity (%) [Gaps excluded]:** 22.64
- **1D identity - Alignment Gaps:** 354
- **1D aligned content (<aminoacid>:%):** {'R': 4.86, 'L': 11.81, 'H': 3.47, 'K': 11.81, 'D': 10.42, 'E': 5.56, 'N': 5.56, 'G': 6.25, 'P': 7.64, 'V': 5.56, 'Y': 2.78, 'F': 2.78, 'M': 1.39, 'A': 5.56, 'T': 2.08, 'C': 1.39, 'Q': 5.56, 'S': 2.08, 'I': 2.78, 'W': 0.69}
- **Common reported functions (%):** 0.0
- **Common reported locations (%):** 25.0
- **Common reported processes (%):** 0.0

- **PDB ID:** 1SEU
- **Chain:** A
- **Crystallized protein length:** 564 aa
- **Resolution:** 3.0 Å
- **b-phipsi:** 0.017751
- **w-rdist:** 0.365916
- **t-alpha:** 0.004694
- **Chemical similarity (Tanimoto Index) (%):** 92.02
- **1D identity (%) [PDB]:** 3.12
- **1D identity (%) [Gaps excluded][PDB]:** 60.87
- **1D identity - Alignment Gaps [PDB]:** 1277
- **1D aligned content [PDB] (<aminoacid>:%):** {'Q': 14.29, 'A': 14.29, 'E': 9.52, 'V': 2.38, 'Y': 4.76, 'F': 2.38, 'K': 14.29, 'L': 11.9, 'P': 4.76, 'H': 2.38, 'G': 2.38, 'N': 4.76, 'I': 2.38, 'T': 2.38, 'R': 4.76, 'D': 2.38}
- **2D identity (%) [PDB]:** 31.61
- **2D identity (%) [Gaps excluded][PDB]:** 83.98
- **2D identity - Alignment Gaps [PDB]:** 641
- **2D aligned content [PDB] (<2D-fold>:%):** {'.': 20.62, 'T': 22.46, 'E': 13.54, 'H': 42.15, 'B': 0.31, 'G': 0.92}
- **3D similarity (TM-Score) (%) [PDB]:** 17.88

- **Gene name:** TOP1
- **Entrez ID:** 7150
- **RefSeq ID:** NM\_003286
- **Transcript sequence length:** 3734
- **5-UTR|CDS|3-UTR identity (%):** 34.85 | 41.02 | 15.37
- **5-UTR|CDS|3-UTR identity (%) [Gaps excluded]:** 73.56 | 75.99 | 75.32
- **5-UTR|CDS|3-UTR identity [Alignment Gaps]:** 231 | 1460 | 3619
- **5-UTR aligned content (<base>:%):** {'C': 54.9, 'A': 2.61, 'G': 32.03, 'T': 10.46}
- **CDS aligned content (<base>:%):** {'A': 36.59, 'T': 19.22, 'G': 25.44, 'C': 18.75}
- **3-UTR aligned content (<base>:%):** {'C': 12.59, 'A': 31.04, 'G': 17.45, 'T': 38.91}

**Uniprot Description:**  
  
 Releases the supercoiling and torsional tension of DNA introduced during the DNA replication and transcription by transiently cleaving and rejoining one strand of the DNA duplex. Introduces a single-strand break via transesterification at a target site in duplex DNA. The scissile phosphodiester is attacked by the catalytic tyrosine of the enzyme, resulting in the formation of a DNA-(3'-phosphotyrosyl)-enzyme intermediate and the expulsion of a 5'-OH DNA strand. The free DNA strand then rotates around the intact phosphodiester bond on the opposing strand, thus removing DNA supercoils. Finally, in the religation step, the DNA 5'-OH attacks the covalent intermediate to expel the active-site tyrosine and restore the DNA phosphodiester backbone (By similarity). Regulates the alternative splicing of tissue factor (F3) pre-mRNA in endothelial cells. Involved in the circadian transcription of the core circadian clock component ARNTL/BMAL1 by altering the chromatin structure around the ROR response elements (ROREs) on the ARNTL/BMAL1 promoter.   
  
Monomer. Interacts with ERCC6 (PubMed:26030138).   
  
 **Gene Ontology Information:**

Molecular Function

- ATP binding
- chromatin binding
- DNA binding
- DNA binding, bending
- DNA topoisomerase type I (single strand cut, ATP-independent) activity
- double-stranded DNA binding
- protein domain specific binding
- protein serine/threonine kinase activity
- RNA binding
- RNA polymerase II cis-regulatory region sequence-specific DNA binding
- single-stranded DNA binding
- supercoiled DNA binding

Location

- chromosome
- fibrillar center
- male germ cell nucleus
- nucleolus
- nucleoplasm
- nucleus
- P-body
- perikaryon
- protein-DNA complex

Biological process

- chromatin remodeling
- chromosome segregation
- circadian regulation of gene expression
- circadian rhythm
- DNA replication
- DNA topological change
- embryonic cleavage
- peptidyl-serine phosphorylation
- phosphorylation
- programmed cell death
- response to xenobiotic stimulus

---

47

- **Protein name:** Endoplasmic reticulum aminopeptidase 1
- **Organism:** Homo sapiens
- **Uniprot Accession Number:** Q9NZ08
- **Protein sequence length:** 941 aa
- **1D identity (%):** 15.54
- **1D identity (%) [Gaps excluded]:** 21.02
- **1D identity - Alignment Gaps:** 270
- **1D aligned content (<aminoacid>:%):** {'P': 9.94, 'L': 13.04, 'T': 10.56, 'V': 8.7, 'N': 3.73, 'H': 3.11, 'I': 3.73, 'G': 9.94, 'S': 6.83, 'E': 3.73, 'Q': 4.35, 'R': 1.86, 'F': 3.11, 'A': 3.73, 'K': 3.73, 'Y': 3.11, 'M': 1.24, 'D': 3.11, 'C': 2.48}
- **Common reported functions (%):** 0.0
- **Common reported locations (%):** 37.5
- **Common reported processes (%):** 0.0

- **PDB ID:** 6MGQ
- **Chain:** A
- **Crystallized protein length:** 861 aa
- **Resolution:** 2.92 Å
- **b-phipsi:** 0.006772
- **w-rdist:** 0.226983
- **t-alpha:** 0.064163
- **Chemical similarity (Tanimoto Index) (%):** N/A
- **1D identity (%) [PDB]:** 3.03
- **1D identity (%) [Gaps excluded][PDB]:** 79.37
- **1D identity - Alignment Gaps [PDB]:** 1588
- **1D aligned content [PDB] (<aminoacid>:%):** {'D': 4.0, 'L': 12.0, 'R': 2.0, 'K': 10.0, 'A': 4.0, 'G': 10.0, 'P': 10.0, 'Q': 8.0, 'C': 4.0, 'F': 4.0, 'E': 4.0, 'H': 2.0, 'M': 4.0, 'T': 6.0, 'Y': 2.0, 'V': 10.0, 'I': 4.0}
- **2D identity (%) [PDB]:** 34.67
- **2D identity (%) [Gaps excluded][PDB]:** 87.09
- **2D identity - Alignment Gaps [PDB]:** 738
- **2D aligned content [PDB] (<2D-fold>:%):** {'.': 14.59, 'E': 27.06, 'T': 10.82, 'H': 45.41, 'G': 2.12}
- **3D similarity (TM-Score) (%) [PDB]:** 25.42

- **Gene name:** ERAP1
- **Entrez ID:** 51752
- **RefSeq ID:** N/A
- **Sequence length:** N/A
- **5-UTR|CDS|3-UTR identity (%):** N/A | N/A | N/A
- **5-UTR|CDS|3-UTR identity (%) [Gaps excluded]:** N/A | N/A | N/A
- **5-UTR|CDS|3-UTR identity [Alignment Gaps]:** N/A | N/A | N/A
- **5-UTR aligned content (<base>:%):** N/A
- **CDS aligned content (<base>:%):** N/A
- **3-UTR aligned content (<base>:%):** N/A

**Uniprot Description:**  
  
 Aminopeptidase that plays a central role in peptide trimming, a step required for the generation of most HLA class I-binding peptides. Peptide trimming is essential to customize longer precursor peptides to fit them to the correct length required for presentation on MHC class I molecules. Strongly prefers substrates 9-16 residues long. Rapidly degrades 13-mer to a 9-mer and then stops. Preferentially hydrolyzes the residue Leu and peptides with a hydrophobic C-terminus, while it has weak activity toward peptides with charged C-terminus. May play a role in the inactivation of peptide hormones. May be involved in the regulation of blood pressure through the inactivation of angiotensin II and/or the generation of bradykinin in the kidney.   
  
Monomer. May also exist as a heterodimer; with ERAP2. Interacts with RBMX.   
  
 **Gene Ontology Information:**

Molecular Function

- aminopeptidase activity
- endopeptidase activity
- interleukin-1, type II receptor binding
- interleukin-6 receptor binding
- metalloaminopeptidase activity
- metalloexopeptidase activity
- peptide binding
- zinc ion binding

Location

- cytoplasm
- cytosol
- endoplasmic reticulum
- endoplasmic reticulum lumen
- endoplasmic reticulum membrane
- extracellular exosome
- extracellular region
- extracellular space
- membrane

Biological process

- adaptive immune response
- angiogenesis
- antigen processing and presentation of endogenous peptide antigen via MHC class I
- antigen processing and presentation of peptide antigen via MHC class I
- fat cell differentiation
- membrane protein ectodomain proteolysis
- peptide catabolic process
- positive regulation of angiogenesis
- proteolysis
- regulation of blood pressure
- regulation of innate immune response
- response to bacterium

---

48

- **Protein name:** Transforming growth factor-beta-induced protein ig-h3
- **Organism:** Homo sapiens
- **Uniprot Accession Number:** Q15582
- **Protein sequence length:** 683 aa
- **1D identity (%):** 13.66
- **1D identity (%) [Gaps excluded]:** 22.84
- **1D identity - Alignment Gaps:** 388
- **1D aligned content (<aminoacid>:%):** {'M': 0.76, 'A': 8.33, 'L': 13.64, 'G': 12.12, 'P': 9.09, 'H': 1.52, 'N': 6.82, 'R': 6.06, 'I': 3.03, 'Y': 2.27, 'T': 5.3, 'D': 5.3, 'K': 3.79, 'S': 3.79, 'E': 3.03, 'V': 9.85, 'Q': 2.27, 'C': 1.52, 'F': 1.52}
- **Common reported functions (%):** 0.0
- **Common reported locations (%):** 0.0
- **Common reported processes (%):** 0.0

- **PDB ID:** 7ASG
- **Chain:** A
- **Crystallized protein length:** 592 aa
- **Resolution:** 2.0 Å
- **b-phipsi:** 0.002925
- **w-rdist:** 0.883423
- **t-alpha:** 0.003928
- **Chemical similarity (Tanimoto Index) (%):** 96.82
- **1D identity (%) [PDB]:** 2.37
- **1D identity (%) [Gaps excluded][PDB]:** 67.35
- **1D identity - Alignment Gaps [PDB]:** 1344
- **1D aligned content [PDB] (<aminoacid>:%):** {'P': 3.03, 'L': 9.09, 'G': 6.06, 'V': 18.18, 'K': 12.12, 'I': 9.09, 'N': 6.06, 'H': 9.09, 'Y': 3.03, 'D': 3.03, 'R': 15.15, 'E': 3.03, 'T': 3.03}
- **2D identity (%) [PDB]:** 36.41
- **2D identity (%) [Gaps excluded][PDB]:** 87.71
- **2D identity - Alignment Gaps [PDB]:** 596
- **2D aligned content [PDB] (<2D-fold>:%):** {'.': 14.02, 'E': 35.04, 'T': 11.32, 'H': 37.74, 'B': 0.27, 'G': 1.62}
- **3D similarity (TM-Score) (%) [PDB]:** 16.41

- **Gene name:** TGFBI
- **Entrez ID:** 7045
- **RefSeq ID:** NM\_000358
- **Transcript sequence length:** 2712
- **5-UTR|CDS|3-UTR identity (%):** 10.87 | 42.51 | 8.03
- **5-UTR|CDS|3-UTR identity (%) [Gaps excluded]:** 81.82 | 74.44 | 75.8
- **5-UTR|CDS|3-UTR identity [Alignment Gaps]:** 359 | 1266 | 3943
- **5-UTR aligned content (<base>:%):** {'C': 62.22, 'A': 2.22, 'G': 22.22, 'T': 13.33}
- **CDS aligned content (<base>:%):** {'A': 27.57, 'T': 20.56, 'G': 25.58, 'C': 26.29}
- **3-UTR aligned content (<base>:%):** {'C': 18.64, 'T': 27.97, 'G': 23.73, 'A': 29.66}

**Uniprot Description:**  
  
 Plays a role in cell adhesion (PubMed:8024701). May play a role in cell-collagen interactions (By similarity).   
  
Binds to type I, II, and IV collagens.   
  
 **Gene Ontology Information:**

Molecular Function

- cell adhesion molecule binding
- collagen binding
- extracellular matrix binding
- extracellular matrix structural constituent
- identical protein binding
- integrin binding

Location

- basement membrane
- collagen-containing extracellular matrix
- extracellular exosome
- extracellular matrix
- extracellular region
- extracellular space
- plasma membrane
- trans-Golgi network

Biological process

- angiogenesis
- cell adhesion
- cell population proliferation
- chondrocyte differentiation
- extracellular matrix organization
- localization
- negative regulation of cell adhesion
- response to stimulus
- visual perception

---

49

- **Protein name:** Ubiquitin carboxyl-terminal hydrolase 7
- **Organism:** Homo sapiens
- **Uniprot Accession Number:** Q93009
- **Protein sequence length:** 1102 aa
- **1D identity (%):** 16.87
- **1D identity (%) [Gaps excluded]:** 24.16
- **1D identity - Alignment Gaps:** 349
- **1D aligned content (<aminoacid>:%):** {'M': 0.51, 'G': 7.18, 'P': 8.21, 'R': 8.21, 'L': 10.77, 'H': 4.62, 'D': 8.72, 'E': 3.08, 'V': 8.21, 'I': 4.1, 'Q': 5.64, 'T': 5.13, 'F': 4.62, 'W': 1.03, 'S': 3.08, 'A': 3.08, 'C': 3.08, 'Y': 4.1, 'K': 5.13, 'N': 1.54}
- **Common reported functions (%):** 0.0
- **Common reported locations (%):** 25.0
- **Common reported processes (%):** 0.0

- **PDB ID:** 5FWI
- **Chain:** C
- **Crystallized protein length:** 662 aa
- **Resolution:** 3.4 Å
- **b-phipsi:** 0.001437
- **w-rdist:** 0.973743
- **t-alpha:** 0.004717
- **Chemical similarity (Tanimoto Index) (%):** 99.54
- **1D identity (%) [PDB]:** 4.01
- **1D identity (%) [Gaps excluded][PDB]:** 61.29
- **1D identity - Alignment Gaps [PDB]:** 1328
- **1D aligned content [PDB] (<aminoacid>:%):** {'F': 5.26, 'M': 1.75, 'C': 3.51, 'V': 5.26, 'L': 12.28, 'D': 3.51, 'I': 5.26, 'Q': 8.77, 'N': 3.51, 'E': 8.77, 'K': 10.53, 'T': 8.77, 'R': 5.26, 'G': 1.75, 'Y': 5.26, 'P': 3.51, 'A': 5.26, 'S': 1.75}
- **2D identity (%) [PDB]:** 36.56
- **2D identity (%) [Gaps excluded][PDB]:** 83.51
- **2D identity - Alignment Gaps [PDB]:** 592
- **2D aligned content [PDB] (<2D-fold>:%):** {'.': 18.44, 'T': 15.06, 'H': 35.58, 'E': 30.13, 'G': 0.78}
- **3D similarity (TM-Score) (%) [PDB]:** 17.19

- **Gene name:** USP7
- **Entrez ID:** 7874
- **RefSeq ID:** NM\_003470
- **Transcript sequence length:** 5831
- **5-UTR|CDS|3-UTR identity (%):** 44.31 | 46.18 | 26.93
- **5-UTR|CDS|3-UTR identity (%) [Gaps excluded]:** 80.44 | 75.84 | 75.28
- **5-UTR|CDS|3-UTR identity [Alignment Gaps]:** 296 | 1433 | 2926
- **5-UTR aligned content (<base>:%):** {'G': 42.47, 'T': 2.74, 'C': 53.42, 'A': 1.37}
- **CDS aligned content (<base>:%):** {'A': 32.15, 'T': 24.53, 'G': 24.0, 'C': 19.33}
- **3-UTR aligned content (<base>:%):** {'T': 36.1, 'C': 17.6, 'A': 23.63, 'G': 22.66}

**Uniprot Description:**  
  
 Hydrolase that deubiquitinates target proteins such as FOXO4, p53/TP53, MDM2, ERCC6, DNMT1, UHRF1, PTEN, KMT2E/MLL5 and DAXX (PubMed:11923872, PubMed:15053880, PubMed:16964248, PubMed:18716620, PubMed:25283148, PubMed:26678539, PubMed:28655758). Together with DAXX, prevents MDM2 self-ubiquitination and enhances the E3 ligase activity of MDM2 towards p53/TP53, thereby promoting p53/TP53 ubiquitination and proteasomal degradation (PubMed:15053880, PubMed:16845383, PubMed:18566590, PubMed:20153724). Deubiquitinates p53/TP53, preventing degradation of p53/TP53, and enhances p53/TP53-dependent transcription regulation, cell growth repression and apoptosis (PubMed:25283148). Deubiquitinates p53/TP53 and MDM2 and strongly stabilizes p53/TP53 even in the presence of excess MDM2, and also induces p53/TP53-dependent cell growth repression and apoptosis (PubMed:11923872). Deubiquitination of FOXO4 in presence of hydrogen peroxide is not dependent on p53/TP53 and inhibits FOXO4-induced transcriptional activity (PubMed:16964248). In association with DAXX, is involved in the deubiquitination and translocation of PTEN from the nucleus to the cytoplasm, both processes that are counteracted by PML (PubMed:18716620). Deubiquitinates KMT2E/MLL5 preventing KMT2E/MLL5 proteasomal-mediated degradation (PubMed:26678539). Involved in cell proliferation during early embryonic development. Involved in transcription-coupled nucleotide excision repair (TC-NER) in response to UV damage: recruited to DNA damage sites following interaction with KIAA1530/UVSSA and promotes deubiquitination of ERCC6, preventing UV-induced degradation of ERCC6 (PubMed:22466611, PubMed:22466612). Involved in maintenance of DNA methylation via its interaction with UHRF1 and DNMT1: acts by mediating deubiquitination of UHRF1 and DNMT1, preventing their degradation and promoting DNA methylation by DNMT1 (PubMed:21745816, PubMed:22411829). Deubiquitinates alkylation repair enzyme ALKBH3. OTUD4 recruits USP7 and USP9X to stabilize ALKBH3, thereby promoting the repair of alkylated DNA lesions (PubMed:25944111). Acts as a chromatin regulator via its association with the Polycomb group (PcG) multiprotein PRC1-like complex; may act by deubiquitinating components of the PRC1-like complex (PubMed:20601937). Able to mediate deubiquitination of histone H2B; it is however unsure whether this activity takes place in vivo (PubMed:20601937). Exhibits a preference towards 'Lys-48'-linked ubiquitin chains (PubMed:22689415). Increases regulatory T-cells (Treg) suppressive capacity by deubiquitinating and stabilizing the transcription factor FOXP3 which is crucial for Treg cell function (PubMed:23973222). Plays a role in the maintenance of the circadian clock periodicity via deubiquitination and stabilization of the CRY1 and CRY2 proteins (PubMed:27123980). Deubiquitinates REST, thereby stabilizing REST and promoting the maintenance of neural progenitor cells (PubMed:21258371). Deubiquitinates SIRT7, inhibiting SIRT7 histone deacetylase activity and regulating gluconeogenesis (PubMed:28655758).   
  
Monomer. Homodimer. Part of a complex with DAXX, MDM2, RASSF1 and USP7 (PubMed:18566590). Part of a complex with DAXX, MDM2 and USP7 (PubMed:16845383). Interacts with MDM2; the interaction is independent of p53/TP53. Interacts with DAXX; the interaction is direct and independent of MDM2 and p53/TP53 (PubMed:16845383). Component of a complex composed of KMT2E/MLL5 (isoform 3), OGT (isoform 1) and USP7; the complex stabilizes KMT2E/MLL5, preventing KMT2E/MLL5 ubiquitination and proteosomal-mediated degradation (PubMed:26678539). Interacts (via MATH domain) with KMT2E/MLL5 isoform 3 (PubMed:26678539). Interacts with OGT isoform 1 (PubMed:26678539). Interacts with FOXO4; the interaction is enhanced in presence of hydrogen peroxide and occurs independently of p53/TP53 (PubMed:16964248). Interacts with p53/TP53; the interaction is enhanced in response to DNA damage (PubMed:25283148). Interacts with TSPYL5; this impairs interaction with p53/TP53 (PubMed:21170034). Interacts with PTEN; the interaction is direct (PubMed:18716620). Interacts with ATXN1 and the strength of interaction is influenced by the length of the poly-Gln region in ATXN1 (PubMed:12093161). A weaker interaction seen with mutants having longer poly-Gln regions (PubMed:12093161). Interacts with KIAA1530/UVSSA (PubMed:22466611, PubMed:22466612). Interacts with ABRAXAS2; the interaction is direct (PubMed:25283148). Identified in a complex with TP53/p53 and ABRAXAS2 (PubMed:25283148). Interacts with MEX3C and antagonizes its ability to degrade mRNA (PubMed:22863774). Interacts with DNMT1 and UHRF1 (PubMed:21745816, PubMed:22411829). Interacts with FOXP3 (PubMed:23973222). Interacts (via MATH domain) with RNF220. Associated component of the Polycomb group (PcG) multiprotein PRC1-like complex (PubMed:20601937). Interacts with EPOP (By similarity). Interacts with OTUD4 and USP9X; the interaction is direct (PubMed:25944111). Interacts with CRY2 (PubMed:27123980). Interacts with REST (PubMed:21258371). Interacts with ERCC6 (PubMed:26030138).   
  
 **Gene Ontology Information:**

Molecular Function

- thiol-dependent ubiquitin-specific protease activity
- cysteine-type endopeptidase activity
- ubiquitinyl hydrolase activity
- Lys48-specific deubiquitinase activity
- p53 binding

Location

- chromosome
- cytosol
- nuclear body
- nucleoplasm
- nucleus
- PML body
- protein-containing complex

Biological process

- symbiont-mediated disruption of host cell PML body
- monoubiquitinated protein deubiquitination
- hypermethylation of CpG island
- negative regulation of gluconeogenesis
- negative regulation of NF-kappaB transcription factor activity
- negative regulation of proteasomal ubiquitin-dependent protein catabolic process
- positive regulation of DNA demethylation
- protein deubiquitination
- protein stabilization
- protein ubiquitination
- regulation of circadian rhythm
- regulation of DNA-binding transcription factor activity
- regulation of protein stability
- regulation of retrograde transport, endosome to Golgi
- regulation of signal transduction by p53 class mediator
- regulation of telomere capping
- rhythmic process
- transcription-coupled nucleotide-excision repair
- ubiquitin-dependent protein catabolic process

---

50

- **Protein name:** UBX domain-containing protein 6
- **Organism:** Homo sapiens
- **Uniprot Accession Number:** Q9BZV1
- **Protein sequence length:** 441 aa
- **1D identity (%):** 10.78
- **1D identity (%) [Gaps excluded]:** 30.86
- **1D identity - Alignment Gaps:** 628
- **1D aligned content (<aminoacid>:%):** {'E': 6.73, 'K': 5.77, 'S': 5.77, 'G': 10.58, 'P': 11.54, 'L': 8.65, 'H': 3.85, 'A': 8.65, 'Q': 6.73, 'R': 8.65, 'D': 4.81, 'V': 6.73, 'Y': 2.88, 'T': 1.92, 'C': 1.92, 'I': 1.92, 'N': 1.92, 'F': 0.96}
- **Common reported functions (%):** 0.0
- **Common reported locations (%):** 37.5
- **Common reported processes (%):** 0.0

- **PDB ID:** 8FCN
- **Chain:** E
- **Crystallized protein length:** 721 aa
- **Resolution:** 2.95 Å
- **b-phipsi:** 0.006247
- **w-rdist:** 0.156726
- **t-alpha:** 0.212832
- **Chemical similarity (Tanimoto Index) (%):** 79.39
- **1D identity (%) [PDB]:** 2.19
- **1D identity (%) [Gaps excluded][PDB]:** 76.74
- **1D identity - Alignment Gaps [PDB]:** 1467
- **1D aligned content [PDB] (<aminoacid>:%):** {'L': 18.18, 'Q': 6.06, 'I': 6.06, 'V': 15.15, 'P': 6.06, 'G': 9.09, 'K': 12.12, 'T': 12.12, 'A': 3.03, 'R': 3.03, 'M': 3.03, 'N': 3.03, 'S': 3.03}
- **2D identity (%) [PDB]:** 42.41
- **2D identity (%) [Gaps excluded][PDB]:** 87.75
- **2D identity - Alignment Gaps [PDB]:** 541
- **2D aligned content [PDB] (<2D-fold>:%):** {'.': 15.09, 'E': 25.68, 'T': 16.89, 'H': 40.77, 'G': 1.35, 'B': 0.23}
- **3D similarity (TM-Score) (%) [PDB]:** 2.35

- **Gene name:** UBXN6
- **Entrez ID:** 80700
- **RefSeq ID:** N/A
- **Sequence length:** N/A
- **5-UTR|CDS|3-UTR identity (%):** N/A | N/A | N/A
- **5-UTR|CDS|3-UTR identity (%) [Gaps excluded]:** N/A | N/A | N/A
- **5-UTR|CDS|3-UTR identity [Alignment Gaps]:** N/A | N/A | N/A
- **5-UTR aligned content (<base>:%):** N/A
- **CDS aligned content (<base>:%):** N/A
- **3-UTR aligned content (<base>:%):** N/A

**Uniprot Description:**  
  
 May negatively regulate the ATPase activity of VCP, an ATP-driven segregase that associates with different cofactors to control a wide variety of cellular processes (PubMed:26475856). As a cofactor of VCP, it may play a role in the transport of CAV1 to lysosomes for degradation (PubMed:21822278, PubMed:23335559). It may also play a role in endoplasmic reticulum-associated degradation (ERAD) of misfolded proteins (PubMed:19275885). Together with VCP and other cofactors, it may play a role in macroautophagy, regulating for instance the clearance of damaged lysosomes (PubMed:27753622).   
  
Interacts with VCP through the PUB domain (via C-terminus) and VIM motif (via N-terminus); the interaction is direct (PubMed:18656546, PubMed:19174149, PubMed:21896481, PubMed:21822278, PubMed:26475856). Forms a ternary complex with CAV1 and VCP (PubMed:21822278). Interacts with SYVN1 (PubMed:18656546). Interacts with HERPUD1 (PubMed:18656546). Interacts with VCPKMT (PubMed:23349634). May interact with DERL1 (PubMed:19275885). Interacts with PLAA, VCP and YOD1; may form a complex involved in macroautophagy (PubMed:27753622). Interacts with LMAN1 (PubMed:22337587).   
  
 **Gene Ontology Information:**

Molecular Function

- ADP binding
- ATP binding
- ATPase activity
- BAT3 complex binding
- deubiquitinase activator activity
- identical protein binding
- K48-linked polyubiquitin modification-dependent protein binding
- lipid binding
- MHC class I protein binding
- polyubiquitin modification-dependent protein binding
- protein domain specific binding
- protein phosphatase binding
- RNA binding
- ubiquitin protein ligase binding
- ubiquitin-like protein ligase binding
- ubiquitin-specific protease binding

Location

- ATPase complex
- azurophil granule lumen
- cytoplasm
- cytoplasmic stress granule
- cytosol
- Derlin-1 retrotranslocation complex
- endoplasmic reticulum
- endoplasmic reticulum membrane
- extracellular exosome
- extracellular region
- ficolin-1-rich granule lumen
- glutamatergic synapse
- intracellular membrane-bounded organelle
- lipid droplet
- nucleoplasm
- nucleus
- perinuclear region of cytoplasm
- proteasome complex
- protein-containing complex
- secretory granule lumen
- site of double-strand break
- VCP-NPL4-UFD1 AAA ATPase complex
- VCP-NSFL1C complex

Biological process

- activation of cysteine-type endopeptidase activity involved in apoptotic process
- aggresome assembly
- ATP metabolic process
- autophagosome maturation
- autophagy
- cellular response to arsenite ion
- cellular response to DNA damage stimulus
- cellular response to heat
- DNA repair
- double-strand break repair
- endoplasmic reticulum stress-induced pre-emptive quality control
- endoplasmic reticulum to Golgi vesicle-mediated transport
- endoplasmic reticulum unfolded protein response
- endosome to lysosome transport via multivesicular body sorting pathway
- ER-associated misfolded protein catabolic process
- ERAD pathway
- establishment of protein localization
- flavin adenine dinucleotide catabolic process
- interstrand cross-link repair
- macroautophagy
- mitotic spindle disassembly
- NADH metabolic process
- negative regulation of smoothened signaling pathway
- positive regulation of ATP biosynthetic process
- positive regulation of canonical Wnt signaling pathway
- positive regulation of Lys63-specific deubiquitinase activity
- positive regulation of mitochondrial membrane potential
- positive regulation of oxidative phosphorylation
- positive regulation of proteasomal ubiquitin-dependent protein catabolic process
- positive regulation of protein catabolic process
- positive regulation of protein K63-linked deubiquitination
- positive regulation of protein-containing complex assembly
- proteasomal protein catabolic process
- proteasome-mediated ubiquitin-dependent protein catabolic process
- protein ubiquitination
- protein-DNA covalent cross-linking repair
- regulation of aerobic respiration
- regulation of apoptotic process
- regulation of protein localization to chromatin
- regulation of synapse organization
- retrograde protein transport, ER to cytosol
- stress granule disassembly
- translesion synthesis
- ubiquitin-dependent ERAD pathway
- viral genome replication

---

51

- **Protein name:** DNA polymerase alpha catalytic subunit
- **Organism:** Homo sapiens
- **Uniprot Accession Number:** P09884
- **Protein sequence length:** 1462 aa
- **1D identity (%):** 14.62
- **1D identity (%) [Gaps excluded]:** 26.2
- **1D identity - Alignment Gaps:** 659
- **1D aligned content (<aminoacid>:%):** {'P': 10.09, 'R': 4.13, 'G': 10.09, 'L': 8.72, 'V': 9.17, 'D': 9.63, 'I': 4.59, 'F': 4.59, 'K': 7.34, 'T': 3.67, 'A': 5.05, 'H': 1.83, 'E': 5.05, 'Y': 3.67, 'C': 2.75, 'Q': 4.13, 'M': 0.46, 'S': 1.83, 'N': 3.21}
- **Common reported functions (%):** 0.0
- **Common reported locations (%):** 25.0
- **Common reported processes (%):** 0.0

- **PDB ID:** 8D96
- **Chain:** C
- **Crystallized protein length:** 869 aa
- **Resolution:** 3.35 Å
- **b-phipsi:** 0.004875
- **w-rdist:** 0.439294
- **t-alpha:** 0.028169
- **Chemical similarity (Tanimoto Index) (%):** 81.3
- **1D identity (%) [PDB]:** 2.65
- **1D identity (%) [Gaps excluded][PDB]:** 76.79
- **1D identity - Alignment Gaps [PDB]:** 1566
- **1D aligned content [PDB] (<aminoacid>:%):** {'Q': 11.63, 'C': 2.33, 'V': 16.28, 'K': 9.3, 'N': 9.3, 'S': 6.98, 'P': 9.3, 'L': 13.95, 'I': 2.33, 'A': 2.33, 'G': 6.98, 'R': 2.33, 'F': 2.33, 'D': 2.33, 'T': 2.33}
- **2D identity (%) [PDB]:** 45.86
- **2D identity (%) [Gaps excluded][PDB]:** 90.11
- **2D identity - Alignment Gaps [PDB]:** 546
- **2D aligned content [PDB] (<2D-fold>:%):** {'.': 14.12, 'E': 26.47, 'T': 18.82, 'H': 40.59}
- **3D similarity (TM-Score) (%) [PDB]:** 23.38

- **Gene name:** POLA1
- **Entrez ID:** 5422
- **RefSeq ID:** NM\_016937
- **Transcript sequence length:** 5469
- **5-UTR|CDS|3-UTR identity (%):** 6.98 | 39.25 | 14.75
- **5-UTR|CDS|3-UTR identity (%) [Gaps excluded]:** 63.64 | 79.15 | 77.35
- **5-UTR|CDS|3-UTR identity [Alignment Gaps]:** 357 | 2351 | 3617
- **5-UTR aligned content (<base>:%):** {'G': 46.43, 'C': 25.0, 'T': 17.86, 'A': 10.71}
- **CDS aligned content (<base>:%):** {'A': 31.26, 'T': 24.7, 'G': 24.54, 'C': 19.51}
- **3-UTR aligned content (<base>:%):** {'G': 20.33, 'A': 28.53, 'T': 33.99, 'C': 17.15}

**Uniprot Description:**  
  
 Catalytic subunit of the DNA polymerase alpha complex (also known as the alpha DNA polymerase-primase complex) which plays an essential role in the initiation of DNA synthesis. During the S phase of the cell cycle, the DNA polymerase alpha complex (composed of a catalytic subunit POLA1, a regulatory subunit POLA2 and two primase subunits PRIM1 and PRIM2) is recruited to DNA at the replicative forks via direct interactions with MCM10 and WDHD1. The primase subunit of the polymerase alpha complex initiates DNA synthesis by oligomerising short RNA primers on both leading and lagging strands. These primers are initially extended by the polymerase alpha catalytic subunit and subsequently transferred to polymerase delta and polymerase epsilon for processive synthesis on the lagging and leading strand, respectively. The reason this transfer occurs is because the polymerase alpha has limited processivity and lacks intrinsic 3' exonuclease activity for proofreading error, and therefore is not well suited for replicating long complexes. In the cytosol, responsible for a substantial proportion of the physiological concentration of cytosolic RNA:DNA hybrids, which are necessary to prevent spontaneous activation of type I interferon responses (PubMed:27019227).   
  
Component of the alpha DNA polymerase complex (also known as the alpha DNA polymerase-primase complex) consisting of four subunits: the catalytic subunit POLA1, the regulatory subunit POLA2, and the primase complex subunits PRIM1 and PRIM2 respectively (PubMed:9705292). Interacts with PARP1; this interaction functions as part of the control of replication fork progression (PubMed:9518481). Interacts with MCM10 and WDHD1; these interactions recruit the polymerase alpha complex to the pre-replicative complex bound to DNA (PubMed:19608746). Interacts with RPA1; this interaction stabilizes the replicative complex and reduces the misincorporation rate of DNA polymerase alpha by acting as a fidelity clamp (PubMed:9214288).   
  
 **Gene Ontology Information:**

Molecular Function

- chromatin binding
- DNA binding
- DNA replication origin binding
- DNA-directed DNA polymerase activity
- nucleotide binding
- protein kinase binding
- single-stranded DNA binding
- zinc ion binding

Location

- alpha DNA polymerase:primase complex
- cytosol
- nuclear envelope
- nuclear matrix
- nucleolus
- nucleoplasm
- nucleus

Biological process

- DNA repair
- DNA replication
- DNA replication initiation
- DNA replication, synthesis of RNA primer
- DNA strand elongation involved in DNA replication
- DNA synthesis involved in DNA repair
- double-strand break repair via nonhomologous end joining
- lagging strand elongation
- leading strand elongation
- mitotic DNA replication initiation
- regulation of type I interferon production

---

52

- **Protein name:** Ubiquitin-like modifier-activating enzyme 6
- **Organism:** Homo sapiens
- **Uniprot Accession Number:** A0AVT1
- **Protein sequence length:** 1052 aa
- **1D identity (%):** 17.5
- **1D identity (%) [Gaps excluded]:** 25.64
- **1D identity - Alignment Gaps:** 361
- **1D aligned content (<aminoacid>:%):** {'M': 2.01, 'E': 5.53, 'P': 10.55, 'G': 8.54, 'A': 5.03, 'S': 4.02, 'K': 7.04, 'D': 5.53, 'R': 4.52, 'V': 8.04, 'T': 7.54, 'F': 3.52, 'N': 2.01, 'H': 3.52, 'Q': 4.52, 'L': 8.54, 'I': 4.52, 'C': 3.02, 'Y': 1.51, 'W': 0.5}
- **Common reported functions (%):** 0.0
- **Common reported locations (%):** 25.0
- **Common reported processes (%):** 0.0

- **PDB ID:** 7SOL
- **Chain:** C
- **Crystallized protein length:** 970 aa
- **Resolution:** 2.25 Å
- **b-phipsi:** 0.004814
- **w-rdist:** 0.373023
- **t-alpha:** 0.143975
- **Chemical similarity (Tanimoto Index) (%):** 99.17
- **1D identity (%) [PDB]:** 4.23
- **1D identity (%) [Gaps excluded][PDB]:** 72.0
- **1D identity - Alignment Gaps [PDB]:** 1602
- **1D aligned content [PDB] (<aminoacid>:%):** {'M': 4.17, 'K': 9.72, 'L': 12.5, 'T': 5.56, 'G': 6.94, 'Q': 2.78, 'I': 5.56, 'V': 13.89, 'P': 11.11, 'A': 5.56, 'E': 1.39, 'D': 2.78, 'S': 5.56, 'N': 2.78, 'H': 2.78, 'R': 2.78, 'F': 2.78, 'Y': 1.39}
- **2D identity (%) [PDB]:** 41.04
- **2D identity (%) [Gaps excluded][PDB]:** 92.93
- **2D identity - Alignment Gaps [PDB]:** 698
- **2D aligned content [PDB] (<2D-fold>:%):** {'.': 16.76, 'T': 25.93, 'E': 17.74, 'H': 38.79, 'B': 0.19, 'G': 0.58}
- **3D similarity (TM-Score) (%) [PDB]:** 22.33

- **Gene name:** UBA6
- **Entrez ID:** 55236
- **RefSeq ID:** NM\_018227
- **Transcript sequence length:** 9540
- **5-UTR|CDS|3-UTR identity (%):** 5.94 | 44.72 | 42.38
- **5-UTR|CDS|3-UTR identity (%) [Gaps excluded]:** 72.73 | 77.5 | 76.07
- **5-UTR|CDS|3-UTR identity [Alignment Gaps]:** 371 | 1541 | 3024
- **5-UTR aligned content (<base>:%):** {'C': 37.5, 'T': 8.33, 'G': 54.17}
- **CDS aligned content (<base>:%):** {'A': 31.92, 'T': 26.58, 'G': 21.85, 'C': 19.64}
- **3-UTR aligned content (<base>:%):** {'G': 18.49, 'A': 29.69, 'T': 37.4, 'C': 14.41}

**Uniprot Description:**  
  
 Activates ubiquitin by first adenylating its C-terminal glycine residue with ATP, and thereafter linking this residue to the side chain of a cysteine residue in E1, yielding a ubiquitin-E1 thioester and free AMP. Specific for ubiquitin, does not activate ubiquitin-like peptides. Differs from UBE1 in its specificity for substrate E2 charging. Does not charge cell cycle E2s, such as CDC34. Essential for embryonic development. Required for UBD/FAT10 conjugation. Isoform 2 may play a key role in ubiquitin system and may influence spermatogenesis and male fertility.   
  
Forms a thioester with UBD in cells stimulated with tumor necrosis factor-alpha (TNFa) and interferon-gamma (IFNg) (PubMed:17889673, PubMed:25422469).   
  
 **Gene Ontology Information:**

Molecular Function

- ATP binding
- FAT10 activating enzyme activity
- nucleotidyltransferase activity
- thiosulfate sulfurtransferase activity
- ubiquitin activating enzyme activity

Location

- cytoplasm
- cytosol

Biological process

- amygdala development
- dendritic spine development
- hippocampus development
- learning
- locomotory behavior
- protein modification by small protein conjugation
- protein ubiquitination
- ubiquitin-dependent protein catabolic process

---

53

- **Protein name:** Tapasin
- **Organism:** Homo sapiens
- **Uniprot Accession Number:** O15533
- **Protein sequence length:** 448 aa
- **1D identity (%):** 10.25
- **1D identity (%) [Gaps excluded]:** 24.87
- **1D identity - Alignment Gaps:** 545
- **1D aligned content (<aminoacid>:%):** {'K': 2.11, 'L': 16.84, 'A': 8.42, 'T': 5.26, 'S': 8.42, 'E': 5.26, 'P': 14.74, 'V': 5.26, 'H': 2.11, 'R': 6.32, 'W': 1.05, 'C': 3.16, 'Q': 3.16, 'G': 8.42, 'D': 3.16, 'N': 1.05, 'F': 2.11, 'Y': 3.16}
- **Common reported functions (%):** 0.0
- **Common reported locations (%):** 12.5
- **Common reported processes (%):** 0.0

- **PDB ID:** 7QNG
- **Chain:** B
- **Crystallized protein length:** 464 aa
- **Resolution:** 2.7 Å
- **b-phipsi:** 0.008522
- **w-rdist:** 0.416694
- **t-alpha:** 0.010277
- **Chemical similarity (Tanimoto Index) (%):** 96.82
- **1D identity (%) [PDB]:** 1.71
- **1D identity (%) [Gaps excluded][PDB]:** 75.86
- **1D identity - Alignment Gaps [PDB]:** 1255
- **1D aligned content [PDB] (<aminoacid>:%):** {'S': 4.55, 'N': 18.18, 'C': 4.55, 'L': 9.09, 'K': 9.09, 'I': 9.09, 'A': 4.55, 'G': 4.55, 'V': 13.64, 'P': 9.09, 'Q': 9.09, 'R': 4.55}
- **2D identity (%) [PDB]:** 36.34
- **2D identity (%) [Gaps excluded][PDB]:** 91.94
- **2D identity - Alignment Gaps [PDB]:** 569
- **2D aligned content [PDB] (<2D-fold>:%):** {'E': 20.18, '.': 15.5, 'T': 25.15, 'H': 38.3, 'G': 0.88}
- **3D similarity (TM-Score) (%) [PDB]:** 17.39

- **Gene name:** TAPBP
- **Entrez ID:** 2923
- **RefSeq ID:** NM\_172209
- **Transcript sequence length:** 3189
- **5-UTR|CDS|3-UTR identity (%):** 6.73 | 23.48 | 25.9
- **5-UTR|CDS|3-UTR identity (%) [Gaps excluded]:** 72.97 | 75.63 | 73.43
- **5-UTR|CDS|3-UTR identity [Alignment Gaps]:** 364 | 1932 | 3039
- **5-UTR aligned content (<base>:%):** {'G': 51.85, 'A': 14.81, 'T': 7.41, 'C': 25.93}
- **CDS aligned content (<base>:%):** {'T': 20.21, 'A': 19.45, 'G': 30.09, 'C': 30.24}
- **3-UTR aligned content (<base>:%):** {'G': 25.0, 'A': 23.6, 'C': 21.71, 'T': 29.69}

**Uniprot Description:**  
  
 Involved in the association of MHC class I with transporter associated with antigen processing (TAP) and in the assembly of MHC class I with peptide (peptide loading).   
  
Heterodimer with PDIA3; disulfide-linked. Obligatory mediator for the interaction between newly assembled MHC class I molecules, calreticulin, PDIA3 and TAP. Up to 4 MHC class I/tapasin complexes bind to 1 TAP (PubMed:19119025, PubMed:21263072, PubMed:26611325). Interacts with HLA-G-B2M complex; this interaction is required for loading of high affinity peptides.   
  
 **Gene Ontology Information:**

Molecular Function

- cysteine-type endopeptidase activity
- disulfide oxidoreductase activity
- identical protein binding
- phospholipase C activity
- protein disulfide isomerase activity
- protein disulfide oxidoreductase activity
- RNA binding

Location

- cell surface
- endoplasmic reticulum
- endoplasmic reticulum lumen
- extracellular exosome
- extracellular space
- focal adhesion
- melanosome
- MHC class I peptide loading complex
- nucleus
- phagocytic vesicle
- recycling endosome membrane
- Tapasin-ERp57 complex

Biological process

- cellular response to interleukin-7
- extrinsic apoptotic signaling pathway
- peptide antigen assembly with MHC class I protein complex
- platelet aggregation
- positive regulation of extrinsic apoptotic signaling pathway
- protein folding
- protein folding in endoplasmic reticulum
- response to endoplasmic reticulum stress

---

54

- **Protein name:** Protein transport protein Sec24A
- **Organism:** Homo sapiens
- **Uniprot Accession Number:** O95486
- **Protein sequence length:** 1093 aa
- **1D identity (%):** 16.45
- **1D identity (%) [Gaps excluded]:** 22.6
- **1D identity - Alignment Gaps:** 308
- **1D aligned content (<aminoacid>:%):** {'M': 1.08, 'G': 10.22, 'P': 15.05, 'A': 4.84, 'S': 6.45, 'L': 11.83, 'Q': 5.91, 'V': 5.91, 'Y': 2.69, 'R': 6.99, 'T': 5.91, 'F': 5.38, 'N': 4.84, 'H': 3.23, 'C': 1.08, 'K': 4.3, 'D': 2.69, 'E': 0.54, 'I': 1.08}
- **Common reported functions (%):** 0.0
- **Common reported locations (%):** 12.5
- **Common reported processes (%):** 0.0

- **PDB ID:** 3EGD
- **Chain:** B
- **Crystallized protein length:** 731 aa
- **Resolution:** 2.7 Å
- **b-phipsi:** 0.00635
- **w-rdist:** 0.652761
- **t-alpha:** 0.003912
- **Chemical similarity (Tanimoto Index) (%):** 95.89
- **1D identity (%) [PDB]:** 2.68
- **1D identity (%) [Gaps excluded][PDB]:** 78.85
- **1D identity - Alignment Gaps [PDB]:** 1480
- **1D aligned content [PDB] (<aminoacid>:%):** {'V': 14.63, 'T': 7.32, 'S': 4.88, 'P': 7.32, 'Q': 9.76, 'L': 12.2, 'N': 4.88, 'K': 7.32, 'I': 4.88, 'G': 9.76, 'R': 2.44, 'F': 4.88, 'A': 4.88, 'D': 2.44, 'H': 2.44}
- **2D identity (%) [PDB]:** 34.04
- **2D identity (%) [Gaps excluded][PDB]:** 83.44
- **2D identity - Alignment Gaps [PDB]:** 666
- **2D aligned content [PDB] (<2D-fold>:%):** {'.': 20.1, 'H': 39.69, 'E': 24.8, 'T': 15.4}
- **3D similarity (TM-Score) (%) [PDB]:** 22.86

- **Gene name:** SEC24A
- **Entrez ID:** 10802
- **RefSeq ID:** NM\_021982
- **Transcript sequence length:** 6389
- **5-UTR|CDS|3-UTR identity (%):** 36.42 | 44.94 | 39.81
- **5-UTR|CDS|3-UTR identity (%) [Gaps excluded]:** 73.8 | 75.25 | 75.65
- **5-UTR|CDS|3-UTR identity [Alignment Gaps]:** 235 | 1480 | 2204
- **5-UTR aligned content (<base>:%):** {'A': 4.14, 'C': 54.44, 'G': 32.54, 'T': 8.88}
- **CDS aligned content (<base>:%):** {'A': 28.65, 'T': 25.44, 'G': 21.62, 'C': 24.29}
- **3-UTR aligned content (<base>:%):** {'A': 32.07, 'T': 39.04, 'G': 16.52, 'C': 12.37}

**Uniprot Description:**  
  
 Component of the coat protein complex II (COPII) which promotes the formation of transport vesicles from the endoplasmic reticulum (ER). The coat has two main functions, the physical deformation of the endoplasmic reticulum membrane into vesicles and the selection of cargo molecules for their transport to the Golgi complex (PubMed:20427317, PubMed:17499046, PubMed:18843296). Plays a central role in cargo selection within the COPII complex and together with SEC24B may have a different specificity compared to SEC24C and SEC24D. May package preferentially cargos with cytoplasmic DxE or LxxLE motifs and may also recognize conformational epitopes (PubMed:17499046, PubMed:18843296).   
  
COPII is composed of at least five proteins: the Sec23/24 complex, the Sec13/31 complex and Sar1 (PubMed:17499046). Interacts with TMED2 (PubMed:20427317). Interacts (as part of the Sec23/24 complex) with SEC22B; recruits SEC22B into COPII-coated vesicles for its transport from the endoplasmic reticulum to the Golgi (PubMed:17499046). Interacts with TMEM39A (PubMed:31806350). Interacts with SACM1L; this interaction is reduced in the absence of TMEM39A (PubMed:31806350).   
  
 **Gene Ontology Information:**

Molecular Function

- SNARE binding
- zinc ion binding

Location

- COPII vesicle coat
- cytosol
- endoplasmic reticulum exit site
- endoplasmic reticulum membrane
- ER to Golgi transport vesicle membrane

Biological process

- cholesterol homeostasis
- COPII-coated vesicle cargo loading
- endoplasmic reticulum to Golgi vesicle-mediated transport
- intracellular protein transport
- positive regulation of protein secretion
- regulation of cholesterol transport

---

55

- **Protein name:** Myosin-7
- **Organism:** Homo sapiens
- **Uniprot Accession Number:** P12883
- **Protein sequence length:** 1935 aa
- **1D identity (%):** 11.25
- **1D identity (%) [Gaps excluded]:** 26.19
- **1D identity - Alignment Gaps:** 1116
- **1D aligned content (<aminoacid>:%):** {'G': 7.27, 'A': 8.18, 'L': 11.82, 'P': 3.64, 'F': 2.73, 'K': 9.55, 'Y': 2.73, 'V': 4.55, 'H': 5.0, 'D': 5.91, 'R': 5.91, 'I': 2.27, 'E': 9.55, 'Q': 6.36, 'N': 2.27, 'M': 1.82, 'T': 5.45, 'C': 1.82, 'S': 3.18}
- **Common reported functions (%):** 0.0
- **Common reported locations (%):** 12.5
- **Common reported processes (%):** 0.0

- **PDB ID:** 8ENC
- **Chain:** M
- **Crystallized protein length:** 742 aa
- **Resolution:** 3.6 Å
- **b-phipsi:** 0.010077
- **w-rdist:** 0.435175
- **t-alpha:** 0.006299
- **Chemical similarity (Tanimoto Index) (%):** 99.47
- **1D identity (%) [PDB]:** 2.59
- **1D identity (%) [Gaps excluded][PDB]:** 66.1
- **1D identity - Alignment Gaps [PDB]:** 1444
- **1D aligned content [PDB] (<aminoacid>:%):** {'G': 7.69, 'K': 15.38, 'T': 7.69, 'P': 2.56, 'V': 17.95, 'Y': 2.56, 'E': 2.56, 'R': 2.56, 'L': 12.82, 'M': 2.56, 'A': 5.13, 'Q': 7.69, 'N': 5.13, 'S': 5.13, 'H': 2.56}
- **2D identity (%) [PDB]:** 43.24
- **2D identity (%) [Gaps excluded][PDB]:** 90.5
- **2D identity - Alignment Gaps [PDB]:** 552
- **2D aligned content [PDB] (<2D-fold>:%):** {'.': 19.26, 'H': 44.86, 'T': 14.88, 'E': 21.01}
- **3D similarity (TM-Score) (%) [PDB]:** 18.67

- **Gene name:** MYH7
- **Entrez ID:** 4625
- **RefSeq ID:** N/A
- **Sequence length:** N/A
- **5-UTR|CDS|3-UTR identity (%):** N/A | N/A | N/A
- **5-UTR|CDS|3-UTR identity (%) [Gaps excluded]:** N/A | N/A | N/A
- **5-UTR|CDS|3-UTR identity [Alignment Gaps]:** N/A | N/A | N/A
- **5-UTR aligned content (<base>:%):** N/A
- **CDS aligned content (<base>:%):** N/A
- **3-UTR aligned content (<base>:%):** N/A

**Uniprot Description:**  
  
 Myosins are actin-based motor molecules with ATPase activity essential for muscle contraction. Forms regular bipolar thick filaments that, together with actin thin filaments, constitute the fundamental contractile unit of skeletal and cardiac muscle.   
  
Muscle myosin is a hexameric protein that consists of 2 heavy chain subunits (MHC), 2 alkali light chain subunits (MLC) and 2 regulatory light chain subunits (MLC-2). Interacts with ECPAS (PubMed:20682791). Interacts (via C-terminus) with LRRC39 (PubMed:20847312).   
  
 **Gene Ontology Information:**

Molecular Function

- actin filament binding
- ATP binding
- calmodulin binding
- microfilament motor activity

Location

- cytoplasm
- muscle myosin complex
- myofibril
- myosin complex
- myosin filament
- myosin II complex
- sarcomere
- stress fiber
- Z disc

Biological process

- adult heart development
- ATP metabolic process
- cardiac muscle contraction
- cardiac muscle hypertrophy in response to stress
- muscle contraction
- muscle filament sliding
- regulation of heart rate
- regulation of slow-twitch skeletal muscle fiber contraction
- regulation of the force of heart contraction
- regulation of the force of skeletal muscle contraction
- sarcomere organization
- skeletal muscle contraction
- striated muscle contraction
- transition between fast and slow fiber
- ventricular cardiac muscle tissue morphogenesis

---

56

- **Protein name:** Phosphatidylinositol 4,5-bisphosphate 3-kinase catalytic subunit alpha isoform
- **Organism:** Homo sapiens
- **Uniprot Accession Number:** P42336
- **Protein sequence length:** 1068 aa
- **1D identity (%):** 15.05
- **1D identity (%) [Gaps excluded]:** 24.19
- **1D identity - Alignment Gaps:** 449
- **1D aligned content (<aminoacid>:%):** {'P': 8.38, 'H': 2.23, 'R': 6.7, 'E': 7.26, 'L': 12.29, 'G': 7.82, 'T': 5.03, 'A': 3.91, 'D': 6.15, 'S': 3.35, 'F': 3.35, 'V': 6.15, 'M': 1.12, 'I': 4.47, 'N': 1.68, 'K': 7.26, 'Y': 4.47, 'C': 3.35, 'Q': 4.47, 'W': 0.56}
- **Common reported functions (%):** 0.0
- **Common reported locations (%):** 25.0
- **Common reported processes (%):** 0.0

- **PDB ID:** 4OVV
- **Chain:** A
- **Crystallized protein length:** 1042 aa
- **Resolution:** 3.5 Å
- **b-phipsi:** 0.00723
- **w-rdist:** 0.428532
- **t-alpha:** 0.012519
- **Chemical similarity (Tanimoto Index) (%):** 88.81
- **1D identity (%) [PDB]:** 1.97
- **1D identity (%) [Gaps excluded][PDB]:** 69.23
- **1D identity - Alignment Gaps [PDB]:** 1774
- **1D aligned content [PDB] (<aminoacid>:%):** {'R': 11.11, 'L': 5.56, 'N': 5.56, 'F': 2.78, 'Q': 8.33, 'V': 8.33, 'I': 11.11, 'K': 8.33, 'Y': 5.56, 'H': 5.56, 'D': 8.33, 'P': 5.56, 'E': 5.56, 'M': 5.56, 'G': 2.78}
- **2D identity (%) [PDB]:** 36.19
- **2D identity (%) [Gaps excluded][PDB]:** 87.61
- **2D identity - Alignment Gaps [PDB]:** 780
- **2D aligned content [PDB] (<2D-fold>:%):** {'.': 14.35, 'E': 21.41, 'T': 14.76, 'H': 48.23, 'G': 1.25}
- **3D similarity (TM-Score) (%) [PDB]:** 27.42

- **Gene name:** PIK3CA
- **Entrez ID:** 5290
- **RefSeq ID:** N/A
- **Sequence length:** N/A
- **5-UTR|CDS|3-UTR identity (%):** N/A | N/A | N/A
- **5-UTR|CDS|3-UTR identity (%) [Gaps excluded]:** N/A | N/A | N/A
- **5-UTR|CDS|3-UTR identity [Alignment Gaps]:** N/A | N/A | N/A
- **5-UTR aligned content (<base>:%):** N/A
- **CDS aligned content (<base>:%):** N/A
- **3-UTR aligned content (<base>:%):** N/A

**Uniprot Description:**  
  
 Phosphoinositide-3-kinase (PI3K) phosphorylates phosphatidylinositol (PI) and its phosphorylated derivatives at position 3 of the inositol ring to produce 3-phosphoinositides (PubMed:15135396, PubMed:23936502, PubMed:28676499). Uses ATP and PtdIns(4,5)P2 (phosphatidylinositol 4,5-bisphosphate) to generate phosphatidylinositol 3,4,5-trisphosphate (PIP3) (PubMed:15135396, PubMed:28676499). PIP3 plays a key role by recruiting PH domain-containing proteins to the membrane, including AKT1 and PDPK1, activating signaling cascades involved in cell growth, survival, proliferation, motility and morphology. Participates in cellular signaling in response to various growth factors. Involved in the activation of AKT1 upon stimulation by receptor tyrosine kinases ligands such as EGF, insulin, IGF1, VEGFA and PDGF. Involved in signaling via insulin-receptor substrate (IRS) proteins. Essential in endothelial cell migration during vascular development through VEGFA signaling, possibly by regulating RhoA activity. Required for lymphatic vasculature development, possibly by binding to RAS and by activation by EGF and FGF2, but not by PDGF. Regulates invadopodia formation through the PDPK1-AKT1 pathway. Participates in cardiomyogenesis in embryonic stem cells through a AKT1 pathway. Participates in vasculogenesis in embryonic stem cells through PDK1 and protein kinase C pathway. In addition to its lipid kinase activity, it displays a serine-protein kinase activity that results in the autophosphorylation of the p85alpha regulatory subunit as well as phosphorylation of other proteins such as 4EBP1, H-Ras, the IL-3 beta c receptor and possibly others (PubMed:23936502, PubMed:28676499). Plays a role in the positive regulation of phagocytosis and pinocytosis (By similarity).   
  
Heterodimer of a catalytic subunit PIK3CA and a p85 regulatory subunit (PIK3R1, PIK3R2 or PIK3R3) (PubMed:26593112). Interacts with IRS1 in nuclear extracts (By similarity). Interacts with RUFY3 (By similarity). Interacts with RASD2 (By similarity). Interacts with APPL1. Interacts with HRAS and KRAS (By similarity). Interaction with HRAS/KRAS is required for PI3K pathway signaling and cell proliferation stimulated by EGF and FGF2 (By similarity). Interacts with FAM83B; activates the PI3K/AKT signaling cascade (PubMed:23676467).   
  
 **Gene Ontology Information:**

Molecular Function

- phosphatidylinositol-3,4-bisphosphate 5-kinase activity
- 1-phosphatidylinositol-3-kinase activity
- phosphatidylinositol-4,5-bisphosphate 3-kinase activity
- 1-phosphatidylinositol-4-phosphate 3-kinase activity
- ATP binding
- insulin receptor substrate binding
- kinase activity
- protein kinase activator activity
- protein serine kinase activity
- protein serine/threonine kinase activity

Location

- cytoplasm
- cytosol
- intercalated disc
- lamellipodium
- perinuclear region of cytoplasm
- phosphatidylinositol 3-kinase complex
- phosphatidylinositol 3-kinase complex, class IA
- phosphatidylinositol 3-kinase complex, class IB
- plasma membrane

Biological process

- actin cytoskeleton organization
- adipose tissue development
- angiogenesis
- anoikis
- cardiac muscle cell contraction
- cardiac muscle contraction
- cellular response to glucose stimulus
- cellular response to hydrostatic pressure
- endothelial cell migration
- energy homeostasis
- epidermal growth factor receptor signaling pathway
- genetic imprinting
- glucose metabolic process
- insulin receptor signaling pathway via phosphatidylinositol 3-kinase
- insulin-like growth factor receptor signaling pathway
- liver development
- negative regulation of actin filament depolymerization
- negative regulation of anoikis
- negative regulation of fibroblast apoptotic process
- negative regulation of gene expression
- negative regulation of macroautophagy
- negative regulation of neuron apoptotic process
- phagocytosis
- phosphatidylinositol 3-kinase signaling
- phosphatidylinositol phosphorylation
- phosphatidylinositol-3-phosphate biosynthetic process
- phosphorylation
- platelet activation
- hypomethylation of CpG island
- positive regulation of lamellipodium assembly
- positive regulation of peptidyl-serine phosphorylation
- positive regulation of protein kinase B signaling
- positive regulation of smooth muscle cell proliferation
- positive regulation of TOR signaling
- protein kinase B signaling
- regulation of actin filament organization
- regulation of cellular respiration
- regulation of multicellular organism growth
- relaxation of cardiac muscle
- response to activity
- response to butyrate
- response to dexamethasone
- response to leucine
- response to muscle inactivity
- response to muscle stretch
- T cell costimulation
- T cell receptor signaling pathway
- vascular endothelial growth factor signaling pathway
- vasculature development

---

57

- **Protein name:** Xanthine dehydrogenase/oxidase
- **Organism:** Homo sapiens
- **Uniprot Accession Number:** P47989
- **Protein sequence length:** 1333 aa
- **1D identity (%):** 16.12
- **1D identity (%) [Gaps excluded]:** 26.54
- **1D identity - Alignment Gaps:** 536
- **1D aligned content (<aminoacid>:%):** {'M': 1.82, 'A': 8.18, 'P': 10.91, 'L': 10.91, 'R': 5.0, 'G': 13.18, 'Q': 3.18, 'H': 2.27, 'V': 8.64, 'K': 5.0, 'T': 7.27, 'D': 3.64, 'F': 4.09, 'W': 0.45, 'S': 2.73, 'E': 3.18, 'I': 3.64, 'C': 2.73, 'N': 1.36, 'Y': 1.82}
- **Common reported functions (%):** 0.0
- **Common reported locations (%):** 12.5
- **Common reported processes (%):** 0.0

- **PDB ID:** 2E1Q
- **Chain:** A
- **Crystallized protein length:** 1307 aa
- **Resolution:** 2.6 Å
- **b-phipsi:** 0.004978
- **w-rdist:** 0.377056
- **t-alpha:** 0.102504
- **Chemical similarity (Tanimoto Index) (%):** 65.37
- **1D identity (%) [PDB]:** 4.09
- **1D identity (%) [Gaps excluded][PDB]:** 64.84
- **1D identity - Alignment Gaps [PDB]:** 1902
- **1D aligned content [PDB] (<aminoacid>:%):** {'A': 9.64, 'G': 9.64, 'I': 6.02, 'E': 3.61, 'K': 10.84, 'V': 16.87, 'F': 3.61, 'Q': 4.82, 'R': 2.41, 'D': 3.61, 'L': 9.64, 'M': 1.2, 'P': 2.41, 'C': 2.41, 'T': 7.23, 'S': 2.41, 'N': 3.61}
- **2D identity (%) [PDB]:** 36.78
- **2D identity (%) [Gaps excluded][PDB]:** 87.05
- **2D identity - Alignment Gaps [PDB]:** 876
- **2D aligned content [PDB] (<2D-fold>:%):** {'.': 14.87, 'E': 31.36, 'T': 13.8, 'H': 38.71, 'G': 0.54, 'B': 0.72}
- **3D similarity (TM-Score) (%) [PDB]:** 28.33

- **Gene name:** XDH
- **Entrez ID:** 7498
- **RefSeq ID:** NM\_000379
- **Transcript sequence length:** 5715
- **5-UTR|CDS|3-UTR identity (%):** 9.69 | 41.48 | 24.1
- **5-UTR|CDS|3-UTR identity (%) [Gaps excluded]:** 62.5 | 77.24 | 75.84
- **5-UTR|CDS|3-UTR identity [Alignment Gaps]:** 349 | 1984 | 3066
- **5-UTR aligned content (<base>:%):** {'C': 25.0, 'G': 42.5, 'A': 12.5, 'T': 20.0}
- **CDS aligned content (<base>:%):** {'A': 27.84, 'T': 22.05, 'G': 26.27, 'C': 23.85}
- **3-UTR aligned content (<base>:%):** {'A': 29.55, 'G': 21.7, 'T': 31.39, 'C': 17.36}

**Uniprot Description:**  
  
 Key enzyme in purine degradation. Catalyzes the oxidation of hypoxanthine to xanthine. Catalyzes the oxidation of xanthine to uric acid. Contributes to the generation of reactive oxygen species. Has also low oxidase activity towards aldehydes (in vitro).   
  
Homodimer. Interacts with BTN1A1 (By similarity).   
  
 **Gene Ontology Information:**

Molecular Function

- 2 iron, 2 sulfur cluster binding
- FAD binding
- flavin adenine dinucleotide binding
- hypoxanthine dehydrogenase activity
- hypoxanthine oxidase activity
- iron ion binding
- molybdopterin cofactor binding
- protein homodimerization activity
- xanthine dehydrogenase activity
- xanthine oxidase activity

Location

- cytosol
- extracellular space
- peroxisome
- sarcoplasmic reticulum

Biological process

- activation of cysteine-type endopeptidase activity involved in apoptotic process
- adenosine catabolic process
- allantoin metabolic process
- cellular amide catabolic process
- AMP catabolic process
- dAMP catabolic process
- deoxyadenosine catabolic process
- deoxyguanosine catabolic process
- deoxyinosine catabolic process
- dGMP catabolic process
- GMP catabolic process
- guanine catabolic process
- hypoxanthine catabolic process
- IMP catabolic process
- inosine catabolic process
- iron-sulfur cluster assembly
- lactation
- negative regulation of endothelial cell differentiation
- negative regulation of endothelial cell proliferation
- negative regulation of gene expression
- negative regulation of protein kinase B signaling
- negative regulation of protein phosphorylation
- negative regulation of vascular endothelial growth factor signaling pathway
- negative regulation of vasculogenesis
- positive regulation of p38MAPK cascade
- positive regulation of reactive oxygen species metabolic process
- xanthine catabolic process

---

58

- **Protein name:** Transitional endoplasmic reticulum ATPase
- **Organism:** Homo sapiens
- **Uniprot Accession Number:** P55072
- **Protein sequence length:** 806 aa
- **1D identity (%):** 17.39
- **1D identity (%) [Gaps excluded]:** 25.74
- **1D identity - Alignment Gaps:** 323
- **1D aligned content (<aminoacid>:%):** {'M': 1.16, 'G': 12.14, 'R': 6.94, 'P': 9.25, 'V': 9.83, 'I': 2.89, 'K': 6.94, 'D': 6.36, 'Q': 3.47, 'H': 2.31, 'L': 6.94, 'E': 7.51, 'F': 4.62, 'N': 2.89, 'Y': 1.73, 'S': 3.47, 'A': 5.78, 'T': 4.62, 'C': 1.16}
- **Common reported functions (%):** 0.0
- **Common reported locations (%):** 37.5
- **Common reported processes (%):** 0.0

- **PDB ID:** 5C1B
- **Chain:** C
- **Crystallized protein length:** 724 aa
- **Resolution:** 3.08 Å
- **b-phipsi:** 0.007085
- **w-rdist:** 0.189552
- **t-alpha:** 0.083724
- **Chemical similarity (Tanimoto Index) (%):** 74.7
- **1D identity (%) [PDB]:** 2.72
- **1D identity (%) [Gaps excluded][PDB]:** 59.42
- **1D identity - Alignment Gaps [PDB]:** 1438
- **1D aligned content [PDB] (<aminoacid>:%):** {'R': 14.63, 'P': 4.88, 'S': 7.32, 'Q': 7.32, 'V': 12.2, 'L': 12.2, 'D': 12.2, 'C': 2.44, 'F': 4.88, 'A': 4.88, 'E': 4.88, 'T': 2.44, 'H': 2.44, 'I': 2.44, 'K': 2.44, 'G': 2.44}
- **2D identity (%) [PDB]:** 37.13
- **2D identity (%) [Gaps excluded][PDB]:** 89.8
- **2D identity - Alignment Gaps [PDB]:** 654
- **2D aligned content [PDB] (<2D-fold>:%):** {'.': 15.46, 'E': 24.15, 'T': 8.21, 'H': 50.24, 'B': 0.48, 'G': 1.45}
- **3D similarity (TM-Score) (%) [PDB]:** 22.53

- **Gene name:** VCP
- **Entrez ID:** N/A
- **RefSeq ID:** NM\_007126
- **Transcript sequence length:** 3746
- **5-UTR|CDS|3-UTR identity (%):** 41.86 | 45.64 | 15.31
- **5-UTR|CDS|3-UTR identity (%) [Gaps excluded]:** 74.07 | 75.33 | 75.19
- **5-UTR|CDS|3-UTR identity [Alignment Gaps]:** 187 | 1229 | 3532
- **5-UTR aligned content (<base>:%):** {'G': 41.11, 'C': 46.11, 'T': 8.33, 'A': 4.44}
- **CDS aligned content (<base>:%):** {'T': 22.56, 'G': 26.99, 'C': 21.78, 'A': 28.67}
- **3-UTR aligned content (<base>:%):** {'T': 29.01, 'G': 25.92, 'C': 20.32, 'A': 24.74}

**Uniprot Description:**  
  
 Necessary for the fragmentation of Golgi stacks during mitosis and for their reassembly after mitosis. Involved in the formation of the transitional endoplasmic reticulum (tER). The transfer of membranes from the endoplasmic reticulum to the Golgi apparatus occurs via 50-70 nm transition vesicles which derive from part-rough, part-smooth transitional elements of the endoplasmic reticulum (tER). Vesicle budding from the tER is an ATP-dependent process. The ternary complex containing UFD1, VCP and NPLOC4 binds ubiquitinated proteins and is necessary for the export of misfolded proteins from the ER to the cytoplasm, where they are degraded by the proteasome. The NPLOC4-UFD1-VCP complex regulates spindle disassembly at the end of mitosis and is necessary for the formation of a closed nuclear envelope. Regulates E3 ubiquitin-protein ligase activity of RNF19A. Component of the VCP/p97-AMFR/gp78 complex that participates in the final step of the sterol-mediated ubiquitination and endoplasmic reticulum-associated degradation (ERAD) of HMGCR. Involved in endoplasmic reticulum stress-induced pre-emptive quality control, a mechanism that selectively attenuates the translocation of newly synthesized proteins into the endoplasmic reticulum and reroutes them to the cytosol for proteasomal degradation (PubMed:26565908). Plays a role in the regulation of stress granules (SGs) clearance process upon arsenite-induced response (PubMed:29804830). Also involved in DNA damage response: recruited to double-strand breaks (DSBs) sites in a RNF8- and RNF168-dependent manner and promotes the recruitment of TP53BP1 at DNA damage sites (PubMed:22020440, PubMed:22120668). Recruited to stalled replication forks by SPRTN: may act by mediating extraction of DNA polymerase eta (POLH) to prevent excessive translesion DNA synthesis and limit the incidence of mutations induced by DNA damage (PubMed:23042607, PubMed:23042605). Together with SPRTN metalloprotease, involved in the repair of covalent DNA-protein cross-links (DPCs) during DNA synthesis (PubMed:32152270). Involved in interstrand cross-link repair in response to replication stress by mediating unloading of the ubiquitinated CMG helicase complex (By similarity). Required for cytoplasmic retrotranslocation of stressed/damaged mitochondrial outer-membrane proteins and their subsequent proteasomal degradation (PubMed:16186510, PubMed:21118995). Essential for the maturation of ubiquitin-containing autophagosomes and the clearance of ubiquitinated protein by autophagy (PubMed:20104022, PubMed:27753622). Acts as a negative regulator of type I interferon production by interacting with DDX58/RIG-I: interaction takes place when DDX58/RIG-I is ubiquitinated via 'Lys-63'-linked ubiquitin on its CARD domains, leading to recruit RNF125 and promote ubiquitination and degradation of DDX58/RIG-I (PubMed:26471729). May play a role in the ubiquitin-dependent sorting of membrane proteins to lysosomes where they undergo degradation (PubMed:21822278). May more particularly play a role in caveolins sorting in cells (PubMed:21822278, PubMed:23335559). By controlling the steady-state expression of the IGF1R receptor, indirectly regulates the insulin-like growth factor receptor signaling pathway (PubMed:26692333).   
  
Homohexamer. Forms a ring-shaped particle of 12.5 nm diameter, that displays 6-fold radial symmetry. Part of a ternary complex containing STX5A, NSFL1C and VCP. NSFL1C forms a homotrimer that binds to one end of a VCP homohexamer. The complex binds to membranes enriched in phosphatidylethanolamine-containing lipids and promotes Golgi membrane fusion. Binds to a heterodimer of NPLOC4 and UFD1, binding to this heterodimer inhibits Golgi-membrane fusion (PubMed:26471729). Interaction with VCIP135 leads to dissociation of the complex via ATP hydrolysis by VCP. Part of a ternary complex containing NPLOC4, UFD1 and VCP. Interacts with NSFL1C-like protein p37; the complex has membrane fusion activity and is required for Golgi and endoplasmic reticulum biogenesis. Interacts with SELENOS and SYVN1, as well as with DERL1, DERL2 and DERL3; which probably transfer misfolded proteins from the ER to VCP. Interacts with SVIP. Component of a complex required to couple retrotranslocation, ubiquitination and deglycosylation composed of NGLY1, SAKS1, AMFR, VCP and RAD23B. Directly interacts with UBXN4 and RNF19A. Interacts with CASR. Interacts with UBE4B and YOD1. Interacts with clathrin. Interacts with RNF103. Interacts with TRIM13 and TRIM21. Component of a VCP/p97-AMFR/gp78 complex that participates in the final step of the endoplasmic reticulum-associated degradation (ERAD) of HMGCR. Interacts directly with AMFR/gp78 (via its VIM). Interacts with RHBDD1 (via C-terminal domain). Interacts with SPRTN; leading to recruitment to stalled replication forks (PubMed:23042607, PubMed:23042605). Interacts with WASHC5. Interacts with UBOX5. Interacts (via N-terminus) with UBXN7, UBXN8, and probably several other UBX domain-containing proteins (via UBX domains); the interactions are mutually exclusive with VIM-dependent interactions such as those with AMFR and SELENOS. Forms a complex with UBQLN1 and UBXN4. Interacts (via the PIM motif) with RNF31 (via the PUB domain) (PubMed:24726327). Interacts with DDX58/RIG-I and RNF125; interaction takes place when DDX58/RIG-I is ubiquitinated via 'Lys-63'-linked ubiquitin on its CARD domains, leading to recruit RNF125 and promote ubiquitination and degradation of DDX58/RIG-I (PubMed:26471729). Interacts with BAG6 (PubMed:21636303). Interacts with UBXN10 (PubMed:26389662). Interacts with UBXN6; the interaction with UBXN6 is direct and competitive with UFD1 (PubMed:19174149, PubMed:19275885). Forms a ternary complex with CAV1 and UBXN6 (PubMed:21822278, PubMed:18656546, PubMed:19174149). Interacts with PLAA, UBXN6 and YOD1; may form a complex involved in macroautophagy (PubMed:27753622). Interacts with ANKZF1 (PubMed:28302725). Interacts with ubiquitin-binding protein FAF1 (PubMed:26842564). Interacts with ZFAND2B (via VIM motif); the interaction is direct (PubMed:24160817, PubMed:26337389). Interacts with ZFAND1 (via its ubiquitin-like region); this interaction occurs in an arsenite-dependent manner (PubMed:29804830). Interacts with CCDC47 (By similarity). Interacts with UBAC2 (By similarity). Interacts with LMBR1L (PubMed:31073040). Interacts with ATXN3 (PubMed:30455355). Interacts with TEX264; bridging VCP to covalent DNA-protein cross-links (DPCs) (PubMed:32152270).   
  
 **Gene Ontology Information:**

Molecular Function

- ADP binding
- ATP binding
- ATPase activity
- BAT3 complex binding
- deubiquitinase activator activity
- identical protein binding
- K48-linked polyubiquitin modification-dependent protein binding
- lipid binding
- MHC class I protein binding
- polyubiquitin modification-dependent protein binding
- protein domain specific binding
- protein phosphatase binding
- RNA binding
- ubiquitin protein ligase binding
- ubiquitin-dependent protein binding
- ubiquitin-like protein ligase binding
- ubiquitin-specific protease binding

Location

- ATPase complex
- azurophil granule lumen
- cytoplasm
- cytoplasmic stress granule
- cytosol
- Derlin-1 retrotranslocation complex
- endoplasmic reticulum
- endoplasmic reticulum membrane
- extracellular exosome
- extracellular region
- ficolin-1-rich granule lumen
- glutamatergic synapse
- intracellular membrane-bounded organelle
- lipid droplet
- nucleoplasm
- nucleus
- perinuclear region of cytoplasm
- proteasome complex
- protein-containing complex
- secretory granule lumen
- site of double-strand break
- VCP-NPL4-UFD1 AAA ATPase complex
- VCP-NSFL1C complex

Biological process

- activation of cysteine-type endopeptidase activity involved in apoptotic process
- aggresome assembly
- ATP metabolic process
- autophagosome maturation
- autophagy
- cellular response to arsenite ion
- cellular response to heat
- cellular response to DNA damage stimulus
- DNA repair
- double-strand break repair
- endoplasmic reticulum stress-induced pre-emptive quality control
- endoplasmic reticulum to Golgi vesicle-mediated transport
- endoplasmic reticulum unfolded protein response
- endosome to lysosome transport via multivesicular body sorting pathway
- ER-associated misfolded protein catabolic process
- ERAD pathway
- establishment of protein localization
- flavin adenine dinucleotide catabolic process
- interstrand cross-link repair
- macroautophagy
- mitotic spindle disassembly
- NADH metabolic process
- negative regulation of protein localization to chromatin
- negative regulation of smoothened signaling pathway
- positive regulation of ATP biosynthetic process
- positive regulation of canonical Wnt signaling pathway
- positive regulation of Lys63-specific deubiquitinase activity
- positive regulation of mitochondrial membrane potential
- positive regulation of oxidative phosphorylation
- positive regulation of proteasomal ubiquitin-dependent protein catabolic process
- positive regulation of protein catabolic process
- positive regulation of protein K63-linked deubiquitination
- positive regulation of protein-containing complex assembly
- proteasomal protein catabolic process
- proteasome-mediated ubiquitin-dependent protein catabolic process
- protein ubiquitination
- protein-DNA covalent cross-linking repair
- regulation of aerobic respiration
- regulation of apoptotic process
- regulation of protein localization to chromatin
- regulation of synapse organization
- retrograde protein transport, ER to cytosol
- stress granule disassembly
- translesion synthesis
- ubiquitin-dependent ERAD pathway
- viral genome replication

---

59

- **Protein name:** DNA polymerase theta
- **Organism:** Homo sapiens
- **Uniprot Accession Number:** O75417
- **Protein sequence length:** 2590 aa
- **1D identity (%):** 10.35
- **1D identity (%) [Gaps excluded]:** 31.54
- **1D identity - Alignment Gaps:** 1745
- **1D aligned content (<aminoacid>:%):** {'M': 0.74, 'L': 9.67, 'G': 10.41, 'S': 4.83, 'P': 9.29, 'K': 5.95, 'R': 3.72, 'H': 4.83, 'F': 3.35, 'V': 9.29, 'Q': 8.55, 'I': 2.97, 'Y': 3.35, 'D': 5.95, 'T': 6.69, 'E': 2.97, 'A': 2.23, 'W': 0.37, 'C': 3.35, 'N': 1.49}
- **Common reported functions (%):** 0.0
- **Common reported locations (%):** 12.5
- **Common reported processes (%):** 0.0

- **PDB ID:** 4X0P
- **Chain:** A
- **Crystallized protein length:** 625 aa
- **Resolution:** 3.91 Å
- **b-phipsi:** 0.052322
- **w-rdist:** 0.222112
- **t-alpha:** 0.005508
- **Chemical similarity (Tanimoto Index) (%):** 72.34
- **1D identity (%) [PDB]:** 2.01
- **1D identity (%) [Gaps excluded][PDB]:** 74.36
- **1D identity - Alignment Gaps [PDB]:** 1402
- **1D aligned content [PDB] (<aminoacid>:%):** {'Q': 6.9, 'T': 3.45, 'F': 3.45, 'K': 3.45, 'S': 13.79, 'V': 17.24, 'L': 20.69, 'E': 3.45, 'A': 6.9, 'G': 3.45, 'H': 3.45, 'N': 3.45, 'P': 3.45, 'D': 6.9}
- **2D identity (%) [PDB]:** 34.11
- **2D identity (%) [Gaps excluded][PDB]:** 89.02
- **2D identity - Alignment Gaps [PDB]:** 660
- **2D aligned content [PDB] (<2D-fold>:%):** {'.': 20.0, 'E': 15.34, 'T': 10.14, 'H': 53.15, 'B': 0.55, 'G': 0.82}
- **3D similarity (TM-Score) (%) [PDB]:** 22.94

- **Gene name:** POLQ
- **Entrez ID:** 10721
- **RefSeq ID:** NM\_199420
- **Transcript sequence length:** 8757
- **5-UTR|CDS|3-UTR identity (%):** 14.81 | 26.74 | 13.44
- **5-UTR|CDS|3-UTR identity (%) [Gaps excluded]:** 80.0 | 83.11 | 75.8
- **5-UTR|CDS|3-UTR identity [Alignment Gaps]:** 352 | 5315 | 3604
- **5-UTR aligned content (<base>:%):** {'G': 51.56, 'A': 10.94, 'T': 1.56, 'C': 35.94}
- **CDS aligned content (<base>:%):** {'A': 30.25, 'T': 25.05, 'G': 23.14, 'C': 21.56}
- **3-UTR aligned content (<base>:%):** {'C': 14.77, 'T': 37.69, 'G': 16.47, 'A': 31.07}

**Uniprot Description:**  
  
 DNA polymerase that promotes microhomology-mediated end-joining (MMEJ), an alternative non-homologous end-joining (NHEJ) machinery triggered in response to double-strand breaks in DNA (PubMed:25642963, PubMed:25643323). MMEJ is an error-prone repair pathway that produces deletions of sequences from the strand being repaired and promotes genomic rearrangements, such as telomere fusions, some of them leading to cellular transformation (PubMed:25642963, PubMed:25643323). POLQ acts as an inhibitor of homology-recombination repair (HR) pathway by limiting RAD51 accumulation at resected ends (PubMed:25642963). POLQ-mediated MMEJ may be required to promote the survival of cells with a compromised HR repair pathway, thereby preventing genomic havoc by resolving unrepaired lesions (By similarity). The polymerase acts by binding directly the 2 ends of resected double-strand breaks, allowing microhomologous sequences in the overhangs to form base pairs. It then extends each strand from the base-paired region using the opposing overhang as a template. Requires partially resected DNA containing 2 to 6 base pairs of microhomology to perform MMEJ (PubMed:25643323). The polymerase activity is highly promiscuous: unlike most polymerases, promotes extension of ssDNA and partial ssDNA (pssDNA) substrates (PubMed:18503084, PubMed:21050863, PubMed:22135286). Also exhibits low-fidelity DNA synthesis, translesion synthesis and lyase activity, and it is implicated in interstrand-cross-link repair, base excision repair and DNA end-joining (PubMed:14576298, PubMed:18503084, PubMed:19188258, PubMed:24648516). Involved in somatic hypermutation of immunoglobulin genes, a process that requires the activity of DNA polymerases to ultimately introduce mutations at both A/T and C/G base pairs (By similarity).   
  
Homomultimer; forms dimers and multimers (PubMed:25643323). Interacts with RAD51 (PubMed:25642963). Interacts with ORC2 and ORC4 (PubMed:24989122).   
  
 **Gene Ontology Information:**

Molecular Function

- 5'-deoxyribose-5-phosphate lyase activity
- ATP binding
- chromatin binding
- damaged DNA binding
- DNA-directed DNA polymerase activity
- identical protein binding
- single-stranded DNA helicase activity

Location

- chromosome
- cytosol
- Golgi apparatus
- nucleoplasm

Biological process

- base-excision repair
- cellular response to DNA damage stimulus
- DNA repair
- DNA-dependent DNA replication
- double-strand break repair
- double-strand break repair via alternative nonhomologous end joining
- negative regulation of double-strand break repair via homologous recombination
- protein homooligomerization
- somatic hypermutation of immunoglobulin genes

---

60

- **Protein name:** Protein arginine N-methyltransferase 5
- **Organism:** Homo sapiens
- **Uniprot Accession Number:** O14744
- **Protein sequence length:** 637 aa
- **1D identity (%):** 11.92
- **1D identity (%) [Gaps excluded]:** 23.8
- **1D identity - Alignment Gaps:** 498
- **1D aligned content (<aminoacid>:%):** {'G': 10.08, 'R': 10.08, 'P': 13.45, 'I': 4.2, 'D': 5.04, 'E': 5.04, 'L': 7.56, 'V': 9.24, 'N': 3.36, 'T': 3.36, 'H': 2.52, 'M': 3.36, 'W': 0.84, 'A': 3.36, 'Q': 4.2, 'F': 2.52, 'K': 5.04, 'C': 4.2, 'Y': 1.68, 'S': 0.84}
- **Common reported functions (%):** 0.0
- **Common reported locations (%):** 0.0
- **Common reported processes (%):** 0.0

- **PDB ID:** 8G1U
- **Chain:** M
- **Crystallized protein length:** 609 aa
- **Resolution:** 2.83 Å
- **b-phipsi:** 0.000924
- **w-rdist:** 0.552582
- **t-alpha:** 0.03231
- **Chemical similarity (Tanimoto Index) (%):** 81.65
- **1D identity (%) [PDB]:** 3.02
- **1D identity (%) [Gaps excluded][PDB]:** 76.36
- **1D identity - Alignment Gaps [PDB]:** 1338
- **1D aligned content [PDB] (<aminoacid>:%):** {'S': 7.14, 'P': 9.52, 'Q': 4.76, 'T': 4.76, 'L': 11.9, 'K': 4.76, 'I': 4.76, 'A': 7.14, 'G': 14.29, 'V': 9.52, 'H': 4.76, 'R': 7.14, 'F': 2.38, 'D': 2.38, 'Y': 2.38, 'C': 2.38}
- **2D identity (%) [PDB]:** 39.25
- **2D identity (%) [Gaps excluded][PDB]:** 91.71
- **2D identity - Alignment Gaps [PDB]:** 580
- **2D aligned content [PDB] (<2D-fold>:%):** {'E': 22.61, 'T': 29.4, 'H': 36.93, '.': 11.06}
- **3D similarity (TM-Score) (%) [PDB]:** 0.7

- **Gene name:** PRMT5
- **Entrez ID:** N/A
- **RefSeq ID:** NM\_006109
- **Transcript sequence length:** 2304
- **5-UTR|CDS|3-UTR identity (%):** 3.49 | 38.41 | 5.43
- **5-UTR|CDS|3-UTR identity (%) [Gaps excluded]:** 93.33 | 74.79 | 75.64
- **5-UTR|CDS|3-UTR identity [Alignment Gaps]:** 386 | 1446 | 4036
- **5-UTR aligned content (<base>:%):** {'A': 42.86, 'G': 42.86, 'C': 14.29}
- **CDS aligned content (<base>:%):** {'T': 23.2, 'G': 25.04, 'A': 27.85, 'C': 23.91}
- **3-UTR aligned content (<base>:%):** {'C': 21.61, 'T': 31.36, 'G': 20.34, 'A': 26.69}

**Uniprot Description:**  
  
 Arginine methyltransferase that can both catalyze the formation of omega-N monomethylarginine (MMA) and symmetrical dimethylarginine (sDMA), with a preference for the formation of MMA (PubMed:10531356, PubMed:11152681, PubMed:11747828, PubMed:12411503, PubMed:15737618, PubMed:17709427, PubMed:20159986, PubMed:20810653, PubMed:21258366, PubMed:21917714, PubMed:22269951, PubMed:21081503). Specifically mediates the symmetrical dimethylation of arginine residues in the small nuclear ribonucleoproteins Sm D1 (SNRPD1) and Sm D3 (SNRPD3); such methylation being required for the assembly and biogenesis of snRNP core particles (PubMed:12411503, PubMed:11747828, PubMed:17709427). Methylates SUPT5H and may regulate its transcriptional elongation properties (PubMed:12718890). Mono- and dimethylates arginine residues of myelin basic protein (MBP) in vitro. May play a role in cytokine-activated transduction pathways. Negatively regulates cyclin E1 promoter activity and cellular proliferation. Methylates histone H2A and H4 'Arg-3' during germ cell development. Methylates histone H3 'Arg-8', which may repress transcription. Methylates the Piwi proteins (PIWIL1, PIWIL2 and PIWIL4), methylation of Piwi proteins being required for the interaction with Tudor domain-containing proteins and subsequent localization to the meiotic nuage (By similarity). Methylates RPS10. Attenuates EGF signaling through the MAPK1/MAPK3 pathway acting at 2 levels. First, monomethylates EGFR; this enhances EGFR 'Tyr-1197' phosphorylation and PTPN6 recruitment, eventually leading to reduced SOS1 phosphorylation (PubMed:21917714, PubMed:21258366). Second, methylates RAF1 and probably BRAF, hence destabilizing these 2 signaling proteins and reducing their catalytic activity (PubMed:21917714). Required for induction of E-selectin and VCAM-1, on the endothelial cells surface at sites of inflammation. Methylates HOXA9 (PubMed:22269951). Methylates and regulates SRGAP2 which is involved in cell migration and differentiation (PubMed:20810653). Acts as a transcriptional corepressor in CRY1-mediated repression of the core circadian component PER1 by regulating the H4R3 dimethylation at the PER1 promoter (By similarity). Methylates GM130/GOLGA2, regulating Golgi ribbon formation (PubMed:20421892). Methylates H4R3 in genes involved in glioblastomagenesis in a CHTOP- and/or TET1-dependent manner (PubMed:25284789). Symmetrically methylates POLR2A, a modification that allows the recruitment to POLR2A of proteins including SMN1/SMN2 and SETX. This is required for resolving RNA-DNA hybrids created by RNA polymerase II, that form R-loop in transcription terminal regions, an important step in proper transcription termination (PubMed:26700805). Along with LYAR, binds the promoter of gamma-globin HBG1/HBG2 and represses its expression (PubMed:25092918). Symmetrically methylates NCL (PubMed:21081503). Methylates TP53; methylation might possibly affect TP53 target gene specificity (PubMed:19011621). Involved in spliceosome maturation and mRNA splicing in prophase I spermatocytes through the catalysis of the symmetrical arginine dimethylation of SNRPB (small nuclear ribonucleoprotein-associated protein) and the interaction with tudor domain-containing protein TDRD6 (By similarity).   
  
Forms, at least, homodimers and homotetramers (PubMed:11152681). Component of the methylosome complex, composed of PRMT5, WDR77 and CLNS1A (PubMed:21081503). Found in a complex composed of PRMT5, WDR77 and RIOK1 (PubMed:21081503). RIOK1 and CLNS1A associate with PRMT5 in a mutually exclusive fashion, which allows the recruitment of distinct methylation substrates, such as nucleolin/NCL and Sm proteins, respectively (PubMed:21081503). Interacts with PRDM1 (By similarity). Identified in a complex composed of methylosome and PRMT1 and ERH (PubMed:25284789). Interacts with EGFR; methylates EGFR and stimulates EGFR-mediated ERK activation. Interacts with HOXA9. Interacts with SRGAP2. Found in a complex with COPRS, RUNX1 and CBFB. Interacts with CHTOP; the interaction symmetrically methylates CHTOP, but seems to require the presence of PRMT1 (PubMed:25284789). Interacts with EPB41L3; this modulates methylation of target proteins. Component of a high molecular weight E2F-pocket protein complex, CERC (cyclin E1 repressor complex). Associates with SWI/SNF remodeling complexes containing SMARCA2 and SMARCA4. Interacts with JAK2, SSTR1, SUPT5H, BRAF and with active RAF1. Interacts with LSM11, PRMT7 and SNRPD3 (PubMed:17709427, PubMed:16087681). Interacts with COPRS; promoting its recruitment on histone H4. Interacts with CLNS1A/pICln (PubMed:21081503, PubMed:9556550). Identified in a complex with CLNS1A/pICln and Sm proteins. Interacts with RPS10 (PubMed:20159986). Interacts with WDR77. Interacts with IWS1. Interacts with CRY1. Interacts with POLR2A (PubMed:26700805). Interacts with SMN1/SMN2 (PubMed:26700805). Interacts with LYAR; this interaction is direct (PubMed:25092918). Interacts with STRAP (PubMed:19011621). Interacts with TP53 in response to DNA damage; the interaction is STRAP dependent (PubMed:19011621). Interacts with TDRD6 (By similarity).   
  
 **Gene Ontology Information:**

Molecular Function   
  
N/A

Location   
  
N/A

Biological process   
  
N/A

---

61

- **Protein name:** Cap-specific mRNA (nucleoside-2'-O-)-methyltransferase 1
- **Organism:** Homo sapiens
- **Uniprot Accession Number:** Q8N1G2
- **Protein sequence length:** 835 aa
- **1D identity (%):** 15.08
- **1D identity (%) [Gaps excluded]:** 22.09
- **1D identity - Alignment Gaps:** 320
- **1D aligned content (<aminoacid>:%):** {'R': 5.92, 'D': 7.89, 'E': 7.89, 'T': 5.92, 'P': 6.58, 'K': 5.26, 'Q': 5.26, 'V': 8.55, 'S': 4.61, 'L': 7.89, 'G': 11.84, 'H': 1.97, 'A': 3.29, 'M': 1.32, 'Y': 4.61, 'F': 4.61, 'C': 3.29, 'N': 0.66, 'I': 2.63}
- **Common reported functions (%):** 0.0
- **Common reported locations (%):** 25.0
- **Common reported processes (%):** 0.0

- **PDB ID:** 8P4E
- **Chain:** O
- **Crystallized protein length:** 695 aa
- **Resolution:** 3.9 Å
- **b-phipsi:** 0.001486
- **w-rdist:** 0.510894
- **t-alpha:** 0.044601
- **Chemical similarity (Tanimoto Index) (%):** N/A
- **1D identity (%) [PDB]:** 3.98
- **1D identity (%) [Gaps excluded][PDB]:** 67.44
- **1D identity - Alignment Gaps [PDB]:** 1373
- **1D aligned content [PDB] (<aminoacid>:%):** {'V': 6.9, 'Q': 10.34, 'S': 10.34, 'E': 12.07, 'L': 10.34, 'D': 3.45, 'M': 1.72, 'R': 10.34, 'F': 3.45, 'Y': 3.45, 'P': 5.17, 'T': 1.72, 'I': 6.9, 'G': 5.17, 'K': 5.17, 'A': 3.45}
- **2D identity (%) [PDB]:** 43.57
- **2D identity (%) [Gaps excluded][PDB]:** 88.43
- **2D identity - Alignment Gaps [PDB]:** 525
- **2D aligned content [PDB] (<2D-fold>:%):** {'.': 16.63, 'E': 20.84, 'T': 25.28, 'H': 36.59, 'G': 0.67}
- **3D similarity (TM-Score) (%) [PDB]:** 5.29

- **Gene name:** CMTR1
- **Entrez ID:** 23070
- **RefSeq ID:** NM\_015050
- **Transcript sequence length:** 4034
- **5-UTR|CDS|3-UTR identity (%):** 23.89 | 46.9 | 18.1
- **5-UTR|CDS|3-UTR identity (%) [Gaps excluded]:** 74.45 | 75.68 | 74.75
- **5-UTR|CDS|3-UTR identity [Alignment Gaps]:** 290 | 1196 | 3446
- **5-UTR aligned content (<base>:%):** {'G': 42.16, 'C': 43.14, 'T': 9.8, 'A': 4.9}
- **CDS aligned content (<base>:%):** {'A': 26.24, 'T': 21.83, 'G': 28.34, 'C': 23.59}
- **3-UTR aligned content (<base>:%):** {'A': 21.63, 'G': 26.12, 'C': 24.3, 'T': 27.95}

**Uniprot Description:**  
  
 S-adenosyl-L-methionine-dependent methyltransferase that mediates mRNA cap1 2'-O-ribose methylation to the 5'-cap structure of mRNAs. Methylates the ribose of the first nucleotide of a m(7)GpppG-capped mRNA and small nuclear RNA (snRNA) to produce m(7)GpppRm (cap1). Displays a preference for cap0 transcripts. Cap1 modification is linked to higher levels of translation. May be involved in the interferon response pathway.   
  
Interacts with POLR2A (via C-terminus).   
  
 **Gene Ontology Information:**

Molecular Function

- mRNA (nucleoside-2'-O-)-methyltransferase activity
- nucleic acid binding

Location

- cytoplasm
- intracellular membrane-bounded organelle
- nucleoplasm
- nucleus

Biological process

- 7-methylguanosine mRNA capping
- cap1 mRNA methylation
- mRNA methylation

---

62

- **Protein name:** DNA ligase 1
- **Organism:** Homo sapiens
- **Uniprot Accession Number:** P18858
- **Protein sequence length:** 919 aa
- **1D identity (%):** 16.54
- **1D identity (%) [Gaps excluded]:** 24.58
- **1D identity - Alignment Gaps:** 348
- **1D aligned content (<aminoacid>:%):** {'R': 5.11, 'M': 1.14, 'H': 2.27, 'P': 13.07, 'G': 9.66, 'E': 7.39, 'K': 5.68, 'W': 1.14, 'V': 6.82, 'S': 7.95, 'A': 6.25, 'L': 9.09, 'I': 5.68, 'T': 4.55, 'Q': 5.68, 'F': 0.57, 'Y': 2.84, 'D': 3.41, 'C': 1.7}
- **Common reported functions (%):** 0.0
- **Common reported locations (%):** 12.5
- **Common reported processes (%):** 0.0

- **PDB ID:** 6P0D
- **Chain:** A
- **Crystallized protein length:** 641 aa
- **Resolution:** 1.75 Å
- **b-phipsi:** 0.005344
- **w-rdist:** 0.73861
- **t-alpha:** 0.004694
- **Chemical similarity (Tanimoto Index) (%):** 99.24
- **1D identity (%) [PDB]:** 2.43
- **1D identity (%) [Gaps excluded][PDB]:** 70.0
- **1D identity - Alignment Gaps [PDB]:** 1391
- **1D aligned content [PDB] (<aminoacid>:%):** {'A': 5.71, 'R': 8.57, 'S': 5.71, 'P': 14.29, 'D': 5.71, 'Q': 2.86, 'E': 11.43, 'L': 11.43, 'V': 11.43, 'N': 5.71, 'M': 2.86, 'G': 5.71, 'I': 2.86, 'H': 2.86, 'T': 2.86}
- **2D identity (%) [PDB]:** 34.02
- **2D identity (%) [Gaps excluded][PDB]:** 83.95
- **2D identity - Alignment Gaps [PDB]:** 631
- **2D aligned content [PDB] (<2D-fold>:%):** {'T': 14.13, '.': 18.28, 'H': 44.04, 'E': 21.61, 'B': 0.28, 'G': 1.66}
- **3D similarity (TM-Score) (%) [PDB]:** 24.03

- **Gene name:** LIG1
- **Entrez ID:** 3978
- **RefSeq ID:** NM\_001320971
- **Transcript sequence length:** 3035
- **5-UTR|CDS|3-UTR identity (%):** 23.24 | 45.4 | 3.06
- **5-UTR|CDS|3-UTR identity (%) [Gaps excluded]:** 72.79 | 74.15 | 75.43
- **5-UTR|CDS|3-UTR identity [Alignment Gaps]:** 290 | 1264 | 4139
- **5-UTR aligned content (<base>:%):** {'G': 47.47, 'C': 32.32, 'A': 14.14, 'T': 6.06}
- **CDS aligned content (<base>:%):** {'A': 24.93, 'T': 16.89, 'G': 30.41, 'C': 27.77}
- **3-UTR aligned content (<base>:%):** {'G': 29.55, 'C': 18.18, 'T': 30.3, 'A': 21.97}

**Uniprot Description:**  
  
 DNA ligase that seals nicks in double-stranded DNA during DNA replication, DNA recombination and DNA repair.   
  
Interacts with PCNA.   
  
 **Gene Ontology Information:**

Molecular Function

- ATP binding
- DNA binding
- DNA ligase (ATP) activity
- DNA ligase activity
- metal ion binding

Location

- intracellular membrane-bounded organelle
- mitochondrion
- nucleoplasm
- nucleus

Biological process

- anatomical structure morphogenesis
- base-excision repair
- base-excision repair, gap-filling
- cell division
- DNA biosynthetic process
- DNA ligation
- DNA recombination
- DNA repair
- lagging strand elongation
- mismatch repair
- Okazaki fragment processing involved in mitotic DNA replication

---

63

- **Protein name:** Alpha-actinin A
- **Organism:** Dictyostelium discoideum
- **Uniprot Accession Number:** P05095
- **Protein sequence length:** 861 aa
- **1D identity (%):** 15.88
- **1D identity (%) [Gaps excluded]:** 22.74
- **1D identity - Alignment Gaps:** 306
- **1D aligned content (<aminoacid>:%):** {'P': 5.59, 'S': 3.11, 'F': 4.35, 'L': 12.42, 'G': 6.83, 'I': 3.73, 'V': 6.83, 'D': 8.07, 'K': 9.32, 'R': 6.21, 'E': 6.21, 'T': 4.35, 'Q': 8.07, 'Y': 4.35, 'N': 2.48, 'A': 5.59, 'H': 1.24, 'M': 0.62, 'C': 0.62}
- **Common reported functions (%):** 0.0
- **Common reported locations (%):** 12.5
- **Common reported processes (%):** 0.0

- **PDB ID:** 5JLH
- **Chain:** G
- **Crystallized protein length:** 737 aa
- **Resolution:** 3.9 Å
- **b-phipsi:** 0.012089
- **w-rdist:** 0.525566
- **t-alpha:** 0.001565
- **Chemical similarity (Tanimoto Index) (%):** N/A
- **1D identity (%) [PDB]:** 2.6
- **1D identity (%) [Gaps excluded][PDB]:** 78.43
- **1D identity - Alignment Gaps [PDB]:** 1487
- **1D aligned content [PDB] (<aminoacid>:%):** {'W': 2.5, 'D': 5.0, 'M': 5.0, 'R': 5.0, 'G': 10.0, 'K': 12.5, 'F': 7.5, 'Y': 2.5, 'A': 10.0, 'E': 2.5, 'I': 5.0, 'V': 5.0, 'Q': 10.0, 'C': 2.5, 'L': 7.5, 'S': 5.0, 'T': 2.5}
- **2D identity (%) [PDB]:** 37.73
- **2D identity (%) [Gaps excluded][PDB]:** 86.9
- **2D identity - Alignment Gaps [PDB]:** 627
- **2D aligned content [PDB] (<2D-fold>:%):** {'.': 17.7, 'E': 20.33, 'T': 8.85, 'H': 52.39, 'G': 0.72}
- **3D similarity (TM-Score) (%) [PDB]:** 19.84

- **Gene name:** abpA
- **Entrez ID:** 8616670
- **RefSeq ID:** N/A
- **Sequence length:** N/A
- **5-UTR|CDS|3-UTR identity (%):** N/A | N/A | N/A
- **5-UTR|CDS|3-UTR identity (%) [Gaps excluded]:** N/A | N/A | N/A
- **5-UTR|CDS|3-UTR identity [Alignment Gaps]:** N/A | N/A | N/A
- **5-UTR aligned content (<base>:%):** N/A
- **CDS aligned content (<base>:%):** N/A
- **3-UTR aligned content (<base>:%):** N/A

**Uniprot Description:**  
  
 F-actin cross-linking protein which is thought to anchor actin to a variety of intracellular structures. This is a bundling protein. Increases the actin-stimulated ATPase activity of myosin. Involved in vegetative cell growth, phagocytosis, motility and development, probably through stabilization of the actin network in the cortical cytoskeleton.   
  
Homodimer; antiparallel.   
  
 **Gene Ontology Information:**

Molecular Function

- actin filament binding
- calcium ion binding
- protein-macromolecule adaptor activity
- structural constituent of cytoskeleton

Location

- actin filament
- cell cortex
- cell junction
- cell leading edge
- cell projection
- contractile vacuole
- cortical actin cytoskeleton
- cytosol
- extracellular matrix
- macropinocytic cup
- phagocytic vesicle
- pseudopodium

Biological process

- actin crosslink formation
- actin cytoskeleton organization
- actin filament bundle assembly
- cell motility
- cellular response to starvation
- hyperosmotic response
- phagocytosis
- sorocarp development

---

64

- **Protein name:** Exostosin-like 3
- **Organism:** Homo sapiens
- **Uniprot Accession Number:** O43909
- **Protein sequence length:** 919 aa
- **1D identity (%):** 18.2
- **1D identity (%) [Gaps excluded]:** 22.76
- **1D identity - Alignment Gaps:** 198
- **1D aligned content (<aminoacid>:%):** {'R': 7.22, 'G': 11.11, 'I': 2.22, 'L': 12.22, 'F': 3.89, 'V': 6.67, 'P': 11.67, 'Y': 1.67, 'H': 4.44, 'E': 6.67, 'K': 2.22, 'Q': 4.44, 'A': 7.22, 'D': 3.89, 'T': 3.89, 'N': 2.22, 'M': 1.67, 'W': 1.11, 'S': 5.0, 'C': 0.56}
- **Common reported functions (%):** 0.0
- **Common reported locations (%):** 0.0
- **Common reported processes (%):** 0.0

- **PDB ID:** 8OG4
- **Chain:** A
- **Crystallized protein length:** 709 aa
- **Resolution:** 2.1 Å
- **b-phipsi:** 0.006707
- **w-rdist:** 0.402141
- **t-alpha:** 0.028986
- **Chemical similarity (Tanimoto Index) (%):** 75.21
- **1D identity (%) [PDB]:** 3.31
- **1D identity (%) [Gaps excluded][PDB]:** 77.42
- **1D identity - Alignment Gaps [PDB]:** 1389
- **1D aligned content [PDB] (<aminoacid>:%):** {'S': 6.25, 'L': 8.33, 'E': 4.17, 'A': 8.33, 'G': 12.5, 'H': 4.17, 'N': 2.08, 'V': 12.5, 'P': 12.5, 'D': 2.08, 'Q': 4.17, 'R': 6.25, 'T': 2.08, 'F': 8.33, 'Y': 2.08, 'W': 2.08, 'M': 2.08}
- **2D identity (%) [PDB]:** 35.57
- **2D identity (%) [Gaps excluded][PDB]:** 91.06
- **2D identity - Alignment Gaps [PDB]:** 663
- **2D aligned content [PDB] (<2D-fold>:%):** {'.': 18.6, 'H': 37.98, 'T': 24.29, 'E': 18.09, 'G': 0.78, 'B': 0.26}
- **3D similarity (TM-Score) (%) [PDB]:** 24.53

- **Gene name:** EXTL3
- **Entrez ID:** N/A
- **RefSeq ID:** N/A
- **Sequence length:** N/A
- **5-UTR|CDS|3-UTR identity (%):** N/A | N/A | N/A
- **5-UTR|CDS|3-UTR identity (%) [Gaps excluded]:** N/A | N/A | N/A
- **5-UTR|CDS|3-UTR identity [Alignment Gaps]:** N/A | N/A | N/A
- **5-UTR aligned content (<base>:%):** N/A
- **CDS aligned content (<base>:%):** N/A
- **3-UTR aligned content (<base>:%):** N/A

**Uniprot Description:**  
  
 Glycosyltransferase which regulates the biosynthesis of heparan sulfate (HS). Important for both skeletal development and hematopoiesis, through the formation of HS proteoglycans (HSPGs) (PubMed:28132690, PubMed:28148688). Required for the function of REG3A in regulating keratinocyte proliferation and differentiation (PubMed:22727489).   
  
Interacts with REG3A.   
  
 **Gene Ontology Information:**

Molecular Function

- glucuronyl-galactosyl-proteoglycan 4-alpha-N-acetylglucosaminyltransferase activity
- transferase activity, transferring glycosyl groups
- metal ion binding

Location

- endoplasmic reticulum
- endoplasmic reticulum membrane
- Golgi apparatus

Biological process

- heparan sulfate proteoglycan biosynthetic process
- positive regulation of cell growth
- protein glycosylation

---

65

- **Protein name:** Transferrin receptor protein 1
- **Organism:** Homo sapiens
- **Uniprot Accession Number:** P02786
- **Protein sequence length:** 760 aa
- **1D identity (%):** 14.74
- **1D identity (%) [Gaps excluded]:** 23.99
- **1D identity - Alignment Gaps:** 387
- **1D aligned content (<aminoacid>:%):** {'R': 6.76, 'A': 8.11, 'N': 4.73, 'S': 5.41, 'T': 7.43, 'F': 2.03, 'E': 2.7, 'V': 10.14, 'K': 7.43, 'C': 2.03, 'P': 7.43, 'G': 9.46, 'Y': 4.73, 'L': 8.11, 'Q': 4.73, 'D': 4.05, 'M': 0.68, 'I': 2.03, 'H': 2.03}
- **Common reported functions (%):** 25.0
- **Common reported locations (%):** 12.5
- **Common reported processes (%):** 8.33

- **PDB ID:** 6WRV
- **Chain:** A
- **Crystallized protein length:** 639 aa
- **Resolution:** 2.47 Å
- **b-phipsi:** 0.006762
- **w-rdist:** 0.899291
- **t-alpha:** 0.000782
- **Chemical similarity (Tanimoto Index) (%):** N/A
- **1D identity (%) [PDB]:** 2.0
- **1D identity (%) [Gaps excluded][PDB]:** 69.05
- **1D identity - Alignment Gaps [PDB]:** 1405
- **1D aligned content [PDB] (<aminoacid>:%):** {'R': 3.45, 'V': 13.79, 'D': 6.9, 'P': 3.45, 'G': 6.9, 'K': 6.9, 'Q': 6.9, 'T': 3.45, 'F': 3.45, 'S': 10.34, 'W': 3.45, 'L': 13.79, 'E': 6.9, 'A': 6.9, 'N': 3.45}
- **2D identity (%) [PDB]:** 38.15
- **2D identity (%) [Gaps excluded][PDB]:** 84.27
- **2D identity - Alignment Gaps [PDB]:** 561
- **2D aligned content [PDB] (<2D-fold>:%):** {'.': 16.11, 'H': 48.34, 'T': 12.79, 'E': 21.74, 'G': 0.77, 'B': 0.26}
- **3D similarity (TM-Score) (%) [PDB]:** 21.88

- **Gene name:** TFRC
- **Entrez ID:** 7037
- **RefSeq ID:** NM\_003234
- **Transcript sequence length:** 5224
- **5-UTR|CDS|3-UTR identity (%):** 35.26 | 45.94 | 36.98
- **5-UTR|CDS|3-UTR identity (%) [Gaps excluded]:** 76.39 | 73.86 | 74.93
- **5-UTR|CDS|3-UTR identity [Alignment Gaps]:** 252 | 1135 | 2355
- **5-UTR aligned content (<base>:%):** {'G': 53.94, 'C': 34.55, 'T': 4.24, 'A': 7.27}
- **CDS aligned content (<base>:%):** {'T': 26.76, 'G': 23.71, 'A': 29.3, 'C': 20.23}
- **3-UTR aligned content (<base>:%):** {'A': 29.78, 'G': 19.02, 'T': 35.78, 'C': 15.42}

**Uniprot Description:**  
  
 Cellular uptake of iron occurs via receptor-mediated endocytosis of ligand-occupied transferrin receptor into specialized endosomes (PubMed:26214738). Endosomal acidification leads to iron release. The apotransferrin-receptor complex is then recycled to the cell surface with a return to neutral pH and the concomitant loss of affinity of apotransferrin for its receptor. Transferrin receptor is necessary for development of erythrocytes and the nervous system (By similarity). A second ligand, the heditary hemochromatosis protein HFE, competes for binding with transferrin for an overlapping C-terminal binding site. Positively regulates T and B cell proliferation through iron uptake (PubMed:26642240). Acts as a lipid sensor that regulates mitochondrial fusion by regulating activation of the JNK pathway (PubMed:26214738). When dietary levels of stearate (C18:0) are low, promotes activation of the JNK pathway, resulting in HUWE1-mediated ubiquitination and subsequent degradation of the mitofusin MFN2 and inhibition of mitochondrial fusion (PubMed:26214738). When dietary levels of stearate (C18:0) are high, TFRC stearoylation inhibits activation of the JNK pathway and thus degradation of the mitofusin MFN2 (PubMed:26214738).   
  
Homodimer; disulfide-linked. Binds one transferrin or HFE molecule per subunit. Binds the HLA class II histocompatibility antigen, DR1. Interacts with SH3BP3. Interacts with STEAP3; facilitates TFRC endocytosis in erythroid precursor cells (PubMed:26642240).   
  
 **Gene Ontology Information:**

Molecular Function

- double-stranded RNA binding
- Hsp70 protein binding
- identical protein binding
- protein homodimerization activity
- protein kinase binding
- protein-containing complex binding
- RNA binding
- transferrin receptor activity
- virus receptor activity

Location

- basolateral plasma membrane
- blood microparticle
- cell surface
- clathrin-coated endocytic vesicle membrane
- clathrin-coated pit
- cytoplasmic vesicle
- early endosome
- endosome
- endosome membrane
- external side of plasma membrane
- extracellular exosome
- extracellular region
- extracellular space
- extracellular vesicle
- HFE-transferrin receptor complex
- intracellular membrane-bounded organelle
- melanosome
- membrane
- perinuclear region of cytoplasm
- plasma membrane
- recycling endosome
- recycling endosome membrane

Biological process

- acute-phase response
- cellular response to leukemia inhibitory factor
- cellular response to xenobiotic stimulus
- cellular iron ion homeostasis
- intracellular signal transduction
- iron ion transport
- negative regulation of apoptotic process
- negative regulation of mitochondrial fusion
- osteoclast differentiation
- positive regulation of B cell proliferation
- positive regulation of bone resorption
- positive regulation of gene expression
- positive regulation of I-kappaB kinase/NF-kappaB signaling
- positive regulation of isotype switching
- positive regulation of NF-kappaB transcription factor activity
- positive regulation of peptidyl-serine phosphorylation
- positive regulation of protein localization to nucleus
- positive regulation of protein phosphorylation
- positive regulation of protein-containing complex assembly
- positive regulation of T cell proliferation
- receptor internalization
- response to copper ion
- response to hypoxia
- response to iron ion
- response to manganese ion
- response to nutrient
- response to retinoic acid
- transferrin transport
- transport across blood-brain barrier

---

66

- **Protein name:** 1-phosphatidylinositol 4,5-bisphosphate phosphodiesterase beta-3
- **Organism:** Homo sapiens
- **Uniprot Accession Number:** Q01970
- **Protein sequence length:** 1234 aa
- **1D identity (%):** 16.56
- **1D identity (%) [Gaps excluded]:** 25.39
- **1D identity - Alignment Gaps:** 441
- **1D aligned content (<aminoacid>:%):** {'M': 1.43, 'P': 11.43, 'G': 10.48, 'A': 5.71, 'R': 11.43, 'N': 3.81, 'F': 2.38, 'V': 7.62, 'D': 3.33, 'K': 3.81, 'I': 2.38, 'Y': 1.43, 'T': 3.81, 'L': 12.86, 'E': 9.05, 'Q': 4.76, 'W': 0.48, 'S': 3.33, 'H': 0.48}
- **Common reported functions (%):** 0.0
- **Common reported locations (%):** 0.0
- **Common reported processes (%):** 0.0

- **PDB ID:** 3OHM
- **Chain:** B
- **Crystallized protein length:** 759 aa
- **Resolution:** 2.7 Å
- **b-phipsi:** 0.002054
- **w-rdist:** 0.506625
- **t-alpha:** 0.055326
- **Chemical similarity (Tanimoto Index) (%):** 87.27
- **1D identity (%) [PDB]:** 3.37
- **1D identity (%) [Gaps excluded][PDB]:** 78.79
- **1D identity - Alignment Gaps [PDB]:** 1479
- **1D aligned content [PDB] (<aminoacid>:%):** {'R': 3.85, 'V': 11.54, 'D': 5.77, 'M': 3.85, 'E': 9.62, 'T': 3.85, 'L': 17.31, 'K': 1.92, 'S': 9.62, 'Q': 3.85, 'A': 5.77, 'G': 7.69, 'P': 7.69, 'F': 1.92, 'Y': 3.85, 'H': 1.92}
- **2D identity (%) [PDB]:** 37.22
- **2D identity (%) [Gaps excluded][PDB]:** 83.67
- **2D identity - Alignment Gaps [PDB]:** 619
- **2D aligned content [PDB] (<2D-fold>:%):** {'.': 18.31, 'E': 27.23, 'T': 12.29, 'H': 40.48, 'B': 0.24, 'G': 1.45}
- **3D similarity (TM-Score) (%) [PDB]:** 23.02

- **Gene name:** PLCB3
- **Entrez ID:** N/A
- **RefSeq ID:** NM\_001316314
- **Transcript sequence length:** 4734
- **5-UTR|CDS|3-UTR identity (%):** 21.78 | 43.15 | 13.13
- **5-UTR|CDS|3-UTR identity (%) [Gaps excluded]:** 75.21 | 74.51 | 74.55
- **5-UTR|CDS|3-UTR identity [Alignment Gaps]:** 287 | 1677 | 3638
- **5-UTR aligned content (<base>:%):** {'G': 43.18, 'C': 45.45, 'T': 9.09, 'A': 2.27}
- **CDS aligned content (<base>:%):** {'A': 25.71, 'T': 18.56, 'G': 29.2, 'C': 26.53}
- **3-UTR aligned content (<base>:%):** {'A': 20.69, 'C': 27.76, 'T': 22.76, 'G': 28.79}

**Uniprot Description:**  
  
 The production of the second messenger molecules diacylglycerol (DAG) and inositol 1,4,5-trisphosphate (IP3) is mediated by activated phosphatidylinositol-specific phospholipase C enzymes.   
  
Interacts with SHANK2 (By similarity). Interacts with LPAR2 (PubMed:15143197).   
  
 **Gene Ontology Information:**

Molecular Function   
  
N/A

Location   
  
N/A

Biological process   
  
N/A

---

67

- **Protein name:** Mediator of RNA polymerase II transcription subunit 16
- **Organism:** Homo sapiens
- **Uniprot Accession Number:** Q9Y2X0
- **Protein sequence length:** 877 aa
- **1D identity (%):** 15.18
- **1D identity (%) [Gaps excluded]:** 23.39
- **1D identity - Alignment Gaps:** 370
- **1D aligned content (<aminoacid>:%):** {'R': 5.62, 'P': 10.62, 'G': 7.5, 'Y': 2.5, 'L': 11.88, 'D': 7.5, 'T': 7.5, 'W': 1.25, 'S': 8.75, 'A': 5.0, 'H': 3.12, 'V': 5.62, 'F': 3.75, 'E': 2.5, 'I': 3.12, 'N': 1.25, 'C': 5.0, 'M': 0.62, 'K': 3.75, 'Q': 3.12}
- **Common reported functions (%):** 0.0
- **Common reported locations (%):** 25.0
- **Common reported processes (%):** 0.0

- **PDB ID:** 7EMF
- **Chain:** P
- **Crystallized protein length:** 766 aa
- **Resolution:** 3.5 Å
- **b-phipsi:** 0.006474
- **w-rdist:** 0.455105
- **t-alpha:** 0.016432
- **Chemical similarity (Tanimoto Index) (%):** 95.54
- **1D identity (%) [PDB]:** 2.29
- **1D identity (%) [Gaps excluded][PDB]:** 72.0
- **1D identity - Alignment Gaps [PDB]:** 1520
- **1D aligned content [PDB] (<aminoacid>:%):** {'V': 16.67, 'S': 19.44, 'Q': 5.56, 'L': 13.89, 'A': 8.33, 'H': 5.56, 'D': 2.78, 'I': 2.78, 'R': 2.78, 'P': 8.33, 'M': 2.78, 'Y': 2.78, 'T': 2.78, 'G': 2.78, 'F': 2.78}
- **2D identity (%) [PDB]:** 32.68
- **2D identity (%) [Gaps excluded][PDB]:** 84.7
- **2D identity - Alignment Gaps [PDB]:** 718
- **2D aligned content [PDB] (<2D-fold>:%):** {'.': 20.94, 'E': 29.84, 'T': 11.52, 'H': 36.13, 'G': 1.57}
- **3D similarity (TM-Score) (%) [PDB]:** 6.99

- **Gene name:** MED16
- **Entrez ID:** 10025
- **RefSeq ID:** NM\_005481
- **Transcript sequence length:** 2892
- **5-UTR|CDS|3-UTR identity (%):** 17.99 | 45.28 | 1.78
- **5-UTR|CDS|3-UTR identity (%) [Gaps excluded]:** 82.8 | 74.34 | 70.64
- **5-UTR|CDS|3-UTR identity [Alignment Gaps]:** 335 | 1268 | 4205
- **5-UTR aligned content (<base>:%):** {'A': 12.99, 'G': 44.16, 'C': 40.26, 'T': 2.6}
- **CDS aligned content (<base>:%):** {'A': 21.38, 'T': 18.72, 'G': 29.88, 'C': 30.02}
- **3-UTR aligned content (<base>:%):** {'G': 27.27, 'C': 33.77, 'T': 19.48, 'A': 19.48}

**Uniprot Description:**  
  
 Component of the Mediator complex, a coactivator involved in the regulated transcription of nearly all RNA polymerase II-dependent genes. Mediator functions as a bridge to convey information from gene-specific regulatory proteins to the basal RNA polymerase II transcription machinery. Mediator is recruited to promoters by direct interactions with regulatory proteins and serves as a scaffold for the assembly of a functional preinitiation complex with RNA polymerase II and the general transcription factors.   
  
Component of the Mediator complex, which is composed of MED1, MED4, MED6, MED7, MED8, MED9, MED10, MED11, MED12, MED13, MED13L, MED14, MED15, MED16, MED17, MED18, MED19, MED20, MED21, MED22, MED23, MED24, MED25, MED26, MED27, MED29, MED30, MED31, CCNC, CDK8 and CDC2L6/CDK11. The MED12, MED13, CCNC and CDK8 subunits form a distinct module termed the CDK8 module. Mediator containing the CDK8 module is less active than Mediator lacking this module in supporting transcriptional activation. Individual preparations of the Mediator complex lacking one or more distinct subunits have been variously termed ARC, CRSP, DRIP, PC2, SMCC and TRAP.   
  
 **Gene Ontology Information:**

Molecular Function

- thyroid hormone receptor binding
- vitamin D receptor binding
- transcription coactivator activity

Location

- core mediator complex
- mediator complex
- membrane
- nucleoplasm
- nucleus

Biological process

- positive regulation of transcription, DNA-templated
- positive regulation of transcription elongation from RNA polymerase II promoter
- positive regulation of transcription initiation from RNA polymerase II promoter
- regulation of transcription by RNA polymerase II
- RNA polymerase II preinitiation complex assembly
- transcription by RNA polymerase II

---

68

- **Protein name:** Maltose/maltodextrin-binding periplasmic protein
- **Organism:** Escherichia coli (strain K12)
- **Uniprot Accession Number:** P0AEX9
- **Protein sequence length:** 396 aa
- **1D identity (%):** 7.6
- **1D identity (%) [Gaps excluded]:** 20.83
- **1D identity - Alignment Gaps:** 585
- **1D aligned content (<aminoacid>:%):** {'G': 12.86, 'A': 7.14, 'M': 5.71, 'S': 2.86, 'E': 5.71, 'K': 17.14, 'F': 4.29, 'V': 8.57, 'H': 2.86, 'P': 10.0, 'T': 7.14, 'Y': 4.29, 'L': 4.29, 'N': 2.86, 'D': 2.86, 'I': 1.43}
- **Common reported functions (%):** 0.0
- **Common reported locations (%):** 0.0
- **Common reported processes (%):** 0.0

- **PDB ID:** 4BLD
- **Chain:** D
- **Crystallized protein length:** 730 aa
- **Resolution:** 2.8 Å
- **b-phipsi:** 0.002775
- **w-rdist:** 0.630586
- **t-alpha:** 0.013482
- **Chemical similarity (Tanimoto Index) (%):** 84.52
- **1D identity (%) [PDB]:** 2.15
- **1D identity (%) [Gaps excluded][PDB]:** 73.33
- **1D identity - Alignment Gaps [PDB]:** 1493
- **1D aligned content [PDB] (<aminoacid>:%):** {'L': 9.09, 'V': 12.12, 'A': 9.09, 'F': 3.03, 'R': 6.06, 'Y': 3.03, 'D': 12.12, 'K': 6.06, 'S': 9.09, 'E': 3.03, 'G': 9.09, 'H': 9.09, 'Q': 3.03, 'N': 3.03, 'I': 3.03}
- **2D identity (%) [PDB]:** 36.76
- **2D identity (%) [Gaps excluded][PDB]:** 83.47
- **2D identity - Alignment Gaps [PDB]:** 615
- **2D aligned content [PDB] (<2D-fold>:%):** {'E': 27.23, 'T': 10.4, '.': 14.85, 'H': 46.53, 'B': 0.25, 'G': 0.74}
- **3D similarity (TM-Score) (%) [PDB]:** 20.76

- **Gene name:** malE
- **Entrez ID:** 75204178; 948538
- **RefSeq ID:** N/A
- **Sequence length:** N/A
- **5-UTR|CDS|3-UTR identity (%):** N/A | N/A | N/A
- **5-UTR|CDS|3-UTR identity (%) [Gaps excluded]:** N/A | N/A | N/A
- **5-UTR|CDS|3-UTR identity [Alignment Gaps]:** N/A | N/A | N/A
- **5-UTR aligned content (<base>:%):** N/A
- **CDS aligned content (<base>:%):** N/A
- **3-UTR aligned content (<base>:%):** N/A

**Uniprot Description:**  
  
 Part of the ABC transporter complex MalEFGK involved in maltose/maltodextrin import. Binds maltose and higher maltodextrins such as maltotriose.   
  
The complex is composed of two ATP-binding proteins (MalK), two transmembrane proteins (MalG and MalF) and a solute-binding protein (MalE).   
  
 **Gene Ontology Information:**

Molecular Function

- carbohydrate transmembrane transporter activity
- maltose binding

Location

- ATP-binding cassette (ABC) transporter complex
- ATP-binding cassette (ABC) transporter complex, substrate-binding subunit-containing
- maltose transport complex
- outer membrane-bounded periplasmic space
- periplasmic space

Biological process

- carbohydrate transport
- cell chemotaxis
- detection of maltose stimulus
- cellular response to DNA damage stimulus
- maltodextrin transport
- maltose transport

---

69

- **Protein name:** Non-homologous end-joining factor 1
- **Organism:** Homo sapiens
- **Uniprot Accession Number:** Q9H9Q4
- **Protein sequence length:** 299 aa
- **1D identity (%):** 6.26
- **1D identity (%) [Gaps excluded]:** 21.05
- **1D identity - Alignment Gaps:** 628
- **1D aligned content (<aminoacid>:%):** {'Q': 8.93, 'L': 14.29, 'F': 3.57, 'G': 8.93, 'Y': 3.57, 'V': 7.14, 'W': 1.79, 'H': 3.57, 'N': 1.79, 'A': 1.79, 'P': 8.93, 'C': 5.36, 'D': 5.36, 'E': 10.71, 'T': 3.57, 'R': 1.79, 'K': 5.36, 'I': 1.79, 'S': 1.79}
- **Common reported functions (%):** 0.0
- **Common reported locations (%):** 0.0
- **Common reported processes (%):** 0.0

- **PDB ID:** 8EZA
- **Chain:** J
- **Crystallized protein length:** 497 aa
- **Resolution:** 4.39 Å
- **b-phipsi:** 0.004266
- **w-rdist:** 0.422758
- **t-alpha:** 0.091549
- **Chemical similarity (Tanimoto Index) (%):** 99.47
- **1D identity (%) [PDB]:** 2.39
- **1D identity (%) [Gaps excluded][PDB]:** 73.81
- **1D identity - Alignment Gaps [PDB]:** 1257
- **1D aligned content [PDB] (<aminoacid>:%):** {'S': 12.9, 'L': 19.35, 'A': 9.68, 'G': 6.45, 'H': 3.23, 'E': 3.23, 'V': 9.68, 'D': 6.45, 'I': 3.23, 'T': 6.45, 'R': 9.68, 'P': 6.45, 'Y': 3.23}
- **2D identity (%) [PDB]:** 41.48
- **2D identity (%) [Gaps excluded][PDB]:** 90.95
- **2D identity - Alignment Gaps [PDB]:** 501
- **2D aligned content [PDB] (<2D-fold>:%):** {'T': 23.04, 'E': 23.04, 'H': 30.1, '.': 22.25, 'B': 0.79, 'G': 0.79}
- **3D similarity (TM-Score) (%) [PDB]:** 28.87

- **Gene name:** NHEJ1
- **Entrez ID:** 79840
- **RefSeq ID:** NM\_024782
- **Transcript sequence length:** 8020
- **5-UTR|CDS|3-UTR identity (%):** 14.08 | 21.29 | 38.47
- **5-UTR|CDS|3-UTR identity (%) [Gaps excluded]:** 75.64 | 77.39 | 78.13
- **5-UTR|CDS|3-UTR identity [Alignment Gaps]:** 341 | 1982 | 3847
- **5-UTR aligned content (<base>:%):** {'G': 55.93, 'A': 10.17, 'C': 30.51, 'T': 3.39}
- **CDS aligned content (<base>:%):** {'A': 26.8, 'G': 27.66, 'T': 21.99, 'C': 23.54}
- **3-UTR aligned content (<base>:%):** {'T': 32.86, 'C': 18.77, 'G': 22.09, 'A': 26.28}

**Uniprot Description:**  
  
 DNA repair protein involved in DNA non-homologous end joining (NHEJ); required for double-strand break (DSB) repair and V(D)J recombination (PubMed:16439204, PubMed:16439205, PubMed:17717001, PubMed:17317666, PubMed:17470781, PubMed:18644470, PubMed:20558749, PubMed:26100018, PubMed:18158905). Plays a key role in NHEJ by promoting the ligation of various mismatched and non-cohesive ends (PubMed:17717001, PubMed:17470781, PubMed:19056826). Together with PAXX, collaborates with DNA polymerase lambda (POLL) to promote joining of non-cohesive DNA ends (PubMed:30250067, PubMed:25670504). May act in concert with XRCC5-XRCC6 (Ku) to stimulate XRCC4-mediated joining of blunt ends and several types of mismatched ends that are non-complementary or partially complementary (PubMed:16439204, PubMed:16439205, PubMed:17317666, PubMed:17470781). Associates with XRCC4 to form alternating helical filaments that bridge DNA and act like a bandage, holding together the broken DNA until it is repaired (PubMed:22228831, PubMed:26100018, PubMed:28500754, PubMed:27437582, PubMed:21775435, PubMed:22287571, PubMed:21768349). The XRCC4-NHEJ1/XLF subcomplex binds to the DNA fragments of a DSB in a highly diffusive manner and robustly bridges two independent DNA molecules, holding the broken DNA fragments in close proximity to one other (PubMed:28500754, PubMed:27437582). The mobility of the bridges ensures that the ends remain accessible for further processing by other repair factors (PubMed:27437582). Binds DNA in a length-dependent manner (PubMed:17317666, PubMed:18158905).   
  
Homodimer; mainly exists as a homodimer when not associated with XRCC4 (PubMed:18046455, PubMed:25574025, PubMed:25670504, PubMed:25941166, PubMed:18158905). Interacts with XRCC4; the interaction is direct and is mediated via a head-to-head interaction between N-terminal head regions (PubMed:16439205, PubMed:20558749, PubMed:22228831, PubMed:26100018, PubMed:18158905, PubMed:21936820, PubMed:21775435, PubMed:22287571, PubMed:21768349, PubMed:27437582). Component of the core long-range non-homologous end joining (NHEJ) complex (also named DNA-PK complex) composed of PRKDC, LIG4, XRCC4, XRCC6/Ku70, XRCC5/Ku86 and NHEJ1/XLF (PubMed:16571728, PubMed:17317666, PubMed:33854234). Additional component of the NHEJ complex includes PAXX (PubMed:25574025, PubMed:25941166). Following autophosphorylation, PRKDC dissociates from DNA, leading to formation of the short-range NHEJ complex, composed of LIG4, XRCC4, XRCC6/Ku70, XRCC5/Ku86 and NHEJ1/XLF (PubMed:33854234). Interacts with POLL (DNA polymerase lambda); promoting POLL recruitment to double-strand breaks (DSBs) and stimulation of the end-filling activity of POLL (PubMed:30250067).   
  
 **Gene Ontology Information:**

Molecular Function   
  
N/A

Location   
  
N/A

Biological process   
  
N/A

---

70

- **Protein name:** DNA topoisomerase 3-alpha
- **Organism:** Homo sapiens
- **Uniprot Accession Number:** Q13472
- **Protein sequence length:** 1001 aa
- **1D identity (%):** 15.74
- **1D identity (%) [Gaps excluded]:** 23.66
- **1D identity - Alignment Gaps:** 374
- **1D aligned content (<aminoacid>:%):** {'P': 11.36, 'R': 7.95, 'L': 8.52, 'V': 6.82, 'E': 5.68, 'K': 5.68, 'D': 4.55, 'M': 1.14, 'G': 12.5, 'Y': 1.7, 'F': 2.84, 'W': 0.57, 'S': 2.84, 'T': 6.82, 'H': 3.41, 'N': 2.27, 'C': 3.41, 'Q': 6.82, 'A': 3.41, 'I': 1.7}
- **Common reported functions (%):** 0.0
- **Common reported locations (%):** 12.5
- **Common reported processes (%):** 0.0

- **PDB ID:** 4CGY
- **Chain:** A
- **Crystallized protein length:** 619 aa
- **Resolution:** 2.85 Å
- **b-phipsi:** 0.006907
- **w-rdist:** 0.446361
- **t-alpha:** 0.017516
- **Chemical similarity (Tanimoto Index) (%):** 96.82
- **1D identity (%) [PDB]:** 3.28
- **1D identity (%) [Gaps excluded][PDB]:** 69.7
- **1D identity - Alignment Gaps [PDB]:** 1337
- **1D aligned content [PDB] (<aminoacid>:%):** {'S': 6.52, 'T': 10.87, 'M': 4.35, 'I': 6.52, 'K': 2.17, 'A': 2.17, 'R': 8.7, 'P': 10.87, 'D': 2.17, 'Q': 4.35, 'E': 10.87, 'L': 10.87, 'V': 4.35, 'G': 6.52, 'F': 2.17, 'H': 2.17, 'Y': 2.17, 'N': 2.17}
- **2D identity (%) [PDB]:** 35.62
- **2D identity (%) [Gaps excluded][PDB]:** 89.26
- **2D identity - Alignment Gaps [PDB]:** 631
- **2D aligned content [PDB] (<2D-fold>:%):** {'.': 10.43, 'T': 10.96, 'E': 20.86, 'H': 57.75}
- **3D similarity (TM-Score) (%) [PDB]:** 19.4

- **Gene name:** TOP3A
- **Entrez ID:** 7156
- **RefSeq ID:** NM\_004618
- **Transcript sequence length:** 6596
- **5-UTR|CDS|3-UTR identity (%):** 32.02 | 46.07 | 40.03
- **5-UTR|CDS|3-UTR identity (%) [Gaps excluded]:** 74.19 | 74.26 | 74.67
- **5-UTR|CDS|3-UTR identity [Alignment Gaps]:** 245 | 1310 | 2313
- **5-UTR aligned content (<base>:%):** {'G': 49.28, 'T': 10.14, 'C': 34.78, 'A': 5.8}
- **CDS aligned content (<base>:%):** {'A': 26.98, 'T': 20.75, 'G': 27.92, 'C': 24.34}
- **3-UTR aligned content (<base>:%):** {'G': 23.55, 'C': 18.74, 'T': 31.61, 'A': 26.1}

**Uniprot Description:**  
  
 Releases the supercoiling and torsional tension of DNA introduced during the DNA replication and transcription by transiently cleaving and rejoining one strand of the DNA duplex. Introduces a single-strand break via transesterification at a target site in duplex DNA. The scissile phosphodiester is attacked by the catalytic tyrosine of the enzyme, resulting in the formation of a DNA-(5'-phosphotyrosyl)-enzyme intermediate and the expulsion of a 3'-OH DNA strand. The free DNA strand then undergoes passage around the unbroken strand thus removing DNA supercoils. Finally, in the religation step, the DNA 3'-OH attacks the covalent intermediate to expel the active-site tyrosine and restore the DNA phosphodiester backbone. As an essential component of the RMI complex it is involved in chromosome separation and the processing of homologous recombination intermediates to limit DNA crossover formation in cells. Has DNA decatenation activity (PubMed:30057030). It is required for mtDNA decatenation and segregation after completion of replication, in a process that does not require BLM, RMI1 and RMI2 (PubMed:29290614).   
  
Binds ssDNA (PubMed:29290614). Interacts (via N-terminal region) with BLM; the interaction is direct. Directly interacts with RMI1. Component of the RMI complex, containing at least TOP3A, RMI1 and RMI2. The RMI complex interacts with BLM.   
  
 **Gene Ontology Information:**

Molecular Function

- DNA binding
- DNA topoisomerase activity
- DNA topoisomerase type I (single strand cut, ATP-independent) activity
- single-stranded DNA binding
- zinc ion binding

Location

- mitochondrial matrix
- nucleoplasm
- nucleus
- PML body
- RecQ family helicase-topoisomerase III complex

Biological process

- chromosome separation
- DNA topological change
- double-strand break repair via homologous recombination
- meiotic cell cycle
- mitochondrial DNA metabolic process
- resolution of recombination intermediates

---

71

- **Protein name:** Exostosin-1
- **Organism:** Homo sapiens
- **Uniprot Accession Number:** Q16394
- **Protein sequence length:** 746 aa
- **1D identity (%):** 14.37
- **1D identity (%) [Gaps excluded]:** 22.12
- **1D identity - Alignment Gaps:** 341
- **1D aligned content (<aminoacid>:%):** {'A': 5.71, 'S': 7.86, 'L': 9.29, 'R': 8.57, 'G': 4.29, 'H': 2.86, 'P': 6.43, 'W': 3.57, 'F': 5.0, 'N': 5.71, 'D': 5.0, 'Y': 4.29, 'C': 0.71, 'Q': 5.0, 'I': 3.57, 'E': 4.29, 'T': 6.43, 'K': 3.57, 'V': 7.14, 'M': 0.71}
- **Common reported functions (%):** 0.0
- **Common reported locations (%):** 12.5
- **Common reported processes (%):** 0.0

- **PDB ID:** 7UQX
- **Chain:** A
- **Crystallized protein length:** 583 aa
- **Resolution:** 3.3 Å
- **b-phipsi:** 0.012784
- **w-rdist:** 0.621529
- **t-alpha:** 0.000782
- **Chemical similarity (Tanimoto Index) (%):** 77.59
- **1D identity (%) [PDB]:** 2.91
- **1D identity (%) [Gaps excluded][PDB]:** 60.0
- **1D identity - Alignment Gaps [PDB]:** 1274
- **1D aligned content [PDB] (<aminoacid>:%):** {'S': 12.82, 'F': 5.13, 'D': 5.13, 'L': 12.82, 'C': 2.56, 'A': 7.69, 'I': 5.13, 'G': 2.56, 'T': 2.56, 'P': 7.69, 'Q': 5.13, 'V': 12.82, 'Y': 7.69, 'R': 7.69, 'H': 2.56}
- **2D identity (%) [PDB]:** 34.61
- **2D identity (%) [Gaps excluded][PDB]:** 91.93
- **2D identity - Alignment Gaps [PDB]:** 636
- **2D aligned content [PDB] (<2D-fold>:%):** {'H': 36.54, '.': 21.81, 'T': 23.51, 'E': 17.0, 'G': 0.85, 'B': 0.28}
- **3D similarity (TM-Score) (%) [PDB]:** 23.09

- **Gene name:** EXT1
- **Entrez ID:** 2131
- **RefSeq ID:** NM\_000127
- **Transcript sequence length:** 8243
- **5-UTR|CDS|3-UTR identity (%):** 36.54 | 45.19 | 44.72
- **5-UTR|CDS|3-UTR identity (%) [Gaps excluded]:** 79.78 | 74.4 | 76.12
- **5-UTR|CDS|3-UTR identity [Alignment Gaps]:** 439 | 1179 | 2471
- **5-UTR aligned content (<base>:%):** {'T': 8.45, 'C': 41.55, 'G': 42.23, 'A': 7.77}
- **CDS aligned content (<base>:%):** {'A': 27.78, 'T': 24.69, 'G': 24.54, 'C': 22.99}
- **3-UTR aligned content (<base>:%):** {'G': 18.63, 'C': 16.28, 'T': 33.98, 'A': 31.11}

**Uniprot Description:**  
  
 Glycosyltransferase forming with EXT2 the heterodimeric heparan sulfate polymerase which catalyzes the elongation of the heparan sulfate glycan backbone (PubMed:10639137, PubMed:22660413, PubMed:36402845, PubMed:36593275, PubMed:9620772). Glycan backbone extension consists in the alternating transfer of (1->4)-beta-D-GlcA and (1->4)-alpha-D-GlcNAc residues from their respective UDP-sugar donors. Both EXT1 and EXT2 are required for the full activity of the polymerase since EXT1 bears the N-acetylglucosaminyl-proteoglycan 4-beta-glucuronosyltransferase activity within the complex while EXT2 carries the glucuronosyl-N-acetylglucosaminyl-proteoglycan 4-alpha-N-acetylglucosaminyltransferase activity (PubMed:36402845, PubMed:36593275). Heparan sulfate proteoglycans are ubiquitous components of the extracellular matrix and play an important role in tissue homeostasis and signaling (PubMed:10639137, PubMed:11391482, PubMed:22660413, PubMed:9620772).   
  
Part of the heparan sulfate polymerase, a dimeric complex composed of EXT1 and EXT2 (PubMed:10639137, PubMed:10679296, PubMed:36402845, PubMed:36593275). Could also form homooligomeric complexes (PubMed:10679296). Interacts with NDST1 (PubMed:35137078).   
  
 **Gene Ontology Information:**

Molecular Function

- acetylglucosaminyltransferase activity
- glucuronosyl-N-acetylglucosaminyl-proteoglycan 4-alpha-N-acetylglucosaminyltransferase activity
- glucuronosyltransferase activity
- transferase activity, transferring glycosyl groups
- heparan sulfate N-acetylglucosaminyltransferase activity
- metal ion binding
- N-acetylglucosaminyl-proteoglycan 4-beta-glucuronosyltransferase activity
- protein heterodimerization activity
- protein homodimerization activity

Location

- endoplasmic reticulum
- endoplasmic reticulum membrane
- Golgi apparatus
- Golgi membrane
- membrane
- synapse

Biological process

- antigen processing and presentation
- axon guidance
- basement membrane organization
- blood vessel remodeling
- BMP signaling pathway
- bone resorption
- canonical Wnt signaling pathway
- cartilage development involved in endochondral bone morphogenesis
- cell adhesion mediated by integrin
- cell fate commitment
- cellular response to virus
- chondrocyte hypertrophy
- chondrocyte proliferation
- chondroitin sulfate metabolic process
- collagen fibril organization
- cranial skeletal system development
- dendrite self-avoidance
- dendritic cell migration
- developmental growth involved in morphogenesis
- embryonic skeletal joint development
- endochondral bone growth
- endochondral ossification
- endoderm development
- epithelial tube branching involved in lung morphogenesis
- fear response
- fibroblast growth factor receptor signaling pathway
- fluid transport
- gastrulation
- gene expression
- glandular epithelial cell differentiation
- glomerular basement membrane development
- glycosaminoglycan biosynthetic process
- hair follicle morphogenesis
- heart contraction
- heart field specification
- hematopoietic stem cell differentiation
- hematopoietic stem cell homeostasis
- hematopoietic stem cell migration to bone marrow
- heparan sulfate proteoglycan biosynthetic process
- heparan sulfate proteoglycan biosynthetic process, polysaccharide chain biosynthetic process
- heparin biosynthetic process
- hypersensitivity
- leukocyte tethering or rolling
- limb joint morphogenesis
- lymphocyte adhesion to endothelial cell of high endothelial venule
- lymphocyte migration into lymphoid organs
- mesenchymal cell differentiation involved in bone development
- mesoderm development
- motor behavior
- multicellular organism growth
- multicellular organismal water homeostasis
- neural crest cell differentiation
- olfactory bulb development
- optic nerve development
- ossification
- ossification involved in bone maturation
- perichondral bone morphogenesis
- glomerular visceral epithelial cell differentiation
- polysaccharide biosynthetic process
- protein catabolic process
- protein glycosylation
- protein-containing complex assembly
- regulation of blood pressure
- response to heparin
- response to leukemia inhibitory factor
- response to light intensity
- sebaceous gland development
- signal transduction
- skeletal system development
- smoothened signaling pathway involved in lung development
- social behavior
- sodium ion homeostasis
- stem cell division
- stomach development
- sulfation
- sweat gland development
- synaptic transmission, glutamatergic
- tight junction organization
- TNFSF11-mediated signaling pathway
- vacuole organization
- vasodilation
- vocalization behavior
- wound healing

---

72

- **Protein name:** Epidermal growth factor receptor
- **Organism:** Homo sapiens
- **Uniprot Accession Number:** P00533
- **Protein sequence length:** 1210 aa
- **1D identity (%):** 17.48
- **1D identity (%) [Gaps excluded]:** 27.72
- **1D identity - Alignment Gaps:** 469
- **1D aligned content (<aminoacid>:%):** {'M': 2.25, 'G': 8.56, 'P': 6.31, 'A': 5.41, 'S': 6.31, 'T': 5.41, 'K': 8.56, 'L': 8.11, 'N': 4.5, 'V': 7.21, 'Y': 3.15, 'D': 4.05, 'R': 6.31, 'Q': 5.41, 'I': 3.6, 'W': 0.9, 'H': 3.15, 'C': 3.6, 'F': 2.25, 'E': 4.95}
- **Common reported functions (%):** 0.0
- **Common reported locations (%):** 37.5
- **Common reported processes (%):** 8.33

- **PDB ID:** 1NQL
- **Chain:** A
- **Crystallized protein length:** 612 aa
- **Resolution:** 2.8 Å
- **b-phipsi:** 0.013315
- **w-rdist:** 0.628362
- **t-alpha:** 0.0
- **Chemical similarity (Tanimoto Index) (%):** N/A
- **1D identity (%) [PDB]:** 1.25
- **1D identity (%) [Gaps excluded][PDB]:** 75.0
- **1D identity - Alignment Gaps [PDB]:** 1414
- **1D aligned content [PDB] (<aminoacid>:%):** {'K': 5.56, 'V': 22.22, 'S': 16.67, 'Q': 5.56, 'W': 5.56, 'L': 16.67, 'E': 5.56, 'A': 11.11, 'G': 5.56, 'H': 5.56}
- **2D identity (%) [PDB]:** 21.61
- **2D identity (%) [Gaps excluded][PDB]:** 85.14
- **2D identity - Alignment Gaps [PDB]:** 870
- **2D aligned content [PDB] (<2D-fold>:%):** {'E': 27.78, '.': 32.94, 'T': 25.79, 'H': 10.71, 'B': 1.59, 'G': 1.19}
- **3D similarity (TM-Score) (%) [PDB]:** 17.65

- **Gene name:** EGFR
- **Entrez ID:** 1956
- **RefSeq ID:** NM\_201284
- **Transcript sequence length:** 2872
- **5-UTR|CDS|3-UTR identity (%):** 41.49 | 43.97 | 7.05
- **5-UTR|CDS|3-UTR identity (%) [Gaps excluded]:** 76.39 | 75.65 | 75.68
- **5-UTR|CDS|3-UTR identity [Alignment Gaps]:** 196 | 1246 | 3964
- **5-UTR aligned content (<base>:%):** {'G': 36.52, 'C': 52.81, 'A': 5.06, 'T': 5.62}
- **CDS aligned content (<base>:%):** {'A': 27.14, 'T': 21.94, 'G': 26.07, 'C': 24.85}
- **3-UTR aligned content (<base>:%):** {'G': 19.16, 'C': 26.62, 'T': 26.3, 'A': 27.92}

**Uniprot Description:**  
  
 Receptor tyrosine kinase binding ligands of the EGF family and activating several signaling cascades to convert extracellular cues into appropriate cellular responses (PubMed:2790960, PubMed:10805725, PubMed:27153536). Known ligands include EGF, TGFA/TGF-alpha, AREG, epigen/EPGN, BTC/betacellulin, epiregulin/EREG and HBEGF/heparin-binding EGF (PubMed:2790960, PubMed:7679104, PubMed:8144591, PubMed:9419975, PubMed:15611079, PubMed:12297049, PubMed:27153536, PubMed:20837704, PubMed:17909029). Ligand binding triggers receptor homo- and/or heterodimerization and autophosphorylation on key cytoplasmic residues. The phosphorylated receptor recruits adapter proteins like GRB2 which in turn activates complex downstream signaling cascades. Activates at least 4 major downstream signaling cascades including the RAS-RAF-MEK-ERK, PI3 kinase-AKT, PLCgamma-PKC and STATs modules (PubMed:27153536). May also activate the NF-kappa-B signaling cascade (PubMed:11116146). Also directly phosphorylates other proteins like RGS16, activating its GTPase activity and probably coupling the EGF receptor signaling to the G protein-coupled receptor signaling (PubMed:11602604). Also phosphorylates MUC1 and increases its interaction with SRC and CTNNB1/beta-catenin (PubMed:11483589). Positively regulates cell migration via interaction with CCDC88A/GIV which retains EGFR at the cell membrane following ligand stimulation, promoting EGFR signaling which triggers cell migration (PubMed:20462955). Plays a role in enhancing learning and memory performance (By similarity).   
  
Binding of the ligand triggers homo- and/or heterodimerization of the receptor triggering its autophosphorylation. Heterodimer with ERBB2 (PubMed:10805725). Forms a complex with CCDC88A/GIV (via SH2-like regions) and GNAI3 which leads to enhanced EGFR signaling and triggering of cell migration; binding to CCDC88A requires autophosphorylation of the EGFR C-terminal region, and ligand stimulation is required for recruitment of GNAI3 to the complex (PubMed:20462955, PubMed:25187647). Interacts with ERRFI1; inhibits dimerization of the kinase domain and autophosphorylation (PubMed:18046415). Part of a complex with ERBB2 and either PIK3C2A or PIK3C2B (PubMed:10805725). Interacts with GRB2; an adapter protein coupling the receptor to downstream signaling pathways. Interacts with GAB2; involved in signaling downstream of EGFR. Interacts with STAT3; mediates EGFR downstream signaling in cell proliferation. Interacts with RIPK1; involved in NF-kappa-B activation. Interacts (autophosphorylated) with CBL, CBLB and CBLC; involved in EGFR ubiquitination and regulation. Interacts with SOCS5; regulates EGFR degradation through ELOC- and ELOB-mediated ubiquitination and proteasomal degradation. Interacts with PRMT5; methylates EGFR and enhances interaction with PTPN6. Interacts (phosphorylated) with PTPN6; inhibits EGFR-dependent activation of MAPK/ERK. Interacts with COPG1; essential for regulation of EGF-dependent nuclear transport of EGFR by retrograde trafficking from the Golgi to the ER. Interacts with TNK2; this interaction is dependent on EGF stimulation and kinase activity of EGFR. Interacts with PCNA; positively regulates PCNA (PubMed:17115032). Interacts with PELP1. Interacts with MUC1. Interacts with AP2M1. Interacts with FER. May interact with EPS8; mediates EPS8 phosphorylation. Interacts (via SH2 domains) with GRB2, NCK1 and NCK2 (PubMed:10026169). Interacts with ATXN2. Interacts with GAREM1. Interacts (ubiquitinated) with ANKRD13A/B/D; the interaction is direct and may regulate EGFR internalization after EGF stimulation. Interacts with GPER1; the interaction occurs in an estrogen-dependent manner. Interacts (via C-terminal cytoplasmic kinase domain) with ZPR1 (via zinc fingers). Interacts with RNF115 and RNF126 (PubMed:23418353). Interacts with GPRC5A (via its transmembrane domain) (PubMed:25311788). Interacts with FAM83B; positively regulates EGFR inducing its autophosphorylation in absence of stimulation by EGF (PubMed:23912460). Interacts with LAPTM4B; positively correlates with EGFR activation (PubMed:28479384). Interacts with STX19 (PubMed:16420529). Interacts with CD44 (PubMed:23589287). Interacts with PGRMC1; the interaction requires PGRMC1 homodimerization (PubMed:26988023). Interacts with PIKFYVE (PubMed:17909029). Interacts with NEU3.   
  
 **Gene Ontology Information:**

Molecular Function

- actin filament binding
- ATP binding
- ATPase binding
- cadherin binding
- chromatin binding
- double-stranded DNA binding
- enzyme binding
- epidermal growth factor binding
- epidermal growth factor-activated receptor activity
- identical protein binding
- kinase binding
- MAP kinase kinase kinase activity
- protein phosphatase binding
- protein tyrosine kinase activator activity
- protein tyrosine kinase activity
- transmembrane receptor protein tyrosine kinase activator activity
- transmembrane receptor protein tyrosine kinase activity
- transmembrane signaling receptor activity
- ubiquitin protein ligase binding
- virus receptor activity

Location

- basal plasma membrane
- basolateral plasma membrane
- cell junction
- cell surface
- clathrin-coated endocytic vesicle membrane
- cytoplasm
- early endosome membrane
- endoplasmic reticulum membrane
- endosome
- endosome membrane
- extracellular space
- focal adhesion
- Golgi membrane
- intracellular vesicle
- membrane
- membrane raft
- multivesicular body, internal vesicle lumen
- nuclear membrane
- nucleus
- perinuclear region of cytoplasm
- plasma membrane
- protein-containing complex
- receptor complex
- ruffle membrane
- Shc-EGFR complex

Biological process

- activation of phospholipase C activity
- cell morphogenesis
- cell surface receptor signaling pathway
- cell-cell adhesion
- cellular response to amino acid stimulus
- cellular response to cadmium ion
- cellular response to epidermal growth factor stimulus
- cellular response to estradiol stimulus
- cellular response to reactive oxygen species
- cerebral cortex cell migration
- digestive tract morphogenesis
- embryonic placenta development
- epidermal growth factor receptor signaling pathway
- epithelial cell proliferation
- ERBB2-EGFR signaling pathway
- eyelid development in camera-type eye
- hair follicle development
- learning or memory
- morphogenesis of an epithelial fold
- negative regulation of apoptotic process
- negative regulation of cardiocyte differentiation
- negative regulation of epidermal growth factor receptor signaling pathway
- negative regulation of protein catabolic process
- neurogenesis
- ossification
- peptidyl-tyrosine autophosphorylation
- peptidyl-tyrosine phosphorylation
- positive regulation of canonical Wnt signaling pathway
- positive regulation of cell growth
- positive regulation of cell migration
- positive regulation of cell population proliferation
- positive regulation of cyclin-dependent protein serine/threonine kinase activity
- positive regulation of DNA repair
- positive regulation of DNA replication
- positive regulation of transcription, DNA-templated
- positive regulation of epithelial cell proliferation
- positive regulation of ERK1 and ERK2 cascade
- positive regulation of fibroblast proliferation
- positive regulation of G1/S transition of mitotic cell cycle
- positive regulation of kinase activity
- positive regulation of MAP kinase activity
- positive regulation of pri-miRNA transcription by RNA polymerase II
- positive regulation of NIK/NF-kappaB signaling
- positive regulation of nitric oxide mediated signal transduction
- positive regulation of peptidyl-serine phosphorylation
- positive regulation of phosphorylation
- positive regulation of protein kinase B signaling
- positive regulation of protein kinase C activity
- positive regulation of protein localization to plasma membrane
- positive regulation of protein phosphorylation
- positive regulation of transcription by RNA polymerase II
- protein autophosphorylation
- protein insertion into membrane
- regulation of ERK1 and ERK2 cascade
- regulation of JNK cascade
- regulation of nitric-oxide synthase activity
- regulation of peptidyl-tyrosine phosphorylation
- regulation of phosphatidylinositol 3-kinase signaling
- response to UV-A
- salivary gland morphogenesis
- signal transduction
- transmembrane receptor protein tyrosine kinase signaling pathway

---

73

- **Protein name:** Dipeptidyl peptidase 8
- **Organism:** Homo sapiens
- **Uniprot Accession Number:** Q6V1X1
- **Protein sequence length:** 898 aa
- **1D identity (%):** 16.73
- **1D identity (%) [Gaps excluded]:** 23.2
- **1D identity - Alignment Gaps:** 285
- **1D aligned content (<aminoacid>:%):** {'G': 9.94, 'V': 7.02, 'I': 5.85, 'R': 7.02, 'P': 10.53, 'D': 4.68, 'T': 4.68, 'H': 3.51, 'M': 1.17, 'E': 5.85, 'S': 4.09, 'L': 11.11, 'Y': 5.26, 'F': 4.09, 'Q': 4.68, 'N': 1.75, 'A': 3.51, 'K': 4.09, 'W': 0.58, 'C': 0.58}
- **Common reported functions (%):** 0.0
- **Common reported locations (%):** 25.0
- **Common reported processes (%):** 0.0

- **PDB ID:** 6EOS
- **Chain:** C
- **Crystallized protein length:** 829 aa
- **Resolution:** 3.1 Å
- **b-phipsi:** 0.01153
- **w-rdist:** 0.397313
- **t-alpha:** 0.011076
- **Chemical similarity (Tanimoto Index) (%):** 99.17
- **1D identity (%) [PDB]:** 2.52
- **1D identity (%) [Gaps excluded][PDB]:** 70.69
- **1D identity - Alignment Gaps [PDB]:** 1566
- **1D aligned content [PDB] (<aminoacid>:%):** {'L': 9.76, 'V': 12.2, 'K': 12.2, 'N': 4.88, 'D': 4.88, 'P': 7.32, 'Y': 4.88, 'G': 12.2, 'H': 2.44, 'E': 7.32, 'M': 4.88, 'T': 2.44, 'R': 7.32, 'A': 2.44, 'I': 4.88}
- **2D identity (%) [PDB]:** 36.84
- **2D identity (%) [Gaps excluded][PDB]:** 86.11
- **2D identity - Alignment Gaps [PDB]:** 674
- **2D aligned content [PDB] (<2D-fold>:%):** {'.': 21.43, 'E': 42.63, 'T': 15.67, 'H': 19.35, 'G': 0.69, 'B': 0.23}
- **3D similarity (TM-Score) (%) [PDB]:** 27.32

- **Gene name:** DPP8
- **Entrez ID:** 54878
- **RefSeq ID:** N/A
- **Sequence length:** N/A
- **5-UTR|CDS|3-UTR identity (%):** N/A | N/A | N/A
- **5-UTR|CDS|3-UTR identity (%) [Gaps excluded]:** N/A | N/A | N/A
- **5-UTR|CDS|3-UTR identity [Alignment Gaps]:** N/A | N/A | N/A
- **5-UTR aligned content (<base>:%):** N/A
- **CDS aligned content (<base>:%):** N/A
- **3-UTR aligned content (<base>:%):** N/A

**Uniprot Description:**  
  
 Dipeptidyl peptidase that cleaves off N-terminal dipeptides from proteins having a Pro or Ala residue at position 2.   
  
Homodimer.   
  
 **Gene Ontology Information:**

Molecular Function

- aminopeptidase activity
- dipeptidyl-peptidase activity
- serine-type peptidase activity

Location

- cytoplasm
- cytosol

Biological process

- apoptotic process
- immune response
- negative regulation of programmed cell death
- proteolysis

---

74

- **Protein name:** Fatty acid synthase
- **Organism:** Homo sapiens
- **Uniprot Accession Number:** P49327
- **Protein sequence length:** 2511 aa
- **1D identity (%):** 10.96
- **1D identity (%) [Gaps excluded]:** 32.78
- **1D identity - Alignment Gaps:** 1682
- **1D aligned content (<aminoacid>:%):** {'M': 0.72, 'E': 4.33, 'G': 9.75, 'P': 12.27, 'A': 6.14, 'L': 11.91, 'R': 6.14, 'T': 4.33, 'N': 1.44, 'Q': 5.42, 'V': 9.39, 'K': 3.97, 'F': 2.53, 'D': 2.89, 'Y': 2.89, 'H': 4.69, 'I': 2.89, 'W': 1.44, 'S': 4.69, 'C': 2.17}
- **Common reported functions (%):** 0.0
- **Common reported locations (%):** 37.5
- **Common reported processes (%):** 0.0

- **PDB ID:** 3HHD
- **Chain:** D
- **Crystallized protein length:** 854 aa
- **Resolution:** 2.15 Å
- **b-phipsi:** 0.003839
- **w-rdist:** 0.784533
- **t-alpha:** 0.007092
- **Chemical similarity (Tanimoto Index) (%):** 99.17
- **1D identity (%) [PDB]:** 2.29
- **1D identity (%) [Gaps excluded][PDB]:** 84.44
- **1D identity - Alignment Gaps [PDB]:** 1612
- **1D aligned content [PDB] (<aminoacid>:%):** {'L': 21.05, 'P': 2.63, 'I': 2.63, 'G': 7.89, 'V': 15.79, 'D': 2.63, 'M': 2.63, 'E': 7.89, 'T': 5.26, 'K': 5.26, 'Q': 7.89, 'F': 2.63, 'S': 5.26, 'A': 5.26, 'H': 2.63, 'N': 2.63}
- **2D identity (%) [PDB]:** 35.27
- **2D identity (%) [Gaps excluded][PDB]:** 85.51
- **2D identity - Alignment Gaps [PDB]:** 708
- **2D aligned content [PDB] (<2D-fold>:%):** {'E': 23.06, 'H': 42.59, '.': 18.59, 'T': 14.12, 'G': 1.41, 'B': 0.24}
- **3D similarity (TM-Score) (%) [PDB]:** 23.13

- **Gene name:** FASN
- **Entrez ID:** 2194
- **RefSeq ID:** N/A
- **Sequence length:** N/A
- **5-UTR|CDS|3-UTR identity (%):** N/A | N/A | N/A
- **5-UTR|CDS|3-UTR identity (%) [Gaps excluded]:** N/A | N/A | N/A
- **5-UTR|CDS|3-UTR identity [Alignment Gaps]:** N/A | N/A | N/A
- **5-UTR aligned content (<base>:%):** N/A
- **CDS aligned content (<base>:%):** N/A
- **3-UTR aligned content (<base>:%):** N/A

**Uniprot Description:**  
  
 Fatty acid synthetase is a multifunctional enzyme that catalyzes the de novo biosynthesis of long-chain saturated fatty acids starting from acetyl-CoA and malonyl-CoA in the presence of NADPH. This multifunctional protein contains 7 catalytic activities and a site for the binding of the prosthetic group 4'-phosphopantetheine of the acyl carrier protein ([ACP]) domain.   
  
Homodimer which is arranged in a head to tail fashion (PubMed:17618296, PubMed:18022563, Ref.33). Interacts with CEACAM1; this interaction is insulin and phosphorylation-dependent; reduces fatty-acid synthase activity.   
  
 **Gene Ontology Information:**

Molecular Function

- 3-hydroxydecanoyl-[acyl-carrier-protein] dehydratase activity
- 3-hydroxyoctanoyl-[acyl-carrier-protein] dehydratase activity
- (3R)-hydroxybutanoyl-[acyl-carrier-protein] hydratase activity
- (3R)-hydroxymyristoyl-[acyl-carrier-protein] dehydratase activity
- 3-hydroxypalmitoyl-[acyl-carrier-protein] dehydratase activity
- 3-oxoacyl-[acyl-carrier-protein] reductase (NADPH) activity
- 3-oxoacyl-[acyl-carrier-protein] synthase activity
- [acyl-carrier-protein] S-acetyltransferase activity
- [acyl-carrier-protein] S-malonyltransferase activity
- cadherin binding
- enoyl-[acyl-carrier-protein] reductase (NADPH, A-specific) activity
- fatty acid synthase activity
- myristoyl-[acyl-carrier-protein] hydrolase activity
- oleoyl-[acyl-carrier-protein] hydrolase activity
- palmitoyl-[acyl-carrier-protein] hydrolase activity
- phosphopantetheine binding
- RNA binding

Location

- cytoplasm
- cytosol
- extracellular exosome
- glycogen granule
- Golgi apparatus
- melanosome
- membrane
- plasma membrane

Biological process

- cellular response to interleukin-4
- establishment of endothelial intestinal barrier
- ether lipid biosynthetic process
- fatty acid biosynthetic process
- fatty acid metabolic process
- glandular epithelial cell development
- inflammatory response
- mammary gland development
- modulation by host of viral process
- monocyte differentiation
- neutrophil differentiation
- osteoblast differentiation

---

75

- **Protein name:** Dipeptidyl peptidase 4
- **Organism:** Homo sapiens
- **Uniprot Accession Number:** P27487
- **Protein sequence length:** 766 aa
- **1D identity (%):** 14.26
- **1D identity (%) [Gaps excluded]:** 21.32
- **1D identity - Alignment Gaps:** 323
- **1D aligned content (<aminoacid>:%):** {'P': 9.35, 'L': 10.79, 'G': 10.07, 'D': 7.19, 'R': 5.04, 'Y': 5.76, 'N': 3.6, 'V': 7.91, 'F': 3.6, 'S': 7.19, 'E': 2.16, 'I': 5.04, 'T': 5.04, 'W': 0.72, 'A': 2.16, 'K': 5.76, 'C': 4.32, 'Q': 1.44, 'M': 1.44, 'H': 1.44}
- **Common reported functions (%):** 0.0
- **Common reported locations (%):** 12.5
- **Common reported processes (%):** 0.0

- **PDB ID:** 3OPM
- **Chain:** B
- **Crystallized protein length:** 733 aa
- **Resolution:** 2.72 Å
- **b-phipsi:** 0.006203
- **w-rdist:** 0.768939
- **t-alpha:** 0.004694
- **Chemical similarity (Tanimoto Index) (%):** 80.22
- **1D identity (%) [PDB]:** 3.37
- **1D identity (%) [Gaps excluded][PDB]:** 72.86
- **1D identity - Alignment Gaps [PDB]:** 1443
- **1D aligned content [PDB] (<aminoacid>:%):** {'D': 13.73, 'G': 7.84, 'R': 3.92, 'Y': 1.96, 'T': 7.84, 'A': 5.88, 'L': 17.65, 'P': 3.92, 'V': 13.73, 'E': 5.88, 'K': 3.92, 'F': 1.96, 'S': 5.88, 'Q': 1.96, 'N': 1.96, 'H': 1.96}
- **2D identity (%) [PDB]:** 32.89
- **2D identity (%) [Gaps excluded][PDB]:** 82.3
- **2D identity - Alignment Gaps [PDB]:** 679
- **2D aligned content [PDB] (<2D-fold>:%):** {'.': 16.67, 'E': 43.28, 'T': 15.32, 'H': 23.12, 'G': 1.61}
- **3D similarity (TM-Score) (%) [PDB]:** 25.7

- **Gene name:** DPP4
- **Entrez ID:** 1803
- **RefSeq ID:** NM\_001935
- **Transcript sequence length:** 3573
- **5-UTR|CDS|3-UTR identity (%):** 38.68 | 43.27 | 15.47
- **5-UTR|CDS|3-UTR identity (%) [Gaps excluded]:** 77.73 | 73.5 | 77.08
- **5-UTR|CDS|3-UTR identity [Alignment Gaps]:** 213 | 1265 | 3543
- **5-UTR aligned content (<base>:%):** {'G': 35.98, 'T': 9.15, 'C': 50.61, 'A': 4.27}
- **CDS aligned content (<base>:%):** {'A': 32.53, 'T': 25.24, 'G': 22.61, 'C': 19.61}
- **3-UTR aligned content (<base>:%):** {'C': 14.43, 'A': 35.13, 'T': 32.94, 'G': 17.49}

**Uniprot Description:**  
  
 Cell surface glycoprotein receptor involved in the costimulatory signal essential for T-cell receptor (TCR)-mediated T-cell activation (PubMed:10951221, PubMed:10900005, PubMed:11772392, PubMed:17287217). Acts as a positive regulator of T-cell coactivation, by binding at least ADA, CAV1, IGF2R, and PTPRC (PubMed:10951221, PubMed:10900005, PubMed:11772392, PubMed:14691230). Its binding to CAV1 and CARD11 induces T-cell proliferation and NF-kappa-B activation in a T-cell receptor/CD3-dependent manner (PubMed:17287217). Its interaction with ADA also regulates lymphocyte-epithelial cell adhesion (PubMed:11772392). In association with FAP is involved in the pericellular proteolysis of the extracellular matrix (ECM), the migration and invasion of endothelial cells into the ECM (PubMed:16651416, PubMed:10593948). May be involved in the promotion of lymphatic endothelial cells adhesion, migration and tube formation (PubMed:18708048). When overexpressed, enhanced cell proliferation, a process inhibited by GPC3 (PubMed:17549790). Acts also as a serine exopeptidase with a dipeptidyl peptidase activity that regulates various physiological processes by cleaving peptides in the circulation, including many chemokines, mitogenic growth factors, neuropeptides and peptide hormones such as brain natriuretic peptide 32 (PubMed:16254193, PubMed:10570924). Removes N-terminal dipeptides sequentially from polypeptides having unsubstituted N-termini provided that the penultimate residue is proline (PubMed:10593948).   
  
Monomer. Homodimer (PubMed:12832764, PubMed:15448155, PubMed:17287217, PubMed:12646248, PubMed:12483204, PubMed:12906826). Heterodimer with Seprase (FAP) (PubMed:16651416). Requires homodimerization for optimal dipeptidyl peptidase activity and T-cell costimulation. Found in a membrane raft complex, at least composed of BCL10, CARD11, DPP4 and IKBKB (PubMed:17287217). Associates with collagen (PubMed:8526932). Interacts with PTPRC; the interaction is enhanced in an interleukin-12-dependent manner in activated lymphocytes (PubMed:12676959). Interacts (via extracellular domain) with ADA; does not inhibit its dipeptidyl peptidase activity (PubMed:15016824, PubMed:10951221, PubMed:14691230, PubMed:7907293, PubMed:8101391). Interacts with CAV1 (via the N-terminus); the interaction is direct (PubMed:17287217). Interacts (via cytoplasmic tail) with CARD11 (via PDZ domain); its homodimerization is necessary for interaction with CARD11 (PubMed:17287217). Interacts with IGF2R; the interaction is direct (PubMed:10900005). Interacts with GPC3 (PubMed:17549790). Interacts with human coronavirus-EMC spike protein and acts as a receptor for this virus (PubMed:23486063).   
  
 **Gene Ontology Information:**

Molecular Function

- aminopeptidase activity
- chemorepellent activity
- dipeptidyl-peptidase activity
- identical protein binding
- protease binding
- protein homodimerization activity
- serine-type endopeptidase activity
- serine-type peptidase activity
- signaling receptor binding
- virus receptor activity

Location

- apical plasma membrane
- cell surface
- endocytic vesicle
- extracellular exosome
- extracellular region
- focal adhesion
- intercellular canaliculus
- lamellipodium
- lamellipodium membrane
- lysosomal membrane
- membrane
- membrane raft
- plasma membrane

Biological process

- behavioral fear response
- cell adhesion
- endothelial cell migration
- glucagon processing
- locomotory exploration behavior
- membrane fusion
- negative regulation of extracellular matrix disassembly
- negative regulation of neutrophil chemotaxis
- peptide hormone processing
- positive regulation of cell population proliferation
- proteolysis
- psychomotor behavior
- receptor-mediated endocytosis of virus by host cell
- receptor-mediated virion attachment to host cell
- regulation of cell-cell adhesion mediated by integrin
- response to hypoxia
- T cell activation
- T cell costimulation
- viral entry into host cell

---

76

- **Protein name:** Anthrax toxin receptor 2
- **Organism:** Homo sapiens
- **Uniprot Accession Number:** P58335
- **Protein sequence length:** 489 aa
- **1D identity (%):** 10.5
- **1D identity (%) [Gaps excluded]:** 23.5
- **1D identity - Alignment Gaps:** 516
- **1D aligned content (<aminoacid>:%):** {'R': 4.08, 'P': 12.24, 'G': 10.2, 'A': 7.14, 'D': 5.1, 'L': 11.22, 'V': 8.16, 'W': 3.06, 'Q': 5.1, 'E': 4.08, 'S': 4.08, 'I': 6.12, 'F': 2.04, 'C': 4.08, 'N': 3.06, 'T': 2.04, 'Y': 3.06, 'K': 4.08, 'H': 1.02}
- **Common reported functions (%):** 0.0
- **Common reported locations (%):** 0.0
- **Common reported processes (%):** 0.0

- **PDB ID:** 1TZN
- **Chain:** H
- **Crystallized protein length:** 552 aa
- **Resolution:** 4.3 Å
- **b-phipsi:** 0.008614
- **w-rdist:** 0.74963
- **t-alpha:** 0.001565
- **Chemical similarity (Tanimoto Index) (%):** 75.32
- **1D identity (%) [PDB]:** 2.36
- **1D identity (%) [Gaps excluded][PDB]:** 65.31
- **1D identity - Alignment Gaps [PDB]:** 1306
- **1D aligned content [PDB] (<aminoacid>:%):** {'V': 12.5, 'N': 6.25, 'T': 9.38, 'L': 9.38, 'G': 12.5, 'R': 6.25, 'P': 6.25, 'A': 9.38, 'Q': 3.12, 'Y': 6.25, 'K': 6.25, 'D': 3.12, 'F': 3.12, 'E': 3.12, 'I': 3.12}
- **2D identity (%) [PDB]:** 23.51
- **2D identity (%) [Gaps excluded][PDB]:** 82.64
- **2D identity - Alignment Gaps [PDB]:** 782
- **2D aligned content [PDB] (<2D-fold>:%):** {'.': 25.68, 'E': 37.35, 'T': 14.01, 'H': 19.84, 'G': 2.33, 'B': 0.78}
- **3D similarity (TM-Score) (%) [PDB]:** 12.62

- **Gene name:** ANTXR2
- **Entrez ID:** 118429
- **RefSeq ID:** NM\_058172
- **Transcript sequence length:** 8419
- **5-UTR|CDS|3-UTR identity (%):** 42.32 | 34.79 | 42.31
- **5-UTR|CDS|3-UTR identity (%) [Gaps excluded]:** 76.84 | 76.42 | 75.61
- **5-UTR|CDS|3-UTR identity [Alignment Gaps]:** 310 | 1517 | 2984
- **5-UTR aligned content (<base>:%):** {'C': 47.26, 'G': 39.73, 'A': 4.79, 'T': 8.22}
- **CDS aligned content (<base>:%):** {'A': 29.41, 'G': 25.39, 'T': 24.87, 'C': 20.33}
- **3-UTR aligned content (<base>:%):** {'A': 31.22, 'G': 19.36, 'C': 14.72, 'T': 34.71}

**Uniprot Description:**  
  
 Necessary for cellular interactions with laminin and the extracellular matrix.   
  
Binds laminin, and possibly also collagen type IV.   
  
 **Gene Ontology Information:**

Molecular Function

- metal ion binding
- transmembrane signaling receptor activity

Location

- cell surface
- endoplasmic reticulum membrane
- endosome membrane
- extracellular region
- plasma membrane

Biological process

- toxin transport

---

77

- **Protein name:** Protein transport protein Sec24B
- **Organism:** Homo sapiens
- **Uniprot Accession Number:** O95487
- **Protein sequence length:** 1268 aa
- **1D identity (%):** 15.88
- **1D identity (%) [Gaps excluded]:** 23.85
- **1D identity - Alignment Gaps:** 427
- **1D aligned content (<aminoacid>:%):** {'M': 1.48, 'A': 6.9, 'P': 13.3, 'S': 8.87, 'G': 6.4, 'H': 2.96, 'Q': 7.39, 'I': 3.45, 'D': 4.43, 'T': 6.4, 'V': 6.9, 'L': 12.81, 'E': 2.46, 'N': 2.96, 'Y': 2.46, 'R': 4.43, 'F': 1.97, 'W': 0.49, 'C': 1.97, 'K': 1.97}
- **Common reported functions (%):** 0.0
- **Common reported locations (%):** 12.5
- **Common reported processes (%):** 0.0

- **PDB ID:** 3EH1
- **Chain:** A
- **Crystallized protein length:** 737 aa
- **Resolution:** 1.8 Å
- **b-phipsi:** 0.001058
- **w-rdist:** 0.738124
- **t-alpha:** 0.018779
- **Chemical similarity (Tanimoto Index) (%):** 95.84
- **1D identity (%) [PDB]:** 3.8
- **1D identity (%) [Gaps excluded][PDB]:** 64.04
- **1D identity - Alignment Gaps [PDB]:** 1411
- **1D aligned content [PDB] (<aminoacid>:%):** {'Q': 8.77, 'T': 5.26, 'L': 17.54, 'N': 3.51, 'C': 1.75, 'I': 1.75, 'A': 8.77, 'K': 5.26, 'G': 5.26, 'R': 5.26, 'S': 10.53, 'V': 10.53, 'F': 1.75, 'H': 1.75, 'P': 3.51, 'D': 1.75, 'Y': 3.51, 'E': 3.51}
- **2D identity (%) [PDB]:** 33.86
- **2D identity (%) [Gaps excluded][PDB]:** 85.97
- **2D identity - Alignment Gaps [PDB]:** 691
- **2D aligned content [PDB] (<2D-fold>:%):** {'.': 17.36, 'H': 38.6, 'E': 26.42, 'T': 15.8, 'G': 1.55, 'B': 0.26}
- **3D similarity (TM-Score) (%) [PDB]:** 22.73

- **Gene name:** SEC24B
- **Entrez ID:** 10427
- **RefSeq ID:** NM\_006323
- **Transcript sequence length:** 5083
- **5-UTR|CDS|3-UTR identity (%):** 8.72 | 41.91 | 19.51
- **5-UTR|CDS|3-UTR identity (%) [Gaps excluded]:** 83.72 | 76.12 | 78.85
- **5-UTR|CDS|3-UTR identity [Alignment Gaps]:** 370 | 1853 | 3322
- **5-UTR aligned content (<base>:%):** {'C': 69.44, 'T': 8.33, 'G': 19.44, 'A': 2.78}
- **CDS aligned content (<base>:%):** {'A': 29.69, 'T': 25.46, 'G': 22.11, 'C': 22.74}
- **3-UTR aligned content (<base>:%):** {'A': 32.75, 'G': 21.49, 'T': 31.01, 'C': 14.75}

**Uniprot Description:**  
  
 Component of the coat protein complex II (COPII) which promotes the formation of transport vesicles from the endoplasmic reticulum (ER). The coat has two main functions, the physical deformation of the endoplasmic reticulum membrane into vesicles and the selection of cargo molecules for their transport to the Golgi complex (PubMed:17499046, PubMed:20427317, PubMed:18843296). Plays a central role in cargo selection within the COPII complex and together with SEC24A may have a different specificity compared to SEC24C and SEC24D. May package preferentially cargos with cytoplasmic DxE or LxxLE motifs and may also recognize conformational epitopes (PubMed:17499046, PubMed:18843296).   
  
COPII is composed of at least five proteins: the Sec23/24 complex, the Sec13/31 complex and SAR1 (PubMed:10075675, PubMed:17499046). Interacts with RNF139 (PubMed:19706601). Interacts with TMED2 and TMED10 (PubMed:20427317). Interacts with CNIH4 (PubMed:24405750).   
  
 **Gene Ontology Information:**

Molecular Function

- SNARE binding
- zinc ion binding

Location

- COPII vesicle coat
- cytosol
- endoplasmic reticulum exit site
- endoplasmic reticulum membrane
- ER to Golgi transport vesicle membrane

Biological process

- aorta morphogenesis
- auditory receptor cell stereocilium organization
- cochlear nucleus development
- COPII-coated vesicle cargo loading
- coronary artery morphogenesis
- endoplasmic reticulum to Golgi vesicle-mediated transport
- intracellular protein transport
- lung lobe morphogenesis
- neural tube closure
- outflow tract morphogenesis
- pulmonary artery morphogenesis
- regulation of cargo loading into COPII-coated vesicle
- regulation of establishment of planar polarity involved in neural tube closure

---

78

- **Protein name:** Ribonucleases P/MRP protein subunit POP1
- **Organism:** Homo sapiens
- **Uniprot Accession Number:** Q99575
- **Protein sequence length:** 1024 aa
- **1D identity (%):** 17.11
- **1D identity (%) [Gaps excluded]:** 27.2
- **1D identity - Alignment Gaps:** 429
- **1D aligned content (<aminoacid>:%):** {'K': 7.58, 'V': 5.56, 'D': 3.54, 'H': 4.55, 'F': 2.02, 'Q': 8.08, 'P': 10.1, 'G': 10.1, 'R': 8.08, 'L': 9.09, 'E': 5.56, 'T': 5.05, 'S': 4.55, 'W': 2.02, 'M': 1.52, 'A': 4.55, 'Y': 2.53, 'C': 3.54, 'I': 1.01, 'N': 1.01}
- **Common reported functions (%):** 0.0
- **Common reported locations (%):** 0.0
- **Common reported processes (%):** 0.0

- **PDB ID:** 6AHR
- **Chain:** B
- **Crystallized protein length:** 774 aa
- **Resolution:** 3.92 Å
- **b-phipsi:** 0.01735
- **w-rdist:** 0.257468
- **t-alpha:** 0.013302
- **Chemical similarity (Tanimoto Index) (%):** N/A
- **1D identity (%) [PDB]:** 2.21
- **1D identity (%) [Gaps excluded][PDB]:** 74.47
- **1D identity - Alignment Gaps [PDB]:** 1534
- **1D aligned content [PDB] (<aminoacid>:%):** {'T': 5.71, 'L': 20.0, 'P': 5.71, 'G': 8.57, 'E': 8.57, 'K': 5.71, 'Q': 11.43, 'F': 2.86, 'V': 17.14, 'S': 5.71, 'D': 5.71, 'A': 2.86}
- **2D identity (%) [PDB]:** 35.46
- **2D identity (%) [Gaps excluded][PDB]:** 84.06
- **2D identity - Alignment Gaps [PDB]:** 662
- **2D aligned content [PDB] (<2D-fold>:%):** {'.': 18.47, 'H': 46.55, 'T': 11.58, 'E': 23.15, 'B': 0.25}
- **3D similarity (TM-Score) (%) [PDB]:** 25.25

- **Gene name:** POP1
- **Entrez ID:** 10940
- **RefSeq ID:** NM\_015029
- **Transcript sequence length:** 4667
- **5-UTR|CDS|3-UTR identity (%):** 4.46 | 45.35 | 22.14
- **5-UTR|CDS|3-UTR identity (%) [Gaps excluded]:** 72.0 | 75.52 | 76.35
- **5-UTR|CDS|3-UTR identity [Alignment Gaps]:** 379 | 1413 | 3219
- **5-UTR aligned content (<base>:%):** {'T': 16.67, 'G': 27.78, 'A': 16.67, 'C': 38.89}
- **CDS aligned content (<base>:%):** {'A': 28.43, 'T': 22.82, 'G': 26.37, 'C': 22.38}
- **3-UTR aligned content (<base>:%):** {'A': 29.58, 'T': 36.16, 'G': 19.22, 'C': 15.04}

**Uniprot Description:**  
  
 Component of ribonuclease P, a ribonucleoprotein complex that generates mature tRNA molecules by cleaving their 5'-ends (PubMed:8918471, PubMed:30454648). Also a component of the MRP ribonuclease complex, which cleaves pre-rRNA sequences (PubMed:28115465).   
  
Component of nuclear RNase P and RNase MRP ribonucleoproteins (PubMed:8918471, PubMed:16723659). RNase P consists of a catalytic RNA moiety and 10 different protein chains; POP1, POP4, POP5, POP7, RPP14, RPP21, RPP25, RPP30, RPP38 and RPP40 (PubMed:16723659, PubMed:30454648). Within the RNase P complex, POP1, POP7 and RPP25 form the 'finger' subcomplex, POP5, RPP14, RPP40 and homodimeric RPP30 form the 'palm' subcomplex, and RPP21, POP4 and RPP38 form the 'wrist' subcomplex. All subunits of the RNase P complex interact with the catalytic RNA (PubMed:30454648). Several subunits of RNase P are also part of the RNase MRP complex. RNase MRP consists of a catalytic RNA moiety and about 8 protein subunits; POP1, POP7, RPP25, RPP30, RPP38, RPP40 and possibly also POP4 and POP5 (PubMed:16723659, PubMed:28115465).   
  
 **Gene Ontology Information:**

Molecular Function

- ribonuclease P activity
- ribonuclease P RNA binding
- RNA binding

Location

- extracellular space
- multimeric ribonuclease P complex
- nucleolar ribonuclease P complex
- nucleolus
- nucleoplasm
- ribonuclease MRP complex

Biological process

- RNA phosphodiester bond hydrolysis, endonucleolytic
- tRNA 5'-leader removal
- tRNA catabolic process
- tRNA processing

---

79

- **Protein name:** Toll-like receptor 2
- **Organism:** Homo sapiens
- **Uniprot Accession Number:** O60603
- **Protein sequence length:** 784 aa
- **1D identity (%):** 14.23
- **1D identity (%) [Gaps excluded]:** 22.75
- **1D identity - Alignment Gaps:** 379
- **1D aligned content (<aminoacid>:%):** {'W': 1.39, 'V': 6.25, 'L': 17.36, 'G': 5.56, 'N': 5.56, 'P': 6.94, 'D': 4.17, 'R': 6.25, 'E': 6.94, 'F': 2.78, 'K': 7.64, 'M': 2.08, 'T': 4.86, 'Q': 3.47, 'S': 6.25, 'H': 2.08, 'C': 2.78, 'I': 2.78, 'A': 2.78, 'Y': 2.08}
- **Common reported functions (%):** 0.0
- **Common reported locations (%):** 12.5
- **Common reported processes (%):** 0.0

- **PDB ID:** 6NIG
- **Chain:** A
- **Crystallized protein length:** 546 aa
- **Resolution:** 2.35 Å
- **b-phipsi:** 0.005428
- **w-rdist:** 0.466349
- **t-alpha:** 0.0484
- **Chemical similarity (Tanimoto Index) (%):** 77.27
- **1D identity (%) [PDB]:** 1.83
- **1D identity (%) [Gaps excluded][PDB]:** 75.76
- **1D identity - Alignment Gaps [PDB]:** 1331
- **1D aligned content [PDB] (<aminoacid>:%):** {'L': 12.0, 'D': 4.0, 'N': 8.0, 'I': 8.0, 'T': 16.0, 'K': 12.0, 'P': 4.0, 'Q': 4.0, 'R': 4.0, 'V': 12.0, 'E': 8.0, 'G': 4.0, 'C': 4.0}
- **2D identity (%) [PDB]:** 28.19
- **2D identity (%) [Gaps excluded][PDB]:** 83.05
- **2D identity - Alignment Gaps [PDB]:** 689
- **2D aligned content [PDB] (<2D-fold>:%):** {'.': 26.53, 'T': 22.11, 'E': 30.27, 'H': 21.09}
- **3D similarity (TM-Score) (%) [PDB]:** 18.88

- **Gene name:** TLR2
- **Entrez ID:** 7097
- **RefSeq ID:** N/A
- **Sequence length:** N/A
- **5-UTR|CDS|3-UTR identity (%):** N/A | N/A | N/A
- **5-UTR|CDS|3-UTR identity (%) [Gaps excluded]:** N/A | N/A | N/A
- **5-UTR|CDS|3-UTR identity [Alignment Gaps]:** N/A | N/A | N/A
- **5-UTR aligned content (<base>:%):** N/A
- **CDS aligned content (<base>:%):** N/A
- **3-UTR aligned content (<base>:%):** N/A

**Uniprot Description:**  
  
 Cooperates with LY96 to mediate the innate immune response to bacterial lipoproteins and other microbial cell wall components. Cooperates with TLR1 or TLR6 to mediate the innate immune response to bacterial lipoproteins or lipopeptides (PubMed:21078852, PubMed:17889651). Acts via MYD88 and TRAF6, leading to NF-kappa-B activation, cytokine secretion and the inflammatory response. May also activate immune cells and promote apoptosis in response to the lipid moiety of lipoproteins (PubMed:10426995, PubMed:10426996). Recognizes mycoplasmal macrophage-activating lipopeptide-2kD (MALP-2), soluble tuberculosis factor (STF), phenol-soluble modulin (PSM) and B.burgdorferi outer surface protein A lipoprotein (OspA-L) cooperatively with TLR6 (PubMed:11441107). Stimulation of monocytes in vitro with M.tuberculosis PstS1 induces p38 MAPK and ERK1/2 activation primarily via this receptor, but also partially via TLR4 (PubMed:16622205). MAPK activation in response to bacterial peptidoglycan also occurs via this receptor (PubMed:16622205). Acts as a receptor for M.tuberculosis lipoproteins LprA, LprG, LpqH and PstS1, some lipoproteins are dependent on other coreceptors (TLR1, CD14 and/or CD36); the lipoproteins act as agonists to modulate antigen presenting cell functions in response to the pathogen (PubMed:19362712). M.tuberculosis HSP70 (dnaK) but not HSP65 (groEL-2) acts via this protein to stimulate NF-kappa-B expression (PubMed:15809303). Recognizes M.tuberculosis major T-antigen EsxA (ESAT-6) which inhibits downstream MYD88-dependent signaling (shown in mouse) (By similarity). Forms activation clusters composed of several receptors depending on the ligand, these clusters trigger signaling from the cell surface and subsequently are targeted to the Golgi in a lipid-raft dependent pathway. Forms the cluster TLR2:TLR6:CD14:CD36 in response to diacylated lipopeptides and TLR2:TLR1:CD14 in response to triacylated lipopeptides (PubMed:16880211). Required for normal uptake of M.tuberculosis, a process that is inhibited by M.tuberculosis LppM (By similarity).   
  
Interacts with LY96, TLR1 and TLR6 (via extracellular domain) (PubMed:17889651). TLR2 seems to exist in heterodimers with either TLR1 or TLR6 before stimulation by the ligand. The heterodimers form bigger oligomers in response to their corresponding ligands as well as further heterotypic associations with other receptors such as CD14 and/or CD36 (PubMed:16880211). Binds MYD88 (via TIR domain). Interacts with TICAM1 (PubMed:12471095). Interacts with CNPY3 (By similarity). Interacts with ATG16L1 (PubMed:23376921). Interacts with PPP1R11 (By similarity). Interacts with TICAM2 (PubMed:25385819).   
  
 **Gene Ontology Information:**

Molecular Function

- amyloid-beta binding
- identical protein binding
- lipopolysaccharide binding
- lipopolysaccharide immune receptor activity
- NAD(P)+ nucleosidase activity
- NAD+ nucleotidase, cyclic ADP-ribose generating
- pattern recognition receptor activity
- peptidoglycan binding
- protein-containing complex binding
- signaling receptor activity
- Toll-like receptor binding
- transmembrane signaling receptor activity
- triacyl lipopeptide binding

Location

- cell body
- cell projection
- cell surface
- cytoplasm
- Golgi apparatus
- membrane raft
- phagocytic vesicle membrane
- plasma membrane
- receptor complex
- secretory granule membrane
- Toll-like receptor 1-Toll-like receptor 2 protein complex
- Toll-like receptor 2-Toll-like receptor 6 protein complex

Biological process

- apoptotic process
- cellular response to bacterial lipopeptide
- cellular response to diacyl bacterial lipopeptide
- cellular response to lipoteichoic acid
- cellular response to triacyl bacterial lipopeptide
- cellular response to interferon-gamma
- central nervous system myelin formation
- defense response to Gram-positive bacterium
- defense response to virus
- detection of diacyl bacterial lipopeptide
- detection of triacyl bacterial lipopeptide
- I-kappaB phosphorylation
- immune response
- inflammatory response
- innate immune response
- learning
- leukotriene metabolic process
- microglia development
- microglial cell activation
- negative regulation of cell population proliferation
- negative regulation of phagocytosis
- negative regulation of synapse assembly
- nitric oxide metabolic process
- positive regulation of cellular response to macrophage colony-stimulating factor stimulus
- positive regulation of chemokine production
- positive regulation of gene expression
- positive regulation of inflammatory response
- positive regulation of interferon-beta production
- positive regulation of interleukin-10 production
- positive regulation of interleukin-12 production
- positive regulation of interleukin-18 production
- positive regulation of interleukin-6 production
- positive regulation of interleukin-8 production
- positive regulation of matrix metallopeptidase secretion
- positive regulation of NF-kappaB transcription factor activity
- positive regulation of NIK/NF-kappaB signaling
- positive regulation of nitric-oxide synthase biosynthetic process
- positive regulation of oligodendrocyte differentiation
- positive regulation of transcription by RNA polymerase II
- positive regulation of tumor necrosis factor production
- positive regulation of Wnt signaling pathway
- response to fatty acid
- response to hypoxia
- response to insulin
- response to progesterone
- response to toxic substance
- signal transduction
- toll-like receptor 2 signaling pathway
- toll-like receptor signaling pathway
- toll-like receptor TLR6:TLR2 signaling pathway

---

80

- **Protein name:** Antiviral innate immune response receptor RIG-I
- **Organism:** Homo sapiens
- **Uniprot Accession Number:** O95786
- **Protein sequence length:** 925 aa
- **1D identity (%):** 14.08
- **1D identity (%) [Gaps excluded]:** 22.63
- **1D identity - Alignment Gaps:** 416
- **1D aligned content (<aminoacid>:%):** {'T': 5.81, 'Q': 7.74, 'F': 5.81, 'L': 7.74, 'A': 6.45, 'E': 5.81, 'V': 5.81, 'M': 1.29, 'G': 7.1, 'R': 3.87, 'P': 7.1, 'I': 7.1, 'C': 5.16, 'D': 5.81, 'N': 3.23, 'K': 8.39, 'Y': 1.94, 'H': 1.94, 'S': 1.94}
- **Common reported functions (%):** 50.0
- **Common reported locations (%):** 25.0
- **Common reported processes (%):** 0.0

- **PDB ID:** 6KYV
- **Chain:** H
- **Crystallized protein length:** 672 aa
- **Resolution:** 3.0 Å
- **b-phipsi:** 0.011076
- **w-rdist:** 0.613021
- **t-alpha:** 0.002353
- **Chemical similarity (Tanimoto Index) (%):** 95.89
- **1D identity (%) [PDB]:** 3.23
- **1D identity (%) [Gaps excluded][PDB]:** 67.14
- **1D identity - Alignment Gaps [PDB]:** 1384
- **1D aligned content [PDB] (<aminoacid>:%):** {'K': 8.51, 'A': 10.64, 'T': 6.38, 'P': 4.26, 'D': 6.38, 'E': 6.38, 'S': 2.13, 'R': 8.51, 'L': 8.51, 'N': 4.26, 'Y': 2.13, 'F': 4.26, 'G': 10.64, 'I': 6.38, 'M': 2.13, 'Q': 2.13, 'V': 4.26, 'W': 2.13}
- **2D identity (%) [PDB]:** 32.82
- **2D identity (%) [Gaps excluded][PDB]:** 84.31
- **2D identity - Alignment Gaps [PDB]:** 670
- **2D aligned content [PDB] (<2D-fold>:%):** {'.': 16.11, 'H': 53.06, 'T': 10.83, 'E': 18.33, 'G': 1.67}
- **3D similarity (TM-Score) (%) [PDB]:** 28.61

- **Gene name:** DDX58
- **Entrez ID:** 23586
- **RefSeq ID:** NM\_014314
- **Transcript sequence length:** 4628
- **5-UTR|CDS|3-UTR identity (%):** 5.46 | 46.22 | 23.41
- **5-UTR|CDS|3-UTR identity (%) [Gaps excluded]:** 78.57 | 75.91 | 75.5
- **5-UTR|CDS|3-UTR identity [Alignment Gaps]:** 375 | 1304 | 3215
- **5-UTR aligned content (<base>:%):** {'G': 45.45, 'A': 13.64, 'C': 36.36, 'T': 4.55}
- **CDS aligned content (<base>:%):** {'G': 23.1, 'A': 33.16, 'C': 20.18, 'T': 23.56}
- **3-UTR aligned content (<base>:%):** {'A': 27.13, 'T': 38.68, 'C': 15.86, 'G': 18.33}

**Uniprot Description:**  
  
 Innate immune receptor that senses cytoplasmic viral nucleic acids and activates a downstream signaling cascade leading to the production of type I interferons and proinflammatory cytokines. Forms a ribonucleoprotein complex with viral RNAs on which it homooligomerizes to form filaments. The homooligomerization allows the recruitment of RNF135 an E3 ubiquitin-protein ligase that activates and amplifies the RIG-I-mediated antiviral signaling in an RNA length-dependent manner through ubiquitination-dependent and -independent mechanisms (PubMed:28469175, PubMed:31006531). Upon activation, associates with mitochondria antiviral signaling protein (MAVS/IPS1) that activates the IKK-related kinases TBK1 and IKBKE which in turn phosphorylate the interferon regulatory factors IRF3 and IRF7, activating transcription of antiviral immunological genes including the IFN-alpha and IFN-beta interferons (PubMed:28469175, PubMed:31006531). Ligands include 5'-triphosphorylated ssRNAs and dsRNAs but also short dsRNAs (<1 kb in length). In addition to the 5'-triphosphate moiety, blunt-end base pairing at the 5'-end of the RNA is very essential. Overhangs at the non-triphosphorylated end of the dsRNA RNA have no major impact on its activity. A 3'overhang at the 5'triphosphate end decreases and any 5'overhang at the 5' triphosphate end abolishes its activity. Detects both positive and negative strand RNA viruses including members of the families Paramyxoviridae: Human respiratory syncytial virus and measles virus (MeV), Rhabdoviridae: vesicular stomatitis virus (VSV), Orthomyxoviridae: influenza A and B virus, Flaviviridae: Japanese encephalitis virus (JEV), hepatitis C virus (HCV), dengue virus (DENV) and west Nile virus (WNV). It also detects rotaviruses and reoviruses. Also involved in antiviral signaling in response to viruses containing a dsDNA genome such as Epstein-Barr virus (EBV). Detects dsRNA produced from non-self dsDNA by RNA polymerase III, such as Epstein-Barr virus-encoded RNAs (EBERs). May play important roles in granulocyte production and differentiation, bacterial phagocytosis and in the regulation of cell migration.   
  
Monomer; maintained as a monomer in an autoinhibited state. Upon binding of viral RNAs and conformational shift, homooligomerizes and forms filaments on these molecules (PubMed:26471729). Interacts (via tandem CARD domain) with MAVS/IPS1 promoting its filamentation. Interacts with DHX58/LGP2, IKBKE, TBK1 and STING1. Interacts (via CARD domain) with TRIM25 (via SPRY domain). Interacts (double-stranded RNA-bound oligomeric form) with RNF135 (homodimer); involved in RNA length-dependent activation of the RIG-I signaling pathway (PubMed:19017631, PubMed:19484123, PubMed:23950712, PubMed:28469175, PubMed:31006531). Interacts with CYLD. Interacts with NLRC5; blocks the interaction of MAVS/IPS1 to DDX58. Interacts with SRC. Interacts with DDX60. Interacts with isoform 2 of ZC3HAV1 (via zinc-fingers) in an RNA-dependent manner. Interacts (via tandem CARD domain) with SEC14L1; the interaction is direct and impairs the interaction of DDX58 with MAVS/IPS1. Interacts with VCP/p97; interaction is direct and allows the recruitment of RNF125 and subsequent ubiquitination and degradation (PubMed:26471729). Interacts with NOP53; may regulate DDX58 through USP15-mediated 'Lys-63'-linked deubiquitination (PubMed:27824081). Interacts with SIGLEC10, CBL and PTPN11; within a negative feedback loop leading to DDX58 degradation (By similarity). Interacts with LRRC25 (PubMed:29288164). Interacts with ZCCHC3; leading to activation of DDX58/RIG-I (PubMed:30193849). Interacts with RNF123 (PubMed:27312109). Interacts with UBE2D3 and UBE2N; E2 ubiquitin ligases involved in RNF135-mediated ubiquitination of DDX58 and activation of the RIG-I signaling pathway (PubMed:28469175). Interacts with IFIT3 (PubMed:21813773). Interacts with DDX3X (PubMed:20127681).   
  
 **Gene Ontology Information:**

Molecular Function

- ATP binding
- ATPase activity
- double-stranded DNA binding
- double-stranded RNA binding
- GTP binding
- identical protein binding
- pattern recognition receptor activity
- RNA helicase activity
- single-stranded RNA binding
- ubiquitin protein ligase binding
- zinc ion binding

Location

- actin cytoskeleton
- bicellular tight junction
- cytoplasm
- cytosol
- ribonucleoprotein complex
- ruffle membrane

Biological process

- antiviral innate immune response
- cellular response to exogenous dsRNA
- cytoplasmic pattern recognition receptor signaling pathway
- defense response to virus
- detection of virus
- gene expression
- innate immune response
- positive regulation of defense response to virus by host
- positive regulation of DNA-binding transcription factor activity
- positive regulation of gene expression
- positive regulation of granulocyte macrophage colony-stimulating factor production
- positive regulation of interferon-alpha production
- positive regulation of interferon-beta production
- positive regulation of interleukin-6 production
- positive regulation of interleukin-8 production
- positive regulation of myeloid dendritic cell cytokine production
- positive regulation of response to cytokine stimulus
- positive regulation of transcription by RNA polymerase II
- positive regulation of tumor necrosis factor production
- regulation of cell migration
- regulation of type III interferon production
- response to exogenous dsRNA
- response to virus
- RIG-I signaling pathway

---

81

- **Protein name:** DNA replication licensing factor MCM6
- **Organism:** Homo sapiens
- **Uniprot Accession Number:** Q14566
- **Protein sequence length:** 821 aa
- **1D identity (%):** 15.42
- **1D identity (%) [Gaps excluded]:** 23.28
- **1D identity - Alignment Gaps:** 342
- **1D aligned content (<aminoacid>:%):** {'L': 13.46, 'E': 4.49, 'A': 3.85, 'G': 8.33, 'H': 2.56, 'V': 9.62, 'D': 7.05, 'S': 6.41, 'Y': 4.49, 'P': 7.69, 'F': 3.21, 'N': 3.85, 'R': 5.13, 'C': 3.21, 'I': 5.13, 'T': 4.49, 'K': 3.21, 'Q': 2.56, 'M': 0.64, 'W': 0.64}
- **Common reported functions (%):** 0.0
- **Common reported locations (%):** 12.5
- **Common reported processes (%):** 0.0

- **PDB ID:** 6XTY
- **Chain:** 6
- **Crystallized protein length:** 609 aa
- **Resolution:** 6.77 Å
- **b-phipsi:** 0.009871
- **w-rdist:** 0.491915
- **t-alpha:** 0.007824
- **Chemical similarity (Tanimoto Index) (%):** N/A
- **1D identity (%) [PDB]:** 2.34
- **1D identity (%) [Gaps excluded][PDB]:** 68.75
- **1D identity - Alignment Gaps [PDB]:** 1365
- **1D aligned content [PDB] (<aminoacid>:%):** {'H': 3.03, 'L': 15.15, 'K': 12.12, 'Y': 3.03, 'V': 24.24, 'G': 9.09, 'I': 3.03, 'P': 3.03, 'A': 6.06, 'E': 3.03, 'R': 3.03, 'D': 3.03, 'T': 6.06, 'Q': 6.06}
- **2D identity (%) [PDB]:** 34.55
- **2D identity (%) [Gaps excluded][PDB]:** 85.92
- **2D identity - Alignment Gaps [PDB]:** 623
- **2D aligned content [PDB] (<2D-fold>:%):** {'.': 16.94, 'H': 42.5, 'E': 27.78, 'T': 12.78}
- **3D similarity (TM-Score) (%) [PDB]:** 18.79

- **Gene name:** MCM6
- **Entrez ID:** 4175
- **RefSeq ID:** NM\_005915
- **Transcript sequence length:** 3753
- **5-UTR|CDS|3-UTR identity (%):** 11.35 | 47.43 | 17.51
- **5-UTR|CDS|3-UTR identity (%) [Gaps excluded]:** 72.31 | 74.76 | 75.61
- **5-UTR|CDS|3-UTR identity [Alignment Gaps]:** 349 | 1130 | 3428
- **5-UTR aligned content (<base>:%):** {'G': 40.43, 'A': 12.77, 'C': 34.04, 'T': 12.77}
- **CDS aligned content (<base>:%):** {'A': 29.88, 'T': 24.56, 'G': 24.83, 'C': 20.74}
- **3-UTR aligned content (<base>:%):** {'G': 15.11, 'A': 29.19, 'T': 40.08, 'C': 15.62}

**Uniprot Description:**  
  
 Acts as component of the MCM2-7 complex (MCM complex) which is the putative replicative helicase essential for 'once per cell cycle' DNA replication initiation and elongation in eukaryotic cells. The active ATPase sites in the MCM2-7 ring are formed through the interaction surfaces of two neighboring subunits such that a critical structure of a conserved arginine finger motif is provided in trans relative to the ATP-binding site of the Walker A box of the adjacent subunit. The six ATPase active sites, however, are likely to contribute differentially to the complex helicase activity.   
  
Component of the MCM2-7 complex (PubMed:16899510, PubMed:17296731, PubMed:9305914). The complex forms a toroidal hexameric ring with the proposed subunit order MCM2-MCM6-MCM4-MCM7-MCM3-MCM5 (PubMed:16899510, PubMed:17296731, PubMed:9305914). May interact with MCM10 (PubMed:11095689). Interacts with TIPIN (PubMed:17116885). Interacts with CDT1 (PubMed:20202939). Interacts with MCMBP (PubMed:17296731). Interacts with DDI2 (PubMed:29290612). Component of the CMG helicase complex, composed of the MCM2-7 complex, the GINS complex and CDC45 (By similarity).   
  
 **Gene Ontology Information:**

Molecular Function

- ATP binding
- ATPase activity
- DNA helicase activity
- identical protein binding
- single-stranded DNA binding

Location

- chromosome, telomeric region
- CMG complex
- MCM complex
- nucleoplasm
- nucleus

Biological process

- DNA replication
- DNA replication initiation
- DNA unwinding involved in DNA replication
- double-strand break repair via break-induced replication
- mitotic DNA replication
- regulation of DNA-dependent DNA replication initiation

---

82

- **Protein name:** Cytoplasmic aconitate hydratase
- **Organism:** Homo sapiens
- **Uniprot Accession Number:** P21399
- **Protein sequence length:** 889 aa
- **1D identity (%):** 18.37
- **1D identity (%) [Gaps excluded]:** 25.55
- **1D identity - Alignment Gaps:** 286
- **1D aligned content (<aminoacid>:%):** {'P': 12.83, 'A': 7.49, 'L': 9.09, 'G': 13.37, 'I': 4.28, 'R': 5.35, 'F': 3.21, 'V': 10.16, 'H': 1.6, 'D': 2.14, 'K': 4.28, 'N': 2.14, 'Q': 5.35, 'S': 3.74, 'Y': 5.35, 'T': 3.74, 'E': 3.74, 'C': 0.53, 'M': 1.07, 'W': 0.53}
- **Common reported functions (%):** 0.0
- **Common reported locations (%):** 25.0
- **Common reported processes (%):** 0.0

- **PDB ID:** 2B3X
- **Chain:** A
- **Crystallized protein length:** 888 aa
- **Resolution:** 2.54 Å
- **b-phipsi:** 0.006221
- **w-rdist:** 0.771249
- **t-alpha:** 0.004717
- **Chemical similarity (Tanimoto Index) (%):** 64.57
- **1D identity (%) [PDB]:** 2.13
- **1D identity (%) [Gaps excluded][PDB]:** 75.0
- **1D identity - Alignment Gaps [PDB]:** 1642
- **1D aligned content [PDB] (<aminoacid>:%):** {'S': 5.56, 'K': 13.89, 'A': 5.56, 'G': 13.89, 'P': 11.11, 'I': 8.33, 'Q': 8.33, 'Y': 5.56, 'V': 8.33, 'E': 2.78, 'M': 2.78, 'F': 2.78, 'T': 2.78, 'L': 8.33}
- **2D identity (%) [PDB]:** 38.94
- **2D identity (%) [Gaps excluded][PDB]:** 83.21
- **2D identity - Alignment Gaps [PDB]:** 630
- **2D aligned content [PDB] (<2D-fold>:%):** {'.': 18.22, 'T': 16.7, 'E': 23.21, 'H': 41.87}
- **3D similarity (TM-Score) (%) [PDB]:** 24.1

- **Gene name:** ACO1
- **Entrez ID:** 48
- **RefSeq ID:** N/A
- **Sequence length:** N/A
- **5-UTR|CDS|3-UTR identity (%):** N/A | N/A | N/A
- **5-UTR|CDS|3-UTR identity (%) [Gaps excluded]:** N/A | N/A | N/A
- **5-UTR|CDS|3-UTR identity [Alignment Gaps]:** N/A | N/A | N/A
- **5-UTR aligned content (<base>:%):** N/A
- **CDS aligned content (<base>:%):** N/A
- **3-UTR aligned content (<base>:%):** N/A

**Uniprot Description:**  
  
 Iron sensor. Binds a 4Fe-4S cluster and functions as aconitase when cellular iron levels are high. Functions as mRNA binding protein that regulates uptake, sequestration and utilization of iron when cellular iron levels are low. Binds to iron-responsive elements (IRES) in target mRNA species when iron levels are low. Binding of a 4Fe-4S cluster precludes RNA binding.   
  
Interacts (when associated with the 4Fe-4S) with FBXL5. Interacts with frataxin(81-210).   
  
 **Gene Ontology Information:**

Molecular Function

- 3 iron, 4 sulfur cluster binding
- 4 iron, 4 sulfur cluster binding
- aconitate hydratase activity
- citrate dehydratase activity
- iron-responsive element binding
- metal ion binding
- RNA binding

Location

- cytoplasm
- cytosol
- endoplasmic reticulum
- extracellular exosome
- Golgi apparatus
- mitochondrion

Biological process

- citrate metabolic process
- intestinal absorption
- cellular iron ion homeostasis
- post-embryonic development
- regulation of translation
- response to iron(II) ion
- tricarboxylic acid cycle

---

83

- **Protein name:** Protein O-linked-mannose beta-1,2-N-acetylglucosaminyltransferase 1
- **Organism:** Homo sapiens
- **Uniprot Accession Number:** Q8WZA1
- **Protein sequence length:** 660 aa
- **1D identity (%):** 14.42
- **1D identity (%) [Gaps excluded]:** 26.49
- **1D identity - Alignment Gaps:** 449
- **1D aligned content (<aminoacid>:%):** {'M': 0.7, 'P': 11.97, 'F': 4.23, 'L': 11.27, 'T': 4.23, 'V': 11.97, 'I': 2.82, 'Y': 4.23, 'D': 4.23, 'R': 7.04, 'A': 5.63, 'E': 4.93, 'Q': 2.82, 'H': 1.41, 'N': 2.11, 'S': 4.23, 'G': 6.34, 'W': 2.82, 'C': 2.11, 'K': 4.93}
- **Common reported functions (%):** 0.0
- **Common reported locations (%):** 12.5
- **Common reported processes (%):** 0.0

- **PDB ID:** 5GGF
- **Chain:** C
- **Crystallized protein length:** 536 aa
- **Resolution:** 2.49 Å
- **b-phipsi:** 0.003248
- **w-rdist:** 0.656784
- **t-alpha:** 0.017516
- **Chemical similarity (Tanimoto Index) (%):** 97.18
- **1D identity (%) [PDB]:** 2.16
- **1D identity (%) [Gaps excluded][PDB]:** 65.91
- **1D identity - Alignment Gaps [PDB]:** 1299
- **1D aligned content [PDB] (<aminoacid>:%):** {'R': 3.45, 'V': 20.69, 'D': 6.9, 'E': 13.79, 'T': 6.9, 'G': 3.45, 'K': 6.9, 'S': 6.9, 'Q': 3.45, 'L': 17.24, 'A': 6.9, 'P': 3.45}
- **2D identity (%) [PDB]:** 32.56
- **2D identity (%) [Gaps excluded][PDB]:** 82.65
- **2D identity - Alignment Gaps [PDB]:** 603
- **2D aligned content [PDB] (<2D-fold>:%):** {'.': 17.59, 'E': 31.48, 'T': 17.28, 'H': 33.64}
- **3D similarity (TM-Score) (%) [PDB]:** 19.04

- **Gene name:** POMGNT1
- **Entrez ID:** 55624
- **RefSeq ID:** N/A
- **Sequence length:** N/A
- **5-UTR|CDS|3-UTR identity (%):** N/A | N/A | N/A
- **5-UTR|CDS|3-UTR identity (%) [Gaps excluded]:** N/A | N/A | N/A
- **5-UTR|CDS|3-UTR identity [Alignment Gaps]:** N/A | N/A | N/A
- **5-UTR aligned content (<base>:%):** N/A
- **CDS aligned content (<base>:%):** N/A
- **3-UTR aligned content (<base>:%):** N/A

**Uniprot Description:**  
  
 Participates in O-mannosyl glycosylation by catalyzing the addition of N-acetylglucosamine to O-linked mannose on glycoproteins (PubMed:11709191, PubMed:27493216, PubMed:28512129). Catalyzes the synthesis of the GlcNAc(beta1-2)Man(alpha1-)O-Ser/Thr moiety on alpha-dystroglycan and other O-mannosylated proteins, providing the necessary basis for the addition of further carbohydrate moieties (PubMed:11709191, PubMed:27493216). Is specific for alpha linked terminal mannose and does not have MGAT3, MGAT4, MGAT5, MGAT7 or MGAT8 activity.   
  
Interacts with DAG1 (via O-linked mannose moiety) (PubMed:27493216). Interacts (via transmembrane domain) with FKTN; the interaction is direct and is required for normal location in Golgi membranes (PubMed:17034757).   
  
 **Gene Ontology Information:**

Molecular Function

- acetylglucosaminyltransferase activity
- beta-1,3-galactosyl-O-glycosyl-glycoprotein beta-1,3-N-acetylglucosaminyltransferase activity
- manganese ion binding

Location

- Golgi membrane
- membrane

Biological process

- basement membrane organization
- dentate gyrus development
- gene expression
- localization of cell
- myelination
- O-glycan processing
- protein O-linked glycosylation
- protein O-linked mannosylation
- reactive gliosis
- sensory perception of sound

---

84

- **Protein name:** Dipeptidyl peptidase 9
- **Organism:** Homo sapiens
- **Uniprot Accession Number:** Q86TI2
- **Protein sequence length:** 863 aa
- **1D identity (%):** 16.21
- **1D identity (%) [Gaps excluded]:** 22.02
- **1D identity - Alignment Gaps:** 262
- **1D aligned content (<aminoacid>:%):** {'T': 8.07, 'G': 10.56, 'P': 12.42, 'D': 4.97, 'R': 6.21, 'V': 6.21, 'F': 5.59, 'Y': 4.97, 'K': 1.86, 'S': 3.73, 'L': 8.07, 'H': 4.97, 'A': 2.48, 'N': 4.35, 'C': 4.35, 'Q': 4.35, 'E': 4.35, 'W': 0.62, 'I': 1.86}
- **Common reported functions (%):** 0.0
- **Common reported locations (%):** 25.0
- **Common reported processes (%):** 0.0

- **PDB ID:** 7A3F
- **Chain:** A
- **Crystallized protein length:** 794 aa
- **Resolution:** 2.9 Å
- **b-phipsi:** 0.015749
- **w-rdist:** 0.42745
- **t-alpha:** 0.006259
- **Chemical similarity (Tanimoto Index) (%):** 98.79
- **1D identity (%) [PDB]:** 2.44
- **1D identity (%) [Gaps excluded][PDB]:** 73.58
- **1D identity - Alignment Gaps [PDB]:** 1544
- **1D aligned content [PDB] (<aminoacid>:%):** {'D': 5.13, 'L': 12.82, 'R': 2.56, 'I': 5.13, 'K': 12.82, 'A': 5.13, 'G': 10.26, 'M': 7.69, 'P': 10.26, 'Q': 10.26, 'F': 5.13, 'Y': 2.56, 'E': 2.56, 'H': 2.56, 'V': 5.13}
- **2D identity (%) [PDB]:** 35.52
- **2D identity (%) [Gaps excluded][PDB]:** 84.08
- **2D identity - Alignment Gaps [PDB]:** 670
- **2D aligned content [PDB] (<2D-fold>:%):** {'.': 20.87, 'B': 0.49, 'E': 46.12, 'T': 13.83, 'H': 17.96, 'G': 0.73}
- **3D similarity (TM-Score) (%) [PDB]:** 27.12

- **Gene name:** DPP9
- **Entrez ID:** 91039
- **RefSeq ID:** N/A
- **Sequence length:** N/A
- **5-UTR|CDS|3-UTR identity (%):** N/A | N/A | N/A
- **5-UTR|CDS|3-UTR identity (%) [Gaps excluded]:** N/A | N/A | N/A
- **5-UTR|CDS|3-UTR identity [Alignment Gaps]:** N/A | N/A | N/A
- **5-UTR aligned content (<base>:%):** N/A
- **CDS aligned content (<base>:%):** N/A
- **3-UTR aligned content (<base>:%):** N/A

**Uniprot Description:**  
  
 Dipeptidyl peptidase that cleaves off N-terminal dipeptides from proteins having a Pro or Ala residue at position 2 (PubMed:12662155, PubMed:16475979, PubMed:19667070, PubMed:30291141, PubMed:29382749). Acts as an inhibitor of caspase-1-dependent monocyte and macrophage pyroptosis: inhibits pyroptosis by preventing activation of NLRP1 and CARD8 via an unknown mechanism (PubMed:27820798, PubMed:30291141, PubMed:29967349, PubMed:31525884, PubMed:32796818).   
  
Homodimer.   
  
 **Gene Ontology Information:**

Molecular Function

- aminopeptidase activity
- dipeptidyl-peptidase activity
- identical protein binding
- serine-type peptidase activity

Location

- cell leading edge
- cytosol
- microtubule
- nucleus

Biological process

- negative regulation of programmed cell death
- proteolysis
- pyroptosis

---

85

- **Protein name:** Voltage-dependent calcium channel subunit alpha-2/delta-1
- **Organism:** Homo sapiens
- **Uniprot Accession Number:** P54289
- **Protein sequence length:** 1103 aa
- **1D identity (%):** 13.24
- **1D identity (%) [Gaps excluded]:** 24.17
- **1D identity - Alignment Gaps:** 574
- **1D aligned content (<aminoacid>:%):** {'L': 12.5, 'G': 13.69, 'E': 3.57, 'P': 7.74, 'S': 5.36, 'D': 7.14, 'T': 4.17, 'Y': 5.36, 'V': 6.55, 'I': 4.17, 'R': 4.76, 'Q': 6.55, 'N': 2.98, 'F': 2.38, 'K': 6.55, 'M': 1.19, 'A': 1.79, 'C': 1.79, 'W': 1.19, 'H': 0.6}
- **Common reported functions (%):** 0.0
- **Common reported locations (%):** 0.0
- **Common reported processes (%):** 0.0

- **PDB ID:** 8IF4
- **Chain:** A
- **Crystallized protein length:** 915 aa
- **Resolution:** 3.2 Å
- **b-phipsi:** 0.005101
- **w-rdist:** 0.507571
- **t-alpha:** 0.040688
- **Chemical similarity (Tanimoto Index) (%):** 83.61
- **1D identity (%) [PDB]:** 1.96
- **1D identity (%) [Gaps excluded][PDB]:** 78.05
- **1D identity - Alignment Gaps [PDB]:** 1592
- **1D aligned content [PDB] (<aminoacid>:%):** {'L': 15.62, 'E': 6.25, 'A': 9.38, 'H': 3.12, 'N': 3.12, 'V': 12.5, 'D': 6.25, 'S': 6.25, 'Q': 3.12, 'I': 3.12, 'T': 6.25, 'P': 6.25, 'R': 6.25, 'Y': 3.12, 'G': 3.12, 'F': 6.25}
- **2D identity (%) [PDB]:** 37.46
- **2D identity (%) [Gaps excluded][PDB]:** 91.56
- **2D identity - Alignment Gaps [PDB]:** 702
- **2D aligned content [PDB] (<2D-fold>:%):** {'.': 14.38, 'H': 37.53, 'T': 23.15, 'E': 24.94}
- **3D similarity (TM-Score) (%) [PDB]:** 24.3

- **Gene name:** CACNA2D1
- **Entrez ID:** 781
- **RefSeq ID:** N/A
- **Sequence length:** N/A
- **5-UTR|CDS|3-UTR identity (%):** N/A | N/A | N/A
- **5-UTR|CDS|3-UTR identity (%) [Gaps excluded]:** N/A | N/A | N/A
- **5-UTR|CDS|3-UTR identity [Alignment Gaps]:** N/A | N/A | N/A
- **5-UTR aligned content (<base>:%):** N/A
- **CDS aligned content (<base>:%):** N/A
- **3-UTR aligned content (<base>:%):** N/A

**Uniprot Description:**  
  
 The alpha-2/delta subunit of voltage-dependent calcium channels regulates calcium current density and activation/inactivation kinetics of the calcium channel. Plays an important role in excitation-contraction coupling (By similarity).   
  
Dimer formed of alpha-2-1 and delta-1 chains; disulfide-linked. Voltage-dependent calcium channels are multisubunit complexes, consisting of alpha-1 (CACNA1), alpha-2 (CACNA2D), beta (CACNB) and delta (CACNA2D) subunits in a 1:1:1:1 ratio (By similarity).   
  
 **Gene Ontology Information:**

Molecular Function

- metal ion binding
- voltage-gated calcium channel activity

Location

- extracellular exosome
- L-type voltage-gated calcium channel complex
- neuronal dense core vesicle
- plasma membrane
- sarcoplasmic reticulum
- voltage-gated calcium channel complex

Biological process

- calcium ion import across plasma membrane
- calcium ion transmembrane transport
- calcium ion transmembrane transport via high voltage-gated calcium channel
- calcium ion transport
- calcium ion transport into cytosol
- cardiac muscle cell action potential involved in contraction
- cellular response to amyloid-beta
- membrane depolarization during bundle of His cell action potential
- positive regulation of high voltage-gated calcium channel activity
- positive regulation of muscle contraction
- regulation of calcium ion transmembrane transport via high voltage-gated calcium channel
- regulation of calcium ion transport
- regulation of heart rate by cardiac conduction
- regulation of membrane repolarization during action potential
- regulation of ventricular cardiac muscle cell membrane repolarization

---

86

- **Protein name:** Transcription initiation factor TFIID subunit 2
- **Organism:** Homo sapiens
- **Uniprot Accession Number:** Q6P1X5
- **Protein sequence length:** 1199 aa
- **1D identity (%):** 15.72
- **1D identity (%) [Gaps excluded]:** 25.74
- **1D identity - Alignment Gaps:** 498
- **1D aligned content (<aminoacid>:%):** {'P': 9.45, 'R': 6.97, 'G': 8.46, 'L': 9.45, 'H': 3.98, 'V': 8.46, 'I': 6.47, 'K': 4.48, 'F': 4.48, 'D': 3.48, 'N': 3.48, 'Y': 3.98, 'S': 7.96, 'W': 1.99, 'A': 4.48, 'T': 3.48, 'E': 1.99, 'C': 2.49, 'Q': 3.48, 'M': 1.0}
- **Common reported functions (%):** 0.0
- **Common reported locations (%):** 12.5
- **Common reported processes (%):** 0.0

- **PDB ID:** 5FUR
- **Chain:** I
- **Crystallized protein length:** 914 aa
- **Resolution:** 8.5 Å
- **b-phipsi:** 0.014339
- **w-rdist:** 0.235211
- **t-alpha:** 0.022692
- **Chemical similarity (Tanimoto Index) (%):** N/A
- **1D identity (%) [PDB]:** 2.16
- **1D identity (%) [Gaps excluded][PDB]:** 71.15
- **1D identity - Alignment Gaps [PDB]:** 1660
- **1D aligned content [PDB] (<aminoacid>:%):** {'K': 2.7, 'Q': 8.11, 'V': 13.51, 'S': 13.51, 'L': 18.92, 'E': 5.41, 'A': 8.11, 'N': 2.7, 'P': 5.41, 'D': 2.7, 'I': 2.7, 'T': 5.41, 'R': 8.11, 'Y': 2.7}
- **2D identity (%) [PDB]:** 38.09
- **2D identity (%) [Gaps excluded][PDB]:** 86.64
- **2D identity - Alignment Gaps [PDB]:** 687
- **2D aligned content [PDB] (<2D-fold>:%):** {'.': 17.56, 'E': 27.19, 'T': 16.27, 'H': 38.33, 'G': 0.64}
- **3D similarity (TM-Score) (%) [PDB]:** 12.02

- **Gene name:** TAF2
- **Entrez ID:** 6873
- **RefSeq ID:** NM\_003184
- **Transcript sequence length:** 5027
- **5-UTR|CDS|3-UTR identity (%):** 31.98 | 42.67 | 16.02
- **5-UTR|CDS|3-UTR identity (%) [Gaps excluded]:** 71.77 | 75.81 | 75.92
- **5-UTR|CDS|3-UTR identity [Alignment Gaps]:** 260 | 1730 | 3541
- **5-UTR aligned content (<base>:%):** {'G': 48.0, 'T': 8.0, 'C': 34.67, 'A': 9.33}
- **CDS aligned content (<base>:%):** {'A': 30.61, 'T': 26.29, 'G': 22.08, 'C': 21.02}
- **3-UTR aligned content (<base>:%):** {'G': 13.49, 'A': 31.99, 'C': 14.33, 'T': 40.19}

**Uniprot Description:**  
  
 Transcription factor TFIID is one of the general factors required for accurate and regulated initiation by RNA polymerase II. TFIID is a multimeric protein complex that plays a central role in mediating promoter responses to various activators and repressors. It requires core promoter-specific cofactors for productive transcription stimulation. TAF2 stabilizes TFIID binding to core promoter.   
  
Component of transcription factor TFIID which is composed of TBP and a number of TBP-associated factors. Interacts with TAF2C1. Component of the TFTC-HAT complex.   
  
 **Gene Ontology Information:**

Molecular Function

- chromatin binding
- RNA polymerase II general transcription initiation factor activity
- transcription regulatory region sequence-specific DNA binding

Location

- nucleoplasm
- nucleus
- transcription factor TFIID complex
- transcription factor TFTC complex

Biological process

- G2/M transition of mitotic cell cycle
- histone H3 acetylation
- monoubiquitinated histone deubiquitination
- monoubiquitinated histone H2A deubiquitination
- mRNA transcription by RNA polymerase II
- positive regulation of transcription, DNA-templated
- positive regulation of transcription initiation from RNA polymerase II promoter
- protein phosphorylation
- regulation of DNA repair
- regulation of transcription by RNA polymerase II
- response to organic cyclic compound
- RNA polymerase II preinitiation complex assembly
- transcription initiation from RNA polymerase II promoter

---

87

- **Protein name:** Protein zwilch homolog
- **Organism:** Homo sapiens
- **Uniprot Accession Number:** Q9H900
- **Protein sequence length:** 591 aa
- **1D identity (%):** 12.89
- **1D identity (%) [Gaps excluded]:** 23.03
- **1D identity - Alignment Gaps:** 410
- **1D aligned content (<aminoacid>:%):** {'R': 4.17, 'N': 3.33, 'D': 6.67, 'Q': 5.0, 'F': 2.5, 'G': 6.67, 'Y': 2.5, 'A': 5.83, 'V': 9.17, 'L': 13.33, 'K': 8.33, 'P': 8.33, 'T': 5.0, 'H': 0.83, 'S': 5.0, 'E': 5.83, 'M': 1.67, 'C': 2.5, 'I': 3.33}
- **Common reported functions (%):** 0.0
- **Common reported locations (%):** 12.5
- **Common reported processes (%):** 0.0

- **PDB ID:** 7QPG
- **Chain:** C
- **Crystallized protein length:** 591 aa
- **Resolution:** 3.9 Å
- **b-phipsi:** 0.004606
- **w-rdist:** 0.532902
- **t-alpha:** 0.034818
- **Chemical similarity (Tanimoto Index) (%):** 99.09
- **1D identity (%) [PDB]:** 2.44
- **1D identity (%) [Gaps excluded][PDB]:** 73.91
- **1D identity - Alignment Gaps [PDB]:** 1349
- **1D aligned content [PDB] (<aminoacid>:%):** {'S': 11.76, 'R': 5.88, 'Y': 2.94, 'A': 2.94, 'V': 11.76, 'Q': 17.65, 'T': 5.88, 'I': 8.82, 'E': 5.88, 'L': 11.76, 'D': 2.94, 'F': 5.88, 'K': 5.88}
- **2D identity (%) [PDB]:** 32.92
- **2D identity (%) [Gaps excluded][PDB]:** 87.79
- **2D identity - Alignment Gaps [PDB]:** 655
- **2D aligned content [PDB] (<2D-fold>:%):** {'.': 20.0, 'H': 48.41, 'T': 8.41, 'E': 23.19}
- **3D similarity (TM-Score) (%) [PDB]:** 20.18

- **Gene name:** ZWILCH
- **Entrez ID:** 55055
- **RefSeq ID:** NM\_017975
- **Transcript sequence length:** 3595
- **5-UTR|CDS|3-UTR identity (%):** 2.49 | 39.6 | 24.34
- **5-UTR|CDS|3-UTR identity (%) [Gaps excluded]:** 76.92 | 76.09 | 75.44
- **5-UTR|CDS|3-UTR identity [Alignment Gaps]:** 388 | 1376 | 3119
- **5-UTR aligned content (<base>:%):** {'C': 20.0, 'T': 20.0, 'G': 60.0}
- **CDS aligned content (<base>:%):** {'A': 32.04, 'T': 26.76, 'G': 21.57, 'C': 19.63}
- **3-UTR aligned content (<base>:%):** {'A': 32.65, 'G': 17.22, 'T': 37.02, 'C': 13.11}

**Uniprot Description:**  
  
 Essential component of the mitotic checkpoint, which prevents cells from prematurely exiting mitosis. Required for the assembly of the dynein-dynactin and MAD1-MAD2 complexes onto kinetochores. Its function related to the spindle assembly machinery is proposed to depend on its association in the mitotic RZZ complex (PubMed:15824131).   
  
Component of the RZZ complex composed of KNTC1/ROD, ZW10 and ZWILCH; in the complex interacts directly with KNTC1/ROD.   
  
 **Gene Ontology Information:**

Molecular Function   
  
N/A

Location

- cytosol
- kinetochore
- kinetochore microtubule
- RZZ complex

Biological process

- cell division
- mitotic spindle assembly checkpoint
- protein localization to kinetochore

---

88

- **Protein name:** Ubiquitin carboxyl-terminal hydrolase 5
- **Organism:** Homo sapiens
- **Uniprot Accession Number:** P45974
- **Protein sequence length:** 858 aa
- **1D identity (%):** 15.65
- **1D identity (%) [Gaps excluded]:** 23.68
- **1D identity - Alignment Gaps:** 351
- **1D aligned content (<aminoacid>:%):** {'M': 3.09, 'L': 8.02, 'S': 4.94, 'R': 9.88, 'P': 11.11, 'G': 6.79, 'H': 2.47, 'F': 3.7, 'K': 8.64, 'V': 8.64, 'D': 5.56, 'Y': 3.09, 'I': 3.09, 'Q': 3.7, 'W': 1.85, 'T': 3.09, 'E': 6.79, 'N': 1.85, 'A': 2.47, 'C': 1.23}
- **Common reported functions (%):** 0.0
- **Common reported locations (%):** 25.0
- **Common reported processes (%):** 0.0

- **PDB ID:** 3IHP
- **Chain:** A
- **Crystallized protein length:** 673 aa
- **Resolution:** 2.8 Å
- **b-phipsi:** 0.007379
- **w-rdist:** 0.405363
- **t-alpha:** 0.053208
- **Chemical similarity (Tanimoto Index) (%):** 84.52
- **1D identity (%) [PDB]:** 2.93
- **1D identity (%) [Gaps excluded][PDB]:** 66.15
- **1D identity - Alignment Gaps [PDB]:** 1401
- **1D aligned content [PDB] (<aminoacid>:%):** {'M': 6.98, 'G': 11.63, 'D': 4.65, 'P': 6.98, 'L': 6.98, 'E': 6.98, 'F': 2.33, 'I': 4.65, 'V': 16.28, 'H': 2.33, 'T': 4.65, 'R': 2.33, 'Q': 4.65, 'K': 6.98, 'A': 9.3, 'N': 2.33}
- **2D identity (%) [PDB]:** 33.33
- **2D identity (%) [Gaps excluded][PDB]:** 87.03
- **2D identity - Alignment Gaps [PDB]:** 683
- **2D aligned content [PDB] (<2D-fold>:%):** {'.': 24.12, 'H': 37.67, 'T': 16.26, 'E': 19.78, 'B': 0.54, 'G': 1.63}
- **3D similarity (TM-Score) (%) [PDB]:** 22.02

- **Gene name:** USP5
- **Entrez ID:** 8078
- **RefSeq ID:** NM\_003481
- **Transcript sequence length:** 3093
- **5-UTR|CDS|3-UTR identity (%):** 4.99 | 45.33 | 8.41
- **5-UTR|CDS|3-UTR identity (%) [Gaps excluded]:** 66.67 | 74.01 | 73.29
- **5-UTR|CDS|3-UTR identity [Alignment Gaps]:** 371 | 1224 | 3844
- **5-UTR aligned content (<base>:%):** {'G': 55.0, 'C': 25.0, 'A': 10.0, 'T': 10.0}
- **CDS aligned content (<base>:%):** {'A': 25.49, 'T': 20.04, 'G': 28.91, 'C': 25.56}
- **3-UTR aligned content (<base>:%):** {'G': 30.68, 'A': 20.82, 'T': 25.48, 'C': 23.01}

**Uniprot Description:**  
  
 Cleaves linear and branched multiubiquitin polymers with a marked preference for branched polymers. Involved in unanchored 'Lys-48'-linked polyubiquitin disassembly. Binds linear and 'Lys-63'-linked polyubiquitin with a lower affinity. Knock-down of USP5 causes the accumulation of p53/TP53 and an increase in p53/TP53 transcriptional activity because the unanchored polyubiquitin that accumulates is able to compete with ubiquitinated p53/TP53 but not with MDM2 for proteasomal recognition.   
  
Interacts with TRIML1.   
  
 **Gene Ontology Information:**

Molecular Function

- thiol-dependent ubiquitin-specific protease activity
- cysteine-type endopeptidase activity
- ubiquitin binding
- zinc ion binding

Location

- cytosol
- lysosome
- nucleus

Biological process

- positive regulation of proteasomal ubiquitin-dependent protein catabolic process
- protein deubiquitination
- protein K48-linked deubiquitination
- protein ubiquitination
- ubiquitin-dependent protein catabolic process

---

89

- **Protein name:** ATPase MORC2
- **Organism:** Homo sapiens
- **Uniprot Accession Number:** Q9Y6X9
- **Protein sequence length:** 1032 aa
- **1D identity (%):** 16.89
- **1D identity (%) [Gaps excluded]:** 23.48
- **1D identity - Alignment Gaps:** 309
- **1D aligned content (<aminoacid>:%):** {'R': 11.83, 'L': 9.68, 'G': 3.76, 'N': 2.69, 'I': 3.76, 'D': 6.99, 'Y': 4.3, 'K': 6.45, 'T': 4.3, 'M': 1.08, 'F': 3.23, 'P': 9.68, 'V': 6.45, 'S': 7.53, 'E': 5.91, 'H': 1.08, 'Q': 4.3, 'A': 4.84, 'C': 1.61, 'W': 0.54}
- **Common reported functions (%):** 0.0
- **Common reported locations (%):** 37.5
- **Common reported processes (%):** 0.0

- **PDB ID:** 5OFB
- **Chain:** A
- **Crystallized protein length:** 534 aa
- **Resolution:** 2.02 Å
- **b-phipsi:** 0.008048
- **w-rdist:** 0.633217
- **t-alpha:** 0.005477
- **Chemical similarity (Tanimoto Index) (%):** 75.37
- **1D identity (%) [PDB]:** 3.5
- **1D identity (%) [Gaps excluded][PDB]:** 64.79
- **1D identity - Alignment Gaps [PDB]:** 1244
- **1D aligned content [PDB] (<aminoacid>:%):** {'A': 6.52, 'F': 6.52, 'R': 13.04, 'Q': 6.52, 'E': 10.87, 'M': 2.17, 'V': 6.52, 'L': 6.52, 'D': 4.35, 'I': 4.35, 'T': 8.7, 'K': 13.04, 'P': 2.17, 'S': 4.35, 'H': 2.17, 'C': 2.17}
- **2D identity (%) [PDB]:** 35.51
- **2D identity (%) [Gaps excluded][PDB]:** 85.71
- **2D identity - Alignment Gaps [PDB]:** 574
- **2D aligned content [PDB] (<2D-fold>:%):** {'E': 25.57, 'H': 47.7, '.': 15.23, 'T': 10.06, 'B': 0.57, 'G': 0.86}
- **3D similarity (TM-Score) (%) [PDB]:** 21.6

- **Gene name:** MORC2
- **Entrez ID:** 22880
- **RefSeq ID:** N/A
- **Sequence length:** N/A
- **5-UTR|CDS|3-UTR identity (%):** N/A | N/A | N/A
- **5-UTR|CDS|3-UTR identity (%) [Gaps excluded]:** N/A | N/A | N/A
- **5-UTR|CDS|3-UTR identity [Alignment Gaps]:** N/A | N/A | N/A
- **5-UTR aligned content (<base>:%):** N/A
- **CDS aligned content (<base>:%):** N/A
- **3-UTR aligned content (<base>:%):** N/A

**Uniprot Description:**  
  
 Essential for epigenetic silencing by the HUSH (human silencing hub) complex. Recruited by HUSH to target site in heterochromatin, the ATPase activity and homodimerization are critical for HUSH-mediated silencing (PubMed:28581500, PubMed:29440755). Represses germ cell-related genes and L1 retrotransposons in collaboration with SETDB1 and the HUSH complex, the silencing is dependent of repressive epigenetic modifications, such as H3K9me3 mark. Silencing events often occur within introns of transcriptionally active genes, and lead to the down-regulation of host gene expression (PubMed:29211708). During DNA damage response, regulates chromatin remodeling through ATP hydrolysis. Upon DNA damage, is phosphorylated by PAK1, both colocalize to chromatin and induce H2AX expression. ATPase activity is required and dependent of phosphorylation by PAK1 and presence of DNA (PubMed:23260667). Recruits histone deacetylases, such as HDAC4, to promoter regions, causing local histone H3 deacetylation and transcriptional repression of genes such as CA9 (PubMed:20225202, PubMed:20110259). Exhibits a cytosolic function in lipogenesis, adipogenic differentiation, and lipid homeostasis by increasing the activity of ACLY, possibly preventing its dephosphorylation (PubMed:24286864).   
  
Homodimerizes upon ATP-binding and dissociate upon ATP hydrolysis; homodimerization is required for gene silencing (PubMed:29440755). Interacts with HDAC4 (PubMed:20110259). Interacts with ACLY (PubMed:24286864). Interacts with TASOR and MPHOSPH8; the interactions associate MORC2 with the HUSH complex which recruits MORC2 to heterochromatic loci (PubMed:28581500).   
  
 **Gene Ontology Information:**

Molecular Function

- ATP binding
- ATPase activity
- chromatin binding
- identical protein binding
- magnesium ion binding
- protein homodimerization activity
- zinc ion binding

Location

- cytoplasm
- cytosol
- heterochromatin
- nuclear matrix
- nucleoplasm
- nucleus

Biological process

- chromatin remodeling
- cellular response to DNA damage stimulus
- fatty acid metabolic process
- negative regulation of gene expression, epigenetic
- negative regulation of single stranded viral RNA replication via double stranded DNA intermediate
- positive regulation of DNA methylation-dependent heterochromatin assembly

---

90

- **Protein name:** Puromycin-sensitive aminopeptidase
- **Organism:** Homo sapiens
- **Uniprot Accession Number:** P55786
- **Protein sequence length:** 919 aa
- **1D identity (%):** 16.16
- **1D identity (%) [Gaps excluded]:** 21.74
- **1D identity - Alignment Gaps:** 262
- **1D aligned content (<aminoacid>:%):** {'M': 1.21, 'L': 11.52, 'G': 10.3, 'P': 7.88, 'F': 5.45, 'R': 4.24, 'Y': 3.03, 'K': 5.45, 'V': 12.73, 'D': 5.45, 'T': 4.24, 'E': 4.85, 'A': 6.67, 'I': 3.64, 'H': 3.64, 'Q': 1.82, 'N': 1.21, 'C': 1.82, 'S': 3.64, 'W': 1.21}
- **Common reported functions (%):** 0.0
- **Common reported locations (%):** 0.0
- **Common reported processes (%):** 0.0

- **PDB ID:** 8SW0
- **Chain:** A
- **Crystallized protein length:** 864 aa
- **Resolution:** 2.3 Å
- **b-phipsi:** 0.009129
- **w-rdist:** 0.246422
- **t-alpha:** 0.13928
- **Chemical similarity (Tanimoto Index) (%):** 84.82
- **1D identity (%) [PDB]:** 2.79
- **1D identity (%) [Gaps excluded][PDB]:** 67.65
- **1D identity - Alignment Gaps [PDB]:** 1578
- **1D aligned content [PDB] (<aminoacid>:%):** {'Y': 4.35, 'S': 8.7, 'L': 15.22, 'K': 8.7, 'P': 6.52, 'V': 4.35, 'G': 4.35, 'E': 8.7, 'H': 2.17, 'T': 8.7, 'I': 4.35, 'A': 8.7, 'Q': 4.35, 'D': 4.35, 'N': 2.17, 'R': 4.35}
- **2D identity (%) [PDB]:** 39.1
- **2D identity (%) [Gaps excluded][PDB]:** 87.36
- **2D identity - Alignment Gaps [PDB]:** 654
- **2D aligned content [PDB] (<2D-fold>:%):** {'.': 9.72, 'E': 28.29, 'T': 20.95, 'H': 40.39, 'G': 0.65}
- **3D similarity (TM-Score) (%) [PDB]:** 26.21

- **Gene name:** NPEPPS
- **Entrez ID:** N/A
- **RefSeq ID:** N/A
- **Sequence length:** N/A
- **5-UTR|CDS|3-UTR identity (%):** N/A | N/A | N/A
- **5-UTR|CDS|3-UTR identity (%) [Gaps excluded]:** N/A | N/A | N/A
- **5-UTR|CDS|3-UTR identity [Alignment Gaps]:** N/A | N/A | N/A
- **5-UTR aligned content (<base>:%):** N/A
- **CDS aligned content (<base>:%):** N/A
- **3-UTR aligned content (<base>:%):** N/A

**Uniprot Description:**  
  
 Aminopeptidase with broad substrate specificity for several peptides. Involved in proteolytic events essential for cell growth and viability. May act as regulator of neuropeptide activity. Plays a role in the antigen-processing pathway for MHC class I molecules. Involved in the N-terminal trimming of cytotoxic T-cell epitope precursors. Digests the poly-Q peptides found in many cellular proteins. Digests tau from normal brain more efficiently than tau from Alzheimer disease brain.   
  
Monomer.   
  
 **Gene Ontology Information:**

Molecular Function   
  
N/A

Location   
  
N/A

Biological process   
  
N/A

---

91

- **Protein name:** Complement C4-A
- **Organism:** Homo sapiens
- **Uniprot Accession Number:** P0C0L4
- **Protein sequence length:** 1744 aa
- **1D identity (%):** 13.93
- **1D identity (%) [Gaps excluded]:** 28.96
- **1D identity - Alignment Gaps:** 913
- **1D aligned content (<aminoacid>:%):** {'M': 0.82, 'L': 10.61, 'A': 6.94, 'S': 4.9, 'F': 5.31, 'Q': 4.49, 'P': 7.76, 'R': 4.49, 'G': 11.84, 'N': 1.63, 'K': 5.31, 'D': 5.31, 'V': 6.94, 'E': 4.08, 'Y': 4.9, 'H': 2.45, 'T': 6.53, 'I': 2.86, 'C': 2.86}
- **Common reported functions (%):** 0.0
- **Common reported locations (%):** 0.0
- **Common reported processes (%):** 0.0

- **PDB ID:** 5JPN
- **Chain:** A
- **Crystallized protein length:** 651 aa
- **Resolution:** 3.6 Å
- **b-phipsi:** 0.026917
- **w-rdist:** 0.278939
- **t-alpha:** 0.011076
- **Chemical similarity (Tanimoto Index) (%):** 78.73
- **1D identity (%) [PDB]:** 3.96
- **1D identity (%) [Gaps excluded][PDB]:** 65.12
- **1D identity - Alignment Gaps [PDB]:** 1329
- **1D aligned content [PDB] (<aminoacid>:%):** {'K': 3.57, 'N': 3.57, 'V': 10.71, 'P': 5.36, 'A': 10.71, 'D': 10.71, 'T': 5.36, 'S': 10.71, 'E': 3.57, 'F': 3.57, 'L': 8.93, 'G': 3.57, 'I': 1.79, 'Q': 5.36, 'R': 3.57, 'H': 5.36, 'Y': 3.57}
- **2D identity (%) [PDB]:** 33.68
- **2D identity (%) [Gaps excluded][PDB]:** 84.15
- **2D identity - Alignment Gaps [PDB]:** 643
- **2D aligned content [PDB] (<2D-fold>:%):** {'.': 17.73, 'E': 58.45, 'T': 12.47, 'H': 11.08, 'B': 0.28}
- **3D similarity (TM-Score) (%) [PDB]:** 24.19

- **Gene name:** C4A
- **Entrez ID:** 720; 721
- **RefSeq ID:** NM\_007293
- **Transcript sequence length:** 5427
- **5-UTR|CDS|3-UTR identity (%):** 7.04 | 35.16 | 2.11
- **5-UTR|CDS|3-UTR identity (%) [Gaps excluded]:** 72.5 | 79.56 | 74.59
- **5-UTR|CDS|3-UTR identity [Alignment Gaps]:** 372 | 3027 | 4182
- **5-UTR aligned content (<base>:%):** {'G': 34.48, 'A': 13.79, 'C': 41.38, 'T': 10.34}
- **CDS aligned content (<base>:%):** {'A': 26.53, 'T': 21.55, 'G': 26.69, 'C': 25.22}
- **3-UTR aligned content (<base>:%):** {'G': 38.46, 'T': 14.29, 'C': 24.18, 'A': 23.08}

**Uniprot Description:**  
  
 Non-enzymatic component of C3 and C5 convertases and thus essential for the propagation of the classical complement pathway. Covalently binds to immunoglobulins and immune complexes and enhances the solubilization of immune aggregates and the clearance of IC through CR1 on erythrocytes. C4A isotype is responsible for effective binding to form amide bonds with immune aggregates or protein antigens, while C4B isotype catalyzes the transacylation of the thioester carbonyl group to form ester bonds with carbohydrate antigens.   
  
Circulates in blood as a disulfide-linked trimer of an alpha, beta and gamma chain.   
  
 **Gene Ontology Information:**

Molecular Function

- complement component C1q complex binding
- endopeptidase inhibitor activity

Location

- axon
- blood microparticle
- classical-complement-pathway C3/C5 convertase complex
- dendrite
- endoplasmic reticulum lumen
- extracellular exosome
- extracellular region
- extracellular space
- neuronal cell body
- plasma membrane
- synapse

Biological process

- complement activation
- complement activation, classical pathway
- inflammatory response
- innate immune response
- positive regulation of apoptotic cell clearance
- positive regulation of immune response
- positive regulation of opsonization

---

92

- **Protein name:** Pyruvate carboxylase, mitochondrial
- **Organism:** Homo sapiens
- **Uniprot Accession Number:** P11498
- **Protein sequence length:** 1178 aa
- **1D identity (%):** 14.97
- **1D identity (%) [Gaps excluded]:** 22.11
- **1D identity - Alignment Gaps:** 393
- **1D aligned content (<aminoacid>:%):** {'T': 1.65, 'V': 9.89, 'R': 4.95, 'L': 9.89, 'P': 10.44, 'K': 3.3, 'N': 2.2, 'F': 5.49, 'I': 2.2, 'G': 10.44, 'A': 10.99, 'S': 3.85, 'Q': 6.04, 'D': 4.95, 'E': 7.14, 'C': 2.2, 'H': 2.2, 'M': 0.55, 'W': 0.55, 'Y': 1.1}
- **Common reported functions (%):** 0.0
- **Common reported locations (%):** 25.0
- **Common reported processes (%):** 0.0

- **PDB ID:** 7WTA
- **Chain:** B
- **Crystallized protein length:** 684 aa
- **Resolution:** 3.9 Å
- **b-phipsi:** 0.01005
- **w-rdist:** 0.713915
- **t-alpha:** 0.002347
- **Chemical similarity (Tanimoto Index) (%):** 87.99
- **1D identity (%) [PDB]:** 3.06
- **1D identity (%) [Gaps excluded][PDB]:** 71.43
- **1D identity - Alignment Gaps [PDB]:** 1408
- **1D aligned content [PDB] (<aminoacid>:%):** {'N': 4.44, 'M': 4.44, 'V': 13.33, 'G': 13.33, 'P': 6.67, 'D': 2.22, 'H': 2.22, 'E': 4.44, 'L': 6.67, 'T': 6.67, 'A': 11.11, 'Q': 6.67, 'Y': 2.22, 'R': 4.44, 'K': 6.67, 'I': 4.44}
- **2D identity (%) [PDB]:** 37.2
- **2D identity (%) [Gaps excluded][PDB]:** 90.18
- **2D identity - Alignment Gaps [PDB]:** 638
- **2D aligned content [PDB] (<2D-fold>:%):** {'H': 47.77, '.': 18.32, 'E': 8.42, 'T': 24.75, 'G': 0.74}
- **3D similarity (TM-Score) (%) [PDB]:** 20.77

- **Gene name:** PC
- **Entrez ID:** 5091
- **RefSeq ID:** N/A
- **Sequence length:** N/A
- **5-UTR|CDS|3-UTR identity (%):** N/A | N/A | N/A
- **5-UTR|CDS|3-UTR identity (%) [Gaps excluded]:** N/A | N/A | N/A
- **5-UTR|CDS|3-UTR identity [Alignment Gaps]:** N/A | N/A | N/A
- **5-UTR aligned content (<base>:%):** N/A
- **CDS aligned content (<base>:%):** N/A
- **3-UTR aligned content (<base>:%):** N/A

**Uniprot Description:**  
  
 Pyruvate carboxylase catalyzes a 2-step reaction, involving the ATP-dependent carboxylation of the covalently attached biotin in the first step and the transfer of the carboxyl group to pyruvate in the second. Catalyzes in a tissue specific manner, the initial reactions of glucose (liver, kidney) and lipid (adipose tissue, liver, brain) synthesis from pyruvate.   
  
Homotetramer (PubMed:18297087). Interacts (via the biotin carboxylation domain) with SIRT4 (PubMed:23438705).   
  
 **Gene Ontology Information:**

Molecular Function

- ATP binding
- biotin binding
- identical protein binding
- metal ion binding
- pyruvate carboxylase activity

Location

- cytoplasm
- cytosol
- mitochondrial matrix
- mitochondrion

Biological process

- gluconeogenesis
- lipid metabolic process
- NADH metabolic process
- NADP metabolic process
- negative regulation of gene expression
- positive regulation by host of viral process
- pyruvate metabolic process
- viral release from host cell
- viral RNA genome packaging

---

93

- **Protein name:** DNA replication licensing factor MCM3
- **Organism:** Homo sapiens
- **Uniprot Accession Number:** P25205
- **Protein sequence length:** 808 aa
- **1D identity (%):** 13.71
- **1D identity (%) [Gaps excluded]:** 19.74
- **1D identity - Alignment Gaps:** 301
- **1D aligned content (<aminoacid>:%):** {'G': 11.11, 'R': 5.19, 'D': 6.67, 'L': 16.3, 'E': 6.67, 'V': 5.93, 'Q': 5.19, 'N': 1.48, 'Y': 3.7, 'F': 1.48, 'H': 2.22, 'S': 5.93, 'T': 5.93, 'P': 5.19, 'C': 2.22, 'I': 3.7, 'K': 4.44, 'A': 6.67}
- **Common reported functions (%):** 0.0
- **Common reported locations (%):** 25.0
- **Common reported processes (%):** 0.0

- **PDB ID:** 6XTY
- **Chain:** 4
- **Crystallized protein length:** 598 aa
- **Resolution:** 6.77 Å
- **b-phipsi:** 0.013804
- **w-rdist:** 0.39031
- **t-alpha:** 0.014867
- **Chemical similarity (Tanimoto Index) (%):** N/A
- **1D identity (%) [PDB]:** 2.95
- **1D identity (%) [Gaps excluded][PDB]:** 67.21
- **1D identity - Alignment Gaps [PDB]:** 1328
- **1D aligned content [PDB] (<aminoacid>:%):** {'N': 7.32, 'A': 12.2, 'K': 7.32, 'L': 7.32, 'G': 7.32, 'I': 7.32, 'V': 14.63, 'H': 4.88, 'Q': 4.88, 'P': 7.32, 'S': 9.76, 'D': 4.88, 'Y': 2.44, 'R': 2.44}
- **2D identity (%) [PDB]:** 26.87
- **2D identity (%) [Gaps excluded][PDB]:** 82.58
- **2D identity - Alignment Gaps [PDB]:** 738
- **2D aligned content [PDB] (<2D-fold>:%):** {'H': 46.94, '.': 17.69, 'E': 21.43, 'T': 12.93, 'B': 0.34, 'G': 0.68}
- **3D similarity (TM-Score) (%) [PDB]:** 18.79

- **Gene name:** MCM3
- **Entrez ID:** 4173
- **RefSeq ID:** NM\_002388
- **Transcript sequence length:** 3068
- **5-UTR|CDS|3-UTR identity (%):** 10.73 | 46.49 | 8.89
- **5-UTR|CDS|3-UTR identity (%) [Gaps excluded]:** 74.58 | 75.85 | 74.95
- **5-UTR|CDS|3-UTR identity [Alignment Gaps]:** 351 | 1203 | 3828
- **5-UTR aligned content (<base>:%):** {'G': 38.64, 'C': 34.09, 'T': 15.91, 'A': 11.36}
- **CDS aligned content (<base>:%):** {'A': 28.72, 'T': 21.66, 'G': 27.06, 'C': 22.56}
- **3-UTR aligned content (<base>:%):** {'G': 22.8, 'C': 16.84, 'T': 34.97, 'A': 25.39}

**Uniprot Description:**  
  
 Acts as component of the MCM2-7 complex (MCM complex) which is the putative replicative helicase essential for 'once per cell cycle' DNA replication initiation and elongation in eukaryotic cells. The active ATPase sites in the MCM2-7 ring are formed through the interaction surfaces of two neighboring subunits such that a critical structure of a conserved arginine finger motif is provided in trans relative to the ATP-binding site of the Walker A box of the adjacent subunit. The six ATPase active sites, however, are likely to contribute differentially to the complex helicase activity. Required for DNA replication and cell proliferation.   
  
Component of the MCM2-7 complex (PubMed:16899510, PubMed:17296731). The complex forms a toroidal hexameric ring with the proposed subunit order MCM2-MCM6-MCM4-MCM7-MCM3-MCM5 (PubMed:16899510, PubMed:17296731). Associated with the replication-specific DNA polymerase alpha (By similarity). Interacts with MCMBP (PubMed:17296731). Interacts with ANKRD17 (PubMed:23711367). Interacts with MCM3AP isoform MCM3AP; this interaction leads to MCM3 acetylation (PubMed:9712829, PubMed:11258703, PubMed:12226073). Component of the CMG helicase complex, composed of the MCM2-7 complex, the GINS complex and CDC45 (By similarity).   
  
 **Gene Ontology Information:**

Molecular Function

- ATP binding
- ATPase activity
- DNA helicase activity
- single-stranded DNA binding

Location

- chromosome, telomeric region
- CMG complex
- MCM complex
- membrane
- nucleoplasm
- nucleus

Biological process

- DNA replication
- DNA strand elongation involved in DNA replication
- DNA unwinding involved in DNA replication
- double-strand break repair via break-induced replication
- mitotic DNA replication initiation
- regulation of DNA-dependent DNA replication initiation

---

94

- **Protein name:** RuvB-like 2
- **Organism:** Homo sapiens
- **Uniprot Accession Number:** Q9Y230
- **Protein sequence length:** 463 aa
- **1D identity (%):** 8.89
- **1D identity (%) [Gaps excluded]:** 23.1
- **1D identity - Alignment Gaps:** 588
- **1D aligned content (<aminoacid>:%):** {'T': 5.88, 'V': 10.59, 'I': 7.06, 'P': 4.71, 'R': 11.76, 'M': 1.18, 'K': 5.88, 'Q': 9.41, 'G': 9.41, 'D': 4.71, 'A': 1.18, 'S': 2.35, 'F': 4.71, 'Y': 3.53, 'L': 7.06, 'E': 7.06, 'N': 2.35, 'C': 1.18}
- **Common reported functions (%):** 0.0
- **Common reported locations (%):** 50.0
- **Common reported processes (%):** 0.0

- **PDB ID:** 6HTS
- **Chain:** D
- **Crystallized protein length:** 434 aa
- **Resolution:** 4.8 Å
- **b-phipsi:** 0.011312
- **w-rdist:** 0.51336
- **t-alpha:** 0.005508
- **Chemical similarity (Tanimoto Index) (%):** 79.21
- **1D identity (%) [PDB]:** 2.85
- **1D identity (%) [Gaps excluded][PDB]:** 62.5
- **1D identity - Alignment Gaps [PDB]:** 1173
- **1D aligned content [PDB] (<aminoacid>:%):** {'T': 8.57, 'R': 11.43, 'I': 17.14, 'G': 8.57, 'V': 8.57, 'S': 2.86, 'E': 8.57, 'Q': 2.86, 'K': 8.57, 'P': 5.71, 'L': 8.57, 'A': 2.86, 'D': 2.86, 'F': 2.86}
- **2D identity (%) [PDB]:** 33.08
- **2D identity (%) [Gaps excluded][PDB]:** 87.01
- **2D identity - Alignment Gaps [PDB]:** 577
- **2D aligned content [PDB] (<2D-fold>:%):** {'.': 13.96, 'B': 0.32, 'E': 26.3, 'T': 8.77, 'H': 48.7, 'G': 1.95}
- **3D similarity (TM-Score) (%) [PDB]:** 16.71

- **Gene name:** RUVBL2
- **Entrez ID:** 10856
- **RefSeq ID:** N/A
- **Sequence length:** N/A
- **5-UTR|CDS|3-UTR identity (%):** N/A | N/A | N/A
- **5-UTR|CDS|3-UTR identity (%) [Gaps excluded]:** N/A | N/A | N/A
- **5-UTR|CDS|3-UTR identity [Alignment Gaps]:** N/A | N/A | N/A
- **5-UTR aligned content (<base>:%):** N/A
- **CDS aligned content (<base>:%):** N/A
- **3-UTR aligned content (<base>:%):** N/A

**Uniprot Description:**  
  
 Possesses single-stranded DNA-stimulated ATPase and ATP-dependent DNA helicase (5' to 3') activity; hexamerization is thought to be critical for ATP hydrolysis and adjacent subunits in the ring-like structure contribute to the ATPase activity (PubMed:10428817, PubMed:17157868). Component of the NuA4 histone acetyltransferase complex which is involved in transcriptional activation of select genes principally by acetylation of nucleosomal histones H4 and H2A (PubMed:14966270). This modification may both alter nucleosome -DNA interactions and promote interaction of the modified histones with other proteins which positively regulate transcription (PubMed:14966270). This complex may be required for the activation of transcriptional programs associated with oncogene and proto-oncogene mediated growth induction, tumor suppressor mediated growth arrest and replicative senescence, apoptosis, and DNA repair (PubMed:14966270). The NuA4 complex ATPase and helicase activities seem to be, at least in part, contributed by the association of RUVBL1 and RUVBL2 with EP400 (PubMed:14966270). NuA4 may also play a direct role in DNA repair when recruited to sites of DNA damage (PubMed:14966270). Component of a SWR1-like complex that specifically mediates the removal of histone H2A.Z/H2AZ1 from the nucleosome (PubMed:24463511). Proposed core component of the chromatin remodeling INO80 complex which exhibits DNA- and nucleosome-activated ATPase activity and catalyzes ATP-dependent nucleosome sliding (PubMed:16230350, PubMed:21303910). Plays an essential role in oncogenic transformation by MYC and also modulates transcriptional activation by the LEF1/TCF1-CTNNB1 complex (PubMed:10882073, PubMed:16014379). May also inhibit the transcriptional activity of ATF2 (PubMed:11713276). Involved in the endoplasmic reticulum (ER)-associated degradation (ERAD) pathway where it negatively regulates expression of ER stress response genes (PubMed:25652260). May play a role in regulating the composition of the U5 snRNP complex (PubMed:28561026).   
  
Forms homohexameric rings (Probable). Can form a dodecamer with RUVBL1 made of two stacked hexameric rings; however, even though RUVBL1 and RUVBL2 are present in equimolar ratio, the oligomeric status of each hexamer is not known. Oligomerization may regulate binding to nucleic acids and conversely, binding to nucleic acids may affect the dodecameric assembly. Interacts with the transcriptional activation domain of MYC. Interacts With ATF2. Component of the RNA polymerase II holoenzyme complex. May also act to bridge the LEF1/TCF1-CTNNB1 complex and TBP. Component of the NuA4 histone acetyltransferase complex which contains the catalytic subunit KAT5/TIP60 and the subunits EP400, TRRAP/PAF400, BRD8/SMAP, EPC1, DMAP1/DNMAP1, RUVBL1/TIP49, RUVBL2, ING3, actin, ACTL6A/BAF53A, MORF4L1/MRG15, MORF4L2/MRGX, MRGBP, YEATS4/GAS41, VPS72/YL1 and MEAF6. The NuA4 complex interacts with MYC and the adenovirus E1A protein. RUVBL2 interacts with EP400. Component of a NuA4-related complex which contains EP400, TRRAP/PAF400, SRCAP, BRD8/SMAP, EPC1, DMAP1/DNMAP1, RUVBL1/TIP49, RUVBL2, actin, ACTL6A/BAF53A, VPS72 and YEATS4/GAS41. Interacts with NPAT. Component of the chromatin-remodeling INO80 complex; specifically part of a complex module associated with the helicase ATP-binding and the helicase C-terminal domain of INO80. Component of some MLL1/MLL complex, at least composed of the core components KMT2A/MLL1, ASH2L, HCFC1/HCF1, WDR5 and RBBP5, as well as the facultative components BAP18, CHD8, E2F6, HSP70, INO80C, KANSL1, LAS1L, MAX, MCRS1, MGA, MYST1/MOF, PELP1, PHF20, PRP31, RING2, RUVB1/TIP49A, RUVB2/TIP49B, SENP3, TAF1, TAF4, TAF6, TAF7, TAF9 and TEX10. Interacts with IGHMBP2. Interacts with TELO2. Interacts with HINT1. Component of a SWR1-like complex. Component of the R2TP complex composed at least of PIHD1, RUVBL1, RUVBL2 and RPAP3 (PubMed:20864032). Interacts with ITFG1 (PubMed:25437307). Interacts with ZMYND10 (PubMed:29601588). Interacts with WAC; WAC positively regulates MTOR activity by promoting the assembly of the TTT complex composed of TELO2, TTI1 and TTI2 and the RUVBL complex composed of RUVBL1 and RUVBL2 into the TTT-RUVBL complex which leads to the dimerization of the mTORC1 complex and its subsequent activation (PubMed:26812014). Forms a complex with APPL1 and APPL2 (PubMed:19433865). Interacts with ZNHIT2 (via HIT-type zinc finger) in the presence of ATP or ADP; shows a stronger interaction in the presence of ADP (PubMed:28561026). The RUVBL1/RUVBL2 complex interacts with ZNHIT1 (via HIT-type zinc finger), ZNHIT3 (via HIT-type zinc finger), ZNHIT6 (via HIT-type zinc finger) and DDX59/ZNHIT5 (via HIT-type zinc finger) in the presence of ADP (PubMed:28561026). Interacts with NOPCHAP1; the interaction is direct and disrupted upon ATP binding (PubMed:33367824).   
  
 **Gene Ontology Information:**

Molecular Function

- ADP binding
- ATP binding
- ATPase activity
- ATPase binding
- beta-catenin binding
- chromatin DNA binding
- DNA helicase activity
- identical protein binding
- promoter-enhancer loop anchoring activity
- protein homodimerization activity
- RNA polymerase II cis-regulatory region sequence-specific DNA binding
- RNA polymerase II core promoter sequence-specific DNA binding
- TBP-class protein binding
- TFIID-class transcription factor complex binding
- transcription corepressor activity
- unfolded protein binding

Location

- centrosome
- cytoplasm
- cytosol
- dynein axonemal particle
- euchromatin
- extracellular exosome
- Ino80 complex
- membrane
- MLL1 complex
- NuA4 histone acetyltransferase complex
- nuclear matrix
- nucleoplasm
- nucleosome
- nucleus
- chaperone complex
- R2TP complex
- ribonucleoprotein complex
- RPAP3/R2TP/prefoldin-like complex
- Swr1 complex

Biological process

- box C/D snoRNP assembly
- cellular response to estradiol stimulus
- cellular response to UV
- chromatin remodeling
- DNA recombination
- DNA repair
- establishment of protein localization to chromatin
- histone acetylation
- histone H2A acetylation
- histone H4 acetylation
- negative regulation of canonical Wnt signaling pathway
- positive regulation of DNA repair
- positive regulation of transcription, DNA-templated
- positive regulation of double-strand break repair via homologous recombination
- positive regulation of telomerase RNA localization to Cajal body
- positive regulation of telomere maintenance in response to DNA damage
- positive regulation of transcription by RNA polymerase II
- protein folding
- protein stabilization
- regulation of apoptotic process
- regulation of cell cycle
- regulation of chromosome organization
- regulation of DNA repair
- regulation of DNA replication
- regulation of DNA strand elongation
- regulation of transcription, DNA-templated
- regulation of double-strand break repair
- regulation of embryonic development
- regulation of transcription by RNA polymerase II
- telomere maintenance

---

95

- **Protein name:** Insulin-degrading enzyme
- **Organism:** Homo sapiens
- **Uniprot Accession Number:** P14735
- **Protein sequence length:** 1019 aa
- **1D identity (%):** 16.9
- **1D identity (%) [Gaps excluded]:** 22.96
- **1D identity - Alignment Gaps:** 286
- **1D aligned content (<aminoacid>:%):** {'M': 1.09, 'L': 12.02, 'P': 11.48, 'R': 3.28, 'I': 4.37, 'N': 4.37, 'H': 4.37, 'D': 5.46, 'G': 4.92, 'E': 8.2, 'F': 6.56, 'V': 6.01, 'S': 2.19, 'A': 3.83, 'T': 3.28, 'Q': 4.37, 'K': 8.2, 'C': 2.19, 'Y': 3.28, 'W': 0.55}
- **Common reported functions (%):** 0.0
- **Common reported locations (%):** 37.5
- **Common reported processes (%):** 0.0

- **PDB ID:** 7RZI
- **Chain:** B
- **Crystallized protein length:** 938 aa
- **Resolution:** 3.0 Å
- **b-phipsi:** 0.006721
- **w-rdist:** 0.417726
- **t-alpha:** 0.071987
- **Chemical similarity (Tanimoto Index) (%):** 98.02
- **1D identity (%) [PDB]:** 0.35
- **1D identity (%) [Gaps excluded][PDB]:** 50.0
- **1D identity - Alignment Gaps [PDB]:** 844
- **1D aligned content [PDB] (<aminoacid>:%):** {'K': 33.33, 'R': 33.33, 'G': 33.33}
- **2D identity (%) [PDB]:** 0.59
- **2D identity (%) [Gaps excluded][PDB]:** 83.33
- **2D identity - Alignment Gaps [PDB]:** 844
- **2D aligned content [PDB] (<2D-fold>:%):** {'.': 100.0}
- **3D similarity (TM-Score) (%) [PDB]:** 24.83

- **Gene name:** IDE
- **Entrez ID:** 3416
- **RefSeq ID:** NM\_004969
- **Transcript sequence length:** 5894
- **5-UTR|CDS|3-UTR identity (%):** 12.04 | 47.02 | 40.41
- **5-UTR|CDS|3-UTR identity (%) [Gaps excluded]:** 72.06 | 74.43 | 76.75
- **5-UTR|CDS|3-UTR identity [Alignment Gaps]:** 339 | 1274 | 2185
- **5-UTR aligned content (<base>:%):** {'T': 6.12, 'G': 44.9, 'C': 34.69, 'A': 14.29}
- **CDS aligned content (<base>:%):** {'A': 31.22, 'T': 24.95, 'G': 22.43, 'C': 21.39}
- **3-UTR aligned content (<base>:%):** {'A': 31.15, 'G': 16.14, 'T': 36.94, 'C': 15.76}

**Uniprot Description:**  
  
 Plays a role in the cellular breakdown of insulin, APP peptides, IAPP peptides, natriuretic peptides, glucagon, bradykinin, kallidin, and other peptides, and thereby plays a role in intercellular peptide signaling (PubMed:2293021, PubMed:10684867, PubMed:26968463, PubMed:17051221, PubMed:17613531, PubMed:18986166, PubMed:19321446, PubMed:23922390, PubMed:24847884, PubMed:26394692, PubMed:29596046, PubMed:21098034). Substrate binding induces important conformation changes, making it possible to bind and degrade larger substrates, such as insulin (PubMed:23922390, PubMed:26394692, PubMed:29596046). Contributes to the regulation of peptide hormone signaling cascades and regulation of blood glucose homeostasis via its role in the degradation of insulin, glucagon and IAPP (By similarity). Plays a role in the degradation and clearance of APP-derived amyloidogenic peptides that are secreted by neurons and microglia (PubMed:9830016, PubMed:26394692) (Probable). Degrades the natriuretic peptides ANP, BNP and CNP, inactivating their ability to raise intracellular cGMP (PubMed:21098034). Also degrades an aberrant frameshifted 40-residue form of NPPA (fsNPPA) which is associated with familial atrial fibrillation in heterozygous patients (PubMed:21098034). Involved in antigen processing. Produces both the N terminus and the C terminus of MAGEA3-derived antigenic peptide (EVDPIGHLY) that is presented to cytotoxic T lymphocytes by MHC class I.   
  
Homodimer (PubMed:17051221, PubMed:19321446, PubMed:23922390, PubMed:26394692, PubMed:29596046) (Probable). Can also form homotetramers (By similarity).   
  
 **Gene Ontology Information:**

Molecular Function

- ATP binding
- endopeptidase activity
- identical protein binding
- insulin binding
- metalloendopeptidase activity
- peptide binding
- protein homodimerization activity
- ubiquitin-dependent protein binding
- virus receptor activity
- zinc ion binding

Location

- basolateral plasma membrane
- cell surface
- cytoplasm
- cytosol
- external side of plasma membrane
- extracellular exosome
- extracellular space
- mitochondrion
- nucleus
- peroxisomal matrix
- peroxisome

Biological process

- amyloid-beta clearance
- amyloid-beta clearance by cellular catabolic process
- amyloid-beta metabolic process
- antigen processing and presentation of endogenous peptide antigen via MHC class I
- bradykinin catabolic process
- hormone catabolic process
- insulin catabolic process
- insulin metabolic process
- insulin receptor signaling pathway
- peptide catabolic process
- positive regulation of protein binding
- positive regulation of protein catabolic process
- protein catabolic process
- proteolysis
- proteolysis involved in cellular protein catabolic process
- regulation of aerobic respiration
- ubiquitin recycling

---

96

- **Protein name:** Ectonucleotide pyrophosphatase/phosphodiesterase family member 1
- **Organism:** Homo sapiens
- **Uniprot Accession Number:** P22413
- **Protein sequence length:** 925 aa
- **1D identity (%):** 15.8
- **1D identity (%) [Gaps excluded]:** 23.24
- **1D identity - Alignment Gaps:** 340
- **1D aligned content (<aminoacid>:%):** {'M': 1.79, 'G': 10.71, 'R': 5.36, 'H': 4.76, 'P': 10.12, 'T': 5.36, 'K': 6.55, 'V': 6.55, 'S': 5.36, 'L': 9.52, 'E': 7.74, 'W': 0.6, 'F': 1.19, 'D': 6.55, 'C': 1.79, 'I': 2.98, 'N': 4.76, 'Y': 3.57, 'A': 1.19, 'Q': 3.57}
- **Common reported functions (%):** 0.0
- **Common reported locations (%):** 0.0
- **Common reported processes (%):** 0.0

- **PDB ID:** 8GHR
- **Chain:** A
- **Crystallized protein length:** 735 aa
- **Resolution:** 3.2 Å
- **b-phipsi:** 0.001692
- **w-rdist:** 0.631486
- **t-alpha:** 0.050987
- **Chemical similarity (Tanimoto Index) (%):** 67.06
- **1D identity (%) [PDB]:** 3.97
- **1D identity (%) [Gaps excluded][PDB]:** 60.82
- **1D identity - Alignment Gaps [PDB]:** 1391
- **1D aligned content [PDB] (<aminoacid>:%):** {'E': 5.08, 'K': 8.47, 'R': 5.08, 'P': 8.47, 'V': 20.34, 'D': 6.78, 'M': 3.39, 'H': 5.08, 'F': 1.69, 'Q': 1.69, 'I': 3.39, 'Y': 1.69, 'L': 15.25, 'G': 1.69, 'T': 3.39, 'S': 5.08, 'W': 1.69, 'A': 1.69}
- **2D identity (%) [PDB]:** 42.2
- **2D identity (%) [Gaps excluded][PDB]:** 91.04
- **2D identity - Alignment Gaps [PDB]:** 581
- **2D aligned content [PDB] (<2D-fold>:%):** {'.': 20.57, 'E': 19.47, 'T': 31.51, 'H': 28.01, 'B': 0.44}
- **3D similarity (TM-Score) (%) [PDB]:** 22.35

- **Gene name:** ENPP1
- **Entrez ID:** N/A
- **RefSeq ID:** NM\_006208
- **Transcript sequence length:** 7438
- **5-UTR|CDS|3-UTR identity (%):** 3.24 | 46.4 | 45.42
- **5-UTR|CDS|3-UTR identity (%) [Gaps excluded]:** 81.25 | 74.78 | 74.85
- **5-UTR|CDS|3-UTR identity [Alignment Gaps]:** 385 | 1256 | 2185
- **5-UTR aligned content (<base>:%):** {'G': 53.85, 'C': 46.15}
- **CDS aligned content (<base>:%):** {'A': 30.73, 'T': 25.78, 'G': 22.14, 'C': 21.35}
- **3-UTR aligned content (<base>:%):** {'T': 35.9, 'C': 17.47, 'A': 27.02, 'G': 19.61}

**Uniprot Description:**  
  
 Nucleotide pyrophosphatase that generates diphosphate (PPi) and functions in bone mineralization and soft tissue calcification by regulating pyrophosphate levels (By similarity). PPi inhibits bone mineralization and soft tissue calcification by binding to nascent hydroxyapatite crystals, thereby preventing further growth of these crystals (PubMed:11004006). Preferentially hydrolyzes ATP, but can also hydrolyze other nucleoside 5' triphosphates such as GTP, CTP and UTP to their corresponding monophosphates with release of pyrophosphate, as well as diadenosine polyphosphates, and also 3',5'-cAMP to AMP (PubMed:25344812, PubMed:27467858, PubMed:28011303, PubMed:35147247, PubMed:8001561). May also be involved in the regulation of the availability of nucleotide sugars in the endoplasmic reticulum and Golgi, and the regulation of purinergic signaling (PubMed:27467858, PubMed:8001561). Inhibits ectopic joint calcification and maintains articular chondrocytes by repressing hedgehog signaling; it is however unclear whether hedgehog inhibition is direct or indirect (By similarity). Appears to modulate insulin sensitivity and function (PubMed:10615944). Also involved in melanogenesis (PubMed:28964717). Also able to hydrolyze 2',3'-cGAMP (cyclic GMP-AMP), a second messenger that activates TMEM173/STING and triggers type-I interferon production (PubMed:25344812). 2',3'-cGAMP degradation takes place in the lumen or extracellular space, and not in the cytosol where it is produced; the role of 2',3'-cGAMP hydrolysis is therefore unclear (PubMed:25344812). Not able to hydrolyze the 2',3'-cGAMP linkage isomer 3'-3'-cGAMP (PubMed:25344812).   
  
Ectonucleotide pyrophosphatase/phosphodiesterase family member 1
Homodimer (PubMed:28964717). Interacts with INSR; leading to inhibit INSR autophosphorylation and subsequent activation of INSR kinase activity (PubMed:10615944).   
  
 **Gene Ontology Information:**

Molecular Function   
  
N/A

Location   
  
N/A

Biological process   
  
N/A

---

97

- **Protein name:** Islet amyloid polypeptide
- **Organism:** Homo sapiens
- **Uniprot Accession Number:** P10997
- **Protein sequence length:** 89 aa
- **1D identity (%):** 1.95
- **1D identity (%) [Gaps excluded]:** 21.25
- **1D identity - Alignment Gaps:** 790
- **1D aligned content (<aminoacid>:%):** {'M': 5.88, 'L': 11.76, 'Q': 11.76, 'T': 11.76, 'P': 11.76, 'V': 5.88, 'A': 5.88, 'N': 17.65, 'S': 5.88, 'G': 5.88, 'R': 5.88}
- **Common reported functions (%):** 0.0
- **Common reported locations (%):** 0.0
- **Common reported processes (%):** 0.0

- **PDB ID:** 7BG0
- **Chain:** E
- **Crystallized protein length:** 401 aa
- **Resolution:** 2.89 Å
- **b-phipsi:** 0.012284
- **w-rdist:** 0.377591
- **t-alpha:** 0.020344
- **Chemical similarity (Tanimoto Index) (%):** 97.18
- **1D identity (%) [PDB]:** 2.23
- **1D identity (%) [Gaps excluded][PDB]:** 69.23
- **1D identity - Alignment Gaps [PDB]:** 1173
- **1D aligned content [PDB] (<aminoacid>:%):** {'I': 14.81, 'E': 3.7, 'K': 11.11, 'V': 3.7, 'W': 3.7, 'A': 7.41, 'F': 7.41, 'Q': 7.41, 'D': 7.41, 'L': 11.11, 'S': 3.7, 'T': 3.7, 'G': 7.41, 'M': 3.7, 'P': 3.7}
- **2D identity (%) [PDB]:** 29.18
- **2D identity (%) [Gaps excluded][PDB]:** 87.82
- **2D identity - Alignment Gaps [PDB]:** 627
- **2D aligned content [PDB] (<2D-fold>:%):** {'.': 16.79, 'T': 9.85, 'H': 48.54, 'E': 22.63, 'G': 2.19}
- **3D similarity (TM-Score) (%) [PDB]:** N/A

- **Gene name:** IAPP
- **Entrez ID:** 3375
- **RefSeq ID:** NM\_001329201
- **Transcript sequence length:** 1894
- **5-UTR|CDS|3-UTR identity (%):** 8.47 | 7.13 | 22.62
- **5-UTR|CDS|3-UTR identity (%) [Gaps excluded]:** 78.26 | 75.0 | 77.99
- **5-UTR|CDS|3-UTR identity [Alignment Gaps]:** 379 | 2360 | 3213
- **5-UTR aligned content (<base>:%):** {'A': 27.78, 'G': 19.44, 'C': 38.89, 'T': 13.89}
- **CDS aligned content (<base>:%):** {'A': 28.49, 'G': 24.19, 'C': 24.73, 'T': 22.58}
- **3-UTR aligned content (<base>:%):** {'A': 30.76, 'G': 21.39, 'T': 34.47, 'C': 13.38}

**Uniprot Description:**  
  
 Selectively inhibits insulin-stimulated glucose utilization and glycogen deposition in muscle, while not affecting adipocyte glucose metabolism.   
  
Interacts with IDE and INS. Can form homodimers. Interaction with INS inhibits homodimerization and fibril formation.   
  
 **Gene Ontology Information:**

Molecular Function

- amyloid-beta binding
- hormone activity
- identical protein binding
- lipid binding
- signaling receptor binding

Location

- extracellular region
- extracellular space
- inclusion body

Biological process

- adenylate cyclase-activating G protein-coupled receptor signaling pathway
- amylin receptor signaling pathway
- amyloid fibril formation
- apoptotic process
- bone resorption
- cell-cell signaling
- eating behavior
- negative regulation of amyloid fibril formation
- negative regulation of bone resorption
- negative regulation of cell population proliferation
- negative regulation of mitochondrion organization
- negative regulation of osteoclast differentiation
- negative regulation of protein-containing complex assembly
- osteoclast differentiation
- positive regulation of apoptotic process
- positive regulation of calcium ion import across plasma membrane
- positive regulation of cytosolic calcium ion concentration
- positive regulation of ERK1 and ERK2 cascade
- positive regulation of gene expression
- positive regulation of MAPK cascade
- positive regulation of peptidyl-serine phosphorylation
- positive regulation of protein kinase A signaling
- positive regulation of protein kinase B signaling
- protein destabilization
- protein homooligomerization
- sensory perception of pain
- signal transduction

---

98

- **Protein name:** Macrophage mannose receptor 1
- **Organism:** Homo sapiens
- **Uniprot Accession Number:** P22897
- **Protein sequence length:** 1456 aa
- **1D identity (%):** 14.56
- **1D identity (%) [Gaps excluded]:** 27.66
- **1D identity - Alignment Gaps:** 719
- **1D aligned content (<aminoacid>:%):** {'P': 6.33, 'L': 7.69, 'A': 7.24, 'D': 5.43, 'K': 6.33, 'R': 4.52, 'V': 2.26, 'Q': 3.17, 'M': 1.81, 'G': 10.86, 'T': 8.14, 'F': 2.71, 'W': 3.17, 'E': 5.88, 'Y': 5.43, 'S': 5.88, 'N': 3.62, 'I': 2.71, 'C': 3.62, 'H': 3.17}
- **Common reported functions (%):** 0.0
- **Common reported locations (%):** 0.0
- **Common reported processes (%):** 0.0

- **PDB ID:** 6INN
- **Chain:** C
- **Crystallized protein length:** 587 aa
- **Resolution:** 3.0 Å
- **b-phipsi:** 0.017363
- **w-rdist:** 0.504348
- **t-alpha:** 0.003928
- **Chemical similarity (Tanimoto Index) (%):** 88.48
- **1D identity (%) [PDB]:** 2.74
- **1D identity (%) [Gaps excluded][PDB]:** 64.41
- **1D identity - Alignment Gaps [PDB]:** 1330
- **1D aligned content [PDB] (<aminoacid>:%):** {'T': 7.89, 'S': 7.89, 'P': 7.89, 'N': 7.89, 'L': 10.53, 'C': 2.63, 'A': 10.53, 'K': 7.89, 'G': 5.26, 'I': 7.89, 'H': 5.26, 'Q': 7.89, 'R': 2.63, 'F': 2.63, 'V': 2.63, 'D': 2.63}
- **2D identity (%) [PDB]:** 24.38
- **2D identity (%) [Gaps excluded][PDB]:** 85.94
- **2D identity - Alignment Gaps [PDB]:** 808
- **2D aligned content [PDB] (<2D-fold>:%):** {'.': 17.45, 'E': 43.27, 'T': 17.45, 'H': 21.09, 'B': 0.73}
- **3D similarity (TM-Score) (%) [PDB]:** 23.16

- **Gene name:** MRC1
- **Entrez ID:** 4360
- **RefSeq ID:** NM\_002438
- **Transcript sequence length:** 5188
- **5-UTR|CDS|3-UTR identity (%):** 15.19 | 39.85 | 10.93
- **5-UTR|CDS|3-UTR identity (%) [Gaps excluded]:** 71.43 | 77.88 | 76.81
- **5-UTR|CDS|3-UTR identity [Alignment Gaps]:** 337 | 2247 | 3742
- **5-UTR aligned content (<base>:%):** {'C': 23.08, 'G': 46.15, 'T': 26.15, 'A': 4.62}
- **CDS aligned content (<base>:%):** {'A': 31.35, 'T': 24.92, 'G': 23.77, 'C': 19.96}
- **3-UTR aligned content (<base>:%):** {'T': 42.56, 'A': 29.35, 'C': 13.63, 'G': 14.47}

**Uniprot Description:**  
  
 Mediates the endocytosis of glycoproteins by macrophages. Binds both sulfated and non-sulfated polysaccharide chains.   
  
(Microbial infection) Interacts with Dengue virus.   
  
 **Gene Ontology Information:**

Molecular Function

- cargo receptor activity
- mannose binding
- signaling receptor activity
- transmembrane signaling receptor activity
- virus receptor activity

Location

- cell surface
- endosome membrane
- plasma membrane

Biological process

- cellular response to interleukin-4
- cellular response to lipopolysaccharide
- cellular response to interferon-gamma
- receptor-mediated endocytosis

---

99

- **Protein name:** N/A
- **Organism:** N/A
- **Uniprot Accession Number:** N/A
- **Protein sequence length:** N/A
- **1D identity (%):** N/A
- **1D identity (%) [Gaps excluded]:** N/A
- **1D identity - Alignment Gaps:** N/A
- **1D aligned content (<aminoacid>:%):** N/A
- **Common reported functions (%):** 0.0
- **Common reported locations (%):** 12.5
- **Common reported processes (%):** 0.0

- **PDB ID:** 3ZUS
- **Chain:** C
- **Crystallized protein length:** 852 aa
- **Resolution:** 2.95 Å
- **b-phipsi:** 0.010624
- **w-rdist:** 0.263934
- **t-alpha:** 0.076664
- **Chemical similarity (Tanimoto Index) (%):** 81.22
- **1D identity (%) [PDB]:** 2.18
- **1D identity (%) [Gaps excluded][PDB]:** 69.23
- **1D identity - Alignment Gaps [PDB]:** 1599
- **1D aligned content [PDB] (<aminoacid>:%):** {'M': 2.78, 'E': 8.33, 'V': 13.89, 'T': 8.33, 'L': 19.44, 'G': 8.33, 'K': 5.56, 'F': 2.78, 'S': 8.33, 'Q': 2.78, 'A': 8.33, 'N': 2.78, 'D': 5.56, 'I': 2.78}
- **2D identity (%) [PDB]:** 32.26
- **2D identity (%) [Gaps excluded][PDB]:** 86.39
- **2D identity - Alignment Gaps [PDB]:** 777
- **2D aligned content [PDB] (<2D-fold>:%):** {'E': 18.0, 'H': 47.75, '.': 19.0, 'T': 12.5, 'G': 2.25, 'B': 0.5}
- **3D similarity (TM-Score) (%) [PDB]:** 23.47

- **Gene name:** N/A
- **Entrez ID:** 5185061
- **RefSeq ID:** N/A
- **Sequence length:** N/A
- **5-UTR|CDS|3-UTR identity (%):** N/A | N/A | N/A
- **5-UTR|CDS|3-UTR identity (%) [Gaps excluded]:** N/A | N/A | N/A
- **5-UTR|CDS|3-UTR identity [Alignment Gaps]:** N/A | N/A | N/A
- **5-UTR aligned content (<base>:%):** N/A
- **CDS aligned content (<base>:%):** N/A
- **3-UTR aligned content (<base>:%):** N/A

**Uniprot Description:**  
  
 N/A N/A   
  
 **Gene Ontology Information:**

Molecular Function

- ganglioside GT1b binding
- metalloendopeptidase activity
- protein transmembrane transporter activity
- toxin activity
- zinc ion binding

Location

- extracellular region
- host cell cytoplasmic vesicle
- host cell cytosol
- host cell plasma membrane
- host cell presynaptic membrane
- membrane

Biological process

- negative regulation of neurotransmitter secretion
- proteolysis

---

100

- **Protein name:** Toll-like receptor 1
- **Organism:** Homo sapiens
- **Uniprot Accession Number:** Q15399
- **Protein sequence length:** 786 aa
- **1D identity (%):** 16.49
- **1D identity (%) [Gaps excluded]:** 23.28
- **1D identity - Alignment Gaps:** 281
- **1D aligned content (<aminoacid>:%):** {'T': 3.77, 'H': 5.66, 'F': 4.4, 'Q': 5.66, 'D': 3.77, 'N': 4.4, 'P': 5.66, 'S': 8.81, 'W': 0.63, 'L': 15.72, 'E': 5.03, 'Y': 4.4, 'V': 6.92, 'A': 3.77, 'I': 4.4, 'C': 2.52, 'K': 6.29, 'G': 5.03, 'M': 0.63, 'R': 2.52}
- **Common reported functions (%):** 0.0
- **Common reported locations (%):** 12.5
- **Common reported processes (%):** 0.0

- **PDB ID:** 6NIH
- **Chain:** A
- **Crystallized protein length:** 519 aa
- **Resolution:** 2.3 Å
- **b-phipsi:** 0.004674
- **w-rdist:** 0.519624
- **t-alpha:** 0.06767
- **Chemical similarity (Tanimoto Index) (%):** 79.79
- **1D identity (%) [PDB]:** 2.03
- **1D identity (%) [Gaps excluded][PDB]:** 72.97
- **1D identity - Alignment Gaps [PDB]:** 1295
- **1D aligned content [PDB] (<aminoacid>:%):** {'L': 22.22, 'P': 7.41, 'G': 3.7, 'E': 7.41, 'D': 7.41, 'Q': 11.11, 'F': 3.7, 'V': 18.52, 'S': 11.11, 'A': 3.7, 'N': 3.7}
- **2D identity (%) [PDB]:** 25.79
- **2D identity (%) [Gaps excluded][PDB]:** 81.21
- **2D identity - Alignment Gaps [PDB]:** 709
- **2D aligned content [PDB] (<2D-fold>:%):** {'E': 33.96, '.': 29.48, 'T': 22.76, 'H': 13.81}
- **3D similarity (TM-Score) (%) [PDB]:** 19.85

- **Gene name:** TLR1
- **Entrez ID:** 7096
- **RefSeq ID:** N/A
- **Sequence length:** N/A
- **5-UTR|CDS|3-UTR identity (%):** N/A | N/A | N/A
- **5-UTR|CDS|3-UTR identity (%) [Gaps excluded]:** N/A | N/A | N/A
- **5-UTR|CDS|3-UTR identity [Alignment Gaps]:** N/A | N/A | N/A
- **5-UTR aligned content (<base>:%):** N/A
- **CDS aligned content (<base>:%):** N/A
- **3-UTR aligned content (<base>:%):** N/A

**Uniprot Description:**  
  
 Participates in the innate immune response to microbial agents. Specifically recognizes diacylated and triacylated lipopeptides. Cooperates with TLR2 to mediate the innate immune response to bacterial lipoproteins or lipopeptides (PubMed:21078852). Forms the activation cluster TLR2:TLR1:CD14 in response to triacylated lipopeptides, this cluster triggers signaling from the cell surface and subsequently is targeted to the Golgi in a lipid-raft dependent pathway (PubMed:16880211). Acts via MYD88 and TRAF6, leading to NF-kappa-B activation, cytokine secretion and the inflammatory response.   
  
Interacts (via extracellular domain) with TLR2. TLR2 seems to exist in heterodimers with either TLR1 or TLR6 before stimulation by the ligand. The heterodimers form bigger oligomers in response to their corresponding ligands as well as further heterotypic associations with other receptors such as CD14 and/or CD36 (PubMed:16880211, PubMed:17889651). The activation cluster TLR2:TLR1:CD14 forms in response to triacylated lipopeptides (PubMed:16880211). Binds MYD88 (via TIR domain). Interacts with CNPY3 (By similarity).   
  
 **Gene Ontology Information:**

Molecular Function

- identical protein binding
- lipopeptide binding
- NAD(P)+ nucleosidase activity
- NAD+ nucleotidase, cyclic ADP-ribose generating
- signaling receptor activity
- Toll-like receptor 2 binding
- transmembrane signaling receptor activity

Location

- Golgi apparatus
- membrane
- membrane raft
- phagocytic vesicle membrane
- plasma membrane
- receptor complex
- Toll-like receptor 1-Toll-like receptor 2 protein complex

Biological process

- cellular response to triacyl bacterial lipopeptide
- detection of triacyl bacterial lipopeptide
- immune response
- inflammatory response
- innate immune response
- macrophage activation
- positive regulation of interleukin-6 production
- positive regulation of interleukin-8 production
- positive regulation of toll-like receptor 2 signaling pathway
- positive regulation of tumor necrosis factor production
- signal transduction
- toll-like receptor signaling pathway

---
