# Supplementary material for: Comparative structural insights and functional analysis for the distinct unbound states of Human AGO proteins
Source: Sci Rep. 2025 Mar 19;15:9432. doi: 10.1038/s41598-025-91849-5 (PMC11923369; doi:10.1038/s41598-025-91849-5)
Supplement: Supplementary file 24 — Supplementary Information 12. [file 41598_2025_91849_MOESM24_ESM.zip › 6OONp_A_mdwhole_HL2REF/candidates/6OONp_A-merged-h-enriched_report.html]

 

# Structural Comparison Report for 6OONp\_A - whole structures (total: 100)

---

0

- **PDB ID:** 3W1G | **Chain:** A
- **b-phipsi:** 0.0023238374000606
- **w-rdist:** 0.4106362131659046
- **t-alpha:** 0.0046944792707916

---

---

1

- **PDB ID:** 7XE1 | **Chain:** A
- **b-phipsi:** 0.0044679535027972
- **w-rdist:** 0.4870877684197067
- **t-alpha:** 0.0015647720218643

---

---

2

- **PDB ID:** 6EA4 | **Chain:** A
- **b-phipsi:** 0.0084718657265788
- **w-rdist:** 0.3952255499116299
- **t-alpha:** 0.0007832732121422

---

---

3

- **PDB ID:** 7Q1L | **Chain:** A
- **b-phipsi:** 0.0065623935063617
- **w-rdist:** 0.4288361632251922
- **t-alpha:** 0.0015647720218643

---

---

4

- **PDB ID:** 6GYR | **Chain:** A
- **b-phipsi:** 0.007294947568491
- **w-rdist:** 0.4147895216706464
- **t-alpha:** 0.0015647720218643

---

---

5

- **PDB ID:** 4Z4F | **Chain:** A
- **b-phipsi:** 0.0057184073904979
- **w-rdist:** 0.2328505602246997
- **t-alpha:** 0.0109543395722921

---

---

6

- **PDB ID:** 6EJE | **Chain:** A
- **b-phipsi:** 0.00116070975571
- **w-rdist:** 0.5300813980245378
- **t-alpha:** 0.0046944792707916

---

---

7

- **PDB ID:** 5GVE | **Chain:** A
- **b-phipsi:** 0.0074371829919015
- **w-rdist:** 0.4569401312283956
- **t-alpha:** 0.0007823187912934

---

---

8

- **PDB ID:** 4KXT | **Chain:** A
- **b-phipsi:** 0.0017965762972204
- **w-rdist:** 0.0702434826768162
- **t-alpha:** 0.1032863364414424

---

---

9

- **PDB ID:** 4A5T | **Chain:** S
- **b-phipsi:** 0.0181458910174755
- **w-rdist:** 0.1668655672154557
- **t-alpha:** 0.0015647720218643

---

---

10

- **PDB ID:** 6C02 | **Chain:** B
- **b-phipsi:** 0.0039304427080214
- **w-rdist:** 0.5069805192861813
- **t-alpha:** 0.0054771144938834

---

---

11

- **PDB ID:** 5KYY | **Chain:** A
- **b-phipsi:** 0.002210133098811
- **w-rdist:** 0.6505033039014648
- **t-alpha:** 0.0023530945443304

---

---

12

- **PDB ID:** 3L4G | **Chain:** B
- **b-phipsi:** 0.0011268959941118
- **w-rdist:** 0.7367258406568182
- **t-alpha:** 0.0023530945443304

---

---

13

- **PDB ID:** 6U7C | **Chain:** A
- **b-phipsi:** 0.0135346537567003
- **w-rdist:** 0.3419216050152358
- **t-alpha:** 0.0015676375257613

---

---

14

- **PDB ID:** 5VM9 | **Chain:** A
- **b-phipsi:** 0.0090134243764787
- **w-rdist:** 0.1454663547765022
- **t-alpha:** 0.0086070234951727

---

---

15

- **PDB ID:** 4ZG7 | **Chain:** A
- **b-phipsi:** 0.0072376268804837
- **w-rdist:** 0.4387712530820633
- **t-alpha:** 0.0039119745263893

---

---

16

- **PDB ID:** 4ZG4 | **Chain:** B
- **b-phipsi:** 0.0116470669372398
- **w-rdist:** 0.3677458890390915
- **t-alpha:** 0.0031401242085145

---

---

17

- **PDB ID:** 3BJU | **Chain:** C
- **b-phipsi:** 0.0033017651641294
- **w-rdist:** 0.629328915425099
- **t-alpha:** 0.0039119745263893

---

---

18

- **PDB ID:** 4AH6 | **Chain:** B
- **b-phipsi:** 0.003407544599048
- **w-rdist:** 0.3542023398493669
- **t-alpha:** 0.050987106591174

---

---

19

- **PDB ID:** 5KYX | **Chain:** B
- **b-phipsi:** 0.0056585083846093
- **w-rdist:** 0.671238537747282
- **t-alpha:** 0.0007832732121422

---

---

20

- **PDB ID:** 8ASC | **Chain:** E
- **b-phipsi:** 0.0018472389570859
- **w-rdist:** 0.3961220592200525
- **t-alpha:** 0.0571204636616826

---

---

21

- **PDB ID:** 5O9Z | **Chain:** B
- **b-phipsi:** 0.0064436252669846
- **w-rdist:** 0.298490508738384
- **t-alpha:** 0.0203438662057466

---

---

22

- **PDB ID:** 5MHL | **Chain:** B
- **b-phipsi:** 0.0105545411599149
- **w-rdist:** 0.3031500842078742
- **t-alpha:** 0.0070418134049574

---

---

23

- **PDB ID:** 3CDZ | **Chain:** B
- **b-phipsi:** 0.050916853571692
- **w-rdist:** 0.3908767644222933
- **t-alpha:** 0.0

---

---

24

- **PDB ID:** 2WJY | **Chain:** A
- **b-phipsi:** 0.0032792118045918
- **w-rdist:** 0.5020873594289996
- **t-alpha:** 0.0126786930545719

---

---

25

- **PDB ID:** 6LID | **Chain:** A
- **b-phipsi:** 0.0058944064880765
- **w-rdist:** 0.7294161647635636
- **t-alpha:** 0.0015647720218643

---

---

26

- **PDB ID:** 2BJJ | **Chain:** X
- **b-phipsi:** 0.0060212641702569
- **w-rdist:** 0.5132179820019266
- **t-alpha:** 0.0062593997286479

---

---

27

- **PDB ID:** 6SVP | **Chain:** B
- **b-phipsi:** 0.0049383390779993
- **w-rdist:** 0.7898441237960858
- **t-alpha:** 0.0015647720218643

---

---

28

- **PDB ID:** 7ZSC | **Chain:** D
- **b-phipsi:** 0.00432033287123
- **w-rdist:** 0.4266467913565103
- **t-alpha:** 0.0266038120788794

---

---

29

- **PDB ID:** 6UZT | **Chain:** A
- **b-phipsi:** 0.004141863187263
- **w-rdist:** 0.818659446845925
- **t-alpha:** 0.0015676375257613

---

---

30

- **PDB ID:** 6U23 | **Chain:** P
- **b-phipsi:** 0.0100937067521591
- **w-rdist:** 0.3515654233643923
- **t-alpha:** 0.0086070234951727

---

---

31

- **PDB ID:** 6VXK | **Chain:** B
- **b-phipsi:** 0.0261367129126431
- **w-rdist:** 0.3440356988989443
- **t-alpha:** 0.0031401242085145

---

---

32

- **PDB ID:** 6ZYM | **Chain:** B
- **b-phipsi:** 0.0053349565739671
- **w-rdist:** 0.4054037548862557
- **t-alpha:** 0.0305161718713362

---

---

33

- **PDB ID:** 2VXO | **Chain:** A
- **b-phipsi:** 0.006129306963229
- **w-rdist:** 0.3699230234762833
- **t-alpha:** 0.040688156290789

---

---

34

- **PDB ID:** 1PL0 | **Chain:** C
- **b-phipsi:** 0.0064129196837788
- **w-rdist:** 0.7674077215646232
- **t-alpha:** 0.0007832732121422

---

---

35

- **PDB ID:** 5IZ5 | **Chain:** B
- **b-phipsi:** 0.0027647002593068
- **w-rdist:** 0.4201662817567533
- **t-alpha:** 0.0712045469065545

---

---

36

- **PDB ID:** 3BDL | **Chain:** A
- **b-phipsi:** 0.0008255547107562
- **w-rdist:** 0.4723719878199611
- **t-alpha:** 0.0597019506692302

---

---

37

- **PDB ID:** 6ID0 | **Chain:** C
- **b-phipsi:** 0.007730454128927
- **w-rdist:** 0.492456465099749
- **t-alpha:** 0.0054771144938834

---

---

38

- **PDB ID:** 5J68 | **Chain:** A
- **b-phipsi:** 0.0048411897295105
- **w-rdist:** 0.4912759876779642
- **t-alpha:** 0.0175161413598659

---

---

39

- **PDB ID:** 6SY1 | **Chain:** A
- **b-phipsi:** 0.0128927269796181
- **w-rdist:** 0.4085310560226737
- **t-alpha:** 0.0046944792707916

---

---

40

- **PDB ID:** 1SEU | **Chain:** A
- **b-phipsi:** 0.0177511789347224
- **w-rdist:** 0.3659161993710003
- **t-alpha:** 0.0046944792707916

---

---

41

- **PDB ID:** 6MGQ | **Chain:** A
- **b-phipsi:** 0.0067715505009976
- **w-rdist:** 0.2269831450070442
- **t-alpha:** 0.064162546478931

---

---

42

- **PDB ID:** 7ASG | **Chain:** A
- **b-phipsi:** 0.0029249406982109
- **w-rdist:** 0.8834231838634882
- **t-alpha:** 0.0039277718342689

---

---

43

- **PDB ID:** 5FWI | **Chain:** C
- **b-phipsi:** 0.0014374710596665
- **w-rdist:** 0.9737432233076684
- **t-alpha:** 0.0047169960791435

---

---

44

- **PDB ID:** 8FCN | **Chain:** E
- **b-phipsi:** 0.0062467510970357
- **w-rdist:** 0.1567259432879207
- **t-alpha:** 0.2128317056828612

---

---

45

- **PDB ID:** 8D96 | **Chain:** C
- **b-phipsi:** 0.0048747580704967
- **w-rdist:** 0.4392936317393059
- **t-alpha:** 0.0281690295641283

---

---

46

- **PDB ID:** 7SOL | **Chain:** C
- **b-phipsi:** 0.0048138052560862
- **w-rdist:** 0.37302252606716
- **t-alpha:** 0.1439745083193748

---

---

47

- **PDB ID:** 7QNG | **Chain:** B
- **b-phipsi:** 0.008521842387543
- **w-rdist:** 0.4166935813044224
- **t-alpha:** 0.0102767895279776

---

---

48

- **PDB ID:** 3EGD | **Chain:** B
- **b-phipsi:** 0.0063504376936264
- **w-rdist:** 0.6527605096252984
- **t-alpha:** 0.0039119745263893

---

---

49

- **PDB ID:** 8ENC | **Chain:** M
- **b-phipsi:** 0.0100772665378872
- **w-rdist:** 0.4351750467326469
- **t-alpha:** 0.0062992257321734

---

---

50

- **PDB ID:** 4OVV | **Chain:** A
- **b-phipsi:** 0.0072298579746153
- **w-rdist:** 0.4285324554914744
- **t-alpha:** 0.0125193542574237

---

---

51

- **PDB ID:** 2E1Q | **Chain:** A
- **b-phipsi:** 0.0049782386227229
- **w-rdist:** 0.3770563029348425
- **t-alpha:** 0.1025037952669631

---

---

52

- **PDB ID:** 5C1B | **Chain:** C
- **b-phipsi:** 0.0070853387447647
- **w-rdist:** 0.1895517914108304
- **t-alpha:** 0.0837240757413027

---

---

53

- **PDB ID:** 4X0P | **Chain:** A
- **b-phipsi:** 0.0523218583973104
- **w-rdist:** 0.222111564680877
- **t-alpha:** 0.0055077996884944

---

---

54

- **PDB ID:** 8P4E | **Chain:** O
- **b-phipsi:** 0.0014860432707068
- **w-rdist:** 0.5108936140773341
- **t-alpha:** 0.0446008926005814

---

---

55

- **PDB ID:** 6P0D | **Chain:** A
- **b-phipsi:** 0.005344391633651
- **w-rdist:** 0.7386102252414369
- **t-alpha:** 0.0046944792707916

---

---

56

- **PDB ID:** 8OG4 | **Chain:** A
- **b-phipsi:** 0.0067068219906576
- **w-rdist:** 0.4021412225881235
- **t-alpha:** 0.0289856519083209

---

---

57

- **PDB ID:** 6WRV | **Chain:** A
- **b-phipsi:** 0.0067618924963164
- **w-rdist:** 0.8992907155126769
- **t-alpha:** 0.0007823187912934

---

---

58

- **PDB ID:** 7EMF | **Chain:** P
- **b-phipsi:** 0.0064736855012348
- **w-rdist:** 0.4551048310041758
- **t-alpha:** 0.0164318357763795

---

---

59

- **PDB ID:** 4CGY | **Chain:** A
- **b-phipsi:** 0.0069073668142136
- **w-rdist:** 0.4463612471428552
- **t-alpha:** 0.0175161413598659

---

---

60

- **PDB ID:** 7UQX | **Chain:** A
- **b-phipsi:** 0.0127835076307609
- **w-rdist:** 0.6215294661890312
- **t-alpha:** 0.0007823187912934

---

---

61

- **PDB ID:** 1NQL | **Chain:** A
- **b-phipsi:** 0.0133146291097237
- **w-rdist:** 0.6283622458201444
- **t-alpha:** 0.0

---

---

62

- **PDB ID:** 6EOS | **Chain:** C
- **b-phipsi:** 0.0115298644098794
- **w-rdist:** 0.3973133667891223
- **t-alpha:** 0.0110763082596549

---

---

63

- **PDB ID:** 3HHD | **Chain:** D
- **b-phipsi:** 0.0038390782486622
- **w-rdist:** 0.7845331247984992
- **t-alpha:** 0.0070922351376341

---

---

64

- **PDB ID:** 3OPM | **Chain:** B
- **b-phipsi:** 0.0062028689028108
- **w-rdist:** 0.76893890861525
- **t-alpha:** 0.0046944792707916

---

---

65

- **PDB ID:** 1TZN | **Chain:** H
- **b-phipsi:** 0.0086135778188221
- **w-rdist:** 0.7496299739417438
- **t-alpha:** 0.0015647720218643

---

---

66

- **PDB ID:** 3EH1 | **Chain:** A
- **b-phipsi:** 0.0010581647807447
- **w-rdist:** 0.7381240575364476
- **t-alpha:** 0.0187789021729365

---

---

67

- **PDB ID:** 6AHR | **Chain:** B
- **b-phipsi:** 0.0173502931548928
- **w-rdist:** 0.2574680773934314
- **t-alpha:** 0.0133018035296799

---

---

68

- **PDB ID:** 6NIG | **Chain:** A
- **b-phipsi:** 0.0054279347037922
- **w-rdist:** 0.4663486833879318
- **t-alpha:** 0.0484003534843218

---

---

69

- **PDB ID:** 6KYV | **Chain:** H
- **b-phipsi:** 0.0110755833130507
- **w-rdist:** 0.6130211882084395
- **t-alpha:** 0.0023530945443304

---

---

70

- **PDB ID:** 6XTY | **Chain:** 6
- **b-phipsi:** 0.0098707633038234
- **w-rdist:** 0.4919150153942814
- **t-alpha:** 0.0078243548762237

---

---

71

- **PDB ID:** 2B3X | **Chain:** A
- **b-phipsi:** 0.0062213805658245
- **w-rdist:** 0.771248985616488
- **t-alpha:** 0.0047169960791435

---

---

72

- **PDB ID:** 5GGF | **Chain:** C
- **b-phipsi:** 0.0032479718450972
- **w-rdist:** 0.6567835717303646
- **t-alpha:** 0.0175161413598659

---

---

73

- **PDB ID:** 7A3F | **Chain:** A
- **b-phipsi:** 0.0157485811309944
- **w-rdist:** 0.4274498941356251
- **t-alpha:** 0.0062593997286479

---

---

74

- **PDB ID:** 8IF4 | **Chain:** A
- **b-phipsi:** 0.0051005393578669
- **w-rdist:** 0.5075706174497258
- **t-alpha:** 0.040688156290789

---

---

75

- **PDB ID:** 5FUR | **Chain:** I
- **b-phipsi:** 0.0143393253669885
- **w-rdist:** 0.2352107910298121
- **t-alpha:** 0.0226916757534991

---

---

76

- **PDB ID:** 7QPG | **Chain:** C
- **b-phipsi:** 0.0046057124015895
- **w-rdist:** 0.5329024028549533
- **t-alpha:** 0.0348178944724737

---

---

77

- **PDB ID:** 3IHP | **Chain:** A
- **b-phipsi:** 0.0073788971062627
- **w-rdist:** 0.4053632317088315
- **t-alpha:** 0.0532080396597527

---

---

78

- **PDB ID:** 5OFB | **Chain:** A
- **b-phipsi:** 0.0080476730964714
- **w-rdist:** 0.6332165892448953
- **t-alpha:** 0.0054771144938834

---

---

79

- **PDB ID:** 5JPN | **Chain:** A
- **b-phipsi:** 0.0269167288251761
- **w-rdist:** 0.2789393745722386
- **t-alpha:** 0.0110763082596549

---

---

80

- **PDB ID:** 7WTA | **Chain:** B
- **b-phipsi:** 0.0100502622657405
- **w-rdist:** 0.7139147157733248
- **t-alpha:** 0.0023473590503262

---

---

81

- **PDB ID:** 6XTY | **Chain:** 4
- **b-phipsi:** 0.0138036313467483
- **w-rdist:** 0.3903095404181558
- **t-alpha:** 0.0148665808060715

---

---

82

- **PDB ID:** 6HTS | **Chain:** D
- **b-phipsi:** 0.0113120982206179
- **w-rdist:** 0.5133601455611831
- **t-alpha:** 0.0055077996884944

---

---

83

- **PDB ID:** 7RZI | **Chain:** B
- **b-phipsi:** 0.0067213165344621
- **w-rdist:** 0.4177260002416145
- **t-alpha:** 0.0719873632833352

---

---

84

- **PDB ID:** 7BG0 | **Chain:** E
- **b-phipsi:** 0.01228358443566
- **w-rdist:** 0.3775914080613909
- **t-alpha:** 0.0203438662057466

---

---

85

- **PDB ID:** 6INN | **Chain:** C
- **b-phipsi:** 0.0173627904452047
- **w-rdist:** 0.5043480200145796
- **t-alpha:** 0.0039277718342689

---

---

86

- **PDB ID:** 6NIH | **Chain:** A
- **b-phipsi:** 0.0046741373117304
- **w-rdist:** 0.5196244373000412
- **t-alpha:** 0.0676695952069212

---

---

87

- **PDB ID:** 5KZW | **Chain:** A
- **b-phipsi:** 0.0071985067234394
- **w-rdist:** 0.6065227628273057
- **t-alpha:** 0.0102767895279776

---

---

88

- **PDB ID:** 6X9K | **Chain:** A
- **b-phipsi:** 0.0058560432011054
- **w-rdist:** 0.4374508665744716
- **t-alpha:** 0.1768381479464797

---

---

89

- **PDB ID:** 8EOJ | **Chain:** B
- **b-phipsi:** 0.0069758053938971
- **w-rdist:** 0.4614407916311441
- **t-alpha:** 0.0348178944724737

---

---

90

- **PDB ID:** 1X6V | **Chain:** B
- **b-phipsi:** 0.0087791110099076
- **w-rdist:** 0.5752210802451104
- **t-alpha:** 0.0078868306657793

---

---

91

- **PDB ID:** 4BTX | **Chain:** B
- **b-phipsi:** 0.0162815773435785
- **w-rdist:** 0.6459486527626612
- **t-alpha:** 0.0007832732121422

---

---

92

- **PDB ID:** 7KTR | **Chain:** B
- **b-phipsi:** 0.0110514828325867
- **w-rdist:** 0.5369110027608828
- **t-alpha:** 0.0062992257321734

---

---

93

- **PDB ID:** 5WJ5 | **Chain:** C
- **b-phipsi:** 0.0372232381226745
- **w-rdist:** 0.4443311955665889
- **t-alpha:** 0.0047169960791435

---

---

94

- **PDB ID:** 3LPO | **Chain:** D
- **b-phipsi:** 0.005843599129802
- **w-rdist:** 0.5998247333204332
- **t-alpha:** 0.0242563010013117

---

---

95

- **PDB ID:** 4ZAJ | **Chain:** A
- **b-phipsi:** 0.0258999440076874
- **w-rdist:** 0.5596772718330456
- **t-alpha:** 0.0015676375257613

---

---

96

- **PDB ID:** 5L46 | **Chain:** A
- **b-phipsi:** 0.0038512903577665
- **w-rdist:** 0.7159599407782519
- **t-alpha:** 0.0215828567205209

---

---

97

- **PDB ID:** 6HZZ | **Chain:** A
- **b-phipsi:** 0.0055447919213696
- **w-rdist:** 0.7867884470293735
- **t-alpha:** 0.0086070234951727

---

---

98

- **PDB ID:** 6I33 | **Chain:** B
- **b-phipsi:** 0.0119756318861142
- **w-rdist:** 0.6529612107620126
- **t-alpha:** 0.0031296009050172

---

---

99

- **PDB ID:** 5LST | **Chain:** A
- **b-phipsi:** 0.0092060115873493
- **w-rdist:** 0.4952992468486997
- **t-alpha:** 0.0191389373786337

---

---
