# Supplementary material for: Comparative structural insights and functional analysis for the distinct unbound states of Human AGO proteins
Source: Sci Rep. 2025 Mar 19;15:9432. doi: 10.1038/s41598-025-91849-5 (PMC11923369; doi:10.1038/s41598-025-91849-5)
Supplement: Supplementary file 24 — Supplementary Information 12. [file 41598_2025_91849_MOESM24_ESM.zip › 6OONp_A_mdwhole_HL2REF/candidates/6OONp_A-merged-notenriched_report.html]

 

# Structural Comparison Report for 6OONp\_A - whole structures (total: 800)

---

1

- **PDB ID:** 7AD7 | **Chain:** A
- **b-phipsi:** 0.0018735163276026
- **w-rdist:** 0.1802022299999945
- **t-alpha:** 0.0007823187912934

---

---

2

- **PDB ID:** 3W1G | **Chain:** A
- **b-phipsi:** 0.0023238374000606
- **w-rdist:** 0.4106362131659046
- **t-alpha:** 0.0046944792707916

---

---

3

- **PDB ID:** 7AUA | **Chain:** A
- **b-phipsi:** 0.0045808803872446
- **w-rdist:** 0.4047591611164471
- **t-alpha:** 0.0039119745263893

---

---

4

- **PDB ID:** 4Z4F | **Chain:** A
- **b-phipsi:** 0.0057184073904979
- **w-rdist:** 0.2328505602246997
- **t-alpha:** 0.0109543395722921

---

---

5

- **PDB ID:** 7Q1L | **Chain:** A
- **b-phipsi:** 0.0065623935063617
- **w-rdist:** 0.4288361632251922
- **t-alpha:** 0.0015647720218643

---

---

6

- **PDB ID:** 6GYR | **Chain:** A
- **b-phipsi:** 0.007294947568491
- **w-rdist:** 0.4147895216706464
- **t-alpha:** 0.0015647720218643

---

---

7

- **PDB ID:** 6EA4 | **Chain:** A
- **b-phipsi:** 0.0084718657265788
- **w-rdist:** 0.3952255499116299
- **t-alpha:** 0.0007832732121422

---

---

8

- **PDB ID:** 5VM9 | **Chain:** A
- **b-phipsi:** 0.0090134243764787
- **w-rdist:** 0.1454663547765022
- **t-alpha:** 0.0086070234951727

---

---

9

- **PDB ID:** 3W5O | **Chain:** A
- **b-phipsi:** 0.0042606472750949
- **w-rdist:** 0.4625133812800811
- **t-alpha:** 0.0039119745263893

---

---

10

- **PDB ID:** 7YBU | **Chain:** H
- **b-phipsi:** 0.0062228200398352
- **w-rdist:** 0.4185658782133471
- **t-alpha:** 0.0070418134049574

---

---

11

- **PDB ID:** 7XE1 | **Chain:** A
- **b-phipsi:** 0.0044679535027972
- **w-rdist:** 0.4870877684197067
- **t-alpha:** 0.0015647720218643

---

---

12

- **PDB ID:** 7XE2 | **Chain:** A
- **b-phipsi:** 0.0047607372443765
- **w-rdist:** 0.4839240244006763
- **t-alpha:** 0.0015647720218643

---

---

13

- **PDB ID:** 7YBU | **Chain:** A
- **b-phipsi:** 0.0064722575233535
- **w-rdist:** 0.4312693311111949
- **t-alpha:** 0.0054771144938834

---

---

14

- **PDB ID:** 7AU2 | **Chain:** A
- **b-phipsi:** 0.0057069859458481
- **w-rdist:** 0.4032468630180043
- **t-alpha:** 0.0094788657373643

---

---

15

- **PDB ID:** 7AU2 | **Chain:** B
- **b-phipsi:** 0.0047661781903498
- **w-rdist:** 0.4007316407750638
- **t-alpha:** 0.0110763082596549

---

---

16

- **PDB ID:** 8P4F | **Chain:** O
- **b-phipsi:** 0.0008886315694299
- **w-rdist:** 0.5098088501771442
- **t-alpha:** 0.0031401242085145

---

---

17

- **PDB ID:** 4ZG7 | **Chain:** A
- **b-phipsi:** 0.0072376268804837
- **w-rdist:** 0.4387712530820633
- **t-alpha:** 0.0039119745263893

---

---

18

- **PDB ID:** 6ERF | **Chain:** A
- **b-phipsi:** 0.0013186054131382
- **w-rdist:** 0.4765773097899994
- **t-alpha:** 0.0070922351376341

---

---

19

- **PDB ID:** 5GVE | **Chain:** A
- **b-phipsi:** 0.0074371829919015
- **w-rdist:** 0.4569401312283956
- **t-alpha:** 0.0007823187912934

---

---

20

- **PDB ID:** 5MHL | **Chain:** B
- **b-phipsi:** 0.0105545411599149
- **w-rdist:** 0.3031500842078742
- **t-alpha:** 0.0070418134049574

---

---

21

- **PDB ID:** 5VM9 | **Chain:** C
- **b-phipsi:** 0.0094707872066082
- **w-rdist:** 0.1932229707703219
- **t-alpha:** 0.0110763082596549

---

---

22

- **PDB ID:** 2HAV | **Chain:** B
- **b-phipsi:** 0.0079355311890881
- **w-rdist:** 0.402547754619158
- **t-alpha:** 0.0078868306657793

---

---

23

- **PDB ID:** 6U7C | **Chain:** A
- **b-phipsi:** 0.0135346537567003
- **w-rdist:** 0.3419216050152358
- **t-alpha:** 0.0015676375257613

---

---

24

- **PDB ID:** 4ZG4 | **Chain:** B
- **b-phipsi:** 0.0116470669372398
- **w-rdist:** 0.3677458890390915
- **t-alpha:** 0.0031401242085145

---

---

25

- **PDB ID:** 6EJE | **Chain:** A
- **b-phipsi:** 0.00116070975571
- **w-rdist:** 0.5300813980245378
- **t-alpha:** 0.0046944792707916

---

---

26

- **PDB ID:** 7Q1L | **Chain:** B
- **b-phipsi:** 0.0061574540047039
- **w-rdist:** 0.4306953092475838
- **t-alpha:** 0.0110763082596549

---

---

27

- **PDB ID:** 5CU5 | **Chain:** B
- **b-phipsi:** 0.0093959153386434
- **w-rdist:** 0.4442111069913361
- **t-alpha:** 0.0023473590503262

---

---

28

- **PDB ID:** 4A5T | **Chain:** S
- **b-phipsi:** 0.0181458910174755
- **w-rdist:** 0.1668655672154557
- **t-alpha:** 0.0015647720218643

---

---

29

- **PDB ID:** 6C02 | **Chain:** B
- **b-phipsi:** 0.0039304427080214
- **w-rdist:** 0.5069805192861813
- **t-alpha:** 0.0054771144938834

---

---

30

- **PDB ID:** 4PNK | **Chain:** A
- **b-phipsi:** 0.0149982519952556
- **w-rdist:** 0.3212952701437429
- **t-alpha:** 0.0015676375257613

---

---

31

- **PDB ID:** 6U23 | **Chain:** P
- **b-phipsi:** 0.0100937067521591
- **w-rdist:** 0.3515654233643923
- **t-alpha:** 0.0086070234951727

---

---

32

- **PDB ID:** 4Z4I | **Chain:** A
- **b-phipsi:** 0.0027245063929017
- **w-rdist:** 0.215233675494717
- **t-alpha:** 0.0352112405915889

---

---

33

- **PDB ID:** 5O9Z | **Chain:** B
- **b-phipsi:** 0.0064436252669846
- **w-rdist:** 0.298490508738384
- **t-alpha:** 0.0203438662057466

---

---

34

- **PDB ID:** 6U2J | **Chain:** P
- **b-phipsi:** 0.011515848678646
- **w-rdist:** 0.2998582950293862
- **t-alpha:** 0.0086070234951727

---

---

35

- **PDB ID:** 6EJC | **Chain:** A
- **b-phipsi:** 0.0022827781835694
- **w-rdist:** 0.5357556720728862
- **t-alpha:** 0.0039119745263893

---

---

36

- **PDB ID:** 6U23 | **Chain:** H
- **b-phipsi:** 0.0095149918681021
- **w-rdist:** 0.3462860862013999
- **t-alpha:** 0.0102767895279776

---

---

37

- **PDB ID:** 4GNK | **Chain:** B
- **b-phipsi:** 0.0042113787377948
- **w-rdist:** 0.5440645646419113
- **t-alpha:** 0.0023473590503262

---

---

38

- **PDB ID:** 6CBD | **Chain:** A
- **b-phipsi:** 0.0018386003588102
- **w-rdist:** 0.2453097168335564
- **t-alpha:** 0.0461656134308179

---

---

39

- **PDB ID:** 4QJ5 | **Chain:** B
- **b-phipsi:** 0.0028732381645955
- **w-rdist:** 0.5820599064275895
- **t-alpha:** 0.0023530945443304

---

---

40

- **PDB ID:** 6U2J | **Chain:** K
- **b-phipsi:** 0.0154001780877989
- **w-rdist:** 0.2989990115219112
- **t-alpha:** 0.0047169960791435

---

---

41

- **PDB ID:** 4Z4E | **Chain:** A
- **b-phipsi:** 0.0005065410693065
- **w-rdist:** 0.2069705598639037
- **t-alpha:** 0.064162546478931

---

---

42

- **PDB ID:** 6OON | **Chain:** A
- **b-phipsi:** 0.0062542736104153
- **w-rdist:** 0.0607349431203927
- **t-alpha:** 0.0297336873714129

---

---

43

- **PDB ID:** 4Z4D | **Chain:** A
- **b-phipsi:** 0.003790717136874
- **w-rdist:** 0.2135337881355463
- **t-alpha:** 0.0446008926005814

---

---

44

- **PDB ID:** 7QNG | **Chain:** B
- **b-phipsi:** 0.008521842387543
- **w-rdist:** 0.4166935813044224
- **t-alpha:** 0.0102767895279776

---

---

45

- **PDB ID:** 6U23 | **Chain:** J
- **b-phipsi:** 0.0137812620843743
- **w-rdist:** 0.3421941860466048
- **t-alpha:** 0.0062593997286479

---

---

46

- **PDB ID:** 4OVV | **Chain:** A
- **b-phipsi:** 0.0072298579746153
- **w-rdist:** 0.4285324554914744
- **t-alpha:** 0.0125193542574237

---

---

47

- **PDB ID:** 3CIK | **Chain:** A
- **b-phipsi:** 0.0188487260639411
- **w-rdist:** 0.3051048671241019
- **t-alpha:** 0.0015676375257613

---

---

48

- **PDB ID:** 3W1B | **Chain:** A
- **b-phipsi:** 0.002251340218094
- **w-rdist:** 0.3973823967901769
- **t-alpha:** 0.0234741774196856

---

---

49

- **PDB ID:** 4W5R | **Chain:** A
- **b-phipsi:** 0.0036242171319629
- **w-rdist:** 0.1763501277032972
- **t-alpha:** 0.0532080396597527

---

---

50

- **PDB ID:** 6R1U | **Chain:** K
- **b-phipsi:** 0.0040765909271687
- **w-rdist:** 0.5199923630573219
- **t-alpha:** 0.0078243548762237

---

---

51

- **PDB ID:** 8ENC | **Chain:** M
- **b-phipsi:** 0.0100772665378872
- **w-rdist:** 0.4351750467326469
- **t-alpha:** 0.0062992257321734

---

---

52

- **PDB ID:** 4W5N | **Chain:** A
- **b-phipsi:** 0.002413823700694
- **w-rdist:** 0.1015148340824598
- **t-alpha:** 0.0657276023874584

---

---

53

- **PDB ID:** 2BJJ | **Chain:** X
- **b-phipsi:** 0.0060212641702569
- **w-rdist:** 0.5132179820019266
- **t-alpha:** 0.0062593997286479

---

---

54

- **PDB ID:** 2HAU | **Chain:** B
- **b-phipsi:** 0.0085355246179955
- **w-rdist:** 0.4544951951634903
- **t-alpha:** 0.0070922351376341

---

---

55

- **PDB ID:** 5WG4 | **Chain:** A
- **b-phipsi:** 0.0191440639663638
- **w-rdist:** 0.3402956432584525
- **t-alpha:** 0.0015647720218643

---

---

56

- **PDB ID:** 5DYH | **Chain:** B
- **b-phipsi:** 0.0056857699139683
- **w-rdist:** 0.3952230723251259
- **t-alpha:** 0.0211265191128235

---

---

57

- **PDB ID:** 5MHP | **Chain:** A
- **b-phipsi:** 0.0054565678303645
- **w-rdist:** 0.5150907990473976
- **t-alpha:** 0.0078243548762237

---

---

58

- **PDB ID:** 5KYY | **Chain:** A
- **b-phipsi:** 0.002210133098811
- **w-rdist:** 0.6505033039014648
- **t-alpha:** 0.0023530945443304

---

---

59

- **PDB ID:** 6SY1 | **Chain:** A
- **b-phipsi:** 0.0128927269796181
- **w-rdist:** 0.4085310560226737
- **t-alpha:** 0.0046944792707916

---

---

60

- **PDB ID:** 6EA4 | **Chain:** B
- **b-phipsi:** 0.0091858549646167
- **w-rdist:** 0.4230868304687527
- **t-alpha:** 0.0102767895279776

---

---

61

- **PDB ID:** 7XE3 | **Chain:** A
- **b-phipsi:** 0.0066803239553043
- **w-rdist:** 0.505928276308808
- **t-alpha:** 0.0062992257321734

---

---

62

- **PDB ID:** 6ID0 | **Chain:** C
- **b-phipsi:** 0.007730454128927
- **w-rdist:** 0.492456465099749
- **t-alpha:** 0.0054771144938834

---

---

63

- **PDB ID:** 8D71 | **Chain:** A
- **b-phipsi:** 0.0029803538987865
- **w-rdist:** 0.1832843690627844
- **t-alpha:** 0.0696396085969683

---

---

64

- **PDB ID:** 5MHM | **Chain:** B
- **b-phipsi:** 0.0162226701188196
- **w-rdist:** 0.3142504543835793
- **t-alpha:** 0.0070418134049574

---

---

65

- **PDB ID:** 5RVY | **Chain:** A
- **b-phipsi:** 0.0146708136068518
- **w-rdist:** 0.4075721685518305
- **t-alpha:** 0.0031296009050172

---

---

66

- **PDB ID:** 3BJU | **Chain:** C
- **b-phipsi:** 0.0033017651641294
- **w-rdist:** 0.629328915425099
- **t-alpha:** 0.0039119745263893

---

---

67

- **PDB ID:** 7NFC | **Chain:** B
- **b-phipsi:** 0.0022784357930952
- **w-rdist:** 0.3408962370987046
- **t-alpha:** 0.0532080396597527

---

---

68

- **PDB ID:** 2WJY | **Chain:** A
- **b-phipsi:** 0.0032792118045918
- **w-rdist:** 0.5020873594289996
- **t-alpha:** 0.0126786930545719

---

---

69

- **PDB ID:** 6U23 | **Chain:** F
- **b-phipsi:** 0.0154867254442015
- **w-rdist:** 0.3537839316135293
- **t-alpha:** 0.0062992257321734

---

---

70

- **PDB ID:** 6CE9 | **Chain:** A
- **b-phipsi:** 0.0180375040673948
- **w-rdist:** 0.4042159813069065
- **t-alpha:** 0.0007823187912934

---

---

71

- **PDB ID:** 6CHD | **Chain:** A
- **b-phipsi:** 0.0015317732148241
- **w-rdist:** 0.6295283397687905
- **t-alpha:** 0.0070418134049574

---

---

72

- **PDB ID:** 1B0L | **Chain:** A
- **b-phipsi:** 0.007855906143306
- **w-rdist:** 0.5166455582892483
- **t-alpha:** 0.0039119745263893

---

---

73

- **PDB ID:** 5RVZ | **Chain:** A
- **b-phipsi:** 0.0142151252883432
- **w-rdist:** 0.4062873668188362
- **t-alpha:** 0.0054771144938834

---

---

74

- **PDB ID:** 4W5T | **Chain:** A
- **b-phipsi:** 0.003043606385232
- **w-rdist:** 0.1719632280353145
- **t-alpha:** 0.0837240757413027

---

---

75

- **PDB ID:** 4E36 | **Chain:** B
- **b-phipsi:** 0.0098101825802796
- **w-rdist:** 0.4705273903781445
- **t-alpha:** 0.0054771144938834

---

---

76

- **PDB ID:** 5KYX | **Chain:** A
- **b-phipsi:** 0.001685932035597
- **w-rdist:** 0.6537317150203802
- **t-alpha:** 0.0055077996884944

---

---

77

- **PDB ID:** 4OLA | **Chain:** A
- **b-phipsi:** 0.0020494162576051
- **w-rdist:** 0.1756359116197856
- **t-alpha:** 0.0899840291479125

---

---

78

- **PDB ID:** 4JBS | **Chain:** B
- **b-phipsi:** 0.0097213938907277
- **w-rdist:** 0.4589681439987007
- **t-alpha:** 0.0070922351376341

---

---

79

- **PDB ID:** 3L4G | **Chain:** B
- **b-phipsi:** 0.0011268959941118
- **w-rdist:** 0.7367258406568182
- **t-alpha:** 0.0023530945443304

---

---

80

- **PDB ID:** 6U23 | **Chain:** B
- **b-phipsi:** 0.0197783861351457
- **w-rdist:** 0.3389265862434261
- **t-alpha:** 0.0039277718342689

---

---

81

- **PDB ID:** 6EJB | **Chain:** A
- **b-phipsi:** 0.0004361270336706
- **w-rdist:** 0.5199968858568976
- **t-alpha:** 0.0156493910563146

---

---

82

- **PDB ID:** 4W5Q | **Chain:** A
- **b-phipsi:** 0.0034332823310346
- **w-rdist:** 0.2326129669757892
- **t-alpha:** 0.0774646231830327

---

---

83

- **PDB ID:** 1SEU | **Chain:** A
- **b-phipsi:** 0.0177511789347224
- **w-rdist:** 0.3659161993710003
- **t-alpha:** 0.0046944792707916

---

---

84

- **PDB ID:** 4AH6 | **Chain:** B
- **b-phipsi:** 0.003407544599048
- **w-rdist:** 0.3542023398493669
- **t-alpha:** 0.050987106591174

---

---

85

- **PDB ID:** 3W5O | **Chain:** B
- **b-phipsi:** 0.0025036325118681
- **w-rdist:** 0.4466725202739381
- **t-alpha:** 0.0234741774196856

---

---

86

- **PDB ID:** 4KXT | **Chain:** A
- **b-phipsi:** 0.0017965762972204
- **w-rdist:** 0.0702434826768162
- **t-alpha:** 0.1032863364414424

---

---

87

- **PDB ID:** 5KYX | **Chain:** B
- **b-phipsi:** 0.0056585083846093
- **w-rdist:** 0.671238537747282
- **t-alpha:** 0.0007832732121422

---

---

88

- **PDB ID:** 7EMF | **Chain:** P
- **b-phipsi:** 0.0064736855012348
- **w-rdist:** 0.4551048310041758
- **t-alpha:** 0.0164318357763795

---

---

89

- **PDB ID:** 5KXA | **Chain:** A
- **b-phipsi:** 0.0103263334631654
- **w-rdist:** 0.4375048580100619
- **t-alpha:** 0.0101717762082627

---

---

90

- **PDB ID:** 7NFC | **Chain:** G
- **b-phipsi:** 0.0010468280107053
- **w-rdist:** 0.3762134820674212
- **t-alpha:** 0.0625977621599711

---

---

91

- **PDB ID:** 6N4O | **Chain:** A
- **b-phipsi:** 0.0052866640141058
- **w-rdist:** 0.3489264767811766
- **t-alpha:** 0.0469481037213646

---

---

92

- **PDB ID:** 4CGY | **Chain:** A
- **b-phipsi:** 0.0069073668142136
- **w-rdist:** 0.4463612471428552
- **t-alpha:** 0.0175161413598659

---

---

93

- **PDB ID:** 4Z4H | **Chain:** A
- **b-phipsi:** 0.0010060791997721
- **w-rdist:** 0.2064635098825256
- **t-alpha:** 0.1087633091707751

---

---

94

- **PDB ID:** 4QJ4 | **Chain:** B
- **b-phipsi:** 0.0034830725427008
- **w-rdist:** 0.5808183953633894
- **t-alpha:** 0.0086820531296027

---

---

95

- **PDB ID:** 6EOS | **Chain:** C
- **b-phipsi:** 0.0115298644098794
- **w-rdist:** 0.3973133667891223
- **t-alpha:** 0.0110763082596549

---

---

96

- **PDB ID:** 1T8I | **Chain:** A
- **b-phipsi:** 0.0196669132257783
- **w-rdist:** 0.3564541012893857
- **t-alpha:** 0.0039277718342689

---

---

97

- **PDB ID:** 3V83 | **Chain:** B
- **b-phipsi:** 0.0063789213635878
- **w-rdist:** 0.5571018619251384
- **t-alpha:** 0.0062593997286479

---

---

98

- **PDB ID:** 4AH6 | **Chain:** C
- **b-phipsi:** 0.0057076699022438
- **w-rdist:** 0.3688911907542483
- **t-alpha:** 0.0373379936293052

---

---

99

- **PDB ID:** 7AUA | **Chain:** B
- **b-phipsi:** 0.0096341108188549
- **w-rdist:** 0.4024857623709362
- **t-alpha:** 0.0156493910563146

---

---

100

- **PDB ID:** 6ZYM | **Chain:** B
- **b-phipsi:** 0.0053349565739671
- **w-rdist:** 0.4054037548862557
- **t-alpha:** 0.0305161718713362

---

---

101

- **PDB ID:** 1FCK | **Chain:** A
- **b-phipsi:** 0.0050840968224733
- **w-rdist:** 0.5156810903718647
- **t-alpha:** 0.0125193542574237

---

---

102

- **PDB ID:** 4Z4G | **Chain:** A
- **b-phipsi:** 0.0026249310905526
- **w-rdist:** 0.2160275817753176
- **t-alpha:** 0.0978086738095724

---

---

103

- **PDB ID:** 7ZSC | **Chain:** D
- **b-phipsi:** 0.00432033287123
- **w-rdist:** 0.4266467913565103
- **t-alpha:** 0.0266038120788794

---

---

104

- **PDB ID:** 3EFO | **Chain:** B
- **b-phipsi:** 0.0062931854624415
- **w-rdist:** 0.6809908991830158
- **t-alpha:** 0.0007823187912934

---

---

105

- **PDB ID:** 6U2W | **Chain:** P
- **b-phipsi:** 0.0124594673595001
- **w-rdist:** 0.3904958600271014
- **t-alpha:** 0.0110763082596549

---

---

106

- **PDB ID:** 3EG9 | **Chain:** B
- **b-phipsi:** 0.0063278237872656
- **w-rdist:** 0.6551647555768091
- **t-alpha:** 0.0015676375257613

---

---

107

- **PDB ID:** 8ASC | **Chain:** L
- **b-phipsi:** 0.0023742540352553
- **w-rdist:** 0.4454165048009388
- **t-alpha:** 0.027386367694415

---

---

108

- **PDB ID:** 8ASC | **Chain:** E
- **b-phipsi:** 0.0018472389570859
- **w-rdist:** 0.3961220592200525
- **t-alpha:** 0.0571204636616826

---

---

109

- **PDB ID:** 6U2J | **Chain:** H
- **b-phipsi:** 0.0099090334737821
- **w-rdist:** 0.3034802926114401
- **t-alpha:** 0.0215828567205209

---

---

110

- **PDB ID:** 6ICZ | **Chain:** c
- **b-phipsi:** 0.0066246548365756
- **w-rdist:** 0.4176368718170567
- **t-alpha:** 0.0226916757534991

---

---

111

- **PDB ID:** 6ICZ | **Chain:** C
- **b-phipsi:** 0.0066246548365756
- **w-rdist:** 0.4176368718170567
- **t-alpha:** 0.0226916757534991

---

---

112

- **PDB ID:** 7LT3 | **Chain:** A
- **b-phipsi:** 0.0056426706952516
- **w-rdist:** 0.3412648444719649
- **t-alpha:** 0.0555554643417337

---

---

113

- **PDB ID:** 7AXZ | **Chain:** A
- **b-phipsi:** 0.0009189635484794
- **w-rdist:** 0.5604607741336565
- **t-alpha:** 0.0140843739056586

---

---

114

- **PDB ID:** 7K7L | **Chain:** A
- **b-phipsi:** 0.0157628397188559
- **w-rdist:** 0.3112998990079151
- **t-alpha:** 0.0117370267412904

---

---

115

- **PDB ID:** 2VXO | **Chain:** A
- **b-phipsi:** 0.006129306963229
- **w-rdist:** 0.3699230234762833
- **t-alpha:** 0.040688156290789

---

---

116

- **PDB ID:** 3V5W | **Chain:** A
- **b-phipsi:** 0.0120193846520181
- **w-rdist:** 0.3395038155505887
- **t-alpha:** 0.0164318357763795

---

---

117

- **PDB ID:** 1RRJ | **Chain:** A
- **b-phipsi:** 0.0180339226820997
- **w-rdist:** 0.3590212892420929
- **t-alpha:** 0.0070922351376341

---

---

118

- **PDB ID:** 4F3T | **Chain:** A
- **b-phipsi:** 0.0030790536303714
- **w-rdist:** 0.0467910375888013
- **t-alpha:** 0.1158056523096886

---

---

119

- **PDB ID:** 7EA9 | **Chain:** A
- **b-phipsi:** 0.002510923853111
- **w-rdist:** 0.6289363923133782
- **t-alpha:** 0.0093893372993878

---

---

120

- **PDB ID:** 1BKA | **Chain:** A
- **b-phipsi:** 0.006895837178018
- **w-rdist:** 0.5236155111548314
- **t-alpha:** 0.0086820531296027

---

---

121

- **PDB ID:** 5J68 | **Chain:** A
- **b-phipsi:** 0.0048411897295105
- **w-rdist:** 0.4912759876779642
- **t-alpha:** 0.0175161413598659

---

---

122

- **PDB ID:** 8D6J | **Chain:** A
- **b-phipsi:** 0.0016931908944261
- **w-rdist:** 0.1158567849905897
- **t-alpha:** 0.1275429598762953

---

---

123

- **PDB ID:** 4W5O | **Chain:** A
- **b-phipsi:** 0.0026163125717997
- **w-rdist:** 0.2161748204018165
- **t-alpha:** 0.1095460606668756

---

---

124

- **PDB ID:** 5MHM | **Chain:** A
- **b-phipsi:** 0.0131845188437627
- **w-rdist:** 0.3259466338528004
- **t-alpha:** 0.0156493910563146

---

---

125

- **PDB ID:** 6MGQ | **Chain:** A
- **b-phipsi:** 0.0067715505009976
- **w-rdist:** 0.2269831450070442
- **t-alpha:** 0.064162546478931

---

---

126

- **PDB ID:** 3EGD | **Chain:** B
- **b-phipsi:** 0.0063504376936264
- **w-rdist:** 0.6527605096252984
- **t-alpha:** 0.0039119745263893

---

---

127

- **PDB ID:** 6AHR | **Chain:** B
- **b-phipsi:** 0.0173502931548928
- **w-rdist:** 0.2574680773934314
- **t-alpha:** 0.0133018035296799

---

---

128

- **PDB ID:** 8D96 | **Chain:** C
- **b-phipsi:** 0.0048747580704967
- **w-rdist:** 0.4392936317393059
- **t-alpha:** 0.0281690295641283

---

---

129

- **PDB ID:** 4P8Q | **Chain:** B
- **b-phipsi:** 0.0093168523294578
- **w-rdist:** 0.5478632629449096
- **t-alpha:** 0.0015647720218643

---

---

130

- **PDB ID:** 6LID | **Chain:** A
- **b-phipsi:** 0.0058944064880765
- **w-rdist:** 0.7294161647635636
- **t-alpha:** 0.0015647720218643

---

---

131

- **PDB ID:** 7A3F | **Chain:** A
- **b-phipsi:** 0.0157485811309944
- **w-rdist:** 0.4274498941356251
- **t-alpha:** 0.0062593997286479

---

---

132

- **PDB ID:** 6VXK | **Chain:** B
- **b-phipsi:** 0.0261367129126431
- **w-rdist:** 0.3440356988989443
- **t-alpha:** 0.0031401242085145

---

---

133

- **PDB ID:** 6EOS | **Chain:** D
- **b-phipsi:** 0.012827129939269
- **w-rdist:** 0.4723204911235214
- **t-alpha:** 0.0039119745263893

---

---

134

- **PDB ID:** 4QCL | **Chain:** A
- **b-phipsi:** 0.0064580931961407
- **w-rdist:** 0.2240040359465484
- **t-alpha:** 0.0766819142571693

---

---

135

- **PDB ID:** 5MQF | **Chain:** B
- **b-phipsi:** 0.0135462931009897
- **w-rdist:** 0.4421910929915723
- **t-alpha:** 0.0078243548762237

---

---

136

- **PDB ID:** 6X6C | **Chain:** A
- **b-phipsi:** 0.0063078100465426
- **w-rdist:** 0.6583965744643434
- **t-alpha:** 0.0039277718342689

---

---

137

- **PDB ID:** 7PWD | **Chain:** A
- **b-phipsi:** 0.0164925577542016
- **w-rdist:** 0.2907889526072083
- **t-alpha:** 0.0140843739056586

---

---

138

- **PDB ID:** 5RVW | **Chain:** A
- **b-phipsi:** 0.0139975872433236
- **w-rdist:** 0.4067256735536844
- **t-alpha:** 0.0101717762082627

---

---

139

- **PDB ID:** 6U2J | **Chain:** G
- **b-phipsi:** 0.0148883527675815
- **w-rdist:** 0.2998481957539909
- **t-alpha:** 0.0156493910563146

---

---

140

- **PDB ID:** 8OG4 | **Chain:** A
- **b-phipsi:** 0.0067068219906576
- **w-rdist:** 0.4021412225881235
- **t-alpha:** 0.0289856519083209

---

---

141

- **PDB ID:** 3V83 | **Chain:** A
- **b-phipsi:** 0.0088491436144987
- **w-rdist:** 0.5813281447062477
- **t-alpha:** 0.0023473590503262

---

---

142

- **PDB ID:** 7YBU | **Chain:** J
- **b-phipsi:** 0.0057458686748834
- **w-rdist:** 0.4269974556317505
- **t-alpha:** 0.0323103167719089

---

---

143

- **PDB ID:** 1LCF | **Chain:** A
- **b-phipsi:** 0.0101563129420483
- **w-rdist:** 0.5062752449085652
- **t-alpha:** 0.0054771144938834

---

---

144

- **PDB ID:** 6C01 | **Chain:** B
- **b-phipsi:** 0.0033718083095234
- **w-rdist:** 0.4943390068063057
- **t-alpha:** 0.0203438662057466

---

---

145

- **PDB ID:** 6XTY | **Chain:** 6
- **b-phipsi:** 0.0098707633038234
- **w-rdist:** 0.4919150153942814
- **t-alpha:** 0.0078243548762237

---

---

146

- **PDB ID:** 4AH6 | **Chain:** D
- **b-phipsi:** 0.0055941784989986
- **w-rdist:** 0.3528756677561013
- **t-alpha:** 0.0650001391403227

---

---

147

- **PDB ID:** 5KI6 | **Chain:** A
- **b-phipsi:** 0.0037501151470687
- **w-rdist:** 0.2051749574740583
- **t-alpha:** 0.1181528955465753

---

---

148

- **PDB ID:** 6LI9 | **Chain:** A
- **b-phipsi:** 0.0037819652970019
- **w-rdist:** 0.733399716346699
- **t-alpha:** 0.0047169960791435

---

---

149

- **PDB ID:** 6C2Y | **Chain:** A
- **b-phipsi:** 0.0125331913643933
- **w-rdist:** 0.3185897942929342
- **t-alpha:** 0.0187789021729365

---

---

150

- **PDB ID:** 8ASC | **Chain:** K
- **b-phipsi:** 0.0017620031405804
- **w-rdist:** 0.55356238155521
- **t-alpha:** 0.0156493910563146

---

---

151

- **PDB ID:** 7EA9 | **Chain:** C
- **b-phipsi:** 0.0030961521928042
- **w-rdist:** 0.6265352124043417
- **t-alpha:** 0.0109543395722921

---

---

152

- **PDB ID:** 4JBS | **Chain:** A
- **b-phipsi:** 0.0073465217936748
- **w-rdist:** 0.3724345748101726
- **t-alpha:** 0.0336461458262049

---

---

153

- **PDB ID:** 5JS1 | **Chain:** A
- **b-phipsi:** 0.0028084443233339
- **w-rdist:** 0.0494754780646928
- **t-alpha:** 0.1377147492711152

---

---

154

- **PDB ID:** 4Z4C | **Chain:** A
- **b-phipsi:** 0.0053759968279778
- **w-rdist:** 0.2247762676556628
- **t-alpha:** 0.1032863364414424

---

---

155

- **PDB ID:** 6RYF | **Chain:** A
- **b-phipsi:** 0.0086341470426358
- **w-rdist:** 0.4603337324478931
- **t-alpha:** 0.0142860356016105

---

---

156

- **PDB ID:** 8ASC | **Chain:** O
- **b-phipsi:** 0.0011331662700114
- **w-rdist:** 0.3939305564199463
- **t-alpha:** 0.0845067327212063

---

---

157

- **PDB ID:** 4X1D | **Chain:** B
- **b-phipsi:** 0.0086738947452022
- **w-rdist:** 0.6230692551333818
- **t-alpha:** 0.0015647720218643

---

---

158

- **PDB ID:** 6SVP | **Chain:** B
- **b-phipsi:** 0.0049383390779993
- **w-rdist:** 0.7898441237960858
- **t-alpha:** 0.0015647720218643

---

---

159

- **PDB ID:** 7ZXS | **Chain:** B
- **b-phipsi:** 0.006917200738688
- **w-rdist:** 0.6478111608776872
- **t-alpha:** 0.0039277718342689

---

---

160

- **PDB ID:** 2NUP | **Chain:** B
- **b-phipsi:** 0.0060284814015517
- **w-rdist:** 0.6576191631871163
- **t-alpha:** 0.0062593997286479

---

---

161

- **PDB ID:** 6ID1 | **Chain:** c
- **b-phipsi:** 0.0089285035166262
- **w-rdist:** 0.4723043835476377
- **t-alpha:** 0.0125193542574237

---

---

162

- **PDB ID:** 7ZWA | **Chain:** A
- **b-phipsi:** 0.0032209126280489
- **w-rdist:** 0.4091028204399985
- **t-alpha:** 0.0625977621599711

---

---

163

- **PDB ID:** 6U2K | **Chain:** B
- **b-phipsi:** 0.0070049416722219
- **w-rdist:** 0.3785606600192233
- **t-alpha:** 0.0398701801688734

---

---

164

- **PDB ID:** 6MGQ | **Chain:** C
- **b-phipsi:** 0.0060378922747851
- **w-rdist:** 0.3053202398689337
- **t-alpha:** 0.0821594238081062

---

---

165

- **PDB ID:** 6U2J | **Chain:** O
- **b-phipsi:** 0.0117839000673718
- **w-rdist:** 0.2928087022669318
- **t-alpha:** 0.0219087977837508

---

---

166

- **PDB ID:** 6GYR | **Chain:** B
- **b-phipsi:** 0.0069866686063712
- **w-rdist:** 0.4046637355255162
- **t-alpha:** 0.0312987592118472

---

---

167

- **PDB ID:** 5RVX | **Chain:** A
- **b-phipsi:** 0.013660845059638
- **w-rdist:** 0.4075335224133684
- **t-alpha:** 0.0117370267412904

---

---

168

- **PDB ID:** 4AH6 | **Chain:** A
- **b-phipsi:** 0.0038693661295055
- **w-rdist:** 0.3632835827686018
- **t-alpha:** 0.0821341394260981

---

---

169

- **PDB ID:** 5JS2 | **Chain:** A
- **b-phipsi:** 0.0032999482118413
- **w-rdist:** 0.0574199907576052
- **t-alpha:** 0.1392798657818292

---

---

170

- **PDB ID:** 6ERF | **Chain:** C
- **b-phipsi:** 0.0030878769092505
- **w-rdist:** 0.4449846024696902
- **t-alpha:** 0.0469481037213646

---

---

171

- **PDB ID:** 6U23 | **Chain:** N
- **b-phipsi:** 0.0110491533981144
- **w-rdist:** 0.3470100634417222
- **t-alpha:** 0.0211265191128235

---

---

172

- **PDB ID:** 6CEB | **Chain:** B
- **b-phipsi:** 0.0215428178146608
- **w-rdist:** 0.4071446468602999
- **t-alpha:** 0.0039277718342689

---

---

173

- **PDB ID:** 5WG3 | **Chain:** A
- **b-phipsi:** 0.0178766648128227
- **w-rdist:** 0.3405276826551395
- **t-alpha:** 0.0117370267412904

---

---

174

- **PDB ID:** 1LPQ | **Chain:** A
- **b-phipsi:** 0.020583489291055
- **w-rdist:** 0.4029858687570244
- **t-alpha:** 0.0054771144938834

---

---

175

- **PDB ID:** 4ZG9 | **Chain:** B
- **b-phipsi:** 0.0196246593431509
- **w-rdist:** 0.4373667484127421
- **t-alpha:** 0.0031401242085145

---

---

176

- **PDB ID:** 1PL0 | **Chain:** C
- **b-phipsi:** 0.0064129196837788
- **w-rdist:** 0.7674077215646232
- **t-alpha:** 0.0007832732121422

---

---

177

- **PDB ID:** 6P0D | **Chain:** A
- **b-phipsi:** 0.005344391633651
- **w-rdist:** 0.7386102252414369
- **t-alpha:** 0.0046944792707916

---

---

178

- **PDB ID:** 7KI3 | **Chain:** D
- **b-phipsi:** 0.0091254853499247
- **w-rdist:** 0.2028342062104213
- **t-alpha:** 0.0492955855664358

---

---

179

- **PDB ID:** 5WG5 | **Chain:** A
- **b-phipsi:** 0.019074527106623
- **w-rdist:** 0.3323869771697254
- **t-alpha:** 0.0110763082596549

---

---

180

- **PDB ID:** 4ZGA | **Chain:** A
- **b-phipsi:** 0.0148426416810927
- **w-rdist:** 0.446276467226317
- **t-alpha:** 0.0078243548762237

---

---

181

- **PDB ID:** 5UKL | **Chain:** A
- **b-phipsi:** 0.0130477528699735
- **w-rdist:** 0.3501829614529059
- **t-alpha:** 0.0179965923641238

---

---

182

- **PDB ID:** 7YBU | **Chain:** B
- **b-phipsi:** 0.0060202052124583
- **w-rdist:** 0.433529063021687
- **t-alpha:** 0.0356565880801509

---

---

183

- **PDB ID:** 7ZT6 | **Chain:** B
- **b-phipsi:** 0.002621803206812
- **w-rdist:** 0.3956387679903319
- **t-alpha:** 0.0821594238081062

---

---

184

- **PDB ID:** 7K7Z | **Chain:** A
- **b-phipsi:** 0.0178721131236585
- **w-rdist:** 0.3340913606045823
- **t-alpha:** 0.0126786930545719

---

---

185

- **PDB ID:** 6ERG | **Chain:** E
- **b-phipsi:** 0.0018436084131454
- **w-rdist:** 0.2954278877285707
- **t-alpha:** 0.1369327264299646

---

---

186

- **PDB ID:** 5C1B | **Chain:** C
- **b-phipsi:** 0.0070853387447647
- **w-rdist:** 0.1895517914108304
- **t-alpha:** 0.0837240757413027

---

---

187

- **PDB ID:** 6FF7 | **Chain:** b
- **b-phipsi:** 0.011592876732646
- **w-rdist:** 0.4686405544243389
- **t-alpha:** 0.0086820531296027

---

---

188

- **PDB ID:** 7SGL | **Chain:** B
- **b-phipsi:** 0.0057687206901072
- **w-rdist:** 0.4221287172834136
- **t-alpha:** 0.0469481037213646

---

---

189

- **PDB ID:** 6UZT | **Chain:** A
- **b-phipsi:** 0.004141863187263
- **w-rdist:** 0.818659446845925
- **t-alpha:** 0.0015676375257613

---

---

190

- **PDB ID:** 5IZ5 | **Chain:** B
- **b-phipsi:** 0.0027647002593068
- **w-rdist:** 0.4201662817567533
- **t-alpha:** 0.0712045469065545

---

---

191

- **PDB ID:** 5JLH | **Chain:** G
- **b-phipsi:** 0.0120888200485844
- **w-rdist:** 0.5255662488433073
- **t-alpha:** 0.0015647720218643

---

---

192

- **PDB ID:** 4KRF | **Chain:** A
- **b-phipsi:** 0.0010717834225373
- **w-rdist:** 0.0471997097913125
- **t-alpha:** 0.1932703499952912

---

---

193

- **PDB ID:** 6XTY | **Chain:** 4
- **b-phipsi:** 0.0138036313467483
- **w-rdist:** 0.3903095404181558
- **t-alpha:** 0.0148665808060715

---

---

194

- **PDB ID:** 5DYH | **Chain:** A
- **b-phipsi:** 0.0066909841438141
- **w-rdist:** 0.4392591352558052
- **t-alpha:** 0.0265064016545928

---

---

195

- **PDB ID:** 5VNJ | **Chain:** A
- **b-phipsi:** 0.008547876000901
- **w-rdist:** 0.5974933953496158
- **t-alpha:** 0.0047169960791435

---

---

196

- **PDB ID:** 6R4F | **Chain:** B
- **b-phipsi:** 0.0056311544628519
- **w-rdist:** 0.7999997844770734
- **t-alpha:** 0.0015676375257613

---

---

197

- **PDB ID:** 7NFE | **Chain:** B
- **b-phipsi:** 0.008885274370178
- **w-rdist:** 0.3530189063867367
- **t-alpha:** 0.0336461458262049

---

---

198

- **PDB ID:** 1SQY | **Chain:** A
- **b-phipsi:** 0.0072755924337122
- **w-rdist:** 0.507001435885411
- **t-alpha:** 0.0142860356016105

---

---

199

- **PDB ID:** 4E36 | **Chain:** A
- **b-phipsi:** 0.0074917440156619
- **w-rdist:** 0.3962263707076277
- **t-alpha:** 0.0359934414192668

---

---

200

- **PDB ID:** 6ERG | **Chain:** B
- **b-phipsi:** 0.0031934752270833
- **w-rdist:** 0.2924129359592112
- **t-alpha:** 0.1345850125084049

---

---

201

- **PDB ID:** 4BLD | **Chain:** D
- **b-phipsi:** 0.0027745732764858
- **w-rdist:** 0.6305856690779167
- **t-alpha:** 0.0134815624146233

---

---

202

- **PDB ID:** 4BLB | **Chain:** A
- **b-phipsi:** 0.0035973851919402
- **w-rdist:** 0.6394173752845445
- **t-alpha:** 0.011876942219535

---

---

203

- **PDB ID:** 5W6V | **Chain:** A
- **b-phipsi:** 0.0044610639426911
- **w-rdist:** 0.0672020003439749
- **t-alpha:** 0.1471044374308914

---

---

204

- **PDB ID:** 5MHN | **Chain:** B
- **b-phipsi:** 0.0171188698421125
- **w-rdist:** 0.2848503799812544
- **t-alpha:** 0.0172143982372043

---

---

205

- **PDB ID:** 6I2X | **Chain:** A
- **b-phipsi:** 0.0022392256797014
- **w-rdist:** 0.190619900326
- **t-alpha:** 0.1658839749938661

---

---

206

- **PDB ID:** 6ID1 | **Chain:** C
- **b-phipsi:** 0.0115269858644001
- **w-rdist:** 0.4748331728076611
- **t-alpha:** 0.0093893372993878

---

---

207

- **PDB ID:** 8AG4 | **Chain:** B
- **b-phipsi:** 0.0022267627213781
- **w-rdist:** 0.3668108072010785
- **t-alpha:** 0.1095460606668756

---

---

208

- **PDB ID:** 3EG9 | **Chain:** A
- **b-phipsi:** 0.0016159537770892
- **w-rdist:** 0.6616127451669458
- **t-alpha:** 0.0133018035296799

---

---

209

- **PDB ID:** 6ERF | **Chain:** E
- **b-phipsi:** 0.0022325025986767
- **w-rdist:** 0.4058391433285797
- **t-alpha:** 0.0876367934858521

---

---

210

- **PDB ID:** 6TRX | **Chain:** A
- **b-phipsi:** 0.0044888567111578
- **w-rdist:** 0.5770624621157481
- **t-alpha:** 0.0156493910563146

---

---

211

- **PDB ID:** 6FOA | **Chain:** A
- **b-phipsi:** 0.0010716898211541
- **w-rdist:** 0.5244476623423365
- **t-alpha:** 0.0226916757534991

---

---

212

- **PDB ID:** 6HTS | **Chain:** D
- **b-phipsi:** 0.0113120982206179
- **w-rdist:** 0.5133601455611831
- **t-alpha:** 0.0055077996884944

---

---

213

- **PDB ID:** 7Z6O | **Chain:** B
- **b-phipsi:** 0.0020421889680792
- **w-rdist:** 0.3850813482718379
- **t-alpha:** 0.1064162560472814

---

---

214

- **PDB ID:** 2VXO | **Chain:** B
- **b-phipsi:** 0.0088055578785537
- **w-rdist:** 0.3817376062828241
- **t-alpha:** 0.0289856519083209

---

---

215

- **PDB ID:** 5KYW | **Chain:** B
- **b-phipsi:** 0.0062483936239403
- **w-rdist:** 0.6863991090417906
- **t-alpha:** 0.0070922351376341

---

---

216

- **PDB ID:** 6R4G | **Chain:** B
- **b-phipsi:** 0.0046095764851992
- **w-rdist:** 0.7969705878722231
- **t-alpha:** 0.0046944792707916

---

---

217

- **PDB ID:** 6MFN | **Chain:** A
- **b-phipsi:** 0.0020063049858797
- **w-rdist:** 0.3565682408831353
- **t-alpha:** 0.1228477492952864

---

---

218

- **PDB ID:** 5DXH | **Chain:** A
- **b-phipsi:** 0.0070277959104479
- **w-rdist:** 0.587632790804862
- **t-alpha:** 0.0101717762082627

---

---

219

- **PDB ID:** 7SVN | **Chain:** C
- **b-phipsi:** 0.0065034933617422
- **w-rdist:** 0.6416185511858987
- **t-alpha:** 0.0086070234951727

---

---

220

- **PDB ID:** 6U23 | **Chain:** K
- **b-phipsi:** 0.0090366534174712
- **w-rdist:** 0.3453119867886006
- **t-alpha:** 0.0422534769714337

---

---

221

- **PDB ID:** 5OFB | **Chain:** A
- **b-phipsi:** 0.0080476730964714
- **w-rdist:** 0.6332165892448953
- **t-alpha:** 0.0054771144938834

---

---

222

- **PDB ID:** 7ZSC | **Chain:** C
- **b-phipsi:** 0.0048149945627589
- **w-rdist:** 0.415496327143554
- **t-alpha:** 0.0703518669333997

---

---

223

- **PDB ID:** 7SQ2 | **Chain:** B
- **b-phipsi:** 0.0032056400092801
- **w-rdist:** 0.5838493644482348
- **t-alpha:** 0.0172143982372043

---

---

224

- **PDB ID:** 5RW0 | **Chain:** A
- **b-phipsi:** 0.014500725131292
- **w-rdist:** 0.4072672368567593
- **t-alpha:** 0.0140843739056586

---

---

225

- **PDB ID:** 4OLB | **Chain:** A
- **b-phipsi:** 0.0048838801673317
- **w-rdist:** 0.0649692015424923
- **t-alpha:** 0.1557116053874756

---

---

226

- **PDB ID:** 4DPG | **Chain:** A
- **b-phipsi:** 0.0020148635796626
- **w-rdist:** 0.627613602924042
- **t-alpha:** 0.0158983514403059

---

---

227

- **PDB ID:** 6U23 | **Chain:** E
- **b-phipsi:** 0.0101212699056756
- **w-rdist:** 0.3537896748513264
- **t-alpha:** 0.0265064016545928

---

---

228

- **PDB ID:** 6ID0 | **Chain:** c
- **b-phipsi:** 0.0062533761094026
- **w-rdist:** 0.4843523772414969
- **t-alpha:** 0.0219087977837508

---

---

229

- **PDB ID:** 6U2J | **Chain:** C
- **b-phipsi:** 0.019322822870581
- **w-rdist:** 0.2976116486644782
- **t-alpha:** 0.0156493910563146

---

---

230

- **PDB ID:** 3OPM | **Chain:** B
- **b-phipsi:** 0.0062028689028108
- **w-rdist:** 0.76893890861525
- **t-alpha:** 0.0046944792707916

---

---

231

- **PDB ID:** 7YBU | **Chain:** D
- **b-phipsi:** 0.0059583394800585
- **w-rdist:** 0.4312685533393399
- **t-alpha:** 0.0524253871955315

---

---

232

- **PDB ID:** 1N76 | **Chain:** A
- **b-phipsi:** 0.0083434673519799
- **w-rdist:** 0.5211652548461903
- **t-alpha:** 0.0117370267412904

---

---

233

- **PDB ID:** 5FUR | **Chain:** I
- **b-phipsi:** 0.0143393253669885
- **w-rdist:** 0.2352107910298121
- **t-alpha:** 0.0226916757534991

---

---

234

- **PDB ID:** 6MGQ | **Chain:** B
- **b-phipsi:** 0.0083716809555236
- **w-rdist:** 0.0715853655798192
- **t-alpha:** 0.0852894374233532

---

---

235

- **PDB ID:** 6P09 | **Chain:** A
- **b-phipsi:** 0.0073481598062142
- **w-rdist:** 0.7288630127169745
- **t-alpha:** 0.0031296009050172

---

---

236

- **PDB ID:** 7ZWA | **Chain:** B
- **b-phipsi:** 0.0028588772830298
- **w-rdist:** 0.4144765987374171
- **t-alpha:** 0.0892015743287577

---

---

237

- **PDB ID:** 6X6A | **Chain:** D
- **b-phipsi:** 0.0071324557960113
- **w-rdist:** 0.6571462112647187
- **t-alpha:** 0.0070418134049574

---

---

238

- **PDB ID:** 7SVL | **Chain:** A
- **b-phipsi:** 0.0082872328168262
- **w-rdist:** 0.6304791218947065
- **t-alpha:** 0.0055077996884944

---

---

239

- **PDB ID:** 4TUU | **Chain:** A
- **b-phipsi:** 0.008310368847312
- **w-rdist:** 0.1804094354811458
- **t-alpha:** 0.0837240757413027

---

---

240

- **PDB ID:** 3KRX | **Chain:** A
- **b-phipsi:** 0.0107102147631507
- **w-rdist:** 0.3066791607624438
- **t-alpha:** 0.0352112405915889

---

---

241

- **PDB ID:** 6PYS | **Chain:** A
- **b-phipsi:** 0.0086576927209455
- **w-rdist:** 0.1650889009102419
- **t-alpha:** 0.0798120464585645

---

---

242

- **PDB ID:** 4X0P | **Chain:** A
- **b-phipsi:** 0.0523218583973104
- **w-rdist:** 0.222111564680877
- **t-alpha:** 0.0055077996884944

---

---

243

- **PDB ID:** 7BG0 | **Chain:** E
- **b-phipsi:** 0.01228358443566
- **w-rdist:** 0.3775914080613909
- **t-alpha:** 0.0203438662057466

---

---

244

- **PDB ID:** 8ASC | **Chain:** P
- **b-phipsi:** 0.0035941748173674
- **w-rdist:** 0.4597637756310818
- **t-alpha:** 0.0485128406736701

---

---

245

- **PDB ID:** 3HHD | **Chain:** D
- **b-phipsi:** 0.0038390782486622
- **w-rdist:** 0.7845331247984992
- **t-alpha:** 0.0070922351376341

---

---

246

- **PDB ID:** 2B3X | **Chain:** A
- **b-phipsi:** 0.0062213805658245
- **w-rdist:** 0.771248985616488
- **t-alpha:** 0.0047169960791435

---

---

247

- **PDB ID:** 8FCQ | **Chain:** A
- **b-phipsi:** 0.0058257948802049
- **w-rdist:** 0.2025423539745311
- **t-alpha:** 0.1392798657818292

---

---

248

- **PDB ID:** 6R4J | **Chain:** B
- **b-phipsi:** 0.0068308389287973
- **w-rdist:** 0.8050711314632585
- **t-alpha:** 0.0007823187912934

---

---

249

- **PDB ID:** 5JPN | **Chain:** A
- **b-phipsi:** 0.0269167288251761
- **w-rdist:** 0.2789393745722386
- **t-alpha:** 0.0110763082596549

---

---

250

- **PDB ID:** 6U3J | **Chain:** A
- **b-phipsi:** 0.0130435159395587
- **w-rdist:** 0.3981638557272486
- **t-alpha:** 0.0183267305342325

---

---

251

- **PDB ID:** 4DPG | **Chain:** G
- **b-phipsi:** 0.0018944792557148
- **w-rdist:** 0.6298861727906253
- **t-alpha:** 0.0172143982372043

---

---

252

- **PDB ID:** 3BDL | **Chain:** A
- **b-phipsi:** 0.0008255547107562
- **w-rdist:** 0.4723719878199611
- **t-alpha:** 0.0597019506692302

---

---

253

- **PDB ID:** 2RD0 | **Chain:** A
- **b-phipsi:** 0.0134784391184478
- **w-rdist:** 0.3147062879002164
- **t-alpha:** 0.0226916757534991

---

---

254

- **PDB ID:** 5CU5 | **Chain:** A
- **b-phipsi:** 0.008417112394888
- **w-rdist:** 0.3807537486595509
- **t-alpha:** 0.0446008926005814

---

---

255

- **PDB ID:** 5KYN | **Chain:** B
- **b-phipsi:** 0.0034584394427758
- **w-rdist:** 0.6157205049720994
- **t-alpha:** 0.0167066822187644

---

---

256

- **PDB ID:** 1X6V | **Chain:** B
- **b-phipsi:** 0.0087791110099076
- **w-rdist:** 0.5752210802451104
- **t-alpha:** 0.0078868306657793

---

---

257

- **PDB ID:** 7JKQ | **Chain:** A
- **b-phipsi:** 0.0098277870326416
- **w-rdist:** 0.6322584938434367
- **t-alpha:** 0.0031296009050172

---

---

258

- **PDB ID:** 2E1Q | **Chain:** A
- **b-phipsi:** 0.0049782386227229
- **w-rdist:** 0.3770563029348425
- **t-alpha:** 0.1025037952669631

---

---

259

- **PDB ID:** 5KZW | **Chain:** A
- **b-phipsi:** 0.0071985067234394
- **w-rdist:** 0.6065227628273057
- **t-alpha:** 0.0102767895279776

---

---

260

- **PDB ID:** 8AYH | **Chain:** A
- **b-phipsi:** 0.0043352391070108
- **w-rdist:** 0.1903500115290752
- **t-alpha:** 0.1776206652520329

---

---

261

- **PDB ID:** 7ASG | **Chain:** A
- **b-phipsi:** 0.0029249406982109
- **w-rdist:** 0.8834231838634882
- **t-alpha:** 0.0039277718342689

---

---

262

- **PDB ID:** 6LI9 | **Chain:** C
- **b-phipsi:** 0.0046490771359054
- **w-rdist:** 0.7323185957113989
- **t-alpha:** 0.0094788657373643

---

---

263

- **PDB ID:** 8EZA | **Chain:** A
- **b-phipsi:** 0.004528382407939
- **w-rdist:** 0.413691878797555
- **t-alpha:** 0.0868544688919852

---

---

264

- **PDB ID:** 8AG5 | **Chain:** A
- **b-phipsi:** 0.0012578758446509
- **w-rdist:** 0.5062098620030341
- **t-alpha:** 0.0352112405915889

---

---

265

- **PDB ID:** 3CDZ | **Chain:** B
- **b-phipsi:** 0.050916853571692
- **w-rdist:** 0.3908767644222933
- **t-alpha:** 0.0

---

---

266

- **PDB ID:** 4YCU | **Chain:** A
- **b-phipsi:** 0.0018813300371044
- **w-rdist:** 0.6334922348224425
- **t-alpha:** 0.0175161413598659

---

---

267

- **PDB ID:** 6LID | **Chain:** C
- **b-phipsi:** 0.0057687232596794
- **w-rdist:** 0.7329538107471079
- **t-alpha:** 0.0078868306657793

---

---

268

- **PDB ID:** 7N2M | **Chain:** A
- **b-phipsi:** 0.0081278108595523
- **w-rdist:** 0.1818222297680874
- **t-alpha:** 0.0962440731859939

---

---

269

- **PDB ID:** 7A3I | **Chain:** A
- **b-phipsi:** 0.0122964617179222
- **w-rdist:** 0.47340128632967
- **t-alpha:** 0.0109543395722921

---

---

270

- **PDB ID:** 3IHP | **Chain:** A
- **b-phipsi:** 0.0073788971062627
- **w-rdist:** 0.4053632317088315
- **t-alpha:** 0.0532080396597527

---

---

271

- **PDB ID:** 6Q1V | **Chain:** A
- **b-phipsi:** 0.0068340334629046
- **w-rdist:** 0.750500258800803
- **t-alpha:** 0.0047169960791435

---

---

272

- **PDB ID:** 8OG1 | **Chain:** A
- **b-phipsi:** 0.0082634434550424
- **w-rdist:** 0.4144189574369748
- **t-alpha:** 0.0381807098839799

---

---

273

- **PDB ID:** 7ZYF | **Chain:** A
- **b-phipsi:** 0.0086159870038571
- **w-rdist:** 0.6220380731270017
- **t-alpha:** 0.0070922351376341

---

---

274

- **PDB ID:** 5O9Z | **Chain:** b
- **b-phipsi:** 0.0094002282172069
- **w-rdist:** 0.3546190674568155
- **t-alpha:** 0.0453832094967918

---

---

275

- **PDB ID:** 6U23 | **Chain:** I
- **b-phipsi:** 0.0102790259830899
- **w-rdist:** 0.3395013090728908
- **t-alpha:** 0.0432653326797045

---

---

276

- **PDB ID:** 6U23 | **Chain:** M
- **b-phipsi:** 0.0122212977468514
- **w-rdist:** 0.3487454210708531
- **t-alpha:** 0.0240384962174629

---

---

277

- **PDB ID:** 6KYV | **Chain:** H
- **b-phipsi:** 0.0110755833130507
- **w-rdist:** 0.6130211882084395
- **t-alpha:** 0.0023530945443304

---

---

278

- **PDB ID:** 6ERG | **Chain:** A
- **b-phipsi:** 0.0012908163963175
- **w-rdist:** 0.5531439273594112
- **t-alpha:** 0.0226916757534991

---

---

279

- **PDB ID:** 8ASC | **Chain:** F
- **b-phipsi:** 0.0020950857826345
- **w-rdist:** 0.4646975889663044
- **t-alpha:** 0.0625977621599711

---

---

280

- **PDB ID:** 5GVC | **Chain:** B
- **b-phipsi:** 0.0053079504495563
- **w-rdist:** 0.4650862739003178
- **t-alpha:** 0.0441178429367157

---

---

281

- **PDB ID:** 7KTR | **Chain:** B
- **b-phipsi:** 0.0110514828325867
- **w-rdist:** 0.5369110027608828
- **t-alpha:** 0.0062992257321734

---

---

282

- **PDB ID:** 8EZA | **Chain:** J
- **b-phipsi:** 0.0042664323486032
- **w-rdist:** 0.4227584729104549
- **t-alpha:** 0.0915490640026019

---

---

283

- **PDB ID:** 5HE1 | **Chain:** A
- **b-phipsi:** 0.0116240610176986
- **w-rdist:** 0.3062651901277243
- **t-alpha:** 0.0375586271097019

---

---

284

- **PDB ID:** 7YBU | **Chain:** F
- **b-phipsi:** 0.0069861913235594
- **w-rdist:** 0.4295096269992772
- **t-alpha:** 0.0516428148679497

---

---

285

- **PDB ID:** 1TZN | **Chain:** H
- **b-phipsi:** 0.0086135778188221
- **w-rdist:** 0.7496299739417438
- **t-alpha:** 0.0015647720218643

---

---

286

- **PDB ID:** 6QZV | **Chain:** A
- **b-phipsi:** 0.0097050853843132
- **w-rdist:** 0.6349488746632227
- **t-alpha:** 0.0046944792707916

---

---

287

- **PDB ID:** 7ZVT | **Chain:** B
- **b-phipsi:** 0.0021107164878308
- **w-rdist:** 0.3747250137972794
- **t-alpha:** 0.1392798657818292

---

---

288

- **PDB ID:** 4FWJ | **Chain:** A
- **b-phipsi:** 0.0055404152896364
- **w-rdist:** 0.499946331675325
- **t-alpha:** 0.0242563010013117

---

---

289

- **PDB ID:** 7ZXS | **Chain:** D
- **b-phipsi:** 0.0053072394949378
- **w-rdist:** 0.6401599699466314
- **t-alpha:** 0.0148665808060715

---

---

290

- **PDB ID:** 3EFO | **Chain:** A
- **b-phipsi:** 0.0016814011801616
- **w-rdist:** 0.6558503265463671
- **t-alpha:** 0.0179965923641238

---

---

291

- **PDB ID:** 5C18 | **Chain:** C
- **b-phipsi:** 0.0074560146027887
- **w-rdist:** 0.1878914196430602
- **t-alpha:** 0.1212827505141209

---

---

292

- **PDB ID:** 5MJ6 | **Chain:** B
- **b-phipsi:** 0.0105702616462473
- **w-rdist:** 0.5966470615440866
- **t-alpha:** 0.0054771144938834

---

---

293

- **PDB ID:** 6YDX | **Chain:** B
- **b-phipsi:** 0.009288275177811
- **w-rdist:** 0.5902875913883389
- **t-alpha:** 0.0078868306657793

---

---

294

- **PDB ID:** 2A73 | **Chain:** A
- **b-phipsi:** 0.0339099311059032
- **w-rdist:** 0.4300863245847651
- **t-alpha:** 0.0023473590503262

---

---

295

- **PDB ID:** 7A3K | **Chain:** A
- **b-phipsi:** 0.0116002326173593
- **w-rdist:** 0.5032789762615867
- **t-alpha:** 0.0101717762082627

---

---

296

- **PDB ID:** 6ILH | **Chain:** A
- **b-phipsi:** 0.0018023424434679
- **w-rdist:** 0.6254070557729657
- **t-alpha:** 0.0199522783818248

---

---

297

- **PDB ID:** 6EJ9 | **Chain:** A
- **b-phipsi:** 0.0004704418478247
- **w-rdist:** 0.5305884729346535
- **t-alpha:** 0.0328637472319743

---

---

298

- **PDB ID:** 7A3J | **Chain:** A
- **b-phipsi:** 0.0112743526499578
- **w-rdist:** 0.4368475677103787
- **t-alpha:** 0.0203438662057466

---

---

299

- **PDB ID:** 1TZN | **Chain:** o
- **b-phipsi:** 0.00707785810512
- **w-rdist:** 0.7551050316561638
- **t-alpha:** 0.0054771144938834

---

---

300

- **PDB ID:** 3O9V | **Chain:** B
- **b-phipsi:** 0.006788862891184
- **w-rdist:** 0.7614113876990394
- **t-alpha:** 0.0062593997286479

---

---

301

- **PDB ID:** 6ERF | **Chain:** B
- **b-phipsi:** 0.0032789281956604
- **w-rdist:** 0.4063961741617864
- **t-alpha:** 0.1158056523096886

---

---

302

- **PDB ID:** 8ASC | **Chain:** B
- **b-phipsi:** 0.0034085200225017
- **w-rdist:** 0.4786526889750196
- **t-alpha:** 0.050077913367889

---

---

303

- **PDB ID:** 6ZHA | **Chain:** C
- **b-phipsi:** 0.0076099207119751
- **w-rdist:** 0.3768828893711976
- **t-alpha:** 0.0704223021796839

---

---

304

- **PDB ID:** 4TV3 | **Chain:** A
- **b-phipsi:** 0.0094881262451742
- **w-rdist:** 0.1330234224413027
- **t-alpha:** 0.0868544688919852

---

---

305

- **PDB ID:** 8AG5 | **Chain:** B
- **b-phipsi:** 0.0065053529783651
- **w-rdist:** 0.426238764707922
- **t-alpha:** 0.0657276023874584

---

---

306

- **PDB ID:** 6EJD | **Chain:** A
- **b-phipsi:** 0.000532346949614
- **w-rdist:** 0.5100127621794044
- **t-alpha:** 0.0477306796865031

---

---

307

- **PDB ID:** 5J67 | **Chain:** D
- **b-phipsi:** 0.0025245667486587
- **w-rdist:** 0.507681677435749
- **t-alpha:** 0.0356565880801509

---

---

308

- **PDB ID:** 6NIG | **Chain:** A
- **b-phipsi:** 0.0054279347037922
- **w-rdist:** 0.4663486833879318
- **t-alpha:** 0.0484003534843218

---

---

309

- **PDB ID:** 5AB0 | **Chain:** C
- **b-phipsi:** 0.0090486432011604
- **w-rdist:** 0.5621845087417421
- **t-alpha:** 0.0101717762082627

---

---

310

- **PDB ID:** 6HP8 | **Chain:** C
- **b-phipsi:** 0.0080964490437191
- **w-rdist:** 0.5763780496505369
- **t-alpha:** 0.0117370267412904

---

---

311

- **PDB ID:** 8FCN | **Chain:** D
- **b-phipsi:** 0.0064000680417159
- **w-rdist:** 0.1572018850916145
- **t-alpha:** 0.1658839749938661

---

---

312

- **PDB ID:** 7UQX | **Chain:** A
- **b-phipsi:** 0.0127835076307609
- **w-rdist:** 0.6215294661890312
- **t-alpha:** 0.0007823187912934

---

---

313

- **PDB ID:** 6U2W | **Chain:** L
- **b-phipsi:** 0.0154976879357914
- **w-rdist:** 0.400954517419998
- **t-alpha:** 0.0175161413598659

---

---

314

- **PDB ID:** 6C01 | **Chain:** A
- **b-phipsi:** 0.0023317226426018
- **w-rdist:** 0.508385679838474
- **t-alpha:** 0.0391232045333622

---

---

315

- **PDB ID:** 6ERF | **Chain:** G
- **b-phipsi:** 0.0019923439892283
- **w-rdist:** 0.4470164486522582
- **t-alpha:** 0.0985915493938487

---

---

316

- **PDB ID:** 5FWI | **Chain:** C
- **b-phipsi:** 0.0014374710596665
- **w-rdist:** 0.9737432233076684
- **t-alpha:** 0.0047169960791435

---

---

317

- **PDB ID:** 8EFI | **Chain:** M
- **b-phipsi:** 0.0094906529298006
- **w-rdist:** 0.4264208873421263
- **t-alpha:** 0.025682454261255

---

---

318

- **PDB ID:** 3MDJ | **Chain:** B
- **b-phipsi:** 0.0099541139807152
- **w-rdist:** 0.4155740055433165
- **t-alpha:** 0.0258213633826065

---

---

319

- **PDB ID:** 6ERH | **Chain:** A
- **b-phipsi:** 0.0032119297449784
- **w-rdist:** 0.5290900739159067
- **t-alpha:** 0.0242563010013117

---

---

320

- **PDB ID:** 6P0B | **Chain:** A
- **b-phipsi:** 0.0075655669526444
- **w-rdist:** 0.7275335114258847
- **t-alpha:** 0.0062593997286479

---

---

321

- **PDB ID:** 6T6R | **Chain:** A
- **b-phipsi:** 0.0122075080113508
- **w-rdist:** 0.63092110301953
- **t-alpha:** 0.0007832732121422

---

---

322

- **PDB ID:** 4ZG9 | **Chain:** A
- **b-phipsi:** 0.0114935421440708
- **w-rdist:** 0.4225647999725577
- **t-alpha:** 0.0219087977837508

---

---

323

- **PDB ID:** 1PL0 | **Chain:** A
- **b-phipsi:** 0.0057669316049882
- **w-rdist:** 0.7706085726698763
- **t-alpha:** 0.0086820531296027

---

---

324

- **PDB ID:** 2E1Q | **Chain:** B
- **b-phipsi:** 0.0050365809465604
- **w-rdist:** 0.3723690102792388
- **t-alpha:** 0.1259778598996253

---

---

325

- **PDB ID:** 4ZG6 | **Chain:** A
- **b-phipsi:** 0.0071891178276969
- **w-rdist:** 0.4448615956681502
- **t-alpha:** 0.0469481037213646

---

---

326

- **PDB ID:** 5C1B | **Chain:** F
- **b-phipsi:** 0.0076038058138808
- **w-rdist:** 0.1982815897553808
- **t-alpha:** 0.1244127877301322

---

---

327

- **PDB ID:** 7RZI | **Chain:** B
- **b-phipsi:** 0.0067213165344621
- **w-rdist:** 0.4177260002416145
- **t-alpha:** 0.0719873632833352

---

---

328

- **PDB ID:** 7NFC | **Chain:** C
- **b-phipsi:** 0.0102420185719627
- **w-rdist:** 0.2747657848641191
- **t-alpha:** 0.0696396085969683

---

---

329

- **PDB ID:** 4BLB | **Chain:** B
- **b-phipsi:** 0.0055249998250164
- **w-rdist:** 0.5833945957094261
- **t-alpha:** 0.0191389373786337

---

---

330

- **PDB ID:** 3ZIM | **Chain:** A
- **b-phipsi:** 0.0107579741631532
- **w-rdist:** 0.1632515271434906
- **t-alpha:** 0.0743346359382088

---

---

331

- **PDB ID:** 6GYR | **Chain:** C
- **b-phipsi:** 0.0045731539019585
- **w-rdist:** 0.4853922907956186
- **t-alpha:** 0.0449715481085233

---

---

332

- **PDB ID:** 7MDO | **Chain:** C
- **b-phipsi:** 0.0209839218133229
- **w-rdist:** 0.4556020880358483
- **t-alpha:** 0.0062593997286479

---

---

333

- **PDB ID:** 8P4E | **Chain:** O
- **b-phipsi:** 0.0014860432707068
- **w-rdist:** 0.5108936140773341
- **t-alpha:** 0.0446008926005814

---

---

334

- **PDB ID:** 3SE6 | **Chain:** B
- **b-phipsi:** 0.0089830859269574
- **w-rdist:** 0.4701006991344834
- **t-alpha:** 0.0207667552249124

---

---

335

- **PDB ID:** 5C18 | **Chain:** A
- **b-phipsi:** 0.0080556790624609
- **w-rdist:** 0.1906837401903535
- **t-alpha:** 0.1189353396200536

---

---

336

- **PDB ID:** 4CHT | **Chain:** A
- **b-phipsi:** 0.0083722400946996
- **w-rdist:** 0.4489487612604834
- **t-alpha:** 0.0281577534344037

---

---

337

- **PDB ID:** 6SVM | **Chain:** B
- **b-phipsi:** 0.0047229682626158
- **w-rdist:** 0.785685241746943
- **t-alpha:** 0.0094788657373643

---

---

338

- **PDB ID:** 4Q5V | **Chain:** E
- **b-phipsi:** 0.0055080013746098
- **w-rdist:** 0.226317354642319
- **t-alpha:** 0.191705500952412

---

---

339

- **PDB ID:** 6P0E | **Chain:** A
- **b-phipsi:** 0.0077679400137791
- **w-rdist:** 0.7413645999845199
- **t-alpha:** 0.0054771144938834

---

---

340

- **PDB ID:** 7KR4 | **Chain:** A
- **b-phipsi:** 0.0063782581162522
- **w-rdist:** 0.7347439917883669
- **t-alpha:** 0.0093893372993878

---

---

341

- **PDB ID:** 4EKZ | **Chain:** A
- **b-phipsi:** 0.0082233079162149
- **w-rdist:** 0.5215020332775099
- **t-alpha:** 0.0164318357763795

---

---

342

- **PDB ID:** 6R4H | **Chain:** A
- **b-phipsi:** 0.0051538692895735
- **w-rdist:** 0.7424924476960042
- **t-alpha:** 0.0110763082596549

---

---

343

- **PDB ID:** 6NIG | **Chain:** B
- **b-phipsi:** 0.0042380324211701
- **w-rdist:** 0.465928256209883
- **t-alpha:** 0.0641133266895068

---

---

344

- **PDB ID:** 5GGF | **Chain:** C
- **b-phipsi:** 0.0032479718450972
- **w-rdist:** 0.6567835717303646
- **t-alpha:** 0.0175161413598659

---

---

345

- **PDB ID:** 6HZZ | **Chain:** A
- **b-phipsi:** 0.0055447919213696
- **w-rdist:** 0.7867884470293735
- **t-alpha:** 0.0086070234951727

---

---

346

- **PDB ID:** 5WOB | **Chain:** H
- **b-phipsi:** 0.0060509704134388
- **w-rdist:** 0.3827470620437535
- **t-alpha:** 0.1103283058684481

---

---

347

- **PDB ID:** 8EOJ | **Chain:** B
- **b-phipsi:** 0.0069758053938971
- **w-rdist:** 0.4614407916311441
- **t-alpha:** 0.0348178944724737

---

---

348

- **PDB ID:** 6U2J | **Chain:** J
- **b-phipsi:** 0.0126725594623855
- **w-rdist:** 0.2953801212137002
- **t-alpha:** 0.041564883073162

---

---

349

- **PDB ID:** 5JPN | **Chain:** B
- **b-phipsi:** 0.0030370752583735
- **w-rdist:** 0.3856356372091528
- **t-alpha:** 0.1431919985610661

---

---

350

- **PDB ID:** 3CCB | **Chain:** B
- **b-phipsi:** 0.0066209068659679
- **w-rdist:** 0.7792979364793722
- **t-alpha:** 0.0070418134049574

---

---

351

- **PDB ID:** 5VNO | **Chain:** B
- **b-phipsi:** 0.0063830530769211
- **w-rdist:** 0.6630667125437874
- **t-alpha:** 0.0125193542574237

---

---

352

- **PDB ID:** 2HAU | **Chain:** A
- **b-phipsi:** 0.0075515106428643
- **w-rdist:** 0.4612124273117927
- **t-alpha:** 0.0273315008079859

---

---

353

- **PDB ID:** 7WUB | **Chain:** G
- **b-phipsi:** 0.0075718583461467
- **w-rdist:** 0.3243947528400316
- **t-alpha:** 0.104850978155695

---

---

354

- **PDB ID:** 6B3Q | **Chain:** B
- **b-phipsi:** 0.0067394023085177
- **w-rdist:** 0.4251479451750908
- **t-alpha:** 0.0712045469065545

---

---

355

- **PDB ID:** 6X6C | **Chain:** D
- **b-phipsi:** 0.0057979548423186
- **w-rdist:** 0.6521749306143002
- **t-alpha:** 0.0148665808060715

---

---

356

- **PDB ID:** 5GVC | **Chain:** A
- **b-phipsi:** 0.0059678448551479
- **w-rdist:** 0.447875534941718
- **t-alpha:** 0.0696396085969683

---

---

357

- **PDB ID:** 6INN | **Chain:** C
- **b-phipsi:** 0.0173627904452047
- **w-rdist:** 0.5043480200145796
- **t-alpha:** 0.0039277718342689

---

---

358

- **PDB ID:** 7AXZ | **Chain:** B
- **b-phipsi:** 0.0064910252987187
- **w-rdist:** 0.4537908595265926
- **t-alpha:** 0.0516428148679497

---

---

359

- **PDB ID:** 6ERH | **Chain:** B
- **b-phipsi:** 0.0074926695492732
- **w-rdist:** 0.3235118818378183
- **t-alpha:** 0.1079811098823984

---

---

360

- **PDB ID:** 5VNK | **Chain:** B
- **b-phipsi:** 0.004623148331591
- **w-rdist:** 0.679550407552643
- **t-alpha:** 0.0156493910563146

---

---

361

- **PDB ID:** 8EZA | **Chain:** B
- **b-phipsi:** 0.0065479431578758
- **w-rdist:** 0.6412425737840316
- **t-alpha:** 0.0134815624146233

---

---

362

- **PDB ID:** 5Z58 | **Chain:** c
- **b-phipsi:** 0.0068423785429519
- **w-rdist:** 0.5244144468902061
- **t-alpha:** 0.0199522783818248

---

---

363

- **PDB ID:** 1NQL | **Chain:** A
- **b-phipsi:** 0.0133146291097237
- **w-rdist:** 0.6283622458201444
- **t-alpha:** 0.0

---

---

364

- **PDB ID:** 2BGN | **Chain:** D
- **b-phipsi:** 0.0086605014483359
- **w-rdist:** 0.7792246624150068
- **t-alpha:** 0.0015676375257613

---

---

365

- **PDB ID:** 4FWJ | **Chain:** B
- **b-phipsi:** 0.0061990330900151
- **w-rdist:** 0.4598777865710443
- **t-alpha:** 0.0518519454679657

---

---

366

- **PDB ID:** 6WRV | **Chain:** A
- **b-phipsi:** 0.0067618924963164
- **w-rdist:** 0.8992907155126769
- **t-alpha:** 0.0007823187912934

---

---

367

- **PDB ID:** 7MD5 | **Chain:** B
- **b-phipsi:** 0.0234677688751114
- **w-rdist:** 0.4108009795237712
- **t-alpha:** 0.0101717762082627

---

---

368

- **PDB ID:** 7NDL | **Chain:** B
- **b-phipsi:** 0.0046828176316837
- **w-rdist:** 0.7844193225769436
- **t-alpha:** 0.0102767895279776

---

---

369

- **PDB ID:** 6C02 | **Chain:** A
- **b-phipsi:** 0.0026842329172068
- **w-rdist:** 0.5099124015400336
- **t-alpha:** 0.0438181656914413

---

---

370

- **PDB ID:** 5J67 | **Chain:** B
- **b-phipsi:** 0.0038308499546466
- **w-rdist:** 0.5113967587799477
- **t-alpha:** 0.0356565880801509

---

---

371

- **PDB ID:** 7WTA | **Chain:** B
- **b-phipsi:** 0.0100502622657405
- **w-rdist:** 0.7139147157733248
- **t-alpha:** 0.0023473590503262

---

---

372

- **PDB ID:** 8EZB | **Chain:** A
- **b-phipsi:** 0.0040240148548625
- **w-rdist:** 0.4060015231786715
- **t-alpha:** 0.1275429598762953

---

---

373

- **PDB ID:** 2NSU | **Chain:** A
- **b-phipsi:** 0.0070886323458353
- **w-rdist:** 0.8858419004080916
- **t-alpha:** 0.0007823187912934

---

---

374

- **PDB ID:** 6ERF | **Chain:** F
- **b-phipsi:** 0.0032192630036347
- **w-rdist:** 0.4250425614321773
- **t-alpha:** 0.1205008021373417

---

---

375

- **PDB ID:** 8EZB | **Chain:** J
- **b-phipsi:** 0.004738139770166
- **w-rdist:** 0.3964982400036798
- **t-alpha:** 0.129107543323707

---

---

376

- **PDB ID:** 7A3F | **Chain:** C
- **b-phipsi:** 0.0176963492776603
- **w-rdist:** 0.4363815597978561
- **t-alpha:** 0.0133018035296799

---

---

377

- **PDB ID:** 6INN | **Chain:** D
- **b-phipsi:** 0.0183798156007132
- **w-rdist:** 0.5006889466559995
- **t-alpha:** 0.0039119745263893

---

---

378

- **PDB ID:** 8EFH | **Chain:** A
- **b-phipsi:** 0.0108564715931869
- **w-rdist:** 0.420898236915716
- **t-alpha:** 0.0248596569180252

---

---

379

- **PDB ID:** 8G1U | **Chain:** M
- **b-phipsi:** 0.0009238807459245
- **w-rdist:** 0.5525817746624264
- **t-alpha:** 0.0323103167719089

---

---

380

- **PDB ID:** 4BL9 | **Chain:** D
- **b-phipsi:** 0.0044338339456679
- **w-rdist:** 0.8013087893367605
- **t-alpha:** 0.0101717762082627

---

---

381

- **PDB ID:** 3SE6 | **Chain:** A
- **b-phipsi:** 0.0089730954409474
- **w-rdist:** 0.4214585883715082
- **t-alpha:** 0.0446008926005814

---

---

382

- **PDB ID:** 4PJ6 | **Chain:** B
- **b-phipsi:** 0.0095495207888696
- **w-rdist:** 0.5597947587755634
- **t-alpha:** 0.0109543395722921

---

---

383

- **PDB ID:** 6R4H | **Chain:** B
- **b-phipsi:** 0.0047991490802496
- **w-rdist:** 0.7955059477425193
- **t-alpha:** 0.0101717762082627

---

---

384

- **PDB ID:** 4BPB | **Chain:** A
- **b-phipsi:** 0.0113451961839694
- **w-rdist:** 0.3438888550359984
- **t-alpha:** 0.0485128406736701

---

---

385

- **PDB ID:** 4XVM | **Chain:** A
- **b-phipsi:** 0.0181487266503988
- **w-rdist:** 0.4104992437807707
- **t-alpha:** 0.0150916313901026

---

---

386

- **PDB ID:** 3ZUS | **Chain:** C
- **b-phipsi:** 0.0106235270490874
- **w-rdist:** 0.2639342989510883
- **t-alpha:** 0.076663945339064

---

---

387

- **PDB ID:** 6EOR | **Chain:** C
- **b-phipsi:** 0.0087814760064689
- **w-rdist:** 0.6389326191031292
- **t-alpha:** 0.0086820531296027

---

---

388

- **PDB ID:** 6U2J | **Chain:** I
- **b-phipsi:** 0.0123085973232871
- **w-rdist:** 0.2988548785135003
- **t-alpha:** 0.0501234795277865

---

---

389

- **PDB ID:** 6CE9 | **Chain:** B
- **b-phipsi:** 0.0310687538092934
- **w-rdist:** 0.4058450883282085
- **t-alpha:** 0.0078243548762237

---

---

390

- **PDB ID:** 3V83 | **Chain:** C
- **b-phipsi:** 0.0097847893211826
- **w-rdist:** 0.564776473742193
- **t-alpha:** 0.0102767895279776

---

---

391

- **PDB ID:** 2E1Q | **Chain:** C
- **b-phipsi:** 0.0057611413583471
- **w-rdist:** 0.3675549994383446
- **t-alpha:** 0.1345850125084049

---

---

392

- **PDB ID:** 6I01 | **Chain:** A
- **b-phipsi:** 0.0053157422131081
- **w-rdist:** 0.7832516150770061
- **t-alpha:** 0.0102767895279776

---

---

393

- **PDB ID:** 2E1Q | **Chain:** D
- **b-phipsi:** 0.006351021413625
- **w-rdist:** 0.3715693632814474
- **t-alpha:** 0.1205008021373417

---

---

394

- **PDB ID:** 6ZHA | **Chain:** B
- **b-phipsi:** 0.0094346581624854
- **w-rdist:** 0.369425027681568
- **t-alpha:** 0.0610327490560462

---

---

395

- **PDB ID:** 7SOL | **Chain:** C
- **b-phipsi:** 0.0048138052560862
- **w-rdist:** 0.37302252606716
- **t-alpha:** 0.1439745083193748

---

---

396

- **PDB ID:** 6GVH | **Chain:** A
- **b-phipsi:** 0.0092666364152908
- **w-rdist:** 0.1205295041622025
- **t-alpha:** 0.1134580270465441

---

---

397

- **PDB ID:** 7T39 | **Chain:** A
- **b-phipsi:** 0.0147327277811967
- **w-rdist:** 0.5234722189660805
- **t-alpha:** 0.0055077996884944

---

---

398

- **PDB ID:** 3KRW | **Chain:** A
- **b-phipsi:** 0.0125925634635209
- **w-rdist:** 0.3111438353913046
- **t-alpha:** 0.0446008926005814

---

---

399

- **PDB ID:** 6EJ7 | **Chain:** A
- **b-phipsi:** 0.0005051671085082
- **w-rdist:** 0.5348734175311263
- **t-alpha:** 0.0438181656914413

---

---

400

- **PDB ID:** 6YDX | **Chain:** A
- **b-phipsi:** 0.0115774134258404
- **w-rdist:** 0.6031298123849264
- **t-alpha:** 0.0055077996884944

---

---

401

- **PDB ID:** 4KTY | **Chain:** A
- **b-phipsi:** 0.0147004407985297
- **w-rdist:** 0.3187064810060805
- **t-alpha:** 0.0266038120788794

---

---

402

- **PDB ID:** 7SVL | **Chain:** C
- **b-phipsi:** 0.0061043897628699
- **w-rdist:** 0.6430119590739872
- **t-alpha:** 0.0158983514403059

---

---

403

- **PDB ID:** 1PKX | **Chain:** C
- **b-phipsi:** 0.0058116296051721
- **w-rdist:** 0.7720937458320603
- **t-alpha:** 0.0102767895279776

---

---

404

- **PDB ID:** 6ZHE | **Chain:** G
- **b-phipsi:** 0.0103548960974216
- **w-rdist:** 0.3768379133508756
- **t-alpha:** 0.0485128406736701

---

---

405

- **PDB ID:** 5LST | **Chain:** A
- **b-phipsi:** 0.0092060115873493
- **w-rdist:** 0.4952992468486997
- **t-alpha:** 0.0191389373786337

---

---

406

- **PDB ID:** 5C1B | **Chain:** D
- **b-phipsi:** 0.0094427601140387
- **w-rdist:** 0.2403480280541579
- **t-alpha:** 0.1009388027447895

---

---

407

- **PDB ID:** 7ZVT | **Chain:** A
- **b-phipsi:** 0.0032344097609886
- **w-rdist:** 0.5334995207964599
- **t-alpha:** 0.0281690295641283

---

---

408

- **PDB ID:** 7R9V | **Chain:** A
- **b-phipsi:** 0.014630842461662
- **w-rdist:** 0.2356644517696003
- **t-alpha:** 0.0422534769714337

---

---

409

- **PDB ID:** 5VNJ | **Chain:** B
- **b-phipsi:** 0.0080814111048346
- **w-rdist:** 0.6646161930047251
- **t-alpha:** 0.0094788657373643

---

---

410

- **PDB ID:** 8FCN | **Chain:** E
- **b-phipsi:** 0.0062467510970357
- **w-rdist:** 0.1567259432879207
- **t-alpha:** 0.2128317056828612

---

---

411

- **PDB ID:** 1TZN | **Chain:** i
- **b-phipsi:** 0.0073382069467716
- **w-rdist:** 0.7521100518191188
- **t-alpha:** 0.0078243548762237

---

---

412

- **PDB ID:** 3OHM | **Chain:** B
- **b-phipsi:** 0.0020539924136123
- **w-rdist:** 0.5066245721878374
- **t-alpha:** 0.0553264749125952

---

---

413

- **PDB ID:** 5F9F | **Chain:** A
- **b-phipsi:** 0.0088223994250082
- **w-rdist:** 0.6172252768721694
- **t-alpha:** 0.0109543395722921

---

---

414

- **PDB ID:** 5K1V | **Chain:** A
- **b-phipsi:** 0.0094367682073854
- **w-rdist:** 0.4805014859049065
- **t-alpha:** 0.0203438662057466

---

---

415

- **PDB ID:** 6U23 | **Chain:** D
- **b-phipsi:** 0.0131437677161906
- **w-rdist:** 0.3511409809068979
- **t-alpha:** 0.0323103167719089

---

---

416

- **PDB ID:** 7ZYF | **Chain:** B
- **b-phipsi:** 0.0133246115310428
- **w-rdist:** 0.6084817997060507
- **t-alpha:** 0.0031296009050172

---

---

417

- **PDB ID:** 1YGR | **Chain:** B
- **b-phipsi:** 0.0083738605271368
- **w-rdist:** 0.7115122650389253
- **t-alpha:** 0.0070922351376341

---

---

418

- **PDB ID:** 6U23 | **Chain:** L
- **b-phipsi:** 0.0138654999166085
- **w-rdist:** 0.3437812688952333
- **t-alpha:** 0.0289856519083209

---

---

419

- **PDB ID:** 6U2W | **Chain:** K
- **b-phipsi:** 0.0167345924754706
- **w-rdist:** 0.396781943015843
- **t-alpha:** 0.0191389373786337

---

---

420

- **PDB ID:** 7A3G | **Chain:** B
- **b-phipsi:** 0.0098637168386431
- **w-rdist:** 0.4388007889793685
- **t-alpha:** 0.0297336873714129

---

---

421

- **PDB ID:** 5C1B | **Chain:** B
- **b-phipsi:** 0.0067320761699778
- **w-rdist:** 0.2325163583691451
- **t-alpha:** 0.1760561506159503

---

---

422

- **PDB ID:** 4EL1 | **Chain:** B
- **b-phipsi:** 0.0055050355964534
- **w-rdist:** 0.443434885143065
- **t-alpha:** 0.0932424929789836

---

---

423

- **PDB ID:** 6U2J | **Chain:** M
- **b-phipsi:** 0.0125466925540266
- **w-rdist:** 0.2913766342381133
- **t-alpha:** 0.0544557624307391

---

---

424

- **PDB ID:** 6KYV | **Chain:** B
- **b-phipsi:** 0.0100496039843204
- **w-rdist:** 0.6200618571322394
- **t-alpha:** 0.0086070234951727

---

---

425

- **PDB ID:** 5C1B | **Chain:** A
- **b-phipsi:** 0.0097910153687367
- **w-rdist:** 0.2356145393138133
- **t-alpha:** 0.099373934821642

---

---

426

- **PDB ID:** 6ERF | **Chain:** H
- **b-phipsi:** 0.0029764419899908
- **w-rdist:** 0.4237708528576455
- **t-alpha:** 0.1361501576045134

---

---

427

- **PDB ID:** 1EX0 | **Chain:** B
- **b-phipsi:** 0.017430243619298
- **w-rdist:** 0.5337992145807008
- **t-alpha:** 0.0015676375257613

---

---

428

- **PDB ID:** 1W1I | **Chain:** D
- **b-phipsi:** 0.0076752385708935
- **w-rdist:** 0.7795120330176664
- **t-alpha:** 0.0062593997286479

---

---

429

- **PDB ID:** 8IF4 | **Chain:** A
- **b-phipsi:** 0.0051005393578669
- **w-rdist:** 0.5075706174497258
- **t-alpha:** 0.040688156290789

---

---

430

- **PDB ID:** 3EH1 | **Chain:** A
- **b-phipsi:** 0.0010581647807447
- **w-rdist:** 0.7381240575364476
- **t-alpha:** 0.0187789021729365

---

---

431

- **PDB ID:** 3N56 | **Chain:** B
- **b-phipsi:** 0.0068196919956653
- **w-rdist:** 0.4264152119362403
- **t-alpha:** 0.0821594238081062

---

---

432

- **PDB ID:** 6U23 | **Chain:** C
- **b-phipsi:** 0.0130989255841998
- **w-rdist:** 0.3396980502993744
- **t-alpha:** 0.0399058818911266

---

---

433

- **PDB ID:** 7WUB | **Chain:** C
- **b-phipsi:** 0.0203937559383238
- **w-rdist:** 0.4437477631940199
- **t-alpha:** 0.0110763082596549

---

---

434

- **PDB ID:** 6I33 | **Chain:** B
- **b-phipsi:** 0.0119756318861142
- **w-rdist:** 0.6529612107620126
- **t-alpha:** 0.0031296009050172

---

---

435

- **PDB ID:** 7SVL | **Chain:** B
- **b-phipsi:** 0.0072226377499323
- **w-rdist:** 0.6556927980858017
- **t-alpha:** 0.0126786930545719

---

---

436

- **PDB ID:** 1XJQ | **Chain:** B
- **b-phipsi:** 0.0109219988137214
- **w-rdist:** 0.5706433108170236
- **t-alpha:** 0.0093893372993878

---

---

437

- **PDB ID:** 4KRE | **Chain:** A
- **b-phipsi:** 0.0066703761911117
- **w-rdist:** 0.0835134355254047
- **t-alpha:** 0.2097023944897873

---

---

438

- **PDB ID:** 6XTY | **Chain:** 7
- **b-phipsi:** 0.0153232179043597
- **w-rdist:** 0.4982721454961324
- **t-alpha:** 0.0086820531296027

---

---

439

- **PDB ID:** 8EXL | **Chain:** A
- **b-phipsi:** 0.0083118363070728
- **w-rdist:** 0.1675153131835193
- **t-alpha:** 0.1392798657818292

---

---

440

- **PDB ID:** 4OVU | **Chain:** A
- **b-phipsi:** 0.0087542750179024
- **w-rdist:** 0.4446987351595933
- **t-alpha:** 0.0438181656914413

---

---

441

- **PDB ID:** 6U2J | **Chain:** N
- **b-phipsi:** 0.0155849402585096
- **w-rdist:** 0.2915767583755247
- **t-alpha:** 0.0339808661347131

---

---

442

- **PDB ID:** 7MDO | **Chain:** F
- **b-phipsi:** 0.0218810640365981
- **w-rdist:** 0.467896185337986
- **t-alpha:** 0.0062992257321734

---

---

443

- **PDB ID:** 8SZP | **Chain:** B
- **b-phipsi:** 0.0101387341646412
- **w-rdist:** 0.3418722251908371
- **t-alpha:** 0.0743346359382088

---

---

444

- **PDB ID:** 3V83 | **Chain:** E
- **b-phipsi:** 0.0078840150188578
- **w-rdist:** 0.588080463831781
- **t-alpha:** 0.0156493910563146

---

---

445

- **PDB ID:** 6HTS | **Chain:** F
- **b-phipsi:** 0.0046932921029073
- **w-rdist:** 0.4945609005565129
- **t-alpha:** 0.0544557624307391

---

---

446

- **PDB ID:** 6RQX | **Chain:** A
- **b-phipsi:** 0.0091420914557503
- **w-rdist:** 0.4591669379241991
- **t-alpha:** 0.0248596569180252

---

---

447

- **PDB ID:** 5KYW | **Chain:** A
- **b-phipsi:** 0.0028424116570293
- **w-rdist:** 0.6652715755110938
- **t-alpha:** 0.0203438662057466

---

---

448

- **PDB ID:** 1CX8 | **Chain:** D
- **b-phipsi:** 0.0054380940874311
- **w-rdist:** 0.8816412050750969
- **t-alpha:** 0.0070922351376341

---

---

449

- **PDB ID:** 6NIG | **Chain:** D
- **b-phipsi:** 0.0070557182378002
- **w-rdist:** 0.4603734490713682
- **t-alpha:** 0.0492615627855226

---

---

450

- **PDB ID:** 5C1B | **Chain:** E
- **b-phipsi:** 0.006992609628081
- **w-rdist:** 0.2889637589893564
- **t-alpha:** 0.1643189523680888

---

---

451

- **PDB ID:** 5F9H | **Chain:** E
- **b-phipsi:** 0.0111179051780762
- **w-rdist:** 0.6659138152326235
- **t-alpha:** 0.0046944792707916

---

---

452

- **PDB ID:** 6ATK | **Chain:** B
- **b-phipsi:** 0.0125539087747471
- **w-rdist:** 0.5781790022730819
- **t-alpha:** 0.0070418134049574

---

---

453

- **PDB ID:** 2Z7X | **Chain:** A
- **b-phipsi:** 0.0065698128003559
- **w-rdist:** 0.4710243533871525
- **t-alpha:** 0.050987106591174

---

---

454

- **PDB ID:** 5Z58 | **Chain:** C
- **b-phipsi:** 0.0053356852318361
- **w-rdist:** 0.5242704745250683
- **t-alpha:** 0.0323103167719089

---

---

455

- **PDB ID:** 4GNK | **Chain:** D
- **b-phipsi:** 0.0044537561864281
- **w-rdist:** 0.4758585654957951
- **t-alpha:** 0.0748531911117893

---

---

456

- **PDB ID:** 4BLD | **Chain:** B
- **b-phipsi:** 0.0043265089520832
- **w-rdist:** 0.5854089742487391
- **t-alpha:** 0.0240384962174629

---

---

457

- **PDB ID:** 1A36 | **Chain:** A
- **b-phipsi:** 0.0190721700792091
- **w-rdist:** 0.4159674764752381
- **t-alpha:** 0.0158983514403059

---

---

458

- **PDB ID:** 1JEQ | **Chain:** B
- **b-phipsi:** 0.0076890958879963
- **w-rdist:** 0.4030987871936304
- **t-alpha:** 0.0860716712560181

---

---

459

- **PDB ID:** 4YCW | **Chain:** F
- **b-phipsi:** 0.0021777605940589
- **w-rdist:** 0.6339786400206409
- **t-alpha:** 0.0232189692826079

---

---

460

- **PDB ID:** 6U23 | **Chain:** G
- **b-phipsi:** 0.012753526399926
- **w-rdist:** 0.3358811285852101
- **t-alpha:** 0.0485128406736701

---

---

461

- **PDB ID:** 5J67 | **Chain:** A
- **b-phipsi:** 0.0038129615329598
- **w-rdist:** 0.4970717373731709
- **t-alpha:** 0.061461872795004

---

---

462

- **PDB ID:** 6QZW | **Chain:** C
- **b-phipsi:** 0.0079029193379392
- **w-rdist:** 0.598496350233068
- **t-alpha:** 0.0156493910563146

---

---

463

- **PDB ID:** 5M7M | **Chain:** A
- **b-phipsi:** 0.0016187778652867
- **w-rdist:** 0.4881624309325305
- **t-alpha:** 0.083969564498129

---

---

464

- **PDB ID:** 5KYY | **Chain:** B
- **b-phipsi:** 0.006337560552302
- **w-rdist:** 0.6766178905773976
- **t-alpha:** 0.0156493910563146

---

---

465

- **PDB ID:** 8FCN | **Chain:** A
- **b-phipsi:** 0.0080586807001388
- **w-rdist:** 0.1604871490084604
- **t-alpha:** 0.1588415973392283

---

---

466

- **PDB ID:** 3ZUS | **Chain:** A
- **b-phipsi:** 0.0119331275435353
- **w-rdist:** 0.2758917565348686
- **t-alpha:** 0.0721478806100059

---

---

467

- **PDB ID:** 8FCL | **Chain:** A
- **b-phipsi:** 0.0098105486401696
- **w-rdist:** 0.1670465512128081
- **t-alpha:** 0.1189353396200536

---

---

468

- **PDB ID:** 6U23 | **Chain:** A
- **b-phipsi:** 0.0142162601478629
- **w-rdist:** 0.336793383583176
- **t-alpha:** 0.0381807098839799

---

---

469

- **PDB ID:** 6GVG | **Chain:** A
- **b-phipsi:** 0.0101681484980527
- **w-rdist:** 0.1890483508876564
- **t-alpha:** 0.1095460606668756

---

---

470

- **PDB ID:** 1CX8 | **Chain:** C
- **b-phipsi:** 0.0064775595952048
- **w-rdist:** 0.8907086301745559
- **t-alpha:** 0.0047169960791435

---

---

471

- **PDB ID:** 5WJ5 | **Chain:** C
- **b-phipsi:** 0.0372232381226745
- **w-rdist:** 0.4443311955665889
- **t-alpha:** 0.0047169960791435

---

---

472

- **PDB ID:** 4EL1 | **Chain:** A
- **b-phipsi:** 0.0043345542511321
- **w-rdist:** 0.4528576031828909
- **t-alpha:** 0.1036272940880995

---

---

473

- **PDB ID:** 4RAL | **Chain:** B
- **b-phipsi:** 0.0063022370848819
- **w-rdist:** 0.432943411721321
- **t-alpha:** 0.1009388027447895

---

---

474

- **PDB ID:** 4Q5V | **Chain:** A
- **b-phipsi:** 0.0064416227436934
- **w-rdist:** 0.2844439906439542
- **t-alpha:** 0.1995297300002283

---

---

475

- **PDB ID:** 5MHL | **Chain:** A
- **b-phipsi:** 0.0147063035516076
- **w-rdist:** 0.3253139326614497
- **t-alpha:** 0.0375586271097019

---

---

476

- **PDB ID:** 6ERF | **Chain:** D
- **b-phipsi:** 0.0078553183905625
- **w-rdist:** 0.3914643682777552
- **t-alpha:** 0.09467909251505

---

---

477

- **PDB ID:** 6ATK | **Chain:** A
- **b-phipsi:** 0.0122421612214049
- **w-rdist:** 0.5834375891900245
- **t-alpha:** 0.0078243548762237

---

---

478

- **PDB ID:** 7K6O | **Chain:** A
- **b-phipsi:** 0.0102929186370286
- **w-rdist:** 0.2312271781122108
- **t-alpha:** 0.1040683801301172

---

---

479

- **PDB ID:** 6MZC | **Chain:** B
- **b-phipsi:** 0.0136708339196361
- **w-rdist:** 0.158805428105616
- **t-alpha:** 0.0665099761701244

---

---

480

- **PDB ID:** 8FCQ | **Chain:** C
- **b-phipsi:** 0.0062814398663553
- **w-rdist:** 0.2992187039254598
- **t-alpha:** 0.2018771433123252

---

---

481

- **PDB ID:** 1LFI | **Chain:** A
- **b-phipsi:** 0.0081183196783988
- **w-rdist:** 0.5183488213271504
- **t-alpha:** 0.0207667552249124

---

---

482

- **PDB ID:** 5MHO | **Chain:** A
- **b-phipsi:** 0.0198749610976065
- **w-rdist:** 0.303857401438114
- **t-alpha:** 0.0226916757534991

---

---

483

- **PDB ID:** 5IUD | **Chain:** J
- **b-phipsi:** 0.0049077946333632
- **w-rdist:** 0.3835610099079592
- **t-alpha:** 0.1697965373475096

---

---

484

- **PDB ID:** 3VE1 | **Chain:** B
- **b-phipsi:** 0.0370868659100026
- **w-rdist:** 0.3455827733624655
- **t-alpha:** 0.0125193542574237

---

---

485

- **PDB ID:** 6GVI | **Chain:** A
- **b-phipsi:** 0.009963519097438
- **w-rdist:** 0.2010330584693108
- **t-alpha:** 0.1150232700097437

---

---

486

- **PDB ID:** 6U2J | **Chain:** D
- **b-phipsi:** 0.0163374511097376
- **w-rdist:** 0.297018522596804
- **t-alpha:** 0.0356565880801509

---

---

487

- **PDB ID:** 4FXK | **Chain:** B
- **b-phipsi:** 0.005717294912187
- **w-rdist:** 0.3814113686204847
- **t-alpha:** 0.1525818235009972

---

---

488

- **PDB ID:** 4KLN | **Chain:** B
- **b-phipsi:** 0.0031070915500497
- **w-rdist:** 0.882343950282197
- **t-alpha:** 0.0102767895279776

---

---

489

- **PDB ID:** 8EXV | **Chain:** A
- **b-phipsi:** 0.009170998382938
- **w-rdist:** 0.1814679397290893
- **t-alpha:** 0.1338024377331197

---

---

490

- **PDB ID:** 7JIU | **Chain:** A
- **b-phipsi:** 0.0092679345768012
- **w-rdist:** 0.1670986440593257
- **t-alpha:** 0.133019862469073

---

---

491

- **PDB ID:** 7QPG | **Chain:** C
- **b-phipsi:** 0.0046057124015895
- **w-rdist:** 0.5329024028549533
- **t-alpha:** 0.0348178944724737

---

---

492

- **PDB ID:** 3IHP | **Chain:** B
- **b-phipsi:** 0.0091833853328648
- **w-rdist:** 0.423275091276137
- **t-alpha:** 0.0571204636616826

---

---

493

- **PDB ID:** 5VNL | **Chain:** B
- **b-phipsi:** 0.0073332587784358
- **w-rdist:** 0.6780983184674926
- **t-alpha:** 0.0133018035296799

---

---

494

- **PDB ID:** 7QPG | **Chain:** B
- **b-phipsi:** 0.0050688122426536
- **w-rdist:** 0.5325579464048177
- **t-alpha:** 0.0314772841117727

---

---

495

- **PDB ID:** 8FCQ | **Chain:** D
- **b-phipsi:** 0.0080837588929995
- **w-rdist:** 0.2605693125226374
- **t-alpha:** 0.1478868884570592

---

---

496

- **PDB ID:** 4PV7 | **Chain:** A
- **b-phipsi:** 0.0075077724991585
- **w-rdist:** 0.7733742266174187
- **t-alpha:** 0.0086070234951727

---

---

497

- **PDB ID:** 1JEY | **Chain:** A
- **b-phipsi:** 0.0012322881086067
- **w-rdist:** 0.5298849992608003
- **t-alpha:** 0.0586852591928757

---

---

498

- **PDB ID:** 5J7T | **Chain:** A
- **b-phipsi:** 0.0010041145313084
- **w-rdist:** 1.0581362426321417
- **t-alpha:** 0.0078868306657793

---

---

499

- **PDB ID:** 5VNF | **Chain:** B
- **b-phipsi:** 0.0074653751477117
- **w-rdist:** 0.6556360693920837
- **t-alpha:** 0.0140843739056586

---

---

500

- **PDB ID:** 5RW1 | **Chain:** A
- **b-phipsi:** 0.014697187392583
- **w-rdist:** 0.4074778333721218
- **t-alpha:** 0.0226916757534991

---

---

501

- **PDB ID:** 7KI3 | **Chain:** A
- **b-phipsi:** 0.0031241086174033
- **w-rdist:** 0.4625678514430112
- **t-alpha:** 0.1040683801301172

---

---

502

- **PDB ID:** 1K4T | **Chain:** A
- **b-phipsi:** 0.01782223611532
- **w-rdist:** 0.3611283673827926
- **t-alpha:** 0.0224000982062968

---

---

503

- **PDB ID:** 1R9M | **Chain:** B
- **b-phipsi:** 0.0085947737153542
- **w-rdist:** 0.7671588942530425
- **t-alpha:** 0.0070418134049574

---

---

504

- **PDB ID:** 5VNN | **Chain:** B
- **b-phipsi:** 0.0106432053220489
- **w-rdist:** 0.6819342121997162
- **t-alpha:** 0.0062593997286479

---

---

505

- **PDB ID:** 6SOY | **Chain:** C
- **b-phipsi:** 0.0068740550948315
- **w-rdist:** 0.6522743758478752
- **t-alpha:** 0.0158983514403059

---

---

506

- **PDB ID:** 8FCM | **Chain:** A
- **b-phipsi:** 0.0086328871436429
- **w-rdist:** 0.2105622055309825
- **t-alpha:** 0.1424100240607699

---

---

507

- **PDB ID:** 3ZUS | **Chain:** B
- **b-phipsi:** 0.0105863788525495
- **w-rdist:** 0.2622533736147685
- **t-alpha:** 0.1017244358474924

---

---

508

- **PDB ID:** 6ERH | **Chain:** C
- **b-phipsi:** 0.0054272594070743
- **w-rdist:** 0.514899784019333
- **t-alpha:** 0.0446008926005814

---

---

509

- **PDB ID:** 5C18 | **Chain:** D
- **b-phipsi:** 0.0091832248484229
- **w-rdist:** 0.2067431606692463
- **t-alpha:** 0.1345850125084049

---

---

510

- **PDB ID:** 1JEY | **Chain:** B
- **b-phipsi:** 0.00538178513261
- **w-rdist:** 0.4498598378750613
- **t-alpha:** 0.104850978155695

---

---

511

- **PDB ID:** 7L34 | **Chain:** A
- **b-phipsi:** 0.0046835230794115
- **w-rdist:** 0.7274579513737489
- **t-alpha:** 0.0175161413598659

---

---

512

- **PDB ID:** 4DPG | **Chain:** E
- **b-phipsi:** 0.0008097873433804
- **w-rdist:** 0.6302146893353893
- **t-alpha:** 0.0331450074819834

---

---

513

- **PDB ID:** 5HMP | **Chain:** B
- **b-phipsi:** 0.0124437462213876
- **w-rdist:** 0.3494167787525358
- **t-alpha:** 0.0544557624307391

---

---

514

- **PDB ID:** 8FCP | **Chain:** A
- **b-phipsi:** 0.0082745580671725
- **w-rdist:** 0.2201875451065
- **t-alpha:** 0.1572766468480066

---

---

515

- **PDB ID:** 7ZYG | **Chain:** B
- **b-phipsi:** 0.0047572796520252
- **w-rdist:** 0.4452498785603958
- **t-alpha:** 0.1228477492952864

---

---

516

- **PDB ID:** 4KTY | **Chain:** B
- **b-phipsi:** 0.017828335328011
- **w-rdist:** 0.3061503014420109
- **t-alpha:** 0.027386367694415

---

---

517

- **PDB ID:** 6BFC | **Chain:** b
- **b-phipsi:** 0.0075597472720069
- **w-rdist:** 0.4310616754085786
- **t-alpha:** 0.0813769138896134

---

---

518

- **PDB ID:** 6BFC | **Chain:** B
- **b-phipsi:** 0.0078489275667005
- **w-rdist:** 0.428843752923242
- **t-alpha:** 0.0790296903641281

---

---

519

- **PDB ID:** 5Y3R | **Chain:** A
- **b-phipsi:** 0.0127656957296317
- **w-rdist:** 0.355961559107461
- **t-alpha:** 0.0485128406736701

---

---

520

- **PDB ID:** 5WOB | **Chain:** c
- **b-phipsi:** 0.0067905476056853
- **w-rdist:** 0.4314726391459773
- **t-alpha:** 0.0970263561161528

---

---

521

- **PDB ID:** 3E4Z | **Chain:** B
- **b-phipsi:** 0.0069806615394134
- **w-rdist:** 0.4391930292406338
- **t-alpha:** 0.0884191622029941

---

---

522

- **PDB ID:** 6P0A | **Chain:** A
- **b-phipsi:** 0.007729479174058
- **w-rdist:** 0.7311353812557716
- **t-alpha:** 0.0102767895279776

---

---

523

- **PDB ID:** 8FCO | **Chain:** C
- **b-phipsi:** 0.0099306473557856
- **w-rdist:** 0.1520334940960089
- **t-alpha:** 0.1322378273556061

---

---

524

- **PDB ID:** 3ZUS | **Chain:** D
- **b-phipsi:** 0.0119651232167903
- **w-rdist:** 0.2610184088912719
- **t-alpha:** 0.083969564498129

---

---

525

- **PDB ID:** 6LF3 | **Chain:** A
- **b-phipsi:** 0.0140293987970079
- **w-rdist:** 0.3776594209860338
- **t-alpha:** 0.0320809565897601

---

---

526

- **PDB ID:** 7ZYG | **Chain:** A
- **b-phipsi:** 0.0062340196813247
- **w-rdist:** 0.4643115974986988
- **t-alpha:** 0.0774646231830327

---

---

527

- **PDB ID:** 7ENJ | **Chain:** A
- **b-phipsi:** 0.0076633947594602
- **w-rdist:** 0.5776496755286973
- **t-alpha:** 0.0191389373786337

---

---

528

- **PDB ID:** 4FWE | **Chain:** A
- **b-phipsi:** 0.0049828481418492
- **w-rdist:** 0.4666984753648409
- **t-alpha:** 0.0907665259446537

---

---

529

- **PDB ID:** 7R9Y | **Chain:** A
- **b-phipsi:** 0.0131394686601537
- **w-rdist:** 0.2284481370440268
- **t-alpha:** 0.0758997739191269

---

---

530

- **PDB ID:** 6U23 | **Chain:** O
- **b-phipsi:** 0.0121113539038216
- **w-rdist:** 0.3457262523563207
- **t-alpha:** 0.0625977621599711

---

---

531

- **PDB ID:** 6UGH | **Chain:** A
- **b-phipsi:** 0.0008420755744489
- **w-rdist:** 0.5417123012029482
- **t-alpha:** 0.0588235890382062

---

---

532

- **PDB ID:** 4AY2 | **Chain:** A
- **b-phipsi:** 0.0113472091789858
- **w-rdist:** 0.3227174016318995
- **t-alpha:** 0.0805944545366483

---

---

533

- **PDB ID:** 3B2D | **Chain:** B
- **b-phipsi:** 0.012551064449496
- **w-rdist:** 0.4374750439557631
- **t-alpha:** 0.0242563010013117

---

---

534

- **PDB ID:** 5WOB | **Chain:** F
- **b-phipsi:** 0.0070480586060399
- **w-rdist:** 0.3898177936285663
- **t-alpha:** 0.1298901102190239

---

---

535

- **PDB ID:** 4DUR | **Chain:** B
- **b-phipsi:** 0.0181609966590569
- **w-rdist:** 0.1860644723393902
- **t-alpha:** 0.0430355279269489

---

---

536

- **PDB ID:** 8FCR | **Chain:** A
- **b-phipsi:** 0.0097682813215021
- **w-rdist:** 0.1637148246006657
- **t-alpha:** 0.1377147492711152

---

---

537

- **PDB ID:** 8SW0 | **Chain:** A
- **b-phipsi:** 0.0091293667200648
- **w-rdist:** 0.2464217824059729
- **t-alpha:** 0.1392798657818292

---

---

538

- **PDB ID:** 6B3Q | **Chain:** b
- **b-phipsi:** 0.0082388431933221
- **w-rdist:** 0.4295542013515036
- **t-alpha:** 0.0774646231830327

---

---

539

- **PDB ID:** 3G0G | **Chain:** D
- **b-phipsi:** 0.006778565327137
- **w-rdist:** 0.8197567863781623
- **t-alpha:** 0.0086070234951727

---

---

540

- **PDB ID:** 4BLB | **Chain:** D
- **b-phipsi:** 0.0045430948657538
- **w-rdist:** 0.6264971161732193
- **t-alpha:** 0.0240384962174629

---

---

541

- **PDB ID:** 4R08 | **Chain:** D
- **b-phipsi:** 0.009320303622419
- **w-rdist:** 0.4051700525875285
- **t-alpha:** 0.0758997739191269

---

---

542

- **PDB ID:** 6SH7 | **Chain:** A
- **b-phipsi:** 0.0105238089223799
- **w-rdist:** 0.7898674819024852
- **t-alpha:** 0.0023473590503262

---

---

543

- **PDB ID:** 5J67 | **Chain:** C
- **b-phipsi:** 0.0040230268211539
- **w-rdist:** 0.5092948136187248
- **t-alpha:** 0.0650001391403227

---

---

544

- **PDB ID:** 1LFG | **Chain:** A
- **b-phipsi:** 0.00719321903125
- **w-rdist:** 0.5096895319804263
- **t-alpha:** 0.0298147080807067

---

---

545

- **PDB ID:** 6U2J | **Chain:** F
- **b-phipsi:** 0.0181190435782957
- **w-rdist:** 0.2959640355211625
- **t-alpha:** 0.0339808661347131

---

---

546

- **PDB ID:** 4RE9 | **Chain:** B
- **b-phipsi:** 0.0060609650016021
- **w-rdist:** 0.4510033600715988
- **t-alpha:** 0.1025037952669631

---

---

547

- **PDB ID:** 3HHD | **Chain:** C
- **b-phipsi:** 0.0039945335430144
- **w-rdist:** 0.7843618812138509
- **t-alpha:** 0.0164318357763795

---

---

548

- **PDB ID:** 2HAV | **Chain:** A
- **b-phipsi:** 0.0097979199498341
- **w-rdist:** 0.4600418102567308
- **t-alpha:** 0.0306454151252038

---

---

549

- **PDB ID:** 4XAM | **Chain:** B
- **b-phipsi:** 0.0262305779679825
- **w-rdist:** 0.3403805606663582
- **t-alpha:** 0.0187789021729365

---

---

550

- **PDB ID:** 6U2J | **Chain:** B
- **b-phipsi:** 0.0160812345680304
- **w-rdist:** 0.3009238875661407
- **t-alpha:** 0.0466830494629222

---

---

551

- **PDB ID:** 4MK0 | **Chain:** A
- **b-phipsi:** 0.0166932278196344
- **w-rdist:** 0.3382885476484403
- **t-alpha:** 0.0348178944724737

---

---

552

- **PDB ID:** 4BTX | **Chain:** B
- **b-phipsi:** 0.0162815773435785
- **w-rdist:** 0.6459486527626612
- **t-alpha:** 0.0007832732121422

---

---

553

- **PDB ID:** 7BG0 | **Chain:** A
- **b-phipsi:** 0.0128808055231004
- **w-rdist:** 0.3621130519333335
- **t-alpha:** 0.0508603233634601

---

---

554

- **PDB ID:** 7WUB | **Chain:** J
- **b-phipsi:** 0.0088851436187898
- **w-rdist:** 0.3230204023287145
- **t-alpha:** 0.1275429598762953

---

---

555

- **PDB ID:** 6U3J | **Chain:** B
- **b-phipsi:** 0.0138646659242359
- **w-rdist:** 0.3970126986438045
- **t-alpha:** 0.0314772841117727

---

---

556

- **PDB ID:** 6EOQ | **Chain:** C
- **b-phipsi:** 0.0181106127092874
- **w-rdist:** 0.4404812763397754
- **t-alpha:** 0.0175161413598659

---

---

557

- **PDB ID:** 8G1U | **Chain:** E
- **b-phipsi:** 0.0018242599127816
- **w-rdist:** 0.5393165182476312
- **t-alpha:** 0.056198410016208

---

---

558

- **PDB ID:** 6GVF | **Chain:** A
- **b-phipsi:** 0.0120896593992308
- **w-rdist:** 0.1635691465266833
- **t-alpha:** 0.1017212837931069

---

---

559

- **PDB ID:** 7MXC | **Chain:** A
- **b-phipsi:** 0.0018386322921656
- **w-rdist:** 0.5451225541493845
- **t-alpha:** 0.0535862707318639

---

---

560

- **PDB ID:** 7B2Q | **Chain:** A
- **b-phipsi:** 0.0322268672478081
- **w-rdist:** 0.334353126202579
- **t-alpha:** 0.0172143982372043

---

---

561

- **PDB ID:** 5Y7J | **Chain:** B
- **b-phipsi:** 0.0056195666328074
- **w-rdist:** 0.7828504498034614
- **t-alpha:** 0.0148665808060715

---

---

562

- **PDB ID:** 3QYT | **Chain:** A
- **b-phipsi:** 0.0058039592271336
- **w-rdist:** 0.6524836532058591
- **t-alpha:** 0.0207667552249124

---

---

563

- **PDB ID:** 5C19 | **Chain:** C
- **b-phipsi:** 0.0087557612514759
- **w-rdist:** 0.3151065119294457
- **t-alpha:** 0.1353675860480128

---

---

564

- **PDB ID:** 1R49 | **Chain:** A
- **b-phipsi:** 0.0155711551177067
- **w-rdist:** 0.3633978852616503
- **t-alpha:** 0.0328637472319743

---

---

565

- **PDB ID:** 8OW2 | **Chain:** A
- **b-phipsi:** 0.0109892787814314
- **w-rdist:** 0.2568999521125089
- **t-alpha:** 0.1118933907565391

---

---

566

- **PDB ID:** 5KYU | **Chain:** A
- **b-phipsi:** 0.0017403553758637
- **w-rdist:** 0.6442666955050833
- **t-alpha:** 0.0305161718713362

---

---

567

- **PDB ID:** 4PJ6 | **Chain:** A
- **b-phipsi:** 0.0128122944414122
- **w-rdist:** 0.5205622751263852
- **t-alpha:** 0.0133018035296799

---

---

568

- **PDB ID:** 6D03 | **Chain:** C
- **b-phipsi:** 0.0080156581676423
- **w-rdist:** 0.4954417093263102
- **t-alpha:** 0.0323103167719089

---

---

569

- **PDB ID:** 7MHS | **Chain:** C
- **b-phipsi:** 0.0218581293275327
- **w-rdist:** 0.5355470516326678
- **t-alpha:** 0.0015647720218643

---

---

570

- **PDB ID:** 6B70 | **Chain:** B
- **b-phipsi:** 0.0093603604569246
- **w-rdist:** 0.4166778501354887
- **t-alpha:** 0.0735521531070955

---

---

571

- **PDB ID:** 6MFR | **Chain:** A
- **b-phipsi:** 0.0053009505897122
- **w-rdist:** 0.4662913481998456
- **t-alpha:** 0.1001563528463682

---

---

572

- **PDB ID:** 8OG4 | **Chain:** B
- **b-phipsi:** 0.0114015149080354
- **w-rdist:** 0.4118769774219927
- **t-alpha:** 0.0492615627855226

---

---

573

- **PDB ID:** 7ABI | **Chain:** R
- **b-phipsi:** 0.0124950260715316
- **w-rdist:** 0.2612555306920382
- **t-alpha:** 0.0868544688919852

---

---

574

- **PDB ID:** 3F8U | **Chain:** C
- **b-phipsi:** 0.0055379733476177
- **w-rdist:** 0.4941438306757615
- **t-alpha:** 0.0712489865698182

---

---

575

- **PDB ID:** 5MV9 | **Chain:** A
- **b-phipsi:** 0.0122628730061078
- **w-rdist:** 0.6296732511102481
- **t-alpha:** 0.0078243548762237

---

---

576

- **PDB ID:** 7OR4 | **Chain:** B
- **b-phipsi:** 0.0100048232457588
- **w-rdist:** 0.4641815914607015
- **t-alpha:** 0.0289513063799407

---

---

577

- **PDB ID:** 8BFU | **Chain:** A
- **b-phipsi:** 0.0112901250569846
- **w-rdist:** 0.1549908375530363
- **t-alpha:** 0.1212827505141209

---

---

578

- **PDB ID:** 5MHN | **Chain:** A
- **b-phipsi:** 0.0187618329482759
- **w-rdist:** 0.3138108408149561
- **t-alpha:** 0.0312987592118472

---

---

579

- **PDB ID:** 5WOB | **Chain:** g
- **b-phipsi:** 0.0074726407891577
- **w-rdist:** 0.4021376771441877
- **t-alpha:** 0.1205008021373417

---

---

580

- **PDB ID:** 7MXA | **Chain:** A
- **b-phipsi:** 0.0015047871291723
- **w-rdist:** 0.5394162388722863
- **t-alpha:** 0.0623444493098053

---

---

581

- **PDB ID:** 2YD0 | **Chain:** A
- **b-phipsi:** 0.0085127583801902
- **w-rdist:** 0.4731081019066795
- **t-alpha:** 0.0424145126110251

---

---

582

- **PDB ID:** 3LPO | **Chain:** D
- **b-phipsi:** 0.005843599129802
- **w-rdist:** 0.5998247333204332
- **t-alpha:** 0.0242563010013117

---

---

583

- **PDB ID:** 8EXU | **Chain:** A
- **b-phipsi:** 0.0100675116229489
- **w-rdist:** 0.1578622388937746
- **t-alpha:** 0.1424100240607699

---

---

584

- **PDB ID:** 7BG0 | **Chain:** D
- **b-phipsi:** 0.0113685743858011
- **w-rdist:** 0.5888003133601328
- **t-alpha:** 0.0117370267412904

---

---

585

- **PDB ID:** 5C18 | **Chain:** F
- **b-phipsi:** 0.011354617641472
- **w-rdist:** 0.1941261475047281
- **t-alpha:** 0.1165880511557739

---

---

586

- **PDB ID:** 5WOB | **Chain:** f
- **b-phipsi:** 0.0084202687596384
- **w-rdist:** 0.3897708439116104
- **t-alpha:** 0.111111102563624

---

---

587

- **PDB ID:** 6V0P | **Chain:** A
- **b-phipsi:** 0.0028663493919389
- **w-rdist:** 0.5388150628375941
- **t-alpha:** 0.0553264749125952

---

---

588

- **PDB ID:** 4B3G | **Chain:** B
- **b-phipsi:** 0.008773713502562
- **w-rdist:** 0.7813404710754661
- **t-alpha:** 0.0078243548762237

---

---

589

- **PDB ID:** 4DTT | **Chain:** A
- **b-phipsi:** 0.0062616990055108
- **w-rdist:** 0.4521355673809182
- **t-alpha:** 0.1040683801301172

---

---

590

- **PDB ID:** 5WOB | **Chain:** B
- **b-phipsi:** 0.0065043973816181
- **w-rdist:** 0.4279556685862579
- **t-alpha:** 0.1259778598996253

---

---

591

- **PDB ID:** 7W9V | **Chain:** K
- **b-phipsi:** 0.0053179297038968
- **w-rdist:** 0.5374525804512612
- **t-alpha:** 0.0432653326797045

---

---

592

- **PDB ID:** 6XTX | **Chain:** 4
- **b-phipsi:** 0.0131924009205745
- **w-rdist:** 0.4215534843971995
- **t-alpha:** 0.0320809565897601

---

---

593

- **PDB ID:** 8CYI | **Chain:** A
- **b-phipsi:** 0.0020275387829122
- **w-rdist:** 0.5389944922178918
- **t-alpha:** 0.0605810409571925

---

---

594

- **PDB ID:** 7K6N | **Chain:** A
- **b-phipsi:** 0.012733119251101
- **w-rdist:** 0.1659821799107949
- **t-alpha:** 0.1009388027447895

---

---

595

- **PDB ID:** 8FCR | **Chain:** D
- **b-phipsi:** 0.0080345142633185
- **w-rdist:** 0.2950000222192106
- **t-alpha:** 0.1823154324464593

---

---

596

- **PDB ID:** 5WEA | **Chain:** A
- **b-phipsi:** 0.0118375499722426
- **w-rdist:** 0.1127996482655781
- **t-alpha:** 0.1181528955465753

---

---

597

- **PDB ID:** 4GU0 | **Chain:** B
- **b-phipsi:** 0.0043520607239427
- **w-rdist:** 0.51551696580199
- **t-alpha:** 0.068561919033538

---

---

598

- **PDB ID:** 7ABI | **Chain:** r
- **b-phipsi:** 0.0133251474746146
- **w-rdist:** 0.2754764274487131
- **t-alpha:** 0.0805944545366483

---

---

599

- **PDB ID:** 5Y7K | **Chain:** D
- **b-phipsi:** 0.0061639645292726
- **w-rdist:** 0.7806458394917417
- **t-alpha:** 0.0148665808060715

---

---

600

- **PDB ID:** 5Y3R | **Chain:** B
- **b-phipsi:** 0.0102416452908982
- **w-rdist:** 0.4681455643297053
- **t-alpha:** 0.0266038120788794

---

---

601

- **PDB ID:** 1TZN | **Chain:** O
- **b-phipsi:** 0.0093116958546701
- **w-rdist:** 0.752653325285725
- **t-alpha:** 0.0078868306657793

---

---

602

- **PDB ID:** 6X6A | **Chain:** A
- **b-phipsi:** 0.0075776141674287
- **w-rdist:** 0.6615361254408718
- **t-alpha:** 0.0164318357763795

---

---

603

- **PDB ID:** 3G0G | **Chain:** B
- **b-phipsi:** 0.0083751106046943
- **w-rdist:** 0.7602918918393182
- **t-alpha:** 0.0101717762082627

---

---

604

- **PDB ID:** 5VNH | **Chain:** B
- **b-phipsi:** 0.0052100597622525
- **w-rdist:** 0.6708073254974979
- **t-alpha:** 0.0219087977837508

---

---

605

- **PDB ID:** 5WOB | **Chain:** e
- **b-phipsi:** 0.0079749454482219
- **w-rdist:** 0.4087025473727788
- **t-alpha:** 0.1095460606668756

---

---

606

- **PDB ID:** 6EJA | **Chain:** A
- **b-phipsi:** 0.0010157157203845
- **w-rdist:** 0.5522732618474475
- **t-alpha:** 0.06807491933535

---

---

607

- **PDB ID:** 2C11 | **Chain:** A
- **b-phipsi:** 0.0146638206221744
- **w-rdist:** 0.7269237765252895
- **t-alpha:** 0.0

---

---

608

- **PDB ID:** 8CSW | **Chain:** X
- **b-phipsi:** 0.0133000932014886
- **w-rdist:** 0.5605292236880106
- **t-alpha:** 0.0101717762082627

---

---

609

- **PDB ID:** 5WOB | **Chain:** b
- **b-phipsi:** 0.0067886939077216
- **w-rdist:** 0.4297645850891991
- **t-alpha:** 0.1205008021373417

---

---

610

- **PDB ID:** 2WJV | **Chain:** A
- **b-phipsi:** 0.0047821043798929
- **w-rdist:** 0.5238984074650974
- **t-alpha:** 0.0597019506692302

---

---

611

- **PDB ID:** 6GYR | **Chain:** D
- **b-phipsi:** 0.0067097167476587
- **w-rdist:** 0.4880542540638688
- **t-alpha:** 0.0632282666494634

---

---

612

- **PDB ID:** 1NUD | **Chain:** B
- **b-phipsi:** 0.0158169858082127
- **w-rdist:** 0.5925047554538789
- **t-alpha:** 0.0062593997286479

---

---

613

- **PDB ID:** 6VXK | **Chain:** D
- **b-phipsi:** 0.0294943727305611
- **w-rdist:** 0.3442807395548221
- **t-alpha:** 0.0187789021729365

---

---

614

- **PDB ID:** 5L46 | **Chain:** A
- **b-phipsi:** 0.0038512903577665
- **w-rdist:** 0.7159599407782519
- **t-alpha:** 0.0215828567205209

---

---

615

- **PDB ID:** 6U2J | **Chain:** A
- **b-phipsi:** 0.0174091065314781
- **w-rdist:** 0.3008169708916642
- **t-alpha:** 0.0475413495623815

---

---

616

- **PDB ID:** 7SYE | **Chain:** B
- **b-phipsi:** 0.0145691837786676
- **w-rdist:** 0.7267653250652991
- **t-alpha:** 0.0007823187912934

---

---

617

- **PDB ID:** 3HII | **Chain:** B
- **b-phipsi:** 0.0166358494707084
- **w-rdist:** 0.6090401984490711
- **t-alpha:** 0.0046944792707916

---

---

618

- **PDB ID:** 3HHM | **Chain:** A
- **b-phipsi:** 0.0096805331493701
- **w-rdist:** 0.4596497498875679
- **t-alpha:** 0.0430355279269489

---

---

619

- **PDB ID:** 6SVO | **Chain:** B
- **b-phipsi:** 0.0051074719543362
- **w-rdist:** 0.7853042946183744
- **t-alpha:** 0.0167066822187644

---

---

620

- **PDB ID:** 5C19 | **Chain:** E
- **b-phipsi:** 0.0103060852113859
- **w-rdist:** 0.1857797103205393
- **t-alpha:** 0.1416274957110146

---

---

621

- **PDB ID:** 4DUR | **Chain:** A
- **b-phipsi:** 0.0167033006820677
- **w-rdist:** 0.0917469695134396
- **t-alpha:** 0.0704223021796839

---

---

622

- **PDB ID:** 5F9H | **Chain:** C
- **b-phipsi:** 0.0089036761817346
- **w-rdist:** 0.5990408987299138
- **t-alpha:** 0.0175161413598659

---

---

623

- **PDB ID:** 8EXO | **Chain:** A
- **b-phipsi:** 0.0100557867753142
- **w-rdist:** 0.1386887724260992
- **t-alpha:** 0.1557116053874756

---

---

624

- **PDB ID:** 6R25 | **Chain:** K
- **b-phipsi:** 0.0049875057231736
- **w-rdist:** 0.5216482122781241
- **t-alpha:** 0.0618152204296507

---

---

625

- **PDB ID:** 6NIH | **Chain:** A
- **b-phipsi:** 0.0046741373117304
- **w-rdist:** 0.5196244373000412
- **t-alpha:** 0.0676695952069212

---

---

626

- **PDB ID:** 7MYO | **Chain:** A
- **b-phipsi:** 0.0094159923368194
- **w-rdist:** 0.3204887293802201
- **t-alpha:** 0.1345850125084049

---

---

627

- **PDB ID:** 3B2D | **Chain:** A
- **b-phipsi:** 0.0116235306482838
- **w-rdist:** 0.4401596403125096
- **t-alpha:** 0.0414710191602802

---

---

628

- **PDB ID:** 5KBY | **Chain:** B
- **b-phipsi:** 0.0072728857365447
- **w-rdist:** 0.7674698756860664
- **t-alpha:** 0.0133018035296799

---

---

629

- **PDB ID:** 5IZ5 | **Chain:** A
- **b-phipsi:** 0.009075957785093
- **w-rdist:** 0.4693253432146369
- **t-alpha:** 0.0458269486756497

---

---

630

- **PDB ID:** 6ZHE | **Chain:** B
- **b-phipsi:** 0.0166384049786174
- **w-rdist:** 0.3766537381146942
- **t-alpha:** 0.0328637472319743

---

---

631

- **PDB ID:** 3V83 | **Chain:** F
- **b-phipsi:** 0.0105941506101835
- **w-rdist:** 0.5662547091427745
- **t-alpha:** 0.0156493910563146

---

---

632

- **PDB ID:** 3QZ2 | **Chain:** B
- **b-phipsi:** 0.0070339658573034
- **w-rdist:** 0.4350368442716393
- **t-alpha:** 0.1181528955465753

---

---

633

- **PDB ID:** 5WOB | **Chain:** G
- **b-phipsi:** 0.0079782399444809
- **w-rdist:** 0.402285119322496
- **t-alpha:** 0.1236302639257407

---

---

634

- **PDB ID:** 7XE3 | **Chain:** B
- **b-phipsi:** 0.0058845690297403
- **w-rdist:** 0.4797373586686068
- **t-alpha:** 0.0885862926148752

---

---

635

- **PDB ID:** 8G7N | **Chain:** C
- **b-phipsi:** 0.0196030723950026
- **w-rdist:** 0.6256830384885966
- **t-alpha:** 0.0007832732121422

---

---

636

- **PDB ID:** 1CX8 | **Chain:** H
- **b-phipsi:** 0.006691624755727
- **w-rdist:** 0.8879225166575143
- **t-alpha:** 0.0086820531296027

---

---

637

- **PDB ID:** 7PFS | **Chain:** B
- **b-phipsi:** 0.0111687529303657
- **w-rdist:** 0.5869286659615902
- **t-alpha:** 0.0140843739056586

---

---

638

- **PDB ID:** 4DSA | **Chain:** A
- **b-phipsi:** 0.0048589359764914
- **w-rdist:** 0.7867249340635689
- **t-alpha:** 0.0179965923641238

---

---

639

- **PDB ID:** 5CJO | **Chain:** A
- **b-phipsi:** 0.0061929510815643
- **w-rdist:** 0.4513454310521109
- **t-alpha:** 0.1212827505141209

---

---

640

- **PDB ID:** 3N57 | **Chain:** B
- **b-phipsi:** 0.0072316227698881
- **w-rdist:** 0.4366268444661699
- **t-alpha:** 0.1150232700097437

---

---

641

- **PDB ID:** 6NIH | **Chain:** B
- **b-phipsi:** 0.0053807913509295
- **w-rdist:** 0.4997647878630735
- **t-alpha:** 0.0803043978200703

---

---

642

- **PDB ID:** 7AAV | **Chain:** r
- **b-phipsi:** 0.013945833365013
- **w-rdist:** 0.2619288602035129
- **t-alpha:** 0.0852894374233532

---

---

643

- **PDB ID:** 7XE1 | **Chain:** B
- **b-phipsi:** 0.0063306573588328
- **w-rdist:** 0.4778189562342764
- **t-alpha:** 0.0848899960610365

---

---

644

- **PDB ID:** 2JBU | **Chain:** B
- **b-phipsi:** 0.0065069204808699
- **w-rdist:** 0.4440658698492238
- **t-alpha:** 0.1275429598762953

---

---

645

- **PDB ID:** 7MXN | **Chain:** A
- **b-phipsi:** 0.0008157458730964
- **w-rdist:** 0.54633373189351
- **t-alpha:** 0.0784813498720293

---

---

646

- **PDB ID:** 3F8U | **Chain:** A
- **b-phipsi:** 0.0050203394971012
- **w-rdist:** 0.4700024402997592
- **t-alpha:** 0.1151833185379382

---

---

647

- **PDB ID:** 8EFD | **Chain:** A
- **b-phipsi:** 0.0128266077785902
- **w-rdist:** 0.4023330930544201
- **t-alpha:** 0.0516428148679497

---

---

648

- **PDB ID:** 4GU0 | **Chain:** D
- **b-phipsi:** 0.0075798642646678
- **w-rdist:** 0.490583184929165
- **t-alpha:** 0.0535862707318639

---

---

649

- **PDB ID:** 7A3L | **Chain:** A
- **b-phipsi:** 0.0060995448779456
- **w-rdist:** 0.5210384283083658
- **t-alpha:** 0.0563379260275018

---

---

650

- **PDB ID:** 6SY1 | **Chain:** B
- **b-phipsi:** 0.0136060237316925
- **w-rdist:** 0.4153029956899035
- **t-alpha:** 0.0407169385382113

---

---

651

- **PDB ID:** 2YKG | **Chain:** A
- **b-phipsi:** 0.0113505350950905
- **w-rdist:** 0.3207193978542634
- **t-alpha:** 0.1071984065049926

---

---

652

- **PDB ID:** 5MQF | **Chain:** b
- **b-phipsi:** 0.0119629218684887
- **w-rdist:** 0.4514821925704729
- **t-alpha:** 0.0312987592118472

---

---

653

- **PDB ID:** 6MDZ | **Chain:** B
- **b-phipsi:** 0.004570649877134
- **w-rdist:** 0.4462142196636648
- **t-alpha:** 0.1690136447929693

---

---

654

- **PDB ID:** 6INN | **Chain:** B
- **b-phipsi:** 0.0188293758385569
- **w-rdist:** 0.4954021574397481
- **t-alpha:** 0.0110763082596549

---

---

655

- **PDB ID:** 8AG4 | **Chain:** A
- **b-phipsi:** 0.0042740143966255
- **w-rdist:** 0.5378734190004808
- **t-alpha:** 0.0594675149611743

---

---

656

- **PDB ID:** 3CWW | **Chain:** A
- **b-phipsi:** 0.0064548543104835
- **w-rdist:** 0.4539723073040182
- **t-alpha:** 0.1118933907565391

---

---

657

- **PDB ID:** 2OQV | **Chain:** A
- **b-phipsi:** 0.0074343722972013
- **w-rdist:** 0.7819877499114491
- **t-alpha:** 0.0125193542574237

---

---

658

- **PDB ID:** 6RLL | **Chain:** A
- **b-phipsi:** 0.0016226744998368
- **w-rdist:** 0.5445483207515949
- **t-alpha:** 0.0730480405888041

---

---

659

- **PDB ID:** 4GUU | **Chain:** A
- **b-phipsi:** 0.0032421889147837
- **w-rdist:** 0.5363704143129306
- **t-alpha:** 0.0650001391403227

---

---

660

- **PDB ID:** 6U2J | **Chain:** E
- **b-phipsi:** 0.0161224133514292
- **w-rdist:** 0.3010788673030444
- **t-alpha:** 0.0641133266895068

---

---

661

- **PDB ID:** 6MQ3 | **Chain:** B
- **b-phipsi:** 0.006515696996469
- **w-rdist:** 0.447678563364902
- **t-alpha:** 0.1236302639257407

---

---

662

- **PDB ID:** 7AAV | **Chain:** R
- **b-phipsi:** 0.0130546949314243
- **w-rdist:** 0.2665338332141586
- **t-alpha:** 0.0985915493938487

---

---

663

- **PDB ID:** 6FF7 | **Chain:** B
- **b-phipsi:** 0.0096626370809052
- **w-rdist:** 0.4928041997265275
- **t-alpha:** 0.0265064016545928

---

---

664

- **PDB ID:** 6QZV | **Chain:** D
- **b-phipsi:** 0.012260867954785
- **w-rdist:** 0.6405072323523251
- **t-alpha:** 0.0093893372993878

---

---

665

- **PDB ID:** 7L5W | **Chain:** B
- **b-phipsi:** 0.0256514334804128
- **w-rdist:** 0.5256344413846677
- **t-alpha:** 0.0023530945443304

---

---

666

- **PDB ID:** 3BJU | **Chain:** A
- **b-phipsi:** 0.0014274077876579
- **w-rdist:** 0.6249996069123085
- **t-alpha:** 0.0535862707318639

---

---

667

- **PDB ID:** 6CKC | **Chain:** A
- **b-phipsi:** 0.0017019596303325
- **w-rdist:** 0.5527912035718929
- **t-alpha:** 0.0712489865698182

---

---

668

- **PDB ID:** 5IUD | **Chain:** A
- **b-phipsi:** 0.0061314364352505
- **w-rdist:** 0.3991331725423456
- **t-alpha:** 0.1956180565138197

---

---

669

- **PDB ID:** 8FCM | **Chain:** D
- **b-phipsi:** 0.0086116280368057
- **w-rdist:** 0.2905377167643054
- **t-alpha:** 0.1924876687885701

---

---

670

- **PDB ID:** 5WOB | **Chain:** E
- **b-phipsi:** 0.0070068307832573
- **w-rdist:** 0.4113399278488603
- **t-alpha:** 0.1439745083193748

---

---

671

- **PDB ID:** 6R4E | **Chain:** B
- **b-phipsi:** 0.0050862948753762
- **w-rdist:** 0.7850167605450117
- **t-alpha:** 0.0183267305342325

---

---

672

- **PDB ID:** 2G48 | **Chain:** A
- **b-phipsi:** 0.0066288580302473
- **w-rdist:** 0.4491871819311738
- **t-alpha:** 0.1197177972971978

---

---

673

- **PDB ID:** 2Z7X | **Chain:** B
- **b-phipsi:** 0.003581412067976
- **w-rdist:** 0.50559387494116
- **t-alpha:** 0.0951160800426831

---

---

674

- **PDB ID:** 6P0C | **Chain:** A
- **b-phipsi:** 0.0103850334185682
- **w-rdist:** 0.7473970966602478
- **t-alpha:** 0.0078868306657793

---

---

675

- **PDB ID:** 5NN5 | **Chain:** A
- **b-phipsi:** 0.0090975207873412
- **w-rdist:** 0.6060616902675687
- **t-alpha:** 0.0183267305342325

---

---

676

- **PDB ID:** 7W5A | **Chain:** C
- **b-phipsi:** 0.0072867564252945
- **w-rdist:** 0.5283605104322855
- **t-alpha:** 0.0339808661347131

---

---

677

- **PDB ID:** 5C18 | **Chain:** E
- **b-phipsi:** 0.0092660649363475
- **w-rdist:** 0.3197568539393779
- **t-alpha:** 0.1517994675171843

---

---

678

- **PDB ID:** 4PF7 | **Chain:** B
- **b-phipsi:** 0.006922685306422
- **w-rdist:** 0.4228464630571138
- **t-alpha:** 0.1392798657818292

---

---

679

- **PDB ID:** 7RLI | **Chain:** B
- **b-phipsi:** 0.0086129412928387
- **w-rdist:** 0.3262849671360508
- **t-alpha:** 0.1705788378564454

---

---

680

- **PDB ID:** 5CJO | **Chain:** a
- **b-phipsi:** 0.0064143882542205
- **w-rdist:** 0.4502644627503598
- **t-alpha:** 0.1236302639257407

---

---

681

- **PDB ID:** 7CYN | **Chain:** B
- **b-phipsi:** 0.0156028223306585
- **w-rdist:** 0.6161400152018434
- **t-alpha:** 0.0070418134049574

---

---

682

- **PDB ID:** 6XOV | **Chain:** A
- **b-phipsi:** 0.011022163957216
- **w-rdist:** 0.3711894697073185
- **t-alpha:** 0.0915490640026019

---

---

683

- **PDB ID:** 6AHU | **Chain:** B
- **b-phipsi:** 0.0169623514644486
- **w-rdist:** 0.2795776169310317
- **t-alpha:** 0.0649447869300658

---

---

684

- **PDB ID:** 7AYQ | **Chain:** B
- **b-phipsi:** 0.015291784885854
- **w-rdist:** 0.3884271386106038
- **t-alpha:** 0.0438181656914413

---

---

685

- **PDB ID:** 7WUB | **Chain:** K
- **b-phipsi:** 0.0084081241968126
- **w-rdist:** 0.3233664157929356
- **t-alpha:** 0.1791861366113638

---

---

686

- **PDB ID:** 7WUB | **Chain:** I
- **b-phipsi:** 0.0073756608114964
- **w-rdist:** 0.3143301011837121
- **t-alpha:** 0.237871300622795

---

---

687

- **PDB ID:** 8GHR | **Chain:** A
- **b-phipsi:** 0.0016924254137125
- **w-rdist:** 0.6314856440406776
- **t-alpha:** 0.050987106591174

---

---

688

- **PDB ID:** 5WOB | **Chain:** a
- **b-phipsi:** 0.0065168276823758
- **w-rdist:** 0.4479492863246825
- **t-alpha:** 0.1275429598762953

---

---

689

- **PDB ID:** 3DDU | **Chain:** A
- **b-phipsi:** 0.0079688917765618
- **w-rdist:** 0.8597185897229398
- **t-alpha:** 0.0078243548762237

---

---

690

- **PDB ID:** 5MHO | **Chain:** B
- **b-phipsi:** 0.0208169946527437
- **w-rdist:** 0.2873674057611014
- **t-alpha:** 0.0422534769714337

---

---

691

- **PDB ID:** 4GUS | **Chain:** A
- **b-phipsi:** 0.0036044671230816
- **w-rdist:** 0.5175017449233714
- **t-alpha:** 0.083969564498129

---

---

692

- **PDB ID:** 6BKF | **Chain:** A
- **b-phipsi:** 0.0098775645880973
- **w-rdist:** 0.4515826331565547
- **t-alpha:** 0.0579025721066006

---

---

693

- **PDB ID:** 4XYJ | **Chain:** B
- **b-phipsi:** 0.0107141149947009
- **w-rdist:** 0.7494566336457501
- **t-alpha:** 0.0078243548762237

---

---

694

- **PDB ID:** 5TE1 | **Chain:** A
- **b-phipsi:** 0.0157509718074664
- **w-rdist:** 0.3907350390958517
- **t-alpha:** 0.0414710191602802

---

---

695

- **PDB ID:** 7LT3 | **Chain:** J
- **b-phipsi:** 0.0124725453768814
- **w-rdist:** 0.3286407059184899
- **t-alpha:** 0.0931137397963648

---

---

696

- **PDB ID:** 4FWF | **Chain:** A
- **b-phipsi:** 0.0060859727225137
- **w-rdist:** 0.4882240077190212
- **t-alpha:** 0.0884191622029941

---

---

697

- **PDB ID:** 7MDM | **Chain:** A
- **b-phipsi:** 0.0092968403031018
- **w-rdist:** 0.3381276089477105
- **t-alpha:** 0.1463219731492844

---

---

698

- **PDB ID:** 7S1R | **Chain:** A
- **b-phipsi:** 0.0036789157932038
- **w-rdist:** 0.5391780497963833
- **t-alpha:** 0.0650001391403227

---

---

699

- **PDB ID:** 5VNG | **Chain:** B
- **b-phipsi:** 0.0059077392373533
- **w-rdist:** 0.6451919700060154
- **t-alpha:** 0.0242563010013117

---

---

700

- **PDB ID:** 1PKX | **Chain:** A
- **b-phipsi:** 0.0059277885618883
- **w-rdist:** 0.775178272511944
- **t-alpha:** 0.0183267305342325

---

---

701

- **PDB ID:** 4GS8 | **Chain:** B
- **b-phipsi:** 0.0065118021404973
- **w-rdist:** 0.4480341260029064
- **t-alpha:** 0.1306726803524212

---

---

702

- **PDB ID:** 4R07 | **Chain:** D
- **b-phipsi:** 0.01048694544118
- **w-rdist:** 0.4040718011208947
- **t-alpha:** 0.0860716712560181

---

---

703

- **PDB ID:** 5EMJ | **Chain:** A
- **b-phipsi:** 0.0025052406251305
- **w-rdist:** 0.5399821225693459
- **t-alpha:** 0.0748531911117893

---

---

704

- **PDB ID:** 5F9F | **Chain:** G
- **b-phipsi:** 0.0076036195881908
- **w-rdist:** 0.6153395224763116
- **t-alpha:** 0.0219087977837508

---

---

705

- **PDB ID:** 7KLU | **Chain:** B
- **b-phipsi:** 0.0090844608718605
- **w-rdist:** 0.9318511925394516
- **t-alpha:** 0.0023530945443304

---

---

706

- **PDB ID:** 1XFD | **Chain:** A
- **b-phipsi:** 0.011904464418553
- **w-rdist:** 0.8144003608620283
- **t-alpha:** 0.0015676375257613

---

---

707

- **PDB ID:** 6MQ3 | **Chain:** A
- **b-phipsi:** 0.0062484105793604
- **w-rdist:** 0.4479798310147159
- **t-alpha:** 0.1392798657818292

---

---

708

- **PDB ID:** 5A9J | **Chain:** C
- **b-phipsi:** 0.0245466951444146
- **w-rdist:** 0.5881417031893963
- **t-alpha:** 0.0007823187912934

---

---

709

- **PDB ID:** 3U1K | **Chain:** C
- **b-phipsi:** 0.0030026567519387
- **w-rdist:** 0.8059023546383817
- **t-alpha:** 0.0199522783818248

---

---

710

- **PDB ID:** 3U7U | **Chain:** E
- **b-phipsi:** 0.0148038819705035
- **w-rdist:** 0.6616927868368495
- **t-alpha:** 0.0055077996884944

---

---

711

- **PDB ID:** 4GU0 | **Chain:** A
- **b-phipsi:** 0.0049505876709364
- **w-rdist:** 0.5204938220643069
- **t-alpha:** 0.0784813498720293

---

---

712

- **PDB ID:** 3L4G | **Chain:** D
- **b-phipsi:** 0.0012449927822047
- **w-rdist:** 0.7217649190351114
- **t-alpha:** 0.0328637472319743

---

---

713

- **PDB ID:** 6MSU | **Chain:** B
- **b-phipsi:** 0.0173681444169352
- **w-rdist:** 0.6974154214755615
- **t-alpha:** 0.0015647720218643

---

---

714

- **PDB ID:** 5EMM | **Chain:** A
- **b-phipsi:** 0.0053617356023849
- **w-rdist:** 0.5354826022611352
- **t-alpha:** 0.061461872795004

---

---

715

- **PDB ID:** 5UKK | **Chain:** A
- **b-phipsi:** 0.0148945521840174
- **w-rdist:** 0.3029505843357534
- **t-alpha:** 0.0803043978200703

---

---

716

- **PDB ID:** 1TL8 | **Chain:** A
- **b-phipsi:** 0.0219591957154218
- **w-rdist:** 0.3887873806530471
- **t-alpha:** 0.0226916757534991

---

---

717

- **PDB ID:** 6GPG | **Chain:** A
- **b-phipsi:** 0.0137114962707358
- **w-rdist:** 0.5870067095596581
- **t-alpha:** 0.0110763082596549

---

---

718

- **PDB ID:** 7K1E | **Chain:** B
- **b-phipsi:** 0.0059773225160368
- **w-rdist:** 0.4471405200747086
- **t-alpha:** 0.1533641609305081

---

---

719

- **PDB ID:** 5WOB | **Chain:** A
- **b-phipsi:** 0.0066788296589217
- **w-rdist:** 0.4480460856211898
- **t-alpha:** 0.1322378273556061

---

---

720

- **PDB ID:** 4X61 | **Chain:** A
- **b-phipsi:** 0.0038591209416259
- **w-rdist:** 0.5350165291352532
- **t-alpha:** 0.0739499805270313

---

---

721

- **PDB ID:** 8EZB | **Chain:** K
- **b-phipsi:** 0.0083549484984016
- **w-rdist:** 0.393703127502549
- **t-alpha:** 0.1392798657818292

---

---

722

- **PDB ID:** 4GSC | **Chain:** B
- **b-phipsi:** 0.0068047054892105
- **w-rdist:** 0.4398033492007041
- **t-alpha:** 0.1377147492711152

---

---

723

- **PDB ID:** 6X9K | **Chain:** A
- **b-phipsi:** 0.0058560432011054
- **w-rdist:** 0.4374508665744716
- **t-alpha:** 0.1768381479464797

---

---

724

- **PDB ID:** 3O3U | **Chain:** N
- **b-phipsi:** 5.5956568732293496e-05
- **w-rdist:** 0.7094462687511035
- **t-alpha:** 0.0441178429367157

---

---

725

- **PDB ID:** 6ZMK | **Chain:** B
- **b-phipsi:** 0.004744973017079
- **w-rdist:** 0.7884591031996572
- **t-alpha:** 0.0199522783818248

---

---

726

- **PDB ID:** 6EOS | **Chain:** E
- **b-phipsi:** 0.0129513549552054
- **w-rdist:** 0.3947914247406626
- **t-alpha:** 0.0649447869300658

---

---

727

- **PDB ID:** 7RZE | **Chain:** A
- **b-phipsi:** 0.0061455381963766
- **w-rdist:** 0.4483019236630743
- **t-alpha:** 0.1463219731492844

---

---

728

- **PDB ID:** 7WUB | **Chain:** L
- **b-phipsi:** 0.0077159788623139
- **w-rdist:** 0.323040975445352
- **t-alpha:** 0.2308294991072663

---

---

729

- **PDB ID:** 5HMP | **Chain:** A
- **b-phipsi:** 0.015501858670193
- **w-rdist:** 0.404493811343108
- **t-alpha:** 0.041564883073162

---

---

730

- **PDB ID:** 7TNY | **Chain:** A
- **b-phipsi:** 0.0173893459205722
- **w-rdist:** 0.633428944575588
- **t-alpha:** 0.0047169960791435

---

---

731

- **PDB ID:** 2Z63 | **Chain:** A
- **b-phipsi:** 0.0104024705183454
- **w-rdist:** 0.4529255009519983
- **t-alpha:** 0.0570720736347449

---

---

732

- **PDB ID:** 4X60 | **Chain:** A
- **b-phipsi:** 0.0059052827795714
- **w-rdist:** 0.5365730331241674
- **t-alpha:** 0.0570720736347449

---

---

733

- **PDB ID:** 7KCL | **Chain:** B
- **b-phipsi:** 0.0086755886081695
- **w-rdist:** 0.8768742315440627
- **t-alpha:** 0.0070418134049574

---

---

734

- **PDB ID:** 6SH6 | **Chain:** A
- **b-phipsi:** 0.0120752754544121
- **w-rdist:** 0.8003306056555134
- **t-alpha:** 0.0031401242085145

---

---

735

- **PDB ID:** 6OFB | **Chain:** A
- **b-phipsi:** 0.0071425054052548
- **w-rdist:** 0.613358270417007
- **t-alpha:** 0.0240384962174629

---

---

736

- **PDB ID:** 7XE2 | **Chain:** B
- **b-phipsi:** 0.00836959196245
- **w-rdist:** 0.4721254247442282
- **t-alpha:** 0.068561919033538

---

---

737

- **PDB ID:** 5WOB | **Chain:** D
- **b-phipsi:** 0.0065436996197349
- **w-rdist:** 0.4305248447665109
- **t-alpha:** 0.1596243130423287

---

---

738

- **PDB ID:** 1YGR | **Chain:** A
- **b-phipsi:** 0.0129413589419828
- **w-rdist:** 0.7577478682890091
- **t-alpha:** 0.0046944792707916

---

---

739

- **PDB ID:** 6NIG | **Chain:** C
- **b-phipsi:** 0.0079807150716755
- **w-rdist:** 0.465676770558382
- **t-alpha:** 0.0803043978200703

---

---

740

- **PDB ID:** 1Z68 | **Chain:** B
- **b-phipsi:** 0.0060884230936487
- **w-rdist:** 0.8017958350848148
- **t-alpha:** 0.0172143982372043

---

---

741

- **PDB ID:** 7RL6 | **Chain:** C
- **b-phipsi:** 0.0088281877595354
- **w-rdist:** 0.3054234148379385
- **t-alpha:** 0.1964005103742734

---

---

742

- **PDB ID:** 6ZMJ | **Chain:** A
- **b-phipsi:** 0.0063787500583585
- **w-rdist:** 0.7402641950053752
- **t-alpha:** 0.0199522783818248

---

---

743

- **PDB ID:** 6EOS | **Chain:** F
- **b-phipsi:** 0.0118864695046268
- **w-rdist:** 0.458450477811542
- **t-alpha:** 0.0352112405915889

---

---

744

- **PDB ID:** 4ZG6 | **Chain:** B
- **b-phipsi:** 0.0110246834435159
- **w-rdist:** 0.4813874759069687
- **t-alpha:** 0.0289856519083209

---

---

745

- **PDB ID:** 6I02 | **Chain:** B
- **b-phipsi:** 0.0088793288521318
- **w-rdist:** 0.7707878873769045
- **t-alpha:** 0.0117370267412904

---

---

746

- **PDB ID:** 7L5W | **Chain:** A
- **b-phipsi:** 0.0299741982326759
- **w-rdist:** 0.4853585343766013
- **t-alpha:** 0.0078243548762237

---

---

747

- **PDB ID:** 7NVR | **Chain:** 0
- **b-phipsi:** 0.0162710152385725
- **w-rdist:** 0.7044243990190602
- **t-alpha:** 0.0031296009050172

---

---

748

- **PDB ID:** 7RLF | **Chain:** D
- **b-phipsi:** 0.0099724468465831
- **w-rdist:** 0.3092779028690372
- **t-alpha:** 0.1572766468480066

---

---

749

- **PDB ID:** 7NDL | **Chain:** A
- **b-phipsi:** 0.0069921258993412
- **w-rdist:** 0.7354086218945171
- **t-alpha:** 0.0183267305342325

---

---

750

- **PDB ID:** 1R9N | **Chain:** B
- **b-phipsi:** 0.0065297015962755
- **w-rdist:** 0.7672808734239771
- **t-alpha:** 0.0183267305342325

---

---

751

- **PDB ID:** 6MCK | **Chain:** L
- **b-phipsi:** 0.0207624696335213
- **w-rdist:** 0.5016763320251527
- **t-alpha:** 0.0109543395722921

---

---

752

- **PDB ID:** 5A9J | **Chain:** D
- **b-phipsi:** 0.025135757489889
- **w-rdist:** 0.5688496776423525
- **t-alpha:** 0.0015676375257613

---

---

753

- **PDB ID:** 6U2W | **Chain:** C
- **b-phipsi:** 0.0193867804832934
- **w-rdist:** 0.4000939863029545
- **t-alpha:** 0.0248596569180252

---

---

754

- **PDB ID:** 7A3L | **Chain:** C
- **b-phipsi:** 0.004787918149777
- **w-rdist:** 0.517371917238011
- **t-alpha:** 0.0868544688919852

---

---

755

- **PDB ID:** 6EOO | **Chain:** B
- **b-phipsi:** 0.0100920751784244
- **w-rdist:** 0.5048238038202518
- **t-alpha:** 0.0250390222700223

---

---

756

- **PDB ID:** 5F9F | **Chain:** C
- **b-phipsi:** 0.011019083373212
- **w-rdist:** 0.6236537760069392
- **t-alpha:** 0.0150916313901026

---

---

757

- **PDB ID:** 4GSC | **Chain:** A
- **b-phipsi:** 0.0067784057828396
- **w-rdist:** 0.4492314989986009
- **t-alpha:** 0.1322378273556061

---

---

758

- **PDB ID:** 4ZAJ | **Chain:** A
- **b-phipsi:** 0.0258999440076874
- **w-rdist:** 0.5596772718330456
- **t-alpha:** 0.0015676375257613

---

---

759

- **PDB ID:** 3H44 | **Chain:** B
- **b-phipsi:** 0.0081123984464733
- **w-rdist:** 0.4412557226272072
- **t-alpha:** 0.1142409049902133

---

---

760

- **PDB ID:** 7K71 | **Chain:** A
- **b-phipsi:** 0.0135229851232763
- **w-rdist:** 0.3572015745943324
- **t-alpha:** 0.0790296903641281

---

---

761

- **PDB ID:** 5XDR | **Chain:** A
- **b-phipsi:** 0.010214187716467
- **w-rdist:** 0.8055739136989782
- **t-alpha:** 0.0070418134049574

---

---

762

- **PDB ID:** 8FCT | **Chain:** C
- **b-phipsi:** 0.008908693898895
- **w-rdist:** 0.3178369512676494
- **t-alpha:** 0.1924876687885701

---

---

763

- **PDB ID:** 6XI2 | **Chain:** C
- **b-phipsi:** 0.0054831316469888
- **w-rdist:** 0.507781133708285
- **t-alpha:** 0.0904441004412783

---

---

764

- **PDB ID:** 4RIB | **Chain:** B
- **b-phipsi:** 0.0376886790037467
- **w-rdist:** 0.4498370114309438
- **t-alpha:** 0.0102767895279776

---

---

765

- **PDB ID:** 3BG3 | **Chain:** A
- **b-phipsi:** 0.0123082944283016
- **w-rdist:** 0.6603866543765977
- **t-alpha:** 0.0101717762082627

---

---

766

- **PDB ID:** 3ALA | **Chain:** E
- **b-phipsi:** 0.0142706949456098
- **w-rdist:** 0.6242205793171816
- **t-alpha:** 0.0094788657373643

---

---

767

- **PDB ID:** 8FE7 | **Chain:** C
- **b-phipsi:** 0.0217419485057035
- **w-rdist:** 0.6486080237797702
- **t-alpha:** 0.0

---

---

768

- **PDB ID:** 6MFR | **Chain:** B
- **b-phipsi:** 0.0058951350696006
- **w-rdist:** 0.457244907466078
- **t-alpha:** 0.1384973100154414

---

---

769

- **PDB ID:** 4IOF | **Chain:** B
- **b-phipsi:** 0.006585966285045
- **w-rdist:** 0.4297517748202181
- **t-alpha:** 0.16666588421628

---

---

770

- **PDB ID:** 6SVM | **Chain:** A
- **b-phipsi:** 0.0046237257945563
- **w-rdist:** 0.7478603357434583
- **t-alpha:** 0.0232189692826079

---

---

771

- **PDB ID:** 4GUR | **Chain:** A
- **b-phipsi:** 0.0064140033078569
- **w-rdist:** 0.5109558299855206
- **t-alpha:** 0.0739499805270313

---

---

772

- **PDB ID:** 7ZGJ | **Chain:** A
- **b-phipsi:** 0.0368244225758621
- **w-rdist:** 0.2850225075087542
- **t-alpha:** 0.0226916757534991

---

---

773

- **PDB ID:** 5RVW | **Chain:** B
- **b-phipsi:** 0.014136148198923
- **w-rdist:** 0.4195475871210823
- **t-alpha:** 0.0475413495623815

---

---

774

- **PDB ID:** 7SFC | **Chain:** A
- **b-phipsi:** 0.0056490189724978
- **w-rdist:** 0.4595075477195142
- **t-alpha:** 0.1400624158202974

---

---

775

- **PDB ID:** 5I04 | **Chain:** A
- **b-phipsi:** 0.0006950868193692
- **w-rdist:** 0.6202067642386755
- **t-alpha:** 0.0775720075441686

---

---

776

- **PDB ID:** 3ZD6 | **Chain:** A
- **b-phipsi:** 0.0168010894692226
- **w-rdist:** 0.308503059883656
- **t-alpha:** 0.0719873632833352

---

---

777

- **PDB ID:** 3MDJ | **Chain:** A
- **b-phipsi:** 0.008157237100001
- **w-rdist:** 0.375469959765447
- **t-alpha:** 0.1737087976681894

---

---

778

- **PDB ID:** 5GGI | **Chain:** B
- **b-phipsi:** 0.0015051248250539
- **w-rdist:** 0.7693882260861002
- **t-alpha:** 0.025682454261255

---

---

779

- **PDB ID:** 4DSZ | **Chain:** A
- **b-phipsi:** 0.0062420453733924
- **w-rdist:** 0.7834100743463668
- **t-alpha:** 0.0187789021729365

---

---

780

- **PDB ID:** 5IUD | **Chain:** D
- **b-phipsi:** 0.0080687686678545
- **w-rdist:** 0.3991694071096539
- **t-alpha:** 0.1525818235009972

---

---

781

- **PDB ID:** 4WJL | **Chain:** B
- **b-phipsi:** 0.0095249380451329
- **w-rdist:** 0.8261967476900586
- **t-alpha:** 0.0070922351376341

---

---

782

- **PDB ID:** 6BYZ | **Chain:** B
- **b-phipsi:** 0.0078873020985611
- **w-rdist:** 0.4445661728906921
- **t-alpha:** 0.1181528955465753

---

---

783

- **PDB ID:** 2YPU | **Chain:** A
- **b-phipsi:** 0.0074212963461456
- **w-rdist:** 0.4513629888159851
- **t-alpha:** 0.1150232700097437

---

---

784

- **PDB ID:** 6EOS | **Chain:** A
- **b-phipsi:** 0.0125917520608083
- **w-rdist:** 0.4563246149634548
- **t-alpha:** 0.0336461458262049

---

---

785

- **PDB ID:** 7BP9 | **Chain:** F
- **b-phipsi:** 0.0097834779119415
- **w-rdist:** 0.346423369847454
- **t-alpha:** 0.1478868884570592

---

---

786

- **PDB ID:** 6WRV | **Chain:** B
- **b-phipsi:** 0.0063705326921139
- **w-rdist:** 0.894005434627646
- **t-alpha:** 0.0125193542574237

---

---

787

- **PDB ID:** 4DWK | **Chain:** A
- **b-phipsi:** 0.0072682172068608
- **w-rdist:** 0.451458140153171
- **t-alpha:** 0.1189353396200536

---

---

788

- **PDB ID:** 8SZP | **Chain:** A
- **b-phipsi:** 0.0104636654262299
- **w-rdist:** 0.4605052161998032
- **t-alpha:** 0.0524253871955315

---

---

789

- **PDB ID:** 3HIZ | **Chain:** A
- **b-phipsi:** 0.0115111690430452
- **w-rdist:** 0.4159486050166758
- **t-alpha:** 0.0782468728132206

---

---

790

- **PDB ID:** 4L3T | **Chain:** B
- **b-phipsi:** 0.0094296682698199
- **w-rdist:** 0.4773665206371689
- **t-alpha:** 0.0532080396597527

---

---

791

- **PDB ID:** 5WTD | **Chain:** A
- **b-phipsi:** 0.0142224786257596
- **w-rdist:** 0.6134440491558697
- **t-alpha:** 0.0102767895279776

---

---

792

- **PDB ID:** 7PFS | **Chain:** A
- **b-phipsi:** 0.0121125678041604
- **w-rdist:** 0.4479333203434661
- **t-alpha:** 0.0492955855664358

---

---

793

- **PDB ID:** 4G8A | **Chain:** B
- **b-phipsi:** 0.0112948471905882
- **w-rdist:** 0.5068339804806944
- **t-alpha:** 0.0232189692826079

---

---

794

- **PDB ID:** 6P8I | **Chain:** A
- **b-phipsi:** 0.0364876768711797
- **w-rdist:** 0.3608537966120016
- **t-alpha:** 0.0195618131710275

---

---

795

- **PDB ID:** 6UXY | **Chain:** A
- **b-phipsi:** 0.0014065636375894
- **w-rdist:** 0.539569876085997
- **t-alpha:** 0.0969960165657513

---

---

796

- **PDB ID:** 7K1D | **Chain:** A
- **b-phipsi:** 0.0063032257403459
- **w-rdist:** 0.451798557139922
- **t-alpha:** 0.1439745083193748

---

---

797

- **PDB ID:** 4GU0 | **Chain:** C
- **b-phipsi:** 0.0074528670384574
- **w-rdist:** 0.5173728645937117
- **t-alpha:** 0.0527184999556347

---

---

798

- **PDB ID:** 4PES | **Chain:** B
- **b-phipsi:** 0.0085729825427294
- **w-rdist:** 0.4255043138184484
- **t-alpha:** 0.1236302639257407

---

---

799

- **PDB ID:** 8EFE | **Chain:** A
- **b-phipsi:** 0.0199361152597242
- **w-rdist:** 0.3180223048920458
- **t-alpha:** 0.0501234795277865

---

---

800

- **PDB ID:** 7L5X | **Chain:** B
- **b-phipsi:** 0.0348012570314495
- **w-rdist:** 0.4799975740012425
- **t-alpha:** 0.0078243548762237

---

---
